# Pan-cancer multi-omics analysis and orthogonal experimental assessment of epigenetic driver genes

**DOI:** 10.1101/gr.268292.120

**Published:** 2020-10

**Authors:** Andrea Halaburkova, Vincent Cahais, Alexei Novoloaca, Mariana Gomes da Silva Araujo, Rita Khoueiry, Akram Ghantous, Zdenko Herceg

**Affiliations:** Epigenetics Group, International Agency for Research on Cancer (IARC), 69008 Lyon, France

## Abstract

The recent identification of recurrently mutated epigenetic regulator genes (ERGs) supports their critical role in tumorigenesis. We conducted a pan-cancer analysis integrating (epi)genome, transcriptome, and DNA methylome alterations in a curated list of 426 ERGs across 33 cancer types, comprising 10,845 tumor and 730 normal tissues. We found that, in addition to mutations, copy number alterations in ERGs were more frequent than previously anticipated and tightly linked to expression aberrations. Novel bioinformatics approaches, integrating the strengths of various driver prediction and multi-omics algorithms, and an orthogonal in vitro screen (CRISPR-Cas9) targeting all ERGs revealed genes with driver roles within and across malignancies and shared driver mechanisms operating across multiple cancer types and hallmarks. This is the largest and most comprehensive analysis thus far; it is also the first experimental effort to specifically identify ERG drivers (epidrivers) and characterize their deregulation and functional impact in oncogenic processes.

Although it has long been known that human cancers harbor both genetic and epigenetic changes, with an intricate interplay between the two mechanisms underpinning the hallmarks of cancer (Hanahan and Weinberg 2011), it is only with the fruition of large-scale international sequencing efforts that major enigmas of the cancer (epi)genome have started to be solved (Jones et al. 2016; Ng et al. 2018). One of the most remarkable findings of the international high-resolution cancer genome sequencing efforts, spearheaded by The Cancer Genome Atlas (TCGA), is the high frequency of genetic alterations in the genes encoding proteins that directly regulate the epigenome (referred to here as epigenetic regulator genes [ERGs]) (Gonzalez-Perez et al. 2013; Plass et al. 2013; Shen and Laird 2013; Timp and Feinberg 2013; Vogelstein et al. 2013; Yang et al. 2015). This high rate of ERG genetic deregulation constitutes a “genetic smoking gun,” indicating that epigenetic mechanisms lie at the very heart of cancer biology. These discoveries have sparked a debate on the role of ERG deregulation (either through mutational or nongenetic events) in ERG expression and in the mechanisms underlying tumorigenesis and epigenome alterations that are rampant in virtually all human malignancies (Plass et al. 2013; Timp and Feinberg 2013). We also still lack a systematic understanding of the functional importance of ERG disruption in tumor development and progression, as well as its impact on cancer cell phenotype.

ERGs are a group of more than 400 coding genes in the human genome, most of which encode enzymes that add (“writers”), modify/revert (“editors”), or recognize (“readers”) epigenetic modifications (Plass et al. 2013; Vogelstein et al. 2013) controlling a range of critical cellular processes. Based on the observation that many ERGs are frequently disrupted across different malignancies, they are candidates to be drivers of cancer development and progression, potentially acting as oncogenes or tumor suppressors (Plass et al. 2013; Vogelstein et al. 2013). Although several distinct definitions of “driver gene” exist in the literature (Sawan et al. 2008; Vogelstein et al. 2013), we define “driver genes” as those genes that, when deregulated (through somatic mutations, copy number variations, or aberrant expression), assume primary importance in tumor development such as conferring a selective growth advantage, immortalization, and invasiveness. This definition relies on inference models for driver prediction and functional data (based on the impact of the gene on cellular processes) compared to other methods that are mostly based on statistical models (largely driven by the mutation frequency of a gene) (Parmigiani et al. 2009; Meyerson et al. 2010; Lahouel et al. 2020). In line with this physiological definition, we refer to those ERGs that make a net contribution to tumorigenesis as “epigenetic driver genes” (henceforth called “epidrivers”). Our definition is different from that used by other investigators (Vogelstein et al. 2013), who define epidrivers as the genes (not necessarily among ERGs) that are aberrantly expressed through changes in DNA methylation and chromatin modifications and confer a selective growth advantage.

The products of ERGs are involved in processes such as DNA methylation, histone modification, chromatin remodeling, and other chromatin-based modifications, and many ERGs may have both histone and nonhistone substrates. All of these processes, in turn, are involved in the proper control of not only gene expression programs, required for the establishment and maintenance of cell identity and function, but also DNA repair, recombination, and genome integrity (Murr et al. 2006; Bell et al. 2011). Because common cancers represent the final outcome of a multistep process, epidriver-based disruption of cellular processes may not only assume a primary role at different stages of tumorigenesis but also constitute critical mechanisms underpinning cancer cell plasticity and emergence of cancer resilience. Here, we conducted a systematic and comprehensive pan-cancer investigation of (epi)genetics- and transcriptome-based deregulation of all ERGs using in silico data curation in clinical samples and characterization of the driver potential by different computational tools. We also developed and tested a conceptual framework for experimental identification and functional characterization of the mechanistically important epidrivers that reshape the epigenome and contribute to cancer phenotypes. This framework builds on the latest knowledge of the cancer (epi)genome and genomic databases and includes powerful new experimental models including state-of-the-art genome-editing screens, phenotyping, and functional genomics.

## Results

**Figure 1.**
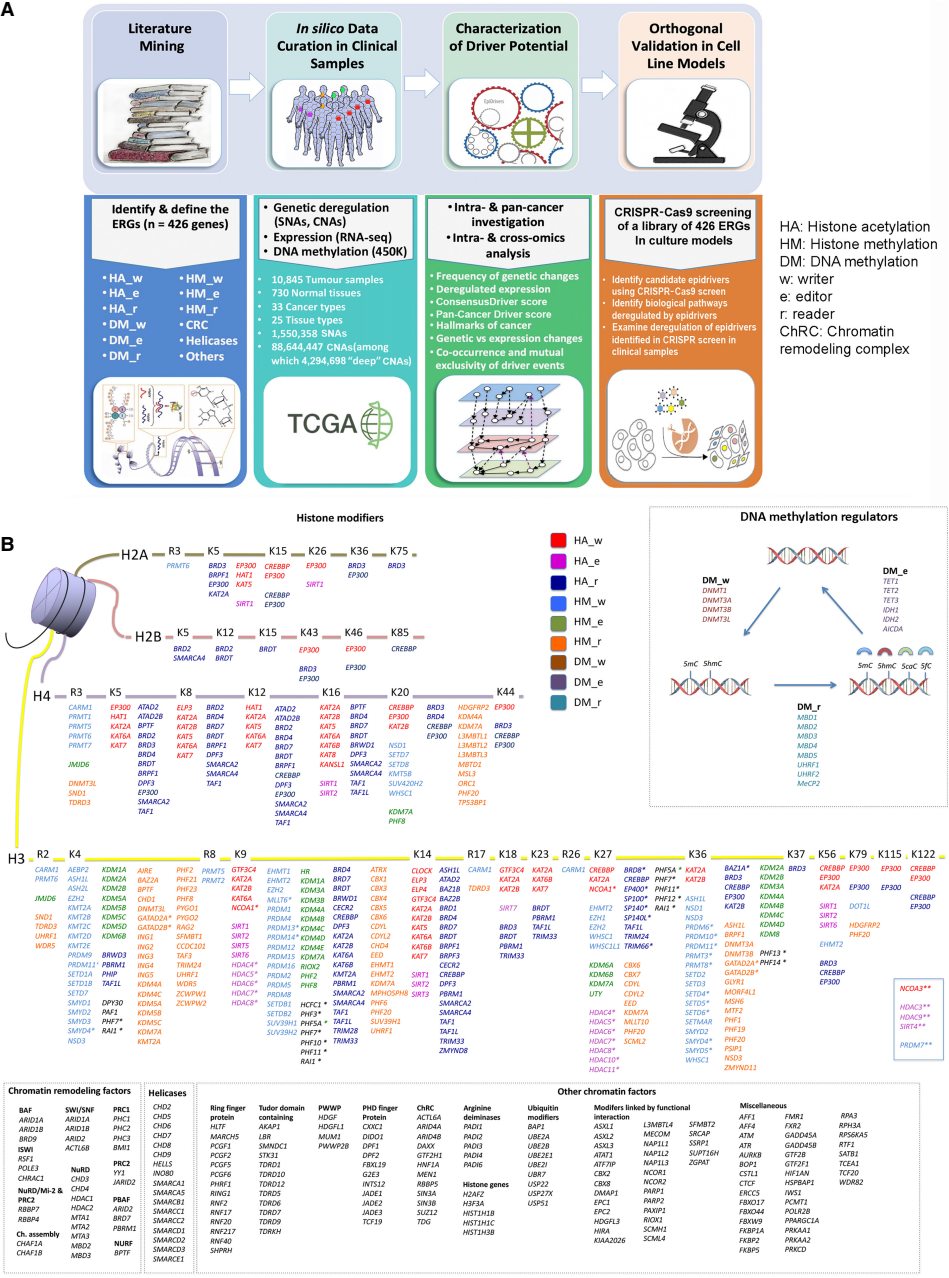
Study design. (*A*) A four-stage approach to identify and characterize ERGs with cancer driver potential. (*B*) The compendium of ERGs curated and analyzed, comprising 426 genes classified into histone modifiers, DNA methylation regulators, chromatin remodeling factors (ChRC), helicases, and other chromatin modifiers (some of which were further divided into subgroups based on function or their presence in molecular complexes). Histone acetylation, histone methylation, and DNA methylation modifiers are further stratified each into “writers” (w), “editors” (e), and “readers” (r). (*) The histone modifying genes whose functions are not well characterized and which were, therefore, assigned based on ENCODE ChIP sequencing data; (**) the histone modifying genes without assignment of residues in the histone tails.

A four-stage strategy was used to identify and characterize ERGs with cancer driver potential (Fig. 1A). We assembled a comprehensive compendium of ERG genes by literature mining and manual curation, resulting in a list of 426 genes coding for histone modifiers, DNA methylation regulators, chromatin remodelers, helicases, and other epigenetic entities (Fig. 1B; Supplemental Tables S1, S2). To identify the candidate epidrivers across different cancer types, we first used comprehensive in silico data mining of genetic and RNA expression alterations of ERGs using data from TCGA (Fig. 1A). Collectively, the data encompassed 33 different cancer types from 25 tissue types, with sequencing information from 10,845 tumor samples and 730 normal tissues, including a total of 90,144,805 genetic alterations, which encompassed 1,500,358 somatic mutations (single-nucleotide alterations [SNAs]) and 88,644,447 somatic copy number alterations (CNAs) (of which 4,294,698 were deep deletions/amplifications). We subsequently characterized, across various cancer types, the driver potential of ERGs based on ConsensusDriver scores (Bertrand et al. 2018), which we complemented with our proposed Pan-Cancer Driver and Multi-Omics Driver scores, and the implications of these ERGs in cancer hallmark pathways. Finally, we performed an orthogonal validation of the driver potential of 426 ERGs in cell line models in comparison with the findings from the clinical samples (Fig. 1A).

### Pan-cancer analysis of genetic alterations in ERGs

**Figure 2.**
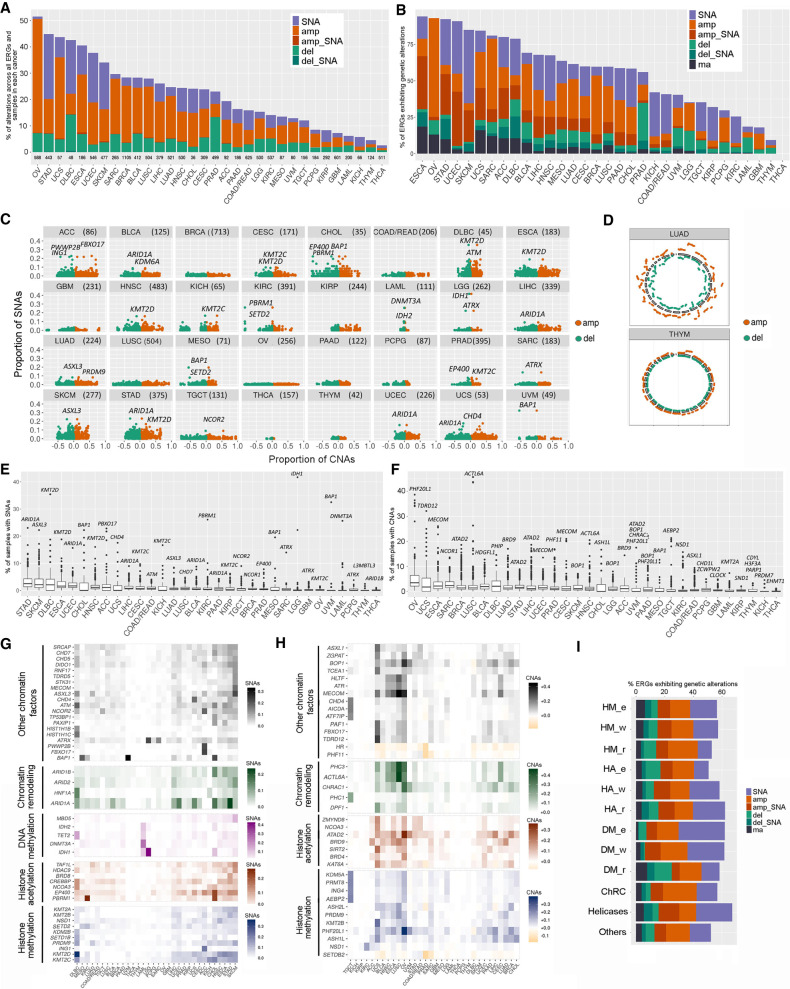
Pan-cancer analysis of genetic alterations across ERG categories and classes. (*A*,*B*) The percentage of samples with genetic deregulation in ERGs (*A*) and the percentage of ERGs showing different types of genetic deregulation (*B*), by cancer type. ERGs are considered altered if at least 1% of samples harbor these genetic aberrations. (*C*) Proportion of samples with SNAs versus that with deletions (−1, −2) or amplifications (+1, +2) in ERGs for each cancer type. Each gene is represented by two dots (red and green) depicting amplified and deleted CNAs, respectively. (*D*) Circos plots showing the relative amount of deregulation in CNAs by chromosomal distribution in two representative cancer types (LUAD and THYM, characterized by high and low CNA burden, respectively). The level of CNAs for each ERG was calculated as the proportion of samples considering all types of CNAs (amplification = +1, +2 and deletion = −1, −2) in ERGs in each cancer type. (*E*,*F*) Box plots showing the percentage of samples with SNAs (*E*) and deep CNAs (*F*) by gene and by cancer type. The most deregulated ERGs are highlighted for each cancer type. (*G*,*H*) Heatmaps representing the top genetically deregulated genes showing SNAs (*G*) and CNAs (*H*) in at least 10% and 15%, respectively, of the samples for any cancer type. Only samples with deep CNAs were included. ERGs are grouped into functional categories as indicated. (*I*) The percentages of ERGs that show genetic alteration among all cancer types by functional groups. Genetic alterations: (SNA) single-nucleotide alteration, (amp) deep copy number amplification, (amp_SNA) deep amplification co-occurring with SNA, (del) deep copy number deletion, (del_SNA) deep deletion co-occurring with SNA, and (ma) multiple alterations. In cases in which both types of CNAs (amplification and deletion) of one gene were present in the samples, we reported in *B* and *H* the alteration that was at least twice as prevalent as the other; otherwise, the alteration was reported under the multiple alteration category.

To identify potential epidrivers, we first analyzed the frequency of genetic disruption of ERGs (vs. all genes) across malignancies from different anatomical sites. Our analysis revealed that the predominant genetic alterations in ERGs were deep amplifications or SNAs, depending on the cancer type (Fig. 2A,B). In comparison, the predominant genetic alteration in all human genes was deep amplification in most cancer types (Supplemental Fig. S1A,B). Overall, higher proportions of amplifications than deletions were observed in ERGs or in all genes across all cancer types except a few (mainly, DLBC and PRAD) (Fig. 2A,B; Supplemental Fig. S1A–C). Some cancer types (e.g., OV) had predominately CNAs with almost no SNAs (Fig. 2A–D; Supplemental Fig. S1A,B). In most cancer types, the CNAs (Supplemental Fig. S1D) and SNAs (Supplemental Fig. S2) were uniformly distributed across chromosomes, with the exception of GBM, KIRP, and UVM, which showed CNAs in specific chromosomes (Supplemental Fig. S1D).

Many specific ERGs were identified as being genetically altered at noticeably high levels in different malignancies (Fig. 2E,F). In particular, SNAs in *IDH1* (Fig. 2E,G; Supplemental Fig. S2) and deep CNAs in *ACTL6A* (Fig. 2F,H) had high proportions of alterations, exceeding 40% of samples in LGG and LUSC, respectively. Several ERGs had the highest mutation frequency repeatedly in many cancer types, namely the *KMT2C/D* family (seven cancers), *ARID1A* (five cancers), *BAP1* (three cancers), and *ATRX* (three cancers) (Fig. 2E,G; Supplemental Table S3). A similar observation was made for deep CNAs in ERGs, namely *BOP1* (four cancers), *ATAD2* (four cancers), *MECOM* (three cancers), and *PHF20L1* (three cancers) (Fig. 2F,H). A larger percentage of ERG alterations was also observed when both deep and shallow CNAs were included (Fig. 2C,D; Supplemental Fig. S1C,D). Among the top ERGs altered by deep CNAs, the majority showed amplifications, with the exception of *HR*, *PHF11*, and *SETB2*, which were commonly deleted in many cancer types (Fig. 2H). Frequently amplified ERGs often co-occurred in the same tumor sample in many cancer types; in particular, the aforementioned pan-cancer recurrent genes *BOP1*, *ATAD2*, and *PHF20L1* highly co-occurred (Supplemental Fig. S3A). These co-occurrences remained prominent even when the analysis was focused only on deep amplifications/deletions across tumors (Supplemental Fig. S3B,C). Moreover, the family of TDRs (*TDRKH*, *TDRD10*, and *TDRD5*) highly co-occurred together. Generally little overlap was observed between the genes with a high frequency of SNAs and those with a high frequency of CNAs (except for a few ERGs) (Fig. 2E–H).

When ERGs were stratified by functional groups, similar total proportions of genetic alterations were seen among ERG classes (Fig. 2I). DNA methylation writers and editors were characterized by a prominent proportion of SNAs in several cancer types, compared with other ERG classes (Fig. 2I; Supplemental Fig. S4A,B). Indeed, DNA methylation modulators appeared among the top SNA profiles (Fig. 2G) but not among the top CNA profiles (Fig. 2H) of ERGs. Moreover, in many cancer types, DNA methylation writers or editors, which are among the smallest ERG classes, were the group showing the largest percentage of genetically altered ERGs (Supplemental Fig. S4A,B). Among ERGs that could be classified as tumor suppressors, *KMT2D*, *KMT2C*, *ARID1A*, *ATRX*, *CREBBP*, and *PBRM1* were frequently mutated in many cancer types, whereas oncogenic ERGs were each mutated in specific cancer types, mainly *IDH1* in LGG and *DNMT3A* in LAML (Supplemental Fig. S5A,B).

### Pan-cancer analysis of RNA expression in relation to genetic and DNA methylome aberrations of ERGs

**Figure 3.**
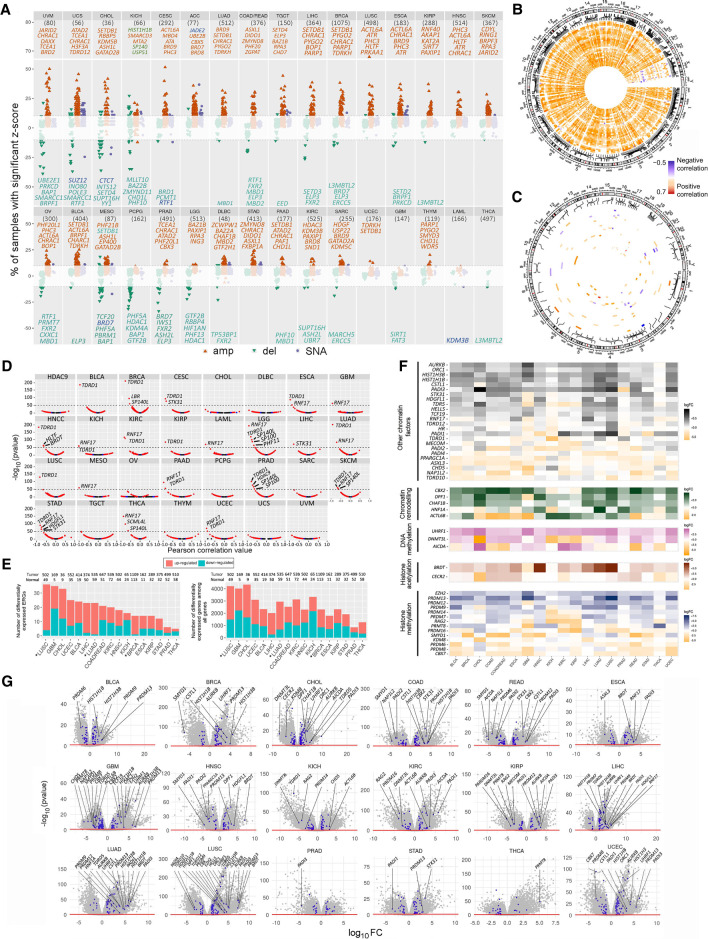
RNA expression alterations of ERGs across cancer types, in relation to genetic and DNA methylome variations. (*A*) Multi-omics plot of SNA, CNA, and RNA expression alterations across ERGs and cancer types. Amplifications, deletions, and SNAs were annotated as described in Methods. The most deregulated ERGs in RNA expression (with the *y*-axis value above 10) are highlighted for each cancer type. (*B*,*C*) Circos plots showing Pearson's correlation between CNAs (*B*) or SNAs (*C*) and expression *Z*-scores in different cancer types across the chromosomal regions. Positive and negative correlations are indicated in orange and blue, respectively. Only ERGs with correlation (*R*^2^) > 30% and FDR < 0.05 in at least in one cancer type were considered for the analysis in *B*; the *R*^2^ limit was set to 10% in *C*. (*D*) Expression quantitative trait methylation (eQTMs) analysis showing Pearson correlation values (*x*-axis) between RNA (RSEM counts) and methylation (beta) levels of promoter CpGs for each ERG in different cancer types. The line bar indicates highly significant CpGs [−log(*P*-value) > 50]. Red, blue, and black dots represent CpGs with FDR < 0.05, *P* < 0.05, and *P* > 0.05, respectively. (*E*) Number of ERGs or all genes with differential RNA expression in tumor relative to adjacent normal tissues for each cancer type (|log FC| > 2 and FDR < 0.05). The star denotes a *P*-value < 0.05 by a two-sample test of proportions of up- versus down-regulation. (*F*) Heatmaps showing the most differentially expressed ERGs comparing tumor samples with adjacent normal tissues among cancer types. Only the top differently expressed ERGs with |log FC| > 3 and FDR < 0.05 are annotated. (*G*) Volcano plots showing differentially expressed ERGs in tumors relative to adjacent normal tissues. ERGs are shown in blue (|log FC| > 1), and the most deregulated ERGs with |log FC| > 3 are highlighted for each cancer type (FDR < 0.05). Sample sizes for each cancer type are indicated in *A* and *E*.

The second approach in our analysis focused on RNA expression deregulation (by RNA-seq) of ERGs across cancer types. For each cancer type, the analysis consisted of two parts: expression variation across tumor samples relative to one another (independent of the corresponding normal tissue) and expression changes in tumor relative to adjacent normal tissue. By integrating genetic and transcriptomic information matched to the same samples (using the TCGA database) a higher proportion of tumor samples showed significantly increased ERG expression (*Z*-score > 2) relative to down-regulation (*Z*-score < −2) (Fig. 3A), in line with the observed higher proportion of samples with ERG amplifications than with deletions (Figs. 2A, 3A).

Amplifications and deletions significantly correlated (false discovery rate [FDR] < 0.05) positively with increased and decreased expression, respectively, in all cancer types and chromosomes (Fig. 3A,B), except for Chromosome X, because of a statistical artifact (Methods; Supplemental Fig. S6A,B). SNAs significantly correlated (FDR < 0.05) negatively or positively with expression across tumor samples and chromosomes, so the correlation was not unidirectional (Fig. 3A,C). ERG-specific analysis revealed some ERGs with noticeably high expression aberrations within tumor samples (Fig. 3A). Among them, several ERGs were repeatedly up- or down-regulated in several cancer types; the primary genes were *CHRAC1*, *PHC3*, *and BOP1*, which were among the top 10 most up-regulated ERGs recurrently observed in 18, 9, and 7, respectively, out of 33 cancer types (Fig. 3A, only the top 5 ERGs are shown; see Supplemental Table S3 for the top 10 ERGs). Many of the ERGs with the highest deregulated expression are the same ERGs with the highest CNAs of the corresponding cancer type (Fig. 3A vs. Fig. 2F).

Because epigenetic inactivation could be an additional mechanism for aberrant expression of ERGs, we performed concurrent analyses of transcriptomic and DNA methylome data available for the tumor samples in the TCGA. We correlated the methylation with RNA-seq levels by limiting the comparison to CpGs in promoter regions (−1000 to +500 bp of TSS) and RNA transcripts of the overlapping gene. The most significantly correlated CpG for each gene is shown in Figure 3D, and results for all analyzed CpGs are provided in Supplemental Table S4 and the figshare repository (https://figshare.com/articles/Supplemental_data/12613220). All CpGs that were highly significant [−log(*P-*value) > 50] were negatively correlated with the expression of their corresponding gene, and many of them were recurrent in several tumor types, namely CpGs in *TDRD1* (*n* = 16 tumor types), *RNF17* (*n* = 13 tumor types), *SP140L* (*n* = 5 tumor types), and *STK31* (*n* = 3 tumor types) (Fig. 3D).

Comparing the expression levels of ERGs in tumor relative to adjacent normal tissue in each cancer type also revealed a predominant pattern of overexpressed ERGs in most cancer types, except GBM and KICH (Fig. 3E). Similar observations were made when all human genes were analyzed (Fig. 3E). ERG-specific analysis revealed several ERGs with significant deregulation of expression (FDR < 0.05) (Fig. 3F,G) and recurrence in several cancer types. Several genes had similar recurrence across several cancer types, namely *PADI3* (in 11 of 18 cancers), *PRDM13* (in 10 of 18 cancers), *AURKB* (in 9 of 18 cancers), and *HIST1H1B* and *HIST1H3B* (each in 8 of 18 cancers), based on the selection of only the top genes (FDR < 0.05 and log_10_ fold change [log_10_FC] > 3) (Fig. 3G).

### Characterizing the driver potential of deregulated ERGs across cancer types

**Figure 4.**
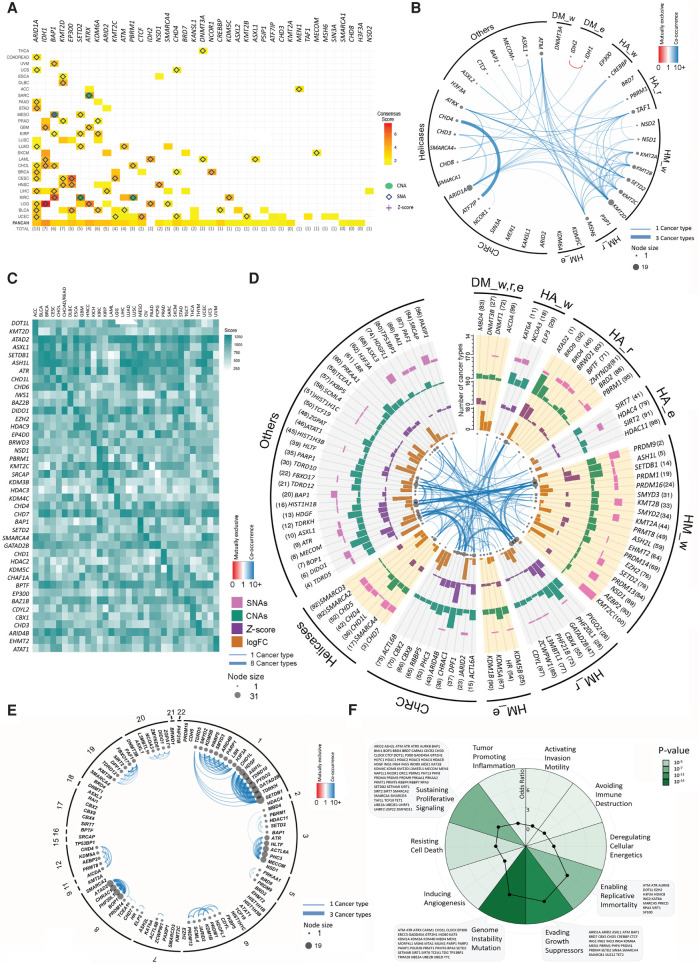
Characterization of ERG driver potentials. (*A*) Heatmap showing the ConsensusDriver scores (with values ranging from 1.5 to 7.5) as obtained by Bailey et al. (2018). ERGs with a score ≥ 1.5 in at least one cancer type are shown. The top 10 deep amplifications or deletions (green circles), SNAs (blue empty diamonds), or significant *Z*-score (purple crosses) of each cancer are overlapped onto the heatmap. (*B*) Significant (FDR < 0.05) co-occurrence and mutual exclusivity for ConsensusDriver ERGs in a pan-cancer analysis. The node size is proportional to both the number and thickness of its connections with other nodes. Blue and red edges represent co-occurrence (odds ratio [OR] > 1) and mutual exclusivity (OR < 1), respectively. The transparency of the edges indicates the average OR across cancer types, and their thickness is proportional to the number of cancer types in which the OR is significant. The co-occurrence filter was set to at least 5% of the samples per cancer type (Methods). (*C*) Heatmap of the Multi-Omics Driver scores of ERGs per cancer type. The ERGs shown represent a pooled set of the top three ERGs in each cancer type, as ranked by the mutli-omics driver score. (*D*) Top 100 ERGs by Pan-Cancer Driver score using SNA (5% of samples), CNA (5% of samples), and expression data (15% of samples with significant *Z*-score or FDR < 0,05 with log_10_FC > 1). Results are represented as bar plots counting the number of cancers in which a given gene has a particular genomic or expression alteration. From outer to inner track: (1, pink) SNAs; (2, green) CNAs; (3, purple) *Z*-score; (4, orange) log_10_FC. Inside the last track, co-occurrence or mutual exclusivity was calculated as in *B*, except that the co-occurrence filter was set to at least 10% of the samples per cancer type. Genes are aggregated by their functional features. (*E*) Significant co-occurrence for the top 100 ERGs by Pan-Cancer Driver score. Co-occurrence or mutual exclusivity was calculated as in *D* but ordered instead by chromosome number. (*F*) Spider pie chart showing enrichment of the 426 ERGs in pathways affecting the 10 hallmarks of cancer; the corresponding *P*-values and ORs are illustrated by green gradients and black spots, respectively. The names of ERGs overlapping with the four significantly enriched hallmarks are indicated.

The third strategy in our analysis to characterize the potential driver roles of ERGs was based on ConsensusDriver, a novel approach that provides a systematic way to integrate the strengths of various driver prediction algorithms (Bertrand et al. 2018). The ERGs with a potential driver role (ConsensusDriver score > 1.5) are shown for each cancer type (Fig. 4A) and are significantly enriched relative to the 233 total genes (Bailey et al. 2018) that have a driver score > 1.5 (*P* = 4.0 × 10^−22^, Fisher's exact test). Six additional ERGs would still be classified as drivers at a score < 1.5 but with manual curation by Bailey et al. (2018), and these are *ATR*, *EZH2*, *HIST1H1C*, *PHF6*, *SMARCB1*, and *TET2.* The ConsensusDriver score matched to a high extent with the driver potential predicted based on SNA frequencies in each cancer type, and to a lesser extent with that predicted based on CNA, FC, or *Z*-scores (Fig. 4A); the latter three, if matching with ConsensusDriver score, never occurred without SNAs, further emphasizing the importance of SNAs in the derivation of ConsensusDriver score (Fig. 4A). *IDH1* had the highest ConsensusDriver score, as evident in LGG, and this ERG showed a driver role in six other cancer types, which explains why it additionally had the highest pan-cancer ConsensusDriver score (PANCAN) (Fig. 4A).

In comparison, *ARID1A* was the ERG with the most frequent driver score, appearing in 13 cancer types, although with a relatively weak to modest driver role in individual cancer categories. ConsensusDriver ERGs were enriched in several gene families (namely *ARID(1A/2)*, *ASXL1/2*, *CHD3/4/8*, *IDH1/2*, *KDM(5C/6A)*, *KMT2A/B/C/D*, *NSD1/2*, and *SMARCA1/4*) as well as in UCEC (*n* = 14 ERGs) and BLCA (*n* = 10 ERGs) (Fig. 4A). Genetic deregulation of ConsensusDriver ERGs often co-occurred in the same sample across several cancer types, with *KMT2D* and *ARID1A* having the highest co-occurrence scores. The ERGs within the same *KMT2A/B/C/D* family highly co-occurred together even though they were not mapped to the same chromosomes (Supplemental Fig. S3C), whereas *IDH1* and *IDH2* were mutually exclusive (Fig. 4B).

We complemented ConsensusDriver (weighted for SNAs) with our Multi-Omics Driver score that is weighted for each of SNAs, CNAs, and expression aberrations. The Multi-Omics Driver scores for all ERGs across cancer types is shown in Figure 4C and Supplemental Table S5 (the figshare repository: https://figshare.com/articles/Supplemental_data/12613220). This score revealed ERGs with a high Multi-Omics Driver score in most cancer types (such as ATAD2) against those showing single driver score (such as IDH1, which has a high SNA driver score in LGG but relatively low CNA and expression driver scores). We next formulated another score, the Pan-Cancer Driver score, that additionally weights for pan-cancer coverage on top of SNAs, CNAs, and expression aberrations (Fig. 4D). *ATAD2* had the highest Pan-Cancer Driver score, showing all the SNA, CNA, and expression *Z*-score alterations in many cancer types. When we considered the top 39 Pan-Cancer Driver genes, representing a sample size identical to that identified from ConsensusDriver, we found several driver ERGs to be similarly represented in both sets, namely, *SMARCA4* (score = 11), *ASXL1* (score = 12), *BAP1* (score = 26), *KMT2B* (score = 38), and *MECOM* (score = 37). HM and HA, but not DM, modulators were highly represented among the top 100 Pan-Cancer Driver ERGs, probably because DM modulators are mostly altered by SNAs (Fig. 2G vs. 2H), and hence are characterized by ConsensusDriver (e.g., *IDH1* and *DNMT3A*) (Fig. 4A) rather than Pan-Cancer Driver (Fig. 4D; Supplemental Fig. S5B) profiles. Similarly, *ARID1A*, which is characterized predominantly by the SNA type of genetic alterations, showed ConsensusDriver potential in many cancer types (Fig. 4A) but did not appear among the top 100 Pan-Cancer Driver ERGs (Fig. 4D; Supplemental Fig. S5B). Genetic deregulation of Pan-Cancer Driver ERGs often co-occurred in the same sample across several cancer types (Fig. 4E).

Next, we investigated whether epidrivers are enriched in pathways affecting the 10 hallmarks of cancer. Our compendium of 426 ERGs was significantly enriched in four hallmarks, namely genome instability and mutation, evading growth suppressors, sustaining proliferative signaling, and enabling replicative immortality (Fig. 4F; Supplemental Fig. S7A,B), further supporting a driver role of ERGs in tumorigenesis and characterizing the nature of biological pathways in which ERGs play a functional role in cancer. These four hallmarks were also topmost significant in the 39 ConsensusDriver (*P* < 0.05), top 39 Pan-Cancer Driver (*P* < 0.1), and 42 multi-omic driver (*P* < 0.05) ERGs (Supplemental Fig. 7C).

### Orthogonal CRISPR-Cas9 screen to assess the driver potential of ERGs in epithelial-to-mesenchymal transition (EMT)

**Figure 5.**
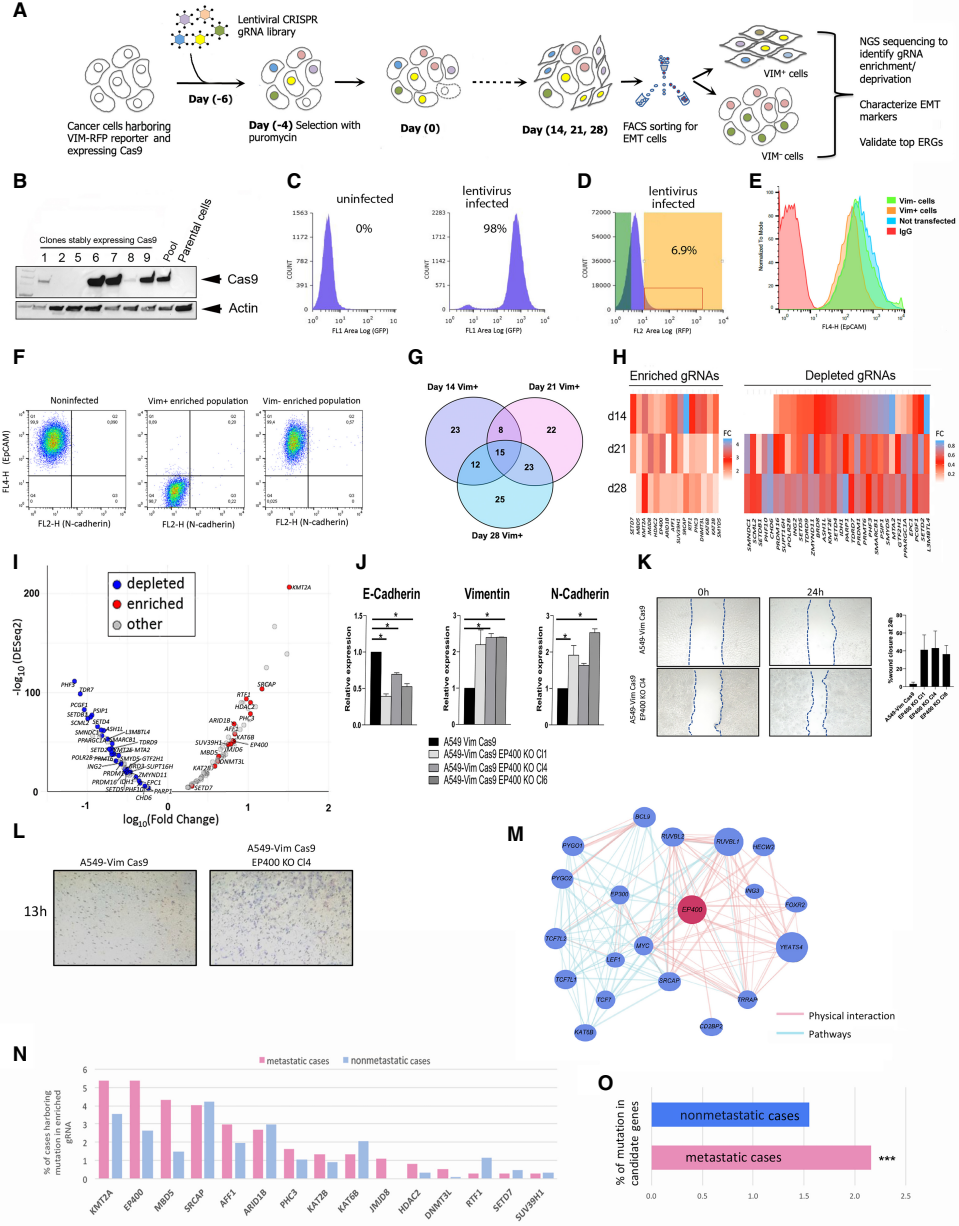
CRISPR-Cas9 screen to perform orthogonal assessment of the driver potential of ERGs in EMT. (*A*) The screening strategy used to identify positive and negative regulators of EMT among ERGs. (*B*) Western blot analysis of Cas9 expression in A549 lung cancer cells. “Pool” represents a heterogeneous population of transduced and stably Cas9 expressing cells derived from the parental cells. Individual cell clones derived by cloning rings are numbered 1, 2, 5, 6, 7, 8, and 9. Actin beta was used to normalize for equal loading. (*C*) Validation of the transduction efficiency of the lentiviral CRISPR ERG library 10 d after puromycin selection using FACS compared with uninfected A549 cells. (*D*) Enrichment of vimentin-positive (VIM^+^) population analyzed by FACS after CRISPR ERG library transduction at day 14 after puromycin selection. (*E*) Validation of cell sorting for the enrichment of VIM^+^ population by FACS based on the fluorescent antibody EPCAM (EPCAM loss is associated with the mesenchymal cell state) of VIM^+^, vimentin-negative (VIM^−^), uninfected cell line, and negative control antibody IgG. (*F*) Confirmation of cell enrichment for VIM^+^ and VIM^−^ fractions after sorting. FACS-sorted VIM^+^ and VIM^−^ populations were grown in culture for 2 wk and analyzed by FACS after staining with cadherin 2 (also known as N-cadherin) antibodies. (*G*) The overlap of the top EMT-associated ERG gRNAs after Illumina MiSeq deep sequencing; the numbers are derived from two statistical methods (DESeq2 and edgeR) at days 14, 21, and 28 after transduction. (*H*) Heatmap showing the top ERGs based on enriched and depleted gRNAs at days 14, 21, and 28 after transduction compared with day 0. (*I*) Volcano plot of ERG gRNAs at day 28 after transduction. (*J*) Expression analysis by qRT-PCR of EMT markers (cadherin 1 [also known as E-cadherin], vimentin, and cadherin 2) on single targeted A549-VimCas9 clones following *EP400* loss of function, relative to expression in the parental A549 Vim Cas9 cell line. (*) *P* < 0.05, indicates results of one-way ANOVA test. Error bars are SEM of *n* = 2. (*K*) Representative image of scratch assay performed on the parental cell line and three generated *EP400* KO clones at day 0 and after 24 h (*left*). On the *right*, a graph plot showing percentage area closure 24 h after the scratch as averaged of at least six areas analyzed for each clone and for the parental cell line. Experiments were performed in duplicates. (*L*) Transwell migration assay showing increase of migration at 13 h for A549-Vim Cas9 *EP400* KO Cl4 compared to the parental cell line. (KO) Knockout; (Cl) clone; (vim) vimentin. (*M*) An example of network analysis of selected top ERGs (*EP400*) associated with the EMT population, obtained with the GeneMANIA package. (*N*,*O*) The bar plots show the mutation frequency of EMT-specific ERGs (identified in the CRISPR-Cas9 screen) in clinical samples from nonmetastatic (M0) and metastatic (M1) subsets (based on the annotation of TCGA samples).

We conducted a CRISPR-Cas9 screen using a custom-made lentiviral CRISPR library consisting of 1649 gRNAs targeting all 426 ERGs (Fig. 5A; Supplemental Fig. S8) and A549 lung cancer cells stably expressing Cas9 (Fig. 5B,C), aimed at an orthogonal in vitro assessment of driver potential of ERGs. The screen was conducted in A549 lung cancer cells, considered a gold standard for studying the epithelial-to-mesenchymal transition (EMT), a cellular program conferring on cancer cells multiple traits associated with higher-grade malignancy, which may have an underlying epigenetic mechanism (Tam and Weinberg 2013). In the A549 cells, a red fluorescent protein (RFP)-tagged reporter is under the control of the endogenous vimentin promoter, thereby permitting the real-time monitoring of the transition from epithelial to mesenchymal status of the cells (activation of vimentin-RFP expression, mesenchymal marker) (Fig. 5D–F). The transduced A549-Vim Cas9 cells were further grown and collected at days 14, 21, and 28 after transduction, followed by flow cytometry (FACS) sorting to enrich for vimentin-positive (VIM^+^) and vimentin-negative (VIM^−^) fractions (Fig. 5C,D).

VIM^+^ cells were further confirmed to express additional markers that are associated with the mesenchymal cell state, including cadherin 2^high^, cadherin 1^low^, and EPCAM^low^ (Fig. 5E,F; Supplemental Fig. S9). These EMT markers remained stable after a prolonged culturing of VIM^+^ cells (Fig. 5F), showing RFP fluorescence in A549 cells that provides a quantitative readout of EMT. To identify potential regulators of EMT among ERGs, we subjected the cells to deep sequencing followed by enrichment or depletion analysis of gRNA-targeting ERGs across the three different time points (days 14, 21, and 28) (Fig. 5G–I; Supplemental Fig. S10A–C). The top most significant hits identified belonged predominantly to histone writers, histone readers, and chromatin remodelers (Supplemental Table S6) and several biological pathways (Supplemental Fig. S11). Among the top most significant EMT-specific hits, one-third belonged to the category of histone methylation writers, whereas several ERGs (including *KMT2A*, *EP400*, *MBD5*, and *SRCAP*) were found to be frequently targeted by genetic alterations in several cancer types (Fig. 2E).

To validate our findings, we knocked out individually several of the identified targets in A549 cells by using three to four distinct gRNAs for each gene and analyzed the changes in expression of the epithelial marker cadherin 1 and the EMT markers cadherin 2 and vimentin in the mutant clones (Fig. 5J; Supplemental Fig. S12). Moreover, because EMT is associated with an increased tumor invasiveness, we evaluated whether an increase in the migration capacity was observed in cells upon loss of the candidate epidrivers of EMT by performing scratch and transwell migration assays. Indeed, loss of several of the epidrivers candidates led to a significant gain in EMT markers and was accompanied by a gain in the migration capacity of cells (Fig. 5K,L; Supplemental Fig. S12). This was significant in all knockout clones of *EP400*, *KAT2B*, *ARID1B* clone 2, and *MBD5* clone 10 (Supplemental Fig. S12).

Next, we assessed the potential link of EMT-specific, enriched, or depleted ERGs to biological pathways using different gene set enrichment bioinformatics tools and found that the *NOTCH1*, *WNT*, and *TP53* pathways were highly correlated with EMT-associated ERGs identified in the CRISPR-Cas9 screen (Supplemental Fig. S11A–C). We further applied the GeneMANIA prediction tool (Warde-Farley et al. 2010) to the top ERGs identified in our screen and found several directional dependencies (predominantly through physical interactions and common pathways) (Fig. 5M; Supplemental Figs. S11D, S13). *EP400*, *KMT2A*, *SRCAP*, and *KAT2B* were found to have direct interactions with several genes known to be involved in EMT, including *PYGO1*, *PYGO2*, and *TWIST1*, and a substantial number of genes known to form multiprotein complexes, thereby, connecting previously uncharacterized complexes/pathways to the EMT process. Finally, the top ERGs identified in our CRISPR-Cas9 screen (including *MBD5* and *JMJD8*) were significantly more frequently mutated in metastatic cancer cases compared with their nonmetastatic counterparts across 21 different cancer types (Fig. 5N,O), further corroborating the findings of the CRISPR-Cas9 screen that EMT-specific ERGs may be involved in conferring on cancer cells invasiveness and metastatic potential.

### Identifying epidrivers involved in sustaining proliferation of cancer cells

**Figure 6.**
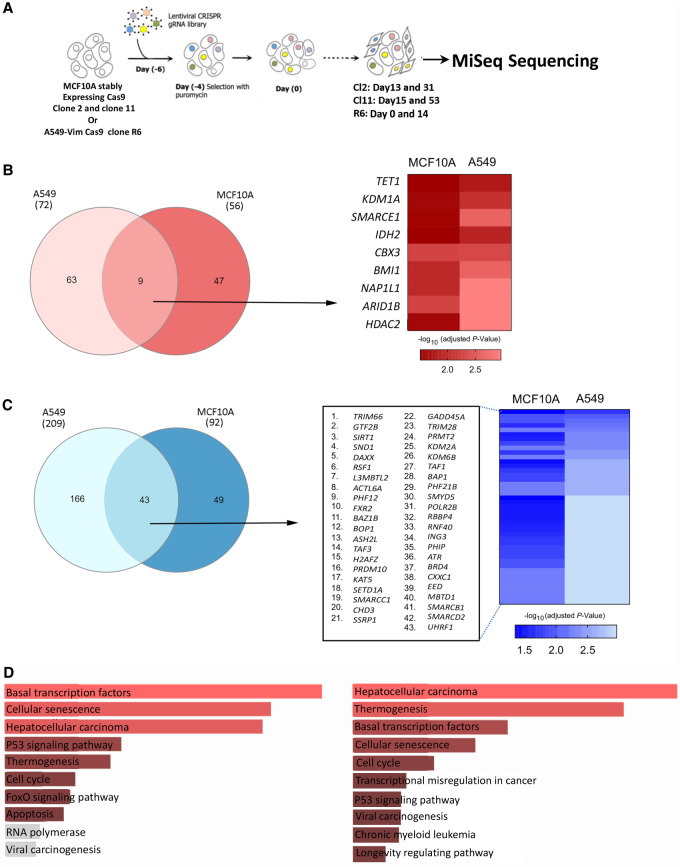
CRISPR-Cas9 screen to perform orthogonal assessment of the driver potential of ERGs in cancer cell proliferation. (*A*) The screening strategy used to identify regulators of cell proliferation among ERGs in both A549 and MCF10A cell lines/clones. (*B*,*C*, *left*) Venn diagrams showing the genes associated with significantly enriched (*B*) or depleted (*C*) gRNAs in the screens performed on A549 and MCF10A cells using edgeR analysis in CRISPRAnalyzeR. (*B*,*C*, *right*) Heatmaps showing the adjusted *P*-values of the commonly enriched (*B*) or depleted (*C*) gRNAs in both cell lines. Data are presented as –log_10_ (adjusted *P*-value). (*D*) KEGG pathway analysis performed on genes associated with commonly depleted gRNAs (*left*) and with commonly depleted and enriched gRNAs (*right*) in both cell lines. All pathways in red show *P* < 0.05.

To further expand our finding on the involvement of ERGs in hallmarks of cancer, we next applied the CRISPR library targeting all 426 ERGs on the A549-Vim and an independent cell line (MCF10A cells, the human mammary epithelial cell line widely used in vitro model for studying oncogenic transformation) and analyzed the ERGs involved in sustaining cell proliferative capacity. To this end, the cells A549-Vim and MCF10A cells expressing Cas9 were infected with the CRISPR library. Following selection, the cells were collected at different time points, subjected to deep sequencing, and analyzed for significantly enriched and depleted gRNAs (Fig. 6A; Supplemental Figs. S14, S15). We revealed 56 gRNAs that are enriched in MCF10A cells over the passages, 15 of which were also detected using DESeq2 (an independent statistical analysis method) (Supplemental Fig. S14B,C). The cell cycle was found to be among the top pathways enriched on the list of genes associated with enriched gRNAs (Supplemental Fig. S14D), consistent with the notion that the loss of function of the ERGs is linked with an increase in proliferation of MCF10A cells. KEGG analysis also revealed NOTCH and FOXO signaling pathways, two major pathways involved in breast cancer development, and several of the identified putative epidrivers (i.e., *ATRX*, *PHF11*, *NAP1L2*, and *PRDM5*) show high mutation rate, copy number depletion, and/or decrease in expression in breast cancer (Supplemental Table S3). Based on the analysis of depleted gRNAs, we identified ERGs associated with cell cycle and cell senescence (Supplemental Fig. S14F), and these ERGs showed higher rate of copy number amplification or up-regulation in breast cancer (i.e., *ARID4B* and *EZH2*). Similarly, when the analysis was performed on A549 cells (e.g., comparison D0 vs. D14), we revealed 69 ERGs associated with enriched gRNAs (Supplemental Fig. S15), among which several genes showed high mutation, copy number alteration, or decrease in expression rates in lung cancer.

Finally, by overlapping the genes associated with enriched gRNAs or depleted gRNAs in A549 and MCF10A, we identified ERGs consistently implicated in proliferation in both cell types. Nine genes (*TET1*, *KDM1A*, *SMARCE1*, *IDH2*, *CBX3*, *BMI1*, *NAP1L1*, *ARID1B*, and *HDAC2*) were associated with enriched gRNAs in both cancer types (Fig. 6B). Three genes (*TET1*, *ARID1B*, and *BAZ1A*) are highly mutated in breast and lung cancer types (Supplemental Table S3), whereas *TET1*, *IDH2*, and *ARID1B* were also among the top genes altered in several cancer types (Supplemental Table S3), further corroborating their putative role as cancer drivers. A higher number of genes were identified when depleted gRNAs were overlapped between the two cell lines (Fig. 6C,D) several of which (i.e., *BOP1*, *RSF1*, *ACTL6A*, *ASH2L*, and *ATR*) showed high copy number amplification or increase in expression in breast and lung cancer, suggesting that those genes might play essential roles in cancer proliferation and viability.

## Discussion

In the present study, we performed a pan-cancer analysis of (epi)genomic and transcriptomic alterations in a comprehensive panel of ERGs using available cancer genome data sets and a range of novel and powerful bioinformatics tools, revealing candidate epidrivers across cancer types. We cataloged recurrent pan-cancer mutations or CNAs in specific ERGs or classes of ERGs. Application of driver prediction algorithms and orthogonal CRISPR-Cas9 in vitro screens revealed the ERGs with a potential driver role conferring on cancer cells the traits associated with the hallmarks of cancer. This is the largest and most comprehensive analysis thus far of the cancer-associated disruption of ERGs and the first experimental effort to identify epidrivers in oncogenic processes through an ERG-wide screen.

Our finding that the predominant genetic alterations in ERGs across tumor samples of most cancer types are CNAs, notably amplifications, reveals that in addition to recurrent mutations, both amplifications and deletions in ERGs may play more important roles than previously anticipated. These results extend the previous studies on a limited set of cancer types showing that mutations in ERGs are recurrent (Gonzalez-Perez et al. 2013; Plass et al. 2013; Timp and Feinberg 2013; Vogelstein et al. 2013) and that amplified regions are enriched for genes involved in epigenetic regulation (Zack et al. 2013). The finding that some cancer types (such as OV and SARC) show frequent deep CNAs with virtually no SNAs argues that the roles of epidrivers in those cancer types may be driven primarily by CNAs, with a relatively minor role of mutations. Our results that different ERG classes showed similar patterns of genetic alterations, with the exception of DNA methylation writers, which showed a markedly high ratio of amplifications to deletions, suggest that amplification of this class may be the principal mechanism of their genetic deregulation across cancer types. These different patterns of alterations may reflect distinct mechanisms by which these CNAs are generated and/or positive/negative selection during tumor development.

The importance of CNAs highlighted herein further suggests that any driver prediction model, particularly for ERGs, would need to account for genetic amplifications and deletions. The inclusion of other omics phenotypes, such as RNA expression, is also important particularly given the high level of RNA expression aberrations observed in ERGs across many cancer types. Recent evidence questions the conventional interpretation of hotspot mutations as being evidence of positive selection and driver events (Hess et al. 2019), further reinforcing the need to integrate multiple omics in driver prediction models. Accordingly, we proposed the Multi-Omics and Pan-Cancer Driver prediction tools, which account for the SNAs, CNAs, and RNA expression aberrations and complement (rather than overlap with) the ConsensusDriver approach, which seems to be heavily weighted by SNA frequencies, at least for ERG driver prediction based on our results. Whereas our Multi-Omics Driver score highlighted the epidrivers within each class of malignancy, our Pan-Cancer Driver score reflects the recurrence of driver potential across cancer types.

We observed both cancer type–specific and cancer-wide genetic deregulation of ERGs. A subset of ERGs was frequently genetically altered across many malignancies (SNAs in the *KMT2A/B/C/D* family members and *ARID1A*, and deep CNAs in *BOP1* and *ATAD2* were each seen in several cancer types), consistent with the notion that disruption of some ERGs represents a shared driver mechanism operating across multiple cancer types. Little overlap was observed between the ERGs showing a high frequency of SNAs and those with a high frequency of CNAs (except for a few ERGs) (Figs. 2E–H, 4C). Similarly, in expression analysis, we observed both cancer type–specific and cancer-wide deregulation of ERGs. Whereas ERG expression correlated highly with CNAs, it correlated negatively with some SNAs and positively with others, a finding consistent with recent evidence indicating that interaction between driver mutations and transcription may be context dependent (Ding et al. 2018). Furthermore, some discrepancy between mutation and expression alterations in ERGs may be explained by the impact of nonmutational mechanisms on gene expression. This is supported by our observation that some cancer types, such as GBM, have a low burden of SNAs (Fig. 2E) and CNAs (Fig. 2F) in ERGs, in line with a low within-tumor variation in ERG expression (Fig. 3A), but a high number of ERGs with deregulated expression in tumor samples relative to adjacent normal tissue (Fig. 3E–G) and vice versa (e.g., STAD). Our analysis of DNA methylation and RNA-seq levels revealed that tumor-specific differentially methylated CpGs in promoter regions were negatively correlated with the expression of their corresponding genes, underscoring the notion that epigenetic inactivation could be an additional mechanism for aberrant expression of ERGs.

ERGs, including top predicted driver genes, were commonly enriched in four of ten cancer hallmarks: genome instability and mutation, evading growth suppressors, sustaining proliferative signaling, and enabling replicative immortality (Fig. 4F). The latter two hallmarks overlapped, respectively, with cell cycle (accelerator of proliferation) and cellular senescence (defined as irreversible cell cycle arrest, hence, a decelerator of proliferation), which were found to be among the top pathways deregulated in the genes associated with enriched gRNAs (Supplemental Figs. 14D and 15D). Although it has been proposed that the hallmarks of cancer are acquired through distinct mechanisms in different cancer types (Hanahan and Weinberg 2011), our results suggest that many of these functional capabilities may be acquired through the shared mechanism involving the disruption of ERGs.

Our orthogonal in vitro CRISPR-Cas9 screen also identified a set of specific ERGs affecting markers of tumorigenesis such as cell proliferative potential and EMT. Our results that five epidriver candidates (*SRCAP*, *EP400*, *ARID1B*, *MBD5*, and *KMT2A*) among the top 15 ERGs enriched in EMT fraction (Fig. 5H; Supplemental Tables S6, S9) were found among the most mutated ERGs in clinical samples across cancer types (Fig. 2G) support the driver role of EMT-specific ERG tumorigenesis. In addition, an analysis of the interaction networks of the top ERG hits associated with EMT (*KAT2B*, *EP400, SRCAP*, *ARID1B*, and *SUV39H1*) uncovered several directional dependencies involving genes known to play a role in EMT and multiprotein complexes regulating chromatin structure and function, connecting previously uncharacterized complexes/pathways to the EMT process.

This study contributes to a greater understanding of the deregulation of ERGs and their functional impact in cancer. This insight should prove instrumental in the clinical application of ERGs, especially considering a growing interest in developing epigenetics-based prognostic and therapeutic strategies. Developing “epigenetic drugs” capable of modulating specific ERGs (epidrivers) can circumvent the high toxicity and off-target effects of broad epigenome reprogrammers (DNMT inhibitors, histone deacetylase inhibitors) and offer a potent tool for precision medicine (Ahuja et al. 2016; Brien et al. 2016; Jones et al. 2016). Therefore, the results of our study may provide the basis for translational approaches aimed at developing epigenetics-based early detection and personalized cancer treatment and prevention.

## Methods

### Generating the compendium of epigenetic regulator genes

A compendium of genome-wide ERGs was generated by integrating the different available gene databases (The Human Gene Database—GeneCards, https://www.genecards.org/; the NCBI Eukaryotic Genome Annotation Pipeline https://www.ncbi.nlm.nih.gov/genome/annotation_euk/process/; Cytoscape version 3.6.1) and the most relevant publications (Gonzalez-Perez et al. 2013; Plass et al. 2013; Timp and Feinberg 2013; Vogelstein et al. 2013; Yang et al. 2015; Xu et al. 2017). This resulted in a comprehensive list of 426 genes, which we categorized into 12 groups based on their functional features: (1) Histone methylation editor (HM_e) = histone demethylases (HDMs); (2) histone methylation writer (HM_w) = histone methyltransferases (HMTs); (3) histone methylation reader (HM_r); (4) DNA methylation writer (DM_w); (5) DNA methylation editor (DM_e); (6) DNA methylation reader (DM_r); (7) histone acetylation editor (HA_e) = histone deacetylases (HDACs); (8) histone acetylation writer (HA_w) = histone acetyltransferases (HATs); (9) histone acetylation reader (HA_r); (10) chromatin remodeling complex (ChRC); (11) helicases; and (12) other chromatin modifiers = the remaining ERGs included in the study (Fig. 1B). To classify ERGs based on their potential function as a tumor suppressor or an oncogene, we used the TSGene (https://bioinfo.uth.edu/TSGene/) (Zhao et al. 2013) and OncoKB (https://oncokb.org/) (Chakravarty et al. 2017) databases, respectively.

### Data resource

We downloaded the data sets using the publicly available TCGA (provisional) database from cBioPortal (https://www.cbioportal.org/datasets), which consists of data sets with genetic alterations, including single-nucleotide alterations (SNAs) and copy number alterations (CNAs), and gene expression (expression median and *Z*-scores) of 426 ERGs. For reproducibility, analyses were repeated using the TCGA expression and genetic data downloaded on May 16, 2019, and March 26, 2019, respectively.

We used TCGA abbreviations for 33 cancer types as follows: (LAML) acute myeloid leukemia; (ACC) adrenocortical carcinoma; (BLCA) bladder urothelial carcinoma; (LGG) brain lower grade glioma; (BRCA) breast invasive carcinoma; (CESC) cervical squamous cell carcinoma and endocervical adenocarcinoma; (CHOL) cholangiocarcinoma; (COAD) colon adenocarcinoma; (ESCA) esophageal carcinoma; (GBM) glioblastoma multiforme; (HNSC) head and neck squamous cell carcinoma; (KICH) kidney chromophobe; (KIRC) kidney renal clear cell carcinoma; (KIRP) kidney renal papillary cell carcinoma; (LIHC) liver hepatocellular carcinoma; (LUAD) lung adenocarcinoma; (LUSC) lung squamous cell carcinoma; (DLBC) lymphoid neoplasm diffuse large B cell lymphoma; (MESO) mesothelioma; (OV) ovarian serous cystadenocarcinoma; (PAAD) pancreatic adenocarcinoma; (PCPG) pheochromocytoma and paraganglioma; (PRAD) prostate adenocarcinoma; (READ) rectum adenocarcinoma; (SARC) sarcoma; (SKCM) skin cutaneous melanoma; (STAD) stomach adenocarcinoma; (TGCT) testicular germ cell tumors; (THYM) thymoma; (THCA) thyroid carcinoma; (UCS) uterine carcinosarcoma; (UCEC) uterine corpus endometrial carcinoma; (UVM) uveal melanoma.

### Pan-cancer analysis of genetic alterations in ERGs

The proportions of each of the CNAs and SNAs detected in ERGs were calculated among tumor samples within each of the 33 cancer types. The raw SNA data set contained somatic, nonsynonymous mutations, which were transformed into data with mutation status indicating the number of SNAs for a gene in each sample. The raw CNA data set was used to characterize CNAs by genomic position and amplitude as follows: CNA = 0 indicates diploid with no alteration; amplification = 1 indicates a shallow gain (a few additional copies, often broad); deep amplification = 2 indicates a high-level amplification (more copies, often focal); shallow deletion = −1 indicates a shallow loss, possibly a heterozygous deletion; deep deletion= −2 indicates a deep loss, possibly a homozygous deletion (https://www.cbioportal.org/). For more robust analyses, we regrouped CNAs into three groups: (1) no alteration, (2) deep amplification (amp), and (3) deep deletion (del). We then pooled together the resulting merged data sets of SNAs and CNAs matched to the same sample ID. The same criteria were used for calculating genetic deregulation in all genes and for ERG functional classes. Circos plots were generated as described previously (Krzywinski et al. 2009; Gu et al. 2014).

### Multi-omics analysis of genomic and transcriptomic aberrations in ERGs

To effectively visualize multidimensional data sets of the deregulation of ERGs across different cancer types, we integrated SNAs, CNAs, and RNA expression alterations. For each ERG, the proportions of CNAs versus SNAs were calculated among tumor samples within each of 33 cancer types. The analysis included only genetically deregulated genes that show SNAs and CNAs (amplification = 1, 2, and deletion = −1, −2) in at least 1% and 10% of the samples in any cancer, respectively. ERGs were then classified based on their mutation profiles such that those harboring mostly SNAs, with CNAs not passing the threshold of 10%, were considered as only mutated; whereas genes with CNAs in at least 10% of the samples were considered as amplified or deleted. Finally, pooled results of SNAs and CNAs were integrated with RNA expression data, expressed as *Z*-score values for each corresponding ERG in each cancer type. The percentage of samples with significant *Z*-scores (*Z* > 2 or *Z* < −2) was reported for each ERG in each cancer type.

### Differential expression analysis of ERGs in tumor samples and adjacent normal tissues

We downloaded HTSeq count files from the Genomic Data Commons Data Portal (https://portal.gdc.cancer.gov) for each cancer type and divided adjacent normal samples and tumor samples based on the ID annotation of TCGA samples. Of the total of 33 cancer types, we focused on 18 cancer types that had available ID annotation for adjacent normal samples. We used DESeq2 analysis to calculate expression changes comparing tumor samples with adjacent normal tissues. Genes with coverage of fewer than 10 reads were excluded. Only ERGs with absolute values of log FC > 1 and FDR < 0.05 were considered to be significantly differentially expressed.

### Co-occurrence and mutual exclusivity analysis

Co-occurrence and mutual exclusivity analysis was performed in each cancer type separately and then meta-analyzed across cancer types. The data set was limited to the samples that had information about both SNAs and CNAs from cBioPortal (nonsynonymous mutations, fusions, deep amplifications, and deep deletions). For each gene pair and cancer type, we calculated an odds ratio (OR) quantifying how strongly the presence or absence of SNAs and/or CNAs in the first gene was associated with the presence or absence of the alterations in the second gene. The *P*-values were derived from the ORs using the Fisher's exact test and were further adjusted for multiple testing using the Benjamini–Hochberg FDR correction. The Haldane-Anscombe correction was used to avoid a division-by-zero error. The significant ORs (FDR < 0.05) were averaged across cancer types for each gene pair. ORs greater or less than 1 indicate tendencies toward co-occurrence and mutual exclusivity, respectively. Specifically, within each co-occurrent gene pair, the proportion of samples with both genes mutated needed to represent at least 5%–10% of the samples per cancer type.

### ERG driver prediction models

The characterization of potential driver roles for ERGs was based on ConsensusDriver, a novel approach that provides a systematic way to integrate the strengths of various driver prediction algorithms (Bertrand et al. 2018). ConsensusDriver scores for ERGs overlapping with the ConsensusDriver genes (Bailey et al. 2018) were shown as a heatmap. The Pan-Cancer Driver score was generated for each of the 426 ERGs using a ranking method that accounts for SNAs, CNAs, and RNA expression aberrations within each cancer type and across multiple cancer types (the script provided on GitHub: https://github.com/IARCbioinfo/EPIDRIVER2020 and as Supplemental Code). First, in the SNA and CNA data, we focused on ERGs that had a SNA or CNA (sharing the same direction) in at least 5% of samples in any cancer type. Then, for each gene, we counted the number of cancer types in which that gene had a SNA or CNA; the number of cancer types was used as a primary ranking method, and the percentage of samples showing alterations was used as the secondary ranking for genes having identical primary ranks. The gene with the lowest rank was given a score of 1, the second rank a score of 2, and so on. Genes (*n* = x) with equal ranks “y” (based on both the primary and secondary methods) were all assigned the same ranking score “y”; a subsequent gene with rank “y + 1” was then assigned a ranking score of “y + x.” Two rankings were obtained, one for SNA and, independently, another for CNA. For expression data, we used both the *Z*-score and log_10_FC data and similarly calculated the ranking by counting for each gene the number of cancer types in which that gene had a |*Z*-score| > 2 or |log_10_FC| > 2 (and then using a secondary ranking based on the exact proportion of samples with |*Z*-score| > 2 or on the exact values of |log_10_FC|). Two rankings were obtained, one for *Z*-score data and one for log_10_FC data. The resulting four rankings were combined into one, such that SNA and CNA rankings each had a weight of 1, whereas *Z*-score and log_10_FC each had a weight of 0.5 (because both *Z*-score and log_10_FC reflect expression aberration).

### Hallmarks of cancer enrichment of ERGs

To investigate whether deregulated ERGs are enriched in pathways affecting the 10 hallmarks of cancer (Hanahan and Weinberg 2011), an analysis was done separately for all ERGs or for genetically altered ERGs (by SNAs and independently by CNAs). For each cancer type, we included only genes with CNA > 1% or SNA > 1% across samples within a given cancer type. Then, for each cancer type, we calculated the enrichment of its genetically deregulated genes in every hallmark using the Fisher's exact test and further adjusted for multiple testing using the Bonferroni correction.

### Generation of cell clones stably expressing Cas9

A549 Vim RFP cells (ATCC CCL-185EMT) and MCF10A cells (ATCC CRL-10317) were cultured according to the recommendation by ATCC. To generate stably expressing Cas9 cell lines, A549 Vim RFP and MCF10A cells were transfected with lentiviral particles containing Cas9 nuclease (GeneCopoeia 217LPP-CP-LVC9NU-01-100-C) and Lenti-Cas9-2A-Blast plasmid (Addgene 73310), respectively, at a multiplicity of infection (MOI) of 5. Briefly, cells were cultured for 5 h in cell culture media supplemented with 8 µg/mL of polybrene. Spinoculation was then applied at 800*g* for 90 min at 37°C. At 48 h after transfection, 500 µg/mL G418 (cells transfected with 217LPP-CP-LVC9NU-01-100-C) or 10 µg/mL blasticidin (cells transfected with Lenti-2A-Cas9-blast) were used for positive selection of Cas9-transduced cells. For each cell type, we then generated single clones stably expressing Cas9 using cloning rings or serial dilutions followed by cultures of single cell. The expression of Cas9 protein in individual cell clones was determined by western blot analysis using Anti-CRISPR-Cas9 antibody (Abcam 7A9-3A3). A549 Vim RFP Cas9 clone R6 and MCF10A Cas9 clones 2 and 11 were used for the CRISPR-Cas9 library screenings.

### Construction of the CRISPR-Cas9 sgRNA library and titration

We generated a CRISPR library comprising 1649 different gRNAs targeting 426 human ERGs. Each candidate gene was targeted by 1–4 sgRNA (Supplemental Table S7). Lentiviral plasmids containing sgRNAs were obtained in bacterial glycerol stock from a commercial source (Thermo Fisher Scientific). We pooled and amplified together 10 µL of glycerol stock of each plasmid gRNA to obtain a homogeneous representation of the library, followed by DNA extraction using Maxi prep (Qiagen). The library was packaged in human embryonic kidney HEK293T cells using a third-generation lentivirus expression system consisting of the mixture of 20 µg of the transfer vector consisting of the pool of sgRNAs lentiviral plasmid constructs (20 μg), 12.5 μg of the packaging plasmid I pMDLg/pRRE (Addgene 12251), 7.5 μg of the packaging plasmid II pRSV-Rev (Addgene 12253), and 7.5 μg of envelope plasmid VSV-G - pMD2.G (Addgene 12259) in Opti-MEM diluent. The library lentiviral particles were produced using the polyethylenimine method (Tebu-Bio). Two collected harvests were pooled together and concentrated using Lenti Concentrator (OriGene) according to the manufacturer's instructions. The resulting lentivirus CRISPR library was aliquoted and stored at −80°C. The virus titer and optimal transduction efficiency (considered to be 40%) of the lentivirus library were determined by colony formation assay in A549 Vim Cas9 cells.

### Evaluation of sgRNAs representation in the generated library

To evaluate the relative representation of sgRNAs in the library, we performed deep sequencing using MiSeq (Illumina) (Supplemental Fig. S8). Briefly, we designed primers (forward, CGATACAAGGCTGTTAGAGAGATA; reverse, GTTGCTATTATGTCTACTATTCTTTCCC) to obtain a 430-bp amplicon of plasmid DNA containing the sgRNA sequences. We followed the suggestion of Illumina to have an amplicon length of >300 bp for the targeting sequencing, using the Nextera XT DNA Sample Preparation Kit (Illumina) according to the manufacturer's instructions. A single gRNA of the *AKAP1* gene was chosen from the candidate gene list to be evaluated as a positive control for library distribution. Targeted sequencing of the pooled library and a single gRNA of the *AKAP1* gene was performed using the MiSeq Reagent Kit v2 (500 cycles, Illumina) according to the manufacturer's instructions. The FastQC generated from MiSeq was analyzed in Galaxy using the BLASTN tool (2.5.0+ Package: blast 2.5.0). The sgRNAs were mapped against all sgRNA sequences present in the custom-made CRISPR library. The representation of genes in the pooled library was calculated relative to the abundance of gRNA for each gene (Supplemental Fig. S8).

### CRISPR-Cas9 library screening for epidrivers of epithelial-to-mesenchymal transition

A549 Vim RFP Cas9 cells were transduced with the lentivirus CRISPR library at a MOI of 0.3. For a negative control, we used the nontargeting sgRNA LentiArray CRISPR Negative Control Lentivirus (Thermo Fisher Scientific). The baseline time point (day 0) was designated as 4 d after puromycin selection (the time point when untransduced A549 Vim RFP Cas9 cells were dead). The library was applied in two technical duplicates and in two independent experiments. During cell passaging, ∼2 × 10^6^ cells were maintained in culture to achieve on average 1000-fold coverage of all 1649 sgRNAs in the library. We isolated the EMT population at days 14, 21, and 28 using FACS sorting (S3e, Bio-Rad) for RFP (Vimentin)-positive cells (Vimentin positive [VIM^+^]).

### Validation of the isolated EMT population

RNA of control A549 Vim RFP Cas9 cells and the sorted VIM^+^ cells (from all time points) were extracted using the AllPrep DNA/RNA Mini Kit (Qiagen). To validate the EMT transition in VIM^+^ cells, mRNA expression levels of several EMT markers were analyzed by quantitative RT-PCR. Cadherin 1/cadherin 2 ratio and expression levels of SNAI1 (also known as SNAIL) and ZEB2 were determined and confirmed the EMT state of VIM^+^ cells (Supplemental Fig. S16). Fluorescence microscopy was used to verify RFP Vimentin-positive (red) cells (Supplemental Fig. S17) in VIM^+^ sorted cells compared to the parental cell lines. For additional validation of the isolated EMT population, intensity levels of the epithelial marker EPCAM (Fig. 5E; Supplemental Fig. S9) and the mesenchymal marker cadherin 2 (Fig. 5F) were determined using flow cytometry (S3e, Bio-Rad) using EPCAM-APC antibody (Miltenyi Biotec 130-111-000) and cadherin 2 antibody (R&D systems IC1388P).

### CRISPR-Cas9 library screening for epidrivers of cell proliferation

A549 Vim Cas9 Clone R6 and MCF10A Cas9 Clone 2 and Clone 11 cells were transduced with the lentiviral CRISPR library of 1649 sgRNAs (MOI 0.3 and 0.1, respectively) and maintained in culture for several passages. Cells were collected at two different time points per cell line throughout their culture. The A549 cells were collected at day 0 (the day following the end of puromycin selection, early time point) and day 14 (late time point). Because the proliferation rate of MCF10A cells is lower than A549, we collected MCF10A cells at later passages. Two independent clones expressing Cas9 were used for MCF10A: Clone 2 and Clone 11 that were collected at early time points (days 13 and 15, respectively) and later time points (days 31 and 53, respectively) following transduction and puromycin selection. To achieve 1000-fold coverage of all sgRNAs, ∼2 × 10^6^ cells were kept in culture at each passage, and 2 × 10^6^ were collected at each time point.

### Identification of enriched and depleted sgRNAs and their associated candidate epidrivers

To identify the enriched and depleted sgRNAs in the CRISPR-Cas9 screens, genomic DNA was isolated from 2 × 10^6^ cells of each of the cell populations analyzed (VIM^+^ cells and transduced parental cell lines at different time points) using AllPrep DNA/RNA Mini Kit. DNA was subsequently subjected to PCR to amplify the same region as that used for the validation of library representation (see above), containing sgRNA sequences using NEBNext High-Fidelity 2X PCR Master Mix (Illumina). PCR products were used for library preparation using the Nextera XT DNA Sample Preparation Kit or Nextera DNA Flex library Prep (Illumina) according to the manufacturer's instructions and sequenced on an Illumina MiSeq platform. The FastQC data generated by MiSeq were first analyzed in Galaxy using the BLASTN tool (2.5.0+ Package: blast 2.5.0). To obtain read counts, we performed mapping of sgRNA against all sgRNA sequences present in the custom-made CRISPR library. To identify the enriched and depleted sgRNAs in EMT epidriver screening, we performed paired analysis comparing the sorted VIM^+^ populations at different time points (days 14, 21, and 28) with cells collected at day 0. The differential sgRNA abundance of the read counts was analyzed using CRISPRAnalyzeR software (DKFZ, Version: 1.50) (Winter et al. 2016). The list of enriched and depleted sgRNAs for each time point was defined by hit candidate overlapping the list of hit candidates identified by DESeq2 (Love et al. 2014) and edgeR (Robinson et al. 2010; McCarthy et al. 2012) analyses (two independent analysis methods) with statistically significant changes of 0.001 and 0.01, respectively. The EMT candidate genes were identified as the genes commonly associated with enriched or depleted sgRNAs across different time points (days 14, 21, and 28) (Fig. 5G). Significantly enriched and depleted sgRNAs in the screening for epidrivers of cell proliferation were analyzed by paired analyses comparing sgRNAs detected at late time points with sgRNAs at early time point for each cell type using edgeR and/or DESeq2 statistical analyses methods in CRISPRAnalyzeR. Statistical significance was set at *P* < 0.001 and *P* < 0.01 for DESeq2 and edgeR, respectively.

### Generation of single targeted knockout clones for EMT-identified epidrivers candidates

A549 Vim RFP Cas9 clone R6 was transfected with a pool of four sgRNAs targeting the genes of interests: *EP400*, *MBD5*, *ARID1B*, or *KAT2B*. Sequences of sgRNAs used are available in Supplemental Table S7. Transfection was performed using Xfect Transfection Reagent (Takara Bio) and 2 µg of pooled sgRNAs according to the manufacturer's instructions. Cells were subjected to puromycin (1 µg/mL) selection 36 h after transfection. After antibiotic selection, single clones were generated from the heterogeneous population of transfected resistant cells by amplification of single cell sorted by flow cytometry (FACSAria). To validate the alterations in the generated single clones, we designed PCR primer amplifying the regions surrounding the Cas9 cutting sites for each of the targeted genes. Targeted regions were amplified from genomic DNA extracted from the generated single clones, and PCR products were sequenced (Sanger sequencing) (Supplemental Fig. S18; Supplemental Table S8). Several alignment tools were used to analyse the sequencing data (CRISPR-ID: http://crispid.gbiomed.kuleuven.be; DSDecodeM: http://skl.scau.edu.cn/dsdecode; and https://blast.ncbi.nlm.nih.gov/Blast.cgi).

### Quantitative RT-PCR

Total RNA extraction was performed by using AllPrep DNA/RNA Mini Kit (Qiagen) according to the manufacturer's protocol using primers shown in Supplemental Table S8.

### Scratch wound healing assay

A549 Vim Cas9 R6 parental cell clone and all generated A549 single targeted knockout clones were analyzed by scratch wound healing assay using a standard protocol. Briefly, cells were plated in 12-well plates (2 × 10^5^ cells/well); after reaching 90%–100% confluence (∼24 h after plating), two scratches per well were performed (in the form of a cross) using a 200-μL tip. Experiments were performed in duplicates. To measure cell migration and wound healing capacity, four different pictures were taken per well using a Zeiss TELAVAL 31 microscope and a Nikon D40 camera, both at 0-h (time of scratch) and 24-h time points. Closure of the wound by cell migration was calculated by comparing the scratched areas at both time points, using the ImageJ software (version 1.52b).

### Transwell migration assay

The migratory properties of A549 Vim Cas9 R6 parental cell clone and all generated A549 single targeted knockout clones were analyzed by transwell migration assay. Experiments were performed in duplicates for each clone using cell culture polycarbonate 8-µm inserts (Millicell) in 24-well plated (corning). Briefly, 1 × 10^4^ cells in 300 µL of serum free F-12K medium were added to the upper part of cell inserts. To stimulate the migration, 500 µL of F-12K medium containing FBS were added to the lower part of the inserts. Cells were incubated for 13 h at 37°C and 5% CO_2_. Thirteen hours is an insufficient time for A549 Vim Cas9 R6 parental cells to migrate. After incubation, the medium was carefully aspirated from the inside of the insert. The interior of the inserts was then gently swabbed with cotton-tipped swabs to remove nonmigratory cells. To stain migratory cells, the insert was transferred to a clean well containing 400 µL of crystal violet Cell Stain Solution and incubated for 20 min. After 2× washes with PBS, inserts were air dried and images taken by Zeiss TELAVAL 31 microscope and Nikon D40 camera.

### Assessing disruption of EMT-specific driver candidates in clinical samples and pathway enrichment

To assess the disruption of EMT-specific driver candidates identified in the CRISPR-Cas9 screen in clinical samples, we analyzed the TCGA data using the Genomic Data Commons Data Portal (https://portal.gdc.cancer.gov/). We divided samples into nonmetastatic (M0) and metastatic (M1) subsets based on the American Joint Committee on Cancer metastasis stage code. For more robust analysis, we further selected samples based on tumor stage using the American Joint Committee on Cancer neoplasm disease stage code. Only stage I and IV were considered to be M0 and M1, respectively. Overall, we collected 873 M0 and 371 M1 cases across 21 different cancer types available in TCGA (Supplemental Table S9). Based on these data sets, we calculated the mean percentage of mutations of each of the EMT-specific ERGs in the M1 versus M0 subsets.

We performed the pathway enrichment analyses using bioinformatics mapping tools of different databases, including the NCI-Nature 2016, Panther 2016, KEGG 2016, and Reactome 2016 databases. We used Enrichr (Kuleshov et al. 2016) score of each database to define the pathway enrichment of EMT-specific epidrivers. The network of EMT-specific ERGs was constructed with the GeneMANIA package (https://genemania.org/) (Warde-Farley et al. 2010).

## Data access

All raw sequencing data generated in this study have been deposited in the NCBI BioProject database (https://www.ncbi.nlm.nih.gov/bioproject/) under accession number: PRJNA655831. The scripts used to generate Driver Scores were provided on GitHub (https://github.com/IARCbioinfo/EPIDRIVER2020) and as Supplemental Code.

## Competing interest statement

The authors declare no competing interests.

## Supplementary Material

Supplemental Material
