# Supplementary material for: Pan-cancer multi-omics analysis and orthogonal experimental assessment of epigenetic driver genes
Source: Genome Res. 2020 Oct;30(10):1517–32. doi: 10.1101/gr.268292.120 (PMC7605261; doi:10.1101/gr.268292.120)
Supplement: Supplemental Material [file supp_30_10_1517__index.html]

Pan-cancer multi-omics analysis and orthogonal experimental assessment of epigenetic driver genes — Pan-cancer multi-omics analysis and orthogonal experimental assessment of epigenetic driver genes — Supplemental Material 

# Pan-cancer multi-omics analysis and orthogonal experimental assessment of epigenetic driver genes

## Supplemental Material

- Supplemental\_Fig\_S1.tif
- Supplemental\_Fig\_S2.tif
- Supplemental\_Fig\_S3.tif
- Supplemental\_Fig\_S4.tif
- Supplemental\_Fig\_S5.tif
- Supplemental\_Fig\_S6.tif
- Supplemental\_Fig\_S7.tif
- Supplemental\_Fig\_S8.tif
- Supplemental\_Fig\_S9.tif
- Supplemental\_Fig\_S10.tif
- Supplemental\_Fig\_S11.tif
- Supplemental\_Fig\_S12.tif
- Supplemental\_Fig\_S13.tif
- Supplemental\_Fig\_S14.tif
- Supplemental\_Fig\_S15.tif
- Supplemental\_Fig\_S16.pdf
- Supplemental\_Fig\_S17.pdf
- Supplemental\_Table\_S1.docx
- Supplemental\_Table\_S2.docx
- Supplemental\_Table\_S3.docx
- Supplemental\_Table\_S4.pdf
- Supplemental\_Table\_S5.pdf
- Supplemental\_Table\_S6.docx
- Supplemental\_Table\_S7.pdf
- Supplemental\_Table\_S8.docx
- Supplemental\_Table\_S9.docx
- Supplemental\_Code.pdf
- Supplemental\_Fig\_S18edited.pdf
