# Supplementary figures and images for: Pan-cancer multi-omics analysis and orthogonal experimental assessment of epigenetic driver genes
Source: Genome Res. 2020 Oct;30(10):1517–32. doi: 10.1101/gr.268292.120 (PMC7605261; doi:10.1101/gr.268292.120)

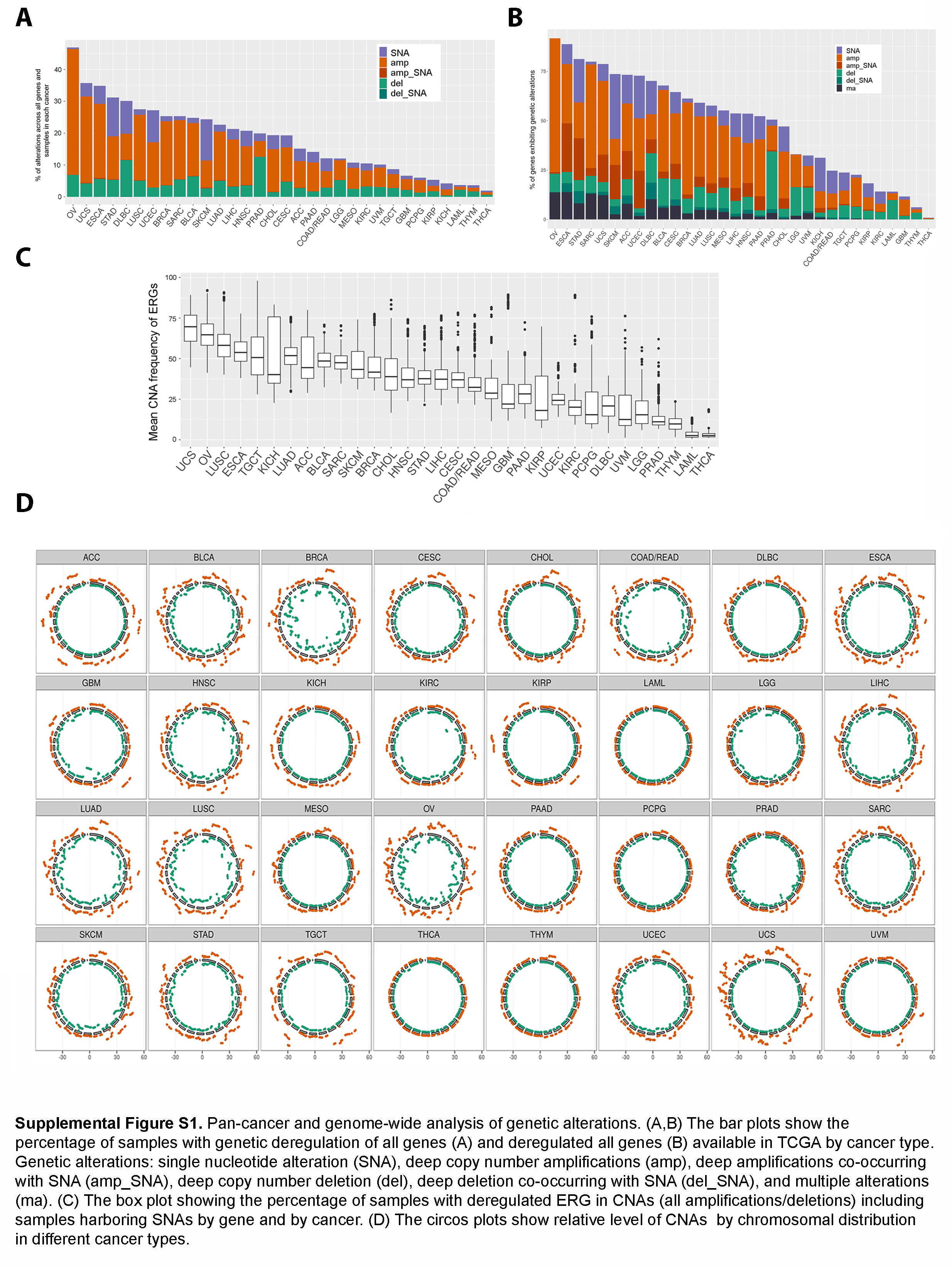

Supplement: Supplemental Material [file supp_gr.268292.120_Supplemental_Fig_S1.tif]

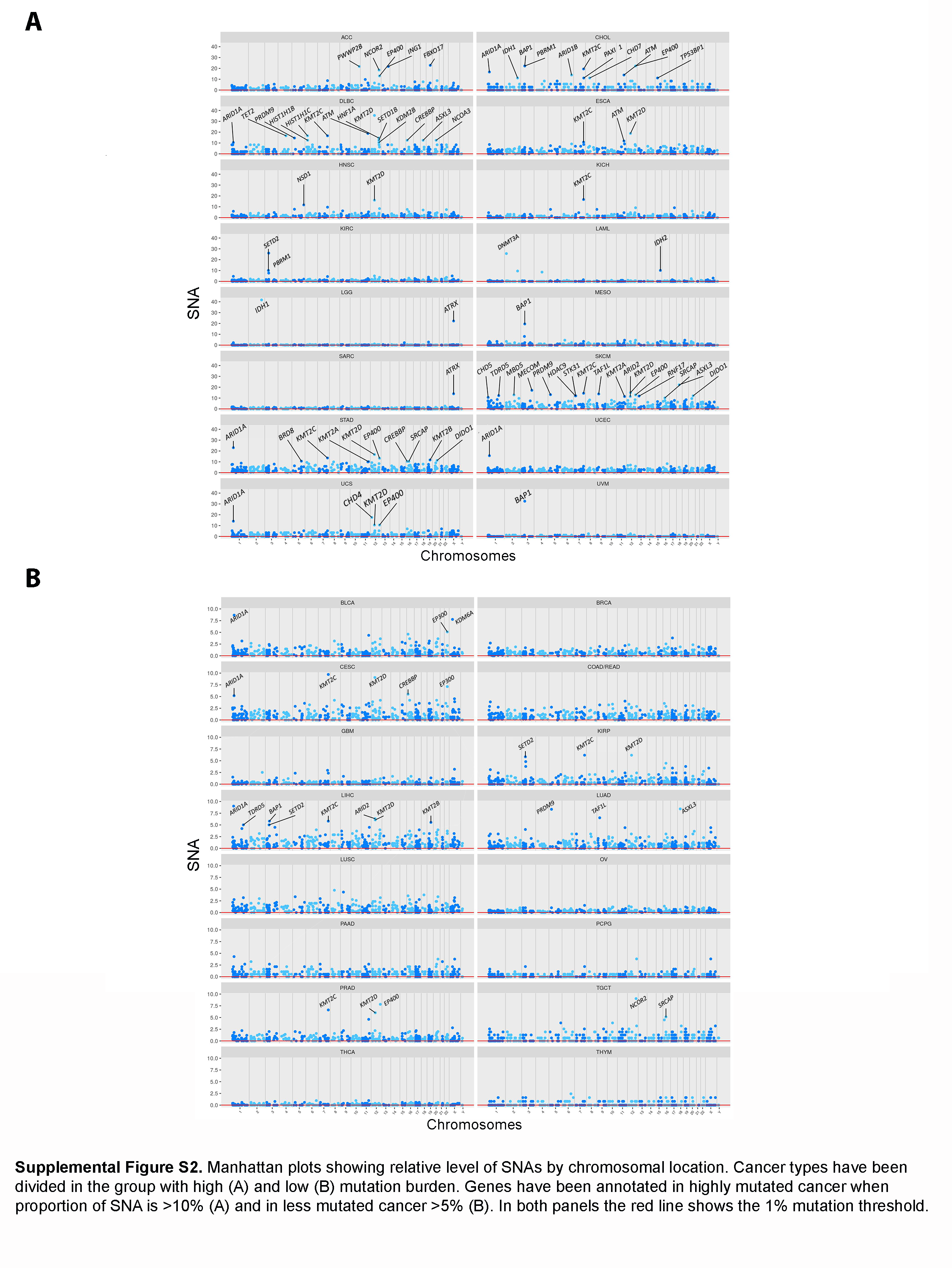

Supplement: Supplemental Material [file supp_gr.268292.120_Supplemental_Fig_S2.tif]

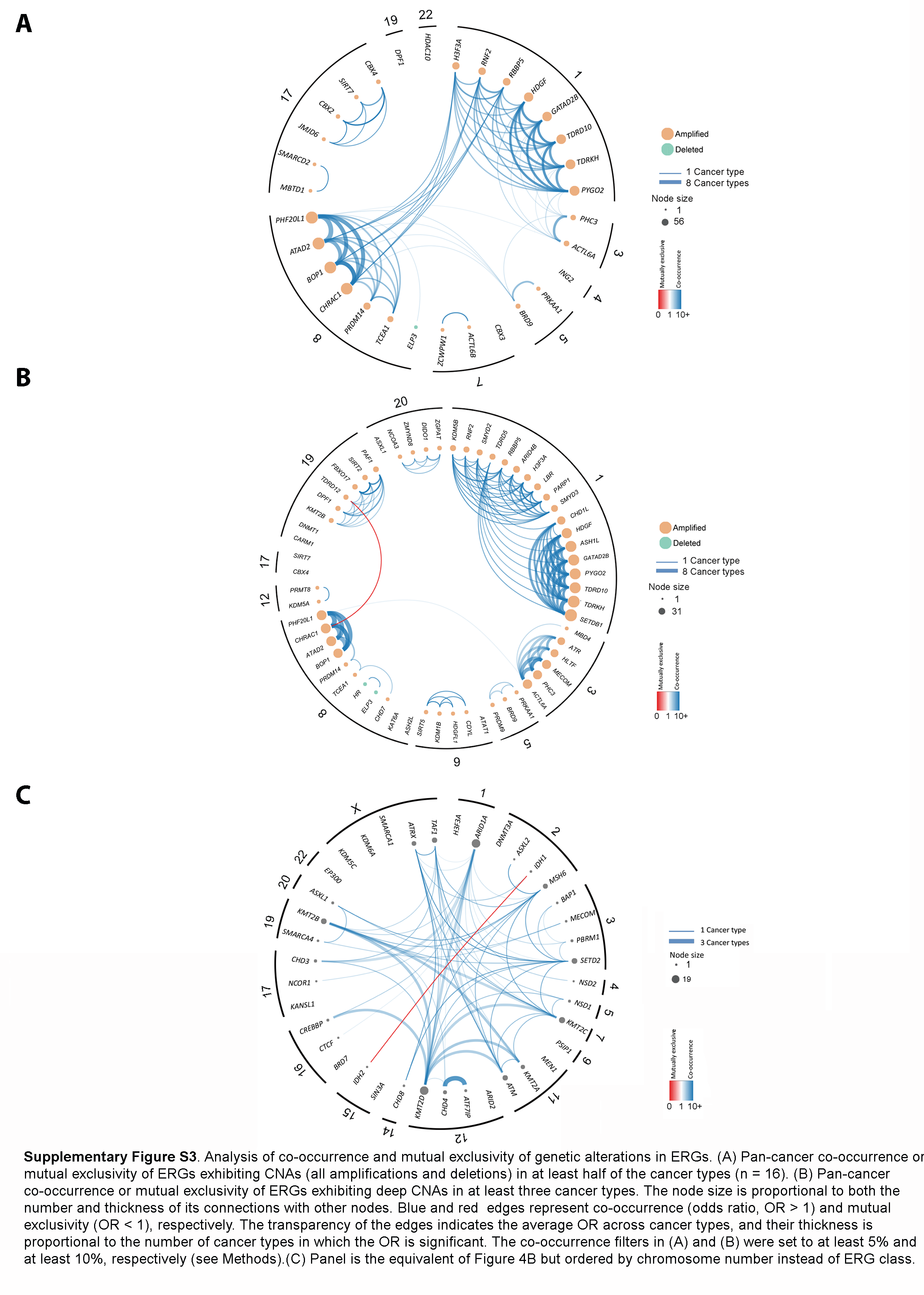

Supplement: Supplemental Material [file supp_gr.268292.120_Supplemental_Fig_S3.tif]

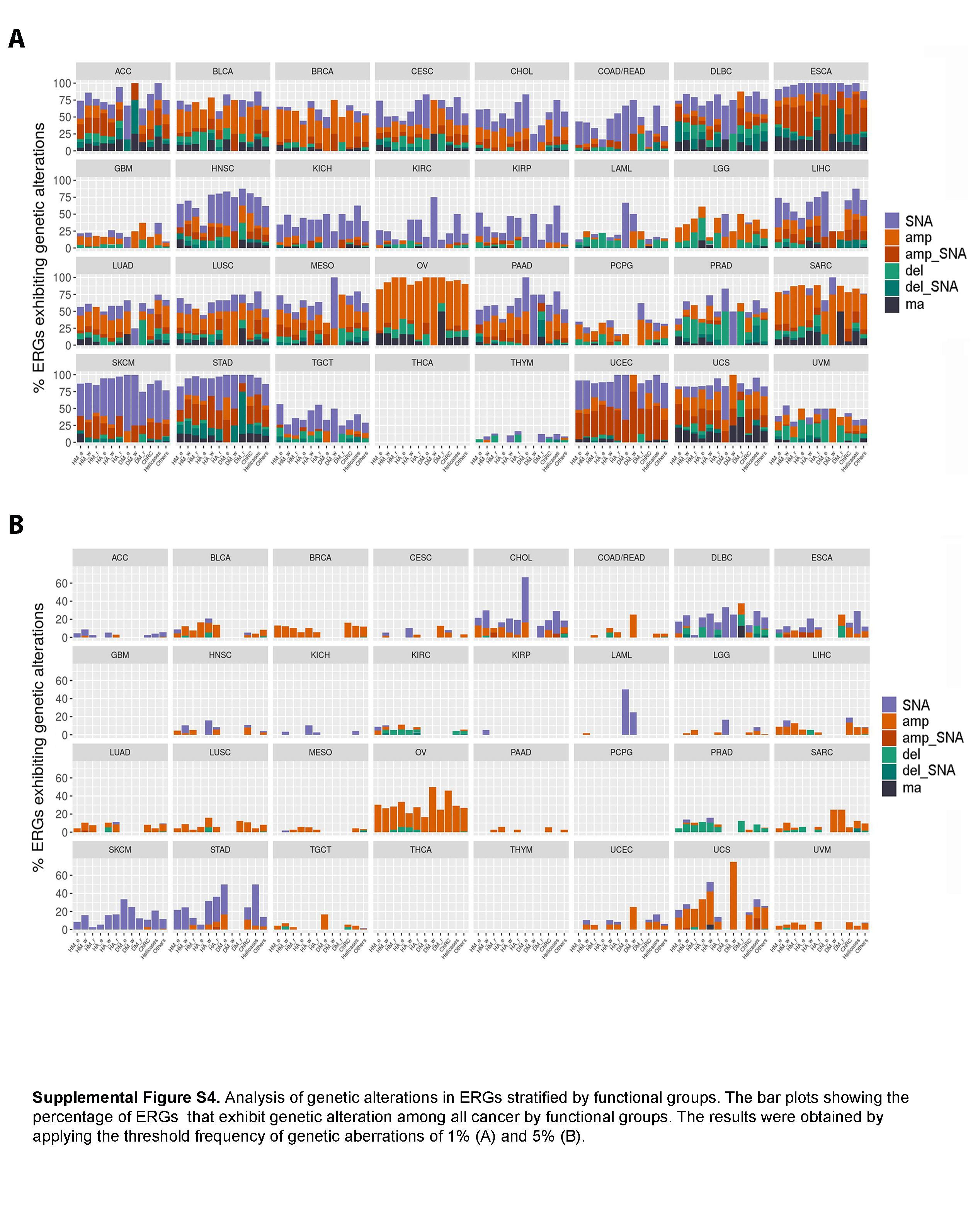

Supplement: Supplemental Material [file supp_gr.268292.120_Supplemental_Fig_S4.tif]

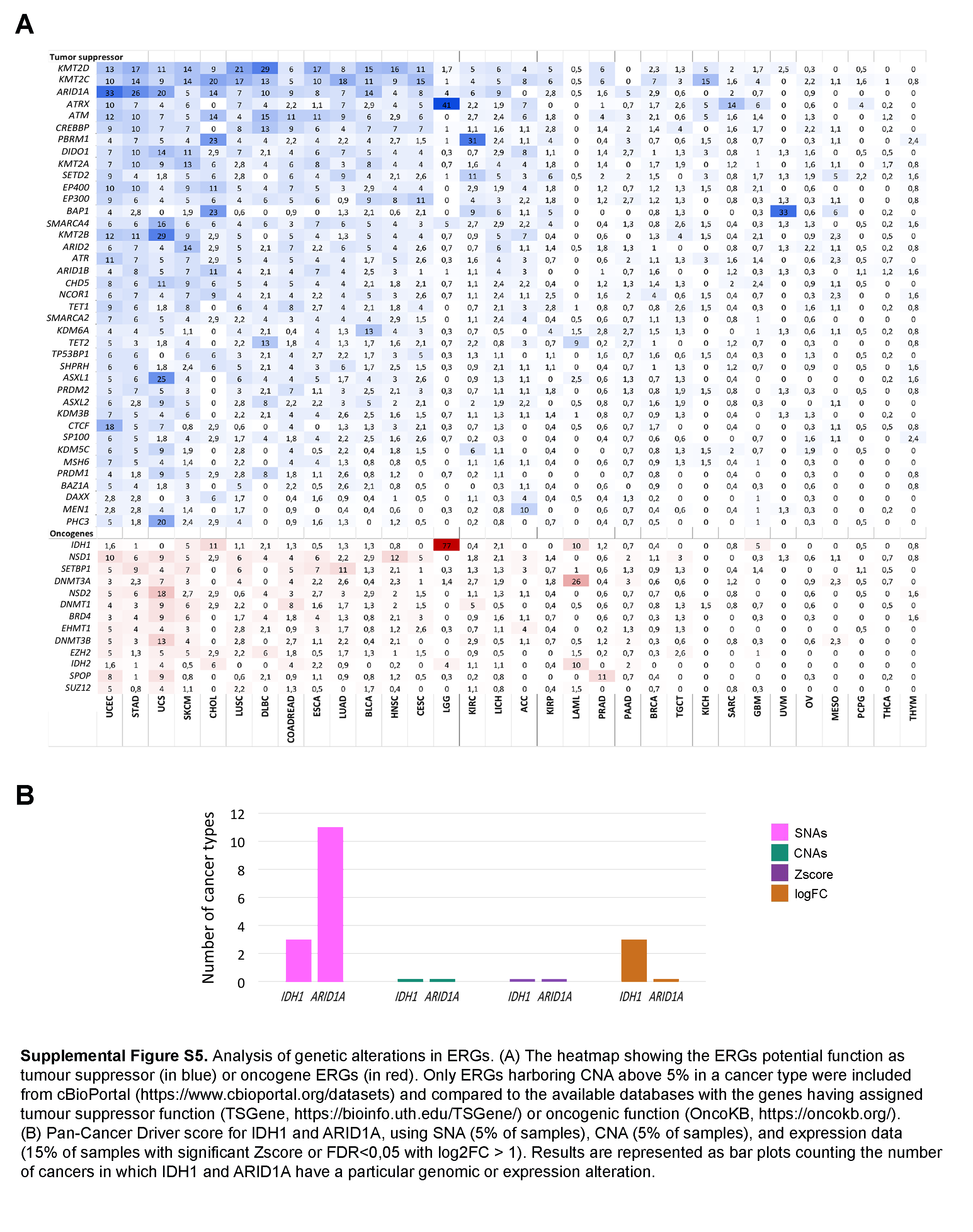

Supplement: Supplemental Material [file supp_gr.268292.120_Supplemental_Fig_S5.tif]

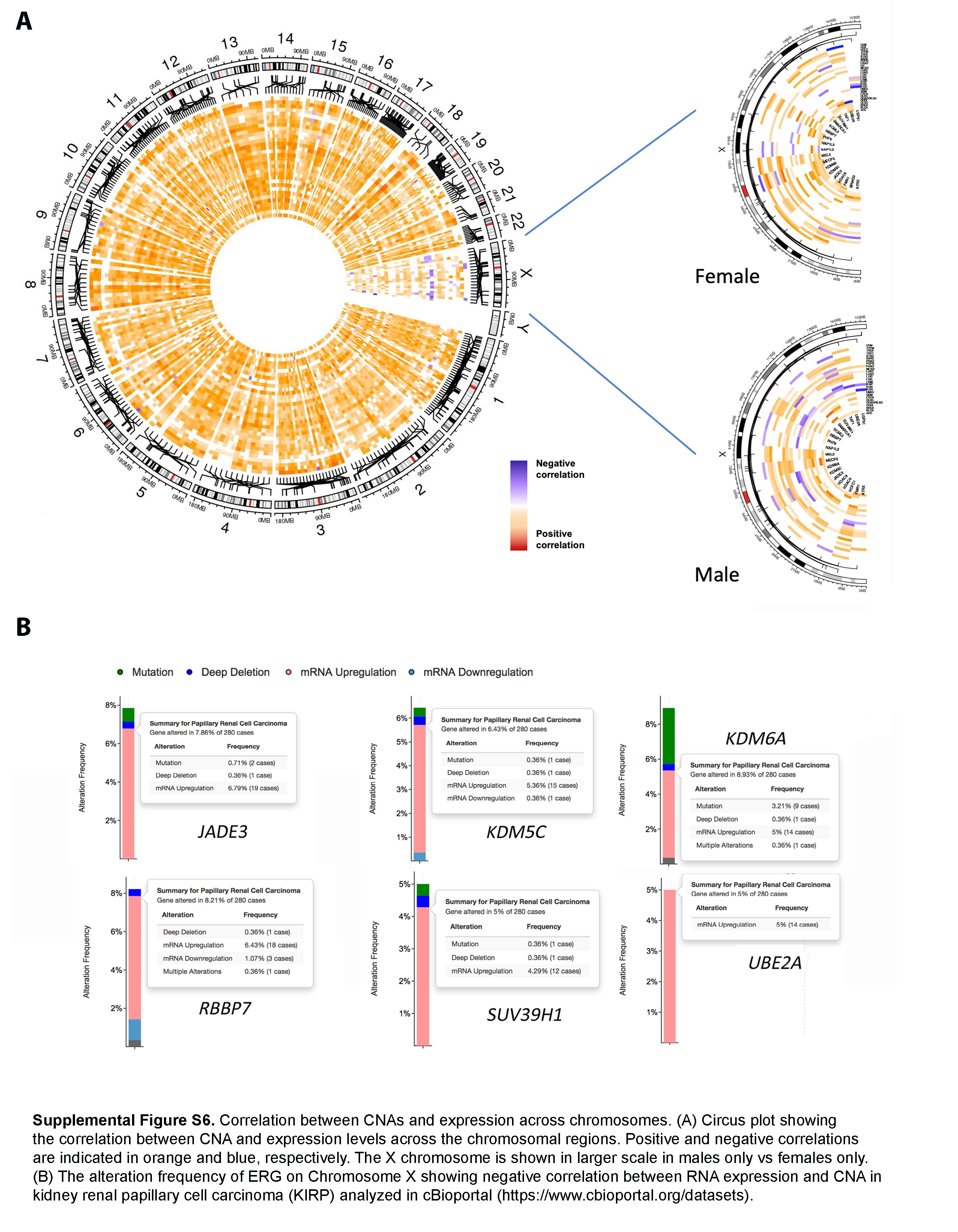

Supplement: Supplemental Material [file supp_gr.268292.120_Supplemental_Fig_S6.tif]

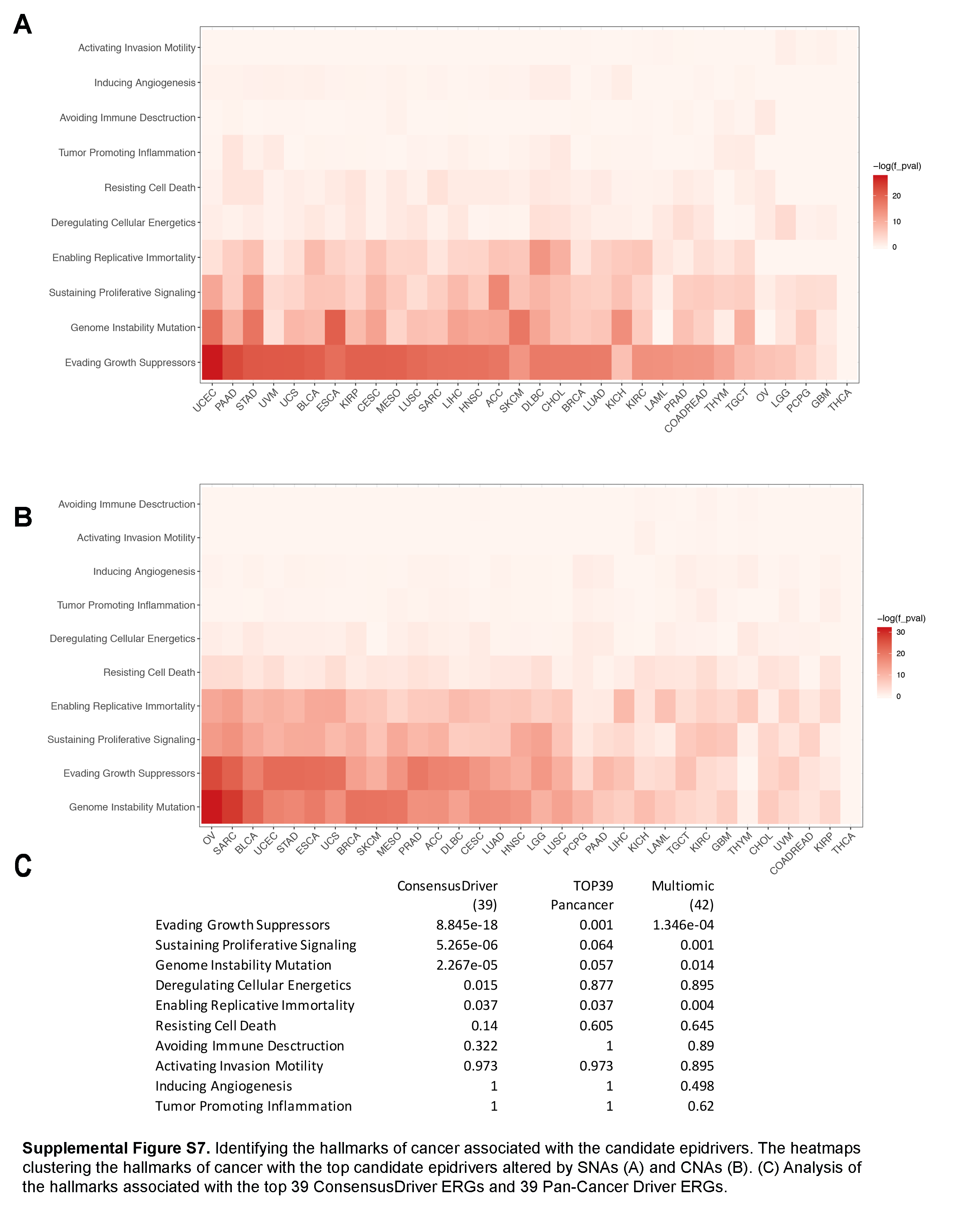

Supplement: Supplemental Material [file supp_gr.268292.120_Supplemental_Fig_S7.tif]

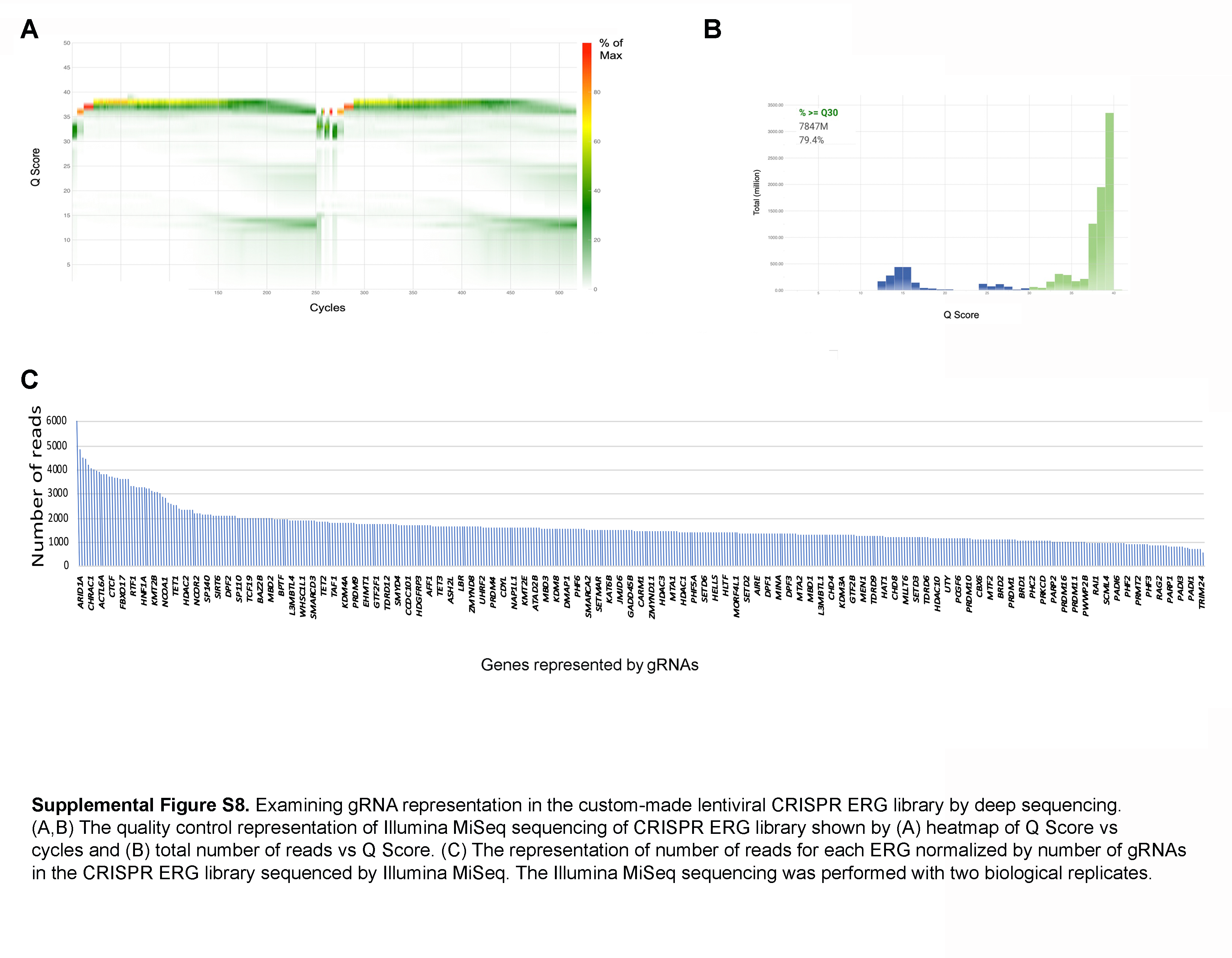

Supplement: Supplemental Material [file supp_gr.268292.120_Supplemental_Fig_S8.tif]

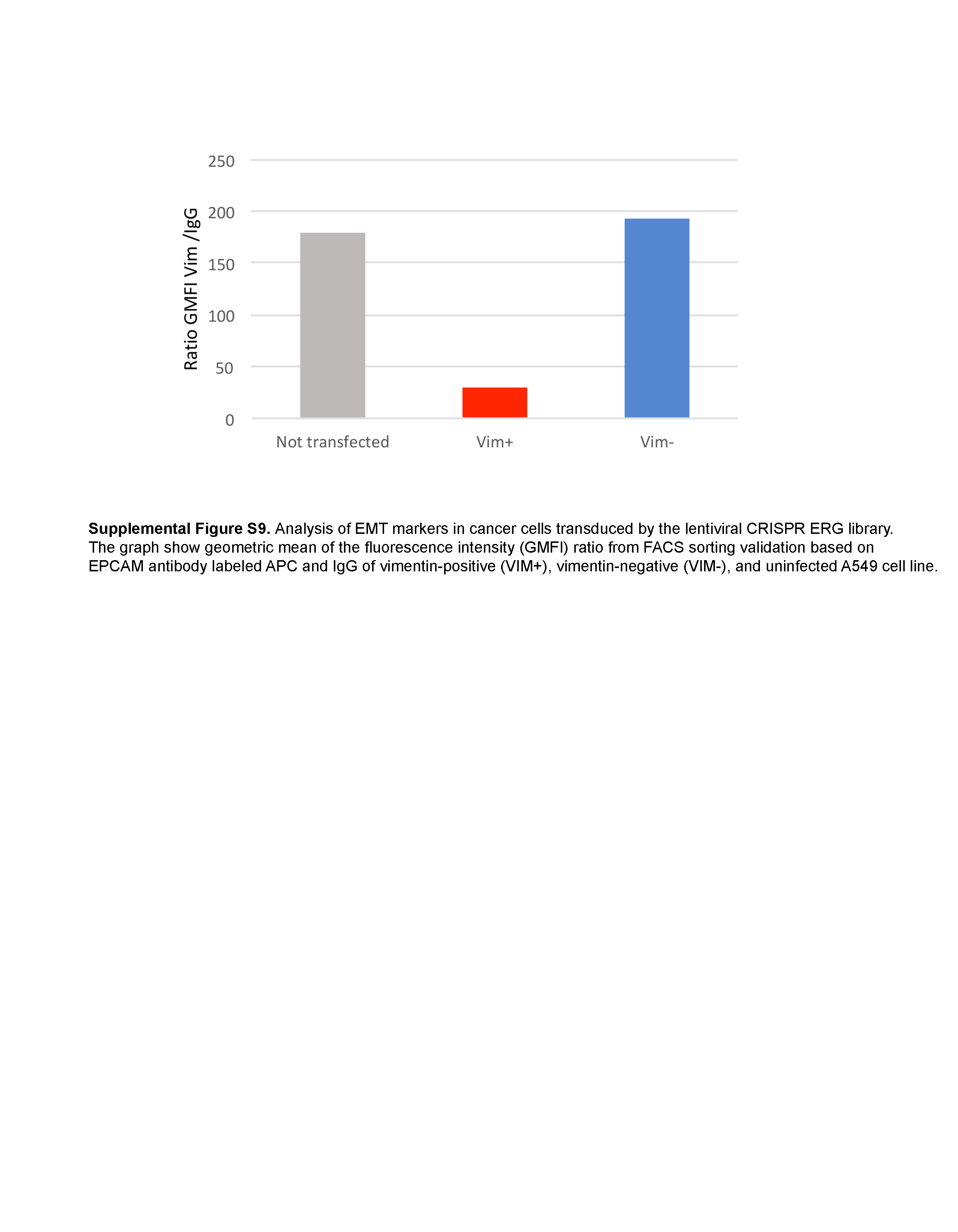

Supplement: Supplemental Material [file supp_gr.268292.120_Supplemental_Fig_S9.tif]

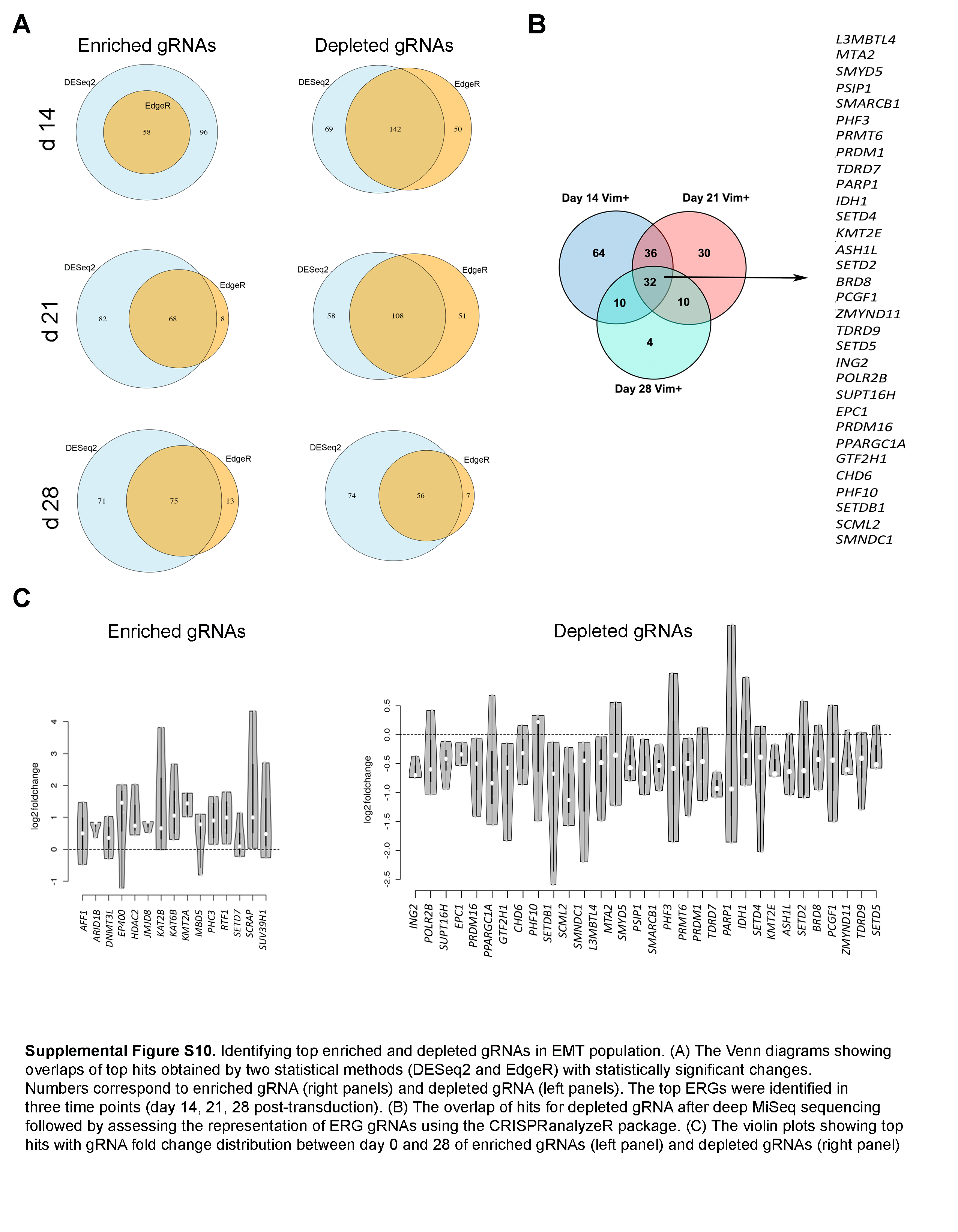

Supplement: Supplemental Material [file supp_gr.268292.120_Supplemental_Fig_S10.tif]

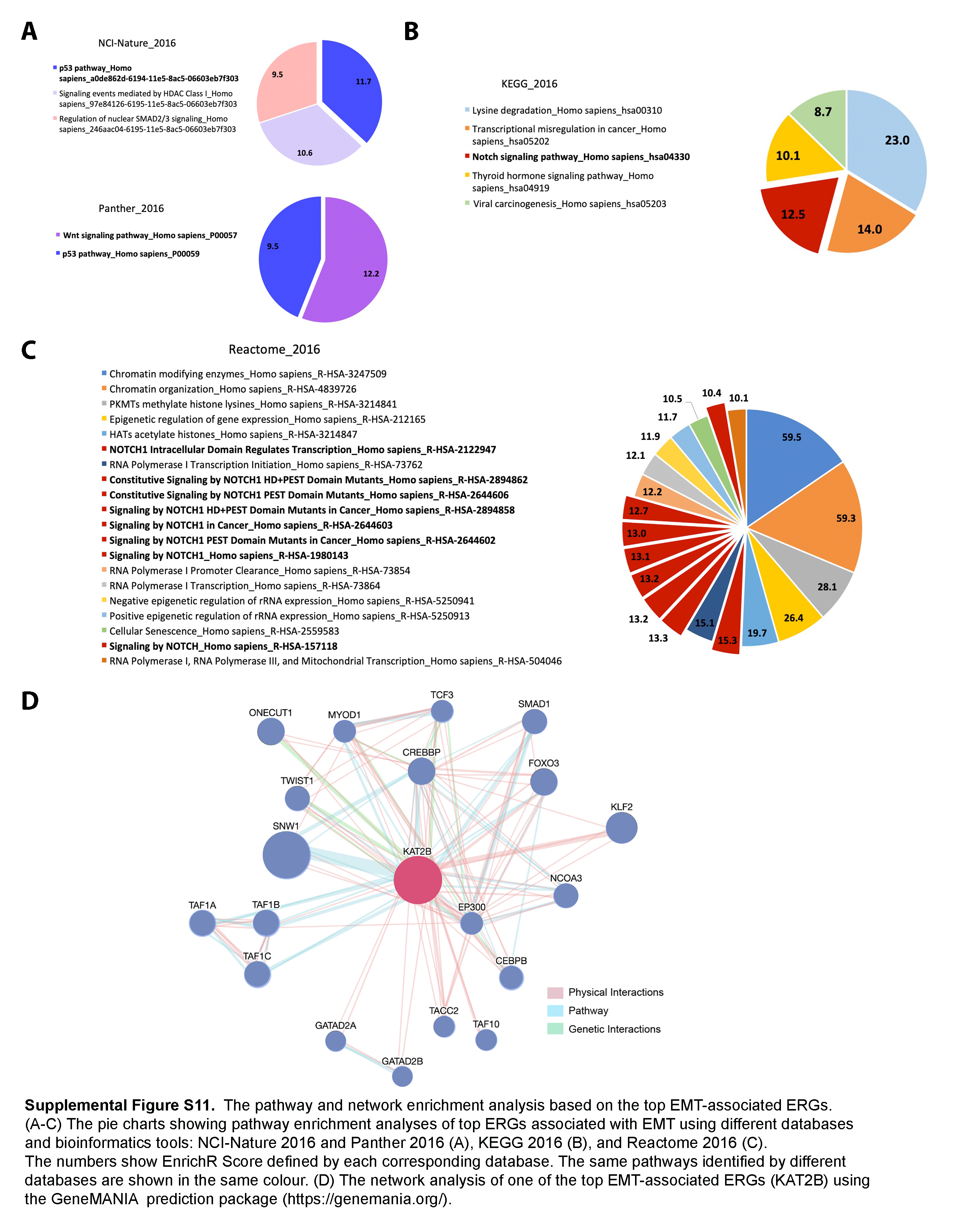

Supplement: Supplemental Material [file supp_gr.268292.120_Supplemental_Fig_S11.tif]

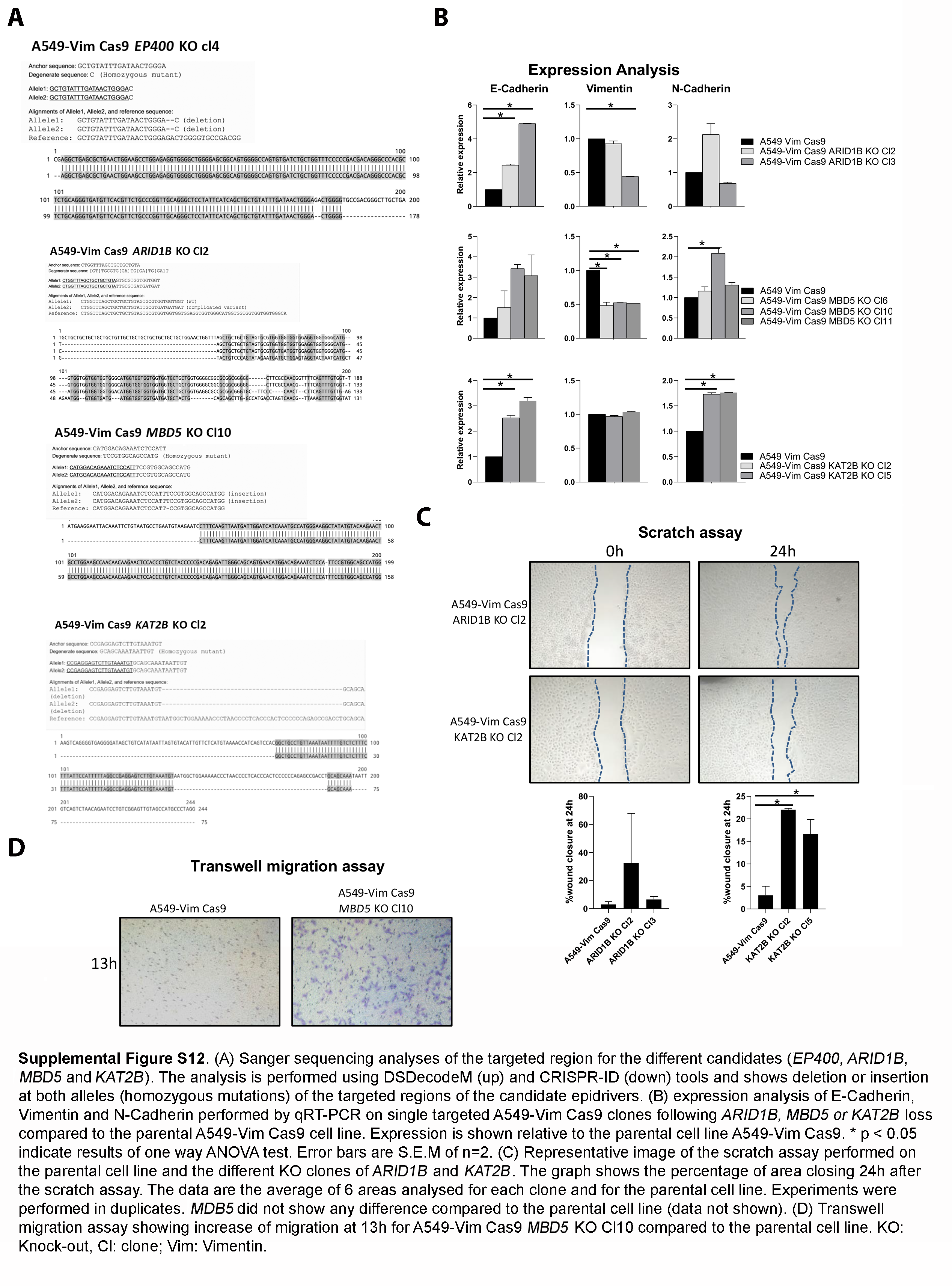

Supplement: Supplemental Material [file supp_gr.268292.120_Supplemental_Fig_S12.tif]

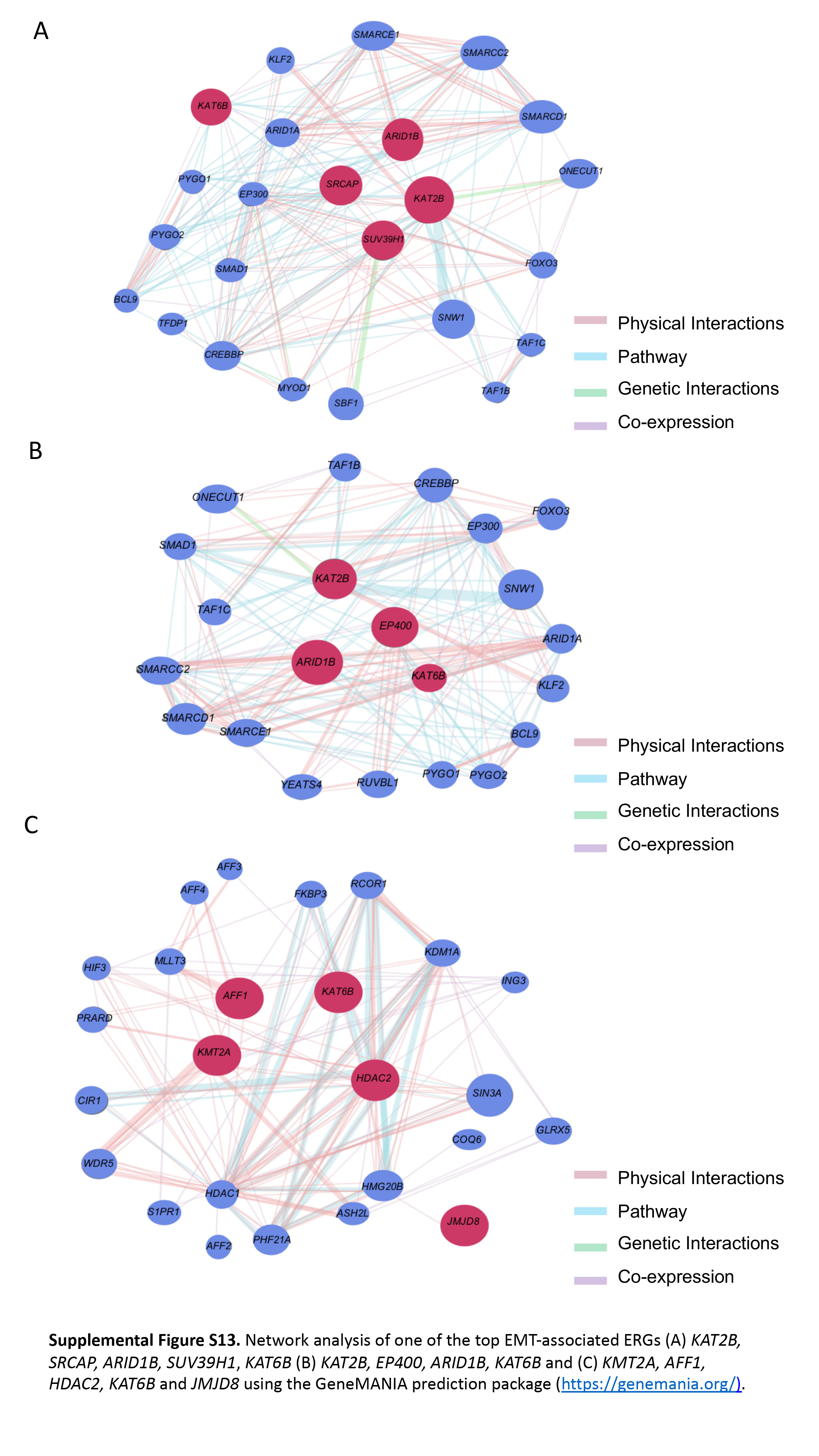

Supplement: Supplemental Material [file supp_gr.268292.120_Supplemental_Fig_S13.tif]

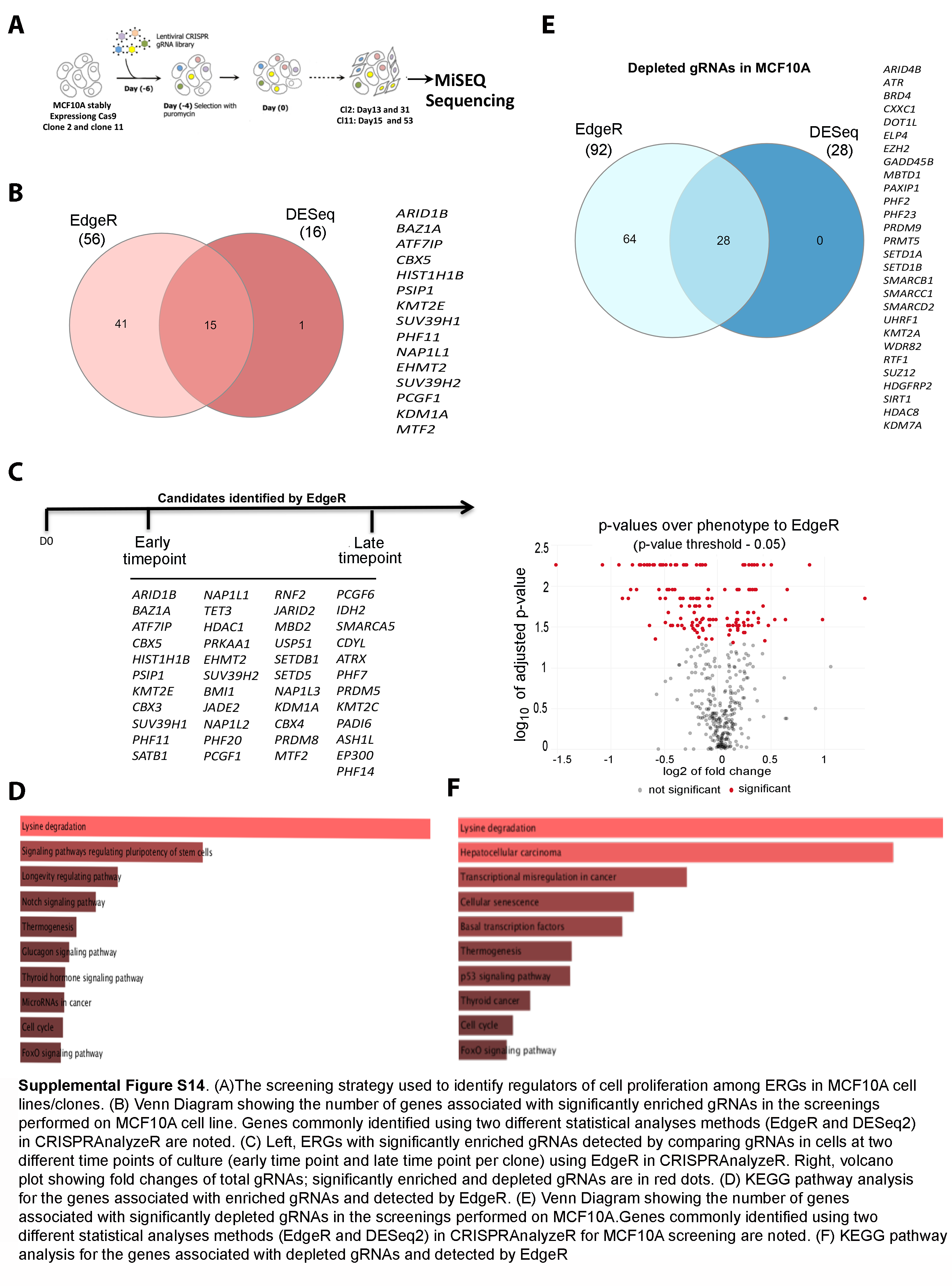

Supplement: Supplemental Material [file supp_gr.268292.120_Supplemental_Fig_S14.tif]

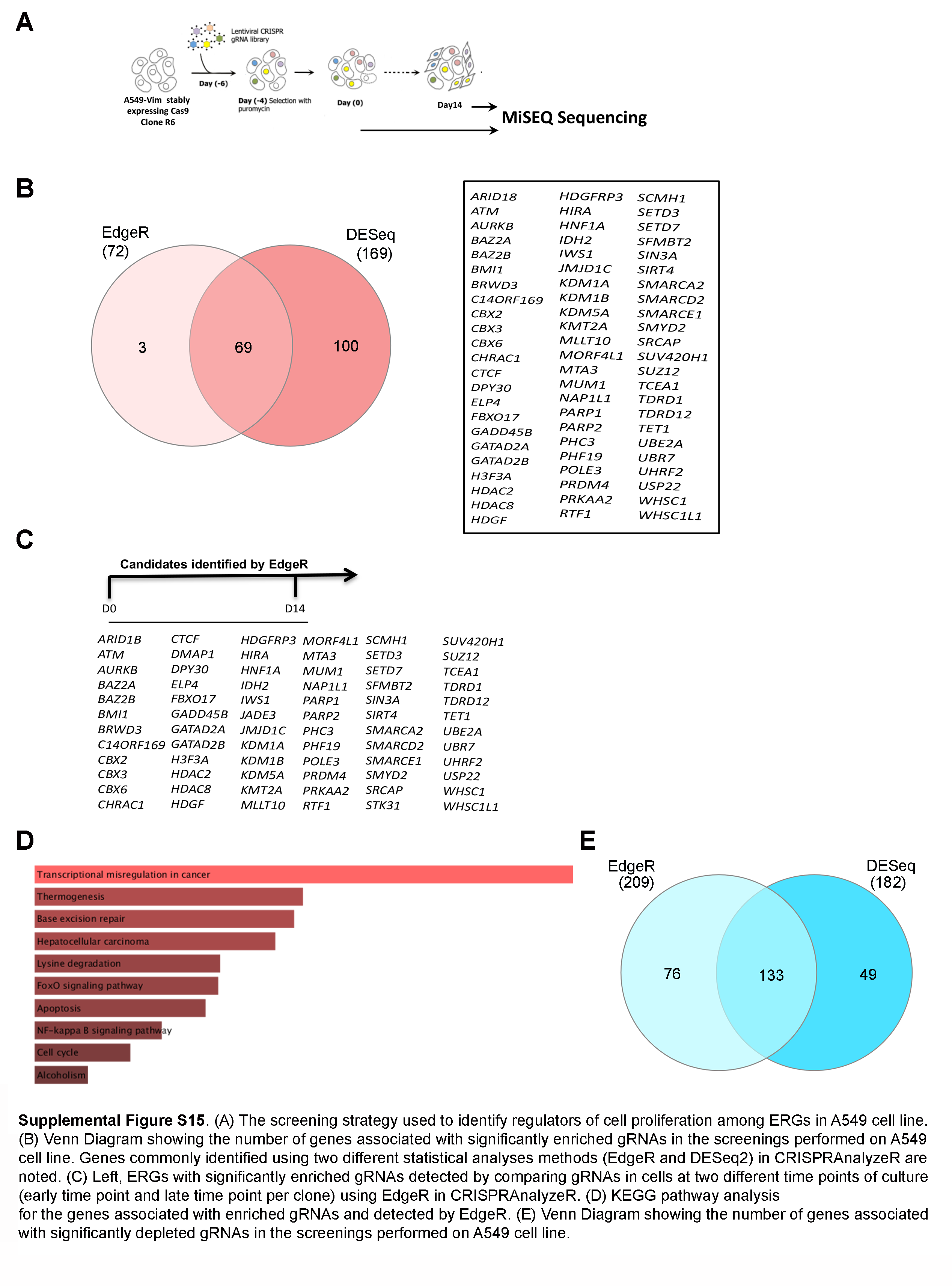

Supplement: Supplemental Material [file supp_gr.268292.120_Supplemental_Fig_S15.tif]
