# Supplementary material for: Pan-cancer multi-omics analysis and orthogonal experimental assessment of epigenetic driver genes
Source: Genome Res. 2020 Oct;30(10):1517–32. doi: 10.1101/gr.268292.120 (PMC7605261; doi:10.1101/gr.268292.120)
Supplement: Supplemental Material [file supp_gr.268292.120_Supplemental_Fig_S16.pdf]

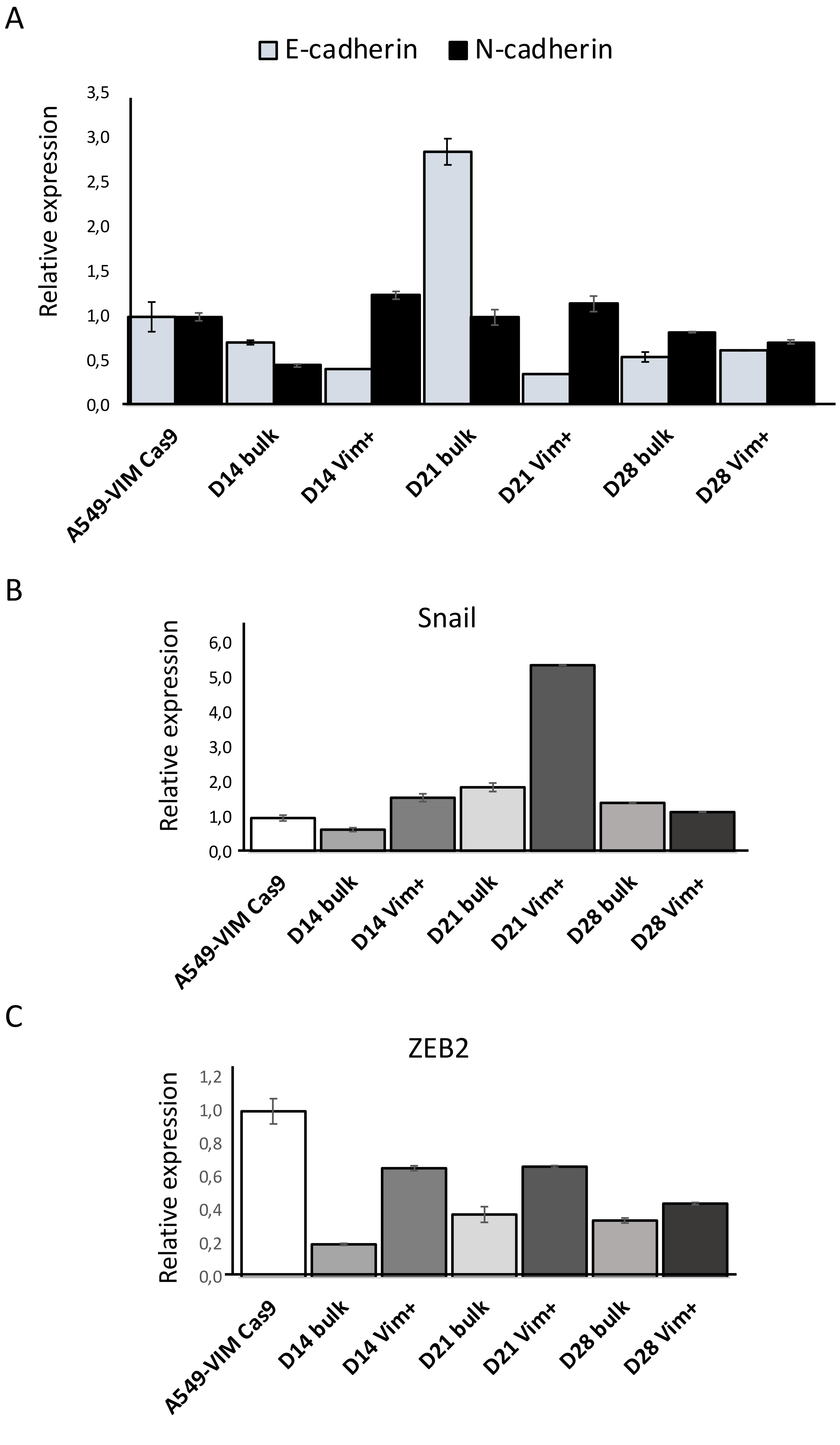

Supplemental Figure S16. Expression analysis of (A) E-Cadherin and N-Cadherin (B) Snail and (C) ZEB2 performed by qRT-PCR on A549-Vim Cas9 transfected cells by CRISPR ERG library (bulk) and A549-Vim Cas9 transfected by CRISPR ERG library after sorting for Vimentin positive (Vim+) cell populations at different time points compared to the parental A549-Vim Cas9 cell line.
