# Supplementary material for: Pan-cancer multi-omics analysis and orthogonal experimental assessment of epigenetic driver genes
Source: Genome Res. 2020 Oct;30(10):1517–32. doi: 10.1101/gr.268292.120 (PMC7605261; doi:10.1101/gr.268292.120)
Supplement: Supplemental Material [file supp_gr.268292.120_Supplemental_Fig_S17.pdf]

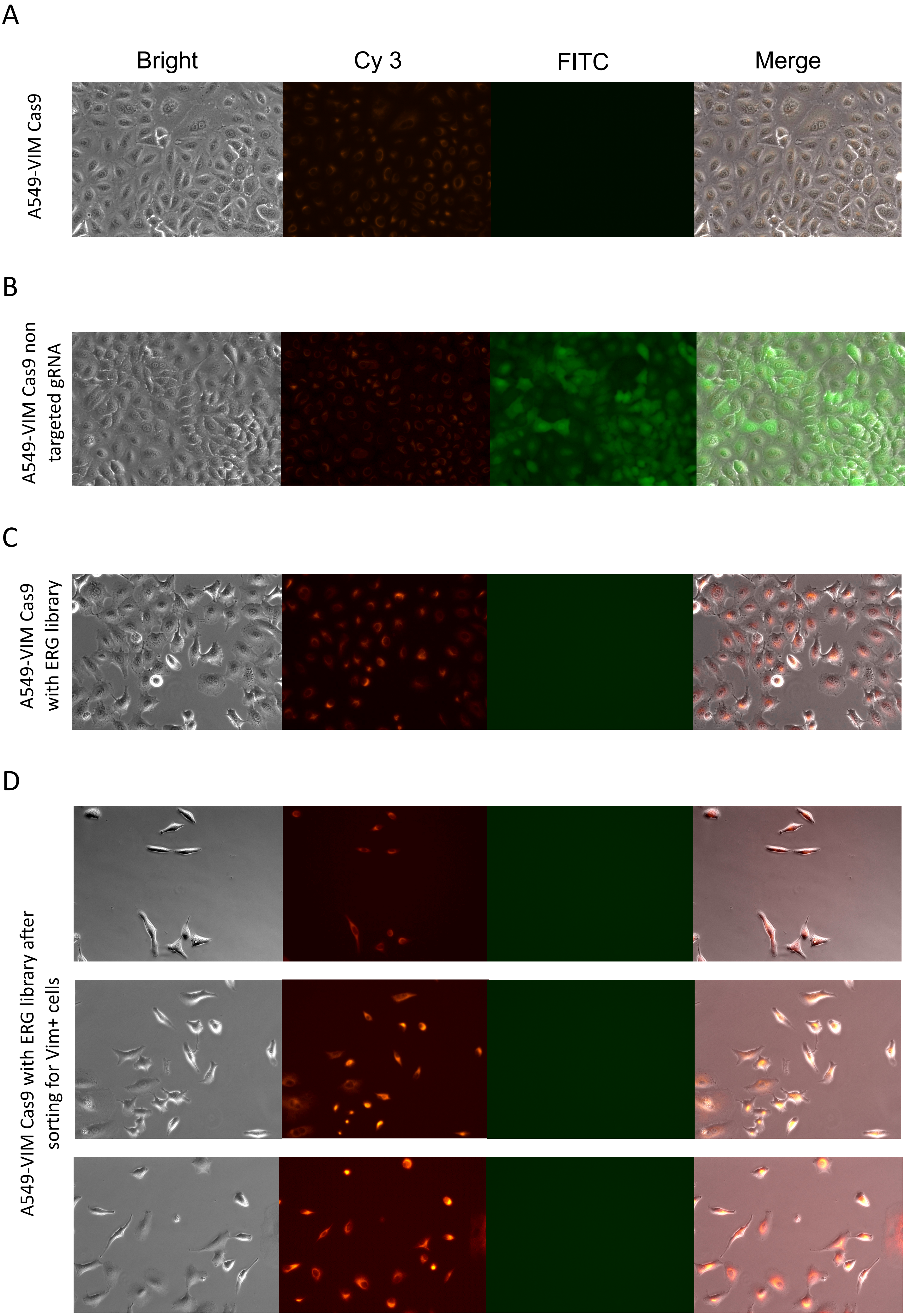

Supplemental Figure S17. Fluorescence microscopy in bright, red and green field and merge images of (A) non transfected A549-VIM Cas9 the parental cell lines, (B) A549-VIM cas9 transfected with non targeted gRNA sequence (control negative), (C) A549-Vim Cas9 transfected cells by CRISPR ERG library (bulk), (D) A549-Vim Cas9 transfected by CRISPR ERG library after sorting for Vimentin positive (Vim+) cell populations.
