# Supplementary material for: Pan-cancer multi-omics analysis and orthogonal experimental assessment of epigenetic driver genes
Source: Genome Res. 2020 Oct;30(10):1517–32. doi: 10.1101/gr.268292.120 (PMC7605261; doi:10.1101/gr.268292.120)
Supplement: Supplemental Material [file supp_gr.268292.120_Supplemental_Table_S1.docx]

**Supplemental Table S1.** A compendium of 426 ERGs included in the study

| Group | Gene Symbol | Gene name | additional function |
| --- | --- | --- | --- |
| DNA methylation | DM_w |  |  |
|  | *DNMT1* | DNA (cytosine-5-)-methyltransferase 1 |  |
|  | *DNMT3A* | DNA (cytosine-5-)-methyltransferase 3 alpha |  |
|  | *DNMT3B* | DNA (cytosine-5-)-methyltransferase 3 beta |  |
|  | *DNMT3L* | DNA (cytosine-5-)-methyltransferase 3-like |  |
|  | DM_e |  |  |
|  | *AICDA* | activation-induced cytidine deaminase |  |
|  | *TET1* | tet methylcytosine dioxygenase 1 |  |
|  | *TET2* | tet methylcytosine dioxygenase 2 |  |
|  | *TET3* | tet methylcytosine dioxygenase 3 |  |
|  | *IDH1* | isocitrate dehydrogenase 1 (NADP+), soluble |  |
|  | *IDH2* | isocitrate dehydrogenase 2 (NADP+), mitochondrial |  |
|  | DM_r |  |  |
|  | *MBD1* | methyl-CpG binding domain protein 1 |  |
|  | *MBD2* | methyl-CpG binding domain protein 2 |  |
|  | *MBD3* | methyl-CpG binding domain protein 3 |  |
|  | *MBD4* | methyl-CpG binding domain protein 4 |  |
|  | *MBD5* | methyl-CpG binding domain protein 5 |  |
|  | *MECP2* | methyl CpG binding protein 2 |  |
|  | *UHRF1* | ubiquitin-like with PHD and ring finger domains 1 | HM_r |
|  | *UHRF2* | ubiquitin-like with PHD and ring finger domains 2, E3 ubiquitin protein ligase |  |
| Histone modifiers | HA_w |  |  |
|  | *CLOCK* | clock circadian regulator |  |
|  | *CREBBP* | CREB binding protein | HA_r |
|  | *ELP3* | elongator acetyltransferase complex subunit 3 |  |
|  | *ELP4* | elongator acetyltransferase complex subunit 4 |  |
|  | *EP300* | E1A binding protein p300 | HA_r |
|  | *GTF3C4* | general transcription factor IIIC, polypeptide 4, 90kDa |  |
|  | *HAT1* | histone acetyltransferase 1 |  |
|  | *KANSL1* | KAT8 regulatory NSL complex subunit 1 | HA_r |
|  | *KAT2A* | K(lysine) acetyltransferase 2A | HA_r |
|  | *KAT2B* | K(lysine) acetyltransferase 2B |  |
|  | *KAT5* | K(lysine) acetyltransferase 5 | HA_r |
|  | *KAT6A* | K(lysine) acetyltransferase 6A |  |
|  | *KAT6B* | K(lysine) acetyltransferase 6B |  |
|  | *KAT7* | K(lysine) acetyltransferase 7 |  |
|  | *KAT8* | K(lysine) acetyltransferase 8 |  |
|  | *MSL3* | male-specific lethal 3 homolog (Drosophila) |  |
|  | *NCOA1* | nuclear receptor coactivator 1 |  |
|  | *NCOA3* | nuclear receptor coactivator 3 |  |
|  | HM_w |  |  |
|  | *AEBP2* | AE binding protein 2 | HA_r, HM_r |
|  | *ASH1L* | ash1 (absent, small, or homeotic)-like (Drosophila) |  |
|  | *ASH2L* | ash2 (absent, small, or homeotic)-like (Drosophila) |  |
|  | *CARM1* | coactivator-associated arginine methyltransferase 1 |  |
|  | *DOT1L* | DOT1-like histone H3K79 methyltransferase | HM_r |
|  | *EHMT1* | euchromatic histone-lysine N-methyltransferase 1 | HM_r |
|  | *EHMT2* | euchromatic histone-lysine N-methyltransferase 2 |  |
|  | *EZH1* | enhancer of zeste 1 polycomb repressive complex 2 subunit |  |
|  | *EZH2* | enhancer of zeste 2 polycomb repressive complex 2 subunit | HM_r |
|  | *KMT2A* | lysine (K)-specific methyltransferase 2A |  |
|  | *KMT2B* | lysine (K)-specific methyltransferase 2B |  |
|  | *KMT2C* | lysine (K)-specific methyltransferase 2C |  |
|  | *KMT2D* | lysine (K)-specific methyltransferase 2D |  |
|  | *KMT2E* | lysine (K)-specific methyltransferase 2E |  |
|  | *KMT5A* | lysine (K)-specific methyltransferase 5A |  |
|  | *KMT5B* | lysine (K)-specific methyltransferase 5B |  |
|  | *KMT5C* | lysine (K)-specific methyltransferase 5C |  |
|  | *MLLT10* | myeloid/lymphoid or mixed-lineage leukemia (trithorax homolog, Drosophila); translocated to, 10 |  |
|  | *MLLT6* | myeloid/lymphoid or mixed-lineage leukemia (trithorax homolog, Drosophila); translocated to, 6 |  |
|  | *NSD1* | nuclear receptor binding SET domain protein 1 |  |
|  | *NSD2* | Nuclear Receptor Binding SET Domain Protein 2 |  |
|  | *NSD3* | Nuclear Receptor Binding SET Domain Protein 3 |  |
|  | *PRDM1* | PR domain containing 1, with ZNF domain |  |
|  | *PRDM10* | PR domain containing 10 |  |
|  | *PRDM11* | PR domain containing 11 |  |
|  | *PRDM12* | PR domain containing 12 |  |
|  | *PRDM13* | PR domain containing 13 |  |
|  | *PRDM14* | PR domain containing 14 |  |
|  | *PRDM15* | PR domain containing 15 |  |
|  | *PRDM16* | PR domain containing 16 |  |
|  | *PRDM2* | PR domain containing 2, with ZNF domain |  |
|  | *PRDM4* | PR domain containing 4 |  |
|  | *PRDM5* | PR domain containing 5 |  |
|  | *PRDM6* | PR domain containing 6 |  |
|  | *PRDM7* | PR domain containing 7 |  |
|  | *PRDM8* | PR domain containing 8 |  |
|  | *PRDM9* | PR domain containing 9 |  |
|  | *PRMT1* | protein arginine methyltransferase 1 |  |
|  | *PRMT2* | protein arginine methyltransferase 2 |  |
|  | *PRMT3* | protein arginine methyltransferase 3 |  |
|  | *PRMT5* | protein arginine methyltransferase 5 |  |
|  | *PRMT6* | protein arginine methyltransferase 6 |  |
|  | *PRMT7* | protein arginine methyltransferase 7 |  |
|  | *PRMT8* | protein arginine methyltransferase 8 |  |
|  | *SETD1A* | SET domain containing 1A |  |
|  | *SETD1B* | SET domain containing 1B |  |
|  | *SETD2* | SET domain containing 2 |  |
|  | *SETD3* | SET domain containing 3 |  |
|  | *SETD4* | SET domain containing 4 |  |
|  | *SETD5* | SET domain containing 5 | HM_r |
|  | *SETD6* | SET domain containing 6 |  |
|  | *SETD7* | SET domain containing (lysine methyltransferase) 7 |  |
|  | *SETDB1* | SET domain, bifurcated 1 | HM_r |
|  | *SETDB2* | SET domain, bifurcated 2 |  |
|  | *SETMAR* | SET domain and mariner transposase fusion gene |  |
|  | *SMYD1* | SET and MYND domain containing 1 |  |
|  | *SMYD2* | SET and MYND domain containing 2 |  |
|  | *SMYD3* | SET and MYND domain containing 3 |  |
|  | *SMYD4* | SET and MYND domain containing 4 |  |
|  | *SMYD5* | SMYD family member 5 |  |
|  | *SUV39H1* | suppressor of variegation 3-9 homolog 1 (Drosophila) |  |
|  | *SUV39H2* | suppressor of variegation 3-9 homolog 2 (Drosophila) | HM_r |
|  | HA_e |  |  |
|  | *HDAC1* | histone deacetylase 1 |  |
|  | *HDAC2* | histone deacetylase 2 |  |
|  | *HDAC3* | histone deacetylase 3 |  |
|  | *HDAC4* | histone deacetylase 4 |  |
|  | *HDAC5* | histone deacetylase 5 |  |
|  | *HDAC6* | histone deacetylase 6 |  |
|  | *HDAC7* | histone deacetylase 7 |  |
|  | *HDAC8* | histone deacetylase 8 |  |
|  | *HDAC9* | histone deacetylase 9 |  |
|  | *HDAC10* | histone deacetylase 10 |  |
|  | *HDAC11* | histone deacetylase 11 |  |
|  | *SIRT1* | sirtuin 1 |  |
|  | *SIRT2* | sirtuin 2 |  |
|  | *SIRT3* | sirtuin 3 |  |
|  | *SIRT4* | sirtuin 4 |  |
|  | *SIRT5* | sirtuin 5 |  |
|  | *SIRT6* | sirtuin 6 |  |
|  | *SIRT7* | sirtuin 7 |  |
| Histone modifiers | HM_e |  |  |
|  | *HR* | hair growth associated |  |
|  | *JMJD1C* | jumonji domain containing 1C |  |
|  | *JMJD6* | jumonji domain containing 6 |  |
|  | *JMJD8* | jumonji domain containing 8 |  |
|  | *KDM1A* | lysine (K)-specific demethylase 1A |  |
|  | *KDM1B* | lysine (K)-specific demethylase 1B |  |
|  | *KDM2A* | lysine (K)-specific demethylase 2A |  |
|  | *KDM2B* | lysine (K)-specific demethylase 2B |  |
|  | *KDM3A* | lysine (K)-specific demethylase 3A |  |
|  | *KDM3B* | lysine (K)-specific demethylase 3B | HM_r |
|  | *KDM4A* | lysine (K)-specific demethylase 4A |  |
|  | *KDM4B* | lysine (K)-specific demethylase 4B | HM_r |
|  | *KDM4C* | lysine (K)-specific demethylase 4C |  |
|  | *KDM4D* | lysine (K)-specific demethylase 4D |  |
|  | *KDM4E* | lysine (K)-specific demethylase 4E | HM_r |
|  | *KDM5A* | lysine (K)-specific demethylase 5A | HM_r |
|  | *KDM5B* | lysine (K)-specific demethylase 5B | HM_r |
|  | *KDM5C* | lysine (K)-specific demethylase 5C |  |
|  | *KDM5D* | lysine (K)-specific demethylase 5D |  |
|  | *KDM6A* | lysine (K)-specific demethylase 6A |  |
|  | *KDM6B* | lysine (K)-specific demethylase 6B | HM_r |
|  | *KDM7A* | lysine (K)-specific demethylase 7A |  |
|  | *KDM8* | lysine (K)-specific demethylase 8 |  |
|  | *UTY* | ubiquitously transcribed tetratricopeptide repeat containing, Y-linked |  |
|  | HA_r |  |  |
|  | *ATAD2* | ATPase family, AAA domain containing 2 |  |
|  | *ATAD2B* | ATPase family, AAA domain containing 2B |  |
|  | *BAZ1A* | bromodomain adjacent to zinc finger domain, 1A |  |
|  | *BAZ1B* | bromodomain adjacent to zinc finger domain, 1B |  |
|  | *BAZ2A* | bromodomain adjacent to zinc finger domain, 2A | HM_r |
|  | *BAZ2B* | bromodomain adjacent to zinc finger domain, 2B |  |
|  | *BPTF* | bromodomain PHD finger transcription factor |  |
|  | *BRD1* | bromodomain containing 1 |  |
|  | *BRD2* | bromodomain containing 2 |  |
|  | *BRD3* | bromodomain containing 3 |  |
|  | *BRD4* | bromodomain containing 4 |  |
|  | *BRD7* | bromodomain containing 7 |  |
|  | *BRD8* | bromodomain containing 8 |  |
|  | *BRD9* | bromodomain containing 9 |  |
|  | *BRDT* | bromodomain, testis-specific |  |
|  | *BRPF1* | bromodomain and PHD finger containing, 1 | HM_r |
|  | *BRPF3* | bromodomain and PHD finger containing, 3 |  |
|  | *BRWD1* | bromodomain and WD repeat domain containing 1 |  |
|  | *BRWD3* | bromodomain and WD repeat domain containing 3 |  |
|  | *CECR2* | cat eye syndrome chromosome region, candidate 2 |  |
|  | *DPF3* | D4, zinc and double PHD fingers, family 3 |  |
|  | *EP400* | E1A binding protein p400 |  |
|  | *PBRM1* | polybromo 1 |  |
|  | *PHIP* | pleckstrin homology domain interacting protein |  |
|  | *SP100* | SP100 nuclear antigen |  |
|  | *SP110* | SP110 nuclear body protein |  |
|  | *SP140* | SP140 nuclear body protein |  |
|  | *SP140L* | SP140 nuclear body protein-like |  |
|  | *TAF1* | TAF1 RNA polymerase II, TATA box binding protein (TBP)-associated factor, 250kDa |  |
|  | *TAF1L* | TAF1 RNA polymerase II, TATA box binding protein (TBP)-associated factor, 210kDa-like | HM_r |
|  | *TAF3* | TAF3 RNA polymerase II, TATA box binding protein (TBP)-associated factor, 140kDa | HM_r |
|  | *TRIM24* | tripartite motif containing 24 |  |
|  | *TRIM28* | tripartite motif containing 28 |  |
|  | *TRIM33* | tripartite motif containing 33 |  |
|  | *TRIM66* | tripartite motif containing 66 | HM_r |
|  | *ZMYND11* | zinc finger, MYND-type containing 11 |  |
|  | *ZMYND8* | zinc finger, MYND-type containing 8 |  |
|  | HM_r |  |  |
|  | *AIRE* | autoimmune regulator |  |
|  | *CBX1* | chromobox homolog 1 |  |
|  | *CBX3* | chromobox homolog 3 |  |
|  | *CBX4* | chromobox homolog 4 |  |
|  | *CBX5* | chromobox homolog 5 |  |
|  | *CBX6* | chromobox homolog 6 |  |
|  | *CBX7* | chromobox homolog 7 |  |
|  | *CDYL* | chromodomain protein, Y-like |  |
|  | *CDYL2* | chromodomain protein, Y-like 2 |  |
|  | *GATAD2A* | GATA zinc finger domain containing 2A |  |
|  | *GATAD2B* | GATA zinc finger domain containing 2B |  |
|  | *GLYR1* | glyoxylate reductase 1 homolog (Arabidopsis) |  |
|  | *HDGFL2* | HDGF Like 2 |  |
|  | *ING1* | inhibitor of growth family, member 1 |  |
|  | *ING2* | inhibitor of growth family, member 2 |  |
|  | *ING3* | inhibitor of growth family, member 3 |  |
|  | *ING4* | inhibitor of growth family, member 4 |  |
|  | *ING5* | inhibitor of growth family, member 5 |  |
|  | *L3MBTL1* | l(3)mbt-like 1 (Drosophila) |  |
|  | *MORF4L1* | mortality factor 4 like 1 |  |
|  | *MPHOSPH8* | M-phase phosphoprotein 8 |  |
|  | *MSH6* | mutS homolog 6 |  |
|  | *MTF2* | metal response element binding transcription factor 2 |  |
|  | *PHF1* | PHD finger protein 1 |  |
|  | *PHF19* | PHD finger protein 19 |  |
|  | *PHF20* | PHD finger protein 20 |  |
|  | *PHF20L1* | PHD finger protein 20-like 1 |  |
|  | *PHF21A* | PHD finger protein 21A |  |
|  | *PHF21B* | PHD finger protein 21B |  |
|  | *PHF23* | PHD finger protein 23 |  |
|  | *PHF6* | PHD finger protein 6 |  |
|  | *PSIP1* | PC4 and SFRS1 interacting protein 1 |  |
|  | *PYGO1* | pygopus family PHD finger 1 |  |
|  | *PYGO2* | pygopus family PHD finger 2 |  |
|  | *RAG2* | recombination activating gene 2 |  |
|  | *SCML2* | sex comb on midleg-like 2 (Drosophila) |  |
|  | *SFMBT1* | Scm-like with four mbt domains 1 |  |
|  | *SGF29* | SAGA Complex Associated Factor 29 |  |
|  | *SND1* | staphylococcal nuclease and tudor domain containing 1 |  |
|  | *TDRD3* | tudor domain containing 3 |  |
|  | *ZCWPW1* | zinc finger, CW type with PWWP domain 1 |  |
|  | *ZCWPW2* | zinc finger, CW type with PWWP domain 2 |  |
|  | H_r |  |  |
|  | *HCFC1* | host cell factor C1 |  |
|  | *PHF10* | PHD finger protein 10 |  |
|  | *PHF11* | PHD finger protein 11 |  |
|  | *PHF12* | PHD finger protein 12 | HM_r, H_e |
|  | *PHF13* | PHD finger protein 13 |  |
|  | *PHF14* | PHD finger protein 14 |  |
|  | *PHF2* | PHD finger protein 2 |  |
|  | *PHF3* | PHD finger protein 3 |  |
|  | *PHF5A* | PHD finger protein 5A |  |
|  | *PHF7* | PHD finger protein 7 | HM_r, H_e |
|  | *PHF8* | PHD finger protein 8 |  |
|  | *RAI1* | retinoic acid induced 1 |  |
| Helicases | Helicases |  |  |
|  | *ATRX* | alpha thalassemia/mental retardation syndrome X-linked | HM_r |
|  | *CHD1* | chromodomain helicase DNA binding protein 1 |  |
|  | *CHD1L* | chromodomain helicase DNA binding protein 1-like |  |
|  | *CHD2* | chromodomain helicase DNA binding protein 2 | HA_r |
|  | *CHD3* | chromodomain helicase DNA binding protein 3 | HA_r |
|  | *CHD4* | chromodomain helicase DNA binding protein 4 |  |
|  | *CHD5* | chromodomain helicase DNA binding protein 5 |  |
|  | *CHD6* | chromodomain helicase DNA binding protein 6 |  |
|  | *CHD7* | chromodomain helicase DNA binding protein 7 |  |
|  | *CHD8* | chromodomain helicase DNA binding protein 8 |  |
|  | *CHD9* | chromodomain helicase DNA binding protein 9 |  |
|  | *HELLS* | helicase, lymphoid-specific |  |
|  | *INO80* | INO80 complex subunit |  |
|  | *SMARCA1* | SWI/SNF related, matrix associated, actin dependent regulator of chromatin, subfamily a, member 1 | |
|  | *SMARCA2* | SWI/SNF related, matrix associated, actin dependent regulator of chromatin, subfamily a, member 2 | HM_r |
|  | *SMARCA4* | SWI/SNF related, matrix associated, actin dependent regulator of chromatin, subfamily a, member 4 | |
|  | *SMARCA5* | SWI/SNF related, matrix associated, actin dependent regulator of chromatin, subfamily a, member 5 | |
|  | *SMARCB1* | SWI/SNF related, matrix associated, actin dependent regulator of chromatin, subfamily b, member 1 | |
|  | *SMARCC1* | SWI/SNF related, matrix associated, actin dependent regulator of chromatin, subfamily c, member 1 | HM_r |
|  | *SMARCC2* | SWI/SNF related, matrix associated, actin dependent regulator of chromatin, subfamily c, member 2 |  |
|  | *SMARCD1* | SWI/SNF related, matrix associated, actin dependent regulator of chromatin, subfamily d, member 1 | |
|  | *SMARCD2* | SWI/SNF related, matrix associated, actin dependent regulator of chromatin, subfamily d, member 2 | |
|  | *SMARCD3* | SWI/SNF related, matrix associated, actin dependent regulator of chromatin, subfamily d, member 3 | |
|  | *SMARCE1* | SWI/SNF related, matrix associated, actin dependent regulator of chromatin, subfamily e, member 1 |  |
| Others Chromatin modifiers | Tudor domain containing | |  |
|  | *AKAP1* | A kinase (PRKA) anchor protein 1 |  |
|  | *LBR* | lamin B receptor |  |
|  | *SMNDC1* | survival motor neuron domain containing 1 |  |
|  | *STK31* | serine/threonine kinase 31 |  |
|  | *TDRD1* | tudor domain containing 1 |  |
|  | *TDRD10* | tudor domain containing 10 |  |
|  | *TDRD12* | tudor domain containing 12 |  |
|  | *TDRD5* | tudor domain containing 5 |  |
|  | *TDRD6* | tudor domain containing 6 |  |
|  | *TDRD7* | tudor domain containing 7 |  |
|  | *TDRD9* | tudor domain containing 9 |  |
|  | *TDRKH* | tudor and KH domain containing |  |
|  | *TP53BP1* | tumor protein p53 binding protein 1 |  |
|  | PHD Finger proteins | |  |
|  | *CXXC1* | CXXC finger protein 1 |  |
|  | *DIDO1* | death inducer-obliterator 1 |  |
|  | *DPF1* | D4, zinc and double PHD fingers family 1 |  |
|  | *DPF2* | D4, zinc and double PHD fingers family 2 |  |
|  | *FBXL19* | F-box and leucine-rich repeat protein 19 |  |
|  | *G2E3* | G2/M-phase specific E3 ubiquitin protein ligase |  |
|  | *INTS12* | integrator complex subunit 12 |  |
|  | *JADE1* | jade family PHD finger 1 |  |
|  | *JADE2* | jade family PHD finger 2 |  |
|  | *JADE3* | jade family PHD finger 3 |  |
|  | *TCF19* | transcription factor 19 |  |
|  | PWWP domain containing | |  |
|  | *HDGF* | hepatoma-derived growth factor |  |
|  | *HDGFL1* | hepatoma derived growth factor-like 1 |  |
|  | *MUM1* | melanoma associated antigen (mutated) 1 |  |
|  | *PWWP2B* | PWWP domain containing 2B |  |
|  | Histones |  |  |
|  | *H2AFZ* | H2A histone family, member Z |  |
|  | *H3F3A* | H3 histone, family 3A |  |
|  | *HIST1H1B* | histone cluster 1, H1b |  |
|  | *HIST1H1C* | histone cluster 1, H1c |  |
|  | *HIST1H3B* | histone cluster 1, H3b |  |
|  | Arginine deiminases | |  |
|  | *PADI1* | peptidyl arginine deiminase, type I |  |
|  | *PADI2* | peptidyl arginine deiminase, type II |  |
|  | *PADI3* | peptidyl arginine deiminase, type III |  |
|  | *PADI4* | peptidyl arginine deiminase, type IV |  |
|  | *PADI6* | peptidyl arginine deiminase, type VI |  |
|  | Ubiquitin modifiers | |  |
|  | *BAP1* | BRCA1 associated protein-1 (ubiquitin carboxy-terminal hydrolase) |  |
|  | *UBE2A* | ubiquitin-conjugating enzyme E2A |  |
|  | *UBE2B* | ubiquitin-conjugating enzyme E2B |  |
|  | *UBE2E1* | ubiquitin-conjugating enzyme E2E 1 |  |
|  | *UBE2I* | ubiquitin-conjugating enzyme E2I |  |
|  | *UBR7* | ubiquitin protein ligase E3 component n-recognin 7 (putative) |  |
|  | *USP22* | ubiquitin specific peptidase 22 |  |
|  | *USP27X* | ubiquitin specific peptidase 27, X-linked |  |
|  | *USP51* | ubiquitin specific peptidase 51 |  |
|  | Ring Finger proteins | |  |
|  | *HLTF* | helicase-like transcription factor |  |
|  | *MARCH5* | membrane-associated ring finger (C3HC4) 5 |  |
|  | *PCGF1* | polycomb group ring finger 1 |  |
|  | *PCGF2* | polycomb group ring finger 2 |  |
|  | *PCGF5* | polycomb group ring finger 5 |  |
|  | *PCGF6* | polycomb group ring finger 6 |  |
|  | *PHRF1* | PHD and ring finger domains 1 |  |
|  | *RING1* | ring finger protein 1 |  |
|  | *RNF2* | ring finger protein 2 |  |
|  | *RNF17* | ring finger protein 17 |  |
|  | *RNF20* | ring finger protein 20, E3 ubiquitin protein ligase |  |
|  | *RNF217* | ring finger protein 217 |  |
|  | *RNF40* | ring finger protein 40, E3 ubiquitin protein ligase |  |
|  | *SHPRH* | SNF2 histone linker PHD RING helicase, E3 ubiquitin protein ligase |  |
|  | ATP-dependent ChRC | |  |
|  | *ACTL6A* | actin-like 6A |  |
|  | *ACTL6B* | actin-like 6B |  |
|  | *ARID1A* | AT rich interactive domain 1A (SWI-like) |  |
|  | *ARID1B* | AT rich interactive domain 1B (SWI1-like) |  |
|  | *ARID2* | AT rich interactive domain 2 (ARID, RFX-like) |  |
|  | *ARID4A* | AT rich interactive domain 4A (RBP1-like) |  |
|  | *ARID4B* | AT rich interactive domain 4B (RBP1-like) |  |
|  | *BMI1* | BMI1 proto-oncogene, polycomb ring finger |  |
|  | *CBX2* | chromobox homolog 2 |  |
|  | *CBX8* | chromobox homolog 8 |  |
|  | *CHAF1A* | chromatin assembly factor 1, subunit A (p150) |  |
|  | *CHAF1B* | chromatin assembly factor 1, subunit B (p60) |  |
|  | *CHRAC1* | chromatin accessibility complex 1 |  |
|  | *DAXX* | death-domain associated protein |  |
|  | *DPY30* | dpy-30 homolog (C. elegans) |  |
|  | *EED* | embryonic ectoderm development |  |
|  | *GTF2H1* | general transcription factor IIH, polypeptide 1, 62kDa |  |
|  | *HNF1A* | HNF1 homeobox A |  |
|  | *JARID2* | jumonji, AT rich interactive domain 2 |  |
|  | *MBTD1* | mbt domain containing 1 |  |
|  | *MEN1* | multiple endocrine neoplasia I |  |
|  | *MTA1* | metastasis associated 1 |  |
|  | *MTA2* | metastasis associated 1 family, member 2 |  |
|  | *MTA3* | metastasis associated 1 family, member 3 |  |
|  | *PHC1* | polyhomeotic homolog 1 (Drosophila) | HM_r |
|  | *PHC2* | polyhomeotic homolog 2 (Drosophila) |  |
|  | *PHC3* | polyhomeotic homolog 3 (Drosophila) |  |
|  | *POLE3* | polymerase (DNA directed), epsilon 3, accessory subunit |  |
|  | *RBBP4* | retinoblastoma binding protein 4 |  |
|  | *RBBP5* | retinoblastoma binding protein 5 |  |
|  | *RBBP7* | retinoblastoma binding protein 7 |  |
|  | *RSF1* | remodeling and spacing factor 1 |  |
|  | *SIN3A* | SIN3 transcription regulator family member A |  |
|  | *SIN3B* | SIN3 transcription regulator family member B |  |
|  | *SUZ12* | SUZ12 polycomb repressive complex 2 subunit | HM_r |
|  | *TDG* | thymine-DNA glycosylase |  |
|  | *WDR5* | WD repeat domain 5 |  |
|  | *YY1* | YY1 transcription factor |  |
|  | ERGs linked by functional interaction | |  |
|  | *ASXL1* | additional sex combs like transcriptional regulator 1 |  |
|  | *ASXL2* | additional sex combs like transcriptional regulator 2 |  |
|  | *ASXL3* | additional sex combs like transcriptional regulator 3 |  |
|  | *ATAT1* | alpha tubulin acetyltransferase 1 |  |
|  | *ATF7IP* | activating transcription factor 7 interacting protein |  |
|  | *DMAP1* | DNA methyltransferase 1 associated protein 1 |  |
|  | *EPC1* | enhancer of polycomb homolog 1 (Drosophila) |  |
|  | *EPC2* | enhancer of polycomb homolog 2 (Drosophila) |  |
|  | *HDGFL3* | HDGF Like 3 |  |
|  | *HIRA* | histone cell cycle regulator |  |
|  | *KIAA2026* | KIAA2026 |  |
|  | *L3MBTL2* | l(3)mbt-like 2 (Drosophila) |  |
|  | *L3MBTL3* | l(3)mbt-like 3 (Drosophila) |  |
|  | *L3MBTL4* | l(3)mbt-like 4 (Drosophila) |  |
|  | *MECOM* | MDS1 and EVI1 complex locus |  |
|  | *L3MBTL2* | nucleosome assembly protein 1-like 1 |  |
|  | *NAP1L2* | nucleosome assembly protein 1-like 2 |  |
|  | *NAP1L3* | nucleosome assembly protein 1-like 3 |  |
|  | *NCOR1* | nuclear receptor corepressor 1 |  |
|  | *NCOR2* | nuclear receptor corepressor 2 |  |
|  | *ORC1* | origin recognition complex, subunit 1 |  |
|  | *PAF1* | Paf1, RNA polymerase II associated factor, homolog (S. cerevisiae) |  |
|  | *PARP1* | poly (ADP-ribose) polymerase 1 |  |
|  | *PARP2* | poly (ADP-ribose) polymerase 2 |  |
|  | *PAXIP1* | PAX interacting (with transcription-activation domain) protein 1 |  |
|  | *RIOX1* | Ribosomal Oxygenase 1 |  |
|  | *RIOX2* | Ribosomal Oxygenase 2 |  |
|  | *SCMH1* | sex comb on midleg homolog 1 (Drosophila) |  |
|  | *SCML4* | sex comb on midleg-like 4 (Drosophila) |  |
|  | *SFMBT2* | Scm-like with four mbt domains 2 |  |
|  | *SRCAP* | Snf2-related CREBBP activator protein |  |
|  | *SSRP1* | structure specific recognition protein 1 |  |
|  | *SUPT16H* | suppressor of Ty 16 homolog (S. cerevisiae) |  |
|  | *ZGPAT* | zinc finger, CCCH-type with G patch domain |  |
| Miscellaneous | Miscellaneous |  |  |
|  | *AFF1* | AF4/FMR2 family, member 1 |  |
|  | *AFF4* | AF4/FMR2 family, member 4 |  |
|  | *ATM* | ATM serine/threonine kinase |  |
|  | *ATR* | ATR serine/threonine kinase |  |
|  | *AURKB* | aurora kinase B |  |
|  | *BOP1* | block of proliferation 1 |  |
|  | *CSTL1* | cystatin-like 1 |  |
|  | *CTCF* | CCCTC-binding factor (zinc finger protein) |  |
|  | *ERCC5* | excision repair cross-complementation group 5 |  |
|  | *FBXO17* | F-box protein 17 |  |
|  | *FBXO44* | F-box protein 44 |  |
|  | *FBXW9* | F-box and WD repeat domain containing 9 |  |
|  | *FKBP1A* | FK506 binding protein 1A, 12kDa |  |
|  | *FKBP2* | FK506 binding protein 2, 13kDa |  |
|  | *FKBP5* | FK506 binding protein 5 |  |
|  | *FMR1* | fragile X mental retardation 1 |  |
|  | *FXR2* | fragile X mental retardation, autosomal homolog 2 |  |
|  | *GADD45A* | growth arrest and DNA-damage-inducible, alpha |  |
|  | *GADD45B* | growth arrest and DNA-damage-inducible, beta |  |
|  | *GTF2B* | general transcription factor IIB |  |
|  | *GTF2F1* | general transcription factor IIF, polypeptide 1, 74kDa |  |
|  | *HIF1AN* | hypoxia inducible factor 1, alpha subunit inhibitor |  |
|  | *HSPBAP1* | HSPB (heat shock 27kDa) associated protein 1 |  |
|  | *IWS1* | IWS1 homolog (S. cerevisiae) |  |
|  | *PCMT1* | protein-L-isoaspartate (D-aspartate) O-methyltransferase |  |
|  | *POLR2B* | polymerase (RNA) II (DNA directed) polypeptide B, 140kDa |  |
|  | *PPARGC1A* | peroxisome proliferator-activated receptor gamma, coactivator 1 alpha |  |
|  | *PRKAA1* | protein kinase, AMP-activated, alpha 1 catalytic subunit |  |
|  | *PRKAA2* | protein kinase, AMP-activated, alpha 2 catalytic subunit |  |
|  | *PRKCD* | protein kinase C, delta |  |
|  | *RPA3* | replication protein A3, 14kDa |  |
|  | *RPH3A* | rabphilin 3A |  |
|  | *RPS6KA5* | ribosomal protein S6 kinase, 90kDa, polypeptide 5 |  |
|  | *RTF1* | Rtf1, Paf1/RNA polymerase II complex component, homolog (S. cerevisiae) |  |
|  | *SATB1* | SATB homeobox 1 |  |
|  | *TCEA1* | transcription elongation factor A (SII), 1 |  |
|  | *TCF20* | transcription factor 20 (AR1) |  |
|  | *WDR82* | WD repeat domain 82 |  |
