# Supplementary material for: Pan-cancer multi-omics analysis and orthogonal experimental assessment of epigenetic driver genes
Source: Genome Res. 2020 Oct;30(10):1517–32. doi: 10.1101/gr.268292.120 (PMC7605261; doi:10.1101/gr.268292.120)
Supplement: Supplemental Material [file supp_gr.268292.120_Supplemental_Table_S2.docx]

| **Group** | **Function** | **Modification** | **Number of genes** | **% of total** |
| --- | --- | --- | --- | --- |
| DNA modifiers | Writers | methylation, 5mC | 4 | 0,9 |
|  | Editors | 5hmC, 5caC, 5fC | 6 | 1,4 |
|  | Readers | methylation, 5mC | 8 | 1,9 |
| Histone modifiers | Writers | acetylation | 18 | 4,2 |
|  |  | methylation | 62 | 14,6 |
|  | Editors | acetylation | 18 | 4,2 |
|  |  | methylation | 24 | 5,6 |
|  | Readers | acetylation, | 37 | 8,7 |
|  |  | methylation | 42 | 9,9 |
|  |  | acetylation, methylation,  phosphorylation | 12 | 2,8 |
| Chromatin remodeling helicase | |  | 24 | 5,6 |
| Others chromatin modifiers | Bind methylated histones | Tudor domain containing | 13 | 3,1 |
|  | Histone binding proteins | PHD finger proteins | 11 | 2,6 |
|  |  | PWWP domain containing | 4 | 0,9 |
|  | Histones |  | 5 | 1,2 |
|  | Peptidyl arginine deiminases | Deiminates histones | 5 | 1,2 |
|  | Ubiquitin modifiers |  | 9 | 2,1 |
|  | Ring finger proteins | Modulates ubiquitination | 14 | 3,3 |
|  | ERG in ATP-dependent chromatin remodeling complex | BAF  ISWI  NRD/Mi-2  SWI/SNF  PRC1  PRC2  SIN3  NURF | 38 | 8,9 |
|  | ERG linked by functional interaction | \| Histone deacetylases \| \| --- \| \| Histone methyltransferases \| \| Chromatin remodeling \| \| DNA demethylases \| | 34 | 8 |
| Miscellaneous | | | 38 | 8,9 |

**Supplemental Table S2.** Distribution of epigenetic regulator genes across their functional categories
