# Supplementary material for: Pan-cancer multi-omics analysis and orthogonal experimental assessment of epigenetic driver genes
Source: Genome Res. 2020 Oct;30(10):1517–32. doi: 10.1101/gr.268292.120 (PMC7605261; doi:10.1101/gr.268292.120)
Supplement: Supplemental Material [file supp_gr.268292.120_Supplemental_Table_S3.docx]

| **Supplemental Table S3** Top 10 ERGs exhibiting genetic and expression aberrations in each of 33 cancer types | | | | | | |  |  |  |  |  |  |  |  |  |  |  |  |
| --- | --- | --- | --- | --- | --- | --- | --- | --- | --- | --- | --- | --- | --- | --- | --- | --- | --- | --- |
| *Cancer* | *Genes* | *%samples with SNA* | *Genes* | *% samples with CNA del* | *Genes* | *% samples with CNA deep deletion* | *Genes* | *% samples with zsc downregulated* | *Genes* | *% samples with CNA gain* | *Genes* | *% samples with CNA deep gain* | *Genes* | *% samples with zsc upregulated* | *Genes* | *logFC positive* | *Genes* | *logFC negative* |
| *ACC* | *FBXO17* | *22.8%* | *CBX6* | *53.3%* | *PHF13* | *4.3%* | *BRD1* | *6.5%* | *BAZ2A* | *75.0%* | *BRD9* | *14.1%* | *JADE2* | *36.7%* |  |  |  |  |
| *ACC* | *ING1* | *21.7%* | *BRD1* | *52.2%* | *SMARCE1* | *4.3%* | *MEN1* | *6.5%* | *HDAC7* | *73.9%* | *ASH1L* | *5.4%* | *UBE2B* | *35.9%* |  |  |  |  |
| *ACC* | *PWWP2B* | *21.7%* | *HDAC10* | *52.2%* | *CHD5* | *3.3%* | *ERCC5* | *5.4%* | *SMARCC2* | *73.9%* | *PRKAA1* | *5.4%* | *CBX5* | *29.3%* |  |  |  |  |
| *ACC* | *NCOR2* | *18.5%* | *PHF21B* | *52.2%* | *ING2* | *3.3%* | *PRDM2* | *5.4%* | *CBX5* | *72.8%* | *BRD4* | *4.3%* | *BRD7* | *27.2%* |  |  |  |  |
| *ACC* | *EP400* | *13.0%* | *TCF20* | *52.2%* | *ARID1B* | *2.2%* | *TRIM33* | *5.4%* | *SMARCD1* | *72.8%* | *CARM1* | *4.3%* | *BRD8* | *27.2%* |  |  |  |  |
| *ACC* | *MEN1* | *9.8%* | *CBX7* | *51.1%* | *DPF2* | *2.2%* | *CBX7* | *4.3%* | *KMT2D* | *71.7%* | *DNMT1* | *4.3%* | *KDM3B* | *27.2%* |  |  |  |  |
| *ACC* | *DIDO1* | *8.7%* | *EP300* | *48.9%* | *EZH1* | *2.2%* | *EP300* | *3.3%* | *NAP1L1* | *71.7%* | *DOT1L* | *4.3%* | *PRDM4* | *26.1%* |  |  |  |  |
| *ACC* | *KMT2C* | *8.7%* | *HIRA* | *48.9%* | *FBXO44* | *2.2%* | *MPHOSPH8* | *3.3%* | *SETD1B* | *71.7%* | *MUM1* | *4.3%* | *TRIM28* | *25.0%* |  |  |  |  |
| *ACC* | *ASXL3* | *7.6%* | *PHF5A* | *48.9%* | *HDAC4* | *2.2%* | *PRMT2* | *3.3%* | *SIRT4* | *71.7%* | *NSD1* | *4.3%* | *CHAF1A* | *22.8%* |  |  |  |  |
| *ACC* | *KDM6B* | *7.6%* | *SMARCB1* | *48.9%* | *HDAC5* | *2.2%* | *PRMT6* | *3.3%* | *ARID2* | *70.7%* | *RNF20* | *4.3%* | *GTF2F1* | *22.8%* |  |  |  |  |
| *BLCA* | *ARID1A* | *8.7%* | *HR* | *60.2%* | *HR* | *7.0%* | *ELP3* | *11.2%* | *ASXL1* | *62.6%* | *HDGFL1* | *12.4%* | *SETDB1* | *25.2%* | *PRDM13* | *4.51* | *PRDM6* | *-3.33* |
| *BLCA* | *KDM6A* | *7.8%* | *ELP3* | *58.7%* | *PHF11* | *7.0%* | *PHRF1* | *8.5%* | *DNMT3B* | *62.1%* | *SETDB1* | *10.7%* | *ACTL6A* | *22.6%* | *PRDM9* | *4.31* | *PRKAA2* | *-2.78* |
| *BLCA* | *EP300* | *5.1%* | *TRIM66* | *52.9%* | *SETDB2* | *7.0%* | *POLE3* | *8.3%* | *L3MBTL1* | *60.2%* | *BRD9* | *10.2%* | *BRPF1* | *20.9%* | *HIST1H3B* | *4.08* | *GADD45B* | *-2.72* |
| *BLCA* | *CREBBP* | *4.6%* | *SIRT3* | *51.7%* | *ELP3* | *5.8%* | *GLYR1* | *7.8%* | *ZMYND8* | *60.2%* | *TAF3* | *10.0%* | *CHRAC1* | *20.6%* | *HIST1H1B* | *3.33* | *CHD5* | *-2.56* |
| *BLCA* | *ATM* | *4.4%* | *PHRF1* | *51.2%* | *PSIP1* | *4.1%* | *GTF2H1* | *7.3%* | *ATAD2* | *60.0%* | *CHD1L* | *9.7%* | *TDRKH* | *19.7%* | *HDGFL1* | *2.95* | *CBX7* | *-2.51* |
| *BLCA* | *DIDO1* | *3.6%* | *GTF2H1* | *51.0%* | *ING5* | *3.9%* | *RNF20* | *6.8%* | *PHF20* | *60.0%* | *ASH2L* | *9.2%* | *ATAD2* | *18.7%* | *CSTL1* | *2.85* | *HDAC4* | *-2.13* |
| *BLCA* | *EP400* | *3.6%* | *NCOR1* | *50.7%* | *KDM4C* | *3.9%* | *ASH2L* | *6.6%* | *NCOA3* | *59.7%* | *SETD5* | *9.2%* | *BRD9* | *18.4%* | *HIST1H1C* | *2.50* | *NAP1L2* | *-2.10* |
| *BLCA* | *SRCAP* | *3.6%* | *FXR2* | *50.5%* | *KIAA2026* | *3.9%* | *BRD1* | *6.6%* | *CHD6* | *59.2%* | *TDRKH* | *9.2%* | *PYGO2* | *18.4%* | *UHRF1* | *2.42* | *NAP1L3* | *-2.03* |
| *BLCA* | *ASH1L* | *3.2%* | *KDM6B* | *50.5%* | *UHRF2* | *3.9%* | *IWS1* | *6.3%* | *BOP1* | *57.8%* | *BRPF1* | *9.0%* | *TAF3* | *18.0%* | *DNMT3B* | *2.21* | *PPARGC1A* | *-2.03* |
| *BLCA* | *CHD7* | *3.2%* | *CHD3* | *50.2%* | *TDRD3* | *3.6%* | *UBE2I* | *5.6%* | *PHF20L1* | *57.5%* | *HDAC11* | *9.0%* | *ASXL1* | *17.5%* | *STK31* | *2.16* | *PRDM8* | *-1.93* |
| *BRCA* | *NCOR1* | *3.8%* | *PRDM7* | *62.6%* | *HR* | *5.3%* | *ERCC5* | *15.0%* | *RBBP5* | *74.2%* | *ATAD2* | *18.2%* | *SETDB1* | *38.3%* | *HIST1H3B* | *4.42* | *SMYD1* | *-4.97* |
| *BRCA* | *ARID1A* | *2.7%* | *CDYL2* | *62.4%* | *ELP3* | *5.1%* | *ELP3* | *14.3%* | *KDM5B* | *73.7%* | *PHF20L1* | *16.4%* | *PYGO2* | *32.2%* | *PRDM13* | *3.96* | *TDRD10* | *-1.98* |
| *BRCA* | *SRCAP* | *2.5%* | *CTCF* | *61.7%* | *PRDM7* | *2.8%* | *BRD7* | *13.0%* | *LBR* | *72.8%* | *CHRAC1* | *14.8%* | *CHRAC1* | *30.7%* | *AURKB* | *3.39* | *PRDM16* | *-1.62* |
| *BRCA* | *CHD4* | *2.4%* | *PRMT7* | *60.9%* | *CDYL2* | *2.3%* | *ASH2L* | *12.6%* | *H3F3A* | *72.4%* | *BOP1* | *14.0%* | *PARP1* | *28.5%* | *UHRF1* | *3.34* | *NAP1L2* | *-1.57* |
| *BRCA* | *CHD6* | *2.4%* | *SETD6* | *59.8%* | *PHF11* | *2.0%* | *L3MBTL2* | *11.9%* | *PARP1* | *72.4%* | *SMYD3* | *13.5%* | *TDRKH* | *27.3%* | *HIST1H1B* | *3.32* | *CBX7* | *-1.53* |
| *BRCA* | *ATM* | *2.2%* | *FXR2* | *59.5%* | *SETDB2* | *2.0%* | *MBD1* | *9.7%* | *ARID4B* | *72.2%* | *ARID4B* | *13.4%* | *ATAD2* | *27.1%* | *CSTL1* | *3.09* | *UTY* | *-1.49* |
| *BRCA* | *TAF1L* | *2.0%* | *PHF23* | *59.4%* | *ING2* | *1.6%* | *SETD3* | *8.1%* | *ASH1L* | *72.2%* | *SETDB1* | *13.0%* | *RBBP5* | *25.7%* | *CBX2* | *2.69* | *ASXL3* | *-1.45* |
| *BRCA* | *ASXL3* | *1.9%* | *KDM6B* | *59.1%* | *CTCF* | *1.5%* | *RTF1* | *7.7%* | *PYGO2* | *71.9%* | *ASH2L* | *12.7%* | *GATAD2B* | *25.6%* | *EZH2* | *2.31* | *KDM4E* | *-1.43* |
| *BRCA* | *ATRX* | *1.9%* | *CHD3* | *59.0%* | *PRDM10* | *1.5%* | *SMYD4* | *7.5%* | *RNF2* | *71.9%* | *H3F3A* | *12.5%* | *KDM5B* | *25.2%* | *ORC1* | *2.29* | *KAT2B* | *-1.43* |
| *BRCA* | *BAZ2B* | *1.9%* | *AURKB* | *58.9%* | *PRMT7* | *1.5%* | *PHF2* | *7.1%* | *SMYD3* | *71.9%* | *LBR* | *12.5%* | *ASH2L* | *24.9%* | *HIST1H1C* | *2.28* | *PRDM5* | *-1.38* |
| *CESC* | *KMT2C* | *9.7%* | *KMT2A* | *52.1%* | *HDAC4* | *4.5%* | *EED* | *12.6%* | *MECOM* | *73.8%* | *MECOM* | *20.1%* | *ACTL6A* | *51.5%* |  |  |  |  |
| *CESC* | *KMT2D* | *9.1%* | *PRDM10* | *51.8%* | *ATM* | *4.2%* | *RTF1* | *12.0%* | *PHC3* | *73.1%* | *PHC3* | *19.4%* | *MBD4* | *39.8%* |  |  |  |  |
| *CESC* | *EP300* | *7.1%* | *ATM* | *50.2%* | *PRDM10* | *4.2%* | *PCMT1* | *11.3%* | *ACTL6A* | *72.5%* | *ACTL6A* | *19.1%* | *ATR* | *29.1%* |  |  |  |  |
| *CESC* | *CREBBP* | *5.5%* | *PPARGC1A* | *43.4%* | *ING5* | *3.9%* | *BRD1* | *10.7%* | *HLTF* | *67.0%* | *HLTF* | *11.7%* | *BRD9* | *28.5%* |  |  |  |  |
| *CESC* | *ARID1A* | *5.2%* | *BAP1* | *40.1%* | *KMT2A* | *3.9%* | *CHAF1A* | *8.4%* | *ATR* | *65.0%* | *ATR* | *10.4%* | *PHC3* | *27.5%* |  |  |  |  |
| *CESC* | *ATRX* | *4.5%* | *PHF7* | *40.1%* | *MUM1* | *3.6%* | *BRPF1* | *8.1%* | *MBD4* | *60.8%* | *BRD9* | *8.4%* | *ASXL1* | *21.7%* |  |  |  |  |
| *CESC* | *CHD6* | *4.2%* | *SP100* | *40.1%* | *MBD3* | *3.2%* | *PHRF1* | *7.8%* | *HSPBAP1* | *58.9%* | *MBD4* | *6.5%* | *SETDB1* | *21.4%* |  |  |  |  |
| *CESC* | *CHD7* | *4.2%* | *SP110* | *40.1%* | *PHF11* | *3.2%* | *ASH2L* | *7.4%* | *PYGO2* | *52.1%* | *PRDM9* | *5.8%* | *PYGO2* | *20.4%* |  |  |  |  |
| *CESC* | *EP400* | *4.2%* | *SP140* | *40.1%* | *SETDB2* | *3.2%* | *POLR2B* | *7.4%* | *HDGF* | *51.8%* | *PRKAA1* | *5.5%* | *HLTF* | *19.7%* |  |  |  |  |
| *CESC* | *SRCAP* | *4.2%* | *SP140L* | *40.1%* | *SP100* | *3.2%* | *CTCF* | *6.8%* | *TDRD10* | *51.5%* | *HSPBAP1* | *5.2%* | *ASH1L* | *18.8%* |  |  |  |  |
| *CHOL* | *BAP1* | *22.2%* | *CHD5* | *83.3%* | *PADI1* | *5.6%* | *YY1* | *36.1%* | *ASH1L* | *66.7%* | *ASH1L* | *16.7%* | *SETDB1* | *41.7%* | *PADI3* | *9.62* | *DNMT3L* | *-7.32* |
| *CHOL* | *EP400* | *22.2%* | *PHF13* | *83.3%* | *PADI2* | *5.6%* | *SUPT16H* | *25.0%* | *GATAD2B* | *63.9%* | *GATAD2B* | *13.9%* | *RBBP5* | *38.9%* | *TDRD5* | *5.66* | *CECR2* | *-4.68* |
| *CHOL* | *PBRM1* | *22.2%* | *PRDM2* | *83.3%* | *PADI3* | *5.6%* | *CTCF* | *22.2%* | *PYGO2* | *63.9%* | *HDGF* | *13.9%* | *KDM5B* | *36.1%* | *AICDA* | *4.97* | *KDM8* | *-4.25* |
| *CHOL* | *KMT2C* | *19.4%* | *SETD2* | *83.3%* | *PADI4* | *5.6%* | *INTS12* | *22.2%* | *RNF2* | *63.9%* | *PYGO2* | *13.9%* | *ASH1L* | *33.3%* | *UHRF1* | *4.30* | *ASXL3* | *-2.70* |
| *CHOL* | *ARID1A* | *16.7%* | *SMARCC1* | *83.3%* | *PADI6* | *5.6%* | *SETD4* | *22.2%* | *TDRD10* | *63.9%* | *RNF2* | *13.9%* | *GATAD2B* | *33.3%* | *AURKB* | *3.98* | *GADD45B* | *-2.67* |
| *CHOL* | *ARID1B* | *13.9%* | *ARID1A* | *80.6%* | *PHF10* | *5.6%* | *CHD8* | *19.4%* | *TDRD5* | *63.9%* | *TDRD10* | *13.9%* | *TDRD5* | *30.6%* | *DPF1* | *3.56* | *DPF3* | *-2.48* |
| *CHOL* | *ATM* | *13.9%* | *BAP1* | *80.6%* | *PRDM2* | *5.6%* | *BRD7* | *16.7%* | *ARID4B* | *61.1%* | *TDRD5* | *13.9%* | *LBR* | *25.0%* | *ORC1* | *3.39* | *IDH1* | *-2.30* |
| *CHOL* | *CHD7* | *11.1%* | *FBXO44* | *80.6%* | *ARID1A* | *2.8%* | *PHF3* | *16.7%* | *H3F3A* | *61.1%* | *ARID4B* | *11.1%* | *SMYD2* | *25.0%* | *CHAF1B* | *3.01* | *KAT2B* | *-2.30* |
| *CHOL* | *IDH1* | *11.1%* | *KDM1A* | *80.6%* | *CHD3* | *2.8%* | *PRMT2* | *16.7%* | *HDGF* | *61.1%* | *CHD1L* | *11.1%* | *ARID4B* | *22.2%* | *CBX2* | *2.93* | *GADD45A* | *-2.22* |
| *CHOL* | *PAXIP1* | *11.1%* | *PADI1* | *80.6%* | *CHD5* | *2.8%* | *ARID4A* | *13.9%* | *KDM5B* | *61.1%* | *H3F3A* | *11.1%* | *BRD9* | *19.4%* | *HIST1H1B* | *2.76* | *HDAC6* | *-2.11* |
| *COAD/READ* | *ATM* | *4.0%* | *MBD2* | *69.3%* | *HR* | *6.3%* | *MBD2* | *20.1%* | *NCOA3* | *76.2%* | *ASXL1* | *9.3%* | *ASXL1* | *30.5%* | *PADI3* | *4.49* | *SMYD1* | *-4.99* |
| *COAD/READ* | *ARID1A* | *3.9%* | *CXXC1* | *67.8%* | *ELP3* | *5.5%* | *ELP3* | *19.9%* | *ZMYND8* | *76.2%* | *PHF20* | *8.5%* | *DIDO1* | *29.7%* | *PRDM13* | *4.32* | *AICDA* | *-3.95* |
| *COAD/READ* | *CREBBP* | *3.5%* | *MBD1* | *67.8%* | *MBD2* | *2.9%* | *MBD1* | *18.6%* | *L3MBTL1* | *75.9%* | *DNMT3B* | *8.2%* | *ZMYND8* | *27.3%* | *CSTL1* | *3.71* | *NAP1L2* | *-3.89* |
| *COAD/READ* | *CHD4* | *3.4%* | *ASXL3* | *65.3%* | *ING2* | *2.6%* | *FXR2* | *18.5%* | *CHD6* | *75.7%* | *L3MBTL1* | *7.9%* | *PHF20* | *27.0%* | *CBX2* | *3.69* | *PADI2* | *-3.19* |
| *COAD/READ* | *TET1* | *3.4%* | *L3MBTL4* | *60.8%* | *CXXC1* | *1.9%* | *RTF1* | *10.3%* | *PHF20* | *75.7%* | *DIDO1* | *7.7%* | *ZGPAT* | *26.4%* | *STK31* | *3.68* | *ACTL6B* | *-2.69* |
| *COAD/READ* | *SFMBT2* | *3.2%* | *CHD3* | *59.2%* | *MBD1* | *1.9%* | *SMYD4* | *8.7%* | *ZGPAT* | *75.7%* | *CHD6* | *7.6%* | *MPHOSPH8* | *20.6%* | *HIST1H1B* | *2.98* | *RPH3A* | *-2.56* |
| *COAD/READ* | *TAF1L* | *3.2%* | *KDM6B* | *59.1%* | *FXR2* | *1.4%* | *USP22* | *7.6%* | *DIDO1* | *75.6%* | *ZGPAT* | *7.6%* | *SETDB2* | *20.6%* | *PRDM12* | *2.62* | *PRDM6* | *-2.54* |
| *COAD/READ* | *CHD6* | *3.1%* | *PHF23* | *59.0%* | *INO80* | *1.4%* | *ASH2L* | *7.2%* | *ASXL1* | *75.4%* | *ZMYND8* | *7.6%* | *CHRAC1* | *20.1%* | *DNMT3L* | *2.39* | *DPF3* | *-2.30* |
| *COAD/READ* | *DNMT1* | *3.1%* | *AURKB* | *58.8%* | *PHF23* | *1.4%* | *PHF23* | *7.1%* | *DNMT3B* | *75.2%* | *NCOA3* | *7.4%* | *L3MBTL1* | *17.8%* | *HIST1H3B* | *2.29* | *NAP1L3* | *-2.22* |
| *COAD/READ* | *EP400* | *2.9%* | *FXR2* | *58.8%* | *RTF1* | *1.4%* | *SETD3* | *6.3%* | *RNF17* | *62.1%* | *ATAD2* | *5.6%* | *NCOA3* | *17.8%* | *BOP1* | *2.06* | *PRKAA2* | *-2.09* |
| *DLBC* | *KMT2D* | *35.4%* | *PHIP* | *39.6%* | *PHIP* | *14.6%* | *FXR2* | *22.9%* | *KMT2A* | *35.4%* | *MBD2* | *8.3%* | *ZCWPW1* | *18.8%* |  |  |  |  |
| *DLBC* | *ATM* | *18.8%* | *PRDM1* | *39.6%* | *PRDM1* | *12.5%* | *ASH2L* | *10.4%* | *PRDM10* | *35.4%* | *TDRD5* | *8.3%* | *BAP1* | *16.7%* |  |  |  |  |
| *DLBC* | *HIST1H1C* | *16.7%* | *SCML4* | *39.6%* | *PRDM13* | *12.5%* | *SND1* | *10.4%* | *TDRD5* | *35.4%* | *HCFC1* | *6.3%* | *BAZ2A* | *16.7%* |  |  |  |  |
| *DLBC* | *KMT2C* | *16.7%* | *PRDM13* | *37.5%* | *RNF217* | *12.5%* | *TP53BP1* | *10.4%* | *KDM5B* | *33.3%* | *KDM5B* | *6.3%* | *CHAF1B* | *16.7%* |  |  |  |  |
| *DLBC* | *TET2* | *16.7%* | *RNF217* | *35.4%* | *SCML4* | *12.5%* | *BAP1* | *8.3%* | *MBD2* | *33.3%* | *MECP2* | *6.3%* | *MBD2* | *16.7%* |  |  |  |  |
| *DLBC* | *HNF1A* | *14.6%* | *HDAC2* | *31.3%* | *HDAC2* | *10.4%* | *BRD7* | *8.3%* | *RBBP5* | *33.3%* | *RBBP5* | *6.3%* | *GTF2H1* | *14.6%* |  |  |  |  |
| *DLBC* | *PRDM9* | *14.6%* | *L3MBTL3* | *29.2%* | *INO80* | *10.4%* | *SETDB2* | *8.3%* | *RNF2* | *33.3%* | *RNF2* | *6.3%* | *HDAC7* | *14.6%* |  |  |  |  |
| *DLBC* | *ASXL3* | *12.5%* | *FXR2* | *27.1%* | *L3MBTL3* | *10.4%* | *BRD8* | *6.3%* | *ACTL6A* | *31.3%* | *SMYD2* | *6.3%* | *PYGO2* | *14.6%* |  |  |  |  |
| *DLBC* | *CREBBP* | *12.5%* | *SHPRH* | *27.1%* | *MBD5* | *10.4%* | *ERCC5* | *6.3%* | *ATM* | *31.3%* | *ACTL6A* | *4.2%* | *SETDB1* | *14.6%* |  |  |  |  |
| *DLBC* | *HIST1H1B* | *12.5%* | *CHD3* | *25.0%* | *BRD2* | *8.3%* | *MORF4L1* | *6.3%* | *CXXC1* | *31.3%* | *ARID2* | *4.2%* | *ATAD2* | *12.5%* |  |  |  |  |
| *ESCA* | *KMT2D* | *18.8%* | *BAP1* | *67.2%* | *KDM6A* | *8.6%* | *BRPF1* | *15.1%* | *ATAD2* | *72.6%* | *MECOM* | *25.3%* | *ACTL6A* | *37.1%* | *PADI3* | *3.82* | *ASXL3* | *-3.37* |
| *ESCA* | *ATM* | *11.8%* | *PRKCD* | *67.2%* | *MBD2* | *5.9%* | *PRKCD* | *15.1%* | *PHF20L1* | *69.9%* | *PHC3* | *25.3%* | *CHRAC1* | *33.9%* | *RNF17* | *3.37* | *CHD5* | *-2.51* |
| *ESCA* | *KMT2C* | *10.8%* | *SFMBT1* | *67.2%* | *CXXC1* | *4.8%* | *SETD2* | *12.4%* | *MECOM* | *68.8%* | *ACTL6A* | *22.6%* | *BRD9* | *32.3%* | *BRDT* | *3.26* | *PRDM16* | *-2.42* |
| *ESCA* | *ARID1B* | *9.1%* | *WDR82* | *67.2%* | *KMT2C* | *4.8%* | *ELP3* | *11.8%* | *CHRAC1* | *67.7%* | *ATAD2* | *17.2%* | *PHC3* | *26.9%* | *HDGFL1* | *3.14* | *FKBP5* | *-2.05* |
| *ESCA* | *KMT2A* | *9.1%* | *PBRM1* | *66.7%* | *MBD1* | *4.8%* | *FXR2* | *8.6%* | *PHC3* | *67.7%* | *CHRAC1* | *16.7%* | *ATR* | *24.2%* | *HIST1H3B* | *2.98* | *JADE1* | *-2.02* |
| *ESCA* | *ARID1A* | *8.6%* | *PHF7* | *66.7%* | *CDYL* | *4.3%* | *CTCF* | *7.5%* | *PHF14* | *67.2%* | *BOP1* | *15.1%* | *ATAD2* | *23.7%* | *CBX2* | *2.72* | *ACTL6B* | *-1.95* |
| *ESCA* | *ASXL3* | *8.6%* | *CXXC1* | *64.0%* | *PAXIP1* | *4.3%* | *SETD5* | *7.5%* | *DNMT3B* | *66.7%* | *BRD9* | *15.1%* | *DIDO1* | *23.1%* | *CSTL1* | *2.67* | *PRKAA2* | *-1.80* |
| *ESCA* | *NCOR2* | *8.6%* | *MBD1* | *64.0%* | *SMARCA2* | *4.3%* | *PHRF1* | *7.0%* | *RPA3* | *66.7%* | *PHF20L1* | *15.1%* | *ZGPAT* | *23.1%* | *PRDM9* | *2.53* | *CBX7* | *-1.80* |
| *ESCA* | *PAXIP1* | *8.6%* | *SMARCC1* | *64.0%* | *ING2* | *3.8%* | *GTF2F1* | *6.5%* | *STK31* | *66.7%* | *HLTF* | *14.5%* | *PHF20L1* | *21.5%* | *DNMT3L* | *2.44* | *TDRD1* | *-1.71* |
| *ESCA* | *EP400* | *7.5%* | *SETD2* | *63.4%* | *KIAA2026* | *3.8%* | *USP22* | *6.5%* | *HDAC9* | *66.1%* | *ATR* | *11.8%* | *BOP1* | *21.0%* | *AURKB* | *2.44* | *ZCWPW2* | *-1.65* |
| *GBM* | *ATRX* | *3.2%* | *PWWP2B* | *86.2%* | *KIAA2026* | *2.0%* | *TAF3* | *12.1%* | *KMT2C* | *80.2%* | *CLOCK* | *4.7%* | *KDM7A* | *9.5%* | *AURKB* | *6.55* | *CHD5* | *-5.48* |
| *GBM* | *KDM7A* | *3.0%* | *HELLS* | *85.9%* | *PSIP1* | *1.8%* | *SIRT1* | *10.1%* | *SMARCD3* | *80.0%* | *RBBP5* | *4.4%* | *BAZ1B* | *7.9%* | *PRDM13* | *6.40* | *PRMT8* | *-4.54* |
| *GBM* | *IDH1* | *2.5%* | *TDRD1* | *85.7%* | *KDM4C* | *1.7%* | *Mar-05* | *8.2%* | *SND1* | *80.0%* | *POLR2B* | *3.0%* | *JADE3* | *7.2%* | *HIST1H3B* | *5.78* | *NAP1L2* | *-3.80* |
| *GBM* | *KMT2C* | *2.3%* | *HIF1AN* | *85.6%* | *SMARCA2* | *1.7%* | *EPC1* | *7.2%* | *EZH2* | *79.7%* | *ACTL6A* | *2.5%* | *PAXIP1* | *6.2%* | *EZH2* | *4.46* | *SMYD1* | *-3.39* |
| *GBM* | *PRDM9* | *2.3%* | *SMNDC1* | *85.6%* | *UHRF2* | *1.7%* | *PCGF6* | *7.0%* | *TRIM24* | *79.7%* | *PRMT8* | *2.2%* | *PHF14* | *6.2%* | *ORC1* | *4.20* | *PRDM8* | *-3.22* |
| *GBM* | *CHD8* | *1.7%* | *PCGF6* | *85.4%* | *PHF11* | *1.3%* | *L3MBTL2* | *6.0%* | *PAXIP1* | *79.5%* | *BAZ2A* | *2.0%* | *JADE1* | *5.4%* | *UHRF1* | *4.09* | *TDRD5* | *-3.13* |
| *GBM* | *CHD9* | *1.7%* | *Mar-05* | *85.2%* | *SETDB2* | *1.3%* | *HIF1AN* | *5.9%* | *BAZ1B* | *79.4%* | *KMT2C* | *2.0%* | *ZCWPW1* | *5.0%* | *HIST1H1B* | *3.82* | *CBX7* | *-3.07* |
| *GBM* | *TAF1L* | *1.7%* | *PCGF5* | *84.9%* | *ZMYND11* | *1.3%* | *SUPT16H* | *3.4%* | *ZCWPW1* | *79.2%* | *SND1* | *2.0%* | *CARM1* | *4.7%* | *TCF19* | *3.20* | *ACTL6B* | *-3.02* |
| *GBM* | *CHD5* | *1.5%* | *KAT6B* | *84.6%* | *MSL3* | *1.3%* | *CHD8* | *2.9%* | *ACTL6B* | *79.0%* | *DOT1L* | *1.8%* | *HDAC9* | *4.5%* | *CBX2* | *3.17* | *NAP1L3* | *-2.85* |
| *GBM* | *KMT2B* | *1.3%* | *SIRT1* | *84.6%* | *BRD1* | *1.2%* | *KDM4C* | *2.7%* | *ING3* | *78.9%* | *MUM1* | *1.7%* | *CBX3* | *3.9%* | *PADI3* | *2.79* | *RPH3A* | *-2.73* |
| *HNSC* | *KMT2D* | *16.0%* | *PBRM1* | *71.1%* | *KDM6A* | *3.0%* | *ASH2L* | *11.7%* | *ATAD2* | *72.1%* | *ACTL6A* | *20.2%* | *PHC3* | *30.4%* | *BRDT* | *7.10* | *SMYD1* | *-4.39* |
| *HNSC* | *NSD1* | *11.7%* | *PRKCD* | *70.9%* | *HR* | *2.8%* | *ERCC5* | *9.1%* | *PHF20L1* | *72.1%* | *MECOM* | *18.9%* | *ACTL6A* | *30.2%* | *HDGFL1* | *4.04* | *PADI1* | *-3.80* |
| *HNSC* | *KMT2C* | *9.6%* | *SFMBT1* | *70.9%* | *MBD2* | *2.8%* | *PHRF1* | *7.5%* | *CHRAC1* | *71.3%* | *PHC3* | *18.7%* | *HLTF* | *24.0%* | *PRDM13* | *3.30* | *PADI2* | *-3.29* |
| *HNSC* | *EP400* | *8.3%* | *WDR82* | *70.9%* | *TRIM33* | *2.6%* | *PRKCD* | *7.4%* | *ACTL6A* | *70.9%* | *HLTF* | *14.0%* | *ATR* | *23.6%* | *DPF1* | *3.17* | *PPARGC1A* | *-3.00* |
| *HNSC* | *PRDM9* | *7.7%* | *BAP1* | *70.8%* | *ING2* | *2.3%* | *MBD2* | *6.4%* | *MECOM* | *70.9%* | *ATR* | *11.7%* | *CHRAC1* | *22.5%* | *PADI3* | *2.55* | *PRKAA2* | *-2.95* |
| *HNSC* | *CREBBP* | *7.5%* | *PHF7* | *70.8%* | *KMT2C* | *2.3%* | *BRPF1* | *6.2%* | *PHC3* | *70.9%* | *CHRAC1* | *10.4%* | *MBD4* | *19.8%* | *HNF1A* | *2.49* | *DPF3* | *-2.44* |
| *HNSC* | *EP300* | *7.4%* | *SETD2* | *70.6%* | *MSL3* | *2.3%* | *KIAA2026* | *5.5%* | *BOP1* | *70.8%* | *BOP1* | *9.8%* | *BRD9* | *19.4%* | *PRDM9* | *2.49* | *KAT2B* | *-2.03* |
| *HNSC* | *ASXL3* | *7.0%* | *SMARCC1* | *70.4%* | *EED* | *1.9%* | *GLYR1* | *5.3%* | *HLTF* | *65.7%* | *PHF20L1* | *9.6%* | *BOP1* | *17.9%* | *HIST1H3B* | *2.42* | *ZCWPW2* | *-1.86* |
| *HNSC* | *TAF1L* | *6.2%* | *ZCWPW2* | *69.1%* | *ELP3* | *1.9%* | *RBBP5* | *5.1%* | *PRDM14* | *62.6%* | *ATAD2* | *9.1%* | *PHF20L1* | *17.9%* | *PRMT8* | *2.41* | *GADD45B* | *-1.81* |
| *HNSC* | *ARID1A* | *5.8%* | *UBE2E1* | *68.3%* | *SMARCA2* | *1.9%* | *FXR2* | *4.9%* | *ATR* | *61.7%* | *BRD9* | *7.0%* | *ASXL1* | *15.7%* | *TDRD5* | *2.32* | *SMARCD3* | *-1.67* |
| *KICH* | *KMT2C* | *16.7%* | *TRIM33* | *81.8%* | *ATRX* | *3.0%* | *CHD1L* | *34.8%* | *ACTL6B* | *37.9%* | *PRDM7* | *4.5%* | *HIST1H1B* | *27.3%* | *ACTL6B* | *3.22* | *TDRD1* | *-6.34* |
| *KICH* | *BPTF* | *9.1%* | *ARID1A* | *80.3%* | *BRWD3* | *1.5%* | *PHF10* | *34.8%* | *ING3* | *37.9%* | *ATAD2* | *3.0%* | *SMARCD3* | *21.2%* | *CHD5* | *3.20* | *DNMT3L* | *-4.98* |
| *KICH* | *EP400* | *9.1%* | *BRDT* | *80.3%* | *DAXX* | *1.5%* | *ZMYND11* | *21.2%* | *KMT2E* | *37.9%* | *PHF20L1* | *3.0%* | *MTA2* | *19.7%* | *PRKCD* | *2.85* | *RAG2* | *-4.20* |
| *KICH* | *PRDM9* | *7.6%* | *CHD5* | *80.3%* | *HDAC8* | *1.5%* | *BAZ2B* | *19.7%* | *SND1* | *37.9%* | *ATM* | *1.5%* | *SP140* | *18.2%* | *PRMT8* | *2.49* | *PRDM14* | *-3.17* |
| *KICH* | *SMARCA2* | *7.6%* | *DMAP1* | *80.3%* | *ING5* | *1.5%* | *ASH2L* | *16.7%* | *TRIM24* | *37.9%* | *BOP1* | *1.5%* | *USP51* | *18.2%* | *UHRF1* | *2.40* | *PADI2* | *-2.61* |
| *KICH* | *NCOA3* | *6.1%* | *FBXO44* | *80.3%* | *JMJD1C* | *1.5%* | *HDAC3* | *12.1%* | *ZCWPW1* | *37.9%* | *BRD3* | *1.5%* | *BRPF3* | *16.7%* | *PADI3* | *1.90* | *NAP1L3* | *-2.57* |
| *KICH* | *ATM* | *4.5%* | *GADD45A* | *80.3%* | *MTA1* | *1.5%* | *MLLT10* | *12.1%* | *BAZ1B* | *36.4%* | *CHD7* | *1.5%* | *HIST1H1C* | *16.7%* | *SETMAR* | *1.80* | *AIRE* | *-2.40* |
| *KICH* | *ATRX* | *4.5%* | *GTF2B* | *80.3%* | *NAP1L2* | *1.5%* | *PHF20L1* | *12.1%* | *CBX3* | *36.4%* | *CHRAC1* | *1.5%* | *DNMT3B* | *15.2%* | *HIST1H3B* | *1.59* | *PRDM7* | *-2.38* |
| *KICH* | *BAZ1B* | *4.5%* | *HDAC1* | *80.3%* | *NAP1L3* | *1.5%* | *YY1* | *12.1%* | *CLOCK* | *36.4%* | *EHMT1* | *1.5%* | *HDAC10* | *15.2%* | *AURKB* | *1.57* | *TDRD9* | *-2.25* |
| *KICH* | *BRD1* | *4.5%* | *KDM1A* | *80.3%* | *RPS6KA5* | *1.5%* | *FXR2* | *10.6%* | *HDAC9* | *36.4%* | *EP400* | *1.5%* | *BRD1* | *13.6%* | *PRDM4* | *1.55* | *LBR* | *-2.04* |
| *KIRC* | *PBRM1* | *26.1%* | *SETD2* | *86.6%* | *KAT2B* | *10.8%* | *UBR7* | *11.4%* | *NSD1* | *61.8%* | *NSD1* | *15.5%* | *JADE2* | *18.4%* | *PADI1* | *5.73* | *RAG2* | *-3.88* |
| *KIRC* | *SETD2* | *10.1%* | *SMARCC1* | *86.6%* | *SATB1* | *10.8%* | *ASH2L* | *10.6%* | *HDAC3* | *60.0%* | *HDAC3* | *13.8%* | *HDAC3* | *16.4%* | *AICDA* | *4.52* | *PRDM16* | *-3.69* |
| *KIRC* | *BAP1* | *7.6%* | *PBRM1* | *86.4%* | *SETD2* | *10.8%* | *SUPT16H* | *10.1%* | *KDM3B* | *59.8%* | *KDM3B* | *13.6%* | *KDM3B* | *16.2%* | *PADI3* | *3.64* | *DNMT3L* | *-3.02* |
| *KIRC* | *KDM5C* | *5.0%* | *UBE2E1* | *86.4%* | *UBE2E1* | *10.8%* | *CHD8* | *7.4%* | *BRD8* | *59.4%* | *AFF4* | *13.4%* | *PAXIP1* | *14.0%* | *ACTL6B* | *3.14* | *TDRD5* | *-2.82* |
| *KIRC* | *KMT2D* | *5.0%* | *ZCWPW2* | *86.4%* | *ZCWPW2* | *10.8%* | *PRMT5* | *7.3%* | *AFF4* | *58.7%* | *BRD8* | *13.4%* | *BRD8* | *13.4%* | *AURKB* | *3.03* | *MECOM* | *-2.55* |
| *KIRC* | *ARID1A* | *4.7%* | *BAP1* | *86.2%* | *BRPF1* | *10.6%* | *TDRD7* | *6.7%* | *UBE2B* | *58.5%* | *UBE2B* | *13.4%* | *SND1* | *11.0%* | *HIST1H3B* | *2.91* | *PADI2* | *-2.48* |
| *KIRC* | *KMT2C* | *4.5%* | *KAT2B* | *86.2%* | *HDAC11* | *10.6%* | *PBRM1* | *6.5%* | *PRDM6* | *56.6%* | *PRDM6* | *12.1%* | *BAZ1B* | *10.4%* | *PADI6* | *2.83* | *PHF21B* | *-2.26* |
| *KIRC* | *DNMT1* | *3.9%* | *PHF7* | *86.2%* | *SETD5* | *10.6%* | *RNF20* | *6.3%* | *CHD1* | *42.6%* | *CHD1* | *4.5%* | *BRD9* | *9.1%* | *UHRF1* | *2.76* | *PRDM7* | *-2.25* |
| *KIRC* | *EP300* | *3.7%* | *WDR82* | *86.2%* | *SMARCC1* | *10.6%* | *ARID1B* | *6.1%* | *KMT2C* | *32.8%* | *MECOM* | *1.9%* | *NSD1* | *9.1%* | *HIST1H1B* | *2.73* | *NAP1L2* | *-2.24* |
| *KIRC* | *EP400* | *3.7%* | *BRPF1* | *86.0%* | *PBRM1* | *10.2%* | *YY1* | *6.1%* | *PAXIP1* | *32.8%* | *ACTL6A* | *1.7%* | *CBX3* | *8.8%* | *BRDT* | *2.30* | *TDRD1* | *-1.93* |
| *KIRP* | *KMT2C* | *6.2%* | *SMARCB1* | *21.3%* | *ATM* | *1.7%* | *L3MBTL2* | *12.0%* | *CBX2* | *68.7%* | *SND1* | *2.1%* | *RNF40* | *37.1%* | *PADI3* | *3.55* | *PRDM16* | *-5.08* |
| *KIRP* | *KMT2D* | *6.2%* | *BRD1* | *20.6%* | *HDAC4* | *1.4%* | *SMARCB1* | *7.2%* | *CBX4* | *68.7%* | *ING3* | *1.7%* | *AKAP1* | *31.3%* | *AICDA* | *3.37* | *DNMT3L* | *-4.04* |
| *KIRP* | *SETD2* | *5.8%* | *EP300* | *20.6%* | *ING5* | *1.4%* | *PRMT2* | *6.9%* | *CBX8* | *68.7%* | *TRIM24* | *1.7%* | *KAT2A* | *22.3%* | *AURKB* | *3.25* | *RAG2* | *-3.55* |
| *KIRP* | *BAP1* | *4.8%* | *HDAC10* | *20.6%* | *KMT2A* | *1.4%* | *SUPT16H* | *6.2%* | *JMJD6* | *68.7%* | *AKAP1* | *1.4%* | *SIRT7* | *21.6%* | *PADI1* | *3.16* | *PRMT8* | *-3.31* |
| *KIRP* | *SRCAP* | *4.5%* | *HIRA* | *20.6%* | *SP100* | *1.4%* | *FXR2* | *5.8%* | *SIRT7* | *68.7%* | *EZH2* | *1.4%* | *PAXIP1* | *20.3%* | *PRDM12* | *3.02* | *MECOM* | *-3.09* |
| *KIRP* | *PBRM1* | *3.8%* | *CBX6* | *20.3%* | *SP110* | *1.4%* | *MBD1* | *4.8%* | *BPTF* | *68.4%* | *MBTD1* | *1.4%* | *BAZ1B* | *19.9%* | *UHRF1* | *2.64* | *TDRD5* | *-1.89* |
| *KIRP* | *SMARCA4* | *3.8%* | *CBX7* | *20.3%* | *SP140* | *1.4%* | *CHD8* | *4.5%* | *SMARCD2* | *68.4%* | *NSD1* | *1.4%* | *EZH1* | *19.2%* | *HIST1H3B* | *2.51* | *UTY* | *-1.84* |
| *KIRP* | *CREBBP* | *3.4%* | *TCF20* | *20.3%* | *SP140L* | *1.4%* | *EHMT1* | *4.5%* | *AKAP1* | *68.0%* | *SIRT7* | *1.4%* | *RPA3* | *18.2%* | *ACTL6B* | *2.17* | *PADI2* | *-1.79* |
| *KIRP* | *KDM6A* | *3.4%* | *CECR2* | *19.9%* | *SUPT16H* | *1.4%* | *BRD1* | *4.1%* | *MBTD1* | *67.7%* | *SMARCD2* | *1.4%* | *FXR2* | *16.8%* | *PRDM13* | *2.05* | *NAP1L2* | *-1.52* |
| *KIRP* | *NCOR1* | *3.4%* | *L3MBTL2* | *19.9%* | *CDYL2* | *1.0%* | *ASH2L* | *3.8%* | *KAT7* | *67.4%* | *ACTL6B* | *1.0%* | *JMJD6* | *16.8%* | *EZH2* | *1.95* | *PRDM7* | *-1.43* |
| *LAML* | *DNMT3A* | *25.6%* | *EZH2* | *12.6%* | *AFF4* | *3.0%* | *KDM3B* | *10.1%* | *ATAD2* | *12.1%* | *KMT2A* | *5.6%* | *BOP1* | *9.1%* |  |  |  |  |
| *LAML* | *IDH2* | *10.1%* | *KMT2C* | *12.6%* | *BRD8* | *3.0%* | *PAXIP1* | *8.1%* | *BOP1* | *12.1%* | *BRWD1* | *4.0%* | *TCEA1* | *8.1%* |  |  |  |  |
| *LAML* | *IDH1* | *9.5%* | *PAXIP1* | *12.6%* | *HDAC3* | *3.0%* | *NCOR1* | *5.1%* | *CHRAC1* | *12.1%* | *PRDM15* | *3.5%* | *ASH2L* | *7.6%* |  |  |  |  |
| *LAML* | *TET2* | *8.5%* | *SMARCD3* | *12.6%* | *KDM3B* | *3.0%* | *BAZ1B* | *4.5%* | *PHF20L1* | *12.1%* | *AIRE* | *2.5%* | *BRDT* | *7.6%* |  |  |  |  |
| *LAML* | *PHF6* | *3.0%* | *SND1* | *12.1%* | *KMT2C* | *3.0%* | *SMYD4* | *4.5%* | *PRDM14* | *12.1%* | *DNMT3L* | *2.5%* | *KAT5* | *7.6%* |  |  |  |  |
| *LAML* | *ASXL1* | *2.5%* | *ING3* | *11.6%* | *PAXIP1* | *3.0%* | *CBX3* | *4.0%* | *TCEA1* | *12.1%* | *PRMT2* | *2.5%* | *ATM* | *7.1%* |  |  |  |  |
| *LAML* | *EZH2* | *1.5%* | *TRIM24* | *11.6%* | *PRDM6* | *3.0%* | *KMT2E* | *4.0%* | *CHD7* | *11.6%* | *CHAF1B* | *2.0%* | *HDAC1* | *7.1%* |  |  |  |  |
| *LAML* | *KDM6A* | *1.5%* | *ACTL6B* | *10.1%* | *SMARCD3* | *3.0%* | *RNF20* | *4.0%* | *ASH2L* | *11.1%* | *SETD4* | *2.0%* | *KAT6A* | *7.1%* |  |  |  |  |
| *LAML* | *SUZ12* | *1.5%* | *ZCWPW1* | *10.1%* | *UBE2B* | *3.0%* | *AFF4* | *3.5%* | *ELP3* | *11.1%* | *ARID4B* | *1.0%* | *CHAF1B* | *6.6%* |  |  |  |  |
| *LAML* | *CECR2* | *1.0%* | *KMT2E* | *9.6%* | *EZH2* | *2.5%* | *EPC1* | *3.5%* | *HR* | *11.1%* | *ATM* | *1.0%* | *CHRAC1* | *6.6%* |  |  |  |  |
| *LGG* | *IDH1* | *41.7%* | *TRIM28* | *50.6%* | *ING5* | *4.9%* | *HDAC1* | *22.1%* | *SND1* | *33.6%* | *ATAD2* | *6.6%* | *BAZ1B* | *11.3%* |  |  |  |  |
| *LGG* | *ATRX* | *22.3%* | *PRMT1* | *48.9%* | *PHRF1* | *4.9%* | *PHF13* | *17.7%* | *EZH2* | *33.2%* | *BOP1* | *6.2%* | *PAXIP1* | *10.8%* |  |  |  |  |
| *LGG* | *SMARCA4* | *2.8%* | *PAF1* | *42.3%* | *SIRT3* | *4.9%* | *ZMYND11* | *13.4%* | *SMARCD3* | *33.2%* | *CHRAC1* | *6.2%* | *RPA3* | *10.2%* |  |  |  |  |
| *LGG* | *ARID1A* | *2.3%* | *FBXO17* | *42.1%* | *TRIM28* | *4.5%* | *HIF1AN* | *12.5%* | *TRIM24* | *33.2%* | *PHF20L1* | *6.2%* | *ING3* | *10.0%* |  |  |  |  |
| *LGG* | *IDH2* | *2.3%* | *SIRT2* | *42.1%* | *HDAC4* | *4.3%* | *RBBP4* | *12.3%* | *KMT2C* | *32.8%* | *CHD4* | *5.8%* | *CBX3* | *9.8%* |  |  |  |  |
| *LGG* | *KMT2D* | *1.1%* | *DPF1* | *41.7%* | *TRIM66* | *3.6%* | *GTF2B* | *12.1%* | *ING3* | *32.6%* | *ING4* | *5.8%* | *PHF14* | *9.8%* |  |  |  |  |
| *LGG* | *KAT6B* | *0.9%* | *KMT2B* | *40.2%* | *ING2* | *3.0%* | *Mar-05* | *10.9%* | *PAXIP1* | *32.6%* | *PRMT8* | *5.5%* | *SMARCD3* | *9.6%* |  |  |  |  |
| *LGG* | *PBRM1* | *0.9%* | *CHD5* | *40.0%* | *PRMT1* | *3.0%* | *SCMH1* | *10.4%* | *KMT2E* | *31.9%* | *KDM5A* | *4.7%* | *FBXO17* | *8.9%* |  |  |  |  |
| *LGG* | *DNMT3A* | *0.8%* | *PHF13* | *39.8%* | *KDM4C* | *2.8%* | *SIRT3* | *9.4%* | *ACTL6B* | *31.5%* | *SMARCD3* | *4.7%* | *TRIM24* | *8.3%* |  |  |  |  |
| *LGG* | *PHF3* | *0.8%* | *TDRD12* | *39.1%* | *KIAA2026* | *2.6%* | *ARID1A* | *7.5%* | *ZCWPW1* | *31.1%* | *AICDA* | *4.3%* | *EZH2* | *7.9%* |  |  |  |  |
| *LIHC* | *ARID1A* | *9.0%* | *ELP3* | *68.3%* | *HR* | *7.4%* | *FXR2* | *26.4%* | *PYGO2* | *74.4%* | *ATAD2* | *17.9%* | *SETDB1* | *38.5%* | *RNF17* | *5.09* | *KDM8* | *-2.43* |
| *LIHC* | *ARID2* | *6.3%* | *HR* | *68.1%* | *ELP3* | *6.1%* | *ELP3* | *25.1%* | *ASH1L* | *74.1%* | *PHF20L1* | *16.6%* | *CHRAC1* | *35.4%* | *PADI3* | *4.93* | *PADI4* | *-2.25* |
| *LIHC* | *KMT2D* | *6.1%* | *FXR2* | *59.6%* | *KAT6A* | *4.2%* | *SETD3* | *14.8%* | *GATAD2B* | *73.4%* | *CHRAC1* | *15.8%* | *PYGO2* | *34.6%* | *HDGFL1* | *4.19* | *GADD45B* | *-1.96* |
| *LIHC* | *BAP1* | *5.8%* | *AURKB* | *59.4%* | *ASH2L* | *3.7%* | *PHF23* | *9.8%* | *HDGF* | *73.1%* | *BOP1* | *15.6%* | *BOP1* | *31.9%* | *BRDT* | *3.83* | *DPF3* | *-1.80* |
| *LIHC* | *KMT2C* | *5.8%* | *CHD3* | *59.4%* | *ING2* | *3.7%* | *PCMT1* | *9.5%* | *TDRD10* | *73.1%* | *PYGO2* | *13.5%* | *PARP1* | *27.2%* | *PRDM9* | *3.71* | *DNMT3L* | *-1.20* |
| *LIHC* | *KMT2B* | *5.5%* | *KDM6B* | *59.4%* | *NCOR1* | *2.9%* | *RNF40* | *9.5%* | *RBBP5* | *71.8%* | *ASH1L* | *13.2%* | *PHF20L1* | *27.2%* | *AURKB* | *3.56* | *NCOR1* | *-1.16* |
| *LIHC* | *SETD2* | *5.0%* | *PHF23* | *58.3%* | *PRDM16* | *2.9%* | *MBD1* | *9.0%* | *TDRKH* | *71.8%* | *GATAD2B* | *12.1%* | *HDGF* | *26.4%* | *TDRD5* | *3.45* | *ASXL3* | *-1.12* |
| *LIHC* | *TDRD5* | *5.0%* | *ASH2L* | *55.7%* | *CHD5* | *2.6%* | *ASH2L* | *8.7%* | *KDM5B* | *71.5%* | *HDGF* | *12.1%* | *GATAD2B* | *23.7%* | *HIST1H3B* | *3.25* | *SATB1* | *-1.10* |
| *LIHC* | *ATR* | *4.5%* | *SMYD4* | *54.9%* | *PHF10* | *2.6%* | *GLYR1* | *8.2%* | *SETDB1* | *71.5%* | *TDRD10* | *12.1%* | *TDRKH* | *20.6%* | *UHRF1* | *3.23* | *PPARGC1A* | *-1.09* |
| *LIHC* | *KMT2A* | *4.5%* | *NCOR1* | *51.2%* | *AURKB* | *2.4%* | *BRD7* | *7.4%* | *SMYD2* | *70.7%* | *SETDB1* | *11.9%* | *TCEA1* | *20.3%* | *PRDM7* | *3.14* | *KDM6B* | *-1.05* |
| *LUAD* | *ASXL3* | *8.4%* | *RNF17* | *56.2%* | *HR* | *5.4%* | *ASH2L* | *11.5%* | *ASH1L* | *73.3%* | *BRD9* | *16.9%* | *BRD9* | *31.7%* | *BRDT* | *6.12* | *PADI4* | *-2.54* |
| *LUAD* | *PRDM9* | *8.3%* | *MPHOSPH8* | *56.0%* | *ELP3* | *5.2%* | *MBD1* | *10.7%* | *GATAD2B* | *73.3%* | *SETDB1* | *14.2%* | *SETDB1* | *29.8%* | *PADI1* | *5.75* | *RPH3A* | *-1.90* |
| *LUAD* | *TAF1L* | *6.5%* | *SMYD4* | *54.3%* | *KDM4C* | *3.5%* | *BAP1* | *9.4%* | *PYGO2* | *73.3%* | *TDRKH* | *13.4%* | *CHRAC1* | *25.7%* | *PADI3* | *5.70* | *GADD45B* | *-1.78* |
| *LUAD* | *ATM* | *4.4%* | *GADD45B* | *53.6%* | *UHRF2* | *3.3%* | *PRMT2* | *7.1%* | *TDRD10* | *73.3%* | *PYGO2* | *13.2%* | *PYGO2* | *25.1%* | *HIST1H1B* | *5.50* | *CBX7* | *-1.66* |
| *LUAD* | *ATRX* | *4.4%* | *MBD3* | *53.6%* | *SMARCA2* | *3.1%* | *BRPF1* | *6.3%* | *SETDB1* | *72.2%* | *TDRD10* | *13.1%* | *TDRKH* | *23.4%* | *HIST1H3B* | *5.16* | *PRDM11* | *-1.19* |
| *LUAD* | *SETD2* | *4.0%* | *SIRT6* | *53.6%* | *KIAA2026* | *2.9%* | *ERCC5* | *6.3%* | *TDRKH* | *72.2%* | *GATAD2B* | *12.9%* | *KDM5B* | *21.7%* | *PRDM13* | *4.85* | *PRDM5* | *-1.17* |
| *LUAD* | *DIDO1* | *3.6%* | *CHAF1A* | *53.4%* | *MPHOSPH8* | *2.9%* | *L3MBTL2* | *6.3%* | *HDGF* | *71.2%* | *ASH1L* | *12.7%* | *PHF20L1* | *21.7%* | *CSTL1* | *4.54* | *KAT2B* | *-1.13* |
| *LUAD* | *BRWD3* | *3.5%* | *GTF2F1* | *53.4%* | *ING2* | *2.7%* | *KDM3B* | *6.0%* | *RBBP5* | *70.4%* | *CHD1L* | *11.7%* | *ARID4B* | *21.5%* | *HNF1A* | *3.83* | *NAP1L2* | *-0.98* |
| *LUAD* | *ARID1A* | *3.1%* | *PHF11* | *53.4%* | *PRDM7* | *2.7%* | *ARID1B* | *5.6%* | *KDM5B* | *70.2%* | *BAZ1A* | *11.5%* | *BOP1* | *20.2%* | *PRDM9* | *3.79* | *SMARCA2* | *-0.97* |
| *LUAD* | *ARID2* | *3.1%* | *SETDB2* | *53.4%* | *PSIP1* | *2.7%* | *GTF2F1* | *5.2%* | *RNF2* | *69.7%* | *PRDM9* | *10.7%* | *ASH1L* | *17.3%* | *TDRD5* | *3.66* | *PRDM6* | *-0.93* |
| *LUSC* | *CHD7* | *4.8%* | *PBRM1* | *86.5%* | *HR* | *5.6%* | *MPHOSPH8* | *8.5%* | *ACTL6A* | *89.7%* | *ACTL6A* | *45.4%* | *ACTL6A* | *68.7%* | *PRDM13* | *7.58* | *TDRD10* | *-3.05* |
| *LUSC* | *TAF1L* | *4.4%* | *PHF7* | *86.3%* | *ELP3* | *5.2%* | *SETD3* | *7.7%* | *MECOM* | *89.3%* | *MECOM* | *43.5%* | *ATR* | *36.3%* | *PADI3* | *7.44* | *PADI4* | *-3.04* |
| *LUSC* | *ASXL3* | *3.8%* | *SFMBT1* | *86.3%* | *ING2* | *5.0%* | *ZMYND11* | *7.1%* | *PHC3* | *89.3%* | *PHC3* | *43.3%* | *PHC3* | *35.1%* | *PADI1* | *7.31* | *GADD45B* | *-2.74* |
| *LUSC* | *CREBBP* | *3.6%* | *WDR82* | *86.3%* | *KDM6A* | *4.4%* | *KIAA2026* | *6.7%* | *HLTF* | *82.7%* | *HLTF* | *26.0%* | *HLTF* | *29.2%* | *BRDT* | *7.28* | *PRDM16* | *-2.54* |
| *LUSC* | *DIDO1* | *3.4%* | *BAP1* | *86.1%* | *SMARCA2* | *3.6%* | *ELP3* | *6.3%* | *ATR* | *80.6%* | *BRD9* | *19.8%* | *PRKAA1* | *28.0%* | *CSTL1* | *5.18* | *CBX7* | *-1.80* |
| *LUSC* | *PRDM9* | *3.4%* | *PRKCD* | *85.9%* | *KDM4C* | *3.2%* | *KDM4C* | *6.3%* | *BRD9* | *76.8%* | *ATR* | *19.6%* | *CHRAC1* | *27.4%* | *HIST1H3B* | *4.98* | *KAT2B* | *-1.74* |
| *LUSC* | *BRWD3* | *3.2%* | *SMARCC1* | *85.7%* | *UHRF2* | *3.2%* | *INO80* | *6.2%* | *PRDM9* | *74.8%* | *PRDM9* | *16.7%* | *BRD9* | *24.4%* | *PRDM9* | *4.60* | *JADE1* | *-1.61* |
| *LUSC* | *EP400* | *3.2%* | *SETD2* | *85.3%* | *KIAA2026* | *3.0%* | *INTS12* | *4.8%* | *PRKAA1* | *72.6%* | *ASH2L* | *15.7%* | *ASH2L* | *23.8%* | *TDRD5* | *4.52* | *PRDM5* | *-1.59* |
| *LUSC* | *TDRD5* | *3.2%* | *ZCWPW2* | *82.1%* | *TRIM33* | *3.0%* | *RNF20* | *4.8%* | *MBD4* | *71.0%* | *PRKAA1* | *14.9%* | *MBD4* | *22.2%* | *AURKB* | *4.37* | *PRMT8* | *-1.55* |
| *LUSC* | *BAZ2B* | *3.0%* | *UBE2E1* | *81.7%* | *PSIP1* | *2.6%* | *ERCC5* | *4.4%* | *HSPBAP1* | *70.0%* | *DPF1* | *9.7%* | *PHF20L1* | *21.8%* | *HDGFL1* | *4.06* | *PRDM6* | *-1.54* |
| *MESO* | *BAP1* | *19.5%* | *EP300* | *80.5%* | *BAP1* | *12.6%* | *BAP1* | *24.1%* | *BRD9* | *32.2%* | *AKAP1* | *5.7%* | *PHF21B* | *18.4%* |  |  |  |  |
| *MESO* | *SETD2* | *8.0%* | *L3MBTL2* | *80.5%* | *PHF7* | *11.5%* | *PBRM1* | *20.7%* | *GATAD2B* | *31.0%* | *BPTF* | *5.7%* | *SETDB1* | *18.4%* |  |  |  |  |
| *MESO* | *BRD4* | *4.6%* | *PHF5A* | *80.5%* | *WDR82* | *5.7%* | *PHF5A* | *19.5%* | *PRDM9* | *31.0%* | *CBX1* | *5.7%* | *ASH1L* | *17.2%* |  |  |  |  |
| *MESO* | *EP400* | *4.6%* | *TCF20* | *80.5%* | *INO80* | *4.6%* | *BRD7* | *17.2%* | *RBBP5* | *31.0%* | *KAT7* | *5.7%* | *EP400* | *16.1%* |  |  |  |  |
| *MESO* | *KMT2A* | *4.6%* | *BRD1* | *79.3%* | *PBRM1* | *4.6%* | *TCF20* | *16.1%* | *SETDB1* | *31.0%* | *MBTD1* | *5.7%* | *GATAD2B* | *16.1%* |  |  |  |  |
| *MESO* | *TDRD6* | *4.6%* | *CBX6* | *79.3%* | *RTF1* | *4.6%* | *MPHOSPH8* | *14.9%* | *TDRKH* | *31.0%* | *SIRT7* | *5.7%* | *H3F3A* | *14.9%* |  |  |  |  |
| *MESO* | *ARID1A* | *3.4%* | *CBX7* | *79.3%* | *TP53BP1* | *4.6%* | *GTF2B* | *13.8%* | *AKAP1* | *29.9%* | *SMARCD2* | *5.7%* | *KDM2B* | *14.9%* |  |  |  |  |
| *MESO* | *ARID2* | *3.4%* | *HDAC10* | *79.3%* | *SETD2* | *3.4%* | *INO80* | *13.8%* | *ASH1L* | *29.9%* | *CBX2* | *4.6%* | *GLYR1* | *13.8%* |  |  |  |  |
| *MESO* | *CHD2* | *3.4%* | *PHF21B* | *79.3%* | *ARID2* | *2.3%* | *RTF1* | *13.8%* | *HDAC9* | *29.9%* | *CBX4* | *4.6%* | *SMARCD2* | *13.8%* |  |  |  |  |
| *MESO* | *CREBBP* | *3.4%* | *SMARCB1* | *73.6%* | *TRIM33* | *2.3%* | *SETD2* | *13.8%* | *HDGF* | *29.9%* | *CBX8* | *4.6%* | *BRD9* | *12.6%* |  |  |  |  |
| *OV* | *KMT2C* | *1.5%* | *MBD3* | *87.5%* | *BRD1* | *11.4%* | *MBD1* | *19.1%* | *MECOM* | *83.1%* | *PHF20L1* | *37.9%* | *PHF20L1* | *24.2%* |  |  |  |  |
| *OV* | *CHD4* | *1.4%* | *GADD45B* | *87.4%* | *HDAC10* | *11.4%* | *CXXC1* | *15.4%* | *PHC3* | *82.1%* | *MECOM* | *36.0%* | *PHC3* | *20.0%* |  |  |  |  |
| *OV* | *CREBBP* | *1.4%* | *DOT1L* | *87.2%* | *HR* | *6.7%* | *FXR2* | *11.6%* | *ACTL6A* | *78.3%* | *ATAD2* | *34.5%* | *ACTL6A* | *17.4%* |  |  |  |  |
| *OV* | *ARID2* | *1.2%* | *MUM1* | *87.2%* | *ELP3* | *6.3%* | *PRMT7* | *11.3%* | *PHF20L1* | *76.1%* | *CHRAC1* | *34.5%* | *CHRAC1* | *15.2%* |  |  |  |  |
| *OV* | *CHD6* | *1.2%* | *BRD1* | *86.0%* | *PHF21B* | *5.6%* | *RTF1* | *10.4%* | *BOP1* | *74.4%* | *BOP1* | *33.6%* | *BOP1* | *14.3%* |  |  |  |  |
| *OV* | *KAT6B* | *1.2%* | *HDAC10* | *86.0%* | *MUM1* | *4.6%* | *SETD6* | *10.1%* | *CHRAC1* | *73.9%* | *PHC3* | *33.6%* | *ZGPAT* | *14.0%* |  |  |  |  |
| *OV* | *SMARCA4* | *1.2%* | *PHF21B* | *84.8%* | *MBD3* | *4.4%* | *L3MBTL2* | *9.7%* | *ATAD2* | *73.2%* | *ACTL6A* | *27.6%* | *PYGO2* | *13.7%* |  |  |  |  |
| *OV* | *TET1* | *1.2%* | *NCOR1* | *83.6%* | *DOT1L* | *4.1%* | *WDR5* | *8.5%* | *ZGPAT* | *68.4%* | *BRD4* | *16.7%* | *SETDB1* | *13.5%* |  |  |  |  |
| *OV* | *DIDO1* | *1.0%* | *RAI1* | *83.6%* | *GADD45B* | *4.1%* | *SUZ12* | *8.2%* | *DIDO1* | *67.6%* | *ZGPAT* | *14.7%* | *BRD4* | *13.0%* |  |  |  |  |
| *OV* | *JMJD1C* | *1.0%* | *EP300* | *83.1%* | *CDYL2* | *3.2%* | *INTS12* | *8.0%* | *HLTF* | *67.4%* | *BRD9* | *14.0%* | *ASXL1* | *12.5%* |  |  |  |  |
| *PAAD* | *ARID1A* | *4.3%* | *MBD2* | *67.7%* | *MBD2* | *4.3%* | *MBD1* | *11.8%* | *ATAD2* | *36.6%* | *PHF20L1* | *10.2%* | *SETDB1* | *17.7%* |  |  |  |  |
| *PAAD* | *BRWD3* | *3.8%* | *CXXC1* | *67.2%* | *CXXC1* | *3.8%* | *SMARCD1* | *11.3%* | *PHF20L1* | *36.6%* | *BOP1* | *9.7%* | *ATAD2* | *16.1%* |  |  |  |  |
| *PAAD* | *DIDO1* | *3.8%* | *MBD1* | *67.2%* | *MBD1* | *3.8%* | *PHF10* | *10.8%* | *BOP1* | *35.5%* | *ATAD2* | *9.1%* | *CHRAC1* | *16.1%* |  |  |  |  |
| *PAAD* | *BRWD1* | *3.2%* | *ASXL3* | *61.3%* | *ASXL3* | *3.2%* | *ELP3* | *9.7%* | *CHRAC1* | *34.9%* | *CHRAC1* | *9.1%* | *PAF1* | *12.9%* |  |  |  |  |
| *PAAD* | *DNMT3A* | *3.2%* | *SMYD4* | *49.5%* | *KDM6A* | *2.7%* | *PRDM4* | *9.1%* | *TDRD10* | *33.3%* | *PAF1* | *8.6%* | *CHD1L* | *12.4%* |  |  |  |  |
| *PAAD* | *ATM* | *2.7%* | *ARID1B* | *48.9%* | *BRD1* | *1.6%* | *ASH2L* | *8.6%* | *ASH1L* | *32.8%* | *FBXO17* | *8.1%* | *ACTL6A* | *11.3%* |  |  |  |  |
| *PAAD* | *CHD6* | *2.7%* | *FXR2* | *48.9%* | *HDAC10* | *1.6%* | *BRPF1* | *6.5%* | *GATAD2B* | *32.8%* | *SIRT2* | *8.1%* | *HDGF* | *10.8%* |  |  |  |  |
| *PAAD* | *HDAC9* | *2.7%* | *PCMT1* | *48.9%* | *PHF21B* | *1.6%* | *CXXC1* | *6.5%* | *HDGF* | *32.8%* | *DPF1* | *6.5%* | *PHF20L1* | *10.8%* |  |  |  |  |
| *PAAD* | *KDM5B* | *2.7%* | *PHF10* | *48.9%* | *ARID1A* | *1.1%* | *EP400* | *6.5%* | *PYGO2* | *32.8%* | *TDRD12* | *5.4%* | *SIRT2* | *10.8%* |  |  |  |  |
| *PAAD* | *PBRM1* | *2.7%* | *KDM4C* | *48.4%* | *CHD3* | *1.1%* | *RBBP4* | *6.5%* | *RNF2* | *31.7%* | *KMT2B* | *4.8%* | *KDM5B* | *10.2%* |  |  |  |  |
| *PCPG* | *ATRX* | *3.8%* | *BRDT* | *66.8%* | *ZCWPW2* | *3.3%* | *GTF2B* | *31.5%* | *CBX3* | *15.8%* | *GATAD2B* | *3.3%* | *HDGF* | *11.4%* |  |  |  |  |
| *PCPG* | *EP400* | *3.8%* | *GTF2B* | *66.8%* | *PRMT6* | *2.7%* | *BAP1* | *28.8%* | *GATAD2B* | *15.2%* | *HDGF* | *3.3%* | *PRDM9* | *9.2%* |  |  |  |  |
| *PCPG* | *KMT2C* | *2.2%* | *MTF2* | *66.8%* | *TRIM33* | *2.7%* | *KDM4A* | *23.9%* | *HDAC9* | *15.2%* | *KDM5B* | *3.3%* | *STK31* | *9.2%* |  |  |  |  |
| *PCPG* | *SETD2* | *2.2%* | *PRMT6* | *64.7%* | *CHD1L* | *2.2%* | *HDAC1* | *23.4%* | *PHF14* | *15.2%* | *PYGO2* | *3.3%* | *HDAC7* | *8.7%* |  |  |  |  |
| *PCPG* | *BPTF* | *1.6%* | *GADD45A* | *64.1%* | *ARID1A* | *1.6%* | *PHF5A* | *20.7%* | *PYGO2* | *15.2%* | *SETDB1* | *3.3%* | *CHRAC1* | *8.2%* |  |  |  |  |
| *PCPG* | *DIDO1* | *1.6%* | *TRIM33* | *64.1%* | *BRDT* | *1.6%* | *USP22* | *18.5%* | *RPA3* | *15.2%* | *TDRD10* | *3.3%* | *KDM4B* | *8.2%* |  |  |  |  |
| *PCPG* | *MLLT6* | *1.6%* | *PRKAA2* | *63.6%* | *CHD5* | *1.6%* | *ATR* | *16.8%* | *SETDB1* | *15.2%* | *TDRKH* | *3.3%* | *PHF1* | *8.2%* |  |  |  |  |
| *PCPG* | *PADI6* | *1.6%* | *ORC1* | *63.0%* | *DMAP1* | *1.6%* | *PRDM2* | *16.3%* | *STK31* | *15.2%* | *ASH1L* | *2.7%* | *SETDB1* | *8.2%* |  |  |  |  |
| *PCPG* | *ARID1B* | *1.1%* | *DMAP1* | *58.2%* | *FBXO44* | *1.6%* | *SMARCB1* | *16.3%* | *TDRD10* | *15.2%* | *KDM4B* | *2.7%* | *SMARCD1* | *8.2%* |  |  |  |  |
| *PCPG* | *ARID2* | *1.1%* | *KDM4A* | *57.1%* | *GADD45A* | *1.6%* | *MTF2* | *15.2%* | *TDRKH* | *15.2%* | *RBBP5* | *2.7%* | *DAXX* | *7.6%* |  |  |  |  |
| *PRAD* | *EP400* | *7.8%* | *HR* | *58.7%* | *PHF11* | *16.6%* | *ELP3* | *33.7%* | *PRDM14* | *30.5%* | *ATAD2* | *6.2%* | *TCEA1* | *14.6%* | *TDRD1* | *2.96* | *PADI3* | *-4.36* |
| *PRAD* | *KMT2C* | *6.6%* | *ELP3* | *51.7%* | *HR* | *16.4%* | *ASH2L* | *25.7%* | *ATAD2* | *30.1%* | *PHF20L1* | *6.2%* | *CHRAC1* | *14.0%* | *HNF1A* | *2.88* | *PRDM16* | *-2.63* |
| *PRAD* | *KMT2D* | *6.0%* | *PHF11* | *44.1%* | *SETDB2* | *16.4%* | *FXR2* | *16.4%* | *PHF20L1* | *29.5%* | *PRDM14* | *5.8%* | *ATAD2* | *12.0%* | *HIST1H1B* | *2.54* | *PPARGC1A* | *-1.80* |
| *PRAD* | *ATM* | *4.6%* | *SETDB2* | *43.7%* | *PRDM1* | *13.4%* | *BRD7* | *12.4%* | *BOP1* | *27.1%* | *CHRAC1* | *5.4%* | *PHF20L1* | *10.6%* | *HIST1H3B* | *2.46* | *PRDM8* | *-1.69* |
| *PRAD* | *KDM6A* | *2.8%* | *CDYL2* | *40.5%* | *SCML4* | *13.4%* | *IWS1* | *12.4%* | *CHRAC1* | *26.9%* | *BOP1* | *5.2%* | *CBX3* | *10.4%* | *PRDM13* | *1.94* | *L3MBTL4* | *-1.58* |
| *PRAD* | *ARID2* | *2.2%* | *TDRD3* | *38.3%* | *PRDM13* | *13.2%* | *MBD1* | *11.6%* | *CHD7* | *24.6%* | *TCEA1* | *4.0%* | *WDR5* | *10.2%* | *PRDM12* | *1.90* | *MECOM* | *-1.49* |
| *PRAD* | *ASH1L* | *2.2%* | *PRDM7* | *37.7%* | *HDAC2* | *13.0%* | *HDAC2* | *9.2%* | *TCEA1* | *23.8%* | *CHD7* | *3.8%* | *MBD4* | *10.0%* | *AURKB* | *1.52* | *FBXO17* | *-1.34* |
| *PRAD* | *NCOR1* | *2.2%* | *ASH2L* | *33.1%* | *ELP3* | *12.4%* | *SETDB2* | *7.8%* | *STK31* | *21.0%* | *PHC3* | *3.6%* | *POLE3* | *9.0%* | *CBX2* | *1.46* | *PADI1* | *-1.24* |
| *PRAD* | *TAF1L* | *2.2%* | *KAT6A* | *32.9%* | *TDRD3* | *12.4%* | *ERCC5* | *7.6%* | *BAZ1B* | *20.8%* | *MECOM* | *3.4%* | *PRDM14* | *8.4%* | *EZH2* | *1.43* | *CHD5* | *-1.21* |
| *PRAD* | *CHD3* | *2.0%* | *SCML4* | *32.5%* | *BRWD1* | *11.6%* | *CHD1* | *6.4%* | *HDAC9* | *20.8%* | *ASH2L* | *2.8%* | *BRD3* | *7.8%* | *PHF21B* | *1.40* | *ASXL3* | *-1.21* |
| *SARC* | *ATRX* | *14.0%* | *SETDB2* | *67.2%* | *SETDB2* | *9.4%* | *ERCC5* | *18.9%* | *BRD9* | *42.6%* | *NCOR1* | *13.2%* | *HDGF* | *19.2%* |  |  |  |  |
| *SARC* | *CECR2* | *2.6%* | *PHF11* | *66.8%* | *PHF11* | *9.1%* | *FXR2* | *11.3%* | *PRKAA1* | *41.5%* | *RAI1* | *12.8%* | *USP22* | *15.1%* |  |  |  |  |
| *SARC* | *ATM* | *2.3%* | *TDRD3* | *59.2%* | *ING5* | *8.3%* | *Mar-05* | *10.9%* | *DNMT1* | *41.1%* | *USP22* | *12.8%* | *PCMT1* | *14.7%* |  |  |  |  |
| *SARC* | *TAF1L* | *2.3%* | *TET1* | *54.7%* | *ATRX* | *7.2%* | *MBD1* | *10.6%* | *PRDM9* | *41.1%* | *BRD9* | *6.8%* | *BRD9* | *14.0%* |  |  |  |  |
| *SARC* | *ARID1A* | *1.9%* | *CHD9* | *53.6%* | *HDAC4* | *7.2%* | *ELP3* | *8.3%* | *CARM1* | *40.8%* | *GATAD2B* | *6.8%* | *GATAD2A* | *13.6%* |  |  |  |  |
| *SARC* | *ASH1L* | *1.9%* | *SIRT1* | *53.2%* | *FXR2* | *6.4%* | *IWS1* | *7.2%* | *SMARCA4* | *40.4%* | *MBD3* | *6.8%* | *KDM5C* | *13.6%* |  |  |  |  |
| *SARC* | *CHD5* | *1.9%* | *KAT6B* | *52.5%* | *SP100* | *5.7%* | *KDM4C* | *6.8%* | *GATAD2B* | *38.5%* | *PRDM9* | *6.8%* | *PYGO2* | *13.6%* |  |  |  |  |
| *SARC* | *CHD7* | *1.9%* | *BRD7* | *51.3%* | *ZMYND11* | *5.3%* | *GTF2H1* | *6.4%* | *RAI1* | *38.5%* | *CARM1* | *6.4%* | *CARM1* | *13.2%* |  |  |  |  |
| *SARC* | *KDM5C* | *1.9%* | *JMJD1C* | *51.3%* | *SP110* | *4.9%* | *SMARCA2* | *6.0%* | *SETDB1* | *38.5%* | *DNMT1* | *6.4%* | *ORC1* | *13.2%* |  |  |  |  |
| *SARC* | *KMT2C* | *1.9%* | *CDYL2* | *50.6%* | *SP140* | *4.9%* | *PRMT2* | *5.7%* | *TDRKH* | *38.5%* | *KDM4B* | *6.4%* | *PHF5A* | *13.2%* |  |  |  |  |
| *SKCM* | *ASXL3* | *22.2%* | *PSIP1* | *53.0%* | *HDAC4* | *2.8%* | *BRD7* | *9.7%* | *TRIM24* | *47.4%* | *BOP1* | *5.5%* | *CDYL* | *28.4%* |  |  |  |  |
| *SKCM* | *MECOM* | *17.2%* | *KDM4C* | *51.7%* | *PRDM10* | *2.5%* | *MBD1* | *7.6%* | *SND1* | *46.6%* | *CHRAC1* | *5.3%* | *RING1* | *22.5%* |  |  |  |  |
| *SKCM* | *KMT2D* | *15.0%* | *SMARCA2* | *51.7%* | *ARID1B* | *2.3%* | *RTF1* | *7.6%* | *EZH2* | *46.2%* | *ATAD2* | *4.9%* | *BRPF3* | *20.3%* |  |  |  |  |
| *SKCM* | *KMT2C* | *14.4%* | *UHRF2* | *51.3%* | *ATM* | *2.3%* | *HDAC3* | *7.4%* | *ING3* | *46.2%* | *CDYL* | *4.9%* | *RPA3* | *20.1%* |  |  |  |  |
| *SKCM* | *TAF1L* | *13.8%* | *KIAA2026* | *51.0%* | *HDAC2* | *2.3%* | *SUZ12* | *7.4%* | *KMT2C* | *45.6%* | *PHF20L1* | *4.9%* | *JARID2* | *19.3%* |  |  |  |  |
| *SKCM* | *PRDM9* | *13.1%* | *PWWP2B* | *50.0%* | *KMT2A* | *2.3%* | *ELP3* | *7.2%* | *SMARCD3* | *45.6%* | *SMYD3* | *4.9%* | *SETDB1* | *19.1%* |  |  |  |  |
| *SKCM* | *EP400* | *12.9%* | *TDRD1* | *48.7%* | *L3MBTL3* | *2.3%* | *SMNDC1* | *7.2%* | *CDYL* | *44.7%* | *TRIM24* | *4.9%* | *CHRAC1* | *18.0%* |  |  |  |  |
| *SKCM* | *MBD5* | *12.9%* | *HIF1AN* | *48.5%* | *SCML4* | *2.3%* | *CHD8* | *7.0%* | *PAXIP1* | *44.7%* | *RBBP5* | *4.7%* | *CBX3* | *17.8%* |  |  |  |  |
| *SKCM* | *TDRD5* | *12.3%* | *PCGF6* | *48.5%* | *PRDM1* | *2.3%* | *YY1* | *7.0%* | *JARID2* | *44.5%* | *EZH2* | *4.4%* | *PHF5A* | *17.8%* |  |  |  |  |
| *SKCM* | *DIDO1* | *12.1%* | *TAF1L* | *48.5%* | *PHF10* | *2.1%* | *NSD3* | *6.8%* | *RPA3* | *44.3%* | *SIRT5* | *4.4%* | *SIRT5* | *17.8%* |  |  |  |  |
| *STAD* | *ARID1A* | *23.0%* | *MBD2* | *46.5%* | *ING2* | *4.1%* | *MBD1* | *10.6%* | *ATAD2* | *66.6%* | *ATAD2* | *8.4%* | *ZMYND8* | *24.4%* | *STK31* | *3.46* | *PADI1* | *-3.58* |
| *STAD* | *KMT2D* | *16.7%* | *ING2* | *45.6%* | *MBD2* | *3.8%* | *ASH2L* | *7.4%* | *NCOA3* | *65.9%* | *MECOM* | *7.4%* | *CHRAC1* | *23.9%* | *PRDM13* | *3.02* | *SMYD1* | *-2.99* |
| *STAD* | *KMT2C* | *13.5%* | *CXXC1* | *45.1%* | *SMARCA2* | *3.6%* | *CXXC1* | *5.4%* | *ZMYND8* | *65.7%* | *PHC3* | *7.4%* | *DIDO1* | *21.9%* | *PRDM9* | *2.79* | *GADD45B* | *-2.25* |
| *STAD* | *EP400* | *13.3%* | *MBD1* | *45.1%* | *CXXC1* | *3.4%* | *KDM4C* | *5.0%* | *L3MBTL1* | *65.0%* | *CHRAC1* | *7.2%* | *ASXL1* | *19.9%* | *TDRD5* | *2.73* | *NAP1L2* | *-2.21* |
| *STAD* | *KMT2B* | *11.7%* | *BRWD1* | *44.5%* | *MBD1* | *3.4%* | *BAP1* | *4.5%* | *PHF20L1* | *64.8%* | *NCOA3* | *7.2%* | *FKBP1A* | *18.3%* | *CBX2* | *2.40* | *PRKAA2* | *-1.90* |
| *STAD* | *DIDO1* | *11.3%* | *PRDM15* | *44.0%* | *ARID1A* | *3.2%* | *HDAC3* | *4.1%* | *CHD6* | *64.3%* | *SMARCE1* | *7.2%* | *ATAD2* | *16.7%* | *DNMT3B* | *2.35* | *PRDM8* | *-1.85* |
| *STAD* | *BRD8* | *10.6%* | *PRMT2* | *44.0%* | *KDM4C* | *3.2%* | *CDYL* | *3.4%* | *DIDO1* | *64.3%* | *ZGPAT* | *6.8%* | *PHF20* | *15.3%* | *HDGFL1* | *2.34* | *CBX7* | *-1.80* |
| *STAD* | *CREBBP* | *10.4%* | *AIRE* | *43.3%* | *KIAA2026* | *3.2%* | *ELP3* | *3.4%* | *ZGPAT* | *64.1%* | *DIDO1* | *6.5%* | *PHF20L1* | *15.1%* | *CSTL1* | *2.12* | *KAT2B* | *-1.62* |
| *STAD* | *SRCAP* | *10.4%* | *DNMT3L* | *43.3%* | *SETMAR* | *3.2%* | *GTF2F1* | *3.4%* | *CHRAC1* | *63.9%* | *PHF20L1* | *6.5%* | *SETDB1* | *15.1%* | *ATAD2* | *1.95* | *FKBP5* | *-1.59* |
| *STAD* | *KMT2A* | *10.2%* | *CHAF1B* | *42.9%* | *KMT2C* | *2.7%* | *KDM3B* | *3.4%* | *PHF20* | *63.7%* | *ZMYND8* | *6.5%* | *TCEA1* | *14.2%* | *HELLS* | *1.94* | *CHD5* | *-1.37* |
| *TGCT* | *NCOR2* | *9.0%* | *CXXC1* | *78.2%* | *PRDM10* | *10.3%* | *EED* | *10.9%* | *AEBP2* | *94.2%* | *AEBP2* | *20.5%* | *SETD4* | *44.2%* |  |  |  |  |
| *TGCT* | *SRCAP* | *5.1%* | *MBD1* | *78.2%* | *JARID2* | *5.8%* | *KAT5* | *9.0%* | *AICDA* | *94.2%* | *AICDA* | *19.9%* | *ELP3* | *35.3%* |  |  |  |  |
| *TGCT* | *CREBBP* | *4.5%* | *MBD2* | *78.2%* | *KMT2A* | *5.1%* | *RSF1* | *9.0%* | *ATF7IP* | *94.2%* | *PHC1* | *19.9%* | *BAZ1B* | *33.3%* |  |  |  |  |
| *TGCT* | *NSD1* | *3.8%* | *PRDM10* | *78.2%* | *ATM* | *3.8%* | *MBD1* | *8.3%* | *CHD4* | *94.2%* | *PRMT8* | *19.9%* | *RPA3* | *32.1%* |  |  |  |  |
| *TGCT* | *TET1* | *3.8%* | *ASXL3* | *77.6%* | *KDM4D* | *3.2%* | *BRD4* | *5.1%* | *ING4* | *94.2%* | *CHD4* | *19.2%* | *CHD7* | *26.3%* |  |  |  |  |
| *TGCT* | *CXXC1* | *3.2%* | *KMT2A* | *77.6%* | *KDM4E* | *3.2%* | *UBE2B* | *5.1%* | *PHC1* | *94.2%* | *ING4* | *19.2%* | *ATAD2* | *25.0%* |  |  |  |  |
| *TGCT* | *EP400* | *3.2%* | *ATM* | *76.3%* | *ZMYND11* | *3.2%* | *BRD7* | *4.5%* | *KDM5A* | *92.9%* | *KDM5A* | *19.2%* | *CHRAC1* | *22.4%* |  |  |  |  |
| *TGCT* | *HCFC1* | *3.2%* | *KDM4D* | *75.6%* | *EED* | *2.6%* | *KDM4C* | *4.5%* | *PRMT8* | *92.9%* | *ATF7IP* | *17.3%* | *ING3* | *19.9%* |  |  |  |  |
| *TGCT* | *ATRX* | *2.6%* | *KDM4E* | *75.6%* | *L3MBTL4* | *2.6%* | *CTCF* | *3.8%* | *BRWD1* | *82.1%* | *TCEA1* | *3.8%* | *CHAF1B* | *19.2%* |  |  |  |  |
| *TGCT* | *CHD4* | *2.6%* | *EED* | *75.0%* | *SMARCA2* | *2.6%* | *FXR2* | *3.8%* | *CHAF1B* | *82.1%* | *CLOCK* | *3.2%* | *SND1* | *19.2%* |  |  |  |  |
| *THCA* | *ARID1B* | *1.0%* | *BRD1* | *17.6%* | *EHMT1* | *0.8%* | *L3MBTL2* | *10.4%* | *SMYD3* | *5.9%* | *CECR2* | *1.0%* | *SIRT4* | *7.4%* | *PRMT8* | *5.06* | *AICDA* | *-2.73* |
| *THCA* | *ATM* | *1.0%* | *HDAC10* | *17.6%* | *JMJD1C* | *0.8%* | *BRD1* | *10.0%* | *ARID4B* | *5.7%* | *SMYD3* | *1.0%* | *KDM4B* | *6.8%* | *PADI1* | *2.99* | *ASXL3* | *-2.22* |
| *THCA* | *JMJD1C* | *1.0%* | *TCF20* | *17.6%* | *KAT6B* | *0.8%* | *SMARCB1* | *8.8%* | *H3F3A* | *5.5%* | *ARID4B* | *0.8%* | *SETD3* | *6.5%* | *RPH3A* | *1.90* | *CECR2* | *-2.20* |
| *THCA* | *ATR* | *0.8%* | *CBX6* | *17.4%* | *PHF11* | *0.8%* | *PHF5A* | *7.6%* | *KDM5B* | *5.5%* | *H3F3A* | *0.8%* | *MECOM* | *6.3%* | *PRDM1* | *1.70* | *PPARGC1A* | *-1.78* |
| *THCA* | *ASH1L* | *0.6%* | *CBX7* | *17.4%* | *SETDB2* | *0.8%* | *PHF2* | *6.1%* | *LBR* | *5.5%* | *KDM5B* | *0.8%* | *PRMT8* | *6.3%* | *CHD5* | *1.47* | *RAG2* | *-1.73* |
| *THCA* | *BPTF* | *0.6%* | *EP300* | *17.4%* | *SIRT1* | *0.8%* | *EP300* | *5.3%* | *PARP1* | *5.5%* | *LBR* | *0.8%* | *RNF40* | *6.3%* | *CBX2* | *1.43* | *RPS6KA5* | *-1.71* |
| *THCA* | *CHD3* | *0.6%* | *L3MBTL2* | *17.4%* | *TDRD3* | *0.8%* | *KIAA2026* | *5.3%* | *RBBP5* | *5.5%* | *MBTD1* | *0.8%* | *SND1* | *6.3%* | *HDAC9* | *1.27* | *TDRD6* | *-1.51* |
| *THCA* | *CHD9* | *0.6%* | *PHF21B* | *17.4%* | *TET1* | *0.8%* | *RBBP4* | *5.3%* | *RNF2* | *5.5%* | *PARP1* | *0.8%* | *HDAC9* | *6.1%* | *MECOM* | *1.10* | *TDRD9* | *-1.48* |
| *THCA* | *DIDO1* | *0.6%* | *PHF5A* | *17.4%* | *BRD1* | *0.6%* | *POLE3* | *4.9%* | *SMYD2* | *5.5%* | *RBBP5* | *0.8%* | *SMYD3* | *6.1%* | *CHAF1B* | *0.98* | *RNF17* | *-1.42* |
| *THCA* | *DNMT3A* | *0.6%* | *SMARCB1* | *16.8%* | *BRD3* | *0.6%* | *TDRD7* | *4.7%* | *ASH1L* | *5.3%* | *SMYD2* | *0.8%* | *TRIM24* | *6.1%* | *UHRF1* | *0.95* | *SCML4* | *-1.39* |
| *THYM* | *L3MBTL3* | *2.4%* | *CDYL* | *21.0%* | *CDYL* | *3.2%* | *BRD7* | *8.9%* | *ARID4B* | *22.6%* | *H3F3A* | *2.4%* | *PARP1* | *20.2%* |  |  |  |  |
| *THYM* | *ASH1L* | *1.6%* | *PCMT1* | *18.5%* | *PCMT1* | *2.4%* | *PCMT1* | *8.1%* | *H3F3A* | *22.6%* | *LBR* | *2.4%* | *PYGO2* | *13.7%* |  |  |  |  |
| *THYM* | *ASXL1* | *1.6%* | *ARID1B* | *17.7%* | *SP100* | *2.4%* | *MBD1* | *7.3%* | *LBR* | *22.6%* | *PARP1* | *2.4%* | *SMYD3* | *11.3%* |  |  |  |  |
| *THYM* | *ATRX* | *1.6%* | *PHF10* | *17.7%* | *SP110* | *2.4%* | *RING1* | *7.3%* | *PARP1* | *22.6%* | *ARID4B* | *1.6%* | *AKAP1* | *10.5%* |  |  |  |  |
| *THYM* | *BRD4* | *1.6%* | *SHPRH* | *17.7%* | *SP140* | *2.4%* | *BRD2* | *6.5%* | *SMYD3* | *22.6%* | *PHF12* | *1.6%* | *CHD1L* | *10.5%* |  |  |  |  |
| *THYM* | *CHD3* | *1.6%* | *HDGFL1* | *16.9%* | *SP140L* | *2.4%* | *DPF2* | *6.5%* | *SMYD2* | *21.0%* | *SMYD2* | *1.6%* | *WDR5* | *10.5%* |  |  |  |  |
| *THYM* | *EP300* | *1.6%* | *HIST1H1B* | *16.9%* | *ARID1B* | *1.6%* | *CHD3* | *5.6%* | *KDM5B* | *20.2%* | *SMYD3* | *1.6%* | *ZCWPW1* | *10.5%* |  |  |  |  |
| *THYM* | *GATAD2A* | *1.6%* | *HIST1H1C* | *16.9%* | *AURKB* | *1.6%* | *SCMH1* | *5.6%* | *RBBP5* | *20.2%* | *AEBP2* | *0.8%* | *FBXO17* | *9.7%* |  |  |  |  |
| *THYM* | *KAT6A* | *1.6%* | *HIST1H3B* | *16.9%* | *CBX6* | *1.6%* | *HAT1* | *4.8%* | *RNF2* | *20.2%* | *AICDA* | *0.8%* | *TDRD5* | *9.7%* |  |  |  |  |
| *THYM* | *MECP2* | *1.6%* | *JARID2* | *16.9%* | *CBX7* | *1.6%* | *SSRP1* | *4.8%* | *TDRD5* | *20.2%* | *AKAP1* | *0.8%* | *MECP2* | *8.9%* |  |  |  |  |
| *UCEC* | *ARID1A* | *15.7%* | *CDYL2* | *30.2%* | *BRD1* | *3.9%* | *SMARCE1* | *3.7%* | *ASH1L* | *46.8%* | *MECOM* | *13.5%* | *TDRKH* | *14.1%* | *PADI3* | *6.22* | *CBX7* | *-3.30* |
| *UCEC* | *CTCF* | *8.3%* | *CTCF* | *29.7%* | *ELP3* | *3.9%* | *ASH2L* | *3.5%* | *TDRKH* | *45.7%* | *PHC3* | *8.9%* | *SETDB1* | *12.6%* | *PRDM13* | *5.46* | *PRDM8* | *-3.29* |
| *UCEC* | *KMT2D* | *7.0%* | *PRMT7* | *29.1%* | *HDAC10* | *3.9%* | *BRD1* | *3.3%* | *PYGO2* | *45.2%* | *TDRKH* | *8.1%* | *PYGO2* | *8.5%* | *HIST1H3B* | *4.29* | *NAP1L3* | *-2.94* |
| *UCEC* | *CHD4* | *6.7%* | *PRDM7* | *28.9%* | *HR* | *3.7%* | *EHMT1* | *2.6%* | *SETDB1* | *45.0%* | *ACTL6A* | *8.0%* | *GATAD2B* | *8.0%* | *HIST1H1B* | *4.18* | *NAP1L2* | *-2.61* |
| *UCEC* | *TAF1* | *6.7%* | *SIRT6* | *28.7%* | *CDYL2* | *1.7%* | *L3MBTL2* | *2.6%* | *HDGF* | *44.8%* | *SETDB1* | *7.8%* | *JADE3* | *7.4%* | *AURKB* | *3.83* | *SETD7* | *-2.26* |
| *UCEC* | *JADE3* | *6.4%* | *GADD45B* | *28.5%* | *PHF21B* | *1.5%* | *MBD1* | *2.4%* | *GATAD2B* | *44.6%* | *BRD4* | *7.2%* | *CHRAC1* | *7.4%* | *UHRF1* | *3.60* | *PRDM5* | *-2.17* |
| *UCEC* | *KMT2B* | *6.1%* | *MBD3* | *28.3%* | *ASH2L* | *1.3%* | *TDRD3* | *1.9%* | *TDRD10* | *44.1%* | *SMARCA4* | *6.9%* | *ACTL6A* | *7.0%* | *PADI1* | *3.35* | *TDRD10* | *-2.09* |
| *UCEC* | *EP400* | *5.9%* | *CHAF1A* | *28.1%* | *ING2* | *1.3%* | *HIF1AN* | *1.7%* | *TDRD5* | *43.0%* | *CARM1* | *6.5%* | *CHD1L* | *6.9%* | *CSTL1* | *3.33* | *SFMBT2* | *-1.97* |
| *UCEC* | *KMT2C* | *5.9%* | *SETD6* | *28.1%* | *MBD2* | *1.3%* | *INTS12* | *1.5%* | *RBBP5* | *42.6%* | *KAT6A* | *6.3%* | *KDM7A* | *6.5%* | *ORC1* | *3.08* | *PHF21B* | *-1.83* |
| *UCEC* | *JADE2* | *5.9%* | *MUM1* | *27.6%* | *PRDM10* | *1.3%* | *KDM5C* | *1.5%* | *RNF2* | *42.6%* | *ASH1L* | *6.3%* | *KDM5B* | *6.5%* | *DNMT3B* | *2.73* | *PYGO1* | *-1.65* |
| *UCS* | *CHD4* | *17.5%* | *DOT1L* | *78.9%* | *HR* | *7.0%* | *RTF1* | *36.8%* | *ASXL1* | *82.5%* | *TDRD12* | *31.6%* | *ATAD2* | *45.6%* |  |  |  |  |
| *UCS* | *ARID1A* | *14.0%* | *GADD45B* | *78.9%* | *ELP3* | *5.3%* | *ELP3* | *31.6%* | *L3MBTL1* | *80.7%* | *KMT2B* | *24.6%* | *TCEA1* | *40.4%* |  |  |  |  |
| *UCS* | *EP400* | *10.5%* | *SIRT6* | *78.9%* | *PHF21B* | *5.3%* | *POLE3* | *29.8%* | *NCOA3* | *80.7%* | *ASXL1* | *22.8%* | *CHRAC1* | *33.3%* |  |  |  |  |
| *UCS* | *KMT2D* | *10.5%* | *MBD3* | *77.2%* | *ASH2L* | *3.5%* | *SMARCC1* | *29.8%* | *ZGPAT* | *80.7%* | *FBXO17* | *22.8%* | *H3F3A* | *33.3%* |  |  |  |  |
| *UCS* | *BRWD1* | *7.0%* | *MUM1* | *77.2%* | *BRD1* | *3.5%* | *INO80* | *28.1%* | *ZMYND8* | *80.7%* | *PAF1* | *22.8%* | *TDRD12* | *31.6%* |  |  |  |  |
| *UCS* | *CREBBP* | *7.0%* | *RTF1* | *75.4%* | *FMR1* | *3.5%* | *SUZ12* | *24.6%* | *CHD6* | *78.9%* | *SIRT2* | *22.8%* | *SIRT7* | *29.8%* |  |  |  |  |
| *UCS* | *CHD5* | *5.3%* | *AURKB* | *73.7%* | *HDAC10* | *3.5%* | *PHF23* | *22.8%* | *DIDO1* | *78.9%* | *DPF1* | *21.1%* | *PHF20L1* | *28.1%* |  |  |  |  |
| *UCS* | *CTCF* | *5.3%* | *CHAF1A* | *73.7%* | *JMJD8* | *3.5%* | *SETD6* | *22.8%* | *PHF20* | *78.9%* | *MECOM* | *21.1%* | *ACTL6A* | *26.3%* |  |  |  |  |
| *UCS* | *HCFC1* | *5.3%* | *CHD3* | *73.7%* | *KAT6A* | *3.5%* | *TP53BP1* | *22.8%* | *TDRD12* | *77.2%* | *TCEA1* | *21.1%* | *JMJD6* | *26.3%* |  |  |  |  |
| *UCS* | *KDM4B* | *5.3%* | *FXR2* | *73.7%* | *KDM4C* | *3.5%* | *HELLS* | *17.5%* | *DNMT3B* | *73.7%* | *PHC3* | *19.3%* | *SETDB1* | *26.3%* |  |  |  |  |
| *UVM* | *BAP1* | *32.5%* | *ACTL6A* | *55.0%* | *PRDM16* | *8.8%* | *BRPF1* | *38.8%* | *ATAD2* | *76.3%* | *ATAD2* | *17.5%* | *JARID2* | *50.0%* |  |  |  |  |
| *UVM* | *HDAC5* | *2.5%* | *ATR* | *55.0%* | *ARID1B* | *5.0%* | *SMARCC1* | *33.8%* | *BOP1* | *76.3%* | *BOP1* | *17.5%* | *CHRAC1* | *46.3%* |  |  |  |  |
| *UVM* | *KAT6A* | *2.5%* | *BAP1* | *55.0%* | *PCMT1* | *5.0%* | *BAP1* | *32.5%* | *CHRAC1* | *76.3%* | *CHRAC1* | *17.5%* | *DAXX* | *42.5%* |  |  |  |  |
| *UVM* | *KMT2D* | *2.5%* | *HLTF* | *55.0%* | *SHPRH* | *5.0%* | *PRKCD* | *22.5%* | *PHF20L1* | *76.3%* | *PHF20L1* | *17.5%* | *TCEA1* | *41.3%* |  |  |  |  |
| *UVM* | *ARID1B* | *1.3%* | *HSPBAP1* | *55.0%* | *KMT2A* | *3.8%* | *UBE2E1* | *21.3%* | *PRDM14* | *67.5%* | *CDYL* | *11.3%* | *BRD2* | *38.8%* |  |  |  |  |
| *UVM* | *ARID2* | *1.3%* | *MBD4* | *55.0%* | *L3MBTL3* | *3.8%* | *ELP3* | *20.0%* | *CHD7* | *62.5%* | *JARID2* | *11.3%* | *CHD7* | *38.8%* |  |  |  |  |
| *UVM* | *ASXL3* | *1.3%* | *MECOM* | *55.0%* | *PHF10* | *3.8%* | *HDAC1* | *15.0%* | *TCEA1* | *60.0%* | *KDM1B* | *11.3%* | *ATAT1* | *37.5%* |  |  |  |  |
| *UVM* | *ATAD2B* | *1.3%* | *PBRM1* | *55.0%* | *PRDM1* | *3.8%* | *KDM4A* | *12.5%* | *CDYL* | *56.3%* | *SIRT5* | *11.3%* | *BOP1* | *37.5%* |  |  |  |  |
| *UVM* | *ATF7IP* | *1.3%* | *PHC3* | *55.0%* | *PRDM10* | *3.8%* | *PHF13* | *12.5%* | *HDGFL1* | *56.3%* | *HDGFL1* | *10.0%* | *RING1* | *35.0%* |  |  |  |  |
| *UVM* | *BAZ2A* | *1.3%* | *PHF7* | *55.0%* | *RNF217* | *3.8%* | *ASH2L* | *11.3%* | *JARID2* | *56.3%* | *ATAT1* | *8.8%* | *CDYL* | *33.8%* |  |  |  |  |
|  |  |  |  |  |  |  |  |  |  |  |  |  |  |  |  |  |  |  |
| *COAD* | *PADI3* | *4.44* | *SMYD1* | *-4.53* |  |  |  |  |  |  |  |  |  |  |  |  |  |  |
| *COAD* | *HIST1H1B* | *4.32* | *NAP1L2* | *-3.61* |  |  |  |  |  |  |  |  |  |  |  |  |  |  |
| *COAD* | *PRDM13* | *4.11* | *PADI2* | *-3.30* |  |  |  |  |  |  |  |  |  |  |  |  |  |  |
| *COAD* | *STK31* | *3.80* | *ACTL6B* | *-2.91* |  |  |  |  |  |  |  |  |  |  |  |  |  |  |
| *COAD* | *CBX2* | *3.59* | *AICDA* | *-2.80* |  |  |  |  |  |  |  |  |  |  |  |  |  |  |
| *COAD* | *HIST1H3B* | *3.47* | *DPF3* | *-2.34* |  |  |  |  |  |  |  |  |  |  |  |  |  |  |
| *COAD* | *CSTL1* | *3.29* | *RPH3A* | *-2.30* |  |  |  |  |  |  |  |  |  |  |  |  |  |  |
| *COAD* | *PRDM12* | *2.91* | *ASXL3* | *-2.02* |  |  |  |  |  |  |  |  |  |  |  |  |  |  |
| *COAD* | *RAG2* | *2.24* | *NAP1L3* | *-1.99* |  |  |  |  |  |  |  |  |  |  |  |  |  |  |
| *COAD* | *PADI1* | *2.24* | *PRDM6* | *-1.90* |  |  |  |  |  |  |  |  |  |  |  |  |  |  |
| *READ* | *PADI3* | *4.54* | *SMYD1* | *-5.45* |  |  |  |  |  |  |  |  |  |  |  |  |  |  |
| *READ* | *PRDM13* | *4.52* | *AICDA* | *-5.10* |  |  |  |  |  |  |  |  |  |  |  |  |  |  |
| *READ* | *CSTL1* | *4.13* | *NAP1L2* | *-4.17* |  |  |  |  |  |  |  |  |  |  |  |  |  |  |
| *READ* | *CBX2* | *3.78* | *PRDM6* | *-3.17* |  |  |  |  |  |  |  |  |  |  |  |  |  |  |
| *READ* | *STK31* | *3.56* | *PADI2* | *-3.08* |  |  |  |  |  |  |  |  |  |  |  |  |  |  |
| *READ* | *DNMT3L* | *2.70* | *RPH3A* | *-2.81* |  |  |  |  |  |  |  |  |  |  |  |  |  |  |
| *READ* | *PRDM12* | *2.32* | *ACTL6B* | *-2.47* |  |  |  |  |  |  |  |  |  |  |  |  |  |  |
| *READ* | *BOP1* | *2.21* | *NAP1L3* | *-2.46* |  |  |  |  |  |  |  |  |  |  |  |  |  |  |
| *READ* | *DNMT3B* | *2.04* | *PRKAA2* | *-2.40* |  |  |  |  |  |  |  |  |  |  |  |  |  |  |
| *READ* | *CBX8* | *1.88* | *DPF3* | *-2.26* |  |  |  |  |  |  |  |  |  |  |  |  |  |  |
