# Supplementary material for: Pan-cancer multi-omics analysis and orthogonal experimental assessment of epigenetic driver genes
Source: Genome Res. 2020 Oct;30(10):1517–32. doi: 10.1101/gr.268292.120 (PMC7605261; doi:10.1101/gr.268292.120)
Supplement: Supplemental Material [file supp_gr.268292.120_Supplemental_Table_S5.pdf]

Supplemental_Table_S5

genes	cancer	geneclass	score_SNA	score_CNA	score_exp	Total_score	finalrank
**DOT1L**	ACC	HM_w	380	418	389	1187	1
**KMT2D**	ACC	HM_w	413	369	397	1179	2
**EHMT1**	ACC	HM_w	404	404	354	1162	3
**ZMYND8**	ACC	HA_r	362	409	383	1154	4
**BRD4**	ACC	HA_r	353	422	371	1146	5
**BAZ2A**	ACC	HA_r	356	408	381	1145	6
**PRDM12**	ACC	HM_w	411	358	376	1145	7
**SMARCA4**	ACC	Helicases	393	413	336	1142	8
**NSD1**	ACC	HM_w	372	416	350	1138	9
**CHAF1A**	ACC	ChRC	385	322	418	1125	10
**FBXW9**	ACC	Others	345	402	378	1125	11
**CBX5**	ACC	HM_r	292	407	424	1123	12
**SIN3B**	ACC	ChRC	316	386	405	1107	13
**DIDO1**	ACC	Others	420	315	367	1102	14
**HDAC7**	ACC	HA_e	339	399	353	1091	15
**NAP1L1**	ACC	Others	331	394	364	1089	16
**ATAD2**	ACC	HA_r	304	380	403	1087	17
**ARID4B**	ACC	ChRC	408	340	330	1078	18
**HDAC9**	ACC	HA_e	377	398	303	1078	19
**CHD6**	ACC	Helicases	287	406	379	1072	20
**BOP 1**	ACC	Others	354	331	380	1065	21
**CTCF**	ACC	Others	284	405	368	1057	22
**BRD7**	ACC	HA_r	297	329	423	1049	23
**GATAD2B**	ACC	HM_r	343	400	306	1049	24
**AURKB**	ACC	Others	303	334	399	1036	25
**NCOR2**	ACC	Others	423	268	339	1030	26
**TDRD9**	ACC	Others	364	349	315	1028	27
**DNMT1**	ACC	DM_w	282	419	326	1027	28
**BRWD1**	ACC	HA_r	294	327	402	1023	29
**ASH1L**	ACC	HM_w	359	425	227	1011	30
**L3MBTL1**	ACC	HM_r	334	395	275	1004	31
**ATR**	ACC	Others	412	336	255	1003	32

**SMARCC2**	ACC	Helicases	207	412	384	1003	33
**BRD9**	ACC	HA_r	179	426	391	996	34
**CHD9**	ACC	Helicases	286	318	390	994	35
**HDGF**	ACC	Others	273	397	324	994	36
**PRDM4**	ACC	HM_w	321	251	420	992	37
**PHF2**	ACC	Others	327	392	272	991	38
**RPH3A**	ACC	Others	318	353	318	989	39
**SETDB1**	ACC	HM_w	216	414	359	989	40
**BRD3**	ACC	HA_r	298	379	309	986	41
**JMJD8**	ACC	HM_e	264	372	343	979	42
**ARID2**	ACC	ChRC	306	341	331	978	43
**DMAP1**	ACC	Others	382	314	282	978	44
**CHD8**	ACC	Helicases	349	319	307	975	45
**UBE2I**	ACC	Others	195	381	393	969	46
**CBX4**	ACC	HM_r	407	377	176	960	47
**KDM4B**	ACC	HM_e	375	287	298	960	48
**GATAD2A**	ACC	HM_r	149	401	408	958	49
**CHD2**	ACC	Helicases	384	321	252	957	50
**HR**	ACC	HM_e	269	297	387	953	51
**FMR1**	ACC	Others	278	307	366	951	52
**BRWD3**	ACC	HA_r	293	326	329	948	53
**HDAC6**	ACC	HA_e	340	300	304	944	54
**BRD1**	ACC	HA_r	299	330	310	939	55
**PRDM9**	ACC	HM_w	370	250	319	939	56
**HLTF**	ACC	Others	338	299	301	938	57
**PHF20L1**	ACC	HM_r	326	262	348	936	58
**ATRX**	ACC	Helicases	415	335	182	932	59
**TDRD6**	ACC	Others	365	221	346	932	60
**CHD5**	ACC	Helicases	383	420	127	930	61
**GTF3C4**	ACC	HA_w	277	373	278	928	62
**CARM1**	ACC	HM_w	178	421	328	927	63
**TDRD7**	ACC	Others	198	383	345	926	64
**ING2**	ACC	HM_r	131	396	398	925	65
**SIRT4**	ACC	HA_e	211	352	347	910	66
**PRKAA1**	ACC	Others	73	423	411	907	67

**BAZ1B**	ACC	HA_r	181	333	392	906	68
**BRPF3**	ACC	HA_r	351	378	177	906	69
**SND1**	ACC	HM_r	314	230	357	901	70
**KMT2A**	ACC	HM_w	401	282	211	894	71
**KDM5B**	ACC	HM_e	257	285	351	893	72
**PHF19**	ACC	HM_r	237	362	292	891	73
**AKAP1**	ACC	Others	360	342	187	889	74
**ING1**	ACC	HM_r	425	296	167	888	75
**PRMT2**	ACC	HM_w	226	248	414	888	76
**SMARCA2**	ACC	Helicases	394	351	140	885	77
**EHMT2**	ACC	HM_w	403	310	171	884	78
**INO80**	ACC	Helicases	376	294	214	884	79
**EP400**	ACC	HA_w	422	403	57	882	80
**ASXL2**	ACC	Others	358	338	185	881	81
**MLLT10**	ACC	HM_w	332	275	274	881	82
**PRMT1**	ACC	HM_w	71	390	410	871	83
**BRDT**	ACC	HA_r	352	328	179	859	84
**KAT6A**	ACC	HA_w	402	69	386	857	85
**CHRAC1**	ACC	ChRC	168	317	369	854	86
**PHF3**	ACC	Others	325	258	270	853	87
**CHD1L**	ACC	Helicases	169	375	308	852	88
**YY1**	ACC	ChRC	191	346	314	851	89
**AFF4**	ACC	Others	361	69	413	843	90
**DNMT3B**	ACC	DM_w	281	312	250	843	91
**AFF1**	ACC	Others	186	343	312	841	92
**GLYR1**	ACC	HM_r	148	304	388	840	93
**KMT2E**	ACC	HM_w	255	281	297	833	94
**DNMT3A**	ACC	DM_w	347	313	172	832	95
**ASXL1**	ACC	Others	305	339	186	830	96
**KDM7A**	ACC	HM_e	389	69	372	830	97
**POLE3**	ACC	ChRC	77	391	362	830	98
**CBX1**	ACC	HM_r	177	325	327	829	99
**PRMT7**	ACC	HM_w	67	355	406	828	100
**KDM3B**	ACC	HM_e	335	69	421	825	101
**CREBBP**	ACC	HA_w	285	316	223	824	102

**HDAC2**	ACC	HA_e	276	301	245	822	103
**KDM5A**	ACC	HM_e	258	286	277	821	104
**FBXO17**	ACC	Others	426	69	325	820	105
**ACTL6A**	ACC	ChRC	189	344	286	819	106
**NCOA3**	ACC	HA_w	248	365	206	819	107
**TAF1**	ACC	HA_r	391	69	356	816	108
**ASXL3**	ACC	Others	418	337	60	815	109
**BMI1**	ACC	ChRC	300	332	181	813	110
**STK31**	ACC	Others	201	350	261	812	111
**KDM6B**	ACC	HM_e	417	283	110	810	112
**MUM1**	ACC	Others	96	417	296	809	113
**TAF3**	ACC	HA_r	366	69	374	809	114
**NCOA1**	ACC	HA_w	330	270	207	807	115
**MTA1**	ACC	ChRC	99	367	340	806	116
**JADE2**	ACC	Others	308	69	426	803	117
**MBD3**	ACC	DM_r	108	278	416	802	118
**PHRF1**	ACC	Others	399	361	42	802	119
**SIRT6**	ACC	HA_e	210	233	358	801	120
**CBX8**	ACC	ChRC	291	376	129	796	121
**SETD1B**	ACC	HM_w	410	238	144	792	122
**TRIM28**	ACC	HA_r	23	348	419	790	123
**BRD8**	ACC	HA_r	296	69	422	787	124
**PPARGC1A**	ACC	Others	323	360	101	784	125
**CBX3**	ACC	HM_r	175	323	284	782	126
**KDM2B**	ACC	HM_e	336	69	377	782	127
**IWS1**	ACC	Others	267	293	213	773	128
**PHF20**	ACC	HM_r	236	263	271	770	129
**SMARCD1**	ACC	Helicases	43	411	316	770	130
**WDR5**	ACC	ChRC	15	347	404	766	131
**KDM5C**	ACC	HM_e	115	284	365	764	132
**KMT2C**	ACC	HM_w	419	69	276	764	133
**PCGF6**	ACC	Others	400	69	295	764	134
**TET2**	ACC	DM_e	363	69	332	764	135
**ARID4A**	ACC	ChRC	409	69	285	763	136
**PCGF2**	ACC	Others	242	364	155	761	137

**SETD1A**	ACC	HM_w	317	69	375	761	138
**PAF1**	ACC	Others	92	266	396	754	139
**DNMT3L**	ACC	DM_w	381	311	58	750	140
**DPF1**	ACC	ChRC	280	69	401	750	141
**SCML2**	ACC	HM_r	218	240	290	748	142
**PWWP2B**	ACC	Others	424	245	73	742	143
**TDRKH**	ACC	Others	25	382	333	740	144
**MEN1**	ACC	ChRC	421	276	42	739	145
**ATAD2B**	ACC	HA_r	357	69	311	737	146
**FXR2**	ACC	Others	344	306	82	732	147
**SFMBT2**	ACC	Others	395	69	264	728	148
**CHD4**	ACC	Helicases	288	69	370	727	149
**PRDM1**	ACC	HM_w	76	359	291	726	150
**DAXX**	ACC	ChRC	405	69	251	725	151
**PYGO2**	ACC	HM_r	64	389	266	719	152
**SMARCA5**	ACC	Helicases	315	69	335	719	153
**PBRM1**	ACC	HA_r	328	69	320	717	154
**PHF14**	ACC	Others	83	393	240	716	155
**SRCAP**	ACC	Others	313	69	334	716	156
**KDM1B**	ACC	HM_e	261	289	165	715	157
**EZH2**	ACC	HM_w	157	309	248	714	158
**KANSL1**	ACC	HA_w	337	292	81	710	159
**PHC3**	ACC	ChRC	88	264	349	701	160
**SETD2**	ACC	HM_w	396	69	236	701	161
**CHD3**	ACC	Helicases	289	320	86	695	162
**PCMT1**	ACC	Others	89	265	337	691	163
**RNF17**	ACC	Others	397	243	47	687	164
**HELLS**	ACC	Helicases	272	69	344	685	165
**RPA3**	ACC	Others	59	388	237	684	166
**ATF7IP**	ACC	Others	387	69	226	682	167
**MTF2**	ACC	HM_r	250	271	159	680	168
**BPTF**	ACC	HA_r	386	69	224	679	169
**PRMT5**	ACC	HM_w	69	247	361	677	170
**SETD3**	ACC	HM_w	54	237	385	676	171
**KAT8**	ACC	HA_w	262	69	342	673	172

**NCOR1**	ACC	ChRC	247	269	157	673	173
**RNF217**	ACC	Others	221	354	95	670	174
**SMARCD3**	ACC	Helicases	206	69	394	669	175
**ATM**	ACC	Others	416	69	183	668	176
**HCFC1**	ACC	Others	378	69	219	666	177
**AEBP2**	ACC	HM_w	187	69	409	665	178
**SIRT7**	ACC	HA_e	45	385	233	663	179
**SP140**	ACC	HA_r	392	227	44	663	180
**RBBP7**	ACC	ChRC	223	244	195	662	181
**POLR2B**	ACC	Others	324	69	268	661	182
**RTF1**	ACC	Others	57	241	360	658	183
**SMARCE1**	ACC	Helicases	205	410	42	657	184
**KDM4C**	ACC	HM_e	374	69	212	655	185
**SUPT16H**	ACC	Others	200	225	230	655	186
**KDM2A**	ACC	HM_e	118	370	164	652	187
**PHF10**	ACC	Others	87	363	201	651	188
**PAXIP1**	ACC	Others	243	69	338	650	189
**BAZ2B**	ACC	HA_r	355	69	225	649	190
**KIAA2026**	ACC	Others	256	69	322	647	191
**L3MBTL2**	ACC	Others	254	280	109	643	192
**LBR**	ACC	Others	333	69	241	643	193
**SHPRH**	ACC	Others	213	387	42	642	194
**ING3**	ACC	HM_r	130	295	216	641	195
**TRIM33**	ACC	HA_r	310	69	259	638	196
**FBXL19**	ACC	Others	346	69	220	635	197
**ASH2L**	ACC	HM_w	183	69	382	634	198
**GTF2F1**	ACC	Others	146	69	417	632	199
**MLLT6**	ACC	HM_w	103	368	160	631	200
**MSH6**	ACC	HM_r	251	273	107	631	201
**SETD5**	ACC	HM_w	368	69	194	631	202
**CBX2**	ACC	ChRC	176	324	130	630	203
**PRDM7**	ACC	HM_w	74	357	199	630	204
**HDAC1**	ACC	HA_e	341	69	218	628	205
**BAP1**	ACC	Others	302	69	254	625	206
**GADD45B**	ACC	Others	150	305	170	625	207

**ZGPAT**	ACC	Others	12	213	400	625	208
**RNF20**	ACC	Others	60	415	148	623	209
**HDAC3**	ACC	HA_e	140	69	412	621	210
**PRDM16**	ACC	HM_w	398	69	152	619	211
**PRDM14**	ACC	HM_w	322	253	42	617	212
**ZMYND11**	ACC	HA_r	11	345	258	614	213
**CDYL**	ACC	HM_r	290	69	253	612	214
**INTS12**	ACC	Others	127	69	415	611	215
**KAT2A**	ACC	HA_w	123	371	115	609	216
**PHF5A**	ACC	Others	79	257	269	605	217
**PHC1**	ACC	ChRC	240	69	294	603	218
**CHD7**	ACC	Helicases	406	69	126	601	219
**SCML4**	ACC	Others	217	239	145	601	220
**TDRD10**	ACC	Others	28	384	189	601	221
**CECR2**	ACC	HA_r	350	69	175	594	222
**CLOCK**	ACC	HA_w	348	69	174	591	223
**ARID1B**	ACC	ChRC	388	69	132	589	224
**KDM4A**	ACC	HM_e	259	288	42	589	225
**PRDM5**	ACC	HM_w	320	69	200	589	226
**UBR7**	ACC	Others	18	216	355	589	227
**KDM8**	ACC	HM_e	112	69	407	588	228
**PADI2**	ACC	Others	245	69	273	587	229
**PRDM11**	ACC	HM_w	231	255	100	586	230
**PHF13**	ACC	Others	84	424	75	583	231
**PRDM13**	ACC	HM_w	230	254	99	583	232
**EZH1**	ACC	HM_w	158	374	42	574	233
**PRKAA2**	ACC	Others	227	249	98	574	234
**ACTL6B**	ACC	ChRC	188	69	313	570	235
**PRDM15**	ACC	HM_w	75	252	239	566	236
**UHRF1**	ACC	DM_r	309	215	42	566	237
**PRDM2**	ACC	HM_w	229	69	267	565	238
**HIRA**	ACC	Others	271	69	217	557	239
**HIST1H1B**	ACC	Others	136	69	352	557	240
**TET3**	ACC	DM_e	197	220	136	553	241
**L3MBTL3**	ACC	Others	111	279	161	551	242

**SUV39H1**	ACC	HM_w	34	224	288	546	243
**PARP1**	ACC	Others	371	69	105	545	244
**BRPF1**	ACC	HA_r	295	69	178	542	245
**MTA2**	ACC	ChRC	98	366	76	540	246
**H3F3A**	ACC	Others	342	69	122	533	247
**SMYD3**	ACC	HM_w	38	231	262	531	248
**NAP1L2**	ACC	Others	249	69	208	526	249
**CXXC1**	ACC	Others	283	69	173	525	250
**KMT2B**	ACC	HM_w	414	69	42	525	251
**PRKCD**	ACC	Others	72	356	97	525	252
**KAT7**	ACC	HA_w	120	291	112	523	253
**PARP2**	ACC	Others	91	69	363	523	254
**CHD1**	ACC	Helicases	170	69	283	522	255
**TET1**	ACC	DM_e	390	69	63	522	256
**ING4**	ACC	HM_r	129	69	323	521	257
**MARCH5**	ACC	Others	110	69	341	520	258
**FKBP1A**	ACC	Others	155	308	56	519	259
**H2AFZ**	ACC	Others	144	69	305	518	260
**SETMAR**	ACC	HM_w	214	69	235	518	261
**ORC1**	ACC	Others	94	267	156	517	262
**UBE2B**	ACC	Others	20	69	425	514	263
**PCGF5**	ACC	Others	241	69	203	513	264
**ELP3**	ACC	HA_w	162	69	281	512	265
**SIRT2**	ACC	HA_e	48	69	395	512	266
**TCF19**	ACC	Others	30	222	260	512	267
**RAI1**	ACC	Others	369	69	72	510	268
**EPC1**	ACC	Others	160	69	280	509	269
**PHF1**	ACC	HM_r	238	69	202	509	270
**HIF1AN**	ACC	Others	137	69	302	508	271
**SMYD4**	ACC	HM_w	204	69	232	505	272
**PADI3**	ACC	Others	329	69	106	504	273
**TRIM24**	ACC	HA_r	196	218	89	503	274
**TAF1L**	ACC	HA_r	367	69	66	502	275
**BAZ1A**	ACC	HA_r	301	69	131	501	276
**FKBP5**	ACC	Others	153	69	279	501	277

**JMJD1C**	ACC	HM_e	265	69	166	500	278
**HDAC10**	ACC	HA_e	142	302	55	499	279
**MBD4**	ACC	DM_r	107	69	321	497	280
**PRDM8**	ACC	HM_w	228	69	198	495	281
**JMJD6**	ACC	HM_e	124	69	300	493	282
**MECP2**	ACC	DM_r	373	69	51	493	283
**SP140L**	ACC	HA_r	36	226	231	493	284
**GTF2B**	ACC	Others	147	303	42	492	285
**KDM3A**	ACC	HM_e	260	69	163	492	286
**EP300**	ACC	HA_w	379	69	42	490	287
**KAT6B**	ACC	HA_w	121	69	299	489	288
**KDM1A**	ACC	HM_e	119	290	80	489	289
**ZCWPW2**	ACC	HM_r	190	69	228	487	290
**HNF1A**	ACC	ChRC	134	298	54	486	291
**EED**	ACC	ChRC	163	69	249	481	292
**SP110**	ACC	HA_r	202	228	45	475	293
**TCEA1**	ACC	Others	31	69	373	473	294
**SMARCA1**	ACC	Helicases	209	69	193	471	295
**TDRD5**	ACC	Others	311	69	91	471	296
**G2E3**	ACC	Others	152	69	247	468	297
**TRIM66**	ACC	HA_r	22	217	229	468	298
**GADD45A**	ACC	Others	151	69	246	466	299
**PRMT6**	ACC	HM_w	68	246	151	465	300
**AICDA**	ACC	DM_e	307	69	88	464	301
**HDAC4**	ACC	HA_e	275	69	120	464	302
**HDAC5**	ACC	HA_e	274	69	119	462	303
**PHC2**	ACC	ChRC	239	69	154	462	304
**MBTD1**	ACC	Others	105	277	78	460	305
**DPF3**	ACC	HA_r	165	69	222	456	306
**HIST1H3B**	ACC	Others	270	69	117	456	307
**PHF7**	ACC	Others	234	69	153	456	308
**JADE1**	ACC	Others	126	69	257	452	309
**ELP4**	ACC	HA_w	161	69	221	451	310
**HDGFL1**	ACC	Others	138	69	244	451	311
**JARID2**	ACC	ChRC	266	69	116	451	312

**JADE3**	ACC	Others	125	69	256	450	313
**USP27X**	ACC	Others	193	214	42	449	314
**PHF12**	ACC	Others	85	69	293	447	315
**PHF21A**	ACC	HM_r	82	261	103	446	316
**RPS6KA5**	ACC	Others	58	242	146	446	317
**SUZ12**	ACC	ChRC	32	223	191	446	318
**IDH1**	ACC	DM_e	133	69	243	445	319
**KAT5**	ACC	HA_w	263	69	113	445	320
**IDH2**	ACC	DM_e	132	69	242	443	321
**RNF40**	ACC	Others	220	69	147	436	322
**ATAT1**	ACC	Others	182	69	184	435	323
**SIRT5**	ACC	HA_e	46	69	317	432	324
**SETD6**	ACC	HM_w	52	236	143	431	325
**PRDM6**	ACC	HM_w	319	69	42	430	326
**BRD2**	ACC	HA_r	180	69	180	429	327
**TDRD1**	ACC	Others	312	69	43	424	328
**SIRT3**	ACC	HA_e	47	234	141	422	329
**MPHOSPH8**	ACC	HM_r	101	274	42	417	330
**SMARCD2**	ACC	Helicases	42	232	139	413	331
**ING5**	ACC	HM_r	128	69	215	412	332
**MTA3**	ACC	ChRC	97	272	42	411	333
**PHF8**	ACC	Others	233	69	102	404	334
**SP100**	ACC	HA_r	37	229	138	404	335
**SMNDC1**	ACC	Others	41	69	289	399	336
**USP51**	ACC	Others	192	69	133	394	337
**ERCC5**	ACC	Others	279	69	42	390	338
**PHF21B**	ACC	HM_r	81	260	49	390	339
**SUV39H2**	ACC	HM_w	33	69	287	389	340
**MBD2**	ACC	DM_r	109	69	210	388	341
**PHF23**	ACC	HM_r	80	259	48	387	342
**RNF2**	ACC	ChRC	222	69	96	387	343
**SETD4**	ACC	HM_w	53	69	265	387	344
**MECOM**	ACC	Others	104	69	209	382	345
**RSF1**	ACC	ChRC	219	69	94	382	346
**HAT1**	ACC	HA_w	143	69	169	381	347

**HSPBAP1**	ACC	Others	268	69	42	379	348
**SFMBT1**	ACC	HM_r	50	235	92	377	349
**HDAC8**	ACC	HA_e	139	69	168	376	350
**PHIP**	ACC	HA_r	78	256	42	376	351
**L3MBTL4**	ACC	Others	253	69	53	375	352
**PRDM10**	ACC	HM_w	232	69	74	375	353
**MBD1**	ACC	DM_r	252	69	52	373	354
**PRMT8**	ACC	HM_w	66	69	238	373	355
**SMYD2**	ACC	HM_w	39	69	263	371	356
**CHAF1B**	ACC	ChRC	171	69	128	368	357
**PADI6**	ACC	Others	93	69	205	367	358
**PADI1**	ACC	Others	246	69	50	365	359
**RBBP4**	ACC	ChRC	224	69	71	364	360
**PCGF1**	ACC	Others	90	69	204	363	361
**CSTL1**	ACC	Others	167	69	125	361	362
**DPY30**	ACC	Others	164	69	124	357	363
**PADI4**	ACC	Others	244	69	42	355	364
**SETDB2**	ACC	HM_w	215	69	69	353	365
**SIN3A**	ACC	ChRC	49	69	234	352	366
**SIRT1**	ACC	HA_e	212	69	68	349	367
**KDM4E**	ACC	HM_e	116	69	162	347	368
**FKBP2**	ACC	Others	154	69	123	346	369
**PHF6**	ACC	HM_r	235	69	42	346	370
**SMARCC1**	ACC	Helicases	208	69	67	344	371
**PSIP1**	ACC	HM_r	225	69	42	336	372
**TCF20**	ACC	Others	199	69	65	333	373
**TP53BP1**	ACC	Others	24	219	90	333	374
**HDAC11**	ACC	HA_e	141	69	121	331	375
**PYGO1**	ACC	HM_r	65	69	197	331	376
**CDYL2**	ACC	HM_r	172	69	87	328	377
**RBBP5**	ACC	ChRC	62	69	196	327	378
**HIST1H1C**	ACC	Others	135	69	118	322	379
**NAP1L3**	ACC	Others	95	69	158	322	380
**DPF2**	ACC	Others	166	69	85	320	381
**AIRE**	ACC	HM_r	185	69	61	315	382

**SMYD5**	ACC	HM_w	203	69	42	314	383
**EPC2**	ACC	Others	159	69	84	312	384
**FBXO44**	ACC	Others	156	69	83	308	385
**KAT2B**	ACC	HA_w	122	69	114	305	386
**UHRF2**	ACC	DM_r	194	69	42	305	387
**CBX6**	ACC	HM_r	174	69	59	302	388
**SMYD1**	ACC	HM_w	40	69	192	301	389
**KDM4D**	ACC	HM_e	117	69	111	297	390
**ARID1A**	ACC	ChRC	184	69	42	295	391
**TDG**	ACC	ChRC	29	69	190	288	392
**CBX7**	ACC	HM_r	173	69	42	284	393
**TDRD12**	ACC	Others	27	69	188	284	394
**MBD5**	ACC	DM_r	106	69	108	283	395
**RAG2**	ACC	HM_r	63	69	150	282	396
**RING1**	ACC	Others	61	69	149	279	397
**SETD7**	ACC	HM_w	51	69	142	262	398
**KDM6A**	ACC	HM_e	113	69	79	261	399
**PHF11**	ACC	Others	86	69	104	259	400
**GTF2H1**	ACC	Others	145	69	42	256	401
**MSL3**	ACC	HA_w	100	69	77	246	402
**SSRP1**	ACC	Others	35	69	137	241	403
**KDM5D**	ACC	HM_e	114	69	42	225	404
**UBE2A**	ACC	Others	21	69	135	225	405
**UBE2E1**	ACC	Others	19	69	134	222	406
**SATB1**	ACC	Others	56	69	93	218	407
**MORF4L1**	ACC	HM_r	102	69	42	213	408
**SCMH1**	ACC	Others	55	69	70	194	409
**PRMT3**	ACC	HM_w	70	69	42	181	410
**SMARCB1**	ACC	Helicases	44	69	46	159	411
**TDRD3**	ACC	HM_r	26	69	64	159	412
**ZCWPW1**	ACC	HM_r	13	69	62	144	413
**USP22**	ACC	Others	17	69	42	128	414
**UTY**	ACC	HM_e	16	69	42	127	415
**WDR82**	ACC	Others	14	69	42	125	416
**ATAD2**	BLCA	HA_r	406	414	419	1239	1

**ASXL1**	BLCA	Others	411	394	416	1221	2
**BRPF1**	BLCA	HA_r	353	416	423	1192	3
**SETDB1**	BLCA	HM_w	339	424	426	1189	4
**ASH1L**	BLCA	HM_w	418	400	368	1186	5
**PHF20L1**	BLCA	HM_r	362	403	415	1180	6
**CHD7**	BLCA	Helicases	417	352	387	1156	7
**ACTL6A**	BLCA	ChRC	330	381	425	1136	8
**BPTF**	BLCA	HA_r	400	341	394	1135	9
**KDM1B**	BLCA	HM_e	334	413	382	1129	10
**ATR**	BLCA	Others	371	333	405	1109	11
**NCOA3**	BLCA	HA_w	390	301	406	1097	12
**GATAD2B**	BLCA	HM_r	274	407	414	1095	13
**NCOA1**	BLCA	HA_w	391	322	380	1093	14
**PAF1**	BLCA	Others	308	368	412	1088	15
**PHF20**	BLCA	HM_r	388	287	403	1078	16
**SETD5**	BLCA	HM_w	245	418	411	1074	17
**JARID2**	BLCA	ChRC	317	384	354	1055	18
**PHF12**	BLCA	Others	306	404	344	1054	19
**CHD6**	BLCA	Helicases	414	274	363	1051	20
**AKAP1**	BLCA	Others	286	363	398	1047	21
**CHRAC1**	BLCA	ChRC	221	393	422	1036	22
**KDM2A**	BLCA	HM_e	315	349	370	1034	23
**ZMYND8**	BLCA	HA_r	386	296	349	1031	24
**TAF3**	BLCA	HA_r	184	422	417	1023	25
**SUZ12**	BLCA	ChRC	298	358	364	1020	26
**ASH2L**	BLCA	HM_w	167	425	424	1016	27
**KDM6A**	BLCA	HM_e	425	370	219	1014	28
**PYGO2**	BLCA	HM_r	197	399	418	1014	29
**SRCAP**	BLCA	Others	419	261	331	1011	30
**BRD9**	BLCA	HA_r	165	423	420	1008	31
**RNF40**	BLCA	Others	340	264	404	1008	32
**MLLT6**	BLCA	HM_w	363	347	296	1006	33
**SMARCA4**	BLCA	Helicases	416	239	351	1006	34
**DPF1**	BLCA	ChRC	279	406	319	1004	35
**ASXL2**	BLCA	Others	410	335	243	988	36

**CTCF**	BLCA	Others	351	291	345	987	37
**EHMT2**	BLCA	HM_w	322	306	359	987	38
**TRIM28**	BLCA	HA_r	238	344	395	977	39
**MLLT10**	BLCA	HM_w	260	323	392	975	40
**CHD4**	BLCA	Helicases	408	275	289	972	41
**ELP3**	BLCA	HA_w	157	405	409	971	42
**DIDO1**	BLCA	Others	421	154	393	968	43
**BRD1**	BLCA	HA_r	285	332	348	965	44
**KDM4A**	BLCA	HM_e	312	371	279	962	45
**MBTD1**	BLCA	Others	261	324	377	962	46
**PHF3**	BLCA	Others	395	299	268	962	47
**CHD1L**	BLCA	Helicases	162	421	365	948	48
**SIRT5**	BLCA	HA_e	187	402	352	941	49
**EP400**	BLCA	HA_w	420	229	288	937	50
**RPA3**	BLCA	Others	194	360	383	937	51
**KAT2B**	BLCA	HA_w	140	397	397	934	52
**PRMT7**	BLCA	HM_w	341	286	304	931	53
**SMYD4**	BLCA	HM_w	337	318	276	931	54
**KDM5A**	BLCA	HM_e	367	252	307	926	55
**SMARCC2**	BLCA	Helicases	377	238	310	925	56
**SMARCB1**	BLCA	Helicases	243	320	361	924	57
**CARM1**	BLCA	HM_w	283	293	347	923	58
**AFF1**	BLCA	Others	373	342	197	912	59
**SMARCE1**	BLCA	Helicases	242	281	389	912	60
**PHRF1**	BLCA	Others	253	266	390	909	61
**NCOR1**	BLCA	ChRC	412	354	137	903	62
**PRDM14**	BLCA	HM_w	292	377	233	902	63
**ATAD2B**	BLCA	HA_r	227	334	340	901	64
**ARID4B**	BLCA	ChRC	375	234	291	900	65
**BOP 1**	BLCA	Others	100	387	413	900	66
**SUV39H2**	BLCA	HM_w	110	382	407	899	67
**TDRD5**	BLCA	Others	240	365	292	897	68
**HIRA**	BLCA	Others	270	362	263	895	69
**GLYR1**	BLCA	HM_r	216	305	371	892	70
**SATB1**	BLCA	Others	123	398	369	890	71

**STK31**	BLCA	Others	299	314	275	888	72
**RNF2**	BLCA	ChRC	124	383	379	886	73
**HIST1H1C**	BLCA	Others	346	374	165	885	74
**SMARCD2**	BLCA	Helicases	116	359	410	885	75
**BAZ2A**	BLCA	HA_r	370	157	355	882	76
**HLTF**	BLCA	Others	146	350	386	882	77
**ERCC5**	BLCA	Others	219	329	325	873	78
**HDAC9**	BLCA	HA_e	347	386	139	872	79
**MECOM**	BLCA	Others	380	396	96	872	80
**HDGF**	BLCA	Others	86	385	400	871	81
**KAT6A**	BLCA	HA_w	76	420	373	869	82
**ASXL3**	BLCA	Others	382	277	209	868	83
**BAZ1B**	BLCA	HA_r	328	216	320	864	84
**PRDM9**	BLCA	HM_w	384	412	68	864	85
**CBX2**	BLCA	ChRC	164	311	388	863	86
**ARID2**	BLCA	ChRC	415	192	254	861	87
**HDAC11**	BLCA	HA_e	214	419	227	860	88
**ING1**	BLCA	HM_r	83	375	402	860	89
**TDRKH**	BLCA	Others	20	417	421	858	90
**BRWD1**	BLCA	HA_r	405	69	381	855	91
**RSF1**	BLCA	ChRC	193	367	295	855	92
**ARID1B**	BLCA	ChRC	383	295	176	854	93
**KDM2B**	BLCA	HM_e	314	206	334	854	94
**DNMT3A**	BLCA	DM_w	160	331	362	853	95
**NSD1**	BLCA	HM_w	389	271	185	845	96
**CDYL2**	BLCA	HM_r	222	340	281	843	97
**DNMT1**	BLCA	DM_w	350	212	280	842	98
**CBX4**	BLCA	HM_r	324	309	208	841	99
**FBXO17**	BLCA	Others	91	392	358	841	100
**CBX8**	BLCA	ChRC	223	308	309	840	101
**PRKAA1**	BLCA	Others	40	410	384	834	102
**MTA2**	BLCA	ChRC	206	251	375	832	103
**SP100**	BLCA	HA_r	359	317	153	829	104
**SIRT2**	BLCA	HA_e	31	388	408	827	105
**ZCWPW2**	BLCA	HM_r	236	343	247	826	106

**BAZ2B**	BLCA	HA_r	401	294	129	824	107
**KDM1A**	BLCA	HM_e	345	181	298	824	108
**DOT1L**	BLCA	HM_w	407	187	229	823	109
**CREBBP**	BLCA	HA_w	423	357	42	822	110
**MEN1**	BLCA	ChRC	310	202	306	818	111
**PRDM2**	BLCA	HM_w	361	170	286	817	112
**CBX1**	BLCA	HM_r	224	214	376	814	113
**PSIP1**	BLCA	HM_r	378	391	42	811	114
**CBX3**	BLCA	HM_r	99	310	401	810	115
**L3MBTL4**	BLCA	Others	137	355	317	809	116
**RAI1**	BLCA	Others	360	353	94	807	117
**SFMBT2**	BLCA	Others	188	415	202	805	118
**CHAF1B**	BLCA	ChRC	163	292	346	801	119
**PHF14**	BLCA	Others	49	361	391	801	120
**TDRD9**	BLCA	Others	239	313	249	801	121
**SCMH1**	BLCA	Others	122	390	285	797	122
**SETD1A**	BLCA	HM_w	302	198	294	794	123
**CHD3**	BLCA	Helicases	399	276	112	787	124
**SSRP1**	BLCA	Others	241	196	350	787	125
**PHC3**	BLCA	ChRC	52	380	353	785	126
**TCEA1**	BLCA	Others	23	366	396	785	127
**DPY30**	BLCA	Others	158	290	335	783	128
**KDM5B**	BLCA	HM_e	366	179	237	782	129
**KDM3A**	BLCA	HM_e	313	205	262	780	130
**PRDM4**	BLCA	HM_w	252	169	357	778	131
**ARID1A**	BLCA	ChRC	426	69	282	777	132
**PBRM1**	BLCA	HA_r	396	69	312	777	133
**SETD3**	BLCA	HM_w	191	283	303	777	134
**SETMAR**	BLCA	HM_w	32	389	356	777	135
**SFMBT1**	BLCA	HM_r	189	321	267	777	136
**SUPT16H**	BLCA	Others	376	69	330	775	137
**HDAC4**	BLCA	HA_e	319	356	99	774	138
**SMARCA2**	BLCA	Helicases	402	69	302	773	139
**ZMYND11**	BLCA	HA_r	11	395	366	772	140
**TCF20**	BLCA	Others	394	162	215	771	141

**GATAD2A**	BLCA	HM_r	320	185	264	769	142
**DNMT3B**	BLCA	DM_w	159	378	230	767	143
**KAT5**	BLCA	HA_w	139	304	318	761	144
**IDH1**	BLCA	DM_e	266	255	238	759	145
**EZH1**	BLCA	HM_w	277	153	324	754	146
**HDAC10**	BLCA	HA_e	294	328	132	754	147
**HIST1H3B**	BLCA	Others	268	373	111	752	148
**RAG2**	BLCA	HM_r	290	285	177	752	149
**SMARCC1**	BLCA	Helicases	338	69	343	750	150
**MTF2**	BLCA	HM_r	259	177	313	749	151
**AEBP2**	BLCA	HM_w	169	279	299	747	152
**MSH6**	BLCA	HM_r	208	221	316	745	153
**SND1**	BLCA	HM_r	300	69	374	743	154
**ATAT1**	BLCA	Others	102	312	328	742	155
**SETD4**	BLCA	HM_w	190	241	311	742	156
**BRDT**	BLCA	HA_r	357	233	151	741	157
**SMYD5**	BLCA	HM_w	112	262	367	741	158
**PRKAA2**	BLCA	Others	251	265	224	740	159
**PWWP2B**	BLCA	Others	249	346	142	737	160
**DPF2**	BLCA	Others	93	307	336	736	161
**KIAA2026**	BLCA	Others	365	69	297	731	162
**KAT7**	BLCA	HA_w	74	253	399	726	163
**BRPF3**	BLCA	HA_r	325	156	242	723	164
**DAXX**	BLCA	ChRC	280	69	372	721	165
**KDM4D**	BLCA	HM_e	210	224	287	721	166
**ING4**	BLCA	HM_r	142	254	323	719	167
**MPHOSPH8**	BLCA	HM_r	343	69	305	717	168
**KAT2A**	BLCA	HA_w	316	182	218	716	169
**TAF1L**	BLCA	HA_r	297	195	222	714	170
**TDRD6**	BLCA	Others	393	237	84	714	171
**ZGPAT**	BLCA	Others	179	193	342	714	172
**PAXIP1**	BLCA	Others	255	69	385	709	173
**POLR2B**	BLCA	Others	126	246	337	709	174
**HIST1H1B**	BLCA	Others	269	339	98	706	175
**BAZ1A**	BLCA	HA_r	354	217	130	701	176

**HCFC1**	BLCA	Others	374	69	258	701	177
**PRDM16**	BLCA	HM_w	387	171	143	701	178
**CDYL**	BLCA	HM_r	323	69	308	700	179
**ATRX**	BLCA	Helicases	385	191	120	696	180
**EP300**	BLCA	HA_w	424	230	42	696	181
**HR**	BLCA	HM_e	144	408	144	696	182
**PRMT8**	BLCA	HM_w	333	298	63	694	183
**TDRD10**	BLCA	Others	108	401	181	690	184
**MBD4**	BLCA	DM_r	133	222	333	688	185
**SIRT7**	BLCA	HA_e	27	336	321	684	186
**KDM8**	BLCA	HM_e	70	303	300	673	187
**BRD2**	BLCA	HA_r	327	69	274	670	188
**ING3**	BLCA	HM_r	211	184	271	666	189
**CHD5**	BLCA	Helicases	398	188	78	664	190
**PCGF2**	BLCA	Others	56	337	269	662	191
**GTF2H1**	BLCA	Others	232	69	360	661	192
**G2E3**	BLCA	Others	275	69	314	658	193
**GTF2F1**	BLCA	Others	273	131	253	657	194
**L3MBTL1**	BLCA	HM_r	64	348	245	657	195
**TET3**	BLCA	DM_e	107	219	329	655	196
**EED**	BLCA	ChRC	278	330	42	650	197
**PRDM7**	BLCA	HM_w	291	69	290	650	198
**FBXW9**	BLCA	Others	276	133	240	649	199
**KDM6B**	BLCA	HM_e	264	288	97	649	200
**RPH3A**	BLCA	Others	303	284	62	649	201
**FMR1**	BLCA	Others	177	257	214	648	202
**CHD1**	BLCA	Helicases	392	213	42	647	203
**MECP2**	BLCA	DM_r	62	338	244	644	204
**TDRD7**	BLCA	Others	182	260	201	643	205
**UBE2E1**	BLCA	Others	17	364	260	641	206
**RBBP5**	BLCA	ChRC	196	167	277	640	207
**MBD1**	BLCA	DM_r	364	149	125	638	208
**SMYD1**	BLCA	HM_w	331	164	140	635	209
**BMI1**	BLCA	ChRC	226	69	339	634	210
**FKBP5**	BLCA	Others	348	186	100	634	211

**PRMT5**	BLCA	HM_w	250	69	315	634	212
**SMARCA5**	BLCA	Helicases	117	282	235	634	213
**CLOCK**	BLCA	HA_w	161	231	241	633	214
**SP140**	BLCA	HA_r	185	345	103	633	215
**TDRD3**	BLCA	HM_r	183	376	73	632	216
**NCOR2**	BLCA	Others	404	69	157	630	217
**ATF7IP**	BLCA	Others	329	69	231	629	218
**UBR7**	BLCA	Others	104	259	266	629	219
**TDRD1**	BLCA	Others	287	280	60	627	220
**KDM5C**	BLCA	HM_e	230	150	246	626	221
**BRWD3**	BLCA	HA_r	234	189	200	623	222
**FBXL19**	BLCA	Others	218	211	194	623	223
**SP110**	BLCA	HA_r	186	316	121	623	224
**ARID4A**	BLCA	ChRC	356	69	196	621	225
**EZH2**	BLCA	HM_w	321	69	228	618	226
**CECR2**	BLCA	HA_r	352	69	195	616	227
**CHAF1A**	BLCA	ChRC	282	69	265	616	228
**CHD9**	BLCA	Helicases	397	69	148	614	229
**PADI1**	BLCA	Others	309	148	156	613	230
**HDGFL1**	BLCA	Others	85	426	101	612	231
**KANSL1**	BLCA	HA_w	77	208	327	612	232
**TCF19**	BLCA	Others	22	297	293	612	233
**NAP1L1**	BLCA	Others	257	137	217	611	234
**TP53BP1**	BLCA	Others	335	134	141	610	235
**JMJD1C**	BLCA	HM_e	368	69	171	608	236
**KAT8**	BLCA	HA_w	73	207	326	606	237
**KDM3B**	BLCA	HM_e	381	180	42	603	238
**ING5**	BLCA	HM_r	141	372	88	601	239
**PARP1**	BLCA	Others	307	69	225	601	240
**FKBP1A**	BLCA	Others	90	132	378	600	241
**HDAC7**	BLCA	HA_e	212	227	160	599	242
**ACTL6B**	BLCA	ChRC	295	235	61	591	243
**PADI2**	BLCA	Others	379	147	65	591	244
**BRD8**	BLCA	HA_r	225	190	174	589	245
**HIF1AN**	BLCA	Others	271	273	42	586	246

**PHC1**	BLCA	ChRC	131	270	184	585	247
**CXXC1**	BLCA	Others	281	155	147	583	248
**PARP2**	BLCA	Others	173	69	341	583	249
**PHF11**	BLCA	Others	130	411	42	583	250
**BRD7**	BLCA	HA_r	326	69	187	582	251
**PHF6**	BLCA	HM_r	293	247	42	582	252
**GTF3C4**	BLCA	HA_w	272	69	239	580	253
**ING2**	BLCA	HM_r	143	327	110	580	254
**ZCWPW1**	BLCA	HM_r	180	218	180	578	255
**RBBP4**	BLCA	ChRC	247	69	261	577	256
**JMJD6**	BLCA	HM_e	79	326	170	575	257
**DMAP1**	BLCA	Others	94	351	128	573	258
**KDM4C**	BLCA	HM_e	311	69	193	573	259
**ATM**	BLCA	Others	422	69	81	572	260
**SETDB2**	BLCA	HM_w	120	409	42	571	261
**GADD45A**	BLCA	Others	153	209	206	568	262
**KDM4B**	BLCA	HM_e	138	204	226	568	263
**PRDM5**	BLCA	HM_w	305	168	95	568	264
**RPS6KA5**	BLCA	Others	246	69	251	566	265
**PRKCD**	BLCA	Others	304	69	191	564	266
**HELLS**	BLCA	Helicases	147	256	159	562	267
**JMJD8**	BLCA	HM_e	78	272	212	562	268
**PYGO1**	BLCA	HM_r	248	130	183	561	269
**UBE2A**	BLCA	Others	170	159	232	561	270
**PRMT1**	BLCA	HM_w	39	242	278	559	271
**H3F3A**	BLCA	Others	151	69	338	558	272
**MUM1**	BLCA	Others	258	176	124	558	273
**TET1**	BLCA	DM_e	336	69	152	557	274
**FKBP2**	BLCA	Others	217	210	127	554	275
**PRDM11**	BLCA	HM_w	200	245	107	552	276
**USP51**	BLCA	Others	228	69	255	552	277
**KAT6B**	BLCA	HA_w	75	325	150	550	278
**PCGF6**	BLCA	Others	132	300	117	549	279
**IWS1**	BLCA	Others	318	183	42	543	280
**PHF8**	BLCA	Others	229	135	178	542	281

**BRD4**	BLCA	HA_r	284	215	42	541	282
**AURKB**	BLCA	Others	101	258	175	534	283
**HSPBAP1**	BLCA	Others	267	225	42	534	284
**AFF4**	BLCA	Others	372	69	89	530	285
**PHF13**	BLCA	Others	50	143	332	525	286
**INO80**	BLCA	Helicases	413	69	42	524	287
**TDG**	BLCA	ChRC	109	194	221	524	288
**MBD5**	BLCA	DM_r	344	69	109	522	289
**CHD2**	BLCA	Helicases	409	69	42	520	290
**TAF1**	BLCA	HA_r	288	69	163	520	291
**ORC1**	BLCA	Others	58	250	211	519	292
**PCMT1**	BLCA	Others	54	249	216	519	293
**CBX5**	BLCA	HM_r	98	232	186	516	294
**PRDM15**	BLCA	HM_w	342	69	105	516	295
**RING1**	BLCA	Others	125	69	322	516	296
**SETD2**	BLCA	HM_w	403	69	42	514	297
**HDAC6**	BLCA	HA_e	231	69	213	513	298
**PCGF1**	BLCA	Others	57	220	236	513	299
**TET2**	BLCA	DM_e	358	69	83	510	300
**AICDA**	BLCA	DM_e	168	278	59	505	301
**TDRD12**	BLCA	Others	21	379	102	502	302
**MSL3**	BLCA	HA_w	175	69	257	501	303
**SP140L**	BLCA	HA_r	111	315	74	500	304
**ELP4**	BLCA	HA_w	156	69	273	498	305
**RBBP7**	BLCA	ChRC	172	69	256	497	306
**PHIP**	BLCA	HA_r	254	200	42	496	307
**HDAC2**	BLCA	HA_e	149	228	118	495	308
**HDAC1**	BLCA	HA_e	150	69	272	491	309
**HDAC5**	BLCA	HA_e	213	151	126	490	310
**SMARCA1**	BLCA	Helicases	289	69	131	489	311
**CHD8**	BLCA	Helicases	369	69	42	480	312
**MTA1**	BLCA	ChRC	207	69	204	480	313
**KMT2B**	BLCA	HM_w	68	369	42	479	314
**LBR**	BLCA	Others	136	69	270	475	315
**SMARCD1**	BLCA	Helicases	26	165	284	475	316

**PADI3**	BLCA	Others	205	146	123	474	317
**SIRT1**	BLCA	HA_e	118	240	115	473	318
**FBXO44**	BLCA	Others	154	152	166	472	319
**PRMT6**	BLCA	HM_w	198	69	203	470	320
**L3MBTL3**	BLCA	Others	262	69	138	469	321
**RNF17**	BLCA	Others	332	69	67	468	322
**BAP1**	BLCA	Others	355	69	42	466	323
**HDAC8**	BLCA	HA_e	176	69	220	465	324
**PHF1**	BLCA	HM_r	204	69	192	465	325
**PRDM8**	BLCA	HM_w	199	199	64	462	326
**EHMT1**	BLCA	HM_w	349	69	42	460	327
**PHF19**	BLCA	HM_r	129	174	155	458	328
**SETD1B**	BLCA	HM_w	34	197	223	454	329
**PPARGC1A**	BLCA	Others	203	142	108	453	330
**PHF21A**	BLCA	HM_r	48	268	136	452	331
**JADE3**	BLCA	Others	80	69	301	450	332
**PHF23**	BLCA	HM_r	46	267	135	448	333
**PHF7**	BLCA	Others	127	69	252	448	334
**INTS12**	BLCA	Others	265	138	42	445	335
**PADI4**	BLCA	Others	256	145	42	443	336
**SETD6**	BLCA	HM_w	121	69	250	440	337
**HNF1A**	BLCA	ChRC	145	226	66	437	338
**UHRF1**	BLCA	DM_r	237	158	42	437	339
**SIN3B**	BLCA	ChRC	119	128	189	436	340
**EPC1**	BLCA	Others	220	69	146	435	341
**TRIM33**	BLCA	HA_r	181	141	113	435	342
**SMYD2**	BLCA	HM_w	114	163	154	431	343
**TRIM24**	BLCA	HA_r	19	161	248	428	344
**KMT2C**	BLCA	HM_w	67	302	56	425	345
**BRD3**	BLCA	HA_r	166	69	188	423	346
**DNMT3L**	BLCA	DM_w	233	69	119	421	347
**FXR2**	BLCA	Others	89	289	42	420	348
**SIN3A**	BLCA	ChRC	244	129	42	415	349
**SHPRH**	BLCA	Others	301	69	42	412	350
**PADI6**	BLCA	Others	174	144	91	409	351

**SIRT3**	BLCA	HA_e	30	263	114	407	352
**UTY**	BLCA	HM_e	296	69	42	407	353
**PRDM1**	BLCA	HM_w	202	69	134	405	354
**GTF2B**	BLCA	Others	152	69	173	394	355
**KMT2E**	BLCA	HM_w	65	178	149	392	356
**PHF10**	BLCA	Others	51	269	71	391	357
**PRDM10**	BLCA	HM_w	201	69	116	386	358
**SMNDC1**	BLCA	Others	25	319	42	386	359
**UBE2I**	BLCA	Others	105	236	42	383	360
**MBD2**	BLCA	DM_r	135	203	42	380	361
**KDM4E**	BLCA	HM_e	72	223	80	375	362
**L3MBTL2**	BLCA	Others	263	69	42	374	363
**CBX6**	BLCA	HM_r	97	69	207	373	364
**EPC2**	BLCA	Others	155	69	145	369	365
**PRDM13**	BLCA	HM_w	42	244	79	365	366
**SMYD3**	BLCA	HM_w	113	69	182	364	367
**YY1**	BLCA	ChRC	12	69	283	364	368
**MBD3**	BLCA	DM_r	134	69	158	361	369
**PHF2**	BLCA	Others	128	69	164	361	370
**PHC2**	BLCA	ChRC	53	136	169	358	371
**PHF21B**	BLCA	HM_r	47	248	57	352	372
**RTF1**	BLCA	Others	192	69	86	347	373
**KDM5D**	BLCA	HM_e	235	69	42	346	374
**WDR5**	BLCA	ChRC	13	69	259	341	375
**MARCH5**	BLCA	Others	63	69	205	337	376
**KMT2D**	BLCA	HM_w	66	69	199	334	377
**SCML2**	BLCA	HM_r	171	69	90	330	378
**CBX7**	BLCA	HM_r	96	69	162	327	379
**NAP1L2**	BLCA	Others	60	69	198	327	380
**HAT1**	BLCA	HA_w	215	69	42	326	381
**NAP1L3**	BLCA	Others	59	175	92	326	382
**PRDM6**	BLCA	HM_w	41	243	42	326	383
**IDH2**	BLCA	DM_e	84	69	172	325	384
**PRDM12**	BLCA	HM_w	43	172	106	321	385
**MORF4L1**	BLCA	HM_r	209	69	42	320	386

**KDM7A**	BLCA	HM_e	71	69	179	319	387
**USP22**	BLCA	Others	15	69	234	318	388
**H2AFZ**	BLCA	Others	87	69	161	317	389
**SMARCD3**	BLCA	Helicases	115	69	133	317	390
**AIRE**	BLCA	HM_r	178	69	69	316	391
**CSTL1**	BLCA	Others	95	139	77	311	392
**RNF20**	BLCA	Others	195	69	42	306	393
**MTA3**	BLCA	ChRC	61	201	42	304	394
**SUV39H1**	BLCA	HM_w	24	69	210	303	395
**PRMT3**	BLCA	HM_w	37	69	190	296	396
**UBE2B**	BLCA	Others	106	140	42	288	397
**PHF5A**	BLCA	Others	45	69	168	282	398
**SIRT4**	BLCA	HA_e	29	166	85	280	399
**SCML4**	BLCA	Others	35	69	167	271	400
**TRIM66**	BLCA	HA_r	18	160	82	260	401
**HDAC3**	BLCA	HA_e	148	69	42	259	402
**POLE3**	BLCA	ChRC	44	173	42	259	403
**JADE1**	BLCA	Others	82	69	93	244	404
**GADD45B**	BLCA	Others	88	69	76	233	405
**KMT2A**	BLCA	HM_w	69	69	87	225	406
**SETD7**	BLCA	HM_w	33	69	122	224	407
**JADE2**	BLCA	Others	81	69	70	220	408
**DPF3**	BLCA	HA_r	92	69	58	219	409
**WDR82**	BLCA	Others	103	69	42	214	410
**SIRT6**	BLCA	HA_e	28	69	104	201	411
**PCGF5**	BLCA	Others	55	69	72	196	412
**RNF217**	BLCA	Others	36	69	75	180	413
**PRMT2**	BLCA	HM_w	38	69	42	149	414
**UHRF2**	BLCA	DM_r	16	69	42	127	415
**USP27X**	BLCA	Others	14	69	42	125	416
**SETDB1**	BRCA	HM_w	396	419	426	1241	1
**ATAD2**	BRCA	HA_r	376	426	420	1222	2
**ASH1L**	BRCA	HM_w	412	410	399	1221	3
**ARID4B**	BRCA	ChRC	383	420	410	1213	4
**CHD7**	BRCA	Helicases	408	399	400	1207	5

**SRCAP**	BRCA	Others	424	374	409	1207	6
**KDM5B**	BRCA	HM_e	371	411	417	1199	7
**PARP1**	BRCA	Others	359	415	423	1197	8
**BPTF**	BRCA	HA_r	404	402	389	1195	9
**PHF20L1**	BRCA	HM_r	342	425	414	1181	10
**DIDO1**	BRCA	Others	388	386	403	1177	11
**CREBBP**	BRCA	HA_w	399	381	391	1171	12
**KDM2A**	BRCA	HM_e	380	393	398	1171	13
**CHD1L**	BRCA	Helicases	350	414	390	1154	14
**GLYR1**	BRCA	HM_r	361	376	416	1153	15
**KAT6A**	BRCA	HA_w	349	406	388	1143	16
**PHF12**	BRCA	Others	358	380	381	1119	17
**RNF40**	BRCA	Others	299	375	418	1092	18
**SMYD2**	BRCA	HM_w	271	407	411	1089	19
**MBTD1**	BRCA	Others	304	398	385	1087	20
**KDM5A**	BRCA	HM_e	347	367	361	1075	21
**TDRD5**	BRCA	Others	318	403	354	1075	22
**NCOA3**	BRCA	HA_w	322	356	394	1072	23
**AKAP1**	BRCA	Others	294	397	374	1065	24
**SETD1A**	BRCA	HM_w	298	377	384	1059	25
**ASXL1**	BRCA	Others	389	296	372	1057	26
**CHD6**	BRCA	Helicases	422	302	315	1039	27
**TDRKH**	BRCA	Others	198	412	421	1031	28
**CBX2**	BRCA	ChRC	262	391	377	1030	29
**CHD4**	BRCA	Helicases	423	353	232	1008	30
**SMYD3**	BRCA	HM_w	205	421	382	1008	31
**LBR**	BRCA	Others	282	416	309	1007	32
**SUZ12**	BRCA	ChRC	295	339	373	1007	33
**MLLT10**	BRCA	HM_w	345	319	342	1006	34
**HCFC1**	BRCA	Others	397	320	285	1002	35
**SMARCA4**	BRCA	Helicases	355	306	341	1002	36
**ERCC5**	BRCA	Others	260	327	408	995	37
**BRD9**	BRCA	HA_r	263	348	380	991	38
**BAZ1A**	BRCA	HA_r	364	304	322	990	39
**KAT8**	BRCA	HA_w	219	370	396	985	40

**DNMT1**	BRCA	DM_w	373	286	321	980	41
**RBBP5**	BRCA	ChRC	124	418	422	964	42
**TCEA1**	BRCA	Others	155	394	413	962	43
**SIRT7**	BRCA	HA_e	206	384	370	960	44
**SFMBT2**	BRCA	Others	336	363	255	954	45
**CARM1**	BRCA	HM_w	291	295	362	948	46
**STK31**	BRCA	Others	377	293	275	945	47
**KAT7**	BRCA	HA_w	139	395	407	941	48
**PRDM14**	BRCA	HM_w	279	401	260	940	49
**GATAD2B**	BRCA	HM_r	105	409	419	933	50
**BAZ1B**	BRCA	HA_r	331	248	353	932	51
**BRPF1**	BRCA	HA_r	292	288	345	925	52
**HDGF**	BRCA	Others	103	405	415	923	53
**NSD1**	BRCA	HM_w	391	274	256	921	54
**BRWD1**	BRCA	HA_r	416	259	245	920	55
**RNF2**	BRCA	ChRC	122	404	393	919	56
**KDM5C**	BRCA	HM_e	387	244	287	918	57
**ZMYND8**	BRCA	HA_r	265	360	293	918	58
**MSH6**	BRCA	HM_r	344	228	339	911	59
**PHC3**	BRCA	ChRC	320	366	225	911	60
**BRD4**	BRCA	HA_r	192	349	368	909	61
**HDAC6**	BRCA	HA_e	414	232	263	909	62
**ATR**	BRCA	Others	382	220	306	908	63
**BOP 1**	BRCA	Others	67	423	412	902	64
**TDRD6**	BRCA	Others	354	292	254	900	65
**L3MBTL1**	BRCA	HM_r	325	338	236	899	66
**RSF1**	BRCA	ChRC	120	396	383	899	67
**ELP3**	BRCA	HA_w	108	383	406	897	68
**SETD5**	BRCA	HM_w	273	297	325	895	69
**PRMT2**	BRCA	HM_w	244	279	367	890	70
**KAT6B**	BRCA	HA_w	406	333	148	887	71
**ACTL6A**	BRCA	ChRC	113	372	401	886	72
**CHRAC1**	BRCA	ChRC	38	424	424	886	73
**PYGO2**	BRCA	HM_r	48	413	425	886	74
**PHF20**	BRCA	HM_r	167	340	378	885	75

**H3F3A**	BRCA	Others	60	417	405	882	76
**SMARCD2**	BRCA	Helicases	78	400	404	882	77
**TRIM28**	BRCA	HA_r	235	335	311	881	78
**MECOM**	BRCA	Others	386	368	125	879	79
**CHAF1A**	BRCA	ChRC	288	258	331	877	80
**HDAC2**	BRCA	HA_e	224	318	335	877	81
**PRMT5**	BRCA	HM_w	277	225	371	873	82
**EHMT2**	BRCA	HM_w	261	256	349	866	83
**HIST1H1C**	BRCA	Others	308	277	280	865	84
**JMJD6**	BRCA	HM_e	99	387	379	865	85
**HIST1H3B**	BRCA	Others	381	276	207	864	86
**KDM8**	BRCA	HM_e	136	362	366	864	87
**PHF8**	BRCA	Others	370	281	211	862	88
**FBXL19**	BRCA	Others	145	378	338	861	89
**AEBP2**	BRCA	HM_w	195	315	350	860	90
**CBX8**	BRCA	ChRC	290	392	176	858	91
**CBX1**	BRCA	HM_r	111	369	376	856	92
**CBX4**	BRCA	HM_r	228	390	234	852	93
**POLR2B**	BRCA	Others	340	165	346	851	94
**JMJD1C**	BRCA	HM_e	360	290	200	850	95
**FBXO17**	BRCA	Others	226	326	291	843	96
**SMARCE1**	BRCA	Helicases	77	371	392	840	97
**BRPF3**	BRCA	HA_r	363	205	258	826	98
**ATM**	BRCA	Others	421	305	99	825	99
**SIN3B**	BRCA	ChRC	272	268	282	822	100
**FMR1**	BRCA	Others	311	235	272	818	101
**JMJD8**	BRCA	HM_e	53	373	387	813	102
**TDRD10**	BRCA	Others	202	408	203	813	103
**ATF7IP**	BRCA	Others	293	314	204	811	104
**TET3**	BRCA	DM_e	366	118	324	808	105
**UBE2I**	BRCA	Others	42	379	386	807	106
**TDRD12**	BRCA	Others	268	336	202	806	107
**MLLT6**	BRCA	HM_w	51	389	365	805	108
**MTA2**	BRCA	ChRC	256	212	337	805	109
**KDM1B**	BRCA	HM_e	138	323	343	804	110

**EED**	BRCA	ChRC	227	284	292	803	111
**SIRT5**	BRCA	HA_e	81	346	375	802	112
**JARID2**	BRCA	ChRC	176	334	290	800	113
**ZGPAT**	BRCA	Others	114	388	297	799	114
**SUPT16H**	BRCA	Others	296	161	340	797	115
**PRDM1**	BRCA	HM_w	339	355	102	796	116
**SMYD4**	BRCA	HM_w	270	162	359	791	117
**SND1**	BRCA	HM_r	204	250	336	790	118
**ATAD2B**	BRCA	HA_r	375	145	268	788	119
**HLTF**	BRCA	Others	178	312	298	788	120
**BAP1**	BRCA	Others	316	143	326	785	121
**PCGF2**	BRCA	Others	50	385	348	783	122
**EP300**	BRCA	HA_w	392	69	320	781	123
**SSRP1**	BRCA	Others	237	180	363	780	124
**ARID2**	BRCA	ChRC	384	178	215	777	125
**SMARCA2**	BRCA	Helicases	378	357	42	777	126
**CHD2**	BRCA	Helicases	374	359	42	775	127
**SUV39H2**	BRCA	HM_w	43	337	395	775	128
**MSL3**	BRCA	HA_w	281	213	279	773	129
**ING1**	BRCA	HM_r	284	331	157	772	130
**MEN1**	BRCA	ChRC	257	202	310	769	131
**L3MBTL3**	BRCA	Others	346	265	155	766	132
**KDM4C**	BRCA	HM_e	372	351	42	765	133
**TAF3**	BRCA	HA_r	269	365	131	765	134
**HIRA**	BRCA	Others	286	231	247	764	135
**KIAA2026**	BRCA	Others	379	342	42	763	136
**PAF1**	BRCA	Others	131	341	289	761	137
**DOT1L**	BRCA	HM_w	362	218	180	760	138
**PHF14**	BRCA	Others	250	240	270	760	139
**G2E3**	BRCA	Others	183	246	330	759	140
**ING4**	BRCA	HM_r	100	352	304	756	141
**BRD2**	BRCA	HA_r	193	206	356	755	142
**ELP4**	BRCA	HA_w	107	301	347	755	143
**DNMT3B**	BRCA	DM_w	188	285	281	754	144
**L3MBTL2**	BRCA	Others	283	69	397	749	145

**PRDM15**	BRCA	HM_w	300	226	223	749	146
**SMARCC1**	BRCA	Helicases	334	163	252	749	147
**BAZ2A**	BRCA	HA_r	351	92	305	748	148
**CTCF**	BRCA	Others	415	291	42	748	149
**EZH2**	BRCA	HM_w	185	255	301	741	150
**MTF2**	BRCA	HM_r	255	171	308	734	151
**HDAC1**	BRCA	HA_e	330	158	244	732	152
**SETD1B**	BRCA	HM_w	369	152	210	731	153
**PHC2**	BRCA	ChRC	321	169	239	729	154
**PARP2**	BRCA	Others	168	189	364	721	155
**SIRT2**	BRCA	HA_e	82	321	318	721	156
**GATAD2A**	BRCA	HM_r	143	233	344	720	157
**PRKAA1**	BRCA	Others	246	252	222	720	158
**CDYL**	BRCA	HM_r	289	69	358	716	159
**KDM4B**	BRCA	HM_e	258	289	168	715	160
**ORC1**	BRCA	Others	216	170	328	714	161
**PRMT7**	BRCA	HM_w	211	309	191	711	162
**CBX3**	BRCA	HM_r	110	287	313	710	163
**INO80**	BRCA	Helicases	307	69	334	710	164
**TCF20**	BRCA	Others	390	69	249	708	165
**DPF1**	BRCA	ChRC	64	347	295	706	166
**PRDM9**	BRCA	HM_w	247	322	137	706	167
**HDAC9**	BRCA	HA_e	259	282	163	704	168
**TET1**	BRCA	DM_e	385	125	194	704	169
**ZMYND11**	BRCA	HA_r	41	364	299	704	170
**KDM2B**	BRCA	HM_e	328	156	219	703	171
**L3MBTL4**	BRCA	Others	305	69	329	703	172
**BRD7**	BRCA	HA_r	230	69	402	701	173
**MECP2**	BRCA	DM_r	172	316	213	701	174
**ACTL6B**	BRCA	ChRC	352	249	95	696	175
**ASH2L**	BRCA	HM_w	232	422	42	696	176
**CECR2**	BRCA	HA_r	402	69	221	692	177
**PRDM11**	BRCA	HM_w	212	253	224	689	178
**PSIP1**	BRCA	HM_r	319	328	42	689	179
**SCML4**	BRCA	Others	208	361	120	689	180

**PRMT8**	BRCA	HM_w	243	354	91	688	181
**ZCWPW1**	BRCA	HM_r	266	221	198	685	182
**KDM4A**	BRCA	HM_e	348	294	42	684	183
**SETMAR**	BRCA	HM_w	118	330	235	683	184
**SHPRH**	BRCA	Others	368	269	42	679	185
**DNMT3A**	BRCA	DM_w	313	69	296	678	186
**IDH2**	BRCA	DM_e	32	345	300	677	187
**TRIM33**	BRCA	HA_r	234	179	262	675	188
**PHC1**	BRCA	ChRC	95	332	246	673	189
**KAT2B**	BRCA	HA_w	329	214	129	672	190
**SMARCA1**	BRCA	Helicases	335	183	153	671	191
**CHAF1B**	BRCA	ChRC	149	160	360	669	192
**TCF19**	BRCA	Others	75	238	355	668	193
**SUV39H1**	BRCA	HM_w	156	209	302	667	194
**KDM3A**	BRCA	HM_e	327	101	237	665	195
**PCMT1**	BRCA	Others	96	262	307	665	196
**KAT2A**	BRCA	HA_w	140	266	257	663	197
**SIN3A**	BRCA	ChRC	367	251	42	660	198
**SP100**	BRCA	HA_r	332	129	195	656	199
**USP51**	BRCA	Others	154	260	242	656	200
**PAXIP1**	BRCA	Others	251	263	141	655	201
**BRWD3**	BRCA	HA_r	403	69	182	654	202
**BRD3**	BRCA	HA_r	231	69	352	652	203
**NCOR2**	BRCA	Others	343	69	240	652	204
**PHRF1**	BRCA	Others	356	254	42	652	205
**SATB1**	BRCA	Others	337	224	90	651	206
**BAZ2B**	BRCA	HA_r	417	112	121	650	207
**DAXX**	BRCA	ChRC	189	194	267	650	208
**NAP1L1**	BRCA	Others	170	227	253	650	209
**PRMT3**	BRCA	HM_w	161	186	303	650	210
**PRMT1**	BRCA	HM_w	17	298	332	647	211
**CDYL2**	BRCA	HM_r	191	343	112	646	212
**PRDM10**	BRCA	HM_w	301	299	42	642	213
**HDAC11**	BRCA	HA_e	180	245	214	639	214
**SIRT3**	BRCA	HA_e	207	261	171	639	215

**FBXW9**	BRCA	Others	184	267	187	638	216
**HDAC4**	BRCA	HA_e	309	191	138	638	217
**PHF3**	BRCA	Others	357	239	42	638	218
**POLE3**	BRCA	ChRC	165	153	319	637	219
**MBD4**	BRCA	DM_r	135	141	357	633	220
**PHF2**	BRCA	Others	213	69	351	633	221
**TAF1L**	BRCA	HA_r	420	126	84	630	222
**CHD8**	BRCA	Helicases	407	176	42	625	223
**HR**	BRCA	HM_e	57	382	186	625	224
**NCOA1**	BRCA	HA_w	253	210	160	623	225
**ATAT1**	BRCA	Others	112	236	273	621	226
**ASXL3**	BRCA	Others	419	113	88	620	227
**CHD1**	BRCA	Helicases	314	195	111	620	228
**PADI2**	BRCA	Others	252	139	229	620	229
**AURKB**	BRCA	Others	152	144	323	619	230
**BMI1**	BRCA	ChRC	151	303	165	619	231
**TRIM24**	BRCA	HA_r	267	208	143	618	232
**ATRX**	BRCA	Helicases	418	69	122	609	233
**CHD3**	BRCA	Helicases	401	159	42	602	234
**PRDM4**	BRCA	HM_w	248	69	284	601	235
**RNF217**	BRCA	Others	121	271	209	601	236
**SMYD5**	BRCA	HM_w	158	105	333	596	237
**SCML2**	BRCA	HM_r	209	69	317	595	238
**MBD3**	BRCA	DM_r	218	243	133	594	239
**CHD9**	BRCA	Helicases	400	69	123	592	240
**ARID1A**	BRCA	ChRC	425	124	42	591	241
**ASXL2**	BRCA	Others	409	69	113	591	242
**MPHOSPH8**	BRCA	HM_r	303	242	42	587	243
**FXR2**	BRCA	Others	310	234	42	586	244
**KANSL1**	BRCA	HA_w	220	324	42	586	245
**SCMH1**	BRCA	Others	274	270	42	586	246
**PRDM13**	BRCA	HM_w	164	273	144	581	247
**CLOCK**	BRCA	HA_w	190	204	181	575	248
**EPC1**	BRCA	Others	312	217	42	571	249
**ING2**	BRCA	HM_r	56	317	197	570	250

**CHD5**	BRCA	Helicases	411	69	89	569	251
**SETD2**	BRCA	HM_w	410	115	42	567	252
**RPA3**	BRCA	Others	85	198	283	566	253
**HIST1H1B**	BRCA	Others	101	230	233	564	254
**TET2**	BRCA	DM_e	353	69	140	562	255
**PHF19**	BRCA	HM_r	127	120	312	559	256
**FKBP5**	BRCA	Others	225	216	116	557	257
**HDAC5**	BRCA	HA_e	287	69	201	557	258
**PPARGC1A**	BRCA	Others	249	200	108	557	259
**RTF1**	BRCA	Others	119	69	369	557	260
**AFF1**	BRCA	Others	365	147	42	554	261
**PHF23**	BRCA	HM_r	92	201	261	554	262
**PRKCD**	BRCA	Others	245	132	177	554	263
**TDRD9**	BRCA	Others	199	196	158	553	264
**PHIP**	BRCA	HA_r	341	166	42	549	265
**PCGF6**	BRCA	Others	214	69	265	548	266
**SETD4**	BRCA	HM_w	47	184	316	547	267
**PRDM7**	BRCA	HM_w	89	358	98	545	268
**H2AFZ**	BRCA	Others	181	69	294	544	269
**RBBP7**	BRCA	ChRC	123	272	149	544	270
**HNF1A**	BRCA	ChRC	285	108	145	538	271
**AFF4**	BRCA	Others	317	69	151	537	272
**NCOR1**	BRCA	ChRC	426	69	42	537	273
**PRKAA2**	BRCA	Others	88	280	166	534	274
**PBRM1**	BRCA	HA_r	302	188	42	532	275
**TDRD1**	BRCA	Others	236	69	227	532	276
**GTF3C4**	BRCA	HA_w	182	121	226	529	277
**PADI3**	BRCA	Others	280	138	107	525	278
**TP53BP1**	BRCA	Others	413	69	42	524	279
**MBD5**	BRCA	DM_r	323	69	128	520	280
**DPY30**	BRCA	Others	37	203	277	517	281
**SFMBT1**	BRCA	HM_r	239	119	159	517	282
**ARID1B**	BRCA	ChRC	405	69	42	516	283
**PRDM2**	BRCA	HM_w	338	133	42	513	284
**PHF1**	BRCA	HM_r	129	187	196	512	285

**EZH1**	BRCA	HM_w	186	283	42	511	286
**MUM1**	BRCA	Others	133	211	167	511	287
**CBX5**	BRCA	HM_r	150	110	250	510	288
**DMAP1**	BRCA	Others	65	257	188	510	289
**EP400**	BRCA	HA_w	398	69	42	509	290
**KDM1A**	BRCA	HM_e	175	102	231	508	291
**HDAC7**	BRCA	HA_e	223	104	179	506	292
**KDM6A**	BRCA	HM_e	395	69	42	506	293
**SMARCB1**	BRCA	Helicases	80	182	243	505	294
**TAF1**	BRCA	HA_r	394	69	42	505	295
**EHMT1**	BRCA	HM_w	393	69	42	504	296
**FKBP1A**	BRCA	Others	106	122	276	504	297
**BRDT**	BRCA	HA_r	229	177	96	502	298
**MTA1**	BRCA	ChRC	20	264	218	502	299
**DPF2**	BRCA	Others	146	313	42	501	300
**NAP1L2**	BRCA	Others	254	69	174	497	301
**UBE2A**	BRCA	Others	74	149	274	497	302
**SP110**	BRCA	HA_r	157	150	189	496	303
**RAG2**	BRCA	HM_r	86	308	101	495	304
**SETD7**	BRCA	HM_w	240	97	154	491	305
**PHF21A**	BRCA	HM_r	93	168	228	489	306
**MORF4L1**	BRCA	HM_r	171	275	42	488	307
**SMARCD3**	BRCA	Helicases	117	181	190	488	308
**TDG**	BRCA	ChRC	116	94	278	488	309
**PRMT6**	BRCA	HM_w	87	131	269	487	310
**KDM3B**	BRCA	HM_e	326	117	42	485	311
**TDRD7**	BRCA	Others	200	69	216	485	312
**AICDA**	BRCA	DM_e	40	344	100	484	313
**SMYD1**	BRCA	HM_w	297	95	92	484	314
**HDAC3**	BRCA	HA_e	58	109	314	481	315
**WDR5**	BRCA	ChRC	153	69	259	481	316
**PHF11**	BRCA	Others	128	310	42	480	317
**PCGF1**	BRCA	Others	97	106	271	474	318
**BRD8**	BRCA	HA_r	315	111	42	468	319
**SMARCC2**	BRCA	Helicases	333	93	42	468	320

**HAT1**	BRCA	HA_w	59	142	266	467	321
**SIRT6**	BRCA	HA_e	45	223	199	467	322
**YY1**	BRCA	ChRC	69	69	327	465	323
**HELLS**	BRCA	Helicases	141	103	220	464	324
**UHRF2**	BRCA	DM_r	72	350	42	464	325
**CSTL1**	BRCA	Others	148	219	94	461	326
**HDGFL1**	BRCA	Others	33	325	103	461	327
**PADI6**	BRCA	Others	215	136	110	461	328
**SMARCD1**	BRCA	Helicases	79	96	286	461	329
**UHRF1**	BRCA	DM_r	197	222	42	461	330
**RPH3A**	BRCA	Others	275	98	87	460	331
**RAI1**	BRCA	Others	242	69	146	457	332
**HDAC8**	BRCA	HA_e	179	69	208	456	333
**IDH1**	BRCA	DM_e	222	69	162	453	334
**PRDM16**	BRCA	HM_w	278	69	104	451	335
**SP140**	BRCA	HA_r	203	128	119	450	336
**TRIM66**	BRCA	HA_r	233	69	142	444	337
**USP22**	BRCA	Others	71	329	42	442	338
**DNMT3L**	BRCA	DM_w	109	247	85	441	339
**PRDM8**	BRCA	HM_w	162	164	114	440	340
**RBBP4**	BRCA	ChRC	210	185	42	437	341
**INTS12**	BRCA	Others	221	173	42	436	342
**MBD1**	BRCA	DM_r	324	69	42	435	343
**ZCWPW2**	BRCA	HM_r	196	69	170	435	344
**SETDB2**	BRCA	HM_w	84	307	42	433	345
**UBR7**	BRCA	Others	115	69	248	432	346
**SMARCA5**	BRCA	Helicases	238	151	42	431	347
**UBE2E1**	BRCA	Others	73	69	288	430	348
**RING1**	BRCA	Others	15	199	212	426	349
**SMNDC1**	BRCA	Others	44	114	264	422	350
**IWS1**	BRCA	Others	306	69	42	417	351
**MTA3**	BRCA	ChRC	134	241	42	417	352
**KDM6B**	BRCA	HM_e	137	172	105	414	353
**ING5**	BRCA	HM_r	54	229	130	413	354
**NAP1L3**	BRCA	Others	217	69	127	413	355

**PRDM5**	BRCA	HM_w	163	99	150	412	356
**AIRE**	BRCA	HM_r	68	237	106	411	357
**SETD6**	BRCA	HM_w	14	278	117	409	358
**CXXC1**	BRCA	Others	147	69	192	408	359
**KAT5**	BRCA	HA_w	52	311	42	405	360
**MBD2**	BRCA	DM_r	21	154	230	405	361
**KDM4E**	BRCA	HM_e	174	69	161	404	362
**PADI1**	BRCA	Others	169	140	93	402	363
**FKBP2**	BRCA	Others	62	175	164	401	364
**PYGO1**	BRCA	HM_r	125	69	206	400	365
**RNF17**	BRCA	Others	241	69	86	396	366
**HSPBAP1**	BRCA	Others	177	174	42	393	367
**RNF20**	BRCA	Others	276	69	42	387	368
**ARID4A**	BRCA	ChRC	194	146	42	382	369
**KMT2D**	BRCA	HM_w	23	116	241	380	370
**SP140L**	BRCA	HA_r	76	127	173	376	371
**BRD1**	BRCA	HA_r	264	69	42	375	372
**ING3**	BRCA	HM_r	55	157	156	368	373
**KMT2B**	BRCA	HM_w	25	300	42	367	374
**TDRD3**	BRCA	HM_r	201	69	97	367	375
**GADD45A**	BRCA	Others	36	193	134	363	376
**PRDM12**	BRCA	HM_w	49	134	178	361	377
**RPS6KA5**	BRCA	Others	160	69	132	361	378
**SIRT4**	BRCA	HA_e	46	130	183	359	379
**PHF5A**	BRCA	Others	18	100	238	356	380
**KDM4D**	BRCA	HM_e	98	69	185	352	381
**PHF7**	BRCA	Others	91	135	124	350	382
**FBXO44**	BRCA	Others	144	69	135	348	383
**KDM7A**	BRCA	HM_e	27	69	251	347	384
**PCGF5**	BRCA	Others	130	69	147	346	385
**PHF21B**	BRCA	HM_r	166	69	109	344	386
**PHF6**	BRCA	HM_r	126	167	42	335	387
**KMT2A**	BRCA	HM_w	26	190	115	331	388
**MARCH5**	BRCA	Others	173	107	42	322	389
**SIRT1**	BRCA	HA_e	83	197	42	322	390

**HDAC10**	BRCA	HA_e	104	69	139	312	391
**PADI4**	BRCA	Others	132	137	42	311	392
**CBX7**	BRCA	HM_r	66	123	118	307	393
**DPF3**	BRCA	HA_r	63	69	175	307	394
**PHF10**	BRCA	Others	19	69	217	305	395
**JADE2**	BRCA	Others	30	69	205	304	396
**EPC2**	BRCA	Others	187	69	42	298	397
**GTF2H1**	BRCA	Others	61	192	42	295	398
**GTF2B**	BRCA	Others	34	215	42	291	399
**UBE2B**	BRCA	Others	13	69	193	275	400
**SETD3**	BRCA	HM_w	159	69	42	270	401
**JADE1**	BRCA	Others	31	69	169	269	402
**PWWP2B**	BRCA	Others	16	69	184	269	403
**KMT2C**	BRCA	HM_w	24	69	172	265	404
**USP27X**	BRCA	Others	12	207	42	261	405
**WDR82**	BRCA	Others	70	148	42	260	406
**GTF2F1**	BRCA	Others	142	69	42	253	407
**JADE3**	BRCA	Others	29	69	152	250	408
**CBX6**	BRCA	HM_r	39	69	136	244	409
**GADD45B**	BRCA	Others	35	69	126	230	410
**KMT2E**	BRCA	HM_w	22	155	42	219	411
**HIF1AN**	BRCA	Others	102	69	42	213	412
**PHF13**	BRCA	Others	94	69	42	205	413
**PRDM6**	BRCA	HM_w	90	69	42	201	414
**KDM5D**	BRCA	HM_e	28	69	42	139	415
**UTY**	BRCA	HM_e	11	69	42	122	416
**ASH1L**	CESC	HM_w	402	399	417	1218	1
**ATR**	CESC	Others	372	422	424	1218	2
**ASXL1**	CESC	Others	351	415	420	1186	3
**SETDB1**	CESC	HM_w	375	378	421	1174	4
**ACTL6A**	CESC	ChRC	321	424	426	1171	5
**ATAD2**	CESC	HA_r	385	374	411	1170	6
**FMR1**	CESC	Others	380	401	373	1154	7
**DIDO1**	CESC	Others	408	324	415	1147	8
**KMT2A**	CESC	HM_w	400	407	333	1140	9

**PAF1**	CESC	Others	338	403	397	1138	10
**CHD6**	CESC	Helicases	420	340	364	1124	11
**CHD8**	CESC	Helicases	395	339	370	1104	12
**CREBBP**	CESC	HA_w	423	325	354	1102	13
**BPTF**	CESC	HA_r	413	342	336	1091	14
**ARID4B**	CESC	ChRC	317	375	398	1090	15
**PHF20**	CESC	HM_r	294	381	408	1083	16
**PHF20L1**	CESC	HM_r	293	380	399	1072	17
**EHMT2**	CESC	HM_w	347	358	356	1061	18
**BRD1**	CESC	HA_r	274	373	404	1051	19
**HLTF**	CESC	Others	210	423	418	1051	20
**HCFC1**	CESC	Others	346	411	290	1047	21
**MBD4**	CESC	DM_r	199	420	425	1044	22
**MORF4L1**	CESC	HM_r	297	335	406	1038	23
**HDGF**	CESC	Others	259	383	372	1014	24
**KDM5B**	CESC	HM_e	363	269	381	1013	25
**KDM2A**	CESC	HM_e	407	295	305	1007	26
**ATAT1**	CESC	Others	275	388	342	1005	27
**PRDM2**	CESC	HM_w	390	229	386	1005	28
**KAT6A**	CESC	HA_w	344	336	321	1001	29
**BRD9**	CESC	HA_r	155	421	423	999	30
**SUPT16H**	CESC	Others	283	326	384	993	31
**CHD7**	CESC	Helicases	419	277	294	990	32
**TDRD10**	CESC	Others	282	389	310	981	33
**MUM1**	CESC	Others	358	404	215	977	34
**PRDM9**	CESC	HM_w	399	419	158	976	35
**ATM**	CESC	Others	414	414	144	972	36
**KMT2C**	CESC	HM_w	426	320	217	963	37
**RTF1**	CESC	Others	329	223	409	961	38
**PHC3**	CESC	ChRC	112	425	422	959	39
**PHF3**	CESC	Others	291	317	350	958	40
**TDRD5**	CESC	Others	281	312	359	952	41
**CHRAC1**	CESC	ChRC	149	385	410	944	42
**NCOA1**	CESC	HA_w	296	291	355	942	43
**INO80**	CESC	Helicases	368	238	322	928	44

**PRMT5**	CESC	HM_w	241	315	371	927	45
**GATAD2B**	CESC	HM_r	138	384	403	925	46
**GLYR1**	CESC	HM_r	264	298	363	925	47
**CHD1**	CESC	Helicases	370	251	295	916	48
**BRPF3**	CESC	HA_r	310	254	341	905	49
**PARP2**	CESC	Others	193	318	392	903	50
**SP100**	CESC	HA_r	353	393	155	901	51
**SMARCD2**	CESC	Helicases	176	347	376	899	52
**EP400**	CESC	HA_w	418	201	277	896	53
**SIRT2**	CESC	HA_e	85	402	405	892	54
**JMJD6**	CESC	HM_e	206	370	314	890	55
**ERCC5**	CESC	Others	220	300	369	889	56
**MECP2**	CESC	DM_r	121	405	362	888	57
**DAXX**	CESC	ChRC	221	249	416	886	58
**PHRF1**	CESC	Others	335	174	377	886	59
**SND1**	CESC	HM_r	398	220	268	886	60
**KDM4B**	CESC	HM_e	365	352	164	881	61
**CECR2**	CESC	HA_r	412	204	264	880	62
**HDAC6**	CESC	HA_e	345	355	179	879	63
**CHD1L**	CESC	Helicases	223	372	279	874	64
**SMARCC2**	CESC	Helicases	354	221	298	873	65
**BAZ2A**	CESC	HA_r	349	257	266	872	66
**POLR2B**	CESC	Others	334	172	366	872	67
**PHC2**	CESC	ChRC	392	232	246	870	68
**ORC1**	CESC	Others	249	267	352	868	69
**ASXL2**	CESC	Others	350	307	208	865	70
**MBD3**	CESC	DM_r	253	406	206	865	71
**TDRKH**	CESC	Others	74	376	413	863	72
**DNMT3A**	CESC	DM_w	268	302	292	862	73
**TRIM28**	CESC	HA_r	166	281	412	859	74
**KDM5A**	CESC	HM_e	406	69	382	857	75
**PRKAA1**	CESC	Others	32	418	407	857	76
**BOP 1**	CESC	Others	68	398	388	854	77
**KAT5**	CESC	HA_w	301	237	312	850	78
**RSF1**	CESC	ChRC	289	330	231	850	79

**MSL3**	CESC	HA_w	196	366	286	848	80
**PYGO2**	CESC	HM_r	29	400	419	848	81
**DNMT3B**	CESC	DM_w	63	409	374	846	82
**HDAC4**	CESC	HA_e	305	416	125	846	83
**ATAD2B**	CESC	HA_r	157	306	379	842	84
**DPF3**	CESC	HA_r	267	323	249	839	85
**FBXO17**	CESC	Others	59	412	368	839	86
**ARID1A**	CESC	ChRC	422	69	347	838	87
**KAT7**	CESC	HA_w	205	236	394	835	88
**ATRX**	CESC	Helicases	421	213	198	832	89
**BRD3**	CESC	HA_r	313	211	308	832	90
**MSH6**	CESC	HM_r	250	187	393	830	91
**KDM4A**	CESC	HM_e	299	270	258	827	92
**BAZ2B**	CESC	HA_r	383	256	186	825	93
**EP300**	CESC	HA_w	424	357	42	823	94
**L3MBTL1**	CESC	HM_r	200	319	304	823	95
**KDM3A**	CESC	HM_e	366	235	218	819	96
**KMT2B**	CESC	HM_w	377	397	42	816	97
**KIAA2026**	CESC	Others	405	368	42	815	98
**TP53BP1**	CESC	Others	403	218	189	810	99
**PCMT1**	CESC	Others	41	365	402	808	100
**SMARCA4**	CESC	Helicases	410	69	329	808	101
**PHF13**	CESC	Others	190	265	351	806	102
**RNF2**	CESC	ChRC	93	313	396	802	103
**PHF12**	CESC	Others	336	177	285	798	104
**TRIM33**	CESC	HA_r	279	156	357	792	105
**PRDM16**	CESC	HM_w	391	287	111	789	106
**AEBP2**	CESC	HM_w	229	216	343	788	107
**CHD5**	CESC	Helicases	382	278	128	788	108
**RNF40**	CESC	Others	330	69	389	788	109
**MECOM**	CESC	Others	198	426	163	787	110
**DPF1**	CESC	ChRC	62	408	316	786	111
**IDH2**	CESC	DM_e	50	382	353	785	112
**DPY30**	CESC	Others	144	301	339	784	113
**EZH1**	CESC	HM_w	369	69	346	784	114

**KDM1B**	CESC	HM_e	203	321	259	783	115
**ZGPAT**	CESC	Others	70	311	401	782	116
**KDM4C**	CESC	HM_e	364	369	42	775	117
**ATF7IP**	CESC	Others	373	214	187	774	118
**NCOA3**	CESC	HA_w	115	290	367	772	119
**PRKAA2**	CESC	Others	242	286	244	772	120
**HIST1H1B**	CESC	Others	379	271	115	765	121
**CXXC1**	CESC	Others	269	202	293	764	122
**CBX1**	CESC	HM_r	225	208	327	760	123
**BRD4**	CESC	HA_r	371	341	42	754	124
**KANSL1**	CESC	HA_w	302	69	383	754	125
**SMARCA2**	CESC	Helicases	325	69	360	754	126
**SP140**	CESC	HA_r	171	391	191	753	127
**RNF17**	CESC	Others	331	314	107	752	128
**CBX3**	CESC	HM_r	271	207	273	751	129
**ING5**	CESC	HM_r	208	410	133	751	130
**BRWD1**	CESC	HA_r	401	305	42	748	131
**PADI3**	CESC	Others	247	181	318	746	132
**BAP1**	CESC	Others	315	387	42	744	133
**G2E3**	CESC	Others	139	274	326	739	134
**CHD2**	CESC	Helicases	309	386	42	737	135
**NSD1**	CESC	HM_w	411	69	257	737	136
**UBE2I**	CESC	Others	278	69	390	737	137
**ARID4A**	CESC	ChRC	386	308	42	736	138
**BRWD3**	CESC	HA_r	416	69	250	735	139
**NCOR1**	CESC	ChRC	376	184	175	735	140
**ZMYND8**	CESC	HA_r	231	310	188	729	141
**HDAC10**	CESC	HA_e	135	371	221	727	142
**RBBP5**	CESC	ChRC	28	284	414	726	143
**UHRF2**	CESC	DM_r	323	361	42	726	144
**CHD9**	CESC	Helicases	348	250	127	725	145
**SFMBT1**	CESC	HM_r	355	328	42	725	146
**L3MBTL4**	CESC	Others	124	367	233	724	147
**BMI1**	CESC	ChRC	314	212	197	723	148
**ZCWPW1**	CESC	HM_r	232	280	209	721	149

**ARID1B**	CESC	ChRC	318	360	42	720	150
**HSPBAP1**	CESC	Others	257	417	42	716	151
**DOT1L**	CESC	HM_w	381	69	263	713	152
**HR**	CESC	HM_e	52	354	307	713	153
**MLLT6**	CESC	HM_w	251	268	194	713	154
**PARP1**	CESC	Others	337	69	302	708	155
**TET3**	CESC	DM_e	280	69	358	707	156
**MBD5**	CESC	DM_r	341	188	176	705	157
**TAF1**	CESC	HA_r	352	69	283	704	158
**TCF19**	CESC	Others	76	346	282	704	159
**LBR**	CESC	Others	254	69	378	701	160
**SIRT6**	CESC	HA_e	84	364	253	701	161
**KAT6B**	CESC	HA_w	367	191	141	699	162
**SETD2**	CESC	HM_w	328	329	42	699	163
**MBTD1**	CESC	Others	340	69	288	697	164
**ING1**	CESC	HM_r	209	322	165	696	165
**KMT2E**	CESC	HM_w	360	294	42	696	166
**PRMT1**	CESC	HM_w	101	263	331	695	167
**DMAP1**	CESC	Others	147	275	272	694	168
**SCMH1**	CESC	Others	238	222	230	690	169
**KMT2D**	CESC	HM_w	425	69	195	689	170
**PHF1**	CESC	HM_r	111	231	344	686	171
**SMARCA1**	CESC	Helicases	177	282	227	686	172
**AFF1**	CESC	Others	320	260	105	685	173
**BAZ1B**	CESC	HA_r	384	258	42	684	174
**FXR2**	CESC	Others	140	198	345	683	175
**GTF3C4**	CESC	HA_w	263	197	223	683	176
**KDM2B**	CESC	HM_e	343	69	271	683	177
**FKBP1A**	CESC	Others	218	69	395	682	178
**GATAD2A**	CESC	HM_r	56	299	325	680	179
**HDAC1**	CESC	HA_e	53	240	387	680	180
**KDM6A**	CESC	HM_e	361	69	247	677	181
**TAF1L**	CESC	HA_r	397	69	210	676	182
**CHAF1A**	CESC	ChRC	270	359	42	671	183
**DPF2**	CESC	Others	145	248	278	671	184

**USP51**	CESC	Others	374	69	226	669	185
**AFF4**	CESC	Others	228	215	225	668	186
**RING1**	CESC	Others	94	224	349	667	187
**SUV39H2**	CESC	HM_w	169	160	337	666	188
**MTF2**	CESC	HM_r	118	186	361	665	189
**CHAF1B**	CESC	ChRC	64	304	296	664	190
**HDAC8**	CESC	HA_e	213	196	248	657	191
**SP110**	CESC	HA_r	22	392	243	657	192
**PHF11**	CESC	Others	110	395	151	656	193
**SIN3A**	CESC	ChRC	287	327	42	656	194
**NCOR2**	CESC	Others	393	69	193	655	195
**SIN3B**	CESC	ChRC	286	69	300	655	196
**FKBP2**	CESC	Others	217	199	238	654	197
**JARID2**	CESC	ChRC	378	69	207	654	198
**SMARCB1**	CESC	Helicases	284	69	299	652	199
**SCML2**	CESC	HM_r	90	350	211	651	200
**PRKCD**	CESC	Others	102	332	212	646	201
**PADI1**	CESC	Others	248	183	214	645	202
**PRDM5**	CESC	HM_w	243	228	172	643	203
**TDRD12**	CESC	Others	75	377	190	642	204
**MEN1**	CESC	ChRC	120	234	287	641	205
**PRDM10**	CESC	HM_w	186	413	42	641	206
**SETD4**	CESC	HM_w	26	283	330	639	207
**KAT2A**	CESC	HA_w	256	69	313	638	208
**NAP1L3**	CESC	Others	339	185	114	638	209
**PADI2**	CESC	Others	295	182	161	638	210
**EZH2**	CESC	HM_w	306	69	262	637	211
**PRDM12**	CESC	HM_w	105	230	301	636	212
**RAI1**	CESC	Others	332	170	132	634	213
**SETD1A**	CESC	HM_w	180	69	385	634	214
**CSTL1**	CESC	Others	148	303	182	633	215
**CLOCK**	CESC	HA_w	222	276	134	632	216
**SHPRH**	CESC	Others	237	349	42	628	217
**CBX4**	CESC	HM_r	224	69	334	627	218
**TDRD6**	CESC	Others	404	69	154	627	219

**KDM7A**	CESC	HM_e	276	69	280	625	220
**SMARCC1**	CESC	Helicases	235	348	42	625	221
**CBX6**	CESC	HM_r	153	206	265	624	222
**HDGFL1**	CESC	Others	131	273	219	623	223
**FKBP5**	CESC	Others	141	244	237	622	224
**PBRM1**	CESC	HA_r	246	334	42	622	225
**BRD8**	CESC	HA_r	312	69	239	620	226
**UHRF1**	CESC	DM_r	233	345	42	620	227
**TCEA1**	CESC	Others	20	219	380	619	228
**CHD3**	CESC	Helicases	409	69	135	613	229
**SMYD3**	CESC	HM_w	80	363	170	613	230
**GTF2B**	CESC	Others	55	242	315	612	231
**SMARCD1**	CESC	Helicases	82	164	365	611	232
**PHF14**	CESC	Others	189	176	245	610	233
**TET1**	CESC	DM_e	388	69	153	610	234
**PRMT7**	CESC	HM_w	184	171	254	609	235
**H3F3A**	CESC	Others	215	69	323	607	236
**PRDM11**	CESC	HM_w	333	69	204	606	237
**HDAC7**	CESC	HA_e	132	239	234	605	238
**ARID2**	CESC	ChRC	387	69	145	601	239
**HAT1**	CESC	HA_w	262	297	42	601	240
**SFMBT2**	CESC	Others	288	166	147	601	241
**CHD4**	CESC	Helicases	415	69	116	600	242
**POLE3**	CESC	ChRC	36	173	391	600	243
**TET2**	CESC	DM_e	324	157	119	600	244
**SETMAR**	CESC	HM_w	326	69	202	597	245
**SIRT5**	CESC	HA_e	178	69	348	595	246
**BRD7**	CESC	HA_r	273	210	104	587	247
**TDRD1**	CESC	Others	389	69	129	587	248
**BRDT**	CESC	HA_r	272	209	102	583	249
**BAZ1A**	CESC	HA_r	156	259	166	581	250
**FBXL19**	CESC	Others	219	69	291	579	251
**HDAC9**	CESC	HA_e	260	195	124	579	252
**PRDM13**	CESC	HM_w	35	289	255	579	253
**SETDB2**	CESC	HM_w	86	394	93	573	254

**ACTL6B**	CESC	ChRC	160	309	103	572	255
**KDM1A**	CESC	HM_e	204	190	177	571	256
**KDM5C**	CESC	HM_e	362	69	140	571	257
**TDRD3**	CESC	HM_r	167	362	42	571	258
**PHC1**	CESC	ChRC	245	69	256	570	259
**MTA1**	CESC	ChRC	195	69	303	567	260
**RAG2**	CESC	HM_r	97	331	138	566	261
**PHF7**	CESC	Others	37	379	149	565	262
**PYGO1**	CESC	HM_r	98	261	203	562	263
**UBE2A**	CESC	Others	15	217	328	560	264
**CARM1**	CESC	HM_w	154	69	335	558	265
**FBXW9**	CESC	Others	265	69	224	558	266
**ZCWPW2**	CESC	HM_r	162	279	117	558	267
**SETD3**	CESC	HM_w	88	69	400	557	268
**SIRT4**	CESC	HA_e	285	69	201	555	269
**SMARCE1**	CESC	Helicases	174	69	311	554	270
**CTCF**	CESC	Others	308	203	42	553	271
**MBD1**	CESC	DM_r	45	189	319	553	272
**ASXL3**	CESC	Others	316	69	167	552	273
**PRDM14**	CESC	HM_w	104	288	159	551	274
**HDAC3**	CESC	HA_e	261	69	220	550	275
**PHF10**	CESC	Others	40	396	113	549	276
**KDM4D**	CESC	HM_e	202	69	276	547	277
**KAT2B**	CESC	HA_w	126	296	122	544	278
**PSIP1**	CESC	HM_r	240	262	42	544	279
**CBX7**	CESC	HM_r	152	205	184	541	280
**HIST1H1C**	CESC	Others	211	69	261	541	281
**KDM8**	CESC	HM_e	201	69	270	540	282
**RBBP7**	CESC	ChRC	95	351	94	540	283
**JADE3**	CESC	Others	230	69	240	539	284
**IWS1**	CESC	Others	304	192	42	538	285
**PHF8**	CESC	Others	357	69	112	538	286
**JADE1**	CESC	Others	322	69	146	537	287
**CDYL**	CESC	HM_r	150	69	317	536	288
**GADD45A**	CESC	Others	57	243	236	536	289

**GTF2F1**	CESC	Others	137	356	42	535	290
**MPHOSPH8**	CESC	HM_r	197	293	42	532	291
**GTF2H1**	CESC	Others	136	69	324	529	292
**L3MBTL3**	CESC	Others	359	69	101	529	293
**PRDM7**	CESC	HM_w	103	316	110	529	294
**SRCAP**	CESC	Others	417	69	42	528	295
**HIRA**	CESC	Others	212	272	42	526	296
**RPS6KA5**	CESC	Others	182	69	274	525	297
**BRD2**	CESC	HA_r	226	255	42	523	298
**KDM6B**	CESC	HM_e	342	69	109	520	299
**H2AFZ**	CESC	Others	54	241	222	517	300
**JADE2**	CESC	Others	277	69	169	515	301
**PCGF1**	CESC	Others	114	69	332	515	302
**RPH3A**	CESC	Others	356	69	90	515	303
**KAT8**	CESC	HA_w	125	69	320	514	304
**PCGF2**	CESC	Others	42	266	205	513	305
**NAP1L2**	CESC	Others	116	233	162	511	306
**PHF21A**	CESC	HM_r	292	69	150	511	307
**SP140L**	CESC	HA_r	21	390	99	510	308
**EPC1**	CESC	Others	266	200	42	508	309
**TCF20**	CESC	Others	396	69	42	507	310
**EHMT1**	CESC	HM_w	394	69	42	505	311
**CBX5**	CESC	HM_r	66	253	185	504	312
**HELLS**	CESC	Helicases	130	194	178	502	313
**ING4**	CESC	HM_r	127	69	306	502	314
**CDYL2**	CESC	HM_r	65	252	183	500	315
**AKAP1**	CESC	Others	159	69	267	495	316
**JMJD1C**	CESC	HM_e	303	69	123	495	317
**ELP3**	CESC	HA_w	60	338	96	494	318
**PPARGC1A**	CESC	Others	107	264	121	492	319
**AIRE**	CESC	HM_r	319	69	98	486	320
**HNF1A**	CESC	ChRC	128	69	289	486	321
**PRDM4**	CESC	HM_w	185	69	232	486	322
**SMYD5**	CESC	HM_w	79	69	338	486	323
**TDRD7**	CESC	Others	18	158	309	485	324

**FBXO44**	CESC	Others	58	245	181	484	325
**PHF6**	CESC	HM_r	108	333	42	483	326
**SETD6**	CESC	HM_w	87	167	228	482	327
**IDH1**	CESC	DM_e	51	337	92	480	328
**RPA3**	CESC	Others	27	168	284	479	329
**CBX2**	CESC	ChRC	67	69	340	476	330
**USP22**	CESC	Others	164	69	241	474	331
**SMYD1**	CESC	HM_w	173	163	136	472	332
**GADD45B**	CESC	Others	216	69	180	465	333
**KDM4E**	CESC	HM_e	298	69	95	462	334
**STK31**	CESC	Others	170	161	131	462	335
**NAP1L1**	CESC	Others	117	69	275	461	336
**TAF3**	CESC	HA_r	168	159	130	457	337
**WDR82**	CESC	Others	71	343	42	456	338
**WDR5**	CESC	ChRC	11	69	375	455	339
**PRDM8**	CESC	HM_w	33	226	192	451	340
**ING2**	CESC	HM_r	49	353	42	444	341
**SMARCD3**	CESC	Helicases	175	69	200	444	342
**INTS12**	CESC	Others	207	193	42	442	343
**AURKB**	CESC	Others	227	69	143	439	344
**SETD5**	CESC	HM_w	327	69	42	438	345
**HDAC5**	CESC	HA_e	133	69	235	437	346
**PRMT6**	CESC	HM_w	99	69	269	437	347
**ELP4**	CESC	HA_w	143	247	42	432	348
**PHF21B**	CESC	HM_r	188	69	174	431	349
**EPC2**	CESC	Others	142	246	42	430	350
**PHF19**	CESC	HM_r	39	175	213	427	351
**PRMT2**	CESC	HM_w	100	285	42	427	352
**UBE2B**	CESC	Others	72	155	199	426	353
**TRIM24**	CESC	HA_r	73	69	281	423	354
**BRPF1**	CESC	HA_r	311	69	42	422	355
**SCML4**	CESC	Others	181	69	171	421	356
**DNMT1**	CESC	DM_w	307	69	42	418	357
**PADI4**	CESC	Others	194	180	42	416	358
**RNF217**	CESC	Others	239	69	106	414	359

**KDM3B**	CESC	HM_e	300	69	42	411	360
**MTA2**	CESC	ChRC	119	69	216	404	361
**SIRT7**	CESC	HA_e	83	69	252	404	362
**PRDM15**	CESC	HM_w	290	69	42	401	363
**USP27X**	CESC	Others	13	344	42	399	364
**SUV39H1**	CESC	HM_w	78	69	251	398	365
**SETD1B**	CESC	HM_w	89	69	229	387	366
**TDRD9**	CESC	Others	17	69	297	383	367
**MTA3**	CESC	ChRC	44	292	42	378	368
**JMJD8**	CESC	HM_e	47	69	260	376	369
**SMYD4**	CESC	HM_w	172	162	42	376	370
**CBX8**	CESC	ChRC	151	69	152	372	371
**HIF1AN**	CESC	Others	258	69	42	369	372
**PCGF5**	CESC	Others	191	69	108	368	373
**L3MBTL2**	CESC	Others	255	69	42	366	374
**MLLT10**	CESC	HM_w	252	69	42	363	375
**RBBP4**	CESC	ChRC	96	225	42	363	376
**PHF2**	CESC	Others	244	69	42	355	377
**PRMT8**	CESC	HM_w	183	69	100	352	378
**PHF23**	CESC	HM_r	109	69	173	351	379
**SMARCA5**	CESC	Helicases	236	69	42	347	380
**SIRT3**	CESC	HA_e	24	165	156	345	381
**SSRP1**	CESC	Others	234	69	42	345	382
**HIST1H3B**	CESC	Others	129	69	142	340	383
**PRDM1**	CESC	HM_w	106	69	160	335	384
**PCGF6**	CESC	Others	113	178	42	333	385
**MBD2**	CESC	DM_r	122	69	139	330	386
**TDG**	CESC	ChRC	19	69	242	330	387
**HDAC11**	CESC	HA_e	134	69	126	329	388
**HDAC2**	CESC	HA_e	214	69	42	325	389
**ING3**	CESC	HM_r	48	69	196	313	390
**PADI6**	CESC	Others	43	179	91	313	391
**DNMT3L**	CESC	DM_w	146	69	97	312	392
**AICDA**	CESC	DM_e	69	69	168	306	393
**PAXIP1**	CESC	Others	192	69	42	303	394

**PRDM6**	CESC	HM_w	34	227	42	303	395
**RNF20**	CESC	Others	92	169	42	303	396
**PHIP**	CESC	HA_r	187	69	42	298	397
**SATB1**	CESC	Others	91	69	137	297	398
**SIRT1**	CESC	HA_e	179	69	42	290	399
**UBE2E1**	CESC	Others	165	69	42	276	400
**YY1**	CESC	ChRC	163	69	42	274	401
**ZMYND11**	CESC	HA_r	161	69	42	272	402
**ASH2L**	CESC	HM_w	158	69	42	269	403
**PWWP2B**	CESC	Others	30	69	157	256	404
**SETD7**	CESC	HM_w	25	69	148	242	405
**MARCH5**	CESC	Others	123	69	42	234	406
**SMYD2**	CESC	HM_w	23	69	120	212	407
**TRIM66**	CESC	HA_r	16	69	118	203	408
**SMNDC1**	CESC	Others	81	69	42	192	409
**SUZ12**	CESC	ChRC	77	69	42	188	410
**EED**	CESC	ChRC	61	69	42	172	411
**KDM5D**	CESC	HM_e	46	69	42	157	412
**PHF5A**	CESC	Others	38	69	42	149	413
**PRMT3**	CESC	HM_w	31	69	42	142	414
**UBR7**	CESC	Others	14	69	42	125	415
**UTY**	CESC	HM_e	12	69	42	123	416
**CHD1L**	CHOL	Helicases	403	418	399	1220	1
**ASH1L**	CHOL	HM_w	368	426	422	1216	2
**HDGF**	CHOL	Others	397	424	385	1206	3
**ARID4B**	CHOL	ChRC	369	419	415	1203	4
**PHF20L1**	CHOL	HM_r	388	405	405	1198	5
**AFF4**	CHOL	Others	415	386	377	1178	6
**LBR**	CHOL	Others	320	415	418	1153	7
**BRD9**	CHOL	HA_r	359	378	413	1150	8
**CTCF**	CHOL	Others	348	365	412	1125	9
**SMYD2**	CHOL	HM_w	293	411	416	1120	10
**KMT2D**	CHOL	HM_w	413	337	362	1112	11
**PRDM9**	CHOL	HM_w	384	314	404	1102	12
**JMJD1C**	CHOL	HM_e	331	403	364	1098	13

**CHD3**	CHOL	Helicases	353	369	373	1095	14
**GATAD2B**	CHOL	HM_r	212	425	421	1058	15
**BOP 1**	CHOL	Others	260	407	389	1056	16
**AKAP1**	CHOL	Others	371	384	297	1052	17
**ATF7IP**	CHOL	Others	363	382	294	1039	18
**ATAT1**	CHOL	Others	268	404	341	1013	19
**BRD8**	CHOL	HA_r	256	379	375	1010	20
**KDM5B**	CHOL	HM_e	165	416	424	1005	21
**TDRD10**	CHOL	Others	282	421	300	1003	22
**ATAD2**	CHOL	HA_r	365	408	222	995	23
**ZCWPW1**	CHOL	HM_r	373	390	227	990	24
**AICDA**	CHOL	DM_e	372	385	224	981	25
**CREBBP**	CHOL	HA_w	240	366	372	978	26
**GLYR1**	CHOL	HM_r	211	353	408	972	27
**DAXX**	CHOL	ChRC	401	364	206	971	28
**FXR2**	CHOL	Others	216	354	398	968	29
**CBX8**	CHOL	ChRC	246	373	339	958	30
**CDYL2**	CHOL	HM_r	244	371	338	953	31
**PARP1**	CHOL	Others	130	414	397	941	32
**CECR2**	CHOL	HA_r	356	370	212	938	33
**PRMT7**	CHOL	HM_w	382	312	240	934	34
**DPF1**	CHOL	ChRC	234	363	336	933	35
**BPTF**	CHOL	HA_r	259	381	292	932	36
**CHD4**	CHOL	Helicases	352	368	209	929	37
**RBBP5**	CHOL	ChRC	84	413	426	923	38
**CBX2**	CHOL	ChRC	252	376	290	918	39
**CBX5**	CHOL	HM_r	249	374	289	912	40
**HDGFL1**	CHOL	Others	196	350	366	912	41
**CDYL**	CHOL	HM_r	245	372	287	904	42
**SETDB1**	CHOL	HM_w	65	412	425	902	43
**H3F3A**	CHOL	Others	206	417	275	898	44
**MUM1**	CHOL	Others	317	329	250	896	45
**ING4**	CHOL	HM_r	184	345	365	894	46
**KDM3B**	CHOL	HM_e	168	339	384	891	47
**ACTL6B**	CHOL	ChRC	275	388	226	889	48

**HDAC3**	CHOL	HA_e	199	351	333	883	49
**PHC3**	CHOL	ChRC	312	324	242	878	50
**INTS12**	CHOL	Others	395	69	411	875	51
**TDRD5**	CHOL	Others	34	420	420	874	52
**KDM2A**	CHOL	HM_e	169	340	363	872	53
**ELP3**	CHOL	HA_w	229	361	281	871	54
**HIST1H3B**	CHOL	Others	191	347	330	868	55
**PHF3**	CHOL	Others	387	69	410	866	56
**ERCC5**	CHOL	Others	226	359	278	863	57
**PYGO2**	CHOL	HM_r	88	423	351	862	58
**DOT1L**	CHOL	HM_w	400	69	388	857	59
**SMYD3**	CHOL	HM_w	46	410	395	851	60
**FKBP5**	CHOL	Others	218	355	276	849	61
**JMJD6**	CHOL	HM_e	179	343	326	848	62
**ARID1A**	CHOL	ChRC	422	383	42	847	63
**BRD7**	CHOL	HA_r	360	69	414	843	64
**TDRKH**	CHOL	Others	32	409	402	843	65
**CBX4**	CHOL	HM_r	250	375	214	839	66
**PRMT8**	CHOL	HM_w	91	394	353	838	67
**BRD2**	CHOL	HA_r	407	380	42	829	68
**CHD8**	CHOL	Helicases	350	69	409	828	69
**PADI6**	CHOL	Others	389	397	42	828	70
**SRCAP**	CHOL	Others	408	69	346	823	71
**PAF1**	CHOL	Others	131	327	360	818	72
**IDH1**	CHOL	DM_e	418	69	329	816	73
**HIST1H1B**	CHOL	Others	193	349	271	813	74
**CHD5**	CHOL	Helicases	402	367	42	811	75
**HIST1H1C**	CHOL	Others	192	348	270	810	76
**PRDM4**	CHOL	HM_w	385	69	356	810	77
**MECOM**	CHOL	Others	149	335	324	808	78
**HR**	CHOL	HM_e	188	346	269	803	79
**KDM4A**	CHOL	HM_e	326	69	407	802	80
**EP300**	CHOL	HA_w	399	360	42	801	81
**MLLT6**	CHOL	HM_w	145	333	322	800	82
**NCOA3**	CHOL	HA_w	411	69	320	800	83

**HDAC4**	CHOL	HA_e	398	69	332	799	84
**PRKAA1**	CHOL	Others	96	313	382	791	85
**JADE2**	CHOL	Others	394	69	327	790	86
**PHF5A**	CHOL	Others	112	320	358	790	87
**FBXO17**	CHOL	Others	223	358	202	783	88
**SMARCA5**	CHOL	Helicases	409	69	305	783	89
**NCOR1**	CHOL	ChRC	410	328	42	780	90
**NCOR2**	CHOL	Others	391	69	319	779	91
**ASXL3**	CHOL	Others	366	69	342	777	92
**NSD1**	CHOL	HM_w	390	69	318	777	93
**FKBP2**	CHOL	Others	219	356	201	776	94
**EP400**	CHOL	HA_w	425	69	279	773	95
**PRDM7**	CHOL	HM_w	98	316	355	769	96
**KAT6B**	CHOL	HA_w	174	402	190	766	97
**CHD1**	CHOL	Helicases	404	69	286	759	98
**SETD7**	CHOL	HM_w	299	307	153	759	99
**PADI2**	CHOL	Others	315	400	42	757	100
**DMAP1**	CHOL	Others	346	69	337	752	101
**SIRT7**	CHOL	HA_e	53	392	306	751	102
**EHMT2**	CHOL	HM_w	343	362	42	747	103
**EPC1**	CHOL	Others	342	69	335	746	104
**EZH2**	CHOL	HM_w	341	69	334	744	105
**RNF2**	CHOL	ChRC	80	422	239	741	106
**ARID4A**	CHOL	ChRC	271	69	400	740	107
**HDAC9**	CHOL	HA_e	336	69	331	736	108
**MECP2**	CHOL	DM_r	148	334	254	736	109
**ARID2**	CHOL	ChRC	370	69	296	735	110
**IDH2**	CHOL	DM_e	396	69	268	733	111
**PAXIP1**	CHOL	Others	417	69	247	733	112
**NAP1L1**	CHOL	Others	412	69	249	730	113
**KDM4B**	CHOL	HM_e	393	69	264	726	114
**BAZ1A**	CHOL	HA_r	265	69	391	725	115
**KAT2A**	CHOL	HA_w	330	69	325	724	116
**PRDM8**	CHOL	HM_w	97	315	311	723	117
**UBE2B**	CHOL	Others	25	297	401	723	118

**MTA2**	CHOL	ChRC	140	331	251	722	119
**TET1**	CHOL	DM_e	31	391	299	721	120
**BMI1**	CHOL	ChRC	261	69	390	720	121
**TAF1L**	CHOL	HA_r	376	302	42	720	122
**TDRD12**	CHOL	Others	281	299	136	716	123
**ATM**	CHOL	Others	420	69	221	710	124
**BAZ2A**	CHOL	HA_r	263	69	376	708	125
**USP22**	CHOL	Others	20	296	392	708	126
**ACTL6A**	CHOL	ChRC	276	389	42	707	127
**CHD6**	CHOL	Helicases	351	69	285	705	128
**AEBP2**	CHOL	HM_w	274	387	42	703	129
**KMT2B**	CHOL	HM_w	322	338	42	702	130
**ORC1**	CHOL	Others	316	69	317	702	131
**DIDO1**	CHOL	Others	347	69	284	700	132
**KDM1B**	CHOL	HM_e	170	341	189	700	133
**PARP2**	CHOL	Others	314	69	316	699	134
**CBX1**	CHOL	HM_r	253	69	374	696	135
**CHD7**	CHOL	Helicases	419	69	208	696	136
**PHC1**	CHOL	ChRC	125	325	244	694	137
**TRIM33**	CHOL	HA_r	278	69	345	692	138
**CHRAC1**	CHOL	ChRC	242	406	42	690	139
**BRWD3**	CHOL	HA_r	405	69	215	689	140
**EHMT1**	CHOL	HM_w	230	69	387	686	141
**ASXL1**	CHOL	Others	270	69	343	682	142
**CSTL1**	CHOL	Others	239	69	371	679	143
**HIRA**	CHOL	Others	335	69	272	676	144
**BRPF3**	CHOL	HA_r	255	377	42	674	145
**TET3**	CHOL	DM_e	375	69	230	674	146
**SIRT2**	CHOL	HA_e	58	306	309	673	147
**BAZ2B**	CHOL	HA_r	262	69	340	671	148
**KDM6B**	CHOL	HM_e	414	69	185	668	149
**SIRT5**	CHOL	HA_e	55	304	308	667	150
**EPC2**	CHOL	Others	227	69	370	666	151
**SND1**	CHOL	HM_r	291	69	303	663	152
**FBXL19**	CHOL	Others	224	69	369	662	153

**HDAC1**	CHOL	HA_e	203	69	386	658	154
**FKBP1A**	CHOL	Others	220	69	368	657	155
**KDM4C**	CHOL	HM_e	325	69	263	657	156
**STK31**	CHOL	Others	286	69	302	657	157
**TAF3**	CHOL	HA_r	284	69	301	654	158
**ATR**	CHOL	Others	362	69	220	651	159
**KMT2E**	CHOL	HM_w	321	69	259	649	160
**MBD5**	CHOL	DM_r	319	69	256	644	161
**BRDT**	CHOL	HA_r	358	69	216	643	162
**MPHOSPH8**	CHOL	HM_r	318	69	252	639	163
**SMARCD2**	CHOL	Helicases	294	303	42	639	164
**ASXL2**	CHOL	Others	269	69	295	633	165
**CHD2**	CHOL	Helicases	354	69	210	633	166
**HDAC8**	CHOL	HA_e	197	69	367	633	167
**PCGF2**	CHOL	Others	128	326	178	632	168
**MBD1**	CHOL	DM_r	154	69	406	629	169
**AURKB**	CHOL	Others	266	69	293	628	170
**PCGF5**	CHOL	Others	313	69	245	627	171
**PHF1**	CHOL	HM_r	123	323	176	622	172
**FBXO44**	CHOL	Others	222	357	42	621	173
**DPF3**	CHOL	HA_r	344	69	205	618	174
**PRDM13**	CHOL	HM_w	308	69	241	618	175
**PHF12**	CHOL	Others	120	322	174	616	176
**PRMT3**	CHOL	HM_w	383	69	164	616	177
**CARM1**	CHOL	HM_w	254	69	291	614	178
**RPH3A**	CHOL	Others	302	69	238	609	179
**GATAD2A**	CHOL	HM_r	340	69	198	607	180
**CBX6**	CHOL	HM_r	248	69	288	605	181
**GTF3C4**	CHOL	HA_w	339	69	197	605	182
**SETD1B**	CHOL	HM_w	381	69	155	605	183
**SFMBT2**	CHOL	Others	298	69	237	604	184
**HDAC5**	CHOL	HA_e	338	69	195	602	185
**HDAC6**	CHOL	HA_e	337	69	194	600	186
**SMARCD1**	CHOL	Helicases	295	69	235	599	187
**HCFC1**	CHOL	Others	204	352	42	598	188

**SIRT3**	CHOL	HA_e	57	305	236	598	189
**SMARCA4**	CHOL	Helicases	378	69	148	595	190
**IWS1**	CHOL	Others	332	69	191	592	191
**MTF2**	CHOL	HM_r	138	69	383	590	192
**L3MBTL1**	CHOL	HM_r	159	69	361	589	193
**DNMT3B**	CHOL	DM_w	236	69	283	588	194
**DNMT3L**	CHOL	DM_w	235	69	282	586	195
**KDM2B**	CHOL	HM_e	328	69	188	585	196
**TDRD6**	CHOL	Others	280	69	232	581	197
**ING5**	CHOL	HM_r	183	69	328	580	198
**TDRD9**	CHOL	Others	279	69	231	579	199
**ELP4**	CHOL	HA_w	228	69	280	577	200
**PADI1**	CHOL	Others	134	401	42	577	201
**PADI3**	CHOL	Others	133	399	42	574	202
**PADI4**	CHOL	Others	132	398	42	572	203
**TCF19**	CHOL	Others	38	301	233	572	204
**AFF1**	CHOL	Others	273	69	225	567	205
**FBXW9**	CHOL	Others	221	69	277	567	206
**JARID2**	CHOL	ChRC	180	344	42	566	207
**PRMT2**	CHOL	HM_w	94	69	403	566	208
**AIRE**	CHOL	HM_r	272	69	223	564	209
**PHF10**	CHOL	Others	122	396	42	560	210
**RAI1**	CHOL	Others	86	311	162	559	211
**ATRX**	CHOL	Helicases	267	69	219	555	212
**KDM1A**	CHOL	HM_e	171	342	42	555	213
**PCGF6**	CHOL	Others	127	69	359	555	214
**SETD4**	CHOL	HM_w	68	69	417	554	215
**TRIM66**	CHOL	HA_r	27	298	228	553	216
**PHF21A**	CHOL	HM_r	311	69	172	552	217
**BAZ1B**	CHOL	HA_r	264	69	218	551	218
**HDAC10**	CHOL	HA_e	202	69	274	545	219
**BRD3**	CHOL	HA_r	258	69	217	544	220
**RNF20**	CHOL	Others	79	69	396	544	221
**PRDM15**	CHOL	HM_w	307	69	167	543	222
**HDAC2**	CHOL	HA_e	200	69	273	542	223

**MEN1**	CHOL	ChRC	147	69	323	539	224
**PRKAA2**	CHOL	Others	305	69	165	539	225
**BAP1**	CHOL	Others	426	69	42	537	226
**PRDM2**	CHOL	HM_w	100	395	42	537	227
**L3MBTL2**	CHOL	Others	158	336	42	536	228
**SCML4**	CHOL	Others	71	308	157	536	229
**PBRM1**	CHOL	HA_r	424	69	42	535	230
**KMT2C**	CHOL	HM_w	423	69	42	534	231
**ARID1B**	CHOL	ChRC	421	69	42	532	232
**POLR2B**	CHOL	Others	106	69	357	532	233
**RNF17**	CHOL	Others	81	69	381	531	234
**SUPT16H**	CHOL	Others	43	69	419	531	235
**CBX7**	CHOL	HM_r	247	69	213	529	236
**TP53BP1**	CHOL	Others	416	69	42	527	237
**NCOA1**	CHOL	HA_w	135	69	321	525	238
**CHAF1B**	CHOL	ChRC	243	69	211	523	239
**JADE1**	CHOL	Others	182	69	267	518	240
**PRMT1**	CHOL	HM_w	95	69	354	518	241
**SETD3**	CHOL	HM_w	69	69	380	518	242
**BRWD1**	CHOL	HA_r	406	69	42	517	243
**MTA1**	CHOL	ChRC	141	332	42	515	244
**CXXC1**	CHOL	Others	238	69	207	514	245
**JMJD8**	CHOL	HM_e	178	69	266	513	246
**MTA3**	CHOL	ChRC	139	330	42	511	247
**PYGO1**	CHOL	HM_r	89	69	352	510	248
**SIN3A**	CHOL	ChRC	61	69	379	509	249
**SMYD4**	CHOL	HM_w	292	69	146	507	250
**KAT8**	CHOL	HA_w	172	69	265	506	251
**YY1**	CHOL	ChRC	14	69	423	506	252
**EED**	CHOL	ChRC	231	69	204	504	253
**RBBP4**	CHOL	ChRC	85	69	350	504	254
**KMT2A**	CHOL	HM_w	392	69	42	503	255
**SP100**	CHOL	HA_r	290	69	144	503	256
**PHF2**	CHOL	Others	116	69	315	500	257
**SP140**	CHOL	HA_r	289	69	142	500	258

**KDM4D**	CHOL	HM_e	167	69	262	498	259
**PHF20**	CHOL	HM_r	115	69	314	498	260
**SP140L**	CHOL	HA_r	288	69	141	498	261
**TDRD3**	CHOL	HM_r	35	69	394	498	262
**EZH1**	CHOL	HM_w	225	69	203	497	263
**PHIP**	CHOL	HA_r	386	69	42	497	264
**SSRP1**	CHOL	Others	287	69	140	496	265
**PHF23**	CHOL	HM_r	113	69	313	495	266
**RPA3**	CHOL	Others	76	69	349	494	267
**SIRT1**	CHOL	HA_e	59	393	42	494	268
**KDM7A**	CHOL	HM_e	161	69	261	491	269
**SETD2**	CHOL	HM_w	380	69	42	491	270
**SHPRH**	CHOL	Others	379	69	42	490	271
**KDM8**	CHOL	HM_e	160	69	260	489	272
**SMARCC1**	CHOL	Helicases	377	69	42	488	273
**UBE2A**	CHOL	Others	26	69	393	488	274
**PPARGC1A**	CHOL	Others	105	69	312	486	275
**UHRF1**	CHOL	DM_r	374	69	42	485	276
**GADD45A**	CHOL	Others	214	69	200	483	277
**TDRD1**	CHOL	Others	36	69	378	483	278
**PHF13**	CHOL	Others	119	321	42	482	279
**GADD45B**	CHOL	Others	213	69	199	481	280
**MBD3**	CHOL	DM_r	152	69	258	479	281
**ASH2L**	CHOL	HM_w	367	69	42	478	282
**MBD4**	CHOL	DM_r	151	69	257	477	283
**ATAD2B**	CHOL	HA_r	364	69	42	475	284
**MBTD1**	CHOL	Others	150	69	255	474	285
**BRD1**	CHOL	HA_r	361	69	42	472	286
**HAT1**	CHOL	HA_w	205	69	196	470	287
**PHRF1**	CHOL	Others	108	319	42	469	288
**BRPF1**	CHOL	HA_r	357	69	42	468	289
**CHAF1A**	CHOL	ChRC	355	69	42	466	290
**MORF4L1**	CHOL	HM_r	144	69	253	466	291
**SMNDC1**	CHOL	Others	48	69	348	465	292
**SMYD1**	CHOL	HM_w	47	69	347	463	293

**PRDM14**	CHOL	HM_w	102	318	42	462	294
**CHD9**	CHOL	Helicases	349	69	42	460	295
**PRDM16**	CHOL	HM_w	101	317	42	460	296
**HELLS**	CHOL	Helicases	195	69	193	457	297
**DNMT1**	CHOL	DM_w	345	69	42	456	298
**RNF40**	CHOL	Others	77	69	310	456	299
**NAP1L3**	CHOL	Others	136	69	248	453	300
**HNF1A**	CHOL	ChRC	189	69	192	450	301
**HSPBAP1**	CHOL	Others	334	69	42	445	302
**INO80**	CHOL	Helicases	333	69	42	444	303
**PCGF1**	CHOL	Others	129	69	246	444	304
**KAT6A**	CHOL	HA_w	329	69	42	440	305
**KDM3A**	CHOL	HM_e	327	69	42	438	306
**ZMYND8**	CHOL	HA_r	11	295	132	438	307
**PHC2**	CHOL	ChRC	124	69	243	436	308
**KDM5A**	CHOL	HM_e	324	69	42	435	309
**KIAA2026**	CHOL	Others	323	69	42	434	310
**RING1**	CHOL	Others	82	310	42	434	311
**SIRT6**	CHOL	HA_e	54	69	307	430	312
**WDR5**	CHOL	ChRC	16	69	344	429	313
**SCMH1**	CHOL	Others	73	309	42	424	314
**KDM4E**	CHOL	HM_e	166	69	187	422	315
**SMARCE1**	CHOL	Helicases	49	69	304	422	316
**PRDM1**	CHOL	HM_w	310	69	42	421	317
**PRDM10**	CHOL	HM_w	309	69	42	420	318
**KDM6A**	CHOL	HM_e	162	69	186	417	319
**PRDM6**	CHOL	HM_w	306	69	42	417	320
**PRKCD**	CHOL	Others	304	69	42	415	321
**PSIP1**	CHOL	HM_r	303	69	42	414	322
**RTF1**	CHOL	Others	301	69	42	412	323
**SATB1**	CHOL	Others	300	69	42	411	324
**L3MBTL3**	CHOL	Others	157	69	184	410	325
**L3MBTL4**	CHOL	Others	156	69	183	408	326
**SMARCA2**	CHOL	Helicases	297	69	42	408	327
**SMARCC2**	CHOL	Helicases	296	69	42	407	328

**MBD2**	CHOL	DM_r	153	69	182	404	329
**MLLT10**	CHOL	HM_w	146	69	181	396	330
**TAF1**	CHOL	HA_r	285	69	42	396	331
**TDG**	CHOL	ChRC	283	69	42	394	332
**MSL3**	CHOL	HA_w	142	69	180	391	333
**UBR7**	CHOL	Others	22	69	298	389	334
**ZCWPW2**	CHOL	HM_r	277	69	42	388	335
**NAP1L2**	CHOL	Others	137	69	179	385	336
**TCF20**	CHOL	Others	37	300	42	379	337
**PCMT1**	CHOL	Others	126	69	177	372	338
**BRD4**	CHOL	HA_r	257	69	42	368	339
**PHF11**	CHOL	Others	121	69	175	365	340
**CBX3**	CHOL	HM_r	251	69	42	362	341
**PHF19**	CHOL	HM_r	117	69	173	359	342
**PHF21B**	CHOL	HM_r	114	69	171	354	343
**SMARCD3**	CHOL	Helicases	50	69	234	353	344
**CLOCK**	CHOL	HA_w	241	69	42	352	345
**DNMT3A**	CHOL	DM_w	237	69	42	348	346
**PHF8**	CHOL	Others	109	69	170	348	347
**DPF2**	CHOL	Others	233	69	42	344	348
**DPY30**	CHOL	Others	232	69	42	343	349
**PRDM11**	CHOL	HM_w	104	69	169	342	350
**PRDM12**	CHOL	HM_w	103	69	168	340	351
**PRDM5**	CHOL	HM_w	99	69	166	334	352
**FMR1**	CHOL	Others	217	69	42	328	353
**TRIM24**	CHOL	HA_r	29	69	229	327	354
**G2E3**	CHOL	Others	215	69	42	326	355
**PWWP2B**	CHOL	Others	90	69	163	322	356
**GTF2B**	CHOL	Others	210	69	42	321	357
**GTF2F1**	CHOL	Others	209	69	42	320	358
**GTF2H1**	CHOL	Others	208	69	42	319	359
**H2AFZ**	CHOL	Others	207	69	42	318	360
**RBBP7**	CHOL	ChRC	83	69	161	313	361
**HDAC11**	CHOL	HA_e	201	69	42	312	362
**HDAC7**	CHOL	HA_e	198	69	42	309	363

**RNF217**	CHOL	Others	78	69	160	307	364
**HIF1AN**	CHOL	Others	194	69	42	305	365
**RPS6KA5**	CHOL	Others	75	69	159	303	366
**HLTF**	CHOL	Others	190	69	42	301	367
**SCML2**	CHOL	HM_r	72	69	158	299	368
**ING1**	CHOL	HM_r	187	69	42	298	369
**ING2**	CHOL	HM_r	186	69	42	297	370
**ING3**	CHOL	HM_r	185	69	42	296	371
**SETD1A**	CHOL	HM_w	70	69	156	295	372
**JADE3**	CHOL	Others	181	69	42	292	373
**SETD6**	CHOL	HM_w	66	69	154	289	374
**KANSL1**	CHOL	HA_w	177	69	42	288	375
**KAT2B**	CHOL	HA_w	176	69	42	287	376
**KAT5**	CHOL	HA_w	175	69	42	286	377
**SETDB2**	CHOL	HM_w	64	69	152	285	378
**KAT7**	CHOL	HA_w	173	69	42	284	379
**SIN3B**	CHOL	ChRC	60	69	151	280	380
**KDM5C**	CHOL	HM_e	164	69	42	275	381
**SIRT4**	CHOL	HA_e	56	69	150	275	382
**KDM5D**	CHOL	HM_e	163	69	42	274	383
**SMARCA1**	CHOL	Helicases	52	69	149	270	384
**SMARCB1**	CHOL	Helicases	51	69	147	267	385
**MARCH5**	CHOL	Others	155	69	42	266	386
**SMYD5**	CHOL	HM_w	45	69	145	259	387
**SP110**	CHOL	HA_r	44	69	143	256	388
**MSH6**	CHOL	HM_r	143	69	42	254	389
**SUV39H1**	CHOL	HM_w	42	69	139	250	390
**SUV39H2**	CHOL	HM_w	41	69	138	248	391
**TCEA1**	CHOL	Others	39	69	137	245	392
**TRIM28**	CHOL	HA_r	28	69	135	232	393
**PHF14**	CHOL	Others	118	69	42	229	394
**PHF6**	CHOL	HM_r	111	69	42	222	395
**PHF7**	CHOL	Others	110	69	42	221	396
**USP51**	CHOL	Others	18	69	134	221	397
**POLE3**	CHOL	ChRC	107	69	42	218	398

**ZGPAT**	CHOL	Others	13	69	133	215	399
**PRMT5**	CHOL	HM_w	93	69	42	204	400
**PRMT6**	CHOL	HM_w	92	69	42	203	401
**RAG2**	CHOL	HM_r	87	69	42	198	402
**RSF1**	CHOL	ChRC	74	69	42	185	403
**SETD5**	CHOL	HM_w	67	69	42	178	404
**SETMAR**	CHOL	HM_w	63	69	42	174	405
**SFMBT1**	CHOL	HM_r	62	69	42	173	406
**SUZ12**	CHOL	ChRC	40	69	42	151	407
**TDRD7**	CHOL	Others	33	69	42	144	408
**TET2**	CHOL	DM_e	30	69	42	141	409
**UBE2E1**	CHOL	Others	24	69	42	135	410
**UBE2I**	CHOL	Others	23	69	42	134	411
**UHRF2**	CHOL	DM_r	21	69	42	132	412
**USP27X**	CHOL	Others	19	69	42	130	413
**UTY**	CHOL	HM_e	17	69	42	128	414
**WDR82**	CHOL	Others	15	69	42	126	415
**ZMYND11**	CHOL	HA_r	12	69	42	123	416
**CHD6**	COAD/R	Helicases	419	421	409	1249	1
**ASXL1**	COAD/R	Others	390	426	426	1242	2
**DIDO1**	COAD/R	Others	387	423	425	1235	3
**ZMYND8**	COAD/R	HA_r	372	419	424	1215	4
**DNMT3B**	COAD/R	DM_w	378	424	408	1210	5
**CHD7**	COAD/R	Helicases	396	407	399	1202	6
**KAT6A**	COAD/R	HA_w	391	409	394	1194	7
**PHF20L1**	COAD/R	HM_r	364	412	411	1187	8
**SRCAP**	COAD/R	Others	415	375	380	1170	9
**ATAD2**	COAD/R	HA_r	359	414	395	1168	10
**NCOA3**	COAD/R	HA_w	326	418	413	1157	11
**KDM5A**	COAD/R	HM_e	355	394	373	1122	12
**NCOR1**	COAD/R	ChRC	392	377	353	1122	13
**L3MBTL1**	COAD/R	HM_r	283	422	414	1119	14
**ERCC5**	COAD/R	Others	301	399	410	1110	15
**FXR2**	COAD/R	Others	299	389	416	1104	16
**KIAA2026**	COAD/R	Others	365	386	346	1097	17

**TDRD3**	COAD/R	HM_r	287	395	412	1094	18
**ING1**	COAD/R	HM_r	269	406	405	1080	19
**ASH1L**	COAD/R	HM_w	413	283	382	1078	20
**KDM4C**	COAD/R	HM_e	296	387	385	1068	21
**INO80**	COAD/R	Helicases	331	388	347	1066	22
**KDM5B**	COAD/R	HM_e	366	346	354	1066	23
**SETD1A**	COAD/R	HM_w	320	369	375	1064	24
**SMARCA2**	COAD/R	Helicases	350	368	341	1059	25
**KMT2E**	COAD/R	HM_w	383	312	344	1039	26
**BRD1**	COAD/R	HA_r	304	374	359	1037	27
**BRD9**	COAD/R	HA_r	303	390	338	1031	28
**SETDB1**	COAD/R	HM_w	352	297	381	1030	29
**MBD1**	COAD/R	DM_r	208	396	415	1019	30
**RNF17**	COAD/R	Others	401	415	198	1014	31
**KMT2C**	COAD/R	HM_w	412	217	374	1003	32
**PHF20**	COAD/R	HM_r	155	425	423	1003	33
**RNF40**	COAD/R	Others	239	376	388	1003	34
**ELP3**	COAD/R	HA_w	165	416	421	1002	35
**BAZ1B**	COAD/R	HA_r	370	238	392	1000	36
**CHD9**	COAD/R	Helicases	388	351	258	997	37
**MBD5**	COAD/R	DM_r	394	309	294	997	38
**SND1**	COAD/R	HM_r	349	245	401	995	39
**ARID4B**	COAD/R	ChRC	253	364	372	989	40
**MBD2**	COAD/R	DM_r	160	403	418	981	41
**PHF23**	COAD/R	HM_r	201	384	386	971	42
**ARID1A**	COAD/R	ChRC	425	284	259	968	43
**PHF11**	COAD/R	Others	156	405	404	965	44
**CREBBP**	COAD/R	HA_w	424	233	307	964	45
**SETDB2**	COAD/R	HM_w	143	404	417	964	46
**TCEA1**	COAD/R	Others	139	400	406	945	47
**BRD7**	COAD/R	HA_r	220	353	371	944	48
**MPHOSPH8**	COAD/R	HM_r	107	413	420	940	49
**BRD4**	COAD/R	HA_r	279	332	316	927	50
**RBBP5**	COAD/R	ChRC	198	340	387	925	51
**PHF12**	COAD/R	Others	265	342	312	919	52

**PRDM14**	COAD/R	HM_w	223	408	288	919	53
**PRKAA1**	COAD/R	Others	241	370	303	914	54
**SETD3**	COAD/R	HM_w	319	200	390	909	55
**ATM**	COAD/R	Others	426	354	128	908	56
**CDYL**	COAD/R	HM_r	277	279	348	904	57
**DNMT1**	COAD/R	DM_w	418	277	209	904	58
**PARP1**	COAD/R	Others	325	303	275	903	59
**GATAD2B**	COAD/R	HM_r	225	324	351	900	60
**CXXC1**	COAD/R	Others	128	397	370	895	61
**MSH6**	COAD/R	HM_r	403	216	276	895	62
**JARID2**	COAD/R	ChRC	395	268	230	893	63
**MLLT6**	COAD/R	HM_w	206	392	293	891	64
**TP53BP1**	COAD/R	Others	373	382	133	888	65
**ATF7IP**	COAD/R	Others	358	239	287	884	66
**KAT7**	COAD/R	HA_w	257	267	360	884	67
**RTF1**	COAD/R	Others	92	383	403	878	68
**ZGPAT**	COAD/R	Others	34	420	422	876	69
**HDAC6**	COAD/R	HA_e	342	320	212	874	70
**RAI1**	COAD/R	Others	263	356	251	870	71
**CHD1L**	COAD/R	Helicases	169	362	336	867	72
**FBXL19**	COAD/R	Others	124	379	363	866	73
**SMARCE1**	COAD/R	Helicases	89	393	383	865	74
**CBX3**	COAD/R	HM_r	182	280	400	862	75
**CHRAC1**	COAD/R	ChRC	30	411	419	860	76
**CHD5**	COAD/R	Helicases	397	361	101	859	77
**TDRKH**	COAD/R	Others	188	288	379	855	78
**CHD3**	COAD/R	Helicases	368	373	107	848	79
**SIN3B**	COAD/R	ChRC	316	249	282	847	80
**USP22**	COAD/R	Others	83	366	397	846	81
**BOP 1**	COAD/R	Others	32	410	402	844	82
**PRMT8**	COAD/R	HM_w	337	398	109	844	83
**ARID2**	COAD/R	ChRC	409	69	365	843	84
**JMJD1C**	COAD/R	HM_e	330	316	194	840	85
**LBR**	COAD/R	Others	209	311	319	839	86
**KAT8**	COAD/R	HA_w	179	347	310	836	87

**ARID4A**	COAD/R	ChRC	400	186	242	828	88
**L3MBTL4**	COAD/R	Others	295	378	153	826	89
**BPTF**	COAD/R	HA_r	389	391	42	822	90
**CHD4**	COAD/R	Helicases	423	352	42	817	91
**KDM3A**	COAD/R	HM_e	329	168	320	817	92
**TET1**	COAD/R	DM_e	422	193	201	816	93
**NCOA1**	COAD/R	HA_w	386	161	267	814	94
**SCML4**	COAD/R	Others	289	251	272	812	95
**HELLS**	COAD/R	Helicases	297	350	163	810	96
**CECR2**	COAD/R	HA_r	334	278	196	808	97
**SUZ12**	COAD/R	ChRC	140	290	377	807	98
**FMR1**	COAD/R	Others	258	360	187	805	99
**HDAC9**	COAD/R	HA_e	404	270	131	805	100
**CSTL1**	COAD/R	Others	168	402	233	803	101
**SMYD4**	COAD/R	HM_w	38	367	396	801	102
**PRDM16**	COAD/R	HM_w	291	358	150	799	103
**ASXL3**	COAD/R	Others	336	333	129	798	104
**L3MBTL2**	COAD/R	Others	266	165	367	798	105
**GTF2H1**	COAD/R	Others	249	227	321	797	106
**STK31**	COAD/R	Others	362	196	236	794	107
**CHD1**	COAD/R	Helicases	369	380	42	791	108
**CARM1**	COAD/R	HM_w	131	331	328	790	109
**RNF2**	COAD/R	ChRC	147	298	345	790	110
**UBE2I**	COAD/R	Others	87	335	366	788	111
**MECP2**	COAD/R	DM_r	178	307	297	782	112
**KDM2B**	COAD/R	HM_e	356	169	256	781	113
**PADI6**	COAD/R	Others	338	208	235	781	114
**SMARCC1**	COAD/R	Helicases	194	292	291	777	115
**PCMT1**	COAD/R	Others	184	302	289	775	116
**PHC1**	COAD/R	ChRC	293	207	274	774	117
**RNF20**	COAD/R	Others	290	149	334	773	118
**BAZ2B**	COAD/R	HA_r	251	281	240	772	119
**SMARCC2**	COAD/R	Helicases	233	197	340	770	120
**IDH2**	COAD/R	DM_e	332	318	119	769	121
**TRIM24**	COAD/R	HA_r	312	134	323	769	122

**SMARCA1**	COAD/R	Helicases	381	142	245	768	123
**SUPT16H**	COAD/R	Others	314	69	384	767	124
**PHF2**	COAD/R	Others	322	206	237	765	125
**KAT6B**	COAD/R	HA_w	407	314	42	763	126
**PHF3**	COAD/R	Others	376	69	318	763	127
**KANSL1**	COAD/R	HA_w	341	69	350	760	128
**PHF14**	COAD/R	Others	203	157	398	758	129
**PRDM2**	COAD/R	HM_w	416	299	42	757	130
**SUV39H1**	COAD/R	HM_w	222	291	244	757	131
**ZCWPW1**	COAD/R	HM_r	186	242	329	757	132
**KAT2B**	COAD/R	HA_w	248	315	193	756	133
**PAXIP1**	COAD/R	Others	294	69	391	754	134
**ATR**	COAD/R	Others	399	69	281	749	135
**HCFC1**	COAD/R	Others	181	321	246	748	136
**SIRT5**	COAD/R	HA_e	235	248	263	746	137
**CBX8**	COAD/R	ChRC	219	328	197	744	138
**SMARCA4**	COAD/R	Helicases	363	339	42	744	139
**SIN3A**	COAD/R	ChRC	317	69	357	743	140
**H3F3A**	COAD/R	Others	64	322	355	741	141
**PRDM10**	COAD/R	HM_w	385	204	151	740	142
**HR**	COAD/R	HM_e	115	417	207	739	143
**SFMBT2**	COAD/R	Others	421	143	172	736	144
**SMYD2**	COAD/R	HM_w	39	338	356	733	145
**KAT2A**	COAD/R	HA_w	57	348	327	732	146
**MUM1**	COAD/R	Others	246	305	181	732	147
**SIRT2**	COAD/R	HA_e	142	295	292	729	148
**SMARCD2**	COAD/R	Helicases	40	355	333	728	149
**ACTL6A**	COAD/R	ChRC	221	188	317	726	150
**TRIM33**	COAD/R	HA_r	346	336	42	724	151
**PCGF2**	COAD/R	Others	103	385	229	717	152
**JADE2**	COAD/R	Others	345	69	298	712	153
**TDRD7**	COAD/R	Others	226	195	290	711	154
**TDRD5**	COAD/R	Others	286	289	134	709	155
**SMARCB1**	COAD/R	Helicases	234	140	331	705	156
**BRD3**	COAD/R	HA_r	172	236	296	704	157

**CHAF1A**	COAD/R	ChRC	218	327	159	704	158
**TAF1L**	COAD/R	HA_r	420	137	145	702	159
**JMJD6**	COAD/R	HM_e	110	221	369	700	160
**MECOM**	COAD/R	Others	393	265	42	700	161
**PHF21A**	COAD/R	HM_r	354	156	190	700	162
**PYGO2**	COAD/R	HM_r	94	254	342	690	163
**SETD5**	COAD/R	HM_w	318	146	226	690	164
**ATAD2B**	COAD/R	HA_r	280	69	339	688	165
**CHD8**	COAD/R	Helicases	276	69	343	688	166
**SFMBT1**	COAD/R	HM_r	237	199	249	685	167
**CTCF**	COAD/R	Others	357	69	257	683	168
**DNMT3A**	COAD/R	DM_w	333	69	280	682	169
**BRPF3**	COAD/R	HA_r	302	69	308	679	170
**MLLT10**	COAD/R	HM_w	327	69	283	679	171
**HDAC4**	COAD/R	HA_e	272	174	232	678	172
**ING3**	COAD/R	HM_r	114	171	393	678	173
**TET3**	COAD/R	DM_e	347	69	261	677	174
**BRWD1**	COAD/R	HA_r	398	235	42	675	175
**TDRD6**	COAD/R	Others	361	135	178	674	176
**TET2**	COAD/R	DM_e	313	192	169	674	177
**GTF3C4**	COAD/R	HA_w	162	226	284	672	178
**EED**	COAD/R	ChRC	125	231	315	671	179
**FBXO17**	COAD/R	Others	68	325	277	670	180
**KMT2A**	COAD/R	HM_w	410	218	42	670	181
**CBX2**	COAD/R	ChRC	171	330	166	667	182
**KDM6A**	COAD/R	HM_e	136	345	185	666	183
**UBR7**	COAD/R	Others	86	190	389	665	184
**PRMT3**	COAD/R	HM_w	151	202	311	664	185
**EPC2**	COAD/R	Others	69	230	364	663	186
**GTF2F1**	COAD/R	Others	298	323	42	663	187
**ING4**	COAD/R	HM_r	113	317	231	661	188
**ORC1**	COAD/R	Others	360	160	140	660	189
**DOT1L**	COAD/R	HM_w	167	326	165	658	190
**MORF4L1**	COAD/R	HM_r	159	163	335	657	191
**PHF7**	COAD/R	Others	244	261	152	657	192

**BRWD3**	COAD/R	HA_r	384	69	200	653	193
**INTS12**	COAD/R	Others	112	222	313	647	194
**PAF1**	COAD/R	Others	105	304	238	647	195
**HDGF**	COAD/R	Others	62	269	314	645	196
**PHC2**	COAD/R	ChRC	323	69	253	645	197
**PYGO1**	COAD/R	HM_r	240	255	148	643	198
**SHPRH**	COAD/R	Others	351	250	42	643	199
**NAP1L3**	COAD/R	Others	339	162	141	642	200
**EZH2**	COAD/R	HM_w	300	69	269	638	201
**EP400**	COAD/R	HA_w	417	178	42	637	202
**CBX4**	COAD/R	HM_r	73	329	234	636	203
**ZMYND11**	COAD/R	HA_r	260	334	42	636	204
**KDM1B**	COAD/R	HM_e	256	69	309	634	205
**KDM5C**	COAD/R	HM_e	340	69	224	633	206
**PBRM1**	COAD/R	HA_r	324	263	42	629	207
**BMI1**	COAD/R	ChRC	174	184	270	628	208
**SCMH1**	COAD/R	Others	196	252	180	628	209
**SCML2**	COAD/R	HM_r	382	69	177	628	210
**EP300**	COAD/R	HA_w	406	179	42	627	211
**TAF1**	COAD/R	HA_r	380	69	176	625	212
**SMYD3**	COAD/R	HM_w	193	69	361	623	213
**SMYD5**	COAD/R	HM_w	230	69	324	623	214
**PHIP**	COAD/R	HA_r	375	205	42	622	215
**EZH1**	COAD/R	HM_w	273	69	278	620	216
**PRDM7**	COAD/R	HM_w	177	258	183	618	217
**ASXL2**	COAD/R	Others	306	69	241	616	218
**MBTD1**	COAD/R	Others	53	308	254	615	219
**RPS6KA5**	COAD/R	Others	238	253	123	614	220
**AURKB**	COAD/R	Others	133	363	117	613	221
**NAP1L2**	COAD/R	Others	282	213	118	613	222
**PRDM11**	COAD/R	HM_w	242	154	217	613	223
**KMT2B**	COAD/R	HM_w	402	166	42	610	224
**JADE1**	COAD/R	Others	408	69	130	607	225
**PRKAA2**	COAD/R	Others	321	69	216	606	226
**TDRD10**	COAD/R	Others	227	244	135	606	227

**SIRT1**	COAD/R	HA_e	236	198	171	605	228
**HDGFL1**	COAD/R	Others	180	319	104	603	229
**ING5**	COAD/R	HM_r	58	223	322	603	230
**MBD4**	COAD/R	DM_r	207	69	326	602	231
**SMARCD1**	COAD/R	Helicases	232	69	301	602	232
**JADE3**	COAD/R	Others	284	69	247	600	233
**RPH3A**	COAD/R	Others	353	148	98	599	234
**KDM6B**	COAD/R	HM_e	185	371	42	598	235
**PSIP1**	COAD/R	HM_r	199	357	42	598	236
**PCGF6**	COAD/R	Others	48	343	206	597	237
**SP100**	COAD/R	HA_r	192	139	262	593	238
**BRD2**	COAD/R	HA_r	173	69	349	591	239
**AFF1**	COAD/R	Others	307	241	42	590	240
**SETMAR**	COAD/R	HM_w	90	296	204	590	241
**HDAC5**	COAD/R	HA_e	214	69	306	589	242
**FBXW9**	COAD/R	Others	29	273	285	587	243
**CBX5**	COAD/R	HM_r	130	234	221	585	244
**BAZ2A**	COAD/R	HA_r	305	237	42	584	245
**AFF4**	COAD/R	Others	255	285	42	582	246
**PRDM9**	COAD/R	HM_w	414	69	99	582	247
**RPA3**	COAD/R	Others	17	201	362	580	248
**GLYR1**	COAD/R	HM_r	309	228	42	579	249
**PRDM15**	COAD/R	HM_w	285	152	142	579	250
**SIRT3**	COAD/R	HA_e	16	294	265	575	251
**PRDM13**	COAD/R	HM_w	97	300	173	570	252
**PRDM5**	COAD/R	HM_w	200	259	111	570	253
**BRD8**	COAD/R	HA_r	278	69	222	569	254
**CLOCK**	COAD/R	HA_w	275	182	112	569	255
**SP110**	COAD/R	HA_r	229	138	202	569	256
**TRIM28**	COAD/R	HA_r	137	133	299	569	257
**PHC3**	COAD/R	ChRC	101	158	304	563	258
**ACTL6B**	COAD/R	ChRC	183	286	93	562	259
**PHF8**	COAD/R	Others	308	69	184	561	260
**BRPF1**	COAD/R	HA_r	335	183	42	560	261
**HDAC7**	COAD/R	HA_e	271	69	219	559	262

**RSF1**	COAD/R	ChRC	262	69	227	558	263
**HDAC1**	COAD/R	HA_e	120	69	368	557	264
**MBD3**	COAD/R	DM_r	54	310	192	556	265
**AICDA**	COAD/R	DM_e	254	187	113	554	266
**TCF20**	COAD/R	Others	374	136	42	552	267
**PADI1**	COAD/R	Others	245	212	94	551	268
**PRDM1**	COAD/R	HM_w	264	69	218	551	269
**HDAC10**	COAD/R	HA_e	24	372	154	550	270
**MTA1**	COAD/R	ChRC	52	306	191	549	271
**RBBP7**	COAD/R	ChRC	281	69	199	549	272
**DPF2**	COAD/R	Others	274	232	42	548	273
**ATRX**	COAD/R	Helicases	310	69	168	547	274
**HLTF**	COAD/R	Others	270	69	208	547	275
**PRDM4**	COAD/R	HM_w	153	69	325	547	276
**NCOR2**	COAD/R	Others	259	69	213	541	277
**PADI3**	COAD/R	Others	204	210	126	540	278
**IWS1**	COAD/R	Others	111	69	358	538	279
**KDM4A**	COAD/R	HM_e	328	167	42	537	280
**PHF19**	COAD/R	HM_r	202	69	266	537	281
**TDG**	COAD/R	ChRC	138	69	330	537	282
**NAP1L1**	COAD/R	Others	51	214	268	533	283
**SMARCD3**	COAD/R	Helicases	261	69	203	533	284
**SP140L**	COAD/R	HA_r	191	69	271	531	285
**TDRD1**	COAD/R	Others	348	69	114	531	286
**KAT5**	COAD/R	HA_w	268	220	42	530	287
**CDYL2**	COAD/R	HM_r	250	69	210	529	288
**DAXX**	COAD/R	ChRC	127	69	332	528	289
**ELP4**	COAD/R	HA_w	164	69	295	528	290
**KDM7A**	COAD/R	HM_e	81	69	378	528	291
**SIRT4**	COAD/R	HA_e	195	69	264	528	292
**SIRT7**	COAD/R	HA_e	41	293	189	523	293
**KMT2D**	COAD/R	HM_w	411	69	42	522	294
**DPF1**	COAD/R	ChRC	70	275	175	520	295
**SP140**	COAD/R	HA_r	315	69	136	520	296
**DPF3**	COAD/R	HA_r	217	180	121	518	297

**SETD2**	COAD/R	HM_w	405	69	42	516	298
**EHMT2**	COAD/R	HM_w	166	69	279	514	299
**KDM8**	COAD/R	HM_e	78	344	92	514	300
**BRDT**	COAD/R	HA_r	343	69	100	512	301
**ZCWPW2**	COAD/R	HM_r	311	69	132	512	302
**SUV39H2**	COAD/R	HM_w	141	69	300	510	303
**FKBP5**	COAD/R	Others	123	229	156	508	304
**HIF1AN**	COAD/R	Others	117	349	42	508	305
**KDM1A**	COAD/R	HM_e	247	219	42	508	306
**UHRF2**	COAD/R	DM_r	84	381	42	507	307
**FKBP1A**	COAD/R	Others	28	69	407	504	308
**UBE2E1**	COAD/R	Others	187	69	248	504	309
**WDR5**	COAD/R	ChRC	82	69	352	503	310
**ING2**	COAD/R	HM_r	59	401	42	502	311
**FBXO44**	COAD/R	Others	67	274	158	499	312
**PRMT5**	COAD/R	HM_w	150	69	273	492	313
**CHD2**	COAD/R	Helicases	379	69	42	490	314
**NSD1**	COAD/R	HM_w	377	69	42	488	315
**TDRD9**	COAD/R	Others	189	194	102	485	316
**AEBP2**	COAD/R	HM_w	75	365	42	482	317
**ARID1B**	COAD/R	ChRC	371	69	42	482	318
**DPY30**	COAD/R	Others	126	69	286	481	319
**BAZ1A**	COAD/R	HA_r	252	185	42	479	320
**KDM2A**	COAD/R	HM_e	267	170	42	479	321
**PRKCD**	COAD/R	Others	152	203	124	479	322
**AKAP1**	COAD/R	Others	33	69	376	478	323
**KDM3B**	COAD/R	HM_e	367	69	42	478	324
**PCGF1**	COAD/R	Others	104	69	305	478	325
**SMARCA5**	COAD/R	Helicases	288	141	42	471	326
**PPARGC1A**	COAD/R	Others	292	69	108	469	327
**UBE2B**	COAD/R	Others	88	191	188	467	328
**RING1**	COAD/R	Others	93	69	302	464	329
**IDH1**	COAD/R	DM_e	211	69	182	462	330
**MTA2**	COAD/R	ChRC	205	215	42	462	331
**DNMT3L**	COAD/R	DM_w	79	276	105	460	332

**PHF21B**	COAD/R	HM_r	20	301	139	460	333
**BAP1**	COAD/R	Others	132	282	42	456	334
**USP51**	COAD/R	Others	176	69	211	456	335
**KDM5D**	COAD/R	HM_e	344	69	42	455	336
**GADD45B**	COAD/R	Others	25	272	155	452	337
**PHF13**	COAD/R	Others	46	359	42	447	338
**PRMT6**	COAD/R	HM_w	149	256	42	447	339
**TDRD12**	COAD/R	Others	13	337	97	447	340
**UBE2A**	COAD/R	Others	134	69	243	446	341
**TAF3**	COAD/R	HA_r	228	69	144	441	342
**CBX1**	COAD/R	HM_r	31	69	337	437	343
**SETD6**	COAD/R	HM_w	42	145	250	437	344
**GATAD2A**	COAD/R	HM_r	122	271	42	435	345
**HDAC8**	COAD/R	HA_e	23	224	186	433	346
**KDM4D**	COAD/R	HM_e	109	69	255	433	347
**PRDM12**	COAD/R	HM_w	154	153	125	432	348
**SMNDC1**	COAD/R	Others	14	246	170	430	349
**HAT1**	COAD/R	HA_w	121	69	239	429	350
**HIRA**	COAD/R	Others	213	173	42	428	351
**MSL3**	COAD/R	HA_w	135	69	223	427	352
**SATB1**	COAD/R	Others	197	69	161	427	353
**HNF1A**	COAD/R	ChRC	212	172	42	426	354
**SMYD1**	COAD/R	HM_w	231	69	122	422	355
**PHF10**	COAD/R	Others	100	69	252	421	356
**CBX7**	COAD/R	HM_r	129	69	220	418	357
**AIRE**	COAD/R	HM_r	74	240	103	417	358
**PHF5A**	COAD/R	Others	99	155	162	416	359
**UHRF1**	COAD/R	DM_r	85	287	42	414	360
**MTA3**	COAD/R	ChRC	106	264	42	412	361
**KDM4B**	COAD/R	HM_e	56	313	42	411	362
**ATAT1**	COAD/R	Others	80	69	260	409	363
**SIRT6**	COAD/R	HA_e	15	247	146	408	364
**PCGF5**	COAD/R	Others	102	262	42	406	365
**H2AFZ**	COAD/R	Others	65	175	164	404	366
**PRDM6**	COAD/R	HM_w	19	341	42	402	367

**POLE3**	COAD/R	ChRC	98	69	228	395	368
**L3MBTL3**	COAD/R	Others	210	69	115	394	369
**PRMT2**	COAD/R	HM_w	95	257	42	394	370
**SETD4**	COAD/R	HM_w	145	69	179	393	371
**DMAP1**	COAD/R	Others	71	181	138	390	372
**PADI2**	COAD/R	Others	50	211	127	388	373
**HDAC2**	COAD/R	HA_e	119	225	42	386	374
**HIST1H1C**	COAD/R	Others	116	69	195	380	375
**MARCH5**	COAD/R	Others	55	266	42	363	376
**RNF217**	COAD/R	Others	146	69	147	362	377
**FKBP2**	COAD/R	Others	27	177	157	361	378
**PARP2**	COAD/R	Others	157	159	42	358	379
**TRIM66**	COAD/R	HA_r	12	132	214	358	380
**PRDM8**	COAD/R	HM_w	96	151	110	357	381
**CHAF1B**	COAD/R	ChRC	170	69	116	355	382
**POLR2B**	COAD/R	Others	243	69	42	354	383
**PRMT7**	COAD/R	HM_w	148	69	137	354	384
**PHRF1**	COAD/R	Others	45	260	42	347	385
**PWWP2B**	COAD/R	Others	43	150	149	342	386
**UTY**	COAD/R	HM_e	224	69	42	335	387
**SETD7**	COAD/R	HM_w	144	144	42	330	388
**PRMT1**	COAD/R	HM_w	44	69	215	328	389
**EHMT1**	COAD/R	HM_w	216	69	42	327	390
**G2E3**	COAD/R	Others	215	69	42	326	391
**HIST1H1B**	COAD/R	Others	161	69	96	326	392
**PHF1**	COAD/R	HM_r	47	69	205	321	393
**WDR82**	COAD/R	Others	36	243	42	321	394
**KDM4E**	COAD/R	HM_e	21	69	225	315	395
**MEN1**	COAD/R	ChRC	108	164	42	314	396
**RAG2**	COAD/R	HM_r	76	69	167	312	397
**HDAC11**	COAD/R	HA_e	63	69	174	306	398
**CBX6**	COAD/R	HM_r	72	69	160	301	399
**SSRP1**	COAD/R	Others	190	69	42	301	400
**PADI4**	COAD/R	Others	49	209	42	300	401
**ASH2L**	COAD/R	HM_w	175	69	42	286	402

**GTF2B**	COAD/R	Others	66	176	42	284	403
**SETD1B**	COAD/R	HM_w	91	147	42	280	404
**EPC1**	COAD/R	Others	163	69	42	274	405
**MTF2**	COAD/R	HM_r	158	69	42	269	406
**YY1**	COAD/R	ChRC	35	189	42	266	407
**TCF19**	COAD/R	Others	37	69	143	249	408
**HDAC3**	COAD/R	HA_e	118	69	42	229	409
**HIST1H3B**	COAD/R	Others	61	69	95	225	410
**GADD45A**	COAD/R	Others	26	69	120	215	411
**JMJD8**	COAD/R	HM_e	22	69	106	197	412
**PHF6**	COAD/R	HM_r	77	69	42	188	413
**HSPBAP1**	COAD/R	Others	60	69	42	171	414
**RBBP4**	COAD/R	ChRC	18	69	42	129	415
**USP27X**	COAD/R	Others	11	69	42	122	416
**IWS1**	DLBC	Others	384	400	417	1201	1
**BAZ2B**	DLBC	HA_r	411	404	383	1198	2
**PRDM1**	DLBC	HM_w	401	425	349	1175	3
**ERCC5**	DLBC	Others	404	379	380	1163	4
**FXR2**	DLBC	Others	348	378	426	1152	5
**SMARCA2**	DLBC	Helicases	399	350	387	1136	6
**BAZ2A**	DLBC	HA_r	361	332	424	1117	7
**KDM4C**	DLBC	HM_e	380	369	365	1114	8
**ARID2**	DLBC	ChRC	369	390	334	1093	9
**CXXC1**	DLBC	Others	392	380	318	1090	10
**ATR**	DLBC	Others	364	388	331	1083	11
**FKBP5**	DLBC	Others	388	316	379	1083	12
**KDM2B**	DLBC	HM_e	413	303	367	1083	13
**HDAC4**	DLBC	HA_e	387	314	377	1078	14
**MBD1**	DLBC	DM_r	339	366	360	1065	15
**SRCAP**	DLBC	Others	373	348	341	1062	16
**JMJD1C**	DLBC	HM_e	382	307	370	1059	17
**BRD2**	DLBC	HA_r	303	421	327	1051	18
**PBRM1**	DLBC	HA_r	332	363	354	1049	19
**SETD1B**	DLBC	HM_w	415	355	274	1044	20
**CBX3**	DLBC	HM_r	393	326	321	1040	21

**CBX5**	DLBC	HM_r	299	325	412	1036	22
**HNF1A**	DLBC	ChRC	421	309	305	1035	23
**AURKB**	DLBC	Others	362	334	330	1026	24
**MBD5**	DLBC	DM_r	250	417	359	1026	25
**EP400**	DLBC	HA_w	390	317	314	1021	26
**KAT6A**	DLBC	HA_w	344	306	369	1019	27
**HIST1H1C**	DLBC	Others	424	373	216	1013	28
**ING1**	DLBC	HM_r	270	371	372	1013	29
**EPC2**	DLBC	Others	285	414	313	1012	30
**TP53BP1**	DLBC	Others	193	405	414	1012	31
**PHC3**	DLBC	ChRC	238	362	408	1008	32
**ASXL2**	DLBC	Others	412	337	251	1000	33
**CHD1L**	DLBC	Helicases	293	383	319	995	34
**RNF217**	DLBC	Others	218	423	345	986	35
**HIST1H3B**	DLBC	Others	385	372	215	972	36
**BAZ1B**	DLBC	HA_r	304	333	329	966	37
**SCML4**	DLBC	Others	376	422	167	965	38
**HDAC8**	DLBC	HA_e	274	312	375	961	39
**ARID1B**	DLBC	ChRC	308	391	253	952	40
**KDM4D**	DLBC	HM_e	257	299	396	952	41
**ASH1L**	DLBC	HM_w	307	389	252	948	42
**CHD5**	DLBC	Helicases	408	416	124	948	43
**ING3**	DLBC	HM_r	269	308	371	948	44
**MBD2**	DLBC	DM_r	114	413	421	948	45
**KDM3A**	DLBC	HM_e	343	302	298	943	46
**PRDM8**	DLBC	HM_w	325	269	347	941	47
**ACTL6B**	DLBC	ChRC	188	341	402	931	48
**DAXX**	DLBC	ChRC	291	322	317	930	49
**BRD1**	DLBC	HA_r	395	403	131	929	50
**CDYL2**	DLBC	HM_r	296	384	238	918	51
**ATAD2B**	DLBC	HA_r	184	336	384	904	52
**AKAP1**	DLBC	Others	309	339	254	902	53
**PRDM16**	DLBC	HM_w	400	412	86	898	54
**KIAA2026**	DLBC	Others	120	367	410	897	55
**ATM**	DLBC	Others	425	335	133	893	56

**HDGF**	DLBC	Others	140	374	374	888	57
**ELP4**	DLBC	HA_w	158	318	411	887	58
**HR**	DLBC	HM_e	273	401	213	887	59
**CARM1**	DLBC	HM_w	175	385	323	883	60
**GATAD2B**	DLBC	HM_r	281	376	223	880	61
**SMYD3**	DLBC	HM_w	204	408	266	878	62
**TDRD6**	DLBC	Others	372	244	262	878	63
**EED**	DLBC	ChRC	159	319	399	877	64
**NCOR2**	DLBC	Others	403	284	189	876	65
**RBBP7**	DLBC	ChRC	220	265	390	875	66
**SMARCC2**	DLBC	Helicases	206	255	405	866	67
**CHD7**	DLBC	Helicases	354	382	123	859	68
**CHD3**	DLBC	Helicases	409	323	126	858	69
**KMT2D**	DLBC	HM_w	426	69	363	858	70
**SETD2**	DLBC	HM_w	57	394	406	857	71
**HDAC10**	DLBC	HA_e	145	402	307	854	72
**ZCWPW1**	DLBC	HM_r	192	236	425	853	73
**RNF2**	DLBC	ChRC	65	395	389	849	74
**ATAD2**	DLBC	HA_r	366	69	413	848	75
**ARID4A**	DLBC	ChRC	368	338	135	841	76
**PYGO2**	DLBC	HM_r	69	356	415	840	77
**KDM6B**	DLBC	HM_e	342	297	199	838	78
**PHF7**	DLBC	Others	85	358	394	837	79
**DPF3**	DLBC	HA_r	287	320	229	836	80
**HDAC7**	DLBC	HA_e	347	69	418	834	81
**BRD9**	DLBC	HA_r	177	329	325	831	82
**HDAC6**	DLBC	HA_e	141	313	376	830	83
**MSH6**	DLBC	HM_r	247	288	293	828	84
**SETDB1**	DLBC	HM_w	54	354	420	828	85
**KDM5C**	DLBC	HM_e	254	368	201	823	86
**HELLS**	DLBC	Helicases	138	311	373	822	87
**BRPF3**	DLBC	HA_r	359	328	130	817	88
**EHMT2**	DLBC	HM_w	352	415	42	809	89
**EHMT1**	DLBC	HM_w	353	69	381	803	90
**PHF20L1**	DLBC	HM_r	90	359	352	801	91

**SMARCD1**	DLBC	Helicases	44	349	404	797	92
**FMR1**	DLBC	Others	349	69	378	796	93
**PHIP**	DLBC	HA_r	328	426	42	796	94
**CHD9**	DLBC	Helicases	292	381	121	794	95
**PHF2**	DLBC	Others	329	279	184	792	96
**KMT2C**	DLBC	HM_w	423	69	297	789	97
**SMARCA4**	DLBC	Helicases	375	256	158	789	98
**CHAF1B**	DLBC	ChRC	295	69	423	787	99
**PRDM10**	DLBC	HM_w	230	273	284	787	100
**PRKAA1**	DLBC	Others	324	69	393	786	101
**PHF10**	DLBC	Others	237	361	186	784	102
**AFF1**	DLBC	Others	186	340	255	781	103
**AFF4**	DLBC	Others	310	69	401	780	104
**PCMT1**	DLBC	Others	94	398	287	779	105
**DPF2**	DLBC	Others	288	69	419	776	106
**KMT2E**	DLBC	HM_w	119	294	362	775	107
**MBD4**	DLBC	DM_r	112	365	296	773	108
**PRMT7**	DLBC	HM_w	225	268	280	773	109
**SHPRH**	DLBC	Others	320	410	42	772	110
**ASXL1**	DLBC	Others	367	69	333	769	111
**GLYR1**	DLBC	HM_r	280	375	112	767	112
**ATF7IP**	DLBC	Others	365	69	332	766	113
**BRD4**	DLBC	HA_r	394	330	42	766	114
**CDYL**	DLBC	HM_r	297	69	400	766	115
**KDM4B**	DLBC	HM_e	258	300	204	762	116
**KDM5B**	DLBC	HM_e	255	399	104	758	117
**PRKCD**	DLBC	Others	226	357	175	758	118
**BPTF**	DLBC	HA_r	360	69	328	757	119
**BRWD3**	DLBC	HA_r	300	327	129	756	120
**RNF40**	DLBC	Others	323	262	169	754	121
**KAT2A**	DLBC	HA_w	381	69	301	751	122
**KDM6A**	DLBC	HM_e	253	298	200	751	123
**MSL3**	DLBC	HA_w	106	287	357	750	124
**PHRF1**	DLBC	Others	327	69	350	746	125
**GTF3C4**	DLBC	HA_w	278	69	397	744	126

**TDRD5**	DLBC	Others	194	406	144	744	127
**SFMBT2**	DLBC	Others	212	257	271	740	128
**ASXL3**	DLBC	Others	419	69	250	738	129
**HDAC2**	DLBC	HA_e	275	420	42	737	130
**SETD1A**	DLBC	HM_w	322	69	343	734	131
**KAT8**	DLBC	HA_w	127	305	299	731	132
**BRD7**	DLBC	HA_r	302	386	42	730	133
**FBXW9**	DLBC	Others	350	69	311	730	134
**INO80**	DLBC	Helicases	267	419	42	728	135
**MLLT6**	DLBC	HM_w	338	290	99	727	136
**PARP1**	DLBC	Others	241	69	416	726	137
**SMYD1**	DLBC	HM_w	398	253	74	725	138
**MTA2**	DLBC	ChRC	246	69	409	724	139
**RAI1**	DLBC	Others	377	69	278	724	140
**BRWD1**	DLBC	HA_r	410	69	243	722	141
**CREBBP**	DLBC	HA_w	418	69	234	721	142
**NCOA1**	DLBC	HA_w	245	285	191	721	143
**PHF21B**	DLBC	HM_r	89	278	351	718	144
**ING2**	DLBC	HM_r	135	370	212	717	145
**DNMT3A**	DLBC	DM_w	162	321	232	715	146
**MECOM**	DLBC	Others	248	364	101	713	147
**SUV39H1**	DLBC	HM_w	198	249	264	711	148
**TDRKH**	DLBC	Others	27	345	337	709	149
**GATAD2A**	DLBC	HM_r	282	377	42	701	150
**PHF14**	DLBC	Others	236	280	185	701	151
**TET1**	DLBC	DM_e	371	69	261	701	152
**UBE2A**	DLBC	Others	397	241	63	701	153
**PRDM12**	DLBC	HM_w	80	272	348	700	154
**SMARCD2**	DLBC	Helicases	43	254	403	700	155
**UHRF2**	DLBC	DM_r	312	344	42	698	156
**KDM3B**	DLBC	HM_e	260	69	366	695	157
**NSD1**	DLBC	HM_w	334	69	291	694	158
**DNMT3L**	DLBC	DM_w	391	69	231	691	159
**PHF3**	DLBC	Others	232	276	183	691	160
**SMYD2**	DLBC	HM_w	205	409	73	687	161

**EZH2**	DLBC	HM_w	389	69	227	685	162
**PRDM13**	DLBC	HM_w	79	424	179	682	163
**PYGO1**	DLBC	HM_r	222	69	391	682	164
**HDAC1**	DLBC	HA_e	146	315	220	681	165
**LBR**	DLBC	Others	251	69	361	681	166
**ATRX**	DLBC	Helicases	363	69	248	680	167
**KMT2B**	DLBC	HM_w	340	295	42	677	168
**PRDM15**	DLBC	HM_w	326	69	282	677	169
**NCOA3**	DLBC	HA_w	416	69	190	675	170
**DNMT3B**	DLBC	DM_w	290	69	315	674	171
**HDAC9**	DLBC	HA_e	386	69	218	673	172
**SETD4**	DLBC	HM_w	215	69	388	672	173
**KDM1A**	DLBC	HM_e	261	304	105	670	174
**CBX2**	DLBC	ChRC	358	69	242	669	175
**PADI1**	DLBC	Others	243	69	356	668	176
**PCGF5**	DLBC	Others	96	283	288	667	177
**PRDM9**	DLBC	HM_w	420	69	177	666	178
**PARP2**	DLBC	Others	240	69	355	664	179
**GADD45B**	DLBC	Others	283	69	309	661	180
**PHC1**	DLBC	ChRC	239	69	353	661	181
**CHD6**	DLBC	Helicases	355	69	236	660	182
**JARID2**	DLBC	ChRC	383	69	208	660	183
**PADI2**	DLBC	Others	402	69	188	659	184
**RNF17**	DLBC	Others	219	263	170	652	185
**TAF1L**	DLBC	HA_r	196	69	386	651	186
**SSRP1**	DLBC	Others	316	69	265	650	187
**L3MBTL4**	DLBC	Others	252	293	102	647	188
**FBXO44**	DLBC	Others	351	69	225	645	189
**BMI1**	DLBC	ChRC	181	331	132	644	190
**IDH1**	DLBC	DM_e	271	69	304	644	191
**TET3**	DLBC	DM_e	314	69	260	643	192
**SMYD4**	DLBC	HM_w	318	252	72	642	193
**TDRD10**	DLBC	Others	32	347	263	642	194
**GTF2H1**	DLBC	Others	149	69	422	640	195
**NAP1L3**	DLBC	Others	379	69	192	640	196

**PWWP2B**	DLBC	Others	223	69	346	638	197
**L3MBTL3**	DLBC	Others	116	418	103	637	198
**SETD6**	DLBC	HM_w	213	258	166	637	199
**JADE2**	DLBC	Others	264	69	302	635	200
**RBBP5**	DLBC	ChRC	67	396	171	634	201
**PRDM14**	DLBC	HM_w	78	271	283	632	202
**KAT2B**	DLBC	HA_w	262	69	300	631	203
**HLTF**	DLBC	Others	345	69	214	628	204
**ACTL6A**	DLBC	ChRC	189	392	42	623	205
**BAZ1A**	DLBC	HA_r	305	69	247	621	206
**CHAF1A**	DLBC	ChRC	169	324	128	621	207
**FKBP1A**	DLBC	Others	153	69	398	620	208
**ARID1A**	DLBC	ChRC	414	69	136	619	209
**CHRAC1**	DLBC	ChRC	168	69	382	619	210
**KDM8**	DLBC	HM_e	121	296	198	615	211
**BRDT**	DLBC	HA_r	301	69	244	614	212
**MBTD1**	DLBC	Others	249	69	295	613	213
**BAP1**	DLBC	Others	182	387	42	611	214
**CBX6**	DLBC	HM_r	298	69	241	608	215
**KMT2A**	DLBC	HM_w	341	69	197	607	216
**KDM4A**	DLBC	HM_e	259	301	42	602	217
**CHD1**	DLBC	Helicases	294	69	237	600	218
**MTA1**	DLBC	ChRC	337	69	194	600	219
**ARID4B**	DLBC	ChRC	396	69	134	599	220
**CHD8**	DLBC	Helicases	407	69	122	598	221
**HIST1H1B**	DLBC	Others	417	69	109	595	222
**SMARCC1**	DLBC	Helicases	45	393	156	594	223
**DIDO1**	DLBC	Others	406	69	118	593	224
**EP300**	DLBC	HA_w	405	69	116	590	225
**PHF19**	DLBC	HM_r	235	69	286	590	226
**AICDA**	DLBC	DM_e	185	69	335	589	227
**TCF19**	DLBC	Others	36	407	146	589	228
**DOT1L**	DLBC	HM_w	289	69	230	588	229
**SETD3**	DLBC	HM_w	56	259	273	588	230
**PHC2**	DLBC	ChRC	331	69	187	587	231

**PHF21A**	DLBC	HM_r	233	69	285	587	232
**PHF13**	DLBC	Others	91	397	91	579	233
**PRDM4**	DLBC	HM_w	228	69	281	578	234
**AIRE**	DLBC	HM_r	370	69	137	576	235
**BRD8**	DLBC	HA_r	178	69	326	573	236
**PRMT8**	DLBC	HM_w	224	69	279	572	237
**BRPF1**	DLBC	HA_r	176	69	324	569	238
**RBBP4**	DLBC	ChRC	221	266	82	569	239
**SFMBT1**	DLBC	HM_r	52	352	164	568	240
**H3F3A**	DLBC	Others	277	69	221	567	241
**KAT6B**	DLBC	HA_w	129	69	368	566	242
**CBX1**	DLBC	HM_r	174	69	322	565	243
**PADI3**	DLBC	Others	101	69	395	565	244
**RPS6KA5**	DLBC	Others	217	69	276	562	245
**RSF1**	DLBC	ChRC	216	69	275	560	246
**CECR2**	DLBC	HA_r	170	69	320	559	247
**TET2**	DLBC	DM_e	422	69	66	557	248
**KDM7A**	DLBC	HM_e	122	69	364	555	249
**SETMAR**	DLBC	HM_w	321	69	165	555	250
**CHD2**	DLBC	Helicases	357	69	127	553	251
**RTF1**	DLBC	Others	61	411	79	551	252
**CHD4**	DLBC	Helicases	356	69	125	550	253
**PRMT3**	DLBC	HM_w	73	69	407	549	254
**DNMT1**	DLBC	DM_w	163	69	316	548	255
**JADE1**	DLBC	Others	265	69	210	544	256
**PHF5A**	DLBC	Others	87	275	182	544	257
**SMARCA1**	DLBC	Helicases	207	69	267	543	258
**KANSL1**	DLBC	HA_w	263	69	207	539	259
**PHF8**	DLBC	Others	84	274	181	539	260
**MEN1**	DLBC	ChRC	110	69	358	537	261
**SP140L**	DLBC	HA_r	317	69	151	537	262
**FBXL19**	DLBC	Others	155	69	312	536	263
**PRMT1**	DLBC	HM_w	74	69	392	535	264
**FKBP2**	DLBC	Others	152	69	310	531	265
**TAF1**	DLBC	HA_r	315	69	147	531	266

**PRMT2**	DLBC	HM_w	378	69	83	530	267
**KDM4E**	DLBC	HM_e	256	69	203	528	268
**TRIM28**	DLBC	HA_r	25	242	259	526	269
**H2AFZ**	DLBC	Others	148	69	308	525	270
**STK31**	DLBC	Others	200	250	70	520	271
**ZCWPW2**	DLBC	HM_r	311	69	140	520	272
**HDAC5**	DLBC	HA_e	142	69	306	517	273
**ZGPAT**	DLBC	Others	191	69	256	516	274
**SP100**	DLBC	HA_r	374	69	71	514	275
**IDH2**	DLBC	DM_e	136	69	303	508	276
**MUM1**	DLBC	Others	336	69	98	503	277
**ATAT1**	DLBC	Others	183	69	249	501	278
**NCOR1**	DLBC	ChRC	335	69	97	501	279
**MLLT10**	DLBC	HM_w	109	291	100	500	280
**PADI6**	DLBC	Others	333	69	95	497	281
**SND1**	DLBC	HM_r	203	251	42	496	282
**BOP 1**	DLBC	Others	180	69	246	495	283
**PHF11**	DLBC	Others	92	360	42	494	284
**BRD3**	DLBC	HA_r	179	69	245	493	285
**ZMYND11**	DLBC	HA_r	11	342	139	492	286
**HIF1AN**	DLBC	Others	137	310	42	489	287
**TAF3**	DLBC	HA_r	195	248	42	485	288
**CBX7**	DLBC	HM_r	172	69	240	481	289
**CBX8**	DLBC	ChRC	171	69	239	479	290
**PRDM2**	DLBC	HM_w	229	69	178	476	291
**MECP2**	DLBC	DM_r	111	69	294	474	292
**SATB1**	DLBC	Others	60	69	344	473	293
**CLOCK**	DLBC	HA_w	167	69	235	471	294
**WDR5**	DLBC	ChRC	14	69	385	468	295
**DMAP1**	DLBC	Others	164	69	233	466	296
**G2E3**	DLBC	Others	284	69	113	466	297
**PHF1**	DLBC	HM_r	93	281	92	466	298
**NAP1L1**	DLBC	Others	103	69	292	464	299
**SMARCA5**	DLBC	Helicases	319	69	75	463	300
**DPY30**	DLBC	Others	160	69	228	457	301

**HIRA**	DLBC	Others	346	69	42	457	302
**PCGF1**	DLBC	Others	98	69	290	457	303
**HCFC1**	DLBC	Others	276	69	111	456	304
**PCGF2**	DLBC	Others	97	69	289	455	305
**SMARCE1**	DLBC	Helicases	41	69	342	452	306
**FBXO17**	DLBC	Others	154	69	226	449	307
**MARCH5**	DLBC	Others	115	292	42	449	308
**SETDB2**	DLBC	HM_w	53	353	42	448	309
**TRIM66**	DLBC	HA_r	313	69	64	446	310
**ING4**	DLBC	HM_r	268	69	108	445	311
**TDRD12**	DLBC	Others	31	346	68	445	312
**GADD45A**	DLBC	Others	151	69	224	444	313
**SIN3A**	DLBC	ChRC	51	351	42	444	314
**TCF20**	DLBC	Others	35	69	340	444	315
**SIN3B**	DLBC	ChRC	211	69	163	443	316
**TDG**	DLBC	ChRC	34	69	339	442	317
**GTF2B**	DLBC	Others	150	69	222	441	318
**PHF12**	DLBC	Others	330	69	42	441	319
**SIRT1**	DLBC	HA_e	210	69	162	441	320
**SIRT4**	DLBC	HA_e	209	69	161	439	321
**MPHOSPH8**	DLBC	HM_r	107	289	42	438	322
**SIRT7**	DLBC	HA_e	208	69	159	436	323
**TDRD7**	DLBC	Others	29	69	338	436	324
**MTA3**	DLBC	ChRC	105	286	42	433	325
**HDAC3**	DLBC	HA_e	143	69	219	431	326
**TRIM24**	DLBC	HA_r	26	69	336	431	327
**HDGFL1**	DLBC	Others	139	69	217	425	328
**SP110**	DLBC	HA_r	202	69	153	424	329
**TDRD1**	DLBC	Others	33	246	145	424	330
**SP140**	DLBC	HA_r	201	69	152	422	331
**PCGF6**	DLBC	Others	95	282	42	419	332
**SUPT16H**	DLBC	Others	199	69	150	418	333
**ASH2L**	DLBC	HM_w	306	69	42	417	334
**SUV39H2**	DLBC	HM_w	197	69	149	415	335
**ING5**	DLBC	HM_r	134	69	211	414	336

**JADE3**	DLBC	Others	133	69	209	411	337
**RING1**	DLBC	Others	66	264	81	411	338
**RNF20**	DLBC	Others	64	69	277	410	339
**ORC1**	DLBC	Others	244	69	96	409	340
**PHF23**	DLBC	HM_r	88	277	42	407	341
**KDM1B**	DLBC	HM_e	126	69	206	401	342
**KDM2A**	DLBC	HM_e	125	69	205	399	343
**SCMH1**	DLBC	Others	59	261	78	398	344
**WDR82**	DLBC	Others	13	343	42	398	345
**ELP3**	DLBC	HA_w	286	69	42	397	346
**SETD7**	DLBC	HM_w	55	69	272	396	347
**KDM5A**	DLBC	HM_e	124	69	202	395	348
**SCML2**	DLBC	HM_r	58	260	77	395	349
**AEBP2**	DLBC	HM_w	187	69	138	394	350
**PHF20**	DLBC	HM_r	234	69	90	393	351
**GTF2F1**	DLBC	Others	279	69	42	390	352
**YY1**	DLBC	ChRC	12	237	141	390	353
**POLR2B**	DLBC	Others	231	69	89	389	354
**PRDM6**	DLBC	HM_w	77	270	42	389	355
**SIRT2**	DLBC	HA_e	50	69	270	389	356
**SIRT3**	DLBC	HA_e	49	69	269	387	357
**SIRT5**	DLBC	HA_e	48	69	268	385	358
**HSPBAP1**	DLBC	Others	272	69	42	383	359
**L3MBTL1**	DLBC	HM_r	118	69	196	383	360
**L3MBTL2**	DLBC	Others	117	69	195	381	361
**PRDM5**	DLBC	HM_w	227	69	85	381	362
**PSIP1**	DLBC	HM_r	70	267	42	379	363
**INTS12**	DLBC	Others	266	69	42	377	364
**NAP1L2**	DLBC	Others	102	69	193	364	365
**SETD5**	DLBC	HM_w	214	69	76	359	366
**CSTL1**	DLBC	Others	166	69	120	355	367
**CTCF**	DLBC	Others	165	69	119	353	368
**PADI4**	DLBC	Others	242	69	42	353	369
**TCEA1**	DLBC	Others	37	247	69	353	370
**UBE2E1**	DLBC	Others	22	69	258	349	371

**DPF1**	DLBC	ChRC	161	69	117	347	372
**USP22**	DLBC	Others	18	69	257	344	373
**EPC1**	DLBC	Others	157	69	115	341	374
**EZH1**	DLBC	HM_w	156	69	114	339	375
**TDRD9**	DLBC	Others	28	243	67	338	376
**POLE3**	DLBC	ChRC	83	69	180	332	377
**HDAC11**	DLBC	HA_e	144	69	110	323	378
**PRKAA2**	DLBC	Others	75	69	176	320	379
**ZMYND8**	DLBC	HA_r	190	69	61	320	380
**TDRD3**	DLBC	HM_r	30	245	42	317	381
**USP51**	DLBC	Others	16	238	62	316	382
**PRMT5**	DLBC	HM_w	72	69	174	315	383
**PRMT6**	DLBC	HM_w	71	69	173	313	384
**RAG2**	DLBC	HM_r	68	69	172	309	385
**JMJD6**	DLBC	HM_e	132	69	107	308	386
**JMJD8**	DLBC	HM_e	131	69	106	306	387
**UHRF1**	DLBC	DM_r	19	240	42	301	388
**RPA3**	DLBC	Others	63	69	168	300	389
**USP27X**	DLBC	Others	17	239	42	298	390
**CBX4**	DLBC	HM_r	173	69	42	284	391
**SIRT6**	DLBC	HA_e	47	69	160	276	392
**SMARCB1**	DLBC	Helicases	46	69	157	272	393
**SMARCD3**	DLBC	Helicases	42	69	155	266	394
**PAF1**	DLBC	Others	100	69	94	263	395
**SMNDC1**	DLBC	Others	40	69	154	263	396
**PAXIP1**	DLBC	Others	99	69	93	261	397
**HAT1**	DLBC	HA_w	147	69	42	258	398
**SUZ12**	DLBC	ChRC	38	69	148	255	399
**KAT5**	DLBC	HA_w	130	69	42	241	400
**KAT7**	DLBC	HA_w	128	69	42	239	401
**PPARGC1A**	DLBC	Others	82	69	88	239	402
**PRDM11**	DLBC	HM_w	81	69	87	237	403
**UBE2B**	DLBC	Others	23	69	143	235	404
**KDM5D**	DLBC	HM_e	123	69	42	234	405
**UBR7**	DLBC	Others	20	69	142	231	406

**PRDM7**	DLBC	HM_w	76	69	84	229	407
**MBD3**	DLBC	DM_r	113	69	42	224	408
**MORF4L1**	DLBC	HM_r	108	69	42	219	409
**MTF2**	DLBC	HM_r	104	69	42	215	410
**RPH3A**	DLBC	Others	62	69	80	211	411
**PHF6**	DLBC	HM_r	86	69	42	197	412
**TRIM33**	DLBC	HA_r	24	69	65	158	413
**SMYD5**	DLBC	HM_w	39	69	42	150	414
**UBE2I**	DLBC	Others	21	69	42	132	415
**UTY**	DLBC	HM_e	15	69	42	126	416
**ATAD2**	ESCA	HA_r	402	423	421	1246	1
**DIDO1**	ESCA	Others	410	408	420	1238	2
**ATR**	ESCA	Others	391	417	422	1230	3
**KAT6A**	ESCA	HA_w	399	413	415	1227	4
**KDM2A**	ESCA	HM_e	404	399	387	1190	5
**PHF20L1**	ESCA	HM_r	350	418	418	1186	6
**HDAC9**	ESCA	HA_e	408	400	362	1170	7
**CHD7**	ESCA	Helicases	413	384	365	1162	8
**BRD9**	ESCA	HA_r	312	421	424	1157	9
**ASXL1**	ESCA	Others	414	327	406	1147	10
**BPTF**	ESCA	HA_r	389	354	389	1132	11
**KDM5A**	ESCA	HM_e	353	341	411	1105	12
**KMT2E**	ESCA	HM_w	407	411	285	1103	13
**PRKAA1**	ESCA	Others	293	407	403	1103	14
**PHC3**	ESCA	ChRC	253	425	423	1101	15
**ARID4B**	ESCA	ChRC	362	372	334	1068	16
**PHF14**	ESCA	Others	299	392	376	1067	17
**ZGPAT**	ESCA	Others	233	405	419	1057	18
**SETDB1**	ESCA	HM_w	290	373	393	1056	19
**CHD2**	ESCA	Helicases	401	353	292	1046	20
**ASH1L**	ESCA	HM_w	344	310	390	1044	21
**HCFC1**	ESCA	Others	306	380	355	1041	22
**STK31**	ESCA	Others	240	406	395	1041	23
**ZCWPW1**	ESCA	HM_r	234	415	391	1040	24
**PRDM16**	ESCA	HM_w	349	375	310	1034	25

**ACTL6A**	ESCA	ChRC	178	424	426	1028	26
**HDGF**	ESCA	Others	271	344	408	1023	27
**MBD4**	ESCA	DM_r	207	402	413	1022	28
**EP400**	ESCA	HA_w	417	212	388	1017	29
**EHMT1**	ESCA	HM_w	396	281	332	1009	30
**BAZ1B**	ESCA	HA_r	279	325	401	1005	31
**KDM6A**	ESCA	HM_e	375	414	215	1004	32
**SMARCA2**	ESCA	Helicases	384	389	228	1001	33
**CHD6**	ESCA	Helicases	341	352	291	984	34
**L3MBTL1**	ESCA	HM_r	303	366	315	984	35
**NCOA3**	ESCA	HA_w	371	397	211	979	36
**HLTF**	ESCA	Others	153	419	404	976	37
**KMT2C**	ESCA	HM_w	424	377	170	971	38
**PHF20**	ESCA	HM_r	250	361	359	970	39
**KMT2D**	ESCA	HM_w	426	267	267	960	40
**SRCAP**	ESCA	Others	406	249	305	960	41
**PCGF2**	ESCA	Others	255	363	338	956	42
**G2E3**	ESCA	Others	338	242	373	953	43
**TDRKH**	ESCA	Others	238	387	320	945	44
**EHMT2**	ESCA	HM_w	308	382	254	944	45
**BRD2**	ESCA	HA_r	278	386	277	941	46
**ZMYND8**	ESCA	HA_r	179	404	357	940	47
**TET3**	ESCA	DM_e	365	188	386	939	48
**PARP1**	ESCA	Others	301	263	372	936	49
**GATAD2A**	ESCA	HM_r	272	346	316	934	50
**CHD1L**	ESCA	Helicases	170	385	378	933	51
**ASH2L**	ESCA	HM_w	105	410	409	924	52
**DNMT1**	ESCA	DM_w	340	283	299	922	53
**CBX3**	ESCA	HM_r	99	403	416	918	54
**MECOM**	ESCA	Others	335	426	151	912	55
**DNMT3B**	ESCA	DM_w	223	323	364	910	56
**MPHOSPH8**	ESCA	HM_r	334	340	235	909	57
**MECP2**	ESCA	DM_r	259	365	284	908	58
**PRDM9**	ESCA	HM_w	416	409	80	905	59
**PRDM14**	ESCA	HM_w	249	360	283	892	60

**BRPF3**	ESCA	HA_r	228	370	293	891	61
**RSF1**	ESCA	ChRC	121	390	379	890	62
**ATF7IP**	ESCA	Others	382	326	181	889	63
**CHRAC1**	ESCA	ChRC	41	422	425	888	64
**ELP4**	ESCA	HA_w	222	322	340	884	65
**BAZ2A**	ESCA	HA_r	361	220	301	882	66
**BOP 1**	ESCA	Others	45	420	417	882	67
**CHD8**	ESCA	Helicases	412	214	256	882	68
**ATAD2B**	ESCA	HA_r	315	182	375	872	69
**RNF40**	ESCA	Others	246	254	369	869	70
**RPA3**	ESCA	Others	65	391	410	866	71
**SMARCD2**	ESCA	Helicases	188	311	367	866	72
**SMARCE1**	ESCA	Helicases	55	412	399	866	73
**EPC1**	ESCA	Others	273	303	289	865	74
**SMARCA5**	ESCA	Helicases	243	312	307	862	75
**IDH2**	ESCA	DM_e	266	379	216	861	76
**SMARCC2**	ESCA	Helicases	287	251	323	861	77
**PHF2**	ESCA	Others	394	69	394	857	78
**PHF3**	ESCA	Others	330	293	234	857	79
**KAT7**	ESCA	HA_w	262	298	296	856	80
**JMJD1C**	ESCA	HM_e	357	342	156	855	81
**MBD2**	ESCA	DM_r	208	398	249	855	82
**PAXIP1**	ESCA	Others	418	393	42	853	83
**EED**	ESCA	ChRC	167	350	333	850	84
**PRMT8**	ESCA	HM_w	328	316	206	850	85
**TDRD6**	ESCA	Others	366	388	96	850	86
**DAXX**	ESCA	ChRC	224	351	273	848	87
**CHD5**	ESCA	Helicases	378	396	73	847	88
**JARID2**	ESCA	ChRC	377	273	196	846	89
**EZH2**	ESCA	HM_w	164	302	377	843	90
**KMT2A**	ESCA	HM_w	422	268	153	843	91
**FMR1**	ESCA	Others	163	347	330	840	92
**KDM4A**	ESCA	HM_e	387	297	155	839	93
**SETD2**	ESCA	HM_w	369	69	400	838	94
**CSTL1**	ESCA	Others	169	324	342	835	95

**TCEA1**	ESCA	Others	184	328	321	833	96
**HDAC6**	ESCA	HA_e	216	345	271	832	97
**KMT2B**	ESCA	HM_w	395	394	42	831	98
**KAT2A**	ESCA	HA_w	212	319	297	828	99
**KDM3A**	ESCA	HM_e	305	236	287	828	100
**MBTD1**	ESCA	Others	206	294	327	827	101
**ASXL2**	ESCA	Others	280	285	258	823	102
**PYGO2**	ESCA	HM_r	128	315	380	823	103
**JMJD6**	ESCA	HM_e	150	320	352	822	104
**DPF1**	ESCA	ChRC	96	368	356	820	105
**BRPF1**	ESCA	HA_r	343	69	405	817	106
**ING3**	ESCA	HM_r	214	274	328	816	107
**TRIM28**	ESCA	HA_r	111	355	345	811	108
**PHF12**	ESCA	Others	202	260	348	810	109
**POLR2B**	ESCA	Others	198	230	381	809	110
**SETD1A**	ESCA	HM_w	291	253	262	806	111
**ING4**	ESCA	HM_r	151	300	354	805	112
**PHF13**	ESCA	Others	251	362	189	802	113
**CHD4**	ESCA	Helicases	275	304	220	799	114
**NCOR2**	ESCA	Others	419	69	311	799	115
**KAT6B**	ESCA	HA_w	355	271	172	798	116
**KDM5B**	ESCA	HM_e	261	269	268	798	117
**KANSL1**	ESCA	HA_w	263	172	361	796	118
**TAF1L**	ESCA	HA_r	368	69	358	795	119
**SETD1B**	ESCA	HM_w	325	159	309	793	120
**PRMT7**	ESCA	HM_w	130	331	325	786	121
**RPH3A**	ESCA	Others	326	228	231	785	122
**PCMT1**	ESCA	Others	138	261	382	781	123
**GATAD2B**	ESCA	HM_r	33	381	363	777	124
**AKAP1**	ESCA	Others	231	286	259	776	125
**KDM2B**	ESCA	HM_e	336	170	269	775	126
**IWS1**	ESCA	Others	264	238	270	772	127
**CBX6**	ESCA	HM_r	277	215	276	768	128
**EP300**	ESCA	HA_w	409	69	290	768	129
**BRD4**	ESCA	HA_r	380	69	318	767	130

**RNF17**	ESCA	Others	385	256	124	765	131
**PRMT5**	ESCA	HM_w	195	198	370	763	132
**NSD1**	ESCA	HM_w	403	317	42	762	133
**PAF1**	ESCA	Others	77	334	349	760	134
**MLLT6**	ESCA	HM_w	82	364	313	759	135
**KDM4D**	ESCA	HM_e	210	296	250	756	136
**SMYD2**	ESCA	HM_w	53	289	414	756	137
**BAZ1A**	ESCA	HA_r	101	309	344	754	138
**CBX8**	ESCA	ChRC	227	306	221	754	139
**ATAT1**	ESCA	Others	104	371	278	753	140
**MSH6**	ESCA	HM_r	372	69	312	753	141
**DNMT3A**	ESCA	DM_w	309	69	374	752	142
**ARID2**	ESCA	ChRC	363	69	319	751	143
**CHD9**	ESCA	Helicases	400	213	138	751	144
**H3F3A**	ESCA	Others	89	278	383	750	145
**MTA1**	ESCA	ChRC	143	265	339	747	146
**TCF20**	ESCA	Others	367	155	225	747	147
**PHF21B**	ESCA	HM_r	298	69	371	738	148
**PARP2**	ESCA	Others	76	262	397	735	149
**SIRT1**	ESCA	HA_e	323	225	186	734	150
**PADI3**	ESCA	Others	204	337	191	732	151
**PADI6**	ESCA	Others	331	335	66	732	152
**TDRD5**	ESCA	Others	346	287	97	730	153
**HNF1A**	ESCA	ChRC	358	174	197	729	154
**RBBP5**	ESCA	ChRC	125	197	407	729	155
**PRKCD**	ESCA	Others	247	69	412	728	156
**SIN3B**	ESCA	ChRC	193	226	308	727	157
**CHD1**	ESCA	Helicases	379	305	42	726	158
**NCOR1**	ESCA	ChRC	332	264	130	726	159
**LBR**	ESCA	Others	145	266	314	725	160
**USP51**	ESCA	Others	236	246	242	724	161
**SMYD1**	ESCA	HM_w	347	193	183	723	162
**SMARCD3**	ESCA	Helicases	187	290	243	720	163
**DPF2**	ESCA	Others	95	282	341	718	164
**FKBP1A**	ESCA	Others	91	243	384	718	165

**CBX2**	ESCA	ChRC	43	308	366	717	166
**KDM1B**	ESCA	HM_e	337	270	107	714	167
**SP100**	ESCA	HA_r	383	250	78	711	168
**DMAP1**	ESCA	Others	274	178	255	707	169
**MBD1**	ESCA	DM_r	260	376	71	707	170
**KAT5**	ESCA	HA_w	83	272	351	706	171
**CDYL**	ESCA	HM_r	172	369	163	704	172
**FKBP5**	ESCA	Others	220	348	136	704	173
**PADI1**	ESCA	Others	256	338	105	699	174
**SMYD5**	ESCA	HM_w	114	192	392	698	175
**SUPT16H**	ESCA	Others	185	191	322	698	176
**L3MBTL3**	ESCA	Others	209	318	169	696	177
**NAP1L2**	ESCA	Others	140	206	350	696	178
**PRDM15**	ESCA	HM_w	393	200	101	694	179
**SIRT2**	ESCA	HA_e	192	313	185	690	180
**SMARCB1**	ESCA	Helicases	189	194	306	689	181
**TDRD3**	ESCA	HM_r	320	247	118	685	182
**SUV39H1**	ESCA	HM_w	51	329	304	684	183
**SHPRH**	ESCA	Others	348	291	42	681	184
**YY1**	ESCA	ChRC	235	149	294	678	185
**HR**	ESCA	HM_e	267	301	108	676	186
**PHF1**	ESCA	HM_r	252	333	91	676	187
**FBXO17**	ESCA	Others	37	321	317	675	188
**SUZ12**	ESCA	ChRC	50	288	336	674	189
**CBX1**	ESCA	HM_r	44	284	343	671	190
**TAF3**	ESCA	HA_r	321	69	280	670	191
**ING1**	ESCA	HM_r	152	343	173	668	192
**PHC1**	ESCA	ChRC	254	204	210	668	193
**RNF217**	ESCA	Others	122	314	232	668	194
**TDRD10**	ESCA	Others	239	356	69	664	195
**FBXL19**	ESCA	Others	93	279	288	660	196
**TDRD12**	ESCA	Others	183	401	76	660	197
**CREBBP**	ESCA	HA_w	411	69	176	656	198
**PRDM1**	ESCA	HM_w	295	259	102	656	199
**MBD5**	ESCA	DM_r	373	69	213	655	200

**CECR2**	ESCA	HA_r	310	69	275	654	201
**CXXC1**	ESCA	Others	225	383	42	650	202
**HDAC1**	ESCA	HA_e	157	240	252	649	203
**AEBP2**	ESCA	HM_w	177	69	402	648	204
**NCOA1**	ESCA	HA_w	351	166	131	648	205
**SETD3**	ESCA	HM_w	119	158	368	645	206
**SND1**	ESCA	HM_r	241	358	42	641	207
**PRMT1**	ESCA	HM_w	132	161	347	640	208
**SIN3A**	ESCA	ChRC	324	69	245	638	209
**TET1**	ESCA	DM_e	284	189	165	638	210
**HAT1**	ESCA	HA_w	31	277	329	637	211
**MTF2**	ESCA	HM_r	302	69	266	637	212
**ING5**	ESCA	HM_r	213	69	353	635	213
**ARID1B**	ESCA	ChRC	423	69	142	634	214
**BRWD3**	ESCA	HA_r	342	69	222	633	215
**PRDM2**	ESCA	HM_w	134	332	167	633	216
**BRD3**	ESCA	HA_r	174	219	239	632	217
**PHC2**	ESCA	ChRC	300	203	129	632	218
**TCF19**	ESCA	Others	49	357	226	632	219
**TAF1**	ESCA	HA_r	322	190	119	631	220
**ATRX**	ESCA	Helicases	381	69	180	630	221
**SMARCA1**	ESCA	Helicases	190	195	244	629	222
**CHD3**	ESCA	Helicases	397	69	161	627	223
**JADE2**	ESCA	Others	317	69	241	627	224
**PSIP1**	ESCA	HM_r	327	258	42	627	225
**TRIM66**	ESCA	HA_r	237	187	203	627	226
**DOT1L**	ESCA	HM_w	339	244	42	625	227
**JADE3**	ESCA	Others	316	69	240	625	228
**BRDT**	ESCA	HA_r	311	218	95	624	229
**KAT8**	ESCA	HA_w	149	237	238	624	230
**SCMH1**	ESCA	Others	18	374	230	622	231
**GTF3C4**	ESCA	HA_w	158	210	253	621	232
**BRWD1**	ESCA	HA_r	360	217	42	619	233
**CHAF1A**	ESCA	ChRC	276	69	274	619	234
**SETDB2**	ESCA	HM_w	289	69	261	619	235

**MEN1**	ESCA	ChRC	24	234	360	618	236
**WDR5**	ESCA	ChRC	46	184	385	615	237
**KIAA2026**	ESCA	Others	374	69	171	614	238
**NAP1L3**	ESCA	Others	333	69	212	614	239
**KDM4B**	ESCA	HM_e	304	69	237	610	240
**SSRP1**	ESCA	Others	16	248	346	610	241
**L3MBTL4**	ESCA	Others	386	69	152	607	242
**RNF20**	ESCA	Others	123	160	324	607	243
**TET2**	ESCA	DM_e	392	69	143	604	244
**ASXL3**	ESCA	Others	420	69	114	603	245
**HIST1H1B**	ESCA	Others	359	69	174	602	246
**HDAC5**	ESCA	HA_e	217	275	109	601	247
**BAZ2B**	ESCA	HA_r	390	69	141	600	248
**AFF1**	ESCA	Others	364	69	164	597	249
**KDM4E**	ESCA	HM_e	148	295	154	597	250
**DPF3**	ESCA	HA_r	388	69	137	594	251
**PHRF1**	ESCA	Others	199	69	326	594	252
**EZH1**	ESCA	HM_w	221	211	160	592	253
**SIRT4**	ESCA	HA_e	58	196	337	591	254
**ACTL6B**	ESCA	ChRC	106	416	68	590	255
**KDM3B**	ESCA	HM_e	376	169	42	587	256
**RNF2**	ESCA	ChRC	66	255	264	585	257
**SP140L**	ESCA	HA_r	286	222	77	585	258
**CBX4**	ESCA	HM_r	98	307	179	584	259
**HDAC4**	ESCA	HA_e	405	69	110	584	260
**POLE3**	ESCA	ChRC	20	165	396	581	261
**PHF10**	ESCA	Others	137	233	209	579	262
**ATM**	ESCA	Others	425	69	83	577	263
**PRDM7**	ESCA	HM_w	133	162	282	577	264
**MLLT10**	ESCA	HM_w	258	69	248	575	265
**PHF8**	ESCA	Others	297	69	208	574	266
**MSL3**	ESCA	HA_w	81	339	150	570	267
**PRDM11**	ESCA	HM_w	135	202	233	570	268
**SIRT7**	ESCA	HA_e	56	252	260	568	269
**HDAC8**	ESCA	HA_e	30	239	298	567	270

**EPC2**	ESCA	Others	165	69	331	565	271
**PHF19**	ESCA	HM_r	201	69	295	565	272
**FBXO44**	ESCA	Others	36	367	159	562	273
**HDAC7**	ESCA	HA_e	155	209	198	562	274
**PBRM1**	ESCA	HA_r	398	69	93	560	275
**PHF21A**	ESCA	HM_r	200	232	128	560	276
**ELP3**	ESCA	HA_w	166	349	42	557	277
**HIST1H3B**	ESCA	Others	268	69	218	555	278
**KDM6B**	ESCA	HM_e	352	69	132	553	279
**JADE1**	ESCA	Others	281	69	202	552	280
**HDAC11**	ESCA	HA_e	156	276	111	543	281
**MTA2**	ESCA	ChRC	142	208	193	543	282
**CARM1**	ESCA	HM_w	173	69	300	542	283
**SETMAR**	ESCA	HM_w	60	292	187	539	284
**PRMT3**	ESCA	HM_w	131	199	207	537	285
**BRD7**	ESCA	HA_r	313	181	42	536	286
**PCGF1**	ESCA	Others	139	205	190	534	287
**UBR7**	ESCA	Others	48	150	335	533	288
**ARID1A**	ESCA	ChRC	421	69	42	532	289
**TDRD9**	ESCA	Others	319	69	144	532	290
**RING1**	ESCA	Others	67	359	100	526	291
**SMARCA4**	ESCA	Helicases	415	69	42	526	292
**GTF2F1**	ESCA	Others	307	176	42	525	293
**TDRD1**	ESCA	Others	285	153	85	523	294
**RPS6KA5**	ESCA	Others	245	69	205	519	295
**JMJD8**	ESCA	HM_e	84	299	134	517	296
**KDM1A**	ESCA	HM_e	211	171	133	515	297
**PRDM13**	ESCA	HM_w	197	69	247	513	298
**CDYL2**	ESCA	HM_r	171	179	162	512	299
**UHRF1**	ESCA	DM_r	282	185	42	509	300
**RAI1**	ESCA	Others	292	69	147	508	301
**SIRT5**	ESCA	HA_e	57	330	120	507	302
**ING2**	ESCA	HM_r	85	378	42	505	303
**TRIM24**	ESCA	HA_r	318	69	116	503	304
**KDM5C**	ESCA	HM_e	147	69	286	502	305

**PRDM6**	ESCA	HM_w	294	163	42	499	306
**SMARCD1**	ESCA	Helicases	115	156	227	498	307
**KDM8**	ESCA	HM_e	26	235	236	497	308
**SP110**	ESCA	HA_r	186	224	86	496	309
**HIST1H1C**	ESCA	Others	269	69	157	495	310
**KDM4C**	ESCA	HM_e	354	69	72	495	311
**SIRT3**	ESCA	HA_e	191	157	146	494	312
**CBX5**	ESCA	HM_r	97	216	178	491	313
**TDRD7**	ESCA	Others	112	152	224	488	314
**ARID4A**	ESCA	ChRC	230	183	74	487	315
**KAT2B**	ESCA	HA_w	356	69	62	487	316
**PPARGC1A**	ESCA	Others	329	69	89	487	317
**SMYD3**	ESCA	HM_w	17	69	398	484	318
**AICDA**	ESCA	DM_e	176	221	84	481	319
**PRDM10**	ESCA	HM_w	370	69	42	481	320
**RBBP4**	ESCA	ChRC	126	229	125	480	321
**UBE2A**	ESCA	Others	14	186	279	479	322
**SFMBT2**	ESCA	Others	288	69	121	478	323
**RBBP7**	ESCA	ChRC	124	69	281	474	324
**RAG2**	ESCA	HM_r	127	257	87	471	325
**TDG**	ESCA	ChRC	113	154	204	471	326
**TP53BP1**	ESCA	Others	283	69	117	469	327
**HSPBAP1**	ESCA	Others	29	395	42	466	328
**PADI4**	ESCA	Others	78	336	42	456	329
**UTY**	ESCA	HM_e	345	69	42	456	330
**SCML2**	ESCA	HM_r	120	69	263	452	331
**PRDM8**	ESCA	HM_w	248	69	126	443	332
**HIRA**	ESCA	Others	215	69	158	442	333
**NAP1L1**	ESCA	Others	80	167	192	439	334
**BRD8**	ESCA	HA_r	229	69	140	438	335
**CHAF1B**	ESCA	ChRC	226	69	139	434	336
**MORF4L1**	ESCA	HM_r	257	69	106	432	337
**FKBP2**	ESCA	Others	35	177	219	431	338
**ZMYND11**	ESCA	HA_r	180	69	182	431	339
**HDAC2**	ESCA	HA_e	88	69	272	429	340

**L3MBTL2**	ESCA	Others	146	69	214	429	341
**GLYR1**	ESCA	HM_r	160	69	199	428	342
**BMI1**	ESCA	ChRC	100	69	257	426	343
**BRD1**	ESCA	HA_r	314	69	42	425	344
**HELLS**	ESCA	Helicases	270	69	81	420	345
**ERCC5**	ESCA	Others	94	280	42	416	346
**MTA3**	ESCA	ChRC	205	168	42	415	347
**PRDM5**	ESCA	HM_w	196	69	149	414	348
**SETD7**	ESCA	HM_w	61	227	123	411	349
**HIF1AN**	ESCA	Others	87	69	251	407	350
**MBD3**	ESCA	DM_r	144	69	194	407	351
**PHIP**	ESCA	HA_r	296	69	42	407	352
**HDAC10**	ESCA	HA_e	219	69	112	400	353
**ZCWPW2**	ESCA	HM_r	108	69	223	400	354
**HDGFL1**	ESCA	Others	154	69	175	398	355
**MUM1**	ESCA	Others	141	207	42	390	356
**SUV39H2**	ESCA	HM_w	15	69	303	387	357
**PWWP2B**	ESCA	Others	68	69	246	383	358
**UBE2I**	ESCA	Others	12	69	302	383	359
**GTF2H1**	ESCA	Others	159	175	42	376	360
**INO80**	ESCA	Helicases	265	69	42	376	361
**IDH1**	ESCA	DM_e	86	69	217	372	362
**PCGF6**	ESCA	Others	203	69	92	364	363
**PYGO1**	ESCA	HM_r	129	69	166	364	364
**PRDM4**	ESCA	HM_w	72	164	127	363	365
**FBXW9**	ESCA	Others	92	69	200	361	366
**SETD6**	ESCA	HM_w	62	69	229	360	367
**PHF5A**	ESCA	Others	21	69	265	355	368
**SETD5**	ESCA	HM_w	244	69	42	355	369
**SMYD4**	ESCA	HM_w	242	69	42	353	370
**PHF6**	ESCA	HM_r	73	231	42	346	371
**AFF4**	ESCA	Others	232	69	42	343	372
**SP140**	ESCA	HA_r	52	223	65	340	373
**SCML4**	ESCA	Others	194	69	70	333	374
**SIRT6**	ESCA	HA_e	117	69	145	331	375

**HDAC3**	ESCA	HA_e	218	69	42	329	376
**PRKAA2**	ESCA	Others	71	69	188	328	377
**GTF2B**	ESCA	Others	32	241	42	315	378
**AIRE**	ESCA	HM_r	175	69	67	311	379
**DPY30**	ESCA	Others	38	69	201	308	380
**PHF7**	ESCA	Others	136	69	103	308	381
**PRDM12**	ESCA	HM_w	19	201	88	308	382
**SMNDC1**	ESCA	Others	54	69	184	307	383
**CBX7**	ESCA	HM_r	42	180	82	304	384
**DNMT3L**	ESCA	DM_w	168	69	64	301	385
**WDR82**	ESCA	Others	11	245	42	298	386
**H2AFZ**	ESCA	Others	90	69	135	294	387
**GADD45B**	ESCA	Others	161	69	63	293	388
**TRIM33**	ESCA	HA_r	182	69	42	293	389
**UHRF2**	ESCA	DM_r	181	69	42	292	390
**KDM7A**	ESCA	HM_e	107	69	115	291	391
**MARCH5**	ESCA	Others	25	69	195	289	392
**CLOCK**	ESCA	HA_w	40	69	177	286	393
**PRMT6**	ESCA	HM_w	69	69	148	286	394
**AURKB**	ESCA	Others	103	69	113	285	395
**SMARCC1**	ESCA	Helicases	116	69	98	283	396
**FXR2**	ESCA	Others	162	69	42	273	397
**ORC1**	ESCA	Others	23	69	168	260	398
**PADI2**	ESCA	Others	79	69	104	252	399
**SFMBT1**	ESCA	HM_r	59	69	122	250	400
**INTS12**	ESCA	Others	28	173	42	243	401
**UBE2B**	ESCA	Others	13	151	75	239	402
**RTF1**	ESCA	Others	64	69	99	232	403
**SETD4**	ESCA	HM_w	118	69	42	229	404
**UBE2E1**	ESCA	Others	110	69	42	221	405
**USP22**	ESCA	Others	109	69	42	220	406
**BAP1**	ESCA	Others	102	69	42	213	407
**SATB1**	ESCA	Others	63	69	79	211	408
**GADD45A**	ESCA	Others	34	69	94	197	409
**PCGF5**	ESCA	Others	75	69	42	186	410

**PHF11**	ESCA	Others	74	69	42	185	411
**PHF23**	ESCA	HM_r	22	69	90	181	412
**PRMT2**	ESCA	HM_w	70	69	42	181	413
**USP27X**	ESCA	Others	47	69	42	158	414
**CTCF**	ESCA	Others	39	69	42	150	415
**KDM5D**	ESCA	HM_e	27	69	42	138	416
**EZH2**	GBM	HM_w	399	405	381	1185	1
**HDAC9**	GBM	HA_e	364	409	411	1184	2
**KMT2C**	GBM	HM_w	423	416	327	1166	3
**ZCWPW1**	GBM	HM_r	387	363	412	1162	4
**CARM1**	GBM	HM_w	373	371	413	1157	5
**DNMT1**	GBM	DM_w	368	382	390	1140	6
**DOT1L**	GBM	HM_w	335	417	388	1140	7
**CHAF1A**	GBM	ChRC	372	393	364	1129	8
**MECOM**	GBM	Others	416	379	333	1128	9
**CLOCK**	GBM	HA_w	405	426	288	1119	10
**SND1**	GBM	HM_r	292	414	404	1110	11
**RBBP5**	GBM	ChRC	299	425	385	1109	12
**ATAD2**	GBM	HA_r	379	348	360	1087	13
**PHRF1**	GBM	Others	395	367	324	1086	14
**BAZ1B**	GBM	HA_r	267	396	422	1085	15
**AICDA**	GBM	DM_e	403	384	296	1083	16
**ARID1B**	GBM	ChRC	382	362	320	1064	17
**KDM5B**	GBM	HM_e	319	380	352	1051	18
**MSL3**	GBM	HA_w	385	403	262	1050	19
**SMARCA4**	GBM	Helicases	277	400	368	1045	20
**DIDO1**	GBM	Others	369	280	394	1043	21
**PHF20L1**	GBM	HM_r	305	386	350	1041	22
**MUM1**	GBM	Others	216	415	399	1030	23
**KIAA2026**	GBM	Others	358	418	245	1021	24
**ERCC5**	GBM	Others	333	341	342	1016	25
**ARID4B**	GBM	ChRC	381	285	346	1012	26
**KDM5A**	GBM	HM_e	398	408	201	1007	27
**MBD3**	GBM	DM_r	224	389	391	1004	28
**TAF3**	GBM	HA_r	287	290	426	1003	29

**BRD4**	GBM	HA_r	264	345	393	1002	30
**EHMT1**	GBM	HM_w	251	369	382	1002	31
**ZGPAT**	GBM	Others	279	349	374	1002	32
**KDM2B**	GBM	HM_e	360	333	308	1001	33
**CHRAC1**	GBM	ChRC	256	370	373	999	34
**ARID2**	GBM	ChRC	343	361	294	998	35
**STK31**	GBM	Others	391	407	184	982	36
**POLR2B**	GBM	Others	206	424	349	979	37
**BRD3**	GBM	HA_r	340	346	291	977	38
**SMYD3**	GBM	HM_w	294	375	298	967	39
**KMT2A**	GBM	HM_w	397	358	199	954	40
**CBX3**	GBM	HM_r	152	394	407	953	41
**HDGF**	GBM	Others	238	336	379	953	42
**DPF1**	GBM	ChRC	253	342	357	952	43
**PARP1**	GBM	Others	309	256	386	951	44
**CDYL2**	GBM	HM_r	401	215	330	946	45
**NCOA3**	GBM	HA_w	312	257	372	941	46
**TRIM24**	GBM	HA_r	170	364	406	940	47
**KDM4D**	GBM	HM_e	320	332	282	934	48
**ATM**	GBM	Others	402	309	219	930	49
**DPF2**	GBM	Others	252	279	392	923	50
**FXR2**	GBM	Others	332	276	314	922	51
**PRMT8**	GBM	HM_w	199	421	302	922	52
**TDRD3**	GBM	HM_r	173	352	395	920	53
**ACTL6A**	GBM	ChRC	164	423	332	919	54
**KDM7A**	GBM	HM_e	425	69	424	918	55
**KDM4C**	GBM	HM_e	97	419	400	916	56
**KMT2E**	GBM	HM_w	227	301	378	906	57
**ACTL6B**	GBM	ChRC	163	374	367	904	58
**ATF7IP**	GBM	Others	378	347	177	902	59
**JADE3**	GBM	Others	413	69	419	901	60
**PAXIP1**	GBM	Others	81	402	417	900	61
**JADE1**	GBM	Others	414	69	414	897	62
**CHD8**	GBM	Helicases	421	69	402	892	63
**GTF3C4**	GBM	HA_w	241	339	311	891	64

**AEBP2**	GBM	HM_w	162	397	331	890	65
**CHD6**	GBM	Helicases	400	343	146	889	66
**KDM4B**	GBM	HM_e	98	404	387	889	67
**ASXL2**	GBM	Others	341	284	261	886	68
**HDAC2**	GBM	HA_e	365	272	249	886	69
**PADI6**	GBM	Others	356	164	362	882	70
**DMAP1**	GBM	Others	255	307	317	879	71
**AKAP1**	GBM	Others	273	310	295	878	72
**GATAD2A**	GBM	HM_r	243	304	329	876	73
**BOP 1**	GBM	Others	158	372	345	875	74
**CREBBP**	GBM	HA_w	412	208	255	875	75
**EPC1**	GBM	Others	249	204	421	874	76
**RAG2**	GBM	HM_r	351	248	271	870	77
**FBXO44**	GBM	Others	247	306	316	869	78
**PHF14**	GBM	Others	73	378	416	867	79
**ASH1L**	GBM	HM_w	272	373	221	866	80
**ING3**	GBM	HM_r	112	391	363	866	81
**TDRD7**	GBM	Others	346	289	227	862	82
**PHC3**	GBM	ChRC	355	368	137	860	83
**HIST1H1C**	GBM	Others	327	190	341	858	84
**JADE2**	GBM	Others	384	69	403	856	85
**USP22**	GBM	Others	282	311	263	856	86
**SCML2**	GBM	HM_r	349	316	188	853	87
**CHD5**	GBM	Helicases	418	281	151	850	88
**ING1**	GBM	HM_r	236	303	310	849	89
**GTF2H1**	GBM	Others	331	197	312	840	90
**RBBP4**	GBM	ChRC	197	294	347	838	91
**CHD7**	GBM	Helicases	371	210	256	837	92
**PAF1**	GBM	Others	83	356	398	837	93
**ASXL1**	GBM	Others	380	69	383	832	94
**SMARCD3**	GBM	Helicases	181	376	266	823	95
**GADD45B**	GBM	Others	244	410	168	822	96
**IDH1**	GBM	DM_e	424	187	209	820	97
**FBXW9**	GBM	Others	134	305	380	819	98
**SIRT6**	GBM	HA_e	34	377	408	819	99

**PADI3**	GBM	Others	310	166	339	815	100
**PRDM15**	GBM	HM_w	408	69	338	815	101
**CBX2**	GBM	ChRC	338	219	257	814	102
**HDAC4**	GBM	HA_e	329	337	143	809	103
**TDRKH**	GBM	Others	276	233	297	806	104
**TDRD1**	GBM	Others	174	235	396	805	105
**BRWD3**	GBM	HA_r	374	69	359	802	106
**GTF2F1**	GBM	Others	127	274	401	802	107
**CHD9**	GBM	Helicases	420	209	172	801	108
**SMARCA2**	GBM	Helicases	347	412	42	801	109
**LBR**	GBM	Others	226	259	307	792	110
**AURKB**	GBM	Others	270	226	292	788	111
**RNF17**	GBM	Others	415	247	126	788	112
**FMR1**	GBM	Others	130	340	315	785	113
**HLTF**	GBM	Others	362	69	354	785	114
**RAI1**	GBM	Others	300	295	190	785	115
**JMJD1C**	GBM	HM_e	361	69	353	783	116
**KDM1A**	GBM	HM_e	231	266	283	780	117
**CBX5**	GBM	HM_r	150	308	319	777	118
**NCOR1**	GBM	ChRC	311	327	138	776	119
**BRD8**	GBM	HA_r	376	222	175	773	120
**PRDM12**	GBM	HM_w	352	320	99	771	121
**ATR**	GBM	Others	406	69	293	768	122
**ARID1A**	GBM	ChRC	386	229	152	767	123
**SUPT16H**	GBM	Others	288	69	410	767	124
**CBX8**	GBM	ChRC	261	216	289	766	125
**DNMT3B**	GBM	DM_w	336	69	358	763	126
**KDM6A**	GBM	HM_e	359	262	141	762	127
**ASXL3**	GBM	Others	271	227	260	758	128
**BPTF**	GBM	HA_r	409	224	125	758	129
**JMJD6**	GBM	HM_e	233	184	340	757	130
**MECP2**	GBM	DM_r	91	357	306	754	131
**PRDM7**	GBM	HM_w	202	249	303	754	132
**USP51**	GBM	Others	281	287	180	748	133
**SMARCD2**	GBM	Helicases	182	238	322	742	134

**SIRT2**	GBM	HA_e	165	353	223	741	135
**AFF4**	GBM	Others	275	286	178	739	136
**SETD2**	GBM	HM_w	404	292	42	738	137
**SIN3B**	GBM	ChRC	37	291	409	737	138
**SMARCC2**	GBM	Helicases	295	399	42	736	139
**TDRD10**	GBM	Others	390	234	112	736	140
**EP400**	GBM	HA_w	411	69	254	734	141
**SETDB1**	GBM	HM_w	187	244	300	731	142
**BAZ2A**	GBM	HA_r	266	420	42	728	143
**TAF1**	GBM	HA_r	410	136	182	728	144
**HDAC10**	GBM	HA_e	123	392	212	727	145
**PHF20**	GBM	HM_r	306	69	351	726	146
**SETDB2**	GBM	HM_w	41	385	299	725	147
**HDAC7**	GBM	HA_e	239	271	211	721	148
**ING5**	GBM	HM_r	110	302	309	721	149
**MLLT6**	GBM	HM_w	221	258	242	721	150
**KMT2B**	GBM	HM_w	417	261	42	720	151
**GATAD2B**	GBM	HM_r	129	275	313	717	152
**ING2**	GBM	HM_r	235	269	207	711	153
**L3MBTL1**	GBM	HM_r	316	69	326	711	154
**HCFC1**	GBM	Others	330	338	42	710	155
**TDRD12**	GBM	Others	25	312	369	706	156
**CHD4**	GBM	Helicases	257	406	42	705	157
**L3MBTL4**	GBM	Others	94	330	281	705	158
**CHAF1B**	GBM	ChRC	145	213	344	702	159
**KDM4E**	GBM	HM_e	229	331	142	702	160
**PRDM2**	GBM	HM_w	63	401	237	701	161
**SETD5**	GBM	HM_w	189	354	158	701	162
**PCGF6**	GBM	Others	211	69	420	700	163
**CHD2**	GBM	Helicases	337	212	147	696	164
**KDM3A**	GBM	HM_e	230	264	202	696	165
**KDM6B**	GBM	HM_e	317	177	200	694	166
**L3MBTL2**	GBM	Others	96	175	418	689	167
**TDG**	GBM	ChRC	285	69	335	689	168
**BRD9**	GBM	HA_r	263	383	42	688	169

**HAT1**	GBM	HA_w	240	195	250	685	170
**MPHOSPH8**	GBM	HM_r	314	329	42	685	171
**MBD5**	GBM	DM_r	315	172	197	684	172
**TET2**	GBM	DM_e	284	133	264	681	173
**PHC2**	GBM	ChRC	77	299	304	680	174
**PRKCD**	GBM	Others	394	154	132	680	175
**PHF1**	GBM	HM_r	354	163	162	679	176
**RNF217**	GBM	Others	195	293	189	677	177
**TDRD6**	GBM	Others	389	134	154	677	178
**HDAC1**	GBM	HA_e	124	194	356	674	179
**HDAC8**	GBM	HA_e	119	270	285	674	180
**PHF2**	GBM	Others	307	325	42	674	181
**ELP4**	GBM	HA_w	250	381	42	673	182
**JARID2**	GBM	ChRC	323	185	165	673	183
**RPA3**	GBM	Others	47	355	270	672	184
**CHD1L**	GBM	Helicases	259	69	343	671	185
**PYGO2**	GBM	HM_r	51	296	323	670	186
**HDAC3**	GBM	HA_e	121	193	355	669	187
**PHF8**	GBM	Others	303	323	42	668	188
**CXXC1**	GBM	Others	142	207	318	667	189
**NAP1L3**	GBM	Others	88	300	279	667	190
**CBX4**	GBM	HM_r	151	218	290	659	191
**SCMH1**	GBM	Others	191	69	397	657	192
**SP140**	GBM	HA_r	289	139	229	657	193
**KMT2D**	GBM	HM_w	407	69	179	655	194
**PBRM1**	GBM	HA_r	308	69	278	655	195
**AIRE**	GBM	HM_r	274	230	149	653	196
**SMARCA1**	GBM	Helicases	297	313	42	652	197
**PRDM13**	GBM	HM_w	302	156	193	651	198
**PHF12**	GBM	Others	353	252	42	647	199
**HDAC6**	GBM	HA_e	328	69	248	645	200
**SUV39H1**	GBM	HM_w	178	237	228	643	201
**EED**	GBM	ChRC	138	360	144	642	202
**HNF1A**	GBM	ChRC	326	69	247	642	203
**PRDM11**	GBM	HM_w	205	321	116	642	204

**PRDM9**	GBM	HM_w	422	69	150	641	205
**HDAC5**	GBM	HA_e	120	192	328	640	206
**PHC1**	GBM	ChRC	210	388	42	640	207
**UBR7**	GBM	Others	18	232	389	639	208
**PRDM10**	GBM	HM_w	64	297	275	636	209
**AFF1**	GBM	Others	344	69	222	635	210
**HR**	GBM	HM_e	237	188	210	635	211
**KAT5**	GBM	HA_w	322	268	42	632	212
**CBX1**	GBM	HM_r	153	220	258	631	213
**KDM1B**	GBM	HM_e	321	265	42	628	214
**FKBP5**	GBM	Others	131	277	215	623	215
**UHRF2**	GBM	DM_r	168	413	42	623	216
**MSH6**	GBM	HM_r	357	69	196	622	217
**NAP1L2**	GBM	Others	313	170	139	622	218
**RTF1**	GBM	Others	166	69	384	619	219
**SFMBT1**	GBM	HM_r	39	242	336	617	220
**EP300**	GBM	HA_w	366	205	42	613	221
**ARID4A**	GBM	ChRC	342	228	42	612	222
**H3F3A**	GBM	Others	125	273	213	611	223
**YY1**	GBM	ChRC	167	69	375	611	224
**TDRD9**	GBM	Others	388	69	153	610	225
**INO80**	GBM	Helicases	109	334	166	609	226
**MEN1**	GBM	ChRC	396	171	42	609	227
**NAP1L1**	GBM	Others	215	69	325	609	228
**ING4**	GBM	HM_r	111	390	107	608	229
**SIRT1**	GBM	HA_e	36	145	425	606	230
**CBX7**	GBM	HM_r	148	282	174	604	231
**BRPF3**	GBM	HA_r	339	221	42	602	232
**KAT6B**	GBM	HA_w	232	267	103	602	233
**PPARGC1A**	GBM	Others	66	298	238	602	234
**GADD45A**	GBM	Others	245	69	287	601	235
**HIF1AN**	GBM	Others	116	69	415	600	236
**RPH3A**	GBM	Others	194	69	337	600	237
**PHF11**	GBM	Others	75	387	136	598	238
**PHF21B**	GBM	HM_r	70	251	277	598	239

**PHF13**	GBM	Others	74	162	361	597	240
**ASH2L**	GBM	HM_w	161	69	366	596	241
**BRD1**	GBM	HA_r	157	395	42	594	242
**SETD1B**	GBM	HM_w	44	315	235	594	243
**TAF1L**	GBM	HA_r	419	69	106	594	244
**BAZ1A**	GBM	HA_r	268	283	42	593	245
**EPC2**	GBM	Others	137	203	253	593	246
**SIRT3**	GBM	HA_e	186	365	42	593	247
**CHD3**	GBM	Helicases	258	211	123	592	248
**PHF19**	GBM	HM_r	72	326	194	592	249
**SMYD5**	GBM	HM_w	293	69	230	592	250
**BRD2**	GBM	HA_r	156	69	365	590	251
**MTA1**	GBM	ChRC	219	328	42	589	252
**SP110**	GBM	HA_r	290	140	156	586	253
**MARCH5**	GBM	Others	93	69	423	585	254
**CBX6**	GBM	HM_r	149	217	217	583	255
**SETD1A**	GBM	HM_w	393	148	42	583	256
**SHPRH**	GBM	Others	298	241	42	581	257
**PRMT2**	GBM	HM_w	57	153	370	580	258
**SIN3A**	GBM	ChRC	348	69	157	574	259
**H2AFZ**	GBM	Others	126	196	251	573	260
**PCGF2**	GBM	Others	79	254	239	572	261
**MBTD1**	GBM	Others	222	69	280	571	262
**KAT2B**	GBM	HA_w	105	181	284	570	263
**RPS6KA5**	GBM	Others	193	245	131	569	264
**BRDT**	GBM	HA_r	375	69	124	568	265
**RNF2**	GBM	ChRC	196	69	301	566	266
**PRKAA2**	GBM	Others	59	155	348	562	267
**SP100**	GBM	HA_r	291	141	129	561	268
**TET1**	GBM	DM_e	171	288	97	556	269
**L3MBTL3**	GBM	Others	95	260	198	553	270
**RSF1**	GBM	ChRC	192	317	42	551	271
**BRWD1**	GBM	HA_r	262	69	218	549	272
**PRDM16**	GBM	HM_w	204	69	274	547	273
**PRDM8**	GBM	HM_w	201	69	273	543	274

**RNF40**	GBM	Others	350	150	42	542	275
**BRPF1**	GBM	HA_r	154	344	42	540	276
**PYGO1**	GBM	HM_r	198	151	191	540	277
**ATRX**	GBM	Helicases	426	69	42	537	278
**BAP1**	GBM	Others	269	225	42	536	279
**FBXO17**	GBM	Others	135	359	42	536	280
**MBD4**	GBM	DM_r	223	69	243	535	281
**KAT2A**	GBM	HA_w	106	182	246	534	282
**CECR2**	GBM	HA_r	146	214	173	533	283
**PRDM14**	GBM	HM_w	301	69	161	531	284
**G2E3**	GBM	Others	246	69	214	529	285
**RBBP7**	GBM	ChRC	50	318	159	527	286
**DPF3**	GBM	HA_r	140	278	108	526	287
**KDM8**	GBM	HM_e	228	176	121	525	288
**NCOA1**	GBM	HA_w	214	69	241	524	289
**TET3**	GBM	DM_e	345	69	110	524	290
**POLE3**	GBM	ChRC	67	322	134	523	291
**PHF10**	GBM	Others	76	69	377	522	292
**SIRT5**	GBM	HA_e	185	69	267	521	293
**PSIP1**	GBM	HM_r	53	422	42	517	294
**DPY30**	GBM	Others	139	206	171	516	295
**SUV39H2**	GBM	HM_w	177	236	98	511	296
**BAZ2B**	GBM	HA_r	265	69	176	510	297
**MBD2**	GBM	DM_r	92	173	244	509	298
**EZH1**	GBM	HM_w	136	202	170	508	299
**SIRT4**	GBM	HA_e	35	240	232	507	300
**PHF3**	GBM	Others	304	160	42	506	301
**SMARCB1**	GBM	Helicases	32	69	405	506	302
**PHF7**	GBM	Others	69	159	276	504	303
**SRCAP**	GBM	Others	392	69	42	503	304
**NSD1**	GBM	HM_w	213	169	120	502	305
**FKBP2**	GBM	Others	132	199	169	500	306
**PHF6**	GBM	HM_r	207	250	42	499	307
**PRMT1**	GBM	HM_w	58	69	371	498	308
**KANSL1**	GBM	HA_w	107	183	206	496	309

**HELLS**	GBM	Helicases	117	335	42	494	310
**KDM5D**	GBM	HM_e	383	69	42	494	311
**FBXL19**	GBM	Others	248	201	42	491	312
**ATAD2B**	GBM	HA_r	160	69	259	488	313
**BMI1**	GBM	ChRC	377	69	42	488	314
**KAT8**	GBM	HA_w	102	180	203	485	315
**TRIM66**	GBM	HA_r	23	351	109	483	316
**GLYR1**	GBM	HM_r	242	198	42	482	317
**DAXX**	GBM	ChRC	370	69	42	481	318
**EHMT2**	GBM	HM_w	367	69	42	478	319
**SIRT7**	GBM	HA_e	33	69	376	478	320
**CHD1**	GBM	Helicases	260	69	148	477	321
**HDAC11**	GBM	HA_e	122	69	286	477	322
**HDGFL1**	GBM	Others	118	191	167	476	323
**HIRA**	GBM	Others	363	69	42	474	324
**NCOR2**	GBM	Others	278	69	127	474	325
**DNMT3L**	GBM	DM_w	254	69	145	468	326
**TCEA1**	GBM	Others	175	135	155	465	327
**ZMYND11**	GBM	HA_r	12	411	42	465	328
**SATB1**	GBM	Others	46	149	269	464	329
**SMARCD1**	GBM	Helicases	183	239	42	464	330
**INTS12**	GBM	Others	234	186	42	462	331
**PRMT3**	GBM	HM_w	200	69	192	461	332
**PWWP2B**	GBM	Others	52	366	42	460	333
**UHRF1**	GBM	DM_r	17	398	42	457	334
**FKBP1A**	GBM	Others	133	200	122	455	335
**PCGF1**	GBM	Others	80	69	305	454	336
**SFMBT2**	GBM	Others	38	314	102	454	337
**MORF4L1**	GBM	HM_r	220	69	163	452	338
**GTF2B**	GBM	Others	128	69	252	449	339
**PCMT1**	GBM	Others	78	253	118	449	340
**ATAT1**	GBM	Others	159	69	220	448	341
**PADI2**	GBM	Others	85	167	195	447	342
**ELP3**	GBM	HA_w	334	69	42	445	343
**KDM3B**	GBM	HM_e	100	178	164	442	344

**MBD1**	GBM	DM_r	225	174	42	441	345
**SMARCC1**	GBM	Helicases	184	69	187	440	346
**PHF21A**	GBM	HM_r	71	324	42	437	347
**HSPBAP1**	GBM	Others	325	69	42	436	348
**IWS1**	GBM	Others	324	69	42	435	349
**SMYD2**	GBM	HM_w	180	69	186	435	350
**CSTL1**	GBM	Others	144	69	216	429	351
**KDM5C**	GBM	HM_e	318	69	42	429	352
**SUZ12**	GBM	ChRC	176	69	183	428	353
**SETD4**	GBM	HM_w	43	147	234	424	354
**PRDM4**	GBM	HM_w	62	319	42	423	355
**UBE2I**	GBM	Others	19	69	334	422	356
**SETD7**	GBM	HM_w	42	146	233	421	357
**BRD7**	GBM	HA_r	155	223	42	420	358
**TRIM28**	GBM	HA_r	24	69	321	414	359
**SETMAR**	GBM	HM_w	40	243	130	413	360
**PHF23**	GBM	HM_r	209	161	42	412	361
**PHF5A**	GBM	Others	208	69	135	412	362
**SMARCA5**	GBM	Helicases	296	69	42	407	363
**TRIM33**	GBM	HA_r	169	132	105	406	364
**WDR5**	GBM	ChRC	14	350	42	406	365
**PRDM5**	GBM	HM_w	203	69	133	405	366
**HIST1H3B**	GBM	Others	114	189	101	404	367
**KDM4A**	GBM	HM_e	99	263	42	404	368
**SMYD1**	GBM	HM_w	29	143	231	403	369
**TCF20**	GBM	Others	286	69	42	397	370
**ORC1**	GBM	Others	87	69	240	396	371
**PRMT7**	GBM	HM_w	54	69	272	395	372
**TP53BP1**	GBM	Others	283	69	42	394	373
**WDR82**	GBM	Others	280	69	42	391	374
**IDH2**	GBM	DM_e	113	69	208	390	375
**SCML4**	GBM	Others	45	69	268	382	376
**PCGF5**	GBM	Others	212	69	100	381	377
**PARP2**	GBM	Others	82	255	42	379	378
**KAT6A**	GBM	HA_w	104	69	205	378	379

**KAT7**	GBM	HA_w	103	69	204	376	380
**UBE2E1**	GBM	Others	20	130	225	375	381
**PADI1**	GBM	Others	86	168	119	373	382
**SETD6**	GBM	HM_w	188	69	115	372	383
**PRMT6**	GBM	HM_w	55	152	160	367	384
**PRKAA1**	GBM	Others	60	69	236	365	385
**ZMYND8**	GBM	HA_r	11	128	224	363	386
**TCF19**	GBM	Others	26	69	265	360	387
**SSRP1**	GBM	Others	179	137	42	358	388
**SMYD4**	GBM	HM_w	28	142	185	355	389
**TDRD5**	GBM	Others	172	69	111	352	390
**PRDM1**	GBM	HM_w	65	157	117	339	391
**RNF20**	GBM	Others	48	246	42	336	392
**UBE2B**	GBM	Others	21	131	181	333	393
**MTA2**	GBM	ChRC	218	69	42	329	394
**MTA3**	GBM	ChRC	217	69	42	328	395
**KDM2A**	GBM	HM_e	101	179	42	322	396
**UBE2A**	GBM	Others	22	69	226	317	397
**SETD3**	GBM	HM_w	190	69	42	301	398
**MTF2**	GBM	HM_r	89	69	140	298	399
**PADI4**	GBM	Others	84	165	42	291	400
**USP27X**	GBM	Others	16	231	42	289	401
**HIST1H1B**	GBM	Others	115	69	104	288	402
**SP140L**	GBM	HA_r	27	138	113	278	403
**ZCWPW2**	GBM	HM_r	13	129	128	270	404
**PHIP**	GBM	HA_r	68	158	42	268	405
**CDYL**	GBM	HM_r	147	69	42	258	406
**CTCF**	GBM	Others	143	69	42	254	407
**DNMT3A**	GBM	DM_w	141	69	42	252	408
**JMJD8**	GBM	HM_e	108	69	42	219	409
**SMARCE1**	GBM	Helicases	31	144	42	217	410
**SMNDC1**	GBM	Others	30	69	114	213	411
**MLLT10**	GBM	HM_w	90	69	42	201	412
**PRDM6**	GBM	HM_w	61	69	42	172	413
**PRMT5**	GBM	HM_w	56	69	42	167	414

**RING1**	GBM	Others	49	69	42	160	415
**UTY**	GBM	HM_e	15	69	42	126	416
**ATR**	HNSC	Others	412	422	424	1258	1
**ASXL1**	HNSC	Others	398	379	417	1194	2
**CHD7**	HNSC	Helicases	414	395	385	1194	3
**PHF20L1**	HNSC	HM_r	341	419	419	1179	4
**EHMT1**	HNSC	HM_w	371	391	393	1155	5
**KMT2E**	HNSC	HM_w	391	393	369	1153	6
**BRD9**	HNSC	HA_r	314	416	420	1150	7
**PHF3**	HNSC	Others	404	382	360	1146	8
**HIRA**	HNSC	Others	364	385	392	1141	9
**CHD8**	HNSC	Helicases	394	346	400	1140	10
**RSF1**	HNSC	ChRC	359	407	367	1133	11
**PHC3**	HNSC	ChRC	278	424	426	1128	12
**ATAD2**	HNSC	HA_r	353	418	354	1125	13
**KDM5A**	HNSC	HM_e	322	400	403	1125	14
**CHD4**	HNSC	Helicases	387	362	362	1111	15
**HDAC9**	HNSC	HA_e	393	374	344	1111	16
**DNMT3B**	HNSC	DM_w	365	392	349	1106	17
**SUPT16H**	HNSC	Others	358	339	408	1105	18
**MECOM**	HNSC	Others	380	425	294	1099	19
**SETDB1**	HNSC	HM_w	337	352	398	1087	20
**HLTF**	HNSC	Others	237	423	423	1083	21
**PHF20**	HNSC	HM_r	330	355	394	1079	22
**PRKAA1**	HNSC	Others	254	408	414	1076	23
**KDM2A**	HNSC	HM_e	236	415	416	1067	24
**ACTL6A**	HNSC	ChRC	215	426	425	1066	25
**STK31**	HNSC	Others	316	364	386	1066	26
**KAT5**	HNSC	HA_w	264	404	395	1063	27
**SMARCA4**	HNSC	Helicases	411	299	351	1061	28
**KDM4C**	HNSC	HM_e	235	414	406	1055	29
**BRD4**	HNSC	HA_r	373	313	356	1042	30
**MTA2**	HNSC	ChRC	301	356	375	1032	31
**KDM6A**	HNSC	HM_e	389	399	228	1016	32
**TAF1L**	HNSC	HA_r	418	381	215	1014	33

**KDM5C**	HNSC	HM_e	321	367	321	1009	34
**ARID4A**	HNSC	ChRC	355	316	333	1004	35
**KAT6A**	HNSC	HA_w	346	403	243	992	36
**PRDM11**	HNSC	HM_w	256	373	359	988	37
**ASH1L**	HNSC	HM_w	382	315	289	986	38
**SETD3**	HNSC	HM_w	193	377	415	985	39
**PRDM14**	HNSC	HM_w	319	396	262	977	40
**BAZ1B**	HNSC	HA_r	244	336	396	976	41
**DIDO1**	HNSC	Others	410	167	399	976	42
**CHRAC1**	HNSC	ChRC	132	421	422	975	43
**GTF3C4**	HNSC	HA_w	284	345	345	974	44
**L3MBTL4**	HNSC	Others	280	398	295	973	45
**KMT2A**	HNSC	HM_w	415	343	213	971	46
**PHF14**	HNSC	Others	196	365	409	970	47
**HCFC1**	HNSC	Others	308	394	267	969	48
**DAXX**	HNSC	ChRC	309	251	407	967	49
**PAXIP1**	HNSC	Others	257	328	382	967	50
**DNMT1**	HNSC	DM_w	372	277	305	954	51
**KMT2C**	HNSC	HM_w	424	384	146	954	52
**MBD4**	HNSC	DM_r	123	409	421	953	53
**DPF2**	HNSC	Others	175	406	371	952	54
**ARID4B**	HNSC	ChRC	292	281	378	951	55
**DNMT3A**	HNSC	DM_w	401	208	342	951	56
**RNF20**	HNSC	Others	276	284	391	951	57
**EHMT2**	HNSC	HM_w	350	289	311	950	58
**ARID2**	HNSC	ChRC	403	256	290	949	59
**SRCAP**	HNSC	Others	416	142	390	948	60
**PRDM9**	HNSC	HM_w	422	402	117	941	61
**MEN1**	HNSC	ChRC	161	366	405	932	62
**ATM**	HNSC	Others	390	347	189	926	63
**BAZ1A**	HNSC	HA_r	213	337	373	923	64
**BRWD1**	HNSC	HA_r	377	278	256	911	65
**ELP4**	HNSC	HA_w	286	274	348	908	66
**ATAD2B**	HNSC	HA_r	366	213	328	907	67
**MECP2**	HNSC	DM_r	122	397	388	907	68

**TDRD6**	HNSC	Others	396	318	180	894	69
**TDRD5**	HNSC	Others	326	229	336	891	70
**CTCF**	HNSC	Others	385	252	246	883	71
**SSRP1**	HNSC	Others	190	319	374	883	72
**BRD3**	HNSC	HA_r	135	370	377	882	73
**HDAC4**	HNSC	HA_e	349	361	166	876	74
**RNF40**	HNSC	Others	318	148	410	876	75
**EP400**	HNSC	HA_w	423	166	286	875	76
**SETD2**	HNSC	HM_w	338	261	275	874	77
**TCEA1**	HNSC	Others	56	401	413	870	78
**IWS1**	HNSC	Others	265	223	381	869	79
**PARP2**	HNSC	Others	157	329	383	869	80
**BOP 1**	HNSC	Others	30	420	418	868	81
**ATF7IP**	HNSC	Others	315	314	235	864	82
**AKAP1**	HNSC	Others	334	172	357	863	83
**PHF21A**	HNSC	HM_r	229	354	278	861	84
**GATAD2B**	HNSC	HM_r	170	309	376	855	85
**SETD1A**	HNSC	HM_w	368	146	338	852	86
**SIN3B**	HNSC	ChRC	328	183	337	848	87
**KIAA2026**	HNSC	Others	392	412	42	846	88
**PARP1**	HNSC	Others	258	266	318	842	89
**KDM4A**	HNSC	HM_e	263	268	309	840	90
**MLLT6**	HNSC	HM_w	303	221	303	827	91
**TDRKH**	HNSC	Others	186	293	346	825	92
**CREBBP**	HNSC	HA_w	421	69	331	821	93
**TET3**	HNSC	DM_e	295	174	350	819	94
**WDR5**	HNSC	ChRC	53	363	402	818	95
**KDM2B**	HNSC	HM_e	375	157	285	817	96
**RAI1**	HNSC	Others	329	262	225	816	97
**BRPF3**	HNSC	HA_r	312	211	288	811	98
**HAT1**	HNSC	HA_w	82	375	353	810	99
**HDAC6**	HNSC	HA_e	282	360	165	807	100
**HR**	HNSC	HM_e	281	390	136	807	101
**NCOA3**	HNSC	HA_w	370	69	368	807	102
**KMT2D**	HNSC	HM_w	426	193	186	805	103

**PRMT5**	HNSC	HM_w	108	285	411	804	104
**BAZ2B**	HNSC	HA_r	407	69	327	803	105
**NCOA1**	HNSC	HA_w	342	190	264	796	106
**RBBP7**	HNSC	ChRC	222	378	195	795	107
**PRMT8**	HNSC	HM_w	225	387	182	794	108
**CXXC1**	HNSC	Others	352	335	105	792	109
**MBD2**	HNSC	DM_r	24	388	380	792	110
**KDM5B**	HNSC	HM_e	345	246	198	789	111
**HDGF**	HNSC	Others	166	270	352	788	112
**PAF1**	HNSC	Others	259	330	197	786	113
**KMT2B**	HNSC	HM_w	409	332	42	783	114
**IDH1**	HNSC	DM_e	163	344	272	779	115
**TCF20**	HNSC	Others	327	176	273	776	116
**TET1**	HNSC	DM_e	356	215	205	776	117
**PHC1**	HNSC	ChRC	116	342	317	775	118
**PRDM15**	HNSC	HM_w	255	324	196	775	119
**PHF19**	HNSC	HM_r	155	327	292	774	120
**KAT6B**	HNSC	HA_w	306	247	218	771	121
**BAZ2A**	HNSC	HA_r	291	171	306	768	122
**PSIP1**	HNSC	HM_r	339	386	42	767	123
**CDYL2**	HNSC	HM_r	289	254	214	757	124
**RAG2**	HNSC	HM_r	251	372	133	756	125
**MTA1**	HNSC	ChRC	302	69	384	755	126
**PHF12**	HNSC	Others	230	241	283	754	127
**AEBP2**	HNSC	HM_w	31	317	404	752	128
**CLOCK**	HNSC	HA_w	287	311	154	752	129
**BRDT**	HNSC	HA_r	313	169	268	750	130
**CBX3**	HNSC	HM_r	49	312	389	750	131
**SMARCC2**	HNSC	Helicases	298	143	307	748	132
**PRKCD**	HNSC	Others	153	236	358	747	133
**PBRM1**	HNSC	HA_r	379	265	102	746	134
**L3MBTL2**	HNSC	Others	233	192	320	745	135
**DOT1L**	HNSC	HM_w	351	69	323	743	136
**SCML2**	HNSC	HM_r	150	353	239	742	137
**JMJD1C**	HNSC	HM_e	406	288	42	736	138

**POLE3**	HNSC	ChRC	23	325	387	735	139
**NSD1**	HNSC	HM_w	425	267	42	734	140
**ARID1B**	HNSC	ChRC	399	292	42	733	141
**BRWD3**	HNSC	HA_r	408	69	255	732	142
**FBXO17**	HNSC	Others	88	333	310	731	143
**BRD8**	HNSC	HA_r	333	170	223	726	144
**NCOR2**	HNSC	Others	405	69	252	726	145
**TDRD7**	HNSC	Others	55	259	412	726	146
**ING4**	HNSC	HM_r	25	358	341	724	147
**KAT2A**	HNSC	HA_w	205	196	322	723	148
**PYGO2**	HNSC	HM_r	35	303	379	717	149
**SMARCB1**	HNSC	Helicases	60	260	397	717	150
**ARID1A**	HNSC	ChRC	417	257	42	716	151
**NCOR1**	HNSC	ChRC	388	286	42	716	152
**SP100**	HNSC	HA_r	296	320	100	716	153
**JARID2**	HNSC	ChRC	348	69	297	714	154
**CHD3**	HNSC	Helicases	397	69	247	713	155
**ING5**	HNSC	HM_r	207	357	147	711	156
**RNF2**	HNSC	ChRC	64	302	343	709	157
**CHD1L**	HNSC	Helicases	267	69	372	708	158
**HDAC10**	HNSC	HA_e	209	201	298	708	159
**ACTL6B**	HNSC	ChRC	214	410	82	706	160
**PHC2**	HNSC	ChRC	279	243	184	706	161
**TAF1**	HNSC	HA_r	367	69	269	705	162
**FBXL19**	HNSC	Others	173	165	366	704	163
**BRPF1**	HNSC	HA_r	270	69	363	702	164
**RING1**	HNSC	Others	152	234	316	702	165
**ING3**	HNSC	HM_r	125	308	266	699	166
**MSH6**	HNSC	HM_r	160	245	293	698	167
**PRDM12**	HNSC	HM_w	112	341	241	694	168
**CHD6**	HNSC	Helicases	402	69	222	693	169
**HDAC5**	HNSC	HA_e	239	200	254	693	170
**PHF1**	HNSC	HM_r	231	242	216	689	171
**SMARCE1**	HNSC	Helicases	144	230	315	689	172
**LBR**	HNSC	Others	40	287	361	688	173

**YY1**	HNSC	ChRC	218	69	401	688	174
**PCGF1**	HNSC	Others	156	189	340	685	175
**SP140L**	HNSC	HA_r	248	296	141	685	176
**TRIM28**	HNSC	HA_r	93	228	364	685	177
**CECR2**	HNSC	HA_r	381	69	233	683	178
**SETD6**	HNSC	HM_w	149	233	301	683	179
**SCMH1**	HNSC	Others	105	301	276	682	180
**SMARCA1**	HNSC	Helicases	191	321	170	682	181
**SETDB2**	HNSC	HM_w	147	322	210	679	182
**SUV39H1**	HNSC	HM_w	189	350	140	679	183
**ASXL3**	HNSC	Others	419	69	190	678	184
**KDM3A**	HNSC	HM_e	200	194	284	678	185
**ATAT1**	HNSC	Others	137	291	248	676	186
**EZH2**	HNSC	HM_w	210	334	130	674	187
**FMR1**	HNSC	Others	86	368	220	674	188
**SP140**	HNSC	HA_r	249	297	126	672	189
**CHD2**	HNSC	Helicases	376	253	42	671	190
**SMARCD3**	HNSC	Helicases	219	351	101	671	191
**TDRD1**	HNSC	Others	357	175	139	671	192
**RPA3**	HNSC	Others	21	340	308	669	193
**PHF2**	HNSC	Others	300	326	42	668	194
**PRMT2**	HNSC	HM_w	67	323	277	667	195
**FKBP2**	HNSC	Others	87	376	200	663	196
**ZMYND11**	HNSC	HA_r	272	349	42	663	197
**ZMYND8**	HNSC	HA_r	294	69	300	663	198
**SFMBT2**	HNSC	Others	384	69	209	662	199
**CBX6**	HNSC	HM_r	179	168	312	659	200
**TP53BP1**	HNSC	Others	395	69	193	657	201
**KDM4B**	HNSC	HM_e	305	69	279	653	202
**H3F3A**	HNSC	Others	26	271	355	652	203
**CBX2**	HNSC	ChRC	269	69	313	651	204
**HELLS**	HNSC	Helicases	238	248	164	650	205
**ERCC5**	HNSC	Others	332	273	42	647	206
**PRDM10**	HNSC	HM_w	299	304	42	645	207
**CHAF1A**	HNSC	ChRC	288	69	287	644	208

**ASH2L**	HNSC	HM_w	183	417	42	642	209
**DPF1**	HNSC	ChRC	129	310	201	640	210
**ATRX**	HNSC	Helicases	413	69	156	638	211
**EZH1**	HNSC	HM_w	242	205	188	635	212
**TET2**	HNSC	DM_e	336	141	158	635	213
**HDGFL1**	HNSC	Others	165	269	199	633	214
**NAP1L2**	HNSC	Others	261	69	302	632	215
**KDM6B**	HNSC	HM_e	344	69	217	630	216
**KDM7A**	HNSC	HM_e	325	69	236	630	217
**AFF1**	HNSC	Others	335	69	224	628	218
**MBD1**	HNSC	DM_r	199	331	97	627	219
**DMAP1**	HNSC	Others	131	250	245	626	220
**ING1**	HNSC	HM_r	162	359	103	624	221
**HNF1A**	HNSC	ChRC	324	162	137	623	222
**CARM1**	HNSC	HM_w	290	290	42	622	223
**ELP3**	HNSC	HA_w	211	369	42	622	224
**POLR2B**	HNSC	Others	340	240	42	622	225
**ZCWPW1**	HNSC	HM_r	32	411	178	621	226
**PYGO1**	HNSC	HM_r	223	149	240	612	227
**MPHOSPH8**	HNSC	HM_r	262	307	42	611	228
**HIST1H1B**	HNSC	Others	363	69	177	609	229
**PHF5A**	HNSC	Others	70	188	347	605	230
**SMARCD2**	HNSC	Helicases	59	180	365	604	231
**UBE2I**	HNSC	Others	92	173	335	600	232
**PCMT1**	HNSC	Others	117	219	263	599	233
**PRDM8**	HNSC	HM_w	226	238	135	599	234
**ZCWPW2**	HNSC	HM_r	217	283	99	599	235
**PCGF2**	HNSC	Others	39	220	339	598	236
**PHIP**	HNSC	HA_r	369	187	42	598	237
**FKBP5**	HNSC	Others	128	224	244	596	238
**MBD3**	HNSC	DM_r	198	222	175	595	239
**AFF4**	HNSC	Others	293	258	42	593	240
**TDRD10**	HNSC	Others	187	295	111	593	241
**KDM1A**	HNSC	HM_e	202	158	229	589	242
**DPF3**	HNSC	HA_r	212	275	98	585	243

**SMARCA2**	HNSC	Helicases	383	69	132	584	244
**TDRD3**	HNSC	HM_r	247	294	42	583	245
**PRDM16**	HNSC	HM_w	361	69	152	582	246
**SP110**	HNSC	HA_r	142	298	142	582	247
**ASXL2**	HNSC	Others	354	69	157	580	248
**CBX8**	HNSC	ChRC	177	69	332	578	249
**PRKAA2**	HNSC	Others	253	237	84	574	250
**HSPBAP1**	HNSC	Others	126	405	42	573	251
**PRDM5**	HNSC	HM_w	227	218	127	572	252
**G2E3**	HNSC	Others	171	69	330	570	253
**CDYL**	HNSC	HM_r	176	69	324	569	254
**PRMT1**	HNSC	HM_w	109	185	270	564	255
**SMYD1**	HNSC	HM_w	297	178	88	563	256
**CHD1**	HNSC	Helicases	311	209	42	562	257
**TRIM33**	HNSC	HA_r	140	380	42	562	258
**PPARGC1A**	HNSC	Others	228	239	94	561	259
**ORC1**	HNSC	Others	232	153	173	558	260
**AIRE**	HNSC	HM_r	184	282	87	553	261
**SIRT2**	HNSC	HA_e	102	300	150	552	262
**KANSL1**	HNSC	HA_w	347	161	42	550	263
**MBTD1**	HNSC	Others	75	155	319	549	264
**SMYD5**	HNSC	HM_w	96	177	274	547	265
**UHRF2**	HNSC	DM_r	91	413	42	546	266
**MSL3**	HNSC	HA_w	120	383	42	545	267
**CBX7**	HNSC	HM_r	178	210	155	543	268
**EED**	HNSC	ChRC	174	69	299	542	269
**FBXO44**	HNSC	Others	241	69	232	542	270
**KAT8**	HNSC	HA_w	79	159	304	542	271
**USP22**	HNSC	Others	139	69	334	542	272
**ING2**	HNSC	HM_r	42	389	109	540	273
**BPTF**	HNSC	HA_r	271	226	42	539	274
**HDAC2**	HNSC	HA_e	283	69	187	539	275
**HIST1H1C**	HNSC	Others	331	69	138	538	276
**RNF17**	HNSC	Others	374	69	93	536	277
**USP51**	HNSC	Others	185	69	282	536	278

**KDM1B**	HNSC	HM_e	201	69	265	535	279
**TRIM24**	HNSC	HA_r	274	69	192	535	280
**JMJD8**	HNSC	HM_e	41	197	296	534	281
**MBD5**	HNSC	DM_r	343	69	120	532	282
**EP300**	HNSC	HA_w	420	69	42	531	283
**GLYR1**	HNSC	HM_r	285	203	42	530	284
**L3MBTL1**	HNSC	HM_r	234	69	227	530	285
**CBX1**	HNSC	HM_r	134	69	326	529	286
**AICDA**	HNSC	DM_e	90	348	89	527	287
**SIRT1**	HNSC	HA_e	103	216	208	527	288
**HDAC1**	HNSC	HA_e	43	202	280	525	289
**PRDM4**	HNSC	HM_w	111	150	261	522	290
**FBXW9**	HNSC	Others	28	272	221	521	291
**KDM3B**	HNSC	HM_e	323	156	42	521	292
**JADE1**	HNSC	Others	246	69	204	519	293
**SFMBT1**	HNSC	HM_r	192	232	95	519	294
**L3MBTL3**	HNSC	Others	320	69	129	518	295
**RPH3A**	HNSC	Others	275	147	96	518	296
**JADE3**	HNSC	Others	245	69	203	517	297
**NAP1L3**	HNSC	Others	362	69	86	517	298
**SATB1**	HNSC	Others	220	184	112	516	299
**PHRF1**	HNSC	Others	400	69	42	511	300
**CHD5**	HNSC	Helicases	310	69	131	510	301
**IDH2**	HNSC	DM_e	81	198	230	509	302
**KAT2B**	HNSC	HA_w	204	195	108	507	303
**PRMT3**	HNSC	HM_w	252	69	183	504	304
**BAP1**	HNSC	Others	181	280	42	503	305
**CHD9**	HNSC	Helicases	386	69	42	497	306
**SMARCD1**	HNSC	Helicases	99	69	329	497	307
**RBBP4**	HNSC	ChRC	106	217	172	495	308
**MUM1**	HNSC	Others	74	244	174	492	309
**EPC2**	HNSC	Others	243	206	42	491	310
**SMYD3**	HNSC	HM_w	97	69	325	491	311
**DNMT3L**	HNSC	DM_w	130	276	83	489	312
**SHPRH**	HNSC	Others	378	69	42	489	313

**CHAF1B**	HNSC	ChRC	268	69	149	486	314
**CBX4**	HNSC	HM_r	133	69	281	483	315
**HIST1H3B**	HNSC	Others	266	69	148	483	316
**JADE2**	HNSC	Others	216	69	191	476	317
**PRDM1**	HNSC	HM_w	277	69	128	474	318
**PRDM2**	HNSC	HM_w	360	69	42	471	319
**FKBP1A**	HNSC	Others	27	69	370	466	320
**SMARCC1**	HNSC	Helicases	145	231	90	466	321
**PRMT6**	HNSC	HM_w	66	263	134	463	322
**PHF7**	HNSC	Others	113	264	85	462	323
**PHF11**	HNSC	Others	36	306	118	460	324
**TDRD12**	HNSC	Others	16	338	106	460	325
**RPS6KA5**	HNSC	Others	194	69	194	457	326
**PADI3**	HNSC	Others	260	69	124	453	327
**PHF21B**	HNSC	HM_r	195	151	107	453	328
**SIRT4**	HNSC	HA_e	62	182	207	451	329
**USP27X**	HNSC	Others	33	371	42	446	330
**GTF2B**	HNSC	Others	44	164	231	439	331
**PWWP2B**	HNSC	Others	224	69	145	438	332
**PHF13**	HNSC	Others	115	69	251	435	333
**BRD1**	HNSC	HA_r	180	212	42	434	334
**PHF23**	HNSC	HM_r	114	69	250	433	335
**RNF217**	HNSC	Others	151	69	211	431	336
**SIRT7**	HNSC	HA_e	101	69	259	429	337
**SIN3A**	HNSC	ChRC	317	69	42	428	338
**TAF3**	HNSC	HA_r	188	69	169	426	339
**SETD1B**	HNSC	HM_w	20	145	260	425	340
**SIRT5**	HNSC	HA_e	61	69	291	421	341
**JMJD6**	HNSC	HM_e	80	69	271	420	342
**AURKB**	HNSC	Others	182	69	168	419	343
**INO80**	HNSC	Helicases	307	69	42	418	344
**PHF6**	HNSC	HM_r	69	305	42	416	345
**HDAC8**	HNSC	HA_e	127	69	219	415	346
**MLLT10**	HNSC	HM_w	304	69	42	415	347
**BMI1**	HNSC	ChRC	136	69	202	407	348

**SIRT6**	HNSC	HA_e	19	181	206	406	349
**HIF1AN**	HNSC	Others	164	199	42	405	350
**KAT7**	HNSC	HA_w	203	160	42	405	351
**PRDM7**	HNSC	HM_w	110	69	226	405	352
**DPY30**	HNSC	Others	29	207	167	403	353
**SUZ12**	HNSC	ChRC	95	69	238	402	354
**UBE2A**	HNSC	Others	14	69	314	397	355
**NAP1L1**	HNSC	Others	73	69	253	395	356
**CSTL1**	HNSC	Others	47	225	121	393	357
**SMYD4**	HNSC	HM_w	143	69	181	393	358
**MTA3**	HNSC	ChRC	159	191	42	392	359
**TRIM66**	HNSC	HA_r	15	140	237	392	360
**PRMT7**	HNSC	HM_w	107	69	212	388	361
**BRD2**	HNSC	HA_r	89	255	42	386	362
**SIRT3**	HNSC	HA_e	146	69	171	386	363
**UHRF1**	HNSC	DM_r	273	69	42	384	364
**PRDM13**	HNSC	HM_w	154	69	160	383	365
**ZGPAT**	HNSC	Others	51	69	257	377	366
**PCGF6**	HNSC	Others	38	152	185	375	367
**HDAC3**	HNSC	HA_e	167	163	42	372	368
**BRD7**	HNSC	HA_r	50	279	42	371	369
**SETD5**	HNSC	HM_w	250	69	42	361	370
**SETD7**	HNSC	HM_w	148	69	144	361	371
**MTF2**	HNSC	HM_r	158	154	42	354	372
**CBX5**	HNSC	HM_r	48	69	234	351	373
**GTF2F1**	HNSC	Others	240	69	42	351	374
**PHF10**	HNSC	Others	37	69	242	348	375
**TCF19**	HNSC	Others	17	69	258	344	376
**MORF4L1**	HNSC	HM_r	121	69	153	343	377
**RBBP5**	HNSC	ChRC	65	235	42	342	378
**HDAC11**	HNSC	HA_e	168	69	104	341	379
**EPC1**	HNSC	Others	46	249	42	337	380
**RTF1**	HNSC	Others	221	69	42	332	381
**TDG**	HNSC	ChRC	141	69	122	332	382
**UBE2B**	HNSC	Others	13	69	249	331	383

**GATAD2A**	HNSC	HM_r	84	204	42	330	384
**SCML4**	HNSC	Others	104	69	151	324	385
**KDM8**	HNSC	HM_e	77	69	176	322	386
**WDR82**	HNSC	Others	52	227	42	321	387
**HDAC7**	HNSC	HA_e	208	69	42	319	388
**INTS12**	HNSC	Others	206	69	42	317	389
**PADI1**	HNSC	Others	119	69	125	313	390
**KDM4D**	HNSC	HM_e	78	69	163	310	391
**SMYD2**	HNSC	HM_w	98	69	143	310	392
**PADI4**	HNSC	Others	197	69	42	308	393
**PCGF5**	HNSC	Others	71	69	162	302	394
**UBR7**	HNSC	Others	54	69	179	302	395
**PHF8**	HNSC	Others	68	69	161	298	396
**SMARCA5**	HNSC	Helicases	100	144	42	286	397
**KDM4E**	HNSC	HM_e	124	69	92	285	398
**SUV39H2**	HNSC	HM_w	57	69	159	285	399
**FXR2**	HNSC	Others	172	69	42	283	400
**GTF2H1**	HNSC	Others	169	69	42	280	401
**PADI2**	HNSC	Others	118	69	91	278	402
**TDRD9**	HNSC	Others	94	69	115	278	403
**UBE2E1**	HNSC	Others	12	214	42	268	404
**H2AFZ**	HNSC	Others	83	69	113	265	405
**GADD45A**	HNSC	Others	85	69	110	264	406
**PADI6**	HNSC	Others	72	69	119	260	407
**PRDM6**	HNSC	HM_w	22	186	42	250	408
**KDM5D**	HNSC	HM_e	138	69	42	249	409
**SETMAR**	HNSC	HM_w	63	69	116	248	410
**SMNDC1**	HNSC	Others	18	179	42	239	411
**GADD45B**	HNSC	Others	45	69	114	228	412
**SETD4**	HNSC	HM_w	34	69	123	226	413
**MARCH5**	HNSC	Others	76	69	42	187	414
**SND1**	HNSC	HM_r	58	69	42	169	415
**UTY**	HNSC	HM_e	11	69	42	122	416
**EP400**	KICH	HA_w	424	414	345	1183	1
**BRWD3**	KICH	HA_r	410	419	352	1181	2

**GTF3C4**	KICH	HA_w	359	411	342	1112	3
**BOP 1**	KICH	Others	260	421	408	1089	4
**ATM**	KICH	Others	420	422	246	1088	5
**ZMYND11**	KICH	HA_r	282	382	423	1087	6
**MECP2**	KICH	DM_r	338	402	335	1075	7
**SMARCC1**	KICH	Helicases	386	392	290	1068	8
**FMR1**	KICH	Others	361	412	277	1050	9
**CHD7**	KICH	Helicases	370	418	239	1027	10
**ATRX**	KICH	Helicases	419	424	182	1025	11
**ATAD2**	KICH	HA_r	268	425	317	1010	12
**BRD3**	KICH	HA_r	258	420	315	993	13
**ZCWPW2**	KICH	HM_r	284	383	319	986	14
**JMJD1C**	KICH	HM_e	350	406	226	982	15
**JMJD8**	KICH	HM_e	170	405	368	943	16
**FKBP1A**	KICH	Others	212	413	310	935	17
**KMT2E**	KICH	HM_w	149	403	365	917	18
**BRD1**	KICH	HA_r	417	69	417	903	19
**RPS6KA5**	KICH	Others	309	394	200	903	20
**KMT2C**	KICH	HM_w	426	69	384	879	21
**NCOA3**	KICH	HA_w	421	69	383	873	22
**HDAC7**	KICH	HA_e	403	69	399	871	23
**CHD1L**	KICH	Helicases	372	69	426	867	24
**MTA1**	KICH	ChRC	397	69	397	863	25
**SMARCD3**	KICH	Helicases	53	391	419	863	26
**HCFC1**	KICH	Others	404	410	42	856	27
**PRDM9**	KICH	HM_w	423	69	360	852	28
**CXXC1**	KICH	Others	367	69	414	850	29
**DOT1L**	KICH	HM_w	406	69	371	846	30
**SMARCC2**	KICH	Helicases	385	69	392	846	31
**PRDM7**	KICH	HM_w	91	426	328	845	32
**CECR2**	KICH	HA_r	374	69	400	843	33
**HNF1A**	KICH	ChRC	416	69	341	826	34
**SETD2**	KICH	HM_w	391	393	42	826	35
**DAXX**	KICH	ChRC	231	416	177	824	36
**MTA2**	KICH	ChRC	335	69	420	824	37

**PHF10**	KICH	Others	330	69	425	824	38
**ARID4A**	KICH	ChRC	380	69	374	823	39
**FXR2**	KICH	Others	360	69	389	818	40
**BRD4**	KICH	HA_r	376	69	372	817	41
**MLLT10**	KICH	HM_w	337	69	411	817	42
**SND1**	KICH	HM_r	46	390	379	815	43
**ING5**	KICH	HM_r	178	407	228	813	44
**KDM5C**	KICH	HM_e	401	69	338	808	45
**UBE2I**	KICH	Others	20	386	401	807	46
**WDR5**	KICH	ChRC	15	384	405	804	47
**ASXL3**	KICH	Others	378	69	354	801	48
**FBXW9**	KICH	Others	362	69	369	800	49
**ATAT1**	KICH	Others	412	69	316	797	50
**NAP1L3**	KICH	Others	130	400	266	796	51
**PHF20L1**	KICH	HM_r	328	423	42	793	52
**PHF8**	KICH	Others	395	69	329	793	53
**PRDM10**	KICH	HM_w	99	398	296	793	54
**CHAF1A**	KICH	ChRC	373	69	349	791	55
**CHD4**	KICH	Helicases	408	69	314	791	56
**PRDM12**	KICH	HM_w	97	397	295	789	57
**PPARGC1A**	KICH	Others	321	69	396	786	58
**DNMT1**	KICH	DM_w	365	69	346	780	59
**SIRT3**	KICH	HA_e	387	69	324	780	60
**KDM3A**	KICH	HM_e	343	69	367	779	61
**SMARCB1**	KICH	Helicases	299	69	410	778	62
**KMT2A**	KICH	HM_w	150	404	221	775	63
**MARCH5**	KICH	Others	340	69	363	772	64
**PRDM8**	KICH	HM_w	317	69	381	767	65
**SMARCA4**	KICH	Helicases	301	69	393	763	66
**ASH2L**	KICH	HM_w	270	69	422	761	67
**EHMT2**	KICH	HM_w	405	69	281	755	68
**ING3**	KICH	HM_r	180	408	167	755	69
**SETD1A**	KICH	HM_w	392	69	293	754	70
**BAZ2B**	KICH	HA_r	262	69	421	752	71
**HR**	KICH	HM_e	402	69	271	742	72

**BRPF3**	KICH	HA_r	252	69	418	739	73
**SCML4**	KICH	Others	306	69	358	733	74
**ARID2**	KICH	ChRC	273	69	390	732	75
**ZGPAT**	KICH	Others	283	69	378	730	76
**TAF1**	KICH	HA_r	296	389	42	727	77
**ACTL6A**	KICH	ChRC	281	69	376	726	78
**INO80**	KICH	Helicases	351	69	303	723	79
**CLOCK**	KICH	HA_w	369	69	283	721	80
**ARID1A**	KICH	ChRC	275	69	375	719	81
**TDG**	KICH	ChRC	293	69	357	719	82
**PHF19**	KICH	HM_r	106	399	209	714	83
**SIN3B**	KICH	ChRC	389	69	255	713	84
**RING1**	KICH	Others	312	69	327	708	85
**BAZ1A**	KICH	HA_r	264	69	373	706	86
**DNMT3B**	KICH	DM_w	229	69	407	705	87
**KMT2D**	KICH	HM_w	415	69	220	704	88
**TRIM24**	KICH	HA_r	27	387	288	702	89
**H2AFZ**	KICH	Others	358	69	274	701	90
**TDRD1**	KICH	Others	382	69	250	701	91
**PRDM14**	KICH	HM_w	96	396	207	699	92
**ARID1B**	KICH	ChRC	274	69	355	698	93
**HIRA**	KICH	Others	353	69	273	695	94
**SIRT6**	KICH	HA_e	303	69	322	694	95
**ATF7IP**	KICH	Others	377	69	247	693	96
**CHRAC1**	KICH	ChRC	234	417	42	693	97
**PHF5A**	KICH	Others	324	69	297	690	98
**KAT2B**	KICH	HA_w	349	69	269	687	99
**NAP1L2**	KICH	Others	131	401	155	687	100
**BAZ2A**	KICH	HA_r	263	69	353	685	101
**MPHOSPH8**	KICH	HM_r	399	69	216	684	102
**KDM1A**	KICH	HM_e	346	69	268	683	103
**MSH6**	KICH	HM_r	398	69	215	682	104
**HIST1H1B**	KICH	Others	188	69	424	681	105
**CHD2**	KICH	Helicases	371	69	240	680	106
**EHMT1**	KICH	HM_w	222	415	42	679	107

**PYGO1**	KICH	HM_r	81	395	203	679	108
**HDAC3**	KICH	HA_e	195	69	413	677	109
**DMAP1**	KICH	Others	366	69	238	673	110
**HDAC10**	KICH	HA_e	197	69	406	672	111
**CARM1**	KICH	HM_w	251	69	351	671	112
**RTF1**	KICH	Others	307	69	294	670	113
**HIST1H1C**	KICH	Others	187	69	412	668	114
**AICDA**	KICH	DM_e	413	69	185	667	115
**DPF1**	KICH	ChRC	227	69	370	666	116
**SETD1B**	KICH	HM_w	305	69	292	666	117
**FBXO17**	KICH	Others	363	69	233	665	118
**CBX6**	KICH	HM_r	245	69	350	664	119
**HELLS**	KICH	Helicases	190	69	404	663	120
**RPH3A**	KICH	Others	393	69	201	663	121
**GTF2B**	KICH	Others	203	69	388	660	122
**SMARCA5**	KICH	Helicases	300	69	291	660	123
**PHF21A**	KICH	HM_r	327	69	263	659	124
**CHAF1B**	KICH	ChRC	240	69	348	657	125
**ASXL1**	KICH	Others	269	69	318	656	126
**CHD3**	KICH	Helicases	409	69	178	656	127
**HDAC9**	KICH	HA_e	355	69	231	655	128
**HDGF**	KICH	Others	354	69	230	653	129
**HIST1H3B**	KICH	Others	186	69	398	653	130
**DIDO1**	KICH	Others	407	69	176	652	131
**PRDM1**	KICH	HM_w	320	69	262	651	132
**CTCF**	KICH	Others	232	69	347	648	133
**STK31**	KICH	Others	383	69	194	646	134
**HDAC8**	KICH	HA_e	192	409	42	643	135
**KAT6B**	KICH	HA_w	348	69	225	642	136
**KAT7**	KICH	HA_w	347	69	224	640	137
**AFF1**	KICH	Others	381	69	186	636	138
**AEBP2**	KICH	HM_w	279	69	286	634	139
**ASXL2**	KICH	Others	379	69	183	631	140
**EZH2**	KICH	HM_w	215	69	344	628	141
**JARID2**	KICH	ChRC	172	69	387	628	142

**MBD5**	KICH	DM_r	400	69	157	626	143
**MECOM**	KICH	Others	339	69	218	626	144
**CHD8**	KICH	Helicases	236	69	313	618	145
**KDM3B**	KICH	HM_e	163	69	386	618	146
**GTF2F1**	KICH	Others	202	69	343	614	147
**PCGF1**	KICH	Others	331	69	212	612	148
**TDRD6**	KICH	Others	292	69	249	610	149
**BRD9**	KICH	HA_r	255	69	285	609	150
**EPC2**	KICH	Others	364	69	174	607	151
**KDM8**	KICH	HM_e	152	69	385	606	152
**EED**	KICH	ChRC	223	69	312	604	153
**EPC1**	KICH	Others	218	69	311	598	154
**KDM4B**	KICH	HM_e	161	69	366	596	155
**CHD5**	KICH	Helicases	238	69	284	591	156
**PAXIP1**	KICH	Others	119	69	403	591	157
**ARID4B**	KICH	ChRC	272	69	248	589	158
**ING1**	KICH	HM_r	352	69	168	589	159
**PRKAA1**	KICH	Others	316	69	204	589	160
**FKBP5**	KICH	Others	210	69	309	588	161
**G2E3**	KICH	Others	209	69	308	586	162
**INTS12**	KICH	Others	177	69	340	586	163
**RNF17**	KICH	Others	311	69	202	582	164
**AURKB**	KICH	Others	266	69	245	580	165
**DNMT3L**	KICH	DM_w	228	69	282	579	166
**GTF2H1**	KICH	Others	201	69	307	577	167
**LBR**	KICH	Others	144	69	364	577	168
**PADI1**	KICH	Others	126	69	382	577	169
**H3F3A**	KICH	Others	200	69	306	575	170
**KDM2B**	KICH	HM_e	344	69	160	573	171
**ELP3**	KICH	HA_w	221	69	280	570	172
**HDAC5**	KICH	HA_e	194	69	305	568	173
**BRDT**	KICH	HA_r	254	69	244	567	174
**SMARCA1**	KICH	Helicases	302	69	196	567	175
**KDM4E**	KICH	HM_e	158	69	339	566	176
**FBXL19**	KICH	Others	214	69	279	562	177

**SRCAP**	KICH	Others	298	69	195	562	178
**MLLT6**	KICH	HM_w	336	69	156	561	179
**CBX4**	KICH	HM_r	247	69	243	559	180
**FKBP2**	KICH	Others	211	69	278	558	181
**KIAA2026**	KICH	Others	151	69	337	557	182
**TAF1L**	KICH	HA_r	295	69	193	557	183
**NCOR2**	KICH	Others	334	69	153	556	184
**IDH2**	KICH	DM_e	182	69	304	555	185
**CDYL**	KICH	HM_r	242	69	242	553	186
**GADD45B**	KICH	Others	207	69	276	552	187
**CDYL2**	KICH	HM_r	241	69	241	551	188
**PRMT2**	KICH	HM_w	87	69	395	551	189
**TDRD9**	KICH	Others	291	69	191	551	190
**GATAD2A**	KICH	HM_r	206	69	275	550	191
**MBD2**	KICH	DM_r	142	69	336	547	192
**TET3**	KICH	DM_e	289	69	189	547	193
**PHF21B**	KICH	HM_r	326	69	146	541	194
**PHF14**	KICH	Others	107	69	362	538	195
**RNF20**	KICH	Others	75	69	394	538	196
**ACTL6B**	KICH	ChRC	280	69	187	536	197
**BPTF**	KICH	HA_r	425	69	42	536	198
**DNMT3A**	KICH	DM_w	230	69	237	536	199
**KAT8**	KICH	HA_w	165	69	302	536	200
**MUM1**	KICH	Others	133	69	334	536	201
**NAP1L1**	KICH	Others	132	69	333	534	202
**SMARCA2**	KICH	Helicases	422	69	42	533	203
**AIRE**	KICH	HM_r	277	69	184	530	204
**DPF3**	KICH	HA_r	225	69	236	530	205
**SIRT2**	KICH	HA_e	59	69	402	530	206
**BAZ1B**	KICH	HA_r	418	69	42	529	207
**KDM4D**	KICH	HM_e	159	69	301	529	208
**DPY30**	KICH	Others	224	69	235	528	209
**PRDM11**	KICH	HM_w	98	69	361	528	210
**SP140**	KICH	HA_r	43	69	416	528	211
**HLTF**	KICH	Others	185	69	272	526	212

**PRDM2**	KICH	HM_w	414	69	42	525	213
**ATR**	KICH	Others	411	69	42	522	214
**KDM7A**	KICH	HM_e	153	69	300	522	215
**ING2**	KICH	HM_r	181	69	270	520	216
**EZH1**	KICH	HM_w	216	69	234	519	217
**PHC1**	KICH	ChRC	114	69	332	515	218
**SETD7**	KICH	HM_w	66	69	380	515	219
**SMARCD1**	KICH	Helicases	55	69	391	515	220
**L3MBTL3**	KICH	Others	146	69	299	514	221
**PHF1**	KICH	HM_r	111	69	331	511	222
**SIN3A**	KICH	ChRC	304	69	135	508	223
**NCOR1**	KICH	ChRC	396	69	42	507	224
**RAG2**	KICH	HM_r	79	69	359	507	225
**GATAD2B**	KICH	HM_r	205	69	232	506	226
**RAI1**	KICH	Others	394	69	42	505	227
**PHF20**	KICH	HM_r	105	69	330	504	228
**SETD6**	KICH	HM_w	390	69	42	501	229
**USP51**	KICH	Others	17	69	415	501	230
**CBX1**	KICH	HM_r	250	69	181	500	231
**SIRT1**	KICH	HA_e	388	69	42	499	232
**CBX2**	KICH	ChRC	249	69	180	498	233
**SMYD1**	KICH	HM_w	384	69	42	495	234
**CBX8**	KICH	ChRC	243	69	179	491	235
**YY1**	KICH	ChRC	13	69	409	491	236
**HIF1AN**	KICH	Others	189	69	229	487	237
**BRWD1**	KICH	HA_r	375	69	42	486	238
**PCGF5**	KICH	Others	117	69	298	484	239
**CREBBP**	KICH	HA_w	368	69	42	479	240
**MBD3**	KICH	DM_r	141	69	267	477	241
**JADE1**	KICH	Others	175	69	227	471	242
**HDAC11**	KICH	HA_e	357	69	42	468	243
**HDAC4**	KICH	HA_e	356	69	42	467	244
**TCEA1**	KICH	Others	36	388	42	466	245
**ELP4**	KICH	HA_w	220	69	175	464	246
**SETD3**	KICH	HM_w	69	69	326	464	247

**NSD1**	KICH	HM_w	128	69	265	462	248
**ORC1**	KICH	Others	127	69	264	460	249
**ERCC5**	KICH	Others	217	69	173	459	250
**TDRD12**	KICH	Others	33	69	356	458	251
**ZMYND8**	KICH	HA_r	11	69	377	457	252
**KDM2A**	KICH	HM_e	345	69	42	456	253
**SFMBT1**	KICH	HM_r	62	69	325	456	254
**FBXO44**	KICH	Others	213	69	172	454	255
**KDM5A**	KICH	HM_e	342	69	42	453	256
**KMT2B**	KICH	HM_w	341	69	42	452	257
**SIRT4**	KICH	HA_e	58	69	323	450	258
**KDM5B**	KICH	HM_e	157	69	223	449	259
**KDM6A**	KICH	HM_e	155	69	222	446	260
**UBR7**	KICH	Others	19	385	42	446	261
**PADI4**	KICH	Others	333	69	42	444	262
**PBRM1**	KICH	HA_r	332	69	42	443	263
**PHF2**	KICH	Others	329	69	42	440	264
**HDAC2**	KICH	HA_e	196	69	171	436	265
**L3MBTL1**	KICH	HM_r	148	69	219	436	266
**PHF3**	KICH	Others	325	69	42	436	267
**PHIP**	KICH	HA_r	323	69	42	434	268
**PHRF1**	KICH	Others	322	69	42	433	269
**HDAC6**	KICH	HA_e	193	69	170	432	270
**PRDM13**	KICH	HM_w	319	69	42	430	271
**PRDM16**	KICH	HM_w	318	69	42	429	272
**PRMT5**	KICH	HM_w	315	69	42	426	273
**PRMT7**	KICH	HM_w	314	69	42	425	274
**MEN1**	KICH	ChRC	138	69	217	424	275
**RBBP5**	KICH	ChRC	313	69	42	424	276
**IDH1**	KICH	DM_e	183	69	169	421	277
**RNF40**	KICH	Others	310	69	42	421	278
**TDRD5**	KICH	Others	31	69	321	421	279
**PRKCD**	KICH	Others	89	69	261	419	280
**RSF1**	KICH	ChRC	308	69	42	419	281
**MTF2**	KICH	HM_r	134	69	214	417	282

**PRMT1**	KICH	HM_w	88	69	260	417	283
**TRIM28**	KICH	HA_r	26	69	320	415	284
**PRMT8**	KICH	HM_w	84	69	259	412	285
**IWS1**	KICH	Others	176	69	166	411	286
**SUPT16H**	KICH	Others	297	69	42	408	287
**JADE3**	KICH	Others	173	69	165	407	288
**TCF20**	KICH	Others	294	69	42	405	289
**JMJD6**	KICH	HM_e	171	69	164	404	290
**PAF1**	KICH	Others	122	69	213	404	291
**KANSL1**	KICH	HA_w	169	69	163	401	292
**TET1**	KICH	DM_e	290	69	42	401	293
**RPA3**	KICH	Others	73	69	258	400	294
**KAT2A**	KICH	HA_w	168	69	162	399	295
**TP53BP1**	KICH	Others	288	69	42	399	296
**PCGF2**	KICH	Others	118	69	211	398	297
**SATB1**	KICH	Others	72	69	257	398	298
**UHRF1**	KICH	DM_r	287	69	42	398	299
**SUV39H2**	KICH	HM_w	39	69	289	397	300
**UHRF2**	KICH	DM_r	286	69	42	397	301
**USP27X**	KICH	Others	285	69	42	396	302
**PCGF6**	KICH	Others	116	69	210	395	303
**KDM1B**	KICH	HM_e	164	69	161	394	304
**AFF4**	KICH	Others	278	69	42	389	305
**AKAP1**	KICH	Others	276	69	42	387	306
**SFMBT2**	KICH	Others	61	69	256	386	307
**ASH1L**	KICH	HM_w	271	69	42	382	308
**KDM6B**	KICH	HM_e	154	69	159	382	309
**PHF7**	KICH	Others	102	69	208	379	310
**ATAD2B**	KICH	HA_r	267	69	42	378	311
**BAP1**	KICH	Others	265	69	42	376	312
**BMI1**	KICH	ChRC	261	69	42	372	313
**L3MBTL4**	KICH	Others	145	69	158	372	314
**SMYD3**	KICH	HM_w	49	69	254	372	315
**BRD2**	KICH	HA_r	259	69	42	370	316
**PRDM15**	KICH	HM_w	95	69	206	370	317

**BRD7**	KICH	HA_r	257	69	42	368	318
**ZCWPW1**	KICH	HM_r	12	69	287	368	319
**BRD8**	KICH	HA_r	256	69	42	367	320
**PRDM5**	KICH	HM_w	93	69	205	367	321
**BRPF1**	KICH	HA_r	253	69	42	364	322
**SP140L**	KICH	HA_r	42	69	253	364	323
**CBX3**	KICH	HM_r	248	69	42	359	324
**TAF3**	KICH	HA_r	37	69	252	358	325
**CBX5**	KICH	HM_r	246	69	42	357	326
**CBX7**	KICH	HM_r	244	69	42	355	327
**TCF19**	KICH	Others	35	69	251	355	328
**NCOA1**	KICH	HA_w	129	69	154	352	329
**CHD1**	KICH	Helicases	239	69	42	350	330
**CHD6**	KICH	Helicases	237	69	42	348	331
**CHD9**	KICH	Helicases	235	69	42	346	332
**PADI2**	KICH	Others	125	69	152	346	333
**CSTL1**	KICH	Others	233	69	42	344	334
**PADI3**	KICH	Others	124	69	151	344	335
**PADI6**	KICH	Others	123	69	150	342	336
**DPF2**	KICH	Others	226	69	42	337	337
**SETD5**	KICH	HM_w	67	69	199	335	338
**PCMT1**	KICH	Others	115	69	149	333	339
**SETDB2**	KICH	HM_w	64	69	198	331	340
**EP300**	KICH	HA_w	219	69	42	330	341
**PHC2**	KICH	ChRC	113	69	148	330	342
**PHF13**	KICH	Others	108	69	147	324	343
**SIRT7**	KICH	HA_e	56	69	197	322	344
**GADD45A**	KICH	Others	208	69	42	319	345
**PHF23**	KICH	HM_r	104	69	145	318	346
**GLYR1**	KICH	HM_r	204	69	42	315	347
**HAT1**	KICH	HA_w	199	69	42	310	348
**HDAC1**	KICH	HA_e	198	69	42	309	349
**PRKAA2**	KICH	Others	90	69	144	303	350
**HDGFL1**	KICH	Others	191	69	42	302	351
**HSPBAP1**	KICH	Others	184	69	42	295	352

**TDRD10**	KICH	Others	34	69	192	295	353
**PWWP2B**	KICH	Others	82	69	143	294	354
**PYGO2**	KICH	HM_r	80	69	142	291	355
**ING4**	KICH	HM_r	179	69	42	290	356
**RBBP7**	KICH	ChRC	77	69	141	287	357
**TET2**	KICH	DM_e	28	69	190	287	358
**JADE2**	KICH	Others	174	69	42	285	359
**RNF2**	KICH	ChRC	76	69	140	285	360
**RNF217**	KICH	Others	74	69	139	282	361
**TRIM66**	KICH	HA_r	24	69	188	281	362
**KAT5**	KICH	HA_w	167	69	42	278	363
**KAT6A**	KICH	HA_w	166	69	42	277	364
**SCML2**	KICH	HM_r	70	69	138	277	365
**KDM4A**	KICH	HM_e	162	69	42	273	366
**KDM4C**	KICH	HM_e	160	69	42	271	367
**SETDB1**	KICH	HM_w	65	69	137	271	368
**SETMAR**	KICH	HM_w	63	69	136	268	369
**KDM5D**	KICH	HM_e	156	69	42	267	370
**L3MBTL2**	KICH	Others	147	69	42	258	371
**SMARCE1**	KICH	Helicases	52	69	134	255	372
**MBD1**	KICH	DM_r	143	69	42	254	373
**MBD4**	KICH	DM_r	140	69	42	251	374
**MBTD1**	KICH	Others	139	69	42	250	375
**SMYD4**	KICH	HM_w	48	69	133	250	376
**MORF4L1**	KICH	HM_r	137	69	42	248	377
**MSL3**	KICH	HA_w	136	69	42	247	378
**MTA3**	KICH	ChRC	135	69	42	246	379
**SP100**	KICH	HA_r	45	69	132	246	380
**SUV39H1**	KICH	HM_w	40	69	131	240	381
**PARP1**	KICH	Others	121	69	42	232	382
**PARP2**	KICH	Others	120	69	42	231	383
**TDRKH**	KICH	Others	29	69	130	228	384
**PHC3**	KICH	ChRC	112	69	42	223	385
**TRIM33**	KICH	HA_r	25	69	129	223	386
**PHF11**	KICH	Others	110	69	42	221	387

**PHF12**	KICH	Others	109	69	42	220	388
**UBE2A**	KICH	Others	23	69	128	220	389
**PHF6**	KICH	HM_r	103	69	42	214	390
**POLE3**	KICH	ChRC	101	69	42	212	391
**POLR2B**	KICH	Others	100	69	42	211	392
**PRDM4**	KICH	HM_w	94	69	42	205	393
**PRDM6**	KICH	HM_w	92	69	42	203	394
**PRMT3**	KICH	HM_w	86	69	42	197	395
**PRMT6**	KICH	HM_w	85	69	42	196	396
**PSIP1**	KICH	HM_r	83	69	42	194	397
**RBBP4**	KICH	ChRC	78	69	42	189	398
**SCMH1**	KICH	Others	71	69	42	182	399
**SETD4**	KICH	HM_w	68	69	42	179	400
**SHPRH**	KICH	Others	60	69	42	171	401
**SIRT5**	KICH	HA_e	57	69	42	168	402
**SMARCD2**	KICH	Helicases	54	69	42	165	403
**SMNDC1**	KICH	Others	51	69	42	162	404
**SMYD2**	KICH	HM_w	50	69	42	161	405
**SMYD5**	KICH	HM_w	47	69	42	158	406
**SP110**	KICH	HA_r	44	69	42	155	407
**SSRP1**	KICH	Others	41	69	42	152	408
**SUZ12**	KICH	ChRC	38	69	42	149	409
**TDRD3**	KICH	HM_r	32	69	42	143	410
**TDRD7**	KICH	Others	30	69	42	141	411
**UBE2B**	KICH	Others	22	69	42	133	412
**UBE2E1**	KICH	Others	21	69	42	132	413
**USP22**	KICH	Others	18	69	42	129	414
**UTY**	KICH	HM_e	16	69	42	127	415
**WDR82**	KICH	Others	14	69	42	125	416
**NSD1**	KIRC	HM_w	402	426	415	1243	1
**PBRM1**	KIRC	HA_r	426	410	377	1213	2
**BAP1**	KIRC	Others	424	408	350	1182	3
**BRD8**	KIRC	HA_r	335	422	422	1179	4
**KMT2C**	KIRC	HM_w	420	394	364	1178	5
**KDM3B**	KIRC	HM_e	323	424	425	1172	6

**ATR**	KIRC	Others	391	396	366	1153	7
**KDM5C**	KIRC	HM_e	423	381	347	1151	8
**BRD9**	KIRC	HA_r	334	385	413	1132	9
**BAZ1B**	KIRC	HA_r	338	372	420	1130	10
**CHD1**	KIRC	Helicases	370	403	348	1121	11
**SETD2**	KIRC	HM_w	425	417	260	1102	12
**ARID1B**	KIRC	ChRC	354	342	404	1100	13
**EZH2**	KIRC	HM_w	300	392	407	1099	14
**AKAP1**	KIRC	Others	342	375	373	1090	15
**ZCWPW1**	KIRC	HM_r	372	376	324	1072	16
**KAT6A**	KIRC	HA_w	380	359	330	1069	17
**ASH1L**	KIRC	HM_w	409	374	285	1068	18
**AFF4**	KIRC	Others	228	423	391	1042	19
**ASH2L**	KIRC	HM_w	226	397	419	1042	20
**KAT2B**	KIRC	HA_w	325	418	292	1035	21
**SND1**	KIRC	HM_r	239	350	417	1006	22
**DIDO1**	KIRC	Others	350	288	365	1003	23
**PHF20**	KIRC	HM_r	360	234	409	1003	24
**SMARCA2**	KIRC	Helicases	312	306	376	994	25
**PHF20L1**	KIRC	HM_r	291	380	318	989	26
**MECOM**	KIRC	Others	320	402	266	988	27
**CHD3**	KIRC	Helicases	412	291	283	986	28
**HAT1**	KIRC	HA_w	213	383	389	985	29
**BAZ2B**	KIRC	HA_r	404	339	238	981	30
**PHF14**	KIRC	Others	250	316	410	976	31
**MSH6**	KIRC	HM_r	319	252	395	966	32
**TRIM24**	KIRC	HA_r	235	345	382	962	33
**SETD5**	KIRC	HM_w	384	412	160	956	34
**BRWD1**	KIRC	HA_r	403	295	255	953	35
**PAXIP1**	KIRC	Others	131	390	423	944	36
**CHRAC1**	KIRC	ChRC	154	384	402	940	37
**KDM5A**	KIRC	HM_e	348	324	267	939	38
**RNF40**	KIRC	Others	315	215	406	936	39
**PRKCD**	KIRC	Others	396	404	131	931	40
**HDAC9**	KIRC	HA_e	262	330	332	924	41

**PCGF2**	KIRC	Others	377	240	305	922	42
**ATRX**	KIRC	Helicases	405	373	143	921	43
**MECP2**	KIRC	DM_r	199	391	328	918	44
**TDRD10**	KIRC	Others	310	348	258	916	45
**HDAC3**	KIRC	HA_e	62	425	424	911	46
**SMARCC1**	KIRC	Helicases	171	411	325	907	47
**BRD1**	KIRC	HA_r	305	296	298	899	48
**USP51**	KIRC	Others	308	377	212	897	49
**GLYR1**	KIRC	HM_r	215	277	403	895	50
**NAP1L2**	KIRC	Others	193	357	340	890	51
**SUPT16H**	KIRC	Others	400	69	418	887	52
**DNMT1**	KIRC	DM_w	419	287	180	886	53
**SIRT7**	KIRC	HA_e	174	352	358	884	54
**CHAF1A**	KIRC	ChRC	332	337	211	880	55
**EP400**	KIRC	HA_w	417	69	390	876	56
**BRWD3**	KIRC	HA_r	333	370	171	874	57
**ACTL6A**	KIRC	ChRC	91	401	380	872	58
**SETD3**	KIRC	HM_w	177	308	384	869	59
**SMARCC2**	KIRC	Helicases	410	69	388	867	60
**JMJD6**	KIRC	HM_e	326	360	178	864	61
**DNMT3B**	KIRC	DM_w	408	69	385	862	62
**KDM4B**	KIRC	HM_e	204	326	329	859	63
**NCOR2**	KIRC	Others	379	69	411	859	64
**HLTF**	KIRC	Others	259	382	209	850	65
**IDH2**	KIRC	DM_e	328	270	251	849	66
**BRD2**	KIRC	HA_r	390	69	386	845	67
**KDM5B**	KIRC	HM_e	363	262	218	843	68
**PRDM14**	KIRC	HM_w	288	378	177	843	69
**AFF1**	KIRC	Others	229	299	314	842	70
**PCGF1**	KIRC	Others	378	69	394	841	71
**GTF2F1**	KIRC	Others	149	332	359	840	72
**NCOA3**	KIRC	HA_w	387	249	204	840	73
**HDGF**	KIRC	Others	60	363	416	839	74
**KAT8**	KIRC	HA_w	205	265	369	839	75
**EZH1**	KIRC	HM_w	218	285	334	837	76

**KMT2D**	KIRC	HM_w	422	69	346	837	77
**PAF1**	KIRC	Others	251	242	344	837	78
**TDRD5**	KIRC	Others	276	347	213	836	79
**KDM6A**	KIRC	HM_e	295	323	217	835	80
**TDRD6**	KIRC	Others	393	197	241	831	81
**UBE2B**	KIRC	Others	18	421	392	831	82
**BRPF1**	KIRC	HA_r	159	414	256	829	83
**PHF2**	KIRC	Others	190	235	393	818	84
**TDRKH**	KIRC	Others	166	346	300	812	85
**PADI6**	KIRC	Others	362	243	203	808	86
**ARID1A**	KIRC	ChRC	421	343	42	806	87
**HR**	KIRC	HM_e	209	398	197	804	88
**HCFC1**	KIRC	Others	366	395	42	803	89
**HDAC5**	KIRC	HA_e	211	272	320	803	90
**SFMBT1**	KIRC	HM_r	281	405	117	803	91
**CHD5**	KIRC	Helicases	369	290	142	801	92
**BAZ2A**	KIRC	HA_r	352	69	379	800	93
**ATAD2**	KIRC	HA_r	89	387	323	799	94
**BAZ1A**	KIRC	HA_r	160	340	299	799	95
**PRDM10**	KIRC	HM_w	376	379	42	797	96
**ING3**	KIRC	HM_r	52	361	383	796	97
**ATM**	KIRC	Others	414	69	312	795	98
**ORC1**	KIRC	Others	318	247	230	795	99
**PADI3**	KIRC	Others	292	318	185	795	100
**PRDM9**	KIRC	HM_w	346	221	228	795	101
**SETDB1**	KIRC	HM_w	395	353	42	790	102
**HDAC10**	KIRC	HA_e	263	274	252	789	103
**HDAC7**	KIRC	HA_e	146	271	370	787	104
**CHD8**	KIRC	Helicases	304	69	412	785	105
**BPTF**	KIRC	HA_r	371	371	42	784	106
**KDM7A**	KIRC	HM_e	355	69	357	781	107
**RING1**	KIRC	Others	358	69	353	780	108
**BRD7**	KIRC	HA_r	336	69	372	777	109
**KAT2A**	KIRC	HA_w	257	267	250	774	110
**DPF3**	KIRC	HA_r	221	393	159	773	111

**MBTD1**	KIRC	Others	200	321	249	770	112
**GATAD2B**	KIRC	HM_r	216	365	187	768	113
**JADE2**	KIRC	Others	272	69	426	767	114
**KDM6B**	KIRC	HM_e	294	261	207	762	115
**ASXL1**	KIRC	Others	339	69	351	759	116
**FBXL19**	KIRC	Others	151	334	272	757	117
**PHF8**	KIRC	Others	359	356	42	757	118
**MLLT6**	KIRC	HM_w	252	254	248	754	119
**PARP1**	KIRC	Others	317	241	196	754	120
**RNF20**	KIRC	Others	285	69	400	754	121
**SIRT6**	KIRC	HA_e	104	307	343	754	122
**ARID2**	KIRC	ChRC	341	69	342	752	123
**MTA2**	KIRC	ChRC	195	251	306	752	124
**JMJD8**	KIRC	HM_e	49	327	375	751	125
**PYGO2**	KIRC	HM_r	32	355	363	750	126
**CBX1**	KIRC	HM_r	158	294	297	749	127
**CHD7**	KIRC	Helicases	407	69	273	749	128
**CDYL2**	KIRC	HM_r	155	292	296	743	129
**RBBP7**	KIRC	ChRC	31	354	354	739	130
**ATF7IP**	KIRC	Others	353	69	313	735	131
**TAF1**	KIRC	HA_r	344	349	42	735	132
**CBX4**	KIRC	HM_r	82	368	284	734	133
**ASXL3**	KIRC	Others	383	69	274	726	134
**RPA3**	KIRC	Others	109	214	401	724	135
**ARID4A**	KIRC	ChRC	340	341	42	723	136
**KMT2E**	KIRC	HM_w	321	358	42	721	137
**EED**	KIRC	ChRC	74	335	311	720	138
**SETMAR**	KIRC	HM_w	176	409	135	720	139
**RTF1**	KIRC	Others	244	211	261	716	140
**SATB1**	KIRC	Others	179	419	118	716	141
**SMARCD3**	KIRC	Helicases	24	389	301	714	142
**STK31**	KIRC	Others	374	201	138	713	143
**JADE3**	KIRC	Others	307	69	336	712	144
**DNMT3A**	KIRC	DM_w	406	69	236	711	145
**FBXO44**	KIRC	Others	72	283	356	711	146

**CHD1L**	KIRC	Helicases	79	336	295	710	147
**YY1**	KIRC	ChRC	12	300	398	710	148
**PRMT8**	KIRC	HM_w	248	313	148	709	149
**UBE2A**	KIRC	Others	165	304	240	709	150
**IWS1**	KIRC	Others	296	69	341	706	151
**MSL3**	KIRC	HA_w	135	320	247	702	152
**UHRF1**	KIRC	DM_r	356	303	42	701	153
**RBBP5**	KIRC	ChRC	345	312	42	699	154
**PRDM15**	KIRC	HM_w	184	314	200	698	155
**CBX8**	KIRC	ChRC	156	367	170	693	156
**TP53BP1**	KIRC	Others	343	196	154	693	157
**HDAC6**	KIRC	HA_e	329	69	294	692	158
**PHC1**	KIRC	ChRC	127	238	327	692	159
**PHC3**	KIRC	ChRC	126	400	166	692	160
**KMT2A**	KIRC	HM_w	389	69	232	690	161
**PHF21A**	KIRC	HM_r	189	233	265	687	162
**PRDM2**	KIRC	HM_w	287	223	176	686	163
**HDAC11**	KIRC	HA_e	147	413	124	684	164
**SUZ12**	KIRC	ChRC	311	200	173	684	165
**PRDM4**	KIRC	HM_w	385	69	229	683	166
**GADD45B**	KIRC	Others	67	333	282	682	167
**ELP3**	KIRC	HA_w	152	399	128	679	168
**ACTL6B**	KIRC	ChRC	162	388	126	676	169
**ARID4B**	KIRC	ChRC	227	298	151	676	170
**NCOR1**	KIRC	ChRC	386	248	42	676	171
**PHF12**	KIRC	Others	397	237	42	676	172
**CBX2**	KIRC	ChRC	83	369	222	674	173
**ERCC5**	KIRC	Others	266	366	42	674	174
**CTCF**	KIRC	Others	269	69	335	673	175
**JMJD1C**	KIRC	HM_e	327	69	277	673	176
**BOP 1**	KIRC	Others	86	386	198	670	177
**SMARCD2**	KIRC	Helicases	102	351	214	667	178
**SETD1A**	KIRC	HM_w	314	309	42	665	179
**TET3**	KIRC	DM_e	309	69	287	665	180
**CHAF1B**	KIRC	ChRC	224	69	371	664	181

**CREBBP**	KIRC	HA_w	331	289	42	662	182
**KIAA2026**	KIRC	Others	254	259	149	662	183
**PRDM11**	KIRC	HM_w	118	227	317	662	184
**TET2**	KIRC	DM_e	392	69	199	660	185
**ING2**	KIRC	HM_r	53	328	278	659	186
**KANSL1**	KIRC	HA_w	349	268	42	659	187
**SRCAP**	KIRC	Others	415	202	42	659	188
**TET1**	KIRC	DM_e	275	69	315	659	189
**ASXL2**	KIRC	Others	399	69	190	658	190
**PRDM12**	KIRC	HM_w	186	226	246	658	191
**MBD4**	KIRC	DM_r	293	322	42	657	192
**GADD45A**	KIRC	Others	68	278	310	656	193
**TRIM28**	KIRC	HA_r	234	69	352	655	194
**RAI1**	KIRC	Others	245	69	338	652	195
**ING1**	KIRC	HM_r	54	362	233	649	196
**PHF7**	KIRC	Others	121	407	119	647	197
**PHF23**	KIRC	HM_r	122	231	290	643	198
**TDRD7**	KIRC	Others	167	69	405	641	199
**HDAC8**	KIRC	HA_e	61	364	210	635	200
**CBX3**	KIRC	HM_r	157	69	408	634	201
**SCML4**	KIRC	Others	178	210	245	633	202
**CECR2**	KIRC	HA_r	413	69	150	632	203
**KDM2B**	KIRC	HM_e	256	69	307	632	204
**KDM2A**	KIRC	HM_e	324	263	42	629	205
**RSF1**	KIRC	ChRC	375	212	42	629	206
**SMYD2**	KIRC	HM_w	278	205	146	629	207
**ZMYND8**	KIRC	HA_r	273	69	286	628	208
**CHD9**	KIRC	Helicases	367	69	189	625	209
**SIRT4**	KIRC	HA_e	313	69	243	625	210
**TDG**	KIRC	ChRC	169	69	387	625	211
**HIST1H1C**	KIRC	Others	260	69	293	622	212
**KMT2B**	KIRC	HM_w	322	258	42	622	213
**FXR2**	KIRC	Others	299	280	42	621	214
**PHF1**	KIRC	HM_r	347	69	202	618	215
**UHRF2**	KIRC	DM_r	274	302	42	618	216

**ATAD2B**	KIRC	HA_r	306	69	239	614	217
**ELP4**	KIRC	HA_w	220	69	321	610	218
**KDM3A**	KIRC	HM_e	143	69	397	609	219
**INO80**	KIRC	Helicases	297	269	42	608	220
**FKBP1A**	KIRC	Others	71	281	254	606	221
**KDM8**	KIRC	HM_e	140	260	206	606	222
**PRDM16**	KIRC	HM_w	117	224	264	605	223
**PYGO1**	KIRC	HM_r	247	69	289	605	224
**FBXW9**	KIRC	Others	150	282	168	600	225
**USP22**	KIRC	Others	164	69	367	600	226
**PRMT5**	KIRC	HM_w	113	69	414	596	227
**SIRT2**	KIRC	HA_e	26	208	362	596	228
**SETD4**	KIRC	HM_w	282	69	244	595	229
**FMR1**	KIRC	Others	265	69	253	587	230
**MEN1**	KIRC	ChRC	198	69	319	586	231
**PRMT1**	KIRC	HM_w	181	219	184	584	232
**UBR7**	KIRC	Others	93	69	421	583	233
**SMARCA1**	KIRC	Helicases	173	69	337	579	234
**UBE2I**	KIRC	Others	16	195	368	579	235
**PRDM6**	KIRC	HM_w	116	420	42	578	236
**H2AFZ**	KIRC	Others	65	276	235	576	237
**WDR5**	KIRC	ChRC	233	301	42	576	238
**CXXC1**	KIRC	Others	268	69	237	574	239
**H3F3A**	KIRC	Others	64	275	234	573	240
**SMARCD1**	KIRC	Helicases	103	69	399	571	241
**PRDM7**	KIRC	HM_w	182	222	164	568	242
**UBE2E1**	KIRC	Others	17	416	134	567	243
**SUV39H2**	KIRC	HM_w	237	69	259	565	244
**TAF1L**	KIRC	HA_r	357	69	137	563	245
**HDAC4**	KIRC	HA_e	212	69	280	561	246
**POLR2B**	KIRC	Others	290	228	42	560	247
**AURKB**	KIRC	Others	88	297	172	557	248
**MUM1**	KIRC	Others	133	69	355	557	249
**IDH1**	KIRC	DM_e	208	69	279	556	250
**SCML2**	KIRC	HM_r	29	310	216	555	251

**BRDT**	KIRC	HA_r	351	69	133	553	252
**DOT1L**	KIRC	HM_w	303	69	179	551	253
**HNF1A**	KIRC	ChRC	210	69	270	549	254
**TDRD3**	KIRC	HM_r	168	198	183	549	255
**PRDM13**	KIRC	HM_w	185	225	136	546	256
**ZCWPW2**	KIRC	HM_r	11	415	120	546	257
**INTS12**	KIRC	Others	207	69	269	545	258
**PHF6**	KIRC	HM_r	188	315	42	545	259
**KAT5**	KIRC	HA_w	144	69	331	544	260
**ZGPAT**	KIRC	Others	92	69	381	542	261
**SETD7**	KIRC	HM_w	243	69	227	539	262
**G2E3**	KIRC	Others	217	279	42	538	263
**RNF2**	KIRC	ChRC	30	311	195	536	264
**SIRT1**	KIRC	HA_e	241	69	226	536	265
**SIRT3**	KIRC	HA_e	25	207	302	534	266
**SHPRH**	KIRC	Others	280	209	42	531	267
**SP140**	KIRC	HA_r	238	69	224	531	268
**EP300**	KIRC	HA_w	418	69	42	529	269
**TDRD9**	KIRC	Others	95	305	129	529	270
**CBX5**	KIRC	HM_r	81	69	378	528	271
**SMYD4**	KIRC	HM_w	100	203	225	528	272
**SUV39H1**	KIRC	HM_w	98	69	360	527	273
**TCF20**	KIRC	Others	416	69	42	527	274
**PWWP2B**	KIRC	Others	33	217	275	525	275
**PRMT3**	KIRC	HM_w	114	69	339	522	276
**SMARCA4**	KIRC	Helicases	411	69	42	522	277
**ATAT1**	KIRC	Others	271	69	181	521	278
**GTF3C4**	KIRC	HA_w	148	331	42	521	279
**PADI2**	KIRC	Others	132	245	144	521	280
**PHIP**	KIRC	HA_r	249	230	42	521	281
**KDM1A**	KIRC	HM_e	48	264	208	520	282
**CBX6**	KIRC	HM_r	225	69	221	515	283
**PRDM5**	KIRC	HM_w	183	69	263	515	284
**KAT7**	KIRC	HA_w	206	266	42	514	285
**PPARGC1A**	KIRC	Others	289	69	156	514	286

**PHF3**	KIRC	Others	401	69	42	512	287
**CHD4**	KIRC	Helicases	398	69	42	509	288
**KDM4C**	KIRC	HM_e	142	325	42	509	289
**PRKAA2**	KIRC	Others	286	69	152	507	290
**RNF17**	KIRC	Others	316	69	122	507	291
**TAF3**	KIRC	HA_r	394	69	42	505	292
**GTF2H1**	KIRC	Others	214	69	220	503	293
**CARM1**	KIRC	HM_w	84	69	349	502	294
**L3MBTL1**	KIRC	HM_r	139	69	291	499	295
**MLLT10**	KIRC	HM_w	388	69	42	499	296
**SMYD3**	KIRC	HM_w	101	204	194	499	297
**PHF19**	KIRC	HM_r	123	69	304	496	298
**MTA1**	KIRC	ChRC	134	319	42	495	299
**BRPF3**	KIRC	HA_r	382	69	42	493	300
**CHD2**	KIRC	Helicases	381	69	42	492	301
**NAP1L3**	KIRC	Others	192	69	231	492	302
**AEBP2**	KIRC	HM_w	230	69	192	491	303
**MPHOSPH8**	KIRC	HM_r	196	253	42	491	304
**SIRT5**	KIRC	HA_e	105	69	316	490	305
**DPF1**	KIRC	ChRC	76	286	127	489	306
**HELLS**	KIRC	Helicases	261	69	158	488	307
**SMARCE1**	KIRC	Helicases	240	206	42	488	308
**MTA3**	KIRC	ChRC	194	250	42	486	309
**SMYD5**	KIRC	HM_w	21	69	396	486	310
**PHF10**	KIRC	Others	125	317	42	484	311
**TRIM33**	KIRC	HA_r	373	69	42	484	312
**JADE1**	KIRC	Others	232	69	182	483	313
**RPH3A**	KIRC	Others	284	69	130	483	314
**SP140L**	KIRC	HA_r	170	69	242	481	315
**CHD6**	KIRC	Helicases	368	69	42	479	316
**KDM4E**	KIRC	HM_e	141	69	268	478	317
**SIN3B**	KIRC	ChRC	106	69	303	478	318
**KAT6B**	KIRC	HA_w	365	69	42	476	319
**L3MBTL4**	KIRC	Others	202	69	205	476	320
**KDM4A**	KIRC	HM_e	364	69	42	475	321

**PHC2**	KIRC	ChRC	361	69	42	472	322
**FKBP5**	KIRC	Others	69	69	333	471	323
**JARID2**	KIRC	ChRC	258	69	141	468	324
**DPY30**	KIRC	Others	75	69	322	466	325
**BRD3**	KIRC	HA_r	85	338	42	465	326
**SETD6**	KIRC	HM_w	107	69	288	464	327
**RAG2**	KIRC	HM_r	246	69	147	462	328
**TCEA1**	KIRC	Others	19	69	374	462	329
**WDR82**	KIRC	Others	13	406	42	461	330
**DMAP1**	KIRC	Others	222	69	169	460	331
**SFMBT2**	KIRC	Others	175	69	215	459	332
**PHRF1**	KIRC	Others	187	229	42	458	333
**NAP1L1**	KIRC	Others	43	69	345	457	334
**MARCH5**	KIRC	Others	201	69	186	456	335
**PRMT7**	KIRC	HM_w	111	69	276	456	336
**CLOCK**	KIRC	HA_w	223	69	162	454	337
**SP110**	KIRC	HA_r	20	69	361	450	338
**TDRD1**	KIRC	Others	236	69	145	450	339
**BRD4**	KIRC	HA_r	337	69	42	448	340
**PRMT6**	KIRC	HM_w	112	69	262	443	341
**DAXX**	KIRC	ChRC	330	69	42	441	342
**RNF217**	KIRC	Others	180	216	42	438	343
**L3MBTL3**	KIRC	Others	138	257	42	437	344
**LBR**	KIRC	Others	137	256	42	435	345
**MBD3**	KIRC	DM_r	45	255	132	432	346
**PRKAA1**	KIRC	Others	36	220	175	431	347
**ING5**	KIRC	HM_r	50	69	309	428	348
**HSPBAP1**	KIRC	Others	55	329	42	426	349
**KDM1B**	KIRC	HM_e	47	69	308	424	350
**SETD1B**	KIRC	HM_w	28	69	326	423	351
**AICDA**	KIRC	DM_e	161	69	191	421	352
**AIRE**	KIRC	HM_r	90	69	257	416	353
**GTF2B**	KIRC	Others	66	69	281	416	354
**TDRD12**	KIRC	Others	96	199	121	416	355
**CBX7**	KIRC	HM_r	80	293	42	415	356

**EHMT1**	KIRC	HM_w	302	69	42	413	357
**EPC2**	KIRC	Others	301	69	42	412	358
**PADI1**	KIRC	Others	42	246	123	411	359
**FKBP2**	KIRC	Others	70	69	271	410	360
**GATAD2A**	KIRC	HM_r	298	69	42	409	361
**PCMT1**	KIRC	Others	128	239	42	409	362
**PHF21B**	KIRC	HM_r	38	232	139	409	363
**PHF13**	KIRC	Others	124	236	42	402	364
**USP27X**	KIRC	Others	15	344	42	401	365
**FBXO17**	KIRC	Others	73	284	42	399	366
**ZMYND11**	KIRC	HA_r	163	194	42	399	367
**SCMH1**	KIRC	Others	283	69	42	394	368
**SMARCB1**	KIRC	Helicases	279	69	42	390	369
**SSRP1**	KIRC	Others	277	69	42	388	370
**TRIM66**	KIRC	HA_r	94	69	223	386	371
**CDYL**	KIRC	HM_r	270	69	42	381	372
**HDGFL1**	KIRC	Others	145	69	167	381	373
**EHMT2**	KIRC	HM_w	267	69	42	378	374
**HDAC2**	KIRC	HA_e	63	273	42	378	375
**HDAC1**	KIRC	HA_e	264	69	42	375	376
**KDM4D**	KIRC	HM_e	255	69	42	366	377
**MBD1**	KIRC	DM_r	253	69	42	364	378
**RPS6KA5**	KIRC	Others	108	213	42	363	379
**TCF19**	KIRC	Others	97	69	193	359	380
**PRDM1**	KIRC	HM_w	119	69	165	353	381
**SIN3A**	KIRC	ChRC	242	69	42	353	382
**PRDM8**	KIRC	HM_w	115	69	161	345	383
**KDM5D**	KIRC	HM_e	231	69	42	342	384
**ING4**	KIRC	HM_r	51	69	219	339	385
**PCGF5**	KIRC	Others	130	69	140	339	386
**CSTL1**	KIRC	Others	78	69	188	335	387
**EPC1**	KIRC	Others	219	69	42	330	388
**PADI4**	KIRC	Others	41	244	42	327	389
**BMI1**	KIRC	ChRC	87	69	163	319	390
**L3MBTL2**	KIRC	Others	203	69	42	314	391

**MORF4L1**	KIRC	HM_r	197	69	42	308	392
**PHF5A**	KIRC	Others	37	69	201	307	393
**NCOA1**	KIRC	HA_w	191	69	42	302	394
**PRMT2**	KIRC	HM_w	35	218	42	295	395
**SMARCA5**	KIRC	Helicases	172	69	42	283	396
**HIST1H3B**	KIRC	Others	56	69	153	278	397
**DNMT3L**	KIRC	DM_w	77	69	125	271	398
**SETDB2**	KIRC	HM_w	27	69	174	270	399
**PHF11**	KIRC	Others	39	69	157	265	400
**DPF2**	KIRC	Others	153	69	42	264	401
**MBD5**	KIRC	DM_r	136	69	42	247	402
**SMYD1**	KIRC	HM_w	22	69	155	246	403
**HIST1H1B**	KIRC	Others	57	69	116	242	404
**PCGF6**	KIRC	Others	129	69	42	240	405
**POLE3**	KIRC	ChRC	120	69	42	231	406
**RBBP4**	KIRC	ChRC	110	69	42	221	407
**SP100**	KIRC	HA_r	99	69	42	210	408
**HIF1AN**	KIRC	Others	59	69	42	170	409
**HIRA**	KIRC	Others	58	69	42	169	410
**MBD2**	KIRC	DM_r	46	69	42	157	411
**MTF2**	KIRC	HM_r	44	69	42	155	412
**PARP2**	KIRC	Others	40	69	42	151	413
**PSIP1**	KIRC	HM_r	34	69	42	145	414
**SMNDC1**	KIRC	Others	23	69	42	134	415
**UTY**	KIRC	HM_e	14	69	42	125	416
**KMT2C**	KIRP	HM_w	426	396	408	1230	1
**SRCAP**	KIRP	Others	422	354	396	1172	2
**CREBBP**	KIRP	HA_w	418	383	364	1165	3
**SIRT7**	KIRP	HA_e	326	410	422	1158	4
**PAXIP1**	KIRP	Others	337	394	421	1152	5
**BPTF**	KIRP	HA_r	384	406	361	1151	6
**BAZ1B**	KIRP	HA_r	386	337	420	1143	7
**NSD1**	KIRP	HM_w	404	421	302	1127	8
**EP400**	KIRP	HA_w	408	323	388	1119	9
**SETD1A**	KIRP	HM_w	371	358	389	1118	10

**ATR**	KIRP	Others	387	341	387	1115	11
**KAT7**	KIRP	HA_w	362	399	351	1112	12
**BAZ2B**	KIRP	HA_r	385	387	337	1109	13
**FXR2**	KIRP	Others	365	319	424	1108	14
**KANSL1**	KIRP	HA_w	390	301	416	1107	15
**CBX1**	KIRP	HM_r	307	386	401	1094	16
**NCOR1**	KIRP	ChRC	417	281	383	1081	17
**DIDO1**	KIRP	Others	352	328	400	1080	18
**KDM6B**	KIRP	HM_e	379	292	406	1077	19
**AKAP1**	KIRP	Others	236	414	425	1075	20
**GLYR1**	KIRP	HM_r	319	376	375	1070	21
**HAT1**	KIRP	HA_w	296	402	372	1070	22
**PHC3**	KIRP	ChRC	336	367	360	1063	23
**FBXL19**	KIRP	Others	300	378	373	1051	24
**ING4**	KIRP	HM_r	344	302	393	1039	25
**JMJD1C**	KIRP	HM_e	415	372	246	1033	26
**KMT2A**	KIRP	HM_w	400	412	221	1033	27
**SMARCB1**	KIRP	Helicases	402	242	384	1028	28
**KDM3B**	KIRP	HM_e	393	297	331	1021	29
**JMJD6**	KIRP	HM_e	201	401	415	1017	30
**AFF4**	KIRP	Others	312	388	315	1015	31
**KDM2B**	KIRP	HM_e	342	299	374	1015	32
**MBTD1**	KIRP	Others	193	411	411	1015	33
**KAT8**	KIRP	HA_w	247	371	394	1012	34
**HDGF**	KIRP	Others	346	311	353	1010	35
**PRMT7**	KIRP	HM_w	271	362	377	1010	36
**ACTL6A**	KIRP	ChRC	237	389	380	1006	37
**ATM**	KIRP	Others	411	425	165	1001	38
**CBX4**	KIRP	HM_r	410	404	186	1000	39
**ARID4A**	KIRP	ChRC	388	344	267	999	40
**BRD2**	KIRP	HA_r	397	334	264	995	41
**HDAC6**	KIRP	HA_e	364	312	316	992	42
**DAXX**	KIRP	ChRC	353	329	309	991	43
**L3MBTL2**	KIRP	Others	287	290	414	991	44
**HCFC1**	KIRP	Others	318	375	290	983	45

**HNF1A**	KIRP	ChRC	345	305	332	982	46
**PBRM1**	KIRP	HA_r	421	277	279	977	47
**KDM5A**	KIRP	HM_e	340	295	329	964	48
**MBD4**	KIRP	DM_r	286	289	385	960	49
**NCOR2**	KIRP	Others	376	280	303	959	50
**KDM6A**	KIRP	HM_e	419	293	245	957	51
**RNF40**	KIRP	Others	167	361	426	954	52
**CHD4**	KIRP	Helicases	305	330	311	946	53
**ZGPAT**	KIRP	Others	321	227	395	943	54
**UBE2I**	KIRP	Others	141	390	410	941	55
**ATF7IP**	KIRP	Others	309	342	287	938	56
**KAT6A**	KIRP	HA_w	406	300	222	928	57
**ARID1A**	KIRP	ChRC	416	345	166	927	58
**MBD5**	KIRP	DM_r	378	288	259	925	59
**SP140L**	KIRP	HA_r	151	417	340	908	60
**JMJD8**	KIRP	HM_e	90	400	413	903	61
**DPF3**	KIRP	HA_r	304	381	212	897	62
**MLLT10**	KIRP	HM_w	285	287	322	894	63
**DOT1L**	KIRP	HM_w	409	326	145	880	64
**SMYD4**	KIRP	HM_w	263	240	369	872	65
**EZH2**	KIRP	HM_w	111	413	347	871	66
**CBX8**	KIRP	ChRC	123	403	336	862	67
**ACTL6B**	KIRP	ChRC	254	415	191	860	68
**ING5**	KIRP	HM_r	93	422	343	858	69
**SETD2**	KIRP	HM_w	424	69	362	855	70
**KMT2D**	KIRP	HM_w	425	69	357	851	71
**SP100**	KIRP	HA_r	153	420	276	849	72
**G2E3**	KIRP	Others	348	318	182	848	73
**CTCF**	KIRP	Others	222	382	242	846	74
**ATAT1**	KIRP	Others	253	343	248	844	75
**RBBP7**	KIRP	ChRC	240	255	346	841	76
**TDRD6**	KIRP	Others	256	391	193	840	77
**EZH1**	KIRP	HM_w	349	69	419	837	78
**ING3**	KIRP	HM_r	204	423	209	836	79
**SETD3**	KIRP	HM_w	161	357	318	836	80

**KDM3A**	KIRP	HM_e	394	298	141	833	81
**CHD8**	KIRP	Helicases	405	384	42	831	82
**FMR1**	KIRP	Others	320	320	189	829	83
**CHD5**	KIRP	Helicases	356	385	85	826	84
**PHF14**	KIRP	Others	64	365	397	826	85
**KMT2E**	KIRP	HM_w	399	69	356	824	86
**RPA3**	KIRP	Others	46	360	418	824	87
**ZCWPW1**	KIRP	HM_r	12	407	402	821	88
**PHF20L1**	KIRP	HM_r	403	69	345	817	89
**GATAD2B**	KIRP	HM_r	212	317	283	812	90
**KDM5C**	KIRP	HM_e	246	294	270	810	91
**TDRD5**	KIRP	Others	257	233	317	807	92
**BAP1**	KIRP	Others	423	339	42	804	93
**PHF2**	KIRP	Others	375	271	157	803	94
**ARID4B**	KIRP	ChRC	366	69	367	802	95
**RAI1**	KIRP	Others	407	257	135	799	96
**L3MBTL1**	KIRP	HM_r	316	69	412	797	97
**SFMBT2**	KIRP	Others	265	356	176	797	98
**SMARCC2**	KIRP	Helicases	323	69	403	795	99
**PRDM10**	KIRP	HM_w	275	364	155	794	100
**HDAC3**	KIRP	HA_e	104	313	371	788	101
**BMI1**	KIRP	ChRC	232	336	216	784	102
**BAZ2A**	KIRP	HA_r	358	69	355	782	103
**SETD6**	KIRP	HM_w	160	246	376	782	104
**SMARCD3**	KIRP	Helicases	36	392	352	780	105
**SUV39H2**	KIRP	HM_w	150	353	275	778	106
**CHD6**	KIRP	Helicases	396	69	310	775	107
**FBXO44**	KIRP	Others	110	377	284	771	108
**GTF3C4**	KIRP	HA_w	210	315	237	762	109
**INO80**	KIRP	Helicases	343	373	42	758	110
**SIRT4**	KIRP	HA_e	157	243	358	758	111
**CHD9**	KIRP	Helicases	354	69	334	757	112
**KDM4E**	KIRP	HM_e	87	397	271	755	113
**NCOA3**	KIRP	HA_w	281	69	405	755	114
**SND1**	KIRP	HM_r	154	426	175	755	115

**SMYD3**	KIRP	HM_w	264	355	133	752	116
**PARP1**	KIRP	Others	338	69	342	749	117
**SMYD1**	KIRP	HM_w	239	241	269	749	118
**AEBP2**	KIRP	HM_w	313	69	365	747	119
**SCMH1**	KIRP	Others	268	249	229	746	120
**HIST1H3B**	KIRP	Others	100	306	339	745	121
**MLLT6**	KIRP	HM_w	284	69	392	745	122
**PHF12**	KIRP	Others	335	69	341	745	123
**SMARCD2**	KIRP	Helicases	37	409	297	743	124
**HIST1H1C**	KIRP	Others	292	307	143	742	125
**RING1**	KIRP	Others	168	254	320	742	126
**BRWD1**	KIRP	HA_r	357	69	313	739	127
**AURKB**	KIRP	Others	132	340	266	738	128
**ARID2**	KIRP	ChRC	398	69	268	735	129
**PHF23**	KIRP	HM_r	62	269	404	735	130
**EED**	KIRP	ChRC	113	380	241	734	131
**DMAP1**	KIRP	Others	221	327	185	733	132
**MSH6**	KIRP	HM_r	191	284	258	733	133
**HDAC4**	KIRP	HA_e	207	424	101	732	134
**BOP 1**	KIRP	Others	131	335	265	731	135
**PCGF2**	KIRP	Others	279	69	382	730	136
**HIST1H1B**	KIRP	Others	249	308	171	728	137
**TP53BP1**	KIRP	Others	401	231	96	728	138
**RPS6KA5**	KIRP	Others	166	359	200	725	139
**EHMT2**	KIRP	HM_w	303	379	42	724	140
**PAF1**	KIRP	Others	186	278	257	721	141
**RSF1**	KIRP	ChRC	269	251	199	719	142
**EPC2**	KIRP	Others	112	321	285	718	143
**ERCC5**	KIRP	Others	301	69	348	718	144
**EPC1**	KIRP	Others	350	322	42	714	145
**JADE3**	KIRP	Others	317	69	327	713	146
**RNF17**	KIRP	Others	389	253	71	713	147
**SUPT16H**	KIRP	Others	261	408	42	711	148
**TDRD1**	KIRP	Others	367	69	274	710	149
**CBX3**	KIRP	HM_r	226	69	409	704	150

**CHD3**	KIRP	Helicases	224	331	149	704	151
**PHF21B**	KIRP	HM_r	180	270	254	704	152
**KDM1A**	KIRP	HM_e	291	370	42	703	153
**KDM8**	KIRP	HM_e	85	291	326	702	154
**MTA1**	KIRP	ChRC	377	282	42	701	155
**BAZ1A**	KIRP	HA_r	233	338	128	699	156
**CHD1**	KIRP	Helicases	382	69	244	695	157
**PRDM12**	KIRP	HM_w	177	264	253	694	158
**KAT2A**	KIRP	HA_w	200	69	423	692	159
**TAF3**	KIRP	HA_r	260	237	195	692	160
**TRIM24**	KIRP	HA_r	144	416	132	692	161
**BRD3**	KIRP	HA_r	231	333	127	691	162
**SFMBT1**	KIRP	HM_r	266	245	177	688	163
**KDM7A**	KIRP	HM_e	245	69	366	680	164
**PRDM4**	KIRP	HM_w	56	263	359	678	165
**BRD7**	KIRP	HA_r	230	69	378	677	166
**PHF3**	KIRP	Others	374	69	232	675	167
**HDAC9**	KIRP	HA_e	206	69	398	673	168
**HDAC11**	KIRP	HA_e	295	69	308	672	169
**ASH1L**	KIRP	HM_w	412	69	188	669	170
**DPF1**	KIRP	ChRC	219	325	125	669	171
**FKBP1A**	KIRP	Others	214	69	386	669	172
**LBR**	KIRP	Others	339	69	260	668	173
**TRIM28**	KIRP	HA_r	143	350	173	666	174
**PHF13**	KIRP	Others	65	366	233	664	175
**UBE2B**	KIRP	Others	23	349	292	664	176
**BRD1**	KIRP	HA_r	308	69	286	663	177
**KDM5B**	KIRP	HM_e	288	69	305	662	178
**SP140**	KIRP	HA_r	152	418	90	660	179
**GTF2B**	KIRP	Others	297	316	42	655	180
**NAP1L1**	KIRP	Others	282	69	304	655	181
**TDG**	KIRP	ChRC	27	235	391	653	182
**BRPF1**	KIRP	HA_r	228	69	354	651	183
**RNF20**	KIRP	Others	329	69	252	650	184
**PHF20**	KIRP	HM_r	181	69	399	649	185

**PHF7**	KIRP	Others	179	266	203	648	186
**SETD4**	KIRP	HM_w	328	69	251	648	187
**AFF1**	KIRP	Others	360	69	218	647	188
**SP110**	KIRP	HA_r	32	419	196	647	189
**ATAD2B**	KIRP	HA_r	359	69	217	645	190
**EP300**	KIRP	HA_w	414	69	161	644	191
**HIRA**	KIRP	Others	293	309	42	644	192
**SUV39H1**	KIRP	HM_w	238	238	167	643	193
**SMYD5**	KIRP	HM_w	33	239	368	640	194
**ORC1**	KIRP	Others	72	279	288	639	195
**SETD1B**	KIRP	HM_w	162	247	228	637	196
**DPY30**	KIRP	Others	218	69	349	636	197
**SETD5**	KIRP	HM_w	267	69	300	636	198
**IWS1**	KIRP	Others	203	69	363	635	199
**PHC2**	KIRP	ChRC	277	274	84	635	200
**PRKCD**	KIRP	Others	273	262	98	633	201
**FBXW9**	KIRP	Others	380	69	183	632	202
**PRMT5**	KIRP	HM_w	331	259	42	632	203
**GADD45B**	KIRP	Others	299	69	262	630	204
**PSIP1**	KIRP	HM_r	330	258	42	630	205
**ASXL2**	KIRP	Others	235	69	324	628	206
**KDM4A**	KIRP	HM_e	289	296	42	627	207
**ATAD2**	KIRP	HA_r	234	69	323	626	208
**TET3**	KIRP	DM_e	145	232	249	626	209
**NCOA1**	KIRP	HA_w	392	69	158	619	210
**KDM4B**	KIRP	HM_e	341	69	208	618	211
**ELP3**	KIRP	HA_w	216	324	77	617	212
**MECP2**	KIRP	DM_r	79	368	170	617	213
**BRD4**	KIRP	HA_r	383	69	164	616	214
**DPF2**	KIRP	Others	381	69	162	612	215
**GTF2H1**	KIRP	Others	251	69	291	611	216
**CBX2**	KIRP	ChRC	127	405	78	610	217
**DNMT3A**	KIRP	DM_w	395	69	146	610	218
**JADE1**	KIRP	Others	248	69	289	606	219
**PHF5A**	KIRP	Others	61	268	277	606	220

**RPH3A**	KIRP	Others	270	252	83	605	221
**DNMT3B**	KIRP	DM_w	351	69	184	604	222
**CBX5**	KIRP	HM_r	126	69	407	602	223
**TDRD9**	KIRP	Others	25	351	224	600	224
**PHC1**	KIRP	ChRC	66	275	256	597	225
**ZMYND8**	KIRP	HA_r	138	69	390	597	226
**KDM4C**	KIRP	HM_e	197	69	330	596	227
**SIN3B**	KIRP	ChRC	327	69	198	594	228
**SMARCE1**	KIRP	Helicases	155	69	370	594	229
**SMARCC1**	KIRP	Helicases	324	69	197	590	230
**MECOM**	KIRP	Others	80	369	138	587	231
**ASXL1**	KIRP	Others	134	69	379	582	232
**HR**	KIRP	HM_e	98	304	180	582	233
**PRDM6**	KIRP	HM_w	176	363	42	581	234
**PHF1**	KIRP	HM_r	185	273	122	580	235
**PRDM2**	KIRP	HM_w	391	69	120	580	236
**HDAC1**	KIRP	HA_e	105	314	160	579	237
**MSL3**	KIRP	HA_w	76	283	220	579	238
**CHD7**	KIRP	Helicases	355	69	148	572	239
**PRDM7**	KIRP	HM_w	175	69	328	572	240
**SIN3A**	KIRP	ChRC	368	69	134	571	241
**TET1**	KIRP	DM_e	413	69	88	570	242
**AICDA**	KIRP	DM_e	137	346	86	569	243
**ASXL3**	KIRP	Others	310	69	187	566	244
**TRIM33**	KIRP	HA_r	322	69	172	563	245
**KAT6B**	KIRP	HA_w	363	69	130	562	246
**PWWP2B**	KIRP	Others	170	69	321	560	247
**SSRP1**	KIRP	Others	262	69	226	557	248
**PRDM15**	KIRP	HM_w	332	69	154	555	249
**PRMT1**	KIRP	HM_w	172	261	119	552	250
**PCGF1**	KIRP	Others	69	276	206	551	251
**CECR2**	KIRP	HA_r	120	332	94	546	252
**MPHOSPH8**	KIRP	HM_r	77	285	178	540	253
**SIRT1**	KIRP	HA_e	43	244	250	537	254
**CHRAC1**	KIRP	ChRC	117	69	350	536	255

**CLOCK**	KIRP	HA_w	223	69	243	535	256
**PRDM16**	KIRP	HM_w	372	69	92	533	257
**SMARCA4**	KIRP	Helicases	420	69	42	531	258
**KDM4D**	KIRP	HM_e	88	398	42	528	259
**PHF19**	KIRP	HM_r	182	272	73	527	260
**FBXO17**	KIRP	Others	215	69	240	524	261
**HDAC5**	KIRP	HA_e	347	69	107	523	262
**CHD1L**	KIRP	Helicases	118	69	335	522	263
**PRDM9**	KIRP	HM_w	55	393	74	522	264
**TDRD10**	KIRP	Others	259	69	194	522	265
**H2AFZ**	KIRP	Others	107	69	344	520	266
**MORF4L1**	KIRP	HM_r	192	286	42	520	267
**GTF2F1**	KIRP	Others	211	69	238	518	268
**BRD8**	KIRP	HA_r	229	69	215	513	269
**BRD9**	KIRP	HA_r	130	69	314	513	270
**HSPBAP1**	KIRP	Others	97	374	42	513	271
**BRWD3**	KIRP	HA_r	252	69	190	511	272
**TAF1L**	KIRP	HA_r	148	69	294	511	273
**CHAF1A**	KIRP	ChRC	225	69	214	508	274
**PARP2**	KIRP	Others	70	395	42	507	275
**PHF11**	KIRP	Others	183	69	255	507	276
**USP22**	KIRP	Others	19	69	417	505	277
**CDYL**	KIRP	HM_r	122	69	312	503	278
**HIF1AN**	KIRP	Others	101	69	333	503	279
**PPARGC1A**	KIRP	Others	276	69	156	501	280
**JADE2**	KIRP	Others	91	69	338	498	281
**TCF19**	KIRP	Others	29	352	117	498	282
**TAF1**	KIRP	HA_r	314	69	111	494	283
**FKBP5**	KIRP	Others	213	69	211	493	284
**HDGFL1**	KIRP	Others	103	310	80	493	285
**PHF8**	KIRP	Others	59	265	168	492	286
**SMARCA2**	KIRP	Helicases	325	69	97	491	287
**ARID1B**	KIRP	ChRC	311	69	109	489	288
**SMARCD1**	KIRP	Helicases	38	69	381	488	289
**HDAC7**	KIRP	HA_e	294	69	124	487	290

**PHIP**	KIRP	HA_r	373	69	42	484	291
**SETDB1**	KIRP	HM_w	370	69	42	481	292
**SHPRH**	KIRP	Others	369	69	42	480	293
**ZMYND11**	KIRP	HA_r	139	69	272	480	294
**PRMT2**	KIRP	HM_w	171	260	42	473	295
**SMARCA1**	KIRP	Helicases	361	69	42	472	296
**ING1**	KIRP	HM_r	95	69	307	471	297
**HDAC8**	KIRP	HA_e	250	69	150	469	298
**PYGO2**	KIRP	HM_r	169	69	230	468	299
**INTS12**	KIRP	Others	92	69	306	467	300
**NAP1L3**	KIRP	Others	73	69	325	467	301
**WDR5**	KIRP	ChRC	15	228	223	466	302
**KDM1B**	KIRP	HM_e	290	69	106	465	303
**MUM1**	KIRP	Others	189	69	207	465	304
**RAG2**	KIRP	HM_r	315	69	79	463	305
**CARM1**	KIRP	HM_w	128	69	263	460	306
**BRPF3**	KIRP	HA_r	227	69	163	459	307
**PRMT6**	KIRP	HM_w	272	69	118	459	308
**H3F3A**	KIRP	Others	106	69	282	457	309
**PHF10**	KIRP	Others	184	69	204	457	310
**RTF1**	KIRP	Others	165	250	42	457	311
**ATRX**	KIRP	Helicases	133	69	247	449	312
**HLTF**	KIRP	Others	99	69	281	449	313
**PHRF1**	KIRP	Others	334	69	42	445	314
**POLR2B**	KIRP	Others	333	69	42	444	315
**PRKAA1**	KIRP	Others	173	69	202	444	316
**SUZ12**	KIRP	ChRC	149	69	225	443	317
**TDRD3**	KIRP	HM_r	258	69	116	443	318
**PRDM5**	KIRP	HM_w	274	69	99	442	319
**PRDM13**	KIRP	HM_w	242	69	129	440	320
**ING2**	KIRP	HM_r	94	303	42	439	321
**KDM2A**	KIRP	HM_e	89	69	280	438	322
**TCF20**	KIRP	Others	28	236	174	438	323
**DNMT1**	KIRP	DM_w	220	69	147	436	324
**RNF2**	KIRP	ChRC	48	69	319	436	325

**TET2**	KIRP	DM_e	255	69	103	427	326
**IDH1**	KIRP	DM_e	96	69	261	426	327
**PRDM14**	KIRP	HM_w	241	69	113	423	328
**HDAC10**	KIRP	HA_e	209	69	144	422	329
**PYGO1**	KIRP	HM_r	51	69	301	421	330
**CHD2**	KIRP	Helicases	306	69	42	417	331
**FKBP2**	KIRP	Others	109	69	239	417	332
**ELP4**	KIRP	HA_w	302	69	42	413	333
**UBR7**	KIRP	Others	21	348	42	411	334
**KAT2B**	KIRP	HA_w	199	69	142	410	335
**PHF21A**	KIRP	HM_r	63	69	278	410	336
**SIRT2**	KIRP	HA_e	42	69	299	410	337
**GATAD2A**	KIRP	HM_r	298	69	42	409	338
**TDRKH**	KIRP	Others	146	69	192	407	339
**SIRT6**	KIRP	HA_e	39	69	298	406	340
**KIAA2026**	KIRP	Others	196	69	140	405	341
**SCML2**	KIRP	HM_r	45	248	112	405	342
**YY1**	KIRP	ChRC	13	347	42	402	343
**CHAF1B**	KIRP	ChRC	119	69	213	401	344
**SMNDC1**	KIRP	Others	35	69	296	400	345
**PADI6**	KIRP	Others	243	69	87	399	346
**SMYD2**	KIRP	HM_w	34	69	295	398	347
**MTA3**	KIRP	ChRC	283	69	42	394	348
**TCEA1**	KIRP	Others	30	69	293	392	349
**PADI4**	KIRP	Others	280	69	42	391	350
**PCMT1**	KIRP	Others	278	69	42	389	351
**MARCH5**	KIRP	Others	83	69	236	388	352
**GADD45A**	KIRP	Others	108	69	210	387	353
**L3MBTL3**	KIRP	Others	195	69	123	387	354
**PRDM11**	KIRP	HM_w	178	69	136	383	355
**PADI2**	KIRP	Others	71	69	235	375	356
**IDH2**	KIRP	DM_e	205	69	100	374	357
**PCGF5**	KIRP	Others	68	69	234	371	358
**PHF6**	KIRP	HM_r	60	267	42	369	359
**JARID2**	KIRP	ChRC	202	69	93	364	360

**UBE2E1**	KIRP	Others	22	69	273	364	361
**TRIM66**	KIRP	HA_r	142	69	151	362	362
**USP51**	KIRP	Others	17	229	110	356	363
**KMT2B**	KIRP	HM_w	244	69	42	355	364
**PRMT3**	KIRP	HM_w	53	69	231	353	365
**HELLS**	KIRP	Helicases	102	69	181	352	366
**RBBP4**	KIRP	ChRC	50	256	42	348	367
**PCGF6**	KIRP	Others	67	69	205	341	368
**SATB1**	KIRP	Others	164	69	105	338	369
**SIRT3**	KIRP	HA_e	41	69	227	337	370
**MTA2**	KIRP	ChRC	190	69	76	335	371
**L3MBTL4**	KIRP	Others	84	69	179	332	372
**SETDB2**	KIRP	HM_w	159	69	104	332	373
**TDRD12**	KIRP	Others	26	234	70	330	374
**EHMT1**	KIRP	HM_w	217	69	42	328	375
**PADI1**	KIRP	Others	188	69	69	326	376
**PADI3**	KIRP	Others	187	69	68	324	377
**SCML4**	KIRP	Others	163	69	91	323	378
**CBX7**	KIRP	HM_r	124	69	126	319	379
**HDAC2**	KIRP	HA_e	208	69	42	319	380
**RBBP5**	KIRP	ChRC	49	69	201	319	381
**PRDM8**	KIRP	HM_w	174	69	75	318	382
**CXXC1**	KIRP	Others	115	69	131	315	383
**BRDT**	KIRP	HA_r	129	69	114	312	384
**NAP1L2**	KIRP	Others	74	69	169	312	385
**UBE2A**	KIRP	Others	24	69	219	312	386
**MBD2**	KIRP	DM_r	82	69	159	310	387
**KAT5**	KIRP	HA_w	198	69	42	309	388
**SETMAR**	KIRP	HM_w	158	69	82	309	389
**MBD1**	KIRP	DM_r	194	69	42	305	390
**AIRE**	KIRP	HM_r	136	69	95	300	391
**CDYL2**	KIRP	HM_r	121	69	108	298	392
**CBX6**	KIRP	HM_r	125	69	102	296	393
**USP27X**	KIRP	Others	18	230	42	290	394
**MBD3**	KIRP	DM_r	81	69	139	289	395

**MTF2**	KIRP	HM_r	75	69	137	281	396
**RNF217**	KIRP	Others	47	69	153	269	397
**SMARCA5**	KIRP	Helicases	156	69	42	267	398
**CSTL1**	KIRP	Others	116	69	81	266	399
**SETD7**	KIRP	HM_w	44	69	152	265	400
**TDRD7**	KIRP	Others	147	69	42	258	401
**UHRF2**	KIRP	DM_r	140	69	42	251	402
**DNMT3L**	KIRP	DM_w	114	69	67	250	403
**PRDM1**	KIRP	HM_w	57	69	121	247	404
**ASH2L**	KIRP	HM_w	135	69	42	246	405
**KDM5D**	KIRP	HM_e	86	69	42	197	406
**ZCWPW2**	KIRP	HM_r	11	69	115	195	407
**PRMT8**	KIRP	HM_w	52	69	72	193	408
**MEN1**	KIRP	ChRC	78	69	42	189	409
**STK31**	KIRP	Others	31	69	89	189	410
**POLE3**	KIRP	ChRC	58	69	42	169	411
**PRKAA2**	KIRP	Others	54	69	42	165	412
**SIRT5**	KIRP	HA_e	40	69	42	151	413
**UHRF1**	KIRP	DM_r	20	69	42	131	414
**UTY**	KIRP	HM_e	16	69	42	127	415
**WDR82**	KIRP	Others	14	69	42	125	416
**KDM3B**	LAML	HM_e	415	419	426	1260	1
**HDAC3**	LAML	HA_e	400	420	402	1222	2
**SUZ12**	LAML	ChRC	418	401	403	1222	3
**ARID4B**	LAML	ChRC	406	400	390	1196	4
**EZH2**	LAML	HM_w	419	412	337	1168	5
**ATM**	LAML	Others	348	399	419	1166	6
**DNMT1**	LAML	DM_w	378	355	421	1154	7
**ASH2L**	LAML	HM_w	352	372	422	1146	8
**CHAF1B**	LAML	ChRC	318	408	413	1139	9
**BOP 1**	LAML	Others	339	368	424	1131	10
**ATAD2**	LAML	HA_r	351	370	398	1119	11
**DNMT3A**	LAML	DM_w	426	354	339	1119	12
**BRDT**	LAML	HA_r	331	364	415	1110	13
**DNMT3B**	LAML	DM_w	377	353	362	1092	14

**BRD4**	LAML	HA_r	335	366	384	1085	15
**TET2**	LAML	DM_e	423	300	354	1077	16
**CHRAC1**	LAML	ChRC	308	358	408	1074	17
**DOT1L**	LAML	HM_w	402	352	319	1073	18
**KDM6A**	LAML	HM_e	420	338	313	1071	19
**BRWD1**	LAML	HA_r	329	425	304	1058	20
**ARID2**	LAML	ChRC	407	373	264	1044	21
**KMT2C**	LAML	HM_w	396	424	203	1023	22
**CHAF1A**	LAML	ChRC	319	362	340	1021	23
**PAXIP1**	LAML	Others	171	418	423	1012	24
**ELP4**	LAML	HA_w	293	397	318	1008	25
**CARM1**	LAML	HM_w	328	363	303	994	26
**KDM8**	LAML	HM_e	397	337	256	990	27
**CHD9**	LAML	Helicases	309	359	320	988	28
**KMT2A**	LAML	HM_w	212	426	350	988	29
**KAT6A**	LAML	HA_w	230	341	414	985	30
**NCOA3**	LAML	HA_w	369	330	286	985	31
**TRIM24**	LAML	HA_r	363	409	213	985	32
**CHD1**	LAML	Helicases	317	405	261	983	33
**CDYL2**	LAML	HM_r	320	398	262	980	34
**PADI2**	LAML	Others	368	326	285	979	35
**EZH1**	LAML	HM_w	288	351	300	939	36
**AIRE**	LAML	HM_r	357	414	165	936	37
**FBXW9**	LAML	Others	284	350	299	933	38
**L3MBTL1**	LAML	HM_r	209	335	367	911	39
**H3F3A**	LAML	Others	268	395	236	899	40
**HDAC7**	LAML	HA_e	260	345	293	898	41
**RNF40**	LAML	Others	106	382	407	895	42
**PARP1**	LAML	Others	172	390	330	892	43
**PRMT2**	LAML	HM_w	124	411	357	892	44
**JMJD8**	LAML	HM_e	233	343	314	890	45
**PHF20L1**	LAML	HM_r	152	317	417	886	46
**MBD3**	LAML	DM_r	201	392	288	881	47
**KAT2A**	LAML	HA_w	232	342	292	866	48
**SETD4**	LAML	HM_w	94	406	366	866	49

**NCOA1**	LAML	HA_w	182	331	349	862	50
**KANSL1**	LAML	HA_w	416	69	370	855	51
**TET1**	LAML	DM_e	408	69	377	854	52
**KDM4B**	LAML	HM_e	223	340	290	853	53
**AFF4**	LAML	Others	388	422	42	852	54
**CBX7**	LAML	HM_r	381	69	397	847	55
**KDM5B**	LAML	HM_e	218	339	289	846	56
**MTA2**	LAML	ChRC	411	69	360	840	57
**DNMT3L**	LAML	DM_w	301	413	124	838	58
**CHD5**	LAML	Helicases	313	361	161	835	59
**PHF13**	LAML	Others	157	319	358	834	60
**IDH1**	LAML	DM_e	424	69	335	828	61
**KAT8**	LAML	HA_w	228	394	205	827	62
**CTCF**	LAML	Others	380	404	42	826	63
**GADD45A**	LAML	Others	277	349	195	821	64
**ASXL2**	LAML	Others	405	371	42	818	65
**FBXL19**	LAML	Others	287	396	133	816	66
**GATAD2A**	LAML	HM_r	275	347	194	816	67
**BRPF1**	LAML	HA_r	383	69	363	815	68
**RNF2**	LAML	ChRC	109	310	393	812	69
**SMARCD3**	LAML	Helicases	68	416	325	809	70
**CHD6**	LAML	Helicases	312	360	135	807	71
**MTF2**	LAML	HM_r	187	332	287	806	72
**ARID1A**	LAML	ChRC	387	69	342	798	73
**BRD8**	LAML	HA_r	333	421	42	796	74
**ATAD2B**	LAML	HA_r	350	69	375	794	75
**BPTF**	LAML	HA_r	384	367	42	793	76
**BRWD3**	LAML	HA_r	403	69	321	793	77
**ARID4A**	LAML	ChRC	354	69	365	788	78
**HCFC1**	LAML	Others	401	69	317	787	79
**SMARCB1**	LAML	Helicases	72	305	410	787	80
**PHRF1**	LAML	Others	144	387	253	784	81
**ELP3**	LAML	HA_w	294	69	420	783	82
**PRMT6**	LAML	HM_w	121	313	347	781	83
**HDAC2**	LAML	HA_e	375	69	336	780	84

**BAZ2A**	LAML	HA_r	342	69	364	775	85
**BRD9**	LAML	HA_r	332	69	374	775	86
**PRMT7**	LAML	HM_w	120	402	252	774	87
**SMARCE1**	LAML	Helicases	67	303	404	774	88
**KMT2D**	LAML	HM_w	395	336	42	773	89
**AFF1**	LAML	Others	359	69	343	771	90
**SMARCC2**	LAML	Helicases	391	69	309	769	91
**ATRX**	LAML	Helicases	346	69	351	766	92
**PRDM16**	LAML	HM_w	367	69	329	765	93
**TCEA1**	LAML	Others	46	301	416	763	94
**HDAC1**	LAML	HA_e	266	69	425	760	95
**SMARCA4**	LAML	Helicases	74	306	379	759	96
**PARP2**	LAML	Others	394	322	42	758	97
**GADD45B**	LAML	Others	276	348	132	756	98
**BAZ1B**	LAML	HA_r	343	369	42	754	99
**DMAP1**	LAML	Others	302	69	383	754	100
**MLLT6**	LAML	HM_w	193	333	228	754	101
**RAI1**	LAML	Others	114	312	328	754	102
**CHD7**	LAML	Helicases	311	69	373	753	103
**RBBP4**	LAML	ChRC	409	69	275	753	104
**IDH2**	LAML	DM_e	425	69	258	752	105
**AEBP2**	LAML	HM_w	360	69	322	751	106
**PADI6**	LAML	Others	174	323	254	751	107
**PRMT3**	LAML	HM_w	123	314	310	747	108
**BRD7**	LAML	HA_r	334	365	42	741	109
**GATAD2B**	LAML	HM_r	376	69	296	741	110
**ASXL1**	LAML	Others	421	69	247	737	111
**SIN3B**	LAML	ChRC	83	308	346	737	112
**FBXO44**	LAML	Others	285	69	382	736	113
**HIRA**	LAML	Others	254	69	412	735	114
**SCML2**	LAML	HM_r	414	69	251	734	115
**AKAP1**	LAML	Others	356	69	307	732	116
**CDYL**	LAML	HM_r	321	69	341	731	117
**ARID1B**	LAML	ChRC	355	69	306	730	118
**MUM1**	LAML	Others	186	391	152	729	119

**KAT6B**	LAML	HA_w	399	69	257	725	120
**HIF1AN**	LAML	Others	255	69	396	720	121
**KAT5**	LAML	HA_w	231	69	418	718	122
**BMI1**	LAML	ChRC	340	69	305	714	123
**EP400**	LAML	HA_w	390	69	248	707	124
**DPF2**	LAML	Others	299	69	338	706	125
**PADI1**	LAML	Others	177	327	202	706	126
**CREBBP**	LAML	HA_w	306	357	42	705	127
**GTF2B**	LAML	Others	273	69	361	703	128
**ATF7IP**	LAML	Others	386	69	246	701	129
**DIDO1**	LAML	Others	303	356	42	701	130
**PHF12**	LAML	Others	158	320	223	701	131
**SRCAP**	LAML	Others	55	377	268	700	132
**AURKB**	LAML	Others	385	69	245	699	133
**HNF1A**	LAML	ChRC	249	69	381	699	134
**SETD1A**	LAML	HM_w	97	381	219	697	135
**KDM2A**	LAML	HM_e	226	69	401	696	136
**CBX4**	LAML	HM_r	324	69	302	695	137
**NCOR2**	LAML	Others	180	328	187	695	138
**ING3**	LAML	HM_r	244	407	42	693	139
**CBX5**	LAML	HM_r	382	69	241	692	140
**CECR2**	LAML	HA_r	417	69	206	692	141
**L3MBTL2**	LAML	Others	208	69	411	688	142
**KDM4D**	LAML	HM_e	221	69	395	685	143
**ING5**	LAML	HM_r	242	69	372	683	144
**SIRT3**	LAML	HA_e	80	380	218	678	145
**HDGF**	LAML	Others	374	69	234	677	146
**CXXC1**	LAML	Others	304	69	301	674	147
**JMJD6**	LAML	HM_e	234	69	371	674	148
**BAZ2B**	LAML	HA_r	341	69	263	673	149
**JMJD1C**	LAML	HM_e	373	69	231	673	150
**SETD6**	LAML	HM_w	92	309	272	673	151
**PRDM15**	LAML	HM_w	134	423	115	672	152
**L3MBTL4**	LAML	Others	206	69	394	669	153
**KAT7**	LAML	HA_w	229	69	369	667	154

**JADE1**	LAML	Others	238	69	353	660	155
**GTF2H1**	LAML	Others	271	346	42	659	156
**MSL3**	LAML	HA_w	190	69	400	659	157
**JADE2**	LAML	Others	237	69	352	658	158
**BAZ1A**	LAML	HA_r	344	69	244	657	159
**PHF20**	LAML	HM_r	153	318	186	657	160
**KDM5C**	LAML	HM_e	217	69	368	654	161
**BRD1**	LAML	HA_r	338	69	243	650	162
**FKBP2**	LAML	Others	282	69	298	649	163
**BRD2**	LAML	HA_r	337	69	242	648	164
**FKBP5**	LAML	Others	281	69	297	647	165
**HDAC6**	LAML	HA_e	261	69	316	646	166
**PHF23**	LAML	HM_r	149	388	108	645	167
**HDAC8**	LAML	HA_e	259	69	315	643	168
**IWS1**	LAML	Others	239	69	334	642	169
**LBR**	LAML	Others	205	393	42	640	170
**RAG2**	LAML	HM_r	115	384	139	638	171
**CBX6**	LAML	HM_r	323	69	240	632	172
**HAT1**	LAML	HA_w	267	69	295	631	173
**ATAT1**	LAML	Others	349	69	209	627	174
**DPF3**	LAML	HA_r	298	69	260	627	175
**HDAC11**	LAML	HA_e	264	69	294	627	176
**INO80**	LAML	Helicases	241	344	42	627	177
**KDM4A**	LAML	HM_e	224	69	333	626	178
**NSD1**	LAML	HM_w	179	403	42	624	179
**BAP1**	LAML	Others	345	69	208	622	180
**PRDM7**	LAML	HM_w	129	385	107	621	181
**CHD4**	LAML	Helicases	413	69	136	618	182
**PHF11**	LAML	Others	159	69	389	617	183
**SMARCA2**	LAML	Helicases	364	69	177	610	184
**SMYD3**	LAML	HM_w	63	379	168	610	185
**FMR1**	LAML	Others	280	69	259	608	186
**BRPF3**	LAML	HA_r	330	69	207	606	187
**PADI3**	LAML	Others	176	325	105	606	188
**MBD1**	LAML	DM_r	203	69	332	604	189

**BRD3**	LAML	HA_r	336	69	198	603	190
**PRDM9**	LAML	HM_w	393	69	141	603	191
**PHF8**	LAML	Others	145	69	388	602	192
**PRKAA2**	LAML	Others	392	69	140	601	193
**AICDA**	LAML	DM_e	358	69	173	600	194
**PHF10**	LAML	Others	160	321	116	597	195
**KAT2B**	LAML	HA_w	372	69	154	595	196
**MEN1**	LAML	ChRC	195	69	331	595	197
**MSH6**	LAML	HM_r	370	69	153	592	198
**PRDM13**	LAML	HM_w	136	69	387	592	199
**FKBP1A**	LAML	Others	283	69	239	591	200
**PHC2**	LAML	ChRC	163	69	359	591	201
**SMARCD1**	LAML	Helicases	70	304	216	590	202
**PRDM6**	LAML	HM_w	130	417	42	589	203
**L3MBTL3**	LAML	Others	207	69	312	588	204
**KDM1B**	LAML	HM_e	227	69	291	587	205
**ASXL3**	LAML	Others	404	69	112	585	206
**PHF2**	LAML	Others	154	389	42	585	207
**PRDM11**	LAML	HM_w	138	316	129	583	208
**GTF2F1**	LAML	Others	272	69	238	579	209
**TDRD10**	LAML	Others	389	69	121	579	210
**TRIM66**	LAML	HA_r	30	298	250	578	211
**RSF1**	LAML	ChRC	102	69	406	577	212
**ACTL6B**	LAML	ChRC	361	69	146	576	213
**H2AFZ**	LAML	Others	269	69	237	575	214
**MLLT10**	LAML	HM_w	194	334	42	570	215
**PRDM10**	LAML	HM_w	139	386	42	567	216
**DPF1**	LAML	ChRC	300	69	197	566	217
**HDAC5**	LAML	HA_e	262	69	235	566	218
**SMYD2**	LAML	HM_w	64	302	199	565	219
**SIRT6**	LAML	HA_e	77	307	179	563	220
**TDRD7**	LAML	Others	37	376	147	560	221
**CBX2**	LAML	ChRC	326	69	164	559	222
**ORC1**	LAML	Others	178	69	311	558	223
**CHD3**	LAML	Helicases	314	69	172	555	224

**SIRT2**	LAML	HA_e	81	69	405	555	225
**CBX8**	LAML	ChRC	322	69	163	554	226
**PRDM14**	LAML	HM_w	135	315	104	554	227
**SMARCA1**	LAML	Helicases	75	69	409	553	228
**NCOR1**	LAML	ChRC	181	329	42	552	229
**SETD7**	LAML	HM_w	91	69	392	552	230
**PYGO2**	LAML	HM_r	366	69	114	549	231
**CHD2**	LAML	Helicases	315	69	162	546	232
**ING4**	LAML	HM_r	243	69	233	545	233
**PRDM8**	LAML	HM_w	128	69	348	545	234
**UBE2B**	LAML	Others	28	415	101	544	235
**G2E3**	LAML	Others	278	69	196	543	236
**TRIM33**	LAML	HA_r	31	299	212	542	237
**PADI4**	LAML	Others	175	324	42	541	238
**SETDB2**	LAML	HM_w	89	69	380	538	239
**JARID2**	LAML	ChRC	235	69	232	536	240
**UBE2I**	LAML	Others	26	297	211	534	241
**PHF6**	LAML	HM_r	422	69	42	533	242
**RNF20**	LAML	Others	108	383	42	533	243
**HDAC10**	LAML	HA_e	265	69	193	527	244
**EHMT2**	LAML	HM_w	295	69	160	524	245
**EED**	LAML	ChRC	412	69	42	523	246
**PHIP**	LAML	HA_r	410	69	42	521	247
**PCGF5**	LAML	Others	167	69	284	520	248
**PCGF6**	LAML	Others	166	69	283	518	249
**HELLS**	LAML	Helicases	256	69	192	517	250
**PCMT1**	LAML	Others	165	69	282	516	251
**FBXO17**	LAML	Others	286	69	159	514	252
**PHC1**	LAML	ChRC	164	69	281	514	253
**SSRP1**	LAML	Others	54	69	391	514	254
**SND1**	LAML	HM_r	60	410	42	512	255
**KDM2B**	LAML	HM_e	398	69	42	509	256
**HR**	LAML	HM_e	248	69	191	508	257
**NAP1L2**	LAML	Others	184	69	255	508	258
**SIRT1**	LAML	HA_e	82	69	356	507	259

**SP100**	LAML	HA_r	59	69	378	506	260
**ING2**	LAML	HM_r	245	69	190	504	261
**RNF217**	LAML	Others	107	69	327	503	262
**MBD2**	LAML	DM_r	202	69	230	501	263
**DPY30**	LAML	Others	297	69	134	500	264
**PHF21A**	LAML	HM_r	151	69	280	500	265
**GTF3C4**	LAML	HA_w	270	69	158	497	266
**MBTD1**	LAML	Others	198	69	229	496	267
**SCMH1**	LAML	Others	99	69	326	494	268
**KDM7A**	LAML	HM_e	214	69	210	493	269
**TDRD6**	LAML	Others	38	69	386	493	270
**DAXX**	LAML	ChRC	379	69	42	490	271
**HDAC4**	LAML	HA_e	263	69	157	489	272
**PPARGC1A**	LAML	Others	141	69	279	489	273
**KDM6B**	LAML	HM_e	215	69	204	488	274
**USP51**	LAML	Others	20	69	399	488	275
**CSTL1**	LAML	Others	305	69	111	485	276
**MTA1**	LAML	ChRC	189	69	227	485	277
**HDAC9**	LAML	HA_e	258	69	156	483	278
**KDM1A**	LAML	HM_e	371	69	42	482	279
**SMYD4**	LAML	HM_w	62	378	42	482	280
**ZMYND8**	LAML	HA_r	11	296	175	482	281
**NAP1L1**	LAML	Others	185	69	226	480	282
**PRDM2**	LAML	HM_w	133	69	278	480	283
**JADE3**	LAML	Others	236	69	174	479	284
**SMYD1**	LAML	HM_w	65	69	345	479	285
**STK31**	LAML	Others	53	69	355	477	286
**TRIM28**	LAML	HA_r	32	69	376	477	287
**SETD2**	LAML	HM_w	365	69	42	476	288
**USP22**	LAML	Others	22	69	385	476	289
**EHMT1**	LAML	HM_w	296	69	110	475	290
**ACTL6A**	LAML	ChRC	362	69	42	473	291
**PRKCD**	LAML	Others	126	69	277	472	292
**HDGFL1**	LAML	Others	257	69	145	471	293
**ING1**	LAML	HM_r	246	69	155	470	294

**PRMT1**	LAML	HM_w	125	69	276	470	295
**PAF1**	LAML	Others	173	69	225	467	296
**RBBP5**	LAML	ChRC	113	311	42	466	297
**ASH1L**	LAML	HM_w	353	69	42	464	298
**SUV39H1**	LAML	HM_w	51	69	344	464	299
**MARCH5**	LAML	Others	204	69	189	462	300
**PCGF2**	LAML	Others	168	69	224	461	301
**ATR**	LAML	Others	347	69	42	458	302
**MECOM**	LAML	Others	197	69	188	454	303
**HLTF**	LAML	Others	250	69	131	450	304
**RPS6KA5**	LAML	Others	103	69	274	446	305
**HIST1H1B**	LAML	Others	253	69	120	442	306
**SATB1**	LAML	Others	100	69	273	442	307
**UHRF1**	LAML	DM_r	24	375	42	441	308
**HIST1H3B**	LAML	Others	251	69	119	439	309
**CBX1**	LAML	HM_r	327	69	42	438	310
**CBX3**	LAML	HM_r	325	69	42	436	311
**MECP2**	LAML	DM_r	196	69	171	436	312
**KDM4E**	LAML	HM_e	220	69	144	433	313
**PRDM1**	LAML	HM_w	140	69	222	431	314
**TDRD9**	LAML	Others	36	69	324	429	315
**ZMYND11**	LAML	HA_r	12	374	42	428	316
**CHD1L**	LAML	Helicases	316	69	42	427	317
**PHF19**	LAML	HM_r	155	69	201	425	318
**TAF3**	LAML	HA_r	47	69	308	424	319
**HIST1H1C**	LAML	Others	252	69	102	423	320
**CHD8**	LAML	Helicases	310	69	42	421	321
**SIRT4**	LAML	HA_e	79	69	271	419	322
**CLOCK**	LAML	HA_w	307	69	42	418	323
**MBD5**	LAML	DM_r	199	69	143	411	324
**PWWP2B**	LAML	Others	117	69	221	407	325
**ZGPAT**	LAML	Others	13	69	323	405	326
**EP300**	LAML	HA_w	292	69	42	403	327
**EPC1**	LAML	Others	291	69	42	402	328
**PHF3**	LAML	Others	148	69	185	402	329

**EPC2**	LAML	Others	290	69	42	401	330
**ERCC5**	LAML	Others	289	69	42	400	331
**RING1**	LAML	Others	111	69	220	400	332
**SMYD5**	LAML	HM_w	61	69	270	400	333
**SP110**	LAML	HA_r	58	69	269	396	334
**PHF14**	LAML	Others	156	69	170	395	335
**NAP1L3**	LAML	Others	183	69	142	394	336
**FXR2**	LAML	Others	279	69	42	390	337
**GLYR1**	LAML	HM_r	274	69	42	385	338
**TCF19**	LAML	Others	45	69	267	381	339
**TDRD3**	LAML	HM_r	40	69	266	375	340
**PYGO1**	LAML	HM_r	116	69	184	369	341
**PHF7**	LAML	Others	146	69	151	366	342
**SIRT5**	LAML	HA_e	78	69	217	364	343
**POLE3**	LAML	ChRC	143	69	150	362	344
**HSPBAP1**	LAML	Others	247	69	42	358	345
**PCGF1**	LAML	Others	169	69	118	356	346
**PRDM12**	LAML	HM_w	137	69	149	355	347
**SFMBT2**	LAML	Others	86	69	200	355	348
**SMARCD2**	LAML	Helicases	69	69	215	353	349
**WDR5**	LAML	ChRC	18	69	265	352	350
**INTS12**	LAML	Others	240	69	42	351	351
**SCML4**	LAML	Others	98	69	183	350	352
**PHF1**	LAML	HM_r	161	69	117	347	353
**SETD1B**	LAML	HM_w	96	69	182	347	354
**PHF5A**	LAML	Others	147	69	130	346	355
**RPA3**	LAML	Others	105	69	169	343	356
**SP140**	LAML	HA_r	57	69	214	340	357
**SETMAR**	LAML	HM_w	88	69	181	338	358
**KDM3A**	LAML	HM_e	225	69	42	336	359
**SFMBT1**	LAML	HM_r	87	69	180	336	360
**KDM4C**	LAML	HM_e	222	69	42	333	361
**ZCWPW2**	LAML	HM_r	14	69	249	332	362
**KDM5A**	LAML	HM_e	219	69	42	330	363
**PHF21B**	LAML	HM_r	150	69	109	328	364

**PRDM5**	LAML	HM_w	131	69	128	328	365
**KDM5D**	LAML	HM_e	216	69	42	327	366
**KIAA2026**	LAML	Others	213	69	42	324	367
**SIRT7**	LAML	HA_e	76	69	178	323	368
**KMT2B**	LAML	HM_w	211	69	42	322	369
**KMT2E**	LAML	HM_w	210	69	42	321	370
**MBD4**	LAML	DM_r	200	69	42	311	371
**PRMT8**	LAML	HM_w	119	69	123	311	372
**MORF4L1**	LAML	HM_r	192	69	42	303	373
**MPHOSPH8**	LAML	HM_r	191	69	42	302	374
**MTA3**	LAML	ChRC	188	69	42	299	375
**RTF1**	LAML	Others	101	69	127	297	376
**RPH3A**	LAML	Others	104	69	113	286	377
**SMNDC1**	LAML	Others	66	69	148	283	378
**RNF17**	LAML	Others	110	69	103	282	379
**PBRM1**	LAML	HA_r	170	69	42	281	380
**PHC3**	LAML	ChRC	162	69	42	273	381
**TDRKH**	LAML	Others	35	69	167	271	382
**TET3**	LAML	DM_e	34	69	166	269	383
**SMARCC1**	LAML	Helicases	71	69	126	266	384
**ZCWPW1**	LAML	HM_r	15	69	176	260	385
**POLR2B**	LAML	Others	142	69	42	253	386
**TDRD1**	LAML	Others	42	69	138	249	387
**PRDM4**	LAML	HM_w	132	69	42	243	388
**PRKAA1**	LAML	Others	127	69	42	238	389
**UBE2A**	LAML	Others	29	69	137	235	390
**PRMT5**	LAML	HM_w	122	69	42	233	391
**TDRD5**	LAML	Others	39	69	122	230	392
**PSIP1**	LAML	HM_r	118	69	42	229	393
**RBBP7**	LAML	ChRC	112	69	42	223	394
**UBE2E1**	LAML	Others	27	69	125	221	395
**TDRD12**	LAML	Others	41	69	106	216	396
**SETD3**	LAML	HM_w	95	69	42	206	397
**SETD5**	LAML	HM_w	93	69	42	204	398
**SETDB1**	LAML	HM_w	90	69	42	201	399

**SHPRH**	LAML	Others	85	69	42	196	400
**SIN3A**	LAML	ChRC	84	69	42	195	401
**SMARCA5**	LAML	Helicases	73	69	42	184	402
**SP140L**	LAML	HA_r	56	69	42	167	403
**SUPT16H**	LAML	Others	52	69	42	163	404
**SUV39H2**	LAML	HM_w	50	69	42	161	405
**TAF1**	LAML	HA_r	49	69	42	160	406
**TAF1L**	LAML	HA_r	48	69	42	159	407
**TCF20**	LAML	Others	44	69	42	155	408
**TDG**	LAML	ChRC	43	69	42	154	409
**TP53BP1**	LAML	Others	33	69	42	144	410
**UBR7**	LAML	Others	25	69	42	136	411
**UHRF2**	LAML	DM_r	23	69	42	134	412
**USP27X**	LAML	Others	21	69	42	132	413
**UTY**	LAML	HM_e	19	69	42	130	414
**WDR82**	LAML	Others	17	69	42	128	415
**YY1**	LAML	ChRC	16	69	42	127	416
**KDM4C**	LGG	HM_e	410	395	402	1207	1
**CHD4**	LGG	Helicases	390	422	389	1201	2
**KMT2C**	LGG	HM_w	408	409	365	1182	3
**KMT2E**	LGG	HM_w	375	394	410	1179	4
**ATAD2**	LGG	HA_r	394	425	354	1173	5
**ARID1B**	LGG	ChRC	416	353	378	1147	6
**CHD8**	LGG	Helicases	414	308	408	1130	7
**KMT2A**	LGG	HM_w	377	390	353	1120	8
**ATF7IP**	LGG	Others	393	380	337	1110	9
**BMI1**	LGG	ChRC	348	333	396	1077	10
**USP51**	LGG	Others	357	400	319	1076	11
**DOT1L**	LGG	HM_w	331	397	334	1062	12
**EHMT1**	LGG	HM_w	330	345	383	1058	13
**PWWP2B**	LGG	Others	404	385	268	1057	14
**BRWD3**	LGG	HA_r	392	346	315	1053	15
**JMJD6**	LGG	HM_e	315	376	361	1052	16
**SND1**	LGG	HM_r	266	405	379	1050	17
**TAF3**	LGG	HA_r	262	368	417	1047	18

**BOP 1**	LGG	Others	237	426	375	1038	19
**CHRAC1**	LGG	ChRC	217	424	381	1022	20
**PBRM1**	LGG	HA_r	419	330	271	1020	21
**SMARCA4**	LGG	Helicases	424	382	201	1007	22
**EZH2**	LGG	HM_w	202	410	392	1004	23
**HCFC1**	LGG	Others	385	377	241	1003	24
**SFMBT2**	LGG	Others	271	373	358	1002	25
**SP100**	LGG	HA_r	401	355	246	1002	26
**ARID1A**	LGG	ChRC	423	173	405	1001	27
**BRD4**	LGG	HA_r	235	364	397	996	28
**MECOM**	LGG	Others	407	260	322	989	29
**AEBP2**	LGG	HM_w	251	399	338	988	30
**CHD5**	LGG	Helicases	415	196	374	985	31
**ING3**	LGG	HM_r	168	403	412	983	32
**HDAC4**	LGG	HA_e	411	411	160	982	33
**ACTL6B**	LGG	ChRC	252	389	339	980	34
**BPTF**	LGG	HA_r	236	365	377	978	35
**CHAF1A**	LGG	ChRC	220	379	376	975	36
**ING4**	LGG	HM_r	167	421	386	974	37
**CARM1**	LGG	HM_w	230	363	367	960	38
**BRPF1**	LGG	HA_r	343	274	336	953	39
**KDM5A**	LGG	HM_e	151	416	385	952	40
**CLOCK**	LGG	HA_w	335	398	215	948	41
**DNMT1**	LGG	DM_w	212	388	347	947	42
**PAXIP1**	LGG	Others	121	408	416	945	43
**CBX5**	LGG	HM_r	341	309	294	944	44
**KDM4B**	LGG	HM_e	309	361	272	942	45
**GTF2F1**	LGG	Others	187	362	390	939	46
**PHF20L1**	LGG	HM_r	107	423	409	939	47
**FBXW9**	LGG	Others	198	378	362	938	48
**NCOR2**	LGG	Others	406	340	190	936	49
**DIDO1**	LGG	Others	333	214	387	934	50
**HIRA**	LGG	Others	321	209	399	929	51
**SCML2**	LGG	HM_r	274	325	328	927	52
**ASXL3**	LGG	Others	395	248	279	922	53

**CBX4**	LGG	HM_r	226	351	335	912	54
**HDAC8**	LGG	HA_e	178	344	388	910	55
**AICDA**	LGG	DM_e	355	413	136	904	56
**KIAA2026**	LGG	Others	147	387	370	904	57
**PHF13**	LGG	Others	290	186	425	901	58
**GATAD2A**	LGG	HM_r	191	315	391	897	59
**ACTL6A**	LGG	ChRC	253	287	355	895	60
**ARID4B**	LGG	ChRC	396	219	280	895	61
**ARID2**	LGG	ChRC	397	335	162	894	62
**SIRT3**	LGG	HA_e	58	418	418	894	63
**KDM6A**	LGG	HM_e	307	332	254	893	64
**HDAC2**	LGG	HA_e	384	284	224	892	65
**PHC1**	LGG	ChRC	116	407	369	892	66
**PCMT1**	LGG	Others	292	329	270	891	67
**FKBP5**	LGG	Others	326	267	290	883	68
**SETD5**	LGG	HM_w	272	252	359	883	69
**DAXX**	LGG	ChRC	214	270	394	878	70
**CHD1**	LGG	Helicases	339	245	292	876	71
**SMARCD3**	LGG	Helicases	50	415	411	876	72
**PHRF1**	LGG	Others	286	419	169	874	73
**SMARCA1**	LGG	Helicases	362	279	232	873	74
**KDM4E**	LGG	HM_e	308	190	371	869	75
**RNF17**	LGG	Others	403	358	108	869	76
**TRIM28**	LGG	HA_r	257	414	198	869	77
**CBX8**	LGG	ChRC	223	350	293	866	78
**KAT6A**	LGG	HA_w	313	208	344	865	79
**SMARCD2**	LGG	Helicases	267	347	247	861	80
**SP140**	LGG	HA_r	360	370	128	858	81
**CBX3**	LGG	HM_r	227	216	414	857	82
**HDGF**	LGG	Others	176	305	373	854	83
**SMARCC1**	LGG	Helicases	269	320	265	854	84
**MTA2**	LGG	ChRC	374	189	286	849	85
**AKAP1**	LGG	Others	247	317	281	845	86
**HNF1A**	LGG	ChRC	171	343	331	845	87
**PRDM10**	LGG	HM_w	284	326	235	845	88

**MSL3**	LGG	HA_w	297	341	206	844	89
**ASH2L**	LGG	HM_w	245	199	398	842	90
**CREBBP**	LGG	HA_w	413	215	214	842	91
**ATAD2B**	LGG	HA_r	352	172	317	841	92
**BAZ1B**	LGG	HA_r	350	69	421	840	93
**KDM7A**	LGG	HM_e	378	69	393	840	94
**CSTL1**	LGG	Others	216	271	348	835	95
**KDM5C**	LGG	HM_e	150	342	343	835	96
**MUM1**	LGG	Others	132	392	307	831	97
**SETD3**	LGG	HM_w	273	230	327	830	98
**TRIM24**	LGG	HA_r	27	404	395	826	99
**ING1**	LGG	HM_r	318	283	223	824	100
**BRPF3**	LGG	HA_r	231	273	316	820	101
**IDH1**	LGG	DM_e	426	153	239	818	102
**ZCWPW1**	LGG	HM_r	255	366	196	817	103
**SCMH1**	LGG	Others	275	121	420	816	104
**PRDM9**	LGG	HM_w	405	234	167	806	105
**POLR2B**	LGG	Others	285	374	146	805	106
**TDG**	LGG	ChRC	398	226	181	805	107
**H3F3A**	LGG	Others	386	160	256	802	108
**ASXL2**	LGG	Others	353	198	244	795	109
**JARID2**	LGG	ChRC	162	303	330	795	110
**HDAC10**	LGG	HA_e	324	314	153	791	111
**HIST1H1C**	LGG	Others	382	263	140	785	112
**KDM2B**	LGG	HM_e	310	349	126	785	113
**TDRD10**	LGG	Others	261	225	297	783	114
**GADD45B**	LGG	Others	192	396	194	782	115
**KDM5B**	LGG	HM_e	409	239	130	778	116
**SIRT7**	LGG	HA_e	54	371	350	775	117
**ATRX**	LGG	Helicases	425	69	278	772	118
**EP400**	LGG	HA_w	387	69	312	768	119
**SETD2**	LGG	HM_w	402	324	42	768	120
**HDAC1**	LGG	HA_e	182	158	426	766	121
**KMT2D**	LGG	HM_w	421	302	42	765	122
**CHD7**	LGG	Helicases	336	168	259	763	123

**ING2**	LGG	HM_r	169	401	193	763	124
**HR**	LGG	HM_e	319	154	288	761	125
**PHF14**	LGG	Others	111	235	415	761	126
**CBX2**	LGG	ChRC	228	352	178	758	127
**ING5**	LGG	HM_r	166	417	175	758	128
**TAF1L**	LGG	HA_r	399	177	182	758	129
**PRMT8**	LGG	HM_w	88	420	249	757	130
**ATM**	LGG	Others	242	218	296	756	131
**EPC2**	LGG	Others	412	69	275	756	132
**MLLT10**	LGG	HM_w	138	331	287	756	133
**STK31**	LGG	Others	264	227	263	754	134
**CECR2**	LGG	HA_r	221	272	260	753	135
**HIF1AN**	LGG	Others	174	156	423	753	136
**IDH2**	LGG	DM_e	422	152	176	750	137
**ATAT1**	LGG	Others	243	275	230	748	138
**PRDM5**	LGG	HM_w	366	257	125	748	139
**MBD3**	LGG	DM_r	140	393	209	742	140
**EED**	LGG	ChRC	206	163	366	735	141
**CHAF1B**	LGG	ChRC	340	69	325	734	142
**PRDM1**	LGG	HM_w	369	129	236	734	143
**RPH3A**	LGG	Others	74	357	300	731	144
**TAF1**	LGG	HA_r	400	289	42	731	145
**KANSL1**	LGG	HA_w	314	69	345	728	146
**SHPRH**	LGG	Others	363	322	42	727	147
**HELLS**	LGG	Helicases	322	69	332	723	148
**PARP1**	LGG	Others	293	69	360	722	149
**MEN1**	LGG	ChRC	300	69	352	721	150
**PYGO2**	LGG	HM_r	85	255	380	720	151
**G2E3**	LGG	Others	194	266	258	718	152
**MECP2**	LGG	DM_r	301	375	42	718	153
**MBD4**	LGG	DM_r	303	206	208	717	154
**SUV39H2**	LGG	HM_w	40	319	357	716	155
**ATR**	LGG	Others	351	69	295	715	156
**KDM4D**	LGG	HM_e	152	191	372	715	157
**DNMT3A**	LGG	DM_w	418	165	131	714	158

**GATAD2B**	LGG	HM_r	190	212	311	713	159
**KDM4A**	LGG	HM_e	153	147	413	713	160
**SETD7**	LGG	HM_w	66	348	299	713	161
**HDAC3**	LGG	HA_e	180	242	289	711	162
**KAT5**	LGG	HA_w	158	150	403	711	163
**SP140L**	LGG	HA_r	42	369	298	709	164
**GTF3C4**	LGG	HA_w	185	265	257	707	165
**PRKCD**	LGG	Others	94	311	302	707	166
**MARCH5**	LGG	Others	142	145	419	706	167
**BRD1**	LGG	HA_r	347	316	42	705	168
**UBR7**	LGG	Others	20	278	406	704	169
**SIN3B**	LGG	ChRC	61	321	321	703	170
**PRMT5**	LGG	HM_w	365	295	42	702	171
**MPHOSPH8**	LGG	HM_r	298	360	42	700	172
**EHMT2**	LGG	HM_w	388	268	42	698	173
**ZMYND11**	LGG	HA_r	254	402	42	698	174
**HDAC9**	LGG	HA_e	177	210	310	697	175
**MBD5**	LGG	DM_r	302	142	252	696	176
**CTCF**	LGG	Others	334	69	291	694	177
**HDAC6**	LGG	HA_e	383	69	240	692	178
**SUPT16H**	LGG	Others	359	290	42	691	179
**PRKAA1**	LGG	Others	279	69	341	689	180
**CDYL2**	LGG	HM_r	391	69	228	688	181
**PRDM11**	LGG	HM_w	368	69	251	688	182
**PHF11**	LGG	Others	113	386	186	685	183
**GTF2B**	LGG	Others	188	69	422	679	184
**UHRF2**	LGG	DM_r	256	381	42	679	185
**PRDM15**	LGG	HM_w	282	127	269	678	186
**EP300**	LGG	HA_w	328	307	42	677	187
**HAT1**	LGG	HA_w	183	159	333	675	188
**RSF1**	LGG	ChRC	277	69	329	675	189
**CHD9**	LGG	Helicases	389	69	216	674	190
**BRD3**	LGG	HA_r	345	286	42	673	191
**JADE1**	LGG	Others	381	69	222	672	192
**PHF10**	LGG	Others	291	339	42	672	193

**FBXO17**	LGG	Others	200	69	400	669	194
**PPARGC1A**	LGG	Others	370	130	168	668	195
**NAP1L1**	LGG	Others	296	237	133	666	196
**POLE3**	LGG	ChRC	101	183	382	666	197
**SETMAR**	LGG	HM_w	364	69	233	666	198
**BRD9**	LGG	HA_r	233	69	363	665	199
**PHF23**	LGG	HM_r	288	69	303	660	200
**BRDT**	LGG	HA_r	232	246	179	657	201
**SATB1**	LGG	Others	276	231	150	657	202
**BRD8**	LGG	HA_r	344	69	243	656	203
**SETD1B**	LGG	HM_w	69	337	248	654	204
**RBBP7**	LGG	ChRC	81	338	234	653	205
**SFMBT1**	LGG	HM_r	63	323	267	653	206
**L3MBTL2**	LGG	Others	305	301	42	648	207
**SRCAP**	LGG	Others	265	118	264	647	208
**HDAC7**	LGG	HA_e	179	306	159	644	209
**DNMT3B**	LGG	DM_w	332	69	242	643	210
**WDR5**	LGG	ChRC	16	277	349	642	211
**JMJD1C**	LGG	HM_e	316	151	174	641	212
**INTS12**	LGG	Others	164	69	404	637	213
**BAZ1A**	LGG	HA_r	239	217	180	636	214
**KAT8**	LGG	HA_w	156	207	273	636	215
**KDM8**	LGG	HM_e	148	146	342	636	216
**BRD2**	LGG	HA_r	346	247	42	635	217
**NAP1L2**	LGG	Others	131	313	191	635	218
**RAG2**	LGG	HM_r	278	204	151	633	219
**ASXL1**	LGG	Others	244	69	318	631	220
**RBBP4**	LGG	ChRC	83	123	424	630	221
**PADI2**	LGG	Others	372	135	122	629	222
**SIRT6**	LGG	HA_e	55	372	202	629	223
**HDGFL1**	LGG	Others	175	304	148	627	224
**DPF3**	LGG	HA_r	208	269	142	619	225
**BAP1**	LGG	Others	240	334	42	616	226
**SP110**	LGG	HA_r	43	354	219	616	227
**ELP4**	LGG	HA_w	329	244	42	615	228

**RING1**	LGG	Others	80	232	301	613	229
**FKBP2**	LGG	Others	196	69	346	611	230
**KAT6B**	LGG	HA_w	420	149	42	611	231
**CXXC1**	LGG	Others	215	167	226	608	232
**CDYL**	LGG	HM_r	222	69	314	605	233
**PHF21B**	LGG	HM_r	106	328	170	604	234
**SMARCC2**	LGG	Helicases	268	293	42	603	235
**TRIM33**	LGG	HA_r	26	175	401	602	236
**PHC2**	LGG	ChRC	371	188	42	601	237
**UBE2B**	LGG	Others	23	222	356	601	238
**PHF19**	LGG	HM_r	110	185	305	600	239
**PRDM14**	LGG	HM_w	283	128	185	596	240
**TRIM66**	LGG	HA_r	25	406	164	595	241
**SUV39H1**	LGG	HM_w	263	69	262	594	242
**MBD2**	LGG	DM_r	141	143	309	593	243
**TDRD9**	LGG	Others	260	224	109	593	244
**TDRKH**	LGG	Others	29	200	364	593	245
**TET2**	LGG	DM_e	358	69	165	592	246
**FKBP1A**	LGG	Others	197	69	324	590	247
**PHF3**	LGG	Others	417	131	42	590	248
**DPY30**	LGG	Others	207	69	313	589	249
**FBXL19**	LGG	Others	201	162	225	588	250
**AURKB**	LGG	Others	241	69	277	587	251
**FXR2**	LGG	Others	195	69	323	587	252
**NAP1L3**	LGG	Others	130	299	158	587	253
**HDAC11**	LGG	HA_e	181	243	161	585	254
**KDM6B**	LGG	HM_e	306	69	210	585	255
**PHC3**	LGG	ChRC	115	282	188	585	256
**PRMT2**	LGG	HM_w	92	124	368	584	257
**JADE3**	LGG	Others	380	69	134	583	258
**MSH6**	LGG	HM_r	136	139	308	583	259
**EZH1**	LGG	HM_w	327	213	42	582	260
**PRDM16**	LGG	HM_w	281	182	118	581	261
**JADE2**	LGG	Others	317	69	192	578	262
**CBX6**	LGG	HM_r	225	69	276	570	263

**FBXO44**	LGG	Others	199	194	177	570	264
**PHF7**	LGG	Others	103	327	137	567	265
**SUZ12**	LGG	ChRC	39	202	326	567	266
**L3MBTL3**	LGG	Others	145	300	120	565	267
**ASH1L**	LGG	HM_w	246	276	42	564	268
**SMYD4**	LGG	HM_w	45	178	340	563	269
**KAT7**	LGG	HA_w	157	148	255	560	270
**PHF1**	LGG	HM_r	114	259	187	560	271
**SMNDC1**	LGG	Others	48	291	220	559	272
**PHF8**	LGG	Others	102	412	42	556	273
**BRWD1**	LGG	HA_r	342	171	42	555	274
**KDM1A**	LGG	HM_e	312	69	173	554	275
**BAZ2B**	LGG	HA_r	349	69	135	553	276
**HIST1H1B**	LGG	Others	173	264	115	552	277
**PAF1**	LGG	Others	294	69	189	552	278
**MBTD1**	LGG	Others	139	205	207	551	279
**RPA3**	LGG	Others	75	69	407	551	280
**PRDM13**	LGG	HM_w	367	69	114	550	281
**CHD6**	LGG	Helicases	337	169	42	548	282
**SMYD1**	LGG	HM_w	361	69	116	546	283
**HIST1H3B**	LGG	Others	172	262	111	545	284
**KDM2A**	LGG	HM_e	311	192	42	545	285
**SSRP1**	LGG	Others	41	117	384	542	286
**SMARCB1**	LGG	Helicases	270	229	42	541	287
**AFF4**	LGG	Others	249	249	42	540	288
**KAT2B**	LGG	HA_w	159	240	138	537	289
**EPC1**	LGG	Others	204	285	42	531	290
**PRMT1**	LGG	HM_w	93	391	42	526	291
**TP53BP1**	LGG	Others	258	223	42	523	292
**CBX7**	LGG	HM_r	224	69	229	522	293
**HDAC5**	LGG	HA_e	323	157	42	522	294
**NCOA3**	LGG	HA_w	295	69	157	521	295
**HLTF**	LGG	Others	320	155	42	517	296
**CHD3**	LGG	Helicases	218	69	227	514	297
**DMAP1**	LGG	Others	213	166	127	506	298

**IWS1**	LGG	Others	163	69	274	506	299
**TCF19**	LGG	Others	37	251	218	506	300
**SMARCD1**	LGG	Helicases	51	292	155	498	301
**RPS6KA5**	LGG	Others	73	280	144	497	302
**GADD45A**	LGG	Others	193	161	141	495	303
**PCGF1**	LGG	Others	120	69	306	495	304
**KAT2A**	LGG	HA_w	160	193	139	492	305
**KDM3B**	LGG	HM_e	379	69	42	490	306
**MBD1**	LGG	DM_r	304	144	42	490	307
**SETDB2**	LGG	HM_w	64	384	42	490	308
**PSIP1**	LGG	HM_r	87	359	42	488	309
**KMT2B**	LGG	HM_w	376	69	42	487	310
**NCOR1**	LGG	ChRC	373	69	42	484	311
**MLLT6**	LGG	HM_w	299	141	42	482	312
**TET1**	LGG	DM_e	259	69	154	482	313
**PHF20**	LGG	HM_r	108	69	304	481	314
**TDRD1**	LGG	Others	35	336	110	481	315
**AIRE**	LGG	HM_r	248	69	163	480	316
**PRDM12**	LGG	HM_w	100	258	121	479	317
**SMARCA2**	LGG	Helicases	53	383	42	478	318
**PARP2**	LGG	Others	122	312	42	476	319
**SIRT5**	LGG	HA_e	56	69	351	476	320
**TDRD3**	LGG	HM_r	33	318	124	475	321
**DPF1**	LGG	ChRC	210	69	195	474	322
**SMYD3**	LGG	HM_w	46	228	200	474	323
**SETD1A**	LGG	HM_w	70	120	283	473	324
**PRDM7**	LGG	HM_w	97	125	250	472	325
**INO80**	LGG	Helicases	165	261	42	468	326
**NSD1**	LGG	HM_w	128	298	42	468	327
**UTY**	LGG	HM_e	356	69	42	467	328
**H2AFZ**	LGG	Others	184	69	213	466	329
**ARID4A**	LGG	ChRC	354	69	42	465	330
**LBR**	LGG	Others	143	69	253	465	331
**PYGO1**	LGG	HM_r	86	233	145	464	332
**KDM1B**	LGG	HM_e	155	69	238	462	333

**CHD1L**	LGG	Helicases	219	197	42	458	334
**HSPBAP1**	LGG	Others	170	241	42	453	335
**L3MBTL1**	LGG	HM_r	146	69	237	452	336
**PRDM8**	LGG	HM_w	96	69	285	450	337
**SMARCA5**	LGG	Helicases	52	356	42	450	338
**CHD2**	LGG	Helicases	338	69	42	449	339
**PRDM2**	LGG	HM_w	280	126	42	448	340
**PHF5A**	LGG	Others	105	297	42	444	341
**AFF1**	LGG	Others	250	69	123	442	342
**ELP3**	LGG	HA_w	205	195	42	442	343
**GLYR1**	LGG	HM_r	189	211	42	442	344
**JMJD8**	LGG	HM_e	161	69	212	442	345
**PHF6**	LGG	HM_r	104	296	42	442	346
**CBX1**	LGG	HM_r	229	170	42	441	347
**UBE2E1**	LGG	Others	22	221	197	440	348
**FMR1**	LGG	Others	325	69	42	436	349
**ORC1**	LGG	Others	127	137	172	436	350
**SMYD2**	LGG	HM_w	47	69	320	436	351
**KDM3A**	LGG	HM_e	154	69	211	434	352
**RNF2**	LGG	ChRC	79	69	284	432	353
**UHRF1**	LGG	DM_r	19	367	42	428	354
**PRDM4**	LGG	HM_w	99	281	42	422	355
**PCGF5**	LGG	Others	118	132	171	421	356
**DPF2**	LGG	Others	209	164	42	415	357
**MTA1**	LGG	ChRC	135	238	42	415	358
**PADI1**	LGG	Others	126	136	152	414	359
**RNF217**	LGG	Others	77	294	42	413	360
**UBE2I**	LGG	Others	21	174	217	412	361
**SMARCE1**	LGG	Helicases	49	179	183	411	362
**PHF21A**	LGG	HM_r	289	69	42	400	363
**PHIP**	LGG	HA_r	287	69	42	398	364
**TDRD6**	LGG	Others	31	201	166	398	365
**PCGF6**	LGG	Others	117	236	42	395	366
**SIRT4**	LGG	HA_e	57	69	266	392	367
**PRMT3**	LGG	HM_w	91	256	42	389	368

**PADI3**	LGG	Others	125	134	129	388	369
**DNMT3L**	LGG	DM_w	211	69	107	387	370
**PRKAA2**	LGG	Others	95	69	221	385	371
**PCGF2**	LGG	Others	119	133	132	384	372
**RBBP5**	LGG	ChRC	82	254	42	378	373
**RTF1**	LGG	Others	72	253	42	367	374
**WDR82**	LGG	Others	15	310	42	367	375
**TCF20**	LGG	Others	36	288	42	366	376
**PRMT7**	LGG	HM_w	89	69	205	363	377
**ZGPAT**	LGG	Others	12	69	282	363	378
**RAI1**	LGG	Others	84	69	204	357	379
**BAZ2A**	LGG	HA_r	238	69	42	349	380
**USP22**	LGG	Others	18	69	261	348	381
**BRD7**	LGG	HA_r	234	69	42	345	382
**PRMT6**	LGG	HM_w	90	69	184	343	383
**TET3**	LGG	DM_e	28	69	245	342	384
**PHF12**	LGG	Others	112	187	42	341	385
**SETD4**	LGG	HM_w	68	69	203	340	386
**PADI6**	LGG	Others	123	69	147	339	387
**PHF2**	LGG	Others	109	184	42	335	388
**L3MBTL4**	LGG	Others	144	69	119	332	389
**PRDM6**	LGG	HM_w	98	181	42	321	390
**MORF4L1**	LGG	HM_r	137	140	42	319	391
**UBE2A**	LGG	Others	24	250	42	316	392
**ERCC5**	LGG	Others	203	69	42	314	393
**MTA3**	LGG	ChRC	134	138	42	314	394
**ZCWPW2**	LGG	HM_r	13	69	231	313	395
**SETDB1**	LGG	HM_w	65	203	42	310	396
**RNF20**	LGG	Others	78	180	42	300	397
**TDRD5**	LGG	Others	32	69	199	300	398
**GTF2H1**	LGG	Others	186	69	42	297	399
**SETD6**	LGG	HM_w	67	69	156	292	400
**YY1**	LGG	ChRC	14	220	42	276	401
**KDM5D**	LGG	HM_e	149	69	42	260	402
**SMYD5**	LGG	HM_w	44	69	143	256	403

**SCML4**	LGG	Others	71	69	113	253	404
**TDRD7**	LGG	Others	30	176	42	248	405
**SIRT2**	LGG	HA_e	59	69	117	245	406
**MTF2**	LGG	HM_r	133	69	42	244	407
**NCOA1**	LGG	HA_w	129	69	42	240	408
**RNF40**	LGG	Others	76	122	42	240	409
**PADI4**	LGG	Others	124	69	42	235	410
**ZMYND8**	LGG	HA_r	11	69	149	229	411
**SIN3A**	LGG	ChRC	62	119	42	223	412
**TDRD12**	LGG	Others	34	69	112	215	413
**TCEA1**	LGG	Others	38	116	42	196	414
**SIRT1**	LGG	HA_e	60	69	42	171	415
**USP27X**	LGG	Others	17	69	42	128	416
**ASH1L**	LIHC	HM_w	416	421	402	1239	1
**CHD7**	LIHC	Helicases	397	402	410	1209	2
**BPTF**	LIHC	HA_r	414	390	369	1173	3
**ATAD2**	LIHC	HA_r	399	426	344	1169	4
**CHD1L**	LIHC	Helicases	349	414	401	1164	5
**ELP3**	LIHC	HA_w	334	397	421	1152	6
**PARP1**	LIHC	Others	324	404	419	1147	7
**PYGO2**	LIHC	HM_r	293	422	424	1139	8
**JARID2**	LIHC	ChRC	383	386	367	1136	9
**SETDB1**	LIHC	HM_w	289	417	426	1132	10
**EHMT2**	LIHC	HM_w	335	388	406	1129	11
**DIDO1**	LIHC	Others	413	328	376	1117	12
**BRD9**	LIHC	HA_r	311	389	411	1111	13
**PHF3**	LIHC	Others	322	372	396	1090	14
**SMARCD2**	LIHC	Helicases	316	392	370	1078	15
**CDYL**	LIHC	HM_r	310	394	368	1072	16
**TDRD10**	LIHC	Others	239	418	414	1071	17
**LBR**	LIHC	Others	300	405	359	1064	18
**BRD2**	LIHC	HA_r	274	377	408	1059	19
**PRDM16**	LIHC	HM_w	295	371	371	1037	20
**KMT2C**	LIHC	HM_w	422	352	252	1026	21
**DNMT3A**	LIHC	DM_w	371	340	313	1024	22

**NSD1**	LIHC	HM_w	380	335	307	1022	23
**KDM2A**	LIHC	HM_e	303	354	360	1017	24
**BAZ2A**	LIHC	HA_r	398	274	343	1015	25
**JMJD6**	LIHC	HM_e	216	395	399	1010	26
**EP400**	LIHC	HA_w	404	293	312	1009	27
**PAXIP1**	LIHC	Others	299	334	365	998	28
**EZH1**	LIHC	HM_w	333	307	357	997	29
**AKAP1**	LIHC	Others	279	370	345	994	30
**KANSL1**	LIHC	HA_w	330	291	372	993	31
**SMYD3**	LIHC	HM_w	178	411	404	993	32
**BRPF3**	LIHC	HA_r	228	369	395	992	33
**GATAD2B**	LIHC	HM_r	155	420	417	992	34
**PHF20L1**	LIHC	HM_r	135	425	422	982	35
**ASXL2**	LIHC	Others	364	342	275	981	36
**BRD7**	LIHC	HA_r	387	206	382	975	37
**ARID2**	LIHC	ChRC	425	277	260	962	38
**KDM5B**	LIHC	HM_e	146	413	394	953	39
**NCOA1**	LIHC	HA_w	326	349	278	953	40
**TDRD5**	LIHC	Others	419	410	124	953	41
**ASH2L**	LIHC	HM_w	167	393	392	952	42
**ASXL1**	LIHC	Others	365	208	379	952	43
**HIST1H1C**	LIHC	Others	331	381	239	951	44
**BOP 1**	LIHC	Others	102	423	423	948	45
**KDM1B**	LIHC	HM_e	213	383	347	943	46
**RBBP5**	LIHC	ChRC	126	412	403	941	47
**TCF20**	LIHC	Others	400	213	327	940	48
**CHD3**	LIHC	Helicases	385	358	194	937	49
**ING1**	LIHC	HM_r	264	379	291	934	50
**TCEA1**	LIHC	Others	114	400	416	930	51
**MECOM**	LIHC	Others	391	351	187	929	52
**SMYD2**	LIHC	HM_w	116	407	405	928	53
**ATR**	LIHC	Others	418	312	196	926	54
**CBX4**	LIHC	HM_r	350	399	176	925	55
**TDRD6**	LIHC	Others	368	374	181	923	56
**BAZ1B**	LIHC	HA_r	231	311	378	920	57

**MBD5**	LIHC	DM_r	343	326	251	920	58
**ARID4B**	LIHC	ChRC	104	408	407	919	59
**HDAC5**	LIHC	HA_e	266	327	325	918	60
**ATAD2B**	LIHC	HA_r	376	360	179	915	61
**BRPF1**	LIHC	HA_r	352	297	259	908	62
**ZGPAT**	LIHC	Others	283	329	294	906	63
**ARID1B**	LIHC	ChRC	415	363	122	900	64
**RING1**	LIHC	Others	124	364	409	897	65
**PRDM14**	LIHC	HM_w	296	415	185	896	66
**ACTL6A**	LIHC	ChRC	169	344	380	893	67
**CBX8**	LIHC	ChRC	98	398	397	893	68
**CHRAC1**	LIHC	ChRC	44	424	425	893	69
**HCFC1**	LIHC	Others	307	356	227	890	70
**PHC3**	LIHC	ChRC	255	348	286	889	71
**TDRKH**	LIHC	Others	55	416	415	886	72
**SMARCC2**	LIHC	Helicases	288	317	277	882	73
**CHD8**	LIHC	Helicases	271	268	342	881	74
**MLLT6**	LIHC	HM_w	260	325	288	873	75
**KMT2E**	LIHC	HM_w	392	256	224	872	76
**HDGF**	LIHC	Others	34	419	418	871	77
**BRD8**	LIHC	HA_r	273	271	326	870	78
**KAT7**	LIHC	HA_w	148	337	385	870	79
**FXR2**	LIHC	Others	92	357	420	869	80
**KIAA2026**	LIHC	Others	393	288	188	869	81
**PHF23**	LIHC	HM_r	133	346	387	866	82
**BRD1**	LIHC	HA_r	230	272	363	865	83
**ARID1A**	LIHC	ChRC	426	313	123	862	84
**BRD4**	LIHC	HA_r	336	309	215	860	85
**FMR1**	LIHC	Others	222	264	374	860	86
**KAT2A**	LIHC	HA_w	215	290	353	858	87
**CBX2**	LIHC	ChRC	100	396	361	857	88
**CHD2**	LIHC	Helicases	386	295	174	855	89
**H3F3A**	LIHC	Others	36	406	413	855	90
**NCOR1**	LIHC	ChRC	381	375	99	855	91
**FKBP5**	LIHC	Others	157	384	310	851	92

**NCOA3**	LIHC	HA_w	390	140	321	851	93
**SETD1B**	LIHC	HM_w	377	176	296	849	94
**TAF3**	LIHC	HA_r	315	299	234	848	95
**MLLT10**	LIHC	HM_w	208	286	352	846	96
**L3MBTL1**	LIHC	HM_r	344	190	308	842	97
**PHF12**	LIHC	Others	198	305	339	842	98
**EP300**	LIHC	HA_w	411	201	229	841	99
**SFMBT2**	LIHC	Others	403	332	106	841	100
**DAXX**	LIHC	ChRC	95	367	377	839	101
**HELLS**	LIHC	Helicases	332	150	356	838	102
**PHF1**	LIHC	HM_r	72	366	400	838	103
**PPARGC1A**	LIHC	Others	320	251	267	838	104
**SIRT5**	LIHC	HA_e	61	385	389	835	105
**SMARCA1**	LIHC	Helicases	317	318	199	834	106
**BMI1**	LIHC	ChRC	275	273	283	831	107
**ING3**	LIHC	HM_r	151	355	323	829	108
**MECP2**	LIHC	DM_r	210	350	269	829	109
**CBX3**	LIHC	HM_r	227	270	331	828	110
**ATAT1**	LIHC	Others	50	391	386	827	111
**CHD6**	LIHC	Helicases	407	162	258	827	112
**SRCAP**	LIHC	Others	408	175	244	827	113
**GATAD2A**	LIHC	HM_r	267	263	292	822	114
**L3MBTL3**	LIHC	Others	328	287	207	822	115
**POLR2B**	LIHC	Others	321	182	318	821	116
**TAF1**	LIHC	HA_r	401	115	303	819	117
**PRDM2**	LIHC	HM_w	294	321	203	818	118
**SUPT16H**	LIHC	Others	241	239	333	813	119
**SND1**	LIHC	HM_r	177	280	355	812	120
**SIRT7**	LIHC	HA_e	17	401	391	809	121
**SIN3B**	LIHC	ChRC	318	243	247	808	122
**PRKAA1**	LIHC	Others	250	219	337	806	123
**AFF4**	LIHC	Others	312	343	147	802	124
**CHD9**	LIHC	Helicases	396	294	112	802	125
**PRMT5**	LIHC	HM_w	249	217	336	802	126
**CHD5**	LIHC	Helicases	372	368	60	800	127

**CHAF1A**	LIHC	ChRC	272	296	231	799	128
**PRDM10**	LIHC	HM_w	251	250	298	799	129
**SMARCA4**	LIHC	Helicases	402	69	328	799	130
**RAI1**	LIHC	Others	291	320	184	795	131
**SUZ12**	LIHC	ChRC	174	316	304	794	132
**ZCWPW1**	LIHC	HM_r	236	315	243	794	133
**KDM3B**	LIHC	HM_e	345	259	189	793	134
**CHD1**	LIHC	Helicases	373	204	214	791	135
**TDG**	LIHC	ChRC	240	212	332	784	136
**SIRT2**	LIHC	HA_e	183	242	358	783	137
**SETD3**	LIHC	HM_w	246	123	412	781	138
**SUV39H2**	LIHC	HM_w	175	300	305	780	139
**HR**	LIHC	HM_e	305	403	71	779	140
**CBX1**	LIHC	HM_r	48	341	388	777	141
**PHF14**	LIHC	Others	253	184	338	775	142
**CHD4**	LIHC	Helicases	348	233	193	774	143
**PARP2**	LIHC	Others	202	252	320	774	144
**CREBBP**	LIHC	HA_w	395	232	146	773	145
**EPC1**	LIHC	Others	270	308	191	769	146
**RNF2**	LIHC	ChRC	22	409	335	766	147
**PCGF2**	LIHC	Others	201	324	236	761	148
**PRDM1**	LIHC	HM_w	339	323	98	760	149
**HDAC6**	LIHC	HA_e	347	195	211	753	150
**TCF19**	LIHC	Others	15	382	354	751	151
**PHF10**	LIHC	Others	341	365	42	748	152
**ATM**	LIHC	Others	388	276	81	745	153
**TRIM28**	LIHC	HA_r	171	210	364	745	154
**WDR5**	LIHC	ChRC	109	330	302	741	155
**BAP1**	LIHC	Others	423	275	42	740	156
**HDAC4**	LIHC	HA_e	362	152	225	739	157
**KMT2D**	LIHC	HM_w	424	143	169	736	158
**FBXO17**	LIHC	Others	159	231	341	731	159
**SHPRH**	LIHC	Others	369	319	42	730	160
**KMT2B**	LIHC	HM_w	421	257	42	720	161
**ATRX**	LIHC	Helicases	375	166	178	719	162

**IWS1**	LIHC	Others	359	69	290	718	163
**PHIP**	LIHC	HA_r	370	304	42	716	164
**CBX6**	LIHC	HM_r	166	234	314	714	165
**KDM4C**	LIHC	HM_e	358	258	90	706	166
**KAT6B**	LIHC	HA_w	410	192	102	704	167
**NCOR2**	LIHC	Others	412	69	222	703	168
**ING2**	LIHC	HM_r	152	378	171	701	169
**MBTD1**	LIHC	Others	75	336	289	700	170
**PRDM9**	LIHC	HM_w	194	303	202	699	171
**ACTL6B**	LIHC	ChRC	168	314	216	698	172
**PRMT1**	LIHC	HM_w	130	218	350	698	173
**HDAC2**	LIHC	HA_e	219	306	172	697	174
**ASXL3**	LIHC	Others	406	168	121	695	175
**SIN3A**	LIHC	ChRC	185	244	265	694	176
**KMT2A**	LIHC	HM_w	417	144	132	693	177
**PAF1**	LIHC	Others	73	253	366	692	178
**PRDM11**	LIHC	HM_w	338	132	220	690	179
**DOT1L**	LIHC	HM_w	363	69	256	688	180
**HIST1H3B**	LIHC	Others	217	380	91	688	181
**EZH2**	LIHC	HM_w	268	69	348	685	182
**KDM3A**	LIHC	HM_e	329	145	210	684	183
**SFMBT1**	LIHC	HM_r	120	245	317	682	184
**HDAC9**	LIHC	HA_e	384	194	103	681	185
**PRDM13**	LIHC	HM_w	297	322	58	677	186
**PRDM4**	LIHC	HM_w	196	130	351	677	187
**ELP4**	LIHC	HA_w	223	159	293	675	188
**TRIM66**	LIHC	HA_r	366	112	197	675	189
**ERCC5**	LIHC	Others	269	362	42	673	190
**JMJD1C**	LIHC	HM_e	382	147	144	673	191
**SMARCE1**	LIHC	Helicases	16	281	375	672	192
**MBD1**	LIHC	DM_r	212	69	390	671	193
**SETD5**	LIHC	HM_w	187	216	266	669	194
**SIRT6**	LIHC	HA_e	181	241	245	667	195
**PHF13**	LIHC	Others	71	373	221	665	196
**HDAC3**	LIHC	HA_e	85	196	383	664	197

**SATB1**	LIHC	Others	357	177	129	663	198
**TET1**	LIHC	DM_e	354	172	137	663	199
**HDGFL1**	LIHC	Others	218	387	56	661	200
**AEBP2**	LIHC	HM_w	105	209	346	660	201
**GTF3C4**	LIHC	HA_w	220	199	240	659	202
**IDH2**	LIHC	DM_e	265	261	133	659	203
**PHC2**	LIHC	ChRC	323	185	151	659	204
**DPY30**	LIHC	Others	41	267	349	657	205
**KAT6A**	LIHC	HA_w	394	69	190	653	206
**PHRF1**	LIHC	Others	389	222	42	653	207
**UBE2B**	LIHC	Others	13	236	398	647	208
**UBE2A**	LIHC	Others	14	237	393	644	209
**BRD3**	LIHC	HA_r	101	310	232	643	210
**PHF11**	LIHC	Others	254	347	42	643	211
**AURKB**	LIHC	Others	103	359	177	639	212
**PCGF6**	LIHC	Others	200	138	299	637	213
**MORF4L1**	LIHC	HM_r	29	223	384	636	214
**BAZ2B**	LIHC	HA_r	405	69	156	630	215
**SMYD5**	LIHC	HM_w	243	69	316	628	216
**HLTF**	LIHC	Others	82	292	253	627	217
**PHC1**	LIHC	ChRC	199	137	287	623	218
**RSF1**	LIHC	ChRC	247	246	130	623	219
**GTF2H1**	LIHC	Others	90	230	300	620	220
**TRIM24**	LIHC	HA_r	54	279	284	617	221
**EHMT1**	LIHC	HM_w	308	266	42	616	222
**SMARCA2**	LIHC	Helicases	409	69	138	616	223
**USP22**	LIHC	Others	314	69	233	616	224
**BRWD3**	LIHC	HA_r	351	69	195	615	225
**PHF21A**	LIHC	HM_r	340	133	140	613	226
**G2E3**	LIHC	Others	156	200	255	611	227
**FBXO44**	LIHC	Others	158	339	110	607	228
**MUM1**	LIHC	Others	259	69	279	607	229
**AFF1**	LIHC	Others	353	170	82	605	230
**BAZ1A**	LIHC	HA_r	276	207	120	603	231
**TDRD9**	LIHC	Others	367	173	63	603	232

**FBXL19**	LIHC	Others	160	158	282	600	233
**PBRM1**	LIHC	HA_r	379	69	152	600	234
**PRKCD**	LIHC	Others	23	248	329	600	235
**KAT2B**	LIHC	HA_w	263	193	143	599	236
**PHF20**	LIHC	HM_r	197	134	268	599	237
**KDM6B**	LIHC	HM_e	144	353	101	598	238
**SMNDC1**	LIHC	Others	244	69	285	598	239
**YY1**	LIHC	ChRC	107	110	381	598	240
**HDAC8**	LIHC	HA_e	84	151	362	597	241
**MSH6**	LIHC	HM_r	342	69	186	597	242
**MTF2**	LIHC	HM_r	204	141	249	594	243
**POLE3**	LIHC	ChRC	67	221	306	594	244
**HIST1H1B**	LIHC	Others	83	376	134	593	245
**GTF2F1**	LIHC	Others	154	153	281	588	246
**ARID4A**	LIHC	ChRC	278	169	136	583	247
**NAP1L1**	LIHC	Others	74	187	322	583	248
**BRWD1**	LIHC	HA_r	374	164	42	580	249
**TDRD3**	LIHC	HM_r	173	331	76	580	250
**ATF7IP**	LIHC	Others	277	167	135	579	251
**IDH1**	LIHC	DM_e	360	148	70	578	252
**HDAC10**	LIHC	HA_e	87	262	226	575	253
**PRMT6**	LIHC	HM_w	193	180	201	574	254
**PHF5A**	LIHC	Others	70	183	319	572	255
**TET3**	LIHC	DM_e	237	69	263	569	256
**MBD4**	LIHC	DM_r	211	189	168	568	257
**KDM7A**	LIHC	HM_e	281	69	217	567	258
**DPF2**	LIHC	Others	224	69	272	565	259
**FBXW9**	LIHC	Others	93	157	311	561	260
**MTA1**	LIHC	ChRC	327	188	42	557	261
**PHF7**	LIHC	Others	68	285	204	557	262
**HDAC11**	LIHC	HA_e	86	197	271	554	263
**MTA2**	LIHC	ChRC	205	69	280	554	264
**UBE2I**	LIHC	Others	12	278	261	551	265
**STK31**	LIHC	Others	242	240	68	550	266
**MSL3**	LIHC	HA_w	206	255	88	549	267

**NAP1L2**	LIHC	Others	140	186	223	549	268
**SIRT4**	LIHC	HA_e	182	120	246	548	269
**PRDM15**	LIHC	HM_w	337	131	79	547	270
**HDAC7**	LIHC	HA_e	153	69	324	546	271
**DNMT3B**	LIHC	DM_w	225	202	118	545	272
**KDM6A**	LIHC	HM_e	145	289	108	542	273
**RNF17**	LIHC	Others	378	69	95	542	274
**KDM4B**	LIHC	HM_e	262	69	208	539	275
**KDM5A**	LIHC	HM_e	78	191	270	539	276
**SETD6**	LIHC	HM_w	121	122	295	538	277
**EPC2**	LIHC	Others	161	265	111	537	278
**RNF20**	LIHC	Others	248	126	163	537	279
**FKBP1A**	LIHC	Others	40	156	340	536	280
**GLYR1**	LIHC	HM_r	91	69	373	533	281
**SUV39H1**	LIHC	HM_w	176	174	182	532	282
**SETD2**	LIHC	HM_w	420	69	42	531	283
**ING5**	LIHC	HM_r	150	69	309	528	284
**L3MBTL4**	LIHC	Others	261	225	42	528	285
**MEN1**	LIHC	ChRC	209	69	250	528	286
**PRMT3**	LIHC	HM_w	129	181	218	528	287
**CARM1**	LIHC	HM_w	49	163	315	527	288
**RNF217**	LIHC	Others	62	302	162	526	289
**RAG2**	LIHC	HM_r	292	69	164	525	290
**CDYL2**	LIHC	HM_r	46	205	273	524	291
**KDM2B**	LIHC	HM_e	302	69	153	524	292
**CBX7**	LIHC	HM_r	99	269	155	523	293
**SETD1A**	LIHC	HM_w	356	124	42	522	294
**GADD45A**	LIHC	Others	38	338	145	521	295
**PHF19**	LIHC	HM_r	136	136	248	520	296
**PHF2**	LIHC	Others	298	135	87	520	297
**SP110**	LIHC	HA_r	287	118	115	520	298
**SP140**	LIHC	HA_r	286	117	114	517	299
**TDRD1**	LIHC	Others	355	69	93	517	300
**KDM4A**	LIHC	HM_e	79	228	209	516	301
**H2AFZ**	LIHC	Others	89	198	228	515	302

**ORC1**	LIHC	Others	139	139	237	515	303
**DMAP1**	LIHC	Others	94	161	257	512	304
**PCMT1**	LIHC	Others	137	333	42	512	305
**TP53BP1**	LIHC	Others	284	69	159	512	306
**TET2**	LIHC	DM_e	238	211	62	511	307
**RPH3A**	LIHC	Others	290	125	94	509	308
**MBD3**	LIHC	DM_r	76	224	206	506	309
**SMYD4**	LIHC	HM_w	59	361	84	504	310
**ZCWPW2**	LIHC	HM_r	235	109	158	502	311
**UBE2E1**	LIHC	Others	112	111	276	499	312
**PRKAA2**	LIHC	Others	24	249	219	492	313
**KDM1A**	LIHC	HM_e	304	69	117	490	314
**TRIM33**	LIHC	HA_r	113	113	262	488	315
**CLOCK**	LIHC	HA_w	165	203	119	487	316
**DNMT1**	LIHC	DM_w	226	69	192	487	317
**PWWP2B**	LIHC	Others	63	127	297	487	318
**BRDT**	LIHC	HA_r	229	165	92	486	319
**JADE1**	LIHC	Others	313	69	104	486	320
**PRMT8**	LIHC	HM_w	192	129	165	486	321
**SIRT3**	LIHC	HA_e	119	215	150	484	322
**SMARCC1**	LIHC	Helicases	180	69	235	484	323
**TDRD7**	LIHC	Others	172	114	198	484	324
**JADE3**	LIHC	Others	233	69	180	482	325
**PYGO1**	LIHC	HM_r	128	247	107	482	326
**PRDM12**	LIHC	HM_w	25	284	166	475	327
**RPS6KA5**	LIHC	Others	319	69	86	474	328
**EED**	LIHC	ChRC	162	69	241	472	329
**HNF1A**	LIHC	ChRC	361	69	42	472	330
**KDM8**	LIHC	HM_e	143	227	100	470	331
**SETMAR**	LIHC	HM_w	186	121	160	467	332
**TAF1L**	LIHC	HA_r	285	69	113	467	333
**RPA3**	LIHC	Others	21	282	161	464	334
**SMARCD1**	LIHC	Helicases	60	69	334	463	335
**JADE2**	LIHC	Others	234	69	157	460	336
**PADI1**	LIHC	Others	325	69	66	460	337

**KDM5C**	LIHC	HM_e	301	69	89	459	338
**INO80**	LIHC	Helicases	346	69	42	457	339
**PRDM6**	LIHC	HM_w	132	283	42	457	340
**SCMH1**	LIHC	Others	188	69	200	457	341
**SMYD1**	LIHC	HM_w	179	214	64	457	342
**INTS12**	LIHC	Others	149	260	42	451	343
**SMARCB1**	LIHC	Helicases	118	69	264	451	344
**WDR82**	LIHC	Others	108	298	42	448	345
**HIF1AN**	LIHC	Others	33	149	254	436	346
**SETDB2**	LIHC	HM_w	18	345	73	436	347
**HAT1**	LIHC	HA_w	35	69	330	434	348
**AIRE**	LIHC	HM_r	280	69	75	424	349
**ZMYND11**	LIHC	HA_r	51	69	301	421	350
**CTCF**	LIHC	Others	309	69	42	420	351
**SMARCA5**	LIHC	Helicases	245	69	105	419	352
**GTF2B**	LIHC	Others	221	154	42	417	353
**HIRA**	LIHC	Others	306	69	42	417	354
**ING4**	LIHC	HM_r	32	229	154	415	355
**NAP1L3**	LIHC	Others	203	69	141	413	356
**L3MBTL2**	LIHC	Others	142	226	42	410	357
**RNF40**	LIHC	Others	190	178	42	410	358
**CHAF1B**	LIHC	ChRC	97	69	242	408	359
**FKBP2**	LIHC	Others	39	155	213	407	360
**PHF8**	LIHC	Others	252	69	80	401	361
**SCML4**	LIHC	Others	20	301	77	398	362
**CXXC1**	LIHC	Others	96	69	230	395	363
**KDM5D**	LIHC	HM_e	282	69	42	393	364
**CBX5**	LIHC	HM_r	47	69	274	390	365
**DPF1**	LIHC	ChRC	164	160	61	385	366
**PADI2**	LIHC	Others	258	69	57	384	367
**PADI3**	LIHC	Others	257	69	55	381	368
**SIRT1**	LIHC	HA_e	184	69	127	380	369
**PCGF5**	LIHC	Others	138	69	167	374	370
**HDAC1**	LIHC	HA_e	88	69	212	369	371
**AICDA**	LIHC	DM_e	232	69	67	368	372

**PADI4**	LIHC	Others	256	69	42	367	373
**TDRD12**	LIHC	Others	56	238	72	366	374
**ZMYND8**	LIHC	HA_r	106	108	148	362	375
**PSIP1**	LIHC	HM_r	191	128	42	361	376
**KDM4D**	LIHC	HM_e	147	69	142	358	377
**PRDM5**	LIHC	HM_w	195	69	78	342	378
**PRDM7**	LIHC	HM_w	66	220	54	340	379
**PRMT7**	LIHC	HM_w	64	179	96	339	380
**KDM4E**	LIHC	HM_e	30	69	238	337	381
**SETD4**	LIHC	HM_w	122	69	139	330	382
**UHRF2**	LIHC	DM_r	52	235	42	329	383
**KAT5**	LIHC	HA_w	214	69	42	325	384
**RBBP7**	LIHC	ChRC	125	69	131	325	385
**MTA3**	LIHC	ChRC	28	254	42	324	386
**USP27X**	LIHC	Others	111	171	42	324	387
**SCML2**	LIHC	HM_r	123	69	128	320	388
**JMJD8**	LIHC	HM_e	80	69	170	319	389
**MPHOSPH8**	LIHC	HM_r	207	69	42	318	390
**SMARCD3**	LIHC	Helicases	117	69	126	312	391
**SSRP1**	LIHC	Others	57	69	183	309	392
**PCGF1**	LIHC	Others	26	69	205	300	393
**RTF1**	LIHC	Others	189	69	42	300	394
**SP140L**	LIHC	HA_r	58	116	125	299	395
**PRDM8**	LIHC	HM_w	131	69	97	297	396
**DPF3**	LIHC	HA_r	163	69	59	291	397
**CECR2**	LIHC	HA_r	45	69	175	289	398
**CSTL1**	LIHC	Others	43	69	173	285	399
**UTY**	LIHC	HM_e	170	69	42	281	400
**SP100**	LIHC	HA_r	115	119	42	276	401
**UBR7**	LIHC	Others	53	69	149	271	402
**PHF21B**	LIHC	HM_r	134	69	65	268	403
**USP51**	LIHC	Others	110	69	83	262	404
**MBD2**	LIHC	DM_r	77	142	42	261	405
**MARCH5**	LIHC	Others	141	69	42	252	406
**PRMT2**	LIHC	HM_w	65	69	116	250	407

**RBBP4**	LIHC	ChRC	127	69	42	238	408
**KAT8**	LIHC	HA_w	31	146	42	219	409
**GADD45B**	LIHC	Others	37	69	109	215	410
**HSPBAP1**	LIHC	Others	81	69	42	192	411
**DNMT3L**	LIHC	DM_w	42	69	74	185	412
**PHF6**	LIHC	HM_r	69	69	42	180	413
**SETD7**	LIHC	HM_w	19	69	85	173	414
**PADI6**	LIHC	Others	27	69	69	165	415
**UHRF1**	LIHC	DM_r	11	69	42	122	416
**ASH1L**	LUAD	HM_w	416	419	415	1250	1
**DIDO1**	LUAD	Others	420	402	389	1211	2
**KDM5B**	LUAD	HM_e	378	404	419	1201	3
**CHD7**	LUAD	Helicases	414	384	402	1200	4
**ATAD2**	LUAD	HA_r	367	414	413	1194	5
**BAZ1A**	LUAD	HA_r	353	418	417	1188	6
**PHF20L1**	LUAD	HM_r	332	412	421	1165	7
**STK31**	LUAD	Others	385	367	397	1149	8
**G2E3**	LUAD	Others	308	410	411	1129	9
**PHF14**	LUAD	Others	335	386	407	1128	10
**CHD8**	LUAD	Helicases	409	326	379	1114	11
**KDM5A**	LUAD	HM_e	361	378	375	1114	12
**GATAD2B**	LUAD	HM_r	277	420	416	1113	13
**BPTF**	LUAD	HA_r	383	373	341	1097	14
**SRCAP**	LUAD	Others	411	279	398	1088	15
**ARID4B**	LUAD	ChRC	243	405	420	1068	16
**ZGPAT**	LUAD	Others	245	403	409	1057	17
**CDYL**	LUAD	HM_r	391	275	388	1054	18
**CBX2**	LUAD	ChRC	363	381	305	1049	19
**CHD1L**	LUAD	Helicases	236	421	392	1049	20
**PYGO2**	LUAD	HM_r	202	423	423	1048	21
**TDRD5**	LUAD	Others	384	406	256	1046	22
**ARID1B**	LUAD	ChRC	392	338	310	1040	23
**BRD9**	LUAD	HA_r	186	426	426	1038	24
**ATR**	LUAD	Others	397	277	361	1035	25
**BAZ2A**	LUAD	HA_r	318	347	370	1035	26

**PRDM14**	LUAD	HM_w	408	359	266	1033	27
**PHF20**	LUAD	HM_r	333	316	374	1023	28
**ZMYND8**	LUAD	HA_r	355	343	324	1022	29
**NCOA3**	LUAD	HA_w	359	345	312	1016	30
**SETD1A**	LUAD	HM_w	372	283	360	1015	31
**FMR1**	LUAD	Others	347	335	329	1011	32
**EHMT2**	LUAD	HM_w	279	336	395	1010	33
**HDAC9**	LUAD	HA_e	413	390	205	1008	34
**SND1**	LUAD	HM_r	371	300	337	1008	35
**PARP1**	LUAD	Others	213	394	399	1006	36
**CHD6**	LUAD	Helicases	415	252	335	1002	37
**JARID2**	LUAD	ChRC	362	290	347	999	38
**MBD1**	LUAD	DM_r	268	321	410	999	39
**CBX3**	LUAD	HM_r	239	365	393	997	40
**SETDB1**	LUAD	HM_w	146	425	425	996	41
**DAXX**	LUAD	ChRC	314	271	391	976	42
**LBR**	LUAD	Others	167	395	412	974	43
**PAF1**	LUAD	Others	263	354	353	970	44
**BRD2**	LUAD	HA_r	284	298	385	967	45
**EZH2**	LUAD	HM_w	278	355	330	963	46
**RNF2**	LUAD	ChRC	201	399	362	962	47
**BOP 1**	LUAD	Others	132	411	418	961	48
**RSF1**	LUAD	ChRC	199	382	372	953	49
**PRDM9**	LUAD	HM_w	425	417	109	951	50
**ACTL6A**	LUAD	ChRC	188	356	401	945	51
**EED**	LUAD	ChRC	280	361	304	945	52
**PRKAA1**	LUAD	Others	155	416	373	944	53
**ATF7IP**	LUAD	Others	366	351	225	942	54
**JMJD6**	LUAD	HM_e	169	364	408	941	55
**HLTF**	LUAD	Others	272	333	334	939	56
**HDGF**	LUAD	Others	173	415	348	936	57
**CBX4**	LUAD	HM_r	238	380	317	935	58
**TDRKH**	LUAD	Others	80	424	422	926	59
**PRDM16**	LUAD	HM_w	329	301	294	924	60
**SUPT16H**	LUAD	Others	250	312	357	919	61

**RBBP5**	LUAD	ChRC	95	409	414	918	62
**CHD9**	LUAD	Helicases	404	272	238	914	63
**PCGF2**	LUAD	Others	212	318	384	914	64
**SMARCC2**	LUAD	Helicases	196	313	405	914	65
**DNMT3A**	LUAD	DM_w	350	179	382	911	66
**CHRAC1**	LUAD	ChRC	72	408	424	904	67
**KDM1B**	LUAD	HM_e	303	245	354	902	68
**ASXL1**	LUAD	Others	319	211	368	898	69
**AEBP2**	LUAD	HM_w	136	391	364	891	70
**PAXIP1**	LUAD	Others	160	362	369	891	71
**RNF40**	LUAD	Others	200	285	406	891	72
**PHF2**	LUAD	Others	334	289	267	890	73
**ELP4**	LUAD	HA_w	310	294	285	889	74
**TDRD10**	LUAD	Others	289	422	177	888	75
**MECOM**	LUAD	Others	302	383	202	887	76
**SMARCA2**	LUAD	Helicases	386	368	126	880	77
**AKAP1**	LUAD	Others	321	327	226	874	78
**SMYD3**	LUAD	HM_w	194	413	265	872	79
**PARP2**	LUAD	Others	161	344	366	871	80
**PHC3**	LUAD	ChRC	211	370	289	870	81
**NCOA1**	LUAD	HA_w	394	195	278	867	82
**SMARCA1**	LUAD	Helicases	356	282	229	867	83
**KDM2A**	LUAD	HM_e	222	244	400	866	84
**MECP2**	LUAD	DM_r	218	387	259	864	85
**MLLT10**	LUAD	HM_w	341	217	302	860	86
**KDM4D**	LUAD	HM_e	270	323	260	853	87
**SMARCD2**	LUAD	Helicases	254	357	240	851	88
**TRIM24**	LUAD	HA_r	246	339	264	849	89
**BRPF3**	LUAD	HA_r	185	308	352	845	90
**PRMT5**	LUAD	HM_w	151	348	345	844	91
**TCEA1**	LUAD	Others	82	366	394	842	92
**HDAC6**	LUAD	HA_e	274	306	261	841	93
**SFMBT2**	LUAD	Others	407	225	208	840	94
**KAT6A**	LUAD	HA_w	51	407	381	839	95
**HCFC1**	LUAD	Others	403	388	42	833	96

**ASXL2**	LUAD	Others	368	185	279	832	97
**ATAD2B**	LUAD	HA_r	285	184	363	832	98
**BAZ2B**	LUAD	HA_r	405	154	273	832	99
**HR**	LUAD	HM_e	345	396	91	832	100
**ING3**	LUAD	HM_r	170	332	327	829	101
**MLLT6**	LUAD	HM_w	217	319	283	819	102
**KIAA2026**	LUAD	Others	395	377	42	814	103
**GLYR1**	LUAD	HM_r	231	204	378	813	104
**CLOCK**	LUAD	HA_w	282	296	232	810	105
**PRDM7**	LUAD	HM_w	328	353	129	810	106
**BRWD3**	LUAD	HA_r	419	140	247	806	107
**RPA3**	LUAD	Others	27	393	386	806	108
**ARID2**	LUAD	ChRC	417	69	319	805	109
**TDRD6**	LUAD	Others	370	329	106	805	110
**PPARGC1A**	LUAD	Others	377	216	210	803	111
**PRDM2**	LUAD	HM_w	376	69	358	803	112
**SIRT7**	LUAD	HA_e	144	376	282	802	113
**DNMT3B**	LUAD	DM_w	312	178	309	799	114
**INO80**	LUAD	Helicases	271	331	196	798	115
**MSL3**	LUAD	HA_w	340	242	213	795	116
**RNF17**	LUAD	Others	400	315	80	795	117
**HAT1**	LUAD	HA_w	275	203	315	793	118
**RAI1**	LUAD	Others	357	286	150	793	119
**CBX8**	LUAD	ChRC	127	379	286	792	120
**H3F3A**	LUAD	Others	63	401	328	792	121
**RAG2**	LUAD	HM_r	327	287	178	792	122
**DPF2**	LUAD	Others	311	139	340	790	123
**MBD5**	LUAD	DM_r	342	243	203	788	124
**ARID4A**	LUAD	ChRC	320	299	166	785	125
**ATM**	LUAD	Others	423	69	292	784	126
**POLR2B**	LUAD	Others	208	230	346	784	127
**KDM4A**	LUAD	HM_e	221	201	359	781	128
**BAP1**	LUAD	Others	242	141	396	779	129
**FBXO17**	LUAD	Others	69	360	349	778	130
**CREBBP**	LUAD	HA_w	390	69	316	775	131

**KDM4C**	LUAD	HM_e	343	389	42	774	132
**ATAT1**	LUAD	Others	75	342	356	773	133
**CXXC1**	LUAD	Others	179	325	262	766	134
**CHD5**	LUAD	Helicases	410	273	81	764	135
**SCML4**	LUAD	Others	373	258	128	759	136
**RING1**	LUAD	Others	94	260	404	758	137
**TAF1L**	LUAD	HA_r	424	255	78	757	138
**TAF3**	LUAD	HA_r	393	69	295	757	139
**CBX1**	LUAD	HM_r	73	297	383	753	140
**PHF12**	LUAD	Others	103	263	387	753	141
**BRD8**	LUAD	HA_r	365	69	318	752	142
**NSD1**	LUAD	HM_w	339	371	42	752	143
**SMARCD1**	LUAD	Helicases	293	160	296	749	144
**SMARCE1**	LUAD	Helicases	143	214	390	747	145
**SMYD5**	LUAD	HM_w	252	145	350	747	146
**ZCWPW1**	LUAD	HM_r	137	375	233	745	147
**IDH1**	LUAD	DM_e	225	219	291	735	148
**IWS1**	LUAD	Others	224	134	376	734	149
**PRMT6**	LUAD	HM_w	204	288	241	733	150
**RPS6KA5**	LUAD	Others	257	227	249	733	151
**BRD1**	LUAD	HA_r	352	337	42	731	152
**HIRA**	LUAD	Others	346	341	42	729	153
**SMARCA4**	LUAD	Helicases	406	281	42	729	154
**DPF1**	LUAD	ChRC	235	372	120	727	155
**MSH6**	LUAD	HM_r	215	167	343	725	156
**PRDM1**	LUAD	HM_w	358	229	137	724	157
**HDAC7**	LUAD	HA_e	229	248	244	721	158
**SMYD2**	LUAD	HM_w	84	398	239	721	159
**FBXL19**	LUAD	Others	123	293	303	719	160
**TET3**	LUAD	DM_e	287	143	288	718	161
**KMT2C**	LUAD	HM_w	41	363	313	717	162
**MTA1**	LUAD	ChRC	267	69	380	716	163
**KMT2E**	LUAD	HM_w	39	350	326	715	164
**SMARCD3**	LUAD	Helicases	195	352	168	715	165
**CBX5**	LUAD	HM_r	129	254	331	714	166

**HDGFL1**	LUAD	Others	305	292	117	714	167
**ATRX**	LUAD	Helicases	422	142	142	706	168
**SMYD4**	LUAD	HM_w	142	280	281	703	169
**KAT7**	LUAD	HA_w	49	304	344	697	170
**SCMH1**	LUAD	Others	92	284	321	697	171
**MBTD1**	LUAD	Others	108	320	268	696	172
**SETD3**	LUAD	HM_w	256	69	371	696	173
**HIST1H1C**	LUAD	Others	226	247	222	695	174
**PRDM15**	LUAD	HM_w	330	165	200	695	175
**ERCC5**	LUAD	Others	381	269	42	692	176
**PADI2**	LUAD	Others	338	239	113	690	177
**ELP3**	LUAD	HA_w	125	397	165	687	178
**PADI6**	LUAD	Others	300	236	146	682	179
**NCOR2**	LUAD	Others	401	69	211	681	180
**KDM2B**	LUAD	HM_e	379	169	130	678	181
**SHPRH**	LUAD	Others	412	224	42	678	182
**UBE2A**	LUAD	Others	139	278	255	672	183
**SUV39H1**	LUAD	HM_w	83	311	275	669	184
**HDAC5**	LUAD	HA_e	174	249	245	668	185
**TP53BP1**	LUAD	Others	286	340	42	668	186
**GTF2F1**	LUAD	Others	176	221	270	667	187
**KAT8**	LUAD	HA_w	48	268	351	667	188
**NAP1L2**	LUAD	Others	301	116	250	667	189
**HIST1H3B**	LUAD	Others	304	246	116	666	190
**HIST1H1B**	LUAD	Others	273	291	101	665	191
**SP100**	LUAD	HA_r	324	124	217	665	192
**TCF19**	LUAD	Others	81	310	274	665	193
**PADI3**	LUAD	Others	337	238	88	663	194
**TDG**	LUAD	ChRC	192	144	325	661	195
**CHAF1A**	LUAD	ChRC	237	208	215	660	196
**TRIM28**	LUAD	HA_r	141	158	355	654	197
**HDAC2**	LUAD	HA_e	306	220	125	651	198
**NCOR1**	LUAD	ChRC	402	69	180	651	199
**L3MBTL1**	LUAD	HM_r	38	303	307	648	200
**PHF1**	LUAD	HM_r	210	265	173	648	201

**DNMT1**	LUAD	DM_w	313	180	148	641	202
**ING1**	LUAD	HM_r	171	305	163	639	203
**SETDB2**	LUAD	HM_w	295	257	87	639	204
**PHF13**	LUAD	Others	102	234	297	633	205
**CHD3**	LUAD	Helicases	316	274	42	632	206
**SETD6**	LUAD	HM_w	147	226	257	630	207
**UBR7**	LUAD	Others	138	156	336	630	208
**PHF10**	LUAD	Others	34	317	277	628	209
**BRD7**	LUAD	HA_r	130	309	188	627	210
**TRIM33**	LUAD	HA_r	140	328	156	624	211
**ZMYND11**	LUAD	HA_r	244	69	311	624	212
**EPC1**	LUAD	Others	309	270	42	621	213
**DOT1L**	LUAD	HM_w	349	176	95	620	214
**PHF3**	LUAD	Others	331	69	219	619	215
**SUV39H2**	LUAD	HM_w	249	69	301	619	216
**MTF2**	LUAD	HM_r	214	69	333	616	217
**PADI4**	LUAD	Others	336	237	42	615	218
**PSIP1**	LUAD	HM_r	203	369	42	614	219
**AIRE**	LUAD	HM_r	322	186	105	613	220
**SCML2**	LUAD	HM_r	296	69	248	613	221
**CHD2**	LUAD	Helicases	317	253	42	612	222
**SUZ12**	LUAD	ChRC	21	213	377	611	223
**CECR2**	LUAD	HA_r	351	69	187	607	224
**MPHOSPH8**	LUAD	HM_r	216	349	42	607	225
**SP140**	LUAD	HA_r	292	122	192	606	226
**L3MBTL3**	LUAD	Others	220	266	114	600	227
**AURKB**	LUAD	Others	134	276	189	599	228
**GATAD2A**	LUAD	HM_r	307	250	42	599	229
**DMAP1**	LUAD	Others	71	295	231	597	230
**GADD45A**	LUAD	Others	120	206	271	597	231
**JMJD1C**	LUAD	HM_e	380	69	147	596	232
**SATB1**	LUAD	Others	326	162	108	596	233
**SMARCB1**	LUAD	Helicases	86	190	320	596	234
**ASXL3**	LUAD	Others	426	69	99	594	235
**JMJD8**	LUAD	HM_e	53	202	339	594	236

**PADI1**	LUAD	Others	264	240	89	593	237
**AFF1**	LUAD	Others	323	69	198	590	238
**CSTL1**	LUAD	Others	180	307	103	590	239
**KANSL1**	LUAD	HA_w	52	170	367	589	240
**SETD2**	LUAD	HM_w	421	126	42	589	241
**SIRT5**	LUAD	HA_e	24	223	342	589	242
**TET1**	LUAD	DM_e	398	69	122	589	243
**BRDT**	LUAD	HA_r	364	69	154	587	244
**TDRD1**	LUAD	Others	290	69	228	587	245
**MTA2**	LUAD	ChRC	164	130	290	584	246
**HDAC4**	LUAD	HA_e	389	69	124	582	247
**RBBP4**	LUAD	ChRC	96	163	322	581	248
**KAT2A**	LUAD	HA_w	115	151	314	580	249
**PRMT8**	LUAD	HM_w	375	69	135	579	250
**HDAC10**	LUAD	HA_e	61	334	182	577	251
**CHD1**	LUAD	Helicases	181	181	214	576	252
**SIRT2**	LUAD	HA_e	89	358	127	574	253
**CBX6**	LUAD	HM_r	128	182	263	573	254
**USP51**	LUAD	Others	369	69	134	572	255
**UBE2I**	LUAD	Others	16	188	365	569	256
**HDAC11**	LUAD	HA_e	230	137	197	564	257
**FKBP5**	LUAD	Others	121	324	118	563	258
**PHRF1**	LUAD	Others	259	262	42	563	259
**PHF21B**	LUAD	HM_r	298	148	112	558	260
**SIRT1**	LUAD	HA_e	90	192	276	558	261
**PRDM4**	LUAD	HM_w	156	69	332	557	262
**DPF3**	LUAD	HA_r	281	69	206	556	263
**TDRD7**	LUAD	Others	248	212	96	556	264
**PRDM5**	LUAD	HM_w	297	114	144	555	265
**PCGF1**	LUAD	Others	106	149	298	553	266
**PHF23**	LUAD	HM_r	209	232	111	552	267
**AFF4**	LUAD	Others	354	155	42	551	268
**TAF1**	LUAD	HA_r	291	69	191	551	269
**EZH1**	LUAD	HM_w	233	174	141	548	270
**NAP1L1**	LUAD	Others	266	69	212	547	271

**BAZ1B**	LUAD	HA_r	74	69	403	546	272
**DNMT3L**	LUAD	DM_w	178	177	186	541	273
**SP110**	LUAD	HA_r	251	123	167	541	274
**KDM3A**	LUAD	HM_e	113	117	308	538	275
**MBD2**	LUAD	DM_r	166	330	42	538	276
**PRMT1**	LUAD	HM_w	153	127	258	538	277
**PHF21A**	LUAD	HM_r	262	233	42	537	278
**MORF4L1**	LUAD	HM_r	165	150	221	536	279
**KDM6A**	LUAD	HM_e	269	69	195	533	280
**PHIP**	LUAD	HA_r	260	231	42	533	281
**ACTL6B**	LUAD	ChRC	76	374	82	532	282
**PRKAA2**	LUAD	Others	29	261	242	532	283
**EPC2**	LUAD	Others	124	222	185	531	284
**HIF1AN**	LUAD	Others	227	69	235	531	285
**ARID1A**	LUAD	ChRC	418	69	42	529	286
**HNF1A**	LUAD	ChRC	58	172	299	529	287
**GTF3C4**	LUAD	HA_w	276	69	183	528	288
**RPH3A**	LUAD	Others	374	69	85	528	289
**HELLS**	LUAD	Helicases	228	135	164	527	290
**PWWP2B**	LUAD	Others	98	215	209	522	291
**SMARCC1**	LUAD	Helicases	255	110	157	522	292
**IDH2**	LUAD	DM_e	172	218	131	521	293
**KDM1A**	LUAD	HM_e	168	69	284	521	294
**SP140L**	LUAD	HA_r	193	121	207	521	295
**CHAF1B**	LUAD	ChRC	126	69	323	518	296
**ING2**	LUAD	HM_r	57	346	115	518	297
**SIN3B**	LUAD	ChRC	198	125	193	516	298
**MEN1**	LUAD	ChRC	107	69	338	514	299
**PRDM8**	LUAD	HM_w	206	128	179	513	300
**SIRT3**	LUAD	HA_e	88	256	169	513	301
**RBBP7**	LUAD	ChRC	149	69	293	511	302
**TCF20**	LUAD	Others	399	69	42	510	303
**EP400**	LUAD	HA_w	396	69	42	507	304
**ORC1**	LUAD	Others	36	241	230	507	305
**PRDM10**	LUAD	HM_w	388	69	42	499	306

**RNF20**	LUAD	Others	387	69	42	498	307
**GTF2H1**	LUAD	Others	175	69	253	497	308
**CBX7**	LUAD	HM_r	183	209	104	496	309
**KDM8**	LUAD	HM_e	44	199	252	495	310
**CHD4**	LUAD	Helicases	382	69	42	493	311
**TDRD12**	LUAD	Others	20	392	79	491	312
**FBXW9**	LUAD	Others	232	152	102	486	313
**KMT2B**	LUAD	HM_w	42	400	42	484	314
**SIN3A**	LUAD	ChRC	294	147	42	483	315
**CARM1**	LUAD	HM_w	283	153	42	478	316
**SMNDC1**	LUAD	Others	85	159	234	478	317
**KDM6B**	LUAD	HM_e	112	267	98	477	318
**TET2**	LUAD	DM_e	247	108	121	476	319
**CDYL2**	LUAD	HM_r	182	69	224	475	320
**HDAC1**	LUAD	HA_e	62	173	237	472	321
**NAP1L3**	LUAD	Others	265	69	138	472	322
**BMI1**	LUAD	ChRC	133	183	155	471	323
**KDM5C**	LUAD	HM_e	360	69	42	471	324
**FXR2**	LUAD	Others	177	251	42	470	325
**PRDM11**	LUAD	HM_w	258	69	136	463	326
**UBE2B**	LUAD	Others	18	157	287	462	327
**EHMT1**	LUAD	HM_w	348	69	42	459	328
**FBXO44**	LUAD	Others	68	207	184	459	329
**KMT2D**	LUAD	HM_w	40	168	251	459	330
**ING4**	LUAD	HM_r	118	69	269	456	331
**KDM3B**	LUAD	HM_e	344	69	42	455	332
**DPY30**	LUAD	Others	70	138	246	454	333
**KDM4E**	LUAD	HM_e	46	322	84	452	334
**EP300**	LUAD	HA_w	234	175	42	451	335
**KAT2B**	LUAD	HA_w	223	133	94	450	336
**L3MBTL4**	LUAD	Others	219	69	162	450	337
**SMARCA5**	LUAD	Helicases	197	146	107	450	338
**RTF1**	LUAD	Others	93	314	42	449	339
**MBD3**	LUAD	DM_r	110	198	140	448	340
**PHC1**	LUAD	ChRC	158	69	220	447	341

**PRMT3**	LUAD	HM_w	205	69	172	446	342
**PHF6**	LUAD	HM_r	100	302	42	444	343
**PRDM13**	LUAD	HM_w	99	194	151	444	344
**TDRD9**	LUAD	Others	288	69	86	443	345
**UHRF2**	LUAD	DM_r	14	385	42	441	346
**PHF8**	LUAD	Others	261	69	110	440	347
**SMYD1**	LUAD	HM_w	253	109	77	439	348
**PRMT7**	LUAD	HM_w	150	69	218	437	349
**BRWD1**	LUAD	HA_r	184	210	42	436	350
**SETD5**	LUAD	HM_w	325	69	42	436	351
**PYGO1**	LUAD	HM_r	97	193	143	433	352
**AICDA**	LUAD	DM_e	135	69	227	431	353
**HDAC8**	LUAD	HA_e	59	136	236	431	354
**PCMT1**	LUAD	Others	35	235	161	431	355
**KAT5**	LUAD	HA_w	114	132	181	427	356
**CTCF**	LUAD	Others	315	69	42	426	357
**SFMBT1**	LUAD	HM_r	145	111	170	426	358
**YY1**	LUAD	ChRC	77	69	280	426	359
**JADE3**	LUAD	Others	54	69	300	423	360
**PRMT2**	LUAD	HM_w	152	228	42	422	361
**PHC2**	LUAD	ChRC	104	166	145	415	362
**MARCH5**	LUAD	Others	37	131	243	411	363
**PBRM1**	LUAD	HA_r	299	69	42	410	364
**PHF7**	LUAD	Others	157	129	123	409	365
**FKBP1A**	LUAD	Others	67	69	272	408	366
**BRD4**	LUAD	HA_r	241	119	42	402	367
**MTA3**	LUAD	ChRC	163	197	42	402	368
**PCGF6**	LUAD	Others	159	69	174	402	369
**MUM1**	LUAD	Others	162	196	42	400	370
**SSRP1**	LUAD	Others	22	69	306	397	371
**ING5**	LUAD	HM_r	117	69	204	390	372
**SETD4**	LUAD	HM_w	148	69	171	388	373
**RNF217**	LUAD	Others	28	259	92	379	374
**SIRT6**	LUAD	HA_e	23	191	158	372	375
**PRDM12**	LUAD	HM_w	207	69	93	369	376

**KDM7A**	LUAD	HM_e	45	69	254	368	377
**PHF19**	LUAD	HM_r	101	69	194	364	378
**SETD7**	LUAD	HM_w	91	113	160	364	379
**GADD45B**	LUAD	Others	66	205	90	361	380
**GTF2B**	LUAD	Others	65	69	223	357	381
**BRPF1**	LUAD	HA_r	240	69	42	351	382
**TDRD3**	LUAD	HM_r	191	69	83	343	383
**JADE2**	LUAD	Others	55	69	216	340	384
**PHF11**	LUAD	Others	33	264	42	339	385
**INTS12**	LUAD	Others	116	69	153	338	386
**HSPBAP1**	LUAD	Others	119	171	42	332	387
**JADE1**	LUAD	Others	56	69	199	324	388
**WDR5**	LUAD	ChRC	78	69	176	323	389
**MBD4**	LUAD	DM_r	109	69	139	317	390
**PCGF5**	LUAD	Others	105	115	97	317	391
**SIRT4**	LUAD	HA_e	87	69	159	315	392
**H2AFZ**	LUAD	Others	64	118	132	314	393
**UBE2E1**	LUAD	Others	17	107	190	314	394
**FKBP2**	LUAD	Others	122	69	119	310	395
**TRIM66**	LUAD	HA_r	19	189	100	308	396
**KDM5D**	LUAD	HM_e	190	69	42	301	397
**POLE3**	LUAD	ChRC	31	69	201	301	398
**UTY**	LUAD	HM_e	189	69	42	300	399
**ASH2L**	LUAD	HM_w	187	69	42	298	400
**KAT6B**	LUAD	HA_w	50	69	175	294	401
**KDM4B**	LUAD	HM_e	47	200	42	289	402
**SETMAR**	LUAD	HM_w	25	112	149	286	403
**PRKCD**	LUAD	Others	154	69	42	265	404
**ZCWPW2**	LUAD	HM_r	11	120	133	264	405
**PHF5A**	LUAD	Others	32	69	152	253	406
**UHRF1**	LUAD	DM_r	15	187	42	244	407
**BRD3**	LUAD	HA_r	131	69	42	242	408
**PRDM6**	LUAD	HM_w	30	164	42	236	409
**SETD1B**	LUAD	HM_w	26	161	42	229	410
**L3MBTL2**	LUAD	Others	111	69	42	222	411

**USP22**	LUAD	Others	79	69	42	190	412
**HDAC3**	LUAD	HA_e	60	69	42	171	413
**WDR82**	LUAD	Others	12	106	42	160	414
**KMT2A**	LUAD	HM_w	43	69	42	154	415
**USP27X**	LUAD	Others	13	69	42	124	416
**ATR**	LUSC	Others	395	422	425	1242	1
**ATAD2**	LUSC	HA_r	396	402	415	1213	2
**PHC3**	LUSC	ChRC	365	424	424	1213	3
**KDM5A**	LUSC	HM_e	392	403	416	1211	4
**HLTF**	LUSC	Others	355	423	423	1201	5
**ASXL1**	LUSC	Others	408	396	384	1188	6
**PHF20L1**	LUSC	HM_r	364	405	417	1186	7
**ASH1L**	LUSC	HM_w	384	387	397	1168	8
**PHF3**	LUSC	Others	379	385	382	1146	9
**ACTL6A**	LUSC	ChRC	287	426	426	1139	10
**TDRD5**	LUSC	Others	418	373	343	1134	11
**ASH2L**	LUSC	HM_w	286	419	420	1125	12
**HIRA**	LUSC	Others	307	400	400	1107	13
**SETDB1**	LUSC	HM_w	295	401	410	1106	14
**ARID4B**	LUSC	ChRC	377	334	389	1100	15
**POLR2B**	LUSC	Others	323	374	403	1100	16
**CHD1L**	LUSC	Helicases	283	408	406	1097	17
**BPTF**	LUSC	HA_r	414	371	308	1093	18
**BRD9**	LUSC	HA_r	243	421	419	1083	19
**MSH6**	LUSC	HM_r	302	369	407	1078	20
**KDM6A**	LUSC	HM_e	351	394	330	1075	21
**CHD6**	LUSC	Helicases	405	332	331	1068	22
**DIDO1**	LUSC	Others	422	271	367	1060	23
**ARID2**	LUSC	ChRC	409	343	302	1054	24
**MLLT10**	LUSC	HM_w	380	324	350	1054	25
**BAZ1B**	LUSC	HA_r	375	300	375	1050	26
**CHD4**	LUSC	Helicases	357	383	301	1041	27
**BRD4**	LUSC	HA_r	337	342	361	1040	28
**SIRT2**	LUSC	HA_e	218	415	405	1038	29
**FMR1**	LUSC	Others	332	331	374	1037	30

**CHD7**	LUSC	Helicases	426	366	244	1036	31
**ATF7IP**	LUSC	Others	407	357	267	1031	32
**STK31**	LUSC	Others	398	306	325	1029	33
**PYGO2**	LUSC	HM_r	222	376	413	1011	34
**ATRX**	LUSC	Helicases	406	287	315	1008	35
**NCOA1**	LUSC	HA_w	389	226	391	1006	36
**MBD4**	LUSC	DM_r	175	412	418	1005	37
**AEBP2**	LUSC	HM_w	208	384	411	1003	38
**PHF20**	LUSC	HM_r	263	380	356	999	39
**HDAC9**	LUSC	HA_e	416	330	248	994	40
**TAF1L**	LUSC	HA_r	425	397	172	994	41
**LBR**	LUSC	Others	271	328	387	986	42
**PRDM9**	LUSC	HM_w	421	420	142	983	43
**KDM2A**	LUSC	HM_e	273	339	366	978	44
**UBR7**	LUSC	Others	339	317	321	977	45
**TAF1**	LUSC	HA_r	411	318	247	976	46
**NCOA3**	LUSC	HA_w	268	311	393	972	47
**CLOCK**	LUSC	HA_w	282	404	285	971	48
**SRCAP**	LUSC	Others	412	241	316	969	49
**HDGF**	LUSC	Others	183	382	402	967	50
**GATAD2B**	LUSC	HM_r	188	389	386	963	51
**BOP 1**	LUSC	Others	135	409	412	956	52
**TDRKH**	LUSC	Others	142	399	408	949	53
**PARP1**	LUSC	Others	325	338	281	944	54
**SMARCA2**	LUSC	Helicases	316	391	237	944	55
**L3MBTL4**	LUSC	Others	303	329	310	942	56
**TET3**	LUSC	DM_e	315	302	324	941	57
**DNMT3A**	LUSC	DM_w	393	204	341	938	58
**RSF1**	LUSC	ChRC	297	292	347	936	59
**PAF1**	LUSC	Others	103	414	414	931	60
**FBXO17**	LUSC	Others	128	416	381	925	61
**MECOM**	LUSC	Others	402	425	95	922	62
**MTA1**	LUSC	ChRC	173	364	377	914	63
**BAZ2B**	LUSC	HA_r	417	286	208	911	64
**PARP2**	LUSC	Others	169	361	370	900	65

**RBBP5**	LUSC	ChRC	154	337	409	900	66
**SMYD2**	LUSC	HM_w	216	289	394	899	67
**CHRAC1**	LUSC	ChRC	69	406	422	897	68
**ASXL3**	LUSC	Others	424	367	105	896	69
**SFMBT2**	LUSC	Others	413	308	174	895	70
**ATAD2B**	LUSC	HA_r	285	239	369	893	71
**RPS6KA5**	LUSC	Others	257	336	293	886	72
**SMARCA4**	LUSC	Helicases	361	320	203	884	73
**EP300**	LUSC	HA_w	382	201	299	882	74
**CBX4**	LUSC	HM_r	201	350	327	878	75
**TDRD6**	LUSC	Others	385	303	190	878	76
**SETD1A**	LUSC	HM_w	387	217	269	873	77
**TCF20**	LUSC	Others	341	183	349	873	78
**KDM3A**	LUSC	HM_e	305	267	296	868	79
**PRKAA1**	LUSC	Others	29	418	421	868	80
**KAT6A**	LUSC	HA_w	53	413	401	867	81
**FBXW9**	LUSC	Others	279	299	284	862	82
**HAT1**	LUSC	HA_w	185	326	351	862	83
**JMJD1C**	LUSC	HM_e	369	297	196	862	84
**GATAD2A**	LUSC	HM_r	189	284	388	861	85
**RNF40**	LUSC	Others	258	245	358	861	86
**KDM4D**	LUSC	HM_e	353	266	241	860	87
**NAP1L2**	LUSC	Others	326	322	212	860	88
**KDM5B**	LUSC	HM_e	352	312	195	859	89
**MECP2**	LUSC	DM_r	174	365	319	858	90
**AKAP1**	LUSC	Others	207	301	342	850	91
**JMJD6**	LUSC	HM_e	111	362	373	846	92
**MSL3**	LUSC	HA_w	269	346	230	845	93
**PRDM11**	LUSC	HM_w	262	249	329	840	94
**EED**	LUSC	ChRC	196	269	371	836	95
**PHC1**	LUSC	ChRC	100	377	359	836	96
**SMARCB1**	LUSC	Helicases	85	354	395	834	97
**CBX2**	LUSC	ChRC	202	351	279	832	98
**DPF1**	LUSC	ChRC	197	417	217	831	99
**FKBP1A**	LUSC	Others	67	363	399	829	100

**INO80**	LUSC	Helicases	331	162	336	829	101
**SIRT7**	LUSC	HA_e	151	344	334	829	102
**CARM1**	LUSC	HM_w	133	327	368	828	103
**CECR2**	LUSC	HA_r	394	69	364	827	104
**DPF2**	LUSC	Others	280	236	306	822	105
**SMYD3**	LUSC	HM_w	215	368	236	819	106
**CBX8**	LUSC	ChRC	200	349	266	815	107
**ARID1A**	LUSC	ChRC	415	210	188	813	108
**ASXL2**	LUSC	Others	312	208	292	812	109
**H3F3A**	LUSC	Others	122	348	340	810	110
**ARID1B**	LUSC	ChRC	397	209	201	807	111
**DNMT3B**	LUSC	DM_w	310	386	111	807	112
**SMYD5**	LUSC	HM_w	81	319	404	804	113
**HCFC1**	LUSC	Others	121	370	312	803	114
**ELP4**	LUSC	HA_w	193	255	354	802	115
**TCEA1**	LUSC	Others	80	360	362	802	116
**DNMT1**	LUSC	DM_w	281	270	243	794	117
**HR**	LUSC	HM_e	274	398	120	792	118
**CBX3**	LUSC	HM_r	72	333	383	788	119
**KDM4B**	LUSC	HM_e	328	228	232	788	120
**ZGPAT**	LUSC	Others	210	258	320	788	121
**BAZ1A**	LUSC	HA_r	404	69	314	787	122
**TP53BP1**	LUSC	Others	314	213	259	786	123
**BRD1**	LUSC	HA_r	338	69	378	785	124
**GTF3C4**	LUSC	HA_w	236	233	313	782	125
**RAG2**	LUSC	HM_r	259	246	276	781	126
**CHD8**	LUSC	Helicases	372	69	326	767	127
**KAT5**	LUSC	HA_w	179	230	357	766	128
**SIN3A**	LUSC	ChRC	294	153	317	764	129
**YY1**	LUSC	ChRC	11	353	398	762	130
**TRIM28**	LUSC	HA_r	78	359	323	760	131
**KDM5C**	LUSC	HM_e	327	69	363	759	132
**L3MBTL2**	LUSC	Others	176	193	390	759	133
**ZMYND8**	LUSC	HA_r	248	288	220	756	134
**SMARCC2**	LUSC	Helicases	342	152	260	754	135

**ING3**	LUSC	HM_r	232	298	223	753	136
**RNF2**	LUSC	ChRC	28	345	379	752	137
**CREBBP**	LUSC	HA_w	423	285	42	750	138
**PRMT7**	LUSC	HM_w	299	189	261	749	139
**SMARCD2**	LUSC	Helicases	20	335	392	747	140
**ZCWPW1**	LUSC	HM_r	138	388	221	747	141
**IWS1**	LUSC	Others	180	177	385	742	142
**CHD2**	LUSC	Helicases	358	341	42	741	143
**UBE2A**	LUSC	Others	141	275	322	738	144
**CBX6**	LUSC	HM_r	132	273	332	737	145
**NSD1**	LUSC	HM_w	399	295	42	736	146
**PHF14**	LUSC	Others	227	279	229	735	147
**KDM2B**	LUSC	HM_e	330	161	242	733	148
**CHAF1B**	LUSC	ChRC	242	272	218	732	149
**DMAP1**	LUSC	Others	198	256	278	732	150
**PRMT8**	LUSC	HM_w	224	392	114	730	151
**KDM1B**	LUSC	HM_e	177	347	205	729	152
**PRKAA2**	LUSC	Others	300	154	272	726	153
**GTF2H1**	LUSC	Others	186	234	305	725	154
**BRWD3**	LUSC	HA_r	420	69	233	722	155
**PYGO1**	LUSC	HM_r	260	172	289	721	156
**KMT2E**	LUSC	HM_w	42	381	295	718	157
**SMARCE1**	LUSC	Helicases	82	307	328	717	158
**MEN1**	LUSC	ChRC	231	143	339	713	159
**CSTL1**	LUSC	Others	199	315	198	712	160
**PRDM10**	LUSC	HM_w	348	321	42	711	161
**ING1**	LUSC	HM_r	114	313	283	710	162
**KDM4A**	LUSC	HM_e	272	253	185	710	163
**SND1**	LUSC	HM_r	293	69	346	708	164
**NCOR1**	LUSC	ChRC	401	264	42	707	165
**TAF3**	LUSC	HA_r	360	305	42	707	166
**ACTL6B**	LUSC	ChRC	247	390	68	705	167
**SUPT16H**	LUSC	Others	291	69	344	704	168
**TRIM24**	LUSC	HA_r	289	276	139	704	169
**AICDA**	LUSC	DM_e	246	379	67	692	170

**ARID4A**	LUSC	ChRC	378	69	245	692	171
**PCGF1**	LUSC	Others	36	280	376	692	172
**PHF21A**	LUSC	HM_r	165	262	263	690	173
**BRWD1**	LUSC	HA_r	373	274	42	689	174
**SETD4**	LUSC	HM_w	220	259	210	689	175
**PPARGC1A**	LUSC	Others	322	250	116	688	176
**SUZ12**	LUSC	ChRC	253	69	365	687	177
**HSPBAP1**	LUSC	Others	233	411	42	686	178
**KANSL1**	LUSC	HA_w	54	296	335	685	179
**TDRD10**	LUSC	Others	143	375	166	684	180
**IDH1**	LUSC	DM_e	182	197	304	683	181
**BRDT**	LUSC	HA_r	336	167	179	682	182
**PHIP**	LUSC	HA_r	388	251	42	681	183
**HDAC8**	LUSC	HA_e	116	314	250	680	184
**MPHOSPH8**	LUSC	HM_r	230	69	380	679	185
**NCOR2**	LUSC	Others	400	142	136	678	186
**ING2**	LUSC	HM_r	113	395	161	669	187
**TET1**	LUSC	DM_e	410	69	189	668	188
**MTA3**	LUSC	ChRC	301	323	42	666	189
**CXXC1**	LUSC	Others	240	69	355	664	190
**RNF17**	LUSC	Others	344	137	182	663	191
**MBTD1**	LUSC	Others	107	281	273	661	192
**BRPF3**	LUSC	HA_r	335	69	256	660	193
**ING4**	LUSC	HM_r	181	378	101	660	194
**PBRM1**	LUSC	HA_r	366	252	42	660	195
**TDRD9**	LUSC	Others	212	372	75	659	196
**EZH2**	LUSC	HM_w	370	69	216	655	197
**FBXL19**	LUSC	Others	191	200	264	655	198
**SETD3**	LUSC	HM_w	256	355	42	653	199
**KAT7**	LUSC	HA_w	51	229	372	652	200
**EHMT2**	LUSC	HM_w	195	202	253	650	201
**PRDM14**	LUSC	HM_w	225	356	69	650	202
**TRIM33**	LUSC	HA_r	250	358	42	650	203
**MBD2**	LUSC	DM_r	108	325	214	647	204
**PRDM5**	LUSC	HM_w	345	174	125	644	205

**CTCF**	LUSC	Others	241	148	254	643	206
**MBD5**	LUSC	DM_r	390	69	184	643	207
**SIN3B**	LUSC	ChRC	219	243	181	643	208
**RPA3**	LUSC	Others	26	218	396	640	209
**PRDM1**	LUSC	HM_w	321	191	126	638	210
**EPC2**	LUSC	Others	192	180	265	637	211
**PADI3**	LUSC	Others	229	224	183	636	212
**SETD2**	LUSC	HM_w	296	187	151	634	213
**SUV39H2**	LUSC	HM_w	144	277	209	630	214
**ELP3**	LUSC	HA_w	194	393	42	629	215
**EZH1**	LUSC	HM_w	308	69	252	629	216
**PRDM15**	LUSC	HM_w	261	278	86	625	217
**SSRP1**	LUSC	Others	146	133	345	624	218
**G2E3**	LUSC	Others	278	69	274	621	219
**HDAC4**	LUSC	HA_e	381	69	171	621	220
**PRDM13**	LUSC	HM_w	347	69	204	620	221
**SCMH1**	LUSC	Others	90	260	270	620	222
**DPF3**	LUSC	HA_r	309	203	107	619	223
**PCGF2**	LUSC	Others	35	223	360	618	224
**CBX1**	LUSC	HM_r	73	206	337	616	225
**DAXX**	LUSC	ChRC	239	69	307	615	226
**PHF10**	LUSC	Others	266	69	277	612	227
**PRMT3**	LUSC	HM_w	158	261	193	612	228
**BRD7**	LUSC	HA_r	204	69	338	611	229
**HDAC7**	LUSC	HA_e	117	268	224	609	230
**RNF217**	LUSC	Others	27	310	271	608	231
**PRDM2**	LUSC	HM_w	363	69	175	607	232
**L3MBTL1**	LUSC	HM_r	41	282	282	605	233
**L3MBTL3**	LUSC	Others	391	69	144	604	234
**DPY30**	LUSC	Others	68	235	300	603	235
**SP140**	LUSC	HA_r	292	214	94	600	236
**TDRD3**	LUSC	HM_r	251	304	42	597	237
**TDRD1**	LUSC	Others	340	150	106	596	238
**PRMT6**	LUSC	HM_w	156	247	192	595	239
**USP51**	LUSC	Others	249	69	275	593	240

**ERCC5**	LUSC	Others	371	179	42	592	241
**ATM**	LUSC	Others	376	69	146	591	242
**RPH3A**	LUSC	Others	320	136	133	589	243
**IDH2**	LUSC	DM_e	60	340	186	586	244
**PHRF1**	LUSC	Others	324	219	42	585	245
**SMARCA1**	LUSC	Helicases	362	69	150	581	246
**EHMT1**	LUSC	HM_w	333	69	178	580	247
**MBD1**	LUSC	DM_r	270	69	240	579	248
**PRMT5**	LUSC	HM_w	157	69	352	578	249
**SCML2**	LUSC	HM_r	318	69	191	578	250
**SP100**	LUSC	HA_r	214	215	149	578	251
**GTF2B**	LUSC	Others	187	165	225	577	252
**BAP1**	LUSC	Others	206	238	129	573	253
**TDRD12**	LUSC	Others	18	407	147	572	254
**BRD3**	LUSC	HA_r	134	237	200	571	255
**PADI1**	LUSC	Others	349	69	153	571	256
**DOT1L**	LUSC	HM_w	356	69	145	570	257
**HIST1H1B**	LUSC	Others	235	231	102	568	258
**PCGF6**	LUSC	Others	168	263	135	566	259
**NAP1L3**	LUSC	Others	367	69	127	563	260
**KAT2B**	LUSC	HA_w	306	176	80	562	261
**CHD5**	LUSC	Helicases	403	69	89	561	262
**SMARCC1**	LUSC	Helicases	255	216	90	561	263
**PHF11**	LUSC	Others	167	294	99	560	264
**KAT8**	LUSC	HA_w	50	195	311	556	265
**TDG**	LUSC	ChRC	252	69	235	556	266
**TDRD7**	LUSC	Others	213	182	156	551	267
**NAP1L1**	LUSC	Others	38	158	353	549	268
**PRMT1**	LUSC	HM_w	94	190	262	546	269
**SETD5**	LUSC	HM_w	317	186	42	545	270
**CHD1**	LUSC	Helicases	334	166	42	542	271
**JARID2**	LUSC	ChRC	354	69	119	542	272
**KAT6B**	LUSC	HA_w	52	283	207	542	273
**ATAT1**	LUSC	Others	75	207	257	539	274
**FXR2**	LUSC	Others	237	178	123	538	275

**SMARCD1**	LUSC	Helicases	84	151	303	538	276
**AFF4**	LUSC	Others	313	181	42	536	277
**PHC2**	LUSC	ChRC	228	155	152	535	278
**SATB1**	LUSC	Others	319	135	79	533	279
**ZMYND11**	LUSC	HA_r	137	352	42	531	280
**EP400**	LUSC	HA_w	419	69	42	530	281
**PRDM16**	LUSC	HM_w	346	69	115	530	282
**HNF1A**	LUSC	ChRC	275	144	110	529	283
**SP140L**	LUSC	HA_r	147	242	140	529	284
**PHF2**	LUSC	Others	264	69	194	527	285
**BRD2**	LUSC	HA_r	205	69	249	523	286
**SCML4**	LUSC	Others	221	188	113	522	287
**PAXIP1**	LUSC	Others	102	69	348	519	288
**CDYL2**	LUSC	HM_r	284	69	165	518	289
**GLYR1**	LUSC	HM_r	277	199	42	518	290
**CHD3**	LUSC	Helicases	311	69	137	517	291
**PHF8**	LUSC	Others	226	69	222	517	292
**RING1**	LUSC	Others	153	69	294	516	293
**CBX7**	LUSC	HM_r	131	257	124	512	294
**TCF19**	LUSC	Others	79	184	246	509	295
**PCMT1**	LUSC	Others	34	156	318	508	296
**RAI1**	LUSC	Others	298	69	141	508	297
**HIST1H1C**	LUSC	Others	276	69	162	507	298
**ING5**	LUSC	HM_r	112	196	197	505	299
**ZCWPW2**	LUSC	HM_r	211	211	83	505	300
**PHF19**	LUSC	HM_r	265	141	98	504	301
**SUV39H1**	LUSC	HM_w	145	69	287	501	302
**KMT2B**	LUSC	HM_w	45	410	42	497	303
**SHPRH**	LUSC	Others	386	69	42	497	304
**MORF4L1**	LUSC	HM_r	106	159	231	496	305
**CHD9**	LUSC	Helicases	383	69	42	494	306
**KDM6B**	LUSC	HM_e	350	69	74	493	307
**PHF5A**	LUSC	Others	33	221	238	492	308
**PCGF5**	LUSC	Others	101	222	167	490	309
**HDAC10**	LUSC	HA_e	120	69	298	487	310

**BRPF1**	LUSC	HA_r	374	69	42	485	311
**SP110**	LUSC	HA_r	148	185	148	481	312
**PRKCD**	LUSC	Others	160	248	72	480	313
**KIAA2026**	LUSC	Others	368	69	42	479	314
**KAT2A**	LUSC	HA_w	110	69	297	476	315
**CBX5**	LUSC	HM_r	71	205	199	475	316
**PHF13**	LUSC	Others	166	69	239	474	317
**HIST1H3B**	LUSC	Others	234	69	169	472	318
**AFF1**	LUSC	Others	359	69	42	470	319
**PADI2**	LUSC	Others	171	225	71	467	320
**MTA2**	LUSC	ChRC	105	69	290	464	321
**PRDM4**	LUSC	HM_w	97	140	227	464	322
**MTF2**	LUSC	HM_r	172	69	213	454	323
**RNF20**	LUSC	Others	343	69	42	454	324
**KDM1A**	LUSC	HM_e	178	69	206	453	325
**HDAC1**	LUSC	HA_e	184	146	122	452	326
**H2AFZ**	LUSC	Others	123	164	163	450	327
**RBBP7**	LUSC	ChRC	92	69	288	449	328
**FBXO44**	LUSC	Others	190	69	187	446	329
**TET2**	LUSC	DM_e	290	69	85	444	330
**RBBP4**	LUSC	ChRC	93	138	211	442	331
**SETMAR**	LUSC	HM_w	21	309	112	442	332
**USP22**	LUSC	Others	139	69	234	442	333
**KDM3B**	LUSC	HM_e	329	69	42	440	334
**BAZ2A**	LUSC	HA_r	244	149	42	435	335
**SIRT5**	LUSC	HA_e	86	69	280	435	336
**MLLT6**	LUSC	HM_w	39	227	168	434	337
**PHF12**	LUSC	Others	98	293	42	433	338
**GADD45A**	LUSC	Others	126	147	154	427	339
**PWWP2B**	LUSC	Others	223	69	134	426	340
**EPC1**	LUSC	Others	129	254	42	425	341
**HDAC2**	LUSC	HA_e	64	69	291	424	342
**KDM8**	LUSC	HM_e	47	160	215	422	343
**UHRF1**	LUSC	DM_r	140	240	42	422	344
**CDYL**	LUSC	HM_r	130	69	219	418	345

**SIRT6**	LUSC	HA_e	217	69	131	417	346
**UBE2I**	LUSC	Others	14	69	333	416	347
**KDM4C**	LUSC	HM_e	304	69	42	415	348
**SMNDC1**	LUSC	Others	19	170	226	415	349
**PHF23**	LUSC	HM_r	163	175	73	411	350
**KDM4E**	LUSC	HM_e	49	265	96	410	351
**SFMBT1**	LUSC	HM_r	89	244	76	409	352
**AIRE**	LUSC	HM_r	245	69	93	407	353
**KDM7A**	LUSC	HM_e	48	69	286	403	354
**SMYD1**	LUSC	HM_w	254	69	78	401	355
**DNMT3L**	LUSC	DM_w	238	69	92	399	356
**KDM5D**	LUSC	HM_e	288	69	42	399	357
**PHF6**	LUSC	HM_r	162	192	42	396	358
**CHAF1A**	LUSC	ChRC	70	69	255	394	359
**SETDB2**	LUSC	HM_w	22	290	81	393	360
**SMYD4**	LUSC	HM_w	149	69	173	391	361
**WDR5**	LUSC	ChRC	12	69	309	390	362
**BMI1**	LUSC	ChRC	136	69	180	385	363
**HDAC6**	LUSC	HA_e	63	69	251	383	364
**JADE3**	LUSC	Others	56	69	258	383	365
**HELLS**	LUSC	Helicases	62	198	121	381	366
**PADI4**	LUSC	Others	267	69	42	378	367
**SIRT4**	LUSC	HA_e	87	134	157	378	368
**BRD8**	LUSC	HA_r	203	69	104	376	369
**FKBP2**	LUSC	Others	127	69	177	373	370
**USP27X**	LUSC	Others	13	316	42	371	371
**KMT2D**	LUSC	HM_w	43	194	128	365	372
**SETD6**	LUSC	HM_w	24	69	268	361	373
**HDAC5**	LUSC	HA_e	118	69	170	357	374
**SETD7**	LUSC	HM_w	23	291	42	356	375
**ORC1**	LUSC	Others	37	157	160	354	376
**SMARCD3**	LUSC	Helicases	83	69	202	354	377
**PHF21B**	LUSC	HM_r	164	69	117	350	378
**PRDM7**	LUSC	HM_w	96	139	109	344	379
**PHF7**	LUSC	Others	32	220	91	343	380

**TRIM66**	LUSC	HA_r	17	169	155	341	381
**HIF1AN**	LUSC	Others	61	232	42	335	382
**WDR82**	LUSC	Others	76	212	42	330	383
**POLE3**	LUSC	ChRC	31	69	228	328	384
**PHF1**	LUSC	HM_r	99	69	159	327	385
**HDAC3**	LUSC	HA_e	119	163	42	324	386
**PADI6**	LUSC	Others	170	69	82	321	387
**UTY**	LUSC	HM_e	209	69	42	320	388
**SIRT1**	LUSC	HA_e	88	69	158	315	389
**RTF1**	LUSC	Others	91	171	42	304	390
**PRDM12**	LUSC	HM_w	161	69	70	300	391
**FKBP5**	LUSC	Others	66	69	164	299	392
**HDAC11**	LUSC	HA_e	65	145	87	297	393
**MUM1**	LUSC	Others	104	69	118	291	394
**KMT2C**	LUSC	HM_w	44	69	176	289	395
**HDGFL1**	LUSC	Others	115	69	103	287	396
**GADD45B**	LUSC	Others	125	69	88	282	397
**MARCH5**	LUSC	Others	109	69	100	278	398
**AURKB**	LUSC	Others	74	69	130	273	399
**PRDM8**	LUSC	HM_w	95	69	108	272	400
**PRMT2**	LUSC	HM_w	159	69	42	270	401
**UBE2B**	LUSC	Others	16	168	84	268	402
**PSIP1**	LUSC	HM_r	155	69	42	266	403
**JADE2**	LUSC	Others	57	69	138	264	404
**SIRT3**	LUSC	HA_e	152	69	42	263	405
**SMARCA5**	LUSC	Helicases	150	69	42	261	406
**MBD3**	LUSC	DM_r	40	69	143	252	407
**PRDM6**	LUSC	HM_w	30	173	42	245	408
**UBE2E1**	LUSC	Others	15	132	97	244	409
**GTF2F1**	LUSC	Others	124	69	42	235	410
**SETD1B**	LUSC	HM_w	25	69	132	226	411
**JADE1**	LUSC	Others	58	69	77	204	412
**UHRF2**	LUSC	DM_r	77	69	42	188	413
**INTS12**	LUSC	Others	59	69	42	170	414
**JMJD8**	LUSC	HM_e	55	69	42	166	415

**KMT2A**	LUSC	HM_w	46	69	42	157	416
**BAP1**	MESO	Others	426	426	426	1278	1
**SETD2**	MESO	HM_w	425	400	413	1238	2
**EP400**	MESO	HA_w	423	389	417	1229	3
**PBRM1**	MESO	HA_r	394	409	424	1227	4
**INO80**	MESO	Helicases	401	412	405	1218	5
**ASH1L**	MESO	HM_w	389	395	418	1202	6
**SETDB1**	MESO	HM_w	411	359	423	1193	7
**BRD4**	MESO	HA_r	424	394	372	1190	8
**KAT8**	MESO	HA_w	399	374	391	1164	9
**CBX4**	MESO	HM_r	382	414	359	1155	10
**BRD7**	MESO	HA_r	385	336	425	1146	11
**ATR**	MESO	Others	407	341	395	1143	12
**RTF1**	MESO	Others	321	407	414	1142	13
**FBXW9**	MESO	Others	372	403	357	1132	14
**CBX1**	MESO	HM_r	274	422	399	1095	15
**AKAP1**	MESO	Others	298	424	361	1083	16
**RNF40**	MESO	Others	322	362	398	1082	17
**DPF1**	MESO	ChRC	376	321	384	1081	18
**BPTF**	MESO	HA_r	284	423	373	1080	19
**BRD9**	MESO	HA_r	279	406	394	1079	20
**CBX8**	MESO	ChRC	268	413	393	1074	21
**CHD2**	MESO	Helicases	418	391	255	1064	22
**SMARCA4**	MESO	Helicases	317	397	348	1062	23
**CTCF**	MESO	Others	378	325	358	1061	24
**ARID4B**	MESO	ChRC	296	344	412	1052	25
**DNMT1**	MESO	DM_w	252	404	392	1048	26
**HDAC1**	MESO	HA_e	368	380	290	1038	27
**GATAD2B**	MESO	HM_r	229	383	416	1028	28
**CBX2**	MESO	ChRC	273	415	338	1026	29
**SIN3B**	MESO	ChRC	318	398	308	1024	30
**GLYR1**	MESO	HM_r	228	382	411	1021	31
**CARM1**	MESO	HM_w	275	405	339	1019	32
**KDM5A**	MESO	HM_e	350	303	365	1018	33
**KDM4A**	MESO	HM_e	398	373	245	1016	34

**HDAC3**	MESO	HA_e	366	313	332	1011	35
**BOP 1**	MESO	Others	285	339	386	1010	36
**ACTL6A**	MESO	ChRC	303	349	343	995	37
**KMT2C**	MESO	HM_w	414	297	283	994	38
**SETD1A**	MESO	HM_w	320	360	311	991	39
**ARID1A**	MESO	ChRC	420	346	223	989	40
**KDM4B**	MESO	HM_e	397	306	284	987	41
**KANSL1**	MESO	HA_w	193	410	383	986	42
**KAT7**	MESO	HA_w	190	421	366	977	43
**HCFC1**	MESO	Others	222	381	369	972	44
**BRPF1**	MESO	HA_r	277	334	360	971	45
**CHRAC1**	MESO	ChRC	258	327	385	970	46
**KDM5B**	MESO	HM_e	185	402	382	969	47
**BMI1**	MESO	ChRC	286	340	341	967	48
**FBXO17**	MESO	Others	373	386	206	965	49
**CHD5**	MESO	Helicases	381	329	254	964	50
**KMT2A**	MESO	HM_w	422	299	243	964	51
**TDRD6**	MESO	Others	421	242	301	964	52
**CHD1L**	MESO	Helicases	261	330	371	962	53
**HDGF**	MESO	Others	215	378	367	960	54
**CREBBP**	MESO	HA_w	417	326	214	957	55
**BRD8**	MESO	HA_r	280	335	340	955	56
**PADI6**	MESO	Others	395	283	277	955	57
**H3F3A**	MESO	Others	224	315	410	949	58
**GATAD2A**	MESO	HM_r	230	384	333	947	59
**NSD1**	MESO	HM_w	338	289	319	946	60
**CHAF1A**	MESO	ChRC	264	332	337	933	61
**ATAD2**	MESO	HA_r	293	342	297	932	62
**TRIM28**	MESO	HA_r	307	352	264	923	63
**CHD7**	MESO	Helicases	379	328	215	922	64
**MORF4L1**	MESO	HM_r	162	371	389	922	65
**CDYL**	MESO	HM_r	267	393	257	917	66
**DAXX**	MESO	ChRC	254	323	335	912	67
**RBBP5**	MESO	ChRC	97	408	407	912	68
**IDH2**	MESO	DM_e	207	376	328	911	69

**KAT6B**	MESO	HA_w	356	308	246	910	70
**MBTD1**	MESO	Others	167	420	321	908	71
**MECP2**	MESO	DM_r	166	372	364	902	72
**HDAC5**	MESO	HA_e	365	379	156	900	73
**GTF2F1**	MESO	Others	370	316	203	889	74
**SETD5**	MESO	HM_w	319	260	309	888	75
**DOT1L**	MESO	HM_w	402	322	160	884	76
**FBXL19**	MESO	Others	239	387	252	878	77
**MBD1**	MESO	DM_r	172	295	409	876	78
**SMARCD2**	MESO	Helicases	61	418	397	876	79
**SIN3A**	MESO	ChRC	74	399	401	874	80
**CECR2**	MESO	HA_r	265	392	216	873	81
**MBD4**	MESO	DM_r	169	293	404	866	82
**PYGO2**	MESO	HM_r	99	364	402	865	83
**ARID2**	MESO	ChRC	419	396	42	857	84
**JMJD8**	MESO	HM_e	194	309	354	857	85
**DMAP1**	MESO	Others	253	390	213	856	86
**KAT2A**	MESO	HA_w	192	375	286	853	87
**CHD1**	MESO	Helicases	262	331	256	849	88
**BRD1**	MESO	HA_r	283	338	219	840	89
**KDM2B**	MESO	HM_e	355	69	415	839	90
**LBR**	MESO	Others	346	296	196	838	91
**EZH1**	MESO	HM_w	240	388	207	835	92
**PADI2**	MESO	Others	151	286	381	818	93
**CDYL2**	MESO	HM_r	266	333	217	816	94
**MPHOSPH8**	MESO	HM_r	344	69	403	816	95
**RNF2**	MESO	ChRC	94	401	314	809	96
**HDAC9**	MESO	HA_e	364	69	368	801	97
**KDM6A**	MESO	HM_e	348	302	150	800	98
**PHRF1**	MESO	Others	412	69	318	799	99
**SRCAP**	MESO	Others	50	356	388	794	100
**JMJD1C**	MESO	HM_e	196	310	287	793	101
**PARP1**	MESO	Others	148	282	363	793	102
**SIRT7**	MESO	HA_e	67	419	307	793	103
**PHC3**	MESO	ChRC	336	69	379	784	104

**PHF7**	MESO	Others	126	425	233	784	105
**DNMT3L**	MESO	DM_w	377	69	334	780	106
**FKBP5**	MESO	Others	235	385	159	779	107
**SND1**	MESO	HM_r	314	69	396	779	108
**SETD1B**	MESO	HM_w	393	69	310	772	109
**RAI1**	MESO	Others	324	262	183	769	110
**ASXL2**	MESO	Others	294	343	131	768	111
**FBXO44**	MESO	Others	238	320	205	763	112
**HNF1A**	MESO	ChRC	361	69	330	760	113
**ACTL6B**	MESO	ChRC	391	69	299	759	114
**HR**	MESO	HM_e	360	69	329	758	115
**FMR1**	MESO	Others	234	319	204	757	116
**PAF1**	MESO	Others	149	369	238	756	117
**ATRX**	MESO	Helicases	387	69	296	752	118
**ASH2L**	MESO	HM_w	295	69	387	751	119
**TDRKH**	MESO	Others	33	353	362	748	120
**MBD3**	MESO	DM_r	170	294	281	745	121
**ASXL1**	MESO	Others	409	69	262	740	122
**ATM**	MESO	Others	408	69	261	738	123
**PRDM8**	MESO	HM_w	329	268	140	737	124
**EP300**	MESO	HA_w	375	69	292	736	125
**MLLT6**	MESO	HM_w	163	292	280	735	126
**EZH2**	MESO	HM_w	374	69	291	734	127
**MSH6**	MESO	HM_r	343	69	320	732	128
**BAZ1B**	MESO	HA_r	287	69	374	730	129
**PHF3**	MESO	Others	129	367	234	730	130
**PRKAA1**	MESO	Others	109	267	352	728	131
**JMJD6**	MESO	HM_e	195	411	121	727	132
**RAG2**	MESO	HM_r	325	263	138	726	133
**MTA2**	MESO	ChRC	160	370	195	725	134
**NAP1L1**	MESO	Others	156	290	279	725	135
**KIAA2026**	MESO	Others	180	300	244	724	136
**FXR2**	MESO	Others	233	69	420	722	137
**PADI4**	MESO	Others	396	284	42	722	138
**SCMH1**	MESO	Others	86	361	270	717	139

**SMARCC1**	MESO	Helicases	64	252	400	716	140
**BAZ2A**	MESO	HA_r	386	69	260	715	141
**BRD3**	MESO	HA_r	281	337	97	715	142
**SETD6**	MESO	HM_w	81	259	375	715	143
**PRMT1**	MESO	HM_w	328	69	316	713	144
**HSPBAP1**	MESO	Others	359	311	42	712	145
**BRWD3**	MESO	HA_r	383	69	259	711	146
**GADD45A**	MESO	Others	232	318	158	708	147
**KDM3A**	MESO	HM_e	354	69	285	708	148
**GTF2B**	MESO	Others	227	69	406	702	149
**ARID1B**	MESO	ChRC	410	69	222	701	150
**AURKB**	MESO	Others	289	69	342	700	151
**KDM4C**	MESO	HM_e	352	305	42	699	152
**PHC2**	MESO	ChRC	140	368	191	699	153
**DNMT3B**	MESO	DM_w	415	69	212	696	154
**TDRD10**	MESO	Others	38	355	303	696	155
**PHF1**	MESO	HM_r	139	280	276	695	156
**AEBP2**	MESO	HM_w	302	348	42	692	157
**AFF4**	MESO	Others	301	347	42	690	158
**KDM4D**	MESO	HM_e	186	304	200	690	159
**MUM1**	MESO	Others	157	291	240	688	160
**NCOR2**	MESO	Others	339	69	278	686	161
**EHMT2**	MESO	HM_w	246	69	370	685	162
**SUV39H2**	MESO	HM_w	311	69	305	685	163
**ARID4A**	MESO	ChRC	297	345	42	684	164
**KDM8**	MESO	HM_e	181	301	198	680	165
**ASXL3**	MESO	Others	388	69	221	678	166
**PHF2**	MESO	Others	335	69	274	678	167
**GADD45B**	MESO	Others	231	317	127	675	168
**JARID2**	MESO	ChRC	358	69	248	675	169
**PRDM10**	MESO	HM_w	121	366	187	674	170
**KAT2B**	MESO	HA_w	357	69	247	673	171
**AICDA**	MESO	DM_e	300	69	298	667	172
**CHD4**	MESO	Helicases	259	69	336	664	173
**PCGF2**	MESO	Others	145	281	237	663	174

**TAF1L**	MESO	HA_r	310	246	106	662	175
**SMARCE1**	MESO	Helicases	59	251	347	657	176
**MECOM**	MESO	Others	345	69	241	655	177
**PHF20L1**	MESO	HM_r	333	277	42	652	178
**POLR2B**	MESO	Others	332	274	42	648	179
**KDM1A**	MESO	HM_e	189	307	151	647	180
**DNMT3A**	MESO	DM_w	416	69	161	646	181
**KDM2A**	MESO	HM_e	187	69	390	646	182
**BAZ2B**	MESO	HA_r	406	69	167	642	183
**BRPF3**	MESO	HA_r	276	69	295	640	184
**HIF1AN**	MESO	Others	213	69	356	638	185
**CHD9**	MESO	Helicases	404	69	164	637	186
**HLTF**	MESO	Others	209	69	355	633	187
**ORC1**	MESO	Others	153	288	192	633	188
**HIRA**	MESO	Others	212	377	42	631	189
**TDG**	MESO	ChRC	40	244	346	630	190
**TET1**	MESO	DM_e	308	241	81	630	191
**HDAC10**	MESO	HA_e	221	314	93	628	192
**NCOR1**	MESO	ChRC	413	69	146	628	193
**CHAF1B**	MESO	ChRC	263	69	294	626	194
**CXXC1**	MESO	Others	255	324	42	621	195
**KAT6A**	MESO	HA_w	400	69	152	621	196
**PCMT1**	MESO	Others	142	69	408	619	197
**PHF21B**	MESO	HM_r	131	69	419	619	198
**PHF5A**	MESO	Others	128	69	422	619	199
**HDAC6**	MESO	HA_e	218	69	331	618	200
**KDM5C**	MESO	HM_e	349	69	199	617	201
**KAT5**	MESO	HA_w	191	69	353	613	202
**KMT2E**	MESO	HM_w	347	69	197	613	203
**EED**	MESO	ChRC	248	69	293	610	204
**UBE2I**	MESO	Others	24	239	345	608	205
**NAP1L3**	MESO	Others	341	69	194	604	206
**PHF13**	MESO	Others	135	278	190	603	207
**NCOA3**	MESO	HA_w	340	69	193	602	208
**ING3**	MESO	HM_r	204	69	327	600	209

**CBX5**	MESO	HM_r	271	69	258	598	210
**ING4**	MESO	HM_r	203	69	326	598	211
**PRMT7**	MESO	HM_w	102	264	232	598	212
**SUZ12**	MESO	ChRC	46	247	304	597	213
**INTS12**	MESO	Others	201	69	325	595	214
**PAXIP1**	MESO	Others	146	69	380	595	215
**JADE2**	MESO	Others	198	69	324	591	216
**HELLS**	MESO	Helicases	363	69	155	587	217
**PRDM9**	MESO	HM_w	110	365	112	587	218
**PRDM14**	MESO	HM_w	330	69	186	585	219
**PPARGC1A**	MESO	Others	122	273	188	583	220
**ATAD2B**	MESO	HA_r	292	69	220	581	221
**PADI3**	MESO	Others	150	285	145	580	222
**TDRD5**	MESO	Others	35	243	302	580	223
**PYGO1**	MESO	HM_r	326	69	184	579	224
**HDAC11**	MESO	HA_e	220	69	289	578	225
**SMYD2**	MESO	HM_w	56	249	267	572	226
**HDAC8**	MESO	HA_e	216	312	42	570	227
**KMT2D**	MESO	HM_w	178	69	323	570	228
**CHD8**	MESO	Helicases	405	69	95	569	229
**G2E3**	MESO	Others	371	69	128	568	230
**L3MBTL2**	MESO	Others	176	69	322	567	231
**ELP4**	MESO	HA_w	244	69	253	566	232
**UBE2B**	MESO	Others	26	240	300	566	233
**BRDT**	MESO	HA_r	278	69	218	565	234
**IDH1**	MESO	DM_e	208	69	288	565	235
**HDAC2**	MESO	HA_e	367	69	126	562	236
**PRDM4**	MESO	HM_w	114	69	378	561	237
**AFF1**	MESO	Others	390	69	99	558	238
**PADI1**	MESO	Others	152	287	117	556	239
**PRMT2**	MESO	HM_w	106	69	377	552	240
**TDRD7**	MESO	Others	309	69	173	551	241
**PCGF1**	MESO	Others	337	69	144	550	242
**PRMT3**	MESO	HM_w	105	69	376	550	243
**SMARCA1**	MESO	Helicases	66	254	227	547	244

**GTF3C4**	MESO	HA_w	226	69	251	546	245
**TRIM66**	MESO	HA_r	306	69	171	546	246
**PRDM1**	MESO	HM_w	331	69	143	543	247
**HAT1**	MESO	HA_w	223	69	250	542	248
**KDM4E**	MESO	HM_e	351	69	120	540	249
**AIRE**	MESO	HM_r	299	69	169	537	250
**HDAC7**	MESO	HA_e	217	69	249	535	251
**PWWP2B**	MESO	Others	327	69	139	535	252
**DPF2**	MESO	Others	251	69	211	531	253
**TCF20**	MESO	Others	41	69	421	531	254
**ATAT1**	MESO	Others	291	69	168	528	255
**DPY30**	MESO	Others	249	69	210	528	256
**HIST1H1C**	MESO	Others	362	69	92	523	257
**MBD2**	MESO	DM_r	171	69	282	522	258
**PRDM7**	MESO	HM_w	111	269	141	521	259
**EPC2**	MESO	Others	242	69	209	520	260
**KMT2B**	MESO	HM_w	179	298	42	519	261
**PHF20**	MESO	HM_r	334	69	116	519	262
**ERCC5**	MESO	Others	241	69	208	518	263
**SP100**	MESO	HA_r	313	69	135	517	264
**SETMAR**	MESO	HM_w	78	258	180	516	265
**DIDO1**	MESO	Others	403	69	42	514	266
**POLE3**	MESO	ChRC	123	69	317	509	267
**PHF10**	MESO	Others	138	279	90	507	268
**SATB1**	MESO	Others	87	69	351	507	269
**ZGPAT**	MESO	Others	305	69	133	507	270
**CBX6**	MESO	HM_r	270	69	166	505	271
**ZMYND8**	MESO	HA_r	304	69	132	505	272
**SCML2**	MESO	HM_r	85	69	350	504	273
**RBBP4**	MESO	ChRC	98	363	42	503	274
**SMYD4**	MESO	HM_w	392	69	42	503	275
**PRDM16**	MESO	HM_w	116	272	114	502	276
**SIRT6**	MESO	HA_e	68	255	179	502	277
**SETD4**	MESO	HM_w	82	69	349	500	278
**PRDM2**	MESO	HM_w	115	271	113	499	279

**H2AFZ**	MESO	Others	225	69	202	496	280
**BRWD1**	MESO	HA_r	384	69	42	495	281
**CHD3**	MESO	Helicases	260	69	165	494	282
**TDRD12**	MESO	Others	37	354	103	494	283
**CHD6**	MESO	Helicases	380	69	42	491	284
**ATF7IP**	MESO	Others	290	69	130	489	285
**CLOCK**	MESO	HA_w	257	69	163	489	286
**STK31**	MESO	Others	312	69	108	489	287
**TP53BP1**	MESO	Others	30	417	42	489	288
**L3MBTL1**	MESO	HM_r	177	69	242	488	289
**CSTL1**	MESO	Others	256	69	162	487	290
**PRKAA2**	MESO	Others	108	266	111	485	291
**PRKCD**	MESO	Others	107	265	110	482	292
**SMYD3**	MESO	HM_w	55	248	178	481	293
**GTF2H1**	MESO	Others	369	69	42	480	294
**RBBP7**	MESO	ChRC	96	69	315	480	295
**RNF17**	MESO	Others	323	69	87	479	296
**PHF14**	MESO	Others	134	69	275	478	297
**RPA3**	MESO	Others	91	69	313	473	298
**SIRT1**	MESO	HA_e	73	358	42	473	299
**WDR82**	MESO	Others	15	416	42	473	300
**SIRT2**	MESO	HA_e	72	357	42	471	301
**RSF1**	MESO	ChRC	88	69	312	469	302
**PHF8**	MESO	Others	125	69	273	467	303
**KDM3B**	MESO	HM_e	353	69	42	464	304
**NCOA1**	MESO	HA_w	154	69	239	462	305
**RNF217**	MESO	Others	92	261	109	462	306
**PRDM13**	MESO	HM_w	118	69	272	459	307
**KDM1B**	MESO	HM_e	188	69	201	458	308
**PRDM15**	MESO	HM_w	117	69	271	457	309
**BAZ1A**	MESO	HA_r	288	69	98	455	310
**MTA1**	MESO	ChRC	342	69	42	453	311
**PHC1**	MESO	ChRC	141	69	236	446	312
**HDAC4**	MESO	HA_e	219	69	157	445	313
**PHF6**	MESO	HM_r	127	276	42	445	314

**PHIP**	MESO	HA_r	124	275	42	441	315
**SMARCC2**	MESO	Helicases	63	69	306	438	316
**PHF19**	MESO	HM_r	133	69	235	437	317
**CBX7**	MESO	HM_r	269	69	96	434	318
**FKBP2**	MESO	Others	236	69	129	434	319
**HIST1H3B**	MESO	Others	210	69	154	433	320
**ING2**	MESO	HM_r	205	69	153	427	321
**SMARCA5**	MESO	Helicases	316	69	42	427	322
**YY1**	MESO	ChRC	14	69	344	427	323
**SMARCB1**	MESO	Helicases	315	69	42	426	324
**PRDM6**	MESO	HM_w	112	270	42	424	325
**TRIM33**	MESO	HA_r	28	351	42	421	326
**SFMBT1**	MESO	HM_r	77	257	85	419	327
**DPF3**	MESO	HA_r	250	69	94	413	328
**HDGFL1**	MESO	Others	214	69	125	408	329
**SIRT4**	MESO	HA_e	70	69	269	408	330
**SIRT5**	MESO	HA_e	69	69	268	406	331
**HIST1H1B**	MESO	Others	211	69	124	404	332
**ZMYND11**	MESO	HA_r	11	350	42	403	333
**SMARCA2**	MESO	Helicases	65	253	84	402	334
**ING1**	MESO	HM_r	206	69	123	398	335
**BRD2**	MESO	HA_r	282	69	42	393	336
**L3MBTL3**	MESO	Others	175	69	149	393	337
**RNF20**	MESO	Others	93	69	231	393	338
**SMYD1**	MESO	HM_w	57	250	83	390	339
**RPH3A**	MESO	Others	90	69	230	389	340
**JADE3**	MESO	Others	197	69	122	388	341
**PHF23**	MESO	HM_r	130	69	189	388	342
**RPS6KA5**	MESO	Others	89	69	229	387	343
**CBX3**	MESO	HM_r	272	69	42	383	344
**MSL3**	MESO	HA_w	161	69	148	378	345
**TDRD1**	MESO	Others	39	69	266	374	346
**SFMBT2**	MESO	Others	76	69	228	373	347
**SHPRH**	MESO	Others	75	256	42	373	348
**NAP1L2**	MESO	Others	155	69	147	371	349

**KDM7A**	MESO	HM_e	182	69	119	370	350
**TET3**	MESO	DM_e	31	69	265	365	351
**L3MBTL4**	MESO	Others	174	69	118	361	352
**EHMT1**	MESO	HM_w	247	69	42	358	353
**SMARCD1**	MESO	Helicases	62	69	226	357	354
**ELP3**	MESO	HA_w	245	69	42	356	355
**PRMT8**	MESO	HM_w	101	69	185	355	356
**EPC1**	MESO	Others	243	69	42	354	357
**SMARCD3**	MESO	Helicases	60	69	225	354	358
**USP51**	MESO	Others	18	69	263	350	359
**FKBP1A**	MESO	Others	237	69	42	348	360
**RING1**	MESO	Others	95	69	182	346	361
**KDM6B**	MESO	HM_e	183	69	91	343	362
**SCML4**	MESO	Others	84	69	181	334	363
**TCEA1**	MESO	Others	43	245	42	330	364
**PRDM5**	MESO	HM_w	113	69	142	324	365
**ING5**	MESO	HM_r	202	69	42	313	366
**IWS1**	MESO	Others	200	69	42	311	367
**JADE1**	MESO	Others	199	69	42	310	368
**ZCWPW2**	MESO	HM_r	12	69	224	305	369
**PRDM12**	MESO	HM_w	119	69	115	303	370
**UHRF1**	MESO	DM_r	22	238	42	302	371
**UHRF2**	MESO	DM_r	21	237	42	300	372
**SP110**	MESO	HA_r	53	69	177	299	373
**SP140**	MESO	HA_r	52	69	176	297	374
**USP27X**	MESO	Others	19	236	42	297	375
**KDM5D**	MESO	HM_e	184	69	42	295	376
**PHF11**	MESO	Others	137	69	89	295	377
**SP140L**	MESO	HA_r	51	69	175	295	378
**SETD7**	MESO	HM_w	80	69	137	286	379
**MARCH5**	MESO	Others	173	69	42	284	380
**MBD5**	MESO	DM_r	168	69	42	279	381
**TDRD3**	MESO	HM_r	36	69	174	279	382
**PRDM11**	MESO	HM_w	120	69	88	277	383
**MEN1**	MESO	ChRC	165	69	42	276	384

**SIRT3**	MESO	HA_e	71	69	136	276	385
**MLLT10**	MESO	HM_w	164	69	42	275	386
**MTA3**	MESO	ChRC	159	69	42	270	387
**TRIM24**	MESO	HA_r	29	69	172	270	388
**MTF2**	MESO	HM_r	158	69	42	269	389
**PARP2**	MESO	Others	147	69	42	258	390
**PCGF5**	MESO	Others	144	69	42	255	391
**PCGF6**	MESO	Others	143	69	42	254	392
**ZCWPW1**	MESO	HM_r	13	69	170	252	393
**SUV39H1**	MESO	HM_w	47	69	134	250	394
**PHF12**	MESO	Others	136	69	42	247	395
**PHF21A**	MESO	HM_r	132	69	42	243	396
**SETDB2**	MESO	HM_w	79	69	86	234	397
**SUPT16H**	MESO	Others	48	69	107	224	398
**TAF3**	MESO	HA_r	44	69	105	218	399
**PRMT5**	MESO	HM_w	104	69	42	215	400
**TCF19**	MESO	Others	42	69	104	215	401
**PRMT6**	MESO	HM_w	103	69	42	214	402
**PSIP1**	MESO	HM_r	100	69	42	211	403
**SMYD5**	MESO	HM_w	54	69	82	205	404
**TDRD9**	MESO	Others	34	69	102	205	405
**TET2**	MESO	DM_e	32	69	101	202	406
**SETD3**	MESO	HM_w	83	69	42	194	407
**WDR5**	MESO	ChRC	16	69	100	185	408
**SMNDC1**	MESO	Others	58	69	42	169	409
**SSRP1**	MESO	Others	49	69	42	160	410
**TAF1**	MESO	HA_r	45	69	42	156	411
**UBE2A**	MESO	Others	27	69	42	138	412
**UBE2E1**	MESO	Others	25	69	42	136	413
**UBR7**	MESO	Others	23	69	42	134	414
**USP22**	MESO	Others	20	69	42	131	415
**UTY**	MESO	HM_e	17	69	42	128	416
**DIDO1**	OV	Others	418	415	411	1244	1
**SMARCA4**	OV	Helicases	420	410	413	1243	2
**PHF20L1**	OV	HM_r	357	426	426	1209	3

**ATAD2**	OV	HA_r	375	424	404	1203	4
**KMT2C**	OV	HM_w	426	411	343	1180	5
**CHRAC1**	OV	ChRC	334	423	420	1177	6
**SETDB1**	OV	HM_w	352	408	414	1174	7
**BRD4**	OV	HA_r	336	419	417	1172	8
**NCOA3**	OV	HA_w	406	384	359	1149	9
**ASH1L**	OV	HM_w	376	380	376	1132	10
**ATR**	OV	Others	340	406	381	1127	11
**CHD4**	OV	Helicases	425	383	311	1119	12
**ARID4B**	OV	ChRC	343	388	377	1108	13
**ACTL6A**	OV	ChRC	265	420	422	1107	14
**ZGPAT**	OV	Others	268	418	419	1105	15
**JARID2**	OV	ChRC	363	386	345	1094	16
**CHD7**	OV	Helicases	395	374	324	1093	17
**SIN3B**	OV	ChRC	280	414	397	1091	18
**RSF1**	OV	ChRC	283	412	394	1089	19
**BRD9**	OV	HA_r	253	417	395	1065	20
**ARID2**	OV	ChRC	423	268	369	1060	21
**EED**	OV	ChRC	331	376	353	1060	22
**CHD1L**	OV	Helicases	371	375	312	1058	23
**PHC1**	OV	ChRC	299	373	382	1054	24
**HDAC6**	OV	HA_e	391	347	308	1046	25
**DNMT1**	OV	DM_w	332	394	319	1045	26
**KAT6A**	OV	HA_w	362	320	356	1038	27
**MECOM**	OV	Others	309	425	304	1038	28
**AEBP2**	OV	HM_w	263	377	396	1036	29
**KDM5B**	OV	HM_e	317	323	388	1028	30
**PRDM9**	OV	HM_w	414	366	248	1028	31
**PHC3**	OV	ChRC	181	421	424	1026	32
**TAF3**	OV	HA_r	347	343	335	1025	33
**BAZ1B**	OV	HA_r	398	251	373	1022	34
**EHMT2**	OV	HM_w	330	325	365	1020	35
**HIST1H1C**	OV	Others	390	352	277	1019	36
**ATAT1**	OV	Others	341	327	346	1014	37
**EPC1**	OV	Others	393	256	364	1013	38

**KDM5A**	OV	HM_e	203	398	410	1011	39
**HCFC1**	OV	Others	367	362	278	1007	40
**KAT6B**	OV	HA_w	421	294	287	1002	41
**ASXL1**	OV	Others	259	328	409	996	42
**DNMT3B**	OV	DM_w	369	306	318	993	43
**MBD4**	OV	DM_r	312	319	362	993	44
**BAZ2B**	OV	HA_r	374	295	313	982	45
**ING4**	OV	HM_r	211	382	389	982	46
**SND1**	OV	HM_r	278	356	347	981	47
**BOP 1**	OV	Others	140	422	418	980	48
**HIST1H3B**	OV	Others	323	357	299	979	49
**PHF20**	OV	HM_r	296	292	390	978	50
**GATAD2A**	OV	HM_r	224	370	371	965	51
**PAXIP1**	OV	Others	184	413	368	965	52
**TDRD5**	OV	Others	275	331	357	963	53
**ATF7IP**	OV	Others	258	365	332	955	54
**KDM2A**	OV	HM_e	389	270	294	953	55
**KDM5C**	OV	HM_e	416	265	270	951	56
**PRMT8**	OV	HM_w	384	397	167	948	57
**DNMT3A**	OV	DM_w	394	192	361	947	58
**TDRKH**	OV	Others	150	389	407	946	59
**KMT2E**	OV	HM_w	360	322	261	943	60
**INO80**	OV	Helicases	364	218	360	942	61
**CHD6**	OV	Helicases	422	163	354	939	62
**CDYL**	OV	HM_r	132	387	412	931	63
**ZMYND8**	OV	HA_r	267	396	268	931	64
**RING1**	OV	Others	284	297	348	929	65
**HLTF**	OV	Others	106	416	406	928	66
**CARM1**	OV	HM_w	137	405	385	927	67
**FBXW9**	OV	Others	228	390	309	927	68
**HAT1**	OV	HA_w	326	299	301	926	69
**LBR**	OV	Others	313	349	252	914	70
**RTF1**	OV	Others	282	225	402	909	71
**PAF1**	OV	Others	82	403	421	906	72
**ATAD2B**	OV	HA_r	342	222	340	904	73

**FMR1**	OV	Others	328	312	264	904	74
**SETD5**	OV	HM_w	412	233	256	901	75
**KDM1B**	OV	HM_e	96	399	405	900	76
**ATM**	OV	Others	399	296	203	898	77
**JMJD1C**	OV	HM_e	417	205	276	898	78
**PARP1**	OV	Others	186	361	349	896	79
**DPF1**	OV	ChRC	235	393	267	895	80
**FBXO17**	OV	Others	123	409	363	895	81
**CTCF**	OV	Others	333	220	338	891	82
**GATAD2B**	OV	HM_r	119	369	400	888	83
**ING3**	OV	HM_r	212	335	334	881	84
**BRD1**	OV	HA_r	139	401	339	879	85
**JMJD6**	OV	HM_e	321	305	253	879	86
**SFMBT2**	OV	Others	405	346	126	877	87
**FKBP1A**	OV	Others	122	353	401	876	88
**BRPF3**	OV	HA_r	251	318	305	874	89
**PHF8**	OV	Others	386	278	208	872	90
**RBBP4**	OV	ChRC	169	348	352	869	91
**ASXL3**	OV	Others	400	313	153	866	92
**AICDA**	OV	DM_e	262	372	230	864	93
**CBX8**	OV	ChRC	246	355	254	855	94
**ARID1B**	OV	ChRC	344	230	280	854	95
**HDAC1**	OV	HA_e	221	330	300	851	96
**L3MBTL2**	OV	Others	387	73	387	847	97
**TRIM24**	OV	HA_r	149	391	303	843	98
**KMT2B**	OV	HM_w	415	385	42	842	99
**DAXX**	OV	ChRC	237	300	302	839	100
**EZH2**	OV	HM_w	124	404	310	838	101
**SCMH1**	OV	Others	50	395	393	838	102
**PYGO2**	OV	HM_r	56	363	415	834	103
**PRDM14**	OV	HM_w	356	367	101	824	104
**DMAP1**	OV	Others	129	354	337	820	105
**MSH6**	OV	HM_r	308	254	258	820	106
**BMI1**	OV	ChRC	256	275	288	819	107
**TET1**	OV	DM_e	419	167	231	817	108

**SMARCC2**	OV	Helicases	158	277	374	809	109
**PRKAA2**	OV	Others	288	301	219	808	110
**KDM4A**	OV	HM_e	95	368	344	807	111
**SUPT16H**	OV	Others	153	285	367	805	112
**HR**	OV	HM_e	322	341	138	801	113
**MECP2**	OV	DM_r	87	364	350	801	114
**PHF1**	OV	HM_r	180	298	323	801	115
**NCOA1**	OV	HA_w	305	209	285	799	116
**CBX4**	OV	HM_r	248	350	200	798	117
**MBD5**	OV	DM_r	311	188	297	796	118
**PHF21B**	OV	HM_r	295	316	177	788	119
**RNF2**	OV	ChRC	54	329	403	786	120
**SMARCA2**	OV	Helicases	279	360	143	782	121
**ARID1A**	OV	ChRC	378	69	333	780	122
**STK31**	OV	Others	348	237	193	778	123
**SMARCD3**	OV	Helicases	156	402	218	776	124
**CHD5**	OV	Helicases	370	288	115	773	125
**PRDM7**	OV	HM_w	355	263	155	773	126
**TET3**	OV	DM_e	346	138	289	773	127
**NSD1**	OV	HM_w	304	259	209	772	128
**BPTF**	OV	HA_r	397	244	130	771	129
**PCGF1**	OV	Others	358	158	250	766	130
**SIRT2**	OV	HA_e	45	407	314	766	131
**RBBP5**	OV	ChRC	55	315	392	762	132
**MBD3**	OV	DM_r	199	286	275	760	133
**PRKAA1**	OV	Others	63	311	386	760	134
**USP51**	OV	Others	270	195	295	760	135
**SP100**	OV	HA_r	411	109	238	758	136
**BAZ2A**	OV	HA_r	257	194	306	757	137
**TCEA1**	OV	Others	29	371	355	755	138
**HDGF**	OV	Others	110	324	320	754	139
**HDAC11**	OV	HA_e	366	174	213	753	140
**PRDM16**	OV	HM_w	290	304	156	750	141
**TAF1**	OV	HA_r	402	116	232	750	142
**PARP2**	OV	Others	185	280	284	749	143

**SIRT5**	OV	HA_e	42	378	327	747	144
**BRWD3**	OV	HA_r	396	103	245	744	145
**ORC1**	OV	Others	188	302	251	741	146
**PHF3**	OV	Others	294	140	307	741	147
**HDAC9**	OV	HA_e	218	321	199	738	148
**MLLT10**	OV	HM_w	86	281	370	737	149
**HDAC10**	OV	HA_e	113	400	223	736	150
**ZMYND11**	OV	HA_r	11	359	366	736	151
**H3F3A**	OV	Others	114	358	263	735	152
**SUV39H2**	OV	HM_w	152	257	326	735	153
**TDRD6**	OV	Others	274	269	192	735	154
**TDG**	OV	ChRC	345	139	249	733	155
**SMARCA1**	OV	Helicases	382	169	181	732	156
**CXXC1**	OV	Others	238	69	423	730	157
**DPF2**	OV	Others	234	229	266	729	158
**HDGFL1**	OV	Others	109	379	241	729	159
**MTA1**	OV	ChRC	84	309	336	729	160
**FKBP5**	OV	Others	227	310	189	726	161
**CHD9**	OV	Helicases	335	69	321	725	162
**CHD8**	OV	Helicases	409	273	42	724	163
**BRD2**	OV	HA_r	373	307	42	722	164
**KDM2B**	OV	HM_e	205	217	298	720	165
**PADI6**	OV	Others	301	185	234	720	166
**GTF2B**	OV	Others	223	219	273	715	167
**TDRD9**	OV	Others	272	252	191	715	168
**SMYD4**	OV	HM_w	154	168	391	713	169
**KDM7A**	OV	HM_e	266	69	372	707	170
**MBTD1**	OV	Others	310	160	235	705	171
**FBXO44**	OV	Others	229	260	214	703	172
**ERCC5**	OV	Others	408	250	42	700	173
**KDM4C**	OV	HM_e	318	339	42	699	174
**TDRD10**	OV	Others	151	342	206	699	175
**SMYD3**	OV	HM_w	35	381	282	698	176
**SUV39H1**	OV	HM_w	32	344	322	698	177
**KMT2A**	OV	HM_w	407	248	42	697	178

**MUM1**	OV	Others	193	293	211	697	179
**MBD1**	OV	DM_r	200	69	425	694	180
**PRMT7**	OV	HM_w	59	226	408	693	181
**CBX2**	OV	ChRC	135	351	201	687	182
**EP300**	OV	HA_w	231	191	265	687	183
**TCF20**	OV	Others	410	231	42	683	184
**PRDM15**	OV	HM_w	385	184	113	682	185
**PADI2**	OV	Others	359	187	135	681	186
**GADD45B**	OV	Others	225	266	188	679	187
**IDH2**	OV	DM_e	104	303	271	678	188
**SMARCB1**	OV	Helicases	404	232	42	678	189
**SETDB2**	OV	HM_w	162	198	315	675	190
**BRWD1**	OV	HA_r	250	177	246	673	191
**ZCWPW1**	OV	HM_r	145	290	236	671	192
**SMYD2**	OV	HM_w	155	276	239	670	193
**EPC2**	OV	Others	230	122	317	669	194
**PCMT1**	OV	Others	183	157	328	668	195
**CLOCK**	OV	HA_w	239	147	279	665	196
**GLYR1**	OV	HM_r	118	161	384	663	197
**SP140**	OV	HA_r	381	107	173	661	198
**L3MBTL1**	OV	HM_r	201	173	286	660	199
**ACTL6B**	OV	ChRC	264	289	103	656	200
**PBRM1**	OV	HA_r	300	159	197	656	201
**PPARGC1A**	OV	Others	291	208	157	656	202
**SETD6**	OV	HM_w	164	91	398	653	203
**ASXL2**	OV	Others	142	179	330	651	204
**PADI3**	OV	Others	303	203	145	651	205
**HIRA**	OV	Others	324	282	42	648	206
**KDM4D**	OV	HM_e	93	334	221	648	207
**KMT2D**	OV	HM_w	314	74	260	648	208
**GADD45A**	OV	Others	226	242	179	647	209
**MTF2**	OV	HM_r	306	142	198	646	210
**PHF13**	OV	Others	76	279	291	646	211
**KAT5**	OV	HA_w	97	228	316	641	212
**SIRT7**	OV	HA_e	41	345	255	641	213

**HDAC7**	OV	HA_e	219	190	229	638	214
**PWWP2B**	OV	Others	57	291	290	638	215
**ATRX**	OV	Helicases	339	148	149	636	216
**RPH3A**	OV	Others	354	171	110	635	217
**KDM8**	OV	HM_e	315	75	244	634	218
**BAZ1A**	OV	HA_r	337	123	172	632	219
**L3MBTL3**	OV	Others	401	69	161	631	220
**BRPF1**	OV	HA_r	372	211	42	625	221
**TCF19**	OV	Others	28	314	281	623	222
**FXR2**	OV	Others	120	86	416	622	223
**KDM6A**	OV	HM_e	316	69	228	613	224
**MTA3**	OV	ChRC	307	264	42	613	225
**RBBP7**	OV	ChRC	285	69	257	611	226
**AIRE**	OV	HM_r	143	262	204	609	227
**CHD2**	OV	Helicases	241	326	42	609	228
**SETD2**	OV	HM_w	413	154	42	609	229
**NCOR2**	OV	Others	189	234	184	607	230
**HDAC4**	OV	HA_e	365	69	170	604	231
**TP53BP1**	OV	Others	380	181	42	603	232
**SSRP1**	OV	Others	276	82	243	601	233
**SETD3**	OV	HM_w	47	153	399	599	234
**ZCWPW2**	OV	HM_r	269	124	205	598	235
**TRIM33**	OV	HA_r	148	151	296	595	236
**TRIM28**	OV	HA_r	24	245	325	594	237
**INTS12**	OV	Others	102	114	375	591	238
**AKAP1**	OV	Others	261	149	180	590	239
**ING2**	OV	HM_r	213	240	137	590	240
**MARCH5**	OV	Others	89	120	380	589	241
**NCOR1**	OV	ChRC	190	130	269	589	242
**ASH2L**	OV	HM_w	260	284	42	586	243
**CBX5**	OV	HM_r	134	236	216	586	244
**CHAF1B**	OV	ChRC	243	193	148	584	245
**DPY30**	OV	Others	127	267	190	584	246
**PRDM2**	OV	HM_w	289	253	42	584	247
**HIST1H1B**	OV	Others	107	336	140	583	248

**TDRD12**	OV	Others	26	392	163	581	249
**SP110**	OV	HA_r	277	108	194	579	250
**PRDM10**	OV	HM_w	70	332	176	578	251
**CHAF1A**	OV	ChRC	244	235	97	576	252
**PHF19**	OV	HM_r	298	119	158	575	253
**KDM3A**	OV	HM_e	204	77	293	574	254
**PRMT5**	OV	HM_w	286	246	42	574	255
**BRDT**	OV	HA_r	252	212	109	573	256
**EP400**	OV	HA_w	368	162	42	572	257
**HDAC8**	OV	HA_e	111	189	272	572	258
**MEN1**	OV	ChRC	198	132	240	570	259
**MPHOSPH8**	OV	HM_r	196	143	227	566	260
**CBX3**	OV	HM_r	249	274	42	565	261
**HNF1A**	OV	ChRC	216	210	139	565	262
**SMARCD1**	OV	Helicases	157	125	283	565	263
**KDM3B**	OV	HM_e	388	133	42	563	264
**CBX1**	OV	HM_r	136	221	202	559	265
**BAP1**	OV	Others	338	178	42	558	266
**EHMT1**	OV	HM_w	232	283	42	557	267
**HIF1AN**	OV	Others	108	69	379	556	268
**MSL3**	OV	HA_w	195	69	292	556	269
**DOT1L**	OV	HM_w	236	272	42	550	270
**G2E3**	OV	Others	327	175	42	544	271
**PRDM1**	OV	HM_w	175	216	151	542	272
**L3MBTL4**	OV	Others	90	239	212	541	273
**PHC2**	OV	ChRC	182	317	42	541	274
**PHF21A**	OV	HM_r	178	141	220	539	275
**SMYD1**	OV	HM_w	349	72	118	539	276
**GTF2F1**	OV	Others	117	69	351	537	277
**CREBBP**	OV	HA_w	424	69	42	535	278
**POLR2B**	OV	Others	292	201	42	535	279
**SRCAP**	OV	Others	403	89	42	534	280
**IDH1**	OV	DM_e	105	241	187	533	281
**PRMT1**	OV	HM_w	287	69	175	531	282
**DNMT3L**	OV	DM_w	128	261	141	530	283

**KDM4E**	OV	HM_e	92	333	105	530	284
**PADI4**	OV	Others	302	186	42	530	285
**HSPBAP1**	OV	Others	215	271	42	528	286
**PADI1**	OV	Others	187	204	136	527	287
**SMARCC1**	OV	Helicases	39	213	274	526	288
**GTF2H1**	OV	Others	116	80	329	525	289
**KAT2B**	OV	HA_w	208	145	168	521	290
**SCML4**	OV	Others	353	69	99	521	291
**RAG2**	OV	HM_r	171	183	166	520	292
**SETD1B**	OV	HM_w	48	224	247	519	293
**MORF4L1**	OV	HM_r	85	247	185	517	294
**EZH1**	OV	HM_w	392	81	42	515	295
**JADE1**	OV	Others	101	69	342	512	296
**ING1**	OV	HM_r	214	255	42	511	297
**CBX7**	OV	HM_r	247	164	98	509	298
**JADE3**	OV	Others	99	69	341	509	299
**SMYD5**	OV	HM_w	34	117	358	509	300
**ELP3**	OV	HA_w	126	337	42	505	301
**KDM1A**	OV	HM_e	319	144	42	505	302
**JMJD8**	OV	HM_e	209	249	42	500	303
**CDYL2**	OV	HM_r	131	243	124	498	304
**SUZ12**	OV	ChRC	31	88	378	497	305
**RNF20**	OV	Others	383	70	42	495	306
**SMARCD2**	OV	Helicases	38	223	233	494	307
**TDRD7**	OV	Others	273	69	150	492	308
**CSTL1**	OV	Others	130	146	215	491	309
**AFF4**	OV	Others	379	69	42	490	310
**ARID4A**	OV	ChRC	377	69	42	488	311
**CECR2**	OV	HA_r	245	69	171	485	312
**KDM6B**	OV	HM_e	202	97	186	485	313
**SETMAR**	OV	HM_w	161	197	119	477	314
**PRMT2**	OV	HM_w	173	258	42	473	315
**FBXL19**	OV	Others	329	101	42	472	316
**KIAA2026**	OV	Others	361	69	42	472	317
**CHD1**	OV	Helicases	242	69	160	471	318

**TET2**	OV	DM_e	271	104	96	471	319
**NAP1L1**	OV	Others	192	96	178	466	320
**WDR5**	OV	ChRC	13	69	383	465	321
**SHPRH**	OV	Others	351	69	42	462	322
**SATB1**	OV	Others	166	69	226	461	323
**SIN3A**	OV	ChRC	350	69	42	461	324
**PHF12**	OV	Others	179	238	42	459	325
**USP22**	OV	Others	147	150	162	459	326
**SFMBT1**	OV	HM_r	160	152	144	456	327
**ELP4**	OV	HA_w	125	287	42	454	328
**HDAC3**	OV	HA_e	325	85	42	452	329
**CBX6**	OV	HM_r	133	176	142	451	330
**PYGO1**	OV	HM_r	172	83	196	451	331
**CHD3**	OV	Helicases	240	87	123	450	332
**RNF217**	OV	Others	53	215	182	450	333
**DPF3**	OV	HA_r	233	102	112	447	334
**HELLS**	OV	Helicases	217	84	146	447	335
**PHF6**	OV	HM_r	177	227	42	446	336
**SIRT3**	OV	HA_e	44	69	331	444	337
**HDAC2**	OV	HA_e	112	69	262	443	338
**KAT2A**	OV	HA_w	320	79	42	441	339
**UBE2A**	OV	Others	22	180	237	439	340
**SIRT4**	OV	HA_e	43	170	225	438	341
**BRD8**	OV	HA_r	254	134	42	430	342
**SETD7**	OV	HM_w	163	127	127	417	343
**AFF1**	OV	Others	144	165	107	416	344
**MBD2**	OV	DM_r	88	69	259	416	345
**SETD1A**	OV	HM_w	281	92	42	415	346
**SIRT6**	OV	HA_e	159	214	42	415	347
**GTF3C4**	OV	HA_w	222	69	122	413	348
**PHF11**	OV	Others	77	202	134	413	349
**NAP1L3**	OV	Others	191	69	152	412	350
**PHF7**	OV	Others	72	156	183	411	351
**PHF2**	OV	Others	297	69	42	408	352
**PSIP1**	OV	HM_r	58	308	42	408	353

**RPS6KA5**	OV	Others	167	199	42	408	354
**PHRF1**	OV	Others	293	69	42	404	355
**USP27X**	OV	Others	15	340	42	397	356
**UBR7**	OV	Others	18	136	242	396	357
**UHRF2**	OV	DM_r	16	338	42	396	358
**ING5**	OV	HM_r	103	69	222	394	359
**PHIP**	OV	HA_r	176	172	42	390	360
**SETD4**	OV	HM_w	165	182	42	389	361
**NAP1L2**	OV	Others	83	95	210	388	362
**PRDM11**	OV	HM_w	69	112	207	388	363
**MLLT6**	OV	HM_w	197	69	121	387	364
**JADE2**	OV	Others	100	69	217	386	365
**RNF17**	OV	Others	168	118	100	386	366
**RAI1**	OV	Others	170	110	104	384	367
**UBE2E1**	OV	Others	20	137	224	381	368
**KAT7**	OV	HA_w	207	121	42	370	369
**PRDM13**	OV	HM_w	68	200	102	370	370
**FKBP2**	OV	Others	121	100	147	368	371
**MTA2**	OV	ChRC	194	131	42	367	372
**BRD3**	OV	HA_r	255	69	42	366	373
**KANSL1**	OV	HA_w	98	98	169	365	374
**PRMT6**	OV	HM_w	60	128	174	362	375
**HDAC5**	OV	HA_e	220	99	42	361	376
**PRDM12**	OV	HM_w	174	69	114	357	377
**TDRD1**	OV	Others	27	207	117	351	378
**H2AFZ**	OV	Others	115	115	108	338	379
**SIRT1**	OV	HA_e	46	126	165	337	380
**KAT8**	OV	HA_w	206	78	42	326	381
**WDR82**	OV	Others	146	135	42	323	382
**IWS1**	OV	Others	210	69	42	321	383
**AURKB**	OV	Others	141	69	106	316	384
**SCML2**	OV	HM_r	49	69	195	313	385
**PRDM8**	OV	HM_w	64	111	133	308	386
**PCGF6**	OV	Others	79	69	159	307	387
**SP140L**	OV	HA_r	33	106	164	303	388

**PRMT3**	OV	HM_w	61	94	129	284	389
**SMARCA5**	OV	Helicases	40	196	42	278	390
**PCGF2**	OV	Others	81	69	120	270	391
**UBE2I**	OV	Others	19	206	42	267	392
**TAF1L**	OV	HA_r	30	105	131	266	393
**PRKCD**	OV	Others	62	155	42	259	394
**BRD7**	OV	HA_r	138	69	42	249	395
**RPA3**	OV	Others	51	69	128	248	396
**PRDM5**	OV	HM_w	66	69	111	246	397
**UBE2B**	OV	Others	21	69	154	244	398
**PRDM4**	OV	HM_w	67	129	42	238	399
**SMARCE1**	OV	Helicases	37	69	132	238	400
**PHF23**	OV	HM_r	74	113	42	229	401
**YY1**	OV	ChRC	12	166	42	220	402
**TRIM66**	OV	HA_r	23	69	125	217	403
**TDRD3**	OV	HM_r	25	69	116	210	404
**KDM4B**	OV	HM_e	94	69	42	205	405
**KDM5D**	OV	HM_e	91	69	42	202	406
**PCGF5**	OV	Others	80	69	42	191	407
**PHF10**	OV	Others	78	69	42	189	408
**POLE3**	OV	ChRC	71	76	42	189	409
**RNF40**	OV	Others	52	93	42	187	410
**PHF14**	OV	Others	75	69	42	186	411
**PHF5A**	OV	Others	73	71	42	186	412
**PRDM6**	OV	HM_w	65	69	42	176	413
**SMNDC1**	OV	Others	36	90	42	168	414
**UHRF1**	OV	DM_r	17	69	42	128	415
**UTY**	OV	HM_e	14	69	42	125	416
**GATAD2B**	PAAD	HM_r	392	404	410	1206	1
**CHD4**	PAAD	Helicases	396	390	406	1192	2
**KDM6A**	PAAD	HM_e	409	401	378	1188	3
**KDM5B**	PAAD	HM_e	418	340	418	1176	4
**MBD1**	PAAD	DM_r	332	412	423	1167	5
**PHF20L1**	PAAD	HM_r	322	425	416	1163	6
**KDM5A**	PAAD	HM_e	335	413	413	1161	7

**MECP2**	PAAD	DM_r	385	363	393	1141	8
**ARID1A**	PAAD	ChRC	426	331	374	1131	9
**ATAD2**	PAAD	HA_r	281	423	424	1128	10
**ASH1L**	PAAD	HM_w	416	407	293	1116	11
**CHRAC1**	PAAD	ChRC	260	424	425	1109	12
**CHD1L**	PAAD	Helicases	263	415	421	1099	13
**AEBP2**	PAAD	HM_w	285	410	398	1093	14
**HDAC5**	PAAD	HA_e	391	371	315	1077	15
**IDH2**	PAAD	DM_e	388	402	285	1075	16
**PRMT8**	PAAD	HM_w	314	395	351	1060	17
**ACTL6A**	PAAD	ChRC	286	349	422	1057	18
**ASXL1**	PAAD	Others	364	330	361	1055	19
**CHD7**	PAAD	Helicases	261	389	405	1055	20
**DIDO1**	PAAD	Others	424	268	360	1052	21
**HDGF**	PAAD	Others	234	403	414	1051	22
**DNMT3B**	PAAD	DM_w	411	267	359	1037	23
**TDRD5**	PAAD	Others	293	351	388	1032	24
**HDAC9**	PAAD	HA_e	419	319	287	1025	25
**HR**	PAAD	HM_e	389	315	313	1017	26
**ATF7IP**	PAAD	Others	280	391	342	1013	27
**CHD6**	PAAD	Helicases	420	273	319	1012	28
**MECOM**	PAAD	Others	407	400	202	1009	29
**BAZ1B**	PAAD	HA_r	278	329	397	1004	30
**ARID1B**	PAAD	ChRC	368	288	343	999	31
**CBX1**	PAAD	HM_r	268	406	321	995	32
**ELP3**	PAAD	HA_w	251	322	420	993	33
**EZH1**	PAAD	HM_w	247	345	396	988	34
**HCFC1**	PAAD	Others	237	372	379	988	35
**SIRT2**	PAAD	HA_e	153	420	415	988	36
**BAZ2B**	PAAD	HA_r	276	328	373	977	37
**MBD2**	PAAD	DM_r	331	411	232	974	38
**SETD2**	PAAD	HM_w	375	208	390	973	39
**SETDB1**	PAAD	HM_w	160	383	426	969	40
**IDH1**	PAAD	DM_e	226	343	395	964	41
**PYGO2**	PAAD	HM_r	166	394	404	964	42

**MORF4L1**	PAAD	HM_r	208	384	367	959	43
**CLOCK**	PAAD	HA_w	259	324	371	954	44
**ING1**	PAAD	HM_r	344	251	357	952	45
**MBTD1**	PAAD	Others	330	364	257	951	46
**SMYD4**	PAAD	HM_w	300	300	347	947	47
**CHD5**	PAAD	Helicases	358	376	209	943	48
**STK31**	PAAD	Others	372	295	274	941	49
**TRIM28**	PAAD	HA_r	290	350	296	936	50
**FBXO17**	PAAD	Others	245	421	266	932	51
**TAF1L**	PAAD	HA_r	297	354	273	924	52
**BOP 1**	PAAD	Others	114	426	381	921	53
**KDM1B**	PAAD	HM_e	340	244	336	920	54
**PHF5A**	PAAD	Others	183	357	366	906	55
**SMARCC2**	PAAD	Helicases	150	355	400	905	56
**KAT2A**	PAAD	HA_w	221	342	337	900	57
**KDM6B**	PAAD	HM_e	386	311	203	900	58
**NCOR1**	PAAD	ChRC	383	235	282	900	59
**PAF1**	PAAD	Others	58	422	417	897	60
**TDRKH**	PAAD	Others	133	381	382	896	61
**BRD7**	PAAD	HA_r	271	282	341	894	62
**L3MBTL4**	PAAD	Others	334	385	174	893	63
**ATR**	PAAD	Others	415	69	403	887	64
**SMARCD2**	PAAD	Helicases	148	334	399	881	65
**EHMT2**	PAAD	HM_w	252	388	239	879	66
**PHF21B**	PAAD	HM_r	321	358	200	879	67
**EZH2**	PAAD	HM_w	246	262	370	878	68
**CDYL2**	PAAD	HM_r	360	274	243	877	69
**PRDM16**	PAAD	HM_w	380	338	155	873	70
**CBX2**	PAAD	ChRC	267	280	320	867	71
**TAF1**	PAAD	HA_r	371	193	299	863	72
**SMYD3**	PAAD	HM_w	144	393	324	861	73
**JARID2**	PAAD	ChRC	342	313	204	859	74
**KAT7**	PAAD	HA_w	77	386	394	857	75
**DPF1**	PAAD	ChRC	255	419	179	853	76
**SP140**	PAAD	HA_r	373	297	183	853	77

**FXR2**	PAAD	Others	241	321	289	851	78
**TCF20**	PAAD	Others	136	352	363	851	79
**HIST1H1B**	PAAD	Others	390	253	205	848	80
**MLLT6**	PAAD	HM_w	64	399	383	846	81
**SETD6**	PAAD	HM_w	307	206	328	841	82
**PARP2**	PAAD	Others	195	234	409	838	83
**CHD9**	PAAD	Helicases	357	271	208	836	84
**KIAA2026**	PAAD	Others	214	310	311	835	85
**BPTF**	PAAD	HA_r	414	377	42	833	86
**PCGF2**	PAAD	Others	57	398	377	832	87
**ING4**	PAAD	HM_r	87	387	356	830	88
**SFMBT2**	PAAD	Others	306	303	221	830	89
**BRD8**	PAAD	HA_r	113	327	387	827	90
**EP300**	PAAD	HA_w	410	375	42	827	91
**HELLS**	PAAD	Helicases	233	254	338	825	92
**ASH2L**	PAAD	HM_w	365	416	42	823	93
**KAT2B**	PAAD	HA_w	220	341	260	821	94
**TCEA1**	PAAD	Others	24	382	411	817	95
**L3MBTL2**	PAAD	Others	408	366	42	816	96
**PHF10**	PAAD	Others	325	69	419	813	97
**PRDM11**	PAAD	HM_w	403	223	186	812	98
**HIST1H1C**	PAAD	Others	230	318	263	811	99
**NSD1**	PAAD	HM_w	406	362	42	810	100
**PARP1**	PAAD	Others	196	361	253	810	101
**RNF2**	PAAD	ChRC	42	356	412	810	102
**HIST1H3B**	PAAD	Others	229	317	262	808	103
**GLYR1**	PAAD	HM_r	100	320	386	806	104
**SMARCA1**	PAAD	Helicases	305	200	300	805	105
**DNMT1**	PAAD	DM_w	394	69	340	803	106
**ACTL6B**	PAAD	ChRC	121	408	268	797	107
**H3F3A**	PAAD	Others	97	373	317	787	108
**BRD2**	PAAD	HA_r	398	346	42	786	109
**FKBP5**	PAAD	Others	349	259	177	785	110
**ING5**	PAAD	HM_r	224	249	312	785	111
**PHF1**	PAAD	HM_r	326	231	227	784	112

**TDRD1**	PAAD	Others	294	192	298	784	113
**RNF40**	PAAD	Others	311	69	401	781	114
**CHD3**	PAAD	Helicases	413	325	42	780	115
**PWWP2B**	PAAD	Others	313	215	252	780	116
**PRMT5**	PAAD	HM_w	170	216	392	778	117
**SMARCD3**	PAAD	Helicases	147	301	325	773	118
**RBBP5**	PAAD	ChRC	43	336	391	770	119
**PRDM13**	PAAD	HM_w	381	221	165	767	120
**ZMYND8**	PAAD	HA_r	287	69	408	764	121
**KDM3A**	PAAD	HM_e	338	69	355	762	122
**TCF19**	PAAD	Others	137	353	272	762	123
**AFF1**	PAAD	Others	370	69	322	761	124
**CBX5**	PAAD	HM_r	110	278	372	760	125
**PCGF5**	PAAD	Others	192	232	331	755	126
**NCOR2**	PAAD	Others	328	69	353	750	127
**BRWD3**	PAAD	HA_r	425	69	245	739	128
**ATM**	PAAD	Others	421	69	247	737	129
**PRMT1**	PAAD	HM_w	48	337	352	737	130
**H2AFZ**	PAAD	Others	347	69	318	734	131
**ZCWPW1**	PAAD	HM_r	123	392	217	732	132
**NAP1L2**	PAAD	Others	329	69	333	731	133
**KAT6B**	PAAD	HA_w	78	368	284	730	134
**PRDM14**	PAAD	HM_w	178	396	156	730	135
**SMYD2**	PAAD	HM_w	145	332	250	727	136
**SND1**	PAAD	HM_r	143	195	389	727	137
**CREBBP**	PAAD	HA_w	412	270	42	724	138
**TRIM33**	PAAD	HA_r	289	69	362	720	139
**HLTF**	PAAD	Others	89	316	314	719	140
**CXXC1**	PAAD	Others	257	414	42	713	141
**CHD2**	PAAD	Helicases	262	405	42	709	142
**CHD8**	PAAD	Helicases	395	272	42	709	143
**ARID4B**	PAAD	ChRC	284	379	42	705	144
**HDAC6**	PAAD	HA_e	345	69	288	702	145
**RPA3**	PAAD	Others	162	210	330	702	146
**EHMT1**	PAAD	HM_w	393	266	42	701	147

**MPHOSPH8**	PAAD	HM_r	207	238	256	701	148
**TET1**	PAAD	DM_e	292	189	218	699	149
**BAZ2A**	PAAD	HA_r	277	378	42	697	150
**JMJD6**	PAAD	HM_e	82	246	369	697	151
**ARID2**	PAAD	ChRC	367	287	42	696	152
**SUV39H2**	PAAD	HM_w	138	194	364	696	153
**ARID4A**	PAAD	ChRC	366	286	42	694	154
**GTF3C4**	PAAD	HA_w	98	257	339	694	155
**JMJD8**	PAAD	HM_e	81	245	368	694	156
**PPARGC1A**	PAAD	Others	319	69	306	694	157
**AICDA**	PAAD	DM_e	119	380	194	693	158
**ELP4**	PAAD	HA_w	250	265	178	693	159
**NCOA1**	PAAD	HA_w	201	236	255	692	160
**PBRM1**	PAAD	HA_r	417	233	42	692	161
**PRDM10**	PAAD	HM_w	318	69	305	692	162
**KDM3B**	PAAD	HM_e	337	312	42	691	163
**DOT1L**	PAAD	HM_w	354	69	267	690	164
**SATB1**	PAAD	Others	308	209	172	689	165
**PYGO1**	PAAD	HM_r	167	214	304	685	166
**HNF1A**	PAAD	ChRC	228	69	385	682	167
**RPS6KA5**	PAAD	Others	310	69	302	681	168
**RING1**	PAAD	Others	163	212	303	678	169
**CECR2**	PAAD	HA_r	397	69	211	677	170
**MTA1**	PAAD	ChRC	205	69	402	676	171
**SMARCE1**	PAAD	Helicases	301	333	42	676	172
**KDM2A**	PAAD	HM_e	387	243	42	672	173
**PRKCD**	PAAD	Others	379	69	224	672	174
**PHF14**	PAAD	Others	55	309	307	671	175
**CHAF1B**	PAAD	ChRC	359	69	242	670	176
**KDM5C**	PAAD	HM_e	215	69	384	668	177
**POLR2B**	PAAD	Others	320	306	42	668	178
**DAXX**	PAAD	ChRC	356	269	42	667	179
**MEN1**	PAAD	ChRC	384	239	42	665	180
**KDM4C**	PAAD	HM_e	336	69	259	664	181
**SMARCA2**	PAAD	Helicases	304	199	161	664	182

**BRD1**	PAAD	HA_r	274	347	42	663	183
**ING3**	PAAD	HM_r	88	314	261	663	184
**HDAC8**	PAAD	HA_e	235	69	358	662	185
**PADI3**	PAAD	Others	405	69	187	661	186
**FMR1**	PAAD	Others	242	374	42	658	187
**EPC2**	PAAD	Others	351	264	42	657	188
**TDRD10**	PAAD	Others	23	409	219	651	189
**HDAC4**	PAAD	HA_e	346	69	235	650	190
**PADI1**	PAAD	Others	327	69	254	650	191
**GATAD2A**	PAAD	HM_r	348	258	42	648	192
**SMARCA4**	PAAD	Helicases	303	302	42	647	193
**ATAT1**	PAAD	Others	117	348	181	646	194
**ASXL2**	PAAD	Others	283	69	292	644	195
**HDAC10**	PAAD	HA_e	94	344	206	644	196
**KDM4B**	PAAD	HM_e	217	69	354	640	197
**KAT6A**	PAAD	HA_w	79	369	189	637	198
**SP140L**	PAAD	HA_r	298	296	42	636	199
**CBX3**	PAAD	HM_r	266	326	42	634	200
**INO80**	PAAD	Helicases	343	248	42	633	201
**MTA2**	PAAD	ChRC	62	237	334	633	202
**TAF3**	PAAD	HA_r	296	292	42	630	203
**CBX7**	PAAD	HM_r	108	276	244	628	204
**PHC3**	PAAD	ChRC	187	397	42	626	205
**CARM1**	PAAD	HM_w	362	69	193	624	206
**SETD5**	PAAD	HM_w	374	207	42	623	207
**AKAP1**	PAAD	Others	118	289	215	622	208
**CSTL1**	PAAD	Others	106	323	191	620	209
**KDM4D**	PAAD	HM_e	216	69	335	620	210
**LBR**	PAAD	Others	213	365	42	620	211
**AURKB**	PAAD	Others	116	285	214	615	212
**MARCH5**	PAAD	Others	333	240	42	615	213
**PRKAA1**	PAAD	Others	173	217	225	615	214
**PRDM5**	PAAD	HM_w	315	69	226	610	215
**EPC1**	PAAD	Others	249	69	291	609	216
**RPH3A**	PAAD	Others	377	69	163	609	217

**AIRE**	PAAD	HM_r	369	69	170	608	218
**PRMT3**	PAAD	HM_w	171	69	365	605	219
**DMAP1**	PAAD	Others	355	69	180	604	220
**FBXW9**	PAAD	Others	244	69	290	603	221
**NCOA3**	PAAD	HA_w	200	69	332	601	222
**RAG2**	PAAD	HM_r	45	305	251	601	223
**BMI1**	PAAD	ChRC	275	283	42	600	224
**RNF17**	PAAD	Others	378	69	153	600	225
**CBX6**	PAAD	HM_r	109	277	213	599	226
**ZMYND11**	PAAD	HA_r	122	183	294	599	227
**TDRD12**	PAAD	Others	22	418	158	598	228
**PHF12**	PAAD	Others	324	229	42	595	229
**CBX8**	PAAD	ChRC	107	275	212	594	230
**BRPF1**	PAAD	HA_r	269	281	42	592	231
**PHF2**	PAAD	Others	323	227	42	592	232
**PHC1**	PAAD	ChRC	189	360	42	591	233
**TRIM24**	PAAD	HA_r	131	188	271	590	234
**MBD4**	PAAD	DM_r	211	69	309	589	235
**FBXO44**	PAAD	Others	350	69	169	588	236
**PRDM12**	PAAD	HM_w	179	222	185	586	237
**BRDT**	PAAD	HA_r	270	69	246	585	238
**SETMAR**	PAAD	HM_w	159	204	222	585	239
**RBBP7**	PAAD	ChRC	164	69	350	583	240
**CBX4**	PAAD	HM_r	111	279	192	582	241
**PRDM7**	PAAD	HM_w	176	219	184	579	242
**KDM1A**	PAAD	HM_e	341	69	167	577	243
**RAI1**	PAAD	Others	165	213	199	577	244
**SIRT7**	PAAD	HA_e	28	201	348	577	245
**UBE2I**	PAAD	Others	16	186	375	577	246
**PAXIP1**	PAAD	Others	194	339	42	575	247
**PHF7**	PAAD	Others	182	226	166	574	248
**FKBP2**	PAAD	Others	103	260	207	570	249
**SMYD5**	PAAD	HM_w	299	69	198	566	250
**SUZ12**	PAAD	ChRC	25	293	248	566	251
**RNF20**	PAAD	Others	312	211	42	565	252

**DPF3**	PAAD	HA_r	253	69	241	563	253
**BRD9**	PAAD	HA_r	112	69	380	561	254
**SIRT3**	PAAD	HA_e	32	202	326	560	255
**NAP1L3**	PAAD	Others	202	69	283	554	256
**SFMBT1**	PAAD	HM_r	158	69	327	554	257
**ERCC5**	PAAD	Others	248	263	42	553	258
**KDM7A**	PAAD	HM_e	73	69	407	549	259
**PRDM8**	PAAD	HM_w	175	218	154	547	260
**FKBP1A**	PAAD	Others	243	261	42	546	261
**CHAF1A**	PAAD	ChRC	265	69	210	544	262
**SMARCC1**	PAAD	Helicases	302	198	42	542	263
**USP51**	PAAD	Others	127	69	346	542	264
**MBD3**	PAAD	DM_r	212	69	258	539	265
**BRWD1**	PAAD	HA_r	423	69	42	534	266
**HIRA**	PAAD	Others	231	69	234	534	267
**PHF21A**	PAAD	HM_r	184	308	42	534	268
**DNMT3A**	PAAD	DM_w	422	69	42	533	269
**HDAC7**	PAAD	HA_e	236	255	42	533	270
**SIN3A**	PAAD	ChRC	156	335	42	533	271
**SETD4**	PAAD	HM_w	161	69	301	531	272
**PRDM1**	PAAD	HM_w	180	69	281	530	273
**KMT2B**	PAAD	HM_w	70	417	42	529	274
**UBE2A**	PAAD	Others	18	187	323	528	275
**PRDM15**	PAAD	HM_w	177	69	280	526	276
**TDRD6**	PAAD	Others	135	191	197	523	277
**HSPBAP1**	PAAD	Others	227	252	42	521	278
**ING2**	PAAD	HM_r	225	250	42	517	279
**PRMT7**	PAAD	HM_w	168	69	279	516	280
**DNMT3L**	PAAD	DM_w	256	69	190	515	281
**PHIP**	PAAD	HA_r	404	69	42	515	282
**SETDB2**	PAAD	HM_w	33	205	277	515	283
**SRCAP**	PAAD	Others	402	69	42	513	284
**TET2**	PAAD	DM_e	401	69	42	512	285
**ATAD2B**	PAAD	HA_r	400	69	42	511	286
**JMJD1C**	PAAD	HM_e	222	247	42	511	287

**ATRX**	PAAD	Helicases	399	69	42	510	288
**ASXL3**	PAAD	Others	282	69	157	508	289
**SIN3B**	PAAD	ChRC	155	69	276	500	290
**JADE1**	PAAD	Others	85	69	345	499	291
**PHF20**	PAAD	HM_r	54	69	376	499	292
**JADE2**	PAAD	Others	84	69	344	497	293
**ZCWPW2**	PAAD	HM_r	12	290	195	497	294
**PADI6**	PAAD	Others	197	69	229	495	295
**SMARCB1**	PAAD	Helicases	151	69	275	495	296
**PHF3**	PAAD	Others	382	69	42	493	297
**KANSL1**	PAAD	HA_w	80	370	42	492	298
**WDR5**	PAAD	ChRC	126	69	295	490	299
**KDM8**	PAAD	HM_e	72	242	175	489	300
**SETD1A**	PAAD	HM_w	376	69	42	487	301
**PHC2**	PAAD	ChRC	188	69	228	485	302
**SP100**	PAAD	HA_r	142	299	42	483	303
**SP110**	PAAD	HA_r	141	298	42	481	304
**HDAC3**	PAAD	HA_e	91	69	316	476	305
**KMT2E**	PAAD	HM_w	67	367	42	476	306
**SUPT16H**	PAAD	Others	139	294	42	475	307
**BRPF3**	PAAD	HA_r	363	69	42	474	308
**CDYL**	PAAD	HM_r	361	69	42	472	309
**EED**	PAAD	ChRC	353	69	42	464	310
**EP400**	PAAD	HA_w	352	69	42	463	311
**PCGF1**	PAAD	Others	193	69	201	463	312
**UBE2E1**	PAAD	Others	130	291	42	463	313
**PHF11**	PAAD	Others	186	230	42	458	314
**SSRP1**	PAAD	Others	140	69	249	458	315
**PHF13**	PAAD	Others	56	359	42	457	316
**PADI2**	PAAD	Others	199	69	188	456	317
**PHF19**	PAAD	HM_r	185	228	42	455	318
**KDM2B**	PAAD	HM_e	339	69	42	450	319
**SIRT4**	PAAD	HA_e	31	69	349	449	320
**PHRF1**	PAAD	Others	181	225	42	448	321
**HDGFL1**	PAAD	Others	90	69	286	445	322

**L3MBTL1**	PAAD	HM_r	66	69	310	445	323
**BAP1**	PAAD	Others	115	284	42	441	324
**ORC1**	PAAD	Others	59	69	308	436	325
**SCML2**	PAAD	HM_r	38	69	329	436	326
**GTF2B**	PAAD	Others	99	69	265	433	327
**HDAC1**	PAAD	HA_e	95	69	264	428	328
**PRDM2**	PAAD	HM_w	317	69	42	428	329
**PRDM4**	PAAD	HM_w	316	69	42	427	330
**JADE3**	PAAD	Others	83	69	269	421	331
**RTF1**	PAAD	Others	309	69	42	420	332
**DPY30**	PAAD	Others	105	69	240	414	333
**PRKAA2**	PAAD	Others	172	69	173	414	334
**FBXL19**	PAAD	Others	104	69	238	411	335
**ZGPAT**	PAAD	Others	11	184	216	411	336
**GADD45A**	PAAD	Others	102	69	237	408	337
**PRDM9**	PAAD	HM_w	174	69	164	407	338
**TDG**	PAAD	ChRC	295	69	42	406	339
**TDRD7**	PAAD	Others	20	190	196	406	340
**TET3**	PAAD	DM_e	291	69	42	402	341
**PHF6**	PAAD	HM_r	52	307	42	401	342
**SIRT1**	PAAD	HA_e	154	203	42	399	343
**UHRF1**	PAAD	DM_r	288	69	42	399	344
**HDAC11**	PAAD	HA_e	93	69	236	398	345
**HAT1**	PAAD	HA_w	96	256	42	394	346
**BAZ1A**	PAAD	HA_r	279	69	42	390	347
**SMARCD1**	PAAD	Helicases	149	197	42	388	348
**TDRD3**	PAAD	HM_r	21	69	297	387	349
**SCMH1**	PAAD	Others	39	304	42	385	350
**TDRD9**	PAAD	Others	134	69	182	385	351
**BRD3**	PAAD	HA_r	273	69	42	384	352
**BRD4**	PAAD	HA_r	272	69	42	383	353
**SETD7**	PAAD	HM_w	34	69	278	381	354
**KDM4E**	PAAD	HM_e	75	69	233	377	355
**CHD1**	PAAD	Helicases	264	69	42	375	356
**SMYD1**	PAAD	HM_w	146	69	159	374	357

**CTCF**	PAAD	Others	258	69	42	369	358
**DPF2**	PAAD	Others	254	69	42	365	359
**MSL3**	PAAD	HA_w	63	69	231	363	360
**MUM1**	PAAD	Others	60	69	230	359	361
**TRIM66**	PAAD	HA_r	19	69	270	358	362
**G2E3**	PAAD	Others	240	69	42	351	363
**KMT2D**	PAAD	HM_w	68	241	42	351	364
**GTF2F1**	PAAD	Others	239	69	42	350	365
**GTF2H1**	PAAD	Others	238	69	42	349	366
**HIF1AN**	PAAD	Others	232	69	42	343	367
**PRMT2**	PAAD	HM_w	47	69	223	339	368
**GADD45B**	PAAD	Others	101	69	168	338	369
**IWS1**	PAAD	Others	223	69	42	334	370
**KAT5**	PAAD	HA_w	219	69	42	330	371
**KDM4A**	PAAD	HM_e	218	69	42	329	372
**KAT8**	PAAD	HA_w	76	69	176	321	373
**MBD5**	PAAD	DM_r	210	69	42	321	374
**MLLT10**	PAAD	HM_w	209	69	42	320	375
**MSH6**	PAAD	HM_r	206	69	42	317	376
**POLE3**	PAAD	ChRC	50	224	42	316	377
**MTF2**	PAAD	HM_r	204	69	42	315	378
**SUV39H1**	PAAD	HM_w	26	69	220	315	379
**NAP1L1**	PAAD	Others	203	69	42	314	380
**PRDM6**	PAAD	HM_w	49	220	42	311	381
**PADI4**	PAAD	Others	198	69	42	309	382
**PCGF6**	PAAD	Others	191	69	42	302	383
**PCMT1**	PAAD	Others	190	69	42	301	384
**PRMT6**	PAAD	HM_w	169	69	42	280	385
**SIRT5**	PAAD	HA_e	30	69	171	270	386
**SCML4**	PAAD	Others	37	69	162	268	387
**SHPRH**	PAAD	Others	157	69	42	268	388
**SMNDC1**	PAAD	Others	27	196	42	265	389
**SMARCA5**	PAAD	Helicases	152	69	42	263	390
**SIRT6**	PAAD	HA_e	29	69	160	258	391
**TP53BP1**	PAAD	Others	132	69	42	243	392

**USP22**	PAAD	Others	15	185	42	242	393
**UBR7**	PAAD	Others	129	69	42	240	394
**UHRF2**	PAAD	DM_r	128	69	42	239	395
**WDR82**	PAAD	Others	125	69	42	236	396
**YY1**	PAAD	ChRC	124	69	42	235	397
**AFF4**	PAAD	Others	120	69	42	231	398
**HDAC2**	PAAD	HA_e	92	69	42	203	399
**INTS12**	PAAD	Others	86	69	42	197	400
**KDM5D**	PAAD	HM_e	74	69	42	185	401
**KMT2A**	PAAD	HM_w	71	69	42	182	402
**KMT2C**	PAAD	HM_w	69	69	42	180	403
**L3MBTL3**	PAAD	Others	65	69	42	176	404
**MTA3**	PAAD	ChRC	61	69	42	172	405
**PHF23**	PAAD	HM_r	53	69	42	164	406
**PHF8**	PAAD	Others	51	69	42	162	407
**PSIP1**	PAAD	HM_r	46	69	42	157	408
**RBBP4**	PAAD	ChRC	44	69	42	155	409
**RNF217**	PAAD	Others	41	69	42	152	410
**RSF1**	PAAD	ChRC	40	69	42	151	411
**SETD1B**	PAAD	HM_w	36	69	42	147	412
**SETD3**	PAAD	HM_w	35	69	42	146	413
**UBE2B**	PAAD	Others	17	69	42	128	414
**USP27X**	PAAD	Others	14	69	42	125	415
**UTY**	PAAD	HM_e	13	69	42	124	416
**GATAD2B**	PCPG	HM_r	413	425	391	1229	1
**EP400**	PCPG	HA_w	425	409	392	1226	2
**KDM4B**	PCPG	HM_e	365	416	380	1161	3
**SETDB1**	PCPG	HM_w	332	421	400	1153	4
**BAZ2A**	PCPG	HA_r	394	366	383	1143	5
**PRDM2**	PCPG	HM_w	343	376	417	1136	6
**ASH1L**	PCPG	HM_w	399	417	310	1126	7
**LBR**	PCPG	Others	358	406	345	1109	8
**PARP1**	PCPG	Others	352	404	323	1079	9
**ELP4**	PCPG	HA_w	380	290	402	1072	10
**CARM1**	PCPG	HM_w	389	307	368	1064	11

**KDM5B**	PCPG	HM_e	363	423	272	1058	12
**GTF2B**	PCPG	Others	231	392	426	1049	13
**KDM5C**	PCPG	HM_e	362	341	346	1049	14
**SETD2**	PCPG	HM_w	423	221	404	1048	15
**HDGF**	PCPG	Others	215	424	408	1047	16
**BAP1**	PCPG	Others	297	316	425	1038	17
**HDAC1**	PCPG	HA_e	224	390	423	1037	18
**DPF2**	PCPG	Others	256	354	415	1025	19
**CHRAC1**	PCPG	ChRC	267	357	397	1021	20
**CBX5**	PCPG	HM_r	281	360	379	1020	21
**BPTF**	PCPG	HA_r	422	365	228	1015	22
**KDM4A**	PCPG	HM_e	184	407	424	1015	23
**CHD5**	PCPG	Helicases	386	398	225	1009	24
**AKAP1**	PCPG	Others	308	367	332	1007	25
**BRPF1**	PCPG	HA_r	287	310	409	1006	26
**CHAF1A**	PCPG	ChRC	275	399	330	1004	27
**UBR7**	PCPG	Others	317	328	355	1000	28
**CBX3**	PCPG	HM_r	282	362	352	996	29
**KDM6B**	PCPG	HM_e	410	260	326	996	30
**GTF2F1**	PCPG	Others	230	391	374	995	31
**PWWP2B**	PCPG	Others	340	336	318	994	32
**FXR2**	PCPG	Others	237	352	399	988	33
**H3F3A**	PCPG	Others	226	408	349	983	34
**NAP1L2**	PCPG	Others	406	252	325	983	35
**CBX1**	PCPG	HM_r	284	364	331	979	36
**PADI6**	PCPG	Others	419	380	180	979	37
**CBX4**	PCPG	HM_r	388	361	227	976	38
**AIRE**	PCPG	HM_r	309	325	333	967	39
**BAZ1B**	PCPG	HA_r	295	315	353	963	40
**MTF2**	PCPG	HM_r	157	387	416	960	41
**HDAC7**	PCPG	HA_e	218	351	389	958	42
**DAXX**	PCPG	ChRC	263	296	393	952	43
**ARID1A**	PCPG	ChRC	307	411	231	949	44
**ATAD2**	PCPG	HA_r	398	321	230	949	45
**PADI2**	PCPG	Others	353	383	212	948	46

**KAT5**	PCPG	HA_w	194	344	406	944	47
**KDM1A**	PCPG	HM_e	190	389	363	942	48
**HELLS**	PCPG	Helicases	213	350	373	936	49
**CSTL1**	PCPG	Others	384	298	253	935	50
**DNMT1**	PCPG	DM_w	261	295	377	933	51
**CXXC1**	PCPG	Others	264	297	367	928	52
**ATAT1**	PCPG	Others	299	319	309	927	53
**KAT2A**	PCPG	HA_w	366	345	216	927	54
**ORC1**	PCPG	Others	355	385	181	921	55
**EHMT2**	PCPG	HM_w	252	292	376	920	56
**BRDT**	PCPG	HA_r	391	400	128	919	57
**JARID2**	PCPG	ChRC	370	268	273	911	58
**PADI3**	PCPG	Others	404	382	116	902	59
**CBX2**	PCPG	ChRC	283	363	255	901	60
**SMARCC2**	PCPG	Helicases	327	208	358	893	61
**ING1**	PCPG	HM_r	374	269	248	891	62
**JMJD6**	PCPG	HM_e	196	347	347	890	63
**HIF1AN**	PCPG	Others	212	349	328	889	64
**CDYL2**	PCPG	HM_r	387	305	195	887	65
**ATR**	PCPG	Others	395	69	418	882	66
**CHAF1B**	PCPG	ChRC	274	304	304	882	67
**CHD7**	PCPG	Helicases	270	358	254	882	68
**MUM1**	PCPG	Others	156	386	340	882	69
**KMT2C**	PCPG	HM_w	424	69	388	881	70
**CHD3**	PCPG	Helicases	414	303	162	879	71
**HDAC6**	PCPG	HA_e	219	278	382	879	72
**TCF19**	PCPG	Others	321	199	357	877	73
**GADD45B**	PCPG	Others	234	393	249	876	74
**RBBP4**	PCPG	ChRC	96	374	405	875	75
**CHD9**	PCPG	Helicases	268	300	302	870	76
**DNMT3A**	PCPG	DM_w	382	294	194	870	77
**DPF1**	PCPG	ChRC	257	355	251	863	78
**PCGF6**	PCPG	Others	142	379	337	858	79
**HDAC2**	PCPG	HA_e	377	69	411	857	80
**PADI1**	PCPG	Others	354	384	117	855	81

**SMARCA4**	PCPG	Helicases	329	210	315	854	82
**SMYD3**	PCPG	HM_w	56	413	384	853	83
**BRPF3**	PCPG	HA_r	286	309	256	851	84
**HIRA**	PCPG	Others	211	276	364	851	85
**PRKAA2**	PCPG	Others	341	375	133	849	86
**TRIM33**	PCPG	HA_r	28	418	403	849	87
**IWS1**	PCPG	Others	372	69	407	848	88
**PRDM16**	PCPG	HM_w	344	377	118	839	89
**HAT1**	PCPG	HA_w	378	69	390	837	90
**BOP 1**	PCPG	Others	293	314	229	836	91
**NAP1L1**	PCPG	Others	155	339	339	833	92
**PHC3**	PCPG	ChRC	350	69	412	831	93
**DPF3**	PCPG	HA_r	255	353	222	830	94
**PHRF1**	PCPG	Others	346	69	413	828	95
**RPS6KA5**	PCPG	Others	337	224	267	828	96
**SIRT7**	PCPG	HA_e	68	370	386	824	97
**ASXL2**	PCPG	Others	302	322	197	821	98
**JADE1**	PCPG	Others	371	69	381	821	99
**SMARCB1**	PCPG	Helicases	328	69	419	816	100
**KAT6A**	PCPG	HA_w	411	265	135	811	101
**JMJD8**	PCPG	HM_e	368	69	372	809	102
**H2AFZ**	PCPG	Others	227	282	298	807	103
**PRDM9**	PCPG	HM_w	342	69	396	807	104
**SUV39H1**	PCPG	HM_w	324	201	281	806	105
**DNMT3L**	PCPG	DM_w	259	293	252	804	106
**CBX8**	PCPG	ChRC	278	359	164	801	107
**BRD3**	PCPG	HA_r	291	311	196	798	108
**ARID2**	PCPG	ChRC	417	69	311	797	109
**DMAP1**	PCPG	Others	262	397	138	797	110
**FBXO44**	PCPG	Others	243	395	159	797	111
**RBBP5**	PCPG	ChRC	339	414	42	795	112
**ACTL6A**	PCPG	ChRC	314	69	410	793	113
**DOT1L**	PCPG	HM_w	258	396	137	791	114
**JMJD1C**	PCPG	HM_e	369	267	154	790	115
**CLOCK**	PCPG	HA_w	266	299	224	789	116

**CHD2**	PCPG	Helicases	415	69	303	787	117
**TDRKH**	PCPG	Others	34	419	334	787	118
**SETDB2**	PCPG	HM_w	331	217	237	785	119
**CTCF**	PCPG	Others	383	356	42	781	120
**AURKB**	PCPG	Others	298	317	165	780	121
**AFF1**	PCPG	Others	311	326	140	777	122
**KDM2B**	PCPG	HM_e	187	263	327	777	123
**PPARGC1A**	PCPG	Others	345	69	362	776	124
**FKBP2**	PCPG	Others	240	285	250	775	125
**ATF7IP**	PCPG	Others	397	69	308	774	126
**HDAC5**	PCPG	HA_e	220	279	275	774	127
**KDM6A**	PCPG	HM_e	361	261	151	773	128
**KMT2A**	PCPG	HM_w	409	69	295	773	129
**PHF1**	PCPG	HM_r	138	246	387	771	130
**MARCH5**	PCPG	Others	357	69	344	770	131
**MBTD1**	PCPG	Others	167	388	214	769	132
**PHF23**	PCPG	HM_r	126	239	401	766	133
**HDGFL1**	PCPG	Others	214	277	274	765	134
**BRWD3**	PCPG	HA_r	390	69	305	764	135
**MBD3**	PCPG	DM_r	169	405	184	758	136
**ARID4B**	PCPG	ChRC	305	410	42	757	137
**ATM**	PCPG	Others	396	318	42	756	138
**MTA1**	PCPG	ChRC	160	254	342	756	139
**GADD45A**	PCPG	Others	235	394	126	755	140
**KANSL1**	PCPG	HA_w	367	346	42	755	141
**MTA2**	PCPG	ChRC	159	253	341	753	142
**MBD2**	PCPG	DM_r	170	340	242	752	143
**BRD1**	PCPG	HA_r	392	313	42	747	144
**CDYL**	PCPG	HM_r	277	306	163	746	145
**MECP2**	PCPG	DM_r	407	69	270	746	146
**ARID1B**	PCPG	ChRC	418	69	257	744	147
**FKBP5**	PCPG	Others	239	284	221	744	148
**RNF17**	PCPG	Others	401	227	115	743	149
**EED**	PCPG	ChRC	253	69	420	742	150
**NCOA1**	PCPG	HA_w	153	251	338	742	151

**RNF2**	PCPG	ChRC	93	373	268	734	152
**CHD6**	PCPG	Helicases	385	302	42	729	153
**AFF4**	PCPG	Others	400	69	258	727	154
**YY1**	PCPG	ChRC	316	368	42	726	155
**ASXL3**	PCPG	Others	301	69	354	724	156
**RNF20**	PCPG	Others	338	69	317	724	157
**L3MBTL1**	PCPG	HM_r	359	69	294	722	158
**CHD1L**	PCPG	Helicases	272	69	378	719	159
**SCML2**	PCPG	HM_r	334	69	316	719	160
**MBD5**	PCPG	DM_r	408	69	241	718	161
**MSH6**	PCPG	HM_r	356	69	293	718	162
**DIDO1**	PCPG	Others	421	69	223	713	163
**RSF1**	PCPG	ChRC	336	335	42	713	164
**SMARCD2**	PCPG	Helicases	62	334	314	710	165
**SMYD2**	PCPG	HM_w	57	369	284	710	166
**HDAC10**	PCPG	HA_e	223	281	192	696	167
**DNMT3B**	PCPG	DM_w	260	69	366	695	168
**HR**	PCPG	HM_e	205	271	218	694	169
**MECOM**	PCPG	Others	166	257	271	694	170
**HDAC11**	PCPG	HA_e	222	280	191	693	171
**TDRD10**	PCPG	Others	39	420	233	692	172
**FBXO17**	PCPG	Others	244	287	160	691	173
**KDM4C**	PCPG	HM_e	183	262	245	690	174
**FBXW9**	PCPG	Others	242	286	158	686	175
**PRDM15**	PCPG	HM_w	114	235	336	685	176
**RING1**	PCPG	Others	94	228	361	683	177
**FMR1**	PCPG	Others	238	69	375	682	178
**AEBP2**	PCPG	HM_w	312	327	42	681	179
**FBXL19**	PCPG	Others	245	69	365	679	180
**KDM5A**	PCPG	HM_e	364	69	243	676	181
**PHC2**	PCPG	ChRC	139	403	134	676	182
**SIRT6**	PCPG	HA_e	69	371	236	676	183
**ARID4A**	PCPG	ChRC	306	324	42	672	184
**BAZ1A**	PCPG	HA_r	296	69	307	672	185
**ASH2L**	PCPG	HM_w	304	323	42	669	186

**GTF2H1**	PCPG	Others	229	283	157	669	187
**JADE2**	PCPG	Others	412	69	188	669	188
**HNF1A**	PCPG	ChRC	206	272	190	668	189
**PHF14**	PCPG	Others	133	243	292	668	190
**TDRD7**	PCPG	Others	320	69	279	668	191
**SETD1B**	PCPG	HM_w	85	222	360	667	192
**HDAC4**	PCPG	HA_e	376	69	220	665	193
**BRD8**	PCPG	HA_r	289	69	306	664	194
**ATAD2B**	PCPG	HA_r	300	320	42	662	195
**FKBP1A**	PCPG	Others	241	69	351	661	196
**PRDM14**	PCPG	HM_w	115	338	207	660	197
**TDRD9**	PCPG	Others	319	196	144	659	198
**L3MBTL2**	PCPG	Others	174	69	414	657	199
**NCOA3**	PCPG	HA_w	405	69	183	657	200
**SETD6**	PCPG	HM_w	81	372	202	655	201
**GATAD2A**	PCPG	HM_r	233	69	350	652	202
**SSRP1**	PCPG	Others	48	204	398	650	203
**BRD2**	PCPG	HA_r	292	312	42	646	204
**STK31**	PCPG	Others	47	203	395	645	205
**EHMT1**	PCPG	HM_w	381	69	193	643	206
**ACTL6B**	PCPG	ChRC	313	69	259	641	207
**MLLT6**	PCPG	HM_w	420	69	149	638	208
**BRD4**	PCPG	HA_r	290	69	277	636	209
**TDRD5**	PCPG	Others	36	401	199	636	210
**BRWD1**	PCPG	HA_r	285	308	42	635	211
**HDAC9**	PCPG	HA_e	216	69	348	633	212
**KAT8**	PCPG	HA_w	191	69	371	631	213
**PAXIP1**	PCPG	Others	351	69	211	631	214
**PHF7**	PCPG	Others	348	238	42	628	215
**TET2**	PCPG	DM_e	32	329	263	624	216
**HCFC1**	PCPG	Others	225	69	329	623	217
**KDM7A**	PCPG	HM_e	179	69	370	618	218
**EZH2**	PCPG	HM_w	246	69	301	616	219
**KMT2D**	PCPG	HM_w	360	69	186	615	220
**PHF5A**	PCPG	Others	124	69	422	615	221

**KAT2B**	PCPG	HA_w	195	266	153	614	222
**NCOR2**	PCPG	Others	151	250	213	614	223
**CHD8**	PCPG	Helicases	269	301	42	612	224
**UBE2E1**	PCPG	Others	24	194	394	612	225
**PRDM8**	PCPG	HM_w	109	233	269	611	226
**CREBBP**	PCPG	HA_w	265	69	276	610	227
**HIST1H1B**	PCPG	Others	210	275	121	606	228
**G2E3**	PCPG	Others	236	69	300	605	229
**KDM1B**	PCPG	HM_e	189	264	152	605	230
**ZCWPW2**	PCPG	HM_r	13	426	166	605	231
**PRMT1**	PCPG	HM_w	403	69	132	604	232
**RPA3**	PCPG	Others	90	226	288	604	233
**HIST1H1C**	PCPG	Others	209	274	120	603	234
**RAI1**	PCPG	Others	402	69	131	602	235
**GLYR1**	PCPG	HM_r	232	69	299	600	236
**HIST1H3B**	PCPG	Others	208	273	119	600	237
**IDH1**	PCPG	DM_e	375	69	156	600	238
**SATB1**	PCPG	Others	335	223	42	600	239
**INTS12**	PCPG	Others	198	348	42	588	240
**ELP3**	PCPG	HA_w	251	291	42	584	241
**PCGF1**	PCPG	Others	145	69	369	583	242
**L3MBTL4**	PCPG	Others	172	259	150	581	243
**NSD1**	PCPG	HM_w	150	249	182	581	244
**ERCC5**	PCPG	Others	248	289	42	579	245
**EZH1**	PCPG	HM_w	247	288	42	577	246
**KAT7**	PCPG	HA_w	192	343	42	577	247
**PHF21A**	PCPG	HM_r	128	240	209	577	248
**MORF4L1**	PCPG	HM_r	163	69	343	575	249
**SMYD4**	PCPG	HM_w	326	207	42	575	250
**KDM2A**	PCPG	HM_e	188	342	42	572	251
**PADI4**	PCPG	Others	149	381	42	572	252
**SND1**	PCPG	HM_r	54	205	313	572	253
**ASXL1**	PCPG	Others	303	69	198	570	254
**IDH2**	PCPG	DM_e	203	69	297	569	255
**SUPT16H**	PCPG	Others	325	202	42	569	256

**CHD4**	PCPG	Helicases	271	69	226	566	257
**PYGO2**	PCPG	HM_r	98	422	42	562	258
**PRMT6**	PCPG	HM_w	103	415	42	560	259
**TAF1L**	PCPG	HA_r	322	69	169	560	260
**PHF11**	PCPG	Others	136	245	178	559	261
**SMARCC1**	PCPG	Helicases	64	209	286	559	262
**SIN3A**	PCPG	ChRC	76	214	265	555	263
**PHF13**	PCPG	Others	134	378	42	554	264
**TP53BP1**	PCPG	Others	318	69	167	554	265
**PHF19**	PCPG	HM_r	132	242	177	551	266
**ZMYND8**	PCPG	HA_r	315	188	42	545	267
**TDRD6**	PCPG	Others	35	197	312	544	268
**KDM8**	PCPG	HM_e	178	69	296	543	269
**PAF1**	PCPG	Others	148	69	324	541	270
**ATRX**	PCPG	Helicases	426	69	42	537	271
**PCMT1**	PCPG	Others	141	69	322	532	272
**SCMH1**	PCPG	Others	87	402	42	531	273
**PHC1**	PCPG	ChRC	140	69	321	530	274
**BRD7**	PCPG	HA_r	416	69	42	527	275
**SMYD5**	PCPG	HM_w	55	206	264	525	276
**RPH3A**	PCPG	Others	89	225	205	519	277
**SMARCD1**	PCPG	Helicases	63	69	385	517	278
**HSPBAP1**	PCPG	Others	204	270	42	516	279
**INO80**	PCPG	Helicases	199	69	247	515	280
**TDRD1**	PCPG	Others	40	330	145	515	281
**TDRD3**	PCPG	HM_r	37	198	280	515	282
**USP22**	PCPG	Others	20	69	421	510	283
**PRDM1**	PCPG	HM_w	120	69	320	509	284
**AICDA**	PCPG	DM_e	310	69	129	508	285
**KAT6B**	PCPG	HA_w	193	69	246	508	286
**PRDM10**	PCPG	HM_w	119	69	319	507	287
**HDAC8**	PCPG	HA_e	217	69	219	505	288
**PRMT7**	PCPG	HM_w	102	229	174	505	289
**PRMT8**	PCPG	HM_w	101	69	335	505	290
**SETD4**	PCPG	HM_w	83	219	203	505	291

**BAZ2B**	PCPG	HA_r	393	69	42	504	292
**SIN3B**	PCPG	ChRC	75	69	359	503	293
**KDM4D**	PCPG	HM_e	182	69	244	495	294
**EPC1**	PCPG	Others	379	69	42	490	295
**PRKAA1**	PCPG	Others	108	337	42	487	296
**PRKCD**	PCPG	Others	107	232	148	487	297
**ING5**	PCPG	HM_r	200	69	217	486	298
**DPY30**	PCPG	Others	254	69	161	484	299
**ING3**	PCPG	HM_r	373	69	42	484	300
**SIRT5**	PCPG	HA_e	70	212	201	483	301
**POLE3**	PCPG	ChRC	122	69	291	482	302
**CHD1**	PCPG	Helicases	273	69	139	481	303
**UHRF1**	PCPG	DM_r	22	412	42	476	304
**PRDM13**	PCPG	HM_w	116	69	290	475	305
**CECR2**	PCPG	HA_r	276	69	127	472	306
**CBX6**	PCPG	HM_r	280	69	122	471	307
**MBD1**	PCPG	DM_r	171	258	42	471	308
**USP51**	PCPG	Others	18	191	262	471	309
**KDM3A**	PCPG	HM_e	186	69	215	470	310
**MSL3**	PCPG	HA_w	161	69	240	470	311
**PRDM7**	PCPG	HM_w	110	234	124	468	312
**MEN1**	PCPG	ChRC	165	256	42	463	313
**NAP1L3**	PCPG	Others	154	69	239	462	314
**ZGPAT**	PCPG	Others	12	189	260	461	315
**ING2**	PCPG	HM_r	202	69	189	460	316
**PHF6**	PCPG	HM_r	349	69	42	460	317
**MPHOSPH8**	PCPG	HM_r	162	255	42	459	318
**PHIP**	PCPG	HA_r	347	69	42	458	319
**SIRT4**	PCPG	HA_e	71	213	172	456	320
**RAG2**	PCPG	HM_r	97	69	289	455	321
**TRIM28**	PCPG	HA_r	29	69	356	454	322
**SMARCA2**	PCPG	Helicases	66	211	171	448	323
**SETD1A**	PCPG	HM_w	333	69	42	444	324
**SHPRH**	PCPG	Others	330	69	42	441	325
**KDM4E**	PCPG	HM_e	181	69	187	437	326

**PARP2**	PCPG	Others	147	248	42	437	327
**SETD7**	PCPG	HM_w	80	69	287	436	328
**PBRM1**	PCPG	HA_r	146	247	42	435	329
**SMNDC1**	PCPG	Others	59	333	42	434	330
**TAF1**	PCPG	HA_r	323	69	42	434	331
**GTF3C4**	PCPG	HA_w	228	69	136	433	332
**L3MBTL3**	PCPG	Others	173	69	185	427	333
**PCGF5**	PCPG	Others	143	69	210	422	334
**JADE3**	PCPG	Others	197	69	155	421	335
**PHF12**	PCPG	Others	135	244	42	421	336
**TCEA1**	PCPG	Others	43	332	42	417	337
**SMARCD3**	PCPG	Helicases	61	69	285	415	338
**TCF20**	PCPG	Others	42	331	42	415	339
**PHF20L1**	PCPG	HM_r	129	241	42	412	340
**SFMBT2**	PCPG	Others	77	69	266	412	341
**BMI1**	PCPG	ChRC	294	69	42	405	342
**PHF21B**	PCPG	HM_r	127	69	208	404	343
**SP110**	PCPG	HA_r	52	69	283	404	344
**PHF8**	PCPG	Others	123	237	42	402	345
**SP140L**	PCPG	HA_r	50	69	282	401	346
**BRD9**	PCPG	HA_r	288	69	42	399	347
**POLR2B**	PCPG	Others	121	236	42	399	348
**RNF217**	PCPG	Others	92	69	238	399	349
**PCGF2**	PCPG	Others	144	69	179	392	350
**CBX7**	PCPG	HM_r	279	69	42	390	351
**TAF3**	PCPG	HA_r	44	200	146	390	352
**PRMT2**	PCPG	HM_w	106	231	42	379	353
**PRMT3**	PCPG	HM_w	105	230	42	377	354
**PYGO1**	PCPG	HM_r	99	69	206	374	355
**TRIM66**	PCPG	HA_r	27	69	278	374	356
**SMARCA1**	PCPG	Helicases	67	69	235	371	357
**TRIM24**	PCPG	HA_r	30	195	142	367	358
**PRDM11**	PCPG	HM_w	118	69	176	363	359
**EP300**	PCPG	HA_w	250	69	42	361	360
**EPC2**	PCPG	Others	249	69	42	360	361

**SCML4**	PCPG	Others	86	69	204	359	362
**PRDM5**	PCPG	HM_w	112	69	175	356	363
**SETD3**	PCPG	HM_w	84	220	42	346	364
**TDG**	PCPG	ChRC	41	69	234	344	365
**ZCWPW1**	PCPG	HM_r	14	69	261	344	366
**SETD5**	PCPG	HM_w	82	218	42	342	367
**SETMAR**	PCPG	HM_w	79	216	42	337	368
**SFMBT1**	PCPG	HM_r	78	215	42	335	369
**TET1**	PCPG	DM_e	33	69	232	334	370
**HDAC3**	PCPG	HA_e	221	69	42	332	371
**SP140**	PCPG	HA_r	51	69	200	320	372
**HLTF**	PCPG	Others	207	69	42	318	373
**SIRT2**	PCPG	HA_e	73	69	173	315	374
**ING4**	PCPG	HM_r	201	69	42	312	375
**PRDM12**	PCPG	HM_w	117	69	125	311	376
**KDM3B**	PCPG	HM_e	185	69	42	296	377
**SP100**	PCPG	HA_r	53	69	170	292	378
**KDM5D**	PCPG	HM_e	180	69	42	291	379
**KIAA2026**	PCPG	Others	177	69	42	288	380
**SIRT3**	PCPG	HA_e	72	69	147	288	381
**KMT2B**	PCPG	HM_w	176	69	42	287	382
**RBBP7**	PCPG	ChRC	95	69	123	287	383
**KMT2E**	PCPG	HM_w	175	69	42	286	384
**MBD4**	PCPG	DM_r	168	69	42	279	385
**MLLT10**	PCPG	HM_w	164	69	42	275	386
**TDRD12**	PCPG	Others	38	69	168	275	387
**SIRT1**	PCPG	HA_e	74	69	130	273	388
**MTA3**	PCPG	ChRC	158	69	42	269	389
**NCOR1**	PCPG	ChRC	152	69	42	263	390
**UHRF2**	PCPG	DM_r	21	193	42	256	391
**USP27X**	PCPG	Others	19	192	42	253	392
**PHF10**	PCPG	Others	137	69	42	248	393
**WDR82**	PCPG	Others	15	190	42	247	394
**TET3**	PCPG	DM_e	31	69	143	243	395
**PHF2**	PCPG	Others	131	69	42	242	396

**PHF20**	PCPG	HM_r	130	69	42	241	397
**SMYD1**	PCPG	HM_w	58	69	114	241	398
**PHF3**	PCPG	Others	125	69	42	236	399
**UBE2A**	PCPG	Others	26	69	141	236	400
**PRDM4**	PCPG	HM_w	113	69	42	224	401
**PRDM6**	PCPG	HM_w	111	69	42	222	402
**PRMT5**	PCPG	HM_w	104	69	42	215	403
**PSIP1**	PCPG	HM_r	100	69	42	211	404
**RNF40**	PCPG	Others	91	69	42	202	405
**RTF1**	PCPG	Others	88	69	42	199	406
**SMARCA5**	PCPG	Helicases	65	69	42	176	407
**SMARCE1**	PCPG	Helicases	60	69	42	171	408
**SRCAP**	PCPG	Others	49	69	42	160	409
**SUV39H2**	PCPG	HM_w	46	69	42	157	410
**SUZ12**	PCPG	ChRC	45	69	42	156	411
**UBE2B**	PCPG	Others	25	69	42	136	412
**UBE2I**	PCPG	Others	23	69	42	134	413
**UTY**	PCPG	HM_e	17	69	42	128	414
**WDR5**	PCPG	ChRC	16	69	42	127	415
**ZMYND11**	PCPG	HA_r	11	69	42	122	416
**CHD1**	PRAD	Helicases	411	416	408	1235	1
**HDAC2**	PRAD	HA_e	385	421	417	1223	2
**IWS1**	PRAD	Others	383	398	420	1201	3
**FXR2**	PRAD	Others	342	410	424	1176	4
**ATAD2**	PRAD	HA_r	346	405	416	1167	5
**ARID4B**	PRAD	ChRC	393	374	364	1131	6
**ATR**	PRAD	Others	373	366	385	1124	7
**KAT6A**	PRAD	HA_w	339	412	370	1121	8
**MBD4**	PRAD	DM_r	306	385	415	1106	9
**PHF3**	PRAD	Others	379	335	387	1101	10
**PRDM14**	PRAD	HM_w	295	399	405	1099	11
**HLTF**	PRAD	Others	340	369	388	1097	12
**PHC3**	PRAD	ChRC	298	389	401	1088	13
**ACTL6A**	PRAD	ChRC	272	387	409	1068	14
**KDM2A**	PRAD	HM_e	310	359	397	1066	15

**ASH1L**	PRAD	HM_w	420	340	305	1065	16
**ATF7IP**	PRAD	Others	374	392	276	1042	17
**CHD7**	PRAD	Helicases	403	397	231	1031	18
**BRD3**	PRAD	HA_r	321	303	406	1030	19
**ASH2L**	PRAD	HM_w	188	415	425	1028	20
**ERCC5**	PRAD	Others	255	361	412	1028	21
**MBD1**	PRAD	DM_r	335	271	419	1025	22
**RNF20**	PRAD	Others	352	265	407	1024	23
**ELP3**	PRAD	HA_w	174	418	426	1018	24
**INO80**	PRAD	Helicases	364	348	302	1014	25
**CREBBP**	PRAD	HA_w	410	249	354	1013	26
**EP400**	PRAD	HA_w	426	299	287	1012	27
**HDAC5**	PRAD	HA_e	341	401	268	1010	28
**CHRAC1**	PRAD	ChRC	179	403	421	1003	29
**KMT2C**	PRAD	HM_w	425	324	244	993	30
**ATM**	PRAD	Others	423	380	175	978	31
**NCOA3**	PRAD	HA_w	408	210	356	974	32
**TDRKH**	PRAD	Others	278	314	378	970	33
**DIDO1**	PRAD	Others	416	192	361	969	34
**SETDB1**	PRAD	HM_w	286	284	396	966	35
**BAZ1B**	PRAD	HA_r	371	254	337	962	36
**KMT2A**	PRAD	HM_w	402	358	200	960	37
**ASXL1**	PRAD	Others	392	229	338	959	38
**ASXL2**	PRAD	Others	347	351	257	955	39
**MECOM**	PRAD	Others	401	384	169	954	40
**BAZ2B**	PRAD	HA_r	397	395	161	953	41
**KAT6B**	PRAD	HA_w	362	364	226	952	42
**CHD9**	PRAD	Helicases	368	377	206	951	43
**AKAP1**	PRAD	Others	375	305	259	939	44
**AURKB**	PRAD	Others	267	391	274	932	45
**ZCWPW1**	PRAD	HM_r	275	309	347	931	46
**TCEA1**	PRAD	Others	104	396	423	923	47
**KAT7**	PRAD	HA_w	242	272	403	917	48
**CHD1L**	PRAD	Helicases	318	222	374	914	49
**KAT5**	PRAD	HA_w	243	326	345	914	50

**LBR**	PRAD	Others	307	357	243	907	51
**HDGFL1**	PRAD	Others	246	339	320	905	52
**CHAF1A**	PRAD	ChRC	390	223	290	903	53
**PAXIP1**	PRAD	Others	300	208	394	902	54
**TDRD1**	PRAD	Others	279	279	340	898	55
**BRD7**	PRAD	HA_r	88	379	422	889	56
**PPARGC1A**	PRAD	Others	355	290	240	885	57
**BOP 1**	PRAD	Others	89	400	395	884	58
**PRKAA2**	PRAD	Others	353	334	194	881	59
**KDM6B**	PRAD	HM_e	337	409	133	879	60
**EHMT1**	PRAD	HM_w	175	300	399	874	61
**RPS6KA5**	PRAD	Others	290	286	297	873	62
**HDGF**	PRAD	Others	156	327	389	872	63
**PHF20L1**	PRAD	HM_r	46	408	418	872	64
**TCF20**	PRAD	Others	394	198	277	869	65
**GLYR1**	PRAD	HM_r	250	219	398	867	66
**PHF11**	PRAD	Others	48	426	393	867	67
**CHD3**	PRAD	Helicases	417	406	42	865	68
**TET3**	PRAD	DM_e	327	278	260	865	69
**DPF2**	PRAD	Others	176	329	353	858	70
**HDAC6**	PRAD	HA_e	312	188	358	858	71
**BPTF**	PRAD	HA_r	370	253	233	856	72
**PRDM15**	PRAD	HM_w	215	289	352	856	73
**HELLS**	PRAD	Helicases	155	386	312	853	74
**HIF1AN**	PRAD	Others	72	376	402	850	75
**BRD9**	PRAD	HA_r	266	195	384	845	76
**CBX4**	PRAD	HM_r	345	226	272	843	77
**CHD4**	PRAD	Helicases	405	251	185	841	78
**SSRP1**	PRAD	Others	329	132	380	841	79
**SND1**	PRAD	HM_r	106	316	410	832	80
**ARID1A**	PRAD	ChRC	412	230	189	831	81
**BRWD1**	PRAD	HA_r	369	417	42	828	82
**NCOR1**	PRAD	ChRC	419	269	136	824	83
**PRDM7**	PRAD	HM_w	294	411	119	824	84
**CXXC1**	PRAD	Others	259	274	289	822	85

**PRDM5**	PRAD	HM_w	214	383	220	817	86
**BRPF1**	PRAD	HA_r	396	378	42	816	87
**GTF3C4**	PRAD	HA_w	248	298	269	815	88
**EED**	PRAD	ChRC	257	173	383	813	89
**BAZ1A**	PRAD	HA_r	322	196	292	810	90
**HNF1A**	PRAD	ChRC	365	69	372	806	91
**PRDM2**	PRAD	HM_w	332	207	262	801	92
**TET2**	PRAD	DM_e	194	313	294	801	93
**PRDM12**	PRAD	HM_w	216	321	263	800	94
**IDH1**	PRAD	DM_e	384	187	228	799	95
**TAF3**	PRAD	HA_r	280	281	235	796	96
**KANSL1**	PRAD	HA_w	363	390	42	795	97
**DNMT3B**	PRAD	DM_w	388	69	336	793	98
**KMT2D**	PRAD	HM_w	424	169	199	792	99
**PHC1**	PRAD	ChRC	299	323	168	790	100
**PHIP**	PRAD	HA_r	333	413	42	788	101
**WDR5**	PRAD	ChRC	97	277	414	788	102
**PADI6**	PRAD	Others	380	183	222	785	103
**SCML2**	PRAD	HM_r	124	331	330	785	104
**TDRD7**	PRAD	Others	100	315	367	782	105
**TP53BP1**	PRAD	Others	398	342	42	782	106
**RNF17**	PRAD	Others	331	344	106	781	107
**GATAD2A**	PRAD	HM_r	252	146	382	780	108
**KIAA2026**	PRAD	Others	409	325	42	776	109
**POLE3**	PRAD	ChRC	43	322	411	776	110
**SMARCA1**	PRAD	Helicases	399	69	308	776	111
**KDM6A**	PRAD	HM_e	422	69	283	774	112
**PYGO2**	PRAD	HM_r	34	333	404	771	113
**DAXX**	PRAD	ChRC	316	149	304	769	114
**BRWD3**	PRAD	HA_r	319	174	273	766	115
**PRDM16**	PRAD	HM_w	354	69	343	766	116
**CLOCK**	PRAD	HA_w	260	250	255	765	117
**USP22**	PRAD	Others	276	311	177	764	118
**NSD1**	PRAD	HM_w	357	209	195	761	119
**KDM7A**	PRAD	HM_e	324	69	365	758	120

**HCFC1**	PRAD	Others	366	69	322	757	121
**SIRT5**	PRAD	HA_e	203	202	351	756	122
**MEN1**	PRAD	ChRC	305	69	381	755	123
**PBRM1**	PRAD	HA_r	334	241	179	754	124
**CBX3**	PRAD	HM_r	86	252	413	751	125
**PRMT3**	PRAD	HM_w	293	139	318	750	126
**DNMT3A**	PRAD	DM_w	367	69	313	749	127
**ARID2**	PRAD	ChRC	421	69	258	748	128
**DOT1L**	PRAD	HM_w	387	221	140	748	129
**KAT2A**	PRAD	HA_w	148	373	227	748	130
**SMARCA2**	PRAD	Helicases	351	353	42	746	131
**CECR2**	PRAD	HA_r	344	194	207	745	132
**JADE2**	PRAD	Others	348	69	326	743	133
**TRIM33**	PRAD	HA_r	376	177	190	743	134
**CBX8**	PRAD	ChRC	262	224	256	742	135
**SETD2**	PRAD	HM_w	414	285	42	741	136
**SMARCD2**	PRAD	Helicases	200	234	307	741	137
**CTCF**	PRAD	Others	317	381	42	740	138
**CBX1**	PRAD	HM_r	87	276	375	738	139
**GADD45A**	PRAD	Others	75	360	303	738	140
**SMYD2**	PRAD	HM_w	199	261	278	738	141
**BRD1**	PRAD	HA_r	391	304	42	737	142
**TET1**	PRAD	DM_e	350	69	315	734	143
**ZMYND8**	PRAD	HA_r	326	69	339	734	144
**EPC1**	PRAD	Others	256	247	230	733	145
**MBD3**	PRAD	DM_r	235	186	310	731	146
**RSF1**	PRAD	ChRC	208	204	317	729	147
**JADE3**	PRAD	Others	273	69	386	728	148
**BRDT**	PRAD	HA_r	265	350	111	726	149
**PCGF5**	PRAD	Others	51	393	282	726	150
**PYGO1**	PRAD	HM_r	128	266	332	726	151
**AIRE**	PRAD	HM_r	91	258	376	725	152
**PHF7**	PRAD	Others	133	237	355	725	153
**NAP1L1**	PRAD	Others	230	270	224	724	154
**TRIM28**	PRAD	HA_r	349	69	306	724	155

**AFF4**	PRAD	Others	92	306	325	723	156
**CARM1**	PRAD	HM_w	181	151	390	722	157
**CBX2**	PRAD	ChRC	263	227	232	722	158
**CDYL**	PRAD	HM_r	261	275	186	722	159
**BRD2**	PRAD	HA_r	183	175	363	721	160
**L3MBTL1**	PRAD	HM_r	309	213	198	720	161
**AICDA**	PRAD	DM_e	269	341	108	718	162
**ATRX**	PRAD	Helicases	372	69	275	716	163
**TDG**	PRAD	ChRC	103	280	327	710	164
**HDAC10**	PRAD	HA_e	160	297	252	709	165
**RBBP7**	PRAD	ChRC	33	332	342	707	166
**ARID1B**	PRAD	ChRC	407	257	42	706	167
**PARP1**	PRAD	Others	301	363	42	706	168
**UBE2B**	PRAD	Others	15	312	377	704	169
**KDM1A**	PRAD	HM_e	241	215	246	702	170
**SETD5**	PRAD	HM_w	287	372	42	701	171
**PHRF1**	PRAD	Others	297	69	333	699	172
**FKBP5**	PRAD	Others	167	246	285	698	173
**GTF2B**	PRAD	Others	165	349	184	698	174
**HDAC11**	PRAD	HA_e	159	217	321	697	175
**HR**	PRAD	HM_e	153	425	118	696	176
**MSL3**	PRAD	HA_w	57	292	344	693	177
**RPH3A**	PRAD	Others	291	287	114	692	178
**PRMT7**	PRAD	HM_w	131	388	167	686	179
**SCML4**	PRAD	Others	123	422	141	686	180
**AFF1**	PRAD	Others	323	69	293	685	181
**MBTD1**	PRAD	Others	58	293	334	685	182
**SMARCA4**	PRAD	Helicases	202	133	350	685	183
**ACTL6B**	PRAD	ChRC	271	308	105	684	184
**MBD5**	PRAD	DM_r	234	407	42	683	185
**PRDM8**	PRAD	HM_w	395	69	219	683	186
**ING4**	PRAD	HM_r	66	242	371	679	187
**PHF21B**	PRAD	HM_r	219	336	123	678	188
**RING1**	PRAD	Others	209	138	331	678	189
**GTF2F1**	PRAD	Others	249	69	359	677	190

**FBXO44**	PRAD	Others	169	220	286	675	191
**SETD1A**	PRAD	HM_w	288	69	316	673	192
**SUPT16H**	PRAD	Others	282	155	236	673	193
**UBE2E1**	PRAD	Others	14	259	400	673	194
**ASXL3**	PRAD	Others	268	256	146	670	195
**CDYL2**	PRAD	HM_r	82	414	174	670	196
**SIRT7**	PRAD	HA_e	111	263	295	669	197
**DPY30**	PRAD	Others	79	301	288	668	198
**G2E3**	PRAD	Others	253	69	346	668	199
**EZH1**	PRAD	HM_w	254	371	42	667	200
**PHF23**	PRAD	HM_r	218	404	42	664	201
**RAI1**	PRAD	Others	377	69	217	663	202
**SRCAP**	PRAD	Others	413	69	178	660	203
**JMJD6**	PRAD	HM_e	64	294	301	659	204
**JARID2**	PRAD	ChRC	150	171	335	656	205
**HDAC4**	PRAD	HA_e	158	245	251	654	206
**NAP1L2**	PRAD	Others	139	142	369	650	207
**SETMAR**	PRAD	HM_w	117	317	216	650	208
**TAF1L**	PRAD	HA_r	418	69	162	649	209
**PRDM10**	PRAD	HM_w	296	69	279	644	210
**SIRT6**	PRAD	HA_e	112	201	328	641	211
**DMAP1**	PRAD	Others	178	148	314	640	212
**PRKCD**	PRAD	Others	36	236	368	640	213
**DNMT1**	PRAD	DM_w	315	69	254	638	214
**RAG2**	PRAD	HM_r	211	319	107	637	215
**PHF2**	PRAD	Others	356	238	42	636	216
**JMJD1C**	PRAD	HM_e	382	69	181	632	217
**FBXW9**	PRAD	Others	168	191	270	629	218
**SMARCC1**	PRAD	Helicases	109	283	237	629	219
**UBE2I**	PRAD	Others	193	69	366	628	220
**HIST1H1C**	PRAD	Others	70	243	311	624	221
**CHD6**	PRAD	Helicases	404	69	150	623	222
**GATAD2B**	PRAD	HM_r	251	328	42	621	223
**H3F3A**	PRAD	Others	73	365	183	621	224
**ORC1**	PRAD	Others	138	355	128	621	225

**PWWP2B**	PRAD	Others	212	267	142	621	226
**AEBP2**	PRAD	HM_w	270	307	42	619	227
**NCOA1**	PRAD	HA_w	229	167	223	619	228
**EPC2**	PRAD	Others	172	402	42	616	229
**SMARCD1**	PRAD	Helicases	108	158	349	615	230
**EZH2**	PRAD	HM_w	171	69	373	613	231
**MLLT10**	PRAD	HM_w	358	212	42	612	232
**CHAF1B**	PRAD	ChRC	180	69	362	611	233
**TDRD6**	PRAD	Others	328	69	213	610	234
**PHF14**	PRAD	Others	221	69	319	609	235
**HDAC1**	PRAD	HA_e	161	218	229	608	236
**PHF1**	PRAD	HM_r	224	141	241	606	237
**H2AFZ**	PRAD	Others	163	189	253	605	238
**KDM2B**	PRAD	HM_e	361	69	172	602	239
**CBX5**	PRAD	HM_r	85	225	291	601	240
**HIST1H3B**	PRAD	Others	245	216	139	600	241
**FBXL19**	PRAD	Others	170	69	360	599	242
**CHD2**	PRAD	Helicases	406	150	42	598	243
**KDM5A**	PRAD	HM_e	359	69	170	598	244
**PHF20**	PRAD	HM_r	220	69	309	598	245
**SMYD5**	PRAD	HM_w	23	232	341	596	246
**PHF12**	PRAD	Others	135	180	280	595	247
**UHRF2**	PRAD	DM_r	191	362	42	595	248
**SFMBT1**	PRAD	HM_r	206	264	124	594	249
**PCGF1**	PRAD	Others	52	240	300	592	250
**PRDM1**	PRAD	HM_w	42	423	127	592	251
**RTF1**	PRAD	Others	207	343	42	592	252
**FKBP1A**	PRAD	Others	77	190	324	591	253
**PADI3**	PRAD	Others	303	166	120	589	254
**EHMT2**	PRAD	HM_w	314	69	205	588	255
**PRDM9**	PRAD	HM_w	415	69	104	588	256
**RNF40**	PRAD	Others	126	69	392	587	257
**UBR7**	PRAD	Others	192	352	42	586	258
**SETDB2**	PRAD	HM_w	118	424	42	584	259
**SMARCE1**	PRAD	Helicases	107	262	215	584	260

**MARCH5**	PRAD	Others	143	394	42	579	261
**SUV39H1**	PRAD	HM_w	22	178	379	579	262
**CHD8**	PRAD	Helicases	343	193	42	578	263
**KDM4B**	PRAD	HM_e	338	69	171	578	264
**SIRT3**	PRAD	HA_e	114	134	329	577	265
**EP300**	PRAD	HA_w	386	147	42	575	266
**PHC2**	PRAD	ChRC	225	69	281	575	267
**SHPRH**	PRAD	Others	330	203	42	575	268
**CHD5**	PRAD	Helicases	389	69	116	574	269
**HAT1**	PRAD	HA_w	162	370	42	574	270
**KDM5B**	PRAD	HM_e	238	69	266	573	271
**BAZ2A**	PRAD	HA_r	185	176	208	569	272
**PRDM13**	PRAD	HM_w	40	420	109	569	273
**SIN3B**	PRAD	ChRC	204	69	296	569	274
**TDRD10**	PRAD	Others	102	330	134	566	275
**MTA1**	PRAD	ChRC	231	69	265	565	276
**HDAC9**	PRAD	HA_e	311	69	182	562	277
**MSH6**	PRAD	HM_r	304	211	42	557	278
**KDM4C**	PRAD	HM_e	145	368	42	555	279
**KDM3A**	PRAD	HM_e	240	69	245	554	280
**GADD45B**	PRAD	Others	74	273	203	550	281
**PRDM11**	PRAD	HM_w	41	354	155	550	282
**RNF217**	PRAD	Others	31	382	135	548	283
**HIRA**	PRAD	Others	154	244	149	547	284
**DNMT3L**	PRAD	DM_w	177	248	121	546	285
**PADI1**	PRAD	Others	228	185	132	545	286
**SP140L**	PRAD	HA_r	283	69	192	544	287
**L3MBTL3**	PRAD	Others	237	168	138	543	288
**MLLT6**	PRAD	HM_w	232	69	242	543	289
**PHF19**	PRAD	HM_r	47	337	157	541	290
**HIST1H1B**	PRAD	Others	71	296	173	540	291
**PADI2**	PRAD	Others	227	184	125	536	292
**PRMT8**	PRAD	HM_w	130	288	117	535	293
**BRPF3**	PRAD	HA_r	264	228	42	534	294
**MBD2**	PRAD	DM_r	59	338	137	534	295

**MPHOSPH8**	PRAD	HM_r	141	347	42	530	296
**PRMT6**	PRAD	HM_w	132	179	218	529	297
**SETD6**	PRAD	HM_w	120	367	42	529	298
**ATAD2B**	PRAD	HA_r	187	153	187	527	299
**MECP2**	PRAD	DM_r	233	69	225	527	300
**HDAC8**	PRAD	HA_e	247	69	202	518	301
**SP110**	PRAD	HA_r	197	156	163	516	302
**PHF8**	PRAD	Others	217	140	156	513	303
**SATB1**	PRAD	Others	125	235	153	513	304
**STK31**	PRAD	Others	105	282	126	513	305
**PHF13**	PRAD	Others	222	69	221	512	306
**NCOR2**	PRAD	Others	400	69	42	511	307
**PADI4**	PRAD	Others	302	165	42	509	308
**PCGF2**	PRAD	Others	136	69	299	504	309
**PHF10**	PRAD	Others	223	239	42	504	310
**ZCWPW2**	PRAD	HM_r	95	197	210	502	311
**PRKAA1**	PRAD	Others	37	163	298	498	312
**TCF19**	PRAD	Others	195	69	234	498	313
**DPF3**	PRAD	HA_r	80	302	115	497	314
**PRMT5**	PRAD	HM_w	292	162	42	496	315
**ING2**	PRAD	HM_r	68	69	357	494	316
**KMT2E**	PRAD	HM_w	381	69	42	492	317
**PSIP1**	PRAD	HM_r	129	320	42	491	318
**JADE1**	PRAD	Others	274	69	147	490	319
**RPA3**	PRAD	Others	30	69	391	490	320
**POLR2B**	PRAD	Others	378	69	42	489	321
**INTS12**	PRAD	Others	151	295	42	488	322
**JMJD8**	PRAD	HM_e	149	69	267	485	323
**BAP1**	PRAD	Others	186	255	42	483	324
**SETD3**	PRAD	HM_w	122	318	42	482	325
**TDRD3**	PRAD	HM_r	18	419	42	479	326
**HDAC7**	PRAD	HA_e	157	69	250	476	327
**PAF1**	PRAD	Others	226	69	180	475	328
**ING1**	PRAD	HM_r	244	69	160	473	329
**MTA2**	PRAD	ChRC	140	69	264	473	330

**KDM3B**	PRAD	HM_e	360	69	42	471	331
**SUZ12**	PRAD	ChRC	20	260	191	471	332
**FKBP2**	PRAD	Others	76	69	323	468	333
**SCMH1**	PRAD	Others	289	137	42	468	334
**PARP2**	PRAD	Others	137	182	148	467	335
**PCGF6**	PRAD	Others	50	375	42	467	336
**L3MBTL4**	PRAD	Others	236	69	158	463	337
**WDR82**	PRAD	Others	190	231	42	463	338
**KDM1B**	PRAD	HM_e	146	170	143	459	339
**MTF2**	PRAD	HM_r	55	356	42	453	340
**YY1**	PRAD	ChRC	96	310	42	448	341
**KMT2B**	PRAD	HM_w	336	69	42	447	342
**ARID4A**	PRAD	ChRC	189	69	188	446	343
**SETD4**	PRAD	HM_w	121	159	166	446	344
**SIRT4**	PRAD	HA_e	113	69	261	443	345
**SMARCC2**	PRAD	Helicases	201	200	42	443	346
**SFMBT2**	PRAD	Others	205	69	164	438	347
**DPF1**	PRAD	ChRC	258	69	110	437	348
**KDM5D**	PRAD	HM_e	325	69	42	436	349
**PRMT1**	PRAD	HM_w	213	69	154	436	350
**SUV39H2**	PRAD	HM_w	21	199	214	434	351
**PHF21A**	PRAD	HM_r	45	346	42	433	352
**UBE2A**	PRAD	Others	16	69	348	433	353
**BRD4**	PRAD	HA_r	320	69	42	431	354
**RNF2**	PRAD	ChRC	32	160	239	431	355
**KDM4A**	PRAD	HM_e	239	145	42	426	356
**PRDM6**	PRAD	HM_w	38	345	42	425	357
**HDAC3**	PRAD	HA_e	313	69	42	424	358
**CBX7**	PRAD	HM_r	83	69	271	423	359
**SIRT2**	PRAD	HA_e	115	69	238	422	360
**SETD7**	PRAD	HM_w	119	136	165	420	361
**L3MBTL2**	PRAD	Others	308	69	42	419	362
**KDM4D**	PRAD	HM_e	62	69	284	415	363
**KDM4E**	PRAD	HM_e	144	69	201	414	364
**RBBP5**	PRAD	ChRC	210	161	42	413	365

**SIRT1**	PRAD	HA_e	285	69	42	396	366
**SP140**	PRAD	HA_r	196	69	131	396	367
**SMARCA5**	PRAD	Helicases	284	69	42	395	368
**TAF1**	PRAD	HA_r	281	69	42	392	369
**MTA3**	PRAD	ChRC	56	291	42	389	370
**UHRF1**	PRAD	DM_r	277	69	42	388	371
**IDH2**	PRAD	DM_e	69	69	249	387	372
**ING5**	PRAD	HM_r	65	69	248	382	373
**KAT8**	PRAD	HA_w	63	69	247	379	374
**TRIM24**	PRAD	HA_r	98	69	212	379	375
**BMI1**	PRAD	ChRC	184	152	42	378	376
**GTF2H1**	PRAD	Others	164	172	42	378	377
**KAT2B**	PRAD	HA_w	147	69	159	375	378
**RBBP4**	PRAD	ChRC	127	205	42	374	379
**ZGPAT**	PRAD	Others	94	69	209	372	380
**FBXO17**	PRAD	Others	78	69	204	351	381
**PRDM4**	PRAD	HM_w	39	268	42	349	382
**PHF5A**	PRAD	Others	134	164	42	340	383
**ATAT1**	PRAD	Others	90	69	176	335	384
**MORF4L1**	PRAD	HM_r	142	143	42	327	385
**TDRD5**	PRAD	Others	101	69	151	321	386
**MUM1**	PRAD	Others	54	69	197	320	387
**NAP1L3**	PRAD	Others	53	69	196	318	388
**KDM8**	PRAD	HM_e	60	214	42	316	389
**SP100**	PRAD	HA_r	198	69	42	309	390
**SMYD4**	PRAD	HM_w	24	233	42	299	391
**CBX6**	PRAD	HM_r	84	69	145	298	392
**TRIM66**	PRAD	HA_r	17	69	211	297	393
**CSTL1**	PRAD	Others	81	69	144	294	394
**BRD8**	PRAD	HA_r	182	69	42	293	395
**SIN3A**	PRAD	ChRC	116	135	42	293	396
**SMARCD3**	PRAD	Helicases	28	69	193	290	397
**TDRD9**	PRAD	Others	99	69	122	290	398
**TDRD12**	PRAD	Others	19	154	112	285	399
**ELP4**	PRAD	HA_w	173	69	42	284	400

**PRMT2**	PRAD	HM_w	35	206	42	283	401
**FMR1**	PRAD	Others	166	69	42	277	402
**USP51**	PRAD	Others	12	131	130	273	403
**PCMT1**	PRAD	Others	49	181	42	272	404
**ING3**	PRAD	HM_r	67	69	129	265	405
**HSPBAP1**	PRAD	Others	152	69	42	263	406
**KDM5C**	PRAD	HM_e	61	144	42	247	407
**SMYD3**	PRAD	HM_w	25	69	152	246	408
**SMNDC1**	PRAD	Others	27	157	42	226	409
**SMARCB1**	PRAD	Helicases	110	69	42	221	410
**SMYD1**	PRAD	HM_w	26	69	113	208	411
**ZMYND11**	PRAD	HA_r	93	69	42	204	412
**PHF6**	PRAD	HM_r	44	69	42	155	413
**SETD1B**	PRAD	HM_w	29	69	42	140	414
**USP27X**	PRAD	Others	13	69	42	124	415
**UTY**	PRAD	HM_e	11	69	42	122	416
**KDM5C**	SARC	HM_e	418	370	418	1206	1
**CHAF1A**	SARC	ChRC	409	395	392	1196	2
**BRD9**	SARC	HA_r	351	418	422	1191	3
**ASH1L**	SARC	HM_w	421	404	353	1178	4
**KDM4B**	SARC	HM_e	403	408	357	1168	5
**DNMT1**	SARC	DM_w	346	409	412	1167	6
**NCOR1**	SARC	ChRC	328	426	411	1165	7
**SCML2**	SARC	HM_r	397	355	393	1145	8
**RAI1**	SARC	Others	316	425	394	1135	9
**ATRX**	SARC	Helicases	426	423	281	1130	10
**SETDB1**	SARC	HM_w	311	407	383	1101	11
**ERCC5**	SARC	Others	342	332	426	1100	12
**RBBP7**	SARC	ChRC	315	358	414	1087	13
**GATAD2A**	SARC	HM_r	383	275	420	1078	14
**BPTF**	SARC	HA_r	353	375	348	1076	15
**PHF20L1**	SARC	HM_r	325	345	396	1066	16
**HDGF**	SARC	Others	253	386	425	1064	17
**EP400**	SARC	HA_w	384	364	313	1061	18
**CBX8**	SARC	ChRC	389	335	326	1050	19

**BAZ2A**	SARC	HA_r	410	282	349	1041	20
**PCMT1**	SARC	Others	217	400	423	1040	21
**KDM3A**	SARC	HM_e	378	347	308	1033	22
**ATAD2**	SARC	HA_r	288	389	351	1028	23
**HDAC6**	SARC	HA_e	341	318	366	1025	24
**AURKB**	SARC	Others	286	367	369	1022	25
**ARID1B**	SARC	ChRC	412	351	258	1021	26
**KMT2C**	SARC	HM_w	417	328	272	1017	27
**NCOR2**	SARC	Others	374	267	373	1014	28
**CHD6**	SARC	Helicases	407	320	277	1004	29
**PAF1**	SARC	Others	221	392	390	1003	30
**PYGO2**	SARC	HM_r	201	381	419	1001	31
**HDAC7**	SARC	HA_e	255	363	377	995	32
**HIRA**	SARC	Others	250	385	358	993	33
**CARM1**	SARC	HM_w	162	410	417	989	34
**PRKAA1**	SARC	Others	206	391	388	985	35
**KDM5A**	SARC	HM_e	237	371	375	983	36
**CHD1L**	SARC	Helicases	274	415	278	967	37
**PRKAA2**	SARC	Others	319	382	266	967	38
**CHD2**	SARC	Helicases	408	366	187	961	39
**BAZ1A**	SARC	HA_r	284	308	368	960	40
**SIRT2**	SARC	HA_e	192	380	387	959	41
**GATAD2B**	SARC	HM_r	136	412	405	953	42
**MBD3**	SARC	DM_r	331	417	201	949	43
**CHD7**	SARC	Helicases	419	334	185	938	44
**CHD8**	SARC	Helicases	387	190	360	937	45
**CHRAC1**	SARC	ChRC	156	374	407	937	46
**MECOM**	SARC	Others	375	327	224	926	47
**KAT6A**	SARC	HA_w	241	316	365	922	48
**SETD5**	SARC	HM_w	312	288	320	920	49
**SMARCA5**	SARC	Helicases	308	285	327	920	50
**CREBBP**	SARC	HA_w	405	189	325	919	51
**PRDM2**	SARC	HM_w	368	219	330	917	52
**RSF1**	SARC	ChRC	314	314	288	916	53
**ING1**	SARC	HM_r	245	361	309	915	54

**CBX3**	SARC	HM_r	159	349	399	907	55
**GTF2F1**	SARC	Others	135	387	385	907	56
**BRD8**	SARC	HA_r	390	280	234	904	57
**SUV39H1**	SARC	HM_w	182	311	408	901	58
**BRPF1**	SARC	HA_r	164	321	415	900	59
**ARID2**	SARC	ChRC	394	309	191	894	60
**ASH2L**	SARC	HM_w	169	390	335	894	61
**HDAC9**	SARC	HA_e	254	362	274	890	62
**DNMT3B**	SARC	DM_w	345	157	386	888	63
**BRD4**	SARC	HA_r	165	322	400	887	64
**KMT2A**	SARC	HM_w	402	329	156	887	65
**ASXL1**	SARC	Others	289	245	352	886	66
**HDAC1**	SARC	HA_e	258	302	324	884	67
**ATM**	SARC	Others	424	350	108	882	68
**SIRT3**	SARC	HA_e	309	287	285	881	69
**GTF2B**	SARC	Others	260	274	346	880	70
**AEBP2**	SARC	HM_w	291	352	235	878	71
**SP140**	SARC	HA_r	413	378	85	876	72
**KMT2E**	SARC	HM_w	376	269	225	870	73
**CXXC1**	SARC	Others	348	188	333	869	74
**PRDM9**	SARC	HM_w	398	413	57	868	75
**BOP 1**	SARC	Others	166	338	361	865	76
**PBRM1**	SARC	HA_r	371	224	269	864	77
**ING2**	SARC	HM_r	380	348	135	863	78
**UBE2I**	SARC	Others	296	203	362	861	79
**USP22**	SARC	Others	16	424	421	861	80
**ATR**	SARC	Others	411	194	255	860	81
**G2E3**	SARC	Others	138	319	398	855	82
**ATF7IP**	SARC	Others	287	376	189	852	83
**NSD1**	SARC	HM_w	327	325	199	851	84
**SETD1B**	SARC	HM_w	396	212	243	851	85
**PHF8**	SARC	Others	72	383	395	850	86
**ORC1**	SARC	Others	87	346	416	849	87
**TDRD6**	SARC	Others	360	205	283	848	88
**KDM6A**	SARC	HM_e	104	369	374	847	89

**DIDO1**	SARC	Others	347	333	165	845	90
**L3MBTL3**	SARC	Others	332	393	115	840	91
**KDM3B**	SARC	HM_e	335	300	204	839	92
**KANSL1**	SARC	HA_w	379	135	323	837	93
**DPF2**	SARC	Others	344	240	252	836	94
**GLYR1**	SARC	HM_r	261	238	332	831	95
**DPF1**	SARC	ChRC	151	365	298	814	96
**FBXW9**	SARC	Others	142	304	367	813	97
**NAP1L1**	SARC	Others	90	401	322	813	98
**ARID1A**	SARC	ChRC	422	246	144	812	99
**PPARGC1A**	SARC	Others	321	315	174	810	100
**SMYD4**	SARC	HM_w	306	284	220	810	101
**MSL3**	SARC	HA_w	93	360	356	809	102
**RPH3A**	SARC	Others	366	289	152	807	103
**SHPRH**	SARC	Others	363	399	42	804	104
**HDAC2**	SARC	HA_e	128	330	345	803	105
**HNF1A**	SARC	ChRC	404	237	162	803	106
**ELP4**	SARC	HA_w	148	305	347	800	107
**GADD45A**	SARC	Others	262	373	164	799	108
**SATB1**	SARC	Others	365	170	264	799	109
**TAF1L**	SARC	HA_r	423	252	123	798	110
**PHF13**	SARC	Others	326	295	175	796	111
**PYGO1**	SARC	HM_r	317	171	306	794	112
**SETD1A**	SARC	HM_w	364	102	328	794	113
**SIRT7**	SARC	HA_e	191	341	261	793	114
**MPHOSPH8**	SARC	HM_r	228	384	179	791	115
**SETDB2**	SARC	HM_w	49	422	319	790	116
**TDRKH**	SARC	Others	25	411	354	790	117
**TAF1**	SARC	HA_r	361	69	355	785	118
**CECR2**	SARC	HA_r	425	278	81	784	119
**CHD5**	SARC	Helicases	420	306	55	781	120
**JMJD6**	SARC	HM_e	114	372	294	780	121
**NCOA3**	SARC	HA_w	224	152	403	779	122
**BRD2**	SARC	HA_r	352	145	280	777	123
**KMT2B**	SARC	HM_w	333	402	42	777	124

**PRDM11**	SARC	HM_w	320	262	195	777	125
**TDRD12**	SARC	Others	300	405	71	776	126
**BAZ1B**	SARC	HA_r	354	69	350	773	127
**PRMT8**	SARC	HM_w	318	343	111	772	128
**PHF14**	SARC	Others	79	359	331	769	129
**DOT1L**	SARC	HM_w	152	403	212	767	130
**CBX5**	SARC	HM_r	158	307	300	765	131
**PRMT5**	SARC	HM_w	64	291	409	764	132
**MEN1**	SARC	ChRC	231	131	397	759	133
**EP300**	SARC	HA_w	385	142	231	758	134
**HDAC3**	SARC	HA_e	257	273	228	758	135
**CLOCK**	SARC	HA_w	155	243	359	757	136
**CBX4**	SARC	HM_r	279	336	140	755	137
**MBTD1**	SARC	Others	232	178	344	754	138
**RPA3**	SARC	Others	56	356	340	752	139
**IDH2**	SARC	DM_e	124	317	310	751	140
**HCFC1**	SARC	Others	382	69	297	748	141
**PADI1**	SARC	Others	415	229	103	747	142
**RNF217**	SARC	Others	197	357	193	747	143
**SFMBT2**	SARC	Others	414	69	263	746	144
**PHF3**	SARC	Others	323	174	244	741	145
**ZMYND8**	SARC	HA_r	293	146	302	741	146
**FBXO17**	SARC	Others	144	388	208	740	147
**KDM5B**	SARC	HM_e	334	180	226	740	148
**PHC2**	SARC	ChRC	215	177	342	734	149
**CHAF1B**	SARC	ChRC	275	144	314	733	150
**UHRF1**	SARC	DM_r	295	396	42	733	151
**KAT7**	SARC	HA_w	337	234	160	731	152
**SND1**	SARC	HM_r	305	207	219	731	153
**PHF20**	SARC	HM_r	76	265	389	730	154
**MUM1**	SARC	Others	225	414	89	728	155
**SUPT16H**	SARC	Others	183	166	379	728	156
**ZGPAT**	SARC	Others	11	339	378	728	157
**BRWD1**	SARC	HA_r	280	193	254	727	158
**HIST1H3B**	SARC	Others	340	137	249	726	159

**HLTF**	SARC	Others	247	184	295	726	160
**SMARCD1**	SARC	Helicases	187	254	284	725	161
**RNF20**	SARC	Others	58	324	341	723	162
**SFMBT1**	SARC	HM_r	194	210	318	722	163
**FXR2**	SARC	Others	263	416	42	721	164
**HR**	SARC	HM_e	381	112	227	720	165
**CHD4**	SARC	Helicases	273	277	166	716	166
**CBX7**	SARC	HM_r	278	158	279	715	167
**KDM2B**	SARC	HM_e	108	231	376	715	168
**PARP2**	SARC	Others	219	151	343	713	169
**ACTL6A**	SARC	ChRC	177	199	336	712	170
**CBX2**	SARC	ChRC	160	337	214	711	171
**PRMT6**	SARC	HM_w	203	217	290	710	172
**KDM1A**	SARC	HM_e	110	233	364	707	173
**POLR2B**	SARC	Others	70	221	410	701	174
**PRMT2**	SARC	HM_w	367	292	42	701	175
**CDYL**	SARC	HM_r	277	191	232	700	176
**PHF11**	SARC	Others	81	421	196	698	177
**PRDM1**	SARC	HM_w	211	263	222	696	178
**TP53BP1**	SARC	Others	358	120	218	696	179
**SMARCA4**	SARC	Helicases	189	406	99	694	180
**PRDM4**	SARC	HM_w	68	261	363	692	181
**SUZ12**	SARC	ChRC	303	69	317	689	182
**KMT2D**	SARC	HM_w	416	69	203	688	183
**SP110**	SARC	HA_r	185	379	124	688	184
**CBX1**	SARC	HM_r	161	192	334	687	185
**HDGFL1**	SARC	Others	252	139	296	687	186
**PHF21B**	SARC	HM_r	324	69	293	686	187
**TET3**	SARC	DM_e	395	121	170	686	188
**IDH1**	SARC	DM_e	339	136	207	682	189
**AFF4**	SARC	Others	174	247	259	680	190
**ATAD2B**	SARC	HA_r	355	69	256	680	191
**PHC1**	SARC	ChRC	216	266	198	680	192
**SMYD1**	SARC	HM_w	307	312	60	679	193
**RNF2**	SARC	ChRC	59	290	329	678	194

**PADI2**	SARC	Others	373	228	75	676	195
**SIN3B**	SARC	ChRC	47	256	370	673	196
**KAT6B**	SARC	HA_w	240	271	161	672	197
**FKBP1A**	SARC	Others	141	276	251	668	198
**GADD45B**	SARC	Others	137	394	136	667	199
**KDM4A**	SARC	HM_e	239	270	158	667	200
**PRMT3**	SARC	HM_w	204	172	291	667	201
**RNF17**	SARC	Others	198	342	127	667	202
**TCF19**	SARC	Others	302	123	238	663	203
**TCEA1**	SARC	Others	30	251	381	662	204
**ING4**	SARC	HM_r	244	236	181	661	205
**TDRD10**	SARC	Others	28	397	236	661	206
**PRDM14**	SARC	HM_w	209	344	101	654	207
**PADI3**	SARC	Others	372	227	54	653	208
**BRWD3**	SARC	HA_r	350	69	233	652	209
**HDAC4**	SARC	HA_e	127	419	105	651	210
**SIN3A**	SARC	ChRC	310	168	172	650	211
**BAZ2B**	SARC	HA_r	392	69	188	649	212
**CSTL1**	SARC	Others	154	242	253	649	213
**SMARCA1**	SARC	Helicases	190	286	171	647	214
**TET2**	SARC	DM_e	359	96	192	647	215
**MLLT10**	SARC	HM_w	230	326	90	646	216
**POLE3**	SARC	ChRC	71	173	402	646	217
**FMR1**	SARC	Others	264	69	312	645	218
**CHD3**	SARC	Helicases	388	69	186	643	219
**SIRT6**	SARC	HA_e	44	354	242	640	220
**SMARCB1**	SARC	Helicases	43	209	382	634	221
**AKAP1**	SARC	Others	290	196	145	631	222
**MORF4L1**	SARC	HM_r	94	230	307	631	223
**PRDM13**	SARC	HM_w	210	293	128	631	224
**ZCWPW1**	SARC	HM_r	178	147	303	628	225
**PHF5A**	SARC	Others	74	129	424	627	226
**ARID4B**	SARC	ChRC	170	195	257	622	227
**EHMT1**	SARC	HM_w	149	69	404	622	228
**EHMT2**	SARC	HM_w	268	143	211	622	229

**JMJD8**	SARC	HM_e	113	301	206	620	230
**KAT5**	SARC	HA_w	112	235	273	620	231
**DMAP1**	SARC	Others	271	241	107	619	232
**DPF3**	SARC	HA_r	343	156	120	619	233
**MBD4**	SARC	DM_r	96	132	391	619	234
**BMI1**	SARC	ChRC	167	281	168	616	235
**ASXL2**	SARC	Others	356	69	190	615	236
**CBX6**	SARC	HM_r	157	159	299	615	237
**EED**	SARC	ChRC	269	69	276	614	238
**KDM2A**	SARC	HM_e	336	232	42	610	239
**PARP1**	SARC	Others	220	69	321	610	240
**RTF1**	SARC	Others	196	127	287	610	241
**RBBP4**	SARC	ChRC	61	259	289	609	242
**L3MBTL4**	SARC	Others	235	296	76	607	243
**MTA1**	SARC	ChRC	92	268	246	606	244
**ING5**	SARC	HM_r	122	420	63	605	245
**KDM6B**	SARC	HM_e	377	69	157	603	246
**STK31**	SARC	Others	184	353	65	602	247
**AICDA**	SARC	DM_e	173	368	59	600	248
**PHF2**	SARC	Others	77	150	372	599	249
**YY1**	SARC	ChRC	357	200	42	599	250
**PHRF1**	SARC	Others	400	69	129	598	251
**DNMT3L**	SARC	DM_w	270	187	138	595	252
**SMARCD2**	SARC	Helicases	41	313	241	595	253
**SMARCC2**	SARC	Helicases	188	255	149	592	254
**FBXL19**	SARC	Others	265	115	209	589	255
**AFF1**	SARC	Others	175	198	215	588	256
**PHC3**	SARC	ChRC	214	176	197	587	257
**TDRD7**	SARC	Others	299	165	122	586	258
**PRDM15**	SARC	HM_w	369	128	88	585	259
**BRPF3**	SARC	HA_r	163	279	141	583	260
**TDG**	SARC	ChRC	29	250	304	583	261
**PRDM12**	SARC	HM_w	69	220	292	581	262
**MARCH5**	SARC	Others	98	69	413	580	263
**SETD3**	SARC	HM_w	195	258	126	579	264

**L3MBTL1**	SARC	HM_r	101	297	180	578	265
**ZCWPW2**	SARC	HM_r	12	248	316	576	266
**BAP1**	SARC	Others	285	244	42	571	267
**H3F3A**	SARC	Others	132	185	250	567	268
**JADE2**	SARC	Others	117	69	380	566	269
**MSH6**	SARC	HM_r	227	69	270	566	270
**RNF40**	SARC	Others	57	103	406	566	271
**SETD2**	SARC	HM_w	313	211	42	566	272
**EPC2**	SARC	Others	147	140	275	562	273
**CHD1**	SARC	Helicases	349	69	139	557	274
**CHD9**	SARC	Helicases	406	69	80	555	275
**USP51**	SARC	Others	15	323	216	554	276
**HDAC5**	SARC	HA_e	256	114	183	553	277
**SCMH1**	SARC	Others	54	213	286	553	278
**SRCAP**	SARC	Others	304	99	148	551	279
**SETD4**	SARC	HM_w	52	126	371	549	280
**DNMT3A**	SARC	DM_w	386	69	93	548	281
**PHF6**	SARC	HM_r	212	294	42	548	282
**MTA2**	SARC	ChRC	91	69	384	544	283
**JARID2**	SARC	ChRC	338	69	134	541	284
**MBD5**	SARC	DM_r	233	153	155	541	285
**PRDM10**	SARC	HM_w	370	69	102	541	286
**TRIM66**	SARC	HA_r	297	148	96	541	287
**KAT2B**	SARC	HA_w	242	181	117	540	288
**RBBP5**	SARC	ChRC	60	215	265	540	289
**KDM4E**	SARC	HM_e	106	298	133	537	290
**FKBP5**	SARC	Others	139	303	92	534	291
**PRDM16**	SARC	HM_w	399	69	66	534	292
**USP27X**	SARC	Others	179	310	42	531	293
**ASXL3**	SARC	Others	393	69	64	526	294
**SUV39H2**	SARC	HM_w	32	253	239	524	295
**KDM4D**	SARC	HM_e	107	299	116	522	296
**RAG2**	SARC	HM_r	200	260	62	522	297
**PHF19**	SARC	HM_r	78	175	268	521	298
**TDRD1**	SARC	Others	301	122	97	520	299

**HDAC11**	SARC	HA_e	129	331	58	518	300
**PHF7**	SARC	Others	73	222	223	518	301
**UBE2E1**	SARC	Others	19	161	337	517	302
**TCF20**	SARC	Others	181	97	237	515	303
**PADI6**	SARC	Others	222	225	67	514	304
**TET1**	SARC	DM_e	298	164	52	514	305
**CDYL2**	SARC	HM_r	276	69	167	512	306
**PAXIP1**	SARC	Others	401	69	42	512	307
**NCOA1**	SARC	HA_w	329	69	113	511	308
**PRKCD**	SARC	Others	205	218	87	510	309
**SP140L**	SARC	HA_r	34	377	98	509	310
**HDAC8**	SARC	HA_e	126	69	311	506	311
**HIST1H1C**	SARC	Others	248	138	119	505	312
**SMARCE1**	SARC	Helicases	40	125	339	504	313
**PCGF6**	SARC	Others	218	108	177	503	314
**BRD7**	SARC	HA_r	391	69	42	502	315
**UBR7**	SARC	Others	18	202	282	502	316
**SMYD5**	SARC	HM_w	36	124	338	498	317
**KAT2A**	SARC	HA_w	243	134	118	495	318
**BRD3**	SARC	HA_r	282	69	142	493	319
**PADI4**	SARC	Others	223	226	42	491	320
**PWWP2B**	SARC	Others	202	216	73	491	321
**GTF2H1**	SARC	Others	259	186	42	487	322
**ING3**	SARC	HM_r	123	182	182	487	323
**KDM7A**	SARC	HM_e	103	69	315	487	324
**EPC1**	SARC	Others	266	141	79	486	325
**JADE3**	SARC	Others	116	69	301	486	326
**WDR5**	SARC	ChRC	14	69	401	484	327
**DAXX**	SARC	ChRC	153	116	213	482	328
**MTA3**	SARC	ChRC	330	109	42	481	329
**PHF10**	SARC	Others	82	223	176	481	330
**SP100**	SARC	HA_r	35	398	42	475	331
**SMARCA2**	SARC	Helicases	362	69	42	473	332
**HSPBAP1**	SARC	Others	246	183	42	471	333
**SMARCC1**	SARC	Helicases	42	208	221	471	334

**TDRD3**	SARC	HM_r	180	206	84	470	335
**AIRE**	SARC	HM_r	172	197	94	463	336
**PCGF1**	SARC	Others	86	130	245	461	337
**SIRT4**	SARC	HA_e	46	149	262	457	338
**RING1**	SARC	Others	199	104	153	456	339
**MBD1**	SARC	DM_r	234	179	42	455	340
**SETMAR**	SARC	HM_w	48	257	150	455	341
**FBXO44**	SARC	Others	143	239	70	452	342
**PHF21A**	SARC	HM_r	75	264	112	451	343
**TRIM33**	SARC	HA_r	22	163	260	445	344
**DPY30**	SARC	Others	150	155	137	442	345
**HIST1H1B**	SARC	Others	249	113	77	439	346
**PHF23**	SARC	HM_r	213	69	154	436	347
**SSRP1**	SARC	Others	33	98	305	436	348
**MECP2**	SARC	DM_r	95	69	271	435	349
**ARID4A**	SARC	ChRC	171	119	143	433	350
**GTF3C4**	SARC	HA_w	134	69	230	433	351
**IWS1**	SARC	Others	119	272	42	433	352
**PHIP**	SARC	HA_r	322	69	42	433	353
**BRDT**	SARC	HA_r	281	69	82	432	354
**ACTL6B**	SARC	ChRC	176	160	95	431	355
**CTCF**	SARC	Others	272	117	42	431	356
**HAT1**	SARC	HA_w	131	69	229	429	357
**TAF3**	SARC	HA_r	31	340	56	427	358
**KAT8**	SARC	HA_w	111	110	205	426	359
**EZH2**	SARC	HM_w	145	69	210	424	360
**KDM8**	SARC	HM_e	102	69	248	419	361
**LBR**	SARC	Others	99	69	247	415	362
**MTF2**	SARC	HM_r	226	69	114	409	363
**ATAT1**	SARC	Others	168	118	121	407	364
**ZMYND11**	SARC	HA_r	294	69	42	405	365
**UTY**	SARC	HM_e	292	69	42	403	366
**MLLT6**	SARC	HM_w	229	69	104	402	367
**PRDM8**	SARC	HM_w	66	69	267	402	368
**SIRT1**	SARC	HA_e	193	167	42	402	369

**HELLS**	SARC	Helicases	251	69	78	398	370
**BRD1**	SARC	HA_r	283	69	42	394	371
**TDRD5**	SARC	Others	27	283	83	393	372
**TDRD9**	SARC	Others	26	249	110	385	373
**HDAC10**	SARC	HA_e	130	69	184	383	374
**ELP3**	SARC	HA_w	267	69	42	378	375
**SCML4**	SARC	Others	53	169	151	373	376
**UBE2A**	SARC	Others	21	204	147	372	377
**MBD2**	SARC	DM_r	97	69	202	368	378
**H2AFZ**	SARC	Others	133	69	163	365	379
**PRMT1**	SARC	HM_w	65	106	194	365	380
**JMJD1C**	SARC	HM_e	115	154	91	360	381
**NAP1L2**	SARC	Others	89	69	200	358	382
**JADE1**	SARC	Others	118	69	169	356	383
**KDM4C**	SARC	HM_e	238	69	42	349	384
**KIAA2026**	SARC	Others	236	69	42	347	385
**SMYD2**	SARC	HM_w	38	69	240	347	386
**KDM1B**	SARC	HM_e	109	69	159	337	387
**NAP1L3**	SARC	Others	88	69	178	335	388
**RPS6KA5**	SARC	Others	55	214	61	330	389
**PRDM7**	SARC	HM_w	207	69	53	329	390
**UBE2B**	SARC	Others	20	162	146	328	391
**SMARCD3**	SARC	Helicases	186	69	72	327	392
**EZH1**	SARC	HM_w	146	69	106	321	393
**PHF1**	SARC	HM_r	83	107	130	320	394
**PRDM6**	SARC	HM_w	208	69	42	319	395
**TRIM24**	SARC	HA_r	24	69	217	310	396
**SETD7**	SARC	HM_w	50	69	173	292	397
**PCGF2**	SARC	Others	85	69	132	286	398
**PCGF5**	SARC	Others	84	69	131	284	399
**FKBP2**	SARC	Others	140	69	69	278	400
**L3MBTL2**	SARC	Others	100	133	42	275	401
**INTS12**	SARC	Others	120	111	42	273	402
**HIF1AN**	SARC	Others	125	69	68	262	403
**WDR82**	SARC	Others	13	201	42	256	404

**PRMT7**	SARC	HM_w	63	105	74	242	405
**SIRT5**	SARC	HA_e	45	69	125	239	406
**PRDM5**	SARC	HM_w	67	69	100	236	407
**INO80**	SARC	Helicases	121	69	42	232	408
**KDM5D**	SARC	HM_e	105	69	42	216	409
**TRIM28**	SARC	HA_r	23	69	109	201	410
**SETD6**	SARC	HM_w	51	101	42	194	411
**SMYD3**	SARC	HM_w	37	69	86	192	412
**PHF12**	SARC	Others	80	69	42	191	413
**SMNDC1**	SARC	Others	39	100	42	181	414
**PSIP1**	SARC	HM_r	62	69	42	173	415
**UHRF2**	SARC	DM_r	17	69	42	128	416
**BPTF**	SKCM	HA_r	402	395	369	1166	1
**EP300**	SKCM	HA_w	355	407	403	1165	2
**ASH1L**	SKCM	HM_w	407	362	393	1162	3
**DIDO1**	SKCM	Others	417	324	418	1159	4
**KDM5B**	SKCM	HM_e	349	402	408	1159	5
**BRPF3**	SKCM	HA_r	345	388	424	1157	6
**TCF20**	SKCM	Others	369	390	394	1153	7
**TRIM24**	SKCM	HA_r	306	420	415	1141	8
**JARID2**	SKCM	ChRC	295	417	422	1134	9
**EZH2**	SKCM	HM_w	312	419	395	1126	10
**ATAD2**	SKCM	HA_r	315	424	378	1117	11
**KMT2A**	SKCM	HM_w	413	355	344	1112	12
**EHMT2**	SKCM	HM_w	305	397	401	1103	13
**CHD6**	SKCM	Helicases	408	317	375	1100	14
**BRD9**	SKCM	HA_r	290	409	392	1091	15
**PHF20L1**	SKCM	HM_r	250	422	409	1081	16
**DAXX**	SKCM	ChRC	280	383	413	1076	17
**CHD7**	SKCM	Helicases	404	346	322	1072	18
**SND1**	SKCM	HM_r	266	415	388	1069	19
**AKAP1**	SKCM	Others	316	377	374	1067	20
**HIRA**	SKCM	Others	320	333	399	1052	21
**TDRD5**	SKCM	Others	418	328	305	1051	22
**TP53BP1**	SKCM	Others	388	308	346	1042	23

**HR**	SKCM	HM_e	330	345	363	1038	24
**PHF21B**	SKCM	HM_r	382	373	283	1038	25
**PRDM10**	SKCM	HM_w	337	371	327	1035	26
**STK31**	SKCM	Others	416	293	325	1034	27
**PAXIP1**	SKCM	Others	215	392	417	1024	28
**TDRKH**	SKCM	Others	323	348	349	1020	29
**BAZ1B**	SKCM	HA_r	314	306	397	1017	30
**CECR2**	SKCM	HA_r	378	335	304	1017	31
**BRD2**	SKCM	HA_r	222	384	410	1016	32
**SETDB1**	SKCM	HM_w	228	367	421	1016	33
**SMYD3**	SKCM	HM_w	211	421	384	1016	34
**HDAC9**	SKCM	HA_e	415	375	223	1013	35
**PYGO2**	SKCM	HM_r	229	378	398	1005	36
**ACTL6B**	SKCM	ChRC	303	325	376	1004	37
**PARP1**	SKCM	Others	261	352	389	1002	38
**KMT2C**	SKCM	HM_w	423	401	163	987	39
**PHF14**	SKCM	Others	259	342	385	986	40
**KDM1B**	SKCM	HM_e	163	410	406	979	41
**CHD1L**	SKCM	Helicases	198	408	368	974	42
**L3MBTL2**	SKCM	Others	165	405	400	970	43
**JMJD6**	SKCM	HM_e	153	411	405	969	44
**RBBP5**	SKCM	ChRC	144	418	407	969	45
**SIRT5**	SKCM	HA_e	139	416	414	969	46
**GATAD2B**	SKCM	HM_r	178	376	412	966	47
**PHF20**	SKCM	HM_r	277	312	377	966	48
**DNMT3B**	SKCM	DM_w	340	285	338	963	49
**CDYL**	SKCM	HM_r	112	423	426	961	50
**ASXL1**	SKCM	Others	297	291	372	960	51
**CBX8**	SKCM	ChRC	199	412	339	950	52
**BRD7**	SKCM	HA_r	273	289	387	949	53
**SMARCD3**	SKCM	Helicases	138	398	404	940	54
**ZMYND8**	SKCM	HA_r	334	249	357	940	55
**ATR**	SKCM	Others	397	174	366	937	56
**L3MBTL4**	SKCM	Others	384	266	274	924	57
**CBX4**	SKCM	HM_r	221	413	286	920	58

**KDM5A**	SKCM	HM_e	329	300	291	920	59
**KMT2D**	SKCM	HM_w	424	314	178	916	60
**ZGPAT**	SKCM	Others	207	326	383	916	61
**CBX6**	SKCM	HM_r	201	347	360	908	62
**BRD4**	SKCM	HA_r	380	218	309	907	63
**RING1**	SKCM	Others	99	381	425	905	64
**KMT2E**	SKCM	HM_w	301	331	270	902	65
**PHF1**	SKCM	HM_r	147	382	370	899	66
**CHRAC1**	SKCM	ChRC	51	425	420	896	67
**BOP 1**	SKCM	Others	53	426	411	890	68
**KANSL1**	SKCM	HA_w	302	241	345	888	69
**KDM2A**	SKCM	HM_e	173	374	341	888	70
**L3MBTL1**	SKCM	HM_r	352	283	247	882	71
**SMYD2**	SKCM	HM_w	119	366	396	881	72
**ING3**	SKCM	HM_r	107	406	359	872	73
**HDAC4**	SKCM	HA_e	292	380	198	870	74
**BRWD3**	SKCM	HA_r	367	288	210	865	75
**ASH2L**	SKCM	HM_w	204	278	380	862	76
**TRIM33**	SKCM	HA_r	166	338	358	862	77
**SIRT7**	SKCM	HA_e	78	399	379	856	78
**ARID4B**	SKCM	ChRC	254	389	212	855	79
**CHD4**	SKCM	Helicases	401	275	175	851	80
**TCF19**	SKCM	Others	95	391	365	851	81
**PRDM1**	SKCM	HM_w	395	372	82	849	82
**SIN3B**	SKCM	ChRC	309	187	353	849	83
**HCFC1**	SKCM	Others	386	303	158	847	84
**PHF5A**	SKCM	Others	45	386	416	847	85
**HDGF**	SKCM	Others	86	357	402	845	86
**CHD2**	SKCM	Helicases	350	318	176	844	87
**PHF12**	SKCM	Others	291	233	319	843	88
**SETD1A**	SKCM	HM_w	399	127	317	843	89
**PRDM9**	SKCM	HM_w	421	330	85	836	90
**ATAT1**	SKCM	Others	75	404	352	831	91
**KAT7**	SKCM	HA_w	164	315	351	830	92
**ATM**	SKCM	Others	372	361	94	827	93

**SMARCD2**	SKCM	Helicases	120	385	318	823	94
**RNF2**	SKCM	ChRC	56	370	391	817	95
**TDRD6**	SKCM	Others	373	364	80	817	96
**PPARGC1A**	SKCM	Others	374	296	146	816	97
**RPA3**	SKCM	Others	43	349	423	815	98
**ZCWPW1**	SKCM	HM_r	317	292	200	809	99
**SETD5**	SKCM	HM_w	268	256	282	806	100
**CXXC1**	SKCM	Others	253	215	334	802	101
**ARID1B**	SKCM	ChRC	396	363	42	801	102
**HNF1A**	SKCM	ChRC	359	204	235	798	103
**NCOR2**	SKCM	Others	405	261	132	798	104
**BRD1**	SKCM	HA_r	346	69	382	797	105
**RSF1**	SKCM	ChRC	354	400	42	796	106
**CBX7**	SKCM	HM_r	200	336	258	794	107
**CBX2**	SKCM	ChRC	202	414	177	793	108
**PHRF1**	SKCM	Others	327	138	328	793	109
**TDRD10**	SKCM	Others	244	365	183	792	110
**ORC1**	SKCM	Others	264	235	292	791	111
**PRKAA2**	SKCM	Others	400	193	193	786	112
**DNMT1**	SKCM	DM_w	360	166	256	782	113
**MBD5**	SKCM	DM_r	419	149	214	782	114
**ATF7IP**	SKCM	Others	347	307	126	780	115
**SCML2**	SKCM	HM_r	341	228	211	780	116
**FKBP5**	SKCM	Others	289	387	102	778	117
**TCEA1**	SKCM	Others	58	339	381	778	118
**NAP1L2**	SKCM	Others	385	263	127	775	119
**ARID2**	SKCM	ChRC	412	319	42	773	120
**TAF1L**	SKCM	HA_r	422	69	281	772	121
**ATRX**	SKCM	Helicases	371	290	107	768	122
**CLOCK**	SKCM	HA_w	220	304	244	768	123
**H3F3A**	SKCM	Others	23	359	386	768	124
**HDAC11**	SKCM	HA_e	176	269	321	766	125
**HDAC1**	SKCM	HA_e	131	270	364	765	126
**HIST1H1C**	SKCM	Others	67	394	303	764	127
**CBX3**	SKCM	HM_r	52	287	419	758	128

**LBR**	SKCM	Others	172	353	233	758	129
**SRCAP**	SKCM	Others	410	120	226	756	130
**MORF4L1**	SKCM	HM_r	66	298	390	754	131
**NCOA3**	SKCM	HA_w	375	262	115	752	132
**HIST1H1B**	SKCM	Others	252	396	100	748	133
**AEBP2**	SKCM	HM_w	72	320	355	747	134
**CDYL2**	SKCM	HM_r	365	276	104	745	135
**PRDM14**	SKCM	HM_w	322	340	83	745	136
**SMARCA2**	SKCM	Helicases	348	309	87	744	137
**PAF1**	SKCM	Others	193	234	312	739	138
**KDM7A**	SKCM	HM_e	333	69	335	737	139
**HIST1H3B**	SKCM	Others	155	393	186	734	140
**ING5**	SKCM	HM_r	106	323	302	731	141
**SIN3A**	SKCM	ChRC	294	69	367	730	142
**NSD1**	SKCM	HM_w	363	322	42	727	143
**PRMT5**	SKCM	HM_w	230	231	264	725	144
**BAZ2B**	SKCM	HA_r	393	172	159	724	145
**PYGO1**	SKCM	HM_r	169	258	297	724	146
**TDRD9**	SKCM	Others	307	252	164	723	147
**YY1**	SKCM	ChRC	117	250	356	723	148
**MECP2**	SKCM	DM_r	115	299	307	721	149
**TET3**	SKCM	DM_e	381	115	224	720	150
**HDGFL1**	SKCM	Others	239	403	77	719	151
**CREBBP**	SKCM	HA_w	392	69	257	718	152
**JMJD1C**	SKCM	HM_e	338	267	111	716	153
**L3MBTL3**	SKCM	Others	262	354	99	715	154
**BRPF1**	SKCM	HA_r	281	69	361	711	155
**NCOA1**	SKCM	HA_w	287	236	184	707	156
**PHF3**	SKCM	Others	285	379	42	706	157
**MSH6**	SKCM	HM_r	127	265	313	705	158
**KAT5**	SKCM	HA_w	47	356	301	704	159
**PHF8**	SKCM	Others	282	232	190	704	160
**HDAC6**	SKCM	HA_e	227	205	271	703	161
**KAT8**	SKCM	HA_w	225	152	324	701	162
**PRDM7**	SKCM	HM_w	189	259	252	700	163

**BAP1**	SKCM	Others	181	173	340	694	164
**RNF217**	SKCM	Others	248	369	76	693	165
**KDM4A**	SKCM	HM_e	151	240	300	691	166
**KDM6B**	SKCM	HM_e	358	200	133	691	167
**ASXL2**	SKCM	Others	366	176	148	690	168
**SMARCA4**	SKCM	Helicases	394	69	227	690	169
**HDAC8**	SKCM	HA_e	182	284	222	688	170
**KMT2B**	SKCM	HM_w	406	239	42	687	171
**SMARCA1**	SKCM	Helicases	263	225	199	687	172
**KDM5C**	SKCM	HM_e	238	201	246	685	173
**RPH3A**	SKCM	Others	357	229	96	682	174
**HDAC7**	SKCM	HA_e	218	302	161	681	175
**BAZ2A**	SKCM	HA_r	332	305	42	679	176
**PWWP2B**	SKCM	Others	269	311	97	677	177
**DOT1L**	SKCM	HM_w	339	69	268	676	178
**SHPRH**	SKCM	Others	267	321	88	676	179
**CHD9**	SKCM	Helicases	356	274	42	672	180
**IDH1**	SKCM	DM_e	343	156	173	672	181
**KIAA2026**	SKCM	Others	265	69	336	670	182
**SMARCB1**	SKCM	Helicases	121	254	295	670	183
**PBRM1**	SKCM	HA_r	328	146	195	669	184
**EPC2**	SKCM	Others	234	164	267	665	185
**INO80**	SKCM	Helicases	304	316	42	662	186
**PADI1**	SKCM	Others	275	69	315	659	187
**BRDT**	SKCM	HA_r	362	217	79	658	188
**TET1**	SKCM	DM_e	403	69	182	654	189
**PHF7**	SKCM	Others	214	139	299	652	190
**CHAF1A**	SKCM	ChRC	296	69	285	650	191
**GTF2F1**	SKCM	Others	158	159	333	650	192
**RNF40**	SKCM	Others	247	128	273	648	193
**TDRD1**	SKCM	Others	389	181	75	645	194
**PRDM13**	SKCM	HM_w	146	351	145	642	195
**SCML4**	SKCM	Others	123	368	151	642	196
**AFF4**	SKCM	Others	351	248	42	641	197
**PARP2**	SKCM	Others	125	195	320	640	198

**CHD8**	SKCM	Helicases	377	216	42	635	199
**SMARCA5**	SKCM	Helicases	122	224	288	634	200
**ING4**	SKCM	HM_r	130	268	234	632	201
**ASXL3**	SKCM	Others	426	69	136	631	202
**SFMBT2**	SKCM	Others	310	188	129	627	203
**SUZ12**	SKCM	ChRC	137	119	371	627	204
**SMYD1**	SKCM	HM_w	319	222	84	625	205
**MBTD1**	SKCM	Others	82	344	196	622	206
**RNF17**	SKCM	Others	414	130	78	622	207
**CHD1**	SKCM	Helicases	293	286	42	621	208
**AFF1**	SKCM	Others	298	279	42	619	209
**BAZ1A**	SKCM	HA_r	274	220	125	619	210
**EHMT1**	SKCM	HM_w	331	246	42	619	211
**EZH1**	SKCM	HM_w	233	211	174	618	212
**FBXW9**	SKCM	Others	179	162	275	616	213
**PRDM12**	SKCM	HM_w	190	136	290	616	214
**ZMYND11**	SKCM	HA_r	40	337	239	616	215
**MEN1**	SKCM	ChRC	128	282	205	615	216
**POLR2B**	SKCM	Others	276	297	42	615	217
**PHIP**	SKCM	HA_r	231	341	42	614	218
**SIRT2**	SKCM	HA_e	97	226	289	612	219
**MECOM**	SKCM	Others	425	69	116	610	220
**HDAC5**	SKCM	HA_e	195	206	208	609	221
**KDM2B**	SKCM	HM_e	364	202	42	608	222
**PRMT8**	SKCM	HM_w	240	294	74	608	223
**RAI1**	SKCM	Others	387	69	152	608	224
**FBXL19**	SKCM	Others	160	163	284	607	225
**SMYD5**	SKCM	HM_w	185	121	294	600	226
**PRDM15**	SKCM	HM_w	336	69	194	599	227
**KDM3A**	SKCM	HM_e	194	151	253	598	228
**PRDM16**	SKCM	HM_w	398	69	131	598	229
**PRDM2**	SKCM	HM_w	326	69	203	598	230
**SMARCC1**	SKCM	Helicases	246	185	167	598	231
**BRD3**	SKCM	HA_r	236	171	188	595	232
**ELP3**	SKCM	HA_w	219	334	42	595	233

**PRDM5**	SKCM	HM_w	257	194	144	595	234
**SCMH1**	SKCM	Others	213	190	192	595	235
**RAG2**	SKCM	HM_r	368	131	93	592	236
**SETD7**	SKCM	HM_w	142	281	169	592	237
**PHC2**	SKCM	ChRC	191	69	331	591	238
**DPF3**	SKCM	HA_r	271	214	103	588	239
**FKBP1A**	SKCM	Others	35	210	342	587	240
**MTF2**	SKCM	HM_r	16	238	332	586	241
**PRKCD**	SKCM	Others	188	192	202	582	242
**SATB1**	SKCM	Others	342	69	170	581	243
**SUPT16H**	SKCM	Others	255	183	143	581	244
**DNMT3A**	SKCM	DM_w	272	69	237	578	245
**PRDM11**	SKCM	HM_w	270	137	171	578	246
**ING1**	SKCM	HM_r	154	69	354	577	247
**RBBP7**	SKCM	ChRC	39	230	306	575	248
**HSPBAP1**	SKCM	Others	175	69	329	573	249
**SP140**	SKCM	HA_r	409	69	95	573	250
**KDM4C**	SKCM	HM_e	150	332	90	572	251
**CHD3**	SKCM	Helicases	344	69	157	570	252
**GATAD2A**	SKCM	HM_r	110	243	217	570	253
**UBE2E1**	SKCM	Others	27	280	262	569	254
**KDM3B**	SKCM	HM_e	376	150	42	568	255
**TET2**	SKCM	DM_e	318	69	181	568	256
**ATAD2B**	SKCM	HA_r	203	175	189	567	257
**MTA3**	SKCM	ChRC	104	196	266	566	258
**IDH2**	SKCM	DM_e	48	301	216	565	259
**DPF2**	SKCM	Others	161	360	42	563	260
**SMARCC2**	SKCM	Helicases	335	184	42	561	261
**DMAP1**	SKCM	Others	36	247	276	559	262
**ERCC5**	SKCM	Others	242	272	42	556	263
**PRMT3**	SKCM	HM_w	171	133	251	555	264
**SMYD4**	SKCM	HM_w	245	69	241	555	265
**SFMBT1**	SKCM	HM_r	256	69	228	553	266
**SSRP1**	SKCM	Others	168	69	316	553	267
**CHD5**	SKCM	Helicases	411	69	72	552	268

**NAP1L3**	SKCM	Others	224	69	259	552	269
**PADI4**	SKCM	Others	383	69	98	550	270
**CARM1**	SKCM	HM_w	235	69	245	549	271
**SETD4**	SKCM	HM_w	79	125	343	547	272
**PHF10**	SKCM	Others	64	343	139	546	273
**SMARCD1**	SKCM	Helicases	60	223	263	546	274
**KDM4B**	SKCM	HM_e	279	69	197	545	275
**KAT6B**	SKCM	HA_w	353	69	118	540	276
**PADI3**	SKCM	Others	390	69	81	540	277
**HDAC2**	SKCM	HA_e	87	358	91	536	278
**JMJD8**	SKCM	HM_e	20	154	362	536	279
**MLLT6**	SKCM	HM_w	278	148	110	536	280
**PADI6**	SKCM	Others	361	69	106	536	281
**KDM8**	SKCM	HM_e	205	69	260	534	282
**SETD6**	SKCM	HM_w	7	255	272	534	283
**KAT2A**	SKCM	HA_w	174	153	206	533	284
**PRMT7**	SKCM	HM_w	170	132	231	533	285
**FKBP2**	SKCM	Others	34	244	254	532	286
**PRMT1**	SKCM	HM_w	100	134	298	532	287
**EP400**	SKCM	HA_w	420	69	42	531	288
**PCMT1**	SKCM	Others	12	313	204	529	289
**PHC1**	SKCM	ChRC	192	69	265	526	290
**BRD8**	SKCM	HA_r	313	170	42	525	291
**PCGF1**	SKCM	Others	32	145	348	525	292
**PRMT6**	SKCM	HM_w	29	295	201	525	293
**USP51**	SKCM	Others	54	251	220	525	294
**CBX1**	SKCM	HM_r	26	277	219	522	295
**PHF13**	SKCM	Others	102	69	350	521	296
**CSTL1**	SKCM	Others	135	69	314	518	297
**CHAF1B**	SKCM	ChRC	136	168	213	517	298
**PSIP1**	SKCM	HM_r	124	350	42	516	299
**SIRT3**	SKCM	HA_e	96	123	296	515	300
**SP100**	SKCM	HA_r	308	69	138	515	301
**KAT2B**	SKCM	HA_w	288	69	155	512	302
**PRDM6**	SKCM	HM_w	10	260	242	512	303

**UHRF2**	SKCM	DM_r	41	327	142	510	304
**TDRD7**	SKCM	Others	243	116	150	509	305
**TDRD3**	SKCM	HM_r	210	69	225	504	306
**KAT6A**	SKCM	HA_w	391	69	42	502	307
**TRIM28**	SKCM	HA_r	183	69	250	502	308
**AURKB**	SKCM	Others	71	221	209	501	309
**PADI2**	SKCM	Others	311	69	121	501	310
**SP140L**	SKCM	HA_r	324	69	108	501	311
**RPS6KA5**	SKCM	Others	187	191	120	498	312
**TDRD12**	SKCM	Others	4	253	240	497	313
**RTF1**	SKCM	Others	143	310	42	495	314
**HLTF**	SKCM	Others	217	69	207	493	315
**SETD2**	SKCM	HM_w	325	126	42	493	316
**KDM4D**	SKCM	HM_e	251	69	172	492	317
**ARID1A**	SKCM	ChRC	379	69	42	490	318
**SIRT4**	SKCM	HA_e	140	186	160	486	319
**SETDB2**	SKCM	HM_w	186	69	229	484	320
**MSL3**	SKCM	HA_w	114	264	105	483	321
**FBXO17**	SKCM	Others	50	245	187	482	322
**NCOR1**	SKCM	ChRC	370	69	42	481	323
**SETD3**	SKCM	HM_w	212	227	42	481	324
**DPF1**	SKCM	ChRC	70	273	134	477	325
**AIRE**	SKCM	HM_r	283	69	119	471	326
**PHF2**	SKCM	Others	286	141	42	469	327
**RBBP4**	SKCM	ChRC	162	257	42	461	328
**KMT5B**	SKCM	Others	18	69	373	460	329
**UHRF1**	SKCM	DM_r	209	69	180	458	330
**BMI1**	SKCM	ChRC	113	219	124	456	331
**DPY30**	SKCM	Others	25	213	218	456	332
**SIRT6**	SKCM	HA_e	61	69	326	456	333
**UBE2I**	SKCM	Others	94	69	293	456	334
**JADE3**	SKCM	Others	206	69	179	454	335
**UBE2A**	SKCM	Others	55	69	330	454	336
**MARCH5**	SKCM	Others	46	69	337	452	337
**PHF6**	SKCM	HM_r	74	140	238	452	338

**MBD3**	SKCM	DM_r	149	69	232	450	339
**MBD4**	SKCM	DM_r	129	198	122	449	340
**TDG**	SKCM	ChRC	167	117	165	449	341
**USP22**	SKCM	Others	118	69	261	448	342
**USP27X**	SKCM	Others	1	178	269	448	343
**KDM6A**	SKCM	HM_e	91	69	287	447	344
**SETD1B**	SKCM	HM_w	28	189	230	447	345
**TAF3**	SKCM	HA_r	284	118	42	444	346
**G2E3**	SKCM	Others	159	161	123	443	347
**PHF23**	SKCM	HM_r	63	69	311	443	348
**DNMT3L**	SKCM	DM_w	299	69	73	441	349
**PRDM4**	SKCM	HM_w	62	69	310	441	350
**SUV39H1**	SKCM	HM_w	38	182	221	441	351
**MTA1**	SKCM	ChRC	126	69	243	438	352
**JADE2**	SKCM	Others	226	69	141	436	353
**EPC1**	SKCM	Others	180	212	42	434	354
**BRWD1**	SKCM	HA_r	321	69	42	432	355
**FMR1**	SKCM	Others	116	271	42	429	356
**ZCWPW2**	SKCM	HM_r	208	69	149	426	357
**JADE1**	SKCM	Others	241	69	113	423	358
**MTA2**	SKCM	ChRC	232	147	42	421	359
**SMNDC1**	SKCM	Others	5	69	347	421	360
**RNF20**	SKCM	Others	249	129	42	420	361
**SP110**	SKCM	HA_r	184	69	166	419	362
**ARID4A**	SKCM	ChRC	237	69	112	418	363
**PRKAA1**	SKCM	Others	44	329	42	415	364
**HELLS**	SKCM	Helicases	156	157	101	414	365
**FBXO44**	SKCM	Others	89	69	255	413	366
**IWS1**	SKCM	Others	216	155	42	413	367
**TAF1**	SKCM	HA_r	300	69	42	411	368
**KDM1A**	SKCM	HM_e	152	69	185	406	369
**NAP1L1**	SKCM	Others	15	237	153	405	370
**ELP4**	SKCM	HA_w	197	165	42	404	371
**RIOX2**	SKCM	Others	8	69	323	400	372
**KMT5A**	SKCM	Others	19	69	308	396	373

**GTF2B**	SKCM	Others	109	242	42	393	374
**ING2**	SKCM	HM_r	108	69	215	392	375
**SIRT1**	SKCM	HA_e	98	124	168	390	376
**MLLT10**	SKCM	HM_w	148	197	42	387	377
**PCGF2**	SKCM	Others	103	144	140	387	378
**AICDA**	SKCM	DM_e	223	69	92	384	379
**FXR2**	SKCM	Others	132	209	42	383	380
**GTF3C4**	SKCM	HA_w	157	69	156	382	381
**GLYR1**	SKCM	HM_r	177	160	42	379	382
**HDAC10**	SKCM	HA_e	68	69	236	373	383
**PHC3**	SKCM	ChRC	260	69	42	371	384
**PHF21A**	SKCM	HM_r	258	69	42	369	385
**KMT5C**	SKCM	Others	17	69	280	366	386
**NSD2**	SKCM	Others	14	69	279	362	387
**GADD45A**	SKCM	Others	33	208	117	358	388
**RIOX1**	SKCM	Others	9	69	278	356	389
**SGF29**	SKCM	Others	6	69	277	352	390
**INTS12**	SKCM	Others	105	203	42	350	391
**CTCF**	SKCM	Others	133	167	42	342	392
**CBX5**	SKCM	HM_r	37	169	135	341	393
**HDGFL2**	SKCM	Others	22	69	249	340	394
**SETMAR**	SKCM	HM_w	141	69	130	340	395
**HDGFL3**	SKCM	Others	21	69	248	338	396
**PHF19**	SKCM	HM_r	80	142	114	336	397
**MBD1**	SKCM	DM_r	84	199	42	325	398
**PRDM8**	SKCM	HM_w	101	135	86	322	399
**GTF2H1**	SKCM	Others	69	207	42	318	400
**TRIM66**	SKCM	HA_r	3	114	191	308	401
**HDAC3**	SKCM	HA_e	196	69	42	307	402
**MBD2**	SKCM	DM_r	83	69	154	306	403
**WDR82**	SKCM	Others	76	177	42	295	404
**KDM4E**	SKCM	HM_e	92	69	128	289	405
**HAT1**	SKCM	HA_w	88	158	42	288	406
**GADD45B**	SKCM	Others	49	69	147	265	407
**SUV39H2**	SKCM	HM_w	59	69	137	265	408

**UBR7**	SKCM	Others	42	179	42	263	409
**PRMT2**	SKCM	HM_w	145	69	42	256	410
**H2AFZ**	SKCM	Others	24	69	162	255	411
**KDM5D**	SKCM	HM_e	134	69	42	245	412
**SMARCE1**	SKCM	Helicases	77	122	42	241	413
**UBE2B**	SKCM	Others	2	180	42	224	414
**PCGF5**	SKCM	Others	65	69	89	223	415
**EED**	SKCM	ChRC	111	69	42	222	416
**PCGF6**	SKCM	Others	31	143	42	216	417
**WDR5**	SKCM	ChRC	57	113	42	212	418
**PHF11**	SKCM	Others	30	69	109	208	419
**MPHOSPH8**	SKCM	HM_r	93	69	42	204	420
**ACTL6A**	SKCM	ChRC	90	69	42	201	421
**HIF1AN**	SKCM	Others	85	69	42	196	422
**MUM1**	SKCM	Others	81	69	42	192	423
**UTY**	SKCM	HM_e	73	69	42	184	424
**NSD3**	SKCM	Others	13	69	42	124	425
**POLE3**	SKCM	ChRC	11	69	42	122	426
**DIDO1**	STAD	Others	421	420	424	1265	1
**ATAD2**	STAD	HA_r	392	426	421	1239	2
**CHD6**	STAD	Helicases	412	406	411	1229	3
**CHD7**	STAD	Helicases	415	377	396	1188	4
**SETDB1**	STAD	HM_w	367	391	418	1176	5
**PHF20L1**	STAD	HM_r	337	417	420	1174	6
**ASXL1**	STAD	Others	383	363	423	1169	7
**ZMYND8**	STAD	HA_r	320	418	425	1163	8
**KAT6A**	STAD	HA_w	401	382	374	1157	9
**ATR**	STAD	Others	405	362	381	1148	10
**PHF3**	STAD	Others	360	386	387	1133	11
**ZGPAT**	STAD	Others	300	419	407	1126	12
**L3MBTL1**	STAD	HM_r	305	409	405	1119	13
**ASH1L**	STAD	HM_w	411	364	341	1116	14
**KDM5A**	STAD	HM_e	381	348	373	1102	15
**ARID4B**	STAD	ChRC	350	407	342	1099	16
**KDM2A**	STAD	HM_e	307	388	403	1098	17

**NCOA3**	STAD	HA_w	343	423	332	1098	18
**KMT2E**	STAD	HM_w	373	408	313	1094	19
**DNMT3B**	STAD	DM_w	324	369	390	1083	20
**SMARCC2**	STAD	Helicases	389	341	351	1081	21
**MECOM**	STAD	Others	315	425	340	1080	22
**KAT6B**	STAD	HA_w	375	403	290	1068	23
**MLLT6**	STAD	HM_w	264	402	395	1061	24
**PHF20**	STAD	HM_r	242	387	419	1048	25
**PHC3**	STAD	ChRC	282	422	337	1041	26
**CHD4**	STAD	Helicases	413	295	317	1025	27
**CHD2**	STAD	Helicases	340	400	284	1024	28
**FMR1**	STAD	Others	269	376	368	1013	29
**TCEA1**	STAD	Others	208	390	415	1013	30
**SMARCE1**	STAD	Helicases	173	421	417	1011	31
**PHF14**	STAD	Others	261	392	353	1006	32
**KMT2C**	STAD	HM_w	424	381	197	1002	33
**BPTF**	STAD	HA_r	403	300	296	999	34
**BRD9**	STAD	HA_r	170	413	412	995	35
**CHD1L**	STAD	Helicases	217	384	393	994	36
**PYGO2**	STAD	HM_r	238	343	413	994	37
**ACTL6A**	STAD	ChRC	171	415	406	992	38
**LBR**	STAD	Others	293	332	366	991	39
**ING1**	STAD	HM_r	201	404	380	985	40
**BAZ1B**	STAD	HA_r	273	302	402	977	41
**CREBBP**	STAD	HA_w	419	351	205	975	42
**HDGF**	STAD	Others	250	315	410	975	43
**KDM5B**	STAD	HM_e	345	241	384	970	44
**TRIM28**	STAD	HA_r	221	339	409	969	45
**EHMT1**	STAD	HM_w	353	319	293	965	46
**ERCC5**	STAD	Others	189	383	389	961	47
**BRD3**	STAD	HA_r	370	271	319	960	48
**HCFC1**	STAD	Others	368	399	188	955	49
**BAZ2A**	STAD	HA_r	298	301	350	949	50
**RSF1**	STAD	ChRC	291	395	252	938	51
**KANSL1**	STAD	HA_w	347	289	301	937	52

**ARID1B**	STAD	ChRC	394	353	189	936	53
**BRPF1**	STAD	HA_r	333	249	349	931	54
**STK31**	STAD	Others	235	371	323	929	55
**TAF1L**	STAD	HA_r	408	340	176	924	56
**CTCF**	STAD	Others	354	270	294	918	57
**PRKAA1**	STAD	Others	107	416	394	917	58
**TDRD6**	STAD	Others	407	379	129	915	59
**MPHOSPH8**	STAD	HM_r	263	311	339	913	60
**PAF1**	STAD	Others	161	347	400	908	61
**HDAC4**	STAD	HA_e	387	360	158	905	62
**SETD1A**	STAD	HM_w	386	186	330	902	63
**SETDB2**	STAD	HM_w	223	355	324	902	64
**BOP 1**	STAD	Others	80	414	401	895	65
**BRPF3**	STAD	HA_r	219	299	376	894	66
**KAT2A**	STAD	HA_w	200	333	354	887	67
**KMT2A**	STAD	HM_w	417	312	155	884	68
**PCGF2**	STAD	Others	110	401	372	883	69
**CBX3**	STAD	HM_r	145	337	397	879	70
**KDM6A**	STAD	HM_e	286	373	215	874	71
**CHRAC1**	STAD	ChRC	23	424	426	873	72
**IWS1**	STAD	Others	265	227	379	871	73
**PRDM14**	STAD	HM_w	225	372	268	865	74
**PWWP2B**	STAD	Others	278	283	299	860	75
**PHF12**	STAD	Others	304	219	336	859	76
**CHAF1A**	STAD	ChRC	289	361	206	856	77
**TDRKH**	STAD	Others	84	370	399	853	78
**HDAC6**	STAD	HA_e	268	268	315	851	79
**ATM**	STAD	Others	416	352	79	847	80
**SRCAP**	STAD	Others	418	180	249	847	81
**ARID1A**	STAD	ChRC	426	378	42	846	82
**CBX8**	STAD	ChRC	190	321	334	845	83
**IDH2**	STAD	DM_e	74	410	361	845	84
**PRDM9**	STAD	HM_w	379	405	59	843	85
**HIRA**	STAD	Others	294	266	282	842	86
**RNF40**	STAD	Others	277	187	378	842	87

**TDRD5**	STAD	Others	207	276	359	842	88
**TET1**	STAD	DM_e	384	275	175	834	89
**KMT2B**	STAD	HM_w	422	367	42	831	90
**RAI1**	STAD	Others	404	307	120	831	91
**ELP3**	STAD	HA_w	167	247	414	828	92
**KDM2B**	STAD	HM_e	346	201	280	827	93
**MECP2**	STAD	DM_r	216	396	213	825	94
**PHF11**	STAD	Others	109	346	365	820	95
**PHF21A**	STAD	HM_r	281	356	180	817	96
**RPA3**	STAD	Others	25	385	404	814	97
**SND1**	STAD	HM_r	371	69	369	809	98
**EP400**	STAD	HA_w	423	69	316	808	99
**ATAT1**	STAD	Others	98	389	320	807	100
**MSH6**	STAD	HM_r	352	171	279	802	101
**KDM5C**	STAD	HM_e	374	240	186	800	102
**SMARCD2**	STAD	Helicases	86	329	382	797	103
**WDR5**	STAD	ChRC	149	250	398	797	104
**NCOR1**	STAD	ChRC	395	358	42	795	105
**DOT1L**	STAD	HM_w	348	320	124	792	106
**CBX4**	STAD	HM_r	191	322	275	788	107
**CBX2**	STAD	ChRC	146	323	318	787	108
**DNMT3A**	STAD	DM_w	252	207	328	787	109
**MTA1**	STAD	ChRC	226	221	338	785	110
**PHRF1**	STAD	Others	342	69	371	782	111
**H3F3A**	STAD	Others	77	349	355	781	112
**EHMT2**	STAD	HM_w	363	69	347	779	113
**KAT7**	STAD	HA_w	186	226	367	779	114
**PBRM1**	STAD	HA_r	380	357	42	779	115
**TDRD10**	STAD	Others	233	354	190	777	116
**BAZ2B**	STAD	HA_r	365	272	138	775	117
**JARID2**	STAD	ChRC	414	69	292	775	118
**KDM6B**	STAD	HM_e	377	223	169	769	119
**PSIP1**	STAD	HM_r	359	365	42	766	120
**GATAD2B**	STAD	HM_r	59	368	333	760	121
**SMARCB1**	STAD	Helicases	313	183	264	760	122

**HLTF**	STAD	Others	164	314	281	759	123
**AEBP2**	STAD	HM_w	46	326	386	758	124
**CHD3**	STAD	Helicases	391	231	136	758	125
**HDAC2**	STAD	HA_e	140	316	302	758	126
**MBD3**	STAD	DM_r	285	288	185	758	127
**CHD9**	STAD	Helicases	390	230	135	755	128
**PRDM15**	STAD	HM_w	302	309	144	755	129
**TET3**	STAD	DM_e	358	148	248	754	130
**GTF3C4**	STAD	HA_w	308	244	201	753	131
**PRDM13**	STAD	HM_w	279	366	108	753	132
**AFF4**	STAD	Others	311	338	101	750	133
**PRMT1**	STAD	HM_w	179	217	352	748	134
**SFMBT1**	STAD	HM_r	276	281	191	748	135
**BRWD1**	STAD	HA_r	378	324	42	744	136
**EZH1**	STAD	HM_w	204	335	204	743	137
**PCMT1**	STAD	Others	69	262	408	739	138
**SMARCA1**	STAD	Helicases	236	278	225	739	139
**CSTL1**	STAD	Others	45	336	357	738	140
**FBXO17**	STAD	Others	44	318	375	737	141
**TDRD7**	STAD	Others	222	252	263	737	142
**ZCWPW1**	STAD	HM_r	102	412	223	737	143
**BRD8**	STAD	HA_r	420	69	247	736	144
**EED**	STAD	ChRC	168	293	274	735	145
**PHF2**	STAD	Others	410	69	254	733	146
**SHPRH**	STAD	Others	385	306	42	733	147
**ELP4**	STAD	HA_w	121	246	362	729	148
**HDAC9**	STAD	HA_e	295	375	58	728	149
**TAF3**	STAD	HA_r	275	211	239	725	150
**CXXC1**	STAD	Others	288	394	42	724	151
**BRD1**	STAD	HA_r	355	325	42	722	152
**JMJD1C**	STAD	HM_e	396	69	257	722	153
**PHIP**	STAD	HA_r	336	344	42	722	154
**KAT8**	STAD	HA_w	247	173	300	720	155
**PARP1**	STAD	Others	321	287	112	720	156
**POLE3**	STAD	ChRC	180	191	346	717	157

**NAP1L2**	STAD	Others	284	197	234	715	158
**BRD4**	STAD	HA_r	369	69	276	714	159
**HELLS**	STAD	Helicases	287	267	157	711	160
**NCOR2**	STAD	Others	409	69	233	711	161
**FKBP1A**	STAD	Others	43	245	422	710	162
**RAG2**	STAD	HM_r	176	342	192	710	163
**KMT2D**	STAD	HM_w	425	69	214	708	164
**SMARCA4**	STAD	Helicases	398	69	240	707	165
**TDRD3**	STAD	HM_r	274	69	364	707	166
**ING5**	STAD	HM_r	73	359	272	704	167
**RBBP5**	STAD	ChRC	105	216	383	704	168
**SETD3**	STAD	HM_w	174	215	309	698	169
**CHD8**	STAD	Helicases	382	69	246	697	170
**SIRT2**	STAD	HA_e	88	280	329	697	171
**EPC1**	STAD	Others	142	269	283	694	172
**TAF1**	STAD	HA_r	334	69	286	689	173
**DAXX**	STAD	ChRC	271	69	348	688	174
**IDH1**	STAD	DM_e	115	313	258	686	175
**CHD1**	STAD	Helicases	296	296	91	683	176
**MBD1**	STAD	DM_r	245	393	42	680	177
**PRDM2**	STAD	HM_w	400	69	210	679	178
**PRDM16**	STAD	HM_w	397	69	211	677	179
**KDM4B**	STAD	HM_e	331	224	121	676	180
**SIRT1**	STAD	HA_e	210	237	228	675	181
**BRD2**	STAD	HA_r	310	69	295	674	182
**KAT5**	STAD	HA_w	138	242	291	671	183
**MBD4**	STAD	DM_r	112	199	360	671	184
**KDM4A**	STAD	HM_e	227	225	216	668	185
**SIN3A**	STAD	ChRC	290	69	308	667	186
**TRIM24**	STAD	HA_r	206	69	391	666	187
**CBX5**	STAD	HM_r	253	297	115	665	188
**YY1**	STAD	ChRC	148	209	307	664	189
**L3MBTL4**	STAD	Others	185	151	327	663	190
**CBX1**	STAD	HM_r	60	298	304	662	191
**PRDM11**	STAD	HM_w	108	285	269	662	192

**MBTD1**	STAD	Others	71	263	326	660	193
**DMAP1**	STAD	Others	230	208	220	658	194
**EZH2**	STAD	HM_w	120	334	203	657	195
**SETD5**	STAD	HM_w	301	69	287	657	196
**UBE2I**	STAD	Others	31	327	297	655	197
**BRWD3**	STAD	HA_r	364	69	221	654	198
**SUV39H2**	STAD	HM_w	33	234	385	652	199
**RNF17**	STAD	Others	341	256	54	651	200
**RNF20**	STAD	Others	237	69	345	651	201
**PRMT3**	STAD	HM_w	178	162	310	650	202
**JMJD6**	STAD	HM_e	114	264	271	649	203
**MBD5**	STAD	DM_r	330	222	96	648	204
**G2E3**	STAD	Others	317	69	261	647	205
**PRKCD**	STAD	Others	211	258	178	647	206
**SUZ12**	STAD	ChRC	48	277	321	646	207
**GLYR1**	STAD	HM_r	316	69	260	645	208
**HR**	STAD	HM_e	339	69	236	644	209
**RBBP4**	STAD	ChRC	131	282	231	644	210
**SMARCA5**	STAD	Helicases	195	184	265	644	211
**RNF2**	STAD	ChRC	35	238	370	643	212
**GTF2H1**	STAD	Others	119	165	356	640	213
**PAXIP1**	STAD	Others	183	69	388	640	214
**TDG**	STAD	ChRC	151	154	335	640	215
**HNF1A**	STAD	ChRC	249	203	187	639	216
**MLLT10**	STAD	HM_w	244	69	325	638	217
**ING3**	STAD	HM_r	57	265	314	636	218
**SIN3B**	STAD	ChRC	314	161	161	636	219
**SMARCC1**	STAD	Helicases	258	235	141	634	220
**KIAA2026**	STAD	Others	322	69	242	633	221
**SETD2**	STAD	HM_w	329	254	42	625	222
**SUPT16H**	STAD	Others	234	69	322	625	223
**MBD2**	STAD	DM_r	184	397	42	623	224
**UBE2A**	STAD	Others	47	232	344	623	225
**TDRD1**	STAD	Others	257	233	130	620	226
**SP100**	STAD	HA_r	328	159	131	618	227

**PRDM10**	STAD	HM_w	280	260	76	616	228
**JMJD8**	STAD	HM_e	39	374	199	612	229
**PHF19**	STAD	HM_r	157	261	194	612	230
**TP53BP1**	STAD	Others	357	153	102	612	231
**NSD1**	STAD	HM_w	361	69	181	611	232
**SMYD3**	STAD	HM_w	126	69	416	611	233
**SP140**	STAD	HA_r	366	157	85	608	234
**ATRX**	STAD	Helicases	388	69	149	606	235
**PRMT8**	STAD	HM_w	177	331	94	602	236
**BAP1**	STAD	Others	254	303	42	599	237
**SMYD5**	STAD	HM_w	153	69	377	599	238
**MTF2**	STAD	HM_r	344	69	184	597	239
**MEN1**	STAD	ChRC	215	69	312	596	240
**KDM1A**	STAD	HM_e	137	202	256	595	241
**EP300**	STAD	HA_w	376	69	147	592	242
**FBXO44**	STAD	Others	141	206	244	591	243
**ASXL2**	STAD	Others	299	69	222	590	244
**SCMH1**	STAD	Others	155	255	177	587	245
**SMARCD1**	STAD	Helicases	34	160	392	586	246
**POLR2B**	STAD	Others	241	190	153	584	247
**AKAP1**	STAD	Others	99	178	306	583	248
**DPF2**	STAD	Others	96	248	237	581	249
**BMI1**	STAD	ChRC	147	69	363	579	250
**PRDM5**	STAD	HM_w	212	188	179	579	251
**UHRF1**	STAD	DM_r	327	210	42	579	252
**SIRT7**	STAD	HA_e	63	236	277	576	253
**DPF1**	STAD	ChRC	143	350	82	575	254
**HDAC10**	STAD	HA_e	118	317	133	568	255
**ACTL6B**	STAD	ChRC	100	411	55	566	256
**PRMT2**	STAD	HM_w	239	284	42	565	257
**SMYD4**	STAD	HM_w	172	253	140	565	258
**SMYD2**	STAD	HM_w	127	181	251	559	259
**AIRE**	STAD	HM_r	193	304	61	558	260
**RPH3A**	STAD	Others	259	69	230	558	261
**PPARGC1A**	STAD	Others	303	189	64	556	262

**PADI3**	STAD	Others	292	194	69	555	263
**SIRT6**	STAD	HA_e	50	279	226	555	264
**ARID4A**	STAD	ChRC	356	69	127	552	265
**NCOA1**	STAD	HA_w	338	69	145	552	266
**SETMAR**	STAD	HM_w	64	380	105	549	267
**ING4**	STAD	HM_r	40	290	217	547	268
**KDM3A**	STAD	HM_e	306	69	170	545	269
**RING1**	STAD	Others	198	69	278	545	270
**SFMBT2**	STAD	Others	372	69	104	545	271
**ATF7IP**	STAD	Others	349	69	126	544	272
**CARM1**	STAD	HM_w	169	69	305	543	273
**CECR2**	STAD	HA_r	325	69	148	542	274
**CHD5**	STAD	Helicases	402	69	71	542	275
**ASXL3**	STAD	Others	406	69	66	541	276
**PARP2**	STAD	Others	160	69	311	540	277
**HDAC1**	STAD	HA_e	251	69	218	538	278
**RNF217**	STAD	Others	260	69	209	538	279
**FXR2**	STAD	Others	203	292	42	537	280
**ING2**	STAD	HM_r	41	398	98	537	281
**PRKAA2**	STAD	Others	224	149	164	537	282
**NAP1L3**	STAD	Others	283	69	182	534	283
**SIRT4**	STAD	HA_e	51	185	298	534	284
**SMARCD3**	STAD	Helicases	85	328	118	531	285
**SP140L**	STAD	HA_r	125	156	250	531	286
**USP51**	STAD	Others	312	69	150	531	287
**DNMT3L**	STAD	DM_w	144	294	90	528	288
**SCML2**	STAD	HM_r	104	330	93	527	289
**CDYL2**	STAD	HM_r	297	69	159	525	290
**MORF4L1**	STAD	HM_r	92	198	235	525	291
**PADI2**	STAD	Others	262	195	68	525	292
**UBR7**	STAD	Others	30	147	343	520	293
**BRD7**	STAD	HA_r	205	176	137	518	294
**UBE2B**	STAD	Others	62	305	151	518	295
**TCF20**	STAD	Others	351	69	92	512	296
**SATB1**	STAD	Others	335	69	107	511	297

**SMARCA2**	STAD	Helicases	399	69	42	510	298
**USP22**	STAD	Others	194	274	42	510	299
**PRMT5**	STAD	HM_w	89	257	163	509	300
**MSL3**	STAD	HA_w	243	69	196	508	301
**EPC2**	STAD	Others	166	168	172	506	302
**TCF19**	STAD	Others	152	69	285	506	303
**HDAC11**	STAD	HA_e	202	204	99	505	304
**KDM8**	STAD	HM_e	135	172	198	505	305
**MARCH5**	STAD	Others	16	200	289	505	306
**ARID2**	STAD	ChRC	393	69	42	504	307
**GATAD2A**	STAD	HM_r	229	69	202	500	308
**RTF1**	STAD	Others	197	170	132	499	309
**FBXL19**	STAD	Others	78	175	245	498	310
**HDAC5**	STAD	HA_e	228	69	200	497	311
**RBBP7**	STAD	ChRC	175	69	253	497	312
**CBX6**	STAD	HM_r	218	69	207	494	313
**DPF3**	STAD	HA_r	309	69	114	492	314
**PRMT7**	STAD	HM_w	156	69	267	492	315
**TET2**	STAD	DM_e	256	169	67	492	316
**BAZ1A**	STAD	HA_r	319	69	100	488	317
**PHF8**	STAD	Others	213	218	56	487	318
**GTF2B**	STAD	Others	94	166	219	479	319
**INTS12**	STAD	Others	139	243	97	479	320
**AFF1**	STAD	Others	326	69	80	475	321
**PRDM8**	STAD	HM_w	240	69	165	474	322
**DNMT1**	STAD	DM_w	270	69	134	473	323
**KDM3B**	STAD	HM_e	362	69	42	473	324
**KDM4D**	STAD	HM_e	246	69	156	471	325
**SSRP1**	STAD	Others	103	155	208	466	326
**SETD4**	STAD	HM_w	129	69	266	464	327
**PHC2**	STAD	ChRC	159	192	111	462	328
**PRDM12**	STAD	HM_w	36	259	166	461	329
**KDM1B**	STAD	HM_e	136	69	255	460	330
**HDAC7**	STAD	HA_e	267	69	123	459	331
**SMNDC1**	STAD	Others	49	182	224	455	332

**FKBP2**	STAD	Others	28	152	273	453	333
**SP110**	STAD	HA_r	209	158	86	453	334
**ORC1**	STAD	Others	214	69	168	451	335
**SIRT5**	STAD	HA_e	87	212	152	451	336
**PRDM4**	STAD	HM_w	67	150	232	449	337
**AURKB**	STAD	Others	97	177	173	447	338
**HDAC8**	STAD	HA_e	117	69	259	445	339
**KDM7A**	STAD	HM_e	17	69	358	444	340
**INO80**	STAD	Helicases	332	69	42	443	341
**PHC1**	STAD	ChRC	132	69	241	442	342
**PHF6**	STAD	HM_r	53	345	42	440	343
**KAT2B**	STAD	HA_w	248	69	122	439	344
**PCGF1**	STAD	Others	38	69	331	438	345
**ATAD2B**	STAD	HA_r	192	69	174	435	346
**KDM4C**	STAD	HM_e	323	69	42	434	347
**TDRD9**	STAD	Others	150	179	103	432	348
**CDYL**	STAD	HM_r	318	69	42	429	349
**PHF13**	STAD	Others	90	69	270	429	350
**FBXW9**	STAD	Others	188	69	171	428	351
**CLOCK**	STAD	HA_w	231	69	125	425	352
**PADI6**	STAD	Others	91	239	95	425	353
**PADI4**	STAD	Others	162	220	42	424	354
**PHF7**	STAD	Others	26	286	109	421	355
**TRIM33**	STAD	HA_r	232	69	117	418	356
**HIST1H1B**	STAD	Others	266	69	81	416	357
**MTA2**	STAD	ChRC	134	69	212	415	358
**BRDT**	STAD	HA_r	272	69	72	413	359
**HIST1H1C**	STAD	Others	93	229	89	411	360
**PRDM7**	STAD	HM_w	52	69	288	409	361
**MTA3**	STAD	ChRC	56	310	42	408	362
**SCML4**	STAD	Others	196	69	142	407	363
**ZMYND11**	STAD	HA_r	220	69	116	405	364
**HDGFL1**	STAD	Others	58	291	53	402	365
**DPY30**	STAD	Others	29	69	303	401	366
**WDR82**	STAD	Others	83	273	42	398	367

**HDAC3**	STAD	HA_e	187	164	42	393	368
**HAT1**	STAD	HA_w	76	69	243	388	369
**SETD6**	STAD	HM_w	128	214	42	384	370
**SETD7**	STAD	HM_w	65	213	106	384	371
**GTF2F1**	STAD	Others	165	174	42	381	372
**PCGF5**	STAD	Others	133	193	42	368	373
**UHRF2**	STAD	DM_r	255	69	42	366	374
**PRDM6**	STAD	HM_w	15	308	42	365	375
**JADE3**	STAD	Others	18	69	262	349	376
**HIST1H3B**	STAD	Others	21	228	88	337	377
**PHF1**	STAD	HM_r	158	69	110	337	378
**PHF23**	STAD	HM_r	181	69	87	337	379
**L3MBTL3**	STAD	Others	113	69	154	336	380
**PHF10**	STAD	Others	68	69	195	332	381
**SUV39H1**	STAD	HM_w	124	69	139	332	382
**PADI1**	STAD	Others	70	196	65	331	383
**PRDM1**	STAD	HM_w	199	69	63	331	384
**HSPBAP1**	STAD	Others	116	163	42	321	385
**PYGO1**	STAD	HM_r	106	69	143	318	386
**RPS6KA5**	STAD	Others	130	69	119	318	387
**TRIM66**	STAD	HA_r	11	69	238	318	388
**USP27X**	STAD	Others	24	251	42	317	389
**SETD1B**	STAD	HM_w	14	69	229	312	390
**PHF21B**	STAD	HM_r	182	69	60	311	391
**FKBP5**	STAD	Others	95	69	146	310	392
**SIRT3**	STAD	HA_e	13	69	227	309	393
**CBX7**	STAD	HM_r	79	69	160	308	394
**NAP1L1**	STAD	Others	55	69	183	307	395
**GADD45B**	STAD	Others	27	205	70	302	396
**PHF5A**	STAD	Others	37	69	193	299	397
**SMYD1**	STAD	HM_w	154	69	75	298	398
**PRMT6**	STAD	HM_w	66	69	162	297	399
**PCGF6**	STAD	Others	54	69	167	290	400
**L3MBTL2**	STAD	Others	163	69	42	274	401
**CHAF1B**	STAD	ChRC	122	69	78	269	402

**GADD45A**	STAD	Others	22	167	77	266	403
**ZCWPW2**	STAD	HM_r	101	69	74	244	404
**ASH2L**	STAD	HM_w	123	69	42	234	405
**UBE2E1**	STAD	Others	32	69	128	229	406
**H2AFZ**	STAD	Others	42	69	113	224	407
**MUM1**	STAD	Others	111	69	42	222	408
**AICDA**	STAD	DM_e	81	69	62	212	409
**KDM4E**	STAD	HM_e	72	69	57	198	410
**UTY**	STAD	HM_e	82	69	42	193	411
**HIF1AN**	STAD	Others	75	69	42	186	412
**JADE1**	STAD	Others	20	69	83	172	413
**KDM5D**	STAD	HM_e	61	69	42	172	414
**TDRD12**	STAD	Others	12	69	84	165	415
**JADE2**	STAD	Others	19	69	73	161	416
**BAZ1B**	TGCT	HA_r	376	408	424	1208	1
**ATAD2**	TGCT	HA_r	406	359	421	1186	2
**BRWD1**	TGCT	HA_r	402	350	410	1162	3
**ATRX**	TGCT	Helicases	418	358	370	1146	4
**BRD1**	TGCT	HA_r	374	353	407	1134	5
**HDAC9**	TGCT	HA_e	359	378	385	1122	6
**ASXL1**	TGCT	Others	380	386	315	1081	7
**BAP1**	TGCT	Others	377	357	347	1081	8
**JARID2**	TGCT	ChRC	414	417	245	1076	9
**KDM5C**	TGCT	HM_e	393	400	282	1075	10
**HIRA**	TGCT	Others	292	406	375	1073	11
**RSF1**	TGCT	ChRC	338	370	362	1070	12
**DOT1L**	TGCT	HM_w	399	339	309	1047	13
**AIRE**	TGCT	HM_r	324	363	358	1045	14
**FBXL19**	TGCT	Others	397	333	285	1015	15
**CHD7**	TGCT	Helicases	185	407	422	1014	16
**STK31**	TGCT	Others	225	368	411	1004	17
**DNMT3B**	TGCT	DM_w	305	341	357	1003	18
**ARID1B**	TGCT	ChRC	409	362	228	999	19
**SND1**	TGCT	HM_r	331	250	418	999	20
**CHD5**	TGCT	Helicases	367	382	249	998	21

**CBX3**	TGCT	HM_r	192	383	412	987	22
**SETD3**	TGCT	HM_w	335	261	388	984	23
**HDAC8**	TGCT	HA_e	293	321	368	982	24
**EED**	TGCT	ChRC	177	409	395	981	25
**ORC1**	TGCT	Others	275	294	409	978	26
**KAT8**	TGCT	HA_w	356	316	304	976	27
**DNMT3L**	TGCT	DM_w	304	340	330	974	28
**BRWD3**	TGCT	HA_r	371	349	252	972	29
**PBRM1**	TGCT	HA_r	344	286	341	971	30
**CLOCK**	TGCT	HA_w	307	410	248	965	31
**CHD9**	TGCT	Helicases	308	345	310	963	32
**HDAC4**	TGCT	HA_e	361	380	219	960	33
**PADI6**	TGCT	Others	388	289	278	955	34
**CHRAC1**	TGCT	ChRC	184	344	420	948	35
**ARID2**	TGCT	ChRC	204	361	382	947	36
**BAZ2B**	TGCT	HA_r	317	356	267	940	37
**BRD3**	TGCT	HA_r	315	385	226	926	38
**BRD9**	TGCT	HA_r	403	384	134	921	39
**KDM4E**	TGCT	HM_e	124	412	384	920	40
**PRMT2**	TGCT	HM_w	257	270	389	916	41
**BRPF1**	TGCT	HA_r	372	352	189	913	42
**ACTL6A**	TGCT	ChRC	209	387	316	912	43
**EZH2**	TGCT	HM_w	415	69	416	900	44
**MSH6**	TGCT	HM_r	348	302	242	892	45
**PADI3**	TGCT	Others	273	291	326	890	46
**PRDM16**	TGCT	HM_w	340	373	175	888	47
**SCML2**	TGCT	HM_r	245	369	274	888	48
**ATM**	TGCT	Others	379	414	94	887	49
**BOP 1**	TGCT	Others	198	354	332	884	50
**EP400**	TGCT	HA_w	420	69	394	883	51
**BAZ2A**	TGCT	HA_r	405	69	408	882	52
**EPC2**	TGCT	Others	301	334	247	882	53
**CHD4**	TGCT	Helicases	417	422	42	881	54
**CHD8**	TGCT	Helicases	309	346	224	879	55
**KDM6A**	TGCT	HM_e	351	377	148	876	56

**SMARCA2**	TGCT	Helicases	332	402	141	875	57
**RPA3**	TGCT	Others	56	392	423	871	58
**PHF14**	TGCT	Others	85	395	390	870	59
**SMARCD1**	TGCT	Helicases	234	254	378	866	60
**JMJD1C**	TGCT	HM_e	395	319	149	863	61
**PRMT7**	TGCT	HM_w	255	268	340	863	62
**PHF20L1**	TGCT	HM_r	386	69	402	857	63
**KDM5A**	TGCT	HM_e	394	420	42	856	64
**TCF20**	TGCT	Others	382	69	404	855	65
**NAP1L1**	TGCT	Others	101	397	355	853	66
**TCEA1**	TGCT	Others	32	415	405	852	67
**CDYL2**	TGCT	HM_r	370	348	132	850	68
**PRDM10**	TGCT	HM_w	385	418	42	845	69
**H3F3A**	TGCT	Others	160	326	356	842	70
**SRCAP**	TGCT	Others	425	249	165	839	71
**HDAC11**	TGCT	HA_e	294	323	221	838	72
**EP300**	TGCT	HA_w	364	69	399	832	73
**MBD1**	TGCT	DM_r	391	69	372	832	74
**SMYD2**	TGCT	HM_w	231	252	349	832	75
**IWS1**	TGCT	Others	139	320	367	826	76
**CECR2**	TGCT	HA_r	369	69	386	824	77
**FMR1**	TGCT	Others	167	329	328	824	78
**KAT5**	TGCT	HA_w	131	317	373	821	79
**KMT2D**	TGCT	HM_w	118	311	391	820	80
**KMT2C**	TGCT	HM_w	119	312	383	814	81
**ARID4B**	TGCT	ChRC	408	69	335	812	82
**TRIM24**	TGCT	HA_r	329	69	413	811	83
**ASXL2**	TGCT	Others	407	69	334	810	84
**PHC3**	TGCT	ChRC	91	396	323	810	85
**FKBP2**	TGCT	Others	169	331	307	807	86
**PADI2**	TGCT	Others	274	292	240	806	87
**MECOM**	TGCT	Others	107	398	300	805	88
**KDM2B**	TGCT	HM_e	355	69	380	804	89
**POLR2B**	TGCT	Others	78	404	322	804	90
**LBR**	TGCT	Others	281	308	211	800	91

**BRPF3**	TGCT	HA_r	195	351	253	799	92
**AEBP2**	TGCT	HM_w	326	426	42	794	93
**RBBP7**	TGCT	ChRC	60	371	363	794	94
**ASH2L**	TGCT	HM_w	320	69	400	789	95
**PRDM2**	TGCT	HM_w	411	274	101	786	96
**ZMYND8**	TGCT	HA_r	327	69	387	783	97
**HDAC6**	TGCT	HA_e	155	379	246	780	98
**NCOR1**	TGCT	ChRC	276	296	208	780	99
**DMAP1**	TGCT	Others	306	69	403	778	100
**EHMT1**	TGCT	HM_w	398	336	42	776	101
**GTF3C4**	TGCT	HA_w	296	327	153	776	102
**ING3**	TGCT	HM_r	287	69	419	775	103
**ASXL3**	TGCT	Others	319	360	95	774	104
**NCOR2**	TGCT	Others	426	69	279	774	105
**DPF1**	TGCT	ChRC	303	338	131	772	106
**KAT6A**	TGCT	HA_w	286	69	415	770	107
**KIAA2026**	TGCT	Others	282	399	89	770	108
**FBXO44**	TGCT	Others	172	332	265	769	109
**KDM2A**	TGCT	HM_e	413	314	42	769	110
**SUPT16H**	TGCT	Others	224	248	294	766	111
**PRDM14**	TGCT	HM_w	75	393	297	765	112
**SIRT6**	TGCT	HA_e	333	69	361	763	113
**MBD3**	TGCT	DM_r	111	306	345	762	114
**NSD1**	TGCT	HM_w	423	295	42	760	115
**DNMT3A**	TGCT	DM_w	400	69	286	755	116
**MUM1**	TGCT	Others	278	298	179	755	117
**HR**	TGCT	HM_e	289	69	392	750	118
**GATAD2B**	TGCT	HM_r	298	69	381	748	119
**KDM1A**	TGCT	HM_e	129	315	303	747	120
**MTA1**	TGCT	ChRC	102	301	343	746	121
**SETD1B**	TGCT	HM_w	336	69	338	743	122
**PRDM13**	TGCT	HM_w	262	276	203	741	123
**EPC1**	TGCT	Others	363	335	42	740	124
**FBXO17**	TGCT	Others	300	69	369	738	125
**CHAF1A**	TGCT	ChRC	401	69	266	736	126

**SMYD3**	TGCT	HM_w	230	251	255	736	127
**INO80**	TGCT	Helicases	358	69	305	732	128
**KDM5B**	TGCT	HM_e	284	69	379	732	129
**MTA2**	TGCT	ChRC	390	300	42	732	130
**MEN1**	TGCT	ChRC	280	304	146	730	131
**KDM3A**	TGCT	HM_e	354	69	301	724	132
**DIDO1**	TGCT	Others	365	69	287	721	133
**ATAD2B**	TGCT	HA_r	318	69	333	720	134
**SETD1A**	TGCT	HM_w	337	263	120	720	135
**PRMT8**	TGCT	HM_w	254	423	42	719	136
**TDRD1**	TGCT	Others	220	245	254	719	137
**PHF13**	TGCT	Others	86	375	257	718	138
**NAP1L3**	TGCT	Others	389	69	258	716	139
**ATR**	TGCT	Others	378	69	268	715	140
**BMI1**	TGCT	ChRC	199	355	160	714	141
**CSTL1**	TGCT	Others	183	343	188	714	142
**HDAC10**	TGCT	HA_e	295	324	91	710	143
**SMARCA1**	TGCT	Helicases	235	69	406	710	144
**PARP2**	TGCT	Others	97	287	325	709	145
**DNMT1**	TGCT	DM_w	416	69	223	708	146
**PRDM7**	TGCT	HM_w	261	273	174	708	147
**SUV39H1**	TGCT	HM_w	223	367	117	707	148
**L3MBTL3**	TGCT	Others	115	309	281	705	149
**KDM4B**	TGCT	HM_e	352	69	283	704	150
**PHF21A**	TGCT	HM_r	343	282	78	703	151
**SETD5**	TGCT	HM_w	244	260	199	703	152
**PHRF1**	TGCT	Others	265	394	42	701	153
**SETD6**	TGCT	HM_w	243	259	198	700	154
**MTF2**	TGCT	HM_r	346	69	280	695	155
**PHF8**	TGCT	Others	80	374	238	692	156
**MTA3**	TGCT	ChRC	347	299	42	688	157
**UBE2E1**	TGCT	Others	215	242	231	688	158
**RPS6KA5**	TGCT	Others	248	265	173	686	159
**TET1**	TGCT	DM_e	422	69	192	683	160
**ARID4A**	TGCT	ChRC	322	69	290	681	161

**PAXIP1**	TGCT	Others	270	69	342	681	162
**ASH1L**	TGCT	HM_w	321	69	289	679	163
**MBD5**	TGCT	DM_r	109	305	260	674	164
**AICDA**	TGCT	DM_e	206	425	42	673	165
**CHAF1B**	TGCT	ChRC	187	69	417	673	166
**NCOA1**	TGCT	HA_w	277	69	327	673	167
**RING1**	TGCT	Others	251	69	353	673	168
**PHF10**	TGCT	Others	89	284	299	672	169
**ELP3**	TGCT	HA_w	176	69	425	670	170
**HAT1**	TGCT	HA_w	159	325	186	670	171
**PRDM15**	TGCT	HM_w	74	275	321	670	172
**SIRT3**	TGCT	HA_e	46	390	234	670	173
**TDRKH**	TGCT	Others	330	69	270	669	174
**ACTL6B**	TGCT	ChRC	208	364	96	668	175
**PPARGC1A**	TGCT	Others	264	279	125	668	176
**SETMAR**	TGCT	HM_w	241	257	170	668	177
**PSIP1**	TGCT	HM_r	253	372	42	667	178
**HDAC2**	TGCT	HA_e	157	322	185	664	179
**ATF7IP**	TGCT	Others	202	419	42	663	180
**PHC2**	TGCT	ChRC	387	69	206	662	181
**TAF1L**	TGCT	HA_r	222	246	194	662	182
**SIN3B**	TGCT	ChRC	240	69	352	661	183
**SIRT2**	TGCT	HA_e	238	69	351	658	184
**NCOA3**	TGCT	HA_w	345	69	241	655	185
**PRDM9**	TGCT	HM_w	259	272	123	654	186
**FKBP5**	TGCT	Others	168	330	154	652	187
**TAF3**	TGCT	HA_r	221	389	42	652	188
**ZCWPW1**	TGCT	HM_r	13	235	401	649	189
**CREBBP**	TGCT	HA_w	424	69	155	648	190
**PHF11**	TGCT	Others	88	283	277	648	191
**RNF40**	TGCT	Others	339	266	42	647	192
**CHD1L**	TGCT	Helicases	186	347	113	646	193
**RBBP5**	TGCT	ChRC	252	69	319	640	194
**ELP4**	TGCT	HA_w	175	381	80	636	195
**BPTF**	TGCT	HA_r	375	69	190	634	196

**CBX6**	TGCT	HM_r	312	69	251	632	197
**L3MBTL1**	TGCT	HM_r	350	69	213	632	198
**UBR7**	TGCT	Others	20	241	371	632	199
**SHPRH**	TGCT	Others	334	255	42	631	200
**CHD3**	TGCT	Helicases	310	69	250	629	201
**KDM4D**	TGCT	HM_e	125	413	90	628	202
**DPY30**	TGCT	Others	178	69	377	624	203
**MSL3**	TGCT	HA_w	103	376	145	624	204
**TDRD9**	TGCT	Others	218	244	162	624	205
**HDAC7**	TGCT	HA_e	154	69	398	621	206
**HDAC1**	TGCT	HA_e	158	69	393	620	207
**RNF217**	TGCT	Others	57	267	296	620	208
**MLLT10**	TGCT	HM_w	105	303	210	618	209
**KMT2A**	TGCT	HM_w	121	416	79	616	210
**TDRD6**	TGCT	Others	381	69	163	613	211
**GADD45A**	TGCT	Others	165	69	376	610	212
**SMNDC1**	TGCT	Others	39	253	318	610	213
**KDM6B**	TGCT	HM_e	392	69	147	608	214
**CBX4**	TGCT	HM_r	313	69	225	607	215
**L3MBTL4**	TGCT	Others	114	405	88	607	216
**KDM7A**	TGCT	HM_e	123	69	414	606	217
**NAP1L2**	TGCT	Others	100	297	209	606	218
**ING4**	TGCT	HM_r	142	421	42	605	219
**PADI4**	TGCT	Others	272	290	42	604	220
**CXXC1**	TGCT	Others	421	69	112	602	221
**GADD45B**	TGCT	Others	164	328	110	602	222
**BRDT**	TGCT	HA_r	196	69	331	596	223
**RNF20**	TGCT	Others	250	69	276	595	224
**CHD1**	TGCT	Helicases	368	69	157	594	225
**EHMT2**	TGCT	HM_w	302	69	222	593	226
**PHF19**	TGCT	HM_r	268	69	256	593	227
**SMYD5**	TGCT	HM_w	229	69	295	593	228
**CHD6**	TGCT	Helicases	366	69	156	591	229
**PHIP**	TGCT	HA_r	266	280	42	588	230
**AURKB**	TGCT	Others	201	69	314	584	231

**BAZ1A**	TGCT	HA_r	200	69	313	582	232
**UBE2A**	TGCT	Others	22	243	317	582	233
**HDAC5**	TGCT	HA_e	360	69	152	581	234
**JADE3**	TGCT	Others	136	69	374	579	235
**TET3**	TGCT	DM_e	217	69	293	579	236
**DPF3**	TGCT	HA_r	179	69	329	577	237
**TP53BP1**	TGCT	Others	216	69	292	577	238
**PHF3**	TGCT	Others	267	69	239	575	239
**ING1**	TGCT	HM_r	288	69	216	573	240
**CBX8**	TGCT	ChRC	189	69	312	570	241
**KDM4C**	TGCT	HM_e	126	401	42	569	242
**CDYL**	TGCT	HM_r	188	69	311	568	243
**PWWP2B**	TGCT	Others	65	403	99	567	244
**SP140L**	TGCT	HA_r	226	69	272	567	245
**CTCF**	TGCT	Others	182	342	42	566	246
**KAT7**	TGCT	HA_w	130	69	366	565	247
**PARP1**	TGCT	Others	98	288	178	564	248
**HNF1A**	TGCT	ChRC	147	69	346	562	249
**DPF2**	TGCT	Others	180	337	42	559	250
**ZMYND11**	TGCT	HA_r	11	411	137	559	251
**PHC1**	TGCT	ChRC	92	424	42	558	252
**TRIM28**	TGCT	HA_r	328	69	161	558	253
**KAT2B**	TGCT	HA_w	132	318	107	557	254
**PCMT1**	TGCT	Others	93	285	177	555	255
**KANSL1**	TGCT	HA_w	357	69	128	554	256
**CARM1**	TGCT	HM_w	194	69	288	551	257
**FBXW9**	TGCT	Others	171	69	308	548	258
**SETD4**	TGCT	HM_w	52	69	426	547	259
**PCGF6**	TGCT	Others	269	69	207	545	260
**HIST1H1B**	TGCT	Others	291	69	184	544	261
**SIRT1**	TGCT	HA_e	239	69	235	543	262
**HIST1H1C**	TGCT	Others	290	69	183	542	263
**SFMBT1**	TGCT	HM_r	49	256	236	541	264
**PRDM4**	TGCT	HM_w	73	69	397	539	265
**RAI1**	TGCT	Others	384	69	83	536	266

**SMARCA5**	TGCT	Helicases	383	69	81	533	267
**KMT2E**	TGCT	HM_w	117	310	105	532	268
**HCFC1**	TGCT	Others	419	69	42	530	269
**PCGF2**	TGCT	Others	95	69	365	529	270
**HDGF**	TGCT	Others	153	69	306	528	271
**KDM3B**	TGCT	HM_e	353	69	106	528	272
**MLLT6**	TGCT	HM_w	279	69	180	528	273
**ARID1A**	TGCT	ChRC	323	69	135	527	274
**PRMT3**	TGCT	HM_w	256	69	202	527	275
**GATAD2A**	TGCT	HM_r	362	69	93	524	276
**USP51**	TGCT	Others	16	239	269	524	277
**MBD2**	TGCT	DM_r	112	307	104	523	278
**PHF2**	TGCT	Others	412	69	42	523	279
**SFMBT2**	TGCT	Others	48	391	82	521	280
**SMARCA4**	TGCT	Helicases	410	69	42	521	281
**PADI1**	TGCT	Others	99	293	126	518	282
**MORF4L1**	TGCT	HM_r	104	69	344	517	283
**SATB1**	TGCT	Others	247	69	200	516	284
**SETDB1**	TGCT	HM_w	51	69	396	516	285
**BRD4**	TGCT	HA_r	404	69	42	515	286
**PHF21B**	TGCT	HM_r	342	69	102	513	287
**PRDM11**	TGCT	HM_w	263	69	176	508	288
**GTF2H1**	TGCT	Others	396	69	42	507	289
**MPHOSPH8**	TGCT	HM_r	349	69	86	504	290
**SIRT5**	TGCT	HA_e	237	69	197	503	291
**SIRT7**	TGCT	HA_e	236	69	196	501	292
**ATAT1**	TGCT	Others	203	69	227	499	293
**G2E3**	TGCT	Others	166	69	264	499	294
**KDM1B**	TGCT	HM_e	128	69	302	499	295
**IDH2**	TGCT	DM_e	144	69	284	497	296
**GTF2F1**	TGCT	Others	162	69	263	494	297
**RBBP4**	TGCT	ChRC	61	69	364	494	298
**SP100**	TGCT	HA_r	228	69	195	492	299
**WDR5**	TGCT	ChRC	211	238	42	491	300
**PCGF1**	TGCT	Others	96	69	324	489	301

**SCML4**	TGCT	Others	54	264	171	489	302
**SCMH1**	TGCT	Others	246	69	172	487	303
**PYGO2**	TGCT	HM_r	63	69	354	486	304
**BRD8**	TGCT	HA_r	373	69	42	484	305
**PRKCD**	TGCT	Others	69	271	143	483	306
**KAT6B**	TGCT	HA_w	285	69	127	481	307
**YY1**	TGCT	ChRC	14	236	230	480	308
**KMT2B**	TGCT	HM_w	120	313	42	475	309
**ZCWPW2**	TGCT	HM_r	12	234	229	475	310
**SMARCB1**	TGCT	Helicases	44	69	360	473	311
**PRKAA2**	TGCT	Others	258	69	144	471	312
**SMARCE1**	TGCT	Helicases	233	69	169	471	313
**SMYD1**	TGCT	HM_w	232	69	168	469	314
**RNF2**	TGCT	ChRC	58	69	339	466	315
**JMJD8**	TGCT	HM_e	134	69	262	465	316
**SIRT4**	TGCT	HA_e	45	69	350	464	317
**SP140**	TGCT	HA_r	227	69	166	462	318
**KDM4A**	TGCT	HM_e	127	69	261	457	319
**PRMT6**	TGCT	HM_w	66	69	320	455	320
**SIN3A**	TGCT	ChRC	47	69	337	453	321
**PHF23**	TGCT	HM_r	341	69	42	452	322
**TDRD12**	TGCT	Others	219	69	164	452	323
**TRIM33**	TGCT	HA_r	24	69	359	452	324
**PHF20**	TGCT	HM_r	84	69	298	451	325
**UHRF2**	TGCT	DM_r	19	388	42	449	326
**JMJD6**	TGCT	HM_e	135	69	244	448	327
**TDG**	TGCT	ChRC	30	69	348	447	328
**SMARCD2**	TGCT	Helicases	41	69	336	446	329
**HDAC3**	TGCT	HA_e	156	69	220	445	330
**KAT2A**	TGCT	HA_w	133	69	243	445	331
**PAF1**	TGCT	Others	271	69	103	443	332
**TAF1**	TGCT	HA_r	33	366	42	441	333
**PRDM1**	TGCT	HM_w	77	278	85	440	334
**HDGFL1**	TGCT	Others	152	69	218	439	335
**PHF7**	TGCT	Others	81	281	77	439	336

**PRDM12**	TGCT	HM_w	76	277	84	437	337
**AFF4**	TGCT	Others	325	69	42	436	338
**HLTF**	TGCT	Others	148	69	217	434	339
**MECP2**	TGCT	DM_r	106	69	259	434	340
**SETD7**	TGCT	HM_w	242	69	119	430	341
**PRDM8**	TGCT	HM_w	260	69	100	429	342
**BRD2**	TGCT	HA_r	316	69	42	427	343
**FKBP1A**	TGCT	Others	170	69	187	426	344
**CBX1**	TGCT	HM_r	314	69	42	425	345
**INTS12**	TGCT	Others	140	69	215	424	346
**USP27X**	TGCT	Others	17	365	42	424	347
**CHD2**	TGCT	Helicases	311	69	42	422	348
**CBX2**	TGCT	ChRC	193	69	159	421	349
**CBX5**	TGCT	HM_r	191	69	158	418	350
**ZGPAT**	TGCT	Others	210	69	138	417	351
**AKAP1**	TGCT	Others	205	69	136	410	352
**FXR2**	TGCT	Others	299	69	42	410	353
**GLYR1**	TGCT	HM_r	297	69	42	408	354
**SETDB2**	TGCT	HM_w	50	258	98	406	355
**KDM8**	TGCT	HM_e	122	69	214	405	356
**RTF1**	TGCT	Others	55	69	275	399	357
**UBE2I**	TGCT	Others	214	69	115	398	358
**L3MBTL2**	TGCT	Others	116	69	212	397	359
**ING2**	TGCT	HM_r	143	69	182	394	360
**KDM5D**	TGCT	HM_e	283	69	42	394	361
**RPH3A**	TGCT	Others	249	69	76	394	362
**CBX7**	TGCT	HM_r	190	69	133	392	363
**ING5**	TGCT	HM_r	141	69	181	391	364
**AFF1**	TGCT	Others	207	69	114	390	365
**POLE3**	TGCT	ChRC	79	69	237	385	366
**SMARCC2**	TGCT	Helicases	42	69	273	384	367
**UBE2B**	TGCT	Others	21	69	291	381	368
**PRMT5**	TGCT	HM_w	67	269	42	378	369
**SUZ12**	TGCT	ChRC	34	69	271	374	370
**ERCC5**	TGCT	Others	174	69	130	373	371

**HIST1H3B**	TGCT	Others	149	69	151	369	372
**IDH1**	TGCT	DM_e	145	69	150	364	373
**PHF1**	TGCT	HM_r	90	69	205	364	374
**DAXX**	TGCT	ChRC	181	69	111	361	375
**PHF12**	TGCT	Others	87	69	204	360	376
**SETD2**	TGCT	HM_w	53	262	42	357	377
**JADE1**	TGCT	Others	138	69	129	336	378
**PYGO1**	TGCT	HM_r	64	69	201	334	379
**TDRD10**	TGCT	Others	29	69	233	331	380
**HELLS**	TGCT	Helicases	151	69	109	329	381
**TDRD5**	TGCT	Others	27	69	232	328	382
**SUV39H2**	TGCT	HM_w	35	247	42	324	383
**UHRF1**	TGCT	DM_r	213	69	42	324	384
**UTY**	TGCT	HM_e	212	69	42	323	385
**H2AFZ**	TGCT	Others	161	69	92	322	386
**JADE2**	TGCT	Others	137	69	108	314	387
**BRD7**	TGCT	HA_r	197	69	42	308	388
**USP22**	TGCT	Others	18	240	42	300	389
**WDR82**	TGCT	Others	15	237	42	294	390
**TCF19**	TGCT	Others	31	69	193	293	391
**EZH1**	TGCT	HM_w	173	69	42	284	392
**TRIM66**	TGCT	HA_r	23	69	191	283	393
**PRMT1**	TGCT	HM_w	68	69	142	279	394
**GTF2B**	TGCT	Others	163	69	42	274	395
**SMYD4**	TGCT	HM_w	38	69	167	274	396
**PRDM5**	TGCT	HM_w	72	69	124	265	397
**MBTD1**	TGCT	Others	108	69	87	264	398
**HIF1AN**	TGCT	Others	150	69	42	261	399
**HSPBAP1**	TGCT	Others	146	69	42	257	400
**RAG2**	TGCT	HM_r	62	69	122	253	401
**RNF17**	TGCT	Others	59	69	121	249	402
**SMARCD3**	TGCT	Helicases	40	69	140	249	403
**SP110**	TGCT	HA_r	37	69	139	245	404
**SMARCC1**	TGCT	Helicases	43	69	118	230	405
**MARCH5**	TGCT	Others	113	69	42	224	406

**MBD4**	TGCT	DM_r	110	69	42	221	407
**TDRD7**	TGCT	Others	26	69	116	211	408
**PCGF5**	TGCT	Others	94	69	42	205	409
**PHF5A**	TGCT	Others	83	69	42	194	410
**PHF6**	TGCT	HM_r	82	69	42	193	411
**TET2**	TGCT	DM_e	25	69	97	191	412
**PRDM6**	TGCT	HM_w	71	69	42	182	413
**PRKAA1**	TGCT	Others	70	69	42	181	414
**TDRD3**	TGCT	HM_r	28	69	75	172	415
**SSRP1**	TGCT	Others	36	69	42	147	416
**CDYL2**	THCA	HM_r	405	398	378	1181	1
**CBX1**	THCA	HM_r	367	402	410	1179	2
**KAT6B**	THCA	HA_w	396	419	347	1162	3
**CHAF1A**	THCA	ChRC	363	373	377	1113	4
**KDM4B**	THCA	HM_e	337	364	412	1113	5
**HDAC10**	THCA	HA_e	349	396	360	1105	6
**BRD8**	THCA	HA_r	370	375	353	1098	7
**NSD1**	THCA	HM_w	415	359	320	1094	8
**BRD1**	THCA	HA_r	257	403	424	1084	9
**BAZ1B**	THCA	HA_r	372	349	354	1075	10
**CHD3**	THCA	Helicases	420	344	311	1075	11
**HDAC9**	THCA	HA_e	347	329	391	1067	12
**KAT2A**	THCA	HA_w	397	392	278	1067	13
**MECP2**	THCA	DM_r	393	361	302	1056	14
**BPTF**	THCA	HA_r	421	404	221	1046	15
**AFF4**	THCA	Others	275	376	392	1043	16
**HDAC5**	THCA	HA_e	398	395	242	1035	17
**PRDM2**	THCA	HM_w	387	306	330	1023	18
**ATRX**	THCA	Helicases	374	351	289	1014	19
**SP140**	THCA	HA_r	381	290	343	1014	20
**HELLS**	THCA	Helicases	346	328	337	1011	21
**KDM5B**	THCA	HM_e	161	418	417	996	22
**HDAC3**	THCA	HA_e	198	367	422	987	23
**DPF3**	THCA	HA_r	357	341	284	982	24
**TRIM24**	THCA	HA_r	286	280	400	966	25

**CBX2**	THCA	ChRC	366	401	195	962	26
**PCGF2**	THCA	Others	392	389	180	961	27
**CBX4**	THCA	HM_r	247	400	312	959	28
**FXR2**	THCA	Others	213	336	409	958	29
**AKAP1**	THCA	Others	272	405	264	941	30
**GTF2H1**	THCA	Others	206	333	402	941	31
**CBX8**	THCA	ChRC	245	399	287	931	32
**GTF2F1**	THCA	Others	207	334	383	924	33
**EPC1**	THCA	Others	222	339	362	923	34
**DOT1L**	THCA	HM_w	358	371	193	922	35
**KDM6B**	THCA	HM_e	394	321	204	919	36
**ZCWPW1**	THCA	HM_r	282	276	355	913	37
**H3F3A**	THCA	Others	203	421	281	905	38
**FKBP5**	THCA	Others	214	337	350	901	39
**NCOR1**	THCA	ChRC	416	313	170	899	40
**POLE3**	THCA	ChRC	103	385	411	899	41
**PCGF5**	THCA	Others	121	388	389	898	42
**STK31**	THCA	Others	411	288	197	896	43
**CHRAC1**	THCA	ChRC	238	372	285	895	44
**RBBP5**	THCA	ChRC	84	412	394	890	45
**SMARCA1**	THCA	Helicases	383	296	210	889	46
**GADD45B**	THCA	Others	211	369	308	888	47
**PARP1**	THCA	Others	126	415	346	887	48
**JMJD1C**	THCA	HM_e	424	420	42	886	49
**HDAC8**	THCA	HA_e	348	330	206	884	50
**TET1**	THCA	DM_e	287	407	187	881	51
**KMT2E**	THCA	HM_w	152	317	407	876	52
**SP140L**	THCA	HA_r	291	289	295	875	53
**SMYD2**	THCA	HM_w	48	409	413	870	54
**HDAC6**	THCA	HA_e	196	331	338	865	55
**KDM5A**	THCA	HM_e	335	323	205	863	56
**TDRD5**	THCA	Others	288	282	293	863	57
**PRDM9**	THCA	HM_w	413	303	146	862	58
**PHF11**	THCA	Others	113	414	332	859	59
**NAP1L3**	THCA	Others	326	314	216	856	60

**SMYD3**	THCA	HM_w	47	424	385	856	61
**ASH1L**	THCA	HM_w	422	69	364	855	62
**UBE2A**	THCA	Others	284	279	291	854	63
**TAF1L**	THCA	HA_r	379	285	188	852	64
**HDAC4**	THCA	HA_e	197	332	322	851	65
**JMJD6**	THCA	HM_e	172	394	279	845	66
**EZH1**	THCA	HM_w	399	397	42	838	67
**PHF2**	THCA	Others	108	311	419	838	68
**BRD3**	THCA	HA_r	371	423	42	836	69
**MLLT6**	THCA	HM_w	140	390	301	831	70
**SMARCE1**	THCA	Helicases	51	379	401	831	71
**CECR2**	THCA	HA_r	243	425	162	830	72
**GATAD2B**	THCA	HM_r	351	69	408	828	73
**PRDM4**	THCA	HM_w	386	69	369	824	74
**KDM2B**	THCA	HM_e	340	69	414	823	75
**KDM6A**	THCA	HM_e	334	322	166	822	76
**LBR**	THCA	Others	147	417	257	821	77
**CARM1**	THCA	HM_w	249	346	220	815	78
**CBX5**	THCA	HM_r	365	69	379	813	79
**MUM1**	THCA	Others	132	316	359	807	80
**ACTL6B**	THCA	ChRC	278	353	175	806	81
**SIRT7**	THCA	HA_e	57	381	365	803	82
**ATAD2**	THCA	HA_r	407	352	42	801	83
**GTF3C4**	THCA	HA_w	205	422	173	800	84
**HIRA**	THCA	Others	191	366	241	798	85
**HIST1H1C**	THCA	Others	345	69	375	789	86
**PRDM16**	THCA	HM_w	388	69	331	788	87
**USP22**	THCA	Others	18	377	393	788	88
**CTCF**	THCA	Others	402	343	42	787	89
**MPHOSPH8**	THCA	HM_r	328	69	390	787	90
**TDRD6**	THCA	Others	377	69	341	787	91
**DNMT1**	THCA	DM_w	401	342	42	785	92
**FBXW9**	THCA	Others	217	338	230	785	93
**KAT8**	THCA	HA_w	341	69	373	783	94
**BAP1**	THCA	Others	373	69	339	781	95

**BRWD3**	THCA	HA_r	250	347	184	781	96
**MBD3**	THCA	DM_r	144	362	275	781	97
**SIRT4**	THCA	HA_e	295	69	416	780	98
**KANSL1**	THCA	HA_w	342	393	42	777	99
**DPY30**	THCA	Others	228	340	208	776	100
**PRDM15**	THCA	HM_w	389	69	318	776	101
**FBXO44**	THCA	Others	354	69	351	774	102
**MEN1**	THCA	ChRC	330	69	372	771	103
**PHF10**	THCA	Others	114	312	345	771	104
**SETDB1**	THCA	HM_w	298	69	404	771	105
**RBBP7**	THCA	ChRC	83	358	329	770	106
**SIRT6**	THCA	HA_e	58	356	356	770	107
**RBBP4**	THCA	ChRC	304	69	395	768	108
**AURKB**	THCA	Others	262	350	155	767	109
**RNF20**	THCA	Others	80	299	387	766	110
**FMR1**	THCA	Others	353	370	42	765	111
**HCFC1**	THCA	Others	350	368	42	760	112
**SMARCD2**	THCA	Helicases	52	380	327	759	113
**JMJD8**	THCA	HM_e	171	325	258	754	114
**SETD6**	THCA	HM_w	299	69	386	754	115
**MSL3**	THCA	HA_w	137	360	256	753	116
**IDH1**	THCA	DM_e	184	327	240	751	117
**SETDB2**	THCA	HM_w	66	411	270	747	118
**KDM3B**	THCA	HM_e	338	365	42	745	119
**PRMT3**	THCA	HM_w	307	69	368	744	120
**SMYD4**	THCA	HM_w	46	293	403	742	121
**PHRF1**	THCA	Others	390	307	42	739	122
**ARID4B**	THCA	ChRC	269	426	42	737	123
**CHD2**	THCA	Helicases	404	69	262	735	124
**PRMT1**	THCA	HM_w	309	69	357	735	125
**EPC2**	THCA	Others	355	69	310	734	126
**ATAD2B**	THCA	HA_r	265	69	399	733	127
**TP53BP1**	THCA	Others	410	69	249	728	128
**RNF2**	THCA	ChRC	303	382	42	727	129
**SETD1A**	THCA	HM_w	385	69	271	725	130

**INO80**	THCA	Helicases	344	69	307	720	131
**NAP1L2**	THCA	Others	131	315	274	720	132
**PRDM8**	THCA	HM_w	414	69	237	720	133
**CHD4**	THCA	Helicases	362	69	286	717	134
**EP300**	THCA	HA_w	224	69	420	713	135
**EHMT2**	THCA	HM_w	356	69	283	708	136
**TCF20**	THCA	Others	378	283	42	703	137
**KDM4C**	THCA	HM_e	336	324	42	702	138
**POLR2B**	THCA	Others	313	69	319	701	139
**SCML2**	THCA	HM_r	72	357	272	701	140
**TDRD7**	THCA	Others	376	281	42	699	141
**ASXL3**	THCA	Others	266	69	363	698	142
**TET3**	THCA	DM_e	375	69	250	694	143
**TRIM28**	THCA	HA_r	285	69	340	694	144
**EP400**	THCA	HA_w	223	69	398	690	145
**PRMT7**	THCA	HM_w	90	302	298	690	146
**CREBBP**	THCA	HA_w	359	69	260	688	147
**SMARCD3**	THCA	Helicases	292	69	326	687	148
**HDGF**	THCA	Others	194	69	421	684	149
**PHF23**	THCA	HM_r	319	309	42	670	150
**BRD9**	THCA	HA_r	253	374	42	669	151
**PHF20L1**	THCA	HM_r	106	310	253	669	152
**PHF6**	THCA	HM_r	317	308	42	667	153
**SMARCA2**	THCA	Helicases	56	295	316	667	154
**DNMT3B**	THCA	DM_w	232	69	352	653	155
**PRDM12**	THCA	HM_w	100	413	139	652	156
**SFMBT2**	THCA	Others	384	69	199	652	157
**ASH2L**	THCA	HM_w	268	69	314	651	158
**HAT1**	THCA	HA_w	202	69	376	647	159
**RAI1**	THCA	Others	305	300	42	647	160
**HDAC7**	THCA	HA_e	195	69	382	646	161
**BRD4**	THCA	HA_r	255	348	42	645	162
**L3MBTL2**	THCA	Others	150	69	425	644	163
**BAZ2A**	THCA	HA_r	260	69	313	642	164
**CBX6**	THCA	HM_r	364	69	209	642	165

**PHF7**	THCA	Others	316	69	252	637	166
**SIN3B**	THCA	ChRC	297	298	42	637	167
**AFF1**	THCA	Others	276	69	290	635	168
**SIRT2**	THCA	HA_e	296	69	269	634	169
**HDAC1**	THCA	HA_e	201	69	361	631	170
**SMARCA4**	THCA	Helicases	294	294	42	630	171
**HR**	THCA	HM_e	186	69	374	629	172
**CHD1**	THCA	Helicases	241	345	42	628	173
**UBE2B**	THCA	Others	24	355	248	627	174
**BRDT**	THCA	HA_r	369	69	185	623	175
**CHD7**	THCA	Helicases	360	69	194	623	176
**PRDM7**	THCA	HM_w	96	384	140	620	177
**NCOR2**	THCA	Others	323	69	227	619	178
**TAF1**	THCA	HA_r	290	286	42	618	179
**BRD2**	THCA	HA_r	256	69	288	613	180
**BRWD1**	THCA	HA_r	368	69	174	611	181
**RNF17**	THCA	Others	81	383	145	609	182
**SIRT3**	THCA	HA_e	60	297	251	608	183
**MECOM**	THCA	Others	141	69	397	607	184
**HNF1A**	THCA	ChRC	187	69	349	605	185
**PRDM5**	THCA	HM_w	98	305	201	604	186
**GTF2B**	THCA	Others	208	69	323	600	187
**KAT7**	THCA	HA_w	167	391	42	600	188
**MBTD1**	THCA	Others	142	416	42	600	189
**PAXIP1**	THCA	Others	124	69	406	599	190
**ATAT1**	THCA	Others	264	69	263	596	191
**FBXO17**	THCA	Others	218	69	309	596	192
**ING4**	THCA	HM_r	179	69	348	596	193
**PHF5A**	THCA	Others	104	69	423	596	194
**PPARGC1A**	THCA	Others	312	69	214	595	195
**SUV39H1**	THCA	HM_w	39	287	268	594	196
**HLTF**	THCA	Others	188	69	336	593	197
**CHD5**	THCA	Helicases	361	69	161	591	198
**MORF4L1**	THCA	HM_r	139	69	381	589	199
**GATAD2A**	THCA	HM_r	210	335	42	587	200

**PYGO1**	THCA	HM_r	306	69	212	587	201
**NCOA1**	THCA	HA_w	325	69	190	584	202
**ZGPAT**	THCA	Others	281	69	233	583	203
**USP51**	THCA	Others	283	69	224	576	204
**PADI6**	THCA	Others	320	69	181	570	205
**CLOCK**	THCA	HA_w	237	69	261	567	206
**KDM4D**	THCA	HM_e	163	69	335	567	207
**NAP1L1**	THCA	Others	327	69	171	567	208
**RNF40**	THCA	Others	78	69	418	565	209
**ATF7IP**	THCA	Others	263	69	232	564	210
**JARID2**	THCA	ChRC	343	69	151	563	211
**PCGF1**	THCA	Others	122	69	371	562	212
**ASXL2**	THCA	Others	267	69	223	559	213
**DPF1**	THCA	ChRC	230	69	259	558	214
**KDM1A**	THCA	HM_e	166	69	321	556	215
**H2AFZ**	THCA	Others	204	69	282	555	216
**PADI2**	THCA	Others	321	69	165	555	217
**PHF1**	THCA	HM_r	115	69	370	554	218
**PRMT8**	THCA	HM_w	89	69	396	554	219
**BAZ1A**	THCA	HA_r	261	69	222	552	220
**KDM7A**	THCA	HM_e	158	69	324	551	221
**L3MBTL4**	THCA	Others	148	69	334	551	222
**MARCH5**	THCA	Others	146	363	42	551	223
**SMARCB1**	THCA	Helicases	55	69	426	550	224
**CXXC1**	THCA	Others	235	69	245	549	225
**HDAC11**	THCA	HA_e	200	69	280	549	226
**DAXX**	THCA	ChRC	234	69	244	547	227
**ING5**	THCA	HM_r	178	326	42	546	228
**PADI1**	THCA	Others	322	69	153	544	229
**PYGO2**	THCA	HM_r	86	69	388	543	230
**SETD3**	THCA	HM_w	69	69	405	543	231
**PHF12**	THCA	Others	112	387	42	541	232
**KDM1B**	THCA	HM_e	165	69	306	540	233
**ARID1B**	THCA	ChRC	426	69	42	537	234
**PHF19**	THCA	HM_r	109	386	42	537	235

**ATM**	THCA	Others	425	69	42	536	236
**KDM4E**	THCA	HM_e	162	69	305	536	237
**ATR**	THCA	Others	423	69	42	534	238
**DMAP1**	THCA	Others	233	69	231	533	239
**SP110**	THCA	HA_r	42	291	198	531	240
**CHAF1B**	THCA	ChRC	242	69	219	530	241
**CHD9**	THCA	Helicases	419	69	42	530	242
**DIDO1**	THCA	Others	418	69	42	529	243
**KMT2A**	THCA	HM_w	156	69	304	529	244
**DNMT3A**	THCA	DM_w	417	69	42	528	245
**FKBP1A**	THCA	Others	216	69	243	528	246
**SND1**	THCA	HM_r	44	69	415	528	247
**PARP2**	THCA	Others	125	69	333	527	248
**PRDM13**	THCA	HM_w	310	69	147	526	249
**SETD5**	THCA	HM_w	412	69	42	523	250
**PRKAA2**	THCA	Others	94	69	358	521	251
**ARID2**	THCA	ChRC	409	69	42	520	252
**ASXL1**	THCA	Others	408	69	42	519	253
**KIAA2026**	THCA	Others	157	320	42	519	254
**BAZ2B**	THCA	HA_r	406	69	42	517	255
**MBD2**	THCA	DM_r	145	69	303	517	256
**KAT2B**	THCA	HA_w	170	69	277	516	257
**KMT2B**	THCA	HM_w	155	319	42	516	258
**PRDM1**	THCA	HM_w	102	69	344	515	259
**CHD6**	THCA	Helicases	403	69	42	514	260
**KMT2C**	THCA	HM_w	154	318	42	514	261
**BOP 1**	THCA	Others	258	69	186	513	262
**SIRT1**	THCA	HA_e	61	410	42	513	263
**ZMYND8**	THCA	HA_r	280	69	164	513	264
**EED**	THCA	ChRC	400	69	42	511	265
**RPS6KA5**	THCA	Others	75	69	367	511	266
**GADD45A**	THCA	Others	212	69	229	510	267
**EZH2**	THCA	HM_w	220	69	218	507	268
**KDM2A**	THCA	HM_e	395	69	42	506	269
**SETD1B**	THCA	HM_w	70	69	366	505	270

**AICDA**	THCA	DM_e	274	69	160	503	271
**PHF21A**	THCA	HM_r	391	69	42	502	272
**KMT2D**	THCA	HM_w	153	69	276	498	273
**TDRD1**	THCA	Others	289	69	138	496	274
**SMARCA5**	THCA	Helicases	382	69	42	493	275
**FKBP2**	THCA	Others	215	69	207	491	276
**SRCAP**	THCA	Others	380	69	42	491	277
**JADE1**	THCA	Others	175	69	246	490	278
**AIRE**	THCA	HM_r	273	69	144	486	279
**PHC2**	THCA	ChRC	117	69	300	486	280
**TDG**	THCA	ChRC	33	69	384	486	281
**FBXL19**	THCA	Others	219	69	192	480	282
**TDRD3**	THCA	HM_r	30	408	42	480	283
**RING1**	THCA	Others	82	69	328	479	284
**CBX7**	THCA	HM_r	246	69	163	478	285
**ELP3**	THCA	HA_w	226	69	183	478	286
**TAF3**	THCA	HA_r	36	284	156	476	287
**PHF21B**	THCA	HM_r	105	69	299	473	288
**G2E3**	THCA	Others	352	69	42	463	289
**WDR5**	THCA	ChRC	15	406	42	463	290
**ZMYND11**	THCA	HA_r	11	69	380	460	291
**SATB1**	THCA	Others	73	69	317	459	292
**SUZ12**	THCA	ChRC	37	378	42	457	293
**KAT6A**	THCA	HA_w	168	69	217	454	294
**ORC1**	THCA	Others	130	69	255	454	295
**KDM3A**	THCA	HM_e	339	69	42	450	296
**PAF1**	THCA	Others	127	69	254	450	297
**CSTL1**	THCA	Others	236	69	142	447	298
**TCEA1**	THCA	Others	35	69	342	446	299
**KDM8**	THCA	HM_e	333	69	42	444	300
**DNMT3L**	THCA	DM_w	231	69	143	443	301
**MBD1**	THCA	DM_r	332	69	42	443	302
**MTA2**	THCA	ChRC	135	69	239	443	303
**PRDM6**	THCA	HM_w	97	304	42	443	304
**MBD5**	THCA	DM_r	331	69	42	442	305

**MLLT10**	THCA	HM_w	329	69	42	440	306
**HIST1H3B**	THCA	Others	189	69	179	437	307
**SMARCD1**	THCA	Helicases	53	69	315	437	308
**NCOA3**	THCA	HA_w	324	69	42	435	309
**MTA1**	THCA	ChRC	136	69	228	433	310
**PSIP1**	THCA	HM_r	88	301	42	431	311
**HDGFL1**	THCA	Others	193	69	168	430	312
**PHF3**	THCA	Others	318	69	42	429	313
**ING2**	THCA	HM_r	181	69	178	428	314
**HIST1H1B**	THCA	Others	190	69	167	426	315
**PHF8**	THCA	Others	315	69	42	426	316
**PHIP**	THCA	HA_r	314	69	42	425	317
**TDRD9**	THCA	Others	29	69	325	423	318
**PRDM10**	THCA	HM_w	311	69	42	422	319
**SMARCC1**	THCA	Helicases	54	69	297	420	320
**PRMT2**	THCA	HM_w	308	69	42	419	321
**RPA3**	THCA	Others	77	69	273	419	322
**PHF13**	THCA	Others	111	69	238	418	323
**UHRF1**	THCA	DM_r	20	354	42	416	324
**RSF1**	THCA	ChRC	302	69	42	413	325
**SCMH1**	THCA	Others	301	69	42	412	326
**SETD2**	THCA	HM_w	300	69	42	411	327
**SMYD5**	THCA	HM_w	45	69	296	410	328
**L3MBTL3**	THCA	Others	149	69	191	409	329
**IDH2**	THCA	DM_e	183	69	154	406	330
**SMARCC2**	THCA	Helicases	293	69	42	404	331
**L3MBTL1**	THCA	HM_r	151	69	182	402	332
**PHC1**	THCA	ChRC	118	69	215	402	333
**PBRM1**	THCA	HA_r	123	69	203	395	334
**TDRD10**	THCA	Others	32	69	294	395	335
**JADE3**	THCA	Others	173	69	152	394	336
**ACTL6A**	THCA	ChRC	279	69	42	390	337
**MBD4**	THCA	DM_r	143	69	177	389	338
**AEBP2**	THCA	HM_w	277	69	42	388	339
**KDM5C**	THCA	HM_e	160	69	159	388	340

**TRIM66**	THCA	HA_r	25	69	292	386	341
**ARID1A**	THCA	ChRC	271	69	42	382	342
**ARID4A**	THCA	ChRC	270	69	42	381	343
**SP100**	THCA	HA_r	43	292	42	377	344
**MTF2**	THCA	HM_r	133	69	172	374	345
**SETD4**	THCA	HM_w	68	69	236	373	346
**BMI1**	THCA	ChRC	259	69	42	370	347
**PRDM14**	THCA	HM_w	99	69	202	370	348
**TCF19**	THCA	Others	34	69	267	370	349
**PWWP2B**	THCA	Others	87	69	213	369	350
**SETMAR**	THCA	HM_w	65	69	235	369	351
**SCML4**	THCA	Others	71	69	226	366	352
**BRD7**	THCA	HA_r	254	69	42	365	353
**BRPF1**	THCA	HA_r	252	69	42	363	354
**BRPF3**	THCA	HA_r	251	69	42	362	355
**SIRT5**	THCA	HA_e	59	69	234	362	356
**CBX3**	THCA	HM_r	248	69	42	359	357
**UBE2I**	THCA	Others	22	69	266	357	358
**CDYL**	THCA	HM_r	244	69	42	355	359
**CHD1L**	THCA	Helicases	240	69	42	351	360
**CHD8**	THCA	Helicases	239	69	42	350	361
**YY1**	THCA	ChRC	13	69	265	347	362
**PADI3**	THCA	Others	129	69	148	346	363
**PRDM11**	THCA	HM_w	101	69	176	346	364
**SFMBT1**	THCA	HM_r	64	69	211	344	365
**DPF2**	THCA	Others	229	69	42	340	366
**UHRF2**	THCA	DM_r	19	278	42	339	367
**EHMT1**	THCA	HM_w	227	69	42	338	368
**PHF14**	THCA	Others	110	69	158	337	369
**RNF217**	THCA	Others	79	69	189	337	370
**ELP4**	THCA	HA_w	225	69	42	336	371
**SETD7**	THCA	HM_w	67	69	200	336	372
**USP27X**	THCA	Others	17	277	42	336	373
**ERCC5**	THCA	Others	221	69	42	332	374
**SUV39H2**	THCA	HM_w	38	69	225	332	375

**ZCWPW2**	THCA	HM_r	12	69	247	328	376
**GLYR1**	THCA	HM_r	209	69	42	320	377
**PRKCD**	THCA	Others	93	69	157	319	378
**RTF1**	THCA	Others	74	69	169	312	379
**HDAC2**	THCA	HA_e	199	69	42	310	380
**RAG2**	THCA	HM_r	85	69	150	304	381
**HIF1AN**	THCA	Others	192	69	42	303	382
**HSPBAP1**	THCA	Others	185	69	42	296	383
**TDRD12**	THCA	Others	31	69	196	296	384
**RPH3A**	THCA	Others	76	69	149	294	385
**ING1**	THCA	HM_r	182	69	42	293	386
**ING3**	THCA	HM_r	180	69	42	291	387
**INTS12**	THCA	Others	177	69	42	288	388
**IWS1**	THCA	Others	176	69	42	287	389
**JADE2**	THCA	Others	174	69	42	285	390
**KAT5**	THCA	HA_w	169	69	42	280	391
**KDM4A**	THCA	HM_e	164	69	42	275	392
**KDM5D**	THCA	HM_e	159	69	42	270	393
**SMYD1**	THCA	HM_w	49	69	141	259	394
**MSH6**	THCA	HM_r	138	69	42	249	395
**MTA3**	THCA	ChRC	134	69	42	245	396
**PADI4**	THCA	Others	128	69	42	239	397
**PCGF6**	THCA	Others	120	69	42	231	398
**PCMT1**	THCA	Others	119	69	42	230	399
**PHC3**	THCA	ChRC	116	69	42	227	400
**PHF20**	THCA	HM_r	107	69	42	218	401
**PRKAA1**	THCA	Others	95	69	42	206	402
**PRMT5**	THCA	HM_w	92	69	42	203	403
**PRMT6**	THCA	HM_w	91	69	42	202	404
**SHPRH**	THCA	Others	63	69	42	174	405
**SIN3A**	THCA	ChRC	62	69	42	173	406
**SMNDC1**	THCA	Others	50	69	42	161	407
**SSRP1**	THCA	Others	41	69	42	152	408
**SUPT16H**	THCA	Others	40	69	42	151	409
**TDRKH**	THCA	Others	28	69	42	139	410

**TET2**	THCA	DM_e	27	69	42	138	411
**TRIM33**	THCA	HA_r	26	69	42	137	412
**UBE2E1**	THCA	Others	23	69	42	134	413
**UBR7**	THCA	Others	21	69	42	132	414
**UTY**	THCA	HM_e	16	69	42	127	415
**WDR82**	THCA	Others	14	69	42	125	416
**CHD3**	THYM	Helicases	421	412	398	1231	1
**ARID4B**	THYM	ChRC	408	416	390	1214	2
**ASH1L**	THYM	HM_w	425	399	389	1213	3
**MECP2**	THYM	DM_r	417	353	415	1185	4
**AKAP1**	THYM	Others	342	400	421	1163	5
**CHD1L**	THYM	Helicases	300	387	420	1107	6
**BPTF**	THYM	HA_r	323	393	388	1104	7
**CBX6**	THYM	HM_r	309	414	364	1087	8
**CDYL**	THYM	HM_r	306	426	327	1059	9
**BAZ1A**	THYM	HA_r	328	396	333	1057	10
**PRMT5**	THYM	HM_w	370	339	346	1055	11
**BAZ2B**	THYM	HA_r	326	395	332	1053	12
**CBX7**	THYM	HM_r	308	413	329	1050	13
**CHRAC1**	THYM	ChRC	296	385	363	1044	14
**FXR2**	THYM	Others	265	411	362	1038	15
**AICDA**	THYM	DM_e	344	401	276	1021	16
**HDAC5**	THYM	HA_e	247	371	396	1014	17
**BRPF1**	THYM	HA_r	402	391	217	1010	18
**PHF10**	THYM	Others	415	349	245	1009	19
**ATF7IP**	THYM	Others	332	397	274	1003	20
**PARP1**	THYM	Others	153	424	426	1003	21
**GATAD2B**	THYM	HM_r	261	377	361	999	22
**HAT1**	THYM	HA_w	255	375	360	990	23
**ARID1B**	THYM	ChRC	340	417	225	982	24
**PCMT1**	THYM	Others	146	422	410	978	25
**HDGF**	THYM	Others	242	369	358	969	26
**KDM5B**	THYM	HM_e	198	359	411	968	27
**CDYL2**	THYM	HM_r	305	390	271	966	28
**GADD45A**	THYM	Others	263	379	317	959	29

**ATAD2**	THYM	HA_r	335	398	223	956	30
**EPC1**	THYM	Others	392	383	171	946	31
**HDAC11**	THYM	HA_e	251	373	315	939	32
**BOP 1**	THYM	Others	324	394	219	937	33
**ING2**	THYM	HM_r	228	368	312	908	34
**CHD1**	THYM	Helicases	301	388	216	905	35
**BRD7**	THYM	HA_r	403	69	418	890	36
**HDAC4**	THYM	HA_e	248	372	264	884	37
**SMYD3**	THYM	HM_w	55	403	422	880	38
**KDM1A**	THYM	HM_e	208	361	309	878	39
**SETD5**	THYM	HM_w	364	330	184	878	40
**AURKB**	THYM	Others	330	415	131	876	41
**CHD8**	THYM	Helicases	399	69	405	873	42
**AFF1**	THYM	Others	410	69	391	870	43
**CHD2**	THYM	Helicases	401	69	399	869	44
**KDM5A**	THYM	HM_e	199	360	307	866	45
**PYGO2**	THYM	HM_r	102	336	425	863	46
**BRD2**	THYM	HA_r	404	69	387	860	47
**RAI1**	THYM	Others	368	335	156	859	48
**PHF21B**	THYM	HM_r	133	346	378	857	49
**KMT2A**	THYM	HM_w	190	357	305	852	50
**IWS1**	THYM	Others	223	365	261	849	51
**GTF3C4**	THYM	HA_w	258	376	212	846	52
**PRDM12**	THYM	HM_w	124	344	377	845	53
**BRD3**	THYM	HA_r	322	392	130	844	54
**JMJD1C**	THYM	HM_e	218	364	260	842	55
**CECR2**	THYM	HA_r	304	389	146	839	56
**GATAD2A**	THYM	HM_r	419	378	42	839	57
**HCFC1**	THYM	Others	254	374	211	839	58
**PRDM4**	THYM	HM_w	371	69	394	834	59
**PRDM7**	THYM	HM_w	116	341	376	833	60
**L3MBTL3**	THYM	Others	426	356	42	824	61
**PRMT1**	THYM	HM_w	110	340	374	824	62
**H3F3A**	THYM	Others	256	425	142	823	63
**SETDB1**	THYM	HM_w	79	329	407	815	64

**PBRM1**	THYM	HA_r	416	351	42	809	65
**KDM6A**	THYM	HM_e	195	358	255	808	66
**BRD1**	THYM	HA_r	405	69	331	805	67
**EPC2**	THYM	Others	277	382	144	803	68
**MBTD1**	THYM	Others	383	69	351	803	69
**KAT6A**	THYM	HA_w	418	69	311	798	70
**NSD1**	THYM	HM_w	380	69	348	797	71
**RNF217**	THYM	Others	93	406	298	797	72
**ING5**	THYM	HM_r	388	366	42	796	73
**AEBP2**	THYM	HM_w	346	402	42	790	74
**DMAP1**	THYM	Others	396	69	323	788	75
**MBD5**	THYM	DM_r	178	354	254	786	76
**EP400**	THYM	HA_w	393	69	321	783	77
**SETMAR**	THYM	HM_w	413	328	42	783	78
**ASH2L**	THYM	HM_w	338	69	367	774	79
**RNF2**	THYM	ChRC	95	333	345	773	80
**ASXL2**	THYM	Others	337	69	366	772	81
**BRWD1**	THYM	HA_r	317	69	386	772	82
**LBR**	THYM	Others	183	423	166	772	83
**NCOR1**	THYM	ChRC	161	409	201	771	84
**CSTL1**	THYM	Others	293	69	404	766	85
**TDRD9**	THYM	Others	356	69	341	766	86
**TDRD5**	THYM	Others	33	320	412	765	87
**KDM2B**	THYM	HM_e	386	69	308	763	88
**TET2**	THYM	DM_e	354	69	340	763	89
**USP22**	THYM	Others	22	317	424	763	90
**FBXO17**	THYM	Others	272	69	417	758	91
**TDRD10**	THYM	Others	36	321	400	757	92
**WDR5**	THYM	ChRC	18	316	423	757	93
**SMARCD2**	THYM	Helicases	61	324	371	756	94
**ACTL6B**	THYM	ChRC	347	69	337	753	95
**ARID2**	THYM	ChRC	409	69	275	753	96
**CHD7**	THYM	Helicases	297	69	385	751	97
**ELP3**	THYM	HA_w	279	69	403	751	98
**BRWD3**	THYM	HA_r	316	69	365	750	99

**DPF2**	THYM	Others	283	69	397	749	100
**SMYD2**	THYM	HM_w	56	404	287	747	101
**KAT2B**	THYM	HA_w	213	362	169	744	102
**ASXL3**	THYM	Others	336	69	336	741	103
**MBD3**	THYM	DM_r	180	355	205	740	104
**CHD6**	THYM	Helicases	400	69	270	739	105
**ATAD2B**	THYM	HA_r	334	69	335	738	106
**SETD2**	THYM	HM_w	365	331	42	738	107
**ATAT1**	THYM	Others	333	69	334	736	108
**CHD9**	THYM	Helicases	398	69	269	736	109
**CTCF**	THYM	Others	397	69	268	734	110
**PHF23**	THYM	HM_r	132	407	195	734	111
**CHD4**	THYM	Helicases	299	386	42	727	112
**SETDB2**	THYM	HM_w	363	69	293	725	113
**FBXW9**	THYM	Others	270	69	384	723	114
**MUM1**	THYM	Others	167	352	203	722	115
**SIRT3**	THYM	HA_e	362	69	291	722	116
**IDH1**	THYM	DM_e	389	69	262	720	117
**ATRX**	THYM	Helicases	423	69	222	714	118
**CARM1**	THYM	HM_w	315	69	330	714	119
**GADD45B**	THYM	Others	262	69	383	714	120
**TAF1L**	THYM	HA_r	360	69	285	714	121
**DOT1L**	THYM	HM_w	285	384	42	711	122
**TDRD6**	THYM	Others	357	69	283	709	123
**SP100**	THYM	HA_r	52	421	234	707	124
**TET1**	THYM	DM_e	355	69	282	706	125
**CBX8**	THYM	ChRC	307	69	328	704	126
**EP300**	THYM	HA_w	420	69	214	703	127
**TET3**	THYM	DM_e	353	69	281	703	128
**MPHOSPH8**	THYM	HM_r	381	69	252	702	129
**EZH1**	THYM	HM_w	275	381	42	698	130
**ACTL6A**	THYM	ChRC	348	69	278	695	131
**CHD5**	THYM	Helicases	298	69	326	693	132
**AFF4**	THYM	Others	345	69	277	691	133
**CLOCK**	THYM	HA_w	295	69	325	689	134

**FMR1**	THYM	Others	266	380	42	688	135
**CREBBP**	THYM	HA_w	294	69	324	687	136
**PPARGC1A**	THYM	Others	373	69	243	685	137
**PRMT8**	THYM	HM_w	105	338	240	683	138
**DNMT3A**	THYM	DM_w	288	69	322	679	139
**KAT2A**	THYM	HA_w	214	69	395	678	140
**PHF20L1**	THYM	HM_r	135	347	196	678	141
**RTF1**	THYM	Others	367	69	239	675	142
**ATM**	THYM	Others	331	69	273	673	143
**HDAC8**	THYM	HA_e	244	69	359	672	144
**MBD1**	THYM	DM_r	181	69	416	666	145
**HIF1AN**	THYM	Others	239	69	357	665	146
**PRDM10**	THYM	HM_w	126	345	193	664	147
**HIST1H1C**	THYM	Others	236	69	356	661	148
**JADE1**	THYM	Others	222	69	369	660	149
**BRPF3**	THYM	HA_r	318	69	272	659	150
**FKBP1A**	THYM	Others	269	69	320	658	151
**HDAC6**	THYM	HA_e	246	370	42	658	152
**JADE2**	THYM	Others	221	69	368	658	153
**MLLT6**	THYM	HM_w	175	69	414	658	154
**SATB1**	THYM	Others	87	332	238	657	155
**HR**	THYM	HM_e	232	69	355	656	156
**FKBP5**	THYM	Others	267	69	319	655	157
**KDM4C**	THYM	HM_e	202	69	382	653	158
**BAZ2A**	THYM	HA_r	406	69	176	651	159
**G2E3**	THYM	Others	264	69	318	651	160
**UBE2B**	THYM	Others	350	69	230	649	161
**PADI3**	THYM	Others	379	69	199	647	162
**KDM6B**	THYM	HM_e	194	410	42	646	163
**PHF2**	THYM	Others	378	69	197	644	164
**JMJD6**	THYM	HM_e	217	69	354	640	165
**BRD4**	THYM	HA_r	422	69	148	639	166
**HDAC10**	THYM	HA_e	252	69	316	637	167
**POLE3**	THYM	ChRC	374	69	194	637	168
**ARID1A**	THYM	ChRC	341	69	226	636	169

**EHMT2**	THYM	HM_w	394	69	172	635	170
**ING4**	THYM	HM_r	226	367	42	635	171
**L3MBTL4**	THYM	Others	184	69	381	634	172
**ARID4A**	THYM	ChRC	339	69	224	632	173
**KAT8**	THYM	HA_w	209	69	353	631	174
**KDM3B**	THYM	HM_e	205	69	352	626	175
**SP110**	THYM	HA_r	51	420	154	625	176
**HDGFL1**	THYM	Others	241	69	314	624	177
**DNMT3B**	THYM	DM_w	287	69	267	623	178
**KMT2C**	THYM	HM_w	385	69	167	621	179
**KANSL1**	THYM	HA_w	215	363	42	620	180
**SCML2**	THYM	HM_r	366	69	185	620	181
**HIST1H1B**	THYM	Others	237	69	313	619	182
**PRDM2**	THYM	HM_w	119	342	158	619	183
**RBBP5**	THYM	ChRC	99	334	186	619	184
**PCGF6**	THYM	Others	147	69	402	618	185
**BAZ1B**	THYM	HA_r	327	69	221	617	186
**MECOM**	THYM	Others	382	69	165	616	187
**BMI1**	THYM	ChRC	325	69	220	614	188
**SMYD4**	THYM	HM_w	54	323	236	613	189
**FBXL19**	THYM	Others	273	69	266	608	190
**BRD9**	THYM	HA_r	320	69	218	607	191
**FBXO44**	THYM	Others	271	69	265	605	192
**ZMYND11**	THYM	HA_r	12	314	279	605	193
**GTF2B**	THYM	Others	391	69	143	603	194
**SP140**	THYM	HA_r	50	419	134	603	195
**KDM7A**	THYM	HM_e	193	69	338	600	196
**PWWP2B**	THYM	Others	104	337	157	598	197
**PHC3**	THYM	ChRC	143	69	380	592	198
**KAT6B**	THYM	HA_w	211	69	310	590	199
**PHF12**	THYM	Others	140	408	42	590	200
**NAP1L2**	THYM	Others	165	69	350	584	201
**PHF20**	THYM	HM_r	136	69	379	584	202
**NAP1L3**	THYM	Others	164	69	349	582	203
**TAF3**	THYM	HA_r	359	69	152	580	204

**RING1**	THYM	Others	97	69	413	579	205
**TRIM66**	THYM	HA_r	351	69	149	569	206
**HIST1H3B**	THYM	Others	235	69	263	567	207
**PAXIP1**	THYM	Others	151	69	347	567	208
**KIAA2026**	THYM	Others	191	69	306	566	209
**DPY30**	THYM	Others	281	69	215	565	210
**SCML4**	THYM	Others	85	69	409	563	211
**PRDM1**	THYM	HM_w	372	69	121	562	212
**RNF40**	THYM	Others	92	69	401	562	213
**KMT2D**	THYM	HM_w	188	69	304	561	214
**L3MBTL1**	THYM	HM_r	186	69	303	558	215
**SETD6**	THYM	HM_w	81	69	408	558	216
**PRKCD**	THYM	Others	111	69	375	555	217
**FKBP2**	THYM	Others	268	69	213	550	218
**PYGO1**	THYM	HM_r	103	69	373	545	219
**SETD3**	THYM	HM_w	83	69	393	545	220
**AIRE**	THYM	HM_r	343	69	132	544	221
**PHC1**	THYM	ChRC	145	350	42	537	222
**CXXC1**	THYM	Others	292	69	175	536	223
**ASXL1**	THYM	Others	424	69	42	535	224
**BRDT**	THYM	HA_r	319	69	147	535	225
**RNF20**	THYM	Others	94	69	372	535	226
**SIRT2**	THYM	HA_e	73	69	392	534	227
**SIRT7**	THYM	HA_e	69	327	137	533	228
**HDAC1**	THYM	HA_e	253	69	210	532	229
**KDM4B**	THYM	HM_e	203	69	259	531	230
**KDM4D**	THYM	HM_e	201	69	258	528	231
**PHF14**	THYM	Others	138	348	42	528	232
**DPF1**	THYM	ChRC	284	69	174	527	233
**SMARCB1**	THYM	Helicases	65	326	136	527	234
**KDM4E**	THYM	HM_e	200	69	257	526	235
**SETD1A**	THYM	HM_w	414	69	42	525	236
**DPF3**	THYM	HA_r	282	69	173	524	237
**SHPRH**	THYM	Others	76	405	42	523	238
**SIN3A**	THYM	ChRC	412	69	42	523	239

**KDM5C**	THYM	HM_e	197	69	256	522	240
**SMARCA4**	THYM	Helicases	411	69	42	522	241
**HDAC9**	THYM	HA_e	243	69	209	521	242
**SUPT16H**	THYM	Others	45	69	406	520	243
**SUV39H1**	THYM	HM_w	44	322	153	519	244
**ATR**	THYM	Others	407	69	42	518	245
**HELLS**	THYM	Helicases	240	69	208	517	246
**JADE3**	THYM	Others	220	69	227	516	247
**PHF1**	THYM	HM_r	142	69	302	513	248
**CBX2**	THYM	ChRC	313	69	129	511	249
**CBX3**	THYM	HM_r	312	69	128	509	250
**SP140L**	THYM	HA_r	49	418	42	509	251
**EED**	THYM	ChRC	395	69	42	506	252
**PRDM15**	THYM	HM_w	121	343	42	506	253
**ZCWPW2**	THYM	HM_r	14	315	177	506	254
**ZCWPW1**	THYM	HM_r	15	69	419	503	255
**GTF2F1**	THYM	Others	390	69	42	501	256
**PHF5A**	THYM	Others	131	69	301	501	257
**DNMT3L**	THYM	DM_w	286	69	145	500	258
**CHAF1A**	THYM	ChRC	303	69	127	499	259
**KDM2A**	THYM	HM_e	387	69	42	498	260
**CHAF1B**	THYM	ChRC	302	69	126	497	261
**MORF4L1**	THYM	HM_r	174	69	253	496	262
**MBD2**	THYM	DM_r	384	69	42	495	263
**PRDM11**	THYM	HM_w	125	69	300	494	264
**MSH6**	THYM	HM_r	173	69	251	493	265
**JMJD8**	THYM	HM_e	216	69	207	492	266
**SFMBT2**	THYM	Others	77	69	344	490	267
**MTA2**	THYM	ChRC	170	69	250	489	268
**HDAC3**	THYM	HA_e	249	69	170	488	269
**PHF3**	THYM	Others	377	69	42	488	270
**PHIP**	THYM	HA_r	376	69	42	487	271
**MTF2**	THYM	HM_r	168	69	249	486	272
**PHRF1**	THYM	Others	375	69	42	486	273
**PRDM5**	THYM	HM_w	118	69	299	486	274

**SIRT4**	THYM	HA_e	72	69	343	484	275
**KDM3A**	THYM	HM_e	206	69	206	481	276
**NCOA1**	THYM	HA_w	163	69	248	480	277
**PSIP1**	THYM	HM_r	369	69	42	480	278
**PADI2**	THYM	Others	157	69	247	473	279
**SND1**	THYM	HM_r	361	69	42	472	280
**TDRD7**	THYM	Others	32	69	370	471	281
**PAF1**	THYM	Others	154	69	246	469	282
**TCF20**	THYM	Others	358	69	42	469	283
**TP53BP1**	THYM	Others	352	69	42	463	284
**UHRF1**	THYM	DM_r	349	69	42	460	285
**RPA3**	THYM	Others	91	69	297	457	286
**RPS6KA5**	THYM	Others	89	69	296	454	287
**SUV39H2**	THYM	HM_w	43	69	342	454	288
**PHF13**	THYM	Others	139	69	244	452	289
**RSF1**	THYM	ChRC	88	69	295	452	290
**H2AFZ**	THYM	Others	257	69	125	451	291
**MSL3**	THYM	HA_w	172	69	204	445	292
**SETD4**	THYM	HM_w	82	69	294	445	293
**KDM1B**	THYM	HM_e	207	69	168	444	294
**BAP1**	THYM	Others	329	69	42	440	295
**SFMBT1**	THYM	HM_r	78	69	292	439	296
**HDAC7**	THYM	HA_e	245	69	124	438	297
**NCOA3**	THYM	HA_w	162	69	202	433	298
**BRD8**	THYM	HA_r	321	69	42	432	299
**SMARCC1**	THYM	Helicases	64	325	42	431	300
**NCOR2**	THYM	Others	160	69	200	429	301
**SIRT6**	THYM	HA_e	70	69	290	429	302
**CBX1**	THYM	HM_r	314	69	42	425	303
**SMARCA2**	THYM	Helicases	67	69	289	425	304
**PRKAA1**	THYM	Others	113	69	242	424	305
**CBX4**	THYM	HM_r	311	69	42	422	306
**IDH2**	THYM	DM_e	230	69	123	422	307
**CBX5**	THYM	HM_r	310	69	42	421	308
**ZGPAT**	THYM	Others	13	69	339	421	309

**SMARCD1**	THYM	Helicases	62	69	288	419	310
**PCGF2**	THYM	Others	149	69	198	416	311
**PRMT7**	THYM	HM_w	106	69	241	416	312
**SRCAP**	THYM	Others	48	69	286	403	313
**DAXX**	THYM	ChRC	291	69	42	402	314
**DIDO1**	THYM	Others	290	69	42	401	315
**DNMT1**	THYM	DM_w	289	69	42	400	316
**KMT2E**	THYM	HM_w	187	69	141	397	317
**TDRKH**	THYM	Others	31	319	42	392	318
**EHMT1**	THYM	HM_w	280	69	42	391	319
**PADI1**	THYM	Others	158	69	164	391	320
**ELP4**	THYM	HA_w	278	69	42	389	321
**TDRD12**	THYM	Others	35	69	284	388	322
**ERCC5**	THYM	Others	276	69	42	387	323
**PADI6**	THYM	Others	155	69	163	387	324
**UBE2E1**	THYM	Others	26	318	42	386	325
**EZH2**	THYM	HM_w	274	69	42	385	326
**PRDM14**	THYM	HM_w	122	69	192	383	327
**TRIM33**	THYM	HA_r	28	69	280	377	328
**PRDM8**	THYM	HM_w	115	69	191	375	329
**PRDM9**	THYM	HM_w	114	69	190	373	330
**PHF11**	THYM	Others	141	69	162	372	331
**SMARCA5**	THYM	Helicases	66	69	237	372	332
**GLYR1**	THYM	HM_r	260	69	42	371	333
**GTF2H1**	THYM	Others	259	69	42	370	334
**PRKAA2**	THYM	Others	112	69	189	370	335
**HDAC2**	THYM	HA_e	250	69	42	361	336
**PCGF1**	THYM	Others	150	69	140	359	337
**PHF7**	THYM	Others	129	69	161	359	338
**RAG2**	THYM	HM_r	101	69	188	358	339
**PHF8**	THYM	Others	128	69	160	357	340
**SMYD5**	THYM	HM_w	53	69	235	357	341
**RBBP4**	THYM	ChRC	100	69	187	356	342
**ORC1**	THYM	Others	159	69	122	350	343
**HIRA**	THYM	Others	238	69	42	349	344

**PRDM16**	THYM	HM_w	120	69	159	348	345
**HLTF**	THYM	Others	234	69	42	345	346
**HNF1A**	THYM	ChRC	233	69	42	344	347
**TAF1**	THYM	HA_r	41	69	233	343	348
**HSPBAP1**	THYM	Others	231	69	42	342	349
**TCEA1**	THYM	Others	40	69	232	341	350
**ING1**	THYM	HM_r	229	69	42	340	351
**ING3**	THYM	HM_r	227	69	42	338	352
**TDG**	THYM	ChRC	38	69	231	338	353
**INO80**	THYM	Helicases	225	69	42	336	354
**INTS12**	THYM	Others	224	69	42	335	355
**JARID2**	THYM	ChRC	219	69	42	330	356
**SIN3B**	THYM	ChRC	75	69	183	327	357
**KAT5**	THYM	HA_w	212	69	42	323	358
**KAT7**	THYM	HA_w	210	69	42	321	359
**SMARCA1**	THYM	Helicases	68	69	182	319	360
**USP51**	THYM	Others	20	69	229	318	361
**KDM4A**	THYM	HM_e	204	69	42	315	362
**SMARCC2**	THYM	Helicases	63	69	181	313	363
**PRDM13**	THYM	HM_w	123	69	120	312	364
**SMARCD3**	THYM	Helicases	60	69	180	309	365
**ZMYND8**	THYM	HA_r	11	69	228	308	366
**KDM5D**	THYM	HM_e	196	69	42	307	367
**SETD7**	THYM	HM_w	80	69	155	304	368
**KDM8**	THYM	HM_e	192	69	42	303	369
**KMT2B**	THYM	HM_w	189	69	42	300	370
**RPH3A**	THYM	Others	90	69	139	298	371
**L3MBTL2**	THYM	Others	185	69	42	296	372
**MARCH5**	THYM	Others	182	69	42	293	373
**MBD4**	THYM	DM_r	179	69	42	290	374
**SUZ12**	THYM	ChRC	42	69	179	290	375
**MEN1**	THYM	ChRC	177	69	42	288	376
**MLLT10**	THYM	HM_w	176	69	42	287	377
**RNF17**	THYM	Others	96	69	119	284	378
**MTA1**	THYM	ChRC	171	69	42	282	379

**SIRT1**	THYM	HA_e	74	69	138	281	380
**MTA3**	THYM	ChRC	169	69	42	280	381
**NAP1L1**	THYM	Others	166	69	42	277	382
**UBR7**	THYM	Others	24	69	178	271	383
**PADI4**	THYM	Others	156	69	42	267	384
**PARP2**	THYM	Others	152	69	42	263	385
**SMYD1**	THYM	HM_w	57	69	135	261	386
**PCGF5**	THYM	Others	148	69	42	259	387
**TDRD1**	THYM	Others	37	69	151	257	388
**PHC2**	THYM	ChRC	144	69	42	255	389
**PHF19**	THYM	HM_r	137	69	42	248	390
**TRIM28**	THYM	HA_r	29	69	150	248	391
**PHF21A**	THYM	HM_r	134	69	42	245	392
**PHF6**	THYM	HM_r	130	69	42	241	393
**POLR2B**	THYM	Others	127	69	42	238	394
**STK31**	THYM	Others	46	69	118	233	395
**TRIM24**	THYM	HA_r	30	69	133	232	396
**PRDM6**	THYM	HM_w	117	69	42	228	397
**PRMT2**	THYM	HM_w	109	69	42	220	398
**PRMT3**	THYM	HM_w	108	69	42	219	399
**PRMT6**	THYM	HM_w	107	69	42	218	400
**RBBP7**	THYM	ChRC	98	69	42	209	401
**SCMH1**	THYM	Others	86	69	42	197	402
**SETD1B**	THYM	HM_w	84	69	42	195	403
**SIRT5**	THYM	HA_e	71	69	42	182	404
**SMARCE1**	THYM	Helicases	59	69	42	170	405
**SMNDC1**	THYM	Others	58	69	42	169	406
**SSRP1**	THYM	Others	47	69	42	158	407
**TCF19**	THYM	Others	39	69	42	150	408
**TDRD3**	THYM	HM_r	34	69	42	145	409
**UBE2A**	THYM	Others	27	69	42	138	410
**UBE2I**	THYM	Others	25	69	42	136	411
**UHRF2**	THYM	DM_r	23	69	42	134	412
**USP27X**	THYM	Others	21	69	42	132	413
**UTY**	THYM	HM_e	19	69	42	130	414

**WDR82**	THYM	Others	17	69	42	128	415
**YY1**	THYM	ChRC	16	69	42	127	416
**ASH1L**	UCEC	HM_w	408	417	414	1239	1
**SETDB1**	UCEC	HM_w	364	421	425	1210	2
**SMARCA4**	UCEC	Helicases	386	420	400	1206	3
**ATAD2**	UCEC	HA_r	353	416	415	1184	4
**CHD7**	UCEC	Helicases	401	365	403	1169	5
**KDM5B**	UCEC	HM_e	368	379	417	1164	6
**ASXL1**	UCEC	Others	347	405	411	1163	7
**BPTF**	UCEC	HA_r	389	366	399	1154	8
**DIDO1**	UCEC	Others	387	373	389	1149	9
**ATR**	UCEC	Others	414	376	354	1144	10
**PHC3**	UCEC	ChRC	313	425	406	1144	11
**KAT6A**	UCEC	HA_w	350	418	372	1140	12
**KAT6B**	UCEC	HA_w	370	396	362	1128	13
**PHF20L1**	UCEC	HM_r	312	406	402	1120	14
**BRD4**	UCEC	HA_r	265	422	410	1097	15
**KDM5A**	UCEC	HM_e	378	319	396	1093	16
**SIN3B**	UCEC	ChRC	288	411	394	1093	17
**SRCAP**	UCEC	Others	380	337	373	1090	18
**DNMT1**	UCEC	DM_w	321	413	327	1061	19
**BRD1**	UCEC	HA_r	266	400	391	1057	20
**ARID4B**	UCEC	ChRC	306	386	363	1055	21
**CHD1L**	UCEC	Helicases	217	414	419	1050	22
**MECOM**	UCEC	Others	329	426	289	1044	23
**NCOA3**	UCEC	HA_w	279	398	360	1037	24
**PARP1**	UCEC	Others	253	368	409	1030	25
**BRD9**	UCEC	HA_r	238	393	398	1029	26
**GATAD2B**	UCEC	HM_r	188	415	423	1026	27
**ZMYND8**	UCEC	HA_r	322	401	302	1025	28
**HLTF**	UCEC	Others	358	390	274	1022	29
**DNMT3B**	UCEC	DM_w	332	347	341	1020	30
**KDM1B**	UCEC	HM_e	331	331	349	1011	31
**KDM2A**	UCEC	HM_e	295	342	374	1011	32
**ACTL6A**	UCEC	ChRC	165	424	421	1010	33

**RNF40**	UCEC	Others	250	351	408	1009	34
**PAF1**	UCEC	Others	227	395	383	1005	35
**HDAC6**	UCEC	HA_e	383	309	300	992	36
**PHF12**	UCEC	Others	220	378	392	990	37
**SETD1A**	UCEC	HM_w	289	358	332	979	38
**GATAD2A**	UCEC	HM_r	213	372	387	972	39
**SMARCC2**	UCEC	Helicases	223	377	358	958	40
**LBR**	UCEC	Others	183	361	413	957	41
**BAP1**	UCEC	Others	239	355	361	955	42
**ARID2**	UCEC	ChRC	336	276	342	954	43
**PYGO2**	UCEC	HM_r	121	408	424	953	44
**L3MBTL1**	UCEC	HM_r	269	270	412	951	45
**ASXL2**	UCEC	Others	335	314	298	947	46
**CDYL**	UCEC	HM_r	264	323	352	939	47
**CHD4**	UCEC	Helicases	422	354	161	937	48
**CARM1**	UCEC	HM_w	161	419	353	933	49
**EHMT1**	UCEC	HM_w	299	292	340	931	50
**TDRKH**	UCEC	Others	78	423	426	927	51
**ING1**	UCEC	HM_r	314	321	291	926	52
**CHD6**	UCEC	Helicases	396	312	212	920	53
**MSH6**	UCEC	HM_r	357	235	326	918	54
**DNMT3A**	UCEC	DM_w	236	311	370	917	55
**NSD1**	UCEC	HM_w	413	326	178	917	56
**JARID2**	UCEC	ChRC	344	259	310	913	57
**HDAC7**	UCEC	HA_e	158	369	385	912	58
**JADE3**	UCEC	Others	421	69	422	912	59
**NCOR2**	UCEC	Others	363	307	240	910	60
**RSF1**	UCEC	ChRC	249	325	333	907	61
**PRDM15**	UCEC	HM_w	311	267	324	902	62
**PHF20**	UCEC	HM_r	206	305	384	895	63
**DPF2**	UCEC	Others	191	322	381	894	64
**HCFC1**	UCEC	Others	411	345	138	894	65
**TDRD5**	UCEC	Others	200	336	357	893	66
**HDGF**	UCEC	Others	68	407	416	891	67
**ZMYND11**	UCEC	HA_r	171	356	364	891	68

**RBBP5**	UCEC	ChRC	120	388	382	890	69
**BRPF1**	UCEC	HA_r	346	224	317	887	70
**CHD8**	UCEC	Helicases	388	221	278	887	71
**PHF14**	UCEC	Others	293	254	336	883	72
**FBXO17**	UCEC	Others	140	391	350	881	73
**TCEA1**	UCEC	Others	80	394	407	881	74
**JADE1**	UCEC	Others	400	69	405	874	75
**KDM7A**	UCEC	HM_e	384	69	418	871	76
**MLLT6**	UCEC	HM_w	255	328	288	871	77
**PRDM14**	UCEC	HM_w	282	374	215	871	78
**FMR1**	UCEC	Others	351	174	345	870	79
**KMT2B**	UCEC	HM_w	420	402	42	864	80
**CBX3**	UCEC	HM_r	166	293	404	863	81
**JADE2**	UCEC	Others	417	69	377	863	82
**CHRAC1**	UCEC	ChRC	38	404	420	862	83
**MBD5**	UCEC	DM_r	377	136	347	860	84
**BAZ2B**	UCEC	HA_r	398	226	235	859	85
**EED**	UCEC	ChRC	261	247	351	859	86
**HR**	UCEC	HM_e	258	389	209	856	87
**ATAD2B**	UCEC	HA_r	318	324	213	855	88
**H3F3A**	UCEC	Others	98	371	386	855	89
**KDM4A**	UCEC	HM_e	184	362	309	855	90
**DAXX**	UCEC	ChRC	216	248	390	854	91
**MBD4**	UCEC	DM_r	154	352	348	854	92
**SMARCA1**	UCEC	Helicases	399	156	299	854	93
**FBXL19**	UCEC	Others	101	364	388	853	94
**MECP2**	UCEC	DM_r	168	341	344	853	95
**ELP3**	UCEC	HA_w	190	397	257	844	96
**PBRM1**	UCEC	HA_r	270	375	194	839	97
**EP300**	UCEC	HA_w	404	272	160	836	98
**ASXL3**	UCEC	Others	410	144	281	835	99
**HDAC4**	UCEC	HA_e	298	243	294	835	100
**PRDM9**	UCEC	HM_w	392	359	84	835	101
**SUV39H1**	UCEC	HM_w	195	296	343	834	102
**MTA1**	UCEC	ChRC	181	257	395	833	103

**POLR2B**	UCEC	Others	367	131	335	833	104
**SMYD3**	UCEC	HM_w	112	383	329	824	105
**ZGPAT**	UCEC	Others	56	387	378	821	106
**SIRT2**	UCEC	HA_e	147	384	286	817	107
**SMARCB1**	UCEC	Helicases	170	300	346	816	108
**DMAP1**	UCEC	Others	215	334	258	807	109
**SUPT16H**	UCEC	Others	273	205	328	806	110
**TRIM28**	UCEC	HA_r	221	316	268	805	111
**KMT2A**	UCEC	HM_w	382	286	136	804	112
**SIRT7**	UCEC	HA_e	114	357	331	802	113
**BOP 1**	UCEC	Others	19	403	376	798	114
**SMYD2**	UCEC	HM_w	202	350	246	798	115
**RNF2**	UCEC	ChRC	29	367	401	797	116
**BAZ2A**	UCEC	HA_r	374	227	195	796	117
**EP400**	UCEC	HA_w	418	219	159	796	118
**BAZ1B**	UCEC	HA_r	302	228	260	790	119
**CBX1**	UCEC	HM_r	71	348	371	790	120
**GTF2H1**	UCEC	Others	243	191	356	790	121
**NCOA1**	UCEC	HA_w	328	308	153	789	122
**AKAP1**	UCEC	Others	337	277	165	779	123
**HDAC5**	UCEC	HA_e	283	190	301	774	124
**FBXW9**	UCEC	Others	48	412	313	773	125
**PRMT5**	UCEC	HM_w	203	211	359	773	126
**SIRT5**	UCEC	HA_e	115	279	379	773	127
**CREBBP**	UCEC	HA_w	407	69	296	772	128
**TDRD6**	UCEC	Others	403	148	218	769	129
**SATB1**	UCEC	Others	381	185	200	766	130
**TAF1**	UCEC	HA_r	423	150	191	764	131
**MBD1**	UCEC	DM_r	256	137	369	762	132
**ING4**	UCEC	HM_r	46	333	380	759	133
**MEN1**	UCEC	ChRC	182	269	308	759	134
**PARP2**	UCEC	Others	153	268	338	759	135
**DPF1**	UCEC	ChRC	50	392	316	758	136
**HDAC1**	UCEC	HA_e	139	344	275	758	137
**BRWD3**	UCEC	HA_r	406	69	282	757	138

**KIAA2026**	UCEC	Others	385	329	42	756	139
**SETD1B**	UCEC	HM_w	326	282	147	755	140
**STK31**	UCEC	Others	287	297	171	755	141
**TAF1L**	UCEC	HA_r	409	204	142	755	142
**G2E3**	UCEC	Others	281	194	276	751	143
**L3MBTL4**	UCEC	Others	210	285	255	750	144
**TET3**	UCEC	DM_e	245	278	217	740	145
**HDAC9**	UCEC	HA_e	259	353	126	738	146
**KDM4D**	UCEC	HM_e	155	239	339	733	147
**PCGF2**	UCEC	Others	21	340	368	729	148
**SFMBT1**	UCEC	HM_r	224	281	220	725	149
**TET1**	UCEC	DM_e	402	180	141	723	150
**SMARCD3**	UCEC	Helicases	222	252	247	721	151
**TAF3**	UCEC	HA_r	272	203	244	719	152
**CBX2**	UCEC	ChRC	39	381	297	717	153
**KMT2D**	UCEC	HM_w	424	188	103	715	154
**TDRD12**	UCEC	Others	108	409	198	715	155
**MTA2**	UCEC	ChRC	132	256	325	713	156
**SMARCD2**	UCEC	Helicases	81	299	330	710	157
**PHF3**	UCEC	Others	343	114	252	709	158
**KMT2C**	UCEC	HM_w	419	69	216	704	159
**KDM4C**	UCEC	HM_e	330	330	42	702	160
**BRPF3**	UCEC	HA_r	237	275	187	699	161
**PHF7**	UCEC	Others	88	360	251	699	162
**ATRX**	UCEC	Helicases	412	118	168	698	163
**KAT8**	UCEC	HA_w	145	288	264	697	164
**CHAF1B**	UCEC	ChRC	193	222	279	694	165
**PRMT2**	UCEC	HM_w	62	266	366	694	166
**RING1**	UCEC	Others	150	210	334	694	167
**PRKAA1**	UCEC	Others	151	339	202	692	168
**CXXC1**	UCEC	Others	316	141	234	691	169
**PRMT1**	UCEC	HM_w	87	317	287	691	170
**SCML2**	UCEC	HM_r	304	69	318	691	171
**PRMT3**	UCEC	HM_w	204	163	323	690	172
**SFMBT2**	UCEC	Others	375	207	108	690	173

**HDAC11**	UCEC	HA_e	138	370	180	688	174
**CTCF**	UCEC	Others	425	220	42	687	175
**HDAC2**	UCEC	HA_e	137	173	375	685	176
**SP140L**	UCEC	HA_r	201	181	303	685	177
**TDRD9**	UCEC	Others	307	295	83	685	178
**SMARCC1**	UCEC	Helicases	247	265	172	684	179
**PRDM2**	UCEC	HM_w	365	69	249	683	180
**CECR2**	UCEC	HA_r	333	69	280	682	181
**PHC2**	UCEC	ChRC	225	306	151	682	182
**FXR2**	UCEC	Others	189	195	295	679	183
**KMT2E**	UCEC	HM_w	371	69	238	678	184
**TCF20**	UCEC	Others	390	69	219	678	185
**TDRD10**	UCEC	Others	173	410	93	676	186
**ATM**	UCEC	Others	416	69	190	675	187
**SCMH1**	UCEC	Others	248	385	42	675	188
**ARID1B**	UCEC	ChRC	369	69	236	674	189
**KDM8**	UCEC	HM_e	198	237	239	674	190
**CBX4**	UCEC	HM_r	105	382	186	673	191
**JMJD1C**	UCEC	HM_e	391	69	208	668	192
**SIN3A**	UCEC	ChRC	394	230	42	666	193
**SSRP1**	UCEC	Others	246	151	269	666	194
**SETD4**	UCEC	HM_w	177	183	305	665	195
**SMARCA5**	UCEC	Helicases	309	155	199	663	196
**ARID4A**	UCEC	ChRC	379	119	164	662	197
**BRWD1**	UCEC	HA_r	397	223	42	662	198
**RPS6KA5**	UCEC	Others	276	162	221	659	199
**KDM2B**	UCEC	HM_e	280	287	91	658	200
**SUV39H2**	UCEC	HM_w	109	229	320	658	201
**BRDT**	UCEC	HA_r	320	143	193	656	202
**L3MBTL3**	UCEC	Others	230	169	256	655	203
**PSIP1**	UCEC	HM_r	310	302	42	654	204
**IWS1**	UCEC	Others	187	69	397	653	205
**CHD2**	UCEC	Helicases	360	249	42	651	206
**SETD5**	UCEC	HM_w	305	301	42	648	207
**BAZ1A**	UCEC	HA_r	334	199	114	647	208

**JMJD8**	UCEC	HM_e	23	332	290	645	209
**PHF8**	UCEC	Others	339	69	237	645	210
**EZH1**	UCEC	HM_w	235	176	233	644	211
**EHMT2**	UCEC	HM_w	37	291	315	643	212
**RNF217**	UCEC	Others	119	253	270	642	213
**USP51**	UCEC	Others	218	69	355	642	214
**CBX8**	UCEC	ChRC	142	380	113	635	215
**YY1**	UCEC	ChRC	41	201	393	635	216
**AIRE**	UCEC	HM_r	164	335	133	632	217
**ERCC5**	UCEC	Others	345	245	42	632	218
**CSTL1**	UCEC	Others	169	197	265	631	219
**JMJD6**	UCEC	HM_e	134	343	154	631	220
**MBTD1**	UCEC	Others	54	258	319	631	221
**MLLT10**	UCEC	HM_w	209	216	206	631	222
**UHRF2**	UCEC	DM_r	271	315	42	628	223
**HDAC10**	UCEC	HA_e	47	399	181	627	224
**SND1**	UCEC	HM_r	274	206	144	624	225
**ZCWPW1**	UCEC	HM_r	76	264	283	623	226
**BRD8**	UCEC	HA_r	317	117	188	622	227
**PHF21A**	UCEC	HM_r	205	213	204	622	228
**RAG2**	UCEC	HM_r	338	69	214	621	229
**SETD2**	UCEC	HM_w	395	184	42	621	230
**SP100**	UCEC	HA_r	325	153	143	621	231
**ATF7IP**	UCEC	Others	352	69	196	617	232
**EZH2**	UCEC	HM_w	315	69	232	616	233
**SMARCD1**	UCEC	Helicases	175	154	285	614	234
**DPY30**	UCEC	Others	26	310	277	613	235
**ARID1A**	UCEC	ChRC	426	69	117	612	236
**KDM6A**	UCEC	HM_e	349	69	192	610	237
**CHD1**	UCEC	Helicases	300	142	162	604	238
**USP27X**	UCEC	Others	267	294	42	603	239
**CHD3**	UCEC	Helicases	415	69	116	600	240
**ORC1**	UCEC	Others	240	214	135	589	241
**PRKCD**	UCEC	Others	122	318	149	589	242
**HIST1H3B**	UCEC	Others	35	240	312	587	243

**KANSL1**	UCEC	HA_w	373	171	42	586	244
**PRMT7**	UCEC	HM_w	178	232	175	585	245
**PRDM5**	UCEC	HM_w	251	130	203	584	246
**SP140**	UCEC	HA_r	308	182	94	584	247
**FKBP2**	UCEC	Others	36	363	184	583	248
**EPC2**	UCEC	Others	141	127	314	582	249
**KAT2B**	UCEC	HA_w	232	170	179	581	250
**IDH2**	UCEC	DM_e	135	289	155	579	251
**MPHOSPH8**	UCEC	HM_r	229	125	225	579	252
**SMYD1**	UCEC	HM_w	196	122	261	579	253
**HIST1H1B**	UCEC	Others	67	218	293	578	254
**PRMT6**	UCEC	HM_w	86	186	306	578	255
**CHD5**	UCEC	Helicases	405	69	99	573	256
**KAT2A**	UCEC	HA_w	296	69	207	572	257
**PCGF1**	UCEC	Others	65	234	273	572	258
**SP110**	UCEC	HA_r	174	152	245	571	259
**CHAF1A**	UCEC	ChRC	263	177	130	570	260
**FBXO44**	UCEC	Others	49	290	231	570	261
**PHF21B**	UCEC	HM_r	180	304	86	570	262
**SMARCA2**	UCEC	Helicases	362	69	139	570	263
**PRDM4**	UCEC	HM_w	252	69	248	569	264
**MTF2**	UCEC	HM_r	208	135	224	567	265
**PRDM13**	UCEC	HM_w	152	165	250	567	266
**UBR7**	UCEC	Others	146	179	241	566	267
**AFF1**	UCEC	Others	361	69	134	564	268
**TET2**	UCEC	DM_e	355	69	140	564	269
**KDM3B**	UCEC	HM_e	393	126	42	561	270
**EPC1**	UCEC	Others	102	246	210	558	271
**CHD9**	UCEC	Helicases	359	69	129	557	272
**PRDM16**	UCEC	HM_w	366	69	121	556	273
**ATAT1**	UCEC	Others	27	262	266	555	274
**PRDM1**	UCEC	HM_w	292	166	96	554	275
**HIST1H1C**	UCEC	Others	17	241	292	550	276
**PHF1**	UCEC	HM_r	90	255	205	550	277
**PHIP**	UCEC	HA_r	376	132	42	550	278

**PHC1**	UCEC	ChRC	129	167	253	549	279
**RNF17**	UCEC	Others	348	112	89	549	280
**KAT5**	UCEC	HA_w	186	320	42	548	281
**SIRT1**	UCEC	HA_e	116	128	304	548	282
**TRIM33**	UCEC	HA_r	354	147	42	543	283
**AFF4**	UCEC	Others	319	120	101	540	284
**SHPRH**	UCEC	Others	340	158	42	540	285
**BMI1**	UCEC	ChRC	53	225	259	537	286
**NCOR1**	UCEC	ChRC	356	69	112	537	287
**L3MBTL2**	UCEC	Others	257	236	42	535	288
**PCMT1**	UCEC	Others	64	134	337	535	289
**PRDM7**	UCEC	HM_w	31	233	271	535	290
**FKBP5**	UCEC	Others	160	244	128	532	291
**HIRA**	UCEC	Others	233	69	229	531	292
**RPH3A**	UCEC	Others	149	208	174	531	293
**NAP1L3**	UCEC	Others	197	69	263	529	294
**PWWP2B**	UCEC	Others	44	283	201	528	295
**PYGO1**	UCEC	HM_r	277	129	120	526	296
**DNMT3L**	UCEC	DM_w	75	346	104	525	297
**PHF10**	UCEC	Others	89	69	367	525	298
**CLOCK**	UCEC	HA_w	192	116	211	519	299
**KDM4E**	UCEC	HM_e	144	238	137	519	300
**PRKAA2**	UCEC	Others	179	164	176	519	301
**SETD6**	UCEC	HM_w	83	69	365	517	302
**DPF3**	UCEC	HA_r	262	69	185	516	303
**TDRD3**	UCEC	HM_r	324	149	42	515	304
**SETD3**	UCEC	HM_w	117	69	322	508	305
**SETMAR**	UCEC	HM_w	176	159	173	508	306
**SIRT4**	UCEC	HA_e	82	280	145	507	307
**TRIM24**	UCEC	HA_r	59	202	243	504	308
**HNF1A**	UCEC	ChRC	157	189	157	503	309
**BRD3**	UCEC	HA_r	301	69	132	502	310
**KAT7**	UCEC	HA_w	242	217	42	501	311
**SMYD5**	UCEC	HM_w	110	69	321	500	312
**INTS12**	UCEC	Others	94	172	228	494	313

**KDM4B**	UCEC	HM_e	294	69	125	488	314
**HDGFL1**	UCEC	Others	74	242	167	483	315
**KDM5C**	UCEC	HM_e	372	69	42	483	316
**KDM3A**	UCEC	HM_e	185	69	227	481	317
**NAP1L2**	UCEC	Others	241	69	166	476	318
**RPA3**	UCEC	Others	43	209	222	474	319
**PRDM10**	UCEC	HM_w	291	69	111	471	320
**PHF13**	UCEC	Others	127	69	272	468	321
**BRD2**	UCEC	HA_r	162	261	42	465	322
**CDYL2**	UCEC	HM_r	51	313	100	464	323
**MTA3**	UCEC	ChRC	92	327	42	461	324
**TDRD1**	UCEC	Others	285	69	107	461	325
**HDAC3**	UCEC	HA_e	260	69	127	456	326
**ACTL6B**	UCEC	ChRC	106	263	85	454	327
**PADI1**	UCEC	Others	131	69	254	454	328
**PHRF1**	UCEC	Others	342	69	42	453	329
**SETDB2**	UCEC	HM_w	275	69	109	453	330
**RAI1**	UCEC	Others	341	69	42	452	331
**SMYD4**	UCEC	HM_w	111	298	42	451	332
**WDR82**	UCEC	Others	57	349	42	448	333
**PCGF5**	UCEC	Others	226	69	152	447	334
**TDRD7**	UCEC	Others	284	69	90	443	335
**RBBP4**	UCEC	ChRC	61	338	42	441	336
**HELLS**	UCEC	Helicases	212	69	158	439	337
**RBBP7**	UCEC	ChRC	268	69	102	439	338
**PAXIP1**	UCEC	Others	327	69	42	438	339
**TP53BP1**	UCEC	Others	323	69	42	434	340
**TCF19**	UCEC	Others	12	250	170	432	341
**FKBP1A**	UCEC	Others	25	175	230	430	342
**UBE2I**	UCEC	Others	42	146	242	430	343
**BRD7**	UCEC	HA_r	194	69	163	426	344
**PRMT8**	UCEC	HM_w	85	231	110	426	345
**AURKB**	UCEC	Others	163	69	189	421	346
**CBX5**	UCEC	HM_r	104	274	42	420	347
**MBD2**	UCEC	DM_r	93	284	42	419	348

**PHF19**	UCEC	HM_r	126	69	223	418	349
**CBX7**	UCEC	HM_r	52	273	92	417	350
**MBD3**	UCEC	DM_r	22	168	226	416	351
**ASH2L**	UCEC	HM_w	303	69	42	414	352
**PCGF6**	UCEC	Others	34	69	307	410	353
**AICDA**	UCEC	DM_e	143	178	88	409	354
**INO80**	UCEC	Helicases	297	69	42	408	355
**POLE3**	UCEC	ChRC	63	303	42	408	356
**MSL3**	UCEC	HA_w	167	124	115	406	357
**PRDM11**	UCEC	HM_w	124	187	95	406	358
**SMARCE1**	UCEC	Helicases	113	251	42	406	359
**GTF3C4**	UCEC	HA_w	234	69	98	401	360
**RNF20**	UCEC	Others	290	69	42	401	361
**CBX6**	UCEC	HM_r	70	198	131	399	362
**HSPBAP1**	UCEC	Others	96	260	42	398	363
**SUZ12**	UCEC	ChRC	286	69	42	397	364
**ING5**	UCEC	HM_r	16	69	311	396	365
**GADD45A**	UCEC	Others	18	193	183	394	366
**UBE2E1**	UCEC	Others	58	69	267	394	367
**SCML4**	UCEC	Others	84	160	148	392	368
**PHF2**	UCEC	Others	278	69	42	389	369
**PADI3**	UCEC	Others	228	69	87	384	370
**ZCWPW2**	UCEC	HM_r	40	145	197	382	371
**ING2**	UCEC	HM_r	66	271	42	379	372
**PHF23**	UCEC	HM_r	125	212	42	379	373
**PPARGC1A**	UCEC	Others	199	69	105	373	374
**SMNDC1**	UCEC	Others	20	69	284	373	375
**PADI4**	UCEC	Others	254	69	42	365	376
**PHF11**	UCEC	Others	128	115	122	365	377
**SETD7**	UCEC	HM_w	148	69	146	363	378
**IDH1**	UCEC	DM_e	136	69	156	361	379
**USP22**	UCEC	Others	244	69	42	355	380
**TRIM66**	UCEC	HA_r	172	69	106	347	381
**PADI6**	UCEC	Others	14	69	262	345	382
**KDM6B**	UCEC	HM_e	231	69	42	342	383

**ELP4**	UCEC	HA_w	103	196	42	341	384
**GTF2F1**	UCEC	Others	159	139	42	340	385
**GLYR1**	UCEC	HM_r	100	192	42	334	386
**PHF6**	UCEC	HM_r	219	69	42	330	387
**DOT1L**	UCEC	HM_w	214	69	42	325	388
**HIF1AN**	UCEC	Others	211	69	42	322	389
**PADI2**	UCEC	Others	130	69	123	322	390
**RTF1**	UCEC	Others	118	161	42	321	391
**PRDM8**	UCEC	HM_w	30	113	177	320	392
**MUM1**	UCEC	Others	207	69	42	318	393
**WDR5**	UCEC	ChRC	77	69	169	315	394
**AEBP2**	UCEC	HM_w	72	200	42	314	395
**MORF4L1**	UCEC	HM_r	45	215	42	302	396
**PRDM6**	UCEC	HM_w	123	123	42	288	397
**SIRT6**	UCEC	HA_e	13	157	118	288	398
**NAP1L1**	UCEC	Others	91	69	124	284	399
**GTF2B**	UCEC	Others	99	140	42	281	400
**HAT1**	UCEC	HA_w	97	138	42	277	401
**GADD45B**	UCEC	Others	24	69	182	275	402
**KDM1A**	UCEC	HM_e	156	69	42	267	403
**PRDM12**	UCEC	HM_w	32	69	150	251	404
**SIRT3**	UCEC	HA_e	60	69	119	248	405
**MARCH5**	UCEC	Others	133	69	42	244	406
**UBE2A**	UCEC	Others	73	121	42	236	407
**H2AFZ**	UCEC	Others	69	69	97	235	408
**UHRF1**	UCEC	DM_r	107	69	42	218	409
**PHF5A**	UCEC	Others	33	133	42	208	410
**ING3**	UCEC	HM_r	95	69	42	206	411
**TDG**	UCEC	ChRC	79	69	42	190	412
**HDAC8**	UCEC	HA_e	55	69	42	166	413
**UBE2B**	UCEC	Others	28	69	42	139	414
**KDM5D**	UCEC	HM_e	15	69	42	126	415
**UTY**	UCEC	HM_e	11	69	42	122	416
**ATAD2**	UCS	HA_r	401	411	426	1238	1
**SETDB1**	UCS	HM_w	410	396	407	1213	2

**SMARCC2**	UCS	Helicases	407	386	401	1194	3
**ACTL6A**	UCS	ChRC	357	403	410	1170	4
**CHD7**	UCS	Helicases	391	410	337	1138	5
**DNMT3B**	UCS	DM_w	336	401	399	1136	6
**BPTF**	UCS	HA_r	397	366	369	1132	7
**SMARCA4**	UCS	Helicases	408	395	322	1125	8
**ERCC5**	UCS	Others	328	382	414	1124	9
**AEBP2**	UCS	HM_w	356	368	381	1105	10
**PHC3**	UCS	ChRC	280	416	403	1099	11
**HDAC6**	UCS	HA_e	389	356	350	1095	12
**ATR**	UCS	Others	399	330	356	1085	13
**CHD4**	UCS	Helicases	426	363	281	1070	14
**CTCF**	UCS	Others	419	273	366	1058	15
**DPF2**	UCS	Others	334	362	353	1049	16
**HDAC7**	UCS	HA_e	388	355	306	1049	17
**SETD1A**	UCS	HM_w	366	338	345	1049	18
**ARID2**	UCS	ChRC	354	333	357	1044	19
**NCOA1**	UCS	HA_w	284	388	348	1020	20
**CHRAC1**	UCS	ChRC	190	405	423	1018	21
**BOP 1**	UCS	Others	208	406	400	1014	22
**HAT1**	UCS	HA_w	321	358	333	1012	23
**BRD4**	UCS	HA_r	206	393	405	1004	24
**EZH2**	UCS	HM_w	327	323	351	1001	25
**HIST1H1B**	UCS	Others	315	353	332	1000	26
**KAT6A**	UCS	HA_w	304	418	274	996	27
**FBXL19**	UCS	Others	326	381	279	986	28
**HLTF**	UCS	Others	314	316	330	960	29
**ARID4B**	UCS	ChRC	217	332	409	958	30
**HDAC2**	UCS	HA_e	319	260	378	957	31
**KDM1B**	UCS	HM_e	301	350	301	952	32
**PRDM8**	UCS	HM_w	369	222	361	952	33
**ARID1B**	UCS	ChRC	405	289	257	951	34
**SUZ12**	UCS	ChRC	237	295	419	951	35
**BAZ1B**	UCS	HA_r	350	285	314	949	36
**CHD2**	UCS	Helicases	342	324	282	948	37

**HDGF**	UCS	Others	155	389	404	948	38
**CBX4**	UCS	HM_r	199	365	380	944	39
**ATF7IP**	UCS	Others	214	331	394	939	40
**EHMT1**	UCS	HM_w	332	269	334	935	41
**DNMT1**	UCS	DM_w	186	391	355	932	42
**JARID2**	UCS	ChRC	309	314	304	927	43
**NSD1**	UCS	HM_w	412	307	208	927	44
**BRD9**	UCS	HA_r	205	328	393	926	45
**ING1**	UCS	HM_r	147	379	397	923	46
**DNMT3A**	UCS	DM_w	185	383	354	922	47
**GATAD2B**	UCS	HM_r	167	390	365	922	48
**SUV39H1**	UCS	HM_w	239	336	343	918	49
**MEN1**	UCS	ChRC	288	238	391	917	50
**ASH1L**	UCS	HM_w	216	415	285	916	51
**PHF20L1**	UCS	HM_r	89	409	418	916	52
**PHF8**	UCS	Others	275	344	296	915	53
**CBX8**	UCS	ChRC	197	325	392	914	54
**HDAC5**	UCS	HA_e	318	319	277	914	55
**KAT6B**	UCS	HA_w	136	378	396	910	56
**MBD4**	UCS	DM_r	290	240	375	905	57
**H3F3A**	UCS	Others	161	321	420	902	58
**FMR1**	UCS	Others	325	322	252	899	59
**CBX2**	UCS	ChRC	201	326	368	895	60
**AKAP1**	UCS	Others	218	334	340	892	61
**KDM5A**	UCS	HM_e	130	377	385	892	62
**NCOR2**	UCS	Others	413	235	243	891	63
**RTF1**	UCS	Others	256	210	424	890	64
**PRDM1**	UCS	HM_w	411	343	134	888	65
**SCMH1**	UCS	Others	254	340	292	886	66
**USP51**	UCS	Others	226	370	289	885	67
**DPY30**	UCS	Others	182	361	336	879	68
**TCEA1**	UCS	Others	34	420	425	879	69
**ACTL6B**	UCS	ChRC	222	369	287	878	70
**TDRD12**	UCS	Others	32	426	417	875	71
**KMT2E**	UCS	HM_w	292	309	271	872	72

**L3MBTL3**	UCS	Others	383	243	245	871	73
**ATAT1**	UCS	Others	215	367	283	865	74
**PRDM15**	UCS	HM_w	371	224	268	863	75
**TAF1**	UCS	HA_r	406	193	261	860	76
**BAZ2B**	UCS	HA_r	349	283	227	859	77
**HIST1H3B**	UCS	Others	151	317	386	854	78
**ASH2L**	UCS	HM_w	404	407	42	853	79
**PYGO2**	UCS	HM_r	69	397	384	850	80
**CBX3**	UCS	HM_r	200	278	367	845	81
**KDM4D**	UCS	HM_e	297	248	299	844	82
**SRCAP**	UCS	Others	243	371	230	844	83
**KMT2A**	UCS	HM_w	293	376	174	843	84
**SIRT7**	UCS	HA_e	48	373	422	843	85
**BRPF3**	UCS	HA_r	204	327	311	842	86
**ATM**	UCS	Others	400	69	370	839	87
**ASXL3**	UCS	Others	402	288	148	838	88
**FKBP5**	UCS	Others	171	359	308	838	89
**AFF4**	UCS	Others	221	292	317	830	90
**CHD5**	UCS	Helicases	420	69	338	827	91
**RNF2**	UCS	ChRC	261	303	263	827	92
**AIRE**	UCS	HM_r	219	290	316	825	93
**ELP4**	UCS	HA_w	179	267	379	825	94
**L3MBTL1**	UCS	HM_r	384	399	42	825	95
**PARP1**	UCS	Others	103	346	374	823	96
**SCML2**	UCS	HM_r	367	69	383	819	97
**ASXL1**	UCS	Others	352	424	42	818	98
**KDM6A**	UCS	HM_e	295	310	211	816	99
**BRD8**	UCS	HA_r	346	281	188	815	100
**ING2**	UCS	HM_r	312	256	247	815	101
**KDM5B**	UCS	HM_e	296	246	272	814	102
**TDRD10**	UCS	Others	232	385	192	809	103
**BAZ2A**	UCS	HA_r	210	284	313	807	104
**PHF3**	UCS	Others	374	228	201	803	105
**CBX5**	UCS	HM_r	393	69	339	801	106
**HIST1H1C**	UCS	Others	152	318	331	801	107

**JMJD6**	UCS	HM_e	139	253	408	800	108
**CREBBP**	UCS	HA_w	421	69	309	799	109
**AICDA**	UCS	DM_e	220	291	286	797	110
**DPF1**	UCS	ChRC	335	419	42	796	111
**KDM3B**	UCS	HM_e	299	250	246	795	112
**FKBP2**	UCS	Others	172	400	222	794	113
**PADI6**	UCS	Others	378	69	347	794	114
**RNF40**	UCS	Others	63	341	390	794	115
**KAT5**	UCS	HA_w	137	352	302	791	116
**ING4**	UCS	HM_r	145	315	329	789	117
**IDH2**	UCS	DM_e	313	257	217	787	118
**PRDM14**	UCS	HM_w	83	408	295	786	119
**SIN3A**	UCS	ChRC	250	301	234	785	120
**PHC1**	UCS	ChRC	282	232	270	784	121
**DIDO1**	UCS	Others	337	404	42	783	122
**EHMT2**	UCS	HM_w	331	268	184	783	123
**KDM4A**	UCS	HM_e	132	349	300	781	124
**HDAC4**	UCS	HA_e	157	258	364	779	125
**PBRM1**	UCS	HA_r	376	234	167	777	126
**CHD6**	UCS	Helicases	341	392	42	775	127
**BRWD3**	UCS	HA_r	394	69	310	773	128
**SCML4**	UCS	Others	61	339	373	773	129
**KDM5C**	UCS	HM_e	416	311	42	769	130
**PHF10**	UCS	Others	96	375	297	768	131
**BRD1**	UCS	HA_r	396	329	42	767	132
**SIN3B**	UCS	ChRC	53	300	412	765	133
**PPARGC1A**	UCS	Others	372	227	165	764	134
**TDRD9**	UCS	Others	230	189	342	761	135
**ASXL2**	UCS	Others	403	69	284	756	136
**SND1**	UCS	HM_r	362	198	195	755	137
**PHC2**	UCS	ChRC	281	231	241	753	138
**EPC1**	UCS	Others	329	69	352	750	139
**CDYL**	UCS	HM_r	343	364	42	749	140
**SIRT5**	UCS	HA_e	50	337	359	746	141
**BRWD1**	UCS	HA_r	422	279	42	743	142

**IWS1**	UCS	Others	310	69	363	742	143
**TDRD5**	UCS	Others	231	190	320	741	144
**ARID4A**	UCS	ChRC	353	69	315	737	145
**NCOA3**	UCS	HA_w	283	412	42	737	146
**SMARCA2**	UCS	Helicases	409	203	124	736	147
**DNMT3L**	UCS	DM_w	184	271	280	735	148
**HDAC11**	UCS	HA_e	159	357	219	735	149
**L3MBTL4**	UCS	Others	125	308	298	731	150
**SMARCE1**	UCS	Helicases	42	299	389	730	151
**SFMBT2**	UCS	Others	365	207	156	728	152
**NAP1L2**	UCS	Others	414	69	244	727	153
**TRIM28**	UCS	HA_r	26	294	406	726	154
**PHIP**	UCS	HA_r	373	304	42	719	155
**BRPF1**	UCS	HA_r	395	280	42	717	156
**EP400**	UCS	HA_w	424	69	223	716	157
**DMAP1**	UCS	Others	187	272	253	712	158
**SIRT2**	UCS	HA_e	248	421	42	711	159
**KDM4B**	UCS	HM_e	417	249	42	708	160
**ZCWPW1**	UCS	HM_r	225	293	190	708	161
**MECOM**	UCS	Others	119	417	170	706	162
**HDAC8**	UCS	HA_e	387	69	249	705	163
**KDM4E**	UCS	HM_e	131	247	327	705	164
**TCF20**	UCS	Others	361	191	153	705	165
**ING5**	UCS	HM_r	144	254	305	703	166
**KMT2D**	UCS	HM_w	423	69	210	702	167
**KAT8**	UCS	HA_w	135	351	213	699	168
**CDYL2**	UCS	HM_r	196	276	224	696	169
**STK31**	UCS	Others	241	297	154	692	170
**TAF3**	UCS	HA_r	235	69	388	692	171
**PRDM7**	UCS	HM_w	267	223	200	690	172
**TDRD1**	UCS	Others	233	69	387	689	173
**HDGFL1**	UCS	Others	154	354	179	687	174
**HDAC10**	UCS	HA_e	320	320	42	682	175
**SMYD3**	UCS	HM_w	39	298	344	681	176
**JMJD1C**	UCS	HM_e	308	69	303	680	177

**ZMYND8**	UCS	HA_r	223	413	42	678	178
**ATAD2B**	UCS	HA_r	351	69	256	676	179
**FBXW9**	UCS	Others	174	360	142	676	180
**ING3**	UCS	HM_r	146	255	275	676	181
**RBBP7**	UCS	ChRC	263	214	197	674	182
**SMYD2**	UCS	HM_w	40	372	262	674	183
**PHF1**	UCS	HM_r	279	69	325	673	184
**SMYD1**	UCS	HM_w	246	69	358	673	185
**KDM6B**	UCS	HM_e	385	245	42	672	186
**PHF21B**	UCS	HM_r	87	345	240	672	187
**BRD7**	UCS	HA_r	347	282	42	671	188
**PHF12**	UCS	Others	94	374	203	671	189
**ZMYND11**	UCS	HA_r	224	183	258	665	190
**KAT7**	UCS	HA_w	303	313	42	658	191
**KDM2A**	UCS	HM_e	134	312	212	658	192
**PRMT1**	UCS	HM_w	77	342	239	658	193
**TDRD6**	UCS	Others	360	69	228	657	194
**CHD9**	UCS	Helicases	339	274	42	655	195
**CARM1**	UCS	HM_w	203	402	42	647	196
**CHD1**	UCS	Helicases	392	69	186	647	197
**GTF2B**	UCS	Others	165	262	220	647	198
**KDM1A**	UCS	HM_e	302	69	273	644	199
**ARID1A**	UCS	ChRC	425	69	149	643	200
**BAZ1A**	UCS	HA_r	211	286	146	643	201
**G2E3**	UCS	Others	323	69	251	643	202
**FBXO17**	UCS	Others	176	423	42	641	203
**FKBP1A**	UCS	Others	173	69	398	640	204
**BRDT**	UCS	HA_r	345	69	225	639	205
**EP300**	UCS	HA_w	330	266	42	638	206
**FXR2**	UCS	Others	324	265	42	631	207
**RPH3A**	UCS	Others	259	211	158	628	208
**PRDM13**	UCS	HM_w	270	225	132	627	209
**INO80**	UCS	Helicases	143	69	413	625	210
**KMT2C**	UCS	HM_w	415	69	139	623	211
**SMARCB1**	UCS	Helicases	47	202	372	621	212

**CHD1L**	UCS	Helicases	192	384	42	618	213
**PRMT8**	UCS	HM_w	72	387	159	618	214
**TET1**	UCS	DM_e	229	69	319	617	215
**ATRX**	UCS	Helicases	398	69	147	614	216
**AFF1**	UCS	Others	355	69	189	613	217
**SHPRH**	UCS	Others	364	206	42	612	218
**PRDM11**	UCS	HM_w	271	69	269	609	219
**TCF19**	UCS	Others	33	192	382	607	220
**GTF2F1**	UCS	Others	164	261	181	606	221
**PHF11**	UCS	Others	95	305	204	604	222
**PRDM4**	UCS	HM_w	268	69	267	604	223
**PRDM5**	UCS	HM_w	370	69	162	601	224
**KANSL1**	UCS	HA_w	306	252	42	600	225
**PRDM9**	UCS	HM_w	368	69	161	598	226
**HDAC3**	UCS	HA_e	158	259	180	597	227
**RPA3**	UCS	Others	62	212	323	597	228
**PRKAA1**	UCS	Others	80	221	294	595	229
**SP110**	UCS	HA_r	244	196	155	595	230
**KMT2B**	UCS	HM_w	126	425	42	593	231
**BMI1**	UCS	ChRC	209	69	312	590	232
**KAT2B**	UCS	HA_w	305	69	214	588	233
**DPF3**	UCS	HA_r	333	69	185	587	234
**EED**	UCS	ChRC	181	69	335	585	235
**PADI1**	UCS	Others	379	69	137	585	236
**PHF19**	UCS	HM_r	375	69	136	580	237
**L3MBTL2**	UCS	Others	291	244	42	577	238
**TRIM33**	UCS	HA_r	358	69	150	577	239
**SUV39H2**	UCS	HM_w	238	296	42	576	240
**HIRA**	UCS	Others	153	380	42	575	241
**KDM8**	UCS	HM_e	127	69	377	573	242
**POLE3**	UCS	ChRC	85	69	416	570	243
**RNF217**	UCS	Others	64	213	293	570	244
**RNF17**	UCS	Others	262	69	238	569	245
**MARCH5**	UCS	Others	123	69	376	568	246
**PAF1**	UCS	Others	104	422	42	568	247

**SMARCD2**	UCS	Helicases	44	200	321	565	248
**NAP1L3**	UCS	Others	285	69	209	563	249
**RSF1**	UCS	ChRC	257	69	237	563	250
**TDG**	UCS	ChRC	234	69	260	563	251
**BRD3**	UCS	HA_r	348	69	145	562	252
**PRMT2**	UCS	HM_w	76	219	266	561	253
**KDM3A**	UCS	HM_e	133	251	176	560	254
**JMJD8**	UCS	HM_e	307	69	178	554	255
**CLOCK**	UCS	HA_w	338	69	144	551	256
**MLLT10**	UCS	HM_w	118	69	362	549	257
**PHF23**	UCS	HM_r	278	229	42	549	258
**KDM2B**	UCS	HM_e	300	69	177	546	259
**RBBP5**	UCS	ChRC	66	215	264	545	260
**GATAD2A**	UCS	HM_r	168	69	307	544	261
**TDRKH**	UCS	Others	29	394	121	544	262
**AURKB**	UCS	Others	213	287	42	542	263
**SSRP1**	UCS	Others	242	69	229	540	264
**LBR**	UCS	Others	124	242	173	539	265
**JADE3**	UCS	Others	140	69	328	537	266
**SMARCC1**	UCS	Helicases	46	69	421	536	267
**MBTD1**	UCS	Others	120	239	171	530	268
**PHF20**	UCS	HM_r	90	398	42	530	269
**HCFC1**	UCS	Others	418	69	42	529	270
**SETD6**	UCS	HM_w	58	69	402	529	271
**NAP1L1**	UCS	Others	110	69	349	528	272
**SMARCD3**	UCS	Helicases	43	69	415	527	273
**UBE2E1**	UCS	Others	22	186	318	526	274
**PWWP2B**	UCS	Others	265	217	42	524	275
**MLLT6**	UCS	HM_w	117	237	169	523	276
**MTF2**	UCS	HM_r	286	69	168	523	277
**FBXO44**	UCS	Others	175	69	278	522	278
**RAI1**	UCS	Others	264	216	42	522	279
**SMNDC1**	UCS	Others	41	69	411	521	280
**SP140L**	UCS	HA_r	35	194	291	520	281
**CBX7**	UCS	HM_r	198	277	42	517	282

**CHAF1B**	UCS	ChRC	193	69	255	517	283
**PAXIP1**	UCS	Others	102	69	346	517	284
**DAXX**	UCS	ChRC	188	69	254	511	285
**CHD3**	UCS	Helicases	191	275	42	508	286
**MPHOSPH8**	UCS	HM_r	115	348	42	505	287
**PRDM10**	UCS	HM_w	272	69	164	505	288
**BRD2**	UCS	HA_r	207	69	226	502	289
**CXXC1**	UCS	Others	390	69	42	501	290
**MTA3**	UCS	ChRC	112	347	42	501	291
**PRDM2**	UCS	HM_w	269	69	163	501	292
**SETD7**	UCS	HM_w	57	209	235	501	293
**TAF1L**	UCS	HA_r	236	69	193	498	294
**HR**	UCS	HM_e	386	69	42	497	295
**SIRT1**	UCS	HA_e	249	205	42	496	296
**DOT1L**	UCS	HM_w	183	270	42	495	297
**HNF1A**	UCS	ChRC	150	69	276	495	298
**RING1**	UCS	Others	65	69	360	494	299
**MBD1**	UCS	DM_r	382	69	42	493	300
**PCGF6**	UCS	Others	98	69	326	493	301
**MBD5**	UCS	DM_r	381	69	42	492	302
**TP53BP1**	UCS	Others	28	69	395	492	303
**MSH6**	UCS	HM_r	380	69	42	491	304
**PARP2**	UCS	Others	377	69	42	488	305
**PHF14**	UCS	Others	92	230	166	488	306
**PHF13**	UCS	Others	93	69	324	486	307
**SMYD4**	UCS	HM_w	245	199	42	486	308
**RPS6KA5**	UCS	Others	258	69	157	484	309
**PHF7**	UCS	Others	276	69	135	480	310
**HDAC1**	UCS	HA_e	160	69	250	479	311
**SMARCD1**	UCS	Helicases	45	201	232	478	312
**SMYD5**	UCS	HM_w	38	69	371	478	313
**GADD45B**	UCS	Others	169	264	42	475	314
**SMARCA5**	UCS	Helicases	363	69	42	474	315
**GLYR1**	UCS	HM_r	166	263	42	471	316
**TET2**	UCS	DM_e	359	69	42	470	317

**UBE2B**	UCS	Others	23	187	259	469	318
**ZGPAT**	UCS	Others	11	414	42	467	319
**IDH1**	UCS	DM_e	148	69	248	465	320
**PRMT3**	UCS	HM_w	266	69	130	465	321
**SP100**	UCS	HA_r	37	197	231	465	322
**GADD45A**	UCS	Others	170	69	221	460	323
**PRKCD**	UCS	Others	78	220	160	458	324
**RNF20**	UCS	Others	260	69	128	457	325
**CBX6**	UCS	HM_r	344	69	42	455	326
**CECR2**	UCS	HA_r	195	69	187	451	327
**CHD8**	UCS	Helicases	340	69	42	451	328
**SATB1**	UCS	Others	255	69	127	451	329
**TET3**	UCS	DM_e	228	69	152	449	330
**PCGF2**	UCS	Others	100	306	42	448	331
**SETD4**	UCS	HM_w	252	69	126	447	332
**HDAC9**	UCS	HA_e	156	69	218	443	333
**PRDM12**	UCS	HM_w	84	226	133	443	334
**GTF2H1**	UCS	Others	322	69	42	433	335
**SUPT16H**	UCS	Others	240	69	123	432	336
**EPC2**	UCS	Others	178	69	183	430	337
**EZH1**	UCS	HM_w	177	69	182	428	338
**HELLS**	UCS	Helicases	317	69	42	428	339
**USP22**	UCS	Others	18	69	341	428	340
**HIF1AN**	UCS	Others	316	69	42	427	341
**JADE2**	UCS	Others	141	69	216	426	342
**SP140**	UCS	HA_r	36	195	194	425	343
**INTS12**	UCS	Others	311	69	42	422	344
**KAT2A**	UCS	HA_w	138	69	215	422	345
**ORC1**	UCS	Others	108	69	242	419	346
**UBR7**	UCS	Others	227	69	120	416	347
**KDM4C**	UCS	HM_e	298	69	42	409	348
**PRMT6**	UCS	HM_w	74	69	265	408	349
**KIAA2026**	UCS	Others	294	69	42	405	350
**MBD3**	UCS	DM_r	121	241	42	404	351
**CSTL1**	UCS	Others	189	69	143	401	352

**MECP2**	UCS	DM_r	289	69	42	400	353
**SETDB2**	UCS	HM_w	56	302	42	400	354
**MTA2**	UCS	ChRC	287	69	42	398	355
**USP27X**	UCS	Others	17	335	42	394	356
**MTA1**	UCS	ChRC	113	236	42	391	357
**PHF6**	UCS	HM_r	277	69	42	388	358
**SFMBT1**	UCS	HM_r	54	208	125	387	359
**PHRF1**	UCS	Others	274	69	42	385	360
**POLR2B**	UCS	Others	273	69	42	384	361
**PADI2**	UCS	Others	107	69	207	383	362
**UBE2A**	UCS	Others	24	69	290	383	363
**PADI3**	UCS	Others	106	69	206	381	364
**PCGF1**	UCS	Others	101	69	205	375	365
**GTF3C4**	UCS	HA_w	163	69	141	373	366
**KDM7A**	UCS	HM_e	128	69	175	372	367
**PCMT1**	UCS	Others	97	233	42	372	368
**H2AFZ**	UCS	Others	162	69	140	371	369
**ZCWPW2**	UCS	HM_r	12	69	288	369	370
**TRIM24**	UCS	HA_r	27	188	151	366	371
**SETD2**	UCS	HM_w	253	69	42	364	372
**SETD3**	UCS	HM_w	59	69	236	364	373
**MBD2**	UCS	DM_r	122	69	172	363	374
**PHF2**	UCS	Others	91	69	202	362	375
**SETD5**	UCS	HM_w	251	69	42	362	376
**SMARCA1**	UCS	Helicases	247	69	42	358	377
**SIRT4**	UCS	HA_e	51	69	233	353	378
**PRKAA2**	UCS	Others	79	69	199	347	379
**PRMT5**	UCS	HM_w	75	218	42	335	380
**RAG2**	UCS	HM_r	68	69	198	335	381
**SETD1B**	UCS	HM_w	60	69	196	325	382
**BAP1**	UCS	Others	212	69	42	323	383
**MSL3**	UCS	HA_w	114	69	138	321	384
**CBX1**	UCS	HM_r	202	69	42	313	385
**CHAF1A**	UCS	ChRC	194	69	42	305	386
**SIRT6**	UCS	HA_e	49	204	42	295	387

**ELP3**	UCS	HA_w	180	69	42	291	388
**TRIM66**	UCS	HA_r	25	69	191	285	389
**PRDM16**	UCS	HM_w	82	69	131	282	390
**PYGO1**	UCS	HM_r	70	69	129	268	391
**HSPBAP1**	UCS	Others	149	69	42	260	392
**JADE1**	UCS	Others	142	69	42	253	393
**UHRF1**	UCS	DM_r	20	185	42	247	394
**WDR5**	UCS	ChRC	15	184	42	241	395
**KDM5D**	UCS	HM_e	129	69	42	240	396
**MORF4L1**	UCS	HM_r	116	69	42	227	397
**MUM1**	UCS	Others	111	69	42	222	398
**TDRD7**	UCS	Others	30	69	122	221	399
**NCOR1**	UCS	ChRC	109	69	42	220	400
**PADI4**	UCS	Others	105	69	42	216	401
**PCGF5**	UCS	Others	99	69	42	210	402
**PHF21A**	UCS	HM_r	88	69	42	199	403
**PHF5A**	UCS	Others	86	69	42	197	404
**PRDM6**	UCS	HM_w	81	69	42	192	405
**PRMT7**	UCS	HM_w	73	69	42	184	406
**PSIP1**	UCS	HM_r	71	69	42	182	407
**RBBP4**	UCS	ChRC	67	69	42	178	408
**SETMAR**	UCS	HM_w	55	69	42	166	409
**SIRT3**	UCS	HA_e	52	69	42	163	410
**TDRD3**	UCS	HM_r	31	69	42	142	411
**UBE2I**	UCS	Others	21	69	42	132	412
**UHRF2**	UCS	DM_r	19	69	42	130	413
**UTY**	UCS	HM_e	16	69	42	127	414
**WDR82**	UCS	Others	14	69	42	125	415
**YY1**	UCS	ChRC	13	69	42	124	416
**EHMT2**	UVM	HM_w	408	408	408	1224	1
**ATAT1**	UVM	Others	358	417	419	1194	2
**BOP 1**	UVM	Others	349	425	418	1192	3
**ATAD2**	UVM	HA_r	359	426	406	1191	4
**TCF19**	UVM	Others	376	404	402	1182	5
**BRD2**	UVM	HA_r	347	411	421	1179	6

**CBX2**	UVM	ChRC	414	394	371	1179	7
**CDYL**	UVM	HM_r	330	422	415	1167	8
**CHRAC1**	UVM	ChRC	317	424	425	1166	9
**BRPF3**	UVM	HA_r	340	402	413	1155	10
**CHD7**	UVM	Helicases	320	410	420	1150	11
**DAXX**	UVM	ChRC	311	409	424	1144	12
**CBX4**	UVM	HM_r	334	393	401	1128	13
**BRD4**	UVM	HA_r	415	362	348	1125	14
**CBX8**	UVM	ChRC	331	392	389	1112	15
**KAT6A**	UVM	HA_w	424	337	341	1102	16
**JARID2**	UVM	ChRC	240	421	426	1087	17
**MUM1**	UVM	Others	392	325	358	1075	18
**KDM3B**	UVM	HM_e	397	334	339	1070	19
**HIST1H1C**	UVM	Others	257	415	393	1065	20
**AFF4**	UVM	Others	370	369	323	1062	21
**ELP3**	UVM	HA_w	299	349	412	1060	22
**HIST1H3B**	UVM	Others	256	414	386	1056	23
**BRD7**	UVM	HA_r	345	361	347	1053	24
**CTCF**	UVM	Others	313	352	388	1053	25
**KDM1B**	UVM	HM_e	230	420	400	1050	26
**AIRE**	UVM	HM_r	368	388	285	1041	27
**EZH1**	UVM	HM_w	294	386	343	1023	28
**PCGF2**	UVM	Others	389	378	254	1021	29
**HDAC5**	UVM	HA_e	425	383	198	1006	30
**ATF7IP**	UVM	Others	418	365	219	1002	31
**CHAF1A**	UVM	ChRC	327	356	319	1002	32
**ING5**	UVM	HM_r	246	382	365	993	33
**PHF20L1**	UVM	HM_r	155	423	410	988	34
**HDAC3**	UVM	HA_e	268	342	366	976	35
**CARM1**	UVM	HM_w	337	358	280	975	36
**PHF1**	UVM	HM_r	164	407	404	975	37
**CBX1**	UVM	HM_r	336	357	279	972	38
**KAT7**	UVM	HA_w	232	336	392	960	39
**HIST1H1B**	UVM	Others	258	416	269	943	40
**ATM**	UVM	Others	357	364	218	939	41

**RING1**	UVM	Others	115	406	414	935	42
**DOT1L**	UVM	HM_w	305	350	273	928	43
**HDAC10**	UVM	HA_e	271	343	312	926	44
**KDM5B**	UVM	HM_e	220	331	361	912	45
**BAP1**	UVM	Others	426	69	416	911	46
**SIRT5**	UVM	HA_e	82	419	403	904	47
**DNMT3L**	UVM	DM_w	306	387	209	902	48
**LBR**	UVM	Others	207	330	360	897	49
**FKBP5**	UVM	Others	286	401	206	893	50
**H3F3A**	UVM	Others	274	344	272	890	51
**CHD4**	UVM	Helicases	322	354	213	889	52
**KAT2A**	UVM	HA_w	399	380	105	884	53
**CHD9**	UVM	Helicases	318	353	212	883	54
**TCEA1**	UVM	Others	48	405	422	875	55
**ARID1B**	UVM	ChRC	422	403	42	867	56
**KMT2A**	UVM	HM_w	213	391	263	867	57
**IDH1**	UVM	DM_e	251	339	268	858	58
**BRWD1**	UVM	HA_r	339	359	158	856	59
**SMARCA4**	UVM	Helicases	377	311	168	856	60
**AICDA**	UVM	DM_e	369	368	117	854	61
**HDAC4**	UVM	HA_e	267	384	199	850	62
**PHRF1**	UVM	Others	386	69	394	849	63
**PRMT2**	UVM	HM_w	128	321	396	845	64
**FBXW9**	UVM	Others	289	348	207	844	65
**FXR2**	UVM	Others	405	69	367	841	66
**SIRT7**	UVM	HA_e	80	376	384	840	67
**PRDM15**	UVM	HM_w	138	323	375	836	68
**BRPF1**	UVM	HA_r	341	69	423	833	69
**GATAD2A**	UVM	HM_r	281	346	203	830	70
**HDGFL1**	UVM	Others	261	418	151	830	71
**MBTD1**	UVM	Others	200	327	303	830	72
**KMT2D**	UVM	HM_w	423	69	335	827	73
**ACTL6A**	UVM	ChRC	374	69	381	824	74
**GTF2F1**	UVM	Others	277	345	202	824	75
**BRD1**	UVM	HA_r	416	363	42	821	76

**MEN1**	UVM	ChRC	393	69	359	821	77
**DNMT1**	UVM	DM_w	309	351	155	815	78
**ATAD2B**	UVM	HA_r	419	69	322	810	79
**ARID1A**	UVM	ChRC	366	69	373	808	80
**KANSL1**	UVM	HA_w	236	381	191	808	81
**RAI1**	UVM	Others	380	69	357	806	82
**SMARCD2**	UVM	Helicases	72	375	352	799	83
**MBD3**	UVM	DM_r	203	329	261	793	84
**RBBP5**	UVM	ChRC	117	318	356	791	85
**HNF1A**	UVM	ChRC	254	341	195	790	86
**BRD3**	UVM	HA_r	346	69	372	787	87
**HR**	UVM	HM_e	253	340	194	787	88
**H2AFZ**	UVM	Others	404	69	313	786	89
**GADD45B**	UVM	Others	282	347	153	782	90
**ASXL1**	UVM	Others	361	69	349	779	91
**AKAP1**	UVM	Others	367	367	42	776	92
**CHD3**	UVM	Helicases	323	69	380	772	93
**KDM6A**	UVM	HM_e	396	69	307	772	94
**ASXL3**	UVM	Others	420	69	282	771	95
**ASH2L**	UVM	HM_w	362	366	42	770	96
**KMT2C**	UVM	HM_w	395	69	306	770	97
**SETD4**	UVM	HM_w	98	316	355	769	98
**TET3**	UVM	DM_e	375	69	325	769	99
**L3MBTL2**	UVM	Others	394	69	304	767	100
**PADI2**	UVM	Others	390	69	301	760	101
**CHD1**	UVM	Helicases	412	69	276	757	102
**CLOCK**	UVM	HA_w	316	69	370	755	103
**PRDM11**	UVM	HM_w	385	69	300	754	104
**PRDM2**	UVM	HM_w	384	69	299	752	105
**DPY30**	UVM	Others	302	69	379	750	106
**PRDM4**	UVM	HM_w	383	69	298	750	107
**L3MBTL3**	UVM	Others	209	399	140	748	108
**BRD8**	UVM	HA_r	344	360	42	746	109
**HDAC1**	UVM	HA_e	272	69	405	746	110
**ATRX**	UVM	Helicases	355	69	321	745	111

**PHC1**	UVM	ChRC	167	324	252	743	112
**PHF10**	UVM	Others	163	397	183	743	113
**BAZ2B**	UVM	HA_r	351	69	320	740	114
**PRMT7**	UVM	HM_w	124	320	295	739	115
**EP400**	UVM	HA_w	297	69	369	735	116
**IWS1**	UVM	Others	400	69	266	735	117
**GTF2B**	UVM	Others	278	69	387	734	118
**KDM1A**	UVM	HM_e	398	69	264	731	119
**CREBBP**	UVM	HA_w	315	69	346	730	120
**PRDM16**	UVM	HM_w	137	412	179	728	121
**FKBP1A**	UVM	Others	288	69	368	725	122
**CHAF1B**	UVM	ChRC	326	355	42	723	123
**DNMT3A**	UVM	DM_w	308	69	345	722	124
**SIRT6**	UVM	HA_e	81	312	328	721	125
**ASH1L**	UVM	HM_w	363	69	284	716	126
**DPF2**	UVM	Others	303	69	344	716	127
**PRDM1**	UVM	HM_w	143	390	180	713	128
**ASXL2**	UVM	Others	360	69	283	712	129
**CHD1L**	UVM	Helicases	325	69	318	712	130
**MTA1**	UVM	ChRC	191	326	186	703	131
**BMI1**	UVM	ChRC	350	69	281	700	132
**HDAC2**	UVM	HA_e	269	385	42	696	133
**KDM4A**	UVM	HM_e	226	69	398	693	134
**SIN3B**	UVM	ChRC	87	314	291	692	135
**PRDM7**	UVM	HM_w	134	377	178	689	136
**EPC2**	UVM	Others	406	69	208	683	137
**FBXL19**	UVM	Others	292	69	317	678	138
**CDYL2**	UVM	HM_r	329	69	278	676	139
**FBXO17**	UVM	Others	291	69	316	676	140
**MECOM**	UVM	Others	199	69	407	675	141
**CECR2**	UVM	HA_r	328	69	277	674	142
**JADE3**	UVM	Others	241	69	364	674	143
**SP100**	UVM	HA_r	62	373	237	672	144
**FKBP2**	UVM	Others	287	69	315	671	145
**JMJD8**	UVM	HM_e	237	69	363	669	146

**CHD2**	UVM	Helicases	324	69	275	668	147
**SMYD2**	UVM	HM_w	67	310	288	665	148
**SP140L**	UVM	HA_r	59	370	236	665	149
**GATAD2B**	UVM	HM_r	280	69	314	663	150
**ING3**	UVM	HM_r	401	69	193	663	151
**AFF1**	UVM	Others	371	69	222	662	152
**KDM4B**	UVM	HM_e	225	333	103	661	153
**KDM2A**	UVM	HM_e	229	69	362	660	154
**MORF4L1**	UVM	HM_r	195	69	395	659	155
**ARID4A**	UVM	ChRC	365	69	221	655	156
**ARID4B**	UVM	ChRC	364	69	220	653	157
**DNMT3B**	UVM	DM_w	307	69	274	650	158
**ARID2**	UVM	ChRC	421	69	159	649	159
**HDAC6**	UVM	HA_e	266	69	311	646	160
**KAT5**	UVM	HA_w	234	69	342	645	161
**HDAC9**	UVM	HA_e	263	69	310	642	162
**AURKB**	UVM	Others	354	69	217	640	163
**KAT8**	UVM	HA_w	231	69	340	640	164
**PARP1**	UVM	Others	174	69	397	640	165
**RNF217**	UVM	Others	111	396	128	635	166
**BPTF**	UVM	HA_r	348	69	216	633	167
**KDM4D**	UVM	HM_e	223	69	338	630	168
**MBD5**	UVM	DM_r	201	328	101	630	169
**PHF13**	UVM	Others	160	69	399	628	170
**ING4**	UVM	HM_r	247	338	42	627	171
**ING2**	UVM	HM_r	248	69	309	626	172
**PADI1**	UVM	Others	179	69	378	626	173
**KDM5C**	UVM	HM_e	219	69	337	625	174
**PRDM10**	UVM	HM_w	142	389	94	625	175
**KDM8**	UVM	HM_e	215	69	336	620	176
**PHC2**	UVM	ChRC	166	69	385	620	177
**CBX3**	UVM	HM_r	335	69	215	619	178
**MLLT6**	UVM	HM_w	196	379	42	617	179
**JMJD1C**	UVM	HM_e	239	69	308	616	180
**PCGF1**	UVM	Others	170	69	377	616	181

**CBX7**	UVM	HM_r	332	69	214	615	182
**HCFC1**	UVM	Others	273	69	271	613	183
**PRKCD**	UVM	Others	130	69	411	610	184
**PCMT1**	UVM	Others	168	398	42	608	185
**PHF12**	UVM	Others	161	69	376	606	186
**KDM2B**	UVM	HM_e	228	335	42	605	187
**AEBP2**	UVM	HM_w	372	69	160	601	188
**HDGF**	UVM	Others	262	69	270	601	189
**SP110**	UVM	HA_r	61	372	166	599	190
**SP140**	UVM	HA_r	60	371	165	596	191
**KDM5A**	UVM	HM_e	221	332	42	595	192
**PRDM14**	UVM	HM_w	139	413	42	594	193
**CHD5**	UVM	Helicases	411	69	113	593	194
**MTA2**	UVM	ChRC	190	69	334	593	195
**CXXC1**	UVM	Others	312	69	211	592	196
**PHF3**	UVM	Others	387	69	135	591	197
**DMAP1**	UVM	Others	310	69	210	589	198
**DPF3**	UVM	HA_r	409	69	110	588	199
**IDH2**	UVM	DM_e	250	69	267	586	200
**EP300**	UVM	HA_w	407	69	109	585	201
**L3MBTL1**	UVM	HM_r	210	69	305	584	202
**PRDM8**	UVM	HM_w	382	69	131	582	203
**HAT1**	UVM	HA_w	403	69	107	579	204
**JADE2**	UVM	Others	242	69	265	576	205
**SMARCE1**	UVM	Helicases	70	374	126	570	206
**RBBP4**	UVM	ChRC	118	69	374	561	207
**SMARCC1**	UVM	Helicases	75	69	417	561	208
**ACTL6B**	UVM	ChRC	373	69	118	560	209
**CBX6**	UVM	HM_r	333	69	157	559	210
**FMR1**	UVM	Others	285	69	205	559	211
**NAP1L1**	UVM	Others	187	69	302	558	212
**GADD45A**	UVM	Others	283	69	204	556	213
**TDRD6**	UVM	Others	40	395	120	555	214
**PHF23**	UVM	HM_r	152	69	333	554	215
**PCGF6**	UVM	Others	388	69	96	553	216

**CHD6**	UVM	Helicases	321	69	156	546	217
**GTF3C4**	UVM	HA_w	275	69	201	545	218
**PPARGC1A**	UVM	Others	144	69	332	545	219
**ATR**	UVM	Others	356	69	116	541	220
**PRDM12**	UVM	HM_w	141	69	331	541	221
**HDAC11**	UVM	HA_e	270	69	200	539	222
**RPH3A**	UVM	Others	379	69	91	539	223
**BAZ1A**	UVM	HA_r	353	69	115	537	224
**MBD2**	UVM	DM_r	204	69	262	535	225
**PRMT8**	UVM	HM_w	123	319	92	534	226
**HDAC7**	UVM	HA_e	265	69	197	531	227
**SHPRH**	UVM	Others	89	400	42	531	228
**BAZ2A**	UVM	HA_r	417	69	42	528	229
**SMARCB1**	UVM	Helicases	76	69	383	528	230
**HELLS**	UVM	Helicases	260	69	196	525	231
**CBX5**	UVM	HM_r	413	69	42	524	232
**BRWD3**	UVM	HA_r	338	69	114	521	233
**DIDO1**	UVM	Others	410	69	42	521	234
**SETD5**	UVM	HM_w	97	69	354	520	235
**EZH2**	UVM	HM_w	293	69	154	516	236
**HIRA**	UVM	Others	402	69	42	513	237
**NCOA1**	UVM	HA_w	184	69	260	513	238
**NCOA3**	UVM	HA_w	183	69	259	511	239
**NCOR2**	UVM	Others	181	69	258	508	240
**RPA3**	UVM	Others	109	69	330	508	241
**SSRP1**	UVM	Others	57	69	382	508	242
**SIRT3**	UVM	HA_e	84	69	353	506	243
**UBE2E1**	UVM	Others	27	69	409	505	244
**NSD1**	UVM	HM_w	391	69	42	502	245
**PADI6**	UVM	Others	176	69	257	502	246
**GLYR1**	UVM	HM_r	279	69	152	500	247
**PAF1**	UVM	Others	175	69	256	500	248
**JMJD6**	UVM	HM_e	238	69	192	499	249
**PRDM6**	UVM	HM_w	135	322	42	499	250
**PRDM9**	UVM	HM_w	133	69	297	499	251

**PARP2**	UVM	Others	173	69	255	497	252
**CSTL1**	UVM	Others	314	69	112	495	253
**PSIP1**	UVM	HM_r	381	69	42	492	254
**PCGF5**	UVM	Others	169	69	253	491	255
**PRMT6**	UVM	HM_w	125	69	296	490	256
**SETD2**	UVM	HM_w	378	69	42	489	257
**SFMBT1**	UVM	HM_r	91	69	329	489	258
**PWWP2B**	UVM	Others	122	69	294	485	259
**DPF1**	UVM	ChRC	304	69	111	484	260
**SMYD5**	UVM	HM_w	64	69	351	484	261
**PHF11**	UVM	Others	162	69	251	482	262
**USP22**	UVM	Others	22	69	391	482	263
**PHF19**	UVM	HM_r	158	69	250	477	264
**RNF2**	UVM	ChRC	113	69	293	475	265
**HLTF**	UVM	Others	255	69	150	474	266
**ZGPAT**	UVM	Others	13	69	390	472	267
**L3MBTL4**	UVM	Others	208	69	190	467	268
**TDRD9**	UVM	Others	38	308	119	465	269
**SCML2**	UVM	HM_r	103	69	292	464	270
**SMYD1**	UVM	HM_w	68	69	327	464	271
**BAZ1B**	UVM	HA_r	352	69	42	463	272
**INO80**	UVM	Helicases	245	69	149	463	273
**G2E3**	UVM	Others	284	69	108	461	274
**JADE1**	UVM	Others	243	69	148	460	275
**TDRD5**	UVM	Others	41	69	350	460	276
**SETD1B**	UVM	HM_w	100	317	42	459	277
**MECP2**	UVM	DM_r	198	69	189	456	278
**BRD9**	UVM	HA_r	343	69	42	454	279
**MLLT10**	UVM	HM_w	197	69	188	454	280
**PRDM5**	UVM	HM_w	136	69	249	454	281
**BRDT**	UVM	HA_r	342	69	42	453	282
**SETD6**	UVM	HM_w	96	315	42	453	283
**SRCAP**	UVM	Others	58	69	326	453	284
**MPHOSPH8**	UVM	HM_r	194	69	187	450	285
**KAT6B**	UVM	HA_w	233	69	147	449	286

**PRKAA1**	UVM	Others	132	69	248	449	287
**SUZ12**	UVM	ChRC	52	309	88	449	288
**KDM3A**	UVM	HM_e	227	69	146	442	289
**NAP1L2**	UVM	Others	186	69	185	440	290
**KDM4C**	UVM	HM_e	224	69	145	438	291
**SIRT4**	UVM	HA_e	83	313	42	438	292
**SMARCA5**	UVM	Helicases	77	69	290	436	293
**KDM4E**	UVM	HM_e	222	69	144	435	294
**HIF1AN**	UVM	Others	259	69	106	434	295
**ORC1**	UVM	Others	180	69	184	433	296
**SMARCC2**	UVM	Helicases	74	69	289	432	297
**CHD8**	UVM	Helicases	319	69	42	430	298
**KDM6B**	UVM	HM_e	217	69	143	429	299
**KDM7A**	UVM	HM_e	216	69	142	427	300
**RNF40**	UVM	Others	110	69	247	426	301
**KMT2E**	UVM	HM_w	211	69	141	421	302
**SETD1A**	UVM	HM_w	101	69	246	416	303
**EED**	UVM	ChRC	301	69	42	412	304
**EHMT1**	UVM	HM_w	300	69	42	411	305
**WDR5**	UVM	ChRC	18	69	324	411	306
**MBD4**	UVM	DM_r	202	69	139	410	307
**PHF14**	UVM	Others	159	69	182	410	308
**ELP4**	UVM	HA_w	298	69	42	409	309
**SETD7**	UVM	HM_w	95	69	245	409	310
**KAT2B**	UVM	HA_w	235	69	104	408	311
**EPC1**	UVM	Others	296	69	42	407	312
**ERCC5**	UVM	Others	295	69	42	406	313
**SFMBT2**	UVM	Others	90	69	244	403	314
**FBXO44**	UVM	Others	290	69	42	401	315
**PHF5A**	UVM	Others	151	69	181	401	316
**SIRT2**	UVM	HA_e	85	69	243	397	317
**SMARCA1**	UVM	Helicases	79	69	242	390	318
**GTF2H1**	UVM	Others	276	69	42	387	319
**KIAA2026**	UVM	Others	214	69	102	385	320
**SMARCD3**	UVM	Helicases	71	69	241	381	321

**SMNDC1**	UVM	Others	69	69	240	378	322
**PRKAA2**	UVM	Others	131	69	177	377	323
**UBE2B**	UVM	Others	28	307	42	377	324
**HDAC8**	UVM	HA_e	264	69	42	375	325
**SMYD3**	UVM	HM_w	66	69	239	374	326
**PHC3**	UVM	ChRC	165	69	138	372	327
**UHRF1**	UVM	DM_r	24	306	42	372	328
**ZCWPW1**	UVM	HM_r	15	69	287	371	329
**SND1**	UVM	HM_r	63	69	238	370	330
**PYGO1**	UVM	HM_r	121	69	176	366	331
**ZMYND8**	UVM	HA_r	11	69	286	366	332
**PYGO2**	UVM	HM_r	120	69	175	364	333
**HSPBAP1**	UVM	Others	252	69	42	363	334
**ING1**	UVM	HM_r	249	69	42	360	335
**PHF21A**	UVM	HM_r	154	69	137	360	336
**SUPT16H**	UVM	Others	55	69	235	359	337
**ZMYND11**	UVM	HA_r	12	305	42	359	338
**PHF21B**	UVM	HM_r	153	69	136	358	339
**MTF2**	UVM	HM_r	188	69	100	357	340
**RNF17**	UVM	Others	114	69	174	357	341
**SUV39H1**	UVM	HM_w	54	69	234	357	342
**INTS12**	UVM	Others	244	69	42	355	343
**NAP1L3**	UVM	Others	185	69	99	353	344
**PHF7**	UVM	Others	149	69	134	352	345
**PHF8**	UVM	Others	148	69	133	350	346
**RSF1**	UVM	ChRC	107	69	173	349	347
**TCF20**	UVM	Others	47	69	233	349	348
**TDG**	UVM	ChRC	46	69	232	347	349
**SATB1**	UVM	Others	105	69	172	346	350
**PADI3**	UVM	Others	178	69	98	345	351
**SCML4**	UVM	Others	102	69	171	342	352
**TDRD3**	UVM	HM_r	42	69	231	342	353
**PRDM13**	UVM	HM_w	140	69	132	341	354
**TDRD7**	UVM	Others	39	69	230	338	355
**PBRM1**	UVM	HA_r	171	69	97	337	356

**TET1**	UVM	DM_e	36	69	229	334	357
**SETDB1**	UVM	HM_w	94	69	170	333	358
**TET2**	UVM	DM_e	35	69	228	332	359
**SETDB2**	UVM	HM_w	93	69	169	331	360
**TP53BP1**	UVM	Others	34	69	227	330	361
**KDM5D**	UVM	HM_e	218	69	42	329	362
**PRMT1**	UVM	HM_w	129	69	130	328	363
**TRIM28**	UVM	HA_r	32	69	226	327	364
**KMT2B**	UVM	HM_w	212	69	42	323	365
**UBE2A**	UVM	Others	29	69	225	323	366
**UBE2I**	UVM	Others	26	69	224	319	367
**MARCH5**	UVM	Others	206	69	42	317	368
**RAG2**	UVM	HM_r	119	69	129	317	369
**MBD1**	UVM	DM_r	205	69	42	316	370
**USP51**	UVM	Others	20	69	223	312	371
**POLE3**	UVM	ChRC	146	69	95	310	372
**MSH6**	UVM	HM_r	193	69	42	304	373
**MSL3**	UVM	HA_w	192	69	42	303	374
**SMYD4**	UVM	HM_w	65	69	167	301	375
**MTA3**	UVM	ChRC	189	69	42	300	376
**NCOR1**	UVM	ChRC	182	69	42	293	377
**PRMT3**	UVM	HM_w	127	69	93	289	378
**PADI4**	UVM	Others	177	69	42	288	379
**PAXIP1**	UVM	Others	172	69	42	283	380
**SIRT1**	UVM	HA_e	86	69	127	282	381
**TDRD10**	UVM	Others	44	69	164	277	382
**TDRKH**	UVM	Others	37	69	163	269	383
**PHF2**	UVM	Others	157	69	42	268	384
**PHF20**	UVM	HM_r	156	69	42	267	385
**RPS6KA5**	UVM	Others	108	69	90	267	386
**RTF1**	UVM	Others	106	69	89	264	387
**TRIM33**	UVM	HA_r	31	69	162	262	388
**PHF6**	UVM	HM_r	150	69	42	261	389
**TRIM66**	UVM	HA_r	30	69	161	260	390
**PHIP**	UVM	HA_r	147	69	42	258	391

**POLR2B**	UVM	Others	145	69	42	256	392
**STK31**	UVM	Others	56	69	125	250	393
**TAF1**	UVM	HA_r	51	69	124	244	394
**TAF1L**	UVM	HA_r	50	69	123	242	395
**TAF3**	UVM	HA_r	49	69	122	240	396
**PRMT5**	UVM	HM_w	126	69	42	237	397
**TDRD1**	UVM	Others	45	69	121	235	398
**RBBP7**	UVM	ChRC	116	69	42	227	399
**RNF20**	UVM	Others	112	69	42	223	400
**SCMH1**	UVM	Others	104	69	42	215	401
**SETD3**	UVM	HM_w	99	69	42	210	402
**SETMAR**	UVM	HM_w	92	69	42	203	403
**SIN3A**	UVM	ChRC	88	69	42	199	404
**TDRD12**	UVM	Others	43	69	87	199	405
**SMARCA2**	UVM	Helicases	78	69	42	189	406
**SMARCD1**	UVM	Helicases	73	69	42	184	407
**ZCWPW2**	UVM	HM_r	14	69	86	169	408
**SUV39H2**	UVM	HM_w	53	69	42	164	409
**TRIM24**	UVM	HA_r	33	69	42	144	410
**UBR7**	UVM	Others	25	69	42	136	411
**UHRF2**	UVM	DM_r	23	69	42	134	412
**USP27X**	UVM	Others	21	69	42	132	413
**UTY**	UVM	HM_e	19	69	42	130	414
**WDR82**	UVM	Others	17	69	42	128	415
**YY1**	UVM	ChRC	16	69	42	127	416
