# Supplementary material for: Pan-cancer multi-omics analysis and orthogonal experimental assessment of epigenetic driver genes
Source: Genome Res. 2020 Oct;30(10):1517–32. doi: 10.1101/gr.268292.120 (PMC7605261; doi:10.1101/gr.268292.120)
Supplement: Supplemental Material [file supp_gr.268292.120_Supplemental_Table_S6.docx]

**Supplemental Table S6.** EMT-specific ERGs and their functional categories identified in CRISPR screen

| **Group** | **Function** | **Modification** | **EMT-specific ERGs** |
| --- | --- | --- | --- |
| DNA modifiers | Writers | methylation, 5mC | *DNMT3L* |
|  | Editors | 5hmC, 5caC, 5fC | *IDH1* |
|  | Readers | methylation, 5mC | *MBD5* |
| Histone modifiers | Writers | acetylation | *KAT2B, KAT6B* |
|  |  | methylation | *ASH1L, KMT2A, KMT2E, PRDM1, PRDM16, PRMT6, SETD2, SETD4, SETD5, SETD7, SETDB1, SMYD5, SUV39H1* |
|  | Editors | acetylation | *HDAC2* |
|  |  | methylation | *JMJD8* |
|  | Readers | acetylation | *BRD8, EP400, ZMYND11* |
|  |  | methylation | *ING2, SCML2, PSIP1* |
|  |  | acetylation, methylation and phosphorylation | *PHF3, PHF10* |
| Chromatin remodeling helicase | | | *CHD6*  *SMARCB1* |
| Chromatin modifying enzymes | Bind methylated histones - Tudor domain containing | | *SMNDC1, TDRD7, TDRD9* |
|  | Link by functional interaction | | *SRCAP, EPC1 L3MBTL4, PARP1, SUPT16H* |
|  | Modulates ubiquitination | | *PCGF1* |
|  | ERG in ATP-dependent chromatin remodeling complex | | *ARID1B, MTA2, PHC3* |
| Miscellaneous | Essential genes | | *GTF2H1*  *POLR2B* |
|  | | | *AFF1, PPARGC1A, RTF1* |

*ERGs highlighted in red are enriched in VIM-positive fraction*
