# Supplementary material for: Pan-cancer multi-omics analysis and orthogonal experimental assessment of epigenetic driver genes
Source: Genome Res. 2020 Oct;30(10):1517–32. doi: 10.1101/gr.268292.120 (PMC7605261; doi:10.1101/gr.268292.120)
Supplement: Supplemental Material [file supp_gr.268292.120_Supplemental_Table_S7.pdf]

Supplemental Table S7. sgRNA target sites and sequences

gene_name	sequence	crispr_pam	crispr_gc	chromosome	chr_directior	chr_start	chr_stop	exon	transcript_id	direction	crispr_start	crispr_stop
ACTL6A	GGCGATAAA	CGG	50	chr3	(+)	179570136	179570158	[exon3, exor	[NM_004301	(+)	7257	7279
ACTL6A	ACCACCATAC	AGG	45	chr3	(-)	179570076	179570098	[exon3]	[NM_004301	(+)	7197	7219
ACTL6A	GCGTGTTCC	TGG	55	chr3	(+)	179570189	179570211	[exon3, exor	[NM_004301	(+)	7310	7332
ACTL6A	GGATCCTAT	TGG	50	chr3	(+)	179569853	179569875	[exon2]	[NM_004301	(+)	6974	6996
ACTL6B	AGACTTGAC	AGG	47,37	chr7	(+)	100655058	100655080	exon4	NM_016188	(-)	11956	11978
ACTL6B	AGGTGTGAT	CGG	57,89	chr7	(+)	100655078	100655100	exon4	NM_016188	(-)	11976	11998
ACTL6B	GTCATGTCG	TGG	57,89	chr7	(-)	100655426	100655448	exon3	NM_016188	(-)	12324	12346
AEBP2	ATACATGTA	AGG	45	chr12	(+)	19462691	19462713	[exon2, exor	[NM_001114	(+)	23018	23040
AEBP2	CCAGTCAAA	AGG	40	chr12	(+)	19473297	19473319	[exon3, exor	[NM_001114	(+)	33624	33646
AEBP2	GGATGTGAT	GGG	50	chr12	(-)	19462663	19462685	[exon2, exor	[NM_001114	(+)	22990	23012
AEBP2	TGCTGGAGT	TGG	60	chr12	(-)	19462548	19462570	[exon2, exor	[NM_001114	(+)	22875	22897
AFF1	CCTGCTTCGA	AGG	47,37	chr4	(+)	87046187	87046209	exon2, exon3	NM_001313	(+)	39187	39209
AFF1	TAGTCGAAT	TGG	36,84	chr4	(+)	87046718	87046740	exon3, exon4	NM_001313	(+)	39718	39740
AFF1	GAGTGTGAC	AGG	42,11	chr4	(-)	87046766	87046788	exon3, exon4	NM_001313	(+)	39766	39788
AFF1	CTTGTAGGG	GGG	47,37	chr4	(-)	87046264	87046286	exon2, exon3	NM_001313	(+)	39264	39286
AFF4	TGCGTATGA	AGG	47,37	chr5	(-)	132937142	132937164	exon2	NM_014423	(-)	61764	61786
AFF4	TTGTATGGC	AGG	42,11	chr5	(+)	132937071	132937093	exon2	NM_014423	(-)	61693	61715
AFF4	CAGTCTCAG	AGG	52,63	chr5	(-)	132934688	132934710	exon3	NM_014423	(-)	59310	59332
AFF4	GCAGCACAT	CGG	57,89	chr5	(+)	132937162	132937184	exon2	NM_014423	(-)	61784	61806
AICDA	ATGTGGCCG	GGG	55	chr12	(-)	8605342	8605364	[exon3]	[NM_020661	(-)	3177	3199
AICDA	TCCTCCGCTA	TGG	60	chr12	(-)	8605438	8605460	[exon3]	[NM_020661	(-)	3273	3295
AICDA	ACCTGTGCT	AGG	50	chr12	(-)	8606916	8606938	[exon2]	[NM_020661	(-)	4751	4773
AICDA	ACTTCCTCCG	AGG	50	chr12	(+)	8606990	8607012	[exon2]	[NM_020661	(-)	4825	4847
AIRE	GTGGTTGGT	GGG	52,63	chr21	(+)	44290809	44290831				0	0
AIRE	ACCGGGTTT	GGG	47,37	chr21	(+)	44290859	44290881				0	0
AIRE	GACTACAAC	TGG	57,89	chr21	(+)	44286674	44286696	exon2	NM_000383	(+)	837	859
AIRE	AAGAGACGC	GGG	57,89	chr21	(-)	44291061	44291083				0	0
AKAP1	GACTCTGAT	TGG	52,63	chr17	(-)	57105762	57105784	exon3, exon3	NM_001242	(+)	20571	20593
AKAP1	GGTGTTAGC	AGG	52,63	chr17	(-)	57105784	57105806	exon3, exon3	NM_001242	(+)	20593	20615
AKAP1	TGGTCGCAA	TGG	47,37	chr17	(-)	57105805	57105827	exon3, exon3	NM_001242	(+)	20614	20636
AKAP1	GTGGACAGT	AGG	47,37	chr17	(-)	57105693	57105715	exon3, exon3	NM_001242	(+)	20502	20524
ARID1A	GAAGAACTC	CGG	55	chr1	(+)	26696703	26696725	[exon1, exor	[NM_006015	(+)	673	695
ARID1A	AAGAACTCG	GGG	55	chr1	(+)	26696704	26696726	[exon1, exor	[NM_006015	(+)	674	696
ARID1A	CGGACCTGA	GGG	55	chr1	(+)	26696696	26696718	[exon1, exor	[NM_006015	(+)	666	688
ARID1A	CGTTCCCGTT	AGG	55	chr1	(-)	26696700	26696722	[exon1, exor	[NM_006015	(+)	670	692
ARID1B	CGCCAACGG	TGG	50	chr6	(-)	156778127	156778149	[exon1, exor	[NM_017519	(+)	198	220
ARID1B	CGGTTTTCA	GGG	45	chr6	(-)	156778121	156778143	[exon1, exor	[NM_017519	(+)	192	214
ARID1B	ACGGTTTTC	GGG	40	chr6	(-)	156778122	156778144	[exon1, exor	[NM_017519	(+)	193	215
ARID1B	AACCACAAA	TGG	40	chr6	(+)	156778125	156778147	[exon1, exor	[NM_017519	(+)	196	218
ARID2	GAACTTTCT	AGG	35	chr12	(-)	45811421	45811443	[exon4]	[NM_152641	(+)	81585	81607
ARID2	GCAGCGTTA	GGG	45	chr12	(-)	45731265	45731287	[exon3]	[NM_152641	(+)	1429	1451
ARID2	ACCAGAGTC	CGG	45	chr12	(+)	45730105	45730127	[exon2]	[NM_152641	(+)	269	291
ARID2	ATTGCACCA	TGG	45	chr12	(-)	45811491	45811513	[exon4]	[NM_152641	(+)	81655	81677
ARID4A	TCGGTACTT	CGG	52,63	chr14	(-)	58301614	58301636	exon3, exon3	NM_023001,	(+)	3111	3133
ARID4A	GCAAGTTGA	TGG	47,37	chr14	(+)	58306080	58306102	exon5, exon5	NM_023001,	(+)	7577	7599
ARID4A	GTCATCTTG	TGG	36,84	chr14	(-)	58304966	58304988	exon4, exon4	NM_023001,	(+)	6463	6485
ARID4A	GTACCGAGG	AGG	57,89	chr14	(+)	58301630	58301652	exon3, exon3	NM_023001,	(+)	3127	3149
ARID4B	GTGCCCACT	AGG	52,63	chr1	(+)	235260718	235260740	exon3, exon3	NM_001206	(-)	93824	93846
ARID4B	CGATCTTCA	AGG	52,63	chr1	(-)	235252755	235252777	exon6, exon6	NM_001206	(-)	85861	85883
ARID4B	AGTGCCAAA	TGG	52,63	chr1	(+)	235246467	235246489	exon7, exon7	NM_001206	(-)	79573	79595
ARID4B	GTTCAGGAT	GGG	42,11	chr1	(-)	235257170	235257192	exon4, exon4	NM_001206	(-)	90276	90298
ASH1L	TTTCTCGATT	AGG	47,37	chr1	(+)	155521354	155521376	exon2	NM_018489	(-)	186094	186116
ASH1L	CGAGAAAGA	TGG	42,11	chr1	(-)	155521338	155521360	exon2	NM_018489	(-)	186078	186100
ASH1L	GACCAATGT	TGG	52,63	chr1	(+)	155521424	155521446	exon2	NM_018489	(-)	186164	186186
ASH1L	GGATTGGGT	AGG	52,63	chr1	(-)	155521470	155521492	exon2	NM_018489	(-)	186210	186232
ASH2L	CGGATGTTC	GGG	47,37	chr8	(-)	38110787	38110809	exon5, exon4	NM_001261	(+)	4995	5017
ASH2L	GCAAACTTG	AGG	42,11	chr8	(+)	38106382	38106404	exon2	NM_004674	(+)	890	912
ASH2L	GTGGGAATA	CGG	42,11	chr8	(+)	38110435	38110457	exon4, exon3	NM_001261	(+)	4643	4665
ASH2L	GCTCTACCTC	CGG	57,89	chr8	(-)	38107086	38107108	exon3, exon3	NM_001261	(+)	1294	1316
ASXL1	TAGCATTGA	GGG	52,63	chr20	(-)	32369024	32369046	exon3, exon3	NM_015338,	(+)	10681	10703
ASXL1	GTGAAAAGC	AGG	52,63	chr20	(-)	32369094	32369116	exon3, exon3	NM_015338,	(+)	10751	10773
ASXL1	CTACATTCCA	AGG	36,84	chr20	(+)	32369049	32369071	exon3, exon3	NM_015338,	(+)	10706	10728
ASXL1	GTATGAACC	TGG	47,37	chr20	(+)	32372227	32372249				0	0
ASXL2	CTATAAGGT	TGG	42,11	chr2	(-)	25806246	25806268	exon3	NM_018263	(-)	66863	66885
ASXL2	ATGCTTCAC	AGG	42,11	chr2	(-)	25806284	25806306	exon3	NM_018263	(-)	66901	66923
ASXL2	ATTGCATTC	AGG	42,11	chr2	(+)	25806305	25806327	exon3	NM_018263	(-)	66922	66944
ASXL2	CTTCAAGTT	AGG	42,11	chr2	(-)	25845496	25845518	exon2	NM_018263	(-)	106113	106135
ASXL3	GTGAAGCAT	AGG	42,11	chr18	(-)	33644911	33644933	exon3	NM_030632	(+)	66335	66357
ASXL3	AATCCAACG	GGG	52,63	chr18	(-)	33646262	33646284	exon4	NM_030632	(+)	67686	67708
ASXL3	TTCACACTAA	GGG	36,84	chr18	(+)	33644929	33644951	exon3	NM_030632	(+)	66353	66375
ASXL3	GGATGGTAC	AGG	52,63	chr18	(+)	33646304	33646326	exon4	NM_030632	(+)	67728	67750
ATAD2	AGGAAGTTC	CGG	42,11	chr8	(-)	123380639	123380661	exon2	NM_014109	(-)	60789	60811
ATAD2	ATAACGGAG	TGG	42,11	chr8	(-)	123380544	123380566	exon2	NM_014109	(-)	60694	60716
ATAD2	GTCCAGTGA	TGG	52,63	chr8	(-)	123396264	123396286	exon1	NM_014109	(-)	76414	76436
ATAD2	GAAGTCACT	AGG	57,89	chr8	(+)	123396274	123396296	exon1	NM_014109	(-)	76424	76446
ATAD2B	ACGAAGTAC	AGG	52,63	chr2	(-)	23895847	23895869	exon2, exon2	NM_001242	(-)	147184	147206
ATAD2B	GATGAAATG	CGG	52,63	chr2	(+)	23926654	23926676	exon1, exon1	NM_001242	(-)	177991	178013
ATAD2B	TTCAAAGTG	AGG	47,37	chr2	(+)	23895906	23895928	exon2, exon2	NM_001242	(-)	147243	147265
ATAD2B	CTTCTCGGG	TGG	57,89	chr2	(-)	23926718	23926740	exon1, exon1	NM_001242	(-)	178055	178077
ATAT1	TGATGCACT	CGG	42,11	chr6	(-)	30627649	30627671	exon3, exon3	NM_001318	(+)	808	830
ATAT1	TGATCGTGA	AGG	42,11	chr6	(+)	30628034	30628056	exon5, exon5	NM_001318	(+)	1193	1215
ATAT1	AACAGCGCG	CGG	57,89	chr6	(-)	30626895	30626917	exon1, exon1	NM_001318	(+)	54	76
ATAT1	CATGAGTCT	TGG	57,89	chr6	(+)	30628089	30628111	exon5, exon5	NM_001318	(+)	1248	1270
ATF7IP	GCAACTTGA	AGG	45	chr12	(+)	14423987	14424009	[exon2, exor	[NM_181352	(+)	58356	58378
ATF7IP	GGCAGTTTA	TGG	50	chr12	(-)	14424270	14424292	[exon2, exor	[NM_181352	(+)	58639	58661

ATF7IP	GTTATATTA	AGG	35	chr12	(-)	14424246	14424268	[exon2, exo	[NM_18135	(+)	58615	58637
ATF7IP	TTTGGACCC	TGG	50	chr12	(+)	14424071	14424093	[exon2, exo	[NM_18135	(+)	58440	58462
ATM	AATAACGTC	AGG	36,84	chr11	(-)	108289750	108289772	exon29	NM_000051	(+)	66919	66941
ATM	ATCACATGC	AGG	36,84	chr11	(-)	108289002	108289024	exon28	NM_000051	(+)	66171	66193
ATM	TGACTGGCA	TGG	42,11	chr11	(-)	108287666	108287688	exon27	NM_000051	(+)	64835	64857
ATM	TTCAGCTGC	TGG	47,37	chr11	(-)	108289627	108289649	exon29	NM_000051	(+)	66796	66818
ATR	GGATCATAA	CGG	42,11	chr3	(-)	142563075	142563097	exon4	NM_001184	(-)	113841	113863
ATR	GAAATCAAG	AGG	42,11	chr3	(+)	142566200	142566222	exon3	NM_001184	(-)	116966	116988
ATR	TTGTGTAAC	GGG	36,84	chr3	(+)	142563040	142563062	exon4	NM_001184	(-)	113806	113828
ATR	AGTGGAAG	AGG	57,89	chr3	(-)	142566132	142566154	exon3	NM_001184	(-)	116898	116920
ATRX	TCGTGACGA	TGG	52,63	chrX	(+)	77697591	77697613	exon4, exon	NM_000489,	(-)	192714	192736
ATRX	TGATTCATT	AGG	42,11	chrX	(+)	77717139	77717161	exon2, exon	NM_000489,	(-)	212262	212284
ATRX	TGGAAGTAA	TGG	36,84	chrX	(-)	77698591	77698613	exon3, exon	NM_000489,	(-)	193714	193736
ATRX	ATTCTTCTG	AGG	42,11	chrX	(+)	77717179	77717201	exon2, exon	NM_000489,	(-)	212302	212324
AURKB	GCGCAGAG	AGG	52,63	chr17	(-)	8207186	8207208	exon5, exon	NM_00128	(-)	2456	2478
AURKB	ATTCTAGAG	CGG	52,63	chr17	(-)	8206808	8206830	exon6, exon	NM_00128	(-)	2078	2100
AURKB	TTTGAGATT	GGG	52,63	chr17	(-)	8207323	8207345	exon5, exon	NM_00128	(-)	2593	2615
AURKB	TTGGAAACG	CGG	47,37	chr17	(-)	8207289	8207311	exon5, exon	NM_00128	(-)	2559	2581
BAP1	TCAAATGGA	CGG	50	chr3	(-)	52408558	52408580	[exon4]	[NM_00465	(-)	7555	7577
BAP1	CACGGACGT	AGG	55	chr3	(+)	52408516	52408538	[exon4]	[NM_00465	(-)	7513	7535
BAP1	CGACCTTCA	AGG	50	chr3	(-)	52409555	52409577	[exon3]	[NM_00465	(-)	8552	8574
BAP1	CGCATGAAG	GGG	50	chr3	(-)	52407971	52407993	[exon5]	[NM_00465	(-)	6968	6990
BAZ1A	TTCATGTAA	GGG	47,37	chr14	(+)	34862127	34862149	exon3, exon	NM_013448,	(-)	109397	109419
BAZ1A	AACGGCTTT	CGG	52,63	chr14	(+)	34874579	34874601	exon2, exon	NM_013448,	(-)	121849	121871
BAZ1A	GCTTCCTGA	AGG	52,63	chr14	(+)	34862236	34862258	exon3, exon	NM_013448,	(-)	109506	109528
BAZ1A	GTAGTGGC	TGG	57,89	chr14	(+)	34874494	34874516	exon2, exon	NM_013448,	(-)	121764	121786
BAZ1B	GTTAGTAGA	TGG	42,11	chr7	(-)	73508371	73508393	exon3	NM_032408	(-)	67974	67996
BAZ1B	ACAAGCTTC	AGG	47,37	chr7	(+)	73508433	73508455	exon3	NM_032408	(-)	68036	68058
BAZ1B	ATTTGGACG	TGG	42,11	chr7	(-)	73510793	73510815	exon2	NM_032408	(-)	70396	70418
BAZ2A	GGGGGACA	CGG	57,89	chr12	(-)	56615376	56615398	exon3, exon	NM_013449,	(-)	19781	19803
BAZ2A	GTATTGTCC	GGG	57,89	chr12	(+)	56615387	56615409	exon3, exon	NM_013449,	(-)	19792	19814
BAZ2A	GTAGTTCCA	AGG	52,63	chr12	(+)	56615465	56615487	exon3, exon	NM_013449,	(-)	19870	19892
BAZ2A	TGGGAGAC	AGG	52,63	chr12	(+)	56617419	56617441	exon2, exon	NM_013449,	(-)	21824	21846
BAZ2B	GAATCATGA	GGG	50	chr2	(+)	159448298	159448320	exon5, exon	NM_013450,	(-)	129320	129342
BAZ2B	GTCTACATT	GGG	50	chr2	(-)	159453692	159453714	exon4, exon	NM_013450,	(-)	134714	134736
BAZ2B	ACTGATGCT	AGG	45	chr2	(-)	159448367	159448389	exon5, exon	NM_013450,	(-)	129389	129411
BAZ2B	AGGCACTCG	AGG	60	chr2	(+)	159453742	159453764	exon4, exon	NM_013450,	(-)	134764	134786
BMI1	AACGTGTAT	NGG	40			0	0	Not available			0	0
BMI1	ACAAATAGG	NGG	35			0	0	Not available			0	0
BMI1	GTGGTCTGG	NGG	50			0	0	Not available			0	0
BMI1	TGAACTTGG	NGG	35			0	0	Not available			0	0
BOP1	CCGGATTGG	CGG	52,63	chr8	(-)	144276244	144276266	exon3	NM_015201	(-)	14199	14221
BOP1	ACTGTGGCC	GGG	57,89	chr8	(+)	144289128	144289150	exon2	NM_015201	(-)	27083	27105
BOP1	TGTCACTGC	AGG	52,63	chr8	(+)	144289196	144289218	exon2	NM_015201	(-)	27151	27173
BOP1	GGATGATG	AGG	57,89	chr8	(-)	144289166	144289188	exon2	NM_015201	(-)	27121	27143
BPTF	GTACGATGA	AGG	60	chr17	(+)	67826099	67826121	exon1, exon1	NM_004459,	(+)	436	458
BPTF	ATCGTACAC	CGG	40	chr17	(-)	67826083	67826105	exon1, exon1	NM_004459,	(+)	420	442
BRD1	TGACCTACG	AGG	52,63	chr22	(-)	49824204	49824226	exon2, exon	NM_001304	(-)	50927	50949
BRD1	AGGTCAGCG	GGG	57,89	chr22	(+)	49824221	49824243	exon2, exon	NM_001304	(-)	50944	50966
BRD1	TACTCCACG	GGG	57,89	chr22	(+)	49823938	49823960	exon2, exon	NM_001304	(-)	50661	50683
BRD1	CGCTTAGTT	AGG	42,11	chr22	(+)	49824046	49824068	exon2, exon	NM_001304	(-)	50769	50791
BRD2	ATCAGTTCG	CGG	47,37	chr6	(+)	32974710	32974732	exon3, exon	NM_005104,	(+)	3823	3845
BRD2	GTAACTCGT	GGG	47,37	chr6	(-)	32974637	32974659	exon3, exon	NM_005104,	(+)	3750	3772
BRD2	CCTACACAA	AGG	47,37	chr6	(+)	32974675	32974697	exon3, exon	NM_005104,	(+)	3788	3810
BRD2	AAAGCCCTC	AGG	42,11	chr6	(-)	32974539	32974561	exon3, exon	NM_005104,	(+)	3652	3674
BRD3	ATTTGATTG	TGG	52,63	chr9	(+)	134053276	134053298	exon2	NM_007371	(-)	22953	22975
BRD3	GAGTGCAA	AGG	47,37	chr9	(-)	134052347	134052369	exon3	NM_007371	(-)	22024	22046
BRD3	CATTAGCAC	TGG	36,84	chr9	(+)	134051686	134051708	exon4	NM_007371	(-)	21363	21385
BRD3	GCAGTACAT	TGG	47,37	chr9	(-)	134053339	134053361	exon2	NM_007371	(-)	23016	23038
BRD4	TTCAGCTTG	AGG	52,63	chr19	(+)	15272822	15272844	exon2, exon	NM_058243,	(-)	25787	25809
BRD4	GATTTCTCA	GGG	47,37	chr19	(+)	15273060	15273082	exon2, exon	NM_058243,	(-)	26025	26047
BRD4	ACTGCAATA	TGG	47,37	chr19	(-)	15272892	15272914	exon2, exon	NM_058243,	(-)	25857	25879
BRD4	GAATGCTCA	AGG	42,11	chr19	(-)	15268946	15268968	exon3, exon	NM_058243,	(-)	21911	21933
BRD7	GTCGGACAA	AGG	52,63	chr16	(-)	50368726	50368748	exon1, exon1	NM_013263,	(-)	49709	49731
BRD7	GGAGGCAA	AGG	47,37	chr16	(+)	50354831	50354853	exon3, exon	NM_013263,	(-)	35814	35836
BRD7	GATCGTTTT	AGG	36,84	chr16	(+)	50368185	50368207	exon2, exon	NM_013263,	(-)	49168	49190
BRD7	GAAGTCACC	GGG	52,63	chr16	(-)	50368229	50368251	exon2, exon	NM_013263,	(-)	49212	49234
BRD8	CTCTAAAAG	GGG	47,37	chr5	(+)	138171378	138171400	exon4, exon	NM_139199,	(-)	14495	14517
BRD8	CCGGAGGTT	TGG	63,16	chr5	(-)	138169311	138169333	exon8, exon	NM_139199,	(-)	12428	12450
BRD8	GGACTTGAC	TGG	52,63	chr5	(-)	138169239	138169261	exon8, exon	NM_139199,	(-)	12356	12378
BRD8	AGCTTCTCT	TGG	57,89	chr5	(+)	138177613	138177635	exon2, exon	NM_139199,	(-)	20730	20752
BRD9	GCCTCTAAA	AGG	52,63	chr5	(-)	891807	891829	exon2	NM_023924	(-)	28073	28095
BRD9	CGACTTTGA	AGG	52,63	chr5	(-)	891209	891231	exon3, exon	NM_001009	(-)	27475	27497
BRD9	GTCACGGAT	TGG	52,63	chr5	(-)	889112	889134	exon5, exon	NM_001317	(-)	25378	25400
BRDT	TCTCCCTTGA	GGG	52,63	chr1	(+)	91976379	91976401	exon5, exon	NM_001242	(+)	24228	24250
BRDT	TTGGGCCGC	TGG	52,63	chr1	(-)	91976416	91976438	exon5, exon	NM_001242	(+)	24265	24287
BRDT	ATGGCCCTT	TGG	52,63	chr1	(+)	91962901	91962923	exon2, exon	NM_207189,	(+)	10750	10772
BRDT	AGAGCAAGT	AGG	47,37	chr1	(+)	91968223	91968245	exon4, exon	NM_001242	(+)	16072	16094
BRPF1	GCTTAGTCG	TGG	57,89	chr3	(-)	9734173	9734195	exon2, exon	NM_004634,	(+)	2445	2467
BRPF1	CTCCGCAAG	GGG	47,37	chr3	(+)	9734312	9734334	exon2, exon	NM_004634,	(+)	2584	2606
BRPF1	GTGCATAGC	CGG	52,63	chr3	(-)	9734398	9734420	exon2, exon	NM_004634,	(+)	2670	2692
BRPF1	TGTCGTGGT	AGG	47,37	chr3	(-)	9734263	9734285	exon2, exon	NM_004634,	(+)	2535	2557
BRPF3	GTCTACCTC	GGG	57,89	chr6	(-)	36200432	36200454	exon2	NM_015695	(+)	3660	3682
BRPF3	GAGTCCACC	GGG	57,89	chr6	(-)	36200671	36200693	exon2	NM_015695	(+)	3899	3921
BRPF3	CACCGAATG	AGG	42,11	chr6	(+)	36200535	36200557	exon2	NM_015695	(+)	3763	3785
BRPF3	GTCCAAGAA	AGG	52,63	chr6	(+)	36200589	36200611	exon2	NM_015695	(+)	3817	3839
BRWD1	CCAGCGCAT	TGG	63,16	chr21	(-)	39298513	39298535	exon5, exon	NM_033656,	(-)	2077	2099

BRWD1	GTTGCCGAA	GGG	52,63	chr21	(-)	39312876	39312898	exon4, exon4	NM_033656,	(-)	16440	16462
BRWD1	CCTTATCGCC	CGG	57,89	chr21	(-)	39313267	39313289	exon2, exon2	NM_033656,	(-)	16831	16853
BRWD1	CAGATTTGC	AGG	31,58	chr21	(+)	39298536	39298558	exon5, exon5	NM_033656,	(-)	2100	2122
BRWD3	GATTCCGCG	GGG	57,89	chrX	(-)	80808574	80808596	exon4	NM_153252	(-)	139087	139109
BRWD3	AGCTGTATT	AGG	47,37	chrX	(-)	80809282	80809304	exon2	NM_153252	(-)	139795	139817
BRWD3	CCTAATAAT	AGG	42,11	chrX	(+)	80793657	80793679	exon5	NM_153252	(-)	124170	124192
BRWD3	GCTTTTGCG	AGG	52,63	chrX	(-)	80791901	80791923	exon6	NM_153252	(-)	122414	122436
C14orf169	GCGAAGTGT	TGG	55	chr14	(+)	73491146	73491168	exon1	NM_024644	(+)	213	235
C14orf169	GATTCCTCCG	AGG	55	chr14	(-)	73491192	73491214	exon1	NM_024644	(+)	259	281
C14orf169	TCGTATCTTC	AGG	50	chr14	(-)	73491115	73491137	exon1	NM_024644	(+)	182	204
C14orf169	GCGAGAACT	AGG	60	chr14	(+)	73491196	73491218	exon1	NM_024644	(+)	263	285
CARM1	AACATCCTG	GGG	47,37	chr19	(+)	10908056	10908078	exon3	NM_199141	(+)	36480	36502
CARM1	GCAGTCCTT	TGG	52,63	chr19	(+)	10905015	10905037	exon2	NM_199141	(+)	33439	33461
CARM1	TGAAGGACT	CGG	57,89	chr19	(-)	10905003	10905025	exon2	NM_199141	(+)	33427	33449
CARM1	TGGGTGTGC	AGG	57,89	chr19	(-)	10905051	10905073	exon2	NM_199141	(+)	33475	33497
CBX1	CTCGACCGT	GGG	57,89	chr17	(-)	48076904	48076926	exon2, exon2	NM_006807,	(-)	6853	6875
CBX1	CAGAAACTC	CGG	47,37	chr17	(+)	48076115	48076137	exon3, exon3	NM_006807,	(-)	6064	6086
CBX1	CTTTGCTCG	CGG	52,63	chr17	(-)	48075061	48075083	exon4, exon4	NM_006807,	(-)	5010	5032
CBX1	ACATGAGAC	AGG	31,58	chr17	(-)	48076075	48076097	exon3, exon3	NM_006807,	(-)	6024	6046
CBX2	ACATAACAG	AGG	57,89	chr17	(+)	79779362	79779384	exon3, exon3	NM_032647,	(+)	1185	1207
CBX2	GAGGTGCAC	AGG	57,89	chr17	(+)	79781709	79781731	exon4, exon4	NM_032647,	(+)	3532	3554
CBX2	AGCTGGAGT	TGG	52,63	chr17	(+)	79778388	79778410	exon2, exon2	NM_032647,	(+)	211	233
CBX2	GACATGGCA	TGG	57,89	chr17	(-)	79781751	79781773	exon4, exon4	NM_032647,	(+)	3574	3596
CBX3	AGAAACGCT	TGG	31,58	chr7	(-)	26208433	26208455	exon4, exon4	NM_007276,	(+)	6730	6752
CBX3	CTAGATCGA	TGG	47,37	chr7	(+)	26206449	26206471	exon3, exon3	NM_007276,	(+)	4746	4768
CBX3	TTTTCCACGA	AGG	31,58	chr7	(-)	26206422	26206444	exon3, exon3	NM_007276,	(+)	4719	4741
CBX3	TTTCTTAACT	TGG	31,58	chr7	(+)	26208451	26208473	exon4, exon4	NM_007276,	(+)	6748	6770
CBX4	ATATAACAC	AGG	47,37	chr17	(-)	79837880	79837902	exon3	NM_003655	(-)	4725	4747
CBX4	GACATTGGA	AGG	57,89	chr17	(+)	79835366	79835388	exon5	NM_003655	(-)	2211	2233
CBX4	CTGATGGGA	AGG	47,37	chr17	(-)	79835674	79835696	exon4	NM_003655	(-)	2519	2541
CBX4	CTTGGCACG	AGG	57,89	chr17	(+)	79835324	79835346	exon5	NM_003655	(-)	2169	2191
CBX5	CAGAGCAAT	GGG	47,37	chr12	(-)	54246193	54246215	exon4, exon4	NM_012117,	(-)	15248	15270
CBX5	GCGCGTGGT	TGG	57,89	chr12	(-)	54257545	54257567	exon2, exon2	NM_012117,	(-)	26600	26622
CBX5	GGGTGAAA	GGG	36,84	chr12	(-)	54252118	54252140	exon3, exon3	NM_012117,	(-)	21173	21195
CBX5	AGAACTGTC	TGG	57,89	chr12	(+)	54257615	54257637	exon2, exon2	NM_012117,	(-)	26670	26692
CBX6	CCGAATCCA	CGG	47,37	chr22	(-)	38872131	38872153	exon1, exon1	NM_001303	(-)	10705	10727
CBX6	CGAGTCCAG	CGG	57,89	chr22	(+)	38871718	38871740	exon3, exon3	NM_001303	(-)	10292	10314
CBX6	GCATCGAGT	TGG	52,63	chr22	(-)	38871919	38871941	exon2, exon2	NM_001303	(-)	10493	10515
CBX6	AATGCACAT	AGG	47,37	chr22	(+)	38867159	38867181	exon5	NM_014292	(-)	5733	5755
CBX7	TCTTCCTATA	CGG	52,63	chr22	(+)	39138668	39138690	exon4	NM_175709	(-)	7895	7917
CBX7	AGAGAGGT	CGG	52,63	chr22	(-)	39138648	39138670	exon4	NM_175709	(-)	7875	7897
CBX7	AAGTCGAGT	TGG	42,11	chr22	(-)	39149806	39149828	exon2	NM_175709	(-)	19033	19055
CBX8	GTTTCGAAG	GGG	52,63	chr17	(-)	79795504	79795526	exon5	NM_020649	(-)	1128	1150
CBX8	GTACAGCAC	AGG	57,89	chr17	(-)	79796293	79796315	exon3	NM_020649	(-)	1917	1939
CBX8	GGCTTGGGT	GGG	57,89	chr17	(+)	79796076	79796098	exon4	NM_020649	(-)	1700	1722
CCDC101	ACGCAGCTT	AGG	52,63	chr16	(-)	28585654	28585676				0	0
CCDC101	ACATCCAGA	CGG	47,37	chr16	(+)	28584947	28584969				0	0
CCDC101	CGCGGAAAT	TGG	52,63	chr16	(+)	28589127	28589149				0	0
CCDC101	TGCCGGTCT	CGG	52,63	chr16	(+)	28590106	28590128				0	0
CDYL	AAAGCCGGT	TGG	52,63	chr6	(+)	4892240	4892262	exon2, exon2	NM_001143	(+)	2249	2271
CDYL	TAACGGGG	AGG	52,63	chr6	(-)	4892114	4892136	exon2, exon2	NM_001143	(+)	2123	2145
CDYL	AATCACGAG	AGG	47,37	chr6	(-)	4891969	4891991	exon2	NM_004824	(+)	115524	115546
CDYL	CGTTAAGAG	TGG	57,89	chr6	(+)	4892132	4892154	exon2, exon2	NM_001143	(+)	2141	2163
CDYL2	GAGTATCTT	AGG	42,11	chr16	(-)	80685062	80685084	exon2	NM_152342	(-)	81284	81306
CDYL2	GCAAGGGG	CGG	57,89	chr16	(-)	80684836	80684858	exon2	NM_152342	(-)	81058	81080
CDYL2	TAAACTCCTC	AGG	42,11	chr16	(+)	80685000	80685022	exon2	NM_152342	(-)	81222	81244
CDYL2	GGGACAGTT	GGG	52,63	chr16	(+)	80684883	80684905	exon2	NM_152342	(-)	81105	81127
CECR2	TCACCGAGA	AGG	52,63	chr22	(+)	17504913	17504935	exon7, exon7	NM_001290	(+)	144965	144987
CECR2	CCGACTCTG	TGG	57,89	chr22	(+)	17497535	17497557	exon3	NM_001290	(+)	128170	128192
CECR2	ATCTCCACCC	AGG	57,89	chr22	(-)	17497506	17497528	exon3	NM_001290	(+)	128141	128163
CECR2	CACGTCATCT	CGG	47,37	chr22	(-)	17477598	17477620	exon2	NM_001290	(+)	108233	108255
CHAF1A	CTGATCGTC	CGG	47,37	chr19	(-)	4408948	4408970	exon3	NM_005483	(+)	6286	6308
CHAF1A	TCAGGCGCT	CGG	57,89	chr19	(-)	4408904	4408926	exon3	NM_005483	(+)	6242	6264
CHAF1A	CCGAAACTT	GGG	52,63	chr19	(+)	4409064	4409086	exon3	NM_005483	(+)	6402	6424
CHAF1A	GTCCAAAGA	CGG	47,37	chr19	(-)	4408996	4409018	exon3	NM_005483	(+)	6334	6356
CHAF1B	ACGCACAAC	TGG	52,63	chr21	(-)	36387668	36387690	exon3	NM_005441	(+)	2278	2300
CHAF1B	GCAGATCGC	AGG	47,37	chr21	(+)	36391597	36391619	exon4	NM_005441	(+)	6207	6229
CHAF1B	TCCACGATG	TGG	47,37	chr21	(-)	36387619	36387641	exon3	NM_005441	(+)	2229	2251
CHAF1B	TGTGCGTTT	GGG	42,11	chr21	(+)	36387684	36387706	exon3	NM_005441	(+)	2294	2316
CHD1	CATCAAGCC	CGG	36,84	chr5	(-)	98903802	98903824	exon3	NM_001270	(-)	48599	48621
CHD1	ACCCAGAAT	TGG	47,37	chr5	(+)	98905076	98905098	exon2	NM_001270	(-)	49873	49895
CHD1	ATCGGAATC	AGG	42,11	chr5	(+)	98902938	98902960	exon4	NM_001270	(-)	47735	47757
CHD1	GCAAAACCA	TGG	47,37	chr5	(-)	98904904	98904926	exon2	NM_001270	(-)	49701	49723
CHD1L	CATTCTGAC	TGG	47,37	chr1	(-)	147252669	147252691	exon2	NM_004284	(+)	10029	10051
CHD1L	TCACCTACG	TGG	52,63	chr1	(+)	147252627	147252649	exon2	NM_004284	(+)	9987	10009
CHD1L	GTCGCCTGC	AGG	47,37	chr1	(-)	147255830	147255852	exon4, exon4	NM_024568,	(+)	13190	13212
CHD1L	GATCAGGCT	AGG	47,37	chr1	(-)	147264493	147264515	exon7, exon7	NM_024568,	(+)	21853	21875
CHD2	GCGATGGTT	CGG	52,63	chr15	(-)	92927288	92927310	exon4, exon4	NM_001271,	(+)	26968	26990
CHD2	TGAACCCGA	AGG	47,37	chr15	(-)	92924329	92924351	exon3, exon3	NM_001271,	(+)	24009	24031
CHD2	AGCTATCCG	AGG	42,11	chr15	(-)	92924518	92924540	exon3, exon3	NM_001271,	(+)	24198	24220
CHD2	CAGTCAGTC	AGG	52,63	chr15	(+)	92924360	92924382	exon3, exon3	NM_001271,	(+)	24040	24062
CHD3	CAGTGAATA	CGG	47,37	chr17	(+)	7890633	7890655	exon3, exon3	NM_005852,	(+)	5829	5851
CHD3	GTTCTGAGC	CGG	47,37	chr17	(+)	7890590	7890612	exon3, exon3	NM_005852,	(+)	5786	5808
CHD3	GACAAAGCG	GGG	52,63	chr17	(+)	7890699	7890721	exon3, exon3	NM_005852,	(+)	5895	5917
CHD3	TTGCGTTTTC	TGG	47,37	chr17	(-)	7889747	7889769	exon2, exon2	NM_005852,	(+)	4943	4965
CHD4	ATGCTCTTAT	GGG	57,89	chr12	(-)	6602150	6602172	exon4	NM_001273,	(-)	32069	32091
CHD4	CGCTTGCTCT	AGG	47,37	chr12	(+)	6602389	6602411	exon3, exon2	NM_001273,	(-)	32308	32330

CHD4	GAGGGCAG(	TGG	57,89	chr12	(-)	6602060	6602082	exon4, exon3	NM_001273,	(-)	31979	32001
CHD4	GAAGAGCA/	AGG	52,63	chr12	(-)	6601998	6602020	exon4, exon3	NM_001273,	(-)	31917	31939
CHD5	CGCCGAGG/	AGG	57,89	chr1	(-)	6179957	6179979	exon1	NM_015557	(-)	78171	78193
CHD5	CTTCGATGA(	TGG	42,11	chr1	(-)	6168230	6168252	exon2	NM_015557	(-)	66444	66466
CHD5	TGAGCTATC/	AGG	36,84	chr1	(-)	6159482	6159504	exon3	NM_015557	(-)	57696	57718
CHD5	TGGGTTTCT'	AGG	42,11	chr1	(+)	6168197	6168219	exon2	NM_015557	(-)	66411	66433
CHD6	GGACCTGTA	AGG	47,37	chr20	(-)	41533405	41533427	exon3	NM_032221	(-)	131303	131325
CHD6	GACATCCCA1	AGG	47,37	chr20	(-)	41533354	41533376	exon3	NM_032221	(-)	131252	131274
CHD6	TTCGGTTCT(	TGG	47,37	chr20	(+)	41533230	41533252	exon3	NM_032221	(-)	131128	131150
CHD6	TAGTCACTG'	AGG	52,63	chr20	(-)	41533426	41533448	exon3	NM_032221	(-)	131324	131346
CHD7	TGCTGACCC/	TGG	42,11	chr8	(-)	60741529	60741551	exon1, exon2	NM_001316(	(+)	62765	62787
CHD7	GAATGATGA	GGG	52,63	chr8	(+)	60741695	60741717	exon1, exon2	NM_001316(	(+)	62931	62953
CHD7	CAGCAAATG	AGG	47,37	chr8	(+)	60741547	60741569	exon1, exon2	NM_001316(	(+)	62783	62805
CHD7	AGTGAAGG1	CGG	52,63	chr8	(+)	60741484	60741506	exon1, exon2	NM_001316(	(+)	62720	62742
CHD8	TGGATAGTT	AGG	42,11	chr14	(+)	21429157	21429179	exon2, exon3	NM_001170(	(-)	43964	43986
CHD8	TGAATCGAA	TGG	47,37	chr14	(-)	21429311	21429333	exon2, exon3	NM_001170(	(-)	44118	44140
CHD8	GGACATCGG	AGG	57,89	chr14	(-)	21429256	21429278	exon2, exon3	NM_001170(	(-)	44063	44085
CHD8	GTACAGCAG	GGG	47,37	chr14	(-)	21429025	21429047	exon2, exon3	NM_001170(	(-)	43832	43854
CHD9	TCAACTAGT(	TGG	42,11	chr16	(-)	53156177	53156199	exon2, exon1	NM_025134,	(+)	252	274
CHD9	TATCAGATG(	TGG	42,11	chr16	(+)	53156442	53156464	exon2, exon1	NM_025134,	(+)	517	539
CHD9	ACAAACCAT1	TGG	42,11	chr16	(-)	53156410	53156432	exon2, exon1	NM_025134,	(+)	485	507
CHD9	GTGTTGGAC	TGG	47,37	chr16	(-)	53156251	53156273	exon2, exon1	NM_025134,	(+)	326	348
CHRAC1	GTCGTGGG1	CGG	55	chr8	(+)	140511512	140511534	[exon1]	[NM_017444	(+)	215	237
CHRAC1	TATTCCTACA	TGG	50	chr8	(+)	140514396	140514418	[exon2]	[NM_017444	(+)	3099	3121
CHRAC1	CTTCATGAT(	GGG	55	chr8	(-)	140511564	140511586	[exon1]	[NM_017444	(+)	267	289
CHRAC1	CATCATGAA(	AGG	60	chr8	(+)	140511577	140511599	[exon1]	[NM_017444	(+)	280	302
CLOCK	CTAGTGAAA	TGG	42,11	chr4	(-)	55476011	55476033	exon8, exon7	NM_001267(	(-)	48111	48133
CLOCK	TCCTGGTAA'	TGG	42,11	chr4	(-)	55478866	55478888	exon7, exon6	NM_001267(	(-)	50966	50988
CLOCK	GTTGGTGG/	AGG	47,37	chr4	(-)	55479653	55479675	exon6, exon5	NM_001267(	(-)	51753	51775
CLOCK	GCTCATTTTA	CGG	36,84	chr4	(+)	55482753	55482775	exon5, exon4	NM_001267(	(-)	54853	54875
CREBBP	AGCGGCTCT/	AGG	52,63	chr16	(-)	3850841	3850863	exon2, exon2	NM_004380,	(-)	125787	125809
CREBBP	GCTGTCATT(	CGG	52,63	chr16	(+)	3879836	3879858	exon1, exon1	NM_004380,	(-)	154782	154804
CREBBP	GATGAGCTG	AGG	47,37	chr16	(-)	3850949	3850971	exon2, exon2	NM_004380,	(-)	125895	125917
CREBBP	CTGTCGGAG	AGG	57,89	chr16	(-)	3850865	3850887	exon2, exon2	NM_004380,	(-)	125811	125833
CSTL1	CCTACTTATA	AGG	42,11	chr20	(+)	23440443	23440465	exon2	NM_138283	(+)	759	781
CSTL1	GTCGTTGCT(	AGG	52,63	chr20	(-)	23440419	23440441	exon2	NM_138283	(+)	735	757
CSTL1	CGTGTCATT(	TGG	47,37	chr20	(-)	23443980	23444002	exon3	NM_138283	(+)	4296	4318
CSTL1	GAGCTGGA/	AGG	36,84	chr20	(-)	23444805	23444827	exon4	NM_138283	(+)	5121	5143
CTCF	AGTTTCGGA	TGG	50	chr16	(-)	67610855	67610877	[exon3]	[NM_006565	(+)	48449	48471
CTCF	GTAGCAACA	AGG	50	chr16	(-)	67611193	67611215	[exon3]	[NM_006565	(+)	48787	48809
CTCF	TACCCCAGA/	GGG	55	chr16	(+)	67610951	67610973	[exon3]	[NM_006565	(+)	48545	48567
CTCF	ACTTACCAG/	AGG	60	chr16	(+)	67610902	67610924	[exon3]	[NM_006565	(+)	48496	48518
CXXC1	CGATAGCGA	GGG	47,37	chr18	(+)	50286227	50286249	exon4, exon4	NM_014593,	(-)	3885	3907
CXXC1	GCTATCGGC	CGG	52,63	chr18	(-)	50286211	50286233	exon4, exon4	NM_014593,	(-)	3869	3891
CXXC1	CTACTGCAT(	CGG	52,63	chr18	(-)	50286759	50286781	exon2, exon2	NM_014593,	(-)	4417	4439
CXXC1	GGACAGCAA	GGG	52,63	chr18	(-)	50286795	50286817	exon2, exon2	NM_014593,	(-)	4453	4475
DAXX	ATGCGAGG1	CGG	45	chr6	(-)	33322892	33322914	[exon1]	[NM_001141	(-)	4335	4357
DAXX	CACGTTGCT(	AGG	55	chr6	(+)	33321492	33321514	[exon3, exor	[NM_00135C	(-)	2935	2957
DAXX	TCTATGTGG(	CGG	55	chr6	(-)	33321163	33321185	[exon3, exor	[NM_00135C	(-)	2606	2628
DAXX	CGGCAAGA/	TGG	50	chr6	(-)	33321742	33321764	[exon2, exor	[NM_00135C	(-)	3185	3207
DIDO1	GACCACTAT(	AGG	57,89	chr20	(-)	62911498	62911520	exon3, exon3	NM_001193	(-)	6501	6523
DIDO1	CAAAGGCGA	AGG	57,89	chr20	(-)	62911582	62911604	exon3, exon3	NM_001193	(-)	6585	6607
DIDO1	AGCGCTTCT(	CGG	52,63	chr20	(-)	62911242	62911264	exon3, exon3	NM_001193	(-)	6245	6267
DIDO1	GGAAAACAT	AGG	47,37	chr20	(-)	62911520	62911542	exon3, exon3	NM_001193	(-)	6523	6545
DMAP1	CGGGACATT	GGG	52,63	chr1	(+)	44213775	44213797	exon2, exon2	NM_001034(	(+)	323	345
DMAP1	AGAACATTG	CGG	47,37	chr1	(-)	44214817	44214839	exon4, exon4	NM_001034(	(+)	1365	1387
DMAP1	CCGTACAGT(	TGG	57,89	chr1	(+)	44214745	44214767	exon4, exon4	NM_001034(	(+)	1293	1315
DMAP1	ACTGACTTT(	AGG	52,63	chr1	(+)	44214376	44214398	exon3, exon3	NM_001034(	(+)	924	946
DNMT1	ACGGTGCTC	CGG	47,37	chr19	(-)	10180492	10180514	exon4, exon4	NM_001318	(-)	47149	47171
DNMT1	TGAAGCCCG	TGG	57,89	chr19	(-)	10180428	10180450	exon4, exon4	NM_001318	(-)	47085	47107
DNMT1	AGCATGAGC	AGG	57,89	chr19	(+)	10180501	10180523	exon4, exon4	NM_001318	(-)	47158	47180
DNMT3A	CTCGTCATCC	NGG	60			0	0	Not available			0	0
DNMT3A	GAGATCACC	NGG	60			0	0	Not available			0	0
DNMT3A	GGAAAGCG(	NGG	60			0	0	Not available			0	0
DNMT3A	CCCATTGGG'	NGG	50			0	0	Not available			0	0
DNMT3B	AGAGTCGCG	CGG	52,63	chr20	(-)	32781354	32781376	exon3, exon3	NM_175848,	(+)	1503	1525
DNMT3B	AGACTCGAT	GGG	52,63	chr20	(+)	32780377	32780399	exon2, exon2	NM_175848,	(+)	526	548
DNMT3B	TGCGGATAG	GGG	57,89	chr20	(-)	32780428	32780450	exon2, exon2	NM_175848,	(+)	577	599
DNMT3B	AAGCTCGCG	GGG	57,89	chr20	(+)	32781363	32781385	exon3, exon3	NM_175848,	(+)	1512	1534
DNMT3L	CGTCATCGT(	AGG	52,63	chr21	(+)	44259510	44259532	exon5, exon5	NM_013369,	(-)	13172	13194
DNMT3L	AGCATGGAC	GGG	47,37	chr21	(-)	44261198	44261220	exon2, exon2	NM_013369,	(-)	14860	14882
DNMT3L	ATGCTACTG(	TGG	47,37	chr21	(-)	44258672	44258694	exon6, exon6	NM_013369,	(-)	12334	12356
DNMT3L	CTCTCAAGCT	CGG	52,63	chr21	(-)	44261165	44261187	exon2, exon2	NM_013369,	(-)	14827	14849
DOT1L	CGTAGTTGT	GGG	47,37	chr19	(-)	2191110	2191132	exon5	NM_032482	(+)	26962	26984
DOT1L	CCCGGATCT(	TGG	63,16	chr19	(+)	2185873	2185895	exon3	NM_032482	(+)	21725	21747
DOT1L	GATATGGCG	TGG	57,89	chr19	(-)	2191055	2191077	exon5	NM_032482	(+)	26907	26929
DOT1L	GTTCAGCTT(	TGG	57,89	chr19	(-)	2191022	2191044	exon5	NM_032482	(+)	26874	26896
DPF1	GGGTACGTG	CGG	47,37	chr19	(+)	38222434	38222456	exon3, exon3	NM_001289(	(-)	11429	11451
DPF1	GTTTCCGCA1	TGG	57,89	chr19	(-)	38218623	38218645	exon5, exon5	NM_001289(	(-)	7618	7640
DPF1	TCTACTGTG1	GGG	52,63	chr19	(-)	38218978	38219000	exon4, exon4	NM_001289(	(-)	7973	7995
DPF1	CCCGCTGTT(	CGG	57,89	chr19	(-)	38222409	38222431	exon3, exon3	NM_001289(	(-)	11404	11426
DPF2	GAAAGTCGT	GGG	52,63	chr11	(-)	65341031	65341053	exon3	NM_006268	(+)	7278	7300
DPF2	GTAGGAGT/	AGG	57,89	chr11	(-)	65340972	65340994	exon3	NM_006268	(+)	7219	7241
DPF2	ACAATTGCT(	CGG	47,37	chr11	(-)	65340489	65340511	exon2	NM_006268	(+)	6736	6758
DPF2	CTTTGTAGT/	AGG	52,63	chr11	(-)	65340385	65340407	exon2	NM_006268	(+)	6632	6654
DPF3	GCTCGGGG/	AGG	63,16	chr14	(-)	72771871	72771893	exon2, exon3	NM_001280	(-)	110829	110851
DPF3	ACAGCCGTG	CGG	47,37	chr14	(+)	72771832	72771854	exon2, exon3	NM_001280	(-)	110790	110812

DPF3	CGCAGTTTT(	TGG	47,37	chr14	(+)	72753284	72753306	exon3, exon4	NM_001280!	(-)	92242	92264
DPF3	GGTATGTAT	GGG	52,63	chr14	(+)	72753342	72753364	exon3, exon4	NM_001280!	(-)	92300	92322
DPY30	GTGAGACCG	AGG	52,63	chr2	(+)	32039292	32039314	exon3	NM_032574	(-)	15390	15412
DPY30	GAAGATCGA	TGG	36,84	chr2	(-)	32024176	32024198				0	0
DPY30	GCTAGAAAT	TGG	36,84	chr2	(+)	32024230	32024252	exon5	NM_032574	(-)	328	350
DPY30	CCATGATCAT	TGG	42,11	chr2	(-)	32024092	32024114				0	0
EED	GGCGTGTTT	AGG	47,37	chr11	(-)	86250338	86250360	exon2, exon2	NM_003797,	(+)	5955	5977
EED	GACGCTGTC,	TGG	42,11	chr11	(+)	86250299	86250321	exon2, exon2	NM_003797,	(+)	5916	5938
EED	TTCTCGTCTC	TGG	52,63	chr11	(-)	86245320	86245342	exon1, exon1	NM_003797,	(+)	937	959
EED	ACAAACACG(	TGG	47,37	chr11	(+)	86250350	86250372	exon2, exon2	NM_003797,	(+)	5967	5989
EHMT1	TAACTCGGA	GGG	42,11	chr9	(+)	137716803	137716825	exon3, exon3	NM_024757,	(+)	97812	97834
EHMT1	CGGATACAT(	CGG	31,58	chr9	(+)	137716909	137716931	exon3, exon3	NM_024757,	(+)	97918	97940
EHMT1	CAAAGTCGT(	TGG	57,89	chr9	(-)	137716861	137716883	exon3, exon3	NM_024757,	(+)	97870	97892
EHMT1	ACACCTATG(	AGG	57,89	chr9	(+)	137716628	137716650	exon3, exon3	NM_024757,	(+)	97637	97659
EHMT2	ATGAGTGGT	AGG	52,63	chr6	(+)	31896668	31896690	exon2, exon3	NM_001289(	(-)	16909	16931
EHMT2	GGCAGGGT	AGG	52,63	chr6	(+)	31896779	31896801	exon2, exon3	NM_001289(	(-)	17020	17042
EHMT2	CTGAGAATC	GGG	47,37	chr6	(-)	31896634	31896656	exon2, exon3	NM_001289(	(-)	16875	16897
EHMT2	ATTGACATC	AGG	57,89	chr6	(+)	31896444	31896466	exon3, exon4	NM_001289(	(-)	16685	16707
ELP3	CGGATAGCT	TGG	57,89	chr8	(-)	28110370	28110392	exon6, exon5	NM_001284	(+)	17304	17326
ELP3	CGATGTCTT	TGG	57,89	chr8	(-)	28097286	28097308	exon2, exon2	NM_001284	(+)	4220	4242
ELP3	CAGCAATGA	CGG	47,37	chr8	(-)	28099873	28099895	exon3, exon3	NM_001284	(+)	6807	6829
ELP3	ATAGCCAGT(	TGG	47,37	chr8	(-)	28107951	28107973	exon5, exon4	NM_001284	(+)	14885	14907
ELP4	GTAGTGTTG	GGG	57,89	chr11	(+)	31509807	31509829	exon1, exon1	NM_001288	(+)	79	101
ELP4	GCAACGTCA(	AGG	52,63	chr11	(+)	31509855	31509877	exon1, exon1	NM_001288	(+)	127	149
ELP4	TGTCAATGG	TGG	42,11	chr11	(+)	31539723	31539745	exon3, exon3	NM_001288	(+)	29995	30017
ELP4	GGACAGCTG	CGG	52,63	chr11	(+)	31509959	31509981	exon1, exon1	NM_001288	(+)	231	253
EP300	TAGTTCCCCT	TGG	42,11	chr22	(+)	41117356	41117378	exon2	NM_001429	(+)	24747	24769
EP300	AGTTTAGGC	AGG	57,89	chr22	(-)	41093035	41093057	exon1	NM_001429	(+)	426	448
EP300	GCGGCCTAA	CGG	52,63	chr22	(+)	41093046	41093068	exon1	NM_001429	(+)	437	459
EP300	GAATTGGGA	TGG	42,11	chr22	(+)	41117246	41117268	exon2	NM_001429	(+)	24637	24659
EP400	GGTGATGTT	CGG	55	chr12	(-)	131960822	131960844	exon2	NM_015409	(+)	10903	10925
EP400	GAACATCAC(	TGG	60	chr12	(+)	131960835	131960857	exon2	NM_015409	(+)	10916	10938
EP400	GTATTTGAT,	TGG	40	chr12	(-)	131960772	131960794	exon2	NM_015409	(+)	10853	10875
EP400	AGCTGATGC	GGG	55	chr12	(-)	131960638	131960660	exon2	NM_015409	(+)	10719	10741
EPC1	TATGGTTAT,	AGG	47,37	chr10	(-)	32305850	32305872	exon2, exon2	NM_001282	(-)	38135	38157
EPC1	GCGGGCTAT	AGG	57,89	chr10	(-)	32305895	32305917	exon2, exon2	NM_001282	(-)	38180	38202
EPC1	GACCACTGC(	CGG	47,37	chr10	(+)	32293597	32293619	exon3, exon3	NM_001282	(-)	25882	25904
EPC1	CGCTTCTCTT	TGG	42,11	chr10	(+)	32293058	32293080	exon4, exon4	NM_001282	(-)	25343	25365
EPC2	GTAAACTCT(	CGG	52,63	chr2	(+)	148645022	148645044	exon1	NM_015630	(+)	32	54
EPC2	GAGACGCAG	AGG	52,63	chr2	(-)	148645093	148645115	exon1	NM_015630	(+)	103	125
EPC2	ACGACTGCG	CGG	52,63	chr2	(+)	148645103	148645125	exon1	NM_015630	(+)	113	135
ERCC5	GATCTTCCCT	CGG	55	chr13	(-)	102846322	102846344	exon1	NM_000123	(+)	482	504
ERCC5	AGCGCTGGA	TGG	60	chr13	(+)	102846326	102846348	exon1	NM_000123	(+)	486	508
EZH1	TGCGACTTC(	CGG	42,11	chr17	(-)	42728849	42728871	exon3	NM_001991	(-)	28575	28597
EZH1	ATATGTTGG	CGG	36,84	chr17	(+)	42724381	42724403	exon5	NM_001991	(-)	24107	24129
EZH1	ACAGGCTTC,	AGG	42,11	chr17	(+)	42727660	42727682	exon4	NM_001991	(-)	27386	27408
EZH1	AGTGATACA	GGG	36,84	chr17	(+)	42728903	42728925	exon3	NM_001991	(-)	28629	28651
EZH2	TGCGACTGA	AGG	52,63	chr7	(-)	148847206	148847228	exon2, exon2	NM_001203	(-)	39835	39857
EZH2	ACACGCTTC(	TGG	52,63	chr7	(+)	148847243	148847265	exon2, exon2	NM_001203	(-)	39872	39894
EZH2	CGGAAATCT	TGG	36,84	chr7	(-)	148846536	148846558	exon3, exon3	NM_001203	(-)	39165	39187
EZH2	TTATGATGG	GGG	36,84	chr7	(-)	148829734	148829756	exon5, exon5	NM_001203	(-)	22363	22385
FBXL19	TTCGGGCAC,	AGG	57,89	chr16	(+)	30930231	30930253	exon7, exon7	NM_001282	(+)	5657	5679
FBXL19	GGTATGAAA	GGG	52,63	chr16	(+)	30924736	30924758	exon1	NM_001099	(+)	162	184
FBXL19	ATGCAGATA	CGG	52,63	chr16	(-)	30930535	30930557	exon7, exon7	NM_001282	(+)	5961	5983
FBXL19	CCCCGAACTT	CGG	52,63	chr16	(-)	30925862	30925884	exon2	NM_001099	(+)	1288	1310
FBXO17	AAGATGAGA	GGG	50	chr19	(+)	38949988	38950010	exon2, exon2	NM_024907,	(-)	8588	8610
FBXO17	GAAGATGAC	AGG	55	chr19	(+)	38949987	38950009	exon2, exon2	NM_024907,	(-)	8587	8609
FBXO17	GCCCAGCAA(	AGG	60	chr19	(-)	38950052	38950074	exon2, exon2	NM_024907,	(-)	8652	8674
FBXO17	CCTTGTCTTC	AGG	60	chr19	(+)	38950055	38950077	exon2, exon2	NM_024907,	(-)	8655	8677
FBXO44	AGGATCTCT(	AGG	52,63	chr1	(+)	11658321	11658343	exon3, exon4	NM_033182,	(+)	3947	3969
FBXO44	TGGTACTTG(	TGG	52,63	chr1	(-)	11658749	11658771	exon5, exon6	NM_033182,	(+)	4375	4397
FBXO44	CAGTGATGA	AGG	57,89	chr1	(-)	11655988	11656010	exon2, exon3	NM_033182,	(+)	1614	1636
FBXO44	ATGTGAATG	TGG	47,37	chr1	(+)	11658291	11658313	exon3, exon4	NM_033182,	(+)	3917	3939
FBXW9	TTTTGGCGG	GGG	52,63	chr19	(+)	12696456	12696478	exon1	NM_032301	(-)	7541	7563
FBXW9	GCTCGAGAT	TGG	57,89	chr19	(-)	12696299	12696321	exon1	NM_032301	(-)	7384	7406
FBXW9	CTCACTTACG	TGG	57,89	chr19	(+)	12696354	12696376	exon1	NM_032301	(-)	7439	7461
FBXW9	TCGTGTCTG,	TGG	52,63	chr19	(-)	12696222	12696244	exon1	NM_032301	(-)	7307	7329
FKBP1A	TCCCTTAGCT	GGG	50	chr20	(-)	1372075	1372097	exon3	NM_001199	(-)	3099	3121
FKBP1A	GTTTATGCT,	AGG	50	chr20	(-)	1375523	1375545	exon3, exon3	NM_054014,	(-)	6547	6569
FKBP1A	TAAGGAAAT	GGG	30	chr20	(-)	1372052	1372074				0	0
FKBP1A	GTGATCCGA	AGG	60	chr20	(-)	1375501	1375523	exon3, exon3	NM_054014,	(-)	6525	6547
FKBP2	GTCCCATCA	GGG	52,63	chr11	(+)	64242512	64242534	exon2, exon2	NM_004470,	(+)	397	419
FKBP2	CAAAAGGAA	GGG	47,37	chr11	(+)	64242468	64242490	exon2, exon2	NM_004470,	(+)	353	375
FKBP2	TGGACAGTA	CGG	52,63	chr11	(-)	64242405	64242427	exon2, exon2	NM_004470,	(+)	290	312
FKBP2	GAAGACAAA	GGG	52,63	chr11	(-)	64243239	64243261	exon3, exon3	NM_004470,	(+)	1124	1146
FKBP5	GTTGCATTC(	AGG	42,11	chr6	(+)	35620145	35620167	exon4, exon4	NM_004117,	(-)	39589	39611
FKBP5	GCCGAGCCA	TGG	47,37	chr6	(+)	35620178	35620200	exon4, exon4	NM_004117,	(-)	39622	39644
FKBP5	GGTGAGGA,	TGG	57,89	chr6	(-)	35637115	35637137	exon3, exon3	NM_004117,	(-)	56559	56581
FKBP5	TCATCAAGG(	GGG	47,37	chr6	(-)	35620246	35620268	exon4, exon4	NM_004117,	(-)	39690	39712
FMR1	GTCAGATTC(	AGG	57,89	chrX	(+)	147925574	147925596	exon3, exon3	NM_001185(	(+)	13624	13646
FMR1	GCACATCCA(	TGG	47,37	chrX	(-)	147928766	147928788	exon5, exon5	NM_001185(	(+)	16816	16838
FMR1	TTAGCTAAC(	AGG	47,37	chrX	(-)	147928349	147928371	exon4, exon4	NM_001185(	(+)	16399	16421
FMR1	AGCTAAAGT	AGG	36,84	chrX	(+)	147928366	147928388	exon4, exon4	NM_001185(	(+)	16416	16438
FXR2	GGTAGCCGG	AGG	63,16	chr17	(+)	7605694	7605716	exon3	NM_004860	(-)	14465	14487
FXR2	TCCTTATTAT	AGG	36,84	chr17	(+)	7605670	7605692	exon3	NM_004860	(-)	14441	14463
FXR2	TTATTCTCGA	AGG	36,84	chr17	(-)	7604056	7604078	exon4	NM_004860	(-)	12827	12849
FXR2	TAACTGGCC(	AGG	57,89	chr17	(+)	7603830	7603852	exon5	NM_004860	(-)	12601	12623

G2E3	TGATAACTT(	GGG	31,58	chr14	(-)	30592371	30592393	exon5, exon4	NM_017769,	(+)	33249	33271
G2E3	CGAAGTTAT(	TGG	36,84	chr14	(+)	30592383	30592405	exon5, exon4	NM_017769,	(+)	33261	33283
G2E3	GATGACTGT	CGG	31,58	chr14	(+)	30586741	30586763	exon3	NM_017769	(+)	27619	27641
G2E3	TGCTGTGTT	TGG	36,84	chr14	(+)	30592326	30592348	exon5, exon4	NM_017769,	(+)	33204	33226
GADD45A	GGCACAACA	GGG	52,63	chr1	(-)	67686353	67686375	exon2, exon3	NM_001199	(+)	1177	1199
GADD45A	AGTCAGCGC	CGG	57,89	chr1	(+)	67686074	67686096	exon2, exon2	NM_001199	(+)	898	920
GADD45A	AAGTCGCTA(	GGG	42,11	chr1	(+)	67687723	67687745	exon3, exon4	NM_001199	(+)	2547	2569
GADD45A	GTTGAACTC/	TGG	57,89	chr1	(-)	67687887	67687909				0	0
GADD45B	CATCAACATC	CGG	57,89	chr19	(+)	2477140	2477162	exon3	NM_015675	(+)	1016	1038
GADD45B	GATGTCGTT(	AGG	52,63	chr19	(-)	2477121	2477143	exon3	NM_015675	(+)	997	1019
GADD45B	GAGCGTGAA	GGG	52,63	chr19	(-)	2477091	2477113	exon3	NM_015675	(+)	967	989
GADD45B	CCTCCTCGTC	AGG	57,89	chr19	(-)	2477053	2477075	exon3	NM_015675	(+)	929	951
GATAD2A	CTCGTTTCTG	CGG	50	chr19	(-)	19465363	19465385	[exon2, exor	[NM_01766(	(+)	24	46
GATAD2A	TCGGAGAGC	AGG	55	chr19	(-)	19492337	19492359	[exon3, exor	[NM_01766(	(+)	26998	27020
GATAD2A	GTCAGAAAC	CGG	50	chr19	(+)	19465374	19465396	[exon2, exor	[NM_01766(	(+)	35	57
GATAD2A	TTCTGAGTG(	CGG	55	chr19	(-)	19494333	19494355	[exon5, exor	[NM_01766(	(+)	28994	29016
GATAD2B	GCAAAGCGA	GGG	45	chr1	(-)	153828241	153828263	[exon2]	[NM_020699	(-)	23515	23537
GATAD2B	CGTCTAGCA(	AGG	50	chr1	(+)	153828016	153828038	[exon2]	[NM_020699	(-)	23290	23312
GATAD2B	GGAGTTAGC	TGG	60	chr1	(+)	153819709	153819731	[exon3]	[NM_020699	(-)	14983	15005
GATAD2B	ACATGAGTT.	AGG	45	chr1	(-)	153828146	153828168	[exon2]	[NM_020699	(-)	23420	23442
GLYR1	GAATCGACG	AGG	42,11	chr16	(-)	4832170	4832192	exon5, exon5	NM_032569,	(-)	28968	28990
GLYR1	CGCAAACTT/	AGG	52,63	chr16	(-)	4832115	4832137	exon5, exon5	NM_032569,	(-)	28913	28935
GLYR1	CGACAGCAT(	TGG	57,89	chr16	(+)	4832809	4832831	exon4, exon4	NM_032569,	(-)	29607	29629
GLYR1	ATGCTGTCG.	AGG	47,37	chr16	(-)	4832795	4832817	exon4, exon4	NM_032569,	(-)	29593	29615
GTF2B	CCACTAAAA1	TGG	47,37	chr1	(+)	88887312	88887334	exon2	NM_001514	(-)	34675	34697
GTF2B	TGAGAATCT(	TGG	47,37	chr1	(+)	88864027	88864049	exon3	NM_001514	(-)	11390	11412
GTF2B	GATATGATC	TGG	36,84	chr1	(-)	88887272	88887294	exon2	NM_001514	(-)	34635	34657
GTF2B	GCAATTCTA/	CGG	36,84	chr1	(-)	88860233	88860255	exon4	NM_001514	(-)	7596	7618
GTF2F1	CGACGTATT(	TGG	42,11	chr19	(+)	6392870	6392892	exon2	NM_002096	(-)	13302	13324
GTF2F1	GAAGTACGC	AGG	57,89	chr19	(-)	6389502	6389524	exon4	NM_002096	(-)	9934	9956
GTF2F1	CGTAGCAAA	CGG	42,11	chr19	(+)	6391911	6391933	exon3	NM_002096	(-)	12343	12365
GTF2H1	TAGTTGTGT(	AGG	52,63	chr11	(-)	18335803	18335825	exon3, exon4	NM_005316,	(+)	13535	13557
GTF2H1	TTCCTTCTGG	TGG	31,58	chr11	(-)	18335755	18335777	exon3, exon4	NM_005316,	(+)	13487	13509
GTF2H1	TTTCACTGCT	TGG	42,11	chr11	(-)	18335838	18335860	exon3, exon4	NM_005316,	(+)	13570	13592
GTF2H1	GGATGGAG(	TGG	57,89	chr11	(+)	18333134	18333156	exon2, exon3	NM_005316,	(+)	10866	10888
GTF3C4	CACCGCATA(	CGG	57,89	chr9	(-)	132670768	132670790	exon1	NM_012204	(+)	734	756
GTF3C4	CATTCCGCCT	CGG	57,89	chr9	(+)	132670735	132670757	exon1	NM_012204	(+)	701	723
GTF3C4	TGCGGTGGA	TGG	57,89	chr9	(-)	132670895	132670917	exon1	NM_012204	(+)	861	883
GTF3C4	TTGAGGAGA	CGG	47,37	chr9	(-)	132670932	132670954	exon1	NM_012204	(+)	898	920
H2AFZ	GACTGGTCG	AGG	47,37	chr4	(+)	99949338	99949360	exon3	NM_002106	(-)	1252	1274
H2AFZ	GTCGATGAA	GGG	57,89	chr4	(+)	99949362	99949384	exon3	NM_002106	(-)	1276	1298
H2AFZ	GACGACCAG	TGG	57,89	chr4	(-)	99949326	99949348	exon3	NM_002106	(-)	1240	1262
H2AFZ	GGTAAGGCT	CGG	52,63	chr4	(-)	99949712	99949734	exon2	NM_002106	(-)	1626	1648
H3F3A	CTTTACCACC	NGG	50			0	0	Not available			0	0
H3F3A	TTTACCACCG	NGG	50			0	0	Not available			0	0
H3F3A	TTTGCGAAT(	NGG	40			0	0	Not available			0	0
H3F3A	AAGAGTGCC	NGG	60			0	0	Not available			0	0
HAT1	CAACACGGA	AGG	42,11	chr2	(-)	171952938	171952960	exon4	NM_003642	(+)	30514	30536
HAT1	GTATGAAGT	TGG	42,11	chr2	(-)	171965440	171965462	exon5	NM_003642	(+)	43016	43038
HAT1	AACTGCTAG(	TGG	36,84	chr2	(+)	171965858	171965880	exon6	NM_003642	(+)	43434	43456
HAT1	GAGTATACC(	TGG	42,11	chr2	(+)	171946755	171946777	exon3	NM_003642	(+)	24331	24353
HCFC1	CGAACTGCA(	CGG	52,63	chrX	(-)	153970648	153970670	exon1	NM_005334	(-)	23092	23114
HCFC1	GCCTATGGC	CGG	57,89	chrX	(-)	153964648	153964670	exon2	NM_005334	(-)	17092	17114
HCFC1	GAGCTCATC(	CGG	52,63	chrX	(-)	153970692	153970714	exon1	NM_005334	(-)	23136	23158
HDAC1	ACTCCGACA1	CGG	47,37	chr1	(-)	32316739	32316761	exon3	NM_004964	(+)	24633	24655
HDAC1	GCAGCAAAT	CGG	36,84	chr1	(-)	32302676	32302698	exon2	NM_004964	(+)	10570	10592
HDAC1	AGTAGTAAC	CGG	36,84	chr1	(-)	32292190	32292212	exon1	NM_004964	(+)	84	106
HDAC1	CTATGGTCT(	TGG	42,11	chr1	(+)	32302703	32302725	exon2	NM_004964	(+)	10597	10619
HDAC10	GACCGCGCT	AGG	57,89	chr22	(-)	50251005	50251027	exon1, exon1	NM_032019,	(-)	5822	5844
HDAC10	GTAGATGG(	CGG	52,63	chr22	(+)	50250436	50250458	exon3, exon3	NM_032019,	(-)	5253	5275
HDAC10	AGAGTATGT	GGG	47,37	chr22	(-)	50250498	50250520	exon3, exon3	NM_032019,	(-)	5315	5337
HDAC10	TCACTGGAG	GGG	47,37	chr22	(-)	50250077	50250099	exon4, exon4	NM_032019,	(-)	4894	4916
HDAC11	ACGATTGGC	TGG	57,89	chr3	(-)	13481277	13481299	exon2	NM_024827	(+)	1063	1085
HDAC11	GCGCTACAA	TGG	52,63	chr3	(+)	13481309	13481331	exon2	NM_024827	(+)	1095	1117
HDAC11	GCCCCATTTT	AGG	57,89	chr3	(-)	13481350	13481372	exon2, exon2	NM_001136(	(+)	1136	1158
HDAC11	TGGCACATG	GGG	57,89	chr3	(-)	13481257	13481279	exon2	NM_024827	(+)	1043	1065
HDAC2	TCCAACATCC	CGG	40	chr6	(+)	113946050	113946072	exon9	NM_001527	(-)	9895	9917
HDAC2	CGGAAACTG	CGG	50	chr6	(-)	113939097	113939119				0	0
HDAC2	CTAACTGGC	GGG	40	chr6	(+)	113940500	113940522				0	0
HDAC2	GAAACTTAG	GGG	40	chr6	(-)	113939535	113939557				0	0
HDAC3	TATTTCTACG	GGG	52,63	chr5	(-)	141636750	141636772	exon1	NM_003883	(-)	15875	15897
HDAC3	AGTCAATGT.	TGG	47,37	chr5	(+)	141634887	141634909	exon3	NM_003883	(-)	14012	14034
HDAC3	GGTGAAGCC	TGG	47,37	chr5	(+)	141634846	141634868	exon3	NM_003883	(-)	13971	13993
HDAC3	CATAGCCTG(	CGG	52,63	chr5	(-)	141636567	141636589	exon2	NM_003883	(-)	15692	15714
HDAC4	GCAGGATTC	TGG	57,89	chr2	(+)	239236619	239236641	exon3	NM_006037	(-)	188452	188474
HDAC4	GCCTCTGGA	AGG	57,89	chr2	(+)	239189886	239189908	exon4	NM_006037	(-)	141719	141741
HDAC4	GCAGTGAGA	AGG	57,89	chr2	(+)	239189994	239190016	exon4	NM_006037	(-)	141827	141849
HDAC4	CAGCTCCTG(	TGG	63,16	chr2	(+)	239176522	239176544	exon5	NM_006037	(-)	128355	128377
HDAC5	AGTCCGCGG	AGG	63,16	chr17	(+)	44110754	44110776	exon3, exon3	NM_005474,	(-)	34002	34024
HDAC5	AACAGCATG	AGG	52,63	chr17	(-)	44093608	44093630	exon4, exon4	NM_005474,	(-)	16856	16878
HDAC5	GTTTCTGGA	AGG	47,37	chr17	(+)	44093628	44093650	exon4, exon4	NM_005474,	(-)	16876	16898
HDAC5	AGAGCCATG	GGG	57,89	chr17	(-)	44093789	44093811	exon4, exon4	NM_005474,	(-)	17037	17059
HDAC6	CCGCTCTATC	CGG	52,63	chrX	(+)	48802900	48802922	exon3	NM_006044	(+)	821	843
HDAC6	ACCTAATCG1	GGG	52,63	chrX	(+)	48802974	48802996	exon3	NM_006044	(+)	895	917
HDAC6	GAAAGGACA	AGG	57,89	chrX	(-)	48805498	48805520	exon5	NM_006044	(+)	3419	3441
HDAC7	CGTGGTCAA	CGG	52,63	chr12	(-)	47798181	47798203	exon5, exon5	NM_001098	(-)	15471	15493
HDAC7	GGGATGGA(	GGG	47,37	chr12	(+)	47798131	47798153	exon5, exon5	NM_001098	(-)	15421	15443

HDAC7	TTCGCTTGCT	TGG	47,37	chr12	(+)	47798562	47798584	exon4, exon4	NM_0010984	(-)	15852	15874
HDAC7	AAAAGCTGC	CGG	47,37	chr12	(+)	47797444	47797466	exon6, exon6	NM_0010984	(-)	14734	14756
HDAC8	CATCCGGACT	TGG	45	chrX	(-)	72568759	72568781	exon3, exon3	NM_018486,	(-)	1178	1200
HDAC8	GTAGCAATT.	AGG	45	chrX	(-)	72567907	72567929	exon4, exon4	NM_018486,	(-)	326	348
HDAC8	CATTCCGTCA	GGG	45	chrX	(+)	72567936	72567958	exon4, exon4	NM_018486,	(-)	355	377
HDAC8	TCGGGACTA	CGG	35	chrX	(+)	72572694	72572716	exon1, exon1	NM_0011664	(-)	5113	5135
HDAC9	GAACTTGAC.	AGG	57,89	chr7	(+)	18585477	18585499	exon3, exon2	NM_178423,	(+)	76201	76223
HDAC9	CCATCTCACC	AGG	47,37	chr7	(+)	18585317	18585339	exon3, exon2	NM_178423,	(+)	89572	89594
HDAC9	CCGTGAGAA	AGG	52,63	chr7	(+)	18585381	18585403	exon3, exon2	NM_178423,	(+)	76105	76127
HDAC9	ACGAAAGAC	TGG	42,11	chr7	(+)	18591578	18591600	exon5, exon4	NM_178423,	(+)	82302	82324
HDGF	AGCCGGAAG	GGG	57,89	chr1	(+)	156745013	156745035	exon3, exon3	NM_001126(	(-)	2907	2929
HDGF	CTTCCCTTAC	AGG	52,63	chr1	(-)	156745100	156745122	exon3, exon3	NM_001126(	(-)	2994	3016
HDGF	CTCGTAAGG	TGG	52,63	chr1	(+)	156745110	156745132	exon3, exon3	NM_001126(	(-)	3004	3026
HDGFL1	GGAAATCGA	CGG	52,63	chr6	(+)	22569815	22569837	exon1	NM_138574	(+)	367	389
HDGFL1	GTTCCCGTA(	AGG	57,89	chr6	(+)	22569743	22569765	exon1	NM_138574	(+)	295	317
HDGFL1	TTGTACGGG	GGG	52,63	chr6	(-)	22569732	22569754	exon1	NM_138574	(+)	284	306
HDGFL1	GGCATGCCC.	CGG	52,63	chr6	(+)	22569588	22569610	exon1	NM_138574	(+)	140	162
HDGFRP2	AGTACGGGA	AGG	52,63	chr19	(+)	4475495	4475517	exon3, exon3	NM_0010015	(+)	3300	3322
HDGFRP2	TTAGCGAAC.	GGG	52,63	chr19	(-)	4472369	4472391	exon1, exon1	NM_0010015	(+)	174	196
HDGFRP2	TCGTAGGGG	GGG	57,89	chr19	(-)	4475458	4475480	exon3, exon3	NM_0010015	(+)	3263	3285
HDGFRP2	TTGGTGTTC(	GGG	42,11	chr19	(+)	4472378	4472400	exon1, exon1	NM_0010015	(+)	183	205
HDGFRP3	ATAGGATAC	AGG	36,84	chr15	(+)	83164023	83164045	exon2	NM_016073	(-)	25972	25994
HDGFRP3	TACTGCAGA	AGG	47,37	chr15	(-)	83157504	83157526	exon4	NM_016073	(-)	19453	19475
HDGFRP3	GGAAAGTCA	AGG	42,11	chr15	(-)	83157964	83157986	exon3	NM_016073	(-)	19913	19935
HDGFRP3	CAGAGCTCT	GGG	47,37	chr15	(-)	83157539	83157561	exon4	NM_016073	(-)	19488	19510
HELLS	CGTCGTTTT(	AGG	57,89	chr10	(-)	94590434	94590456	exon12, exor	NM_001289(	(+)	44668	44690
HELLS	ACGACTACT	AGG	42,11	chr10	(-)	94588283	94588305	exon11, exor	NM_001289(	(+)	42517	42539
HELLS	GACCATTAC.	GGG	52,63	chr10	(-)	94574082	94574104	exon8, exon8	NM_001289(	(+)	28316	28338
HELLS	TACCCCGGC(	AGG	57,89	chr10	(+)	94546423	94546445	exon2, exon2	NM_001289(	(+)	657	679
HIF1AN	GGAAGCTAT	TGG	47,37	chr10	(-)	100536048	100536070	exon1	NM_017902	(+)	165	187
HIF1AN	CTTTAAGCC(	GGG	52,63	chr10	(+)	100536572	100536594	exon2	NM_017902	(+)	689	711
HIF1AN	TTTGTGTCG(	AGG	52,63	chr10	(-)	100536414	100536436	exon2	NM_017902	(+)	531	553
HIF1AN	TCAATAAGC	GGG	52,63	chr10	(-)	100536106	100536128	exon1	NM_017902	(+)	223	245
HIRA	TTGCGAACT	GGG	57,89	chr22	(+)	19410728	19410750	exon2	NM_003325	(-)	80028	80050
HIRA	TCTATCCTC(	AGG	42,11	chr22	(-)	19405788	19405810	exon5	NM_003325	(-)	75088	75110
HIRA	CTTACCACTG	CGG	57,89	chr22	(+)	19405841	19405863	exon5	NM_003325	(-)	75141	75163
HIRA	TGTGTGCGG	TGG	52,63	chr22	(-)	19407241	19407263	exon4	NM_003325	(-)	76541	76563
HIST1H1B	GGCCGCTAA	GGG	55	chr6	(+)	27867317	27867339	[exon1]	[NM_005322	(-)	526	548
HIST1H1B	GAAATCCCC(	AGG	50	chr6	(-)	27867460	27867482	[exon1]	[NM_005322	(-)	669	691
HIST1H1B	TTTAGCGGC(	TGG	60	chr6	(+)	27867140	27867162	[exon1]	[NM_005322	(-)	349	371
HIST1H1B	TGGTGATCA	GGG	50	chr6	(+)	27867385	27867407	[exon1]	[NM_005322	(-)	594	616
HIST1H1C	AACTCCGCTA	AGG	50	chr6	(+)	26056258	26056280	[exon1]	[NM_005319	(-)	519	541
HIST1H1C	TCCGCCCGC(	TGG	55	chr6	(+)	26056054	26056076	[exon1]	[NM_005319	(-)	315	337
HIST1H1C	GGCCGCCTT(	GGG	55	chr6	(+)	26056354	26056376	[exon1]	[NM_005319	(-)	615	637
HIST1H1C	GGCTGCTCT(	TGG	50	chr6	(-)	26056230	26056252	[exon1]	[NM_005319	(-)	491	513
HIST1H3B	CTCGTACTAA	CGG	45	chr6	(-)	26032034	26032056	[exon1]	[NM_003537	(-)	446	468
HIST1H3B	GAAGATCGC	TGG	45	chr6	(+)	26031811	26031833	[exon1]	[NM_003537	(-)	223	245
HIST1H3B	ACTCGGTCG.	CGG	50	chr6	(+)	26031880	26031902	[exon1]	[NM_003537	(-)	292	314
HIST1H3B	AGTCGACCG	CGG	50	chr6	(-)	26031869	26031891	[exon1]	[NM_003537	(-)	281	303
HLTF	GTTGGACTA	GGG	42,11	chr3	(-)	149084686	149084708	exon2, exon2	NM_139048,	(-)	54560	54582
HLTF	GTCTGCAAG	TGG	42,11	chr3	(+)	149084863	149084885	exon2, exon2	NM_139048,	(-)	54737	54759
HLTF	CATCTGGAG	TGG	47,37	chr3	(+)	149084771	149084793	exon2, exon2	NM_139048,	(-)	54645	54667
HLTF	AGGTGCTTT(	TGG	47,37	chr3	(-)	149075906	149075928	exon3, exon3	NM_139048,	(-)	45780	45802
HNF1A	GATGTTGTG	AGG	55	chr12	(-)	120988868	120988890	[exon2, exor	[NM_000545	(+)	10301	10323
HNF1A	TGAGGTTCT(	AGG	50	chr12	(-)	120979023	120979045	[exon1, exor	[NM_000545	(+)	456	478
HNF1A	AGCAGCACA	CGG	55	chr12	(+)	120988877	120988899	[exon2, exor	[NM_000545	(+)	10310	10332
HNF1A	TAAACTGAG	CGG	50	chr12	(+)	120978777	120978799	[exon1, exor	[NM_000545	(+)	210	232
HR	TCTCACGAT(	GGG	52,63	chr8	(+)	22129096	22129118	exon2, exon2	NM_005144,	(-)	14678	14700
HR	CTCGAGATG	GGG	57,89	chr8	(-)	22129054	22129076	exon2, exon2	NM_005144,	(-)	14636	14658
HR	AGTACGCCC.	GGG	57,89	chr8	(-)	22129142	22129164	exon2, exon2	NM_005144,	(-)	14724	14746
HR	CACTATGCT(	GGG	52,63	chr8	(+)	22128789	22128811	exon2, exon2	NM_005144,	(-)	14371	14393
HSPBAP1	ATCGTATCT(	AGG	47,37	chr3	(+)	122777751	122777773	exon2, exon2	NM_024610,	(-)	37755	37777
HSPBAP1	AGCCGCAAC(	TGG	57,89	chr3	(+)	122793636	122793658	exon1, exon1	NM_024610,	(-)	53640	53662
HSPBAP1	AGCAGATAC	GGG	36,84	chr3	(-)	122777740	122777762	exon2, exon2	NM_024610,	(-)	37744	37766
HSPBAP1	CACTCGAAG.	TGG	47,37	chr3	(-)	122768821	122768843	exon3, exon3	NM_024610,	(-)	28825	28847
IDH1	GCATGACGA	AGG	45	chr2	(+)	208248382	208248404	[exon4, exor	[NM_005896	(-)	12156	12178
IDH1	ACGAAATAT	CGG	45	chr2	(-)	208248464	208248486	[exon4, exor	[NM_005896	(-)	12238	12260
IDH1	ATGTAGATC(	AGG	40	chr2	(+)	208251432	208251454	[exon3, exor	[NM_005896	(-)	15206	15228
IDH1	CTATCACTCC	AGG	45	chr2	(-)	208248537	208248559	[exon4, exor	[NM_005896	(-)	12311	12333
IDH2	GAAGTGTAC	AGG	55	chr15	(-)	90088393	90088415	[exon5, exor	[NM_001289	(-)	4414	4436
IDH2	ACTATCCGG.	GGG	55	chr15	(-)	90088687	90088709	[exon4, exor	[NM_001289	(-)	4708	4730
IDH2	CGCCACCTTC	CGG	50	chr15	(+)	90091619	90091641	[exon2]	[NM_002168	(-)	7640	7662
IDH2	ACCATACTGA	TGG	45	chr15	(-)	90087475	90087497	[exon6, exor	[NM_001289	(-)	3496	3518
ING1	TTGAACGTC	CGG	57,89	chr13	(+)	110715603	110715625	exon1	NM_005537	(+)	592	614
ING1	CGTTCCGCA(	AGG	57,89	chr13	(-)	110715462	110715484	exon1, exon1	NM_005537,	(+)	26	48
ING1	AAATCGGCG	CGG	47,37	chr13	(+)	110715720	110715742	exon1	NM_005537	(+)	709	731
ING1	TGCGGAACG	AGG	57,89	chr13	(+)	110715476	110715498	exon1, exon1	NM_005537,	(+)	40	62
ING2	TGCTCGAAT	CGG	42,11	chr4	(+)	183510429	183510451	exon2, exon2	NM_0012919	(+)	5380	5402
ING2	AAGGTAGTC	AGG	52,63	chr4	(-)	183505269	183505291	exon1	NM_001564	(+)	220	242
ING2	GCAGCAGCA	CGG	57,89	chr4	(+)	183505210	183505232	exon1	NM_001564	(+)	161	183
ING2	ACGTTCCTCT	GGG	52,63	chr4	(-)	183505310	183505332	exon1	NM_001564	(+)	261	283
ING3	TCTCGCGCA	CGG	52,63	chr7	(-)	120951195	120951217	exon2, exon2	NM_198267,	(+)	433	455
ING3	AATGCGCGA	AGG	57,89	chr7	(+)	120951207	120951229	exon2, exon2	NM_198267,	(+)	445	467
ING3	GTTGTACCTA	TGG	36,84	chr7	(+)	120950899	120950921	exon1, exon1	NM_198267,	(+)	137	159
ING3	GAAAAATAA	GGG	36,84	chr7	(+)	120953356	120953378	exon3, exon3	NM_198267,	(+)	2594	2616
ING4	CCGTTTTGA(	AGG	52,63	chr12	(-)	6652990	6653012	exon4, exon3	NM_0011275	(-)	2453	2475
ING4	GCTGCTCGT(	AGG	63,16	chr12	(-)	6652722	6652744	exon5, exon4	NM_0011275	(-)	2185	2207

ING4	GGCACTACT(	TGG	42,11	chr12	(+)	6653347	6653369	exon3, exon2	NM_001127!	(-)	2810	2832
ING4	GGACAAACA	TGG	57,89	chr12	(-)	6653026	6653048	exon4, exon3	NM_001127!	(-)	2489	2511
ING5	GATCTGCTT(	AGG	57,89	chr2	(-)	241711409	241711431	exon4	NM_032329	(+)	9369	9391
ING5	GAAGTTCCT(	AGG	47,37	chr2	(-)	241704668	241704690	exon2	NM_032329	(+)	2628	2650
ING5	ATAGTGCTC(	CGG	42,11	chr2	(-)	241702073	241702095	exon1	NM_032329	(+)	33	55
ING5	GAACGCCTA(	AGG	57,89	chr2	(+)	241709313	241709335	exon3	NM_032329	(+)	7273	7295
INO80	GTCGCAGAA	CGG	47,37	chr15	(+)	41096202	41096224	exon2	NM_017553	(-)	117322	117344
INO80	AATCCATTAT	TGG	42,11	chr15	(-)	41095875	41095897	exon3	NM_017553	(-)	116995	117017
INO80	TCTCCAAGT/	AGG	36,84	chr15	(+)	41096232	41096254	exon2	NM_017553	(-)	117352	117374
INO80	GGATCCCTT/	AGG	36,84	chr15	(-)	41095852	41095874	exon3	NM_017553	(-)	116972	116994
INTS12	TGCTTGATG,	CGG	42,11	chr4	(-)	105699883	105699905	exon3, exon2	NM_020395,	(-)	17256	17278
INTS12	CTTTTGAGA	TGG	42,11	chr4	(+)	105699850	105699872	exon3, exon2	NM_020395,	(-)	17223	17245
INTS12	CAAGGTCCT(	AGG	47,37	chr4	(-)	105695551	105695573	exon4, exon3	NM_020395,	(-)	12924	12946
INTS12	TTCTCCAATC	TGG	36,84	chr4	(+)	105693431	105693453	exon5, exon4	NM_020395,	(-)	10804	10826
IWS1	GAATCCCGT	TGG	47,37	chr2	(+)	127523752	127523774	exon2	NM_017969	(-)	42946	42968
IWS1	CAACACTCCC	TGG	52,63	chr2	(-)	127523698	127523720	exon2	NM_017969	(-)	42892	42914
IWS1	GAACGGGA1	CGG	57,89	chr2	(-)	127523740	127523762	exon2	NM_017969	(-)	42934	42956
IWS1	ATGGACTCG	CGG	36,84	chr2	(-)	127526186	127526208	exon1	NM_017969	(-)	45380	45402
JADE1	GCCAACACA	CGG	42,11	chr4	(-)	128846432	128846454	exon4, exon4	NM_001287(	(+)	36810	36832
JADE1	CTCATCCGG/	AGG	57,89	chr4	(-)	128846418	128846440	exon4, exon4	NM_001287(	(+)	36796	36818
JADE1	GCAGGAGCT	GGG	57,89	chr4	(-)	128842986	128843008	exon3, exon3	NM_001287(	(+)	33364	33386
JADE1	GATGACACG	GGG	42,11	chr4	(-)	128849017	128849039	exon5, exon5	NM_001287(	(+)	39395	39417
JADE2	AGACCCATG(	GGG	57,89	chr5	(+)	134552132	134552154	exon4, exon4	NM_015288,	(+)	27758	27780
JADE2	GGCTTCTTTT	GGG	47,37	chr5	(-)	134538054	134538076	exon3, exon3	NM_015288,	(+)	13680	13702
JADE2	CAGTTTGGA	CGG	42,11	chr5	(-)	134538007	134538029	exon3, exon3	NM_015288,	(+)	13633	13655
JADE2	GCCAGGATG	CGG	47,37	chr5	(-)	134552109	134552131	exon4, exon4	NM_015288,	(+)	27735	27757
JADE3	AGTGAATAT	CGG	47,37	chrX	(-)	47024760	47024782	exon5, exon5	NM_001077(	(+)	112328	112350
JADE3	TCCATGATG	AGG	42,11	chrX	(-)	47024806	47024828	exon5, exon5	NM_001077(	(+)	112374	112396
JADE3	GATTATAGC	AGG	36,84	chrX	(+)	47024724	47024746	exon5, exon5	NM_001077(	(+)	112292	112314
JADE3	CTGTGGAAC	TGG	57,89	chrX	(-)	46998244	46998266	exon4, exon4	NM_001077(	(+)	85812	85834
JARID2	ATCGGTCGG	CGG	57,89	chr6	(+)	15487514	15487536	exon6, exon6	NM_001267(	(+)	241540	241562
JARID2	GATTCCGTG(	GGG	47,37	chr6	(+)	15374131	15374153	exon2	NM_004973	(+)	128157	128179
JARID2	ATCGCTGTG(	CGG	47,37	chr6	(-)	15487379	15487401	exon6, exon6	NM_001267(	(+)	241405	241427
JARID2	ACTAACATC(	AGG	42,11	chr6	(-)	15410308	15410330	exon3	NM_004973	(+)	164334	164356
JMJD1C	TTGGCATTA(	AGG	47,37	chr10	(-)	63215586	63215608	exon5, exon6	NM_001282!	(-)	48366	48388
JMJD1C	ATCCGTCCA/	GGG	42,11	chr10	(-)	63215331	63215353	exon6, exon6	NM_001282!	(-)	48111	48133
JMJD1C	ATTCATAACC	GGG	36,84	chr10	(+)	63217213	63217235	exon4, exon5	NM_001282!	(-)	49993	50015
JMJD1C	ACAGAATGT	TGG	42,11	chr10	(-)	63215663	63215685	exon5, exon6	NM_001282!	(-)	48443	48465
JMJD6	TTGCGCATT(	CGG	42,11	chr17	(+)	76725757	76725779	exon2, exon2	NM_001081(	(-)	7315	7337
JMJD6	GGACTCTGG	AGG	57,89	chr17	(-)	76725709	76725731	exon2, exon2	NM_001081(	(-)	7267	7289
JMJD6	AGCTCTCGT/	AGG	47,37	chr17	(+)	76726373	76726395	exon1, exon1	NM_001081(	(-)	7931	7953
JMJD6	GGATGCGCT	TGG	52,63	chr17	(+)	76726448	76726470	exon1, exon1	NM_001081(	(-)	8006	8028
JMJD8	GACAGGTTG	TGG	55	chr16	(-)	683735	683757	exon4	NM_001005!	(-)	2069	2091
JMJD8	ACAGGTTGC	GGG	50	chr16	(-)	683734	683756	exon4	NM_001005!	(-)	2068	2090
JMJD8	GGCTTCGTT	TGG	60	chr16	(-)	683724	683746	exon4	NM_001005!	(-)	2058	2080
JMJD8	TTTGTGGTA(	TGG	45	chr16	(+)	683686	683708	exon4	NM_001005!	(-)	2020	2042
KANSL1	ACACCATAT(	TGG	42,11	chr17	(-)	46172077	46172099	exon2, exon2	NM_015443,	(-)	142162	142184
KANSL1	GGAGTCTTT(	GGG	42,11	chr17	(-)	46171843	46171865	exon2, exon2	NM_015443,	(-)	141928	141950
KANSL1	GAACTCCGA(	GGG	57,89	chr17	(-)	46171767	46171789	exon2, exon2	NM_015443,	(-)	141852	141874
KANSL1	CGTTATTTTC	GGG	52,63	chr17	(+)	46172032	46172054	exon2, exon2	NM_015443,	(-)	142117	142139
KAT2A	TTCTCAAGCT	CGG	52,63	chr17	(+)	42120991	42121013	exon1	NM_021078	(-)	7881	7903
KAT2A	TTTCCAGCCA	AGG	36,84	chr17	(+)	42120797	42120819	exon2	NM_021078	(-)	7687	7709
KAT2A	GACTTGCGC(	TGG	57,89	chr17	(+)	42121023	42121045	exon1	NM_021078	(-)	7913	7935
KAT2A	TGAGAAGCT	CGG	52,63	chr17	(-)	42120974	42120996	exon1	NM_021078	(-)	7864	7886
KAT2B	GCGCTTTCTT	CGG	57,89	chr3	(-)	20040696	20040718	exon1	NM_003884	(+)	665	687
KAT2B	GACAATTAT	CGG	42,11	chr3	(-)	20072397	20072419	exon2	NM_003884	(+)	32366	32388
KAT2B	TTCTCCAGTT	CGG	52,63	chr3	(-)	20040733	20040755	exon1	NM_003884	(+)	702	724
KAT2B	GAGGAGTCT	TGG	36,84	chr3	(+)	20072336	20072358	exon2	NM_003884	(+)	32305	32327
KAT5	GTCCTTCACC	CGG	57,89	chr11	(-)	65712772	65712794	exon3, exon2	NM_006388,	(+)	771	793
KAT5	GATCCAGTT(	AGG	47,37	chr11	(+)	65712974	65712996	exon4, exon3	NM_006388,	(+)	973	995
KAT5	GCCAAGACC	CGG	57,89	chr11	(+)	65712996	65713018	exon4, exon3	NM_006388,	(+)	995	1017
KAT5	GATTGGCAA	TGG	57,89	chr11	(-)	65713364	65713386	exon5, exon4	NM_006388,	(+)	1363	1385
KAT6A	GAATAGCAC	CGG	47,37	chr8	(-)	42048720	42048742	exon2, exon2	NM_006766,	(-)	106672	106694
KAT6A	CTGATAAAG	GGG	52,63	chr8	(-)	42048652	42048674	exon2, exon2	NM_006766,	(-)	106604	106626
KAT6A	AACAGCGTC(	AGG	47,37	chr8	(-)	42048885	42048907	exon2, exon2	NM_006766,	(-)	106837	106859
KAT6A	GCTATTCGC(	AGG	47,37	chr8	(+)	42048736	42048758	exon2, exon2	NM_006766,	(-)	106688	106710
KAT6B	TAACTGATG,	GGG	47,37	chr10	(-)	74843085	74843107	exon3, exon3	NM_012330,	(+)	16673	16695
KAT6B	GTTGTCTGG	AGG	47,37	chr10	(-)	74843063	74843085	exon3, exon3	NM_012330,	(+)	16651	16673
KAT6B	ATGCGGTCA	GGG	52,63	chr10	(+)	74842958	74842980	exon3, exon3	NM_012330,	(+)	16546	16568
KAT6B	GTTTATTCCA	CGG	31,58	chr10	(-)	74843163	74843185	exon3, exon3	NM_012330,	(+)	16751	16773
KAT7	GATGAACGA	AGG	52,63	chr17	(-)	49796863	49796885	exon3, exon3	NM_001199:	(+)	8245	8267
KAT7	TCACCGCCTC	TGG	57,89	chr17	(+)	49798348	49798370	exon4, exon4	NM_001199:	(+)	9730	9752
KAT7	CTGACTACG(	TGG	52,63	chr17	(-)	49796807	49796829	exon3, exon3	NM_001199:	(+)	8189	8211
KAT7	CTCGAGATC	CGG	36,84	chr17	(-)	49791923	49791945	exon2, exon2	NM_001199:	(+)	3305	3327
KAT8	ACGAGTGGC	CGG	47,37	chr16	(+)	31120354	31120376	exon3, exon3	NM_182958,	(+)	2691	2713
KAT8	GTCTCGAGT(	AGG	57,89	chr16	(+)	31120205	31120227	exon2, exon2	NM_182958,	(+)	2542	2564
KAT8	TCGGAGAAA	CGG	52,63	chr16	(+)	31117851	31117873	exon1, exon1	NM_182958,	(+)	188	210
KDM1A	GAATAGCAG	GGG	55	chr1	(+)	23019895	23019917	exon1, exon1	NM_015013,	(+)	448	470
KDM1A	GGAATAGCA	GGG	55	chr1	(+)	23019894	23019916	exon1, exon1	NM_015013,	(+)	447	469
KDM1A	TGGAATAGC	AGG	55	chr1	(+)	23019893	23019915	exon1, exon1	NM_015013,	(+)	446	468
KDM1A	CTGCTATTCC	GGG	45	chr1	(-)	23019881	23019903	exon1, exon1	NM_015013,	(+)	434	456
KDM1B	AAAATGGCT	TGG	52,63	chr6	(+)	18162843	18162865	exon5	NM_153042	(+)	7456	7478
KDM1B	GCCATGAAA	AGG	52,63	chr6	(-)	18166334	18166356	exon6	NM_153042	(+)	10947	10969
KDM1B	TACCGGAGC	AGG	57,89	chr6	(-)	18159955	18159977	exon3	NM_153042	(+)	4568	4590
KDM1B	ATGTGAAAA	CGG	47,37	chr6	(+)	18161395	18161417	exon4	NM_153042	(+)	6008	6030
KDM2A	GTACTATAG(	AGG	42,11	chr11	(-)	67243050	67243072	exon13, exor	NM_012308,	(+)	3016	3038
KDM2A	CGTCTGGCT(	CGG	57,89	chr11	(-)	67245300	67245322	exon14, exor	NM_012308,	(+)	5266	5288

KDM2A	CTCCACGGT	AGG	52,63	chr11	(+)	67245262	67245284	exon14, exor	NM_012308,	(+)	5228	5250
KDM2A	TAAATTCCCC	AGG	52,63	chr11	(+)	67245191	67245213	exon14, exor	NM_012308,	(+)	5157	5179
KDM2B	TCGTATCGC	CGG	57,89	chr12	(+)	121578921	121578943	exon2, exon2	NM_032590,	(-)	149826	149848
KDM2B	CCAGCGATA	AGG	57,89	chr12	(-)	121578910	121578932	exon2, exon2	NM_032590,	(-)	149815	149837
KDM2B	TCTCGTAGT	TGG	42,11	chr12	(+)	121549552	121549574	exon5, exon5	NM_032590,	(-)	120457	120479
KDM2B	ACGTCTCGG	AGG	47,37	chr12	(+)	121574561	121574583	exon4, exon4	NM_032590,	(-)	145466	145488
KDM3A	CAGATCCTTC	CGG	47,37	chr2	(-)	86442208	86442230	exon2, exon2	NM_018433,	(+)	748	770
KDM3A	TTTCTGAACC	TGG	31,58	chr2	(+)	86449931	86449953	exon3, exon3	NM_018433,	(+)	8471	8493
KDM3A	GGTGTGGG	TGG	57,89	chr2	(-)	86442187	86442209	exon2, exon2	NM_018433,	(+)	727	749
KDM3A	TGCTCACGC	TGG	57,89	chr2	(+)	86442052	86442074	exon2, exon2	NM_018433,	(+)	592	614
KDM3B	GTTATCGTG	AGG	52,63	chr5	(+)	138372740	138372762	exon2	NM_016604	(+)	20145	20167
KDM3B	AGACTCGAA	AGG	57,89	chr5	(-)	138372796	138372818	exon2	NM_016604	(+)	20201	20223
KDM3B	TACTCCCCTT	TGG	36,84	chr5	(+)	138375098	138375120	exon3	NM_016604	(+)	22503	22525
KDM3B	TAACTGGAA	GGG	47,37	chr5	(+)	138372697	138372719	exon2	NM_016604	(+)	20102	20124
KDM4A	TTCAAACTCA	GGG	36,84	chr1	(-)	43660306	43660328	exon4	NM_014663	(+)	10181	10203
KDM4A	TATCGCTATT	CGG	36,84	chr1	(-)	43655743	43655765	exon3	NM_014663	(+)	5618	5640
KDM4A	CCATTCAACA	GGG	52,63	chr1	(+)	43655661	43655683	exon3	NM_014663	(+)	5536	5558
KDM4A	TTGAATCCCA	CGG	47,37	chr1	(+)	43653276	43653298	exon2	NM_014663	(+)	3151	3173
KDM4B	ATGTCATCA	CGG	47,37	chr19	(-)	5039860	5039882	exon4	NM_015015	(+)	70748	70770
KDM4B	GTATGATGA	TGG	47,37	chr19	(+)	5039871	5039893	exon4	NM_015015	(+)	70759	70781
KDM4B	GGATATTGT	AGG	42,11	chr19	(-)	5039937	5039959	exon4	NM_015015	(+)	70825	70847
KDM4B	CGTGGCCTA	AGG	57,89	chr19	(+)	5032983	5033005	exon3	NM_015015	(+)	63871	63893
KDM4C	CTTTGCAAG	GGG	57,89	chr9	(-)	6793110	6793132	exon2, exon2	NM_015061,	(+)	35470	35492
KDM4C	GGCCACCTC	GGG	57,89	chr9	(-)	6792978	6793000	exon2	NM_001146	(+)	72116	72138
KDM4C	CGATGACTG	AGG	47,37	chr9	(+)	6805732	6805754	exon3, exon3	NM_015061,	(+)	48092	48114
KDM4C	GAAGGTCAT	TGG	36,84	chr9	(-)	6793023	6793045	exon2, exon2	NM_015061,	(+)	35383	35405
KDM4D	GCGATACTC	TGG	63,16	chr11	(-)	94997656	94997678	exon3	NM_018039	(+)	23976	23998
KDM4D	GATATTATC	TGG	31,58	chr11	(-)	94997548	94997570	exon3	NM_018039	(+)	23868	23890
KDM4D	TCTTCGAAA	TGG	42,11	chr11	(-)	94997709	94997731	exon3	NM_018039	(+)	24029	24051
KDM4D	ATTTGGATT	TGG	47,37	chr11	(-)	94997395	94997417	exon3	NM_018039	(+)	23715	23737
KDM4E	TGCCAAGGT	AGG	52,63	chr11	(+)	95025692	95025714	exon1	NM_001161	(+)	435	457
KDM4E	TCCCAAGGC	TGG	57,89	chr11	(+)	95025666	95025688	exon1	NM_001161	(+)	409	431
KDM4E	GATGGTATG	GGG	47,37	chr11	(-)	95025580	95025602	exon1	NM_001161	(+)	323	345
KDM4E	CCCGGTAAT	TGG	47,37	chr11	(+)	95025936	95025958	exon1	NM_001161	(+)	679	701
KDM5A	TCAGCTTTAT	CGG	52,63	chr12	(-)	388972	388994	exon1	NM_001042	(-)	108916	108938
KDM5A	CTCCCAACTC	CGG	57,89	chr12	(+)	389011	389033	exon1	NM_001042	(-)	108955	108977
KDM5A	CGGCCGAAT	CGG	52,63	chr12	(+)	388933	388955	exon1	NM_001042	(-)	108877	108899
KDM5B	ATGCCAGTC	GGG	57,89	chr1	(+)	202808124	202808146	exon1, exon1	NM_006618,	(-)	82940	82962
KDM5B	ACTCCCAGT	TGG	42,11	chr1	(+)	202774681	202774703	exon3, exon3	NM_006618,	(-)	49497	49519
KDM5B	TGAAAATTC	AGG	36,84	chr1	(-)	202774643	202774665	exon3, exon3	NM_006618,	(-)	49459	49481
KDM5B	TCTTGTGGA	GGG	42,11	chr1	(+)	202808152	202808174	exon1, exon1	NM_006618,	(-)	82968	82990
KDM5C	TTCGTAGTG	AGG	57,89	chrX	(+)	53217844	53217866	exon2, exon4	NM_001146	(-)	26524	26546
KDM5C	ATGCCCGAT	GGG	52,63	chrX	(+)	53224762	53224784	exon1, exon1	NM_001146	(-)	33442	33464
KDM5C	TGGTGGATA	GGG	57,89	chrX	(+)	53217883	53217905	exon2, exon4	NM_001146	(-)	26563	26585
KDM5C	CACACGTCC	AGG	36,84	chrX	(-)	53217247	53217269	exon3, exon5	NM_001146	(-)	25927	25949
KDM5D	GATTTACTCC	AGG	52,63	chrY	(-)	19743177	19743199	exon3, exon3	NM_001146	(-)	37763	37785
KDM5D	CACACACCC	AGG	47,37	chrY	(-)	19739632	19739654	exon5, exon6	NM_001146	(-)	34218	34240
KDM5D	GATGCCAGA	TGG	52,63	chrY	(+)	19744406	19744428	exon2, exon2	NM_001146	(-)	38992	39014
KDM5D	GCTGAATTC	TGG	57,89	chrY	(-)	19744449	19744471	exon2, exon2	NM_001146	(-)	39035	39057
KDM6A	GAAATCTCA	AGG	42,11	chrX	(-)	44873927	44873949	exon2, exon2	NM_001291	(+)	753	775
KDM6A	GATTTCATG	AGG	47,37	chrX	(+)	44873944	44873966	exon2, exon2	NM_001291	(+)	770	792
KDM6A	TTCCTCATCA	CGG	52,63	chrX	(-)	44873595	44873617	exon1, exon1	NM_001291	(+)	421	443
KDM6A	ACTGTAAAC	TGG	36,84	chrX	(-)	44974679	44974701	exon4, exon4	NM_001291	(+)	101505	101527
KDM6B	CGAGTCAGA	AGG	52,63	chr17	(+)	7846192	7846214	exon6	NM_001080	(+)	6276	6298
KDM6B	GAACTACTG	GGG	47,37	chr17	(-)	7845915	7845937	exon5	NM_001080	(+)	5999	6021
KDM6B	AGCAAACCA	AGG	36,84	chr17	(+)	7845948	7845970	exon5	NM_001080	(+)	6032	6054
KDM6B	GATTCCAGC	GGG	52,63	chr17	(-)	7846094	7846116	exon6	NM_001080	(+)	6178	6200
KDM7A	AGTGCGATA	TGG	47,37	chr7	(-)	140176755	140176777	exon1	NM_030647	(-)	92010	92032
KDM7A	TATCGCACTC	TGG	42,11	chr7	(+)	140176769	140176791	exon1	NM_030647	(-)	92024	92046
KDM7A	GGTTCCAAA	TGG	52,63	chr7	(-)	140133584	140133606	exon3	NM_030647	(-)	48839	48861
KDM7A	CACAGTTGG	AGG	47,37	chr7	(+)	140139129	140139151	exon2	NM_030647	(-)	54384	54406
KDM8	ACGTAGACA	CGG	52,63	chr16	(+)	27210380	27210402	exon2, exon2	NM_001145	(+)	6895	6917
KDM8	GGTCTTCTTT	AGG	52,63	chr16	(-)	27210198	27210220	exon2, exon2	NM_001145	(+)	6713	6735
KDM8	GGTGATCCT	GGG	57,89	chr16	(+)	27210330	27210352	exon2, exon2	NM_001145	(+)	6845	6867
KIAA2026	GTAGCCCTG	AGG	60	chr9	(+)	6007539	6007561	exon1	NM_001017	(-)	88532	88554
KIAA2026	CTGACCTACC	GGG	55	chr9	(-)	6007543	6007565	exon1	NM_001017	(-)	88536	88558
KIAA2026	GCTGACCTA	AGG	60	chr9	(-)	6007544	6007566	exon1	NM_001017	(-)	88537	88559
KIAA2026	GAGATGGA	CGG	55	chr9	(-)	6007591	6007613	exon1	NM_001017	(-)	88584	88606
KMT2A	TCATCGTCCT	AGG	55	chr11	(+)	118436795	118436817	exon1, exon1	NM_001197	(+)	306	328
KMT2A	TGACGAAGA	CGG	55	chr11	(-)	118436775	118436797	exon1, exon1	NM_001197	(+)	286	308
KMT2A	CATCGTCCTC	GGG	55	chr11	(+)	118436796	118436818	exon1, exon1	NM_001197	(+)	307	329
KMT2A	CGATGACGA	AGG	55	chr11	(-)	118436778	118436800	exon1, exon1	NM_001197	(+)	289	311
KMT2B	CAGAGCTAC	CGG	55	chr19	(-)	35718137	35718159	exon1	NM_014727	(+)	119	141
KMT2C	GCGATCTGT	CGG	52,63	chr7	(-)	152330641	152330663	exon3	NM_170606	(-)	195717	195739
KMT2C	GACACAGAT	TGG	52,63	chr7	(+)	152330652	152330674	exon3	NM_170606	(-)	195728	195750
KMT2C	GCAGTTTTC	AGG	52,63	chr7	(+)	152358652	152358674	exon2	NM_170606	(-)	223728	223750
KMT2C	CAAAGAACA	AGG	36,84	chr7	(-)	152330713	152330735	exon3	NM_170606	(-)	195789	195811
KMT2D	AGTTGCCAT	CGG	47,37	chr12	(-)	49054649	49054671	exon3	NM_003482	(-)	35675	35697
KMT2D	AATCTGTGA	TGG	42,11	chr12	(+)	49054574	49054596	exon3	NM_003482	(-)	35600	35622
KMT2D	CAGAGACCT	GGG	57,89	chr12	(+)	49054949	49054971	exon2	NM_003482	(-)	35975	35997
KMT2E	GAGCATAGT	GGG	47,37	chr7	(+)	105040955	105040977	exon3, exon2	NM_182931,	(+)	26766	26788
KMT2E	GTTATGGTA	AGG	36,84	chr7	(+)	105063502	105063524	exon5, exon4	NM_182931,	(+)	49313	49335
KMT2E	GAGGACGA	TGG	47,37	chr7	(-)	105063353	105063375	exon5, exon4	NM_182931,	(+)	49164	49186
KMT2E	TACCACAGG	CGG	52,63	chr7	(-)	105062172	105062194	exon4, exon3	NM_182931,	(+)	47983	48005
L3MBTL1	GCGGCTTAT	CGG	47,37	chr20	(-)	43514637	43514659	exon1, exon4	NM_015478,	(+)	6958	6980
L3MBTL1	GACCGAATA	GGG	52,63	chr20	(+)	43514715	43514737	exon1, exon4	NM_015478,	(+)	7036	7058
L3MBTL1	TACGGGTCC	CGG	57,89	chr20	(-)	43514576	43514598	exon1	NM_015478	(+)	141	163

L3MBTL1	GTATAAGCC	CGG	47,37	chr20	(+)	43514649	43514671	exon1, exon4	NM_015478,	(+)	6970	6992
L3MBTL2	CGGAGTTAT	GGG	47,37	chr22	(+)	41209771	41209793	exon2	NM_031488	(+)	4463	4485
L3MBTL2	GTGTGGTAT	GGG	52,63	chr22	(+)	41213906	41213928	exon3	NM_031488	(+)	8598	8620
L3MBTL2	TTCACTTGAC	AGG	47,37	chr22	(-)	41209808	41209830	exon2	NM_031488	(+)	4500	4522
L3MBTL2	CTGTAACCTA	TGG	42,11	chr22	(-)	41214004	41214026	exon3	NM_031488	(+)	8696	8718
L3MBTL3	AGTGCCGGC	AGG	60	chr6	(+)	130057473	130057495	exon9, exon8	NM_032438,	(+)	38891	38913
L3MBTL3	GGATTCGGC	AGG	50	chr6	(+)	130055233	130055255	exon8, exon7	NM_032438,	(+)	36651	36673
L3MBTL3	CTCTTGACCA	AGG	50	chr6	(-)	130042713	130042735	exon3, exon3	NM_032438,	(+)	24131	24153
L3MBTL3	TGGGATGCC	AGG	50	chr6	(+)	130051265	130051287	exon6, exon5	NM_032438,	(+)	32683	32705
L3MBTL4	CATTGATCCC	CGG	50	chr18	(-)	6244510	6244532	exon6	NM_173464	(-)	289805	289827
L3MBTL4	GTCCTGATCC	TGG	50	chr18	(+)	6311563	6311585	exon3	NM_173464	(-)	356858	356880
L3MBTL4	ACATGAACT	AGG	45	chr18	(-)	6243294	6243316	exon7	NM_173464	(-)	288589	288611
L3MBTL4	GTCGCCACA	AGG	60	chr18	(+)	6239787	6239809	exon9	NM_173464	(-)	285082	285104
LBR	GATCGGCGC	TGG	57,89	chr1	(+)	225422153	225422175	exon3, exon3	NM_194442,	(-)	20652	20674
LBR	CTTCCTTAAT	TGG	52,63	chr1	(+)	225422118	225422140	exon3, exon3	NM_194442,	(-)	20617	20639
LBR	CCATCGGCA	TGG	47,37	chr1	(+)	225424050	225424072	exon2, exon2	NM_194442,	(-)	22549	22571
LBR	GCCGATGGT	AGG	52,63	chr1	(-)	225424035	225424057	exon2, exon2	NM_194442,	(-)	22534	22556
Mar-05	ATCCACCCAG	AGG	52,63	chr10	(-)	92311233	92311255	exon2	NM_017824	(+)	20071	20093
Mar-05	GTGCAGAGC	GGG	42,11	chr10	(+)	92311201	92311223	exon2	NM_017824	(+)	20039	20061
Mar-05	AGATCCAAG	TGG	42,11	chr10	(-)	92340675	92340697	exon3	NM_017824	(+)	49513	49535
Mar-05	GCTGCAGCA	CGG	52,63	chr10	(+)	92340732	92340754	exon3	NM_017824	(+)	49570	49592
MBD1	CGCGAAGTC	AGG	52,63	chr18	(-)	50279919	50279941	exon2, exon2	NM_015847,	(-)	8451	8473
MBD1	AACCTGACG	TGG	52,63	chr18	(+)	50276924	50276946	exon4, exon4	NM_015847,	(-)	5456	5478
MBD1	ACCCTCTTCG	AGG	47,37	chr18	(-)	50277115	50277137	exon3, exon3	NM_015847,	(-)	5647	5669
MBD1	GTTGAGCTG	GGG	52,63	chr18	(-)	50277154	50277176	exon3, exon3	NM_015847,	(-)	5686	5708
MBD2	GGTTCTGCC	GGG	55	chr18	(+)	54202913	54202935	exon3	NM_015832	(-)	234	256
MBD2	AATACAATG	GGG	40	chr18	(+)	54202868	54202890				0	0
MBD2	GAGGAAGT	TGG	45	chr18	(-)	54224054	54224076	exon1, exon1	NM_015832,	(-)	72454	72476
MBD2	CCTCAGTTG	GGG	55	chr18	(-)	54205107	54205129	exon2, exon2	NM_015832,	(-)	53507	53529
MBD3	TGGTTGGTA	CGG	42,11	chr19	(+)	1584595	1584617	exon3, exon3	NM_00128	(-)	7925	7947
MBD3	TCATCAGCA	CGG	52,63	chr19	(+)	1585111	1585133	exon2, exon2	NM_00128	(-)	8441	8463
MBD3	CAGCAATGT	AGG	52,63	chr19	(+)	1582664	1582686	exon4, exon4	NM_00128	(-)	5994	6016
MBD3	TCGCTCTTGA	GGG	52,63	chr19	(+)	1584571	1584593	exon3, exon3	NM_00128	(-)	7901	7923
MBD4	TCTTAAAGG	GGG	42,11	chr3	(+)	129433952	129433974	exon5, exon5	NM_00127	(-)	3009	3031
MBD4	GCGATGGGT	GGG	52,63	chr3	(+)	129437846	129437868	exon2, exon2	NM_00127	(-)	6903	6925
MBD4	ACGAGATTA	AGG	42,11	chr3	(+)	129433918	129433940	exon5, exon5	NM_00127	(-)	2975	2997
MBD4	TGCCGTAAG	TGG	52,63	chr3	(-)	129437789	129437811	exon2, exon2	NM_00127	(-)	6846	6868
MBD5	GGACAGAAA	TGG	50	chr2	(+)	148468568	148468590	exon9	NM_018328	(+)	447558	447580
MBD5	CAGCGTCGT	TGG	50	chr2	(+)	148458834	148458856	exon6	NM_018328	(+)	437824	437846
MBD5	GTCTACCCCC	GGG	55	chr2	(+)	148468532	148468554	exon9	NM_018328	(+)	447522	447544
MBD5	GAGAACCGC	AGG	45	chr2	(+)	148463774	148463796	exon8	NM_018328	(+)	442764	442786
MBTD1	GCGGCTAGC	AGG	52,63	chr17	(+)	51218980	51219002	exon5	NM_017643	(-)	41556	41578
MBTD1	CGGTTATGA	AGG	47,37	chr17	(-)	51225131	51225153	exon3	NM_017643	(-)	47707	47729
MBTD1	TTGATAATC	AGG	36,84	chr17	(+)	51225055	51225077	exon3	NM_017643	(-)	47631	47653
MBTD1	TTACTCGTCA	AGG	42,11	chr17	(-)	51220353	51220375	exon4	NM_017643	(-)	42929	42951
MECOM	TATCCGGCG	TGG	52,63	chr3	(+)	169131435	169131457	exon4, exon3	NM_004991,	(-)	47937	47959
MECOM	GTAGTTGCA	GGG	52,63	chr3	(-)	169131497	169131519	exon4, exon4	NM_004991,	(-)	47999	48021
MECOM	GCTTCCAAC	TGG	47,37	chr3	(+)	169143770	169143792	exon3, exon3	NM_004991,	(-)	60272	60294
MECOM	CTCAAGTAC	TGG	36,84	chr3	(-)	169143744	169143766	exon3, exon3	NM_004991,	(-)	60246	60268
MECP2	GATGATGGA	TGG	63,16	chrX	(+)	154032320	154032342	exon3, exon2	NM_004992,	(-)	2086	2108
MECP2	GGTCATCAT	CGG	52,63	chrX	(+)	154032292	154032314	exon3, exon2	NM_004992,	(-)	2058	2080
MECP2	GGACACGGA	AGG	52,63	chrX	(-)	154032251	154032273	exon3, exon4	NM_004992,	(-)	2017	2039
MECP2	CATCATACTT	CGG	47,37	chrX	(+)	154032220	154032242	exon3, exon4	NM_004992,	(-)	1986	2008
MEN1	AGAGGGCG	AGG	57,89	chr11	(+)	64809842	64809864	exon3, exon2	NM_130804,	(-)	6329	6351
MEN1	AGAAGGTCT	TGG	47,37	chr11	(-)	64809732	64809754	exon3, exon2	NM_130804,	(-)	6219	6241
MEN1	GTCCCTCTAT	GGG	52,63	chr11	(-)	64809779	64809801	exon3, exon2	NM_130804,	(-)	6266	6288
MEN1	GTGAGCTCG	AGG	63,16	chr11	(+)	64809925	64809947	exon3, exon2	NM_130804,	(-)	6412	6434
MINA	GATGAAGTT	GGG	42,11	chr3	(-)	97967509	97967531	exon2, exon2	NM_00104	(-)	25693	25715
MINA	CATAGTATG	GGG	52,63	chr3	(+)	97967362	97967384	exon2, exon2	NM_00104	(-)	25546	25568
MINA	GGTCATCTC	AGG	47,37	chr3	(+)	97967383	97967405	exon2, exon2	NM_00104	(-)	25567	25589
MINA	CTCTTCCATA	CGG	47,37	chr3	(+)	97967299	97967321	exon2, exon2	NM_00104	(-)	25483	25505
MLLT10	AACGTTAGA	GGG	47,37	chr10	(-)	21556731	21556753	exon4, exon4	NM_001195	(+)	22560	22582
MLLT10	GGCATTGTT	TGG	47,37	chr10	(+)	21538841	21538863	exon3, exon2	NM_004641,	(+)	4670	4692
MLLT10	TGCCCGTCG	CGG	52,63	chr10	(-)	21534756	21534778	exon2, exon1	NM_004641,	(+)	585	607
MLLT10	CGAGGTCTC	AGG	52,63	chr10	(+)	21534680	21534702	exon2, exon1	NM_004641,	(+)	509	531
MLLT6	CACGTTGGC	CGG	52,63	chr17	(-)	38707796	38707818	exon4	NM_005937	(+)	2177	2199
MLLT6	CTGTTACATC	AGG	52,63	chr17	(+)	38709175	38709197	exon5	NM_005937	(+)	3556	3578
MLLT6	TGGCATCGT	CGG	57,89	chr17	(+)	38706957	38706979	exon2	NM_005937	(+)	1338	1360
MLLT6	CTGCGTATG	GGG	57,89	chr17	(+)	38705656	38705678	exon1	NM_005937	(+)	37	59
MORF4L1	TTAGGCTTC	CGG	57,89	chr15	(-)	78873024	78873046	exon1, exon1	NM_006791,	(+)	244	266
MORF4L1	TTTTCTTCCG	GGG	52,63	chr15	(-)	78891521	78891543	exon5, exon7	NM_001265	(+)	18741	18763
MORF4L1	CAAAGATTT	TGG	42,11	chr15	(-)	78884981	78885003	exon4	NM_206839	(+)	12201	12223
MORF4L1	CTCCTTCAGC	AGG	47,37	chr15	(-)	78885027	78885049	exon4	NM_206839	(+)	12247	12269
MPHOSPH8	ATCCTGGAC	GGG	52,63	chr13	(+)	19633938	19633960	exon1	NM_017520	(+)	291	313
MPHOSPH8	GATAATGAC	AGG	57,89	chr13	(+)	19633857	19633879	exon1	NM_017520	(+)	210	232
MPHOSPH8	AAGTTCTTTA	TGG	31,58	chr13	(+)	19642119	19642141	exon2	NM_017520	(+)	8472	8494
MPHOSPH8	ACAGTCCTCC	CGG	52,63	chr13	(-)	19642176	19642198	exon2	NM_017520	(+)	8529	8551
MSH6	GGAACATTC	AGG	52,63	chr2	(+)	47791015	47791037	exon2	NM_000179	(+)	7934	7956
MSH6	GCATCAAAA	AGG	47,37	chr2	(-)	47799139	47799161	exon4, exon3	NM_00128	(+)	16058	16080
MSH6	GGGTGGTTC	AGG	52,63	chr2	(-)	47790985	47791007	exon2	NM_000179	(+)	7904	7926
MSH6	CGCCTAGAT	CGG	52,63	chr2	(-)	47798680	47798702	exon2, exon4	NM_00128	(+)	15599	15621
MSL3	CTGTAATCTA	CGG	31,58	chrX	(-)	11760444	11760466	exon3, exon3	NM_078628,	(+)	2286	2308
MSL3	TTTAAGACA	AGG	42,11	chrX	(-)	11760874	11760896	exon4, exon4	NM_078628,	(+)	2716	2738
MSL3	CATCTTTTTC	AGG	42,11	chrX	(-)	11760900	11760922	exon4, exon4	NM_078628,	(+)	2742	2764
MSL3	CTTGTGGCA	TGG	42,11	chrX	(-)	11763907	11763929	exon8, exon7	NM_078628,	(+)	5749	5771
MTA1	ATACCTGAT	AGG	47,37	chr14	(+)	105438706	105438728	exon2, exon2	NM_004689,	(+)	18858	18880
MTA1	AGTCTGCTA	CGG	52,63	chr14	(+)	105449366	105449388	exon4, exon4	NM_004689,	(+)	29518	29540

MTA1	TAAAGCACC/	CGG	57,89	chr14	(+)	105450112	105450134	exon5, exon5	NM_004689,	(+)	30264	30286
MTA1	TCCGACCCTC	CGG	52,63	chr14	(-)	105420040	105420062	exon1, exon1	NM_004689,	(+)	192	214
MTA2	TACTAGAAA	CGG	47,37	chr11	(+)	62600196	62600218	exon3	NM_004739	(-)	6994	7016
MTA2	GTTCCCGGT(	TGG	57,89	chr11	(+)	62598568	62598590	exon4	NM_004739	(-)	5366	5388
MTA2	AGATATCTT(	TGG	42,11	chr11	(-)	62598335	62598357	exon5	NM_004739	(-)	5133	5155
MTA2	TCAATCCGT(	AGG	47,37	chr11	(+)	62600635	62600657	exon2	NM_004739	(-)	7433	7455
MTA3	GCAACCCAT/	AGG	42,11	chr2	(+)	42570464	42570486	exon2	NM_020744	(+)	1948	1970
MTA3	TGCGAGCAT	TGG	42,11	chr2	(-)	42579165	42579187	exon3	NM_020744	(+)	10649	10671
MTA3	GGATCCAAA	CGG	47,37	chr2	(+)	42656246	42656268	exon7, exon7	NM_001282	(+)	161678	161700
MTF2	ACAGCTTGG	GGG	47,37	chr1	(-)	93110296	93110318	exon2, exon2	NM_001164	(+)	31062	31084
MTF2	TATGACCTG(	AGG	47,37	chr1	(-)	93115550	93115572	exon4, exon6	NM_001164	(+)	36316	36338
MTF2	GACGTAAAG	TGG	52,63	chr1	(-)	93110260	93110282	exon2, exon2	NM_001164	(+)	31026	31048
MTF2	GCAGACATT,	TGG	42,11	chr1	(+)	93115523	93115545	exon4, exon6	NM_001164	(+)	36289	36311
MUM1	GCCGATGGC	TGG	50	chr19	(+)	1356418	1356440	exon2	NM_032853	(+)	1442	1464
MUM1	GACGACACG	GGG	55	chr19	(+)	1360630	1360652	exon5	NM_032853	(+)	5654	5676
MUM1	TGCAAAGTG	GGG	45	chr19	(+)	1360435	1360457	exon5	NM_032853	(+)	5459	5481
MUM1	TTTTGTTGA/	GGG	45	chr19	(-)	1357016	1357038	exon3	NM_032853	(+)	2040	2062
NAP1L1	GGTCTGGTA	AGG	47,37	chr12	(-)	76067383	76067405	exon4, exon4	NM_004537,	(-)	18385	18407
NAP1L1	ACTCGTCTTT	AGG	42,11	chr12	(+)	76060253	76060275	exon5, exon5	NM_004537,	(-)	11255	11277
NAP1L1	TCTGTGCAC/	AGG	36,84	chr12	(+)	76060215	76060237	exon5, exon5	NM_004537,	(-)	11217	11239
NAP1L1	ACCATCAAG	GGG	36,84	chr12	(+)	76067403	76067425	exon4, exon4	NM_004537,	(-)	18405	18427
NAP1L2	ACGGTGAAG	GGG	52,63	chrX	(-)	73214289	73214311	exon1	NM_021963	(-)	1989	2011
NAP1L2	CTTGGAGAC	CGG	52,63	chrX	(-)	73214350	73214372	exon1	NM_021963	(-)	2050	2072
NAP1L2	GACCGTCCA	CGG	47,37	chrX	(-)	73214215	73214237	exon1	NM_021963	(-)	1915	1937
NAP1L3	TAGCTCGAC	GGG	42,11	chrX	(-)	93673214	93673236	exon1	NM_004538	(-)	2289	2311
NAP1L3	CGAGCTAGC	CGG	57,89	chrX	(+)	93673230	93673252	exon1	NM_004538	(-)	2305	2327
NAP1L3	GCCGCTTGT/	AGG	42,11	chrX	(-)	93673068	93673090	exon1	NM_004538	(-)	2143	2165
NAP1L3	CACGAAATT	GGG	42,11	chrX	(+)	93673014	93673036	exon1	NM_004538	(-)	2089	2111
NCOA1	GTATTGCAC(	CGG	50	chr2	(+)	24693273	24693295	exon8, exon8	NM_003743,	(+)	108797	108819
NCOA1	TTTCGTCGT(	AGG	50	chr2	(-)	24691498	24691520	exon7, exon7	NM_003743,	(+)	107022	107044
NCOA1	GTCTACAGC	GGG	50	chr2	(+)	24683062	24683084	exon6, exon6	NM_003743,	(+)	98586	98608
NCOA1	CTAACCCAG/	AGG	45	chr2	(+)	24658712	24658734	exon3, exon3	NM_003743,	(+)	74236	74258
NCOA3	AAAGCCGAT	AGG	36,84	chr20	(+)	47625413	47625435	exon5, exon5	NM_006534,	(+)	123557	123579
NCOA3	CGTCTCGATT	AGG	47,37	chr20	(-)	47627016	47627038	exon6, exon6	NM_006534,	(+)	125160	125182
NCOA3	TTTCGTGAA	TGG	47,37	chr20	(-)	47622275	47622297	exon3, exon3	NM_006534,	(+)	120419	120441
NCOA3	CCATGTGAT/	AGG	52,63	chr20	(+)	47622308	47622330	exon3, exon3	NM_006534,	(+)	120452	120474
NCOR1	CGGTGTTTC	AGG	57,89	chr17	(+)	16158794	16158816	exon5, exon3	NM_001190	(-)	128701	128723
NCOR1	GTATACTGG	AGG	47,37	chr17	(+)	16194487	16194509	exon1, exon1	NM_001190	(-)	164394	164416
NCOR1	TGGTTGCCC(	AGG	52,63	chr17	(+)	16165105	16165127	exon4, exon2	NM_001190	(-)	135012	135034
NCOR1	GGAAGAGTT	TGG	36,84	chr17	(-)	16165041	16165063	exon4, exon2	NM_001190	(-)	134948	134970
NCOR2	TGGTCAGCG	GGG	55	chr12	(+)	124426739	124426761	exon13, exor	NM_001206	(-)	102329	102351
NCOR2	GCTTATGGC	AGG	60	chr12	(-)	124426686	124426708	exon13, exor	NM_001206	(-)	102276	102298
NSD1	ATGCAGTTA	TGG	42,11	chr5	(+)	177135245	177135267	exon2	NM_022455	(+)	1414	1436
NSD1	TGTAGGACC	AGG	57,89	chr5	(-)	177135447	177135469	exon2	NM_022455	(+)	1616	1638
NSD1	ACTGCGGAG	TGG	52,63	chr5	(+)	177135316	177135338	exon2	NM_022455	(+)	1485	1507
NSD1	TGCATAGTA	TGG	36,84	chr5	(-)	177135227	177135249	exon2	NM_022455	(+)	1396	1418
ORC1	GTGCAGTTT	AGG	47,37	chr1	(+)	52402143	52402165	exon2, exon2	NM_001190	(-)	29315	29337
ORC1	GCCTGTAAA	GGG	47,37	chr1	(-)	52397776	52397798	exon4, exon4	NM_001190	(-)	24948	24970
ORC1	CGAGCACGT	AGG	52,63	chr1	(+)	52397833	52397855	exon4, exon4	NM_001190	(-)	25005	25027
ORC1	ACCGAGATT	TGG	47,37	chr1	(-)	52401436	52401458	exon3, exon3	NM_001190	(-)	28608	28630
PADI1	CAGTGAGGT	CGG	52,63	chr1	(-)	17223665	17223687	exon3	NM_013358	(+)	18540	18562
PADI1	CATCAGTGT(	CGG	57,89	chr1	(-)	17222395	17222417	exon2	NM_013358	(+)	17270	17292
PADI1	TGCAGACAT	TGG	47,37	chr1	(+)	17222416	17222438	exon2	NM_013358	(+)	17291	17313
PADI1	ATTTCCCTTG	AGG	42,11	chr1	(+)	17224369	17224391	exon4	NM_013358	(+)	19244	19266
PADI2	GTAGACATC	AGG	57,89	chr1	(+)	17119282	17119304	exon1	NM_007365	(-)	52522	52544
PADI2	AACCTTCAG(	CGG	52,63	chr1	(-)	17105018	17105040	exon2	NM_007365	(-)	38258	38280
PADI3	GATTGCGAC	GGG	52,63	chr1	(+)	17265670	17265692	exon4	NM_016233	(+)	16573	16595
PADI3	CGCGACTTT(	TGG	52,63	chr1	(+)	17259698	17259720	exon2	NM_016233	(+)	10601	10623
PADI3	GAGATGTAG	AGG	52,63	chr1	(-)	17259621	17259643	exon2	NM_016233	(+)	10524	10546
PADI3	GCTGTCGTT(	TGG	52,63	chr1	(-)	17259733	17259755	exon2	NM_016233	(+)	10636	10658
PADI4	GTGGTCGTG	CGG	57,89	chr1	(+)	17331018	17331040	exon2	NM_012387	(+)	22824	22846
PADI4	GCTTTGACT(	GGG	52,63	chr1	(-)	17333964	17333986	exon3	NM_012387	(+)	25770	25792
PADI4	AGCTCTACT(	GGG	52,63	chr1	(+)	17333984	17334006	exon3	NM_012387	(+)	25790	25812
PADI4	GGTGACCCT	TGG	52,63	chr1	(+)	17331101	17331123	exon2	NM_012387	(+)	22907	22929
PADI6	ATCTTCACTG	GGG	52,63	chr1	(-)	17373177	17373199	exon2	NM_207421	(+)	982	1004
PADI6	CTTGATCGA	CGG	52,63	chr1	(+)	17373107	17373129	exon2	NM_207421	(+)	912	934
PADI6	GATGATACT	TGG	47,37	chr1	(-)	17372268	17372290	exon1	NM_207421	(+)	73	95
PADI6	GTGAAGCAC	GGG	52,63	chr1	(-)	17373063	17373085	exon2	NM_207421	(+)	868	890
PAF1	GGTGATGA/	AGG	57,89	chr19	(+)	39390089	39390111	exon2, exon3	NM_001256	(-)	4460	4482
PAF1	TGAGATCGA	AGG	57,89	chr19	(+)	39389673	39389695	exon3, exon4	NM_001256	(-)	4044	4066
PAF1	TACTCTGTCT	TGG	47,37	chr19	(+)	39389336	39389358	exon5, exon6	NM_001256	(-)	3707	3729
PAF1	GGCTATTGC	CGG	47,37	chr19	(+)	39390124	39390146	exon2, exon3	NM_001256	(-)	4495	4517
PARP1	AAGTACGTG	TGG	52,63	chr1	(-)	226392213	226392235	exon3	NM_001618	(-)	31523	31545
PARP1	ACCCTGACG	GGG	57,89	chr1	(-)	226402281	226402303	exon2	NM_001618	(-)	41591	41613
PARP1	GGGACTTTT	GGG	42,11	chr1	(+)	226402354	226402376	exon2	NM_001618	(-)	41664	41686
PARP1	GCACGTACT	TGG	52,63	chr1	(+)	226392226	226392248	exon3	NM_001618	(-)	31536	31558
PARP2	TCTACGAGT	GGG	42,11	chr14	(-)	20344986	20345008	exon2, exon2	NM_005484,	(+)	1373	1395
PARP2	CCAGAGTGT	GGG	57,89	chr14	(+)	20345438	20345460	exon3, exon3	NM_005484,	(+)	1825	1847
PARP2	GTTAAAGGC	TGG	47,37	chr14	(+)	20345413	20345435	exon3, exon3	NM_005484,	(+)	1800	1822
PARP2	GGAGTCGA/	TGG	47,37	chr14	(+)	20345020	20345042	exon2, exon2	NM_005484,	(+)	1407	1429
PAXIP1	GAGGTCAAC	GGG	52,63	chr7	(-)	155002865	155002887	exon1	NM_007349	(-)	59176	59198
PAXIP1	GGGCTTTGG	GGG	47,37	chr7	(-)	154983282	154983304	exon5	NM_007349	(-)	39593	39615
PAXIP1	ACGGACAGA	AGG	52,63	chr7	(+)	154993747	154993769	exon3	NM_007349	(-)	50058	50080
PAXIP1	GTGATTCTG	TGG	47,37	chr7	(-)	154993738	154993760	exon3	NM_007349	(-)	50049	50071
PBRM1	TAATACCATC	AGG	36,84	chr3	(-)	52678552	52678574	exon3	NM_018313	(-)	133201	133223
PBRM1	GGCCTGGTG	TGG	57,89	chr3	(+)	52679621	52679643	exon2	NM_018313	(-)	134270	134292
PBRM1	GGATGGTAT	TGG	36,84	chr3	(+)	52678564	52678586	exon3	NM_018313	(-)	133213	133235

PBRM1	CACCAGGCC(	AGG	63,16	chr3	(-)	52679607	52679629	exon2	NM_018313	(-)	134256	134278
PCGF1	GCATGACCC(	AGG	57,89	chr2	(+)	74506771	74506793	exon3	NM_032673	(-)	1729	1751
PCGF1	TTGCGATCG(	CGG	57,89	chr2	(-)	74507621	74507643	exon1	NM_032673	(-)	2579	2601
PCGF1	GCAGGACAT	TGG	47,37	chr2	(-)	74506750	74506772	exon3	NM_032673	(-)	1708	1730
PCGF1	TGTACAAGA	CGG	52,63	chr2	(-)	74507582	74507604	exon1	NM_032673	(-)	2540	2562
PCGF2	CATCGACGC(	TGG	57,89	chr17	(-)	38740306	38740328	exon3	NM_007144	(-)	6410	6432
PCGF2	ACGTGCAGG	CGG	47,37	chr17	(-)	38739603	38739625	exon4	NM_007144	(-)	5707	5729
PCGF2	AACCTGCAT(	TGG	57,89	chr17	(-)	38739653	38739675	exon4	NM_007144	(-)	5757	5779
PCGF2	TTGTCTACA/	GGG	42,11	chr17	(-)	38739208	38739230	exon5	NM_007144	(-)	5312	5334
PCGF5	GATCAAGCC.	CGG	47,37	chr10	(+)	91222946	91222968	exon2, exon2	NM_00125€	(+)	2345	2367
PCGF5	ATACCCTTTA	AGG	31,58	chr10	(-)	91222921	91222943	exon2, exon2	NM_00125€	(+)	2320	2342
PCGF5	ATTATATTTA	TGG	31,58	chr10	(+)	91248527	91248549	exon4, exon4	NM_00125€	(+)	27926	27948
PCGF5	TGAACTTGG	TGG	52,63	chr10	(-)	91240531	91240553	exon3, exon3	NM_00125€	(+)	19930	19952
PCGF6	CACTTCTCGT	GGG	55	chr10	(-)	103350738	103350760	exon1, exon1	NM_032154,	(-)	47943	47965
PCGF6	ACATGAGTC.	AGG	45	chr10	(-)	103350746	103350768	exon1, exon1	NM_032154,	(-)	47951	47973
PCGF6	GAGTCACTT(	TGG	55	chr10	(-)	103350742	103350764	exon1, exon1	NM_032154,	(-)	47947	47969
PCMT1	GATTGTGGA	TGG	47,37	chr6	(-)	149749928	149749950	exon1, exon1	NM_005389,	(+)	234	256
PCMT1	TCGCTAAGC.	AGG	57,89	chr6	(-)	149749880	149749902	exon1, exon1	NM_005389,	(+)	186	208
PCMT1	TACATTTTGC	CGG	36,84	chr6	(-)	149771214	149771236	exon2, exon2	NM_005389,	(+)	21520	21542
PHC1	CAATGGGAG	GGG	47,37	chr12	(+)	8917713	8917735	exon2	NM_004426	(+)	2994	3016
PHC1	AAGTGACAT	GGG	36,84	chr12	(-)	8917748	8917770	exon2	NM_004426	(+)	3029	3051
PHC1	TAGCACAGA	TGG	47,37	chr12	(-)	8921671	8921693	exon5	NM_004426	(+)	6952	6974
PHC1	CACAATTGCT	AGG	57,89	chr12	(+)	8920987	8921009	exon4	NM_004426	(+)	6268	6290
PHC2	ACTTATAGG(	CGG	42,11	chr1	(+)	33331391	33331413	exon11, exor	NM_198040,	(-)	7769	7791
PHC2	ACCCTTCGAT	AGG	47,37	chr1	(+)	33334122	33334144	exon9, exon2	NM_198040,	(-)	10500	10522
PHC2	CGAAGGGTT	AGG	52,63	chr1	(-)	33334107	33334129	exon9, exon2	NM_198040,	(-)	10485	10507
PHC2	AGCTCACACT	GGG	42,11	chr1	(+)	33331420	33331442	exon11, exor	NM_198040,	(-)	7798	7820
PHC3	AGACCATCT/	AGG	47,37	chr3	(-)	170171410	170171432	exon4, exon4	NM_024947,	(-)	83831	83853
PHC3	ATGGATACT(	GGG	42,11	chr3	(-)	170178894	170178916	exon2, exon2	NM_024947,	(-)	91315	91337
PHC3	TAAGGACCA	TGG	42,11	chr3	(-)	170178913	170178935	exon2	NM_024947	(-)	91334	91356
PHC3	CAGCATCAA(	GGG	47,37	chr3	(+)	170172623	170172645	exon3, exon3	NM_024947,	(-)	85044	85066
PHF1	ACTGCGAAT(	TGG	42,11	chr6	(-)	33412537	33412559	exon3, exon3	NM_024165,	(+)	1542	1564
PHF1	GTCAAGATG	TGG	57,89	chr6	(+)	33412364	33412386	exon2, exon2	NM_024165,	(+)	1369	1391
PHF1	ACTGATGGG	GGG	47,37	chr6	(+)	33412387	33412409	exon2, exon2	NM_024165,	(+)	1392	1414
PHF1	TGTCTGTCG(	TGG	52,63	chr6	(+)	33412729	33412751	exon4, exon4	NM_024165,	(+)	1734	1756
PHF10	GCGACGAGA	AGG	52,63	chr6	(-)	169717877	169717899	exon4, exon4	NM_018288,	(-)	13972	13994
PHF10	CCCATTCGCC	TGG	57,89	chr6	(+)	169721053	169721075	exon2, exon2	NM_018288,	(-)	17148	17170
PHF10	TAATGACATT	AGG	31,58	chr6	(+)	169717844	169717866	exon4, exon4	NM_018288,	(-)	13939	13961
PHF10	TGGGTGTGA	AGG	47,37	chr6	(-)	169718798	169718820	exon3, exon3	NM_018288,	(-)	14893	14915
PHF11	TCATAAAAG.	TGG	47,37	chr13	(+)	49518032	49518054	exon4, exon4	NM_001040	(+)	20977	20999
PHF11	TTCAGGACTT	AGG	42,11	chr13	(+)	49513067	49513089	exon3, exon3	NM_001040	(+)	16012	16034
PHF11	CATTATATTC	GGG	26,32	chr13	(-)	49506684	49506706	exon2, exon2	NM_001040	(+)	9629	9651
PHF11	TCTCTGATTG	AGG	36,84	chr13	(-)	49506708	49506730	exon2, exon2	NM_001040	(+)	9653	9675
PHF12	GTGTACGAC	AGG	47,37	chr17	(-)	28950908	28950930	exon1, exon1	NM_020889,	(-)	45656	45678
PHF12	AATGGACTG	TGG	42,11	chr17	(-)	28924247	28924269	exon4, exon4	NM_020889,	(-)	18995	19017
PHF12	CGGTGACAC	AGG	57,89	chr17	(+)	28927010	28927032	exon3, exon3	NM_020889,	(-)	21758	21780
PHF12	CAAGTCGTA(	TGG	52,63	chr17	(+)	28950919	28950941	exon1, exon1	NM_020889,	(-)	45667	45689
PHF13	TTGGCTCGCT	AGG	57,89	chr1	(-)	6619944	6619966	exon3	NM_153812	(+)	6249	6271
PHF13	TACCATTGA(	GGG	57,89	chr1	(+)	6619850	6619872	exon3	NM_153812	(+)	6155	6177
PHF13	TGGCTACAT(	AGG	52,63	chr1	(+)	6616834	6616856	exon2	NM_153812	(+)	3139	3161
PHF13	TACCAGCAGT	GGG	52,63	chr1	(-)	6619829	6619851	exon3	NM_153812	(+)	6134	6156
PHF14	TCCAGCAGA(	AGG	55	chr7	(-)	10974867	10974889	exon2	NM_014660	(+)	996	1018
PHF14	GCCTTTGGC/	TGG	60	chr7	(+)	10974866	10974888	exon2	NM_014660	(+)	995	1017
PHF14	TGGATCGCA	AGG	60	chr7	(+)	10974835	10974857	exon2	NM_014660	(+)	964	986
PHF14	GAGGAGGC/	TGG	60	chr7	(+)	10974851	10974873	exon2	NM_014660	(+)	980	1002
PHF19	TTGATGTCC/	NGG	45			0	0	Not available			0	0
PHF19	GTTGTTCTTC	NGG	50			0	0	Not available			0	0
PHF19	TCCACCGGC/	NGG	60			0	0	Not available			0	0
PHF2	TGAAGCGGC	GGG	52,63	chr9	(-)	93576809	93576831	exon1	NM_005392	(+)	183	205
PHF2	AAGCGGACC	CGG	57,89	chr9	(+)	93636419	93636441	exon3	NM_005392	(+)	59793	59815
PHF2	GACGTCAAG	TGG	63,16	chr9	(+)	93636458	93636480	exon3	NM_005392	(+)	59832	59854
PHF2	TGAGCTGAC	CGG	57,89	chr9	(-)	93645647	93645669	exon4	NM_005392	(+)	69021	69043
PHF20	AAGCATCCA(	AGG	47,37	chr20	(+)	35801529	35801551	exon2	NM_016436	(+)	29529	29551
PHF20	GAAGACATT	AGG	47,37	chr20	(+)	35842589	35842611	exon3	NM_016436	(+)	70589	70611
PHF20	GGAACCATC	TGG	42,11	chr20	(+)	35842638	35842660	exon3	NM_016436	(+)	70638	70660
PHF20	TTCTCTAAAG	AGG	36,84	chr20	(-)	35842676	35842698	exon3	NM_016436	(+)	70676	70698
PHF20L1	GTGATTCCA(	GGG	52,63	chr8	(-)	132777841	132777863	exon2, exon2	NM_001277	(+)	2484	2506
PHF20L1	GGAGTCATC	TGG	42,11	chr8	(+)	132794475	132794497	exon3, exon3	NM_001277	(+)	19118	19140
PHF20L1	CTTAGTGCT(	GGG	47,37	chr8	(-)	132794525	132794547	exon3, exon3	NM_001277	(+)	19168	19190
PHF20L1	GATGACTCC/	TGG	52,63	chr8	(-)	132794461	132794483	exon3, exon3	NM_001277	(+)	19104	19126
PHF21A	ACTGGTTGCT	AGG	42,11	chr11	(-)	46079142	46079164	exon4, exon4	NM_001101	(-)	149824	149846
PHF21A	GGCTGTATTT	CGG	42,11	chr11	(+)	45979893	45979915	exon6, exon6	NM_001101	(-)	50575	50597
PHF21A	GGAGTTGCA	AGG	52,63	chr11	(-)	46084195	46084217	exon3, exon3	NM_001101	(-)	154877	154899
PHF21A	CTCACTCAAA	TGG	42,11	chr11	(+)	46076760	46076782	exon5, exon5	NM_001101	(-)	147442	147464
PHF21B	ATCGCCTTCA	AGG	57,89	chr22	(-)	44896058	44896080	exon6, exon7	NM_001242	(-)	14896	14918
PHF21B	GATCACTGC/	CGG	57,89	chr22	(-)	44920457	44920479	exon3, exon4	NM_001242	(-)	39295	39317
PHF21B	CGGTGGCCT	GGG	57,89	chr22	(+)	44916537	44916559	exon4, exon5	NM_001242	(-)	35375	35397
PHF21B	TCAAGAAGC	AGG	52,63	chr22	(-)	45008578	45008600	exon2, exon3	NM_001242	(-)	127416	127438
PHF23	GAAACGGCG	AGG	47,37	chr17	(-)	7237447	7237469	exon3, exon3	NM_024297,	(-)	2420	2442
PHF23	GACCGAAAG	GGG	47,37	chr17	(-)	7236361	7236383	exon4, exon4	NM_024297,	(-)	1334	1356
PHF23	TTTCGGTCCT	AGG	47,37	chr17	(+)	7236376	7236398	exon4, exon4	NM_024297,	(-)	1349	1371
PHF23	CAAGCAAGG	GGG	52,63	chr17	(+)	7236542	7236564	exon4, exon4	NM_024297,	(-)	1515	1537
PHF3	TGGTGCTGC	TGG	52,63	chr6	(-)	63684162	63684184	exon3, exon5	NM_015153,	(+)	47682	47704
PHF3	AGCGATAAG	AGG	47,37	chr6	(+)	63646699	63646721	exon1, exon2	NM_015153,	(+)	10219	10241
PHF3	CCAGGTTGG	AGG	47,37	chr6	(-)	63646608	63646630	exon1, exon2	NM_015153,	(+)	10128	10150
PHF5A	TGCAGCAGA	GGG	52,63	chr22	(+)	41460355	41460377				0	0
PHF5A	CATATGCGC/	GGG	57,89	chr22	(+)	41467554	41467576	exon3	NM_032758	(-)	7838	7860

PHF5A	GTAACTATG(	GGG	36,84	chr22	(-)	41467523	41467545	exon3	NM_032758	(-)	7807	7829
PHF5A	CTCCCCAGAT	TGG	47,37	chr22	(+)	41460453	41460475	exon4	NM_032758	(-)	737	759
PHF6	ATACGAGAG	AGG	42,11	chrX	(+)	134393603	134393625	exon4, exon4	NM_032458,	(+)	20292	20314
PHF6	CCTACAAGA(	TGG	47,37	chrX	(+)	134377648	134377670	exon2, exon2	NM_032458,	(+)	4337	4359
PHF6	TATGGTGCG	TGG	57,89	chrX	(-)	134377725	134377747	exon2, exon2	NM_032458,	(+)	4414	4436
PHF7	TATCTAGTA/	AGG	42,11	chr3	(+)	52419837	52419859	exon4, exon5	NM_001278;	(+)	9277	9299
PHF7	TGGCTATGC	TGG	52,63	chr3	(+)	52414501	52414523	exon3, exon4	NM_001278;	(+)	3941	3963
PHF7	AGACTAGGA	AGG	52,63	chr3	(+)	52414007	52414029	exon2, exon3	NM_001278;	(+)	3447	3469
PHF7	TGGGGATCC	GGG	42,11	chr3	(+)	52414521	52414543	exon3, exon4	NM_001278;	(+)	3961	3983
PHF8	CACGGTCAG	TGG	47,37	chrX	(+)	54017776	54017798	exon5, exon5	NM_001184(	(-)	81097	81119
PHF8	CAGGTTCGT(	GGG	52,63	chrX	(+)	54042762	54042784	exon2	NM_001184(	(-)	106083	106105
PHF8	AAAACGCCG	AGG	47,37	chrX	(-)	54022341	54022363	exon4, exon4	NM_001184(	(-)	85662	85684
PHF8	GGAGCTCTC	GGG	57,89	chrX	(+)	54022278	54022300	exon4, exon4	NM_001184(	(-)	85599	85621
PHIP	TGCAAGCAT(	AGG	47,37	chr6	(-)	79060552	79060574	exon6	NM_017934	(-)	126134	126156
PHIP	CTGCAAATA	AGG	42,11	chr6	(-)	79060760	79060782	exon5	NM_017934	(-)	126342	126364
PHIP	ATCGCCCGG	TGG	63,16	chr6	(-)	79077877	79077899	exon2	NM_017934	(-)	143459	143481
PHIP	CATATTTGC/	AGG	36,84	chr6	(+)	79060772	79060794	exon5	NM_017934	(-)	126354	126376
PHRF1	TCCGAGGAT	CGG	52,63	chr11	(+)	587261	587283	exon4, exon4	NM_001286	(+)	10816	10838
PHRF1	TCTCAACGC/	AGG	47,37	chr11	(+)	587377	587399	exon4, exon4	NM_001286	(+)	10932	10954
PHRF1	AAGTAATGG	CGG	42,11	chr11	(-)	587411	587433	exon4, exon4	NM_001286	(+)	10966	10988
PHRF1	GACGACAGT	TGG	63,16	chr11	(+)	581997	582019	exon3, exon3	NM_001286	(+)	5552	5574
POLE3	TCAATGGGG	TGG	55	chr9	(+)	113409625	113409647	[exon4, exor	[NM_017443	(-)	2387	2409
POLE3	GATCACCAG(	AGG	50	chr9	(-)	113410230	113410252	[exon2, exor	[NM_017443	(-)	2992	3014
POLE3	GATCCTGGT(	TGG	55	chr9	(+)	113410240	113410262	[exon2, exor	[NM_017443	(-)	3002	3024
POLE3	CGGTGTCAA	AGG	55	chr9	(-)	113410110	113410132	[exon3, exor	[NM_017443	(-)	2872	2894
POLR2B	CGCTCCTCCT	AGG	52,63	chr4	(+)	56990838	56990860	exon3, exon4	NM_000938,	(+)	12199	12221
POLR2B	TGCTTCTTG(	GGG	47,37	chr4	(-)	56986387	56986409	exon2, exon3	NM_000938,	(+)	7748	7770
POLR2B	CAAGCCTTT(	AGG	42,11	chr4	(-)	56990750	56990772	exon3, exon4	NM_000938,	(+)	12111	12133
POLR2B	ATCTTTCCAA	TGG	47,37	chr4	(+)	56994435	56994457	exon4, exon3	NM_000938,	(+)	15796	15818
PPARGC1A	ATCTGTCAG(	AGG	42,11	chr4	(+)	23831644	23831666	exon3	NM_013261	(-)	39624	39646
PPARGC1A	TGAACTAGA	TGG	42,11	chr4	(-)	23884847	23884869	exon2	NM_013261	(-)	92827	92849
PPARGC1A	GCAATCCGT(	GGG	52,63	chr4	(+)	23831667	23831689	exon3	NM_013261	(-)	39647	39669
PPARGC1A	GCTTTCTGG(	TGG	52,63	chr4	(-)	23884815	23884837	exon2	NM_013261	(-)	92795	92817
PRDM1	ATTGTCAGC	AGG	52,63	chr6	(-)	106099519	106099541	exon2, exon4	NM_182907,	(+)	13200	13222
PRDM1	AACCTGGCT(	CGG	57,89	chr6	(+)	106099402	106099424	exon2, exon4	NM_182907,	(+)	13083	13105
PRDM1	CATTAAAGC(	TGG	36,84	chr6	(-)	106099323	106099345	exon2, exon4	NM_182907,	(+)	13004	13026
PRDM1	AATACCACA(	TGG	36,84	chr6	(-)	106099467	106099489	exon2, exon4	NM_182907,	(+)	13148	13170
PRDM10	GGACCCATC	TGG	57,89	chr11	(+)	129947314	129947336	exon1, exon5	NM_199438,	(-)	47609	47631
PRDM10	GCTTCAGTG	CGG	57,89	chr11	(-)	129944964	129944986	exon2, exon6	NM_199438,	(-)	45259	45281
PRDM10	CTGTCTATG1	GGG	47,37	chr11	(+)	129944886	129944908	exon2, exon6	NM_199438,	(-)	45181	45203
PRDM10	TGTAGATGG	TGG	57,89	chr11	(-)	129947304	129947326	exon1, exon5	NM_199438,	(-)	47599	47621
PRDM11	GGAAGCTTT	AGG	52,63	chr11	(-)	45182309	45182331	exon3, exon3	NM_001256(	(+)	34967	34989
PRDM11	ATTCATCCAC	TGG	42,11	chr11	(-)	45182874	45182896	exon4, exon4	NM_001256(	(+)	35532	35554
PRDM11	GAGGTGGT(	TGG	52,63	chr11	(+)	45182989	45183011	exon4, exon4	NM_001256(	(+)	35647	35669
PRDM11	CCATACTCCT	TGG	57,89	chr11	(-)	45181854	45181876	exon2, exon2	NM_001256(	(+)	34512	34534
PRDM12	TCCCGCCTTG	TGG	63,16	chr9	(-)	130666695	130666717	exon2	NM_021619	(+)	2102	2124
PRDM12	GATGTCGGA	CGG	52,63	chr9	(-)	130664727	130664749	exon1	NM_021619	(+)	134	156
PRDM12	ATCAAGGCG	GGG	57,89	chr9	(+)	130666706	130666728	exon2	NM_021619	(+)	2113	2135
PRDM12	ACGTCTTGG,	AGG	52,63	chr9	(-)	130666681	130666703	exon2	NM_021619	(+)	2088	2110
PRDM13	GCTATTGCA(	AGG	52,63	chr6	(+)	99608846	99608868	exon2	NM_021620	(+)	2073	2095
PRDM13	GATGTCGAA	AGG	57,89	chr6	(-)	99609251	99609273	exon3	NM_021620	(+)	2478	2500
PRDM13	CAAGTACCT(	GGG	57,89	chr6	(+)	99607136	99607158	exon1	NM_021620	(+)	363	385
PRDM13	TGGAGTGG/	CGG	42,11	chr6	(+)	99608790	99608812	exon2	NM_021620	(+)	2017	2039
PRDM14	ACGTGGGG/	CGG	57,89	chr8	(+)	70069541	70069563	exon2	NM_024504	(-)	17891	17913
PRDM14	TACCAGGGC	AGG	52,63	chr8	(+)	70069577	70069599	exon2	NM_024504	(-)	17927	17949
PRDM14	AGGTTGGA/	CGG	52,63	chr8	(+)	70069705	70069727	exon2	NM_024504	(-)	18055	18077
PRDM15	GTTTTGACT(	CGG	50	chr21	(+)	41879053	41879075	exon2	NM_022115	(-)	78060	78082
PRDM15	TTTGTTACAT	CGG	50	chr21	(-)	41879079	41879101	exon2	NM_022115	(-)	78086	78108
PRDM15	TGGCGATCG	CGG	55	chr21	(-)	41879049	41879071	exon2	NM_022115	(-)	78056	78078
PRDM15	CGAATGTAA	CGG	35	chr21	(+)	41879089	41879111	exon2	NM_022115	(-)	78096	78118
PRDM16	ATGTAGACA	CGG	57,89	chr1	(-)	3186286	3186308	exon2, exon2	NM_022114,	(+)	117109	117131
PRDM16	AGCTCGAAG	CGG	57,89	chr1	(-)	3186322	3186344	exon2, exon2	NM_022114,	(+)	117145	117167
PRDM16	GCGGCAAA(	CGG	57,89	chr1	(+)	3186445	3186467	exon2, exon2	NM_022114,	(+)	117268	117290
PRDM16	TAATATGTA	GGG	36,84	chr1	(+)	3186144	3186166	exon2, exon2	NM_022114,	(+)	116967	116989
PRDM2	GCCGCTTCT/	TGG	57,89	chr1	(-)	13778549	13778571	exon8, exon3	NM_012231,	(+)	73695	73717
PRDM2	ATGATGAGT	GGG	42,11	chr1	(+)	13778652	13778674	exon8, exon3	NM_012231,	(+)	73798	73820
PRDM2	ATTAGAAGC	AGG	47,37	chr1	(+)	13778560	13778582	exon8, exon3	NM_012231,	(+)	73706	73728
PRDM2	GGCTTCTCTT	AGG	47,37	chr1	(-)	13778420	13778442	exon8, exon3	NM_012231,	(+)	73566	73588
PRDM4	TAATGCTAC(	GGG	36,84	chr12	(-)	107754024	107754046	exon4	NM_012406	(-)	21159	21181
PRDM4	TTAGGGTGC	AGG	47,37	chr12	(+)	107752158	107752180	exon5	NM_012406	(-)	19293	19315
PRDM4	GGTTTGGAA	AGG	52,63	chr12	(+)	107754086	107754108	exon4	NM_012406	(-)	21221	21243
PRDM4	AATAACTGC	GGG	47,37	chr12	(+)	107753942	107753964	exon4	NM_012406	(-)	21077	21099
PRDM5	TTTTGGATG(	CGG	47,37	chr4	(-)	120853487	120853509	exon3, exon3	NM_018699,	(-)	161575	161597
PRDM5	CTGGCTTCG(	AGG	52,63	chr4	(-)	120853456	120853478	exon3, exon3	NM_018699,	(-)	161544	161566
PRDM5	GAAAAGTTC	TGG	47,37	chr4	(-)	120907532	120907554	exon2, exon2	NM_018699,	(-)	215620	215642
PRDM5	GACTTCAGG	CGG	52,63	chr4	(+)	120922568	120922590	exon1, exon1	NM_018699,	(-)	230656	230678
PRDM6	GGTAGGCTC	AGG	60	chr5	(-)	123090050	123090072	exon2	NM_001136;	(+)	905	927
PRDM6	GGCTGGGT(	AGG	60	chr5	(-)	123090046	123090068	exon2	NM_001136;	(+)	901	923
PRDM6	CAGCAACTC	AGG	60	chr5	(+)	123090084	123090106	exon2	NM_001136;	(+)	939	961
PRDM7	TTCTGTAATT	AGG	47,37	chr16	(+)	90061957	90061979	exon7	NM_001098:	(-)	5392	5414
PRDM7	CCTAGGTGT(	CGG	47,37	chr16	(+)	90074923	90074945	exon3	NM_001098:	(-)	18358	18380
PRDM7	TAGCCAGGA	TGG	47,37	chr16	(+)	90061921	90061943	exon7	NM_001098:	(-)	5356	5378
PRDM7	TCTCCTCTGA	AGG	57,89	chr16	(+)	90066880	90066902	exon4	NM_001098:	(-)	10315	10337
PRDM8	AACTAGTAA(	TGG	42,11	chr4	(-)	80201432	80201454	exon9, exon3	NM_020226,	(+)	3930	3952
PRDM8	GGTCCAATC(	AGG	57,89	chr4	(+)	80201343	80201365	exon9, exon3	NM_020226,	(+)	3841	3863
PRDM8	GAACAGTAC	CGG	36,84	chr4	(+)	80200277	80200299	exon8, exon2	NM_020226,	(+)	2775	2797
PRDM8	GTCGCAGGT	TGG	57,89	chr4	(-)	80200160	80200182	exon8, exon2	NM_020226,	(+)	2658	2680

PRDM9	CTCGCTATA(	AGG	40	chr5	(+)	23509546	23509568	exon3, exon3	NM_020227,	(+)	1892	1914
PRDM9	ACTCTTAAG(	AGG	50	chr5	(-)	23517886	23517908	exon5, exon5	NM_020227,	(+)	10232	10254
PRDM9	TCCACTCTTA	AGG	50	chr5	(-)	23517889	23517911	exon5, exon5	NM_020227,	(+)	10235	10257
PRKAA1	CGGCACCTT(	AGG	63,16	chr5	(-)	40798063	40798085	exon1, exon1	NM_206907,	(-)	38685	38707
PRKAA1	ATTCGGAGC	AGG	52,63	chr5	(-)	40777505	40777527	exon2, exon2	NM_206907,	(-)	18127	18149
PRKAA1	TACATTCTG(	GGG	52,63	chr5	(-)	40798089	40798111	exon1, exon1	NM_206907,	(-)	38711	38733
PRKAA1	CCCGTCGTG	CGG	57,89	chr5	(+)	40798130	40798152	exon1, exon1	NM_206907,	(-)	38752	38774
PRKAA2	AAGATCGGA	GGG	52,63	chr1	(+)	56645421	56645443	exon1	NM_006252	(+)	105	127
PRKAA2	ACTACATCT(	CGG	42,11	chr1	(+)	56691465	56691487	exon3	NM_006252	(+)	46149	46171
PRKAA2	AATCTGCTG/	TGG	52,63	chr1	(-)	56692374	56692396	exon4	NM_006252	(+)	47058	47080
PRKAA2	GTAATGGAA	AGG	36,84	chr1	(+)	56691431	56691453	exon3	NM_006252	(+)	46115	46137
PRKCD	TCGAACGTC(	AGG	52,63	chr3	(-)	53179618	53179640	exon4, exon4	NM_006254,	(+)	18412	18434
PRKCD	GTTCGATGC	AGG	47,37	chr3	(+)	53179635	53179657	exon4, exon4	NM_006254,	(+)	18429	18451
PRKCD	ATTGTTCTTC	CGG	47,37	chr3	(-)	53179736	53179758	exon4, exon4	NM_006254,	(+)	18530	18552
PRKCD	TTGAAGGCG	CGG	57,89	chr3	(-)	53178429	53178451	exon3, exon3	NM_006254,	(+)	17223	17245
PRMT1	ACATGGAGT	AGG	47,37	chr19	(-)	49681936	49681958	exon4, exon3	NM_001536,	(+)	4785	4807
PRMT1	GAGGTGCCG	TGG	52,63	chr19	(-)	49681953	49681975	exon4, exon3	NM_001536,	(+)	4802	4824
PRMT1	CCGGCACCT(	AGG	63,16	chr19	(+)	49681966	49681988	exon4, exon3	NM_001536,	(+)	4815	4837
PRMT1	GATGTCATG	GGG	52,63	chr19	(-)	49680520	49680542	exon3, exon2	NM_001536,	(+)	3369	3391
PRMT2	ACATTCCGG(	GGG	47,37	chr21	(+)	46644394	46644416	exon5, exon4	NM_206962,	(+)	8800	8822
PRMT2	GAAGAGTAC	TGG	52,63	chr21	(+)	46644459	46644481	exon5, exon4	NM_206962,	(+)	8865	8887
PRMT2	GGTATTTAG	TGG	42,11	chr21	(-)	46648484	46648506	exon6, exon5	NM_206962,	(+)	12890	12912
PRMT2	GACAAACCA	TGG	47,37	chr21	(+)	46644343	46644365	exon5, exon4	NM_206962,	(+)	8749	8771
PRMT3	ACTGTCTGC	TGG	57,89	chr11	(+)	20395909	20395931	exon6, exon5	NM_005788,	(+)	8380	8402
PRMT3	GTACCCTTCT	TGG	52,63	chr11	(+)	20395832	20395854	exon6, exon5	NM_005788,	(+)	8303	8325
PRMT3	GTCATCTACT	CGG	36,84	chr11	(+)	20397625	20397647	exon7, exon6	NM_005788,	(+)	10096	10118
PRMT3	TATGAGAAG	CGG	42,11	chr11	(-)	20395823	20395845	exon6, exon5	NM_005788,	(+)	8294	8316
PRMT5	CTTCCATCCG	GGG	52,63	chr14	(-)	22928569	22928591	exon2, exon2	NM_00128(	(-)	8059	8081
PRMT5	GAACCTGCT/	CGG	52,63	chr14	(-)	22928535	22928557	exon2, exon2	NM_00128(	(-)	8025	8047
PRMT5	AGTTCATAG	AGG	42,11	chr14	(+)	22924922	22924944	exon4, exon7	NM_00128(	(-)	4412	4434
PRMT5	CTATTTCGG(	AGG	47,37	chr14	(+)	22929283	22929305	exon1, exon1	NM_00128(	(-)	8773	8795
PRMT6	CGAGTGCTA	CGG	57,89	chr1	(+)	107056859	107056881	exon1	NM_018137	(+)	215	237
PRMT6	AGGCAAGA(	TGG	57,89	chr1	(+)	107056961	107056983	exon1	NM_018137	(+)	317	339
PRMT6	AGTTCCGAA	CGG	52,63	chr1	(-)	107056925	107056947	exon1	NM_018137	(+)	281	303
PRMT6	AGTAGCACT	TGG	47,37	chr1	(-)	107056847	107056869	exon1	NM_018137	(+)	203	225
PRMT7	GTTTGACAC	GGG	47,37	chr16	(+)	68337502	68337524	exon7, exon5	NM_019023,	(+)	26389	26411
PRMT7	CTCGATGGC	CGG	52,63	chr16	(-)	68324810	68324832	exon5, exon5	NM_019023,	(+)	13697	13719
PRMT7	AGTGCCAAT	AGG	52,63	chr16	(-)	68324747	68324769	exon5, exon5	NM_019023,	(+)	13634	13656
PRMT7	CATTCCACCG	AGG	57,89	chr16	(+)	68329147	68329169	exon6, exon4	NM_019023,	(+)	18034	18056
PRMT8	GGATCCACG	AGG	52,63	chr12	(-)	3576930	3576952	exon7, exon7	NM_00125(	(+)	85733	85755
PRMT8	TAAAATAGG	TGG	47,37	chr12	(-)	3583141	3583163	exon8, exon8	NM_00125(	(+)	91944	91966
PRMT8	AGTCGTTGC	AGG	52,63	chr12	(-)	3583114	3583136	exon8, exon8	NM_00125(	(+)	91917	91939
PRMT8	GTCGAAGTA	AGG	36,84	chr12	(-)	3540742	3540764	exon2, exon2	NM_00125(	(+)	49545	49567
PSIP1	GGTGGCTTT	AGG	52,63	chr9	(+)	15506603	15506625	exon3, exon3	NM_033222,	(-)	35960	35982
PSIP1	AGATGAAAC	TGG	42,11	chr9	(-)	15510126	15510148	exon2, exon2	NM_033222,	(-)	39483	39505
PSIP1	ATGACTCGC(	TGG	42,11	chr9	(-)	15510166	15510188	exon2, exon2	NM_033222,	(-)	39523	39545
PSIP1	GCTGGCTTT	CGG	47,37	chr9	(+)	15486833	15486855	exon5, exon5	NM_033222,	(-)	16190	16212
PWWP2B	CTTCGAAGT/	GGG	52,63	chr10	(-)	132404845	132404867	exon2, exon2	NM_138499,	(+)	7648	7670
PWWP2B	TCCCCTGTCA	TGG	63,16	chr10	(+)	132404678	132404700	exon2, exon2	NM_138499,	(+)	7481	7503
PWWP2B	TGACAGGG(	TGG	57,89	chr10	(-)	132404665	132404687	exon2, exon2	NM_138499,	(+)	7468	7490
PWWP2B	GATGCAGAC	GGG	57,89	chr10	(+)	132404720	132404742	exon2, exon2	NM_138499,	(+)	7523	7545
PYGO1	TGACGAAGG	AGG	42,11	chr15	(+)	55547022	55547044	exon3	NM_015617	(-)	8136	8158
PYGO1	GGATAACCA	TGG	52,63	chr15	(+)	55546996	55547018	exon3	NM_015617	(-)	8110	8132
PYGO1	TGGTCAGAG	TGG	47,37	chr15	(+)	55547089	55547111	exon3	NM_015617	(-)	8203	8225
PYGO1	GATAGTGG/	AGG	52,63	chr15	(-)	55548968	55548990	exon2	NM_015617	(-)	10082	10104
PYGO2	GGTGATCCA	GGG	57,89	chr1	(+)	154959786	154959808	exon3	NM_138300	(-)	2761	2783
PYGO2	GCTCCGAAG	AGG	47,37	chr1	(+)	154959749	154959771	exon3	NM_138300	(-)	2724	2746
PYGO2	GTTGCAGCC	TGG	63,16	chr1	(-)	154959713	154959735	exon3	NM_138300	(-)	2688	2710
RAG2	TCGCTGCAC/	TGG	52,63	chr11	(-)	36593781	36593803	exon3, exon3	NM_00124?	(-)	1839	1861
RAG2	CCAGCCACTT	AGG	52,63	chr11	(-)	36593924	36593946	exon3, exon3	NM_00124?	(-)	1982	2004
RAG2	CATCAATAC/	AGG	36,84	chr11	(-)	36593882	36593904	exon3, exon3	NM_00124?	(-)	1940	1962
RAG2	GGTTATGCT	TGG	42,11	chr11	(+)	36594009	36594031	exon3, exon3	NM_00124?	(-)	2067	2089
RAI1	TAGGCGTGA	AGG	52,63	chr17	(-)	17793007	17793029	exon3	NM_030665	(+)	111535	111557
RAI1	CATCACGCCT	AGG	42,11	chr17	(+)	17793019	17793041	exon3	NM_030665	(+)	111547	111569
RAI1	CTGCGGGTT	TGG	47,37	chr17	(-)	17793088	17793110	exon3	NM_030665	(+)	111616	111638
RAI1	CCTTACCCGA	TGG	57,89	chr17	(+)	17793111	17793133	exon3	NM_030665	(+)	111639	111661
RBBP4	GCGATGATA	GGG	47,37	chr1	(-)	32668314	32668336	exon4, exon4	NM_005610,	(+)	16957	16979
RBBP4	TTCTTCCACT	AGG	57,89	chr1	(-)	32651917	32651939	exon2, exon2	NM_005610,	(+)	770	792
RBBP4	GGAAGAAC(	AGG	47,37	chr1	(+)	32651933	32651955	exon2, exon2	NM_005610,	(+)	786	808
RBBP4	CAAACTGAG	GGG	36,84	chr1	(-)	32657520	32657542	exon3, exon3	NM_005610,	(+)	6163	6185
RBBP5	GTCAATTGA	AGG	52,63	chr1	(-)	205101604	205101626	exon6, exon6	NM_005057,	(-)	15463	15485
RBBP5	TGACTCCGA	TGG	42,11	chr1	(-)	205103910	205103932	exon5, exon5	NM_005057,	(-)	17769	17791
RBBP5	GGCATCTTTT	GGG	47,37	chr1	(-)	205103889	205103911	exon5, exon5	NM_005057,	(-)	17748	17770
RBBP5	TTTCAGGCG.	AGG	52,63	chr1	(-)	205105078	205105100	exon4, exon4	NM_005057,	(-)	18937	18959
RBBP7	TACACCGTTT	TGG	36,84	chrX	(-)	16869137	16869159	exon2, exon2	NM_00119?	(-)	24486	24508
RBBP7	ACCCTTGTCA	GGG	47,37	chrX	(+)	16862956	16862978	exon3, exon3	NM_00119?	(-)	18305	18327
RBBP7	GGCCCAGTC	TGG	57,89	chrX	(-)	16869093	16869115	exon2, exon2	NM_00119?	(-)	24442	24464
RBBP7	GCCCTTCATT	GGG	57,89	chrX	(-)	16863059	16863081	exon3, exon3	NM_00119?	(-)	18408	18430
RING1	TAAGATCTA	AGG	47,37	chr6	(+)	33209999	33210021	exon4	NM_002931	(+)	1491	1513
RING1	ATTGTCACA(	CGG	52,63	chr6	(+)	33209764	33209786	exon3	NM_002931	(+)	1256	1278
RING1	GATAAGCAC	GGG	47,37	chr6	(-)	33210031	33210053	exon4	NM_002931	(+)	1523	1545
RING1	CCACGTTTTG	GGG	52,63	chr6	(-)	33208842	33208864	exon2	NM_002931	(+)	334	356
RNF17	GGTCTTCCT/	GGG	52,63	chr13	(+)	24764238	24764260	exon1, exon1	NM_00118(	(+)	76	98
RNF17	CAACGCTAC	TGG	52,63	chr13	(+)	24774837	24774859	exon3, exon3	NM_00118(	(+)	10675	10697
RNF17	ACTGCTGAT(	TGG	42,11	chr13	(+)	24778326	24778348	exon4, exon4	NM_00118(	(+)	14164	14186
RNF17	ACAATCAGG	TGG	31,58	chr13	(-)	24767341	24767363	exon2, exon2	NM_00118(	(+)	3179	3201
RNF2	ACTAGTTTTT	AGG	42,11	chr1	(-)	185093074	185093096	exon4	NM_007212	(+)	47656	47678

RNF2	AATTCACTG1	AGG	36,84	chr1	(-)	185091615	185091637	exon3	NM_007212	(+)	46197	46219
RNF2	ATCATCACAC	TGG	42,11	chr1	(+)	185091717	185091739	exon3	NM_007212	(+)	46299	46321
RNF2	GTCTGGCCT	TGG	47,37	chr1	(-)	185093099	185093121	exon4	NM_007212	(+)	47681	47703
RNF20	TCGGTTGAC	AGG	36,84	chr9	(-)	101540336	101540358	exon3	NM_019592	(+)	6488	6510
RNF20	AAACGTTAT	GGG	42,11	chr9	(+)	101540517	101540539	exon4	NM_019592	(+)	6669	6691
RNF20	GTTCTGGTT	AGG	52,63	chr9	(-)	101540570	101540592	exon4	NM_019592	(+)	6722	6744
RNF20	GTGGAAACA	AGG	36,84	chr9	(+)	101535517	101535539	exon2	NM_019592	(+)	1669	1691
RNF217	ACCAGACGA	GGG	52,63	chr6	(-)	125057950	125057972	exon5, exon3	NM_152553,	(+)	95406	95428
RNF217	GGGCCAGCG	GGG	57,89	chr6	(+)	125058051	125058073	exon5, exon3	NM_152553,	(+)	95507	95529
RNF217	GCATGAAGC	AGG	47,37	chr6	(+)	125057995	125058017	exon5, exon3	NM_152553,	(+)	95451	95473
RNF217	GCTTGGTGC	CGG	47,37	chr6	(-)	125045339	125045361	exon4, exon2	NM_152553,	(+)	82795	82817
RNF40	ATCGAGCCC	AGG	52,63	chr16	(+)	30762642	30762664	exon2, exon2	NM_00128€	(+)	1031	1053
RNF40	GCGATTGAC	TGG	52,63	chr16	(-)	30763251	30763273	exon3, exon3	NM_00128€	(+)	1640	1662
RNF40	ACGACTCAG	GGG	47,37	chr16	(-)	30762595	30762617	exon2, exon2	NM_00128€	(+)	984	1006
RNF40	CCGAGAACG	TGG	47,37	chr16	(+)	30763204	30763226	exon3, exon3	NM_00128€	(+)	1593	1615
RPA3	TGTACACAA	TGG	52,63	chr7	(+)	7637901	7637923	exon7	NM_002947	(-)	1339	1361
RPA3	GATGAATTG	CGG	42,11	chr7	(+)	7640356	7640378	exon5	NM_002947	(-)	3794	3816
RPA3	ACAAGCCTG	GGG	52,63	chr7	(-)	7640332	7640354	exon5	NM_002947	(-)	3770	3792
RPA3	GATGGCTAT	TGG	31,58	chr7	(+)	7637870	7637892	exon7	NM_002947	(-)	1308	1330
RPH3A	TGGACCGCC	AGG	47,37	chr12	(+)	112865422	112865444	exon5, exon6	NM_014954,	(+)	73679	73701
RPH3A	GTGAACCGC	TGG	52,63	chr12	(+)	112865466	112865488	exon5, exon6	NM_014954,	(+)	73723	73745
RPH3A	GTTGGATGT	CGG	52,63	chr12	(+)	112828353	112828375	exon3, exon3	NM_014954,	(+)	36610	36632
RPH3A	ACAGTCCTCA	AGG	42,11	chr12	(-)	112865515	112865537	exon5, exon6	NM_014954,	(+)	73772	73794
RPS6KA5	GCGACTGCC	NGG	55	chr14		90868121	91060648	Not available	NM_004755.2,	NM_1823	0	0
RPS6KA5	GCAGCTCGT	NGG	60	chr14		90868121	91060648	Not available	NM_004755.2,	NM_1823	0	0
RPS6KA5	CATTAGGCA	NGG	45	chr14		90868121	91060648	Not available	NM_004755.2,	NM_1823	0	0
RPS6KA5	AAGGACAGA	NGG	50	chr14		90868121	91060648	Not available	NM_004755.2,	NM_1823	0	0
RSF1	GGAGCAGA	TGG	63,16	chr11	(+)	77820631	77820653	exon1	NM_016578	(-)	154403	154425
RSF1	GTTTAACAG	GGG	47,37	chr11	(-)	77747095	77747117	exon3	NM_016578	(-)	80867	80889
RSF1	AACTCAGGC	CGG	52,63	chr11	(+)	77820590	77820612	exon1	NM_016578	(-)	154362	154384
RSF1	ATCTGTTACT	GGG	42,11	chr11	(-)	77764615	77764637	exon2	NM_016578	(-)	98387	98409
RTF1	AGACGTCTC	AGG	60	chr15	(-)	41438394	41438416	[exon2]	[NM_015138	(+)	21291	21313
RTF1	GGACCACCA	CGG	50	chr15	(+)	41417228	41417250	[exon1]	[NM_015138	(+)	125	147
RTF1	CGTGATCGA	AGG	60	chr15	(+)	41417262	41417284	[exon1]	[NM_015138	(+)	159	181
RTF1	GCGAAAGCG	AGG	60	chr15	(+)	41438341	41438363	[exon2]	[NM_015138	(+)	21238	21260
SATB1	GCAATGCCA	TGG	47,37	chr3	(+)	18416944	18416966	exon3, exon3	NM_002971,	(-)	69304	69326
SATB1	CGCCATTGA	AGG	42,11	chr3	(-)	18417013	18417035	exon3, exon3	NM_002971,	(-)	69373	69395
SATB1	GGGAGTGCC	CGG	47,37	chr3	(-)	18420787	18420809	exon2, exon2	NM_002971,	(-)	73147	73169
SATB1	ATTTGAACG	GGG	42,11	chr3	(-)	18420938	18420960	exon2, exon2	NM_002971,	(-)	73298	73320
SCMH1	ATCTTGAAC	AGG	42,11	chr1	(+)	41143081	41143103	exon6, exon6	NM_012236,	(-)	115883	115905
SCMH1	TTCACATCG	GGG	52,63	chr1	(+)	41075412	41075434	exon9, exon1	NM_012236,	(-)	48214	48236
SCMH1	GCGTAAACC	GGG	47,37	chr1	(-)	41075323	41075345	exon9, exon1	NM_012236,	(-)	48125	48147
SCMH1	CTGTCTTGG	GGG	47,37	chr1	(-)	41075354	41075376	exon9, exon1	NM_012236,	(-)	48156	48178
SCML2	GCTACGGTT	TGG	36,84	chrX	(-)	18323987	18324009	exon5	NM_006089	(-)	84675	84697
SCML2	ATACTGAAG	GGG	47,37	chrX	(+)	18324015	18324037	exon5	NM_006089	(-)	84703	84725
SCML2	AGGTTACGG	TGG	52,63	chrX	(-)	18323960	18323982	exon5	NM_006089	(-)	84648	84670
SCML2	CCCCAGACA	GGG	52,63	chrX	(-)	18323899	18323921	exon5	NM_006089	(-)	84587	84609
SCML4	CCTCTTGCAG	AGG	57,89	chr6	(+)	107720863	107720885	exon5, exon6	NM_001286	(-)	18707	18729
SCML4	TTTCTGAAG	AGG	47,37	chr6	(+)	107707870	107707892	exon6, exon7	NM_001286	(-)	5714	5736
SCML4	CATCCTGCG	GGG	57,89	chr6	(+)	107707982	107708004	exon6, exon7	NM_001286	(-)	5826	5848
SCML4	GCCTCCACCT	GGG	52,63	chr6	(-)	107720900	107720922	exon5, exon6	NM_001286	(-)	18744	18766
SETD1A	TATGTGCCG	AGG	47,37	chr16	(+)	30961356	30961378	exon4	NM_014712	(+)	4063	4085
SETD1A	TAGCGGTAC	AGG	52,63	chr16	(-)	30958834	30958856	exon2	NM_014712	(+)	1541	1563
SETD1A	CAAGGCTGA	CGG	47,37	chr16	(+)	30961316	30961338	exon4	NM_014712	(+)	4023	4045
SETD1A	TAGTTCCGC	CGG	57,89	chr16	(-)	30958768	30958790	exon2	NM_014712	(+)	1475	1497
SETD1B	CTTCGACAA	CGG	52,63	chr12	(-)	121805129	121805151	exon2	NM_015048	(+)	398	420
SETD1B	GGAAATTGT	GGG	47,37	chr12	(+)	121805138	121805160	exon2	NM_015048	(+)	407	429
SETD1B	GATTGACCC	AGG	52,63	chr12	(+)	121804842	121804864	exon1	NM_015048	(+)	111	133
SETD1B	ATAAACTGT	GGG	42,11	chr12	(+)	121804871	121804893	exon1	NM_015048	(+)	140	162
SETD2	GGAGTCGAC	AGG	52,63	chr3	(+)	47124295	47124317	exon3	NM_014159	(-)	107888	107910
SETD2	TCTAGTCGA	AGG	42,11	chr3	(-)	47124463	47124485	exon3	NM_014159	(-)	108056	108078
SETD2	ATCAAAGGA	AGG	36,84	chr3	(-)	47124493	47124515	exon3	NM_014159	(-)	108086	108108
SETD2	TCGTAGAAA	CGG	47,37	chr3	(+)	47163875	47163897	exon1	NM_014159	(-)	147468	147490
SETD3	GTATGTGCA	TGG	52,63	chr14	(-)	99463513	99463535	exon3, exon3	NM_199123,	(-)	65768	65790
SETD3	TGAAAATGG	AGG	47,37	chr14	(-)	99461254	99461276	exon4, exon4	NM_199123,	(-)	63509	63531
SETD3	TACAGCAAC	AGG	47,37	chr14	(-)	99465736	99465758	exon2, exon2	NM_199123,	(-)	67991	68013
SETD3	GAGGCCCAT	AGG	42,11	chr14	(+)	99461277	99461299	exon4, exon4	NM_199123,	(-)	63532	63554
SETD4	GCTTCGAAT	TGG	52,63	chr21	(+)	36048331	36048353	exon6, exon5	NM_00128	(-)	4648	4670
SETD4	CAGTGATTC	GGG	42,11	chr21	(-)	36048322	36048344	exon6, exon5	NM_00128	(-)	4639	4661
SETD4	GGAAGAAG	CGG	47,37	chr21	(+)	36045853	36045875	exon7, exon6	NM_00128	(-)	2170	2192
SETD4	ACAGGGCAG	GGG	57,89	chr21	(+)	36045886	36045908	exon7, exon6	NM_00128	(-)	2203	2225
SETD5	GTACCTACCT	TGG	52,63	chr3	(+)	9434421	9434443	exon5	NM_00108	(+)	36722	36744
SETD5	TAGACTTCA	AGG	52,63	chr3	(+)	9434848	9434870	exon6, exon7	NM_00108	(+)	37149	37171
SETD5	ATGAGCATT	GGG	47,37	chr3	(+)	9428939	9428961	exon3	NM_00108	(+)	31240	31262
SETD5	GACTAGCCT	GGG	52,63	chr3	(-)	9433845	9433867	exon4	NM_00108	(+)	36146	36168
SETD6	AAAGTAGGC	AGG	60	chr16	(-)	58516277	58516299	exon3, exon4	NM_00116	(+)	799	821
SETD6	CACCACGAA	CGG	55	chr16	(-)	58516017	58516039	exon2, exon3	NM_00116	(+)	539	561
SETD7	GACGATGAC	CGG	57,89	chr4	(-)	139547019	139547041	exon2, exon2	NM_030648,	(-)	14441	14463
SETD7	CACGGAGAA	GGG	52,63	chr4	(-)	139546944	139546966	exon2, exon2	NM_030648,	(-)	14366	14388
SETD7	CATCCACATA	AGG	47,37	chr4	(+)	139533341	139533363	exon3, exon3	NM_030648,	(-)	763	785
SETD7	GGATAGCGA	TGG	57,89	chr4	(-)	139556113	139556135	exon1, exon1	NM_030648,	(-)	23535	23557
SETD8	GCATTTGTT	AGG	52,63	chr12	(-)	123390664	123390686				0	0
SETD8	ACTGAGTTC	GGG	42,11	chr12	(-)	123390702	123390724				0	0
SETD8	GAAATGCTG	CGG	52,63	chr12	(+)	123395053	123395075				0	0
SETD8	AAGATCAAA	AGG	47,37	chr12	(+)	123395100	123395122				0	0
SETDB1	CCGGTATTG	TGG	57,89	chr1	(-)	150930008	150930030	exon3, exon3	NM_001243	(+)	3670	3692
SETDB1	ACTTCGGCA	AGG	42,11	chr1	(+)	150927831	150927853	exon2, exon2	NM_001243	(+)	1493	1515

SETDB1	ATACCGGGA	AGG	52,63	chr1	(+)	150930024	150930046	exon3, exon3	NM_001243	(+)	3686	3708
SETDB1	CCAAACCAA1	AGG	57,89	chr1	(-)	150927723	150927745	exon2, exon2	NM_001243	(+)	1385	1407
SETDB2	GGAGTTTTC	AGG	36,84	chr13	(-)	49467915	49467937	exon6, exon5	NM_031915,	(+)	23623	23645
SETDB2	TCCTATGCCT	AGG	52,63	chr13	(+)	49467865	49467887	exon6, exon5	NM_031915,	(+)	23573	23595
SETDB2	TGGATGGAC	TGG	47,37	chr13	(+)	49460124	49460146	exon3, exon3	NM_031915,	(+)	15832	15854
SETDB2	CATTCACTAC	TGG	36,84	chr13	(-)	49461104	49461126	exon4, exon4	NM_031915,	(+)	16812	16834
SETMAR	CAAAGACGA	GGG	57,89	chr3	(+)	4303387	4303409	exon1, exon1	NM_006515,	(+)	84	106
SETMAR	CATCATAGT1	CGG	42,11	chr3	(-)	4313011	4313033	exon2, exon2	NM_006515,	(+)	9708	9730
SETMAR	CCGGGAAAC	GGG	47,37	chr3	(-)	4312940	4312962	exon2, exon2	NM_006515,	(+)	9637	9659
SETMAR	GTGGGATCA	AGG	52,63	chr3	(-)	4312922	4312944	exon2, exon2	NM_006515,	(+)	9619	9641
SFMBT1	GGTCCACAG	GGG	57,89	chr3	(-)	52954329	52954351	exon3	NM_016329	(-)	50763	50785
SFMBT1	GTGGACACA	TGG	47,37	chr3	(-)	52943571	52943593	exon4	NM_016329	(-)	40005	40027
SFMBT1	TACCTCTTCC	CGG	47,37	chr3	(+)	52954386	52954408	exon3	NM_016329	(-)	50820	50842
SFMBT1	TTGCTCCTTC	TGG	52,63	chr3	(-)	52943460	52943482	exon4	NM_016329	(-)	39894	39916
SFMBT2	GACTACGTC	CGG	52,63	chr10	(+)	7367713	7367735	exon4, exon4	NM_001029	(-)	209090	209112
SFMBT2	GAACAACCC	GGG	57,89	chr10	(-)	7367808	7367830	exon4, exon4	NM_001029	(-)	209185	209207
SFMBT2	CCACGATCA1	GGG	52,63	chr10	(-)	7367782	7367804	exon4, exon4	NM_001029	(-)	209159	209181
SFMBT2	TTCTGCGCTA	GGG	52,63	chr10	(-)	7367749	7367771	exon4, exon4	NM_001029	(-)	209126	209148
SHPRH	GCTTCATTG	AGG	36,84	chr6	(-)	145955241	145955263	exon2, exon2	NM_173082,	(-)	70433	70455
SHPRH	GAAACGTGC	GGG	57,89	chr6	(-)	145955286	145955308	exon2, exon2	NM_173082,	(-)	70478	70500
SHPRH	TGGCTCACA	AGG	42,11	chr6	(-)	145955107	145955129	exon2, exon2	NM_173082,	(-)	70299	70321
SHPRH	AGAGGTATC	AGG	52,63	chr6	(+)	145955173	145955195	exon2, exon2	NM_173082,	(-)	70365	70387
SIN3A	GCTATGAAC	CGG	42,11	chr15	(+)	75422764	75422786	exon3, exon3	NM_001145	(-)	53386	53408
SIN3A	TGCGCTATC1	AGG	42,11	chr15	(-)	75414281	75414303	exon4, exon4	NM_001145	(-)	44903	44925
SIN3A	TTTGGATGA	CGG	47,37	chr15	(-)	75430342	75430364	exon2, exon2	NM_001145	(-)	60964	60986
SIN3B	AAGCGTTGA	AGG	36,84	chr19	(-)	16831551	16831573	exon3, exon3	NM_015260,	(+)	2165	2187
SIN3B	GATCTTCACC	AGG	52,63	chr19	(-)	16829807	16829829	exon2, exon2	NM_015260,	(+)	421	443
SIN3B	GAGACACGT	AGG	52,63	chr19	(-)	16831504	16831526	exon3, exon3	NM_015260,	(+)	2118	2140
SIN3B	GATCTCCAG	AGG	57,89	chr19	(-)	16829852	16829874	exon2, exon2	NM_015260,	(+)	466	488
SIRT1	TCGTACAAG	NGG	55			0	0	Not available			0	0
SIRT1	TACAAGTTG	NGG	60			0	0	Not available			0	0
SIRT1	GTCTTCGTC	NGG	45			0	0	Not available			0	0
SIRT2	TAGGTTGTC	TGG	52,63	chr19	(+)	38889909	38889931	exon6, exon5	NM_012237,	(-)	11355	11377
SIRT2	GCGCATGAA	TGG	52,63	chr19	(+)	38889129	38889151	exon8, exon7	NM_012237,	(-)	10575	10597
SIRT2	AGCGTTCGC	CGG	52,63	chr19	(+)	38893414	38893436	exon4, exon3	NM_012237,	(-)	14860	14882
SIRT3	CGTTGGGCT	GGG	52,63	chr11	(+)	233067	233089	exon3, exon3	NM_012239,	(-)	18038	18060
SIRT3	TCTACACGCA	GGG	47,37	chr11	(-)	232993	233015	exon3, exon3	NM_012239,	(-)	17964	17986
SIRT3	ATGAGCTTC	AGG	42,11	chr11	(+)	230515	230537	exon4, exon4	NM_012239,	(-)	15486	15508
SIRT3	GGTACGGG	TGG	57,89	chr11	(+)	233163	233185	exon3, exon3	NM_012239,	(-)	18134	18156
SIRT4	GTCTGGTAT	TGG	57,89	chr12	(-)	120303758	120303780	exon2	NM_012240	(+)	1438	1460
SIRT4	CGTGCTCGA	GGG	57,89	chr12	(+)	120303629	120303651	exon2	NM_012240	(+)	1309	1331
SIRT4	TACTGGGCG	AGG	52,63	chr12	(+)	120303874	120303896	exon2	NM_012240	(+)	1554	1576
SIRT4	GAGACTCCT	GGG	47,37	chr12	(+)	120303726	120303748	exon2	NM_012240	(+)	1406	1428
SIRT5	GTGCAGCTC	GGG	52,63	chr6	(-)	13591835	13591857	exon4, exon5	NM_001242	(+)	17209	17231
SIRT5	AACTTGGCC	AGG	47,37	chr6	(-)	13584200	13584222	exon3, exon3	NM_031244,	(+)	9574	9596
SIRT5	TCAATCGAC1	TGG	42,11	chr6	(-)	13584122	13584144	exon3, exon3	NM_031244,	(+)	9496	9518
SIRT5	AGTGGTAGA	CGG	52,63	chr6	(-)	13591707	13591729	exon5, exon5	NM_031244,	(+)	17081	17103
SIRT6	ATGTCGGTG	GGG	52,63	chr19	(-)	4182517	4182539	exon1, exon1	NM_016539,	(-)	8409	8431
SIRT6	AAAGGTGG1	GGG	47,37	chr19	(+)	4179223	4179245	exon3, exon3	NM_016539,	(-)	5115	5137
SIRT6	GGTCTGGCA	TGG	57,89	chr19	(-)	4180834	4180856	exon2, exon2	NM_016539,	(-)	6726	6748
SIRT6	CTTCCTGGT	TGG	57,89	chr19	(-)	4179135	4179157	exon3, exon3	NM_016539,	(-)	5027	5049
SIRT7	CGACCAAGT	CGG	47,37	chr17	(+)	81917641	81917663	exon3	NM_016538	(-)	5703	5725
SIRT7	CAGCGTCTA	CGG	52,63	chr17	(-)	81915658	81915680	exon4	NM_016538	(-)	3720	3742
SIRT7	AAATACTTG	AGG	36,84	chr17	(-)	81917631	81917653	exon3	NM_016538	(-)	5693	5715
SIRT7	GTGTGGACA	AGG	52,63	chr17	(-)	81915623	81915645	exon4	NM_016538	(-)	3685	3707
SMARCA1	TTCAAATCTC	CGG	42,11	chrX	(+)	129516471	129516493	exon3, exon3	NM_003069,	(-)	69971	69993
SMARCA1	GTTGGAGAT	AGG	47,37	chrX	(+)	129516406	129516428	exon3, exon3	NM_003069,	(-)	69906	69928
SMARCA1	TAAAGCGCC	AGG	36,84	chrX	(-)	129518390	129518412	exon2, exon2	NM_003069,	(-)	71890	71912
SMARCA2	CATCGAAGA	AGG	47,37	chr9	(+)	2161688	2161710	exon28, exor	NM_139045,	(+)	4009	4031
SMARCA2	GGATTCGTA	CGG	31,58	chr9	(+)	2182145	2182167	exon30, exor	NM_139045,	(+)	24466	24488
SMARCA2	GATAGCCGG	TGG	57,89	chr9	(+)	2159883	2159905	exon2, exon2	NM_001289	(+)	1432	1454
SMARCA2	GGTGACAGT	AGG	57,89	chr9	(-)	2161815	2161837	exon28, exor	NM_139045,	(+)	4136	4158
SMARCA4	CCGCGCTAC	AGG	55	chr19	(+)	10985312	10985334	exon2, exon3	NM_001128	(+)	24391	24413
SMARCA4	TTCTCATGCA	GGG	45	chr19	(-)	10985273	10985295	exon2, exon3	NM_001128	(+)	24352	24374
SMARCA4	ATCTGGTGC	AGG	50	chr19	(-)	10984344	10984366	exon1, exon2	NM_001128	(+)	23423	23445
SMARCA4	ATGGAGTCC	GGG	45	chr19	(+)	10985276	10985298	exon2, exon3	NM_001128	(+)	24355	24377
SMARCA5	ATATTTGAT	TGG	36,84	chr4	(+)	143517358	143517380	exon2	NM_003601	(+)	3896	3918
SMARCA5	TTTTTTATTC	TGG	47,37	chr4	(-)	143521540	143521562	exon3	NM_003601	(+)	8078	8100
SMARCA5	GTTGGAGTC	AGG	52,63	chr4	(-)	143521498	143521520	exon3	NM_003601	(+)	8036	8058
SMARCB1	AACTACCTCC	AGG	50	chr22	(+)	23791762	23791784	[exon2, exor	[NM_001007	(+)	4832	4854
SMARCB1	GACGGCGAC	CGG	50	chr22	(+)	23787233	23787255	[exon1, exor	[NM_001007	(+)	303	325
SMARCB1	CCACTTCCGA	AGG	50	chr22	(-)	23793593	23793615	[exon3, exor	[NM_001007	(+)	6663	6685
SMARCB1	ATGGCGCTG	CGG	55	chr22	(+)	23787179	23787201	[exon1, exor	[NM_001007	(+)	249	271
SMARCC1	CCTAGCTGT1	AGG	50	chr3	(-)	47781695	47781717	exon1	NM_003074	(-)	195808	195830
SMARCC1	GCTGTTTAT	TGG	50	chr3	(-)	47781691	47781713	exon1	NM_003074	(-)	195804	195826
SMARCC1	CCTTCCGTCG	AGG	50	chr3	(+)	47781695	47781717	exon1	NM_003074	(-)	195808	195830
SMARCC1	TCGATAAAC	CGG	50	chr3	(+)	47781702	47781724	exon1	NM_003074	(-)	195815	195837
SMARCC2	AGTTCGACA	TGG	57,89	chr12	(-)	56189372	56189394	exon1, exon1	NM_001130	(-)	27521	27543
SMARCC2	ACTGTACAA	AGG	47,37	chr12	(+)	56187252	56187274	exon2, exon2	NM_001130	(-)	25401	25423
SMARCC2	ATCACGCAT	TGG	52,63	chr12	(-)	56185065	56185087	exon4, exon4	NM_001130	(-)	23214	23236
SMARCC2	GGCCTCGTA	TGG	57,89	chr12	(+)	56189411	56189433	exon1, exon1	NM_001130	(-)	27560	27582
SMARCD1	GGACTGATC	GGG	57,89	chr12	(-)	50086258	50086280	exon2, exon2	NM_003076,	(+)	1059	1081
SMARCD1	AGGCAGCCG	AGG	57,89	chr12	(+)	50086178	50086200	exon2, exon2	NM_003076,	(+)	979	1001
SMARCD1	TGGACCTGC	AGG	57,89	chr12	(-)	50086296	50086318	exon2, exon2	NM_003076,	(+)	1097	1119
SMARCD1	GATTTTGGA	TGG	57,89	chr12	(-)	50086316	50086338	exon2, exon2	NM_003076,	(+)	1117	1139
SMARCD2	TCGGAATGG	TGG	50	chr17	(+)	63837494	63837516	[exon2]	[NM_001098	(-)	5414	5436
SMARCD2	CGGAATGGA	GGG	50	chr17	(+)	63837495	63837517	[exon2]	[NM_001098	(-)	5415	5437

SMARCD2	TTTTCGGAA	TGG	40	chr17	(+)	63837491	63837513	[exon2]	[NM_001098	(-)	5411	5433
SMARCD2	ACAAGCAGG	TGG	50	chr17	(+)	63837480	63837502	[exon2]	[NM_001098	(-)	5400	5422
SMARCD3	ATCAAACCA	CGG	42,11	chr7	(-)	151242757	151242779	exon5, exon5	NM_003078,	(-)	3785	3807
SMARCD3	TGGACCTCT	AGG	47,37	chr7	(-)	151242787	151242809	exon5, exon5	NM_003078,	(-)	3815	3837
SMARCD3	GTCCATGTA	CGG	57,89	chr7	(+)	151242805	151242827	exon5, exon5	NM_003078,	(-)	3833	3855
SMARCD3	CTGACAAAA	AGG	47,37	chr7	(-)	151243659	151243681	exon4, exon4	NM_003078,	(-)	4687	4709
SMARCE1	TTTTGGAAT	AGG	36,84	chr17	(+)	40637546	40637568	exon5	NM_003079	(-)	9819	9841
SMARCE1	GCGGCTTAT	GGG	57,89	chr17	(+)	40637521	40637543	exon5	NM_003079	(-)	9794	9816
SMARCE1	TTGTAGGCG	TGG	47,37	chr17	(+)	40642501	40642523	exon4	NM_003079	(-)	14774	14796
SMARCE1	ATCTCGCCTA	AGG	42,11	chr17	(-)	40642491	40642513	exon4	NM_003079	(-)	14764	14786
SMNDC1	GTGTTATGA	AGG	42,11	chr10	(-)	110297706	110297728	exon4	NM_005871	(-)	4667	4689
SMNDC1	ACCGCTGCA	TGG	52,63	chr10	(-)	110297663	110297685	exon4	NM_005871	(-)	4624	4646
SMNDC1	GAAGCTGCA	TGG	42,11	chr10	(-)	110303508	110303530	exon2	NM_005871	(-)	10469	10491
SMNDC1	TTCCTTCTTC	AGG	42,11	chr10	(+)	110297607	110297629	exon4	NM_005871	(-)	4568	4590
SMYD1	GTTCGGCCA	GGG	47,37	chr2	(+)	88084447	88084469	exon2	NM_198274	(+)	16668	16690
SMYD1	TGGCCGAAC	TGG	42,11	chr2	(-)	88084433	88084455	exon2	NM_198274	(+)	16654	16676
SMYD1	TGAGCGGG	TGG	57,89	chr2	(+)	88067969	88067991	exon1	NM_198274	(+)	190	212
SMYD2	GTTAGTCTT	GGG	47,37	chr1	(-)	214314828	214314850	exon3	NM_020197	(+)	33607	33629
SMYD2	GACCGTGAG	AGG	63,16	chr1	(-)	214281367	214281389	exon1	NM_020197	(+)	146	168
SMYD2	TGGTGAAG	TGG	52,63	chr1	(-)	214281404	214281426	exon1	NM_020197	(+)	183	205
SMYD3	CACTACAGT	CGG	47,37	chr1	(+)	246355042	246355064	exon2, exon2	NM_022743,	(-)	605703	605725
SMYD3	AACTCTGCC	CGG	47,37	chr1	(+)	246335379	246335401	exon3, exon3	NM_022743,	(-)	586040	586062
SMYD3	AAGCTTGGC	CGG	52,63	chr1	(-)	246335448	246335470	exon3, exon3	NM_022743,	(-)	586109	586131
SMYD3	ATCTGGGTT	AGG	42,11	chr1	(+)	246335414	246335436	exon3, exon3	NM_022743,	(-)	586075	586097
SMYD4	TTGTGACCT	GGG	47,37	chr17	(+)	1827916	1827938	exon2	NM_052928	(-)	48382	48404
SMYD4	ACTGTGTCA	CGG	52,63	chr17	(-)	1804655	1804677	exon4	NM_052928	(-)	25121	25143
SMYD4	TTACTTGGT	CGG	47,37	chr17	(-)	1812054	1812076	exon3	NM_052928	(-)	32520	32542
SMYD4	ACATTCAAG	AGG	42,11	chr17	(-)	1804685	1804707	exon4	NM_052928	(-)	25151	25173
SMYD5	GCTCACGAA	CGG	57,89	chr2	(-)	73214331	73214353	exon1	NM_006062	(+)	94	116
SMYD5	CATCTTCGTA	TGG	57,89	chr2	(+)	73218905	73218927	exon2	NM_006062	(+)	4668	4690
SMYD5	GACGTGTTC	GGG	57,89	chr2	(+)	73214285	73214307	exon1	NM_006062	(+)	48	70
SMYD5	TGTACTGCA	CGG	47,37	chr2	(+)	73220665	73220687	exon4	NM_006062	(+)	6428	6450
SND1	AGGTTGATC	AGG	57,89	chr7	(-)	127686664	127686686	exon2	NM_014390	(+)	34517	34539
SND1	CGCCACACA	AGG	57,89	chr7	(+)	127686726	127686748	exon2	NM_014390	(+)	34579	34601
SND1	TTTCGAAGG	TGG	47,37	chr7	(-)	127694840	127694862	exon3	NM_014390	(+)	42693	42715
SND1	GATAGAAAA	AGG	42,11	chr7	(+)	127694893	127694915	exon3	NM_014390	(+)	42746	42768
SP100	TTGTGATGA	CGG	42,11	chr2	(-)	230443063	230443085	exon4, exon3	NM_001206	(+)	26908	26930
SP100	AAGACCAGG	AGG	47,37	chr2	(+)	230442948	230442970	exon4, exon3	NM_001206	(+)	26793	26815
SP100	TGCAAATCG	AGG	52,63	chr2	(-)	230417640	230417662	exon2, exon2	NM_003113,	(+)	1485	1507
SP100	TCTTCTTCAC	AGG	36,84	chr2	(-)	230446836	230446858	exon6, exon5	NM_001206	(+)	30681	30703
SP110	GGTTAAAAG	TGG	47,37	chr2	(+)	230215034	230215056	exon4, exon3	NM_001185	(-)	46109	46131
SP110	CCAGATTGG	AGG	47,37	chr2	(+)	230214977	230214999	exon4, exon3	NM_001185	(-)	46052	46074
SP110	TTGTGCACC	AGG	47,37	chr2	(+)	230215066	230215088	exon4, exon3	NM_001185	(-)	46141	46163
SP110	GTCTAGGAG	AGG	52,63	chr2	(+)	230216811	230216833	exon3, exon2	NM_001185	(-)	47886	47908
SP140	CGTAGAGG	AGG	57,89	chr2	(+)	230237104	230237126	exon2, exon2	NM_007237,	(+)	11375	11397
SP140	TTAAATCAG	AGG	36,84	chr2	(-)	230238332	230238354	exon3, exon3	NM_007237,	(+)	12603	12625
SP140L	TTGTGATGA	CGG	47,37	chr2	(-)	230357931	230357953	exon3, exon2	NM_138402,	(+)	30752	30774
SP140L	TTTGGAAGC	AGG	47,37	chr2	(+)	230359056	230359078	exon4, exon3	NM_138402,	(+)	31877	31899
SP140L	GGAAGACCA	AGG	47,37	chr2	(+)	230357814	230357836	exon3, exon2	NM_138402,	(+)	30635	30657
SRCAP	ATGGTGTCG	AGG	57,89	chr16	(+)	30704064	30704086	exon4	NM_006662	(+)	4924	4946
SRCAP	CCATGCCGA	AGG	47,37	chr16	(+)	30704285	30704307	exon4	NM_006662	(+)	5145	5167
SRCAP	GATAAGCTG	GGG	52,63	chr16	(-)	30704217	30704239	exon4	NM_006662	(+)	5077	5099
SSRP1	GTTCAACGA	AGG	47,37	chr11	(-)	57335082	57335104	exon2	NM_003146	(-)	9098	9120
SSRP1	GGCCATGTC	TGG	47,37	chr11	(-)	57334476	57334498	exon3	NM_003146	(-)	8492	8514
SSRP1	TCGACTGAG	AGG	57,89	chr11	(-)	57334618	57334640	exon3	NM_003146	(-)	8634	8656
SSRP1	TCGCCTTGA	AGG	52,63	chr11	(-)	57333480	57333502	exon4	NM_003146	(-)	7496	7518
STK31	ACTGTCTGA	AGG	52,63	chr7	(+)	23717522	23717544	exon4, exon4	NM_032944,	(+)	7356	7378
STK31	CTAGAAAAC	AGG	47,37	chr7	(-)	23729157	23729179	exon6, exon6	NM_032944,	(+)	18991	19013
STK31	GCCAAAAAG	GGG	26,32	chr7	(+)	23729184	23729206	exon6, exon6	NM_032944,	(+)	19018	19040
STK31	CTGGTGAGC	TGG	47,37	chr7	(+)	23729094	23729116	exon6, exon6	NM_032944,	(+)	18928	18950
SUPT16H	CGTATTAGC	AGG	47,37	chr14	(+)	21371881	21371903	exon3	NM_007192	(-)	20410	20432
SUPT16H	GATGCCATT	GGG	42,11	chr14	(-)	21373381	21373403	exon2	NM_007192	(-)	21910	21932
SUPT16H	AGAGACTGT	CGG	42,11	chr14	(-)	21383862	21383884	exon1	NM_007192	(-)	32391	32413
SUPT16H	AAACAGATT	GGG	36,84	chr14	(-)	21371926	21371948	exon3	NM_007192	(-)	20455	20477
SUV39H1	GGTTCCTCTT	AGG	47,37	chrX	(-)	48698978	48699000	exon2, exon2	NM_001282	(+)	2246	2268
SUV39H1	GATATCCAC	AGG	47,37	chrX	(-)	48700105	48700127	exon3, exon3	NM_001282	(+)	3373	3395
SUV39H1	ACTCGACTT	AGG	36,84	chrX	(-)	48698999	48699021	exon2, exon2	NM_001282	(+)	2267	2289
SUV39H2	AACGAATAC	TGG	42,11	chr10	(+)	14897185	14897207	exon2, exon3	NM_024670,	(+)	18403	18425
SUV39H2	GGGCACTTC	AGG	47,37	chr10	(-)	14896906	14896928	exon3, exon3	NM_001193	(+)	18124	18146
SUV39H2	AATCACGTA	TGG	31,58	chr10	(+)	14899592	14899614	exon4, exon4	NM_001193	(+)	20810	20832
SUV39H2	GTATCAAGT	AGG	42,11	chr10	(-)	14881511	14881533	exon2, exon2	NM_001193	(+)	2729	2751
SUV420H1	TTCCTTGGC	AGG	52,63	chr11	(+)	68185861	68185883				0	0
SUV420H1	AATCAAAAT	GGG	31,58	chr11	(-)	68189969	68189991				0	0
SUV420H1	TGATGACCT	TGG	47,37	chr11	(-)	68185827	68185849				0	0
SUV420H1	AATGGTAAC	AGG	42,11	chr11	(-)	68185904	68185926				0	0
SUV420H2	ACTGTGCGA	TGG	57,89	chr19	(+)	55341966	55341988				0	0
SUV420H2	TTCTCGTGCT	CGG	52,63	chr19	(-)	55341944	55341966				0	0
SUV420H2	CGCTACTCCA	CGG	57,89	chr19	(+)	55342802	55342824				0	0
SUV420H2	AAGTTTCCA	AGG	57,89	chr19	(-)	55342245	55342267				0	0
SUZ12	ACCGGTGAA	TGG	50	chr17	(+)	31937453	31937475	exon1	NM_015355	(+)	429	451
SUZ12	GCCTGGACG	CGG	55	chr17	(-)	31937466	31937488	exon1	NM_015355	(+)	442	464
SUZ12	GCCGAAAAT	AGG	60	chr17	(+)	31937465	31937487	exon1	NM_015355	(+)	441	463
SUZ12	TCCATTTTCG	CGG	45	chr17	(-)	31937454	31937476	exon1	NM_015355	(+)	430	452
TAF1	CGAAGATAC	GGG	52,63	chrX	(+)	71368122	71368144	exon3, exon3	NM_001286	(+)	1884	1906
TAF1	AGCGACGAA	AGG	57,89	chrX	(+)	71366390	71366412	exon1, exon1	NM_001286	(+)	152	174
TAF1	GGCGGCCCA	GGG	52,63	chrX	(+)	71366411	71366433	exon1, exon1	NM_001286	(+)	173	195
TAF1	TCGGCAACA	GGG	52,63	chrX	(+)	71366442	71366464	exon1, exon1	NM_001286	(+)	204	226

TAF1L	TTTCGGCAA(	CGG	47,37	chr9	(-)	32635435	32635457	exon1	NM_153809	(-)	5982	6004
TAF1L	GGATACCCG	GGG	42,11	chr9	(+)	32635459	32635481	exon1	NM_153809	(-)	6006	6028
TAF1L	CATGTCAGA(	AGG	57,89	chr9	(-)	32635501	32635523	exon1	NM_153809	(-)	6048	6070
TAF1L	CCACCCTTCA	AGG	47,37	chr9	(+)	32635283	32635305	exon1	NM_153809	(-)	5830	5852
TAF3	TGACGGAAT	AGG	52,63	chr10	(-)	7824423	7824445	exon2	NM_031923	(+)	5920	5942
TAF3	CATCCAAAAT	CGG	42,11	chr10	(-)	7824322	7824344	exon2	NM_031923	(+)	5819	5841
TAF3	GTAGACTAA	TGG	52,63	chr10	(-)	7824361	7824383	exon2	NM_031923	(+)	5858	5880
TAF3	GTCGTTGTT(	TGG	57,89	chr10	(+)	7818730	7818752	exon1	NM_031923	(+)	227	249
TCEA1	TCGCAAGCA(	AGG	47,37	chr8	(-)	53999996	54000018	exon3, exon2	NM_006756,	(-)	33443	33465
TCEA1	AACTGACAT	TGG	36,84	chr8	(+)	54000027	54000049	exon3, exon2	NM_006756,	(-)	33474	33496
TCEA1	CTGTTCTGC(	AGG	42,11	chr8	(+)	53993689	53993711	exon4, exon3	NM_006756,	(-)	27136	27158
TCEA1	GGTCCGCTT	TGG	57,89	chr8	(-)	54022089	54022111	exon1, exon1	NM_006756,	(-)	55536	55558
TCF19	ACAAGTACG	AGG	47,37	chr6	(+)	31161574	31161596	exon3, exon3	NM_007109,	(+)	3051	3073
TCF19	TGCTTCCAA(	AGG	57,89	chr6	(+)	31159479	31159501	exon2, exon2	NM_007109,	(+)	956	978
TCF19	AATAATGTC(	AGG	42,11	chr6	(+)	31161458	31161480	exon3, exon3	NM_007109,	(+)	2935	2957
TCF19	GAACATGAA	AGG	42,11	chr6	(-)	31161549	31161571	exon3, exon3	NM_007109,	(+)	3026	3048
TCF20	GTAACTGCT	AGG	52,63	chr22	(+)	42215276	42215298	exon1, exon2	NM_181492,	(-)	55264	55286
TCF20	AGAAGAGTT	AGG	52,63	chr22	(-)	42215203	42215225	exon1, exon2	NM_181492,	(-)	55191	55213
TCF20	GGCAATCAG	GGG	52,63	chr22	(-)	42214905	42214927	exon1, exon2	NM_181492,	(-)	54893	54915
TCF20	TGAAACCCT(	TGG	47,37	chr22	(+)	42215059	42215081	exon1, exon2	NM_181492,	(-)	55047	55069
TDG	CCTGTTGAG	TGG	31,58	chr12	(+)	103979899	103979921	exon3	NM_003211	(+)	14085	14107
TDG	GATTGAAGC	GGG	31,58	chr12	(-)	103980036	103980058	exon3	NM_003211	(+)	14222	14244
TDG	GATGGCTGA	TGG	47,37	chr12	(+)	103976969	103976991	exon2	NM_003211	(+)	11155	11177
TDG	TTGGGTTCC/	TGG	47,37	chr12	(-)	103979872	103979894	exon3	NM_003211	(+)	14058	14080
TDRD1	TCAAACCCG/	CGG	47,37	chr10	(+)	114188090	114188112	exon2	NM_198795	(+)	8821	8843
TDRD1	GAATTACTTT	TGG	36,84	chr10	(-)	114190975	114190997	exon3	NM_198795	(+)	11706	11728
TDRD1	GGACTTCTT/	TGG	36,84	chr10	(-)	114187940	114187962	exon2	NM_198795	(+)	8671	8693
TDRD1	CAAACAATA	TGG	31,58	chr10	(+)	114188050	114188072	exon2	NM_198795	(+)	8781	8803
TDRD10	GATCATATC/	TGG	52,63	chr1	(-)	154521436	154521458	exon6, exon6	NM_182499,	(+)	19218	19240
TDRD10	GAGGGTTG/	AGG	47,37	chr1	(-)	154520318	154520340	exon5, exon5	NM_182499,	(+)	18100	18122
TDRD10	ACAGTTTAT(	GGG	36,84	chr1	(-)	154507259	154507281	exon3, exon3	NM_182499,	(+)	5041	5063
TDRD10	GTATGTTGG	TGG	42,11	chr1	(+)	154508448	154508470	exon4, exon4	NM_182499,	(+)	6230	6252
TDRD12	TAAACGATT(	CGG	31,58	chr19	(-)	32742784	32742806	exon4	NM_00111(	(+)	23012	23034
TDRD12	GCTGTTGTA(	TGG	42,11	chr19	(-)	32731813	32731835	exon2	NM_00111(	(+)	12041	12063
TDRD12	TGTTCTTGG(	AGG	47,37	chr19	(-)	32738951	32738973	exon3	NM_00111(	(+)	19179	19201
TDRD12	AAACCCTTA/	AGG	36,84	chr19	(+)	32731857	32731879	exon2	NM_00111(	(+)	12085	12107
TDRD3	CTGCGATTA(	TGG	47,37	chr13	(+)	60460470	60460492	exon4, exon4	NM_030794,	(+)	64014	64036
TDRD3	CACTATTGAT	AGG	47,37	chr13	(-)	60444712	60444734	exon3	NM_001146(	(+)	47420	47442
TDRD3	TCGCAATGT	AGG	52,63	chr13	(+)	60460415	60460437	exon4	NM_001146(	(+)	63123	63145
TDRD3	GACTCTAAC/	TGG	47,37	chr13	(+)	60467314	60467336	exon5, exon5	NM_030794,	(+)	70858	70880
TDRD5	CTACATCCG(	GGG	47,37	chr1	(+)	179635825	179635847	exon9, exon9	NM_001199(	(+)	44213	44235
TDRD5	CGGGACATC	AGG	52,63	chr1	(+)	179639901	179639923	exon10, exon	NM_001199(	(+)	48289	48311
TDRD5	GGGAACTG(	GGG	52,63	chr1	(-)	179635759	179635781	exon9, exon9	NM_001199(	(+)	44147	44169
TDRD5	GCGCTGTTA	TGG	42,11	chr1	(+)	179639839	179639861	exon10, exon	NM_001199(	(+)	48227	48249
TDRD6	ATCGGGATG	AGG	63,16	chr6	(-)	46688184	46688206	exon1, exon1	NM_00116{	(+)	310	332
TDRD6	AATTTGCCCT	GGG	52,63	chr6	(+)	46688489	46688511	exon1, exon1	NM_00116{	(+)	615	637
TDRD6	ACAAAAGCC(	AGG	57,89	chr6	(-)	46688347	46688369	exon1, exon1	NM_00116{	(+)	473	495
TDRD6	ATGTGATCC(	TGG	57,89	chr6	(+)	46688205	46688227	exon1, exon1	NM_00116{	(+)	331	353
TDRD7	GTAGCATTA(	AGG	63,16	chr9	(+)	97428529	97428551	exon2	NM_014290	(+)	16510	16532
TDRD7	TGGATCCCCT	AGG	52,63	chr9	(+)	97428577	97428599	exon2	NM_014290	(+)	16558	16580
TDRD7	GGAGAGTA(	TGG	47,37	chr9	(+)	97428550	97428572	exon2	NM_014290	(+)	16531	16553
TDRD7	CTCGTCAAA(	AGG	42,11	chr9	(+)	97430988	97431010	exon3, exon2	NM_014290,	(+)	18969	18991
TDRD9	CCAGTCGTT(	TGG	57,89	chr14	(-)	103928526	103928548	exon1	NM_153046	(+)	47	69
TDRD9	ATCAACGAC	CGG	47,37	chr14	(+)	103928537	103928559	exon1	NM_153046	(+)	58	80
TDRD9	ACTCTACTTC	TGG	47,37	chr14	(-)	103955673	103955695	exon2	NM_153046	(+)	27194	27216
TDRD9	CGGCAAGAC	TGG	57,89	chr14	(+)	103928557	103928579	exon1	NM_153046	(+)	78	100
TDRKH	TCGGATTGA	AGG	47,37	chr1	(-)	151780098	151780120	exon4, exon4	NM_00108{	(-)	8534	8556
TDRKH	TATCCTATAC	GGG	42,11	chr1	(-)	151782908	151782930	exon2, exon2	NM_00108{	(-)	11344	11366
TDRKH	GATATAGGC	TGG	42,11	chr1	(+)	151782927	151782949	exon2, exon2	NM_00108{	(-)	11363	11385
TDRKH	GTTTAATATT	CGG	36,84	chr1	(+)	151781483	151781505	exon3, exon3	NM_00108{	(-)	9919	9941
TET1	GGTCGTAGC	AGG	47,37	chr10	(+)	68572698	68572720	exon2	NM_030625	(+)	12339	12361
TET1	TACGAAGCA	AGG	47,37	chr10	(+)	68572655	68572677	exon2	NM_030625	(+)	12296	12318
TET1	CGCCATTGT/	AGG	47,37	chr10	(-)	68572631	68572653	exon2	NM_030625	(+)	12272	12294
TET1	CAACTACGA/	GGG	42,11	chr10	(+)	68572414	68572436	exon2	NM_030625	(+)	12055	12077
TET2	CAGGACTCA(	TGG	47,37	chr4	(-)	105234149	105234171	exon3, exon3	NM_00112(	(+)	88275	88297
TET2	TGGAGAAA(	GGG	42,11	chr4	(+)	105234299	105234321	exon3, exon3	NM_00112(	(+)	88425	88447
TET2	GACTTTACA(	AGG	31,58	chr4	(+)	105234171	105234193	exon3, exon3	NM_00112(	(+)	88297	88319
TET3	CGAAAAGG(	GGG	57,89	chr2	(-)	74046507	74046529	exon3	NM_00128(	(+)	60104	60126
TET3	CGTGAGTCC(	TGG	57,89	chr2	(-)	74046335	74046357	exon3	NM_00128(	(+)	59932	59954
TET3	TTTCATACCA	GGG	57,89	chr2	(-)	74046699	74046721	exon3	NM_00128(	(+)	60296	60318
TET3	ACGACTCAT(	AGG	52,63	chr2	(-)	74046585	74046607	exon3	NM_00128(	(+)	60182	60204
TP53BP1	AACGAGGA(	GGG	47,37	chr15	(-)	43492036	43492058	exon3, exon3	NM_00114(	(-)	84823	84845
TP53BP1	CAGGTTCTA(	TGG	42,11	chr15	(-)	43492351	43492373	exon2, exon2	NM_00114(	(-)	85138	85160
TP53BP1	GAATCCAAC	AGG	47,37	chr15	(+)	43492432	43492454	exon2, exon2	NM_00114(	(-)	85219	85241
TP53BP1	TGTGCGTCT(	AGG	52,63	chr15	(+)	43492301	43492323	exon2, exon2	NM_00114(	(-)	85088	85110
TRIM24	AGCGCTGGC	AGG	60	chr7	(-)	138460767	138460789	exon1, exon1	NM_003852,	(+)	434	456
TRIM24	ACACGGCGC	AGG	60	chr7	(-)	138460701	138460723	exon1, exon1	NM_003852,	(+)	368	390
TRIM24	AGTGTCCAA(	GGG	55	chr7	(-)	138460691	138460713	exon1, exon1	NM_003852,	(+)	358	380
TRIM24	AAGTGTCCA,	CGG	50	chr7	(-)	138460692	138460714	exon1, exon1	NM_003852,	(+)	359	381
TRIM28	GAAGCACTG	CGG	52,63	chr19	(-)	58545437	58545459	exon2	NM_005762	(+)	969	991
TRIM28	TCGGCCTGT/	AGG	63,16	chr19	(+)	58545013	58545035	exon1	NM_005762	(+)	545	567
TRIM28	CTACAGGCC(	GGG	57,89	chr19	(-)	58545001	58545023	exon1	NM_005762	(+)	533	555
TRIM28	GTGCTTCTC(	TGG	47,37	chr19	(+)	58545453	58545475	exon2	NM_005762	(+)	985	1007
TRIM33	CTGCAAGCT(	CGG	55	chr1	(+)	114510678	114510700	exon1, exon1	NM_015906,	(-)	117902	117924
TRIM33	TTCATCTCCT	TGG	60	chr1	(-)	114510821	114510843	exon1, exon1	NM_015906,	(-)	118045	118067
TRIM33	ATGGCGGA/	CGG	55	chr1	(-)	114511054	114511076	exon1, exon1	NM_015906,	(-)	118278	118300
TRIM66	AGCGATTGC	AGG	52,63	chr11	(+)	8649827	8649849	exon3	NM_014818	(-)	37791	37813

TRIM66	ACAATACAA(	CGG	36,84	chr11	(+)	8648511	8648533	exon4	NM_014818	(-)	36475	36497
TRIM66	GTATTGTCCT	AGG	42,11	chr11	(-)	8648495	8648517	exon4	NM_014818	(-)	36459	36481
TRIM66	GGAAGGTG	TGG	52,63	chr11	(-)	8648019	8648041	exon5	NM_014818	(-)	35983	36005
UBE2A	TCCGTCCGA(	TGG	47,37	chrX	(+)	119574937	119574959	exon2, exon2	NM_181762,	(+)	471	493
UBE2A	GTGTGGAA(	CGG	52,63	chrX	(+)	119574959	119574981	exon2, exon2	NM_00128:	(+)	493	515
UBE2A	CGGGTACAG	TGG	42,11	chrX	(+)	119583259	119583281				0	0
UBE2A	GAGACAAAT	TGG	36,84	chrX	(-)	119581554	119581576	exon4, exon4	NM_00128:	(+)	7088	7110
UBE2B	TTGGACTCC/	AGG	47,37	chr5	(-)	134388356	134388378	exon5	NM_003337	(+)	17181	17203
UBE2B	TTACAAGAG	GGG	52,63	chr5	(+)	134374384	134374406	exon2	NM_003337	(+)	3209	3231
UBE2B	TTGGCTGGA	CGG	47,37	chr5	(-)	134390240	134390262	exon6	NM_003337	(+)	19065	19087
UBE2B	TCTTCAAAA(	TGG	42,11	chr5	(-)	134376670	134376692	exon3	NM_003337	(+)	5495	5517
UBE2E1	GAATACACC/	CGG	47,37	chr3	(-)	23887629	23887651	exon3, exon4	NM_182666,	(+)	77187	77209
UBE2E1	GAAGTGAG(	AGG	42,11	chr3	(-)	23889219	23889241	exon4, exon5	NM_182666,	(+)	78777	78799
UBE2E1	AATGGAGAT	GGG	36,84	chr3	(+)	23887596	23887618	exon3, exon4	NM_182666,	(+)	77154	77176
UBE2E1	TTCATAGAT(	TGG	36,84	chr3	(-)	23887575	23887597	exon3, exon4	NM_182666,	(+)	77133	77155
UBE2I	ACCCGAATG	GGG	52,63	chr16	(+)	1320198	1320220	exon5, exon5	NM_003345,	(+)	10572	10594
UBE2I	GCACGATGA	TGG	47,37	chr16	(+)	1314327	1314349	exon3, exon3	NM_003345,	(+)	4701	4723
UBE2I	GTGCCTGTC(	AGG	52,63	chr16	(+)	1320226	1320248	exon5, exon5	NM_003345,	(+)	10600	10622
UBE2I	TGCGCCATT(	AGG	52,63	chr16	(+)	1314353	1314375	exon3, exon3	NM_003345,	(+)	4727	4749
UBR7	ATCGTTGGT(	AGG	52,63	chr14	(+)	93207345	93207367	exon1	NM_175748	(+)	290	312
UBR7	GTGAAGACT	AGG	52,63	chr14	(+)	93214960	93214982	exon5	NM_175748	(+)	7905	7927
UBR7	GTGGGCTTA	TGG	52,63	chr14	(+)	93215256	93215278	exon6	NM_175748	(+)	8201	8223
UBR7	ACGCACTGG	TGG	47,37	chr14	(-)	93214932	93214954	exon5	NM_175748	(+)	7877	7899
UHRF1	CCATACCCTC	AGG	52,63	chr19	(+)	4929233	4929255	exon3, exon2	NM_001290(	(+)	18867	18889
UHRF1	ATCCAGGTT(	CGG	57,89	chr19	(+)	4910892	4910914	exon2, exon1	NM_001290(	(+)	526	548
UHRF1	TCAGACAAG	CGG	57,89	chr19	(+)	4929378	4929400	exon3, exon2	NM_001290(	(+)	19012	19034
UHRF1	ATGTGGGAT	GGG	47,37	chr19	(+)	4929444	4929466	exon3, exon2	NM_001290(	(+)	19078	19100
UHRF2	CGTGCCCGT(	TGG	52,63	chr9	(+)	6421104	6421126	exon2	NM_152896	(+)	7954	7976
UHRF2	GCACGTCTT(	TGG	57,89	chr9	(-)	6413510	6413532	exon1	NM_152896	(+)	360	382
UHRF2	GTCCTCAAT(	TGG	57,89	chr9	(-)	6413522	6413544	exon1	NM_152896	(+)	372	394
UHRF2	TGATTGGAA	CGG	52,63	chr9	(-)	6421065	6421087	exon2	NM_152896	(+)	7915	7937
USP22	GCCTCCGTA(	TGG	52,63	chr17	(+)	21021204	21021226	exon3	NM_015276	(-)	21612	21634
USP22	GCATATTCA(	AGG	52,63	chr17	(-)	21028560	21028582	exon2	NM_015276	(-)	28968	28990
USP22	CATGGAAAT	AGG	42,11	chr17	(-)	21021140	21021162	exon3	NM_015276	(-)	21548	21570
USP22	CCACACGAA(	TGG	57,89	chr17	(+)	21042695	21042717	exon1	NM_015276	(-)	43103	43125
USP27X	GTGAGATGT	CGG	52,63	chrX	(+)	49880678	49880700	exon1	NM_001145(	(+)	812	834
USP27X	AGCTTTACG/	AGG	36,84	chrX	(+)	49880521	49880543	exon1	NM_001145(	(+)	655	677
USP27X	AATCTCTCA(	TGG	47,37	chrX	(-)	49880598	49880620	exon1	NM_001145(	(+)	732	754
USP27X	AGGTTGGG/	AGG	47,37	chrX	(-)	49880433	49880455	exon1	NM_001145(	(+)	567	589
USP51	GTCTTCGAG,	AGG	57,89	chrX	(-)	55488777	55488799	exon2	NM_201286	(-)	4162	4184
USP51	CTCCACGTCA	CGG	52,63	chrX	(+)	55488716	55488738	exon2	NM_201286	(-)	4101	4123
USP51	GCAAAGAA(	TGG	42,11	chrX	(+)	55488912	55488934	exon2	NM_201286	(-)	4297	4319
USP51	GCGGGCTCA	TGG	63,16	chrX	(+)	55488740	55488762	exon2	NM_201286	(-)	4125	4147
UTY	AATTCTTGC/	AGG	45	chrY	(+)	13359779	13359801	exon12, exor	NM_00125&	(-)	111401	111423
UTY	CCTTGGCTC(	GGG	55	chrY	(+)	13411055	13411077	exon6, exon6	NM_00125&	(-)	162677	162699
UTY	GTCTGTTAG(	AGG	55	chrY	(-)	13479542	13479564	exon1, exon1	NM_00125&	(-)	231164	231186
UTY	CGCTGTTGC(	AGG	60	chrY	(-)	13479611	13479633	exon1, exon1	NM_00125&	(-)	231233	231255
WDR5	ATCTGACGA(	CGG	52,63	chr9	(-)	134141956	134141978	exon4, exon5	NM_052821,	(+)	5869	5891
WDR5	GTGAACTTT/	TGG	36,84	chr9	(-)	134140718	134140740	exon2, exon3	NM_052821,	(+)	4631	4653
WDR5	AAATTTGGG	GGG	47,37	chr9	(+)	134141528	134141550	exon3, exon4	NM_052821,	(+)	5441	5463
WDR5	CATCTGAGG	AGG	47,37	chr9	(-)	134141984	134142006	exon4, exon5	NM_052821,	(+)	5897	5919
WDR82	TAAGGTGTT	CGG	47,37	chr3	(-)	52278298	52278320	exon1	NM_025222	(-)	23877	23899
WDR82	CTGCATGAG	AGG	42,11	chr3	(+)	52270748	52270770	exon2	NM_025222	(-)	16327	16349
WDR82	TGCTTCGATT	CGG	52,63	chr3	(-)	52278264	52278286	exon1	NM_025222	(-)	23843	23865
WDR82	CGTGCTCTA1	AGG	57,89	chr3	(-)	52278205	52278227	exon1	NM_025222	(-)	23784	23806
WHSC1	CTTACTTCCC	TGG	47,37	chr4	(+)	1900898	1900920	exon2, exon4	NM_133335,	(+)	8117	8139
WHSC1	GAAATCCTC(	CGG	57,89	chr4	(+)	1900730	1900752	exon2, exon4	NM_133335,	(+)	7949	7971
WHSC1	CAGCTTGTC(	AGG	52,63	chr4	(-)	1900869	1900891	exon2, exon4	NM_133335,	(+)	8088	8110
WHSC1	GGGGTCATC	CGG	47,37	chr4	(+)	1900832	1900854	exon2, exon4	NM_133335,	(+)	8051	8073
WHSC1L1	GGATACCCA	AGG	47,37	chr8	(+)	38347951	38347973	exon2, exon2	NM_023034,	(-)	31535	31557
WHSC1L1	GAAGATCTT(	GGG	42,11	chr8	(+)	38347973	38347995	exon2, exon2	NM_023034,	(-)	31557	31579
WHSC1L1	TATCCTAATC	TGG	47,37	chr8	(-)	38347876	38347898	exon2, exon2	NM_023034,	(-)	31460	31482
WHSC1L1	TTGGCGGAC	AGG	47,37	chr8	(+)	38348092	38348114	exon2, exon2	NM_023034,	(-)	31676	31698
YY1	ACCATCGAG,	GGG	55	chr14	(+)	100239347	100239369	[exon1]	[NM_003403	(+)	583	605
YY1	GACCATCGA(	TGG	60	chr14	(+)	100239346	100239368	[exon1]	[NM_003403	(+)	582	604
YY1	GGAGACCAT	TGG	60	chr14	(+)	100239343	100239365	[exon1]	[NM_003403	(+)	579	601
ZCWPW1	TTGTAGCCCT	AGG	52,63	chr7	(-)	100419797	100419819	exon4, exon4	NM_001258(	(-)	18926	18948
ZCWPW1	GGGACTTCT(	TGG	47,37	chr7	(+)	100419126	100419148	exon5, exon5	NM_001258(	(-)	18255	18277
ZCWPW1	AGTTAGGG(	AGG	42,11	chr7	(+)	100419806	100419828	exon4, exon4	NM_001258(	(-)	18935	18957
ZCWPW1	GGTGGGGC/	TGG	52,63	chr7	(+)	100419847	100419869	exon4, exon4	NM_001258(	(-)	18976	18998
ZCWPW2	GCGATCCCC/	TGG	50	chr3	(+)	28435205	28435227	exon4	NM_001040(	(+)	86057	86079
ZCWPW2	TTTGCCCTG/	GGG	40	chr3	(+)	28435124	28435146	exon4	NM_001040(	(+)	85976	85998
ZCWPW2	AGGTTGATC	TGG	40	chr3	(+)	28413220	28413242	exon3	NM_001040(	(+)	64072	64094
ZCWPW2	TTGCTGCACT	AGG	45	chr3	(+)	28492151	28492173	exon6	NM_001040(	(+)	143003	143025
ZGPAT	AGAGACCGT	AGG	47,37	chr20	(+)	63708883	63708905	exon2, exon2	NM_001195(	(+)	1442	1464
ZGPAT	TTTCCGGGA(	AGG	57,89	chr20	(+)	63708823	63708845	exon2, exon2	NM_001195(	(+)	1382	1404
ZGPAT	GGTACTCAG	CGG	52,63	chr20	(-)	63708796	63708818	exon2, exon2	NM_001195(	(+)	1355	1377
ZGPAT	CAGCCTGCT(	AGG	57,89	chr20	(-)	63708661	63708683	exon2, exon2	NM_001195(	(+)	1220	1242
ZMYND11	CTGGTTCCG	TGG	42,11	chr10	(-)	180074	180096	exon2, exon1	NM_006624,	(+)	81	103
ZMYND11	GCTGACGGC	GGG	57,89	chr10	(-)	209916	209938	exon3, exon2	NM_006624,	(+)	29923	29945
ZMYND11	ATTCATTGT(	AGG	47,37	chr10	(+)	236888	236910	exon5, exon4	NM_006624,	(+)	56895	56917
ZMYND11	ACTCTAACA(	AGG	52,63	chr10	(+)	209974	209996	exon3, exon2	NM_006624,	(+)	29981	30003
ZMYND8	GTTGATGTT(	TGG	52,63	chr20	(-)	47298868	47298890	exon4, exon4	NM_012408,	(-)	89655	89677
ZMYND8	TTGATGTGT(	TGG	57,89	chr20	(+)	47310115	47310137	exon3, exon3	NM_012408,	(-)	100902	100924
ZMYND8	GACTGACAT(	GGG	52,63	chr20	(-)	47298753	47298775	exon4, exon4	NM_012408,	(-)	89540	89562
