# Supplementary material for: Pan-cancer multi-omics analysis and orthogonal experimental assessment of epigenetic driver genes
Source: Genome Res. 2020 Oct;30(10):1517–32. doi: 10.1101/gr.268292.120 (PMC7605261; doi:10.1101/gr.268292.120)
Supplement: Supplemental Material [file supp_gr.268292.120_Supplemental_Table_S8.docx]

**Supplemental Table S8.** The list of primers and primer sequences used in the study

| **Primers used for amplification of the targeted regions by gRNAS** | |  |  |  |
| --- | --- | --- | --- | --- |
| Gene/region | Forward | Reverse |  |  |
| EP400 | GCACGCACATTCTGCAAG | CAGAAGGGAGGTGATCATGC | | |
| ARID1B | GAAATGGGATGTTGCTGTTGC | CATCCTCTTCCTCCTCGTCG | | |
| KAT2B first targeted region | CGAGAGGGAGACCCTGGC | CGAGCGCGTACCTTGCAG | |  |
| KAT2B second targeted region | AAGTCAGGGGTGAGGGGATA | CCTAGGGCATGGCTACAACT | | |
| MBD5 first targeted region | GGCAGCAACTCCAAGATCAG | GAGCTCCATGAAAACCAGGA | | |
| MBD5 second targeted region | GAAGGCCATCATGCTCTGTA | CCATGCTTGGTTCTCTCCTT | | |
| MBD5 third targeted region | TGGCTTAATCTATGCACAACTTTT | GGCTTCCATGCTTTTATGAA | | |
|  |  |  |  |  |
| **Primers used for q-RT-PCR** |  |  |  |  |
| Gene/region | Forward | Reverse |  |  |
| *GAPDH* | GTCTCCTCTGACTTCAACAGCG | ACCACCCTGTTGCTGTAGCCAA | | |
| *Vimentin* | AAAGTGTGGCTGCCAAGAAC | AGCCTCAGAGAGGTCAGCAA | | |
| *N-Cadherin* | CCACAATCCTGTCCACATCT | TTCGGGTAATCCTCCCAAAT | | |
| *E-Cadherin* | TCCTGGGCAGAGTGAATTTTG | CTGTAATCACACCATCTGTGC | | |
