# Supplementary material for: Pan-cancer multi-omics analysis and orthogonal experimental assessment of epigenetic driver genes
Source: Genome Res. 2020 Oct;30(10):1517–32. doi: 10.1101/gr.268292.120 (PMC7605261; doi:10.1101/gr.268292.120)
Supplement: Supplemental Material [file supp_gr.268292.120_Supplemental_Table_S9.docx]

**Supplemental Table S9.** The percentage of mutations in EMT-specific epidriver candidates in metastatic and non-metastatic cases from the TCGA

|  | metastatic cases | | | non-metastatic cases | | |
| --- | --- | --- | --- | --- | --- | --- |
|  | % SNA | % CNA down | % CNA up | % SNA | % CNA down | % CNA up |
| ***EP400*** | 10,36 | 5,09 | 2,78 | 7,62 | 6,95 | 2,72 |
| ***KMT2A*** | 10,36 | 9,26 | 3,7 | 10,26 | 9,97 | 2,11 |
| ***MBD5*** | 8,29 | 3,7 | 3,24 | 4,3 | 4,53 | 2,72 |
| ***SRCAP*** | 7,77 | 0,46 | 5,56 | 12,25 | 0,6 | 6,65 |
| ***AFF1*** | 5,7 | 4,63 | 2,31 | 5,63 | 3,32 | 4,23 |
| ***ARID1B*** | 5,18 | 11,57 | 5,09 | 8,61 | 10,57 | 4,23 |
| ***PHC3*** | 3,11 | 1,39 | 13,89 | 2,98 | 1,21 | 9,97 |
| ***KAT2B*** | 2,59 | 11,57 | 5,56 | 2,65 | 14,2 | 0,91 |
| ***KAT6B*** | 2,59 | 3,7 | 6,94 | 5,96 | 2,11 | 3,63 |
| ***JMJD8*** | 2,07 | 3,7 | 5,56 | 0 | 3,93 | 2,72 |
| ***HDAC2*** | 1,55 | 8,33 | 2,78 | 0,99 | 8,16 | 3,63 |
| ***DNMT3L*** | 1,04 | 3,7 | 3,7 | 0,33 | 2,11 | 3,02 |
| ***RTF1*** | 0,52 | 8,8 | 2,31 | 3,31 | 7,85 | 1,21 |
| ***SETD7*** | 0,52 | 6,02 | 4,63 | 1,32 | 5,14 | 3,93 |
| ***SUV39H1*** | 0,52 | 0,46 | 5,09 | 0,99 | 0 | 1,81 |
