# Supplementary material for: Pan-cancer multi-omics analysis and orthogonal experimental assessment of epigenetic driver genes
Source: Genome Res. 2020 Oct;30(10):1517–32. doi: 10.1101/gr.268292.120 (PMC7605261; doi:10.1101/gr.268292.120)
Supplement: Supplemental Material [file supp_gr.268292.120_Supplemental_Code.pdf]

```
---
title: "Figure4"
author: "cahaisv"
date: "22 mars 2019"
output:
  html_document:
    code_folding: "show"
---

```{r include = TRUE}
library(data.table)
library(ggplot2)
library(knitr)
```

```{r include = TRUE}
cs<-fread("consensus_score_ERG.tsv.csv")

cs$Cancer<-factor(cs$Cancer)
levels(cs$Cancer)[6]<-"COAD/READ"
orderf<-
c("KIRC","SARC","CESC","DLBC","GBM","LGG","MESO","CHOL","UVM","LAML","HNSC","SKCM","BRCA","STA
D","LUAD","KIRP","BLCA","PRAD","LUSC","COAD/READ","PAAD","LIHC","ACC","UCEC","ESCA","THCA","UCS",
"PANCAN")
cs$Cancer<-factor(cs$Cancer,levels=orderf)

fig4<-ggplot( data=cs, aes(x=Cancer, y=Consensus_Score)) +
  geom_jitter(aes(shape=prediction2,size=5)) +
  theme(axis.text.x=element_text(angle=50,hjust=1, size=14), panel.grid.major = element_blank(),
panel.grid.minor = element_blank(), panel.background=element_rect(fill = "white")) +
  scale_shape_manual(values=c(16,1)) +
  guides(shape=F, size=F)
```



```{r echo = FALSE, fig.height = 10, fig.width = 17, fig.align = "center"}
fig4
```

```{r include = FALSE}
jpeg("Fig4.jpeg", width=3000, height=1700, res = 200)
plot(fig4)
dev.off()
```
```
