# Supplementary material for: Pan-cancer multi-omics analysis and orthogonal experimental assessment of epigenetic driver genes
Source: Genome Res. 2020 Oct;30(10):1517–32. doi: 10.1101/gr.268292.120 (PMC7605261; doi:10.1101/gr.268292.120)
Supplement: Supplemental Material [file supp_gr.268292.120_Supplemental_Fig_S18edited.pdf]

A

A549-Vim-Cas9 *KAT2B* targeted region

AAGTCAGGGGTGAGGGGATAGCTGTCATATAATTAGTGTACATTGTTCTCATGTAAAACCATCAGTCCACGGCTGCCTGTTAAATAATTTTGTCTCTTTCTTTATTC
CATTTTTAGGCCGAGGAGTCTTGTAAATGTAATGGCTGGAAAAACCCTAACCCCTCACCCACTCCCCCCAGAGCCGACCTGCAGCAAATAATTGTCAGTCTAACAG
AATCCTGTCGG**AGTTGTAGCCATGCCCTAGG**

A549-Vim-Cas9 *KAT2B* Clone 2

tgtTCTcnTGTaaAccaTCaGTCcaCGGCTGCCTGTTAAA
TAATTTTGTCTCTTTCTTTATTCCATTTTTAGGCCGAGG
AGTCTTGTAAATGTGCAGCAAATAATTGTCAGTCTAA
CAGAAtCCtGTCGGAGTTGTAGCCaTgCCCTAGGa

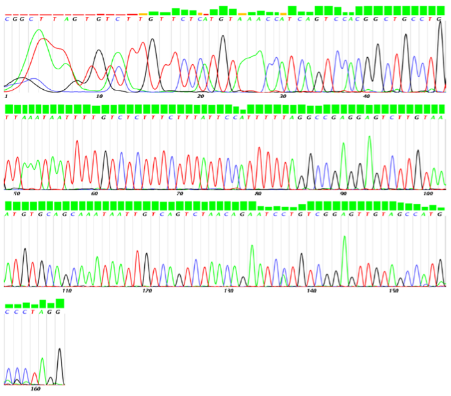

A549-Vim-Cas9 *KAT2B* Clone 5

TGTnnccaTCAGTCcaCGGCTGCCTGTTAAATAATT
TTGTCTCTTTCTTTATTCCaTTTTTAGGCCGAGGAG
TCTTGTAAATGTGCAGCAAATAATTGTCAGTCTAA
CAGAATCCTGTCGGAGTTGTAGCCATGCCCTAGG
a

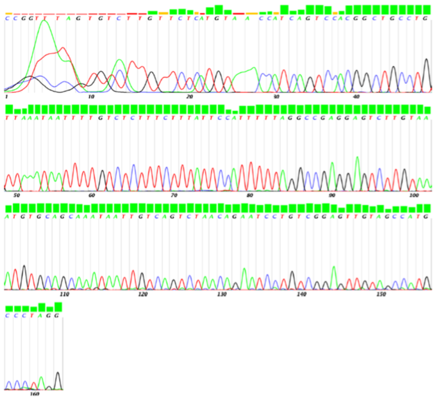

B

A549-Vim-Cas9 *ARID1B* targeted region

GAAATGGGATGTTGCTGTTGCTGCTGCTGCTGCTGCTGCTGTTGCTGCTGCTGCTGCTGCTGGAACTGGTTTAGCTGCTGCTGTAGTGCGTGGTGGTGG
TGGAGGTGGTGGGCATGGTGGTGGTGGTGGTGGGCATGGTGGTGGTGGTGGTGCTGCTGGTGGGGCGGCGCGGCGGGGGGCTTCGCCAACGGTTTTCAGTT
TGTGGTTGGGGAGCAGCCCCGTCTCCATGGCCGAGCCCGGGCC**CGACGAGGAGGAAGAGGATG**

A549-Vim-Cas9 *ARID1B* Clone 2

CtGctGcTGCTgctggaACTGGTTTAGCTGCTGCTGTAGT
GCGTGGTGgTGgTGgAGGTGGTGGGCATGgTGGTGgT
GGTGGTGGGCATGGTGGTGGTGGTGGTGCTGCTGGT
GGGGCGGCGCGGCGGggGCTTCgCCAACGTTTCAgtT
TGTGGTTGGggAGCAGCCCCgtCTCCATGGcCgAGCCC
GGgcCCgACgngGAgGAAGAGGAtGa

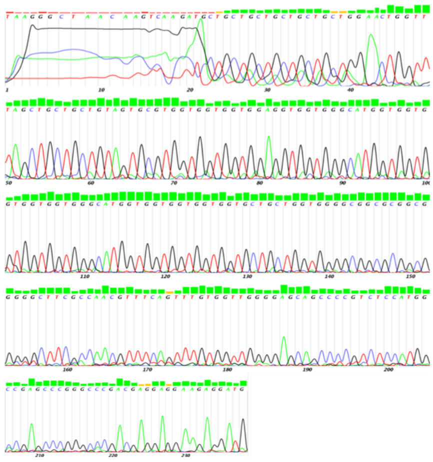

A549-Vim-Cas9 *ARID1B* Clone 3

GcTGCTGCTGCTGCTGGnncTGGTTTAGCTGCTGCTGT
AGTGCGTGGTGGTGGTGGAGGTGGTGGGCATGGTG
GTGGTGGTGGTGGGCATGGTGGTGGTGGTGGTGCT
GCTGGTGGGGCGGCGCGGCGGGGGGCTTCGCCAACG
GTTTTCAGTTGTTGGGGAGCAGCCCCGTCTCCATGGC
CGAGCCCGGGCCCGACGAGGAgGAAGAGGATGa

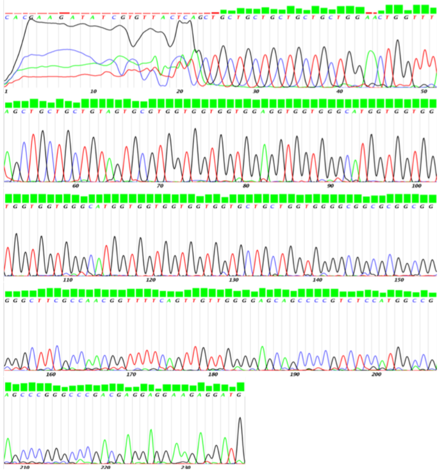

C

A549-Vim-Cas9 *EP400* targeted region

GCACGCACATTCTGCAAGGCCTGCCCCGGCCCCGGACTGGGCTGCGTGGGACTCTGGGTCTGGACCTGCTGGGACAAGGGGGACGTGACCTGAATGTATGATG
GAGACCCATGCTCAAACCGCCGAGGCTGAGCGCTGAACTGGAAGCCTGGAGAGGTGGGGCTGGGGAGCGGCAGTGGGGCCAGTGTGATCTGCTGGTTTCCCC
CGACGACAGGGCCCACGCTCTGCAGGGTGATGTTCACGTTCTGCCCGGTTGCAGGGCTCCTATTCATCAGCTGCTGTATTTGATAACTGGGAGACTGGGGTGCC
GACGGGCTTGCTGAGGGAGCGAAGGGAGCTGCGGGGGACGGGGGTGGGTTGGGGTGGGCCGGCTGCTCCTCACCCTCGCTGCCAGGGCAGGCCCTGGACCTC
TGCAGCTGATGCTGGACGTTCTGGGGGCCAGTGCCATGGT**GCATGATCACCTCCCTTCTG**

A549-Vim-Cas9 *EP400* Clone 1

gCgtgGGnnTCTGGGTCTGGanCTGCTGGGAcAAGGGG
GAcGTGACCTGAATGTATGATGGAGACCCATGCTCAA
ACCGCCGAGGCTGAGCGCTGAACTGGAAGCCTGGAG
AGGTGGGGCTGGGGAGCGGCAGTGGGGCCAGTGTGA
TCTGCTGGTTTCCCCCGACGACAGGGCCCACGCTCTGC
AGGGTGATGTTCACGTTCTAGACTGGGGTGCCAGTGC
CATGGTGCATGATCacCTCCCTTCTGa

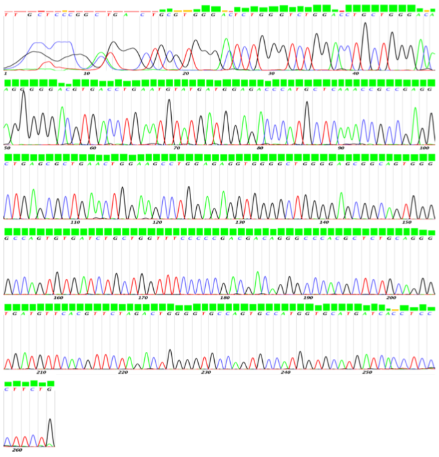

A549-Vim-Cas9 *EP400* Clone 4

cngCgtgGGanTCTGGGTCTGGacCTGCTGGGAcAAG
GGGGAcGTGACCTGAATGTATGATGGAGACCCATG
CTCAAACCGCCGAGGCTGAGCGCTGAACTGGAAGC
CTGGAGAGGTGGGGCTGGGGAGCGGCAGTGGGGC
CAGTGTGATCTGCTGGTTTCCCCCGACGACAGGGCC
CACGCTCTGCAGGGTGATGTTCACGTTCTGCCCGGT
TGCAGGGCTCCTATTCATCAGCTGCTGTATTTGATAA
CTGGGACTGGGGGCCAGTGCCATGGTGCATGATCAc
CTCCCTTCTGa

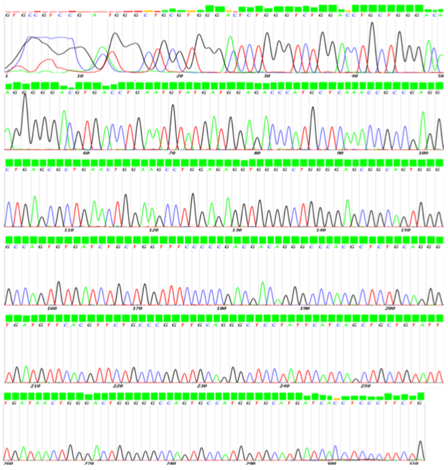

A549-Vim-Cas9 *EP400* Clone6

GGtCTGGacCTGCTGGGAcAAGGGGGacGTGACCTG
AATGTATGATGGAGACCCATGCTCAAACCGCCGAGG
CTGAGCGCTGAACTGGAAGCCTGGAGAGGTGGGGC
TGGGGAGCGGCAGTGGGGCCAGTGTGATCTGCTGG
TTTCCCCCGACGACAGGGCCCACGCTCTGCAGGGTG
ATGTTCACGTTCTAGACTGGGGTGCCAGTGCCATGG
TGCATGATCacCTCCCTTCTGA

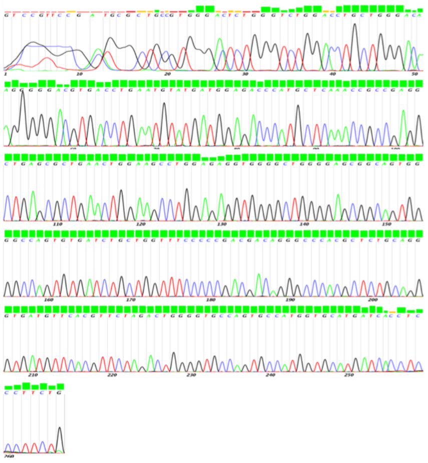

D

A549-Vim-Cas9 *MBD5* targeted region cut1

GGCAGCAACTCCAAGATCAGTAAGAAATAAGTCTCATGAAGGAATTACAAATTCTGTAATGCCTGAATGTAAGAATCCTTTCAAGTTAATGATTGGATCATCAAAT
GCCATGGGAAGGCTATATGTACAAGAACTGCCTGGAAGCCAACAACAAGAACTCCACCCTGTCTACCCCCGACAGAGATTGGGCAGCAGTGAACATGGACAGAAA
TCTCCATTCCGTGGCAGCCATGGAGGCCTGCCCAGCCCAGCGTCATCAGGTTCCCAGATATATGGAGATGGTTCAATCTCTCCAAGGACTGACCCACTTGGAAGTCC
TGATGTTTTCACAAGAAGTAAT**TCCTGGTTTTCATGGAGCTC**

A549-Vim-Cas9 *MBD5* cut1 Clone 6

tGTaATGCCTGaATGTAAGAATCCTTTcaAGTTAATGAT
TGGATCATCAAATGCCATGGGAAGGCTATATGTACAA
GAACTGCCTGGAAGCCAACAACAAGAACTCCACCCTG
TCTACCCCCGACAGAGTCCGTGGCAGCCATGGAGGCC
TGCCCAGCCCAGCGTCATCAGGTTCCCAGATATATGG
AGATGGTTCAATCTCTCCAAGGACTGACCCACTTGGA
AGTCCTGATGTTTTCACAAGAAGTAATCCTGGTTTTcat
GGAGCTC

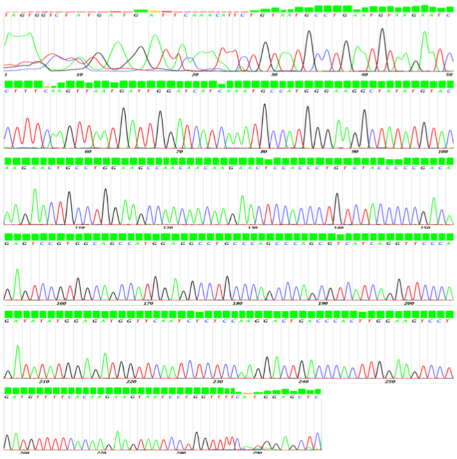

A549-Vim-Cas9 *MBD5* cut1 Clone 10

tGTAATGCCTGAATGTAAGAATCCTTTCaAGTTAATG
ATTGGATCATCAAATGCCATGGGAAGGCTATATGTA
CAAGAACTGCCTGGAAGCCAACAACAAGAACTCCAC
CCTGTCTACCCCCGACAGAGATTGGGCAGCAGTGAA
CATGGACAGAAATCTCCATTTCCGTGGCAGCCATGG
AGGCCTGCCCAGCCCAGCGTCATCAGGTTCCCAGAT
ATATGGAGATGGTTCAATCTCTCCAAGGACTGACCC
ACTTGGAAGTCCTGATGTTTTCACAAGAAGTAATCCT
GGTTTTcntGGAG

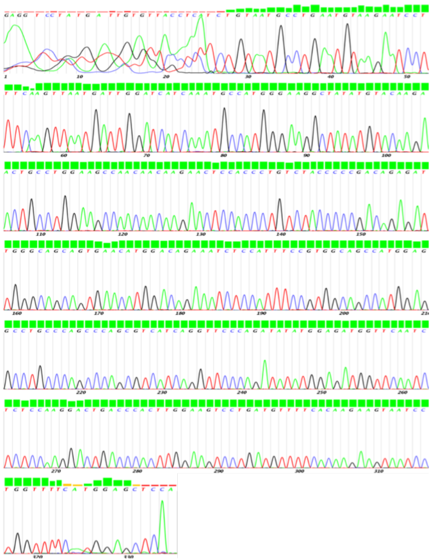

Sequence of A549-Vim-Cas9 *MBD5* targeted region cut2

GAAGGCCATCATGCTCTGTAATATAAGGATATCATCTTATTGCTGATATCTTTGGAGAGTCCCTAGCAGACACAGAAAATGAATGGAGGCAAAGAGTGTGACGGAGGGGACA
AGGAAGGAGGTCTTCCAGCTATACAAGTTCCTGTGGGTTGGCAGCGTCGTGTGGATCAAAATGGAGTGCTTTATGTCAGGTAAGTTCTTATTATTACCTGTGGTACCTGCAAAG
TTGTACTCCAAAGACT**AAGGAGAGAACCAAGCATGG**

A549-Vim-Cas9 *MBD5* cut2 Clone6

TGataTnntTTGgAGAGTcCCTAGCAGACACAGAAAA
TGAATGGAGGCAAAGAGTGTGACGGAGGGGACAA
GGAAGGAGGTCTTCCAGCTATACAAGTTCCTGTGG
GTTGGCAGCGTCGTGTGGAGTGCTTTATGTCAGGT
AAGTTCTTATTATTACCTGTGGTACCTGCAAAGTTG
TACTCCAAAGACTAAGGAGAGAACCAAGCATGGA

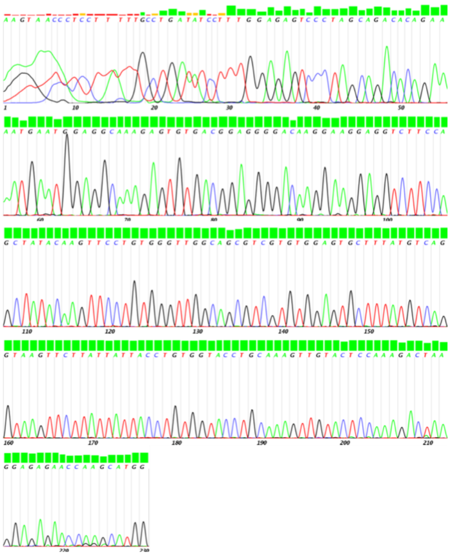

A549-Vim-Cas9 *MBD5* cut2 Clone10

tGgAgAGTcCCTAGCAGACACAGAAAATGAATGG
AGGCAAAGAGTGTGACGGAGGGGACAAGGAA
GGAGGTCTTCCAGCTATACAAGTTCCTGTGGGTT
GGCAGCGTCGTGTGGATCAAAATGGAGTGCTTT
ATGTCAGGTAAGTTCTTATTATTACCTGTGGTAC
CTGCAAAGTTGTACTCCAAAGACTAAGGAGAGA
AcCAAGCATGGA

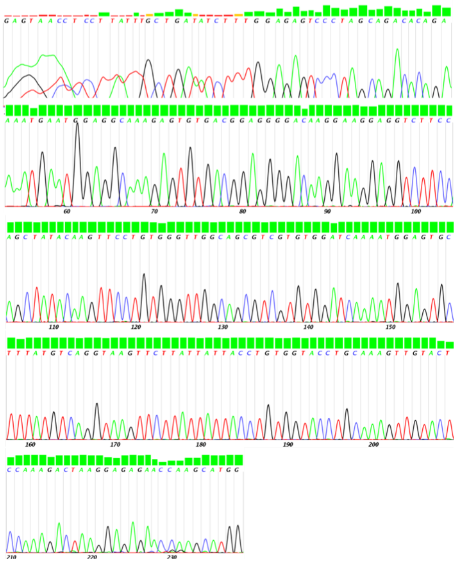

A549-Vim-Cas9 *MBD5* cut2 Clone11

AGACaCAGAAAATGAATGGAGGCAAAGAGTGTGA
CGGAGGGGACAAGGAAGGAGGTCTTCCAGCTATA
CAAGTTCCTGTGGGTTGGCAGCGTCGTGTGGAGTG
CTTTATGTCAGGTAAGTTCTTATTATTACCTGTGGT
ACCTGCAAAGTTGTACTCCAAAGACTAAGGAGAGA
AcCAAGCATGGA

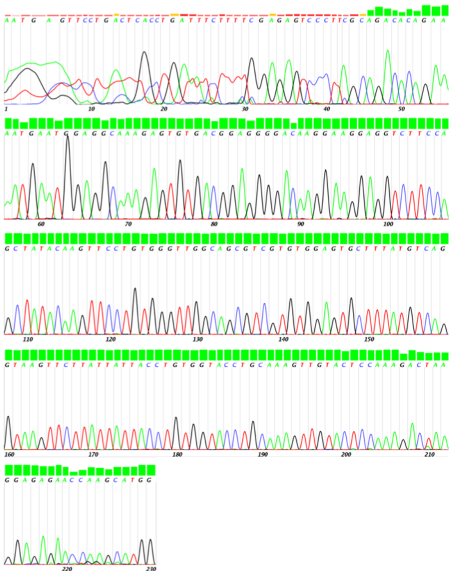

Supplemental Figure S18. Sequence of A549-Vim Cas9 CRISPR knock out targeted regions of (A) *KAT2B*, (B) *ARID1B*, (C) *EP400* and (D) *MBD5*. In bold are shown forward and reverse complement sequences of primers used for amplification of the targeted region. sgRNA sequences are underlined in corresponding sequence. Result of sanger sequencing for each A549-Vim-Cas9 single cell clone are shown as sequence and chromatogram.
