# Supplementary material for: Pan-cancer multi-omics analysis and orthogonal experimental assessment of epigenetic driver genes
Source: Genome Res. 2020 Oct;30(10):1517–32. doi: 10.1101/gr.268292.120 (PMC7605261; doi:10.1101/gr.268292.120)
Supplement: Supplemental Material [file supp_gr.268292.120_Supplemental_Table_S4.pdf]

Supplemental Table S4

cancer	genes	cpg	cor	N	t	pvalue	R2	qvalue
**ACC**	ACTL6A	cg03667091	0.00160038443231363	80	0.0140433343838073	0.988831731899424	2.56123033119181e-06	0.994109351796744
**ACC**	ACTL6A	cg03839554	-0.388542072446699	80	-3.7001608461201	0.000402408823457845	0.150964942061176	0.00311958066886692
**ACC**	ACTL6A	cg07261848	-0.397786422212673	80	-3.80451794927386	0.000283120195215998	0.158234037696759	0.00232872197786354
**ACC**	ACTL6A	cg10112003	0.222425267396934	80	2.00192247370236	0.048815061184108	0.0494729995765974	0.141740775968815
**ACC**	ACTL6A	cg20132173	-0.0762012364244825	80	-0.670612970683909	0.50447364562561	0.00580662843261987	0.687712872600124
**ACC**	ACTL6A	cg22619386	0.200228429327867	80	1.79331329119005	0.0768493675592617	0.0400914239111047	0.197320605317354
**ACC**	ACTL6A	cg25198545	0.0980356052002976	80	0.864422949316744	0.390040542622359	0.00961097988698861	0.58753802905979
**ACC**	ACTL6A	cg25575835	-0.103082185103512	80	-0.909386950999543	0.365983656123311	0.0106259368857146	0.564858638177635
**ACC**	ACTL6B	cg05729836	0.149282592758375	80	1.32479432123247	0.189157652293084	0.0222852925006629	0.368953224413003
**ACC**	ACTL6B	cg08572611	-0.0982886230262131	80	-0.866675653092476	0.388812434833506	0.00966065341638902	0.586508757078576
**ACC**	ACTL6B	cg09375063	-0.0752824595722112	80	-0.662480857307256	0.509641205870549	0.00566744871924162	0.692092455117765
**ACC**	ACTL6B	cg14088161	0.0807881825226282	80	0.711238254333046	0.479085687531998	0.00652673043530949	0.666477283216739
**ACC**	ACTL6B	cg16024950	-0.063023676221918	80	-0.554132111254091	0.581094241133658	0.00397198376452515	0.746736551512566
**ACC**	ACTL6B	cg20497359	0.0405386456567659	80	0.356017828846142	0.722800798470535	0.00164338179168483	0.842920868804593
**ACC**	ACTL6B	cg24471254	-0.181202261542292	80	-1.61680827827284	0.110009543189081	0.0328342595880411	0.255008414864463
**ACC**	ACTL6B	cg26990587	-0.112595933720297	80	-0.994348511430741	0.323167764374199	0.0126778442903455	0.522596418738839
**ACC**	AEBP2	cg02664216	0.0985709076404855	80	0.869189129355	0.387444998020109	0.00971622383306912	0.585373784734441
**ACC**	AEBP2	cg07849438	-0.202844762394885	80	-1.81774483068053	0.0729929025337798	0.0411459976310373	0.190135940388845
**ACC**	AEBP2	cg08161947	-0.512013768870678	80	-5.23052576186368	1.41342836287383e-06	0.262158099513156	2.31975202661195e-05
**ACC**	AEBP2	cg24304000	-0.0743487552233144	80	-0.654218358684635	0.514920415692789	0.00552773740325632	0.696401641459724
**ACC**	AEBP2	cg26259627	-0.257836340307414	80	-2.34168017592904	0.0217842999895084	0.0664795783831204	0.07769876820796
**ACC**	AFF4	cg00931577	0.0601631260647492	80	0.528887337139091	0.598404928431018	0.0036196017378829	0.75891023951945
**ACC**	AFF4	cg01907658	-0.327382640826214	80	-3.0403172776363	0.00322871287394268	0.107179393514346	0.0174863888412348
**ACC**	AFF4	cg05619425	0.26933466864396	80	2.45408895118033	0.0163826234192143	0.072541163733552	0.0624311651941978
**ACC**	AFF4	cg06638012	0.183857805675413	80	1.6413256195369	0.104808464768435	0.0338036927077778	0.246343274765715
**ACC**	AFF4	cg13393914	-0.141998075272605	80	-1.25878338901515	0.211911651018506	0.0201634533811244	0.398026340705336
**ACC**	AFF4	cg13565585	-0.273093702785782	80	-2.49107984520424	0.0148875931140239	0.0745801705012489	0.0580359500256752
**ACC**	AICDA	cg07999668	0.0752238805218063	80	0.66196243066056	0.509971596025308	0.00565863220075899	0.6923705809495
**ACC**	AIRE	cg08089567	-0.145656929749975	80	-1.29191241048237	0.200251655169004	0.0212159411841891	0.383222485602261
**ACC**	AIRE	cg09510531	-0.153238180715745	80	-1.36073078413031	0.177568460517419	0.0234819400290713	0.353935812280409
**ACC**	AIRE	cg11923631	-0.160103909097442	80	-1.42326599527504	0.1586983367767	0.025633261708282	0.327909121147586
**ACC**	AIRE	cg12843033	-0.107693529820765	80	-0.950535068094278	0.34481451789019	0.0115978963652561	0.544052339615646
**ACC**	AIRE	cg15955655	-0.196792619712272	80	-1.76129001630169	0.0821591364975495	0.0387273351732189	0.206924626543977
**ACC**	AIRE	cg16501323	-0.130503375103085	80	-1.15504053147681	0.25164568506817	0.0170311309132965	0.445417042255995
**ACC**	AIRE	cg16717549	-0.143960547918163	80	-1.27654591380609	0.205599248990935	0.0207246393568976	0.389907315180049
**ACC**	AIRE	cg17356252	-0.129283235257394	80	-1.14405704692882	0.256144089588038	0.0167141549186187	0.450407389195811
**ACC**	AIRE	cg18689454	-0.125022525111172	80	-1.10574397944803	0.272280250525648	0.0156306317851737	0.468633593871374
**ACC**	AIRE	cg18876487	0.0981008394720347	80	0.865003736017594	0.389723684980611	0.00962377470511793	0.587345054134751
**ACC**	AIRE	cg23272544	-0.175689334556917	80	-1.56602615709488	0.121443224633996	0.0308667422770522	0.272916756544306
**ACC**	AIRE	cg27251412	-0.162631090213948	80	-1.44633719069982	0.152141095636558	0.0264488715041771	0.318816408444655
**ACC**	ARID1A	cg04081153	0.139734281771302	80	1.23831238226611	0.219362361362199	0.0195256695021415	0.407310202157933
**ACC**	ARID1A	cg04360147	0.232723986784968	80	2.09979925218468	0.0390217829414851	0.0541604540250898	0.120291951765909
**ACC**	ARID1A	cg14851949	0.229411804924144	80	2.06824155211532	0.0419751240149386	0.0526297762385536	0.126812147480316
**ACC**	ARID1A	cg17385674	0.165123767495316	80	1.4691220327599	0.14587358594491	0.0272658585918472	0.309861566072433
**ACC**	ARID1A	cg20413923	0.00757090656753177	80	0.0664363395575075	0.947202638141335	5.73186262542956e-05	0.971876205589193
**ACC**	ARID1A	cg21294655	-0.25005782783176	80	-2.2662450927673	0.0262445306623219	0.0625289172599384	0.0893642083303655
**ACC**	ARID1A	cg24542126	0.0830923039460034	80	0.731662201882922	0.466595741718524	0.00690433097505501	0.655879114754464
**ACC**	ARID1A	cg25601319	0.333207443734097	80	3.10110035736007	0.00269396682915006	0.111027200559811	0.0151120893789227
**ACC**	ARID2	cg00035453	-0.250653553155689	80	-2.27200556671319	0.0258775405540561	0.0628272037095716	0.0884124864416034
**ACC**	ARID2	cg01183821	-0.0689822613503872	80	-0.606762263243074	0.54579362776809	0.00475855238101312	0.7195967797385
**ACC**	ARID2	cg01353809	-0.390701958004852	80	-3.72442322322936	0.000371012469353024	0.152648019988825	0.00291953712733655
**ACC**	ARID2	cg01751433	-0.0423126645179345	80	-0.371624944825995	0.711192791315628	0.00179036157860727	0.835571510671579
**ACC**	ARID2	cg06613666	0.0348404649730145	80	0.305909561139422	0.760499055811797	0.00121385799953585	0.866752256127108
**ACC**	ARID2	cg07276957	-0.336264120185068	80	-3.13315652220618	0.00244636878676272	0.113073558523838	0.0139833073767391
**ACC**	ARID2	cg23465749	-0.355103749293348	80	-3.33326176652239	0.00132150479496564	0.126098672762193	0.00845715235435647

**ACC**	ARID4A	cg02136725	-0.0780052946859669	80	-0.686585747619654	0.494406289834727	0.00608482599904454	0.679523992589059
**ACC**	ARID4A	cg06154646	0.446492376856238	80	4.37864295379547	3.71581673215751e-05	0.199355442590733	0.000410316886957362
**ACC**	ARID4A	cg08066755	-0.236863951235475	80	-2.13935266123206	0.035575623239118	0.0561045313948814	0.112220412283728
**ACC**	ARID4A	cg08790491	-0.341598965918885	80	-3.18937290454409	0.00206270812420677	0.116689853516852	0.0121953424391656
**ACC**	ARID4A	cg10064886	0.0400751124682028	80	0.351940409014188	0.725844313474767	0.00160601463933911	0.844672139453343
**ACC**	ARID4A	cg10242170	-0.347466457331293	80	-3.25160548092614	0.00170396317398002	0.120732938970359	0.0104368247505394
**ACC**	ARID4A	cg10844118	-0.296990122855438	80	-2.72921859009453	0.00786321052456957	0.0882031330736881	0.0354510589191401
**ACC**	ARID4A	cg12203852	0.227276505699382	80	2.04793721257186	0.0439756449921003	0.0516546100429212	0.131320436070269
**ACC**	ARID4A	cg14272338	-0.00371671493196831	80	-0.0326142664331356	0.974066586644343	1.38139698855162e-05	0.986465403984912
**ACC**	ARID4A	cg14460816	-0.286511929063103	80	-2.62414485251453	0.0104704629319426	0.0820890854954607	0.0441748030439578
**ACC**	ARID4A	cg17323045	0.188013705824161	80	1.67976990980186	0.0970553926977568	0.035349153577734	0.233049176477847
**ACC**	ARID4A	cg19548283	-0.353807389739199	80	-3.31934832196356	0.0013803802753559	0.125179669034066	0.00878048267710847
**ACC**	ARID4A	cg20996579	-0.233562048249991	80	-2.10779629736417	0.0383025858416516	0.054551230382731	0.118538861557284
**ACC**	ARID4A	cg26429530	-0.232935720160151	80	-2.10181921061071	0.0388390270751566	0.0542590497265281	0.119836722019781
**ACC**	ARID4A	cg27026909	-0.354000391095812	80	-3.32141835980658	0.00137146730616138	0.125316276895988	0.00873235616430828
**ACC**	ARID4B	cg05652840	0.210757760867403	80	1.89188680274475	0.0622657719006457	0.0444188337658416	0.169148425436981
**ACC**	ARID4B	cg06212607	-0.372228218141859	80	-3.51917276395062	0.000730266819640828	0.138553846381063	0.00516457972428532
**ACC**	ARID4B	cg06891548	-0.404501474422276	80	-3.88118216091397	0.000217887221207677	0.163621442809795	0.00186431131617136
**ACC**	ARID4B	cg07814876	-0.32380160277614	80	-3.00314178650883	0.00360274076115906	0.104847477960397	0.0190583173380666
**ACC**	ARID4B	cg10107779	0.191616172220443	80	1.7131702350344	0.0907050998742861	0.0367167574564146	0.222001338355206
**ACC**	ARID4B	cg11732257	-0.347594138607665	80	-3.25296449591681	0.00169682460481324	0.120821685194405	0.0104031380482808
**ACC**	ARID4B	cg22596515	0.291376020280071	80	2.67279031320101	0.00917881296500422	0.0848999851942523	0.039920193790862
**ACC**	ARID4B	cg23687319	-0.273328928300832	80	-2.49339866407008	0.0147980907522604	0.0747087030460812	0.05777371740796
**ACC**	ARID4B	cg26020585	0.268386323459343	80	2.4447759278776	0.0167796117704777	0.0720312186200229	0.0635982030729621
**ACC**	ASH1L	cg03119126	-0.0789782401942713	80	-0.695202822218299	0.489020923600983	0.00623756242418401	0.675037682503046
**ACC**	ASH1L	cg08095278	0.203153407249026	80	1.82062964745171	0.072548384756093	0.0412713068768885	0.189218796517098
**ACC**	ASH1L	cg09410084	0.18067505824117	80	1.61194523817933	0.111065498168332	0.0326434766704503	0.256664038078521
**ACC**	ASH1L	cg10558228	0.116293669499881	80	1.02744414815441	0.307428182572389	0.0135242175657476	0.505914556021167
**ACC**	ASH1L	cg17038116	-0.225258539427204	80	-2.02877721356459	0.0459381393900129	0.050741409584877	0.135629876492719
**ACC**	ASH1L	cg20064047	-0.049736849318984	80	-0.436979906624704	0.663348967182988	0.00247375418017932	0.804310622709373
**ACC**	ASH1L	cg21852905	-0.273777170021263	80	-2.49781870445804	0.0146288228611934	0.0749539388248518	0.0572604649340252
**ACC**	ASH1L	cg23821590	-0.358975012337585	80	-3.37494241058599	0.00115895580094083	0.128863059482769	0.00759304426296034
**ACC**	ASH1L	cg24312492	-0.00337884351272654	80	-0.0296494007426588	0.976423381212389	1.14165834834942e-05	0.987543624613701
**ACC**	ASH1L	cg26185843	-0.203207809818184	80	-1.82113819163926	0.0724702585425686	0.0412934139711033	0.189065739731734
**ACC**	ASH2L	cg00590869	0.373552951811211	80	3.5337241802446	0.000696564823034415	0.139541807806869	0.00496641589617261
**ACC**	ASH2L	cg01642070	-0.186434012979091	80	-1.66514602233932	0.0999476534655911	0.0347576411954877	0.238163621579385
**ACC**	ASH2L	cg02897270	0.00788857399095268	80	0.0692241097657446	0.94499069041749	6.22295996107351e-05	0.970553908117211
**ACC**	ASH2L	cg03189282	-0.29920731156322	80	-2.75158894226107	0.00739113251958967	0.0895250152928898	0.0337928747122515
**ACC**	ASH2L	cg03705346	0.0230370249152297	80	0.202202735295707	0.840291524917378	0.000530704516944913	0.914424968625297
**ACC**	ASH2L	cg10062221	0.0463832141085669	80	0.407449582001178	0.684808390879073	0.00215140255104116	0.818629991549189
**ACC**	ASH2L	cg21453287	0.088692302769458	80	0.781351046251429	0.436991887298594	0.0078663245705492	0.629986784783863
**ACC**	ASH2L	cg24318412	0.0400458698816767	80	0.351683187406868	0.72603646049894	0.00160367169458018	0.844819917554962
**ACC**	ASH2L	cg27255459	-0.0040775537282826	80	-0.0357806862073893	0.971549830619737	1.66264444070314e-05	0.984945104295661
**ACC**	ASXL1	cg00174466	-0.304685320266868	80	-2.8070704074106	0.00633004139558777	0.092833144386124	0.0299179684747155
**ACC**	ASXL1	cg04218270	-0.152969410430048	80	-1.35828691054218	0.178339104515833	0.0233996405273164	0.354935333010252
**ACC**	ASXL1	cg09065199	0.0357154351365297	80	0.313601749217432	0.754671498390322	0.00127559230699166	0.862990253175548
**ACC**	ASXL1	cg10421984	-0.232093113869766	80	-2.09378252904587	0.0395705654704552	0.0538672135057643	0.121544303320906
**ACC**	ASXL1	cg12592359	-0.0910354232117033	80	-0.802163456190378	0.424927232483583	0.00828744827933392	0.619814069872357
**ACC**	ASXL1	cg16926526	-0.0433036148030543	80	-0.380344456622351	0.704736933237507	0.0018752030550113	0.831520213405087
**ACC**	ASXL1	cg18793288	-0.0155114844029803	80	-0.136129100966279	0.892074497441077	0.000240606148383902	0.942829013359791
**ACC**	ASXL1	cg24727216	-0.244826452167492	80	-2.21577630090428	0.029660796146503	0.0599399916809211	0.097838872770567
**ACC**	ASXL2	cg05165989	-0.0155448863285466	80	-0.136422307716256	0.89184349345127	0.000241643490967434	0.942722001470544
**ACC**	ASXL2	cg05496738	0.0408366859025182	80	0.35863962925407	0.720846159456212	0.00166763491550093	0.841852911318329
**ACC**	ASXL2	cg06583696	-0.0376029543953851	80	-0.330198115386539	0.742146982015107	0.00141398217926141	0.854959829390088
**ACC**	ASXL2	cg09476286	-0.176296484075579	80	-1.57161134210225	0.120141081542418	0.031080450297411	0.270935600147241
**ACC**	ASXL2	cg19084174	-0.121269502445164	80	-1.07204768573737	0.287047182990145	0.0147062922232977	0.484467241306388
**ACC**	ASXL2	cg20667782	0.133063969647358	80	1.17810797748846	0.242381753129626	0.0177060200183131	0.434777242312311

**ACC**	ASXL2	cg24738909	-0.134490207911758	80	-1.19096681044553	0.23732503446109	0.0180876160241478	0.428747750527187
**ACC**	ASXL3	cg09963640	-0.0602681746221888	80	-0.529814170862306	0.597765184437937	0.00363225287229064	0.758506960888707
**ACC**	ASXL3	cg12361352	-0.0520895829177343	80	-0.457705608795762	0.648452911021149	0.00271332464854352	0.794233578839317
**ACC**	ASXL3	cg17880622	-0.0447923399841869	80	-0.393446082368742	0.695077356557616	0.00200635372125899	0.825174317875187
**ACC**	ASXL3	cg20034091	-0.0835056345511897	80	-0.735327243089115	0.464374096398895	0.00697319100179685	0.653608495272151
**ACC**	ASXL3	cg20329085	-0.00962456072087461	80	-0.0844590894748898	0.932910634911325	9.26321690698024e-05	0.964334497299879
**ACC**	ASXL3	cg24880056	-0.0440863016864611	80	-0.387232222959747	0.699652542519779	0.00194360199638966	0.827851763468808
**ACC**	ASXL3	cg27109877	-0.0527335587183497	80	-0.463379838176856	0.644399266715916	0.00278082821510163	0.791399288881314
**ACC**	ATAD2B	cg08192560	-0.161772936674784	80	-1.43849966453987	0.154344663881392	0.0261704830403836	0.321852235719006
**ACC**	ATAD2B	cg14009731	-0.219414035854721	80	-1.97343944529648	0.0520335729961402	0.0481425191300568	0.14854132977365
**ACC**	ATAD2B	cg15717719	0.214883490621014	80	1.93069664850358	0.057202033032048	0.0461749145414714	0.159001499185162
**ACC**	ATAD2B	cg16929802	0.159061272212649	80	1.41375593158261	0.161463922527123	0.0253004883179065	0.331729153283376
**ACC**	ATAD2B	cg17933838	0.108067848803268	80	0.953877890645606	0.343130430136802	0.011678659944966	0.542440330868978
**ACC**	ATAD2B	cg19614157	0.0168977542423191	80	0.148298365334833	0.882494982395385	0.000285534098433812	0.937636606078567
**ACC**	ATAD2B	cg20806130	-0.453884779101672	80	-4.46975631023863	2.65503489007039e-05	0.206011392700174	0.000306447751095157
**ACC**	ATAD2B	cg27610867	-0.383611588373382	80	-3.64504381198393	0.000483396998992327	0.147157850734349	0.00364111019941993
**ACC**	ATAT1	cg01245721	-0.0384418996983706	80	-0.337575824398417	0.736601409877718	0.00147777965241958	0.851458693611103
**ACC**	ATAT1	cg05093836	-0.225832201038747	80	-2.03422115085713	0.045373027946512	0.051000183026005	0.134428583433757
**ACC**	ATAT1	cg06814856	-0.128779869314369	80	-1.13952737806751	0.258015754616357	0.016584254740626	0.452542670663861
**ACC**	ATAT1	cg07142893	-0.167025371903685	80	-1.48652348347384	0.141224058506559	0.0278974748595641	0.303201443358119
**ACC**	ATAT1	cg07816366	-0.130329740443895	80	-1.15347717821023	0.252282522078423	0.016985841244173	0.446148363777423
**ACC**	ATAT1	cg08081524	-0.197681890002625	80	-1.76957187948509	0.0807576462545719	0.0390781296350098	0.204360820139489
**ACC**	ATAT1	cg08188471	-0.179443904959961	80	-1.6005943925214	0.113561959687181	0.0322001150272797	0.260741369810062
**ACC**	ATAT1	cg08635134	-0.110553314656601	80	-0.97608460727234	0.332079176604704	0.0122220353815615	0.531758844708098
**ACC**	ATAT1	cg09532691	-0.0453696926157025	80	-0.398527814881955	0.691344129465194	0.00205840900804333	0.82259066924966
**ACC**	ATAT1	cg12907138	-0.158726029137865	80	-1.41069915679476	0.16236068810185	0.0251939523258744	0.333023686582831
**ACC**	ATAT1	cg13542073	0.0552784005043015	80	0.485808807069148	0.628482338951361	0.00305570156231396	0.780627089193184
**ACC**	ATAT1	cg13964084	-0.0951853166727533	80	-0.839057445610008	0.40403447693532	0.00906024451009232	0.600536083966188
**ACC**	ATAT1	cg15381130	-0.20808772712759	80	-1.86682692556404	0.065732403678807	0.0433005021811263	0.175978615589136
**ACC**	ATAT1	cg15729859	-0.156428784339589	80	-1.38976608807438	0.168605089122296	0.0244699645699616	0.341779302790211
**ACC**	ATAT1	cg18298197	0.0225722570487027	80	0.198121230231881	0.843472347468098	0.000509506788272709	0.916388578835445
**ACC**	ATAT1	cg20629923	-0.0986506393032611	80	-0.869899102397926	0.387059284946455	0.00973194863494212	0.584995531806118
**ACC**	ATAT1	cg22759096	-0.160355441690568	80	-1.42556099636012	0.158036443068036	0.025713867679777	0.327062704230774
**ACC**	ATAT1	cg23328390	-0.225615543411976	80	-2.032164850378	0.0455857776030352	0.0509023734290814	0.134876202139372
**ACC**	ATAT1	cg26934609	-0.189180094805121	80	-1.69057633316165	0.0949622725562346	0.0357891082704747	0.229482325771612
**ACC**	ATF7IP	cg00236831	0.140507653746964	80	1.24530357688452	0.216796547354829	0.0197424007614768	0.404089191962086
**ACC**	ATF7IP	cg11409060	-0.280669726980338	80	-2.56600911048133	0.0122290484246101	0.0787754956432174	0.0499080155882969
**ACC**	ATF7IP	cg12526346	-0.242849117290568	80	-2.19675422941016	0.0310462049781817	0.0589756937688082	0.101232646075642
**ACC**	ATF7IP	cg12948116	-0.169849669763642	80	-1.51239999933232	0.134525786677512	0.0288489103188182	0.292948691322091
**ACC**	ATF7IP	cg17439009	-0.141874325277617	80	-1.25766382631146	0.212314251137807	0.0201283241729791	0.398532353368752
**ACC**	ATF7IP	cg19789919	0.019029603010216	80	0.16701433155747	0.867796600317148	0.00036212579072642	0.929778656967169
**ACC**	ATF7IP	cg21275967	-0.316726471433764	80	-2.93011485283645	0.0044570293170021	0.100315657706883	0.0226280844930406
**ACC**	ATF7IP	cg26637901	-0.0931041180579926	80	-0.820549476977507	0.414435989089275	0.00866837679935662	0.610005739224095
**ACC**	ATM	cg01756564	-0.320381023086932	80	-2.96776672384651	0.00399559506173419	0.102643999954229	0.020723603055707
**ACC**	ATM	cg01800843	-0.178571786308801	80	-1.59255843867524	0.115356450860114	0.0318878828655159	0.263446446048958
**ACC**	ATM	cg01991180	-0.108868535289994	80	-0.961029730515965	0.339545417259057	0.0118523579761887	0.538808779858729
**ACC**	ATM	cg02105423	0.020514556747454	80	0.180052396127914	0.857584445253251	0.00042084703854451	0.924234239360958
**ACC**	ATM	cg03165700	-0.413951291868374	80	-3.99034571707817	0.000149297726238568	0.171355672039496	0.00135714738431914
**ACC**	ATM	cg03569616	-0.144512087238851	80	-1.28154070677563	0.203849557970464	0.0208837433581292	0.38778023016883
**ACC**	ATM	cg04709886	-0.34512764064381	80	-3.22674782886832	0.00183960614514605	0.119113088336363	0.0111032912138639
**ACC**	ATM	cg04826772	0.204093799336909	80	1.82942272243621	0.0712073889587369	0.0416542789277746	0.186666249381857
**ACC**	ATM	cg05033322	-0.433429693252053	80	-4.22035437959123	6.60565521437372e-05	0.187861298992569	0.000676247324315027
**ACC**	ATM	cg05637308	-0.00837093982676707	80	-0.073457272586244	0.941632732764352	7.00726335833551e-05	0.968883115264264
**ACC**	ATM	cg06053805	-0.407911000837771	80	-3.92039370530898	0.000190351432661229	0.166391384604472	0.00166402959348777
**ACC**	ATM	cg06081482	-0.299104110129478	80	-2.75054660808894	0.00741253993341383	0.0894632686963471	0.0338748241543791
**ACC**	ATM	cg06750635	-0.192718080496734	80	-1.72340087715132	0.0888295705776764	0.0371402585503455	0.218833768860692
**ACC**	ATM	cg09808176	-0.037995667363892	80	-0.333651556474562	0.739549428333802	0.00144367073842753	0.853281545833145

**ACC**	ATM	cg10610482	-0.336229398262473	80	-3.13279176057591	0.00244906266943431	0.113050208255945	0.0139945924273522
**ACC**	ATM	cg10976287	-0.270486495416257	80	-2.46541057826127	0.0159113568541535	0.0731629442025688	0.0610419220392554
**ACC**	ATM	cg11588932	-0.180941486553243	80	-1.61440265215024	0.110530882877159	0.0327398215560974	0.255873813496375
**ACC**	ATM	cg12019961	-0.272593006955417	80	-2.48614566561263	0.015079663900428	0.0743069474409961	0.0586172674496527
**ACC**	ATM	cg12106634	-0.0443651652428986	80	-0.389686437593189	0.697844195515358	0.0019682678870297	0.826728359955793
**ACC**	ATM	cg12848864	-0.352872772097525	80	-3.30933092006167	0.00142428693880496	0.124519193287792	0.00900894203735208
**ACC**	ATM	cg14761454	-0.180198878278307	80	-1.60755408334593	0.11202598316495	0.0324716357327602	0.258237110395979
**ACC**	ATM	cg15370815	0.390958556129381	80	3.72731044515574	0.000367435648352067	0.15284859261077	0.00289642081733712
**ACC**	ATM	cg15504467	-0.27163765294083	80	-2.4767370401213	0.0154520864211786	0.0737870144952029	0.0597425381963705
**ACC**	ATM	cg16693212	-0.0782459033115982	80	-0.688716558412967	0.493071606240299	0.00612242138504797	0.678454442425107
**ACC**	ATM	cg16788234	0.275244683639879	80	2.51230195429463	0.0140863042240576	0.0757596358720173	0.0556061359676226
**ACC**	ATM	cg17114932	-0.370981651159126	80	-3.50550276594234	0.000763329258720415	0.137627385496751	0.00535362751107969
**ACC**	ATM	cg17420659	-0.0731522499919943	80	-0.643632819199714	0.521725994216784	0.00535125167889122	0.701656045289641
**ACC**	ATM	cg18042593	-0.0495174100045276	80	-0.435047198881674	0.664745111179567	0.00245197389355649	0.805388359561794
**ACC**	ATM	cg18391757	-0.129144552228136	80	-1.14280898215093	0.256658829265972	0.0166783153702059	0.451008803124427
**ACC**	ATM	cg18457775	-0.200460745361829	80	-1.79548105180578	0.076500479989823	0.0401845104310201	0.19671696493995
**ACC**	ATM	cg19288979	0.222107409100871	80	1.99891304679798	0.0491468696092127	0.0493317011775016	0.142475557085116
**ACC**	ATM	cg20342375	-0.394371105717479	80	-3.76580623223182	0.000322777749180689	0.155528569024827	0.00259778318019476
**ACC**	ATM	cg22477592	-0.0649873786111838	80	-0.571469971319251	0.569345503269496	0.00422335937875335	0.737604317901465
**ACC**	ATM	cg22678707	-0.224165855786584	80	-2.01841400119216	0.0470306158069311	0.0502503309005315	0.137965222145797
**ACC**	ATM	cg22837512	-0.306504824645326	80	-2.82556593923061	0.00600863135395048	0.093945207530862	0.0287051994319101
**ACC**	ATM	cg23275463	-0.354746933938564	80	-3.32942998365548	0.00133747907649552	0.125845387138812	0.008547428512026
**ACC**	ATM	cg23389069	-0.191370263709489	80	-1.71088802261824	0.0911278859746046	0.0366225778322394	0.222804788531913
**ACC**	ATM	cg24030675	-0.252946721981163	80	-2.29420556143731	0.0245051911703643	0.063982044161016	0.0849232377898843
**ACC**	ATM	cg24568245	-0.321148339948266	80	-2.97569085399356	0.0039042950702736	0.103136256251527	0.0203709410259466
**ACC**	ATM	cg25400013	-0.370035731400979	80	-3.49514437108123	0.00078932050572482	0.136926442513457	0.00550712102442661
**ACC**	ATM	cg25740661	0.160479408546993	80	1.42669218351491	0.157710986377867	0.0257536405675928	0.326519453739981
**ACC**	ATM	cg25918541	-0.0954795713269009	80	-0.841675123816487	0.40257630078088	0.00911634854076876	0.59911276624991
**ACC**	ATM	cg27453365	-0.0426869445902255	80	-0.374918157625531	0.708752009478655	0.00182217523844898	0.834039054658778
**ACC**	ATM	cg27599391	-0.111316385410281	80	-0.982906061018238	0.328732052494949	0.0123913376608103	0.528397481131288
**ACC**	ATR	cg00911981	-0.00507385230839215	80	-0.0445234464220128	0.964602425928874	2.57439772473764e-05	0.981419038761078
**ACC**	ATR	cg00969988	-0.260557057400997	80	-2.36817948835824	0.0203851223511235	0.0678899801614666	0.0738733967570366
**ACC**	ATR	cg11458642	0.185751373307028	80	1.65883068504395	0.101218160782202	0.0345035726854468	0.24032980099776
**ACC**	ATR	cg11936145	-0.0178706648366601	80	-0.156839493702809	0.875781860522937	0.000319360661704239	0.934340215710097
**ACC**	ATR	cg15135999	0.105141463031835	80	0.927754883485568	0.356433803358845	0.0110547272484746	0.555245625633232
**ACC**	ATR	cg15291273	-0.17482745195646	80	-1.55810084040714	0.123310237748823	0.0305646379575882	0.275892914450157
**ACC**	ATR	cg15480357	-0.0430745329984805	80	-0.378328634405047	0.706227526968569	0.00185541539303718	0.8325021112784
**ACC**	ATR	cg16031872	-0.0256508389784097	80	-0.225159284255801	0.822451854744913	0.000657965540296302	0.904585595761027
**ACC**	ATR	cg17945282	-0.214347798528371	80	-1.92565144203789	0.0578399050614353	0.0459449787339592	0.160242078858593
**ACC**	ATR	cg20032934	-0.244567498883456	80	-2.2132834890355	0.0298392103266823	0.0598132615101091	0.0982690512299517
**ACC**	ATR	cg20636078	-0.182092722897539	79	-1.61443876948891	0.110576774566382	0.03315775973224	0.255934354842673
**ACC**	ATR	cg24631870	-0.173769971241217	80	-1.54838197492503	0.12563087835292	0.0301960029051734	0.279381640574783
**ACC**	ATR	cg24856922	0.132840628972484	80	1.17609503546911	0.243180283362155	0.0176466327058052	0.435771046524237
**ACC**	AURKB	cg02245743	0.0465911783336678	80	0.409280392207241	0.683470160734519	0.00217073789851963	0.817696199621389
**ACC**	AURKB	cg04612488	0.0361820049499024	80	0.317703832003513	0.751569571550491	0.00130913748219477	0.860883844336197
**ACC**	AURKB	cg05617798	0.00188646715295612	80	0.0165537115405305	0.986835470400218	3.55875831918237e-06	0.99309063676284
**ACC**	AURKB	cg05811247	0.0177218914088333	80	0.155533391738889	0.876807847923817	0.000314065435106479	0.934798675054619
**ACC**	AURKB	cg11009596	-0.0297945030792357	80	-0.26156182507625	0.794357662149045	0.000887712413738584	0.887431032802797
**ACC**	AURKB	cg12844497	0.0504746277990677	80	0.443478343913109	0.658663403038876	0.00254768805145442	0.801379747756277
**ACC**	AURKB	cg13678641	0.0483615375955457	80	0.42486790964656	0.672117882799567	0.00233883831860538	0.810169800342581
**ACC**	AURKB	cg21724796	0.0382494766787879	80	0.335883588243475	0.737872182676643	0.00146302246620114	0.852429913168073
**ACC**	BAP1	cg00794529	-0.161951896345125	80	-1.4401338280435	0.153883180200703	0.0262284167297822	0.321227577227399
**ACC**	BAP1	cg02503646	0.101992042015269	80	0.899668112538652	0.371101922517574	0.0104023766344444	0.569798206015871
**ACC**	BAP1	cg03075736	-0.125764453869364	80	-1.11241100230788	0.269422468119883	0.0158166978570595	0.465375077432947
**ACC**	BAP1	cg05442719	0.0711187031089933	80	0.625648316709741	0.533395702512261	0.00505786993190513	0.710445413755866
**ACC**	BAP1	cg05908979	-0.118217939092941	80	-1.04468387032464	0.299437712925858	0.0139754811233823	0.497734181734455
**ACC**	BAP1	cg09096430	-0.180873528892977	80	-1.61377580661415	0.110667057107391	0.0327152334541985	0.256044285052713

**ACC**	BAP1	cg10449522	-0.108616205414502	80	-0.958775682401301	0.340672654987433	0.0117974800786453	0.539947937697495
**ACC**	BAP1	cg10956904	-0.190865256098538	80	-1.7062022274231	0.092001006071193	0.0364295459855606	0.224326164121686
**ACC**	BAP1	cg11234564	0.0857474259462011	80	0.755212125568995	0.45242561329359	0.00735262105639924	0.64283754014651
**ACC**	BAP1	cg12859382	0.00710079851231578	80	0.0623108249850511	0.950476771450277	5.0421339512506e-05	0.973809955988447
**ACC**	BAP1	cg16841708	0.0103862775419469	80	0.0911441317564645	0.927614759864022	0.00010787476117835	0.96181242507946
**ACC**	BAP1	cg24291822	-0.168326458721511	80	-1.49843945813704	0.138107762282231	0.0283337967057245	0.29843491177978
**ACC**	BAP1	cg24747396	0.0346619110378618	80	0.304339914204987	0.76168992801492	0.00120144807679665	0.867555471515684
**ACC**	BAP1	cg25749966	-0.372953318586038	80	-3.52713446059828	0.000711640300953097	0.139094177844339	0.00505490737512283
**ACC**	BAZ1A	cg05294307	0.0936433033355951	80	0.825343366279337	0.411726431534732	0.00876906825960228	0.607373585186554
**ACC**	BAZ1A	cg07414384	-0.102973426276212	80	-0.908417196690265	0.366492335301134	0.0106035265190624	0.565389430997963
**ACC**	BAZ1A	cg23422263	-0.214465660224869	80	-1.92676131985665	0.0576990669655231	0.0459955194156891	0.159965830916461
**ACC**	BAZ1A	cg26967526	0.125652724047566	80	1.11140686944214	0.269851536698838	0.0157886070605738	0.465915022572802
**ACC**	BAZ1B	cg00525989	0.103874725516715	80	0.916454680707899	0.362289895557266	0.0107899586011729	0.561187928297996
**ACC**	BAZ1B	cg04742505	0.106538497417013	80	0.94022272488773	0.350043601122977	0.0113504514318749	0.549048614504467
**ACC**	BAZ1B	cg05166819	0.138948629894112	80	1.23121254530007	0.221990724475629	0.0193067217494509	0.410442330399103
**ACC**	BAZ1B	cg06059318	-0.0766715796306718	80	-0.674776651950912	0.501838773979112	0.00587853112306244	0.685628639842959
**ACC**	BAZ1B	cg06394887	0.343519364139116	80	3.2096942225111	0.00193844417110462	0.118005553538542	0.0115901599080286
**ACC**	BAZ1B	cg14093368	0.0429782554550547	80	0.377481450929537	0.706854317736767	0.00184713044195994	0.832851568254662
**ACC**	BAZ1B	cg15636799	-0.381679082800993	80	-3.6235408509121	0.000519015187945037	0.145678922247807	0.00386952414547862
**ACC**	BAZ1B	cg19160975	-0.310503169017062	80	-2.86633023378965	0.00535249662597013	0.0964122179696379	0.0261898309655148
**ACC**	BAZ1B	cg20612831	0.150903049880001	80	1.33950817663508	0.18434560286642	0.0227717304630862	0.362782164914238
**ACC**	BAZ1B	cg21340129	-0.104429239144176	80	-0.92140077980913	0.359719160541732	0.0109054659882315	0.558540425036595
**ACC**	BAZ1B	cg25710357	0.147942596665452	80	1.31263532251831	0.1932049056486	0.0218870119081167	0.3741736769655
**ACC**	BAZ2A	cg14634319	0.245299247872583	80	2.22032896694663	0.0293373679879151	0.0601717210068547	0.0970890034990371
**ACC**	BAZ2A	cg18206859	0.189161712150337	80	1.69040596313352	0.0949949835413008	0.0357821533436471	0.229547112803999
**ACC**	BAZ2A	cg18955060	-0.349842462643813	80	-3.27692906403023	0.00157546193059189	0.122389748668688	0.00979473231267207
**ACC**	BAZ2A	cg25683012	0.132966888463843	80	1.17723297429867	0.242728633888359	0.0176801934277559	0.43520861401104
**ACC**	BRD7	cg03021120	-0.406616727942406	80	-3.9054858205941	0.000200402759048301	0.165337163442589	0.00173977594435928
**ACC**	BRD7	cg16433860	-0.487027348019461	80	-4.89319053952725	5.32648285826148e-06	0.237195637718869	7.52302845816256e-05
**ACC**	BRD7	cg16834187	-0.255421555957501	80	-2.31821060683134	0.023093980406295	0.065240171247751	0.0811863668366086
**ACC**	BRD8	cg01096096	0.264478083637632	80	2.40647630505296	0.0185042236639631	0.0699486567246342	0.0685572872630835
**ACC**	BRD8	cg03204906	-0.527063193103019	80	-5.44224374922348	6.03310156647805e-07	0.27779560952395	1.09199419093205e-05
**ACC**	BRD8	cg03684156	-0.0931910969503321	80	-0.82132275580297	0.413998197690211	0.00868458055080619	0.609568956016131
**ACC**	BRD8	cg07430605	0.438206038093682	80	4.27784094137599	5.36703715920198e-05	0.192024531821761	0.000563992015078967
**ACC**	BRD8	cg07632946	0.156582580904643	80	1.391166794438	0.168181597780218	0.0245181046427592	0.341265607421838
**ACC**	BRD8	cg08976717	0.245407332795916	80	2.22136997981801	0.0292638488893518	0.0602247589900055	0.0969180685916022
**ACC**	BRD8	cg11110536	0.169377838109283	80	1.50807437768857	0.135627734098605	0.0286888520425744	0.294630810266869
**ACC**	BRD8	cg11346339	0.270291974973523	80	2.46349778268845	0.0159901144331369	0.0730577517350878	0.0612573864205519
**ACC**	BRD8	cg12964860	-0.517664261822279	80	-5.30922305327609	1.0316451784496e-06	0.267976287968005	1.7561445298098e-05
**ACC**	BRD8	cg14473243	0.252747023770503	80	2.29227066323327	0.0246221910090943	0.063881058024847	0.0852148201513248
**ACC**	BRD8	cg22106577	0.333879585365278	80	3.10813979097711	0.00263767388570972	0.11147557752369	0.0148530729109592
**ACC**	BRD8	cg22818036	0.0538716221074104	80	0.473409017002404	0.637260827566553	0.00290215166848363	0.78662402128338
**ACC**	BRD8	cg23298160	-0.208215612714769	80	-1.86802620881946	0.0655628813301488	0.0433537413781865	0.175657762439647
**ACC**	BRD8	cg26008208	-0.00455123756730263	80	-0.0399373612003089	0.968246435452748	2.07137633940268e-05	0.983351782655271
**ACC**	BRD8	cg26630792	0.404525348797031	80	3.88145605367648	0.000217682280722575	0.163640757819359	0.00186276266049192
**ACC**	BRD8	cg27119451	0.130892506637737	80	1.15854454548798	0.25022246843634	0.0171328482939099	0.443865477007747
**ACC**	BRD9	cg02364331	-0.0472776494448022	80	-0.415324111129737	0.67905971951721	0.0022351761370256	0.814782010724367
**ACC**	BRD9	cg02822723	0.496023869386471	80	5.01272284645047	3.34354508847323e-06	0.246039679001126	4.97623391262074e-05
**ACC**	BRD9	cg05193570	-0.022321175605722	80	-0.195916333281129	0.845191767874556	0.000498234880421481	0.91726735835963
**ACC**	BRD9	cg07308931	-0.314675207289616	80	-2.9090449464116	0.00473625822696984	0.0990204860827631	0.0237573326783733
**ACC**	BRD9	cg09497020	-0.094289864054928	80	-0.831092895625516	0.408490906777617	0.0088905784634968	0.604480447430293
**ACC**	BRD9	cg10551270	-0.443211611455455	80	-4.33856791539339	4.30298933351863e-05	0.196436532528941	0.000466156179100053
**ACC**	BRD9	cg10600960	-0.207119839390924	80	-1.85775352409266	0.0670269292387185	0.0428986278693223	0.178620612948646
**ACC**	BRD9	cg13722821	-0.357756652009379	80	-3.36180333155149	0.00120804459929516	0.12798982205696	0.00786168826815856
**ACC**	BRD9	cg17977043	-0.441824400200029	80	-4.32168839420928	4.57631208865247e-05	0.195208800612116	0.000491291267916343
**ACC**	BRD9	cg20079193	0.227302749863173	80	2.04818657578969	0.0439505867341693	0.05166654009536	0.131261207939111
**ACC**	BRD9	cg21160852	0.399084153883776	80	3.81927648895397	0.000269263097290359	0.159268161881129	0.00223054556390226

**ACC**	BRD9	cg21758832	-0.301691050349562	80	-2.77670681469099	0.00689206035192868	0.091017489861022	0.0320249040149289
**ACC**	BRD9	cg21910709	-0.0629495676180808	80	-0.553477921694342	0.581539798552731	0.00396264806330332	0.746946473964554
**ACC**	BRD9	cg24953233	-0.324856545524018	80	-3.0140782685194	0.00348873283379994	0.105531775169798	0.0185968536434584
**ACC**	BRD9	cg25523559	-0.195335625133772	80	-1.74773064256438	0.0844970819262429	0.0381560064464014	0.211111089423199
**ACC**	BRD9	cg25951128	-0.340497159162195	80	-3.17773422276229	0.00213719418093271	0.115938315397525	0.0125271913887492
**ACC**	BRD9	cg26504197	0.0445988186072063	80	0.391742837875299	0.696330316638474	0.00198905462115849	0.825905573382327
**ACC**	BRDT	cg00308185	0.0995933550234582	80	0.878294829997799	0.382516194137789	0.00991883636482858	0.580875454624984
**ACC**	BRDT	cg01515515	-0.0650455161904002	80	-0.571983378428403	0.568999370208814	0.00423091917647562	0.737337028637249
**ACC**	BRDT	cg02935097	-0.261810131962204	80	-2.3804045240624	0.0197670079168105	0.0685445451980665	0.0721747213874193
**ACC**	BRDT	cg03421440	-0.191151531185729	80	-1.7088583044587	0.0915052527932098	0.0365389078746487	0.223480294406625
**ACC**	BRDT	cg06442199	-0.181523661332413	80	-1.61977364798398	0.109369625679706	0.0329508396235245	0.253888179163495
**ACC**	BRDT	cg09250199	0.161846496943929	80	1.4391713597217	0.154154849235117	0.0261942885730211	0.321596416815
**ACC**	BRDT	cg13299824	-0.038963756563596	80	-0.342165408268568	0.733158598811535	0.00151817432554717	0.849435401533168
**ACC**	BRDT	cg14732540	-0.00486288476148789	80	-0.0426721451529693	0.966073345710377	2.36476482035112e-05	0.982181816619244
**ACC**	BRDT	cg19245011	0.112276846675937	80	0.991494586617902	0.324549690696399	0.0126060902994918	0.523922671414774
**ACC**	BRPF1	cg04879506	-0.134094450831722	80	-1.1873979491125	0.238720804794852	0.0179813217438611	0.430483224112442
**ACC**	BRPF1	cg13694772	0.0380644003302962	80	0.334255996620812	0.739095100635851	0.00144889857250505	0.853023029180247
**ACC**	BRPF1	cg14712964	-0.0939889423338388	80	-0.828416823198036	0.409994931403164	0.00883392128103368	0.605910822446386
**ACC**	BRPF1	cg17738494	0.0413002630330934	80	0.36271781534727	0.717809433414834	0.0017057117266027	0.839730929233125
**ACC**	BRPF1	cg20952324	0.253153911849972	80	2.29621337828605	0.0243843015938535	0.0640869030849433	0.0846136026670341
**ACC**	BRPF1	cg25467642	-0.280199407478077	80	-2.56134255057347	0.0123811760859602	0.0785117079510652	0.0503808563882826
**ACC**	BRPF1	cg26227975	-0.0237482291757148	80	-0.208448653838551	0.835429079933086	0.000563978388982271	0.911827125972916
**ACC**	BRPF3	cg01499736	0.0222591972236661	80	0.195372069607849	0.845616311061601	0.000495471861042064	0.917538988266099
**ACC**	BRPF3	cg05882415	-0.054772139026114	80	-0.481346127813359	0.631635634411915	0.00299998721349596	0.782705403772607
**ACC**	BRPF3	cg10562781	0.0574959301318614	80	0.505360737014883	0.614749018932449	0.00330578198172789	0.770818608448417
**ACC**	BRPF3	cg14654948	0.0402194225068802	80	0.353209792224333	0.724896329790619	0.00161760194678695	0.844107163370787
**ACC**	BRPF3	cg18779695	-0.0178383570608406	80	-0.156555858503972	0.876004647564011	0.000318206982630043	0.934385407882598
**ACC**	BRPF3	cg23517469	-0.0837896242538019	80	-0.737845627779994	0.462851007711828	0.00702070113259331	0.652218582678536
**ACC**	BRWD3	cg03164831	-0.0134685387475892	80	-0.118196668875172	0.906219625006018	0.000181401535995311	0.950382851045688
**ACC**	BRWD3	cg05835545	-0.167270655305543	80	-1.4887692928236	0.140632551746877	0.0279794721263457	0.302245178622443
**ACC**	BRWD3	cg09523186	-0.0887400305683749	80	-0.781774849481593	0.436744225236964	0.00787479302527611	0.629758118627725
**ACC**	BRWD3	cg11957438	-0.0658367638977565	80	-0.578971391039378	0.564298352934157	0.00433447948052893	0.734011504809801
**ACC**	BRWD3	cg15511898	-0.10054791850548	80	-0.88679850666014	0.377948813391648	0.0101098839157847	0.576548735068672
**ACC**	BRWD3	cg22618269	-0.0797607862339571	80	-0.702135037563525	0.484712026663902	0.006361783020659	0.671430444688784
**ACC**	BRWD3	cg25063710	0.0340831277572068	80	0.299252097323593	0.765553937017665	0.00116165959771408	0.869983167275306
**ACC**	BRWD3	cg25708850	-0.13123540530015	80	-1.161632697698	0.248972920785323	0.0172227316042945	0.442457423259996
**ACC**	CARM1	cg12317676	0.0908338690157797	80	0.800372658822093	0.425957467401768	0.00825079176037582	0.620619014903655
**ACC**	CARM1	cg17918830	-0.30218814539849	80	-2.78174134620369	0.00679581912386787	0.091317675219379	0.0316495422008009
**ACC**	CARM1	cg22378614	-0.448901056348258	80	-4.40820554129155	3.33317237372431e-05	0.201512158390582	0.000373524649440376
**ACC**	CARM1	cg26504915	-0.263223842096853	80	-2.39421224548412	0.0190889831663917	0.0692867910482291	0.0701899303514805
**ACC**	CBX1	cg01544580	0.0195541996365117	80	0.1716202195019	0.864186343304957	0.000382366723424553	0.927885207302341
**ACC**	CBX1	cg01553295	0.0584786637462455	80	0.514027868969259	0.608704583997767	0.00341975411354645	0.766424931739456
**ACC**	CBX1	cg02835499	-0.121332986410332	80	-1.0726172804304	0.286793070335922	0.0147216935912498	0.484174915662168
**ACC**	CBX1	cg04864609	-0.182242401623252	80	-1.6264069923004	0.107949032700392	0.0332122929494108	0.251611548714475
**ACC**	CBX1	cg11729481	-0.167725822439966	80	-1.49293754685941	0.139539856769407	0.0281319515131631	0.300635970353364
**ACC**	CBX1	cg12245530	-0.0801611719513802	80	-0.705682374834334	0.482515226548264	0.00642581348861874	0.669505863203625
**ACC**	CBX1	cg13342109	-0.337396713665199	80	-3.14506258511408	0.00235994162544404	0.113836542392076	0.0135901472210965
**ACC**	CBX1	cg17778721	-0.0438651847829641	80	-0.385286287957762	0.701087608224461	0.00192415443604358	0.828730465480668
**ACC**	CBX1	cg21511817	0.0701171786829126	80	0.616793822416568	0.539190204894942	0.00491641874645149	0.714606138335238
**ACC**	CBX4	cg04398978	0.253721201368543	80	2.30171254498003	0.0240558936795463	0.064374448023897	0.0837204935761026
**ACC**	CBX4	cg04925705	0.088962798482885	80	0.783753008804329	0.435589319703037	0.00791437951390641	0.628768773996216
**ACC**	CBX4	cg05949524	-0.108130466555027	80	-0.954437132973889	0.342849212313446	0.0116921977974077	0.542105927290898
**ACC**	CBX4	cg07159673	0.136761545993136	80	1.21146058251541	0.22942373165553	0.0187037204624326	0.419454518928562
**ACC**	CBX4	cg10756274	-0.1960976640098	80	-1.75482097333515	0.0832677902185445	0.0384542938301006	0.208824451072258
**ACC**	CBX4	cg21365914	0.0421999508345023	80	0.3706332312534	0.711928398165399	0.00178083585043441	0.836042731904469
**ACC**	CBX4	cg22353329	0.195328715652804	80	1.7476663689212	0.0845082935261767	0.0381533071585741	0.211130336949179
**ACC**	CBX4	cg27487227	-0.00328679558159498	80	-0.0288416699661981	0.977065489884924	1.08030251951923e-05	0.987974689284562

**ACC**	CBX5	cg03689456	-0.433341042676008	80	-4.21929158142278	6.63096919226701e-05	0.18778445926753	0.000678392447847475
**ACC**	CBX5	cg05754589	-0.365962857621611	80	-3.45068707793511	0.000910680873476159	0.133928813158575	0.00620721918769326
**ACC**	CBX5	cg06399839	-0.388773909073298	80	-3.70276165303769	0.000398925922268605	0.151145152376133	0.00309782822888439
**ACC**	CBX5	cg06621757	-0.163666128147705	80	-1.45579469125295	0.14951466327799	0.0267866015028611	0.314987059944719
**ACC**	CBX5	cg08288469	0.136800486086376	80	1.21181209896814	0.229289890485735	0.0187143729934687	0.419337934984772
**ACC**	CBX5	cg09060496	-0.419654043713525	80	-4.056972198303	0.00011819421273038	0.176109516405113	0.00111249855839651
**ACC**	CBX5	cg09158487	-0.339727917191653	80	-3.1696172951353	0.00219062159685027	0.115415057719379	0.0127835283553649
**ACC**	CBX5	cg09653879	-0.371035348299534	80	-3.50609116116141	0.000761877443739031	0.137667229687756	0.00534585495248797
**ACC**	CBX5	cg11005091	-0.333731757928692	80	-3.10659111638033	0.00264996345436978	0.111376886250175	0.0149136408138061
**ACC**	CBX5	cg11338003	-0.314467040614737	80	-2.9069092631475	0.00476544460906347	0.0988895196329904	0.023868336203804
**ACC**	CBX5	cg12263485	-0.0729508525089973	80	-0.641851321974042	0.522875962198119	0.00532182688178948	0.702520035979352
**ACC**	CBX5	cg12600134	-0.262788027038986	80	-2.38995387408461	0.0192958451559886	0.0690575471550428	0.0707899541648185
**ACC**	CBX5	cg13445218	-0.494471027294516	80	-4.99194092385192	3.62680556060488e-06	0.244501596833694	5.35987294418115e-05
**ACC**	CBX5	cg14017196	-0.424750777040348	80	-4.11701143284692	9.5581841244528e-05	0.18041322259638	0.000926797177835824
**ACC**	CBX5	cg14394617	0.0563433440970965	80	0.495197482866997	0.621870945222004	0.00317457242404382	0.775989024757792
**ACC**	CBX5	cg15708001	-0.381313644692573	80	-3.61948089602873	0.000526014281570914	0.145400095628734	0.00391347964052171
**ACC**	CBX5	cg20545544	-0.448982796283257	80	-4.40921086778602	3.32085486184062e-05	0.201585551358333	0.000372305206977993
**ACC**	CBX5	cg20927027	0.0143907394827851	80	0.126291304193495	0.899830529557411	0.000207093382861389	0.947184115359405
**ACC**	CBX5	cg21826008	-0.313300945499587	80	-2.8949543175105	0.00493190854943701	0.0981574824509354	0.0245127072383659
**ACC**	CBX5	cg24505978	-0.155431052263901	80	-1.3806817279505	0.17137149507537	0.0241588120078636	0.345514424981037
**ACC**	CBX5	cg25817259	-0.33765414551005	80	-3.14777090992505	0.00234068364303952	0.114010321980122	0.0135022351569491
**ACC**	CBX6	cg03028106	0.00233893365200619	80	0.0205241156406267	0.983678366496654	5.470610628487e-06	0.99146197920873
**ACC**	CBX6	cg04134624	-0.234694270827472	80	-2.11860821486086	0.0373485233685815	0.0550814007592388	0.116433879415542
**ACC**	CBX6	cg04215348	-0.10273163858631	80	-0.906261400841117	0.367624755475024	0.0105537895666281	0.566554324625736
**ACC**	CBX6	cg04801716	-0.115837492257342	80	-1.02335893848927	0.309342548808888	0.0134183246124697	0.507957494132866
**ACC**	CBX6	cg06060673	0.257534788853534	80	2.33874680608555	0.0219443061617788	0.0663241674698344	0.0781047423502504
**ACC**	CBX6	cg09913544	0.26652266741436	80	2.42649657020612	0.0175839274621177	0.0710343322456658	0.0659367614474108
**ACC**	CBX6	cg14498989	-0.127794386274382	80	-1.13066184331814	0.261706939836652	0.016331405163246	0.457068911973765
**ACC**	CBX6	cg24649346	0.00930480014788426	80	0.0816528247284376	0.93513466787733	8.6579305792067e-05	0.965478856426084
**ACC**	CBX6	cg25836955	0.062782978567345	80	0.552007399695049	0.582541940250999	0.00394170239778771	0.74768085727211
**ACC**	CBX7	cg00545295	0.136559731721547	80	1.20963887480859	0.230118261508335	0.018648560327861	0.420289713006044
**ACC**	CBX7	cg01525244	-0.0643773671463653	80	-0.566083372815271	0.572983249728428	0.00414444540069791	0.740500505124587
**ACC**	CBX7	cg02523424	-0.299980117560255	80	-2.75939765494976	0.00723254726361247	0.0899880709314641	0.0332515557596289
**ACC**	CBX7	cg03804568	-0.263646321410069	80	-2.39834181596372	0.0188902681942384	0.0695093827930615	0.0696799205264381
**ACC**	CBX7	cg10339854	-0.30272126865871	80	-2.78714354368739	0.00669392014754227	0.0916401664983389	0.0312591886642597
**ACC**	CBX7	cg22268271	0.123539814400035	80	1.09242587303777	0.278052142897187	0.015262085741995	0.47486573290094
**ACC**	CBX7	cg26672614	-0.318059157772064	80	-2.94382822482137	0.00428354785561398	0.101161627842675	0.0219420768999562
**ACC**	CBX7	cg27062573	0.00754299953459623	80	0.0661914353588018	0.947396976709283	5.6896841978919e-05	0.971983246866634
**ACC**	CDYL2	cg01442398	-0.0561560912968318	80	-0.493546516846294	0.623031304468238	0.00315350658973811	0.776712272032758
**ACC**	CDYL2	cg02461391	-0.053777283295977	80	-0.472577586839682	0.637851321575847	0.00289199619869576	0.786985435999991
**ACC**	CDYL2	cg06919916	0.340607479876886	80	3.1788989018036	0.002129628693775	0.116013455348083	0.0124922586791986
**ACC**	CDYL2	cg10112854	0.183914775813625	80	1.64185200227482	0.104699019339667	0.0338246447625761	0.246151343529529
**ACC**	CECR2	cg03927893	-0.124521254077578	80	-1.10124059089834	0.274222532445505	0.0155055427170528	0.470683776948975
**ACC**	CECR2	cg12632097	0.0124193715211844	80	0.108987948329886	0.913495576028676	0.000154240788981206	0.954311957625298
**ACC**	CECR2	cg13466481	-0.0169383677181544	80	-0.148654900199591	0.882214578848618	0.000286908300955415	0.937492329243779
**ACC**	CECR2	cg26796679	-0.0827783820379524	80	-0.728878881068233	0.468286922866264	0.0068522605328212	0.657259021460269
**ACC**	CHAF1A	cg03006338	-0.152918289738494	80	-1.35782211543518	0.178485958430416	0.0233840033365459	0.355091339542638
**ACC**	CHAF1A	cg04346879	0.168258499235761	80	1.49781685381337	0.138269238426101	0.0283109225650706	0.298650695623114
**ACC**	CHAF1A	cg05297461	0.195839002822362	80	1.7524139107486	0.0836834501401296	0.038352915026457	0.209566743569577
**ACC**	CHAF1A	cg07783855	0.352530698786235	80	3.30566734721181	0.00144066979720418	0.124277893586711	0.00909334411389062
**ACC**	CHAF1A	cg09700233	-0.173838687304365	80	-1.54901334748067	0.125479074771918	0.0302198892037047	0.279147792124641
**ACC**	CHAF1A	cg18804734	0.180237947121738	80	1.60791431767679	0.111946937796368	0.0324857175826586	0.258099700045364
**ACC**	CHAF1A	cg23811775	0.204690745915683	80	1.83500717267186	0.0703665308374673	0.0418983014635188	0.185104450004055
**ACC**	CHAF1A	cg24085258	-0.259076957636063	80	-2.35375612074855	0.0211364448623901	0.0671208699779586	0.0759802500635264
**ACC**	CHAF1B	cg00660452	0.233713299576604	80	2.10924011353194	0.0381739709913511	0.0546219063989832	0.118281989058875
**ACC**	CHAF1B	cg02544117	0.0548365892752173	80	0.481914234007527	0.631233833445457	0.00300705152333887	0.782406909151186
**ACC**	CHAF1B	cg02908490	-0.130699456538688	80	-1.15680611863937	0.250927842979891	0.0170823479395084	0.444551037707582

**ACC**	CHAF1B	cg04634639	-0.037294274269412	80	-0.327483750310722	0.744190735042597	0.00139086289328213	0.856287147491551
**ACC**	CHAF1B	cg07872789	0.0781511550692982	80	0.687877461105719	0.493596959600448	0.0061076030386655	0.678893263470541
**ACC**	CHAF1B	cg13854874	0.0749891955056002	80	0.659885529115363	0.511296337701792	0.00562337944257713	0.693501652779587
**ACC**	CHAF1B	cg25942896	-0.133333236872333	80	-1.18053509281122	0.241421423826155	0.0177777520548536	0.433674423190982
**ACC**	CHD1	cg03255775	0.245174321550085	80	2.21912585699673	0.0294225365821843	0.0601104479475444	0.097279816858959
**ACC**	CHD1	cg03833628	-0.314443130204944	80	-2.90666398441952	0.00476880720638041	0.0988744821330834	0.0238821029878433
**ACC**	CHD1	cg08865521	-0.0887888591103473	80	-0.782208432474395	0.436490933407814	0.0078834615021171	0.629567926311322
**ACC**	CHD1	cg11365324	-0.261500132645333	80	-2.37737896086811	0.0199184106555928	0.0683823193735266	0.0725810500979523
**ACC**	CHD1	cg12662497	-0.403347340186034	80	-3.86795294020853	0.000228008845467236	0.162689076835148	0.00193793096350548
**ACC**	CHD1	cg16890600	0.253554721417818	80	2.300098465976	0.0241518780224727	0.0642899967532671	0.0839831574682745
**ACC**	CHD1	cg21082921	-0.472024097202132	80	-4.6983462816057	1.12521080125218e-05	0.222806748339487	0.000145189333343195
**ACC**	CHD4	cg00079216	-0.0955722984517641	80	-0.842500066897993	0.402117434104055	0.00913406423135308	0.598670385790115
**ACC**	CHD4	cg00824109	-0.206769514249226	80	-1.85447083006405	0.0675005207665771	0.042753632022861	0.179550554349829
**ACC**	CHD4	cg04344119	0.0325112160846576	80	0.285435652986004	0.776076804240609	0.0010569791713033	0.876079155310879
**ACC**	CHD4	cg05147525	-0.314915070299948	80	-2.91150639551546	0.00470282456113256	0.0991715015020214	0.0236216124682816
**ACC**	CHD4	cg11565429	-0.017202848632721	80	-0.150976725642072	0.880388905293109	0.000295938001080312	0.936524517624155
**ACC**	CHD4	cg19509514	-0.189910330937522	80	-1.69734563841133	0.0936699959559598	0.0360659337967991	0.227294967729286
**ACC**	CHD4	cg20308811	-0.265360126675254	80	-2.41510884049626	0.018102256021046	0.0704159968291067	0.0674350861897837
**ACC**	CHRAC1	cg00296189	-0.570561379516965	80	-6.09634768646831	4.02761347572927e-08	0.325540287796302	9.65262362079122e-07
**ACC**	CHRAC1	cg07270064	-0.54344956728362	80	-5.68085898294985	2.27579121439876e-07	0.295337432180754	4.54538900469672e-06
**ACC**	CHRAC1	cg08877624	-0.313114363717118	80	-2.89304280860343	0.00495901682894007	0.0980406047659758	0.0246128464457422
**ACC**	CHRAC1	cg12002314	-0.0290000394520461	80	-0.254581387706635	0.799725113983864	0.000841002288220231	0.890600886355656
**ACC**	CHRAC1	cg18770186	0.0898658042258307	80	0.791772837195928	0.430925555118266	0.00807586276915533	0.624965086780545
**ACC**	CHRAC1	cg19486048	0.244557120856845	80	2.21318359554185	0.0298463794112488	0.0598081853617896	0.0982885001870502
**ACC**	CHRAC1	cg22610305	-0.0854183040591643	80	-0.752292069906182	0.454169054021934	0.00729628666834385	0.64446723799468
**ACC**	CHRAC1	cg23548819	-0.401790187985799	80	-3.85013905689643	0.000242350897754849	0.161435355161663	0.00203953329077679
**ACC**	CLOCK	cg05960024	-0.203750075580715	80	-1.82620814462023	0.0716952091159623	0.0415140932991469	0.18760998008568
**ACC**	CLOCK	cg06085476	-0.068637446321584	80	-0.603714906317784	0.547807663980548	0.00471109903754832	0.721085438438287
**ACC**	CLOCK	cg07236904	-0.101080928738322	80	-0.891547873994881	0.375412867478877	0.0102173541546018	0.574040037493458
**ACC**	CLOCK	cg07500410	-0.084415041163075	80	-0.743392384982763	0.459506432455827	0.00712589917456364	0.649411270895968
**ACC**	CLOCK	cg11774413	-0.194755757017068	80	-1.74233751111254	0.0854421123389016	0.037929804891291	0.212810479041572
**ACC**	CLOCK	cg18928811	0.053137452032803	80	0.46693893700394	0.641862143142935	0.00282358880853844	0.789630463145797
**ACC**	CLOCK	cg22523077	-0.0241466374867332	80	-0.211947682149248	0.832707875262637	0.000583060101915711	0.910232945375207
**ACC**	CLOCK	cg23506964	-0.119546638721107	80	-1.05659477598336	0.29400033237752	0.0142913988295148	0.491774788358492
**ACC**	CREBBP	cg00039463	-0.184212442200821	80	-1.64460260683998	0.104128617992657	0.0339342238615907	0.245204676962125
**ACC**	CREBBP	cg00747661	0.04619000631718	80	0.405748727438565	0.686052534569869	0.00213351668358113	0.819266852299599
**ACC**	CREBBP	cg03039399	-0.104010277779716	80	-0.917663687212615	0.361660433367657	0.0108181378838137	0.560570068806606
**ACC**	CREBBP	cg03264209	0.0102194210199774	80	0.0896797385543081	0.928774577562822	0.000104436565983556	0.962340907690556
**ACC**	CREBBP	cg08506924	0.0992362342448146	80	0.875114069917893	0.384233429920503	0.0098478301870917	0.582376484573815
**ACC**	CREBBP	cg24567591	-0.0209551853561066	80	-0.183921391426812	0.854558616058078	0.000439119793308786	0.922430039928155
**ACC**	CTCF	cg01866162	-0.278131432026408	80	-2.54084756508238	0.0130697339663024	0.0773570934810601	0.0525396281611476
**ACC**	CTCF	cg04487155	-0.16561539416728	80	-1.47361925121527	0.144660666490566	0.0274284587851834	0.308118822937441
**ACC**	CTCF	cg06241380	-0.395450426521181	80	-3.77801993303109	0.000309725997377698	0.156381039835784	0.00250982773096057
**ACC**	CTCF	cg07967402	-0.0619497808697537	80	-0.544653254025877	0.587565986856077	0.0038377753498105	0.751350024546853
**ACC**	CTCF	cg08324636	0.267148287173	80	2.43262957582379	0.0173103056564006	0.0713682073394675	0.0651620012694294
**ACC**	CTCF	cg10218542	-0.371632679209531	80	-3.51263926524625	0.000745895969359282	0.138110848256454	0.00525742783730506
**ACC**	CTCF	cg10481400	0.00739387641673285	80	0.0648827758173364	0.948435489717448	5.46694084659182e-05	0.972602397636192
**ACC**	CTCF	cg23858565	-0.126358199928511	80	-1.11774781083552	0.267150047106441	0.0159663946891734	0.462842890568378
**ACC**	CXXC1	cg02159718	-0.0268550685503013	80	-0.235737291725409	0.814262412261438	0.000721194706841383	0.900077157302333
**ACC**	CXXC1	cg07675399	0.0717803667987647	80	0.631499138103689	0.529584530371344	0.00515242105776521	0.707719907163492
**ACC**	CXXC1	cg11871295	0.228499958424971	80	2.05956706358951	0.0428199802417669	0.0522122310002137	0.128709769441985
**ACC**	CXXC1	cg13423076	-0.268833708880008	80	-2.44916842503741	0.01659131124494	0.0722715630301811	0.0630165250946616
**ACC**	CXXC1	cg15632775	0.353970088436129	80	3.32109331625492	0.00137286325688455	0.125294823507481	0.00873909861763169
**ACC**	CXXC1	cg20016845	-0.0498275693111686	80	-0.43777893981311	0.66277211155963	0.00248278666345931	0.80389647267199
**ACC**	CXXC1	cg27419474	-0.126154376096417	80	-1.11591562977469	0.267928672996262	0.0159149266082762	0.463622134800695
**ACC**	DAXX	cg00221327	-0.174566945110184	80	-1.55570611550813	0.123878850564924	0.0304736183251021	0.276727388394765
**ACC**	DAXX	cg02173004	-0.282951618837385	80	-2.58867885445347	0.0115137829306842	0.080061618602697	0.0476101300077855

**ACC**	DAXX	cg02328295	-0.0145451948395738	80	-0.12764707011813	0.898761058650979	0.000211562692921165	0.946503557556441
**ACC**	DAXX	cg03085224	-0.274259802049709	80	-2.50257982945269	0.0144484414900092	0.0752184390203456	0.0567248073315054
**ACC**	DAXX	cg03369465	-0.261094290240539	80	-2.37341917193656	0.0201181220798202	0.0681702283962106	0.0731682650340129
**ACC**	DAXX	cg03382121	0.0864468958784638	80	0.761418834559351	0.4487326873581	0.00747306580702196	0.640033564598585
**ACC**	DAXX	cg03902329	0.0400583952310627	80	0.351793361903808	0.725954156932979	0.00160467502848803	0.844749419398153
**ACC**	DAXX	cg04086445	-0.346761281527881	80	-3.24410342684241	0.00174387902236987	0.120243386366858	0.0106335887024801
**ACC**	DAXX	cg04152866	-0.0462112901294699	80	-0.405936091706732	0.685915438461896	0.00213548333543004	0.81919808434077
**ACC**	DAXX	cg04271391	0.126276037681018	80	1.1170092336187	0.267463730770018	0.0159456376924179	0.463213194227895
**ACC**	DAXX	cg04488476	0.0648888511289955	80	0.570599898384453	0.569932330160149	0.00421056300084094	0.738078191575348
**ACC**	DAXX	cg04973788	-0.1430632320607	80	-1.26842232897286	0.208468689464356	0.0204670883676537	0.393688722342819
**ACC**	DAXX	cg05431670	-0.249834395446628	80	-2.26408528124862	0.0263833061281328	0.0624172251481819	0.0896741924559579
**ACC**	DAXX	cg05532204	-0.251002517946969	80	-2.27538122360551	0.025664591923889	0.0630022640157185	0.087911200040818
**ACC**	DAXX	cg06171013	-0.185291230472035	80	-1.65457514773676	0.102081655161082	0.0343328400898407	0.241738159617704
**ACC**	DAXX	cg07689821	-0.348696918416663	80	-3.26471085190598	0.00163628219893446	0.121589540913277	0.0101003399129389
**ACC**	DAXX	cg08375286	0.185993203610392	80	1.6610676562802	0.100766639263597	0.0345934717892567	0.239551285459878
**ACC**	DAXX	cg08567382	-0.196295922754973	80	-1.75666619763808	0.0829503063332384	0.0385320892902262	0.208269950002838
**ACC**	DAXX	cg09097215	-0.196506986547789	80	-1.75863084673192	0.0826133744856664	0.0386149957620931	0.207671982897791
**ACC**	DAXX	cg09432361	0.0630860429517476	80	0.55468265720793	0.580719399871872	0.00397984881530974	0.746452445640983
**ACC**	DAXX	cg09615909	-0.252357012483847	80	-2.28849270256008	0.0248520605312538	0.0636840617497725	0.0858270974415501
**ACC**	DAXX	cg11111423	-0.346954079831919	80	-3.24615390089859	0.00173288297589028	0.120377133512014	0.0105804422213792
**ACC**	DAXX	cg12025215	0.239123421358695	80	2.16099164435975	0.0338047219666539	0.0571800106422879	0.107911552756417
**ACC**	DAXX	cg12832355	-0.253812761697902	80	-2.30260034627679	0.0240032427342561	0.064420918000716	0.0835896085441926
**ACC**	DAXX	cg12923297	-0.324025182178522	80	-3.00545856384184	0.00357830480412678	0.104992318685824	0.0189755040171942
**ACC**	DAXX	cg13443893	0.106576314118157	80	0.940560298196489	0.349871619656545	0.0113585107310122	0.548925702515736
**ACC**	DAXX	cg13826470	-0.340401468184658	80	-3.17672411317128	0.002143775955851	0.11587315954227	0.01256103839041
**ACC**	DAXX	cg14539675	-0.0328026981650049	80	-0.287997495012912	0.774122423318411	0.00107601700690442	0.874888569596893
**ACC**	DAXX	cg14622330	-0.121537169723506	80	-1.07444935698814	0.285976778381018	0.0147712836244004	0.483261204763224
**ACC**	DAXX	cg14746369	-0.0712218433997969	80	-0.626560289304107	0.532800723099659	0.00507255097726519	0.7099933101426
**ACC**	DAXX	cg14910283	-0.209714286417443	80	-1.88208792895396	0.063602485194865	0.0439800819275773	0.171760049059233
**ACC**	DAXX	cg16922466	0.0283209840065081	80	0.248615350542085	0.804320233430045	0.000802078135096887	0.893565880852544
**ACC**	DAXX	cg17079757	0.154851793711995	80	1.37540956196308	0.172992809750943	0.0239790780158222	0.347872683703791
**ACC**	DAXX	cg17504991	0.146524515008801	80	1.29977583060587	0.197555526969995	0.0214694334985645	0.379894565940028
**ACC**	DAXX	cg18187593	-0.0379831248385983	80	-0.333541257573847	0.739632344555132	0.00144271777250455	0.853326589632383
**ACC**	DAXX	cg18279148	-0.33668877982095	80	-3.13761883372528	0.00241363617581019	0.11335933445732	0.0138348373608148
**ACC**	DAXX	cg18486541	0.0898327357672117	80	0.791479113032396	0.431095841667718	0.00806992041542168	0.625101023424016
**ACC**	DAXX	cg18944910	-0.26146013032268	80	-2.37698859964186	0.0199380197742816	0.068361399748353	0.0726286903128142
**ACC**	DAXX	cg18967278	-0.0743022889698111	80	-0.653807216777806	0.515183863708806	0.00552083014615331	0.696564184670088
**ACC**	DAXX	cg20223162	-0.359727792678096	80	-3.38307045866129	0.00112953611129808	0.129404084825055	0.00742970594760486
**ACC**	DAXX	cg20763184	-0.0956132137258532	80	-0.842864075000752	0.401915059835647	0.00914188663898569	0.598472172208375
**ACC**	DAXX	cg21286626	0.110385760137804	80	0.974586991873654	0.332817016039201	0.0121850160412008	0.532499011676887
**ACC**	DAXX	cg24328964	-0.158627646354301	80	-1.40980218928373	0.162624557146186	0.0251627301879051	0.333251186645691
**ACC**	DAXX	cg24496586	-0.197041286091136	80	-1.76360541466648	0.0817653049311706	0.0388252684244487	0.206180609177916
**ACC**	DAXX	cg24513883	-0.295060246589613	80	-2.70978650916657	0.00829545984113349	0.0870605491175231	0.0369481852101877
**ACC**	DAXX	cg24728112	-0.203271605335202	80	-1.82173456161071	0.0723787293761969	0.04131934553555	0.188859566610928
**ACC**	DAXX	cg25642825	-0.24639149711398	80	-2.23085295605555	0.0286015440298041	0.0607087698500686	0.0952653302902821
**ACC**	DAXX	cg26521092	-0.345821646039916	80	-3.23411677309263	0.0017983755482235	0.119592610869757	0.0108984051880111
**ACC**	DAXX	cg27052777	-0.312081219541746	80	-2.8824650779352	0.00511152553841461	0.0973946875906635	0.025222781834749
**ACC**	DAXX	cg27393652	-0.0404607471264558	80	-0.355332586780662	0.723311973888006	0.001637072058031	0.843251535264181
**ACC**	DIDO1	cg00080059	-0.0971449559300968	80	-0.856494539341519	0.394381969000432	0.00943714246266045	0.591136026211819
**ACC**	DIDO1	cg01488865	-0.209882855121153	80	-1.88367043724757	0.0633849846334025	0.0440508128738068	0.171315411686444
**ACC**	DIDO1	cg06646488	-0.115379747559621	80	-1.01926035152988	0.311271238323448	0.0133124861469219	0.510185322886137
**ACC**	DIDO1	cg08610063	-0.127995411098001	80	-1.13247001167935	0.260951099889464	0.0163828252621463	0.456220191581476
**ACC**	DIDO1	cg13815390	0.296747487688101	80	2.72677346828029	0.00791644525600655	0.0880590714491994	0.0356437054199429
**ACC**	DIDO1	cg20969846	-0.0421897963224265	80	-0.370543887274781	0.711994682796441	0.00177997891372784	0.836084324862129
**ACC**	DIDO1	cg21212389	-0.00678951498228769	80	-0.0595791254178406	0.952645203656482	4.6097513694709e-05	0.975031922660075
**ACC**	DIDO1	cg21687703	0.333986256381883	80	3.10925746165712	0.00262883758509141	0.111546819451985	0.0148183313596552
**ACC**	DIDO1	cg22394521	-0.340902735521663	80	-3.18201670882052	0.00210949916807745	0.116214675086153	0.0124041102253528

ACC	DMAP1	cg01604592	0.116880571387878	80	1.03270100775761	0.304976559475695	0.0136610679679569	0.503466311600293
ACC	DMAP1	cg11683482	0.00661125620682074	80	0.0580148056607502	0.953887127918608	4.37087086322257e-05	0.975563901850235
ACC	DMAP1	cg15990015	-0.112475195288467	80	-0.993268586411696	0.323690224601434	0.0126506695551787	0.523122948088305
ACC	DMAP1	cg18017761	0.140578316649983	80	1.24594247721204	0.21656316926315	0.0197622631121428	0.403818676645673
ACC	DMAP1	cg18167340	-0.0970120914028354	80	-0.855311980211102	0.395032058381381	0.00941134587835209	0.591631672212254
ACC	DMAP1	cg21437596	0.0580343844281481	80	0.510109402742358	0.611433972551891	0.00336798977595407	0.768394010963957
ACC	DMAP1	cg23021241	-0.00256513978092427	80	-0.0225090842806507	0.98210009470274	6.57994209568019e-06	0.990670436940716
ACC	DMAP1	cg23451311	0.0362087120571774	80	0.317938647216109	0.751392131411829	0.00131107082883959	0.860766606356327
ACC	DMAP1	cg24485408	-0.187692384374604	80	-1.67679421022309	0.0976383214315457	0.0352284311522243	0.234051569267731
ACC	DNMT1	cg04160663	-0.103614099335324	80	-0.914130270274983	0.363502050741011	0.0107358815810705	0.562438413129921
ACC	DNMT1	cg04762346	-0.243274802876088	80	-2.20084687991376	0.030743439163493	0.0591826297143997	0.100468603498625
ACC	DNMT1	cg06128182	0.0196302834237693	80	0.172288236622612	0.863662966059384	0.000385348027297513	0.927572642427917
ACC	DNMT1	cg06716436	0.00656496301862057	80	0.0576085581344362	0.954209669435302	4.30987394358557e-05	0.975701242131492
ACC	DNMT1	cg10635912	-0.149838671628206	80	-1.32984231624087	0.187496216579685	0.0224516275153054	0.366825457651586
ACC	DNMT1	cg11380607	0.028179467118415	80	0.247372056819815	0.805278708859128	0.000794082367077834	0.894238125429834
ACC	DNMT1	cg13393778	-0.279686730136532	80	-2.55625799174922	0.0125488738838439	0.0782246670144655	0.05092531381884
ACC	DNMT1	cg15043801	0.090346448612862	80	0.79604236620617	0.428454791416432	0.00816248077695651	0.622652519912759
ACC	DNMT1	cg21893394	-0.0313509549830532	80	-0.275238810428747	0.783870004388959	0.000982882378349427	0.880757276588506
ACC	DNMT1	cg23401624	-0.0180792785677459	80	-0.158670959320819	0.874343540967517	0.000326860313530157	0.933472232499887
ACC	DNMT1	cg24477899	-0.0854304652871345	80	-0.75239996308888	0.454104566966922	0.0072983643991763	0.644395557885039
ACC	DNMT1	cg27429799	0.0613661720232356	80	0.539502763916953	0.591096714433859	0.00376580706878534	0.753881857870478
ACC	DNMT3A	cg02208653	-0.0521012916275927	80	-0.457808771989964	0.648379116329967	0.00271454458926346	0.79415569757492
ACC	DNMT3A	cg03463641	0.15335542903568	80	1.36179699611677	0.177233037516583	0.0235178876147174	0.353466478338889
ACC	DNMT3A	cg06112956	-0.0168385543946302	80	-0.147778666986909	0.882903735814794	0.000283536914100919	0.937827537944457
ACC	DNMT3A	cg07150430	0.118663667126046	80	1.04867888701796	0.297606396045423	0.014081065895801	0.495737088563475
ACC	DNMT3A	cg12066181	-0.129124069473082	80	-1.1426246553198	0.256734913244956	0.0166730253172893	0.451048686562288
ACC	DNMT3A	cg13076778	0.00122150207512125	80	0.0107186452047877	0.991475645037207	1.49206731952553e-06	0.995493114974775
ACC	DNMT3A	cg13344237	-0.0148473504896785	80	-0.130299334426929	0.896669412730438	0.000220443816563358	0.945222096722834
ACC	DNMT3A	cg15990840	-0.186524619327226	80	-1.66598444080205	0.0997799612226017	0.0347914336151667	0.237887903872236
ACC	DNMT3A	cg15998962	-0.0726006732746428	80	-0.638753937152223	0.524878499038038	0.00527085775993143	0.703958312429576
ACC	DNMT3A	cg21629895	-0.182538767247853	80	-1.6291429723521	0.107367451729062	0.0333204015483658	0.250676576825049
ACC	DNMT3A	cg21708767	0.015137032978434	80	0.132842145233341	0.894664771813839	0.000229129767390198	0.944029938671375
ACC	DNMT3A	cg22731525	0.120889911566646	80	1.06864217570318	0.288569711979129	0.0146143707185915	0.48610883639818
ACC	DNMT3A	cg25044635	-0.126544527683002	80	-1.11942284477849	0.266439593791372	0.016013517486514	0.462128818322617
ACC	DNMT3A	cg26470599	-0.42423751812468	80	-4.11094384209485	9.76627785442301e-05	0.179977471784589	0.000944619942070103
ACC	DNMT3A	cg26995204	-0.161380317542131	80	-1.43491498803375	0.155360722197825	0.0260436068899992	0.323283994771204
ACC	DNMT3B	cg00300969	0.0970745467107225	80	0.855867857175926	0.394726393361038	0.00942346761909224	0.59142894414553
ACC	DNMT3B	cg13636640	0.0139836188516661	80	0.122717756229797	0.902650340462179	0.000195541596188671	0.948530579521868
ACC	DNMT3B	cg14224313	0.132909145964681	80	1.1767125512804	0.242935115819924	0.0176648410810608	0.435480480761967
ACC	DNMT3B	cg16523653	0.0425913654070903	80	0.374077161218906	0.709375029719478	0.00181402440724028	0.834418789494331
ACC	DNMT3B	cg16953106	0.203671191329106	80	1.82547050174393	0.0718075411819223	0.0414819541774172	0.187783913496923
ACC	DNMT3B	cg17475857	0.0741807942543078	80	0.652732229549657	0.515873021986768	0.00550279023619995	0.697044993664375
ACC	DNMT3B	cg17721173	-0.0769548241318115	80	-0.677284273072989	0.500255484889674	0.00592204495715803	0.684204630733791
ACC	DNMT3B	cg22605822	0.0250177377964083	80	0.2195984909158	0.826764977887541	0.000625887204449836	0.906964037083598
ACC	DNMT3B	cg24403338	0.0897583173409382	80	0.790818115459333	0.431479200425227	0.00805655553187657	0.625402169222148
ACC	DNMT3B	cg25803642	0.0207523430007063	80	0.182140295255799	0.855951291768347	0.000430659740018963	0.92333118116154
ACC	DNMT3L	cg09247319	-0.189223305923857	80	-1.69097682009129	0.0948854154401315	0.0358054595047537	0.229360718663568
ACC	DNMT3L	cg15635600	0.190396094703842	80	1.70185029709403	0.0928180417601221	0.0362506728784744	0.225822884775197
ACC	DNMT3L	cg27187798	-0.112289507213701	80	-0.991607816951613	0.324494787734	0.0126089334302959	0.523881366309277
ACC	DPF1	cg06168449	-0.163231764807489	80	-1.45182516327788	0.150612709541943	0.0266446090421672	0.316498247598755
ACC	DPF1	cg06615189	-0.0403622524526711	80	-0.354466178007176	0.723958475850186	0.00162911142305316	0.843600978424491
ACC	DPF1	cg07480327	-0.216922318327126	80	-1.94991530711543	0.054826566402989	0.0470552921884151	0.154272720611789
ACC	DPF1	cg08057062	-0.00126356182116604	80	-0.0110877188332659	0.991182139082601	1.59658847590843e-06	0.99526328916156
ACC	DPF1	cg13322582	0.0756676791004778	80	0.665890235701609	0.507471278851019	0.00572559766045289	0.690214883069674
ACC	DPF1	cg13917712	-0.128541127532885	80	-1.13737931166001	0.258906710467589	0.0165228214674255	0.453594014821089
ACC	DPF1	cg17121541	-0.130728996874414	80	-1.15707212232041	0.250819819188872	0.0170900706237907	0.444430507789248
ACC	DPF1	cg22145401	-0.165795350238624	80	-1.47526570483243	0.144218586196765	0.0274880981607479	0.307429733506636

ACC	DPF1	cg27400772	0.00209992570317461	79	0.0183067682217756	0.985442104123701	4.40968795885338e-06	0.992381657581867
ACC	DPF2	cg02186298	0.179006801151204	80	1.59656630540436	0.114458641103601	0.0320434348583868	0.262096238954862
ACC	DPF2	cg14699126	-0.0385743835689107	80	-0.338740956306797	0.735726888800258	0.00148798306772145	0.851001765378241
ACC	DPF2	cg15918224	-0.121802597451027	80	-1.07683116858349	0.284917944145874	0.0148358727458169	0.482139499421624
ACC	DPF2	cg15929437	-0.278691647674183	80	-2.54639586886711	0.0128800009865679	0.0776690344833508	0.0519783574596184
ACC	DPF2	cg18224556	-0.0365410028004622	80	-0.320860283334478	0.749185495409234	0.00133524488566339	0.859491762717386
ACC	DPF2	cg19517014	-0.124994017907814	80	-1.105487849429	0.27239045961085	0.015623504512739	0.468761053311944
ACC	DPF3	cg00888802	0.409826487451139	80	3.94250902780008	0.000176325610617241	0.167957749816538	0.00156177553823951
ACC	DPF3	cg05151458	0.339969168008422	80	3.17216216289009	0.00217373786219169	0.115579035196339	0.012701198982375
ACC	DPF3	cg07171024	-0.148083972291178	80	-1.31391780162554	0.192774983219204	0.0219288628495342	0.373601480893718
ACC	DPF3	cg10266394	0.305132616774264	80	2.81161409053668	0.00624965285361075	0.0931059138195099	0.0296201714447298
ACC	DPF3	cg12913429	-0.0541183312073952	80	-0.475583382557063	0.635717671308683	0.00292879377267333	0.785485594690545
ACC	DPF3	cg13819165	0.0327460304661892	80	0.287499435696915	0.774502269520404	0.00107230251129259	0.875034462497555
ACC	DPF3	cg14238615	0.247064594834578	80	2.23734285935998	0.0281558998339632	0.0610409140207743	0.094196880904973
ACC	DPF3	cg14242696	-0.0169354604242413	80	-0.148629377817838	0.882234650905844	0.000286809819781045	0.937500852810748
ACC	DPF3	cg15392845	0.0613945270064281	80	0.539752990663586	0.590924951086224	0.00376928794634303	0.753767019704569
ACC	DPF3	cg15740492	0.110793842333577	80	0.978234609792583	0.331021807024588	0.0122752754990375	0.530675207611857
ACC	DPF3	cg16696021	0.444392779456198	80	4.35297087667685	4.0823091927923e-05	0.197484942432805	0.00044597551094521
ACC	DPF3	cg19710444	0.0583637407563911	80	0.513014238919563	0.609410092709463	0.00340632623507923	0.766940851771252
ACC	DPF3	cg19839347	0.209125255505103	80	1.87655953537109	0.0643672495798167	0.0437333724900746	0.173289929879992
ACC	DPF3	cg20548765	0.0582967340373598	80	0.512423243695136	0.60982160933023	0.00339850919942266	0.767184599640089
ACC	DPF3	cg21035356	0.0329280963974473	80	0.289099645262221	0.773282061735705	0.00108425953235958	0.874232343362702
ACC	DPF3	cg22784589	0.0825043313491171	80	0.726449248449743	0.469766023244611	0.00680696469136491	0.658503667086352
ACC	DPF3	cg23515460	0.1022527285432	80	0.901991884476668	0.369874044374542	0.0104556204945294	0.568536237702792
ACC	DPF3	cg24383902	-0.232365558710381	80	-2.09638053037649	0.0393327889258021	0.0539937528747873	0.121005061165573
ACC	DPF3	cg25495195	0.0691089779566631	80	0.607882195979272	0.545054392184344	0.00477605083421454	0.719079508633454
ACC	DPF3	cg26057784	-0.00818306240979298	80	-0.0718084855042192	0.942940511209562	6.6962510402567e-05	0.969613161617848
ACC	EED	cg00080012	-0.107529713142498	80	-0.949072246492755	0.34555316396218	0.0115626392085079	0.544853111947663
ACC	EED	cg01739327	0.151964821109277	80	1.34915511177746	0.181241163646824	0.0230933068547745	0.358518958596919
ACC	EED	cg02172616	-0.269906261371414	80	-2.45970587221708	0.0161472765106057	0.0728493899274942	0.061746029731914
ACC	EED	cg04607174	-0.477154363312281	80	-4.76436008233346	8.74759598754843e-06	0.227676286427948	0.00011618755032531
ACC	EED	cg06358433	-0.192866126402337	80	-1.72477592094234	0.0885799371359504	0.0371973427134422	0.218435592276734
ACC	EED	cg10086030	-0.405658962792032	80	-3.8944720965319	0.000208152951013817	0.164559194093507	0.00179498252626716
ACC	EED	cg10535675	-0.572901028536499	80	-6.13352307584927	3.44301483816694e-08	0.328215588498179	8.34670914055342e-07
ACC	EED	cg12672785	0.408507054686881	80	3.92726878467255	0.000185880495908648	0.166878013728951	0.00163154544722073
ACC	EED	cg16265078	-0.39465921577546	80	-3.76906470444982	0.000319245268844843	0.155755896596501	0.00257494727262993
ACC	EED	cg18574463	-0.0499317120182266	80	-0.438696209450678	0.662110147437359	0.00249317586507112	0.803422139878365
ACC	EED	cg19325359	-0.44159311904655	80	-4.31887792980753	4.6234212452076e-05	0.195004482789261	0.000495555845067071
ACC	ELP3	cg01209934	0.0858464820575163	80	0.756091026072805	0.45190161509247	0.00736961848165148	0.642456766164174
ACC	ELP3	cg02516513	0.0136615925058865	80	0.119891176427525	0.904881623690048	0.000186639109796894	0.949594110875931
ACC	ELP3	cg04720865	-0.185351011511074	80	-1.65512795673255	0.101969147552986	0.0343549974681782	0.241604113688575
ACC	ELP3	cg05621091	-0.405187701628829	80	-3.88905851715374	0.000212066810320396	0.164177073551253	0.00182172321577572
ACC	ELP3	cg15692687	-0.286578357027764	80	-2.62480769298407	0.0104518073537137	0.0821271547167327	0.0441224170433473
ACC	ELP3	cg17572663	-0.00011216462830457	80	-0.0009842406250890	0.999217235232148	1.25809038427026e-08	0.999615570356721
ACC	ELP3	cg18022570	-0.128900555054183	80	-1.14061331819714	0.257566163900592	0.0166153530932763	0.452080279646285
ACC	ELP3	cg18249244	-0.427701383568778	80	-4.15198694748982	8.43938942497244e-05	0.182928473506647	0.000831371230753838
ACC	ELP3	cg19781013	-0.00942309873491651	80	-0.0826910271654595	0.934311806128179	8.87947897679851e-05	0.965090391379119
ACC	ELP3	cg22414525	0.246220937960035	80	2.22920899753106	0.0287154087385346	0.0606247502899196	0.0955428128568309
ACC	ELP3	cg22487209	-0.479334485666163	80	-4.79260283603525	7.85031284234046e-06	0.229761549148845	0.000105805134659916
ACC	ELP3	cg25374552	0.274378752828745	80	2.5037535829394	0.0144042807048586	0.0752837000038573	0.0565999905623685
ACC	ELP4	cg04972224	-0.157159131586286	80	-1.39641866967378	0.166600986343078	0.0246989926409557	0.339084813508421
ACC	ELP4	cg05195750	0.140705989604582	80	1.24709688454393	0.216141953865239	0.0197981755106048	0.403392172276326
ACC	ELP4	cg05635455	-0.218066015401382	80	-1.96070787419326	0.0535297334213262	0.0475527870730358	0.151649383895799
ACC	ELP4	cg08111895	0.0184688629284172	80	0.162091261405583	0.871658584593907	0.000341098897868662	0.93191424234372
ACC	ELP4	cg08626560	-0.0656499837367242	80	-0.577321718032608	0.565406410122091	0.00430992036463215	0.734789366516395
ACC	ELP4	cg09616795	0.287221615662559	80	2.63122847847734	0.0102726494488525	0.0824962565038108	0.0435219742524795
ACC	ELP4	cg10583382	-0.225602079053961	80	-2.03203707046223	0.0455990262482325	0.0508962980734694	0.134895567201499

**ACC**	ELP4	cg11385473	-0.00720228641400311	80	-0.0632014460340138	0.949769873516411	5.18729295893338e-05	0.973367728570581
**ACC**	ELP4	cg13263383	-0.235415969452498	80	-2.12550468016144	0.0367507977993814	0.0554206786732594	0.115003683186045
**ACC**	ELP4	cg19328333	-0.0669111287214835	80	-0.588461549733239	0.557944759104219	0.00447709914678293	0.72914854505029
**ACC**	ELP4	cg24001477	0.216066893212372	80	1.94184854697379	0.0558132247604786	0.0466849023424468	0.156156590793622
**ACC**	ELP4	cg24531794	-0.0702803001562023	80	-0.618235853596011	0.538244332080197	0.00493932059004589	0.713969675684125
**ACC**	ELP4	cg25130734	-0.191367999003942	80	-1.71086700603475	0.0911317868482579	0.0366217110427729	0.222807323488274
**ACC**	EP300	cg00500400	-0.275537291415225	80	-2.51519203931696	0.0139802379720144	0.0759207989604384	0.0552996711646356
**ACC**	EP300	cg02046995	0.254021983326746	80	2.30462928146572	0.0238832992908151	0.0645271680132537	0.0832837592464777
**ACC**	EP300	cg07519229	0.00393446606232833	80	0.0345250668057857	0.972547797724171	1.54800231956134e-05	0.985558207323099
**ACC**	EP300	cg08131204	0.225254308300072	80	2.0287370692563	0.0459423289454758	0.0507395034077437	0.135633295103038
**ACC**	EP300	cg09331127	-0.185517967324516	80	-1.65667193628977	0.101655449995411	0.0344169162002201	0.241081122974819
**ACC**	EP300	cg24349919	-0.0128431133228033	80	-0.112707157686786	0.910556046230715	0.000164945559822367	0.952606200382898
**ACC**	EPC1	cg02900516	-0.421888656164961	80	-4.08323777385453	0.000107731791811364	0.177990038200676	0.00102681781575267
**ACC**	EPC1	cg16942190	-0.316949265080742	80	-2.93240605827656	0.0044275998628367	0.100456836635223	0.0225154074865681
**ACC**	EPC1	cg26211455	-0.23022088617744	80	-2.075943236399	0.0412371070013432	0.0530016564323257	0.125141388806807
**ACC**	EPC2	cg00066942	-0.0424425788486485	80	-0.372768016270245	0.710345254320973	0.00180137249932375	0.834995386106653
**ACC**	EPC2	cg03664802	-0.0709007478728597	80	-0.623721209841674	0.534654090334563	0.00502691604893082	0.711317707377542
**ACC**	EPC2	cg04098458	-0.00060961586890101	80	-0.0053493585335906	0.995745684805916	3.71631507615936e-07	0.997823575378599
**ACC**	EPC2	cg05586854	-0.0368316917977147	80	-0.3234162272454	0.747256771997949	0.00135657352068185	0.858087766892588
**ACC**	EPC2	cg10458926	-0.136860453566445	80	-1.21235344312361	0.229083882542688	0.0187307837504129	0.419058510447409
**ACC**	EPC2	cg11721798	0.119489702404093	80	1.0560842622652	0.294231990597169	0.0142777889806187	0.491993024600553
**ACC**	EPC2	cg12432635	0.183089708254074	80	1.63423035586342	0.106292766638864	0.0335218412685618	0.248727312013952
**ACC**	EPC2	cg16434205	0.058734004141203	80	0.516280065327296	0.607138334733716	0.00344968324245885	0.76529513120007
**ACC**	EPC2	cg21347644	-0.0454522042858819	80	-0.39925409725076	0.690811197489607	0.00206590287444554	0.822292434869065
**ACC**	EPC2	cg21650422	0.0618915418793439	80	0.544139255659879	0.587917892153666	0.00383056295620258	0.7516761085725
**ACC**	EZH1	cg00728999	-0.272369338083255	80	-2.48394219373936	0.0151661540012619	0.0741850563279107	0.0588916733739725
**ACC**	EZH1	cg01716316	0.102141321399703	80	0.900998773023831	0.370398487926854	0.0104328495372775	0.569068430330437
**ACC**	EZH1	cg03623386	-0.332602909857157	80	-3.09477353262272	0.00274551420010398	0.110624695645448	0.0153448187141957
**ACC**	EZH1	cg05375741	-0.259706852743137	80	-2.35989218079254	0.0208138578952591	0.0674476493617455	0.075073871527438
**ACC**	EZH1	cg05979501	-0.24919157286851	80	-2.25787357968501	0.0267860391136603	0.0620964399886819	0.0907061823361377
**ACC**	EZH1	cg06975499	-0.145031257803375	80	-1.28624347974033	0.20221228392968	0.0210340657400291	0.385730320049171
**ACC**	EZH1	cg12812489	-0.171932788944743	80	-1.53151030643808	0.129741699083493	0.0295608839143175	0.285681453113536
**ACC**	EZH1	cg19395400	0.0670792420956951	80	0.589946726381933	0.556953663434214	0.00449962472013287	0.728306017602683
**ACC**	EZH1	cg22227424	0.134248048292483	80	1.18878299271274	0.238178418954004	0.0180225384703409	0.429799188205941
**ACC**	EZH1	cg27088844	-0.0397333512126766	80	-0.348934288799848	0.728091009878766	0.00157873919858991	0.846262086279239
**ACC**	EZH2	cg01893439	-0.0498445012035185	80	-0.437928071803508	0.662664469477376	0.00248447430022756	0.803844002721351
**ACC**	EZH2	cg02303805	-0.104932835343939	80	-0.925893463039955	0.357394238646837	0.0110108999333182	0.556296192426661
**ACC**	EZH2	cg06816161	-0.128673153635849	80	-1.13856718458114	0.25841374684789	0.0165567804665949	0.453046650492072
**ACC**	EZH2	cg13751213	0.0419324979447656	80	0.368280098373195	0.713674935402883	0.00175833438388777	0.837088610816936
**ACC**	EZH2	cg18494018	0.0105201240538106	80	0.0923188226654865	0.926684503206706	0.000110673010107565	0.961321948533324
**ACC**	EZH2	cg20101066	-0.00454541718710272	80	-0.0398862859864306	0.968287022723986	2.06608174048088e-05	0.983336732739507
**ACC**	FBXL19	cg00026853	-0.0955373370013488	80	-0.842189031183099	0.402290407161084	0.00912738276130929	0.598836224616624
**ACC**	FBXL19	cg02291855	0.0530709244441726	80	0.466352681623335	0.642279766498876	0.00281652302135908	0.789972095617376
**ACC**	FBXL19	cg03181514	0.0416860690687345	80	0.366112011453859	0.715285483738817	0.00173772835440331	0.838088996490361
**ACC**	FBXL19	cg03627896	0.316547505830742	80	2.92827476457697	0.00448079511016943	0.100202323447664	0.0227190884964167
**ACC**	FBXL19	cg04477789	-0.0257798310991534	80	-0.226292309205611	0.821573711416198	0.000664599691500876	0.904204699928634
**ACC**	FBXL19	cg05643349	-0.252086615715065	80	-2.2858741229584	0.0250124975069084	0.0635476618226746	0.0862242314464673
**ACC**	FBXL19	cg05768582	-0.106784406958537	80	-0.942417926741446	0.34892620304625	0.0114029095694865	0.547983547790047
**ACC**	FBXL19	cg06862768	-0.202224376126635	80	-1.81194796267284	0.0738930063292378	0.0408946982998067	0.191757402443473
**ACC**	FBXL19	cg07697662	0.0372572723100933	80	0.327158381529281	0.744435841761224	0.00138810433998844	0.856441239556409
**ACC**	FBXL19	cg08535091	-0.1204165598328	80	-1.06439615390758	0.290475776670557	0.0145001478819662	0.488083126059641
**ACC**	FBXL19	cg08593668	0.0826507047561938	80	0.727746920563745	0.468975705153665	0.00683113899669551	0.657874436399265
**ACC**	FBXL19	cg09011601	-0.238490014775962	80	-2.15492176279338	0.034293547245616	0.0568774871478385	0.109104867267522
**ACC**	FBXL19	cg11940219	-0.0574351754137902	80	-0.504824964806785	0.615123547709922	0.00329879937481285	0.770994531039068
**ACC**	FBXL19	cg26666512	-0.183468340980953	80	-1.63772756986074	0.105559052804097	0.0336606321423033	0.247576380169493
**ACC**	FBXL19	cg27602786	-0.105420046608544	80	-0.930240658373453	0.355153809384267	0.0111133862269475	0.554006044539706
**ACC**	FBXW9	cg02753619	0.0829809503592479	80	0.730674884292837	0.467195252640793	0.00688583812252396	0.656290827331119

**ACC**	FBXW9	cg03160342	-0.0427003300698971	80	-0.375035936608046	0.708664773238939	0.00182331818807815	0.834012092967687
**ACC**	FBXW9	cg05868316	0.170250700013562	80	1.51607737520805	0.133594539666478	0.0289853008551078	0.291595393371148
**ACC**	FBXW9	cg06489804	0.160814585707427	80	1.42975099411208	0.156833520972137	0.0258613309762515	0.325422729570688
**ACC**	FBXW9	cg12110584	-0.279901857008709	80	-2.5583912573881	0.0124782668911067	0.0783450495569237	0.0506831459576441
**ACC**	FBXW9	cg14342428	0.184048328177108	80	1.64308604322911	0.104442800382832	0.0338737871047886	0.245743677614918
**ACC**	FBXW9	cg15828427	0.0634830195889264	80	0.558187144890944	0.57833606668466	0.00403009377612802	0.744614146696011
**ACC**	FBXW9	cg19721801	0.0511625804174873	80	0.449538564897716	0.65430607942462	0.00261760963497585	0.798310678043928
**ACC**	FBXW9	cg21564527	-0.144326055916397	80	-1.27985585319484	0.204438528192163	0.0208300104163829	0.3885760858312
**ACC**	FBXW9	cg22303873	0.153986226817704	80	1.36753424529204	0.175436394399782	0.0237117580495535	0.351116513144756
**ACC**	FKBP1A	cg04613791	0.279120565483026	80	2.55064572020496	0.0127363548839167	0.0779082900755641	0.0515457284278125
**ACC**	FKBP1A	cg07516946	0.208152231368959	80	1.86743181981416	0.0656468540432514	0.0433273514238767	0.175828244566731
**ACC**	FKBP1A	cg10996143	0.0583929417679455	80	0.513271792258183	0.609230794817819	0.00340973564831468	0.766814445808038
**ACC**	FKBP1A	cg21332426	-0.100248986240829	80	-0.884135220006246	0.379375584669912	0.0100498592423139	0.577884596616693
**ACC**	FKBP1A	cg21775445	0.133880278242557	80	1.18546682322289	0.239478519954869	0.0179239289023045	0.431417453093898
**ACC**	FKBP1A	cg24641773	-0.144819170173885	80	-1.28432220765301	0.202879991871967	0.0209725920498526	0.386663875929438
**ACC**	FKBP1A	cg26779912	-0.0615345416623496	80	-0.540988609716085	0.590077127454204	0.00378649981759544	0.753100043527453
**ACC**	FKBP2	cg03764062	0.0387042972926863	80	0.339883502383093	0.734869659082611	0.00149802262892064	0.850518481591774
**ACC**	FKBP2	cg04446112	0.17393184998016	80	1.54986937610697	0.125273488975763	0.0302522884375208	0.278833961871778
**ACC**	FKBP2	cg06615912	-0.259640368884021	80	-2.35924438266084	0.0208477063598219	0.0674131211542303	0.075185502227805
**ACC**	FKBP2	cg09854726	0.139016065239055	80	1.23182185679767	0.221764258831857	0.0193254663945493	0.410262033044767
**ACC**	FKBP2	cg10998122	-0.0184636474217399	80	-0.162045472100818	0.871694519572674	0.000340906276114324	0.931939853715888
**ACC**	FKBP2	cg12423493	0.0288932923262752	80	0.253643506936399	0.800447018306613	0.000834822341451596	0.890983922250206
**ACC**	FKBP2	cg15010817	-0.194127921805392	80	-1.73650039685342	0.0864747399173181	0.0376856500244802	0.214728076770016
**ACC**	FKBP2	cg16977872	0.0342727791040192	80	0.300919201389867	0.764287172393001	0.00117462338751289	0.869160524504906
**ACC**	FKBP2	cg19924313	-0.0356572894111492	80	-0.313090546320748	0.755058345577681	0.00127144228815045	0.863275755208109
**ACC**	FKBP2	cg21809711	-0.111850414298833	80	-0.987681046488007	0.3264023952227	0.0125105151788205	0.525869774550692
**ACC**	FKBP2	cg22074794	0.0441338981616501	80	0.387651102260516	0.699343775028374	0.0019478009669429	0.827587119283185
**ACC**	FKBP2	cg24677501	0.19754013600732	80	1.76825140637493	0.0809797667029709	0.0390221053337905	0.204676564244807
**ACC**	FKBP5	cg00130530	-0.300637838433794	80	-2.76604820754459	0.00709994424529176	0.0903831098981437	0.0327638547050793
**ACC**	FKBP5	cg00140191	-0.0158088236278343	80	-0.138739202198853	0.890018450537428	0.000249918904495971	0.941711034866837
**ACC**	FKBP5	cg00610228	-0.10092817591948	80	-0.890186696730818	0.376138576317695	0.0101864966944336	0.574686733430069
**ACC**	FKBP5	cg01294490	-0.238010772519301	80	-2.15033116319329	0.0346673206420179	0.0566491278352347	0.110046498634069
**ACC**	FKBP5	cg07485685	-0.113522591000181	80	-1.00263833368458	0.319175880529766	0.0128873786673945	0.518576647311723
**ACC**	FKBP5	cg07843056	-0.00081442773062535	80	-0.0071465767024779	0.994316388706782	6.63292528411563e-07	0.997058888261977
**ACC**	FKBP5	cg08636224	-0.0241019969616069	80	-0.211555620921467	0.833012681433185	0.000580906257537306	0.91045405874669
**ACC**	FKBP5	cg08915438	-0.135752028464321	80	-1.20234955032012	0.232912583271036	0.0184286132321778	0.423629003169012
**ACC**	FKBP5	cg10913456	-0.163441004365865	80	-1.45373723803793	0.150083013708323	0.0267129619081228	0.315729837640977
**ACC**	FKBP5	cg16012111	-0.0371928148448567	80	-0.326591591871319	0.74486287860961	0.00138330547608379	0.856667742619949
**ACC**	FKBP5	cg17030679	-0.413327524470527	80	-3.98309279587515	0.000153123200002989	0.170839642484934	0.00138609101943595
**ACC**	FKBP5	cg20813374	-0.433143665393311	80	-4.21692584148359	6.68765337864448e-05	0.187613434870353	0.000683129412139329
**ACC**	FKBP5	cg25114611	-0.367654895186075	80	-3.46912838797218	0.000858341357476623	0.135170121954283	0.00591389669325449
**ACC**	FMR1	cg02921434	-0.241486563843784	80	-2.18366334219685	0.0320322294030438	0.058315760517078	0.103644106566585
**ACC**	FMR1	cg03513471	-0.200484429714335	80	-1.79570207022577	0.0764649825395532	0.0401940065578822	0.196656816133852
**ACC**	FMR1	cg04552106	-0.254249962114533	80	-2.30684050870939	0.0237531832268693	0.0646430432352413	0.0829674355805853
**ACC**	FMR1	cg04744025	-0.223338962448698	80	-2.01057692322787	0.0478715192541895	0.049880292147661	0.13978368624204
**ACC**	FMR1	cg05288927	-0.215185421508062	80	-1.93354107116794	0.0568450456820301	0.0463047656296024	0.158297661933986
**ACC**	FMR1	cg07147350	-0.107662651796515	80	-0.950259332911826	0.34495367070579	0.0115912465918576	0.544184396236374
**ACC**	FMR1	cg08434396	-0.257817859738395	80	-2.34150038339971	0.0217940772513629	0.0664700488000868	0.077730077282804
**ACC**	FMR1	cg14470409	-0.156633826086831	80	-1.39163353435341	0.168040664036672	0.0245341554745997	0.341070638125361
**ACC**	FMR1	cg16783314	-0.185458273453119	80	-1.65611988025125	0.101767523709534	0.034394771192212	0.241251302396987
**ACC**	FMR1	cg22625568	-0.286293481894289	80	-2.62196540418267	0.0105320163907692	0.0819639577751558	0.0443719297646088
**ACC**	FXR2	cg01807919	0.0780804954432251	80	0.687251706540744	0.493988939256135	0.0060965637686595	0.679255896243229
**ACC**	FXR2	cg01933235	-0.220717312647752	80	-1.98575971687823	0.050619846890759	0.0487161321024457	0.145638535782378
**ACC**	FXR2	cg05529097	-0.30771885544384	80	-2.83792573709328	0.00580227609955297	0.0946908939956667	0.0279347994601739
**ACC**	FXR2	cg05913233	-0.436256606863485	80	-4.25432464354805	5.84391719158833e-05	0.190319827032041	0.000607701769820108
**ACC**	FXR2	cg09625173	-0.364383443144234	80	-3.43350889527811	0.00096213555988014	0.132775293637648	0.00650176978329705
**ACC**	FXR2	cg17777561	-0.39795232788251	80	-3.8064032065874	0.000281312870396001	0.158366055267109	0.0023160602645345

**ACC**	FXR2	cg19178740	-0.19322978705761	80	-1.72815410453364	0.0879690892405973	0.0373377506063294	0.217332049925578
**ACC**	FXR2	cg22521096	-0.289233426722804	80	-2.65133478296296	0.00972950866530284	0.0836559751338157	0.0416931473564266
**ACC**	FXR2	cg26904212	0.0573137231896019	80	0.503753941782846	0.615872547330518	0.00328486286585431	0.771628560452084
**ACC**	G2E3	cg02259031	0.266423441546819	80	2.42552415390863	0.0176276647012975	0.0709814502056514	0.0660721054094308
**ACC**	G2E3	cg05076167	-0.360275248882588	80	-3.38898631029105	0.00110856762831657	0.12979825495741	0.00731156507649504
**ACC**	G2E3	cg07906495	0.216934644906899	80	1.95003158261854	0.0548124536690317	0.0470606401608822	0.154249747530338
**ACC**	G2E3	cg08810065	-0.197504848666265	80	-1.76792271411614	0.0810351354758572	0.0390081652466841	0.204789894721399
**ACC**	G2E3	cg09837748	-0.437348264090538	80	-4.26748431648469	5.5722341708406e-05	0.191273504103007	0.000582956408131218
**ACC**	G2E3	cg18580322	0.131279754375094	80	1.16203213731116	0.248811622452911	0.0172343739087849	0.442231975989896
**ACC**	G2E3	cg18755078	0.0625352312646698	80	0.549820559732687	0.584033761445762	0.00391065514932574	0.748752841758597
**ACC**	G2E3	cg18956956	-0.385669869542798	80	-3.66800803692833	0.000447929686262725	0.148741248273159	0.0034166332006199
**ACC**	G2E3	cg19554565	0.0775987655135102	80	0.682985831768565	0.496665650035472	0.00602156840922073	0.681295097666446
**ACC**	G2E3	cg21061067	-0.356790519518267	80	-3.35139833759828	0.0012483009248215	0.127299474818115	0.00807771769635364
**ACC**	G2E3	cg22926526	-0.280258257676835	80	-2.56192635881615	0.0123620511390816	0.0785446909960553	0.0503210142197769
**ACC**	GADD45/	cg01810953	-0.00768861620020317	80	-0.0674693275931287	0.946382967629875	5.91148190740267e-05	0.97141955407511
**ACC**	GADD45/	cg02639608	0.166790930136519	80	1.48437720527468	0.141791170642912	0.0278192143758051	0.303987828060264
**ACC**	GADD45/	cg04718306	-0.181309264472243	80	-1.6177954727986	0.109796175587588	0.0328730493834657	0.254634711899227
**ACC**	GADD45/	cg07000785	0.116785592572523	80	1.03185021160415	0.305372442619662	0.0136388746325154	0.503914074686269
**ACC**	GADD45/	cg22224769	-0.0944457853623152	80	-0.832479581501515	0.407712871371139	0.00892000637270452	0.603834506357614
**ACC**	GADD45/	cg23369529	-0.101151728449862	80	-0.892178790384944	0.375076795008994	0.0102316721683946	0.573796983224448
**ACC**	GADD45/	cg23723105	-0.0438458225519407	80	-0.385115893726336	0.701213320480983	0.00192245615525627	0.828816096958196
**ACC**	GATAD2A	cg02887458	0.16776145488243	80	1.49326389715093	0.139454587063105	0.0281439057442698	0.300481854395376
**ACC**	GATAD2A	cg08505228	0.117578054213792	80	1.03894976382344	0.302079600097556	0.0138245988327014	0.500446790961907
**ACC**	GATAD2A	cg08870822	0.21582366272627	80	1.93955571216348	0.0560964023221825	0.0465798533925826	0.156779720576679
**ACC**	GATAD2A	cg09334331	-0.0941345365528478	80	-0.829711552111645	0.409266840266558	0.00886131097201944	0.605271114559786
**ACC**	GATAD2A	cg11795262	-0.0295719257589435	80	-0.259606132853784	0.795860459482239	0.000874498793092468	0.88820429496688
**ACC**	GATAD2A	cg14948912	0.131037228787196	80	1.15984786439581	0.249694567740929	0.0171707553282278	0.443339751562772
**ACC**	GATAD2A	cg15264323	0.0565247852646386	80	0.49679725902855	0.620747477512879	0.00319505134921351	0.775239268363598
**ACC**	GATAD2A	cg22191731	-0.108693513028074	80	-0.959466246873022	0.340327047883221	0.0118142797743841	0.539554553038115
**ACC**	GATAD2A	cg26631329	-0.172596342285132	80	-1.53760210386981	0.128245303394864	0.0297894973702066	0.283405253310799
**ACC**	GATAD2E	cg02134200	0.378692561439	80	3.59041908906358	0.000578796639967806	0.143408056089231	0.00424443933857389
**ACC**	GATAD2E	cg02940454	0.237448503787087	80	2.14494738595077	0.0351101911970527	0.0563817919507264	0.111107766097853
**ACC**	GATAD2E	cg09805553	0.393199156071153	80	3.75256522975472	0.000337519604717607	0.154605576335067	0.00269745224259313
**ACC**	GATAD2E	cg10226922	-0.116430737277201	80	-1.02867176155826	0.306854477623866	0.0135561165829126	0.505343944118529
**ACC**	GATAD2E	cg10489353	0.441788227976388	80	4.32124876877937	4.58365037681283e-05	0.195176838378517	0.000491903017538664
**ACC**	GATAD2E	cg15064304	-0.271938998023158	80	-2.47970392552421	0.0153337665097159	0.073950818645839	0.0593913384046222
**ACC**	GATAD2E	cg20773694	-0.00143076481496204	80	-0.0125549231485481	0.990015356677805	2.04708795573336e-06	0.994592368632072
**ACC**	GATAD2E	cg23046518	0.0239092417704298	80	0.209862737850375	0.8343290987665	0.000571651842036865	0.911179244504998
**ACC**	GATAD2E	cg23323627	0.358176884449413	80	3.36633298505261	0.00119090435372487	0.128290680553888	0.00776768787571668
**ACC**	GATAD2E	cg26398333	0.434284789498431	80	4.23061355988451	6.36604515944924e-05	0.188603278389697	0.000655513060402639
**ACC**	GLYR1	cg00636946	-0.0198934963660233	80	-0.17459927450744	0.861852788831462	0.000395751197664983	0.926633599258153
**ACC**	GLYR1	cg00885266	0.0239774299025252	80	0.210461601185241	0.833863357188648	0.000574917144730507	0.9108847170458
**ACC**	GLYR1	cg01261535	-0.187117953316638	80	-1.67147589822391	0.0986872628784794	0.0350131284534077	0.235919025516822
**ACC**	GLYR1	cg01565912	0.2825349703325	80	2.58453605748458	0.0116415990101805	0.0798260094607864	0.047991444724603
**ACC**	GLYR1	cg02454264	0.132266571804838	80	1.17092196486865	0.245241073268013	0.0174944460170045	0.438156947748023
**ACC**	GLYR1	cg06399529	-0.178663441004768	80	-1.59340278888426	0.115166838396798	0.0319206251516641	0.263142082411772
**ACC**	GLYR1	cg06976003	-0.458448422963536	80	-4.52658203707081	2.14906910652759e-05	0.210174956517753	0.000254893554007024
**ACC**	GLYR1	cg11289281	0.0695586679036466	80	0.611856835083204	0.542434944611705	0.00483840828052979	0.717022090687198
**ACC**	GLYR1	cg15663121	0.283803429985633	80	2.59715349843259	0.0112562655637403	0.0805443868716102	0.0467642277884974
**ACC**	GLYR1	cg18354686	-0.0749046632731058	80	-0.659137467806142	0.511773933470205	0.00561070858005737	0.69379362412231
**ACC**	GTF2B	cg02705666	-0.315143408921331	80	-2.913850157892	0.00467119199416313	0.099315368186557	0.0234900265025163
**ACC**	GTF2B	cg04270788	0.342123396489627	80	3.19491783043043	0.00202808165534465	0.117048418425598	0.0120280997404804
**ACC**	GTF2B	cg05306856	-0.30474546359108	80	-2.80768123014072	0.00631917956757365	0.0928697975793423	0.0298896002480589
**ACC**	GTF2B	cg06356032	0.0400892128638027	80	0.352064438257656	0.72575166871786	0.00160714498803928	0.844640136979194
**ACC**	GTF2B	cg07167781	-0.100629956723199	80	-0.88752945473644	0.377557821332362	0.0101263881901128	0.576151203131744
**ACC**	GTF2B	cg10430205	0.0413322158081328	80	0.362998919283064	0.717600282652921	0.00170835206361006	0.839599885527167
**ACC**	GTF2B	cg12074130	-0.354178478664314	80	-3.32332887001888	0.00136328925810676	0.125442394748968	0.00868761119932486

ACC	GTF2B	cg13868361	-0.131911707795214	80	-1.16772473615153	0.24652097885262	0.0174006986534499	0.439697657484885
ACC	GTF2B	cg14010405	-0.148043813598292	80	-1.3135534964794	0.192897035420719	0.0219169707447258	0.373749211762258
ACC	GTF2B	cg17811845	-0.0475789332234161	80	-0.417976811198276	0.677127416309288	0.00226375488667829	0.813486604890018
ACC	GTF2B	cg22607980	-0.156902613752004	80	-1.39408183490167	0.167302871703271	0.0246184302022104	0.340060426499629
ACC	GTF2H1	cg05849260	-0.344272477263757	80	-3.21767597169748	0.00189158093024169	0.118523538601324	0.0113671975103912
ACC	GTF2H1	cg07583091	-0.308022141534828	80	-2.84101581698241	0.00575171310911294	0.0948776396757014	0.0277281260215859
ACC	GTF2H1	cg08690812	-0.413741115396697	80	-3.98790111128126	0.000150576743010804	0.171181710569703	0.00136653839778144
ACC	GTF2H1	cg10797557	-0.0218253490429189	80	-0.191562290980826	0.848589334011356	0.000476345860845239	0.919213537882109
ACC	GTF2H1	cg11063328	-0.00878507752804785	80	-0.0770917173828687	0.938750549804426	7.71775871738113e-05	0.967222402408952
ACC	GTF2H1	cg11176342	0.0752219830922746	80	0.661945638458054	0.509982299504129	0.00565834674033444	0.692371215295094
ACC	GTF2H1	cg11347316	0.0251841263437969	80	0.221059925461237	0.825630922032538	0.000634240219700323	0.906256711986219
ACC	GTF2H1	cg11662851	-0.0414484892123287	80	-0.36402184178652	0.716839376241582	0.00171797725798453	0.839091445386494
ACC	GTF2H1	cg13777411	0.171686880372203	80	1.52925327180121	0.130299615927754	0.0294763848919391	0.286666524928837
ACC	GTF2H1	cg14132868	0.0738106882620479	80	0.649457705593666	0.517975271663957	0.00544801770171722	0.698868644140031
ACC	GTF2H1	cg17174493	-0.122356227384886	80	-1.0817999135145	0.282717800373563	0.0149710463798619	0.479966309400473
ACC	GTF2H1	cg18321761	0.125439954721129	80	1.10949479800465	0.270669890671914	0.0157351822404388	0.466853501428527
ACC	H2AFZ	cg02758870	-0.228470228961905	80	-2.05928434060975	0.0428477610871699	0.0521986455219052	0.128763422799679
ACC	H2AFZ	cg09308803	-0.184860532527957	80	-1.65059294673564	0.102895091440204	0.0341734164865199	0.243124187325333
ACC	H2AFZ	cg16267491	-0.0849825535982647	80	-0.748426359068251	0.456483034765071	0.00722203441608193	0.646748036834595
ACC	H2AFZ	cg17709884	-0.332217361663195	80	-3.09074077094689	0.00277884864655042	0.110368375390454	0.0154881945110982
ACC	H2AFZ	cg20934652	0.336728663301642	80	3.13803803907163	0.00241058224946034	0.11338619268891	0.0138224246712256
ACC	H2AFZ	cg23752380	-0.027148573835976	80	-0.238315609439314	0.812269387131255	0.000737045061327442	0.898819178448841
ACC	H2AFZ	cg24061702	-0.265702289625185	80	-2.41845933603451	0.0179483548539157	0.0705977067120657	0.0669900838413626
ACC	H3F3A	cg06319713	-0.151183653821374	80	-1.34205719721993	0.183521438085508	0.022856497182781	0.361769378532942
ACC	H3F3A	cg10632229	0.0428056012224195	80	0.375962225692008	0.70797882767517	0.0018323194960128	0.833532673287763
ACC	H3F3A	cg10983539	0.022319842414963	80	0.195904625831719	0.845200899580109	0.000498175365428781	0.91726735835963
ACC	H3F3A	cg11978873	-0.0337064820997353	80	-0.29594134129074	0.768071539169392	0.00113612693553978	0.871477601926643
ACC	H3F3A	cg15538837	-0.089426806402832	80	-0.787873738975106	0.433189296404574	0.00799715370340959	0.626816363009828
ACC	H3F3A	cg15624314	0.0265721599003139	80	0.23325211865327	0.816184598177933	0.000706079681767849	0.90104963501574
ACC	H3F3A	cg16724613	-0.293066834071614	80	-2.68975281355982	0.00876368312685077	0.0858881692327591	0.0385151150044099
ACC	H3F3A	cg16727519	0.37502968024124	80	3.5499747941227	0.000660670524666138	0.140647261061847	0.00474967219257717
ACC	H3F3A	cg18947521	0.152789333009618	80	1.35664967585726	0.178856803046264	0.0233445802815241	0.355556337009202
ACC	H3F3A	cg19410789	0.0465303151440431	80	0.40874457844169	0.683861708933828	0.00216507022740397	0.817887922159675
ACC	H3F3A	cg21266749	-0.183251040951147	80	-1.63572040162752	0.105979653242735	0.0335809440096789	0.248241031686298
ACC	H3F3A	cg21789564	-0.152938842553253	80	-1.35800898260872	0.178426905959718	0.0233902895615289	0.355012071410433
ACC	H3F3A	cg22519151	-0.284382387890431	80	-2.60291734897905	0.0110841116997195	0.0808733425422638	0.0462025068621323
ACC	H3F3A	cg27530209	-0.00303080541471874	80	-0.0265953317290053	0.978851303410674	9.18578146188844e-06	0.989094294612233
ACC	H3F3A	cg27560444	-0.261408896472911	80	-2.37648865516403	0.0199631587085822	0.0683346111551852	0.0726998393678234
ACC	HAT1	cg00414306	-0.215335498238996	80	-1.93495512639037	0.0566682800875254	0.0463693768018366	0.157946765831713
ACC	HAT1	cg01583365	0.137198806185549	80	1.21540810688031	0.227923950559944	0.0188235124187398	0.417735404237327
ACC	HAT1	cg04507121	0.0118833199398639	80	0.104283072614285	0.917215865351386	0.000141213292793167	0.956319824013855
ACC	HAT1	cg04950839	0.110588223472662	80	0.976396635602852	0.331925583431108	0.0122297551708395	0.531620272008804
ACC	HAT1	cg05421799	0.00936270291969437	80	0.0821609858934454	0.934731899020984	8.76602059624536e-05	0.965306429741649
ACC	HAT1	cg09545452	-0.162046090308245	80	-1.44099401528106	0.153640695437651	0.026258935384188	0.320939714327447
ACC	HAT1	cg10190161	-0.130430924548167	80	-1.15438819574438	0.251911277018791	0.0170122260784896	0.445707121904563
ACC	HAT1	cg12941622	0.341197846143582	80	3.18513404909064	0.00208955061118777	0.11641597021302	0.0123175343067926
ACC	HAT1	cg13293756	-0.0296279606410262	80	-0.26009848387187	0.795482053062514	0.000877816051746198	0.888075008767493
ACC	HAT1	cg14446129	-0.104675687408291	80	-0.923599304703931	0.358580236626846	0.0109569995343983	0.557535073494813
ACC	HAT1	cg16017358	0.142694347783403	80	1.26508367128464	0.209656524762295	0.0203616768893307	0.395123426326562
ACC	HDAC1	cg03762702	-0.208043974137268	80	-1.86641664250485	0.0657904829443912	0.043282295174828	0.176085626855294
ACC	HDAC1	cg04967775	-0.21213504200281	80	-1.90483069714875	0.0605364220516403	0.0450012760455341	0.165706207497724
ACC	HDAC1	cg06473843	-0.214216313533135	80	-1.92441338063206	0.0579973526469308	0.0458886289837265	0.160603924529788
ACC	HDAC1	cg10842753	-0.191383147506525	80	-1.71100758545443	0.0911056966028174	0.0366275091495041	0.222771540516095
ACC	HDAC1	cg14172124	-0.2075258285691	80	-1.86155874774633	0.0664814458868666	0.0430669695232916	0.17747092173917
ACC	HDAC1	cg20002354	-0.243119714106405	80	-2.19935566030912	0.0308534558741102	0.0591071953871801	0.100756181104464
ACC	HDAC1	cg20793809	-0.204541046479131	80	-1.83360653022251	0.0705766432881898	0.041837039694778	0.185481550892248
ACC	HDAC1	cg21930229	-0.177210713153636	80	-1.58002485535694	0.118200603935524	0.0314036368564204	0.267950799846641

**ACC**	HDAC1	cg23018091	-0.392290859695871	80	-3.7423179406605	0.000349366940752293	0.153892118600925	0.00277474750269361
**ACC**	HDAC1	cg26115470	-0.290960303228811	80	-2.66862397828288	0.00928347776803304	0.0846578980550014	0.0402524156624906
**ACC**	HDAC10	cg00711351	-0.141847672949655	80	-1.2574227113324	0.212401030921819	0.0201207623212322	0.398637534367479
**ACC**	HDAC10	cg01120165	-0.18530030719149	80	-1.65465908102082	0.102064566587951	0.0343362038452604	0.241719766595928
**ACC**	HDAC10	cg04321608	-0.175177393921733	80	-1.56131823438193	0.122549560393159	0.0306871193412099	0.27464912713832
**ACC**	HDAC10	cg04586023	-0.126395681906868	80	-1.11808475357981	0.267007028708055	0.0159758684047022	0.462731288095401
**ACC**	HDAC10	cg07482373	-0.148383690831886	80	-1.31663694989921	0.191865821183225	0.0220177197048928	0.372349690493635
**ACC**	HDAC10	cg07744063	-0.160712948930468	80	-1.42882340819929	0.157099213229546	0.0258286519539273	0.325790424492291
**ACC**	HDAC10	cg09151829	-0.148433698582618	80	-1.31709067326802	0.191714429807025	0.0220325628749154	0.372199251823447
**ACC**	HDAC10	cg10508940	-0.151226471697396	80	-1.34244618618023	0.18339591294541	0.0228694457420432	0.361586072222299
**ACC**	HDAC10	cg11645658	-0.119791606128513	80	-1.05879137212824	0.293004995484823	0.0143500288988488	0.490690173266447
**ACC**	HDAC10	cg12464820	-0.256504435290423	80	-2.32872946139383	0.0224986072803398	0.0657945253236588	0.079553504602825
**ACC**	HDAC10	cg26943378	-0.129676325056229	80	-1.14759499260176	0.254688909040007	0.0168159492800887	0.448934443505814
**ACC**	HDAC11	cg01409735	-0.278669692736996	80	-2.54617837698287	0.0128873914260156	0.0776567976501317	0.0520027868974251
**ACC**	HDAC11	cg01712016	0.106183168770855	80	0.937051061349206	0.351662119547793	0.01127486533022	0.55064616819172
**ACC**	HDAC11	cg02762546	-0.162971807224546	80	-1.44944989695264	0.151272744043302	0.0265598099500345	0.317528640056089
**ACC**	HDAC11	cg02941903	-0.217587310807883	80	-1.95618952028093	0.0540694454356911	0.0473442378246064	0.152783351066948
**ACC**	HDAC11	cg05987698	-0.270512805258686	80	-2.46566931823417	0.0159007302206375	0.0731771778089238	0.0610101803592736
**ACC**	HDAC11	cg07450135	-0.225894441180854	80	-2.03481193168737	0.04531206238204	0.0510282985564105	0.134296197079717
**ACC**	HDAC11	cg08051076	-0.160883668331888	80	-1.43038150188693	0.156653120416983	0.0258835547359249	0.325166071172625
**ACC**	HDAC11	cg08448040	0.116619859790479	80	1.03036568807565	0.306064036797159	0.0136001916975509	0.50454817873242
**ACC**	HDAC11	cg10455785	-0.455476601427865	80	-4.48952657694842	2.46712753526178e-05	0.207458934448278	0.000287661450057187
**ACC**	HDAC11	cg11432034	-0.333638470929707	80	-3.10561395283542	0.00265774520624389	0.111314629284313	0.014948738239789
**ACC**	HDAC11	cg13397067	-0.197121264222389	80	-1.76435018741906	0.0816389571618982	0.038856792808633	0.205941211889254
**ACC**	HDAC11	cg13706066	-0.303552115067775	80	-2.79556834785716	0.00653779901865294	0.0921438865621199	0.0306865674580245
**ACC**	HDAC11	cg16254764	-0.244349847450898	80	-2.21118865876356	0.0299898670683923	0.0597068479492771	0.0986649631531129
**ACC**	HDAC11	cg18521047	-0.247407752217306	80	-2.24065285604868	0.0279309693179597	0.06121059585722	0.0936336217840797
**ACC**	HDAC11	cg19779799	-0.29876090425106	80	-2.7470809918718	0.00748412489697821	0.089258077908911	0.0341176485300045
**ACC**	HDAC11	cg20894727	-0.187803518548822	80	-1.67782334044519	0.0974363966952693	0.0352701615793179	0.233675536238726
**ACC**	HDAC11	cg20922324	-0.273276734617908	80	-2.49288410463443	0.014817910008904	0.0746801736834265	0.0578121902196652
**ACC**	HDAC11	cg21655280	-0.236897824439863	80	-2.13967679280085	0.0355485126123888	0.0561205792243403	0.112143592464241
**ACC**	HDAC11	cg25127083	-0.269772717257833	80	-2.45839331163103	0.0162019994175714	0.0727773189766749	0.0619157215056516
**ACC**	HDAC11	cg25150878	-0.325518142095824	80	-3.02094338488309	0.00341888668967745	0.105962060833517	0.0182947359428235
**ACC**	HDAC11	cg27025856	-0.344136280662432	80	-3.2162319925975	0.0018999795926274	0.118429779668172	0.0114066223513548
**ACC**	HDAC2	cg00161229	-0.0574663660812928	80	-0.505100022458323	0.614931257306031	0.00330238323058916	0.7709075376875
**ACC**	HDAC2	cg01129566	0.207959711918188	80	1.86562652696487	0.065902452415345	0.0432472417810956	0.176312517477108
**ACC**	HDAC2	cg08198132	-0.278815988299395	80	-2.54762770433588	0.0128382149454091	0.0777383553313686	0.0518544538210632
**ACC**	HDAC2	cg10999867	0.22821955825928	80	2.05690073934765	0.0430825960394091	0.0520841667720607	0.129264404361686
**ACC**	HDAC2	cg13393754	-0.0925149598824978	80	-0.815312111724558	0.417408461701934	0.00855901780206018	0.612850313666892
**ACC**	HDAC2	cg15005908	-0.158159778234777	80	-1.40553716915387	0.163883751449716	0.0250145154512737	0.33513412965352
**ACC**	HDAC2	cg15242771	-0.30259647410121	80	-2.78587872860191	0.00671765139271133	0.0915646261384842	0.0313567643430849
**ACC**	HDAC2	cg18387216	-0.26011692372057	80	-2.36388858130903	0.0206061176824445	0.0676608140058527	0.0745249151204299
**ACC**	HDAC2	cg18674783	0.235548878024791	80	2.12677514394153	0.0366415958963601	0.0554832739387377	0.114745041106249
**ACC**	HDAC2	cg22867643	-0.281879927233355	80	-2.57802608113958	0.0118450431845799	0.0794562933770816	0.0486882219962597
**ACC**	HDAC2	cg24023618	0.175538610431949	80	1.56463992773412	0.12176815049989	0.0308138037523797	0.273433538274692
**ACC**	HDAC3	cg01947930	0.198310650859877	80	1.77543033458809	0.0797782542803026	0.0393271142444682	0.202663560866684
**ACC**	HDAC3	cg01961137	-0.255613837285156	80	-2.32007770284444	0.0229872941684601	0.0653384338116422	0.0808880750199558
**ACC**	HDAC3	cg04284814	-0.213799310815114	80	-1.92048761205802	0.0584990064188703	0.0457101453050178	0.161664572301529
**ACC**	HDAC3	cg08520975	0.131032682698297	80	1.15980692270391	0.249711138822795	0.0171695639351126	0.443354672575783
**ACC**	HDAC3	cg10414058	0.179048411628798	80	1.5969497205566	0.114373046200283	0.0320583337067953	0.261950840974656
**ACC**	HDAC3	cg11064797	0.103430383969652	80	0.912491908031184	0.364357991612028	0.0106978443281097	0.56339300748278
**ACC**	HDAC3	cg11153694	0.0247958212906459	80	0.217649368049303	0.828278047169926	0.000614832753477648	0.907676716132601
**ACC**	HDAC3	cg12082871	0.156608489139233	80	1.39140276459014	0.168110334467061	0.0245262188704731	0.3411853423999
**ACC**	HDAC3	cg14098966	-0.0621231338777079	80	-0.546183244301638	0.586519078525414	0.00385928376278762	0.750613060550005
**ACC**	HDAC3	cg17940889	-0.0197437343535383	80	-0.173284343540709	0.862882650766599	0.000389815046223089	0.927067353520838
**ACC**	HDAC3	cg18671773	-0.0581942072343088	80	-0.511518976918586	0.610451503980543	0.00338656575562968	0.767686371774891
**ACC**	HDAC3	cg19549288	0.0363261035033358	80	0.318970788709601	0.750612343830155	0.00131958579573507	0.860202652739612

**ACC**	HDAC3	cg20642138	-0.323053648167078	80	-2.9953954033659	0.00368557295715283	0.104363659594058	0.0194159587342553
**ACC**	HDAC3	cg23421926	-0.00098661792998374	80	-0.0086575414132610	0.993114759161008	9.73414939765415e-07	0.996437637722882
**ACC**	HDAC3	cg24385322	0.0140730648497802	80	0.123502873427198	0.902030712506477	0.000198051154266118	0.948253223954811
**ACC**	HDAC6	cg07153944	-0.324135830106339	80	-3.00660532930093	0.00356626637067526	0.105064036358726	0.01893360581726
**ACC**	HDAC6	cg07674520	-0.378176832538637	80	-3.58471275197378	0.000589734631721495	0.143017716668956	0.00431101446533941
**ACC**	HDAC6	cg10783042	-0.33494256215174	80	-3.11928339490805	0.00255079724258726	0.112186519940772	0.0144728116614275
**ACC**	HDAC6	cg12510923	-0.220883224438207	80	-1.98732893205265	0.0504421598878156	0.0487893988382193	0.145218613955767
**ACC**	HDAC6	cg13178935	-0.230489958339025	80	-2.07850555384237	0.0409940687870697	0.0531256208951255	0.124578795547533
**ACC**	HDAC6	cg17152375	-0.231118939658575	80	-2.0844971758509	0.040430582675919	0.0534159642689038	0.123341459569949
**ACC**	HDAC6	cg24616736	-0.277603929599132	80	-2.53562581785124	0.0132505979304486	0.0770639417288796	0.0531047867395456
**ACC**	HDAC6	cg26680189	-0.217470711928976	80	-1.95508920183367	0.054201576047469	0.0472935105468957	0.153067753549817
**ACC**	HDAC9	cg00332048	-0.353386374434134	80	-3.31483439961806	0.00140000508443672	0.124881929635702	0.00887845402614089
**ACC**	HDAC9	cg02301805	-0.190101733943575	80	-1.69912043137459	0.0933335736457797	0.0361386692483539	0.226687231601617
**ACC**	HDAC9	cg03260621	-0.021975535940341	80	-0.192881124373971	0.847559909999476	0.000482924179865218	0.918692542496394
**ACC**	HDAC9	cg07513622	-0.141647471368903	80	-1.25561164164715	0.213053688822926	0.0200640061452041	0.399438440938225
**ACC**	HDAC9	cg08146609	-0.200325587926928	80	-1.79421984795634	0.0767033023526603	0.0401303411782692	0.197056131013601
**ACC**	HDAC9	cg08285151	-0.468119020892998	80	-4.64851019851875	1.35923845534303e-05	0.219135417721819	0.000171111824540074
**ACC**	HDAC9	cg08725366	0.129408421575539	80	1.14518370796272	0.25568004877	0.0167465395746724	0.449928741488109
**ACC**	HDAC9	cg10068437	-0.090075820453134	80	-0.793638327226326	0.429844966528246	0.00811365343030524	0.623880283701158
**ACC**	HDAC9	cg11321459	-0.18910691225325	80	-1.68989808986534	0.0950925496419603	0.0357614242619585	0.229718653754205
**ACC**	HDAC9	cg11765288	-0.106426016804028	80	-0.939218683569718	0.350555447229817	0.0113264970527712	0.549576939978823
**ACC**	HDAC9	cg12081743	-0.16765537076535	80	-1.4922923120226	0.139708565924664	0.0281083233464669	0.300893949537237
**ACC**	HDAC9	cg16475478	0.0848045760578857	80	0.746847576032519	0.457430020510213	0.00719181612035772	0.647623114786147
**ACC**	HDAC9	cg18856632	-0.170489829847993	80	-1.51827052222958	0.133041579495519	0.0290667820815978	0.290752307442283
**ACC**	HDAC9	cg19585556	-0.163671803204185	80	-1.45584655979651	0.14950035678991	0.0267884591641095	0.314978872209738
**ACC**	HDAC9	cg20648975	0.113212206689645	80	0.999861359555514	0.320509425103652	0.0128170037435389	0.519919390744804
**ACC**	HDAC9	cg21433125	-0.171503479200345	80	-1.52757014366164	0.130716902908696	0.0294134433778231	0.287249939244397
**ACC**	HDAC9	cg23074799	-0.0536963195346044	80	-0.471864044263428	0.63835827698657	0.00288329473156234	0.787311240406703
**ACC**	HDAC9	cg23385718	-0.09966214102086	80	-0.878907524118507	0.382185963262463	0.00993254235286178	0.580629684754749
**ACC**	HDAC9	cg24350771	0.0317234295115263	80	0.278512143452371	0.781365823184304	0.00100637597997277	0.879204663910782
**ACC**	HDAC9	cg25620356	-0.241479024531422	80	-2.1835909456441	0.0320377576503413	0.0583121192886472	0.103657682525813
**ACC**	HDGF	cg00692559	0.204766486839182	80	1.83571588352943	0.0702604155754302	0.0419293141324608	0.184887824959409
**ACC**	HDGF	cg00746981	0.309466752010658	80	2.85574762620034	0.00551617953658982	0.0957696706000261	0.0268270611946404
**ACC**	HDGF	cg01910231	-0.313069639210619	80	-2.89258466637289	0.00496553440104814	0.0980125989954673	0.0246323926837329
**ACC**	HDGF	cg02914429	-0.0018026601664786	80	-0.0158183044648929	0.987420260780331	3.24958367580864e-06	0.993306502620421
**ACC**	HDGF	cg04402095	-0.100914485250033	80	-0.890064702560814	0.376203660337917	0.0101837333332791	0.574709297173866
**ACC**	HDGF	cg05762852	-0.00484797388951832	80	-0.0425412981553599	0.966177312469544	2.35028508334514e-05	0.982241937479196
**ACC**	HDGF	cg05967123	0.268836343649561	80	2.44919429865724	0.0165902077046279	0.0722729796668647	0.0630154102911881
**ACC**	HDGF	cg06106793	0.352142716389628	80	3.30151392367052	0.00145945730237831	0.124004492706266	0.00919501877435961
**ACC**	HDGF	cg06119628	-0.422803965352672	80	-4.09402237149565	0.00010369907606243	0.178763193117943	0.000993860530146694
**ACC**	HDGF	cg07313589	-0.104683533218957	80	-0.923669298728635	0.358544014934907	0.0109586421272044	0.557505808355743
**ACC**	HDGF	cg07892339	0.348322231164397	80	3.2607181104666	0.00165663030526107	0.121328376723344	0.0101966847034955
**ACC**	HDGF	cg08597205	0.0908281937577933	80	0.800322235919891	0.425986496886784	0.00824976078130325	0.620619014903655
**ACC**	HDGF	cg08708935	-0.110635161587124	80	-0.976816192163772	0.331719134235781	0.0122401389794091	0.531431008232013
**ACC**	HDGF	cg09063372	0.326345572848881	80	3.02953628128493	0.00333329081434933	0.106501432918064	0.0179192886194107
**ACC**	HDGF	cg09477492	-0.270547435398377	80	-2.46600989193747	0.0158867523174937	0.073195914800639	0.0609806093544731
**ACC**	HDGF	cg11588596	0.288801249704029	80	2.64701232882003	0.00984402171671578	0.0834061618306089	0.0420864929341717
**ACC**	HDGF	cg12220178	-0.324700242961044	80	-3.01245710391194	0.00350541876932363	0.105430247778961	0.0186678907270511
**ACC**	HDGF	cg12421458	0.0980674979143297	80	0.864706891667	0.389885613303695	0.00961723414717707	0.587420324575462
**ACC**	HDGF	cg13170934	0.147960625272875	80	1.31279886319194	0.19315004238283	0.0218923466311402	0.3740767376302
**ACC**	HDGF	cg13235992	0.299518946876979	80	2.75473710189011	0.00732681855999668	0.0897115995382947	0.0335639219795302
**ACC**	HDGF	cg14155849	-0.0231667684658706	80	-0.203342142361041	0.839404027637164	0.000536699161151257	0.914062107026953
**ACC**	HDGF	cg15503799	0.129866397643608	80	1.14930590964172	0.253987304472497	0.0168652812369277	0.448052979979896
**ACC**	HDGF	cg17170583	0.0837617282299805	80	0.737598241367542	0.463000498128759	0.00701602711607311	0.652311077230358
**ACC**	HDGF	cg17218029	-0.394261441681117	80	-3.76456629760375	0.000324131721958782	0.155442084396473	0.00260706194975821
**ACC**	HDGF	cg17971171	0.246141512642277	80	2.22844352062415	0.0287685632559594	0.0605856442458283	0.0956787486611988
**ACC**	HDGF	cg18593717	-0.19039787497355	80	-1.70186680854587	0.0928149306972711	0.0362513507944437	0.225822378140474

ACC	HDGF	cg19599803	0.00573644574203529	80	0.0503379353338112	0.959983445355199	3.29068097513148e-05	0.979061311921597
ACC	HDGF	cg20284773	0.352559048468782	80	3.30597091209337	0.00143930560834566	0.124297882657213	0.00908842049477618
ACC	HDGF	cg23747104	0.316034291248622	80	2.92299992184836	0.00454957297915852	0.0998776732450189	0.0229824337883544
ACC	HDGF	cg26431298	0.340280953463933	80	3.17545212410985	0.00215209106320258	0.115791127290323	0.012596476726415
ACC	HDGFL1	cg02685945	0.0185230109438215	80	0.162566652238293	0.871285518694824	0.00034310193442493	0.931694647990998
ACC	HDGFL1	cg03930485	-0.086797606130208	80	-0.764531269617959	0.446887408081765	0.00753382442993473	0.638537221495322
ACC	HDGFL1	cg05276066	-0.287118193313553	80	-2.63019589087868	0.0103012721955381	0.082436856931639	0.0436076165413121
ACC	HDGFL1	cg06566039	-0.454257463790364	80	-4.47438016686059	2.60988199128044e-05	0.206349843409254	0.000302087724489913
ACC	HDGFL1	cg08671671	-0.186352541864478	80	-1.66439217360028	0.100098626469566	0.0347272698593521	0.238406531781324
ACC	HDGFL1	cg09081892	-0.576770716066798	80	-6.1955010819351	2.6492158981608e-08	0.332664458912207	6.62465771718165e-07
ACC	HDGFL1	cg11894257	-0.135284415443893	80	-1.19813057442351	0.234541074020696	0.0183018730619958	0.425530302541305
ACC	HDGFL1	cg15317945	-0.14652979194212	80	-1.29982366782101	0.19753920832794	0.0214709799266008	0.379881966051066
ACC	HDGFL1	cg20209681	-0.124713910892996	80	-1.10297130650277	0.273474947379422	0.015553559570226	0.469847836935397
ACC	HDGFL1	cg21898884	-0.0471224401173993	80	-0.413957590757604	0.680055973252713	0.00222052436261788	0.81536703013411
ACC	HIST1H1	cg00667898	-0.170218692667107	80	-1.51578384508858	0.133668685054295	0.0289744033332991	0.291716769304768
ACC	HIST1H1	cg00700007	-0.117161533961682	80	-1.03521796755595	0.303807429177623	0.0137268250402544	0.502335432413059
ACC	HIST1H1	cg03821311	0.0869405145952276	80	0.765799616051095	0.446136703585895	0.00755865307808298	0.638027073529075
ACC	HIST1H1	cg04676561	-0.130763995753525	80	-1.15738728294133	0.250691875659975	0.0170992225854279	0.444303961582474
ACC	HIST1H1	cg07138603	-0.109011798936716	80	-0.962309579987305	0.338906457924896	0.0118835723074189	0.538245697238009
ACC	HIST1H1	cg11206041	-0.115260520456005	80	-1.01819291557167	0.311774870681166	0.0132849875757892	0.510647289087113
ACC	HIST1H1	cg14334286	-0.102105269334453	80	-0.900677403135392	0.37056829813382	0.0104254860258612	0.569231551585055
ACC	HIST1H1	cg18798248	-0.136771764259425	80	-1.21155282336155	0.229388605133615	0.0187065154986358	0.419439585092594
ACC	HIST1H1	cg23460835	-0.10597861095738	80	-0.935225343035682	0.352595982460863	0.0112314659804556	0.551608261215685
ACC	HIST1H1	cg00903962	-0.09954997905788	80	-0.877908475938988	0.382724523532741	0.00991019833042435	0.58100637298675
ACC	HIST1H1	cg04290376	0.0723534767354019	80	0.636567591883397	0.526294432625676	0.00523502559570034	0.704994388147772
ACC	HIST1H1	cg07750111	-0.0117494196107443	80	-0.103107855863488	0.91814543709429	0.000138048861189343	0.956882161533155
ACC	HIST1H1	cg08283682	-0.0377573777208621	80	-0.331556065561623	0.741125221732974	0.00142561957235585	0.854392899923449
ACC	HIST1H1	cg10717387	-0.0994441011003457	80	-0.876965431617661	0.383233328465416	0.00988912924365578	0.581487693343062
ACC	HIST1H1	cg12633985	0.066991373713927	80	0.589170457828317	0.557471577833082	0.00448784415207903	0.728726037201686
ACC	HIST1H1	cg15022984	0.119393854907688	80	1.05522487927116	0.29462223862593	0.014254892589718	0.492454290536307
ACC	HIST1H1	cg16481032	-0.00268587266641781	80	-0.0235685220076612	0.981257752204367	7.21391198021032e-06	0.99035020835348
ACC	HIST1H1	cg16482183	0.0943487742441928	80	0.831616806032639	0.40819684761518	0.00890169120138165	0.604174971735948
ACC	HIST1H1	cg17691542	0.00457695728234276	80	0.0401630578360468	0.968067085068911	2.09485379643904e-05	0.983292326313032
ACC	HIST1H1	cg20855557	-0.0636682255631936	80	-0.559822223842765	0.577225688082684	0.0040536429463657	0.743836988830431
ACC	HIST1H1	cg23407813	0.0440451041929935	80	0.386869660907481	0.699919837899319	0.00193997120337165	0.828067278070711
ACC	HIST1H3	cg00958605	-0.133369766226276	80	-1.18086438083384	0.241291346109361	0.0177874945432515	0.433500819362871
ACC	HIST1H3	cg01043409	-0.16794683651317	80	-1.49496186699141	0.13901159653252	0.0282061398947813	0.299755194186071
ACC	HIST1H3	cg08041469	-0.206119739853679	80	-1.8483841420644	0.068386070559164	0.0424853471573483	0.181275520300475
ACC	HIST1H3	cg08401836	-0.062617458808423	80	-0.550546362471688	0.583538431403321	0.00392094614762455	0.748500201694833
ACC	HIST1H3	cg10075390	-0.0937794202691059	80	-0.826553695815009	0.411044038189692	0.0087945796660096	0.606917215009959
ACC	HIST1H3	cg15479139	-0.185713781982035	80	-1.65848298697502	0.101288489224106	0.0344896088180708	0.240460091266139
ACC	HIST1H3	cg15797414	-0.199834432713955	80	-1.78963759553243	0.0774439694542773	0.039933800498108	0.198374291994482
ACC	HIST1H3	cg21209604	-0.0857832201416115	80	-0.755529716053302	0.452236226293132	0.00735876085786417	0.642704216656563
ACC	HIST1H3	cg23099349	-0.0894065760386727	80	-0.787694067763002	0.433293778831245	0.00799353583895897	0.626850951786174
ACC	HIST1H3	cg25701114	-0.122553967775572	80	-1.08357485183648	0.281934717049768	0.015019475017536	0.479039628222554
ACC	HLTF	cg00906762	-0.00169141566465436	80	-0.0148421334524673	0.988196515489786	2.86088695063813e-06	0.993791816097166
ACC	HLTF	cg02059813	-0.275254399536163	80	-2.51239790600497	0.0140827712040825	0.0757649844640135	0.0555978230423951
ACC	HLTF	cg02398045	-0.218180859455659	80	-1.96179207477952	0.0534009114265897	0.0476028874328099	0.151367116178543
ACC	HLTF	cg02611507	0.0745586068357471	80	0.656075217533412	0.513731482939707	0.00555898585328751	0.69532555602783
ACC	HLTF	cg02710346	0.0508379518563809	80	0.446678810607308	0.656360769869021	0.0025844973489517	0.799713851919225
ACC	HLTF	cg03678609	0.0252342860171147	80	0.221500494717029	0.825289118162296	0.00063676919079355	0.906124448278398
ACC	HLTF	cg04120413	0.0994757650988709	80	0.87724745651382	0.383081122051037	0.00989542784200574	0.581293306852562
ACC	HLTF	cg04731810	-0.055137962823341	80	-0.484570815740901	0.629356405111317	0.00304019494430813	0.781299803386735
ACC	HLTF	cg04836786	-0.179457752689282	80	-1.60072202089997	0.113533641144677	0.0322050850002875	0.260694721414436
ACC	HLTF	cg05555455	-0.0303710993219398	80	-0.266628312793588	0.790468091454965	0.000922403674023131	0.884905426659435
ACC	HLTF	cg07275648	0.00997536025243464	80	0.0875377864216784	0.930471307581893	9.9507812165853e-05	0.963241790958822
ACC	HLTF	cg09352720	0.235232695223162	80	2.12375298162607	0.0369018273681514	0.0553344209019529	0.115366603104686

**ACC**	HLTF	cg09392355	0.14977816191478	80	1.3292929562439	0.187676491864191	0.0224334977865702	0.367030468540759
**ACC**	HLTF	cg15438497	-0.123350168603318	80	-1.09072296143632	0.278796241852536	0.0152152640944669	0.475714038438201
**ACC**	HLTF	cg16724124	0.411489322839821	80	3.96175816335006	0.000164928865057217	0.169323462811174	0.00147663438005077
**ACC**	HLTF	cg20117402	-0.175160041975815	80	-1.56115868485185	0.12258719384614	0.0306810403049692	0.2747018135173
**ACC**	HLTF	cg21089667	-0.343208654851343	80	-3.20640326693122	0.00195808096771449	0.117792180764868	0.0116895825955323
**ACC**	HLTF	cg21111416	-0.0338224316801305	80	-0.296960537118128	0.767296243652102	0.00114395688475709	0.871097320889139
**ACC**	HLTF	cg21835021	-0.14313585483313	80	-1.26907967879393	0.208235403182268	0.0204878729388108	0.393410519347241
**ACC**	HLTF	cg21926402	-0.174849310551408	80	-1.55830179220081	0.123262617614973	0.0305722814003029	0.275810166961176
**ACC**	HLTF	cg24041269	0.140518058331346	80	1.24539764896399	0.216762173041562	0.0197453247172116	0.404044482798496
**ACC**	HLTF	cg24710191	0.379989722162164	80	3.60478894375918	0.000552098956455774	0.144392188948879	0.00407882110337327
**ACC**	HLTF	cg25951608	0.167679379436227	80	1.49251219387379	0.139651055584225	0.0281163742881182	0.300805773712383
**ACC**	HLTF	cg26151310	-0.303924757123819	80	-2.79934922797001	0.00646882635249737	0.0923702579927721	0.0304290650179091
**ACC**	HLTF	cg27554556	0.110089069597429	80	0.971935354878333	0.334126059441154	0.0121196032448275	0.533715302619299
**ACC**	IDH1	cg00707300	0.200369775583684	80	1.79463216752466	0.0766369454517945	0.0401480469674561	0.196935378125378
**ACC**	IDH1	cg01046174	0.185305285094048	80	1.65470511234323	0.10205519572993	0.0343380486837862	0.241705837385184
**ACC**	IDH1	cg02241586	0.238531140156771	80	2.15531577520483	0.0342616303843314	0.0568971048244892	0.109029939169641
**ACC**	IDH1	cg03448938	-0.181154314221756	80	-1.6163659420582	0.110105255976107	0.0328168855611546	0.255184659578302
**ACC**	IDH1	cg05268564	-0.041597979178056	80	-0.365337010829408	0.71586150169029	0.00173039187169798	0.838452965650507
**ACC**	IDH1	cg07129696	0.110120521583246	80	0.972216440846438	0.333987134512702	0.0121265292737661	0.533570081985443
**ACC**	IDH1	cg07915542	0.112378330682501	80	0.992402229112855	0.324109767669494	0.0126288892069855	0.523487911680279
**ACC**	IDH1	cg09406631	0.119904591295357	80	1.0598045639414	0.292546669040122	0.0143771110137065	0.49023922562754
**ACC**	IDH1	cg09615745	-0.165851648583072	80	-1.47578082060989	0.144080492324018	0.0275067693377229	0.307265179536263
**ACC**	IDH1	cg10356455	0.247460004038398	80	2.24115694072594	0.0278968532238548	0.0612364535986838	0.093534493594375
**ACC**	IDH1	cg14772913	0.0244222151139445	80	0.21436800674057	0.830826774678087	0.00059644459107178	0.909252969856889
**ACC**	IDH1	cg15399923	-0.0996160706945241	80	-0.878497162766934	0.382407120775452	0.00992336154061642	0.580762524473012
**ACC**	IDH1	cg16085695	-0.240281771084804	80	-2.17209958720414	0.0329258816636714	0.05773532951565	0.105774530812011
**ACC**	IDH1	cg16863783	0.102393557260952	80	0.903247316370216	0.369211747938034	0.0104844405685519	0.567895658482936
**ACC**	IDH1	cg24324379	-0.223951712214829	80	-2.01638396507735	0.047247209966483	0.0501543694039537	0.138464850334624
**ACC**	IDH1	cg24830471	0.290480103770927	80	2.66381345299627	0.00940567537398492	0.0843786906867687	0.0406245422606465
**ACC**	IDH1	cg24987806	-0.0555214856003814	80	-0.487951728003435	0.626970615134424	0.00308263536327336	0.779445027386976
**ACC**	IDH1	cg25795625	0.180110131147733	80	1.60673581950827	0.112205699632028	0.0324396593420537	0.25858133712042
**ACC**	IDH1	cg26105702	-0.106853469092496	80	-0.943034466694372	0.348612788827092	0.0114176638571009	0.547767790224496
**ACC**	ING2	cg02075087	-0.344701090307211	80	-3.22222170320065	0.00186536785879265	0.11881884165898	0.0112308975471647
**ACC**	ING2	cg02552216	-0.380917804797591	80	-3.61508542252448	0.000533692840961447	0.145098374011816	0.00396051711053657
**ACC**	ING2	cg10348863	-0.0129186594321162	80	-0.1133702371237	0.910032101891717	0.000166891761523006	0.952265517025831
**ACC**	ING2	cg17985608	-0.116023757955556	80	-1.02502692654054	0.308559946447926	0.0134615124101295	0.507154137330079
**ACC**	ING3	cg01382559	-0.245169122009133	80	-2.21907578507621	0.0294260858872752	0.060107898386729	0.097283282712791
**ACC**	ING3	cg01416111	-0.250566427501889	80	-2.27116291768521	0.0259309402443119	0.0627835345910593	0.0885467283542271
**ACC**	ING3	cg10268125	-0.1177658608906	80	-1.04063259185367	0.301302632636752	0.0138687979913042	0.499670368862577
**ACC**	ING3	cg14303833	-0.403416672716756	80	-3.86874703809348	0.000227388880221019	0.162745011825858	0.00193433400537338
**ACC**	ING3	cg16130600	0.148461861616261	80	1.31734620273529	0.191629208139353	0.0220409243545659	0.372085426631544
**ACC**	ING3	cg18485114	0.103426939108617	80	0.912461187876215	0.364374053212432	0.0106971317333775	0.563395446417585
**ACC**	ING3	cg19478983	0.045105460603813	80	0.396202052727654	0.693051777000917	0.00203450257628212	0.8238263326131
**ACC**	ING3	cg19960827	-0.00763777569300066	80	-0.0670231646523308	0.946736988502934	5.83356175365917e-05	0.971563715369525
**ACC**	ING3	cg20553930	-0.188235596674404	80	-1.68182512696279	0.0966544419837902	0.0354326398553687	0.23243796283307
**ACC**	ING3	cg24859794	-0.177286157795544	80	-1.5807193493702	0.118041553346154	0.0314303817459065	0.267700335277111
**ACC**	ING4	cg00369908	-5.16289438047517e-05	80	-0.0004530421438491	0.999639696365676	2.6655478383942e-09	0.999832480991931
**ACC**	ING4	cg00443163	-0.194080896855411	80	-1.73606328550434	0.0865524798795791	0.0376673945242007	0.214866248197083
**ACC**	ING4	cg00591160	-0.321503869951384	80	-2.97936464876386	0.00386262525430722	0.103364738393716	0.0201914439614014
**ACC**	ING4	cg03930474	-0.0592922597584744	80	-0.521204440344829	0.603720175758734	0.00351557206726641	0.762691946166306
**ACC**	ING4	cg04375284	-0.294206429171828	80	-2.70120096428775	0.00849325297408991	0.0865574229660379	0.0376245157944508
**ACC**	ING4	cg08733494	-0.229398933434708	80	-2.06811906439808	0.0419869529290672	0.0526238706609817	0.126838032625188
**ACC**	ING4	cg12392916	-0.221775086295242	80	-1.99576738925954	0.0494957591141974	0.0491841889012618	0.143236895983469
**ACC**	ING4	cg18123790	0.130789264083724	80	1.15761482389727	0.250599531346762	0.0171058315995622	0.4442931685223
**ACC**	ING4	cg23354319	-0.283585481191862	80	-2.59498449684885	0.0113216733493189	0.0804207251428201	0.0469737736819687
**ACC**	ING4	cg27116036	-0.277779884346672	80	-2.53736731760428	0.0131900289770404	0.0771616641476504	0.0529165651198949
**ACC**	INO80	cg00734724	-0.118116696719218	80	-1.04377653419424	0.299854701992623	0.0139515540438598	0.498139930956139

**ACC**	INO80	cg06380007	0.189786964641552	80	1.69620182531418	0.09388733741412	0.0360190919478539	0.227645463536673
**ACC**	INO80	cg12580230	-0.139270968868511	80	-1.23412519435884	0.220909691327221	0.0193964027695737	0.40914812101778
**ACC**	INO80	cg18952654	0.157353826094972	80	1.39819249765982	0.166069712680223	0.0247602265867265	0.33835910188689
**ACC**	INO80	cg21007089	-0.107682962843862	80	-0.950440706584889	0.344862134362196	0.0115956204868326	0.544096773735974
**ACC**	INO80	cg21598047	-0.107879342143159	80	-0.952194397383531	0.343977889987974	0.0116379524612407	0.54326118061848
**ACC**	INO80	cg22835709	-0.460874410698205	80	-4.55697392988332	1.91824510155648e-05	0.212405222436418	0.000230994357175645
**ACC**	INO80	cg26409855	0.0830034438460498	80	0.730874320775817	0.467074117360083	0.00688957169030435	0.656184001848668
**ACC**	INO80	cg27441689	-0.488729032835415	80	-4.91564042040598	4.88232092511651e-06	0.23885606753624	6.97292590505808e-05
**ACC**	INTS12	cg00093890	-0.09617816566103	80	-0.847890685611765	0.399126835448157	0.00925023954992053	0.595722895509563
**ACC**	INTS12	cg01852585	0.0732641827649943	80	0.644622974645722	0.521087416419503	0.00536764047622249	0.70121907062686
**ACC**	INTS12	cg03607115	0.0986396027183572	80	0.869800825779777	0.387112662273655	0.00972977122443534	0.585053472605912
**ACC**	INTS12	cg19703969	-0.167594694420708	80	-1.49173662384121	0.139853989606467	0.0280879815979703	0.301128497740333
**ACC**	INTS12	cg26533745	-0.224570796595055	80	-2.0222535968105	0.046623279191	0.0504320426833376	0.13708562179245
**ACC**	IWS1	cg02372421	-0.0937977950412862	80	-0.826717084868128	0.410951970631251	0.00879802635460714	0.606817963770547
**ACC**	IWS1	cg02592214	-0.341904994922813	80	-3.1926082097137	0.00204243797336476	0.116899025553169	0.0120984862331484
**ACC**	IWS1	cg10180764	-0.329077364830569	80	-3.05796155148285	0.00306411304202781	0.108291912043832	0.0167382634640483
**ACC**	IWS1	cg10488833	0.14141062179 4937	80	1.25346924876951	0.213827650600388	0.0199969639564308	0.400416634590824
**ACC**	IWS1	cg15239145	-0.112454237568309	80	-0.993081138075275	0.323780967925356	0.0126459555470697	0.523196818745901
**ACC**	IWS1	cg17144508	0.0402154734628737	80	0.353175055260303	0.724922265869332	0.0016172843058431	0.844107163370787
**ACC**	IWS1	cg18451256	0.191347696451302	80	1.71067859857304	0.0911667631423536	0.0366139409372197	0.22287182364076
**ACC**	IWS1	cg18530950	-0.261671852805709	80	-2.3790548352921	0.0198344208994706	0.0684721585507725	0.0723393783147406
**ACC**	IWS1	cg19311602	0.0607930883913315	80	0.534445701923152	0.594573042782672	0.00369579959615624	0.75615154398485
**ACC**	IWS1	cg19409088	-0.273746309580606	80	-2.49751433820448	0.0146404227072329	0.0749370420090007	0.0572877141032383
**ACC**	IWS1	cg21581038	-0.0696379327286484	80	-0.612557465631673	0.541973865033437	0.00484944167471976	0.71674143402314
**ACC**	IWS1	cg21679674	-0.29669805839507	80	-2.72627542254821	0.0079273288745096	0.0880297378554043	0.035680318355913
**ACC**	IWS1	cg22409775	-0.383400179769944	80	-3.64268874585949	0.000487181615579177	0.146995697847625	0.00366642223105025
**ACC**	IWS1	cg23570261	-0.109384596053621	80	-0.965640257249598	0.337247321778441	0.0119649898538138	0.536816200160253
**ACC**	IWS1	cg23623107	-0.0881075461148849	80	-0.776159087043515	0.440032649587442	0.00776293968238657	0.632647063187372
**ACC**	JADE1	cg09863040	-0.335600179953841	79	-3.12618417336852	0.00249834350054031	0.11262748078505	0.0142315865225488
**ACC**	JADE1	cg12832492	-0.253315437887742	79	-2.29777891437971	0.0242904074290975	0.0641687110722583	0.0843630499370157
**ACC**	JADE1	cg14617908	0.0715820583220165	79	0.629745489766474	0.530725358059436	0.00512399107361658	0.708502050740909
**ACC**	JADE1	cg15389440	0.0173286698392909	79	0.152081296133646	0.87952059781147	0.00030028279839915	0.936148382503975
**ACC**	JADE1	cg15836763	-0.156271855451831	79	-1.38833696081127	0.169038013713922	0.024420892806358	0.342353576810312
**ACC**	JADE1	cg20039387	0.200312753449834	79	1.79410009026893	0.0767225845764097	0.040125199194654	0.197073127667477
**ACC**	JADE1	cg22922695	-0.080247491744294	79	-0.706447195899603	0.482042310999306	0.00643965993125054	0.66917475719514
**ACC**	JADE1	cg26766900	-0.293191453767473	79	-2.69100410676685	0.00873374763900579	0.085961228562284	0.0384099250284819
**ACC**	JADE2	cg04590931	-0.331063013602367	79	-3.07867690676247	0.00288083083009606	0.109602718975481	0.0159351076220487
**ACC**	JADE2	cg04842522	-0.056049958514076	79	-0.492610791069462	0.623689390977644	0.00314159784942964	0.777098533798108
**ACC**	JADE2	cg17378563	-0.27544439665143	79	-2.51427443644831	0.0140138359082618	0.07586961564667	0.0553931380848189
**ACC**	JADE2	cg18145011	-0.138022817877617	79	-1.22284914032971	0.225116273199291	0.0190502982548779	0.41445261761367
**ACC**	JADE2	cg21194015	-0.120736593308354	79	-1.06726681169473	0.289186177711174	0.0145773249637069	0.486720149884811
**ACC**	JADE2	cg25669900	-0.207650854664803	79	-1.8627307849049	0.0663141857534048	0.0431188774430232	0.177139673702007
**ACC**	JADE2	cg26133206	-0.23965748012547	79	-2.16611175745203	0.0333971185193893	0.05743570778009	0.106901695756425
**ACC**	JADE3	cg10860619	0.0339939332592229	79	0.298468056564423	0.766149919057072	0.0011555874984325	0.870360630846355
**ACC**	JADE3	cg17843048	-0.0367820936001885	79	-0.322980118903584	0.747585746982425	0.00135292240961303	0.858350397706137
**ACC**	JADE3	cg19194595	-0.0893371339935227	79	-0.787077342077768	0.43365253027261	0.00798112351017663	0.62719317443455
**ACC**	JARID2	cg01769037	-0.299000335192128	80	-2.74949858841988	0.00743412126529928	0.0894012004450049	0.0339435397178479
**ACC**	JARID2	cg03223185	0.250847978792621	80	2.27388619631658	0.0257587122623686	0.0629247084643431	0.0881017041872367
**ACC**	JARID2	cg05197191	-0.352064900844974	80	-3.30068112786306	0.00146325195243963	0.123949694406981	0.00921121721865098
**ACC**	JARID2	cg06047828	-0.114672165283301	80	-1.01292604504867	0.314267874267228	0.0131497054907606	0.513325529862153
**ACC**	JARID2	cg14862454	0.118728503401274	80	1.04926006170929	0.297340622571365	0.0140964575199064	0.49538091387152
**ACC**	JARID2	cg15109221	0.08874525933533	80	0.781821279112073	0.436717097708209	0.00787572105449498	0.629748637410527
**ACC**	JARID2	cg21397480	-0.153967228689718	80	-1.36736142802556	0.175490309725481	0.0237059075103919	0.351180767768257
**ACC**	JARID2	cg21901688	-0.348117704753868	80	-3.25853938728658	0.00166783353224377	0.121185936363101	0.01025590402398
**ACC**	JARID2	cg25338254	-0.176871727316901	80	-1.57690472822057	0.118917280172232	0.0312836079240643	0.269090168234055
**ACC**	JMJD6	cg02318866	0.0945688572351093	80	0.833574164634384	0.407099364049024	0.00894326875875448	0.603293417026951
**ACC**	JMJD6	cg04510639	0.0664572173521035	80	0.584451777425316	0.56062494154314	0.00441656173818472	0.731275263286503

ACC	JMJD6	cg07312083	-0.402198926826815	80	-3.85481117805534	0.000238508035900764	0.161763976740642	0.00201198158152939
ACC	JMJD6	cg08857478	-0.0418636814750635	80	-0.367674642731213	0.714124563398745	0.00175256782664557	0.837334111339502
ACC	JMJD6	cg10712116	0.0690583057094448	80	0.607434346567416	0.545349943953738	0.00476904958745913	0.719258834049045
ACC	JMJD6	cg12504415	0.136159977929096	80	1.20603088827184	0.2314983105106	0.0185395395896519	0.422003792708404
ACC	JMJD6	cg15457598	0.00568817699618783	80	0.0499143580757141	0.960319887194008	3.23553575399605e-05	0.979174862435392
ACC	JMJD6	cg21102709	-0.04482662768439	80	-0.393747864177532	0.694855444797691	0.00200942654955492	0.825048764522763
ACC	JMJD6	cg21807925	-0.0552114644027547	80	-0.485218745921453	0.628898877094763	0.00304830580149665	0.780881350369655
ACC	JMJD6	cg23630158	-0.152116763524317	80	-1.35053600520531	0.180800040218653	0.023139509745113	0.357955827469775
ACC	JMJD6	cg25004679	0.0658137759172313	80	0.578768354131351	0.564434671823255	0.00433145310048354	0.734151995649863
ACC	JMJD6	cg25908504	-0.102666898273935	80	-0.905684199288057	0.367928331008203	0.0105404920011906	0.566840293793027
ACC	JMJD6	cg26612088	-0.140283593184456	80	-1.24327785626967	0.217537724241118	0.019679486516742	0.405063063252197
ACC	JMJD8	cg00873473	-0.373255583949791	80	-3.53045558861241	0.000704004248520356	0.139319730949699	0.00501027412948427
ACC	JMJD8	cg02851105	-0.363468182835631	80	-3.42356990413153	0.00099314742221196	0.132109119933836	0.0066782704264463
ACC	JMJD8	cg03899598	-0.357910316132295	80	-3.36345939354527	0.00120175128152415	0.128099794393919	0.00782805845812713
ACC	JMJD8	cg05787952	-0.27372358831941	80	-2.49729025129844	0.0146489682719334	0.0749246028024536	0.0573153869007085
ACC	JMJD8	cg06191810	-0.365649976171584	80	-3.44728135980925	0.000920670954670502	0.13369990507428	0.00626620089655617
ACC	JMJD8	cg06646636	-0.202429755337608	80	-1.81386676672326	0.0735940467155896	0.0409778058460438	0.191221618910908
ACC	JMJD8	cg08398290	-0.382122407986975	80	-3.6284687883342	0.00051063861138362	0.146017534685764	0.00381527277506505
ACC	JMJD8	cg26750188	-0.162352116399089	80	-1.44378895076795	0.152854854081121	0.0263582096992633	0.319905047439852
ACC	JMJD8	cg27579097	-0.134150571471313	80	-1.1879039990144	0.238522531706129	0.0179963758260799	0.430215432787243
ACC	KAT2B	cg00770008	0.0378712745144664	80	0.332557653393424	0.740371896626876	0.00143423343335007	0.853901222015851
ACC	KAT2B	cg04371528	-0.152911622227495	80	-1.35776149449427	0.178505118625224	0.0233819642122442	0.355102214451253
ACC	KAT2B	cg05072819	-0.0732811488971797	80	-0.644773058905048	0.5209906588772	0.00537012678369062	0.701140149031883
ACC	KAT2B	cg12062464	0.00190425259075195	80	0.0167097789647908	0.986711367631595	3.62617792938551e-06	0.993007258731262
ACC	KAT2B	cg14937543	-0.121092757805501	80	-1.07046195703989	0.287755438431417	0.0146634559929418	0.485259572080199
ACC	KAT2B	cg18053505	-0.0661908142292342	80	-0.582098589506599	0.56220079693439	0.00438122388832899	0.732471090214603
ACC	KAT5	cg00582417	-0.359141288649911	80	-3.37673710597389	0.00115239840664856	0.128982465213118	0.00755772059069449
ACC	KAT5	cg02561205	-0.299426321005853	80	-2.75380128839578	0.00734588268762229	0.0896561217111001	0.0336386683909181
ACC	KAT5	cg05785768	-0.299424964832326	80	-2.75378758740038	0.00734616213709678	0.0896553095648399	0.0336386683909181
ACC	KAT5	cg07564927	-0.158995193467258	80	-1.41315338031452	0.161640390492574	0.0252794715456907	0.331995261853926
ACC	KAT5	cg14726348	-0.357688479676798	80	-3.3610687280954	0.00121084614291176	0.127941048493499	0.00787794303267354
ACC	KAT5	cg18896539	0.104597609528374	80	0.922902766033745	0.35894082107819	0.0109406599190501	0.557944376984769
ACC	KAT5	cg20108992	0.0227131357103518	80	0.199358386771748	0.842507921828648	0.000515886533796856	0.915788417761011
ACC	KAT5	cg20423942	-0.223276212335725	80	-2.0099823809032	0.0479358350125728	0.0498522669949879	0.139908469609368
ACC	KAT5	cg22048492	-0.23060259558176	80	-2.07957832401554	0.0408926835808411	0.0531775570890449	0.124358134024782
ACC	KAT6A	cg10163397	-0.251386371598368	80	-2.27909546608295	0.0254320710897802	0.0631951078253927	0.0873217073240079
ACC	KAT6A	cg12468056	0.110409751010026	80	0.97480141900346	0.33271130675059	0.0121903131180958	0.532362726451704
ACC	KAT6A	cg15251374	-0.58011883826294	80	-6.24962934317257	2.1059183459168e-08	0.336537866507543	5.40240466396453e-07
ACC	KAT6A	cg20966972	0.320475175593062	80	2.96873869190173	0.00398429088843794	0.102704338171404	0.0206863749532912
ACC	KAT6B	cg04396765	-0.0837328963530715	80	-0.737342557495091	0.463155031363261	0.00701119793167422	0.652434269078742
ACC	KAT6B	cg06972998	-0.184906555609569	80	-1.65101842567129	0.102807929688519	0.0341904343073945	0.242984659948006
ACC	KAT6B	cg09631472	0.0358192712594658	80	0.314514658771815	0.753980820209961	0.00128302019355919	0.862529248721181
ACC	KAT6B	cg10359932	0.00827410668573277	80	0.0726074769318105	0.942306750526678	6.84608414468878e-05	0.969305657429882
ACC	KAT6B	cg10739686	-0.303622261771487	80	-2.79627995840593	0.00652476617853615	0.0921864778432332	0.0306458769602754
ACC	KAT6B	cg11128436	0.0800126250716282	80	0.704366239210748	0.483329640621241	0.00640202017085295	0.67019421456977
ACC	KAT6B	cg11348552	0.0322965650002525	80	0.283549126820818	0.777516925026891	0.00104306811081553	0.877015151839124
ACC	KAT6B	cg15874302	-0.119153458969777	80	-1.05306958493577	0.295602518601007	0.0141975467844623	0.493522183638208
ACC	KAT6B	cg15912797	0.283638938020623	80	2.59551645262864	0.0113056000812645	0.0804510471614671	0.046914591813191
ACC	KAT6B	cg17147776	-0.303503164788957	80	-2.79507179631237	0.00654690728019378	0.092114171036913	0.0307188358382437
ACC	KAT6B	cg18462764	-0.00228267641274059	80	-0.020030456415271	0.984070891082154	5.21061160528223e-06	0.991642628249288
ACC	KAT6B	cg18491775	0.202217791862118	80	1.81188645174321	0.0739026067713592	0.0408920353455909	0.191773554527206
ACC	KAT6B	cg24671245	0.21633804576469	80	1.94440503879075	0.055498916479447	0.0468021500452849	0.155579356008868
ACC	KAT6B	cg26605858	0.12750320185368	80	1.12804296292382	0.262804401946819	0.0162570664829403	0.458183493087354
ACC	KDM1A	cg00062312	0.206292099016589	80	1.84999844774018	0.0681502622710029	0.0425564301166702	0.18081852128756
ACC	KDM1A	cg00541565	-0.129464843221426	80	-1.14569151384394	0.255471092286776	0.0167611456303485	0.449732256644056
ACC	KDM1A	cg04886391	-0.387836804597487	80	-3.6922540425392	0.000413176028098519	0.150417387000389	0.00318970089608973
ACC	KDM1A	cg06958034	0.243569998469773	80	2.20368575962671	0.0305349459361546	0.0593263441545651	0.100009918497693

**ACC**	KDM1A	cg07118078	-0.0531390444597373	80	-0.466952969888024	0.641852148128714	0.00282375804609393	0.789630463145797
**ACC**	KDM1A	cg07785945	-0.0404653781820505	80	-0.355373324134045	0.723281581202136	0.00163744683141637	0.843235653071571
**ACC**	KDM1A	cg10499166	0.0594475620528467	80	0.522574449549289	0.602770780553383	0.00353401263402706	0.762037053747784
**ACC**	KDM1A	cg12578844	-0.0719318853374802	80	-0.632839072209884	0.528713700020215	0.0051741961282044	0.707090608045052
**ACC**	KDM1A	cg22683154	-0.168532193433089	80	-1.50032441109709	0.137619791775235	0.028403100223368	0.297702966502493
**ACC**	KDM1A	cg26662347	0.315363446590944	80	2.91610924740771	0.00464088819606035	0.0994541034457193	0.0233542438918635
**ACC**	KDM1B	cg11007366	-0.0579236252743737	80	-0.509132573741292	0.612115237770176	0.00335514636492607	0.768865647059922
**ACC**	KDM1B	cg15124342	-0.185534895085889	80	-1.65682848970152	0.101623686096723	0.0344231972945317	0.241044187221889
**ACC**	KDM1B	cg20677365	-0.240789454590189	80	-2.17697109565538	0.0325467900814257	0.0579795614418406	0.104864153555371
**ACC**	KDM1B	cg22406543	0.0120428235461592	80	0.105683011601471	0.916108693734175	0.000145029598963927	0.955716236024627
**ACC**	KDM1B	cg23073439	-0.271467087823993	80	-2.47505809422356	0.015519405446922	0.0736943797716394	0.0599447115156937
**ACC**	KDM1B	cg23860886	-0.132058504329071	80	-1.16904727771746	0.245990965151869	0.0174394485656311	0.439003949049678
**ACC**	KDM1B	cg24342628	0.0190884080962821	80	0.167530625380435	0.867391769661055	0.00036436732365021	0.929511334513563
**ACC**	KDM2A	cg05038268	-0.518021235585597	80	-5.31422637538086	1.01113363202122e-06	0.268346000517628	1.72424659731386e-05
**ACC**	KDM2A	cg05295919	-0.307576572072436	80	-2.83647638908693	0.00582613226404424	0.0946033476878301	0.0280226371552527
**ACC**	KDM2A	cg06320126	0.0123506420065851	80	0.108384710497358	0.91397246843322	0.000152538357974823	0.954601374935138
**ACC**	KDM2A	cg07283932	-0.428364446341021	80	-4.1598689430762	8.20526951374436e-05	0.18349609888905	0.00081108098672456
**ACC**	KDM2A	cg08836144	-0.32556353096168	80	-3.021414548272	0.00341414095235475	0.105991612692237	0.0182743691514714
**ACC**	KDM2A	cg09993857	0.00290026351547146	80	0.025449816098466	0.979762017580822	8.41152845917488e-06	0.989607977092735
**ACC**	KDM2A	cg11637544	0.396622192216779	80	3.79130075626637	0.000296106003679253	0.157309163358843	0.00241840109713636
**ACC**	KDM2A	cg16004548	-0.144504796245671	80	-1.28147467089688	0.203872618243016	0.0208816361380029	0.387786913078789
**ACC**	KDM2A	cg18132081	0.20279256294709	80	1.81725699299091	0.0730682969817508	0.0411248235866493	0.190274991477483
**ACC**	KDM2A	cg21293464	-0.485191381984531	80	-4.86905103209533	5.84806732288678e-06	0.235410677152059	8.17510421054185e-05
**ACC**	KDM2A	cg21711868	-0.513280245760869	80	-5.24808360303545	1.31776564996458e-06	0.263456610688338	2.18347733702544e-05
**ACC**	KDM2A	cg25597833	-0.215756754439024	80	-1.93892506140451	0.0561745046841567	0.0465509770860613	0.156952893632126
**ACC**	KDM2B	cg03486832	-0.221863858873022	80	-1.99660760940857	0.0494023623487241	0.0492235718740281	0.143048123245876
**ACC**	KDM2B	cg05067991	0.0346376282075751	80	0.304126448917664	0.761851926086063	0.0011997652878462	0.867651061924506
**ACC**	KDM2B	cg07020001	0.064717267755275	80	0.56908472610743	0.570954950390609	0.00418832474570795	0.738938751446323
**ACC**	KDM2B	cg07529210	0.0730780446897415	80	0.642976413813139	0.522149553763987	0.00534040061567585	0.702005296257744
**ACC**	KDM2B	cg07793808	0.0432038736240277	80	0.379466769400114	0.705385794770709	0.00186657469612095	0.831927692399941
**ACC**	KDM2B	cg09151742	0.0380709461744442	80	0.334313561254241	0.739051836993701	0.00144939694261742	0.853018352990141
**ACC**	KDM2B	cg12251659	-0.0665753538451301	80	-0.585495339006326	0.559926800180328	0.00443227773960428	0.730601673755179
**ACC**	KDM2B	cg12888660	-0.21711428268593	80	-1.95172620052139	0.0546071226772797	0.0471386117462261	0.15388561447817
**ACC**	KDM2B	cg15234492	0.0584857126419858	80	0.514090041465891	0.608661322648907	0.00342057858324094	0.766409160267997
**ACC**	KDM2B	cg15316703	-0.338233100686572	80	-3.15386475146956	0.00229788754339029	0.114401630400053	0.0133066743747584
**ACC**	KDM2B	cg16837338	0.139640004269794	80	1.2374602827193	0.21967660089534	0.0194993307924681	0.407699059809911
**ACC**	KDM2B	cg17452615	-0.0150855324999839	80	-0.132390075531936	0.895021114065726	0.00022757329080807	0.944226641546367
**ACC**	KDM2B	cg20055546	-0.375158235746221	80	-3.55139096414451	0.000657626959954421	0.140743701848217	0.00472953977282927
**ACC**	KDM2B	cg21249371	0.0353523687155634	80	0.310409810668042	0.757087986369745	0.00124978997380114	0.864554383354587
**ACC**	KDM2B	cg21781916	-0.0377232733612552	80	-0.33125616050215	0.741350838849011	0.00142304535308798	0.854447562964576
**ACC**	KDM2B	cg26218726	-0.268897876038527	80	-2.44979856784188	0.0165644535960823	0.0723060677380312	0.0629544727663114
**ACC**	KDM2B	cg26509318	-0.14910934770239	80	-1.32322189324512	0.189677435828678	0.0222335975722322	0.369653105816536
**ACC**	KDM3A	cg01744130	-0.206968471763075	80	-1.85633505418707	0.0672312274521834	0.0428359483039427	0.178984108589811
**ACC**	KDM3A	cg02152982	-0.127384991138808	80	-1.12697987351859	0.263250820970296	0.0162269359674341	0.458677511459408
**ACC**	KDM3A	cg02972551	0.185491756704844	80	1.6564295350159	0.101704647892943	0.0344071918054492	0.241161039325276
**ACC**	KDM3A	cg03037033	-0.138621654451227	80	-1.22825840888034	0.223091096629526	0.0192159630827953	0.411845011181712
**ACC**	KDM3A	cg04793821	-0.354228305195271	80	-3.32386347934312	0.00136100905654794	0.125477692201514	0.00867570784538595
**ACC**	KDM3A	cg07052513	-0.0165863007424427	80	-0.1455642224606	0.884645796911792	0.000275105372318756	0.938851752588101
**ACC**	KDM3A	cg07211384	-0.299702202599835	80	-2.75658881493634	0.00728922928900334	0.0898214102431925	0.0334330701791596
**ACC**	KDM3A	cg12888358	-0.276248145575156	80	-2.52221629486772	0.0137254376872944	0.0763130379337125	0.0545306195723983
**ACC**	KDM3A	cg18611949	-0.251390363351944	80	-2.27913409709255	0.0254296624890084	0.0631971147862225	0.0873217073240079
**ACC**	KDM3A	cg19139691	-0.244040084416459	80	-2.20820789450707	0.0302053876555299	0.0595555628019924	0.0992020064708193
**ACC**	KDM3A	cg20191795	-0.145084206810861	80	-1.28672316561246	0.202045831662602	0.0210494270659366	0.385489611722187
**ACC**	KDM3A	cg20918903	-0.21839451607522	80	-1.96380935628732	0.0531619255576729	0.0476961646517295	0.150882117359854
**ACC**	KDM3A	cg22404242	-0.0406817917382652	80	-0.357277044360262	0.721861780492548	0.00165500817903558	0.842438969412416
**ACC**	KDM3A	cg24749656	-0.122957908068467	80	-1.08720106935415	0.280339535795488	0.0151186471565737	0.477560029072945
**ACC**	KDM3B	cg00435490	-0.0346523147716898	80	-0.304255555369669	0.761753946372295	0.00120078291903627	0.867590280277675

**ACC**	KDM3B	cg03315484	-0.299447896565332	80	-2.75401926196611	0.00734143814307205	0.089669042757402	0.0336229741494258
**ACC**	KDM3B	cg05191700	-0.517357343253738	80	-5.30492433020821	1.04959375571364e-06	0.267658620618566	1.78240769770764e-05
**ACC**	KDM3B	cg05708550	-0.097639670552481	80	-0.860898147943537	0.391966978810131	0.00953350526559702	0.589059773004063
**ACC**	KDM3B	cg05722427	-0.131639392463101	80	-1.16527155859909	0.247506258508106	0.0173289296480544	0.44080819309477
**ACC**	KDM3B	cg15001364	-0.292152199596522	80	-2.68057364891388	0.008986155608354	0.0853529077290861	0.0392978239022031
**ACC**	KDM3B	cg18682923	-0.0661634955306637	80	-0.581857285480285	0.562362513843705	0.00437760814083616	0.732559099162043
**ACC**	KDM3B	cg19076659	-0.306732857226076	80	-2.82788632845741	0.00596938416438055	0.0940850457020725	0.02855101578136
**ACC**	KDM3B	cg24137987	-0.426572576515325	80	-4.13858738605336	8.85224130743304e-05	0.181964163034923	0.000866445974170163
**ACC**	KDM4A	cg00154982	-0.294063316985539	80	-2.69976259606118	0.00852680739189112	0.0864732343965375	0.037728043126196
**ACC**	KDM4A	cg00979347	0.310493214210633	80	2.86622853391238	0.00535404812700732	0.0964060360708501	0.0261957748422898
**ACC**	KDM4A	cg03587511	0.150976267879495	80	1.34017326071928	0.184130294977391	0.022793833462821	0.362502320891462
**ACC**	KDM4A	cg08552254	-0.396725879015943	80	-3.79247700242674	0.000294927680474962	0.157391423080973	0.002410802140892
**ACC**	KDM4A	cg10958452	0.11749421187776	80	1.03819853719413	0.302426882486017	0.013804889824776	0.500768258797759
**ACC**	KDM4A	cg10968649	0.25644477276907	80	2.32814966877427	0.0225310662039996	0.06576392148058	0.0796501756009247
**ACC**	KDM4A	cg12293275	0.0816139392013518	80	0.718556501506816	0.474589053012853	0.00666083507196194	0.662507314860488
**ACC**	KDM4A	cg17737314	0.232940580446107	80	2.10186558195358	0.038834840344455	0.0542613140185692	0.119828629493133
**ACC**	KDM4A	cg19743044	0.02401942898692	80	0.210830460261839	0.833576521621652	0.000576932968857693	0.910712055656693
**ACC**	KDM4A	cg20873046	-0.0686683101241522	80	-0.603987661141556	0.547627244379222	0.00471533681530675	0.720908940675814
**ACC**	KDM4A	cg21451504	-0.336454134202731	80	-3.13515291752096	0.00243167366657931	0.113201384422109	0.013914646650818
**ACC**	KDM4A	cg22094309	-0.341600885618033	80	-3.18939319581221	0.00206258040911598	0.116691165055024	0.0121953424391656
**ACC**	KDM4A	cg24324755	-0.0126333546785056	80	-0.110866084955283	0.912011010723683	0.000159601650432918	0.953299770508344
**ACC**	KDM4A	cg24505556	-0.28410302022209	80	-2.60013570095146	0.0111668936372065	0.080714526099313	0.0464728681117447
**ACC**	KDM4A	cg25841161	0.134373982248537	80	1.18991864962451	0.237734355729858	0.01805636710533	0.429231704914442
**ACC**	KDM4B	cg01098771	-0.0578474003867531	80	-0.508460327120422	0.612584278328687	0.00334632173150532	0.769255550416496
**ACC**	KDM4B	cg12072803	0.0445473576076096	80	0.39128992034922	0.696663639410284	0.00198446706982026	0.826149771562684
**ACC**	KDM4B	cg18115693	0.124881164445423	80	1.10447391687859	0.272827045759082	0.0155953052332448	0.469146967649161
**ACC**	KDM4C	cg00121158	0.313596666320367	80	2.89798470495776	0.00488921265490637	0.0983428691272476	0.0243501503659944
**ACC**	KDM4C	cg00667760	0.287952905618506	80	2.63853267576393	0.0100722334010625	0.08291687585414	0.0428713314659002
**ACC**	KDM4C	cg05545111	0.181035223940406	80	1.61526733066702	0.110343263914586	0.0327737523071528	0.255591599612069
**ACC**	KDM4C	cg14196303	-0.217337473156588	80	-1.95383196385505	0.0543528863412411	0.0472355772380908	0.153350322417964
**ACC**	KDM4C	cg14473811	0.194376208462515	80	1.73880850388982	0.0860651973614179	0.037782110416263	0.21396382345437
**ACC**	KDM4C	cg14497348	0.172163748497749	80	1.533630402415	0.129219356058353	0.0296403562967963	0.284942617001716
**ACC**	KDM4E	cg15555590	0.0686149057328635	80	0.603515707909702	0.547939447021076	0.00470800528872974	0.721197890898529
**ACC**	KDM4E	cg19409026	-0.0991419027748241	80	-0.874273946882815	0.384687799065295	0.00982911688581266	0.582792651226593
**ACC**	KDM5B	cg00528052	0.0568540198663337	80	0.499700263753036	0.618711100299052	0.00323237957496147	0.773688979049231
**ACC**	KDM5B	cg00571448	0.277101387713667	80	2.53065346648285	0.0134249172511912	0.0767851790728398	0.0536348609613379
**ACC**	KDM5B	cg02624558	0.254328028823318	80	2.30759779306896	0.0237087664055606	0.0646827462451543	0.0828596788799222
**ACC**	KDM5B	cg04504182	0.136020343305607	80	1.20477075449182	0.231981718979389	0.0185015337929752	0.422609711862845
**ACC**	KDM5B	cg08915090	0.151054621375075	80	1.34088501867158	0.183900087683281	0.0228174986387672	0.362231255126805
**ACC**	KDM5B	cg09889216	-0.0890280715535443	80	-0.784332650326146	0.435251249087613	0.00792599752454301	0.628642376286097
**ACC**	KDM5B	cg11171004	0.386373074875066	80	3.67586840466979	0.000436368971856585	0.149284152988413	0.00334024282166793
**ACC**	KDM5B	cg11221497	-0.162851310852084	80	-1.44834901110737	0.151579417098467	0.0265205494462421	0.317965619901811
**ACC**	KDM5B	cg13134514	0.0872700090071983	80	0.768724138570296	0.444408540524771	0.00761605447211647	0.636371527943277
**ACC**	KDM5B	cg13253785	-0.0835057427067271	80	-0.73532820216433	0.464373515820954	0.00697320906500211	0.653608495272151
**ACC**	KDM5B	cg13993945	-0.0760707717559258	80	-0.669458120966238	0.505205776086828	0.00578676231554217	0.688361119973705
**ACC**	KDM5B	cg14560895	-0.316747096042417	80	-2.93032693325211	0.00445429767380107	0.100328722851304	0.0226201209762831
**ACC**	KDM5B	cg21105875	0.224875848884011	80	2.02514679107896	0.0463183481607802	0.0505691474113044	0.136405571502867
**ACC**	KDM5B	cg24354901	0.0221476687575763	80	0.194392687135246	0.846380375969444	0.00049051923139532	0.917900305503955
**ACC**	KDM5B	cg27531483	-0.456587677842809	80	-4.50335820124485	2.34340028617153e-05	0.208472307557889	0.000275007515254831
**ACC**	KDM5C	cg01859586	-0.212060599774091	80	-1.90413077494356	0.0606288852167032	0.0449696979765471	0.16590093199255
**ACC**	KDM5C	cg04927657	-0.0732939326434594	80	-0.644886145693413	0.520917759397549	0.00537200056234396	0.701112044542784
**ACC**	KDM5C	cg04927982	-0.479964759136296	80	-4.80078922389026	7.60744356491282e-06	0.230366170012763	0.000102888293582898
**ACC**	KDM5C	cg09554224	-0.00914818298741355	80	-0.080278339211265	0.936224170474086	8.36892519712028e-05	0.965975531418088
**ACC**	KDM5C	cg12234996	-0.440321731741099	80	-4.30344745020906	4.89053716703348e-05	0.19388322744348	0.000520027571258743
**ACC**	KDM5C	cg16417926	-0.222830219249975	80	-2.00575746020365	0.0483950096266372	0.0496533066109917	0.140799632704836
**ACC**	KDM5C	cg16824069	-0.135712699134472	80	-1.20199467521772	0.233049246418149	0.0184179367063639	0.423812078074092
**ACC**	KDM5C	cg19903753	-0.437730641502341	80	-4.27209929684158	5.47987807760847e-05	0.191608114510051	0.000574580673409575

ACC	KDM5C	cg24985300	0.0472386555805003	80	0.414980791882187	0.679309960742417	0.00223149058105313	0.814944832106396
ACC	KDM5C	cg27526317	-0.130805519278217	80	-1.15776120290685	0.250540138273553	0.017110083873644	0.444218240424213
ACC	KDM6A	cg00751785	-0.481940745671817	80	-4.82651733429637	6.89087539340394e-06	0.232266882338707	9.42289963709731e-05
ACC	KDM6A	cg03267667	-0.0903729872485532	80	-0.796278123279256	0.428318604742143	0.00816727682422717	0.622537747663627
ACC	KDM6A	cg03478540	-0.0655226218442026	80	-0.57619687187466	0.566162560817248	0.00429321397333837	0.735244872484184
ACC	KDM6A	cg06877198	0.0459943666046275	80	0.404026510587985	0.687313188997497	0.00211548175936087	0.820086845271726
ACC	KDM6A	cg07167981	-0.429050942612252	80	-4.1680381702668	7.96922623464682e-05	0.184084711356462	0.000792076128399277
ACC	KDM6A	cg11895950	-0.0778719640234591	80	-0.685405033911776	0.495146705030515	0.00606404278087091	0.680027093380728
ACC	KDM6A	cg14384228	-0.0952874049266098	80	-0.839965593650787	0.403528228844392	0.00907968953764771	0.59996703237048
ACC	KDM6A	cg15923127	0.0922045211528915	80	0.812552791461179	0.418979656922542	0.00850167372103402	0.614386911943009
ACC	KDM6A	cg17824914	-0.293501839063788	80	-2.69412130529163	0.00865957758272955	0.086143329533826	0.0381657231018561
ACC	KDM6A	cg20211377	-0.225187452617052	80	-2.02810276847424	0.0460085696462223	0.0507093888161569	0.135775473955004
ACC	KDM6A	cg22133177	0.057363230090548	80	0.504190514284869	0.615567189328355	0.00329054016642115	0.771320511018927
ACC	KDM6A	cg27306636	-0.232615592384106	80	-2.09876528051731	0.0391156194139675	0.0541100138202085	0.120484656980131
ACC	KDM6B	cg01046511	-0.125958260197241	80	-1.1141528712227	0.268679295672983	0.0158654833119158	0.464480825057424
ACC	KDM6B	cg02308232	-0.13194902120429	80	-1.1680608983529	0.246386183266125	0.0174105441967703	0.439517696422951
ACC	KDM6B	cg24893073	-0.0500854108177337	80	-0.440049986171758	0.661133661934698	0.00250854837678116	0.802850864733351
ACC	KDM8	cg00636124	0.0107041169046155	80	0.0939336261597226	0.92540588133422	0.000114578118707674	0.960725773781118
ACC	KDM8	cg01755539	-0.502608179469414	80	-5.10155673388698	2.35785991256474e-06	0.252614982069559	3.65320362553398e-05
ACC	KDM8	cg06329197	-0.0108112188608189	80	-0.0948736051798736	0.924661684267853	0.000116882453256527	0.960235090051439
ACC	KDM8	cg08572679	-0.440126193068198	80	-4.30107710539443	4.93286945552218e-05	0.193711065824704	0.000523887571908385
ACC	KDM8	cg09140090	-0.257951763274831	80	-2.34280315751484	0.0217233193693554	0.0665391121765946	0.0775666180300011
ACC	KDM8	cg10214171	0.246457882322427	80	2.2314928789232	0.0285573286694343	0.060741487758855	0.0951486244440384
ACC	KDM8	cg10221365	-0.302745681018502	80	-2.78739098575826	0.00668928648119156	0.0916549473753564	0.0312431767842109
ACC	KDM8	cg16752029	0.143046058410182	80	1.2682668835994	0.208523883558169	0.0204621748266892	0.393716494284845
ACC	KDM8	cg24206694	-0.169589397212145	80	-1.51001376059323	0.135132802499	0.0287605636467786	0.293908556184501
ACC	KDM8	cg24705286	0.239024777850669	80	2.16004615988262	0.0338804629071204	0.0571328444265617	0.108104537607011
ACC	KIAA202(	cg03495688	0.334683043150852	80	3.11656152000128	0.00257176754166482	0.112012739372715	0.0145546401961496
ACC	KIAA202(	cg25608569	-0.530408682995193	80	-5.49025161153136	4.9648070335074e-07	0.281333370996695	9.16327872751304e-06
ACC	KMT2B	cg09284703	0.285351404157442	80	2.61257138638018	0.010801101316351	0.0814254238546236	0.0452799954628556
ACC	KMT2B	cg13830730	0.236362373984927	80	2.13455405168816	0.0359790915322213	0.0558671718357907	0.113212694521604
ACC	KMT2B	cg15496871	0.169738713981599	80	1.51138269133174	0.134784308036045	0.0288112310241269	0.293380259075435
ACC	KMT2B	cg24471867	0.195291116695144	80	1.74731661917332	0.0845693237796543	0.0381386202600364	0.21124409112877
ACC	L3MBTL1	cg01071811	-0.319490626655075	80	-2.95857970138538	0.00410391773596961	0.102074260520453	0.0211714419624327
ACC	L3MBTL1	cg01877937	-0.375830791661646	80	-3.55880374447899	0.000641911442644304	0.14124878396102	0.00463322126267127
ACC	L3MBTL1	cg02177317	-0.31297048202677	80	-2.89156901087833	0.0049800113762469	0.0979505226200689	0.0246839761072795
ACC	L3MBTL1	cg02472486	-0.149834952056958	80	-1.32980854628542	0.187507294601635	0.0224505128579108	0.366834812234487
ACC	L3MBTL1	cg02611863	-0.327888722017638	80	-3.04558278498978	0.00317875169644793	0.10751101402636	0.017266290285817
ACC	L3MBTL1	cg03486259	-0.172318580586867	80	-1.53505183133789	0.128870082125786	0.0296936932154727	0.284335385837093
ACC	L3MBTL1	cg04984575	-0.224019224086902	80	-2.01702393025043	0.0471788369209927	0.0501846127604977	0.138306155158145
ACC	L3MBTL1	cg06362543	-0.0970781353342186	80	-0.855899797655716	0.394708834396721	0.00942416435996886	0.59142894414553
ACC	L3MBTL1	cg06446163	-0.272872333818802	80	-2.48889806319178	0.0149722497893757	0.0744593105637195	0.0582958558615962
ACC	L3MBTL1	cg06908806	-0.212140491460843	80	-1.90488193551534	0.060529657894501	0.0450035881172479	0.165693522159288
ACC	L3MBTL1	cg07678583	-0.0710221229369442	80	-0.62479436736345	0.533953137300959	0.00504414194647042	0.710909175191217
ACC	L3MBTL1	cg08369943	-0.230157767413883	80	-2.07534224255803	0.0412942916590745	0.0529725979009429	0.125266060648315
ACC	L3MBTL1	cg08633313	-0.419439352374319	80	-4.05445345192438	0.000119247331664332	0.175929370320189	0.00112091915411975
ACC	L3MBTL1	cg09541000	-0.249613791020939	80	-2.26195318467265	0.0265209344363186	0.0623070446678448	0.0900593466995053
ACC	L3MBTL1	cg10360552	-0.311134496758086	80	-2.87278211201728	0.00525491071408238	0.0968046750729073	0.0257950860734051
ACC	L3MBTL1	cg12699433	-0.280657264659463	80	-2.56588543215604	0.0122330584003188	0.0787685002061319	0.0499217618000525
ACC	L3MBTL1	cg14306330	-0.106712728188513	80	-0.941778041825708	0.349251677442607	0.0113876063574355	0.548273437832316
ACC	L3MBTL1	cg15123011	-0.163698089865297	80	-1.45608681499495	0.14943410299907	0.026797064625547	0.314900431010227
ACC	L3MBTL1	cg15302378	-0.361138222267364	80	-3.39831983863242	0.00107622992166584	0.130420815582432	0.00713518242083452
ACC	L3MBTL1	cg15330298	-0.314401944051693	80	-2.90624150135979	0.00477460428271115	0.0988485824234837	0.0239003645562854
ACC	L3MBTL1	cg15388309	-0.344884225662861	80	-3.22416467472255	0.00185426777528541	0.118945129111071	0.0111787745900924
ACC	L3MBTL1	cg16862791	-0.324031799810439	80	-3.00552714572196	0.00357758379194202	0.104996607288393	0.0189729738571366
ACC	L3MBTL1	cg17091610	-0.317745495969054	80	-2.9405988956383	0.00432382932930869	0.10096220020862	0.0220916838857376
ACC	L3MBTL1	cg17774347	-0.18773957314688	80	-1.67723118188861	0.0975525419925703	0.0352461473253728	0.233867563999076

**ACC**	L3MBTL1	cg18911945	-0.251642442104406	80	-2.28157389221263	0.0252779510701054	0.0633239186682692	0.0869312994804731
**ACC**	L3MBTL1	cg20091959	-0.30217451633874	80	-2.78160327910943	0.00679844194180851	0.0913094383245512	0.0316579667437478
**ACC**	L3MBTL1	cg20252111	-0.308247026631234	80	-2.84330771330299	0.00571447278413495	0.0950162294269965	0.0275948126181604
**ACC**	L3MBTL1	cg20529070	-0.350708813926597	80	-3.28618056267007	0.00153082806445651	0.1229966721658	0.00955925818320193
**ACC**	L3MBTL1	cg22330467	-0.277656765211246	80	-2.53614872555321	0.0132323850404258	0.077093279267573	0.0530486725984899
**ACC**	L3MBTL1	cg22457903	-0.278645502567737	80	-2.5459387472621	0.0128955385444621	0.0776433161012269	0.05201308342728
**ACC**	L3MBTL1	cg22601123	-0.279469194235853	80	-2.5541012621683	0.012620623901864	0.0781030305268372	0.0511651892136957
**ACC**	L3MBTL1	cg23626798	-0.305699340918946	80	-2.8173738790663	0.0061490953504012	0.0934520870382781	0.0292558695914079
**ACC**	L3MBTL1	cg24343973	-0.119994952595567	80	-1.06061490683192	0.292180456599285	0.0143987886484124	0.489899916819724
**ACC**	L3MBTL2	cg02358112	-0.494871809812169	80	-4.99729857920673	3.55161290108031e-06	0.244898108146772	5.25673847082652e-05
**ACC**	L3MBTL2	cg06235991	-0.211245330251481	80	-1.89646769979382	0.0616490426799904	0.0446245895530571	0.167948132656331
**ACC**	L3MBTL2	cg08053598	-0.0706923277947686	80	-0.621878494494668	0.535858797779561	0.00499740520904302	0.712153193154011
**ACC**	L3MBTL2	cg09993119	-0.104364003096906	80	-0.920818848380973	0.360021011652584	0.010891845142411	0.558886748170637
**ACC**	L3MBTL2	cg12821546	-0.0318815858560135	80	-0.279902067867267	0.780303193429252	0.00101643551669436	0.878657132119898
**ACC**	L3MBTL2	cg13843727	0.0557044881160753	80	0.489565047299576	0.625833551304998	0.00310298999627397	0.778666058220249
**ACC**	L3MBTL2	cg14679202	-0.209721167624749	80	-1.88215252569035	0.0635935947038302	0.043982968149888	0.17174200188814
**ACC**	L3MBTL2	cg15283028	-0.293380394178514	80	-2.69290152374849	0.00868853222169015	0.08607205568834	0.0382508740823648
**ACC**	L3MBTL2	cg19467356	-0.0744716883072647	80	-0.65530611375939	0.514223757529264	0.00554603235933439	0.695761844184492
**ACC**	L3MBTL2	cg24240981	-0.290886763280353	80	-2.66788712962084	0.00930210114384742	0.0846151090517202	0.0403078959719527
**ACC**	L3MBTL2	cg26947834	0.35669766238068	80	3.35039894053084	0.00125223330462245	0.127233222347842	0.00809778216599372
**ACC**	L3MBTL2	cg27177954	-0.341360590828672	80	-3.18685362494614	0.00207862228606362	0.1165270529709	0.0122721140526482
**ACC**	L3MBTL3	cg03454037	0.0352631299084758	80	0.309625276726654	0.757682297290718	0.00124348833094204	0.864902958700427
**ACC**	L3MBTL3	cg07826275	0.301100129040417	80	2.77072526018266	0.00700802693478162	0.0906612877081556	0.0324223875922927
**ACC**	L3MBTL3	cg08106148	0.00866144662012828	80	0.076006736726081	0.939610875939548	7.50206575533316e-05	0.967773814597199
**ACC**	L3MBTL3	cg08358392	-0.191861290499146	80	-1.71544544683423	0.0902852131907629	0.0368107547919979	0.2212869525823
**ACC**	L3MBTL3	cg09710740	-0.00631137510748351	80	-0.0553831948666467	0.955976637926468	3.98334557473625e-05	0.9766416701345
**ACC**	L3MBTL3	cg10237990	-0.0293488440542162	80	-0.257646047601876	0.797367407533228	0.0008613546473187	0.889159011247222
**ACC**	L3MBTL3	cg16487825	-0.122533023796984	80	-1.08338685030603	0.282017590167869	0.0150143419208324	0.479128115516908
**ACC**	L3MBTL3	cg16800851	-0.0956693378177415	80	-0.84336339735969	0.401637557497277	0.00915262219848515	0.598219263793666
**ACC**	L3MBTL3	cg18411550	0.0223526991181008	80	0.196193158131784	0.844975853155112	0.000499643157864346	0.917197323966269
**ACC**	L3MBTL3	cg18798995	0.0500982430884783	80	0.440163014036354	0.661052160964043	0.00250983396055227	0.802797023674767
**ACC**	L3MBTL3	cg22934935	0.0151612389052127	80	0.133054624519428	0.894497293293511	0.000229863165140935	0.944028921939992
**ACC**	L3MBTL3	cg24516147	-0.122453257125939	80	-1.08267084620958	0.282333366791755	0.0149948001807514	0.479486583626908
**ACC**	LBR	cg01017344	-0.124219940024792	80	-1.09853401612131	0.275394492848454	0.015430593499763	0.471832779294645
**ACC**	LBR	cg05530459	0.149395333661745	80	1.32581766103805	0.188819951488403	0.0223189657199041	0.368571769611301
**ACC**	LBR	cg12994003	-0.208084319687297	80	-1.86679497269824	0.0657369253472753	0.0432990840997251	0.175982778863114
**ACC**	LBR	cg14607642	-0.23426535915202	80	-2.11451134390131	0.0377075842822747	0.0548802584986247	0.117271280549104
**ACC**	LBR	cg18107727	0.0537830314968635	80	0.472628246724537	0.637815335466663	0.00289261447699262	0.786965998529637
**ACC**	LBR	cg18289048	0.0504134265092926	80	0.442939248410636	0.659051590683575	0.00254151357240784	0.801609079554663
**ACC**	LBR	cg18764107	0.188454266598689	80	1.68385077219246	0.0962605832745934	0.0355150105992497	0.231774413998304
**ACC**	LBR	cg19322477	-0.183619542219595	80	-1.63912433989408	0.105267158301459	0.0337161362849338	0.247107824770781
**ACC**	LBR	cg20483690	-0.263184968137272	80	-2.39383234209908	0.0191073573757769	0.0692663274534169	0.0702451718618269
**ACC**	LBR	cg26930584	-0.313660666260961	80	-2.8986406648755	0.00488001566292487	0.0983830135592699	0.0243107030277649
**ACC**	MBD1	cg01719254	-0.111375099114044	80	-0.983431003615944	0.328475402952486	0.0124044127026631	0.52808314900671
**ACC**	MBD1	cg03841029	-0.153587326314711	80	-1.36390595598252	0.176570990671169	0.0235890668045014	0.352561886200891
**ACC**	MBD1	cg05549078	-0.161530981484949	80	-1.43629049150397	0.154970232275559	0.0260922579794908	0.322708770534955
**ACC**	MBD1	cg12090413	-0.242551948178687	80	-2.19389796879225	0.0312590481053368	0.0588314475652767	0.101794154730968
**ACC**	MBD1	cg13478031	-0.176530103713348	80	-1.57376090835502	0.119642906698671	0.0311628775170453	0.27018985960626
**ACC**	MBD1	cg14389183	-0.345571883014135	80	-3.2314640977411	0.00181311698361718	0.119419926329935	0.0109732186368303
**ACC**	MBD1	cg19328682	-0.276587396168228	80	-2.52557015453125	0.0136052598405218	0.0765005877191201	0.0541833067587072
**ACC**	MBD1	cg21282183	0.117102026061234	80	1.03468485421054	0.304054807107436	0.013712884507646	0.502665629680847
**ACC**	MBD2	cg15090185	-0.138584451839322	80	-1.227922319116	0.223216536776597	0.0192056502916052	0.411971557353831
**ACC**	MBD2	cg16852837	-0.108201566665302	80	-0.955072145613453	0.342530075153483	0.0117075790288257	0.541734434260412
**ACC**	MBD2	cg17987217	-0.139737370164154	80	-1.23834029637999	0.219352072689832	0.0195265326203938	0.407300819716268
**ACC**	MBD2	cg22224310	-0.0347964891638181	80	-0.305522972184873	0.760792302608702	0.00121079565812771	0.86688697164586
**ACC**	MBD2	cg22836370	0.216251492094562	80	1.94358893860955	0.055599088250418	0.0467647078331243	0.155736703681742
**ACC**	MBD2	cg23388714	-0.153001932527733	80	-1.35858261094189	0.17824572448923	0.0234095913572209	0.354822105704072

**ACC**	MBD2	cg24881765	-0.24690147826503	80	-2.23576979864259	0.0282633538349128	0.0609603399694571	0.0944669967017489
**ACC**	MBD2	cg25192916	0.252371718450919	80	2.28863513467063	0.0248433600142445	0.06369148427387	0.0858084779535646
**ACC**	MBD3	cg08660999	0.272652584145294	80	2.48673266317894	0.0150566980581289	0.0743394316411064	0.0585426213881486
**ACC**	MBD3	cg09174855	-0.0468474431922833	80	-0.411536489757794	0.681822460900928	0.00219468293365421	0.816528579484711
**ACC**	MBD3	cg12286540	-0.32069865248482	80	-2.9710461118577	0.00395757360930725	0.102847625705579	0.0205936776831069
**ACC**	MBD5	cg03890220	0.0985669503614414	80	0.869153892082488	0.387464147893589	0.00971544370355486	0.58538700753097
**ACC**	MBD5	cg04152151	0.0448917540258175	80	0.394321074882055	0.694434014355656	0.0020152695795145	0.824863721508923
**ACC**	MBD5	cg05218490	0.054948927872143	80	0.482904473529993	0.630533738459653	0.00301938467429798	0.781975220790241
**ACC**	MBD5	cg06394585	0.247997776501543	80	2.24634616001015	0.0275477743259031	0.061502897149709	0.0926244351432469
**ACC**	MBD5	cg06758980	0.131424040291272	80	1.16333172900792	0.248287347873642	0.0172722783664819	0.441588730917956
**ACC**	MBD5	cg20595490	-0.0504525271835759	80	-0.443283668640759	0.65880357246504	0.00254545749920947	0.801497640378226
**ACC**	MBD5	cg21412502	-0.0468774652660303	80	-0.411800802589589	0.681629525837054	0.00219749674976788	0.816385499252811
**ACC**	MBD5	cg23120335	-0.150670520583048	80	-1.3373961196593	0.185030599624321	0.0227016057727668	0.363589122559743
**ACC**	MBD5	cg25686162	0.082745664422174	80	0.728588809978411	0.468463372663028	0.00684684498066703	0.657457611756371
**ACC**	MBD5	cg26664634	-0.0539279165506632	80	-0.47390515924388	0.636908571801053	0.00290822018349529	0.786316921438351
**ACC**	MBTD1	cg01388767	0.0442543648808252	80	0.388711297952092	0.698562502125071	0.00195844881100521	0.827114225505579
**ACC**	MBTD1	cg14577476	-0.161167300031229	80	-1.43297039638286	0.155914068664476	0.025974898599356	0.324127603690019
**ACC**	MBTD1	cg17045523	-0.187335019152068	80	-1.67348537115608	0.0982898572803864	0.0350944094007057	0.235164113709684
**ACC**	MBTD1	cg18711617	-0.0133558654732553	80	-0.117207698061178	0.907000652642034	0.000178379142539694	0.950841639389257
**ACC**	MBTD1	cg22141797	-0.232961424088591	80	-2.10206445026715	0.0388168895483729	0.0542710251133844	0.119792173338865
**ACC**	MBTD1	cg25259117	-0.268859010786369	80	-2.44941689396174	0.0165807164674914	0.0722851676810249	0.0629916617117754
**ACC**	MBTD1	cg25716740	-0.164153087367549	80	-1.460245913117	0.148290789663033	0.026946236092298	0.31328423045712
**ACC**	MBTD1	cg25855326	-0.393908259487636	80	-3.76057427814885	0.000328527795139081	0.155163716892578	0.00263615175812787
**ACC**	MBTD1	cg27207041	-0.238345806930421	80	-2.15354023911332	0.0344056619020876	0.0568087236813135	0.109389981282124
**ACC**	MBTD1	cg27214721	0.00600701696877713	80	0.0527123110262391	0.958097646269028	3.60842528631764e-05	0.977917888758433
**ACC**	MBTD1	cg27353579	-0.186358824966952	80	-1.66445030961269	0.100086976998764	0.034729611643063	0.238393383198366
**ACC**	MLLT10	cg00879184	-0.343942866425886	80	-3.21418177828634	0.0019119643142288	0.118296695365255	0.0114679531118824
**ACC**	MLLT10	cg03524461	-0.360194311804031	80	-3.38811144510398	0.00111164526147038	0.12973994225598	0.00732875690430733
**ACC**	MLLT10	cg04188362	-0.337596968874128	80	-3.14716931138376	0.00234494868935594	0.113971713392999	0.0135218247972542
**ACC**	MLLT10	cg05913198	0.0464678728972156	80	0.408194868065874	0.684263502229816	0.00215926321159178	0.818192359244794
**ACC**	MLLT10	cg06398735	-0.0876560413690664	80	-0.772150803423266	0.442388614728931	0.00768358158849549	0.63475561426505
**ACC**	MLLT10	cg11443787	-0.151912441747544	80	-1.34867909678689	0.18139341424236	0.0230773899577009	0.358665089151337
**ACC**	MLLT10	cg15910013	0.0698600683669508	80	0.614521009251124	0.540682735204841	0.00488042915223505	0.715898143834992
**ACC**	MLLT10	cg19119538	-0.290843080532349	80	-2.66744946578554	0.0093131788971413	0.0845896974935461	0.0403401692162701
**ACC**	MLLT10	cg20569108	-0.177238956159644	80	-1.58028483862358	0.118141043418963	0.0314136475805601	0.267847880737179
**ACC**	MLLT10	cg23344688	-0.289638645146262	80	-2.65538921557401	0.00962319776334857	0.0838905447621622	0.0413423374642113
**ACC**	MLLT10	cg26877631	-0.383555625784766	80	-3.64442033018947	0.000484396211365892	0.147114918071144	0.00364757708518035
**ACC**	MLLT10	cg27470303	-0.276235465309768	80	-2.52209095650568	0.0137299473704509	0.076306032294904	0.0545389778305945
**ACC**	MLLT10	cg27572529	-0.136651822942059	80	-1.21047013138783	0.229801154342252	0.0186737207133877	0.419878126709282
**ACC**	MLLT6	cg05652455	0.00465685138419707	80	0.0408641481514784	0.967509972478831	2.16862648144982e-05	0.982898960496999
**ACC**	MORF4L1	cg03589001	0.0175370542346397	80	0.15391069567292	0.878082824571272	0.000307548271228693	0.935298871724606
**ACC**	MORF4L1	cg07757959	0.0799165937908772	80	0.703515420566519	0.483856524948263	0.00638666196313607	0.670654144279392
**ACC**	MORF4L1	cg08692282	0.0362089287314468	80	0.317940552270392	0.751390691895935	0.001311086519879	0.860766606356327
**ACC**	MORF4L1	cg13747872	0.00139975407398855	80	0.012282804183282	0.990231755522827	1.95931146764754e-06	0.994707003630272
**ACC**	MORF4L1	cg15822222	0.0147866219244251	80	0.129766267892318	0.897089744825007	0.000218644187935888	0.945511443014721
**ACC**	MORF4L1	cg20775702	-0.39948372443962	80	-3.82382612044402	0.000265123689741796	0.15958724609215	0.00220117740872714
**ACC**	MORF4L1	cg20905746	0.0216518692783768	80	0.190038932513172	0.849778729080112	0.000468803443247916	0.919887100119088
**ACC**	MORF4L1	cg23623458	-0.264527802953842	80	-2.40696273391357	0.0184813634670038	0.0699749585355864	0.0684963882587944
**ACC**	MORF4L1	cg24795825	0.174918406778673	80	1.55893702756466	0.123112180317923	0.0305964490299892	0.275560736438385
**ACC**	MPHOSPH8	cg00331616	-0.00317530540962492	80	-0.0278633323558284	0.97784324336507	1.00825644443933e-05	0.98852003171519
**ACC**	MPHOSPH8	cg03134886	0.0197981220923586	80	0.173761873977305	0.862508618450384	0.000391965638383936	0.9268727401554
**ACC**	MPHOSPH8	cg03428000	0.31326354816044	80	2.89457115629445	0.00493733141953128	0.0981340506060685	0.0245286902389052
**ACC**	MPHOSPH8	cg04203901	-0.134061000551684	80	-1.18709632745455	0.238839038178548	0.0179723518689187	0.430634924411637
**ACC**	MPHOSPH8	cg06019692	-0.30637842399036	80	-2.82427995647857	0.00603048447711491	0.0938677386868168	0.0287830854293317
**ACC**	MPHOSPH8	cg09559995	-0.380380921668709	80	-3.60912749555916	0.000544270993525104	0.144689645569537	0.00402911264222653
**ACC**	MPHOSPH8	cg11051893	0.169976868814927	80	1.51356630806724	0.134229881882002	0.0288921359321269	0.292517211920233
**ACC**	MPHOSPH8	cg19693579	0.288997032224979	80	2.64897024491235	0.00979200058325272	0.0835192846348458	0.0419055392987987

**ACC**	MPHOSF	cg20759595	-0.3261559275934	80	-3.02756612153456	0.00335273838972989	0.106377689104311	0.018000202228478
**ACC**	MPHOSF	cg20952532	-0.314575485501466	80	-2.90802179393508	0.00475022004931437	0.098957736078483	0.0238104779971883
**ACC**	MPHOSF	cg21958444	-0.130362953035885	80	-1.15377620585616	0.252160623690024	0.0169944995242365	0.445996953660435
**ACC**	MPHOSF	cg22656705	0.101389882982208	80	0.894301153811132	0.37394766007131	0.0102799083711458	0.572587573664281
**ACC**	MSH6	cg00620552	0.197527573537725	80	1.76813438946987	0.0809994748099578	0.0390171423077013	0.204716880783077
**ACC**	MSH6	cg02905881	-0.0225723082291661	80	-0.198121679682079	0.843471997055551	0.00050950909879248	0.916388578835445
**ACC**	MSH6	cg05039065	0.22705737163248	80	2.04585525796439	0.0441853388611259	0.05155505001265	0.131810501240986
**ACC**	MSH6	cg07652213	-0.0511709186679684	80	-0.449612021026067	0.654253337376368	0.00261846291732384	0.798271367616449
**ACC**	MSH6	cg09898070	0.242145659897659	80	2.18999396364337	0.0315520313499467	0.0586345206072725	0.10248679443999
**ACC**	MSH6	cg11869233	-0.323874151342533	80	-3.00389348856028	0.00359479535915751	0.104894465907846	0.0190294426307429
**ACC**	MSH6	cg16595246	0.0459749606940208	80	0.4038556828543	0.687438282481301	0.00211369701081675	0.820210943809916
**ACC**	MSH6	cg18222961	-0.0258701711186356	80	-0.227085833366176	0.820958831510373	0.000669265753707485	0.903831977165876
**ACC**	MSH6	cg19076255	-0.0734859530491548	80	-0.646584823256344	0.519823380530627	0.00540018529554258	0.700290958880891
**ACC**	MSH6	cg19736286	-0.021220600062949	80	-0.186251950471643	0.852737002263183	0.000450313867031631	0.921544397245133
**ACC**	MSH6	cg21405109	0.1026955937763	80	0.905940036724963	0.367793755281519	0.0105463849810668	0.566700615943482
**ACC**	MTA1	cg01318265	-0.0393312515309984	80	-0.345397590838512	0.730737298247121	0.00154694734699466	0.847816995717239
**ACC**	MTA1	cg01655777	0.029397597369734	80	0.258074410468133	0.797038008642238	0.000864218731112993	0.888951979763185
**ACC**	MTA1	cg05922925	-0.517477539513307	80	-5.30660747422888	1.04252987990161e-06	0.267783003900746	1.77157207245665e-05
**ACC**	MTA1	cg13052453	-0.139297727353019	80	-1.23436700150334	0.220820117607095	0.019403856845716	0.409066604994448
**ACC**	MTA1	cg13981078	0.0804879695868386	80	0.708577991169722	0.480726122290806	0.00647831324821186	0.66794563141851
**ACC**	MTA1	cg16531924	-0.0806044673867423	80	-0.709610284711563	0.480089194722249	0.0064970801627004	0.667346713378408
**ACC**	MTA1	cg20222911	-0.181942936355718	80	-1.62364286277001	0.108539176700322	0.033103232089741	0.252585979605277
**ACC**	MTA1	cg21517792	-0.0116257471406124	80	-0.102022411941082	0.919004101800143	0.000135157996577458	0.957311834274317
**ACC**	MTA1	cg23123838	-0.277484115600188	80	-2.53444012546024	0.0132919793561526	0.0769974344104184	0.0532221849406825
**ACC**	MTA1	cg23883989	-0.283880718179268	80	-2.59792276686245	0.0112331500321749	0.0805882621539769	0.0466861669838131
**ACC**	MTA1	cg23966123	-0.20189452931518	80	-1.80886681199713	0.0743751829135123	0.0407614009673981	0.192666108959322
**ACC**	MTA2	cg00501779	-0.163452427375035	80	-1.4538416296798	0.150054136185397	0.0267166960147909	0.315725298742509
**ACC**	MTA2	cg07654871	0.158541368037202	80	1.40901561508446	0.162856222122522	0.0251353653791076	0.333592522441714
**ACC**	MTA2	cg09086761	0.0320726747908779	80	0.281581441647041	0.779019831029632	0.00102865646824142	0.877796476464647
**ACC**	MTA2	cg13708959	-0.146448212256781	80	-1.29908413259242	0.197791597370597	0.0214470788732072	0.38019412538588
**ACC**	MTA2	cg19570317	-0.0323544849211496	80	-0.284058169734376	0.777128259032743	0.00104681269451289	0.876769912595438
**ACC**	MTA2	cg21002970	-0.00110322617370841	80	-0.0096807762768070	0.992301016901576	1.21710799035531e-06	0.995936850843003
**ACC**	MTF2	cg05396182	0.21788783924776	80	1.95902594773568	0.0537300992529771	0.0474751104920579	0.152078564354597
**ACC**	MTF2	cg05962718	-0.29737596235299	80	-2.73310802777927	0.00777920371729206	0.0884324629853671	0.0351704380683438
**ACC**	MTF2	cg09936581	0.376842614808276	80	3.56996820582669	0.000618912608780582	0.142010356335539	0.00449264602850393
**ACC**	MTF2	cg11404133	-0.141028595290457	80	-1.25001413900386	0.215080198774385	0.0198890646895996	0.402113650167135
**ACC**	MTF2	cg14474880	-0.259165265229817	80	-2.35461616604719	0.0210909643112634	0.0671666347016416	0.0758623302029973
**ACC**	MTF2	cg15463775	-0.242015955924077	80	-2.18874790204641	0.0316460485091449	0.0585717229218448	0.102708287827311
**ACC**	MTF2	cg16813892	-0.125980009046977	80	-1.11434835097163	0.268595983439214	0.0158709626794763	0.464386879936781
**ACC**	MTF2	cg19708337	-0.120026933321197	80	-1.06090171028103	0.292050918838085	0.0144064647224911	0.489798844049712
**ACC**	MTF2	cg22801127	-0.0288806492014515	80	-0.253532424955615	0.800532531704775	0.000834091898297299	0.891040859775671
**ACC**	MTF2	cg23992808	-0.0829573992748735	80	-0.7304660719209	0.467322101753399	0.00688193009445079	0.656426260473502
**ACC**	MTF2	cg26054395	-0.330023674915029	80	-3.06782826605073	0.00297550390792539	0.108915626004421	0.0163561306663012
**ACC**	NAP1L2	cg02379437	-0.188264031806881	80	-1.68208852066758	0.0966031545032337	0.0354433456721822	0.23235053801O9
**ACC**	NAP1L2	cg04297329	-0.0261397089058192	80	-0.229453419109569	0.819124922827383	0.000683284381680964	0.902710918851084
**ACC**	NAP1L2	cg07693661	0.0329900242931255	80	0.289643947012947	0.772867145340977	0.00108834170286101	0.874054002219223
**ACC**	NAP1L2	cg20001978	-0.110410713277949	80	-0.974810019658337	0.332707067220701	0.0121905256065455	0.532362726451704
**ACC**	NAP1L2	cg23483095	-0.171728341909347	80	-1.52963379959157	0.130205420378808	0.0294906234149334	0.286499801243078
**ACC**	NCOA1	cg11380327	-0.226077652923836	80	-2.03655112235219	0.0451329952996221	0.0511111051515505	0.133882792615397
**ACC**	NCOA1	cg13911252	0.187468880009564	80	1.67472470601683	0.0980454092319147	0.0351445809720404	0.234724112325417
**ACC**	NCOA1	cg15477048	0.0900100466370811	80	0.79305407329473	0.430183224017386	0.00810180849560952	0.624208190722558
**ACC**	NCOA1	cg23006163	-0.150497956483503	80	-1.33582887170984	0.185540139865597	0.0226496349057104	0.364271896847977
**ACC**	NCOA3	cg04175879	-0.356226943630868	80	-3.3453344670354	0.00127234065124202	0.126897635368589	0.00820053592566047
**ACC**	NCOA3	cg09568061	0.0745924825647892	80	0.656374973009791	0.513539688399666	0.00556403845517837	0.695162722848207
**ACC**	NCOA3	cg12128876	0.135164223004528	80	1.1970462860978	0.234960925657593	0.0182693671804178	0.426083364428233
**ACC**	NCOA3	cg14341558	-0.197463027643085	80	-1.76753317159448	0.0811007951478621	0.0389916472859737	0.204915887418649
**ACC**	NCOA3	cg14774045	-0.0653649907183292	80	-0.57480473035008	0.567099079001236	0.00427258201160726	0.735845911062184

**ACC**	NCOA3	cg18295930	-0.123862983254084	80	-1.09532802605558	0.276787205794182	0.0153420386206015	0.473478547123878
**ACC**	NCOA3	cg21735648	0.192304850393361	80	1.71956346068982	0.0895292959102667	0.0369811554848128	0.220045016301635
**ACC**	NCOA3	cg22511723	-0.32710894433676	80	-3.03747083178092	0.00325602385303596	0.10700026146511	0.0175889951824929
**ACC**	NCOA3	cg22876277	-0.034473642195211	80	-0.302684896245606	0.762946193675063	0.00118843200620343	0.868122094920758
**ACC**	NCOA3	cg24856420	0.152718567651794	80	1.35600632658759	0.17906054364105	0.0233229609056155	0.35580300335149
**ACC**	NCOA3	cg25373408	-0.0917136366448168	80	-0.808190074617837	0.421471074482809	0.00841139114661748	0.616595595873804
**ACC**	NCOR1	cg00253204	-0.0909282213277895	80	-0.801210961294602	0.425475012166386	0.00826794143383548	0.620148248127892
**ACC**	NCOR1	cg08125503	-0.193286949627407	80	-1.72868517685907	0.0878733757639049	0.0373598448962678	0.217154428023419
**ACC**	NCOR1	cg08206308	-0.113093777909432	80	-0.998801868548072	0.321019185730452	0.0127902026018278	0.520473647090883
**ACC**	NCOR1	cg21211144	0.0816127045486783	80	0.718545558321364	0.474595759271997	0.00666063354374986	0.662507314860488
**ACC**	NCOR1	cg22291359	-0.0621874609052783	80	-0.546750998589854	0.586130812412466	0.00386728029384552	0.750364049527354
**ACC**	NCOR1	cg22625220	-0.130420984251199	80	-1.154298696022	0.251947731474279	0.0170096331330515	0.445748830418004
**ACC**	NCOR2	cg08976554	-0.123719275956556	80	-1.09403744787511	0.277349224719386	0.0153064592432145	0.474106343532486
**ACC**	NCOR2	cg10641483	-0.145535122625754	80	-1.2908086472673	0.200632283882056	0.0211804719176932	0.383658322902529
**ACC**	NCOR2	cg13416396	-0.386137260108885	80	-3.67323164938304	0.000440214986716181	0.149101983644397	0.00336537781806197
**ACC**	NCOR2	cg13419087	-0.187936692819474	80	-1.67905665545164	0.0971948574760087	0.0353202005079215	0.233304845644377
**ACC**	NCOR2	cg13857025	-0.376520973597259	80	-3.5664176038414	0.000626140734645573	0.141768043558628	0.00453580336260524
**ACC**	NCOR2	cg16181859	-0.395144412776027	80	-3.77455517118031	0.000313376403799905	0.156139106948111	0.002535233356615
**ACC**	NCOR2	cg19895345	-0.0140713911238631	80	-0.123488182178189	0.902042306534676	0.000198004048160734	0.948253223954811
**ACC**	NCOR2	cg23611643	-0.528096845768715	80	-5.45703898488185	5.68181373411161e-07	0.278886278510865	1.03636815388389e-05
**ACC**	NCOR2	cg24626542	-0.391972937289572	80	-3.73873423853893	0.000353603016746922	0.153642783567414	0.00280438302424407
**ACC**	NCOR2	cg25113767	0.0337887655915643	80	0.296664611250482	0.767521328076017	0.00114168068020168	0.871185848079126
**ACC**	NCOR2	cg25449924	-0.307308013426977	80	-2.83374132968627	0.00587139688329975	0.0944382151164352	0.028187569484236
**ACC**	ORC1	cg00298020	-0.189478282293002	80	-1.69334018567406	0.0944328979973854	0.0359020194607066	0.228579385382514
**ACC**	ORC1	cg02275620	0.142970416350155	80	1.26758223188635	0.208767111833504	0.0204405399513367	0.393984431643527
**ACC**	ORC1	cg04407474	-0.100469377093867	80	-0.886098731717133	0.378323368793472	0.0100940957336296	0.576914400704093
**ACC**	ORC1	cg07142859	-0.153997180077398	80	-1.36763388278337	0.175405315339974	0.0237151314717906	0.351090136766649
**ACC**	ORC1	cg08613513	0.0313814724795601	80	0.27550699605382	0.783664749466303	0.000984796814985388	0.880621304294228
**ACC**	ORC1	cg10586599	0.0291059929052378	80	0.255512303886122	0.799008742934352	0.000847158822999755	0.890253276914179
**ACC**	ORC1	cg13460942	-0.105730626759232	80	-0.933012196788349	0.353730159688346	0.0111789654349001	0.55274955895057
**ACC**	ORC1	cg16136434	0.096293591751862	80	0.848917780785738	0.398558576928589	0.00927245581247426	0.595157042261088
**ACC**	ORC1	cg16965475	-0.242887303369155	80	-2.19712130506558	0.0310189433662662	0.05899424213794	0.10116616534711
**ACC**	ORC1	cg17146111	0.0406682438739178	80	0.357157866693951	0.72195063498344	0.00165390605978846	0.8424531318734
**ACC**	ORC1	cg19776520	0.00450077528842262	80	0.0394945428936359	0.968598326853443	2.02569781968757e-05	0.983470202724742
**ACC**	ORC1	cg21197365	-0.0525477079346937	80	-0.461742201955418	0.645568087475684	0.00276126160918987	0.792214020177017
**ACC**	ORC1	cg23606758	-0.127451074808187	80	-1.12757416936745	0.263001194886238	0.0162437764697621	0.458387867050583
**ACC**	ORC1	cg25605769	0.0266038714970873	80	0.233530682117654	0.815969083683924	0.000707765978633535	0.900926777599523
**ACC**	PADI1	cg14524955	0.046961286389001	80	0.412538765475638	0.681090962464759	0.00220536241930977	0.816029421438217
**ACC**	PADI1	cg21010178	0.077047050221654	80	0.67810080495588	0.49974051776612	0.00593624794785807	0.683824910981101
**ACC**	PADI1	cg22193702	0.0186968260865242	80	0.164092666617333	0.870088167149673	0.000349571305709731	0.931107602851242
**ACC**	PADI2	cg00569276	-0.368595276716823	80	-3.47939469320809	0.000830449431224126	0.135862478017951	0.00574921981424309
**ACC**	PADI2	cg01603374	-0.0881968439967004	80	-0.776951895951493	0.439567527944286	0.00777868329097831	0.632200337750005
**ACC**	PADI2	cg07014827	-0.101495029710056	80	-0.895238240058317	0.373449796668761	0.0103012410558451	0.572005384564867
**ACC**	PADI2	cg09313801	-0.0746116513118524	80	-0.656544592125341	0.513431176711523	0.00556689851148144	0.695088404035574
**ACC**	PADI2	cg10233913	-0.148600657514335	80	-1.31860557667579	0.191209609200432	0.0220821554136926	0.371680898987204
**ACC**	PADI2	cg15185794	-0.101246139531905	80	-0.893020135730495	0.374628927629601	0.010250780770114	0.573269656477777
**ACC**	PADI2	cg17183414	-0.0959007481228872	80	-0.845422284203285	0.400494553778507	0.00919695349052945	0.596994377453703
**ACC**	PADI2	cg18228326	-0.0957160321931162	80	-0.843778832016414	0.401406765781338	0.00916155881879365	0.597967100784984
**ACC**	PADI2	cg18879300	-0.0741828343555996	80	-0.652750280157833	0.5158614459871	0.00550309291303032	0.697044193096077
**ACC**	PADI2	cg19282714	-0.0740456967593569	80	-0.651536919021563	0.516639889914453	0.00548276520857863	0.697709438700557
**ACC**	PADI3	cg01459162	0.0684655992503916	80	0.602196262095642	0.548812751655122	0.00468753828071523	0.721927736676146
**ACC**	PADI3	cg12858514	-0.211004604528005	80	-1.89420580649088	0.0619529155506839	0.0445229431320197	0.168539834674426
**ACC**	PADI3	cg14664242	-0.169428899150329	80	-1.50854243956448	0.135508153957979	0.0287061518672923	0.294436127507887
**ACC**	PADI3	cg17589397	0.0262446985321906	80	0.230375648197872	0.818410845540622	0.000688784201045567	0.902329499224449
**ACC**	PADI3	cg18768338	-0.216477879683015	80	-1.94572361121355	0.0553373930279833	0.0468626723920541	0.155288837501585
**ACC**	PADI3	cg21966975	-0.201784371297756	80	-1.80783795171149	0.0745367743476802	0.0407169325000306	0.192987879371745
**ACC**	PADI4	cg16091981	-0.334457652318696	80	-3.11419823453891	0.00259010537751017	0.111861921194534	0.0146403151738739

**ACC**	PADI4	cg18333690	-0.328144358657129	79	-3.02838518915443	0.0033572560635712	0.107678720118498	0.0180194824209644
**ACC**	PADI4	cg19159961	0.0218881629539567	80	0.192113876019252	0.848158758570142	0.000479091677498964	0.918964658275845
**ACC**	PADI4	cg21295838	-0.271007273727978	80	-2.47053319959631	0.0157021476889516	0.0734449424134712	0.0604539679953626
**ACC**	PADI4	cg23228178	-0.00934363445154716	80	-0.0819936388052763	0.934864536551595	8.7303504764139e-05	0.965353700791042
**ACC**	PADI4	cg26918754	-0.187241514422267	80	-1.67261972603791	0.0984608924731041	0.0350593847231441	0.235493656430322
**ACC**	PADI6	cg12643633	0.101563642434952	80	0.89584974514814	0.373125136558863	0.0103151734646548	0.571688204070197
**ACC**	PADI6	cg16017904	0.0846817465927369	80	0.745758036308256	0.458084203646927	0.00717099820599651	0.648141621494065
**ACC**	PARP1	cg00653565	0.00184800085185913	80	0.0162161693529357	0.987103880627002	3.41510714847207e-06	0.993204285131626
**ACC**	PARP1	cg02088626	0.28054692115718	80	2.56479042473627	0.0122686132562146	0.0787065749707732	0.0500301147797852
**ACC**	PARP1	cg03149313	-0.0750543887238395	80	-0.660462461117196	0.510928160683978	0.00563316126670921	0.693123120276386
**ACC**	PARP1	cg04960271	0.166386508804966	80	1.48067539883071	0.142773490076889	0.0276844703123051	0.305378485250379
**ACC**	PARP1	cg07113065	-0.276738892309038	80	-2.52706818815668	0.0135518880872531	0.0765844145164335	0.0540283699000548
**ACC**	PARP1	cg17661881	0.151704841905058	80	1.34679257805866	0.181997758052531	0.0230143590574386	0.359563308322211
**ACC**	PARP1	cg25722198	-0.0460474567470121	80	-0.404493858622139	0.686971004666401	0.00212036827286795	0.819842026463013
**ACC**	PARP1	cg26464410	0.0953998399515775	80	0.840965814572051	0.40297110304029	0.00910112946278661	0.599493880324263
**ACC**	PARP2	cg01813761	-0.422397913729823	80	-4.08923620403534	0.000105470527986759	0.178419997523307	0.00100822981742466
**ACC**	PARP2	cg10910545	-0.559633009612662	80	-5.92556952357233	8.24668550969708e-08	0.313189105448126	1.82463780586284e-06
**ACC**	PARP2	cg15078479	-0.20354816961433	80	-1.82432020557519	0.0719830075570493	0.041431857353344	0.188147904777512
**ACC**	PARP2	cg15365367	-0.421068452934083	80	-4.07358666378494	0.000111468204499015	0.177298642056302	0.00105832830226074
**ACC**	PARP2	cg18899035	-0.196362740107735	80	-1.75728812737779	0.0828435245216132	0.0385583257026179	0.208089152647134
**ACC**	PARP2	cg19540080	0.127970275990682	80	1.13224391987437	0.261045525173106	0.0163763915371312	0.45631349059223
**ACC**	PARP2	cg24595957	0.264104190487564	80	2.40281898253073	0.0186769125514592	0.0697510234330917	0.0690533593949061
**ACC**	PARP2	cg26206608	0.0475842531247175	80	0.418023652125021	0.677093315439399	0.00226426114543718	0.813469813770889
**ACC**	PARP2	cg26430074	0.0960743138234458	80	0.846966611041608	0.39963852063048	0.00923027377664594	0.596281540024083
**ACC**	PAXIP1	cg02824291	0.0355036585312881	80	0.311739876768126	0.756080751130831	0.00126050976910631	0.863936438536244
**ACC**	PAXIP1	cg12220104	0.0927037617688601	80	0.816990385762315	0.416454563204176	0.00859398744609757	0.612004199140595
**ACC**	PAXIP1	cg22583711	0.0538106535552168	80	0.472871685341573	0.637642421658189	0.00289558643603957	0.786859254070434
**ACC**	PAXIP1	cg24448952	-0.499392513028741	80	-5.05802591810029	2.79894745330987e-06	0.249392882069161	4.25606563149274e-05
**ACC**	PAXIP1	cg24452135	0.0889578282964101	80	0.783708872697405	0.435615068009385	0.00791349521521359	0.628788900803044
**ACC**	PCGF1	cg03203239	0.0596570819059714	80	0.524422802805329	0.601490987404221	0.00355896742153578	0.761139404816406
**ACC**	PCGF1	cg08092637	0.0443296339356007	80	0.389373729590258	0.698074512219007	0.00196511644486436	0.82680199451243
**ACC**	PCGF1	cg27306986	0.0389174228019898	80	0.34175790462273	0.733464061782687	0.00151456579754883	0.849577691419927
**ACC**	PCGF2	cg09395368	-0.191271276380564	80	-1.7099694416038	0.0912985109639173	0.0365847011682502	0.223135734181466
**ACC**	PCGF2	cg17902383	0.00625735102769165	80	0.0549091074056558	0.956353098578336	3.91544418837537e-05	0.976973814959796
**ACC**	PCGF2	cg20228512	-0.192585272453685	80	-1.72216746595923	0.0890539826640181	0.0370890871660603	0.219187696629374
**ACC**	PCGF2	cg25377665	0.00529326062288702	80	0.0464488241792757	0.963072780289857	2.80186080218063e-05	0.980593736844525
**ACC**	PCGF5	cg03905463	0.0761432884298461	80	0.670100021212941	0.504798765501044	0.00579780037291074	0.687987301390824
**ACC**	PCGF5	cg06001881	0.010292234682275	80	0.0903187766686226	0.928268432277243	0.000105930094755024	0.962135366888748
**ACC**	PCGF5	cg07421809	-0.163482201593101	80	-1.45411373064033	0.149978886082634	0.0267264302377272	0.315601864840875
**ACC**	PCGF5	cg07424295	0.153891800257341	80	1.36667530545746	0.175704489774588	0.0236826861864455	0.351508385473545
**ACC**	PCGF5	cg08633154	0.0881895061283916	80	0.776886747811439	0.439605737914031	0.00777738899116961	0.632243603839533
**ACC**	PCGF5	cg11113644	-0.0654154819084596	80	-0.575250645708891	0.5667990214912	0.00427918527331601	0.735701990153942
**ACC**	PCGF5	cg12783297	-0.0156623185815041	80	-0.137453148004505	0.89103141664584	0.000245308223348527	0.942381675700361
**ACC**	PCGF5	cg15550435	0.256242649258684	80	2.32618567046176	0.0226413266355746	0.0656602952991089	0.0799621627125927
**ACC**	PCGF5	cg17001035	0.23248832932204	80	2.09755142665164	0.0392260300785848	0.0540508232709536	0.120753028528093
**ACC**	PCGF5	cg20515900	0.0551138397937389	80	0.484358168442337	0.629506595046055	0.00303753533680992	0.781407272160917
**ACC**	PCGF5	cg22968329	-0.114382515240579	80	-1.01033353683984	0.315499899535634	0.0130833597927612	0.514700314232168
**ACC**	PCGF5	cg23395902	-0.284748226864727	80	-2.60656107796666	0.0109765151394545	0.0810815527026061	0.0458424340343974
**ACC**	PCGF5	cg25390656	0.188807918019563	80	1.68712736600565	0.0956262676556364	0.0356484299068821	0.230628406064068
**ACC**	PCGF6	cg01917164	-0.270912833917553	80	-2.46960407141945	0.0157399093913862	0.0733937635812396	0.0605484107794837
**ACC**	PCGF6	cg06691148	-0.198165895448219	80	-1.77408137974865	0.0800028902712468	0.0392697221187946	0.203065184696446
**ACC**	PCGF6	cg11612727	0.0839901249946938	80	0.73962375193988	0.461777332403564	0.00705434109662428	0.651342733795132
**ACC**	PCGF6	cg11814370	-0.212797121351531	80	-1.91105724222706	0.0597190976789517	0.045282614855498	0.16411570880445
**ACC**	PCGF6	cg14409890	-0.360322916733976	80	-3.38950160285057	0.00110675867346717	0.12983260432368	0.00730211044387287
**ACC**	PCGF6	cg16113748	-0.240875713390136	80	-2.17779898568324	0.0324827455644259	0.0580211093012069	0.104730412135961
**ACC**	PCGF6	cg16382383	0.2302348024985	80	2.07607574625354	0.0412245078734505	0.0530080642815235	0.125108034856332
**ACC**	PCGF6	cg24418712	0.168806854357247	80	1.50284118448725	0.136970371734672	0.028495754077989	0.296751973117417

**ACC**	PCGF6	cg25481680	0.0311679445157334	80	0.273630543379748	0.785101207105599	0.000971440765335834	0.881304483356512
**ACC**	PCGF6	cg26155979	0.271118447598115	80	2.47162706132314	0.0156577950207774	0.073505212628012	0.060317779429822
**ACC**	PCGF6	cg27217148	0.369954069470246	80	3.49425071485825	0.000791601678868633	0.136866013517595	0.00552089463192542
**ACC**	PHC1	cg02770112	-0.152371151455229	80	-1.35284817045457	0.18006324468753	0.0232169677957924	0.357141084615991
**ACC**	PHC1	cg08021945	-0.225164374984839	80	-2.02788382386036	0.0460314532953014	0.0506989957623133	0.135807002820353
**ACC**	PHC1	cg08867249	-0.107335898522681	80	-0.94734165549329	0.346428345979791	0.0115209951116713	0.545750337370188
**ACC**	PHC1	cg09059548	-0.236806164660799	80	-2.13879972249362	0.0356219129883941	0.0560771596213575	0.112315936408995
**ACC**	PHC1	cg09912793	-0.21037801841379	80	-1.88832000388967	0.0627495683919935	0.044258910631713	0.170124065082831
**ACC**	PHC1	cg12581035	0.303595609505678	80	2.79600957581788	0.00652971531018612	0.0921702941111238	0.0306654194289537
**ACC**	PHC1	cg14199415	0.0982204067485366	80	0.866068283675716	0.38914331821291	0.00964724830184798	0.586800063908803
**ACC**	PHC2	cg06899053	0.166284650157835	80	1.47974317311185	0.14302170506338	0.0276505848781135	0.305776473611353
**ACC**	PHC2	cg13640100	0.487659258570399	80	4.90151860740529	5.15729619727771e-06	0.237811552469431	7.31208865440756e-05
**ACC**	PHC2	cg15586632	-0.416768482219504	80	-4.02318770064224	0.00013309427003571	0.173695967771549	0.00123001751210059
**ACC**	PHC2	cg16558908	-0.153608147631412	80	-1.364095324045	0.176511636119322	0.0235954630187536	0.352506705865034
**ACC**	PHC2	cg17161743	0.66159398104661	80	7.74204004747705	3.18761248573217e-11	0.437706595757101	1.37841648535324e-09
**ACC**	PHC2	cg19746874	0.0838651256958003	80	0.738515195438988	0.462446539703446	0.00703335930797239	0.651897869947585
**ACC**	PHC2	cg21198021	-0.0880800152089963	80	-0.775914664586186	0.440176104319743	0.00775808907921702	0.632783144404511
**ACC**	PHC2	cg21578869	-0.231529901528118	80	-2.08841345407998	0.0400659027742845	0.0536060953016201	0.122634830044959
**ACC**	PHC2	cg23886755	0.0183465053516985	80	0.161017032079223	0.872501699795721	0.000336594258619901	0.932431206334427
**ACC**	PHF1	cg01097444	-0.231413499053602	80	-2.08730407205228	0.0401689174148016	0.0535522075442314	0.1228438069568
**ACC**	PHF1	cg02776641	-0.192289318112856	80	-1.71941924039174	0.0895556814275556	0.0369751818603071	0.220100198477838
**ACC**	PHF1	cg03560363	-0.193364710379236	80	-1.72940764720458	0.0877433047316581	0.0373899112200458	0.216922862575805
**ACC**	PHF1	cg03876174	-0.285112761948951	80	-2.6101930530959	0.0108702081453092	0.0812892870261593	0.0455059448127956
**ACC**	PHF1	cg06618123	0.234401473558329	80	2.11581133742071	0.0375933263544776	0.0549440508063158	0.117009491494768
**ACC**	PHF1	cg06919790	-0.18946195620325	80	-1.69318884917174	0.0944618216971864	0.0358958328483621	0.228628055413817
**ACC**	PHF1	cg07857404	-0.278347616005125	80	-2.54298829000189	0.0129962319150803	0.0774773953357368	0.0523144399048824
**ACC**	PHF1	cg08665801	-0.371430580681039	80	-3.51042324032867	0.000751268590726696	0.137960676265054	0.00528954554683615
**ACC**	PHF1	cg08798692	-0.351181498094466	80	-3.29123226522755	0.00150696004163555	0.123328444603874	0.00943596663546332
**ACC**	PHF1	cg10033570	0.0651285841620311	80	0.57271695347392	0.568504980403113	0.00424173247495077	0.736955296409305
**ACC**	PHF1	cg10797813	-0.21906818612397	80	-1.97017188951785	0.0524141200769026	0.0479908701716465	0.149331772835106
**ACC**	PHF1	cg13303256	0.0805247179289789	80	0.708903617133716	0.480525159638556	0.00648423019754161	0.667797603860834
**ACC**	PHF1	cg13528777	-0.283535180939015	80	-2.59448397658564	0.0113368156641604	0.0803921988301201	0.0470217829046954
**ACC**	PHF1	cg13547657	0.0404828009070623	80	0.355526584366605	0.72316724319732	0.00163885716928084	0.843133346081564
**ACC**	PHF1	cg18443095	0.0158287522730948	80	0.138914141010676	0.889880673335614	0.000250549398523003	0.941612855025446
**ACC**	PHF1	cg20135607	-0.18486119838821	80	-1.65059910246908	0.102893829976563	0.0341736626695253	0.243124187325333
**ACC**	PHF1	cg20872639	-0.11419650511933	80	-1.00866879384942	0.316292730547463	0.0130408417814691	0.515561259695121
**ACC**	PHF1	cg23345642	0.13216932401893	80	1.17004574310195	0.245591366173189	0.017468730211621	0.438522189223215
**ACC**	PHF1	cg23718418	-0.0671221113128757	80	-0.590325457208531	0.556701066852462	0.00450537782709808	0.728049134987315
**ACC**	PHF1	cg24796663	-0.160156010151907	80	-1.42374134558129	0.15856106717414	0.0256499475877777	0.327723660663189
**ACC**	PHF1	cg26673053	-0.319670121405323	80	-2.96043100691102	0.0040818730961235	0.102188986519294	0.021085341011941
**ACC**	PHF10	cg04353997	-0.224885448394621	80	-2.0252378454767	0.0463087792447153	0.0505734648996499	0.136392881254056
**ACC**	PHF10	cg05243705	-0.124691882459525	80	-1.10277340959003	0.27356035723046	0.0155480655513	0.469935159724841
**ACC**	PHF10	cg05736120	-0.141592083111911	80	-1.25511061447291	0.213234505139281	0.0200483179999702	0.399652229571042
**ACC**	PHF10	cg07652237	-0.316536071558912	80	-2.9281572115129	0.00448231734610127	0.100195084597948	0.0227214193411042
**ACC**	PHF10	cg11442717	-0.150292494911789	80	-1.33396300786723	0.186148144124856	0.0225878340268102	0.365046186236227
**ACC**	PHF10	cg15342139	-0.12305477934183	80	-1.08807077518043	0.279957881432497	0.0151424787188666	0.47709793047295
**ACC**	PHF10	cg15360169	-0.477164014970946	80	-4.76448486439233	8.74342002471585e-06	0.227685497183193	0.000116151915540086
**ACC**	PHF10	cg16740543	0.104781624999878	80	0.924544409579854	0.358091345809962	0.010979188937615	0.557080313481187
**ACC**	PHF10	cg27124293	-0.473855528493575	80	-4.72184091894535	1.02896562129212e-05	0.224539061883925	0.000134375275378209
**ACC**	PHF11	cg00092518	-0.461473827794275	80	-4.56450313134871	1.86489234796806e-05	0.2129580937391	0.000225696072367497
**ACC**	PHF11	cg01214340	-0.460199053542984	80	-4.54850034083645	1.98006270668832e-05	0.211783168881858	0.000237272024343979
**ACC**	PHF11	cg02289189	0.0050377611782663	80	0.0442067358972408	0.964854054296634	2.5379037689247e-05	0.981512539573662
**ACC**	PHF11	cg05080330	-0.0225165407430972	80	-0.197631948665629	0.843853833147971	0.000506994607035554	0.916616553265309
**ACC**	PHF11	cg09997676	-0.481509370312887	80	-4.82089250983603	7.04168588992549e-06	0.231851273699113	9.59539169943709e-05
**ACC**	PHF11	cg16956501	-0.405987384651531	80	-3.89824701712632	0.00020546482200083	0.16482575649619	0.00177500614799917
**ACC**	PHF11	cg21320768	-0.42291302880109	80	-4.09530842359536	0.000103227984199616	0.178855429929712	0.000989833329572894
**ACC**	PHF11	cg23137039	-0.088179261172214	80	-0.776795789750156	0.439659088886095	0.00777558210087753	0.632281522510839

**ACC**	PHF11	cg24481868	-0.489396332804845	80	-4.92446401699993	4.71783203191513e-06	0.23950877056283	6.76532765144341e-05
**ACC**	PHF11	cg24984523	-0.390905272062091	80	-3.72671081266408	0.000368175785459809	0.152806931725937	0.00289992241521581
**ACC**	PHF11	cg25162948	-0.499581484075477	80	-5.06057629012795	2.77101560138112e-06	0.249581659231056	4.22021119212691e-05
**ACC**	PHF12	cg01406758	-0.222463324005331	80	-2.00228283202678	0.0487754582253747	0.0494899305275007	0.141641643853253
**ACC**	PHF12	cg02058367	-0.263017881999271	80	-2.3921996026985	0.0191865051733785	0.0691784062513824	0.0704552741778525
**ACC**	PHF12	cg06895197	0.306519803620426	80	2.82571834417951	0.00600604631368559	0.0939543900115046	0.0286946119834709
**ACC**	PHF12	cg08657247	0.135113793924792	80	1.19659136783771	0.235137237858347	0.0182557373087513	0.426303719078801
**ACC**	PHF12	cg08806622	-0.0561500988184346	80	-0.493493683369447	0.623068453551197	0.00315283359731997	0.776728478832505
**ACC**	PHF12	cg13564075	0.0386917381290518	80	0.339773048148492	0.734952516026441	0.00149705059944712	0.850581450752738
**ACC**	PHF14	cg06715026	-0.260934427833451	80	-2.37185977145119	0.0201972579011395	0.0680867756287705	0.0733737499374822
**ACC**	PHF14	cg07287563	-0.326005341097441	80	-3.02600202127285	0.00336825267609478	0.106279482424059	0.018073203880199
**ACC**	PHF14	cg07574654	-0.00974027927733558	80	-0.0854746584984108	0.932105902431976	9.48730404004929e-05	0.963996931661863
**ACC**	PHF14	cg11931558	-0.3405000250637	80	-3.17776447681014	0.00213699734017765	0.11594026706838	0.0125269814695034
**ACC**	PHF14	cg13029706	-0.369508903138887	80	-3.48938074672571	0.000804142917139928	0.136536829498904	0.00559198123679124
**ACC**	PHF14	cg16800461	-0.443215004667259	80	-4.33860925128167	4.30233978318422e-05	0.196439540362198	0.000466150725756313
**ACC**	PHF14	cg20819769	0.223600051646438	80	2.01305096355902	0.0476046785974	0.0499969830962897	0.139239702477954
**ACC**	PHF14	cg20964328	-0.0844836331007319	80	-0.744000773433021	0.459140428883985	0.00713748426189908	0.649059085326466
**ACC**	PHF14	cg22876561	-0.265897345383034	80	-2.42036978246816	0.0178611247628595	0.0707013982817445	0.066744564309823
**ACC**	PHF14	cg23083824	-0.293573943947388	80	-2.6948455878065	0.00864242651355999	0.0861856605648244	0.038126853135754
**ACC**	PHF14	cg24491907	-0.154573132206152	80	-1.37287382110416	0.173776761699903	0.0238928532000206	0.348817973982926
**ACC**	PHF19	cg11054680	-0.350273412113723	80	-3.28152984074602	0.00155311495185581	0.12269146323379	0.00967280638577141
**ACC**	PHF19	cg11594303	-0.0827894258437524	80	-0.728976794875016	0.468227370519941	0.00685408903153818	0.657235034142187
**ACC**	PHF19	cg13501527	-0.0600208495866874	80	-0.527632073022905	0.599271875129207	0.00360250238510775	0.759562259830684
**ACC**	PHF19	cg13793166	-0.287727699323546	80	-2.63628275726825	0.0101335861508471	0.0827872289580211	0.0430853255188828
**ACC**	PHF19	cg13851767	0.44189093352702	80	4.32249708700139	4.56284292660704e-05	0.195267597133381	0.000489940376304304
**ACC**	PHF19	cg13852822	0.234014884638699	80	2.11211947030856	0.0379185958988939	0.0547629662324637	0.117726725430921
**ACC**	PHF19	cg14218513	-0.15844658709825	80	-1.40815156313071	0.163110998564522	0.0251053209630833	0.333940129015432
**ACC**	PHF19	cg27207079	0.394251778365186	80	3.76445704684862	0.000324251280041053	0.155434464744112	0.00260748439937086
**ACC**	PHF20	cg01512265	-0.36271245030204	80	-3.41537186820166	0.00101943470550303	0.13156032160411	0.00681966665060644
**ACC**	PHF20	cg02674305	-0.307303861174212	80	-2.83369904811703	0.00587209916346644	0.0944356630925796	0.028187569484236
**ACC**	PHF20	cg02957488	-0.0286889108165537	80	-0.251847834196644	0.801829665633861	0.000823053603840173	0.891887839931751
**ACC**	PHF20	cg04179482	-0.0496032283874433	80	-0.435803035181844	0.664198970737579	0.00246048026645686	0.804950070363565
**ACC**	PHF20	cg09692677	0.0659651652925367	80	0.580105488653019	0.563537217660745	0.00435140303207168	0.733426143652758
**ACC**	PHF20	cg10372813	-0.454024336829346	80	-4.47148744080933	2.63804206302821e-05	0.206138098433327	0.000304667227213802
**ACC**	PHF20	cg21765224	0.0560752857243544	80	0.492834088351003	0.623532320399661	0.00314443766906798	0.776927085709719
**ACC**	PHF20	cg23538717	0.0797493873783497	80	0.702034051001208	0.484774646557705	0.00635996478722209	0.67148405274487
**ACC**	PHF20	cg23734815	0.167703476773791	80	1.49273289052064	0.139593350814567	0.0281244561220174	0.300709581958531
**ACC**	PHF20	cg25561939	0.0560183318147346	80	0.492331955698814	0.623885552357173	0.00313805349930571	0.777221834237663
**ACC**	PHF20	cg27405321	-0.312403017648205	80	-2.88575855416253	0.00506358332736296	0.0975956454357045	0.0250344074590959
**ACC**	PHF20L1	cg02898212	0.314430996646854	80	2.90653951781388	0.00477051439241586	0.098866851652334	0.0238891146857805
**ACC**	PHF20L1	cg05307234	-0.257702309339915	80	-2.34037628678376	0.0218552948393783	0.0664104802391254	0.0778877093194648
**ACC**	PHF20L1	cg10591701	-0.409305982849727	80	-3.93649334454572	0.000180039519977431	0.167531387596581	0.00158870172608034
**ACC**	PHF20L1	cg17646969	-0.127710424147501	80	-1.12990666679446	0.262023070553797	0.0163099524359346	0.457355165515383
**ACC**	PHF20L1	cg24511731	0.0439750367540057	80	0.386253030979944	0.700374529417491	0.00193380385751615	0.828365428012742
**ACC**	PHF20L1	cg26292018	0.390023956056226	80	3.71679932560261	0.00038061748101197	0.152118686297749	0.00298154831515911
**ACC**	PHF20L1	cg27342122	0.0279419198822897	80	0.2452851238686	0.806888229696204	0.000780750886708297	0.895373619105162
**ACC**	PHF21B	cg01775414	-0.104444771120008	80	-0.921539332968814	0.359647316131522	0.0109087102143108	0.558495381101542
**ACC**	PHF21B	cg01995480	-0.112081481958745	80	-0.989747391665693	0.325397649657264	0.0125622585980686	0.524848847368647
**ACC**	PHF21B	cg05064352	-0.0691142369054817	80	-0.607928675679361	0.545023723220576	0.00477677774302705	0.719051241804116
**ACC**	PHF21B	cg10080155	-0.0622490130613411	80	-0.54729426809585	0.585759404380024	0.00387493962711102	0.750140185570099
**ACC**	PHF21B	cg11428482	-0.0518511025449015	80	-0.455604445151079	0.649956680422689	0.00268853683512189	0.79526156018878
**ACC**	PHF21B	cg14786713	-0.0133153923763879	80	-0.116852453317105	0.907281225269869	0.000177299674137169	0.950929408254256
**ACC**	PHF21B	cg15679813	-0.0673882619292726	80	-0.592676855333794	0.555134064278529	0.00454117784584825	0.72665464571966
**ACC**	PHF21B	cg20495645	-0.0764061814680595	80	-0.672427177230247	0.503324661104714	0.00583790456653004	0.686751742452628
**ACC**	PHF21B	cg20525183	-0.0444672920776141	80	-0.390585255403268	0.697182353377306	0.00197734006471585	0.826399219851686
**ACC**	PHF21B	cg21715212	-0.00623842428548043	80	-0.0547430161915405	0.956484989689488	3.8917937565672e-05	0.977019042895837
**ACC**	PHF21B	cg23080354	-0.0812896170495179	80	-0.715682028057072	0.476352425050824	0.00660800184005726	0.664146110980945

ACC	PHF21B	cg25561581	-0.0577104487663756	80	-0.507252538446491	0.613427384412393	0.00333049589681647	0.769904166351705
ACC	PHF21B	cg25976257	-0.0626544161783624	80	-0.550872579946941	0.583315866636456	0.00392557586665143	0.748325767300733
ACC	PHF23	cg00039960	0.0525744109854698	80	0.461977494653631	0.645400098356902	0.00276406869046909	0.792142909701212
ACC	PHF23	cg01441172	0.0966714516255561	80	0.852280330689998	0.396701664967524	0.00934536955939223	0.593309801489638
ACC	PHF23	cg04379155	-0.238966912947011	80	-2.15949156573744	0.0339249591750942	0.0571051854834243	0.108215445057704
ACC	PHF23	cg11180288	-0.00147491806939218	80	-0.0129423676105505	0.989707248122116	2.17538331141956e-06	0.994436321090902
ACC	PHF23	cg13351208	-0.0464259848787313	80	-0.407826109545494	0.684533085612177	0.00215537207196018	0.818414072387559
ACC	PHF23	cg22033559	-0.200863599218988	80	-1.79924086362563	0.0758984845631061	0.0403461854912064	0.195569762780374
ACC	PHF23	cg26213471	-0.195483678249341	80	-1.7491079330589	0.0842571283941683	0.0382138684618919	0.210660422506699
ACC	PHF23	cg26784276	0.145404659106627	80	1.28962651007528	0.201040536337994	0.0211425148899144	0.3842217782552
ACC	PHIP	cg01492710	0.351471342342228	80	3.2943313277707	0.00149249118489208	0.123532104487847	0.0093642115684728
ACC	PHIP	cg04480383	-0.364063849712719	80	-3.43003706647001	0.0009728630418231	0.132542486667645	0.00656229146584464
ACC	PHIP	cg05801338	-0.444916448092558	80	-4.35936544412121	3.98787924458212e-05	0.197950645783298	0.000436886463812169
ACC	PHIP	cg06848288	-0.0783720345037439	80	-0.689833614541648	0.49237269887887	0.00614217579225603	0.677868575665996
ACC	PHIP	cg08287994	-0.321527455886386	80	-2.97960841875873	0.00385987493057802	0.103379904888772	0.0201824916218166
ACC	PHIP	cg08587415	-0.437042346154695	80	-4.26379420009575	5.64716132079151e-05	0.1910060123324	0.000589684923207591
ACC	PHIP	cg15564972	-0.12841921419055	80	-1.13628248046481	0.259362481635542	0.0164914945733183	0.4542154989171
ACC	PHIP	cg19494004	-0.322991622860626	80	-2.99475330413739	0.00369251756485631	0.104323588438141	0.0194459593447354
ACC	PHIP	cg19950079	-0.154252135973428	80	-1.36995326220158	0.174683034270536	0.0237937214523649	0.350095114593005
ACC	PHIP	cg21607066	0.194149207968044	80	1.73669826236095	0.0864395685757318	0.0376939149546188	0.214661297254053
ACC	PHIP	cg21875336	-0.414969172156236	80	-4.00219580296324	0.000143244125129193	0.172199413840032	0.00130884818772615
ACC	PHIP	cg26363168	-0.237420799322401	80	-2.14468217215231	0.0351321344208951	0.056368635950888	0.11116585600568
ACC	PHRF1	cg06063517	-0.0430108718293422	80	-0.377768453885955	0.706641955173845	0.00184993509552011	0.832719572828109
ACC	PHRF1	cg07530960	-0.306535699163318	80	-2.82588007736498	0.00600330416833808	0.0939641348615441	0.0286885585399406
ACC	PHRF1	cg08482978	-0.255865704397398	80	-2.32252383624788	0.0228481821787803	0.0654672586867766	0.0805224012148244
ACC	PHRF1	cg12469954	-0.253855342123504	80	-2.30301324366105	0.0239787906347851	0.0644425347246414	0.083534370992901
ACC	PHRF1	cg12813709	-0.196483980411109	80	-1.75841668593244	0.0826500474557201	0.0386059545581931	0.207707927857336
ACC	PHRF1	cg15219216	0.103632747855383	80	0.914296581905536	0.363415235171758	0.0107397464280574	0.562371196788505
ACC	PHRF1	cg16143007	-0.133722662204213	80	-1.18404575776086	0.240037206243492	0.017881750386982	0.431985898223477
ACC	PHRF1	cg18793109	-0.160317692697523	80	-1.42521655372323	0.158135646456367	0.0257017625918576	0.327172268833984
ACC	PHRF1	cg20152424	0.0982078865020951	80	0.865956809938283	0.389204065968763	0.00964478897120839	0.586817635008447
ACC	PHRF1	cg21006161	0.0597311253895904	80	0.525076020123601	0.601039001227928	0.00356780734030697	0.760873983947234
ACC	PPARGC	cg04893087	-0.136599604730701	80	-1.20999878251341	0.22998092525124	0.0186594520125838	0.420098058114843
ACC	PPARGC	cg05158538	-0.113638624434708	80	-1.00367654832484	0.318678266505143	0.0129137369634125	0.517995341924286
ACC	PPARGC	cg11270806	0.040733437976132	80	0.357731367084499	0.721523089259063	0.00165921296935539	0.842216701320644
ACC	PPARGC	cg12691631	-0.144058611735476	80	-1.27743390019687	0.20528737408976	0.0207528836151526	0.38954017215178
ACC	PPARGC	cg20723350	-0.0747976121893229	80	-0.658190148207483	0.512379085103478	0.00559468278922434	0.694268171782348
ACC	PPARGC	cg27461259	-0.124740891388591	80	-1.10321369353024	0.273370361474641	0.0155602899844203	0.469751146416289
ACC	PPARGC	cg27514608	-0.10341225828859	80	-0.912330269450251	0.364442507126392	0.0106940951643459	0.56339707700184
ACC	PRDM1	cg00555933	-0.0559800274647618	80	-0.491994249112637	0.624123166196447	0.00313376347495548	0.777368729720794
ACC	PRDM1	cg01761420	0.365292221398986	80	3.44338884805715	0.000932215714978946	0.133438407014605	0.00633258726258052
ACC	PRDM1	cg05170275	0.417969101984907	80	4.03722669927599	0.000126695627161738	0.17469817021407	0.00118037839753536
ACC	PRDM1	cg08742502	-0.115920931713896	80	-1.02410611574965	0.308991815932229	0.0134376624094178	0.507628013613327
ACC	PRDM1	cg14359052	-0.0266820208976092	80	-0.234217171147761	0.815438032522894	0.000711930239180456	0.900665358144551
ACC	PRDM1	cg17143179	-0.08370616283659	80	-0.737105483752054	0.463298342974039	0.00700672169682572	0.652612513685741
ACC	PRDM1	cg17521665	0.0542206960409327	80	0.476485598519954	0.635077837458401	0.00293988387916322	0.785049206170322
ACC	PRDM1	cg17965230	0.243306824042716	80	2.20115479425481	0.0307207651976259	0.0591982106257533	0.100429468606168
ACC	PRDM1	cg19464016	-0.00860426464141384	80	-0.0755049108013352	0.94000881888462	7.40333700194845e-05	0.96808289941897
ACC	PRDM1	cg22186515	-0.028153266827201	80	-0.247141876333247	0.805456191535108	0.000792606433043578	0.894330790509238
ACC	PRDM1	cg22196848	0.251809865569119	80	2.28319460795719	0.0251776136637956	0.063408208397938	0.0866781996442588
ACC	PRDM1	cg24040346	-0.0368308530847424	80	-0.323408852567931	0.747262334639389	0.00135651173894988	0.858087766892588
ACC	PRDM1	cg24793124	-0.231501664109259	80	-2.08814432665459	0.0400908723097832	0.0535930204853562	0.122682295667818
ACC	PRDM1	cg24874254	-0.0797769251342659	80	-0.702278018361078	0.484623374567006	0.00636435778387826	0.671346240024674
ACC	PRDM1	cg26351406	0.370164248814846	80	3.49655097403363	0.000785742565275825	0.137021571100659	0.00548609380031121
ACC	PRDM10	cg04181189	0.0939644319220198	80	0.828198864195816	0.410117577863436	0.0088293144664279	0.606046023332348
ACC	PRDM10	cg17627813	0.0337952393586853	80	0.296721515767444	0.767478044330015	0.00114211820331083	0.871163449049525
ACC	PRDM12	cg09191327	-0.0387545960583067	80	-0.34032586718789	0.734537851868075	0.00150191871564252	0.850335800540741

**ACC**	PRDM12	cg12539975	0.0186377526720748	80	0.163574028439715	0.870495070566802	0.00034736582466543	0.93126169045019
**ACC**	PRDM12	cg13795948	0.194533597741714	80	1.74027179618727	0.0858063842749423	0.0378433206503348	0.213475740362065
**ACC**	PRDM12	cg13928649	0.0484301945652408	80	0.425472494919769	0.671679078589151	0.00234548374562708	0.809891847852761
**ACC**	PRDM12	cg23342516	0.244491801880881	80	2.21255488757428	0.0298915347351976	0.0597762411869598	0.0983997145053301
**ACC**	PRDM13	cg00126261	0.316163394296924	80	2.92432658018988	0.00453218371072726	0.099959291893352	0.0229139338137594
**ACC**	PRDM13	cg00579292	0.275046134352165	80	2.51034131460042	0.0141586714202306	0.075650376022069	0.0558176959520251
**ACC**	PRDM13	cg01422797	0.227426425482642	80	2.04936176328304	0.0438326587148994	0.0517227790078117	0.131004566140339
**ACC**	PRDM13	cg01815538	0.00927034863182628	80	0.0813504747821126	0.935374318672376	8.59393637556034e-05	0.965541585356972
**ACC**	PRDM13	cg02400433	0.116497393723834	80	1.02926877494689	0.306575734710493	0.0135716427444461	0.505034573650434
**ACC**	PRDM13	cg03120185	0.303107532912429	80	2.7910594013933	0.00662093471666365	0.0918741765082593	0.0310002404663335
**ACC**	PRDM13	cg04910970	0.168679629286949	80	1.50167535077267	0.137270899400847	0.0284528173363825	0.297202287375525
**ACC**	PRDM13	cg11809464	0.225960752935552	80	2.03544138898656	0.0452471830784098	0.0510582618672014	0.134158442063484
**ACC**	PRDM13	cg13738144	0.133098624706761	80	1.17842033564059	0.24225800997084	0.0177152438988312	0.434645286616042
**ACC**	PRDM13	cg14344102	0.144779944709242	80	1.28396688949284	0.203003656325381	0.0209612323900113	0.386815729851389
**ACC**	PRDM13	cg17879189	0.115884841030342	80	1.02378293096231	0.30914348937164	0.0134292963806276	0.50776995993027
**ACC**	PRDM13	cg17978124	0.175320618345969	80	1.5626352269941	0.122239268595587	0.0307373192172131	0.274127462125254
**ACC**	PRDM13	cg25273233	0.235717911002835	80	2.12839110092125	0.0365031046515006	0.0555629335675406	0.11445033707709
**ACC**	PRDM13	cg25924096	0.0829799991564243	80	0.730666450563112	0.467200375576489	0.00688568026000018	0.656290827331119
**ACC**	PRDM13	cg26507427	0.236122954838659	80	2.13226415672652	0.03617302450134	0.0557540498017394	0.113659198925448
**ACC**	PRDM14	cg00384539	-0.122672796489712	80	-1.08464153374528	0.281464830940321	0.0150486149986064	0.478633015424867
**ACC**	PRDM14	cg01295203	-0.0555190813123369	80	-0.48793053253442	0.626985559676853	0.00308236838976587	0.779451150254904
**ACC**	PRDM14	cg04838988	-0.052675182626632	80	-0.462865448269415	0.644766302927744	0.00277467486474904	0.791637596786051
**ACC**	PRDM14	cg09050331	0.0291813937360739	80	0.256174787338027	0.798499044812587	0.000851553740379776	0.88994892109098
**ACC**	PRDM14	cg10457247	-0.0133282501544899	80	-0.116965309903864	0.907192089752958	0.00017764225218066	0.9509017960717
**ACC**	PRDM14	cg11229513	0.0507872549006776	80	0.446232218143757	0.656681879732839	0.0025793452603464	0.799954747841977
**ACC**	PRDM14	cg13267264	-0.0957442680476453	80	-0.844030045996514	0.401267245062829	0.00916696486397936	0.597850845796486
**ACC**	PRDM14	cg18035229	-0.0594138543774761	80	-0.522277091885239	0.602976786587517	0.00353000609198793	0.762161247453763
**ACC**	PRDM14	cg21255438	-0.140148600811298	80	-1.24205749088842	0.217985131038539	0.0196416303093646	0.405517606868145
**ACC**	PRDM14	cg27029821	-0.115802894708854	80	-1.02304913345105	0.309488053190345	0.0134103104229498	0.508142791623007
**ACC**	PRDM4	cg00090609	0.0722420036928585	80	0.635581700183547	0.52693357183007	0.00521890709755898	0.705564125061723
**ACC**	PRDM4	cg03909902	-0.201991227318288	80	-1.80977001663671	0.074233567450445	0.0408004559135481	0.192420569980005
**ACC**	PRDM4	cg12900929	-0.188396247666687	80	-1.68331328948145	0.0963649615226084	0.0354931461348876	0.231972507463333
**ACC**	PRDM4	cg18440127	-0.214546510094825	80	-1.92752271641365	0.0576026176236021	0.0460302049938689	0.159813033102672
**ACC**	PRDM4	cg25995916	0.0916120903616393	80	0.807287660284476	0.421987520224502	0.00839277510042917	0.617104294383987
**ACC**	PRDM4	cg27140793	0.128728648471377	80	1.13906650431043	0.258206728846804	0.0165710649372673	0.452775809970799
**ACC**	PRDM5	cg05713693	0.0887461596218865	80	0.781829273349887	0.4367124269995	0.00787588084763335	0.629748637410527
**ACC**	PRDM5	cg06329345	-0.154491340466174	80	-1.37212960316421	0.174007357691982	0.0238675742790353	0.349136706496699
**ACC**	PRDM5	cg07070341	-0.169101156148463	80	-1.50553833105463	0.136277087037953	0.0285952010107469	0.295669893714594
**ACC**	PRDM5	cg10416963	-0.168350042602398	80	-1.49865552474354	0.138051758765682	0.0283417368442291	0.298355331742902
**ACC**	PRDM5	cg11171429	-0.125627229313712	80	-1.11117775042216	0.269949506777182	0.0157822007450401	0.466024275574732
**ACC**	PRDM5	cg13155001	-0.125218117562261	80	-1.10750140326319	0.271524895398124	0.0156795769658363	0.467832906918379
**ACC**	PRDM5	cg18181323	-0.179828236240706	80	-1.60413696875532	0.112778026657086	0.0323381945494431	0.259622166353136
**ACC**	PRDM5	cg19294653	-0.23658762842634	80	-2.13670884603394	0.0357974264036026	0.0559737059244001	0.112741880939104
**ACC**	PRDM5	cg22135105	-0.224273141044244	80	-2.01943115864057	0.0469224110834114	0.0502984417938512	0.137746492370676
**ACC**	PRDM8	cg01304890	-0.023050680388798	80	-0.202322657098442	0.840198106676558	0.000531333866386515	0.914394463222514
**ACC**	PRDM8	cg02895669	0.139796815161846	80	1.23887759092146	0.219154103796692	0.0195431495293953	0.407059531331563
**ACC**	PRDM8	cg04343891	-0.0479120791996754	80	-0.42091018010667	0.674993186043106	0.00229556733323597	0.812062847610495
**ACC**	PRDM8	cg05007549	0.240412604909369	80	2.17335483011279	0.0328278342489696	0.0577982205993084	0.105542362175656
**ACC**	PRDM8	cg07462804	0.213595213956931	80	1.91856659401143	0.058745819336697	0.045622915425307	0.162128291098304
**ACC**	PRDM8	cg08074201	0.110264743562232	80	0.973505388579381	0.33335056688541	0.0121583136728448	0.53294881733097
**ACC**	PRDM8	cg10695325	-0.00433954571980844	80	-0.0380797177025593	0.969722677765623	1.88316570543077e-05	0.984061878304425
**ACC**	PRDM8	cg11571772	0.25831108284616	80	2.34629976406845	0.021534414113502	0.0667246155211557	0.0770583353976898
**ACC**	PRDM8	cg12572049	-0.0262923297029155	80	-0.230794043010609	0.818086935169407	0.000691286601206813	0.902165910116911
**ACC**	PRDM8	cg14489346	0.290370611361232	80	2.66271689169794	0.00943373430477191	0.0843150919422954	0.040696940415096
**ACC**	PRDM8	cg15656813	-0.0196220549637668	80	-0.172215990440764	0.863719566473191	0.000385025041001086	0.92760558619032
**ACC**	PRDM8	cg16117799	-0.00703696655859434	80	-0.0617506598781873	0.950921402440416	4.95188983467751e-05	0.974097688734974
**ACC**	PRDM8	cg18624348	0.0374585346399054	80	0.328928154899936	0.743102955857021	0.001403141817369	0.855592329691577

ACC	PRDM8	cg19595012	-0.0454697600090101	80	-0.399408626898254	0.690697826762882	0.00206749907527697	0.822238021974067
ACC	PRDM8	cg20142271	-0.0193862836085026	80	-0.170145924073635	0.865341637205499	0.000375827992149295	0.928512062161531
ACC	PRDM8	cg26162932	-0.0406775137647282	80	-0.357239411920311	0.72188983744435	0.00165466012607965	0.842440242788694
ACC	PRKAA1	cg00301775	-0.408961646864665	80	-3.93251625169979	0.00018253590726005	0.167249628606259	0.001605993934561
ACC	PRKAA1	cg01883943	-0.0909654405642031	80	-0.801541653163305	0.425284783699509	0.00827471137703956	0.620085211739465
ACC	PRKAA1	cg03125217	0.0123092430227137	80	0.108021353030802	0.914259737578102	0.000151517463792225	0.95474759413586
ACC	PRKAA1	cg05874655	0.208979975737884	80	1.87519632994314	0.0645570120998605	0.0436726302594064	0.173656576739161
ACC	PRKAA1	cg06971720	-0.157895525628379	80	-1.40312870876072	0.164598121477	0.0249309970134621	0.336250262918036
ACC	PRKAA1	cg10786880	-0.239347015869082	80	-2.16313502983302	0.0336335658981054	0.0572869940054347	0.1074798564524
ACC	PRKAA1	cg24728477	0.0726394073905204	80	0.639096534179107	0.524656804715056	0.00527648350604598	0.703806584804853
ACC	PRKCD	cg02121118	-0.258714760587473	80	-2.35022927299344	0.0213238624387587	0.0669333273458336	0.0764666983756244
ACC	PRKCD	cg02559423	-0.110176917421667	80	-0.972720457088887	0.333738122856999	0.0121389531325409	0.533336555660177
ACC	PRKCD	cg02630105	-0.0891434698134978	80	-0.785357441245671	0.434653927037714	0.00794655821039	0.628071170384158
ACC	PRKCD	cg10531918	-0.318957792552691	80	-2.95308614826049	0.00416998288091856	0.101734073430085	0.0214635204603797
ACC	PRKCD	cg13469346	-0.156398974379583	80	-1.38949460505282	0.16868726396038	0.0244606391869854	0.341892426661121
ACC	PRKCD	cg13889007	-0.056102834161627	80	-0.49307697015803	0.623361493597019	0.00314752800096703	0.776845188275126
ACC	PRKCD	cg13908523	-0.064366174772837	80	-0.565984546509387	0.573050095281849	0.0041430044548874	0.74053889173202
ACC	PRKCD	cg14727059	-0.137323562296811	80	-1.2165345204166	0.227497301740904	0.0188577607618862	0.417177594503733
ACC	PRKCD	cg21903826	-0.0217340404361258	80	-0.190760490767745	0.849215313238429	0.000472368513679153	0.919597105763785
ACC	PRMT1	cg07702240	0.308667294775582	80	2.84759225087616	0.00564544863779089	0.0952754988640763	0.0273378170761472
ACC	PRMT1	cg08377944	0.0689683397782949	80	0.606639224939837	0.545874872731265	0.00475663189177433	0.7195967797385
ACC	PRMT1	cg11787855	0.24231948371794	80	2.19166407868061	0.0314264018618197	0.0587187321893292	0.102193934976316
ACC	PRMT1	cg14258555	-0.0276008537761693	80	-0.242288815204115	0.809200552035291	0.000761807129173482	0.896890461974033
ACC	PRMT1	cg14576628	-0.301435412061066	80	-2.77411870475003	0.00694201957242088	0.0908633076444245	0.0322012682974724
ACC	PRMT1	cg18880319	-0.323909886046277	80	-3.00426377056697	0.00359088749806927	0.104917614278512	0.0190150093271255
ACC	PRMT1	cg22292364	0.13518496628822	80	1.19723341299496	0.234888428788388	0.0182749751103471	0.425982502077541
ACC	PRMT1	cg24228123	-0.0545250247675365	80	-0.479167958719832	0.63317720226763	0.00297297832590047	0.783916404443511
ACC	PRMT1	cg25595446	-0.397602063934832	80	-3.80242352711765	0.00028514104207533	0.158087401245238	0.00234237193529126
ACC	PRMT1	cg26846726	0.0514053720545987	80	0.451677485522382	0.652771039752493	0.00264251227607172	0.797165007716528
ACC	PRMT2	cg04493422	0.32048535412409	80	2.96884377418085	0.00398307053643185	0.102710862208043	0.0206848457484099
ACC	PRMT2	cg06725035	-0.403390318374495	80	-3.86844518011665	0.000227624307031057	0.162723748958276	0.0019359139981605
ACC	PRMT2	cg09031657	-0.296919874839333	80	-2.72851061598047	0.00787859068004997	0.088161412074605	0.0354999758681614
ACC	PRMT2	cg10187059	-0.376720877145011	80	-3.56862416510019	0.00062163937596995	0.141918619276906	0.00450833988279916
ACC	PRMT2	cg10657630	-0.102490950750446	80	-0.904115572613954	0.368754144054773	0.0105043949857303	0.567505981969911
ACC	PRMT2	cg12162201	-0.471367826972209	80	-4.6899463626266	1.16170292494084e-05	0.222187628304503	0.000149279017745041
ACC	PRMT2	cg13271076	0.32431314475988	80	3.0084433224814	0.00354704993443694	0.105179015864043	0.0188483061884964
ACC	PRMT2	cg17243659	-0.201725693768962	80	-1.80728994109757	0.0746229634669848	0.0406932555265689	0.193140781641245
ACC	PRMT2	cg23283320	-0.215294540691705	79	-1.9219660104693	0.0583585116137252	0.0463517392516521	0.161362788430003
ACC	PRMT3	cg01769160	-0.0437471997268452	80	-0.384247985999639	0.701853768706203	0.00191381748394048	0.829245509510964
ACC	PRMT3	cg04101203	-0.0880079977613332	80	-0.775275294115963	0.440551488527663	0.00774540766995883	0.63318237660541
ACC	PRMT3	cg06578097	-0.141656069499633	80	-1.25568941908839	0.213025629748384	0.0200664420260849	0.399414712603836
ACC	PRMT3	cg13432154	-0.262428495385034	80	-2.38644205476229	0.0194679416083779	0.0688687151900529	0.0713134442257409
ACC	PRMT3	cg13982511	-0.0736017927541431	80	-0.647609615484115	0.519163739854326	0.00541722389662384	0.699831124706415
ACC	PRMT3	cg19653624	-0.289419568417867	80	-2.65319703939625	0.00968054679033821	0.0837636865831841	0.0415457525574996
ACC	PRMT3	cg23019633	-0.286929336272709	80	-2.62831056636032	0.010353719075519	0.0823284440138974	0.0437962710978697
ACC	PRMT3	cg23071995	-0.0612941511904952	80	-0.538867201150259	0.591533089486145	0.00375697297016329	0.754149080484138
ACC	PRMT6	cg00944433	-0.205524963340655	80	-1.84281487925029	0.0692048510071571	0.0422405105561774	0.182827526717519
ACC	PRMT6	cg02990361	-0.352891597848027	80	-3.30953258605057	0.00142339022729491	0.124532479831733	0.00900400222330297
ACC	PRMT6	cg02993280	-0.290445194392653	80	-2.66346382481967	0.0094146134456588	0.084358410945786	0.0406505210851741
ACC	PRMT6	cg09367891	-0.304555632673806	80	-2.80575341078858	0.00635351892196562	0.092754133393342	0.0300100577422826
ACC	PRMT6	cg14671364	-0.227901945544249	80	-2.05388121324193	0.0433816745909072	0.0519392967828538	0.130005694956297
ACC	PRMT6	cg14828511	-0.29602971449339	80	-2.71954359745339	0.00807578345850221	0.0876335918630379	0.0361940551011794
ACC	PRMT6	cg22528358	0.122706713090068	80	1.08494599934871	0.281330809663495	0.0150569374373684	0.478530610808714
ACC	PRMT7	cg00894222	-0.536441681462149	80	-5.57773206022889	3.47497452023069e-07	0.287769677609937	6.63880569319319e-06
ACC	PRMT7	cg02477175	-0.384140937559248	80	-3.65094364668235	0.000474038248120293	0.147564259908898	0.00358310643939662
ACC	PRMT7	cg06089844	-0.415558212004721	80	-4.009061647179	0.000139845277698179	0.172688627564561	0.00128246180988472
ACC	PRMT7	cg06092312	0.32494705053381	80	3.01501710946437	0.00347910347962961	0.105590585650622	0.0185537791705774

**ACC**	PRMT7	cg06745885	-0.461125749566104	80	-4.56012999980883	1.89570284587871e-05	0.212636956912901	0.00022868676532542
**ACC**	PRMT7	cg09175928	-0.357089251515453	80	-3.35461428429651	0.00123572571289772	0.127512733547867	0.00800966889761417
**ACC**	PRMT7	cg09779635	-0.412948323655156	80	-3.9786869048774	0.000155492774530916	0.170526318009604	0.00140427268421849
**ACC**	PRMT7	cg10233539	0.175486577097683	80	1.56416139676421	0.121880476760229	0.0307955387414609	0.27362258481719
**ACC**	PRMT7	cg10446007	-0.348081581594726	80	-3.25815463937744	0.00166981931451073	0.121160787445486	0.0102656807138492
**ACC**	PRMT7	cg11893236	0.147044815306777	80	1.30449307807201	0.195951175304227	0.0216221777086043	0.377855532102647
**ACC**	PRMT7	cg19328639	0.122395640370089	80	1.08215367812737	0.28256160348948	0.0149806927816042	0.479779932378658
**ACC**	PRMT7	cg21918026	-0.0112674049551157	80	-0.0988773538769649	0.921492610584584	0.000126954414422565	0.958833426887501
**ACC**	PRMT7	cg26492318	-0.185787131265046	80	-1.65916143252706	0.101151297721931	0.0345168581436953	0.240198576288385
**ACC**	PRMT7	cg26561196	0.373147667468136	80	3.52926971095634	0.000706721906371647	0.13923918173691	0.00502685599197915
**ACC**	PRMT7	cg26796563	0.229190836579363	80	2.06613893118022	0.0421785782823438	0.0525284395719484	0.12727852284316
**ACC**	PRMT8	cg01693063	0.33871243493187	80	3.1589130838813	0.00226298843421182	0.114726113577477	0.0131358694412083
**ACC**	PRMT8	cg06029846	-0.0404513950700345	80	-0.355250320935292	0.723373350856462	0.00163631536311201	0.84326516211296
**ACC**	PRMT8	cg07912789	-0.0909235056460902	80	-0.801169062837009	0.425499117611541	0.0082670838789746	0.620152846887594
**ACC**	PRMT8	cg10350722	-0.0313667579421108	80	-0.275377685631532	0.783763714827038	0.000983873503798972	0.880667221758884
**ACC**	PRMT8	cg11596580	-0.0161338358416055	80	-0.141592264421365	0.88777187963797	0.000260300658963874	0.940344778745559
**ACC**	PRMT8	cg19656577	0.0916521357446328	80	0.807643529729531	0.421783813344391	0.0084001139865526	0.616875974794863
**ACC**	PRMT8	cg21667836	0.350857252296839	80	3.28776665459832	0.00152329621565063	0.123100811489287	0.00952139689862809
**ACC**	PRMT8	cg23739862	-0.087559137549265	80	-0.771290591576358	0.442895182220747	0.0076666025683711	0.635175961143891
**ACC**	PRMT8	cg24100636	0.138902116019077	80	1.2307922797479	0.222147024918374	0.0192937978345772	0.410667403082077
**ACC**	PRMT8	cg27316886	0.139262400171951	80	1.23404776260249	0.220938380338248	0.0193940161016525	0.409162291047532
**ACC**	PWWP2E	cg00259755	-0.320438713120132	80	-2.96836226587208	0.00398866527779612	0.102680968866086	0.0207000818292776
**ACC**	PWWP2E	cg05010219	-0.383196689194989	80	-3.64042251962971	0.00049085004856968	0.146839702610001	0.0036911733860251
**ACC**	PWWP2E	cg15012692	-0.181342272441329	80	-1.61810001183497	0.109730421287951	0.0328850197741852	0.254535837841038
**ACC**	PWWP2E	cg15278699	-0.370072972676508	80	-3.49555194707422	0.000788282181812035	0.136954005105627	0.00550185035455638
**ACC**	PWWP2E	cg19919881	0.0416864633919505	80	0.366115480665012	0.71528290562457	0.00173776123012843	0.838088996490361
**ACC**	PWWP2E	cg22633292	-0.306773404923332	80	-2.82829898559256	0.00596242917281578	0.0941099219682546	0.0285282744984335
**ACC**	PWWP2E	cg22664157	0.0637265383431012	80	0.560337046458	0.576876285217709	0.00406107168919475	0.743522212999544
**ACC**	PWWP2E	cg24520806	-0.193704437566933	80	-1.73256442881562	0.0871768249320797	0.0375214091331218	0.21589946258647
**ACC**	PYGO1	cg06364757	-0.0247077976768238	80	-0.216876253605594	0.828878382448778	0.000610475266038861	0.907998049794771
**ACC**	PYGO1	cg09275667	-0.120428648485805	80	-1.06450458139434	0.290426995704029	0.0145030593761175	0.488011895491307
**ACC**	PYGO1	cg11480800	-0.00240097872268167	80	-0.0210685635134671	0.983245462873013	5.76469882677011e-06	0.991324666436562
**ACC**	PYGO1	cg12686317	-0.10945279007121	80	-0.966249566902673	0.336944378561744	0.0119799132543724	0.536535886272602
**ACC**	PYGO1	cg13878116	-0.023946313684356	80	-0.210188322149223	0.83407588147142	0.000573425939069578	0.911012396068489
**ACC**	PYGO1	cg17841803	-0.0936923303754639	80	-0.825779301324747	0.411480568992607	0.00877825277118507	0.607240656676507
**ACC**	PYGO1	cg19850348	-0.0314005777313036	80	-0.275674892062542	0.783536258658471	0.000985996281859638	0.880580552357001
**ACC**	PYGO1	cg22510134	0.0675891684760487	80	0.594451916695377	0.553952597408096	0.0045682956952837	0.725789362074593
**ACC**	PYGO1	cg23030278	-0.126480070005995	80	-1.11884337483462	0.266685221590497	0.0159972081087215	0.462410514162421
**ACC**	PYGO1	cg23412777	0.119134139615669	80	1.05289638345429	0.295681391371225	0.0141929432219657	0.493599668683786
**ACC**	PYGO1	cg25902187	-0.0882080369838153	80	-0.777051271419025	0.439509247083432	0.00778065778853812	0.632139889949869
**ACC**	PYGO2	cg01277844	0.0605528795678905	80	0.532326185231644	0.596032869589847	0.00366665122396345	0.75726241288446
**ACC**	PYGO2	cg02341811	-0.485607948924553	80	-4.87452068937584	5.72568496522369e-06	0.235815080058711	8.02710283266556e-05
**ACC**	PYGO2	cg04602755	-0.233649925750675	80	-2.1086351394318	0.0382278163214073	0.0545922878032961	0.118400665955331
**ACC**	PYGO2	cg10892866	-0.00557563139537232	80	-0.0489267274463957	0.961104377080238	3.10876654570615e-05	0.979633195133639
**ACC**	PYGO2	cg14024356	0.227365253477397	80	2.04878048306162	0.0438909550831289	0.0516949584888411	0.131148567687226
**ACC**	PYGO2	cg18188585	-0.387221432445022	80	-3.68536130218119	0.00042278559136327	0.149940437744775	0.00325323267008054
**ACC**	PYGO2	cg24683561	-0.356925821476669	80	-3.35285476094122	0.00124259106847762	0.127396042036795	0.00804612365416577
**ACC**	PYGO2	cg27105205	-0.111549100922254	80	-0.984986765771484	0.327715554038615	0.0124432019165631	0.527226652445631
**ACC**	RBBP4	cg05797186	-0.288425527985914	80	-2.64325595171815	0.00994453076024947	0.0831892851939532	0.0424369076227563
**ACC**	RBBP4	cg06526778	-0.0333083075370528	80	-0.292441481089775	0.770735656676923	0.00110944335098289	0.872988696827765
**ACC**	RBBP4	cg09036220	-0.076670810356124	80	-0.674769841631077	0.501843077631924	0.00587841316066473	0.685628639842959
**ACC**	RBBP4	cg11229946	-0.329374082523963	80	-3.06105416828005	0.00303607888233511	0.108487286238503	0.0166189642958329
**ACC**	RBBP4	cg13641837	-0.162359830151086	80	-1.44385940596972	0.15283508482286	0.0263607144466895	0.319877529520472
**ACC**	RBBP4	cg16148416	-0.023232698174144	80	-0.203921140589886	0.838953118945295	0.000539758264450876	0.913814129390939
**ACC**	RBBP4	cg16396527	-0.350108588866421	80	-3.27976992273623	0.00156162788400876	0.122576023998037	0.00971804492509217
**ACC**	RBBP4	cg18758482	-0.404214513165162	80	-3.87789081205493	0.000220364463696415	0.163389372653349	0.00188331625388832
**ACC**	RBBP4	cg20740619	0.0230499785534551	80	0.202316493606797	0.840202907937267	0.000531301511314741	0.914394463222514

ACC	RBBP4	cg22358606	0.0518473812215303	80	0.455571658547423	0.649980156816494	0.00268815093953069	0.795262451305531
ACC	RBBP4	cg22857567	-0.0599064107118042	80	-0.526622435432155	0.599969600883497	0.00358877804437137	0.760103966135248
ACC	RBBP4	cg23016766	-0.0471042516386286	80	-0.413797454626685	0.680172756895008	0.00221881052243524	0.815456778601441
ACC	RBBP4	cg26131911	-0.00659325043140746	80	-0.057856795291389	0.95401257984227	4.34709512512547e-05	0.975622803667631
ACC	RBBP5	cg00960017	-0.267840702006272	80	-2.43942124154526	0.0170117456883846	0.0717386416512124	0.0642776115441347
ACC	RBBP5	cg02189925	0.160653766964595	80	1.4282833067258	0.157254076884944	0.0258096328399145	0.325937805589411
ACC	RBBP5	cg03143531	-0.0356791915310084	80	-0.313283103990187	0.754912622313248	0.00127300470830638	0.863159889183057
ACC	RBBP5	cg05290904	-0.0291750292463715	80	-0.256118867770547	0.79854206454283	0.000851182331526631	0.889984117262574
ACC	RBBP5	cg07601985	0.19659705306169	80	1.7594692919559	0.0824699281945114	0.0386504012725409	0.207447222016571
ACC	RBBP5	cg07972983	-0.154967144235984	80	-1.37645931748686	0.172669056478759	0.0240148157926564	0.347374294129887
ACC	RBBP5	cg14717946	-0.145311645397755	80	-1.28878374966362	0.201331961702256	0.0211154742882028	0.384589776026159
ACC	RBBP5	cg18131828	-0.269109768025865	80	-2.45187966734695	0.0164760303156225	0.072420067246935	0.0627072554977269
ACC	RBBP5	cg21214746	-0.155419718181901	80	-1.38057855595753	0.171403111233476	0.0241552887997415	0.345562721305546
ACC	RBBP5	cg23227967	-0.0524432301336821	80	-0.460821610179223	0.646225528522306	0.00275029238685434	0.792578611768502
ACC	RBBP5	cg24103461	-0.104781865540638	80	-0.924546555561058	0.358090236207755	0.0109792393461763	0.557080313481187
ACC	RBBP5	cg26795850	-0.0847109994073555	80	-0.746017515755837	0.45792835806391	0.00717595342059299	0.64798100038639
ACC	RBBP7	cg01947226	-0.0768714771167371	80	-0.676546367912479	0.500721110780714	0.00590922399410903	0.684576687440903
ACC	RBBP7	cg03048084	-0.223304142868932	80	-2.01024701278215	0.0479071988137602	0.0498647402224284	0.139856372880429
ACC	RBBP7	cg03171708	-0.0271644417231076	80	-0.238455003755294	0.8121616713034	0.00073790689412811	0.898789476746567
ACC	RBBP7	cg03811055	0.0429036781937914	80	0.376825223902608	0.707339968249089	0.00184072560255641	0.833196636887628
ACC	RBBP7	cg07096248	-0.271661677579649	80	-2.47697354522754	0.0154426245944218	0.0738000670653891	0.0597183144060041
ACC	RBBP7	cg08363715	0.108857943610517	80	0.960935111881892	0.339592686453345	0.0118500518871106	0.538829538639408
ACC	RBBP7	cg14719055	-0.0311884588452475	80	-0.273810818689889	0.784963170680704	0.000972719965141698	0.88117496248102
ACC	RBBP7	cg15289846	-0.115608854486706	80	-1.02131166295568	0.310304935362377	0.0133654072357285	0.509140154421377
ACC	RBBP7	cg15464143	-0.0687277367212955	80	-0.604512840839566	0.547279937262179	0.00472350179483172	0.720671250052879
ACC	RBBP7	cg16978043	-0.0254814715664997	80	-0.223671632924415	0.823605193091163	0.000649305393194334	0.905058776260372
ACC	RBBP7	cg17878446	-0.0428458099807209	80	-0.376316029002515	0.707716889148419	0.00183576343290405	0.833451325720005
ACC	RBBP7	cg17880859	-0.183336465622639	80	-1.63650942706845	0.105814152252948	0.0336122596270012	0.247975735264023
ACC	RBBP7	cg21122900	-0.0421659716504772	80	-0.370334267513591	0.71215020918901	0.00177796916522885	0.836193677838789
ACC	RING1	cg01241018	-0.0860766715012372	80	-0.75813352765287	0.450685233099035	0.0074091933767319	0.64146599746979
ACC	RING1	cg01878788	-0.382362177259462	80	-3.6311352501606	0.000506159848203019	0.146200834598596	0.00378762978367696
ACC	RING1	cg02475416	-0.00809951735336769	80	-0.071075307716716	0.943522100873273	6.56021813575044e-05	0.969877884716377
ACC	RING1	cg03037740	-0.0138430167234491	80	-0.121483619219066	0.903624464635131	0.000191629112005693	0.948902049105388
ACC	RING1	cg06316695	-0.19594930993475	80	-1.75344036579978	0.0835059892971047	0.0383961320639049	0.209212744561494
ACC	RING1	cg06671577	0.00749618798764135	80	0.0657806308587606	0.947722969126466	5.61928343460585e-05	0.972179287949475
ACC	RING1	cg07946923	-0.317749204255537	80	-2.94063706831082	0.00432335113924618	0.100964556805027	0.0220916838857376
ACC	RING1	cg08892712	-0.0784877133704091	80	-0.690858131004631	0.491732167212438	0.00616032115011549	0.677310366970456
ACC	RING1	cg09478665	-0.0979055312611903	80	-0.863264922688917	0.390672799349745	0.00958549305153591	0.587908206376648
ACC	RING1	cg09724776	0.157844625410402	80	1.40266482773372	0.16473598676594	0.0249149257709502	0.336461218579368
ACC	RING1	cg09832551	-0.427711424530135	80	-4.1521062457109	8.43579840369621e-05	0.182937062673597	0.000831122721652916
ACC	RING1	cg09869033	-0.23223181279739	80	-2.09510508025489	0.0394493667968196	0.0539316148751618	0.121267793124107
ACC	RING1	cg10265934	0.0732324448158033	80	0.644342218789667	0.521268441715252	0.00536299097369967	0.701341355150403
ACC	RING1	cg12065531	-0.216318142171137	80	-1.94421736685513	0.055521938583258	0.0467935386323724	0.155614322856293
ACC	RING1	cg12115664	-0.248573385417926	80	-2.25190293291896	0.027178234669264	0.0617887279381287	0.0916989889344115
ACC	RING1	cg12256228	0.0836586151334549	80	0.736683833046394	0.463553294021957	0.00699876388604753	0.652794340450094
ACC	RING1	cg14046664	-0.355152829322972	80	-3.33378895944713	0.00131932108268677	0.126133532176112	0.00844734685650493
ACC	RING1	cg14363494	-0.122484167202282	80	-1.08294829967853	0.282210973525075	0.0150023712152367	0.47934150945197
ACC	RING1	cg18189601	-0.440120462188666	80	-4.3010076463899	4.93411525708731e-05	0.193706021237165	0.000523948351501297
ACC	RING1	cg20505183	0.0685967681373897	80	0.603355420838618	0.548045499213831	0.00470551659889481	0.721276460257837
ACC	RING1	cg24536938	-0.054339244033754	80	-0.477530467303079	0.634337184154295	0.00295275344215987	0.784652214539458
ACC	RING1	cg24550302	-0.483056133362429	80	-4.84108240404144	6.51484808493787e-06	0.233343227979061	8.9781415751932e-05
ACC	RNF17	cg00202166	-0.592075071573496	80	-6.44688976930747	9.08203783415209e-09	0.35055289037876	2.50364298819996e-07
ACC	RNF17	cg01405147	0.141028644842745	80	1.25001458712581	0.21508003597217	0.019889078666181	0.402113650167135
ACC	RNF17	cg07131398	-0.323099545315596	80	-2.99587056820048	0.00368044162034798	0.104393316183145	0.0193970287490628
ACC	RNF17	cg12020676	-0.235726759578511	80	-2.12847569902635	0.036495866941101	0.0555671051813852	0.114436093221121
ACC	RNF17	cg14855529	-0.162778770141049	80	-1.4476862926227	0.151764262820788	0.0264969280086325	0.318230432934781
ACC	RNF17	cg15523998	-0.00747158930658138	80	-0.0655647601728013	0.947894276104378	5.58246467662213e-05	0.972293178012039

**ACC**	RNF17	cg17806989	-0.631567120466846	80	-7.14797808707783	4.35533315718627e-10	0.398877027654783	1.53589658943857e-08
**ACC**	RNF17	cg20477497	-0.453565615860091	80	-4.46579883673672	2.69428104927283e-05	0.205721767890543	0.000310241118927073
**ACC**	RNF17	cg21831927	0.075894484808357	80	0.667897715623834	0.506195919024141	0.00575997282432592	0.689227361942138
**ACC**	RNF17	cg23678722	-0.243926827692365	80	-2.20711823577529	0.0302845121369591	0.0595002972680609	0.0993863462215817
**ACC**	RNF17	cg24753530	-0.468369572452861	80	-4.65169715963893	1.34295233562587e-05	0.219370056399676	0.000169297737802105
**ACC**	RNF20	cg04845236	0.0785760921133389	80	0.691640881287704	0.491243096143263	0.00617420225180393	0.676888402376439
**ACC**	RNF20	cg14625770	0.0661953166809638	80	0.582138359453001	0.562174146147174	0.00438181995049308	0.732460901811704
**ACC**	RNF20	cg21219529	0.197180183312691	80	1.76489887750946	0.0815459773994452	0.0388800246912264	0.205780062469587
**ACC**	RNF217	cg00991794	-0.26884345939914	80	-2.44926417610804	0.016587227677579	0.0722768056616971	0.0630071674780069
**ACC**	RNF217	cg02312409	-0.332627819688671	80	-3.09503414475067	0.00274337286580655	0.110641266430839	0.0153372582624905
**ACC**	RNF217	cg03113502	-0.0695258791379788	80	-0.611567014965362	0.542625731283353	0.00483384786990884	0.717176794758022
**ACC**	RNF217	cg06993703	-0.388339617085444	80	-3.69789032330833	0.000405473078989512	0.15080765819807	0.00313864405043882
**ACC**	RNF217	cg07439975	-0.00233705352305058	80	-0.0205076174407237	0.983691484690116	5.46181916960314e-06	0.99146197920873
**ACC**	RNF217	cg10621825	-0.199732840792489	80	-1.78868996511105	0.0775978817118253	0.0398932076910379	0.198611539663121
**ACC**	RNF217	cg16203203	-0.500556569414674	80	-5.07375146051905	2.63100642050094e-06	0.250556879184187	4.0275171560845e-05
**ACC**	RNF217	cg17382198	-0.255670310402744	80	-2.32062612578268	0.022956040206576	0.0653673076214354	0.0807927158808529
**ACC**	RNF217	cg18192252	-0.336445240678489	80	-3.13505946746883	0.00243235969483734	0.113195399975207	0.0139175485510574
**ACC**	RNF217	cg19171596	-0.298065157779144	80	-2.74005908849684	0.00763110890475007	0.0888428382819059	0.0346637739900307
**ACC**	RNF217	cg19478500	-0.388777030510238	80	-3.70279667582525	0.000398879217584049	0.151147579452359	0.00309782822888439
**ACC**	RNF217	cg20034100	0.29905011081285	80	2.75000125739929	0.00742376290092814	0.0894309687771781	0.0339161505235379
**ACC**	RNF217	cg23521835	-0.290121388302216	80	-2.66022136031928	0.00949787417056626	0.0841704199504049	0.0409146477227439
**ACC**	RNF217	cg24822696	-0.040605534081753	80	-0.356606224628195	0.722361968857595	0.00164880939806441	0.842708698637653
**ACC**	RPH3A	cg00851732	-0.0900348753987097	80	-0.793274620339016	0.430055518409156	0.00810627878806118	0.624069447277917
**ACC**	RPH3A	cg00854817	-0.0428264712059447	80	-0.37614586379434	0.707842866679069	0.00183410663595361	0.833510967716594
**ACC**	RPH3A	cg01119407	-0.0902470782142727	80	-0.795159620322895	0.428964943653857	0.00814453512621305	0.623056363454222
**ACC**	RPH3A	cg01573562	0.0226249731328679	80	0.198584166578539	0.843111437238931	0.000511889409262994	0.916181865105899
**ACC**	RPH3A	cg05967001	-0.0330966182623267	80	-0.290580839313373	0.772153115725475	0.00109538614040218	0.873666770988549
**ACC**	RPH3A	cg08254200	0.0694394210285475	80	0.610802820708333	0.543128958759477	0.00482183319277988	0.717524950204374
**ACC**	RPH3A	cg09384610	-0.0646845035689828	80	-0.568795406716871	0.571150319093644	0.00418408500196574	0.739093233587354
**ACC**	RPH3A	cg11596863	-0.0470293196254879	80	-0.413137737905789	0.68065395577406	0.0022117569044363	0.815807380449992
**ACC**	RPH3A	cg16403344	0.013533065390783	80	0.118763042715321	0.905772379763742	0.000183143858871209	0.950080313234374
**ACC**	RPH3A	cg17453780	-0.0551982690255217	80	-0.485102425618818	0.628981004569152	0.00304684890341387	0.780933467441029
**ACC**	RPH3A	cg18629535	-0.0395608255451495	80	-0.347416805633516	0.729226044376506	0.00156505891781375	0.846833230113334
**ACC**	RPH3A	cg19712198	-0.0771092005371463	80	-0.6786510681723	0.499393641807107	0.00594582880747785	0.683590740616943
**ACC**	RPH3A	cg20704159	-0.115051603252925	80	-1.01632259489999	0.312658636039826	0.0132368714110686	0.511642355373109
**ACC**	RPH3A	cg21900624	0.032817154677965	80	0.28812455551336	0.774025529127658	0.00107696564115748	0.874804480662439
**ACC**	RPH3A	cg23199238	-0.111717102098037	80	-0.986488962901583	0.326982971314818	0.0124807109011832	0.52648894278102
**ACC**	RPH3A	cg23549179	-0.00071953060433158	80	-0.0063138570630671	0.994978634007531	5.17724290569769e-07	0.997367964782302
**ACC**	RPH3A	cg23596620	-0.0826562273702544	80	-0.727795882176469	0.468945900871048	0.0068320519230832	0.657861978876605
**ACC**	RTF1	cg04910470	-0.40683779929925	80	-3.9080301972503	0.00019865202553341	0.165516994938657	0.00172669672339063
**ACC**	RTF1	cg07091958	-0.145517045350367	80	-1.2906448439669	0.200688816696776	0.0211752104875008	0.383729363979506
**ACC**	RTF1	cg07129439	0.351831205373275	80	3.29818054685165	0.00147470167377187	0.123785197074412	0.00927054076843711
**ACC**	RTF1	cg15581235	0.0415106439842638	80	0.364568658331275	0.716432741264651	0.00172313356398829	0.838801649749722
**ACC**	RTF1	cg18782716	0.171648132301819	80	1.52889765537216	0.130387693664922	0.0294630813227028	0.286795415091861
**ACC**	RTF1	cg22263512	0.200016487257953	80	1.79133591665022	0.0771687659309213	0.0400065951750111	0.197866413211275
**ACC**	RTF1	cg24280607	-0.0638656615952371	80	-0.561565339029024	0.576043071117139	0.00407882273099734	0.74270670856169
**ACC**	RTF1	cg27576415	0.15652967207953	80	1.39068491418657	0.168327198105005	0.0245015382413252	0.341411707714269
**ACC**	SATB1	cg01069104	0.124447375596516	80	1.10057694361452	0.274509573067115	0.0154871492928603	0.470996311849236
**ACC**	SATB1	cg01373248	0.206402824037509	80	1.85103558657871	0.0679991230638414	0.0426021257706589	0.18052529830471
**ACC**	SATB1	cg04004754	-0.356092607556528	80	-3.34388967755764	0.00127813233077002	0.126801945156408	0.00823179707998164
**ACC**	SATB1	cg14415616	-0.0895760118019184	80	-0.78919890194352	0.432419146566348	0.00802386189033742	0.626144533978269
**ACC**	SATB1	cg19818826	0.0207803183662816	80	0.18238593697132	0.855759192164093	0.000431821631404021	0.923264785110995
**ACC**	SATB1	cg26131754	0.0266502996409102	80	0.233938521087268	0.815653579002444	0.000710238470950296	0.900821342223997
**ACC**	SATB1	cg26768584	0.143018435338522	80	1.26801685891465	0.208612682644224	0.020454272846679	0.393797260589442
**ACC**	SCML2	cg00457389	-0.116767474101742	80	-1.03168791419612	0.305448000527068	0.013634643008101	0.503987905171009
**ACC**	SCML2	cg04189389	-0.446918688776819	80	-4.38386653273488	3.64524580719601e-05	0.199736314377991	0.000403793242038598
**ACC**	SCML2	cg05049545	-0.145621236332618	80	-1.29158896603382	0.200363138087638	0.02120554447104	0.383353264082353

ACC	SCML2	cg06131666	-0.380445765683651	80	-3.60984685915006	0.000542983290938338	0.144738980626619	0.00402140538225981
ACC	SCML2	cg08300622	-0.0884726270707325	80	-0.779400486267026	0.438132817793799	0.00782740574079691	0.630913290929613
ACC	SCML2	cg17710066	-0.303591240257296	80	-2.7959652512449	0.00653052696348119	0.0921676411609634	0.0306655287376746
ACC	SCML2	cg17727916	-0.0934313272941132	80	-0.823458602895604	0.41279043941942	0.0087294129199397	0.608389433040834
ACC	SCML2	cg22823767	-0.114621237364724	80	-1.01247019655673	0.31448427091331	0.0131380280550204	0.513528040373418
ACC	SCML2	cg26246138	-0.124549319978836	80	-1.10149271058291	0.27411354049596	0.0155125331071904	0.470577930955743
ACC	SCML4	cg00977377	0.0316081823215759	80	0.277499330488378	0.7821404047512	0.000999077189673981	0.879752696906044
ACC	SCML4	cg03500965	-0.389238899730277	80	-3.70798054439541	0.000392023400569922	0.151506921063236	0.00305428827480586
ACC	SCML4	cg05603896	-0.66452665257303	80	-7.80339118807987	2.42996630478098e-11	0.441595671979917	1.07346279792238e-09
ACC	SCML4	cg08792995	-0.401022797910988	80	-3.84137475306951	0.000249719901292341	0.160819284444357	0.00209046701830801
ACC	SCML4	cg17388996	-0.456075305133053	80	-4.49697645154239	2.3997199799794e-05	0.208004683952208	0.000280558785876942
ACC	SCML4	cg23503775	-0.710234762271126	80	-8.85311857573355	2.30177925294313e-13	0.504433417538322	1.43596323338779e-11
ACC	SCML4	cg27353361	-0.361358643582433	80	-3.40070542365728	0.00106810851628179	0.130580069291736	0.00709161337506403
ACC	SCML4	cg27659622	-0.481348225839122	80	-4.81879247249667	7.09881217380912e-06	0.23169611451847	9.66138898266807e-05
ACC	SETD1A	cg26692825	-0.104466598928082	80	-0.921734049320912	0.359546364776261	0.0109132702916008	0.558439313622387
ACC	SETD6	cg01152986	-0.402634797343939	80	-3.85979645961528	0.000234471891761383	0.162114780032195	0.00198266373344403
ACC	SETD6	cg01472538	-0.457089541551827	80	-4.50961454630282	2.28943287053398e-05	0.20893084899606	0.000269650478017139
ACC	SETD6	cg01773760	-0.134029168668927	80	-1.18680930274521	0.238951588782128	0.0179638180540837	0.430729659098568
ACC	SETD6	cg02549819	-0.188864438339375	80	-1.68765109195771	0.095525196554763	0.0356697760692476	0.230448945352474
ACC	SETD6	cg05502081	-0.230512233082239	80	-2.07871769408097	0.0409740026617754	0.0531358896005605	0.124545127830005
ACC	SETD6	cg09132913	-0.264465221511713	80	-2.40635047182129	0.0185101414247342	0.0699418533892394	0.0685684876207324
ACC	SETD6	cg19824325	-0.132625729430146	80	-1.17415834450797	0.243950342688864	0.0175895841068782	0.436644849074181
ACC	SETD6	cg26666090	-0.249419095917078	80	-2.26007180840924	0.0266429033172171	0.0622098854080924	0.0903561347946503
ACC	SETD7	cg05316864	0.0654871515305505	80	0.575883607022341	0.566373233615883	0.00428856701558528	0.735398000235461
ACC	SETDB1	cg02060408	-0.54032227430073	80	-5.63463392929556	2.75218470774259e-07	0.291948160105513	5.39303418524249e-06
ACC	SETDB1	cg08531528	0.0996245039963478	80	0.878572280130942	0.382366631525235	0.00992504179651831	0.580757721409519
ACC	SETDB1	cg10589385	0.109702846791703	80	0.968483927647031	0.335835001822218	0.0120347145942039	0.53542613293621
ACC	SETDB1	cg10961700	-0.565465563229297	80	-6.01613627233559	5.64380975434878e-08	0.319751303198226	1.30090232897721e-06
ACC	SETDB1	cg12082615	0.220543841202549	80	1.98411919529179	0.0508061794856909	0.0486395858923751	0.146018025968296
ACC	SETDB1	cg15448220	-0.0390918266537585	80	-0.343291791277647	0.732314491207267	0.0015281709111275	0.848748153049345
ACC	SETDB1	cg16370685	-0.569100406918841	80	-6.07324567030255	4.43925303135709e-08	0.32387527315519	1.05064572656341e-06
ACC	SETDB1	cg17899625	-0.00545696478458825	80	-0.0478853846299397	0.96193157293978	2.97784646602363e-05	0.980021802368806
ACC	SETDB1	cg17918089	0.133321461056511	80	1.18042894264578	0.241463366751252	0.0177746119782427	0.433719721843614
ACC	SETDB1	cg19689029	-0.44300302307498	80	-4.33602735062708	4.34309419564625e-05	0.196251678453571	0.000469715931956214
ACC	SETDB1	cg26820223	-0.440677375626775	80	-4.30776055867504	4.81440766403526e-05	0.194196549389302	0.000513338856655646
ACC	SETMAR	cg00300090	-0.298063229029698	80	-2.74003962903496	0.00763151987463372	0.0888416884996101	0.0346637739900307
ACC	SETMAR	cg02520519	-0.291241021401927	80	-2.67143716712709	0.00921268776800198	0.084821332547238	0.0400252335655058
ACC	SETMAR	cg06021447	-0.295136139808108	80	-2.71054999564873	0.00827807571016927	0.087105341020831	0.0368855387935682
ACC	SETMAR	cg06267913	-0.294342756337333	80	-2.70257132374377	0.00846139708781193	0.0866376582082584	0.0375111650928448
ACC	SETMAR	cg14751177	-0.291223708709303	80	-2.67126364791795	0.00921703988512809	0.0848112485144008	0.0400396694674813
ACC	SETMAR	cg14961031	-0.189901923153253	80	-1.69726768145942	0.0936847958561243	0.0360627404173042	0.227298202913076
ACC	SETMAR	cg15232450	-0.295397739979907	80	-2.71318212667297	0.00821839811931383	0.0872598247852364	0.0366763459897832
ACC	SETMAR	cg15384599	-0.167892079550076	80	-1.49446031342159	0.139142333839544	0.0281877503756489	0.299970589405944
ACC	SETMAR	cg16423812	-0.298671397743103	80	-2.74617736765917	0.0075028935474194	0.0892046038298191	0.0341813129906269
ACC	SETMAR	cg21901604	-0.0678105786577044	80	-0.596408217132411	0.552651954728184	0.00459827457789272	0.724865731512584
ACC	SETMAR	cg22149154	-0.301010045058451	80	-2.76981370075282	0.00702585572284896	0.0906070472260909	0.0324932590882865
ACC	SETMAR	cg23182840	-0.269711246427744	80	-2.45778918675244	0.0162272422169679	0.072744156449607	0.061975653248898
ACC	SETMAR	cg23549342	-0.24083465646265	80	-2.17740492490249	0.0325132158652768	0.0580013317534826	0.104788665368351
ACC	SETMAR	cg24390964	-0.294583306308923	80	-2.70498977053896	0.00840544262400652	0.0867793243558967	0.0373266810171597
ACC	SETMAR	cg26797270	-0.28972233298226	80	-2.65622675235944	0.00960136880186417	0.0839390302286834	0.0412667892028852
ACC	SETMAR	cg27265118	-0.306685916891395	80	-2.82740863416952	0.00597744460958035	0.0940562516195158	0.0285761638797313
ACC	SFMBT2	cg09060610	-0.0709687000873905	80	-0.624322017049537	0.534261603483914	0.00503655639209397	0.711047042259837
ACC	SFMBT2	cg12328063	-0.0987743386159487	80	-0.871000620220839	0.38646132687331	0.0097563699690181	0.584443779529602
ACC	SFMBT2	cg20299663	-0.0954189336722174	80	-0.841135675356038	0.402876536817412	0.00910477290314302	0.599399045769081
ACC	SFMBT2	cg25291138	-0.107486817526607	80	-0.948689217629001	0.345746742645463	0.0115534159419981	0.544995421960323
ACC	SHPRH	cg00571935	-0.156509200826352	80	-1.39049847046256	0.168383558032495	0.0244951299433033	0.341508212675129
ACC	SHPRH	cg02758840	0.0332581848293769	80	0.29200092426114	0.771071207782791	0.001106106858145	0.873125535117432

**ACC**	SHPRH	cg06708562	0.0560941306102059	80	0.493000234855839	0.623415461839437	0.00314655148891484	0.776875109140871
**ACC**	SHPRH	cg11387577	0.0571932250315705	80	0.502691354340157	0.616616051004042	0.00327106498951186	0.77223673184166
**ACC**	SHPRH	cg11675148	-0.16025130878034	80	-1.42461084580912	0.15831021382202	0.025680481965812	0.327402827894253
**ACC**	SHPRH	cg12989205	0.00469958684051996	80	0.0412391625700078	0.967211978613217	2.20861164715884e-05	0.982775902722455
**ACC**	SHPRH	cg14102550	-0.198093303226698	80	-1.77340494878377	0.0801157307115901	0.0392409567832645	0.203291902765263
**ACC**	SHPRH	cg15443174	0.0866259734398874	80	0.763008051737096	0.44778993265727	0.00750405927440808	0.639321869423353
**ACC**	SHPRH	cg16339435	0.00756063003554537	80	0.0663461556121021	0.947274201339545	5.71631265343908e-05	0.971923994922365
**ACC**	SHPRH	cg18099523	-0.325002358217232	80	-3.01559088150152	0.00347323065240473	0.105626532846762	0.0185307252844917
**ACC**	SHPRH	cg26814539	-0.105929488089558	80	-0.934786929766084	0.352820470787834	0.0112210564469157	0.551837944920761
**ACC**	SHPRH	cg27398948	-0.107107079434769	80	-0.945298646566471	0.347463372418874	0.0114719264650459	0.546756858973945
**ACC**	SIN3A	cg01088093	0.0783852614351897	80	0.689950758192004	0.492299437123185	0.00614424921026303	0.677837508296998
**ACC**	SIN3A	cg01664094	-0.0129165417371968	80	-0.113351649774042	0.9100467884835	0.000166837050448748	0.952265517025831
**ACC**	SIN3A	cg02028562	0.0782092584670186	80	0.688392026785486	0.493274757151961	0.00611668810996092	0.678624031757402
**ACC**	SIN3A	cg07544511	0.124509067048523	80	1.10113111404858	0.274269868951003	0.0155025077772937	0.470745729402632
**ACC**	SIN3A	cg10483776	0.289321426691377	80	2.65221513859711	0.00970633464909981	0.0837068879427336	0.0416236479627403
**ACC**	SIN3A	cg11184237	-0.340332742320264	80	-3.17599871496916	0.0021485142625279	0.115826375495232	0.0125840625311772
**ACC**	SIN3A	cg15851274	-0.123353121174418	80	-1.09074947291831	0.278784646904018	0.0152159925034706	0.475714038438201
**ACC**	SIN3A	cg22059910	0.278646247538984	80	2.5459461269275	0.0128952875755179	0.0776437312675566	0.05201308342728
**ACC**	SIN3A	cg25697800	0.169625965648974	80	1.51034900879449	0.135047391386686	0.0287729682223471	0.2937947732052
**ACC**	SIN3B	cg06815056	-0.194345510908775	80	-1.73852311638732	0.0861157488199229	0.0377701776103926	0.214055294874815
**ACC**	SIRT1	cg02720207	-0.238234978941356	80	-2.15247859999748	0.0344920338186054	0.0567559051911886	0.109610836555743
**ACC**	SIRT1	cg03211233	-0.213635961544486	80	-1.91895010092808	0.0586964759552981	0.0456403240650371	0.162043850046718
**ACC**	SIRT1	cg07832903	-0.195454454969618	80	-1.74883606844519	0.0843044484498062	0.0382024439674705	0.210744867053478
**ACC**	SIRT1	cg10582690	-0.0841132505340747	80	-0.740715725378117	0.461118675227348	0.00707503891540801	0.650720449519044
**ACC**	SIRT1	cg10705386	-0.184055909216901	80	-1.64315609583275	0.104428270884152	0.0338765777176601	0.245716922907317
**ACC**	SIRT1	cg19192316	0.246559229220315	80	2.23246988089386	0.0284899386591174	0.0607914535137157	0.0949957690646512
**ACC**	SIRT1	cg19681528	0.01061053492243	80	0.0931123076755171	0.926056187383319	0.000112583451340106	0.961054615617427
**ACC**	SIRT1	cg19980381	0.0597589086835258	80	0.525321128727299	0.600869441521236	0.00357112716704598	0.760824657850589
**ACC**	SIRT1	cg25513531	-0.173792887651701	80	-1.54859253143617	0.125580237151504	0.0302039677983168	0.279332903555901
**ACC**	SIRT4	cg04118464	-0.1380026167521	80	-1.22266668818664	0.225184814130957	0.019044722230427	0.414542851415217
**ACC**	SIRT4	cg07106325	0.233044941193295	80	2.1028613141699	0.0387450329162104	0.0543099446157865	0.11961069099677
**ACC**	SIRT4	cg08264848	-0.168678431777123	80	-1.50166437769463	0.137273730493063	0.0284524133467894	0.297202287375525
**ACC**	SIRT4	cg12124973	-0.0673498562843818	80	-0.592337539769179	0.555360052891868	0.00453600314152688	0.726803854267063
**ACC**	SIRT4	cg20010714	0.00243514661959843	80	0.0213683882217684	0.983007067161821	5.92993905894166e-06	0.991201532093721
**ACC**	SIRT4	cg21068152	-0.0564140732484305	80	-0.495821099591172	0.621432893625439	0.00318254766047928	0.77569678171297
**ACC**	SIRT4	cg21123573	-0.0885976970323743	80	-0.780511002235446	0.43748303570907	0.00784955191944039	0.630356083049978
**ACC**	SIRT5	cg01064902	-0.11872307291067	80	-1.04921138375007	0.297362876984691	0.0140951680413522	0.495407380921877
**ACC**	SIRT5	cg02206980	-0.107134544482408	80	-0.945543859936932	0.34733903693567	0.0114778106214531	0.546626596045776
**ACC**	SIRT5	cg02712746	-0.231308508054989	80	-2.08630352915135	0.0402620218580569	0.0535036258986248	0.123046306361252
**ACC**	SIRT5	cg06139021	0.0341007349084126	80	0.299406869197663	0.765436305178082	0.00116286012129383	0.869950209283306
**ACC**	SIRT5	cg07534331	-0.251285093768472	80	-2.278115370746	0.0254932463111022	0.0631441983502298	0.0874616781155888
**ACC**	SIRT5	cg07909422	-0.264617978273819	80	-2.40784501669165	0.0184399640243835	0.0700226744257231	0.0683853058737135
**ACC**	SIRT5	cg08619119	-0.355141995868802	80	-3.33367258923324	0.00131980281344862	0.126125837229676	0.00844904049287538
**ACC**	SIRT5	cg10907941	-0.308530511367145	80	-2.84619757316103	0.00566783235091739	0.0951910764444719	0.0274154915070116
**ACC**	SIRT5	cg11824369	-0.220064585170685	80	-1.97958789317118	0.0513238966668432	0.0484284216463458	0.147061955887344
**ACC**	SIRT5	cg19366958	-0.0164000354621904	80	-0.14392908410128	0.885932494461063	0.000268961163161101	0.939518082566
**ACC**	SIRT5	cg20150972	-0.369590589077645	80	-3.4902741544731	0.000801828206700066	0.136597203534761	0.00557837806403371
**ACC**	SIRT7	cg11691214	-0.33480958555515	80	-3.11788861623458	0.00256152312024901	0.112097458579611	0.0145188436879163
**ACC**	SIRT7	cg12710567	-0.0482989411803738	80	-0.424316698170995	0.672518047929023	0.00233278771914521	0.810438680514141
**ACC**	SIRT7	cg17552093	0.186207701392588	80	1.66305205872701	0.100367468453176	0.0346733080579114	0.238849398618732
**ACC**	SIRT7	cg20397086	-0.141652613529702	80	-1.2556581568517	0.213036907620048	0.020065462919795	0.399426231355298
**ACC**	SMARCA	cg03460558	-0.0392382885100896	80	-0.344579951584676	0.731349552881064	0.00153964328520103	0.848173155783919
**ACC**	SMARCA	cg06675660	-0.129365994047896	80	-1.14480185934293	0.255837254983729	0.0167355604160003	0.450112025108085
**ACC**	SMARCA	cg08617833	-0.171121416988931	80	-1.52406436820567	0.131589459834864	0.0292825393522996	0.288525339477053
**ACC**	SMARCA	cg12935118	-0.0946392978945089	80	-0.834200670846819	0.406748463802894	0.0089565967059656	0.602934204628005
**ACC**	SMARCA	cg17420696	-0.0323899471701328	80	-0.284369839504324	0.776890320043186	0.001049108677684	0.876615884738913
**ACC**	SMARCA	cg18959966	-0.285165117417823	80	-2.61071478792586	0.0108550139698623	0.0813191441919205	0.0454570144141396

ACC	SMARCA	cg24447042	-0.148456288448121	80	-1.31729563588127	0.191646070442506	0.0220392695797917	0.372101168085917
ACC	SMARCA	cg26010110	-0.137202814997918	80	-1.21544430122841	0.227910232285599	0.018824612443353	0.417728032476342
ACC	SMARCA	cg27353825	-0.122259994786245	80	-1.08093616702187	0.283099419054968	0.0149475063251326	0.480435616131821
ACC	SMARCA	cg04518912	-0.260771045508861	80	-2.37026625178528	0.0202784111398794	0.0680015381757844	0.0735929611968546
ACC	SMARCA	cg13529335	0.124023327663986	80	1.0967680981569	0.276161020275691	0.0153817858048484	0.4726983020178
ACC	SMARCA	cg14290704	-0.340445201265512	80	-3.17718574372172	0.00214076566922105	0.115902935064715	0.0125462350814662
ACC	SMARCA	cg21210735	-0.3682781471824	80	-3.47593115747155	0.000839762086555595	0.135628793692101	0.00580078598486112
ACC	SMARCA	cg08405378	-0.182023126665861	80	-1.62438299092357	0.108380904145164	0.0331324186412162	0.252308228059636
ACC	SMARCA	cg17218495	-0.0513557733608302	80	-0.451240529571787	0.653084508497887	0.00263741545748896	0.797393518841982
ACC	SMARCA	cg17747332	0.123243738669502	80	1.08976733421942	0.279214414226214	0.0151890191212365	0.476269560968107
ACC	SMARCA	cg18522266	-0.30871518121792	80	-2.84808055911463	0.00563763082769788	0.095305063114413	0.0273084590666207
ACC	SMARCA	cg23892580	-0.132078926718684	80	-1.16923127640382	0.245917291548504	0.0174448428831596	0.438931739189341
ACC	SMARCA	cg01278696	0.336946597509826	80	3.14032902787838	0.00239395591351101	0.113533009573448	0.013746339410664
ACC	SMARCA	cg01575392	-0.273293121284833	80	-2.49304565254309	0.0148116851105514	0.0746891301416062	0.057800864383759
ACC	SMARCA	cg02782292	-0.0490786312341494	80	-0.431182852012794	0.667540181561627	0.00240871204381763	0.807165496735092
ACC	SMARCA	cg05358385	0.1134772460727	80	1.00223261909974	0.319370479475164	0.012877085376244	0.518686995413172
ACC	SMARCA	cg10878459	-0.329427322875864	80	-3.0616091871939	0.00303107302125216	0.108522361057159	0.0165985706472822
ACC	SMARCA	cg12253543	-0.358125168170946	80	-3.36577541267274	0.00119300178200608	0.128253636077469	0.00777941162023311
ACC	SMARCA	cg13848802	-0.295035176700661	80	-2.70953431819623	0.00830120939089897	0.0870457554907905	0.0369619740720815
ACC	SMARCA	cg13898584	-0.302114818920472	80	-2.78099854571007	0.00680994080561861	0.0912733638113495	0.0317001277544456
ACC	SMARCA	cg16091845	-0.179468410450105	80	-1.60082024942344	0.113511849786604	0.0322089103494874	0.260681101390935
ACC	SMARCA	cg16587443	-0.270638029499668	80	-2.46690089788854	0.0158502355450297	0.0732449430114632	0.0608824976048012
ACC	SMARCA	cg19993723	0.0868762616025197	80	0.765229349652016	0.44647414026077	0.00754748483002944	0.638267564900721
ACC	SMARCA	cg20045045	-0.370888946103277	80	-3.50448703105162	0.00076584166167748	0.1375586103416	0.00536737713770612
ACC	SMARCA	cg22077701	-0.167804306447009	80	-1.49365637388915	0.139352093824828	0.0281582852621619	0.300306290673112
ACC	SMARCA	cg24607315	-0.321170020764291	80	-2.97591484797161	0.00390174256205868	0.103150182237735	0.0203613775802062
ACC	SMARCB	cg01745726	-0.298178192149621	80	-2.74119957469762	0.00760705795277488	0.0889102342736165	0.0345789100372893
ACC	SMARCB	cg01912455	-0.277573226417548	80	-2.53532196360685	0.0132611915584767	0.0770468960238473	0.0531362933865636
ACC	SMARCB	cg03979008	0.135432791349671	80	1.19946918769816	0.234023491857271	0.0183420409727635	0.424967869410471
ACC	SMARCB	cg10122452	-0.172117128078771	80	-1.53320242873868	0.129324664583772	0.029624305778084	0.285118260971205
ACC	SMARCB	cg16142952	-0.185975313046315	80	-1.66090215482977	0.100799988758121	0.0345868170626747	0.23960016882942
ACC	SMARCB	cg19906397	-0.0407742984153099	80	-0.358090811100151	0.721255168212856	0.00166254341126074	0.842052474051549
ACC	SMARCC	cg02564102	0.196355579259473	80	1.75722147364647	0.0828549631530715	0.0385555135063233	0.208103952826494
ACC	SMARCC	cg02627110	-0.0828558358742791	80	-0.729565588449044	0.467869349420118	0.00686508953842547	0.656874718801455
ACC	SMARCC	cg05592996	-0.229157528627593	80	-2.06582201911991	0.0422093170478298	0.0525131729267062	0.127353317209294
ACC	SMARCC	cg07143957	0.0758328172894117	80	0.667351879960751	0.506542520307921	0.00575061617804929	0.689572551001056
ACC	SMARCC	cg07304068	-0.177850392673286	80	-1.58591423971012	0.116857260410094	0.0316307621740421	0.265898204578749
ACC	SMARCC	cg07474652	0.0247547940770466	80	0.217289023984538	0.828557847186543	0.000612799829796981	0.907881025452201
ACC	SMARCC	cg12894336	-0.161538494770044	80	-1.43635908758674	0.154950778532539	0.0260946852925715	0.322694173876599
ACC	SMARCC	cg16250355	0.0324091269816343	80	0.284538407023118	0.776761638925779	0.0010503515117117	0.876521539579229
ACC	SMARCC	cg20685352	0.0977109069015467	80	0.861532297814481	0.391619958604254	0.00954742132752272	0.58868610024075
ACC	SMARCC	cg22984439	0.0635859514521126	80	0.559095865021143	0.57771883039353	0.00404317322207042	0.744200983194333
ACC	SMARCD	cg02553054	0.0641148940573728	80	0.563765846994057	0.574551803146544	0.00411071963998814	0.741708106527758
ACC	SMARCD	cg04550398	-0.15064790990074	80	-1.33719075959955	0.185097305685765	0.0226947927574615	0.363668639942376
ACC	SMARCD	cg09878554	0.0633151146185645	80	0.556704856780668	0.579343569410142	0.00400880373916196	0.745402107689059
ACC	SMARCD	cg13349421	-0.144374951214872	80	-1.2802986760401	0.204283609552099	0.0208441265382967	0.388349521565319
ACC	SMARCD	cg17846064	0.0632568024938353	80	0.556190080455542	0.579693656320274	0.00400142306174409	0.745641123739127
ACC	SMARCD	cg18591973	0.0592678924232956	80	0.520989485775804	0.603869198115556	0.00351268307229934	0.762793498493189
ACC	SMARCD	cg20405918	-0.0851095282790404	80	-0.749552755801066	0.455808086768974	0.00724363180388077	0.646097706025756
ACC	SMARCD	cg22402853	-0.302345914005012	80	-2.7833397334968	0.00676552247132465	0.0914130517155258	0.0315273185057045
ACC	SMARCD	cg25107077	-0.0158497400969567	80	-0.139098377745738	0.889735577000262	0.000251214261141078	0.941534993534505
ACC	SMARCD	cg00945038	-0.146025277716457	80	-1.29525058508688	0.199103769139582	0.0213233817321683	0.381833566287307
ACC	SMARCD	cg02127607	-0.0623087535287515	80	-0.547821553333184	0.58539903059133	0.0038823807663067	0.749909962222071
ACC	SMARCD	cg02360836	-0.0181396245757508	80	-0.159200753991876	0.873927551784639	0.000329045979749183	0.933249378291058
ACC	SMARCD	cg04367190	0.0505809672728705	80	0.444415053932443	0.657989127312237	0.0025584342502592	0.800928479134825
ACC	SMARCD	cg06420487	-0.196625945103703	80	-1.75973826293599	0.0824239546457719	0.0386617622879245	0.207376750572871
ACC	SMARCD	cg07362569	-0.0659219645539038	80	-0.579723917493639	0.563793247994152	0.00434570541064615	0.733624352838623

ACC	SMARCD	cg11697258	-0.0732807879306778	80	-0.644769865750067	0.52099271736814	0.00537007387974096	0.701140149031883
ACC	SMARCD	cg12577610	-0.21047211511924	80	-1.88920374058677	0.062629405005399	0.0442985112427666	0.169898941067406
ACC	SMARCD	cg15868002	-0.24767839270987	80	-2.24326400924511	0.0277546445343461	0.0613445862153448	0.0931795180953123
ACC	SMARCD	cg19016967	0.0993053540540465	80	0.875729671166496	0.383900702963956	0.00986155334379953	0.582076309471779
ACC	SMARCD	cg19143629	-0.0267247707213325	80	-0.234592700926261	0.815147568405383	0.000714213370107793	0.900551269806447
ACC	SMARCD	cg21254277	-0.261716718751427	80	-2.37949273700151	0.0198125266039449	0.0684956408740135	0.0722798581235833
ACC	SMARCD	cg23682543	-0.382835798017932	80	-3.63640488789518	0.000497418182453135	0.146563248244027	0.00373226756228387
ACC	SMARCD	cg24795630	-0.282804341584023	80	-2.58721427334686	0.011558823264075	0.0799782956187726	0.0477412846830608
ACC	SMARCD	cg25945412	-0.143415893165922	80	-1.2716146603087	0.207337572778112	0.0205681184125792	0.39226681428781
ACC	SMARCD	cg00864064	0.151470489667838	80	1.34466317191702	0.182681738619072	0.0229433092402145	0.360546866561747
ACC	SMARCD	cg02917064	-0.249060522455987	80	-2.25660760855823	0.0268687785945793	0.0620311438460493	0.0909349065512356
ACC	SMARCD	cg14795305	-0.199125264958536	80	-1.78302386432896	0.0785234672829705	0.0396508711448073	0.200210019161838
ACC	SMARCD	cg19786287	-0.141766959501017	80	-1.25669253992436	0.212663986520244	0.0200978708061629	0.39894500355338
ACC	SMARCD	cg19902439	-0.142483655715327	80	-1.26317700398928	0.210337120908788	0.0203015921460037	0.396029198122746
ACC	SMARCD	cg22750243	-0.140758733873221	80	-1.24757381198407	0.215968110200086	0.0198130211615922	0.403182624964735
ACC	SMARCD	cg22787816	-0.262884941041667	80	-2.39090068981452	0.0192496791022908	0.0691084922264809	0.070643906787603
ACC	SMNDC1	cg03999743	-0.0594495704134775	80	-0.522592166681294	0.602758507344029	0.00353425142234702	0.762033921414525
ACC	SMNDC1	cg08770590	-0.0214892985827766	80	-0.188611384223916	0.850893634811543	0.000461789953579723	0.920495849101554
ACC	SMNDC1	cg11559952	-0.180807312718203	80	-1.61316504763051	0.110799866626873	0.032691284332378	0.256237249156366
ACC	SMNDC1	cg12486840	0.332090330970213	80	3.08941243534339	0.0027899108015036	0.110283987923906	0.0155315815723609
ACC	SMNDC1	cg17981675	-0.24016774534164	80	-2.17100570646936	0.0330115333515895	0.057680545902487	0.105992786559642
ACC	SMNDC1	cg18209001	0.00203831075432527	80	0.0178861414355531	0.985775950388573	4.15471073119803e-06	0.992553031623472
ACC	SMNDC1	cg21872391	0.240877535531885	80	2.17781647472719	0.0324813938221911	0.0580219871239145	0.104730412135961
ACC	SMNDC1	cg23721301	-0.366905453775125	80	-3.46095542233488	0.000881176736681711	0.13461961200993	0.00604073520033636
ACC	SMNDC1	cg26944434	0.173670551162462	80	1.54746853096529	0.125850759746327	0.0301614603410734	0.279702711302054
ACC	SMYD1	cg00578637	-0.0487542908166493	80	-0.428326530501577	0.669609179699353	0.00237698087303442	0.808523879765497
ACC	SMYD1	cg00666842	-0.0921971407856655	80	-0.812487194320547	0.419017051964003	0.00850031276905183	0.614423548913119
ACC	SMYD1	cg03704645	0.0651969860481295	80	0.573321020978444	0.56809802891313	0.00425064698975999	0.736700382383047
ACC	SMYD1	cg14559139	-0.165403115665684	80	-1.4716772678636	0.14518345785764	0.0273581906719157	0.308927842466554
ACC	SMYD1	cg16090764	0.0205604022073062	80	0.180454943062928	0.857269524675497	0.000422730138926203	0.924074066822857
ACC	SMYD1	cg18148488	-0.0432957305555756	80	-0.380275077608907	0.704788216178987	0.001874520284341	0.831550686121607
ACC	SMYD1	cg19074493	-0.0900854904404821	80	-0.793724224419391	0.429795249091773	0.0081153955879022	0.623831401157627
ACC	SMYD2	cg01770831	-0.119938126346876	80	-1.06010529676202	0.292410724360028	0.0143851541515991	0.490127550867535
ACC	SMYD2	cg04406444	-0.0121511347837324	80	-0.106633647517367	0.915356957480067	0.000147650076532431	0.955252252333548
ACC	SMYD2	cg11523160	-0.175739227101827	80	-1.56648505034112	0.121335814583734	0.0308842759423477	0.272750198740541
ACC	SMYD2	cg14013804	0.306093367156314	80	2.82138043274518	0.00608002512222925	0.09369314941709	0.028983974162553
ACC	SMYD2	cg27570636	0.222420397472317	80	2.00187636104268	0.0488201308972113	0.0494708332117436	0.141744915392164
ACC	SMYD5	cg01329511	-0.150204927389696	80	-1.3331678322718	0.186407713234552	0.0225615202121438	0.365357427209412
ACC	SMYD5	cg05103132	-0.27124946252189	80	-2.47291628015597	0.0156056655168104	0.0735762709184141	0.0601927286040334
ACC	SMYD5	cg06754047	-0.143017005356618	80	-1.26800391580303	0.208617280296836	0.0204538638211748	0.393797260589442
ACC	SMYD5	cg08834152	0.144034616048675	80	1.27721661116954	0.205363657024048	0.0207459706202891	0.389612494440406
ACC	SMYD5	cg15623359	-0.445775671477924	80	-4.36986958759402	3.83733097121574e-05	0.198715949281594	0.000421911554532114
ACC	SMYD5	cg17895873	0.0310749847156883	80	0.272813637916185	0.785726796198477	0.00096565467508026	0.881790423743337
ACC	SMYD5	cg20402064	-0.00567522906838522	80	-0.0498007349684687	0.960410137760194	3.22082249786446e-05	0.979228381196563
ACC	SMYD5	cg25886337	0.0243263466943357	80	0.213526014534727	0.831481067971305	0.000591771143493019	0.909691101041144
ACC	SMYD5	cg26467949	-0.104219634508793	80	-0.919531065998276	0.360689567909729	0.0108617322171463	0.559622741243906
ACC	SP100	cg05876232	-0.086683760192692	80	-0.763520894027561	0.447485949676209	0.00751407428114414	0.639056192261231
ACC	SP100	cg09456094	-0.295335251428363	80	-2.71255332759398	0.00823261891872166	0.0872229107362542	0.0367271680332052
ACC	SP100	cg11702456	-0.29457940207243	80	-2.70495051354371	0.00840634819421341	0.0867770241253501	0.0373266810171597
ACC	SP100	cg11827097	-0.249779688334101	80	-2.26355651316892	0.0264173796771782	0.0623898927042806	0.089774315682614
ACC	SP100	cg23539753	-0.359828585594451	80	-3.38415933251765	0.00112564878770703	0.129476611010903	0.00740789457667547
ACC	SP100	cg27140170	-0.106213261106474	80	-0.937319651450496	0.351524869430059	0.0112812568348721	0.550497739198848
ACC	SP140L	cg04002454	-0.152103033022661	80	-1.35041121469587	0.180839870838627	0.0231353326546928	0.358020273485195
ACC	SP140L	cg05217331	0.104946044124528	80	0.926011310911754	0.35733338357766	0.0110136721773873	0.556223727755577
ACC	SP140L	cg13278004	-0.217198210749869	80	-1.95251800952136	0.0545114063552281	0.0471750627529444	0.153697232648277
ACC	SP140L	cg24044052	-0.339781178588571	80	-3.17017906951399	0.00218688404096842	0.115451249323038	0.0127636324617823
ACC	SP140L	cg25095994	-0.32157949361923	80	-2.98014627125035	0.00385381304906906	0.1034133707164	0.0201588932167168

**ACC**	SP140L	cg26847438	-0.18526737053894	80	-1.6543545147682	0.102126586466307	0.0343239985864129	0.241803298818824
**ACC**	SSRP1	cg01511567	-0.0619561862359769	80	-0.544709786043296	0.587527288681687	0.00383856901290705	0.751349584029839
**ACC**	SSRP1	cg01766191	-0.128353648835647	80	-1.13569262307752	0.259607821851948	0.0164746591694245	0.454513980133549
**ACC**	SSRP1	cg02688348	0.227976565625457	80	2.05459055993496	0.0433112548445404	0.0519733144743785	0.129825290308487
**ACC**	SSRP1	cg04937561	-0.165877163511486	80	-1.47601428071201	0.144017939645152	0.0275152333746164	0.307220894942278
**ACC**	SSRP1	cg08821673	-0.315020232823124	80	-2.91258575754769	0.00468823248345668	0.0992377470879349	0.0235576305788487
**ACC**	SSRP1	cg09381635	-0.130496920018255	80	-1.15498240993686	0.251669340586622	0.0170294461342508	0.445438643132339
**ACC**	SSRP1	cg11948159	-0.0918592368332714	80	-0.809484028457655	0.420731213248506	0.00843811939159105	0.615892290677122
**ACC**	SSRP1	cg12795368	-0.123435258614211	80	-1.09148700641158	0.278462216480992	0.0152362630691572	0.475388558691826
**ACC**	SSRP1	cg12911972	-0.0177922284343735	80	-0.156150888667856	0.876322756725157	0.000316563392660927	0.934555525602937
**ACC**	SSRP1	cg16313264	0.165826427672941	80	1.47555005375299	0.144142344250495	0.0274984041147693	0.307343004758751
**ACC**	SSRP1	cg22636253	0.0794100891822763	80	0.699028212960167	0.486640555582744	0.00630596226393708	0.673076580318331
**ACC**	SSRP1	cg26706201	-0.0799933729038689	80	-0.704195667132732	0.483435244907303	0.00639893970853743	0.670309083561806
**ACC**	STK31	cg00755064	0.118155647908857	80	1.04412561160773	0.299694228008824	0.0139607571327618	0.497956563168941
**ACC**	STK31	cg05000488	0.0679923190287558	80	0.598014075584474	0.551585445490985	0.0046229554469081	0.723974470824711
**ACC**	STK31	cg05407490	0.0808788666430844	80	0.712041867666363	0.478590757832851	0.00654139106946984	0.666090708181388
**ACC**	STK31	cg11755819	0.0326813828307426	80	0.286931243133815	0.774935670230182	0.00106807278372956	0.875282940262973
**ACC**	STK31	cg13313214	-0.0131190439850734	80	-0.115129051583082	0.908642536339251	0.000172109315082291	0.951487225531702
**ACC**	STK31	cg13514718	0.108906452712571	80	0.961368460884805	0.339376230982458	0.0118606154424355	0.538628907153772
**ACC**	STK31	cg13802883	0.0607378371671292	80	0.533958176654605	0.594908680501701	0.0036890848637407	0.756396865505512
**ACC**	STK31	cg14898779	-0.0466834381410553	80	-0.410092617221919	0.682876789347133	0.00217934339666974	0.817250233842606
**ACC**	STK31	cg17102984	0.0892391866591256	80	0.786207476118812	0.434158831817767	0.00796363243558226	0.627650147142781
**ACC**	STK31	cg17331904	0.0572212552575387	80	0.502938530972227	0.616443063077966	0.0032742720532484	0.772078658970608
**ACC**	STK31	cg21519371	0.0736391952497985	80	0.647940507544226	0.518950844621351	0.00542273107703795	0.699718789149953
**ACC**	STK31	cg25570411	0.0347255565124474	80	0.30489941083566	0.761265378495017	0.00120586427509918	0.867258174574761
**ACC**	STK31	cg25612145	-0.0040640795786896	80	-0.0356624480854867	0.971643804442509	1.65167428219219e-05	0.98496147038081
**ACC**	SUV39H1	cg05903800	0.030737533273653	80	0.269848265730991	0.787998871651737	0.000944795951748925	0.883231769894177
**ACC**	SUZ12	cg00160853	-0.126234419795731	80	-1.11663512883946	0.26762271655782	0.0159351287411647	0.46337213370853
**ACC**	SUZ12	cg01109633	0.102658640698222	80	0.905610578522422	0.367967062829046	0.0105387965100066	0.566866602355
**ACC**	SUZ12	cg07482957	-0.287232522845188	80	-2.6313373836241	0.0102696348704922	0.0825025221800116	0.0435115720883979
**ACC**	SUZ12	cg08177746	0.0484629167966691	80	0.42576064520257	0.671469981042895	0.00234865430444087	0.809713647531211
**ACC**	SUZ12	cg17335595	-0.266785725071297	80	-2.42907494471856	0.0174684267308649	0.0711746231018176	0.0656175175809224
**ACC**	SUZ12	cg25098644	-0.144054085369926	80	-1.27739291225595	0.205301761982326	0.0207515795117659	0.38954017215178
**ACC**	TAF3	cg00194465	-0.180847482259645	80	-1.61353555724062	0.110719284024681	0.0327058118396526	0.256142266078332
**ACC**	TAF3	cg02334915	0.179108410055962	80	1.59750258502762	0.114249713294883	0.0320798225527747	0.261741090086349
**ACC**	TAF3	cg03672997	0.0748459682626695	80	0.658618059357937	0.512105686610654	0.00560191896517653	0.693994421813243
**ACC**	TAF3	cg04179819	0.0264192309986712	80	0.231908758629724	0.817224106049539	0.000697975766561151	0.901749341948
**ACC**	TAF3	cg04234156	0.0526429032489561	80	0.462581015613489	0.644969294052913	0.00277127526247896	0.791811846253608
**ACC**	TAF3	cg05976695	-0.184725980605208	80	-1.64934909170711	0.10315024580948	0.0341236879105555	0.243496825062696
**ACC**	TAF3	cg07855525	0.0416864577927831	80	0.366115431404167	0.715282942232317	0.00173776076330948	0.838088996490361
**ACC**	TAF3	cg09871669	0.126969738040936	80	1.12324581719216	0.264823076295378	0.0161213143781838	0.46045430238529
**ACC**	TAF3	cg09994553	-0.0560117292484596	80	-0.492273744658161	0.623926507401306	0.00313731381340275	0.777236374017671
**ACC**	TAF3	cg11104088	0.18689220184553	80	1.6693862877321	0.0991019031647348	0.0349286951106702	0.236750198218811
**ACC**	TAF3	cg12176426	0.168607980489942	80	1.50101882720938	0.137440365574848	0.0284286510848967	0.297422342750278
**ACC**	TAF3	cg12590721	-0.0877215741868208	80	-0.77273254849258	0.442046224312191	0.0076950745778139	0.634463275108256
**ACC**	TAF3	cg12777293	-0.313090300436318	80	-2.89279631010403	0.00496252255766793	0.0980255362273039	0.0246223917246775
**ACC**	TAF3	cg27268107	-0.326977378996681	80	-3.03610285581147	0.00326922546081976	0.106914206375539	0.0176467005548342
**ACC**	TCEA1	cg01677098	-0.254929398850073	80	-2.31343298771434	0.0233689730586684	0.0649889983980597	0.0818866668224875
**ACC**	TCEA1	cg06047473	-0.350473849996086	80	-3.28367050610788	0.00154281902225489	0.122831919531079	0.00961869394272293
**ACC**	TCEA1	cg11530293	-0.165520940774532	80	-1.47275513906229	0.144893107792923	0.027397181834886	0.308488356964507
**ACC**	TCEA1	cg11787876	-0.0817801140141113	80	-0.720029401033402	0.473686907512936	0.00668798704816104	0.661661100026823
**ACC**	TCEA1	cg12850379	-0.0918014147325525	80	-0.808970155213476	0.421024944293045	0.0084274997468981	0.616136769245718
**ACC**	TCEA1	cg23569945	-0.204116620453773	80	-1.82963617536177	0.0711750953958064	0.0416635947454695	0.186606746131753
**ACC**	TCEA1	cg26181865	-0.512828449045105	80	-5.24181478096656	1.35116908948588e-06	0.262993018150008	2.23128911458781e-05
**ACC**	TCEA1	cg26358945	-0.150463233280012	80	-1.33551352649455	0.185642791947069	0.0226391845690754	0.364399862654681
**ACC**	TDRD1	cg00337703	-0.185324667578369	80	-1.65488434599248	0.10201871476843	0.0343452324130328	0.241677392633614
**ACC**	TDRD1	cg02635786	-0.307238296381197	80	-2.83313143847852	0.00588319813245689	0.0943953707632202	0.0282342699269803

**ACC**	TDRD1	cg16550453	-0.43867096355566	80	-4.28346041698328	5.25877216024068e-05	0.192432214266851	0.000553886977299977
**ACC**	TDRD1	cg23504215	-0.223056834842685	80	-2.00790403822076	0.048161245773285	0.0497543515700368	0.140371509261059
**ACC**	TDRD1	cg23825092	-0.330292636067325	80	-3.07063448525865	0.00295074114020116	0.109093225440302	0.0162477206735804
**ACC**	TDRD1	cg24203376	-0.221167704385507	80	-1.99002000241936	0.0501386822289197	0.0489151534631549	0.144548200612088
**ACC**	TDRD1	cg26600802	-0.385194532648354	80	-3.66269901772755	0.000455902224620358	0.148374827982184	0.00347029918416009
**ACC**	TDRD1	cg27547703	-0.389594908986059	80	-3.71197852455812	0.000386812773928395	0.151784193107856	0.00302095301023849
**ACC**	TDRD3	cg01557749	-0.357041797040768	80	-3.35410334272998	0.00123771563037299	0.127478844834101	0.00801855844014296
**ACC**	TDRD3	cg03447530	-0.199526609590838	80	-1.78676646381261	0.0779110741447103	0.0398108679348147	0.199133097940454
**ACC**	TDRD3	cg05666423	-0.361368713714527	80	-3.40081442676222	0.00106773881401554	0.130587347251692	0.00708976385263954
**ACC**	TDRD3	cg10765900	0.243380413197422	80	2.20186245380836	0.0306687105704798	0.0592340255281476	0.100312885558354
**ACC**	TDRD3	cg18522102	0.173063694554228	80	1.54189395821518	0.12719928554886	0.029951042372759	0.281950684408137
**ACC**	TDRD3	cg19937698	0.0941474272660705	80	0.82982618822767	0.409202412434839	0.00886373806082004	0.605210982584381
**ACC**	TDRD3	cg20944895	-0.281825693471736	80	-2.57748726865132	0.0118620248824825	0.0794257215008246	0.048716756165553
**ACC**	TDRD3	cg22045829	0.0682766865447908	80	0.600526867856123	0.549918680290551	0.00466170592553561	0.722723871617849
**ACC**	TDRD3	cg23103367	-0.264119288843077	80	-2.40296664801736	0.0186699124346465	0.0697589987389726	0.0690384537198964
**ACC**	TDRD3	cg23767211	0.294810900846016	80	2.70727848405866	0.00835280068849466	0.0869134672576396	0.0371419135044729
**ACC**	TDRD3	cg26737458	-0.220534626101063	80	-1.98403205350139	0.0508160935264748	0.0486355213095354	0.14602706371257
**ACC**	TDRD5	cg05378938	-0.128732353235186	80	-1.13909983864044	0.258192912606517	0.0165720187694686	0.45277172903186
**ACC**	TDRD5	cg07171496	-0.169206821373886	80	-1.50650680750509	0.136028821135154	0.0286309483994542	0.295263005340071
**ACC**	TDRD5	cg07901138	-0.166937456708959	80	-1.48571860305943	0.141436523809652	0.0278681144524556	0.303485134272298
**ACC**	TDRD5	cg09656934	0.170406778418539	80	1.51750879595444	0.133233429139683	0.0290384701309851	0.291087746412012
**ACC**	TDRD5	cg11853788	-0.0574995019230746	80	-0.505392235426433	0.614727003316903	0.00330619272140166	0.770818608448417
**ACC**	TDRD5	cg12277666	-0.119620456304827	80	-1.05725666961404	0.293700167483015	0.0143090535665749	0.491458337161849
**ACC**	TDRD5	cg13848905	-0.163612734651929	80	-1.4553066958277	0.149649315329893	0.0267691269402824	0.3151036333236
**ACC**	TDRD5	cg15545772	-0.0970263236381663	80	-0.855438651842177	0.394962391489554	0.00941410747873819	0.59160371327798
**ACC**	TDRD7	cg24339935	0.0192725051046389	80	0.169146961948295	0.866124615279438	0.000371429453008334	0.928844354517425
**ACC**	TDRD9	cg07613153	0.141955301834268	80	1.25839641211964	0.212050746109081	0.0201513077188583	0.398229908816383
**ACC**	TDRD9	cg08933517	0.0948472009198311	80	0.836049854741044	0.405713826808108	0.00899599152232681	0.602054430083739
**ACC**	TDRD9	cg16701547	0.07463556143169	80	0.656756167406154	0.513295841157371	0.00557046703022357	0.694977751483658
**ACC**	TRIM24	cg00733782	0.00802951300212629	80	0.0704609620959624	0.944009451159088	6.44730790513152e-05	0.970109658306629
**ACC**	TRIM24	cg02308366	-0.250267381120425	80	-2.26827109279839	0.0261149380860291	0.0626337620528759	0.0890343366405708
**ACC**	TRIM24	cg06386929	-0.138258960843369	80	-1.22498204735419	0.224316140847107	0.0191155402534881	0.413498172149658
**ACC**	TRIM24	cg06650216	0.0283041450155269	80	0.248467411148936	0.804434266635381	0.000801124625059978	0.893606536228711
**ACC**	TRIM24	cg10571650	0.215098356410267	80	1.93272079094537	0.0569477999581325	0.0462673029303983	0.1585270575375
**ACC**	TRIM24	cg10703331	-0.0127130704287605	80	-0.111565756380346	0.911458038101992	0.000161622159726624	0.952960334060511
**ACC**	TRIM24	cg13935558	0.181908633290004	80	1.62332626785843	0.108606935786806	0.033090750865437	0.252698309035405
**ACC**	TRIM24	cg26889928	-6.65969849510956e-05	80	-0.0005843861725494	0.999535238697254	4.43515840457645e-09	0.999766564997096
**ACC**	TRIM33	cg00760200	-0.188393621687099	80	-1.68328896305044	0.0963696878580969	0.0354921566923818	0.231975477976137
**ACC**	TRIM33	cg02852257	-0.15319443976156	80	-1.36033303547069	0.177693712882932	0.0234685363738583	0.354131028564489
**ACC**	TRIM33	cg05792124	-0.0763381851843285	80	-0.671825253679695	0.503705718773494	0.00582751851723684	0.687039233562991
**ACC**	TRIM33	cg16036305	-0.110807193491369	80	-0.978353956757574	0.330963177395808	0.0122782341294336	0.530652284552734
**ACC**	TRIM33	cg17645130	-0.103434247485993	80	-0.912526361658053	0.364339978555202	0.0106986435529936	0.563376352449283
**ACC**	TRIM33	cg18199720	0.166987450151688	80	1.48617629811269	0.141315674773717	0.0278848085081624	0.303330281147507
**ACC**	TRIM33	cg20932765	-0.385170441019597	80	-3.66243003047941	0.000456309733816594	0.148356268635231	0.00347238182847776
**ACC**	TRIM33	cg20992663	0.0342143765751878	80	0.30040581853727	0.764677202809347	0.00117062356442876	0.869443120656977
**ACC**	TRIM33	cg21666217	0.212378098490361	80	1.90711620953882	0.0602353239990512	0.0451044567183815	0.16514931963002
**ACC**	TRIM33	cg21813112	-0.172645674666562	80	-1.53805508926868	0.128134580227937	0.0298065289810722	0.283232981026655
**ACC**	TRIM66	cg06492264	0.203648019704866	80	1.82525383203896	0.071840564681302	0.0414725159297135	0.187857643321587
**ACC**	TRIM66	cg11245569	0.213613336585832	80	1.91873715872836	0.0587238695314723	0.0456306575673318	0.162086249661189
**ACC**	TRIM66	cg12608775	0.186340676210497	80	1.66428238417941	0.100120629413685	0.0347228476105851	0.238451636193001
**ACC**	TRIM66	cg13143389	0.188946481651235	80	1.68841134881702	0.0953786338082469	0.0357007729283805	0.230197454837605
**ACC**	TRIM66	cg27044202	-0.0497838834186137	80	-0.437394166842123	0.663049869900835	0.00247843504823812	0.804085824861893
**ACC**	UBE2A	cg00142683	-0.258340768121836	80	-2.34658868372154	0.0215188705491036	0.06673995247378	0.0770310609008359
**ACC**	UBE2A	cg00428314	-0.139354994142731	80	-1.23488451088774	0.220628503475905	0.0194198143925207	0.408836659493626
**ACC**	UBE2A	cg00935924	-0.309592463224139	80	-2.85703063255896	0.00549609258917733	0.0958474932851896	0.0267477652100382
**ACC**	UBE2A	cg05206294	-0.329261056826098	80	-3.05987600888823	0.00304673049960103	0.108412843542239	0.0166631996967072
**ACC**	UBE2A	cg06206449	-0.40092308559057	80	-3.84023665442659	0.000250692383086798	0.160739320559464	0.00209770498546352

**ACC**	UBE2A	cg06273903	-0.122872556722837	80	-1.08643481505729	0.280676090841152	0.0150976651956069	0.477882204611337
**ACC**	UBE2A	cg09080960	-0.252333108667985	80	-2.28826118988339	0.024866208307454	0.0636719977300493	0.0858607105668032
**ACC**	UBE2A	cg14625604	-0.279986927616297	80	-2.5592349596034	0.012450441087451	0.0783926796360133	0.0505974535905658
**ACC**	UBE2A	cg15661671	-0.293644044179623	80	-2.69554978216037	0.00862578081811781	0.0862268246821642	0.0380675196113745
**ACC**	UBE2A	cg16211147	-0.244677588038735	80	-2.21434319988079	0.0297632506484486	0.0598671220884529	0.0981019541117452
**ACC**	UBE2A	cg17976175	-0.25704615544269	80	-2.33399513237438	0.0222057077759001	0.0660727260278675	0.0787576744195484
**ACC**	UBE2A	cg18990292	-0.306474921017822	80	-2.82526168897317	0.00601379499878914	0.09392687721288	0.0287210490598442
**ACC**	UBE2A	cg25165659	-0.316983881831582	80	-2.93276210467738	0.00442304276498078	0.100478781341018	0.02249958731831
**ACC**	UHRF2	cg06523306	0.130653450306267	80	1.156391849726	0.251096143033125	0.0170703240769322	0.444697798348452
**ACC**	UHRF2	cg08487038	0.175150199533553	80	1.56106818517709	0.122608544382553	0.0306775923966435	0.274739642482551
**ACC**	UHRF2	cg13428749	-0.302271665846912	80	-2.78258747827259	0.00677976565349731	0.091368159973867	0.0315853348151057
**ACC**	UHRF2	cg14078101	0.0214013875127615	80	0.187839435243587	0.851496648615562	0.000458019387471386	0.920824197317084
**ACC**	UHRF2	cg20595300	0.344620187324487	80	3.22136349733592	0.00187029052632631	0.118763073511565	0.0112529040312166
**ACC**	UHRF2	cg24119045	-0.00389660388951518	80	-0.0341928199469694	0.972811875137967	1.51835218717848e-05	0.985688331765356
**ACC**	USP27X	cg00478198	-0.0349063930120205	80	-0.306489134289717	0.760059486721542	0.00121845627310963	0.866484967687329
**ACC**	USP27X	cg01125381	-0.143687923631765	80	-1.27407745108784	0.206468056226576	0.0206462193976078	0.391030674021088
**ACC**	USP27X	cg01290688	-0.0721050298378262	80	-0.634370306563543	0.527719453375325	0.00519913532791381	0.70624657496052
**ACC**	USP27X	cg01855997	-0.0442658835358062	80	-0.388812671473207	0.698487815607038	0.00195946844520556	0.827084419418615
**ACC**	USP27X	cg06455206	-0.0764822338566213	80	-0.673100427023757	0.502898633165555	0.0058495320956989	0.686431839640685
**ACC**	USP27X	cg06624970	-0.0206576247663085	80	-0.18130861136873	0.856601759466371	0.000426737460985601	0.923712640014511
**ACC**	USP27X	cg16810342	-0.0742471383679384	80	-0.65331923972587	0.515496638091112	0.00551263755582779	0.696829634468462
**ACC**	USP27X	cg16867483	0.00422926109530843	80	0.0371119474019947	0.970491793007158	1.78866494122894e-05	0.984385160501246
**ACC**	USP27X	cg19318186	0.011570001439298	80	0.101533146683317	0.919391177583887	0.000133864933305358	0.957522722354088
**ACC**	USP51	cg00834229	0.0208750889151604	80	0.183218086576224	0.855108489638096	0.000435769337215852	0.922844318335637
**ACC**	USP51	cg01084827	-0.152781095093784	80	-1.3565747813743	0.178880512138245	0.0233420630180558	0.355585296059349
**ACC**	USP51	cg02714202	-0.0143234158886622	80	-0.125700359348354	0.900296743638038	0.00020516024271958	0.947402603611416
**ACC**	USP51	cg03728850	0.0676679152061616	80	0.595147684250616	0.553489842624981	0.00457894674834828	0.72542268375847
**ACC**	USP51	cg07386410	0.0368379410576916	80	0.323471176073363	0.747215325099105	0.00135703390136996	0.858079344002476
**ACC**	USP51	cg12064531	0.0772672588647509	80	0.680050512607825	0.498512045120384	0.00597022929247242	0.682780436945988
**ACC**	USP51	cg12517811	0.0878613233841186	80	0.773973157746733	0.441316571164516	0.00771961214680867	0.633918928637917
**ACC**	USP51	cg18770944	-0.0848906864441123	80	-0.747611425306796	0.456971708018553	0.00720642864495259	0.647204650699012
**ACC**	USP51	cg21331369	0.0261062695033752	80	0.229159688928378	0.81935238875985	0.000681537307382859	0.902792839415335
**ACC**	USP51	cg25424556	0.024328436534296	80	0.213544369097493	0.831466803780016	0.000591872824203267	0.909691101041144
**ACC**	WDR82	cg01736403	0.00186602013618267	80	0.0163742887490761	0.986978145451615	3.48203114863921e-06	0.993148441205393
**ACC**	WDR82	cg03869063	-0.118041564511916	80	-1.0431032192254	0.300164395856584	0.0139338109524209	0.498452174339169
**ACC**	WDR82	cg10989431	0.109773972329662	80	0.969119497875338	0.335519874892612	0.0120503250010335	0.535084730368919
**ACC**	WDR82	cg11442381	-0.128551393112071	80	-1.13747167151962	0.258868357606219	0.0165254606710541	0.453573005803112
**ACC**	WDR82	cg11665333	-0.333082377511792	80	-3.09979110865674	0.00270455928671594	0.110943870208908	0.0151613007593935
**ACC**	WDR82	cg12661343	-0.0170323158100829	80	-0.149479648247682	0.881565997974325	0.0002900997818544	0.937135940290712
**ACC**	WDR82	cg13539115	-0.0861050372345907	80	-0.758385229039809	0.450535466942604	0.00741407743717025	0.641311519729548
**ACC**	WDR82	cg15991175	-0.01587693750412	80	-0.139337124138567	0.889547556980845	0.000252077144509733	0.941467059185646
**ACC**	WDR82	cg17632831	-0.0347071336530523	80	-0.304737458204836	0.761388261628159	0.00120458512641084	0.86731435557058
**ACC**	WDR82	cg24007312	-0.0111852828871085	80	-0.0981565994025761	0.922063014650476	0.000125110553264643	0.959085587326271
**ACC**	WDR82	cg26236504	0.0718195239542709	80	0.631845415001512	0.529359411937214	0.00515804402101809	0.707545910620732
**ACC**	WDR82	cg26858096	-0.0986047995290927	80	-0.869490918652946	0.38728101320204	0.00972290649017256	0.585210651711078
**ACC**	ZMYND1	cg00614684	0.13182905364885	80	1.16698011030967	0.246819749315388	0.0173788993859514	0.440026610872973
**ACC**	ZMYND1	cg02813021	-0.136137967899491	80	-1.20583225372309	0.231574461591884	0.0185335463038027	0.422067419466276
**ACC**	ZMYND1	cg02867818	0.190844843173662	80	1.70601285237603	0.0920364365325411	0.0364217541659796	0.224377390899796
**ACC**	ZMYND1	cg03254875	0.229294111313858	80	2.06712159836259	0.0420833875860916	0.0525757894832119	0.12706520682602
**ACC**	ZMYND1	cg05236965	0.115587194771402	80	1.02111772538742	0.310396206230327	0.013360399595122	0.509214729385956
**ACC**	ZMYND1	cg08828193	-0.0535459085696566	80	-0.470538480571524	0.639300516739361	0.00286716432455002	0.788036069769483
**ACC**	ZMYND1	cg12639781	0.0711891534540828	80	0.626271240671905	0.532989264053829	0.00506789556950895	0.710142895408435
**ACC**	ZMYND1	cg13939078	-0.318655711099603	80	-2.94997305459317	0.0042078560460598	0.101541462216394	0.021619837081248
**ACC**	ZMYND1	cg14255089	0.323924435179792	80	3.0044145323988	0.00358929752523365	0.104927039706547	0.0190104719279702
**ACC**	ZMYND1	cg14694828	-0.108290309816833	80	-0.955864753417624	0.342132007463768	0.0117267912002258	0.541312969322874
**ACC**	ZMYND1	cg17632565	0.197469652194445	80	1.76759487540394	0.0810903916756827	0.0389942635377953	0.204902911294314
**ACC**	ZMYND1	cg22593633	0.154604427983683	80	1.37315858708017	0.173688588537185	0.0239025291521619	0.348696943872304

**ACC**	ZMYND1	cg24757747	0.11875153321167	80	1.04946649792596	0.297246257423372	0.0141019266401223	0.495304984623434
**BLCA**	ACTL6A	cg03667091	0.00314467888795961	419	0.0638303256918689	0.949136288051445	9.88900530837891e-06	0.973026044919537
**BLCA**	ACTL6A	cg03839554	-0.150666202322448	419	-3.0935031880046	0.00211280338660347	0.0227003045222689	0.0124188459884722
**BLCA**	ACTL6A	cg07261848	-0.227923182565271	418	-4.74562651477569	2.87374581263288e-06	0.0519489771506819	4.35026623366341e-05
**BLCA**	ACTL6A	cg10112003	0.0568853130037788	419	1.15651847440175	0.248139292579442	0.00323593883553788	0.44147070683175
**BLCA**	ACTL6A	cg20132173	0.0135302511537044	419	0.274659245383192	0.783715715431101	0.000183067696282321	0.880650998280686
**BLCA**	ACTL6A	cg22619386	0.0824403770399384	419	1.67907245949188	0.0938960985909765	0.00679641576648721	0.227655460131722
**BLCA**	ACTL6A	cg25198545	0.0915108115426191	419	1.86529322262862	0.062850910598251	0.00837422862918875	0.170280133004121
**BLCA**	ACTL6A	cg25575835	-0.00263837558602218	419	-0.0535533618545409	0.957316972218594	6.9610257329179e-06	0.977560827701368
**BLCA**	ACTL6B	cg05729836	0.0740991867619704	418	1.50819543866061	0.13227093214934	0.00549068947878537	0.289578569697353
**BLCA**	ACTL6B	cg08572611	-0.14995931963006	418	-3.0786546588162	0.00221845748379787	0.0224877975439106	0.0129139989145893
**BLCA**	ACTL6B	cg09375063	-0.0626644313348647	418	-1.27445378083466	0.203220985013076	0.00392683095452197	0.387076691430777
**BLCA**	ACTL6B	cg14088161	-0.0207254459743666	418	-0.420770987220376	0.674141713076707	0.000429544110836387	0.811627152740914
**BLCA**	ACTL6B	cg16024950	-0.0809991013198087	418	-1.64952223460341	0.0998028678556113	0.00656085441461663	0.237906061037519
**BLCA**	ACTL6B	cg20497359	-0.0497669266651376	418	-1.01141156627738	0.312413095227749	0.00247674698969319	0.511477246672716
**BLCA**	ACTL6B	cg24471254	-0.347219876283717	418	-7.51537202934921	3.56938871207656e-13	0.12056164248648	2.15253508114174e-11
**BLCA**	ACTL6B	cg26990587	-0.159986942022001	418	-3.28975515873853	0.00108895475832084	0.025595821617551	0.00720542200125979
**BLCA**	AEBP2	cg02664216	0.0630423608287328	419	1.28217059517134	0.200503787936552	0.00397433925886014	0.38351630593632
**BLCA**	AEBP2	cg07849438	-0.0282828694371742	419	-0.574309297037587	0.56607193978099	0.000799920703600243	0.735217461502672
**BLCA**	AEBP2	cg08161947	-0.0880480351201893	419	-1.79414798015157	0.0735226187127897	0.0077524564885261	0.191102082952877
**BLCA**	AEBP2	cg24304000	0.0317575593150966	419	0.644933355135987	0.519329324739233	0.00100854257365188	0.699930796222586
**BLCA**	AEBP2	cg26259627	-0.00675123130282944	419	-0.137038151921598	0.891067579776939	4.55791241043041e-05	0.942381675700361
**BLCA**	AFF4	cg00931577	0.0522939753423009	419	1.06290610880787	0.288447134369228	0.00273465985710117	0.48595500421483
**BLCA**	AFF4	cg01907658	-0.0310282490410674	419	-0.63010806195895	0.528973190050355	0.000962752238554501	0.70732820562373
**BLCA**	AFF4	cg05619425	0.204796222173564	419	4.24692443025075	2.68125271175915e-05	0.041941492616564	0.000308878088936164
**BLCA**	AFF4	cg06638012	-0.0492840768917076	419	-1.00157461646193	0.317136959821203	0.00242892023506775	0.516374746794146
**BLCA**	AFF4	cg13393914	-0.0212081049173528	419	-0.43057435777245	0.667003044497992	0.000449783714185444	0.806817204043923
**BLCA**	AFF4	cg13565585	-0.0312523584440682	419	-0.634663610840685	0.526000095569864	0.000976709908316523	0.704769779288361
**BLCA**	AICDA	cg07999668	0.0515942281748472	419	1.04864511231379	0.294956237901422	0.0026619643809582	0.492854470247294
**BLCA**	AIRE	cg08089567	-0.0724268088297613	418	-1.47397471395914	0.141252042014487	0.00524564263726279	0.303249844256403
**BLCA**	AIRE	cg09510531	-0.0317438092860765	418	-0.644653837460291	0.519510305096189	0.0010076694279908	0.700075964045829
**BLCA**	AIRE	cg11923631	-0.0364484838192111	418	-0.74031533490679	0.459530344900697	0.0013284919727193	0.649424056634917
**BLCA**	AIRE	cg12843033	-0.0350288170064633	418	-0.711443942146612	0.477211783187255	0.0012270180208723	0.664903346729042
**BLCA**	AIRE	cg15955655	-0.0560620869297354	418	-1.13972855109767	0.255061576075095	0.00314295759091721	0.44932255572147
**BLCA**	AIRE	cg16501323	-0.011248853066424	418	-0.228341227234773	0.819494263629073	0.000126536695309996	0.902815161705527
**BLCA**	AIRE	cg16717549	-0.030637163546069	418	-0.622158560078195	0.534181761966484	0.000938635790148579	0.711033005435611
**BLCA**	AIRE	cg17356252	-0.0347814485310268	418	-0.706413720246643	0.480330179829825	0.00120974916191647	0.667610119699156
**BLCA**	AIRE	cg18689454	-0.0572697726985442	418	-1.164360444723	0.244951752821912	0.00327982686494291	0.437842393709568
**BLCA**	AIRE	cg18876487	-0.048209338065595	418	-0.979681810136248	0.327818119970156	0.00232414027672283	0.527320682042891
**BLCA**	AIRE	cg23272544	-0.0562607295372909	418	-1.14377970721536	0.253379123522927	0.00316526968806819	0.447406213208856
**BLCA**	AIRE	cg27251412	-0.0273837710145913	418	-0.556038362936446	0.578486469629014	0.000749870914979572	0.744733838921022
**BLCA**	ARID1A	cg04081153	0.067973311184208	419	1.38290599726828	0.167442710131052	0.00462037103334518	0.340214735816102
**BLCA**	ARID1A	cg04360147	0.0784658769637697	419	1.59760910208981	0.110896637074711	0.00615689384769345	0.25639244485824
**BLCA**	ARID1A	cg14851949	0.0404290441433483	419	0.821291448239778	0.411955255698443	0.00163450761034481	0.607607446568418
**BLCA**	ARID1A	cg17385674	0.139391819078978	419	2.8572392684992	0.00448999984841741	0.0194300792261466	0.0227479679718518
**BLCA**	ARID1A	cg20413923	0.0358467569921354	419	0.728077635954499	0.466979647579744	0.00128498998685321	0.656099236117367
**BLCA**	ARID1A	cg21294655	0.0892851465157915	419	1.81955766167954	0.0695520030140241	0.00797183738834636	0.183526508461875
**BLCA**	ARID1A	cg24542126	-0.0379954352538816	419	-0.771780397509006	0.440686943787589	0.00144365310013191	0.633310870461549
**BLCA**	ARID1A	cg25601319	-0.0804568205408192	419	-1.6384066533371	0.102100301198059	0.00647329997153759	0.24176759600042
**BLCA**	ARID2	cg00035453	-0.289001893097759	416	-6.11267936933446	2.28300674627101e-09	0.0835220942140887	7.06460727205277e-08
**BLCA**	ARID2	cg01183821	-0.0806189583195321	419	-1.64172997265462	0.101409033292021	0.00649941644052645	0.24068016156933
**BLCA**	ARID2	cg01353809	-0.268793681582991	419	-5.66437754363005	2.77345504868398e-08	0.0722500432589381	6.902052529025e-07
**BLCA**	ARID2	cg01751433	0.0523879794238983	419	1.06482205118692	0.287580109573979	0.00274450038811879	0.48508254649172
**BLCA**	ARID2	cg06613666	0.214300218358981	419	4.45327863150585	1.09060342514627e-05	0.0459245835887069	0.000141404004759715
**BLCA**	ARID2	cg07276957	-0.0712373787754884	419	-1.44964384394672	0.147918624179647	0.0050747641348024	0.312804526018374
**BLCA**	ARID2	cg23465749	-0.166004401185721	417	-3.4086276025722	0.000717550143681532	0.0275574612130299	0.00508991482425104
**BLCA**	ARID4A	cg02136725	-0.00274124979020748	419	-0.0556415028053445	0.955654373633323	7.51445041231254e-06	0.976401365130885

**BLCA**	ARID4A	cg06154646	0.187621369135699	419	3.87715052001355	0.000122962537061888	0.0352017781563543	0.00115055900988604
**BLCA**	ARID4A	cg08066755	-0.0372242669136584	419	-0.756094117996351	0.450024855876809	0.00138564604725928	0.641040134678076
**BLCA**	ARID4A	cg08790491	-0.117736250840334	419	-2.4065224884699	0.0165445317918948	0.0138618247619381	0.0629007311022508
**BLCA**	ARID4A	cg10064886	0.105424404729976	419	2.15187340559253	0.0319877951209035	0.0111143051126698	0.103517556205981
**BLCA**	ARID4A	cg10242170	0.0313726435714353	419	0.637108727447801	0.524407878263511	0.00098424276466032	0.703581851260397
**BLCA**	ARID4A	cg10844118	-0.265612566548293	419	-5.59221961877076	4.08951172460729e-08	0.0705500355083716	9.76786844040833e-07
**BLCA**	ARID4A	cg12203852	0.138053698794461	419	2.8292750676293	0.00489337992196106	0.0190588237508318	0.0243624927548233
**BLCA**	ARID4A	cg14272338	0.0013689194147187	419	0.0277860554373768	0.977846241620314	1.87394036399379e-06	0.98852003171519
**BLCA**	ARID4A	cg14460816	-0.114201728247545	419	-2.33330738762436	0.0201123907545546	0.0130420347347261	0.0731508404488204
**BLCA**	ARID4A	cg17323045	0.121049556714924	419	2.4752394094084	0.013716000008176	0.0146529951808797	0.0545038823433644
**BLCA**	ARID4A	cg19548283	-0.237960215686669	419	-4.97291224184449	9.69832273701382e-07	0.0566250642496462	1.6673399314989e-05
**BLCA**	ARID4A	cg20996579	-0.0807244847945283	419	-1.64389300707416	0.100961124048683	0.00651644244534203	0.239879526170147
**BLCA**	ARID4A	cg26429530	-0.0967634474158585	419	-1.97334356798175	0.0491243125033458	0.00936316475580162	0.142426087018682
**BLCA**	ARID4A	cg27026909	-0.278486339086414	419	-5.88548394080221	8.23203221135257e-09	0.0775546410577531	2.29042458458498e-07
**BLCA**	ARID4B	cg05652840	0.0716533381384294	418	1.45815195759735	0.1455605170599	0.0051342008663801	0.309493136114515
**BLCA**	ARID4B	cg06212607	-0.144355289666407	417	-2.95751147144289	0.00328033174248931	0.0208384496546722	0.0176834680600938
**BLCA**	ARID4B	cg06891548	-0.339969758316461	418	-7.33769200698517	1.16632352984271e-12	0.115579436569753	6.52755199140891e-11
**BLCA**	ARID4B	cg07814876	-0.252210383630318	418	-5.29033538320926	1.98738735113805e-07	0.0636100776109523	4.03567767148091e-06
**BLCA**	ARID4B	cg10107779	0.0778473966154267	418	1.58493941049357	0.113747321302361	0.00606021715979955	0.26095951610803
**BLCA**	ARID4B	cg11732257	-0.317265119186662	418	-6.7906022614024	3.91289208365194e-11	0.100657155852527	1.664294842841e-09
**BLCA**	ARID4B	cg22596515	0.178996143888377	418	3.69286551746323	0.000251598784689821	0.0320396195269087	0.00210393161949478
**BLCA**	ARID4B	cg23687319	-0.284496889787285	418	-6.02356830506877	3.78393010876699e-09	0.0809384802986384	1.12011818448029e-07
**BLCA**	ARID4B	cg26020585	0.0815057628336071	418	1.65990903029689	0.0976936434164298	0.00664318937508821	0.234140894541968
**BLCA**	ASH1L	cg03119126	0.00754645574975811	419	0.153180684015181	0.878330784418118	5.69489943830572e-05	0.935408422792357
**BLCA**	ASH1L	cg08095278	0.163845212751273	419	3.37125351359139	0.00081893207319253	0.0268452537415098	0.00567902492030128
**BLCA**	ASH1L	cg09410084	0.210542051265193	419	4.37152550718195	1.56413922481613e-05	0.044327955350955	0.000193481709103746
**BLCA**	ASH1L	cg10558228	-0.266168163292571	417	-5.59118763146501	4.12366613024327e-08	0.0708454911505406	9.83434956885791e-07
**BLCA**	ASH1L	cg17038116	0.0693996957150776	419	1.41206455305463	0.158685791307775	0.00481631776534536	0.327891908124972
**BLCA**	ASH1L	cg20064047	-0.0278140725201296	419	-0.564782518518111	0.572529069852675	0.000773622630155029	0.740101802261864
**BLCA**	ASH1L	cg21852905	0.00891520312652157	419	0.180966051434087	0.85648321035378	7.94808467871399e-05	0.923661623409421
**BLCA**	ASH1L	cg23821590	-0.11532175382273	419	-2.35649805652958	0.018915506197764	0.0132991069047503	0.0697416837749207
**BLCA**	ASH1L	cg24312492	-0.0154999021495	419	-0.314651451668466	0.753185592663595	0.000240246966644076	0.861920368332627
**BLCA**	ASH1L	cg26185843	-0.151379361878431	419	-3.10848848696525	0.00201088894886448	0.022915711202721	0.011951510376397
**BLCA**	ASH2L	cg00590869	0.170189908646909	419	3.50562036301499	0.000505571946643079	0.0289646050052434	0.00378359442204442
**BLCA**	ASH2L	cg01642070	-0.0887903761778014	419	-1.8093943017354	0.0711184375042797	0.00788373090179548	0.186502198793458
**BLCA**	ASH2L	cg02897270	-0.0494738082245057	419	-1.00543987016074	0.315275223590679	0.00244765770023516	0.514463245039504
**BLCA**	ASH2L	cg03189282	-0.115985237518811	419	-2.37024009620825	0.018236164661138	0.013452575322295	0.067804052652515
**BLCA**	ASH2L	cg03705346	0.0583092606321642	419	1.18556588697444	0.236477105727189	0.00339996987546965	0.427838826741479
**BLCA**	ASH2L	cg10062221	0.0759338820811943	419	1.54575228173051	0.122932149534473	0.00576595444792072	0.275252810294865
**BLCA**	ASH2L	cg21453287	0.0151990525545997	419	0.308542713006965	0.75782535103992	0.000231011198557483	0.864981129503282
**BLCA**	ASH2L	cg24318412	0.235371541413742	419	4.91562262283565	1.28005248450904e-06	0.0553997625074807	2.12869608764208e-05
**BLCA**	ASH2L	cg27255459	0.101981048221258	419	2.08083797820131	0.0380659475949481	0.0104001341963065	0.118031899525744
**BLCA**	ASXL1	cg00174466	-0.131941923378918	419	-2.70174872436676	0.00718241359333891	0.0174086711449283	0.0330777103410021
**BLCA**	ASXL1	cg04218270	-0.229508499263059	419	-4.78627543597535	2.37212893943375e-06	0.0526741512339817	3.67238604406585e-05
**BLCA**	ASXL1	cg09065199	0.151489889918705	419	3.11081140663253	0.00199550245358697	0.0229491867475814	0.0118765295596622
**BLCA**	ASXL1	cg10421984	-0.336684672556242	419	-7.25767394354492	1.9744560122242e-12	0.113356568734304	1.073381069287e-10
**BLCA**	ASXL1	cg12592359	-0.0757406194746903	419	-1.54179539254844	0.123891011063545	0.00573664143840983	0.276746606405486
**BLCA**	ASXL1	cg16926526	0.0380304574950749	419	0.772492814955453	0.440265510758749	0.0014463156972847	0.632876587160763
**BLCA**	ASXL1	cg18793288	-0.102929312426783	419	-2.10039273354019	0.0363016176400759	0.0105944433566502	0.113976524189575
**BLCA**	ASXL1	cg24727216	-0.001160521020405	419	-0.0235560198529366	0.981218157893482	1.34680903880186e-06	0.990332979256928
**BLCA**	ASXL2	cg05165989	0.219902620161239	419	4.57553646052562	6.29509114566809e-06	0.0483571623537782	8.71233447048751e-05
**BLCA**	ASXL2	cg05496738	0.0917442658772683	419	1.87009213576843	0.0621799317650592	0.00841701032135889	0.16899445428607
**BLCA**	ASXL2	cg06583696	0.279599745674514	419	5.91100570860172	7.1381151626171e-09	0.0781760177812527	2.01368250225852e-07
**BLCA**	ASXL2	cg09476286	0.0774776357550194	419	1.57736566686031	0.115478882981285	0.00600278404218746	0.263664129568152
**BLCA**	ASXL2	cg19084174	0.0847635277850822	419	1.72672602531598	0.084966447606359	0.0071848556425724	0.211977417262815
**BLCA**	ASXL2	cg20667782	0.217609010551495	419	4.52542774906981	7.89708564659311e-06	0.0473536814732005	0.000106325005329018
**BLCA**	ASXL2	cg24738909	0.156883425863135	419	3.22431199901625	0.00136332310359871	0.0246124093105537	0.00868761119932486

**BLCA**	ASXL3	cg09963640	-0.0507765462907997	419	-1.03198254176249	0.302685698968407	0.00257825765322173	0.50104543504869
**BLCA**	ASXL3	cg12361352	-0.00623758538158449	419	-0.12661161842433	0.899309566645617	3.89074713925565e-05	0.946832025424861
**BLCA**	ASXL3	cg17880622	-0.0240739879854516	419	-0.488790248615561	0.625250281543974	0.000579556897523666	0.778174506186395
**BLCA**	ASXL3	cg20034091	0.00403508847240446	419	0.0819040175047947	0.934762813940216	1.62819389801314e-05	0.965308083837773
**BLCA**	ASXL3	cg20329085	-0.00567322021736008	419	-0.115155646803533	0.908377855203047	3.21854276346632e-05	0.951376543090361
**BLCA**	ASXL3	cg24880056	-0.0379743629984041	419	-0.771351750059348	0.440940623481802	0.00144205224513456	0.633486423653126
**BLCA**	ASXL3	cg27109877	-0.00442247278962922	419	-0.0897672714333953	0.928515778586866	1.95582655750109e-05	0.962233661205662
**BLCA**	ATAD2B	cg08192560	0.040858330002556	419	0.830026635377647	0.407004407865322	0.00166940313059777	0.603187169577742
**BLCA**	ATAD2B	cg14009731	0.0495870559615357	419	1.00774703308001	0.314167398114058	0.00245887611893247	0.513236845209682
**BLCA**	ATAD2B	cg15717719	0.102586982336467	419	2.09333266379072	0.0369303732355964	0.0105240889449027	0.115411312555308
**BLCA**	ATAD2B	cg16929802	0.168541180758266	419	3.47066149079533	0.00057401852104384	0.0284061296113905	0.00421315188471407
**BLCA**	ATAD2B	cg17933838	0.0390747073803011	419	0.793736118907354	0.427805971592117	0.00152683275685615	0.622081492252885
**BLCA**	ATAD2B	cg19614157	0.0544983059456468	419	1.10784119881742	0.268576735099334	0.00297006535094532	0.464386080421355
**BLCA**	ATAD2B	cg20806130	-0.149224276418918	419	-3.0632197216067	0.00233341756022428	0.0222678846727496	0.0134673110527552
**BLCA**	ATAD2B	cg27610867	-0.134459161570848	419	-2.75423372360738	0.00614354129422902	0.0180792661303355	0.029234808627554
**BLCA**	ATAT1	cg01245721	-0.166211983060221	418	-3.42132526657177	0.000685446586227451	0.0276264233128111	0.00489881779779314
**BLCA**	ATAT1	cg05093836	0.074853064328094	418	1.52362568520889	0.128369423345558	0.00560298123930577	0.283570945628903
**BLCA**	ATAT1	cg06814856	0.0223971390625715	418	0.454726338339839	0.64954562785079	0.000501631838188167	0.795021201923006
**BLCA**	ATAT1	cg07142893	-0.0872782103380593	418	-1.77834037501858	0.0760853407716804	0.00761748599981452	0.195901843625584
**BLCA**	ATAT1	cg07816366	0.0165638451728225	418	0.336255467971907	0.736849417549901	0.000274360966909235	0.851593528477493
**BLCA**	ATAT1	cg08081524	-0.0914014149103232	418	-1.86304456011986	0.0631673772105442	0.00835421864760906	0.170909787143783
**BLCA**	ATAT1	cg08188471	-0.0915606356421566	417	-1.86405106195511	0.0630272944495391	0.00838334999919576	0.170680730476814
**BLCA**	ATAT1	cg08635134	-0.0712901263997764	418	-1.45072271192497	0.147617993476551	0.0050822821220961	0.312390690800436
**BLCA**	ATAT1	cg09532691	-0.460569157860491	418	-10.532088754741	3.95774081558396e-23	0.212123949172322	6.87251091534684e-21
**BLCA**	ATAT1	cg12907138	-0.0393254026699758	418	-0.79883642909588	0.424845520358939	0.00154648729515574	0.619769377264891
**BLCA**	ATAT1	cg13542073	-0.190063776272666	418	-3.92950125932495	9.98079749615965e-05	0.036124239051026	0.000962090990534973
**BLCA**	ATAT1	cg13964084	0.00598659940401957	418	0.121516873953842	0.903340894414725	3.58393724242075e-05	0.948758505509675
**BLCA**	ATAT1	cg15381130	0.0408855146998279	418	0.830579810017336	0.406692088583898	0.00167162531226985	0.602894417983342
**BLCA**	ATAT1	cg15729859	-0.0933898554025151	418	-1.90392792512536	0.0576159947974896	0.00872166509210268	0.159843753763272
**BLCA**	ATAT1	cg18298197	-0.060521503215008	418	-1.23070836624919	0.219134108926884	0.00366285235140422	0.407032110927319
**BLCA**	ATAT1	cg20629923	-0.0557433697996292	418	-1.13322885519786	0.257777170796182	0.00310732327661821	0.452270867433815
**BLCA**	ATAT1	cg22759096	-0.473905529375091	418	-10.9237987688573	1.44166333503515e-24	0.224586450772285	2.84651668745494e-22
**BLCA**	ATAT1	cg23328390	-0.0415812694922237	418	-0.84473818335936	0.398747294419877	0.00172900197258493	0.595350587711115
**BLCA**	ATAT1	cg26934609	0.00663550524380763	418	0.134689011604514	0.892923534823704	4.40299298405986e-05	0.943222019714917
**BLCA**	ATF7IP	cg00236831	0.0080011785944984	419	0.162411386676033	0.871061528615585	6.40188589010595e-05	0.931528357535337
**BLCA**	ATF7IP	cg11409060	-0.132714189491848	419	-2.71784494394834	0.00684812307739662	0.0176130560924781	0.0318473748614893
**BLCA**	ATF7IP	cg12526346	-0.187236086314949	419	-3.86889917514708	0.000127046378333688	0.0350573520185391	0.0011829451786104
**BLCA**	ATF7IP	cg12948116	-0.183558626267439	418	-3.78563135291192	0.000176155529903665	0.0336937692771894	0.00156080219741751
**BLCA**	ATF7IP	cg17439009	0.0837118659178344	419	1.7051503747257	0.0889201720342539	0.00700767649544548	0.218976127357795
**BLCA**	ATF7IP	cg19789919	0.000126192375016828	419	0.00256142548116921	0.997957520138854	1.59245155123878e-08	0.99895912452625
**BLCA**	ATF7IP	cg21275967	-0.045254238356728	419	-0.919502744464451	0.358370697094042	0.00204794608924755	0.557334703498253
**BLCA**	ATF7IP	cg26637901	-0.137110886525095	400	-2.74752179496063	0.00628052196935033	0.0187993952036976	0.0297411082349428
**BLCA**	ATM	cg01756564	-0.200519140125854	418	-4.15447224506165	3.96625523304739e-05	0.0402079255568121	0.000434578675492519
**BLCA**	ATM	cg01800843	-0.125522184406631	418	-2.56813392861758	0.0105763052431975	0.0157558187782123	0.0445271723178541
**BLCA**	ATM	cg01991180	-0.109380357422596	418	-2.23358032594329	0.0260468017652607	0.0119640625898949	0.0888682468543789
**BLCA**	ATM	cg02105423	0.112278699671372	418	2.29351112864589	0.0223213106006916	0.0126065063998942	0.0790655920764246
**BLCA**	ATM	cg03165700	-0.164584912338525	418	-3.38689623779498	0.000774825665348462	0.0270881933694799	0.00542057444335596
**BLCA**	ATM	cg03569616	-0.219392194139683	418	-4.56437827260225	6.62225002278743e-06	0.0481329348494242	9.10357515944028e-05
**BLCA**	ATM	cg04709886	-0.104632787963555	418	-2.13553575459359	0.0333063717344721	0.0109480203170262	0.106719217542383
**BLCA**	ATM	cg04826772	0.189006845139768	418	3.90683769469881	0.000109273362220453	0.0357235875096882	0.00103998188653999
**BLCA**	ATM	cg05033322	-0.148204458347691	418	-3.04181348405519	0.00250192570211885	0.0219645614741325	0.0142470924549106
**BLCA**	ATM	cg05637308	-0.0103109204957191	418	-0.209299954261252	0.834317535720383	0.00010631508146904	0.911179244504998
**BLCA**	ATM	cg06053805	-0.101195964359605	418	-2.06465260750395	0.0395811434412051	0.0102406232026705	0.121571994348749
**BLCA**	ATM	cg06081482	-0.151767978434833	418	-3.11665640027982	0.00195726503761112	0.023033519278196	0.0116856083143453
**BLCA**	ATM	cg06750635	-0.0586699018455634	418	-1.19292382954806	0.233585739854527	0.00344215738256804	0.424470194960127
**BLCA**	ATM	cg09808176	-0.126370007669991	418	-2.58576059786267	0.0100588139573024	0.0159693788385136	0.0428325920785848
**BLCA**	ATM	cg10610482	-0.254292798582363	418	-5.33702253319926	1.56355339535994e-07	0.0646648274108503	3.24880002239934e-06

**BLCA**	ATM	cg10976287	-0.114623259249541	418	-2.34203432700643	0.0196544192250385	0.0131384915609875	0.0718411825960928
**BLCA**	ATM	cg11588932	-0.106649381055015	418	-2.17716304142488	0.0300353365543683	0.0113740904794177	0.0987853114577656
**BLCA**	ATM	cg12019961	-0.207750756187124	409	-4.27415777102608	2.39468592738772e-05	0.0431603766963216	0.000280096522380394
**BLCA**	ATM	cg12106634	-0.0935921586478332	418	-1.90808866400608	0.057074577706323	0.00875949216036118	0.158779077955922
**BLCA**	ATM	cg12848864	-0.22184052307041	418	-4.61793611757969	5.18785016350941e-06	0.049213217676153	7.34991104753325e-05
**BLCA**	ATM	cg14761454	-0.0951056117334556	417	-1.93686835944683	0.0534464453538536	0.00904507738319481	0.151479625832763
**BLCA**	ATM	cg15370815	0.150401282553901	418	3.08793780906195	0.00215184879679599	0.0226205457938584	0.012596476726415
**BLCA**	ATM	cg15504467	-0.0822060895415868	418	-1.6742681868054	0.0948368007230856	0.00675784115771938	0.229278169603683
**BLCA**	ATM	cg16693212	0.00345317352372628	418	0.0700921852006221	0.944154294739109	1.19244073849642e-05	0.970165960631898
**BLCA**	ATM	cg16788234	0.123466127781083	418	2.52541114390553	0.0119304485380162	0.0152438847092548	0.0489229515347863
**BLCA**	ATM	cg17114932	-0.176116549828245	418	-3.63153895230497	0.0003172504277929	0.0310170391234047	0.00256151321014228
**BLCA**	ATM	cg17420659	-0.128811861834978	418	-2.63656028869325	0.0086916793595022	0.0165924957493935	0.0382616853832681
**BLCA**	ATM	cg18042593	-0.155930190073611	418	-3.20423108643135	0.00145956163858276	0.0243142241763923	0.00919501877435961
**BLCA**	ATM	cg18391757	-0.0245541153978845	418	-0.498544419316457	0.618366415318689	0.000602904582972627	0.773480795508098
**BLCA**	ATM	cg18457775	-0.0906286693596697	418	-1.84716260359063	0.0654403621139054	0.00821355570990434	0.175444309552647
**BLCA**	ATM	cg19288979	0.237591901258212	418	4.9647543047066	1.00908998851025e-06	0.0564499115434919	1.7211389293174e-05
**BLCA**	ATM	cg20342375	-0.102827483176294	418	-2.09829257181723	0.0364876898839935	0.010573491296371	0.11441967699272
**BLCA**	ATM	cg22477592	-0.0383128665731063	418	-0.778237644636419	0.436875625801746	0.00146787574504865	0.62987754014532
**BLCA**	ATM	cg22678707	-0.147714382937497	418	-3.03153021232853	0.00258678511583843	0.0218195389266054	0.0146257984811058
**BLCA**	ATM	cg22837512	-0.090292856254632	418	-1.84026181009928	0.0664488685542154	0.00815279989061964	0.177405250108082
**BLCA**	ATM	cg23275463	-0.159948315254021	417	-3.2849461768642	0.00110744134184924	0.0255834635525997	0.00730537537079794
**BLCA**	ATM	cg23389069	-0.0262178828872845	418	-0.53234789438173	0.594772033220822	0.000687377383091368	0.756275568434328
**BLCA**	ATM	cg24030675	-0.0332861401678044	418	-0.676009456285068	0.499413923832816	0.00110796712727072	0.683593979021762
**BLCA**	ATM	cg24568245	-0.189479041378413	418	-3.91696115922726	0.000104944810112615	0.0359023071216825	0.00100443855289134
**BLCA**	ATM	cg25400013	-0.11562992883566	418	-2.36288056256926	0.0185972706193362	0.0133702804425398	0.0688374206861415
**BLCA**	ATM	cg25740661	0.174464012121789	418	3.59638851141289	0.000361816946188318	0.0304376915256316	0.00285816282613465
**BLCA**	ATM	cg25918541	-0.14792959965632	418	-3.03604583163346	0.00254920128191761	0.0218831664544791	0.0144700886191535
**BLCA**	ATM	cg27453365	-0.110512223696404	418	-2.25697766853591	0.0245325946174625	0.012212951586324	0.0849700296606489
**BLCA**	ATM	cg27599391	-0.228207595822505	418	-4.75765081656246	2.71414434268711e-06	0.052078706791088	4.14740021596938e-05
**BLCA**	ATR	cg00911981	0.0681706678523968	419	1.38693990609668	0.166209974734766	0.00464723995544181	0.338543433533608
**BLCA**	ATR	cg00969988	-0.0301999137365394	419	-0.613271025624516	0.540035550976717	0.00091203478969442	0.715333316068155
**BLCA**	ATR	cg11458642	0.160207740370331	419	3.29441487368731	0.00107151987622603	0.0256665200745673	0.00711183493312242
**BLCA**	ATR	cg11936145	0.00800201540354397	419	0.162428373675938	0.871048161087462	6.4032250518555e-05	0.931528357535337
**BLCA**	ATR	cg15135999	0.066333410639051	419	1.34939320328635	0.177951739901782	0.00440012136700897	0.354487586625127
**BLCA**	ATR	cg15291273	-0.0202517961423884	419	-0.411150888477287	0.681175671786524	0.000410135246992859	0.816080638971276
**BLCA**	ATR	cg15480357	0.100957103267328	419	2.05972899701628	0.0400521402948605	0.01019233670013	0.122599526715728
**BLCA**	ATR	cg16031872	0.0663329672639891	419	1.34938414402897	0.177954645865634	0.00440006254604545	0.354487586625127
**BLCA**	ATR	cg17945282	0.0135625559029113	419	0.275315140708627	0.783212153967838	0.000183942922619595	0.880365361524859
**BLCA**	ATR	cg20032934	-0.114662583040749	419	-2.34284851116885	0.0196121623901798	0.0131475079495766	0.0717406574026259
**BLCA**	ATR	cg20636078	0.0882549735943736	419	1.7983978122652	0.0728458536972078	0.00778894036414357	0.189822379071162
**BLCA**	ATR	cg24631870	-0.0167147417269403	419	-0.339319605956969	0.734541866002825	0.000279382590998319	0.850335800540741
**BLCA**	ATR	cg24856922	0.101178467962081	419	2.06429194446072	0.0396154839039133	0.0102370823791539	0.121634248522754
**BLCA**	AURKB	cg02245743	-0.0424996297986614	419	-0.863428391374763	0.388404329100629	0.00180621853302326	0.586140820272311
**BLCA**	AURKB	cg04612488	-0.203330251249487	419	-4.21520814455849	3.0691710213997e-05	0.0413431910731797	0.000347544527567349
**BLCA**	AURKB	cg05617798	-0.166002510125268	419	-3.41689118694876	0.00069639216859195	0.0275568333678897	0.00496609518410873
**BLCA**	AURKB	cg05811247	-0.199048268006597	419	-4.12273580960988	4.52935928304082e-05	0.0396202129964261	0.000487219270001214
**BLCA**	AURKB	cg11009596	-0.110671262262782	419	-2.26026594001643	0.0243260443308808	0.0122481282908375	0.0844566560751791
**BLCA**	AURKB	cg12844497	0.0328600044108912	419	0.667345633698491	0.504924984007174	0.00107977988988379	0.688100942007841
**BLCA**	AURKB	cg13678641	-0.179872455261737	419	-3.71154764949286	0.000234292024418785	0.0323541001618855	0.00198178903421061
**BLCA**	AURKB	cg21724796	-0.217327090226227	419	-4.51927406033937	8.11890116922578e-06	0.0472310641461984	0.000108895714088668
**BLCA**	BAP1	cg00794529	-0.233802894053605	419	-4.88096108052475	1.51215233072813e-06	0.0546637932678413	2.4615689296391e-05
**BLCA**	BAP1	cg02503646	-0.0833047544121961	419	-1.69679971424003	0.0904898129587199	0.00693968210767631	0.221620844645217
**BLCA**	BAP1	cg03075736	-0.0918515137146143	419	-1.87229683837842	0.0618736725761309	0.00843670057166598	0.168394915633708
**BLCA**	BAP1	cg05442719	0.0270337909299531	419	0.548926647036755	0.583353148229215	0.000730825852044416	0.748359226735243
**BLCA**	BAP1	cg05908979	-0.0135418700774918	419	-0.274895148610498	0.783534591028808	0.000183382245195667	0.880580552357001
**BLCA**	BAP1	cg09096430	-0.0990780077536967	419	-2.0210079416482	0.0439252137246953	0.00981645162044158	0.131225732584445
**BLCA**	BAP1	cg10449522	-0.113043869160174	419	-2.30934283314753	0.0214183994782387	0.0127789163547025	0.0767349621907573

**BLCA**	BAP1	cg10956904	-0.136807274896605	419	-2.80324136042291	0.00529819813640684	0.0187162304646353	0.025974793661686
**BLCA**	BAP1	cg11234564	0.00475227315310643	419	0.0964616990936372	0.923200795370218	2.25841001217361e-05	0.959627512660584
**BLCA**	BAP1	cg12859382	0.0308966539698689	419	0.627433128077046	0.530722926754104	0.000954603226533817	0.708502050740909
**BLCA**	BAP1	cg16841708	0.0304678936452773	419	0.618717939947054	0.53644411260335	0.000928292543179927	0.712547274258077
**BLCA**	BAP1	cg24291822	-0.124967388280349	419	-2.55660253696122	0.0109275815589669	0.0156168481336115	0.0457043161181865
**BLCA**	BAP1	cg24747396	-0.00612032927781657	419	-0.124231443145666	0.901192614249583	3.74584304688987e-05	0.947909386599947
**BLCA**	BAP1	cg25749966	-0.118167347593953	419	-2.41545863486414	0.0161497445910004	0.0139635220373901	0.0617497000588873
**BLCA**	BAZ1A	cg05294307	0.169528931923202	416	3.47886632497426	0.000557623858649518	0.0287400587590216	0.00411143876976406
**BLCA**	BAZ1A	cg07414384	-0.26496446995544	419	-5.57754290619564	4.42345740102443e-08	0.0702061703387672	1.0472259435645e-06
**BLCA**	BAZ1A	cg23422263	-0.136090100863799	419	-2.78826810525774	0.00554455337154385	0.0185205155531189	0.026935630093171
**BLCA**	BAZ1A	cg26967526	0.185394714827878	419	3.82948922821195	0.000148384540906414	0.0343712002861102	0.00134931934252175
**BLCA**	BAZ1B	cg00525989	0.14818543310727	419	3.04141423398932	0.00250517211109663	0.0219589225851891	0.0142617899166228
**BLCA**	BAZ1B	cg04742505	0.200882747050495	419	4.162322152471	3.83763035692886e-05	0.040353878062553	0.000421911554532114
**BLCA**	BAZ1B	cg05166819	0.13441274054938	419	2.75326534649937	0.00616140921764074	0.0180667848219949	0.0293019115220163
**BLCA**	BAZ1B	cg06059318	0.157945559207385	419	3.24669783772922	0.00126298190592379	0.0249467996733337	0.008152374244062
**BLCA**	BAZ1B	cg06394887	0.128850116694378	419	2.6373565180864	0.00867164678073271	0.0166023525721548	0.0382059290740398
**BLCA**	BAZ1B	cg14093368	0.151143774899356	419	3.10353765618774	0.00204404741385038	0.0228444406908273	0.0121070979528762
**BLCA**	BAZ1B	cg15636799	0.0428929640260336	419	0.871434085651925	0.384024688766016	0.00183980636293862	0.582218908595718
**BLCA**	BAZ1B	cg19160975	-0.0225242986126743	419	-0.45730934970088	0.647689754613724	0.000507344027992919	0.793548732505237
**BLCA**	BAZ1B	cg20612831	-0.0510455956326678	419	-1.03746494826703	0.300127723288513	0.00260565283349383	0.49840191490216
**BLCA**	BAZ1B	cg21340129	0.0772502182001119	419	1.5727078403775	0.116554075234803	0.0059675962119649	0.265348035726676
**BLCA**	BAZ1B	cg25710357	0.182464788060809	419	3.76686740307044	0.0001893879963663	0.0332933988820761	0.00165728344086838
**BLCA**	BAZ2A	cg14634319	0.109011534070778	419	2.22595810841877	0.0265572968220782	0.0118835145604644	0.0901434769831473
**BLCA**	BAZ2A	cg18206859	0.0219942693683731	419	0.446542929910272	0.65543972409119	0.000483747885048556	0.799084817300086
**BLCA**	BAZ2A	cg18955060	-0.19803323808607	419	-4.10085183772592	4.9611272486803e-05	0.0392171633868541	0.000526242068698985
**BLCA**	BAZ2A	cg25683012	0.0341732246457208	419	0.694046077828842	0.488044453319723	0.0011678092826869	0.674190639875741
**BLCA**	BRD7	cg03021120	-0.172832108999626	419	-3.56170773997494	0.000411494412901548	0.0298709379012586	0.00318113846340684
**BLCA**	BRD7	cg16433860	-0.162855247838122	419	-3.35032746600641	0.000881573943290761	0.0265218317484163	0.00604239324619924
**BLCA**	BRD7	cg16834187	0.0923417084551553	419	1.88237464972076	0.0604896566956311	0.00852699112041691	0.165613160870695
**BLCA**	BRD8	cg01096096	0.00316906002491836	419	0.0643252161203301	0.9487424734397	1.00429414415355e-05	0.972788970473146
**BLCA**	BRD8	cg03204906	-0.185724130513031	419	-3.83653646387833	0.00014433561062345	0.0344934526548215	0.0013174286627761
**BLCA**	BRD8	cg03684156	-0.100224313360345	419	-2.04462634206813	0.0415267456800443	0.0100449129885526	0.125775030306925
**BLCA**	BRD8	cg07430605	0.092491193775673	419	1.88544816152881	0.0600727223081298	0.00855462092604908	0.164813872658743
**BLCA**	BRD8	cg07632946	-0.279845318634275	419	-5.91663826681572	6.9165357537216e-09	0.078313402361519	1.955177988463e-07
**BLCA**	BRD8	cg08976717	-0.0411248564326894	419	-0.835450195258118	0.403948496533929	0.00169125381660931	0.600431231048024
**BLCA**	BRD8	cg11110536	0.02342137611396	419	0.475532460111062	0.634659523231223	0.000548560859071576	0.784791150863829
**BLCA**	BRD8	cg11346339	0.0665229748835923	419	1.35326654680738	0.176712521965075	0.00442530618736305	0.352744892069974
**BLCA**	BRD8	cg12964860	-0.223586670948846	419	-4.65618961340108	4.35151454177962e-06	0.0499909994259877	6.30042289713761e-05
**BLCA**	BRD8	cg14473243	0.00985248482487287	419	0.199993307325167	0.84158449084087	9.70714572243501e-05	0.915193870031937
**BLCA**	BRD8	cg22106577	0.104717628171471	419	2.13728651818234	0.0331628734497759	0.0109657816498584	0.106382578195159
**BLCA**	BRD8	cg22818036	-0.019510886693208	419	-0.39610314717605	0.692233989644051	0.000380674699555203	0.823293854003505
**BLCA**	BRD8	cg23298160	-0.0871482453716369	419	-1.77567198805853	0.0765250697983556	0.00759481667135503	0.196767188229822
**BLCA**	BRD8	cg26008208	0.0909512235133827	419	1.85379153032759	0.0644835479959867	0.0082721250585813	0.173509490858883
**BLCA**	BRD8	cg26630792	0.0342672240532077	419	0.695957413765106	0.48684789889947	0.00117424264431274	0.673240995573546
**BLCA**	BRD8	cg27119451	-0.0433648553348	419	-0.881039220710066	0.378810258453984	0.00188051067820814	0.577362615273392
**BLCA**	BRD9	cg02364331	-0.153436905093046	419	-3.15175064642173	0.00174152564501078	0.0235428838445323	0.0106246042523818
**BLCA**	BRD9	cg02822723	0.0992512771885357	419	2.02457744922666	0.0435553656586655	0.00985081602355554	0.130371147212398
**BLCA**	BRD9	cg05193570	-0.0776719756518752	419	-1.58134621334798	0.114566247218375	0.0060329358016655	0.262250011361078
**BLCA**	BRD9	cg07308931	-0.220197324890209	419	-4.58198066219747	6.1132772585696e-06	0.0484868618888041	8.49395054568965e-05
**BLCA**	BRD9	cg09497020	-0.227319635632414	419	-4.73812790294227	2.97414775959854e-06	0.0516742167440536	4.48045799400095e-05
**BLCA**	BRD9	cg10551270	-0.404246770751657	419	-8.97098742414545	1.0456678102977e-17	0.163415451663142	1.11893647364923e-15
**BLCA**	BRD9	cg10600960	-0.17873921488494	419	-3.68738975710768	0.000256895140697167	0.0319477069376849	0.00214142166272018
**BLCA**	BRD9	cg13722821	-0.250479119434653	419	-5.25158072484698	2.42207955021977e-07	0.062739789272759	4.80303993193466e-06
**BLCA**	BRD9	cg17977043	-0.171088419451249	419	-3.52468471749267	0.000471541694061823	0.0292712472703267	0.00356952650015316
**BLCA**	BRD9	cg20079193	-0.124265555980257	419	-2.54201849943137	0.0113867318072009	0.0154419284030823	0.0471908483410254
**BLCA**	BRD9	cg21160852	0.0722280843603844	419	1.46990918778068	0.142349564403777	0.00521689617037081	0.304765026784109
**BLCA**	BRD9	cg21758832	-0.112682147375452	419	-2.30185812815355	0.0218412092937261	0.0126972663371431	0.0778515488833974

**BLCA**	BRD9	cg21910709	-0.11607680238699	419	-2.37213683508725	0.0181441029636869	0.0134738240523883	0.0675584518750231
**BLCA**	BRD9	cg24953233	-0.253329684052556	419	-5.3154199680746	1.7474522114165e-07	0.064175928822168	3.60014028959044e-06
**BLCA**	BRD9	cg25523559	-0.154627478516449	419	-3.17680292539052	0.00160118343843253	0.0239096571123547	0.00992529596887814
**BLCA**	BRD9	cg25951128	-0.254590116889809	419	-5.34369477909577	1.51065330186263e-07	0.0648161276179668	3.14813187691137e-06
**BLCA**	BRD9	cg26504197	-0.0505748038088884	419	-1.02787179200943	0.304613207543195	0.00255781078030755	0.503137349133088
**BLCA**	BRDT	cg00308185	-0.22668022834067	418	-4.72407745674653	3.17594021485305e-06	0.0513839259205782	4.75498639480905e-05
**BLCA**	BRDT	cg01515515	-0.334249401568818	418	-7.1985488359649	2.90529249062912e-12	0.111722662449113	1.52815634899258e-10
**BLCA**	BRDT	cg02935097	-0.130834145347753	418	-2.67866821726729	0.00768742335332008	0.017117573588877	0.0348769704524513
**BLCA**	BRDT	cg03421440	-0.529725252299344	418	-12.6770071995441	2.52544360545086e-31	0.280608842923604	7.33075969561358e-29
**BLCA**	BRDT	cg06442199	-0.482725150064913	418	-11.1881305533481	1.48674530155084e-25	0.233023570505193	3.16010557346573e-23
**BLCA**	BRDT	cg09250199	-0.306821397945895	418	-6.5434013598026	1.78821332766299e-10	0.0941393702374734	6.79259119200267e-09
**BLCA**	BRDT	cg13299824	-0.328348601116048	418	-7.05595472113966	7.3036374987154e-12	0.107812803854866	3.56001989708688e-10
**BLCA**	BRDT	cg14732540	-0.3217659253329	418	-6.89797443552862	1.99558843930769e-11	0.103533310705337	8.93752487320104e-10
**BLCA**	BRDT	cg19245011	-0.202800227381744	418	-4.20374836850389	3.22207052871597e-05	0.0411279322260872	0.000362588969638262
**BLCA**	BRPF1	cg04879506	-0.153130804617534	419	-3.14531189678662	0.00177938527212332	0.0234490433228135	0.0108094251022615
**BLCA**	BRPF1	cg13694772	0.092912173316864	419	1.89410445621638	0.0589112963548277	0.00863267195046299	0.162446667681393
**BLCA**	BRPF1	cg14712964	-0.0773539837697613	419	-1.57483307227408	0.116062521008805	0.0059836388050525	0.264589178902239
**BLCA**	BRPF1	cg17738494	0.162232178071939	419	3.33716224506337	0.000923245203908619	0.0263192796019654	0.00628042474579111
**BLCA**	BRPF1	cg20952324	0.099866944987704	419	2.03726227145291	0.0422623398274273	0.00997340670117709	0.127447240552579
**BLCA**	BRPF1	cg25467642	-0.102648871611565	419	-2.09460898605808	0.036816022083131	0.0105367908431276	0.115175357780798
**BLCA**	BRPF1	cg26227975	-0.0662269938401282	419	-1.347218865916	0.178650225668046	0.00438601471310038	0.355254611477722
**BLCA**	BRPF3	cg01499736	-0.00017128980295918	419	-0.0034768033238825	0.997227600699398	2.9340196597795e-08	0.99860101160044
**BLCA**	BRPF3	cg05882415	-0.0568660830425914	419	-1.15612624716845	0.248299483272641	0.00323375140060691	0.441588730917956
**BLCA**	BRPF3	cg10562781	0.06237579765957	419	1.26856063845278	0.205314094034637	0.00389074013366762	0.389546964326876
**BLCA**	BRPF3	cg14654948	-0.0443852395549687	419	-0.901810710466424	0.367684315406394	0.00197004949035196	0.566586800334723
**BLCA**	BRPF3	cg18779695	0.0610372931519929	419	1.24123604095957	0.215224801572923	0.00372555115532232	0.40225832384893
**BLCA**	BRPF3	cg23517469	0.0107110715100221	419	0.217423479137567	0.827985968555002	0.000114727052892808	0.907510044630906
**BLCA**	BRWD3	cg03164831	-0.0821803299305291	418	-1.67373998023047	0.0949406871993813	0.00675360662749062	0.229458674661013
**BLCA**	BRWD3	cg05835545	-0.0809031810694117	418	-1.64755597020609	0.100206226881959	0.00654532470715001	0.23857515574767
**BLCA**	BRWD3	cg09523186	-0.0948483487699751	418	-1.93392988561599	0.0538062954206074	0.00899620926439083	0.152222232378745
**BLCA**	BRWD3	cg11957438	-0.134549964611188	409	-2.72923829948454	0.00662549134654596	0.0181036929768718	0.0310156041641704
**BLCA**	BRWD3	cg15511898	-0.06300747191961	418	-1.28145818720174	0.200753515925448	0.00396994151770044	0.383814672349577
**BLCA**	BRWD3	cg22618269	-0.0982500548819747	418	-2.00395391839245	0.0457291701809335	0.00965307328431104	0.135182211014423
**BLCA**	BRWD3	cg25063710	-0.105923061522906	418	-2.16216697254744	0.0311802918802662	0.0112196949623853	0.101576198766056
**BLCA**	BRWD3	cg25708850	-0.236822843261514	394	-4.80764023735909	2.18616434561049e-06	0.0560850590904675	3.41644172564127e-05
**BLCA**	CARM1	cg12317676	0.071047357384236	419	1.445757362703	0.149005500184704	0.00504772699128336	0.314335056576426
**BLCA**	CARM1	cg17918830	-0.23753990621973	419	-4.96360282962505	1.01475329222105e-06	0.0564252070468781	1.73003983377261e-05
**BLCA**	CARM1	cg22378614	-0.0460613034997088	419	-0.935935737385543	0.349854538539759	0.00212164368009228	0.548925702515736
**BLCA**	CARM1	cg26504915	-0.206021762089787	419	-4.27346187258807	2.39307858645281e-05	0.0424449664545809	0.000279950609856406
**BLCA**	CBX1	cg01544580	-0.0767755797864573	419	-1.56298738300884	0.118823315073944	0.00589448965154667	0.26894315720879
**BLCA**	CBX1	cg01553295	-0.00817549917392222	419	-0.165950055256126	0.868277658836162	6.68387867428029e-05	0.930050700035805
**BLCA**	CBX1	cg02835499	-0.0194934520301193	419	-0.395749060177611	0.692495007926448	0.000379994672050562	0.823465808328061
**BLCA**	CBX1	cg04864609	-0.0405885152598396	419	-0.824536342228565	0.410111973916897	0.00164742757099824	0.606046023332348
**BLCA**	CBX1	cg11729481	-0.066668755880776	419	-1.35624537257263	0.17576389056687	0.0044447230106905	0.35155910084334
**BLCA**	CBX1	cg12245530	-0.0764206916886673	419	-1.55572007623055	0.120542465360278	0.00584012211817434	0.271507465281981
**BLCA**	CBX1	cg13342109	-0.0579153964267159	419	-1.17753064795641	0.2396635323501	0.00335419314326365	0.431591679038705
**BLCA**	CBX1	cg17778721	-0.0160287923564922	419	-0.325390753827652	0.745050533762553	0.000256922184407543	0.856832895609787
**BLCA**	CBX1	cg21511817	-0.0742652634918757	419	-1.5115944466081	0.131403677134208	0.00551532936151772	0.288239606907614
**BLCA**	CBX4	cg04398978	-0.108828935302603	419	-2.22218481030597	0.0268132063238344	0.0118437371590982	0.0907823724923345
**BLCA**	CBX4	cg04925705	0.0557312669052016	419	1.13298204403188	0.25788068448644	0.00310597411085882	0.452364865681606
**BLCA**	CBX4	cg05949524	-0.101076962881245	419	-2.06219960153799	0.0398152070255633	0.0102165524252966	0.122088459414532
**BLCA**	CBX4	cg07159673	0.0504338439643127	419	1.02499963770847	0.305964787897361	0.00254357261701664	0.504487499410511
**BLCA**	CBX4	cg10756274	0.048910870943453	419	0.993971889233565	0.320819956941853	0.00239227329644712	0.520269907451055
**BLCA**	CBX4	cg21365914	-0.0164017528173765	419	-0.33296401122669	0.739330814290698	0.000269017495482317	0.853168501601011
**BLCA**	CBX4	cg22353329	0.0248002617442707	419	0.503545211767299	0.614850119325737	0.000615052982584336	0.770884248100861
**BLCA**	CBX4	cg27487227	-0.0665144450600226	419	-1.35309225478398	0.176768145034877	0.00442417140164276	0.3528197114851
**BLCA**	CBX5	cg03689456	-0.0418612717170314	419	-0.850436479606683	0.395576417197063	0.00175236606976714	0.592275911301981

BLCA	CBX5	cg05754589	0.0182821121142534	419	0.371148377545147	0.710717793490931	0.000334235623358131	0.835247812572851
BLCA	CBX5	cg06399839	-0.0720359213875459	419	-1.46597806475443	0.143417041498902	0.0051891739701527	0.306303857542815
BLCA	CBX5	cg06621757	0.0158990285671135	419	0.322755829379653	0.747043897645039	0.00025277910937789	0.857958471343552
BLCA	CBX5	cg08288469	0.0300237640577792	419	0.609690709204626	0.542402802271896	0.000901426408197197	0.7169917860349
BLCA	CBX5	cg09060496	-0.0570680419124451	419	-1.16024560072842	0.246620706806476	0.0032567614077206	0.439815022228426
BLCA	CBX5	cg09158487	-0.0423230917914869	419	-0.859835371262729	0.39037983387509	0.00179124409879063	0.587768084007713
BLCA	CBX5	cg09653879	-0.0137844664990599	419	-0.279820697403728	0.779755501377505	0.000190011516663705	0.878294667045458
BLCA	CBX5	cg11005091	-0.026236549834292	419	-0.532727183306932	0.594509649842252	0.000688356547207285	0.756103387927405
BLCA	CBX5	cg11338003	-0.0508404064390183	419	-1.03328379673611	0.302077247577885	0.00258474692688457	0.500446790961907
BLCA	CBX5	cg12263485	0.0439765797826008	419	0.893491476620586	0.372115643176336	0.00193393956937546	0.570782269165366
BLCA	CBX5	cg12600134	0.0731708534488185	419	1.4891980286864	0.137200291465988	0.00535397379442847	0.297117709386884
BLCA	CBX5	cg13445218	-0.049623248271831	419	-1.00848437661049	0.313813891931843	0.00246246676904777	0.512863556092816
BLCA	CBX5	cg14017196	-0.0783598601992283	419	-1.59543719641729	0.111381255391726	0.0061402676904426	0.257138421282131
BLCA	CBX5	cg14394617	0.0920341740050455	419	1.8760519522918	0.0613549278205403	0.008470289184791	0.167334098438375
BLCA	CBX5	cg15708001	-0.0552074118339577	419	-1.12229968298015	0.262388661104008	0.00304785832140421	0.457776710067845
BLCA	CBX5	cg20545544	-0.0119305129172147	419	-0.242180200057183	0.808761040235554	0.000142337138467826	0.896620489434164
BLCA	CBX5	cg20927027	0.0963463415895158	419	1.96475745391791	0.050113925197126	0.00928261753768365	0.144514315779561
BLCA	CBX5	cg21826008	0.0291747750554048	419	0.592435441517469	0.553884036620272	0.000851167499533472	0.725731472973194
BLCA	CBX5	cg24505978	0.001419027957907	419	0.0288031507448725	0.977035534665126	2.0136403453217e-06	0.987957241060374
BLCA	CBX5	cg25817259	0.00975551031857339	419	0.198024655996318	0.843123441821512	9.51699815757918e-05	0.916181865105899
BLCA	CBX6	cg03028106	-0.0750163384989569	416	-1.52139839389255	0.128932604582409	0.00562745104179008	0.284455433506819
BLCA	CBX6	cg04134624	0.0448753394235843	419	0.911788448189983	0.362413275889783	0.0020137960883819	0.561356689721444
BLCA	CBX6	cg04215348	0.0292948643974927	419	0.594876117250815	0.552252844933105	0.000858189080067482	0.724546046356792
BLCA	CBX6	cg04801716	-0.00102518154903577	419	-0.0208089236864578	0.983408153030017	1.05099720848337e-06	0.991380470007356
BLCA	CBX6	cg06060673	0.0572433082957232	419	1.16382062363818	0.245170246310437	0.00327679634463922	0.438051771738038
BLCA	CBX6	cg09913544	0.047898522436458	419	0.973351026107518	0.330949985086521	0.00229426845159587	0.530652284552734
BLCA	CBX6	cg14498989	0.00782034567381827	419	0.158740534675907	0.873951081804276	6.11578064580081e-05	0.933249378291058
BLCA	CBX6	cg24649346	0.141066759158722	419	2.89226488560006	0.00402749224067352	0.0198998305395448	0.0208551541316671
BLCA	CBX6	cg25836955	0.0149356521322475	419	0.303194447128658	0.761894716717255	0.00022307370461551	0.867687092364142
BLCA	CBX7	cg00545295	-0.0725048664634954	418	-1.47377984325934	0.141306356604351	0.0052569556608893	0.303324596608231
BLCA	CBX7	cg01525244	-0.00936934676835644	419	-0.190185316635372	0.849257586885569	8.77846588657112e-05	0.919617281175022
BLCA	CBX7	cg02523424	-0.0974445662244591	419	-1.98736664171123	0.0475434610832798	0.009495443486673	0.139128649244383
BLCA	CBX7	cg03804568	-0.0152339294058303	419	-0.309250881711148	0.75728702451229	0.000232072605141822	0.864631994933965
BLCA	CBX7	cg10339854	-0.0953110440152709	419	-1.94345038146027	0.0526423925888407	0.0090841951112809	0.149780708943634
BLCA	CBX7	cg22268271	-0.0933285251169724	419	-1.90266660185027	0.0577809687958816	0.00871021360050935	0.160124418869579
BLCA	CBX7	cg26672614	-0.116888291768049	419	-2.38894928024418	0.0173457765868917	0.0136628727524525	0.0652576334171898
BLCA	CBX7	cg27062573	-0.0484279440472904	419	-0.984134611739509	0.325626901204238	0.00234526576464749	0.525033558951303
BLCA	CDYL2	cg01442398	-0.111309896067002	419	-2.27347206568014	0.0235116754537061	0.0123898929624469	0.0823125548015668
BLCA	CDYL2	cg02461391	-0.132406656961294	419	-2.7114344904483	0.00697953858476258	0.0175315228076657	0.0323210312037139
BLCA	CDYL2	cg06919916	-0.075807213118406	419	-1.54315882158845	0.123559955923141	0.00574673356077943	0.276308589487318
BLCA	CDYL2	cg10112854	0.154288207340192	419	3.16966247569964	0.00164008038283436	0.02380485092425	0.0101180343617935
BLCA	CECR2	cg03927893	-0.259812596510977	419	-5.46116116171028	8.19466595634462e-08	0.0675025853057757	1.81519317599053e-06
BLCA	CECR2	cg12632097	-0.153962321907347	419	-3.16280482974281	0.00167825657941437	0.0237043965671014	0.0103077761202401
BLCA	CECR2	cg13466481	-0.155320006604912	419	-3.19138175158173	0.00152439955806209	0.02412430445175	0.00952676246946354
BLCA	CECR2	cg26796679	-0.198303825796071	419	-4.10668434342553	4.84237325796193e-05	0.0393244073253586	0.000515896446493961
BLCA	CHAF1A	cg03006338	-0.0264111127375811	419	-0.53627410936747	0.592058541643142	0.000697546876037218	0.754488509382889
BLCA	CHAF1A	cg04346879	-0.0183796412507448	419	-0.373129001021754	0.709244333475031	0.000337811212506078	0.834340748198345
BLCA	CHAF1A	cg05297461	0.0676743584392762	419	1.37679580675931	0.169323053876227	0.00457981879016764	0.342797003415583
BLCA	CHAF1A	cg07783855	0.0589292157127226	419	1.19821474160556	0.231522236356868	0.00347265246451659	0.422003792708404
BLCA	CHAF1A	cg09700233	-0.0446530944239108	419	-0.907263774120808	0.364797658307985	0.00199389884163069	0.563725498200536
BLCA	CHAF1A	cg18804734	0.00279523736300593	419	0.0567373434465316	0.954781931431801	7.81335191554436e-06	0.97597192715476
BLCA	CHAF1A	cg23811775	-0.0243304870807742	419	-0.49400118944757	0.621568602367679	0.00059197260158772	0.775779044642888
BLCA	CHAF1A	cg24085258	-0.106829226740076	419	-2.18087679680848	0.0297574576424123	0.0114124836858825	0.098087015797374
BLCA	CHAF1B	cg00660452	0.0337370069152769	419	0.68517648986322	0.493617862578162	0.00113818563560144	0.678910010279083
BLCA	CHAF1B	cg02544117	0.013685054885096	419	0.277802290907951	0.781303482286821	0.000187280727208089	0.879172666141421
BLCA	CHAF1B	cg02908490	-0.11921452102557	419	-2.43717113096011	0.0152249471201761	0.0142121020233562	0.059069848699166
BLCA	CHAF1B	cg04634639	-0.0578703784555828	419	-1.17661226977617	0.240029646846995	0.00334898070259238	0.431982287207123

**BLCA**	CHAF1B	cg07872789	-0.101459713005313	419	-2.07008964078977	0.0390665221693399	0.0102940733631205	0.120376322954871
**BLCA**	CHAF1B	cg13854874	-0.07176020601173	419	-1.46033797713788	0.144959329988881	0.00514952716684593	0.308610068653127
**BLCA**	CHAF1B	cg25942896	-0.0594190034499107	419	-1.20820879358841	0.227659987896132	0.0035306179709805	0.417387375056366
**BLCA**	CHD1	cg03255775	0.137060272828471	419	2.8085245555159	0.00521367028788533	0.018785518387815	0.0256395642187086
**BLCA**	CHD1	cg03833628	-0.235659631117351	419	-4.9219927933136	1.2413155762171e-06	0.0555354617383661	2.07136216079435e-05
**BLCA**	CHD1	cg08865521	0.0110947029216958	419	0.225211735155177	0.821926223809528	0.000123092432920686	0.904386593830712
**BLCA**	CHD1	cg11365324	-0.0554071692499068	419	-1.12637299115	0.260663315581447	0.00306995440428781	0.455860485406629
**BLCA**	CHD1	cg12662497	-0.252866636778673	419	-5.30503988034313	1.84311953966036e-07	0.0639415359957573	3.77126884451178e-06
**BLCA**	CHD1	cg16890600	0.170954000729108	419	3.52183208318307	0.000476493497605407	0.0292252703652878	0.00359851811986361
**BLCA**	CHD1	cg21082921	-0.230399613777053	419	-4.80589881957947	2.16207681911874e-06	0.0530839820286154	3.38083626264368e-05
**BLCA**	CHD4	cg00079216	0.00235772812098445	419	0.0478567870949781	0.96185356824254	5.55888189248088e-06	0.980001978725799
**BLCA**	CHD4	cg00824109	-0.0602299700287348	419	-1.22475838488882	0.221366058476313	0.0036276492896623	0.409739730974
**BLCA**	CHD4	cg04344119	0.146337935400369	419	3.00266034185263	0.0028393378616646	0.0214147913372426	0.0157473882534796
**BLCA**	CHD4	cg05147525	-0.116918761452343	419	-2.38958064573927	0.0173164085631862	0.0136699967795498	0.0651723603175863
**BLCA**	CHD4	cg11565429	0.135988716744597	419	2.78615175499748	0.00558019834290264	0.0184929310818422	0.0270791560032292
**BLCA**	CHD4	cg19509514	-0.139723200837514	419	-2.86416698439421	0.00439485849626185	0.0195225728522801	0.0223854921664502
**BLCA**	CHD4	cg20308811	-0.107695030721918	419	-2.19875840699953	0.0284503276392219	0.0115982196421948	0.0948965170152812
**BLCA**	CHRAC1	cg00296189	-0.264196805698993	419	-5.56016899515947	4.85315371711431e-08	0.0697999521415515	1.13821597910519e-06
**BLCA**	CHRAC1	cg07270064	-0.283724355942923	419	-6.00577758041085	4.18583521740462e-09	0.0804995101552265	1.23112614329972e-07
**BLCA**	CHRAC1	cg08877624	-0.027620742214774	419	-0.560853815391953	0.575202079980566	0.000762905400494998	0.74217656114756
**BLCA**	CHRAC1	cg12002314	0.0226079937757574	419	0.459009474440553	0.646469425722646	0.000511121382564685	0.7927152754432
**BLCA**	CHRAC1	cg18770186	0.157420590889325	419	3.23563201005348	0.00131169506195461	0.0247812424359442	0.00840820745300734
**BLCA**	CHRAC1	cg19486048	0.230922186326327	419	4.81741241352802	2.0473034240055e-06	0.053325056137731	3.22634407982136e-05
**BLCA**	CHRAC1	cg22610305	-0.0461239544701826	419	-0.937211476557723	0.349198840325159	0.00212741917596748	0.548257433609004
**BLCA**	CHRAC1	cg23548819	-0.0723919179630964	419	-1.47326090016661	0.141444268106948	0.00524058978637568	0.303486647905561
**BLCA**	CLOCK	cg05960024	-0.0155760458511421	419	-0.31619755989065	0.752012689891023	0.00024261320435688	0.861122439869029
**BLCA**	CLOCK	cg06085476	-0.0688661743479161	419	-1.40115714950147	0.161919869301031	0.00474254996931757	0.332420085831273
**BLCA**	CLOCK	cg07236904	0.091696065068414	419	1.86910128915917	0.0623179814555263	0.00840816834903082	0.16925264613454
**BLCA**	CLOCK	cg07500410	0.0062934324309422	419	0.127745256474607	0.898412901573785	3.96072917628351e-05	0.946303441312686
**BLCA**	CLOCK	cg11774413	0.118094837720164	419	2.41395549073493	0.0162155644973919	0.0139463906961519	0.0619493014050045
**BLCA**	CLOCK	cg18928811	0.00587646008551227	419	0.119281171962116	0.905110738558659	3.45327831366188e-05	0.949691373297898
**BLCA**	CLOCK	cg22523077	0.098174098743077	419	2.00238959858955	0.0458977351523057	0.00963815366401544	0.135536304602129
**BLCA**	CLOCK	cg23506964	0.0697770341476579	419	1.41977966004346	0.156428036442737	0.00486883449444342	0.324866191490064
**BLCA**	CREBBP	cg00039463	-0.160896365034881	419	-3.30895083031531	0.00101878302792709	0.0258876402814375	0.00681647947661135
**BLCA**	CREBBP	cg00747661	0.0246309230049664	419	0.500104858866885	0.617268248797373	0.000606682368076581	0.772641118068084
**BLCA**	CREBBP	cg03039399	-0.0980999275752342	419	-2.00086207443854	0.0460628417858715	0.0096235957902662	0.135874292416111
**BLCA**	CREBBP	cg03264209	-0.210032685165126	419	-4.36046073985164	1.64167393761948e-05	0.044113728837673	0.00020159807782663
**BLCA**	CREBBP	cg08506924	-0.0557701254506464	419	-1.13377447798162	0.257548436858154	0.00311030689278084	0.452071397030929
**BLCA**	CREBBP	cg24567591	-0.136975408406724	419	-2.80675232332924	0.00524188762239903	0.0187622625081888	0.0257441544000575
**BLCA**	CTCF	cg01866162	-0.0930920720104382	419	-1.8978038938 08	0.0584206762279913	0.00866613387119662	0.161485644005109
**BLCA**	CTCF	cg04487155	0.0201798462287341	419	0.409689569007477	0.682246606681478	0.000407226193815356	0.816810189913185
**BLCA**	CTCF	cg06241380	0.0123287823039726	419	0.25026597026083	0.802506417696742	0.000151998873098747	0.892293174660554
**BLCA**	CTCF	cg07967402	0.0779646774989148	419	1.58734179054659	0.113202380247026	0.0060784909375098	0.260272635460743
**BLCA**	CTCF	cg08324636	0.188621696653179	419	3.89858253752721	0.000112927654430703	0.0355781444483239	0.00106978737807868
**BLCA**	CTCF	cg10218542	-0.152130769896657	419	-3.12428285885319	0.00190838842215826	0.0231437711493495	0.011450036164845
**BLCA**	CTCF	cg10481400	-0.0189464010921521	419	-0.384638982843065	0.700703372389937	0.000358966114344701	0.828515062587782
**BLCA**	CTCF	cg23858565	-0.0428622903415116	419	-0.87080975691314	0.384365143136916	0.00183717593332004	0.582530721706473
**BLCA**	CXXC1	cg02159718	-0.10768256470006	419	-2.19850090812882	0.0284687924707895	0.0115955347403827	0.094949678091001
**BLCA**	CXXC1	cg07675399	-0.0190101591314971	419	-0.385933829193312	0.699744881985121	0.000361386150204842	0.827923244854
**BLCA**	CXXC1	cg11871295	0.113617525171842	419	2.32121476489442	0.0207624233920582	0.0129089420261741	0.0749396576330336
**BLCA**	CXXC1	cg13423076	-0.198129283380718	419	-4.10292197786141	4.91866401559617e-05	0.039255212932957	0.000522592936942487
**BLCA**	CXXC1	cg15632775	0.135181252564728	419	2.76929946214105	0.00587152456089289	0.0182739710449688	0.028187569484236
**BLCA**	CXXC1	cg20016845	-0.0760561448229655	419	-1.54825559890615	0.122328538710772	0.0057845371653319	0.274280215023801
**BLCA**	CXXC1	cg27419474	-0.0954182174387636	419	-1.94565578108692	0.05237581152087	0.00910463621919116	0.149255426259371
**BLCA**	DAXX	cg00221327	-0.060320353999176	417	-1.22362220890601	0.221797521268794	0.00363854510658591	0.410266015112251
**BLCA**	DAXX	cg02173004	-0.241172292067615	419	-5.04415464458134	6.84253623252401e-07	0.0581640744611471	1.22089778063353e-05
**BLCA**	DAXX	cg02328295	-0.0433963145377884	419	-0.881679579552247	0.378464182862029	0.00188324011546266	0.577064050088592

**BLCA**	DAXX	cg03085224	-0.17120103421756	419	-3.52707478430049	0.000467429985185018	0.029309794117162	0.00354417624439408
**BLCA**	DAXX	cg03369465	-0.103324269126863	419	-2.10853908940109	0.0355875198737342	0.0106759045906004	0.1122484398742
**BLCA**	DAXX	cg03382121	-0.0475098974386634	419	-0.965435796476962	0.334892916706033	0.00225719035463232	0.534479311550738
**BLCA**	DAXX	cg03902329	-0.0601268229487269	419	-1.22265330304513	0.222159616724551	0.00361523483790755	0.4106809215947
**BLCA**	DAXX	cg04086445	-0.210420843897694	419	-4.36889222796212	1.58226739844644e-05	0.0442769315466176	0.000195165546210151
**BLCA**	DAXX	cg04152866	-0.0579539790276259	419	-1.17831774866572	0.239350066666036	0.0033586636851345	0.431208149098391
**BLCA**	DAXX	cg04271391	0.0137031448548001	419	0.278169580500837	0.78102173113955	0.000187776178911635	0.879071784832532
**BLCA**	DAXX	cg04488476	-0.023264134113908	419	-0.472338185676244	0.636935506651383	0.000541219936069897	0.786316921438351
**BLCA**	DAXX	cg04973788	-0.111494891068303	419	-2.27729805632141	0.0232802229420506	0.0124311107343327	0.0816934306262746
**BLCA**	DAXX	cg05431670	-0.0671052128696532	416	-1.36184673151277	0.173994051224229	0.00450310959428147	0.349136117939699
**BLCA**	DAXX	cg05532204	-0.0692519961556662	419	-1.40904483612867	0.159576194118001	0.00479583897154441	0.329207097648195
**BLCA**	DAXX	cg06171013	-0.134945416219871	419	-2.76437846919458	0.005959152218581	0.0182102653587541	0.0285213662909001
**BLCA**	DAXX	cg07689821	-0.142518843329955	419	-2.92265073266233	0.00366187841989789	0.0203116207041082	0.0193212269260402
**BLCA**	DAXX	cg08375286	-0.0379803751995025	419	-0.771474048901821	0.44086823671465	0.00144250890029499	0.633430816362331
**BLCA**	DAXX	cg08567382	-0.13258607198407	419	-2.71517426338521	0.00690259895709929	0.017579066484165	0.0320623825691774
**BLCA**	DAXX	cg09097215	-0.142084038495707	419	-2.91355013581036	0.00376806953721445	0.0201878739952495	0.0197889152486371
**BLCA**	DAXX	cg09432361	-0.0032779206310425	419	-0.0665348795385961	0.946984263655433	1.0744763663414e-05	0.971710141185216
**BLCA**	DAXX	cg09615909	-0.192092999604212	419	-3.97305330473818	8.37569075185535e-05	0.0368997204969437	0.000825409787623303
**BLCA**	DAXX	cg11111423	-0.343224046163882	419	-7.4172593369403	6.88104421698112e-13	0.117802745865107	3.98588200905308e-11
**BLCA**	DAXX	cg12025215	0.0014827936844541	419	0.0300974577211703	0.976003902197024	2.19867711065696e-06	0.987381599702393
**BLCA**	DAXX	cg12832355	-0.264910174143454	419	-5.57631369962828	4.45260044361695e-08	0.0701774003647152	1.05284376568613e-06
**BLCA**	DAXX	cg12923297	-0.182288745837043	419	-3.76310814804159	0.000192162037269575	0.0332291868588422	0.00167834888597163
**BLCA**	DAXX	cg13443893	0.0398190630232717	419	0.808880223250735	0.419050895523128	0.00158555778005128	0.614438430456993
**BLCA**	DAXX	cg13826470	-0.119827977258117	418	-2.44989459546744	0.0147050435040809	0.0143587441337718	0.057479859027056
**BLCA**	DAXX	cg14539675	-0.0865681186622765	419	-1.76376217838047	0.0785131371272317	0.00749403916872598	0.200204896737769
**BLCA**	DAXX	cg14622330	0.025851481009161	419	0.524903180585907	0.599932826368106	0.000668299070367009	0.760092086487092
**BLCA**	DAXX	cg14746369	0.0338163980144337	419	0.686790714810254	0.492600988305548	0.0011435487746706	0.678050874679155
**BLCA**	DAXX	cg14910283	-0.274928818026878	419	-5.80410929454959	1.29277454656502e-08	0.0755858549816564	3.45483006099208e-07
**BLCA**	DAXX	cg16922466	-0.0615132175333802	419	-1.25095091375026	0.2116622891557	0.00378387593130895	0.397740431473671
**BLCA**	DAXX	cg17079757	-0.0517173745030104	419	-1.05115474785015	0.293803667576811	0.00267468682548463	0.491522970559761
**BLCA**	DAXX	cg17504991	-0.0159225251396105	419	-0.323232938873487	0.746682830051944	0.000253526806821527	0.857746401870335
**BLCA**	DAXX	cg18187593	-0.120182390149358	419	-2.4572466362225	0.0144118398362104	0.0144438069020125	0.0566211134901343
**BLCA**	DAXX	cg18279148	-0.290343421077841	400	-6.02259040757989	3.93785891239337e-09	0.0842993021631847	1.16170571191024e-07
**BLCA**	DAXX	cg18486541	0.00993413545782635	419	0.201650877519178	0.840289191496313	9.86870472944428e-05	0.914424968625297
**BLCA**	DAXX	cg18944910	-0.386722036917058	419	-8.51185350047071	3.22322677848408e-16	0.149553933837278	2.91567097680686e-14
**BLCA**	DAXX	cg18967278	-0.0532658741200463	419	-1.08271622227289	0.279567658143493	0.00283725334577262	0.476652195967715
**BLCA**	DAXX	cg20223162	-0.224731778560875	419	-4.68130149406991	3.87471383016998e-06	0.0505043722951343	5.67450089804675e-05
**BLCA**	DAXX	cg20763184	0.015691808990849	419	0.31854815674439	0.750230590529928	0.00024623286940529	0.860059300982195
**BLCA**	DAXX	cg21286626	0.00668001763660935	419	0.135592574576478	0.892209598235906	4.4622635625412e-05	0.94289726518997
**BLCA**	DAXX	cg24328964	-0.196066937741656	419	-4.05849753278287	5.91041165227946e-05	0.0384422440753905	0.00061379597393863
**BLCA**	DAXX	cg24496586	-0.0874310557027064	419	-1.78147864994977	0.0755708274257425	0.00764418950128974	0.194938555234373
**BLCA**	DAXX	cg24513883	-0.347845408276628	419	-7.53077310486087	3.21810054704291e-13	0.120996428059134	1.9635208937777e-11
**BLCA**	DAXX	cg24728112	-0.302550392241396	419	-6.44306695017248	3.27261489529174e-10	0.0915367398454225	1.18029579584759e-08
**BLCA**	DAXX	cg25642825	-0.188777277183356	419	-3.9019169791457	0.000111437890984541	0.0356368603807615	0.00105825186660784
**BLCA**	DAXX	cg26521092	-0.141413235523938	419	-2.89951340058219	0.00393737499504136	0.0199977031813489	0.0205132701824436
**BLCA**	DAXX	cg27052777	-0.299709315625728	418	-6.36881835366498	5.10603780687846e-10	0.0898256738728425	1.77250827821644e-08
**BLCA**	DAXX	cg27393652	-0.0212142911353717	419	-0.430700009073248	0.666911741453785	0.00045004614837631	0.806731850144699
**BLCA**	DIDO1	cg00080059	-0.0280332408881762	419	-0.569236358726251	0.569505945646681	0.000785862594694511	0.737713892627513
**BLCA**	DIDO1	cg01488865	-0.17723990767112	419	-3.65545149922556	0.000289937970176358	0.031413984871267	0.00237600700009476
**BLCA**	DIDO1	cg06646488	-0.137150400877566	419	-2.81040677757001	0.00518385259102329	0.0188102324608771	0.0255138952591462
**BLCA**	DIDO1	cg08610063	-0.018142486885701	419	-0.368312884298555	0.712829111844861	0.000329149830397834	0.836561549296933
**BLCA**	DIDO1	cg13815390	0.0734328258290278	419	1.494558634289	0.135795159669478	0.00539237990923633	0.294895699727197
**BLCA**	DIDO1	cg20969846	-0.251001823011862	419	-5.26327585030042	2.2820142391619e-07	0.063001915155278	4.55597187953415e-06
**BLCA**	DIDO1	cg21212389	-0.00914011371426506	419	-0.185531795938085	0.852903247407279	8.35416787096962e-05	0.921640671585758
**BLCA**	DIDO1	cg21687703	0.0829413425449154	419	1.68934614241255	0.0919096580091239	0.00687926630315299	0.224194780357512
**BLCA**	DIDO1	cg22394521	-0.156495790205005	419	-3.21614493595388	0.00140173255139341	0.0244909323518889	0.00888651145816145
**BLCA**	DMAP1	cg01604592	-0.113082616774986	419	-2.31014464937197	0.021373532007743	0.0127876782166784	0.0766059686252181

**BLCA**	DMAP1	cg11683482	-0.126843936314843	419	-2.5956163176476	0.00977943769567956	0.0160893841798439	0.0418599645432115
**BLCA**	DMAP1	cg15990015	-0.0431859778279363	419	-0.877398180443401	0.380781736001743	0.001865028680955	0.57916465033178
**BLCA**	DMAP1	cg18017761	-0.0515995026749937	419	-1.04875260193226	0.294906810191601	0.00266250867630668	0.49278246395836
**BLCA**	DMAP1	cg18167340	-0.158332807453174	419	-3.25486246755329	0.00122812081353304	0.0250692779160039	0.00796834283302666
**BLCA**	DMAP1	cg21437596	-0.0443196127035132	419	-0.900474690604121	0.368393728541479	0.00196422807018941	0.567265527565881
**BLCA**	DMAP1	cg23021241	-0.155699218623402	419	-3.19936681372516	0.00148380075808217	0.0242422466799378	0.00931897044976046
**BLCA**	DMAP1	cg23451311	-0.0169578261663988	419	-0.344255779894368	0.730829577275905	0.0002875678682898	0.847864034448555
**BLCA**	DMAP1	cg24485408	-0.09320135681847	419	-1.90005131864209	0.0581242889831575	0.00868649291280376	0.160858090969611
**BLCA**	DNMT1	cg04160663	-0.0390885085509663	419	-0.794016895105361	0.427642683462686	0.00152791150073896	0.621964243948121
**BLCA**	DNMT1	cg04762346	-0.0892589031171277	419	-1.81901854701522	0.0696343742925169	0.00796715178567279	0.183637971243502
**BLCA**	DNMT1	cg06128182	-0.0134819792935466	419	-0.273679165474229	0.784468337919419	0.000181763765671619	0.881021976229305
**BLCA**	DNMT1	cg06716436	0.0586438564922664	419	1.19239242643379	0.233793712734558	0.00343910190428554	0.424709230535966
**BLCA**	DNMT1	cg10635912	0.0142319899647842	419	0.288907106298405	0.772797774090139	0.000202549538357719	0.874033725284649
**BLCA**	DNMT1	cg11380607	0.027461929102504	419	0.5576265900696	0.577402238478196	0.000754157550030957	0.743953588064738
**BLCA**	DNMT1	cg13393778	-0.0500576248915748	419	-1.01733421417144	0.309591496776584	0.00250576580978561	0.508237547485388
**BLCA**	DNMT1	cg15043801	0.0171355719255957	419	0.347865197957741	0.728119079266335	0.000293627825217263	0.846278237842008
**BLCA**	DNMT1	cg21893394	-0.0512497557742344	419	-1.04162526148218	0.298196288972116	0.00262653746691867	0.496391888051202
**BLCA**	DNMT1	cg23401624	-0.0421124485396036	419	-0.855548323837854	0.392744929057755	0.00177345832200076	0.589613829119029
**BLCA**	DNMT1	cg24477899	-0.0565837877858081	419	-1.15036851141911	0.25065937661752	0.00320172504018937	0.444303961582474
**BLCA**	DNMT1	cg27429799	0.0194009868572161	419	0.393871156931067	0.693879932506782	0.000376398291033871	0.824495119435353
**BLCA**	DNMT3A	cg02208653	-0.0778729040277288	419	-1.58546189835002	0.113628627294942	0.00606418918171185	0.260840550968831
**BLCA**	DNMT3A	cg03463641	-0.0676213225325109	419	-1.3757118633824	0.169658279030578	0.00457264326104586	0.343235149284912
**BLCA**	DNMT3A	cg06112956	-0.0128471972968271	419	-0.260791147301621	0.794383871632533	0.000165050478383603	0.887437827377082
**BLCA**	DNMT3A	cg07150430	0.0125321826325864	419	0.25439550312793	0.799316946054777	0.0001570556015365	0.890414011618606
**BLCA**	DNMT3A	cg12066181	-0.0814697530466633	419	-1.65917077031997	0.0978423704261095	0.0066373206614843	0.234403466522812
**BLCA**	DNMT3A	cg13076778	-0.130149739090576	419	-2.66441375022174	0.00801505240900383	0.016938954585345	0.0359877055930996
**BLCA**	DNMT3A	cg13344237	-0.0908475067500456	419	-1.85165994565406	0.0647899471435452	0.00825326948269959	0.17413690763456
**BLCA**	DNMT3A	cg15990840	-0.0891749372796139	419	-1.81729367358236	0.0698984577245902	0.00795216943882307	0.184178316631444
**BLCA**	DNMT3A	cg15998962	-0.158617221151269	419	-3.26085994457193	0.00120308352701706	0.0251594228457505	0.00783530179160862
**BLCA**	DNMT3A	cg21629895	-0.279890176264927	419	-5.91766727517597	6.87678730106254e-09	0.0783385107696117	1.94535560472313e-07
**BLCA**	DNMT3A	cg21708767	-0.0998713923909556	419	-2.03735391146101	0.0422531184011911	0.00997429501810823	0.127429311606988
**BLCA**	DNMT3A	cg22731525	-0.0390278821663964	419	-0.7927834925633	0.428360252141683	0.00152317558639413	0.622566363841131
**BLCA**	DNMT3A	cg25044635	-0.130774050177361	419	-2.67741642975883	0.00771570462007726	0.0171018521997909	0.0349583881895335
**BLCA**	DNMT3A	cg26470599	-0.252709197344834	419	-5.30151146285402	1.87678151348433e-07	0.0638619384226703	3.83409509086134e-06
**BLCA**	DNMT3A	cg26995204	-0.089999576239846	419	-1.83423556839607	0.0673400422378437	0.00809992372335185	0.179185253995226
**BLCA**	DNMT3B	cg00300969	0.282540143755972	419	5.97853096526193	4.8834095938105e-09	0.0798289328336451	1.4159521652661e-07
**BLCA**	DNMT3B	cg13636640	-0.109039878520236	419	-2.22654385053748	0.0265177615715157	0.0118896951077079	0.0900525032169763
**BLCA**	DNMT3B	cg14224313	0.13250755634983	419	2.71353762987958	0.00693617538155896	0.0175582524898035	0.0321818350112124
**BLCA**	DNMT3B	cg16523653	-0.0292301662934857	419	-0.593561200593212	0.55313135708639	0.000854402621544826	0.725197195516696
**BLCA**	DNMT3B	cg16953106	0.0228370649760459	419	0.4636627153463	0.643134271758597	0.000521531536720141	0.790569597338879
**BLCA**	DNMT3B	cg17475857	0.237286327507998	419	4.95798775755848	1.04281274716098e-06	0.056304801222233	1.7716657534973e-05
**BLCA**	DNMT3B	cg17721173	-0.163185869536758	419	-3.3573150444151	0.000860174014456984	0.0266296280164678	0.00592337587684045
**BLCA**	DNMT3B	cg22605822	-0.111152185569875	419	-2.27021055093452	0.0237105600887723	0.0123548083569599	0.0828596788799222
**BLCA**	DNMT3B	cg24403338	0.232057305622694	419	4.84243714981128	1.81774908154466e-06	0.0538505930928642	2.90134980174523e-05
**BLCA**	DNMT3B	cg25803642	-0.00817045071641162	419	-0.165847572492214	0.868358259132844	6.67562649093112e-05	0.93007300378594
**BLCA**	DNMT3L	cg09247319	-0.0790021358463428	418	-1.60859597264214	0.108470685841748	0.006241337468284	0.252471906602428
**BLCA**	DNMT3L	cg15635600	-0.0879960859258937	418	-1.79308115223113	0.0736933137884398	0.00774331113827727	0.191377275098741
**BLCA**	DNMT3L	cg27187798	-0.102381308580091	418	-2.08909129762355	0.03731255841973	0.0104819323465719	0.116344596784995
**BLCA**	DPF1	cg06168449	-0.108708937309451	419	-2.21970524504227	0.0269825345881563	0.0118176330509501	0.0912106648995906
**BLCA**	DPF1	cg06615189	-0.0255961201680059	419	-0.519714772559116	0.603541494581332	0.000655161367654998	0.762552897899372
**BLCA**	DPF1	cg07480327	-0.0410614207613148	409	-0.823973845905451	0.410443003012493	0.00168604027493774	0.6063146754959
**BLCA**	DPF1	cg08057062	-0.0837210343006812	419	-1.70533844623716	0.0888850761048826	0.00700921158437584	0.21893128199415
**BLCA**	DPF1	cg13322582	-0.0186226845564395	419	-0.378064775373345	0.705577182951869	0.000346804380088652	0.83207772629584
**BLCA**	DPF1	cg13917712	-0.11587255554217	419	-2.36790600680629	0.0183500171326712	0.0134264491278733	0.068128675766187
**BLCA**	DPF1	cg17121541	-0.0720930794858569	419	-1.4671473442173	0.14309888744731	0.00519741210975409	0.305839029920494
**BLCA**	DPF1	cg22145401	-0.0207093192270364	419	-0.420443439142754	0.674380742299384	0.000428875902847298	0.811724790207874
**BLCA**	DPF1	cg27400772	-0.0161665729064492	320	-0.28696570825533	0.774327246393932	0.000261358079539536	0.874929393396702

**BLCA**	DPF2	cg02186298	0.148727458718211	419	3.05279014904679	0.00241417045721485	0.0221198569767771	0.013836880317437
**BLCA**	DPF2	cg14699126	-0.121533234026495	419	-2.4852776889326	0.0133408712160499	0.0147703269729388	0.053359724942332
**BLCA**	DPF2	cg15918224	-0.159606028646319	419	-3.28171757673426	0.00111964850662763	0.0254740843802496	0.00737776858585378
**BLCA**	DPF2	cg15929437	-0.0823078212189399	419	-1.67635425058648	0.0944274115134566	0.00677457743380898	0.228573217103673
**BLCA**	DPF2	cg18224556	-0.037527963728928	419	-0.762271429956181	0.446334187522147	0.00140834806163974	0.638153238345855
**BLCA**	DPF2	cg19517014	-0.201885469638345	419	-4.18397922389565	3.50305221181776e-05	0.0407577428510951	0.000390312867038314
**BLCA**	DPF3	cg00888802	-0.00067863234888971	419	-0.0137747354148126	0.98901636652615	4.60541864959569e-07	0.994255889590719
**BLCA**	DPF3	cg05151458	-0.0405009151487127	419	-0.822753860713476	0.411123913656461	0.00164032412788323	0.606956650453386
**BLCA**	DPF3	cg07171024	-0.0192073943238612	419	-0.389939460129033	0.696782818638091	0.000368923996712296	0.826191873999401
**BLCA**	DPF3	cg10266394	-0.00350225997466665	419	-0.0710885494124967	0.943361775213304	1.22658249301521e-05	0.969792367964991
**BLCA**	DPF3	cg12913429	-0.0253391342494163	418	-0.513868684481955	0.60761995774684	0.000642071724509943	0.765543908639814
**BLCA**	DPF3	cg13819165	0.0382051459843103	419	0.77604634596897	0.438166874849167	0.00145963317968246	0.630913290929613
**BLCA**	DPF3	cg14238615	0.195052436592905	419	4.03666502682375	6.46483534451273e-05	0.0380454530208291	0.000664278696065279
**BLCA**	DPF3	cg14242696	0.0650513726459715	419	1.32320130538008	0.18650229544924	0.00423168108312505	0.365478350853398
**BLCA**	DPF3	cg15392845	-0.0379152580477113	419	-0.770149454910193	0.441652606974566	0.00143756679282454	0.63419083924578
**BLCA**	DPF3	cg15740492	0.0786241580001885	419	1.6008518179477	0.110176199711664	0.00618175822123861	0.25532626174057
**BLCA**	DPF3	cg16696021	0.0126190766992156	419	0.256159678529935	0.797955393966774	0.000159241096740686	0.889585472175973
**BLCA**	DPF3	cg19710444	0.0341747642797312	419	0.694077383811667	0.488024842005396	0.00116791451357519	0.67418908983259
**BLCA**	DPF3	cg19839347	-0.0155666268515643	419	-0.316006305524963	0.752157747467604	0.000242319871535843	0.861201855971137
**BLCA**	DPF3	cg20548765	0.164594431615407	419	3.38709758398013	0.000774272763740852	0.0270913269187988	0.0054186555759676
**BLCA**	DPF3	cg21035356	-0.0346218029925266	419	-0.703167407655514	0.482348576894195	0.00119866924245333	0.669357808536808
**BLCA**	DPF3	cg22784589	-0.00986649249220453	419	-0.200277673382454	0.841362243824427	9.73476740987284e-05	0.915041722299419
**BLCA**	DPF3	cg23515460	0.1296523628531	419	2.65405708183869	0.00826090681775302	0.016809735193392	0.0368385775282475
**BLCA**	DPF3	cg24383902	0.0471480150061863	419	0.958065635020077	0.338591523434931	0.00222293531902357	0.537954190305061
**BLCA**	DPF3	cg25495195	-0.0606294222661913	419	-1.23291099607158	0.218311988170414	0.00367592684433213	0.40597033053806
**BLCA**	DPF3	cg26057784	0.158920441562461	417	3.25931504101881	0.00120995001834883	0.0252557067464077	0.00787277113812556
**BLCA**	EED	cg00080012	0.0965368024396875	419	1.96867795634005	0.0496599968965047	0.00931935422527926	0.143556071784801
**BLCA**	EED	cg01739327	0.0794303864530983	419	1.6173709786627	0.106563556325453	0.00630918629208854	0.249188568617959
**BLCA**	EED	cg02172616	-0.22207544876724	419	-4.62307998955019	5.06700601112095e-06	0.049317504945171	7.20088073818124e-05
**BLCA**	EED	cg04607174	-0.346879664050373	419	-7.50700053546799	3.77592267824555e-13	0.1203255013317	2.26654420394625e-11
**BLCA**	EED	cg06358433	-0.0699131216996649	419	-1.42256227258816	0.155619758200531	0.00488784458579215	0.323706074211933
**BLCA**	EED	cg10086030	-0.269262124346971	419	-5.67502013052677	2.61818426364777e-08	0.0725020916078439	6.551271360766e-07
**BLCA**	EED	cg10535675	-0.187252268782678	418	-3.86454716766701	0.000129297641517122	0.0350634121642602	0.00119987841156901
**BLCA**	EED	cg12672785	0.0974158196836811	419	1.98677474266447	0.0476093087584409	0.00948984192464348	0.139248009833602
**BLCA**	EED	cg16265078	-0.198279509498172	419	-4.10616016679095	4.85293424993609e-05	0.0393147638868355	0.000516667807635867
**BLCA**	EED	cg18574463	0.147994991529799	419	3.03741795364675	0.00253788069598636	0.0219025175179054	0.0144205602851189
**BLCA**	EED	cg19325359	-0.2255204205321	417	-4.68718979358191	3.77590141239096e-06	0.0508594600769754	5.55383767206546e-05
**BLCA**	ELP3	cg01209934	0.0789043729028824	419	1.60659290126598	0.108909803968121	0.00622590006319712	0.253111154583213
**BLCA**	ELP3	cg02516513	0.067085408103434	419	1.36475954532128	0.17307350489336	0.00450045198040429	0.347961560706275
**BLCA**	ELP3	cg04720865	-0.326632810388307	419	-7.01466722545815	9.51351558365404e-12	0.106688992822163	4.53211531729812e-10
**BLCA**	ELP3	cg05621091	-0.239308163383243	419	-5.00278787608293	8.38277987513711e-07	0.0572683970618607	1.46381588688309e-05
**BLCA**	ELP3	cg15692687	0.0150414163176928	419	0.305341949264495	0.760259933285808	0.000226244204842154	0.866611884039389
**BLCA**	ELP3	cg17572663	0.0753957135209822	419	1.5347341674301	0.1256166806427	0.00568451361733802	0.279360940955358
**BLCA**	ELP3	cg18022570	0.00518267142884191	419	0.105198153522698	0.916269771687261	2.68600831393343e-05	0.955798304202007
**BLCA**	ELP3	cg18249244	-0.149477984960624	418	-3.06482045084095	0.0023216003000883	0.0223436679878885	0.0134100515106229
**BLCA**	ELP3	cg19781013	0.0962561080561677	419	1.96290013628114	0.0503301870093193	0.00926523833812063	0.144975958733769
**BLCA**	ELP3	cg22414525	-0.193884359286154	419	-4.01154417186495	7.16385689497921e-05	0.0375911447758025	0.000723397044766371
**BLCA**	ELP3	cg22487209	-0.0978100726529203	419	-1.99489295627408	0.0467128521394704	0.00956681031236955	0.13729709911357
**BLCA**	ELP3	cg25374552	0.0707255853526872	419	1.43917656436561	0.150859793844212	0.00500210842348024	0.316914744067741
**BLCA**	ELP4	cg04972224	-0.217464895177377	419	-4.52228188508525	8.00974468803718e-06	0.0472909806345076	0.000107692720058964
**BLCA**	ELP4	cg05195750	0.122618822789049	419	2.50781464349507	0.0125316435403452	0.0150353757021722	0.050867922950853
**BLCA**	ELP4	cg05635455	-0.361480715625702	419	-7.86938847906578	3.17422960077451e-14	0.13066830776927	2.2387671583196e-12
**BLCA**	ELP4	cg08111895	-0.277947037778946	419	-5.87313120577492	8.818649568697e-09	0.077254555810091	2.44054793506657e-07
**BLCA**	ELP4	cg08626560	-0.366557395158663	419	-7.99692494858705	1.30200131872761e-14	0.134364323945504	9.64984696320063e-13
**BLCA**	ELP4	cg09616795	0.216707075463865	419	4.5057447012554	8.62778842557522e-06	0.0469619565561012	0.000114797534678823
**BLCA**	ELP4	cg10583382	-0.213996799737045	419	-4.44667059539612	1.12308975336255e-05	0.0457946302976968	0.00014499944854572
**BLCA**	ELP4	cg11385473	-0.0584829268602802	419	-1.18910903471575	0.23508164066333	0.00342025273414488	0.426232721618493

**BLCA**	ELP4	cg13263383	-0.223798261470454	418	-4.65516841889895	4.37514435669598e-06	0.0500856618371976	6.3287463756937e-05
**BLCA**	ELP4	cg19328333	0.0525548900215313	419	1.0682240062936	0.286044969447819	0.00276201646517525	0.483320981998251
**BLCA**	ELP4	cg24001477	-0.0324732885982778	419	-0.659483578697399	0.50995373973677	0.00105451447238704	0.6923705809495
**BLCA**	ELP4	cg24531794	-0.154729367995351	419	-3.17894756065254	0.00158966824583694	0.0239411773202407	0.00986413428385091
**BLCA**	ELP4	cg25130734	-0.387523086552839	419	-8.53259668390385	2.76790550190532e-16	0.150174142611439	2.51843790193243e-14
**BLCA**	EP300	cg00500400	-0.0421730738360619	419	-0.856782168483211	0.392063345036666	0.00177856815678192	0.589124929764784
**BLCA**	EP300	cg02046995	0.0764117602955126	419	1.55553718915307	0.120585979763119	0.00583875711145888	0.271531368232374
**BLCA**	EP300	cg07519229	-0.27053057333737	417	-5.69693358552141	2.32815059907994e-08	0.0731867911102464	5.90339464487694e-07
**BLCA**	EP300	cg08131204	-0.169457340136648	418	-3.4858454809029	0.000543421523072534	0.0287157901261877	0.00402426786919607
**BLCA**	EP300	cg09331127	0.00101346904363089	419	0.0205711854213108	0.983597684984046	1.0271195023981e-06	0.991435472157568
**BLCA**	EP300	cg24349919	0.0808441362500632	419	1.6463456363335	0.100455162404772	0.00653577436601878	0.238999599489781
**BLCA**	EPC1	cg02900516	-0.0245303505734695	419	-0.49806160992051	0.618706368189894	0.000601738099257314	0.773688979049231
**BLCA**	EPC1	cg16942190	-0.0378826099174579	419	-0.769485339924908	0.442046170289477	0.00143509213415828	0.634463275108256
**BLCA**	EPC1	cg26211455	0.0113763740725719	419	0.230930117922277	0.817483725345097	0.000129421887039086	0.901906214650616
**BLCA**	EPC2	cg00066942	-0.104275690722174	419	-2.12816720777524	0.0339161814068403	0.0108734196755864	0.108200755233288
**BLCA**	EPC2	cg03664802	-0.0985997949536435	419	-2.01115728628856	0.0449597034870579	0.00972191956490054	0.133485809215792
**BLCA**	EPC2	cg04098458	-0.114122899023121	419	-2.33167553123402	0.0201990568228151	0.0130240360814415	0.0733768585792238
**BLCA**	EPC2	cg05586854	0.183235992608756	419	3.78334034382763	0.000177671693646913	0.0335754289873162	0.00157155107851824
**BLCA**	EPC2	cg10458926	-0.0447759831074893	419	-0.90976564153519	0.363478027650962	0.00200488866324217	0.562412428805993
**BLCA**	EPC2	cg11721798	-0.120869626610495	419	-2.47150558193786	0.0138579003934137	0.0146094666369604	0.0549498701873297
**BLCA**	EPC2	cg12432635	-0.0861801205982698	419	-1.75579771593842	0.0798660097909709	0.00742701318633232	0.202805697148815
**BLCA**	EPC2	cg16434205	0.0743328085919101	419	1.51297689397119	0.131052214672099	0.00552536643316155	0.287736396144763
**BLCA**	EPC2	cg21347644	-0.120526817568211	419	-2.46439244888413	0.0141318318339506	0.0145267137531208	0.0557377674756819
**BLCA**	EPC2	cg21650422	-0.111763465699551	419	-2.2828530333206	0.0229477102689838	0.0124910722651748	0.0807707072964133
**BLCA**	EZH1	cg00728999	-0.238922668617898	419	-4.99424072685714	8.74040923427555e-07	0.0570840415794979	1.51978407682439e-05
**BLCA**	EZH1	cg01716316	0.00997527220036185	419	0.202485986329022	0.839636764345339	9.95060554713119e-05	0.914174783354765
**BLCA**	EZH1	cg03623386	-0.145896829435583	419	-2.99341228177224	0.00292489780430749	0.0212858848393556	0.0161364087503225
**BLCA**	EZH1	cg05375741	-0.263227317534229	419	-5.53824359795165	5.45367873951003e-08	0.0692886206962661	1.26118752634198e-06
**BLCA**	EZH1	cg05979501	-0.158871461664443	419	-3.26622185965005	0.00118110099683289	0.0252401413313965	0.0077121513301677
**BLCA**	EZH1	cg06975499	-0.0623982986251289	419	-1.26902003763198	0.205150362928002	0.00389354767131077	0.389407264630611
**BLCA**	EZH1	cg12812489	-0.166150942912669	419	-3.42003313224693	0.000688619551806003	0.0276061358307689	0.00491923502416861
**BLCA**	EZH1	cg19395400	-0.0600849108913226	419	-1.22179795016257	0.222482644863727	0.00361019651681818	0.411081059932015
**BLCA**	EZH1	cg22227424	-0.121358191065411	419	-2.48164462554616	0.0134755740519463	0.0147278105386688	0.0537860235231927
**BLCA**	EZH1	cg27088844	-0.0421219570972011	419	-0.855741841158033	0.392637981161542	0.00177425926969845	0.589508351735245
**BLCA**	EZH2	cg01893439	-0.0541457056979443	419	-1.10065239889819	0.271690613111751	0.0029317574455284	0.467991487642883
**BLCA**	EZH2	cg02303805	-0.105519626575342	419	-2.15383890390452	0.0318322317503044	0.0111343915925996	0.10312437193502
**BLCA**	EZH2	cg06816161	0.00787642682980662	419	0.159878963002615	0.873054772533464	6.20380996052975e-05	0.932714754243021
**BLCA**	EZH2	cg13751213	0.0408866923885739	419	0.830603774554298	0.406678561585909	0.00167172161447787	0.602894417983342
**BLCA**	EZH2	cg18494018	-0.0148684279882074	419	-0.301829491381073	0.762934342763379	0.00022107015084051	0.868122094920758
**BLCA**	EZH2	cg20101066	-0.0106573974596461	419	-0.216333828328101	0.82883461312881	0.00011358012061287	0.907992450483617
**BLCA**	FBXL19	cg00026853	-0.0512516279323894	419	-1.04166341235619	0.298178615988463	0.00262672936572008	0.496375738588116
**BLCA**	FBXL19	cg02291855	0.0555818021380096	419	1.12993408932166	0.259161395316931	0.00308933672890885	0.453937983232434
**BLCA**	FBXL19	cg03181514	-0.0312963546190066	419	-0.635557947443799	0.525417431634082	0.000979461812438616	0.704365391634357
**BLCA**	FBXL19	cg03627896	-0.0207077786310905	419	-0.420412148334199	0.674403578590137	0.000428812095834248	0.811724790207874
**BLCA**	FBXL19	cg04477789	-0.327597960415387	419	-7.03788227432709	8.20073118630822e-12	0.107320423668321	3.96499491552307e-10
**BLCA**	FBXL19	cg05643349	-0.213260554148746	419	-4.43064179186902	1.2057873351602e-05	0.0454800639558304	0.000154332049936579
**BLCA**	FBXL19	cg05768582	-0.11479882239656	419	-2.34566938162653	0.0194663722232341	0.013178769623637	0.0713113091323355
**BLCA**	FBXL19	cg06862768	-0.0933989476164816	419	-1.90411491814367	0.0575915706456122	0.00872336341586627	0.159798788986152
**BLCA**	FBXL19	cg07697662	-0.140954968209552	419	-2.88992637643529	0.00405696425850232	0.0198683030629558	0.0209761715755636
**BLCA**	FBXL19	cg08535091	-0.0117246187784686	419	-0.238000128356068	0.811999312663252	0.000137466685500419	0.898725481620422
**BLCA**	FBXL19	cg08593668	0.0278556215695962	419	0.565626853182326	0.571955375393073	0.000775935653028552	0.739667466932569
**BLCA**	FBXL19	cg09011601	-0.24096299848539	419	-5.03950728003937	7.00087466469969e-07	0.0580631666390701	1.24567742100408e-05
**BLCA**	FBXL19	cg11940219	-0.202632578238538	418	-4.19502405291555	3.34492309725413e-05	0.0410599617635973	0.000374571681917069
**BLCA**	FBXL19	cg26666512	-0.14100636392711	419	-2.89100148842722	0.00404339040582295	0.0198827946679446	0.0209170123073876
**BLCA**	FBXL19	cg27602786	-0.103654581094723	419	-2.11535286042714	0.0349995045138968	0.0107442721819225	0.110856701850661
**BLCA**	FBXW9	cg02753619	-0.0481939936312096	419	-0.979369263511249	0.327972283675531	0.00232266102212507	0.527503221887493
**BLCA**	FBXW9	cg03160342	-0.0300187427474996	419	-0.609588649793139	0.542470358456606	0.00090112491614056	0.717032351809926

**BLCA**	FBXW9	cg05868316	-0.0457640000859419	419	-0.929882005550449	0.352976685738193	0.00209434370386609	0.552004589211318
**BLCA**	FBXW9	cg06489804	-0.0012256973470727	419	-0.0248789576223829	0.980163556709896	1.50233398662106e-06	0.989801346921421
**BLCA**	FBXW9	cg12110584	-0.124919990395033	419	-2.55561748895441	0.010958065780527	0.0156050040002951	0.0457955892186924
**BLCA**	FBXW9	cg14342428	0.00245856968998029	419	0.0499036652008646	0.960223340213594	6.04456492048977e-06	0.979140587075492
**BLCA**	FBXW9	cg15828427	0.0631219396611352	419	1.28379555886633	0.199935023719085	0.003984379266584	0.382874977965966
**BLCA**	FBXW9	cg19721801	-0.0188258490928463	419	-0.382190734734891	0.702516957496771	0.000354412594066622	0.829788816689175
**BLCA**	FBXW9	cg21564527	-0.0403358137775022	419	-0.81939444009068	0.413035143326125	0.00162697787309334	0.608611723457004
**BLCA**	FBXW9	cg22303873	-0.043643534781783	419	-0.886711892396282	0.375751324598896	0.0019047581282487	0.574296625616555
**BLCA**	FKBP1A	cg04613791	0.194134048843499	419	4.01691254082009	7.00869935434216e-05	0.0376880289203699	0.000710652211107175
**BLCA**	FKBP1A	cg07516946	0.0987311505309276	419	2.01386292559872	0.0446735307183777	0.00974784008516069	0.132879584335301
**BLCA**	FKBP1A	cg10996143	-0.0428090352272756	419	-0.869725814142241	0.384956670866044	0.00183261349709012	0.583063729086737
**BLCA**	FKBP1A	cg21332426	-0.089703221066247	419	-1.82814662778075	0.0682504910243904	0.00804666786965997	0.181031698958417
**BLCA**	FKBP1A	cg21775445	0.0344842151589493	419	0.700369670766432	0.484091777274636	0.00118916109512871	0.670800942649811
**BLCA**	FKBP1A	cg24641773	-0.101243481858969	419	-2.06563212210401	0.0394880070768286	0.0102502426189274	0.121343420071686
**BLCA**	FKBP1A	cg26779912	0.0134271388896202	419	0.272565724520582	0.785323616449374	0.000180288058761151	0.881452393854603
**BLCA**	FKBP2	cg03764062	-0.079097781732114	419	-1.61055571268167	0.108042428820202	0.00625645907494114	0.251761351134906
**BLCA**	FKBP2	cg04446112	-0.0906477504378472	419	-1.8475547293771	0.065383437870042	0.00821701465944222	0.1753460826552
**BLCA**	FKBP2	cg06615912	-0.118013177535937	419	-2.41226270573065	0.0162899715939571	0.0139271100721285	0.0621603006906508
**BLCA**	FKBP2	cg09854726	-0.0559387433539366	419	-1.13721312648981	0.256110151360805	0.00312914300801759	0.450378252067509
**BLCA**	FKBP2	cg10998122	-0.0924261138019083	419	-1.88411006071863	0.0602539462513856	0.0085425865125233	0.165162410712458
**BLCA**	FKBP2	cg12423493	0.0848545178308567	419	1.72859302809415	0.0846311364944752	0.00720028919630717	0.211382048201218
**BLCA**	FKBP2	cg15010817	-0.077644661268293	419	-1.5807867384178	0.11469417513666	0.00602869342346796	0.26243473809763
**BLCA**	FKBP2	cg16977872	-0.0556309342924454	419	-1.13093600653789	0.258739915774676	0.00309480085025037	0.453403906108374
**BLCA**	FKBP2	cg19924313	-0.0586564372457482	419	-1.1926491111313	0.233693238742487	0.0034405776303644	0.424606030800005
**BLCA**	FKBP2	cg21809711	-0.0900348466271791	419	-1.8349602696898	0.067232352382079	0.00810627360717967	0.178984108589811
**BLCA**	FKBP2	cg22074794	-0.0572051505519291	419	-1.16304228603028	0.245485521282243	0.00327242924966887	0.438383505328622
**BLCA**	FKBP2	cg24677501	-0.0278412218085149	419	-0.565334229611221	0.572154171332176	0.000775133631790928	0.739813868835438
**BLCA**	FKBP5	cg00130530	-0.129620498906774	419	-2.65339366104689	0.00827688401520122	0.0168014737368411	0.0368855387935682
**BLCA**	FKBP5	cg00140191	-0.105115771177934	419	-2.14550323312812	0.0324964783180088	0.0110493253503318	0.10474977352578
**BLCA**	FKBP5	cg00610228	-1.56333918353228e-05	419	-0.0003173231971013	0.99974696631081	2.44402940276739e-10	0.99985346304832
**BLCA**	FKBP5	cg01294490	-0.111855563209461	419	-2.28475801911869	0.0228346379279071	0.0125116670209057	0.0804856065861813
**BLCA**	FKBP5	cg07485685	-0.125566205754585	419	-2.569049013369	0.0105488658718025	0.0157668720276028	0.0444260757704095
**BLCA**	FKBP5	cg07843056	-0.114485279329237	419	-2.33917759338025	0.0198033179963103	0.0131068791830934	0.0722564288825554
**BLCA**	FKBP5	cg08636224	0.155592430533329	419	3.19711803194933	0.00149513200680253	0.0242090044392687	0.00937699930161204
**BLCA**	FKBP5	cg08915438	0.345852984483471	419	7.48175775754485	4.47265435587509e-13	0.119614286876124	2.65607383939654e-11
**BLCA**	FKBP5	cg10913456	-0.110357918645487	419	-2.25378743469012	0.0247344426712883	0.0121788702077638	0.0854968112134633
**BLCA**	FKBP5	cg16012111	-0.104075037808992	419	-2.12402718721868	0.0342629884422991	0.010831613494943	0.109029939169641
**BLCA**	FKBP5	cg17030679	0.0158148801674021	419	0.321047159000745	0.748337444241355	0.000250110434709289	0.858873493513203
**BLCA**	FKBP5	cg20813374	-0.219375099962641	419	-4.56400465288413	6.63348224613925e-06	0.0481254344836185	9.11638461316292e-05
**BLCA**	FKBP5	cg25114611	-0.233003885989664	419	-4.86332135718399	1.64536809969926e-06	0.0542908108862845	2.65725069437418e-05
**BLCA**	FMR1	cg02921434	-0.0400677901351962	418	-0.813940938819868	0.416148953459258	0.00160542780631812	0.611728424575971
**BLCA**	FMR1	cg03513471	-0.041063657648972	418	-0.834204842337879	0.404648970287701	0.00168622397951197	0.601093412155078
**BLCA**	FMR1	cg04552106	-0.0426670178898379	418	-0.866835261074814	0.386536828239305	0.00182047441561174	0.584501147145522
**BLCA**	FMR1	cg04744025	-0.084390634783141	418	-1.71907517786687	0.0863518095593514	0.00712177923910149	0.214518686663266
**BLCA**	FMR1	cg05288927	-0.0377844596309681	418	-0.767488821573737	0.443230545822951	0.00142766538960426	0.635401767934566
**BLCA**	FMR1	cg07147350	-0.117081121630996	418	-2.39294504327355	0.0171606501394511	0.0137079890423721	0.0647427554291202
**BLCA**	FMR1	cg08434396	-0.0638868593800593	418	-1.29941613143775	0.194527786010125	0.00408153080144747	0.375921587963152
**BLCA**	FMR1	cg14470409	-0.0522581998030979	418	-1.06217695779042	0.28877756251728	0.0027309194466605	0.48630101520628
**BLCA**	FMR1	cg16783314	-0.0217219166266112	418	-0.4410108087713	0.659436499010122	0.000471841661933449	0.801767207440802
**BLCA**	FMR1	cg22625568	-0.182476172112795	416	-3.75795591862227	0.000196152425446706	0.0332975533889384	0.00170821468545853
**BLCA**	FXR2	cg01807919	0.0357300942238656	419	0.725705083652509	0.468431602625539	0.00127663963324631	0.657426810294988
**BLCA**	FXR2	cg01933235	-0.157910884547371	419	-3.24596684224743	0.00126614739964694	0.0249358474585333	0.00816781280916286
**BLCA**	FXR2	cg05529097	-0.295984137145717	419	-6.28964275986052	8.1313868929589e-10	0.0876066094418943	2.73959771308291e-08
**BLCA**	FXR2	cg05913233	-0.324207161155369	419	-6.95643053345875	1.37851370637752e-11	0.105110283344424	6.38333900440077e-10
**BLCA**	FXR2	cg09625173	-0.287420891894279	419	-6.09102135134098	2.57513620564886e-09	0.0826107690973027	7.8839097198838e-08
**BLCA**	FXR2	cg17777561	-0.309306386400269	419	-6.60197889856104	1.25238321776878e-10	0.0956704406679926	4.91315683525491e-09
**BLCA**	FXR2	cg19178740	-0.113853961058862	419	-2.32610853900215	0.0204971792699285	0.0129627244487929	0.0742103394668785

**BLCA**	FXR2	cg22521096	-0.132194640104261	419	-2.70701550613862	0.00707144770069712	0.017475422872295	0.0326575365050776
**BLCA**	FXR2	cg26904212	0.0170359876631617	419	0.345842972754079	0.729637254504347	0.000290224875659397	0.847121865187839
**BLCA**	G2E3	cg02259031	0.138659221891607	419	2.84192739658192	0.00470696216679653	0.0192263798155859	0.0236347650425205
**BLCA**	G2E3	cg05076167	-0.237076807678984	419	-4.95334910533663	1.06655682617472e-06	0.0562054127392582	1.80923946217698e-05
**BLCA**	G2E3	cg07906495	0.12785318581867	419	2.61661048609496	0.00920734344307628	0.0163464371239833	0.0400109527321347
**BLCA**	G2E3	cg08810065	0.0315629029328561	419	0.64097631394626	0.521894443487451	0.000996216841548899	0.701758816639232
**BLCA**	G2E3	cg09837748	-0.229916435153839	419	-4.79525708064839	2.27365607329387e-06	0.0528615671538497	3.53612156269139e-05
**BLCA**	G2E3	cg18580322	-0.0824077217529382	419	-1.67840281711336	0.0940267659090618	0.00679103260450968	0.227872841966653
**BLCA**	G2E3	cg18755078	0.0692180771747695	419	1.40835137584767	0.159781204382258	0.00479114220777234	0.32947502852729
**BLCA**	G2E3	cg18956956	-0.278905885437303	419	-5.89509785283858	7.80199705855124e-09	0.0777884929315658	2.18248313258876e-07
**BLCA**	G2E3	cg19554565	0.0548691514666955	419	1.11540243118823	0.265328173233419	0.00301062378267517	0.461014026417169
**BLCA**	G2E3	cg21061067	-0.16510392842779	419	-3.39787558401142	0.000745206963914523	0.0272593071822889	0.00525447571383725
**BLCA**	G2E3	cg22926526	-0.054262341841986	419	-1.10303032615499	0.270657862915416	0.00294440174217655	0.46684310646184
**BLCA**	GADD45/	cg01810953	0.026471473134974	419	0.537500577612267	0.591212073582322	0.000700738889935651	0.753967181722647
**BLCA**	GADD45/	cg02639608	-0.0930470919369208	419	-1.89687890579767	0.0585430278243531	0.0086577613179178	0.161757993484913
**BLCA**	GADD45/	cg04718306	-0.0628563380321481	419	-1.27837218969389	0.201837914565873	0.00395091923081167	0.385240808890634
**BLCA**	GADD45/	cg07000785	0.00619245925825916	419	0.125695605086259	0.900034193048967	3.83465516651995e-05	0.947318531937754
**BLCA**	GADD45/	cg22224769	-0.0306235917920521	419	-0.621882695416905	0.53436297600336	0.000937804374246239	0.711124986831438
**BLCA**	GADD45/	cg23369529	-0.252320139341004	419	-5.29279410787934	1.96252441275123e-07	0.0636654527170637	3.99128408049181e-06
**BLCA**	GADD45/	cg23723105	-0.171212828732937	419	-3.52732511210627	0.000467001285835618	0.0293138327227341	0.00354196139517364
**BLCA**	GATAD2A	cg02887458	-0.0274749990236069	419	-0.557892181026118	0.577221021924153	0.000754875571347198	0.743836988830431
**BLCA**	GATAD2A	cg08505228	-0.00017782375539267	419	-0.0036094280794228	0.997121846311232	3.16212879819538e-08	0.998528025129523
**BLCA**	GATAD2A	cg08870822	0.0297794178171815	419	0.604724363002633	0.545695049218043	0.000886813725530268	0.719522045065566
**BLCA**	GATAD2A	cg09334331	-0.123507073400242	419	-2.52626162833238	0.0119020525959702	0.0152539971798927	0.0488399767723865
**BLCA**	GATAD2A	cg11795262	0.0756622368031703	419	1.54019062316895	0.124281555849773	0.00572477407805902	0.277335284433363
**BLCA**	GATAD2A	cg14948912	0.0271487261065388	419	0.551262147019446	0.581752811283828	0.00073705332920786	0.74709970833204
**BLCA**	GATAD2A	cg15264323	0.150672228560719	419	3.09362979388199	0.00211192278345091	0.0227021204594535	0.0124146079050764
**BLCA**	GATAD2A	cg22191731	0.000865118647454917	419	0.0175599972591364	0.985998369767145	7.48430274174224e-07	0.992714095280661
**BLCA**	GATAD2A	cg26631329	-0.0831915486817344	419	-1.69447779348448	0.0909302082540616	0.00692083377206538	0.222447315123159
**BLCA**	GATAD2E	cg02134200	0.281163741832585	419	5.94689972252211	5.83625628299386e-09	0.0790530497213004	1.67105528626877e-07
**BLCA**	GATAD2E	cg02940454	0.118089422089593	419	2.41384322520621	0.016220489880025	0.013945111609454	0.0619559478434068
**BLCA**	GATAD2E	cg09805553	0.102694547297145	419	2.0955509565408	0.0367318211836063	0.0105461700445655	0.114958172633764
**BLCA**	GATAD2E	cg10226922	0.0183864012162658	419	0.373266282918623	0.709142244694101	0.000338059749685501	0.834292720111722
**BLCA**	GATAD2E	cg10489353	0.160587003370395	419	3.30242000212649	0.0010421714967863	0.0257881856514834	0.00694786951932411
**BLCA**	GATAD2E	cg15064304	-0.00265354872893024	419	-0.0538613462530469	0.957071740086499	7.04132085680731e-06	0.977413048221714
**BLCA**	GATAD2E	cg20773694	0.0234869975860983	419	0.476865530351311	0.633710707003357	0.000551639055609387	0.784264886105937
**BLCA**	GATAD2E	cg23046518	0.112458169232282	419	2.29722406340739	0.0221066250443402	0.0126468398270765	0.0785244762629652
**BLCA**	GATAD2E	cg23323627	0.177374340486616	419	3.65831411223543	0.000286820721928695	0.0314616566630619	0.00235378121558521
**BLCA**	GATAD2E	cg26398333	0.235181019367838	419	4.91141059868799	1.306303908952e-06	0.0553101118708955	2.16771771102841e-05
**BLCA**	GLYR1	cg00636946	-0.140346794756087	419	-2.87720621785539	0.00422074392647176	0.0196972227983072	0.0216717413382578
**BLCA**	GLYR1	cg00885266	0.0110728199365849	419	0.224767477206163	0.822271601014889	0.000122607341348031	0.904546729411957
**BLCA**	GLYR1	cg01261535	-0.116888652533599	419	-2.38895675566461	0.0173454286107345	0.0136629570911203	0.0652576334171898
**BLCA**	GLYR1	cg01565912	0.118718138232495	419	2.42687791572816	0.0156573647796209	0.0140939963453898	0.060317779429822
**BLCA**	GLYR1	cg02454264	0.00651481738810348	419	0.132239156809314	0.89485968703247	4.24428456003355e-05	0.944171576311239
**BLCA**	GLYR1	cg06399529	-0.0346889673086457	419	-0.704533154023399	0.481498859771344	0.00120332445294029	0.668744595748267
**BLCA**	GLYR1	cg06976003	-0.224900936299728	419	-4.68501280307033	3.80865740488779e-06	0.0505804311484941	5.59182101405711e-05
**BLCA**	GLYR1	cg11289281	0.173825037649717	419	3.58280557311651	0.00038056237427308	0.0302151437139256	0.00298141685238671
**BLCA**	GLYR1	cg15663121	0.260217975413896	419	5.47030044039528	7.81031413241279e-08	0.0677133947285071	1.74096154044963e-06
**BLCA**	GLYR1	cg18354686	0.0379325663613688	419	0.770501534587703	0.441444041606826	0.00143887959075965	0.634037043904103
**BLCA**	GTF2B	cg02705666	-0.119034913051429	419	-2.43344648621115	0.0153801854060721	0.0141693105251613	0.0595414851196801
**BLCA**	GTF2B	cg04270788	0.11594578155965	419	2.36942279669344	0.01827596016703	0.013443424261478	0.0679227997914167
**BLCA**	GTF2B	cg05306856	-0.0893328670200466	419	-1.82053798747032	0.0694024248929316	0.00798036113002133	0.183222646101388
**BLCA**	GTF2B	cg06356032	-0.0302679146059343	419	-0.614653187221056	0.539123076687265	0.00091614665459213	0.714599428822647
**BLCA**	GTF2B	cg07167781	0.0181997490604659	419	0.369475755408221	0.711962965636061	0.000331230865863929	0.836059700042141
**BLCA**	GTF2B	cg10430205	-0.250160254938775	419	-5.24444876289382	2.51154872854377e-07	0.0625801531510329	4.96779612988391e-06
**BLCA**	GTF2B	cg12074130	-0.119262577745168	419	-2.43816775484969	0.0151836446772722	0.0142235624504221	0.0589419388235385
**BLCA**	GTF2B	cg13868361	-0.250748677587547	419	-5.25761128644566	2.34884530764413e-07	0.0628748993119038	4.66852508591895e-06

**BLCA**	GTF2B	cg14010405	-0.233324695899342	419	-4.87040263262771	1.59058316892303e-06	0.0544404137165203	2.57894595754893e-05
**BLCA**	GTF2B	cg17811845	-0.272529589500085	419	-5.74937529708385	1.7463932599053e-08	0.0742723771530848	4.54557788153879e-07
**BLCA**	GTF2B	cg22607980	0.00382531325170349	419	0.0776459468875804	0.938147386175526	1.46330214736583e-05	0.966895480453875
**BLCA**	GTF2H1	cg05849260	-0.324813932139064	418	-6.97098386615553	1.25676561925792e-11	0.10550409051164	5.8720014765496e-10
**BLCA**	GTF2H1	cg07583091	-0.376441989854693	418	-8.24763211046814	2.19359262142475e-15	0.141708571725761	1.78502452291963e-13
**BLCA**	GTF2H1	cg08690812	-0.42219711764475	418	-9.45353253077869	2.51385540711016e-19	0.178250406147535	3.14916050790222e-17
**BLCA**	GTF2H1	cg10797557	-0.0427361155520044	418	-0.868241635307453	0.385767520316039	0.00182637557247428	0.583825817081997
**BLCA**	GTF2H1	cg11063328	-0.0884636180174068	418	-1.80268291652269	0.0721686607668896	0.00782581171272967	0.18845586907789
**BLCA**	GTF2H1	cg11176342	-0.0401881802561361	418	-0.816390505649299	0.414748596374074	0.00161508983229968	0.610315747480889
**BLCA**	GTF2H1	cg11347316	0.0475899140565695	418	0.967065480367123	0.334078624400222	0.00226479991991167	0.533661447773939
**BLCA**	GTF2H1	cg11662851	-0.0125594255620165	418	-0.254948604781223	0.79889000815625	0.000157739170447833	0.890155107486713
**BLCA**	GTF2H1	cg13777411	0.125233981192923	418	2.56214334383817	0.0107575157010802	0.0156835500454294	0.0451386287189771
**BLCA**	GTF2H1	cg14132868	0.046581102167796	418	0.946520547108455	0.344437942205543	0.00216979907916665	0.543667679989003
**BLCA**	GTF2H1	cg17174493	0.0931380063078798	418	1.89874851695316	0.0582959475067248	0.00867468821900666	0.161224179328741
**BLCA**	GTF2H1	cg18321761	0.0784563190621292	418	1.59741329251289	0.110940259513357	0.00615539400077862	0.256455191174104
**BLCA**	H2AFZ	cg02758870	-0.055800217370974	419	-1.13438813958357	0.257291348836863	0.00311366425864795	0.451740615496409
**BLCA**	H2AFZ	cg09308803	-0.0395663983031605	419	-0.803739545496438	0.422010887081104	0.00156549987468434	0.617126864724001
**BLCA**	H2AFZ	cg16267491	-0.0739498091375724	419	-1.50513831060274	0.133054751928235	0.00546857427148339	0.290770868349489
**BLCA**	H2AFZ	cg17709884	-0.0255821863155089	419	-0.519431668221379	0.603738682246471	0.000654448256681412	0.762702940024065
**BLCA**	H2AFZ	cg20934652	-0.0205657078603951	419	-0.417526583494831	0.676510779060603	0.000422948339799118	0.813108992603176
**BLCA**	H2AFZ	cg23752380	0.124742993406861	419	2.55193920067329	0.0110725690127097	0.0155608144041041	0.0461717106440147
**BLCA**	H2AFZ	cg24061702	-0.0715867471602729	419	-1.45678984149471	0.145936086477081	0.00512466236898884	0.3099467473772
**BLCA**	H3F3A	cg06319713	-0.260134171210468	419	-5.46841081808632	7.8883170784165e-08	0.0676697870313569	1.75533106063596e-06
**BLCA**	H3F3A	cg10632229	0.050693099153291	419	1.03028218896866	0.303481995808717	0.00256979030176539	0.501968370759751
**BLCA**	H3F3A	cg10983539	-0.0186109741095644	419	-0.377826955635419	0.705753720280788	0.000346368357306877	0.832223434981621
**BLCA**	H3F3A	cg11978873	0.0736988088741461	419	1.50000162630143	0.134379870660877	0.00543153442946792	0.292696484998104
**BLCA**	H3F3A	cg15538837	-0.001545046149461	419	-0.0313610490999484	0.974996790565185	2.38716760396425e-06	0.986832077714679
**BLCA**	H3F3A	cg15624314	0.16978032266738	419	3.49693287187106	0.000521829644091002	0.0288253579650396	0.00388694133784139
**BLCA**	H3F3A	cg16724613	-0.0859020565701765	419	-1.75009037219689	0.0808471281301696	0.0073791633229858	0.204487482061268
**BLCA**	H3F3A	cg16727519	-0.041792152963938	419	-0.849029832421531	0.396357736075479	0.00174658404936119	0.592995447359652
**BLCA**	H3F3A	cg18947521	0.0456249528782453	419	0.927050792811804	0.354442905130212	0.0020816363251421	0.553451985341516
**BLCA**	H3F3A	cg19410789	0.00213492756369586	419	0.0433343954439248	0.965455967126812	4.55791570222834e-06	0.98181926373398
**BLCA**	H3F3A	cg21266749	-0.0192140878031768	419	-0.390075398166048	0.69668237692393	0.000369181170108189	0.826159398043051
**BLCA**	H3F3A	cg21789564	-0.0477245579802348	419	-0.969807790782097	0.332711288991736	0.0022776334344088	0.532362726451704
**BLCA**	H3F3A	cg22519151	-0.0712127571657659	419	-1.44914025263048	0.148059112462427	0.00507125678315034	0.313000641183239
**BLCA**	H3F3A	cg27530209	-0.0878811877094015	419	-1.79072165643601	0.0740719941059271	0.00772310315321506	0.192104170871999
**BLCA**	H3F3A	cg27560444	-0.216780284722115	416	-4.49090169658817	9.23847782891621e-06	0.0469936918442012	0.000122144484062323
**BLCA**	HAT1	cg00414306	-0.155707082713411	419	-3.19953242308745	0.00148296942679818	0.024244695607121	0.0093149877745933
**BLCA**	HAT1	cg01583365	0.0917065928955427	419	1.86931770476585	0.0622878076542386	0.00841009918050881	0.169189507042154
**BLCA**	HAT1	cg04507121	0.149502460464448	418	3.06533375604015	0.00231770915363344	0.0223509856849239	0.0133945372481806
**BLCA**	HAT1	cg04950839	-0.134097284409307	419	-2.74668518489668	0.00628407150382748	0.0179820816859505	0.0297524836326916
**BLCA**	HAT1	cg05421799	0.113293983585875	419	2.31451871539829	0.0211302146534729	0.0128355267167565	0.0759613640828222
**BLCA**	HAT1	cg09545452	-0.109478886525708	419	-2.23561671677706	0.0259118649096429	0.0119856265949089	0.0885049007192114
**BLCA**	HAT1	cg10190161	-0.133866122791808	419	-2.74186387925972	0.0063753423393592	0.0179201388313114	0.0300802633398902
**BLCA**	HAT1	cg12941622	-0.00722749549320471	419	-0.146705967862658	0.883435865996267	5.22366911042944e-05	0.938175001833507
**BLCA**	HAT1	cg13293756	-0.108551639826954	419	-2.21645509460913	0.027205888301538	0.0117834585091207	0.0917763702584383
**BLCA**	HAT1	cg14446129	-0.0592669578373987	419	-1.20510623021669	0.228854023708739	0.00351257229129999	0.418816484048028
**BLCA**	HAT1	cg16017358	0.0577252276328237	419	1.17365120470621	0.241212778891836	0.00333220190526131	0.433409712701207
**BLCA**	HDAC1	cg03762702	-0.0948382203827003	419	-1.93372149637067	0.053832010713133	0.00899428804535763	0.152278369565378
**BLCA**	HDAC1	cg04967775	-0.169529716227127	419	-3.49161832417216	0.000532014975735613	0.0287403246840501	0.00395070380129594
**BLCA**	HDAC1	cg06473843	-0.0727996430344657	419	-1.48160267201006	0.139210438249994	0.00529978802594563	0.300075833561098
**BLCA**	HDAC1	cg10842753	-0.158022510786286	419	-3.24832014007725	0.00125598288707447	0.0249711139152018	0.0081179738050076
**BLCA**	HDAC1	cg14172124	-0.117899185363085	419	-2.40989977371162	0.0163943387428774	0.013900217909279	0.0624696898591009
**BLCA**	HDAC1	cg20002354	-0.247727645003648	419	-5.19009888238648	3.30687910139216e-07	0.0613689860990536	6.35355279856019e-06
**BLCA**	HDAC1	cg20793809	0.0608454669424575	419	1.23732060236764	0.216672813453694	0.00370217084744569	0.403984393869635
**BLCA**	HDAC1	cg21930229	-0.0509986813575332	419	-1.0365089618953	0.300572722125978	0.0026008655002072	0.498879584336311
**BLCA**	HDAC1	cg23018091	-0.231458208630006	419	-4.8292269149351	1.93564534216359e-06	0.0535729023422115	3.07058714821114e-05

**BLCA**	HDAC1	cg26115470	-0.0501009937606185	419	-1.01821782805731	0.309171988817687	0.00251010957580153	0.507795327733204
**BLCA**	HDAC10	cg00711351	-0.140158030768679	418	-2.87325882996461	0.0042727793588031	0.0196442735889538	0.0218998944155177
**BLCA**	HDAC10	cg01120165	-0.0506807029998771	418	-1.0300296017543	0.303600404700945	0.00256853365656175	0.502066009108143
**BLCA**	HDAC10	cg04321608	-0.122094549082928	418	-2.49692952198681	0.012916880604038	0.0149070789157634	0.0520731621345563
**BLCA**	HDAC10	cg04586023	-0.195391401618554	418	-4.043958199115	6.27435306044807e-05	0.0381777998264629	0.000647337930953053
**BLCA**	HDAC10	cg07482373	-0.148610971967974	418	-3.05034511360822	0.00243346938260483	0.022085220989266	0.0139228739538398
**BLCA**	HDAC10	cg07744063	-0.0372412301465561	418	-0.756439151237961	0.449818257295066	0.00138690922282876	0.640924736781158
**BLCA**	HDAC10	cg09151829	-0.045310199846373	418	-0.920642141312878	0.357776032541592	0.00205301421011826	0.556812481005494
**BLCA**	HDAC10	cg10508940	-0.0523058385209827	418	-1.06314789639252	0.288337620394344	0.00273590074338312	0.485882934886285
**BLCA**	HDAC10	cg11645658	-0.125625901009915	418	-2.57028994088934	0.0105117574382422	0.0157818670045531	0.044303372962976
**BLCA**	HDAC10	cg12464820	-0.158862963666448	418	-3.2660426266907	0.00118182975011703	0.0252374412248871	0.00771561460058781
**BLCA**	HDAC10	cg26943378	-0.0589444155119253	418	-1.19852487834173	0.231401684649389	0.0034744441200425	0.421890896332574
**BLCA**	HDAC11	cg01409735	0.0695829735294131	419	1.41581179702095	0.157586121907523	0.00484179020519501	0.326321730400177
**BLCA**	HDAC11	cg01712016	0.0106343404449667	419	0.215865742471301	0.829199230560304	0.000113089196699455	0.908212836738775
**BLCA**	HDAC11	cg02762546	-0.0947877927421774	419	-1.93268396824973	0.0539601952847017	0.00898472565293399	0.152535589824785
**BLCA**	HDAC11	cg02941903	-0.0172715401740432	419	-0.350626277656425	0.726047941501832	0.000298306099983587	0.844820640489306
**BLCA**	HDAC11	cg05987698	0.0170093499795336	419	0.345302051730646	0.730043528862941	0.000289317986726259	0.847382300159085
**BLCA**	HDAC11	cg07450135	-0.0377887875165	419	-0.767576856536654	0.443178283352825	0.00142799246196718	0.635401767934566
**BLCA**	HDAC11	cg08051076	-0.0133283191704726	419	-0.270559364715823	0.786865437835735	0.000177644091909987	0.88256504062943
**BLCA**	HDAC11	cg08448040	0.0429565761595945	419	0.872728847763665	0.383319229187642	0.00184526743535504	0.581591405133762
**BLCA**	HDAC11	cg10455785	-0.0721390411533126	419	-1.46808758956873	0.142843446715192	0.00520404125851934	0.30546932469519
**BLCA**	HDAC11	cg11432034	0.0686537507803273	419	1.3968146619175	0.163221238054495	0.00471333749620729	0.334133992735839
**BLCA**	HDAC11	cg13397067	-0.0194877428424317	419	-0.395633110259182	0.692580489375985	0.000379772121092748	0.823520289444961
**BLCA**	HDAC11	cg13706066	0.0634324891156135	419	1.29013707346396	0.197726700327469	0.00402368067540243	0.380139158888768
**BLCA**	HDAC11	cg16254764	0.0524145520741001	419	1.06536364628893	0.287335340851918	0.00274728526912855	0.484827361592758
**BLCA**	HDAC11	cg18521047	0.0314856033060283	419	0.639404961100638	0.522914870744972	0.000991343215544582	0.702550942724217
**BLCA**	HDAC11	cg19779799	-0.0154548210696024	419	-0.313736076789571	0.753880280625003	0.000238851494293426	0.862501430114082
**BLCA**	HDAC11	cg20894727	0.00128262720456017	419	0.0260345102501559	0.979242417697374	1.64513254587783e-06	0.989314363813271
**BLCA**	HDAC11	cg20922324	-0.0571554343016348	419	-1.16202818690479	0.245896722284549	0.00326674367020849	0.438906241129869
**BLCA**	HDAC11	cg21655280	0.0078392932483792	419	0.159125163804815	0.873648236744097	6.14545186340837e-05	0.933118113697488
**BLCA**	HDAC11	cg25127083	0.0100646527427805	419	0.204300486452809	0.838219571326899	0.00010129723483276	0.913422043274069
**BLCA**	HDAC11	cg25150878	0.00602639982188681	419	0.122324777925353	0.902701453144277	3.63174948132373e-05	0.94855468417086
**BLCA**	HDAC11	cg27025856	-0.00262111528938967	419	-0.0532030124624308	0.957595942343849	6.87024536027229e-06	0.977640372428737
**BLCA**	HDAC2	cg00161229	-0.0228611536703487	419	-0.464152045594787	0.642783967734883	0.000522632347139299	0.790228325212499
**BLCA**	HDAC2	cg01129566	-0.0187434749290038	419	-0.380517836441192	0.703757166854022	0.000351317852414194	0.830776037166682
**BLCA**	HDAC2	cg08198132	-0.0916460319868909	419	-1.86807279119717	0.0624615459736302	0.00839899517894223	0.16953817059672
**BLCA**	HDAC2	cg10999867	0.0246338267863141	419	0.50016385287506	0.617226748315926	0.000606825422138127	0.772637823058546
**BLCA**	HDAC2	cg13393754	-0.022284400272674	419	-0.45243627655337	0.651192847051777	0.000496594495512753	0.79606041050876
**BLCA**	HDAC2	cg15005908	0.00302225566363357	419	0.0613453701902498	0.951113905840632	9.13402929636516e-06	0.974102246315845
**BLCA**	HDAC2	cg15242771	-0.0351129363061372	419	-0.713154532432184	0.476153872501462	0.00123291829603885	0.663952578551331
**BLCA**	HDAC2	cg18387216	-0.0625903062169539	419	-1.27294030982723	0.203757041039061	0.00391754643233206	0.387651842718503
**BLCA**	HDAC2	cg18674783	0.150856756741474	419	3.09750673686838	0.00208511984149905	0.0227577610545562	0.0122991217067767
**BLCA**	HDAC2	cg22867643	-0.131972215398094	419	-2.70238000154659	0.00716903096749654	0.0174166656370808	0.0330238983352336
**BLCA**	HDAC2	cg24023618	0.131887689783236	419	2.70061853069103	0.00720642911693444	0.0173943627163591	0.0331647507526501
**BLCA**	HDAC3	cg01947930	-0.0859605079093793	419	-1.75129006841206	0.0806400836469739	0.00738920892003846	0.204143009021565
**BLCA**	HDAC3	cg01961137	-0.205952077234718	419	-4.27195237776471	2.40864420278776e-05	0.0424162581172953	0.000281517528713448
**BLCA**	HDAC3	cg04284814	-0.0746891954016351	419	-1.52027141946837	0.129209814356283	0.00557847590974363	0.284942617001716
**BLCA**	HDAC3	cg08520975	-0.0923233923755123	419	-1.88199806848487	0.0605409066138476	0.0085236087797228	0.165707152899551
**BLCA**	HDAC3	cg10414058	0.158333814305574	419	3.25488369774373	0.00122803133962674	0.025069596752552	0.00796834283302666
**BLCA**	HDAC3	cg11064797	0.0793769358661411	419	1.61627570816006	0.106800131594045	0.00630069794749748	0.249582392442463
**BLCA**	HDAC3	cg11153694	0.041935617795802	419	0.851949522616527	0.394737044475463	0.00175859603991558	0.59142894414553
**BLCA**	HDAC3	cg12082871	0.0166757983715089	419	0.338528811567348	0.73513717085776	0.000278082251327218	0.85064221363309
**BLCA**	HDAC3	cg14098966	-0.155691558698122	419	-3.19920550445406	0.00148461091794041	0.0242398614498506	0.00932304018003358
**BLCA**	HDAC3	cg17940889	-0.0919474632550196	419	-1.87426933665803	0.0616007322575164	0.00845433599903318	0.167864507874843
**BLCA**	HDAC3	cg18671773	-0.151922435723669	419	-3.11990318248289	0.00193631767546694	0.0230804264762124	0.0115827679542695
**BLCA**	HDAC3	cg19549288	0.104167372066065	419	2.12593226387655	0.0341030253303392	0.01085084140315	0.108640898957756
**BLCA**	HDAC3	cg20642138	-0.158763933167598	419	-3.26395400971208	0.00119035277115982	0.0252059864748455	0.00776474119399689

**BLCA**	HDAC3	cg23421926	-0.109767836633773	419	-2.24158909704437	0.0255196113826514	0.0120489779592588	0.0875186319829829
**BLCA**	HDAC3	cg24385322	0.0158393393283111	419	0.321543812348331	0.747961380015619	0.000250884670357382	0.858593885154716
**BLCA**	HDAC6	cg07153944	-0.0913539317502668	418	-1.86206855854492	0.0633051460398515	0.00834554084623241	0.171182888909041
**BLCA**	HDAC6	cg07674520	-0.172744215026962	410	-3.52946978112367	0.000464183279422671	0.0298405638252813	0.00352333632934016
**BLCA**	HDAC6	cg10783042	-0.0265216825993445	418	-0.538520792634327	0.590508380413722	0.000703399647900375	0.753440306800037
**BLCA**	HDAC6	cg12510923	-0.125241931162777	417	-2.5591970958871	0.0108485221140179	0.0156855413213817	0.0454371664336119
**BLCA**	HDAC6	cg13178935	-0.0706982689804583	418	-1.43861791781102	0.151018014768284	0.00499824523683324	0.317152915284503
**BLCA**	HDAC6	cg17152375	-0.0983723995094699	418	-2.00647368539273	0.045458750772872	0.00967712898525075	0.134592504571829
**BLCA**	HDAC6	cg24616736	-0.0690837806740411	418	-1.40560578464414	0.160594856701538	0.00477256875221902	0.330502354090268
**BLCA**	HDAC6	cg26680189	-0.0384349643348232	418	-0.780721442811896	0.435414680856823	0.00147724648341913	0.628668331153967
**BLCA**	HDAC9	cg00332048	-0.16756787490953	419	-3.45003805437238	0.000618362926798523	0.0280789927016959	0.00448949374963736
**BLCA**	HDAC9	cg02301805	-0.123774323774244	419	-2.53181302731589	0.0117181757876114	0.0153200832257714	0.0482559825960215
**BLCA**	HDAC9	cg03260621	-0.0697375393975143	419	-1.41897211699405	0.156663204437451	0.00486332440121986	0.325166071172625
**BLCA**	HDAC9	cg07513622	-0.0906640649292114	419	-1.84789000203525	0.0653347994033776	0.00821997266948826	0.175251892448326
**BLCA**	HDAC9	cg08146609	-0.0579160391707393	419	-1.17754376015184	0.239658307992875	0.00335426759322661	0.431591679038705
**BLCA**	HDAC9	cg08285151	-0.0936085028386175	419	-1.90842482279794	0.0570310210348864	0.00876255180368746	0.158673554250132
**BLCA**	HDAC9	cg08725366	0.11197626142329	419	2.28725468485469	0.0226871816032419	0.0125386831223371	0.0800946863971726
**BLCA**	HDAC9	cg10068437	-0.0323528238139331	419	-0.657034552195662	0.511525550143451	0.0010467052087354	0.693627243983151
**BLCA**	HDAC9	cg11321459	-0.0189863306498652	419	-0.385449901866198	0.700103045778846	0.000360480751546011	0.828221051449506
**BLCA**	HDAC9	cg11765288	0.113400716777526	419	2.3167275938108	0.0210082636955879	0.0128597225656567	0.0756383054535364
**BLCA**	HDAC9	cg12081743	0.0223789122415461	419	0.454356095952995	0.649811823376499	0.00050081571311482	0.795252810521982
**BLCA**	HDAC9	cg16475478	0.0394812067902901	419	0.802006286863109	0.42301166016194	0.00155876568961765	0.618055914105589
**BLCA**	HDAC9	cg18856632	-0.0744809875362261	419	-1.51600974975564	0.130283728957999	0.00554741750437147	0.286639679022556
**BLCA**	HDAC9	cg19585556	0.00390879279543807	417	0.0791476264273006	0.936953816576919	1.52786611176686e-05	0.966360948228465
**BLCA**	HDAC9	cg20648975	-0.0631681838772632	419	-1.28473985609582	0.19960504777882	0.00399021945435174	0.382417807829221
**BLCA**	HDAC9	cg21433125	0.0870240705355983	419	1.77312256700952	0.0769471348185853	0.00757318885258479	0.197499930914152
**BLCA**	HDAC9	cg23074799	-0.0865771932035362	419	-1.76394846176403	0.0784817197648678	0.00749561038300243	0.200164150134028
**BLCA**	HDAC9	cg23385718	0.0294501749533162	419	0.598032661297586	0.550146728635571	0.000867312804780932	0.722794612704784
**BLCA**	HDAC9	cg24350771	0.0662274764795306	419	1.34722872723302	0.178647053196803	0.00438607864084678	0.355254611477722
**BLCA**	HDAC9	cg25620356	-0.0251086974419653	419	-0.509811625220441	0.610456418803077	0.000630446687232154	0.767686371774891
**BLCA**	HDGF	cg00692559	-0.0561017999098204	419	-1.14053845449233	0.254724599726604	0.00314741195312152	0.448976923851991
**BLCA**	HDGF	cg00746981	0.0247775894744289	419	0.503084590444811	0.615173634740994	0.000613928940163328	0.771011481014971
**BLCA**	HDGF	cg01910231	-0.00865691474755422	419	-0.175722762768559	0.860598084711802	7.49421729464217e-05	0.925933556854983
**BLCA**	HDGF	cg02914429	0.149130439768547	419	3.06124965025268	0.0023484783782405	0.0222398880655603	0.0135361581801023
**BLCA**	HDGF	cg04402095	0.0319249957814502	419	0.648337119363936	0.517128100273386	0.00101920535564561	0.698125178874929
**BLCA**	HDGF	cg05762852	0.110361926958419	419	2.25387030387218	0.024729181277901	0.0121797549219754	0.085486221486537
**BLCA**	HDGF	cg05967123	0.0953175719550249	419	1.9435847105345	0.05262612286324	0.00908543952340135	0.149756998863848
**BLCA**	HDGF	cg06106793	0.105552694658274	419	2.15452148462231	0.0317783600164105	0.0111413713496227	0.102987738570141
**BLCA**	HDGF	cg06119628	-0.142270251210955	419	-2.91744741326298	0.00372225455223059	0.0202408243796283	0.01958400315408
**BLCA**	HDGF	cg07313589	0.0547088253346378	419	1.11213345780128	0.266729286509155	0.00299305556949591	0.462455996672385
**BLCA**	HDGF	cg07892339	0.130655057362658	419	2.67493788788603	0.00777197782351059	0.0170707440144393	0.0351517981196852
**BLCA**	HDGF	cg08597205	0.194635726529777	419	4.02770115230165	6.70648198104383e-05	0.0378830660417741	0.000684559887890804
**BLCA**	HDGF	cg08708935	0.0552815044448378	419	1.12381050990122	0.261747793490566	0.00305604473368463	0.457088918194683
**BLCA**	HDGF	cg09063372	0.166677459704464	419	3.43118008584638	0.000661692021235505	0.0277813755735332	0.00475525811547297
**BLCA**	HDGF	cg09477492	0.0292227800050308	419	0.593411083010424	0.553231696220097	0.000853970871222425	0.725273979327693
**BLCA**	HDGF	cg11588596	0.139387538703657	419	2.85714979133479	0.00449124083591394	0.0194288859458635	0.0227498104954479
**BLCA**	HDGF	cg12220178	0.0169332398037909	419	0.343756516207427	0.731204766694493	0.000286734610252689	0.84810893904064
**BLCA**	HDGF	cg12421458	0.164202447692852	419	3.37880737293257	0.000797344549116375	0.0269624438283238	0.00555314430205544
**BLCA**	HDGF	cg13170934	-0.02715206561683	419	-0.551330006694798	0.581706343118563	0.000737234667260642	0.747080519923707
**BLCA**	HDGF	cg13235992	0.0955620266283891	419	1.9486151736511	0.0520198712709428	0.00913210093332495	0.148518220612014
**BLCA**	HDGF	cg14155849	-0.0011506126625262	419	-0.0233549017506943	0.981378485363653	1.32390949916564e-06	0.990430547466757
**BLCA**	HDGF	cg15503799	0.0039549084977169	419	0.0802765028033169	0.9360563247779	1.56413012253133e-05	0.965893462508212
**BLCA**	HDGF	cg17170583	0.0281530265748448	419	0.571670623823316	0.5678568823112	0.000792592905323916	0.736510514144506
**BLCA**	HDGF	cg17218029	-0.115756266213734	419	-2.36549729204699	0.0184681639783993	0.0133995131677449	0.0684560606848704
**BLCA**	HDGF	cg17971171	0.181978441713447	419	3.75648273791074	0.00019714460007188	0.0331161532484545	0.00171551085212437
**BLCA**	HDGF	cg18593717	-0.0323719201870813	419	-0.657422775970301	0.511276214936653	0.00104794121659876	0.693486450200194
**BLCA**	HDGF	cg19599803	0.178243650660989	419	3.67683031970105	0.000267406198554789	0.0317707990009567	0.0022172881912568

**BLCA**	HDGF	cg20284773	0.0491741325261306	419	0.999334852386435	0.318219068098467	0.00241809530969746	0.517540906563505
**BLCA**	HDGF	cg23747104	0.10117195761129	419	2.06415774344694	0.039628268304876	0.0102357650069006	0.1216638977272
**BLCA**	HDGF	cg26431298	0.100766078107653	419	2.05579168096668	0.0404322131450387	0.0101538024971977	0.123341459569949
**BLCA**	HDGFL1	cg02685945	0.0455029840220356	418	0.92456736575842	0.35573218797234	0.00207052155490963	0.554530204010023
**BLCA**	HDGFL1	cg03930485	0.024813659092024	418	0.503817399438295	0.614658984807011	0.000615717677535187	0.770790848282879
**BLCA**	HDGFL1	cg05276066	0.0136422447630953	418	0.276933096935288	0.781970363509102	0.000186110842176201	0.879592285408142
**BLCA**	HDGFL1	cg06566039	-0.00783416121069169	418	-0.159020985219883	0.873730261901647	6.13740818751063e-05	0.933163160380612
**BLCA**	HDGFL1	cg08671671	0.027054927741072	418	0.549356148563195	0.583058689656051	0.000731969115074626	0.748094532641226
**BLCA**	HDGFL1	cg09081892	0.0228520560605891	418	0.463967239482969	0.642916258234179	0.000522216466196309	0.790339070414957
**BLCA**	HDGFL1	cg11894257	0.0142007226162813	418	0.288272255989656	0.773283309498559	0.000201660522824564	0.874232343362702
**BLCA**	HDGFL1	cg15317945	0.0394097452817936	418	0.800552384691858	0.423852209125221	0.00155312802317585	0.61891861893331
**BLCA**	HDGFL1	cg20209681	0.0514892347274167	418	1.04650545759997	0.295941289541655	0.00265114129281501	0.493918440100429
**BLCA**	HDGFL1	cg21898884	0.071108698227905	417	1.44525479185288	0.149148341046699	0.00505644696366726	0.314585203030853
**BLCA**	HIST1H1	cg00667898	-0.170317687908893	419	-3.50833099766661	0.00050059709585449	0.029008114814631	0.00375250052754907
**BLCA**	HIST1H1	cg00700007	-0.157648721092165	419	-3.24044042930592	0.00129031783903809	0.0248531192619954	0.00829372766835735
**BLCA**	HIST1H1	cg03821311	0.000155266929224601	419	0.00315157449467944	0.997486936919408	2.41078193108372e-08	0.998778778729371
**BLCA**	HIST1H1	cg04676561	-0.17223054932133	419	-3.54893119797865	0.000431356224984357	0.0296633621195271	0.00330903522004073
**BLCA**	HIST1H1	cg07138603	-0.14830494413455	419	-3.04392226408544	0.00248484263840651	0.021994356454752	0.0141688666871067
**BLCA**	HIST1H1	cg11206041	-0.154304590010745	419	-3.17000724614108	0.00163818240298826	0.0238099064983841	0.0101111362946738
**BLCA**	HIST1H1	cg14334286	-0.142840150903355	419	-2.92937690683439	0.00358515654977423	0.0204033087100933	0.018996299457372
**BLCA**	HIST1H1	cg18798248	-0.161188897938063	419	-3.31512731324718	0.000997113050419317	0.0259818608184874	0.0067012191010987
**BLCA**	HIST1H1	cg23460835	-0.155430190927195	419	-3.19370175212753	0.0015124992810479	0.0241585442516642	0.00946531808139653
**BLCA**	HIST1H1	cg00903962	0.00912250471125703	419	0.185174327505798	0.853183426789523	8.32200922069068e-05	0.921812856158839
**BLCA**	HIST1H1	cg04290376	-0.136739391266225	419	-2.8018238848602	0.00532108725832609	0.0186976611238578	0.0260591522581544
**BLCA**	HIST1H1	cg07750111	0.0452656377380572	419	0.91973483942571	0.358249513439151	0.00204897795983303	0.557237226546273
**BLCA**	HIST1H1	cg08283682	0.0738876701534398	419	1.50386661847803	0.133381863042347	0.00545938780070352	0.291305689318258
**BLCA**	HIST1H1	cg10717387	-0.130753437821661	419	-2.67698707955356	0.00772542633450644	0.017096461502183	0.0349861343734192
**BLCA**	HIST1H1	cg12633985	0.075023105255076	419	1.52710641809272	0.127501861487603	0.00562846632211422	0.282350125745525
**BLCA**	HIST1H1	cg15022984	-0.0696018123697253	419	-1.41619697853486	0.157473415472811	0.00484441228515045	0.326166481623361
**BLCA**	HIST1H1	cg16481032	0.0329439656787181	419	0.66905263239854	0.503836623173021	0.00108530487464055	0.687151096223535
**BLCA**	HIST1H1	cg16482183	-0.0246577492547051	419	-0.500649868703042	0.616884897730883	0.000608004598307909	0.77241177746783
**BLCA**	HIST1H1	cg17691542	0.0206907736944612	419	0.420066763866959	0.674655663023714	0.000428108116075406	0.811895344490855
**BLCA**	HIST1H1	cg20855557	-0.0266319207981794	419	-0.540760756489607	0.588964724616394	0.000709259205400501	0.752371535794406
**BLCA**	HIST1H1	cg23407813	-0.0858092692386951	419	-1.74818597741632	0.0811766794823721	0.00736323068727887	0.205087639765252
**BLCA**	HIST1H3	cg00958605	0.179270073555262	419	3.6987044616288	0.000246064468864495	0.0321377592725091	0.00206430920854573
**BLCA**	HIST1H3	cg01043409	0.209578606538707	419	4.3506000933805	1.7138574759101e-05	0.0439231923187061	0.000209437368019086
**BLCA**	HIST1H3	cg08041469	0.166949240311161	419	3.4369351681427	0.000648177318065555	0.0278720488404738	0.00467202395484902
**BLCA**	HIST1H3	cg08401836	0.283463152206866	419	5.99976518464629	4.33086264871765e-09	0.0803513586590529	1.26659822892609e-07
**BLCA**	HIST1H3	cg10075390	0.176188330763546	419	3.63306649026259	0.000315435823964197	0.0310423278972449	0.00254924314247566
**BLCA**	HIST1H3	cg15479139	0.234376462128835	419	4.89363020605958	1.42296148564929e-06	0.0549323260000294	2.33441302856602e-05
**BLCA**	HIST1H3	cg15797414	0.167467776773039	419	3.44791765871256	0.000623099201270742	0.0280454562573044	0.00451797737567864
**BLCA**	HIST1H3	cg21209604	0.185956446998562	419	3.8415072482867	0.000141542734844019	0.034579800180329	0.00129634748227639
**BLCA**	HIST1H3	cg23099349	0.250804011984968	419	5.25884939032177	2.33407788650874e-07	0.0629026524277561	4.64273216832169e-06
**BLCA**	HIST1H3	cg25701114	0.113537831598651	419	2.31956534925989	0.0208524964273907	0.0128908392041236	0.0751992910751172
**BLCA**	HLTF	cg00906762	-0.22343284735499	419	-4.65281785534234	4.41967884236072e-06	0.0499222372771582	6.38398374148436e-05
**BLCA**	HLTF	cg02059813	-0.195248354574009	419	-4.04088021498383	6.35408392628824e-05	0.038121919963858	0.00065445466034909
**BLCA**	HLTF	cg02398045	-0.0802048658136012	419	-1.63324262606426	0.103181908996927	0.00643282050017777	0.243531250882331
**BLCA**	HLTF	cg02611507	-0.397557514251727	419	-8.7943943063827	3.96438823424562e-17	0.158051977138012	4.00007287023221e-15
**BLCA**	HLTF	cg02710346	-0.179183854785779	419	-3.69686657081833	0.00024779399218113	0.0321068538158911	0.00207568491738331
**BLCA**	HLTF	cg03678609	-0.157527200877665	419	-3.23787901820686	0.00130166483231504	0.0248148190163524	0.00835699545804376
**BLCA**	HLTF	cg04120413	-0.453816342084494	419	-10.3372403687257	2.00508115772042e-22	0.20594927234295	3.2564359829583e-20
**BLCA**	HLTF	cg04731810	-0.42402867974641	419	-9.50351013631119	1.69671304574783e-19	0.179800321247483	2.16383761771978e-17
**BLCA**	HLTF	cg04836786	-0.0744392852916224	419	-1.51515619795051	0.130499652008015	0.00554120719472756	0.286928097007278
**BLCA**	HLTF	cg05555455	-0.19537975427187	419	-4.04370756979623	6.28080954611556e-05	0.0381732483793362	0.000647851097627305
**BLCA**	HLTF	cg07275648	-0.417602689138626	419	-9.32878211981616	6.66888808473122e-19	0.174392005975812	7.96927004091521e-17
**BLCA**	HLTF	cg09352720	-0.420853123260754	419	-9.41694882079628	3.34935424878862e-19	0.177117351358331	4.12931322393442e-17
**BLCA**	HLTF	cg09392355	-0.187798527041388	419	-3.88094521483664	0.000121126411454708	0.0352682867589148	0.00113570062103261

**BLCA**	HLTF	cg15438497	-0.147313608979151	419	-3.02312245363493	0.00265812139844978	0.0217012993904621	0.014948738239789
**BLCA**	HLTF	cg16724124	0.138761052939417	419	2.84405546230877	0.00467624911080582	0.0192546298128556	0.0235139372584854
**BLCA**	HLTF	cg20117402	-0.270094074473145	419	-5.69393198107334	2.36289978158422e-08	0.0729508090655046	5.98175807382241e-07
**BLCA**	HLTF	cg21089667	-0.192755449730065	419	-3.98728251229645	7.90664735013481e-05	0.0371546634006398	0.000786760966943448
**BLCA**	HLTF	cg21111416	-0.377993333791494	419	-8.28727348204109	1.64947959288266e-15	0.142878960390808	1.36801295787541e-13
**BLCA**	HLTF	cg21835021	-0.240547393860467	419	-5.03028099014245	7.3257854730922e-07	0.0578630486930627	1.29493786660699e-05
**BLCA**	HLTF	cg21926402	-0.0694021710078749	419	-1.41211516120081	0.158670901062144	0.00481666134060631	0.327878558111626
**BLCA**	HLTF	cg24041269	-0.0808779971456347	419	-1.64703973385613	0.100312343402078	0.00654125042228929	0.238754732832225
**BLCA**	HLTF	cg24710191	0.132695055075475	411	2.69426206993119	0.00734787969715707	0.0176079776414834	0.033642572724421
**BLCA**	HLTF	cg25951608	-0.366271062398235	419	-7.98970992142523	1.36969328761485e-14	0.134154491150332	1.00998975940009e-12
**BLCA**	HLTF	cg26151310	-0.113351821515141	419	-2.31571568023645	0.021064054275052	0.0128486354408005	0.075800584618068
**BLCA**	HLTF	cg27554556	-0.292497171717556	419	-6.20856722889288	1.30628880573295e-09	0.0855545954627696	4.23246278022445e-08
**BLCA**	IDH1	cg00707300	0.0579271304901986	419	1.17777002705092	0.239568168040972	0.00335555244682849	0.431502441002754
**BLCA**	IDH1	cg01046174	0.101072154227801	419	2.06210048149378	0.0398246896950781	0.0102155803602483	0.1221030981807
**BLCA**	IDH1	cg02241586	-0.0174999385708277	419	-0.355264361541227	0.722573355097951	0.000306247849982743	0.842819875350326
**BLCA**	IDH1	cg03448938	0.0730065997877583	419	1.48583714894422	0.138086975205272	0.00532996361256991	0.298406570991386
**BLCA**	IDH1	cg05268564	0.033860601834535	419	0.687689497641361	0.492035292666822	0.00114654035659692	0.677583726444483
**BLCA**	IDH1	cg07129696	0.0809131061913661	419	1.64775942264459	0.100164430286305	0.00654693075353528	0.238523598437349
**BLCA**	IDH1	cg07915542	-0.125238951910473	419	-2.56224665945939	0.0107543671534814	0.0156847950756338	0.0451302074132908
**BLCA**	IDH1	cg09406631	0.0209706806483941	419	0.425751954538898	0.670510916450044	0.00043976944685693	0.809085373075718
**BLCA**	IDH1	cg09615745	0.0301106654043093	419	0.611457008551476	0.541234303056014	0.000906652171090266	0.716276673573541
**BLCA**	IDH1	cg10356455	0.0944243695263457	419	1.92520712737418	0.0548915252226444	0.00891596156044789	0.154427575335252
**BLCA**	IDH1	cg14772913	0.0753843215967236	419	1.53450095116886	0.125673994662031	0.00568279594259826	0.279461546442318
**BLCA**	IDH1	cg15399923	0.0497737924110401	419	1.01155144511517	0.312346260350799	0.00247743041097731	0.511400113182011
**BLCA**	IDH1	cg16085695	-0.0902482893556683	419	-1.83934602995261	0.0665836645382131	0.00814475373162444	0.177695011975497
**BLCA**	IDH1	cg16863783	0.100838696473067	419	2.05728842768782	0.0402873706935318	0.0101684427063874	0.123091721120948
**BLCA**	IDH1	cg24324379	-0.0661113177264716	419	-1.34485539204731	0.17941178948207	0.00437070633153047	0.356280181670862
**BLCA**	IDH1	cg24830471	0.0658182601011105	419	1.33886793408335	0.181351919151745	0.00433204336273743	0.358637734467713
**BLCA**	IDH1	cg24987806	0.105284315458106	419	2.14898189611874	0.0322178381702448	0.011084787081482	0.104106106027665
**BLCA**	IDH1	cg25795625	0.115694024396433	419	2.3642081103593	0.0185316721085119	0.0133851072810425	0.0686340027937721
**BLCA**	IDH1	cg26105702	0.0684017767474363	419	1.39166390789939	0.164775073901203	0.00467880306220611	0.336532215780261
**BLCA**	ING2	cg02075087	-0.155537422810887	419	-3.19595970449707	0.00150099980418902	0.0241918898946527	0.00940545942545955
**BLCA**	ING2	cg02552216	-0.189414620487068	419	-3.91557986713562	0.00010552569939759	0.0358778984542601	0.00100863327914192
**BLCA**	ING2	cg10348863	-0.0193736676111486	419	-0.393316324012164	0.694289309893039	0.000375338996707269	0.824779992270395
**BLCA**	ING2	cg17985608	0.0244385590025862	419	0.49619676784378	0.62002019829007	0.000597243166122887	0.774680667085681
**BLCA**	ING3	cg01382559	-0.174955996589859	419	-3.60685001586283	0.000347973816004041	0.0306096007427508	0.00276622471308178
**BLCA**	ING3	cg01416111	-0.155575341158383	419	-3.19675816844724	0.00149695271021601	0.0242036867765473	0.00938463527589205
**BLCA**	ING3	cg10268125	-0.0527265074584497	419	-1.07172198933845	0.284472301133019	0.00278008458876595	0.481772900714969
**BLCA**	ING3	cg14303833	-0.123392366470113	419	-2.52387907541051	0.0119817533301143	0.0152256761030947	0.0490778741362497
**BLCA**	ING3	cg16130600	0.104376977672381	419	2.13025713542618	0.0337422564523949	0.0108945534680206	0.107747520665693
**BLCA**	ING3	cg18485114	0.019980298567582	419	0.405636742878345	0.685220096282913	0.000399212330849721	0.818843126625962
**BLCA**	ING3	cg19478983	0.0407573629100575	419	0.82797209729257	0.408165644853242	0.00166116263138213	0.604174971735948
**BLCA**	ING3	cg19960827	0.0434176255067442	419	0.882113370401085	0.378229855935059	0.00188509020464389	0.576821833465447
**BLCA**	ING3	cg20553930	-0.0231686337708294	419	-0.470398172130376	0.638319486196595	0.000536785590806816	0.787302830283069
**BLCA**	ING3	cg24859794	-0.0876683571411045	419	-1.78635125808303	0.0747776246578446	0.00768574084382025	0.193469819496886
**BLCA**	ING4	cg00369908	0.0040860013196867	419	0.0829374609968622	0.933941546583838	1.66954067844814e-05	0.964887298303339
**BLCA**	ING4	cg00443163	-0.0999702507980606	419	-2.03939094889097	0.042048578805601	0.00999405104462714	0.126994609038082
**BLCA**	ING4	cg00591160	-0.134509441349571	419	-2.7552826181016	0.00612424055945423	0.0180927898121737	0.0291536635712998
**BLCA**	ING4	cg03930474	-0.018749680187744	419	-0.380643855862827	0.703663714289599	0.000351550507142679	0.830715293636109
**BLCA**	ING4	cg04375284	-0.110501582680042	419	-2.25675766181293	0.024546468447981	0.0122105997747941	0.0850094807658031
**BLCA**	ING4	cg08733494	-0.110342092983542	419	-2.25346025082893	0.0247552251965134	0.0121753774839885	0.0855572432776917
**BLCA**	ING4	cg12392916	-0.110705586632153	419	-2.26097565159163	0.0242816633506874	0.0122557269115691	0.084343977438086
**BLCA**	ING4	cg18123790	-0.0231170908724145	419	-0.469351124483109	0.639066962154706	0.00053439989040347	0.787873017129098
**BLCA**	ING4	cg23354319	-0.114604078448902	419	-2.3416371998619	0.019675059395855	0.0131340947971221	0.0719031131977799
**BLCA**	ING4	cg27116036	-0.109543774254151	419	-2.23695784706206	0.0258233296115535	0.0119998384778443	0.0882767521042544
**BLCA**	INO80	cg00734724	-0.00752427615547781	419	-0.152730449073396	0.878685616837774	5.6614731663892e-05	0.935556954441632
**BLCA**	INO80	cg06380007	0.0656202548722022	419	1.33482268793251	0.182671528028462	0.00430601784949277	0.360541642812283

**BLCA**	INO80	cg12580230	-0.0412620014365462	419	-0.838241035455185	0.402381380126514	0.00170255276254954	0.598925802842476
**BLCA**	INO80	cg18952654	0.146639116985738	419	3.00897585165961	0.0027822334442831	0.0215030306303571	0.0154989468761018
**BLCA**	INO80	cg21007089	0.0442481827986289	419	0.899020546478964	0.369166835479963	0.00195790168098087	0.567857024707857
**BLCA**	INO80	cg21598047	0.0213580374463709	419	0.433619724690612	0.664791560872756	0.000456165763560582	0.805398884155155
**BLCA**	INO80	cg22835709	-0.161174374328731	419	-3.31482064343058	0.000998178806665001	0.0259771789402579	0.00670572729582877
**BLCA**	INO80	cg26409855	0.085506622395141	419	1.74197468184074	0.0822591430873015	0.00731138247342523	0.207112298553782
**BLCA**	INO80	cg27441689	-0.127857073028588	419	-2.61669136284241	0.00920519876126504	0.0163474311234376	0.0400061023706063
**BLCA**	INTS12	cg00093890	-0.0049627450206368	419	-0.100733962647157	0.91981065142116	2.46288381398553e-05	0.957776754988605
**BLCA**	INTS12	cg01852585	-0.100034501224504	419	-2.04071490059699	0.0419160917617704	0.0100069014352353	0.126687922705434
**BLCA**	INTS12	cg03607115	-0.0790152144142419	419	-1.60886394382031	0.108412047337902	0.00624340410892862	0.252365296090848
**BLCA**	INTS12	cg19703969	0.0383279001244248	419	0.77854346577881	0.436695592089185	0.00146902792794788	0.629746355447781
**BLCA**	INTS12	cg26533745	-0.107133319076612	419	-2.18715669826079	0.029292615545618	0.0114775480563711	0.0969819815797656
**BLCA**	IWS1	cg02372421	-0.208647512430922	419	-4.33038990915315	1.87140345101663e-05	0.0435337844436119	0.000226272863022051
**BLCA**	IWS1	cg02592214	-0.239719475533029	419	-5.01191021303245	8.01669495800225e-07	0.0574654269498307	1.40556855434489e-05
**BLCA**	IWS1	cg10180764	-0.21732557798519	419	-4.51924105470996	8.12010685074349e-06	0.0472304068465967	0.000108895714088668
**BLCA**	IWS1	cg10488833	0.0382605777453597	419	0.777173959366214	0.437502140219567	0.00146387180940871	0.630371934661449
**BLCA**	IWS1	cg15239145	-0.0723360080230824	419	-1.47211707960255	0.141752713211306	0.00523249805671544	0.303947260868232
**BLCA**	IWS1	cg17144508	-0.00621311935397711	419	-0.126114983431498	0.899702426959012	3.8602852106765e-05	0.947110292325431
**BLCA**	IWS1	cg18451256	0.0271913096564741	419	0.552127456674484	0.581160405039638	0.000739367320834264	0.746748811691668
**BLCA**	IWS1	cg18530950	-0.174587625186389	419	-3.59901674456585	0.000358291341249755	0.030480838868223	0.00283549507641744
**BLCA**	IWS1	cg19311602	-0.0130179282965097	419	-0.264257477704831	0.791713660245286	0.000169466457133067	0.885723751170194
**BLCA**	IWS1	cg19409088	-0.105367982362665	419	-2.15070880541392	0.0320802780033534	0.0111024117071788	0.103760732870151
**BLCA**	IWS1	cg21581038	0.187925755813604	419	3.88367067757983	0.000119823692856903	0.0353160896981143	0.00112538504915006
**BLCA**	IWS1	cg21679674	-0.161922363817378	419	-3.3306175179516	0.000944635871185708	0.0262188519042075	0.00640074932175081
**BLCA**	IWS1	cg22409775	-0.0918855440862793	419	-1.87299641725588	0.0617767550814311	0.00844295321203158	0.168166452684053
**BLCA**	IWS1	cg23570261	0.00955927467316063	419	0.19404095010526	0.846239452517143	9.13797322769302e-05	0.917861143885125
**BLCA**	IWS1	cg23623107	0.0469777789023414	419	0.954598707439611	0.340340416565992	0.00220691171059727	0.539564751708473
**BLCA**	JADE1	cg09863040	-0.051443969843209	414	-1.04558301817299	0.296366642093722	0.002646482033229	0.494329900408212
**BLCA**	JADE1	cg12832492	0.00974540198435693	414	0.197819449957441	0.843283891957619	9.4972859836708e-05	0.916260609108509
**BLCA**	JADE1	cg14617908	0.133154790242119	414	2.72703057056072	0.00666370430618763	0.0177301981644227	0.0311629824343589
**BLCA**	JADE1	cg15389440	0.221672114516445	414	4.61424920821631	5.27616509340979e-06	0.0491385263541921	7.45602156724289e-05
**BLCA**	JADE1	cg15836763	-0.00586045697401029	414	-0.118956327494691	0.905367934089935	3.43449559422259e-05	0.949796033632693
**BLCA**	JADE1	cg20039387	0.0965604924161084	414	1.96916561307052	0.0496037772186625	0.00932392869564133	0.143437916179181
**BLCA**	JADE1	cg22922695	-0.0175584421206336	414	-0.356452401443987	0.721684263412778	0.000308298889703639	0.842318387247239
**BLCA**	JADE1	cg26766900	-0.0528991955272658	413	-1.07393619261584	0.283481379997182	0.0027983248874319	0.480743696850787
**BLCA**	JADE2	cg04590931	-0.06239841283876	414	-1.26902236952124	0.205149532079456	0.00389356192479632	0.389407264630611
**BLCA**	JADE2	cg04842522	0.0464150887401416	414	0.943139884615727	0.346162071373331	0.00215436046275522	0.545459175131174
**BLCA**	JADE2	cg17378563	-0.109423701944576	414	-2.23447615849685	0.0259873665107204	0.0119735465472555	0.0887130805939454
**BLCA**	JADE2	cg18145011	-0.041813638293941	414	-0.849467082619913	0.396114766384927	0.00174838034737653	0.592793485245353
**BLCA**	JADE2	cg21194015	-0.0310409133150327	414	-0.630365490467397	0.528804955596772	0.000963538299431372	0.707171948241404
**BLCA**	JADE2	cg25669900	-0.10476913155132	414	-2.13834936558324	0.0330760183209486	0.0109765709260179	0.106164779851462
**BLCA**	JADE2	cg26133206	-0.0595065811670369	414	-1.20999589704008	0.226974238781065	0.00354103320218916	0.416542437644118
**BLCA**	JADE3	cg10860619	-0.144596298067274	414	-2.96615642964124	0.00319097991872134	0.02090808941476	0.0173239662114533
**BLCA**	JADE3	cg17843048	-0.157806056558259	414	-3.24375698092524	0.00127576171519993	0.0249027514864685	0.00821850007223117
**BLCA**	JADE3	cg19194595	-0.139314061714145	414	-2.85561385039657	0.0045125919792703	0.0194084077912926	0.022837144186531
**BLCA**	JARID2	cg01769037	-0.0408957701809781	419	-0.830788496731024	0.406574302538812	0.00167246401869538	0.602836855389185
**BLCA**	JARID2	cg03223185	0.170327524869813	419	3.50853968076562	0.000500216003214809	0.0290114657282769	0.0037500051801188
**BLCA**	JARID2	cg05197191	-0.201653942656683	419	-4.17897742720701	3.5777611240401e-05	0.0406643125889849	0.000397672511234454
**BLCA**	JARID2	cg06047828	-0.0363741293062158	419	-0.738803096773232	0.460447238986142	0.00132307728278531	0.650221524566846
**BLCA**	JARID2	cg14862454	0.143707110785918	419	2.94753035686239	0.00338535772071129	0.020651733690436	0.0181490261542977
**BLCA**	JARID2	cg15109221	0.220997691473003	419	4.59948864849324	5.64442232978678e-06	0.0488399796363966	7.92460188997352e-05
**BLCA**	JARID2	cg21397480	0.0854482084789385	419	1.74077589465744	0.0824694061770139	0.00730139633226014	0.207447222016571
**BLCA**	JARID2	cg21901688	-0.188722040447752	419	-3.90073309646778	0.000111964683403582	0.0356160085507631	0.00106208762110697
**BLCA**	JARID2	cg25338254	-0.129618140125888	419	-2.6533445505671	0.00827806784782279	0.0168008622496944	0.0368855387935682
**BLCA**	JMJD6	cg02318866	-0.0653176560378018	419	-1.32864090054425	0.184701884084467	0.00426633619027258	0.363185258207593
**BLCA**	JMJD6	cg04510639	-0.139594276604855	419	-2.86147163668215	0.00443165476900835	0.0194865620608329	0.0225236939267108
**BLCA**	JMJD6	cg07312083	-0.148145880770018	419	-3.04058422802418	0.00251193349258822	0.0219472019891244	0.0142950515085887

**BLCA**	JMJD6	cg08857478	-0.0466782396860458	419	-0.948498671513402	0.343431658794676	0.00217885806018794	0.542795198477744
**BLCA**	JMJD6	cg10712116	0.0480188191508075	419	0.97580123120145	0.329735561587332	0.00230580699263796	0.529343326897478
**BLCA**	JMJD6	cg12504415	-0.130255896678517	419	-2.66662449230092	0.00796343157331702	0.0169665986195245	0.0358096644979552
**BLCA**	JMJD6	cg15457598	0.0315170287643715	419	0.640043778882863	0.522499902247313	0.000993323102134223	0.702281611559071
**BLCA**	JMJD6	cg21102709	-0.170541539322747	419	-3.51308009029166	0.000491991313113234	0.029084416634572	0.00369868305105633
**BLCA**	JMJD6	cg21807925	-0.215829240891456	419	-4.48659922825799	9.40047224537273e-06	0.0465822612237823	0.000124012266891051
**BLCA**	JMJD6	cg23630158	-0.258242283851705	419	-5.42578758669578	9.86313321012893e-08	0.0666890771689448	2.14447340678807e-06
**BLCA**	JMJD6	cg25004679	-0.115310375193331	419	-2.35626241088753	0.0189273465701679	0.0132964826272267	0.0697602744759065
**BLCA**	JMJD6	cg25908504	-0.177670269892397	419	-3.66461637967449	0.000280068571009089	0.0315667248036373	0.00230752535618313
**BLCA**	JMJD6	cg26612088	-0.119426753367967	419	-2.44157264085203	0.0150432842668764	0.0142627494200132	0.0585109372660058
**BLCA**	JMJD8	cg00873473	-0.254787136503339	418	-5.34811707205185	1.47655336897553e-07	0.0649164849275711	3.08119615842497e-06
**BLCA**	JMJD8	cg02851105	-0.25702464993286	418	-5.39839035036924	1.1377839142422e-07	0.0660616706731093	2.44038494139943e-06
**BLCA**	JMJD8	cg03899598	-0.270581091902056	418	-5.7050092179938	2.22481071309732e-08	0.0732141272949091	5.66907712462145e-07
**BLCA**	JMJD8	cg05787952	-0.268157194731157	418	-5.64992409060911	2.99875643038257e-08	0.0719082810860837	7.40559632768851e-07
**BLCA**	JMJD8	cg06191810	-0.260520991618378	418	-5.47713397531639	7.53443705862135e-08	0.0678711870738227	1.68429309350112e-06
**BLCA**	JMJD8	cg06646636	-0.302858919282462	418	-6.45030043799233	3.133840481596e-10	0.0917235249889407	1.13550995074653e-08
**BLCA**	JMJD8	cg08398290	-0.254347118188387	418	-5.33824142159317	1.55375720920501e-07	0.0646924565307371	3.22930772997313e-06
**BLCA**	JMJD8	cg26750188	-0.214583393615676	418	-4.45944701417854	1.0610925665198e-05	0.04604603281562	0.000138084033321916
**BLCA**	JMJD8	cg27579097	-0.26569323929851	418	-5.59404709387095	4.04968872501384e-08	0.0705928974089352	9.69556167967491e-07
**BLCA**	KAT2B	cg00770008	-0.0688032489656658	419	-1.39987077187157	0.162304552494165	0.00473388706823139	0.332930424413481
**BLCA**	KAT2B	cg04371528	-0.0622353045687622	419	-1.26569225385251	0.206338550146087	0.0038732331347666	0.390852009302995
**BLCA**	KAT2B	cg05072819	-0.0999007656351929	419	-2.03795915860504	0.0421922573071191	0.00998016297449774	0.127314862299756
**BLCA**	KAT2B	cg12062464	-0.112133951843585	419	-2.29051669596701	0.0224957747944525	0.0125740231560594	0.0795507206854693
**BLCA**	KAT2B	cg14937543	-0.0924834087890849	419	-1.88528809415701	0.0600943769110407	0.00855318090124899	0.16486165523213
**BLCA**	KAT2B	cg18053505	-0.183274322569135	419	-3.78415926060405	0.000177107472890321	0.0335894773131754	0.00156780823236569
**BLCA**	KAT5	cg00582417	-0.255945493031559	419	-5.37413191684281	1.29057797268866e-07	0.0655080954031678	2.73791172095287e-06
**BLCA**	KAT5	cg02561205	-0.0487204594641977	419	-0.990093102021845	0.322709724782505	0.00237368317040253	0.52210465705287
**BLCA**	KAT5	cg05785768	-0.266187535742724	419	-5.60524712515351	3.81374344807327e-08	0.0708558041847839	9.17890810896263e-07
**BLCA**	KAT5	cg07564927	-0.262553676373476	419	-5.52301953042937	5.9125483834076e-08	0.0689344329772282	1.35801827262714e-06
**BLCA**	KAT5	cg14726348	-0.265745841277748	419	-5.59523875153318	4.02392454267617e-08	0.070620852156418	9.64972829448059e-07
**BLCA**	KAT5	cg18896539	0.09964315659512	419	2.03265120713823	0.0427285469349196	0.00992875865623961	0.128494513906805
**BLCA**	KAT5	cg20108992	-0.0142815259276634	419	-0.289912883158784	0.772028735710802	0.000203961982822522	0.873589413516895
**BLCA**	KAT5	cg20423942	-0.171424771549678	419	-3.53182367102127	0.000459359748060061	0.0293864523008592	0.00349205172458575
**BLCA**	KAT5	cg22048492	0.0760679236092623	419	1.54849677206261	0.122270508961065	0.00578632900222457	0.274181712617235
**BLCA**	KAT6A	cg10163397	-0.0275197755384026	418	-0.558802076784787	0.576600390826352	0.000757338045684064	0.743240502451372
**BLCA**	KAT6A	cg12468056	0.162093271077058	418	3.33422775397321	0.000932779921071037	0.0262742285284606	0.00633531353062688
**BLCA**	KAT6A	cg15251374	-0.40049918469236	417	-8.86107123802278	2.41957867841364e-17	0.160399596939245	2.49309541335776e-15
**BLCA**	KAT6A	cg20966972	0.255431591164673	418	5.36258745028164	1.3701157490183e-07	0.0652452977649164	2.89062512476167e-06
**BLCA**	KAT6B	cg04396765	-0.0101735564799104	418	-0.20651133045902	0.836493530773487	0.000103501251449926	0.912469885003328
**BLCA**	KAT6B	cg06972998	-0.0904126257748916	418	-1.84272294186848	0.0660877258416446	0.00817444289951059	0.176704882080251
**BLCA**	KAT6B	cg09631472	0.00647572003069992	418	0.131445516904417	0.895487046413441	4.19349499160081e-05	0.944526056385157
**BLCA**	KAT6B	cg10359932	-0.00345300272188949	418	-0.0700887182399717	0.944157052496709	1.19232277973762e-05	0.970165960631898
**BLCA**	KAT6B	cg10739686	-0.163132017792523	418	-3.35617682676037	0.000863626596711892	0.0266120552290599	0.00594084069896586
**BLCA**	KAT6B	cg11128436	0.000848396560059445	418	0.017220575581967	0.986268983777321	7.19776723120699e-07	0.992812561828328
**BLCA**	KAT6B	cg11348552	0.0972774776586065	418	1.98392631315119	0.0479272684009198	0.0094629076596207	0.139899212711761
**BLCA**	KAT6B	cg15874302	-0.269662880385279	418	-5.68412838406398	2.49204947555102e-08	0.0727180690576852	6.2669911622248e-07
**BLCA**	KAT6B	cg15912797	0.16180548805617	418	3.32814881771013	0.000952823532651753	0.0261810159650953	0.0064461217409436
**BLCA**	KAT6B	cg17147776	-0.0122220264327858	418	-0.248098572861761	0.80418174717425	0.000149377930123714	0.893491885510492
**BLCA**	KAT6B	cg18462764	-0.0940105947737158	418	-1.91669533090542	0.0559681072752704	0.00883799192970781	0.156460505152091
**BLCA**	KAT6B	cg18491775	0.238136405410564	418	4.97681553925105	9.51574734753987e-07	0.0567089475818643	1.63957485969993e-05
**BLCA**	KAT6B	cg24671245	0.0380804268385609	418	0.773509289165717	0.4396646137263	0.00145011890820699	0.632281522510839
**BLCA**	KAT6B	cg26605858	0.0440595884267559	418	0.895181276913899	0.371212874239386	0.00194124733233512	0.569891941242453
**BLCA**	KDM1A	cg00062312	0.0279735792761245	419	0.568023934384376	0.570328142681838	0.00078252113751762	0.738336343137038
**BLCA**	KDM1A	cg00541565	-0.0183722406918021	419	-0.372978709963373	0.709356102442277	0.000337539228037507	0.83440914241165
**BLCA**	KDM1A	cg04886391	-0.0074251076387982	419	-0.150717379319985	0.880272425385195	5.51322234477393e-05	0.93646463720723
**BLCA**	KDM1A	cg06958034	0.0508149696137245	419	1.03276547829288	0.302319509177641	0.00258216113684375	0.500705624682578
**BLCA**	KDM1A	cg07118078	-0.0224606569281364	419	-0.456016583675617	0.648618323001476	0.000504481109643442	0.794358009063455

**BLCA**	KDM1A	cg07785945	0.033938714481419	419	0.689277748216995	0.491036500536102	0.00115183634065128	0.676711607339466
**BLCA**	KDM1A	cg10499166	0.0552482451446374	419	1.12313231519226	0.262035337971902	0.00305236859156195	0.457355165515383
**BLCA**	KDM1A	cg12578844	0.0135398730980946	419	0.274854603204973	0.783565720593502	0.000183328163512505	0.880580552357001
**BLCA**	KDM1A	cg22683154	-0.210652055714356	419	-4.37391558106408	1.54785751603151e-05	0.0443742885766843	0.000191681037252714
**BLCA**	KDM1A	cg26662347	0.0592488501859063	419	1.2047367405492	0.22899652113283	0.00351042624835197	0.418958993556299
**BLCA**	KDM1B	cg11007366	0.0310446627193709	418	0.63044170520401	0.528755153053434	0.000963771083359499	0.707122965192856
**BLCA**	KDM1B	cg15124342	-0.334698506169674	418	-7.20944022809841	2.7062411839678e-12	0.112023090032211	1.43510106793177e-10
**BLCA**	KDM1B	cg20677365	-0.263035672380673	413	-5.50022953296882	6.7146619907893e-08	0.0691877649447529	1.51981499828764e-06
**BLCA**	KDM1B	cg22406543	0.272091148271593	418	5.73938562736004	1.8444849537491e-08	0.0740335929677539	4.7766265809573e-07
**BLCA**	KDM1B	cg23073439	-0.353071068946575	418	-7.65988323843873	1.34210540947973e-13	0.124659179727077	8.73705006067497e-12
**BLCA**	KDM1B	cg23860886	0.0598105724457898	418	1.21619933508724	0.224605324860528	0.00357730457629308	0.413844926510502
**BLCA**	KDM1B	cg24342628	0.21416945013986	418	4.45043051579906	1.10449332396028e-05	0.0458685533732102	0.000142919084571135
**BLCA**	KDM2A	cg05038268	-0.226654403960943	419	-4.72351012280304	3.1843590656546e-06	0.0513722188348903	4.76483995294353e-05
**BLCA**	KDM2A	cg05295919	-0.241206032441555	419	-5.04490391959421	6.81733436505806e-07	0.0581803500861965	1.21723137387038e-05
**BLCA**	KDM2A	cg06320126	-0.193408778257064	419	-4.00132133672512	7.46837986111077e-05	0.0374069555068903	0.000749671152148711
**BLCA**	KDM2A	cg07283932	-0.304762239298609	419	-6.49497372098956	2.39605746623533e-10	0.0928800225023028	8.88036658067227e-09
**BLCA**	KDM2A	cg08836144	-0.145290369844787	417	-2.97345694596097	0.00311847540601622	0.0211092915696349	0.0169863237456677
**BLCA**	KDM2A	cg09993857	-0.176779874364546	419	-3.64565714503097	0.000300846179536554	0.0312511239803448	0.00244912953272627
**BLCA**	KDM2A	cg11637544	0.0941493202525698	419	1.91954895291669	0.0556052277477978	0.00886409450402095	0.155747934058877
**BLCA**	KDM2A	cg16004548	-0.149617748618357	419	-3.07148147062594	0.00227121986772699	0.022385470701626	0.0131739717765034
**BLCA**	KDM2A	cg18132081	0.171035340425324	419	3.52355825011657	0.000473491311099319	0.0292530876744066	0.00358071187476042
**BLCA**	KDM2A	cg21293464	-0.246832663595721	419	-5.1701294088846	3.65647284898049e-07	0.0609263638177583	6.94292528224527e-06
**BLCA**	KDM2A	cg21711868	-0.246669483282056	419	-5.16648993200104	3.72393249713392e-07	0.0608458339826363	7.06412106027886e-06
**BLCA**	KDM2A	cg25597833	-0.263771793597128	419	-5.55055493618629	5.10810924303096e-08	0.0695755590974457	1.18907509466866e-06
**BLCA**	KDM2B	cg03486832	-0.198291286098195	419	-4.10641402922619	4.84781675337166e-05	0.0393194341424763	0.000516334962365546
**BLCA**	KDM2B	cg05067991	-0.201593719282279	419	-4.17767651033137	3.59743956791259e-05	0.0406400276540624	0.000399570550751274
**BLCA**	KDM2B	cg07020001	-0.0566735503705492	419	-1.15219929227434	0.249907306007715	0.00321189131160318	0.443530877730572
**BLCA**	KDM2B	cg07529210	-0.182504670832736	419	-3.76771912302267	0.000188764763073067	0.0333079548757654	0.00165331749363951
**BLCA**	KDM2B	cg07793808	-0.0697571909139188	419	-1.41937392767304	0.156546157855638	0.00486606568420092	0.325042085147503
**BLCA**	KDM2B	cg09151742	-0.0304026033805017	419	-0.617390848803042	0.537318021406655	0.000924318292312095	0.713261736775718
**BLCA**	KDM2B	cg12251659	-0.0879752756326472	419	-1.79265379647969	0.0737617829264711	0.00773964912264025	0.191510334111467
**BLCA**	KDM2B	cg12888660	-0.120425714735007	419	-2.46229478163826	0.0142135248633438	0.0145023527694373	0.0559776719090249
**BLCA**	KDM2B	cg15234492	-0.171677767944871	419	-3.53719426773509	0.000450390276468134	0.0294732560065331	0.00343338179005997
**BLCA**	KDM2B	cg15316703	-0.0771072091198464	419	-1.5697789428778	0.117234200749839	0.00594552169825173	0.266467522150791
**BLCA**	KDM2B	cg16837338	-0.0272109513703437	416	-0.55051128157007	0.582269284008671	0.000740435874479208	0.747489941796081
**BLCA**	KDM2B	cg17452615	-0.150292044767041	419	-3.08564316280005	0.00216813996355228	0.0225876987202582	0.0126761070437837
**BLCA**	KDM2B	cg20055546	-0.0607853975476988	419	-1.23609453114517	0.217127683843485	0.00369486455503178	0.404502874584992
**BLCA**	KDM2B	cg21249371	-0.176811135487893	419	-3.6463226328139	0.000300092962232175	0.0312621776325182	0.00244352437763134
**BLCA**	KDM2B	cg21781916	0.0286478539463296	419	0.581726686956444	0.561068940666076	0.00082069953573023	0.731586590460064
**BLCA**	KDM2B	cg26218726	-0.0996163028973544	419	-2.03209791910691	0.0427847799313513	0.00992340780293745	0.128638755893157
**BLCA**	KDM2B	cg26509318	-0.128048017653196	419	-2.62066428106618	0.00910039463281447	0.0163962948249131	0.0396747422139189
**BLCA**	KDM3A	cg01744130	-0.188022673487276	419	-3.88574695957601	0.000118840179659007	0.0353525257453028	0.00111776725140766
**BLCA**	KDM3A	cg02152982	-0.0945305739647652	419	-1.92739203030025	0.0546179884653112	0.00893602941410795	0.153897344875599
**BLCA**	KDM3A	cg02972551	0.0713264168850415	419	1.4514649826463	0.147411429200731	0.00508745774565873	0.312131865085511
**BLCA**	KDM3A	cg03037033	-0.0982296438939826	419	-2.00353354978224	0.0457744159400716	0.00964906293953864	0.135285119430208
**BLCA**	KDM3A	cg04793821	-0.0896841278109449	419	-1.82775435280696	0.0683094925402148	0.00804324278120989	0.181138789333384
**BLCA**	KDM3A	cg07052513	0.00736536108220318	419	0.149504557179324	0.881228669636619	5.42485438712333e-05	0.937033434831551
**BLCA**	KDM3A	cg07211384	-0.112809314384012	419	-2.30448934725033	0.0216917485381038	0.0127259414117908	0.077478782634217
**BLCA**	KDM3A	cg12888358	-0.143448252963901	419	-2.94210935653168	0.0034439294445614	0.0205774012783954	0.0183945792797947
**BLCA**	KDM3A	cg18611949	-0.0477300123136887	419	-0.969918881021063	0.332655975156127	0.00227815407546487	0.5323398870939
**BLCA**	KDM3A	cg19139691	-0.120042733275622	419	-2.45434944108755	0.0145267581299657	0.014410257812282	0.0569374557619183
**BLCA**	KDM3A	cg20191795	-0.0169224710799223	419	-0.343537840999661	0.731369118269272	0.000286370027450807	0.848179581942523
**BLCA**	KDM3A	cg20918903	-0.0394495936517665	419	-0.801363108175065	0.42338338216423	0.0015562704392895	0.618459644586227
**BLCA**	KDM3A	cg22404242	0.136321458638477	419	2.79309793451177	0.00546397731897675	0.0185835400853219	0.0266231148410671
**BLCA**	KDM3A	cg24749656	-0.0713434463340866	419	-1.45181329801894	0.14731457404872	0.00508988733482469	0.31202869673127
**BLCA**	KDM3B	cg00435490	-0.121327642480672	419	-2.48101060560822	0.0134992047887184	0.0147203968299178	0.0538652614553527
**BLCA**	KDM3B	cg03315484	-0.0315791616120051	419	-0.641306823109104	0.521679943682965	0.000997243448117139	0.701615917896247

**BLCA**	KDM3B	cg05191700	-0.0684347028706093	419	-1.39233695553216	0.16457140197881	0.00468330855698858	0.33622216622563
**BLCA**	KDM3B	cg05708550	-0.135498465751407	419	-2.77591923152784	0.00575548584194061	0.0183598342209851	0.0277428753694732
**BLCA**	KDM3B	cg05722427	-0.169789491222964	419	-3.49712732036025	0.000521460465188038	0.0288284713297529	0.00388456338493136
**BLCA**	KDM3B	cg15001364	-0.0236772783382449	419	-0.480731031869427	0.630962846391302	0.000560613509506723	0.782248219568487
**BLCA**	KDM3B	cg18682923	-0.0510551094727957	419	-1.037658815504	0.300037534480349	0.00260662420327916	0.498314298406215
**BLCA**	KDM3B	cg19076659	-0.190207347246179	419	-3.93258091464576	9.85833865108784e-05	0.0361788349464284	0.000952340242174006
**BLCA**	KDM3B	cg24137987	-0.1065264397149	419	-2.17462446681298	0.0302265689546087	0.0113478823583322	0.099229679084054
**BLCA**	KDM4A	cg00154982	-0.053197039687183	419	-1.0813130762019	0.280190384721513	0.00282992503147973	0.477400034802782
**BLCA**	KDM4A	cg00979347	0.11173382964231	419	2.28224004062121	0.0229841987236634	0.0124844486865368	0.0808808411593013
**BLCA**	KDM4A	cg03587511	0.176534742034078	419	3.64043915483528	0.000306813931715432	0.0311645151450385	0.00249120999936022
**BLCA**	KDM4A	cg08552254	-0.160570705984591	419	-3.30207598131443	0.0010434172374293	0.0257829516203899	0.00695379084798898
**BLCA**	KDM4A	cg10958452	-0.142118310324349	419	-2.91426739299742	0.00375959929116764	0.0201976141294479	0.0197510985451847
**BLCA**	KDM4A	cg10968649	0.119334794093334	419	2.4396654456918	0.0151217630542974	0.0142407930812984	0.0587339575139054
**BLCA**	KDM4A	cg12293275	0.107449237965537	419	2.19368149101343	0.0288163016823818	0.0115453387393745	0.095808312458613
**BLCA**	KDM4A	cg17737314	-0.159014943652775	419	-3.26924817917005	0.00116885886750404	0.0252857523048953	0.00764504848987803
**BLCA**	KDM4A	cg19743044	-0.0683300760783331	419	-1.39019827998525	0.165219248648318	0.00466899929687079	0.337164980702165
**BLCA**	KDM4A	cg20873046	-0.0121881268952308	419	-0.247410333621686	0.804713923571113	0.000148550437214248	0.89384061239475
**BLCA**	KDM4A	cg21451504	-0.163295721640867	419	-3.35963698874996	0.000853170602226167	0.0266654927062115	0.00588295992107627
**BLCA**	KDM4A	cg22094309	-0.0938051337777397	419	-1.91246914749319	0.0565091630137463	0.00879940312305965	0.157633169373615
**BLCA**	KDM4A	cg24324755	0.0824759614124048	419	1.67980217306861	0.0937538760295013	0.00680228421090049	0.227416950885193
**BLCA**	KDM4A	cg24505556	-0.145885471575227	419	-2.99317418130255	0.0029271314898513	0.0212825708167263	0.0161452968747513
**BLCA**	KDM4A	cg25841161	0.0848801103432217	419	1.7291181625224	0.0845370169274815	0.00720463313187749	0.211183735103449
**BLCA**	KDM4B	cg01098771	-0.102781570806567	419	-2.0973456810182	0.0365718498850889	0.0105640512974653	0.114628142222426
**BLCA**	KDM4B	cg12072803	-0.212189802524151	419	-4.40734456141269	1.33645626969761e-05	0.0450245122952384	0.000168506124870107
**BLCA**	KDM4B	cg18115693	-0.111905179759302	419	-2.28578433458033	0.0227739215490764	0.0125227692569616	0.0803158775671242
**BLCA**	KDM4C	cg00121158	0.212409586116252	419	4.41212521597738	1.30858323594288e-05	0.0451178322740777	0.000165501421324891
**BLCA**	KDM4C	cg00667760	0.209760935051221	419	4.35455913499794	1.68451844891521e-05	0.0439996498735627	0.00020624083760609
**BLCA**	KDM4C	cg05545111	0.158993119516904	419	3.26878785118929	0.00117071337520598	0.0252788120537167	0.00765428804440272
**BLCA**	KDM4C	cg14196303	-0.0251073977735481	419	-0.509785219867244	0.610474903571429	0.000630381422959166	0.767697207468814
**BLCA**	KDM4C	cg14473811	0.139412976173628	419	2.85768153978683	0.00448387042449169	0.0194359779255884	0.0227257942408565
**BLCA**	KDM4C	cg14497348	0.0903340536349646	419	1.84110836181636	0.0663244632120442	0.00816024124612466	0.177155203128507
**BLCA**	KDM4E	cg15555590	0.0526568428338258	418	1.07030204246397	0.28510998969486	0.00277274309722624	0.482317369162831
**BLCA**	KDM4E	cg19409026	0.0491423737018921	418	0.998687874912405	0.318532096890611	0.00241497289305642	0.517909249373159
**BLCA**	KDM5B	cg00528052	-0.00147102292771319	419	-0.0298585366718388	0.976194332179127	2.1639084538579e-06	0.987422964433455
**BLCA**	KDM5B	cg00571448	0.0373876380589644	419	0.759417124783188	0.448037354181845	0.00139783547962812	0.639487339116807
**BLCA**	KDM5B	cg02624558	0.0895984005464924	419	1.82599309303424	0.0685749200019385	0.00802787338048969	0.181613580943379
**BLCA**	KDM5B	cg04504182	0.0260309352232024	419	0.528549383247537	0.597402679066733	0.000677609588594561	0.758275077989932
**BLCA**	KDM5B	cg08915090	0.151742106009042	419	3.11611256967485	0.00196079403365602	0.0230256667360592	0.0117012895868546
**BLCA**	KDM5B	cg09889216	-0.0935221345962419	419	-1.90664846344698	0.0572615014659385	0.00874638965943759	0.159124380565817
**BLCA**	KDM5B	cg11171004	0.183819149177031	419	3.79580137397978	0.000169266939064629	0.0337894796041675	0.00150887501519351
**BLCA**	KDM5B	cg11221497	-0.126618548570272	419	-2.59092897697215	0.00991143067913776	0.0160322568420423	0.0423165489409386
**BLCA**	KDM5B	cg13134514	0.0407757404602015	419	0.828346052653653	0.407954135753247	0.00166266101007771	0.604042330353789
**BLCA**	KDM5B	cg13253785	-0.0241373647066621	419	-0.490077777638861	0.624339727433449	0.000582612374982418	0.777416684102018
**BLCA**	KDM5B	cg13993945	-0.145030503270013	419	-2.97525452020449	0.00309979406917528	0.0210338468787532	0.0168987652289714
**BLCA**	KDM5B	cg14560895	-0.110830114484415	419	-2.26355053412401	0.0241212369541871	0.0122833142766285	0.0838953595750002
**BLCA**	KDM5B	cg21105875	-0.092018231083922	416	-1.86888262154365	0.062353687037089	0.00846735485181407	0.169322211999557
**BLCA**	KDM5B	cg24354901	0.089270195392685	419	1.81925052226882	0.069598920967946	0.00796916778544815	0.183588039393069
**BLCA**	KDM5B	cg27531483	-0.114640599533424	419	-2.34239334962335	0.0196357758257719	0.0131424670613828	0.0717873349475363
**BLCA**	KDM5C	cg01859586	0.0183964967699197	418	0.373471304289776	0.708989791658739	0.000338431093405664	0.834222331232297
**BLCA**	KDM5C	cg04927657	0.0889309181396821	418	1.81228111678584	0.070670581545016	0.00790870820116683	0.185640628759513
**BLCA**	KDM5C	cg04927982	-0.152863049922808	418	-3.13968050162717	0.0018131186512818	0.0233671120317028	0.0109732186368303
**BLCA**	KDM5C	cg09554224	0.093012462557253	418	1.89616678152877	0.0586373685993619	0.0086513181909644	0.161943888555054
**BLCA**	KDM5C	cg12234996	-0.199714781471847	418	-4.13711330120318	4.26542493499286e-05	0.0398859939383477	0.000462731093840238
**BLCA**	KDM5C	cg16417926	-0.0128219918707667	418	-0.26027940658878	0.79477828559671	0.000164403475534007	0.887606080844577
**BLCA**	KDM5C	cg16824069	-0.114431869672556	418	-2.33807183770717	0.0198612170578889	0.0130946527967568	0.0724099503140599
**BLCA**	KDM5C	cg19903753	-0.0738318576036414	418	-1.50272441365994	0.13367619891684	0.00545114319720439	0.291719204963437
**BLCA**	KDM5C	cg24985300	-0.163557098716246	418	-3.36516223329178	0.000836718290417324	0.0267509245404759	0.0057833551918903

**BLCA**	KDM5C	cg27526317	-0.0819506707731854	418	-1.6690309170959	0.0958709046347837	0.00671591244017503	0.231089457617846
**BLCA**	KDM6A	cg00751785	-0.0836200749170821	418	-1.70326748521093	0.089272155116192	0.00699231692913842	0.219561536896706
**BLCA**	KDM6A	cg03267667	-0.0530483305142013	418	-1.07828178717182	0.281538919946312	0.00281412537034394	0.47866067157409
**BLCA**	KDM6A	cg03478540	0.0397064884567257	418	0.80658977919985	0.420368211646955	0.00157660522556409	0.615572763803207
**BLCA**	KDM6A	cg06877198	-0.0583835806756246	418	-1.1870821552656	0.235879207139845	0.00340864249250717	0.427141225331403
**BLCA**	KDM6A	cg07167981	-0.0916611886797424	418	-1.86838435610272	0.0624180267336608	0.00840177351018333	0.169442346471528
**BLCA**	KDM6A	cg11895950	-0.064033264723034	418	-1.30240616441106	0.193505148413622	0.00410025899109015	0.374577971426528
**BLCA**	KDM6A	cg14384228	-0.0815891205589367	418	-1.66161802615283	0.0973500513618375	0.00665678459358071	0.233559036450761
**BLCA**	KDM6A	cg15923127	-0.0412134741910781	418	-0.837253522046288	0.402935471276581	0.00169855045489866	0.599452335057474
**BLCA**	KDM6A	cg17824914	-0.130718802732948	418	-2.67626564728564	0.00774178643160282	0.0170874053879353	0.0350446542756153
**BLCA**	KDM6A	cg20211377	-0.0496478360617909	418	-1.00898530429169	0.313573880996741	0.00246490762561846	0.512622491400182
**BLCA**	KDM6A	cg22133177	-0.0460403383445006	418	-0.93550883394486	0.350074131285197	0.00211971275487609	0.549076936414053
**BLCA**	KDM6A	cg27306636	-0.0521258450746932	418	-1.05947943561575	0.290002219147741	0.00271710372475092	0.487508538504644
**BLCA**	KDM6B	cg01046511	-0.250929232670339	419	-5.2616513988854	2.30098873887669e-07	0.0629654798085249	4.58747098801059e-06
**BLCA**	KDM6B	cg02308232	-0.258987266951771	419	-5.44256364567391	9.03437009407137e-08	0.0670744044431481	1.97754582751319e-06
**BLCA**	KDM6B	cg24893073	-0.25454449384574	419	-5.34267082788568	1.51865721451741e-07	0.0647928993471839	3.16142411951211e-06
**BLCA**	KDM8	cg00636124	0.0337760143268263	418	0.685969609098652	0.49311809953388	0.00114081914380597	0.678480166814789
**BLCA**	KDM8	cg01755539	-0.202309688480257	418	-4.19314574022561	3.36998027633467e-05	0.0409292100529787	0.000377269025208202
**BLCA**	KDM8	cg06329197	0.0311287527902924	418	0.632151024416339	0.527638827776107	0.000968999250279136	0.706221525474446
**BLCA**	KDM8	cg08572679	-0.184983878659615	418	-3.82070202600594	0.000153583575782772	0.0342190353639553	0.00139009664856835
**BLCA**	KDM8	cg09140090	-0.18847113827814	418	-3.89535602181471	0.000114387132309283	0.0355213699638578	0.00108124332574342
**BLCA**	KDM8	cg10214171	2.93900912032238e-05	418	0.000596553697677702	0.999524307778736	8.63777460933814e-10	0.999766564997096
**BLCA**	KDM8	cg10221365	-0.0458764397655262	418	-0.932171488127238	0.35179383583394	0.00210464772555995	0.550686045468613
**BLCA**	KDM8	cg16752029	0.0637104030586757	418	1.29581247995689	0.195765572675456	0.00405901545789891	0.377628678140158
**BLCA**	KDM8	cg24206694	0.0382356488288848	418	0.77666684523326	0.437801014839408	0.0014619648413658	0.630685755127901
**BLCA**	KDM8	cg24705286	0.0143774983381071	418	0.291861510485735	0.770539413861193	0.000206712458462274	0.872878760184189
**BLCA**	KIAA2026	cg03495688	0.216734959756986	419	4.50635304216431	8.60426400129342e-06	0.0469740427808622	0.000114557609740993
**BLCA**	KIAA2026	cg25608569	-0.263776846312237	419	-5.55066921156986	5.10500361089858e-08	0.0695782246504292	1.1890378769648e-06
**BLCA**	KMT2B	cg09284703	-0.145843398135814	418	-2.99229218542443	0.0029354193545595	0.0212702967798016	0.0161806854154752
**BLCA**	KMT2B	cg13830730	0.163020291824744	418	3.35381547007055	0.00087083052602193	0.0265756155466247	0.00597982080866361
**BLCA**	KMT2B	cg15496871	0.119176351768295	418	2.43637957050923	0.0152578219631376	0.0142030028208003	0.0591796879792754
**BLCA**	KMT2B	cg24471867	-0.00113632045046611	418	-0.0230648009608736	0.98160974944338	1.2912241661475e-06	0.990518921343635
**BLCA**	L3MBTL1	cg01071811	-0.405337853923595	418	-8.99995323149798	8.38962952692145e-18	0.164298775823385	9.03965151547545e-16
**BLCA**	L3MBTL1	cg01877937	-0.306284813878799	418	-6.5307721552885	1.93032066265361e-10	0.0938103872127708	7.28260745055649e-09
**BLCA**	L3MBTL1	cg02177317	-0.259738037871427	418	-5.45948057239862	8.26731655020352e-08	0.0674638483172988	1.82868246717809e-06
**BLCA**	L3MBTL1	cg02472486	-0.267986783028535	418	-5.64605570632762	3.06201924591596e-08	0.0718169158779832	7.53874324054573e-07
**BLCA**	L3MBTL1	cg02611863	-0.39319414142459	418	-8.6801043047206	9.30488802138383e-17	0.154601632850621	9.03700947235373e-15
**BLCA**	L3MBTL1	cg03486259	-0.00482388834015292	418	-0.0979153786197485	0.922047105376292	2.32698987182632e-05	0.959081862757632
**BLCA**	L3MBTL1	cg04984575	-0.238563902803003	418	-4.98628845904352	9.08635843846149e-07	0.0569127357206005	1.57360678620141e-05
**BLCA**	L3MBTL1	cg06362543	0.0132294905277048	418	0.26855283166292	0.788408231537914	0.00017501941962263	0.88355057067088
**BLCA**	L3MBTL1	cg06446163	-0.278791620819289	418	-5.89247911474014	7.91691206947565e-09	0.077724767839046	2.21224230435628e-07
**BLCA**	L3MBTL1	cg06908806	0.0152397899471633	418	0.30936987914575	0.757196578168426	0.000232251197633659	0.864610871784079
**BLCA**	L3MBTL1	cg07678583	-0.303900192087782	418	-6.47472982396366	2.70646770290368e-10	0.0923553267509909	9.92677739178165e-09
**BLCA**	L3MBTL1	cg08369943	-0.199910542278945	418	-4.14133723092565	4.19071052746197e-05	0.0399642249142618	0.000455718848144178
**BLCA**	L3MBTL1	cg08633313	-0.332160860938003	418	-7.14797124604889	4.03528661087528e-12	0.110330837539075	2.06053155153225e-10
**BLCA**	L3MBTL1	cg09541000	-0.286102407419734	418	-6.0605827576388	3.06482799474236e-09	0.0818545875313672	9.21628252891329e-08
**BLCA**	L3MBTL1	cg10360552	-0.29940353998641	418	-6.36941500762938	5.07655765669565e-10	0.089642479756394	1.76384871078598e-08
**BLCA**	L3MBTL1	cg12699433	-0.235471350324481	418	-4.91782941814575	1.26650270755535e-06	0.0554467568236343	2.10931945261316e-05
**BLCA**	L3MBTL1	cg14306330	-0.189351299189964	418	-3.91422220330135	0.000106099618646204	0.0358539145049274	0.00101362074578936
**BLCA**	L3MBTL1	cg15123011	-0.00881545402048526	418	-0.178941126993982	0.858071890799159	7.77122295872896e-05	0.92459305335166
**BLCA**	L3MBTL1	cg15302378	-0.370018308240206	418	-8.08434188718465	7.0276625345797e-15	0.136913548432944	5.35465209809102e-13
**BLCA**	L3MBTL1	cg15330298	-0.373553635333288	418	-8.17404110316618	3.7145856199663e-15	0.139542318470715	2.92482260849857e-13
**BLCA**	L3MBTL1	cg15388309	-0.389728420825141	418	-8.58982158997119	1.81605175418326e-16	0.151888241998859	1.7103865637401e-14
**BLCA**	L3MBTL1	cg16862791	-0.302877214233842	418	-6.45072943744993	3.12579379888998e-10	0.0917346069020527	1.13457783495876e-08
**BLCA**	L3MBTL1	cg17091610	-0.340004072622733	418	-7.33852942862028	1.15988944181735e-12	0.115602769400045	6.50089619861234e-11
**BLCA**	L3MBTL1	cg17774347	0.060679909987039	418	1.23394146627904	0.217928134922937	0.00368205147603515	0.405485706228303
**BLCA**	L3MBTL1	cg18911945	-0.302972137577601	418	-6.45295542693871	3.08436523012959e-10	0.0917921161483405	1.12228769276287e-08

**BLCA**	L3MBTL1	cg20091959	-0.32696731778154	418	-7.02271044670531	9.0368289446931e-12	0.106907626897255	4.32090393929597e-10
**BLCA**	L3MBTL1	cg20252111	-0.252690949224269	418	-5.30110252841583	1.88072120362749e-07	0.0638527158198619	3.83911900590389e-06
**BLCA**	L3MBTL1	cg20529070	-0.37744852047557	418	-8.27334266208619	1.82348677755252e-15	0.142467385609196	1.4995383760351e-13
**BLCA**	L3MBTL1	cg22330467	-0.268106790959457	418	-5.64877985407293	3.01733530140022e-08	0.0718812513585778	7.4446743557605e-07
**BLCA**	L3MBTL1	cg22457903	-0.366026900434247	418	-7.98355958685492	1.43013158537212e-14	0.133975691841502	1.05355735371628e-12
**BLCA**	L3MBTL1	cg22601123	-0.221547828038215	418	-4.61152852647608	5.34225987667948e-06	0.0490834401084506	7.54259236266571e-05
**BLCA**	L3MBTL1	cg23626798	-0.313744792128527	418	-6.70697678848283	6.57407026884458e-11	0.0984357945877726	2.70165463547013e-09
**BLCA**	L3MBTL1	cg24343973	-0.276781210032854	418	-5.84644838380664	1.02283856109344e-08	0.0766078382272509	2.79195449198957e-07
**BLCA**	L3MBTL2	cg02358112	-0.232925484948043	419	-4.86159105703421	1.6590289419784e-06	0.054254281538281	2.677089763789e-05
**BLCA**	L3MBTL2	cg06235991	-0.00241405011364166	419	-0.049000008449458	0.960943033910165	5.82763795117333e-06	0.97951496193618
**BLCA**	L3MBTL2	cg08053598	-0.106528658302274	419	-2.17467027678741	0.0302231087794247	0.0113483550396826	0.099229679084054
**BLCA**	L3MBTL2	cg09993119	-0.0136261923393757	419	-0.276607177445428	0.782220463796271	0.00018567311766966	0.879759122736588
**BLCA**	L3MBTL2	cg12821546	0.0637676113568117	419	1.29698079404626	0.195363645615125	0.00406630825815338	0.377075212448306
**BLCA**	L3MBTL2	cg13843727	-0.149981484253069	419	-3.07912016761148	0.00221507269779374	0.0224944456187537	0.0128971907381308
**BLCA**	L3MBTL2	cg14679202	-0.0904802628824985	419	-1.84411284556735	0.0658844917002625	0.00818667797128604	0.176276590567142
**BLCA**	L3MBTL2	cg15283028	-0.168671945042506	419	-3.47343305778897	0.000568290918720178	0.0284502250444223	0.00417705556088883
**BLCA**	L3MBTL2	cg19467356	-0.0889324936591919	419	-1.81231347950684	0.0706655740323468	0.0079089884284422	0.185640011693256
**BLCA**	L3MBTL2	cg24240981	-0.0228643984190466	419	-0.464217958473052	0.642736787805496	0.000522780715064901	0.790198879395383
**BLCA**	L3MBTL2	cg26947834	0.0508284529066467	419	1.03304022299591	0.302191077583832	0.00258353162488321	0.500588831173218
**BLCA**	L3MBTL2	cg27177954	-0.137835553873834	419	-2.82471775022526	0.00496215209396084	0.0189986399117067	0.0246221239952538
**BLCA**	L3MBTL3	cg03454037	-0.0679513410477454	419	-1.38245694461936	0.167580362878813	0.00461738475018701	0.340401277469998
**BLCA**	L3MBTL3	cg07826275	-0.0714331822918845	419	-1.45364875215319	0.146805000466062	0.00510269953234561	0.311220520118182
**BLCA**	L3MBTL3	cg08106148	-0.0824579689275767	419	-1.67943320803391	0.0938257663267186	0.00679931663966121	0.227541669231896
**BLCA**	L3MBTL3	cg08358392	-0.309313456984071	419	-6.60214578281727	1.25110870531581e-10	0.095674814671437	4.91063323013816e-09
**BLCA**	L3MBTL3	cg09710740	-0.0911565917462718	419	-1.85801243333558	0.0638803664489835	0.00830952421879647	0.172331006260437
**BLCA**	L3MBTL3	cg10237990	-0.0984821151944901	419	-2.00873342925765	0.045217388491801	0.00969872701318082	0.134087120543233
**BLCA**	L3MBTL3	cg16487825	-0.0762308189987562	419	-1.55183215674121	0.121470179542603	0.00581113776522112	0.272945644185243
**BLCA**	L3MBTL3	cg16800851	-0.208434955577721	419	-4.32577791154074	1.90924844809324e-05	0.0434451307066864	0.000230073675061198
**BLCA**	L3MBTL3	cg18411550	-0.110253473201298	419	-2.25162813440081	0.0248718808691307	0.0121558283529494	0.0858727100290519
**BLCA**	L3MBTL3	cg18798995	-0.106395282445155	419	-2.17191635543518	0.0304317293199518	0.0113199561265843	0.0997642625670598
**BLCA**	L3MBTL3	cg22934935	0.0309954324676465	419	0.629440996189882	0.529409257441895	0.000960716833856433	0.707595075483382
**BLCA**	L3MBTL3	cg24516147	-0.106846385032842	419	-2.18123112132793	0.029731061884022	0.0114161499945864	0.0980166219521807
**BLCA**	LBR	cg01017344	-0.0476582678602301	419	-0.968457644619869	0.333384027562062	0.00227131049543744	0.532989663792911
**BLCA**	LBR	cg05530459	0.123444022907689	419	2.52495200742077	0.01194580321359	0.0152384267916342	0.0489643978695948
**BLCA**	LBR	cg12994003	-0.12161886803967	419	-2.48705513144561	0.0132754053100139	0.0147911490632507	0.0531768127638754
**BLCA**	LBR	cg14607642	-0.244541561655627	419	-5.11907271688551	4.72090034805754e-07	0.059800575376973	8.76300934566423e-06
**BLCA**	LBR	cg18107727	0.0621183446082396	419	1.26330439226927	0.207194223018366	0.00385868873686801	0.392081425090993
**BLCA**	LBR	cg18289048	-0.0614400436593944	419	-1.24945718629351	0.212207249016434	0.00377487896486829	0.398383151725696
**BLCA**	LBR	cg18764107	0.0727371625614173	419	1.48032431657207	0.139550987293154	0.00529069481748604	0.300643298407189
**BLCA**	LBR	cg19322477	-0.102036321151757	419	-2.08197763836685	0.0379611493035321	0.0104114108341845	0.11781495447694
**BLCA**	LBR	cg20483690	-0.111084295133019	419	-2.26880660363975	0.0237966217120812	0.0123397206251996	0.083052238199625
**BLCA**	LBR	cg26930584	-0.175411283282622	419	-3.61653366561808	0.000335604527998555	0.0307691183028563	0.00268380125936674
**BLCA**	MBD1	cg01719254	-0.0208906233594533	419	-0.424125900623432	0.671695358796435	0.000436418144346536	0.80989892500209
**BLCA**	MBD1	cg03841029	-0.0435685477971096	419	-0.885185471208301	0.376572923391849	0.00189821835714902	0.575113157217509
**BLCA**	MBD1	cg05549078	-0.00264185896037169	419	-0.0536240673708966	0.957260672693996	6.97941876649616e-06	0.977547775816274
**BLCA**	MBD1	cg12090413	-0.0276860647717501	419	-0.562181240841258	0.574298264504921	0.000766518182545544	0.741536108763481
**BLCA**	MBD1	cg13478031	0.00472509297250921	419	0.095909983099218	0.923638698716962	2.23265035988559e-05	0.959803374973999
**BLCA**	MBD1	cg14389183	-0.0657355469625131	419	-1.33717808565363	0.181902302243154	0.00432116213446076	0.359433774465428
**BLCA**	MBD1	cg19328682	-0.138395054856484	419	-2.83640726090159	0.00478748702167612	0.0191531912087293	0.0239540626038251
**BLCA**	MBD1	cg21282183	0.085643472953829	419	1.74478323923149	0.0817682400269059	0.00733480445959324	0.206180609177916
**BLCA**	MBD2	cg15090185	-0.036551079564256	419	-0.742401969532035	0.458266871545658	0.00133598141731257	0.648295412067846
**BLCA**	MBD2	cg16852837	-0.213933260486324	419	-4.44528696966147	1.13000833212024e-05	0.0457674399423094	0.000145735855750932
**BLCA**	MBD2	cg17987217	0.00845221544484426	419	0.171567364560124	0.863861900736784	7.14399459260639e-05	0.927709452041929
**BLCA**	MBD2	cg22224310	-0.215976947717737	419	-4.48981989746344	9.26600603104045e-06	0.0466460419454699	0.000122487625009135
**BLCA**	MBD2	cg22836370	0.0709353717709897	419	1.44346701055607	0.149648869110812	0.00503182696828853	0.3151036333236
**BLCA**	MBD2	cg23388714	-0.179896619110893	419	-3.71206292910485	0.000233830955496738	0.0323627935675296	0.00197831923348703
**BLCA**	MBD2	cg24881765	-0.199107108386817	419	-4.12400482680675	4.5054518778032e-05	0.0396436406101595	0.000485117125787989

**BLCA**	MBD2	cg25192916	0.2168269992546	419	4.50836111467514	8.52704815337388e-06	0.0470139476057541	0.000113763193970771
**BLCA**	MBD3	cg08660999	-0.0878078859925498	419	-1.78921640445639	0.0743144080497367	0.00771022484248062	0.192559539680286
**BLCA**	MBD3	cg09174855	-0.0358028748211002	419	-0.727185207677673	0.467525501868117	0.00128184584545537	0.656597294781003
**BLCA**	MBD3	cg12286540	-0.211686640498277	419	-4.3964025953668	1.40240733924967e-05	0.0448112337654467	0.000175937552894031
**BLCA**	MBD5	cg03890220	0.20791438350809	419	4.31448539293591	2.00502555032861e-05	0.0432283908695491	0.000239893813691578
**BLCA**	MBD5	cg04152151	0.169845671249093	418	3.49407070572468	0.00052742170540734	0.0288475520420551	0.00392146078089062
**BLCA**	MBD5	cg05218490	0.0635633682693192	419	1.29280977830985	0.196801361497233	0.00404030178574109	0.3789501526278
**BLCA**	MBD5	cg06394585	0.159177365566151	419	3.27267423571706	0.00115514179577365	0.0253374337085802	0.0075718824452659
**BLCA**	MBD5	cg06758980	0.263147357438947	419	5.5364360715692	5.50629173611367e-08	0.0692465317271011	1.27050072967561e-06
**BLCA**	MBD5	cg20595490	0.182524734246008	419	3.76814759623689	0.000188451966169234	0.0333152786115759	0.00165094957839745
**BLCA**	MBD5	cg21412502	-0.0816738282095154	419	-1.66335473406838	0.0970018817213209	0.00667061421439744	0.232956947457739
**BLCA**	MBD5	cg23120335	-0.109971991342843	419	-2.24580916722776	0.0252455641612642	0.0120938388799104	0.0868530235372428
**BLCA**	MBD5	cg25686162	0.204821176382053	419	4.24746457210977	2.67507026206327e-05	0.041951714294528	0.000308348519731738
**BLCA**	MBD5	cg26664634	-0.118807926894662	419	-2.42873967655039	0.0155783580718733	0.0141153234930073	0.0601291385682615
**BLCA**	MBTD1	cg01388767	0.0935754312422617	419	1.90774462506378	0.0571191841760505	0.00875636133217524	0.158839314165922
**BLCA**	MBTD1	cg14577476	-0.0534672944500642	419	-1.08682213639383	0.277750850552029	0.00285875157580987	0.474542632109559
**BLCA**	MBTD1	cg17045523	-0.0838898293522587	419	-1.70880102583977	0.0882409252007912	0.00703750346875108	0.217875854098148
**BLCA**	MBTD1	cg18711617	0.0717931769983677	419	1.46101242034496	0.144774234205804	0.00515426026351895	0.308296516329195
**BLCA**	MBTD1	cg22141797	-0.0781975938975517	419	-1.59211304364374	0.112126221748474	0.00611486369136641	0.258421144003698
**BLCA**	MBTD1	cg25259117	-0.0299863976736458	419	-0.608931229028316	0.542905625845661	0.000899184045442033	0.717412652359181
**BLCA**	MBTD1	cg25716740	0.0185183742878751	419	0.375946412432905	0.707150236189907	0.000342930186265833	0.833099194212029
**BLCA**	MBTD1	cg25855326	-0.0970017987199335	419	-1.9782504951405	0.0485661808201902	0.00940934895490248	0.141186751521894
**BLCA**	MBTD1	cg27207041	0.0298355843669033	419	0.605865940525911	0.544937404891851	0.000890162094514606	0.718975755566779
**BLCA**	MBTD1	cg27214721	0.0838562506561474	419	1.70811219700349	0.0883687676025408	0.00703187077410662	0.218101519888073
**BLCA**	MBTD1	cg27353579	-0.0914171158990595	419	-1.86336729187503	0.0631218763225368	0.00835708907930208	0.170829822460791
**BLCA**	MLLT10	cg00879184	-0.16904460309294	419	-3.48133263253287	0.000552257717334418	0.0285760778348498	0.00407921922353909
**BLCA**	MLLT10	cg03524461	-0.149287347761054	419	-3.06454393242526	0.00232334416504067	0.0222867122015298	0.0134191280700255
**BLCA**	MLLT10	cg04188362	-0.175880837489113	419	-3.62652328386238	0.000323277885668964	0.0309340689958717	0.00260153924048116
**BLCA**	MLLT10	cg05913198	0.0340073613424671	419	0.690673543089766	0.490159640006972	0.00115650062547713	0.676102041431628
**BLCA**	MLLT10	cg06398735	0.00254170005090272	419	0.0515910430607093	0.958879559960305	6.46023914875888e-06	0.978411569726944
**BLCA**	MLLT10	cg11443787	-0.0856702797832202	419	-1.74533340222266	0.0816723578981321	0.00733939683813523	0.206018787624101
**BLCA**	MLLT10	cg15910013	0.137745625331686	419	2.82283915368816	0.00499075550373294	0.0189738572980173	0.0247293524622548
**BLCA**	MLLT10	cg19119538	-0.221938925934111	419	-4.62009061809044	5.13690149453613e-06	0.0492568868447866	7.28967739631418e-05
**BLCA**	MLLT10	cg20569108	-0.15418931834042	419	-3.16758143082613	0.00165157983958285	0.0237743458902835	0.0101720372132458
**BLCA**	MLLT10	cg23344688	-0.109377144003956	419	-2.23351391241198	0.0260512127553807	0.0119633596304621	0.088872232443399
**BLCA**	MLLT10	cg26877631	-0.052583597611721	419	-1.06880913056569	0.285781491906676	0.00276503473779139	0.483033185186371
**BLCA**	MLLT10	cg27470303	0.0126002081296074	419	0.255776597032957	0.798250995640578	0.000158765244909424	0.889804100304595
**BLCA**	MLLT10	cg27572529	-0.137283222725501	419	-2.8131807408586	0.00514019001241335	0.0188466832418996	0.0253383026615256
**BLCA**	MLLT6	cg05652455	-0.0177740863262036	419	-0.360831550705764	0.718410312262302	0.000315918144731336	0.84013065905226
**BLCA**	MORF4L	cg03589001	0.00659034555384842	419	0.133772309879794	0.893647941802426	4.34326545191297e-05	0.943635361456408
**BLCA**	MORF4L	cg07757959	0.00283584440261044	419	0.0575615861311251	0.95412575490899	8.04201347581694e-06	0.975672160486888
**BLCA**	MORF4L	cg08692282	0.0834026137569254	419	1.69880692427321	0.0901104977496261	0.00695599598148688	0.220991017368129
**BLCA**	MORF4L	cg13747872	0.00597468393180272	419	0.121275003306131	0.903532342883443	3.56968480849416e-05	0.948890126666653
**BLCA**	MORF4L	cg15822222	-0.00455486395413401	419	-0.0924545998013628	0.926381799298248	2.07467856406693e-05	0.961225834217301
**BLCA**	MORF4L	cg20775702	-0.107883909646053	419	-2.20266002947707	0.0281718130267241	0.0116389379605177	0.0942455345810708
**BLCA**	MORF4L	cg20905746	0.126470145287616	419	2.58784288838784	0.00999920063085319	0.0159946976490708	0.0426234418562517
**BLCA**	MORF4L	cg23623458	-0.140540352500259	410	-2.84961040862137	0.00460189975684553	0.0197515906808972	0.0232000641360923
**BLCA**	MORF4L	cg24795825	0.064054718690827	418	1.30126224305495	0.193897686489243	0.00410300698656099	0.375067176138055
**BLCA**	MPHOSF	cg00331616	-0.112200604763166	419	-2.29189554238412	0.0224152920336505	0.0125889757092201	0.0792993499532538
**BLCA**	MPHOSF	cg03134886	0.0767756893070968	419	1.56298962583629	0.118822787505286	0.00589450646857986	0.26894315720879
**BLCA**	MPHOSF	cg03428000	0.127847582870863	419	2.61649391215998	0.00921043552699104	0.0163450044459222	0.040019918531431
**BLCA**	MPHOSF	cg04203901	0.143242008129862	419	2.9377906187266	0.00349125260005934	0.0205182728930754	0.0186064605582665
**BLCA**	MPHOSF	cg06019692	-0.00832622735465924	419	-0.169009815631496	0.865871865425733	6.93260619614758e-05	0.92874776887639
**BLCA**	MPHOSF	cg09559995	-0.104950502602908	419	-2.14209236046276	0.0327716975879152	0.0110146079966029	0.105415997804536
**BLCA**	MPHOSF	cg11051893	0.166768002052941	419	3.43309727327422	0.000657160934758822	0.0278115665087296	0.00472662516259502
**BLCA**	MPHOSF	cg19693579	0.104197834663799	419	2.12656079234708	0.0340503905785122	0.0108571887486243	0.108499880587564
**BLCA**	MPHOSF	cg20759595	-0.128075906164624	419	-2.62124457238472	0.00908517657448577	0.0164034377398896	0.0396194980904505

**BLCA**	MPHOSF	cg20952532	-0.0513467523788049	419	-1.04360187461171	0.297281568303083	0.00263648897985031	0.495346177274033
**BLCA**	MPHOSF	cg21958444	-0.109173653564036	419	-2.22930840524089	0.0263318532162472	0.0119188866325201	0.0895797320783621
**BLCA**	MPHOSF	cg22656705	-0.146406095381077	419	-3.00408952121809	0.00282632208857034	0.021434744764733	0.0156960951283724
**BLCA**	MSH6	cg00620552	-0.0329802266677576	419	-0.669789851246039	0.503366966261603	0.00108769535105667	0.686765379089446
**BLCA**	MSH6	cg02905881	0.0450068276879232	419	0.914465474562345	0.361007177998679	0.00202561453853041	0.559865899356703
**BLCA**	MSH6	cg05039065	0.109695939059457	419	2.24010297484578	0.0256167318265831	0.0120331990461362	0.0877665830933325
**BLCA**	MSH6	cg07652213	-0.0416377538362422	419	-0.845887674291662	0.398106414339736	0.0017337025445275	0.594713311259417
**BLCA**	MSH6	cg09898070	0.0672043637375883	419	1.36719050216549	0.172311048500836	0.00451642650537407	0.346797550179133
**BLCA**	MSH6	cg11869233	-0.0327567110029504	419	-0.665245616764495	0.506265632240089	0.00107300211573081	0.689298153464227
**BLCA**	MSH6	cg16595246	0.0672915710438344	419	1.36897269285086	0.171753678159722	0.00452815553354741	0.346006982565709
**BLCA**	MSH6	cg18222961	0.0980021854327483	419	1.99884916734419	0.0462811787741636	0.00960442834959478	0.136335271067964
**BLCA**	MSH6	cg19076255	-0.0336925422916263	419	-0.684272415540321	0.494187872766944	0.00113518740607303	0.679363041297198
**BLCA**	MSH6	cg19736286	0.086704067111348	419	1.76655299248071	0.0780435307973944	0.00751759525364913	0.199333605355845
**BLCA**	MSH6	cg21405109	-0.0676947525095329	419	-1.37721262216308	0.169194280496546	0.00458257951732691	0.342607628115683
**BLCA**	MTA1	cg01318265	-0.261745524642639	419	-5.50476701008578	6.51233314273939e-08	0.0685107196704502	1.48048055086578e-06
**BLCA**	MTA1	cg01655777	-0.0954767086174795	419	-1.94685943312818	0.0522307967234009	0.00911580188842709	0.148953094216293
**BLCA**	MTA1	cg05922925	-0.226213787719173	419	-4.71383187313825	3.33133029125967e-06	0.0511726777542549	4.96318254876528e-05
**BLCA**	MTA1	cg13052453	-0.192575042375798	419	-3.98340686248552	8.03187107997247e-05	0.0370851469460405	0.000797291316493051
**BLCA**	MTA1	cg13981078	-0.16340966971995	419	-3.36204564537662	0.000845961759306422	0.0267027201579832	0.00583894500075271
**BLCA**	MTA1	cg16531924	-0.13308616007538	419	-2.72559966354319	0.0066921345827309	0.0177119260036098	0.0312546027333234
**BLCA**	MTA1	cg20222911	-0.262523401788456	419	-5.52233553870518	5.93402263676418e-08	0.068918536486583	1.36214622898918e-06
**BLCA**	MTA1	cg21517792	-0.165405471730894	419	-3.40425580957656	0.000728483529902725	0.0273589700785196	0.00515477967302643
**BLCA**	MTA1	cg23123838	-0.133265381888069	419	-2.72933643891895	0.00661811892981042	0.0177596620097728	0.0309907869756018
**BLCA**	MTA1	cg23883989	-0.283639952704491	419	-6.00383462512133	4.23217537878148e-09	0.080451622770206	1.24334536033582e-07
**BLCA**	MTA1	cg23966123	-0.189814573018176	419	-3.92415636980014	0.000101967589017349	0.0360295721300725	0.00098015823573616
**BLCA**	MTA2	cg00501779	-0.148698890846048	419	-3.05219050264715	0.00241889047363192	0.0221113601388448	0.0138567868560914
**BLCA**	MTA2	cg07654871	-0.031412817771279	419	-0.637925381569634	0.523876639980782	0.000986765120331582	0.703087368768479
**BLCA**	MTA2	cg09086761	0.0419569657810492	419	0.852383985537493	0.394496222622081	0.00176038697755213	0.591248061105319
**BLCA**	MTA2	cg13708959	-0.0738459319531113	419	-1.50301244413148	0.133601928550737	0.00545322166602354	0.291598025690206
**BLCA**	MTA2	cg19570317	-0.146609549020649	419	-3.0083557999189	0.00278779294146848	0.0214943598640382	0.0155209020316753
**BLCA**	MTA2	cg21002970	-0.00663481419411301	419	-0.134674983901329	0.892934619288847	4.40207593904035e-05	0.943222019714917
**BLCA**	MTF2	cg05396182	-0.0437729688903295	419	-0.889346664507138	0.374335770220087	0.00191607280547375	0.572990120781959
**BLCA**	MTF2	cg05962718	-0.19403468107951	419	-4.01477601947404	7.07006331668603e-05	0.0376494574616271	0.000715597847590779
**BLCA**	MTF2	cg09936581	0.176246403609025	419	3.63430235626523	0.000313974846426287	0.0310627947851152	0.0025394028790782
**BLCA**	MTF2	cg11404133	-0.0497380446055552	419	-1.01082314104599	0.312694351587792	0.0024738730811842	0.511690037598248
**BLCA**	MTF2	cg14474880	-0.145300161106467	419	-2.98090566168241	0.00304436241803186	0.0211121368175653	0.016652589646208
**BLCA**	MTF2	cg15463775	-0.117426415184567	419	-2.40010079946145	0.0168334628648346	0.0137889629830984	0.0637526123415093
**BLCA**	MTF2	cg16813892	0.0690156171919303	419	1.40421226277714	0.161009025479946	0.00476315541638307	0.331133668740075
**BLCA**	MTF2	cg19708337	-0.0592160430956645	419	-1.20406730940677	0.229254855451309	0.00350653975990759	0.419301698796638
**BLCA**	MTF2	cg22801127	-0.0543198142961971	419	-1.10420206634115	0.270149962591742	0.00295064222517333	0.46619717656461
**BLCA**	MTF2	cg23992808	-0.0511403346078185	419	-1.03939549401385	0.29923042475186	0.00261533382379964	0.497542267020586
**BLCA**	MTF2	cg26054395	-0.07531142466234	419	-1.53300861438785	0.126041227841899	0.00567181068467131	0.28001663728118
**BLCA**	NAP1L2	cg02379437	-0.131026303589073	414	-2.66961665343791	0.00789700934769046	0.0171678922322159	0.0355706065192654
**BLCA**	NAP1L2	cg04297329	-0.0861845598506876	418	-1.7558888362134	0.0798504249191171	0.00742777835665676	0.202779094482199
**BLCA**	NAP1L2	cg07693661	-0.125797176509631	416	-2.56759571338647	0.0105942016366987	0.0158249296177952	0.0445832155212236
**BLCA**	NAP1L2	cg20001978	-0.149208012005951	416	-3.05543503471697	0.00239417746499745	0.022263030846768	0.0137465969231686
**BLCA**	NAP1L2	cg23483095	-0.142086505857854	418	-2.91360177358573	0.00376745915451233	0.0201885751468939	0.0197870454676028
**BLCA**	NCOA1	cg11380327	-0.186588329987117	419	-3.85503077882104	0.000134200706473634	0.0348152048873811	0.00123843532140094
**BLCA**	NCOA1	cg13911252	0.0412973335457081	419	0.838960035938521	0.401978239491579	0.00170546975798547	0.59851511820058
**BLCA**	NCOA1	cg15477048	0.251975676714453	419	5.28507825908396	2.04157736621036e-07	0.0634917416557065	4.12418773375665e-06
**BLCA**	NCOA1	cg23006163	-0.0811105664757819	419	-1.65180721119766	0.0993357621622891	0.00657892399402224	0.23711873542071
**BLCA**	NCOA3	cg04175879	-0.0659055709247821	419	-1.34065174434954	0.180772279603032	0.00434354427892148	0.357919083745343
**BLCA**	NCOA3	cg09568061	0.0364897783102315	419	0.741155196493681	0.459021566962462	0.00133150392112984	0.648926433457109
**BLCA**	NCOA3	cg12128876	0.149114364301391	419	3.06091215929671	0.0023510673770119	0.0222350936410078	0.0135490729333479
**BLCA**	NCOA3	cg14341558	0.0623534825131846	419	1.26810503503647	0.205476566448834	0.00388795678152202	0.389741161806228
**BLCA**	NCOA3	cg14774045	0.114503651206354	419	2.3395579564518	0.0197834358217294	0.0131110861395863	0.0722273287944123
**BLCA**	NCOA3	cg18295930	0.0170184740566917	419	0.345487330714195	0.729904361161766	0.000289628459218288	0.847318170415412

**BLCA**	NCOA3	cg21735648	0.193912570414324	419	4.01215067541758	7.14616559294404e-05	0.0376020849646901	0.00072207923904077
**BLCA**	NCOA3	cg22511723	-0.0117648678604774	419	-0.238817264560627	0.811366027223497	0.000138412115774493	0.898292328036671
**BLCA**	NCOA3	cg22876277	0.0187143700826869	419	0.379926761610598	0.704195551726788	0.000350227647591768	0.831141351362934
**BLCA**	NCOA3	cg24856420	0.168764143745062	419	3.47538733543257	0.000564284450049667	0.0284813362140039	0.00415110586356159
**BLCA**	NCOA3	cg25373408	0.124140165964785	419	2.5394133103315	0.0114705370995106	0.0154107808057644	0.0474698884519273
**BLCA**	NCOR1	cg00253204	0.0181394670533232	419	0.368251558125142	0.712874800008559	0.000329040264978599	0.836591610462752
**BLCA**	NCOR1	cg08125503	-0.0610239745376137	419	-1.24096418514089	0.215325112960593	0.00372392546836732	0.40236847151537
**BLCA**	NCOR1	cg08206308	-0.0531692059899379	419	-1.08074570840183	0.280442455081037	0.00282696446560045	0.477644759698999
**BLCA**	NCOR1	cg21211144	0.197899981941402	419	4.09797986633325	5.02061572565452e-05	0.0391644028524073	0.000531754635482052
**BLCA**	NCOR1	cg22291359	-0.0936518920397238	419	-1.90931723680884	0.0569155240383953	0.00877067688262008	0.158454220271371
**BLCA**	NCOR1	cg22625220	-0.0445656690084104	419	-0.90548392178818	0.365738282526654	0.00198609885416719	0.564584660201975
**BLCA**	NCOR2	cg08976554	-0.264062361264295	419	-5.55712739564043	4.93244485793579e-08	0.0697289306364749	1.15437609891172e-06
**BLCA**	NCOR2	cg10641483	0.0445293225037764	419	0.904743966774391	0.366129783927817	0.00198286056264532	0.56502083735133
**BLCA**	NCOR2	cg13416396	-0.0384612048341556	419	-0.781255249993946	0.435101070601594	0.00147926427729488	0.628472143161284
**BLCA**	NCOR2	cg13419087	0.0434739501055238	419	0.883259879476747	0.377610961787495	0.00188998433777757	0.576189458472361
**BLCA**	NCOR2	cg13857025	-0.183088387450522	419	-3.78018693723055	0.000179860223234604	0.0335213576192326	0.00158765984412944
**BLCA**	NCOR2	cg16181859	-0.217820524004757	419	-4.53004539415419	7.73446923901069e-06	0.0474457806777069	0.000104479128317346
**BLCA**	NCOR2	cg19895345	-0.0651455207539128	419	-1.32512451559083	0.185864264884322	0.00424393887429848	0.364678166471057
**BLCA**	NCOR2	cg23611643	-0.16316155308983	419	-3.35680108493015	0.000861731426076349	0.0266216924066853	0.00593095059366394
**BLCA**	NCOR2	cg24626542	-0.0354950549398985	419	-0.720925216951869	0.471364389372652	0.00125989892518641	0.659627312262025
**BLCA**	NCOR2	cg25113767	0.0609949131417732	419	1.24037099439498	0.215544110261247	0.00372037942917245	0.402651966997971
**BLCA**	NCOR2	cg25449924	-0.0266576485627367	419	-0.541283529686212	0.588604728249055	0.000710630226894379	0.752141169386361
**BLCA**	ORC1	cg00298020	-0.0962265352782221	418	-1.96229143704476	0.0504012331214795	0.00925954609165093	0.145133014859801
**BLCA**	ORC1	cg02275620	0.0919031784255374	418	1.87335893753554	0.0617265822702828	0.00844619420471616	0.168079922404709
**BLCA**	ORC1	cg04407474	0.0127557834552731	418	0.2589351927618	0.795814562205148	0.000162710011557818	0.888198838972216
**BLCA**	ORC1	cg07142859	-0.074988414688247	418	-1.5263962923202	0.127678485429234	0.00562326233745649	0.282578096599767
**BLCA**	ORC1	cg08613513	0.046396349022783	418	0.942758277880866	0.346357035794806	0.0021526212026439	0.545667171117705
**BLCA**	ORC1	cg10586599	0.0134935449070147	418	0.273913985862953	0.784287996301892	0.000182075754157623	0.881003759928425
**BLCA**	ORC1	cg13460942	0.0634897635042894	418	1.29130667499605	0.197321370678896	0.0040309500698306	0.379622581625137
**BLCA**	ORC1	cg16136434	0.0730821627563659	418	1.48738326710246	0.137678521772456	0.00534100251314796	0.297788609635678
**BLCA**	ORC1	cg16965475	-0.0724099519422467	418	-1.4736298471315	0.141344887562263	0.00524320114027848	0.303372586033462
**BLCA**	ORC1	cg17146111	-0.161780623666539	418	-3.32762363990407	0.000954573819775391	0.0261729701939342	0.00645740136831363
**BLCA**	ORC1	cg19776520	-0.0476336468750795	418	-0.967956186604481	0.333634113718955	0.00226896431461977	0.533225110275344
**BLCA**	ORC1	cg21197365	-0.0115843292734367	418	-0.235151982138454	0.814207605472426	0.000134196684715403	0.900052068393165
**BLCA**	ORC1	cg23606758	-0.0124853284486036	418	-0.253444243807336	0.800051361708777	0.000155883426469511	0.890862184140224
**BLCA**	ORC1	cg25605769	0.104382920277956	418	2.13037975562971	0.0337320757475313	0.0108957940457541	0.107719432728683
**BLCA**	PADI1	cg14524955	-0.261820107911761	419	-5.50645098991658	6.45460821471861e-08	0.0685497689069261	1.46778658712604e-06
**BLCA**	PADI1	cg21010178	0.0201948267074581	419	0.409993825486666	0.682023577844445	0.000407831025744264	0.816654757294874
**BLCA**	PADI1	cg22193702	-0.310122176165181	419	-6.62124166878325	1.11337134729521e-10	0.0961757641494273	4.41680829125362e-09
**BLCA**	PADI2	cg00569276	-0.0913861648368587	419	-1.86273109821007	0.0632115972160761	0.0083514311235895	0.171007198283051
**BLCA**	PADI2	cg01603374	0.0124538616813348	419	0.252805389191194	0.800544686320792	0.000155098670777818	0.891041639758483
**BLCA**	PADI2	cg07014827	-0.048120126035798	419	-0.977864684203614	0.328715078337939	0.00231554652970108	0.528392032486603
**BLCA**	PADI2	cg09313801	-0.010595250331228	419	-0.215072165700663	0.829817474833886	0.000112259329581387	0.908616013877552
**BLCA**	PADI2	cg10233913	-0.0202857569010582	419	-0.411840641598125	0.680670406742982	0.00041151193304883	0.815814528820674
**BLCA**	PADI2	cg15185794	0.00551124134145775	419	0.111867680462138	0.910982803068098	3.0373781123793e-05	0.952780078345478
**BLCA**	PADI2	cg17183414	0.0341745567349719	419	0.694073163722302	0.488027485612549	0.00116790032803181	0.67418908983259
**BLCA**	PADI2	cg18228326	0.00620496110776131	419	0.125949379542591	0.899833432470843	3.85015423488304e-05	0.947184115359405
**BLCA**	PADI2	cg18879300	0.132270484736213	419	2.70859626261983	0.00703844547192108	0.0174954811323528	0.0325344617064126
**BLCA**	PADI2	cg19282714	-0.0119670612164686	419	-0.242922208412377	0.808186553218431	0.000143210554158707	0.89616996395458
**BLCA**	PADI3	cg01459162	-0.319617868152505	419	-6.84666556877451	2.75577299101385e-11	0.102155581642352	1.20669750704243e-09
**BLCA**	PADI3	cg12858514	-0.578122255588351	417	-14.3465668669431	4.10465618740764e-38	0.334225342406562	1.58864489275219e-35
**BLCA**	PADI3	cg14664242	-0.326333759604527	419	-7.00747905575762	9.96039290732291e-12	0.106493722657625	4.72763151819572e-10
**BLCA**	PADI3	cg17589397	-0.213800904570156	419	-4.44240498746529	1.1445505230095e-05	0.0457108267950171	0.000147342649986764
**BLCA**	PADI3	cg18768338	-0.460121368282247	419	-10.5190970748148	4.41227858628838e-23	0.211711673549927	7.62775111870486e-21
**BLCA**	PADI3	cg21966975	-0.390530903877108	419	-8.61068788402597	1.55663593683152e-16	0.152514386883071	1.47679190329112e-14
**BLCA**	PADI4	cg16091981	-0.0690656118212371	419	-1.40523434197953	0.160705174146038	0.0047700587362418	0.330650577030333
**BLCA**	PADI4	cg18333690	-0.0876312131197179	419	-1.78558854439024	0.0749013329412752	0.00767922951283341	0.193699697321773

**BLCA**	PADI4	cg19159961	0.0461472102731735	419	0.937685028927182	0.348955645631444	0.00212956501599649	0.548018723680809
**BLCA**	PADI4	cg21295838	-0.0644511829749765	419	-1.31094176453175	0.190607650679255	0.00415395498687391	0.370898010879262
**BLCA**	PADI4	cg23228178	0.091561038119375	419	1.86632565948496	0.062706051393559	0.00838342370149764	0.170053495158284
**BLCA**	PADI4	cg26918754	-0.0650330817609493	419	-1.32282767249599	0.186626437442972	0.00422930172332632	0.365660337943011
**BLCA**	PADI6	cg12643633	0.0962179121429134	418	1.96211394723719	0.0504219652400032	0.0092578866171414	0.145180673198973
**BLCA**	PADI6	cg16017904	0.0977248474665769	418	1.9931379716798	0.0469054295688786	0.00955014581236572	0.137710557667981
**BLCA**	PARP1	cg00653565	0.00951406406518624	419	0.193123149783783	0.846957689835327	9.05174150364681e-05	0.918240276629941
**BLCA**	PARP1	cg02088626	-0.114010942333964	419	-2.32935797457742	0.0203227016610322	0.0129984949718785	0.0737177211530216
**BLCA**	PARP1	cg03149313	0.0914022284021468	419	1.86306128130271	0.063165019077894	0.00835436735687821	0.170909787143783
**BLCA**	PARP1	cg04960271	0.111639696075288	419	2.28029302125165	0.0231004309122801	0.0124634217397827	0.0812017045045375
**BLCA**	PARP1	cg07113065	-0.0282985471047686	419	-0.574627901357094	0.565856601449388	0.000800807768240805	0.735045381139579
**BLCA**	PARP1	cg17661881	-0.0171783266963992	419	-0.348733408346077	0.727467602707092	0.000295094908088221	0.845865251999036
**BLCA**	PARP1	cg25722198	-0.00963243000343359	419	-0.195526046252561	0.845077541817873	9.27837077710476e-05	0.91721025570874
**BLCA**	PARP1	cg26464410	0.0326199028483611	419	0.662464257767586	0.50804413676865	0.00106405806183652	0.690664772043072
**BLCA**	PARP2	cg01813761	-0.25419297052612	415	-5.31532437398613	1.75440915311891e-07	0.0646140662648929	3.61160374855074e-06
**BLCA**	PARP2	cg10910545	-0.120279507802269	418	-2.45926143842151	0.0143323970805056	0.0144671599971562	0.0563604024795016
**BLCA**	PARP2	cg15078479	-0.101475583516962	418	-2.07041681671535	0.0390357373743702	0.0102972940501079	0.12032435824161
**BLCA**	PARP2	cg15365367	-0.185208867304559	418	-3.82551395634774	0.000150715606276333	0.0343023245282379	0.00136716066635507
**BLCA**	PARP2	cg18899035	-0.0513711144728993	385	-1.00141109731891	0.317267024335417	0.00263899140218773	0.516489231195711
**BLCA**	PARP2	cg19540080	-0.119252868480506	418	-2.43796639794224	0.0151919813908156	0.0142212466408289	0.058965474246226
**BLCA**	PARP2	cg24595957	0.0240615905090008	418	0.488538388520749	0.625428466852864	0.000578960137822839	0.778322791329578
**BLCA**	PARP2	cg26206608	-0.0631345643183808	418	-1.28405335090491	0.199844901023296	0.00398597321167176	0.382764217697178
**BLCA**	PARP2	cg26430074	-0.102782417347678	418	-2.09736313985041	0.0365702966368905	0.0105642253158322	0.114627892202356
**BLCA**	PAXIP1	cg02824291	0.0654237076180282	419	1.33080738481724	0.183988421297553	0.00428026151848924	0.362322682422586
**BLCA**	PAXIP1	cg12220104	0.133143620565161	419	2.72679768527712	0.00666832399850102	0.0177272236971996	0.0311714696033763
**BLCA**	PAXIP1	cg22583711	0.00377731646230723	419	0.0766716973484147	0.938921938594157	1.42681196564172e-05	0.967373364911317
**BLCA**	PAXIP1	cg24448952	-0.124152355622527	419	-2.53966656561052	0.0114623661894182	0.0154138074066225	0.0474436454024793
**BLCA**	PAXIP1	cg24452135	0.133593447243808	419	2.73617730913569	0.0064845309617702	0.0178472091464841	0.0304863480775942
**BLCA**	PCGF1	cg03203239	0.0100369901906973	419	0.203738912877481	0.838658125417801	0.000100741172088155	0.913684707290038
**BLCA**	PCGF1	cg08092637	-0.0178506872708722	419	-0.362387120339954	0.717248577214481	0.00031864703604248	0.839365121484796
**BLCA**	PCGF1	cg27306986	-0.00913861948559222	419	-0.185501462608092	0.852927021577408	8.35143661024459e-05	0.921640757793606
**BLCA**	PCGF2	cg09395368	0.00573824806951434	419	0.116475632503153	0.907332350348689	3.29274909072851e-05	0.95094389344564
**BLCA**	PCGF2	cg17902383	0.0102939096473666	419	0.208954616770923	0.834586937803355	0.000105964575828147	0.911367998869655
**BLCA**	PCGF2	cg20228512	-0.0664025832970422	419	-1.35080658290012	0.177498800207507	0.00440930306852063	0.353824159876564
**BLCA**	PCGF2	cg25377665	0.0109778507423513	419	0.222839461557425	0.823770888162462	0.000120513206921343	0.905160136071281
**BLCA**	PCGF5	cg03905463	-0.019779080685496	419	-0.401550043556515	0.688223401866492	0.000391212032763361	0.820800281393116
**BLCA**	PCGF5	cg06001881	-0.0559617752508416	419	-1.1376828267581	0.255914125109582	0.0031317202892257	0.450176000560213
**BLCA**	PCGF5	cg07421809	-0.147029508049118	419	-3.01716327782233	0.00270977043981572	0.0216176762371655	0.0151875995385464
**BLCA**	PCGF5	cg07424295	0.0683688863972478	419	1.39099159606879	0.164978713277035	0.00467430462719977	0.336833163285664
**BLCA**	PCGF5	cg08633154	-0.0613672741735133	419	-1.24797173382777	0.212750198112218	0.00376594233948715	0.399033447901935
**BLCA**	PCGF5	cg11113644	-0.029469583857083	419	-0.598427132438702	0.549883808092563	0.00086845637270965	0.722723871617849
**BLCA**	PCGF5	cg12783297	-0.127188557886015	419	-2.6027841289909	0.00958063512777928	0.0161769292571241	0.0411867776928858
**BLCA**	PCGF5	cg15550435	-0.150977497562598	418	-3.0962792100871	0.00209390398115317	0.0227942047702642	0.0123375873899742
**BLCA**	PCGF5	cg17001035	0.0847305861794631	419	1.72605011519479	0.0850881056717807	0.00717927223431542	0.212170774074377
**BLCA**	PCGF5	cg20515900	-0.148678942682766	419	-3.05177179061112	0.00242219132011685	0.0221054279972653	0.0138716100969648
**BLCA**	PCGF5	cg22968329	-0.124201280789252	419	-2.54068305846506	0.0114296226837217	0.0154259581496907	0.0473383414288156
**BLCA**	PCGF5	cg23395902	-0.0546961508961071	419	-1.11187503572617	0.2668402661626	0.00299166892284972	0.462563717098636
**BLCA**	PCGF5	cg25390656	-0.00246544900826317	419	-0.050043301381058	0.96011213353084	6.07843881234585e-06	0.979105654296394
**BLCA**	PCGF6	cg01917164	-0.195128026186652	419	-4.03829128232121	6.42189097568834e-05	0.0380749466034989	0.000660444221284342
**BLCA**	PCGF6	cg06691148	-0.0575644120787765	419	-1.17037066757712	0.242528365307396	0.00331366153797519	0.434949782164312
**BLCA**	PCGF6	cg11612727	-0.0981538390599317	419	-2.00197235555296	0.045942784266912	0.00963417612220298	0.135633295103038
**BLCA**	PCGF6	cg11814370	-0.0959734787308938	419	-1.95708296491468	0.0510126108193745	0.00921090861970932	0.14641658227872
**BLCA**	PCGF6	cg14409890	-0.217810837363305	419	-4.5298339061045	7.74184632969778e-06	0.0474415608729042	0.000104560623849394
**BLCA**	PCGF6	cg16113748	-0.0525382501430883	419	-1.06788484993139	0.286197764676452	0.00276026772809772	0.4834741678843
**BLCA**	PCGF6	cg16382383	0.106121952193913	419	2.16627305058095	0.0308631136745945	0.0112618687374471	0.100779258949018
**BLCA**	PCGF6	cg24418712	-0.0703769429242454	419	-1.43204672922181	0.152888657274417	0.0049529140953625	0.319949970517189
**BLCA**	PCGF6	cg25481680	0.0421219539629686	419	0.855741777370372	0.392638016411054	0.00177425900565844	0.589508351735245

**BLCA**	PCGF6	cg26155979	-0.00504667072414748	419	-0.102437532381389	0.918459232174727	2.54688853979672e-05	0.956976694729708
**BLCA**	PCGF6	cg27217148	0.0795558811818931	419	1.61994258044632	0.106009736213011	0.00632913823062749	0.24829927669706
**BLCA**	PHC1	cg02770112	-0.0146032590101194	419	-0.296445395323842	0.767039339108427	0.000213255173716632	0.870881496053606
**BLCA**	PHC1	cg08021945	-0.234895978741807	419	-4.90511009506238	1.34654425578208e-06	0.0551761208290712	2.22452885611194e-05
**BLCA**	PHC1	cg08867249	-0.0344900604027819	419	-0.700488528241992	0.484017650456946	0.00118956426658754	0.670769757122985
**BLCA**	PHC1	cg09059548	-0.122642003784007	419	-2.50829598311578	0.0125148466062522	0.0150410610921564	0.0508051963099089
**BLCA**	PHC1	cg09912793	-0.335318546717804	419	-7.22448614169225	2.4531764828376e-12	0.11243852777294	1.31163169282384e-10
**BLCA**	PHC1	cg12581035	-0.00566924123492768	419	-0.115074878384457	0.908441833642988	3.21402961798043e-05	0.951392318508467
**BLCA**	PHC1	cg14199415	-0.0537312995735263	419	-1.09220403086032	0.275381684510906	0.00288705255386002	0.471828858046773
**BLCA**	PHC2	cg06899053	-0.0445220397750803	419	-0.90459570283737	0.366208260159452	0.00198221202573383	0.565108313150267
**BLCA**	PHC2	cg13640100	0.134766593516293	419	2.76064744959928	0.00602637658456204	0.0181620347279857	0.0287723051441893
**BLCA**	PHC2	cg15586632	0.1474595610151	419	3.02618417646588	0.00263193763090147	0.021744322134766	0.0148315071393226
**BLCA**	PHC2	cg16558908	-0.145589376136552	419	-2.98696741189863	0.00298591296380522	0.0211962664438303	0.0163974384516633
**BLCA**	PHC2	cg17161743	0.115540683447924	419	2.36103216117672	0.0186889458102848	0.0133496495316135	0.0690857109899444
**BLCA**	PHC2	cg19746874	-0.0314945150806586	419	-0.63958611976445	0.52279717496274	0.000991904480165834	0.702475598021312
**BLCA**	PHC2	cg21198021	-0.0287547037974196	419	-0.583898184712406	0.559608348985938	0.000826832990477337	0.730349835035069
**BLCA**	PHC2	cg21578869	0.196714788193419	419	4.07244664191343	5.58017127482446e-05	0.0386967078939817	0.000583629798539518
**BLCA**	PHC2	cg23886755	-0.0648732808960785	419	-1.31956343366644	0.187713609312909	0.0042085425742215	0.367093829132439
**BLCA**	PHF1	cg01097444	-0.118314732048894	419	-2.41851406477782	0.0160166789756085	0.0139983758198016	0.0613370839927382
**BLCA**	PHF1	cg02776641	-0.213199395030242	419	-4.42931065283272	1.21291141941026e-05	0.0454539820412613	0.000155167293145209
**BLCA**	PHF1	cg03560363	-0.0760993734408824	419	-1.5491407172539	0.122115672225809	0.00579111463809488	0.273921357663695
**BLCA**	PHF1	cg03876174	-0.135056267802447	419	-2.76669145359546	0.00591781886175751	0.0182401954727262	0.0283706662064191
**BLCA**	PHF1	cg06618123	-0.0840698074038245	419	-1.71249317125809	0.0875582343749488	0.00706773251691615	0.216588415310243
**BLCA**	PHF1	cg06919790	-0.000639204586197868	419	-0.0129744387170282	0.989654466327381	4.08582503016388e-07	0.994436321090902
**BLCA**	PHF1	cg07857404	-0.0829338805921951	418	-1.68714186792625	0.092334820759351	0.00687802855008048	0.224942693957754
**BLCA**	PHF1	cg08665801	-0.211239143639057	419	-4.38667422186481	1.46365011510037e-05	0.044621975805362	0.000182502319368678
**BLCA**	PHF1	cg08798692	-0.0678427770347871	419	-1.38023800955146	0.168261808489618	0.00460264239579184	0.341365987636143
**BLCA**	PHF1	cg10033570	-0.0684200064186425	419	-1.39203654267314	0.164662286826876	0.00468129727832709	0.336354845641476
**BLCA**	PHF1	cg10797813	-0.167325006203153	419	-3.44489351553174	0.000629912787425285	0.0279976577008853	0.00455803510231258
**BLCA**	PHF1	cg13303256	-0.053381744828447	419	-1.08507820782857	0.278521525569341	0.00284961068092943	0.475452110070687
**BLCA**	PHF1	cg13528777	-0.177427134172935	419	-3.65943835905296	0.00028560511167015	0.0314803879408208	0.00234494607464566
**BLCA**	PHF1	cg13547657	-0.123619936822646	419	-2.52860599051891	0.011824090272069	0.015281888780035	0.0486106328628837
**BLCA**	PHF1	cg18443095	-0.11571474141822	419	-2.36463720788802	0.0185105124884053	0.0133899013814855	0.0685684876207324
**BLCA**	PHF1	cg20135607	-0.00168066020774144	419	-0.0341137245914332	0.972802985053906	2.82461873388549e-06	0.985688331765356
**BLCA**	PHF1	cg20872639	-0.0991256233063177	419	-2.02198884672385	0.0438233148005121	0.009825889195866	0.130981671722416
**BLCA**	PHF1	cg23345642	-0.0365101743577708	419	-0.741570019738536	0.458770389006972	0.00133299283163483	0.648736387872331
**BLCA**	PHF1	cg23718418	-0.064587004903851	419	-1.31371595278174	0.189672861447033	0.00417148120245007	0.369653105816536
**BLCA**	PHF1	cg24796663	-0.101788127023398	419	-2.07686033179977	0.0384336520190353	0.0103608228029314	0.118845491699689
**BLCA**	PHF1	cg26673053	-0.163627969230146	419	-3.36666048903136	0.000832308168362166	0.0267741123143817	0.00576157516013226
**BLCA**	PHF10	cg04353997	-0.29424019094471	419	-6.24906006706017	1.03151815053603e-09	0.0865772899671791	3.40457882913875e-08
**BLCA**	PHF10	cg05243705	-0.0914012217116074	419	-1.86304058895443	0.063167937261491	0.0083541833303744	0.170909787143783
**BLCA**	PHF10	cg05736120	-0.0283064204771113	419	-0.574787905575315	0.565748472669025	0.000801253440227023	0.734997458937947
**BLCA**	PHF10	cg07652237	-0.248633690239356	419	-5.21032963972862	2.98581985391075e-07	0.06181871192204	5.79680407051502e-06
**BLCA**	PHF10	cg11442717	-0.0719509036042387	419	-1.46423888839991	0.143891268188967	0.00517693252946645	0.30700960800561
**BLCA**	PHF10	cg15342139	-0.0785776192199894	419	-1.5998983617196	0.110387643797151	0.00617444224228164	0.255641115788152
**BLCA**	PHF10	cg15360169	-0.188843784621568	419	-3.90334247647052	0.000110806701012779	0.0356619749901973	0.00105302913493625
**BLCA**	PHF10	cg16740543	-0.0715865479321432	419	-1.45678576631975	0.145937211220396	0.00512463384484103	0.3099467473772
**BLCA**	PHF10	cg27124293	-0.165969419816039	419	-3.41619078166965	0.000698135965317714	0.0275458483140727	0.00497488222826109
**BLCA**	PHF11	cg00092518	-0.199306999937824	419	-4.12831625769257	4.42512161155905e-05	0.0397232802242158	0.000477326553868033
**BLCA**	PHF11	cg01214340	-0.172858513969904	419	-3.56226864984644	0.000410642406976425	0.0298800658518834	0.00317549854953519
**BLCA**	PHF11	cg02289189	-0.072596451075451	419	-1.47744541254061	0.14032027273866	0.00527024470875035	0.301840876467062
**BLCA**	PHF11	cg05080330	-0.0663825372263871	419	-1.35039698686013	0.177629972930132	0.00440664124861267	0.354022138040291
**BLCA**	PHF11	cg09997676	-0.208940032098779	419	-4.33673796617709	1.82048401088171e-05	0.0436559370134386	0.000220957385718648
**BLCA**	PHF11	cg16956501	-0.163579256475874	419	-3.36563065917128	0.000835337144829646	0.0267581731491997	0.00577842947224591
**BLCA**	PHF11	cg21320768	-0.280685082668313	419	-5.93590895721617	6.208060181073e-09	0.0787841156325177	1.76581292038901e-07
**BLCA**	PHF11	cg23137039	-0.0645407727780193	419	-1.31277164475927	0.18999067369854	0.00416551135078392	0.370039919626063
**BLCA**	PHF11	cg24481868	-0.1589291483168	419	-3.26743855995296	0.00117616495291473	0.0252584741847034	0.00768379042215724

**BLCA**	PHF11	cg24984523	-0.139150697106696	419	-2.85219909798524	0.00456039156392704	0.0193629165052795	0.023032597631899
**BLCA**	PHF11	cg25162948	-0.230606165052907	419	-4.81044914069876	2.11599445519781e-06	0.0531792033604086	3.31811474798747e-05
**BLCA**	PHF12	cg01406758	-0.0936681304400851	419	-1.90965122540681	0.0568723491999605	0.00877371866014079	0.158351021247735
**BLCA**	PHF12	cg02058367	-0.0648902691013623	419	-1.31991044545997	0.187597812837237	0.00421074702404722	0.366931945084857
**BLCA**	PHF12	cg06895197	0.139782145075122	419	2.86539934848805	0.00437812731175006	0.0195390480818026	0.0223163436043955
**BLCA**	PHF12	cg08657247	-0.266207343484825	419	-5.60569603858555	3.80457081059335e-08	0.0708663497252477	9.16892136429056e-07
**BLCA**	PHF12	cg08806622	-0.00945553377010052	419	-0.19193495421306	0.847887718154393	8.94071188775114e-05	0.918841470920135
**BLCA**	PHF12	cg13564075	0.0463042526081053	419	0.940882884570805	0.347316201582559	0.00214408380959522	0.546624786783338
**BLCA**	PHF14	cg06715026	-0.0571033899633369	419	-1.16096660933827	0.246327694029453	0.00326079714530492	0.43945366759912
**BLCA**	PHF14	cg07287563	-0.0902244837871236	419	-1.83885686727567	0.0666557581818638	0.00814045747465292	0.17783860525413
**BLCA**	PHF14	cg07574654	0.00845437563740053	419	0.171611216380426	0.863827445484909	7.14764674182716e-05	0.927685256478596
**BLCA**	PHF14	cg11931558	-0.0917979789765542	419	-1.87119631215787	0.0620263916649838	0.00842686894417989	0.168694279196462
**BLCA**	PHF14	cg13029706	-0.144999704389039	419	-2.97460912006012	0.00310618317472837	0.0210249142729086	0.016930036705305
**BLCA**	PHF14	cg16800461	-0.0633448882000072	419	-1.28834819802326	0.198347823817579	0.00401257486107141	0.380947305420682
**BLCA**	PHF14	cg20819769	0.121955096113425	419	2.49403451696043	0.0130210925866194	0.0148730454680348	0.0523901151633549
**BLCA**	PHF14	cg20964328	0.00945767445227417	419	0.1919784111448	0.847853699649145	8.94476060451994e-05	0.918837291000497
**BLCA**	PHF14	cg22876561	-0.145446099425386	419	-2.98396433185572	0.00301474002473917	0.0211545678380594	0.0165312388443335
**BLCA**	PHF14	cg23083824	-0.159117136451328	419	-3.27140375741657	0.00116021095889643	0.0253182631124706	0.00759829373522613
**BLCA**	PHF14	cg24491907	0.100303380301859	419	2.04625573386772	0.0413654646820201	0.0100607680999794	0.1254232747193
**BLCA**	PHF19	cg11054680	-0.169540698553682	413	-3.46633185693782	0.000583919869425812	0.0287440484660705	0.00427642319701486
**BLCA**	PHF19	cg11594303	-0.0941964948070973	419	-1.92051937243128	0.0554822739586352	0.0088729796339435	0.155555123809351
**BLCA**	PHF19	cg13501527	0.175597836241973	419	3.62050221591312	0.000330655628816371	0.0308346000928626	0.00265002139469596
**BLCA**	PHF19	cg13793166	-0.0860680733492595	321	-1.53567804223229	0.125617979221526	0.00740771325005351	0.279360940955358
**BLCA**	PHF19	cg13851767	0.193347232458542	419	3.99999859551229	7.50866847158794e-05	0.0373831522993776	0.000752550058076156
**BLCA**	PHF19	cg13852822	0.200754988226589	419	4.15956376401594	3.88236837731619e-05	0.0403025652978578	0.000426528690220217
**BLCA**	PHF19	cg14218513	-0.156362852250984	419	-3.21334442843028	0.00141513216705079	0.0244493415640632	0.00895977796073484
**BLCA**	PHF19	cg27207079	0.148938610885892	419	3.05722252553464	0.00237954329837371	0.0221827098126192	0.0136777147446198
**BLCA**	PHF20	cg01512265	-0.0795169349007325	419	-1.61914449619741	0.106181365923412	0.00632294293600732	0.248548871055122
**BLCA**	PHF20	cg02674305	-0.0922432794056698	419	-1.88035095692436	0.0607654909493279	0.00850882259551247	0.166175364815693
**BLCA**	PHF20	cg02957488	-0.187197887249513	419	-3.8680811921708	0.000127458139809769	0.0350430489906814	0.00118620391534408
**BLCA**	PHF20	cg04179482	0.106741269263988	419	2.17906047627009	0.0298930840215728	0.0113936985640873	0.0984006507161273
**BLCA**	PHF20	cg09692677	-0.109400455543684	418	-2.23128289977049	0.0262010782125484	0.0119684596731656	0.0892576054679887
**BLCA**	PHF20	cg10372813	-0.152263078886068	419	-3.12706452760821	0.00189084299222282	0.0231840451918651	0.0113640481873441
**BLCA**	PHF20	cg21765224	-0.043395956500055	419	-0.881672291602408	0.378468120462868	0.00188320904055467	0.577064050088592
**BLCA**	PHF20	cg23538717	0.0983262148639837	419	2.005522471859	0.045560674909082	0.00966804452947829	0.134822440711976
**BLCA**	PHF20	cg23734815	0.00853781337887828	419	0.173305000955236	0.862496804595098	7.28942572925529e-05	0.9268727401554
**BLCA**	PHF20	cg25561939	0.0640628906531826	419	1.30301122437129	0.193298691460472	0.00410405395884163	0.374294248216318
**BLCA**	PHF20	cg27405321	-0.114699133358711	416	-2.33505714877518	0.0200233547493961	0.0131558911932394	0.072881526195411
**BLCA**	PHF20L1	cg02898212	0.286679049250836	419	6.07389050975344	2.84045502254041e-09	0.0821848772793634	8.63165461029329e-08
**BLCA**	PHF20L1	cg05307234	-0.186961939626351	419	-3.86302908561452	0.000130029411256517	0.0349547668688473	0.00120580617705203
**BLCA**	PHF20L1	cg10591701	-0.233008903218682	419	-4.8634320901481	1.6444975594634e-06	0.0542931489791731	2.65705969767007e-05
**BLCA**	PHF20L1	cg17646969	-0.0319334380217356	418	-0.647721237189867	0.51752691087343	0.00101974446388803	0.698402179630342
**BLCA**	PHF20L1	cg24511731	0.0351340364877065	419	0.713583613105818	0.475888711050918	0.00123440051991949	0.663654208868047
**BLCA**	PHF20L1	cg26292018	0.272329445046692	419	5.74481462381317	1.79052837408404e-08	0.0741633266394393	4.64772653765413e-07
**BLCA**	PHF20L1	cg27342122	0.000670871192559444	419	0.0136172010391944	0.989141972714829	4.5006815700613e-07	0.994286077624878
**BLCA**	PHF21B	cg01775414	-0.0559267690281762	419	-1.13696892917198	0.256212106821268	0.00312780349393097	0.450496443315563
**BLCA**	PHF21B	cg01995480	-0.0569067609994138	419	-1.15695594368215	0.247960710078515	0.00323837944744441	0.441298313881979
**BLCA**	PHF21B	cg05064352	-0.0478710557986984	419	-0.972791591217426	0.331227670958513	0.00229163798328209	0.530886136373823
**BLCA**	PHF21B	cg10080155	-0.0353244545902113	419	-0.717455885048552	0.473499420038526	0.0012478170920959	0.66154034408234
**BLCA**	PHF21B	cg11428482	-0.032253713349406	419	-0.65501967651349	0.512820618857913	0.00104030202482565	0.694640966193445
**BLCA**	PHF21B	cg14786713	-0.0476874969166598	419	-0.969052957971216	0.333087291718664	0.00227409736217644	0.532734226397239
**BLCA**	PHF21B	cg15679813	-0.047327391570387	419	-0.961718802329693	0.336754957923796	0.00223988199285674	0.53637620444658
**BLCA**	PHF21B	cg20495645	-0.0466255363944355	419	-0.947425409668307	0.343977399241689	0.00217394064406883	0.54326118061848
**BLCA**	PHF21B	cg20525183	-0.04236096613662	419	-0.860606208567667	0.389955498588621	0.00179445145202787	0.587466799457836
**BLCA**	PHF21B	cg21715212	-0.0635337218045869	419	-1.29220435708765	0.197010689968339	0.00403653380634264	0.379231197490893
**BLCA**	PHF21B	cg23080354	-0.0448449882036649	419	-0.911170521343247	0.362738328007867	0.00201107296698684	0.561735649268526
**BLCA**	PHF21B	cg25561581	-0.0334366923843613	419	-0.679070441354608	0.497474522741031	0.00111801239760641	0.681851598402266

BLCA	PHF21B	cg25976257	-0.0449952089297481	419	-0.914228921329608	0.361131288175484	0.00202456882663169	0.560006526846804
BLCA	PHF23	cg00039960	0.0521471885320127	419	1.059914432903	0.289804496417778	0.00271932927179328	0.487270921464611
BLCA	PHF23	cg01441172	0.121711713528009	419	2.48898232095962	0.0132047462002432	0.0148137412099242	0.052959221833362
BLCA	PHF23	cg04379155	-0.162241053366763	419	-3.33734974775386	0.000922639063441537	0.0263221593975568	0.00627685031057026
BLCA	PHF23	cg11180288	0.144383447319949	419	2.9616971148962	0.00323656369669553	0.0208465798599924	0.0175142763091772
BLCA	PHF23	cg13351208	0.0848934942804192	419	1.72939278977421	0.0844878294881816	0.00720690537113957	0.211101532269266
BLCA	PHF23	cg22033559	-0.305925568378507	419	-6.5223206850631	2.03154332744234e-10	0.0935904533877124	7.63118694423222e-09
BLCA	PHF23	cg26213471	-0.176152403565068	419	-3.6323019321565	0.000316342840860714	0.0310296692817506	0.00255471555713884
BLCA	PHF23	cg26784276	0.123342210791075	419	2.52283733489741	0.0120167504035582	0.0152133009628299	0.0491938683836451
BLCA	PHIP	cg01492710	0.159684483522886	419	3.28337291530359	0.00111326224140759	0.0254991342779708	0.00733879525532261
BLCA	PHIP	cg04480383	-0.101246769472196	419	-2.06569989272111	0.0394815700735122	0.010250908328556	0.121328432245657
BLCA	PHIP	cg05801338	-0.147355166349971	419	-3.02399420680138	0.002650641962291	0.0217135450500277	0.0149153013247353
BLCA	PHIP	cg06848288	0.0613951696829571	419	1.24854116593077	0.212541945582254	0.00376936686039909	0.398796172659268
BLCA	PHIP	cg08287994	-0.266339659443654	419	-5.60869498187816	3.74384200665943e-08	0.0709368141925617	9.03376070304169e-07
BLCA	PHIP	cg08587415	-0.231938571935274	419	-4.83981858085846	1.84055443228762e-06	0.0537955011513742	2.93169720526993e-05
BLCA	PHIP	cg15564972	-0.00398279988684301	419	-0.0808426495477491	0.935606345120551	1.58626949386367e-05	0.96567107164873
BLCA	PHIP	cg19494004	-0.224435578785637	419	-4.67480396499197	3.99302398638518e-06	0.0503713290248438	5.83020472967059e-05
BLCA	PHIP	cg19950079	0.138470376778306	419	2.83798115370636	0.00476440131826383	0.0191740452451261	0.023864647231538
BLCA	PHIP	cg21607066	0.0677358973888131	419	1.37805354930147	0.168934704004495	0.00458815179506782	0.342230001723844
BLCA	PHIP	cg21875336	-0.111713519687021	419	-2.28181995273037	0.0230092336973355	0.0124799104808625	0.0809359887072943
BLCA	PHIP	cg26363168	-0.019873975637821	419	-0.403477337012645	0.686806420103169	0.000394974907652701	0.819721059305093
BLCA	PHRF1	cg06063517	-0.104646109899993	419	-2.13581066311914	0.0332838041060909	0.0109508083172015	0.106671114574613
BLCA	PHRF1	cg07530960	-0.194015012960645	419	-4.01435314749737	7.08226912751584e-05	0.0376418252541193	0.000716367240288607
BLCA	PHRF1	cg08482978	-0.275257816849761	419	-5.81162391156544	1.24025963921554e-08	0.0757668657368967	3.32247790541094e-07
BLCA	PHRF1	cg12469954	-0.275790039239063	419	-5.82378500954239	1.1596500399752e-08	0.0760601457434841	3.1313368694839e-07
BLCA	PHRF1	cg12813709	-0.151368590997269	419	-3.10826212643105	0.00201239413809786	0.0229124503404984	0.0119573606257197
BLCA	PHRF1	cg15219216	0.0976377411922753	419	1.99134429686233	0.0471029448436186	0.00953312850512973	0.138130522133763
BLCA	PHRF1	cg16143007	-0.208531262301473	419	-4.32786746775791	1.89201225966203e-05	0.0434852873570456	0.00022830339960935
BLCA	PHRF1	cg18793109	-0.161043423993581	419	-3.31205569260443	0.00100783562244944	0.0259349844115763	0.00675776283510011
BLCA	PHRF1	cg20152424	0.0612209451264865	419	1.24498476143858	0.213845015369783	0.00374800412218027	0.400429874249053
BLCA	PHRF1	cg21006161	-0.203635681978338	419	-4.2218136920568	2.98420837301319e-05	0.041467490974783	0.00033950498123748
BLCA	PPARGC	cg04893087	0.0874436407917232	419	1.78173705708222	0.0755285894410692	0.00764639031491192	0.194857413856981
BLCA	PPARGC	cg05158538	-0.0538635510193816	419	-1.09490013802043	0.274200048794311	0.00290128212841752	0.470683481284664
BLCA	PPARGC	cg11270806	-0.0437913766948642	419	-0.889721379038445	0.374134720744526	0.0019176846728315	0.572772536959552
BLCA	PPARGC	cg12691631	-0.151751070575086	419	-3.11630100141329	0.00195957060944386	0.0230283874206848	0.0116957830283164
BLCA	PPARGC	cg20723350	-0.044017101871127	419	-0.894316379199279	0.371674771801724	0.00193750525713317	0.570279660242263
BLCA	PPARGC	cg27461259	-0.0229654500056324	419	-0.466270698274158	0.641268177233815	0.000527411893961199	0.789267819017475
BLCA	PPARGC	cg27514608	-0.0461160996956998	419	-0.937051532003446	0.349281004881158	0.00212669465114372	0.548297243451476
BLCA	PRDM1	cg00555933	-0.0631946157878129	419	-1.2852795939169	0.199416620804421	0.00399355946456929	0.382179163353841
BLCA	PRDM1	cg01761420	0.0357940821045013	419	0.727006391395242	0.467634917623527	0.00128121631370378	0.656652240602239
BLCA	PRDM1	cg05170275	-0.0378268881781605	419	-0.768351876668879	0.442718340685586	0.00143087346924306	0.635053022980371
BLCA	PRDM1	cg08742502	0.256609209761261	419	5.38904909240412	1.19441787060212e-07	0.065848286534299	2.55130543178531e-06
BLCA	PRDM1	cg14359052	0.274760835399538	419	5.80027327716565	1.32041170064222e-08	0.0754935166694521	3.51781191232058e-07
BLCA	PRDM1	cg17143179	0.228539283129545	419	4.76494658581644	2.62271670111994e-06	0.0522302039333664	4.01795240344475e-05
BLCA	PRDM1	cg17521665	0.299732826477309	419	6.37711118606433	4.84980858170091e-10	0.0898397672680765	1.69186551033561e-08
BLCA	PRDM1	cg17965230	0.00683813777774112	419	0.138802282871123	0.889674208686969	4.67601282673702e-05	0.941510125344532
BLCA	PRDM1	cg19464016	0.0264369130904674	419	0.53679834829704	0.591696660849014	0.000698910373752929	0.754238968917861
BLCA	PRDM1	cg22186515	0.0773732357492578	419	1.57522737944506	0.115971500057713	0.00598661761031022	0.264447381741404
BLCA	PRDM1	cg22196848	-0.0459501962399284	419	-0.933673327436266	0.351019287980019	0.00211142053448793	0.549974017262514
BLCA	PRDM1	cg24040346	0.0710121502001495	419	1.4450372924189	0.149207541966162	0.00504272547604859	0.314641823743342
BLCA	PRDM1	cg24793124	0.0383198288351642	418	0.777434065967462	0.437349973610615	0.00146840928195628	0.630222721577153
BLCA	PRDM1	cg24874254	0.254934124906095	419	5.35141683068881	1.45159715307611e-07	0.0649914080416364	3.03726597435188e-06
BLCA	PRDM1	cg26351406	-0.0531630780591625	419	-1.08062079574192	0.280497972078131	0.00282631286872461	0.477683484158537
BLCA	PRDM10	cg04181189	-0.0713286561741295	419	-1.45151078433785	0.147398690454135	0.00508777719160718	0.312121882660705
BLCA	PRDM10	cg17627813	0.192498172712606	419	3.98175561661629	8.0857930710258e-05	0.0370555464976922	0.000801685486696494
BLCA	PRDM12	cg09191327	0.0660108372281627	419	1.34280243650377	0.180075261039804	0.00435743063156299	0.357141084615991
BLCA	PRDM12	cg12539975	0.0036287674365177	419	0.0736564194100465	0.941319523234183	1.31679531083312e-05	0.968727408496554

**BLCA**	PRDM12	cg13795948	0.162436218529389	419	3.34147308797457	0.000909402724832097	0.0263855250901275	0.00620307545599195
**BLCA**	PRDM12	cg13928649	0.072289116705153	419	1.47115777518772	0.142011800937951	0.00522571639401123	0.304259599068217
**BLCA**	PRDM12	cg23342516	0.0274418341856135	419	0.557218246296135	0.577680909786916	0.000753054263470708	0.744164740784291
**BLCA**	PRDM13	cg00126261	0.310639143127798	418	6.63345688226795	1.03318560168701e-10	0.0964966772431725	4.12605958406771e-09
**BLCA**	PRDM13	cg00579292	0.141197221754701	418	2.89499412205792	0.00399334268196161	0.0199366554312462	0.0207174458553199
**BLCA**	PRDM13	cg01422797	0.194428936591392	418	4.02325372372247	6.82953546293509e-05	0.0378026113840597	0.000694869058074251
**BLCA**	PRDM13	cg01815538	-0.0431940896676439	418	-0.877563294591757	0.380692196632556	0.00186572938221646	0.579073748994644
**BLCA**	PRDM13	cg02400433	-0.00192218794852601	418	-0.039016226193405	0.968896350267463	3.69480650945865e-06	0.983665109397685
**BLCA**	PRDM13	cg03120185	0.1483408894124	418	3.04467662983658	0.00247875763629566	0.022005019471662	0.0141403880294775
**BLCA**	PRDM13	cg04910970	0.123512181523382	418	2.526367730448	0.0118985142856169	0.0152552589846648	0.0488306087959105
**BLCA**	PRDM13	cg11809464	0.0844443555918899	418	1.72017735183372	0.0861511139208561	0.00713084919132955	0.214115835160509
**BLCA**	PRDM13	cg13738144	-0.0425549123985301	418	-0.864553556625797	0.387786952354861	0.00181092056924657	0.585697054215816
**BLCA**	PRDM13	cg14344102	0.0232883888965768	418	0.472830904361431	0.63658420985122	0.000542349057398204	0.786107783246279
**BLCA**	PRDM13	cg17879189	0.0550023020978125	418	1.11811737483232	0.264168396587105	0.00302525323605903	0.459685864181481
**BLCA**	PRDM13	cg17978124	0.219036583930711	418	4.55660676964905	6.85969020339682e-06	0.0479770251000354	9.38685557929731e-05
**BLCA**	PRDM13	cg25273233	-0.00223622307494663	418	-0.0453904844980763	0.963818057292878	5.00069364092375e-06	0.980956279375411
**BLCA**	PRDM13	cg25924096	0.0714007636116615	418	1.45298565823627	0.14698893761177	0.00509806904432837	0.311485751796204
**BLCA**	PRDM13	cg26507427	0.0481035618740177	418	0.977527297670239	0.32888179258557	0.00231395266496745	0.528590599832477
**BLCA**	PRDM14	cg00384539	0.065971220356548	418	1.341993020308	0.180337349440822	0.00435220191533222	0.357490861278538
**BLCA**	PRDM14	cg01295203	0.05283846991634	418	1.07400410933454	0.283449447237099	0.00279190390309997	0.480720869813885
**BLCA**	PRDM14	cg04838988	0.0333946216976879	418	0.678215067406492	0.498016071214112	0.00111520075833169	0.68229333152582
**BLCA**	PRDM14	cg09050331	-0.00202675263740976	418	-0.0411386699859568	0.967205274673718	4.10772625324742e-06	0.982775902722455
**BLCA**	PRDM14	cg10457247	0.0120095607959341	418	0.243785041662079	0.807518650158677	0.000144229550511238	0.895817612161596
**BLCA**	PRDM14	cg11229513	0.0531483246223326	418	1.08032005997524	0.280631663772835	0.00282474441016084	0.477837936691121
**BLCA**	PRDM14	cg13267264	0.0495892597560497	418	1.00779193072526	0.314145865227412	0.00245909468315297	0.513217433168807
**BLCA**	PRDM14	cg18035229	0.0643413521003697	418	1.30869850192191	0.191366025355059	0.00413980959010375	0.371850351650821
**BLCA**	PRDM14	cg21255438	0.077717697730686	418	1.58228273735134	0.11435235658994	0.00604004054055827	0.26192995991396
**BLCA**	PRDM14	cg27029821	0.0916010890318239	418	1.86714893986408	0.0625907371914879	0.00839075951181614	0.169799965444086
**BLCA**	PRDM4	cg00090609	-0.0225536826568221	419	-0.457906235431162	0.647261208814775	0.000508668601384636	0.793248554256449
**BLCA**	PRDM4	cg03909902	-0.153540759764811	418	-3.15010549846768	0.00175142305251477	0.0235747649091555	0.0106720611692123
**BLCA**	PRDM4	cg12900929	-0.0296825182173241	419	-0.602754905218556	0.547003374169967	0.000881051887721779	0.720503919223481
**BLCA**	PRDM4	cg18440127	-0.233627610028997	419	-4.87709043612899	1.54046113723179e-06	0.0545818601678611	2.50394136460112e-05
**BLCA**	PRDM4	cg25995916	-0.0487564154446116	419	-0.990825536941816	0.322352321272807	0.00237718804700756	0.521766086186385
**BLCA**	PRDM4	cg27140793	0.0819345722025317	419	1.66870083281458	0.0959363823809489	0.00671327412201188	0.231175657630515
**BLCA**	PRDM5	cg05713693	-0.305391391014756	419	-6.50975951718686	2.19167087432749e-10	0.0932639017059273	8.18334721241209e-09
**BLCA**	PRDM5	cg06329345	-0.333512676424897	419	-7.18069420774039	3.2632088632939e-12	0.111230705336098	1.70032197127318e-10
**BLCA**	PRDM5	cg07070341	-0.406931064875839	419	-9.04233213867811	6.07350417561459e-18	0.165592891560984	6.66406465215461e-16
**BLCA**	PRDM5	cg10416963	-0.385980578459853	419	-8.49267362280319	3.70978831245184e-16	0.148981006948202	3.31342447736944e-14
**BLCA**	PRDM5	cg11171429	-0.257593872019021	419	-5.41119463122751	1.0643741232607e-07	0.0663546029017518	2.30005334847063e-06
**BLCA**	PRDM5	cg13155001	-0.438313723607447	419	-9.89828508661173	7.27293804940796e-21	0.192118920302625	1.03814851856081e-18
**BLCA**	PRDM5	cg18181323	-0.406023713334756	419	-9.01818480069513	7.30190155861841e-18	0.164855255790144	7.91147813163176e-16
**BLCA**	PRDM5	cg19294653	-0.406136907885212	419	-9.02119549980276	7.13624106520425e-18	0.164947187946561	7.7753044457493e-16
**BLCA**	PRDM5	cg22135105	-0.443753693735745	419	-10.0510275843252	2.10629387765585e-21	0.196917340704117	3.14504848211822e-19
**BLCA**	PRDM8	cg01304890	-0.0378190300742512	419	-0.768192031696989	0.442813179690645	0.00143027903575712	0.635128558012072
**BLCA**	PRDM8	cg02895669	-0.0260100278040364	419	-0.528124577628688	0.597697206506592	0.000676521546366747	0.758457817192298
**BLCA**	PRDM8	cg04343891	-0.072619827228831	419	-1.47792367369077	0.140192248066395	0.00527363930674526	0.301648893543829
**BLCA**	PRDM8	cg05007549	0.276187427378965	419	5.83286897086067	1.10279419783151e-08	0.0762794950422111	2.99131003577769e-07
**BLCA**	PRDM8	cg07462804	-0.0524187642610496	419	-1.0654494979198	0.287296553989464	0.00274772684665549	0.484793460610307
**BLCA**	PRDM8	cg08074201	-0.142075211297244	419	-2.91336539771988	0.00377025396670316	0.0201853656651565	0.0197968091727669
**BLCA**	PRDM8	cg10695325	-0.0768109044554267	419	-1.56371078510789	0.118653248498779	0.00589991504326068	0.268688094944227
**BLCA**	PRDM8	cg11571772	-0.0656375038236817	419	-1.33517507764855	0.182556290879752	0.00430828190820382	0.360369055106687
**BLCA**	PRDM8	cg12572049	-0.0669664383926404	419	-1.36232835928053	0.17383856201966	0.00448450387099531	0.348915015706221
**BLCA**	PRDM8	cg14489346	-0.061931200870891	419	-1.25948376311982	0.208568688364717	0.00383547364131064	0.393762863239451
**BLCA**	PRDM8	cg15656813	-0.121047054770186	419	-2.47518748849553	0.0137179643469149	0.0146523894685363	0.0545089037901675
**BLCA**	PRDM8	cg16117799	-0.0353780869625514	419	-0.718546547193486	0.472827650654635	0.00125160903712985	0.660913740994101
**BLCA**	PRDM8	cg18624348	-0.0553430154778122	419	-1.12506479925034	0.261216570293874	0.00306284936217736	0.456571444896235
**BLCA**	PRDM8	cg19595012	-0.157742323381916	419	-3.24241348746484	0.00128163978902101	0.0248826405859248	0.00825171444722973

**BLCA**	PRDM8	cg20142271	-0.174110727001349	419	-3.58887800906574	0.000372072091699782	0.0303145452569383	0.00292580373008115
**BLCA**	PRDM8	cg26162932	-0.130787766565189	419	-2.67770214063836	0.00770924140894451	0.0171054398831104	0.0349380646934972
**BLCA**	PRKAA1	cg00301775	-0.263283950712412	419	-5.53952388526449	5.41670864117206e-08	0.0693184387027356	1.25375612029556e-06
**BLCA**	PRKAA1	cg01883943	-0.234274968459926	419	-4.89138799338444	1.43836794383449e-06	0.0548847608468994	2.35571359626654e-05
**BLCA**	PRKAA1	cg03125217	0.102331773980826	419	2.08806984475801	0.0374051037305876	0.0104717919660629	0.116572896387202
**BLCA**	PRKAA1	cg05874655	0.0948121784460201	419	1.93318569227782	0.0538981763691022	0.00898934918167997	0.152421197879165
**BLCA**	PRKAA1	cg06971720	-0.0511029648450226	419	-1.03863398741574	0.299584150680221	0.00261151301595161	0.497854062415635
**BLCA**	PRKAA1	cg10786880	-0.0650829653054454	419	-1.32384666038426	0.186288016237251	0.00423579237294981	0.36523324517152
**BLCA**	PRKAA1	cg24728477	0.0837371989251412	419	1.70567003307699	0.0888232261385616	0.00701191848382867	0.218833768860692
**BLCA**	PRKCD	cg02121118	0.000203310233667658	419	0.00412674711642289	0.996709338922177	4.13350511139975e-08	0.998316892964834
**BLCA**	PRKCD	cg02559423	-0.0761508459473976	419	-1.55019464304435	0.121862587524365	0.00579895133850428	0.27360611082197
**BLCA**	PRKCD	cg02630105	0.106353046078834	419	2.17104429106461	0.0304980499792426	0.0113109704102465	0.0999235895801379
**BLCA**	PRKCD	cg10531918	-0.45433453536918	419	-10.352112084346	1.77259736068683e-22	0.206419870029129	2.89700502263176e-20
**BLCA**	PRKCD	cg13469346	-0.0588588351319958	419	-1.19677870698027	0.232081013754973	0.00346436247309546	0.422708629691948
**BLCA**	PRKCD	cg13889007	-0.316482714458117	419	-6.77198942600873	4.39375301467527e-11	0.100161308550778	1.85563312716421e-09
**BLCA**	PRKCD	cg13908523	-0.0283015221409061	419	-0.574688360612872	0.565815742696016	0.0008009761554922	0.735014358736497
**BLCA**	PRKCD	cg14727059	0.0487438212403169	419	0.990568988498378	0.322477478746068	0.00237596010910797	0.521859148393485
**BLCA**	PRKCD	cg21903826	-0.00754574890677147	419	-0.153166335443959	0.878342092221159	5.69383265640429e-05	0.935408422792357
**BLCA**	PRMT1	cg07702240	0.1293759330083	419	2.64830197415138	0.00840043401982447	0.016738132041768	0.0373131988202709
**BLCA**	PRMT1	cg08377944	0.0572780237500225	419	1.16452875023571	0.24488365893552	0.00328077200470814	0.437811409485823
**BLCA**	PRMT1	cg11787855	0.0613139326671554	419	1.24688287978694	0.213148825145182	0.00375939833911247	0.399568657156661
**BLCA**	PRMT1	cg14258555	0.0432949995836748	419	0.879617301259042	0.379579419391877	0.0018744569889504	0.578081896442349
**BLCA**	PRMT1	cg14576628	-0.106353909199357	419	-2.17106211200177	0.0304966934475246	0.011311154001985	0.0999235895801379
**BLCA**	PRMT1	cg18880319	-0.12345504415499	419	-2.52518092735649	0.0119381453601354	0.0152411479273105	0.0489467734872125
**BLCA**	PRMT1	cg22292364	0.0828559865670775	419	1.68759559114429	0.0922457155921062	0.00686511451000372	0.224753772394134
**BLCA**	PRMT1	cg24228123	-0.0837291810454558	419	-1.70550556119996	0.0888539002662196	0.00701057575854272	0.218875283154754
**BLCA**	PRMT1	cg25595446	-0.174256479010392	419	-3.59197638342184	0.000367808832004142	0.0303653204770991	0.00289813838518487
**BLCA**	PRMT1	cg26846726	0.0807274119069808	419	1.64395300650692	0.100948722282086	0.00651691503319934	0.239858431361676
**BLCA**	PRMT2	cg04493422	0.11402884502298	419	2.32972856270394	0.0203028858727121	0.0130025774972749	0.0736542950746953
**BLCA**	PRMT2	cg06725035	-0.234494993743316	419	-4.89624903781618	1.40516883789377e-06	0.0549879020906779	2.30814409998116e-05
**BLCA**	PRMT2	cg09031657	-0.100880138089866	419	-2.05814260121166	0.0402049091293377	0.0101768022610304	0.122918009580179
**BLCA**	PRMT2	cg10187059	-0.111649247142413	419	-2.28049056869912	0.023088614516498	0.0124655543874676	0.081171171262493
**BLCA**	PRMT2	cg10657630	-0.114055024917064	419	-2.3302704942546	0.0202739386067089	0.013008548708832	0.0735835952211585
**BLCA**	PRMT2	cg12162201	-0.14134453222972	419	-2.89807599584486	0.00395509809387993	0.0199782767912385	0.0205867711099624
**BLCA**	PRMT2	cg13271076	0.117227255114927	419	2.39597336058332	0.0170215071459035	0.0137422293417802	0.0643104129276401
**BLCA**	PRMT2	cg17243659	-0.126982622213298	419	-2.5985007416077	0.00969899742144175	0.0161245863441651	0.0416036504964512
**BLCA**	PRMT2	cg23283320	-0.220875051576874	419	-4.59680527304422	5.71396977970306e-06	0.0487857884090866	8.01212265757425e-05
**BLCA**	PRMT3	cg01769160	-0.107527481580776	419	-2.19529758988989	0.0286993648401312	0.0115621592951041	0.0955057701318888
**BLCA**	PRMT3	cg04101203	-0.151619636841476	419	-3.11353839357002	0.00197757792495761	0.0229885142759412	0.0117860796057728
**BLCA**	PRMT3	cg06578097	-0.178444398154329	419	-3.6811074803153	0.000263100792895885	0.0318424032326607	0.00218601549530519
**BLCA**	PRMT3	cg13432154	-0.0843506149387276	419	-1.71825411235221	0.0865015641537269	0.0071150262405415	0.214774118529779
**BLCA**	PRMT3	cg13982511	-0.12149180549725	419	-2.48441780750295	0.0133726446212962	0.0147602588029817	0.0534727070505841
**BLCA**	PRMT3	cg19653624	-0.19733233500711	419	-4.08574843744302	5.2816548149034e-05	0.0389400504393585	0.000555996014439235
**BLCA**	PRMT3	cg23019633	-0.194701171662867	419	-4.02910879619683	6.66797538005737e-05	0.0379085462468932	0.00068146147755673
**BLCA**	PRMT3	cg23071995	-0.0518976688589642	419	-1.05482910674664	0.292121664415002	0.0026933680329947	0.489867880355664
**BLCA**	PRMT6	cg00944433	-0.281743435961231	419	-5.96021686292686	5.41480618109799e-09	0.0793793637072403	1.55899123631509e-07
**BLCA**	PRMT6	cg02990361	-0.334871501024526	419	-7.21363706191634	2.63317646897393e-12	0.112138922198419	1.39921673652567e-10
**BLCA**	PRMT6	cg02993280	-0.266897956190448	419	-5.62135254726207	3.49775759415151e-08	0.0712345190186384	8.4688625670533e-07
**BLCA**	PRMT6	cg09367891	-0.255914563039651	419	-5.3734369572071	1.29523646895556e-07	0.0654922635757755	2.74704541619217e-06
**BLCA**	PRMT6	cg14671364	-0.270622069943555	419	-5.70594148108911	2.2135545698354e-08	0.0732363047405343	5.64594308871395e-07
**BLCA**	PRMT6	cg14828511	-0.20776799246645	419	-4.3113104935181	2.03276970999524e-05	0.0431675386935388	0.000242951738852926
**BLCA**	PRMT6	cg22528358	-0.0663071570435463	419	-1.3488567782913	0.178123871588523	0.00439663907519751	0.354717851999375
**BLCA**	PRMT7	cg00894222	-0.240893431794183	419	-5.03796271680186	7.0542790051013e-07	0.0580296454815788	1.25349607337334e-05
**BLCA**	PRMT7	cg02477175	-0.0736602088799505	419	-1.49921170842263	0.13458455247133	0.00542582637223794	0.293052052368086
**BLCA**	PRMT7	cg06089844	-0.128441268235065	419	-2.62884744278043	0.00888788832527083	0.0164971593858319	0.0389644623486119
**BLCA**	PRMT7	cg06092312	0.0121914257331572	419	0.247477307645514	0.804662132361131	0.000148630861407087	0.893795846777398
**BLCA**	PRMT7	cg06745885	-0.156590264313297	419	-3.21813526242388	0.00139228089996747	0.0245205108777082	0.00884822321150799

**BLCA**	PRMT7	cg09175928	-0.177914211898505	419	-3.66981226805911	0.000274614502279879	0.0316534667954662	0.00226859515666995
**BLCA**	PRMT7	cg09779635	-0.0469948542522596	419	-0.954946450127887	0.340164735936085	0.00220851632619112	0.53941814724228
**BLCA**	PRMT7	cg10233539	0.0973848376967588	419	1.98613682079814	0.0476803625254696	0.00948380661322404	0.139395989067845
**BLCA**	PRMT7	cg10446007	-0.140386915585523	419	-2.87804525638302	0.00420975813197285	0.0197084860676167	0.0216267532271476
**BLCA**	PRMT7	cg11893236	-0.00469009835883205	419	-0.0951996464069525	0.924202535327257	2.19970226155191e-05	0.960116350285809
**BLCA**	PRMT7	cg19328639	0.0912363503003151	419	1.85965176355222	0.0636473648882489	0.0083240716161218	0.171834861707215
**BLCA**	PRMT7	cg21918026	0.122357594406253	419	2.50239067883598	0.0127223088245304	0.014971380908885	0.0514969193348
**BLCA**	PRMT7	cg26492318	-0.1522245192876	419	-3.12625383151736	0.00189594105828246	0.0231723042723409	0.0113894083928977
**BLCA**	PRMT7	cg26561196	0.071684052256712	419	1.4587802204037	0.145387539811478	0.00513860334794301	0.30925213332497
**BLCA**	PRMT7	cg26796563	0.113600988672193	419	2.320872505697	0.02078108580577	0.0129051846272996	0.0749834781620627
**BLCA**	PRMT8	cg01693063	-0.0933071438441043	418	-1.90222687811799	0.0578385747502776	0.00870622309234437	0.160242078858593
**BLCA**	PRMT8	cg06029846	-0.0490721021314522	418	-0.997256342682972	0.319225437951741	0.00240807120759967	0.518609334416806
**BLCA**	PRMT8	cg07912789	-0.0712058271528274	418	-1.44899851199883	0.148098672689396	0.00507026982051832	0.313063122809309
**BLCA**	PRMT8	cg10350722	-0.0221620897445321	418	-0.449951803681531	0.652981838941444	0.000491158221844697	0.797357775767732
**BLCA**	PRMT8	cg11596580	-0.008768958101926	418	-0.177997253478265	0.858812616455285	7.68946261933337e-05	0.924994028417269
**BLCA**	PRMT8	cg19656577	-0.0542456885917003	418	-1.10269080412222	0.27080515417553	0.00294259473078772	0.466993619523213
**BLCA**	PRMT8	cg21667836	0.0121620684458881	418	0.246881287239333	0.805123066913101	0.000147915908882467	0.894180165426117
**BLCA**	PRMT8	cg23739862	-0.0887852466948188	418	-1.80928894118148	0.0711348270435891	0.00788282003065983	0.18652631572836
**BLCA**	PRMT8	cg24100636	-0.11374932458209	418	-2.32394271319346	0.0206141996563726	0.0129389088428816	0.0745438129534628
**BLCA**	PRMT8	cg27316886	-0.111635060645256	418	-2.28019714555987	0.0231061676625531	0.01246238676527	0.0812182002141992
**BLCA**	PWWP2E	cg00259755	-0.0439211972931847	419	-0.892364070051734	0.372718714582558	0.00192907157166685	0.57134681830648
**BLCA**	PWWP2E	cg05010219	-0.0617865430007171	419	-1.25653059472378	0.20963562182719	0.00381757689597946	0.395105959017938
**BLCA**	PWWP2E	cg15012692	-0.0241540776170472	419	-0.490417309554794	0.624099702713656	0.000583419465530342	0.777365220186951
**BLCA**	PWWP2E	cg15278699	-0.45392289473127	419	-10.3402972611397	1.95494290532854e-22	0.206045994361216	3.18831925318928e-20
**BLCA**	PWWP2E	cg19919881	-0.214807302451774	419	-4.46432523590866	1.03829759576939e-05	0.0461421771866079	0.000135502974610441
**BLCA**	PWWP2E	cg22633292	-0.0300455636385577	418	-0.609392888295684	0.542600767573376	0.000902735894358623	0.717168169367005
**BLCA**	PWWP2E	cg22664157	-0.0425993184277835	419	-0.865457357092148	0.387291473184544	0.00181470193051169	0.585210651711078
**BLCA**	PWWP2E	cg24520806	-0.0504327512561491	419	-1.02497737326418	0.305975280676899	0.00254346239926461	0.504489330898527
**BLCA**	PYGO1	cg06364757	-0.369351135700314	419	-8.06746007155824	7.9192990177426e-15	0.136420261443112	5.9754990085962e-13
**BLCA**	PYGO1	cg09275667	-0.328520708740608	419	-7.06010049883829	7.11187033583635e-12	0.107925856071431	3.47525653835461e-10
**BLCA**	PYGO1	cg11480800	-0.359261347568915	419	-7.81388755346267	4.66322744656466e-14	0.129068715857033	3.21605599271322e-12
**BLCA**	PYGO1	cg12686317	-0.239607917926661	419	-5.00943573626271	8.11444045118593e-07	0.0574119543331497	1.42088724324618e-05
**BLCA**	PYGO1	cg13878116	-0.0297854718135499	419	-0.604847409383788	0.545613360355397	0.000887174331155775	0.719438722592084
**BLCA**	PYGO1	cg17841803	-0.337642278565957	419	-7.28096821638243	1.69465583517194e-12	0.114002308275211	9.29718307767038e-11
**BLCA**	PYGO1	cg19850348	-0.286671631712728	419	-6.0737192820345	2.84323735553379e-09	0.0821806244288377	8.63660543795419e-08
**BLCA**	PYGO1	cg22510134	0.0798997239060705	419	1.62698890389055	0.104503973467132	0.0063839658802663	0.245822103660046
**BLCA**	PYGO1	cg23030278	-0.321454579587694	419	-6.89053024397152	2.0914961185766e-11	0.103333046737901	9.32946382159103e-10
**BLCA**	PYGO1	cg23412777	0.142799225986187	419	2.9285201421323	0.00359484697321888	0.020391618942254	0.0190294426307429
**BLCA**	PYGO1	cg25902187	-0.299913801525938	419	-6.38134205294226	4.72941375960265e-10	0.0899482883457399	1.65431660977756e-08
**BLCA**	PYGO2	cg01277844	0.00633976167981682	419	0.128685693804865	0.897669149728893	4.01925781568738e-05	0.945879443263507
**BLCA**	PYGO2	cg02341811	-0.194108370111241	419	-4.0163604061991	7.02450881718117e-05	0.0376780593472424	0.00071200604420038
**BLCA**	PYGO2	cg04602755	-0.226807834277448	419	-4.72688097660961	3.13465145878837e-06	0.0514417936896262	4.70040623718162e-05
**BLCA**	PYGO2	cg10892866	-0.04956487450282	419	-1.00729513366402	0.314384183084275	0.0024566767844803	0.513416948627518
**BLCA**	PYGO2	cg14024356	0.0859818021046892	419	1.75172712991426	0.08056476272817	0.00739287029316994	0.204032005718968
**BLCA**	PYGO2	cg18188585	-0.201979084952585	419	-4.18600185131924	3.47326562265637e-05	0.0407955507582835	0.000387271357100372
**BLCA**	PYGO2	cg24683561	-0.137440975219109	419	-2.81647557768444	0.00508876237501517	0.0188900216691796	0.025138124219071
**BLCA**	PYGO2	cg27105205	-0.0947835927211231	419	-1.93259755520821	0.0539708829726238	0.00898392944912373	0.152560258347164
**BLCA**	RBBP4	cg05797186	-0.0768832317666433	419	-1.56519197044386	0.118305630231794	0.00591103132688338	0.268124805853148
**BLCA**	RBBP4	cg06526778	-0.039175730828345	419	-0.795791388399643	0.426611552526268	0.00153473788593494	0.621174357326916
**BLCA**	RBBP4	cg09036220	0.0451484402679667	419	0.917348679520884	0.359496632519416	0.00203838165863016	0.558439313622387
**BLCA**	RBBP4	cg11229946	0.0786639484617747	419	1.60166702736904	0.109995668843334	0.00618801678759675	0.254991449490385
**BLCA**	RBBP4	cg13641837	-0.0693239198807776	418	-1.40880247438119	0.159649655784685	0.00480580586763647	0.329280060504634
**BLCA**	RBBP4	cg16148416	0.043564727012083	419	0.885107696416664	0.376614815567018	0.00189788543963732	0.575125456926735
**BLCA**	RBBP4	cg16396527	-0.147811126641259	419	-3.03356000818593	0.00256982849971843	0.0218481291589582	0.0145494200397709
**BLCA**	RBBP4	cg18758482	-0.102727903167664	419	-2.09623886372019	0.0366704345734503	0.0105530220892249	0.114807621829
**BLCA**	RBBP4	cg20740619	0.102942906330009	419	2.1006731039559	0.0362768386184815	0.0105972419636689	0.113914603353764
**BLCA**	RBBP4	cg22358606	0.0760317594742893	418	1.54587681958453	0.122903932541636	0.00578082844875618	0.275221316296603

**BLCA**	RBBP4	cg22857567	0.0488197709229406	419	0.992116117870743	0.321723192320346	0.0023833700329684	0.521202291198854
**BLCA**	RBBP4	cg23016766	0.000785301561926656	419	0.0159398857108353	0.987290065821164	6.16698543164446e-07	0.993269806321138
**BLCA**	RBBP4	cg26131911	0.0237807936395648	419	0.482833938875836	0.629470102040113	0.000565526146127568	0.781403541171069
**BLCA**	RBBP5	cg00960017	0.00455649403221087	419	0.0924876878027198	0.926355527743572	2.07616378655733e-05	0.961211395106017
**BLCA**	RBBP5	cg02189925	0.168932530565701	419	3.47895676687281	0.000557034815635485	0.0285381998833316	0.00410748497133146
**BLCA**	RBBP5	cg03143531	-0.159602033436285	419	-3.28163328253136	0.00111997461995077	0.025472809076997	0.007379292418434
**BLCA**	RBBP5	cg05290904	0.0437565263400405	419	0.889011956020443	0.374515411527775	0.00191463359734666	0.573197425178377
**BLCA**	RBBP5	cg07601985	0.249796811135131	419	5.23632192654196	2.61740600543604e-07	0.0623984468532801	5.15881638679735e-06
**BLCA**	RBBP5	cg07972983	0.115739621995643	419	2.36515254643621	0.0184851282070248	0.0133956600996943	0.068505033453503
**BLCA**	RBBP5	cg14717946	-0.0543196359539442	419	-1.10419843030776	0.270151537644711	0.00295062285016902	0.46619717656461
**BLCA**	RBBP5	cg18131828	-0.137580832756955	419	-2.81939684106781	0.00504355656313288	0.0189284855420973	0.0249529675493469
**BLCA**	RBBP5	cg21214746	-0.0491756005271511	419	-0.999364757962216	0.31820460369823	0.00241823968720594	0.517528201624438
**BLCA**	RBBP5	cg23227967	-0.0296876870303546	419	-0.602859959495475	0.546933546533457	0.000881358761212285	0.720459019574712
**BLCA**	RBBP5	cg24103461	0.0211634449238586	419	0.429667248261668	0.667662331761036	0.000447891401045195	0.807213045877971
**BLCA**	RBBP5	cg26795850	-0.122154270013098	419	-2.49816935785658	0.0128724769817876	0.0149216656824329	0.0519533838817734
**BLCA**	RBBP7	cg01947226	-0.0951834410224975	418	-1.94082467396719	0.0529612624882467	0.00905988744488326	0.150449824868018
**BLCA**	RBBP7	cg03048084	-0.0989696931892546	418	-2.01877665766252	0.0441577532137761	0.00979500016997519	0.131748418098968
**BLCA**	RBBP7	cg03171708	-0.0429192289949238	418	-0.871968681848568	0.38373331393966	0.00184206021751871	0.581924587703891
**BLCA**	RBBP7	cg03811055	-0.064677757083878	418	-1.31556962384834	0.189050141545739	0.00418321226140113	0.368808253163708
**BLCA**	RBBP7	cg07096248	-0.112671252992279	418	-2.30163271724224	0.0218540550028982	0.0126948112508502	0.0778868588462278
**BLCA**	RBBP7	cg08363715	-0.0490614007083095	418	-0.9970383407162	0.319331110826994	0.00240702103946131	0.518663226323326
**BLCA**	RBBP7	cg14719055	-0.123932123306123	418	-2.53509114564022	0.0116107875342033	0.0153591711871642	0.0479029880976831
**BLCA**	RBBP7	cg15289846	-0.0436793728661084	418	-0.88744141111232	0.375359052562682	0.00190788761397652	0.574006941175198
**BLCA**	RBBP7	cg15464143	-0.0733842673127198	418	-1.49356498298173	0.136054771845081	0.00538525068902471	0.295294615989628
**BLCA**	RBBP7	cg16978043	-0.0826858546071595	418	-1.68410649516069	0.0929184802195062	0.00683695055211631	0.225989503226295
**BLCA**	RBBP7	cg17878446	-0.0638449602682183	418	-1.29856044234739	0.194821176093826	0.00407617895165038	0.376348205235607
**BLCA**	RBBP7	cg17880859	-0.111600629592629	418	-2.27948500470111	0.0231488176986033	0.0124547005254711	0.0813497368227469
**BLCA**	RBBP7	cg21122900	-0.0791914102317109	418	-1.61247416076245	0.107624497435878	0.00627127945448712	0.251118447362907
**BLCA**	RING1	cg01241018	0.0268378678814747	419	0.544945511552327	0.586085848688602	0.000720271152423493	0.750320286227184
**BLCA**	RING1	cg01878788	-0.307990486845895	419	-6.57094126534761	1.51296329100964e-10	0.0948581399875716	5.82959218726123e-09
**BLCA**	RING1	cg02475416	0.0619035745297429	419	1.25891976942181	0.208772144710917	0.00383205253955943	0.393984431643527
**BLCA**	RING1	cg03037740	-0.0124702231135546	419	-0.253137567373251	0.80028816778957	0.000155506464501831	0.890909304059132
**BLCA**	RING1	cg06316695	-0.158572552149499	419	-3.25991794712918	0.00120698443933164	0.0251452542952055	0.00785544612008414
**BLCA**	RING1	cg06671577	-0.093587097283791	419	-1.90798456482578	0.0570880716463582	0.00875854477800577	0.158785342702188
**BLCA**	RING1	cg07946923	-0.223314322225993	419	-4.65022007659029	4.47289524252532e-06	0.0498692865112545	6.45095314232508e-05
**BLCA**	RING1	cg08892712	-0.210506301833004	419	-4.37074881434012	1.56946541264894e-05	0.0443129031114078	0.000194017149708584
**BLCA**	RING1	cg09478665	-0.0447868928370791	419	-0.909987752650187	0.363361018346459	0.00200586577	0.562309669012222
**BLCA**	RING1	cg09724776	-0.0189488079271873	419	-0.384687862578875	0.700667181206735	0.000359057321861437	0.828515062587782
**BLCA**	RING1	cg09832551	-0.295145497971155	419	-6.27011854676567	9.11870152583375e-10	0.0871108649726408	3.03933276135694e-08
**BLCA**	RING1	cg09869033	-0.182210755409126	419	-3.76144283824396	0.00019340311761366	0.0332007593867643	0.00168786202957561
**BLCA**	RING1	cg10265934	0.0197543458825887	419	0.401047687496992	0.688592924109601	0.000390234181248949	0.821053338386317
**BLCA**	RING1	cg12065531	-0.0392769162606179	419	-0.797849977875516	0.425417162477474	0.00154267615094359	0.620148248127892
**BLCA**	RING1	cg12115664	-0.223116346360773	419	-4.64588141757032	4.56315071204982e-06	0.0497809040133803	6.56651399358497e-05
**BLCA**	RING1	cg12256228	-0.012199535393714	419	-0.247641952506774	0.804534815662514	0.000148828663822481	0.893705469637446
**BLCA**	RING1	cg14046664	-0.230764148565373	419	-4.81392997831423	2.08138366942175e-06	0.0532520922631016	3.27175512586372e-05
**BLCA**	RING1	cg14363494	-0.079719963975209	419	-1.62330503466899	0.105289056906875	0.00635527265620862	0.247129401792585
**BLCA**	RING1	cg18189601	-0.234983618420741	419	-4.90704712927014	1.33404702580871e-06	0.0552173009261044	2.20810328352687e-05
**BLCA**	RING1	cg20505183	0.0457539744155587	419	0.929677865835421	0.353082275977516	0.0020934261748196	0.552092028006811
**BLCA**	RING1	cg24536938	0.00588067271902274	419	0.119366683516084	0.905043036465571	3.45823116282584e-05	0.949667578244084
**BLCA**	RING1	cg24550302	-0.265126652103644	418	-5.57443746459721	4.50364383867907e-08	0.0702921416556866	1.06362012381967e-06
**BLCA**	RNF17	cg00202166	-0.439669325160988	418	-9.93622003077816	5.35182080410538e-21	0.193309115487519	7.70999162286248e-19
**BLCA**	RNF17	cg01405147	-0.0206773541760571	418	-0.419794202496285	0.674854622217811	0.000427552975722107	0.812034283252064
**BLCA**	RNF17	cg07131398	-0.214411815838205	407	-4.40130310273027	1.38057510404159e-05	0.0459724267710362	0.00017350639683976
**BLCA**	RNF17	cg12020676	-0.188565680671633	418	-3.89738206325597	0.000113468603898167	0.0355570159271562	0.00107373513826226
**BLCA**	RNF17	cg14855529	-0.0822102124796808	418	-1.67435272888497	0.094820181667483	0.00675851903595426	0.229272339994194
**BLCA**	RNF17	cg15523998	-0.186145379177467	418	-3.8455502477606	0.000139308894252396	0.034650102189123	0.00127864796711618
**BLCA**	RNF17	cg17806989	-0.362676331611557	418	-7.89935155705504	2.57695183150763e-14	0.131534121511216	1.84087594839582e-12

**BLCA**	RNF17	cg20477497	-0.160474633304141	418	-3.30004804358763	0.00105078878999865	0.0257521079340986	0.00699596209866534
**BLCA**	RNF17	cg21831927	-0.159549874170268	418	-3.28053279893479	0.00112424021193038	0.0254561623477485	0.00740050288093687
**BLCA**	RNF17	cg23678722	-0.0473332128312683	418	-0.961837359176431	0.336695463477039	0.00224043303693015	0.536310876261446
**BLCA**	RNF17	cg24753530	-0.163966073954815	418	-3.37380901382246	0.000811562837263243	0.026884873408156	0.00563702851446935
**BLCA**	RNF20	cg04845236	0.250881325130831	419	5.2605793595229	2.31359445928446e-07	0.0629414392994017	4.60787934883705e-06
**BLCA**	RNF20	cg14625770	-0.00674199404438939	419	-0.136850643251021	0.891215700480844	4.54544836945821e-05	0.942435829095054
**BLCA**	RNF20	cg21219529	0.115572639907292	419	2.36169401912668	0.0186560741298641	0.0133570350951406	0.0690091118281894
**BLCA**	RNF217	cg00991794	-0.277909972232369	419	-5.87228243800579	8.86042119300838e-09	0.0772339526661961	2.45036475751473e-07
**BLCA**	RNF217	cg02312409	-0.291019548404027	419	-6.17429294213978	1.59383281290024e-09	0.0846923775532841	5.07742136964623e-08
**BLCA**	RNF217	cg03113502	-0.171297327185894	419	-3.52911856021529	0.000463940663217769	0.0293427743010311	0.00352218209762498
**BLCA**	RNF217	cg06993703	-0.257424973940865	419	-5.40739474996192	1.08566561310382e-07	0.0662676172084551	2.34151013878012e-06
**BLCA**	RNF217	cg07439975	-0.217279494700025	419	-4.51823527386697	8.15693007690281e-06	0.047210378817098	0.000109278072330642
**BLCA**	RNF217	cg10621825	-0.270634343814224	419	-5.70622072194909	2.21019382764601e-08	0.0732429480517556	5.64237510550777e-07
**BLCA**	RNF217	cg16203203	-0.235079797248955	419	-4.90917304368963	1.32046006430534e-06	0.0552625110746098	2.18747594213309e-05
**BLCA**	RNF217	cg17382198	-0.301880395750585	419	-6.42736640030451	3.5948754702005e-10	0.09113177333853	1.286383359378e-08
**BLCA**	RNF217	cg18192252	-0.290123356757679	419	-6.15352882373054	1.79723747419907e-09	0.0841715621363437	5.66738111341072e-08
**BLCA**	RNF217	cg19171596	-0.278578350678908	419	-5.8875920751221	8.13579791220534e-09	0.0776058974669804	2.26851707090359e-07
**BLCA**	RNF217	cg19478500	-0.191587498811668	419	-3.96219911101449	8.75110004528209e-05	0.0367057697009108	0.000857625443339222
**BLCA**	RNF217	cg20034100	0.341500354082766	419	7.37507765667495	9.10686617343882e-13	0.116622491838654	5.16746569727571e-11
**BLCA**	RNF217	cg23521835	-0.293862102810196	419	-6.24027075973497	1.08588335899936e-09	0.08635493546803	3.56887241360356e-08
**BLCA**	RNF217	cg24822696	-0.208009971209101	419	-4.31655864324568	1.98710397540661e-05	0.0432681481224109	0.000237969160503051
**BLCA**	RPH3A	cg00851732	-0.0656584806481749	419	-1.33560362805502	0.182416220857374	0.00431103608102676	0.360138247014986
**BLCA**	RPH3A	cg00854817	0.0884040491117543	419	1.80145947593617	0.072361476352145	0.00781527589935347	0.188851606660587
**BLCA**	RPH3A	cg01119407	-0.105697007346063	419	-2.1575004252797	0.031544168676055	0.0111718573619137	0.102469812008728
**BLCA**	RPH3A	cg01573562	0.0339156544118682	419	0.688808870604191	0.491331246576446	0.00115027161418528	0.676980929032296
**BLCA**	RPH3A	cg05967001	-0.0407955582590557	419	-0.828749315842198	0.407726123616279	0.00166427757366801	0.603837719440139
**BLCA**	RPH3A	cg08254200	-0.0451318222386942	419	-0.917010336766863	0.359673687645743	0.00203688137858509	0.558498211409222
**BLCA**	RPH3A	cg09384610	-0.0764150417248454	419	-1.55560438254824	0.120569990975276	0.00583925860180987	0.271525847431991
**BLCA**	RPH3A	cg11596863	0.14785674096461	419	3.03451708082523	0.00256186869871179	0.0218616158486759	0.0145188925955985
**BLCA**	RPH3A	cg16403344	-0.0295882965674564	419	-0.600839892343979	0.548277023495672	0.000875467293763749	0.721446909995134
**BLCA**	RPH3A	cg17453780	0.0487948475049567	419	0.991608414678068	0.321970590063533	0.00238093714303198	0.521406036746802
**BLCA**	RPH3A	cg18629535	-0.0173670733444614	419	-0.352566262039949	0.724593923240337	0.000301615236551903	0.844000560750908
**BLCA**	RPH3A	cg19712198	-0.00641683191049297	419	-0.130250144102136	0.896432092788247	4.11757317675209e-05	0.945125609551526
**BLCA**	RPH3A	cg20704159	-0.118119547742133	419	-2.41446773001319	0.016193107956479	0.0139522275588061	0.0618949298964505
**BLCA**	RPH3A	cg21900624	0.126546879113446	419	2.58943856811739	0.00995373230257211	0.016014112613353	0.0424645270494266
**BLCA**	RPH3A	cg23199238	0.100076506062372	416	2.03413395214381	0.0425828596482831	0.0100153070656521	0.128200115459696
**BLCA**	RPH3A	cg23549179	0.121268195182692	419	2.47977682846225	0.0135452946044506	0.0147059751628674	0.0540131418816287
**BLCA**	RPH3A	cg23596620	0.012510374090539	419	0.253952733947492	0.799658761970755	0.00015650945988523	0.890577652092404
**BLCA**	RTF1	cg04910470	0.00246247289795077	419	0.0499828923892422	0.960160243341973	6.06377277314204e-06	0.979127583605928
**BLCA**	RTF1	cg07091958	0.0382249209337832	419	0.776448614485642	0.437929668374366	0.00146114458039398	0.630801003953403
**BLCA**	RTF1	cg07129439	0.173562034240535	419	3.57721616017445	0.000388537629759134	0.0301237797297125	0.00303198880223513
**BLCA**	RTF1	cg15581235	0.148119682452879	419	3.04003446444099	0.00251642118752659	0.0219394403299418	0.0143174482456259
**BLCA**	RTF1	cg18782716	0.182730705172544	419	3.77254658238156	0.000185268743408307	0.0333905106128553	0.00162712042080654
**BLCA**	RTF1	cg22263512	0.136264520194502	419	2.79190924331943	0.00548370922998008	0.0185680194638378	0.0267025335401271
**BLCA**	RTF1	cg24280607	-0.00207588894146762	419	-0.042136034325076	0.96641066659345	4.30931489730757e-06	0.982359454299184
**BLCA**	RTF1	cg27576415	0.212789701948935	419	4.42039501609482	1.2616764670726e-05	0.0452794572555165	0.000160613416919401
**BLCA**	SATB1	cg01069104	-0.13826840583458	419	-2.83376097333945	0.00482653162475298	0.0191181520520362	0.0241013673031666
**BLCA**	SATB1	cg01373248	0.183375503719085	419	3.78632107421332	0.000175626130500155	0.0336265753642283	0.00155699819898907
**BLCA**	SATB1	cg04004754	-0.168105222262943	419	-3.46142265211169	0.000593503121808741	0.0282593657520736	0.00433611775525819
**BLCA**	SATB1	cg14415616	-0.261074521521708	419	-5.48962150876842	7.05457030738613e-08	0.068159905787789	1.58476246749292e-06
**BLCA**	SATB1	cg19818826	-0.351008795628068	419	-7.60883407379292	1.89897428616344e-13	0.123207174608267	1.20300330307654e-11
**BLCA**	SATB1	cg26131754	-0.147350531210526	419	-3.02389697420188	0.00265147523738161	0.0217121790480241	0.0149189110817927
**BLCA**	SATB1	cg26768584	0.0295711642003934	419	0.600491686373114	0.548508768489116	0.000874453752166626	0.721691464213248
**BLCA**	SCML2	cg00457389	-0.0810831986298437	418	-1.65124617909245	0.0994502885207932	0.00657448510004669	0.237326241938065
**BLCA**	SCML2	cg04189389	0.0168199306663903	418	0.341455608962506	0.73293469787592	0.000282910067622178	0.849276247438057
**BLCA**	SCML2	cg05049545	-0.0379008770367229	418	-0.769856921930227	0.441825941064255	0.00143647648015279	0.634322652699085
**BLCA**	SCML2	cg06131666	-0.00774460968211995	418	-0.157203122267686	0.875161777377793	5.99789791283861e-05	0.933901253883048

**BLCA**	SCML2	cg08300622	-0.0483654345545865	418	-0.982861336760416	0.326252500237541	0.00233921525965399	0.525758938631132
**BLCA**	SCML2	cg17710066	0.0570032410445847	418	1.15892384236576	0.247158496343102	0.00324936948958703	0.440410647865575
**BLCA**	SCML2	cg17727916	-0.0756834410241879	418	-1.54062474542781	0.124175810674348	0.00572798324526173	0.277196256403792
**BLCA**	SCML2	cg22823767	-0.062896716269481	418	-1.27919666284258	0.201547781580009	0.00395599691748359	0.384888629998165
**BLCA**	SCML2	cg26246138	-0.0450318150778141	418	-0.91497420900761	0.360740356029012	0.00202786436920245	0.559634572187631
**BLCA**	SCML4	cg00977377	0.0299537722863799	419	0.608268112771119	0.543344840949124	0.000897228474184298	0.717700459407355
**BLCA**	SCML4	cg03500965	-0.22918857179948	413	-4.74430548808542	2.90272307582057e-06	0.0525274014434856	4.38815466304674e-05
**BLCA**	SCML4	cg05603896	-0.163449590811456	419	-3.36288953785749	0.00084344952360878	0.0267157687364323	0.00582315515083612
**BLCA**	SCML4	cg08792995	-0.0493171396982671	419	-1.00224817231322	0.316812015693324	0.0024321802680184	0.516094104258118
**BLCA**	SCML4	cg17388996	0.0322003886936703	419	0.653935615823855	0.513518111657597	0.00103686503202345	0.695145611092975
**BLCA**	SCML4	cg23503775	-0.12837715857592	419	-2.62751329728339	0.00892222797905263	0.0164806948440269	0.0390885737119119
**BLCA**	SCML4	cg27353361	0.0386036414451086	419	0.784152848347736	0.43340102388862	0.0014902411328225	0.626910665306265
**BLCA**	SCML4	cg27659622	-0.104498998098559	419	-2.13277496103906	0.0335337387325324	0.0109200406036027	0.107248958681082
**BLCA**	SETD1A	cg26692825	-0.24245641104453	419	-5.07268489906632	5.94375374442383e-07	0.0587851112565941	1.07757720529878e-05
**BLCA**	SETD6	cg01152986	-0.258804926206739	419	-5.43845660641636	9.230777935387e-08	0.0669799898288758	2.01656596098146e-06
**BLCA**	SETD6	cg01472538	-0.283095552597528	419	-5.99130622454162	4.54324480968462e-09	0.0801430919004995	1.32522379724261e-07
**BLCA**	SETD6	cg01773760	-0.295636004641922	419	-6.2815360160014	8.52793439781497e-10	0.0874006472406384	2.85957814027422e-08
**BLCA**	SETD6	cg02549819	-0.332485561569644	417	-7.13843699864246	4.32172647168553e-12	0.110546648652281	2.19311408439859e-10
**BLCA**	SETD6	cg05502081	-0.297284985567062	419	-6.31995915260909	6.80216805625605e-10	0.0883783626436081	2.31482004273133e-08
**BLCA**	SETD6	cg09132913	-0.304437732978107	419	-6.48735111133194	2.50861034602613e-10	0.0926823332608492	9.25342974199889e-09
**BLCA**	SETD6	cg19824325	-0.337963186982963	419	-7.28878018576015	1.60986662063527e-12	0.114219115755681	8.86326708320594e-11
**BLCA**	SETD6	cg26666090	-0.316472982786792	419	-6.77175801350752	4.40008257103962e-11	0.100155148833969	1.85729801156515e-09
**BLCA**	SETD7	cg05316864	-0.123577473790651	419	-2.52772395058467	0.0118533692243282	0.0152713920284791	0.0487018593766444
**BLCA**	SETDB1	cg02060408	-0.482111391341219	419	-11.1695943063128	1.74512452547614e-25	0.232431393660966	3.67913868116235e-23
**BLCA**	SETDB1	cg08531528	0.109266337290298	419	2.23122385105133	0.0262037088387426	0.0119391324648373	0.0892596486740519
**BLCA**	SETDB1	cg10589385	-0.350415374088562	419	-7.59416806999074	2.09743634105315e-13	0.122790934397627	1.31799646781817e-11
**BLCA**	SETDB1	cg10961700	-0.39080189731748	419	-8.61773952614473	1.47754283316603e-16	0.152726122946942	1.40346724253135e-14
**BLCA**	SETDB1	cg12082615	0.0934942221625059	419	1.90607439004636	0.0573361528107366	0.00874116957777201	0.159269714216549
**BLCA**	SETDB1	cg15448220	-0.250512773823802	419	-5.25233357043392	2.41281758157467e-07	0.0627566498488953	4.78589319074502e-06
**BLCA**	SETDB1	cg16370685	-0.43116218340677	419	-9.69952829171156	3.58620235321287e-20	0.185900828400093	4.88590237943682e-18
**BLCA**	SETDB1	cg17899625	0.113565896330796	419	2.32014619905313	0.0208207378497503	0.012897212809417	0.0750917237034528
**BLCA**	SETDB1	cg17918089	0.152873012137624	419	3.13989001343705	0.00181185313438484	0.0233701578400301	0.0109672660081181
**BLCA**	SETDB1	cg19689029	-0.449171513712971	419	-10.2045233433754	5.99532462296064e-22	0.201755048731202	9.3843115436338e-20
**BLCA**	SETDB1	cg26820223	-0.315664899490102	419	-6.75255062374179	4.95786871436808e-11	0.0996443287700959	2.07696520606112e-09
**BLCA**	SETMAR	cg00300090	-0.0366410860335475	419	-0.744232577623058	0.457160036427176	0.00134256918571783	0.647367611254257
**BLCA**	SETMAR	cg02520519	-0.0720819819326668	419	-1.46692032116427	0.143160616524416	0.00519581211934131	0.305914137493752
**BLCA**	SETMAR	cg06021447	-0.084132001809671	419	-1.71376909267168	0.0873233086465647	0.00707819372850249	0.216138386027708
**BLCA**	SETMAR	cg06267913	-0.0896376767531959	419	-1.82680001447021	0.0684532089557949	0.00803491309371044	0.181390044877277
**BLCA**	SETMAR	cg14751177	-0.0909602503687858	419	-1.85397705294064	0.064456937400843	0.0082737671471522	0.17346294280292
**BLCA**	SETMAR	cg14961031	-0.324368571690259	419	-6.96030100885511	1.34504713434186e-11	0.105214970300379	6.23951083893803e-10
**BLCA**	SETMAR	cg15232450	-0.0749008734250781	419	-1.52460432252881	0.128125036943639	0.00561014083983957	0.283225822487551
**BLCA**	SETMAR	cg15384599	-0.106569318349964	419	-2.17550984194384	0.0301597541871777	0.0113570196135761	0.0991023407491995
**BLCA**	SETMAR	cg16423812	-0.110916943880556	419	-2.2653459834477	0.0240099191572144	0.0123025684398025	0.0835955550002607
**BLCA**	SETMAR	cg21901604	-0.363736495825906	419	-7.92595766938655	2.14049041256791e-14	0.132304238395709	1.54468748752605e-12
**BLCA**	SETMAR	cg22149154	-0.12746310929287	419	-2.60849523099792	0.00942483213502668	0.0162468442306062	0.0406727168557852
**BLCA**	SETMAR	cg23182840	-0.382404436031128	419	-8.40043713855172	7.2723483285562e-16	0.146233152696285	6.27211824691464e-14
**BLCA**	SETMAR	cg23549342	-0.422975836608003	419	-9.4747640466256	2.12754215078255e-19	0.17890855835424	2.67815556760482e-17
**BLCA**	SETMAR	cg24390964	-0.101783283473217	419	-2.07676047060271	0.0384429223150476	0.0103598367945892	0.118864415682703
**BLCA**	SETMAR	cg26797270	-0.166407597918243	419	-3.42546643138503	0.000675369204239233	0.0276914886449198	0.00483434595827998
**BLCA**	SETMAR	cg27265118	-0.183068647821206	419	-3.77976524386036	0.000180154808247108	0.0335141298150846	0.00158953874932239
**BLCA**	SFMBT2	cg09060610	-0.0943131836166976	419	-1.92291981362369	0.0551791105598371	0.00889497660391692	0.154980921339902
**BLCA**	SFMBT2	cg12328063	-0.165844750400767	419	-3.41355207587297	0.000704742183410996	0.0275044812354926	0.00501460837979283
**BLCA**	SFMBT2	cg20299663	-0.124055116397618	419	-2.53764633243194	0.0115276905800166	0.0153896719044265	0.0476455828807676
**BLCA**	SFMBT2	cg25291138	-0.208170096607361	419	-4.32003197420526	1.95742243565628e-05	0.043334789121518	0.000234957137724802
**BLCA**	SHPRH	cg00571935	-0.154038237986249	419	-3.1644022462818	0.00166929124139487	0.0237277787619082	0.0102640564996106
**BLCA**	SHPRH	cg02758840	0.166539780180112	419	3.42826496415954	0.000668637529076155	0.0277354983824399	0.00479524972588573
**BLCA**	SHPRH	cg06708562	0.124340733673114	419	2.5435805021252	0.0113367463643899	0.0154606180503682	0.0470217829046954

**BLCA**	SHPRH	cg11387577	-0.0253591377789228	419	-0.514899868045833	0.606899115632503	0.000643085868890389	0.765129967936909
**BLCA**	SHPRH	cg11675148	0.0830994842980642	419	1.6925895437624	0.0912896238279215	0.00690552429060422	0.223123095166965
**BLCA**	SHPRH	cg12989205	0.118531530765992	419	2.42300881774981	0.0158226884226209	0.0140497237857292	0.0608097136226345
**BLCA**	SHPRH	cg14102550	0.00540856095937723	419	0.109783403132802	0.912634606861417	2.92525316512996e-05	0.953703409357061
**BLCA**	SHPRH	cg15443174	0.23891325594434	419	4.99403206139458	8.74932200288757e-07	0.0570795438659256	1.5209938679165e-05
**BLCA**	SHPRH	cg16339435	0.0171247648449751	419	0.347645741589325	0.728283783358342	0.000293257570995696	0.846362282612382
**BLCA**	SHPRH	cg18099523	-0.0408382516258265	419	-0.829618066671744	0.407235175615381	0.00166776279585432	0.603414217588338
**BLCA**	SHPRH	cg26814539	0.153404230215011	419	3.15106329398521	0.00174553151742058	0.0235328578478601	0.0106403290131811
**BLCA**	SHPRH	cg27398948	-0.0126374485567675	419	-0.256532675731164	0.797667601690057	0.000159705106024945	0.88938477504299
**BLCA**	SIN3A	cg01088093	0.114219265194442	419	2.33367042883964	0.0200931543887662	0.0130460405415582	0.0731048008848413
**BLCA**	SIN3A	cg01664094	0.209452661678606	419	4.34786563133024	1.73440624883175e-05	0.0438704174842525	0.000211516538206015
**BLCA**	SIN3A	cg02028562	0.0404171273557055	419	0.821048969787351	0.412093194811587	0.00163354418368732	0.607764806194976
**BLCA**	SIN3A	cg07544511	0.130235412464203	419	2.66619789928681	0.00797336919320182	0.0169612626597211	0.0358377792359569
**BLCA**	SIN3A	cg10483776	0.184531363542875	419	3.81102577054741	0.000159507539095035	0.0340518241309927	0.00143436937884166
**BLCA**	SIN3A	cg11184237	-0.0561566355554026	419	-1.14165677726543	0.254259810412623	0.0031535677169023	0.448394388690656
**BLCA**	SIN3A	cg15851274	-0.0963050409010791	419	-1.96390733709892	0.0502128136335485	0.00927466090295852	0.144716772889205
**BLCA**	SIN3A	cg22059910	0.245650079025576	419	5.1437643205106	4.17324808021344e-07	0.0603439613252718	7.83998215774267e-06
**BLCA**	SIN3A	cg25697800	0.0414636102075884	419	0.842343771026066	0.400084260598223	0.00171923097144683	0.596683137731538
**BLCA**	SIN3B	cg06815056	-0.161176732995083	419	-3.31487044713826	0.000998005654054107	0.0259779392589684	0.00670514309106876
**BLCA**	SIRT1	cg02720207	-0.0187704187415549	419	-0.38106502484995	0.703351419461369	0.000352328619733315	0.830523821389293
**BLCA**	SIRT1	cg03211233	-0.031069940592179	419	-0.630955533228851	0.52841945451872	0.000965341208401535	0.706818601503281
**BLCA**	SIRT1	cg07832903	-0.00749174086750032	419	-0.152069999010105	0.87920616508514	5.61261812257744e-05	0.935880634438014
**BLCA**	SIRT1	cg10582690	0.126450854660539	419	2.58744174727	0.0100106601700488	0.0159898186443808	0.0426676143583594
**BLCA**	SIRT1	cg10705386	0.00871900976310163	419	0.176983296644467	0.859608480518572	7.60211312490615e-05	0.925402794424003
**BLCA**	SIRT1	cg19192316	-0.0349548969901822	419	-0.709940769737181	0.478142481491659	0.00122184482359425	0.665735081437584
**BLCA**	SIRT1	cg19681528	0.0370598371334634	419	0.75274963963129	0.452030253471281	0.00137343152835883	0.642579498374312
**BLCA**	SIRT1	cg19980381	-0.0499714330725251	419	-1.0155781262842	0.310426343770367	0.00249714412332186	0.509237726520117
**BLCA**	SIRT1	cg25513531	0.0456985521923195	419	0.928549380106559	0.353666341252612	0.00208835767247415	0.552686686941548
**BLCA**	SIRT4	cg04118464	0.0252006066371053	417	0.510435500119043	0.610021087762234	0.000635070574878114	0.767345889175368
**BLCA**	SIRT4	cg07106325	-0.131454793279687	419	-2.69159812644318	0.00740071139442733	0.0172803626762052	0.0338326933195087
**BLCA**	SIRT4	cg08264848	0.017490572713606	418	0.355074168086443	0.722715724851669	0.00030592013384994	0.842910107781154
**BLCA**	SIRT4	cg12124973	-0.00797878997850856	419	-0.161956903883583	0.871419187089046	6.36610895211487e-05	0.931795167131695
**BLCA**	SIRT4	cg20010714	-0.0145070627861733	419	-0.294492204579731	0.768530140469422	0.000210454870681974	0.871701842571033
**BLCA**	SIRT4	cg21068152	-0.044898598242986	419	-0.912261981080466	0.36216430423512	0.00201588412418507	0.561105111998464
**BLCA**	SIRT4	cg21123573	0.0517623356835378	409	1.04051652035833	0.298723827119126	0.00267933939541525	0.496940395264158
**BLCA**	SIRT5	cg01064902	-0.13407934100807	419	-2.74631092554387	0.00629111401523848	0.0179772696851583	0.0297785776514428
**BLCA**	SIRT5	cg02206980	-0.134622049260377	419	-2.75763182835439	0.00608121138083211	0.0181230961470633	0.0289878528284591
**BLCA**	SIRT5	cg02712746	-0.230977402839101	419	-4.81862922987162	2.03552258166874e-06	0.0533505606222962	3.20939286012035e-05
**BLCA**	SIRT5	cg06139021	0.0298796314550663	419	0.606761195651023	0.544343607297837	0.000892792375890586	0.718495140420916
**BLCA**	SIRT5	cg07534331	-0.12636923057832	419	-2.58574443910835	0.0100592778013182	0.0159691824369565	0.0428325920785848
**BLCA**	SIRT5	cg07909422	-0.0796370636549534	419	-1.62160618955084	0.105652683432529	0.00634206190758309	0.247736228560453
**BLCA**	SIRT5	cg08619119	-0.17843805512166	419	-3.68097232730967	0.000263235834620787	0.0318401395156005	0.00218667042375755
**BLCA**	SIRT5	cg10907941	-0.212639703211224	419	-4.41713139269676	1.27999229913685e-05	0.0452156433817573	0.000162360542832304
**BLCA**	SIRT5	cg11824369	-0.257304263700472	419	-5.4046793274373	1.10113350839832e-07	0.066205484118442	2.37223982698252e-06
**BLCA**	SIRT5	cg19366958	0.103648085500128	419	2.11521886060677	0.0350109876951689	0.0107429256278418	0.110882231914856
**BLCA**	SIRT5	cg20150972	-0.137098050159796	419	-2.80931348356531	0.00520115340313017	0.0187958753576181	0.0255844785231179
**BLCA**	SIRT7	cg11691214	-0.194578685973147	419	-4.02647432605503	6.74021440537486e-05	0.0378608650350364	0.00068766982223178
**BLCA**	SIRT7	cg12710567	-0.171043403618596	416	-3.51087681769671	0.000496340543432218	0.0292558459214339	0.00372633818140957
**BLCA**	SIRT7	cg17552093	-0.0098643936394858	419	-0.200235065104897	0.84139554362467	9.73062618747278e-05	0.915065145471719
**BLCA**	SIRT7	cg20397086	-0.145038228549421	418	-2.97180327121598	0.00313452837665192	0.021036087740754	0.017066594382227
**BLCA**	SMARCA	cg03460558	-0.0752404092938827	418	-1.53155482055556	0.126399782552928	0.005661119190711	0.280618266713921
**BLCA**	SMARCA	cg06675660	-0.0550091448449501	418	-1.11825690017854	0.264108888720928	0.0030260060165727	0.459643917261633
**BLCA**	SMARCA	cg08617833	-0.318654789684634	418	-6.82369955587153	3.18220656771378e-11	0.101540874988958	1.37684414754575e-09
**BLCA**	SMARCA	cg12935118	-0.0412558938660694	418	-0.838116748087573	0.402451092203159	0.00170204877868838	0.598998749105532
**BLCA**	SMARCA	cg17420696	-0.0755073389837413	418	-1.53701940361256	0.125056153797474	0.00570135824040561	0.278493642931345
**BLCA**	SMARCA	cg18959966	-0.218860903282102	418	-4.55276815013019	6.97996497606166e-06	0.0479000949854578	9.52296379073555e-05
**BLCA**	SMARCA	cg24447042	-0.432121450699295	418	-9.72605623851341	2.90162760342666e-20	0.186728948154463	3.98814872404547e-18

**BLCA**	SMARCA	cg26010110	-0.282438901322599	418	-5.97620293793274	4.94802194234149e-09	0.0797717329803166	1.43361794779335e-07
**BLCA**	SMARCA	cg27353825	-0.030874035221761	416	-0.625449728611356	0.532023903913612	0.000953206050874541	0.709572009867982
**BLCA**	SMARCA	cg04518912	0.00225114405451088	419	0.0456933495923541	0.96357680342484	5.0676495541597e-06	0.980854943540968
**BLCA**	SMARCA	cg13529335	0.316880101805495	419	6.78144105677576	4.14273499412598e-11	0.100412998920261	1.75342723685004e-09
**BLCA**	SMARCA	cg14290704	-0.113530830462073	419	-2.31942044910163	0.0208604255981502	0.012889249465408	0.0752165600157106
**BLCA**	SMARCA	cg21210735	-0.222670243655969	419	-4.63610725295942	4.77293920936854e-06	0.0495820374098085	6.83050097228874e-05
**BLCA**	SMARCA	cg08405378	-0.0665033438149879	419	-1.3528654207074	0.176840555837482	0.00442269473857449	0.352918965670995
**BLCA**	SMARCA	cg17218495	0.149463998443636	419	3.06825299559669	0.00229534136949623	0.0223394868307592	0.0132933239495523
**BLCA**	SMARCA	cg17747332	-0.00330347199952624	419	-0.0670535240991339	0.946571619959145	1.09129272516539e-05	0.97151064821961
**BLCA**	SMARCA	cg18522266	-0.15361346226635	419	-3.15546490635639	0.00172002543646631	0.0235970957894553	0.0105161630622808
**BLCA**	SMARCA	cg23892580	0.0534567338071213	419	1.08660685644797	0.277845907904247	0.00285762238932542	0.474673764922325
**BLCA**	SMARCA	cg01278696	0.126281399019853	419	2.58391811100848	0.0101118266888564	0.0159469917384114	0.0430022109786744
**BLCA**	SMARCA	cg01575392	-0.205618452819113	419	-4.26472643407493	2.48450540938443e-05	0.0422789481397259	0.000289340739247983
**BLCA**	SMARCA	cg02782292	0.04194125473753	419	0.852064242563865	0.394673446788999	0.00175906884895838	0.591424781744874
**BLCA**	SMARCA	cg05358385	-0.029460935252587	419	-0.598251356075995	0.550000957825511	0.000867946705957122	0.722734571341638
**BLCA**	SMARCA	cg10878459	-0.316206989743487	419	-6.76543380015266	4.57656978445738e-11	0.0999868603626376	1.92865476604592e-09
**BLCA**	SMARCA	cg12253543	-0.139598807829931	419	-2.86156636598437	0.00443035680962036	0.0194878271475381	0.0225220661077963
**BLCA**	SMARCA	cg13848802	-0.252550800872811	411	-5.25924317352368	2.34516141746989e-07	0.063781907021498	4.66239425787509e-06
**BLCA**	SMARCA	cg13898584	-0.173544994858329	419	-3.57685406228014	0.000389059658357232	0.0301178652403776	0.00303514912377081
**BLCA**	SMARCA	cg16091845	-0.00598952218040102	419	-0.121576203025975	0.903293934389349	3.58743759495157e-05	0.948758505509675
**BLCA**	SMARCA	cg16587443	-0.171926506652074	412	-3.52091396756406	0.000478707921561305	0.0295587236895856	0.00360928513761777
**BLCA**	SMARCA	cg19993723	0.119148337397219	419	2.43579861076212	0.0152819901011422	0.0141963263045215	0.0592586550657093
**BLCA**	SMARCA	cg20045045	-0.0825670566883171	419	-1.68167025459315	0.0933905735074952	0.00681731885017178	0.226783190018794
**BLCA**	SMARCA	cg22077701	-0.0779887763401622	419	-1.58783543909773	0.113090660160761	0.00608224923503585	0.260127578029812
**BLCA**	SMARCA	cg24607315	-0.225223297471991	419	-4.69208662036633	3.68574090178274e-06	0.0507255337241571	5.43355178535695e-05
**BLCA**	SMARCB	cg01745726	-0.0496235954452649	419	-1.00849144957105	0.313810502199619	0.00246250122491532	0.512863556092816
**BLCA**	SMARCB	cg01912455	0.0139362977713241	419	0.282903423941537	0.777392951800907	0.000194220395571013	0.876922452203735
**BLCA**	SMARCB	cg03979008	0.200656016662972	419	4.15742706153582	3.91736436354848e-05	0.0402628370230508	0.00042994842451734
**BLCA**	SMARCB	cg10122452	0.0269692483535921	419	0.547615141787462	0.584252723064776	0.00072734035675773	0.748934819702431
**BLCA**	SMARCB	cg16142952	0.132815753284069	419	2.71996219610512	0.00680521173945128	0.0176400243204147	0.0316838763703906
**BLCA**	SMARCB	cg19906397	0.245351778056866	419	5.13711772682577	4.31429524774758e-07	0.0601974949956657	8.08153827361607e-06
**BLCA**	SMARCC	cg02564102	0.181735795880032	419	3.75130273202611	0.000201124762149991	0.0330278995041485	0.0017442920564879
**BLCA**	SMARCC	cg02627110	-0.107648902788102	419	-2.19780559096358	0.0285187043209512	0.0115882862714823	0.0950590892473576
**BLCA**	SMARCC	cg05592996	-0.250995872948086	419	-5.26314269417927	2.28356385067545e-07	0.0629989282369715	4.5562340651307e-06
**BLCA**	SMARCC	cg07143957	0.0737686682254725	419	1.50143125905455	0.134010041647875	0.00544181641175983	0.292186612696621
**BLCA**	SMARCC	cg07304068	-0.128542056121646	419	-2.63094494338951	0.00883414015958966	0.0165230601919805	0.0387761653058206
**BLCA**	SMARCC	cg07474652	0.0342896471341077	419	0.696413355968003	0.486562700397793	0.00117577990058162	0.673034320508649
**BLCA**	SMARCC	cg12894336	-0.0511189971162744	419	-1.03896068631344	0.299432362146847	0.00261315186617367	0.497734181734455
**BLCA**	SMARCC	cg16250355	0.135607478253478	419	2.77819436165191	0.00571608661294651	0.0183893881582675	0.0275955649791685
**BLCA**	SMARCC	cg20685352	-0.149776309986862	419	-3.07481121072847	0.00224658610461309	0.0224329430332805	0.0130523464841128
**BLCA**	SMARCC	cg22984439	-0.0149683598700398	419	-0.303858564392243	0.761389043949168	0.000224051797199018	0.86731435557058
**BLCA**	SMARCD	cg02553054	-0.0309794874166726	419	-0.629116880280119	0.529621201381677	0.000959728640599773	0.707744597445438
**BLCA**	SMARCD	cg04550398	-0.143522399323249	419	-2.94366206647659	0.00342705913608314	0.020598679107502	0.0183244785220778
**BLCA**	SMARCD	cg09878554	0.0722994833833074	419	1.47136985624997	0.141954490945549	0.00522721529749314	0.30419687142439
**BLCA**	SMARCD	cg13349421	-0.0536978756117351	419	-1.09152265172979	0.275680867082864	0.00288346184521338	0.472124189885083
**BLCA**	SMARCD	cg17846064	-0.14931529233654	419	-3.06513065341869	0.00231889371517255	0.0222950565255465	0.0133993922363614
**BLCA**	SMARCD	cg18591973	0.0213504282048843	419	0.433465168251823	0.664903726324722	0.000455840784531919	0.805475950405089
**BLCA**	SMARCD	cg20405918	-0.0146938181396683	419	-0.298284136700832	0.765636683129591	0.000215908291521644	0.870050835948806
**BLCA**	SMARCD	cg22402853	-0.169691453225162	419	-3.49504816010363	0.000525420638823071	0.0287951892976673	0.00390971806263404
**BLCA**	SMARCD	cg25107077	-0.0681461807991076	419	-1.3864393894752	0.166362555963266	0.00464390195750466	0.338769433777618
**BLCA**	SMARCD	cg00945038	-0.0273191473276546	419	-0.554725172027133	0.579383667224408	0.000746335810710098	0.745411192976893
**BLCA**	SMARCD	cg02127607	0.00390284839262109	419	0.0792197736124402	0.93689627949048	1.5232225575785e-05	0.966360948228465
**BLCA**	SMARCD	cg02360836	-0.0632274216902254	419	-1.28594949199646	0.199182935243828	0.00399770685359358	0.38191006985824
**BLCA**	SMARCD	cg04367190	0.00770115685629346	419	0.156321047319338	0.875856534428034	5.93078169252358e-05	0.934340215710097
**BLCA**	SMARCD	cg06420487	-0.190162074562994	419	-3.9316097690734	9.89680058133616e-05	0.0361616146021019	0.00095534396876097
**BLCA**	SMARCD	cg07362569	0.0465906829013047	419	0.946715649544966	0.344338608603055	0.00217069173320993	0.543599125728845
**BLCA**	SMARCD	cg11697258	-0.0355966480212492	419	-0.722991245714638	0.470095488876438	0.0012671213503487	0.658716244491041

**BLCA**	SMARCD	cg12577610	-0.123695528893041	419	-2.5301762200588	0.0117721264164799	0.0153005838681292	0.0484473788522266
**BLCA**	SMARCD	cg15868002	-0.0534794940431139	419	-1.08707082698523	0.277641068323476	0.00286005628310746	0.474417582295264
**BLCA**	SMARCD	cg19016967	-0.277296894045491	419	-5.8582476166692	9.57978458310082e-09	0.0768935674472765	2.62689376756343e-07
**BLCA**	SMARCD	cg19143629	-0.00700792560692218	419	-0.142248847197743	0.886952984609049	4.91110213121556e-05	0.939908467187156
**BLCA**	SMARCD	cg21254277	-0.163994946355895	419	-3.37441951758413	0.000809813631965483	0.0268943424302729	0.00562688832485913
**BLCA**	SMARCD	cg23682543	-0.192249641596585	419	-3.97641741252288	8.26247332643906e-05	0.0369599246940153	0.000815906130943133
**BLCA**	SMARCD	cg24795630	-0.145861959810644	419	-2.99268129634264	0.00293176034394823	0.0212757113198019	0.0161662435637304
**BLCA**	SMARCD	cg25945412	0.0220140100117453	419	0.446943912395907	0.655150410333384	0.000484616636797221	0.798984016852642
**BLCA**	SMARCD	cg00864064	0.0476546622768055	419	0.968384209238657	0.333420643521359	0.00227096683671639	0.533004388091826
**BLCA**	SMARCD	cg02917064	-0.0493136795433216	419	-1.0021776818447	0.316846012196945	0.00243183899010142	0.516097237169749
**BLCA**	SMARCD	cg14795305	-0.00803095423522557	419	-0.163015824432532	0.870585903024325	6.44962259282875e-05	0.931314701305609
**BLCA**	SMARCD	cg19786287	-0.022510297602036	419	-0.457024943947929	0.647893990533168	0.000506713498132226	0.793736458260429
**BLCA**	SMARCD	cg19902439	-0.0635543677419965	419	-1.29262597490971	0.196864895475063	0.00403915765908492	0.379006822376437
**BLCA**	SMARCD	cg22750243	-0.0660582621645516	419	-1.34377138842579	0.179761888752076	0.00436369400020063	0.356744805448443
**BLCA**	SMARCD	cg22787816	-0.0574846961547726	419	-1.16874454628496	0.243182359086262	0.00330449029200653	0.435771046524237
**BLCA**	SMNDC1	cg03999743	-0.00915000748317154	419	-0.185732642709031	0.852745834336207	8.37226369420952e-05	0.921544397245133
**BLCA**	SMNDC1	cg08770590	-0.0213502519756632	419	-0.433461588742082	0.664906324153195	0.000455833259424312	0.805475950405089
**BLCA**	SMNDC1	cg11559952	0.101092625178771	419	2.06252244711403	0.0397843341922539	0.0102197188655354	0.122013029021217
**BLCA**	SMNDC1	cg12486840	0.23003866239605	419	4.79794871315108	2.24492095561871e-06	0.052917786196964	3.4963239403684e-05
**BLCA**	SMNDC1	cg17981675	-0.144950122668024	419	-2.97357013820569	0.00311649384087279	0.0210105380614751	0.0169790967054316
**BLCA**	SMNDC1	cg18209001	-0.0359758855038453	419	-0.730703736868621	0.465375451245487	0.00129426433778579	0.654625666392873
**BLCA**	SMNDC1	cg21872391	0.138152805833842	419	2.83134567498164	0.00486242027787445	0.0190861977597633	0.0242308066809919
**BLCA**	SMNDC1	cg23721301	-0.0872398269965638	419	-1.77755229460456	0.0762149943872481	0.00761078741439037	0.196131229727493
**BLCA**	SMNDC1	cg26944434	-0.00464432561456549	419	-0.0942705308122752	0.924940087453331	2.15697604141091e-05	0.960447277868396
**BLCA**	SMYD1	cg00578637	-0.0047036866873624	418	-0.0954754684798024	0.923983594338098	2.21246684528703e-05	0.959994871420752
**BLCA**	SMYD1	cg00666842	-0.00637683200388408	418	-0.129438184835508	0.897074101554516	4.06639864057603e-05	0.945507765398962
**BLCA**	SMYD1	cg03704645	0.0379868890025431	418	0.771606551312871	0.440789818330675	0.00144300373609153	0.633377998029958
**BLCA**	SMYD1	cg14559139	-0.0643674342969336	418	-1.30923121862226	0.191185729610733	0.00414316659797007	0.371651031993237
**BLCA**	SMYD1	cg16090764	0.033830869965483	418	0.687084968145271	0.492415746172037	0.00114452776262142	0.677901030431349
**BLCA**	SMYD1	cg18148488	0.0127408170973257	418	0.258631334802827	0.796048861483694	0.000162328420307507	0.888312607657895
**BLCA**	SMYD1	cg19074493	-0.0399198272276248	418	-0.810930399633958	0.417873835641214	0.00159359260588341	0.613245677140378
**BLCA**	SMYD2	cg01770831	-0.0386128694951786	419	-0.784340576891835	0.433291015034149	0.00149095369065169	0.626850951786174
**BLCA**	SMYD2	cg04406444	-0.107331908599132	419	-2.19125816470425	0.0289924189623598	0.0115201386035324	0.0962874078192375
**BLCA**	SMYD2	cg11523160	-0.185309227141537	419	-3.82766059684113	0.000149452571286791	0.0343395096637938	0.00135792026754083
**BLCA**	SMYD2	cg14013804	0.0465747221079452	419	0.94639062346225	0.344504101142037	0.00216920473943232	0.543727978174957
**BLCA**	SMYD2	cg27570636	0.0335661693775664	419	0.681702967952847	0.49580981232579	0.00112668772668347	0.680705376107095
**BLCA**	SMYD5	cg01329511	-0.156606851912585	419	-3.21848472982066	0.00139062745111341	0.0245257060659702	0.00883973645667502
**BLCA**	SMYD5	cg05103132	-0.1741841746161	419	-3.5904393170296	0.000369918022088139	0.030340126686692	0.0029118084195411
**BLCA**	SMYD5	cg06754047	-0.0471519603015522	419	-0.958145983557651	0.33855106034736	0.00222330736027915	0.537919283619602
**BLCA**	SMYD5	cg08834152	0.043696728059421	419	0.887794694017477	0.375169178873378	0.00190940404309899	0.573875503886774
**BLCA**	SMYD5	cg15623359	-0.107926613086503	419	-2.20354217669691	0.028109169436849	0.0116481538123237	0.0940795875555505
**BLCA**	SMYD5	cg17895873	-0.0966069951213964	419	-1.9701228782683	0.0494935743683564	0.0093329115063855	0.143236895983469
**BLCA**	SMYD5	cg20402064	-0.0523434029084663	419	-1.06391351226052	0.287991032053764	0.00273983182803804	0.485515810780463
**BLCA**	SMYD5	cg25886337	-0.0852779275378528	419	-1.73728144336536	0.0830848170267235	0.00727232492515127	0.208513460732206
**BLCA**	SMYD5	cg26467949	-0.202902906755427	419	-4.20596812311668	3.19189791894435e-05	0.0411695895698014	0.000359818152016167
**BLCA**	SP100	cg05876232	0.0459009839205961	419	0.932671257835654	0.351535967667681	0.00210690032487882	0.550502388151326
**BLCA**	SP100	cg09456094	-0.193229462950341	419	-3.99746763204966	7.58633326757813e-05	0.0373376253520771	0.000759844483350036
**BLCA**	SP100	cg11702456	-0.153620830503362	419	-3.15561991979731	0.00171913348065226	0.0235993595645426	0.0105123620495097
**BLCA**	SP100	cg11827097	-0.0585020783459402	419	-1.18949977098383	0.234928108543038	0.00342249317079452	0.426043715784253
**BLCA**	SP100	cg23539753	-0.235386143730625	419	-4.91594547274702	1.27806147497049e-06	0.0554066366603746	2.12629414849987e-05
**BLCA**	SP100	cg27140170	0.0428840482161285	419	0.871252614001408	0.384123628456449	0.00183904159140323	0.582278128451696
**BLCA**	SP140L	cg04002454	-0.155504994431262	419	-3.19527685679131	0.0015044689088129	0.0241818032930667	0.00942188490518359
**BLCA**	SP140L	cg05217331	0.120001884312296	419	2.45350205340977	0.0145605229929941	0.0144004522385017	0.0570353134802107
**BLCA**	SP140L	cg13278004	-0.17715838424645	419	-3.65371564194376	0.00029184372036827	0.0313850931088127	0.00238885631127201
**BLCA**	SP140L	cg24044052	-0.327075891715149	419	-7.02532173912528	8.88717983961454e-12	0.10697863894126	4.2748002164434e-10
**BLCA**	SP140L	cg25095994	-0.277890129450856	419	-5.87182806778965	8.88286203629896e-09	0.0772229240462133	2.45569782960853e-07
**BLCA**	SP140L	cg26847438	-0.181136072758128	419	-3.73850287415761	0.000211287157578662	0.0328102768542377	0.00181783600272887

BLCA	SSRP1	cg01511567	0.145233703175971	419	2.97951285904603	0.00305793941940148	0.0210928285382062	0.0167127539126682
BLCA	SSRP1	cg01766191	-0.103171123355868	419	-2.10538019344027	0.0358629836255178	0.0106442806945117	0.112902146742889
BLCA	SSRP1	cg02688348	0.0355298999359248	419	0.721633833194004	0.470928962438976	0.00126237378945683	0.659349705811019
BLCA	SSRP1	cg04937561	-0.146787957822762	419	-3.01209722718177	0.00275440066399318	0.0215467045617771	0.0153790171000277
BLCA	SSRP1	cg08821673	-0.143914024979026	419	-2.95186399795479	0.00333919075861915	0.0207112465856636	0.0179398484721007
BLCA	SSRP1	cg09381635	-0.0964141339536693	419	-1.96615288698154	0.0499519589747734	0.00929568522603608	0.144170167562645
BLCA	SSRP1	cg11948159	0.0514701040122953	419	1.04611559773614	0.296121010510114	0.00264917160703649	0.494079096871288
BLCA	SSRP1	cg12795368	0.0469592703574128	419	0.954221778414658	0.340530908109593	0.00220517307250059	0.539800747116263
BLCA	SSRP1	cg12911972	-0.0735832986816316	419	-1.49763782317939	0.134993095241852	0.00541450184487021	0.293711448783237
BLCA	SSRP1	cg16313264	0.143172260326946	419	2.93633019799782	0.00350738924773006	0.0204982961271267	0.0186758274563937
BLCA	SSRP1	cg22636253	-0.0787802138938227	419	-1.60404906639432	0.109469502298287	0.00620632210115645	0.254074540897217
BLCA	SSRP1	cg26706201	-0.093782445158505	419	-1.91200247420429	0.0565691757080792	0.008795147019908	0.157732704481517
BLCA	STK31	cg00755064	0.098425400599177	419	2.00756530574402	0.0453420195450463	0.00968755948310847	0.134369266623265
BLCA	STK31	cg05000488	-0.129262094227165	419	-2.64593209994128	0.00845850171792507	0.0167086890039925	0.0375004663272504
BLCA	STK31	cg05407490	-0.0159763538660878	419	-0.324325959735114	0.745855862651705	0.000255243882854458	0.857305246697864
BLCA	STK31	cg11755819	-0.148283952520994	419	-3.04348172918942	0.00248840250201937	0.0219881305752483	0.014182927997809
BLCA	STK31	cg13313214	-0.015770989330492	419	-0.320155938904436	0.74901242369396	0.000248724104462491	0.859365690220954
BLCA	STK31	cg13514718	-0.0673368402378461	419	-1.36989783803511	0.171464878578078	0.00453425005321721	0.345642500720911
BLCA	STK31	cg13802883	0.0655085721843462	419	1.33254108088315	0.183418960492262	0.0042913730296317	0.361604019577675
BLCA	STK31	cg14898779	-0.107574084438403	419	-2.19626017782512	0.0286299094718771	0.0115721836427607	0.0953154096570037
BLCA	STK31	cg17102984	-0.109197350971394	419	-2.22979814149024	0.0262990379847693	0.0119240614591699	0.0894933450412117
BLCA	STK31	cg17331904	0.0265835180044786	419	0.539777242816424	0.589642276161774	0.00070668342949444	0.752746955274238
BLCA	STK31	cg21519371	0.0873001255530672	419	1.77879033816461	0.0760113946410326	0.00762131192158129	0.195779672653587
BLCA	STK31	cg25570411	0.0951803647470826	419	1.94076137414106	0.0529689696672742	0.00905930183338768	0.150460730503375
BLCA	STK31	cg25612145	-0.157951860580107	419	-3.24683068173823	0.00126240742790888	0.0249487902607176	0.00814934224933559
BLCA	SUV39H1	cg05903800	-0.0345165524013672	418	-0.701027218746574	0.483681767464027	0.00119139238967633	0.670514435782333
BLCA	SUZ12	cg00160853	0.010940925171492	419	0.222089819274832	0.824354008826617	0.000119703843608187	0.905608704209611
BLCA	SUZ12	cg01109633	0.0691206973572504	419	1.40636051087724	0.160370882027137	0.0047776708031526	0.330231295130617
BLCA	SUZ12	cg07482957	-0.0293824492766675	419	-0.596656193960517	0.551064649864837	0.000863328325495939	0.7235413556268
BLCA	SUZ12	cg08177746	0.0895633823538572	419	1.82527365884681	0.0686835854884044	0.00802159945866322	0.181802342616023
BLCA	SUZ12	cg17335595	-0.291233640159614	419	-6.17925591945447	1.54865467229657e-09	0.0848170331606194	4.9456503110279e-08
BLCA	SUZ12	cg25098644	-0.00648731066666778	419	-0.131680795945605	0.895301055315346	4.20851996858615e-05	0.944413402040565
BLCA	TAF3	cg00194465	-0.0363161605908836	419	-0.737624125053594	0.46116278019945	0.00131886352006285	0.650757323524856
BLCA	TAF3	cg02334915	0.222714390844918	419	4.63707438650688	4.75177471331887e-06	0.0496016998894229	6.80522021443167e-05
BLCA	TAF3	cg03672997	-0.00804845279455721	419	-0.163371040834488	0.87030640894868	6.47775923862158e-05	0.931198052151149
BLCA	TAF3	cg04179819	0.0426355433672766	419	0.866194650805164	0.386887563016584	0.00181778955822292	0.584845187663011
BLCA	TAF3	cg04234156	0.142128374484349	419	2.91447802257298	0.00375711522187777	0.0202004748335634	0.0197433816246122
BLCA	TAF3	cg05976695	-0.0634713140114461	419	-1.29092991610483	0.197451871140407	0.00402860770233959	0.379760948959079
BLCA	TAF3	cg07855525	-0.19031847310526	419	-3.93496478888403	9.76452566758391e-05	0.0362211212051177	0.000944567905724972
BLCA	TAF3	cg09871669	-0.0799008260315788	419	-1.62701149054112	0.10449917437052	0.00638414200052862	0.245822103660046
BLCA	TAF3	cg09994553	-0.194576171093688	412	-3.99699411628366	7.61955056704392e-05	0.0378598863574801	0.000762556933848405
BLCA	TAF3	cg11104088	-0.0612170399634687	419	-1.24490504758349	0.213874288730861	0.00374752598188893	0.400446133902828
BLCA	TAF3	cg12176426	0.180534679332143	419	3.72567169728183	0.000221958790668781	0.0325927704415595	0.00189435679224215
BLCA	TAF3	cg12590721	-0.143462932441441	419	-2.94241675763839	0.0034405834977297	0.0205816129846975	0.0183804939309425
BLCA	TAF3	cg12777293	-0.110519107864239	419	-2.25712000152448	0.0245236225989285	0.0122144732031074	0.0849543324054779
BLCA	TAF3	cg27268107	-0.138026872771722	419	-2.82871461618892	0.00490179049129148	0.0190514176071412	0.0243988668167848
BLCA	TCEA1	cg01677098	-0.194378142660483	419	-4.02216138460728	6.86008514117986e-05	0.0377828623441392	0.000697521191312177
BLCA	TCEA1	cg06047473	-0.102864941612272	419	-2.09906511981579	0.036419148279733	0.0105811962128961	0.114287665238949
BLCA	TCEA1	cg11530293	0.0725739864377894	419	1.47698580272563	0.140443389702041	0.00526698350747243	0.301954254062867
BLCA	TCEA1	cg11787876	-0.0640123770335137	419	-1.30197957044581	0.193650807334918	0.00409758441348071	0.374732009189629
BLCA	TCEA1	cg12850379	-0.182249383968921	419	-3.76226765488906	0.00019278747704211	0.0332148379570513	0.00168324455544488
BLCA	TCEA1	cg23569945	-0.0119056044582953	419	-0.24167450582041	0.809152624483338	0.000141743417517381	0.896890461974033
BLCA	TCEA1	cg26181865	-0.265038223064391	419	-5.57921269922464	4.38416571415018e-08	0.0702452596851301	1.0391888713181e-06
BLCA	TCEA1	cg26358945	0.0118569025907065	419	0.240685756517168	0.809918400840963	0.000140586139045502	0.897331358916113
BLCA	TDRD1	cg00337703	-0.107467384109029	418	-2.19405629060007	0.0287891455206944	0.0115492386472375	0.0957349229134349
BLCA	TDRD1	cg02635786	-0.430801243152187	414	-9.65421521454741	5.30226109584282e-20	0.18558971110147	7.08735566477657e-18
BLCA	TDRD1	cg16550453	-0.934746470839078	418	-53.3984201452832	2.99477828931286e-187	0.87375096474611	3.88292970398008e-183

**BLCA**	TDRD1	cg23504215	-0.756635366289351	418	-23.4890634499375	4.87543707142822e-78	0.57249707751982	7.74040309254464e-75
**BLCA**	TDRD1	cg23825092	-0.662754385446906	418	-17.9644535715987	1.04490127290583e-53	0.439243375429106	7.13044294951195e-51
**BLCA**	TDRD1	cg24203376	-0.651036561476981	418	-17.4094751167667	2.81677000385333e-51	0.423848604379771	1.75302244543813e-48
**BLCA**	TDRD1	cg26600802	-0.659272808373465	418	-17.7971916331996	5.65814735353513e-53	0.434640635860635	3.76213602752916e-50
**BLCA**	TDRD1	cg27547703	-0.826595091417912	417	-29.7756942538593	1.17918207784873e-104	0.683259445156186	4.36825193162687e-101
**BLCA**	TDRD3	cg01557749	-0.0842118371241837	419	-1.71540694779568	0.0870224913189207	0.00709163351183005	0.21562020860796
**BLCA**	TDRD3	cg03447530	-0.19452343531254	419	-4.02528603730647	6.77304079130594e-05	0.0378393668857918	0.00069020426423743
**BLCA**	TDRD3	cg05666423	-0.184284165333376	419	-3.80574092328574	0.000162833476389882	0.0339606535926191	0.0014603951420653
**BLCA**	TDRD3	cg10765900	0.0998252103879869	419	2.03640232417176	0.0423489567181617	0.00996507262900585	0.127649065788395
**BLCA**	TDRD3	cg18522102	0.128902670681759	419	2.63845038634257	0.00864419334889714	0.0166158985088901	0.0381301701590963
**BLCA**	TDRD3	cg19937698	0.0349844920549013	419	0.710542587531073	0.477769742872081	0.00122391468433945	0.66541856518531
**BLCA**	TDRD3	cg20944895	-0.203193930104569	419	-4.21226034289015	3.10782663052003e-05	0.0412877732313405	0.000351461353241279
**BLCA**	TDRD3	cg22045829	0.0373496916169926	419	0.758645279971927	0.448498550448027	0.00139499946388445	0.639811281917386
**BLCA**	TDRD3	cg23103367	-0.111069673037505	419	-2.26850422871662	0.0238151927743887	0.0123364722686582	0.0831021398892436
**BLCA**	TDRD3	cg23767211	0.0966217328796541	419	1.97042625996086	0.0494586913262968	0.00933575926466723	0.14317144574823
**BLCA**	TDRD3	cg26737458	-0.0956299474321941	419	-1.95001293187079	0.0518524636089801	0.00914508684588421	0.14816567691449
**BLCA**	TDRD5	cg05378938	-0.235222617687445	419	-4.91233019504471	1.30052855130903e-06	0.0553296798717338	2.15905501751035e-05
**BLCA**	TDRD5	cg07171496	-0.274882500778232	419	-5.80305154581389	1.30033841841221e-08	0.0755603892340949	3.47027536610497e-07
**BLCA**	TDRD5	cg07901138	-0.260262125575678	419	-5.47129599427445	7.76952008256139e-08	0.0677363740091698	1.73286136841394e-06
**BLCA**	TDRD5	cg09656934	-0.261541667401347	419	-5.50016475534523	6.6726655318939e-08	0.0684040437870767	1.51118876968895e-06
**BLCA**	TDRD5	cg11853788	-0.30241247245174	419	-6.4398341100663	3.33656735736167e-10	0.0914533034943746	1.20168944906756e-08
**BLCA**	TDRD5	cg12277666	-0.212500465422905	419	-4.41410219522235	1.29722013622001e-05	0.0451564478049514	0.000164331449726591
**BLCA**	TDRD5	cg13848905	-0.167301476908311	419	-3.44439514321071	0.000631042306450398	0.0279897841757021	0.00456382836636308
**BLCA**	TDRD5	cg15545772	-0.198176895690182	419	-4.10394824919392	4.89774130469092e-05	0.0392740819853974	0.0005206543960879
**BLCA**	TDRD7	cg24339935	0.0110331778100778	419	0.223962682442652	0.822897356517444	0.000121731012588793	0.904671034879323
**BLCA**	TDRD9	cg07613153	0.323678922776507	417	6.92689429867424	1.67254486836431e-11	0.10476804504976	7.60011422251944e-10
**BLCA**	TDRD9	cg08933517	0.151213650109501	419	3.10500601845637	0.00203416083138968	0.0228655679794385	0.0120568005879717
**BLCA**	TDRD9	cg16701547	0.231900682805816	419	4.83898301695103	1.84788932263387e-06	0.0537779266858038	2.94217564398238e-05
**BLCA**	TRIM24	cg00733782	-0.00481650586553162	419	-0.0977655255283531	0.922166026367797	2.31987287527005e-05	0.959139116104927
**BLCA**	TRIM24	cg02308366	-0.0502861561411215	419	-1.02199046206607	0.307385121880954	0.00252869749944925	0.50589463965937
**BLCA**	TRIM24	cg06386929	-0.0496978656126655	419	-1.0100045633053	0.313085894886995	0.00246987784645456	0.512094754419779
**BLCA**	TRIM24	cg06650216	-0.0525050938866869	419	-1.06720905707045	0.286502384863981	0.00275678488404981	0.483831166763091
**BLCA**	TRIM24	cg10571650	0.123732027347221	418	2.52786100857461	0.0118497282153706	0.0153096145914533	0.048699024232459
**BLCA**	TRIM24	cg10703331	-0.0514548954343325	419	-1.04580566690844	0.296263937396012	0.00264760626415809	0.494221382922003
**BLCA**	TRIM24	cg13935558	0.0936726358844033	419	1.9097438928754	0.056860374887591	0.00877456271353199	0.158329014389192
**BLCA**	TRIM24	cg26889928	-0.0302746069034475	419	-0.614789212952395	0.539033317260712	0.000916551823158272	0.714565519627277
**BLCA**	TRIM33	cg00760200	0.0553887249390098	419	1.12599688247825	0.260822294398468	0.00306791085036928	0.456066746919181
**BLCA**	TRIM33	cg02852257	0.0514021064557558	419	1.04472990257389	0.296760393498025	0.00264217654808885	0.494753290724472
**BLCA**	TRIM33	cg05792124	0.062874835212725	419	1.27874987822421	0.201704967656615	0.00395324490302732	0.385065920340581
**BLCA**	TRIM33	cg16036305	0.0962513810496331	419	1.96280283951415	0.050341537626762	0.00926432835396166	0.144987952246726
**BLCA**	TRIM33	cg17645130	0.0561543346535756	419	1.1416098522716	0.254279301130609	0.00315330930038576	0.448415558601681
**BLCA**	TRIM33	cg18199720	0.0553639727947019	419	1.12549214892337	0.26103574791746	0.00306516948361249	0.456312232154445
**BLCA**	TRIM33	cg20932765	-0.109831257123883	419	-2.24290002755361	0.0254342055640306	0.0120629050414124	0.0873217073240079
**BLCA**	TRIM33	cg20992663	-0.214067036116468	419	-4.44820012611106	1.11548883650699e-05	0.0458246959516892	0.000144150064031935
**BLCA**	TRIM33	cg21666217	0.00641050335694882	419	0.130121680568303	0.896533663208371	4.10945532894521e-05	0.945181458132972
**BLCA**	TRIM33	cg21813112	-0.112395924205394	418	-2.29314826552988	0.02234361678508	0.0126328437779846	0.0791169469357541
**BLCA**	TRIM66	cg06492264	0.025673730583116	419	0.521291646041657	0.602443703673026	0.000659140442054426	0.761772109349989
**BLCA**	TRIM66	cg11245569	0.220954264500369	406	4.53673486444675	7.55911694780862e-06	0.0488207870008989	0.00010227025110223
**BLCA**	TRIM66	cg12608775	0.0567257996038612	419	1.15326497074838	0.249470262730864	0.00321781634069742	0.443033270182853
**BLCA**	TRIM66	cg13143389	0.0695624488420882	419	1.41539214818023	0.157708983499708	0.00483893428890814	0.326519453739981
**BLCA**	TRIM66	cg27044202	-0.106925078474025	419	-2.18285619262656	0.0296102592760603	0.0114329724066763	0.0977218950501373
**BLCA**	UBE2A	cg00142683	-0.00214195652554808	418	-0.0434770687658849	0.965342306722513	4.58797775733799e-06	0.981774803840441
**BLCA**	UBE2A	cg00428314	0.0075443457360437	418	0.153137851756598	0.878364539681646	5.69171525849607e-05	0.935408422792357
**BLCA**	UBE2A	cg00935924	-0.021974891408151	418	-0.446149314946617	0.65572377257892	0.000482895852400027	0.79923815376427
**BLCA**	UBE2A	cg05206294	-0.0156671408400535	418	-0.318047263266698	0.75061022993749	0.000245459302102072	0.860202652739612
**BLCA**	UBE2A	cg06206449	-0.0163151459185419	418	-0.331205377309483	0.740657750257716	0.000266183986343316	0.854104282823391
**BLCA**	UBE2A	cg06273903	0.0197201925784704	418	0.400354045853848	0.689103274498434	0.000388885995331958	0.821244651021585

**BLCA**	UBE2A	cg09080960	-0.00844282674188398	418	-0.171376774302968	0.864011654329967	7.12813233934712e-05	0.927817284023295
**BLCA**	UBE2A	cg14625604	0.00801746150838849	418	0.162741925536111	0.870801425319918	6.42796890384911e-05	0.931420248330667
**BLCA**	UBE2A	cg15661671	-0.0280893666089366	418	-0.570376933365091	0.56873299163667	0.000789012516491243	0.737120473016143
**BLCA**	UBE2A	cg16211147	0.000967312160884409	418	0.0196343016466615	0.984344605294043	9.35692816594865e-07	0.991815669674707
**BLCA**	UBE2A	cg17976175	-0.013159644839623	418	-0.267134748723749	0.789499077611376	0.000173176252305016	0.884143168286734
**BLCA**	UBE2A	cg18990292	-8.85132085971657e-05	418	-0.0017966219192999	0.998567373457217	7.83458809616536e-09	0.999208692107392
**BLCA**	UBE2A	cg25165659	0.00666680721021953	418	0.135324414293204	0.892421471896675	4.44463183782351e-05	0.943018690365797
**BLCA**	UHRF2	cg06523306	0.034864683213366	419	0.708106277554675	0.479279666383078	0.00121554613556837	0.66666217934854
**BLCA**	UHRF2	cg08487038	-0.0328068044082585	419	-0.666264043068907	0.505615235551081	0.00107628641548173	0.68872847952202
**BLCA**	UHRF2	cg13428749	0.187597377153166	419	3.87663664563595	0.00012321319880589	0.0351927759147471	0.00115221151435334
**BLCA**	UHRF2	cg14078101	0.236759632875257	419	4.94632842516564	1.10349000892265e-06	0.0560551237592266	1.8667571667144e-05
**BLCA**	UHRF2	cg20595300	0.168249830564667	419	3.46448696134824	0.000586973065782189	0.0283080054850391	0.0042956709952455
**BLCA**	UHRF2	cg24119045	0.228356497193195	419	4.76092582364444	2.67272981945606e-06	0.0521466898103458	4.08813101798594e-05
**BLCA**	USP27X	cg00478198	-0.0704633289018541	418	-1.43381329736325	0.152384032096317	0.00496508071993087	0.319151502070344
**BLCA**	USP27X	cg01125381	-0.130073802436988	418	-2.66283241922508	0.00805216075990212	0.0169191940804166	0.036110842370384
**BLCA**	USP27X	cg01290688	-0.177040274024781	418	-3.65120088914206	0.000294625497206579	0.0313432586267694	0.0024088382479967
**BLCA**	USP27X	cg01855997	-0.0782989681101314	418	-1.59418975915391	0.11166035411908	0.00613072840711138	0.257584010566668
**BLCA**	USP27X	cg06455206	-0.261176691436379	418	-5.4919270781705	6.96928242627048e-08	0.0682132641496537	1.5674135792694e-06
**BLCA**	USP27X	cg06624970	-0.0847776191234317	418	-1.72701515923636	0.0849144490976092	0.00718724470423765	0.211912607647717
**BLCA**	USP27X	cg16810342	-0.0814428942713767	418	-1.65862012542197	0.0979534194015095	0.00663294502729864	0.234590041219131
**BLCA**	USP27X	cg16867483	-0.124931728857709	418	-2.55586144225919	0.0109505091148482	0.0156079368753761	0.0457719663892017
**BLCA**	USP27X	cg19318186	-0.0779238515664618	418	-1.58650550517288	0.113391843336683	0.00607212664295197	0.260528253001576
**BLCA**	USP51	cg00834229	-0.0633662020174463	418	-1.28878343917399	0.19819657011381	0.00401527555811581	0.380741405952039
**BLCA**	USP51	cg01084827	-0.0772190612187257	417	-1.57016071081177	0.117147239815327	0.00596278341550131	0.266332116844746
**BLCA**	USP51	cg02714202	-0.0587592731978245	418	-1.19474729105425	0.232873100683627	0.00345265218673658	0.42360142760208
**BLCA**	USP51	cg03728850	-0.0518748051817434	418	-1.05436314436885	0.292334606784834	0.00269099541264383	0.490061441006627
**BLCA**	USP51	cg07386410	-0.042860632770695	418	-0.87077601897269	0.384383546139492	0.00183703384150437	0.582547263610209
**BLCA**	USP51	cg12064531	-0.0532824249922321	418	-1.08305360356806	0.279418066763064	0.00283901681305284	0.476459801976366
**BLCA**	USP51	cg12517811	-0.0657269039405436	418	-1.33700150832668	0.181959885111151	0.00432002590160944	0.359520161083405
**BLCA**	USP51	cg18770944	-0.0636440270961645	418	-1.29445695890194	0.196232665157034	0.00405056218501732	0.378229576899143
**BLCA**	USP51	cg21331369	-0.0520307256062728	418	-1.0575408399851	0.29088449218801	0.00270719640711525	0.488421751856727
**BLCA**	USP51	cg25424556	-0.0313209159354767	418	-0.636057221644818	0.525092296663304	0.000980999775037199	0.704123661988917
**BLCA**	WDR82	cg01736403	0.158363121128189	419	3.25550165725831	0.0012254296166006	0.0250788781334616	0.00795486244941815
**BLCA**	WDR82	cg03869063	-0.0503958694342422	419	-1.02422589090834	0.306329579442233	0.00253974365603319	0.504761711052526
**BLCA**	WDR82	cg10989431	0.229516816467201	419	4.7864585316987	2.37008109207548e-06	0.0526779690412389	3.66994602860111e-05
**BLCA**	WDR82	cg11442381	-0.116564885353446	419	-2.38224836348595	0.0176601821849825	0.0135873724974621	0.0661684830177976
**BLCA**	WDR82	cg11665333	-0.187110479542822	419	-3.86620953897092	0.000128405058364035	0.0350103315547449	0.00119385446796388
**BLCA**	WDR82	cg12661343	0.0151186487956503	419	0.306910132248022	0.759066833946946	0.000228573541406219	0.86583547572717
**BLCA**	WDR82	cg13539115	0.00919111650025872	419	0.186567169895136	0.852091839191496	8.4476622521328e-05	0.921173844440215
**BLCA**	WDR82	cg15991175	-0.20480324527538	419	-4.24707644667158	2.67951135823873e-05	0.0419443692753274	0.00030876482422905
**BLCA**	WDR82	cg17632831	0.0420377619194762	419	0.854028316469055	0.393585581558622	0.00176717342719857	0.590394365648177
**BLCA**	WDR82	cg24007312	0.0135792937748018	419	0.275654976279285	0.782951282161145	0.000184397219422371	0.880186590237632
**BLCA**	WDR82	cg26236504	0.0687777737950645	419	1.39934999014641	0.162460485691001	0.00473038216820507	0.333099231032779
**BLCA**	WDR82	cg26858096	-0.0442813180289526	419	-0.899695099774026	0.368808078182604	0.00196083512638124	0.567544075210917
**BLCA**	ZMYND1	cg00614684	-0.0224929035507759	419	-0.456671614954274	0.648147758285224	0.000505930710144506	0.793934823537462
**BLCA**	ZMYND1	cg02813021	-0.0726478582113037	419	-1.47849717289616	0.140038848259518	0.00527771130268968	0.301393851643361
**BLCA**	ZMYND1	cg02867818	-0.200621812215684	419	-4.15668865070386	3.92952814063621e-05	0.0402491115367052	0.000430979433487457
**BLCA**	ZMYND1	cg03254875	-0.0646916827035588	419	-1.31585406639754	0.188954720306789	0.00418501381101793	0.368706671045965
**BLCA**	ZMYND1	cg05236965	-0.00610916001447325	419	-0.124004719144865	0.901372013553852	3.73218360824388e-05	0.947956498370603
**BLCA**	ZMYND1	cg08828193	-0.291870262045993	419	-6.19401979500618	1.42153724615203e-09	0.0851882498667963	4.57160266751349e-08
**BLCA**	ZMYND1	cg12639781	-0.0825700333996165	419	-1.68173129842375	0.0933787209223842	0.00681781041561378	0.226768565131921
**BLCA**	ZMYND1	cg13939078	-0.23056273526665	419	-4.80949232624555	2.12560507703083e-06	0.0531591748936394	3.33049992673789e-05
**BLCA**	ZMYND1	cg14255089	0.262797337200446	419	5.52852518171616	5.74242649122986e-08	0.0690624404396451	1.3220666660513e-06
**BLCA**	ZMYND1	cg14694828	-0.0898405778184717	419	-1.83096871126102	0.0678272638863016	0.00807132942275687	0.180204028782178
**BLCA**	ZMYND1	cg17632565	-0.236213853469283	419	-4.93425149083868	1.16993099505358e-06	0.0557969845708079	1.96743648571549e-05
**BLCA**	ZMYND1	cg22593633	-0.134046395051577	419	-2.74562375349165	0.00630406323270394	0.0179684360263233	0.029827167931211
**BLCA**	ZMYND1	cg24757747	-0.125560747626131	419	-2.56893555280485	0.0105522646007168	0.015765501344433	0.0444355782368823

**BRCA**	ACTL6A	cg03667091	0.0287193702419351	111	0.955502116504598	0.339532630915429	0.000824802227093347	0.538800185411641
**BRCA**	ACTL6A	cg03839554	-0.236925700191868	793	-6.85007701680815	1.48604856079915e-11	0.0561337874114071	6.81235484612902e-10
**BRCA**	ACTL6A	cg07261848	-0.266806392796462	795	-7.7861006773477	2.16536268598223e-14	0.0711856512370602	1.5597428221602e-12
**BRCA**	ACTL6A	cg10112003	0.0762108050694601	796	2.15101791309053	0.0317766714989984	0.00580808680933525	0.102987738570141
**BRCA**	ACTL6A	cg20132173	0.087980347662341	796	2.48562518641309	0.0131376645895165	0.00774054157478639	0.0527445672228336
**BRCA**	ACTL6A	cg22619386	0.162443477187093	796	4.6331022287989	4.21047637210312e-06	0.0263878832806337	6.11442596399833e-05
**BRCA**	ACTL6A	cg25198545	0.00145957753335758	111	0.048540606222781	0.961294167257704	2.13036657588219e-06	0.979744506644209
**BRCA**	ACTL6A	cg25575835	0.0642480688644601	796	1.81184427379303	0.0703888915106905	0.00412781435281241	0.185131970592422
**BRCA**	ACTL6B	cg05729836	0.103255815740223	796	2.92149209622409	0.00358263616673204	0.0106617634841788	0.0189868245762485
**BRCA**	ACTL6B	cg08572611	-0.161764638800289	111	-5.45153858417514	6.15568230608118e-08	0.0261677983661881	1.40569290178939e-06
**BRCA**	ACTL6B	cg09375063	-0.106914651507468	796	-3.02619051136824	0.00255672798733883	0.0114307427069633	0.0144969458489094
**BRCA**	ACTL6B	cg14088161	-0.0120467703226269	111	-0.400482261873147	0.688878759863702	0.000145124675206125	0.821151757574765
**BRCA**	ACTL6B	cg16024950	-0.135225158152751	796	-3.8408519047102	0.000132405899824158	0.0182858433974365	0.00122536100058536
**BRCA**	ACTL6B	cg20497359	0.00769243153132685	796	0.216490617864245	0.828661086700774	5.91735028641515e-05	0.907891259506928
**BRCA**	ACTL6B	cg24471254	-0.35733291835196	796	-10.7671161623701	2.52343823748021e-25	0.127686814537929	5.27710629694989e-23
**BRCA**	ACTL6B	cg26990587	-0.203495984604349	796	-5.84927617728276	7.22019294785984e-09	0.0414106157500934	2.03510032676018e-07
**BRCA**	AEBP2	cg02664216	0.106415476547875	796	3.01189928103041	0.00267876799565952	0.0113242536489113	0.0150463593829846
**BRCA**	AEBP2	cg07849438	-0.0851246025671974	796	-2.40434568515494	0.0164301039947389	0.00724619796222331	0.062581448793905
**BRCA**	AEBP2	cg08161947	-0.204903440570882	796	-5.89149864648354	5.65663202789449e-09	0.0419854199577851	1.62320926587246e-07
**BRCA**	AEBP2	cg24304000	-0.0659201735109299	796	-1.85920207510208	0.063369174538915	0.0043454692757111	0.17130082363152
**BRCA**	AEBP2	cg26259627	-0.137179787335513	795	-3.89496577502588	0.000106511491385779	0.0188182940534166	0.00101680635180578
**BRCA**	AFF4	cg00931577	0.0617960981587459	796	1.74242649081235	0.0818219965745391	0.00381875774764535	0.206249121946721
**BRCA**	AFF4	cg01907658	-0.195106273389911	796	-5.59836603480297	2.98366422538354e-08	0.0380664579160987	7.37950217865538e-07
**BRCA**	AFF4	cg05619425	0.09523246913058	796	2.69231565699917	0.00724563379997653	0.00906922317670687	0.0332978990923543
**BRCA**	AFF4	cg06638012	0.0530208483948158	111	1.76577572630825	0.07770935119948	0.00281121036450604	0.19878555000008
**BRCA**	AFF4	cg07236381	-0.11181666930488	315	-1.98754032671636	0.0477365017146339	0.012502967534437	0.139473199669054
**BRCA**	AFF4	cg13393914	0.0111413814138135	796	0.313565727937758	0.75393345639237	0.000124130379808068	0.862518083885781
**BRCA**	AFF4	cg13565585	-0.110026473327467	796	-3.11533368638784	0.001903605299125	0.0121058248328798	0.0114257442049325
**BRCA**	AICDA	cg07999668	0.0159016759827367	796	0.447569420339938	0.654586362729473	0.000252863299059945	0.798539932604307
**BRCA**	AIRE	cg08089567	0.0372792335170614	796	1.04986039720289	0.29410254465211	0.00138974125161959	0.491871726511152
**BRCA**	AIRE	cg09510531	0.0191631084428602	796	0.539396724086613	0.589764743122796	0.000367224725192819	0.752824862603288
**BRCA**	AIRE	cg11923631	0.0305554010686786	796	0.860306907362423	0.389880231937835	0.000933632534467806	0.587420324575462
**BRCA**	AIRE	cg12843033	0.0798924959227102	796	2.25558413876902	0.0243688235398737	0.00638281090476026	0.0845749836475993
**BRCA**	AIRE	cg15955655	0.012098474330902	796	0.340506169409462	0.733565673909082	0.000146373081135495	0.849641307150688
**BRCA**	AIRE	cg16501323	0.0376491360188646	796	1.06029233166774	0.289334860490525	0.00141745744296697	0.486860099865892
**BRCA**	AIRE	cg16717549	0.0427924745613477	796	1.20539114186681	0.228412353334644	0.00183119587908359	0.418295920405903
**BRCA**	AIRE	cg17356252	0.0407380807093708	110	1.35470825114621	0.175787687714989	0.00165959121988321	0.351593453608429
**BRCA**	AIRE	cg18689454	0.0466407366718407	796	1.31401668469194	0.189221046407026	0.00217535831729199	0.369030612053152
**BRCA**	AIRE	cg18876487	0.123399687249749	111	4.13545845780804	3.81057970407106e-05	0.0152274828133359	0.000419708675489882
**BRCA**	AIRE	cg23272544	0.0103614777663386	796	0.291613485926161	0.770658523578452	0.000107360221502329	0.872951408778292
**BRCA**	AIRE	cg27251412	0.00896628355131177	796	0.252343729751865	0.800840862406073	8.03942407225241e-05	0.891256531286917
**BRCA**	ARID1A	cg04081153	-0.0668814627044057	796	-1.8864350637081	0.0596019559727044	0.00447313005348081	0.163874834344404
**BRCA**	ARID1A	cg04360147	0.0247195162945615	796	0.695881496063346	0.486707192049767	0.000611054485837091	0.673094277506926
**BRCA**	ARID1A	cg14851949	-0.0193623237821494	796	-0.545006262574518	0.585902643421212	0.000374899582244787	0.750233898052963
**BRCA**	ARID1A	cg17385674	-0.0544441563048314	111	-1.81331582614135	0.0700540826527313	0.00296416615574492	0.184479781135755
**BRCA**	ARID1A	cg20413923	0.0429897413130651	796	1.21095807510295	0.226272695277444	0.00184811785816426	0.415784629072503
**BRCA**	ARID1A	cg21294655	0.122152674519628	796	3.46361890505571	0.00056158069272288	0.0149212758922981	0.00413512621009784
**BRCA**	ARID1A	cg24542126	-0.13251419485632	796	-3.76246074400014	0.000180660760216001	0.0175600118384187	0.00159309943099564
**BRCA**	ARID1A	cg25601319	-0.0793789660517206	796	-2.24099354998408	0.0253028543877174	0.00630102025144021	0.0869929767539555
**BRCA**	ARID2	cg00035453	-0.201317823970598	783	-5.73634152030158	1.3844821500631e-08	0.0405288662482565	3.66840614380138e-07
**BRCA**	ARID2	cg01183821	0.0513936850478957	796	1.44826041946185	0.147939988401409	0.0026413108628023	0.312804526018374
**BRCA**	ARID2	cg01353809	-0.206016358249548	796	-5.92491221302867	4.65814014114442e-09	0.0424427398664063	1.35771957339898e-07
**BRCA**	ARID2	cg01751433	-0.00134365443008662	796	-0.0378138216224979	0.969845653181354	1.8054072274914e-06	0.984089486403587
**BRCA**	ARID2	cg06613666	0.0673540355054696	796	1.89982481668342	0.0578191005407365	0.00453656609887206	0.160206937850507
**BRCA**	ARID2	cg07276957	0.00133196208342727	796	0.037484768936877	0.970107930191019	1.77412299168791e-06	0.984240076180342
**BRCA**	ARID2	cg23465749	0.0042528558900746	760	0.11693526171218	0.906942412067365	1.80867832217422e-05	0.950841639389257
**BRCA**	ARID4A	cg02136725	-0.0737097481591829	796	-2.08003442804297	0.0378434059972716	0.00543312697369017	0.117562092730283

**BRCA**	ARID4A	cg06154646	0.14494949623269	795	4.12017728331216	4.18481516478412e-05	0.0210103564581106	0.000455255923548057
**BRCA**	ARID4A	cg08066755	-0.0507929316375532	796	-1.43128729933907	0.152742358192446	0.00257992190433715	0.319729819535657
**BRCA**	ARID4A	cg08790491	-0.141301654191567	111	-4.7446900660782	2.3616810560638e-06	0.0199661574772731	3.65839538182849e-05
**BRCA**	ARID4A	cg10064886	0.286018680073949	796	8.40020579218115	2.05127314923523e-16	0.0818066853512438	1.9156871953374e-14
**BRCA**	ARID4A	cg10242170	0.159730507484952	796	4.55368120753897	6.10079087080023e-06	0.0255138350214003	8.47811584499881e-05
**BRCA**	ARID4A	cg10844118	-0.262916112079222	796	-7.66891777381465	5.0880177674778e-14	0.0691248819908539	3.4873766890147e-12
**BRCA**	ARID4A	cg12203852	0.106282314499239	111	3.55472006830538	0.000394361102862963	0.0112959303753152	0.00307004179286714
**BRCA**	ARID4A	cg14272338	-0.00726150063063361	796	-0.204362130003749	0.838123024630724	5.27293914086923e-05	0.91337035200844
**BRCA**	ARID4A	cg14460816	-0.0355984046699612	796	-1.00246329510109	0.316426058885801	0.00126724641504632	0.515669665083387
**BRCA**	ARID4A	cg17323045	0.220427269719002	796	6.35980267634944	3.41190994614909e-10	0.0485881812357736	1.22655324561332e-08
**BRCA**	ARID4A	cg19548283	-0.132139858430543	792	-3.74215725936056	0.000195695244269558	0.0174609421860439	0.0017044240744185
**BRCA**	ARID4A	cg20996579	-0.0441578441827933	796	-1.24392525581148	0.21389510724687	0.00194991520287185	0.400465836518086
**BRCA**	ARID4A	cg26429530	-0.241038160592103	796	-6.98949594610233	5.86015141733669e-12	0.0580993948616245	2.90557437450791e-10
**BRCA**	ARID4A	cg27026909	-0.25698739676513	796	-7.48360512996369	1.92594179115025e-13	0.0660425220961183	1.21711385622049e-11
**BRCA**	ARID4B	cg05652840	0.171136684229996	796	4.88832927708852	1.23137966554097e-06	0.0292877646892375	2.05698839813387e-05
**BRCA**	ARID4B	cg06212607	-0.130643592571805	110	-4.37835386163354	1.30933996120176e-05	0.0170677482800678	0.000165543300734161
**BRCA**	ARID4B	cg06891548	-0.250092666220257	796	-7.26923390010669	8.68199427136942e-13	0.0625463416971567	4.94644613172198e-11
**BRCA**	ARID4B	cg07814876	-0.312596057486151	796	-9.26135496701288	1.85531724209519e-19	0.0977162951558852	2.35069298911325e-17
**BRCA**	ARID4B	cg10107779	0.277125408097262	796	8.11690912648381	1.8232087194924e-15	0.0767984918130741	1.4995383760351e-13
**BRCA**	ARID4B	cg11732257	-0.173243871827016	796	-4.95036946750082	9.05401196143156e-07	0.0300134391256156	1.56870335529534e-05
**BRCA**	ARID4B	cg22596515	0.224799321161574	796	6.49259100236577	1.48809424721289e-10	0.0505347347947046	5.74230177915078e-09
**BRCA**	ARID4B	cg23687319	-0.201976409373409	111	-6.85529185978021	1.1810805394754e-11	0.0407944699433749	5.54167548178223e-10
**BRCA**	ARID4B	cg26020585	0.23241131637341	796	6.72477473276711	3.36697839260812e-11	0.0540150199784214	1.45119013236079e-09
**BRCA**	ASH1L	cg03119126	-0.195348769618443	796	-5.60560009356503	2.86623971456014e-08	0.0381611417914394	7.12156666734243e-07
**BRCA**	ASH1L	cg08095278	0.0978047073988574	796	2.76572838039761	0.00581179502953901	0.00956576078937611	0.0279675109815636
**BRCA**	ASH1L	cg09410084	0.192521645922352	796	5.52132837059514	4.56280995464306e-08	0.0370645841486514	1.07661279227025e-06
**BRCA**	ASH1L	cg10558228	-0.0943749671669274	789	-2.65603844790226	0.00806689934299515	0.00890663442775863	0.036162058746627
**BRCA**	ASH1L	cg17038116	-0.0665433411559097	111	-2.21791977568104	0.0267628793115864	0.00442801625219178	0.0906553789587022
**BRCA**	ASH1L	cg20064047	-0.235110046670868	796	-6.80740317704325	1.96291886232786e-11	0.0552767340455779	8.79627361612521e-10
**BRCA**	ASH1L	cg21852905	-0.076034768622056	796	-2.14602043310989	0.0321746161434123	0.00578128603940959	0.104001000883393
**BRCA**	ASH1L	cg23821590	-0.209455561638269	796	-6.02832121833089	2.53844359010274e-09	0.0438716323012028	7.78348189612098e-08
**BRCA**	ASH1L	cg24312492	-0.104378538076678	796	-2.95360609461322	0.00323380704920552	0.0108948792110245	0.0175066656635974
**BRCA**	ASH1L	cg26185843	-0.166981584241895	796	-4.76619564288652	2.23361748829272e-06	0.0278828494759332	3.4829031646471e-05
**BRCA**	ASH2L	cg00590869	0.366830805614577	796	11.0971414733109	1.07004280452985e-26	0.13456483994784	2.44832088045868e-24
**BRCA**	ASH2L	cg01642070	0.00951993542045127	796	0.267926872041682	0.788825370671234	9.06291704095627e-05	0.8838270665687
**BRCA**	ASH2L	cg02897270	0.194110718113463	796	5.56867816262173	3.51646754819025e-08	0.0376789708865243	8.51151451287841e-07
**BRCA**	ASH2L	cg03189282	-0.0904465479786764	796	-2.55586718818704	0.0107779807266751	0.00818057804125902	0.0452025571540764
**BRCA**	ASH2L	cg03705346	0.240542832410982	796	6.97424960944497	6.49106905640539e-12	0.057860854224298	3.19396727497787e-10
**BRCA**	ASH2L	cg10062221	0.339088101202675	796	10.1437556782362	8.15253706346863e-23	0.114980740377235	1.38475648103816e-20
**BRCA**	ASH2L	cg21453287	0.258849023881671	796	7.54169485122124	1.27261320646557e-13	0.0670028171644938	8.32646524674368e-12
**BRCA**	ASH2L	cg24318412	0.166786746848375	111	5.62555367849291	2.34152466963229e-08	0.0278178189242641	5.93150668021407e-07
**BRCA**	ASH2L	cg27255459	0.285744846739698	796	8.39144804971124	2.19659843315943e-16	0.0816501174382933	2.02947955474115e-14
**BRCA**	ASXL1	cg00174466	-0.0438680383695014	796	-1.23574564113926	0.216919526165435	0.00192440479038804	0.404231246550899
**BRCA**	ASXL1	cg04218270	-0.062586491698541	796	-1.76479981476755	0.077982790220669	0.00391706894313154	0.199267912783244
**BRCA**	ASXL1	cg09065199	-0.041973081348023	111	-1.3964805339517	0.162850272988532	0.00176173955784775	0.333590703238009
**BRCA**	ASXL1	cg10421984	-0.176542974119581	796	-5.04764336017014	5.55255331832892e-07	0.0311674217109872	1.01564856065384e-05
**BRCA**	ASXL1	cg12592359	-0.190242956188374	796	-5.45350856283823	6.60389987946015e-08	0.0361923823792914	1.49823210038706e-06
**BRCA**	ASXL1	cg16926526	-0.0225123405850574	796	-0.633714027646021	0.526450433769507	0.000506805478617625	0.70515478993552
**BRCA**	ASXL1	cg18793288	0.027315747648958	796	0.769020235373284	0.442110563552089	0.000746150069621552	0.634511428410718
**BRCA**	ASXL1	cg24727216	-0.129537419540106	796	-3.67648219950245	0.000252426192517547	0.0167799430611094	0.002109943399668
**BRCA**	ASXL2	cg05165989	0.158858502905549	796	4.52817623212062	6.86423804625231e-06	0.0252360239453923	9.39109314252605e-05
**BRCA**	ASXL2	cg05496738	0.0162209500452646	796	0.456558066942578	0.648113904548975	0.00026311922037097	0.793909851717419
**BRCA**	ASXL2	cg06583696	0.0464051962736168	796	1.30736641131481	0.191467702603062	0.0021534422411929	0.371911072566857
**BRCA**	ASXL2	cg09476286	0.0195746169078083	796	0.550984119022674	0.581799950585929	0.000383165627087456	0.747137685309632
**BRCA**	ASXL2	cg19084174	-0.0684333599773824	796	-1.93041094302071	0.0539126554936438	0.00468312475779401	0.152428897745685
**BRCA**	ASXL2	cg20667782	0.0989448813831974	796	2.79828721783735	0.00526222841232322	0.009790089551935	0.0258212310526222
**BRCA**	ASXL2	cg24738909	-0.0134141933742477	796	-0.377542833214445	0.705871388447848	0.000179940583881712	0.832259212371962

**BRCA**	ASXL3	cg09963640	-0.0965776621800844	796	-2.73070112622353	0.00646045548488339	0.0093272448321705	0.0303981132689946
**BRCA**	ASXL3	cg12361352	-0.0800321682950455	784	-2.24236970986523	0.0252177037392407	0.00640514796200649	0.0867816528660751
**BRCA**	ASXL3	cg17880622	-0.101148686752661	796	-2.86125080664145	0.00433089036620041	0.0102310568317879	0.022121949123322
**BRCA**	ASXL3	cg20034091	-0.073587311615358	796	-2.07656053246302	0.038164076660959	0.0054150924307758	0.118270174073324
**BRCA**	ASXL3	cg20329085	-0.0705370756478593	796	-1.99004615751101	0.0469292558201095	0.00497547904095183	0.137756189241174
**BRCA**	ASXL3	cg24880056	-0.0800467219797174	796	-2.259966415369	0.0240941990272493	0.00640747769969817	0.0838262026781335
**BRCA**	ASXL3	cg27109877	-0.0830738182041874	795	-2.34453217940095	0.0192974218522482	0.00690125927102238	0.0707924000553521
**BRCA**	ATAD2B	cg08192560	-0.0128416495282652	796	-0.361425854206357	0.717877518020448	0.000164907962606794	0.83977269310521
**BRCA**	ATAD2B	cg14009731	-0.132426492264288	796	-3.75992615078168	0.000182468312020693	0.0175367758534235	0.00160576242820563
**BRCA**	ATAD2B	cg15717719	0.170812834394989	796	4.87880058382551	1.29054710787638e-06	0.0291770243940499	2.14385696583676e-05
**BRCA**	ATAD2B	cg16929802	0.0509102363535084	796	1.43460139315275	0.151795449968007	0.00259185216557009	0.318266556299516
**BRCA**	ATAD2B	cg17933838	-0.0243080381702973	796	-0.68429102900696	0.493991587858407	0.000590880719688632	0.679255896243229
**BRCA**	ATAD2B	cg19614157	-0.0875617593663803	796	-2.47370761777984	0.0135805562499759	0.00766706170333589	0.0541121590304559
**BRCA**	ATAD2B	cg20806130	-0.120417059516308	796	-3.4136764296016	0.000673497576430178	0.014500268222554	0.00482227983992722
**BRCA**	ATAD2B	cg27610867	-0.0830120997660228	796	-2.34425868257395	0.0193111670481428	0.00689100870756412	0.0708361434054708
**BRCA**	ATAT1	cg01245721	-0.265616150843184	796	-7.75361989699075	2.74413863200459e-14	0.0705519395887492	1.95134845281686e-12
**BRCA**	ATAT1	cg05093836	-0.0373836185142944	796	-1.05280420316718	0.292751829102948	0.00139753493322229	0.490445684237117
**BRCA**	ATAT1	cg06814856	-0.0848776453176972	796	-2.39731968345109	0.0167462132598139	0.0072042146746768	0.0634963646894752
**BRCA**	ATAT1	cg07142893	-0.102576237156085	796	-2.90205913502788	0.00381003872838377	0.0105218844291013	0.0199729213501272
**BRCA**	ATAT1	cg07816366	-0.0881480690925382	796	-2.49040073173288	0.0129638138359553	0.00777008208474289	0.0522217757639967
**BRCA**	ATAT1	cg08081524	-0.12658581619283	796	-3.5913305277471	0.000349279176922466	0.0160239688612048	0.00277461700086861
**BRCA**	ATAT1	cg08188471	-0.0871130436433139	786	-2.4453482105868	0.014690795184938	0.00758868237280191	0.0574472564902518
**BRCA**	ATAT1	cg08635134	-0.0971655302880006	796	-2.74748084095712	0.00614198592662282	0.00944114027614836	0.0292291951535876
**BRCA**	ATAT1	cg09532691	-0.203580199344725	796	-5.85180147990816	7.11587165399637e-09	0.0414448975652381	2.00860710976413e-07
**BRCA**	ATAT1	cg12907138	-0.0628529737433207	796	-1.77234375465379	0.0767218863101737	0.00395049630837856	0.197073127667477
**BRCA**	ATAT1	cg13542073	-0.236752051600659	796	-6.85775869370458	1.40896061118985e-11	0.0560515339371209	6.51656847722374e-10
**BRCA**	ATAT1	cg13964084	-0.085486309212719	111	-2.85342754866969	0.0044056685277601	0.0073079090628126	0.0224317414391367
**BRCA**	ATAT1	cg15381130	-0.0950113329947137	796	-2.68600690438046	0.00738259056482529	0.00902715339743237	0.0337657269915938
**BRCA**	ATAT1	cg15729859	-0.0621704353386508	796	-1.75302227669067	0.0799850557179106	0.00386516303019736	0.203033165547007
**BRCA**	ATAT1	cg18298197	-0.0237232478581096	796	-0.667819322064155	0.504443481778993	0.000562792488937303	0.687712872600124
**BRCA**	ATAT1	cg20629923	-0.0394213689365476	796	-1.11027870740065	0.267215917854917	0.0015540443288314	0.462917986763581
**BRCA**	ATAT1	cg22759096	-0.212672789595179	796	-6.12526766550092	1.42503273705258e-09	0.0452297154341953	4.57905810600035e-08
**BRCA**	ATAT1	cg23328390	-0.094629717120972	796	-2.67512079765197	0.00762439716015755	0.00895478336239517	0.0346435577756729
**BRCA**	ATAT1	cg26934609	-0.0968288718642474	795	-2.73614213373132	0.00635577086822967	0.00937583042650284	0.0300127407731145
**BRCA**	ATF7IP	cg00236831	-0.0596965148060565	796	-1.68301036966634	0.092767197033367	0.00356367387998972	0.225741541055828
**BRCA**	ATF7IP	cg11409060	-0.0650838454676628	792	-1.83087242234526	0.0674970627163317	0.00423590694085862	0.179549291211823
**BRCA**	ATF7IP	cg11697652	-0.0425036547476061	315	-0.751443300140642	0.452952345143275	0.0018065606669037	0.643345219020895
**BRCA**	ATF7IP	cg12526346	-0.240902936276703	796	-6.98533312995688	6.02617658439828e-12	0.0580342247067371	2.98408899558676e-10
**BRCA**	ATF7IP	cg12948116	-0.208060317846077	796	-5.98634147605103	3.25141018879251e-09	0.0432890958622106	9.73595859226037e-08
**BRCA**	ATF7IP	cg17439009	-0.0159513507251751	796	-0.448967923578964	0.653577615720992	0.000254445589957543	0.797809776202712
**BRCA**	ATF7IP	cg19789919	-0.0594585128229869	796	-1.67627657975689	0.094078684503015	0.00353531474712129	0.227970258604147
**BRCA**	ATF7IP	cg21275967	-0.0363550288854389	796	-1.02379799712809	0.306243296444372	0.0013216881252611	0.504711374353187
**BRCA**	ATF7IP	cg26637901	-0.184640988733889	102	-5.98834972752384	2.9377454542647e-09	0.0340922947206282	8.87185442038307e-08
**BRCA**	ATM	cg01756564	-0.205003039706653	796	-5.89448797649842	5.55941147035572e-09	0.0420262462889677	1.59825889107484e-07
**BRCA**	ATM	cg01800843	-0.129245159142435	111	-4.33264750430667	1.60734184887967e-05	0.0167043111617534	0.000197756684788463
**BRCA**	ATM	cg01991180	-0.0985761836840721	794	-2.78423534500436	0.00549369583802129	0.00971726398971593	0.0267411196360298
**BRCA**	ATM	cg02105423	0.153238296165686	796	4.36405055824567	1.44581776411953e-05	0.0234819754117626	0.000180539240998258
**BRCA**	ATM	cg03165700	-0.139039646029674	110	-4.66299997091873	3.49740695241387e-06	0.019332023168057	5.18637583789715e-05
**BRCA**	ATM	cg03569616	-0.178208505396514	791	-5.08070402835082	4.70003449936844e-07	0.0317582713956594	8.73052731241328e-06
**BRCA**	ATM	cg04709886	-0.132507003860783	796	-3.76225292180329	0.000180808331992867	0.0175581060721615	0.00159367815307649
**BRCA**	ATM	cg04826772	0.0841244081230895	796	2.37589262094621	0.0177435181217834	0.00707691604206012	0.0664167468010401
**BRCA**	ATM	cg05033322	-0.156654207499822	795	-4.46093292662142	9.34263071591714e-06	0.0245405407273973	0.000123353804126622
**BRCA**	ATM	cg05637308	-0.0624450147543262	796	-1.76079484512921	0.0786590252139941	0.00389937986766802	0.200465199262816
**BRCA**	ATM	cg06053805	-0.062190626940623	796	-1.75359383033149	0.0798869304308193	0.00386767407926775	0.202830413379085
**BRCA**	ATM	cg06081482	-0.170602128972589	792	-4.86028170872292	1.4147879345991e-06	0.02910508640998	2.32149362126561e-05
**BRCA**	ATM	cg06750635	-0.0120053559680332	796	-0.33788501532831	0.735539378284541	0.000144128571919189	0.850913814862856
**BRCA**	ATM	cg09808176	-0.0772748251315221	796	-2.18122858978832	0.0294599537502751	0.00597139859910731	0.0973745599103034

**BRCA**	ATM	cg10610482	-0.294799959449021	796	-8.68225440725656	2.19425312400645e-17	0.0869070160911444	2.2699431852255e-15
**BRCA**	ATM	cg10976287	-0.115604295667118	796	-3.27535336884819	0.00110117655498922	0.0133643531766904	0.00726897996765648
**BRCA**	ATM	cg11588932	-0.07309955921203	796	-2.06272245506453	0.0394645248452553	0.00534354555699309	0.1212952173289
**BRCA**	ATM	cg12019961	-0.292844175209742	758	-8.41549009050545	1.95395359521231e-16	0.0857577109542739	1.82919212979479e-14
**BRCA**	ATM	cg12106634	-0.0777086781245157	796	-2.19354910532927	0.0285577966442637	0.00603863865585958	0.0951486244440384
**BRCA**	ATM	cg12848864	-0.286977942128684	796	-8.43090262693265	1.61297764595928e-16	0.0823563392684141	1.52652047432794e-14
**BRCA**	ATM	cg14761454	-0.11006111175504	784	-3.09460965154785	0.00204100874282584	0.0121134483207553	0.0120909407660214
**BRCA**	ATM	cg15370815	0.0895754624054889	796	2.53105169433318	0.0115645914635703	0.00802376346515716	0.0477549672656186
**BRCA**	ATM	cg15504467	-0.174982766378497	796	-5.00161904834254	7.00506753121375e-07	0.0306189685294717	1.24588985716334e-05
**BRCA**	ATM	cg16693212	-0.138245760240365	785	-3.90092502203316	0.00010407883535124	0.0191118902244364	0.00099700885572151
**BRCA**	ATM	cg16788234	0.173510704525141	796	4.95823058207518	8.705908242818e-07	0.0301059645848107	1.51446204347447e-05
**BRCA**	ATM	cg17114932	-0.179926842407162	796	-5.14759902842501	3.33076541254094e-07	0.0323736686186116	6.39786579020271e-06
**BRCA**	ATM	cg17420659	-0.135043323428353	796	-3.83559118062893	0.000135219357132963	0.0182366992025749	0.00124665260355555
**BRCA**	ATM	cg18042593	-0.112710631670922	796	-3.19230013906565	0.00146717520021151	0.0127036864916583	0.0092299391497052
**BRCA**	ATM	cg18391757	-0.060608728677689	795	-1.70774317130305	0.0880763908638155	0.00367341799192572	0.21755936847843
**BRCA**	ATM	cg18457775	-0.224064415238598	111	-7.64602092214999	4.48565858915535e-14	0.0502048621762147	3.10460252922377e-12
**BRCA**	ATM	cg19288979	0.116153082593072	796	3.29111398170316	0.00104208783922781	0.013491538595873	0.00694786951932411
**BRCA**	ATM	cg20342375	-0.144676753864138	796	-4.11485728535126	4.27989575234579e-05	0.0209313631086643	0.000464171490531143
**BRCA**	ATM	cg22477592	0.00119337049316698	111	0.0396874475030123	0.968349471830038	1.4241331339616e-06	0.983379398362326
**BRCA**	ATM	cg22678707	-0.0986028845937266	796	-2.78851998633817	0.00542193663415605	0.00972252885020377	0.0264613637714891
**BRCA**	ATM	cg22837512	-0.0663473679241668	796	-1.87130369781922	0.0616714623747312	0.00440197323046476	0.167973872417192
**BRCA**	ATM	cg23275463	-0.237601056604596	789	-6.85333115199395	1.45948610271311e-11	0.0564542620996204	6.70639467657789e-10
**BRCA**	ATM	cg23389069	0.00180370429947753	796	0.0507608210053895	0.95952890982022	3.25334919995373e-06	0.97885588411123
**BRCA**	ATM	cg24030675	0.000340923572919305	796	0.00959444035347167	0.992347277780327	1.16228882572064e-07	0.995970431650253
**BRCA**	ATM	cg24568245	-0.24462631397675	795	-7.09563060013725	2.86367831591427e-12	0.0598420334898517	1.50829664811239e-10
**BRCA**	ATM	cg25400013	-0.00348667552779057	796	-0.098124343515672	0.921858384990958	1.21569062360937e-05	0.959045279896789
**BRCA**	ATM	cg25740661	0.0928020342725926	796	2.62300009217936	0.00888351234864086	0.00861221756513146	0.0389496680183828
**BRCA**	ATM	cg25918541	-0.0390433035721502	796	-1.09961439283603	0.271834295799448	0.00152437955382707	0.468156056040873
**BRCA**	ATM	cg27453365	-0.0895724834785802	794	-2.52776915559622	0.0116728676345741	0.00802322979652052	0.048102960339213
**BRCA**	ATM	cg27599391	-0.214366313856436	793	-6.16467431761754	1.12632063247707e-09	0.0459529165163961	3.69397079607592e-08
**BRCA**	ATR	cg00911981	0.25220503363056	796	7.33478434923856	5.49968321034286e-13	0.0636073789885918	3.23388023934552e-11
**BRCA**	ATR	cg00969988	-0.0566104349743984	796	-1.59571784158477	0.110950725499954	0.0032047413479906	0.256465779134971
**BRCA**	ATR	cg11458642	0.0518796529184103	111	1.72766631137436	0.0843272211822861	0.00269149838693472	0.210781474943121
**BRCA**	ATR	cg11936145	0.0207508881102993	796	0.584107527441018	0.55931429534374	0.000430599357366161	0.730141061734951
**BRCA**	ATR	cg15135999	-0.131399346015546	796	-3.73024843481836	0.000204940172082548	0.0172657881333132	0.00177165415568282
**BRCA**	ATR	cg15291273	-0.169130322041222	796	-4.82932184501038	1.64456673320325e-06	0.0286050658337675	2.65705969767007e-05
**BRCA**	ATR	cg15480357	0.0534496141953799	796	1.5063587446367	0.132373830763106	0.00285686125763495	0.28973046142377
**BRCA**	ATR	cg16031872	-0.0622000661492157	795	-1.75275343683806	0.080031733666966	0.0038688482289668	0.20312514074216
**BRCA**	ATR	cg17945282	-0.142178159521447	796	-4.04231360636962	5.80929033226339e-05	0.020214629044906	0.000604738112394606
**BRCA**	ATR	cg20032934	-0.00486944320890105	796	-0.137039903730473	0.891034082517216	2.37114771647125e-05	0.942381675700361
**BRCA**	ATR	cg20636078	0.0413616122063138	111	1.37672383675942	0.168876261075175	0.00171078296430549	0.34215068505566
**BRCA**	ATR	cg24631870	-0.0326564599950865	796	-0.919524689486494	0.3581011640911	0.00106644437941069	0.55708444736343
**BRCA**	ATR	cg24856922	0.145777802135521	796	4.14685032597771	3.73479309179611e-05	0.0212511675954631	0.000412353808945766
**BRCA**	AURKB	cg02245743	-0.0441492912385465	796	-1.24368384902133	0.213983928887808	0.001949159916866	0.400588353496232
**BRCA**	AURKB	cg04612488	-0.0887286955257742	796	-2.50693460465619	0.0123775545403274	0.00787278140970553	0.0503687544023764
**BRCA**	AURKB	cg05617798	-0.089141215669178	796	-2.51868306014204	0.0119753968792874	0.0079461563309789	0.0490607206712983
**BRCA**	AURKB	cg05811247	-0.0629934680328023	796	-1.77632121316016	0.0760638226846818	0.00396817701479969	0.195872526379746
**BRCA**	AURKB	cg11009596	-0.0494902420050671	111	-1.64789542738666	0.0996581635091098	0.00244928405372011	0.237670360883743
**BRCA**	AURKB	cg12844497	-0.0122311351357251	111	-0.40679613292239	0.68423643490863	0.000149600666708368	0.818172569779462
**BRCA**	AURKB	cg13678641	-0.10239682174754	796	-2.89692932915976	0.0038722206763914	0.0104851091039975	0.0202361638653226
**BRCA**	AURKB	cg21724796	-0.0833356267628999	796	-2.35345884271105	0.018843194774381	0.00694482668796536	0.0695623544003319
**BRCA**	BAP1	cg00794529	-0.132072685751925	796	-3.74970210180542	0.000189934003097736	0.0174431943217266	0.00166094040433737
**BRCA**	BAP1	cg02503646	-0.0768841373556939	795	-2.16876447540132	0.0303978882418781	0.00591117057692921	0.099682726378985
**BRCA**	BAP1	cg03075736	0.0037921767311354	796	0.106722080389285	0.915036491245047	1.43806043601648e-05	0.95509719177144
**BRCA**	BAP1	cg05442719	0.0280177162538172	796	0.788798088271027	0.43046607601235	0.000784992424079415	0.624467197204803
**BRCA**	BAP1	cg05908979	-0.0104966561692335	796	-0.295418364144802	0.767751735896066	0.000110179790735109	0.871313506987396
**BRCA**	BAP1	cg09096430	0.0524824928848093	796	1.47902659936842	0.139530813612492	0.00275441205940406	0.300624812755704

**BRCA**	BAP1	cg10449522	-0.0356954625534785	796	-1.00519996064296	0.31510757995887	0.00127416604690678	0.514249646278044
**BRCA**	BAP1	cg10956904	0.0230671287064397	796	0.649339321365435	0.516307335874375	0.000532092426759455	0.697428641402496
**BRCA**	BAP1	cg11234564	0.0980255542625941	796	2.77203402626699	0.00570147659177278	0.00960900928848879	0.027547174023875
**BRCA**	BAP1	cg12859382	-0.0104536303529667	796	-0.294207311019012	0.768676583303894	0.000109278387556467	0.871773422187701
**BRCA**	BAP1	cg16841708	-0.0345349575612722	796	-0.972479948846484	0.331108742630055	0.00119266329375887	0.530781048943158
**BRCA**	BAP1	cg24291822	0.0196126229372829	796	0.552054319732448	0.581066877849711	0.000384654978480037	0.746733570586279
**BRCA**	BAP1	cg24747396	-0.0423270516053021	111	-1.40891556354657	0.159141210771283	0.00179157929759791	0.328581457754966
**BRCA**	BAP1	cg25749966	-0.00812614213435845	796	-0.228697461851385	0.819163105192861	6.60341859877958e-05	0.902710918851084
**BRCA**	BAZ1A	cg00169548	-0.103831105557743	315	-1.84398985902689	0.06613264438886	0.0107808984813432	0.176770304342598
**BRCA**	BAZ1A	cg05294307	0.129343635215648	790	3.65695732028734	0.000272228176388645	0.0167297759707987	0.00225127232422433
**BRCA**	BAZ1A	cg07414384	-0.203035262227369	111	-6.89588964009846	8.98396602383897e-12	0.0412233177077366	4.30621474342901e-10
**BRCA**	BAZ1A	cg23422263	-0.0520517391631789	796	-1.46685426940145	0.142812653065139	0.00270938354991161	0.305435736379032
**BRCA**	BAZ1A	cg26967526	0.0957200249112685	795	2.70451745373825	0.00698739619203781	0.00916232316901387	0.0323471800317891
**BRCA**	BAZ1B	cg00525989	0.0909039716355074	796	2.56890064209602	0.0103842346895478	0.00826353205910913	0.043889555223225
**BRCA**	BAZ1B	cg04742505	0.126544426961103	796	3.59013717260636	0.000350856249810354	0.0160134919949138	0.00278458591080868
**BRCA**	BAZ1B	cg05166819	-0.0222787293212403	796	-0.627134674590538	0.530751701906427	0.000496341780169091	0.708525060456604
**BRCA**	BAZ1B	cg06059318	0.121693237578123	796	3.45039544739666	0.000589391244880999	0.0148092440722455	0.0043089091724718
**BRCA**	BAZ1B	cg06394887	0.176636389034617	796	5.05040023344852	5.47547816859383e-07	0.0312004139311884	1.00296526641768e-05
**BRCA**	BAZ1B	cg14093368	0.0540275846174558	111	1.79940072454518	0.0722278012348609	0.00291897989959634	0.188584983026171
**BRCA**	BAZ1B	cg15636799	-0.0026306677344979	796	-0.0740338085771865	0.94100217760465	6.92041272932831e-06	0.968477693314672
**BRCA**	BAZ1B	cg19160975	-0.0732697944415317	796	-2.06755204802914	0.0390064405302661	0.00536846277750431	0.120257877961856
**BRCA**	BAZ1B	cg20612831	-0.0500745485944602	796	-1.41099282601047	0.158639404073067	0.00250746041693895	0.327830888576443
**BRCA**	BAZ1B	cg21340129	-0.128281121105256	796	-3.64022686995437	0.000290088008677067	0.0164560460320214	0.00237678603510501
**BRCA**	BAZ1B	cg25710357	0.11121003061892	796	3.14926279338899	0.00169826463785398	0.0123676709102611	0.0104101173459312
**BRCA**	BAZ2A	cg14634319	0.0692432878609783	111	2.30833528352473	0.0211642549473904	0.00479463291379831	0.0760530677684114
**BRCA**	BAZ2A	cg18206859	0.0467522101354177	796	1.31716411516941	0.188164565618185	0.00218576915254626	0.367633779985963
**BRCA**	BAZ2A	cg18955060	-0.0783957300986556	796	-2.21306250886693	0.0271777319756367	0.00614589049770125	0.0916989889344115
**BRCA**	BAZ2A	cg25683012	0.0106095750794953	111	0.35285802427772	0.724262116158155	0.000112563083367447	0.843841862952819
**BRCA**	BRD7	cg03021120	-0.314355327452084	796	-9.31917501250057	1.13678283706117e-19	0.0988192718975069	1.47637535937123e-17
**BRCA**	BRD7	cg16433860	-0.254459917639219	796	-7.40488093423689	3.36243469012734e-13	0.0647498496849579	2.03720595236578e-11
**BRCA**	BRD7	cg16834187	0.137078795601487	110	4.58762885111294	4.99844177683392e-06	0.0187905962035543	7.12568773294883e-05
**BRCA**	BRD8	cg01096096	0.0807756145652164	796	2.28067984549687	0.0228322317035156	0.0065246999083884	0.0804807717781282
**BRCA**	BRD8	cg03204906	-0.00756857874006507	796	-0.213004786916083	0.831378042953921	5.72833841445649e-05	0.909651953567734
**BRCA**	BRD8	cg03684156	-0.0429596038764861	796	-1.21010757746883	0.226598654569928	0.0018455275652246	0.416137856368193
**BRCA**	BRD8	cg07430605	0.0355997939183652	111	1.18467827002416	0.23639918389687	0.00126734532703008	0.427737126242132
**BRCA**	BRD8	cg07632946	-0.166298441490536	796	-4.74614077446036	2.45996819073755e-06	0.0276551716421812	3.78801990162781e-05
**BRCA**	BRD8	cg08976717	0.0924296849001527	796	2.61238496508305	0.00916168097220998	0.00854324665074151	0.0398681998966327
**BRCA**	BRD8	cg11110536	0.0486163181494956	796	1.36980422457124	0.171136238799088	0.00236354639041298	0.345219047352166
**BRCA**	BRD8	cg11346339	0.0714749441041539	796	2.0166409956122	0.044070045000698	0.00510866763469193	0.131552322657776
**BRCA**	BRD8	cg12964860	-0.242032219319241	796	-7.02011085725909	4.76969030020269e-12	0.0585795951885972	2.40631184963663e-10
**BRCA**	BRD8	cg14473243	-0.0138492943265284	796	-0.389791073561683	0.696795841104249	0.000191802953342811	0.826191873999401
**BRCA**	BRD8	cg22106577	0.086536207178675	796	2.44451483271635	0.0147217324112517	0.00748851515287057	0.0575335301517592
**BRCA**	BRD8	cg22818036	-0.050058017885737	796	-1.41052585594723	0.158777097620768	0.00250580515464877	0.328028297233037
**BRCA**	BRD8	cg23298160	0.00022746657227177	796	0.00640147693810868	0.99489400730692	5.17410415010682e-08	0.997348081450852
**BRCA**	BRD8	cg26008208	-0.0329029200532552	796	-0.926471884602999	0.35448302740434	0.0010826021480309	0.553470198372167
**BRCA**	BRD8	cg26630792	0.0696798462257571	796	1.96574261623082	0.0496773417250652	0.00485528097004516	0.143595999039859
**BRCA**	BRD8	cg27119451	-0.081294888139174	111	-2.71256816129948	0.00678000239192547	0.00660885883756082	0.0315853348151057
**BRCA**	BRD9	cg02364331	-0.0299254728489374	796	-0.842554809534808	0.399731834933756	0.000895533925232487	0.596362733331479
**BRCA**	BRD9	cg02822723	0.0180560653835692	111	0.600580861274314	0.548242234147184	0.000326021497135726	0.721420245616303
**BRCA**	BRD9	cg05193570	-0.0527314673051621	796	-1.48606254840003	0.137660520293454	0.00278060764415538	0.297757952391396
**BRCA**	BRD9	cg07308931	-0.090755829606914	796	-2.56467942573977	0.0105103320417244	0.0082366206076392	0.0442997654469258
**BRCA**	BRD9	cg09497020	0.0478793618687443	796	1.34899180491785	0.177725147602383	0.00229243329295817	0.354168727737045
**BRCA**	BRD9	cg10551270	-0.258467004884276	796	-7.52976711437022	1.38592623749299e-13	0.0668051926138485	8.98472880996081e-12
**BRCA**	BRD9	cg10600960	0.0281419339048563	796	0.792298021453226	0.428424172645729	0.0007919684439053	0.622619654152846
**BRCA**	BRD9	cg13722821	-0.0700923764938725	796	-1.97743782289529	0.048338492493661	0.00491294124255878	0.140687817915069
**BRCA**	BRD9	cg17977043	0.00442718900797327	796	0.124593363589392	0.900877098004727	1.96000025123193e-05	0.947756916698398
**BRCA**	BRD9	cg20079193	-0.0934339977208048	796	-2.6410189738812	0.0084285945756795	0.00872991193009136	0.0373955792414972

**BRCA**	BRD9	cg21160852	0.065184887901946	796	1.83837520293703	0.0663814473801894	0.00424906961078927	0.177272785213543
**BRCA**	BRD9	cg21758832	-0.079448637000511	796	-2.24297295960328	0.0251743476293608	0.00631208592123896	0.0866746271068155
**BRCA**	BRD9	cg21910709	-0.0517239682759669	796	-1.4575926005805	0.145349254212986	0.00267536889421323	0.309196059126197
**BRCA**	BRD9	cg24953233	-0.119262781280175	796	-3.38047958859951	0.000759030911672493	0.014223610998683	0.00533020858843202
**BRCA**	BRD9	cg25523559	0.0522343363429854	796	1.47201403856888	0.141414327533324	0.00272842589319213	0.30347057804248
**BRCA**	BRD9	cg25951128	-0.0776163755306505	796	-2.19092780040111	0.0287477167167302	0.00602430175051496	0.0956266678562237
**BRCA**	BRD9	cg26504197	0.036809051218736	796	1.0366010102692	0.300238226370923	0.00135490625162353	0.498542777932677
**BRCA**	BRDT	cg00308185	-0.00304651151366126	796	-0.0857368315696145	0.931697291504133	9.2812324028706e-06	0.963743121433367
**BRCA**	BRDT	cg01515515	0.0719890971372501	794	2.02865665031047	0.0428282284217166	0.00518243010663642	0.128724614682959
**BRCA**	BRDT	cg02935097	0.0251917213630279	796	0.709182948946611	0.478419660648095	0.000634622825232434	0.665955589277644
**BRCA**	BRDT	cg03421440	0.0198213456582867	796	0.557931724816887	0.577048677038805	0.00039288574370528	0.743682798760114
**BRCA**	BRDT	cg06442199	0.0433765559574103	796	1.22187452533073	0.222118623906725	0.00188152560672635	0.410644174723729
**BRCA**	BRDT	cg09250199	-0.0155201272536064	796	-0.43682771025256	0.662355369096	0.000240874349968136	0.803636661897953
**BRCA**	BRDT	cg13299824	0.0505913281121858	796	1.42559173352863	0.154380214187622	0.00255948248015484	0.321885084358818
**BRCA**	BRDT	cg14732540	0.0576563959314803	111	1.91978098944548	0.055142809503017	0.00332425999180762	0.154927217395995
**BRCA**	BRDT	cg19245011	0.0848499495759043	796	2.3965317597528	0.0167819944737049	0.00719951394303351	0.0636010365902176
**BRCA**	BRPF1	cg04879506	-0.0354822662608658	796	-0.999188668161299	0.318008478533391	0.00125899121900697	0.517317376500912
**BRCA**	BRPF1	cg13694772	0.0211615456889311	796	0.5956720736027	0.551564495589799	0.000447811015944717	0.723960390253131
**BRCA**	BRPF1	cg14712964	0.010678867458677	111	0.355162843403722	0.722535316441965	0.00011403821019999	0.842795123584416
**BRCA**	BRPF1	cg17738494	0.179036061606237	111	6.05191047084287	1.95703206028408e-09	0.0320539113554724	6.12164664647125e-08
**BRCA**	BRPF1	cg20952324	0.116106700378632	796	3.28978181280142	0.00104696622178601	0.0134807658728135	0.00697445540825664
**BRCA**	BRPF1	cg25467642	0.0149441965275143	796	0.420613939733259	0.674151113564795	0.000223329009852971	0.811627152740914
**BRCA**	BRPF1	cg26227975	0.00890994163863883	796	0.250757937751149	0.802066301552332	7.93870600039499e-05	0.891983763652846
**BRCA**	BRPF3	cg01499736	0.0400175001952437	796	1.1270951083333	0.260043628865591	0.00160140032187633	0.455124616166163
**BRCA**	BRPF3	cg05882415	0.00114258227197734	796	0.0321551363614439	0.974356435072221	1.3054942482369e-06	0.986610049110127
**BRCA**	BRPF3	cg10562781	0.039969104072896	796	1.12572984650201	0.260620892596527	0.00159752928039	0.45582728295722
**BRCA**	BRPF3	cg14654948	-0.0598109077967175	796	-1.68624699288043	0.0921420789499568	0.00357734469146744	0.224571599668941
**BRCA**	BRPF3	cg18779695	0.0175217549312626	796	0.493181604937494	0.622020987930782	0.000307011895871224	0.776059764289379
**BRCA**	BRPF3	cg23517469	0.0487636886872336	796	1.37396639133914	0.169840832544342	0.00237789733438543	0.34346983796804
**BRCA**	BRWD3	cg03164831	-0.064747335763798	111	-2.15780258048501	0.0311579438103425	0.00419221748850999	0.101528905117776
**BRCA**	BRWD3	cg05835545	-0.134975141689184	796	-3.83361869264314	0.000136288724019484	0.0182182888740152	0.00125547010022164
**BRCA**	BRWD3	cg09523186	-0.15761582445612	796	-4.49184830737033	8.11139473751507e-06	0.0248427481189824	0.000108852482699715
**BRCA**	BRWD3	cg11957438	-0.192730526711984	774	-5.4502376374062	6.77657328957919e-08	0.0371450559266787	1.53026046794517e-06
**BRCA**	BRWD3	cg15511898	-0.0829842513816745	796	-2.34346679102547	0.0193519187629745	0.00688638597737695	0.0709688949345606
**BRCA**	BRWD3	cg22618269	-0.16556833653757	795	-4.721731299165	2.76589181469395e-06	0.0274128740638181	4.21570900925355e-05
**BRCA**	BRWD3	cg25063710	-0.164360230222721	111	-5.53891432880274	3.8023458621308e-08	0.0270142852788657	9.16639894634656e-07
**BRCA**	BRWD3	cg25708850	-0.138661619710609	779	-3.89782986932521	0.00010545824884894	0.0192270447807697	0.00100822981742466
**BRCA**	CARM1	cg12317676	0.0625110051586386	111	2.0829758660886	0.0374827036219784	0.00390762576594334	0.116735235420481
**BRCA**	CARM1	cg17918830	-0.23821590842678	796	-6.90270339686322	1.04618349730502e-11	0.056746819027596	4.9445199872021e-10
**BRCA**	CARM1	cg22378614	-0.145643238415192	796	-4.14293948066117	3.79769758551212e-05	0.0212119528960645	0.000418526825283086
**BRCA**	CARM1	cg26504915	-0.11715835502515	796	-3.31999233856646	0.000941379392971549	0.013726080152199	0.00638368797915173
**BRCA**	CBX1	cg01544580	0.100610134384993	796	2.84586026129907	0.00454356303536607	0.0101223991409663	0.022961020058027
**BRCA**	CBX1	cg01553295	0.0979060528048575	796	2.76862195518263	0.00576093421479173	0.00958559517582754	0.0277622570962961
**BRCA**	CBX1	cg02835499	-0.0589567439516789	796	-1.66208097205317	0.0968922440496296	0.00347589765738383	0.232771986381392
**BRCA**	CBX1	cg04864609	-0.103071976725543	796	-2.91623470142136	0.00364290469682558	0.0106238323861109	0.0192354665027387
**BRCA**	CBX1	cg11729481	0.0673986998792613	796	1.90109039155039	0.05765290990263	0.00454258474541474	0.159906249036124
**BRCA**	CBX1	cg12245530	0.0962683267786798	796	2.72187282255761	0.00663391961023775	0.00926759074076668	0.031047957054436
**BRCA**	CBX1	cg13342109	-0.0113767373700325	796	-0.320190491357338	0.748908383636799	0.000129430153186694	0.859325921069075
**BRCA**	CBX1	cg17778721	0.128676305160398	111	4.3132561962807	1.75249555765106e-05	0.0165575915097318	0.000213459091998472
**BRCA**	CBX1	cg21511817	-0.0422466838158274	796	-1.18998947975111	0.234407112800463	0.0017847822934345	0.425346774892685
**BRCA**	CBX4	cg04398978	0.0813501931893815	111	2.71442581552325	0.00674234974447688	0.00661785393194969	0.0314454486595061
**BRCA**	CBX4	cg04925705	0.0812977172477711	796	2.2955190499562	0.02196389495903	0.00660931882969854	0.078161283600514
**BRCA**	CBX4	cg05949524	-0.123880886095466	796	-3.51338039598999	0.000467528719259054	0.0153464739397977	0.00354457939635892
**BRCA**	CBX4	cg07159673	0.187034229419656	796	5.35816316071759	1.10310180896824e-07	0.0349818029746044	2.37553348750728e-06
**BRCA**	CBX4	cg10756274	-0.0235156525017222	796	-0.661972178534621	0.508181609686666	0.000552985912581755	0.690770389194046
**BRCA**	CBX4	cg21365914	-0.0200720962376417	796	-0.564992685302723	0.572238734495049	0.000402889047373151	0.739874021262264
**BRCA**	CBX4	cg22353329	0.153271151793336	796	4.36500875638222	1.43964335571606e-05	0.0234920459720558	0.000179941541154523

**BRCA**	CBX4	cg27487227	-0.025101686327265	796	-0.706646733130667	0.479993876448844	0.000630094656472402	0.667270977783963
**BRCA**	CBX5	cg03689456	-0.0773676801337996	796	-2.18386537211523	0.0292648388195134	0.00598575792928593	0.0969180685916022
**BRCA**	CBX5	cg05754589	-0.0769191266681126	111	-2.56566817311299	0.0104286026429682	0.00591655204738514	0.0440411874494908
**BRCA**	CBX5	cg06399839	-0.162910352704726	796	-4.64678063616222	3.9477417845851e-06	0.0265397830183783	5.77167119695571e-05
**BRCA**	CBX5	cg06621757	-0.0166251623621278	796	-0.467938213917609	0.63995751223959	0.000276396023567112	0.788471115553549
**BRCA**	CBX5	cg08288469	0.109270388607185	796	3.09366599267401	0.00204644374709456	0.0119400178263653	0.0121166789604593
**BRCA**	CBX5	cg09060496	-0.00345756811873078	796	-0.0973051736011259	0.92250866587917	1.19547772956635e-05	0.959279783369033
**BRCA**	CBX5	cg09158487	-0.128557508044365	796	-3.64820154414155	0.000281377378569103	0.0165270328745769	0.00231634622099521
**BRCA**	CBX5	cg09653879	-0.0433936981314697	796	-1.22235831415923	0.221935800906079	0.00188301303752512	0.410373729439524
**BRCA**	CBX5	cg11005091	-0.0498827590185762	111	-1.66024661683363	0.0971485664662142	0.00248828964730535	0.23320812107485
**BRCA**	CBX5	cg11338003	-0.0489430728434086	796	-1.37903283593878	0.168273940070839	0.0023954243793552	0.341365987636143
**BRCA**	CBX5	cg12263485	0.101122918647912	111	3.3803300381763	0.000749267994596866	0.0102258446758723	0.0052783260320265
**BRCA**	CBX5	cg12600134	0.0946636182341333	796	2.67608782432673	0.00760263285495555	0.00896120061717773	0.0345611731344174
**BRCA**	CBX5	cg13445218	0.0108568390759876	796	0.305556543274703	0.760022602712907	0.000117870954721891	0.866484967687329
**BRCA**	CBX5	cg14017196	-0.0469663918895	796	-1.32321163176682	0.186146877862217	0.0022058419671181	0.365046186236227
**BRCA**	CBX5	cg14394617	0.135144341914566	796	3.83851373305668	0.00013364947215982	0.0182639931515211	0.00123422685626793
**BRCA**	CBX5	cg15708001	-0.18788449562882	796	-5.38341085884749	9.6371023611399e-08	0.0353005836976962	2.09943640739994e-06
**BRCA**	CBX5	cg20545544	-0.0801833054042617	796	-2.26384751944616	0.0238532304555708	0.0064293624655531	0.0832087444536423
**BRCA**	CBX5	cg20927027	0.0902312003148582	796	2.54973181392511	0.0109678945346813	0.00814166951026006	0.0458181332777437
**BRCA**	CBX5	cg21826008	0.0141551084638628	796	0.398399978048241	0.690442750617334	0.00020036709562372	0.822131286509495
**BRCA**	CBX5	cg24505978	0.0215608631016643	796	0.606917558053933	0.544079565956479	0.000464870817688708	0.718305061587074
**BRCA**	CBX5	cg25817259	0.00599557530513821	796	0.168733478154519	0.866049366553165	3.59469232395831e-05	0.92881487270823
**BRCA**	CBX6	cg03028106	-0.173350151757948	774	-4.88421729002675	1.26331430473246e-06	0.0300502751145037	2.10491053806718e-05
**BRCA**	CBX6	cg04134624	-0.0470426357470991	111	-1.56621107892265	0.117585262472393	0.00221300957803424	0.267039203292288
**BRCA**	CBX6	cg04215348	-0.0170115418487843	796	-0.478816511898956	0.632201329531855	0.00028939255607294	0.783162138407161
**BRCA**	CBX6	cg04801716	-0.0149656763340062	796	-0.421218637920511	0.673709720732126	0.000223971468134233	0.811309195272987
**BRCA**	CBX6	cg06060673	-0.0679483849483787	796	-1.91666678159852	0.0556398611713075	0.00461698301709306	0.155805569786967
**BRCA**	CBX6	cg09913544	-0.0722489243959447	796	-2.03859255503611	0.0418223471254008	0.00521990707637093	0.126486507795443
**BRCA**	CBX6	cg14498989	-0.0215011512760378	796	-0.605235949109734	0.545195609826762	0.000462299506195062	0.719190564005349
**BRCA**	CBX6	cg24649346	-0.0534354569761194	111	-1.77962299653498	0.0754122095146922	0.00285534806224671	0.194664944320469
**BRCA**	CBX6	cg25836955	-0.140468988938741	795	-3.99021395168137	7.21452305793265e-05	0.0197315368534722	0.00072738025760603
**BRCA**	CBX7	cg00545295	-0.0829980781394385	796	-2.34385996548159	0.0193316761198932	0.00688868097484033	0.0709013442115399
**BRCA**	CBX7	cg01525244	0.0616139669667766	796	1.73727145094759	0.0827280205949258	0.00379628092538303	0.207859428788891
**BRCA**	CBX7	cg02523424	0.0567906047982218	796	1.60081282027848	0.109817158419328	0.00322517279334781	0.254635574208262
**BRCA**	CBX7	cg03804568	-0.0142620739607287	111	-0.474356025410449	0.63533968334156	0.000203406753661294	0.785219085326449
**BRCA**	CBX7	cg10339854	0.00326704211292194	796	0.091943205568897	0.926766413888481	1.06735641676055e-05	0.961351054857318
**BRCA**	CBX7	cg22268271	-0.113075447332181	796	-3.20276640952187	0.00141554844160942	0.0127860567893728	0.00896095495699922
**BRCA**	CBX7	cg26672614	0.0394903172381857	796	1.11222362450156	0.266379498632239	0.00155948515557254	0.462117314109146
**BRCA**	CBX7	cg27062573	0.0294856325375668	796	0.830160203414175	0.406698373485485	0.00086940252614042	0.602894417983342
**BRCA**	CDYL2	cg01442398	-0.0168425496654854	796	-0.474058605541633	0.635588776723972	0.000283671479234343	0.785423142773521
**BRCA**	CDYL2	cg02461391	-0.0246459283227677	796	-0.6938086529763	0.488005650867952	0.000607421782891002	0.674182870196258
**BRCA**	CDYL2	cg06919916	-0.0577151867386501	796	-1.62696132798363	0.104143068693655	0.00333104278027725	0.245216437723727
**BRCA**	CDYL2	cg10112854	-0.158319415813383	796	-4.51241409307292	7.38095855545965e-06	0.0250650374234909	9.99815932201685e-05
**BRCA**	CECR2	cg03927893	-0.221782028869627	796	-6.40090598868035	2.64321273470613e-10	0.0491872683295281	9.71767918165069e-09
**BRCA**	CECR2	cg12632097	-0.13146937672345	796	-3.7322714684149	0.000203329162028054	0.0172841970160525	0.00176007442203298
**BRCA**	CECR2	cg13466481	-0.124015860021699	796	-3.51726814641759	0.00046083814312671	0.0153799335369216	0.00350067791293812
**BRCA**	CECR2	cg26796679	-0.16867571523977	796	-4.81596040533028	1.75517610678186e-06	0.028451496911648	2.81472212020177e-05
**BRCA**	CHAF1A	cg03006338	0.0472733338249204	796	1.33187859222719	0.183283239097081	0.00223476809092236	0.361436044035159
**BRCA**	CHAF1A	cg04346879	-0.0147861714569311	796	-0.416165245545167	0.677401835706283	0.000218630866353764	0.813564059760622
**BRCA**	CHAF1A	cg05297461	0.100774361230426	796	2.85055320534157	0.00447773071046895	0.0101554718814004	0.0227050308232448
**BRCA**	CHAF1A	cg07783855	0.0136788405242654	796	0.384992714835723	0.700346177069604	0.000187110678088286	0.828357515340156
**BRCA**	CHAF1A	cg09700233	-0.0427582316627654	796	-1.20442481030321	0.228785229812514	0.00182826637492672	0.418720137581393
**BRCA**	CHAF1A	cg18804734	-0.0528436901041908	795	-1.48829355483634	0.137072039437923	0.00279245558382776	0.296922625122763
**BRCA**	CHAF1A	cg23811775	-0.0256765073974183	796	-0.722839286848917	0.469992024190072	0.000659283032129679	0.658642447215781
**BRCA**	CHAF1A	cg24085258	-0.106014817454553	111	-3.54567153158622	0.000407992921276344	0.0112391415199223	0.00315595120988087
**BRCA**	CHAF1B	cg00660452	-0.173433108260301	788	-4.93085086448974	9.99712268244614e-07	0.0300790430408294	1.70964203552037e-05
**BRCA**	CHAF1B	cg02544117	-0.075915257426103	788	-2.13177893968696	0.0333353415614415	0.00576312631007148	0.106772462180121

**BRCA**	CHAF1B	cg02908490	-0.368724300653769	796	-11.1634087446817	5.62611884461922e-27	0.135957609892611	1.30650235641286e-24
**BRCA**	CHAF1B	cg04634639	-0.0332632730275916	796	-0.936629788430011	0.349234534108603	0.0011064453325081	0.548273437832316
**BRCA**	CHAF1B	cg07872789	-0.105434757622328	796	-2.98382828785003	0.00293413742338703	0.011116488114879	0.0161759114672929
**BRCA**	CHAF1B	cg13854874	-0.16791951109623	111	-5.66486544138801	1.87518297941558e-08	0.0281969622067968	4.84644467444039e-07
**BRCA**	CHAF1B	cg25942896	-0.237968622747202	796	-6.89510751025353	1.10029475850312e-11	0.0566290654122003	5.18451426062941e-10
**BRCA**	CHD1	cg03255775	0.12847699112155	796	3.64587827638967	0.00028388936763322	0.0165063372476469	0.00233406145272233
**BRCA**	CHD1	cg03833628	0.0185243740180892	796	0.521411564511349	0.602225862946389	0.000343152432762058	0.761645214385255
**BRCA**	CHD1	cg08865521	0.117469688727503	796	3.32893808363528	0.000912064450612449	0.0137991277697364	0.00621523667404913
**BRCA**	CHD1	cg11365324	-0.0612362820100967	796	-1.72658201340837	0.0846327050328219	0.0037498822344201	0.211382048201218
**BRCA**	CHD1	cg12662497	-0.105148271277673	796	-2.97562989106303	0.00301281367891686	0.0110561589526831	0.0165230050995882
**BRCA**	CHD1	cg16890600	0.176543605108345	796	5.0476619815213	5.55202921437035e-07	0.0311676445046512	1.01564856065384e-05
**BRCA**	CHD1	cg21082921	-0.0698553073496826	796	-1.97071680364141	0.0491041437214915	0.00487976396491862	0.142383531129364
**BRCA**	CHD4	cg00079216	-0.0647618265063583	796	-1.82639340737885	0.0681673299609642	0.00419409417243965	0.180854282347154
**BRCA**	CHD4	cg00824109	-0.20835670560922	111	-7.0847201257115	2.47552541523945e-12	0.0434125167723271	1.3217640641945e-10
**BRCA**	CHD4	cg04344119	-0.0218625866399664	796	-0.615414818878004	0.538457601329687	0.000477972694590036	0.714115491358181
**BRCA**	CHD4	cg05147525	-0.0477710355971101	796	-1.34593274415345	0.17870934186474	0.00228207184202036	0.355335834914392
**BRCA**	CHD4	cg11565429	0.0503408075298934	796	1.41851443963737	0.156433995998369	0.00253419690276177	0.324869895480436
**BRCA**	CHD4	cg19509514	-0.109491535958306	796	-3.10000301607354	0.0020036812376181	0.011988396446509	0.0119170013913809
**BRCA**	CHD4	cg20308811	-0.101520930585495	796	-2.87189013423255	0.00418920162054714	0.010306499346945	0.0215382163022169
**BRCA**	CHRAC1	cg00296189	-0.359732267695145	796	-10.8501183834645	1.14710565542095e-25	0.129407304421091	2.46513639109993e-23
**BRCA**	CHRAC1	cg07270064	-0.40806599867419	796	-12.5789633294884	3.26548835653715e-33	0.166517859273964	1.02433629519537e-30
**BRCA**	CHRAC1	cg08877624	-0.0867333717581918	796	-2.4501265965226	0.0144960045304879	0.0075226777765447	0.0568397454549454
**BRCA**	CHRAC1	cg12002314	0.0445917442476264	111	1.48444542153564	0.137975724985515	0.00198842365504572	0.298249008016982
**BRCA**	CHRAC1	cg18770186	0.10008557101829	796	2.83087191796008	0.00475974365237134	0.0100171215260572	0.0238474591158998
**BRCA**	CHRAC1	cg19486048	0.171890448541535	796	4.91051374992994	1.10358333672514e-06	0.0295463262998102	1.8667571667144e-05
**BRCA**	CHRAC1	cg22610305	-0.0132473741200086	111	-0.440600997521222	0.659587981791803	0.000175492921075474	0.80187509697627
**BRCA**	CHRAC1	cg23548819	-0.19433995340039	796	-5.57551246566944	3.38617297978659e-08	0.0377680174876657	8.2191557188617e-07
**BRCA**	CLOCK	cg05960024	0.0340333599818511	111	1.13248915483621	0.257674296936297	0.00115826959165427	0.452184304218419
**BRCA**	CLOCK	cg06085476	-0.0751892716783766	786	-2.10858136405603	0.0352982097634557	0.00565342657552472	0.111519109368279
**BRCA**	CLOCK	cg07236904	0.0394372767351292	796	1.11072743857321	0.267022778870018	0.00155529879628316	0.462737966617232
**BRCA**	CLOCK	cg07500410	0.01493700880733	796	0.420411591608149	0.674298840472008	0.000223114232110254	0.811724790207874
**BRCA**	CLOCK	cg11774413	-0.0786481547065884	796	-2.2202325774484	0.0266853249358624	0.00618553223875146	0.0904596351937113
**BRCA**	CLOCK	cg18928811	0.109145266454372	796	3.09008079733562	0.00207100679453309	0.0119126891893959	0.0122378961317058
**BRCA**	CLOCK	cg22523077	-0.0544030075916077	796	-1.53330708124689	0.125599551634364	0.00295968723501253	0.279348757512444
**BRCA**	CLOCK	cg23506964	-0.0250845215393383	796	-0.706163216572355	0.480294313481485	0.000629233220857525	0.667572196229741
**BRCA**	CREBBP	cg00039463	-0.162007452743959	796	-4.62033057535451	4.47088912368561e-06	0.0262464147445861	6.44934512990784e-05
**BRCA**	CREBBP	cg00747661	0.00958789250059206	796	0.269839615679946	0.787353935207586	9.19276826029095e-05	0.882833322860923
**BRCA**	CREBBP	cg03039399	-0.23587787175792	796	-6.8309424264682	1.68150660288377e-11	0.0556383703850458	7.6363762209422e-10
**BRCA**	CREBBP	cg03264209	0.0520217456162827	111	1.732411023431	0.0834791847329526	0.00270626201696523	0.209172281356711
**BRCA**	CREBBP	cg08506924	-0.0630533735482244	796	-1.77801719831876	0.0757846312938331	0.00397572791581193	0.195366988331261
**BRCA**	CREBBP	cg24567591	-0.0987369458231738	796	-2.79234857880214	0.00535881839148878	0.00974898447048837	0.0262158167493069
**BRCA**	CTCF	cg01866162	-0.0619709025509045	111	-2.06490901776746	0.0391642564875957	0.0038403927629737	0.1206010437872
**BRCA**	CTCF	cg04487155	-0.0954540834332259	796	-2.69863845469249	0.00711066845283832	0.00911148204407726	0.0328068729865603
**BRCA**	CTCF	cg06241380	-0.205801124753933	796	-5.91844830504773	4.83683744447919e-09	0.0423541029499837	1.40401840356647e-07
**BRCA**	CTCF	cg07967402	-0.0518881083347269	796	-1.46223057030442	0.144074711572554	0.00269237578655635	0.307265179536263
**BRCA**	CTCF	cg08324636	0.135448305445233	796	3.84730839745127	0.000129028455308265	0.018346243447985	0.00119809496923504
**BRCA**	CTCF	cg10218542	-0.236673067586606	795	-6.85100583288883	1.47445433621598e-11	0.0560141409208541	6.77117477164027e-10
**BRCA**	CTCF	cg10481400	-0.149714159595923	796	-4.26135832313316	2.27642190516226e-05	0.0224143295835136	0.000268280511574296
**BRCA**	CTCF	cg23858565	-0.0914457288730867	111	-3.05396798004549	0.00231237302194175	0.00836232132913008	0.013371435927828
**BRCA**	CXXC1	cg02159718	-0.0550080864933849	796	-1.5504122400966	0.121442084126394	0.00302588957966371	0.272916756544306
**BRCA**	CXXC1	cg07675399	-0.0675215995114773	796	-1.90457283897905	0.0571976602807904	0.00455916640058833	0.159001499185162
**BRCA**	CXXC1	cg11871295	0.0555009178956481	796	1.56434550549756	0.118135904008703	0.00308035188725947	0.267844034636659
**BRCA**	CXXC1	cg13423076	-0.193523905048211	111	-6.55698888615818	8.41138438289796e-11	0.0374515018251092	3.4098761682291e-09
**BRCA**	CXXC1	cg15632775	0.024708190253835	796	0.695562460780513	0.486906918483097	0.000610494665619708	0.673275072955273
**BRCA**	CXXC1	cg20016845	-0.0397798237164302	111	-1.32398879949886	0.185780327848498	0.00158243437491026	0.36455025412148
**BRCA**	CXXC1	cg27419474	-0.0911110644874932	796	-2.57480191433949	0.0102102112355431	0.00830122607204414	0.0433014041749435
**BRCA**	DAXX	cg00221327	-0.0231770647021653	795	-0.652435677313215	0.514309467779162	0.000537176328208357	0.695853607715262

**BRCA**	DAXX	cg02173004	-0.0732390540821075	796	-2.06667992708019	0.0390888243719488	0.00536395904284187	0.120421194487224
**BRCA**	DAXX	cg02328295	0.0116681599035816	796	0.328393482082527	0.742700973589998	0.00013614595553555	0.855357368677982
**BRCA**	DAXX	cg03085224	-0.0521054418997434	796	-1.4683717677779	0.142400303523671	0.00271497707556754	0.30483171107896
**BRCA**	DAXX	cg03369465	-0.00375966947717165	795	-0.105807225605989	0.915762095763093	1.41351145775761e-05	0.955482785378139
**BRCA**	DAXX	cg03382121	-0.0371895033724013	796	-1.04732990651525	0.295266958649379	0.00138305916108585	0.493204169393635
**BRCA**	DAXX	cg03902329	-0.027902918635621	796	-0.785563604682904	0.432358134461415	0.000778572868386084	0.626102803601782
**BRCA**	DAXX	cg04086445	-0.155842519251514	796	-4.44004611635217	1.02707754507897e-05	0.0242868908066586	0.000134151226564596
**BRCA**	DAXX	cg04152866	-0.00709164538638492	796	-0.199581610386096	0.841859040539361	5.02914342862346e-05	0.91541067971566
**BRCA**	DAXX	cg04271391	-0.0742535619986283	796	-2.0954652188202	0.0364466223427134	0.00551359146948413	0.114346206586911
**BRCA**	DAXX	cg04488476	-0.0306058638870146	795	-0.86118485871933	0.389397207942951	0.000936718904270465	0.587000860262642
**BRCA**	DAXX	cg04973788	-0.123759058347162	796	-3.50987147438054	0.000473645203685295	0.0153163045229762	0.00358152750539404
**BRCA**	DAXX	cg05431670	-0.0383314840775049	786	-1.0727000486169	0.283736632887791	0.00146930267158401	0.480977242632111
**BRCA**	DAXX	cg05532204	0.0424753640075885	796	1.19644247247213	0.231881972494904	0.00180415654757714	0.422469523133296
**BRCA**	DAXX	cg06171013	-0.0552688528693699	796	-1.55778445096655	0.119683835621211	0.00305464609749606	0.270250908751785
**BRCA**	DAXX	cg07689821	0.00642803482187489	111	0.21377886002466	0.830758913516903	4.13196316712362e-05	0.909217075141514
**BRCA**	DAXX	cg08375286	-0.0596705248923171	796	-1.6822750230693	0.0929096955890831	0.00356057154092463	0.225975202709305
**BRCA**	DAXX	cg08567382	-0.0160309973198778	796	-0.451210237482669	0.651961547563664	0.00025699287506993	0.796567814372712
**BRCA**	DAXX	cg09097215	-0.052755187341027	796	-1.48673288460488	0.137483346981663	0.00278310979138685	0.297507079140236
**BRCA**	DAXX	cg09432361	0.0279429350346	796	0.786691084343261	0.431698051585219	0.000780807618347874	0.625562457401539
**BRCA**	DAXX	cg09615909	-0.239011840774803	796	-6.92716176318235	8.89095142325297e-12	0.0571266600305598	4.2748002164434e-10
**BRCA**	DAXX	cg11111423	-0.220566962296633	796	-6.36403914695575	3.3235263775321e-10	0.0486497848567644	1.19754706351891e-08
**BRCA**	DAXX	cg12025215	0.0185292851861694	796	0.521549848223677	0.602129600230751	0.000343334409510398	0.761612252205131
**BRCA**	DAXX	cg12832355	-0.185553158770485	796	-5.31421419523996	1.39369847788688e-07	0.0344299747297049	2.93506711934845e-06
**BRCA**	DAXX	cg12923297	-0.0608572956253716	796	-1.71585649046001	0.0865793892533808	0.00370361043083388	0.214922853238801
**BRCA**	DAXX	cg13443893	-0.0526199357435364	796	-1.48291066268756	0.13849594500388	0.0027688576376539	0.298974763316364
**BRCA**	DAXX	cg13826470	-0.0962244212426626	796	-2.72061984139882	0.00665887606952339	0.00925913924348539	0.0311460200187892
**BRCA**	DAXX	cg14539675	0.0285570838067993	796	0.803995474499651	0.421640836980745	0.000815507035548561	0.61676902178385
**BRCA**	DAXX	cg14622330	-0.0853625789484278	796	-2.41111660096999	0.0161304527127151	0.00728676988472657	0.0616968601373204
**BRCA**	DAXX	cg14746369	0.126793503369897	796	3.59731895682244	0.000341465013388353	0.016076592496812	0.00272385957538413
**BRCA**	DAXX	cg14910283	-0.250387874915409	796	-7.27838805701189	8.14740177120623e-13	0.0626940879046545	4.67073672357566e-11
**BRCA**	DAXX	cg16922466	-0.0802587710219529	796	-2.26599197755953	0.0237209865395469	0.00644147032595426	0.0828886685018869
**BRCA**	DAXX	cg17079757	-0.0433400441010383	796	-1.22084408622845	0.222508385288105	0.00187835942267994	0.411100967204267
**BRCA**	DAXX	cg17504991	-0.0594862069403057	796	-1.67706011462407	0.0939253200187983	0.00353860881614488	0.227697922890071
**BRCA**	DAXX	cg18187593	-0.0402931800503177	796	-1.13487222998978	0.25677219949399	0.00162354035856732	0.451073446107746
**BRCA**	DAXX	cg18279148	-0.185812345952515	753	-5.17886938646304	2.87077263629142e-07	0.0345262279083771	5.59441098365868e-06
**BRCA**	DAXX	cg18486541	-0.0554463292745867	796	-1.56280212679751	0.118498606377765	0.00307429543002588	0.268438426246516
**BRCA**	DAXX	cg18944910	-0.108351008140353	796	-3.06732592692883	0.0022333293982991	0.0117399409650308	0.0129879365486492
**BRCA**	DAXX	cg18967278	0.0382276088371781	796	1.07660721132034	0.281983609643121	0.0014613500774083	0.479091310574319
**BRCA**	DAXX	cg20223162	-0.150028276293344	795	-4.26780789941319	2.21336582480507e-05	0.022508483687552	0.000261801096206303
**BRCA**	DAXX	cg20763184	-0.0682123785813494	796	-1.92414817441992	0.0546940450421038	0.00465292859172533	0.154061428778529
**BRCA**	DAXX	cg21286626	0.0301319390392866	796	0.848373151230453	0.396486516964114	0.000907933750267281	0.593112221279724
**BRCA**	DAXX	cg24328964	-0.0557546510788863	796	-1.57151946369764	0.116461425581633	0.00310858111692836	0.26519143372256
**BRCA**	DAXX	cg24496586	-0.0138211670614667	796	-0.388999274826949	0.697381242316469	0.000191024658940972	0.826496798719815
**BRCA**	DAXX	cg24513883	-0.218010049587955	795	-6.28259021718412	5.49369127669997e-10	0.0475283817213424	1.90198584414596e-08
**BRCA**	DAXX	cg24728112	-0.148136328552283	796	-4.21543492462149	2.78031086592271e-05	0.0219443718369499	0.000318919940288397
**BRCA**	DAXX	cg25642825	-0.00767748702926914	796	-0.216070005035732	0.828988816118208	5.89438070845959e-05	0.908060845187348
**BRCA**	DAXX	cg26521092	-0.0831106685155234	796	-2.34706160989228	0.0191675279759948	0.00690738322109722	0.0704089591714541
**BRCA**	DAXX	cg27052777	-0.245278036614659	796	-7.12023935797001	2.41954618342274e-12	0.0601613152455418	1.29454041123238e-10
**BRCA**	DAXX	cg27393652	-0.0187000024198302	796	-0.526356755269917	0.59878772159545	0.000349690090501654	0.759197679381267
**BRCA**	DIDO1	cg00080059	-0.00341945185692746	796	-0.096232467886116	0.923360289268864	1.16926510018447e-05	0.959703537080243
**BRCA**	DIDO1	cg01488865	-0.0767378327051972	796	-2.16598085604192	0.0306103715068554	0.00588869496829083	0.100181036643008
**BRCA**	DIDO1	cg06646488	-0.0448485045455869	795	-1.26262218331058	0.207097089550612	0.00201138835997553	0.391973893837291
**BRCA**	DIDO1	cg08610063	-0.0372541776706067	796	-1.04915379168311	0.29442738073365	0.00138787375391313	0.49220180365383
**BRCA**	DIDO1	cg13815390	0.0580567823635433	796	1.63662320276798	0.102106494621821	0.00337058997840783	0.241774902374442
**BRCA**	DIDO1	cg20969846	-0.0877786004002182	111	-2.93052770787803	0.00345340503721928	0.00770508268822119	0.0184388600868522
**BRCA**	DIDO1	cg21212389	0.0352410178891651	796	0.9923865826547	0.321312070125363	0.00124192934186446	0.520774848604786
**BRCA**	DIDO1	cg21687703	0.149569841960486	796	4.25715652327787	2.31863404332413e-05	0.0223711376240848	0.000272635756901991

**BRCA**	DIDO1	cg22394521	-0.197531554548104	796	-5.67076444126048	1.99230308790087e-08	0.0390187150421906	5.1236107907491e-07
**BRCA**	DMAP1	cg01604592	-0.060414319135402	796	-1.70332095615425	0.0889003335639176	0.0036498899565942	0.218961929690404
**BRCA**	DMAP1	cg11683482	-0.00812967508062567	796	-0.228796897625847	0.819085842582839	6.60916169165459e-05	0.902710112188615
**BRCA**	DMAP1	cg15990015	-0.0852688842834276	111	-2.84611695120417	0.0045073306959565	0.00727078262694057	0.0228134862824489
**BRCA**	DMAP1	cg18017761	-0.102560790968443	795	-2.89978508195996	0.0038376225708901	0.0105187158440726	0.020087741238045
**BRCA**	DMAP1	cg18167340	-0.0555601690411274	795	-1.56503176149935	0.117975415493226	0.00308693238387865	0.267612756170871
**BRCA**	DMAP1	cg21437596	-0.147604741471633	796	-4.19997030330511	2.97271411534829e-05	0.0217871597049075	0.000338246777664773
**BRCA**	DMAP1	cg23021241	-0.026297761760858	796	-0.74034066108024	0.459312725760229	0.000691572273630844	0.649196478702603
**BRCA**	DMAP1	cg23451311	-0.0310182682194157	111	-1.03205807369079	0.30227053753178	0.000962132963331613	0.500686183484739
**BRCA**	DMAP1	cg24485408	-0.00277612545228478	796	-0.0781273964953836	0.937746448160002	7.70687252682335e-06	0.96664874103142
**BRCA**	DNMT1	cg04160663	-0.0549915102831789	796	-1.54994361944931	0.121554529229706	0.00302406620322497	0.273079965545102
**BRCA**	DNMT1	cg04762346	-0.0598398092209242	111	-1.99363991735516	0.0464366851968834	0.00358080276759661	0.136653385525182
**BRCA**	DNMT1	cg06128182	0.0402312068491	796	1.13312390005396	0.257505124773616	0.00161855000453507	0.45203433695818
**BRCA**	DNMT1	cg06716436	0.00315511856605825	111	0.104928969900619	0.916451196670952	9.95477316588544e-06	0.955906900953569
**BRCA**	DNMT1	cg10635912	0.0266614051252502	796	0.750585265553418	0.453125223818087	0.000710830523252717	0.643514251372891
**BRCA**	DNMT1	cg11380607	0.0238768190453657	796	0.672144873116237	0.501687498251627	0.000570102487725136	0.685488315429649
**BRCA**	DNMT1	cg13393778	-0.0870467792563972	796	-2.45904750224269	0.0141434607150845	0.00757714177891194	0.055769495811713
**BRCA**	DNMT1	cg15043801	-0.0380118787597124	111	-1.26505929649331	0.206116543611571	0.00144490292684308	0.390545592559577
**BRCA**	DNMT1	cg21893394	-0.0265651273954138	796	-0.747872888946541	0.45475883193856	0.000705705993534562	0.645015556080797
**BRCA**	DNMT1	cg23401624	-0.0719679283107431	796	-2.0306225434219	0.042626920707792	0.00517938270534026	0.128273196253364
**BRCA**	DNMT1	cg24477899	-0.0787085920838186	796	-2.22194935230591	0.0265685767626775	0.00619504246781696	0.0901699616384142
**BRCA**	DNMT1	cg27429799	-0.03581965036095	796	-1.00870162906341	0.313425817827068	0.00128304735198071	0.512477365001449
**BRCA**	DNMT3A	cg02208653	-0.0131248245616687	796	-0.369397121546539	0.711930483176628	0.000172261019774582	0.836042731904469
**BRCA**	DNMT3A	cg03463641	0.00715029101362402	796	0.20123217017604	0.840568706574453	5.11266615795124e-05	0.914600640016406
**BRCA**	DNMT3A	cg06112956	0.0753856019961326	796	2.12759305162371	0.0336791611876492	0.00568298898831931	0.107585786779123
**BRCA**	DNMT3A	cg07150430	0.135657341019825	796	3.85335712598039	0.000125938258328367	0.0184029141725692	0.00117416597176378
**BRCA**	DNMT3A	cg12066181	-0.0464057632942272	111	-1.5449613301246	0.122641684284774	0.00215349486691984	0.274776429561941
**BRCA**	DNMT3A	cg13076778	0.0148380204995927	796	0.417624887284426	0.676334588089777	0.000220166852346333	0.813022116278196
**BRCA**	DNMT3A	cg13344237	-0.00494922572656019	796	-0.139285263975935	0.889260143738681	2.44948352924452e-05	0.941289463670411
**BRCA**	DNMT3A	cg15990840	-0.0400255205326187	796	-1.12732136401432	0.259948048536652	0.00160204229390708	0.454967568571371
**BRCA**	DNMT3A	cg15998962	-0.047032183785217	796	-1.32506933173697	0.185530304816105	0.00221202631160643	0.36426756407237
**BRCA**	DNMT3A	cg21629895	-0.245863156356769	111	-8.43549874976152	1.01956715085287e-16	0.0604486916537131	9.87613011137102e-15
**BRCA**	DNMT3A	cg21708767	-0.025045798485748	796	-0.705072425454335	0.480972462604103	0.000627292021788695	0.668204367380234
**BRCA**	DNMT3A	cg22731525	-0.0527142034403578	796	-1.48557466735048	0.137789580621508	0.00277878724435143	0.297945983124485
**BRCA**	DNMT3A	cg25044635	-0.0244305808418013	796	-0.687742759807036	0.491816146015172	0.00059685328026779	0.677390058126558
**BRCA**	DNMT3A	cg26470599	-0.167856961753598	796	-4.79190422984898	1.97262705952662e-06	0.0281759596091489	3.12288460457497e-05
**BRCA**	DNMT3A	cg26995204	-0.0607713893280589	796	-1.71342539876358	0.0870256312597286	0.00369316176086251	0.215621121033802
**BRCA**	DNMT3B	cg00300969	-0.120492526086765	796	-3.41584732274854	0.000668229397044072	0.0145184488427697	0.00479294096566905
**BRCA**	DNMT3B	cg13636640	-0.0178567945663198	787	-0.499750957704732	0.617390849822866	0.000318865112183747	0.772697058642815
**BRCA**	DNMT3B	cg14224313	-0.0910283099909326	796	-2.57244372009015	0.0102794378854466	0.00828615321980532	0.043541249638486
**BRCA**	DNMT3B	cg16523653	0.0584980120887043	111	1.94878095788538	0.0515741667744197	0.00342201741833019	0.147609018433803
**BRCA**	DNMT3B	cg16953106	-0.0849248813844761	796	-2.39866352808899	0.0166853413575229	0.00721223547816733	0.0633149332016555
**BRCA**	DNMT3B	cg17475857	-0.0953659562646173	796	-2.69612408180353	0.00716406663734892	0.00909466561426491	0.0330068936917928
**BRCA**	DNMT3B	cg17721173	-0.0787445571423273	796	-2.22297098193442	0.0264993115024549	0.00620070527954125	0.0900055640509071
**BRCA**	DNMT3B	cg22605822	-0.177681433766074	796	-5.08125135415012	4.68047956518364e-07	0.0315706919051677	8.69835707821062e-06
**BRCA**	DNMT3B	cg24403338	-0.145076576960978	796	-4.12647308966879	4.07377686282947e-05	0.0210472131827145	0.00044517217348443
**BRCA**	DNMT3B	cg25803642	-0.034820010194238	796	-0.980516533066845	0.327130628913631	0.00121243310992683	0.526672188445923
**BRCA**	DNMT3L	cg09247319	0.0181493435250952	796	0.510851945308904	0.609597096400004	0.000329398670391914	0.767089329321955
**BRCA**	DNMT3L	cg15635600	0.0608875636590935	796	1.71671306501604	0.0864226014017759	0.00370729540834016	0.214632864686814
**BRCA**	DNMT3L	cg27187798	0.0228825163807523	796	0.644139753686389	0.519671309896086	0.000523609555915396	0.70017437111182
**BRCA**	DPF1	cg06168449	-0.0957204422105631	111	-3.19801886355906	0.00142301368938748	0.00916240305698575	0.00900235235847845
**BRCA**	DPF1	cg06615189	0.031266505355505	796	0.880347873620148	0.378937936467157	0.000977594357145822	0.577502978824962
**BRCA**	DPF1	cg07480327	-0.0164712082072541	741	-0.44721681171212	0.654849924164944	0.000271300699806713	0.798723679473317
**BRCA**	DPF1	cg08057062	-0.0761551928777889	796	-2.14943912265791	0.0319019297289085	0.00579961340225323	0.103308442797765
**BRCA**	DPF1	cg13322582	-0.0918251170873611	796	-2.59515198781677	0.00962988446410297	0.00843185212810757	0.0413664954169203
**BRCA**	DPF1	cg13917712	-0.0317590673785933	796	-0.894230472253634	0.371470250573432	0.00100863836075803	0.570093183812734
**BRCA**	DPF1	cg17121541	0.0115985988425391	796	0.326435462828569	0.744181151162706	0.000134527495110149	0.856287147491551

**BRCA**	DPF1	cg22145401	0.0562653036318162	796	1.58595839247719	0.113147904604322	0.00316578439278047	0.260228472750587
**BRCA**	DPF1	cg27400772	-0.0422897460849385	910	-1.27405411569648	0.202970953645852	0.00178842262392857	0.386778761659168
**BRCA**	DPF2	cg02186298	0.192122466420873	111	6.51062250486653	1.13297705496445e-10	0.0369110421036394	4.48771980722527e-09
**BRCA**	DPF2	cg14699126	-0.14045596090088	796	-3.99235764816949	7.15008313559056e-05	0.0197278769525896	0.000722381256428743
**BRCA**	DPF2	cg15918224	-0.143467300881508	796	-4.07973232927516	4.96518147149527e-05	0.0205828664222252	0.000526528526981329
**BRCA**	DPF2	cg15929437	-0.15069145290046	796	-4.28981950957113	2.00920239965008e-05	0.0227079139772514	0.000240356591539871
**BRCA**	DPF2	cg18224556	-0.127391995584989	796	-3.61457850738809	0.000319850266910627	0.0162287205391259	0.00257902484080072
**BRCA**	DPF2	cg19517014	-0.200726254675176	796	-5.76629683259283	1.16125116501595e-08	0.0402910293159237	3.13457228075124e-07
**BRCA**	DPF3	cg00888802	-0.147648475577679	796	-4.20124244973975	2.95641999135433e-05	0.0218000723404124	0.000336589692386095
**BRCA**	DPF3	cg05151458	-0.122267105854707	796	-3.46691280493076	0.00055484629444643	0.014949245174086	0.00409522890229275
**BRCA**	DPF3	cg07171024	-0.0777523306059443	796	-2.19478881359642	0.0284683544492645	0.00604542491465606	0.094949678091001
**BRCA**	DPF3	cg10266394	-0.185029371077277	796	-5.29868043082956	1.51317316962072e-07	0.0342358681612527	3.15253865981453e-06
**BRCA**	DPF3	cg12913429	-0.0504809910025439	795	-1.42247463850398	0.155282230117166	0.00254833045259891	0.323185451595452
**BRCA**	DPF3	cg13819165	-0.0343268475889277	796	-0.966612784972686	0.33403270537586	0.00117833246539347	0.533610010308629
**BRCA**	DPF3	cg14238615	-0.103122320176378	796	-2.9176743842415	0.00362630960471983	0.0106342129185595	0.0191595442399874
**BRCA**	DPF3	cg14242696	0.0722914854825868	796	2.03979977576435	0.0417016083161522	0.00522605887327905	0.126201724085114
**BRCA**	DPF3	cg15392845	-0.0707490015544503	796	-1.99605520674185	0.0462699045929762	0.00500542122095161	0.136322978778507
**BRCA**	DPF3	cg15740492	0.0775336387922932	796	2.18857820474781	0.0289188758499961	0.00601146514437379	0.09609238970932
**BRCA**	DPF3	cg16696021	-0.102469084418063	796	-2.89899541313538	0.00384706597978973	0.0104999132614762	0.0201290456572345
**BRCA**	DPF3	cg19710444	-0.108413208504978	796	-3.06910770290703	0.00222020951068802	0.0117534237783439	0.0129232307276068
**BRCA**	DPF3	cg19839347	-0.133629803126953	796	-3.79470954590349	0.000159086851875351	0.0178569242837481	0.0014311255249798
**BRCA**	DPF3	cg20548765	-0.0910139261531611	796	-2.57203383988838	0.0102915128479818	0.00828353475381306	0.043585276852082
**BRCA**	DPF3	cg21035356	-0.0574995072305102	796	-1.62086122802387	0.105445437792993	0.00330619333175149	0.247406876211487
**BRCA**	DPF3	cg22784589	-0.190217808360208	796	-5.45276060983608	6.63073526533338e-08	0.036182814617361	1.50300530079063e-06
**BRCA**	DPF3	cg23515460	0.100756444590601	796	2.8500412082595	0.00448487065402347	0.0101518611265388	0.0227284718569228
**BRCA**	DPF3	cg24383902	-0.14896810229795	796	-4.23963990283617	2.50278585818202e-05	0.0221914955022525	0.00029112354502216
**BRCA**	DPF3	cg25495195	-0.255923958569434	796	-7.45046197234553	2.43663940161169e-13	0.0654970725698491	1.51281664492402e-11
**BRCA**	DPF3	cg26057784	0.297140516606126	792	8.73569003842019	1.43931243735651e-17	0.0882924866089554	1.51515388026674e-15
**BRCA**	EED	cg00080012	0.0546040045168763	111	1.81865562486586	0.0692343393355039	0.00298159730927905	0.182863682333646
**BRCA**	EED	cg01739327	0.092767196669964	796	2.6220068769182	0.00890921515143686	0.00860575277800377	0.0390381594846727
**BRCA**	EED	cg02172616	-0.246875901384026	796	-7.16962434998056	1.72607159379628e-12	0.0609477106841752	9.46286212598925e-11
**BRCA**	EED	cg04607174	-0.193897549553651	796	-5.56232371538753	3.64197616045209e-08	0.0375962597229105	8.80161209773875e-07
**BRCA**	EED	cg06358433	-0.0362955044576939	796	-1.02211951013125	0.307036439748371	0.00131736364383848	0.505534168182549
**BRCA**	EED	cg10086030	-0.303741710953384	796	-8.97193371551052	2.07796847631769e-18	0.092259026972889	2.36335496559443e-16
**BRCA**	EED	cg10535675	-0.264541637352636	789	-7.68569690297162	4.54601307018192e-14	0.0699822778932134	3.14357814028207e-12
**BRCA**	EED	cg12672785	0.0837102371613841	796	2.36411260156473	0.0183137264933626	0.00700740380561517	0.0680274095795565
**BRCA**	EED	cg16265078	-0.209453596792475	796	-6.02826207354328	2.53933170326182e-09	0.0438708092093047	7.78348189612098e-08
**BRCA**	EED	cg18574463	0.02397069497185	796	0.674789045617536	0.500006730343556	0.000574594217433477	0.684008714726407
**BRCA**	EED	cg19325359	-0.261431297655359	792	-7.60314785777853	8.22734359531196e-14	0.0683463233937651	5.49860796953349e-12
**BRCA**	EED	cg21042619	-0.0134632190884875	314	-0.237447975435344	0.812465619411066	0.000181258268224615	0.898984324075102
**BRCA**	ELP3	cg01209934	0.110875109322818	796	3.13966021491069	0.00175420558177149	0.0122932898673468	0.0106856682349332
**BRCA**	ELP3	cg02516513	0.196906239119837	796	5.65208750204862	2.21202454064947e-08	0.0387720670043183	5.6438910172281e-07
**BRCA**	ELP3	cg04720865	-0.406906167409071	796	-12.5361041768942	5.12972727470402e-33	0.165572629075539	1.58988846059094e-30
**BRCA**	ELP3	cg05621091	-0.144510969247301	796	-4.11004145521636	4.36822555040455e-05	0.0208834202327945	0.000472154422874362
**BRCA**	ELP3	cg15692687	0.0156013534890673	796	0.439114449648764	0.660698381969357	0.000243402230690831	0.802560309899976
**BRCA**	ELP3	cg17572663	0.0191326995148643	796	0.538540470130726	0.59035529629591	0.000366060190726088	0.753337717275829
**BRCA**	ELP3	cg18022570	-0.0867790229216096	796	-2.45142597644551	0.014444176231534	0.00753059881922925	0.0567149396726365
**BRCA**	ELP3	cg18249244	-0.116176014033011	111	-3.88995700132781	0.000106242496544095	0.0134968662365983	0.0010147364979928
**BRCA**	ELP3	cg19781013	0.134604891015145	796	3.82290836111369	0.000142235954329072	0.018118476685199	0.00130162378909255
**BRCA**	ELP3	cg22414525	0.240506463918399	796	6.97313040540474	6.5399263053756e-12	0.0578433591865319	3.21191304924488e-10
**BRCA**	ELP3	cg22487209	-0.145881751277207	796	-4.14987157624268	3.68687725661402e-05	0.0212814853557049	0.000407630223794832
**BRCA**	ELP3	cg25374552	0.0923174929960882	796	2.60918675759507	0.00924700337462853	0.0085225195130828	0.040138454442911
**BRCA**	ELP4	cg04972224	-0.041850114596927	796	-1.17879936825097	0.238832094413368	0.00175143209177593	0.430634924411637
**BRCA**	ELP4	cg05195750	0.0511586659968059	796	1.44162022621613	0.149804813016904	0.00261720910657274	0.315362765163096
**BRCA**	ELP4	cg05635455	-0.238749553116366	796	-6.91910023980017	9.38126063674685e-12	0.0570013491132644	4.4718492032787e-10
**BRCA**	ELP4	cg08111895	-0.101019998014528	796	-2.85757295813809	0.00438087344454535	0.0102050399988553	0.0223287512663545
**BRCA**	ELP4	cg08626560	-0.23249471357232	796	-6.72732567746072	3.31163298179912e-11	0.0540537918390752	1.42966246649616e-09

**BRCA**	ELP4	cg09616795	0.047106887575315	796	1.32717868891217	0.184832042393135	0.00221905885703337	0.363358533961678
**BRCA**	ELP4	cg10583382	-0.118507232661029	111	-3.9691146620463	7.67925040394374e-05	0.0140439641929754	0.000767175556599973
**BRCA**	ELP4	cg11385473	-0.108379471753897	111	-3.62568714794323	0.000301315680377953	0.0117461098976538	0.0024524536555056
**BRCA**	ELP4	cg13263383	-0.0788446883259168	796	-2.22581537155357	0.0263072880581904	0.00621648487721095	0.0895135882079719
**BRCA**	ELP4	cg19328333	0.0758989817043046	796	2.14216573065875	0.0324844784914455	0.00576065542375036	0.104730412135961
**BRCA**	ELP4	cg24001477	-0.0574754633354081	796	-1.62018120460012	0.105591421160531	0.00330342888561984	0.247637364497975
**BRCA**	ELP4	cg24531794	-0.0447878452363573	796	-1.26170778817264	0.207425410426408	0.0020059510809159	0.392356660556617
**BRCA**	ELP4	cg25130734	-0.0344759297538126	796	-0.970815787891275	0.331936404747706	0.00118858973238982	0.531625814154841
**BRCA**	EP300	cg00500400	-0.147818669518515	111	-4.9682996130636	7.82153065549053e-07	0.0218503590582239	1.37413765992148e-05
**BRCA**	EP300	cg02046995	-0.0200038332449704	796	-0.563070436786068	0.573546282754901	0.000400153344492585	0.740924269688389
**BRCA**	EP300	cg07519229	-0.120052335151956	793	-3.39673396669054	0.000716092466629736	0.0144125631754377	0.00508142819930618
**BRCA**	EP300	cg08131204	-0.105308043541067	796	-2.98020201090596	0.00296870252292727	0.0110897840344473	0.0163259751214905
**BRCA**	EP300	cg09331127	-0.0968337138628985	796	-2.73800933880869	0.00631996367598736	0.00937676814048171	0.029889674400253
**BRCA**	EP300	cg24349919	0.00898704230752142	796	0.252928003555747	0.80038948164354	8.076692943718e-05	0.890945373745869
**BRCA**	EPC1	cg02900516	0.00878088214420845	796	0.247125455316851	0.804875178838777	7.71038912304787e-05	0.89396867067266
**BRCA**	EPC1	cg16942190	-0.0350861878616896	796	-0.98802118535617	0.323444015558466	0.00123104057866577	0.522857695668592
**BRCA**	EPC1	cg26211455	0.0767771339750333	796	2.16709673896477	0.0305248881207924	0.00589472830142021	0.0999811859066533
**BRCA**	EPC2	cg00066942	-0.0184652501692143	796	-0.519746817828335	0.603385279199201	0.000340965463811668	0.762423983072819
**BRCA**	EPC2	cg03664802	-0.209481367549503	796	-6.02909802181955	2.52680726832292e-09	0.0438824433504098	7.75731825698159e-08
**BRCA**	EPC2	cg04098458	-0.118877572020411	796	-3.36940413899119	0.000789745542888305	0.0141318771294681	0.00550959239202339
**BRCA**	EPC2	cg05586854	-0.00332219279282551	796	-0.093495308549489	0.925533722585851	1.10369649527018e-05	0.960794384964354
**BRCA**	EPC2	cg10458926	-0.0877075113779017	796	-2.47785715266715	0.0134248699889493	0.00769260755210475	0.0536348609613379
**BRCA**	EPC2	cg11721798	-0.168597254477524	796	-4.81365466969921	1.77497445482789e-06	0.0284250342173591	2.84354124256345e-05
**BRCA**	EPC2	cg12432635	0.00436504577516858	796	0.122844447298776	0.9022614420667	1.9053624619317e-05	0.948328401325905
**BRCA**	EPC2	cg16434205	-0.0246845265565204	796	-0.694895895583252	0.487324353046377	0.000609325851319563	0.673612486156537
**BRCA**	EPC2	cg21347644	-0.127741904259986	796	-3.62467116724676	0.000307812432979998	0.0163179941039675	0.00249775325036466
**BRCA**	EPC2	cg21650422	-0.0347744940509316	796	-0.979233265191898	0.327763752528259	0.00120926543649828	0.527285897553887
**BRCA**	EZH1	cg00728999	-0.27483736697017	796	-8.04439280822127	3.1581292042417e-15	0.075535578283096	2.53281962180185e-13
**BRCA**	EZH1	cg01716316	-0.0194674538989994	796	-0.547966559934755	0.583869265520107	0.000378981761309668	0.74870446821879
**BRCA**	EZH1	cg03623386	-0.193377175500659	796	-5.54681506374161	3.96684424382577e-08	0.0373947320046128	9.52458892296858e-07
**BRCA**	EZH1	cg05375741	-0.238353587667994	796	-6.90693311368973	1.01719326834169e-11	0.0568124327542039	4.81335359594728e-10
**BRCA**	EZH1	cg05979501	-0.191365880332781	796	-5.48691823275229	5.507016557146e-08	0.0366209001555403	1.27050072967561e-06
**BRCA**	EZH1	cg06975499	-0.0458636386165719	111	-1.52687440379228	0.127078156264408	0.00210347334715151	0.281784888640622
**BRCA**	EZH1	cg12812489	-0.109839787294362	796	-3.10998317559772	0.00193799788135368	0.0120647788728706	0.0115901450785692
**BRCA**	EZH1	cg19395400	0.206542428520512	796	5.94071497588724	4.24793803266883e-09	0.0426597747791507	1.24703430684317e-07
**BRCA**	EZH1	cg22227424	0.247833400473385	796	7.19924745179277	1.40820990684598e-12	0.0614213943902012	7.82502010665543e-11
**BRCA**	EZH1	cg27088844	-0.0204360015975525	111	-0.679773448970094	0.496790169766289	0.00041763016129517	0.681395596305592
**BRCA**	EZH2	cg01893439	-0.0583825445734806	796	-1.64583777397826	0.100193913400146	0.00340852151087445	0.238565988789698
**BRCA**	EZH2	cg02303805	-0.0812290913282475	796	-2.29356845549191	0.0220763682791349	0.00659816527801277	0.0784742514922103
**BRCA**	EZH2	cg06816161	0.0202493165721126	111	0.673256515877247	0.500924893533263	0.000410034821637633	0.684734957521861
**BRCA**	EZH2	cg13751213	-0.0576277372149551	796	-1.62448795083315	0.104669582301683	0.00332095609651592	0.246098633468647
**BRCA**	EZH2	cg18494018	-0.0366299311840888	796	-1.03154991334232	0.302597912137868	0.00134175185855108	0.500964076970703
**BRCA**	EZH2	cg20101066	0.043232891828584	796	1.21782005869848	0.223655048916147	0.00186908293586205	0.412556367530597
**BRCA**	FBXL19	cg00026853	-0.0964482556508433	796	-2.72700782069227	0.00653252031879059	0.00930226601809043	0.0306730375229355
**BRCA**	FBXL19	cg02291855	-0.0360954078618233	796	-1.01647720831182	0.309712606715499	0.00130287846871137	0.508414908774542
**BRCA**	FBXL19	cg03181514	-0.104638054655323	796	-2.96103084535124	0.00315770347991479	0.0109491224820503	0.017172344251415
**BRCA**	FBXL19	cg03627896	-0.0690116922874874	111	-2.30057766770193	0.0216009011109599	0.00476261367238284	0.0772216580591892
**BRCA**	FBXL19	cg04477789	-0.0831102922638331	796	-2.34705091056677	0.0191680744907646	0.00690732068017976	0.0704089591714541
**BRCA**	FBXL19	cg05643349	-0.214739295828928	796	-6.18764860814275	9.78723059912096e-10	0.0461129651731039	3.24408954933113e-08
**BRCA**	FBXL19	cg05768582	-0.0304743231968022	796	-0.85802198310127	0.391139902382161	0.000928684374303156	0.588316884165384
**BRCA**	FBXL19	cg06862768	-0.165333049840361	796	-4.71781196448013	2.81762205819308e-06	0.0273350173695153	4.27862756968714e-05
**BRCA**	FBXL19	cg07697662	-0.00074304727176329	796	-0.0209112095753755	0.983321751595029	5.5211924807487e-07	0.991358882722222
**BRCA**	FBXL19	cg08535091	-0.0148093236252371	111	-0.492561446944139	0.622420258502604	0.000219316066237005	0.776345949233976
**BRCA**	FBXL19	cg08593668	-0.0414573827866023	796	-1.16771808810383	0.243271911948244	0.00171871458751486	0.435889212137018
**BRCA**	FBXL19	cg09011601	-0.275527245019692	796	-8.06624189854774	2.67748226781185e-15	0.0759152627481412	2.16294969410338e-13
**BRCA**	FBXL19	cg11940219	-0.0946459197045628	784	-2.6552360400701	0.00808697428705236	0.00895785011672255	0.0362269997516383
**BRCA**	FBXL19	cg26666512	-0.0413052094819224	796	-1.16342452790113	0.245007690938968	0.00170612033034549	0.437882014080732

**BRCA**	FBXL19	cg27602786	-0.0457855892329961	796	-1.28987339898299	0.197471123020046	0.00209632018141265	0.379788586719608
**BRCA**	FBXW9	cg02753619	-0.0290255784681371	795	-0.816680424383489	0.414356967893582	0.000842484205409982	0.609924048444908
**BRCA**	FBXW9	cg03160342	0.0305123119939278	796	0.85909257549403	0.390549380697806	0.000931001183214788	0.587883332791647
**BRCA**	FBXW9	cg05868316	0.0563898595981429	111	1.87747317565372	0.0607165173360309	0.00317981626549827	0.166076465301473
**BRCA**	FBXW9	cg06489804	0.051164081492247	111	1.70377378164663	0.0887042633586261	0.00261776323494529	0.218645146342668
**BRCA**	FBXW9	cg12110584	-0.0799112106386982	796	-2.25611590183267	0.0243353552451207	0.0063858015857424	0.0844852117966317
**BRCA**	FBXW9	cg14342428	0.0268707381773031	796	0.756482756372473	0.449584766259369	0.000722036570193172	0.640778962045754
**BRCA**	FBXW9	cg15828427	-0.0360385957913296	796	-1.01487525013361	0.31047522735026	0.00129878038661084	0.509279927391855
**BRCA**	FBXW9	cg19721801	-0.0405270053761147	796	-1.14146880947589	0.25401988909081	0.00164243816475563	0.44810030049729
**BRCA**	FBXW9	cg21564527	-0.127052749378732	796	-3.60479470235086	0.000331939995248641	0.0161424011246948	0.0026583220084798
**BRCA**	FBXW9	cg22303873	-0.0651684758651234	796	-1.8379103685029	0.0664500036365196	0.00424693024658316	0.177405250108082
**BRCA**	FKBP1A	cg04613791	0.079721501505719	111	2.65972980884716	0.00793338975234525	0.00635551780232635	0.0357034665274758
**BRCA**	FKBP1A	cg07516946	0.119958493071453	796	3.40048646456072	0.000706346734226712	0.0143900400599738	0.00502464684001763
**BRCA**	FKBP1A	cg10996143	0.116762659192005	111	3.9081026590709	9.86785426621592e-05	0.0136335185815883	0.000953022786823093
**BRCA**	FKBP1A	cg21332426	-0.0302387751112746	796	-0.85138389876195	0.394813475300433	0.000914383520230239	0.591497891182104
**BRCA**	FKBP1A	cg21775445	0.0883455870441662	796	2.49602494948417	0.0127616847880322	0.00780494275017835	0.0516240708439591
**BRCA**	FKBP1A	cg24641773	-0.236524715773839	796	-6.85078326480309	1.47536862347848e-11	0.0559439411718953	6.7713762061879e-10
**BRCA**	FKBP1A	cg26779912	0.0113539697784594	796	0.319549630368105	0.74939403248586	0.000128912629730169	0.859593587326487
**BRCA**	FKBP2	cg03764062	-0.0589624273133428	796	-1.66224175367033	0.0968600039024333	0.00347656783468123	0.232716487340125
**BRCA**	FKBP2	cg04446112	-0.0353939877907432	796	-0.996699604389611	0.319214754459862	0.00125273437173128	0.518607313684122
**BRCA**	FKBP2	cg06615912	-0.101423335263888	796	-2.86910058523902	0.00422593798856064	0.0102866929360511	0.0216927050731708
**BRCA**	FKBP2	cg09854726	0.0168485043069484	796	0.474226255362975	0.635469285979138	0.000283872097381258	0.785329356041574
**BRCA**	FKBP2	cg10998122	-0.0578935735975878	796	-1.63200684631157	0.103075554306454	0.00335166586389931	0.243370348850115
**BRCA**	FKBP2	cg12423493	0.0433467465342234	796	1.22103324220426	0.222436800708909	0.0018789404351022	0.411046806839965
**BRCA**	FKBP2	cg15010817	-0.00356454506493571	796	-0.100315827402601	0.920118979846587	1.27059815199575e-05	0.958001373014311
**BRCA**	FKBP2	cg16977872	0.030290616790195	111	1.00782472544872	0.313759048783091	0.000917521465530444	0.512817677286244
**BRCA**	FKBP2	cg19924313	0.00369381964473805	796	0.103954008446192	0.917232164420122	1.36443035678527e-05	0.956324000842992
**BRCA**	FKBP2	cg21809711	-0.0194001613158123	796	-0.546071705426105	0.585170429670037	0.000376366259079539	0.749703535938981
**BRCA**	FKBP2	cg22074794	0.081374654174341	796	2.29770591209042	0.02183839238869	0.00662183434199359	0.0778488703425628
**BRCA**	FKBP2	cg24677501	-0.0522056365554744	796	-1.4712030399163	0.141633411697419	0.00272542848816229	0.303748319737079
**BRCA**	FKBP5	cg00130530	-0.19503913887052	796	-5.59636349110028	3.01698777430242e-08	0.0380402656913539	7.4446743557605e-07
**BRCA**	FKBP5	cg00140191	-0.0666155415775412	796	-1.87890108599465	0.060625042180155	0.00443763037966912	0.165896251410988
**BRCA**	FKBP5	cg00610228	-0.0432612646023985	796	-1.21862078468903	0.223351014889087	0.00187153701499873	0.412168347383091
**BRCA**	FKBP5	cg01294490	-0.0962501024441735	796	-2.72135273248939	0.00664426834387992	0.0092640822205139	0.0310833009527809
**BRCA**	FKBP5	cg07485685	-0.0276104892491334	796	-0.777324392349059	0.437199522979403	0.000762339116576514	0.630134315713936
**BRCA**	FKBP5	cg07843056	-0.0654157368022269	796	-1.84491364343527	0.0654232936403533	0.00427921862137822	0.175422731377577
**BRCA**	FKBP5	cg08636224	-0.180362013061687	111	-6.09823261284355	1.48007543218155e-09	0.0325304557556642	4.74026299592966e-08
**BRCA**	FKBP5	cg08915438	-0.243532356400365	796	-7.06635641045785	3.48972933537204e-12	0.0593080086139142	1.80625418440408e-10
**BRCA**	FKBP5	cg10913456	-0.0732357352160033	796	-2.06658576937284	0.0390977277138577	0.00536347291262855	0.120439084096454
**BRCA**	FKBP5	cg16012111	-0.0860307870683322	796	-2.43013071768384	0.0153145528340863	0.00740129632359671	0.0593405550219109
**BRCA**	FKBP5	cg17030679	-0.120748255740345	796	-3.42320416285878	0.000650660403994206	0.0145801412643357	0.00468724906938579
**BRCA**	FKBP5	cg20813374	-0.155332994540709	796	-4.42516992767429	1.09862521346925e-05	0.0241283391929841	0.000142230736988895
**BRCA**	FKBP5	cg25114611	-0.0659472008041474	796	-1.85996767752369	0.0632606351160868	0.00434903329390254	0.171092262836214
**BRCA**	FMR1	cg02921434	-0.29919202239802	796	-8.82422166638716	6.96558690641281e-18	0.0895158662666175	7.60001217107262e-16
**BRCA**	FMR1	cg03513471	-0.283050150892012	796	-8.3053851429913	4.29084541256435e-16	0.0801173879199905	3.79752022781605e-14
**BRCA**	FMR1	cg04552106	-0.291659918554771	796	-8.58112722322946	4.92387798643245e-17	0.0850655080913755	4.91717797274103e-15
**BRCA**	FMR1	cg04744025	-0.295086579795676	796	-8.69150048214767	2.03717489095402e-17	0.0870760895755097	2.11588762973133e-15
**BRCA**	FMR1	cg05288927	-0.258521383466665	796	-7.53146473957432	1.36921240828727e-13	0.0668333057095184	8.89119449835557e-12
**BRCA**	FMR1	cg07147350	-0.180933035181709	111	-6.11819185146249	1.31147250277805e-09	0.0327367632200656	4.24748925400148e-08
**BRCA**	FMR1	cg08434396	-0.295586634695637	111	-10.2899990141626	8.86921662495392e-24	0.087371458610692	1.58979686203149e-21
**BRCA**	FMR1	cg14470409	-0.306449765607279	796	-9.06017456088127	1.00121533181727e-18	0.0939114588407563	1.16774431069554e-16
**BRCA**	FMR1	cg16783314	-0.254043859697468	796	-7.39193745498542	3.68331753796825e-13	0.0645382826499866	2.21664455887392e-11
**BRCA**	FMR1	cg22625568	-0.284833788889225	774	-8.24536529780911	7.08882314852569e-16	0.0811302872929917	6.12062051072594e-14
**BRCA**	FXR2	cg01807919	0.0383425095609655	796	1.07984792680566	0.280538633719495	0.00147014803943273	0.477731814276085
**BRCA**	FXR2	cg01933235	-0.0814226946868128	796	-2.29907144091353	0.0217603430727375	0.00662965521006193	0.077626631825394
**BRCA**	FXR2	cg05529097	-0.173881006667371	110	-5.86682779755148	5.86572770391137e-09	0.0302346044796581	1.67887572111141e-07
**BRCA**	FXR2	cg05913233	-0.118482815183912	796	-3.35805578064456	0.000822412803419014	0.014038177493905	0.00569942898602687

**BRCA**	FXR2	cg09625173	-0.292232307892378	796	-8.59953867727155	4.25251311509412e-17	0.0853997217761056	4.28523322895896e-15
**BRCA**	FXR2	cg17777561	-0.173666443100519	795	-4.95968520842187	8.64509216050042e-07	0.0301600334591856	1.50556592687255e-05
**BRCA**	FXR2	cg19178740	-0.128104044781506	796	-3.63511808772977	0.000295800591514692	0.0164106462893821	0.00241641407290706
**BRCA**	FXR2	cg22521096	-0.262071324467452	796	-7.64245607269253	6.16335830258451e-14	0.0686813791081247	4.18022925711647e-12
**BRCA**	FXR2	cg26904212	-0.00473105283345908	111	-0.157340389855318	0.875005308535974	2.23828609129812e-05	0.933872894088728
**BRCA**	G2E3	cg02259031	0.073026721382413	796	2.06065609288926	0.0396619113271244	0.00533290203586458	0.121743163264769
**BRCA**	G2E3	cg05076167	-0.230153739268127	796	-6.65577953651773	5.26071056952618e-11	0.0529707436991008	2.19636637545075e-09
**BRCA**	G2E3	cg07906495	0.0927899661081857	111	3.09924784397689	0.00198912123259981	0.00860997781035825	0.011843081062978
**BRCA**	G2E3	cg08810065	-0.0158365901963192	111	-0.52673685812455	0.598481957881343	0.000250797589046152	0.758970811023429
**BRCA**	G2E3	cg09837748	-0.240816026160146	796	-6.98265787141061	6.13530246556796e-12	0.0579923584555644	3.03619414762337e-10
**BRCA**	G2E3	cg18580322	-0.0179502949425078	791	-0.503650111035671	0.614648216182006	0.000322213088523021	0.770790848282879
**BRCA**	G2E3	cg18755078	0.048227983502236	796	1.35883696881987	0.174585100623826	0.00232593839269195	0.34996195000979
**BRCA**	G2E3	cg18956956	-0.0736184583176744	795	-2.0761323176988	0.0382041718752953	0.00541967740507117	0.118342947301266
**BRCA**	G2E3	cg19554565	0.138604848778004	796	3.9387034937886	8.91449918764554e-05	0.0192113041047735	0.000871771904215836
**BRCA**	G2E3	cg21061067	-0.186983233089724	796	-5.3566492794304	1.11205390121872e-07	0.0349627294566862	2.39377756478718e-06
**BRCA**	G2E3	cg22926526	-0.0974135049364928	796	-2.75455973323674	0.006011934100122	0.00948939094401211	0.0287160701449983
**BRCA**	GADD45/	cg01810953	-0.0705039046145011	794	-1.98659255002813	0.04731265107511	0.00497080056589067	0.138573110113968
**BRCA**	GADD45/	cg02639608	-0.120304104059691	796	-3.41042723253488	0.000681454834529166	0.0144730774536049	0.00487252733431636
**BRCA**	GADD45/	cg04718306	-0.0997599465970145	111	-3.33430755430073	0.000883479690129337	0.00995204694503918	0.00605438856711783
**BRCA**	GADD45/	cg07000785	0.00666863758536513	111	0.22178099942555	0.82452531352343	4.44707272449445e-05	0.905707660725515
**BRCA**	GADD45/	cg22224769	-0.112434753195749	796	-3.18438627410977	0.00150735996084331	0.0126415737261891	0.0094377111302893
**BRCA**	GADD45/	cg23369529	-0.132527972679927	796	-3.76285892943955	0.000180378330923704	0.0175636635426514	0.00159133044634596
**BRCA**	GADD45/	cg23723105	-0.131906359962324	796	-3.74489624170194	0.000193541810256158	0.0173992877985103	0.00168850415914182
**BRCA**	GATAD2A	cg02887458	-0.0800371983702218	796	-2.25969580078448	0.0241110794354122	0.00640595312295423	0.0838750307918642
**BRCA**	GATAD2A	cg08505228	0.020096600062765	796	0.565682701535073	0.571769719260958	0.000403873334082727	0.739513425885931
**BRCA**	GATAD2A	cg08870822	0.0943300393291961	796	2.66657291944657	0.00781922604382923	0.00889815631984769	0.0353134930215703
**BRCA**	GATAD2A	cg09334331	-0.0199057807631034	796	-0.56030934633368	0.575426905508844	0.000396240107788737	0.742318265573677
**BRCA**	GATAD2A	cg11795262	-0.116043088993859	111	-3.88544544417952	0.000108207837526878	0.0134659985032367	0.00103084992806343
**BRCA**	GATAD2A	cg14948912	0.0517139831638246	796	1.45731046336054	0.14542706550528	0.00267433605466833	0.309302379471191
**BRCA**	GATAD2A	cg15264323	0.0834678000824642	796	2.3572176802079	0.0186548769751784	0.00696687365060621	0.0690079647839768
**BRCA**	GATAD2A	cg22191731	0.0340726719063134	796	0.959447079058537	0.337626369148301	0.00116094697083528	0.53707888437598
**BRCA**	GATAD2A	cg26631329	-0.0217377556626112	796	-0.611899257925787	0.540780025076795	0.000472530021247383	0.715929232896563
**BRCA**	GATAD2E	cg02134200	0.132076979124454	796	3.74982615988037	0.000189841715348571	0.0174443284146416	0.00166032000043021
**BRCA**	GATAD2E	cg02940454	0.11881005730019	796	3.36746313010243	0.000795245973477374	0.0141158297156745	0.00554150530819589
**BRCA**	GATAD2E	cg09805553	0.0705471126683975	796	1.99033074590969	0.0468978512553421	0.00497689510584757	0.137710557667981
**BRCA**	GATAD2E	cg10226922	-0.117657867699444	796	-3.33434562958948	0.000894757478641152	0.0138433738315798	0.00612244798755682
**BRCA**	GATAD2E	cg10489353	0.16517405099622	796	4.71314758327765	2.88111105537679e-06	0.0272824671225017	4.35971899323053e-05
**BRCA**	GATAD2E	cg15064304	-0.157195604668166	796	-4.47956874459552	8.58001742533571e-06	0.0247104581269902	0.000114332626856212
**BRCA**	GATAD2E	cg20773694	-0.109978887925506	796	-3.11396983439104	0.00191231827332095	0.0120953557893311	0.0114683077208395
**BRCA**	GATAD2E	cg23046518	0.101513459696096	796	2.87167659222501	0.00419200352323527	0.0103049824994709	0.0215511976002223
**BRCA**	GATAD2E	cg23323627	0.155056474623567	796	4.41709811091003	1.13941755842856e-05	0.0240425103226889	0.000146803328155667
**BRCA**	GATAD2E	cg26398333	0.11086389362014	796	3.13933866658527	0.00175610784104933	0.0122908029086177	0.0106939063316314
**BRCA**	GLYR1	cg00636946	-0.314490639727913	781	-9.23491260381457	2.41286301583965e-19	0.0989043624764717	3.0275204105521e-17
**BRCA**	GLYR1	cg00885266	-0.10882623854176	796	-3.08094018361433	0.00213486250347728	0.0118431501951481	0.01251918389835
**BRCA**	GLYR1	cg01261535	-0.113467483490525	111	-3.79806928037628	0.000153759923392813	0.0128748698096725	0.00139136902180068
**BRCA**	GLYR1	cg01565912	0.0632132738564255	796	1.7825442307806	0.0750434964979377	0.00399591799164745	0.193944844575282
**BRCA**	GLYR1	cg02454264	-0.00493673716869325	796	-0.138933791922878	0.889537786528121	2.43713738727574e-05	0.941467059185646
**BRCA**	GLYR1	cg06399529	-0.233255611271876	796	-6.75060718953019	2.84603428320481e-11	0.0544081801898163	1.24175205287513e-09
**BRCA**	GLYR1	cg06976003	-0.0975700381098457	796	-2.75902852705267	0.00593111960599498	0.00951991233675673	0.0284204199956128
**BRCA**	GLYR1	cg11289281	0.0220300946841629	796	0.620132320926299	0.535349056111636	0.000485325071793184	0.711755412833877
**BRCA**	GLYR1	cg15663121	0.0464777633145423	111	1.54736357211494	0.122061676276793	0.00216018248272261	0.273839717507118
**BRCA**	GLYR1	cg18354686	-0.0509549956976654	796	-1.43586595020056	0.151435321540424	0.0025964115865491	0.317751392962929
**BRCA**	GTF2B	cg02705666	-0.0769921691002068	111	-2.56811905401705	0.0103555982211112	0.00592779410275484	0.0438017478210251
**BRCA**	GTF2B	cg04270788	0.117264253591578	111	3.92690050883142	9.13714038744327e-05	0.0137509051703899	0.000891862859850391
**BRCA**	GTF2B	cg05306856	-0.129384689519232	796	-3.67207362885068	0.000256747180204674	0.0167403978819881	0.00214077064703563
**BRCA**	GTF2B	cg06356032	0.0276422279808901	796	0.77821862297585	0.436672562196003	0.000764092767747503	0.629746355447781
**BRCA**	GTF2B	cg07167781	-0.114380699277579	796	-3.24022382429813	0.00124422250985982	0.013082944367228	0.00805400615177525

**BRCA**	GTF2B	cg10430205	-0.0920091695840296	796	-2.60039802740496	0.0094851410414381	0.00846568728754272	0.0408711123395168
**BRCA**	GTF2B	cg12074130	-0.0895755957580618	796	-2.53105549293287	0.0115644672508883	0.00802378735541169	0.0477549672656186
**BRCA**	GTF2B	cg13868361	-0.0282660776368703	796	-0.795795908607753	0.426389114975735	0.000798971144973578	0.620996252184261
**BRCA**	GTF2B	cg14010405	-0.0327057402860059	795	-0.920332216056276	0.357679762365083	0.00106966544765567	0.556684927845496
**BRCA**	GTF2B	cg17811845	-0.0723139484179712	793	-2.03656879815728	0.0420266797034612	0.005229307135797	0.126933397556045
**BRCA**	GTF2B	cg22607980	-0.0180051999980984	795	-0.506473351227715	0.612665529733935	0.000324187226971523	0.769320327024062
**BRCA**	GTF2H1	cg05849260	-0.127430453836757	795	-3.61340436626127	0.000321303622562779	0.0162385205650418	0.00258806109066565
**BRCA**	GTF2H1	cg07583091	-0.359439602463353	796	-10.8399808466314	1.263350058869e-25	0.129196827819013	2.70746706555524e-23
**BRCA**	GTF2H1	cg08690812	-0.31722542035311	796	-9.41373240548936	5.07625104718831e-20	0.100631967318207	6.80865299939599e-18
**BRCA**	GTF2H1	cg10797557	-0.0513934026821183	796	-1.44825244139673	0.147942218240405	0.00264128183924637	0.312804526018374
**BRCA**	GTF2H1	cg11063328	-0.0278056206396191	796	-0.782822206562374	0.433965526313915	0.000773152539154411	0.627519361959604
**BRCA**	GTF2H1	cg11176342	-0.131074287469459	796	-3.72085894971691	0.000212575852529952	0.0171804688356263	0.00182549132042335
**BRCA**	GTF2H1	cg11347316	0.0139232804416914	796	0.391873829801114	0.695256859491879	0.000193857738257986	0.825299257313709
**BRCA**	GTF2H1	cg11662851	0.0650842620341394	796	1.8355252226288	0.066802697339655	0.00423596116452852	0.178138999651771
**BRCA**	GTF2H1	cg13777411	0.155349131133624	111	5.2298731408269	2.02756082728261e-07	0.0241333525439718	4.10439934940471e-06
**BRCA**	GTF2H1	cg14132868	0.104228011607224	796	2.94929982271247	0.00327870978390856	0.0108634784035956	0.0176771743661642
**BRCA**	GTF2H1	cg17174493	0.023104143611752	796	0.650381846441201	0.51563421540552	0.000533801452032462	0.696921338859426
**BRCA**	GTF2H1	cg18321761	0.0373260396552087	796	1.05118039286734	0.293496370067148	0.00139323323634221	0.491238120721266
**BRCA**	H2AFZ	cg02758870	-0.0253544914419571	796	-0.713768097328004	0.475580871235741	0.000642850236280276	0.663367609139231
**BRCA**	H2AFZ	cg09308803	0.0686997030493684	796	1.93795967297617	0.0529832497503042	0.0047196491990714	0.15048480945875
**BRCA**	H2AFZ	cg16267491	0.0126689738601828	111	0.421360537269911	0.673573704953094	0.000160502898669995	0.811189630659013
**BRCA**	H2AFZ	cg17709884	-0.0364320510303529	111	-1.2124102373889	0.225614301868723	0.00132729434227824	0.415025750758465
**BRCA**	H2AFZ	cg20934652	0.108173108925	796	3.0622300719601	0.00227124597612236	0.0117014214944998	0.0131739717765034
**BRCA**	H2AFZ	cg23752380	0.0448565940341521	795	1.26285038621413	0.207015093434878	0.00201211402834473	0.391847299902014
**BRCA**	H2AFZ	cg24061702	-0.0572987471130397	796	-1.61518330034613	0.106669276172687	0.00328314642072408	0.249374922336594
**BRCA**	H3F3A	cg06319713	-0.100789472359253	796	-2.85098503256832	0.00447171678151297	0.0101585177384565	0.0226834073618297
**BRCA**	H3F3A	cg10632229	0.0527435677148599	796	1.48640450973949	0.137570116126082	0.00278188393529201	0.297633272905956
**BRCA**	H3F3A	cg10983539	0.0493065324605286	796	1.38929863482953	0.165132421685576	0.00243113414328116	0.33706737018807
**BRCA**	H3F3A	cg11978873	0.0302890738881933	796	0.852801378788008	0.394027279304977	0.000917427997004431	0.590786511829072
**BRCA**	H3F3A	cg15538837	0.0384922452247749	111	1.28106976932898	0.200437705444292	0.00148165294244421	0.383453641122517
**BRCA**	H3F3A	cg15624314	0.0391301827708688	795	1.10136903617348	0.271071097797448	0.0015311712036816	0.467338274982928
**BRCA**	H3F3A	cg16724613	-0.0618033082372794	796	-1.74263056841203	0.0817862955255698	0.00381964890907217	0.20620590095985
**BRCA**	H3F3A	cg16727519	0.0901269005975451	796	2.54676038685454	0.0110609381636497	0.00812285821131978	0.046130629041064
**BRCA**	H3F3A	cg18947521	0.0734035719134968	111	2.44775489829435	0.0145295611180879	0.0053880843696599	0.0569441318185097
**BRCA**	H3F3A	cg19410789	0.0048439694399913	796	0.13632298296197	0.891600598734888	2.34640399355696e-05	0.942573783113618
**BRCA**	H3F3A	cg21266749	-0.0859770749940338	796	-2.42860219837988	0.0153787697028486	0.00739205742452972	0.0595389673665474
**BRCA**	H3F3A	cg21789564	-0.0779910462437721	796	-2.20156844109238	0.0279834838791706	0.00608260329419819	0.0937571447907402
**BRCA**	H3F3A	cg22519151	-0.139294405821772	796	-3.95868522924838	8.21401018295833e-05	0.0194029314932406	0.000811841834802516
**BRCA**	H3F3A	cg27530209	0.00477352498165336	796	0.134340431408723	0.893167513732337	2.27865407504687e-05	0.943332929162108
**BRCA**	H3F3A	cg27560444	-0.258034921501941	780	-7.439971389081	2.67448224787144e-13	0.0665820207145126	1.65125930151516e-11
**BRCA**	HAT1	cg00414306	-0.182332140131075	796	-5.21876341253053	2.30259375472719e-07	0.0332450093247781	4.58949471061356e-06
**BRCA**	HAT1	cg01583365	0.200907460574493	796	5.77172121488504	1.12593493058453e-08	0.0403638077144915	3.04775757755617e-07
**BRCA**	HAT1	cg04507121	0.0769235870424859	786	2.15750411564999	0.0312703340448629	0.0059172382434829	0.101822626373365
**BRCA**	HAT1	cg04950839	-0.193197115488063	796	-5.54144988790577	4.08565429674401e-08	0.0373251254329078	9.76165203811128e-07
**BRCA**	HAT1	cg05421799	0.0614702955841839	796	1.73320510016944	0.0834484353046724	0.00377859723920694	0.209122207179912
**BRCA**	HAT1	cg09545452	-0.107784807547576	796	-3.05110836077002	0.00235605932777253	0.0116175647380679	0.013571038764409
**BRCA**	HAT1	cg10190161	-0.104188313595538	796	-2.94816417334037	0.00329064597668745	0.0108552046898823	0.0177243310330557
**BRCA**	HAT1	cg12941622	0.0289830807668523	796	0.81599899210382	0.414746056376305	0.000840018970737886	0.610315747480889
**BRCA**	HAT1	cg13293756	-0.159083079986241	796	-4.53474377128447	6.65933167575078e-06	0.0253074263379088	9.14485522300717e-05
**BRCA**	HAT1	cg14446129	-0.0920274628310743	796	-2.60091945363505	0.00947086112794878	0.00846905391512476	0.0408321974389076
**BRCA**	HAT1	cg16017358	0.135467409038313	111	4.54709854565158	6.03845763376903e-06	0.0183514189115536	8.4003745507183e-05
**BRCA**	HAT1	cg24611092	-0.0686373791071128	315	-1.21524377823247	0.225191928693821	0.00471108981069352	0.414542851415217
**BRCA**	HDAC1	cg03762702	-0.0932570224628958	796	-2.6359726364972	0.00855384567597371	0.00869687223864505	0.0378283139398953
**BRCA**	HDAC1	cg04967775	-0.0936499757653743	795	-2.6455060672727	0.00831881231287579	0.00877031796085519	0.0370246401434784
**BRCA**	HDAC1	cg06473843	-0.0868873397591893	796	-2.45450908663225	0.0143218549867023	0.00754940981042879	0.0563358642279173
**BRCA**	HDAC1	cg10842753	-0.0743914485903717	796	-2.0993780675695	0.0360995115333786	0.00553408762337392	0.113490620336539
**BRCA**	HDAC1	cg14172124	-0.0914307826113177	796	-2.58391319322882	0.00994663315248343	0.00835958800891804	0.0424435510649057

**BRCA**	HDAC1	cg20002354	-0.18007987032114	796	-5.15212373347644	3.25392399594229e-07	0.0324287596948786	6.26108739402263e-06
**BRCA**	HDAC1	cg20793809	-0.0870812060550194	795	-2.45847394144984	0.0141661670675585	0.00758313644799675	0.0558384971822788
**BRCA**	HDAC1	cg21930229	-0.0355949874971915	796	-1.00236694426023	0.316472545088867	0.00126700313492522	0.515724688354002
**BRCA**	HDAC1	cg23018091	-0.211985199651574	796	-6.10453075212691	1.61342584387608e-09	0.0449377248713177	5.13353170136999e-08
**BRCA**	HDAC1	cg26115470	-0.0672911781512196	796	-1.89804375914305	0.0580536580786799	0.00452810265697917	0.160702639453895
**BRCA**	HDAC10	cg00711351	0.0421064389278964	796	1.18603208394968	0.235965314566847	0.00177295219918867	0.427247426542845
**BRCA**	HDAC10	cg01120165	0.0359284089213561	111	1.19562788202606	0.232098088493563	0.00129085056762018	0.422708629691948
**BRCA**	HDAC10	cg04321608	0.0146564909502573	796	0.412514525885555	0.680073973989384	0.000214812726974974	0.815376045812285
**BRCA**	HDAC10	cg04586023	-0.0883493137665292	111	-2.94973063432065	0.0032474521696376	0.00780560124301662	0.0175553991106669
**BRCA**	HDAC10	cg07482373	0.0288264068111252	796	0.811584264646771	0.417274062402297	0.000830961729640486	0.612745501076397
**BRCA**	HDAC10	cg07744063	0.000885660453255293	796	0.0249247042622075	0.980121300631117	7.8439443846037e-07	0.989790955439119
**BRCA**	HDAC10	cg09151829	-0.00295979690230128	796	-0.0832964330743225	0.933636884323181	8.76039770287224e-06	0.964662218815246
**BRCA**	HDAC10	cg10508940	0.0301301059236397	796	0.848321492449458	0.396515260584552	0.000907823282969747	0.593122237043372
**BRCA**	HDAC10	cg11645658	-0.0721274501670159	796	-2.03514706999209	0.0421685750380307	0.00520236906759536	0.127268021316654
**BRCA**	HDAC10	cg12464820	0.0220608296061297	796	0.620997909240945	0.534779671820348	0.000486680202910689	0.711436116585874
**BRCA**	HDAC10	cg26943378	-0.0129178904343313	796	-0.36357199750056	0.716274665283926	0.000166871893273388	0.838696167547869
**BRCA**	HDAC11	cg01409735	-0.0665012620171581	796	-1.87566348396066	0.0610691541986111	0.00442241784987472	0.166754727673469
**BRCA**	HDAC11	cg01712016	-0.0553292180908117	111	-1.84288346590089	0.0656135027458838	0.0030613223745406	0.175769174676766
**BRCA**	HDAC11	cg02762546	-0.203536202925242	796	-5.85048216577942	7.17018850738866e-09	0.0414269859012253	2.02173847315619e-07
**BRCA**	HDAC11	cg02941903	-0.0872575949790127	796	-2.465048624933	0.0139105886093316	0.00761388788152141	0.0551060890768669
**BRCA**	HDAC11	cg05987698	-0.0316857786323147	795	-0.891601413595667	0.372877743373377	0.00100398856753605	0.571455471089784
**BRCA**	HDAC11	cg07450135	0.0384990943644998	796	1.08426438928579	0.278577538552643	0.00148218026688666	0.475491233715819
**BRCA**	HDAC11	cg08051076	-0.063622446694483	796	-1.79412919412141	0.073173859879515	0.00404781572339232	0.190473374003446
**BRCA**	HDAC11	cg08448040	-0.0607744183212088	111	-2.02489216060413	0.043118310039888	0.00369352992228128	0.129361581613693
**BRCA**	HDAC11	cg10061496	-0.0655889661652807	315	-1.16103215024854	0.246516348769288	0.00430191248263033	0.439697657484885
**BRCA**	HDAC11	cg10455785	-0.0303806795096885	796	-0.855382952039274	0.392597867897003	0.000922985687470407	0.589473338750687
**BRCA**	HDAC11	cg11432034	-0.0812887997484872	796	-2.29526558082509	0.0219784819625806	0.00660786896454966	0.0781870324582494
**BRCA**	HDAC11	cg13397067	-0.0860747331501125	796	-2.43138133568793	0.0152621873257625	0.00740885968686308	0.0591936684026506
**BRCA**	HDAC11	cg13706066	-0.0430206768431177	796	-1.21183109888724	0.225938451457357	0.00185077863603997	0.415366667281257
**BRCA**	HDAC11	cg16254764	-0.0674028014557643	111	-2.24669587804314	0.0248561032105348	0.00454313764408519	0.085833438084177
**BRCA**	HDAC11	cg18521047	-0.0109814107515737	796	-0.309062928050322	0.757354942893696	0.000120591382094779	0.864631994933965
**BRCA**	HDAC11	cg19779799	-0.099091504501152	796	-2.80247499618958	0.00519506589728341	0.00981912626430183	0.0255609990774313
**BRCA**	HDAC11	cg20894727	-0.0327895806557128	796	-0.923277062387655	0.356144026100352	0.00107515659957749	0.554938676570339
**BRCA**	HDAC11	cg20922324	-0.0852055213641171	796	-2.4066479442161	0.0163276708090858	0.00725998087093102	0.0622617565217171
**BRCA**	HDAC11	cg21655280	-0.0505480328782784	796	-1.4243686088129	0.154733681824382	0.00255510362786351	0.322371506262614
**BRCA**	HDAC11	cg25127083	-0.0404941812525528	796	-1.14054277811609	0.254405013796444	0.0016397787153146	0.448566071559588
**BRCA**	HDAC11	cg25150878	-0.0521622356922479	796	-1.46997662517963	0.141965213025593	0.00272089883241362	0.304201674207766
**BRCA**	HDAC11	cg27025856	-0.0964063708519519	111	-3.21969340603241	0.00132065040211141	0.00929418834084408	0.00845307531527525
**BRCA**	HDAC2	cg00161229	-0.171093546726173	796	-4.88705994022692	1.23910780075731e-06	0.0292730017313411	2.06812169603334e-05
**BRCA**	HDAC2	cg01129566	0.0584759438089934	796	1.64847977952195	0.0996508481780682	0.00341943600435255	0.237667486760826
**BRCA**	HDAC2	cg08198132	-0.267541880242774	796	-7.81415115792883	1.75908889182106e-14	0.0715786576838389	1.28133484316786e-12
**BRCA**	HDAC2	cg10999867	0.00503748525749786	796	0.14176920024717	0.887298366236223	2.53762577195082e-05	0.940108350079743
**BRCA**	HDAC2	cg13393754	-0.22420507188435	796	-6.47451838442194	1.6674464639164e-10	0.0502679142586665	6.39631805788524e-09
**BRCA**	HDAC2	cg15005908	-0.183788529608061	796	-5.26189991682481	1.83697030138006e-07	0.0337782236154932	3.76066493751476e-06
**BRCA**	HDAC2	cg15242771	-0.264020728542654	796	-7.70354682197589	3.95565323298683e-14	0.0697069451001938	2.75740221870052e-12
**BRCA**	HDAC2	cg18387216	-0.223195454701372	111	-7.61481052034565	5.64561921983335e-14	0.0498162109993521	3.84247857907013e-12
**BRCA**	HDAC2	cg18674783	0.0944133893913463	796	2.66895028662277	0.0077645958037898	0.00891388809636199	0.0351287563803445
**BRCA**	HDAC2	cg22867643	-0.0812105042013978	796	-2.29304014841661	0.0221069172702894	0.00659514599264526	0.0785244762629652
**BRCA**	HDAC2	cg24023618	0.0493046292825137	796	1.38924487874698	0.165148756011575	0.0024309464686861	0.337083022646914
**BRCA**	HDAC3	cg01947930	0.0641047554547803	796	1.8077860312742	0.0710190668040382	0.00410941967191719	0.186304410148486
**BRCA**	HDAC3	cg01961137	-0.0555123612272108	796	-1.56466904346595	0.11805998156411	0.00308162224902033	0.267718714096611
**BRCA**	HDAC3	cg04284814	-0.10568166507393	111	-3.53440318125002	0.000425585250692828	0.0111686143327984	0.00327124027369657
**BRCA**	HDAC3	cg08520975	-0.104324810658077	796	-2.95206903328038	0.00324976959752253	0.0108836661188435	0.0175643116499307
**BRCA**	HDAC3	cg10414058	0.0225224385503198	111	0.749209287224329	0.453890385716651	0.000507260238252933	0.644201063023447
**BRCA**	HDAC3	cg11064797	0.0967948444531852	111	3.23278975518018	0.00126212325833082	0.00936924191271632	0.00814818396336828
**BRCA**	HDAC3	cg11153694	0.0850807519053425	796	2.40309808920562	0.0164858485666222	0.00723873434477844	0.0627384846586346
**BRCA**	HDAC3	cg12082871	0.0228240973604434	796	0.642494407911742	0.520738157240442	0.000520939420319001	0.700955205723235

**BRCA**	HDAC3	cg14098966	-0.110989823704388	796	-3.14294908594859	0.00173485753424196	0.012318740965931	0.0105885381310858
**BRCA**	HDAC3	cg17940889	0.0458070697813592	796	1.29047982197768	0.197260721745406	0.00209828764195431	0.379531567731493
**BRCA**	HDAC3	cg18671773	-0.0953234865629801	796	-2.69491239357456	0.00718992802013278	0.00908656709052266	0.0331025188138847
**BRCA**	HDAC3	cg19549288	0.0622127498495392	796	1.75422005499956	0.0797795316860083	0.00387042624384134	0.202663560866684
**BRCA**	HDAC3	cg20642138	-0.0100374769707081	796	-0.28249387215424	0.777638653154252	0.000100750943937495	0.877027768034473
**BRCA**	HDAC3	cg23421926	-0.0348878521027535	796	-0.982429257907713	0.326188428988224	0.0012171622243436	0.525688356253442
**BRCA**	HDAC3	cg24385322	0.0873619084587579	111	2.91650901436299	0.00361119889705546	0.0076321030495564	0.0190900793012729
**BRCA**	HDAC6	cg07153944	-0.0959028595534292	796	-2.71144356560353	0.00684423858807868	0.00919735847052477	0.0318331159106178
**BRCA**	HDAC6	cg07674520	-0.184720090133613	781	-5.23918090246074	2.07925451952232e-07	0.03412151169897	4.19159176189996e-06
**BRCA**	HDAC6	cg10783042	-0.092524116794399	111	-3.0902915137479	0.00204956005349737	0.00856071218858359	0.0121295910841974
**BRCA**	HDAC6	cg12510923	-0.0537139275801898	796	-1.51382933174652	0.130468075663117	0.00288518601608987	0.286891104023305
**BRCA**	HDAC6	cg13178935	-0.11169555767127	796	-3.16318534209989	0.00162007255738006	0.0124758976034959	0.0100184359720846
**BRCA**	HDAC6	cg17152375	-0.0746782509794128	795	-2.10618620872989	0.0355026848437868	0.00557684116934417	0.11202627828091
**BRCA**	HDAC6	cg24616736	-0.074204035063067	111	-2.47459456052643	0.0134878588958993	0.00550623881964088	0.0538310329852037
**BRCA**	HDAC6	cg26680189	-0.0972764859871474	796	-2.7506482114604	0.00608348510394982	0.00946271472600769	0.0289951378615778
**BRCA**	HDAC9	cg00332048	-0.136078857028543	796	-3.86555586004831	0.000119917489178539	0.0185174553301946	0.00112593065071363
**BRCA**	HDAC9	cg02301805	-0.119628439633516	796	-3.39099434756773	0.000730902904778736	0.0143109635691499	0.00516813857247382
**BRCA**	HDAC9	cg03260621	-0.132722595188736	796	-3.76848384676672	0.000176432803648015	0.017615287273633	0.00156254707729892
**BRCA**	HDAC9	cg07513622	-0.0349373395990257	794	-0.982581521837192	0.326114259854081	0.00122061769825765	0.525623269611909
**BRCA**	HDAC9	cg08146609	-0.00827596685959645	796	-0.232914328793467	0.815888110346141	6.84916274611387e-05	0.900888659651445
**BRCA**	HDAC9	cg08285151	-0.098732654118505	110	-3.2936600719278	0.00102035753989215	0.00974813698928434	0.00682466636216746
**BRCA**	HDAC9	cg08725366	-0.131046003479384	796	-3.72004200975238	0.00021325270798213	0.0171730550279187	0.00183049554946065
**BRCA**	HDAC9	cg10068437	-0.137156330538941	796	-3.89674783762221	0.00010573513522292	0.0188118590069072	0.0010102627253171
**BRCA**	HDAC9	cg11321459	0.0326733054509468	796	0.919999522399852	0.357853129860815	0.00106754488909087	0.556872131121134
**BRCA**	HDAC9	cg11765288	0.0417670895683446	796	1.17645669717852	0.239765899621105	0.00174448977101012	0.431677391171382
**BRCA**	HDAC9	cg12081743	-0.0218469937804202	111	-0.726729710728812	0.467545329153026	0.00047729113724172	0.656597294781003
**BRCA**	HDAC9	cg16475478	-0.11181204700551	795	-3.16452632819528	0.0016127951231948	0.0125019338555623	0.00998257491915612
**BRCA**	HDAC9	cg18856632	-0.158591008983933	795	-4.51749991177775	7.2115622890465e-06	0.025151108130542	9.79256897737971e-05
**BRCA**	HDAC9	cg19585556	0.068524986822001	789	1.92444650466954	0.0546598029823958	0.00469567381895541	0.153987278670692
**BRCA**	HDAC9	cg20648975	-0.0172348576065604	796	-0.485103939481717	0.627736766610768	0.000297040316718412	0.779998626720517
**BRCA**	HDAC9	cg21433125	0.0227829470078888	796	0.641335430452658	0.521490319806894	0.000519062674364271	0.701469327629716
**BRCA**	HDAC9	cg23074799	-0.0438563124702893	796	-1.23541469047476	0.217042541007474	0.00192337614349165	0.404387724404687
**BRCA**	HDAC9	cg23385718	-0.169462404108145	796	-4.83908414064162	1.56802860384614e-06	0.0287175064061123	2.54555962453269e-05
**BRCA**	HDAC9	cg24350771	-0.0737186290531199	796	-2.08028640957869	0.0378202355048475	0.00543443626947149	0.117508882533114
**BRCA**	HDAC9	cg25620356	-0.0313565791867181	796	-0.882886508376898	0.377565489770826	0.000983235058292921	0.576151203131744
**BRCA**	HDGF	cg00692559	0.040589207808696	796	1.14322366932747	0.253291180708277	0.00164748379053751	0.447332155372874
**BRCA**	HDGF	cg00746981	0.0872651676189468	111	2.91325460027576	0.00364875718137304	0.00761520947956288	0.0192574288432941
**BRCA**	HDGF	cg01910231	-0.0858016309252106	796	-2.42360962140121	0.0155901778034136	0.00736191986942605	0.0601522003261992
**BRCA**	HDGF	cg02914429	0.00333648602034734	796	0.0938975623154168	0.925214278867403	1.11321389639732e-05	0.960618337451796
**BRCA**	HDGF	cg04402095	-0.122933736963185	796	-3.48610454088333	0.000517084024561447	0.0151127036837335	0.00385787231291198
**BRCA**	HDGF	cg05762852	0.0463106382243154	796	1.30469671054252	0.192375115019639	0.00214467521274342	0.373021677413703
**BRCA**	HDGF	cg05967123	-0.0123713819991481	796	-0.348188196909181	0.727791377437117	0.000153051092568846	0.84605203849885
**BRCA**	HDGF	cg06106793	-0.00206986400173433	111	-0.0688367416971145	0.94513201464839	4.28433698567566e-06	0.970660610808957
**BRCA**	HDGF	cg06119628	-0.0850353521452471	796	-2.40180643465462	0.0165437370440304	0.00723101111446617	0.0629007311022508
**BRCA**	HDGF	cg07313589	0.0258322424599792	796	0.727226424191333	0.467302165521827	0.000667304750511152	0.656410109143856
**BRCA**	HDGF	cg07892339	0.0288657428086611	796	0.81269266089396	0.416638507180177	0.000833231107895768	0.612193487439916
**BRCA**	HDGF	cg08597205	0.0848109921211297	796	2.39542345798578	0.0168324385159612	0.00719290438457033	0.0637518365097706
**BRCA**	HDGF	cg08708935	0.0371271722438394	796	1.04557211373729	0.296077631568778	0.00137842691882372	0.49403850693367
**BRCA**	HDGF	cg09063372	0.0384140300562378	796	1.08186514703462	0.279641740591692	0.00147563770516154	0.476715816443662
**BRCA**	HDGF	cg09477492	-0.0250561186907192	796	-0.705363135376397	0.48079167605717	0.000627809083843405	0.667988992323889
**BRCA**	HDGF	cg11588596	0.0767633503104977	796	2.1667053775306	0.0305548453564055	0.00589261195089218	0.100058243797618
**BRCA**	HDGF	cg12220178	-0.116345820541505	796	-3.29664994742846	0.00102204010806846	0.013536349957476	0.0068335701046049
**BRCA**	HDGF	cg12421458	-0.0286519914453799	111	-0.953258559622197	0.340667366885589	0.000820936613786124	0.539947937697495
**BRCA**	HDGF	cg13170934	0.014335660057443	796	0.403482697392185	0.686702067630481	0.000205511149282565	0.819701903658329
**BRCA**	HDGF	cg13235992	0.0286233188959143	796	0.80586178294774	0.420564435141326	0.000819294384617205	0.615729254505125
**BRCA**	HDGF	cg14155849	-0.00642745153016402	796	-0.180888256193951	0.856501564784704	4.13121331726078e-05	0.923665853835985
**BRCA**	HDGF	cg15503799	-0.0514492556288362	111	-1.71329526917127	0.0869384690509172	0.00264702590476133	0.21547110608516

**BRCA**	HDGF	cg17170583	-0.0349794127536704	796	-0.98501072706611	0.324919613277839	0.00122355931659164	0.524295210539839
**BRCA**	HDGF	cg17218029	-0.073775314072686	796	-2.08189477046341	0.0376726263798142	0.00544279696652346	0.117188159268105
**BRCA**	HDGF	cg17971171	0.0263883737362585	796	0.742893364847311	0.457766531784384	0.000696346268444457	0.647905712350528
**BRCA**	HDGF	cg18593717	-0.153108900611522	795	-4.35752345413698	1.48878419349506e-05	0.0234423354464689	0.000185250283987132
**BRCA**	HDGF	cg19599803	-0.0329771502770922	795	-0.927977912918634	0.35370209825768	0.00108749244039792	0.5527168115995
**BRCA**	HDGF	cg20284773	-0.0337582395874766	796	-0.950582879348519	0.342106147098468	0.00113961874004547	0.5412940713767
**BRCA**	HDGF	cg23747104	-0.0351368459535785	796	-0.98944947084319	0.322745464067205	0.00123459794356551	0.52212990271059
**BRCA**	HDGF	cg26431298	-0.0564591473768853	796	-1.5914397371756	0.111909679844086	0.00318763532252485	0.258036741865225
**BRCA**	HDGFL1	cg02685945	-0.013093966908985	796	-0.368528486312702	0.712577692250114	0.000171451969413593	0.836491157249214
**BRCA**	HDGFL1	cg03930485	0.00190272331200831	796	0.0535474773859166	0.957309206888242	3.62035600205988e-06	0.977560827701368
**BRCA**	HDGFL1	cg05276066	-0.0393831453655487	796	-1.10920049077822	0.267680387898574	0.00155103213888394	0.463392409457066
**BRCA**	HDGFL1	cg06566039	-0.0101316892147436	796	-0.285145644235175	0.775607110939925	0.000102651126344151	0.875770860814279
**BRCA**	HDGFL1	cg08671671	-0.039286260899278	796	-1.10646758004753	0.268860150155307	0.00154341029544614	0.464731514046615
**BRCA**	HDGFL1	cg09081892	-0.0467418851542585	796	-1.31687258936447	0.188262236874998	0.00218480382777389	0.367759955189173
**BRCA**	HDGFL1	cg11894257	-0.0215940856502615	796	-0.607853176789573	0.543459111232548	0.000466304535070828	0.717791988573207
**BRCA**	HDGFL1	cg15317945	-0.0442761530407412	796	-1.24726455061824	0.212669201585233	0.00196037772808713	0.39894500355338
**BRCA**	HDGFL1	cg20209681	-0.10620062000872	796	-3.00574871208127	0.00273291411933904	0.0112785716902365	0.0152919744659326
**BRCA**	HDGFL1	cg21898884	0.0114984072871163	795	0.323410891261	0.746469564231056	0.000132213370140409	0.857615389883488
**BRCA**	HIST1H1I	cg00667898	-0.103587436467325	796	-2.93097649134301	0.00347620454153752	0.0107303569938721	0.0185440482825461
**BRCA**	HIST1H1I	cg00700007	-0.116412670959775	796	-3.29857016406036	0.00101517026511399	0.0135519099599889	0.00679873929100185
**BRCA**	HIST1H1I	cg03821311	-0.184703408903501	111	-6.25014139107607	5.84379675545398e-10	0.034115349260574	2.01423271951168e-08
**BRCA**	HIST1H1I	cg04676561	-0.1154903863382	111	-3.86668864427625	0.000116752992762395	0.0133380293365466	0.00110039766403656
**BRCA**	HIST1H1I	cg07138603	-0.111375604878646	796	-3.15401039882136	0.00167121851982409	0.0124045253620843	0.0102734708440296
**BRCA**	HIST1H1I	cg11206041	-0.109677524890773	796	-3.10533292833238	0.0019683525543727	0.0120291594661662	0.0117391918594656
**BRCA**	HIST1H1I	cg14334286	-0.110605640265276	796	-3.13193501371988	0.00180043636883284	0.0122336076584917	0.0109091943980826
**BRCA**	HIST1H1I	cg18798248	-0.132212466771581	796	-3.75374121977078	0.000186950872180721	0.0174801363698264	0.00163946073164547
**BRCA**	HIST1H1I	cg23460835	-0.107999674669165	796	-3.05726240060734	0.00230877667309363	0.0116639297286454	0.0133558129466572
**BRCA**	HIST1H1(	cg00903962	-0.0530620273592877	796	-1.49540451476962	0.135207240178297	0.0028155787474778	0.294021133852252
**BRCA**	HIST1H1(	cg04290376	0.00277849061514847	796	0.0781939588485418	0.937693517976656	7.72001009846814e-06	0.96664874103142
**BRCA**	HIST1H1(	cg07750111	-0.0209623668589506	111	-0.697289813393426	0.485767984783391	0.00043942082432923	0.672357167658378
**BRCA**	HIST1H1(	cg08283682	-0.00971404759532863	796	-0.273390430833566	0.784624383225956	9.436272068431e-05	0.881048921314666
**BRCA**	HIST1H1(	cg10717387	0.0256393196902313	796	0.721791697685093	0.470635591433434	0.000657374714177882	0.659140625787142
**BRCA**	HIST1H1(	cg12633985	-0.0134176450511254	796	-0.37763999824366	0.705799225073898	0.00018003319871799	0.832223434981621
**BRCA**	HIST1H1(	cg15022984	0.0332964968321293	796	0.937566344663001	0.34875312602524	0.001108656701292	0.547899767478644
**BRCA**	HIST1H1(	cg16481032	0.00313871768199321	796	0.0883317803910337	0.929635310326674	9.85154868725682e-06	0.962701330256825
**BRCA**	HIST1H1(	cg16482183	0.0223572909796269	796	0.629347248533373	0.529303234437428	0.000499848459947706	0.707501990031362
**BRCA**	HIST1H1(	cg17691542	0.013483331651373	796	0.379489084805034	0.704426435169834	0.000181800232420916	0.831312956577701
**BRCA**	HIST1H1(	cg20855557	-0.00385425799034957	796	-0.108469240196963	0.913650953000786	1.48553046561735e-05	0.954410088759573
**BRCA**	HIST1H1(	cg21453677	-0.0647021128359652	315	-1.14526694224231	0.252976325806126	0.00418636340543797	0.446989195279407
**BRCA**	HIST1H1(	cg23407813	0.0109630397875656	796	0.308545829918516	0.757748170363044	0.000120188241383746	0.86495276976791
**BRCA**	HIST1H3I	cg00958605	-0.0171561844837595	795	-0.482583948794829	0.629524698020355	0.000294334666040792	0.781407272160917
**BRCA**	HIST1H3I	cg01043409	-0.0473055636618131	796	-1.3327886713332	0.182984448838741	0.00223781635336185	0.360985246562891
**BRCA**	HIST1H3I	cg08041469	-0.0519864576973694	796	-1.46500960371068	0.143315139439015	0.00270259178392038	0.306163120085041
**BRCA**	HIST1H3I	cg08401836	-0.026125033402738	796	-0.735474640337801	0.462268217683364	0.000682517370294174	0.651739710146428
**BRCA**	HIST1H3I	cg10075390	-0.114189238372008	796	-3.23472832355485	0.00126809590021274	0.0130391821599792	0.00817792029023876
**BRCA**	HIST1H3I	cg15479139	-0.0457252260988747	796	-1.28816928110783	0.198063255460564	0.00209079630179321	0.380616888871575
**BRCA**	HIST1H3I	cg15797414	-0.0482075685307213	111	-1.60508494655469	0.108760587942478	0.00232396966364419	0.252912317112698
**BRCA**	HIST1H3I	cg21209604	-0.097739863054186	796	-2.76387701536107	0.0058445491775461	0.00955308082985103	0.0280921136063035
**BRCA**	HIST1H3I	cg23099349	-0.0462727030097285	111	-1.54052190870638	0.123719229742087	0.00214116304382654	0.276505796327164
**BRCA**	HIST1H3I	cg25701114	-0.108490822355913	796	-3.07133106088638	0.00220393734310827	0.0117702585354622	0.0128438910532448
**BRCA**	HLTF	cg00906762	-0.121469157232823	796	-3.4439468116873	0.000603414628770137	0.0147547561588523	0.00439491036705777
**BRCA**	HLTF	cg02059813	-0.0197224589909006	111	-0.656029103630487	0.511941848114511	0.000388975388647757	0.693840827998921
**BRCA**	HLTF	cg02398045	-0.0837973379684702	796	-2.3665898555713	0.0181924986436346	0.00702199385060202	0.0676708061338295
**BRCA**	HLTF	cg02611507	-0.243352787361185	796	-7.06081790479357	3.62312308326772e-12	0.059220579116458	1.86907982188149e-10
**BRCA**	HLTF	cg02710346	-0.112429909738024	796	-3.18424734108543	0.00150807441810555	0.0126404846037002	0.00944142453582034
**BRCA**	HLTF	cg03678609	-0.0776227537336272	796	-2.19110893341001	0.0287345582221157	0.00602529189719133	0.0955983501830931
**BRCA**	HLTF	cg04120413	-0.232723711177931	796	-6.73433103894464	3.16419217380159e-11	0.0541603257444293	1.37057442076125e-09

**BRCA**	HLTF	cg04731810	-0.185434830059382	796	-5.31070455781825	1.41986540303819e-07	0.0343860761991517	2.98049134279419e-06
**BRCA**	HLTF	cg04836786	-0.0833606743415751	110	-2.76936832213894	0.00571129945934427	0.00694900202668213	0.0275862926946621
**BRCA**	HLTF	cg05555455	-0.116318021365819	796	-3.29585145510867	0.00102490949583412	0.0135298820944592	0.00685098894302451
**BRCA**	HLTF	cg07275648	-0.240778565655193	796	-6.98150482087677	6.1829325251789e-12	0.0579743176789719	3.05256450521162e-10
**BRCA**	HLTF	cg09352720	-0.236732136457341	796	-6.85714758236064	1.41465934953708e-11	0.0560421044316572	6.53903799393272e-10
**BRCA**	HLTF	cg09392355	-0.10683998557779	796	-3.02405270110535	0.00257465378291525	0.0114147825182623	0.0145677952133325
**BRCA**	HLTF	cg15438497	-0.107705817710768	796	-3.04884611039445	0.0023736627618097	0.0116005431687451	0.0136525631021144
**BRCA**	HLTF	cg16724124	0.0992801154122346	796	2.80786228064983	0.00510980753085283	0.00985654131626662	0.0252164658116699
**BRCA**	HLTF	cg20117402	-0.106485287897127	796	-3.01389782035444	0.00266138655807634	0.011339116538534	0.0149649371809896
**BRCA**	HLTF	cg21089667	-0.00674316321866507	111	-0.224259634177299	0.822596703954431	4.54702501935575e-05	0.904623805307196
**BRCA**	HLTF	cg21111416	-0.190886174932846	796	-5.47264312648621	5.95213559615384e-08	0.0364375317804931	1.36590099282358e-06
**BRCA**	HLTF	cg21835021	-0.118507697110549	796	-3.35877103151607	0.000820317360791647	0.0140440742744455	0.00568717304744902
**BRCA**	HLTF	cg21926402	-0.0851123593601993	796	-2.40399735204219	0.0164456513547019	0.0072441137158597	0.0626161414197181
**BRCA**	HLTF	cg24041269	-0.0114603749400804	796	-0.322544721633293	0.747125189504934	0.000131340193767222	0.858013832186992
**BRCA**	HLTF	cg24710191	0.156271951623721	766	4.36744337348441	1.43096546515827e-05	0.0244209228642865	0.000178914380257991
**BRCA**	HLTF	cg25951608	-0.192400251930015	796	-5.51771303815406	4.65409721290765e-08	0.0370178569427334	1.09682168609796e-06
**BRCA**	HLTF	cg26151310	-0.0832115077592927	111	-2.7769607766001	0.00557965728957027	0.00692415502357483	0.0270782195374192
**BRCA**	HLTF	cg27554556	-0.0713954692912985	796	-2.01438714609694	0.0443065036514962	0.00509731303532475	0.132065601941243
**BRCA**	IDH1	cg00707300	-0.0141497789328344	796	-0.398249946677481	0.690553283582335	0.000200216243848085	0.822193967469674
**BRCA**	IDH1	cg01046174	-0.0129161174278759	111	-0.42958170474117	0.667583621968868	0.000166826089410679	0.807180607513927
**BRCA**	IDH1	cg02241586	-0.043726454152884	111	-1.45558445273296	0.145791305714139	0.00191200279278426	0.309772180283661
**BRCA**	IDH1	cg03448938	-0.0591700899493721	796	-1.66811661600734	0.0956878521883329	0.00350109954461679	0.230726862757312
**BRCA**	IDH1	cg05268564	-0.0145924827468945	796	-0.410712597317804	0.681394380490649	0.000212940552718414	0.816222095981819
**BRCA**	IDH1	cg07129696	-0.0496038400977589	796	-1.39769640372979	0.162595609965467	0.00246054095244404	0.333242352432598
**BRCA**	IDH1	cg07915542	-0.0219014625451759	111	-0.728213022723072	0.466637479805214	0.000479674061617744	0.655886584561008
**BRCA**	IDH1	cg09406631	-0.00620416783883815	796	-0.174604120084174	0.861435334242556	3.84916985724736e-05	0.926382366492472
**BRCA**	IDH1	cg09615745	-0.0921542355880723	796	-2.60453302623733	0.00937242566045345	0.00849240313682192	0.040511083555357
**BRCA**	IDH1	cg10356455	0.00559167965978521	796	0.157366274596398	0.874996300351651	3.12668814176556e-05	0.933872894088728
**BRCA**	IDH1	cg14772913	-0.00162787510084134	796	-0.0458125268691816	0.963471222786288	2.6499773439392e-06	0.980806871398951
**BRCA**	IDH1	cg15399923	-0.0363748965351148	796	-1.0243582332768	0.305978868547513	0.00132313309794031	0.504489330898527
**BRCA**	IDH1	cg16085695	-0.0566717312637343	796	-1.59745120688661	0.110564039816158	0.00321168512442892	0.255927722966503
**BRCA**	IDH1	cg16863783	-0.0315950763221996	796	-0.889608399634055	0.373946322872062	0.000998248847805617	0.572587573664281
**BRCA**	IDH1	cg24324379	-0.0956821389373416	111	-3.19672732515174	0.00142934086817965	0.00915507171162475	0.00903503237987872
**BRCA**	IDH1	cg24830471	-0.0198766282793223	796	-0.559488435563243	0.575986603395811	0.000395080351754354	0.742683138988177
**BRCA**	IDH1	cg24987806	0.00459391587299697	796	0.129285616693149	0.897164477167085	2.11040630481735e-05	0.945564587556714
**BRCA**	IDH1	cg25795625	-0.0162787229493008	796	-0.458184584778804	0.64694552026991	0.000264996820860093	0.792974094092729
**BRCA**	IDH1	cg26105702	-0.0583534881456279	796	-1.64501585700343	0.10036334002805	0.00340512957876194	0.238849398618732
**BRCA**	ING2	cg02075087	-0.11062206818362	796	-3.13240595304399	0.00179758627206837	0.0122372419692215	0.0108953195519506
**BRCA**	ING2	cg02552216	-0.0780123643380445	795	-2.20078320229079	0.0280396399812285	0.0060859289896118	0.0938967652145706
**BRCA**	ING2	cg10348863	0.0131031218242849	111	0.435802410336072	0.663065137158847	0.00017169180154205	0.804091804834534
**BRCA**	ING2	cg17985608	0.0174087205102875	796	0.48999907829542	0.624270252831025	0.000303063549805304	0.777416684102018
**BRCA**	ING3	cg01382559	-0.0628521003344417	796	-1.77231902837439	0.0767259917280435	0.00395038651645073	0.197075372288157
**BRCA**	ING3	cg01416111	-0.128410414696782	791	-3.63243670670771	0.000298958229241175	0.0164892346025994	0.00243658004039686
**BRCA**	ING3	cg10268125	0.0343720504518023	796	0.967887161055256	0.333396192588934	0.00118143785226124	0.532998158818429
**BRCA**	ING3	cg14303833	-0.105085561399349	796	-2.97383541875355	0.00303028957595968	0.0110429752146164	0.0165966169580546
**BRCA**	ING3	cg16130600	0.143378002511953	796	4.07713966636334	5.01969381410808e-05	0.0205572516043177	0.000531729385314166
**BRCA**	ING3	cg18485114	0.0808975530463563	796	2.28414541188435	0.0226268082203237	0.00654441408888803	0.0799341500700179
**BRCA**	ING3	cg19478983	0.101197566111972	796	2.8626477880179	0.00431204111186323	0.0102409473869869	0.0220473825998218
**BRCA**	ING3	cg19960827	0.0045313627716553	796	0.127525161405535	0.89855713082058	2.05332485683436e-05	0.946352852298873
**BRCA**	ING3	cg20553930	-0.0277631503104987	796	-0.781625600203324	0.434668228119905	0.000770792515163347	0.628077161426951
**BRCA**	ING3	cg24859794	-0.063087541456895	796	-1.7789845346847	0.0756257649036223	0.00398003788707544	0.195047931403123
**BRCA**	ING4	cg00369908	-0.0382234427843239	796	-1.07648971075855	0.282036095841769	0.00146103157828648	0.479149091304286
**BRCA**	ING4	cg00443163	-0.177643643836417	796	-5.08013543617937	4.7071826596246e-07	0.0315572641954799	8.73963169028249e-06
**BRCA**	ING4	cg00591160	-0.242784207653018	795	-7.03883818122225	4.2076304493844e-12	0.0589441714857037	2.14220159148829e-10
**BRCA**	ING4	cg03930474	0.00631249285975313	111	0.209936095800205	0.833756214905142	3.98475661044343e-05	0.910811347482481
**BRCA**	ING4	cg04375284	-0.264096325848573	796	-7.7059179402605	3.88791891055337e-14	0.0697468693267155	2.71749113861266e-12
**BRCA**	ING4	cg08733494	-0.273463119213988	796	-8.0009090855225	4.38185195577758e-15	0.0747820775702441	3.42594764872122e-13

**BRCA**	ING4	cg12392916	-0.161685890071106	796	-4.6109134188309	4.67273378527663e-06	0.0261423270480857	6.70560140364896e-05
**BRCA**	ING4	cg18123790	-0.0124281032015177	796	-0.349784841259496	0.726593167112867	0.000154457749187575	0.845189581661807
**BRCA**	ING4	cg23354319	-0.336568831468054	796	-10.0587238551597	1.75758656868361e-22	0.113278578315771	2.87851977945626e-20
**BRCA**	ING4	cg27116036	-0.177294075067361	796	-5.06981394239985	4.96124798601739e-07	0.0314331890539909	9.15888290992493e-06
**BRCA**	INO80	cg00734724	0.00553130879056486	796	0.155667208908973	0.876335011546974	3.05953769365801e-05	0.934555525602937
**BRCA**	INO80	cg06380007	-0.0209357542646026	111	-0.696404186626292	0.486322067581962	0.000438305806627825	0.672812842123938
**BRCA**	INO80	cg12580230	-0.00335680930941595	796	-0.0944695199763848	0.92476008819108	1.12681687397816e-05	0.960298822675524
**BRCA**	INO80	cg18952654	0.129391789408329	795	3.66995947217541	0.000258864893597968	0.0167422351662893	0.00215519429928942
**BRCA**	INO80	cg21007089	0.0232301097965042	796	0.653929705021385	0.513346915676358	0.00053963800115764	0.695024808495245
**BRCA**	INO80	cg21598047	-0.0391065331815182	796	-1.10139791496907	0.271058119207755	0.00152932093747718	0.467338274982928
**BRCA**	INO80	cg22835709	-0.150986292229094	796	-4.29840847921856	1.9346260629544e-05	0.0227968604410893	0.000232507801547157
**BRCA**	INO80	cg26409855	0.0330122417971247	796	0.929553490696681	0.352885542220633	0.00108980810847183	0.551911128125655
**BRCA**	INO80	cg27441689	-0.17619676893477	796	-5.03742733417285	5.8474084275611e-07	0.0310453013830528	1.06220440449225e-05
**BRCA**	INTS12	cg00093890	-0.0548325517939664	796	-1.54544982091076	0.122636958152641	0.00300660873623801	0.274773754284916
**BRCA**	INTS12	cg01852585	-0.182113338217233	796	-5.21228584122866	2.38171606361224e-07	0.0331652679566243	4.72902550925601e-06
**BRCA**	INTS12	cg03607115	-0.150991111382467	796	-4.29854887580342	1.93342925144205e-05	0.0227983157165125	0.000232439438426708
**BRCA**	INTS12	cg19703969	-0.200744459828491	796	-5.76684177434096	1.15765507292041e-08	0.0402983381518326	3.12703537301286e-07
**BRCA**	INTS12	cg26533745	0.000100620268407787	796	0.00283170537051999	0.997741342099958	1.0124438414455e-08	0.99888411257093
**BRCA**	IWS1	cg02372421	-0.187295267259708	795	-5.36252441365593	1.07806377221607e-07	0.0350795171378856	2.32898897794438e-06
**BRCA**	IWS1	cg02592214	-0.0756846848022137	795	-2.13473357723105	0.0330895379242719	0.00572817151361044	0.106198687546661
**BRCA**	IWS1	cg10180764	-0.114262753469403	796	-3.23683838354365	0.00125887977033155	0.0130559768304095	0.00813129299677619
**BRCA**	IWS1	cg10488833	-0.161944093215677	796	-4.61847493394552	4.50998845924425e-06	0.0262258893274479	6.49842641597421e-05
**BRCA**	IWS1	cg15239145	-0.231183270793827	796	-6.68722951994664	4.29451725057029e-11	0.0534457046949317	1.81470763167227e-09
**BRCA**	IWS1	cg17144508	-0.0882725273115822	796	-2.49394457823286	0.012836123380466	0.00779203907797402	0.0518497005171593
**BRCA**	IWS1	cg18451256	0.0968149289134736	796	2.7374731612145	0.00633017663436534	0.00937313046052095	0.0299179684747155
**BRCA**	IWS1	cg18530950	-0.1460562769522	796	-4.15494442792339	3.6077364038783e-05	0.0213324360371377	0.000400485510564082
**BRCA**	IWS1	cg19311602	-0.00319937676226614	111	-0.106400868356387	0.915283599431771	1.02360116669286e-05	0.955226622089807
**BRCA**	IWS1	cg19409088	-0.123952006564344	796	-3.51542890496666	0.000463992128577336	0.0153640999313272	0.00352222908377686
**BRCA**	IWS1	cg21581038	-0.0387114679992695	796	-1.09025449939031	0.27593264335063	0.00149857775465847	0.472411453967272
**BRCA**	IWS1	cg21679674	-0.165005010815108	796	-4.70818904429364	2.95011135683854e-06	0.027226653594094	4.4476930793391e-05
**BRCA**	IWS1	cg22409775	-0.111959732026998	796	-3.17076153758947	0.00157893111025101	0.0125349815955572	0.00981159571777837
**BRCA**	IWS1	cg23570261	0.0566219479763753	111	1.88607809900873	0.0595462658599689	0.00320604499263935	0.163768019738048
**BRCA**	IWS1	cg23623107	-0.0365717753193951	796	-1.02990996797833	0.30336668441904	0.00133749475001231	0.501870792814829
**BRCA**	JADE1	cg09863040	-0.114751470643949	794	-3.25086711247566	0.00119916456075549	0.0131679000149492	0.00781435817049863
**BRCA**	JADE1	cg12832492	-0.131472865093124	794	-3.73237224125237	0.000203249225680926	0.0172851142557949	0.00175972572064255
**BRCA**	JADE1	cg14617908	0.116425338360402	794	3.29893402858555	0.00101387332487243	0.0135548594123341	0.00679122278587276
**BRCA**	JADE1	cg15389440	0.0156788720865452	794	0.441296817062754	0.659118575869176	0.000245827029906247	0.801655209204945
**BRCA**	JADE1	cg15836763	-0.0571339493664578	794	-1.61052261277203	0.10768226161275	0.00326428817020896	0.251230621998029
**BRCA**	JADE1	cg20039387	0.0770476519462204	794	2.174777845237	0.0299420242252259	0.00593634067042591	0.0985325648298318
**BRCA**	JADE1	cg22922695	-0.0329309763334748	794	-0.927262743341644	0.354072615384449	0.00108444920227588	0.553077849557978
**BRCA**	JADE1	cg26766900	-0.115528705762548	794	-3.27318274077305	0.00110955351016928	0.0133468818551693	0.00731620662570853
**BRCA**	JADE2	cg04590931	-0.0437018477056751	110	-1.45476377523669	0.146018408537575	0.00190985149289002	0.310031256002526
**BRCA**	JADE2	cg04842522	0.00680683646958786	794	0.191565796271577	0.848131437340868	4.63330227237113e-05	0.918947854935243
**BRCA**	JADE2	cg17378563	-0.225832150156456	794	-6.52402010430741	1.22012241796622e-10	0.0510001600442883	4.80598498143109e-09
**BRCA**	JADE2	cg18145011	-0.164470659604909	794	-4.69251745948716	3.17878836955793e-06	0.0270505978708739	4.75833485513545e-05
**BRCA**	JADE2	cg21194015	-0.0299908210692189	794	-0.844396350061052	0.398702944864525	0.000899449348405906	0.595307222233136
**BRCA**	JADE2	cg25669900	-0.0808085998792681	794	-2.28161729816685	0.0227765040955405	0.00653002981444764	0.0803158775671242
**BRCA**	JADE2	cg26133206	-0.0178933362305614	794	-0.503643750147259	0.614651795228935	0.000320171481459921	0.770790848282879
**BRCA**	JADE3	cg10860619	0.0175242228716486	794	0.493251090937974	0.621971917715731	0.000307098387255213	0.776058307672701
**BRCA**	JADE3	cg17843048	0.00993659540936717	110	0.330473478439146	0.741104819515829	9.87359283294566e-05	0.854392899923449
**BRCA**	JADE3	cg19194595	0.0058028521814145	110	0.19298625716448	0.847005162856566	3.36730934393471e-05	0.918277490931263
**BRCA**	JARID2	cg01769037	-0.018333424044035	111	-0.609809443657713	0.54211330534946	0.0003361144371784	0.716840537060714
**BRCA**	JARID2	cg03223185	0.0929199814672881	796	2.62636282264518	0.00879698230715421	0.00863412295588116	0.0386334222424497
**BRCA**	JARID2	cg05197191	-0.143118550208958	796	-4.06960738019007	5.18130725049977e-05	0.020482919413914	0.000547209633784115
**BRCA**	JARID2	cg06047828	-0.0546823448556023	796	-1.54120353933906	0.123666686324583	0.00299015883890701	0.276436015054728
**BRCA**	JARID2	cg14862454	0.029880438484117	796	0.841285728354761	0.40044181560749	0.0008928406040031	0.59694114519927
**BRCA**	JARID2	cg15109221	0.0961879478496435	796	2.71957897176757	0.00667967203243693	0.00925212131152574	0.0312057654390703

**BRCA**	JARID2	cg21397480	0.042915981469503	796	1.208876531252	0.227071056027147	0.00184178146549072	0.41664148189339
**BRCA**	JARID2	cg21901688	-0.160082514296612	787	-4.53797423850322	6.57140522282363e-06	0.0256264113835249	9.04646784470609e-05
**BRCA**	JARID2	cg25338254	-0.16013231263336	796	-4.56543715169775	5.77696264037883e-06	0.0256423575493081	8.08878746662402e-05
**BRCA**	JMJD6	cg02318866	0.0565622015625693	796	1.59435388720514	0.111255753016763	0.00319928264560472	0.256964242180863
**BRCA**	JMJD6	cg04510639	-0.119209069028888	796	-3.37893517329691	0.000763246194019575	0.0142108021387341	0.00535352758250463
**BRCA**	JMJD6	cg07312083	-0.166207979444432	796	-4.74348562815063	2.49154077779081e-06	0.0276250924310006	3.83284404325605e-05
**BRCA**	JMJD6	cg08857478	-0.00111435957098137	796	-0.0313608776349785	0.974989642021508	1.24179725343779e-06	0.986832077714679
**BRCA**	JMJD6	cg10712116	0.0695732940571362	796	1.96272203400688	0.0500281530009143	0.00484044324596075	0.144341880894304
**BRCA**	JMJD6	cg12504415	-0.127996268793946	796	-3.63200884425699	0.000299328811955703	0.0163830448251721	0.00243883384973628
**BRCA**	JMJD6	cg15457598	0.015993440864469	796	0.45015289852801	0.65272338365911	0.000255790150685267	0.797147505072823
**BRCA**	JMJD6	cg21102709	-0.161752551541624	796	-4.61286551610899	4.63018749818862e-06	0.0261638879302256	6.65682510135069e-05
**BRCA**	JMJD6	cg21807925	-0.161260889556692	796	-4.59846933615941	4.95290475681855e-06	0.0260050745006156	7.06595035121846e-05
**BRCA**	JMJD6	cg23630158	-0.202154199977266	796	-5.80905901930701	9.0968628297267e-09	0.0408663205684486	2.50684146998144e-07
**BRCA**	JMJD6	cg25004679	-0.0476310571786236	796	-1.34197991861193	0.179987096196266	0.00226871760795331	0.357046005750009
**BRCA**	JMJD6	cg25908504	-0.120504790051678	796	-3.41620011717821	0.000667376893632739	0.014521404425399	0.00478770915375003
**BRCA**	JMJD6	cg26612088	-0.130267535460921	796	-3.69756076744646	0.000232694370683457	0.0169696307950623	0.00196998866829349
**BRCA**	JMJD8	cg00873473	-0.0839139818153625	796	-2.36990743551328	0.0180312512791853	0.00704155634410898	0.0672253025022977
**BRCA**	JMJD8	cg02851105	-0.0851019615721627	796	-2.40370152405143	0.0164588653883157	0.00724234386342986	0.0626541874152785
**BRCA**	JMJD8	cg03899598	-0.0866624150732955	796	-2.44810697039123	0.0145768874163182	0.00751037418633616	0.0570889851559774
**BRCA**	JMJD8	cg05787952	-0.125438177266229	796	-3.55824826500912	0.000395554350922858	0.0157347363158739	0.0030768678307862
**BRCA**	JMJD8	cg06191810	-0.082950150197645	796	-2.34249710354154	0.019401922603075	0.00688072741781186	0.0711120455587098
**BRCA**	JMJD8	cg06646636	-0.266947767006016	796	-7.79546574428599	2.01852471459577e-14	0.0712611103094981	1.46345863604159e-12
**BRCA**	JMJD8	cg08398290	-0.0898109271987444	796	-2.53775903768709	0.0113470976247019	0.00806600264429817	0.0470532122210699
**BRCA**	JMJD8	cg26750188	0.0763655302354869	796	2.15541055641438	0.0314303882835322	0.00583169420814707	0.102202626071272
**BRCA**	JMJD8	cg27579097	-0.163886774949683	796	-4.67539799983271	3.44804347972023e-06	0.0268588750034082	5.12000562056414e-05
**BRCA**	KAT2B	cg00770008	-0.137653888881688	796	-3.91115649565511	9.97334549949389e-05	0.0189485931242522	0.000961739429969169
**BRCA**	KAT2B	cg04371528	-0.0374397993722483	796	-1.05438859785659	0.292026586427192	0.0014017385770342	0.489769966626385
**BRCA**	KAT2B	cg05072819	-0.0683299395653579	796	-1.92747990013123	0.0542771841981799	0.00466898064100546	0.153214531278828
**BRCA**	KAT2B	cg12062464	0.00506271968353368	111	0.168370891331315	0.866322290617914	2.56311305940394e-05	0.928979022972216
**BRCA**	KAT2B	cg14937543	-0.120437550185041	796	-3.41426586415149	0.000672063362784968	0.0145052034945742	0.00481378300750334
**BRCA**	KAT2B	cg18053505	-0.0429587762261652	111	-1.42998199917358	0.153004606833729	0.00184545645484974	0.320115116693734
**BRCA**	KAT5	cg00582417	-0.235000492588461	796	-6.80404567348128	2.00665094326681e-11	0.0552252315168194	8.9715749126723e-10
**BRCA**	KAT5	cg02561205	-0.099900187911629	796	-2.8255755393905	0.00483833736389288	0.00998004754477878	0.0241479192202914
**BRCA**	KAT5	cg05785768	-0.230785957841752	796	-6.67508965529391	4.64488568304898e-11	0.053262158336935	1.95426845228292e-09
**BRCA**	KAT5	cg07564927	-0.282676088061217	796	-8.29345533119717	4.70608249287296e-16	0.079905770761593	4.15085012982493e-14
**BRCA**	KAT5	cg14726348	-0.120475647134359	796	-3.41536177271619	0.000669404354088767	0.0145143815524426	0.00479915605215939
**BRCA**	KAT5	cg18896539	0.0175982651680846	111	0.585348731406155	0.558432660968307	0.00030969893692622	0.729454195196321
**BRCA**	KAT5	cg20108992	-0.063816402098229	796	-1.79962099549979	0.0723010001343963	0.00407253317676285	0.188738096186545
**BRCA**	KAT5	cg20423942	-0.159706656122244	796	-4.55298344166666	6.12054883805457e-06	0.0255062160097486	8.50101725241238e-05
**BRCA**	KAT5	cg22048492	0.0319235516194085	111	1.06220948773541	0.28837247132831	0.00101911314799704	0.485889710679232
**BRCA**	KAT6A	cg10163397	-0.147405283345872	796	-4.19416874462963	3.0481130034814e-05	0.0217283175582769	0.000345814354663602
**BRCA**	KAT6A	cg12468056	0.0703284199187445	111	2.3446883070481	0.0192191177645186	0.00494608664826726	0.0705582246137893
**BRCA**	KAT6A	cg15251374	-0.333577051898907	792	-9.93288131747404	5.50392236961197e-22	0.111273649553566	8.68503320124937e-20
**BRCA**	KAT6A	cg20966972	0.0869811831074605	111	2.90370168736447	0.00376106830453286	0.00756572621477357	0.0197561477165988
**BRCA**	KAT6B	cg04396765	-0.0718294747428975	796	-2.02669570478109	0.0430281336976817	0.00515947344184054	0.129140842317803
**BRCA**	KAT6B	cg06972998	-0.0155634416427339	796	-0.438047127006315	0.661471564569971	0.000242220715766784	0.803050694173131
**BRCA**	KAT6B	cg09631472	-0.0143949259204448	796	-0.405151103035729	0.685475854671661	0.000207213892255094	0.819025152257402
**BRCA**	KAT6B	cg10359932	0.0261976597944462	796	0.737520628273691	0.461024245283521	0.000686317378705544	0.650669813816876
**BRCA**	KAT6B	cg10739686	-0.109268427287679	796	-3.09360979273658	0.00204682671168044	0.0119395892019227	0.0121180241406749
**BRCA**	KAT6B	cg11128436	0.0131846858056404	796	0.37108220366022	0.710675543030765	0.000173835939793457	0.835228697816013
**BRCA**	KAT6B	cg11348552	0.0037752655332665	796	0.106246146874708	0.915413962567873	1.425262984667e-05	0.955263066407437
**BRCA**	KAT6B	cg15874302	-0.327657170819159	796	-9.7598671553944	2.51517464080598e-21	0.107359221589216	3.73407435127596e-19
**BRCA**	KAT6B	cg15912797	0.0654766216562655	796	1.84663816293599	0.0651724926385156	0.00428718798351774	0.174952628671689
**BRCA**	KAT6B	cg17147776	-0.0079940556879268	796	-0.22497985747681	0.822052977691192	6.39049263416749e-05	0.904447782348404
**BRCA**	KAT6B	cg18462764	-0.0727126652131645	796	-2.05174690142162	0.0405225894390722	0.00528713168240174	0.123536888581518
**BRCA**	KAT6B	cg18491775	0.0358764570731556	796	1.01030339827051	0.312658505462409	0.00128712017212198	0.511642355373109
**BRCA**	KAT6B	cg24671245	-0.0036065830097159	796	-0.101498902849939	0.919180118293361	1.30074410059714e-05	0.957406991723557

**BRCA**	KAT6B	cg26605858	0.00868423438776743	796	0.244405235203751	0.806980289887953	7.54159269016823e-05	0.895450229239794
**BRCA**	KDM1A	cg00062312	0.0350948101153239	796	0.988264285954284	0.323325049295207	0.00123164569703064	0.522752896945166
**BRCA**	KDM1A	cg00541565	0.00177658198699736	796	0.0499975278048349	0.960136964350894	3.1562435565235e-06	0.979116679400066
**BRCA**	KDM1A	cg04886391	-0.112296516708736	796	-3.18042109532624	0.00152787398597667	0.0126105076649155	0.00954387577204668
**BRCA**	KDM1A	cg06958034	-0.00221941782695961	111	-0.0738104245237127	0.941174578660963	4.92581549062611e-06	0.968642313228964
**BRCA**	KDM1A	cg07118078	-0.0120031090106505	796	-0.337821766671537	0.735587025609099	0.000144074625921559	0.850956281621994
**BRCA**	KDM1A	cg07785945	0.118657100110605	796	3.36306587518536	0.000807838936915262	0.0140795074066581	0.00561517353988437
**BRCA**	KDM1A	cg10499166	-0.00022718659490938	796	-0.0063935976761003	0.994900291935516	5.16137489065199e-08	0.997348081450852
**BRCA**	KDM1A	cg12578844	-0.0506189589886578	796	-1.42637233192783	0.154154952606003	0.00256227900909542	0.321596416815
**BRCA**	KDM1A	cg22683154	-0.116812020124111	796	-3.31004204824639	0.000975012206386905	0.0136450480454757	0.00657564799164828
**BRCA**	KDM1A	cg26662347	0.101428165914379	796	2.86923865685738	0.00422411281976397	0.0102876728407548	0.0216876077547992
**BRCA**	KDM1B	cg11007366	-0.0800897652294121	796	-2.26118950259685	0.0240180332365987	0.00641437049450235	0.0836149144190441
**BRCA**	KDM1B	cg15124342	-0.226117618710075	794	-6.52445744150571	1.21847084160621e-10	0.051129177491115	4.80191087395713e-09
**BRCA**	KDM1B	cg20677365	-0.205323467013109	769	-5.80259562606113	9.55991271277912e-09	0.0421577261062831	2.62329400203858e-07
**BRCA**	KDM1B	cg22406543	0.153184513064959	796	4.36248206542814	1.45597941085336e-05	0.0234654950429487	0.00018169146982343
**BRCA**	KDM1B	cg23073439	-0.245769109512939	796	-7.13541028557329	2.1815577766753e-12	0.0604024551907829	1.18019544978219e-10
**BRCA**	KDM1B	cg23860886	-0.0737845046369483	795	-2.08084063489927	0.0377697212238126	0.00544415312451984	0.117370683585734
**BRCA**	KDM1B	cg24342628	0.0800972650726934	111	2.67234707797039	0.00764302675336949	0.00641557187212531	0.0347059084316849
**BRCA**	KDM2A	cg05038268	-0.215376493192568	796	-6.2069010000473	8.70994228459253e-10	0.0463870338199283	2.91182316324706e-08
**BRCA**	KDM2A	cg05295919	-0.163135670574736	796	-4.65338309181381	3.82662687970265e-06	0.0266132470138686	5.61147241243332e-05
**BRCA**	KDM2A	cg06320126	-0.164862036312081	796	-4.70399543564943	3.00970453701332e-06	0.0271794910169658	4.52788541389313e-05
**BRCA**	KDM2A	cg07283932	-0.30790365485935	796	-9.10764890131029	6.74381284332049e-19	0.0948046606757455	8.04644442228948e-17
**BRCA**	KDM2A	cg08836144	-0.1952181905664	776	-5.53052373388308	4.37211762335809e-08	0.0381101419280192	1.0366489435889e-06
**BRCA**	KDM2A	cg09993857	-0.101350737513507	795	-2.86521502888183	0.00427773632355328	0.0102719719945319	0.0219195244074894
**BRCA**	KDM2A	cg11637544	0.254500760017149	796	7.40615176924492	3.33245360624568e-13	0.0647706368493065	2.02376967872191e-11
**BRCA**	KDM2A	cg16004548	-0.0392171933257388	796	-1.10451934550669	0.269703358465107	0.00153798825234837	0.465764713930663
**BRCA**	KDM2A	cg18132081	0.125458406920534	796	3.55883128640666	0.000394691343795665	0.0157398118670384	0.00307230522305783
**BRCA**	KDM2A	cg21293464	-0.222774357990806	796	-6.43103791440391	2.19013043347737e-10	0.0496284145782157	8.18334721241209e-09
**BRCA**	KDM2A	cg21711868	-0.28527330565203	796	-8.3763724584075	2.47095146141825e-16	0.0813808589176363	2.26948285701973e-14
**BRCA**	KDM2A	cg25597833	-0.201106855717563	796	-5.77769080403573	1.08827751407496e-08	0.0404439674166047	2.95812232459635e-07
**BRCA**	KDM2B	cg03486832	-0.168914720156134	796	-4.82298465177281	1.69616383044046e-06	0.0285321826854251	2.7307816437352e-05
**BRCA**	KDM2B	cg05067991	-0.0996299596342459	796	-2.81785567421549	0.00495500148861697	0.00992612885672146	0.024600764937486
**BRCA**	KDM2B	cg07020001	0.0695709675506023	796	1.96265608205027	0.0500358358331649	0.00484011952592696	0.144358693547146
**BRCA**	KDM2B	cg07529210	-0.0430757653196707	796	-1.21338574552536	0.225344117597184	0.00185552155787535	0.414744494283035
**BRCA**	KDM2B	cg07793808	0.0645182438796888	796	1.81949519522148	0.069213336348321	0.004162603793319	0.182831317075765
**BRCA**	KDM2B	cg09151742	0.091925170178541	796	2.59800376302142	0.00955095827188735	0.00845023691235372	0.0410878870988868
**BRCA**	KDM2B	cg12251659	-0.0361359946076627	796	-1.01762165899155	0.309168544769417	0.00130581010628502	0.507795327733204
**BRCA**	KDM2B	cg12888660	-0.0379536810105851	796	-1.06888139092118	0.285448783702464	0.00144048190225325	0.482670086711794
**BRCA**	KDM2B	cg15234492	-0.0214354095942769	796	-0.603384534395327	0.546425665106504	0.000459476784474417	0.720021650314972
**BRCA**	KDM2B	cg15316703	-0.0569604740390565	795	-1.60460268793797	0.108980428446271	0.00324449560275402	0.253226506886177
**BRCA**	KDM2B	cg16837338	0.0224414028005555	787	0.628116596089722	0.530110716526323	0.00050361655965678	0.708082503415958
**BRCA**	KDM2B	cg17452615	-0.0227855014744075	796	-0.641407375527775	0.521443612010261	0.000519179077440227	0.701467850952398
**BRCA**	KDM2B	cg20055546	-0.0485950109180363	796	-1.36920245465409	0.171324141531148	0.00236147508612407	0.345451985924756
**BRCA**	KDM2B	cg21249371	-0.0492276213171443	796	-1.38706976832354	0.165810707764766	0.00242335870054416	0.338008442949851
**BRCA**	KDM2B	cg21781916	0.021463509840938	796	0.604175891501913	0.545899729452751	0.000460682254692043	0.7195967797385
**BRCA**	KDM2B	cg26218726	-0.0286280732530474	796	-0.805995747045148	0.420487232980218	0.000819566578181845	0.615714046020382
**BRCA**	KDM2B	cg26509318	-0.300482885292131	796	-8.86606352556412	4.95286354170956e-18	0.0902899643534838	5.48084020432081e-16
**BRCA**	KDM3A	cg01744130	-0.140808118431376	796	-4.00256968779846	6.85414861097958e-05	0.0198269262161844	0.000697057204259253
**BRCA**	KDM3A	cg02152982	-0.130395231786986	794	-3.69657176226118	0.000233625767719339	0.0170029164727818	0.00197679823514882
**BRCA**	KDM3A	cg02972551	0.0434752552412559	111	1.44720655774977	0.148122448108534	0.00189009781829235	0.31309199348319
**BRCA**	KDM3A	cg03037033	-0.15252343633253	796	-4.34320612294926	1.5865664967578e-05	0.0232633986306835	0.000195618928983091
**BRCA**	KDM3A	cg04793821	-0.123306965832839	796	-3.49685156929873	0.000497000177598499	0.0152046078229009	0.00373057041838071
**BRCA**	KDM3A	cg07052513	0.0182453451325782	796	0.513555012571188	0.607706372374269	0.000332892619006894	0.765614172415487
**BRCA**	KDM3A	cg07211384	-0.23488198542982	792	-6.78321959347473	2.30762896949656e-11	0.0551695470794542	1.02290420543029e-09
**BRCA**	KDM3A	cg12888358	-0.0672069978063373	796	-1.89565855211775	0.0583690198423473	0.00451678055414102	0.161386107819717
**BRCA**	KDM3A	cg18611949	-0.117814130785481	796	-3.33883631149121	0.000880617278205927	0.0138801694127385	0.00603798270975448
**BRCA**	KDM3A	cg19139691	-0.178202021949794	796	-5.0966264311323	4.32714093445912e-07	0.031755960626995	8.10169920229393e-06

**BRCA**	KDM3A	cg20191795	-0.0177814394139173	796	-0.500493191076931	0.616866894384554	0.000316179587630813	0.772409133268988
**BRCA**	KDM3A	cg20918903	-0.112261304949011	111	-3.75717704425542	0.00018080737892795	0.0126026005888548	0.00159367815307649
**BRCA**	KDM3A	cg22404242	0.0332121079517729	796	0.935187486159608	0.349976731872912	0.00110304411460022	0.549005338045433
**BRCA**	KDM3A	cg24749656	-0.0875347393079064	796	-2.47293837892659	0.0136095922409217	0.00766233058570313	0.0541945443688711
**BRCA**	KDM3B	cg00435490	0.0859074092423717	796	2.42661970671869	0.0154624127482028	0.00738008296273634	0.0597765212609299
**BRCA**	KDM3B	cg03315484	-0.199766689464302	795	-5.73395924268355	1.39588171901385e-08	0.0399067302195267	3.69483574171363e-07
**BRCA**	KDM3B	cg05191700	-0.153464690694923	796	-4.37065340592356	1.40377875990897e-05	0.0235514112900883	0.000176059672202208
**BRCA**	KDM3B	cg05708550	0.106391210543386	796	3.01120461135259	0.00268483388799765	0.0113190896808872	0.0150728128370419
**BRCA**	KDM3B	cg05722427	0.265323578135127	796	7.74443225062706	2.93500959570843e-14	0.0703966011144266	2.07758085976835e-12
**BRCA**	KDM3B	cg15001364	-0.0166544509677233	796	-0.468762810793088	0.639368181105333	0.000277370737036298	0.788094501622618
**BRCA**	KDM3B	cg18682923	0.202270349511296	796	5.81253900374459	8.91728604698057e-09	0.040913294291422	2.46346360347588e-07
**BRCA**	KDM3B	cg19076659	0.179910368044962	796	5.14711194013577	3.33914133385185e-07	0.0323677405300738	6.41237128920442e-06
**BRCA**	KDM3B	cg24137987	-0.0507498456191487	796	-1.43007004770849	0.153091281731983	0.00257554683036743	0.32021033987734
**BRCA**	KDM4A	cg00154982	-0.0778088019099935	796	-2.19639258874782	0.0283530044072387	0.0060542096546686	0.0946690254885071
**BRCA**	KDM4A	cg00979347	0.117453246208248	796	3.32846560562506	0.000913591299077687	0.0137952650448553	0.00622400573784478
**BRCA**	KDM4A	cg03587511	0.129577912644184	796	3.67765108250253	0.00025129204452669	0.0167904354452238	0.00210159248676729
**BRCA**	KDM4A	cg08552254	-0.116953538700169	111	-3.91635091691797	9.54038909307782e-05	0.0136781302144919	0.000925995045672983
**BRCA**	KDM4A	cg10958452	-0.243098066661682	795	-7.0485087614645	3.94150232434245e-12	0.0590966700146476	2.02189316850921e-10
**BRCA**	KDM4A	cg10968649	0.0318537643345298	111	1.05988505680814	0.289428298838392	0.00101466230227976	0.486922538003804
**BRCA**	KDM4A	cg12293275	0.125201053412282	796	3.55141465918038	0.000405802240050742	0.015675303775545	0.00314056699786186
**BRCA**	KDM4A	cg17737314	-0.167783387564091	795	-4.78671823025479	2.02323457244244e-06	0.028151265142482	3.19245310055063e-05
**BRCA**	KDM4A	cg19743044	-0.0122657758120696	796	-0.345215498736688	0.730024029797438	0.000150449256271951	0.847381220144164
**BRCA**	KDM4A	cg20873046	-0.0609726582391521	796	-1.71912123674874	0.0859830389872175	0.00371766505274844	0.213821070069098
**BRCA**	KDM4A	cg21451504	-0.194158549029947	796	-5.57010409067885	3.48888650212335e-08	0.0376975421614142	8.45001359110163e-07
**BRCA**	KDM4A	cg22094309	-0.0936685112861446	796	-2.64770636878994	0.00826514475935437	0.00877379000656259	0.036849026959093
**BRCA**	KDM4A	cg24324755	0.0376679594221597	796	1.06082319807731	0.289093642416114	0.00141887516702947	0.486654205918662
**BRCA**	KDM4A	cg24505556	-0.0846622959850213	796	-2.39119329894625	0.0170262008844599	0.00716770436145536	0.0643197334825268
**BRCA**	KDM4A	cg25841161	0.139360549298678	796	3.96060221924691	8.14961923800286e-05	0.0194213627008294	0.000806779036441825
**BRCA**	KDM4B	cg01098771	-0.0309249372254054	796	-0.870721335128334	0.384170163706322	0.000956351742395266	0.582337322253456
**BRCA**	KDM4B	cg12072803	0.0427804670766794	111	1.42403566218569	0.154718211019083	0.00183016836329885	0.32236517417089
**BRCA**	KDM4B	cg18115693	0.141439969505634	796	4.02089630850078	6.35195441762393e-05	0.0200052649737547	0.00065432195705063
**BRCA**	KDM4C	cg00121158	0.0785073859926876	111	2.6189703973755	0.00894019058339616	0.00616340965540484	0.0391452235180234
**BRCA**	KDM4C	cg00667760	0.175836842381281	796	5.02680845537392	6.1699005476673e-07	0.0309185951386196	1.11493900860681e-05
**BRCA**	KDM4C	cg05545111	0.0907816543822879	795	2.56379518262718	0.0105371513857838	0.00824130877238516	0.0443911596937975
**BRCA**	KDM4C	cg14196303	-0.0683780520563295	796	-1.92884345078857	0.0541073462646389	0.0046755580030181	0.15286823661926
**BRCA**	KDM4C	cg14473811	0.137804147699789	796	3.91550839309238	9.79850598790814e-05	0.0189899831232653	0.00094750152246529
**BRCA**	KDM4C	cg14497348	0.0468179430809899	796	1.31902009457016	0.187543626342605	0.00219191979433481	0.366862854678183
**BRCA**	KDM4E	cg15555590	0.14496733707585	796	4.12329920850647	4.12913943440082e-05	0.021015528818863	0.000450332641468915
**BRCA**	KDM4E	cg19409026	0.190576024376331	796	5.46341581162224	6.25823715029217e-08	0.0363192210670879	1.42772228994085e-06
**BRCA**	KDM5B	cg00528052	-0.120844733109874	111	-4.04855246413822	5.51252471690249e-05	0.0146034495203966	0.000577797558376061
**BRCA**	KDM5B	cg00571448	0.0793325231080692	796	2.23967408567574	0.025388831947136	0.00629364922269233	0.087196979934462
**BRCA**	KDM5B	cg02624558	0.00373298015825576	795	0.104989761532996	0.916410549291481	1.39351408619312e-05	0.955902953493986
**BRCA**	KDM5B	cg04504182	-0.149875895660639	796	-4.26606759067447	2.22998263960417e-05	0.0224627841000789	0.000263406118228617
**BRCA**	KDM5B	cg08915090	0.030349563750021	796	0.854506064432653	0.393083045036045	0.000921096019816588	0.589918584595426
**BRCA**	KDM5B	cg09889216	-0.175892399782622	796	-5.02844742419527	6.11902818343111e-07	0.0309381363012897	1.10651715132924e-05
**BRCA**	KDM5B	cg11171004	0.195141417111574	796	5.59941436327802	2.96636235282828e-08	0.0380801726723132	7.34453191839348e-07
**BRCA**	KDM5B	cg11221497	-0.20648545216512	796	-5.9390031886341	4.29061188466384e-09	0.0426362419558339	1.25671634395911e-07
**BRCA**	KDM5B	cg13134514	-0.121716388690436	796	-3.45106172593362	0.000587959793660846	0.0148148792758414	0.00430046485408536
**BRCA**	KDM5B	cg13253785	-0.00455462174832221	796	-0.12817974729455	0.898039266945514	2.07445792702896e-05	0.946027651130161
**BRCA**	KDM5B	cg13993945	-0.0388156007112966	796	-1.09319167133137	0.274642044048439	0.00150665085857881	0.47107271909832
**BRCA**	KDM5B	cg14560895	-0.0382739201962213	111	-1.27379295082367	0.203004419397931	0.00146489296718671	0.386815729851389
**BRCA**	KDM5B	cg21105875	0.0502515596720934	780	1.40161728339633	0.161429394332094	0.00252521924947796	0.331693254342752
**BRCA**	KDM5B	cg24354901	0.154841314606052	796	4.41081817007607	1.17215219909403e-05	0.0239758327089305	0.000150522298079104
**BRCA**	KDM5B	cg27531483	-0.239672555867248	796	-6.94747625717631	7.76424240270156e-12	0.0574429340359393	3.77271376312158e-10
**BRCA**	KDM5C	cg01859586	-0.0625797049156806	796	-1.76460769014809	0.078015121612871	0.00391621946733366	0.199294268898029
**BRCA**	KDM5C	cg04927657	-0.0832668784833708	796	-2.35150378041477	0.0189418003746809	0.00693337305236444	0.0697938908894013
**BRCA**	KDM5C	cg04927982	-0.128638711661037	111	-4.31392552631618	1.74714942393903e-05	0.0165479181378115	0.000212937086457642

**BRCA**	KDM5C	cg09554224	-0.0689793169454726	796	-1.94588497436658	0.0520219518650788	0.00475814616626396	0.148518220612014
**BRCA**	KDM5C	cg12234996	-0.132519761499343	796	-3.76262162227664	0.000180546600609852	0.0175614871878428	0.00159227323975092
**BRCA**	KDM5C	cg16417926	-0.119801826273791	796	-3.39598068940728	0.000717905838822187	0.0143524775785355	0.00509197363469486
**BRCA**	KDM5C	cg16824069	-0.131658876677561	796	-3.73774599177671	0.000199029175558869	0.0173340598079972	0.0017297816650013
**BRCA**	KDM5C	cg19903753	-0.132728296386385	796	-3.76864862763864	0.000176318455323689	0.017616800661632	0.00156177553823951
**BRCA**	KDM5C	cg24985300	-0.0817447515556828	796	-2.30822617197487	0.0212433414417135	0.0066822044069003	0.0762457912104144
**BRCA**	KDM5C	cg27526317	-0.121095140788077	796	-3.43318447037799	0.000627513640413627	0.0146640331224842	0.0045444792536155
**BRCA**	KDM6A	cg00751785	-0.0556204968844874	796	-1.56772639964922	0.117344424784563	0.00309363967367727	0.266663517123545
**BRCA**	KDM6A	cg03267667	0.0142935510035476	796	0.402297279238082	0.687573806123615	0.000204305600291016	0.820259418395653
**BRCA**	KDM6A	cg03478540	0.0302567486721708	796	0.851890414580244	0.394532430555295	0.000915470840210907	0.591270413658877
**BRCA**	KDM6A	cg06877198	0.0251551981333562	796	0.708154116347507	0.479057910670945	0.000632783993128407	0.666472890710245
**BRCA**	KDM6A	cg07167981	-0.0308776625954702	796	-0.869389001218708	0.384897788537903	0.000953430047359699	0.583042654441888
**BRCA**	KDM6A	cg11895950	-0.013980807643014	796	-0.393493261585736	0.694061103974464	0.000195462982350958	0.824597039090234
**BRCA**	KDM6A	cg14384228	0.0211055138654321	796	0.594094141313603	0.552618800225941	0.000445442715523945	0.724865731512584
**BRCA**	KDM6A	cg15923127	0.00987685308289519	796	0.277972842910424	0.781105754460254	9.75522268210962e-05	0.879089717158936
**BRCA**	KDM6A	cg17824914	-0.08418755242743	796	-2.37768870579181	0.017657962794629	0.00708754398372127	0.0661665409973205
**BRCA**	KDM6A	cg20211377	-0.00855566914490769	796	-0.240786685249354	0.809782766127341	7.31994745171255e-05	0.897228970946709
**BRCA**	KDM6A	cg22133177	0.0396531658738396	796	1.11681737732741	0.264411109535508	0.00157237356381824	0.459984745632749
**BRCA**	KDM6A	cg27306636	-0.00831464027067262	795	-0.233855031697027	0.815158034781538	6.91332428306909e-05	0.900551269806447
**BRCA**	KDM6B	cg01046511	-0.115222268884208	796	-3.26438373650841	0.00114411921492991	0.0132761712468247	0.00750912091506435
**BRCA**	KDM6B	cg02308232	-0.109191696612126	796	-3.09141116937457	0.00206186064523608	0.0119228266090346	0.0121921850893505
**BRCA**	KDM6B	cg24893073	-0.104213310843002	796	-2.94887927319627	0.00328312533621151	0.0108604141568602	0.0176936233048312
**BRCA**	KDM8	cg00636124	-0.132118221323011	796	-3.75101787448351	0.000188957338224071	0.0174552244055561	0.00165463160398507
**BRCA**	KDM8	cg01755539	-0.0915069400566685	796	-2.5860836353459	0.0098847475904386	0.00837352007853473	0.0422362919035213
**BRCA**	KDM8	cg06329197	-0.0956445360861909	796	-2.70407251411731	0.00699648546897845	0.00914787728314267	0.0323766932706983
**BRCA**	KDM8	cg08572679	-0.0895201407147656	796	-2.52947588796463	0.0116162234840906	0.00801385559359143	0.0479185223600052
**BRCA**	KDM8	cg09140090	-0.0818526867749915	796	-2.31129448196442	0.0210724774531097	0.00669986233228488	0.0758145252658635
**BRCA**	KDM8	cg10214171	-0.0218046602873249	796	-0.613783460432054	0.539534673659522	0.000475443210245642	0.714876780315583
**BRCA**	KDM8	cg10221365	-0.0331996740944821	796	-0.934836986851068	0.35015724748089	0.00110221835997983	0.549168935381023
**BRCA**	KDM8	cg16752029	-0.0735720180925977	796	-2.07612661553779	0.0382042931141731	0.00541284184621752	0.118342947301266
**BRCA**	KDM8	cg24206694	-0.0324681166026508	111	-1.0798595382614	0.280440371577871	0.00105417859572333	0.477644759698999
**BRCA**	KDM8	cg24705286	-0.070630768397072	111	-2.35481877189263	0.0187057401873895	0.00498870544436082	0.0691350650662468
**BRCA**	KIAA202	cg03495688	0.220909540041376	796	6.37443027932629	3.11611653739465e-10	0.0488010248812923	1.13225207804801e-08
**BRCA**	KIAA202	cg25608569	-0.103217626578	796	-2.92039994460395	0.00359508083327799	0.0106538784363954	0.0190294426307429
**BRCA**	KMT2B	cg09284703	-0.0626314890437336	796	-1.7660736348089	0.0777687037852839	0.00392270341983532	0.198867124255885
**BRCA**	KMT2B	cg13830730	0.0666390138925181	795	1.87837910908071	0.0606969258215038	0.00444075817256722	0.166034552809806
**BRCA**	KMT2B	cg15496871	0.188159460411573	796	5.39157830999099	9.22393135974054e-08	0.0354039825423743	2.01563628145971e-06
**BRCA**	KMT2B	cg24471867	-0.0288360488048344	111	-0.958953454085922	0.337791963536074	0.000831517710674791	0.537221466039565
**BRCA**	L3MBTL1	cg01071811	-0.402689322838503	111	-14.6241411116756	2.08960444229053e-44	0.162158690728132	9.61885727713311e-42
**BRCA**	L3MBTL1	cg01877937	-0.307884207032745	796	-9.10701340081786	6.77965416764965e-19	0.0947926849401822	8.07682107684743e-17
**BRCA**	L3MBTL1	cg02177317	-0.210565348146562	796	-6.06173967718351	2.08220677708266e-09	0.0443377658400828	6.48711229540922e-08
**BRCA**	L3MBTL1	cg02472486	-0.223150549269775	796	-6.44246639859629	2.03897088717135e-10	0.0497961676394024	7.65539098439229e-09
**BRCA**	L3MBTL1	cg02611863	-0.395168817919576	111	-14.3063802557327	1.00782574288247e-42	0.156158394655955	4.42953648823721e-40
**BRCA**	L3MBTL1	cg03486259	-0.0880975158103608	796	-2.48896130174423	0.0130159994706783	0.00776117229195677	0.0523773109304147
**BRCA**	L3MBTL1	cg04984575	-0.168135226086611	796	-4.80007889563296	1.89597629360665e-06	0.0282694542511958	3.01032037509767e-05
**BRCA**	L3MBTL1	cg06362543	0.102332981963785	796	2.89510410506122	0.00389456732978354	0.0104720391976003	0.0203351883249333
**BRCA**	L3MBTL1	cg06446163	-0.188385650376844	796	-5.39829795736809	8.8969317809023e-08	0.0354891532679064	1.95075510418127e-06
**BRCA**	L3MBTL1	cg06908806	-0.0847660121379629	796	-2.39414383489573	0.0168908460217629	0.00718527681377327	0.0638991332528689
**BRCA**	L3MBTL1	cg07678583	-0.300414032643274	796	-8.86383039584629	5.04399586731008e-18	0.090248591008994	5.57375872871478e-16
**BRCA**	L3MBTL1	cg08369943	-0.0966261612317245	796	-2.73208534134274	0.00643363142299343	0.00933661503437922	0.0302964844382779
**BRCA**	L3MBTL1	cg08633313	-0.301693138447288	796	-8.90534114859543	3.59190073837724e-18	0.0910187497861747	4.03215477693101e-16
**BRCA**	L3MBTL1	cg09541000	-0.182387977567412	796	-5.22041659308477	2.28281165009639e-07	0.0332653743611308	4.55597187953415e-06
**BRCA**	L3MBTL1	cg10360552	-0.314889771228994	796	-9.33676106269052	9.78964847363151e-20	0.0991555680246485	1.27781193516391e-17
**BRCA**	L3MBTL1	cg12699433	-0.194846766435562	796	-5.59062570052467	3.11448386196555e-08	0.0379652623903945	7.64557139658403e-07
**BRCA**	L3MBTL1	cg14306330	-0.199079380514467	795	-5.71341561856555	1.56782686208313e-08	0.039632599746024	4.11315473218129e-07
**BRCA**	L3MBTL1	cg15123011	-0.100043089969711	796	-2.82965821196805	0.00477765105846499	0.0100086198506878	0.0239110001571169
**BRCA**	L3MBTL1	cg15302378	-0.395403793186559	796	-12.114925934792	4.11113830404088e-31	0.156344159666319	1.188928971095e-28

**BRCA**	L3MBTL1	cg15330298	-0.380183123679727	796	-11.5679225050812	1.04836324857285e-28	0.144539207530875	2.70951397207563e-26
**BRCA**	L3MBTL1	cg15388309	-0.357891679627843	796	-10.7864238139495	2.10144497871308e-25	0.128086454346838	4.41837326145959e-23
**BRCA**	L3MBTL1	cg16862791	-0.339362030339915	796	-10.1530162997335	7.49602471900496e-23	0.115166587636429	1.27882839252253e-20
**BRCA**	L3MBTL1	cg17091610	-0.349744607711273	796	-10.506204172985	2.92235225519512e-24	0.122321290623112	5.54491393513778e-22
**BRCA**	L3MBTL1	cg17774347	-0.0998640155294647	796	-2.82454213238117	0.00485380870910933	0.00997282159766916	0.0242016248684306
**BRCA**	L3MBTL1	cg18911945	-0.311480536024361	796	-9.22474709587539	2.52682911030969e-19	0.0970201243220232	3.16032385542496e-17
**BRCA**	L3MBTL1	cg20091959	-0.345355383827166	111	-12.2383391264079	2.17180127645118e-32	0.119270341138409	6.49819648077859e-30
**BRCA**	L3MBTL1	cg20252111	-0.366089964886324	796	-11.0712574805841	1.37445584932722e-26	0.13402186239047	3.10826797507447e-24
**BRCA**	L3MBTL1	cg20529070	-0.315186191609106	796	-9.34651912256633	9.00966734999723e-20	0.0993423353810519	1.17797993584149e-17
**BRCA**	L3MBTL1	cg22330467	-0.165957659741279	793	-4.72716067973088	2.69609238480274e-06	0.0275419448268022	4.12305506159513e-05
**BRCA**	L3MBTL1	cg22457903	-0.427937994736372	796	-13.3249998030728	1.0740503106424e-36	0.183130927338987	3.90442382551939e-34
**BRCA**	L3MBTL1	cg22601123	-0.179731088328445	796	-5.14181156591788	3.43161310975711e-07	0.0323032641117272	6.56402533219682e-06
**BRCA**	L3MBTL1	cg23626798	-0.307874246160317	111	-10.7615648265982	9.35893931590772e-26	0.0947865514487834	2.02241479206035e-23
**BRCA**	L3MBTL1	cg24343973	-0.266567063653872	796	-7.78349730279426	2.20414821614068e-14	0.0710579994250473	1.58474589950507e-12
**BRCA**	L3MBTL2	cg02358112	-0.122124799984599	796	-3.46281655929529	0.000563232605931073	0.0149144667712782	0.00414532803649971
**BRCA**	L3MBTL2	cg06235991	-0.0326420444840283	111	-1.08614150591749	0.277653057929118	0.00106550306809728	0.474427648667584
**BRCA**	L3MBTL2	cg08053598	-0.080809951197094	796	-2.28165570319183	0.0227742236056607	0.00653024821247671	0.0803158775671242
**BRCA**	L3MBTL2	cg09993119	-0.0300221811584507	796	-0.845280094505158	0.398209754410813	0.000901331361510831	0.594800536081713
**BRCA**	L3MBTL2	cg12821546	0.0564289217535138	796	1.5905850314415	0.112102048355325	0.00318422321026418	0.258380740393286
**BRCA**	L3MBTL2	cg13843727	-0.0708491974718322	796	-1.99889630457162	0.0459608923819328	0.00501960878240267	0.135681605265637
**BRCA**	L3MBTL2	cg14679202	-0.0491889888778843	111	-1.6378400833569	0.101739503753485	0.00241955662682863	0.241199578076389
**BRCA**	L3MBTL2	cg15283028	-0.285363798178858	796	-8.37926507171304	2.41580920478555e-16	0.0814324973110639	2.22145935315706e-14
**BRCA**	L3MBTL2	cg19467356	-0.00591431485752937	796	-0.166446484799141	0.867848077208293	3.49791202339927e-05	0.929808198847844
**BRCA**	L3MBTL2	cg24240981	-0.00233197763497912	796	-0.0656278463502554	0.947690680436895	5.43811969004283e-06	0.972174329662575
**BRCA**	L3MBTL2	cg26947834	0.0914810024279677	796	2.58534442314308	0.00990578595893207	0.00836877380522583	0.0423017403444114
**BRCA**	L3MBTL2	cg27177954	-0.0917140560462722	796	-2.59198655002857	0.00971817339748112	0.00841146807645877	0.0416629329485091
**BRCA**	L3MBTL3	cg03454037	0.0644139509994045	796	1.81654174125318	0.069665202197723	0.00414915708335368	0.183702468951156
**BRCA**	L3MBTL3	cg07826275	-0.182285333414977	796	-5.21737764500714	2.31930373099767e-07	0.0332279427782095	4.61688624486266e-06
**BRCA**	L3MBTL3	cg08106148	-0.204800660458978	796	-5.88841404416236	5.758687756676e-09	0.0419433105244335	1.65006024067349e-07
**BRCA**	L3MBTL3	cg08358392	-0.348065282696745	796	-10.448784723073	4.98025451277627e-24	0.121149441018765	9.13759244261597e-22
**BRCA**	L3MBTL3	cg09710740	-0.20240148037252	796	-5.81646815473098	8.71867979362971e-09	0.0409663592569875	2.41718095461735e-07
**BRCA**	L3MBTL3	cg10237990	-0.192224449833992	796	-5.51247780033845	4.7894382338655e-08	0.0369502391139811	1.12462891024851e-06
**BRCA**	L3MBTL3	cg16487825	-0.207875082633651	795	-5.9769941129118	3.43669423811399e-09	0.0432120499799474	1.02670580476129e-07
**BRCA**	L3MBTL3	cg16800851	-0.316156770525512	796	-9.37849153317353	6.86052750905082e-20	0.0999551035491215	9.10764295288565e-18
**BRCA**	L3MBTL3	cg18411550	-0.191828067157966	796	-5.50067588043241	5.10871003217045e-08	0.0367980073495612	1.18907509466866e-06
**BRCA**	L3MBTL3	cg18798995	-0.208776174752527	796	-6.0078752064801	2.86423050678979e-09	0.0435874911442977	8.68355214517556e-08
**BRCA**	L3MBTL3	cg22934935	0.103715748218334	796	2.93464648873036	0.0034358002237992	0.0107569564284889	0.0183587226190147
**BRCA**	L3MBTL3	cg24516147	-0.186302132291776	796	-5.33643436090983	1.23855556281697e-07	0.0347084844964625	2.63881438048228e-06
**BRCA**	LBR	cg01017344	-0.0643409049136388	796	-1.81447319603027	0.0699831234943121	0.0041397520451059	0.184370182163856
**BRCA**	LBR	cg05530459	-0.105097236096661	796	-2.97416949292419	0.0030270291137167	0.0110454290351574	0.0165882714375509
**BRCA**	LBR	cg12994003	-0.142094687809599	111	-4.77402480740112	2.04799875764644e-06	0.0201909003037075	3.22644826553964e-05
**BRCA**	LBR	cg14607642	-0.568944219301049	111	-23.0078885065753	4.92084630269225e-96	0.323697524676081	1.16003732506558e-92
**BRCA**	LBR	cg18107727	-0.00989162642281149	796	-0.278388662484392	0.780786686153682	9.78442732884624e-05	0.878934398290181
**BRCA**	LBR	cg18289048	-0.061262982870348	796	-1.72733769063653	0.0844968981969659	0.00375315307017256	0.211111089423199
**BRCA**	LBR	cg18764107	-0.0318246773006554	796	-0.896079702009724	0.370482467555579	0.00101281008529085	0.569163732395013
**BRCA**	LBR	cg19322477	-0.0836838283117189	796	-2.36336151220538	0.0183506217960856	0.00700298312090525	0.068128675766187
**BRCA**	LBR	cg20483690	-0.143617668147322	796	-4.08409827871461	4.87465307170278e-05	0.0206260346040744	0.000518614899881169
**BRCA**	LBR	cg26930584	-0.071810939713163	796	-2.02617002072747	0.0430820858236321	0.00515681106248754	0.129264404361686
**BRCA**	MBD1	cg01719254	-0.102726210793734	796	-2.90634736788004	0.0037587571760954	0.0105526743840387	0.019748008087875
**BRCA**	MBD1	cg03841029	-0.0579534612286008	111	-1.93057857179594	0.0537905771466984	0.00335860366837493	0.152188833232116
**BRCA**	MBD1	cg05549078	-0.206397772703918	796	-5.93636908612746	4.35709620974221e-09	0.0426000405771381	1.2729542402362e-07
**BRCA**	MBD1	cg12090413	-0.104178672026207	796	-2.94788835708845	0.00329355091041868	0.0108531957051441	0.0177375216009076
**BRCA**	MBD1	cg13478031	-0.0574579320236623	796	-1.61968537562935	0.105697963995593	0.0033014139524358	0.247790122079109
**BRCA**	MBD1	cg14389183	-0.157035263386114	796	-4.47488394775385	8.76554535576498e-06	0.0246600739467463	0.000116406083203547
**BRCA**	MBD1	cg19328682	-0.197905777198934	796	-5.68194517775577	1.87108842058722e-08	0.0391666966487142	4.83907754624874e-07
**BRCA**	MBD1	cg21282183	0.158414270209905	796	4.51518720005972	7.28742604815147e-06	0.0250950810061368	9.88178528830217e-05
**BRCA**	MBD2	cg15090185	-0.039366695822397	796	-1.10873648092749	0.267880443302228	0.00154973673997313	0.463583992308712

BRCA	MBD2	cg16852837	-0.22878818092617	796	-6.61410103872884	6.87528304064622e-11	0.052344031731506	2.82096924506346e-09
BRCA	MBD2	cg17987217	-0.0220760494527998	796	-0.621426546784959	0.534497827243841	0.000487351959442463	0.711216577913113
BRCA	MBD2	cg22224310	-0.0579350449847544	796	-1.63317985115387	0.102828626961734	0.00335646943738552	0.243018811126809
BRCA	MBD2	cg22836370	-0.0585768843589246	111	-1.95141751989656	0.0512595622984343	0.00343125138119882	0.146921850743946
BRCA	MBD2	cg23388714	-0.201140578007576	796	-5.77870047238895	1.08203075094385e-08	0.0404575321212218	2.94422875966863e-07
BRCA	MBD2	cg24881765	-0.186926755365132	796	-5.35497272937465	1.12205010944523e-07	0.0349416118713359	2.41195817115729e-06
BRCA	MBD2	cg25192916	0.150728182512562	796	4.29088941662165	1.99976523560548e-05	0.0227189850035403	0.000239338056521066
BRCA	MBD3	cg08660999	-0.0797394484197393	796	-2.25123551151101	0.0246440220621988	0.00635837963428427	0.08527520026273
BRCA	MBD3	cg09174855	0.033543529405177	796	0.94453012103532	0.345186866159027	0.00112516836495597	0.54440480017558
BRCA	MBD3	cg12286540	-0.0992589381873084	796	-2.80725738136236	0.00511931702376858	0.00985233681007191	0.0252553838890896
BRCA	MBD5	cg03890220	0.277366129423384	796	8.12454712359253	1.72029844718244e-15	0.0769319697513095	1.42371167446926e-13
BRCA	MBD5	cg04152151	0.286335686232526	796	8.41034708817985	1.89482291528165e-16	0.0819881252102514	1.77811645200749e-14
BRCA	MBD5	cg05218490	-0.0354900497812138	111	-1.18102162778804	0.237848035336248	0.00125954363347303	0.42938712663483
BRCA	MBD5	cg06394585	0.157060172655964	796	4.4756117144826	8.73647436983347e-06	0.0246678978347211	0.000116117550409959
BRCA	MBD5	cg06758980	0.217451358158412	796	6.26964910221596	5.94310126065496e-10	0.0472850931649379	2.04574167907696e-08
BRCA	MBD5	cg20595490	0.276269549573237	795	8.084657661557	2.33267092878203e-15	0.0763248640213994	1.89226071150854e-13
BRCA	MBD5	cg21412502	-0.0499537212906993	796	-1.40757964561326	0.159647925089276	0.00249537427078886	0.329280060504634
BRCA	MBD5	cg23120335	-0.165607925791494	796	-4.72587660233818	2.71101079877472e-06	0.0274259850849608	4.14342581689353e-05
BRCA	MBD5	cg25686162	0.156221110709532	796	4.45110190220711	9.76815405216981e-06	0.0244050354313199	0.000128210524098954
BRCA	MBD5	cg26664634	-0.259601691538226	796	-7.5652064161885	1.07531084320602e-13	0.067393038249508	7.10728391982745e-12
BRCA	MBTD1	cg01388767	0.00242162266086069	796	0.0681507023839655	0.945682870735288	5.86425631159401e-06	0.970957162506843
BRCA	MBTD1	cg14577476	-0.181536699007802	796	-5.19521842735337	2.60300020905771e-07	0.0329555730866494	5.13562765060703e-06
BRCA	MBTD1	cg17045523	-0.113213269332942	796	-3.20672075407759	0.00139648358264721	0.0128172443530532	0.00886045541378818
BRCA	MBTD1	cg18711617	-0.107297629191182	796	-3.03715661696795	0.00246656066378816	0.0115127812300483	0.0140807141675933
BRCA	MBTD1	cg22141797	-0.250117689967313	795	-7.26541867781971	8.92429651332383e-13	0.0625588588345849	5.07497604501107e-11
BRCA	MBTD1	cg25259117	-0.136049612154949	796	-3.86470944022164	0.000120326307361582	0.0185094969675121	0.00112915135764619
BRCA	MBTD1	cg25716740	0.0118355244221329	111	0.393636615829327	0.693925215693759	0.000140079638346903	0.824515954518223
BRCA	MBTD1	cg25855326	-0.16422120978921	796	-4.68520298819115	3.29126171522017e-06	0.0269686057446316	4.91063317748059e-05
BRCA	MBTD1	cg27207041	0.0419381182210592	111	1.39594645181445	0.163010761982259	0.00175880575992354	0.333790989128995
BRCA	MBTD1	cg27214721	0.0785318335679213	796	2.21692844635698	0.0269112700136793	0.00616724888353969	0.0910509867979022
BRCA	MBTD1	cg27353579	-0.155482642789023	796	-4.42953871286775	1.07713330430451e-05	0.0241748522086589	0.000139890664899942
BRCA	MLLT10	cg00879184	-0.26870864165645	796	-7.85087485466125	1.34128191647583e-14	0.0722043341008545	9.91860127474532e-13
BRCA	MLLT10	cg03524461	-0.149063646718713	796	-4.24242087532091	2.47264672711819e-05	0.0222199707730814	0.000288045941134221
BRCA	MLLT10	cg04188362	-0.176606206159072	796	-5.04950945704906	5.50026828725344e-07	0.0311897520539008	1.00703193960601e-05
BRCA	MLLT10	cg05913198	-0.0689180220883018	796	-1.9441476099826	0.0522314247516036	0.00474969376856366	0.148953094216293
BRCA	MLLT10	cg06398735	-0.108964881773147	795	-3.08296423446125	0.00212065936790431	0.011873345459836	0.012454671211441
BRCA	MLLT10	cg11443787	-0.0472292574668124	111	-1.5724382204227	0.116134964156804	0.00223060276086646	0.264702306964086
BRCA	MLLT10	cg15910013	0.11844022813433	796	3.35683159674807	0.000826010796282237	0.0140280876405123	0.00572103667076036
BRCA	MLLT10	cg19119538	-0.16635070195205	786	-4.7176068176285	2.8263095369879e-06	0.0276725560399399	4.28847131110662e-05
BRCA	MLLT10	cg20569108	-0.225479831735024	795	-6.50918318849778	1.34108865419081e-10	0.0508411545192548	5.2242689416184e-09
BRCA	MLLT10	cg23344688	-0.140092844880637	796	-3.98182944736593	7.4678337453092e-05	0.0196260051867503	0.000749671152148711
BRCA	MLLT10	cg26877631	-0.151349321640579	796	-4.30898550817466	1.84641361689278e-05	0.0229066171610633	0.00022366848475951
BRCA	MLLT10	cg27470303	-0.124828128775465	796	-3.54066869903572	0.000422421109080973	0.0155820617335842	0.00325074960528689
BRCA	MLLT10	cg27572529	-0.222273661699157	796	-6.41583172454349	2.40836441205595e-10	0.0494055806851513	8.92172862245147e-09
BRCA	MLLT6	cg05652455	-0.114458709984781	796	-3.2424630698253	0.00123461462382865	0.0131007962913802	0.00800513502634821
BRCA	MORF4L	cg03589001	-0.0506309594990892	111	-1.68521248162906	0.0922299354651615	0.00256349405979841	0.224726049608129
BRCA	MORF4L	cg07757959	-0.0163324164171764	796	-0.459696255732386	0.645860415613923	0.000266747826024054	0.792380658459675
BRCA	MORF4L	cg08692282	0.0794671873125665	796	2.24349999497704	0.0251402271295003	0.00631503385937053	0.0865993105434087
BRCA	MORF4L	cg13747872	-0.0958712589117649	796	-2.71054183716615	0.00686270203376044	0.00919129828532666	0.031911082720502
BRCA	MORF4L	cg15822222	-0.0199082344281688	796	-0.560378439656745	0.575379809353128	0.000396337798046926	0.742294437717736
BRCA	MORF4L	cg20775702	-0.255641777043554	796	-7.4416723574262	2.59308953489409e-13	0.0653527181699862	1.60460561357307e-11
BRCA	MORF4L	cg20905746	0.133991291385756	796	3.80516220336899	0.000152632214959687	0.0179536661672227	0.00138225226640913
BRCA	MORF4L	cg23623458	-0.256441109802551	777	-7.37647101994027	4.1968443881759e-13	0.065762042796764	2.50375239519751e-11
BRCA	MORF4L	cg24795825	0.00780418953429631	785	0.218105466659238	0.827403912180492	6.090537428722e-05	0.907292853529911
BRCA	MPHOSF	cg00331616	-0.0305911942006311	795	-0.860771697755241	0.389624626745559	0.000935821162620724	0.58729041021962
BRCA	MPHOSF	cg03134886	0.0668576033755738	796	1.88575907367951	0.0596931630198172	0.00446993912912554	0.164061823846093
BRCA	MPHOSF	cg03428000	0.20403857437976	111	6.93144262803964	7.06454633967953e-12	0.0416317398349247	3.45647369779264e-10

**BRCA**	MPHOSF	cg04203901	0.0593766397677124	796	1.67396021184951	0.094533253383973	0.00352558535010469	0.228765356448589
**BRCA**	MPHOSF	cg06019692	-0.226368028362008	796	-6.54033606405217	1.10027032314745e-10	0.0512424842645029	4.37305949093744e-09
**BRCA**	MPHOSF	cg09559995	-0.124028387295861	796	-3.51762898849734	0.000460221716993709	0.0153830408552121	0.00349736136092689
**BRCA**	MPHOSF	cg11051893	0.0929094567468968	796	2.62606275334496	0.00880467293983586	0.0086321671530035	0.0386584674727165
**BRCA**	MPHOSF	cg19693579	0.199725204177585	796	5.73634157188582	1.376574072196e-08	0.0398901571837779	3.65118320396918e-07
**BRCA**	MPHOSF	cg20759595	-0.217430812596699	796	-6.26902732741875	5.96575071101914e-10	0.0472761582664609	2.05262985764274e-08
**BRCA**	MPHOSF	cg20952532	-0.114561348157895	796	-3.24540932299855	0.00122207776218155	0.0131243024917544	0.00793648955102523
**BRCA**	MPHOSF	cg21958444	-0.0449607141598353	796	-1.26658748287576	0.205675195133623	0.00202146581776241	0.389965784309653
**BRCA**	MPHOSF	cg22656705	-0.133242097638091	796	-3.78350051485153	0.000166294090010818	0.0177534565829986	0.00148709779812079
**BRCA**	MSH6	cg00620552	-0.118008413367146	796	-3.34441994683337	0.000863325090110296	0.0139259856254311	0.00594033202835227
**BRCA**	MSH6	cg02905881	0.0910374323098607	111	3.0402180886736	0.00241967457385337	0.00828781408157247	0.0138592375054005
**BRCA**	MSH6	cg05039065	0.0971212358981456	796	2.74621643057137	0.0061654807940445	0.00943253446238324	0.0293123151556498
**BRCA**	MSH6	cg07652213	0.0800039074032656	111	2.66800526791074	0.00774195218629617	0.0064006251997903	0.0350446542756153
**BRCA**	MSH6	cg09898070	-0.0977352410281345	796	-2.76374505366934	0.00584689018257792	0.00955217733882755	0.0280981575774319
**BRCA**	MSH6	cg11869233	0.0407832217140099	796	1.14869729028794	0.251027626045411	0.00166327117337409	0.444669326038784
**BRCA**	MSH6	cg16595246	-0.0562766266014071	796	-1.5862785686017	0.113075281276151	0.0031670587016342	0.260099894488376
**BRCA**	MSH6	cg18222961	-0.0194261775458407	796	-0.546804281129133	0.584667222434314	0.000377376374042524	0.749256760731009
**BRCA**	MSH6	cg19076255	-0.163207978209146	796	-4.65550207003348	3.78851874984975e-06	0.0266368441511172	5.56692722470352e-05
**BRCA**	MSH6	cg19736286	0.0398136991991117	796	1.12134590543248	0.262480524931662	0.00158513064391735	0.457906172639959
**BRCA**	MSH6	cg21405109	-0.165270202039361	796	-4.71596821972557	2.84255567365013e-06	0.0273142396821311	4.30975981438196e-05
**BRCA**	MTA1	cg01318265	-0.183635204373896	796	-5.25735692450777	1.88134493417133e-07	0.0337218882854425	3.83938477987735e-06
**BRCA**	MTA1	cg01655777	-0.0332907833529151	796	-0.937405285386312	0.348835883533221	0.00110827625625073	0.547963385905488
**BRCA**	MTA1	cg05922925	-0.138346944044803	796	-3.93123152587606	9.19067879640908e-05	0.0191398769265359	0.000895787997425773
**BRCA**	MTA1	cg13052453	-0.0393222673463811	796	-1.10748324391487	0.26842128423635	0.00154624070926027	0.464220474542765
**BRCA**	MTA1	cg13981078	-0.0998563962319915	796	-2.82432445848834	0.00485707326111487	0.00997129986844049	0.0242130232416228
**BRCA**	MTA1	cg16531924	-0.11347060265262	796	-3.21410458108251	0.00136151796346574	0.0128755776663487	0.00867824075787411
**BRCA**	MTA1	cg20222911	-0.0244111076900437	111	-0.812071907967565	0.416925115615025	0.00059590217865491	0.612441179859047
**BRCA**	MTA1	cg21517792	-0.0327490093618797	796	-0.922133443393168	0.356739790329867	0.00107249761418448	0.555588781984779
**BRCA**	MTA1	cg23123838	-0.049324575292665	796	-1.38980826314628	0.164977626523176	0.00243291372780178	0.336833163285664
**BRCA**	MTA1	cg23883989	-0.17760658401999	796	-5.07904110062978	4.73351224748417e-07	0.0315440986872498	8.78013475872159e-06
**BRCA**	MTA1	cg23966123	-0.08237148714075	796	-2.32604373515835	0.0202677796632996	0.00678506189377874	0.0735646736866856
**BRCA**	MTA2	cg00501779	-0.0884988320360228	796	-2.50038872284305	0.0126067852144957	0.00783204327174017	0.0511197419325764
**BRCA**	MTA2	cg07654871	-0.00307432304880007	796	-0.0865195285422749	0.931075300965722	9.45146220838337e-06	0.963538397607218
**BRCA**	MTA2	cg09086761	-0.130526143394088	111	-4.37633013571446	1.32123252801128e-05	0.017037074109334	0.000166802926459119
**BRCA**	MTA2	cg13708959	-0.219238818148582	795	-6.31978344566737	4.37140845921924e-10	0.048065659383187	1.54086701258043e-08
**BRCA**	MTA2	cg19570317	-0.13078650527986	111	-4.38719522178897	1.2579198656293e-05	0.0171051099633187	0.000160292576620419
**BRCA**	MTA2	cg21002970	-0.00361137798352791	796	-0.101633848010784	0.919073036054478	1.30420509399101e-05	0.957345184602084
**BRCA**	MTF2	cg05396182	-0.0829524289353943	796	-2.34256190054864	0.0193985776950019	0.00688110546628164	0.0711043647068768
**BRCA**	MTF2	cg05962718	-0.158462865024033	796	-4.51660793894811	7.23994778367343e-06	0.0251104795916248	9.82768274093684e-05
**BRCA**	MTF2	cg09936581	0.0986535221377504	796	2.78996610243832	0.00539801733355761	0.00973251743018361	0.0263718596517141
**BRCA**	MTF2	cg11404133	0.155260450151051	796	4.4230522013192	1.10919023647789e-05	0.0241058073811071	0.000143431424961038
**BRCA**	MTF2	cg14474880	-0.119767761134918	111	-4.01194415788887	6.42698189797633e-05	0.0143443166072708	0.000660650937858312
**BRCA**	MTF2	cg15463775	-0.170269974548809	796	-4.86283157022736	1.39589862379223e-06	0.0289918642328521	2.29534004521862e-05
**BRCA**	MTF2	cg16813892	0.135482015244992	796	3.84828380091745	0.00012852534237001	0.0183553764548443	0.00119427860539089
**BRCA**	MTF2	cg19708337	-0.0211069521616977	794	-0.593384001422596	0.55309403613512	0.000445503429556194	0.725172707841971
**BRCA**	MTF2	cg22801127	0.0287506405450217	796	0.809449359416406	0.418499830936515	0.000826599331749046	0.613954436295453
**BRCA**	MTF2	cg23992808	-0.0240198232550964	796	-0.676172833009866	0.499128320809091	0.000576951909206071	0.683359824519499
**BRCA**	MTF2	cg26054395	-0.0219016407922425	111	-0.728548388517808	0.466432186713969	0.000479681869392421	0.655709016105144
**BRCA**	NAP1L2	cg02379437	0.0306245945242457	790	0.858984586167985	0.390610904679944	0.000937865789774462	0.587907688948316
**BRCA**	NAP1L2	cg04297329	-0.0575102537139063	111	-1.91576500806986	0.0556526150687452	0.00330742928223787	0.155830527180577
**BRCA**	NAP1L2	cg07693661	0.0386065150284155	776	1.07347798324039	0.283392134523286	0.00149046300263927	0.48069449670658
**BRCA**	NAP1L2	cg20001978	-0.0735905333044886	793	-2.07271515586139	0.0385229636619047	0.00541556659203904	0.11906700416399
**BRCA**	NAP1L2	cg23483095	-0.0981923164600609	796	-2.77679571428299	0.00561942780475669	0.00964173101179274	0.0272355471087933
**BRCA**	NCOA1	cg11380327	-0.281302637590959	796	-8.24968772415568	6.59836108517078e-16	0.0791311739156303	5.70982093726113e-14
**BRCA**	NCOA1	cg13911252	-0.0345709510126032	796	-0.973494711109482	0.33060471261063	0.00119515065391581	0.530271781720416
**BRCA**	NCOA1	cg15477048	0.0270761635713714	796	0.762270254478339	0.44612567070751	0.000733118633743659	0.638023024248475
**BRCA**	NCOA1	cg23006163	-0.187395725246556	795	-5.3655053186144	1.06102473075378e-07	0.0351171578406827	2.29408999178043e-06

**BRCA**	NCOA3	cg04175879	-0.190312976459006	79€	-5.45559118194709	6.52973295554772e-08	0.0362190290086862	1.48313589939819e-06
**BRCA**	NCOA3	cg09568061	-0.028435692614824	79€	-0.800575058085914	0.423617774252383	0.000808588614484757	0.618647851499523
**BRCA**	NCOA3	cg12128876	-0.015261610043604	111	-0.507608047996564	0.611829451418001	0.000232916741123035	0.768692329391818
**BRCA**	NCOA3	cg14341558	-0.0424246655839223	79€	-1.19501182705951	0.232440126523208	0.00179985224990764	0.423050492542554
**BRCA**	NCOA3	cg14774045	-0.0629134735277555	79€	-1.77405651863255	0.076437944606648	0.00395810515132759	0.196595148700022
**BRCA**	NCOA3	cg18295930	-0.196087204487781	79€	-5.62763540261931	2.53557652108536e-08	0.038450191763833	6.36504162250128e-07
**BRCA**	NCOA3	cg21735648	0.207297398438732	79€	5.96340312304944	3.71996891887207e-09	0.0429722113994665	1.10370427946123e-07
**BRCA**	NCOA3	cg22511723	-0.130284549023063	79€	-3.69805202462424	0.000232252277950239	0.0169740637141429	0.00196753062298387
**BRCA**	NCOA3	cg22876277	-0.0945220174395739	79€	-2.67204873471938	0.00769391002018412	0.00893441178084711	0.0348982587668476
**BRCA**	NCOA3	cg24856420	0.120024843936145	79€	3.40239480922193	0.000701503502006245	0.014405963161896	0.00499521862105939
**BRCA**	NCOA3	cg25373408	-0.00218262387888494	79€	-0.0614246269446698	0.951036536819953	4.76384699667874e-06	0.974102246315845
**BRCA**	NCOR1	cg00253204	0.0720369611537942	79€	2.0325805062343	0.0424280587768339	0.00518932377227325	0.127823112248665
**BRCA**	NCOR1	cg08125503	0.102615147710369	79€	2.90317169478377	0.00379667315931322	0.0105298685396209	0.0199162772593131
**BRCA**	NCOR1	cg08206308	0.104820445683905	79€	2.96624940548654	0.0031051960272036	0.0109873258333726	0.0169258421903221
**BRCA**	NCOR1	cg21211144	0.19990028784774	79€	5.74157939205728	1.33630789112574e-08	0.0399601250816092	3.55529193167701e-07
**BRCA**	NCOR1	cg22291359	0.0844673489985908	79€	2.3856476234647	0.0172831893182545	0.00713473304684973	0.0650784341587356
**BRCA**	NCOR1	cg22625220	0.141362806307483	79€	4.01865794846663	6.41136524055626e-05	0.0199834430071269	0.000659655796222501
**BRCA**	NCOR2	cg08976554	-0.0881386388028818	79€	-2.49013221670245	0.012973534587876	0.00776841965002486	0.0522515342580271
**BRCA**	NCOR2	cg10641483	0.00549388646431621	79€	0.154614003287942	0.8771650212531	3.01827884827969e-05	0.934939589130533
**BRCA**	NCOR2	cg13416396	-0.123690664334308	79€	-3.50790163889213	0.000477111565067883	0.0152993804434626	0.00360098372567204
**BRCA**	NCOR2	cg13419087	-0.167518315126633	79€	-4.78195728268338	2.06992305085748e-06	0.0280623859028658	3.25637196801632e-05
**BRCA**	NCOR2	cg13857025	-0.0896792201437127	79€	-2.53400723748753	0.0114683016843965	0.00804236252558448	0.0474631622724871
**BRCA**	NCOR2	cg16181859	-0.109267569215162	79€	-3.09358520541205	0.00204699427839182	0.0119394016821901	0.012118093972545
**BRCA**	NCOR2	cg19895345	-0.0784989735844866	79€	-2.21599506680386	0.0269753959386647	0.00616208885381792	0.0911965560667715
**BRCA**	NCOR2	cg23611643	-0.148475331459808	79€	-4.22529894940207	2.66384771578418e-05	0.0220449240520998	0.000307252288375001
**BRCA**	NCOR2	cg24626542	-0.144798809256067	79€	-4.11840306387488	4.21594861749651e-05	0.0209666951619749	0.000457874503349886
**BRCA**	NCOR2	cg25113767	-0.186601204765196	79€	-5.34193415098689	1.20321958587358e-07	0.0348200096198227	2.56728646361627e-06
**BRCA**	NCOR2	cg25449924	-0.170682410880625	79€	-4.87496356901452	1.31513838133191e-06	0.0291324853840224	2.17958830927427e-05
**BRCA**	ORC1	cg00298020	-0.147164159370883	79€	-4.18715596637014	3.14168492019987e-05	0.0216572898033388	0.000354877648732436
**BRCA**	ORC1	cg02275620	-0.0406011087660515	79€	-1.14355942157595	0.253151925447223	0.00164845003303274	0.447177586018194
**BRCA**	ORC1	cg04407474	-0.0432031260053574	79€	-1.21698002175267	0.223974327861612	0.00186651009663479	0.413024673153801
**BRCA**	ORC1	cg07142859	-0.0769366756737344	79€	-2.17162671881189	0.0301799707150334	0.00591925206372539	0.0991310860414334
**BRCA**	ORC1	cg08613513	0.0193696660041487	111	0.644289688964028	0.519521194365815	0.000375183961112273	0.700075964045829
**BRCA**	ORC1	cg10586599	0.00543732165333257	111	0.180829386369975	0.856534626008792	2.95644667617992e-05	0.923665853835985
**BRCA**	ORC1	cg13460942	-0.00942459165701857	79€	-0.265243299532709	0.790891061207685	8.88229279015441e-05	0.885185365727058
**BRCA**	ORC1	cg16136434	0.013041354364548	79€	0.367047458724089	0.713681665221147	0.000170076923661716	0.837088610816936
**BRCA**	ORC1	cg16965475	-0.0921244377564815	79€	-2.60368364751177	0.00939548057758568	0.00848691203194784	0.0405904379437275
**BRCA**	ORC1	cg17146111	0.0837119664276523	79€	2.36416178349166	0.0183113128309427	0.00700769332318439	0.0680227570165502
**BRCA**	ORC1	cg19776520	-0.0187618458898287	79€	-0.528098101128618	0.597579178301696	0.000352006861193682	0.758383173347344
**BRCA**	ORC1	cg21197365	-0.0467525592415734	79€	-1.31717397218782	0.188161263829895	0.00218580179563683	0.367633779985963
**BRCA**	ORC1	cg23606758	0.0327285749391281	79€	0.921557441948069	0.357040094312694	0.00107115961754612	0.555911798434106
**BRCA**	ORC1	cg25605769	-0.0400531658897282	79€	-1.12810124797989	0.259618778070473	0.00160425609779008	0.454513980133549
**BRCA**	PADI1	cg14524955	0.0144470166530608	79€	0.406617523616603	0.684398777657981	0.000208716290173817	0.818282470092717
**BRCA**	PADI1	cg21010178	0.0506594417851009	79€	1.4275160151547	0.153825365689424	0.00256637904197803	0.321155321269098
**BRCA**	PADI1	cg22193702	-0.0244216958940961	111	-0.812424350526273	0.41672300868211	0.00059641923034371	0.612236779993127
**BRCA**	PADI2	cg00569276	-0.373087145439194	79€	-11.3167113450394	1.25834644517714e-27	0.139194018091966	3.0401181166494e-25
**BRCA**	PADI2	cg01603374	-0.0657793812172774	79€	-1.85521391673702	0.0639370717332999	0.00432692699332791	0.172436143470977
**BRCA**	PADI2	cg07014827	-0.173629889327266	79€	-4.9586088272913	8.69166423110293e-07	0.0301473384677985	1.51266068723584e-05
**BRCA**	PADI2	cg09313801	-0.0207337131491825	79€	-0.58362386908884	0.559639561466332	0.000429886860952582	0.730357323346952
**BRCA**	PADI2	cg10233913	-0.17205720561671	79€	-4.91542287272544	1.0770836720605e-06	0.0296036820046309	1.82550429595369e-05
**BRCA**	PADI2	cg15185794	-0.0704725785727233	111	-2.34951839463416	0.0189728270421099	0.00496638433068866	0.0698519690919971
**BRCA**	PADI2	cg17183414	-0.0275621605947789	79€	-0.775962749425929	0.438002629019463	0.000759672696652384	0.630829687818389
**BRCA**	PADI2	cg18228326	-0.0411086758708073	79€	-1.15787946441226	0.247262279344875	0.00168992323185109	0.440504769261804
**BRCA**	PADI2	cg18879300	0.029938111909414	79€	0.842910982768201	0.399532712116808	0.000896290544700597	0.596168558672964
**BRCA**	PADI2	cg19282714	-0.0415772264960622	111	-1.38391296719398	0.166664311374534	0.00172866576310486	0.339144217605821
**BRCA**	PADI3	cg01459162	-0.0215635992972713	111	-0.717298315624468	0.473341506033311	0.000464988814653279	0.661396490178053
**BRCA**	PADI3	cg12858514	-0.292969539872149	111	-10.1856886997835	2.37876528873387e-23	0.0858311512928986	4.16787537999466e-21

**BRCA**	PADI3	cg14664242	-0.0376143645724584	796	-1.05931169407108	0.289780805242954	0.00141484042218982	0.487252149809154
**BRCA**	PADI3	cg17589397	0.0334397640113688	796	0.941604983733469	0.346682028070611	0.00111821781713604	0.545998594606139
**BRCA**	PADI3	cg18768338	-0.0984713517772733	796	-2.78476375065534	0.00548451473806152	0.00969660712084351	0.0267045770406428
**BRCA**	PADI3	cg21966975	-0.070709613208979	796	-1.99493835009475	0.0463918585735125	0.00499984940016341	0.13655640577962
**BRCA**	PADI4	cg16091981	-0.174705637829873	796	-4.99344815554111	7.29868783380901e-07	0.0305220598895426	1.29197793574163e-05
**BRCA**	PADI4	cg18333690	-0.169095380346258	111	-5.70569755981977	1.48670922485047e-08	0.0285932476544456	3.92323804063832e-07
**BRCA**	PADI4	cg19159961	-0.080105264815932	790	-2.25304686449427	0.0245311526122161	0.0064168534512306	0.0849700296606489
**BRCA**	PADI4	cg21295838	-0.162470127492981	796	-4.63388293698035	4.19503935620945e-06	0.0263965423275853	6.09990465730177e-05
**BRCA**	PADI4	cg23228178	-0.0442717775770743	111	-1.47377283594007	0.140827316042972	0.00195999028983394	0.302580170250145
**BRCA**	PADI4	cg26918754	-0.155562423549992	796	-4.43186793570022	1.06583984764665e-05	0.0241996676207471	0.000138609069053534
**BRCA**	PADI6	cg12643633	0.0509750130869093	796	1.43643149146924	0.151274474352248	0.00259845195921057	0.317528640056089
**BRCA**	PADI6	cg16017904	0.00332080565737311	796	0.0934562704515458	0.925564724739566	1.10277502140413e-05	0.960813746949423
**BRCA**	PARP1	cg00653565	-0.0956132489984046	796	-2.70317979980377	0.00701512960470889	0.00914189338403092	0.032447994871926
**BRCA**	PARP1	cg02088626	-0.0855486115053131	796	-2.41640989793958	0.0158995568780987	0.00731856493048699	0.0610099380213388
**BRCA**	PARP1	cg03149313	0.129176761214937	796	3.66607218020391	0.000262741172410855	0.0166866356379809	0.00218349393937935
**BRCA**	PARP1	cg04960271	-0.0158635367767968	111	-0.527633346996865	0.597859672856787	0.000251651799068786	0.758564992582664
**BRCA**	PARP1	cg07113065	-0.171448688883909	795	-4.89441815158029	1.19523479311875e-06	0.0293946529200115	2.00435644526579e-05
**BRCA**	PARP1	cg17661881	-0.118061454469386	111	-3.95397294251414	8.17481154754772e-05	0.013938507031427	0.000808583966344473
**BRCA**	PARP1	cg25722198	-0.0782155023677192	796	-2.20794343973018	0.0275340750218716	0.00611766481063469	0.0925932219822647
**BRCA**	PARP1	cg26464410	0.017345960398337	796	0.488232051857232	0.625520624800784	0.000300882342140676	0.778376305416962
**BRCA**	PARP2	cg01813761	-0.118344377519381	779	-3.31788409714531	0.000949334656134945	0.0140053916904498	0.00642698983894891
**BRCA**	PARP2	cg10910545	-0.0285330364198872	788	-0.799250434546664	0.424387283897988	0.000814134167338608	0.619319854424039
**BRCA**	PARP2	cg15078479	-0.149526097145241	111	-5.02926648046099	5.74091300261809e-07	0.022358053727488	1.04592174736691e-05
**BRCA**	PARP2	cg15365367	-0.145693372075797	796	-4.14439649518846	3.77414517770084e-05	0.0212265586668167	0.000416049100119114
**BRCA**	PARP2	cg18899035	-0.00201738375179263	717	-0.053906173938301	0.957024996752274	4.06983720199691e-06	0.977378142113404
**BRCA**	PARP2	cg19540080	0.0323996208402196	796	0.912285107934857	0.361896252934903	0.00104973543058999	0.560812675062606
**BRCA**	PARP2	cg24595957	0.0642085934404275	111	2.13977353937896	0.0325914061779309	0.0041227434715981	0.104978771461764
**BRCA**	PARP2	cg26206608	-0.08712175495951	796	-2.46118173747592	0.0140602502719562	0.0075902001872249	0.0555314570572454
**BRCA**	PARP2	cg26430074	0.0291055094148041	796	0.819448803990717	0.412776919251517	0.000847130678295252	0.608381032477976
**BRCA**	PAXIP1	cg02824291	0.131345420445872	796	3.72869068208118	0.000206188841256305	0.0172516194721029	0.00178007487700511
**BRCA**	PAXIP1	cg12220104	0.0858571812850079	796	2.42519038234781	0.0155229657932044	0.00737145557820671	0.0599540065989745
**BRCA**	PAXIP1	cg22583711	0.00828226147940192	796	0.233091493312058	0.815750587060472	6.85958552131849e-05	0.900838957070414
**BRCA**	PAXIP1	cg24448952	-0.0799083523284486	796	-2.25603468517283	0.0243404643044023	0.00638534477184747	0.0844991780131497
**BRCA**	PAXIP1	cg24452135	0.0261205300173045	796	0.735347773999567	0.46234541488563	0.000682282088384904	0.651808871762132
**BRCA**	PCGF1	cg03203239	0.111033383148066	796	3.14419797220009	0.00172756212660807	0.0123284121733052	0.0105531188125126
**BRCA**	PCGF1	cg08092637	0.0170914394462136	796	0.481066010604233	0.630602455322667	0.000292117302343586	0.782023041388971
**BRCA**	PCGF1	cg27306986	-0.0388662667230303	796	-1.09462077202219	0.274015590330227	0.00151058668898573	0.470444227448572
**BRCA**	PCGF2	cg09395368	-0.0219645955531855	796	-0.618287673259136	0.536563486409697	0.000482443457815015	0.712663602495364
**BRCA**	PCGF2	cg17902383	0.141651022851721	796	4.02701895341243	6.19210228212966e-05	0.0200650122749388	0.000640229139999993
**BRCA**	PCGF2	cg20228512	-0.0782447312880596	111	-2.61015429198109	0.00917243894805516	0.00612223797434065	0.0399029597715876
**BRCA**	PCGF2	cg25377665	-0.0740230071058505	796	-2.0889229640368	0.0370333543714673	0.0054794055809928	0.115655269770129
**BRCA**	PCGF5	cg03905463	-0.00755117470730494	796	-0.212514952027037	0.831759996900855	5.70202394602418e-05	0.90977513882858
**BRCA**	PCGF5	cg06001881	-0.0309193023702564	796	-0.870562528531478	0.384256848140741	0.000956003259063345	0.582400632109042
**BRCA**	PCGF5	cg07421809	-0.0757782130030501	796	-2.13873746422767	0.0327622099005151	0.00574233756593563	0.105398269537978
**BRCA**	PCGF5	cg07424295	0.048549603612714	796	1.36792004416158	0.171725090609634	0.00235706401095165	0.345985229951463
**BRCA**	PCGF5	cg08633154	-0.0126999688848696	111	-0.422391574559705	0.672821325924106	0.000161289209676657	0.810678575527607
**BRCA**	PCGF5	cg11113644	-0.080569901666521	795	-2.27339700641502	0.0232695607604086	0.00649150905455287	0.0816633829474095
**BRCA**	PCGF5	cg12783297	-0.0747327194178747	796	-2.10906293233518	0.0352524851031084	0.00558497935159079	0.111435669488469
**BRCA**	PCGF5	cg15550435	-0.103080606761646	794	-2.91279674103346	0.00368307085015664	0.0106256114903491	0.019406719975419
**BRCA**	PCGF5	cg17001035	0.0947796636802901	111	3.16630112331517	0.00158611963220744	0.00898318464734889	0.00984604138748366
**BRCA**	PCGF5	cg20515900	-0.0254333546050236	796	-0.71598965197947	0.474208782630517	0.000646855526464874	0.662146632980032
**BRCA**	PCGF5	cg22968329	-0.0468604363170057	796	-1.32021990648588	0.187143022784395	0.00219590049182014	0.366340542800588
**BRCA**	PCGF5	cg23395902	-0.0358564043917497	796	-1.00973797510281	0.31292922439179	0.00128568173590469	0.511934390728974
**BRCA**	PCGF5	cg25390656	-0.00456181679871059	796	-0.12838224027183	0.897879077061759	2.08101725049982e-05	0.945971707646941
**BRCA**	PCGF6	cg01917164	-0.131120991604923	796	-3.72220794775664	0.000211462596180282	0.0171927144394583	0.00181894307908545
**BRCA**	PCGF6	cg06691148	0.0265922327957889	796	0.748636511550831	0.454298581998257	0.000707146845065433	0.644615764198965
**BRCA**	PCGF6	cg11612727	0.0808927312773269	796	2.28400837229325	0.0226349006596486	0.00654363397350582	0.0799482162050716

**BRCA**	PCGF6	cg11814370	-0.11830054919364	796	-3.35281659459435	0.00083791420835255	0.0139950199395168	0.00579007798228622
**BRCA**	PCGF6	cg14409890	-0.161170154200893	796	-4.59581293102148	5.01476554635923e-06	0.0259758186051395	7.14241433382405e-05
**BRCA**	PCGF6	cg16113748	-0.0543437786145658	796	-1.5316328106869	0.126012387883335	0.00295324627410894	0.279973944793401
**BRCA**	PCGF6	cg16382383	-0.00288349793611882	796	-0.0811491623384452	0.93534383041482	8.31456034760149e-06	0.965530197065828
**BRCA**	PCGF6	cg24418712	-0.0532259747436945	796	-1.50003801879546	0.134003065777644	0.0028330043874164	0.292186612696621
**BRCA**	PCGF6	cg25481680	-0.0380017489265024	796	-1.07023707389214	0.284838657284089	0.00144413292147293	0.482122417678362
**BRCA**	PCGF6	cg26155979	-0.0202837339022475	796	-0.570952336482668	0.568193915588817	0.000411429861017183	0.736762688045945
**BRCA**	PCGF6	cg27217148	0.113186727260599	111	3.78854948425549	0.000159692738831581	0.0128112352279653	0.00143570286890836
**BRCA**	PHC1	cg02770112	-0.00640279484011815	796	-0.180194312591251	0.857046118095224	4.09957817646436e-05	0.923921010896951
**BRCA**	PHC1	cg08021945	-0.266778098509048	796	-7.79013127038133	2.09927460384771e-14	0.0711705538441033	1.51917180029515e-12
**BRCA**	PHC1	cg08867249	-0.0140168175469283	796	-0.394506968075434	0.69331299159501	0.000196471174143877	0.82401024930322
**BRCA**	PHC1	cg09059548	-0.0614392311705712	111	-2.04712597919149	0.0408816154048195	0.00377477912683089	0.124339056564334
**BRCA**	PHC1	cg09912793	-0.194662871246905	796	-5.5851413832452	3.21053147362272e-08	0.0378936334420893	7.84668820166527e-07
**BRCA**	PHC1	cg12581035	0.00266574504508216	111	0.0886538738071231	0.92937304118232	7.1061966453801e-06	0.962647578266925
**BRCA**	PHC1	cg14199415	0.0145282912591158	796	0.408905514086122	0.682719546982759	0.000211071246909701	0.817114313412379
**BRCA**	PHC2	cg06899053	-0.0401540311867451	796	-1.13094671082616	0.258419865359348	0.0016123462205461	0.453047167390029
**BRCA**	PHC2	cg13640100	-0.0304214951889111	796	-0.856533201337104	0.391961992992729	0.000925467369528944	0.589059773004063
**BRCA**	PHC2	cg15586632	0.0464658824494052	796	1.3090798104985	0.190886994817958	0.00215907823180194	0.371233326395681
**BRCA**	PHC2	cg16558908	0.0674525129167183	796	1.90261521106777	0.0574532048672424	0.00454984149878005	0.15950589270341
**BRCA**	PHC2	cg17161743	-0.0321438415950467	796	-0.905075582093682	0.365700582867152	0.00103322655248745	0.564551441401571
**BRCA**	PHC2	cg19746874	0.058386342671165	795	1.64490577575691	0.100386550684392	0.0034089650105147	0.238886745132306
**BRCA**	PHC2	cg21198021	0.041590580401985	111	1.38435822684097	0.166528034607309	0.00172977637817398	0.339026534184053
**BRCA**	PHC2	cg21578869	0.167037678829983	796	4.767842712382	2.21594557716767e-06	0.0279015861489087	3.45811976389532e-05
**BRCA**	PHC2	cg23886755	-0.0661944887968227	110	-2.20124842899541	0.0279253684638487	0.00438171034707269	0.0936188801670607
**BRCA**	PHF1	cg01097444	0.0380005074769791	796	1.07020206058976	0.284854403927484	0.00144403856850794	0.482136623713822
**BRCA**	PHF1	cg02776641	-0.0952134661830689	796	-2.69177350958062	0.0072573129198046	0.0090656041425944	0.0333317916363087
**BRCA**	PHF1	cg03560363	0.00415204573595645	796	0.11684993175322	0.907008598588672	1.72394837934741e-05	0.950841639389257
**BRCA**	PHF1	cg03876174	-0.00350337518299029	796	-0.0985943220825812	0.92148532594181	1.22736376727923e-05	0.958838426887501
**BRCA**	PHF1	cg06618123	0.115280122699056	796	3.26604487395869	0.00113751825061745	0.0132895066895093	0.00747083957691296
**BRCA**	PHF1	cg06919790	-0.140071271660649	796	-3.98120400415789	7.48712514016441e-05	0.0196199611446313	0.000751165093053843
**BRCA**	PHF1	cg07857404	-0.0888913647816381	779	-2.48446605343913	0.0131846955427846	0.00790167473274225	0.0529006243259262
**BRCA**	PHF1	cg08665801	-0.00198442121156917	796	-0.0558466731094161	0.955478038225756	3.93792754492565e-06	0.976310958385669
**BRCA**	PHF1	cg08798692	0.034091869114671	796	0.959988279104318	0.337354086847131	0.00116225553973186	0.536920228159039
**BRCA**	PHF1	cg10033570	0.00524694962708361	796	0.147664283979563	0.882645303020589	2.75304803891528e-05	0.937680628107672
**BRCA**	PHF1	cg10797813	-0.0247664664306613	796	-0.69720400431786	0.485879732247808	0.000613377859461073	0.672440054268488
**BRCA**	PHF1	cg13303256	0.078924996903643	111	2.63298879764169	0.00858171218986568	0.00622915513624006	0.0379170624239456
**BRCA**	PHF1	cg13528777	0.0486939272757315	796	1.37199611774949	0.170453127007609	0.00237109855353423	0.344266690993035
**BRCA**	PHF1	cg13547657	0.00134712309798354	796	0.037911438854955	0.969767846342012	1.81474064112077e-06	0.984061878304425
**BRCA**	PHF1	cg18443095	-0.0695585269835321	796	-1.9623034166022	0.0500769351258401	0.00483838867611877	0.144429062068721
**BRCA**	PHF1	cg20135607	0.100305779126099	796	2.83716363067656	0.00466788552627551	0.0100612493260937	0.02347491671285
**BRCA**	PHF1	cg20872639	-0.00542638990192508	796	-0.152714396697886	0.878662403742594	2.94457073677145e-05	0.9355486044804
**BRCA**	PHF1	cg23345642	0.00473737476225922	796	0.133323039544736	0.893971774786918	2.24427196380906e-05	0.943810767958275
**BRCA**	PHF1	cg23718418	-0.0116761807440496	796	-0.328619254678924	0.742530360458356	0.000136333196767714	0.855253352751616
**BRCA**	PHF1	cg24796663	-0.127081052156578	796	-3.60561090061744	0.000330915277584487	0.016149593817223	0.00265120732280202
**BRCA**	PHF1	cg26673053	-0.0462596294615052	796	-1.30325657134696	0.192865921335694	0.00213995331791576	0.373731168345263
**BRCA**	PHF10	cg04353997	-0.288032521338072	795	-8.45933600283613	1.29261488668478e-16	0.0829627333483671	1.23383659502768e-14
**BRCA**	PHF10	cg05243705	-0.0954657180326264	796	-2.69897040842888	0.0071036455095967	0.00911370331948494	0.0327789903774581
**BRCA**	PHF10	cg05736120	-0.0754333703555649	796	-2.12894892137625	0.0335664393453735	0.00569019336319982	0.10732706873958
**BRCA**	PHF10	cg07652237	-0.223228409969107	795	-6.44076214971358	2.06223289372127e-10	0.0498309230173358	7.73153473417602e-09
**BRCA**	PHF10	cg11442717	-0.14617863778202	111	-4.91418849400307	1.02606065096542e-06	0.0213681941438071	1.74740285204036e-05
**BRCA**	PHF10	cg15342139	-0.059889789884119	796	-1.68847891317482	0.0917129864006397	0.00358678693236392	0.223855423696391
**BRCA**	PHF10	cg15360169	-0.240508862955787	796	-6.97320423242425	6.53669239929732e-12	0.0578445131602857	3.21191304924488e-10
**BRCA**	PHF10	cg16740543	-0.00309447269488811	111	-0.102912066196203	0.918051417059767	9.57576125940808e-06	0.956844747303691
**BRCA**	PHF10	cg27124293	-0.281684360770783	796	-8.26184651736079	6.00792431358938e-16	0.0793460791028449	5.22212808995947e-14
**BRCA**	PHF11	cg00092518	-0.2070754440568	796	-5.95673185815599	3.86818525629841e-09	0.0428802395313211	1.14245103959179e-07
**BRCA**	PHF11	cg01214340	-0.206397238123255	796	-5.93635302649309	4.35750461700002e-09	0.0425998199049078	1.2729542402362e-07
**BRCA**	PHF11	cg02289189	-0.0441301659703683	796	-1.24314403945518	0.214182639338768	0.00194747154857225	0.400820886329568

**BRCA**	PHF11	cg05080330	-0.0867736660086363	796	-2.45127350055171	0.0144502495386383	0.00752966911257837	0.0567277030685897
**BRCA**	PHF11	cg09997676	-0.231787544670365	796	-6.70569983719085	3.8105808655866e-11	0.0537254658643165	1.62611260481319e-09
**BRCA**	PHF11	cg16956501	-0.21017767512241	796	-6.05006308987861	2.23169400649635e-09	0.0441746551198612	6.91682882634969e-08
**BRCA**	PHF11	cg21320768	-0.255638796038435	796	-7.44157951357207	2.59479376542526e-13	0.0653511940399804	1.60460561357307e-11
**BRCA**	PHF11	cg23137039	-0.0335252661638815	796	-0.944015279658904	0.345449724312737	0.0011239434713591	0.544723134755955
**BRCA**	PHF11	cg24481868	-0.205202546240998	796	-5.90047646364877	5.3695254741183e-09	0.0421080849837888	1.54824634815997e-07
**BRCA**	PHF11	cg24984523	-0.21611637893903	796	-6.22926647016446	7.60346470104478e-10	0.0467062892457185	2.57287487147924e-08
**BRCA**	PHF11	cg25162948	-0.243018407859789	796	-7.05050658615481	3.88494392243514e-12	0.0590579465587065	1.99620427676301e-10
**BRCA**	PHF12	cg01406758	0.0507506958440994	796	1.430094067899	0.153084390506552	0.00257563312866029	0.32021033987734
**BRCA**	PHF12	cg02058367	0.0179380340469186	796	0.504902264073752	0.613767939289517	0.000321773065468413	0.770220614483623
**BRCA**	PHF12	cg06895197	0.144236199619697	796	4.1020605287765	4.51843422081183e-05	0.0208040812807332	0.000486178522508763
**BRCA**	PHF12	cg08657247	-0.048953295129699	796	-1.3793215535719	0.16818497728547	0.00239642510405541	0.341265607421838
**BRCA**	PHF12	cg08806622	0.033552077935751	796	0.94477110472049	0.345063873094954	0.00112574193380671	0.544291225360385
**BRCA**	PHF12	cg13564075	-0.0321500399361862	111	-1.0697533252412	0.284963761105764	0.00103362506789837	0.48215567865798
**BRCA**	PHF14	cg06715026	0.0244819462205994	796	0.689189607565364	0.49090580947526	0.000599365690748323	0.676637717077147
**BRCA**	PHF14	cg07287563	-0.0437712225168711	796	-1.23301313503599	0.217936709114042	0.00191591992062144	0.405485706228303
**BRCA**	PHF14	cg07574654	0.0233028148012947	796	0.65597746776721	0.512029139013098	0.000543021177663439	0.693914862296133
**BRCA**	PHF14	cg11931558	-0.202217147744914	796	-5.81094498095066	8.99910929635198e-09	0.0408917748420883	2.48430343719094e-07
**BRCA**	PHF14	cg13029706	-0.148717649264599	796	-4.23235064471	2.58345455537766e-05	0.0221169392027881	0.000299519021879359
**BRCA**	PHF14	cg16800461	0.0189487107190804	796	0.533359749286413	0.593934211971848	0.000359053637915394	0.755716684431435
**BRCA**	PHF14	cg20819769	0.110805534657695	795	3.135684090137	0.00177794288356021	0.0122778665107777	0.0108040375475459
**BRCA**	PHF14	cg20964328	-0.147013105849147	796	-4.18276315930371	3.20168460360295e-05	0.0216128532914124	0.000360660081165201
**BRCA**	PHF14	cg22876561	-0.0707344143704127	796	-1.99564158600342	0.0463150379030655	0.00500335737632524	0.136405571502867
**BRCA**	PHF14	cg23083824	-0.0374025108753978	796	-1.05333699841052	0.292507811697363	0.00139894781978425	0.490205788521859
**BRCA**	PHF14	cg24491907	0.0787213853470541	796	2.22231275893785	0.0265439202960446	0.00619705651095938	0.0901098680184365
**BRCA**	PHF19	cg11054680	-0.0812133663036816	762	-2.24483984922161	0.025065651480982	0.00659561086637597	0.0863858705361623
**BRCA**	PHF19	cg11594303	-0.0654461620615739	796	-1.84577541433576	0.0652978647461945	0.00428320012858979	0.17518647274797
**BRCA**	PHF19	cg13501527	-0.265792741891786	796	-7.75916649220276	2.63488694767409e-14	0.0706457816423537	1.87881205506286e-12
**BRCA**	PHF19	cg13793166	-0.0647713368664746	572	-1.5482885921815	0.122108817712379	0.00419532607947034	0.273913880193679
**BRCA**	PHF19	cg13851767	-0.108650455570229	796	-3.07590415765585	0.00217081180871342	0.0118049214956182	0.0126898206978548
**BRCA**	PHF19	cg13852822	-0.147075373945539	796	-4.18457394963937	3.17682074052345e-05	0.0216311656212201	0.000358326218193825
**BRCA**	PHF19	cg14218513	-0.133497638798086	795	-3.78849432401174	0.000163060818064501	0.0178216195646642	0.00146159157512499
**BRCA**	PHF19	cg27207079	-0.147070859492071	796	-4.18444266515138	3.17861720276793e-05	0.0216298377117365	0.000358476872531354
**BRCA**	PHF20	cg01512265	-0.0552166829909054	795	-1.55532668489348	0.120268276220357	0.00304888208051814	0.27112989104806
**BRCA**	PHF20	cg02674305	-0.0504816217985621	793	-1.41979578815937	0.156062121977171	0.00254839413941306	0.324366045501938
**BRCA**	PHF20	cg02957488	-0.188402083008597	796	-5.39878617238171	8.87361690971385e-08	0.0354953448819782	1.94728957369331e-06
**BRCA**	PHF20	cg04179482	0.110755770273695	796	3.13623888909926	0.00177454362148244	0.0122668406489195	0.0107875944744554
**BRCA**	PHF20	cg09692677	-0.130647916816851	779	-3.66852765662819	0.000260638934830226	0.0170688781685827	0.00216857168943129
**BRCA**	PHF20	cg10372813	-0.232334983171152	796	-6.72243999829356	3.4184271517e-11	0.0539795444051394	1.47168302069369e-09
**BRCA**	PHF20	cg21765224	-0.130821689191917	796	-3.71356336042922	0.000218692682699522	0.0171143143630265	0.00187099731199017
**BRCA**	PHF20	cg23538717	-0.00629604106101814	796	-0.177189813251259	0.85940460037099	3.96401330420264e-05	0.925319273951536
**BRCA**	PHF20	cg23734815	-0.0475112692104922	796	-1.33859731052091	0.181085917326808	0.00225732070199186	0.358339425953799
**BRCA**	PHF20	cg25561939	-0.00837328928493194	796	-0.235653509352733	0.813762469508634	7.01119734491561e-05	0.899677930120865
**BRCA**	PHF20	cg27405321	-0.0418334890590221	793	-1.17609644929803	0.239911068656672	0.00175004080685132	0.431838813370904
**BRCA**	PHF20L1	cg02898212	0.413335878599872	796	12.7746273202345	4.10154517948309e-34	0.170846548537928	1.34631057254307e-31
**BRCA**	PHF20L1	cg05307234	-0.150820994018634	796	-4.29359302973899	1.9761059788729e-05	0.0227469722367687	0.000236907364032114
**BRCA**	PHF20L1	cg10591701	-0.102179572009732	795	-2.88889263012083	0.00397163227396286	0.0104406649360919	0.0206462350617247
**BRCA**	PHF20L1	cg17646969	-0.0451634185114962	796	-1.27230950879992	0.203636582871929	0.00203973437164455	0.387498271315954
**BRCA**	PHF20L1	cg24511731	-0.147915584883416	796	-4.20901272584155	2.85872669449756e-05	0.0218790202514031	0.000326758425612317
**BRCA**	PHF20L1	cg26292018	0.330075465962978	796	9.84067192069267	1.23231211125611e-21	0.108949813230677	1.87605652413028e-19
**BRCA**	PHF20L1	cg27342122	0.017837666812532	111	0.59331416153906	0.553092230168821	0.000318182357314905	0.725172707841971
**BRCA**	PHF21B	cg01775414	-0.0555078498721445	111	-1.84885162084693	0.064745975798774	0.00308112139742853	0.174056549909801
**BRCA**	PHF21B	cg01995480	-0.0731560126793649	796	-2.06432402629073	0.03931211219755	0.00535180219114341	0.120955800359761
**BRCA**	PHF21B	cg05064352	-0.0626421237443793	111	-2.0864182931437	0.0371695882833609	0.00392403566720613	0.11599690913494
**BRCA**	PHF21B	cg10080155	-0.0329050486564617	796	-0.926531886223905	0.354451879327847	0.00108274222708411	0.553452675983059
**BRCA**	PHF21B	cg11428482	-0.0237447855413755	796	-0.668425958706982	0.504056487389661	0.000563814840405915	0.687348952304006
**BRCA**	PHF21B	cg14786713	-0.0858769590579406	796	-2.42575319124203	0.0154990975960394	0.0073748520970392	0.0598856063567243

**BRCA**	PHF21B	cg15679813	-0.0596398128528152	796	-1.68140607525597	0.0930783108067247	0.00355690727711882	0.226243840365516
**BRCA**	PHF21B	cg20495645	-0.0613767660213641	796	-1.73055797830156	0.0839201421196714	0.00376710740724128	0.210054167826825
**BRCA**	PHF21B	cg20525183	-0.0493514889323361	795	-1.38969029273703	0.165013942802077	0.00243556945983849	0.336883377676538
**BRCA**	PHF21B	cg21715212	-0.0688887397514553	796	-1.943317628272	0.0523317442829259	0.00474565846454374	0.149162631984243
**BRCA**	PHF21B	cg23080354	-0.0482105504569963	796	-1.35834464338039	0.17474113218449	0.00232425717536659	0.350163615949338
**BRCA**	PHF21B	cg25561581	-0.0319643949606484	796	-0.900017712957085	0.368384394837514	0.00102172254520033	0.567262383451892
**BRCA**	PHF21B	cg25976257	-0.0574878221877375	796	-1.62053074449549	0.105516363971135	0.00330484969988892	0.247513569496155
**BRCA**	PHF23	cg00039960	0.107431577182223	796	3.04099237924471	0.00243571622571952	0.0115415437758599	0.0139336795399385
**BRCA**	PHF23	cg01441172	0.0346248757172613	796	0.975015015742986	0.329850512283941	0.00119888201843579	0.529495083830979
**BRCA**	PHF23	cg04379155	-0.143243174808412	796	-4.07322531062285	5.10307356577845e-05	0.0205186071291933	0.000539679859945852
**BRCA**	PHF23	cg11180288	0.0537116814899677	796	1.51376584662483	0.130484180454206	0.00288494472847974	0.286910297746028
**BRCA**	PHF23	cg13351208	0.0239973967794778	796	0.675541149954641	0.499529202747345	0.000575875052191691	0.683660050232816
**BRCA**	PHF23	cg22033559	-0.0234312344380581	796	-0.659594478727904	0.509705846276417	0.000549022747291239	0.692116678103499
**BRCA**	PHF23	cg26213471	-0.149504481032549	796	-4.25525363511377	2.3379954033742e-05	0.0223515898488117	0.000274497456097332
**BRCA**	PHF23	cg26784276	0.0630239092302668	796	1.77718303154813	0.0759218461251995	0.00397201313466491	0.195623479645726
**BRCA**	PHIP	cg01492710	0.166518625441598	796	4.7526038961329	2.38471479043711e-06	0.0277284526189591	3.68966790786127e-05
**BRCA**	PHIP	cg04480383	-0.174990852044845	796	-5.00185746554182	6.99667360069008e-07	0.0306217982993808	1.24525701483954e-05
**BRCA**	PHIP	cg05801338	-0.231371827681448	796	-6.69299209647338	4.13740357466663e-11	0.0535329226446538	1.75228535890221e-09
**BRCA**	PHIP	cg06848288	0.0868127505560213	111	2.89672570765239	0.0038451284765514	0.00753645365910198	0.0201202612971574
**BRCA**	PHIP	cg08287994	-0.271989184201401	796	-7.95432992083862	6.21308528609151e-15	0.0739781163225437	4.79029491324284e-13
**BRCA**	PHIP	cg08587415	-0.0695420469479155	796	-1.96183624163423	0.0501314229027963	0.00483609629370609	0.144533264422881
**BRCA**	PHIP	cg15564972	-0.114225535357232	796	-3.23577012753924	0.00126353783098495	0.0130474729276462	0.00815460942621897
**BRCA**	PHIP	cg19494004	-0.230307433016698	796	-6.66047304966815	5.10404638780717e-11	0.0530415137027407	2.13360658083327e-09
**BRCA**	PHIP	cg19950079	0.145119206701395	796	4.12771170592127	4.05236349763455e-05	0.0210595841536421	0.000443264294059311
**BRCA**	PHIP	cg21607066	0.0507810134369038	111	1.69098443710008	0.0911215171595422	0.00257871132567901	0.222796219188152
**BRCA**	PHIP	cg21875336	-0.216424795008284	796	-6.23859267757845	7.18378819281108e-10	0.0468396918943776	2.43828803957917e-08
**BRCA**	PHIP	cg26363168	-0.0502688220296614	796	-1.41648087383867	0.157027945794242	0.00252695446824977	0.32568604071444
**BRCA**	PHRF1	cg06063517	-0.0394953578636072	796	-1.11236581279858	0.266318420882081	0.0015598832927744	0.462116229805483
**BRCA**	PHRF1	cg07530960	-0.327079971913577	796	-9.74061252915482	2.97928498463657e-21	0.106981308026986	4.3730282282041e-19
**BRCA**	PHRF1	cg08482978	-0.0869355279396581	795	-2.4543298146948	0.0143292139194372	0.00755778601814708	0.0563562622673761
**BRCA**	PHRF1	cg12469954	-0.187413408447166	796	-5.36942090955542	1.03868807649332e-07	0.0351237856657844	2.24892012865909e-06
**BRCA**	PHRF1	cg12813709	-0.240376859997572	796	-6.96914222759635	6.71697807660351e-12	0.0577810348222925	3.29678607250028e-10
**BRCA**	PHRF1	cg15219216	-0.00621350449776187	796	-0.174866892151646	0.861228917994565	3.8607638143707e-05	0.926245759579237
**BRCA**	PHRF1	cg16143007	-0.190219350238923	796	-5.45280646845838	6.6290868883431e-08	0.0361834012053179	1.50300530079063e-06
**BRCA**	PHRF1	cg18793109	-0.0270606441164472	796	-0.761833017846672	0.446386468690309	0.000732278459997011	0.638196551289103
**BRCA**	PHRF1	cg20152424	0.190810960894039	796	5.47040527128569	6.02500974495227e-08	0.0364088227973065	1.38099472038544e-06
**BRCA**	PHRF1	cg21006161	-0.12609246703286	796	-3.57710727540969	0.000368515566678871	0.0158993102424328	0.00290194351596479
**BRCA**	PPARGC	cg04893087	-0.00994043783604859	111	-0.330601283338182	0.741008293428173	9.88123043723463e-05	0.854392899923449
**BRCA**	PPARGC	cg05158538	-0.0594727456312593	110	-1.97508707887401	0.0485082036763815	0.00353700747292048	0.141070923244876
**BRCA**	PPARGC	cg11270806	0.0629801214360442	796	1.77594336026093	0.0761261388231	0.00396649569609887	0.195966288562427
**BRCA**	PPARGC	cg12691631	-0.104016701466382	796	-2.94325499288897	0.00334270129515255	0.0108194741839465	0.0179562287360239
**BRCA**	PPARGC	cg20723350	-0.0555131995081005	796	-1.56469274425385	0.118054421370019	0.00308171531962617	0.267716881192058
**BRCA**	PPARGC	cg27461259	0.0259367092421714	796	0.730169337203789	0.465502598161227	0.000672712886312941	0.654757162098692
**BRCA**	PPARGC	cg27514608	0.0548140803644834	796	1.54492763636319	0.122763225410342	0.00300458340620404	0.274961631807565
**BRCA**	PRDM1	cg00555933	-0.133353048916222	795	-3.78431671499808	0.000165774132822225	0.0177830356552523	0.00148300746191033
**BRCA**	PRDM1	cg01761420	-0.0395399944614499	796	-1.11362494634167	0.265777974629113	0.00156341116201149	0.461547241071885
**BRCA**	PRDM1	cg05170275	-0.0720727106630533	796	-2.03359447296564	0.0423253838617431	0.0051944756223202	0.127592843220198
**BRCA**	PRDM1	cg08742502	-0.106588108363332	796	-3.01684141733589	0.00263597390658788	0.0113610248444734	0.0148484290378151
**BRCA**	PRDM1	cg14359052	-0.096266291871361	796	-2.72181474978776	0.00663507441957863	0.00926719895066207	0.0310514936772363
**BRCA**	PRDM1	cg17143179	-0.0671126030812703	796	-1.89298397421802	0.0587243353819138	0.00450410149234414	0.162086249661189
**BRCA**	PRDM1	cg17521665	0.0568604407942686	796	1.60278773761887	0.10938023874994	0.00323310972731852	0.253897663463414
**BRCA**	PRDM1	cg17965230	-0.101685016276773	111	-3.39777845559265	0.000703561604506166	0.0103398425352076	0.00500845867463469
**BRCA**	PRDM1	cg19464016	-0.128020925843119	111	-4.29286190017143	1.91846998703222e-05	0.0163893574537293	0.000230994357175645
**BRCA**	PRDM1	cg22186515	-0.163259320414699	796	-4.65700670228279	3.76168071639482e-06	0.0266536057022695	5.53606109820694e-05
**BRCA**	PRDM1	cg22196848	-0.0911062730725317	796	-2.57466537543178	0.0102142080731592	0.00830035299316672	0.0433121172377272
**BRCA**	PRDM1	cg24040346	-0.0931143993658404	796	-2.63190601265098	0.0086559904507671	0.00867029136926123	0.0381585607257311
**BRCA**	PRDM1	cg24793124	-0.159379245591456	796	-4.54340595193091	6.39802318379561e-06	0.0254017439253016	8.83906616160887e-05

**BRCA**	PRDM1	cg24874254	-0.115591753205294	796	-3.27499319745334	0.00110256247253003	0.0133614534090736	0.00727659082253136
**BRCA**	PRDM1	cg26351406	-0.083615631983458	796	-2.36142197584936	0.0184461981302755	0.00699157391199309	0.0683986433435012
**BRCA**	PRDM10	cg04181189	-0.0597067415232504	796	-1.68329972061363	0.0927111736065603	0.00356489498332424	0.225647562480016
**BRCA**	PRDM10	cg17627813	0.0290555194206788	796	0.818040174363356	0.413580287384461	0.000844223208805442	0.609160853311222
**BRCA**	PRDM12	cg09191327	0.174227893593478	111	5.88422063574938	5.29527585818364e-09	0.0303553589060204	1.52967207616613e-07
**BRCA**	PRDM12	cg12539975	0.0922634219716352	111	3.08150948308227	0.00211045657931761	0.00851253903391601	0.0124078642027988
**BRCA**	PRDM12	cg13795948	0.136857326844877	796	3.88809051700205	0.000109503745148178	0.0187299279111256	0.00104186430018819
**BRCA**	PRDM12	cg13928649	0.166810072677336	796	4.76115995381478	2.2884918994609e-06	0.0278256003466181	3.55706171481841e-05
**BRCA**	PRDM12	cg23342516	0.188638983561168	796	5.40582503245452	8.54398932504578e-08	0.0355846661189905	1.88452255614577e-06
**BRCA**	PRDM13	cg00126261	0.351957244513831	796	10.5820349381524	1.4406042926496e-24	0.123873901965768	2.84651668745494e-22
**BRCA**	PRDM13	cg00579292	0.299805552958385	796	8.84410190851635	5.92461532888519e-18	0.0898833695846831	6.50988029513127e-16
**BRCA**	PRDM13	cg01422797	0.140104143510166	796	3.98215701556822	7.45774891569805e-05	0.0196291710287172	0.000748934648277599
**BRCA**	PRDM13	cg01815538	0.0438534254549057	796	1.23533320770555	0.217072835983244	0.00192312292412897	0.404420064241797
**BRCA**	PRDM13	cg02400433	0.0270313026547713	796	0.761006369065529	0.44687977637674	0.000730691323213843	0.638537221495322
**BRCA**	PRDM13	cg03120185	0.178733753913465	796	5.11233519211428	3.99281536216696e-07	0.0319457547879991	7.53011098871314e-06
**BRCA**	PRDM13	cg04910970	0.116933877910856	796	3.31354291383923	0.000963053884593723	0.013673531803271	0.00650702055705545
**BRCA**	PRDM13	cg11809464	0.169969610217935	796	4.8539978761346	1.45768718238565e-06	0.0288896683976368	2.3833399887875e-05
**BRCA**	PRDM13	cg13738144	-0.0234787457665182	796	-0.660932670161119	0.508847696984412	0.000551251502768799	0.691352007538544
**BRCA**	PRDM13	cg14344102	0.111203093532365	796	3.14906388813784	0.00169940651467603	0.0123661280111679	0.0104146549868211
**BRCA**	PRDM13	cg17879189	0.0432337125180967	796	1.21784321985469	0.223646250509506	0.00186915389809743	0.412556367530597
**BRCA**	PRDM13	cg17978124	0.174563135471529	796	4.98924707486651	7.45424211298316e-07	0.0304722882656514	1.31495535359957e-05
**BRCA**	PRDM13	cg25273233	0.115290227573045	796	3.26633501531829	0.0011363689227558	0.0132918365738445	0.00746518189299651
**BRCA**	PRDM13	cg25924096	0.159361367252339	796	4.54288301488153	6.41351556534494e-06	0.0253960453725348	8.85732344914689e-05
**BRCA**	PRDM13	cg26507427	0.125790380482939	796	3.56839945463299	0.000380777672836833	0.0158232198220425	0.00298250284742938
**BRCA**	PRDM14	cg00384539	-0.0297505984957624	796	-0.837626829315121	0.402493012915168	0.00088509811085606	0.599046116182108
**BRCA**	PRDM14	cg01295203	0.01091905974501	111	0.363152214549415	0.716560575588681	0.000119225865715099	0.838888087544708
**BRCA**	PRDM14	cg04838988	0.0562139050941223	796	1.58450501956975	0.113478026121743	0.00316000312593099	0.260633272242179
**BRCA**	PRDM14	cg09050331	0.0444923449577784	796	1.25336675327678	0.210442138956948	0.00197956875984195	0.396107809291478
**BRCA**	PRDM14	cg10457247	-0.0138580054038968	111	-0.460914101841116	0.644950852274619	0.000192044313774433	0.791811846253608
**BRCA**	PRDM14	cg11229513	0.0391704116751204	796	1.10319975368167	0.270275517693524	0.00153432115079841	0.466359401651326
**BRCA**	PRDM14	cg13267264	-0.00616934821505331	796	-0.173624152747847	0.862205214873708	3.80608573985814e-05	0.926761552161084
**BRCA**	PRDM14	cg18035229	0.0472720073994013	796	1.33184113782507	0.183295543596905	0.00223464268356905	0.36144314513137
**BRCA**	PRDM14	cg21255438	0.0265206039980935	796	0.746618564160552	0.455515408358699	0.000703342436423694	0.645830191133465
**BRCA**	PRDM14	cg27029821	-0.00670004118904052	796	-0.188560105038343	0.850485907793242	4.48905519348395e-05	0.92031966047026
**BRCA**	PRDM4	cg00090609	0.10358538200847	796	2.93091773051931	0.00347685496989339	0.0107299313658407	0.0185462462649401
**BRCA**	PRDM4	cg03909902	-0.19729924481448	795	-5.66024819149918	2.11413665691753e-08	0.0389269920043639	5.41901637852529e-07
**BRCA**	PRDM4	cg12900929	0.0690216639280374	796	1.94708528602961	0.0518776426763913	0.00476399009139494	0.148210405228321
**BRCA**	PRDM4	cg18440127	0.0138432006095435	796	0.389619531835356	0.696922651855651	0.000191634203116066	0.826191873999401
**BRCA**	PRDM4	cg25995916	0.0740501282930561	111	2.46831703620247	0.0137255323270063	0.00548342150021806	0.0545306195723983
**BRCA**	PRDM4	cg27140793	0.116641329534269	796	3.30513851454953	0.000991993521074409	0.0136051997555219	0.00667281832930935
**BRCA**	PRDM5	cg05713693	-0.172292358832382	796	-4.92234623372819	1.04075157033743e-06	0.0296846569120262	1.77009680067403e-05
**BRCA**	PRDM5	cg06329345	-0.112002626473087	796	-3.17199176172848	0.00157234222945646	0.0125445883368699	0.00977768116693333
**BRCA**	PRDM5	cg07070341	-0.274861025518969	796	-8.04514187402618	3.14032275422591e-15	0.0755485833493395	2.52374244155218e-13
**BRCA**	PRDM5	cg10416963	-0.0652623511559825	796	-1.8405691975198	0.0660586529150666	0.00425917447840677	0.176645361091527
**BRCA**	PRDM5	cg11171429	-0.132971580456616	796	-3.77568058616204	0.000171503245570098	0.0176814412091304	0.00152653586794968
**BRCA**	PRDM5	cg13155001	-0.239374363774957	796	-6.93830671057925	8.25430429872852e-12	0.0573000860326654	3.98594257365169e-10
**BRCA**	PRDM5	cg18181323	-0.291103408680319	796	-8.56323620781266	5.67628510248812e-17	0.0847411945453005	5.63240973549695e-15
**BRCA**	PRDM5	cg19294653	-0.296881697765936	796	-8.74946772897896	1.27691426280879e-17	0.0881387424683845	1.35705284372878e-15
**BRCA**	PRDM5	cg22135105	-0.323370782568303	796	-9.61716846933622	8.76924167178898e-21	0.104568663018837	1.23810233505472e-18
**BRCA**	PRDM8	cg01304890	-0.285869308076295	796	-8.39542829669218	2.12933151312892e-16	0.0817212613000197	1.97908262523717e-14
**BRCA**	PRDM8	cg02895669	0.0917997887280328	796	2.59443007580084	0.00964995639605451	0.00842720121051145	0.0414412756210138
**BRCA**	PRDM8	cg04343891	-0.270727838695515	796	-7.91451701390609	8.36276882979888e-15	0.0732935626447446	6.29790162967448e-13
**BRCA**	PRDM8	cg05007549	0.180687351182293	796	5.17008944854275	2.9653945418933e-07	0.0326479188772734	5.76004751540692e-06
**BRCA**	PRDM8	cg07462804	-0.0695419802599246	796	-1.96183435117072	0.0501316434933621	0.00483608701847174	0.144533264422881
**BRCA**	PRDM8	cg08074201	-0.254767562751406	795	-7.40977221779643	3.25218270517133e-13	0.0649065110302917	1.98120831140249e-11
**BRCA**	PRDM8	cg10695325	-0.140136523660164	796	-3.98309578489555	7.4289184937336e-05	0.0196382452635556	0.000746577038239907
**BRCA**	PRDM8	cg11571772	-0.0693177352906153	796	-1.95547762982617	0.0508780077359073	0.00480494842581982	0.146141302048974

**BRCA**	PRDM8	cg12572049	-0.12944291566052	796	-3.67375430214144	0.000255091764037676	0.0167554684146966	0.00212856469929711
**BRCA**	PRDM8	cg14489346	-0.0867465508117781	796	-2.45050171346248	0.0144810254179465	0.00752496407774041	0.0567925434242656
**BRCA**	PRDM8	cg15656813	-0.0174087464074141	796	-0.489999807436603	0.624269737104166	0.000303064451477652	0.777416684102018
**BRCA**	PRDM8	cg16117799	-0.209207372696914	796	-6.02085096535995	2.65304888567707e-09	0.0437677247907454	8.10330918776452e-08
**BRCA**	PRDM8	cg18624348	-0.0802415050023286	796	-2.26550133668216	0.023751186906839	0.00643869912503873	0.0829674355805853
**BRCA**	PRDM8	cg19595012	-0.292628068494683	796	-8.61227460479436	3.84188392800131e-17	0.085631186470929	3.88654770214478e-15
**BRCA**	PRDM8	cg20142271	-0.247299011644937	796	-7.18271176867969	1.57777668570636e-12	0.0611568011605625	8.70507514098158e-11
**BRCA**	PRDM8	cg26162932	-0.2260440289933	796	-6.53047046232151	1.17128480662316e-10	0.0510959030435238	4.62767548229773e-09
**BRCA**	PRKAA1	cg00301775	-0.103301989864755	796	-2.92281262299233	0.00356764168697316	0.0106713011100179	0.0189383225790781
**BRCA**	PRKAA1	cg01883943	-0.0309849026180331	786	-0.866886196119822	0.386270277405835	0.000960064190248995	0.58426843655596
**BRCA**	PRKAA1	cg03125217	-0.123770870637036	796	-3.51021168865674	0.00047304891027289	0.0153192284182498	0.0035780619276392
**BRCA**	PRKAA1	cg05874655	-0.00779049486498943	796	-0.219250612808668	0.826511321818898	6.06918102414266e-05	0.906775283036884
**BRCA**	PRKAA1	cg06971720	-0.0282523826082338	796	-0.795410034201411	0.426613337695769	0.000798197123042032	0.621174357326916
**BRCA**	PRKAA1	cg10786880	-0.072293216720382	111	-2.41053236773267	0.016091597311205	0.00522630918378011	0.0615785194169848
**BRCA**	PRKAA1	cg24728477	-0.105358100563939	796	-2.98163451471446	0.00295500388559573	0.011003293544411	0.0162632877450325
**BRCA**	PRKCD	cg02121118	-0.0277174394699448	796	-0.78033769679865	0.435425278772334	0.000768256450770053	0.628671964826469
**BRCA**	PRKCD	cg02559423	-0.100490952455725	796	-2.84245466732698	0.00459188517602331	0.0100984315254589	0.0231600826882493
**BRCA**	PRKCD	cg02630105	0.0105503755213911	111	0.350888918926589	0.725738508925016	0.000111310423642369	0.844640136979194
**BRCA**	PRKCD	cg10531918	-0.263252043089296	796	-7.679445568202	4.71361004133084e-14	0.0693016381906887	3.2450493765955e-12
**BRCA**	PRKCD	cg13469346	0.0482986990990416	796	1.36083405592703	0.173953237112451	0.00233276433465976	0.349082137128567
**BRCA**	PRKCD	cg13889007	-0.314108562238529	796	-9.31105844081442	1.21787774261907e-19	0.0986641888715555	1.57643229799181e-17
**BRCA**	PRKCD	cg13908523	-0.00441979875963799	111	-0.146988819197711	0.883167634112974	1.95346210756975e-05	0.937954169668051
**BRCA**	PRKCD	cg14727059	-0.0818377263776195	796	-2.31086919294136	0.0210960885394058	0.00669741345865812	0.0758702377067419
**BRCA**	PRKCD	cg21903826	0.0270081238364763	796	0.760353344302583	0.447269692474338	0.000729438753166438	0.638895695201128
**BRCA**	PRMT1	cg07702240	0.0137245298412222	796	0.38627888836487	0.699393881572652	0.0001883627193626	0.827633824506585
**BRCA**	PRMT1	cg08377944	-0.00291849967535988	796	-0.0821342110296654	0.934560740794605	8.51764035507573e-06	0.965222617950599
**BRCA**	PRMT1	cg11787855	0.182998029936178	796	5.23848183853519	2.07708601199391e-07	0.0334882789605224	4.18830557846175e-06
**BRCA**	PRMT1	cg14258555	-0.0742693343500922	796	-2.0959127893946	0.0364067743397895	0.00551593402480578	0.114253443180023
**BRCA**	PRMT1	cg14576628	-0.0839402713972661	111	-2.80145313067217	0.00517601022501883	0.00704596916224669	0.0254817453135752
**BRCA**	PRMT1	cg18880319	-0.10345451033074	796	-2.92717466840788	0.00351851662032675	0.0107028357077733	0.0187184217986527
**BRCA**	PRMT1	cg22292364	0.0140146740913255	796	0.39444662810879	0.693357513936182	0.00019641108988607	0.824050575029812
**BRCA**	PRMT1	cg24228123	-0.119569355378255	796	-3.38929524421461	0.000735381466936822	0.0142968307455714	0.0051946123525727
**BRCA**	PRMT1	cg25595446	-0.0604024800531433	111	-2.01245434103439	0.0444141108031818	0.00364845959657038	0.132290042722365
**BRCA**	PRMT1	cg26846726	-0.0447484729205013	796	-1.26059641355667	0.207825539821481	0.00200242582871684	0.392865162803982
**BRCA**	PRMT2	cg04493422	0.0305536767567026	796	0.860258312887672	0.389906996147122	0.000933527163353068	0.587426118213132
**BRCA**	PRMT2	cg06725035	-0.0742761823359345	110	-2.46916281210894	0.0136941370031568	0.00551695126240099	0.0544496573602878
**BRCA**	PRMT2	cg09031657	-0.127669003026936	796	-3.62256831282403	0.000310284967495531	0.0162993743338918	0.00251257507664696
**BRCA**	PRMT2	cg10187059	-0.0464499955944058	796	-1.3086312633761	0.191038891417816	0.00215760209072031	0.371458409831726
**BRCA**	PRMT2	cg10657630	0.0150285168874984	796	0.422987724658222	0.672419041867272	0.000225856319837825	0.810417443915767
**BRCA**	PRMT2	cg12162201	-0.14778642613107	796	-4.20525534816626	2.90557675242954e-05	0.0218408277485943	0.000331480331248723
**BRCA**	PRMT2	cg13271076	0.0426084400750383	111	1.41829895441796	0.156385201589389	0.00181547916562813	0.324785903477093
**BRCA**	PRMT2	cg17243659	-0.0261441417740492	795	-0.735548147586501	0.462223767612425	0.000683516149101584	0.651726948603099
**BRCA**	PRMT2	cg23283320	-0.110610015621584	794	-3.1281033203236	0.00182394535388593	0.012234575555807	0.0110293046918151
**BRCA**	PRMT3	cg01769160	-0.0021067876925079	796	-0.0592903927552088	0.952735769662668	4.43855438130276e-06	0.975037248149736
**BRCA**	PRMT3	cg04101203	-0.0421857480068227	794	-1.18676871012483	0.235675618160343	0.00177963733489515	0.426941916109921
**BRCA**	PRMT3	cg06578097	-0.0626921894198016	795	-1.76667562285054	0.0776681836921547	0.00393031061424829	0.198734292081291
**BRCA**	PRMT3	cg13432154	-0.0530566484591865	796	-1.49525249759541	0.135246888492302	0.00281500794572169	0.294049815359272
**BRCA**	PRMT3	cg13982511	-0.128837427590683	795	-3.65397004069502	0.000275252198494222	0.0165990827481846	0.00227265649858411
**BRCA**	PRMT3	cg19653624	-0.0853251177660894	796	-2.41005072601369	0.0161773021705011	0.00728037572179702	0.0618426066364599
**BRCA**	PRMT3	cg23019633	-0.136113500230788	796	-3.86655853433779	0.000119434898411724	0.0185268849450768	0.00112213991389392
**BRCA**	PRMT3	cg23071995	-0.0300593225578758	796	-0.846326762660315	0.397626117833056	0.000903562872638423	0.594235447328878
**BRCA**	PRMT6	cg00944433	-0.188824425439158	796	-5.41133561973024	8.29425058880874e-08	0.0356546636424282	1.83359741490704e-06
**BRCA**	PRMT6	cg02990361	-0.287030956370602	796	-8.43259991221171	1.59164847107762e-16	0.0823867699150221	1.50816931984181e-14
**BRCA**	PRMT6	cg02993280	-0.16992523276524	796	-4.85269285651998	1.46703581064897e-06	0.0288745847303211	2.39761730784928e-05
**BRCA**	PRMT6	cg09367891	-0.224685764461362	796	-6.4891368680486	1.52084603241516e-10	0.0504836927515867	5.85416606856533e-09
**BRCA**	PRMT6	cg14671364	-0.250730010551879	795	-7.28439699138025	7.82258813733654e-13	0.0628655381913455	4.50111258547307e-11
**BRCA**	PRMT6	cg14828511	-0.210341566529111	796	-6.05499908184396	2.16726571735714e-09	0.0442435746099202	6.72518026390432e-08

**BRCA**	PRMT6	cg22528358	0.117383806557698	796	3.32647027850195	0.000920065559711953	0.0137789580419751	0.00626262841475472
**BRCA**	PRMT7	cg00894222	-0.14666375583051	796	-4.17260481132212	3.34463875494129e-05	0.0215102572743116	0.000374571681917069
**BRCA**	PRMT7	cg02477175	-0.0965606724664735	796	-2.73021622616016	0.00646987595241572	0.00932396346717758	0.0304303222395544
**BRCA**	PRMT7	cg06089844	-0.0379339569204237	796	-1.06832510489827	0.285699396712888	0.00143898508764056	0.482946890938538
**BRCA**	PRMT7	cg06092312	0.0223143138031352	796	0.62813685791634	0.530095370272754	0.000497928600504789	0.708082503415958
**BRCA**	PRMT7	cg06745885	-0.0573424558368074	796	-1.61641946024581	0.106401874366384	0.00328815724139621	0.248932834317474
**BRCA**	PRMT7	cg09175928	-0.135185091381652	796	-3.83969268499082	0.000133021066952633	0.0182750089318657	0.00122997594291325
**BRCA**	PRMT7	cg09779635	-0.0669939274782834	796	-1.88962149494152	0.0591735894795459	0.00448818631896549	0.163015632156348
**BRCA**	PRMT7	cg10233539	0.0904477915642878	796	2.55590261967903	0.0107768925431665	0.00818080299885686	0.0452006297210879
**BRCA**	PRMT7	cg10446007	-0.0587028879727288	796	-1.65489958214669	0.0983410560031389	0.00344602905633875	0.235243200107875
**BRCA**	PRMT7	cg11893236	-0.0737139222648984	111	-2.45816042960299	0.014117554885167	0.00543374233567549	0.0556927517614951
**BRCA**	PRMT7	cg19328639	-0.0877362651646572	796	-2.4786757869989	0.0133943429820718	0.00769765222504304	0.0535376917546356
**BRCA**	PRMT7	cg21918026	-0.00967836241735495	111	-0.321738743213131	0.747711475396541	9.36706990816688e-05	0.858415717034383
**BRCA**	PRMT7	cg26492318	-0.149784770803108	796	-4.26341426595587	2.25603544611096e-05	0.0224354775645395	0.000266119820310472
**BRCA**	PRMT7	cg26561196	-0.124724495917544	796	-3.53768275111112	0.000427150754827406	0.0155561998818854	0.00328130402103715
**BRCA**	PRMT7	cg26796563	-0.114896535016573	796	-3.25503167557478	0.00118194883260141	0.0132012137588146	0.00771574452323525
**BRCA**	PRMT8	cg01693063	-0.0737415880824574	796	-2.08093783858949	0.0377603905901756	0.00543782181292282	0.117360440494292
**BRCA**	PRMT8	cg06029846	-0.0500443981662875	796	-1.4101411182494	0.158890611728751	0.00250444178782592	0.328219231248712
**BRCA**	PRMT8	cg07912789	-0.0935996708378669	796	-2.64574326673311	0.00831282872571913	0.00876089838095704	0.0370043601446895
**BRCA**	PRMT8	cg10350722	0.0805708113256363	796	2.27485944373683	0.0231808931111408	0.00649165563767128	0.0814392929317841
**BRCA**	PRMT8	cg11596580	-0.0653975285783394	796	-1.84439791266992	0.0654984522948676	0.00427683674415472	0.175557697003409
**BRCA**	PRMT8	cg19656577	-0.0859306643041353	796	-2.42728147648439	0.0154344476120694	0.00738407906774999	0.0596989177872488
**BRCA**	PRMT8	cg21667836	0.0573079017039078	111	1.90900206615554	0.0565204153363277	0.00328419559770476	0.157651594679251
**BRCA**	PRMT8	cg23739862	-0.0610395409332585	111	-2.03375852259	0.042214248305581	0.00372582555734295	0.127356520308864
**BRCA**	PRMT8	cg24100636	-0.139914260332882	796	-3.97665216110932	7.62895274879047e-05	0.0195760002444974	0.000763130706106989
**BRCA**	PRMT8	cg27316886	-0.180628656071597	796	-5.16835332542463	2.99215876464982e-07	0.0326267113942314	5.80623594255844e-06
**BRCA**	PWWP2E	cg00259755	-0.030762872734974	111	-1.02355232196969	0.30627059358678	0.000946354338908206	0.504724284147989
**BRCA**	PWWP2E	cg05010219	-0.0340749163367508	796	-0.959510353091872	0.337594528147886	0.00116109992335657	0.537039214828061
**BRCA**	PWWP2E	cg15012692	0.0276874426658137	796	0.779492539195273	0.435922490116944	0.00076659448137272	0.629061158998646
**BRCA**	PWWP2E	cg15278699	-0.138272027702364	796	-3.92906121644965	9.27240320470466e-05	0.0191191536449233	0.000902687191724183
**BRCA**	PWWP2E	cg17803807	-0.111373531569134	315	-1.97956442691502	0.0486311132569208	0.012404063534181	0.141338544652329
**BRCA**	PWWP2E	cg19919881	0.0434449185943909	796	1.22380387165783	0.221390170588448	0.00188746095167326	0.40975511350299
**BRCA**	PWWP2E	cg22633292	-0.0883641167101785	796	-2.49655258700164	0.0127428661355729	0.00780821712197005	0.0515640326736414
**BRCA**	PWWP2E	cg22664157	-0.0101767613959057	796	-0.286414284054481	0.774635739449171	0.000103566472509197	0.875083946091106
**BRCA**	PWWP2E	cg24520806	-0.242641010961816	796	-7.03887194104377	4.2027185056939e-12	0.0588746602005719	2.14110205259955e-10
**BRCA**	PYGO1	cg06364757	-0.23700284547803	796	-6.86545527899981	1.33908831547842e-11	0.0561703487646828	6.2192857560793e-10
**BRCA**	PYGO1	cg09275667	-0.198995720187962	796	-5.71452441018666	1.55737568364331e-08	0.0395992966531257	4.08891272134148e-07
**BRCA**	PYGO1	cg11480800	-0.223627365610295	796	-6.45695617090614	1.86193500552026e-10	0.0500091986498006	7.05540047829727e-09
**BRCA**	PYGO1	cg12686317	-0.164813667775498	796	-4.7025767991723	3.0301246816444e-06	0.0271635450856121	4.55332276383706e-05
**BRCA**	PYGO1	cg13878116	-0.0967877673643373	796	-2.73669789609927	0.00634496997976967	0.00936787191137308	0.0299738858302541
**BRCA**	PYGO1	cg17841803	-0.175658129417507	796	-5.02153667703619	6.33631727877812e-07	0.0308557784304578	1.14235797540038e-05
**BRCA**	PYGO1	cg19850348	-0.165358025010624	111	-5.57600357364702	3.09188340976186e-08	0.027343276435414	7.60208527114457e-07
**BRCA**	PYGO1	cg22510134	-0.0353454101657375	796	-0.995329939437463	0.319879813754866	0.00124929801978422	0.519275702834732
**BRCA**	PYGO1	cg23030278	-0.213316322990642	796	-6.14468451778517	1.26820784202626e-09	0.0455038536542478	4.11765279059227e-08
**BRCA**	PYGO1	cg23412777	-0.0227129022190284	111	-0.755548320726668	0.450080924274496	0.000515875927211147	0.6410750393286
**BRCA**	PYGO1	cg25902187	-0.166835307016387	796	-4.76190082222045	2.28033798345003e-06	0.0278340196672521	3.54580477882294e-05
**BRCA**	PYGO2	cg01277844	-0.036123825319837	111	-1.20213943553319	0.229566827386897	0.0013049307557381	0.419617522785156
**BRCA**	PYGO2	cg02341811	-0.14965911149849	796	-4.25975556801432	2.2924363309166e-05	0.0223978496545175	0.000269922995128684
**BRCA**	PYGO2	cg04602755	-0.0699215046948278	796	-1.97259349500135	0.0488893338150162	0.00488901681878883	0.141904006844591
**BRCA**	PYGO2	cg10892866	0.00776632593233332	111	0.258289216026001	0.796231763572311	6.0315818487233e-05	0.888445572350281
**BRCA**	PYGO2	cg14024356	0.0464056846537751	796	1.30738020006717	0.191463024107111	0.00215348756818562	0.371911072566857
**BRCA**	PYGO2	cg18188585	-0.242873777240816	796	-7.04604741451679	4.00384483970002e-12	0.0589876716712214	2.04783106811061e-10
**BRCA**	PYGO2	cg24683561	-0.158944290279876	796	-4.53068491559988	6.78526420164257e-06	0.0252632874125736	9.29645726140511e-05
**BRCA**	PYGO2	cg27105205	-0.106158283083927	796	-3.0045368086418	0.00274370045719562	0.0112695810673273	0.0153379874509253
**BRCA**	RBBP4	cg05797186	-0.26782812756152	796	-7.82315732717679	1.64607072999701e-14	0.0717319059131098	1.2046512358362e-12
**BRCA**	RBBP4	cg06526778	-0.203561402141848	796	-5.85123780643902	7.13902915549914e-09	0.0414372444419551	2.01368250225852e-07
**BRCA**	RBBP4	cg09036220	-0.166177070015273	796	-4.74257843550906	2.50241734162572e-06	0.0276148185988609	3.84805405563217e-05

**BRCA**	RBBP4	cg11229946	0.0834514582757287	796	2.3567529336716	0.0186780710522749	0.00696414588834568	0.0690543607756236
**BRCA**	RBBP4	cg13641837	0.00983299699787388	793	0.276213820340515	0.782456176385423	9.66878299601968e-05	0.879807992740259
**BRCA**	RBBP4	cg16148416	-0.0784950371681703	796	-2.21588325437736	0.0269830866390619	0.00616147086003243	0.0912106648995906
**BRCA**	RBBP4	cg16396527	-0.0701736460114951	796	-1.97974192853053	0.0480783345370192	0.00492434059454662	0.140198139177332
**BRCA**	RBBP4	cg18758482	-0.033651268823398	111	-1.11925391346542	0.263275065017324	0.0011324078934246	0.458694724243896
**BRCA**	RBBP4	cg20740619	0.129416855456944	796	3.67300208045413	0.000255831437756298	0.0167487224763636	0.00213382125751189
**BRCA**	RBBP4	cg22358606	0.0914658846485467	796	2.58491357483135	0.00991806655666559	0.00836600805454125	0.0423341180630264
**BRCA**	RBBP4	cg22857567	-0.0334189627727167	796	-0.941018602321026	0.346982248828768	0.00111682707280422	0.546264966717635
**BRCA**	RBBP4	cg23016766	-0.0709656341007846	796	-2.00219799108289	0.0456039739134535	0.00503612122332645	0.13490438613671
**BRCA**	RBBP4	cg26131911	-0.0186830435114891	111	-0.621442567989646	0.534436533337959	0.000349056114852196	0.711174213141978
**BRCA**	RBBP5	cg00960017	-0.0189535207117131	796	-0.533495187434213	0.593840522898692	0.000359235947369339	0.755696356055435
**BRCA**	RBBP5	cg02189925	0.318666958746833	796	9.46133323523732	3.37476315550541e-20	0.101548630596955	4.61399516554285e-18
**BRCA**	RBBP5	cg03143531	-0.0866119525535638	796	-2.44667068848546	0.0146346505753627	0.00750163032514078	0.0572708892227863
**BRCA**	RBBP5	cg05290904	-0.0576671113604646	111	-1.92100761537236	0.0549875728121653	0.00332549573266022	0.154621463342736
**BRCA**	RBBP5	cg07601985	0.326831323554164	796	9.73232169824961	3.20438714235051e-21	0.106818714056166	4.69457802922816e-19
**BRCA**	RBBP5	cg07972983	0.160853419685649	796	4.58654101997078	5.23654598987505e-06	0.0258738226245677	7.41080332429215e-05
**BRCA**	RBBP5	cg14717946	0.00677210077087047	111	0.225222064072173	0.821848132010566	4.58613488508244e-05	0.904386593830712
**BRCA**	RBBP5	cg18131828	-0.0187975345295506	796	-0.529103000037838	0.596882255820597	0.000353347304389647	0.757918194879744
**BRCA**	RBBP5	cg21214746	-0.124796166855224	796	-3.5397477755771	0.000423874571409549	0.0155740832617569	0.00326064455732566
**BRCA**	RBBP5	cg23227967	-0.0418159349272212	796	-1.17783493655398	0.239216211872727	0.00174857241383759	0.431055661802899
**BRCA**	RBBP5	cg24103461	-0.090472916005276	796	-2.55661845269656	0.0107549285964856	0.00818534853049773	0.0451302074132908
**BRCA**	RBBP5	cg26795850	-0.185426343558592	796	-5.31045285683862	1.42176020586516e-07	0.034382928885509	2.98366370259169e-06
**BRCA**	RBBP7	cg01947226	-0.148663491085178	796	-4.23077451883873	2.60122007052672e-05	0.0221008335816329	0.000301191577621492
**BRCA**	RBBP7	cg03048084	-0.0357967464637993	796	-1.00805581453938	0.313735540124556	0.00128140705739353	0.512811333538886
**BRCA**	RBBP7	cg03171708	-0.113906671212936	796	-3.22661846780194	0.00130410132068413	0.0129747297468119	0.00837056585324267
**BRCA**	RBBP7	cg03811055	-0.0903065032881039	796	-2.55187719631265	0.0109011504788172	0.00815526453612432	0.0456109342413329
**BRCA**	RBBP7	cg07096248	-0.0450655674848056	796	-1.26954730816991	0.20461883733467	0.00203090537272757	0.388773475898076
**BRCA**	RBBP7	cg08363715	-0.0497462109521614	796	-1.40171795095297	0.161391254493564	0.00247468550409694	0.331649926092197
**BRCA**	RBBP7	cg14719055	-0.252622710407436	111	-8.68300127478404	1.36175565062895e-17	0.0638182338135992	1.44244235712055e-15
**BRCA**	RBBP7	cg15289846	-0.0792171527267216	796	-2.23639642626034	0.0256035042186435	0.00627535728612874	0.0877328550052924
**BRCA**	RBBP7	cg15464143	-0.202542462390635	796	-5.82069284563394	8.50994192941519e-09	0.0410234490712619	2.36436579448902e-07
**BRCA**	RBBP7	cg16978043	-0.0391222594218008	796	-1.10184150821511	0.270865306964814	0.00153055118226668	0.467066290369517
**BRCA**	RBBP7	cg17878446	-0.137884571289212	796	-3.91783778864177	9.7061167013694e-05	0.0190121549996097	0.000939618768872985
**BRCA**	RBBP7	cg17880859	-0.0421790978969239	111	-1.40398192313721	0.160604960798651	0.00177907629939829	0.330514319887051
**BRCA**	RBBP7	cg21122900	-0.147080961526585	796	-4.18473644204136	3.17459857585117e-05	0.0216328092435847	0.000358127496533883
**BRCA**	RING1	cg01241018	-0.0441735582796583	796	-1.24436887679738	0.213731986806127	0.00195130325108637	0.400285670508832
**BRCA**	RING1	cg01878788	-0.0469564798778449	796	-1.32293175770187	0.186239899796244	0.00220491100251845	0.36515731443277
**BRCA**	RING1	cg02475416	0.0142128129007638	796	0.400024415043677	0.689246399229819	0.000202004050552118	0.821333177251232
**BRCA**	RING1	cg03037740	0.0762810638894458	796	2.15301253971832	0.0316190258768282	0.00581880070810571	0.102644404067016
**BRCA**	RING1	cg06316695	-0.025577421399517	795	-0.719593291075769	0.47198799391009	0.000654204485448473	0.660107759209002
**BRCA**	RING1	cg06671577	-0.132253556286071	796	-3.75492858737043	0.000186082357188448	0.0174910031503128	0.00163313299809546
**BRCA**	RING1	cg07946923	-0.0105130593925873	796	-0.295880068191664	0.767399232562085	0.000110524417792068	0.871162968391531
**BRCA**	RING1	cg08892712	-0.0626598213383807	796	-1.76687569311193	0.0776341508878227	0.00392625321015779	0.198666813623924
**BRCA**	RING1	cg09478665	-0.0372078260653455	796	-1.04784662493708	0.295028937873959	0.001384422320509	0.492901838178996
**BRCA**	RING1	cg09724776	0.000231601827001711	796	0.00651785333104325	0.994801183624547	5.36394062705306e-08	0.99732674302986
**BRCA**	RING1	cg09832551	-0.0737139407157127	796	-2.08015338524366	0.037832466002531	0.0054337450558396	0.117532800615027
**BRCA**	RING1	cg09869033	-0.0645183949837281	796	-1.81949947435684	0.0692126834128791	0.00416262329127635	0.182831317075765
**BRCA**	RING1	cg10265934	0.0446928946668081	796	1.25902759890605	0.208391316034714	0.0019974548336984	0.393586752437441
**BRCA**	RING1	cg12065531	0.276805276648916	796	8.10675404060233	1.96949219222915e-15	0.0766211611806826	1.61448551741069e-13
**BRCA**	RING1	cg12115664	-0.0762401151573698	796	-2.1518500136927	0.0317108244025846	0.00581255515920901	0.102839414439497
**BRCA**	RING1	cg12256228	0.0841004097898266	111	2.8068356814824	0.00509092413751704	0.00707287892681676	0.0251456096732699
**BRCA**	RING1	cg14046664	-0.0640764546911527	796	-1.80698464459098	0.0711440546926621	0.00410579204578735	0.186544224300143
**BRCA**	RING1	cg14363494	0.252693004982627	111	8.68165471430844	1.37844225788628e-17	0.0638537547671499	1.45699099198377e-15
**BRCA**	RING1	cg18189601	0.0621578355194716	111	2.07022532099254	0.0386631214294366	0.00386359651646568	0.119431340076302
**BRCA**	RING1	cg20505183	-0.0726221116769825	796	-2.04917817950914	0.0407736695867713	0.00527397110442412	0.124073806056456
**BRCA**	RING1	cg24536938	-0.0458798353977558	796	-1.29253410051477	0.196549202084099	0.00210495929612517	0.378661432068609
**BRCA**	RING1	cg24550302	-0.0701211611061878	796	-1.97825390279094	0.0482462132501691	0.00491697723487994	0.140534060826228

**BRCA**	RNF17	cg00202166	-0.310192236807016	796	-9.18252174710833	3.60414750833891e-19	0.0962192237753396	4.43640903898287e-17
**BRCA**	RNF17	cg01405147	-0.048396720877506	796	-1.36360233390221	0.17308020711894	0.00234224259169522	0.347961560706275
**BRCA**	RNF17	cg07131398	-0.181083908888664	756	-5.04927125341228	5.56930798701145e-07	0.032791382058398	1.01799517279504e-05
**BRCA**	RNF17	cg12020676	-0.0983815282118312	796	-2.782198707575	0.00552762234682213	0.00967892509329533	0.0268743111585952
**BRCA**	RNF17	cg14855529	-0.0733774153757925	796	-2.07060535997995	0.0387191763876257	0.00538424508723159	0.1195522765588
**BRCA**	RNF17	cg15523998	-0.146166519712623	796	-4.1581490040925	3.55857803257802e-05	0.0213646514849005	0.000396071570531628
**BRCA**	RNF17	cg17806989	-0.298596844820495	111	-10.4049919509347	2.96770991040058e-24	0.0891600757367545	5.60364137790541e-22
**BRCA**	RNF17	cg20477497	-0.124597428274388	796	-3.53402174301371	0.000433017278649228	0.0155245191325912	0.00331850518916738
**BRCA**	RNF17	cg21831927	-0.0937091565003409	796	-2.64886545394635	0.00823710572007722	0.00878140601200538	0.0367429703203949
**BRCA**	RNF17	cg23678722	-0.108009134056277	796	-3.05753333821671	0.00230671515913999	0.0116659730395869	0.0133458723107345
**BRCA**	RNF17	cg24753530	-0.161146052384336	796	-4.59510733656325	5.03132120651661e-06	0.0259680501990552	7.16206041975761e-05
**BRCA**	RNF20	cg04845236	-0.0735267486621004	796	-2.07484221295682	0.0383235462466778	0.00540618276881969	0.118594293993956
**BRCA**	RNF20	cg14625770	0.213082791229683	796	6.13763737143713	1.32307250020325e-09	0.0454042759182327	4.27793441732383e-08
**BRCA**	RNF20	cg21219529	0.148691645055384	796	4.23159385837546	2.5919702963061e-05	0.0221092053092764	0.000300372020305135
**BRCA**	RNF217	cg00991794	-0.354131285535272	796	-10.6567401616196	7.15038855393029e-25	0.125408967394865	1.44482422640118e-22
**BRCA**	RNF217	cg02312409	-0.352999499623561	796	-10.6178247839464	1.03037907910875e-24	0.124608646734485	2.06591005361304e-22
**BRCA**	RNF217	cg03113502	-0.234829737604644	796	-6.79881309334036	2.07671842987729e-11	0.055145005663466	9.26886021422109e-10
**BRCA**	RNF217	cg06993703	-0.350309733927379	796	-10.5255528605463	2.44065503189842e-24	0.122716909684271	4.6765595456036e-22
**BRCA**	RNF217	cg07439975	-0.257365542895505	796	-7.49539745437738	1.77095518282955e-13	0.0662370226698979	1.12925973354952e-11
**BRCA**	RNF217	cg10621825	-0.321151743653376	796	-9.54355477654627	1.66095996656355e-20	0.103138442451604	2.3073699935508e-18
**BRCA**	RNF217	cg16203203	-0.416034915776092	796	-12.8754361529344	1.39710614256423e-34	0.17308505114482	4.66465559032796e-32
**BRCA**	RNF217	cg17382198	-0.376265205017848	796	-11.4289306854873	4.16603653971948e-28	0.141575504507123	1.03543976540236e-25
**BRCA**	RNF217	cg18192252	-0.37464090430456	796	-11.3715173156432	7.34100945571926e-28	0.140355807178138	1.79586946414536e-25
**BRCA**	RNF217	cg19171596	-0.331720551991304	796	-9.89576484495717	7.55700014770245e-22	0.110038524613416	1.1687659433208e-19
**BRCA**	RNF217	cg19478500	-0.330576819351921	796	-9.85745124097899	1.062027378361e-21	0.109281033492832	1.61998740925912e-19
**BRCA**	RNF217	cg20034100	0.337583398656535	110	11.910851179873	7.39588198212778e-31	0.113962551048497	2.12308207718689e-28
**BRCA**	RNF217	cg23521835	-0.379106434603151	796	-11.5296540043313	1.53460647515014e-28	0.143721688757514	3.9400388161e-26
**BRCA**	RNF217	cg24822696	-0.21061364908451	796	-6.06319469547409	2.06427827355392e-09	0.0443581091806931	6.4364113832807e-08
**BRCA**	RPA3	cg26322315	-0.0730392747007997	315	-1.29358589957302	0.19676534852985	0.00533473564881888	0.378915471151295
**BRCA**	RPH3A	cg00851732	0.0189469594442234	796	0.533310437489746	0.593968324992773	0.000358987272181046	0.755735368069213
**BRCA**	RPH3A	cg00854817	-0.0809985032044475	796	-2.28701455536822	0.0224579575911571	0.00656075752136089	0.0794278210968574
**BRCA**	RPH3A	cg01119407	-0.0083488663784795	795	-0.234817731713426	0.814410975992691	6.97035698057054e-05	0.900137522634679
**BRCA**	RPH3A	cg01573562	-0.048387425303211	111	-1.61108735743267	0.107446008022675	0.00234134292747382	0.250829874808425
**BRCA**	RPH3A	cg05967001	-0.045055606654861	796	-1.26926612962102	0.204719019323134	0.00203000769103756	0.388851046570549
**BRCA**	RPH3A	cg08254200	0.014129491945144	796	0.397678848976579	0.690974090201082	0.000199642542627888	0.822433490654255
**BRCA**	RPH3A	cg09384610	-0.0592037313893374	796	-1.6690683667697	0.0954990344198537	0.00350508181042082	0.23041441192377
**BRCA**	RPH3A	cg11596863	-0.0344050245962876	796	-0.968816784268425	0.332932368540745	0.00118370571747116	0.532594714327927
**BRCA**	RPH3A	cg16403344	-0.0465701774546635	796	-1.31202448503954	0.189892017382359	0.00216878142815884	0.369949884683356
**BRCA**	RPH3A	cg17453780	-0.0674375103770566	796	-1.9021901048746	0.0575088228420741	0.00454781780585561	0.159637515224846
**BRCA**	RPH3A	cg18629535	-0.0218534050244882	796	-0.615156240023903	0.538628250842847	0.000477571311164326	0.714284065698454
**BRCA**	RPH3A	cg19712198	-0.0150586617385778	796	-0.42383636403233	0.671800239840869	0.000226763293356907	0.809950063668045
**BRCA**	RPH3A	cg20704159	0.00200116426623988	796	0.0563178672412257	0.95510278969048	4.00465842047541e-06	0.97610554571028
**BRCA**	RPH3A	cg21900624	-0.0376587171285631	796	-1.06056254245621	0.289212063619901	0.00141817897576913	0.48672716662513
**BRCA**	RPH3A	cg23199238	-0.0196650641667354	789	-0.551079412194245	0.581736044145417	0.00038671474868182	0.74709970833204
**BRCA**	RPH3A	cg23549179	0.144593475737974	796	4.11243811855963	4.32405266047119e-05	0.020907273225988	0.000468177247973132
**BRCA**	RPH3A	cg23596620	-0.00458955782001022	796	-0.129162966331753	0.897261492670489	2.1064040983217e-05	0.945641122108747
**BRCA**	RTF1	cg04910470	-0.151678360209173	796	-4.31857373981708	1.76977628329743e-05	0.0230063249557437	0.00021542477888099
**BRCA**	RTF1	cg07091958	-0.0948052094260126	111	-3.16716226815759	0.00158147258136447	0.00898802773431012	0.00982503417941765
**BRCA**	RTF1	cg07129439	0.162754490677051	796	4.64221387167716	4.03365869481116e-06	0.0264890242355463	5.8840135853017e-05
**BRCA**	RTF1	cg15581235	-0.0299731964850562	796	-0.843899680748052	0.398980282319976	0.000898392507531785	0.59560700189592
**BRCA**	RTF1	cg18782716	0.0970047396597466	796	2.74289105298039	0.00622765942583273	0.00940991951645521	0.0295411303276361
**BRCA**	RTF1	cg22263512	0.167490693714087	796	4.78114604561015	2.07805870103496e-06	0.0280531324808261	3.26784917300008e-05
**BRCA**	RTF1	cg24280607	0.0965221627697272	796	2.72911713742256	0.00649127451173388	0.00931652790574572	0.0305162079626435
**BRCA**	RTF1	cg27576415	0.101229744632068	796	2.86356746766874	0.00429967272882615	0.0102474611982738	0.0220043905181436
**BRCA**	SATB1	cg01069104	-0.274441960200729	796	-8.03187596377667	3.47084479584275e-15	0.0753183895188187	2.75521326579378e-13
**BRCA**	SATB1	cg01373248	0.313950636134309	796	9.30586505331379	1.27274464158749e-19	0.0985650019291375	1.6447158911571e-17
**BRCA**	SATB1	cg04004754	-0.07472345005158	796	-2.10879986879046	0.0352752658004809	0.00558359398761097	0.111485030578209

**BRCA**	SATB1	cg14415616	-0.328027938963595	796	-9.77224196777699	2.25550257407795e-21	0.107602328740704	3.3613901771613e-19
**BRCA**	SATB1	cg19818826	-0.24686709771579	795	-7.16482455035149	1.78561710786733e-12	0.0609433639346174	9.77553112522387e-11
**BRCA**	SATB1	cg26131754	-0.301184844232967	796	-8.88883918793606	4.11155907854685e-18	0.0907123103956366	4.59561245627117e-16
**BRCA**	SATB1	cg26768584	0.199191196271394	796	5.72036967122034	1.50678709056482e-08	0.0396771326720289	3.96944283091367e-07
**BRCA**	SCML2	cg00457389	-0.181037397827036	796	-5.18044454808898	2.81050536143457e-07	0.0327745394119846	5.48934105165556e-06
**BRCA**	SCML2	cg04189389	-0.199276180925469	796	-5.72291116283574	1.48529359749917e-08	0.0397109962842404	3.92083237610623e-07
**BRCA**	SCML2	cg05049545	-0.237923745392716	796	-6.89372916274054	1.11040403843751e-11	0.0566077086216979	5.22898134178012e-10
**BRCA**	SCML2	cg06131666	-0.215982033871755	796	-6.22520461542445	7.7936508098097e-10	0.0466482389553798	2.63150725303097e-08
**BRCA**	SCML2	cg08300622	-0.255164888693256	111	-8.77643248333295	6.28748594287039e-18	0.0651091204218415	6.87944699633838e-16
**BRCA**	SCML2	cg17710066	-0.157358088996344	796	-4.48431652677326	8.3958343934697e-06	0.0247615681725815	0.000112127989837868
**BRCA**	SCML2	cg17727916	-0.236342906559567	796	-6.84520560348065	1.53067068063701e-11	0.0558579694810241	6.99218995475487e-10
**BRCA**	SCML2	cg22823767	-0.30968531547539	796	-9.16592223787672	4.14266285508517e-19	0.0959049946210921	5.05132153837767e-17
**BRCA**	SCML2	cg26246138	-0.175491439724306	111	-5.92824550279002	4.08733940083143e-09	0.0307972454165096	1.20397758935358e-07
**BRCA**	SCML4	cg00977377	0.0451947231471026	796	1.27319320358448	0.203323063140174	0.00204256300034326	0.387166847161275
**BRCA**	SCML4	cg03500965	-0.421244234014696	762	-12.7875265621179	4.80830606975695e-34	0.177446704690628	1.57166958987678e-31
**BRCA**	SCML4	cg05603896	-0.31304907612467	796	-9.27623371188387	1.63596067771009e-19	0.0979997240625091	2.09667092194034e-17
**BRCA**	SCML4	cg08792995	-0.228222064473485	796	-6.59683457649004	7.67831648619924e-11	0.0520853107125398	3.13392944767777e-09
**BRCA**	SCML4	cg17388996	-0.0802283404595617	796	-2.26512724704583	0.0237742356563301	0.00643658661289535	0.0830076248215316
**BRCA**	SCML4	cg23503775	-0.348101079708104	796	-10.4500074966529	4.92414705516937e-24	0.121174361693948	9.0560069978687e-22
**BRCA**	SCML4	cg27353361	-0.0629228649253839	795	-1.77320188636754	0.0765800037064059	0.00395928693041811	0.196862891029547
**BRCA**	SCML4	cg27659622	-0.188099341336279	796	-5.38979244325799	9.31277971901648e-08	0.035381362211142	2.0333381573426e-06
**BRCA**	SETD1A	cg26692825	-0.16967049165992	796	-4.84520220457813	1.52182323944668e-06	0.0287880757401189	2.47519793204087e-05
**BRCA**	SETD6	cg01152986	-0.157153658850939	796	-4.47834315096222	8.62818707441551e-06	0.0246972724902372	0.000114797534678823
**BRCA**	SETD6	cg01472538	-0.250507052168612	795	-7.27748552278937	8.20737891977662e-13	0.0627537831862078	4.69819599473953e-11
**BRCA**	SETD6	cg01773760	0.0609538124734272	796	1.71858789941845	0.0860802324425745	0.00371536725504573	0.21399436304086
**BRCA**	SETD6	cg02549819	-0.14264121486798	786	-4.03006603113744	6.12108553556824e-05	0.0203465161790132	0.000633728677340957
**BRCA**	SETD6	cg05502081	0.0171675108718677	796	0.483207792878355	0.629081751161683	0.000294723429535696	0.780996229770387
**BRCA**	SETD6	cg09132913	-0.0590914556042987	796	-1.66589199215843	0.0961303632344679	0.0034918001254348	0.231506840772132
**BRCA**	SETD6	cg19824325	-0.0619280451528244	111	-2.06347548297998	0.0393003860828354	0.00383508277645026	0.120934070445319
**BRCA**	SETD6	cg26666090	-0.0391775857723344	796	-1.10340211631392	0.270187721585857	0.00153488322694862	0.466245834344962
**BRCA**	SETD7	cg05316864	0.000979595856601422	796	0.0275682842917431	0.978013422332607	9.59608042270674e-07	0.988611956561107
**BRCA**	SETDB1	cg02060408	-0.411766670277891	796	-12.7162045375199	7.63697101169727e-34	0.169551790751741	2.45500216067759e-31
**BRCA**	SETDB1	cg08531528	0.0591635133657872	796	1.6679305583944	0.0957247991143823	0.00350032131378368	0.230794490246831
**BRCA**	SETDB1	cg10589385	-0.175963889528867	796	-5.03055647745228	6.0541604816341e-07	0.0309632904181271	1.09529618722847e-05
**BRCA**	SETDB1	cg10961700	-0.186292418792216	795	-5.33277628098616	1.26330774973402e-07	0.0347048652994543	2.68665290002209e-06
**BRCA**	SETDB1	cg12082615	-0.0165158968458912	111	-0.549337144382627	0.5828849944531	0.000272774848624117	0.747984880035243
**BRCA**	SETDB1	cg15448220	-0.224913723531731	796	-6.49607114175956	1.4557945415883e-10	0.050586183032908	5.62807519398397e-09
**BRCA**	SETDB1	cg16370685	-0.294176882474187	788	-8.61830410243591	3.72114545891565e-17	0.0865400381822315	3.7693071592563e-15
**BRCA**	SETDB1	cg17899625	0.0197354981419561	796	0.555514343223499	0.578699777821747	0.000389489886911152	0.744897505102255
**BRCA**	SETDB1	cg17918089	0.0402964217965995	111	1.34121047883067	0.180127475689367	0.00162380160960946	0.357187857657939
**BRCA**	SETDB1	cg19689029	-0.345375092958206	796	-10.3570399476192	1.16203780911952e-23	0.11928395483589	2.07338461749184e-21
**BRCA**	SETDB1	cg26820223	-0.160652075506973	796	-4.58064773830594	5.38236952342288e-06	0.0258090893646982	7.59093645223276e-05
**BRCA**	SETMAR	cg00300090	-0.00624992718965522	796	-0.175891977272334	0.860423770254082	3.90615898759916e-05	0.925844872998134
**BRCA**	SETMAR	cg02520519	0.0173813953321912	796	0.489229730452686	0.624814520877131	0.00030211290369392	0.7778547762551
**BRCA**	SETMAR	cg06021447	-0.0726639137391798	796	-2.05036396754193	0.0406576008593998	0.00528004435989497	0.12379324466756
**BRCA**	SETMAR	cg06267913	-0.142390786822271	796	-4.0484838924662	5.66130278859486e-05	0.0202751361718654	0.000591082256255467
**BRCA**	SETMAR	cg14751177	-0.0922800535089486	796	-2.60811950817027	0.00927563335482557	0.00851560827561441	0.040231301360688
**BRCA**	SETMAR	cg14961031	-0.0830992282718795	796	-2.34673628850828	0.0191841512881555	0.00690548173938194	0.0704505571011961
**BRCA**	SETMAR	cg15232450	0.0315526484146551	111	1.0498558549029	0.294013734756006	0.000995569621978835	0.49178565472597
**BRCA**	SETMAR	cg15384599	-0.0194247038443209	111	-0.645828928510254	0.518524239960889	0.000377319119439576	0.699330363958971
**BRCA**	SETMAR	cg16423812	-0.0191487260524513	796	-0.538991744853038	0.590044020851836	0.000366673709431827	0.753070145163449
**BRCA**	SETMAR	cg21901604	-0.08689917199678	796	-2.45484588253991	0.0143085483589209	0.00755146609372595	0.0562949077547107
**BRCA**	SETMAR	cg22149154	-0.0626016945060704	796	-1.7652301865247	0.0779104054459543	0.00391897215503136	0.199133097940454
**BRCA**	SETMAR	cg23182840	-0.117534785494336	796	-3.33080867527274	0.000906042720779177	0.0138144258011996	0.00618938245699818
**BRCA**	SETMAR	cg23549342	-0.146767494587204	796	-4.17562114473728	3.30156915359613e-05	0.0215406974674049	0.000370409966447732
**BRCA**	SETMAR	cg24390964	-0.0740819037291435	796	-2.09059418999312	0.0368827119078692	0.00548812846013409	0.115314431724169
**BRCA**	SETMAR	cg26797270	-0.0802310206961484	796	-2.2652034097713	0.0237695414740932	0.0064370166819458	0.0830031291597116

**BRCA**	SETMAR	cg27265118	-0.0113563514054298	796	-0.319616668257509	0.749343226047421	0.000128966717243607	0.859583982292538
**BRCA**	SFMBT2	cg09060610	-0.117068384103969	796	-3.31740733305336	0.000950011839004236	0.0137050065567143	0.00643045514691513
**BRCA**	SFMBT2	cg12328063	-0.153093622466472	796	-4.35983148566445	1.47330673516382e-05	0.0234376572399067	0.000183559295572284
**BRCA**	SFMBT2	cg20299663	-0.194591970102378	796	-5.58302705768545	3.24832363065713e-08	0.0378660348283246	7.92908969323315e-07
**BRCA**	SFMBT2	cg25291138	-0.211958982234471	796	-6.10374025341362	1.62106898607144e-09	0.0449266101498728	5.15574164768771e-08
**BRCA**	SHPRH	cg00571935	-0.105447255288163	796	-2.9841859513841	0.00293074824426679	0.011119123647807	0.0161629539851475
**BRCA**	SHPRH	cg02758840	0.0117376215275662	796	0.330348707119538	0.741223858803988	0.000137771759124386	0.854392899923449
**BRCA**	SHPRH	cg06708562	0.0939289683364459	796	2.65513408995067	0.00808693588349166	0.00882265109274906	0.0362269997516383
**BRCA**	SHPRH	cg11387577	-0.0249798209970132	796	-0.703213910464627	0.482129111039284	0.00062399145704282	0.669213718448953
**BRCA**	SHPRH	cg11675148	-0.0209927013825437	796	-0.590917205473267	0.554744488623875	0.000440693511336651	0.726358763885588
**BRCA**	SHPRH	cg12989205	0.0993427582560197	796	2.80965161279686	0.00508177139193692	0.00986898361791397	0.0251084788170438
**BRCA**	SHPRH	cg14102550	-0.00413935315121098	796	-0.116492721547151	0.907291586101507	1.71342445104403e-05	0.950929408254256
**BRCA**	SHPRH	cg15443174	0.189608760479629	796	5.43464955203263	7.31381113112917e-08	0.0359514820506214	1.63826842250234e-06
**BRCA**	SHPRH	cg16339435	0.0420373023941633	796	1.18408123208133	0.236736146859981	0.00176713479257833	0.428076332707046
**BRCA**	SHPRH	cg18099523	-0.0923642947970561	796	-2.61052090468653	0.0092113247438562	0.00853116295335748	0.0400215468932448
**BRCA**	SHPRH	cg26814539	0.0860294226549029	796	2.43009188942255	0.0153161811729522	0.00740106156233592	0.05934390866464
**BRCA**	SHPRH	cg27398948	-0.0080270403754924	796	-0.225908218223649	0.82133109000131	6.44333771897852e-05	0.904115112931239
**BRCA**	SIN3A	cg01088093	-0.0451814602714094	796	-1.27281880700676	0.203455849598948	0.00204136435225694	0.387311531258877
**BRCA**	SIN3A	cg01664094	0.0669937642682375	796	1.88961687070708	0.0591742092765908	0.00448816445082817	0.163015632156348
**BRCA**	SIN3A	cg02028562	-0.159024261872658	796	-4.53302362277346	6.71242609502253e-06	0.0252887158641437	9.20314549940399e-05
**BRCA**	SIN3A	cg07544511	-0.0425470972923026	796	-1.19846671088814	0.231093863072514	0.00181025548800067	0.421556910000073
**BRCA**	SIN3A	cg10483776	0.0534461930197764	796	1.50626205017949	0.132398638489633	0.00285649554830719	0.289766689752641
**BRCA**	SIN3A	cg11184237	-0.210346896874292	796	-6.0551596272376	2.16520093587869e-09	0.0442458170246441	6.72518026390432e-08
**BRCA**	SIN3A	cg15851274	-0.133291152102893	796	-3.78491864047527	0.00016536554262025	0.0177665312289166	0.00148020331637323
**BRCA**	SIN3A	cg22059910	0.125740729464848	796	3.56696833169142	0.000382829131994945	0.0158107310463521	0.00299435044182734
**BRCA**	SIN3A	cg25697800	-0.152520515717377	796	-4.34312097573688	1.58716741554675e-05	0.0232625077146948	0.000195645859491434
**BRCA**	SIN3B	cg06815056	-0.169420828139816	796	-4.83786182914358	1.57742041659937e-06	0.0287034170075812	2.55920425211535e-05
**BRCA**	SIRT1	cg02720207	-0.173246905558581	796	-4.95045883745149	9.04998007722234e-07	0.0300144902856238	1.56835408805399e-05
**BRCA**	SIRT1	cg03211233	-0.0948960336491013	796	-2.68271770952095	0.00745491456817322	0.00900525720233136	0.0340145233968603
**BRCA**	SIRT1	cg07832903	-0.172994581439391	795	-4.93990459656266	9.54065859589731e-07	0.02992712520739	1.64277555291996e-05
**BRCA**	SIRT1	cg10582690	-0.125865898593029	796	-3.57057621349425	0.000377677100993347	0.0158422244286307	0.00296378049455326
**BRCA**	SIRT1	cg10705386	-0.128356743048665	796	-3.64240872649538	0.000287679975239409	0.016475453486061	0.00235899399112202
**BRCA**	SIRT1	cg19192316	0.178603373452264	111	6.03680188819788	2.14283408417049e-09	0.031899165008529	6.66531926205354e-08
**BRCA**	SIRT1	cg19681528	-0.0103423705448795	796	-0.291075674602829	0.771069653237224	0.000106964628487591	0.873125535117432
**BRCA**	SIRT1	cg19980381	-0.0196202596317428	796	-0.552269359475271	0.58091963085737	0.000384954588016998	0.746581341487448
**BRCA**	SIRT1	cg25513531	-0.0340836314182195	796	-0.959756045119331	0.337470908396632	0.00116169393065304	0.536983113990077
**BRCA**	SIRT4	cg04118464	-0.112385162133313	772	-3.13436606061933	0.00178777393514637	0.0126304246677309	0.0108459865484502
**BRCA**	SIRT4	cg07106325	0.00854020286101959	796	0.240351377729985	0.810120066382526	7.29350649073672e-05	0.89741725661673
**BRCA**	SIRT4	cg08264848	-0.0633834284123093	794	-1.78510353231877	0.0746281044213458	0.00401745899729833	0.193140781641245
**BRCA**	SIRT4	cg12124973	-0.0749587380416637	796	-2.11547748507282	0.0347008801825636	0.00561881240879877	0.110117082313781
**BRCA**	SIRT4	cg20010714	-0.104885941397901	796	-2.96812343837827	0.00308653560382134	0.0110010607029239	0.0168418286290017
**BRCA**	SIRT4	cg21068152	-0.0229770660641822	111	-0.764340388286673	0.44482745963937	0.000527945564917795	0.636775125040209
**BRCA**	SIRT4	cg21123573	-0.0784066534046334	108	-2.57987169943825	0.0100153938843145	0.00614760329811431	0.0426807752306965
**BRCA**	SIRT5	cg01064902	-0.0220539700059756	796	-0.620804722011329	0.534906723910846	0.000486377593024472	0.711507799947344
**BRCA**	SIRT5	cg02206980	-0.180333013326576	796	-5.15960947459506	3.13054647450496e-07	0.032519995695443	6.04912400490907e-06
**BRCA**	SIRT5	cg02712746	-0.2415060001476	796	-7.00390150017086	5.31955055689554e-12	0.0583251481072927	2.65615607203551e-10
**BRCA**	SIRT5	cg06139021	0.0818811410701647	796	2.31210337520696	0.0210276331827707	0.00670452126295222	0.075687026133367
**BRCA**	SIRT5	cg07534331	-0.127248719984624	796	-3.61044630545152	0.000324905117083291	0.0161922367377253	0.00261220221975791
**BRCA**	SIRT5	cg07909422	-0.0564749054832856	111	-1.88116441876863	0.0602120643975147	0.00318941494934604	0.165091366360281
**BRCA**	SIRT5	cg08619119	-0.185880189868638	796	-5.32391520512594	1.32377824878612e-07	0.0345514449856007	2.80223143091339e-06
**BRCA**	SIRT5	cg10907941	-0.265379158286293	796	-7.74617745420625	2.89777391831394e-14	0.0704260976527416	2.05496284595547e-12
**BRCA**	SIRT5	cg11824369	-0.232744592543197	796	-6.73496988841301	3.15107118662601e-11	0.054170045358099	1.36565143115534e-09
**BRCA**	SIRT5	cg19366958	0.0737822986902093	796	2.08209295095752	0.0376544721714232	0.00544382760001127	0.117143565868339
**BRCA**	SIRT5	cg20150972	-0.110709132781866	796	-3.13490187828553	0.0017825503182289	0.0122565120813128	0.0108251505992015
**BRCA**	SIRT7	cg11691214	-0.178759652191427	796	-5.11310041341927	3.97718404951466e-07	0.0319550132516	7.50245043520716e-06
**BRCA**	SIRT7	cg12710567	-0.195363638056047	778	-5.54197250329843	4.10257287475909e-08	0.0381669510744941	9.7883987203189e-07
**BRCA**	SIRT7	cg17552093	-0.0703876171512808	111	-2.34667171374727	0.0191176453977383	0.00495441664823528	0.0702720707839563

**BRCA**	SIRT7	cg20397086	-0.179975017457334	792	-5.13600437559575	3.5398300777708e-07	0.0323910069087678	6.75607313714676e-06
**BRCA**	SMARCA	cg03460558	-0.115966650663177	796	-3.28575950081871	0.00106182463139701	0.0134482640660353	0.00705711963903454
**BRCA**	SMARCA	cg06675660	-0.0619998785036009	796	-1.74819449527808	0.080817818174683	0.00384398493446127	0.204453232320292
**BRCA**	SMARCA	cg08617833	-0.324642307014945	796	-9.6594293483599	6.06693497690783e-21	0.105392627503986	8.70795460504737e-19
**BRCA**	SMARCA	cg12935118	-0.0854856664449641	796	-2.41461885555949	0.015977354859777	0.00730779916753967	0.0612226551059743
**BRCA**	SMARCA	cg17420696	-0.0800377875584005	794	-2.25685756669862	0.02428943185454	0.00640604743724365	0.0843630499370157
**BRCA**	SMARCA	cg18959966	-0.258754669470557	796	-7.53874848145112	1.29972491214297e-13	0.0669539789728174	8.48957177290097e-12
**BRCA**	SMARCA	cg24447042	-0.348323257501435	111	-12.3579636335801	5.90341176429214e-33	0.121329091716411	1.81521744976815e-30
**BRCA**	SMARCA	cg26010110	-0.321354103623094	796	-9.55026051021522	1.56732192172827e-20	0.103268459915403	2.18118500141197e-18
**BRCA**	SMARCA	cg27353825	-0.123965546290736	782	-3.48460619400712	0.000520439301117045	0.0153674566671606	0.0038780704014463
**BRCA**	SMARCA	cg04518912	-0.00375675353757517	796	-0.10572516205315	0.915827186790105	1.41131971420836e-05	0.955512253653278
**BRCA**	SMARCA	cg13529335	-0.0461381119419165	796	-1.29982578740278	0.194038861588928	0.00212872537356482	0.375264388192736
**BRCA**	SMARCA	cg14290704	-0.0738375099661229	794	-2.08102697205481	0.0377530212124001	0.0054519778779973	0.117360440494292
**BRCA**	SMARCA	cg21210735	-0.192100796613926	796	-5.50879584212822	4.88690539393995e-08	0.0369027160597051	1.14475133458646e-06
**BRCA**	SMARCA	cg08405378	-0.119134520624822	795	-3.37465920922373	0.0007750768920423	0.0141930340045062	0.00542184441502911
**BRCA**	SMARCA	cg17218495	0.0319715233765447	796	0.900218632278335	0.368277549119923	0.00102217830701695	0.567142759843125
**BRCA**	SMARCA	cg17747332	-0.0642189204636101	111	-2.14011911605885	0.0325634070538287	0.00412406974551148	0.104904658288287
**BRCA**	SMARCA	cg18522266	-0.00337052162804293	796	-0.0948554253780681	0.924453655193319	1.13604160451052e-05	0.960171416536241
**BRCA**	SMARCA	cg23892580	-0.0595708656074577	796	-1.6794553411748	0.0934577377925265	0.00354868802922178	0.226932119789993
**BRCA**	SMARCA	cg01278696	-0.0528855042364389	111	-1.76125564430282	0.0784714332386007	0.00279687655834239	0.200144476488105
**BRCA**	SMARCA	cg01575392	-0.0905794324247437	796	-2.55965331997332	0.0106622518143656	0.00820463357838871	0.0448114110019858
**BRCA**	SMARCA	cg02782292	-0.0775357234942166	792	-2.18310354832866	0.0293225857452543	0.00601178841777161	0.0970524691740262
**BRCA**	SMARCA	cg05358385	-0.0133616061965025	796	-0.376062500875239	0.706971142031708	0.000178532520150413	0.83295647356384
**BRCA**	SMARCA	cg10878459	-0.284424425453085	796	-8.34924967602014	3.0523609105127e-16	0.0808972537943176	2.77401126953767e-14
**BRCA**	SMARCA	cg12253543	-0.142500824201536	796	-4.05167731992586	5.58612391842209e-05	0.0203064848981172	0.00058417384609454
**BRCA**	SMARCA	cg13848802	-0.11949287331675	766	-3.32232707627284	0.000935280008015288	0.0142785467734928	0.00634952203015458
**BRCA**	SMARCA	cg13898584	-0.146572654943883	796	-4.16995605574012	3.38290023760107e-05	0.0214835431772985	0.000378388700336358
**BRCA**	SMARCA	cg16091845	-0.105133173703082	111	-3.51585395153598	0.000456097253359517	0.0110529842128825	0.00347144405907937
**BRCA**	SMARCA	cg16587443	-0.0864078080263391	763	-2.38946815756596	0.0171159369548548	0.00746630928791667	0.0646022609027206
**BRCA**	SMARCA	cg19993723	0.0019315938760748	796	0.0543599715576317	0.956662078722611	3.73105490208966e-06	0.977135782681409
**BRCA**	SMARCA	cg20045045	-0.126995903770524	796	-3.60315541019607	0.000334007081908905	0.0161279595744921	0.00267322499280055
**BRCA**	SMARCA	cg22077701	-0.175876440014352	796	-5.0279765986361	6.13360063938719e-07	0.0309325221521221	1.1088945576121e-05
**BRCA**	SMARCA	cg24607315	-0.120749141112327	795	-3.42106782204755	0.000655758407835711	0.0145803550793645	0.00471784607224371
**BRCA**	SMARCB	cg01745726	-0.0615685892091489	796	-1.73598710637155	0.0829550137271441	0.00379069117720492	0.208275047212827
**BRCA**	SMARCB	cg01912455	-0.161512820869202	796	-4.60584560753948	4.78494615463065e-06	0.0260863913051269	6.84516552323165e-05
**BRCA**	SMARCB	cg03979008	0.0800949240330414	796	2.26133609244874	0.0240089186422868	0.00641519685585866	0.0835955550002607
**BRCA**	SMARCB	cg10122452	-0.0750722012326681	796	-2.11869776637008	0.0344267551779899	0.00563583539791822	0.109443626836523
**BRCA**	SMARCB	cg16142952	-0.0293163917826	796	-0.825391164031671	0.409398090678386	0.000859450827150895	0.605373789512153
**BRCA**	SMARCB	cg19906397	-0.0202611192211325	111	-0.673953845346125	0.50048148252593	0.000410512952092945	0.684345287673742
**BRCA**	SMARCC	cg02564102	0.0877866864220718	796	2.48011131986239	0.0133409601014006	0.00770650231296716	0.053359724942332
**BRCA**	SMARCC	cg02627110	-0.0606271558512739	796	-1.70934377217984	0.0877790205327476	0.00367565202661466	0.216983290118667
**BRCA**	SMARCC	cg05592996	-0.107444748214213	791	-3.03175407400351	0.00251111938684943	0.0115443739188157	0.014292509626907
**BRCA**	SMARCC	cg07143957	0.0702914361462114	796	1.98308151558623	0.0477033527341761	0.00494088599549691	0.139420126970192
**BRCA**	SMARCC	cg07304068	-0.136697330136475	796	-3.88345843458598	0.000111571920156249	0.0186861600664405	0.00105900755937472
**BRCA**	SMARCC	cg07474652	-0.0795801738984691	796	-2.24671012251252	0.0249332670040834	0.00633300407771058	0.0860197158772301
**BRCA**	SMARCC	cg12894336	-0.144012188527592	796	-4.09555464593362	4.64449927483969e-05	0.0207395104445068	0.000497123504725998
**BRCA**	SMARCC	cg16250355	-0.0487483309570743	111	-1.62313243822943	0.104846066343935	0.00237639977110045	0.246386989039394
**BRCA**	SMARCC	cg20685352	-0.134044557587941	796	-3.80670255932131	0.000151702172973547	0.017967943418947	0.00137514784948778
**BRCA**	SMARCC	cg22984439	-0.0615909156694654	796	-1.73661901963499	0.0828432672154309	0.0037934408930032	0.208089152647134
**BRCA**	SMARCC	cg24832353	-0.0661355852818111	315	-1.17075053583054	0.242592474048055	0.0043739156405677	0.435024639852805
**BRCA**	SMARCD	cg02553054	0.0283344026271045	796	0.797721056598555	0.425271485091789	0.000802838372234866	0.620085211739465
**BRCA**	SMARCD	cg04550398	-0.0203414468229033	796	-0.572577527769247	0.567093279522787	0.000413774458849002	0.735845911062184
**BRCA**	SMARCD	cg09878554	0.0164969578397179	796	0.464328733986537	0.642539846658206	0.000272149617965428	0.790101080074741
**BRCA**	SMARCD	cg13349421	-0.066199729073166	796	-1.86712124739266	0.0622538935190809	0.00438240412936057	0.169122062872726
**BRCA**	SMARCD	cg17846064	-0.0699929366540127	796	-1.97461861677897	0.0486584213640704	0.00489901118145263	0.141407345496525
**BRCA**	SMARCD	cg18591973	0.0920046724953924	111	3.07279361731408	0.00217253309112287	0.00846485976098442	0.0126960666534565
**BRCA**	SMARCD	cg20405918	-0.0581381823379729	796	-1.63892564849302	0.10162589070733	0.00338004824556339	0.241044187221889

**BRCA**	SMARCD	cg22402853	-0.117508933694306	796	-3.33006580427487	0.000908429706984531	0.0138083494979728	0.00619970002852483
**BRCA**	SMARCD	cg25107077	-0.0834904577293393	796	-2.3578620468226	0.0186227603797834	0.00697065653185459	0.0689088194912896
**BRCA**	SMARCD	cg00945038	0.168414373051929	796	4.80828067727789	1.82195665350119e-06	0.0283634010504743	2.90683543688417e-05
**BRCA**	SMARCD	cg02127607	0.184484868870441	796	5.28253730091534	1.64783300129911e-07	0.0340346668421437	3.40844244889825e-06
**BRCA**	SMARCD	cg02360836	-0.0430323944629663	111	-1.43243709449305	0.152301333636622	0.00185178697321633	0.319034666547762
**BRCA**	SMARCD	cg04367190	0.102225471948622	796	2.89203040017188	0.00393246480887979	0.0104500471151185	0.0204945513058213
**BRCA**	SMARCD	cg06420487	-0.273224143527937	796	-7.99335287141141	4.63775938906345e-15	0.0746514326065749	3.61512879672146e-13
**BRCA**	SMARCD	cg07362569	0.171439136438437	796	4.89722985802562	1.17848029668061e-06	0.0293913775027571	1.97936075075043e-05
**BRCA**	SMARCD	cg11697258	-0.00850944204967372	796	-0.239485597387908	0.810791025668897	7.24106039967553e-05	0.897962430609695
**BRCA**	SMARCD	cg12577610	-0.0919184182580521	111	-3.06850017208729	0.0022037719690182	0.0084489956150622	0.0128438894634254
**BRCA**	SMARCD	cg15868002	-0.0547006254348288	796	-1.54172031735324	0.123541006857008	0.00299215842296144	0.276290041323389
**BRCA**	SMARCD	cg19016967	-0.0499189322578254	796	-1.40659692190334	0.15993919759116	0.00249189979776136	0.329666905208514
**BRCA**	SMARCD	cg19143629	0.197827849883529	796	5.67961671240562	1.89572555053257e-08	0.0391358581895403	4.89791011219299e-07
**BRCA**	SMARCD	cg21254277	-0.219220265423894	796	-6.32321483389275	4.27727905592923e-10	0.0480575247725226	1.51111102123959e-08
**BRCA**	SMARCD	cg23682543	-0.255671687207383	796	-7.44260392656207	2.57605062454798e-13	0.0653680116394698	1.59555161055801e-11
**BRCA**	SMARCD	cg24795630	-0.20314019136055	796	-5.83860868853238	7.67755411148316e-09	0.0412659373460011	2.15076573478113e-07
**BRCA**	SMARCD	cg25945412	0.0535810601650481	796	1.51007391044543	0.131423404806797	0.00287093000841051	0.288274751974849
**BRCA**	SMARCD	cg00864064	0.00702104355520078	796	0.19759455032722	0.8434129997493	4.92950526040264e-05	0.916362493575468
**BRCA**	SMARCD	cg02917064	-0.0759995094674661	111	-2.53481468866441	0.0113876404812703	0.00577592543929547	0.0471921001278468
**BRCA**	SMARCD	cg14795305	-0.028654288883647	110	-0.952472702117978	0.341065785824373	0.000821068271427496	0.540318326526723
**BRCA**	SMARCD	cg19786287	-0.0185864607211212	796	-0.523159741992208	0.601009425022447	0.00034545652213778	0.760861305292047
**BRCA**	SMARCD	cg19902439	-0.0666922877728653	796	-1.88107538908417	0.0603282939291061	0.00444786124837868	0.165311000279002
**BRCA**	SMARCD	cg22750243	-0.0510700025020045	796	-1.43911520349272	0.150512969447909	0.00260814515555475	0.316348470678699
**BRCA**	SMARCD	cg22787816	-0.0930581119059316	796	-2.63030112876794	0.00869660132229653	0.00865981219149689	0.0382746890284418
**BRCA**	SMNDC1	cg03999743	-0.0397177984792909	796	-1.11864060558509	0.263632661669516	0.00157750351604156	0.45907194811233
**BRCA**	SMNDC1	cg08770590	-0.135000533865323	796	-3.83435327819219	0.00013588954553854	0.0182251441439222	0.00125208945938946
**BRCA**	SMNDC1	cg11559952	0.0895773822545768	796	2.53110638065161	0.011562803313525	0.00802410741158258	0.0477526528094899
**BRCA**	SMNDC1	cg12486840	0.179460287644308	796	5.13380639356492	3.57598458620677e-07	0.0322059948413778	6.81504519596692e-06
**BRCA**	SMNDC1	cg17981675	-0.047051803490653	796	-1.32562331708486	0.185346729577832	0.00221387221172302	0.364011599827772
**BRCA**	SMNDC1	cg18209001	-0.0409818286847749	796	-1.15430062800556	0.248725118199641	0.00167951028234824	0.442148938467686
**BRCA**	SMNDC1	cg21872391	0.0774865588744121	111	2.58470900660322	0.0098732951256526	0.00600416680619774	0.0421994507180076
**BRCA**	SMNDC1	cg23721301	-0.115009419362764	796	-3.25827254824098	0.00116871013145014	0.0132271665421601	0.00764471840292882
**BRCA**	SMNDC1	cg26944434	-0.00243880336098598	796	-0.0686342144271579	0.945298107760862	5.9477618335565e-06	0.97074148234636
**BRCA**	SMYD1	cg00578637	-0.116775462410097	796	-3.30899180727219	0.000978626413148513	0.013636508621092	0.00659716318756286
**BRCA**	SMYD1	cg00666842	-0.125360053231736	796	-3.5559967620004	0.000398903708689213	0.0157151429462636	0.00309782822888439
**BRCA**	SMYD1	cg03704645	-0.232614232583086	796	-6.73098178582877	3.2338622532751e-11	0.0541093812002179	1.39763933406269e-09
**BRCA**	SMYD1	cg14559139	-0.376544388297301	796	-11.438811177903	3.77828355088624e-28	0.141785676358188	9.48154163089174e-26
**BRCA**	SMYD1	cg16090764	-0.290318929183892	796	-8.53803241173008	6.93246304727982e-17	0.0842850806424817	6.77517626005133e-15
**BRCA**	SMYD1	cg18148488	-0.151836194101226	796	-4.32317356362988	1.73409753028011e-05	0.0230542298391451	0.000211512046520243
**BRCA**	SMYD1	cg19074493	-0.350757948125335	796	-10.5409080516146	2.11519447602453e-24	0.123031138173095	4.06294911278648e-22
**BRCA**	SMYD2	cg01770831	-0.0593296410883233	796	-1.67263053167527	0.0947949883525966	0.00352000631166926	0.229241857810373
**BRCA**	SMYD2	cg04406444	-0.0426542143289262	796	-1.20148947642268	0.229920543383611	0.00181938200001797	0.42002412441057
**BRCA**	SMYD2	cg11523160	-0.0872461828811825	796	-2.46472375732837	0.0139231076478176	0.00761189642733674	0.0551437855795907
**BRCA**	SMYD2	cg14013804	0.0774586209594538	796	2.18644785341946	0.0290748228786924	0.00599983796094033	0.0964992862760782
**BRCA**	SMYD2	cg27570636	-0.012859174326426	796	-0.361919167931846	0.717508974930498	0.000165358364357414	0.839556120852808
**BRCA**	SMYD5	cg01329511	-0.0896871698384891	796	-2.53423368826973	0.0114609535458961	0.00804378843363799	0.0474403224512847
**BRCA**	SMYD5	cg05103132	-0.0275531162176855	796	-0.775707927871713	0.438153018838605	0.000759174213305284	0.630913290929613
**BRCA**	SMYD5	cg06754047	-0.0317578164498734	111	-1.05668931042721	0.290884155831793	0.00100855890566385	0.488421751856727
**BRCA**	SMYD5	cg08834152	-0.0191427399072095	796	-0.538823187122082	0.590160277935098	0.000366444491155071	0.753181453206953
**BRCA**	SMYD5	cg15623359	-0.0596158984400709	796	-1.68072945868863	0.0932097753734441	0.00355405534681685	0.226483239209454
**BRCA**	SMYD5	cg17895873	-0.0675593135617878	111	-2.25193669197158	0.0245218182087477	0.00456426084893997	0.0849543324054779
**BRCA**	SMYD5	cg20402064	-0.0915895321054555	796	-2.58843751157724	0.00981802049678248	0.00838864239129627	0.0419937918697326
**BRCA**	SMYD5	cg25886337	0.0186662358333727	796	0.525405981523459	0.599448053804818	0.000348428360187088	0.75968822835696
**BRCA**	SMYD5	cg26467949	-0.156900267711325	796	-4.4709399715485	8.92470730364808e-06	0.0246176940078856	0.000118277458258944
**BRCA**	SP100	cg05876232	-0.0327182862910044	796	-0.921267428018611	0.357191356273328	0.00107048625782012	0.556069407654986
**BRCA**	SP100	cg09456094	-0.228991663705881	796	-6.62030896017748	6.60717014862015e-11	0.0524371820467871	2.71382362482448e-09
**BRCA**	SP100	cg11702456	-0.151691289059318	796	-4.31895051836831	1.76682763298139e-05	0.0230102471764775	0.000215133180278845

**BRCA**	SP100	cg11827097	-0.10722379962193	796	-3.0350424897444	0.00248371362359404	0.0114969432053637	0.014165284360161
**BRCA**	SP100	cg23539753	-0.336635225088607	111	-11.889250218648	9.2046195794737e-31	0.113323274770457	2.62294569804241e-28
**BRCA**	SP100	cg27140170	-0.0394323392517634	796	-1.11058816071344	0.267082715342925	0.00155490937886616	0.462790287718004
**BRCA**	SP140L	cg04002454	-0.474751474733235	796	-15.180534838876	7.09989170465554e-46	0.225388962761382	3.45205609544983e-43
**BRCA**	SP140L	cg05217331	-0.0603460529331133	796	-1.7013892205746	0.0892624187029758	0.00364164610460611	0.219555467528025
**BRCA**	SP140L	cg13278004	-0.511732919607611	796	-16.7625346732478	3.32923638107467e-54	0.261870581010129	2.31245191990467e-51
**BRCA**	SP140L	cg24044052	-0.519296715497466	796	-17.1008745976466	4.89049151409021e-56	0.269669078726456	3.72991075340327e-53
**BRCA**	SP140L	cg25095994	-0.538495578107929	796	-17.9849267255331	6.66328197400871e-61	0.289977487641793	6.02748090565155e-58
**BRCA**	SP140L	cg26847438	-0.516656105931636	796	-16.9821424628752	2.1603187303392e-55	0.266933531796442	1.58547014441517e-52
**BRCA**	SSRP1	cg01511567	0.0117818597390144	111	0.391851538725133	0.695243400706591	0.000138812218909808	0.825298301051116
**BRCA**	SSRP1	cg01766191	-0.203961707934883	796	-5.86324327345956	6.66132026742063e-09	0.0416003783037147	1.88646068031933e-07
**BRCA**	SSRP1	cg02688348	0.0921806236277222	796	2.60528521570409	0.00935205100889453	0.00849726737239577	0.0404432406573985
**BRCA**	SSRP1	cg04937561	-0.100751631348536	796	-2.84990366252752	0.00448679052835847	0.0101508912193913	0.0227331888995128
**BRCA**	SSRP1	cg08821673	-0.22375160996697	796	-6.46073258160321	1.81832394137975e-10	0.0500647829628111	6.90359652004374e-09
**BRCA**	SSRP1	cg09381635	-0.172095507648142	796	-4.91655049881	1.07108392279634e-06	0.0296168637526716	1.81612691129072e-05
**BRCA**	SSRP1	cg11948159	-0.00851709249324892	796	-0.239700923363031	0.810624140098878	7.25408645385571e-05	0.89780316564425
**BRCA**	SSRP1	cg12795368	0.107573043590863	796	3.04504362238056	0.00240352339741155	0.0115719597074018	0.0137910974463958
**BRCA**	SSRP1	cg12911972	-0.152724593831641	796	-4.34907092147712	1.54569509768695e-05	0.0233248015610396	0.000191474210874934
**BRCA**	SSRP1	cg16313264	0.0938566640191046	796	2.6530720584029	0.00813600601484847	0.00880907338079508	0.0363965878776028
**BRCA**	SSRP1	cg22636253	-0.0404551653781011	111	-1.34650268298336	0.178416328925542	0.00163662040576951	0.355012071410433
**BRCA**	SSRP1	cg26706201	-0.0164498187971693	796	-0.463001583546139	0.643490423043681	0.000270596538459704	0.790736297152792
**BRCA**	STK31	cg00755064	0.00314861727738104	796	0.088610383830373	0.929413956177066	9.91379075942238e-06	0.962671899544514
**BRCA**	STK31	cg05000488	0.0100234512958406	796	0.282099095099181	0.77794122544222	0.000100469575880088	0.877216403711437
**BRCA**	STK31	cg05407490	-0.0235362691425971	796	-0.662552864260503	0.507809722429329	0.000553955965152768	0.690482050349871
**BRCA**	STK31	cg11755819	-0.0131645142520149	796	-0.37051437799213	0.711098335277591	0.000173304435491504	0.835511008829255
**BRCA**	STK31	cg13313214	-0.0142365974182113	796	-0.400693973906355	0.688753514544863	0.000202680706048221	0.821150494406263
**BRCA**	STK31	cg13514718	-0.0286881534416185	796	-0.807688638523429	0.419512355350892	0.000823010147889845	0.614856198223501
**BRCA**	STK31	cg13802883	0.00644630463744934	796	0.181418862640072	0.856085232007098	4.15548434788009e-05	0.923417146759222
**BRCA**	STK31	cg14898779	-0.0216557880035113	111	-0.720366345361503	0.471451679913761	0.000468973154053025	0.659689761623338
**BRCA**	STK31	cg17102984	0.0233939028042085	796	0.658543009001592	0.510380660042456	0.000547274688412753	0.692682363352108
**BRCA**	STK31	cg17331904	-0.0169177546580043	796	-0.47617596655123	0.634080347701571	0.000286210422668427	0.784535134299738
**BRCA**	STK31	cg21519371	0.0355245406532034	796	1.00038062799071	0.317431879595669	0.0012619929886211	0.516671108719855
**BRCA**	STK31	cg25570411	0.0192806119981445	796	0.542705400822562	0.587485336528537	0.000371741999022994	0.751335875445259
**BRCA**	STK31	cg25612145	0.00131403875488248	111	0.0437004704653281	0.965151052360271	1.72669784933309e-06	0.981635856389837
**BRCA**	SUV39H1	cg05903800	-0.0337607576746377	796	-0.950653865923666	0.342070121364197	0.00113978875876561	0.541259727301256
**BRCA**	SUZ12	cg00160853	0.011173681955923	796	0.314474915541007	0.753243187752044	0.000124851168452118	0.861968379283349
**BRCA**	SUZ12	cg01109633	0.167541334103406	796	4.78263335570451	2.06316634434346e-06	0.0280700986331492	3.24642843514589e-05
**BRCA**	SUZ12	cg07482957	-0.0952290999727796	796	-2.69221953589378	0.00724770323949748	0.00906858148162565	0.0332995408583432
**BRCA**	SUZ12	cg08177746	0.00250447053862118	796	0.0704822695520512	0.943827604892349	6.27237267882148e-06	0.970012480447531
**BRCA**	SUZ12	cg17335595	-0.331221926066366	796	-9.87905549165672	8.76709542436544e-22	0.109707964307113	1.35054934939225e-19
**BRCA**	SUZ12	cg25098644	0.0386932027838994	111	1.28776788884121	0.198096153096345	0.00149716394167596	0.380633095412776
**BRCA**	TAF3	cg00194465	-0.126439689692336	796	-3.58711742011771	0.000354876847220812	0.0159869951294941	0.00281276509961241
**BRCA**	TAF3	cg02334915	0.0258537569730603	796	0.727832503321895	0.466931237389025	0.000668416749622067	0.656090119136857
**BRCA**	TAF3	cg03672997	-0.00393467973522823	793	-0.110522640473347	0.912023001756747	1.54817046188157e-05	0.953299770508344
**BRCA**	TAF3	cg04179819	-0.0157637548093993	796	-0.443686514531582	0.65739043075933	0.000248495965690858	0.800422764029649
**BRCA**	TAF3	cg04234156	-0.0120072236137755	796	-0.33793758690541	0.735499775136311	0.000144173418911208	0.850899858981718
**BRCA**	TAF3	cg05976695	-0.117940045254706	796	-3.3424550191096	0.000869374058398149	0.013909854274682	0.00597403811492144
**BRCA**	TAF3	cg07855525	-0.0373963044893439	796	-1.05316196812679	0.292587959543618	0.00139848358945972	0.490266175387947
**BRCA**	TAF3	cg09871669	-0.0789575959241726	796	-2.2290227773952	0.0260922037990685	0.00623430195412492	0.0889724249296368
**BRCA**	TAF3	cg09994553	0.0410392609075332	772	1.13827231492567	0.255361744944026	0.00168422093583658	0.449657448232277
**BRCA**	TAF3	cg11104088	0.0463047438211175	796	1.30453029231826	0.19243178409157	0.00214412930033932	0.373112960760172
**BRCA**	TAF3	cg12176426	-0.0648622087918067	796	-1.82923630931089	0.0677400542633217	0.00420710612935192	0.180021514069649
**BRCA**	TAF3	cg12590721	-0.179685650424331	796	-5.14046828616056	3.45543884565568e-07	0.0322869329684147	6.6031051230395e-06
**BRCA**	TAF3	cg12777293	-0.0556751610165195	796	-1.56927195828009	0.116983995842422	0.00309972355421538	0.266069844830002
**BRCA**	TAF3	cg27268107	-0.127417916073039	796	-3.61532610299867	0.000318943661896309	0.0162353253363959	0.00257278133902546
**BRCA**	TCEA1	cg01677098	-0.237460491490244	111	-8.12965432606301	1.14539696243354e-15	0.0563874850187882	9.67481121558687e-14
**BRCA**	TCEA1	cg06047473	-0.343425398401505	796	-10.2907300261327	2.13634811385123e-23	0.117941004267232	3.76006934771363e-21

**BRCA**	TCEA1	cg11530293	-0.0701425274743808	796	-1.97885966936385	0.0481778113602414	0.00491997416049427	0.140408841641174
**BRCA**	TCEA1	cg11787876	0.0806526528849432	796	2.27718530353822	0.0230410145947202	0.00650485041737914	0.081029460213447
**BRCA**	TCEA1	cg12850379	-0.101711026917296	796	-2.87732386306142	0.00411847546147171	0.0103451329965709	0.0212391567815532
**BRCA**	TCEA1	cg23569945	-0.214328401395182	796	-6.17523816712969	1.05495604678553e-09	0.0459366636446143	3.47603772569393e-08
**BRCA**	TCEA1	cg26181865	-0.420045641733709	796	-13.0259982021319	2.76879362345673e-35	0.176438341139483	9.44717241856108e-33
**BRCA**	TCEA1	cg26358945	-0.121729228387944	796	-3.45143124908275	0.000587167298437959	0.0148180050439243	0.00429668825272153
**BRCA**	TDRD1	cg00337703	-0.218855705629489	796	-6.31216996290757	4.57832687831248e-10	0.0478978198865816	1.60797454253472e-08
**BRCA**	TDRD1	cg02635786	-0.648270140154717	776	-23.6562473888997	1.7996552528403e-93	0.420254174616217	3.88895502054051e-90
**BRCA**	TDRD1	cg16550453	-0.786007885179562	796	-35.7807073838684	1.38041059031117e-167	0.617808395564448	1.19319623847408e-163
**BRCA**	TDRD1	cg23504215	-0.715191666244342	796	-28.7973391100207	2.44442133975763e-125	0.511499119465358	1.18850821065691e-121
**BRCA**	TDRD1	cg23825092	-0.332524962477608	796	-9.92274093722617	5.94338124988512e-22	0.110572850670734	9.32176211599926e-20
**BRCA**	TDRD1	cg24203376	-0.444776378370214	796	-13.9755858222824	7.80658048730911e-40	0.197826026756124	3.16304751265482e-37
**BRCA**	TDRD1	cg26600802	-0.7749619093801	796	-34.5080494432272	5.43307189665443e-160	0.600565960990049	4.22660395128335e-156
**BRCA**	TDRD1	cg27547703	-0.764427187071118	110	-39.3606686345202	2.61074209344485e-212	0.584348924333462	2.03100070417449e-207
**BRCA**	TDRD3	cg01557749	-0.00704680784275505	796	-0.198319675463483	0.842845852228154	4.96575007727141e-05	0.916029949852076
**BRCA**	TDRD3	cg03447530	-0.131372841875901	796	-3.72948280316448	0.000205552997936846	0.0172588235825506	0.00177537358959686
**BRCA**	TDRD3	cg05666423	-0.142917599718277	796	-4.0637740773477	5.30984895016878e-05	0.0204254403092338	0.00055866160296109
**BRCA**	TDRD3	cg10765900	0.0460352063541145	796	1.29692051626343	0.195036231239791	0.00211924022406591	0.376622487685225
**BRCA**	TDRD3	cg18522102	0.00793220630311645	796	0.223239095926768	0.823406989805456	6.29198968352003e-05	0.904987433525432
**BRCA**	TDRD3	cg19937698	-0.00715092409936416	796	-0.201249988154602	0.840554779608806	5.11357154748672e-05	0.914598278573451
**BRCA**	TDRD3	cg20944895	-0.211120120427275	796	-6.07845444762559	1.88509716558966e-09	0.044571705249227	5.92043798546152e-08
**BRCA**	TDRD3	cg22045829	0.0674306574458508	796	1.9019959232832	0.0575342431469208	0.00454689356357967	0.159685287073087
**BRCA**	TDRD3	cg23103367	-0.0439918781312306	796	-1.23924091406628	0.215623395548567	0.00193528534151305	0.402751721129084
**BRCA**	TDRD3	cg23767211	0.120587921540346	796	3.41859158189795	0.000661624740564471	0.0145414468214206	0.00475521388280418
**BRCA**	TDRD3	cg26737458	0.0210987128550095	796	0.593902615999933	0.552746836360451	0.000445155684138145	0.724900746604375
**BRCA**	TDRD5	cg05378938	-0.209666384631021	796	-6.03466774862126	2.44488526348992e-09	0.0439599928442434	7.53555484104336e-08
**BRCA**	TDRD5	cg07171496	-0.275348880209541	796	-8.06059162550273	2.79437496776344e-15	0.0758170058326482	2.25270058282061e-13
**BRCA**	TDRD5	cg07901138	-0.291533080852635	796	-8.57704873117107	5.0862149495571e-17	0.0849915372314288	5.05980825813101e-15
**BRCA**	TDRD5	cg09656934	-0.193117154101722	111	-6.54563267385459	9.04692446616934e-11	0.0372942352083481	3.64283872630009e-09
**BRCA**	TDRD5	cg11853788	-0.190107463276178	796	-5.44947883033379	6.74973663507828e-08	0.0361408475933035	1.52642154589907e-06
**BRCA**	TDRD5	cg12277666	-0.204556120952305	111	-6.94979111321822	6.23775686661398e-12	0.041843206619054	3.07570415886802e-10
**BRCA**	TDRD5	cg13848905	-0.232126095855771	796	-6.7160516224268	3.56317533387667e-11	0.0538825243772426	1.52976634615674e-09
**BRCA**	TDRD5	cg15545772	-0.213085321450843	796	-6.13771371847909	1.32246587057196e-09	0.0454053542178092	4.27775093285967e-08
**BRCA**	TDRD7	cg24339935	0.0913983362851178	796	2.58298850602749	0.00997310352738613	0.00835365587568747	0.0425331733901363
**BRCA**	TDRD9	cg07613153	0.200016976314581	109	6.75213999666862	2.35999801439066e-11	0.0400067908140275	1.04492706620095e-09
**BRCA**	TDRD9	cg08933517	0.0868201675057199	111	2.89697507239523	0.00384209685577759	0.00753774148572127	0.0201057502218728
**BRCA**	TDRD9	cg16701547	0.140531785076568	795	3.99203369231328	7.16046036962369e-05	0.0197491826168066	0.000723147934563813
**BRCA**	TRIM24	cg00733782	0.0302586750886319	796	0.851944703282285	0.394502315188752	0.000915587418119392	0.591248061105319
**BRCA**	TRIM24	cg02308366	-0.170001407115795	796	-4.85493295470979	1.45102398220462e-06	0.0289004784213502	2.37344322270029e-05
**BRCA**	TRIM24	cg06386929	-0.129720979943371	796	-3.68178104506711	0.000247323039682625	0.0168275326374684	0.00207330264537394
**BRCA**	TRIM24	cg06650216	-0.181900525684741	796	-5.20598635220667	2.46118376834252e-07	0.0330878012443853	4.87561319262638e-06
**BRCA**	TRIM24	cg10571650	0.136703863464047	796	3.88364757560799	0.000111486752667645	0.0186879462859967	0.00105832830226074
**BRCA**	TRIM24	cg10703331	-0.0757354305937026	111	-2.52595595438383	0.0116771184919616	0.00573585544721355	0.0481174721099455
**BRCA**	TRIM24	cg13935558	0.235116479891965	796	6.80760034448195	1.96037999062485e-11	0.0552797591167887	8.79502889219548e-10
**BRCA**	TRIM24	cg26889928	-0.00287721983744233	111	-0.0956868828682007	0.923786593016603	8.27839399297164e-06	0.959890129523076
**BRCA**	TRIM33	cg00760200	-0.0694923389035838	796	-1.96042713103082	0.050296072644612	0.00482918516629055	0.144905271732651
**BRCA**	TRIM33	cg02852257	-0.0173642425751678	796	-0.48874679046028	0.625156277120404	0.000301516920209271	0.778118721739949
**BRCA**	TRIM33	cg05792124	-0.0444303046231638	796	-1.25161559349995	0.211079506176122	0.00197405196890713	0.396952636051263
**BRCA**	TRIM33	cg16036305	-0.0520615675019379	796	-1.46713199150303	0.142737119128022	0.00271040681075884	0.305326224609496
**BRCA**	TRIM33	cg17645130	-0.0326899510163431	796	-0.920468721652419	0.357608144865031	0.00106863289745091	0.556595734897167
**BRCA**	TRIM33	cg18199720	0.194650119898811	796	5.58476112172604	3.21729673035031e-08	0.0378886691766217	7.86075319851985e-07
**BRCA**	TRIM33	cg20932765	-0.100336288510716	110	-3.33857002388685	0.000870428522510074	0.0100673707921056	0.00597894424627591
**BRCA**	TRIM33	cg20992663	-0.0690361301608168	795	-1.94626546147641	0.0519766177435389	0.00476598726758123	0.14843320732502
**BRCA**	TRIM33	cg21666217	0.0936734104844972	111	3.12901545617178	0.00179982042552403	0.0087747078317971	0.0109063117450706
**BRCA**	TRIM33	cg21813112	-0.0411460366947902	795	-1.15820168344457	0.247131311297341	0.00169299633568902	0.440392468928309
**BRCA**	TRIM66	cg06492264	0.122931195526594	796	3.48603136603027	0.000517223334101484	0.0151120788335977	0.00385817164187275
**BRCA**	TRIM66	cg11245569	0.268437894000141	756	7.64174494868364	6.537197365392e-14	0.0720589029352307	4.42606381064669e-12

**BRCA**	TRIM66	cg12608775	0.141507703005887	796	4.02286119227209	6.30023291179968e-05	0.0200244300100023	0.000649682289422779
**BRCA**	TRIM66	cg13143389	0.133032825739747	796	3.77745094830978	0.000170310646438392	0.0176977327243019	0.00151748326984633
**BRCA**	TRIM66	cg27044202	-0.0261332784259349	796	-0.735706913688606	0.462126899791881	0.000682948241287436	0.651611325353614
**BRCA**	UBE2A	cg00142683	-0.147717404978153	796	-4.20324753309223	2.93091068653357e-05	0.0218204317334798	0.000333978710924554
**BRCA**	UBE2A	cg00428314	-0.136175921410905	796	-3.86836521998553	0.000118569962032602	0.0185438815721088	0.00111563033700584
**BRCA**	UBE2A	cg00935924	-0.227518157250734	796	-6.57537536004214	8.80525125453024e-11	0.0517645118787698	3.55472608248534e-09
**BRCA**	UBE2A	cg05206294	-0.155452044326313	796	-4.42864540529897	1.08149488711322e-05	0.0241653380852298	0.00014041023572778
**BRCA**	UBE2A	cg06206449	-0.14008056464176	796	-3.98147342228942	7.47880932875304e-05	0.0196225645903542	0.000750621201033433
**BRCA**	UBE2A	cg06273903	-0.114828447764195	111	-3.84422954705224	0.000127823251928204	0.0131855724159345	0.00118917508496804
**BRCA**	UBE2A	cg09080960	-0.219038653906125	796	-6.31771230053216	4.42475787119454e-10	0.047977931905007	1.55825990869945e-08
**BRCA**	UBE2A	cg14625604	-0.0356586840513466	111	-1.18664048954516	0.235624285967221	0.00127154174827376	0.426888276451105
**BRCA**	UBE2A	cg15661671	-0.274841543454803	796	-8.04452504095748	3.15497860469188e-15	0.0755378740086183	2.53281962180185e-13
**BRCA**	UBE2A	cg16211147	-0.151110034067557	796	-4.30201355445911	1.90411743535001e-05	0.0228342423958982	0.000229550459887833
**BRCA**	UBE2A	cg17976175	-0.115444773691941	796	-3.27077262786671	0.00111892383646988	0.0133274957727835	0.00737424270877139
**BRCA**	UBE2A	cg18990292	-0.210433957401539	796	-6.05778189512527	2.13174636205283e-09	0.0442824504276728	6.63348305958152e-08
**BRCA**	UBE2A	cg25165659	-0.130749911492932	796	-3.71149039821984	0.000220460688699878	0.0170955393554096	0.00188384433399806
**BRCA**	UHRF2	cg06523306	-0.0286519494935798	796	-0.806668511597645	0.420099651645608	0.00082093420978265	0.615396232066403
**BRCA**	UHRF2	cg08487038	-0.0119142453853312	111	-0.396075980189269	0.692125442319469	0.000141949243101885	0.82321509738863
**BRCA**	UHRF2	cg13428749	-0.0887761326057469	796	-2.50828553282084	0.0123307093744576	0.00788120172043316	0.0502280450872634
**BRCA**	UHRF2	cg14078101	-0.132111710946434	796	-3.7508297524761	0.000189096686115965	0.0174535041691942	0.00165547913568595
**BRCA**	UHRF2	cg20595300	0.0391417126147477	111	1.30212867727527	0.1931437530895	0.0015320736664155	0.374073869789742
**BRCA**	UHRF2	cg24119045	-0.150949046550941	796	-4.29732340998895	1.94389961523097e-05	0.0227856146546381	0.000233514093062504
**BRCA**	USP27X	cg00478198	-0.188204876266584	796	-5.39292745198471	9.15735614809873e-08	0.0354210754505202	2.00221294037435e-06
**BRCA**	USP27X	cg01125381	-0.134808553412931	796	-3.82879955084143	0.000138935050793733	0.0181733460732871	0.00127591941228281
**BRCA**	USP27X	cg01290688	-0.153480275017494	796	-4.37110795213486	1.40092814019128e-05	0.0235561948194456	0.000175780328609743
**BRCA**	USP27X	cg01855997	-0.149309391990061	796	-4.24957424116185	2.39670239606456e-05	0.0222932945364416	0.000280290237822378
**BRCA**	USP27X	cg06455206	-0.244582152568585	796	-7.09875109463171	2.80083616491035e-12	0.0598204293550826	1.4802190802516e-10
**BRCA**	USP27X	cg06624970	-0.171662178506478	796	-4.90379446655421	1.14087527381822e-06	0.02946790352959	1.92314736839469e-05
**BRCA**	USP27X	cg16810342	-0.137553201485777	796	-3.90824047328577	0.000100921445606503	0.0189208832389866	0.000971428228100998
**BRCA**	USP27X	cg16867483	-0.175200983815218	796	-5.00805386143794	6.78189196404822e-07	0.0306953847298202	1.21237819169723e-05
**BRCA**	USP27X	cg19318186	-0.137433977732467	796	-3.90478777644522	0.000102345382059742	0.0188880982353682	0.00098330945436033
**BRCA**	USP51	cg00834229	-0.0541076399570611	796	-1.52495786059786	0.127668810398789	0.00292763670172296	0.28257421756098
**BRCA**	USP51	cg01084827	-0.00445848362979539	795	-0.125394859924693	0.900242819335108	1.98780762771535e-05	0.94738430376683
**BRCA**	USP51	cg02714202	-0.00512858261569022	796	-0.144333006518944	0.885274233954706	2.63023596459599e-05	0.939207899337524
**BRCA**	USP51	cg03728850	-0.0669687223308732	796	-1.88890735865572	0.0592693708276458	0.0044848097706296	0.163187610260905
**BRCA**	USP51	cg07386410	-0.0311029947202438	796	-0.875739555771019	0.381437150570582	0.000967396280567516	0.579878091368089
**BRCA**	USP51	cg12064531	-0.0555611995890547	796	-1.56604986022039	0.117736385742343	0.00308704689977477	0.267265374742918
**BRCA**	USP51	cg12517811	-0.0825731366146861	796	-2.33177704910288	0.0199623048038288	0.00681832289038762	0.0726998393678234
**BRCA**	USP51	cg18770944	-0.0892394618935629	111	-2.97968750893007	0.00294845107244227	0.00796368155905267	0.0162364127365735
**BRCA**	USP51	cg21331369	-0.0887588258863445	796	-2.5077926646	0.0123477819489598	0.00787812917272241	0.0502817917157339
**BRCA**	USP51	cg25424556	-0.0251394145525431	111	-0.836315221662646	0.403158240041985	0.000631990164044615	0.599654237781356
**BRCA**	WDR82	cg01736403	0.0970379090315895	796	2.74383785895957	0.00620989849653939	0.00941635578922304	0.0294700262081316
**BRCA**	WDR82	cg03869063	-0.143550065739594	796	-4.08213539096138	4.9151580494792e-05	0.0206066213738419	0.000522291770661364
**BRCA**	WDR82	cg10989431	0.162473000584726	796	4.63396710347424	4.19337837274134e-06	0.0263974759190045	6.09984437414061e-05
**BRCA**	WDR82	cg11442381	-0.27817424706159	796	-8.15020052682405	1.4148623018087e-15	0.0773809117282824	1.18240108018569e-13
**BRCA**	WDR82	cg11665333	-0.0679636864641426	796	-1.91710040467404	0.0555846711769631	0.00461906267779628	0.15571874786779
**BRCA**	WDR82	cg12661343	-0.0549772338869569	796	-1.54954001712542	0.121651438613124	0.00302249624586116	0.273242443062491
**BRCA**	WDR82	cg13539115	-0.105255448173008	796	-2.97869689083789	0.00298315808890984	0.0110787093701007	0.0163880940871868
**BRCA**	WDR82	cg15991175	0.00682346831756671	796	0.192033890574339	0.847764883149289	4.65597198808366e-05	0.918781032859891
**BRCA**	WDR82	cg17632831	0.056474885193488	796	1.59188476537072	0.111809620738644	0.00318941265761766	0.257859529104176
**BRCA**	WDR82	cg24007312	-0.00143729160543666	796	-0.040449012965564	0.96774534959953	2.06580715905868e-06	0.983035382413374
**BRCA**	WDR82	cg26236504	0.140065564058134	796	3.98103853232023	7.49223690595219e-05	0.0196183622349231	0.000751484112766432
**BRCA**	WDR82	cg26858096	-0.16887249863535	796	-4.82174371671481	1.70644835884415e-06	0.0285179207953463	2.74506707253767e-05
**BRCA**	ZMYND1	cg00614684	-0.0436522858088532	796	-1.22965634314762	0.219190980426971	0.00190552205633781	0.407089147738816
**BRCA**	ZMYND1	cg02813021	-0.00258158004141793	111	-0.0858159828887493	0.931628247032169	6.66455551024737e-06	0.963738834733392
**BRCA**	ZMYND1	cg02867818	-0.0413134814959435	796	-1.16365792016834	0.244913113077802	0.00170680375331566	0.437817202032721
**BRCA**	ZMYND1	cg03254875	-0.00136613802692786	796	-0.038446567866647	0.969341321290592	1.86633310861834e-06	0.983885741199315

**BRCA**	ZMYND1	cg05236965	0.131363312655548	796	3.72920753220231	0.000205773751122503	0.0172563199118392	0.00177688569151116
**BRCA**	ZMYND1	cg08828193	-0.272888836081515	796	-7.98275342240298	5.02174164314432e-15	0.0744683168579241	3.8988160617442e-13
**BRCA**	ZMYND1	cg12639781	-0.0041534886335728	796	-0.116890539541984	0.906976429211178	1.72514678292184e-05	0.950841639389257
**BRCA**	ZMYND1	cg13939078	-0.109969112662931	796	-3.11368966687921	0.00191411266144261	0.0120932057398724	0.0114772992434304
**BRCA**	ZMYND1	cg14255089	0.10342328700989	111	3.45804865875433	0.000564801904351037	0.01069637629593	0.00415412681734751
**BRCA**	ZMYND1	cg14694828	-0.0149631052454763	796	-0.421146256764117	0.673762548640104	0.000223894518587201	0.811347693707753
**BRCA**	ZMYND1	cg17632565	-0.0584918053460988	796	-1.64892846276385	0.0995588552779959	0.00342129129264591	0.237520902462476
**BRCA**	ZMYND1	cg22593633	0.000798591490753676	796	0.0224743638498472	0.982075222389021	6.37748369104179e-07	0.990658193082618
**BRCA**	ZMYND1	cg24757747	-0.0321530783133009	796	-0.905335929895943	0.3655627685214	0.00103382044502126	0.564465950331934
**CESC**	ACTL6A	cg03667091	0.225454971986962	309	4.03482399384936	6.91885536016983e-05	0.0508299443936419	0.000702670279228527
**CESC**	ACTL6A	cg03839554	-0.32486271840772	308	-5.97915350520974	6.30404406431093e-09	0.105535785811254	1.79049581576854e-07
**CESC**	ACTL6A	cg07261848	-0.40503465920619	308	-7.71123875264714	1.80494934580388e-13	0.164053075158275	1.14905261380906e-11
**CESC**	ACTL6A	cg10112003	0.288187379525553	309	5.24734220864356	2.90003651790549e-07	0.0830519657178051	5.64578180365214e-06
**CESC**	ACTL6A	cg20132173	0.0868603166889621	309	1.52020699234882	0.129498328885424	0.00754471461530678	0.28534748611564
**CESC**	ACTL6A	cg22619386	0.289200263864029	309	5.26746495463535	2.62340880170352e-07	0.0836367926190239	5.16802897745565e-06
**CESC**	ACTL6A	cg25198545	0.310263077323459	309	5.69044065505183	2.98099218991866e-08	0.096263177150223	7.3784061858903e-07
**CESC**	ACTL6A	cg25575835	0.0986449079296084	309	1.72836248733696	0.0849386486603713	0.00973081786044091	0.2119420481087
**CESC**	ACTL6B	cg05729836	0.0245118179570559	309	0.427506598113746	0.669313038155198	0.00060082921955985	0.808277037919501
**CESC**	ACTL6B	cg08572611	-0.0584519871427798	309	-1.02089071760254	0.308117945918005	0.00341663480093969	0.506680546517933
**CESC**	ACTL6B	cg09375063	-0.0185948644478307	309	-0.32426860584777	0.745957685618372	0.000345768983833198	0.85731744001272
**CESC**	ACTL6B	cg14088161	0.0138868225772597	309	0.242148374581734	0.80882846897609	0.000192843841292291	0.896660714384775
**CESC**	ACTL6B	cg16024950	-0.0707503381132607	309	-1.23667333164437	0.217162918860713	0.0050056103431407	0.404558828272955
**CESC**	ACTL6B	cg20497359	-0.0654468048943701	309	-1.14355575019333	0.25370762042333	0.00428328427088174	0.447732195073104
**CESC**	ACTL6B	cg24471254	-0.0926663368883016	309	-1.62267480775053	0.105695472297974	0.0085870499922962	0.247790122079109
**CESC**	ACTL6B	cg26990587	-0.0537586768837015	309	-0.938671919635638	0.34864439638726	0.00288999534028622	0.547794440086723
**CESC**	AEBP2	cg02664216	-0.0281519811244613	309	-0.491041185124177	0.623751609400778	0.000792534041232027	0.777142067228808
**CESC**	AEBP2	cg07849438	-0.0379779471789999	309	-0.66264618318589	0.508059260015291	0.00144232447193091	0.690664772043072
**CESC**	AEBP2	cg08161947	-0.180437024855893	309	-3.19852590774011	0.00152704027781476	0.0325575199388462	0.00953966463540945
**CESC**	AEBP2	cg24304000	-0.0896191860873445	309	-1.5688768983289	0.117716878097311	0.00803159851495809	0.267244486377628
**CESC**	AEBP2	cg26259627	-0.0193830113251201	308	-0.33746144479429	0.736002726607429	0.000375701128029734	0.851099060406994
**CESC**	AFF4	cg00931577	-0.0507299688638217	309	-0.885647585102185	0.37650746730082	0.00257352974092432	0.575047058235005
**CESC**	AFF4	cg01907658	-0.0776307413145598	309	-1.35763532372075	0.175586341962953	0.00602653199704811	0.351308160245511
**CESC**	AFF4	cg05619425	0.0374959422389799	309	0.654224155627298	0.513462013101798	0.00140594568438892	0.695105957491365
**CESC**	AFF4	cg06638012	-0.0530976422009819	309	-0.927096858157368	0.354611694154201	0.00281935960730349	0.553553358873608
**CESC**	AFF4	cg13393914	-0.0263755396611962	309	-0.460033291168227	0.645821174244415	0.000695669092419334	0.792357506729068
**CESC**	AFF4	cg13565585	-0.162535821361905	309	-2.8721002205007	0.00436425742073945	0.0264178932257892	0.0222664215617414
**CESC**	AICDA	cg07999668	0.0460548837062347	309	0.803847289334122	0.422113289075067	0.00212105231319481	0.617218186950944
**CESC**	AIRE	cg08089567	-0.120455169594965	309	-2.11561190732132	0.035190830355334	0.0145094478821519	0.11129504255073
**CESC**	AIRE	cg09510531	-0.0931060888160438	309	-1.63044245477567	0.104043612664132	0.00866874377462103	0.245063848964318
**CESC**	AIRE	cg11923631	-0.0693575731905285	309	-1.21220977607559	0.226373357114689	0.00481047295887952	0.415900832330731
**CESC**	AIRE	cg12843033	0.0188101609793329	309	0.328024399566811	0.743119020243362	0.000353822156068418	0.855598162724586
**CESC**	AIRE	cg15955655	-0.00241488734018416	309	-0.0421051222755908	0.966442537324606	5.83168086578171e-06	0.982360933050189
**CESC**	AIRE	cg16501323	-0.0113262901178872	309	-0.197493284220627	0.843573520568175	0.000128284847834549	0.9164116319467
**CESC**	AIRE	cg16717549	-0.0432196395306107	309	-0.754264953438145	0.451273996162421	0.00186793724115593	0.641901758884908
**CESC**	AIRE	cg17356252	-0.00165319755155881	309	-0.028824523633449	0.977023455365705	2.73306214448004e-06	0.987957241060374
**CESC**	AIRE	cg18689454	-0.0652916102491986	309	-1.14083240190113	0.254837599787829	0.00426299436893325	0.44910486912748
**CESC**	AIRE	cg18876487	0.0247349031435824	309	0.43139976143187	0.666483488739425	0.0006118154335224	0.806471593870785
**CESC**	AIRE	cg23272544	-0.0265895676095553	309	-0.463768925136406	0.643145199571374	0.000707005105663112	0.790570538269399
**CESC**	AIRE	cg27251412	-0.0862577598290661	309	-1.50958186534221	0.132188784457111	0.00744040113072884	0.289463893994722
**CESC**	ARID1A	cg04081153	-0.0507000406950797	309	-0.885123749984639	0.376789444492384	0.00257049412648274	0.575297011733636
**CESC**	ARID1A	cg04360147	-0.00525244030453168	309	-0.0915806892563888	0.927091511195124	2.75881291526688e-05	0.961487742090007
**CESC**	ARID1A	cg14851949	0.0498543800072514	309	0.8703230665067	0.384810581382041	0.00248545920590743	0.582944606313837
**CESC**	ARID1A	cg17385674	0.0179694152815052	309	0.313358056871406	0.754223533483932	0.000322899885559192	0.862650928661624
**CESC**	ARID1A	cg20413923	-0.0584516482757196	309	-1.02088477884379	0.308120755288744	0.00341659518614843	0.506680546517933
**CESC**	ARID1A	cg21294655	-0.0167965694550016	309	-0.292899515425229	0.769798784570635	0.000282124745456693	0.872397503778688
**CESC**	ARID1A	cg24542126	-0.00291013920941226	309	-0.0507402257595166	0.959565844839645	8.46891021815859e-06	0.978880726648072
**CESC**	ARID1A	cg25601319	-0.0139171783169983	309	-0.242677798461035	0.808418656446769	0.000193687852307129	0.896343100489146

**CESC**	ARID2	cg00035453	-0.0534523690354279	309	-0.933308156785821	0.351401565140273	0.00285715575549958	0.550348956323933
**CESC**	ARID2	cg01183821	-0.0911428662689178	309	-1.59577205312141	0.111578698373153	0.00830702207171384	0.257473099574792
**CESC**	ARID2	cg01353809	-0.0795683019999901	309	-1.39173335712431	0.16502059912443	0.00633111468316163	0.336883377676538
**CESC**	ARID2	cg01751433	-0.0738366072528305	309	-1.29090896417619	0.197716075846992	0.00545184457060875	0.380128127040529
**CESC**	ARID2	cg06613666	0.00579265985105036	309	0.101000170160067	0.919616904624373	3.35549081499708e-05	0.957668065249702
**CESC**	ARID2	cg07276957	-0.0119352570716823	308	-0.207770567882408	0.835547692362809	0.000142450361367141	0.911892330069336
**CESC**	ARID2	cg23465749	-0.0512320355869461	304	-0.887049604084547	0.375765130222232	0.00262472147038211	0.574296625616555
**CESC**	ARID4A	cg02136725	-0.00688321241818146	309	-0.120015752474742	0.904549946398966	4.73786131938075e-05	0.949386920266611
**CESC**	ARID4A	cg06154646	0.0498265735584819	309	0.869836432380389	0.385076079139944	0.0024826874325788	0.583176713637135
**CESC**	ARID4A	cg08066755	-0.0866225230095313	309	-1.51601368080026	0.130554994459335	0.00750346149253678	0.287017329988399
**CESC**	ARID4A	cg08790491	-0.194790710033691	309	-3.46261914121682	0.000611687204476102	0.0379434207154296	0.00444642070500971
**CESC**	ARID4A	cg10064886	-0.0193390019706787	309	-0.337250092195043	0.736161106125123	0.000373996997221914	0.851190040128682
**CESC**	ARID4A	cg10242170	0.0278020944935872	309	0.484933533748777	0.628072738237933	0.00077295645823035	0.780291459300548
**CESC**	ARID4A	cg10844118	-0.141448734512025	309	-2.49129144301536	0.0132610616445625	0.0200077444950532	0.0531362933865636
**CESC**	ARID4A	cg12203852	0.0150904812588923	309	0.263141494609455	0.792619779897845	0.000227722624624981	0.886316848603895
**CESC**	ARID4A	cg14272338	-0.128594816129357	309	-2.26089898283654	0.0244722966302377	0.0165366267353432	0.0848394761164309
**CESC**	ARID4A	cg14460816	-0.035737675298267	309	-0.623505952736335	0.533419617742824	0.00127718143572436	0.710465103114046
**CESC**	ARID4A	cg17323045	-0.0435236013377628	309	-0.759579697974397	0.448094466775247	0.00189430387340851	0.639557122251419
**CESC**	ARID4A	cg19548283	-0.188423100969455	309	-3.34518821263606	0.000925368303921411	0.0355032649789456	0.00629376655317907
**CESC**	ARID4A	cg20996579	-0.0758541196711842	309	-1.32638317651452	0.185708392312507	0.00575384747109033	0.364455062350131
**CESC**	ARID4A	cg26429530	-0.0971890401863809	309	-1.70260907260741	0.0896634273326395	0.00944570953234996	0.220282595227579
**CESC**	ARID4A	cg27026909	-0.201806998771972	309	-3.59254067039958	0.000381805989729484	0.0407260647533508	0.00298905254755112
**CESC**	ARID4B	cg05652840	0.151655742040202	309	2.67515068101774	0.0078740710125771	0.0229994640937642	0.0354857768713025
**CESC**	ARID4B	cg06212607	0.0116466050595511	308	0.202744984515654	0.839470433444009	0.000135643409413162	0.914083238611266
**CESC**	ARID4B	cg06891548	-0.0793726130613026	309	-1.38828882425222	0.166065580326999	0.00630001170417927	0.33835910188689
**CESC**	ARID4B	cg07814876	-0.0698023335572469	309	-1.22002109576191	0.223402473524933	0.00487236577003716	0.412224194150821
**CESC**	ARID4B	cg10107779	-0.0455539128860085	309	-0.795085003331271	0.427184371349596	0.00207515897922605	0.62156101045095
**CESC**	ARID4B	cg11732257	-0.225898554976206	309	-4.04318896258255	6.6886864440256e-05	0.0510301571403379	0.000683129412139329
**CESC**	ARID4B	cg22596515	0.120464301622241	309	2.1157746592041	0.035176865516438	0.0145116479653343	0.111259923401601
**CESC**	ARID4B	cg23687319	-0.150156676508751	309	-2.64809464599632	0.00851713015895711	0.0225470275001537	0.0377002346279323
**CESC**	ARID4B	cg26020585	0.0706344271107329	309	1.23463710957694	0.217919055186154	0.00498922229326143	0.405485706228303
**CESC**	ASH1L	cg03119126	-0.0874945694368932	309	-1.53139284199334	0.126712264142518	0.00765529968094733	0.281135495442576
**CESC**	ASH1L	cg08095278	0.0365678253989979	309	0.638008539073758	0.523948406593145	0.0013372058544116	0.703136776707939
**CESC**	ASH1L	cg09410084	0.112291457545264	309	1.97033018345877	0.0497077868322399	0.0126093714376399	0.143678664220379
**CESC**	ASH1L	cg10558228	0.0537266493328698	306	0.933470758085014	0.351325156367914	0.00288655284853716	0.550273601000352
**CESC**	ASH1L	cg17038116	-0.00417638187880596	309	-0.0728183412949518	0.94199856190867	1.74421655976188e-05	0.96909004429298
**CESC**	ASH1L	cg20064047	-0.205079197336617	309	-3.65332807913937	0.000304789136094069	0.0420574771802312	0.00247735514087368
**CESC**	ASH1L	cg21852905	-0.0540380850630513	309	-0.943564873569966	0.346141322092472	0.00292011463728157	0.545437785064752
**CESC**	ASH1L	cg23821590	-0.21310046668426	309	-3.80288474939594	0.000172851820060039	0.0454118089010494	0.00153678108454293
**CESC**	ASH1L	cg24312492	0.00340345195831523	309	0.0593415562739825	0.952719051791937	1.15834852325598e-05	0.975037248149736
**CESC**	ASH1L	cg26185843	0.0193451466734966	309	0.337357288899881	0.73608038595776	0.000374234699819096	0.851123637062561
**CESC**	ASH2L	cg00590869	0.115557177438416	309	2.02839680981491	0.0433913173243516	0.0133534612575335	0.130010173314228
**CESC**	ASH2L	cg01642070	-0.130382154483073	309	-2.29286284624454	0.0225385254982748	0.0169995062076479	0.0796729246427404
**CESC**	ASH2L	cg02897270	-0.0161772905694399	309	-0.282097614645391	0.778060612852698	0.000261704730168089	0.877257869682499
**CESC**	ASH2L	cg03189282	-0.230624327280856	309	-4.13247215040832	4.64507642729938e-05	0.0531875803337473	0.000497123504725998
**CESC**	ASH2L	cg03705346	0.0282467563672582	309	0.492695620978475	0.622583333167603	0.000797879245271239	0.776447590824338
**CESC**	ASH2L	cg10062221	0.0879512858390533	309	1.53944877503421	0.124734978805949	0.00773542868074286	0.2779693758409
**CESC**	ASH2L	cg21453287	0.0524428166073556	309	0.915631726157685	0.360585815814095	0.00275024901371273	0.559551044351745
**CESC**	ASH2L	cg24318412	0.0830191028196982	309	1.45250161782241	0.147393525045481	0.00689217143298763	0.312119440547354
**CESC**	ASH2L	cg27255459	0.166816193025715	309	2.94987330258489	0.00342598024427681	0.0278276422555924	0.0183213519710779
**CESC**	ASXL1	cg00174466	-0.0469789791580542	309	-0.820011883466177	0.412851799615102	0.00220702448273289	0.60844528661496
**CESC**	ASXL1	cg04218270	-0.0994690236773855	309	-1.74294554998377	0.082354228676999	0.00989408667133228	0.207241536705003
**CESC**	ASXL1	cg09065199	0.230591316001509	309	4.13184741231116	4.65704563230844e-05	0.0531723550153079	0.000498267374391147
**CESC**	ASXL1	cg10421984	-0.0527403835219452	309	-0.920841582429549	0.357863296136657	0.00278154805404187	0.556872131121134
**CESC**	ASXL1	cg12592359	-0.116450112910386	309	-2.0442853056107	0.0417866588186056	0.0135606287968416	0.126404764791173
**CESC**	ASXL1	cg16926526	0.151774613906553	309	2.6772969576345	0.00782497976644507	0.0230355334264832	0.0353259329126525
**CESC**	ASXL1	cg18793288	-0.0625301228928472	309	-1.09238766491443	0.275527677490449	0.00391001626899457	0.471931408374265

CESC	ASXL1	cg24727216	-0.0603125149712213	309	-1.0535024868879	0.292947365965911	0.00363759946215379	0.490616507674474
CESC	ASXL2	cg05165989	-0.0511801964124413	309	-0.893528243169808	0.372281162402873	0.00261941250481607	0.570928991924353
CESC	ASXL2	cg05496738	-0.055927490997264	309	-0.976657758183346	0.329515152020353	0.00312788424924904	0.529160850161251
CESC	ASXL2	cg06583696	-0.00399069667993042	309	-0.0695807282302758	0.944573123312209	1.59256599912077e-05	0.970330013006246
CESC	ASXL2	cg09476286	-0.0433529043239543	309	-0.75659505030083	0.449878446256982	0.00187947431332193	0.640947083492668
CESC	ASXL2	cg19084174	0.0481381751396071	309	0.840291925197135	0.401405116890948	0.00231728390577149	0.597967100784984
CESC	ASXL2	cg20667782	0.0179683252720572	309	0.31333904269072	0.754237963690636	0.00032286071308245	0.862654750244047
CESC	ASXL2	cg24738909	-0.0555375889428437	309	-0.96982778613458	0.332903311479719	0.00308442378558428	0.53259326724907
CESC	ASXL3	cg09963640	-0.171611035923819	309	-3.03719814212348	0.00259483656108458	0.0294503476508463	0.0146564085844053
CESC	ASXL3	cg12361352	-0.0688052179768504	306	-1.19656163281957	0.232419087695265	0.0047341580208419	0.423031995230937
CESC	ASXL3	cg17880622	-0.120623802691444	309	-2.11861739078509	0.0349337119856065	0.0145501017757445	0.110697889621518
CESC	ASXL3	cg20034091	-0.0913870365483788	309	-1.60008305653974	0.110618848494903	0.00835159044909473	0.255993654801655
CESC	ASXL3	cg20329085	-0.103248544727934	309	-1.80987259562776	0.0713029805513676	0.0106602619884363	0.186866462370741
CESC	ASXL3	cg24880056	-0.122757820826655	309	-2.15666735780941	0.0318154305681988	0.0150694825741091	0.103079821982527
CESC	ASXL3	cg27109877	-0.0903084860698218	309	-1.5810426508199	0.114908125594368	0.0081556226562232	0.262761984788014
CESC	ATAD2B	cg08192560	-0.0496582759541521	308	-0.865464156520934	0.387468799544755	0.00246594437073872	0.58538700753097
CESC	ATAD2B	cg14009731	-0.156118433146928	309	-2.75580867311675	0.00620815598630196	0.0243729651682519	0.029466582477021
CESC	ATAD2B	cg15717719	0.0755445563513414	309	1.32093902699168	0.187515144398239	0.00570697999432099	0.366834812234487
CESC	ATAD2B	cg16929802	0.150705108277495	309	2.65799089338926	0.00827663925930226	0.0227120296609314	0.0368855387935682
CESC	ATAD2B	cg17933838	-0.105236593829422	309	-1.8451082036215	0.065994287802956	0.0110747406808187	0.176500742972729
CESC	ATAD2B	cg19614157	-0.0857882171130162	309	-1.50130338778089	0.134315033914055	0.00735961819543001	0.292604417482778
CESC	ATAD2B	cg20806130	-0.144195626278831	309	-2.54068888080639	0.0115605897357037	0.0207923786379442	0.0477460457580873
CESC	ATAD2B	cg27610867	-0.261483949496412	309	-4.72346817950465	3.5471093561066e-06	0.068373855844242	5.25207128376393e-05
CESC	ATAT1	cg01245721	0.0343423563878424	309	0.599132856023719	0.549530509154792	0.00117939744226958	0.722436441557885
CESC	ATAT1	cg05093836	-0.0706353235182746	309	-1.23465285667956	0.217913200325566	0.00498934892853131	0.405485706228303
CESC	ATAT1	cg06814856	-0.122789502572314	309	-2.15723247702732	0.0317709864234871	0.0150772619419562	0.102974423707556
CESC	ATAT1	cg07142893	-0.14321812947023	309	-2.52310371709863	0.0121422627530198	0.0205114326089516	0.0496163036352781
CESC	ATAT1	cg07816366	-0.131632469915067	309	-2.31523635895233	0.0212656442344401	0.0173271071359409	0.0763142138377171
CESC	ATAT1	cg08081524	-0.182901006949537	309	-3.24370499714848	0.00131120347908942	0.0334527783431547	0.00840759822814272
CESC	ATAT1	cg08188471	-0.019828911687788	302	-0.341792476779467	0.73274878537774	0.000393185738722096	0.849129113992149
CESC	ATAT1	cg08635134	-0.115064045822235	309	-2.01962438459238	0.0442994956993322	0.0132397346409814	0.132049772719513
CESC	ATAT1	cg09532691	-0.341564407304467	309	-6.33646422342305	8.4623856788184e-10	0.116666244337252	2.84372713390064e-08
CESC	ATAT1	cg12907138	-0.12704650136892	309	-2.23322781888794	0.0262614764975481	0.0161408135100831	0.0893983616136934
CESC	ATAT1	cg13542073	-0.125783327217253	309	-2.21066492959218	0.0278033945853717	0.0158214454058426	0.0932773321573574
CESC	ATAT1	cg13964084	-0.102516748867267	309	-1.79690800135409	0.073342739637155	0.0105096837983142	0.190785296841197
CESC	ATAT1	cg15381130	-0.116985327236412	309	-2.05381107690185	0.0408490230527095	0.0136855667886105	0.124265634143286
CESC	ATAT1	cg15729859	-0.110126315047831	309	-1.93186830991196	0.0543048929893958	0.0121278052660142	0.153270454484322
CESC	ATAT1	cg18298197	-0.0626031537946088	309	-1.09366851583044	0.274966228682792	0.00391915486503145	0.471452058409352
CESC	ATAT1	cg20629923	-0.106233814866567	309	-1.86279105816273	0.0634560504742266	0.0112856234211039	0.171471742352704
CESC	ATAT1	cg22759096	-0.228221017813678	309	-4.0870287579557	5.59674425042805e-05	0.0520848329719111	0.000585127163308426
CESC	ATAT1	cg23328390	-0.140327921820675	309	-2.47115277356136	0.0140157966973411	0.0196919256425096	0.0553980737868588
CESC	ATAT1	cg26934609	-0.132348471908093	308	-2.3242215755837	0.0207746229586172	0.0175161180164072	0.0749636355323838
CESC	ATF7IP	cg00236831	-0.112943221433425	309	-1.98191374118967	0.0483892635516529	0.0127561712677597	0.140793446113524
CESC	ATF7IP	cg11409060	0.104064479432239	305	1.81228938627528	0.070941112517261	0.0108294158795029	0.18615013010314
CESC	ATF7IP	cg12526346	-0.12342385699304	308	-2.16497947674815	0.0311696666883006	0.0152334484750384	0.101554343106322
CESC	ATF7IP	cg12948116	-0.084152149867831	309	-1.47246582721856	0.141929662040083	0.00708158432737789	0.304181035897503
CESC	ATF7IP	cg17439009	-0.0652252104106527	309	-1.13966724608213	0.255322122308166	0.00425432807311392	0.449622627282721
CESC	ATF7IP	cg19789919	0.0404325014892407	309	0.705541693251445	0.481013468170886	0.00163478717667745	0.668249401626621
CESC	ATF7IP	cg21275967	-0.102439359837754	309	-1.7955371396383	0.0735611979969748	0.0104938224439688	0.191179237609254
CESC	ATF7IP	cg26637901	0.0237878835319541	269	0.387348296998396	0.698809285456535	0.000565863402929815	0.827258109185456
CESC	ATM	cg01756564	-0.277987932417104	309	-5.04576628510156	7.78736072765692e-07	0.0772772905695363	1.36844350677059e-05
CESC	ATM	cg01800843	-0.196129810662267	309	-3.48737204537995	0.000559756591730353	0.0384669026304168	0.00412442738180253
CESC	ATM	cg01991180	-0.104978448484747	309	-1.84053165580695	0.0666647420792938	0.0110204746462647	0.1778564746842
CESC	ATM	cg02105423	0.119754250293433	309	2.10312167789543	0.0362768283845368	0.0143410804633423	0.113914603353764
CESC	ATM	cg03165700	-0.070148603461405	309	-1.22610313486417	0.221108772975992	0.00492082656758544	0.409391733713704
CESC	ATM	cg03569616	-0.28520834019719	305	-5.15402288664251	4.63290240475022e-07	0.081343797318036	8.61817335425965e-06
CESC	ATM	cg04709886	-0.309180894624821	309	-5.66849074487048	3.34761165554552e-08	0.0955928256010045	8.14079715947195e-07

**CESC**	ATM	cg04826772	0.113110202240053	309	1.98488184547435	0.0480562038763194	0.0127939178507856	0.140149365486575
**CESC**	ATM	cg05033322	-0.0472257021565668	308	-0.822971082365884	0.411171654065519	0.00223026694418076	0.607012187983742
**CESC**	ATM	cg05637308	-0.0736239016457187	309	-1.28716986720357	0.199014246798579	0.00542047889353846	0.381727706707364
**CESC**	ATM	cg06053805	0.0136717963401399	309	0.238398195993416	0.811732882138154	0.000186918015166264	0.898558284116917
**CESC**	ATM	cg06081482	-0.281324443451384	307	-5.09465696523678	6.16709839773929e-07	0.0791434424832311	1.1146915723832e-05
**CESC**	ATM	cg06750635	-0.0884481553357694	309	-1.54821407221581	0.122611138490233	0.00782307618230039	0.274739642482551
**CESC**	ATM	cg09808176	-0.12943808339437	309	-2.2759767399279	0.0235428067794434	0.0167542174328078	0.0823955871243484
**CESC**	ATM	cg10610482	-0.2800496309981	309	-5.08636020680314	6.39808218627508e-07	0.0784277958221722	1.15162518648562e-05
**CESC**	ATM	cg10976287	-0.261141722765789	309	-4.7168334124768	3.65653940544835e-06	0.068194999369084	5.39663871196071e-05
**CESC**	ATM	cg11588932	-0.218838941838497	309	-3.91037083133782	0.000113697163224753	0.0478904824649931	0.00107576018301945
**CESC**	ATM	cg12019961	-0.125669398527759	278	-2.092992107266	0.0372728743682888	0.0157927977263288	0.116253025496975
**CESC**	ATM	cg12106634	-0.0766991271466695	309	-1.34124590759693	0.180841795596071	0.00588275610506098	0.358020273485195
**CESC**	ATM	cg12848864	-0.160484013859533	309	-2.83487884720644	0.0048914523466492	0.0257551187044669	0.0243583180037913
**CESC**	ATM	cg14761454	-0.105949327366736	292	-1.80505513016425	0.0721140253750987	0.0112252599694638	0.188376430946927
**CESC**	ATM	cg15370815	0.167740108553343	309	2.96668291259751	0.00324916879135705	0.0281367440174871	0.0175629403109248
**CESC**	ATM	cg15504467	-0.106668394426754	309	-1.87049885529118	0.0623753024747491	0.0113781463695817	0.169357262345408
**CESC**	ATM	cg16693212	0.0266943854732691	301	0.459431295584712	0.646261708848656	0.000712590215795478	0.792582569344597
**CESC**	ATM	cg16788234	0.0376818603921271	309	0.657472631756431	0.511374552042216	0.00141992260261176	0.693559369960637
**CESC**	ATM	cg17114932	-0.22323253099271	309	-3.99295347029153	8.1886855537023e-05	0.049832762893411	0.000809647437677576
**CESC**	ATM	cg17420659	-0.180023365902364	309	-3.19094726781606	0.00156631611022859	0.0324084122708164	0.00974410199753079
**CESC**	ATM	cg18042593	-0.0944195001694417	309	-1.65364791066593	0.099231270867228	0.00891504201224721	0.236935560168354
**CESC**	ATM	cg18391757	-0.0913845725502625	309	-1.60003955141323	0.110628502124642	0.0083511401001942	0.255994932883755
**CESC**	ATM	cg18457775	-0.296294061888134	309	-5.40894251480767	1.28573761425729e-07	0.0877901711101695	2.7291315678999e-06
**CESC**	ATM	cg19288979	0.133375550863368	309	2.34644635360802	0.019595315933359	0.0177890375681068	0.0716958897431912
**CESC**	ATM	cg20342375	-0.235211396827387	309	-4.21943017169726	3.23675620730934e-05	0.0553244011974903	0.000363978335344641
**CESC**	ATM	cg22477592	-0.226874839759892	309	-4.06160882327692	6.20725430704211e-05	0.0514721929160768	0.000641369559784877
**CESC**	ATM	cg22678707	-0.211683080799726	309	-3.77640004075125	0.000191369907779571	0.0448097266968635	0.00167236919858503
**CESC**	ATM	cg22837512	-0.0322766410402389	309	-0.563055833142571	0.573811892275666	0.00104178155684043	0.741099781144667
**CESC**	ATM	cg23275463	-0.0304283872043977	307	-0.529033961288404	0.597170507514773	0.000925886747860755	0.758135719137755
**CESC**	ATM	cg23389069	-0.0816767581765229	309	-1.42885693359186	0.154072041322627	0.00667109282622621	0.321504717046353
**CESC**	ATM	cg24030675	-0.0196518854097662	309	-0.34270851302665	0.732054599685555	0.000386196600158582	0.84866188105116
**CESC**	ATM	cg24568245	-0.189363001053562	307	-3.35141474579908	0.000906235193324233	0.0358583461680114	0.0061901321842818
**CESC**	ATM	cg25400013	-0.100972565803649	309	-1.76956069403775	0.0778028101681905	0.0101954590449723	0.198940086570627
**CESC**	ATM	cg25740661	0.138500149410054	309	2.43833225964035	0.0153276439779113	0.0191822913866072	0.0593735366039751
**CESC**	ATM	cg25918541	-0.0612381509139206	309	-1.06973133501654	0.285588876477685	0.00375011112735611	0.482823015466805
**CESC**	ATM	cg27453365	-0.102278091882908	309	-1.79268056066464	0.0740181362370652	0.0104608080792085	0.192015702628593
**CESC**	ATM	cg27599391	-0.169906583302427	309	-3.00613113184498	0.00286656121112198	0.0288682470495046	0.015881018576985
**CESC**	ATR	cg00911981	0.0561239904903167	309	0.980100038582332	0.327816070508011	0.00314990230855715	0.527320682042891
**CESC**	ATR	cg00969988	-0.075366578146552	309	-1.31780917914355	0.188559734468152	0.00568012110152033	0.368183930704938
**CESC**	ATR	cg11458642	0.12663598975018	309	2.22589403118656	0.0267543062429128	0.0160366739000078	0.0906342318350966
**CESC**	ATR	cg11936145	-0.191579646396803	309	-3.40334519773387	0.000754915864671331	0.036702760913524	0.00530897891667343
**CESC**	ATR	cg15135999	0.0681220055585809	309	1.19051331704905	0.234773500902181	0.00464060764132333	0.425842750569709
**CESC**	ATR	cg15291273	-0.15209998942227	309	-2.68317234443504	0.00769200003772459	0.0231344067822545	0.0348916297921135
**CESC**	ATR	cg15480357	0.012632075419054	309	0.220265335292398	0.825812360740033	0.000159569329392667	0.906322063580431
**CESC**	ATR	cg16031872	-0.0450436389166902	309	-0.786160617491464	0.432385752464733	0.00202892940685717	0.626109815585199
**CESC**	ATR	cg17945282	-0.164330495150366	309	-2.9046883467376	0.00394576200690909	0.0270045116363644	0.0205473331257438
**CESC**	ATR	cg20032934	-0.115354877439898	309	-2.02479786778979	0.0437619728385831	0.0133067477491739	0.130824221343944
**CESC**	ATR	cg20636078	-0.00887604876543703	309	-0.154765294999201	0.877109053449654	7.87842416864162e-05	0.934920074586898
**CESC**	ATR	cg24631870	-0.0316954965758681	309	-0.552907662708545	0.580732978989758	0.00100460450319087	0.746452445640983
**CESC**	ATR	cg24856922	0.0790538136052733	309	1.38267763559705	0.167778566992422	0.0062495054455373	0.340698664594323
**CESC**	AURKB	cg02245743	-0.00145021632206948	309	-0.0252854121659318	0.979843899214805	2.10312738079674e-06	0.989664496271231
**CESC**	AURKB	cg04612488	-0.0964977315417533	309	-1.69038411742501	0.0919795275900682	0.00931181219270428	0.224301914339418
**CESC**	AURKB	cg05617798	0.0258013312138156	309	0.450011394665066	0.653022948805375	0.000665708692405016	0.797381334352235
**CESC**	AURKB	cg05811247	-0.109115955706141	309	-1.91392971724057	0.056567944758511	0.0119062917896645	0.157732704481517
**CESC**	AURKB	cg11009596	0.0696666391302983	309	1.21763781928335	0.22430591997001	0.00485344060771121	0.413498172149658
**CESC**	AURKB	cg12844497	0.110034257938861	309	1.93023361655505	0.0545079252366179	0.0121075379201559	0.153692987888998
**CESC**	AURKB	cg13678641	-0.0887916752068325	309	-1.55427480237803	0.121159295451215	0.00788396158603563	0.27249107344122

**CESC**	AURKB	cg21724796	-0.14667443041983	309	-2.58531674074582	0.0101944422113114	0.0215133885389815	0.0432589558384748
**CESC**	BAP1	cg00794529	-0.287928077675886	309	-5.24219380900875	2.97523897828637e-07	0.0829025779141308	5.77772693651548e-06
**CESC**	BAP1	cg02503646	-0.0785677252436268	309	-1.3741228163059	0.170415802926879	0.00617288744995803	0.344220983873232
**CESC**	BAP1	cg03075736	-0.0491845461163555	309	-0.858601022533994	0.391237142810933	0.0024191195766719	0.588426109319754
**CESC**	BAP1	cg05442719	-0.0214080520905361	309	-0.373347705870552	0.709149884533352	0.000458304694311109	0.834292720111722
**CESC**	BAP1	cg05908979	-0.0446365012301597	309	-0.779040466008134	0.436561880066502	0.00199241724207005	0.629635234207039
**CESC**	BAP1	cg09096430	-0.19154726422679	309	-3.40274802761015	0.000756506445259915	0.0366903544327678	0.00531680028932603
**CESC**	BAP1	cg10449522	-0.144371098065485	309	-2.54384642001834	0.0114588184153714	0.0208430139566338	0.0474340083970308
**CESC**	BAP1	cg10956904	-0.186392160568605	308	-3.30238163410705	0.00107342187768353	0.0347420375214325	0.00711943908535559
**CESC**	BAP1	cg11234564	-0.154201119964809	309	-2.72113457941418	0.0068807566057694	0.0237779853984013	0.0319857531753346
**CESC**	BAP1	cg12859382	0.13037991015239	309	2.29282269557048	0.0225408683252028	0.0169989209713453	0.0796765473835824
**CESC**	BAP1	cg16841708	-0.0329569629563798	309	-0.574936605858973	0.565759419313796	0.00108616140730819	0.734997458937947
**CESC**	BAP1	cg24291822	-0.211932220404959	309	-3.78105355138306	0.00018798599784983	0.0449152660457761	0.00164723842269989
**CESC**	BAP1	cg24747396	0.0252187839891352	309	0.439844413475057	0.660362329477529	0.00063598706589066	0.802402684338049
**CESC**	BAP1	cg25749966	-0.310009581357026	309	-5.68529680613246	3.06322539256794e-08	0.0961059405331583	7.5387711543635e-07
**CESC**	BAZ1A	cg05294307	0.0920238355928601	306	1.60335723501189	0.109904581931517	0.00846838631722175	0.254793337037758
**CESC**	BAZ1A	cg07414384	-0.129063116291446	309	-2.26927164861335	0.0239522810973372	0.0166572879868592	0.0834719238313065
**CESC**	BAZ1A	cg23422263	-0.109009383417829	309	-1.91203791289888	0.0568111393805792	0.0118830456731353	0.158225897786509
**CESC**	BAZ1A	cg26967526	0.0309973767714544	309	0.540717564696372	0.589098225860113	0.000960837366711501	0.752445814195517
**CESC**	BAZ1B	cg00525989	-0.0463687018725874	309	-0.809336470170433	0.418954627672092	0.00215005651334889	0.61438685830893
**CESC**	BAZ1B	cg04742505	0.11027456734431	309	1.93450098855328	0.0539792420835884	0.0121604802029749	0.15257834309464
**CESC**	BAZ1B	cg05166819	-0.0845079175025861	309	-1.4787356108025	0.140246383831649	0.00714158812062389	0.30171881438527
**CESC**	BAZ1B	cg06059318	0.0626566937803591	309	1.09460753693437	0.27455511671516	0.00392586127548569	0.470996311849236
**CESC**	BAZ1B	cg06394887	0.00517263536024214	309	0.0901891857943812	0.928196270430635	2.67561565700273e-05	0.962133253322862
**CESC**	BAZ1B	cg14093368	-0.0175094927963395	309	-0.305335247392558	0.760319790658236	0.000306582337985064	0.866641549931735
**CESC**	BAZ1B	cg15636799	-0.0733652194085913	309	-1.28262279687545	0.200601358886659	0.00538245541887074	0.383646338550747
**CESC**	BAZ1B	cg19160975	0.01611271350838	309	0.280971234735457	0.778923595327102	0.000259619536603133	0.877741106792967
**CESC**	BAZ1B	cg20612831	-0.0386209440029807	309	-0.673881929098693	0.500898435446722	0.00149157731568137	0.68471082212515
**CESC**	BAZ1B	cg21340129	-0.205476166848418	309	-3.66071120326001	0.000296500768967748	0.0422204551427189	0.00242111691204755
**CESC**	BAZ1B	cg25710357	0.0780588356244811	309	1.36516776718527	0.173209731940912	0.00609318181904976	0.3481109302111
**CESC**	BAZ2A	cg14634319	0.100120687128935	309	1.75447956251775	0.0803558530242797	0.01002415199117	0.203648789098606
**CESC**	BAZ2A	cg18206859	-0.00734352360272846	309	-0.128042161628266	0.898200267902314	5.392733890383e-05	0.946143525445385
**CESC**	BAZ2A	cg18955060	-0.131748739632536	309	-2.31731749318919	0.0211505057673884	0.0173577303947618	0.0760198132864305
**CESC**	BAZ2A	cg25683012	0.0353679270878889	309	0.617046929837993	0.537665633458017	0.00125089026649423	0.713530540587394
**CESC**	BRD7	cg03021120	-0.155553252991679	309	-2.74558424285859	0.00640002337733272	0.0241968145162932	0.0301694632349439
**CESC**	BRD7	cg16433860	-0.133973994979514	309	-2.35716663658308	0.0190487555020128	0.0179490313307707	0.0700685084648724
**CESC**	BRD7	cg16834187	0.0577486253135957	309	1.0085648252255	0.313985463585697	0.00333490372561006	0.513068920227392
**CESC**	BRD8	cg01096096	-0.110175051689092	309	-1.93273376549751	0.0541976586166928	0.0121385420146941	0.153062246947905
**CESC**	BRD8	cg03204906	-0.0785130551741748	309	-1.37316072267025	0.170714334070008	0.00616429983278301	0.344591357152108
**CESC**	BRD8	cg03684156	-0.189114762117746	309	-3.35792228088948	0.000885236782818549	0.0357643932508515	0.00606589538294603
**CESC**	BRD8	cg07430605	-0.0607236033341298	309	-1.06070961475276	0.289663864720208	0.00368735600188074	0.487135008975035
**CESC**	BRD8	cg07632946	-0.279301586966207	309	-5.07162276912225	6.87216908770976e-07	0.0780093764818415	1.2258966338209e-05
**CESC**	BRD8	cg08976717	-0.153999185875651	309	-2.71748449619105	0.00695525724157654	0.0237157492503634	0.0322511344013355
**CESC**	BRD8	cg11110536	-0.087963912153145	309	-1.53967150204155	0.124680657129561	0.00773764984128622	0.277917755822604
**CESC**	BRD8	cg11346339	-0.135065947790532	309	-2.3767342085733	0.0180855870734273	0.0182428102525547	0.0673867403420937
**CESC**	BRD8	cg12964860	-0.1485146834885	309	-2.61848040504494	0.00927474788682037	0.0220566112116895	0.040231301360688
**CESC**	BRD8	cg14473243	-0.0440957878202483	309	-0.769584901097609	0.442143718938592	0.00194443850348836	0.634511428410718
**CESC**	BRD8	cg22106577	-0.0883485102031963	309	-1.54645613478386	0.123034796715029	0.00780545925512428	0.275426211283679
**CESC**	BRD8	cg22818036	-0.178242090715175	309	-3.15833247936445	0.00174624121328851	0.0317702429025168	0.0106438211193737
**CESC**	BRD8	cg23298160	-0.00898801301389961	308	-0.156459720337352	0.875774861148277	8.07843779380288e-05	0.934340215710097
**CESC**	BRD8	cg26008208	-0.0559937413640376	309	-0.977818320135954	0.328941668622917	0.00313529907194273	0.528657952047333
**CESC**	BRD8	cg26630792	-0.013378029237355	309	-0.233274785760089	0.81570503079576	0.000178971666275526	0.900838957070414
**CESC**	BRD8	cg27119451	-0.0709295771786294	309	-1.23982214431453	0.215997367122331	0.00503100491873914	0.403198540561838
**CESC**	BRD9	cg02364331	-0.180787843101365	308	-3.19967896949565	0.0015216387212549	0.0326842442132437	0.00951332979838496
**CESC**	BRD9	cg02822723	0.0263887260920436	309	0.460263444690499	0.645656172950664	0.000696364864760903	0.79224512225408
**CESC**	BRD9	cg05193570	-0.0941094906258719	309	-1.64816985665221	0.100350900680072	0.00885659622586108	0.238831889359853
**CESC**	BRD9	cg07308931	-0.0584152928797898	309	-1.02024764040761	0.308422256939552	0.00341234644223162	0.507002811604165

**CESC**	BRD9	cg09497020	-0.065179621386821	308	-1.13699259837751	0.256439743005261	0.00424838304412934	0.450713328980871
**CESC**	BRD9	cg10551270	-0.209434534809407	309	-3.73443569978856	0.000224593903717653	0.0438628243708328	0.00191265004332907
**CESC**	BRD9	cg10600960	-0.0615173779668408	309	-1.07462746629407	0.283393739775009	0.00378438779191515	0.48069449670658
**CESC**	BRD9	cg13722821	-0.169882662785097	309	-3.00569533166837	0.00287055206709164	0.028860119114955	0.0158974676092637
**CESC**	BRD9	cg17977043	-0.16463783659972	309	-2.91027209106517	0.00387784573217914	0.0271056172402362	0.0202587556839127
**CESC**	BRD9	cg20079193	-0.175211543209632	309	-3.10291726014567	0.00209630930860707	0.0306990848739006	0.0123474680158209
**CESC**	BRD9	cg21160852	0.0724309928979828	309	1.26620329009174	0.206409708735565	0.00524624873218764	0.390958241170981
**CESC**	BRD9	cg21758832	-0.0733479949442408	309	-1.28232003755927	0.200707362877507	0.00537992836234037	0.383735864427555
**CESC**	BRD9	cg21910709	-0.118765938197497	309	-2.0855155878437	0.0378563103277958	0.0141053480759316	0.117588093657039
**CESC**	BRD9	cg24953233	-0.249619150357701	309	-4.49453726293429	9.92372947884305e-06	0.0623097202253004	0.000129989326667304
**CESC**	BRD9	cg25523559	-0.183143076335654	309	-3.2481469290946	0.00129158604247065	0.033541386409687	0.00829984550737732
**CESC**	BRD9	cg25951128	-0.152277936716133	309	-2.6863859606978	0.0076201294059535	0.0231885700105227	0.0346282111692708
**CESC**	BRD9	cg26504197	-0.0472532719864606	309	-0.824810311777992	0.410126008115663	0.00223287171342642	0.606046968854589
**CESC**	BRDT	cg00308185	-0.205513934442477	309	-3.66141373031408	0.000295723257225909	0.0422359772500266	0.002416036029472
**CESC**	BRDT	cg01515515	-0.219588886635679	306	-3.90503699662607	0.000116345479875355	0.0482192791338973	0.00109712040959519
**CESC**	BRDT	cg02935097	-0.152336210969707	309	-2.68743841520147	0.00759672371961646	0.023206321172607	0.0345445565968508
**CESC**	BRDT	cg03421440	-0.413317613682763	309	-7.91405919735345	4.68742682323045e-14	0.170831449780414	3.22988203973773e-12
**CESC**	BRDT	cg06442199	-0.373478375785676	309	-7.0197768645346	1.45453628295817e-11	0.139486097179507	6.687600212556e-10
**CESC**	BRDT	cg09250199	-0.217056738802856	307	-3.86416966790896	0.000136460351192429	0.0471136278597313	0.00125689621317226
**CESC**	BRDT	cg13299824	-0.307819855871721	309	-5.6409193491157	3.87081176132045e-08	0.0947530636688868	9.30837496631106e-07
**CESC**	BRDT	cg14732540	-0.276123082911899	309	-5.00911334820819	9.2893959562739e-07	0.0762439569167713	1.60555269722811e-05
**CESC**	BRDT	cg19245011	-0.159448395060759	309	-2.8161062810994	0.00517874023985008	0.0254237906874517	0.0254919588850226
**CESC**	BRPF1	cg04879506	0.0130124663490023	309	0.226899313880871	0.820654537893175	0.000169324280483917	0.903612057986492
**CESC**	BRPF1	cg13694772	0.189557227695732	309	3.36607116451289	0.000860412663655691	0.0359319425716916	0.00592379190129086
**CESC**	BRPF1	cg14712964	-0.0708122833712231	309	-1.23776155183627	0.21675959465387	0.0050143794762464	0.404044482798496
**CESC**	BRPF1	cg17738494	0.154302044517429	309	2.72295898383775	0.0068437900108713	0.0238091209422586	0.0318329327417472
**CESC**	BRPF1	cg20952324	0.121591046175084	309	2.1358598069498	0.0334895690182682	0.0147843825099515	0.10714305941041
**CESC**	BRPF1	cg25467642	-0.115925728098613	309	-2.03495402960349	0.0427228390613685	0.0134387744351936	0.128492250132997
**CESC**	BRPF1	cg26227975	-0.197776018995022	309	-3.51782957367137	0.000501518760505406	0.0391153536895191	0.00375796093765725
**CESC**	BRPF3	cg01499736	-0.203711213560569	309	-3.62789972487703	0.000335034402489304	0.0414982585303199	0.00268006851488462
**CESC**	BRPF3	cg05882415	-0.0852209925208725	309	-1.49130403082828	0.13691860269852	0.00726261756624261	0.296672862388342
**CESC**	BRPF3	cg10562781	-0.102785660053126	309	-1.80167172438656	0.0725877431141401	0.0105648919125567	0.189277029155373
**CESC**	BRPF3	cg14654948	-0.15200954598046	309	-2.68153909513401	0.00772876018330742	0.0231069020691855	0.0349971577241104
**CESC**	BRPF3	cg18779695	0.052992974732482	309	0.925264188766464	0.355562400334272	0.00280825537099747	0.554357927979322
**CESC**	BRPF3	cg23517469	0.0450570894618078	309	0.786395851519258	0.432248180312175	0.00203014131076935	0.626048461036739
**CESC**	BRWD3	cg03164831	-0.096268414768368	309	-1.68632947464919	0.0927582894467208	0.00926760768201453	0.225726925964033
**CESC**	BRWD3	cg05835545	-0.0680632450095786	309	-1.1894816248678	0.235178390832205	0.00463260532123392	0.426368392831522
**CESC**	BRWD3	cg09523186	-0.0984421162950442	309	-1.72477455882312	0.0855845066754563	0.009690850260647	0.213082140526835
**CESC**	BRWD3	cg11957438	-0.0366718645481632	295	-0.62491969553765	0.532515148812083	0.00134482564943883	0.709906512869688
**CESC**	BRWD3	cg15511898	-0.16830528513952	309	-2.97696963057065	0.00314513017161784	0.0283266690058952	0.0171171300245444
**CESC**	BRWD3	cg22618269	-0.0896561182935784	309	-1.56952867297197	0.117565040367718	0.00803821954747211	0.267031976817282
**CESC**	BRWD3	cg25063710	-0.151646119813444	309	-2.67497695326447	0.0078780567467904	0.0229965456544735	0.0354999758681614
**CESC**	BRWD3	cg25708850	-0.117087926243137	307	-2.04886422276166	0.0413393835231862	0.0137095824719183	0.125373513773449
**CESC**	CARM1	cg12317676	0.121509716370655	309	2.13440975670442	0.0336090096546904	0.0147646111724771	0.107422131067874
**CESC**	CARM1	cg17918830	-0.263286129875618	309	-4.75843877073687	3.02051463623108e-06	0.069319586184881	4.5415136376297e-05
**CESC**	CARM1	cg22378614	-0.183822875477325	309	-3.26062439941373	0.00123792721867448	0.0337908495487522	0.00801859367606684
**CESC**	CARM1	cg26504915	-0.174503678501054	309	-3.08998674326451	0.00218680500537804	0.0304515338103993	0.0127636324617823
**CESC**	CBX1	cg01544580	-0.0217678974606514	309	-0.379626212934829	0.704487955493204	0.000473841359857433	0.831338136143307
**CESC**	CBX1	cg01553295	0.0246005113801618	309	0.429054420594858	0.668187513533609	0.000605185160165469	0.807559337370023
**CESC**	CBX1	cg02835499	-0.0474367314408702	309	-0.828019822042722	0.408308830472668	0.00225024348979325	0.604268484529748
**CESC**	CBX1	cg04864609	-0.0974258339651511	309	-1.70679705456939	0.0888809228741467	0.00949179312380519	0.218927983854332
**CESC**	CBX1	cg11729481	0.0171938317293193	309	0.299829022021849	0.764512481877136	0.000295627849536148	0.869332066757534
**CESC**	CBX1	cg12245530	-0.0204997303797545	309	-0.357500138014764	0.720965488158227	0.000420238945642629	0.841902278313238
**CESC**	CBX1	cg13342109	-0.0867301799605922	309	-1.51791209725822	0.1300757858568	0.0075221241159967	0.286271236984947
**CESC**	CBX1	cg17778721	0.0830573543058769	309	1.45317551335989	0.147206493216505	0.00689852410429197	0.311842222402439
**CESC**	CBX1	cg21511817	-0.0319624700268689	309	-0.557569586511646	0.577548654438856	0.00102159949021849	0.744055974553554
**CESC**	CBX4	cg04398978	0.0107648168139808	309	0.187701870433165	0.851235585286465	0.000115881281038563	0.920644262005244

**CESC**	CBX4	cg04925705	0.00644313174692273	309	0.112342172566704	0.9106262483265	4.15139467082035e-05	0.952641210848294
**CESC**	CBX4	cg05949524	-0.0996350514965448	309	-1.74588391703632	0.0818413243289343	0.00992714348671914	0.206284473329611
**CESC**	CBX4	cg07159673	0.0452929901026572	309	0.790521542787691	0.429839492805878	0.0020514549524394	0.623880283701158
**CESC**	CBX4	cg10756274	-0.0322001460389135	309	-0.561720015259548	0.574720676283305	0.00103684940492736	0.741837765530927
**CESC**	CBX4	cg21365914	-0.063486566783861	309	-1.10916363893322	0.268236257422843	0.00403054416200164	0.463984923790111
**CESC**	CBX4	cg22353329	0.0114928532685938	309	0.200397979171328	0.841303336692217	0.000132085676253428	0.915016032751748
**CESC**	CBX4	cg27487227	-0.0322879211629137	309	-0.56325281664984	0.573677938310191	0.00104250985302253	0.741019662434536
**CESC**	CBX5	cg03689456	-0.0845855143364687	309	-1.48010319513806	0.139881280508423	0.00715470923556495	0.301162435832703
**CESC**	CBX5	cg05754589	-0.0345936912133451	309	-0.603522849418293	0.54661096070659	0.00119672347176427	0.720131637745067
**CESC**	CBX5	cg06399839	-0.122119364559589	309	-2.14528039740331	0.0327224466881138	0.0149131392004377	0.105310825027177
**CESC**	CBX5	cg06621757	0.0149130887354966	309	0.26004750585501	0.795003166683305	0.000222400215632796	0.887764580336702
**CESC**	CBX5	cg08288469	-0.0458385309935504	309	-0.800063073910171	0.424299000992324	0.00210117092364668	0.619239015518477
**CESC**	CBX5	cg09060496	-0.0584797766425606	309	-1.02137773910103	0.307887615006907	0.00341988427616378	0.506466402813316
**CESC**	CBX5	cg09158487	-0.121573376043428	309	-2.13554475786293	0.0335154885366814	0.0147800857625967	0.107203448127624
**CESC**	CBX5	cg09653879	-0.18473345895251	309	-3.27734542445934	0.00116925968656591	0.0341264508565586	0.00764702715903392
**CESC**	CBX5	cg11005091	-0.0547142789612887	309	-0.955407201431928	0.340130926457658	0.00299365232225372	0.539386523419093
**CESC**	CBX5	cg11338003	-0.082885762993842	307	-1.44537444366818	0.149389595319498	0.00687004970707134	0.31484650461327
**CESC**	CBX5	cg12263485	-0.04175813891635	309	-0.728713651998621	0.466737830319789	0.00174374216575719	0.655949032985831
**CESC**	CBX5	cg12600134	-0.0868764082502948	309	-1.52049076430291	0.12942706347036	0.00754751031047189	0.285251797093142
**CESC**	CBX5	cg13445218	0.00603447330711334	309	0.105216553033693	0.916273364687162	3.64148680942634e-05	0.955798304202007
**CESC**	CBX5	cg14017196	-0.126063994362388	309	-2.21567727404708	0.0274542399847179	0.0158921306746001	0.092401797411575
**CESC**	CBX5	cg14394617	-0.128445146474664	309	-2.25822338695207	0.0246405344793575	0.016498155652898	0.0852669249271446
**CESC**	CBX5	cg15708001	-0.112198045965933	309	-1.96867022550835	0.0498991931244238	0.0125884015185737	0.14406597995626
**CESC**	CBX5	cg20545544	-0.0379965466741773	309	-0.662971179681569	0.507851376161239	0.00144373755916293	0.690514549630122
**CESC**	CBX5	cg20927027	-0.0741129941374568	309	-1.29576776260244	0.196038474517845	0.00549273590001871	0.377966495941648
**CESC**	CBX5	cg21826008	-0.0148378864656079	309	-0.258735874142031	0.79601413560929	0.000220162874766271	0.88829222432715
**CESC**	CBX5	cg24505978	-0.059199984057226	309	-1.03400048090575	0.301957785558167	0.00350463811237581	0.50035153722655
**CESC**	CBX5	cg25817259	-0.0658682923780977	309	-1.15095241373375	0.250656281449246	0.00433863194080657	0.444303961582474
**CESC**	CBX6	cg03028106	0.0242246675496351	296	0.41336317760748	0.679644992981943	0.000586834517890343	0.815166956860307
**CESC**	CBX6	cg04134624	-0.122284972188222	309	-2.14823377326527	0.0324850868298423	0.0149536144230743	0.104730412135961
**CESC**	CBX6	cg04215348	-0.154574966290197	309	-2.72789300465628	0.00674471211583818	0.0238934202036155	0.0314491809122222
**CESC**	CBX6	cg04801716	-0.00515673325038993	309	-0.0899119119411178	0.928416423726088	2.65918978156771e-05	0.962169150301036
**CESC**	CBX6	cg06060673	-0.095047211810709	309	-1.6647414389043	0.0969946163864949	0.00903397247298979	0.232953573127442
**CESC**	CBX6	cg09913544	0.0714447090653593	309	1.24887248411808	0.212672548555958	0.00510434645343384	0.39894500355338
**CESC**	CBX6	cg14498989	-0.197659898755167	309	-3.5156801488065	0.000505434871198946	0.0390694355759027	0.00378293249663756
**CESC**	CBX6	cg24649346	-0.17981890101002	309	-3.18720191579613	0.00158606938912194	0.0323348371604513	0.00984604138748366
**CESC**	CBX6	cg25836955	0.0728290989248276	309	1.27319979405388	0.203919883812613	0.00530407765020233	0.387857104748501
**CESC**	CBX7	cg00545295	-0.0107003668765211	309	-0.186577953187397	0.852116001151846	0.000114497851292149	0.921173844440215
**CESC**	CBX7	cg01525244	0.0172283624964226	309	0.300431354135015	0.764053498168398	0.000296816474308142	0.869038801048487
**CESC**	CBX7	cg02523424	-0.0921194159191949	308	-1.61036032315764	0.108360455095727	0.00848598678929361	0.252288888698747
**CESC**	CBX7	cg03804568	-0.111632911844002	309	-1.95862871087124	0.0510703464707191	0.0124619070067708	0.146533638230484
**CESC**	CBX7	cg10339854	-0.103435856414085	308	-1.81020683196416	0.0712542778224749	0.0106989763921153	0.1867702850137
**CESC**	CBX7	cg22268271	-0.172823155566169	309	-3.05930849225027	0.00241609198869009	0.0298678430998481	0.0138438843851305
**CESC**	CBX7	cg26672614	-0.0975755549504118	309	-1.7094452007155	0.0883889894741964	0.00952098892388083	0.21813066799339
**CESC**	CBX7	cg27062573	-0.0969422093050889	309	-1.69824388315043	0.0904849655447335	0.00939779194495167	0.221615949676888
**CESC**	CDYL2	cg01442398	-0.131578772352245	308	-2.31046573951911	0.0215338698751293	0.0173129733337239	0.0770583353976898
**CESC**	CDYL2	cg02461391	-0.118252224191317	308	-2.07294872861423	0.0390221314595532	0.0139835885261935	0.120291951765909
**CESC**	CDYL2	cg06919916	-0.234695162172173	309	-4.20962903968204	3.37227629069231e-05	0.0550818191470227	0.000377471743536859
**CESC**	CDYL2	cg10112854	0.159194826657926	309	2.81151132667129	0.00525135474119323	0.0253429928346472	0.0257832321972369
**CESC**	CECR2	cg03927893	-0.284255429309594	309	-5.1694081588188	4.26387565081666e-07	0.0808011490919814	7.99671992236334e-06
**CESC**	CECR2	cg12632097	-0.22353186019154	309	-3.99858895964661	8.0057194388209e-05	0.0499664925206902	0.000795499984702559
**CESC**	CECR2	cg13466481	-0.228058948838334	309	-4.08396709726951	5.66712770795266e-05	0.052010884145246	0.000591531642174251
**CESC**	CECR2	cg26796679	-0.257903667222437	308	-4.6464899000998	5.04219734255198e-06	0.0665143015667815	7.17491677458367e-05
**CESC**	CHAF1A	cg03006338	0.0871391291008759	309	1.52512397171176	0.128267821826683	0.00759322782045912	0.283414761735542
**CESC**	CHAF1A	cg04346879	0.00136380937623381	309	0.0237788511110824	0.981044610224831	1.85997601470326e-06	0.990222054517542
**CESC**	CHAF1A	cg05297461	0.0460604144495947	309	0.803944028797531	0.422057500741547	0.00212156177926844	0.617171827306163
**CESC**	CHAF1A	cg07783855	0.189315677417203	309	3.36162227245358	0.000873883850702014	0.0358404257159343	0.00599919875410452

**CESC**	CHAF1A	cg09700233	-0.126427344521119	309	-2.22216703112803	0.0270078220240584	0.0159838734426616	0.0912704824734837
**CESC**	CHAF1A	cg18804734	0.0296516990513202	309	0.517222465687056	0.605376987438323	0.000879223256630062	0.764028185606374
**CESC**	CHAF1A	cg23811775	0.0584256673593271	309	1.02042945563466	0.308336199777091	0.00341355860638274	0.506893479120455
**CESC**	CHAF1A	cg24085258	-0.215664443552172	309	-3.85085824579251	0.000143542513939862	0.0465111522126681	0.00131111263701276
**CESC**	CHAF1B	cg00660452	0.0595805211984691	308	1.03895759288091	0.299652815101799	0.00354983850628122	0.497921504966772
**CESC**	CHAF1B	cg02544117	0.125033644273695	296	2.14978462186735	0.0323963848231338	0.0156334122003609	0.104522410456655
**CESC**	CHAF1B	cg02908490	-0.0496395237715911	309	-0.866562970651625	0.386864936597137	0.00246408232027036	0.584835473170592
**CESC**	CHAF1B	cg04634639	-0.0255255833475681	309	-0.445198812626703	0.65649292478289	0.000651555405233644	0.799812237143485
**CESC**	CHAF1B	cg07872789	0.0433508066472515	309	0.756558372709488	0.4499003942705	0.00187929243696739	0.640958726707798
**CESC**	CHAF1B	cg13854874	0.0712299534056854	309	1.24509932540782	0.21405414942462	0.00507370626217612	0.400657535737906
**CESC**	CHAF1B	cg25942896	0.00993338188374603	309	0.173202976554791	0.862607150081962	9.86720756483338e-05	0.926924976980443
**CESC**	CHD1	cg03255775	-0.00226098126416835	309	-0.0394216561375916	0.968580090323232	5.11203627692029e-06	0.983470202724742
**CESC**	CHD1	cg03833628	0.0646850735854481	309	1.1301897207524	0.259287201389711	0.00418415874475483	0.454107218643175
**CESC**	CHD1	cg08865521	-0.132161758272449	309	-2.32471098157198	0.020745863943117	0.0174667303496652	0.0749005228700363
**CESC**	CHD1	cg11365324	0.0999951298274823	309	1.75225710434027	0.0807378050606784	0.00999902598921504	0.204330550990286
**CESC**	CHD1	cg12662497	-0.0752886200276165	309	-1.31643828124084	0.189018629039802	0.00566837630566282	0.368774520427405
**CESC**	CHD1	cg16890600	-0.0201455655142034	309	-0.351321234956505	0.725590729725571	0.000405843809887061	0.844562485903722
**CESC**	CHD1	cg21082921	-0.155954153402365	309	-2.75283647364367	0.00626338736668609	0.0243216979634483	0.0296708048230409
**CESC**	CHD4	cg00079216	-0.00351304775897807	309	-0.0612524586357846	0.951198401805525	1.23415045568608e-05	0.974151583971499
**CESC**	CHD4	cg00824109	-0.215836561812905	309	-3.8540816567845	0.000141752098979757	0.0465854214152159	0.00129795936770613
**CESC**	CHD4	cg04344119	-0.0211352754603734	309	-0.368588452905032	0.71269105318442	0.000446699868785861	0.836505756872285
**CESC**	CHD4	cg05147525	-0.0991428926819736	309	-1.73717409827155	0.0833692836675698	0.00982931316934933	0.209004867830071
**CESC**	CHD4	cg11565429	0.045283468659162	309	0.790355018693202	0.429936561991023	0.00205059253380531	0.623978301248641
**CESC**	CHD4	cg19509514	-0.188071892011619	309	-3.33872413494693	0.000946382101028044	0.03537103656483	0.00640979010685841
**CESC**	CHD4	cg20308811	-0.13891695285464	309	-2.44581436855243	0.0150193776663689	0.0192979197904183	0.0584325598208394
**CESC**	CHRAC1	cg00296189	-0.188205096525791	309	-3.34117563671579	0.000938360867434853	0.0354211583582822	0.0063676592220191
**CESC**	CHRAC1	cg07270064	-0.370519673145828	309	-6.95527564528474	2.16112923578661e-11	0.137284828188091	9.61251502394419e-10
**CESC**	CHRAC1	cg08877624	-0.0247442058742182	309	-0.431562109106286	0.666365597684922	0.000612275724345696	0.806409764580623
**CESC**	CHRAC1	cg12002314	-0.0086505749789348	309	-0.150833572275065	0.880207037355662	7.48324474661727e-05	0.936442099333596
**CESC**	CHRAC1	cg18770186	0.129997689687588	309	2.285985362368	0.0229429480998491	0.0168993993241104	0.0807659474361653
**CESC**	CHRAC1	cg19486048	0.164060366855647	309	2.89978140471982	0.00400634045250772	0.0269158039728096	0.0207668742778775
**CESC**	CHRAC1	cg22610305	-0.0193527304864207	309	-0.337489591486946	0.735980764851929	0.000374528177280039	0.851095368368578
**CESC**	CHRAC1	cg23548819	-0.109802252816848	309	-1.92611403566004	0.0550224096396344	0.0120565347236551	0.154681739242052
**CESC**	CLOCK	cg05960024	-0.116509625579125	309	-2.04534442568265	0.0416815114380615	0.0135744928525878	0.126150462994575
**CESC**	CLOCK	cg06085476	-0.129626757766645	308	-2.27559893438257	0.0235680217014157	0.0168030963290924	0.0824615759755299
**CESC**	CLOCK	cg07236904	-0.0605860728359332	309	-1.05829839446676	0.290759611086885	0.003670672221681	0.488338547569963
**CESC**	CLOCK	cg07500410	-0.13996699804015	309	-2.46466979189212	0.0142667392034738	0.0195907605403713	0.0561559759965109
**CESC**	CLOCK	cg11774413	-0.150055739617109	309	-2.64627355011581	0.00856206293672074	0.0225167249920376	0.0378538942997984
**CESC**	CLOCK	cg18928811	0.00996443013712187	309	0.173744401734212	0.862181977209875	9.92898679575825e-05	0.926749841069233
**CESC**	CLOCK	cg22523077	-0.139939340977307	309	-2.4641730518347	0.0142861302572061	0.019583019153163	0.0562237677558098
**CESC**	CLOCK	cg23506964	-0.111578493562828	309	-1.95766188821287	0.0511843190374527	0.0124497602257501	0.146762993926103
**CESC**	CREBBP	cg00039463	-0.0996606143167477	309	-1.74633634193924	0.0817625834875022	0.00993223804599153	0.206180609177916
**CESC**	CREBBP	cg00747661	-0.0481539390503081	309	-0.840567736830734	0.401250771553014	0.00231880184606079	0.597837751559863
**CESC**	CREBBP	cg03039399	-0.304789913397799	309	-5.57967694530096	5.33402664270195e-08	0.0928968913090376	1.23572146707074e-06
**CESC**	CREBBP	cg03264209	-0.124422768916432	309	-2.18637477654481	0.029550445320538	0.0154810254248318	0.0976035256636913
**CESC**	CREBBP	cg08506924	-0.0977149513057006	309	-1.71191083832129	0.0879329449248893	0.00954821170867544	0.217267127758832
**CESC**	CREBBP	cg24567591	-0.0511402549625712	309	-0.892829097107453	0.372654907481405	0.00261532567763679	0.571271520929482
**CESC**	CTCF	cg01866162	-0.0603969064925725	309	-1.05498197998043	0.29227128255512	0.00364778631387254	0.489995978556737
**CESC**	CTCF	cg04487155	-0.130643427215426	309	-2.29753721387674	0.0222672217723006	0.0170677050745923	0.0789074955839453
**CESC**	CTCF	cg06241380	-0.0248072125351648	309	-0.432661680056759	0.665567346847966	0.000615397793764836	0.805836605429406
**CESC**	CTCF	cg07967402	-0.109923384253132	309	-1.92826485678182	0.0547532935265993	0.0120831504056617	0.154155756816918
**CESC**	CTCF	cg08324636	0.109294736372747	309	1.91710346941798	0.0561619095584622	0.0119453393987883	0.15692897497184
**CESC**	CTCF	cg10218542	-0.0906425729189939	309	-1.58693991672073	0.113565802140173	0.00821607602537513	0.26074163900734
**CESC**	CTCF	cg10481400	-0.0396885126169654	309	-0.692538513553127	0.4891278756244	0.00157517803374702	0.675097827626226
**CESC**	CTCF	cg23858565	-0.131627890506133	309	-2.31515439321404	0.0212701901855275	0.0173259015590944	0.0763234859452457
**CESC**	CXXC1	cg02159718	-0.0231102243533309	309	-0.403048174931946	0.687196091646872	0.000534082469661289	0.820022590670421
**CESC**	CXXC1	cg07675399	-0.040332621897978	309	-0.703795964537294	0.482098561191984	0.00162672038916525	0.669213718448953

**CESC**	CXXC1	cg11871295	0.200078883619885	309	3.56048835371173	0.000429439778393253	0.0400315596705795	0.00329693458209067
**CESC**	CXXC1	cg13423076	-0.235705736764086	309	-4.22881915767226	3.11182244399623e-05	0.0555571943435005	0.000351862086058492
**CESC**	CXXC1	cg15632775	0.117904172188991	309	2.07016896120594	0.0392806867885322	0.0139013938195711	0.120892580133207
**CESC**	CXXC1	cg20016845	-0.221942941576074	308	-3.96215503495015	9.26738370416173e-05	0.0492586693154404	0.000902424393392862
**CESC**	CXXC1	cg27419474	-0.034364523196796	309	-0.599520032737026	0.549272708408566	0.00118092045454313	0.722268404488363
**CESC**	DAXX	cg00221327	-0.0548299430610935	298	-0.939951408480514	0.348016732050348	0.00300632265608276	0.547244278449499
**CESC**	DAXX	cg02173004	-0.154421374086139	309	-2.72511620680404	0.00680031119845942	0.0238459607746513	0.0316647758049292
**CESC**	DAXX	cg02328295	0.0807856379323681	309	1.41316465772353	0.15863037778507	0.00652631929613967	0.327820943826685
**CESC**	DAXX	cg03085224	0.00844186366252272	309	0.147194167394526	0.883076334870869	7.12650620966215e-05	0.937893581467281
**CESC**	DAXX	cg03369465	-0.0852198843827284	308	-1.48882970641276	0.137572270704069	0.00726242869420559	0.297633272905956
**CESC**	DAXX	cg03382121	0.0602542530935215	309	1.05248109365345	0.293414726766434	0.00363057501585815	0.491143738656654
**CESC**	DAXX	cg03902329	-0.0828684720707944	309	-1.4498479435062	0.148131796497225	0.00686718366334804	0.313097181157218
**CESC**	DAXX	cg04086445	-0.161617241873857	308	-2.85073147788671	0.0046612176638791	0.0261201328709129	0.0234444150089747
**CESC**	DAXX	cg04152866	-0.0297953806271675	309	-0.519730963180097	0.603629326206748	0.000887764706717786	0.762640150488998
**CESC**	DAXX	cg04271391	0.00953160314039887	309	0.166196729214833	0.868112612770323	9.08514584260615e-05	0.929965141011083
**CESC**	DAXX	cg04488476	0.050702946356161	309	0.885174607896811	0.376762062257852	0.00257078876919574	0.575266494039006
**CESC**	DAXX	cg04973788	-0.0488236539679856	309	-0.85228591882273	0.394726354347667	0.0023837491867856	0.59142894414553
**CESC**	DAXX	cg05431670	-0.0830609601246644	307	-1.44845075917124	0.148528462684506	0.00689912309683108	0.313600847498398
**CESC**	DAXX	cg05532204	-0.0547972064411651	309	-0.956859618488845	0.339398429141922	0.00300273383375567	0.538642159954847
**CESC**	DAXX	cg06171013	-0.130741752314426	309	-2.29929645064516	0.0221658559855883	0.0170934057982468	0.0786701309613968
**CESC**	DAXX	cg07689821	-0.109990431362555	309	-1.92945538891753	0.054604806052253	0.0120978949913209	0.15388561447817
**CESC**	DAXX	cg08375286	0.0501646475362064	309	0.875753123511339	0.381855687383225	0.00251649186243181	0.580298906922909
**CESC**	DAXX	cg08567382	-0.0895009871233391	309	-1.56679098263739	0.118203853592591	0.00801042669605211	0.267950799846641
**CESC**	DAXX	cg09097215	-0.0885397993606022	309	-1.54983089562005	0.122222500331637	0.00783929607081569	0.274113563894236
**CESC**	DAXX	cg09432361	0.0780843508470902	309	1.36561673887211	0.173068840764804	0.00609716584721148	0.347961560706275
**CESC**	DAXX	cg09615909	-0.168424819503695	308	-2.97424170254642	0.00317321081172525	0.0283669198248523	0.0172422128858947
**CESC**	DAXX	cg11111423	-0.237224879212622	308	-4.25068543060478	2.84105181208137e-05	0.0562756433174433	0.00032502468333685
**CESC**	DAXX	cg12025215	0.0260511021389178	309	0.454370694123757	0.649886294196042	0.000678659922652327	0.795259629164021
**CESC**	DAXX	cg12832355	-0.35402281054333	309	-6.60003829908616	1.8275843444225e-10	0.125332150384999	6.93198910238926e-09
**CESC**	DAXX	cg12923297	-0.1355296270515	309	-2.38504590999549	0.0176896068555034	0.0183682798087188	0.0662595828261849
**CESC**	DAXX	cg13443893	0.0373737653264931	309	0.652089442658129	0.514836196719119	0.00139679833467978	0.696359139734125
**CESC**	DAXX	cg13826470	-0.0728248813382932	309	-1.27312566898905	0.203946146848923	0.00530346334193649	0.387897573418541
**CESC**	DAXX	cg14539675	0.066614510536039	309	1.16404928026136	0.245316788116765	0.00443749301395605	0.438231065343066
**CESC**	DAXX	cg14622330	-0.0605606601589209	309	-1.05785285946284	0.29096238473437	0.00366759355888431	0.488522394047618
**CESC**	DAXX	cg14746369	0.140303462433847	309	2.47071339768664	0.0140326794953857	0.0196850615709259	0.0554535339156777
**CESC**	DAXX	cg14910283	-0.359983931478884	309	-6.72756000566761	8.56936242887378e-11	0.129588430922994	3.46849625802189e-09
**CESC**	DAXX	cg16922466	0.0060228700392749	309	0.105014232116105	0.916433769182551	3.62749635099953e-05	0.955906900953569
**CESC**	DAXX	cg17079757	0.084322747150207	309	1.47547223562605	0.1411205815069	0.00711032568695773	0.303075288603365
**CESC**	DAXX	cg17504991	0.1073952072562	309	1.88339219047127	0.060601688745359	0.0115337305416021	0.165845440641471
**CESC**	DAXX	cg18187593	-0.0720239730991528	309	-1.25905076023917	0.208977915157312	0.00518745270098749	0.394277106486259
**CESC**	DAXX	cg18279148	-0.20262767769436	278	-3.41888045028791	0.000724794941119298	0.0410579757678094	0.00513334829292013
**CESC**	DAXX	cg18486541	0.0462129689168688	309	0.806612422185356	0.4205203875155	0.00213563849611148	0.61571685320456
**CESC**	DAXX	cg18944910	-0.198524141880148	309	-3.53168128088192	0.000476955869230829	0.039411834909249	0.00360026245788309
**CESC**	DAXX	cg18967278	0.0684755646541693	309	1.19672121713718	0.232347668252202	0.00468890295470732	0.422961378355255
**CESC**	DAXX	cg20223162	-0.217569974749332	305	-3.86091170455163	0.00013838583242553	0.0473366939124249	0.00127162620454898
**CESC**	DAXX	cg20763184	0.0550444179717489	309	0.961189474247857	0.33722079239845	0.00302988794984859	0.536795938774427
**CESC**	DAXX	cg21286626	0.128560543381401	309	2.26028628617438	0.0245107336100924	0.0165278133145212	0.0849386614309557
**CESC**	DAXX	cg24328964	-0.0586688342682004	309	-1.02469110882533	0.306323636944895	0.00344203211438957	0.504761711052526
**CESC**	DAXX	cg24496586	-0.0195986834137764	309	-0.341780368366635	0.732752325975524	0.000384108391553435	0.849129113992149
**CESC**	DAXX	cg24513883	-0.415248729891654	308	-7.94561747842062	3.83057989624317e-14	0.172431507676632	2.67982133496709e-12
**CESC**	DAXX	cg24728112	-0.34372619880949	309	-6.38192213456502	6.51756388203146e-10	0.118147699748021	2.22846587574747e-08
**CESC**	DAXX	cg25642825	-0.0758132833543106	309	-1.32566498470314	0.18594599554074	0.00574765393296099	0.364777053136258
**CESC**	DAXX	cg26521092	-0.0946750610354863	308	-1.65543469868868	0.0988716577888827	0.00896336718207306	0.236279966394137
**CESC**	DAXX	cg27052777	-0.395024970201701	309	-7.49724365485394	7.18968417429383e-13	0.156044727082855	4.1492158060461e-11
**CESC**	DAXX	cg27393652	0.0109863879790107	309	0.191565781232968	0.848210234834203	0.00012070072082535	0.918983416757208
**CESC**	DIDO1	cg00080059	-0.145291030747977	309	-2.5604042305748	0.0109381119329343	0.0211094836158095	0.0457336063479893
**CESC**	DIDO1	cg01488865	-0.163420135265382	309	-2.88815412708058	0.00415328659977048	0.0267061406101556	0.0213931522043663

**CESC**	DIDO1	cg06646488	-0.0962123497044125	309	-1.68533820679754	0.0929494859817799	0.00925681623564416	0.226033094799798
**CESC**	DIDO1	cg08610063	-0.183461217435345	309	-3.2539856938362	0.00126621362159683	0.0336580183028589	0.00816781280916286
**CESC**	DIDO1	cg13815390	0.00110886847537261	309	0.0193337943896259	0.98458750803536	1.22958929567518e-06	0.99189844047809
**CESC**	DIDO1	cg20969846	-0.186959852202729	309	-3.31826554275711	0.00101585416839101	0.0349539863356665	0.00680233525262859
**CESC**	DIDO1	cg21212389	0.00205335524687146	309	0.0358015475397418	0.971464095795294	4.21626776985454e-06	0.984886394138455
**CESC**	DIDO1	cg21687703	0.0455422154154047	309	0.794880414624946	0.427303199555854	0.00207409338494313	0.621684793531928
**CESC**	DIDO1	cg22394521	-0.321402447402291	309	-5.91782711441063	8.78916271442975e-09	0.103299533196182	2.43325311105462e-07
**CESC**	DMAP1	cg01604592	-0.0272819264964396	309	-0.475853765111359	0.634520336326195	0.000744303513357131	0.784740980588493
**CESC**	DMAP1	cg11683482	-0.0179028582641598	309	-0.312197035405837	0.755104812071946	0.000320512334026596	0.863290818580719
**CESC**	DMAP1	cg15990015	-0.0356089157114434	309	-0.621256660369774	0.534896316698031	0.00126799487814468	0.711506122378883
**CESC**	DMAP1	cg18017761	-0.0242079039014112	309	-0.422202955333536	0.673175321016453	0.000586022611299961	0.810916706769184
**CESC**	DMAP1	cg18167340	-0.00039665645685536	309	-0.0069159421870062	0.994486456659475	1.57336344765049e-07	0.997126867677569
**CESC**	DMAP1	cg21437596	-0.0414882200690043	309	-0.723995199352182	0.469625451476214	0.00172127240449413	0.658419590290554
**CESC**	DMAP1	cg23021241	0.035120623737007	309	0.612727001989723	0.54051494528838	0.00123345821167642	0.715785507766861
**CESC**	DMAP1	cg23451311	-0.040926873453125	309	-0.714182803809343	0.4756621434922	0.00167500897064811	0.663445285357816
**CESC**	DMAP1	cg24485408	-0.0478361251975906	308	-0.833632762453249	0.405144632233903	0.00228829487391956	0.601588469775424
**CESC**	DNMT1	cg04160663	-0.143257380309601	309	-2.52380969388737	0.0121184177507809	0.0205226770131696	0.0495422875875898
**CESC**	DNMT1	cg04762346	-0.0614059860322232	309	-1.07267422433561	0.284268077483652	0.00377069512058959	0.481542348657852
**CESC**	DNMT1	cg06128182	-0.0248482854844244	309	-0.433378474061454	0.665047183878669	0.000617437291515456	0.805553610317745
**CESC**	DNMT1	cg06716436	0.0774361326595325	309	1.35421137807293	0.176674699837397	0.00599635464126472	0.352714645704068
**CESC**	DNMT1	cg10635912	-0.0550444909710624	309	-0.961190752840973	0.337220150684933	0.00302989598626337	0.536795938774427
**CESC**	DNMT1	cg11380607	-0.0856247884455041	309	-1.49842222412234	0.135061236387294	0.00733160439633732	0.2937947732052
**CESC**	DNMT1	cg13393778	-0.124656703388998	309	-2.19055033100137	0.029243533919708	0.0155392936998127	0.0968897562925795
**CESC**	DNMT1	cg15043801	-0.00561722923750498	309	-0.0979412835517372	0.92204346187562	3.15532643066807e-05	0.959081862757632
**CESC**	DNMT1	cg21893394	-0.129060756055523	309	-2.26922944645615	0.0239548778814508	0.0166566787536233	0.0834734947327921
**CESC**	DNMT1	cg23401624	-0.0153791754174789	309	-0.268176802254036	0.788745109648862	0.000236519036521588	0.883804515337629
**CESC**	DNMT1	cg24477899	-0.106070272280448	309	-1.85989071131213	0.0638667280955621	0.0112509026616483	0.172300188842633
**CESC**	DNMT1	cg27429799	-0.012876505860778	309	-0.224528165823057	0.822497188918177	0.000165804403182649	0.904585595761027
**CESC**	DNMT3A	cg02208653	0.0574506169076259	309	1.003342905356	0.316493395527686	0.00330057338306679	0.515747862579459
**CESC**	DNMT3A	cg03463641	-0.0451080020113098	309	-0.787286255305725	0.431727675340402	0.00203473184545233	0.625562457401539
**CESC**	DNMT3A	cg06112956	0.00702083977092386	309	0.122415541343302	0.902650815802971	4.92921910889862e-05	0.948530579521868
**CESC**	DNMT3A	cg07150430	0.196513490281524	309	3.49446798947279	0.000545648684320985	0.0386175518626268	0.004038454357156
**CESC**	DNMT3A	cg12066181	0.0395855958062739	309	0.690739860521412	0.490256098500788	0.00156701939533769	0.676153446419745
**CESC**	DNMT3A	cg13076778	-0.131988137751265	309	-2.32160283319368	0.0209151376441467	0.0174208685070468	0.0753727807425187
**CESC**	DNMT3A	cg13344237	-0.0066809950646531	309	-0.116489729145492	0.90734134224734	4.4635695053919e-05	0.95094389344564
**CESC**	DNMT3A	cg15990840	-0.0498686452918885	309	-0.870572720688604	0.38467441876462	0.0024868817832482	0.582792651226593
**CESC**	DNMT3A	cg15998962	-0.134363066296472	308	-2.36024612137432	0.0188963442136218	0.0180534335845902	0.0696957235802434
**CESC**	DNMT3A	cg21629895	-0.198533815856195	309	-3.53186043931464	0.000476645687954759	0.0394156760384214	0.00359917409115333
**CESC**	DNMT3A	cg21708767	0.0888589886132239	309	1.55546248001807	0.120876378350985	0.00789591985736506	0.2720041936142
**CESC**	DNMT3A	cg22731525	0.099760101959045	309	1.74809716240589	0.0814567148883976	0.00995207794287905	0.205614837536195
**CESC**	DNMT3A	cg25044635	-0.0596909919243908	308	-1.04089084927369	0.298756040298711	0.00356301451691769	0.496962118566466
**CESC**	DNMT3A	cg26470599	-0.20915773374681	309	-3.72927409486252	0.000229037335922459	0.0437469575861015	0.00194431803914794
**CESC**	DNMT3A	cg26995204	-0.152592819429799	309	-2.69207320014563	0.00749441885734574	0.0232845685415353	0.0341517452931172
**CESC**	DNMT3B	cg00300969	0.0750486564396043	309	1.31221866031571	0.190436298172158	0.00563230083338975	0.370675298627769
**CESC**	DNMT3B	cg13636640	-0.0447683623893998	305	-0.776188993845126	0.438248899764125	0.00200420627102862	0.630973774496373
**CESC**	DNMT3B	cg14224313	0.169602846844898	309	3.00059786152251	0.00291761233359131	0.0287651256578938	0.0161007827111728
**CESC**	DNMT3B	cg16523653	-0.0162390668466649	309	-0.283175145425323	0.777235313775142	0.00026370729205045	0.876814396957952
**CESC**	DNMT3B	cg16953106	0.0114433679550969	309	0.199535003008991	0.841977663972626	0.000130950670155738	0.915480661808666
**CESC**	DNMT3B	cg17475857	0.0524801515161587	309	0.91628537916127	0.360243520906317	0.00275416630315897	0.559153720378811
**CESC**	DNMT3B	cg17721173	0.118241273435289	309	2.07617160974212	0.0387182042509306	0.0139809987435988	0.1195522765588
**CESC**	DNMT3B	cg22605822	0.114337189082708	309	2.0066969294364	0.0456671809261806	0.013072992807335	0.135040013416881
**CESC**	DNMT3B	cg24403338	0.0525492278522197	309	0.917494765640005	0.359610748772589	0.0027614213478645	0.558475598395442
**CESC**	DNMT3B	cg25803642	-0.0623979066434986	309	-1.09006883784116	0.276546111960647	0.00389349875349077	0.473159765854372
**CESC**	DNMT3L	cg09247319	-0.0266926988703688	309	-0.465568996174525	0.641857397425163	0.000712500172984187	0.789630463145797
**CESC**	DNMT3L	cg15635600	0.0210497744787662	309	0.367096696949526	0.713802295502291	0.000443093005606916	0.837124716669098
**CESC**	DNMT3L	cg27187798	0.0375878275283068	309	0.655829623297747	0.512429787489134	0.00141284477829774	0.694300593700879
**CESC**	DPF1	cg06168449	-0.191758362085942	309	-3.40664115946576	0.000746192972646076	0.036771269429883	0.00525856835891193

**CESC**	DPF1	cg06615189	-0.0873551368424797	309	-1.52893361133794	0.127320729395015	0.00763091993276835	0.282114815126152
**CESC**	DPF1	cg07480327	-0.0890738626387006	273	-1.46402142799993	0.144360047041304	0.0079341530053781	0.30765122564941
**CESC**	DPF1	cg08057062	-0.135302296077347	309	-2.38097068027602	0.0178827917470169	0.0183067113238021	0.0667951214249176
**CESC**	DPF1	cg13322582	-0.121145545192329	309	-2.12791738867187	0.0341482859913353	0.0146762431199466	0.10874055507204
**CESC**	DPF1	cg13917712	-0.0467129027118388	309	-0.815357368678942	0.415506118805071	0.00218209527976572	0.611115902431492
**CESC**	DPF1	cg17121541	0.0247582572336524	309	0.431807328235436	0.666187544056747	0.0006129713012477	0.806307177010511
**CESC**	DPF1	cg22145401	-0.113555411841969	309	-1.99279633403939	0.0471775455448912	0.0128948315585991	0.138306155158145
**CESC**	DPF1	cg27400772	-0.096524721439649	193	-1.33322088573983	0.184064065140638	0.00931702184900183	0.362425773457967
**CESC**	DPF2	cg02186298	0.0297786910230551	309	0.519439582275975	0.603832213228461	0.000886770439046581	0.762759165314522
**CESC**	DPF2	cg14699126	-0.1010501401303	309	-1.77093421660734	0.0775736260839136	0.0102111308203533	0.198570717237734
**CESC**	DPF2	cg15918224	-0.0966895102748608	309	-1.69377524350752	0.091332266361278	0.00934886139719241	0.223178236251704
**CESC**	DPF2	cg15929437	-0.0325931610365799	309	-0.568583268109758	0.570058757818828	0.00106231414635643	0.738159575980524
**CESC**	DPF2	cg18224556	-0.161186492820906	309	-2.84761808415977	0.00470483025115196	0.0259810854679041	0.0236286355428093
**CESC**	DPF2	cg19517014	-0.128570564787282	309	-2.26046543879851	0.0244994891949922	0.0165303901297208	0.0849110426104972
**CESC**	DPF3	cg00888802	-0.0529782781955509	309	-0.925006862996064	0.355696018582987	0.00280669796056519	0.554496043717711
**CESC**	DPF3	cg05151458	-0.0947987073634335	309	-1.66034938871135	0.0978752396861317	0.00898679491777791	0.234439738893354
**CESC**	DPF3	cg07171024	-0.0878064058726049	309	-1.53689315937947	0.125359605080085	0.00770996491226462	0.278962312879866
**CESC**	DPF3	cg10266394	-0.0555218908266937	309	-0.969552809249638	0.333040191549114	0.00308268036097129	0.532680798169576
**CESC**	DPF3	cg12913429	-0.0590832684891812	304	-1.02343309673731	0.306930415595726	0.00349083261536468	0.505415506018965
**CESC**	DPF3	cg13819165	-0.0705944091492567	309	-1.23393412106827	0.218180546017588	0.00498357060313266	0.405803504922589
**CESC**	DPF3	cg14238615	-0.092093718218923	309	-1.6125616770251	0.107877373840156	0.00848125293538639	0.251489733908334
**CESC**	DPF3	cg14242696	-0.195624031063353	309	-3.47802047386283	0.000578870861008863	0.0382687615294758	0.00424443933857389
**CESC**	DPF3	cg15392845	-0.0777407740944717	309	-1.35957131006712	0.174973184993096	0.00604362795680768	0.350478390054429
**CESC**	DPF3	cg15740492	-0.0641061453532017	309	-1.12003264828341	0.263583941248202	0.00410959787204582	0.459007658439384
**CESC**	DPF3	cg16696021	-0.0231728269353569	309	-0.404140565641951	0.68639352099469	0.000536979908176004	0.819504858532504
**CESC**	DPF3	cg19710444	-0.0803025473784465	309	-1.40465905195649	0.161143612220548	0.00644849911546764	0.331289803622762
**CESC**	DPF3	cg19839347	-0.0307324670968913	309	-0.536092098598013	0.592286932593742	0.000944484533861506	0.754657521524462
**CESC**	DPF3	cg20548765	-0.0833756424258178	309	-1.45878320524909	0.145657209042227	0.00695149774991782	0.309571808874437
**CESC**	DPF3	cg21035356	-0.0775719247192245	309	-1.3566004878047	0.175914750454854	0.00601740350464503	0.35172357562875
**CESC**	DPF3	cg22784589	-0.101713707780382	309	-1.78268460928753	0.0756354761368806	0.0103456783504329	0.195056189552342
**CESC**	DPF3	cg23515460	-0.0366231629303703	309	-0.638975323371982	0.523320122663995	0.00134125606302445	0.702735882977571
**CESC**	DPF3	cg24383902	-0.0789818834808877	309	-1.38141165269599	0.168166884282861	0.00623813791818851	0.341264499697428
**CESC**	DPF3	cg25495195	-0.0959198447438086	309	-1.68016678570399	0.0939521148255734	0.00920061661567634	0.227748685676825
**CESC**	DPF3	cg26057784	0.0476741247217527	309	0.83217299611024	0.405964530710636	0.00227282216798523	0.602300080139281
**CESC**	EED	cg00080012	-0.0291479516874977	309	-0.508427930131681	0.611522020465753	0.000849603087576699	0.768455006059296
**CESC**	EED	cg01739327	0.054060459597325	309	0.943956702640045	0.345941373939998	0.00292253329187401	0.545222107866455
**CESC**	EED	cg02172616	-0.345996382577493	309	-6.42978318283991	4.94370342013886e-10	0.119713496756711	1.72222716757492e-08
**CESC**	EED	cg04607174	-0.480311636030482	309	-9.5479871724714	4.57747587525106e-19	0.230699267706278	5.52950556272175e-17
**CESC**	EED	cg06358433	-0.334516305737131	309	-6.18904203092773	1.95537408155774e-09	0.111901158804018	6.11892080855602e-08
**CESC**	EED	cg10086030	-0.416729022191171	309	-7.99303951030296	2.76483899052633e-14	0.173663077936409	1.96427291715987e-12
**CESC**	EED	cg10535675	-0.297355521709635	302	-5.367312860058	1.61270870303606e-07	0.088420306291209	3.34379160031949e-06
**CESC**	EED	cg12672785	0.0788613304625247	309	1.37928994694556	0.168819198916741	0.00621910944231952	0.342074663071978
**CESC**	EED	cg16265078	-0.343098586016237	309	-6.36871296326536	7.03233447407864e-10	0.117716639726341	2.38792417318408e-08
**CESC**	EED	cg18574463	0.0535352425276471	309	0.934759330027768	0.350654243049916	0.00286602219249399	0.549661196235371
**CESC**	EED	cg19325359	-0.365249531821685	301	-6.75037641054094	7.77974236293789e-11	0.13340722049596	3.17033670708429e-09
**CESC**	ELP3	cg01209934	-0.048958186782544	309	-0.854640014124204	0.393423467796663	0.00239690405303447	0.59022245210247
**CESC**	ELP3	cg02516513	-0.063776782164146	309	-1.11425460563602	0.266050134080593	0.00406747794321294	0.461865217590503
**CESC**	ELP3	cg04720865	-0.25910516378586	309	-4.67738990057063	4.37739387155015e-06	0.0671354859004973	6.33058533223871e-05
**CESC**	ELP3	cg05621091	-0.0630668148360539	309	-1.10180083745561	0.271419819583577	0.00397742313356511	0.46770109078733
**CESC**	ELP3	cg15692687	-0.031788267206122	309	-0.554527621579536	0.579625530646151	0.00101049393196781	0.745595656713902
**CESC**	ELP3	cg17572663	-0.00640937073472298	309	-0.111753492739549	0.911092614374841	4.10800332151234e-05	0.952780078345478
**CESC**	ELP3	cg18022570	-0.00484762488443221	309	-0.0845222210691399	0.932696869833015	2.34994670201664e-05	0.964292913705755
**CESC**	ELP3	cg18249244	-0.0454354595742197	309	-0.793013270590018	0.428388558834589	0.00206438098672055	0.622579527844309
**CESC**	ELP3	cg19781013	-0.0527267382658866	309	-0.920602673406287	0.357987857942934	0.00278010892815931	0.556963869861457
**CESC**	ELP3	cg22414525	-0.0801326823137659	309	-1.40166853445975	0.162034394811878	0.00642124677479893	0.332602303261144
**CESC**	ELP3	cg22487209	-0.0461062659138058	309	-0.804746029819889	0.421595164946265	0.00212578775651457	0.616716012520068
**CESC**	ELP3	cg25374552	0.0761048719340157	309	1.33079332539148	0.184254303309631	0.00579195153209292	0.36267184352578

**CESC**	ELP4	cg04972224	-0.0549271275525344	309	-0.95913513621546	0.338252863584235	0.00301698934117238	0.537668688209721
**CESC**	ELP4	cg05195750	0.133339664035465	308	2.34194216715889	0.0198311737965546	0.0177794660050908	0.0723365438000455
**CESC**	ELP4	cg05635455	-0.312789657934684	309	-5.7417828029543	2.26965710574119e-08	0.0978373701108965	5.7738948621331e-07
**CESC**	ELP4	cg08111895	0.23418393434938	309	4.19992668119004	3.51175435367458e-05	0.0548421151073545	0.000391226433037033
**CESC**	ELP4	cg08626560	-0.349757756593381	309	-6.50936523838041	3.11203098655587e-10	0.122330488297235	1.13129597461742e-08
**CESC**	ELP4	cg09616795	0.265490351560708	309	4.80128363643029	2.477532398282e-06	0.0704851267718283	3.8135566955273e-05
**CESC**	ELP4	cg10583382	-0.188719618153269	309	-3.35064671212351	0.00090796269462512	0.0356150942759155	0.00619775925733841
**CESC**	ELP4	cg11385473	-0.021347814523057	309	-0.372296707590817	0.709931350108343	0.000455729184910842	0.834808099676957
**CESC**	ELP4	cg13263383	-0.177566607889898	306	-3.13041155242033	0.00191698319601065	0.0315299002375249	0.0114909686200072
**CESC**	ELP4	cg19328333	0.278815019013356	309	5.06204212773696	7.19842673183804e-07	0.077737814827418	1.27532318190984e-05
**CESC**	ELP4	cg24001477	0.302300563955332	308	5.52039903573147	7.27393052274437e-08	0.091385630967712	1.63038405854445e-06
**CESC**	ELP4	cg24531794	0.0832216566449555	309	1.45607018332403	0.146405184714654	0.00692584413473086	0.310618401824305
**CESC**	ELP4	cg25130734	-0.104510117007292	309	-1.83222976810905	0.0678953355829514	0.0109223645568779	0.180347563442008
**CESC**	EP300	cg00500400	-0.0127308418347308	309	-0.221987802086694	0.824472431671305	0.000162074333820931	0.905662360201037
**CESC**	EP300	cg02046995	-0.00848732485790195	309	-0.147986895616948	0.882451217279283	7.20346832435604e-05	0.937603060526493
**CESC**	EP300	cg07519229	-0.152024722504632	309	-2.68181315162105	0.00772258086769599	0.0231115162526105	0.034978359814413
**CESC**	EP300	cg08131204	-0.123934058500195	306	-2.16688121879312	0.0310281319303851	0.0153596508563298	0.101182197157628
**CESC**	EP300	cg09331127	-0.0241927465070064	309	-0.42193844479755	0.673368174104718	0.000585288983552269	0.811073665133348
**CESC**	EP300	cg24349919	0.0514923280330397	309	0.898992028753065	0.369368414092799	0.00265145984626216	0.568066877739158
**CESC**	EPC1	cg02900516	-0.0766788245630222	309	-1.34088877403172	0.180957607287065	0.00587964213636673	0.358176630315496
**CESC**	EPC1	cg16942190	-0.0718580561531065	309	-1.25613529521007	0.210031397399139	0.00516358023410301	0.395621853008926
**CESC**	EPC1	cg26211455	-0.100438973116892	309	-1.7601138422492	0.0793941516813525	0.0100879873207757	0.201908749130407
**CESC**	EPC2	cg00066942	-0.0390444134663928	309	-0.68128210428065	0.496211642015012	0.00152446622293464	0.681049495299463
**CESC**	EPC2	cg03664802	-0.201612102902367	309	-3.58892408470247	0.00038692106760422	0.0406474400367147	0.00302149543597698
**CESC**	EPC2	cg04098458	-0.0137274459519325	308	-0.23897473044154	0.811286732327424	0.000188442772363227	0.898242888215414
**CESC**	EPC2	cg05586854	0.0716770535144719	309	1.2529548664337	0.211185022232639	0.00513760000051647	0.397090511260127
**CESC**	EPC2	cg10458926	-0.08427274686283	309	-1.47459107509653	0.141357348290446	0.00710189586380662	0.303383826327888
**CESC**	EPC2	cg11721798	-0.0499264268410508	309	-0.87158395065787	0.384123191904084	0.00249264809711479	0.582278128451696
**CESC**	EPC2	cg12432635	-0.0912411841266572	309	-1.59750789127712	0.11119142074865	0.00832495368083457	0.256875494022702
**CESC**	EPC2	cg16434205	-0.0287524347736602	309	-0.501523178333203	0.616365961726546	0.000826702505413587	0.77202072547389
**CESC**	EPC2	cg21347644	-0.0937613062305655	309	-1.64201780684365	0.101620325164467	0.00879118254606189	0.241044187221889
**CESC**	EPC2	cg21650422	-0.0929302939984807	309	-1.62733715228502	0.104701493140758	0.00863603954264406	0.246151343529529
**CESC**	EZH1	cg00728999	-0.152614420779419	309	-2.69246338265947	0.00748586317337401	0.0232911614298376	0.0341217108870603
**CESC**	EZH1	cg01716316	-0.0744227789704737	309	-1.30121404383783	0.194170518801242	0.00553875002968798	0.37546222139877
**CESC**	EZH1	cg03623386	-0.101732056095176	309	-1.78300955358113	0.0755824482200012	0.010349411237352	0.194962066939447
**CESC**	EZH1	cg05375741	-0.149321333025778	308	-2.62869178365193	0.00900839615036191	0.0222968604965952	0.0393773765309465
**CESC**	EZH1	cg05979501	-0.167887327252288	309	-2.9693621338593	0.00322177220269927	0.0281861546519167	0.0174585223416542
**CESC**	EZH1	cg06975499	-0.0919340366436969	309	-1.60974179753444	0.108492108016255	0.0084518670936046	0.252506658220391
**CESC**	EZH1	cg12812489	-0.0712612708517548	309	-1.24564954775871	0.213852272735291	0.00507816872340716	0.400433825282079
**CESC**	EZH1	cg19395400	-0.0914901172158301	309	-1.60190310782239	0.110215584414218	0.00837044154816634	0.255390642637089
**CESC**	EZH1	cg22227424	-0.0151890479193189	309	-0.264860654104874	0.791296302370725	0.000230707176695364	0.88544340533514
**CESC**	EZH1	cg27088844	-0.0900003299698154	309	-1.57560358893119	0.116157243770642	0.00810005939467566	0.264739009811424
**CESC**	EZH2	cg01893439	-0.0784017526255421	309	-1.37120203907632	0.171323316649217	0.0061468348147567	0.345451985924756
**CESC**	EZH2	cg02303805	-0.0136148302195689	309	-0.237404680355325	0.812502771679604	0.000185363601907686	0.898992480887438
**CESC**	EZH2	cg06816161	0.0168646741695482	309	0.294087466429339	0.768891762562661	0.000284417234845027	0.87185222756861
**CESC**	EZH2	cg13751213	-0.0351302955694489	309	-0.612895948723307	0.540403370020751	0.00123413766679684	0.715686484183011
**CESC**	EZH2	cg18494018	-0.0968682053074842	309	-1.69693518852736	0.090732447092516	0.00938344919949291	0.222053544960982
**CESC**	EZH2	cg20101066	0.0486162154259637	309	0.848656188441771	0.39674037957646	0.00236353640234371	0.59334793127912
**CESC**	FBXL19	cg00026853	-0.0244975956940273	309	-0.427258400961759	0.669493588358042	0.000600132194788024	0.808421951303462
**CESC**	FBXL19	cg02291855	0.0780023025641609	309	1.36417300815286	0.173522202767405	0.0060843592053109	0.348489350511559
**CESC**	FBXL19	cg03181514	-0.0118791518068125	309	-0.20713470438811	0.836043335951515	0.000141114247649298	0.912215704185421
**CESC**	FBXL19	cg03627896	-0.0245613475756681	309	-0.42837095697845	0.668684413121691	0.000603259794732776	0.807871212349378
**CESC**	FBXL19	cg04477789	-0.297225196203066	309	-5.42758502058998	1.16915547820749e-07	0.0883428172579513	2.50146538150916e-06
**CESC**	FBXL19	cg05643349	-0.12464455446514	309	-2.19033347271568	0.0292594053017753	0.0155362649578133	0.0969180685916022
**CESC**	FBXL19	cg05768582	0.00699799986389292	309	0.122017284606421	0.90296594740312	4.89720020950453e-05	0.948698233430593
**CESC**	FBXL19	cg06862768	0.0363059469791667	309	0.633433424585626	0.52692689771298	0.00131812178605406	0.705564125061723
**CESC**	FBXL19	cg07697662	0.0106827484886592	309	0.186270713419345	0.85235670842043	0.00011412111527195	0.921318833625473

**CESC**	FBXL19	cg08535091	-0.147757901808523	309	-2.60483890604655	0.00964364597508921	0.0218323975468572	0.0414164621279723
**CESC**	FBXL19	cg08593668	0.00997396712567108	309	0.173910709603587	0.862051386245231	9.94800202239674e-05	0.926731466972557
**CESC**	FBXL19	cg09011601	-0.218944572484179	309	-3.91235332672586	0.000112812167763955	0.0479367258202801	0.00106895368806688
**CESC**	FBXL19	cg11940219	-0.16616998226357	307	-2.92844015325565	0.0036659198935781	0.027612463005475	0.0193397566812028
**CESC**	FBXL19	cg26666512	-0.0334049427257691	309	-0.582760317246701	0.560486667832235	0.00111589019851191	0.731149869233017
**CESC**	FBXL19	cg27602786	-0.127830989390481	309	-2.24724594055175	0.0253413650243613	0.0163407618485492	0.0870800896994199
**CESC**	FBXW9	cg02753619	-0.0447344993557361	309	-0.780754252268107	0.43555457769612	0.00200117543260835	0.628730290530912
**CESC**	FBXW9	cg03160342	0.0969275988153162	309	1.69798550743185	0.0905337825672579	0.00939495941210289	0.221707592188033
**CESC**	FBXW9	cg05868316	0.00866400634506292	309	0.151067782475701	0.880022440335217	7.50650059472906e-05	0.936347006362666
**CESC**	FBXW9	cg06489804	0.180491561839688	309	3.19952520984987	0.00152193002944421	0.03257720389533	0.00951438642804426
**CESC**	FBXW9	cg12110584	0.0142743651583636	309	0.248907420502841	0.803600472592771	0.000203757500674303	0.893015475584586
**CESC**	FBXW9	cg14342428	0.0782098729380326	309	1.36782549359445	0.172376967957831	0.0061167842249832	0.3469212460628
**CESC**	FBXW9	cg15828427	0.00582225205592139	309	0.101516153985628	0.919207657862538	3.38986190026808e-05	0.957421314192964
**CESC**	FBXW9	cg19721801	0.0638936654507421	309	1.11630505577981	0.265173137011987	0.00408240048473136	0.460856955022352
**CESC**	FBXW9	cg21564527	0.123768056508051	309	2.174690618449	0.0304240913132584	0.0153185318117801	0.0997434261715043
**CESC**	FBXW9	cg22303873	0.148242440874724	309	2.61357250839011	0.00940598574695689	0.0219758212764962	0.0406245422606465
**CESC**	FKBP1A	cg04613791	0.272434787046983	309	4.93680102455319	1.31167092797311e-06	0.0742207131933351	2.17476829008397e-05
**CESC**	FKBP1A	cg07516946	0.22922349681726	309	4.10597466335764	5.17936746583186e-05	0.0525434114931326	0.000547079039561335
**CESC**	FKBP1A	cg10996143	0.0181401015285491	309	0.316335529040396	0.75196494187739	0.000329063283466071	0.861105248249702
**CESC**	FKBP1A	cg21332426	-0.00587673494149692	309	-0.10246614431319	0.918454240056464	3.45360135726108e-05	0.956976694729708
**CESC**	FKBP1A	cg21775445	0.0605047109506974	309	1.05687196532842	0.291409149914529	0.00366082004722744	0.489011505688452
**CESC**	FKBP1A	cg24641773	-0.100995940400664	309	-1.7699745586703	0.0777336950287222	0.0102001799774146	0.198823444716897
**CESC**	FKBP1A	cg26779912	0.0254606942548604	309	0.444066328812833	0.657310557155372	0.000648246951939479	0.800382192013289
**CESC**	FKBP2	cg03764062	0.143451533790471	309	2.52730197264444	0.0120010752461073	0.0205783425468385	0.0491374551418774
**CESC**	FKBP2	cg04446112	-0.0390700451882249	309	-0.681730033181809	0.495928709209708	0.00152646843100993	0.680812416253619
**CESC**	FKBP2	cg06615912	0.00846161637138265	308	0.147295742481576	0.882996554340519	7.15989516164509e-05	0.93786761001864
**CESC**	FKBP2	cg09854726	-0.103537341630771	309	-1.81498977683016	0.0705108462170834	0.0107199811119669	0.185371253780264
**CESC**	FKBP2	cg10998122	-0.019125947034281	309	-0.33353329041492	0.738961698514222	0.00036580184995812	0.853018352990141
**CESC**	FKBP2	cg12423493	-0.0391533525293946	309	-0.683185885611629	0.495009722711422	0.00153298501429105	0.679994759013836
**CESC**	FKBP2	cg15010817	-0.012051146796896	309	-0.210134183628188	0.833703711530324	0.000145230139120336	0.910811347482481
**CESC**	FKBP2	cg16977872	0.112827971271332	309	1.97986525467625	0.048620264867422	0.0127301511012044	0.141317574634643
**CESC**	FKBP2	cg19924313	-0.0357512045085246	309	-0.623742295382911	0.533264575062725	0.00127814862381035	0.710343733025627
**CESC**	FKBP2	cg21809711	0.0936569154592939	309	1.6401734509293	0.102003386178337	0.00877161781334933	0.241663157033669
**CESC**	FKBP2	cg22074794	0.0740133448369228	309	1.2940159232759	0.196642121075855	0.00547797521394925	0.378784162010971
**CESC**	FKBP2	cg24677501	0.169314374332558	309	2.99534347484348	0.00296686098645642	0.0286673573556256	0.0163193087449898
**CESC**	FKBP5	cg00130530	-0.0837200898282675	309	-1.46485228550552	0.143994621871368	0.00700905344085318	0.307188000160731
**CESC**	FKBP5	cg00140191	0.0572329498820729	309	0.999528955675352	0.318333446322332	0.00327561055220387	0.517676286705648
**CESC**	FKBP5	cg00610228	0.0366276774067963	309	0.639054194604182	0.523268883777844	0.00134158675221633	0.702720265581646
**CESC**	FKBP5	cg01294490	-0.0568242372545282	309	-0.992367901360378	0.321807303124872	0.00322899393955891	0.521275587337622
**CESC**	FKBP5	cg07485685	0.0404618957503527	309	0.706055459955445	0.480694380807038	0.00163716500771241	0.667925387331036
**CESC**	FKBP5	cg07843056	0.0422105805721369	309	0.736623145742189	0.461919622965169	0.00178173311223686	0.651452512625592
**CESC**	FKBP5	cg08636224	0.0721771008332504	309	1.26174158858398	0.208009022622425	0.00520953388469319	0.393126036292913
**CESC**	FKBP5	cg08915438	-0.06561218441188	309	-1.1464579138733	0.252507309767889	0.00430495874329855	0.44642410926965
**CESC**	FKBP5	cg10913456	0.0390377116741349	309	0.68116498687561	0.496285633102978	0.00152394293275288	0.681062033263003
**CESC**	FKBP5	cg16012111	-0.0977867528344915	309	-1.71318090080669	0.0876987792511839	0.00956224902991393	0.21685384549336
**CESC**	FKBP5	cg17030679	-0.0872057204703644	309	-1.52629839314757	0.127975270705189	0.00760483768275533	0.282986827129316
**CESC**	FKBP5	cg20813374	-0.0670617934084139	309	-1.17190047594338	0.24215462109448	0.00449728413515278	0.434519919578908
**CESC**	FKBP5	cg25114611	-0.163219230392124	309	-2.88450625733282	0.00420039135244875	0.0266405171697972	0.0215857606600871
**CESC**	FMR1	cg02921434	-0.197723379727305	309	-3.51685518395352	0.000503290498645347	0.0390945348907879	0.00376905863030575
**CESC**	FMR1	cg03513471	-0.146632051027003	309	-2.5845533356534	0.0102165415402653	0.0215009583883857	0.0433196507648881
**CESC**	FMR1	cg04552106	-0.163699468657117	309	-2.89322665263506	0.00408858572918861	0.0267975160386226	0.0211116048198924
**CESC**	FMR1	cg04744025	-0.148515936080501	309	-2.61850298775075	0.00927414783627076	0.0220569832698673	0.040231301360688
**CESC**	FMR1	cg05288927	-0.253201866936103	309	-4.56343182663574	7.31250771055128e-06	0.0641111854199279	9.91406805218937e-05
**CESC**	FMR1	cg07147350	-0.277799348449828	309	-5.04205693569098	7.92796781983936e-07	0.0771724779991488	1.39063884684686e-05
**CESC**	FMR1	cg08434396	-0.268080413467507	309	-4.85173190225153	1.95842750263181e-06	0.0718671080849095	3.10293093970956e-05
**CESC**	FMR1	cg14470409	-0.33336567327516	309	-6.1650868443763	2.23740903687192e-09	0.111132672118201	6.92902064547827e-08
**CESC**	FMR1	cg16783314	-0.297693746227845	309	-5.43697241360465	1.11440909220307e-07	0.0886215665431687	2.39752049001233e-06

**CESC**	FMR1	cg22625568	-0.326473163084686	304	-5.97250324938207	6.62245390542947e-09	0.10658472621452	1.87682032465931e-07
**CESC**	FXR2	cg01807919	0.0420951126273735	309	0.734604515477169	0.463146648335723	0.00177199850711126	0.652434269078742
**CESC**	FXR2	cg01933235	-0.0173649720652467	309	-0.302814292653864	0.762238490859956	0.000301542254826797	0.867824513134385
**CESC**	FXR2	cg05529097	-0.158202060269892	309	-2.79352672825805	0.00554454097902589	0.0250278918736385	0.026935630093171
**CESC**	FXR2	cg05913233	-0.311262580280408	309	-5.71073531387798	2.67703979554881e-08	0.0968843938828173	6.68563832600077e-07
**CESC**	FXR2	cg09625173	-0.353825859178469	309	-6.59584091100973	1.87339149414049e-10	0.125192738623381	7.09535627532452e-09
**CESC**	FXR2	cg17777561	-0.128919446962366	309	-2.26670284312029	0.0241107926321744	0.0166202238050822	0.0838750307918642
**CESC**	FXR2	cg19178740	-0.0158680064614476	309	-0.276702984621086	0.782196217509398	0.000251793629060544	0.879755152459218
**CESC**	FXR2	cg22521096	-0.328717051032456	308	-6.05863074411036	4.06641190052951e-09	0.108054899639475	1.19826684617346e-07
**CESC**	FXR2	cg26904212	0.0794909191246625	309	1.39037123802409	0.165433234247305	0.00631880622328364	0.337504275281517
**CESC**	G2E3	cg02259031	0.0214195619181711	309	0.373548524685335	0.709000601467245	0.000458797632766367	0.834222331232297
**CESC**	G2E3	cg05076167	-0.172805999507997	309	-3.05899544783488	0.00241854163621766	0.029861913465958	0.0138558088259752
**CESC**	G2E3	cg07906495	-0.0607441436365827	309	-1.06106973785181	0.28950045181167	0.00368985098614179	0.487001797994099
**CESC**	G2E3	cg08810065	-0.0685969519875132	309	-1.19885267787391	0.231518904063013	0.00470554182197719	0.422003792708404
**CESC**	G2E3	cg09837748	-0.100098808673392	308	-1.75120489472135	0.0809224827627665	0.0100197714978324	0.204609965774847
**CESC**	G2E3	cg18580322	0.00774446823456965	309	0.135033467121351	0.892674776037826	5.99767882362584e-05	0.94313688957904
**CESC**	G2E3	cg18755078	-0.122040426698148	309	-2.14387271725788	0.0328361047652766	0.014893865748666	0.105556158745553
**CESC**	G2E3	cg18956956	-0.0855687484157004	309	-1.49743429518057	0.135317843380801	0.00732201070542943	0.29413831925916
**CESC**	G2E3	cg19554565	0.00445717697059114	309	0.077714307909763	0.938106437186333	1.9866426547168e-05	0.966878895778608
**CESC**	G2E3	cg21061067	-0.0618779713946091	309	-1.08095069831432	0.280575810491277	0.00382888334391207	0.477774206005437
**CESC**	G2E3	cg22926526	-0.0644881141934368	309	-1.12673401612237	0.260743560385695	0.00415871687222575	0.455970070059002
**CESC**	GADD45A	cg01810953	0.186673885015345	309	3.31300664298451	0.00103446317848301	0.0348471393467221	0.00690476435065702
**CESC**	GADD45A	cg02639608	0.00640939677499128	309	0.11175394679512	0.911092254649422	4.10803670192686e-05	0.952780078345478
**CESC**	GADD45A	cg04718306	0.138921033971127	309	2.44588763590762	0.0150163863499389	0.019299053679607	0.0584286971703365
**CESC**	GADD45A	cg07000785	0.106543646106132	309	1.86828618132909	0.0626839751579516	0.0113515485255886	0.170011406179189
**CESC**	GADD45A	cg22224769	0.106743981743593	309	1.87183959886992	0.0621888814262859	0.0113942776384765	0.168998562255091
**CESC**	GADD45A	cg23369529	-0.142145814688123	309	-2.50382144721573	0.0128099032802367	0.0202054326333502	0.0517679800406614
**CESC**	GADD45A	cg23723105	-0.0673847028333054	309	-1.17756897440129	0.239889548029252	0.00454069817593288	0.431810067550272
**CESC**	GATAD2A	cg02887458	-0.0248128754070437	309	-0.432760506835218	0.665495620626244	0.000615678785965476	0.805836605429406
**CESC**	GATAD2A	cg08505228	0.0410907040748656	309	0.717046509206319	0.473895954377571	0.00168844596136818	0.661848082202591
**CESC**	GATAD2A	cg08870822	0.162985426854063	309	2.88026150410608	0.00425581638776771	0.0265642493668013	0.0218244548497035
**CESC**	GATAD2A	cg09334331	0.00133917929524915	308	0.0233109745300069	0.981417561585855	1.79340118482402e-06	0.990457134891936
**CESC**	GATAD2A	cg11795262	-0.0493765311615094	309	-0.861960627318655	0.389388592173238	0.00243804182954351	0.586999246962986
**CESC**	GATAD2A	cg14948912	0.0158585579616394	309	0.276538182194425	0.782322655642814	0.000251493860622676	0.879790132860442
**CESC**	GATAD2A	cg15264323	0.0924593919057897	309	1.61901972494224	0.10647995874171	0.0085487391515884	0.249047752964467
**CESC**	GATAD2A	cg22191731	-0.0571840163151144	309	-0.99867156410737	0.318748064842963	0.00327001172192727	0.518087145468085
**CESC**	GATAD2A	cg26631329	-0.0366006678386577	309	-0.638582318115374	0.523575478039761	0.00133960888623575	0.70291129876349
**CESC**	GATAD2B	cg02134200	-0.0250281093079584	309	-0.436516736709405	0.662771719956151	0.000626406255531112	0.80389647267199
**CESC**	GATAD2B	cg02940454	-0.052976443363904	309	-0.92497473645855	0.355712702752219	0.00280650355148893	0.554510940964775
**CESC**	GATAD2B	cg09805553	-0.115999643655818	309	-2.03626923094268	0.0425898192256504	0.0134559173282767	0.128211144525975
**CESC**	GATAD2B	cg10226922	-0.14907315486881	308	-2.62422340799316	0.00912472834036142	0.0222228055025403	0.0397474167932178
**CESC**	GATAD2B	cg10489353	-0.0189544103706712	309	-0.330540819340591	0.74121904461934	0.000359269672499807	0.854392899923449
**CESC**	GATAD2B	cg15064304	-0.14572823583727	309	-2.56827583275134	0.010698060556346	0.021236718720243	0.0449327784753472
**CESC**	GATAD2B	cg20773694	-0.201281011979297	309	-3.58278121351454	0.000395756936488182	0.0405140457834098	0.00307813588453925
**CESC**	GATAD2B	cg23046518	-0.135676013623348	309	-2.387670299716	0.0175661736954304	0.0184079806727229	0.065889243802426
**CESC**	GATAD2B	cg23323627	0.0558526393155819	309	0.975346535519799	0.33016386597336	0.00311951731851649	0.529898781305311
**CESC**	GATAD2B	cg26398333	0.0700935080883486	309	1.22513538540955	0.221472598063751	0.00491309987613139	0.409868667184591
**CESC**	GLYR1	cg00636946	-0.0483329883095219	308	-0.842311689570589	0.400277861405441	0.00233607775892838	0.59682741982656
**CESC**	GLYR1	cg00885266	-0.131311758265892	309	-2.30949638263927	0.0215860555083878	0.01724277785888	0.077175679131372
**CESC**	GLYR1	cg01261535	-0.0555751925678771	309	-0.970486474963079	0.332575572842906	0.00308860202895662	0.532316798280695
**CESC**	GLYR1	cg01565912	-0.0206574806771782	309	-0.360252356517505	0.718908590015388	0.000426731507927989	0.840473307860555
**CESC**	GLYR1	cg02454264	0.0186986872440045	309	0.32607976268929	0.744588360335798	0.000349640904649097	0.856483814331392
**CESC**	GLYR1	cg06399529	-0.12377881694168	309	-2.17488262842759	0.0304095561061278	0.0153211955234819	0.0997040667030811
**CESC**	GLYR1	cg06976003	-0.00097077833117781	308	-0.0168982446217393	0.986528914597564	9.42410568284389e-07	0.992960550623424
**CESC**	GLYR1	cg11289281	0.035437884825678	309	0.618268981472548	0.536860977765988	0.00125584368091802	0.712912669278243
**CESC**	GLYR1	cg15663121	0.017360367117197	309	0.302733966320806	0.76229965150023	0.000301382346443855	0.867841864168719
**CESC**	GLYR1	cg18354686	-0.18999860864276	309	-3.37420219178198	0.000836290765370684	0.0360994712861847	0.00578250259061796

**CESC**	GTF2B	cg02705666	-0.179151840744358	309	-3.17498578814842	0.00165211125523854	0.0320953820420917	0.0101729771029045
**CESC**	GTF2B	cg04270788	0.113827884005947	309	1.99764066973067	0.0466464611513406	0.0129567871772713	0.13714341643263
**CESC**	GTF2B	cg05306856	-0.173422349078067	308	-3.06518980072107	0.00237115075937369	0.030075311159755	0.0136405606873266
**CESC**	GTF2B	cg06356032	-0.0262843247022666	309	-0.458441248739355	0.646963018026006	0.000690865725054184	0.792983047224867
**CESC**	GTF2B	cg07167781	0.0775805692573714	309	1.35675258137533	0.175866454238724	0.00601874472629781	0.351671320831403
**CESC**	GTF2B	cg10430205	-0.0790543336675003	309	-1.38268678886326	0.167775761860248	0.00624958767161247	0.340698664594323
**CESC**	GTF2B	cg12074130	-0.142291940247144	309	-2.50644853298558	0.012717061170672	0.0202469962592969	0.0514810353703105
**CESC**	GTF2B	cg13868361	-0.0488708723869587	309	-0.853112155493937	0.394268770874545	0.0023883621678624	0.591011903605494
**CESC**	GTF2B	cg14010405	-0.0405189778598693	309	-0.707053173126808	0.480075057831769	0.00164178756680858	0.667338986651502
**CESC**	GTF2B	cg17811845	0.0160559316879119	309	0.279980825554557	0.779682629448097	0.000257792942366893	0.878264780350669
**CESC**	GTF2B	cg22607980	-0.00494336290980556	309	-0.0861915305909781	0.931370903623798	2.44368368580413e-05	0.963680928157119
**CESC**	GTF2H1	cg05849260	-0.0207764958521647	309	-0.3623287936501	0.717358096066242	0.000431662779895017	0.839455402839652
**CESC**	GTF2H1	cg07583091	-0.164146761606568	309	-2.90135071207631	0.00398687496366374	0.0269441593459234	0.0206935515694727
**CESC**	GTF2H1	cg08690812	-0.134792644136953	309	-2.37183583823274	0.0183225875113532	0.0181690569134312	0.0680517297034847
**CESC**	GTF2H1	cg10797557	-0.080222233871535	309	-1.40324509698619	0.161564321266885	0.00643560680733926	0.331895998895469
**CESC**	GTF2H1	cg11063328	-0.00243739495600788	309	-0.0424975594318755	0.9661299558841	5.94089417157265e-06	0.982215287350336
**CESC**	GTF2H1	cg11176342	0.0543237841539685	309	0.948568222857923	0.343593700207621	0.00295107352480696	0.542957705503467
**CESC**	GTF2H1	cg11347316	0.166826934198433	309	2.95006868019368	0.00342387557399421	0.0278312259740483	0.0183138735150788
**CESC**	GTF2H1	cg11662851	0.126562800857709	309	2.22458663424614	0.0268430010730075	0.0160181425609481	0.0908674307242306
**CESC**	GTF2H1	cg13777411	0.142338881585454	309	2.50729249285317	0.0126873626191569	0.0202603572109979	0.0513741769516288
**CESC**	GTF2H1	cg14132868	0.192763067864082	309	3.42517699211739	0.000698863981793157	0.0371576003323725	0.00497915785324818
**CESC**	GTF2H1	cg17174493	0.087592403377803	309	1.53311843403533	0.126286676277834	0.00767242912949974	0.280439189722477
**CESC**	GTF2H1	cg18321761	0.165907135944711	309	2.93334182675808	0.0036084092978153	0.0275251777573767	0.0190804892150712
**CESC**	H2AFZ	cg02758870	-0.041131673288795	309	-0.717762646640248	0.473454843834528	0.00169181454753617	0.661493285223837
**CESC**	H2AFZ	cg09308803	-0.00916216208623086	309	-0.15975446000478	0.873180590184462	8.39452140943663e-05	0.932810739111109
**CESC**	H2AFZ	cg16267491	-0.216610971504924	308	-3.86222132694458	0.000137412768841044	0.0469203129763069	0.00126402848991607
**CESC**	H2AFZ	cg17709884	-0.212175992141227	309	-3.78560754629276	0.000184729232296131	0.0450186516411141	0.00162290523966631
**CESC**	H2AFZ	cg20934652	0.192072792920588	309	3.41244090021666	0.000731071728138813	0.0368919577803151	0.00516886249375905
**CESC**	H2AFZ	cg23752380	0.0781259312414613	309	1.36634840277748	0.172839422842201	0.00610366113234554	0.347600177358622
**CESC**	H2AFZ	cg24061702	-0.241840753601541	309	-4.34563361106945	1.89577724449653e-05	0.0584869501025612	0.00022868676532542
**CESC**	H3F3A	cg06319713	-0.12783756596002	309	-2.24736347623943	0.025333770352678	0.0163424432705824	0.0870731980490489
**CESC**	H3F3A	cg10632229	0.0509987179426413	309	0.890351628957935	0.373981176418231	0.00260086923179308	0.572616352509052
**CESC**	H3F3A	cg10983539	-0.0231484435910343	309	-0.40371508520145	0.686706075857015	0.000535850440687297	0.819701903658329
**CESC**	H3F3A	cg11978873	0.0721134837657501	309	1.26062367042648	0.208411155670676	0.0052003545408331	0.393608528132738
**CESC**	H3F3A	cg15538837	0.0763154321551615	309	1.33449678589844	0.183039776954175	0.00582404518502906	0.361066927209804
**CESC**	H3F3A	cg15624314	0.0690691149545652	309	1.20714397698482	0.22831511542915	0.00477054264060694	0.41823313786055
**CESC**	H3F3A	cg16724613	-0.130583943568695	309	-2.29647296347825	0.0223287394648321	0.0170521663179521	0.079082951770076
**CESC**	H3F3A	cg16727519	0.0825089820478347	309	1.44351518214777	0.149905085349897	0.00680773321856991	0.315497016197545
**CESC**	H3F3A	cg18947521	0.148714894573663	309	2.62209013036058	0.00917927380686094	0.0221161198680558	0.039920193790862
**CESC**	H3F3A	cg19410789	0.131713020008862	309	2.31667813035987	0.021185820212209	0.017348319639855	0.0761085060073233
**CESC**	H3F3A	cg21266749	-0.0411381912344006	309	-0.717876579959635	0.473384686632036	0.00169235077803812	0.661442781662701
**CESC**	H3F3A	cg21789564	-0.035365130714975	309	-0.616998081790824	0.537697809933404	0.00125069247048727	0.713561069001881
**CESC**	H3F3A	cg22519151	-0.0615075781888982	309	-1.07445562694564	0.283470587407209	0.00378318217466342	0.480743696850787
**CESC**	H3F3A	cg27530209	-0.0172655465900231	309	-0.301079970342426	0.763559338619515	0.000298099099052257	0.868603737439922
**CESC**	H3F3A	cg27560444	-0.161087572884168	301	-2.80812923711146	0.00531423805497708	0.0259492061377123	0.0260337427738594
**CESC**	HAT1	cg00414306	-0.111011067880694	308	-1.9443758796559	0.0527753669554472	0.0123234571920121	0.150031313609796
**CESC**	HAT1	cg01583365	0.0206525250107461	309	0.360165896196967	0.718973175974768	0.000426526789319493	0.840523552112604
**CESC**	HAT1	cg04507121	0.0400683118868626	308	0.698025459688229	0.485696594401588	0.00160546961746289	0.672282277901129
**CESC**	HAT1	cg04950839	-0.132188588734451	309	-2.32519131896487	0.0207198115154064	0.0174738229916058	0.0748318020905073
**CESC**	HAT1	cg05421799	0.00690263610170595	309	0.120354440106645	0.904281884289716	4.76463851525743e-05	0.94935702997094
**CESC**	HAT1	cg09545452	-0.108107938564317	309	-1.89603869221408	0.0589030315050012	0.0116873263806261	0.162429634996989
**CESC**	HAT1	cg10190161	0.0391508527127801	309	0.683142199409106	0.495037285702251	0.0015327892681378	0.679994759013836
**CESC**	HAT1	cg12941622	0.0448381648825946	309	0.78256717433735	0.434490474777829	0.00201046103003874	0.6279866229724
**CESC**	HAT1	cg13293756	-0.00705765100176424	309	-0.123057414791885	0.902142948237287	4.98104376627037e-05	0.948281470876085
**CESC**	HAT1	cg14446129	0.0565194897440968	309	0.987028745906802	0.324413471897722	0.00319445272093306	0.523804438114846
**CESC**	HAT1	cg16017358	0.0880792414264922	309	1.54170593433601	0.124185329977467	0.00775795277026629	0.277197106629952
**CESC**	HDAC1	cg03762702	0.0774558583311219	309	1.35455842453838	0.176564155814075	0.00599940998981083	0.352557287920948

**CESC**	HDAC1	cg04967775	-0.127922119349082	309	-2.24887463144864	0.0252363019225034	0.0163640686187609	0.0868379720346441
**CESC**	HDAC1	cg06473843	0.00787839079286535	309	0.137368700453224	0.890830305479437	6.20690414851056e-05	0.942271646490915
**CESC**	HDAC1	cg10842753	-0.0782532886848359	309	-1.36858947582753	0.172138141880985	0.00612357718999226	0.3465443489727
**CESC**	HDAC1	cg14172124	0.0813962578264485	309	1.42391706290178	0.155496070708742	0.00662535078814967	0.32349684216853
**CESC**	HDAC1	cg20002354	-0.117098170639502	308	-2.05243563306491	0.0409861236614352	0.0137119815671178	0.124559516529326
**CESC**	HDAC1	cg20793809	-0.00162683752597876	309	-0.0283649190285315	0.977389715072654	2.64660033593269e-06	0.988173495177723
**CESC**	HDAC1	cg21930229	0.120983118312513	309	2.12502195741173	0.0343911758334571	0.0146369149166194	0.109352862453526
**CESC**	HDAC1	cg23018091	-0.0909305063520342	309	-1.59202292072613	0.11241880560322	0.00826835698543733	0.258995722542627
**CESC**	HDAC1	cg26115470	0.0100905460690996	309	0.175943639842689	0.860455363071332	0.000101819119972621	0.925853255436054
**CESC**	HDAC10	cg00711351	0.0374083523822449	309	0.652693755464965	0.514446986298217	0.00139938482795421	0.695979146341643
**CESC**	HDAC10	cg01120165	0.0725591828004594	309	1.26845609360483	0.205605600298817	0.00526483500867049	0.389909854947253
**CESC**	HDAC10	cg04321608	0.062230212479116	309	1.08712787699469	0.277841494556099	0.00387259934519593	0.474673764922325
**CESC**	HDAC10	cg04586023	0.0403203634657598	309	0.703581709039053	0.482231828128996	0.00162573171001098	0.669296584137074
**CESC**	HDAC10	cg07482373	-0.0231521620310215	309	-0.403779970572648	0.686658408050939	0.000536022606710673	0.819670157985495
**CESC**	HDAC10	cg07744063	-0.0594680492697775	309	-1.03869914845316	0.299770113753018	0.00353644888395268	0.498031312958938
**CESC**	HDAC10	cg09151829	0.0159767506121572	309	0.27859972493124	0.780741435743618	0.000255256560123066	0.878908895915476
**CESC**	HDAC10	cg10508940	0.0296497639246491	309	0.517188681057001	0.605400540609777	0.000879108500787423	0.764045516072046
**CESC**	HDAC10	cg11645658	-0.208409771498603	309	-3.71533126933178	0.000241458300334166	0.0434346328561	0.00203356143944961
**CESC**	HDAC10	cg12464820	0.0010391946922054	309	0.0181189883675311	0.985555815817641	1.07992560830787e-06	0.992449308107985
**CESC**	HDAC10	cg26943378	-0.132750181453647	309	-2.33524653392506	0.0201809810874471	0.0176226106759762	0.0733283158672051
**CESC**	HDAC11	cg01409735	-0.0312911680538451	309	-0.54584745270667	0.585571119619808	0.000979137198173977	0.750032395271499
**CESC**	HDAC11	cg01712016	-0.0970518269789335	309	-1.7001824260981	0.0901193793689738	0.00941905711994884	0.220996179384248
**CESC**	HDAC11	cg02762546	-0.137746610101777	309	-2.42480869842587	0.0158990464023084	0.0189741285945309	0.0610099380213388
**CESC**	HDAC11	cg02941903	-0.153933695977331	309	-2.71630080080652	0.00697957314109064	0.0236955827572415	0.0323210312037139
**CESC**	HDAC11	cg05987698	-0.102554522289633	309	-1.7975771280451	0.0732363025018538	0.0105174300420548	0.190604025185816
**CESC**	HDAC11	cg07450135	-0.0817175161963477	309	-1.42957474844453	0.153865946449057	0.00667775245330035	0.321217460231268
**CESC**	HDAC11	cg08051076	-0.0495652382708087	309	-0.8652629638418	0.387576766000552	0.00245671284484204	0.585470532131633
**CESC**	HDAC11	cg08448040	0.174363044428	309	3.08741837924687	0.00220520292984232	0.0304024712622009	0.0128503038744684
**CESC**	HDAC11	cg10455785	-0.0977386796053968	309	-1.7123305537414	0.0878555045918088	0.0095528494910064	0.217130252455739
**CESC**	HDAC11	cg11432034	-0.0105916659575402	309	-0.184682365647897	0.853601320798659	0.000112183387756117	0.921966206568838
**CESC**	HDAC11	cg13397067	-0.00661027464925603	309	-0.115256594878711	0.908317833097297	4.36957309385969e-05	0.951364912458884
**CESC**	HDAC11	cg13706066	0.0491049859733011	309	0.857208803337027	0.3920047482344	0.00241129964743809	0.589082394223205
**CESC**	HDAC11	cg16254764	-0.0761685218872803	309	-1.33191282035466	0.183886541449772	0.0058016437264931	0.362214083582951
**CESC**	HDAC11	cg18521047	0.0116847061179256	309	0.203743721892113	0.838690092481341	0.000136532357062288	0.913698406530171
**CESC**	HDAC11	cg19779799	-0.138783700168344	309	-2.44342218319388	0.0151173346128006	0.0192609154324167	0.0587255532242189
**CESC**	HDAC11	cg20894727	-0.203671468789691	309	-3.62716127346074	0.000335953544396311	0.0414820671989502	0.00268631617152499
**CESC**	HDAC11	cg20922324	-0.107237300461937	309	-1.88059073543121	0.0609834443991985	0.0114998386103637	0.166578162696322
**CESC**	HDAC11	cg21655280	-0.110636118618713	309	-1.94092199958144	0.0531918547219998	0.0122403507430139	0.150930278160718
**CESC**	HDAC11	cg25127083	0.0999008039034508	309	1.75058752053777	0.081025713514418	0.00998017062055572	0.204772735921663
**CESC**	HDAC11	cg25150878	-0.115880993977866	309	-2.03415807882872	0.0428035130903375	0.0134284047652982	0.128680071545394
**CESC**	HDAC11	cg27025856	-0.136154205605873	309	-2.39624434286587	0.0171681826365281	0.0185379677041662	0.064761751443828
**CESC**	HDAC2	cg00161229	0.00635235787730463	309	0.110759378890229	0.911880244619345	4.03524506013542e-05	0.953203412412411
**CESC**	HDAC2	cg01129566	0.110081748837499	309	1.93107692435702	0.0544031053667071	0.0121179914271223	0.153458616298546
**CESC**	HDAC2	cg08198132	-0.0646657573371629	309	-1.12985080620744	0.259429781317821	0.00418166017198883	0.454311831789477
**CESC**	HDAC2	cg10999867	0.122927064470479	309	2.1596862980475	0.0315786241109196	0.0151110631793292	0.102542786295078
**CESC**	HDAC2	cg13393754	-0.063840625761035	309	-1.11537458992677	0.265570857774853	0.00407562549756053	0.461310635648758
**CESC**	HDAC2	cg15005908	-0.0420781704200426	309	-0.734308331407416	0.463326837436756	0.00177057242589815	0.652629017211157
**CESC**	HDAC2	cg15242771	-0.102792662574634	309	-1.8017957783781	0.0725681676882224	0.0105663314791825	0.189257703481095
**CESC**	HDAC2	cg18387216	-0.0547983594413725	309	-0.956879812624406	0.339388251808139	0.00300286019746586	0.538636996575931
**CESC**	HDAC2	cg18674783	0.210494091100595	309	3.75420230091445	0.000208318341712607	0.0443077623882658	0.00179586847021172
**CESC**	HDAC2	cg22867643	-0.312237147186787	309	-5.73054385716204	2.40961069033427e-08	0.0974920360833434	6.08416923219293e-07
**CESC**	HDAC2	cg24023618	0.179100122815973	309	3.17403884733748	0.00165733528349547	0.0320768539926966	0.0101994099394231
**CESC**	HDAC3	cg01947930	-0.0784103689013675	309	-1.37135366501066	0.171276115700882	0.00614818595124854	0.345411643417612
**CESC**	HDAC3	cg01961137	-0.055375313395606	309	-0.966985312675391	0.334320021010586	0.00306642533366159	0.533874292193635
**CESC**	HDAC3	cg04284814	-0.0834344862621295	309	-1.4598199821165	0.145372149078375	0.00696131349782547	0.30922785096257
**CESC**	HDAC3	cg08520975	-0.00640142632329047	309	-0.111614968667952	0.911202360892485	4.09782589725162e-05	0.952782651154854
**CESC**	HDAC3	cg10414058	0.0377721541634596	309	0.659050324616527	0.510362340292965	0.00142673563014816	0.692671706690964

**CESC**	HDAC3	cg11064797	0.0906481379255999	309	1.58703815420012	0.113543547080377	0.00821708490937858	0.260706788511875
**CESC**	HDAC3	cg11153694	-0.00091226228631463	309	-0.0159058430827969	0.987319942196068	8.32222479032004e-07	0.993269806321138
**CESC**	HDAC3	cg12082871	0.0219890891096523	309	0.383485591851948	0.701627795407228	0.00048352003987223	0.82907925443776
**CESC**	HDAC3	cg14098966	-0.0678589547568568	309	-1.18589488210367	0.236589880845827	0.00460483774069314	0.427959941184464
**CESC**	HDAC3	cg17940889	-0.064766651377713	309	-1.13162106351744	0.258685643150595	0.00419471913068222	0.453370075769519
**CESC**	HDAC3	cg18671773	-0.0993943789251278	309	-1.74162453206865	0.0825856698000527	0.00987924256191188	0.207622744973036
**CESC**	HDAC3	cg19549288	-0.0454154595113278	309	-0.792663475447758	0.428592071716168	0.00206256396262506	0.622760969170299
**CESC**	HDAC3	cg20642138	-0.0604906605388045	309	-1.05662563693252	0.291521417205009	0.00365912001242087	0.489132257738519
**CESC**	HDAC3	cg23421926	-0.0716526766742054	309	-1.25252654625671	0.211340736320236	0.00513410607457822	0.397328143292405
**CESC**	HDAC3	cg24385322	-0.0169040794609044	309	-0.294774815066245	0.768367104536045	0.00028574790242057	0.8716636075083
**CESC**	HDAC6	cg07153944	-0.189104309919888	309	-3.35772980854724	0.000885831101871854	0.0357604400302772	0.00606889870004571
**CESC**	HDAC6	cg07674520	-0.210915814396795	308	-3.75588090805777	0.000207108768597212	0.0444854807626634	0.00178682705381519
**CESC**	HDAC6	cg10783042	-0.133165078933877	309	-2.34267666739939	0.0197907542803679	0.0177329382474657	0.0722383143751939
**CESC**	HDAC6	cg12510923	-0.122875503600239	309	-2.15876654348643	0.0316506084629659	0.0150983893850124	0.102708356683267
**CESC**	HDAC6	cg13178935	-0.158196190467401	309	-2.79342041893024	0.00554631732105016	0.0250260346783981	0.0269399481564546
**CESC**	HDAC6	cg17152375	-0.152404106437373	307	-2.67980578231881	0.00777063529108577	0.0232270116589742	0.0351479040545803
**CESC**	HDAC6	cg24616736	-0.142556122747799	309	-2.51119850382984	0.012550713929271	0.0203222481328855	0.0509295414643841
**CESC**	HDAC6	cg26680189	-0.182808823305256	309	-3.24201360352893	0.00131874575509084	0.0334190658782524	0.00844505328214821
**CESC**	HDAC9	cg00332048	-0.357117485025145	309	-6.66612382061291	1.23586310218475e-10	0.127532898110684	4.86060334536707e-09
**CESC**	HDAC9	cg02301805	0.0174192007762005	309	0.303760231703391	0.761518363205999	0.000303428555681583	0.867398160227934
**CESC**	HDAC9	cg03260621	0.0567748073542572	309	0.991501874034335	0.322229095872978	0.00322337875011302	0.521661107477275
**CESC**	HDAC9	cg07513622	0.0644673580373877	309	1.12636985158288	0.260897363039372	0.00415604025232074	0.456146745932912
**CESC**	HDAC9	cg08146609	-0.082956059248538	309	-1.45139096184677	0.147702172920254	0.00688170776604696	0.312492326357309
**CESC**	HDAC9	cg08285151	-0.136017353016547	309	-2.39379038448971	0.0172812707455057	0.0185007203216278	0.0650784341587356
**CESC**	HDAC9	cg08725366	-0.125789402505394	309	-2.21077342049721	0.0277957966747255	0.0158229737826641	0.0932648784349191
**CESC**	HDAC9	cg10068437	-0.102810806487163	309	-1.80211721024936	0.0725174667041394	0.0105700619305409	0.189157182295528
**CESC**	HDAC9	cg11321459	-0.0759835879608317	309	-1.32866019083693	0.184956567028838	0.00577350563940145	0.363511474292392
**CESC**	HDAC9	cg11765288	-0.171605462721434	309	-3.03709651390172	0.00259568548868938	0.0294484348358376	0.0146580108091683
**CESC**	HDAC9	cg12081743	-0.101730564084607	309	-1.78298313035993	0.0755867591071494	0.0103491076689723	0.194966722081617
**CESC**	HDAC9	cg16475478	-0.138819019321085	309	-2.44405622822009	0.0150913163558493	0.0192707201252677	0.058650840015334
**CESC**	HDAC9	cg18856632	-0.111603808364675	309	-1.95811164154466	0.0511312738872146	0.0124554100414992	0.146654364221582
**CESC**	HDAC9	cg19585556	-0.119110524521477	305	-2.07784699169116	0.0385736556341384	0.0141873170517815	0.119188106859521
**CESC**	HDAC9	cg20648975	0.0633279651572442	309	1.10638156402813	0.269436140495984	0.00401043117095713	0.46537555758758
**CESC**	HDAC9	cg21433125	-0.183304239681702	309	-3.25110458793338	0.00127867514214946	0.0336004442852868	0.00823454089473301
**CESC**	HDAC9	cg23074799	0.0622643633998087	309	1.08772679624009	0.277577357064001	0.00387685094958344	0.474360812693575
**CESC**	HDAC9	cg23385718	-0.0907205029650965	309	-1.58831560731958	0.113254462623644	0.00823020965824008	0.260335007692692
**CESC**	HDAC9	cg24350771	-0.0368485731116967	309	-0.642913453413466	0.520764871574419	0.00135781734036806	0.700971078546478
**CESC**	HDAC9	cg25620356	-0.157604094961591	309	-2.78269840106143	0.00572814686887159	0.0248390507486623	0.0276384951632448
**CESC**	HDGF	cg00692559	-0.0156645229846046	309	-0.273153805679698	0.784920457999634	0.000245377280335206	0.881152444653865
**CESC**	HDGF	cg00746981	0.102567210806392	309	1.79780189697792	0.0732005772973279	0.0105200327326028	0.190523795050632
**CESC**	HDGF	cg01910231	0.00716458374554735	309	0.124921992320295	0.900667873253622	5.13312602469613e-05	0.947626510121752
**CESC**	HDGF	cg02914429	0.148187892322719	309	2.61258919886593	0.00943247889145743	0.0219596514310497	0.0406960380945061
**CESC**	HDGF	cg04402095	0.0214599964902622	309	0.374254011914621	0.708476251133967	0.000460531449362068	0.833878530610724
**CESC**	HDGF	cg05762852	0.117211644938568	309	2.05783963594111	0.0404579106694716	0.013738569709205	0.1233897589524
**CESC**	HDGF	cg05967123	0.0310872985254545	309	0.542287673002682	0.588017636189307	0.000966420129610727	0.751717507122451
**CESC**	HDGF	cg06106793	0.0158094370399052	309	0.275681407468925	0.782980075172366	0.000249938299518726	0.88019802760321
**CESC**	HDGF	cg06119628	-0.011564034923095	309	-0.201639321189196	0.840333560610391	0.000133726903702562	0.914442216247234
**CESC**	HDGF	cg07313589	-0.0212919469792534	309	-0.371321959166009	0.710656394555241	0.000453347006167337	0.835218811306962
**CESC**	HDGF	cg07892339	0.070800978874285	309	1.2375629595838	0.21683315794753	0.00501277860955696	0.404138064864279
**CESC**	HDGF	cg08597205	0.0498058534949773	309	0.869473816736027	0.385273988063227	0.00248062304236314	0.583351829072618
**CESC**	HDGF	cg08708935	0.0992112093552778	309	1.73838303302132	0.0831558230992376	0.00984286406173676	0.20861895842554
**CESC**	HDGF	cg09063372	0.0549513311448138	309	0.959559058115002	0.338039724309131	0.00301964879458698	0.537494630930477
**CESC**	HDGF	cg09477492	-0.03636366598045	309	-0.634441785987241	0.526269688842346	0.00132231620353774	0.704973381785334
**CESC**	HDGF	cg11588596	0.125066032298691	309	2.19785743745292	0.0287130770425866	0.0156415124349371	0.09553914095171
**CESC**	HDGF	cg12220178	0.0182782454972504	309	0.31874534989462	0.750138508570875	0.000334094258457756	0.860047091364456
**CESC**	HDGF	cg12421458	0.134283740385969	309	2.36271631954194	0.0187711088138651	0.0180321229320464	0.069329138255036
**CESC**	HDGF	cg13170934	0.0132937035428749	309	0.231804124728593	0.816846109846696	0.000176722553885844	0.901472901071255

**CESC**	HDGF	cg13235992	0.205781334658523	309	3.66638821574488	0.000290272475662826	0.0423459576938431	0.00237799673248882
**CESC**	HDGF	cg14155849	-0.0237874374221386	309	-0.414865534213076	0.678532961147802	0.000565842179112157	0.814447874658634
**CESC**	HDGF	cg15503799	0.0618899789138389	309	1.08116126499462	0.280482302258557	0.00383036948995542	0.477677712338322
**CESC**	HDGF	cg17170583	-0.0412578238672818	309	-0.719967767916162	0.472098008216775	0.00170220803026365	0.660190408973859
**CESC**	HDGF	cg17218029	0.0134190796738401	309	0.233990717355908	0.815149684744113	0.000180071699292867	0.900551269806447
**CESC**	HDGF	cg17971171	0.0891212794357755	309	1.56009054398144	0.119778879253659	0.00794260244826958	0.270387038844501
**CESC**	HDGF	cg18593717	-0.00851836654788357	309	-0.148528184676105	0.882024418009903	7.25625686441019e-05	0.937367079338566
**CESC**	HDGF	cg19599803	-0.103697902673588	308	-1.81484262944644	0.070536776148348	0.010753255018901	0.185408094461568
**CESC**	HDGF	cg20284773	0.108048855677093	309	1.89499023009137	0.0590423325193221	0.0116745552131292	0.162733010310297
**CESC**	HDGF	cg23747104	0.142891037838431	309	2.51722102609234	0.012342586235635	0.0204178486945438	0.0502658964304779
**CESC**	HDGF	cg26431298	0.0762906046870826	309	1.33406009604614	0.183182675767938	0.00582025636352071	0.361282702954626
**CESC**	HDGFL1	cg02685945	-0.0123657475296937	309	-0.215620661465583	0.829428043599657	0.000152911711968125	0.908373927946049
**CESC**	HDGFL1	cg03930485	0.0146562257913486	309	0.255567478527285	0.79845766285255	0.000214804954446993	0.889937412081701
**CESC**	HDGFL1	cg05276066	0.0181867535047188	309	0.317149336731023	0.751347990170374	0.000330758003041401	0.860741389655176
**CESC**	HDGFL1	cg06566039	0.0194247814487234	309	0.33874655165697	0.735034522637647	0.000377322134330669	0.850620353116635
**CESC**	HDGFL1	cg08671671	0.00991785773333328	309	0.172932263739336	0.862819751702985	9.83639020186388e-05	0.927058931643581
**CESC**	HDGFL1	cg09081892	0.0224972754815748	308	0.391706855600615	0.695550164380682	0.000506127404093867	0.825458695349196
**CESC**	HDGFL1	cg11894257	0.0352157819686273	309	0.614389225261861	0.539417688627585	0.0012401512996619	0.714774345311167
**CESC**	HDGFL1	cg15317945	0.0302351891870387	309	0.527409662106311	0.59829382523658	0.000914166665176022	0.758818818012855
**CESC**	HDGFL1	cg20209681	0.0439539374034416	309	0.767104446676406	0.443614755637718	0.00193194861326566	0.635744933264949
**CESC**	HDGFL1	cg21898884	0.103318492828308	309	1.8111119608145	0.0711104585563173	0.010674710960313	0.186487561115499
**CESC**	HIST1H1	cg00667898	-0.0602133279975736	309	-1.05176363926231	0.293743314157248	0.00362564486854338	0.491486259325558
**CESC**	HIST1H1	cg00700007	-0.0587293760648862	309	-1.02575217036017	0.305823914725849	0.00344913961297083	0.504330046682128
**CESC**	HIST1H1	cg03821311	0.0870721193303467	309	1.52394219204966	0.128562733015263	0.00758155396467813	0.283897052206688
**CESC**	HIST1H1	cg04676561	-0.0377990908023838	309	-0.659520988592535	0.510060577054145	0.00142877126548686	0.692453318872487
**CESC**	HIST1H1	cg07138603	-0.0877267234941705	309	-1.53548764095278	0.125704175079637	0.00769597801502265	0.279496632352138
**CESC**	HIST1H1	cg11206041	-0.0831446076059737	309	-1.45471272253575	0.146780541219836	0.00691302577395134	0.311194762449633
**CESC**	HIST1H1	cg14334286	-0.0974143550518142	309	-1.70659402947736	0.088918729188154	0.00948955657016091	0.218976127357795
**CESC**	HIST1H1	cg18798248	-0.062662292635415	309	-1.09470573405398	0.274512149502447	0.00392656291832639	0.470996311849236
**CESC**	HIST1H1	cg23460835	-0.0884334532491263	309	-1.54795469535308	0.122673575492186	0.0078204756535654	0.274828659546643
**CESC**	HIST1H1	cg00903962	0.000705248199283519	309	0.0122964255811314	0.990197184754034	4.97375022592646e-07	0.994699165980107
**CESC**	HIST1H1	cg04290376	-0.117720894067776	309	-2.06690569404398	0.0395893888424082	0.0138582089001165	0.121578119201259
**CESC**	HIST1H1	cg07750111	0.0552666347436185	309	0.965081703579689	0.335270974331305	0.00305440091588453	0.534813525863794
**CESC**	HIST1H1	cg08283682	0.0924605894250436	309	1.61904087503689	0.106475406014114	0.00854896059682648	0.249047752964467
**CESC**	HIST1H1	cg10717387	-0.0816334565702113	309	-1.42809432996101	0.154291226602773	0.00666402123160057	0.321759909991854
**CESC**	HIST1H1	cg12633985	0.0687052362942131	309	1.20075410549133	0.230781364857273	0.00472040949424366	0.421192473430444
**CESC**	HIST1H1	cg15022984	0.0268463750116718	309	0.468251314001814	0.639940428192733	0.000720727851267318	0.788462554178421
**CESC**	HIST1H1	cg16481032	-0.0355254215518835	309	-0.619798123202714	0.535854977479356	0.00126205557643903	0.712153193154011
**CESC**	HIST1H1	cg16482183	-0.0658935047371957	309	-1.15139488378186	0.250474569301772	0.00434195396655084	0.444152598396709
**CESC**	HIST1H1	cg17691542	-0.0610023246837455	309	-1.06559641569295	0.287451704356774	0.00372128361682111	0.48494126240772
**CESC**	HIST1H1	cg20855557	-0.104890349285241	309	-1.83896986913252	0.0668948265377488	0.0110019853731799	0.178329659916306
**CESC**	HIST1H1	cg23407813	-0.0481746285016857	308	-0.839545481227525	0.401825185387975	0.00232079483127543	0.598407069031589
**CESC**	HIST1H3	cg00958605	-0.103180736055368	309	-1.80867116348867	0.0714900196745508	0.0106462642929275	0.187207333647643
**CESC**	HIST1H3	cg01043409	0.0786207465010187	309	1.37505590576855	0.170126647243138	0.00618122178037744	0.343862494715469
**CESC**	HIST1H3	cg08041469	0.135965353991699	309	2.39285800584135	0.0173244102603716	0.018486577486088	0.0651961673662609
**CESC**	HIST1H3	cg08401836	-0.0337456185606933	309	-0.588710261699076	0.556492812385943	0.00113876677204381	0.727874671667234
**CESC**	HIST1H3	cg10075390	0.10030797271876	309	1.75779481495184	0.0797888357865775	0.0100616893909474	0.202668934387534
**CESC**	HIST1H3	cg15479139	0.108511106856936	309	1.90319370444678	0.0579597038169909	0.0117746603113173	0.160516810207867
**CESC**	HIST1H3	cg15797414	-0.0331102021444529	309	-0.577612801679517	0.56395311877367	0.00109628548604653	0.733758741648055
**CESC**	HIST1H3	cg21209604	0.000956191426345858	309	0.0166717748140071	0.98670940073842	9.14302043817326e-07	0.993007258731262
**CESC**	HIST1H3	cg23099349	0.0396461913463634	309	0.691798871935193	0.489591652925524	0.00157182048827246	0.675558151930475
**CESC**	HIST1H3	cg25701114	-0.0145234714349002	309	-0.25325208797139	0.800244593245447	0.000210931222520363	0.890909304059132
**CESC**	HLTF	cg00906762	-0.169270887867989	309	-2.99455145879901	0.00297435020528675	0.0286526334796171	0.0163535656138298
**CESC**	HLTF	cg02059813	-0.135173952724424	309	-2.37867011309385	0.0179926676234386	0.0182719974951447	0.0671219996328994
**CESC**	HLTF	cg02398045	-0.160173736726532	309	-2.82925347724368	0.00497598243250759	0.0256556259369405	0.0246718659881769
**CESC**	HLTF	cg02611507	-0.421812969006959	309	-8.11150259603355	1.24492228822791e-14	0.177926180822466	9.25884172948397e-13
**CESC**	HLTF	cg02710346	-0.181682670978265	309	-3.22135798729832	0.00141415707435453	0.0330085929337966	0.0089545031923529

**CESC**	HLTF	cg03678609	-0.277480400720088	309	-5.03578483557236	8.17132785736142e-07	0.0769953727837804	1.42913731865012e-05
**CESC**	HLTF	cg04120413	-0.461164841946526	309	-9.06181961386956	1.60977783401756e-17	0.212673011447565	1.68321312929519e-15
**CESC**	HLTF	cg04731810	-0.42870449975104	309	-8.27357533624227	4.13034383138596e-15	0.183787548106789	3.23907225825443e-13
**CESC**	HLTF	cg04836786	-0.123863548882217	309	-2.17639461871127	0.0302953073623548	0.0153421787416973	0.099411891542485
**CESC**	HLTF	cg05555455	-0.283196201481369	309	-5.14846226447672	4.72568761222299e-07	0.0802000885334759	8.76980300823653e-06
**CESC**	HLTF	cg07275648	-0.401190172191097	309	-7.63649508397519	2.91849532066255e-13	0.160953554262722	1.79337618464157e-11
**CESC**	HLTF	cg09352720	-0.396441016119361	309	-7.52911992777039	5.85455641684463e-13	0.157165479261751	3.43217303611161e-11
**CESC**	HLTF	cg09392355	-0.139516755492524	308	-2.45254009174699	0.0147488392065054	0.0194649250631608	0.0576278853455993
**CESC**	HLTF	cg15438497	-0.117769250609366	309	-2.06776666150847	0.0395077418240036	0.0138695963890916	0.121384884180748
**CESC**	HLTF	cg16724124	0.0394786562420834	309	0.688870926092153	0.491429894459284	0.00155856429868059	0.677073838367199
**CESC**	HLTF	cg20117402	-0.208405152088324	309	-3.71524517973117	0.000241536929713241	0.0434327074169576	0.00203400345422298
**CESC**	HLTF	cg21089667	-0.16363262884391	309	-2.89201281699973	0.00410398417350291	0.0267756372223688	0.0211714419624327
**CESC**	HLTF	cg21111416	-0.447130423009786	309	-8.71577136551354	1.90555449930737e-16	0.19992561518091	1.78603261107371e-14
**CESC**	HLTF	cg21835021	-0.184002274437657	309	-3.26391800989993	0.00122411238745376	0.0338568369982309	0.0079476380462008
**CESC**	HLTF	cg21926402	-0.124072763419023	309	-2.1801281328581	0.0300147824778677	0.0153940506224329	0.0987344068706176
**CESC**	HLTF	cg24041269	-0.0956704455595458	309	-1.67575781194264	0.0948137879049247	0.00915283415356202	0.229272339994194
**CESC**	HLTF	cg24710191	0.15255289753744	299	2.64671630659135	0.00856568124233374	0.0232723865470686	0.0378655873716395
**CESC**	HLTF	cg25951608	-0.38983506814745	309	-7.38095302387131	1.51381516191036e-12	0.151971380357527	8.36998839414743e-11
**CESC**	HLTF	cg26151310	-0.226733229612137	309	-4.05893622301226	6.27500236177208e-05	0.0514079574103498	0.000647337930953053
**CESC**	HLTF	cg27554556	-0.395679797931313	309	-7.51197642935236	6.53892376780149e-13	0.156562502490965	3.79902192376661e-11
**CESC**	IDH1	cg00707300	-0.00288360684580674	309	-0.0502776123694397	0.959934181810212	8.31518844118347e-06	0.979061311921597
**CESC**	IDH1	cg01046174	-0.0183142847208852	309	-0.319374030883181	0.749662254347731	0.000335413024837648	0.859741573701293
**CESC**	IDH1	cg02241586	0.0672240835511846	309	1.17474934434431	0.24101435990983	0.00451907740929665	0.433213288235335
**CESC**	IDH1	cg03448938	-0.0304310744049172	309	-0.53082975675181	0.595924335859418	0.000926050289437604	0.757204340836808
**CESC**	IDH1	cg05268564	-0.0104408827599588	309	-0.18205293457875	0.855662518626297	0.000109012032807205	0.923211699731133
**CESC**	IDH1	cg07129696	-0.00046209629491927	309	-0.0080569250671603	0.993576858011557	2.13532985778117e-07	0.996651598784732
**CESC**	IDH1	cg07915542	0.00800468939035757	309	0.139571000089194	0.889091375442664	6.40750522361031e-05	0.941174522189533
**CESC**	IDH1	cg09406631	0.0568972152425398	309	0.993646511940542	0.321185226922049	0.0032372931023559	0.520590956395822
**CESC**	IDH1	cg09615745	-0.0973458789763464	309	-1.70538292201709	0.0891445261326854	0.00947622015367748	0.219334216774183
**CESC**	IDH1	cg10356455	0.0615192333611947	309	1.07466000071505	0.283379191775095	0.00378461607334913	0.48069449670658
**CESC**	IDH1	cg14772913	-0.0371877093698835	309	-0.648838671405402	0.516932505826564	0.00138292572817892	0.697994365228446
**CESC**	IDH1	cg15399923	-0.0745149132417759	309	-1.30283391581725	0.193617479155239	0.00555247229542939	0.374711500196599
**CESC**	IDH1	cg16085695	-0.137589318947826	308	-2.41799956839738	0.0161957630790568	0.0189308206885267	0.0618949298964505
**CESC**	IDH1	cg16863783	0.0668017330705736	309	1.16733552785886	0.243989686385601	0.00446247154123217	0.436680868658552
**CESC**	IDH1	cg24324379	-0.11448639686098	309	-2.00935038592846	0.0453835734747317	0.0131071350662097	0.134436437243671
**CESC**	IDH1	cg24830471	0.10893032613065	309	1.91063458069174	0.0569921043688435	0.0118658159509298	0.15860498559311
**CESC**	IDH1	cg24987806	0.144709252693737	309	2.54993205843048	0.0112649227189073	0.0209407678151798	0.0467832264571149
**CESC**	IDH1	cg25795625	0.0581122466806887	309	1.0149368296304	0.310943024463869	0.00337703321427722	0.509832264998993
**CESC**	IDH1	cg26105702	-0.00240527360929516	309	-0.0419374996894782	0.966576052537184	5.78534113557176e-06	0.982427050054405
**CESC**	ING2	cg02075087	-0.0250360075615147	308	-0.435935803548787	0.663193723452056	0.000626801674620221	0.804176435921889
**CESC**	ING2	cg02552216	-0.10051080296669	308	-1.758486043559	0.0796743467005402	0.0101024215130087	0.202442634066754
**CESC**	ING2	cg10348863	-0.0650299212479804	309	-1.1362404838707	0.256750847497603	0.00422889065751853	0.451056410423298
**CESC**	ING2	cg17985608	0.0269501621200689	309	0.470062869753856	0.638647129699697	0.000726311238297998	0.787517670679975
**CESC**	ING3	cg01382559	-0.168939021762666	309	-2.98850779646213	0.00303207171330476	0.0285403930741264	0.0166017023412747
**CESC**	ING3	cg01416111	-0.135934550049362	309	-2.39230567925405	0.0173500101772029	0.0184782018971225	0.0652672481491935
**CESC**	ING3	cg10268125	0.03890471805593	309	0.678840871268353	0.497755158448688	0.00151357708701141	0.682128081391604
**CESC**	ING3	cg14303833	-0.190384455185866	309	-3.3813119144557	0.000815716818266331	0.0362462407764188	0.00566133233653412
**CESC**	ING3	cg16130600	-0.00077468333872241	309	-0.0135070695999711	0.989232105272885	6.00134275294109e-07	0.994314147234511
**CESC**	ING3	cg18485114	0.084444952781737	309	1.47762592608707	0.140543176842258	0.00713095005030979	0.30212309998696
**CESC**	ING3	cg19478983	0.0400196281979122	309	0.698325493177561	0.485507489919575	0.00160157064109913	0.67209227664828
**CESC**	ING3	cg19960827	-0.023824222925152	309	-0.415507456980104	0.678063584591974	0.000567593597987337	0.814107455933389
**CESC**	ING3	cg20553930	-0.0781478711932984	309	-1.36673446912691	0.172718461391604	0.00610708977204437	0.347437746890554
**CESC**	ING3	cg24859794	-0.0584723304654067	309	-1.02124724179534	0.307949320892214	0.00341901343005573	0.506525064899545
**CESC**	ING4	cg00369908	0.0295287668248556	309	0.515076250911671	0.606874053345921	0.000871948070196691	0.765123170394019
**CESC**	ING4	cg00443163	-0.227655298146957	309	-4.07634324891163	5.84606913513777e-05	0.0518269347743798	0.000607844296042379
**CESC**	ING4	cg00591160	-0.234284533333417	309	-4.20183561154613	3.48388428095871e-05	0.054889242559257	0.00038834402314501
**CESC**	ING4	cg03930474	0.0480669637443692	309	0.839045990223526	0.402102793497359	0.00231043300360251	0.598660045880225

**CESC**	ING4	cg04375284	-0.273268277405021	309	-4.95312162619852	1.21382506828981e-06	0.0746755514359073	2.03159008955546e-05
**CESC**	ING4	cg08733494	-0.137959444124492	309	-2.42862793563467	0.0157357972313794	0.019032808223139	0.0605471621923772
**CESC**	ING4	cg12392916	-0.232786992705608	309	-4.17343353392334	3.92120435272058e-05	0.0541897839729206	0.000430187803434699
**CESC**	ING4	cg18123790	0.0588370949952435	309	1.02764009110846	0.304936115058862	0.00346180374747931	0.503452962390737
**CESC**	ING4	cg23354319	-0.24610256528209	309	-4.42710583742993	1.33325088165574e-05	0.0605664726384252	0.000168156483604939
**CESC**	ING4	cg27116036	-0.267790081173045	309	-4.84607130459862	2.0109757744935e-06	0.071711527574666	3.17648425179588e-05
**CESC**	INO80	cg00734724	0.0816457092460753	309	1.42831011639772	0.154229181762063	0.00666602183829467	0.321665012493242
**CESC**	INO80	cg06380007	0.0207985007076314	309	0.362712710235327	0.717071550083056	0.000432577631685345	0.839233701928107
**CESC**	INO80	cg12580230	0.0494136162249882	309	0.862609601166456	0.389032124569796	0.00244170546843042	0.586666507042136
**CESC**	INO80	cg18952654	0.0425420726552646	309	0.742418511344154	0.45840706101583	0.00180982794580581	0.648445812363982
**CESC**	INO80	cg21007089	-0.10382415924924	309	-1.82007234643228	0.0697312648057296	0.0107794560438115	0.183843630809534
**CESC**	INO80	cg21598047	-0.0898401002168957	309	-1.57277565868344	0.116810921298208	0.00807124360698187	0.265816088792863
**CESC**	INO80	cg22835709	-0.0906306756836542	309	-1.58672989871061	0.113613391930545	0.0082139193748757	0.260820970042931
**CESC**	INO80	cg26409855	0.0595558230983941	309	1.04023770379287	0.299056085886405	0.00354689606492721	0.49734298474682
**CESC**	INO80	cg27441689	-0.0597178858200501	309	-1.04307850562354	0.297740698365584	0.00356622588681654	0.495866924036142
**CESC**	INTS12	cg00093890	-0.0929532751800779	309	-1.62774309136185	0.10461530392707	0.00864031136670328	0.245985883442722
**CESC**	INTS12	cg01852585	-0.00931596119963127	309	-0.16243638257147	0.871070138685607	8.67871330730352e-05	0.931528357535337
**CESC**	INTS12	cg03607115	-0.0402480860213737	309	-0.702318434884735	0.483017993781782	0.0016199084283839	0.669955214495904
**CESC**	INTS12	cg19703969	-0.0975419954945082	309	-1.70885161747015	0.0884990643584312	0.00951444088505065	0.218316963773042
**CESC**	INTS12	cg26533745	-0.0939969494036796	308	-1.64347154042006	0.101322616620473	0.00883542649719789	0.240526237599062
**CESC**	IWS1	cg02372421	-0.0223092167935694	309	-0.389071318722917	0.697495772181752	0.000497701153942477	0.826595341881637
**CESC**	IWS1	cg02592214	-0.0891415585004124	309	-1.56044837723588	0.119694350408596	0.00794621745188244	0.270258962549671
**CESC**	IWS1	cg10180764	-0.0590026078861063	309	-1.03054099983087	0.303575307855008	0.00348130773736161	0.502066009108143
**CESC**	IWS1	cg10488833	-0.00324205534300708	309	-0.0565274635168204	0.954958752303106	1.05109228471208e-05	0.976013835942963
**CESC**	IWS1	cg15239145	0.0303740082261717	308	0.528961238490895	0.59721961207726	0.000922580375723549	0.758160154326218
**CESC**	IWS1	cg17144508	-0.0691765844971483	309	-1.20903128254965	0.227590308468292	0.00478539984269109	0.417316502784587
**CESC**	IWS1	cg18451256	-0.00418633501802329	309	-0.0729918847582994	0.941860576484184	1.75254008831281e-05	0.968989389639899
**CESC**	IWS1	cg18530950	-0.0746005960700639	309	-1.30434039092808	0.193104198258203	0.00556524893400883	0.374034509356836
**CESC**	IWS1	cg19311602	-0.0312204805758821	309	-0.544613166144679	0.586418865156504	0.000974718407389032	0.750561346407349
**CESC**	IWS1	cg19409088	-0.0175664771730362	309	-0.306329262525351	0.759563651547239	0.0003085811202708	0.866160872302344
**CESC**	IWS1	cg21581038	-0.0187379277116706	309	-0.326764303359062	0.744071024232957	0.000351109934927793	0.856238055399592
**CESC**	IWS1	cg21679674	-0.149429626787526	309	-2.63497915776389	0.00884552247910959	0.0223292133618592	0.0388093494862022
**CESC**	IWS1	cg22409775	-0.0260134667384535	309	-0.453713830893037	0.650358531150723	0.000676700451752627	0.795465490186616
**CESC**	IWS1	cg23570261	0.0353547660890417	309	0.61681702890618	0.537817078932514	0.00124995948521085	0.713609787454818
**CESC**	IWS1	cg23623107	-0.0243522890213624	309	-0.424722623702982	0.671339329677721	0.000593033980579968	0.809645326919598
**CESC**	JADE1	cg09863040	-0.089810584947805	306	-1.57225475062457	0.116931645502452	0.00806594116866691	0.266005217715523
**CESC**	JADE1	cg12832492	0.0316411326083059	306	0.551958366855402	0.581382406023347	0.0010011612727364	0.746821304242258
**CESC**	JADE1	cg14617908	0.062369078743154	306	1.08956325774779	0.276768505973418	0.00388990198326975	0.47345696969162
**CESC**	JADE1	cg15389440	0.216828028444538	306	3.87265707850209	0.000131839500360959	0.0470143939191454	0.00122040957771066
**CESC**	JADE1	cg15836763	-0.0152013131403109	306	-0.265074579626921	0.791131656178985	0.000231079921189787	0.88534927531134
**CESC**	JADE1	cg20039387	-0.0591479160243474	306	-1.03308785451083	0.302383934300538	0.00349847597002325	0.500761320424151
**CESC**	JADE1	cg22922695	-0.0344798978148187	306	-0.601535238573214	0.547931856897261	0.00118886335332034	0.721197890898529
**CESC**	JADE1	cg26766900	-0.0811344675625118	306	-1.41930700144472	0.156834061458007	0.00658280182665228	0.325422729570688
**CESC**	JADE2	cg04590931	0.0870571326294116	306	1.52367789048488	0.128628761446966	0.00757894434165496	0.283978371257634
**CESC**	JADE2	cg04842522	0.0392344038506509	306	0.684602326252798	0.494116492702529	0.00153933844551597	0.679319579982337
**CESC**	JADE2	cg17378563	-0.083045114196987	306	-1.45295987254448	0.147266321954311	0.00689649099199061	0.311943657757656
**CESC**	JADE2	cg18145011	-0.136492941308731	306	-2.40231894934543	0.0168910427052471	0.0186303230271088	0.0638991332528689
**CESC**	JADE2	cg21194015	0.0434771883884888	306	0.758768158145831	0.448579139076057	0.00189026591016815	0.639885220124772
**CESC**	JADE2	cg25669900	-0.0772684222078263	306	-1.35126080448958	0.17761663460312	0.0059704090704869	0.354004623701453
**CESC**	JADE2	cg26133206	0.0478189424817636	306	0.834706639140249	0.404538366518474	0.00228665126007421	0.600958172325275
**CESC**	JADE3	cg10860619	-0.103774845032508	306	-1.81919843812154	0.0698647992953365	0.010769218461521	0.184127047780385
**CESC**	JADE3	cg17843048	-0.257041109116604	306	-4.63748209514065	5.24508370076669e-06	0.066070131775894	7.42018624145197e-05
**CESC**	JADE3	cg19194595	-0.123376310589313	306	-2.16770080501661	0.0309573208304687	0.0152217140146307	0.101003169091966
**CESC**	JARID2	cg01769037	-0.132590811095399	309	-2.33239280458712	0.020332641347207	0.0175803231869359	0.0737484847521738
**CESC**	JARID2	cg03223185	-0.10918249440591	309	-1.9151109043229	0.0564165431969392	0.0119208170846966	0.157465146435946
**CESC**	JARID2	cg05197191	-0.142823203195873	309	-2.51600113631279	0.012384493621053	0.0203984673711296	0.0503838142849178
**CESC**	JARID2	cg06047828	-0.123621082388964	309	-2.17206807929225	0.0306232194168626	0.0152821720110191	0.100213679823042

**CESC**	JARID2	cg14862454	-0.0934906885700963	309	-1.63723669908188	0.102615713733982	0.00874050884931073	0.242670441215387
**CESC**	JARID2	cg15109221	-0.109562107784442	309	-1.92185025470236	0.0555591812255268	0.0120038554621697	0.155669762083869
**CESC**	JARID2	cg21397480	-0.0954842838885084	309	-1.67246698267197	0.0954610733977335	0.00911724846970126	0.230344253843154
**CESC**	JARID2	cg21901688	-0.152135239211588	308	-2.67939111680145	0.00777868767160849	0.0231451310099672	0.0351704380683438
**CESC**	JARID2	cg25338254	-0.10717373533036	309	-1.87946305476681	0.0611376781032421	0.0114862095446621	0.166870554008968
**CESC**	JMJD6	cg02318866	-0.0233583627455078	309	-0.407378121362209	0.684017004407102	0.000545613110150724	0.818023348821616
**CESC**	JMJD6	cg04510639	-0.0821064962754678	309	-1.43642568251739	0.151909529547414	0.00674147673063342	0.318457784947358
**CESC**	JMJD6	cg07312083	-0.105684356251501	309	-1.85304728613742	0.064844478545663	0.0111691831562941	0.174261135967297
**CESC**	JMJD6	cg08857478	0.0949400413093648	309	1.66284727650516	0.0973736194011672	0.00901361144382389	0.233589791473508
**CESC**	JMJD6	cg10712116	0.19072922594079	309	3.38766613788731	0.000797729461583553	0.0363776376279728	0.00555433327973068
**CESC**	JMJD6	cg12504415	-0.0607794749843971	309	-1.06168918829645	0.289219510032625	0.00369414457937895	0.486729168941246
**CESC**	JMJD6	cg15457598	0.120938502630887	309	2.12422666594267	0.0344581497945642	0.014626121418601	0.109516579038213
**CESC**	JMJD6	cg21102709	-0.0596400107244284	309	-1.04171342064907	0.298372293292385	0.00355693087920994	0.496589238466214
**CESC**	JMJD6	cg21807925	-0.195121531089497	309	-3.46873239853899	0.000598458488577686	0.0380724118947095	0.00436494277708724
**CESC**	JMJD6	cg23630158	-0.0934638164787281	309	-1.63676195936064	0.10271497470654	0.00873548499076936	0.242794468181476
**CESC**	JMJD6	cg25004679	-0.0498016116894261	308	-0.867968470908697	0.386098503679595	0.00248020052686438	0.584156268140543
**CESC**	JMJD6	cg25908504	-0.101225514958902	309	-1.77403950452624	0.0770575187215269	0.0102466048786949	0.197685417867777
**CESC**	JMJD6	cg26612088	-0.0830009754872477	309	-1.45218226181045	0.147482222302779	0.00688916193183469	0.31223077346711
**CESC**	JMJD8	cg00873473	-0.0333157244114708	309	-0.581202142338244	0.561534881922065	0.00110993749306107	0.731946727727709
**CESC**	JMJD8	cg02851105	-0.010857174217301	309	-0.189312459140348	0.84997426241876	0.000117878231984826	0.919985923414824
**CESC**	JMJD8	cg03899598	-0.060445631926158	309	-1.05583621051991	0.291881405137622	0.00365367441895257	0.489613881149155
**CESC**	JMJD8	cg05787952	0.0630262032479381	309	1.10108850732327	0.271729194338158	0.00397230229585041	0.468021986000467
**CESC**	JMJD8	cg06191810	-0.0775044740017982	309	-1.35541375822822	0.17629192925406	0.00600694349029541	0.352203335361384
**CESC**	JMJD8	cg06646636	-0.0883907992004264	309	-1.54720219070698	0.122854859109322	0.0078129333832901	0.275143105410831
**CESC**	JMJD8	cg08398290	0.0396574767947068	309	0.691996105423594	0.489467958405817	0.00157271546572271	0.675447375673576
**CESC**	JMJD8	cg26750188	-0.0581141774545225	309	-1.01497066507855	0.310926921408694	0.00337725762121573	0.509816607104393
**CESC**	JMJD8	cg27579097	-0.0785368868026288	309	-1.37358011438634	0.170584151293017	0.00616804258864893	0.344417946163741
**CESC**	KAT2B	cg00770008	-0.0514477237658338	309	-0.898211226125059	0.369783786364131	0.00264686828068554	0.568444104325943
**CESC**	KAT2B	cg04371528	-0.0504668406086759	309	-0.88104211210537	0.378991045950761	0.0025469020010215	0.57753669884053
**CESC**	KAT2B	cg05072819	0.0228037244191845	309	0.397699938506732	0.691130497954553	0.000520009847386113	0.822498209332381
**CESC**	KAT2B	cg12062464	-0.0631482764167489	309	-1.10322969592154	0.270799977919215	0.00398770481440613	0.466993619523213
**CESC**	KAT2B	cg14937543	-0.174989377566812	309	-3.09885844187992	0.00212433676378433	0.0306212822612204	0.0124708439180686
**CESC**	KAT2B	cg18053505	-0.234807553468767	309	-4.21176255789228	3.34232278319988e-05	0.0551345871659878	0.000374442192678933
**CESC**	KAT5	cg00582417	-0.227619036100386	309	-4.07565846742497	5.86240239229834e-05	0.0518104255952687	0.000609216847056448
**CESC**	KAT5	cg02561205	-0.189634991400168	309	-3.36750355739577	0.00085611674634425	0.0359614299633418	0.00590118254165378
**CESC**	KAT5	cg05785768	-0.0766322682224568	309	-1.3400698288385	0.181223384716217	0.00587250453291856	0.358500547284598
**CESC**	KAT5	cg07564927	-0.0860133013325457	309	-1.50527170713574	0.1332925150467	0.00739828800612332	0.291192303160433
**CESC**	KAT5	cg14726348	-0.0784599589320125	307	-1.36770498633056	0.172421346758638	0.00615596515561308	0.346965680688623
**CESC**	KAT5	cg18896539	0.120306462916699	309	2.11296171902808	0.0354188983840076	0.014473645019527	0.111811783503855
**CESC**	KAT5	cg20108992	0.0775722449427609	309	1.35660612187014	0.175912961220764	0.00601745318545969	0.35172357562875
**CESC**	KAT5	cg20423942	-0.0354993227215991	309	-0.619342212376371	0.536154814789477	0.00126020191369224	0.712376219670923
**CESC**	KAT5	cg22048492	-0.0308841069977233	309	-0.53873979932508	0.590460684777083	0.000953828065046819	0.753404164600837
**CESC**	KAT6A	cg10163397	-0.0383697565973406	308	-0.668390527649233	0.504393187226779	0.00147223822133916	0.687663441003839
**CESC**	KAT6A	cg12468056	0.181160308015677	309	3.21178138663991	0.00146052869855324	0.0328190572003351	0.00919928504374148
**CESC**	KAT6A	cg15251374	-0.14536889722274	308	-2.5575890875419	0.0110267382604385	0.0211321162797555	0.0460027927405243
**CESC**	KAT6A	cg20966972	0.1256606843881	309	2.20847486008481	0.027957156346447	0.0157906076008856	0.0937052572518526
**CESC**	KAT6B	cg04396765	-0.0586152001146405	309	-1.0237511208071	0.30676679188775	0.00343574168447935	0.505231625026265
**CESC**	KAT6B	cg06972998	0.0194783991250064	308	0.33912279100961	0.73475213975375	0.00037940803247305	0.850460940112165
**CESC**	KAT6B	cg09631472	-0.114029832830101	309	-2.00123145183158	0.0462560745702564	0.0130028027752608	0.136294411980855
**CESC**	KAT6B	cg10359932	-0.0941271316008371	309	-1.64848157023306	0.100286920834592	0.00885991690340131	0.238717541634817
**CESC**	KAT6B	cg10739686	-0.150182010230743	309	-2.64855172841845	0.00850588565971422	0.0225546361969471	0.0376718968979111
**CESC**	KAT6B	cg11128436	-0.0301750010471558	309	-0.526358808240227	0.599022733600282	0.000910530688195851	0.759347138420054
**CESC**	KAT6B	cg11348552	0.0986015900354036	309	1.72759605923208	0.0850762785704242	0.0097222735575098	0.212158679091811
**CESC**	KAT6B	cg15874302	-0.312765672343277	309	-5.74129476392391	2.27556626147894e-08	0.0978223657963424	5.78514384789192e-07
**CESC**	KAT6B	cg15912797	-0.0598996709599689	309	-1.04626512076832	0.296269817628193	0.00358797058111255	0.494221382922003
**CESC**	KAT6B	cg17147776	-0.0830109944363924	309	-1.45235876921433	0.147433194446262	0.00689082519731878	0.312154251681225
**CESC**	KAT6B	cg18462764	-0.167479242138489	309	-2.96193592712238	0.00329823379202071	0.0280492965472825	0.0177590531295999

**CESC**	KAT6B	cg18491775	-0.0428241654366644	309	-0.747350438798472	0.455429720726928	0.0018339091453468	0.645749639015613
**CESC**	KAT6B	cg24671245	-0.113503295979103	309	-1.99186980789062	0.0472797006570321	0.0128829981981198	0.138538348647326
**CESC**	KAT6B	cg26605858	-0.0773695540490454	309	-1.35304002943906	0.177048189903791	0.00598604789374816	0.353215543041892
**CESC**	KDM1A	cg00062312	0.0900340152379458	309	1.57619812334113	0.116020185020097	0.00810612389986666	0.264524567809694
**CESC**	KDM1A	cg00541565	0.0390231968635055	309	0.680911333110306	0.496445903835085	0.00152280989344791	0.681173830060613
**CESC**	KDM1A	cg04886391	-0.0110837543848761	308	-0.192945602849855	0.847130834163227	0.00012284961126426	0.918362543379237
**CESC**	KDM1A	cg06958034	0.0946718947146406	309	1.65810823159001	0.0983270678790927	0.00896276764886	0.23521697166624
**CESC**	KDM1A	cg07118078	-0.00391996174509575	309	-0.068347393556133	0.945554029954935	1.53661000830141e-05	0.970914577312033
**CESC**	KDM1A	cg07785945	-0.00519966144386152	309	-0.0906604206752383	0.927822126258319	2.70364791307801e-05	0.961966281493482
**CESC**	KDM1A	cg10499166	0.138150480572797	309	2.43205631453404	0.0155905187440825	0.0190855552824948	0.0601522003261992
**CESC**	KDM1A	cg12578844	0.13960453045135	309	2.45816008854499	0.014522715917676	0.0194894249225419	0.0569244803798906
**CESC**	KDM1A	cg22683154	-0.364924454529268	309	-6.83396510593838	4.51977664925964e-11	0.133169857513484	1.90678690158625e-09
**CESC**	KDM1A	cg26662347	0.0749017329872562	309	1.30963520957586	0.191308136584785	0.00561026960449422	0.371805365680942
**CESC**	KDM1B	cg11007366	-0.130406538939857	309	-2.29329908212159	0.0225130844601729	0.0170058653982725	0.0795943984219925
**CESC**	KDM1B	cg15124342	-0.140642191422077	308	-2.47272152490522	0.0139574830891834	0.0197802260080042	0.0552293204191217
**CESC**	KDM1B	cg20677365	-0.0655497564374731	282	-1.0933167683861	0.275204627200842	0.00429677056901205	0.4717011761842
**CESC**	KDM1B	cg22406543	0.127357487726196	309	2.23878436463617	0.0258933452339271	0.0162199296799282	0.0884532955310289
**CESC**	KDM1B	cg23073439	-0.118466512580015	309	-2.08018277303501	0.0383461808607428	0.0140343146028709	0.118645459725595
**CESC**	KDM1B	cg23860886	-0.0376039456981785	308	-0.655031230828709	0.512944606862603	0.00141405673207156	0.694711038125812
**CESC**	KDM1B	cg24342628	0.0911589311176996	309	1.59605568075305	0.111515346061662	0.0083099507225215	0.257363973879226
**CESC**	KDM2A	cg05038268	-0.0272681053776439	309	-0.475612516514125	0.634692031935219	0.000743549570886293	0.784791150863829
**CESC**	KDM2A	cg05295919	-0.145303246720861	309	-2.56062415063793	0.010931340491327	0.0211130335076234	0.0457143335461269
**CESC**	KDM2A	cg06320126	-0.0647257277232133	309	-1.13090302485354	0.258987295574176	0.00418941982929955	0.453714760885859
**CESC**	KDM2A	cg07283932	-0.103816263153171	309	-1.81993241702813	0.0697526320577169	0.0107778164950885	0.183881269342529
**CESC**	KDM2A	cg08836144	0.00263518244645243	303	0.0454904600432818	0.963746847189406	6.94418652609104e-06	0.980933420081546
**CESC**	KDM2A	cg09993857	-0.0293185033641017	309	-0.511405416176017	0.60943844388079	0.000859574639510842	0.766964124227743
**CESC**	KDM2A	cg11637544	0.00341777626605686	309	0.0595913134713756	0.95252029109384	1.16811946048216e-05	0.974978817805479
**CESC**	KDM2A	cg16004548	-0.224475872147784	308	-4.00975842784423	7.66013222461094e-05	0.0503894171765082	0.000765757294116402
**CESC**	KDM2A	cg18132081	0.0651338753325739	309	1.1380645622364	0.255989638895963	0.00424242171583928	0.450237570217101
**CESC**	KDM2A	cg21293464	-0.050875926089961	309	-0.8882023203455	0.375134145128739	0.00258835985551117	0.573862148231116
**CESC**	KDM2A	cg21711868	-0.156946054939776	309	-2.77078583096645	0.00593648860653305	0.0246320641611591	0.0284391400121086
**CESC**	KDM2A	cg25597833	-0.157525390291732	309	-2.78127340207245	0.00575271522006367	0.0248142485865626	0.0277312385567997
**CESC**	KDM2B	cg03486832	-0.19003500826038	309	-3.37487283256436	0.000834329614289863	0.0361133043645228	0.00577351343302487
**CESC**	KDM2B	cg05067991	-0.0209223961952576	309	-0.364874312685369	0.715458930629223	0.00043774666255133	0.838241721251371
**CESC**	KDM2B	cg07020001	-0.0437388441889844	309	-0.763343327366469	0.445850643232712	0.00191308649098825	0.637770390917284
**CESC**	KDM2B	cg07529210	-0.0892755655223974	309	-1.56281304085683	0.11913693975069	0.00797012659934387	0.269375663865756
**CESC**	KDM2B	cg07793808	-0.0535735904961852	309	-0.935430836530751	0.350308775044094	0.00287012959865294	0.549334210441256
**CESC**	KDM2B	cg09151742	-0.0515834762739452	309	-0.900587605565281	0.368520504308929	0.00266085502450467	0.56735966974488
**CESC**	KDM2B	cg12251659	-0.0621410291774765	309	-1.0855638495102	0.27853207830205	0.00386150750723598	0.475459691032622
**CESC**	KDM2B	cg12888660	-0.0913632908496312	309	-1.59966379623986	0.11071190866266	0.00834725091487431	0.256132820511003
**CESC**	KDM2B	cg15234492	-0.0783057906710397	309	-1.36951335810564	0.171849662395266	0.00613179685261668	0.346128641165526
**CESC**	KDM2B	cg15316703	-0.0493373993033486	309	-0.861275839253748	0.389764948225044	0.00243417897001806	0.587361726017842
**CESC**	KDM2B	cg16837338	-0.0505563106086062	299	-0.867970099559817	0.386118625292033	0.00255594054235387	0.584163989419845
**CESC**	KDM2B	cg17452615	-0.106160667467881	309	-1.86149380635241	0.06363946435495	0.011270087317226	0.171834861707215
**CESC**	KDM2B	cg20055546	-0.0363108954986088	309	-0.633519875867221	0.526870535795271	0.00131848113191089	0.705535278370526
**CESC**	KDM2B	cg21249371	-0.096651784911376	309	-1.69310815000318	0.0914593023549396	0.00934156752655489	0.223383409230485
**CESC**	KDM2B	cg21781916	-0.187459648236231	309	-3.32745882733179	0.000984069079598534	0.035141197168516	0.00662753614217716
**CESC**	KDM2B	cg26218726	-0.200827670008009	309	-3.57437224189609	0.000408159856662525	0.0403317530408457	0.00315692860302292
**CESC**	KDM2B	cg26509318	-0.170677618613623	309	-3.02018131062176	0.00274059186785728	0.0291308494956172	0.0153261162941621
**CESC**	KDM3A	cg01744130	-0.101140398957033	309	-1.77253237062904	0.0773076562042179	0.0102293803011879	0.198124585957863
**CESC**	KDM3A	cg02152982	0.061257159077799	309	1.07006462732524	0.285439082475823	0.00375243953828277	0.482670086711794
**CESC**	KDM3A	cg02972551	0.104754807769031	309	1.83656712989164	0.0672500838735162	0.0109735697507267	0.179000685149222
**CESC**	KDM3A	cg03037033	-0.110866894948418	309	-1.94502090581361	0.0526942790119708	0.0122914683955036	0.149877212583295
**CESC**	KDM3A	cg04793821	-0.116626498017773	309	-2.04742441666886	0.0414756689982152	0.0136017400398897	0.125683943364255
**CESC**	KDM3A	cg07052513	-0.106670573531829	309	-1.87053750709863	0.0623699217147718	0.0113786112576092	0.169348563396704
**CESC**	KDM3A	cg07211384	-0.0393834003663116	309	-0.687206202170604	0.492476710360115	0.00155105222441319	0.677963779963808
**CESC**	KDM3A	cg12888358	-0.0266129563443034	308	-0.463413076756947	0.643401001296929	0.000708249445383796	0.790710059791999

**CESC**	KDM3A	cg18611949	-0.0656784802011022	309	-1.14762133264633	0.252027248044069	0.00431366276152657	0.445845985992499
**CESC**	KDM3A	cg19139691	-0.077914436969188	308	-1.36038393744787	0.174719615724779	0.00607065948822557	0.350150388626241
**CESC**	KDM3A	cg20191795	0.00828626585973935	309	0.144480942282622	0.885216440271769	6.86622018982819e-05	0.939168465796141
**CESC**	KDM3A	cg20918903	-0.121647534358666	309	-2.13686697516732	0.0334068234837941	0.0147981226155429	0.106917777845817
**CESC**	KDM3A	cg22404242	0.0120491198926781	309	0.210098835646759	0.833731274745201	0.000145181290188132	0.910811347482481
**CESC**	KDM3A	cg24749656	-0.145948789387533	309	-2.57224734353513	0.0105787417697892	0.0213010491236865	0.0445326102402046
**CESC**	KDM3B	cg00435490	-0.0786609526055746	309	-1.3757634768325	0.169907624068892	0.00618754546481645	0.343578116160625
**CESC**	KDM3B	cg03315484	-0.0920751678520956	309	-1.61223408239328	0.107948646936289	0.00847783653499158	0.251611548714475
**CESC**	KDM3B	cg05191700	-0.285088628603153	309	-5.18589887550142	3.93137499086259e-07	0.0812755261588264	7.41963576029025e-06
**CESC**	KDM3B	cg05708550	-0.0794393363992694	309	-1.38946327579373	0.165708721946012	0.00631060816755628	0.33786251644786
**CESC**	KDM3B	cg05722427	-0.0469609289962517	309	-0.819696123785433	0.413031547296594	0.00220532885219099	0.608611723457004
**CESC**	KDM3B	cg15001364	-0.0923894415579163	309	-1.61778430283695	0.106746162457862	0.00853580891138364	0.249480591307064
**CESC**	KDM3B	cg18682923	-0.00244594240876649	309	-0.0426465906958025	0.966011251857982	5.9826342670024e-06	0.982158783598508
**CESC**	KDM3B	cg19076659	-0.0468388590350861	309	-0.817560719605396	0.414248358523486	0.00219387871570866	0.609844952084024
**CESC**	KDM3B	cg24137987	-0.252503162085757	309	-4.54998031301679	7.7639669228072e-06	0.0637578468633059	0.000104768437605007
**CESC**	KDM4A	cg00154982	-0.053946150894092	309	-0.941954911776236	0.346963652737819	0.00291018719628814	0.546247507760276
**CESC**	KDM4A	cg00979347	-0.0723167068641744	309	-1.26419488572689	0.207128514947544	0.00522970609167892	0.392009654058211
**CESC**	KDM4A	cg03587511	0.0268424109291495	309	0.468182123084128	0.639989846527427	0.000720515024489327	0.788485978188472
**CESC**	KDM4A	cg08552254	-0.187411160559416	309	-3.32656682429219	0.000987112083436059	0.0351229431022273	0.00664421823975661
**CESC**	KDM4A	cg10958452	-0.13549429738964	308	-2.38048758900442	0.0179078603587626	0.0183587046251122	0.0668710261963988
**CESC**	KDM4A	cg10968649	-0.0371304859126771	309	-0.647838876277859	0.517578131157114	0.00137867298411151	0.698453946975376
**CESC**	KDM4A	cg12293275	-0.0426211913477833	309	-0.743801753312223	0.457570910839243	0.00181656595190436	0.647733279732381
**CESC**	KDM4A	cg17737314	-0.178147789282612	309	-3.15660673630123	0.00175627394114579	0.031736634826282	0.0106940806964226
**CESC**	KDM4A	cg19743044	-0.0872312166887723	309	-1.52674805605526	0.127863396758265	0.00760928516500355	0.282794253920864
**CESC**	KDM4A	cg20873046	-0.164951564660296	309	-2.91597276527154	0.00380960968236698	0.0272090186838797	0.0199720180355857
**CESC**	KDM4A	cg21451504	-0.157283473915052	309	-2.77689368962631	0.00582882459892556	0.0247380911667867	0.0280321204777952
**CESC**	KDM4A	cg22094309	-0.15038779874599	309	-2.65226485536091	0.00841503373659396	0.0226164900116645	0.037351029373395
**CESC**	KDM4A	cg24324755	-0.104133226932714	309	-1.82554971356472	0.0688991029113659	0.0108437289514202	0.18219303211825
**CESC**	KDM4A	cg24505556	-0.114493336580928	309	-2.00947380271875	0.0453704187964861	0.0131087241214336	0.134428182505098
**CESC**	KDM4A	cg25841161	-0.103256042974908	309	-1.81000545092103	0.0712823223936527	0.0106618104108361	0.186824910325848
**CESC**	KDM4B	cg01098771	-0.180279202952602	309	-3.19563424812732	0.00154191730850349	0.0325005910172253	0.0096138426783458
**CESC**	KDM4B	cg12072803	-0.186095969127176	309	-3.30238150596752	0.0010730321782645	0.0346317097253829	0.0071186034095588
**CESC**	KDM4B	cg18115693	-0.131387057240511	309	-2.31084398897303	0.0215104537555962	0.0172625588103214	0.0770157518022164
**CESC**	KDM4C	cg00121158	0.337291436297641	309	6.24694645510649	1.40962412397799e-09	0.113765512999726	4.5351653887818e-08
**CESC**	KDM4C	cg00667760	0.200215970532743	309	3.56302971243972	0.000425468382682499	0.0400864348563684	0.00327119251874546
**CESC**	KDM4C	cg05545111	0.205091995352807	309	3.65356607645247	0.000304518590833136	0.0420627265577959	0.00247567345127735
**CESC**	KDM4C	cg14196303	0.0635479288205372	309	1.11024002998052	0.267773011192826	0.00403833925738006	0.46349086936487
**CESC**	KDM4C	cg14473811	0.257604353489144	309	4.64836546353094	4.99329517730258e-06	0.0663600029365599	7.11965551728514e-05
**CESC**	KDM4C	cg14497348	0.108211588179775	309	1.89787806742196	0.0586593105341682	0.0117097478163891	0.161975735764565
**CESC**	KDM4E	cg15555590	-0.0994065841688575	309	-1.74184053109738	0.0825477907756423	0.00988166897612015	0.207574193864962
**CESC**	KDM4E	cg19409026	-0.0128929380660575	309	-0.224814742473526	0.822274433861442	0.000166227851975194	0.904546729411957
**CESC**	KDM5B	cg00528052	-0.0961698585191956	309	-1.68458694499216	0.093094601613433	0.0092486416876021	0.226269298525804
**CESC**	KDM5B	cg00571448	-0.137932316561811	309	-2.42814112129972	0.0157565228532398	0.0190253239521077	0.0606003331608708
**CESC**	KDM5B	cg02624558	-0.082599053478712	309	-1.445101823377	0.149459276396507	0.00682260363557912	0.314923024628042
**CESC**	KDM5B	cg04504182	-0.0799350327822079	309	-1.39818901907064	0.163075529903212	0.00638960946589265	0.333876300057648
**CESC**	KDM5B	cg08915090	-0.0735851759849885	309	-1.28648913782817	0.199251260210398	0.00541477812474173	0.38199816483482
**CESC**	KDM5B	cg09889216	-0.150384072620008	309	-2.65219762020108	0.00841667104681054	0.0226153692977798	0.0373533291925141
**CESC**	KDM5B	cg11171004	0.096117774026513	309	1.683666082206	0.0932727269956089	0.00923862648381183	0.22657481729575
**CESC**	KDM5B	cg11221497	-0.176357842537418	309	-3.12386717190758	0.00195698677555748	0.0311020886244528	0.0116848437499208
**CESC**	KDM5B	cg13134514	0.00141950381482105	309	0.024749919650548	0.980270675182814	2.01499108029152e-06	0.989801346921421
**CESC**	KDM5B	cg13253785	-0.292656865626166	309	-5.33628245179008	1.85781804124167e-07	0.0856480409981316	3.79734883605765e-06
**CESC**	KDM5B	cg13993945	-0.167420127312095	309	-2.9608603037815	0.00330944530461516	0.0280294990291982	0.0178083273173709
**CESC**	KDM5B	cg14560895	-0.178619948229458	309	-3.16524828159619	0.00170656474521108	0.0319050859054941	0.0104486461348143
**CESC**	KDM5B	cg21105875	-0.123699226892373	299	-2.1374157836309	0.0333890162277421	0.0153014987337709	0.106887170421833
**CESC**	KDM5B	cg24354901	0.103874944423739	308	1.81797484873978	0.0700553691441629	0.0107900040790349	0.184479781135755
**CESC**	KDM5B	cg27531483	-0.181829848611346	309	-3.22405673759492	0.00140133779243183	0.0330620938460251	0.00888545702375432
**CESC**	KDM5C	cg01859586	-0.0749441210380644	309	-1.31038053746579	0.191056308046704	0.00561662127816805	0.371482989957142

**CESC**	KDM5C	cg04927657	-0.0385803523442081	308	-0.672064500780042	0.502054756481335	0.00148844358700324	0.685766567278287
**CESC**	KDM5C	cg04927982	-0.165348031946451	309	-2.92317817495253	0.00372493139984784	0.0273399716685646	0.0195967615689296
**CESC**	KDM5C	cg09554224	-0.0583952930730363	309	-1.01989713996253	0.308588201589	0.0034100102530858	0.507172470205501
**CESC**	KDM5C	cg12234996	-0.124522677122094	309	-2.18815801755195	0.0294190346666146	0.0155058971176533	0.0972765071133004
**CESC**	KDM5C	cg16417926	-0.109988512800069	309	-1.92942132130221	0.0546090503925244	0.012097472947971	0.15388561447817
**CESC**	KDM5C	cg16824069	-0.16020968733335	309	-2.82990522289887	0.00496612138277589	0.0256671439154497	0.0246323926837329
**CESC**	KDM5C	cg19903753	-0.128540806254386	309	-2.25993344770966	0.0245328924622443	0.0165227388725276	0.0849700296606489
**CESC**	KDM5C	cg24985300	-0.230874864721215	309	-4.13721406011605	4.55517827636598e-05	0.0533032031600391	0.000489455164132064
**CESC**	KDM5C	cg27526317	-0.109489093316452	309	-1.92055394859013	0.0557232410788288	0.0119878615552587	0.155983369309719
**CESC**	KDM6A	cg00751785	-0.100029323117361	309	-1.75286234277191	0.0806336421755136	0.0100058654833173	0.204139988264837
**CESC**	KDM6A	cg03267667	0.137870171276638	309	2.42702592173473	0.0158040923084988	0.0190081841278495	0.0607472482359481
**CESC**	KDM6A	cg03478540	-0.00626303989188029	309	-0.109201973653503	0.913114341779971	3.92256686872839e-05	0.954076572171153
**CESC**	KDM6A	cg06877198	0.0462087926875332	309	0.806539373275813	0.42056242290396	0.00213525252163943	0.615729254505125
**CESC**	KDM6A	cg07167981	-0.161746091284958	309	-2.8577694065836	0.00456078264290953	0.0261617980459619	0.0230330774423853
**CESC**	KDM6A	cg11895950	-0.125250437655018	309	-2.20114970907522	0.0284768096745995	0.0156876721327736	0.0949642031818328
**CESC**	KDM6A	cg14384228	0.0642783056011595	309	1.12305301431134	0.26230111166876	0.00413170057095605	0.45768555269064
**CESC**	KDM6A	cg15923127	-0.0106459533263577	309	-0.185629058384434	0.852859458169719	0.000113336322226987	0.921606154781224
**CESC**	KDM6A	cg17824914	-0.0432338202193611	309	-0.754512896812221	0.451125380101233	0.00186916321076004	0.641777936511774
**CESC**	KDM6A	cg20211377	0.0969136678016725	309	1.69773914877868	0.0905803489358031	0.00939225900677293	0.221786719914134
**CESC**	KDM6A	cg22133177	0.00116720105196711	309	0.020350859591862	0.983776834654165	1.36235829571313e-06	0.991486957584084
**CESC**	KDM6A	cg27306636	-0.127227573194468	309	-2.23646303646803	0.0260465885346739	0.0161868553809537	0.0888682468543789
**CESC**	KDM6B	cg01046511	-0.0490509785965406	309	-0.856263740116117	0.392526335262222	0.00240599850127829	0.589430520910073
**CESC**	KDM6B	cg02308232	-0.0280622447113588	309	-0.489474721381631	0.624858641643825	0.000787489578240185	0.777876064841973
**CESC**	KDM6B	cg24893073	-0.150663614132548	309	-2.65724205928718	0.00829462099736855	0.0226995246234814	0.0369481852101877
**CESC**	KDM8	cg00636124	-0.0415742777861913	309	-0.725499557420796	0.468703731126438	0.0017284205734434	0.657663832071953
**CESC**	KDM8	cg01755539	-0.0464641349325728	309	-0.811005794320957	0.417996819882022	0.00215891583503233	0.613401580885498
**CESC**	KDM8	cg06329197	-0.00522292259768362	309	-0.091066009272634	0.927500115798889	2.72789204613942e-05	0.961757647767468
**CESC**	KDM8	cg08572679	-0.0682862948062222	309	-1.19339790234227	0.233644072127446	0.00466301805836229	0.424526613268306
**CESC**	KDM8	cg09140090	-0.0748021433319881	308	-1.30573121148637	0.192634470644232	0.0055953606470593	0.373357068346639
**CESC**	KDM8	cg10214171	0.156391141152762	309	2.76074310758451	0.00611743348593595	0.0244581890310633	0.0291315686948332
**CESC**	KDM8	cg10221365	0.0574843859587044	309	1.00393461766598	0.316208551993089	0.0033044546290493	0.515474459257106
**CESC**	KDM8	cg16752029	-0.0186927464606105	309	-0.325976127260687	0.744666692077747	0.000349418770240666	0.856531801987788
**CESC**	KDM8	cg24206694	0.279137442099773	309	5.06839024365558	6.98061504972646e-07	0.0779177115820043	1.24296170102637e-05
**CESC**	KDM8	cg24705286	0.0716136911245376	309	1.25184154430177	0.211589939403484	0.00512852075648068	0.397652503888356
**CESC**	KIAA2026	cg03495688	0.0602090399166745	309	1.05168846558738	0.293777757347232	0.0036251284876877	0.491508169277615
**CESC**	KIAA2026	cg25608569	-0.184585387325499	309	-3.27462579468398	0.00118018190226612	0.0340717652141044	0.00770744383016207
**CESC**	KMT2B	cg09284703	-0.143698713235633	309	-2.5317484715405	0.0118531358576621	0.0206493201855768	0.0487018593766444
**CESC**	KMT2B	cg13830730	0.122094586112752	309	2.1448385247938	0.03275808759269	0.0149070879580443	0.105398269537978
**CESC**	KMT2B	cg15496871	0.127835359883368	309	2.24732404945991	0.0253363177280003	0.0163418792365103	0.0870742843846994
**CESC**	KMT2B	cg24471867	-0.0491552753083172	309	-0.858088812138854	0.391519445288999	0.00241624109063646	0.588580500247592
**CESC**	L3MBTL1	cg01071811	-0.291759845053392	309	-5.31840184302447	2.03275693661513e-07	0.0851238071855793	4.11277745453933e-06
**CESC**	L3MBTL1	cg01877937	-0.297301510072029	309	-5.42911367437732	1.160065608161e-07	0.0883881878911087	2.4840667195507e-06
**CESC**	L3MBTL1	cg02177317	-0.182961753405759	309	-3.24481962685492	0.00130625500225209	0.0334750032093099	0.00838093209444938
**CESC**	L3MBTL1	cg02472486	-0.217798160176769	309	-3.89084495624581	0.000122772368905734	0.0474360385763853	0.00114905602341827
**CESC**	L3MBTL1	cg02611863	-0.352542279880262	309	-6.56851005117559	2.20046624919733e-10	0.124286059103173	8.20628338399123e-09
**CESC**	L3MBTL1	cg03486259	-0.135545745860461	309	-2.38533487703877	0.0176759785192786	0.0183726492208685	0.0662149117796868
**CESC**	L3MBTL1	cg04984575	-0.236142891102763	309	-4.23712491455773	3.0051531013106e-05	0.0557634650183714	0.000341438411513593
**CESC**	L3MBTL1	cg06362543	-0.151633322834433	309	-2.67474590707692	0.00788336031802871	0.0229926645938113	0.0355153965297818
**CESC**	L3MBTL1	cg06446163	-0.291045091107811	309	-5.30416539894718	2.1833852115986e-07	0.0847072450579538	4.37881591005675e-06
**CESC**	L3MBTL1	cg06908806	-0.0861468126089232	309	-1.50762567077657	0.132688835054995	0.00742127332267692	0.290199472428122
**CESC**	L3MBTL1	cg07678583	-0.217193581322458	309	-3.87950898935766	0.000128351623313789	0.0471730517676753	0.00119366242487422
**CESC**	L3MBTL1	cg08369943	-0.0441265233963242	309	-0.770122362097309	0.441825347159902	0.00194715006704635	0.634322652699085
**CESC**	L3MBTL1	cg08633313	-0.276903305194124	309	-5.02444084755632	8.62996648645434e-07	0.07667544042743	1.50326827775913e-05
**CESC**	L3MBTL1	cg09541000	-0.212257860849429	309	-3.78713713351294	0.000183647366227307	0.0450533994923758	0.0016143122269251
**CESC**	L3MBTL1	cg10360552	-0.276613837248117	309	-5.01875298410223	8.869207556968e-07	0.0765152149571279	1.53908349918976e-05
**CESC**	L3MBTL1	cg12699433	-0.106477530672956	309	-1.86711352118914	0.0628480776764082	0.0113374645382103	0.170280133004121
**CESC**	L3MBTL1	cg14306330	-0.035615357555824	308	-0.620346361130387	0.5354960650904	0.00126845369382919	0.711874249997806

**CESC**	L3MBTL1	cg15123011	-0.126078413279097	309	-2.21593479144259	0.0274364047180387	0.0158957662949748	0.092353756593618
**CESC**	L3MBTL1	cg15302378	-0.341824857802511	309	-6.34193489159726	8.20116715484196e-10	0.116844233411707	2.76191167811158e-08
**CESC**	L3MBTL1	cg15330298	-0.331136695996087	309	-6.11876945609222	2.90010719630574e-09	0.109651511435205	8.78154040425054e-08
**CESC**	L3MBTL1	cg15388309	-0.339348063204976	309	-6.28997737632048	1.10372540840904e-09	0.115157108000968	3.62444974342646e-08
**CESC**	L3MBTL1	cg16862791	-0.298690233160809	309	-5.45695136172259	1.00606534230738e-07	0.0892158553856583	2.1849761931731e-06
**CESC**	L3MBTL1	cg17091610	-0.376535987301393	309	-7.08669597828394	9.62002667525741e-12	0.141779349733035	4.57724987874603e-10
**CESC**	L3MBTL1	cg17774347	-0.119791027204747	309	-2.10377695496525	0.0362191481467598	0.0143498901987683	0.113765591752293
**CESC**	L3MBTL1	cg18911945	-0.279466220092947	309	-5.07486539436203	6.76502409904193e-07	0.0781013681730395	1.21011332435242e-05
**CESC**	L3MBTL1	cg20091959	-0.209000117930006	309	-3.72633539730537	0.000231604058661218	0.0436810492947565	0.0019631080997484
**CESC**	L3MBTL1	cg20252111	-0.236039258695632	309	-4.23515569429298	3.03012298952687e-05	0.0557145316455834	0.000344074423948698
**CESC**	L3MBTL1	cg20529070	-0.282436687582226	309	-5.13345573366375	5.08598609383084e-07	0.0797704824924199	9.36914994514507e-06
**CESC**	L3MBTL1	cg22330467	-0.0795965423660042	306	-1.38534386948498	0.166973076257154	0.0063356095566231	0.339639259886234
**CESC**	L3MBTL1	cg22457903	-0.335172529386651	309	-6.20271799268529	1.81029436040717e-09	0.112340624455445	5.70393031484469e-08
**CESC**	L3MBTL1	cg22601123	-0.0454974973942897	309	-0.79409829966388	0.427757642077559	0.0020700222691434	0.622046097049903
**CESC**	L3MBTL1	cg23626798	-0.277742411221343	309	-5.04093713393787	7.97089592339358e-07	0.0771408469910455	1.3978536462229e-05
**CESC**	L3MBTL1	cg24343973	-0.179047045455752	309	-3.17306704397613	0.00166271234914308	0.0320578444864341	0.0102284552023752
**CESC**	L3MBTL2	cg02358112	-0.167650983280178	309	-2.9650610281748	0.00326585697702754	0.0281068521948104	0.0176310949112339
**CESC**	L3MBTL2	cg06235991	-0.0398768076263494	309	-0.695829358579218	0.487067303716585	0.00159015978646888	0.673400757541196
**CESC**	L3MBTL2	cg08053598	-0.03027732110859	309	-0.528145267133099	0.597783825146408	0.000916716173512668	0.758518241912927
**CESC**	L3MBTL2	cg09993119	0.0260137957789643	309	0.453719573738743	0.650354401849992	0.000676717570829661	0.795465490186616
**CESC**	L3MBTL2	cg12821546	0.0217287433325644	309	0.378943052750472	0.704994680099269	0.000472138286812461	0.831604843636347
**CESC**	L3MBTL2	cg13843727	-0.0422004774437376	309	-0.736446519952542	0.462026912175663	0.0017808802964794	0.651505766024281
**CESC**	L3MBTL2	cg14679202	0.0481195541501408	309	0.83996612579111	0.401587481727578	0.00231549149160834	0.598167583547431
**CESC**	L3MBTL2	cg15283028	-0.0499187136610205	309	-0.871448962444081	0.384196746595707	0.00249187797357095	0.582354922835111
**CESC**	L3MBTL2	cg19467356	0.0245693673589506	309	0.428510913345276	0.668582648547458	0.000603653812419066	0.807869014482548
**CESC**	L3MBTL2	cg24240981	-0.0583980009489205	308	-1.01826567365025	0.309364071036174	0.00341032651483012	0.507982112463603
**CESC**	L3MBTL2	cg26947834	0.0822169754429762	309	1.43837162359671	0.15135731425309	0.00675963105099095	0.317661106691033
**CESC**	L3MBTL2	cg27177954	-0.113185993301686	309	-1.98622909707112	0.0479056647491433	0.0128110690796892	0.139856372880429
**CESC**	L3MBTL3	cg03454037	0.0185278074685996	309	0.323098822989485	0.746842529132281	0.000343279649593495	0.857811760316905
**CESC**	L3MBTL3	cg07826275	-0.125604606499554	309	-2.20747349481767	0.028027705887148	0.0157765171739077	0.0938729647300464
**CESC**	L3MBTL3	cg08106148	-0.141436239915512	309	-2.49106688741662	0.0132692742205008	0.0200042099614382	0.0531577279319038
**CESC**	L3MBTL3	cg08358392	-0.300063641822693	309	-5.48451949546067	8.73234296911963e-08	0.0900381891438973	1.92116484428646e-06
**CESC**	L3MBTL3	cg09710740	-0.142940134006917	309	-2.51810395912143	0.0123123332631197	0.0204318819099153	0.0501673089947087
**CESC**	L3MBTL3	cg10237990	-0.117867058476717	309	-2.06950813420381	0.0393430340231242	0.0138926434739538	0.121031793293061
**CESC**	L3MBTL3	cg16487825	-0.126687922630278	309	-2.22682174748625	0.0266915236125512	0.0160498297403753	0.0904737379473167
**CESC**	L3MBTL3	cg16800851	-0.16805025715572	308	-2.96743479688034	0.00324226173202272	0.0282408889301037	0.017537790931788
**CESC**	L3MBTL3	cg18411550	-0.142636963109849	309	-2.51265211361598	0.0125001958678224	0.0203453032452005	0.0507543332082986
**CESC**	L3MBTL3	cg18798995	-0.139313389453976	309	-2.45293209852152	0.0147312307288827	0.0194082204811553	0.0575648665522756
**CESC**	L3MBTL3	cg22934935	0.0267561590664214	309	0.466676648909553	0.641065500706816	0.000715892047987646	0.789098885474462
**CESC**	L3MBTL3	cg24516147	-0.138848222646917	309	-2.44458048986611	0.0150698330403181	0.0192788289322079	0.0585819803886922
**CESC**	LBR	cg01017344	-0.0533093539451864	309	-0.930803907255042	0.352693581136235	0.00284188721805316	0.551683847084736
**CESC**	LBR	cg05530459	0.167319717409781	309	2.95903337151904	0.00332856788324395	0.0279958878340889	0.0178976092002405
**CESC**	LBR	cg12994003	0.0171154402124883	309	0.298461615661953	0.765554768992446	0.000292938293667263	0.869983167275306
**CESC**	LBR	cg14607642	-0.0937500819311089	309	-1.64181949547586	0.101661457866516	0.00878907786208963	0.241088021377506
**CESC**	LBR	cg18107727	-0.0668256709453476	309	-1.16775570940504	0.243820368433899	0.00446567029729587	0.436512133614405
**CESC**	LBR	cg18289048	-0.00601461888509568	309	-0.104870360509551	0.916547835852806	3.61756403329496e-05	0.955930798674512
**CESC**	LBR	cg18764107	0.0288363840154492	309	0.502988705644668	0.615336425549555	0.000831537043086456	0.771130722960228
**CESC**	LBR	cg19322477	-0.148207074218695	309	-2.61293497503556	0.00942315508644205	0.0219653368484659	0.0406693440662787
**CESC**	LBR	cg20483690	-0.166133262372777	308	-2.93261796601938	0.00361745539780535	0.0276002608666219	0.0191192557386282
**CESC**	LBR	cg26930584	-0.202970337054132	306	-3.59626030218837	0.000377174821734867	0.0411969577238681	0.00296084138062989
**CESC**	MBD1	cg01719254	-0.197165395260098	309	-3.50652844523291	0.000522431534124471	0.0388741930880708	0.00389105205990226
**CESC**	MBD1	cg03841029	-0.276149533147815	309	-5.00963278840417	9.26627094138128e-07	0.0762585646577559	1.60226779642991e-05
**CESC**	MBD1	cg05549078	-0.0126569536472066	309	-0.220699206071248	0.82547479887504	0.000160198475627536	0.906186837758226
**CESC**	MBD1	cg12090413	-0.0507735265394045	309	-0.886409982836486	0.376097307199183	0.00257795099724761	0.574634965752479
**CESC**	MBD1	cg13478031	-0.0925916560351388	309	-1.62135576267964	0.105978044471882	0.00857321476732945	0.248241031686298
**CESC**	MBD1	cg14389183	-0.137891386286536	309	-2.42740662262873	0.0157878390503479	0.0190140344120228	0.0607027702803719
**CESC**	MBD1	cg19328682	-0.345014126276537	309	-6.40905914524728	5.57322107877311e-10	0.119034747330362	1.92609133985818e-08

CESC	MBD1	cg21282183	0.0571606592081951	309	0.998262314114667	0.318946095708308	0.00326734096111542	0.518322384991271
CESC	MBD2	cg15090185	0.0194737812525037	309	0.339601377421322	0.734391238747555	0.000379228156270364	0.850268365685309
CESC	MBD2	cg16852837	-0.181023602181655	309	-3.20927559146657	0.00147289255394573	0.0327695445468219	0.00926066461987019
CESC	MBD2	cg17987217	0.000810374116752249	309	0.0141293601649564	0.988736044914993	6.56706209101988e-07	0.994064539567533
CESC	MBD2	cg22224310	-0.158872189358188	308	-2.80104720596446	0.00542127491341481	0.0252403725514641	0.0264597942539803
CESC	MBD2	cg22836370	0.14621770835631	309	2.57709030135313	0.0104348520440337	0.0213796182369711	0.044062795414078
CESC	MBD2	cg23388714	-0.16438315807488	309	-2.90564505895453	0.0039340487877289	0.0270218226586709	0.0205007181718908
CESC	MBD2	cg24881765	-0.131888571231254	308	-2.31600185788568	0.0212254351608116	0.0173945952214216	0.0761910069629098
CESC	MBD2	cg25192916	0.144719910297485	309	2.55012387403574	0.0112588591266207	0.0209438524365121	0.0467680311243235
CESC	MBD3	cg08660999	0.199664109628119	309	3.55280047359776	0.000441666462876732	0.0398657566735897	0.00337614236150462
CESC	MBD3	cg09174855	-0.113708320262953	309	-1.99551486648732	0.046878887071473	0.0129295820970222	0.137675870770439
CESC	MBD3	cg12286540	-0.115518429488703	309	-2.02770745919861	0.0434621065460605	0.0133445075515364	0.130172046368347
CESC	MBD5	cg03890220	0.24235700524446	309	4.35548835128079	1.81725637601579e-05	0.0587369179910633	0.000220651853466166
CESC	MBD5	cg04152151	0.147915954838548	309	2.60768753093844	0.00956554406061286	0.0218791296957994	0.0411355408873033
CESC	MBD5	cg05218490	-0.00343284011019269	309	-0.0598539651909619	0.952311271934505	1.17843912221478e-05	0.974867793363593
CESC	MBD5	cg06394585	0.00189052478285141	309	0.0329624848202306	0.973726135450518	3.57408395457538e-06	0.986273951638083
CESC	MBD5	cg06758980	0.214357167295633	309	3.82638798727041	0.000157849110183025	0.0459489951710078	0.00142175682269055
CESC	MBD5	cg20595490	0.146737781305955	309	2.58645794489386	0.0101614859653955	0.0215319764625944	0.0431803484564363
CESC	MBD5	cg21412502	-0.0672373120574173	309	-1.17498156388816	0.240921581674052	0.00452085613270652	0.433096557475476
CESC	MBD5	cg23120335	-0.0466368344642838	309	-0.814026727542333	0.416266798327506	0.00217499432884901	0.611855407721914
CESC	MBD5	cg25686162	0.166829640007742	309	2.95011789794636	0.00342334557111636	0.0278321287851128	0.0183126329513263
CESC	MBD5	cg26664634	-0.150944228528263	309	-2.66230651495531	0.00817368974057001	0.0227841601259925	0.0365313121726935
CESC	MBTD1	cg01388767	-0.0476547955372567	309	-0.831834828492605	0.406155110115619	0.00227097953769774	0.602473140051958
CESC	MBTD1	cg14577476	-0.0421309737161907	309	-0.735231441975351	0.462765375420597	0.00177501894627435	0.652133352935837
CESC	MBTD1	cg17045523	-0.105451996074831	309	-1.84892726603617	0.0654390864611582	0.0111201234761662	0.175444309552647
CESC	MBTD1	cg18711617	-0.117740102065531	309	-2.06724768241304	0.0395569402860818	0.0138627316344016	0.12151204788215
CESC	MBTD1	cg22141797	-0.0958884351489014	308	-1.67684670290033	0.0946037814538417	0.00919459199530507	0.22891466794464
CESC	MBTD1	cg25259117	-0.00285002108355239	309	-0.0496920173757619	0.960400450163884	8.12262017669311e-06	0.979228381196563
CESC	MBTD1	cg25716740	-0.134907636906126	309	-2.37389676490118	0.018222541672382	0.0182000704955952	0.0677663562723496
CESC	MBTD1	cg25855326	-0.0826076698758838	308	-1.44287458634082	0.15008876167572	0.006824027122323	0.315729837640977
CESC	MBTD1	cg27207041	-0.0971793983487684	309	-1.70243855133554	0.0896954061463969	0.00944383546342861	0.22032023343774
CESC	MBTD1	cg27214721	0.00511061314793945	309	0.0891077486876219	0.9290549542144	2.61183667478915e-05	0.962460435222498
CESC	MBTD1	cg27353579	-0.21059938884336	309	-3.75616744263782	0.000206762571992053	0.0443521025811969	0.00178423599839709
CESC	MLLT10	cg00879184	-0.0261136313369855	309	-0.455462041405187	0.649102007228189	0.000681921741603993	0.794720653285341
CESC	MLLT10	cg03524461	-0.053127366848816	309	-0.927617325858496	0.35434199337271	0.00282251710828867	0.553365224136135
CESC	MLLT10	cg04188362	-0.202895570477422	309	-3.61274895047122	0.00035437409601543	0.0411666125193584	0.00280963905681047
CESC	MLLT10	cg05913198	-0.23193978986352	309	-4.15737956017023	4.19106430256962e-05	0.053796066121934	0.000455718848144178
CESC	MLLT10	cg06398735	-0.140271483715142	309	-2.47013895535227	0.0140547793609121	0.0196760891436473	0.0555155661378761
CESC	MLLT10	cg11443787	-0.0658072751602373	309	-1.14988158730923	0.251096427619792	0.00433059746401519	0.444697798348452
CESC	MLLT10	cg15910013	0.107959737110799	309	1.89340880482606	0.0592529639894273	0.0116553048370328	0.163180582009116
CESC	MLLT10	cg19119538	-0.22761944761129	309	-4.07566623839869	5.86221679736192e-05	0.0518106129308688	0.000609216847056448
CESC	MLLT10	cg20569108	-0.212617736338817	309	-3.79386182820933	0.000178961651862194	0.0452063018058425	0.00158134288334479
CESC	MLLT10	cg23344688	-0.0561962516398987	309	-0.981365938949953	0.327192671792169	0.00315801869837482	0.52672847258919
CESC	MLLT10	cg26877631	0.0618437129655351	309	1.08034993703699	0.280842712872769	0.00382464483336349	0.478016094060618
CESC	MLLT10	cg27470303	-0.19950854199302	309	-3.54991752410355	0.000446335071204041	0.0398036583281805	0.0034064741027418
CESC	MLLT10	cg27572529	-0.164925013175592	309	-2.91549026966122	0.00381534230903607	0.027200259970969	0.0199926404141959
CESC	MLLT6	cg05652455	-0.0307818395648526	309	-0.53695415997097	0.591692041076201	0.000947521646996327	0.754238968917861
CESC	MORF4L1	cg03589001	0.107151609381559	309	1.87907053364289	0.0611914396199763	0.0114814673930582	0.166982140234195
CESC	MORF4L1	cg07757959	0.0299509278136666	309	0.522446655492296	0.601739886384169	0.000897058076899469	0.761315260235657
CESC	MORF4L1	cg08692282	0.0792520835411551	309	1.38616733408197	0.166711669258251	0.00628089274561423	0.339231712439549
CESC	MORF4L1	cg13747872	0.0249791031106511	309	0.435661482555734	0.663391531284102	0.000623955592212541	0.804347675499022
CESC	MORF4L1	cg15822222	0.127545880712568	309	2.24215080083483	0.0256725008909733	0.0162679516867447	0.0879139876756989
CESC	MORF4L1	cg20775702	-0.243541734165867	309	-4.37811854871706	1.64855568468028e-05	0.0593125762805178	0.000202347335017383
CESC	MORF4L1	cg20905746	0.152861782932301	309	2.69693173654047	0.00738850955667863	0.0233667246812419	0.0337848534915804
CESC	MORF4L1	cg23623458	-0.308135217034842	283	-5.40041088196132	1.42806237402595e-07	0.0949473119771094	2.99527323604678e-06
CESC	MORF4L1	cg24795825	0.140139502380421	302	2.43919095193995	0.015305553534685	0.0196390801274321	0.0593246327076165
CESC	MPHOSP	cg00331616	-0.224176091123095	309	-4.0107219774338	7.62489417602875e-05	0.0502549198312304	0.000762920922868142

CESC	MPHOSP	cg03134886	-0.0314410445698414	309	-0.548464500218015	0.583775546014204	0.000988539283642755	0.748668559621316
CESC	MPHOSP	cg03428000	-0.00492971332854078	309	-0.0859535333098268	0.931559938050029	2.43020735015926e-05	0.963732247571897
CESC	MPHOSP	cg04203901	-0.132834446986612	309	-2.33675549016818	0.0201011898763629	0.017644990306239	0.0731271962795443
CESC	MPHOSP	cg06019692	-0.292873069446353	309	-5.34059441602157	1.817871889359e-07	0.0857746348069281	3.72960962868978e-06
CESC	MPHOSP	cg09559995	-0.340599509682587	309	-6.31621128490609	9.50234684209048e-10	0.116008025996018	3.15503871205116e-08
CESC	MPHOSP	cg11051893	0.0775458055336649	309	1.35614094399093	0.176060735661824	0.00601335195586498	0.351897793697753
CESC	MPHOSP	cg19693579	-0.0876141708257134	309	-1.5335023736905	0.126192136415563	0.00767624292947728	0.280271100807693
CESC	MPHOSP	cg20759595	-0.137208678500173	309	-2.41515721604293	0.0163182762880092	0.0188262214557637	0.0622401092837535
CESC	MPHOSP	cg20952532	-0.10015578946787	309	-1.75510091531936	0.0802493314783609	0.0100311821639323	0.20349140903532
CESC	MPHOSP	cg21958444	-0.366911822117428	308	-6.86563431429157	3.75053997189907e-11	0.134624285209531	1.60224880051574e-09
CESC	MPHOSP	cg22656705	-0.385686756441212	308	-7.27660111384412	2.95222824514581e-12	0.148754274094143	1.55179489258698e-10
CESC	MSH6	cg00620552	-0.158970534629853	309	-2.80744737006283	0.00531634679034612	0.0252716308805011	0.0260407934900004
CESC	MSH6	cg02905881	-0.00925750190023628	309	-0.161416977985036	0.871872218042791	8.57013414328783e-05	0.932056975318238
CESC	MSH6	cg05039065	0.092029053024588	309	1.61141971392184	0.108125987258635	0.00846934660060243	0.25189569828402
CESC	MSH6	cg07652213	0.0355134399218737	309	0.619588820376026	0.535992618414511	0.00126120441508453	0.712258032812997
CESC	MSH6	cg09898070	-0.0451233511282306	309	-0.787554695471104	0.431570824422038	0.00203611681704159	0.625440028224442
CESC	MSH6	cg11869233	-0.0763215873312508	308	-1.33240816732753	0.183727281543926	0.00582498469276174	0.361991696394189
CESC	MSH6	cg16595246	-0.0477088580785991	309	-0.832780664895125	0.405622204799779	0.00227613513916391	0.601975882794293
CESC	MSH6	cg18222961	-0.0230009262333945	309	-0.401140976608875	0.688598143940109	0.000529042607594054	0.821053338386317
CESC	MSH6	cg19076255	-0.0232908160470491	309	-0.406199442695893	0.684881846907105	0.000542462112137481	0.818665482018785
CESC	MSH6	cg19736286	0.0906835123474204	309	1.58766261231629	0.113402160875381	0.00822349941166475	0.260528253001576
CESC	MSH6	cg21405109	-0.112703538421278	309	-1.97765364353502	0.0488707051910151	0.0127020875726765	0.14186528003395
CESC	MTA1	cg01318265	0.0121693060916208	309	0.212194814625696	0.832097254046319	0.00014809201075156	0.90994854024158
CESC	MTA1	cg01655777	-0.0181624244971748	309	-0.316724935727772	0.751669710807024	0.000329873663615575	0.860945133723816
CESC	MTA1	cg05922925	-0.134067106375718	309	-2.35883483177512	0.018964920017213	0.0179739890119581	0.0698360781889174
CESC	MTA1	cg13052453	-0.0125441964202919	309	-0.218732748322618	0.827005008783717	0.000157356863830863	0.907121482038906
CESC	MTA1	cg13981078	-0.00870666644448667	309	-0.151811670896431	0.879436174677641	7.58060405755502e-05	0.936074237181337
CESC	MTA1	cg16531924	-0.0571488146732355	309	-0.998054781512998	0.319046549016442	0.00326598701855582	0.518458450523007
CESC	MTA1	cg20222911	-0.13284746782653	309	-2.33698866101075	0.0200888848841328	0.0176484497079209	0.0730926855935752
CESC	MTA1	cg21517792	0.00350082040501398	309	0.0610392635016891	0.951368048600889	1.22557435081623e-05	0.974229631438141
CESC	MTA1	cg23123838	-0.0511997890651716	309	-0.893871199893082	0.372097912151497	0.00262141840031807	0.570766325773199
CESC	MTA1	cg23883989	-0.206686854825144	309	-3.68324011735015	0.000272501730792392	0.0427194559575102	0.00225329503032136
CESC	MTA1	cg23966123	-0.0532961896329999	309	-0.930573397947674	0.352812659292288	0.00284048382939669	0.551836822023529
CESC	MTA2	cg00501779	-0.0855958552615468	308	-1.49544645891816	0.13583873684792	0.00732665043795567	0.29495070117831
CESC	MTA2	cg07654871	0.0727452058549982	309	1.27172536672519	0.204442748709039	0.00529186497488606	0.3885760858312
CESC	MTA2	cg09086761	0.0524462240564422	309	0.915691383016082	0.360554567129149	0.00275060641777853	0.559524875229304
CESC	MTA2	cg13708959	-0.0981894973420803	309	-1.72030535470072	0.0863945748310115	0.0096411773882904	0.214590662656568
CESC	MTA2	cg19570317	-0.0771661436571083	309	-1.34946144801488	0.178192898975238	0.00595461372690947	0.354771417896291
CESC	MTA2	cg21002970	-0.00056794402546493	309	-0.0099024440474257	0.992105617079542	3.22560416061318e-07	0.995792124159238
CESC	MTF2	cg05396182	-0.185992871105344	309	-3.30048639503984	0.00108005030474973	0.0345933481020091	0.00715868053230811
CESC	MTF2	cg05962718	-0.148685764268747	308	-2.61724956609857	0.00930898373240365	0.0221074564961813	0.040329097633778
CESC	MTF2	cg09936581	-0.0576684837071252	309	-1.00716049922068	0.314658624364786	0.00332565401307897	0.513704917500875
CESC	MTF2	cg11404133	0.0352423874344011	309	0.614853972328313	0.53911110331483	0.00124202587207643	0.714599428822647
CESC	MTF2	cg14474880	-0.209104177200829	309	-3.72827551575171	0.000229906492332113	0.0437245569228357	0.00195041937453483
CESC	MTF2	cg15463775	-0.11668960928322	309	-2.0485476502935	0.0413648704244708	0.0136164649146705	0.1254232747193
CESC	MTF2	cg16813892	0.0456104673441217	309	0.796074145164187	0.426610135832199	0.00208031473134919	0.621174357326916
CESC	MTF2	cg19708337	-0.0282805254245404	309	-0.49328511082619	0.62216729667017	0.000799788118288078	0.776177598337972
CESC	MTF2	cg22801127	-0.0447564700632502	309	-0.781138477986567	0.435328928572431	0.00200314161252261	0.628661196758191
CESC	MTF2	cg23992808	-0.00051828849603717	309	-0.0090366699250314	0.992795805967813	2.68622965124474e-07	0.996246124154723
CESC	MTF2	cg26054395	-0.184664586545436	309	-3.27608041706466	0.00117432831179085	0.0341010095239968	0.00767243610375893
CESC	NAP1L2	cg02379437	-0.147495561401252	309	-2.60011113682312	0.00977453073623588	0.0217549406330704	0.0418468903249537
CESC	NAP1L2	cg04297329	-0.225330875438724	309	-4.03248428804547	6.9845699488527e-05	0.0507740034259818	0.000708696536586731
CESC	NAP1L2	cg07693661	-0.206090022430577	305	-3.64789308719919	0.000311684946759885	0.0424730973454356	0.00252259870456081
CESC	NAP1L2	cg20001978	-0.24727825950402	309	-4.44962919387005	1.20850917753404e-05	0.0611465376233377	0.000154654981011817
CESC	NAP1L2	cg23483095	-0.364562775118253	309	-6.8261531384354	4.73824938281345e-11	0.132906017001922	1.98817353013263e-09
CESC	NCOA1	cg11380327	-0.317016764411641	309	-5.82798395536811	1.43013752357405e-08	0.100499628918026	3.78036420349709e-07
CESC	NCOA1	cg13911252	-0.112643050476062	309	-1.97657859320186	0.048992835195447	0.0126884568205526	0.142140248422265

**CESC**	NCOA1	cg15477048	0.098528704293249	309	1.7263065064305	0.085308256578482	0.00970790556970651	0.212598363411918
**CESC**	NCOA1	cg23006163	-0.201010573289992	308	-3.57187527042569	0.000412111465745957	0.0404052505743712	0.00318403378529661
**CESC**	NCOA3	cg04175879	-0.0951085665641147	308	-1.6630837647764	0.0973296358799895	0.00904563943388064	0.233534680915933
**CESC**	NCOA3	cg09568061	-0.00234963116947956	309	-0.0409673323751156	0.967348831307717	5.52076663258988e-06	0.982850770994717
**CESC**	NCOA3	cg12128876	-0.0298150331998971	309	-0.520074074597322	0.603390458979411	0.000888936204710967	0.762423983072819
**CESC**	NCOA3	cg14341558	-0.028917650257835	309	-0.504407405170424	0.614340510415157	0.000836230496434463	0.77065235293456
**CESC**	NCOA3	cg14774045	-0.0414427309307189	309	-0.723200019174421	0.470113064938595	0.00171749994699596	0.658720428511703
**CESC**	NCOA3	cg18295930	-0.0310766221717995	309	-0.542101254335937	0.588145886210407	0.000965756445608779	0.751757571461354
**CESC**	NCOA3	cg21735648	0.142385846979794	309	2.50813690250516	0.012657709998101	0.0202737294201534	0.0512781279796007
**CESC**	NCOA3	cg22511723	-0.00896447501397959	308	-0.156049947315901	0.876097566401166	8.03618122762644e-05	0.934413650125404
**CESC**	NCOA3	cg22876277	-0.0670224500177564	309	-1.17120984954137	0.242431618115665	0.00449200880638265	0.434828778447225
**CESC**	NCOA3	cg24856420	0.05509529769683	308	0.960496970536752	0.337570986389924	0.00303549182830232	0.537022828241513
**CESC**	NCOA3	cg25373408	0.0766924431366175	309	1.34112833201428	0.180879917035353	0.00588173083426331	0.358068407192433
**CESC**	NCOR1	cg00253204	0.10155832299261	309	1.77993285530012	0.0760857613803598	0.0103140929690714	0.195901843625584
**CESC**	NCOR1	cg08125503	-0.0669953966259697	309	-1.17073496262814	0.242622216051705	0.004488383169071	0.435057917101381
**CESC**	NCOR1	cg08206308	-0.0489265416784872	309	-0.854086274468903	0.393729702274289	0.00239380648061674	0.590476904338019
**CESC**	NCOR1	cg21211144	0.0474982713228569	309	0.829096443204686	0.407700344426864	0.00225608577865973	0.603827449155532
**CESC**	NCOR1	cg22291359	-0.137867380765409	309	-2.42697584658754	0.0158062312662767	0.0190074146791143	0.0607524681387712
**CESC**	NCOR1	cg22625220	-0.117744916157612	309	-2.06733339517722	0.0395488112399423	0.013863865280963	0.121491874174699
**CESC**	NCOR2	cg08976554	-0.0235060593262239	309	-0.409955421628088	0.682127390461878	0.000552534825047959	0.816742877140789
**CESC**	NCOR2	cg10641483	0.029748941375421	309	0.518920189414898	0.604193940135205	0.000884999512958237	0.763116967494328
**CESC**	NCOR2	cg13416396	-0.107287561469974	309	-1.88148241008565	0.0608617188485419	0.0115106208461734	0.166317478270593
**CESC**	NCOR2	cg13419087	0.00249310616391032	309	0.0434689263884824	0.965356271716123	6.21557834452763e-06	0.981774803840441
**CESC**	NCOR2	cg13857025	-0.0516818606662641	309	-0.902309878665735	0.367606633756003	0.00267101472192713	0.566546951411821
**CESC**	NCOR2	cg16181859	0.0683278495482119	309	1.19412753460375	0.233359006698886	0.00466869502388308	0.4241869889743
**CESC**	NCOR2	cg19895345	-0.175282689526896	309	-3.10421715930063	0.00208740539479277	0.0307240212477824	0.0123095524016456
**CESC**	NCOR2	cg23611643	-0.0933246304216919	309	-1.63430306631089	0.103230321312987	0.00870948664334538	0.243620301426481
**CESC**	NCOR2	cg24626542	-0.0467860896874655	309	-0.816637621928548	0.414775023659403	0.00218893818824356	0.610343090974702
**CESC**	NCOR2	cg25113767	-0.00942711633811031	309	-0.164374693982048	0.869545420950408	8.88705224522664e-05	0.930806292173488
**CESC**	NCOR2	cg25449924	0.00743969440385091	309	0.129719094273021	0.896874462291539	5.53490528226906e-05	0.945361388535959
**CESC**	ORC1	cg00298020	-0.151231905803642	309	-2.66749912331946	0.00805134712756507	0.0228710893330015	0.036110842370384
**CESC**	ORC1	cg02275620	0.0550172763349121	309	0.960714085779579	0.337459439942955	0.00302690069531208	0.536983113990077
**CESC**	ORC1	cg04407474	0.0492329435050172	309	0.859447934171987	0.390770643765543	0.00242388272616821	0.588022969188298
**CESC**	ORC1	cg07142859	-0.0341679522625577	309	-0.596086656067632	0.551560903385287	0.00116744896181642	0.723960390253131
**CESC**	ORC1	cg08613513	0.0994836341135315	309	1.74320412016935	0.0823089893196259	0.00989699345643501	0.20716790200372
**CESC**	ORC1	cg10586599	0.145949412230569	309	2.57225855963277	0.0105784064881547	0.0213012309304487	0.0445326102402046
**CESC**	ORC1	cg13460942	-0.0314626735767769	309	-0.548842175106359	0.583516633715462	0.000989899828598815	0.748484583222211
**CESC**	ORC1	cg16136434	0.0545972921448274	309	0.953358292525768	0.341165982436139	0.00298086430954764	0.540438966050104
**CESC**	ORC1	cg16965475	0.0380187109871468	309	0.663358466580271	0.507603706874313	0.0014454223851242	0.690315259663774
**CESC**	ORC1	cg17146111	-0.11928530201774	309	-2.0947669043159	0.037019174509214	0.0142289832774634	0.115620890810316
**CESC**	ORC1	cg19776520	-0.0360684451736934	309	-0.629284291732945	0.529635556437816	0.00130093273724773	0.707751622878993
**CESC**	ORC1	cg21197365	-0.058065479807863	309	-1.01411727669531	0.311333237148185	0.00337159994531735	0.510197697047293
**CESC**	ORC1	cg23606758	0.122396120290316	309	2.15021604328157	0.032326606312073	0.0149808102621214	0.104357872497361
**CESC**	ORC1	cg25605769	0.127703978988385	309	2.24497608447268	0.0254884226042489	0.0163083062494658	0.0874545027603476
**CESC**	PADI1	cg14524955	-0.377895159265432	309	-7.11652932048899	7.99388643267399e-12	0.142804751396246	3.87220673190187e-10
**CESC**	PADI1	cg21010178	-0.209043014353175	309	-3.72713515759693	0.000230902856650553	0.0436989818498619	0.00195759119771939
**CESC**	PADI1	cg22193702	-0.436901763585988	309	-8.46866526381532	1.07559697276869e-15	0.190883151024547	9.13482433401391e-14
**CESC**	PADI2	cg00569276	-0.239473683728643	309	-4.30049857658402	2.29880829635483e-05	0.0573476451985662	0.000270508988967823
**CESC**	PADI2	cg01603374	-0.169769555603793	309	-3.00363474255376	0.00288949110593387	0.0288217020099095	0.0159750601304115
**CESC**	PADI2	cg07014827	-0.131582488886339	306	-2.30289400633349	0.0219665823964493	0.0173139513815235	0.0781628595308901
**CESC**	PADI2	cg09313801	-0.0982250721554433	309	-1.72093470560104	0.0862801264041163	0.00964816479994203	0.214370333887115
**CESC**	PADI2	cg10233913	-0.271771045748387	309	-4.92381287360861	1.39494401835569e-06	0.0738595013071718	2.29474043061879e-05
**CESC**	PADI2	cg15185794	-0.129464042016867	309	-2.2764409638564	0.0235146843275633	0.016760938175345	0.0823193840598712
**CESC**	PADI2	cg17183414	-0.111671432731303	309	-1.95931310318839	0.0509897969749184	0.0124705088882618	0.14637270353752
**CESC**	PADI2	cg18228326	-0.108812540747974	309	-1.90854386081605	0.0572626042360601	0.0118401690240296	0.159124380565817
**CESC**	PADI2	cg18879300	0.109205781308116	309	1.9155242967944	0.0563636357431807	0.0119259026711161	0.157390885494993
**CESC**	PADI2	cg19282714	-0.100307765089215	309	-1.75779113947464	0.0797894625997763	0.0100616477371931	0.202668934387534

**CESC**	PADI3	cg01459162	-0.21728316195949	309	-3.88118834700107	0.000127510201022889	0.047211972471114	0.00118654648066682
**CESC**	PADI3	cg12858514	-0.417685453164533	309	-8.01525568492672	2.38190486342714e-14	0.174461137785261	1.70310576236628e-12
**CESC**	PADI3	cg14664242	-0.0961835567283805	309	-1.68482913400522	0.093047799826019	0.00925127658492159	0.226183812132154
**CESC**	PADI3	cg17589397	-0.0997736418892301	309	-1.74833680818313	0.0814151587327653	0.00995477961584033	0.205529947379827
**CESC**	PADI3	cg18768338	-0.232131740482847	309	-4.16101602881369	4.12842838196108e-05	0.0538851449395958	0.000450318224265676
**CESC**	PADI3	cg21966975	-0.134978454157052	309	-2.37516601644686	0.018161166439231	0.0182191830866273	0.0675865758693809
**CESC**	PADI4	cg16091981	-0.17176692021109	309	-3.04004083838017	0.00257119388240403	0.0295038748788029	0.0145524523017635
**CESC**	PADI4	cg18333690	-0.189460212659852	309	-3.36428425781808	0.000865799971374429	0.0358951721811164	0.00595263305109168
**CESC**	PADI4	cg19159961	-0.109131220730207	309	-1.91420069782962	0.0565331811487427	0.0119096233380651	0.157660594883861
**CESC**	PADI4	cg21295838	-0.166011722527977	309	-2.93524337279443	0.00358698240871737	0.0275598920167061	0.019002091215782
**CESC**	PADI4	cg23228178	-0.036519493490624	309	-0.6371641525809	0.524497465658287	0.00133367340481173	0.703653505844772
**CESC**	PADI4	cg26918754	-0.17312213126149	309	-3.06476432494219	0.00237376363883075	0.0299712723325207	0.0136525631021144
**CESC**	PADI6	cg12643633	-0.00118346848639072	309	-0.0206344925904847	0.98355076192551	1.40059765827993e-06	0.991421409567287
**CESC**	PADI6	cg16017904	-0.0238315936476683	309	-0.415636079444726	0.677969550408703	0.000567944855787585	0.814007118122246
**CESC**	PARP1	cg00653565	-0.0171058068339444	309	-0.298293578177754	0.765682882784287	0.000292608627440219	0.870077753440124
**CESC**	PARP1	cg02088626	-0.0332096246231104	309	-0.579349154931584	0.562782660815024	0.0011028791676079	0.732885505297197
**CESC**	PARP1	cg03149313	0.027636371943268	309	0.482040728925863	0.63012387315332	0.000763769054186653	0.781741086787378
**CESC**	PARP1	cg04960271	0.108866557981153	309	1.90950267005284	0.0571384194168364	0.0118519274466638	0.158875766677867
**CESC**	PARP1	cg07113065	-0.0966435256849972	308	-1.69017533319851	0.0920228813034529	0.00933997105682671	0.224365437932767
**CESC**	PARP1	cg17661881	-0.0315107281032655	309	-0.549681281947544	0.582941582604384	0.000992925985597926	0.748018300351755
**CESC**	PARP1	cg25722198	0.0094554459260002	309	0.164868703275867	0.86915690023802	8.94054576595138e-05	0.930580358895956
**CESC**	PARP1	cg26464410	0.0657089459157327	309	1.14815597781815	0.251806851900822	0.00431766557335669	0.445600924382351
**CESC**	PARP2	cg01813761	-0.116406604662607	303	-2.02324429726546	0.0439404484665121	0.0135504976090765	0.131248579876001
**CESC**	PARP2	cg10910545	-0.158998964016669	308	-2.80334029505323	0.00538385833656162	0.0252806705583739	0.0263151467350135
**CESC**	PARP2	cg15078479	-0.163895871406179	309	-2.89679363589797	0.00404364023049081	0.0268618566639908	0.0209170123073876
**CESC**	PARP2	cg15365367	-0.0501192574870534	309	-0.874958726919603	0.382287101493046	0.00251193997105356	0.580691159155214
**CESC**	PARP2	cg18899035	0.0173939769206808	253	0.273962069274174	0.784341815217341	0.000302550433117177	0.88100871674462
**CESC**	PARP2	cg19540080	-0.0471015416368752	309	-0.822155945897064	0.411632514405942	0.00221855522457029	0.607327172524434
**CESC**	PARP2	cg24595957	0.0577203081138492	309	1.00806861906314	0.314223210144031	0.00333163396875768	0.513306463608096
**CESC**	PARP2	cg26206608	-0.0433002304430199	309	-0.755674057702967	0.450429756408425	0.00187490995641863	0.64125489477412
**CESC**	PARP2	cg26430074	0.0541285101251999	309	0.945148431186324	0.345333696233402	0.00292989560837386	0.544551213458898
**CESC**	PAXIP1	cg02824291	0.127961586096557	309	2.2495800061762	0.0251909175269539	0.0163741675163467	0.0867048154186289
**CESC**	PAXIP1	cg12220104	0.0621706731256648	309	1.08608371915386	0.278302403932628	0.00386519259689825	0.475192757216366
**CESC**	PAXIP1	cg22583711	-0.0399607975756297	309	-0.697297280711756	0.486149682052746	0.00159686534288045	0.672670052569434
**CESC**	PAXIP1	cg24448952	0.0123624421864254	309	0.215563017618874	0.82947293989133	0.000152829976812711	0.908397520741974
**CESC**	PAXIP1	cg24452135	0.0519344358517285	309	0.906731467462093	0.365266958718033	0.0026971856272373	0.564241145805839
**CESC**	PCGF1	cg03203239	0.166346323227187	309	2.9413276162019	0.00351920333767363	0.0276710992512037	0.0187207948886066
**CESC**	PCGF1	cg08092637	0.117482748040383	309	2.06266582365553	0.0399935656080637	0.0138021960871202	0.122456781316712
**CESC**	PCGF1	cg27306986	0.154552737973769	309	2.72749112575258	0.00675273333546565	0.0238865488151885	0.0314809215017208
**CESC**	PCGF2	cg09395368	-0.0221874927138452	309	-0.386947410096051	0.699065872709464	0.000492284832926933	0.827371527484558
**CESC**	PCGF2	cg17902383	0.0764038176995923	309	1.33605141778872	0.182531725811761	0.00583754335907254	0.360329706602724
**CESC**	PCGF2	cg20228512	-0.073648577569494	309	-1.28760362983304	0.198863329251708	0.00542411297800977	0.381560582952457
**CESC**	PCGF2	cg25377665	0.00882757521178888	309	0.153920030368226	0.877774918126125	7.79260841197895e-05	0.935124850810071
**CESC**	PCGF5	cg03905463	0.0138415012263761	309	0.241357941939038	0.809440419292909	0.000191587156199772	0.897002962656305
**CESC**	PCGF5	cg06001881	-0.164935612810593	309	-2.91568288625753	0.00381305284884188	0.0272037563732058	0.0199833355782003
**CESC**	PCGF5	cg07421809	-0.1636443456167	309	-2.89222559495211	0.00410128110336339	0.0267794718523181	0.021164514902491
**CESC**	PCGF5	cg07424295	-0.0326609648754305	309	-0.569767358674738	0.569256291425984	0.00106673862659411	0.73752762155225
**CESC**	PCGF5	cg08633154	-0.00523764524902352	309	-0.0913227180037913	0.927296312246614	2.74329277546186e-05	0.961597586143684
**CESC**	PCGF5	cg11113644	-0.0338758730432038	309	-0.590985226088395	0.554969453151043	0.00114757477443926	0.726554031140525
**CESC**	PCGF5	cg12783297	-0.145878035585247	309	-2.57097323619113	0.0106168904066094	0.0212804012662106	0.0446666146931897
**CESC**	PCGF5	cg15550435	0.0304691076050235	307	0.529742591660791	0.596679632227146	0.000928366518246503	0.757747482932492
**CESC**	PCGF5	cg17001035	-0.0414682840299015	309	-0.723646703668274	0.469839118373335	0.00171961858038459	0.65854679785837
**CESC**	PCGF5	cg20515900	-0.0818321034527708	309	-1.43159285462808	0.153287647731808	0.00669649315550498	0.320465971558095
**CESC**	PCGF5	cg22968329	-0.0686436132131085	309	-1.19967202446305	0.231200884094397	0.00471194563495084	0.421706437839524
**CESC**	PCGF5	cg23395902	-0.147090182445512	309	-2.59280670031575	0.00997987784635057	0.0216355217718541	0.0425573982995667
**CESC**	PCGF5	cg25390656	-0.0245546040349884	309	-0.428253272883236	0.668769987896115	0.000602928579315067	0.807897171757882
**CESC**	PCGF6	cg01917164	-0.172956412427127	309	-3.0617401110296	0.00239714156252669	0.0299139205996623	0.0137595536571387

**CESC**	PCGF6	cg06691148	-0.0375809998985108	309	-0.655710326818626	0.51250645101936	0.00141233155337187	0.69436192274531
**CESC**	PCGF6	cg11612727	-0.0278142222813635	309	-0.485145234466843	0.627922745490246	0.000773630961117097	0.780167410324824
**CESC**	PCGF6	cg11814370	-0.0643914167340013	309	-1.12503748203857	0.261460618457645	0.00414625454901182	0.456799210204584
**CESC**	PCGF6	cg14409890	-0.326975129794175	309	-6.03260048221868	4.68123743542857e-09	0.106912735503917	1.36394076798401e-07
**CESC**	PCGF6	cg16113748	-0.0453744810045845	309	-0.791946776554602	0.42900922784974	0.0020588435264354	0.623078983567098
**CESC**	PCGF6	cg16382383	0.0461306550771056	309	0.805172629699402	0.421349361250133	0.00212803733784289	0.61648396105121
**CESC**	PCGF6	cg24418712	-0.0664367482211073	309	-1.1609291986881	0.246581487705366	0.0044138415141948	0.439785415529169
**CESC**	PCGF6	cg25481680	0.0373701426121491	309	0.652026145915211	0.514876972168831	0.00139652755885236	0.696379206038145
**CESC**	PCGF6	cg26155979	-0.00429663547651127	309	-0.0749150908671777	0.940331542369196	1.84610764180153e-05	0.968279525875538
**CESC**	PCGF6	cg27217148	-0.0188059254281666	309	-0.327950511005976	0.743174832250638	0.000353662831209763	0.855605325839317
**CESC**	PHC1	cg02770112	-0.10850589509499	309	-1.90310120545247	0.0579718180422415	0.011773529270365	0.160544644646974
**CESC**	PHC1	cg08021945	-0.136348188969775	309	-2.39972297546057	0.0170089915241183	0.0185908286353375	0.0642703267256295
**CESC**	PHC1	cg08867249	0.0601516002455171	309	1.05068150533719	0.294239390406572	0.00361821501209649	0.491994822940115
**CESC**	PHC1	cg09059548	-0.146624469180632	309	-2.58441676090003	0.0102204996724313	0.0214987349625021	0.0433317102576229
**CESC**	PHC1	cg09912793	-0.379150190219612	309	-7.14412446098546	6.73238555121893e-12	0.143754866743568	3.3022648270588e-10
**CESC**	PHC1	cg12581035	0.0508404057608841	309	0.887580591604798	0.375468074602379	0.00258474685793133	0.574040037493458
**CESC**	PHC1	cg14199415	0.104787970532618	309	1.83715499535803	0.0671630210774712	0.0109805187683448	0.178875045478679
**CESC**	PHC2	cg06899053	-0.0432912425459249	309	-0.755516906695248	0.450523866075115	0.0018741316811701	0.641311519729548
**CESC**	PHC2	cg13640100	0.138777995370008	309	2.44331977225732	0.0151215408029985	0.0192593319989179	0.0587339575139054
**CESC**	PHC2	cg15586632	-0.0269714653867656	309	-0.470434710255177	0.638381803254713	0.000727459945109497	0.787327774028523
**CESC**	PHC2	cg16558908	-0.0270098767123033	309	-0.471105167105104	0.637903516988813	0.000729533440013823	0.787012389194
**CESC**	PHC2	cg17161743	0.106246308852908	309	1.86301263911246	0.0634247658519794	0.0112882781448675	0.171399111914715
**CESC**	PHC2	cg19746874	0.0108142494442397	309	0.188563908318245	0.850560437710477	0.000116947991042238	0.920323500163412
**CESC**	PHC2	cg21198021	-0.0462417876077484	309	-0.807116508192164	0.420230383005751	0.00213830292116011	0.615483430585511
**CESC**	PHC2	cg21578869	0.0399843794686269	309	0.697709432479972	0.485892208409114	0.00159875060149115	0.672445357948101
**CESC**	PHC2	cg23886755	-0.120827904440488	309	-2.12225526751874	0.0346246503788529	0.0145993824914797	0.1099554268518
**CESC**	PHF1	cg01097444	-0.0966898871112482	309	-1.69378190710159	0.0913309981187247	0.00934893426958593	0.223178236251704
**CESC**	PHF1	cg02776641	-0.0734058015523473	309	-1.28333612533871	0.200351766211989	0.00538841170154259	0.383348056980065
**CESC**	PHF1	cg03560363	-0.0122290566172259	309	-0.213236833239106	0.831285169399541	0.000149549825747317	0.909618208816201
**CESC**	PHF1	cg03876174	-0.093250910187815	309	-1.63300074678779	0.103504103352472	0.00869573225085593	0.244066509539032
**CESC**	PHF1	cg06618123	0.0380953379389359	309	0.66469741094878	0.506747945538893	0.00145125477268173	0.68964102085707
**CESC**	PHF1	cg06919790	-0.121065014739768	309	-2.12648182634792	0.0342685266174899	0.0146567377939402	0.109043102081193
**CESC**	PHF1	cg07857404	-0.114485540581597	304	-1.99274251075422	0.0471984439848629	0.0131069390022604	0.13834768388704
**CESC**	PHF1	cg08665801	-0.0734720583430337	309	-1.28450076104751	0.199944751490288	0.00539814335716214	0.382874977965966
**CESC**	PHF1	cg08798692	-0.0755560980429296	307	-1.31678896533014	0.188907724780713	0.00570872395147279	0.368678103090068
**CESC**	PHF1	cg10033570	0.0230042909070721	308	0.400539276833887	0.689041627342871	0.000529197400137201	0.821210939867652
**CESC**	PHF1	cg10797813	-0.0183050688869152	308	-0.318687812198274	0.750182822686476	0.000335075546954711	0.86005692563356
**CESC**	PHF1	cg13303256	0.120903856770792	309	2.12360909934064	0.0345102342046764	0.0146177425820522	0.109650758034578
**CESC**	PHF1	cg13528777	-0.0693128441541325	309	-1.21142424095243	0.226673680394101	0.00480427036473506	0.416204091293112
**CESC**	PHF1	cg13547657	-0.101225701990119	309	-1.77404281629805	0.0770569698008669	0.0102466427433925	0.197685417867777
**CESC**	PHF1	cg18443095	-0.0730563811275986	309	-1.27719443332598	0.202508210742915	0.00533723482346094	0.386150760378196
**CESC**	PHF1	cg20135607	-0.0616882553995814	309	-1.07762385649045	0.282056014764318	0.00380544085424398	0.479172467081049
**CESC**	PHF1	cg20872639	-0.0095165545634255	309	-0.165934312605592	0.868318944666915	9.05648107586547e-05	0.930066028382407
**CESC**	PHF1	cg23345642	0.0373840124514595	309	0.652268482482448	0.514720869277388	0.00139756438697088	0.696240702889225
**CESC**	PHF1	cg23718418	-0.0692717962224443	309	-1.21070336016183	0.226949536444705	0.00479858175188385	0.416516755642619
**CESC**	PHF1	cg24796663	-0.113008789432942	309	-1.98307920011304	0.0482582523915067	0.012770986489099	0.140554192682324
**CESC**	PHF1	cg26673053	-0.059495438399095	309	-1.0391792398869	0.299547185668812	0.00353970719030051	0.49783362409725
**CESC**	PHF10	cg04353997	-0.127469122755829	309	-2.24077916606629	0.0257622842041319	0.0162483772561406	0.0881100473655251
**CESC**	PHF10	cg05243705	0.0949028008083737	309	1.66218909079564	0.0975055939769308	0.00900654160127385	0.233780855874771
**CESC**	PHF10	cg05736120	0.0917741696878935	309	1.60691876979838	0.10911031541593	0.00842249822190227	0.25346013137674
**CESC**	PHF10	cg07652237	-0.188539190033718	308	-3.34181514574423	0.000936634207445029	0.0355470261785703	0.00635760592740412
**CESC**	PHF10	cg11442717	-0.0720164489186274	309	-1.25891854431578	0.209025606991843	0.00518636891484927	0.39431927034103
**CESC**	PHF10	cg15342139	0.0555285788960746	309	0.96966996108382	0.332981870361489	0.00308342307421758	0.53262036851859
**CESC**	PHF10	cg15360169	-0.254227583653069	309	-4.58319282619982	6.69475976720008e-06	0.0646316642900782	9.18377960376587e-05
**CESC**	PHF10	cg16740543	0.0128785661480852	309	0.224564097097129	0.822469258872197	0.000165857466030605	0.904585595761027
**CESC**	PHF10	cg27124293	-0.00093845554679659	309	-0.016362538771202	0.98695589884134	8.8069881331329e-07	0.993148441205393
**CESC**	PHF11	cg00092518	-0.281239052830131	309	-5.10981407704842	5.70821659626723e-07	0.079095404836789	1.04045220686507e-05

**CESC**	PHF11	cg01214340	-0.269631713056506	309	-4.88200171969475	1.69916836895018e-06	0.0727012606857859	2.73505284697104e-05
**CESC**	PHF11	cg02289189	-0.0813748264574797	309	-1.42353965068663	0.155605279787713	0.00662186238098494	0.323684612433963
**CESC**	PHF11	cg05080330	-0.0788898076071081	309	-1.37979113272766	0.168664938394979	0.00622360174428653	0.341857748219878
**CESC**	PHF11	cg09997676	-0.318388798778856	309	-5.85604574496076	1.22913147035866e-08	0.101371427187843	3.29493637508895e-07
**CESC**	PHF11	cg16956501	-0.225247197204436	309	-4.03090674243229	7.02921244983995e-05	0.0507362998484543	0.000712111672513152
**CESC**	PHF11	cg21320768	-0.36432403083885	309	-6.82099841775671	4.88805983471812e-11	0.132731999446668	2.04882395895507e-09
**CESC**	PHF11	cg23137039	-0.0529681770842597	309	-0.924830000166378	0.355787874326819	0.00280562778362949	0.554583671863841
**CESC**	PHF11	cg24481868	-0.229816700423349	309	-4.11719214015745	4.9464005390488e-05	0.0528157157934751	0.000524823081744084
**CESC**	PHF11	cg24984523	-0.204853976135591	309	-3.64914009057645	0.000309587030859343	0.0419651515385612	0.00250953563391391
**CESC**	PHF11	cg25162948	-0.295156672071357	309	-5.38619344043991	1.44337168476196e-07	0.0871174610682386	3.02141601015382e-06
**CESC**	PHF12	cg01406758	0.0462877904833899	309	0.807921177863072	0.419767695172617	0.0021425595478342	0.61510914425837
**CESC**	PHF12	cg02058367	-0.0571984320568611	309	-0.998924149386947	0.318625882423406	0.00327166062976336	0.517942660368314
**CESC**	PHF12	cg06895197	-0.0429216779558966	309	-0.749055326105934	0.454403052347788	0.0018422704385497	0.644681689025656
**CESC**	PHF12	cg08657247	0.0534071823056946	309	0.93251691345882	0.351809464810098	0.0028523271218337	0.550686045468613
**CESC**	PHF12	cg08806622	-0.139866157016385	309	-2.46285864544322	0.0143375529973989	0.019562541878532	0.0563741931353147
**CESC**	PHF12	cg13564075	-0.160769445599529	309	-2.84005452827633	0.0048148347803284	0.0258468146383801	0.0240600387398603
**CESC**	PHF14	cg06715026	-0.0994472027054862	309	-1.74255937247874	0.0824218318924203	0.00988974612594606	0.207376750572871
**CESC**	PHF14	cg07287563	-0.209454583065022	309	-3.73480958297649	0.000224275220501487	0.0438712223669423	0.00191048511994832
**CESC**	PHF14	cg07574654	-0.022983585329838	309	-0.400838387713181	0.688820687282843	0.000528245194613944	0.821151757574765
**CESC**	PHF14	cg11931558	-0.136543375109438	309	-2.40322346087659	0.0168501157661639	0.0186440932862768	0.0638001511687413
**CESC**	PHF14	cg13029706	-0.3133099358285	309	-5.75237198734343	2.14506764317688e-08	0.0981631158888586	5.49106259405405e-07
**CESC**	PHF14	cg16800461	-0.226214831535196	309	-4.0491548389938	6.52899916089827e-05	0.0511731500064973	0.000670075146072454
**CESC**	PHF14	cg20819769	0.212794745821257	309	3.79717005058142	0.000176698184554181	0.0452816038491335	0.00156471924521434
**CESC**	PHF14	cg20964328	0.0637951442424953	309	1.11457672328626	0.265912228607061	0.00406982042892077	0.461728849432117
**CESC**	PHF14	cg22876561	-0.194703739891636	309	-3.46101222001518	0.000615209596588129	0.0379095463277899	0.0044695195514547
**CESC**	PHF14	cg23083824	-0.0791770002649621	309	-1.38484579306862	0.1671150965387	0.00626899737095781	0.339830400986293
**CESC**	PHF14	cg24491907	0.0543214686148289	309	0.948527670684961	0.343614300202261	0.00295082195247184	0.542962521732505
**CESC**	PHF19	cg11054680	-0.0015936929772291	281	-0.0264764498528561	0.978896450399168	2.53985730566936e-06	0.989094294612233
**CESC**	PHF19	cg11594303	0.0320428060718052	309	0.558972447916895	0.576592050230419	0.00102674142095532	0.743240502451372
**CESC**	PHF19	cg13501527	-0.0378510854355316	309	-0.660429495052381	0.509478359263468	0.00143270466864792	0.691940633389354
**CESC**	PHF19	cg13793166	-0.00577580389434766	190	-0.0789842478171292	0.937129608559885	3.33599106259616e-05	0.966414845035019
**CESC**	PHF19	cg13851767	-0.034711397161987	309	-0.6055788248700l9	0.545246301515503	0.0012048810929372	0.719190564005349
**CESC**	PHF19	cg13852822	0.0178443548125548	309	0.311176503873481	0.755879715801724	0.000318420998676349	0.863833978891164
**CESC**	PHF19	cg14218513	-0.147891680496908	309	-2.60725001581259	0.00957750263115448	0.0218719491601996	0.0411778622575457
**CESC**	PHF19	cg27207079	-0.0589897538068978	309	-1.03031570610692	0.303680846969331	0.00347979105419841	0.50216915313279
**CESC**	PHF20	cg01512265	0.00343746462699461	309	0.0599345978237975	0.9522471048633	1.18161630618392e-05	0.974840590013759
**CESC**	PHF20	cg02674305	-0.0519039173875823	305	-0.900215636014797	0.368727558194505	0.00269401664017697	0.567505981969911
**CESC**	PHF20	cg02957488	-0.0486255650700862	309	-0.848819784824904	0.39664947110602	0.00236444557838519	0.593254550885773
**CESC**	PHF20	cg04179482	0.132733679716391	309	2.33495104073945	0.0201966387769161	0.0176182297310534	0.0733737499374822
**CESC**	PHF20	cg09692677	-0.127299552348193	302	-2.2118355522153	0.0277390945234414	0.0162051760280504	0.0931388976372135
**CESC**	PHF20	cg10372813	-0.110588528771154	309	-1.94007677773809	0.0532949472031654	0.0122298226957684	0.151129905333686
**CESC**	PHF20	cg21765224	-0.213440525145256	309	-3.80924267986017	0.000168665559255023	0.0455568577742828	0.00150472116017032
**CESC**	PHF20	cg23538717	0.00571875589137788	308	0.0995474122138452	0.920769474266873	3.27041689451692e-05	0.958432108341479
**CESC**	PHF20	cg23734815	-0.123604454506127	309	-2.17177138807755	0.030645817701041	0.0152780611737571	0.100259083318676
**CESC**	PHF20	cg25561939	0.00662512972741341	309	0.115515619032026	0.90811270618694	4.38923439050568e-05	0.951298360516742
**CESC**	PHF20	cg27405321	-0.0276554522055215	295	-0.471135583103853	0.637898126797429	0.000764824036691884	0.787012389194
**CESC**	PHF20L1	cg02898212	0.0738146655567307	309	1.29052324857795	0.19784970307918	0.00544860485125201	0.380291053327941
**CESC**	PHF20L1	cg05307234	-0.105976623520272	309	-1.8582299656329	0.0641028746612436	0.0112310447327575	0.172793452231351
**CESC**	PHF20L1	cg10591701	-0.162143617026613	309	-2.8649823892287	0.00446088241967165	0.026290552542473	0.0226417359532809
**CESC**	PHF20L1	cg17646969	-0.193601337426539	309	-3.44065079932714	0.000661516312015777	0.0374814778533446	0.00475487387757141
**CESC**	PHF20L1	cg24511731	-0.152764477852736	309	-2.69517395415254	0.00742666998560747	0.0233369856936192	0.0339234551617842
**CESC**	PHF20L1	cg26292018	0.141815671239816	309	2.49788665172337	0.0130218631723731	0.0201116846091996	0.0523905059801195
**CESC**	PHF20L1	cg27342122	-0.0455856890049058	309	-0.795640769157266	0.426861671863779	0.00207805504205199	0.621300645505366
**CESC**	PHF21B	cg01775414	-0.0559289397176923	309	-0.976683136467548	0.329502604565684	0.00312804629794525	0.529154981618901
**CESC**	PHF21B	cg01995480	-0.0486733822232415	309	-0.849656473998254	0.396184730926092	0.00236909813704976	0.592855809292023
**CESC**	PHF21B	cg05064352	-0.125241771099243	309	-2.20099497597641	0.0284878761709011	0.0156855012280752	0.0949929635164628
**CESC**	PHF21B	cg10080155	-0.0356817056190439	309	-0.622528218445999	0.534061262526005	0.00127318411588411	0.710978880432405

**CESC**	PHF21B	cg11428482	-0.0280310570367355	309	-0.488930303449375	0.625243585519004	0.000785740158596716	0.778174506186395
**CESC**	PHF21B	cg14786713	-0.0508566462379238	309	-0.887864855763136	0.375315373905827	0.00258639846656932	0.573970083894512
**CESC**	PHF21B	cg15679813	-0.0239395815420719	309	-0.417520525167387	0.676592435640607	0.000573103564409511	0.813169446579924
**CESC**	PHF21B	cg20495645	-0.019944710966529	309	-0.347817104469098	0.72821824929825	0.000397791495538383	0.846333275855029
**CESC**	PHF21B	cg20525183	-0.0328822683769451	309	-0.57363214146487	0.566640879655611	0.00108124357361345	0.73561946118297
**CESC**	PHF21B	cg21715212	-0.053146800239382	309	-0.927957598749263	0.354165738016816	0.00282458237568477	0.553174642585986
**CESC**	PHF21B	cg23080354	-0.0375684569982015	309	-0.65549116991448	0.512647303540058	0.00141138896122572	0.694498542971497
**CESC**	PHF21B	cg25561581	-0.0319280696892525	309	-0.556968876828432	0.577958504864866	0.00101940163408176	0.74434977695943
**CESC**	PHF21B	cg25976257	-0.0258561056841294	309	-0.450967376664929	0.652334559056847	0.000668538201148867	0.79690511592576
**CESC**	PHF23	cg00039960	0.143819458984973	308	2.52974967096799	0.011921116539487	0.0206840367827304	0.0488968566475598
**CESC**	PHF23	cg01441172	0.0542414567597206	309	0.947126427789867	0.344326600876632	0.00294213563141664	0.543591200531621
**CESC**	PHF23	cg04379155	0.00958916876413205	309	0.167200557784375	0.867323409836801	9.19521575870059e-05	0.929476489762565
**CESC**	PHF23	cg11180288	0.0536366780761415	309	0.93653556432171	0.349740901156802	0.00287689323504364	0.548814220006338
**CESC**	PHF23	cg13351208	0.0134437123996904	309	0.234420319853422	0.814816488118811	0.00018073340308559	0.900395367566971
**CESC**	PHF23	cg22033559	-0.17650715670859	309	-3.12659702012496	0.00193947540653177	0.0311547763693506	0.011595415752823
**CESC**	PHF23	cg26213471	-0.0606464042554696	309	-1.05935613307086	0.290278591607355	0.00367798634911784	0.48783294588769
**CESC**	PHF23	cg26784276	0.120852084343984	309	2.12268626431456	0.0345881903356323	0.0146052262902855	0.109862554261399
**CESC**	PHIP	cg01492710	0.0966989409719506	309	1.69394200665086	0.0913005315927994	0.00935068518509679	0.223135734181466
**CESC**	PHIP	cg04480383	-0.203979368964573	309	-3.63288249820023	0.000328893937097786	0.0416075829631856	0.00263854542049966
**CESC**	PHIP	cg05801338	-0.117490365049275	309	-2.06280142868545	0.0399805844524176	0.013803985879412	0.122443265180379
**CESC**	PHIP	cg06848288	-0.114266170759261	309	-2.00543401392218	0.0458026904251057	0.0130567577799845	0.135332693947004
**CESC**	PHIP	cg08287994	-0.278979660933344	309	-5.06528349352256	7.0864019617589e-07	0.0778296512144837	1.25834182655346e-05
**CESC**	PHIP	cg08587415	-0.237997972038027	308	-4.26536833489975	2.67041344368885e-05	0.0566430346942133	0.000307857355421355
**CESC**	PHIP	cg15564972	-0.112286992913519	309	-1.97025084411528	0.0497169211852045	0.0126083687775606	0.143699727537871
**CESC**	PHIP	cg19494004	-0.155870658374588	309	-2.75132594577408	0.00629162701845588	0.0242956621421276	0.0297791939811242
**CESC**	PHIP	cg19950079	-0.0565648345883495	309	-0.987823166900391	0.32402482397402	0.00319958051200734	0.523405048925144
**CESC**	PHIP	cg21607066	0.0856669917781165	309	1.49916623556971	0.134868234673096	0.00733883348031188	0.293538299755445
**CESC**	PHIP	cg21875336	-0.145503531330361	309	-2.56422997797785	0.0108208480322978	0.0211712776296054	0.0453432292930017
**CESC**	PHIP	cg26363168	-0.181247726320292	309	-3.2133838505009	0.00145267243793385	0.0328507382962754	0.00915959966561548
**CESC**	PHRF1	cg06063517	-0.090235455030282	309	-1.57975358634424	0.11520320377567	0.00814243734452205	0.263204641248877
**CESC**	PHRF1	cg07530960	-0.264263908076432	309	-4.77743453385388	2.76693865670521e-06	0.069835413111829	4.21647846933839e-05
**CESC**	PHRF1	cg08482978	-0.110923273543588	309	-1.94602231589076	0.0525733115633028	0.0123039726136256	0.149653781688155
**CESC**	PHRF1	cg12469954	-0.274023193341287	309	-4.96791406316883	1.13126609421736e-06	0.0750887104889566	1.90901766884045e-05
**CESC**	PHRF1	cg12813709	-0.127850749790299	309	-2.24759909703202	0.0253185514696076	0.0163458142219417	0.0870324507545693
**CESC**	PHRF1	cg15219216	0.0372673376040004	309	0.65022992829689	0.516034789158444	0.00138885445209054	0.69715732747151
**CESC**	PHRF1	cg16143007	-0.140751451799408	309	-2.47876158138947	0.0137262760750326	0.0198109711836411	0.054530789550663
**CESC**	PHRF1	cg18793109	-0.177960955510714	309	-3.15318789030548	0.00177630708029821	0.0316701016862864	0.0107949404737691
**CESC**	PHRF1	cg20152424	0.124232490495857	309	2.18297872294107	0.0298021110273274	0.0154337116948033	0.0981925977408795
**CESC**	PHRF1	cg21006161	-0.0512967212836448	309	-0.895567950961884	0.371192123117178	0.00263135361445193	0.569871329842472
**CESC**	PPARGC	cg04893087	0.015979142555979	309	0.27864144587252	0.78070944482568	0.0002553329968243	0.87889831918685
**CESC**	PPARGC	cg05158538	-0.131945640429778	305	-2.30552282374339	0.021819346758655	0.0174096520284243	0.0778048840538839
**CESC**	PPARGC	cg11270806	-0.0891480578820009	309	-1.56056306217333	0.119667268999335	0.00794737622413259	0.270222948448396
**CESC**	PPARGC	cg12691631	-0.190285347568096	309	-3.37948557311514	0.000820956318780763	0.0362085134991113	0.00569058860048389
**CESC**	PPARGC	cg20723350	-0.111720968560906	309	-1.96019320876161	0.0508863702007519	0.012481574816187	0.146145183277652
**CESC**	PPARGC	cg27461259	-0.0421917989250118	309	-0.736294799778686	0.462119083929717	0.00178014789652863	0.651611325353614
**CESC**	PPARGC	cg27514608	-0.129305376151703	309	-2.27360358056831	0.0236870302760743	0.0167198803017335	0.0827960475061522
**CESC**	PRDM1	cg00555933	0.00298637395689898	307	0.0518978776325214	0.958644375752979	8.91842941044444e-06	0.978274245632826
**CESC**	PRDM1	cg01761420	-0.00240280460748692	308	-0.0418254886931075	0.966665362953547	5.77346998176037e-06	0.98245233097507
**CESC**	PRDM1	cg05170275	-0.012206515159629	309	-0.212843721477327	0.83159151509807	0.000148999012342254	0.909713206794132
**CESC**	PRDM1	cg08742502	-0.0340957276010651	309	-0.594825172296473	0.552402808016949	0.00116251864064604	0.724676045918766
**CESC**	PRDM1	cg14359052	-0.0114257905117672	309	-0.199228469695641	0.842217216352413	0.000130548688818789	0.915566814810392
**CESC**	PRDM1	cg17143179	-0.0749781733765959	309	-1.31097930000525	0.190854178108402	0.00562172648289087	0.371186814425636
**CESC**	PRDM1	cg17521665	0.00666979527923861	309	0.116294441161078	0.907495977000096	4.44861690669537e-05	0.950952222346011
**CESC**	PRDM1	cg17965230	-0.0485652128293926	309	-0.847763769070369	0.397236508310946	0.0023585798971642	0.593824306832086
**CESC**	PRDM1	cg19464016	0.0427417495149848	309	0.74590951374863	0.456298455508974	0.0018268571516017	0.646568952257065
**CESC**	PRDM1	cg22186515	-0.0300369596069257	309	-0.523948697546882	0.600695995023654	0.000902218942428088	0.760712098923492
**CESC**	PRDM1	cg22196848	-0.0327755575212248	309	-0.571768562255145	0.567901295277004	0.00107423717082712	0.736518294291465

**CESC**	PRDM1	cg24040346	0.0695844550228782	309	1.21619440725753	0.224854359399223	0.00484199638083096	0.414147341027185
**CESC**	PRDM1	cg24793124	-0.127315786713444	309	-2.23803923326644	0.0259424497458602	0.0162093095464631	0.0885821417517206
**CESC**	PRDM1	cg24874254	-0.0428432775644994	309	-0.747684589324703	0.455228394847854	0.00183554643246874	0.645523008125881
**CESC**	PRDM1	cg26351406	-0.0442681907394604	309	-0.772599671875964	0.440359587609571	0.00195967271134524	0.632976732848598
**CESC**	PRDM10	cg04181189	-0.22643890849132	309	-4.05338238511911	6.41804367944708e-05	0.0512745792787406	0.000660168306226241
**CESC**	PRDM10	cg17627813	-0.0602174673994312	309	-1.05183620652367	0.293710067760093	0.00362614338000156	0.491458337161849
**CESC**	PRDM12	cg09191327	0.127482345606177	309	2.2410154503295	0.0257467982790383	0.0162517484412529	0.0880764445415552
**CESC**	PRDM12	cg12539975	0.127936734884551	308	2.24543356455454	0.0254611046093979	0.0163678081329199	0.0873878750373434
**CESC**	PRDM12	cg13795948	0.0790924043562785	309	1.38335684859087	0.167570509853212	0.00625560842685705	0.340391774693365
**CESC**	PRDM12	cg13928649	0.146519549431929	309	2.58252684835823	0.0102754130578937	0.0214679783657355	0.0435313120637031
**CESC**	PRDM12	cg23342516	0.122361634077531	309	2.1496009903865	0.0323757077958278	0.0149723694941236	0.104473030208587
**CESC**	PRDM13	cg00126261	0.263802624458943	309	4.76847098875817	2.88392481335566e-06	0.0695918246714262	4.36312810054823e-05
**CESC**	PRDM13	cg00579292	0.13936642873063	309	2.45388447118613	0.0146930496108794	0.0194230014571299	0.0574531842889546
**CESC**	PRDM13	cg01422797	0.135475141473203	309	2.38406914388903	0.0177357418783898	0.0183535139571844	0.0663920996938595
**CESC**	PRDM13	cg01815538	0.0640306170333033	309	1.11870761700696	0.264148087686619	0.00409991991766555	0.459671088347638
**CESC**	PRDM13	cg02400433	0.175190444524391	309	3.10253178022007	0.00209895645068247	0.0306916918526537	0.0123589326464118
**CESC**	PRDM13	cg03120185	0.124753088829185	309	2.19227084654334	0.0291178767848566	0.0155633331724224	0.0965803746312415
**CESC**	PRDM13	cg04910970	0.19702436307525	309	3.50391889401085	0.000527375234253834	0.038818599645208	0.00392146078089062
**CESC**	PRDM13	cg11809464	0.0246596257690715	309	0.430086053787763	0.667437760413671	0.000608097143070657	0.807165496735092
**CESC**	PRDM13	cg13738144	0.0993819792338738	309	1.74140509267	0.0826241666486135	0.00987677779644212	0.207682068913146
**CESC**	PRDM13	cg14344102	0.145473235471393	309	2.56368452530818	0.0108374978973894	0.0211624622385153	0.0454032156513281
**CESC**	PRDM13	cg17879189	0.0951058360353701	309	1.6657776050968	0.0967877919998247	0.0090451200479867	0.232629205389266
**CESC**	PRDM13	cg17978124	0.201807305003733	309	3.59254635333995	0.000381798002337543	0.0407261883528698	0.00298905254755112
**CESC**	PRDM13	cg25273233	-0.00397498003681461	309	-0.0693066926734595	0.944791064450342	1.58004662930747e-05	0.970464388585564
**CESC**	PRDM13	cg25924096	0.109773782382222	309	1.92560852456928	0.0550858210396723	0.0120502832984995	0.154806240949363
**CESC**	PRDM13	cg26507427	0.0324782019812644	309	0.566575701604889	0.571420536833082	0.00105483360393581	0.739307631093547
**CESC**	PRDM14	cg00384539	-0.126763515214504	309	-2.22817214822914	0.0266003646148905	0.0160689887895378	0.0902502841314837
**CESC**	PRDM14	cg01295203	-0.0171054026837501	309	-0.298286528484432	0.765688257695766	0.000292594800973246	0.870077753440124
**CESC**	PRDM14	cg04838988	0.0129587131533139	309	0.225961857780372	0.821382929345363	0.00016792824658987	0.904145339882179
**CESC**	PRDM14	cg09050331	0.110834218820953	309	1.94444051333391	0.05276449570706	0.0122842240616508	0.150011372255784
**CESC**	PRDM14	cg10457247	0.0205700098918439	309	0.358726278657873	0.720048868009081	0.000423125306950557	0.841216363112428
**CESC**	PRDM14	cg11229513	0.0854635444830019	309	1.49557969510613	0.135800583634785	0.00730401743559805	0.294899246407004
**CESC**	PRDM14	cg13267264	-0.113004794991695	309	-1.98300819891674	0.0482662251788464	0.0127700836911149	0.140561626233039
**CESC**	PRDM14	cg18035229	-0.0455376719988777	309	-0.794800950379422	0.427349358805479	0.00207367957107737	0.621684793531928
**CESC**	PRDM14	cg21255438	-0.129849995104279	309	-2.2833436124666	0.0230999689506503	0.0168610212285812	0.0812017045045375
**CESC**	PRDM14	cg27029821	-0.16693106122318	309	-2.95196276488011	0.00340353311584798	0.027865979201097	0.0182313885019815
**CESC**	PRDM4	cg00090609	-0.136896744941705	309	-2.40956155244743	0.0165657785851612	0.0187407187756344	0.0629564327920876
**CESC**	PRDM4	cg03909902	-0.068093984445932	309	-1.19002133297964	0.234966519244625	0.00463679071772283	0.426083575713667
**CESC**	PRDM4	cg12900929	-0.132826902844535	309	-2.33662039391956	0.0201083222578337	0.0176429861192716	0.0731410887618315
**CESC**	PRDM4	cg18440127	-0.152791760575817	309	-2.69566679894792	0.00741595273089539	0.0233453220998578	0.0338875261959801
**CESC**	PRDM4	cg25995916	-0.184296387405521	309	-3.26931840145138	0.00120177008722282	0.0339651584107258	0.00782805845812713
**CESC**	PRDM4	cg27140793	-0.109985316278209	309	-1.92936456120975	0.0546161225068224	0.0120967697968177	0.153897344875599
**CESC**	PRDM5	cg05713693	-0.298675361328498	309	-5.45665304685253	1.00760505168959e-07	0.0892069714647087	2.18709897854743e-06
**CESC**	PRDM5	cg06329345	-0.349479360112164	309	-6.5034627958282	3.22116486399761e-10	0.122135823144408	1.16357806329911e-08
**CESC**	PRDM5	cg07070341	-0.432052901842775	309	-8.35295698420136	2.39445066060726e-15	0.186669709990762	1.94035306970084e-13
**CESC**	PRDM5	cg10416963	-0.386882523864024	309	-7.31516655316599	2.29888947114443e-12	0.149678087271397	1.23764572676962e-10
**CESC**	PRDM5	cg11171429	-0.32952484559447	309	-6.08534750431478	3.49403132459712e-09	0.108586623864059	1.04263395805795e-07
**CESC**	PRDM5	cg13155001	-0.394286423332858	309	-7.48064355413038	7.99967261480597e-13	0.155461783624617	4.59960481445836e-11
**CESC**	PRDM5	cg18181323	-0.433794699944296	309	-8.39441784044611	1.79885721873161e-15	0.188177841699762	1.48556580121027e-13
**CESC**	PRDM5	cg19294653	-0.425924173283318	309	-8.20797897274343	6.46567300859571e-15	0.181411401387078	4.97517869466563e-13
**CESC**	PRDM5	cg22135105	-0.479255891102125	309	-9.5207336431802	5.60309620624669e-19	0.229686209156092	6.72665534365363e-17
**CESC**	PRDM8	cg01304890	0.00624500875530791	309	0.108887571599319	0.91336350147809	3.90001343538724e-05	0.954223703058324
**CESC**	PRDM8	cg02895669	-0.0913234988644237	309	-1.59896122465646	0.110867992654434	0.00833998144484039	0.256341455761725
**CESC**	PRDM8	cg04343891	0.000433976068842403	309	0.00756663202452805	0.993967721715753	1.88335228327906e-07	0.996872564214446
**CESC**	PRDM8	cg05007549	0.288337249449798	309	5.25031842764506	2.85740786536831e-07	0.0831383694202748	5.57115758091384e-06
**CESC**	PRDM8	cg07462804	-0.117136176216416	309	-2.05649622193277	0.0405879798352011	0.0137208837786032	0.123658921191667
**CESC**	PRDM8	cg08074201	-0.145404212443493	308	-2.55822383436639	0.011007064100697	0.0211423849963126	0.0459305661454498

**CESC**	PRDM8	cg10695325	0.041998520131168	309	0.732915891671239	0.464174480043735	0.00176387569320813	0.653408900921438
**CESC**	PRDM8	cg11571772	-0.109816196217061	309	-1.92636161155345	0.0549913759934531	0.0120595969515841	0.154624610692691
**CESC**	PRDM8	cg12572049	0.0440335070716361	309	0.768495828713416	0.442789249009345	0.00193894974502783	0.635128558012072
**CESC**	PRDM8	cg14489346	-0.116263062810301	309	-2.04095660478756	0.0421185959017865	0.0135170997740321	0.127141905614201
**CESC**	PRDM8	cg15656813	-0.110119237658685	309	-1.93174263221578	0.0543204798816385	0.01212624650253	0.153292248409772
**CESC**	PRDM8	cg16117799	-0.00321975153967979	309	-0.0561385773285501	0.955268290371455	1.03667999772704e-05	0.976175096358221
**CESC**	PRDM8	cg18624348	-0.082216838085932	309	-1.43836920420506	0.151357999867811	0.00675960846484835	0.317661106691033
**CESC**	PRDM8	cg19595012	-0.0643472241786269	308	-1.12241150616169	0.262576134596516	0.00414056525949447	0.458011341393336
**CESC**	PRDM8	cg20142271	-0.125201010808396	309	-2.20026724667029	0.0285399732418848	0.0156752931074441	0.0951178684761916
**CESC**	PRDM8	cg26162932	-0.0390589996246661	309	-0.681537005518102	0.496050623884559	0.00152560545167967	0.680907686672467
**CESC**	PRKAA1	cg00301775	-0.14636620751762	309	-2.57976487072473	0.0103561399037473	0.021423066703091	0.0438017478210251
**CESC**	PRKAA1	cg01883943	-0.337725706924189	308	-6.24572629501898	1.42466907455991e-09	0.114058653117443	4.57905810600035e-08
**CESC**	PRKAA1	cg03125217	0.122662405699757	309	2.15496544272284	0.0319496024536849	0.0150460657720518	0.10343698752682
**CESC**	PRKAA1	cg05874655	0.283899493399558	309	5.16236734725876	4.4140018808517e-07	0.0805989223525257	8.24747218522087e-06
**CESC**	PRKAA1	cg06971720	-0.120705861656052	308	-2.11659011783812	0.0351096548198329	0.01456990503813	0.111107766097853
**CESC**	PRKAA1	cg10786880	-0.021932394395146	309	-0.382496370199084	0.702360498174022	0.000481029923904234	0.829683994408577
**CESC**	PRKAA1	cg24728477	-0.000253887163191111	309	-0.004426674092318	0.99647094003494	6.44586916332326e-08	0.998268379680933
**CESC**	PRKCD	cg02121118	0.037538706302658	309	0.654971349568987	0.512981474039343	0.00140915447087721	0.694711038125812
**CESC**	PRKCD	cg02559423	-0.143600322867911	309	-2.52997847413634	0.0119118295916082	0.0206210527277682	0.0488699852685865
**CESC**	PRKCD	cg02630105	0.174598786068603	309	3.09172377892434	0.00217444238448039	0.0304847360966297	0.012703407243787
**CESC**	PRKCD	cg10531918	-0.273977155890065	309	-4.96701168400654	1.13614292668984e-06	0.0750634819496092	1.91600049509884e-05
**CESC**	PRKCD	cg13469346	0.124869539917109	309	2.19434962537984	0.0289666772289139	0.0155924019991104	0.0962224556277436
**CESC**	PRKCD	cg13889007	-0.439530039302995	309	-8.53176367698002	6.93307305589496e-16	0.193186655449693	5.99279428122547e-14
**CESC**	PRKCD	cg13908523	0.0453865062998081	309	0.792157094095094	0.428886787485981	0.00205993495410252	0.623018747300588
**CESC**	PRKCD	cg14727059	-0.0413429439919536	309	-0.721455693359774	0.471183689074135	0.00170923901792181	0.659552035192047
**CESC**	PRKCD	cg21903826	-0.110804188478254	309	-1.94390712084053	0.0528290955603775	0.0122775681843244	0.150151132951811
**CESC**	PRMT1	cg07702240	0.208956108081839	309	3.72551490171015	0.000232325527341776	0.043662655104709	0.00196772259924074
**CESC**	PRMT1	cg08377944	0.0857957452778646	309	1.50143610840887	0.134280737483501	0.00736090990778423	0.292563467654546
**CESC**	PRMT1	cg11787855	0.155088960626956	309	2.73718699789981	0.00656159527034441	0.0240525857083495	0.0307668460286404
**CESC**	PRMT1	cg14258555	0.0178867264293448	309	0.311915632097752	0.755318460810821	0.000319934982358223	0.863458464668651
**CESC**	PRMT1	cg14576628	-0.00525699543183482	309	-0.0916601139043409	0.927028457659927	2.76360009703322e-05	0.961483612813065
**CESC**	PRMT1	cg18880319	-0.0416239511926264	309	-0.72636789585267	0.468172158391499	0.00173255331288615	0.657181250269006
**CESC**	PRMT1	cg22292364	0.135926735230485	309	2.39216555765652	0.0173565100058158	0.0184760773504183	0.0652885421107505
**CESC**	PRMT1	cg24228123	0.100728312235763	309	1.76523617984495	0.0785280139173351	0.0101461928858653	0.200210019161838
**CESC**	PRMT1	cg25595446	-0.153667341961627	309	-2.71148697004151	0.00707925304663411	0.0236136519855516	0.0326849202527746
**CESC**	PRMT1	cg26846726	0.12035622023828	309	2.11384845714983	0.0353424474100723	0.0144856197500453	0.11162479614385
**CESC**	PRMT2	cg04493422	0.0899495237610203	309	1.5747068856032	0.116364203707741	0.00809091682483435	0.264997305167462
**CESC**	PRMT2	cg06725035	-0.232884318075843	304	-4.14079990299047	4.50715416944738e-05	0.0542351056056503	0.000485233256930514
**CESC**	PRMT2	cg09031657	-0.119555801013321	309	-2.09958593532655	0.0365894187253045	0.0142935895559369	0.114669348600747
**CESC**	PRMT2	cg10187059	-0.0847287390248792	309	-1.48262749103323	0.139209301355933	0.00717895921674608	0.300075833561098
**CESC**	PRMT2	cg10657630	-0.0904991202974204	308	-1.58179955989521	0.114738571101036	0.00819009077460696	0.26249771792242
**CESC**	PRMT2	cg12162201	-0.106899312519841	309	-1.87459490942376	0.0618072286849385	0.0114274630172147	0.168237632901193
**CESC**	PRMT2	cg13271076	-0.0254679890188738	309	-0.444193641179617	0.65721861939162	0.000648618464665477	0.80033894435604
**CESC**	PRMT2	cg17243659	-0.0819808483110674	308	-1.43185178296019	0.1532169588193	0.00672085948980224	0.3203878852461
**CESC**	PRMT2	cg23283320	-0.142905537273693	309	-2.51748177828067	0.0123336448986762	0.0204219925834828	0.0502373721790467
**CESC**	PRMT3	cg01769160	-0.014111708442905	309	-0.246070546596353	0.8057936812869	0.000199140315177557	0.894605549152565
**CESC**	PRMT3	cg04101203	-0.0765403227139582	309	-1.33845249539504	0.181749123290276	0.00585842100115686	0.359210755441994
**CESC**	PRMT3	cg06578097	-0.221295656796998	308	-3.9500036767814	9.72625275514658e-05	0.0489717677172148	0.000941333798001832
**CESC**	PRMT3	cg13432154	-0.206543725892416	309	-3.68057580431296	0.000275241404584534	0.0426603107055213	0.00227265649858411
**CESC**	PRMT3	cg13982511	0.059144629429179	309	1.03303024874876	0.302410846659203	0.00349808719031491	0.500768258797759
**CESC**	PRMT3	cg19653624	-0.100229287184884	309	-1.75640193304384	0.0800266650661571	0.0100459100095899	0.203118903169874
**CESC**	PRMT3	cg23019633	-0.213693542990637	309	-3.81397418949138	0.000165612583729006	0.0456649303158913	0.001481903075525
**CESC**	PRMT3	cg23071995	-0.0481437085455175	309	-0.840388739789798	0.40135093496299	0.00231781667251573	0.597918279448301
**CESC**	PRMT6	cg00944433	-0.251267344885742	309	-4.52620687254677	8.6281181206121e-06	0.0631352786059304	0.000114797534678823
**CESC**	PRMT6	cg02990361	-0.312150217007338	309	-5.72877614636767	2.43237588104395e-08	0.0974377579777284	6.13567604701468e-07
**CESC**	PRMT6	cg02993280	-0.168871082255802	309	-2.98727066923442	0.00304401307566112	0.0285174424222459	0.0166518496032615
**CESC**	PRMT6	cg09367891	-0.207914985274881	309	-3.7061116657464	0.000250017483687258	0.0432286411018538	0.00209228271578814

CESC	PRMT6	cg14671364	-0.304127618613246	309	-5.56631535008383	5.7185194065829e-08	0.092493608403364	1.31734231186174e-06
CESC	PRMT6	cg14828511	-0.327827588772484	309	-6.05021914676222	4.24638321941038e-09	0.107470927960381	1.24703430684317e-07
CESC	PRMT6	cg22528358	0.0971673488418339	309	1.70222544977008	0.0897353832537721	0.00944149368095064	0.220369796225896
CESC	PRMT7	cg00894222	-0.0291543155200772	309	-0.508539028929758	0.611444219153056	0.000849974113444211	0.768394475070962
CESC	PRMT7	cg02477175	-0.0183323239253348	309	-0.319688713625781	0.749423903970666	0.000336074100503403	0.859593587326487
CESC	PRMT7	cg06089844	0.00135737972159333	309	0.0236667459405064	0.981133958454025	1.84247970859279e-06	0.990286541212746
CESC	PRMT7	cg06092312	0.0711123221067609	309	1.24303267109528	0.214813639077984	0.00505696235541572	0.401740804347253
CESC	PRMT7	cg06745885	-0.0279921856452704	309	-0.48825175910649	0.62572351052794	0.000783562457199281	0.778557010702849
CESC	PRMT7	cg09175928	-0.108997933547068	309	-1.9118346659536	0.0568373190487	0.0118805495175309	0.158287601957181
CESC	PRMT7	cg09779635	-0.116119258956257	308	-2.03504226460975	0.0427167714146749	0.0134836823005502	0.128483935796212
CESC	PRMT7	cg10233539	0.0304233350372318	309	0.530694628653351	0.59601787288319	0.000925579314787653	0.75726241288446
CESC	PRMT7	cg10446007	-0.0504974283220974	309	-0.881577472312689	0.378701824682756	0.00254999026714536	0.577242583799407
CESC	PRMT7	cg11893236	-0.0384646950571864	309	-0.6711515537321	0.502633614399925	0.00147953276584234	0.686190499063382
CESC	PRMT7	cg19328639	0.0652872141018643	309	1.1407552597275	0.25486965898409	0.00426242032518266	0.449130844266939
CESC	PRMT7	cg21918026	-0.0984076540950908	309	-1.72416485155676	0.0856946547438175	0.00968406638449904	0.213294831903393
CESC	PRMT7	cg26492318	-0.00903215005889058	309	-0.157487341417181	0.874965333485987	8.1579734686317e-05	0.93385815433554
CESC	PRMT7	cg26561196	0.144349155403309	309	2.5434515578638	0.0114715014315902	0.0208366786656486	0.0474713539214389
CESC	PRMT7	cg26796563	0.0501660717958079	309	0.875778050361801	0.381842155180663	0.00251663475942215	0.580289678064553
CESC	PRMT8	cg01693063	-0.0607188982971269	309	-1.06062712382549	0.289701305378711	0.00368678461041684	0.487181654790995
CESC	PRMT8	cg06029846	-0.0314065213561731	309	-0.547861674746281	0.584188920049607	0.000986369583695759	0.748880532183299
CESC	PRMT8	cg07912789	-0.0789152900003475	309	-1.38023961502571	0.16852698967832	0.00622762299583894	0.341683310790598
CESC	PRMT8	cg10350722	-0.14493539704023	309	-2.55400240007089	0.0111368760910164	0.0210062693152091	0.0463702707463354
CESC	PRMT8	cg11596580	-0.0540847608451652	309	-0.944382274469738	0.345724290845194	0.00292516135567871	0.544995421960323
CESC	PRMT8	cg19656577	-0.080708199845779	309	-1.41180116521782	0.159031243108366	0.0065138135223462	0.328387655315926
CESC	PRMT8	cg21667836	0.076773657746177	309	1.34255695605158	0.180417121442798	0.00589419452372712	0.357543486066004
CESC	PRMT8	cg23739862	-0.0824659646486471	309	-1.44275742748228	0.150118355952923	0.00680063532543192	0.315767188439039
CESC	PRMT8	cg24100636	-0.0926550034661519	309	-1.62247463023888	0.105738316586983	0.00858494966731263	0.247854845141851
CESC	PRMT8	cg27316886	-0.141019176334803	309	-2.48357202657804	0.0135459855272974	0.0198864080941463	0.0540131418816287
CESC	PWWP2E	cg00259755	-0.075636914714938	309	-1.32256325130979	0.186974752741486	0.0057209428675948	0.366090655259518
CESC	PWWP2E	cg05010219	-0.0753760123617848	309	-1.31797508199654	0.18850425618306	0.00568154323956394	0.36810332108803
CESC	PWWP2E	cg15012692	-0.0240063475038801	309	-0.418685633732409	0.675741540945501	0.00057630472047705	0.812548495058648
CESC	PWWP2E	cg15278699	-0.287224103621992	309	-5.22822284407757	3.18897272987452e-07	0.0824976857014565	6.15285080723856e-06
CESC	PWWP2E	cg19919881	-0.103568980932715	309	-1.81555042058156	0.0704245017936405	0.010726533811441	0.185194324190734
CESC	PWWP2E	cg22633292	-0.00095546748926500	309	-0.0166591525224017	0.986719462189337	9.12918123042378e-07	0.993007258731262
CESC	PWWP2E	cg22664157	0.0224717089831279	309	0.391906598727063	0.695401817925798	0.000504977704622389	0.825408355437353
CESC	PWWP2E	cg24520806	-0.142130359496977	309	-2.50354359928214	0.0128197576810028	0.02020103909074	0.0518024220359409
CESC	PYGO1	cg06364757	-0.369548373360491	309	-6.93415449726323	2.45895812173132e-11	0.136566000253385	1.08442283515854e-09
CESC	PYGO1	cg09275667	-0.361475532258766	309	-6.7596156909463	7.07244153650804e-11	0.130664560421758	2.8972802363934e-09
CESC	PYGO1	cg11480800	-0.387369501648817	309	-7.32599871931987	2.14642777032763e-12	0.150055130807653	1.16199862188495e-10
CESC	PYGO1	cg12686317	-0.192255588498792	309	-3.41581310329409	0.000722411635821956	0.0369622113090166	0.0051192649660351
CESC	PYGO1	cg13878116	-0.0227684770145882	309	-0.397084900072362	0.691583488607565	0.00051840354556383	0.822809517377108
CESC	PYGO1	cg17841803	-0.351797663115611	309	-6.55267457534736	2.41497380657875e-10	0.123761595773605	8.93770087102697e-09
CESC	PYGO1	cg19850348	-0.252658211729764	308	-4.54547008491226	7.93047209309002e-06	0.0638361719544825	0.000106719105000838
CESC	PYGO1	cg22510134	-0.00270587043942892	309	-0.0471786359141858	0.962401832710182	7.32173483497526e-06	0.980256828372825
CESC	PYGO1	cg23030278	-0.372176555152074	309	-6.99136605230479	1.7322085467816e-11	0.138515388204865	7.84830702902316e-10
CESC	PYGO1	cg23412777	-0.0695284968924793	309	-1.2152116203116	0.225228330048195	0.0048342118801275	0.414590239642452
CESC	PYGO1	cg25902187	-0.284229472628687	309	-5.16889462800028	4.27465582380862e-07	0.0807863931107815	8.01500542678641e-06
CESC	PYGO2	cg01277844	-0.0527197399906494	309	-0.920480143762524	0.358051752786008	0.00277937098468168	0.557052137996414
CESC	PYGO2	cg02341811	-0.085453253711203	309	-1.4953982857149	0.135847874979019	0.00730225856983122	0.294952542174653
CESC	PYGO2	cg04602755	-0.366032622577446	309	-6.85792284113127	3.909697581753e-11	0.133979880790923	1.66384580784952e-09
CESC	PYGO2	cg10892866	-0.0737818882132892	309	-1.28994705539926	0.198049442988433	0.00544376702831831	0.380606124883705
CESC	PYGO2	cg14024356	0.124482961753255	309	2.18744913801252	0.0294712128207624	0.0154960077668624	0.0974014789029773
CESC	PYGO2	cg18188585	-0.0990082255088112	309	-1.73479109489407	0.0837913545989232	0.00980262871840362	0.209792560254534
CESC	PYGO2	cg24683561	-0.0997662716911355	309	-1.74820636153578	0.0814377768955922	0.00995330896714946	0.205573704192864
CESC	PYGO2	cg27105205	-0.0922975145620004	309	-1.61616077876844	0.107096798450092	0.00851883119432267	0.250164532177189
CESC	RBBP4	cg05797186	-0.18308506901585	309	-3.24708244901216	0.00129626227291836	0.0335201424965385	0.00832712033521147
CESC	RBBP4	cg06526778	-0.0340567747255441	309	-0.594144821022038	0.55285713220279	0.00115986390470646	0.724999001583083

**CESC**	RBBP4	cg09036220	-0.0623944835666572	309	-1.09000880420642	0.276572513082304	0.00389307157954985	0.473191769298874
**CESC**	RBBP4	cg11229946	-0.157994487053537	309	-2.78976750700032	0.00560766891504646	0.0249622579393104	0.0271921084321588
**CESC**	RBBP4	cg13641837	0.0609499989797137	308	1.06292641475787	0.288661720327742	0.0037149023756271	0.486190133176937
**CESC**	RBBP4	cg16148416	-0.0304148280099617	309	-0.530546097453877	0.596120695424975	0.000925061762875549	0.757338918890358
**CESC**	RBBP4	cg16396527	-0.111776270235008	309	-1.96117577386455	0.0507711114210284	0.0124939345876495	0.145949591378593
**CESC**	RBBP4	cg18758482	-0.160179911046818	309	-2.82936541017686	0.00497428759894455	0.0256576039029666	0.0246681247652213
**CESC**	RBBP4	cg20740619	-0.0457234298068737	309	-0.798049890318553	0.425464493409186	0.0020906320333041	0.620148248127892
**CESC**	RBBP4	cg22358606	0.0121275352085211	309	0.211466353134604	0.832665078768267	0.00014707711023392	0.910227599770935
**CESC**	RBBP4	cg22857567	-0.0525414541827337	309	-0.917358663802029	0.359681924573966	0.0027606044076363	0.558498211409222
**CESC**	RBBP4	cg23016766	-0.085720061923257	309	-1.50010183188525	0.13462583865025	0.00734792901612702	0.293109134699771
**CESC**	RBBP4	cg26131911	-0.065585638971142	309	-1.14599207462487	0.252699708555162	0.00430147603925298	0.446662753961199
**CESC**	RBBP5	cg00960017	-0.0603963745978615	309	-1.05497265510352	0.292275540439729	0.00364772206456521	0.489995978556737
**CESC**	RBBP5	cg02189925	0.0145164781505926	309	0.253130117322552	0.80033875478359	0.000210728137896631	0.89091440351484
**CESC**	RBBP5	cg03143531	-0.0714963759550798	309	-1.24978027376926	0.212341115182348	0.00511173177471011	0.398573162371712
**CESC**	RBBP5	cg05290904	-0.111461108944201	309	-1.95557643452321	0.0514308886990165	0.0124235788070711	0.147297429695305
**CESC**	RBBP5	cg07601985	-0.01301624558964	309	-0.226965223937376	0.820603332458515	0.000169422649249822	0.903570777504359
**CESC**	RBBP5	cg07972983	0.00284224411216023	309	0.0495564195986146	0.960508419140846	8.07835159310952e-06	0.979266362863907
**CESC**	RBBP5	cg14717946	9.54821710679863e-06	309	0.00016647885384567	0.999867278284274	9.1168449918562e-11	0.999903533818848
**CESC**	RBBP5	cg18131828	-0.226339584020449	309	-4.05150839712195	6.46700492222816e-05	0.0512296072945501	0.000664413868092733
**CESC**	RBBP5	cg21214746	0.0223898714631198	308	0.389835871622988	0.696931794584903	0.000501306344135027	0.826191873999401
**CESC**	RBBP5	cg23227967	-0.0255893053985515	309	-0.446310934000266	0.655690395849337	0.000654812550780335	0.79923815376427
**CESC**	RBBP5	cg24103461	-0.163928313287156	309	-2.89738286560534	0.00403625921226575	0.026872491897172	0.0208923628840956
**CESC**	RBBP5	cg26795850	-0.156211794979865	309	-2.75749790844793	0.00617696225870147	0.0244021248908313	0.0293507575099818
**CESC**	RBBP7	cg01947226	-0.218959527520828	309	-3.91263401727816	0.000112687395124506	0.0479432746921441	0.00106803158093516
**CESC**	RBBP7	cg03048084	-0.0872555107343625	309	-1.52717651970896	0.127756868206319	0.00761352415351445	0.282671154870374
**CESC**	RBBP7	cg03171708	-0.122175294481165	309	-2.14627780743062	0.032642119470442	0.0149268025815593	0.105110353991621
**CESC**	RBBP7	cg03811055	-0.114544054024252	309	-2.01037577670703	0.0452743777288968	0.0131203403123108	0.134214623843953
**CESC**	RBBP7	cg07096248	-0.0801579936445971	309	-1.40211413905229	0.16190142670251	0.00642530394512727	0.332399254707761
**CESC**	RBBP7	cg08363715	-0.0771260540397129	309	-1.34875617485679	0.17841915178726	0.00594842821173671	0.355012071410433
**CESC**	RBBP7	cg14719055	-0.263989609327731	309	-4.77210400656158	2.83594546936809e-06	0.0696905138330082	4.30057586440587e-05
**CESC**	RBBP7	cg15289846	-0.0913921197941258	309	-1.60017280802069	0.110598935079188	0.00835251956046384	0.255962799891424
**CESC**	RBBP7	cg15464143	-0.261740857824937	309	-4.72845013067323	3.46702129135467e-06	0.0685082766549341	5.14461214469154e-05
**CESC**	RBBP7	cg16978043	-0.0775804171014131	309	-1.35674990431316	0.17586730423458	0.00601872111762923	0.351671320831403
**CESC**	RBBP7	cg17878446	-0.154765565759264	309	-2.73133913967532	0.00667628220605112	0.0239523803447851	0.0311936755518043
**CESC**	RBBP7	cg17880859	-0.0873599520564576	309	-1.52901853775902	0.127299678801185	0.00763176122330656	0.282100874879914
**CESC**	RBBP7	cg21122900	-0.17106293269583	309	-3.02720482998791	0.00267954513476366	0.0292625269424979	0.015046674773067
**CESC**	RING1	cg01241018	0.0442592165543281	309	0.772442740630267	0.440452356510825	0.00195887825000291	0.633071699049967
**CESC**	RING1	cg01878788	-0.00942101550555012	309	-0.164268308159288	0.869629093755588	8.87555331558158e-05	0.930844625878838
**CESC**	RING1	cg02475416	0.0024854379720047	309	0.0433352256513883	0.965462761101396	6.17740191268284e-06	0.98181926373398
**CESC**	RING1	cg03037740	0.0924429064014888	309	1.61872856543666	0.106542648924851	0.00854569094395442	0.249154657321586
**CESC**	RING1	cg06316695	-0.0520851112215541	309	-0.909369278051795	0.363875626784165	0.00271285881096166	0.562803755890954
**CESC**	RING1	cg06671577	0.0788609704177101	309	1.37928361032982	0.168821149951442	0.00621905265522295	0.342074663071978
**CESC**	RING1	cg07946923	-0.031819684696759	309	-0.555076236416073	0.579250708302448	0.00101249233420116	0.745347672792362
**CESC**	RING1	cg08892712	-0.0501356863861914	309	-0.875246257627893	0.38213091710694	0.00251358704941454	0.580581070746192
**CESC**	RING1	cg09478665	0.0436605101801865	308	0.760719327177957	0.447416300723217	0.00190624014919417	0.639022980437356
**CESC**	RING1	cg09724776	0.147533104988964	309	2.60078769439192	0.00975570372346063	0.0217660170676846	0.0417821747524934
**CESC**	RING1	cg09832551	-0.0600507424113045	309	-1.0489134176642	0.295051138320031	0.00360609166414885	0.492917756613591
**CESC**	RING1	cg09869033	-0.045443077258237	309	-0.793146501865557	0.428311058952281	0.0020650732706981	0.622537747663627
**CESC**	RING1	cg10265934	0.097185023082749	309	1.70253802783202	0.0896767496379388	0.00944492871159446	0.220302304017867
**CESC**	RING1	cg12065531	-0.103189145739591	309	-1.80882016465053	0.0714668012274854	0.0106479997984666	0.18717598675861
**CESC**	RING1	cg12115664	-0.113279101389162	309	-1.98788422184188	0.0477212698630081	0.0128321548115361	0.139451979641601
**CESC**	RING1	cg12256228	0.0869436519473362	309	1.52167660847048	0.129129586201634	0.00755919861393953	0.284801174556869
**CESC**	RING1	cg14046664	-0.23516608010373	309	-4.21856964589082	3.24844327704656e-05	0.0553030852313541	0.000365186988864971
**CESC**	RING1	cg14363494	-0.0956710064031678	309	-1.67576772639214	0.0948118431479712	0.00915294146619497	0.229272339994194
**CESC**	RING1	cg18189601	-0.200685812914741	309	-3.57174144799243	0.000412114509912739	0.0402747955052503	0.00318403378529661
**CESC**	RING1	cg20505183	0.0841151507538921	309	1.47181381501481	0.142105602232533	0.00707535858634999	0.304401883968326
**CESC**	RING1	cg24536938	0.0312395481145981	309	0.544946107006094	0.586190134937736	0.000975909366404291	0.750364049527354

**CESC**	RING1	cg24550302	-0.0781648669143112	308	-1.36478327320522	0.173333786124425	0.00610974641973198	0.348270276299486
**CESC**	RNF17	cg00202166	-0.251463712418743	309	-4.52998281587039	8.48494914812216e-06	0.0632339986634163	0.000113259803368053
**CESC**	RNF17	cg01405147	0.0857127087359913	309	1.49997219863052	0.134659404061559	0.00734666843886087	0.293174008719494
**CESC**	RNF17	cg07131398	0.0194999469945943	285	0.326358577885086	0.744396872645734	0.000380247932791988	0.85642068031459
**CESC**	RNF17	cg12020676	0.0139740844901525	309	0.243670281007485	0.807650545267481	0.000195275037337921	0.895874502994859
**CESC**	RNF17	cg14855529	0.0601748854508432	309	1.05108971107996	0.294052192800449	0.0036210168390221	0.491808661808917
**CESC**	RNF17	cg15523998	-0.00466198660142122	309	-0.0812853972249936	0.935268475850647	2.1734119071831e-05	0.965493651574875
**CESC**	RNF17	cg17806989	-0.378687425891648	309	-7.13394403194282	7.17313824529913e-12	0.143404166528443	3.50299508257879e-10
**CESC**	RNF17	cg20477497	-0.00033229500000468	309	-0.0057937616177306	0.995381073018821	1.10419967028115e-07	0.99763811479845
**CESC**	RNF17	cg21831927	0.0343744277956242	309	0.599693031763423	0.549157536774491	0.00118160128627658	0.722166320621439
**CESC**	RNF17	cg23678722	0.0345477701503202	309	0.602720750830724	0.547143816398492	0.00119354842235935	0.720546706979652
**CESC**	RNF17	cg24753530	-0.0599237824142128	309	-1.04668779200847	0.296075088324781	0.00359085969882592	0.49403850693367
**CESC**	RNF20	cg04845236	-0.00290219069384644	309	-0.0506016368993694	0.959676189638662	8.42271082344887e-06	0.978941860693937
**CESC**	RNF20	cg14625770	0.0670109666911925	309	1.17100827444419	0.242512508230677	0.00449046965688811	0.434941397669156
**CESC**	RNF20	cg21219529	-0.00148729398884941	309	-0.0259318854681645	0.979328681692575	2.2120434092676e-06	0.989350121595619
**CESC**	RNF217	cg00991794	-0.19841713676277	309	-3.52969965763433	0.000480399344731818	0.0393693601611356	0.00362063423988249
**CESC**	RNF217	cg02312409	-0.200192936146652	309	-3.56260267877783	0.000426133292765854	0.0400772116830176	0.00327445805782565
**CESC**	RNF217	cg03113502	-0.181223115600033	309	-3.21293270372873	0.00145488026830911	0.0328418176277828	0.0091696472164659
**CESC**	RNF217	cg06993703	-0.184013842694341	309	-3.26413040471772	0.00122322644122503	0.0338610943031378	0.00794321183377798
**CESC**	RNF217	cg07439975	-0.156770525100696	309	-2.76760883515376	0.00599319906119177	0.0245769975403479	0.0286437874157617
**CESC**	RNF217	cg10621825	-0.192272224830287	309	-3.41612002777345	0.000721628202344448	0.0369686084411885	0.00511464507773178
**CESC**	RNF217	cg16203203	-0.290682104066036	309	-5.29693915324178	2.26393455890724e-07	0.0844960856242578	4.52751992482338e-06
**CESC**	RNF217	cg17382198	-0.209742066687097	309	-3.74017145399277	0.000219751641414141	0.0439917345381748	0.00187918433543127
**CESC**	RNF217	cg18192252	-0.206559380332982	309	-3.68086719619455	0.000274940512002355	0.0426667776035454	0.0022710471640169
**CESC**	RNF217	cg19171596	-0.189421396728916	309	-3.36356934111633	0.000867964148964265	0.0358804655387333	0.00596540356961976
**CESC**	RNF217	cg19478500	-0.248200450645446	309	-4.46731070912407	1.11850610247679e-05	0.0616034637006024	0.000144515967008935
**CESC**	RNF217	cg20034100	-0.0109467679761077	309	-0.19087485823975	0.848751047526423	0.000119831729122737	0.919337505621901
**CESC**	RNF217	cg23521835	-0.202859378202778	309	-3.6120768519193	0.000355255861824651	0.0411519273248176	0.00281531938482297
**CESC**	RNF217	cg24822696	-0.135655648771747	309	-2.38730519311933	0.0175833002256255	0.0184024550436835	0.0659367614474108
**CESC**	RPH3A	cg00851732	-0.0560781950973348	309	-0.979297784161164	0.328211545260361	0.00314476396537475	0.527811657922161
**CESC**	RPH3A	cg00854817	-0.0363717097361528	309	-0.634582312360087	0.526178132904601	0.00132290126913095	0.704911427287891
**CESC**	RPH3A	cg01119407	-0.0883477358525698	309	-1.54644247388892	0.123038093447323	0.00780532243027545	0.275426211283679
**CESC**	RPH3A	cg01573562	-0.0520231332887625	309	-0.908284248812603	0.364447529449104	0.00270640639718035	0.56339707700184
**CESC**	RPH3A	cg05967001	-0.0773187851395619	309	-1.35214684050756	0.177333384417422	0.00597819453545773	0.353606850274497
**CESC**	RPH3A	cg08254200	-0.0889655169611186	309	-1.55734210993219	0.120429693031026	0.00791486320815909	0.271375823064498
**CESC**	RPH3A	cg09384610	-0.0736831444933717	309	-1.28821126416524	0.198652058511696	0.00542920578243109	0.381333915014038
**CESC**	RPH3A	cg11596863	-0.113580820235259	309	-1.99324805553817	0.0471278082078914	0.0129006027253143	0.138187807158596
**CESC**	RPH3A	cg16403344	-0.0256374983033129	309	-0.447152033571808	0.655083706787979	0.000657281319252373	0.798946193300474
**CESC**	RPH3A	cg17453780	-0.114605945707068	309	-2.01147649825053	0.0451574076470003	0.0131345227914114	0.133939887543493
**CESC**	RPH3A	cg18629535	-0.0289357275591094	309	-0.504722989704891	0.614119069775343	0.000837276329375002	0.770511239824899
**CESC**	RPH3A	cg19712198	-0.0371592459039646	309	-0.648341363454957	0.517253593818764	0.00138080955615131	0.698221895811923
**CESC**	RPH3A	cg20704159	-0.091182561094309	309	-1.59647287428386	0.111422211724942	0.00831425944771738	0.257202443219197
**CESC**	RPH3A	cg21900624	-0.00429663979890157	309	-0.0749151662327147	0.940331482454546	1.8461113561505e-05	0.968279525875538
**CESC**	RPH3A	cg23199238	0.0297570019477134	297	0.508713149509414	0.611337449013067	0.000885479164916222	0.768304136784698
**CESC**	RPH3A	cg23549179	-0.0584653356516362	309	-1.02112465497369	0.308007293781291	0.00341819547285849	0.506588287541422
**CESC**	RPH3A	cg23596620	-0.0605162250478488	309	-1.05707382814268	0.291317170155342	0.00366221349404188	0.488916098959392
**CESC**	RTF1	cg04910470	-0.029125818432566	309	-0.508041531695843	0.611792645503287	0.0008483132993668	0.768662616321675
**CESC**	RTF1	cg07091958	-0.0740029655821136	309	-1.29383345773691	0.196705073398984	0.0054764389149475	0.378846171265036
**CESC**	RTF1	cg07129439	0.209144400641975	309	3.72902549153285	0.000229253428361324	0.0437413803198908	0.00194551556735473
**CESC**	RTF1	cg15581235	0.13284829056215	309	2.33700339419983	0.0200881075998747	0.0176486683052854	0.0730926855935752
**CESC**	RTF1	cg18782716	0.0792164544335886	309	1.38554022307353	0.166903015834388	0.00627524665302882	0.339541140528776
**CESC**	RTF1	cg22263512	0.0438883673718221	309	0.765957877997325	0.444295678026438	0.00192618879056402	0.636259742215797
**CESC**	RTF1	cg24280607	0.0377279743080675	309	0.658278372979775	0.510857475266677	0.0014234000453902	0.693073188932971
**CESC**	RTF1	cg27576415	0.129943440543279	309	2.28501501312927	0.0230005153148893	0.0168852977402248	0.0809162975809025
**CESC**	SATB1	cg01069104	-0.226093906759368	309	-4.04687369417979	6.58962510176472e-05	0.0511184546737139	0.000674783855688673
**CESC**	SATB1	cg01373248	0.291137070281158	309	5.30599689215523	2.16341622174318e-07	0.0847607936918961	4.34324649172359e-06
**CESC**	SATB1	cg04004754	-0.0885365426208008	309	-1.54977343813257	0.122236294869844	0.00783871937924488	0.274127462125254

**CESC**	SATB1	cg14415616	-0.347895154250362	309	-6.46991241716403	3.91665190747524e-10	0.121031038350883	1.39255949949785e-08
**CESC**	SATB1	cg19818826	-0.372089874308753	309	-6.98947604169972	1.7524283452491e-11	0.138450874563103	7.92607038897145e-10
**CESC**	SATB1	cg26131754	-0.288733820637535	309	-5.2581958393335	2.74749282984473e-07	0.0833672191799483	5.38655386101161e-06
**CESC**	SATB1	cg26768584	0.0224097827470381	309	0.390826061792383	0.696199558320696	0.000502198362769445	0.825851213613703
**CESC**	SCML2	cg00457389	0.0194041962676658	308	0.337830417011385	0.735724920017346	0.000376522832794094	0.851001765378241
**CESC**	SCML2	cg04189389	-0.0525566664985545	309	-0.917625002030604	0.359542648690306	0.00276220319344028	0.558439313622387
**CESC**	SCML2	cg05049545	-0.0316425459635806	309	-0.551983046601642	0.58136551793067	0.00100125071505731	0.746821304242258
**CESC**	SCML2	cg06131666	-0.00069544880355183	309	-0.0121255671526148	0.990333387804435	4.83649038361685e-07	0.994732410190131
**CESC**	SCML2	cg08300622	-0.0859216708260299	309	-1.50365619889201	0.133708050821507	0.00738253351753663	0.291780529765444
**CESC**	SCML2	cg17710066	0.0278386390863228	309	0.485571450948084	0.627620812347384	0.00077498982617854	0.779938640598319
**CESC**	SCML2	cg17727916	-0.062873577973311	309	-1.09841149826582	0.272894025245426	0.00395308680716602	0.469191720996811
**CESC**	SCML2	cg22823767	-0.115783405145309	309	-2.03242173175956	0.0429799507300176	0.0134057969070428	0.129016140110009
**CESC**	SCML2	cg26246138	-0.0689721806047201	309	-1.20544171939128	0.228970271789726	0.00475716169737012	0.418940526920596
**CESC**	SCML4	cg00977377	0.0701314304406381	309	1.22580148978619	0.221222130129954	0.00491841753565007	0.409541535179897
**CESC**	SCML4	cg03500965	-0.225761893245655	297	-3.96005971528912	9.420472416785e-05	0.0509684324418624	0.000915612482747842
**CESC**	SCML4	cg05603896	-0.161847697014528	309	-2.85961287531584	0.00453505859456465	0.0261946770289064	0.022925488875532
**CESC**	SCML4	cg08792995	-0.128557857217514	309	-2.26023826575531	0.0245137483486734	0.0165271226523587	0.0849422663400809
**CESC**	SCML4	cg17388996	-0.0241364695206348	309	-0.42095636193805	0.674084393729329	0.000582569160920531	0.811597303639224
**CESC**	SCML4	cg23503775	-0.132497785762562	309	-2.3307271525933	0.0204216227481728	0.0175556632319817	0.0739884370376003
**CESC**	SCML4	cg27353361	-0.0137340093652137	309	-0.239483222739139	0.810892288288859	0.000188623013243777	0.898036225712058
**CESC**	SCML4	cg27659622	-0.0780307720808469	309	-1.36467395777626	0.173364793051575	0.00608880139153308	0.348301013365884
**CESC**	SETD1A	cg26692825	-0.182107208070096	309	-3.22914320059881	0.00137746894539984	0.0331630352310854	0.00876268044308078
**CESC**	SETD6	cg01152986	-0.167990166323166	309	-2.9712338151063	0.0032027588614498	0.028220695981285	0.0173784908186947
**CESC**	SETD6	cg01472538	-0.179119531779744	308	-3.16916887303387	0.00168497181721227	0.0320838066649948	0.0103424883658049
**CESC**	SETD6	cg01773760	-0.101075854367644	309	-1.77138951729047	0.0774977773052603	0.0102163283361491	0.198467988533608
**CESC**	SETD6	cg02549819	-0.216103685771841	307	-3.84636976329353	0.000146256707287782	0.0467008030041747	0.00133293044596365
**CESC**	SETD6	cg05502081	-0.13642847661848	309	-2.40116282672096	0.0169434820748032	0.0186127292324392	0.0640632439624419
**CESC**	SETD6	cg09132913	-0.153282708605208	309	-2.70453653819882	0.00722544230541502	0.023495588757349	0.0332286627280359
**CESC**	SETD6	cg19824325	-0.208688221873111	309	-3.72052108770642	0.000236762541038281	0.0435507739485608	0.00199899122178555
**CESC**	SETD6	cg26666090	-0.179177453278489	309	-3.17545475655643	0.00164952971892591	0.0321045597633653	0.0101634337837892
**CESC**	SETD7	cg05316864	-0.169869180324445	309	-3.00544970148466	0.00287280367991805	0.0288555384240989	0.015905408118678
**CESC**	SETDB1	cg02060408	-0.293522857056706	308	-5.3447467140525	1.78394221122692e-07	0.0861556676147314	3.66561015267267e-06
**CESC**	SETDB1	cg08531528	-0.0602205146025968	309	-1.05188962664092	0.293685595170545	0.00362651037900157	0.491458337161849
**CESC**	SETDB1	cg10589385	-0.17629664274045	309	-3.12274834606851	0.00196420584616458	0.031080506241554	0.0117198519402153
**CESC**	SETDB1	cg10961700	-0.117765897962197	308	-2.06430333340605	0.0398398553737288	0.0138688067228426	0.122130342788504
**CESC**	SETDB1	cg12082615	-0.114452129960042	309	-2.00874098372827	0.045448575456159	0.0130992900523904	0.134572621285595
**CESC**	SETDB1	cg15448220	-0.176294628289162	309	-3.12271151945509	0.00196444388194536	0.0310797959636136	0.0117203732918212
**CESC**	SETDB1	cg16370685	-0.197539570288063	307	-3.5018765078794	0.000531754352572102	0.0390218818295927	0.00394914540372259
**CESC**	SETDB1	cg17899625	-0.0654646712794079	309	-1.1438692740185	0.253577757591002	0.00428562318572093	0.447574091299703
**CESC**	SETDB1	cg17918089	-0.067974910495024	309	-1.18793070924889	0.235787985425811	0.00462058845680653	0.427075449085344
**CESC**	SETDB1	cg19689029	-0.274544544381726	309	-4.97813563337136	1.07741868503986e-06	0.0753747068497693	1.82567434510979e-05
**CESC**	SETDB1	cg26820223	-0.0671444661918688	309	-1.17335171616806	0.24157328569713	0.00450837934019101	0.433867071165244
**CESC**	SETMAR	cg00300090	0.0137620828566002	309	0.239972839593234	0.810513043125964	0.00018939492455193	0.897718462874856
**CESC**	SETMAR	cg02520519	0.0772538677707435	309	1.35100474993902	0.17769855377679	0.00596816008553952	0.354131603968429
**CESC**	SETMAR	cg06021447	-0.0151480547877249	309	-0.264145667685508	0.79184665422956	0.000229463563851915	0.885770632240052
**CESC**	SETMAR	cg06267913	-0.034642062248061	309	-0.604367745553893	0.546049952929547	0.00120007247679853	0.719721629870238
**CESC**	SETMAR	cg14751177	-0.0235604017963202	309	-0.410903702616754	0.681432636868847	0.000555092532804047	0.816222095981819
**CESC**	SETMAR	cg14961031	-0.250803760897855	309	-4.51729500026644	8.9752793310106e-06	0.0629025264805085	0.000118886919849589
**CESC**	SETMAR	cg15232450	0.115906828452882	309	2.03461774812756	0.0427569072803074	0.0134343928820058	0.128565806027125
**CESC**	SETMAR	cg15384599	-0.122955091218488	309	-2.16018625301858	0.0315395544955559	0.0151179544565468	0.102459101450172
**CESC**	SETMAR	cg16423812	-0.0343513875088624	309	-0.599290598051079	0.549425469743242	0.00118001782378403	0.7223871526487
**CESC**	SETMAR	cg21901604	-0.277660201017953	309	-5.0393203824749	8.03327196348003e-07	0.0770951872293302	1.40784041254104e-05
**CESC**	SETMAR	cg22149154	-0.0537483656026211	309	-0.938491354272939	0.348736988274119	0.00288888680495302	0.547896543860506
**CESC**	SETMAR	cg23182840	-0.359825989225599	309	-6.72416917896363	8.74484372207468e-11	0.129474742522181	3.53584393199105e-09
**CESC**	SETMAR	cg23549342	-0.375635110491381	309	-7.06695145854214	1.08710849601199e-11	0.141101736233872	5.12548595992466e-10
**CESC**	SETMAR	cg24390964	-0.0372756050570576	309	-0.650374377156346	0.515941629271008	0.00138947073236974	0.697067785819882
**CESC**	SETMAR	cg26797270	-0.102309418635545	309	-1.79323544795783	0.0739291944836417	0.0104672171415432	0.191816954796399

**CESC**	SETMAR	cg27265118	-0.178515642991543	309	-3.16333907261954	0.00171743392946524	0.031867834792684	0.0105052724570545
**CESC**	SFMBT2	cg09060610	-0.281466855149633	309	-5.11430895919422	5.58449862814503e-07	0.0792235905478247	1.02005279708362e-05
**CESC**	SFMBT2	cg12328063	-0.308920118557512	307	-5.64454536469842	3.8172956167768e-08	0.0954316396495873	9.18251995088231e-07
**CESC**	SFMBT2	cg20299663	-0.332989085683871	308	-6.14711794873052	2.48339405596828e-09	0.11088173118458	7.63911258165268e-08
**CESC**	SFMBT2	cg25291138	-0.328614841268298	309	-6.0665048250934	3.87971883966349e-09	0.107987713901789	1.14542257082649e-07
**CESC**	SHPRH	cg00571935	0.0508221364449732	309	0.887260816637877	0.375639897039878	0.00258288955283148	0.574194000619284
**CESC**	SHPRH	cg02758840	-0.0824276343315365	309	-1.44208224295475	0.15030858297258	0.0067943149014935	0.316055515413923
**CESC**	SHPRH	cg06708562	-0.135993151610559	309	-2.39335643395637	0.0173013370784302	0.0184941372849724	0.065134543974032
**CESC**	SHPRH	cg11387577	-0.0100870901919205	308	-0.175593855208689	0.860730318813358	0.000101749388539938	0.926007930406676
**CESC**	SHPRH	cg11675148	-0.022281304104076	309	-0.388584281253142	0.69785570017514	0.000496456512578315	0.826728359955793
**CESC**	SHPRH	cg12989205	0.0948036668690483	309	1.66043703960029	0.0978576027706653	0.00898773525181749	0.23442552041452
**CESC**	SHPRH	cg14102550	-0.18196084943286	309	-3.22645904723562	0.00139001730050112	0.0331097507263278	0.00883723141769209
**CESC**	SHPRH	cg15443174	-0.0281192716230712	309	-0.490470197611974	0.62415503204104	0.000790693436612055	0.777373345969495
**CESC**	SHPRH	cg16339435	-0.189547986522028	309	-3.36590094819117	0.000860924496001143	0.035928439194555	0.00592644546871187
**CESC**	SHPRH	cg18099523	-0.024619814679231	309	-0.429391291111767	0.667942651785063	0.00060613527483968	0.80737629007547
**CESC**	SHPRH	cg26814539	-0.0727844325629814	309	-1.27241477583646	0.204198146964362	0.00529757362351518	0.388243979004439
**CESC**	SHPRH	cg27398948	-0.121989718541727	309	-2.14296847252812	0.0329092938024079	0.0148814914298898	0.105734341141722
**CESC**	SIN3A	cg01088093	-0.0317473673301473	309	-0.553813427005947	0.580113650937568	0.00100789533239531	0.745964517191265
**CESC**	SIN3A	cg01664094	0.101977373796645	309	1.7873542485301	0.0748763590625267	0.0103993847664607	0.193667303152249
**CESC**	SIN3A	cg02028562	-0.202667353483614	309	-3.60851116855412	0.000359968483502395	0.0410740561680522	0.00284645133213919
**CESC**	SIN3A	cg07544511	-0.0137454167051141	309	-0.23968217284046	0.810738181052379	0.000188936480397229	0.897916687643809
**CESC**	SIN3A	cg10483776	0.0977246280443734	309	1.71208200389332	0.0879013570419835	0.00955010292641114	0.217202876781884
**CESC**	SIN3A	cg11184237	-0.296376337324638	308	-5.40168276715551	1.33704954000198e-07	0.0878389333259677	2.8280160933908e-06
**CESC**	SIN3A	cg15851274	-0.164026444049408	308	-2.89439294493073	0.00407476333109584	0.0269046743474934	0.0210560880760205
**CESC**	SIN3A	cg22059910	0.149453703411553	309	2.63541341375879	0.00883446993187604	0.0223364094634283	0.0387761653058206
**CESC**	SIN3A	cg25697800	-0.148871298003265	309	-2.62491025065482	0.00910529848418066	0.0221626633691768	0.039687224914744
**CESC**	SIN3B	cg06815056	-0.0663545435446269	309	-1.15948638096246	0.247167871854769	0.00440292544901579	0.440417266155201
**CESC**	SIRT1	cg02720207	-0.0529257306996404	309	-0.924086799140971	0.356174028629963	0.00280113297009086	0.554940965015809
**CESC**	SIRT1	cg03211233	-0.0957932010055735	309	-1.67792788961127	0.0943888842284582	0.0091763373588942	0.228501287059862
**CESC**	SIRT1	cg07832903	-0.0693999175564222	308	-1.21095680565509	0.226855625657271	0.0048163485568382	0.416403335276531
**CESC**	SIRT1	cg10582690	-0.0772188662733095	309	-1.35038897614631	0.177895673158951	0.00596275330853526	0.354413587863525
**CESC**	SIRT1	cg10705386	-0.101546955673796	309	-1.77973155334471	0.0761187876505376	0.0103117842066158	0.195955689019687
**CESC**	SIRT1	cg19192316	-0.0415485788250655	309	-0.725050317231428	0.468978875226804	0.00172628440238268	0.657874436399265
**CESC**	SIRT1	cg19681528	-0.0264841803786751	309	-0.461929493311055	0.644462275014406	0.000701411810330197	0.791439187687983
**CESC**	SIRT1	cg19980381	-0.118459249737863	309	-2.08005342789117	0.0383581294161644	0.0140325938484575	0.118664567492494
**CESC**	SIRT1	cg25513531	-0.060439971678013	309	-1.05573697742101	0.291926677852993	0.00365299017643902	0.489665483784239
**CESC**	SIRT4	cg04118464	-0.0797165820722343	303	-1.38051496126963	0.168462741099252	0.00635473345727927	0.341615561606245
**CESC**	SIRT4	cg07106325	0.0756084910035166	309	1.32206338515659	0.187140938704725	0.00571664391182885	0.366340542800588
**CESC**	SIRT4	cg08264848	-0.0483816475473886	305	-0.838977224511908	0.402150104291974	0.00234078381939973	0.598684652734419
**CESC**	SIRT4	cg12124973	-0.0339255590412563	308	-0.590878782301491	0.555042132393926	0.00115094355626177	0.726606969745001
**CESC**	SIRT4	cg20010714	-0.0405766380591458	309	-0.708060997515684	0.479449902523311	0.00164646355618292	0.666786860418476
**CESC**	SIRT4	cg21068152	0.0119190310107623	309	0.207830169767605	0.835500735226087	0.000142063300235514	0.911866667548341
**CESC**	SIRT4	cg21123573	-0.105759389082046	297	-1.81740972168471	0.0701792449216225	0.0111850483790077	0.184729053232558
**CESC**	SIRT5	cg01064902	-0.154850013684601	309	-2.7328661008378	0.00664616236318084	0.023978526738121	0.0310884225170639
**CESC**	SIRT5	cg02206980	-0.0711070911334169	309	-1.24294076977952	0.214847457826995	0.00505621840945605	0.401772635366915
**CESC**	SIRT5	cg02712746	-0.132881912923751	308	-2.33375758004495	0.0202621481748137	0.0176576027822752	0.0735510967808993
**CESC**	SIRT5	cg06139021	0.104918842940838	309	1.8394749882452	0.0668203399497254	0.0110079636040443	0.178173831227041
**CESC**	SIRT5	cg07534331	-0.213964003993179	309	-3.81903278077229	0.000162406384758745	0.0457805950047932	0.001456732652591
**CESC**	SIRT5	cg07909422	-0.05703056478521	309	-0.995982903419968	0.320050554057803	0.00325248531972003	0.519444479729048
**CESC**	SIRT5	cg08619119	-0.247665201663337	309	-4.45704660849518	1.16993778975549e-05	0.0613380521149412	0.000150262738015913
**CESC**	SIRT5	cg10907941	-0.255559465986263	309	-4.60887696243943	5.96647890939921e-06	0.0653106406551837	8.31523217982448e-05
**CESC**	SIRT5	cg11824369	-0.0739316542896058	309	-1.292579828738	0.197137988223477	0.00546588950599779	0.379382363344972
**CESC**	SIRT5	cg19366958	0.0236091474506387	309	0.411754321767376	0.680809665657463	0.000557391843345998	0.815857196610555
**CESC**	SIRT5	cg20150972	-0.0935084975797072	309	-1.63755132653621	0.102549972102769	0.0087438391196141	0.242561121472705
**CESC**	SIRT7	cg11691214	-0.274735225446473	307	-4.96546050726009	1.14827208780871e-06	0.0754794441011242	1.93435857078802e-05
**CESC**	SIRT7	cg12710567	-0.2783273435483	296	-4.95172064541959	1.2463551072929e-06	0.0774661101666537	2.07843406681123e-05
**CESC**	SIRT7	cg17552093	-0.0887894911570979	309	-1.55423626736745	0.121168483601326	0.00788357373993637	0.272495982113827

**CESC**	SIRT7	cg20397086	-0.173797314378791	299	-3.02605536362838	0.0026968168065659	0.0302055064852803	0.0151269858425256
**CESC**	SMARCA	cg03460558	-0.14592052482487	309	-2.57173836129119	0.0105939667218618	0.0212927995651655	0.0445832155212236
**CESC**	SMARCA	cg06675660	-0.0821421600607482	309	-1.43705384601821	0.151731102303971	0.00674733445944558	0.318172070168619
**CESC**	SMARCA	cg08617833	-0.343415435410125	309	-6.37538038368563	6.76770656824668e-10	0.117934161277926	2.30410050227651e-08
**CESC**	SMARCA	cg12935118	-0.0246828028632792	309	-0.430490529465012	0.667143893313679	0.000609240757187502	0.806937388815467
**CESC**	SMARCA	cg17420696	0.0031777713366281	309	0.0554066162432702	0.955850920879838	1.00982306678952e-05	0.976565972442971
**CESC**	SMARCA	cg18959966	-0.243309205267673	309	-4.37367523972202	1.68044866594322e-05	0.0591993693679868	0.000205774946510919
**CESC**	SMARCA	cg24447042	-0.373137906537998	309	-7.01234185337412	1.52265426875904e-11	0.13923189729556	6.96374874684542e-10
**CESC**	SMARCA	cg26010110	-0.296636414052093	309	-5.41579487562546	1.24162889442716e-07	0.0879931621416847	2.64416310465552e-06
**CESC**	SMARCA	cg27353825	-0.0865075243169571	308	-1.5114937001218	0.131704918203652	0.00748355176344893	0.288681752142232
**CESC**	SMARCA	cg04518912	-0.0200671501316457	309	-0.349953186544129	0.726616156044086	0.000402690514406009	0.845192752613899
**CESC**	SMARCA	cg13529335	0.0994578129525139	309	1.74274714742398	0.0823889548605762	0.00989185655729725	0.20730880597806
**CESC**	SMARCA	cg14290704	0.0176212564552883	307	0.306272342277753	0.759608331861651	0.000310508679063039	0.866180192155704
**CESC**	SMARCA	cg21210735	-0.215557464484313	309	-3.84885494383892	0.00014466599119168	0.0464650204949058	0.00131920596867489
**CESC**	SMARCA	cg08405378	-0.0801753956416259	309	-1.40242050184009	0.161810056281303	0.00642809406629124	0.332273559242627
**CESC**	SMARCA	cg17218495	0.143495571849855	309	2.52809413575108	0.0119745993854586	0.0205909791405168	0.0490600371072451
**CESC**	SMARCA	cg17747332	-0.0375385917861201	309	-0.654969348677079	0.51298276054836	0.00140914587328496	0.694711038125812
**CESC**	SMARCA	cg18522266	-0.114966630194307	309	-2.01789161607531	0.0444807769492074	0.0132173260582346	0.132432835622743
**CESC**	SMARCA	cg23892580	-0.068275963575475	309	-1.19321650405564	0.233714982454044	0.00466160720215958	0.424617012799201
**CESC**	SMARCA	cg01278696	-0.0303087206306362	309	-0.528693490964386	0.597403866872612	0.000918618546265953	0.758275077989932
**CESC**	SMARCA	cg01575392	-0.0940945993484488	309	-1.64790673106474	0.100404933188161	0.00885379362654511	0.238894707989963
**CESC**	SMARCA	cg02782292	0.029489208214325	309	0.514385621085025	0.607356146994019	0.00086961340110781	0.765419958712177
**CESC**	SMARCA	cg05358385	-0.031526600916607	309	-0.549958446816451	0.582751696203395	0.000993926565355003	0.74787332895257
**CESC**	SMARCA	cg10878459	-0.12496562718033	309	-2.19606495711389	0.0288424248298521	0.0156164079765733	0.0958793093416594
**CESC**	SMARCA	cg12253543	0.01840257394807	308	0.320385930556867	0.748896622239424	0.000338654727914185	0.859325552278288
**CESC**	SMARCA	cg13848802	-0.183039062842223	282	-3.09872816801717	0.00214343431428945	0.0335032985261591	0.0125599826036332
**CESC**	SMARCA	cg13898584	-0.0193586839253743	309	-0.337593451602301	0.73590256346325	0.000374758643322546	0.851051497870785
**CESC**	SMARCA	cg16091845	-0.229170479144609	309	-4.10497233477339	5.20068510337613e-05	0.0525191085113695	0.000548929636865349
**CESC**	SMARCA	cg16587443	-0.112033385534402	285	-1.88655401414492	0.0602549602595351	0.0125514794743	0.165162410712458
**CESC**	SMARCA	cg19993723	0.0529693355550533	309	0.924850284100567	0.355777338869221	0.00280575050914383	0.554578361752704
**CESC**	SMARCA	cg20045045	-0.129320550931126	309	-2.2738749397938	0.0236705001081371	0.01672380489313	0.0827484615428499
**CESC**	SMARCA	cg22077701	-0.0165917659311019	309	-0.289327150653132	0.772528252307945	0.000275286696712474	0.873886708933188
**CESC**	SMARCA	cg24607315	-0.158958471799758	309	-2.80722881578491	0.00531986257668978	0.0252677957569146	0.0260563740660457
**CESC**	SMARCB	cg01745726	0.0189361304823957	309	0.330221926926354	0.741459730922468	0.000358577037646316	0.854527714648793
**CESC**	SMARCB	cg01912455	-0.0977343848822226	309	-1.71225458678426	0.0878695169106388	0.00955200998830643	0.217151790036095
**CESC**	SMARCB	cg03979008	0.144934161543413	309	2.55398016141998	0.0111375721400575	0.0210059111822922	0.0463706870250807
**CESC**	SMARCB	cg10122452	-0.0892722631892986	309	-1.56275476745791	0.119150651642768	0.0079695369749394	0.269391007727781
**CESC**	SMARCB	cg16142952	0.145757810778949	309	2.56880836640097	0.0106819917821586	0.0212453394030718	0.0448749794092908
**CESC**	SMARCB	cg19906397	0.097785047630701	309	1.71315073796925	0.0877043346087418	0.00956191554013847	0.216860689293511
**CESC**	SMARCC	cg02564102	0.160045351755754	309	2.82692609074493	0.00501134168318217	0.025614514618623	0.0248139720515227
**CESC**	SMARCC	cg02627110	-0.256236730407078	309	-4.62194814447644	5.6257248156652e-06	0.0656572620097095	7.90262976363052e-05
**CESC**	SMARCC	cg05592996	-0.482103375039822	307	-9.56275115979033	4.23836137149429e-19	0.232423664224788	5.15186069584417e-17
**CESC**	SMARCC	cg07143957	0.100409676964572	309	1.75959522071107	0.079482279086262	0.0100821032281296	0.20208001893054
**CESC**	SMARCC	cg07304068	-0.132100718036199	309	-2.32361821663313	0.020805240004471	0.0174505997056795	0.075049746865799
**CESC**	SMARCC	cg07474652	0.0653015136895661	309	1.14100618451868	0.254765388516397	0.00426428769014859	0.449018297520211
**CESC**	SMARCC	cg12894336	-0.128144919124307	309	-2.25285679277329	0.0249810161259921	0.0164211202973753	0.086153884315531
**CESC**	SMARCC	cg16250355	0.102999407694724	309	1.80545851789821	0.0719921503209935	0.010608877985464	0.188165479843817
**CESC**	SMARCC	cg20685352	-0.144846068445778	309	-2.55239453791572	0.011187300877855	0.0209803835441989	0.046536503360881
**CESC**	SMARCC	cg22984439	-0.171772944882003	309	-3.04015070862877	0.0025702840785481	0.0295059445934356	0.0145494200397709
**CESC**	SMARCD	cg02553054	0.0774513120407284	309	1.35447843857674	0.176589628988995	0.00599870573683028	0.352590051783016
**CESC**	SMARCD	cg04550398	-0.152747137519081	309	-2.69486071548896	0.00743348883342488	0.0233316880202732	0.0339435397178479
**CESC**	SMARCD	cg09878554	-0.048645595494749	309	-0.849170270043793	0.396454753369546	0.00236639396103874	0.593088748194887
**CESC**	SMARCD	cg13349421	-0.0383214845982897	309	-0.668649062975961	0.504226771046976	0.00146853618181695	0.687496987112654
**CESC**	SMARCD	cg17846064	-0.0767817165632771	308	-1.3404885128205	0.181090770698661	0.00589543199840342	0.358339914934416
**CESC**	SMARCD	cg18591973	-0.0564883507600258	309	-0.986483208654622	0.324680537089642	0.00319093377158771	0.524054166258242
**CESC**	SMARCD	cg20405918	-0.0259156790143808	309	-0.452007118059458	0.651586193042435	0.000671622418776417	0.796374322086577
**CESC**	SMARCD	cg22402853	-0.16908751053226	309	-2.99121181775073	0.00300612051893207	0.028590586217997	0.0164909484274594

**CESC**	SMARCD	cg25107077	-0.0742997665459525	309	-1.29905133537996	0.194910703036114	0.00552045530878304	0.376437021648248
**CESC**	SMARCD	cg00945038	0.00689492912149254	309	0.120220054721372	0.904388245380868	4.75400475904058e-05	0.949357680480391
**CESC**	SMARCD	cg02127607	0.0392074294769621	309	0.684130924673621	0.494413669801346	0.00153722252619096	0.679523992589059
**CESC**	SMARCD	cg02360836	0.0497859399873985	309	0.869125317446657	0.385464251398361	0.00247863982042885	0.583485818146525
**CESC**	SMARCD	cg04367190	0.109676586392262	309	1.9238827826658	0.0553027598497023	0.0120289536026593	0.1552359108018
**CESC**	SMARCD	cg06420487	-0.178448791315725	309	-3.16211547369357	0.00172443351907111	0.0318439711220432	0.0105364892540542
**CESC**	SMARCD	cg07362569	0.0538561213486893	309	0.940378327252795	0.347770144984441	0.00290048180672474	0.54708466106365
**CESC**	SMARCD	cg11697258	-0.00648540047275926	309	-0.113079199180866	0.910042402092289	4.20604192920661e-05	0.952265517025831
**CESC**	SMARCD	cg12577610	-0.126017352505382	309	-2.21484427467064	0.0275120011254976	0.0158803731324657	0.0925361500954196
**CESC**	SMARCD	cg15868002	-0.155949918934421	309	-2.75275986544865	0.00626481680895488	0.0243203772156525	0.0296757692769796
**CESC**	SMARCD	cg19016967	-0.118494817127459	309	-2.08068685658164	0.0382996453202834	0.0140410216860699	0.118534476768226
**CESC**	SMARCD	cg19143629	0.0257911458711059	309	0.449833629736859	0.653150987695347	0.000665183205344663	0.797426637843843
**CESC**	SMARCD	cg21254277	-0.0868265223490254	309	-1.51961103880011	0.129648094085951	0.00753884498322581	0.285560413520584
**CESC**	SMARCD	cg23682543	-0.18076365930065	309	-3.20451141214789	0.00149666723404611	0.0326755005237615	0.00938391102485814
**CESC**	SMARCD	cg24795630	-0.0622576632859177	309	-1.08760929318599	0.277629165121934	0.0038760166378227	0.474412777102797
**CESC**	SMARCD	cg25945412	-0.00473306795825799	309	-0.0825247840543173	0.934283720690372	2.24019322974884e-05	0.96508219221312
**CESC**	SMARCD	cg00864064	-0.0716047807447648	309	-1.25168498383139	0.211646925915748	0.00512724462550583	0.397730776498048
**CESC**	SMARCD	cg02917064	-0.0089658939679468	309	-0.156331986655019	0.875875109689891	8.03872546444648e-05	0.934340215710097
**CESC**	SMARCD	cg14795305	-0.0111188568066122	307	-0.193237075467635	0.846903198485847	0.000123628976685947	0.918218132219423
**CESC**	SMARCD	cg19786287	-0.0338997922506395	309	-0.591402991146684	0.554689932518118	0.00114919591463652	0.726346994736731
**CESC**	SMARCD	cg19902439	-0.0853099341844746	309	-1.49287184812679	0.13650781735685	0.00727778487055938	0.296005383639726
**CESC**	SMARCD	cg22750243	-0.0831789453650853	309	-1.45531768675115	0.146613169058033	0.00691873695204785	0.31093247024973
**CESC**	SMARCD	cg22787816	-0.0660672367385203	309	-1.15444389343705	0.249224924943531	0.00436487977026368	0.4427003843595
**CESC**	SMNDC1	cg03999743	-0.0165986656955411	309	-0.289447501870711	0.772436251616239	0.000275515702872333	0.873833463117974
**CESC**	SMNDC1	cg08770590	-0.0907726535229603	309	-1.58923623185593	0.113046489129327	0.00823967462759939	0.260056734048758
**CESC**	SMNDC1	cg11559952	0.131929863732635	309	2.3205596625486	0.0209722205390118	0.0174054889445117	0.0755260126197519
**CESC**	SMNDC1	cg12486840	0.147132141706863	309	2.59356269322413	0.00995844716445027	0.0216478671232485	0.0424799823825873
**CESC**	SMNDC1	cg17981675	-0.00277225134207417	309	-0.0483360395254976	0.96148017251362	7.68537750363204e-06	0.979823418084945
**CESC**	SMNDC1	cg18209001	-0.0211331120553565	309	-0.368550707363681	0.712719163136088	0.000446608425144253	0.836505756872285
**CESC**	SMNDC1	cg21872391	-0.0209670527315746	309	-0.365653438627415	0.714877991862428	0.000439617300248629	0.837841624521711
**CESC**	SMNDC1	cg23721301	-0.106454363753257	309	-1.86670262572795	0.0629056629295206	0.0113325315621108	0.170392867059162
**CESC**	SMNDC1	cg26944434	0.0342652065428417	309	0.597785324650972	0.550428227039732	0.0011741043794236	0.723042340588445
**CESC**	SMYD1	cg00578637	0.0829944878012512	309	1.45206796616665	0.147513976491894	0.00688808500539203	0.312247603871169
**CESC**	SMYD1	cg00666842	0.0909787693401711	309	1.59287496591123	0.112227440198501	0.00827713647065207	0.25861362598466
**CESC**	SMYD1	cg03704645	0.0249216510002474	309	0.434658834673154	0.664118455221023	0.000621088688578133	0.804904904505029
**CESC**	SMYD1	cg14559139	-0.0252271360165142	309	-0.439990175126052	0.660256871979034	0.000636408391595709	0.802362270350178
**CESC**	SMYD1	cg16090764	0.0876285756455017	309	1.53375645069847	0.126129603908022	0.00767876726965941	0.280182280247602
**CESC**	SMYD1	cg18148488	0.0874100051372346	309	1.52990133545761	0.127081022097689	0.00764050899809138	0.281784888640622
**CESC**	SMYD1	cg19074493	0.000924359322297722	309	0.016116762379068	0.987151812908173	8.54440156718705e-07	0.993204285131626
**CESC**	SMYD2	cg01770831	-0.0364578737294602	309	-0.636087626078939	0.525197902173496	0.00132917655687326	0.70420245339856
**CESC**	SMYD2	cg04406444	-0.0106414509940047	309	-0.185550544195744	0.852920979760157	0.000113240479257803	0.921640757793606
**CESC**	SMYD2	cg11523160	-0.0406496217430922	309	-0.70933666640888	0.478659243345781	0.00165239174785648	0.666132686526685
**CESC**	SMYD2	cg14013804	0.0222560471924676	309	0.38814358418395	0.698181440793682	0.000495331636633347	0.826915611232685
**CESC**	SMYD2	cg27570636	-0.0227893263751821	309	-0.397448704214259	0.691315524661908	0.00051935339663457	0.82259066924966
**CESC**	SMYD5	cg01329511	-0.113827951985742	309	-1.99764187841362	0.0466463292770355	0.0129568026532683	0.13714341643263
**CESC**	SMYD5	cg05103132	0.0280706497603425	309	0.489621441747081	0.624754917044074	0.000787961377967818	0.777796725983432
**CESC**	SMYD5	cg06754047	0.0635832887256823	309	1.11086030661542	0.267506313937158	0.00404283460517348	0.463266316120017
**CESC**	SMYD5	cg08834152	0.0719313701104813	309	1.25742354295703	0.209565422976288	0.00517412200597105	0.395031076205896
**CESC**	SMYD5	cg15623359	-0.127920570606271	309	-2.24884695155691	0.0252380843189805	0.016363672384234	0.0868402641209593
**CESC**	SMYD5	cg17895873	-0.0699117448764981	309	-1.22194279699495	0.222675907182987	0.00488765207167655	0.411361628158754
**CESC**	SMYD5	cg20402064	0.137875633671462	309	2.42712394345126	0.015799906055588	0.0190096903603071	0.0607380462598466
**CESC**	SMYD5	cg25886337	0.0187260759700137	309	0.326557552372229	0.744227262405782	0.000350665921234725	0.85631651139009
**CESC**	SMYD5	cg26467949	-0.105408653543695	309	-1.84815878572994	0.0655504932059443	0.0111109842418948	0.175630620577346
**CESC**	SP100	cg05876232	0.215234471244468	309	3.84280741787471	0.000148108211205193	0.0463258776118859	0.00134727902040421
**CESC**	SP100	cg09456094	-0.21167868968684	309	-3.77631802903078	0.000191430057238244	0.0448078676675377	0.00167270693842434
**CESC**	SP100	cg11702456	-0.139193019780951	309	-2.45077082186162	0.014818203610667	0.0193746967557402	0.0578121902196652
**CESC**	SP100	cg11827097	-0.030876009116941	309	-0.538598405752656	0.590558144924859	0.000953327938989421	0.753449822082405

**CESC**	SP100	cg23539753	-0.327919707686784	309	-6.05212405513399	4.2018043231118e-09	0.107531334689386	1.2351585989488e-07
**CESC**	SP100	cg27140170	-0.0530433202414635	309	-0.926145706917986	0.355104906979719	0.00281359382223845	0.554003729901768
**CESC**	SP140L	cg04002454	-0.230859921674883	309	-4.13693120945416	4.56049378594519e-05	0.0532963034357331	0.000489936202906086
**CESC**	SP140L	cg05217331	0.161827263799994	309	2.85924214076849	0.00454022124688949	0.026188063308993	0.0229456227948107
**CESC**	SP140L	cg13278004	-0.275827119261244	309	-5.00330193678805	9.55195156333298e-07	0.0760805997199567	1.6443560962999e-05
**CESC**	SP140L	cg24044052	-0.357039571943464	309	-6.66445694646571	1.24816100371709e-10	0.127477255933572	4.9015364524567e-09
**CESC**	SP140L	cg25095994	-0.350198694212529	309	-6.51871782980336	2.94649161637261e-10	0.12263912542816	1.0751377523644e-08
**CESC**	SP140L	cg26847438	-0.261018594133223	309	-4.71444677850744	3.69669294241375e-06	0.0681307064832842	5.4476327100234e-05
**CESC**	SSRP1	cg01511567	-0.0236561347228321	309	-0.412574259661272	0.680209371649097	0.000559612710024783	0.815488108278289
**CESC**	SSRP1	cg01766191	-0.0817212235507243	309	-1.42964004132792	0.153847210385773	0.00667835837862745	0.321186965213505
**CESC**	SSRP1	cg02688348	0.0605614971309031	309	1.05786753322112	0.290955704833952	0.00366769493473639	0.488522394047618
**CESC**	SSRP1	cg04937561	-0.0345815530789625	309	-0.603310833474392	0.546751783490272	0.0011958838133531	0.72028057042678
**CESC**	SSRP1	cg08821673	-0.0637470484412245	309	-1.11373300276879	0.266273548359296	0.00406368618496783	0.462088079349136
**CESC**	SSRP1	cg09381635	-0.0791465127688464	309	-1.38430918931204	0.167279116035627	0.00626417048346916	0.340041822843836
**CESC**	SSRP1	cg11948159	-0.0804112083906807	309	-1.40657212118604	0.160575720777762	0.00646596243484947	0.330489116413176
**CESC**	SSRP1	cg12795368	-0.00850707618531433	309	-0.148331309078283	0.882179647840186	7.23703452227421e-05	0.937468015245737
**CESC**	SSRP1	cg12911972	-0.0436918718969063	309	-0.762521984173845	0.446339767226731	0.00190897966985567	0.638153238345855
**CESC**	SSRP1	cg16313264	0.0983807559124323	309	1.72368897168135	0.085780705895184	0.00967877313390159	0.213433897345677
**CESC**	SSRP1	cg22636253	-0.0293334322024043	309	-0.511666045510432	0.609256212503369	0.00086045024477305	0.766834031120358
**CESC**	SSRP1	cg26706201	-0.0108787009931087	309	-0.189687857808379	0.849680326524566	0.000118346135297465	0.919822691566618
**CESC**	STK31	cg00755064	-0.827781641325598	309	-25.7247228055273	2.74253249537169e-78	0.685222445715701	4.44484526968636e-75
**CESC**	STK31	cg05000488	-0.453782411422924	309	-8.87875158131399	5.99074201802826e-17	0.205918476916803	5.90676533017098e-15
**CESC**	STK31	cg05407490	-0.454989215029389	309	-8.90851793891637	4.84315222156944e-17	0.20701518579306	4.84277871368603e-15
**CESC**	STK31	cg11755819	-0.714569158701595	309	-17.8095566008274	4.28992347892669e-49	0.510609082567506	2.36688161077747e-46
**CESC**	STK31	cg13313214	-0.725387985842008	309	-18.374012122576	3.09360632824696e-51	0.526187730003925	1.91003183094956e-48
**CESC**	STK31	cg13514718	-0.302572296547394	309	-5.5349721180203	6.72940092469526e-08	0.0915499946379644	1.52270801493817e-06
**CESC**	STK31	cg13802883	-0.287012868574538	308	-5.2154332707896	3.40418352508808e-07	0.0823763867273848	6.51957294807244e-06
**CESC**	STK31	cg14898779	-0.454034975177176	309	-8.88497607788295	5.73037479398116e-17	0.206147758684139	5.67883792003784e-15
**CESC**	STK31	cg17102984	-0.722504851693018	309	-18.220892063617	1.17821412800738e-50	0.52201326071995	7.05061460570813e-48
**CESC**	STK31	cg17331904	-0.805247217680632	309	-23.6785905133319	5.60460106569367e-71	0.6484230815824	6.60612629249354e-68
**CESC**	STK31	cg21519371	-0.660765450997122	309	-15.348994489654	9.14449255686554e-40	0.43661098123143	3.68594121227356e-37
**CESC**	STK31	cg25570411	-0.589081810157349	309	-12.7104695077679	5.65439447563146e-30	0.347017379058259	1.55433909483136e-27
**CESC**	STK31	cg25612145	-0.573904713983352	309	-12.2189531877964	3.34355080191784e-28	0.329366620732313	8.41774081179275e-26
**CESC**	SUV39H1	cg05903800	0.0272340311800233	309	0.475017750172298	0.635115408989278	0.000741692454314482	0.785058686075896
**CESC**	SUZ12	cg00160853	-0.0565695501367305	309	-0.987905781509633	0.323984424623371	0.00320011400267207	0.523394129979245
**CESC**	SUZ12	cg01109633	0.00967911261379225	309	0.168769000752415	0.866090577334896	9.36852209904723e-05	0.928833460251601
**CESC**	SUZ12	cg07482957	-0.0182693256890437	309	-0.318589749791247	0.750256397456899	0.000333768261132353	0.860059300982195
**CESC**	SUZ12	cg08177746	0.0545679086651117	309	0.952843675999848	0.341426271542368	0.00297765665608397	0.540614181847855
**CESC**	SUZ12	cg17335595	-0.10707902672299	309	-1.87778291482368	0.0613680741866815	0.0114659179639428	0.167340879921438
**CESC**	SUZ12	cg25098644	-0.0601855349548734	309	-1.05127640481982	0.293966604341078	0.00362229861780429	0.491750091777439
**CESC**	TAF3	cg00194465	-0.0255773080587184	309	-0.446101547699453	0.655841462687333	0.000654198687530581	0.799331506811925
**CESC**	TAF3	cg02334915	0.120825558866651	309	2.12221345883758	0.0346281889262543	0.0145988156754386	0.109958175000164
**CESC**	TAF3	cg03672997	0.0399426291154454	309	0.696979742835507	0.486348100398721	0.00159541362065403	0.672824927043162
**CESC**	TAF3	cg04179819	0.0725018637165366	309	1.26744876282779	0.205964870509279	0.00525652024237125	0.390358496854468
**CESC**	TAF3	cg04234156	0.012080032364191	309	0.210637930701379	0.833310928158396	0.000145927181919902	0.910590604421767
**CESC**	TAF3	cg05976695	-0.0495696549289872	309	-0.865340255584242	0.387534421850756	0.00245715068977887	0.585440671381421
**CESC**	TAF3	cg07855525	-0.141347293666521	309	-2.48946836123441	0.0133278671178112	0.0199790574268499	0.0533371542917727
**CESC**	TAF3	cg09871669	0.0490711861603312	309	0.856617346953492	0.392331127677633	0.00240798131118188	0.58935733453775
**CESC**	TAF3	cg09994553	-0.0205589778095117	284	-0.343475228230686	0.731499658441009	0.000422671568571996	0.848258719088332
**CESC**	TAF3	cg11104088	0.0678749822911069	309	1.18617627357399	0.236478927822928	0.00460701322101807	0.427838826741479
**CESC**	TAF3	cg12176426	0.0219101210932423	309	0.382107741816043	0.702648425944055	0.000480053406320539	0.82986482165016
**CESC**	TAF3	cg12590721	-0.0302823086335289	309	-0.528232347335864	0.597723464975803	0.000917018216176301	0.758466394283322
**CESC**	TAF3	cg12777293	0.0232184379035586	309	0.404936462293387	0.685809005773633	0.000539095858681408	0.8191581994857
**CESC**	TAF3	cg27268107	-0.0890845375082435	308	-1.55687523623683	0.120543953818731	0.00793605482305765	0.271507465281981
**CESC**	TCEA1	cg01677098	-0.0941287351492896	309	-1.64850990478948	0.100281106730005	0.00886021878080512	0.238717541634817
**CESC**	TCEA1	cg06047473	0.0470998301476058	309	0.822126005484201	0.411649526121217	0.00221839399993331	0.607329235052323
**CESC**	TCEA1	cg11530293	0.0617764495706023	309	1.07917040656244	0.281367250985417	0.00381632972154917	0.478556241351135

CESC	TCEA1	cg11787876	-0.0410965966959272	309	-0.717149511362795	0.473832495405715	0.0016889302599877	0.661806979561065
CESC	TCEA1	cg12850379	-0.112961211119959	309	-1.98223350189047	0.0483532888742503	0.012760235217688	0.140710216040612
CESC	TCEA1	cg23569945	0.0410208066096097	308	0.714646404334317	0.475377780952231	0.001682706574903	0.663155669967325
CESC	TCEA1	cg26181865	-0.120744738068592	309	-2.12077289054269	0.0347503035827825	0.0145792917712529	0.110246935969943
CESC	TCEA1	cg26358945	0.00212183238820749	308	0.0369345971258496	0.970561475326138	4.50217268364628e-06	0.984417781346844
CESC	TDRD1	cg00337703	-0.282818471427414	309	-5.14099773276962	4.90170036384181e-07	0.0799862877805392	9.05758755118836e-06
CESC	TDRD1	cg02635786	-0.714935097554168	303	-17.6513948858732	3.15029682728647e-48	0.511132193714788	1.70190410681892e-45
CESC	TDRD1	cg16550453	-0.867122991769567	309	-30.3533136358586	5.06126555440164e-94	0.751902282855404	1.12496026439749e-90
CESC	TDRD1	cg23504215	-0.680237640936288	309	-16.1807417810025	6.5457014451594e-43	0.462723248146566	2.92653044956742e-40
CESC	TDRD1	cg23825092	-0.283369348840635	309	-5.15188479520671	4.64704800895647e-07	0.0802981878623655	8.64242057874156e-06
CESC	TDRD1	cg24203376	-0.640184822115515	309	-14.5296793914356	1.09548146492429e-36	0.409836606467073	3.96380860848003e-34
CESC	TDRD1	cg26600802	-0.832394395505457	309	-26.1885946409837	6.45548445132098e-80	0.692880429668896	1.09173469001318e-76
CESC	TDRD1	cg27547703	-0.8375801084808	307	-26.6432425060959	2.79032703325645e-81	0.701540438122709	4.93342502784437e-78
CESC	TDRD3	cg01557749	-0.104763984318707	309	-1.83672979896716	0.067225983269613	0.0109754924103303	0.178984108589811
CESC	TDRD3	cg03447530	-0.24536798043994	309	-4.41304359906435	1.41729409108076e-05	0.0602054458251747	0.000177490303479614
CESC	TDRD3	cg05666423	-0.370074709759883	309	-6.9455965337675	2.29292837936803e-11	0.136955290803862	1.0169673337774e-09
CESC	TDRD3	cg10765900	0.00607561325216375	309	0.105933891935839	0.915704670199609	3.69130763898678e-05	0.955461313002245
CESC	TDRD3	cg18522102	-0.127697148998042	309	-2.24485402603762	0.0254963514064013	0.0163065618622281	0.087465853565709
CESC	TDRD3	cg19937698	-0.0897939175045004	309	-1.57196059267354	0.116999862218321	0.00806294762080502	0.266098151773485
CESC	TDRD3	cg20944895	-0.322421382304006	309	-5.93876102591339	7.84038930228071e-09	0.103955547766826	2.19243438311152e-07
CESC	TDRD3	cg22045829	0.0978484996720619	309	1.71427313232752	0.0874978053815034	0.0095743288880735	0.216466982726941
CESC	TDRD3	cg23103367	-0.19743234411004	309	-3.51146848198907	0.000513191453127554	0.0389795305007854	0.00383177041027017
CESC	TDRD3	cg23767211	0.0317553272571773	309	0.553952423216188	0.580018638023491	0.00100840080921043	0.745928649326338
CESC	TDRD3	cg26737458	-0.117222175857779	309	-2.05802709904948	0.0404397887837805	0.013741038512832	0.123347301652438
CESC	TDRD5	cg05378938	-0.393182408822281	309	-7.45586092863098	9.37911722463432e-13	0.154592406607292	5.30260934137501e-11
CESC	TDRD5	cg07171496	-0.397298963771834	309	-7.54846397240707	5.16693529361812e-13	0.157846466614173	3.05518503941204e-11
CESC	TDRD5	cg07901138	-0.392647417097448	309	-7.44386530966051	1.01286028051353e-12	0.154171994153297	5.70560844766617e-11
CESC	TDRD5	cg09656934	-0.43388044464446	309	-8.39646184837872	1.7736327759505e-15	0.188252240244875	1.46629105390323e-13
CESC	TDRD5	cg11853788	-0.498473126499336	309	-10.0255211544458	1.26378660306579e-20	0.248475457842023	1.76508105922621e-18
CESC	TDRD5	cg12277666	-0.362531313905467	309	-6.78234133502326	6.17007577503351e-11	0.131428953562024	2.54234573539702e-09
CESC	TDRD5	cg13848905	-0.317044086721562	309	-5.82854237214557	1.42584107970914e-08	0.10051695292511	3.77028827175026e-07
CESC	TDRD5	cg15545772	-0.383008432468478	309	-7.22924759467932	3.95235699308382e-12	0.146695459341961	2.0254918308298e-10
CESC	TDRD7	cg24339935	-0.0809164007560033	309	-1.41546712498213	0.157955200554892	0.00654746391130613	0.326932965552917
CESC	TDRD9	cg07613153	0.352677334655352	308	6.56056663366044	2.31581311488338e-10	0.124381302379603	8.61579940025048e-09
CESC	TDRD9	cg08933517	0.247762241517361	309	4.45890715182908	1.16044961453282e-05	0.0613861283217072	0.000149191897724287
CESC	TDRD9	cg16701547	0.23589331556117	309	4.23238274618569	3.06562039686308e-05	0.0556456563264418	0.000347294121382797
CESC	TRIM24	cg00733782	-0.198558026451697	309	-3.53230881523485	0.000475870232596613	0.0394252898683929	0.00359450906637741
CESC	TRIM24	cg02308366	-0.177490822984126	309	-3.14458655560692	0.00182764492236973	0.0315029922435824	0.0110439497507248
CESC	TRIM24	cg06386929	-0.17746442044607	309	-3.14410357255409	0.00183056788651235	0.0314936205242593	0.0110564594847315
CESC	TRIM24	cg06650216	-0.177757757819343	309	-3.14947000291229	0.00179833194707596	0.03159782046496	0.0108984051880111
CESC	TRIM24	cg10571650	0.113145151571135	309	1.98550309566504	0.0479867370993805	0.0128018253240551	0.140024913159334
CESC	TRIM24	cg10703331	-0.087196249044313	309	-1.52613135153855	0.128016849325458	0.00760318584739785	0.283037082260692
CESC	TRIM24	cg13935558	0.0715745865125366	309	1.25115445613607	0.211840115814	0.00512292143444059	0.397959237150379
CESC	TRIM24	cg26889928	-0.157552930483415	309	-2.78177202888501	0.00574410757491898	0.0248229259039117	0.0277000436823238
CESC	TRIM33	cg00760200	-0.114405425077968	309	-2.00791039630339	0.0455372971559128	0.0130886012872705	0.134768640909499
CESC	TRIM33	cg02852257	-0.0356126840600987	307	-0.619275292319838	0.536201902857374	0.00126826326596441	0.712400428178735
CESC	TRIM33	cg05792124	-0.030062338590198	309	-0.524391795268418	0.600388207484958	0.000903744201511711	0.760516267360002
CESC	TRIM33	cg16036305	-0.0890345658706394	309	-1.55856046601536	0.120140850698489	0.00792715391977322	0.270935600147241
CESC	TRIM33	cg17645130	-0.0118875807878162	308	-0.206940495153902	0.836195328668031	0.000141314576986858	0.912304771098407
CESC	TRIM33	cg18199720	0.0974383103385807	309	1.70701772230527	0.0888398459981314	0.00949422432163756	0.218847592766898
CESC	TRIM33	cg20932765	-0.224065016327835	309	-4.00862967404612	7.68931411430821e-05	0.0502051315419931	0.000767786551416369
CESC	TRIM33	cg20992663	-0.135338297015733	309	-2.38161602254632	0.0178520762702042	0.0183164546391189	0.0667241343339254
CESC	TRIM33	cg21666217	0.0816847344797954	309	1.42899740866471	0.154031692343148	0.00667239584703467	0.32145996013904
CESC	TRIM33	cg21813112	-0.153748827418525	308	-2.70849381671786	0.00714315753805916	0.0236387019325714	0.0329261597153389
CESC	TRIM66	cg06492264	-0.16111911116595	309	-2.84639594652947	0.00472244874160703	0.0259593679829057	0.0236974522965471
CESC	TRIM66	cg11245569	0.0718795142375877	287	1.21019268336521	0.227218374055604	0.00516666456703157	0.416823311983437
CESC	TRIM66	cg12608775	-0.0566803020483035	309	-0.989846131846688	0.323036522545024	0.00321265664028692	0.522459526712425

**CESC**	TRIM66	cg13143389	-0.0326920037437061	309	-0.570309407765067	0.56888912193412	0.00106876710877849	0.737234055501298
**CESC**	TRIM66	cg27044202	-0.108193953307143	309	-1.89756511299913	0.0587007181226552	0.0117059315322282	0.162049810703827
**CESC**	UBE2A	cg00142683	-0.130718209815409	309	-2.29887522098873	0.0221900901016232	0.0170872503773453	0.0787270159001189
**CESC**	UBE2A	cg00428314	-0.206854389970977	309	-3.68635906038552	0.000269327203741108	0.0427887386502649	0.00223066220052047
**CESC**	UBE2A	cg00935924	-0.214872791338986	309	-3.83603711600701	0.000152053733733426	0.0461703164578075	0.00137785301829449
**CESC**	UBE2A	cg05206294	-0.150374973998447	309	-2.65203344285789	0.00842067029579987	0.0226126328050335	0.0373689460919256
**CESC**	UBE2A	cg06206449	-0.149039830499925	309	-2.62794929644762	0.00902617960185052	0.0222128710754465	0.0394351688164865
**CESC**	UBE2A	cg06273903	-0.0878724025932583	309	-1.53805729883367	0.125074770849699	0.00772155913751167	0.278519986683439
**CESC**	UBE2A	cg09080960	-0.235002967396305	309	-4.21547251615032	3.29084068801415e-05	0.0552263946850689	0.00036936612391195
**CESC**	UBE2A	cg14625604	-0.125656670152945	309	-2.20840317843209	0.0279622014422093	0.0157895987539259	0.0937140918057571
**CESC**	UBE2A	cg15661671	-0.21354066618363	309	-3.81111525123645	0.000167450987655783	0.0455996161141483	0.00149594420460427
**CESC**	UBE2A	cg16211147	-0.143562679131587	309	-2.52930130091927	0.0119343532947537	0.0206102428394391	0.0489338048917972
**CESC**	UBE2A	cg17976175	-0.149250833390682	309	-2.63175451569788	0.00892798415192259	0.022275811267813	0.0391071846348348
**CESC**	UBE2A	cg18990292	-0.172778739289268	309	-3.05849803998746	0.00242243867091562	0.0298524927503888	0.0138720054446234
**CESC**	UBE2A	cg25165659	-0.153486808044215	309	-2.70822451490314	0.00714753702626378	0.0235582002436018	0.032942854512707
**CESC**	UHRF2	cg06523306	0.35465239324407	309	6.61346257820537	1.68834119856864e-10	0.125778320033746	6.46372122054375e-09
**CESC**	UHRF2	cg08487038	0.035174352118556	309	0.613665526899502	0.539895274131889	0.00123723504696016	0.71515968185762
**CESC**	UHRF2	cg13428749	-0.0130463699342566	309	-0.227490593650924	0.820195200921675	0.000170207768461475	0.903333308283207
**CESC**	UHRF2	cg14078101	0.0483110832549985	309	0.843317228612223	0.399714103061034	0.00233396076527139	0.596359152574318
**CESC**	UHRF2	cg20595300	0.311759361919194	309	5.72083014184695	2.53732459394508e-08	0.0971938997442629	6.36737514391496e-07
**CESC**	UHRF2	cg24119045	0.0475975586730965	309	0.830833464578366	0.406719758432216	0.00226552759163886	0.602914630375499
**CESC**	USP27X	cg00478198	-0.124856419105016	309	-2.19411539998059	0.028983679544944	0.0155891253917273	0.0962666026180246
**CESC**	USP27X	cg01125381	-0.164830601935038	309	-2.91377467414576	0.00383578949768088	0.0271691273342668	0.0200806177465036
**CESC**	USP27X	cg01290688	-0.116577171970969	309	-2.04654654535554	0.0415624401558191	0.0135902370247489	0.125853741367864
**CESC**	USP27X	cg01855997	-0.171812936368443	309	-3.04088003116015	0.00256425226483141	0.0295196851035466	0.0145298625506536
**CESC**	USP27X	cg06455206	-0.249950728247079	309	-4.50090508273623	9.649067670132e-06	0.0624753665512451	0.000126797224717947
**CESC**	USP27X	cg06624970	-0.124532672640556	309	-2.18833642922364	0.029405914990954	0.015508386555	0.0972496599416008
**CESC**	USP27X	cg16810342	-0.176756712659328	309	-3.13116004410465	0.00191052712256079	0.0312429354701322	0.0114619840342789
**CESC**	USP27X	cg16867483	-0.120820917458686	309	-2.12213072816703	0.0346351918892914	0.0145976940955586	0.109974931120002
**CESC**	USP27X	cg19318186	-0.194383908782422	309	-3.45510352602824	0.000628325286846936	0.0377851039935328	0.00454954965021823
**CESC**	USP51	cg00834229	-0.0997063535089347	309	-1.74714586812698	0.0816218462272885	0.00994135693004865	0.205924757755981
**CESC**	USP51	cg01084827	-0.0942276901724064	309	-1.65025845617299	0.0999228352442822	0.00887885759522702	0.238119070148375
**CESC**	USP51	cg02714202	-0.152669862542535	309	-2.69346483847335	0.00746394413618584	0.0233080869287565	0.0340457385007588
**CESC**	USP51	cg03728850	-0.0628792747616312	309	-1.09851141711366	0.272850486582906	0.00395380319454871	0.469146967649161
**CESC**	USP51	cg07386410	-0.129008720621184	309	-2.26829903774331	0.0240121901778513	0.0166432499963147	0.0835983138373581
**CESC**	USP51	cg12064531	-0.157059761314185	309	-2.77284399893138	0.0059000094686783	0.0246677686240689	0.0282992377215833
**CESC**	USP51	cg12517811	-0.0665371574747854	309	-1.16269156678723	0.245866563099628	0.00442719332482439	0.438862478713546
**CESC**	USP51	cg18770944	-0.0588100719010428	309	-1.02716647125003	0.30515867436376	0.00345862455700582	0.503713507892261
**CESC**	USP51	cg21331369	-0.124401210703989	309	-2.18598999730849	0.0295788669396918	0.0154756612246182	0.0976426604275136
**CESC**	USP51	cg25424556	-0.0828651809803573	309	-1.44978996521241	0.148147958112197	0.00686663821890737	0.313097181157218
**CESC**	WDR82	cg01736403	-0.0203387910638189	309	-0.354692309261563	0.723066039669786	0.000413666421937678	0.843061472869637
**CESC**	WDR82	cg03869063	-0.0111404645812548	308	-0.193932934160913	0.846358344041231	0.000124109951086193	0.917896750258313
**CESC**	WDR82	cg10989431	0.220715146074865	309	3.94560535348652	9.89073230463068e-05	0.0487151757068489	0.000954911398937764
**CESC**	WDR82	cg11442381	-0.2283582073351	309	-4.08962069856815	5.53781020962863e-05	0.0521474708573006	0.000580135210675801
**CESC**	WDR82	cg11665333	0.0459838211882947	309	0.802604325599983	0.422830475730587	0.00211451181107706	0.617884026391639
**CESC**	WDR82	cg12661343	-0.0970834320045897	309	-1.70074135942229	0.0900141933438036	0.00942519276978979	0.220803561738912
**CESC**	WDR82	cg13539115	0.00204211522956021	309	0.0356055699087407	0.971620234904668	4.17023461080177e-06	0.984960826799615
**CESC**	WDR82	cg15991175	-0.269811271182426	309	-4.88550799790577	1.67137286261559e-06	0.0727981220570768	2.69477265231746e-05
**CESC**	WDR82	cg17632831	0.0607398037464644	309	1.06099364856355	0.28953497361646	0.00368932375915901	0.487017746059783
**CESC**	WDR82	cg24007312	-0.0278874385680751	309	-0.48642329078102	0.627017553974818	0.00077770922988816	0.779478468374141
**CESC**	WDR82	cg26236504	0.118606949112896	309	2.08268389682418	0.0381157582308929	0.0140676083778691	0.11816279970567
**CESC**	WDR82	cg26858096	-0.192131267235011	309	-3.4135195911145	0.000728291076075136	0.0369144238493314	0.00515435552876539
**CESC**	ZMYND1	cg00614684	-0.244663229636127	309	-4.39956007997143	1.5026308214671e-05	0.05986009593598	0.000186793963127535
**CESC**	ZMYND1	cg02813021	-0.126332878433415	309	-2.22047969543731	0.027123280603262	0.015959996173272	0.0915731486524679
**CESC**	ZMYND1	cg02867818	-0.376236427402976	309	-7.08012792842622	1.00195946916993e-11	0.141553849304955	4.75283139906129e-10
**CESC**	ZMYND1	cg03254875	-0.340490059868944	309	-6.31391539072378	9.6278629300802e-10	0.115933480869557	3.19534969617175e-08
**CESC**	ZMYND1	cg05236965	-0.0735848211991771	309	-1.28648290134126	0.199253432561217	0.00541472591091487	0.38199816483482

**CESC**	ZMYND1	cg08828193	-0.289928994887184	309	-5.28195442676091	2.44026522327366e-07	0.0840588220762928	4.83786933688458e-06
**CESC**	ZMYND1	cg12639781	-0.131063645138743	309	-2.30505625365932	0.0218367973079216	0.0171776790770544	0.07784675143307
**CESC**	ZMYND1	cg13939078	-0.0355964013980752	309	-0.621038050343755	0.535039948319532	0.00126710379249289	0.71156334284246
**CESC**	ZMYND1	cg14255089	0.110109099710121	309	1.93156260673946	0.0543428136694882	0.0121240138389734	0.153338036939595
**CESC**	ZMYND1	cg14694828	-0.150290627392253	309	-2.65051150867895	0.00845782496349053	0.0225872726819569	0.0374996030553848
**CESC**	ZMYND1	cg17632565	-0.341472737675927	309	-6.3345391294492	8.55623267305108e-10	0.116603630575892	2.86604183214581e-08
**CESC**	ZMYND1	cg22593633	-0.155321054546673	309	-2.74138444245139	0.00648037679230544	0.0241246299854906	0.0304723423706848
**CESC**	ZMYND1	cg24757747	-0.329736831732871	309	-6.08973964861193	3.409669087054e-09	0.108726378201232	1.01941505364442e-07
**CHOL**	ACTL6A	cg03667091	0.131940245122464	36	0.776122338582986	0.443042486269414	0.017408228282976	0.635318841969452
**CHOL**	ACTL6A	cg03839554	-0.336271931495971	36	-2.08203277534795	0.0449365629155372	0.113078811912031	0.133422196689107
**CHOL**	ACTL6A	cg07261848	-0.308481612393277	35	-1.86294731859701	0.0713911960189394	0.095160905184756	0.187040942413948
**CHOL**	ACTL6A	cg10112003	0.133119046550725	36	0.783181024601253	0.438942386298244	0.0177206805545741	0.631686627073009
**CHOL**	ACTL6A	cg20132173	-0.183767481525059	36	-1.0901040991944	0.283333151758995	0.0337704872660629	0.48067034211312
**CHOL**	ACTL6A	cg22619386	-0.011893952415026	36	-0.0693579704460446	0.945111008580992	0.000141466104050903	0.970651851578936
**CHOL**	ACTL6A	cg25198545	0.113914056740667	36	0.66857943077023	0.508277901255072	0.0129764123231158	0.690816783434729
**CHOL**	ACTL6A	cg25575835	0.00998388650652199	36	0.0582184635651415	0.95391544078783	9.96779897751118e-05	0.975563901850235
**CHOL**	ACTL6B	cg05729836	0.154220915875037	36	0.910143341097123	0.369154349407499	0.0237840908933352	0.567852282004369
**CHOL**	ACTL6B	cg08572611	-0.418283449865046	36	-2.68517696142138	0.0111256681904614	0.174961044431004	0.0463310439060409
**CHOL**	ACTL6B	cg09375063	-0.231285296571171	36	-1.38619887549876	0.174710451418716	0.0534928884100145	0.350141042781966
**CHOL**	ACTL6B	cg14088161	-0.0741074670180349	36	-0.433308560621032	0.667527364774245	0.00549191666782913	0.807165496735092
**CHOL**	ACTL6B	cg16024950	-0.239013859456333	35	-1.41401363995609	0.166723360439418	0.0571276250122117	0.339246628531704
**CHOL**	ACTL6B	cg20497359	0.128641008065329	36	0.756384153456023	0.454628272169523	0.0165485089560641	0.644907127972791
**CHOL**	ACTL6B	cg24471254	-0.648116022958672	36	-4.96247783735754	1.92120204919162e-05	0.420054379215766	0.000231251728631924
**CHOL**	ACTL6B	cg26990587	-0.421331587829231	36	-2.70894935947877	0.0104927002199481	0.177520306902701	0.0442446268584013
**CHOL**	AEBP2	cg02664216	-0.207771554004444	36	-1.23853399670105	0.224001374726395	0.0431690186534214	0.413064757993343
**CHOL**	AEBP2	cg07849438	-0.2087295798068	36	-1.24450434373034	0.221824766116359	0.0435680374863235	0.410296129370839
**CHOL**	AEBP2	cg08161947	-0.531633983500333	36	-3.66000777504714	0.000847813199421526	0.282634692412432	0.00585016675854162
**CHOL**	AEBP2	cg24304000	0.0779366846545974	33	0.43525701199249	0.666391362912422	0.00607412681495016	0.806428399881916
**CHOL**	AEBP2	cg26259627	-0.190257233468528	36	-1.13002143774635	0.266376351987631	0.0361978148870979	0.462117314109146
**CHOL**	AFF4	cg00931577	0.165742511286857	36	0.979990801713877	0.334012819738957	0.027470580047674	0.533600158098662
**CHOL**	AFF4	cg01907658	-0.0166759929975553	36	-0.0972504360334701	0.923098546020239	0.000278088742454512	0.959610982829976
**CHOL**	AFF4	cg05619425	0.193927191299212	36	1.15266235248687	0.257088928555908	0.0376077555252011	0.45152578443028
**CHOL**	AFF4	cg06638012	-0.178943608748779	36	-1.06052922477402	0.296379769655292	0.032020815112036	0.494331577310079
**CHOL**	AFF4	cg13393914	-0.0653624520078571	36	-0.381942062673899	0.704881221865954	0.00427225013247942	0.831604843636347
**CHOL**	AFF4	cg13565585	0.0339629506097056	36	0.198150645789666	0.844107234305197	0.0011534820141173	0.916678944644693
**CHOL**	AICDA	cg07999668	0.108530306606386	36	0.636595275627227	0.528651449490135	0.0117788274520761	0.707043820473052
**CHOL**	AIRE	cg08089567	0.0673553851213342	36	0.393639948151297	0.696304702069314	0.00453674790484324	0.825905573382327
**CHOL**	AIRE	cg09510531	0.169548534381696	36	1.00315316113003	0.322873303590469	0.0287467055109811	0.522254455006837
**CHOL**	AIRE	cg11923631	0.259016405584042	36	1.56367588984561	0.127154787698763	0.0670894983616771	0.281890634185207
**CHOL**	AIRE	cg12843033	0.120628705174465	36	0.708554255640556	0.483431206560235	0.014551284512068	0.670309083561806
**CHOL**	AIRE	cg15955655	0.0723601811510743	36	0.423037700749488	0.674931622648241	0.00523599581621629	0.812061578302013
**CHOL**	AIRE	cg16501323	0.23630496317311	36	1.41804336251214	0.165278963682009	0.0558400356202449	0.337259773734591
**CHOL**	AIRE	cg16717549	0.273602482191762	36	1.65865234659447	0.106384596862813	0.0748583182614933	0.248927221353676
**CHOL**	AIRE	cg17356252	0.23633934884034	36	1.41826191419241	0.165215644749323	0.0558562878102761	0.337164980702165
**CHOL**	AIRE	cg18689454	0.337709223149796	36	2.09207459689002	0.043971836582653	0.114047519400439	0.131314589447636
**CHOL**	AIRE	cg18876487	0.105207439777512	36	0.616883037528558	0.541421062860314	0.0110686053845389	0.716412269767235
**CHOL**	AIRE	cg23272544	0.144181404401388	36	0.849591987622875	0.401494900258317	0.0207882773751566	0.59805258435828
**CHOL**	AIRE	cg27251412	0.212013274996437	36	1.26499664692296	0.214474275930251	0.0449496287747149	0.401248647726799
**CHOL**	ARID1A	cg04081153	-0.00296967076074292	36	-0.0173160837043789	0.986285672550603	8.81894442721145e-06	0.992812561828328
**CHOL**	ARID1A	cg04360147	-0.23756835124591	36	-1.42607703779351	0.162963953025342	0.0564387215137001	0.333744085452566
**CHOL**	ARID1A	cg14851949	-0.0803039312330289	36	-0.469765503271615	0.64152163897344	0.00644872137147903	0.789435514921379
**CHOL**	ARID1A	cg17385674	-0.213541896725069	36	-1.27455144981031	0.211110360475592	0.04560014165694	0.397000565239756
**CHOL**	ARID1A	cg20413923	0.106453282647537	36	0.624271263602937	0.536616164848948	0.0113323013864364	0.712709233405478
**CHOL**	ARID1A	cg21294655	-0.05511449370747	36	-0.321859174215951	0.749529956809542	0.00303760741663075	0.859658746535966
**CHOL**	ARID1A	cg24542126	0.134743569157213	36	0.792914249274964	0.433326257414196	0.0181558294292247	0.626886280903038
**CHOL**	ARID1A	cg25601319	-0.0459739892929605	34	-0.260343434174543	0.79626684519117	0.00211360769150925	0.888456403236562
**CHOL**	ARID2	cg00035453	-0.0285678741237887	26	-0.140010573895366	0.889820031668216	0.000816123431952635	0.941561494900599

**CHOL**	ARID2	cg01183821	0.233046753099558	36	1.39735996999356	0.171358136682293	0.0543107891302462	0.345496446326517
**CHOL**	ARID2	cg01353809	0.0623679679741737	36	0.364373975797122	0.71783522346371	0.00388976342922755	0.839735844184837
**CHOL**	ARID2	cg01751433	0.188370691438741	36	1.11840207352729	0.271235283090167	0.0354835173931094	0.467548805954275
**CHOL**	ARID2	cg06613666	-0.137454138178137	36	-0.809168992592814	0.424044547763549	0.0188936401022943	0.619065091836986
**CHOL**	ARID2	cg07276957	0.196455620163881	36	1.16829006919235	0.250816739006796	0.0385948106939753	0.444430507789248
**CHOL**	ARID2	cg23465749	0.148833570281177	25	0.721820173831167	0.477679888980608	0.022151431642642	0.665388720873754
**CHOL**	ARID4A	cg02136725	-0.0359842306685105	36	-0.209958295842933	0.834953331121962	0.00129486485680457	0.911640132509501
**CHOL**	ARID4A	cg06154646	-0.053487427644501	36	-0.312329710377561	0.756697514968159	0.00286090491602573	0.864325619749879
**CHOL**	ARID4A	cg08066755	-0.175895163174172	36	-1.04188028993891	0.304819321421826	0.0309391084280686	0.503334910228594
**CHOL**	ARID4A	cg08790491	-0.303112701971065	36	-1.85469000263602	0.0723300493587061	0.0918773100961995	0.188797902251049
**CHOL**	ARID4A	cg10064886	0.150178930215523	36	0.885731344419305	0.381984132330147	0.0225537110806789	0.580460084980495
**CHOL**	ARID4A	cg10242170	0.025256684747043	36	0.14731750820732	0.883751547248613	0.000637900124411514	0.938420570918875
**CHOL**	ARID4A	cg10844118	-0.28302475639098	36	-1.7206569203423	0.0944033558716002	0.0801030127301736	0.228529209194525
**CHOL**	ARID4A	cg12203852	0.0880612602044114	36	0.515483594448891	0.609550916436067	0.00775478554878905	0.767043625842794
**CHOL**	ARID4A	cg14272338	-0.189769517796353	36	-1.12701631891048	0.26762698073962	0.0360124698846602	0.46337213370853
**CHOL**	ARID4A	cg14460816	-0.327702649448835	36	-2.02249892909951	0.0510476397373991	0.107389026455786	0.14648469515792
**CHOL**	ARID4A	cg17323045	0.063342133140266	36	0.370088116012645	0.713612314671618	0.00401222583075919	0.837061510043031
**CHOL**	ARID4A	cg19548283	-0.228235280241857	36	-1.36690707559113	0.180625176370945	0.052091343147079	0.357770433642952
**CHOL**	ARID4A	cg20996579	0.0239590270426011	36	0.139744048876277	0.889686766772852	0.000574034976828092	0.941510125344532
**CHOL**	ARID4A	cg26429530	-0.0890172956389264	36	-0.52112439060806	0.605658016674397	0.00792407892286802	0.764279551712728
**CHOL**	ARID4A	cg27026909	-0.410235160824814	36	-2.62293260209305	0.0129545150752945	0.168292887176961	0.0522005358835317
**CHOL**	ARID4B	cg05652840	0.166477236995502	36	0.984458586790199	0.331844193574386	0.0277146704376567	0.531587570679854
**CHOL**	ARID4B	cg06212607	-0.351361088200869	22	-1.67834584360773	0.108837661764862	0.123454614301699	0.25299300981073
**CHOL**	ARID4B	cg06891548	-0.132644332994822	34	-0.757039082943951	0.454564800136273	0.0175945190756414	0.644840607264925
**CHOL**	ARID4B	cg07814876	-0.152223521732235	36	-0.898074104632217	0.375462020412311	0.0231720005685642	0.574040037493458
**CHOL**	ARID4B	cg10107779	0.273495170990474	36	1.65794919094312	0.106527382386664	0.0747996085551084	0.24912644477343
**CHOL**	ARID4B	cg11732257	-0.196041503156264	36	-1.16572884408955	0.251836975358398	0.0384322709597675	0.44563395721473
**CHOL**	ARID4B	cg22596515	0.333985951811192	36	2.06609523165089	0.0465059813747423	0.111546616007228	0.136785363849614
**CHOL**	ARID4B	cg23687319	-0.200686818351556	36	-1.19449668105268	0.240550461998995	0.0402751990600706	0.432624110120762
**CHOL**	ARID4B	cg26020585	0.287231664719808	36	1.74851441726828	0.0893999929624886	0.0825020292177124	0.219823726295083
**CHOL**	ASH1L	cg03119126	-0.0452671587799023	36	-0.26422147426416	0.793204322317735	0.00204911566400489	0.88674106240136
**CHOL**	ASH1L	cg08095278	0.319284109189835	36	1.96455708362537	0.0576810900018929	0.101942342381147	0.159938790832879
**CHOL**	ASH1L	cg09410084	0.216397141872855	36	1.29242478432876	0.204925032669025	0.0468277230107406	0.389118128338185
**CHOL**	ASH1L	cg10558228	-0.485339023574478	15	-2.0014441367376	0.0666677145221922	0.235553967804228	0.177858305333999
**CHOL**	ASH1L	cg17038116	-0.326435675861934	36	-2.01374483534279	0.0520050117048543	0.106560250475437	0.148492489651952
**CHOL**	ASH1L	cg20064047	-0.359574002336865	36	-2.24694188624992	0.0312459436056478	0.129293463156551	0.101760243515626
**CHOL**	ASH1L	cg21852905	-0.077015357793243	36	-0.450410608028823	0.655273499480918	0.00593136533602124	0.799076039050607
**CHOL**	ASH1L	cg23821590	-0.264967797618799	36	-1.60228451824492	0.118344010734433	0.0702079337749567	0.268179788635746
**CHOL**	ASH1L	cg24312492	-0.113320367329239	36	-0.665049526651623	0.510505114493357	0.0128415056516336	0.692822006955233
**CHOL**	ASH1L	cg26185843	-0.218877855901495	36	-1.30798184472991	0.199654168410722	0.0479075158040358	0.382493076990266
**CHOL**	ASH2L	cg00590869	0.0650479636323544	36	0.380096538606171	0.706237923130376	0.00423123757271611	0.8325021112784
**CHOL**	ASH2L	cg01642070	-0.312352096219094	36	-1.9172362731846	0.0636417835539679	0.0975638320124623	0.171834861707215
**CHOL**	ASH2L	cg02897270	-0.312635228878305	36	-1.9191623275987	0.063389168366944	0.0977407863357904	0.171320767229643
**CHOL**	ASH2L	cg03189282	-0.277570439290313	36	-1.68469966639289	0.101206008152022	0.0770453487678172	0.240308280626877
**CHOL**	ASH2L	cg03705346	-0.183854167382602	36	-1.09063630303292	0.283102155424568	0.0338023548639499	0.480435616131821
**CHOL**	ASH2L	cg10062221	-0.096784084536541	36	-0.567005203258995	0.574435299498625	0.00936715901957631	0.741653992916588
**CHOL**	ASH2L	cg21453287	-0.275906092858495	36	-1.67376293935132	0.10335428756112	0.0761241720764407	0.243824097723489
**CHOL**	ASH2L	cg24318412	-0.176017944743067	36	-1.04263080698086	0.304476488910131	0.0309823168715734	0.503046425227769
**CHOL**	ASH2L	cg27255459	0.0193395087009294	36	0.112788839326583	0.910861287269931	0.000374016596793326	0.952746161049252
**CHOL**	ASXL1	cg00174466	-0.1807486938483	36	-1.07158670807025	0.291453528779124	0.0326700903278665	0.489049993914049
**CHOL**	ASXL1	cg04218270	-0.0878305162257693	36	-0.514122375496537	0.610492076310397	0.00771419958048513	0.767706392908378
**CHOL**	ASXL1	cg09065199	-0.158939463237613	36	-0.93870081914863	0.354505468273572	0.0252617529742605	0.553477536834774
**CHOL**	ASXL1	cg10421984	-0.145882507982031	36	-0.859832440426139	0.39590380843668	0.0212817061351274	0.592617822892057
**CHOL**	ASXL1	cg12592359	-0.230642565559712	36	-1.38212994109772	0.175945228784509	0.0531959930479662	0.351772889438758
**CHOL**	ASXL1	cg16926526	-0.200330117273018	36	-1.19228473135828	0.24140485290301	0.0401321558866213	0.433664683679408
**CHOL**	ASXL1	cg18793288	0.0667327251880477	36	0.389984630105395	0.698980375801619	0.0044532566110235	0.827349769345842
**CHOL**	ASXL1	cg24727216	-0.297480342739685	36	-1.8168459197018	0.0780648689726573	0.0884945543165205	0.199356556017886

**CHOL**	ASXL2	cg05165989	0.0451447719973267	36	0.263505649675993	0.793751346310132	0.00203805043869062	0.88698315117374
**CHOL**	ASXL2	cg05496738	-0.098570527929848	36	-0.577572746963532	0.567358129587116	0.00971614897636894	0.736033476825925
**CHOL**	ASXL2	cg06583696	0.0927927187183025	36	0.543414469024153	0.590389908214357	0.00861048864713401	0.75333876005195
**CHOL**	ASXL2	cg09476286	-0.026118117736182	36	-0.152345458658093	0.879814948530077	0.000682156074081063	0.936238665597495
**CHOL**	ASXL2	cg19084174	-0.0206007061304578	36	-0.120147223737683	0.905073706437446	0.000424389093073482	0.949667578244084
**CHOL**	ASXL2	cg20667782	0.0247501580309199	36	0.144361203335939	0.886067572855498	0.000612570322555507	0.93957495890383
**CHOL**	ASXL2	cg24738909	0.0905883973644984	36	0.530397359190477	0.599283835127017	0.00820625773706825	0.759562259830684
**CHOL**	ASXL3	cg09963640	-0.277403278761431	36	-1.68360048362898	0.10142022591022	0.0769525790675919	0.240699382362479
**CHOL**	ASXL3	cg12361352	-0.308712458207232	22	-1.45150210617944	0.162146473196964	0.0953033818523522	0.332736025742141
**CHOL**	ASXL3	cg17880622	-0.16647826047367	36	-0.984464811621074	0.331841178738126	0.0277150112103392	0.531587570679854
**CHOL**	ASXL3	cg20034091	-0.135852598196937	36	-0.799562661842051	0.429515156431063	0.0184559284368583	0.623587743862757
**CHOL**	ASXL3	cg20329085	-0.12911654574925	36	-0.759227537722554	0.452948362395827	0.0166710823862182	0.643345219020895
**CHOL**	ASXL3	cg24880056	-0.166075903298006	36	-0.98201791287943	0.333027696048049	0.0275812056562488	0.532671764014267
**CHOL**	ASXL3	cg27109877	-0.119430779270545	34	-0.680472976292257	0.50109921090908	0.0142637110371697	0.684924064968325
**CHOL**	ATAD2B	cg08192560	0.111047907139999	36	0.651544773547507	0.519074991764264	0.0123316376801738	0.699806247670124
**CHOL**	ATAD2B	cg14009731	0.117675421673677	36	0.690960439431021	0.494281201235225	0.0138475048660778	0.67943795753778
**CHOL**	ATAD2B	cg15717719	0.0699387242595463	36	0.408810395119776	0.685242486997459	0.00489142515105285	0.818843126625962
**CHOL**	ATAD2B	cg16929802	0.301766184998606	36	1.84562344827087	0.0736701335793302	0.0910628304086128	0.191331430410728
**CHOL**	ATAD2B	cg17933838	0.104955011229773	36	0.615386418712156	0.542397108667317	0.0110155543822417	0.7169917860349
**CHOL**	ATAD2B	cg19614157	0.0380903638501971	36	0.222264376799339	0.825437833956315	0.0014508758182404	0.906186347850947
**CHOL**	ATAD2B	cg20806130	-0.044874160230719	36	-0.261922918335523	0.794961225298712	0.00201369025641224	0.887748353658154
**CHOL**	ATAD2B	cg27610867	-0.0552366307178498	36	-0.322574613826625	0.748992742725274	0.0030510853730601	0.859359341438726
**CHOL**	ATAT1	cg01245721	0.0714841133115799	36	0.417889496608988	0.678655423506853	0.00510997845594279	0.814466076583446
**CHOL**	ATAT1	cg05093836	0.0926239551793419	36	0.542417592614551	0.591068774246884	0.00857917907306474	0.753862804205406
**CHOL**	ATAT1	cg06814856	-0.115006681337417	36	-0.675077757662978	0.504191704331869	0.0132265367522461	0.687485135694765
**CHOL**	ATAT1	cg07142893	-0.0889291474304423	36	-0.520604238414179	0.606016503841245	0.00790839326270534	0.764512825541235
**CHOL**	ATAT1	cg07816366	-0.0216293741636728	36	-0.126149351930726	0.900356742687931	0.000467829826712158	0.947433830380797
**CHOL**	ATAT1	cg08081524	-0.0753103570891616	34	-0.427232994284166	0.67207074999798	0.00567164988489703	0.810163199636515
**CHOL**	ATAT1	cg08188471	-0.105788802534646	30	-0.562940599149747	0.577955590022798	0.0111912707417143	0.74434977695943
**CHOL**	ATAT1	cg08635134	-0.0288989559726004	36	-0.168578831067478	0.867126814191979	0.000835149656306293	0.929381031140223
**CHOL**	ATAT1	cg09532691	-0.286238893997384	36	-1.74193058776329	0.0905619120371323	0.0819327044368454	0.221748556388426
**CHOL**	ATAT1	cg12907138	0.302344752020957	36	1.84951764217144	0.0730919748881679	0.0914123490746137	0.190304799171664
**CHOL**	ATAT1	cg13542073	0.0185393048159713	36	0.108120376944233	0.914535751033719	0.000343705823059497	0.954882013984151
**CHOL**	ATAT1	cg13964084	-0.127681412956127	36	-0.750648056109524	0.458028374654983	0.016302543214473	0.648087656928151
**CHOL**	ATAT1	cg15381130	0.0615716330328677	36	0.359703707993124	0.721293389819135	0.00379106599433413	0.842059185851551
**CHOL**	ATAT1	cg15729859	-0.162945200791836	36	-0.962995951908324	0.342349024946313	0.0265511384610917	0.541579226587634
**CHOL**	ATAT1	cg18298197	-0.147651694554091	36	-0.870491013303898	0.390136877787667	0.0218010229046944	0.58759599707493
**CHOL**	ATAT1	cg20629923	-0.254484865114616	36	-1.53440664984134	0.134183654017729	0.0647625465724041	0.292441047223847
**CHOL**	ATAT1	cg22759096	-0.0425558641099147	36	-0.248366194166247	0.805345134917485	0.00181100157014152	0.894273593676252
**CHOL**	ATAT1	cg23328390	-0.271989511641618	36	-1.6480904109232	0.108546102441602	0.0739782944430458	0.252587543921531
**CHOL**	ATAT1	cg26934609	-0.0262574719620565	36	-0.153158863053848	0.879178388002844	0.000689454833838185	0.935875862399966
**CHOL**	ATF7IP	cg00236831	0.254734183709389	36	1.53601416658372	0.133789632391335	0.0648895043500888	0.291877507848988
**CHOL**	ATF7IP	cg11409060	-0.202891488761972	34	-1.17210594538241	0.249807225349004	0.0411649562120495	0.443420539172461
**CHOL**	ATF7IP	cg12526346	0.25963173908852	36	1.56765880860854	0.126221857315117	0.0674086399421291	0.280319254559714
**CHOL**	ATF7IP	cg12948116	0.125416233915264	36	0.737116139261485	0.466108276175442	0.0157292317294883	0.655350537219957
**CHOL**	ATF7IP	cg17439009	0.196867686829796	36	1.17083925883342	0.249804292460205	0.0387568861177146	0.443420539172461
**CHOL**	ATF7IP	cg19789919	0.0863169755678666	36	0.50519566141948	0.616680712640715	0.00745062027118368	0.772264206000853
**CHOL**	ATF7IP	cg21275967	0.200978812169084	36	1.19630774103908	0.239852577826991	0.0403924829408961	0.431773491599512
**CHOL**	ATF7IP	cg26637901	0.64575554730603	14	2.9297096109573	0.0126107823638094	0.417000226876511	0.0511306198577261
**CHOL**	ATM	cg01756564	0.0395906803954471	36	0.231032486574221	0.818674238165003	0.00156742197417444	0.902436199484439
**CHOL**	ATM	cg01800843	0.0718363039584884	36	0.419959023989568	0.677157500339746	0.00516045456641634	0.813494541534955
**CHOL**	ATM	cg01991180	-0.195424835548009	35	-1.14470156513813	0.260567644163743	0.0381908663489663	0.45574439745659
**CHOL**	ATM	cg02105423	0.0496421326047695	36	0.289818212955029	0.773715482337675	0.00246434132954952	0.87454027953204
**CHOL**	ATM	cg03165700	0.065382373167732	27	0.327612864508147	0.745932142534823	0.00427485472104456	0.85731744001272
**CHOL**	ATM	cg03569616	-0.15981577101468	35	-0.930025457734824	0.359111748109418	0.0255410806650168	0.558065108518259
**CHOL**	ATM	cg04709886	-0.164328733882699	36	-0.971398492576442	0.338210234800562	0.0270039327794908	0.537653255354288
**CHOL**	ATM	cg04826772	0.0766403429877844	36	0.448204410366596	0.656848927483344	0.00587374217328523	0.800090727157287

**CHOL**	ATM	cg05033322	0.152829644144306	36	0.901735395824179	0.373541232171036	0.0233569001292752	0.572111641673332
**CHOL**	ATM	cg05637308	0.0890064113870715	36	0.521060163252441	0.605702276586689	0.0079221412680046	0.764292364724371
**CHOL**	ATM	cg06053805	0.0394256621677631	36	0.230068015948001	0.819417537688316	0.00155438283736659	0.902798987663502
**CHOL**	ATM	cg06081482	-0.0257542094320539	33	-0.143440948065495	0.886870312239175	0.000663279303470096	0.939884874129286
**CHOL**	ATM	cg06750635	0.0707743481129467	36	0.413719281699595	0.681677850157044	0.00500900835081256	0.816418238397615
**CHOL**	ATM	cg09808176	-0.129306409596415	36	-0.760362940016941	0.452278575598855	0.0167201475627159	0.642704216656563
**CHOL**	ATM	cg10610482	0.0153210663488651	36	0.089346887897595	0.92933047645276	0.000234735074066328	0.962629123805521
**CHOL**	ATM	cg10976287	0.176542366159559	36	1.04583696148367	0.303014945102004	0.0311672070492157	0.501387740918118
**CHOL**	ATM	cg11588932	-0.0305162914896943	36	-0.178021937927299	0.85976219929258	0.000931244046283989	0.925481304917936
**CHOL**	ATM	cg12019961	-0.451309922186402	12	-1.59930482005209	0.140836270722231	0.203680645863897	0.302591052932094
**CHOL**	ATM	cg12106634	-0.160723291102642	36	-0.949513929495822	0.349060188882437	0.0258319763028646	0.548127577843005
**CHOL**	ATM	cg12848864	0.00466941389189885	36	0.0272274246079726	0.978437529944317	2.1803426093858e-05	0.98883804712013
**CHOL**	ATM	cg14761454	-0.288678057469402	23	-1.38171382112808	0.181583543419763	0.0833350208643074	0.358986281494208
**CHOL**	ATM	cg15370815	0.0265158683481948	36	0.154667134905437	0.877998248722387	0.000703091274258797	0.935265374673582
**CHOL**	ATM	cg15504467	0.100569817463857	36	0.589406054352346	0.559485390312317	0.0101142881847134	0.730254126605758
**CHOL**	ATM	cg16693212	-0.234709288966176	26	-1.18287899517827	0.248443036560749	0.0550884503270079	0.441778728341377
**CHOL**	ATM	cg16788234	0.110838921323918	36	0.650303337764507	0.519866663076951	0.0122852664802496	0.700291028508742
**CHOL**	ATM	cg17114932	-0.0230409976304735	36	-0.134386625622941	0.893889233304161	0.000530887571807486	0.943762048446234
**CHOL**	ATM	cg17420659	-0.141298444874158	36	-0.832254406181837	0.41107308742433	0.0199652505238555	0.606917215009959
**CHOL**	ATM	cg18042593	-0.097563323637558	36	-0.57161403490862	0.571343363000011	0.00951860211920689	0.739232371706464
**CHOL**	ATM	cg18391757	0.0666003808376986	36	0.389207763227157	0.699549543369234	0.00443561072772649	0.827778769130344
**CHOL**	ATM	cg18457775	-0.157156398286964	36	-0.927901758849224	0.359999305917259	0.0246981335225308	0.558875024616719
**CHOL**	ATM	cg19288979	0.11971513681488	36	0.703109770617394	0.486774291536266	0.0143317139826055	0.673175105963633
**CHOL**	ATM	cg20342375	-0.170008485926855	36	-1.00595539991781	0.32154297030638	0.0289028852871416	0.521019723482841
**CHOL**	ATM	cg22477592	0.0299346794092584	36	0.174625933000569	0.862409289616726	0.000896085031335078	0.926864784797352
**CHOL**	ATM	cg22678707	-0.0642331552761693	36	-0.375315495518007	0.709757143284107	0.00412589823673248	0.834678949745943
**CHOL**	ATM	cg22837512	0.0319407036459406	36	0.186339783422834	0.853285684744697	0.00102020854939781	0.921812856158839
**CHOL**	ATM	cg23275463	-0.256062610845019	16	-0.9911430726645	0.338437890432697	0.0655680606727676	0.537808952064574
**CHOL**	ATM	cg23389069	-0.161214031710294	36	-0.952490344267628	0.347571110794878	0.0259899640202877	0.546860023322885
**CHOL**	ATM	cg24030675	0.0327616481123973	36	0.191134196289726	0.849557274269712	0.00107332558704055	0.919783688966055
**CHOL**	ATM	cg24568245	-0.0376869093979881	36	-0.219906778399578	0.827258786714027	0.00142030313997216	0.907237087707667
**CHOL**	ATM	cg25400013	0.0365992980294249	36	0.213551821375722	0.832172010967826	0.00133950861624667	0.909982702499663
**CHOL**	ATM	cg25740661	0.159057528614131	36	0.939416208627149	0.354143489161265	0.025299297408835	0.553150997787645
**CHOL**	ATM	cg25918541	-0.136489006235171	36	-0.803379184841549	0.42733661286023	0.0186292488230645	0.621684793531928
**CHOL**	ATM	cg27453365	-0.30610686279998	36	-1.8748946642943	0.0694185217018758	0.0937014114532459	0.183255097807042
**CHOL**	ATM	cg27599391	-0.12028037690831	35	-0.696011228695762	0.491295058336262	0.0144673690692051	0.676948011268552
**CHOL**	ATR	cg00911981	0.195265398495701	36	1.16093054843868	0.253756454481386	0.0381285758496849	0.447798058704403
**CHOL**	ATR	cg00969988	-0.125255362598611	36	-0.736155560795526	0.46668496433151	0.0156889058597095	0.655886584561008
**CHOL**	ATR	cg11458642	0.147695180228812	36	0.870753102513653	0.389995746759152	0.0218138662628212	0.587504678905937
**CHOL**	ATR	cg11936145	-0.113549269348316	36	-0.666410425608857	0.509645816594839	0.0128934365695365	0.692092455117765
**CHOL**	ATR	cg15135999	-0.00233922980086588	36	-0.0136399737588299	0.989196938414717	5.47199606125902e-06	0.994314147234511
**CHOL**	ATR	cg15291273	-0.0966373064307329	36	-0.566137198076289	0.575018544542663	0.00933876899418738	0.742052882997855
**CHOL**	ATR	cg15480357	-0.130535681766698	36	-0.767716158262507	0.447955006099985	0.0170395642142967	0.639440194952884
**CHOL**	ATR	cg16031872	0.0334840694542567	36	0.195353542471165	0.846278970967534	0.00112118290721748	0.917861143885125
**CHOL**	ATR	cg17945282	-0.00410565398882607	36	-0.0239400726782317	0.98104036634007	1.68563946759634e-05	0.990222054517542
**CHOL**	ATR	cg20032934	-0.182655075902691	36	-1.08327686711894	0.286308257264806	0.0333628767530178	0.483588430016362
**CHOL**	ATR	cg20636078	-0.0235854873959958	36	-0.13756410949737	0.891396369865624	0.000556275215706675	0.942509562080416
**CHOL**	ATR	cg24631870	-0.145246335039756	36	-0.856001843854558	0.397989466957258	0.0210964978424809	0.594618536796807
**CHOL**	ATR	cg24856922	0.336919072013703	36	2.08655206817037	0.0445001068641507	0.113514461086575	0.132475176541778
**CHOL**	AURKB	cg02245743	-0.125331362528027	36	-0.736609356498168	0.466412474253171	0.0157079504331317	0.655693153261853
**CHOL**	AURKB	cg04612488	-0.166210275910417	36	-0.982835031594069	0.332631152031063	0.027625855818217	0.53233301462877
**CHOL**	AURKB	cg05617798	-0.14985635970988	36	-0.8837851218755	0.383019136022784	0.0224569285454971	0.581283469913314
**CHOL**	AURKB	cg05811247	-0.193550587309184	36	-1.15033668938451	0.25803197362412	0.0374618298477302	0.452560914352718
**CHOL**	AURKB	cg11009596	-0.148498566185571	36	-0.875596059532232	0.38739372639041	0.0220518241591704	0.585341792930419
**CHOL**	AURKB	cg12844497	-0.0807029717006091	36	-0.472115092894179	0.639860814658859	0.00651296964130931	0.78841441040882
**CHOL**	AURKB	cg13678641	-0.24866783624307	36	-1.49699248755845	0.143621805578039	0.0618356927818101	0.306602857856206
**CHOL**	AURKB	cg21724796	-0.207709969253	36	-1.2381503326595	0.22414179066639	0.0431434313270823	0.413304094979761

**CHOL**	BAP1	cg00794529	-0.325152841644831	36	-2.00489353082261	0.0529889258242068	0.105724370429708	0.150495436295438
**CHOL**	BAP1	cg02503646	-0.147036012491908	36	-0.866780835680246	0.392138229381932	0.0216195889695204	0.589191930943643
**CHOL**	BAP1	cg03075736	-0.129561426077617	36	-0.76188809458021	0.451379790289158	0.0167861631272657	0.641997137616799
**CHOL**	BAP1	cg05442719	0.00457166892681392	36	0.0266574601654149	0.978888796009323	2.09001567763959e-05	0.989094294612233
**CHOL**	BAP1	cg05908979	-0.131357765321049	36	-0.772635677208006	0.445076155174283	0.0172548625101397	0.636981859937976
**CHOL**	BAP1	cg09096430	-0.128881739232968	36	-0.757823491792265	0.45377743369613	0.0166105027077146	0.64407600352053
**CHOL**	BAP1	cg10449522	-0.0713369791795382	36	-0.417024959985633	0.679281568167884	0.00508896459846187	0.814928372489049
**CHOL**	BAP1	cg10956904	-0.0188620130576157	36	-0.110003060718659	0.913053689811575	0.000355775536585666	0.954026012990768
**CHOL**	BAP1	cg11234564	-0.0723061401421338	36	-0.422720100924607	0.675161109804661	0.00522817790225389	0.812176950303754
**CHOL**	BAP1	cg12859382	0.0412662774122211	36	0.240826818515887	0.811135685523843	0.00170290565146239	0.89815234808833
**CHOL**	BAP1	cg16841708	0.0460426998253151	36	0.268757792810345	0.789740207494338	0.00211993020720407	0.884304421760555
**CHOL**	BAP1	cg24291822	-0.0697312212605316	36	-0.407591549663765	0.686128703340453	0.00486244321848521	0.819327004277514
**CHOL**	BAP1	cg24747396	-0.105720720467644	36	-0.619926589837083	0.539438987422731	0.0111768707361978	0.714774345311167
**CHOL**	BAP1	cg25749966	-0.0170999757642548	36	-0.0997237172151293	0.92114936283485	0.000292409171138102	0.958686433514934
**CHOL**	BAZ1A	cg05294307	0.0668694383029887	35	0.384997403995239	0.702709638538745	0.00447152177895722	0.829916405351194
**CHOL**	BAZ1A	cg07414384	-0.213759294573866	36	-1.2759111114687	0.210634927105152	0.0456930360167169	0.396384371156008
**CHOL**	BAZ1A	cg23422263	-0.252274391097514	36	-1.52016856577966	0.137714629713106	0.0636423684036216	0.297810947638027
**CHOL**	BAZ1A	cg26967526	-0.121978338045733	36	-0.716600849948483	0.478514267270074	0.014878714952399	0.666058688270646
**CHOL**	BAZ1B	cg00525989	-0.0451537500065448	36	-0.263558160495477	0.79371121460737	0.00203886113965355	0.88698315117374
**CHOL**	BAZ1B	cg04742505	0.0582348235587893	36	0.340141704974521	0.735842374841135	0.00339129467492332	0.851015757223425
**CHOL**	BAZ1B	cg05166819	-0.0167521883419352	36	-0.0976949136629325	0.922748219026698	0.000280635814243671	0.959387821271038
**CHOL**	BAZ1B	cg06059318	0.0138844056494788	36	0.0809671060933667	0.935942855809016	0.000192776720239279	0.965838995633229
**CHOL**	BAZ1B	cg06394887	0.181880233012313	36	1.07852392337931	0.288392422059282	0.0330804191606132	0.485894770703245
**CHOL**	BAZ1B	cg14093368	0.0774218532238159	36	0.452802222225918	0.653567475887082	0.0059941433566101	0.797809776202712
**CHOL**	BAZ1B	cg15636799	-0.0536199509171375	36	-0.313105783475729	0.756112965513781	0.00287509913635624	0.863941289251342
**CHOL**	BAZ1B	cg19160975	-0.144991370932798	36	-0.854466929837868	0.398827124572427	0.0210224976449721	0.595435494827708
**CHOL**	BAZ1B	cg20612831	0.10830197145523	36	0.635240040415093	0.529524219911536	0.0117293170210895	0.707700095584765
**CHOL**	BAZ1B	cg21340129	0.0200877684395991	36	0.117154450821735	0.907426991381834	0.000403518440882954	0.9509449957766
**CHOL**	BAZ1B	cg25710357	0.178841943207308	36	1.05990677836175	0.29665879733493	0.0319844406501661	0.494658117669565
**CHOL**	BAZ2A	cg14634319	0.0421548735867722	36	0.246021731629598	0.807144600865433	0.00177703336711675	0.895543137413185
**CHOL**	BAZ2A	cg18206859	0.237147529701489	36	1.42340025406347	0.16373245029683	0.0562389508435184	0.33489515271577
**CHOL**	BAZ2A	cg18955060	-0.0629112854773864	36	-0.367560771888009	0.715478971474892	0.0039578298404172	0.83825011882161
**CHOL**	BAZ2A	cg25683012	0.0753090499457894	36	0.44037400335084	0.662453393048228	0.0056714530037374	0.803687257829896
**CHOL**	BRD7	cg03021120	-0.596518686730002	36	-4.33375938978528	0.000123206301329882	0.355834543618087	0.00115221151435334
**CHOL**	BRD7	cg16433860	-0.258714926120718	36	-1.5617252247346	0.127613737923526	0.0669334129976483	0.282493116939913
**CHOL**	BRD7	cg16834187	0.501002380819733	36	3.37550939350281	0.00185601507248721	0.251003385587041	0.0111875745040345
**CHOL**	BRD8	cg01096096	-0.0401310059709022	36	-0.234190623019757	0.816241516041984	0.00161049764023659	0.901086896351163
**CHOL**	BRD8	cg03204906	-0.209247572752255	36	-1.24773399695124	0.220653947043408	0.0437845467027104	0.408869141230852
**CHOL**	BRD8	cg03684156	-0.109870232724032	36	-0.644550187091625	0.523543959626219	0.012071468038833	0.70291129876349
**CHOL**	BRD8	cg07430605	-0.0288608682192259	36	-0.168356465071431	0.867300383929669	0.000832949714367526	0.929464617754607
**CHOL**	BRD8	cg07632946	-0.0535254563253509	36	-0.312552409462389	0.756529759909079	0.00286497447483704	0.864196002207966
**CHOL**	BRD8	cg08976717	0.255003169751536	36	1.53774885932099	0.133365487717095	0.0650266165833307	0.291302637900486
**CHOL**	BRD8	cg11110536	-0.0218793501492207	36	-0.127607985218501	0.899210977508124	0.000478705962952202	0.946810751922188
**CHOL**	BRD8	cg11346339	-0.250487122569641	36	-1.50867502540136	0.140619226192992	0.0627437985732184	0.302224889005902
**CHOL**	BRD8	cg12964860	-0.205914742058813	36	-1.22697306714087	0.228261416142282	0.0424008809971474	0.418164808839577
**CHOL**	BRD8	cg14473243	-0.0798940665251813	36	-0.467352416106818	0.643229299595953	0.0063830618659301	0.790624049577421
**CHOL**	BRD8	cg22106577	0.092160405920884	36	0.539679676461418	0.592935203023469	0.00849354041950211	0.755140620896296
**CHOL**	BRD8	cg22818036	-0.0387291124186539	36	-0.225997146596736	0.822556740855401	0.00149994414873673	0.904618220989087
**CHOL**	BRD8	cg23298160	-0.281700999773267	36	-1.71191364810951	0.0960212491807389	0.0793554532732581	0.23133716502838
**CHOL**	BRD8	cg26008208	-0.352291993459547	36	-2.19491325134692	0.0350968604839384	0.124109648655702	0.111083655335347
**CHOL**	BRD8	cg26630792	0.0342505850302083	36	0.199830759065762	0.842803348662037	0.00117310257491153	0.916007149025727
**CHOL**	BRD8	cg27119451	0.194313995558633	33	1.1029168157982	0.278552057323784	0.0377579288699605	0.475483362168044
**CHOL**	BRD9	cg02364331	-0.0229535972553699	36	-0.13387659367789	0.894289476496187	0.000526867626961726	0.943984826805381
**CHOL**	BRD9	cg02822723	0.78721668852562	36	7.44348578938983	1.23519938518805e-08	0.619710114693242	3.31006203828176e-07
**CHOL**	BRD9	cg05193570	-0.167309383642618	36	-0.989520814917757	0.329398540798147	0.0279924298548728	0.529087565609454
**CHOL**	BRD9	cg07308931	-0.0883077552527996	36	-0.516937825465602	0.608546192153849	0.00779825963778836	0.766299915382518
**CHOL**	BRD9	cg09497020	-0.103463193883728	36	-0.606544056766446	0.548182448901834	0.0107046324886218	0.721371271036576

**CHOL**	BRD9	cg10551270	-0.246207028958682	36	-1.48121726665104	0.147757514545515	0.0606179011086611	0.312583909024386
**CHOL**	BRD9	cg10600960	-0.110153725560655	36	-0.646233689773205	0.522466458018816	0.0121338432548921	0.702273060251499
**CHOL**	BRD9	cg13722821	-0.111746871079796	36	-0.655697456949146	0.516431532971391	0.0124873631961246	0.697547958607108
**CHOL**	BRD9	cg17977043	-0.00652558279025859	36	-0.0380511695153192	0.969869432126679	4.25832307525191e-05	0.984100778709294
**CHOL**	BRD9	cg20079193	-0.100866773044445	36	-0.591164273640893	0.558320381572205	0.0101741059043995	0.729382118323198
**CHOL**	BRD9	cg21160852	0.566896619922956	36	4.01261384838146	0.000312317949635753	0.321371777680072	0.00252719602391968
**CHOL**	BRD9	cg21758832	-0.125622323737567	36	-0.738346808199329	0.465370042223478	0.0157809682212261	0.654625666392873
**CHOL**	BRD9	cg21910709	-0.0451314396589674	36	-0.263427671258275	0.79381094282724	0.00203684684569102	0.886998585000249
**CHOL**	BRD9	cg24953233	-0.387888244124143	36	-2.45388082628027	0.0194107538756653	0.150457289929711	0.0711343596666435
**CHOL**	BRD9	cg25523559	-0.196304362847149	36	-1.16735449975034	0.251189063109884	0.0385354028728253	0.44480623732849
**CHOL**	BRD9	cg25951128	-0.149750749966419	36	-0.88314799041668	0.383358352962866	0.0224252871155048	0.581628078213422
**CHOL**	BRD9	cg26504197	-0.0968523136119003	36	-0.567408703878353	0.57416427201273	0.0093803706519779	0.741424779709904
**CHOL**	BRDT	cg00308185	0.0459192398918068	36	0.268035614789846	0.790291403174494	0.0021085765922413	0.884768797308225
**CHOL**	BRDT	cg01515515	-0.0312403815240027	35	-0.179549967076514	0.858603840711218	0.000975961437765246	0.924867780186889
**CHOL**	BRDT	cg02935097	-0.0252039450457007	36	-0.147009691672919	0.883992648553687	0.0006352388458667	0.938510052563432
**CHOL**	BRDT	cg03421440	-0.14491521821961	36	-0.85400851525327	0.399077512717595	0.0210004204716371	0.595691460562656
**CHOL**	BRDT	cg06442199	0.00479983196405351	36	0.0279879116857625	0.977835429932328	2.30383868831498e-05	0.98852003171519
**CHOL**	BRDT	cg09250199	0.162065405401003	35	0.943467497280041	0.352298398437085	0.0262651956277914	0.551221733138819
**CHOL**	BRDT	cg13299824	0.0485240088787362	36	0.28327485463342	0.778683866363432	0.00235457943766367	0.877646731475136
**CHOL**	BRDT	cg14732540	-0.0260722662205103	36	-0.152077827353443	0.880024411343921	0.000679763065873164	0.936347006362666
**CHOL**	BRDT	cg19245011	0.064973304371184	36	0.379658429557509	0.70656013399026	0.00422153028091051	0.832696882416647
**CHOL**	BRPF1	cg04879506	-0.272199406763055	36	-1.64946397912165	0.108262963763169	0.074092517042159	0.252139298954943
**CHOL**	BRPF1	cg13694772	0.216334056503397	36	1.29202950720866	0.205060320035019	0.046800424003215	0.38928820273778
**CHOL**	BRPF1	cg14712964	-0.0883592475984358	36	-0.517241623628738	0.60833639614874	0.00780735663616169	0.766155141658452
**CHOL**	BRPF1	cg17738494	0.0109587732866644	36	0.0639039172375377	0.949420972593266	0.000120094711948508	0.97315657784027
**CHOL**	BRPF1	cg20952324	0.00366498077017631	36	0.0213704700918883	0.983075056510303	1.34320840457622e-05	0.991216913306494
**CHOL**	BRPF1	cg25467642	-0.536863917827298	36	-3.71049336161902	0.000736121451801841	0.288222866264876	0.00519747978049305
**CHOL**	BRPF1	cg26227975	-0.402976956651154	36	-2.56743144057483	0.0148155129394667	0.162390427591826	0.0578100021875343
**CHOL**	BRPF3	cg01499736	0.106786125042134	36	0.626245621813324	0.535335953924568	0.0114032765015143	0.711755412833877
**CHOL**	BRPF3	cg05882415	-0.174030526666753	36	-1.03048861966593	0.310055843772536	0.0302866242119073	0.508849506570201
**CHOL**	BRPF3	cg10562781	0.050812372817537	36	0.296667731884997	0.768524987993696	0.00258189723134837	0.871701842571033
**CHOL**	BRPF3	cg14654948	0.0238315669994655	36	0.139000198494314	0.890270066730883	0.000567943585650012	0.941898533350707
**CHOL**	BRPF3	cg18779695	0.0643943165513789	36	0.376261079862657	0.709060603287667	0.00414662800411919	0.834238147822337
**CHOL**	BRPF3	cg23517469	-0.146868261050686	36	-0.865770123082472	0.392684555547681	0.0215702861040525	0.58954594658657
**CHOL**	BRWD3	cg03164831	-0.058532795184422	36	-0.341888084842646	0.734539402544408	0.0034260881121015	0.850335800540741
**CHOL**	BRWD3	cg05835545	0.0369314130415646	36	0.215492301126753	0.830671026437064	0.00136392926924665	0.909184842009187
**CHOL**	BRWD3	cg09523186	-0.0289895577302159	36	-0.169107790138092	0.866713957437504	0.00084039445739352	0.929180459818372
**CHOL**	BRWD3	cg11957438	0.0323785377727096	12	0.10244364015372	0.920429718136173	0.00104836970829878	0.958206665498219
**CHOL**	BRWD3	cg15511898	0.0278200529941512	36	0.162280201691552	0.872045822341445	0.00077395534859738	0.932136092873362
**CHOL**	BRWD3	cg22618269	0.341181192874865	28	1.85073839874523	0.0756009726669361	0.116404606371516	0.194996918790876
**CHOL**	BRWD3	cg25063710	-0.045198882723178	36	-0.263822134900525	0.793509479260947	0.0020429389994236	0.886840720665407
**CHOL**	BRWD3	cg25708850	0.345522419630492	26	1.80380255144344	0.0838301268577243	0.11938574246731	0.209876126822959
**CHOL**	CARM1	cg12317676	-0.0225476435634493	36	-0.131507658209308	0.896148845212478	0.000508396230264359	0.944967851771732
**CHOL**	CARM1	cg17918830	-0.129430881548255	36	-0.761107339245497	0.451839763481641	0.0167523530983585	0.642409465835254
**CHOL**	CARM1	cg22378614	0.0290518439653551	36	0.16947143747861	0.866430149826039	0.000844009637787341	0.928992723803554
**CHOL**	CARM1	cg26504915	-0.0338202361062264	36	-0.197317048540721	0.844754330105514	0.0011438083702809	0.917038574924345
**CHOL**	CBX1	cg01544580	-0.0904107747056475	36	-0.529348794527096	0.600003018252903	0.00817410818287535	0.760119120002058
**CHOL**	CBX1	cg01553295	-0.164371078508492	36	-0.971655754664194	0.338084048386768	0.0270178514500449	0.53751235963506
**CHOL**	CBX1	cg02835499	-0.327372580760627	36	-2.02021716076754	0.0512956835855897	0.107172806633874	0.146995852538305
**CHOL**	CBX1	cg04864609	-0.0319674170316141	36	-0.186495786762273	0.853164313199216	0.00102191575167313	0.921812856158839
**CHOL**	CBX1	cg11729481	-0.318301580049903	36	-1.95782884610472	0.0584979095059577	0.101315895862265	0.161664572301529
**CHOL**	CBX1	cg12245530	-0.0665473438821522	36	-0.388896439870953	0.699777682368796	0.00442854897776942	0.827949461182311
**CHOL**	CBX1	cg13342109	-0.17297810870599	36	-1.02406411187791	0.313036276607077	0.0299214260915012	0.512044914787337
**CHOL**	CBX1	cg17778721	-0.223349852734412	36	-1.33609419622896	0.190392762322807	0.0498851567164833	0.370628194883779
**CHOL**	CBX1	cg21511817	-0.148017427922778	36	-0.872695458123784	0.388950828824378	0.0219091589688749	0.586589395296196
**CHOL**	CBX4	cg04398978	-0.376272248786907	36	-2.36805583099638	0.023709739417995	0.141580805207156	0.0828596788799222
**CHOL**	CBX4	cg04925705	-0.142374040477984	36	-0.838720284964019	0.407484535134124	0.0202703674020267	0.603691714458656

**CHOL**	CBX4	cg05949524	-0.136625233772061	36	-0.804196269477876	0.426871080782787	0.0186664545032704	0.621302715830345
**CHOL**	CBX4	cg07159673	-0.104939674837661	36	-0.615295494947275	0.542456435686807	0.011012335355034	0.717026131746686
**CHOL**	CBX4	cg10756274	0.0646728764530647	36	0.377895548955928	0.707857213637926	0.00418258094871336	0.833510967716594
**CHOL**	CBX4	cg21365914	-0.146535109038272	36	-0.863763088303321	0.393770857289922	0.0214725381808583	0.590504473591106
**CHOL**	CBX4	cg22353329	-0.176032789285583	36	-1.04272154942642	0.304435056332152	0.0309875429036625	0.503031387869914
**CHOL**	CBX4	cg27487227	-0.148643240226863	36	-0.876468369080451	0.386926230269244	0.0220948128651408	0.584862611385489
**CHOL**	CBX5	cg03689456	-0.218425342995684	36	-1.30514206923256	0.200608515417925	0.0477096304627821	0.383646751975092
**CHOL**	CBX5	cg05754589	-0.261122549626974	36	-1.57731700040919	0.123982702097901	0.0681849859236916	0.276911668542509
**CHOL**	CBX5	cg06399839	-0.198711670140958	36	-1.18225471992495	0.245307062785296	0.0394863278502088	0.438225770829663
**CHOL**	CBX5	cg06621757	-0.229637581236175	34	-1.33469442043728	0.191395761068674	0.0527334187160011	0.371873469281862
**CHOL**	CBX5	cg08288469	-0.0454357030046082	36	-0.26520728732965	0.792451150602295	0.00206440310752296	0.886204554487517
**CHOL**	CBX5	cg09060496	-0.0113954519727741	36	-0.0664506469317658	0.947408266408005	0.0001298563256638	0.971983246866634
**CHOL**	CBX5	cg09158487	-0.267533545269582	36	-1.61898976217485	0.11468987770178	0.0715741978445114	0.262432623858234
**CHOL**	CBX5	cg09653879	-0.0458292175505614	36	-0.267509037493541	0.790693377530632	0.00210031718129669	0.885078716101441
**CHOL**	CBX5	cg11005091	-0.209941472474807	36	-1.25206213970847	0.21909216830514	0.0440754218648902	0.407011563773894
**CHOL**	CBX5	cg11338003	-0.1765143660105	36	-1.04566575380744	0.303092867753023	0.0311573214080887	0.501474011654409
**CHOL**	CBX5	cg12263485	-0.0114946013002565	36	-0.0670288955036989	0.946951319078683	0.000132125859051859	0.971695235862017
**CHOL**	CBX5	cg12600134	-0.0284044134302573	36	-0.16569162268779	0.869380967681476	0.000806810702316981	0.930684265190089
**CHOL**	CBX5	cg13445218	-0.288475316128179	36	-1.75677069974614	0.0879606644632059	0.0832180080152527	0.217321813168947
**CHOL**	CBX5	cg14017196	-0.0758362821147156	36	-0.443474794901952	0.660231674868548	0.00575114168498273	0.802344183624522
**CHOL**	CBX5	cg14394617	0.14984100534056	36	0.883692488806054	0.383068443116702	0.0224523268814698	0.581293306852562
**CHOL**	CBX5	cg15708001	-0.195869483591405	36	-1.16466513065436	0.252261581430019	0.0383648546023636	0.446131614662338
**CHOL**	CBX5	cg20545544	-0.221336256001138	36	-1.3234252497413	0.194524751254209	0.0489897382206012	0.375921587963152
**CHOL**	CBX5	cg20927027	0.184901666914468	36	1.09706946451528	0.280320464041358	0.0341886264277488	0.477537997189011
**CHOL**	CBX5	cg21826008	-0.209311675051731	36	-1.24813374704052	0.220509351504179	0.0438113773129613	0.408745314998801
**CHOL**	CBX5	cg24505978	-0.114747509121444	36	-0.673536120944258	0.505159456640669	0.0131669908495759	0.688322119522607
**CHOL**	CBX5	cg25817259	-0.206116959048178	36	-1.22823144140943	0.227794819231387	0.0424842008072684	0.417555847485545
**CHOL**	CBX6	cg03028106	-0.352773252576034	15	-1.35933529439797	0.197154223848639	0.124448967733074	0.379385525109168
**CHOL**	CBX6	cg04134624	0.132078713928027	36	0.776951318170693	0.442559784031327	0.0174447866728816	0.634925415992894
**CHOL**	CBX6	cg04215348	0.219518288044104	36	1.31200243569125	0.19830892090562	0.0481882787858141	0.380900799785981
**CHOL**	CBX6	cg04801716	0.156784427923521	36	0.925650112387906	0.361151793022555	0.0245813568393059	0.560015997655856
**CHOL**	CBX6	cg06060673	-0.146274289049884	36	-0.862192018887699	0.394622518890408	0.0213961676370491	0.591359856578514
**CHOL**	CBX6	cg09913544	-0.260905584271366	36	-1.5759106430158	0.12430672425541	0.068071723903983	0.277358225587078
**CHOL**	CBX6	cg14498989	0.113565201186474	36	0.666505149706988	0.509586035423172	0.0128970549205242	0.69207478989037
**CHOL**	CBX6	cg24649346	0.196158345271728	36	1.16645142181916	0.251548838663186	0.0384780964197424	0.445286148200421
**CHOL**	CBX6	cg25836955	-0.351414659533544	36	-2.18867590072585	0.0355858495371457	0.123492262935076	0.112247722454394
**CHOL**	CBX7	cg00545295	-0.116279712942501	36	-0.682652179281478	0.499451787295908	0.0135209716419905	0.683610189505038
**CHOL**	CBX7	cg01525244	-0.0481452748218459	36	-0.281058712913833	0.780368742915723	0.00231796748767106	0.878671542894224
**CHOL**	CBX7	cg02523424	0.0162105169994842	36	0.0945351666469346	0.925238994379076	0.000262780861390567	0.960618337451796
**CHOL**	CBX7	cg03804568	-0.00217169209815546	36	-0.0126630620158568	0.989970622794953	4.71624656919087e-06	0.994592368632072
**CHOL**	CBX7	cg10339854	-0.0059354921723619	36	-0.0346101789941108	0.972592982392259	3.52300673281694e-05	0.985558207323099
**CHOL**	CBX7	cg22268271	-0.0756156977902564	36	-0.442177434901208	0.661160854547419	0.00571773375230739	0.8028713534905
**CHOL**	CBX7	cg26672614	-0.0231479179962265	36	-0.135010572243176	0.893399634942789	0.000535826107560027	0.94347561529545
**CHOL**	CBX7	cg27062573	-0.135162749888309	36	-0.795426797673832	0.431883581098831	0.0182689689573696	0.625671824577784
**CHOL**	CDYL2	cg01442398	-0.12038946324556	36	-0.707128297924842	0.484305529255564	0.0144936228605539	0.671025421534674
**CHOL**	CDYL2	cg02461391	-0.161872971489574	36	-0.95648805338203	0.34557773791468	0.0262028588988644	0.54486977185518
**CHOL**	CDYL2	cg06919916	-0.0864508831776165	36	-0.505985292298302	0.616132126421973	0.00747375520218989	0.771854339729641
**CHOL**	CDYL2	cg10112854	-0.299911866200397	36	-1.83315756481561	0.0755472325324942	0.089947127487805	0.194897078681242
**CHOL**	CECR2	cg03927893	-0.298280840468251	36	-1.82221167724486	0.077228921507507	0.0889714597904461	0.197988028332674
**CHOL**	CECR2	cg12632097	-0.176910171250726	36	-1.04808615924956	0.301992547829764	0.0312972086919614	0.500378122032076
**CHOL**	CECR2	cg13466481	-0.0908949146896446	36	-0.532206951321553	0.598043643667315	0.00826188551643777	0.758699421331275
**CHOL**	CECR2	cg26796679	-0.223820532592674	36	-1.33905816112248	0.189435875257988	0.0500956308100683	0.36934276841389
**CHOL**	CHAF1A	cg03006338	-0.066772224639715	36	-0.390216497545025	0.698810533526008	0.00445852998333657	0.827258109185456
**CHOL**	CHAF1A	cg04346879	-0.157643261501355	36	-0.930849497666477	0.358494179863717	0.0248513978967845	0.557468706333912
**CHOL**	CHAF1A	cg05297461	0.0144080412544001	36	0.0840213169680349	0.933532256852292	0.000207591652788496	0.964633027821854
**CHOL**	CHAF1A	cg07783855	-0.0273747106660683	36	-0.159680462498227	0.87407764705378	0.000749374784050954	0.933346095957693
**CHOL**	CHAF1A	cg09700233	-0.24728960019297	36	-1.48815340390363	0.145927535005757	0.0611521463635992	0.309943120054547

CHOL	CHAF1A	cg18804734	-0.0925320705420348	36	-0.541874855236272	0.591438532276787	0.00856218407879609	0.754115417949105
CHOL	CHAF1A	cg23811775	-0.192320693453902	36	-1.14274533234754	0.261127662751606	0.0369872491305896	0.456426301393005
CHOL	CHAF1A	cg24085258	-0.00357616466830838	36	-0.0208525774907023	0.983485154134752	1.27889537348572e-05	0.991408097481847
CHOL	CHAF1B	cg00660452	0.108498049408751	34	0.617402386870057	0.541338195187445	0.0117718267255037	0.716351356678439
CHOL	CHAF1B	cg02544117	-0.0793637000335526	30	-0.421282065542007	0.676765623793193	0.00629859688301572	0.813264515460519
CHOL	CHAF1B	cg02908490	-0.0274588111694019	36	-0.160171401927024	0.873693886735227	0.00075398631083687	0.933141248039214
CHOL	CHAF1B	cg04634639	-0.0727911207475685	36	-0.425570473519096	0.673102655992162	0.00529854725968709	0.810871688818933
CHOL	CHAF1B	cg07872789	-0.107627144739585	36	-0.631235342282514	0.532107707525914	0.0115836022847955	0.709628975010216
CHOL	CHAF1B	cg13854874	-0.0775902873134906	36	-0.453793269998969	0.652861079451464	0.00602025268539002	0.797247858328973
CHOL	CHAF1B	cg25942896	0.205724835479708	36	1.22579144867518	0.228700199500991	0.042322707933153	0.418633457727949
CHOL	CHD1	cg03255775	0.136396300495074	36	0.802823167904046	0.427653578802743	0.0186039507887424	0.621964526255013
CHOL	CHD1	cg03833628	-0.154442024688201	36	-0.911480088833234	0.36845999057134	0.023852338989791	0.567322642385093
CHOL	CHD1	cg08865521	-0.0766388473839777	36	-0.448195612170693	0.656855213401476	0.00587351292834462	0.800090727157287
CHOL	CHD1	cg11365324	0.0314140615503244	36	0.183264330626904	0.855679142801695	0.00098684326308757	0.923216831964205
CHOL	CHD1	cg12662497	-0.0532947079303966	36	-0.311201148803957	0.75754782330044	0.00284032589338627	0.864800213744525
CHOL	CHD1	cg16890600	0.0788767596271121	36	0.461364028383896	0.647475578189922	0.00622154320927321	0.793398782876646
CHOL	CHD1	cg21082921	-0.103287097281464	36	-0.605500563907322	0.548867276298323	0.0106682244648306	0.721944349047662
CHOL	CHD4	cg00079216	-0.143625730352646	36	-0.846248563746437	0.403331017097941	0.0206283504193311	0.599793630438851
CHOL	CHD4	cg00824109	-0.17680207092594	36	-1.04742505831252	0.302292809304802	0.0312589722837011	0.500686183484739
CHOL	CHD4	cg04344119	0.0508531073816783	36	0.296906176772477	0.768344490478301	0.00258603853037251	0.871658128591577
CHOL	CHD4	cg05147525	-0.501392747745004	36	-3.37902226996471	0.00183840247596572	0.251394687491285	0.0110976395937017
CHOL	CHD4	cg11565429	-0.344338145288643	36	-2.1386039989105	0.0397362409110745	0.118568758300823	0.121894366144958
CHOL	CHD4	cg19509514	-0.166414745881108	36	-0.984078519793387	0.332028304252138	0.0276938676466738	0.531729211375566
CHOL	CHD4	cg20308811	-0.13062017551297	36	-0.768221711946298	0.447658652760709	0.0170616302510392	0.639204824214724
CHOL	CHRAC1	cg00296189	-0.50910181486467	36	-3.44896764151124	0.00151935601884787	0.259184657898501	0.00949982174330902
CHOL	CHRAC1	cg07270064	-0.4768765988523	36	-3.16352514927732	0.00327597647796115	0.227411290532938	0.0176685603249105
CHOL	CHRAC1	cg08877624	-0.355037190272413	36	-2.21447286264587	0.033602068332128	0.126051406476529	0.107405674411602
CHOL	CHRAC1	cg12002314	-0.0667243872191851	36	-0.389935685269205	0.699016229774639	0.00445214384977575	0.827352632715797
CHOL	CHRAC1	cg18770186	-0.227823682556905	36	-1.36430692721113	0.181434107890935	0.0519036303337893	0.358718199335843
CHOL	CHRAC1	cg19486048	0.208306497776058	36	1.24186726671462	0.222784206055953	0.0433915970157269	0.411463783086604
CHOL	CHRAC1	cg22610305	-0.342222473578252	36	-2.12371490850725	0.0410505295096225	0.117116221422017	0.124721144021542
CHOL	CHRAC1	cg23548819	-0.37986756162243	36	-2.39447738842004	0.0223021907464282	0.14429936437297	0.0790161249291974
CHOL	CLOCK	cg05960024	-0.243857950025784	36	-1.4661867444856	0.151785895338128	0.0594666997907777	0.318266556299516
CHOL	CLOCK	cg06085476	-0.0936814320781277	29	-0.488933218822003	0.628837187238787	0.00877621071620885	0.78081721483838
CHOL	CLOCK	cg07236904	0.104765908633881	36	0.614265330742873	0.543128845437783	0.0109758956118827	0.717524950204374
CHOL	CLOCK	cg07500410	0.110012574478603	36	0.645395453865726	0.523002810000903	0.0121027665434102	0.702578999627156
CHOL	CLOCK	cg11774413	0.0478826341230863	36	0.279521956565826	0.781537733440515	0.00229274665056535	0.879321110278629
CHOL	CLOCK	cg18928811	0.072240987971377	36	0.4223372056098	0.675437819276061	0.00521876034308063	0.812359075365046
CHOL	CLOCK	cg22523077	0.0400544259446914	36	0.233743009746573	0.816586202022684	0.00160435703775876	0.901377873320743
CHOL	CLOCK	cg23506964	0.0833183134985885	36	0.487520193238616	0.629018580667891	0.00694194136424907	0.780934136308588
CHOL	CREBBP	cg00039463	-0.27662312046117	36	-1.67847265372734	0.102424568818069	0.076520350773675	0.242336280615354
CHOL	CREBBP	cg00747661	-0.0318041181064342	36	-0.185542144596506	0.853906309235507	0.00101150192852801	0.922107767766478
CHOL	CREBBP	cg03039399	-0.0876622830135894	36	-0.513129976454048	0.611178654829205	0.00768467586315464	0.768160014763202
CHOL	CREBBP	cg03264209	-0.063605487721783	36	-0.371633051152727	0.712472130266393	0.00404565806832589	0.836430527559548
CHOL	CREBBP	cg08506924	0.112999689396581	36	0.663143169597427	0.511710154534011	0.0127689298037238	0.693757222237238
CHOL	CREBBP	cg24567591	0.00466516599346331	36	0.0272026545071724	0.97845714138023	2.17637737465665e-05	0.98883804712013
CHOL	CTCF	cg01866162	-0.255218632504622	36	-1.53913865541443	0.133026457507817	0.0651365503775294	0.290750279278991
CHOL	CTCF	cg04487155	-0.274180330584357	36	-1.662439844651	0.105618216088708	0.0751748536793474	0.24767033918327
CHOL	CTCF	cg06241380	-0.309545504915209	36	-1.89817449001271	0.0661890393285904	0.0958184196132119	0.176896733733969
CHOL	CTCF	cg07967402	-0.310371076530055	36	-1.90377587029695	0.0654315660645013	0.0963302051464254	0.17543281924597
CHOL	CTCF	cg08324636	0.0578023202288803	36	0.337607011323209	0.737734919345643	0.00334110822384203	0.852388984339447
CHOL	CTCF	cg10218542	-0.299934155415253	36	-1.83330727077102	0.0755244502955947	0.0899604975846612	0.194857413856981
CHOL	CTCF	cg10481400	0.20553966957586	36	1.22463946641892	0.229128581319225	0.0422465557693538	0.419117619848084
CHOL	CTCF	cg23858565	0.0259574642567859	36	0.151407742496307	0.880548894630757	0.000673789950642317	0.93665628447651
CHOL	CXXC1	cg02159718	-0.248897223682117	36	-1.49846456397438	0.143240655335077	0.061949827956666	0.306047038662174
CHOL	CXXC1	cg07675399	-0.242862518159746	36	-1.45982579613024	0.153516814917589	0.058982202726893	0.320772665243989
CHOL	CXXC1	cg11871295	0.118203773817391	36	0.694106651197184	0.492331012416849	0.0139721321446729	0.677856544116987

**CHOL**	CXXC1	cg13423076	-0.056887938343559	36	-0.332248885704924	0.741741054276449	0.00323623752898057	0.854682044855616
**CHOL**	CXXC1	cg15632775	0.0152224443431197	36	0.0887716264880373	0.929784250496859	0.000231722811779378	0.962787361823849
**CHOL**	CXXC1	cg20016845	-0.218506543765488	36	-1.30565158502701	0.200437029827882	0.047745109668339	0.383453641122517
**CHOL**	CXXC1	cg27419474	-0.123010341266387	36	-0.72275643653969	0.474772190174368	0.0151315440584729	0.662607914505028
**CHOL**	DAXX	cg00221327	-0.312167880101398	14	-1.13826376432548	0.277225882630596	0.0974487853670008	0.473987828450444
**CHOL**	DAXX	cg02173004	0.0755353997233193	36	0.441705180480616	0.66149922251609	0.00570559661136162	0.803050694173131
**CHOL**	DAXX	cg02328295	-0.0234652073217567	36	-0.136862179533683	0.891946966489559	0.000550615954653026	0.942747782819607
**CHOL**	DAXX	cg03085224	-0.116884826392654	36	-0.686253742383077	0.497206702555613	0.0136620626408409	0.6817007088229
**CHOL**	DAXX	cg03369465	-0.113890451423453	18	-0.458545416711276	0.652727680754159	0.0129710349254379	0.797147505072823
**CHOL**	DAXX	cg03382121	-0.0598655980649449	36	-0.34970062920176	0.728720289500335	0.00358388983167353	0.846589092679946
**CHOL**	DAXX	cg03902329	-0.123786149730239	36	-0.727385465441559	0.471969221844209	0.0153230108650373	0.660093372240771
**CHOL**	DAXX	cg04086445	-0.133294769334595	36	-0.784233545076293	0.438332976851996	0.0177674955319628	0.631008060718434
**CHOL**	DAXX	cg04152866	0.114143697723032	36	0.669945003497758	0.507417715472202	0.0130287837298868	0.690184171041828
**CHOL**	DAXX	cg04271391	0.0935664268042932	36	0.547985323816713	0.587281998781033	0.00875467622492316	0.751196432252613
**CHOL**	DAXX	cg04488476	-0.117734496144193	36	-0.691312184301151	0.494062957024747	0.013861411582327	0.679319579982337
**CHOL**	DAXX	cg04973788	0.0712376770081312	36	0.416441491779945	0.679704278443598	0.00507480662551482	0.815196636612625
**CHOL**	DAXX	cg05431670	-0.160875376470002	36	-0.950436272452986	0.348598295661124	0.0258808867543648	0.547756080968339
**CHOL**	DAXX	cg05532204	-0.0267112158007818	36	-0.155807407744906	0.877106236126261	0.000713489049555936	0.934920074586898
**CHOL**	DAXX	cg06171013	-0.0647795973149574	36	-0.378521764324664	0.707396359728758	0.00419639622828804	0.833212597222266
**CHOL**	DAXX	cg07689821	-0.211300792956143	36	-1.26054653372471	0.216054720112644	0.0446480251038949	0.403266894513857
**CHOL**	DAXX	cg08375286	-0.0442489412604008	36	-0.258266410558482	0.797758316170179	0.0019579688026664	0.889441926881303
**CHOL**	DAXX	cg08567382	0.0501147477558237	36	0.29258432536641	0.771618063304674	0.00251148794262984	0.873389081192628
**CHOL**	DAXX	cg09097215	0.111034149172688	36	0.651463044580358	0.519127090830265	0.0123285822825026	0.699831124706415
**CHOL**	DAXX	cg09432361	0.0350150127056673	36	0.204296131845067	0.839340103687995	0.00122605111477804	0.914018411164563
**CHOL**	DAXX	cg09615909	-0.0227495541271948	36	-0.132685895464476	0.895223975968772	0.000517542212986164	0.944372396915615
**CHOL**	DAXX	cg11111423	-0.0276971574868553	36	-0.161562774849517	0.872606439403164	0.000767132532851662	0.93249190014739
**CHOL**	DAXX	cg12025215	0.158377781452492	36	0.935298005238735	0.356230594487834	0.0250835216578133	0.554991742310719
**CHOL**	DAXX	cg12832355	-0.111735711445736	36	-0.655631147625693	0.516473685919688	0.0124848692122848	0.697580671257813
**CHOL**	DAXX	cg12923297	0.117088968684566	33	0.65643915020174	0.516385168865372	0.0137098265876152	0.697509555444459
**CHOL**	DAXX	cg13443893	-0.178836146314481	36	-1.05987128799191	0.296674712339177	0.0319823672286145	0.494671466250036
**CHOL**	DAXX	cg13826470	-0.17677204969307	36	-1.04724146668454	0.302376230483756	0.0312483575526893	0.500761320424151
**CHOL**	DAXX	cg14539675	-0.17240261672027	36	-1.02055253969034	0.314673654547841	0.0297226622519962	0.513718674596968
**CHOL**	DAXX	cg14622330	-0.146355837080612	36	-0.862683210011565	0.39435612393392	0.0214200310475665	0.591108676402994
**CHOL**	DAXX	cg14746369	-0.151874741588188	36	-0.89596776362189	0.376569932785858	0.0230659371324789	0.575113157217509
**CHOL**	DAXX	cg14910283	-0.0455710789810231	36	-0.265999116156178	0.791846331360248	0.00207672323949464	0.885770632240052
**CHOL**	DAXX	cg16922466	0.145129721400292	36	0.855299794383746	0.398372463018466	0.0210626360337265	0.594974418861512
**CHOL**	DAXX	cg17079757	-0.0191886856773767	36	-0.111908907753461	0.911553716008673	0.000368205658025162	0.953021943356029
**CHOL**	DAXX	cg17504991	-0.0140751469277812	36	-0.0820796354399706	0.935064696806689	0.000198109761038628	0.965445053796877
**CHOL**	DAXX	cg18187593	-0.0449165858814226	36	-0.262171050085281	0.794771513111996	0.00201749968724321	0.887606080844577
**CHOL**	DAXX	cg18279148	-0.119101910101052	21	-0.522874996144478	0.607105558117684	0.014185264989719	0.76529513120007
**CHOL**	DAXX	cg18486541	-0.199920575366375	36	-1.18974571789898	0.242388324228821	0.0399682364548222	0.434778817040741
**CHOL**	DAXX	cg18944910	0.00921479320975298	36	0.0537332972874365	0.957462145141252	8.49124138985097e-05	0.977600167678701
**CHOL**	DAXX	cg18967278	-0.0908890212015779	36	-0.53217215638823	0.598067478651588	0.00826081417498088	0.758717123406078
**CHOL**	DAXX	cg20223162	-0.11636808087802	31	-0.630947854658508	0.533016192587161	0.0135415302472333	0.710149001158654
**CHOL**	DAXX	cg20763184	0.0718917646179558	36	0.420284933877424	0.676921728203258	0.00516842581988356	0.813389282441758
**CHOL**	DAXX	cg21286626	0.00397708433113036	36	0.0231903708206534	0.981633990576983	1.58171997769226e-05	0.990518921343635
**CHOL**	DAXX	cg24328964	-0.102502641990995	36	-0.600852827331799	0.551922857990539	0.010506791615134	0.724223034350706
**CHOL**	DAXX	cg24496586	-0.0358046354388459	36	-0.208909057573123	0.835765830155019	0.00128197191890866	0.912015247455177
**CHOL**	DAXX	cg24513883	-0.102109507661211	36	-0.598524013851615	0.553457175846386	0.010426351554815	0.725392090603888
**CHOL**	DAXX	cg24728112	0.000446055625693087	36	0.00260092915458927	0.997939962157253	1.98965621212451e-07	0.998954402831646
**CHOL**	DAXX	cg25642825	0.0499003886792171	36	0.291329704586412	0.772569170984119	0.00249004879033694	0.873905312113269
**CHOL**	DAXX	cg26521092	-0.137047917892502	36	-0.806731799756668	0.425428435373119	0.0187821317986699	0.620148248127892
**CHOL**	DAXX	cg27052777	0.0224989759650086	36	0.131223663705891	0.896371791672277	0.000506203919474035	0.945074841246231
**CHOL**	DAXX	cg27393652	-0.144155982800479	36	-0.849439011020574	0.401578796249055	0.0207809473771719	0.598166099438969
**CHOL**	DIDO1	cg00080059	-0.0608456157198304	36	-0.355446433311361	0.724450946360544	0.00370218895232527	0.843921728053969
**CHOL**	DIDO1	cg01488865	-0.0627042744514554	36	-0.366346522651057	0.716376430206264	0.00393182603448344	0.83876086286691
**CHOL**	DIDO1	cg06646488	0.00372780374831868	36	0.0217367953633092	0.982784981568898	1.38965207859788e-05	0.991091426929181

**CHOL**	DIDO1	cg08610063	-0.162407592668226	36	-0.9597325015201	0.343965568702127	0.0263762261562884	0.54326118061848
**CHOL**	DIDO1	cg13815390	0.18404900455038	36	1.09183259301488	0.282583407530717	0.0338740360759857	0.479806482428894
**CHOL**	DIDO1	cg20969846	-0.0274890809371371	36	-0.160348103515051	0.873555769226888	0.000755649570768476	0.933083405572305
**CHOL**	DIDO1	cg21212389	-0.138544153753532	36	-0.815710800478476	0.42034359821527	0.0191944825392822	0.615572763803207
**CHOL**	DIDO1	cg21687703	0.130188932927104	36	0.765641624780218	0.449172307097422	0.0169491582566981	0.64040228829149
**CHOL**	DIDO1	cg22394521	-0.182253085554797	36	-1.08081076852645	0.287388310840943	0.0332161871942441	0.48489570463401
**CHOL**	DMAP1	cg01604592	-0.190036567916104	36	-1.1286616730051	0.266941718759855	0.036113897145332	0.462679947178312
**CHOL**	DMAP1	cg11683482	-0.2122503582667	36	-1.26647792041565	0.213950142116075	0.0450502145843423	0.400539956581266
**CHOL**	DMAP1	cg15990015	-0.0120751733053776	36	-0.0704148884434876	0.944275988796332	0.000145809810354904	0.9701876527382
**CHOL**	DMAP1	cg18017761	-0.103468812369974	36	-0.606577351090347	0.548160605640371	0.0107057951332528	0.721354728925959
**CHOL**	DMAP1	cg18167340	0.0645733376593469	36	0.377311488175206	0.708287145279954	0.00416971593646802	0.833744252813048
**CHOL**	DMAP1	cg21437596	-0.129765343730536	36	-0.763107757509773	0.45066179372759	0.0168390444335042	0.641452605036788
**CHOL**	DMAP1	cg23021241	-0.161414912922915	36	-0.953708927790913	0.346962682014083	0.0260547741139121	0.546247507760276
**CHOL**	DMAP1	cg23451311	-0.205380729444821	36	-1.22365075345722	0.229496724479629	0.0421812440272866	0.419572734262162
**CHOL**	DMAP1	cg24485408	-0.145626482805192	36	-0.858290701884286	0.396742413828135	0.0212070724942109	0.59334793127912
**CHOL**	DNMT1	cg04160663	-0.0551712715055391	36	-0.322191758624399	0.749280207794228	0.00304386919953791	0.859537041733307
**CHOL**	DNMT1	cg04762346	0.045235866067306	36	0.264038446070143	0.793344180019793	0.00204628357885924	0.886808206630646
**CHOL**	DNMT1	cg06128182	-0.075933610210735	36	-0.44404724795096	0.659821853240073	0.00576591315963584	0.802021550459496
**CHOL**	DNMT1	cg06716436	0.071180083035211	36	0.416103093483179	0.679949489006362	0.00506660422089954	0.815329309433833
**CHOL**	DNMT1	cg10635912	0.0811014355557436	36	0.474461515366271	0.638204108336651	0.00657744284920243	0.78724590752452
**CHOL**	DNMT1	cg11380607	0.227992194884146	36	1.36537136135197	0.181102613157349	0.0519804409280906	0.358348820986804
**CHOL**	DNMT1	cg13393778	-0.120959711250042	36	-0.710527363391879	0.482222877384764	0.0146312517456936	0.669296102110086
**CHOL**	DNMT1	cg15043801	0.0152855267566208	36	0.0891395854265631	0.929493996710358	0.000233647328227371	0.962671899544514
**CHOL**	DNMT1	cg21893394	0.139625034181978	36	0.822200767593343	0.416691627113752	0.0194951501703186	0.612213798932695
**CHOL**	DNMT1	cg23401624	0.0501343139541857	36	0.292698846251815	0.77153126470846	0.00251344943565686	0.873324178411476
**CHOL**	DNMT1	cg24477899	0.10135689591976	36	0.59406655027552	0.556400011176318	0.010273220350489	0.727803848724231
**CHOL**	DNMT1	cg27429799	-0.194027787478875	36	-1.1532836591086	0.256837415180861	0.0376467823139474	0.451157447480749
**CHOL**	DNMT3A	cg02208653	0.0344110305928519	36	0.200767965831987	0.842076205838417	0.00118411902646219	0.915500383495578
**CHOL**	DNMT3A	cg03463641	-0.114804067536142	36	-0.673872536035327	0.504948187118914	0.0131799739228431	0.688118615224902
**CHOL**	DNMT3A	cg06112956	0.0387490880969467	36	0.226113886507825	0.822466676637001	0.00150149182834494	0.904585595761027
**CHOL**	DNMT3A	cg07150430	-0.112006919733146	36	-0.657242708756939	0.515449741602982	0.0125455500681075	0.696779560151032
**CHOL**	DNMT3A	cg12066181	-0.0417501406779204	36	-0.243655509335957	0.808961849995326	0.00174307424662615	0.896740879159527
**CHOL**	DNMT3A	cg13076778	-0.131357145554472	36	-0.772631967786374	0.445078321729806	0.0172546996882188	0.636981859937976
**CHOL**	DNMT3A	cg13344237	-0.0931541269086846	36	-0.545549445209495	0.588937266868654	0.00867769136011932	0.752361168858054
**CHOL**	DNMT3A	cg15990840	0.0164104327515857	36	0.0957013311102227	0.924319636065724	0.000269302303094317	0.960149040179687
**CHOL**	DNMT3A	cg15998962	0.0189086161825121	36	0.110274946739491	0.91283968529468	0.000357535765937559	0.953866463542531
**CHOL**	DNMT3A	cg21629895	-0.293124643478983	36	-1.78772278577429	0.0827373760224402	0.0859220566146809	0.207876220982777
**CHOL**	DNMT3A	cg21708767	0.0214065902901173	36	0.124849407119904	0.901378040021986	0.000458242107848943	0.947956498370603
**CHOL**	DNMT3A	cg22731525	-0.343598285765057	36	-2.13339303620978	0.0401919199599813	0.118059781980686	0.122885168266263
**CHOL**	DNMT3A	cg25044635	-0.0120859754046989	36	-0.0704778887559157	0.944226217307367	0.000146070801482987	0.970185463237239
**CHOL**	DNMT3A	cg26470599	-0.444424596168084	36	-2.89280128764393	0.00661672376740945	0.197513221679165	0.030986118996018
**CHOL**	DNMT3A	cg26995204	0.147298896951085	36	0.868364884278311	0.39128296406248	0.0216969650430063	0.588429671491911
**CHOL**	DNMT3B	cg00300969	0.102993969012564	36	0.603763705928221	0.550008125209754	0.010607757652961	0.722734571341638
**CHOL**	DNMT3B	cg13636640	0.0259483445857707	36	0.151354512362383	0.880590560831856	0.000673316586741893	0.936662183154264
**CHOL**	DNMT3B	cg14224313	-0.0222336314923479	36	-0.129675291118241	0.897587472786222	0.000494334369337522	0.945818194670396
**CHOL**	DNMT3B	cg16523653	0.0573040902474691	36	0.334687361750649	0.739916953016233	0.00328375875909008	0.853528365529513
**CHOL**	DNMT3B	cg16953106	0.129632656191503	36	0.762314121694363	0.451128917965383	0.0168046255512644	0.641777936511774
**CHOL**	DNMT3B	cg17475857	0.109593089322728	36	0.642904544013062	0.524598379635898	0.0120106452272995	0.703752480520695
**CHOL**	DNMT3B	cg17721173	0.197731361631754	36	1.1761843517944	0.247691103812347	0.0390976913727474	0.441026337918055
**CHOL**	DNMT3B	cg22605822	0.0770612506479084	36	0.45068060711339	0.655080805151296	0.00593843635141977	0.798946193300474
**CHOL**	DNMT3B	cg24403338	0.0673100662988132	36	0.393373888982421	0.696499323328704	0.00453064502515063	0.826005280104779
**CHOL**	DNMT3B	cg25803642	-0.246697099664661	36	-1.48435646329737	0.146927034941802	0.0608594589829557	0.311428307892282
**CHOL**	DNMT3L	cg09247319	0.00510585370369466	36	0.0297723754099641	0.976422669883437	2.60697420435325e-05	0.987543624613701
**CHOL**	DNMT3L	cg15635600	0.044139983035554	36	0.257629214492027	0.798246025989832	0.001948338102379	0.889804100304595
**CHOL**	DNMT3L	cg27187798	0.082530286175497	36	0.482877437430191	0.632277549358138	0.00681124813620943	0.783225833698999
**CHOL**	DPF1	cg06168449	-0.159674620009676	35	-0.929182548976889	0.3595418605323	0.0254959842752344	0.558439313622387
**CHOL**	DPF1	cg06615189	-0.0222056131985026	36	-0.129511796730966	0.897715852622218	0.000493089257521514	0.945905264825489

**CHOL**	DPF1	cg07480327	0.4878082950537	8	1.3687844036829	0.220098123626139	0.237956932723197	0.408267088613746
**CHOL**	DPF1	cg08057062	-0.185267455443551	36	-1.09931684559558	0.279353284943005	0.0343240300465281	0.476401548739639
**CHOL**	DPF1	cg13322582	-0.226059810972433	36	-1.3531728968357	0.184929802074673	0.0511030381368921	0.363471563152709
**CHOL**	DPF1	cg13917712	-0.0457075798306892	36	-0.266797539358058	0.791236607655484	0.00208918285397883	0.885427553010007
**CHOL**	DPF1	cg17121541	-0.072065979366369	36	-0.421308717655731	0.67618131019238	0.00519350538203392	0.812884395850588
**CHOL**	DPF1	cg22145401	-0.174635660995105	34	-1.00330616393708	0.323240005140232	0.0304976140911971	0.522672589110066
**CHOL**	DPF2	cg02186298	0.206726153779994	36	1.23202338787059	0.226393083748281	0.0427357026566697	0.41591709238429
**CHOL**	DPF2	cg14699126	-0.196446611064475	36	-1.16823434292614	0.250838904847123	0.0385912709987172	0.444454202566384
**CHOL**	DPF2	cg15918224	-0.0425655907576667	36	-0.248423064234377	0.805301498266885	0.00181182951654916	0.894241748949188
**CHOL**	DPF2	cg15929437	-0.100860705865876	36	-0.591128349398614	0.558344172900262	0.0101728819877628	0.729400950236826
**CHOL**	DPF2	cg18224556	-0.153306218485979	36	-0.904614881498166	0.372035066310091	0.0235027966264707	0.57068117812338
**CHOL**	DPF2	cg19517014	-0.232960239210406	36	-1.39681145468064	0.17152170363210 7	0.0542704730529695	0.345700149082156
**CHOL**	DPF3	cg00888802	-0.19641397694423	36	-1.16803248540633	0.250919208232803	0.0385784503390486	0.444551037707582
**CHOL**	DPF3	cg05151458	-0.153487205858335	36	-0.905708585360082	0.371464017192159	0.0235583223621989	0.570093183812734
**CHOL**	DPF3	cg07171024	-0.103425397691078	35	-0.597337067249507	0.554359307432451	0.0106968128875577	0.72604848584801
**CHOL**	DPF3	cg10266394	-0.263429305479123	36	-1.59228518505165	0.120576547360793	0.069394998985213	0.271525847431991
**CHOL**	DPF3	cg12913429	-0.103439055114687	31	-0.560040532303884	0.579752612251352	0.0106996381230193	0.745672817893686
**CHOL**	DPF3	cg13819165	-0.0564732948820366	36	-0.329819421300265	0.743559923018767	0.00318923303483346	0.85593961240725
**CHOL**	DPF3	cg14238615	-0.127073184849005	36	-0.747013408779162	0.460190524019372	0.0161475943076694	0.650041522029776
**CHOL**	DPF3	cg14242696	-0.186858909101757	36	-1.10910015627885	0.275170581747247	0.0349162519106988	0.471682760880506
**CHOL**	DPF3	cg15392845	-0.130418961372908	36	-0.767017810008721	0.448364566223655	0.017009105485588	0.639743095719215
**CHOL**	DPF3	cg15740492	-0.11930146338302	36	-0.700645047028211	0.488291990220306	0.0142328391653301	0.674326973783968
**CHOL**	DPF3	cg16696021	-0.219198203657733	36	-1.30999274458081	0.198980474532487	0.0480478524867771	0.381711954376747
**CHOL**	DPF3	cg19710444	-0.131934164268441	36	-0.776085935026049	0.443063690711875	0.017406623701212	0.635337537653492
**CHOL**	DPF3	cg19839347	-0.240350808322631	36	-1.44379734392287	0.157947961658038	0.0577685110613422	0.326931772276112
**CHOL**	DPF3	cg20548765	-0.114911254617286	36	-0.674510113704532	0.504547920219556	0.0132045964377187	0.687777968872069
**CHOL**	DPF3	cg21035356	-0.115527605216728	36	-0.678176797398468	0.502249397529645	0.0133466275671122	0.685918747808598
**CHOL**	DPF3	cg22784589	-0.237648046649702	36	-1.42658406427411	0.162818707675733	0.0564765940764187	0.333545438728823
**CHOL**	DPF3	cg23515460	-0.165755273115759	36	-0.980068390627507	0.333975077434729	0.0274748105656799	0.533561777096918
**CHOL**	DPF3	cg24383902	-0.21900913414457	36	-1.30880585604221	0.199377897064378	0.0479650008387541	0.382133191855582
**CHOL**	DPF3	cg25495195	-0.337635111735516	36	-2.09155640511486	0.0440211692834425	0.113997468676655	0.131441730376761
**CHOL**	DPF3	cg26057784	0.0454496425477081	36	0.265288820558117	0.792388867390887	0.00206567000771444	0.886183706370009
**CHOL**	EED	cg00080012	0.0514402622521547	36	0.300343327194938	0.765744105854547	0.00264610058057045	0.870078379588329
**CHOL**	EED	cg01739327	0.171863301389694	36	1.01726269014501	0.316212974582456	0.0295369943645648	0.515474459257106
**CHOL**	EED	cg02172616	0.0263142279028903	36	0.153490147504867	0.878919152117107	0.000692438590125248	0.935697233349594
**CHOL**	EED	cg04607174	0.225693818567968	36	1.35086441648915	0.185661057765528	0.0509376997397909	0.364426521530328
**CHOL**	EED	cg06358433	0.0343352670191986	36	0.200325407978009	0.842419552031781	0.00117891056127967	0.915728735408695
**CHOL**	EED	cg10086030	0.200012244274674	36	1.19031397589068	0.242167957679069	0.0400048978597919	0.43453382769428
**CHOL**	EED	cg10535675	0.0900627324506748	21	0.394176244359715	0.697840081610079	0.00811129577648183	0.826728359955793
**CHOL**	EED	cg12672785	-0.0532463104733622	36	-0.310917740052752	0.757761404224732	0.00283516957902568	0.864955184368389
**CHOL**	EED	cg16265078	-0.0340915670943364	36	-0.198901906724342	0.843524147778127	0.00116223494694764	0.916394556290143
**CHOL**	EED	cg18574463	-0.023632268317187	36	-0.137837115018062	0.891182238020191	0.000558484105815519	0.942418343236566
**CHOL**	EED	cg19325359	0.0633812208271504	28	0.32383318430848	0.748654945650021	0.00401717915354	0.859169423049264
**CHOL**	ELP3	cg01209934	-0.086279800511915	36	-0.504976451198349	0.616833045883547	0.00744420397637584	0.772384148139532
**CHOL**	ELP3	cg02516513	0.175007047300001	36	1.03645306691576	0.307306414102817	0.0306274666046647	0.505820518771862
**CHOL**	ELP3	cg04720865	-0.11699689597826	36	-0.686920852109332	0.496791467610106	0.0136882736685478	0.681395596305592
**CHOL**	ELP3	cg05621091	-0.195763659883469	36	-1.1640108065741	0.252523028997961	0.0383234105309704	0.44644175437737
**CHOL**	ELP3	cg15692687	-0.270109052518557	36	-1.63579585607969	0.111107768450386	0.0729589002524727	0.256743234682745
**CHOL**	ELP3	cg17572663	-0.229475483778258	36	-1.37474638172913	0.178203222057591	0.0526589976552654	0.354773811463513
**CHOL**	ELP3	cg18022570	-0.203602397180119	36	-1.21259521864476	0.233643179642093	0.0414539361374908	0.424526613268306
**CHOL**	ELP3	cg18249244	-0.102052190962579	28	-0.523097179445265	0.605336464128349	0.0104146496802628	0.764014226688528
**CHOL**	ELP3	cg19781013	-0.197808689172736	36	-1.17666305371566	0.247502488022508	0.0391282775122362	0.44080819309477
**CHOL**	ELP3	cg22414525	0.268139099511864	36	1.62293783799818	0.11383995015544	0.0718985766870331	0.26110221954102
**CHOL**	ELP3	cg22487209	-0.213515419166149	36	-1.2743858659538	0.211168315568309	0.0455888342216963	0.397071157819807
**CHOL**	ELP3	cg25374552	-0.0582704594250248	36	-0.340350558168261	0.735686507793875	0.00339544644160346	0.850995437933129
**CHOL**	ELP4	cg04972224	-0.148057203013522	36	-0.872935223115131	0.388821967360123	0.0219209353641872	0.586508757078576
**CHOL**	ELP4	cg05195750	0.335298950663413	36	2.0752442382832	0.0455992586921591	0.112425386315986	0.134895567201499

CHOL	ELP4	cg05635455	-0.0445639267425509	36	-0.260108521864122	0.796348828728132	0.00198594356671544	0.888484467824802
CHOL	ELP4	cg08111895	0.00469871225183687	36	0.0273982675579126	0.978302267419956	2.20778968255619e-05	0.988739781322494
CHOL	ELP4	cg08626560	0.0446864938851086	36	0.260825345144173	0.795800540192765	0.00199688273574385	0.888198838972216
CHOL	ELP4	cg09616795	0.356482551035756	36	2.2247973886133	0.0328361961287809	0.127079809192961	0.105556158745553
CHOL	ELP4	cg10583382	0.0607831481853328	36	0.355080158059852	0.724722837041312	0.00369459110332012	0.844052564908
CHOL	ELP4	cg11385473	-0.0689017695722759	36	-0.402719988405506	0.689675284624356	0.00474745385019101	0.82163245163962
CHOL	ELP4	cg13263383	-0.184224557932365	35	-1.07671843242733	0.289414801407778	0.0339386877453752	0.486918784127039
CHOL	ELP4	cg19328333	0.0676699069651934	36	0.395486520415648	0.694954517718603	0.00457921630867793	0.825091443614569
CHOL	ELP4	cg24001477	0.0828111478680453	36	0.484532062117873	0.631115229986319	0.00685768621122327	0.782297294479855
CHOL	ELP4	cg24531794	0.067205555928241	36	0.392760335532312	0.696948214873758	0.00451658674762393	0.826191873999401
CHOL	ELP4	cg25130734	0.08801562912251	36	0.515214398160767	0.60973698788862	0.00774675096983122	0.767178496107123
CHOL	EP300	cg00500400	-0.0759696854213369	36	-0.444259434161051	0.659669975284178	0.00577139310301689	0.801912119028945
CHOL	EP300	cg02046995	0.150139857300522	36	0.885495583396166	0.38210941488348	0.0225419767502212	0.580559740272752
CHOL	EP300	cg07519229	-0.411518178349595	35	-2.59379847518738	0.014046366993285	0.169347211112169	0.0554907106375997
CHOL	EP300	cg08131204	-0.219937833618517	36	-1.31463728091827	0.19743109195156	0.0483726506568065	0.379744208312285
CHOL	EP300	cg09331127	0.28528325552496	36	1.73559878353311	0.091691296594607	0.0813865358829193	0.223816527369967
CHOL	EP300	cg24349919	0.186334489814859	36	1.10587534330074	0.276544340170479	0.0347205420945639	0.473159765854372
CHOL	EPC1	cg02900516	-0.140070934160288	36	-0.824878971883322	0.415190289400594	0.0196198665965357	0.610848275049883
CHOL	EPC1	cg16942190	-0.0259078044140227	36	-0.151117885997719	0.88077578569158	0.000671214329555252	0.936772166165112
CHOL	EPC1	cg26211455	-0.0427730560026004	36	-0.249636095391944	0.804370885261113	0.00182953431980159	0.893586471591677
CHOL	EPC2	cg00066942	-0.260856128417568	36	-1.57559010878959	0.124380671223888	0.068045919733003	0.277447497827705
CHOL	EPC2	cg03664802	0.0178051530086229	36	0.10383745143313	0.917908446664068	0.000317023473660473	0.95678546621179
CHOL	EPC2	cg04098458	-0.151121927925812	36	-0.891422563955719	0.378967828232069	0.0228378371000144	0.577523570550963
CHOL	EPC2	cg05586854	0.124629855916328	36	0.732421158688262	0.468930856677544	0.0155326009857248	0.657861978876605
CHOL	EPC2	cg10458926	-0.00447123103772426	36	-0.0260717937054494	0.979352501407099	1.99919069927088e-05	0.989361337209135
CHOL	EPC2	cg11721798	0.0856769421031628	36	0.501421870900683	0.619305590696625	0.0073405384081487	0.774134476141291
CHOL	EPC2	cg12432635	0.0415757808730008	36	0.242636172799174	0.809745028800275	0.00172854555519978	0.897212715716971
CHOL	EPC2	cg16434205	0.232904656955921	36	1.39645907060385	0.171626848748447	0.0542445792317553	0.345837344780173
CHOL	EPC2	cg21347644	0.155348419620603	36	0.916961309241461	0.365621717049058	0.0241331314786191	0.564494331000346
CHOL	EPC2	cg21650422	0.101162829502797	36	0.592917328290362	0.557160022713642	0.010233918073012	0.728416691432258
CHOL	EZH1	cg00728999	-0.185705654095562	36	-1.10200973286291	0.27819750293399	0.0344865899630604	0.475064981444475
CHOL	EZH1	cg01716316	0.0150058849414443	36	0.0875084462142873	0.930780747789449	0.000225176582875865	0.963372900695398
CHOL	EZH1	cg03623386	-0.285994235904481	36	-1.74030901069227	0.0908500287645695	0.0817927029705879	0.222293109948762
CHOL	EZH1	cg05375741	-0.239682735807355	36	-1.43953924500327	0.159141992509597	0.0574478138440984	0.328581457754966
CHOL	EZH1	cg05979501	-0.283640558744469	36	-1.72472786823777	0.0936578721890935	0.0804519665648746	0.227283292543854
CHOL	EZH1	cg06975499	-0.1251866193307	36	-0.735745105971115	0.46693150902985	0.0156716896594497	0.656090119136857
CHOL	EZH1	cg12812489	-0.17419042132486	36	-1.03146501800917	0.309604595737992	0.0303423028813324	0.508248326000577
CHOL	EZH1	cg19395400	0.0444084159270951	36	0.259199047311749	0.797044624784198	0.00197210740515388	0.888951979763185
CHOL	EZH1	cg22227424	-0.0250328210785914	36	-0.146010930971213	0.884775018041872	0.000626642131152772	0.938895641525258
CHOL	EZH1	cg27088844	-0.194192405471231	36	-1.15430046167414	0.256426184692513	0.037710690342703	0.450699681705551
CHOL	EZH2	cg01893439	-0.0612845767622997	36	-0.358020377816868	0.722541301030144	0.00375579934893421	0.842795123584416
CHOL	EZH2	cg02303805	-0.00644952357607028	36	-0.0376076438953115	0.970220463556484	4.15963543582864e-05	0.984298837253315
CHOL	EZH2	cg06816161	0.237647556362147	36	1.42658094494478	0.16281960094258	0.0564763610449	0.333545438728823
CHOL	EZH2	cg13751213	-0.0162248472714935	36	-0.0946187587502361	0.925173090158424	0.000263245668983291	0.960618337451796
CHOL	EZH2	cg18494018	0.14720084442793	36	0.867774032542426	0.391601840472189	0.0216680886002957	0.58867023975756
CHOL	EZH2	cg20101066	-0.0727827745218581	36	-0.425521417753536	0.673138061028372	0.00529733226709964	0.81088437912698
CHOL	FBXL19	cg00026853	-0.179654718651548	36	-1.06488397167103	0.294432772898098	0.0322758179337667	0.49220180365383
CHOL	FBXL19	cg02291855	0.292622753395532	36	1.78437491675965	0.0832893835602588	0.0856280758047825	0.208858405205389
CHOL	FBXL19	cg03181514	-0.164636329775968	36	-0.97326739758966	0.337294260353915	0.0271051210821013	0.536868945063375
CHOL	FBXL19	cg03627896	0.101105056712718	36	0.592575222752859	0.557386368323733	0.0102222324928819	0.728651382771959
CHOL	FBXL19	cg04477789	-0.165847301636913	36	-0.980627917350805	0.333702986661849	0.0275053274602454	0.533291360635426
CHOL	FBXL19	cg05643349	-0.229663442446646	36	-1.37593508096971	0.177838187548314	0.0527452967964441	0.354328184457255
CHOL	FBXL19	cg05768582	-0.0284495283724829	36	-0.165955004937107	0.86917528886269	0.000809375664616708	0.930580358895956
CHOL	FBXL19	cg06862768	-0.177007337106344	36	-1.04868042249883	0.301722819965962	0.0313315973894788	0.500132639913749
CHOL	FBXL19	cg07697662	-0.201401947497145	36	-1.19893278184978	0.238843677964739	0.0405627444556427	0.430634924411637
CHOL	FBXL19	cg08535091	-0.209807052121759	36	-1.25122354943437	0.219394118159242	0.0440189991200225	0.407350897391208
CHOL	FBXL19	cg08593668	-0.234556922929676	36	-1.40694041404615	0.168520885708643	0.0550169500942379	0.341679840048428

**CHOL**	FBXL19	cg09011601	-0.214053766127496	36	-1.27775312857051	0.209992119526477	0.0458190147933649	0.395567023740684
**CHOL**	FBXL19	cg11940219	0.336855766136548	20	1.51786785149601	0.146415541884294	0.113471807179441	0.310623433018266
**CHOL**	FBXL19	cg26666512	-0.188239998723211	36	-1.11759760374364	0.271574025683933	0.0354342971193145	0.467873541225908
**CHOL**	FBXL19	cg27602786	-0.206225368440319	36	-1.22890612888795	0.227544942079817	0.0425289025883454	0.417255120312967
**CHOL**	FBXW9	cg02753619	-0.315552640940857	36	-1.93904164395427	0.0608320327456345	0.0995734692047491	0.166281347695499
**CHOL**	FBXW9	cg03160342	-0.330200778582919	36	-2.03979551920601	0.049201265255641	0.109032554176766	0.142611991106127
**CHOL**	FBXW9	cg05868316	-0.166748598543968	36	-0.986109132111254	0.331045442108204	0.0278050951163774	0.530690511114525
**CHOL**	FBXW9	cg06489804	-0.141756062213756	36	-0.835004976472946	0.409544135558488	0.0200947811743502	0.605486164344381
**CHOL**	FBXW9	cg12110584	-0.27187266626433	36	-1.64732587677043	0.108703963604027	0.0739147466616759	0.252840882156662
**CHOL**	FBXW9	cg14342428	0.0668459170188037	36	0.390649087293897	0.698493704802216	0.00446837662208478	0.827084419418615
**CHOL**	FBXW9	cg15828427	0.349092847637505	36	2.17220079165104	0.0369067551882596	0.121865816271662	0.11537569271109
**CHOL**	FBXW9	cg19721801	-0.232939987285595	36	-1.39668305850376	0.171560008924737	0.0542610376766129	0.345738314644221
**CHOL**	FBXW9	cg21564527	-0.160197350113535	36	-0.946324821612494	0.35066036572786	0.0256631909833984	0.549661196235371
**CHOL**	FBXW9	cg22303873	0.0434786820320255	36	0.253762072219334	0.801207694164663	0.00189039579124198	0.891475878455677
**CHOL**	FKBP1A	cg04613791	0.0522340080916466	36	0.304990339183238	0.762232782472529	0.0027283916013182	0.867824513134385
**CHOL**	FKBP1A	cg07516946	-0.1877535463203	36	-1.11460382084702	0.272837293013939	0.0352513941558491	0.469146967649161
**CHOL**	FKBP1A	cg10996143	-0.0634719635621157	36	-0.370849739446601	0.713050141892522	0.00402869015843054	0.836730111447121
**CHOL**	FKBP1A	cg21332426	-0.134492629623439	36	-0.791410337786982	0.434191177427071	0.0180882674230277	0.627651405509467
**CHOL**	FKBP1A	cg21775445	-0.0814075073592151	36	-0.476264026906995	0.636932713640674	0.00662718225444066	0.786316921438351
**CHOL**	FKBP1A	cg24641773	-0.120136267238175	36	-0.705619305642717	0.485231738908762	0.0144327227059223	0.671865974239432
**CHOL**	FKBP1A	cg26779912	-0.246671568810685	36	-1.48419289296037	0.146970215474862	0.0608468628595246	0.311468915295069
**CHOL**	FKBP2	cg03764062	0.278364321138902	36	1.68992219109322	0.100193336849407	0.0774866952831215	0.238565988789698
**CHOL**	FKBP2	cg04446112	-0.0715031208516831	36	-0.418001183872735	0.67857455037467	0.0051126962915304	0.814453533846609
**CHOL**	FKBP2	cg06615912	-0.136357440354309	36	-0.80259010462522	0.427786482608856	0.0185933515399789	0.622066978806564
**CHOL**	FKBP2	cg09854726	0.136121585128366	36	0.801175646373254	0.428593612936283	0.018529085937859	0.622760969170299
**CHOL**	FKBP2	cg10998122	-0.109401496370178	36	-0.641766977617087	0.525327921149912	0.0119686874080341	0.704293942335096
**CHOL**	FKBP2	cg12423493	0.109109657603833	36	0.640034349550804	0.526440127349884	0.0119049173824256	0.705153126262214
**CHOL**	FKBP2	cg15010817	-0.0793209810384674	36	-0.463978762851494	0.645620022768791	0.00629181803290491	0.792223162422716
**CHOL**	FKBP2	cg16977872	-0.0336589195759018	36	-0.196374811160678	0.845485891637979	0.00113292286701703	0.917476983247079
**CHOL**	FKBP2	cg19924313	-0.0135068359386543	36	-0.0787648956593654	0.937681377890067	0.000182434617073723	0.96664874103142
**CHOL**	FKBP2	cg21809711	-0.143351306827708	36	-0.844597692111459	0.404239565183716	0.0205495971692118	0.600690083617895
**CHOL**	FKBP2	cg22074794	0.0012767694948198	36	0.00744478757312694	0.994103485645399	1.63014034290241e-06	0.996961320110585
**CHOL**	FKBP2	cg24677501	-0.0379159975489065	36	-0.22124544871	0.826224709963104	0.00143762287012869	0.906537546005327
**CHOL**	FKBP5	cg00130530	-0.155126625410768	36	-0.91561983970924	0.366315033111543	0.0240642699113328	0.565217019435111
**CHOL**	FKBP5	cg00140191	0.167692828806489	36	0.991854172420661	0.328275371187514	0.0281208848331224	0.527881562407736
**CHOL**	FKBP5	cg00610228	0.315998610453289	36	1.94208585025373	0.060448433137405	0.0998551218084097	0.165535029751192
**CHOL**	FKBP5	cg01294490	-0.202258063099887	36	-1.20424608090921	0.236811160845279	0.040908324088918	0.428150020735784
**CHOL**	FKBP5	cg07485685	0.258827070263775	36	1.56245077475475	0.127442874402318	0.0669914523013292	0.2822573941994
**CHOL**	FKBP5	cg07843056	0.192073213175021	36	1.14121847023843	0.261753531334742	0.0368921192193769	0.457088918194683
**CHOL**	FKBP5	cg08636224	-0.108701573916888	36	-0.6376118635937	0.527997270153464	0.0118160321720086	0.706479379767079
**CHOL**	FKBP5	cg08915438	-0.555891664815525	36	-3.89937929610015	0.000431655310432661	0.309015543011376	0.00331100307826843
**CHOL**	FKBP5	cg10913456	0.150700612891224	36	0.888879518940538	0.380313726616088	0.0227106747257905	0.578717584978072
**CHOL**	FKBP5	cg16012111	0.130072068037886	36	0.764942508425223	0.449582977945825	0.0169187428836523	0.640778962045754
**CHOL**	FKBP5	cg17030679	-0.168203651065681	36	-0.99496337041207	0.326782784808943	0.0282924682318254	0.52627554003575
**CHOL**	FKBP5	cg20813374	-0.143730314468158	36	-0.846877771242828	0.402985072767001	0.0206584032971156	0.599503198340812
**CHOL**	FKBP5	cg25114611	-0.37418652413906	36	-2.35278532861787	0.0245594810585228	0.140015554847272	0.0850507598587393
**CHOL**	FMR1	cg02921434	-0.0963969945946893	36	-0.564716140708	0.575974036956219	0.00929238056688856	0.74267924535855
**CHOL**	FMR1	cg03513471	-0.143515438563786	36	-0.845585048642619	0.403696025309217	0.0205966811061559	0.600170630705089
**CHOL**	FMR1	cg04552106	-0.0457466046693255	36	-0.267025806890311	0.791062313423472	0.00209275183877155	0.885300614424145
**CHOL**	FMR1	cg04744025	-0.106001849714097	36	-0.621593787761407	0.538354857087098	0.0112363921428101	0.714043233120791
**CHOL**	FMR1	cg05288927	-0.120255341485448	36	-0.706328944435671	0.484796042475016	0.0144613471557818	0.671501732930372
**CHOL**	FMR1	cg07147350	-0.356748360098884	36	-2.22669810874267	0.0326969142125939	0.127269392433243	0.105250454586561
**CHOL**	FMR1	cg08434396	-0.134753345479181	36	-0.792972843117494	0.43329258037778	0.0181584641178316	0.626850951786174
**CHOL**	FMR1	cg14470409	-0.140290206988184	36	-0.826196175586226	0.414453122721124	0.0196813421767876	0.610019416297104
**CHOL**	FMR1	cg16783314	-0.0930082891652029	36	-0.544687899840674	0.589523256360508	0.008650541853438	0.752652457704518
**CHOL**	FMR1	cg22625568	-0.115490969540866	20	-0.493287504863475	0.627773787632739	0.0133381640454892	0.780032168459826
**CHOL**	FXR2	cg01807919	-0.0838404181626464	36	-0.490596741048509	0.626863152993435	0.00702921571768742	0.779403868697083

**CHOL**	FXR2	cg01933235	-0.165946173304795	36	-0.981229079265715	0.333410815728152	0.0275381324345052	0.532999630083551
**CHOL**	FXR2	cg05529097	-0.325511239580617	36	-2.00736515705607	0.0527125571100828	0.10595756709331	0.149907536750933
**CHOL**	FXR2	cg05913233	-0.219455916913446	36	-1.31161079560942	0.198439654434137	0.0481608994683214	0.381078051168213
**CHOL**	FXR2	cg09625173	-0.40054920664102	36	-2.54899731976883	0.0154861310669594	0.160439666940751	0.0598473959375578
**CHOL**	FXR2	cg17777561	-0.184207912992584	36	-1.0928083811324	0.282160774667265	0.0339325552090835	0.479298104776846
**CHOL**	FXR2	cg19178740	-0.348901612097438	36	-2.17084586050102	0.0370173049044452	0.121732334924191	0.115620890810316
**CHOL**	FXR2	cg22521096	-0.275584326661493	36	-1.67165043846058	0.103773575445016	0.0759467211014683	0.244598137499458
**CHOL**	FXR2	cg26904212	0.104106768431117	36	0.610358167480361	0.545683067674411	0.0108382192331703	0.719518442120428
**CHOL**	G2E3	cg02259031	0.267651263623763	36	1.61975709705423	0.114524281521361	0.0716371989193968	0.262192523739633
**CHOL**	G2E3	cg05076167	-0.590553941545048	36	-4.26703772539776	0.00014967331708799	0.348753957874392	0.00135960836402885
**CHOL**	G2E3	cg07906495	0.0976134980705824	36	0.571910829284506	0.571144535734182	0.00952839500557559	0.739093233587354
**CHOL**	G2E3	cg08810065	-0.115205501282962	36	-0.676260495037474	0.503449941829964	0.0132723075258586	0.686846914782368
**CHOL**	G2E3	cg09837748	-0.104382402760911	36	-0.611991936624359	0.544614270142408	0.0108956860061411	0.718742642228756
**CHOL**	G2E3	cg18580322	-0.24155540404387	36	-1.4514805930871	0.15581141511966	0.0583490132227975	0.323983461038111
**CHOL**	G2E3	cg18755078	-0.339651320853352	36	-2.10566957152928	0.0426948736150309	0.115363019757427	0.128423036693644
**CHOL**	G2E3	cg18956956	-0.572531471249109	36	-4.0718059872308	0.000263455230605774	0.327792285570669	0.00218825925792714
**CHOL**	G2E3	cg19554565	0.13091560534656	36	0.769989499088737	0.446623299347548	0.0171388957232562	0.638405270430775
**CHOL**	G2E3	cg21061067	-0.126882755456916	36	-0.745875617770522	0.46086858800845	0.0160992336323395	0.650591764091046
**CHOL**	G2E3	cg22926526	-0.0840004592538658	36	-0.491539878661213	0.626203056055966	0.00705607715486037	0.778839300102606
**CHOL**	GADD45A	cg01810953	-0.113220421723174	36	-0.664455350300816	0.510880536941108	0.0128188638951733	0.693081900092428
**CHOL**	GADD45A	cg02639608	-0.0513064451008302	36	-0.299559946595881	0.766336533036368	0.00263235130888451	0.870451959453799
**CHOL**	GADD45A	cg04718306	0.0208811055886255	36	0.121783275099728	0.903787608957174	0.000436020570603327	0.949009316765618
**CHOL**	GADD45A	cg07000785	0.131921558065605	36	0.776010467189822	0.443107651351798	0.0174032974824567	0.635377152033284
**CHOL**	GADD45A	cg22224769	0.000416669257474087	36	0.00242957860729586	0.998075678222795	1.73613270124007e-07	0.998970965558325
**CHOL**	GADD45A	cg23369529	-0.273065981439663	36	-1.65513759899861	0.107099901588314	0.0745650302196062	0.250164532177189
**CHOL**	GADD45A	cg23723105	-0.120930882673625	36	-0.710355508391092	0.482328052978093	0.014624278384222	0.669357808536808
**CHOL**	GATAD2A	cg02887458	0.0260434421295208	36	0.151909584135859	0.880156092126274	0.000678260877953697	0.936404904622083
**CHOL**	GATAD2A	cg08505228	-0.00587605112643418	36	-0.0342635629807499	0.972867348660587	3.45279768404684e-05	0.98571558376793
**CHOL**	GATAD2A	cg08870822	-0.218404443991882	36	-1.30501093749003	0.200652667969774	0.0477005011554033	0.383678439977402
**CHOL**	GATAD2A	cg09334331	-0.0729765797287706	36	-0.426660550198563	0.672316109931677	0.00532558118890961	0.810333407536329
**CHOL**	GATAD2A	cg11795262	-0.0847247219097669	36	-0.49580850853558	0.623219382113557	0.00717827850268733	0.776730300263414
**CHOL**	GATAD2A	cg14948912	-0.173475120778085	36	-1.02709768048084	0.311626507880621	0.0300936175289711	0.510479523143083
**CHOL**	GATAD2A	cg15264323	-0.139725275960335	36	-0.822802804205844	0.416353848515924	0.0195231527421919	0.611913989485525
**CHOL**	GATAD2A	cg22191731	-0.271212322524795	36	-1.64300664103668	0.109599366920111	0.0735561238892932	0.25430008202646
**CHOL**	GATAD2A	cg26631329	-0.31623762558383	36	-1.94371796582886	0.0602436342593747	0.100006235834899	0.165159811914208
**CHOL**	GATAD2B	cg02134200	0.32837852590265	36	2.02717386042139	0.050542716954429	0.107832456273997	0.14545428095416
**CHOL**	GATAD2B	cg02940454	0.134975020777113	36	0.794301505041209	0.432529351423047	0.0182182562337821	0.626223290216397
**CHOL**	GATAD2B	cg09805553	-0.135181036201538	36	-0.795536414815994	0.431820706446659	0.0182739125485215	0.625638992016079
**CHOL**	GATAD2B	cg10226922	-0.143507085707231	36	-0.845534799190909	0.403723676593567	0.0205942836481826	0.600200269395351
**CHOL**	GATAD2B	cg10489353	0.172046082048021	36	1.01837755522778	0.315690752011885	0.0295998543480744	0.514923884408458
**CHOL**	GATAD2B	cg15064304	-0.0188961341863485	36	-0.110202125795432	0.912897002824548	0.000357063887188488	0.953887918734911
**CHOL**	GATAD2B	cg20773694	-0.168973134075659	36	-0.999648504815583	0.324542371950498	0.0285519200393506	0.523922671414774
**CHOL**	GATAD2B	cg23046518	0.435017116458614	36	2.81708283760927	0.00801398343610001	0.189239891611968	0.0359862883487103
**CHOL**	GATAD2B	cg23323627	-0.0899147383768501	36	-0.526420799194988	0.602013410667331	0.00808466017737739	0.761555771298972
**CHOL**	GATAD2B	cg26398333	0.274347741594055	36	1.66353750093946	0.105396967852044	0.0752666833177585	0.247308068923265
**CHOL**	GLYR1	cg00636946	0.202996277593154	31	1.11641266976754	0.273410657546155	0.0412074887166767	0.46977888270045
**CHOL**	GLYR1	cg00885266	0.0183619115260359	36	0.107085476786313	0.915350563923861	0.000337159794889971	0.955252252333548
**CHOL**	GLYR1	cg01261535	-0.109529674265088	36	-0.642528014631797	0.524839794576745	0.0119967495446162	0.703918538339453
**CHOL**	GLYR1	cg01565912	0.0839739990414621	36	0.491383943431877	0.626312172717747	0.00705163251501548	0.778925200863352
**CHOL**	GLYR1	cg02454264	-0.291812395911143	36	-1.77897281157219	0.0841866692094665	0.0851544744074015	0.210556176328507
**CHOL**	GLYR1	cg06399529	0.054299340328484	36	0.317084635525522	0.753118333345657	0.00294841836010852	0.861890853149173
**CHOL**	GLYR1	cg06976003	-0.102551013807182	36	-0.601139387544411	0.55173421125667	0.0105167104328808	0.724036558602274
**CHOL**	GLYR1	cg11289281	0.310516167672326	36	1.90476078399698	0.0652991509663069	0.096420290385908	0.17518647274797
**CHOL**	GLYR1	cg15663121	0.140032243352701	36	0.824646563441245	0.41532043961098	0.0196090291783901	0.610960767715458
**CHOL**	GLYR1	cg18354686	-0.213420645977844	36	-1.27379320287511	0.211375848789088	0.0455483721296003	0.397379950078743
**CHOL**	GTF2B	cg02705666	-0.117655480505949	36	-0.690841706174879	0.494354882827889	0.0138428120930858	0.679491214437133
**CHOL**	GTF2B	cg04270788	0.415935525466069	36	2.6669405776444	0.0116350431299959	0.173002361344735	0.0479702892962029

**CHOL**	GTF2B	cg05306856	-0.195508883854528	35	-1.14521343990164	0.260358618637718	0.0382237236660433	0.455510309174539
**CHOL**	GTF2B	cg06356032	0.0018712946145538	35	0.0107497879649502	0.991487801647434	3.50174353445804e-06	0.995493114974775
**CHOL**	GTF2B	cg07167781	0.156774003908183	36	0.925587018566691	0.361184121864971	0.0245780883014029	0.560036128559621
**CHOL**	GTF2B	cg10430205	0.308075707624799	35	1.86023878647187	0.0717839485353126	0.0949106416285209	0.187750517941661
**CHOL**	GTF2B	cg12074130	-0.0323085321005613	36	-0.18848789787814	0.851614758911397	0.001043841246493	0.920863367754122
**CHOL**	GTF2B	cg13868361	-0.00772460389958629	36	-0.0450431376161411	0.964336442421882	5.96695054055037e-05	0.98129845396746
**CHOL**	GTF2B	cg14010405	-0.0249359997478859	35	-0.143290969088048	0.886931793353914	0.000621804083426566	0.939908467187156
**CHOL**	GTF2B	cg17811845	-0.157290987695651	36	-0.928716567990849	0.359582847471461	0.0247404548102734	0.558462528173184
**CHOL**	GTF2B	cg22607980	-0.0952789902574512	36	-0.558106252607625	0.580428663689118	0.00907808598447948	0.746246239555616
**CHOL**	GTF2H1	cg05849260	-0.064730005359511	36	-0.378230767633907	0.707610500509158	0.00418997359384232	0.833376499176574
**CHOL**	GTF2H1	cg07583091	-0.130473352903207	36	-0.767343235220131	0.448173686549825	0.0170232958178049	0.639610890111257
**CHOL**	GTF2H1	cg08690812	-0.0644125546270509	36	-0.37636809030094	0.708981792833938	0.00414897719358281	0.834222331232297
**CHOL**	GTF2H1	cg10797557	-0.256470328021278	36	-1.54721730787168	0.131069508538445	0.0657770291553419	0.287740815193831
**CHOL**	GTF2H1	cg11063328	0.0164178135151003	36	0.0957443853857984	0.924285695777154	0.000269544600616611	0.960126604583895
**CHOL**	GTF2H1	cg11176342	-0.0465512611935226	36	-0.271732749483504	0.787470761048161	0.00216701991870756	0.882906973913391
**CHOL**	GTF2H1	cg11347316	0.206650784170865	36	1.23155416699292	0.226566187219159	0.0427045465984332	0.416118860517654
**CHOL**	GTF2H1	cg11662851	-0.219575677012075	36	-1.31236280676482	0.19818868342855	0.0482134779353109	0.380741405952039
**CHOL**	GTF2H1	cg13777411	0.385216537840953	36	2.43402178023239	0.0203369784397924	0.148391781026171	0.0737538996198412
**CHOL**	GTF2H1	cg14132868	-0.18098670940935	36	-1.0730455622083	0.290807909180784	0.0327561889828245	0.488380744360493
**CHOL**	GTF2H1	cg17174493	0.0834441610487289	36	0.488261723955818	0.628498761559858	0.0069629280131262	0.780627089193184
**CHOL**	GTF2H1	cg18321761	0.294459310521586	36	1.79663358994674	0.0812832057631661	0.0867062855528479	0.205282469802157
**CHOL**	H2AFZ	cg02758870	-0.277506970749109	36	-1.6842823023819	0.10128730310574	0.0770101188143468	0.240460091266139
**CHOL**	H2AFZ	cg09308803	-0.30830501670602	36	-1.89976688232373	0.0673401558706896	0.095051983326099	0.179185253995226
**CHOL**	H2AFZ	cg16267491	-0.335366894415859	36	-2.07571804193233	0.0455527284016361	0.112470953870138	0.134804053304811
**CHOL**	H2AFZ	cg17709884	-0.32244489071362	36	-1.98624974500318	0.055114644281303	0.103970707547318	0.154864864452058
**CHOL**	H2AFZ	cg20934652	0.0545563029497264	36	0.318589654942696	0.751986607515898	0.00297639019154233	0.861105248249702
**CHOL**	H2AFZ	cg23752380	0.2006827554867	36	1.19447148391525	0.240560182136395	0.0402735683497348	0.432624110120762
**CHOL**	H2AFZ	cg24061702	-0.325178945792502	36	-2.00507352069366	0.05296875737368	0.105741346786723	0.150460730503375
**CHOL**	H3F3A	cg06319713	-0.170366359774852	36	-1.00813620209113	0.320510247859071	0.0290246965429344	0.519919390744804
**CHOL**	H3F3A	cg10632229	0.0562387887930025	36	0.328445487337799	0.744589210001853	0.00316280136490395	0.856483814331392
**CHOL**	H3F3A	cg10983539	-0.0312158088493503	36	-0.182106626165139	0.856580482094569	0.000974426722119175	0.92370249960584
**CHOL**	H3F3A	cg11978873	0.114441833294724	36	0.671718045426218	0.506302053963337	0.0130969332078575	0.689323614099369
**CHOL**	H3F3A	cg15538837	-0.306071555378551	36	-1.87465605314301	0.0694523107814456	0.0936797970118453	0.18331941318942
**CHOL**	H3F3A	cg15624314	-0.0156404354230575	36	-0.0912097832640703	0.927861164690885	0.000244623220222832	0.961966281493482
**CHOL**	H3F3A	cg16724613	-0.267976144910821	36	-1.62187520752558	0.114068198370841	0.0718112142412654	0.261456140956429
**CHOL**	H3F3A	cg16727519	0.187073761759051	36	1.11042163384794	0.274609044909067	0.034996592338682	0.471046323946995
**CHOL**	H3F3A	cg18947521	-0.0904218730422427	36	-0.529414310145935	0.599958070854095	0.00817611512446745	0.760103966135248
**CHOL**	H3F3A	cg19410789	-0.0779193010706286	36	-0.455729263823664	0.651482084871939	0.00607141747933527	0.796314173369303
**CHOL**	H3F3A	cg21266749	-0.147734339645925	36	-0.870989121682176	0.389868681799283	0.0218254351106175	0.587420324575462
**CHOL**	H3F3A	cg21789564	-0.216942938804553	36	-1.29584532093753	0.203757151563868	0.047064238697156	0.387651842718503
**CHOL**	H3F3A	cg22519151	-0.338757456689253	36	-2.09940862427524	0.0432788346930239	0.114756614462571	0.129743108520582
**CHOL**	H3F3A	cg27530209	0.0511610779572562	36	0.298708969379039	0.766980241532502	0.00261745589774845	0.870839817119789
**CHOL**	H3F3A	cg27560444	-0.316625227820788	24	-1.56565582624401	0.131701031943848	0.100251534892566	0.288681752142232
**CHOL**	HAT1	cg00414306	0.0862396124916837	36	0.50473947681562	0.616997743152556	0.00743727076271577	0.772460379600739
**CHOL**	HAT1	cg01583365	0.0129172737089709	36	0.0753262861916255	0.940396585415945	0.00016685596007247	0.968306790135694
**CHOL**	HAT1	cg04507121	0.0020492459291083	36	0.0119490795228997	0.990536081483058	4.19940887796694e-06	0.994871395299116
**CHOL**	HAT1	cg04950839	-0.00689873075773692	36	-0.0402271244496734	0.968147347180525	4.75924860677454e-05	0.983312139983066
**CHOL**	HAT1	cg05421799	0.0295593236823848	36	0.172434343464476	0.864118431348628	0.000873753616559993	0.927863518669029
**CHOL**	HAT1	cg09545452	0.0829971144120442	36	0.485627700301493	0.630346104142767	0.00688852100072596	0.781844116175221
**CHOL**	HAT1	cg10190161	0.113130590630814	36	0.663921322141925	0.511218084101604	0.0127985305364768	0.693431783278703
**CHOL**	HAT1	cg12941622	0.137564270954087	36	0.809829825958317	0.423669787584517	0.0189239286431295	0.618702830194553
**CHOL**	HAT1	cg13293756	-0.0447071442021163	36	-0.260946117889148	0.795708173094561	0.00199872874270882	0.88817907416999
**CHOL**	HAT1	cg14446129	0.190980716597261	36	1.13448086592886	0.26452824044182	0.0364736341120031	0.460116488248875
**CHOL**	HAT1	cg16017358	0.233719271438547	36	1.40162506806604	0.170090445731038	0.0546246978417651	0.34380482072389
**CHOL**	HDAC1	cg03762702	-0.369958604886206	36	-2.32195782443497	0.0263593622774666	0.136869369329348	0.0896321044111638
**CHOL**	HDAC1	cg04967775	-0.0510037609934604	36	-0.297788059528481	0.767677038769541	0.00260138363547803	0.871251663474975
**CHOL**	HDAC1	cg06473843	-0.315111959751242	36	-1.93603493768964	0.0612129727992203	0.0992955471782681	0.16702332453939

**CHOL**	HDAC1	cg10842753	-0.317522537995987	36	-1.95249905468869	0.0591520594684366	0.100820562135413	0.162983470790096
**CHOL**	HDAC1	cg14172124	-0.268735884066501	36	-1.6268307653255	0.113006973797231	0.072218975385004	0.2600042742098
**CHOL**	HDAC1	cg20002354	-0.247105709679336	36	-1.48697478225558	0.146237209877293	0.0610612317561282	0.310338193714717
**CHOL**	HDAC1	cg20793809	-0.0814269337321637	36	-0.476378436860694	0.636852052632033	0.00663034553702217	0.786316921438351
**CHOL**	HDAC1	cg21930229	0.0263762282912727	36	0.153852045496151	0.878635976098158	0.000695705418873335	0.935542513544389
**CHOL**	HDAC1	cg23018091	-0.22988922483921	36	-1.37736320211071	0.177400398827031	0.0528490556971729	0.353682384068428
**CHOL**	HDAC1	cg26115470	-0.591776858747296	36	-4.28062844705515	0.000143864183935318	0.350199850548817	0.00131343390741276
**CHOL**	HDAC10	cg00711351	-0.0108867671161426	36	-0.0634839775705073	0.949752887892383	0.000118521698241124	0.973363143856298
**CHOL**	HDAC10	cg01120165	0.110552923841771	36	0.648604572072864	0.520951029834365	0.0122219489699645	0.701140149031883
**CHOL**	HDAC10	cg04321608	0.0164829620970103	36	0.0961244178865883	0.923986117084868	0.000271688039491479	0.959994871420752
**CHOL**	HDAC10	cg04586023	0.0930318697788395	36	0.544827201356426	0.589428489881849	0.00865492879454695	0.752642635773563
**CHOL**	HDAC10	cg07482373	-0.139306553457341	36	-0.820288192607117	0.417765816003537	0.0194063158361631	0.613143550423151
**CHOL**	HDAC10	cg07744063	-0.0670563895900047	36	-0.391884640896002	0.697589087679323	0.00449655938484649	0.826617195273876
**CHOL**	HDAC10	cg09151829	-0.180856405230086	36	-1.07224687268979	0.291161246676653	0.0327090393127492	0.488711032179606
**CHOL**	HDAC10	cg10508940	-0.0932440500419094	36	-0.54608068925638	0.588576075462956	0.0086944528682181	0.752122067324242
**CHOL**	HDAC10	cg11645658	-0.171481424590312	36	-1.0149337933932	0.317305781182781	0.0294058789795229	0.516541515471107
**CHOL**	HDAC10	cg12464820	0.183011271693931	36	1.08546249848488	0.285353433966992	0.0334931255670299	0.482587312447773
**CHOL**	HDAC10	cg26943378	-0.289946544259863	36	-1.76655033339803	0.0862810695470016	0.0840689985282368	0.214370333887115
**CHOL**	HDAC11	cg01409735	-0.242765659747075	36	-1.4592071194191	0.1536860003901	0.0589351655524326	0.320970399266713
**CHOL**	HDAC11	cg01712016	0.097001337807063	36	0.568290047662635	0.573572501935474	0.00940925953635995	0.740930239303547
**CHOL**	HDAC11	cg02762546	-0.373510694632904	36	-2.34784621157663	0.0248401643913923	0.139510239005155	0.0858012498296765
**CHOL**	HDAC11	cg02941903	-0.0115328971847689	36	-0.0672522413748746	0.946774830130369	0.00013300771747445	0.97157821252588
**CHOL**	HDAC11	cg05987698	-0.210126310476798	36	-1.25321538512019	0.218677430319523	0.0441530663545916	0.406465295540988
**CHOL**	HDAC11	cg07450135	-0.0405665065160815	36	-0.236736219710481	0.81428197952377	0.00164564145091928	0.900078333653165
**CHOL**	HDAC11	cg08051076	-0.0328409860128347	36	-0.191597559386679	0.84919712129129	0.0010785303622952	0.919590206625017
**CHOL**	HDAC11	cg08448040	-0.118404127847467	36	-0.695299868143139	0.491592522158919	0.0140195374913193	0.677165979085098
**CHOL**	HDAC11	cg10455785	-0.00448648315151116	36	-0.026160730723361	0.97928208444565	2.01285310687935e-05	0.989341347722826
**CHOL**	HDAC11	cg11432034	-0.22751973863205	36	-1.3623873453138	0.182033108413559	0.0517652314671963	0.359619168975682
**CHOL**	HDAC11	cg13397067	-0.0770705090756389	36	-0.45073507709698	0.655041933642429	0.00593986336917814	0.79892029640316
**CHOL**	HDAC11	cg13706066	-0.193019182666258	36	-1.14705597576554	0.25936654360099	0.0372564048771504	0.4542154989171
**CHOL**	HDAC11	cg16254764	-0.186126202531697	36	-1.10459479360956	0.277091202142935	0.0346429632688702	0.47387253354219
**CHOL**	HDAC11	cg18521047	-0.0846690287413327	36	-0.495480238393137	0.62344860707462	0.00716884442800062	0.776891523791235
**CHOL**	HDAC11	cg19779799	-0.376743594419993	36	-2.37151255328033	0.0235211222585592	0.141935735936496	0.0823336589696063
**CHOL**	HDAC11	cg20894727	-0.221105524563266	36	-1.32197469722173	0.195002203254859	0.0488876529923968	0.376576342965159
**CHOL**	HDAC11	cg20922324	-0.0778772977853634	36	-0.455482098350302	0.65165806987197	0.00606487351035017	0.79640081727855
**CHOL**	HDAC11	cg21655280	-0.13015917301787	36	-0.765463590134583	0.449276866067148	0.0169414103206959	0.640492670175883
**CHOL**	HDAC11	cg25127083	-0.163157353965904	36	-0.964284030685999	0.341712376647787	0.0266203221531553	0.540869043702577
**CHOL**	HDAC11	cg25150878	-0.280758273784303	36	-1.70569352032682	0.0971863062576666	0.0788252082983418	0.233291517804521
**CHOL**	HDAC11	cg27025856	-0.388838022716524	36	-2.46095811401165	0.0190900831462548	0.151195007910096	0.0701906573532349
**CHOL**	HDAC2	cg00161229	-0.169779616871149	36	-1.00456093866421	0.322204508088288	0.0288251183049141	0.521661107477275
**CHOL**	HDAC2	cg01129566	-0.0569290359081377	36	-0.332489692485949	0.741560851169605	0.00324091512943003	0.854587320746039
**CHOL**	HDAC2	cg08198132	-0.17287034063381	36	-1.02340644391237	0.313342487657014	0.0298841546708494	0.512427275274117
**CHOL**	HDAC2	cg10999867	0.222502202706482	36	1.33075885421413	0.192124588434176	0.0495072302092364	0.372647357949743
**CHOL**	HDAC2	cg13393754	-0.271932108554138	36	-1.64771480539278	0.108623633856054	0.0739470716626993	0.252714485680897
**CHOL**	HDAC2	cg15005908	-0.158036411457653	36	-0.933230352558655	0.357281529055019	0.0249755073464127	0.556165268060153
**CHOL**	HDAC2	cg15242771	-0.222040405556545	36	-1.32785351321068	0.193072722575928	0.0493019416997148	0.373982853943323
**CHOL**	HDAC2	cg18387216	-0.214532508386467	36	-1.28074859949747	0.208949960828894	0.0460241971545894	0.394233926385405
**CHOL**	HDAC2	cg18674783	0.0646112165358304	36	0.377533747583494	0.70812352696073	0.00417460930223997	0.833652567439211
**CHOL**	HDAC2	cg22867643	-0.245815894492208	36	-1.4787126838983	0.14842278971518	0.0604254539850046	0.313471317345461
**CHOL**	HDAC2	cg24023618	0.169791873608078	36	1.00463561268857	0.322169058950601	0.0288292803433416	0.521631314587862
**CHOL**	HDAC3	cg01947930	-0.325440892844456	36	-2.00687994729394	0.052766712123534	0.105911774735397	0.150012191307492
**CHOL**	HDAC3	cg01961137	-0.149126573756166	36	-0.879383037613826	0.38536678342598	0.0222387350002533	0.583405046348312
**CHOL**	HDAC3	cg04284814	-0.0642533480661873	36	-0.375433971407644	0.709669857215826	0.00412849273771462	0.834626768746095
**CHOL**	HDAC3	cg08520975	-0.187005350860202	36	-1.11000084633207	0.274787761812521	0.0349710012503473	0.471264661800921
**CHOL**	HDAC3	cg10414058	0.126092164296651	36	0.741152832592825	0.463689350325102	0.0158992338970136	0.652926843433869
**CHOL**	HDAC3	cg11064797	0.00676417387435889	36	0.0394424748065243	0.968768313795569	4.57540482025593e-05	0.983580451165309
**CHOL**	HDAC3	cg11153694	0.128208071364596	36	0.753795954850742	0.456160598427141	0.0164373095630294	0.646387001618259

**CHOL**	HDAC3	cg12082871	0.193413675059798	36	1.14949133873042	0.258375379392092	0.037408849700137	0.452989593987839
**CHOL**	HDAC3	cg14098966	-0.190233677802012	36	-1.12987627674117	0.266436666365291	0.0361888521700797	0.462128818322617
**CHOL**	HDAC3	cg17940889	0.071512284121966	36	0.418055026910144	0.678535563827228	0.00511400678034079	0.814447874658634
**CHOL**	HDAC3	cg18671773	-0.0895421524719649	36	-0.52422176491664	0.603525373595288	0.00801779706931261	0.762544914220524
**CHOL**	HDAC3	cg19549288	0.212763629165823	36	1.2696855904139	0.21281844900653	0.045268361895812	0.399122451773439
**CHOL**	HDAC3	cg20642138	-0.12672270489316	36	-0.744919401170852	0.461438894079176	0.0160586439354389	0.651030619452574
**CHOL**	HDAC3	cg23421926	-0.0591112772424853	36	-0.345278768619524	0.732011898247903	0.00349414309723796	0.848648583149875
**CHOL**	HDAC3	cg24385322	0.118108496355427	36	0.693539252393834	0.492682397399029	0.0139496169113398	0.678114938222255
**CHOL**	HDAC6	cg07153944	0.0624305895852939	36	0.364741262449377	0.717563515684944	0.00389757851596741	0.839577735439971
**CHOL**	HDAC6	cg07674520	-0.167828469305287	24	-0.798511210679775	0.433109928285505	0.0281663951093555	0.626783126740133
**CHOL**	HDAC6	cg10783042	0.0405109369578646	35	0.232908811220218	0.817270816243105	0.00164113601320408	0.901785317024616
**CHOL**	HDAC6	cg12510923	-0.138000600349843	28	-0.710465383682204	0.483740223476105	0.0190441656969171	0.67054165600778
**CHOL**	HDAC6	cg13178935	0.0930461504527993	36	0.544911564306481	0.589371101542672	0.00865758611408497	0.752631124499921
**CHOL**	HDAC6	cg17152375	0.0650531069760086	35	0.374494902257605	0.71043197431611	0.00423190672723202	0.83507926881841
**CHOL**	HDAC6	cg24616736	0.124848473782469	36	0.733726250730505	0.468145257001322	0.0155871414058118	0.657165319490805
**CHOL**	HDAC6	cg26680189	0.11327542113784	36	0.664782319138994	0.510673927371203	0.012831321033955	0.692899058270086
**CHOL**	HDAC9	cg00332048	-0.0597255959176205	36	-0.348879885480292	0.729330854056518	0.00356714680771488	0.846911561679724
**CHOL**	HDAC9	cg02301805	-0.223604269315827	36	-1.33769618583791	0.189875114959983	0.049998869256265	0.369944517461353
**CHOL**	HDAC9	cg03260621	0.0171620632504288	36	0.100085905786972	0.92086396467947	0.000294536415011719	0.958491989139345
**CHOL**	HDAC9	cg07513622	-0.103387553952875	35	-0.597116137444063	0.554505032380882	0.0106889863123586	0.726178214720441
**CHOL**	HDAC9	cg08146609	-0.0750057576913465	36	-0.438590430011825	0.663732732812122	0.00562586368685299	0.804612909111093
**CHOL**	HDAC9	cg08285151	0.0995117042484481	36	0.583142451183862	0.563645686040333	0.0099025792824306	0.733493685176007
**CHOL**	HDAC9	cg08725366	0.0336583655437837	36	0.196371575128435	0.845488404362587	0.00113288557107897	0.917476983247079
**CHOL**	HDAC9	cg10068437	-0.035314408038054	36	-0.20604513475468	0.837984496635932	0.00124710741507818	0.913311608463789
**CHOL**	HDAC9	cg11321459	-0.119282013826275	36	-0.700529172997573	0.488363407325441	0.0142281988224516	0.674373571661913
**CHOL**	HDAC9	cg11765288	-0.0408104701637311	36	-0.238162299975917	0.813184748119655	0.00166549447498479	0.899407754722299
**CHOL**	HDAC9	cg12081743	0.156686472988969	35	0.911351920252592	0.368719564339722	0.0245506508177228	0.567505981969911
**CHOL**	HDAC9	cg16475478	-0.111804972221231	34	-0.636454897057995	0.529006646623058	0.0125003518133903	0.707332747826174
**CHOL**	HDAC9	cg18856632	0.0312295060775147	36	0.18218661097546	0.856518203035502	0.000975282049845528	0.923665853835985
**CHOL**	HDAC9	cg19585556	-0.103136418244404	30	-0.548672577535223	0.587578975680666	0.0106371207682847	0.751350024546853
**CHOL**	HDAC9	cg20648975	-0.045977521947978	36	-0.268376533735387	0.790031186273948	0.0021139325244768	0.884541120074199
**CHOL**	HDAC9	cg21433125	-0.0379406281099123	36	-0.221389378792078	0.826113547636615	0.00143949126137467	0.906466717324083
**CHOL**	HDAC9	cg23074799	-0.0638919681731548	36	-0.373313739711235	0.711232519952807	0.00408218359703942	0.835586189857948
**CHOL**	HDAC9	cg23385718	0.0317577460979158	36	0.185271341471145	0.85411703644018	0.00100855443721969	0.92216741958416
**CHOL**	HDAC9	cg24350771	0.101097796775664	36	0.592532232966284	0.557414814752847	0.0102207645128934	0.728676324968627
**CHOL**	HDAC9	cg25620356	-0.00259405303807023	36	-0.0151258493695114	0.988020190734558	6.72911116432141e-06	0.993665874418299
**CHOL**	HDGF	cg00692559	-0.0891634895617745	36	-0.521987090940047	0.605063665258944	0.00795012787083267	0.763827740111763
**CHOL**	HDGF	cg00746981	-0.0183565056572153	36	-0.107053939481642	0.915375395804364	0.000336961299943376	0.955258679757528
**CHOL**	HDGF	cg01910231	0.0345578788543055	36	0.201625760633275	0.841410798990883	0.00119424699090886	0.915068943922954
**CHOL**	HDGF	cg02914429	-0.0553370706729157	36	-0.323162969896645	0.748551049300828	0.00306219139065927	0.859080627414747
**CHOL**	HDGF	cg04402095	-0.00437535178288182	36	-0.0255127099740524	0.979795166551205	1.91437032239671e-05	0.989628610206105
**CHOL**	HDGF	cg05762852	0.162606794916756	36	0.96094161926762	0.343366041877657	0.0264409697530998	0.542735596680628
**CHOL**	HDGF	cg05967123	0.0694650094722204	36	0.406027934297899	0.687266260565549	0.00482538754097567	0.820086254649351
**CHOL**	HDGF	cg06106793	0.0111747227158816	36	0.0651633393389209	0.948425595617786	0.000124874427776839	0.972602397636192
**CHOL**	HDGF	cg06119628	-0.14654294555718	36	-0.863810295029324	0.393745284979739	0.0214748348925746	0.590477507464363
**CHOL**	HDGF	cg07313589	-0.16364534849196	36	-0.967247390178873	0.340250707345035	0.0267798000830549	0.539466504854678
**CHOL**	HDGF	cg07892339	0.135301526661549	36	0.796258714516916	0.431406545361945	0.0183065031169459	0.625344521469331
**CHOL**	HDGF	cg08597205	0.316235421858978	36	1.94370291580901	0.0602455199975511	0.100004842038326	0.165159811914208
**CHOL**	HDGF	cg08708935	-0.0228796918944947	36	-0.133445315374849	0.894627940389232	0.000523480301187005	0.944029938671375
**CHOL**	HDGF	cg09063372	0.116765363787358	36	0.685542653949068	0.497649524121064	0.0136341501803939	0.682019362262597
**CHOL**	HDGF	cg09477492	-0.0113610512025677	36	-0.0662500187354014	0.947566812350913	0.000129073484427364	0.972096010866623
**CHOL**	HDGF	cg11588596	0.138794643754459	36	0.817214560612504	0.419495673216678	0.0192639531349272	0.614856198223501
**CHOL**	HDGF	cg12220178	-0.0733821396002223	36	-0.42904447434176	0.670597292823301	0.00538493841230651	0.809126728156586
**CHOL**	HDGF	cg12421458	0.0284749952754754	36	0.166103681851615	0.869059189098753	0.000810825355938347	0.930580358895956
**CHOL**	HDGF	cg13170934	-0.126197847473278	36	-0.741784072992785	0.463311750748808	0.0159258967068887	0.652619583141754
**CHOL**	HDGF	cg13235992	0.183704113607414	36	1.08971507143095	0.283502088685488	0.0337472013562856	0.480761301293673
**CHOL**	HDGF	cg14155849	-0.0304167446283973	36	-0.177440675856112	0.860215160858911	0.000925178353789134	0.925684421842778

**CHOL**	HDGF	cg15503799	0.0178449926781851	36	0.104069865389554	0.917725386360801	0.00031844376368448	0.956645924594356
**CHOL**	HDGF	cg17170583	-0.332478396447854	36	-2.05560724024895	0.0475648710435838	0.110541884104536	0.139170361740806
**CHOL**	HDGF	cg17218029	-0.138825306375345	36	-0.817398647710719	0.419391944270651	0.0192724656902085	0.614744996095311
**CHOL**	HDGF	cg17971171	0.140955386086298	36	0.830192762528633	0.41222140558378	0.0198684208667374	0.607908521156823
**CHOL**	HDGF	cg18593717	-0.173588936885057	36	-1.02779248354304	0.311304233367422	0.0301331190088842	0.510195531515157
**CHOL**	HDGF	cg19599803	0.148962707361537	36	0.87839479440232	0.385895077087975	0.0221898881844788	0.58397342048713
**CHOL**	HDGF	cg20284773	0.119358145983885	36	0.700982747897228	0.488083887252751	0.0142463670127105	0.674216371641771
**CHOL**	HDGF	cg23747104	0.182960679399671	36	1.08515203525475	0.285488926927684	0.0334746102063891	0.482675017525748
**CHOL**	HDGF	cg26431298	-0.110928559607877	36	-0.650835804214733	0.51952702625305	0.0123051453366784	0.700075964045829
**CHOL**	HDGFL1	cg02685945	0.0931929912438936	36	0.545779044616392	0.588781149732873	0.00868493361698443	0.752244895421244
**CHOL**	HDGFL1	cg03930485	-0.0395637631737674	36	-0.230875164059521	0.818795472092879	0.00156529135646996	0.902523130141455
**CHOL**	HDGFL1	cg05276066	-0.0250320419600007	36	-0.14600638369472	0.884778580375747	0.000626603124687234	0.938895641525258
**CHOL**	HDGFL1	cg06566039	-0.185388838566934	36	-1.10006272017229	0.279032816041907	0.0343690214651966	0.476038856214278
**CHOL**	HDGFL1	cg08671671	-0.054635600375021	36	-0.319054109479078	0.751637465076744	0.002985048828339	0.860920875722261
**CHOL**	HDGFL1	cg09081892	-0.0273556065045253	36	-0.159568941806004	0.874164825498017	0.000748329207230425	0.93337463838024
**CHOL**	HDGFL1	cg11894257	0.0909104354186005	36	0.532298585395124	0.597980875284781	0.00826470726799954	0.758632162620748
**CHOL**	HDGFL1	cg15317945	0.0929131426292673	36	0.544125834939229	0.589905702256394	0.00863285207324657	0.752967729360492
**CHOL**	HDGFL1	cg20209681	0.183254197421887	36	1.0869533539111	0.284703424675765	0.0335821008727401	0.481975457951091
**CHOL**	HDGFL1	cg21898884	0.0230728482999244	36	0.134572493541778	0.89374338206128	0.000532356328671324	0.943672095660513
**CHOL**	HIST1H1	cg00667898	-0.248554337765508	36	-1.49626421903233	0.143810669147906	0.0617792588220502	0.306904978895898
**CHOL**	HIST1H1	cg00700007	-0.205891925666253	36	-1.22683109352738	0.228314103632978	0.042391485054558	0.41823313786055
**CHOL**	HIST1H1	cg03821311	0.0946399750733145	36	0.554329212881575	0.582981687030063	0.00895672488187759	0.748057422613963
**CHOL**	HIST1H1	cg04676561	-0.290627535130244	36	-1.77108167894681	0.0855120567232976	0.0844643641758811	0.212928907903854
**CHOL**	HIST1H1	cg07138603	-0.140503103230003	36	-0.827475192857395	0.413738101177014	0.019741122017261	0.609345078886526
**CHOL**	HIST1H1	cg11206041	-0.141110878019024	36	-0.831127167886863	0.411700703665272	0.0199122798952998	0.607373585186554
**CHOL**	HIST1H1	cg14334286	-0.116710890410778	36	-0.685218416993318	0.497851511474406	0.0136214319404767	0.682176004573219
**CHOL**	HIST1H1	cg18798248	-0.225042115228628	36	-1.34675531873813	0.186968216547983	0.0506439536265749	0.366087071509244
**CHOL**	HIST1H1	cg23460835	-0.152308071209761	36	-0.898584764610343	0.375193735907147	0.0231977485556377	0.573875503886774
**CHOL**	HIST1H1	cg00903962	-0.250291061052663	36	-1.50741520043967	0.140940574639487	0.0626456152428677	0.302762359772842
**CHOL**	HIST1H1	cg04290376	-0.333985541234086	36	-2.06609237286178	0.0465062671579944	0.111546341753425	0.136785363849614
**CHOL**	HIST1H1	cg07750111	-0.107597478011061	36	-0.631059308062928	0.532221422539486	0.0115772172743409	0.709707628602423
**CHOL**	HIST1H1	cg08283682	-0.206479142783464	36	-1.23048568218732	0.226960737182268	0.0426336364045943	0.4165274856297
**CHOL**	HIST1H1	cg10717387	-0.225415501544803	36	-1.3491093480763	0.186218502402505	0.0508121483366952	0.365133766248278
**CHOL**	HIST1H1	cg12633985	-0.223292797890105	36	-1.33573497740044	0.190508984766495	0.0498596735895913	0.370770938680194
**CHOL**	HIST1H1	cg15022984	-0.33850914639402	36	-2.09767051023065	0.0434421942890878	0.114588442192408	0.130122518963703
**CHOL**	HIST1H1	cg16481032	-0.245326950400988	36	-1.47558286989633	0.149257497071205	0.0601853125930488	0.314706758867826
**CHOL**	HIST1H1	cg16482183	-0.145313315196244	36	-0.85640510118268	0.397769578797541	0.0211159595733231	0.594404168596706
**CHOL**	HIST1H1	cg17691542	-0.128223828674336	36	-0.753890148104512	0.456104778768389	0.0164413502399055	0.646341606271892
**CHOL**	HIST1H1	cg20855557	-0.205599654008929	36	-1.2250126356437	0.228989747491177	0.0422712177285912	0.418958993556299
**CHOL**	HIST1H1	cg23407813	-0.210569769998404	36	-1.25598280128343	0.217684596664263	0.0443396280371806	0.405210459248174
**CHOL**	HIST1H3	cg00958605	0.0922762726665813	36	0.540364000766074	0.592468436878674	0.00851491049723727	0.754752805583041
**CHOL**	HIST1H3	cg01043409	0.0289502073439481	36	0.168878050774814	0.866893266175447	0.000838114505257586	0.929245938612351
**CHOL**	HIST1H3	cg08041469	-0.0349975782580563	36	-0.204194285266679	0.839419057730988	0.00122483048392877	0.914065679051588
**CHOL**	HIST1H3	cg08401836	0.056395457398928	36	0.329363377778205	0.743901516501851	0.0031804476152343	0.856138733401134
**CHOL**	HIST1H3	cg10075390	-0.0406058437988569	36	-0.23696616143301	0.814105035712271	0.00164883455061716	0.899992712067649
**CHOL**	HIST1H3	cg15479139	0.142276528798615	36	0.838133974696132	0.407809129980972	0.020242610646983	0.603908085541273
**CHOL**	HIST1H3	cg15797414	0.157089541191313	36	0.92749702328752	0.360206288537374	0.0246771239516972	0.559108823582496
**CHOL**	HIST1H3	cg21209604	-0.103282009909514	36	-0.60547041860341	0.548887066724593	0.0106671735709489	0.721944349047662
**CHOL**	HIST1H3	cg23099349	-0.0701834745525443	36	-0.41024809486302	0.68419772422078	0.00492572010026763	0.818172569779462
**CHOL**	HIST1H3	cg25701114	-0.219562042046212	36	-1.31227718540154	0.198217245872166	0.0482074903075027	0.380771721988772
**CHOL**	HLTF	cg00906762	-0.0824250589297323	36	-0.482257547931171	0.632713246042132	0.00679389033956985	0.783578933115792
**CHOL**	HLTF	cg02059813	-0.179674781381538	36	-1.06500685826947	0.294377960818188	0.0322830270645035	0.492163054391483
**CHOL**	HLTF	cg02398045	0.075699665083063	36	0.442671278275126	0.660807095547737	0.00573043929368791	0.802617171088396
**CHOL**	HLTF	cg02611507	-0.675730874387609	36	-5.34513015759532	6.11958220294324e-06	0.456612214600643	8.50101725241238e-05
**CHOL**	HLTF	cg02710346	0.117520920361063	36	0.690040533306246	0.494852222281141	0.0138111667225113	0.679906811047169
**CHOL**	HLTF	cg03678609	0.0607411166794085	36	0.354833710792673	0.724905798330776	0.00368948325546152	0.844107163370787
**CHOL**	HLTF	cg04120413	-0.645267862281434	36	-4.92505311659381	2.14791252164771e-05	0.416370614093251	0.000254795222185212

**CHOL**	HLTF	cg04731810	-0.553165223402634	36	-3.8717919751722	0.000466880863403964	0.305991764382086	0.00354139331977847
**CHOL**	HLTF	cg04836786	0.107365480777412	36	0.629682758970564	0.533111092176844	0.0115273464625647	0.710226299629134
**CHOL**	HLTF	cg05555455	0.0319401904642344	36	0.186336786500006	0.853288016404388	0.00102017576689157	0.921812856158839
**CHOL**	HLTF	cg07275648	-0.431153111283217	36	-2.7863151725831	0.00865700409509841	0.185893005369198	0.0381608667596377
**CHOL**	HLTF	cg09352720	-0.644015084953972	36	-4.90870352003785	2.25511375639096e-05	0.414755429648271	0.000266091793667039
**CHOL**	HLTF	cg09392355	-0.164554206620246	36	-0.972768401479204	0.337538661647117	0.0270780869164185	0.537022828241513
**CHOL**	HLTF	cg15438497	-0.0406838661939917	36	-0.237422235954791	0.813754108441034	0.00165517696849062	0.899677930120865
**CHOL**	HLTF	cg16724124	-0.0399498169219446	36	-0.233131572548655	0.817057101006507	0.00159598787209689	0.901616217704051
**CHOL**	HLTF	cg20117402	-0.408843936462883	36	-2.6122485802769	0.0132950642918495	0.167153364382466	0.0532308919979486
**CHOL**	HLTF	cg21089667	-0.442117678989311	36	-2.87412524894101	0.00693839302772949	0.195468042074895	0.0321882847635034
**CHOL**	HLTF	cg21111416	-0.641865983480044	36	-4.88081312146232	2.45038293475778e-05	0.411991940748804	0.000285966231662986
**CHOL**	HLTF	cg21835021	-0.431011419346041	36	-2.78519053267237	0.00868139663416732	0.185770843606688	0.0382358925300579
**CHOL**	HLTF	cg21926402	0.150821374411798	36	0.889608381149871	0.379927665318983	0.0227470869794638	0.578363164507464
**CHOL**	HLTF	cg24041269	0.235071329997734	36	1.41020624428555	0.167562169181302	0.0552585301869035	0.340391774693365
**CHOL**	HLTF	cg24710191	0.70360520261898	15	3.57010468751346	0.00342211336417952	0.495060281152496	0.0183107426269332
**CHOL**	HLTF	cg25951608	-0.0360312099783694	36	-0.210232763559905	0.834740821778033	0.00129824809250535	0.911472068066535
**CHOL**	HLTF	cg26151310	0.140888297370437	36	0.829789622353279	0.412446183304878	0.0198495123359407	0.60811247245266
**CHOL**	HLTF	cg27554556	-0.191436783578905	36	-1.13729297152915	0.263367582117471	0.0366480421070365	0.458795209782264
**CHOL**	IDH1	cg00707300	0.20709831939771	36	1.23434067744226	0.22553964186848	0.0428897138973558	0.414926193732726
**CHOL**	IDH1	cg01046174	0.229302726068909	36	1.37365396047709	0.178539204177003	0.0525797401826331	0.355142776592236
**CHOL**	IDH1	cg02241586	0.392013201275178	36	2.48468534906853	0.0180499795690798	0.153674349974013	0.0672822285863436
**CHOL**	IDH1	cg03448938	0.232812287439693	36	1.39587349219862	0.171801686327647	0.0542015611829024	0.346058889883042
**CHOL**	IDH1	cg05268564	0.0487254128667794	36	0.284453408927938	0.777788283127389	0.00237416585903812	0.877101948463541
**CHOL**	IDH1	cg07129696	0.171047243241597	36	1.0122864869845	0.318551135612729	0.0292571594205502	0.517910785425631
**CHOL**	IDH1	cg07915542	-0.0509268278230869	36	-0.297337712413715	0.768017860889947	0.00259354179212234	0.871477601926643
**CHOL**	IDH1	cg09406631	0.224940723738384	36	1.34611619988425	0.187172165307562	0.0505983291959478	0.366375749891464
**CHOL**	IDH1	cg09615745	0.343069091865467	36	2.12966859303601	0.0405204388171709	0.117696401793396	0.123535173309676
**CHOL**	IDH1	cg10356455	0.280620379301053	36	1.70478413925625	0.0973576223908506	0.0787477972790666	0.233565820960121
**CHOL**	IDH1	cg14772913	0.116303932259429	36	0.682796314935553	0.49936183041231	0.0135266046590058	0.683559223576838
**CHOL**	IDH1	cg15399923	0.334717222146605	36	2.07118906464245	0.0459992142878979	0.112035618801539	0.135763824125986
**CHOL**	IDH1	cg16085695	-0.0733429427831484	36	-0.428814061951211	0.670763342500758	0.00537918725609217	0.809226671626691
**CHOL**	IDH1	cg16863783	0.252003261165453	36	1.51842392216325	0.138152393323245	0.0635056436380234	0.298473319434252
**CHOL**	IDH1	cg24324379	0.210484000748728	36	1.25544749370281	0.217876378202473	0.0443035145711907	0.405459041933867
**CHOL**	IDH1	cg24830471	0.323540497367589	36	1.99378621601242	0.0542465716659221	0.104678453436867	0.153159998841864
**CHOL**	IDH1	cg24987806	0.337442494827461	36	2.09020982059697	0.044149594549546	0.113867437315381	0.131734180668433
**CHOL**	IDH1	cg25795625	0.27192627559391	36	1.64767663965661	0.108631514478212	0.0739438993583751	0.252725262038878
**CHOL**	IDH1	cg26105702	0.394614343942338	36	2.50420108192548	0.0172336215216134	0.155720480445042	0.0649424700955433
**CHOL**	ING2	cg02075087	-0.164701493806764	36	-0.973663361593788	0.337100407310893	0.0271265820621794	0.536659212670233
**CHOL**	ING2	cg02552216	-0.24065671781752	36	-1.44574784796121	0.15740338497454	0.0579156558307015	0.326070956325781
**CHOL**	ING2	cg10348863	-0.245555039737147	36	-1.47704275910665	0.148867686635855	0.0602972775403118	0.314138035429657
**CHOL**	ING2	cg17985608	-0.00752022966429406	36	-0.0438513374092905	0.965279436615299	5.65538542037283e-05	0.981735501268801
**CHOL**	ING3	cg01382559	-0.275025703730354	36	-1.66798433886988	0.104504571204014	0.0756391377123762	0.245822103660046
**CHOL**	ING3	cg01416111	-0.254662347688667	32	-1.44239896716879	0.159549693630725	0.0648529113303037	0.329187345612217
**CHOL**	ING3	cg10268125	-0.279964457522806	36	-1.70046005977266	0.0981756785757132	0.078380097476039	0.23494874147473
**CHOL**	ING3	cg14303833	-0.235162000042925	36	-1.41078201360711	0.167393591001949	0.0553011662641888	0.340165535196845
**CHOL**	ING3	cg16130600	-0.123632822898313	36	-0.726470500642412	0.472522494633901	0.0152850748978057	0.660558409810593
**CHOL**	ING3	cg18485114	-0.25858697971751	36	-1.5608975199274	0.12780888561281	0.0668672260794239	0.282721918999174
**CHOL**	ING3	cg19478983	0.000376595398131012	36	0.00219590980603851	0.998260752777689	1.41824093893455e-07	0.999036231260181
**CHOL**	ING3	cg19960827	-0.00801566358480565	36	-0.0467404503495139	0.962993559239752	6.42508627047794e-05	0.980550489719862
**CHOL**	ING3	cg20553930	-0.142886338499163	36	-0.841800998597917	0.405781626438587	0.0204165057296975	0.602132068957453
**CHOL**	ING3	cg24859794	-0.187307806090979	36	-1.11186134008585	0.273998200024114	0.0350842142226158	0.47043628849893
**CHOL**	ING4	cg00369908	-0.0853151756251164	36	-0.499289086089859	0.620791308130469	0.00727867919194445	0.775256226560608
**CHOL**	ING4	cg00443163	-0.210547255194814	36	-1.2558422777624	0.217734928933856	0.0443301466700702	0.405286501152353
**CHOL**	ING4	cg00591160	-0.116641775359677	35	-0.674661188396784	0.504590543874275	0.0136053037590574	0.687811967026255
**CHOL**	ING4	cg03930474	-0.0888287603662063	36	-0.520011881189999	0.606424875227682	0.00789054866819691	0.764817157781921
**CHOL**	ING4	cg04375284	-0.183413378278582	36	-1.08793037180602	0.284278015232792	0.0336404673315624	0.481548697158842
**CHOL**	ING4	cg08733494	-0.153209843574911	36	-0.904032527463563	0.372339359202468	0.0234732561682487	0.570957721606474

**CHOL**	ING4	cg12392916	-0.193504825354905	36	-1.15005412912147	0.258146720933691	0.0374441174356321	0.452721341967032
**CHOL**	ING4	cg18123790	-0.165123237514549	36	-0.976226370523655	0.335847438776801	0.0272656835672862	0.535431503651989
**CHOL**	ING4	cg23354319	-0.278085036993305	36	-1.68808450255284	0.100548706736607	0.0773312877995677	0.23913439596049
**CHOL**	ING4	cg27116036	-0.178871035673295	36	-1.06008489315389	0.296578934038711	0.0319948474028371	0.494577954868328
**CHOL**	INO80	cg00734724	-0.213062257487872	36	-1.27155236490181	0.212161916740961	0.0453955255658282	0.398342524278281
**CHOL**	INO80	cg06380007	-0.40423661765179	36	-2.57702160641335	0.0144772954482982	0.163407243050559	0.0567807775197836
**CHOL**	INO80	cg12580230	-0.248501603086308	36	-1.49592586674069	0.143898482487447	0.0617530467364648	0.307016580182889
**CHOL**	INO80	cg18952654	-0.0217213852566229	36	-0.126686242508211	0.899934986563873	0.000471818577466636	0.947262255436596
**CHOL**	INO80	cg21007089	-0.446599238069931	36	-2.91047212329256	0.00632530424366561	0.199450879444643	0.0299076592920272
**CHOL**	INO80	cg21598047	0.0301922409588902	36	0.176129800377466	0.861236866254526	0.000911571414119688	0.926245759579237
**CHOL**	INO80	cg22835709	-0.301438870755525	36	-1.84342136970873	0.0739987947160989	0.0908653928023659	0.19197833109265
**CHOL**	INO80	cg26409855	-0.270498284261298	36	-1.63833901294499	0.110573830904501	0.073169321788306	0.255934354842673
**CHOL**	INO80	cg27441689	-0.245497258930876	36	-1.47667290751372	0.148966365219887	0.0602689041425733	0.314286589534778
**CHOL**	INTS12	cg00093890	0.0398328479720951	36	0.232447900968355	0.817583713067036	0.00158665577756804	0.901915873147149
**CHOL**	INTS12	cg01852585	0.0220726491540279	36	0.128735919418001	0.898325128747743	0.000487201840676808	0.946236613171782
**CHOL**	INTS12	cg03607115	-0.0370170559344097	36	-0.21599270651656	0.830284061227612	0.00137026243005122	0.90890196663816
**CHOL**	INTS12	cg19703969	0.25508253398054	36	1.53826075267521	0.133240534056458	0.0650670991419335	0.291095093978546
**CHOL**	INTS12	cg26533745	-0.0979668775701525	36	-0.574001282637318	0.569745083146515	0.00959750910084525	0.737937648773788
**CHOL**	IWS1	cg02372421	-0.0190290876710222	36	-0.110977789503369	0.912286500557186	0.000362106177591448	0.953429289524641
**CHOL**	IWS1	cg02592214	-0.0805323297942864	36	-0.471110308627022	0.640570823600142	0.00648545614209571	0.788851397213396
**CHOL**	IWS1	cg10180764	-0.19806293957405	36	-1.17823717104433	0.246883002631992	0.0392289280327136	0.440064221735393
**CHOL**	IWS1	cg10488833	0.0848893889059361	36	0.496779126391506	0.622541840738515	0.00720620834882327	0.776417251781179
**CHOL**	IWS1	cg15239145	-0.156184081968461	36	-0.922016892414571	0.363016518314077	0.024393467460331	0.561966185434471
**CHOL**	IWS1	cg17144508	-0.0201560618027872	36	-0.117552908110892	0.90711362594592	0.000406266827397776	0.950871768356872
**CHOL**	IWS1	cg18451256	-0.127066584099006	36	-0.746973968747072	0.460214018458222	0.0161459167945898	0.650041522029776
**CHOL**	IWS1	cg18530950	0.0687108631802567	36	0.401598870262739	0.690492490763413	0.00472118271897595	0.822165345166434
**CHOL**	IWS1	cg19311602	-0.162101670650445	36	-0.95787585076699	0.344887525345939	0.0262769516276653	0.544112353412329
**CHOL**	IWS1	cg19409088	-0.00130778803692046	36	-0.0076256556530559	0.993960235056811	1.71030954951226e-06	0.996872564214446
**CHOL**	IWS1	cg21581038	0.0194456550805162	36	0.113408123070922	0.910374007806105	0.000378133501510405	0.952466990737373
**CHOL**	IWS1	cg21679674	-0.0918899028998564	36	-0.538082135900937	0.594025543352331	0.00844375425494504	0.755758726973984
**CHOL**	IWS1	cg22409775	-0.0725633918418213	36	-0.424232006590961	0.67406893938596	0.00526544583558969	0.811597303639224
**CHOL**	IWS1	cg23570261	-0.158775783508068	36	-0.937709108523955	0.355007666364806	0.0252097494286009	0.553933577976687
**CHOL**	IWS1	cg23623107	-0.0882609739707781	36	-0.516661825588142	0.608736820480625	0.00778999952627036	0.766440711030956
**CHOL**	JADE1	cg09863040	-0.505255893809644	36	-3.41393591133037	0.00167185301313221	0.255283518229383	0.0102765592054051
**CHOL**	JADE1	cg12832492	-0.320807246388736	36	-1.97500129508983	0.0564327627212433	0.102917289335523	0.157482166055759
**CHOL**	JADE1	cg14617908	0.0309811055127426	36	0.180736094580617	0.857647772050795	0.000959828898791688	0.924276878881217
**CHOL**	JADE1	cg15389440	0.293318331632485	36	1.78901521909527	0.0825251054779374	0.0860356436716647	0.207537274699381
**CHOL**	JADE1	cg15836763	-0.101700236292051	36	-0.596099910304824	0.555056590367331	0.0103429380618591	0.726606969745001
**CHOL**	JADE1	cg20039387	-0.168190718480741	36	-0.994884644085715	0.326820520876159	0.028288117783068	0.526303626750018
**CHOL**	JADE1	cg22922695	-0.151207693784424	36	-0.891940304978786	0.37869419101236	0.0228637666596042	0.577242583799407
**CHOL**	JADE1	cg26766900	-0.300339466915599	36	-1.83603012938403	0.0751111068389705	0.090203795387146	0.194069391741646
**CHOL**	JADE2	cg04590931	-0.388604653507747	36	-2.45921830529668	0.0191684621730754	0.151013576727876	0.0704089591714541
**CHOL**	JADE2	cg04842522	-0.116097947235794	36	-0.681570482163602	0.500127174932957	0.0134787333523653	0.684113332513969
**CHOL**	JADE2	cg17378563	-0.179884439284633	36	-1.06629111996177	0.293805558963409	0.0323584114967468	0.491522970559761
**CHOL**	JADE2	cg18145011	-0.30858279190527	36	-1.89164862030276	0.0670810360916657	0.0952233394600514	0.178715676552036
**CHOL**	JADE2	cg21194015	-0.235305436798553	36	-1.41169293962931	0.167127154931734	0.0553686485869576	0.339833522677997
**CHOL**	JADE2	cg25669900	-0.197445211155608	36	-1.17441311454111	0.248389911883596	0.0389846114082824	0.441704455278589
**CHOL**	JADE2	cg26133206	-0.31457387380628	36	-1.9323655373288	0.0616806681818148	0.0989567220814895	0.167987183186392
**CHOL**	JADE3	cg10860619	0.00130425716830249	36	0.00760506727532133	0.993976541391189	1.70108676106842e-06	0.996872564214446
**CHOL**	JADE3	cg17843048	-0.0787003067294708	36	-0.460325484697919	0.648213220681633	0.00619373827931278	0.793990006293508
**CHOL**	JADE3	cg19194595	-0.0107851227326079	36	-0.0628911896563418	0.95022143587117	0.000116318872357416	0.973659203175049
**CHOL**	JARID2	cg01769037	-0.322842344118466	36	-1.98898271959116	0.0547984644226297	0.104227179155906	0.154227117010747
**CHOL**	JARID2	cg03223185	-0.189438691555561	36	-1.12497838598553	0.268477494966836	0.035887017858283	0.464278168743817
**CHOL**	JARID2	cg05197191	-0.186028796815335	36	-1.10399599646414	0.277347183908457	0.0346067132445612	0.474106343532486
**CHOL**	JARID2	cg06047828	-0.165083402179546	36	-0.975984260833677	0.335965663932325	0.0272525296751736	0.535532187114811
**CHOL**	JARID2	cg14862454	0.0688054986972769	36	0.402154621878786	0.690087344764187	0.00473419665098097	0.821921962437767
**CHOL**	JARID2	cg15109221	-0.226993361791286	36	-1.35906396368246	0.183073782108378	0.0515259862973096	0.361115692723933

**CHOL**	JARID2	cg21397480	-0.125343726365372	36	-0.736683182236886	0.466368152940302	0.0157110497391572	0.655654542149415
**CHOL**	JARID2	cg21901688	-0.277058541449921	36	-1.6813341477638	0.101863098603605	0.0767614353903576	0.241408553437282
**CHOL**	JARID2	cg25338254	-0.179042053653066	36	-1.06113198630837	0.296109741096857	0.0320560569763074	0.494079096871288
**CHOL**	JMJD6	cg02318866	-0.164025970742444	36	-0.969559235041919	0.339113306281242	0.0269045190780009	0.538431311716118
**CHOL**	JMJD6	cg04510639	-0.0642815025091195	36	-0.375599160976733	0.709548161899241	0.00413211156482994	0.834559346045411
**CHOL**	JMJD6	cg07312083	-0.233287912454619	36	-1.39888915366091	0.170902779016425	0.054423250097434	0.344876213763389
**CHOL**	JMJD6	cg08857478	-0.0833976852101413	36	-0.487987871930704	0.6286907116828	0.00695517389840982	0.780685181085617
**CHOL**	JMJD6	cg10712116	-0.167555075371558	36	-0.991015855867452	0.328678598733879	0.0280747032827684	0.528361075651984
**CHOL**	JMJD6	cg12504415	-0.0779164584364163	36	-0.4557125364591	0.651493994331188	0.00607097449527379	0.796315931794621
**CHOL**	JMJD6	cg15457598	0.0845006469790202	36	0.494487780556571	0.624141853693632	0.007140359339873	0.777369378261967
**CHOL**	JMJD6	cg21102709	-0.204928996800728	36	-1.22084122859438	0.230545238440319	0.0419958937297526	0.420842299533665
**CHOL**	JMJD6	cg21807925	-0.209436038909642	36	-1.24890934386998	0.22022900910944	0.043863454394161	0.408383284102302
**CHOL**	JMJD6	cg23630158	-0.108851212305116	36	-0.638500116225271	0.527426028205893	0.0118485864202934	0.706023925634505
**CHOL**	JMJD6	cg25004679	-0.0255271448001476	36	-0.148896074203887	0.882515292128258	0.000651635121647704	0.937645378801225
**CHOL**	JMJD6	cg25908504	-0.115512635494662	36	-0.678087732793322	0.502305160542338	0.0133431689589225	0.685958776449648
**CHOL**	JMJD6	cg26612088	0.0537845636272783	36	0.314069799500656	0.755387057782887	0.00289277928457675	0.86351059842849
**CHOL**	JMJD8	cg00873473	-0.24587202398807	36	-1.47907205512579	0.148327185812978	0.0604530521799902	0.31333029466213
**CHOL**	JMJD8	cg02851105	-0.0836574547566046	36	-0.489518566854468	0.627618143822048	0.00699856973635335	0.779938640598319
**CHOL**	JMJD8	cg03899598	-0.210637677812817	36	-1.25640665265582	0.217532835848118	0.0443682313143761	0.405063063252197
**CHOL**	JMJD8	cg05787952	-0.0236312168887569	36	-0.13783097905015	0.891187050674833	0.000558434411643468	0.942418343236566
**CHOL**	JMJD8	cg06191810	-0.188435203948803	36	-1.11879919843468	0.271068174998329	0.0355078260872269	0.467338274982928
**CHOL**	JMJD8	cg06646636	-0.153258690234403	36	-0.904327684477304	0.372185112987379	0.0234882261323646	0.570825843647056
**CHOL**	JMJD8	cg08398290	-0.0339112704850776	36	-0.197848780118645	0.84434155063414	0.00114997426591209	0.916839847942843
**CHOL**	JMJD8	cg26750188	-0.0565946710521252	36	-0.330530566512441	0.743027354148894	0.00320295679149826	0.855543270557244
**CHOL**	JMJD8	cg27579097	0.0667171778948265	36	0.389893365772209	0.699047230997465	0.00445118182624993	0.827362051578707
**CHOL**	KAT2B	cg00770008	0.0405512719898565	36	0.236647168273754	0.81435050867998	0.00164440565999532	0.900123378074343
**CHOL**	KAT2B	cg04371528	0.0462909528722511	36	0.270209984671336	0.788632168434295	0.00214285231782097	0.883737877202869
**CHOL**	KAT2B	cg05072819	-0.198050048443893	36	-1.17815735341159	0.246914387101405	0.0392238216886283	0.440106720819492
**CHOL**	KAT2B	cg12062464	0.0375851370007944	36	0.219312085346574	0.827718267140779	0.00141264252336848	0.907395612839786
**CHOL**	KAT2B	cg14937543	0.0905301154767652	36	0.530053296520059	0.599519773755997	0.00819570180823644	0.759719427163052
**CHOL**	KAT2B	cg18053505	-0.0364787938506806	36	-0.212847757780835	0.832716771080136	0.00133070240080045	0.910232945375207
**CHOL**	KAT5	cg00582417	-0.196104159318201	36	-1.16611631736133	0.25168243577768	0.0384568413018985	0.445451686055622
**CHOL**	KAT5	cg02561205	-0.332314135809418	36	-2.05446555938515	0.0476814056237673	0.11043268485876	0.139395989067845
**CHOL**	KAT5	cg05785768	-0.233747100737745	36	-1.40180160672871	0.170038132790249	0.0546377071033015	0.343734805038189
**CHOL**	KAT5	cg07564927	-0.334252017402905	36	-2.06794808643679	0.0463210823425672	0.111724411137912	0.136408459694805
**CHOL**	KAT5	cg14726348	-0.218421625008831	36	-1.30511874041837	0.200616369775905	0.0477080062714985	0.383646751975092
**CHOL**	KAT5	cg18896539	-0.106805550489432	36	-0.626360856467607	0.535261283305376	0.0114074256153506	0.711722670451622
**CHOL**	KAT5	cg20108992	-0.196501974185572	36	-1.16857679933864	0.250702710975451	0.0386130258588271	0.444303961582474
**CHOL**	KAT5	cg20423942	-0.0939033216501599	36	-0.549975918499285	0.58593098989277	0.00881783381693338	0.750247113074301
**CHOL**	KAT5	cg22048492	-0.266359400019442	36	-1.61134048384346	0.116351425563569	0.0709473299787174	0.264997305167462
**CHOL**	KAT6A	cg10163397	-0.0152831677471427	36	-0.0891258253342896	0.929504850785828	0.000233575216387304	0.962671899544514
**CHOL**	KAT6A	cg12468056	-0.408671740531163	36	-2.61092771131032	0.013337735750965	0.16701259150877	0.0533543146198831
**CHOL**	KAT6A	cg15251374	-0.240946342101106	36	-1.44759493759332	0.156889052475655	0.0580551397719031	0.325502105512351
**CHOL**	KAT6A	cg20966972	-0.0664319909421375	36	-0.388219337312286	0.700273963545525	0.00441320942053624	0.828297670635676
**CHOL**	KAT6B	cg04396765	-0.0964878428122852	36	-0.565253349825945	0.575612735545878	0.00930990381056825	0.742410258456396
**CHOL**	KAT6B	cg06972998	-0.299039668294507	19	-1.29209764738484	0.213612640862012	0.0894247232136886	0.400129584725129
**CHOL**	KAT6B	cg09631472	-0.0168458455427959	36	-0.0982412555522732	0.922317626433241	0.000283782512051737	0.959199830761722
**CHOL**	KAT6B	cg10359932	0.066568428386536	36	0.389020204094666	0.699686984059897	0.00443135565785337	0.827879923920965
**CHOL**	KAT6B	cg10739686	-0.0393488897955404	36	-0.22961931620287	0.819763400038817	0.00154833512814158	0.902981385619281
**CHOL**	KAT6B	cg11128436	-0.162790122658716	36	-0.962054488585201	0.342814855314063	0.0265006240352399	0.542062620262652
**CHOL**	KAT6B	cg11348552	0.0246030861309878	36	0.143502850256671	0.886740214452916	0.000605311847168805	0.939854473608295
**CHOL**	KAT6B	cg15874302	-0.314083304335473	36	-1.92902197053349	0.0621095201978	0.0986483220622892	0.168871383135316
**CHOL**	KAT6B	cg15912797	0.0345245043254723	36	0.201430806567431	0.841562017992182	0.00119194139891956	0.915190858192273
**CHOL**	KAT6B	cg17147776	0.07280601385518	36	0.42565800945265	0.673039480530015	0.00530071565348066	0.810866075302411
**CHOL**	KAT6B	cg18462764	-0.266096358269958	36	-1.60962788939896	0.116726122922749	0.0708072718845338	0.265677521479632
**CHOL**	KAT6B	cg18491775	0.283616103867371	36	1.72456615767097	0.0936873907551712	0.0804380943729074	0.227298202913076
**CHOL**	KAT6B	cg24671245	0.354053191990709	36	2.20745435313906	0.0341317714588617	0.12535366275881	0.108701315301539

**CHOL**	KAT6B	cg26605858	0.0478320488017131	36	0.279225979747938	0.781762938471497	0.00228790489256946	0.879452572309566
**CHOL**	KDM1A	cg00062312	-0.182604264275121	36	-1.08296512096397	0.286444631389564	0.0333443173314583	0.483765482219412
**CHOL**	KDM1A	cg00541565	-0.18380603390574	36	-1.09034078651285	0.283230404164335	0.0337846581001579	0.480548429948317
**CHOL**	KDM1A	cg04886391	-0.214885354480428	36	-1.28295696247231	0.208184155980696	0.0461757155701792	0.393332804623249
**CHOL**	KDM1A	cg06958034	0.160196120226236	36	0.94631736501023	0.350664112860942	0.0256627969355388	0.549661196235371
**CHOL**	KDM1A	cg07118078	-0.0135609054371801	36	-0.0790802589459805	0.937432396953512	0.000183898156276142	0.966551912185071
**CHOL**	KDM1A	cg07785945	-0.183214679095755	36	-1.08671081262795	0.284809100753616	0.0335676186361606	0.482091411563062
**CHOL**	KDM1A	cg10499166	-0.140839854279903	36	-0.82949853212644	0.412608532831456	0.0198358645535843	0.608248089954716
**CHOL**	KDM1A	cg12578844	0.088793390986028	36	0.519803179740894	0.606568784733274	0.00788426628279764	0.764913034430821
**CHOL**	KDM1A	cg22683154	0.0461367521344473	36	0.269307959417515	0.789320371658651	0.00212859989751543	0.88408632188378
**CHOL**	KDM1A	cg26662347	-0.0912161740250175	36	-0.534103739842773	0.596745001091818	0.00832039040376228	0.7578057560388
**CHOL**	KDM1B	cg11007366	-0.0545311981430513	36	-0.318442614337636	0.752097152781135	0.00297365157091672	0.861182447566601
**CHOL**	KDM1B	cg15124342	-0.235504297821583	36	-1.41295601095279	0.166758271015979	0.0554622742924371	0.339291040549681
**CHOL**	KDM1B	cg20677365	-0.0785417667208038	19	-0.32483948657914	0.749265869664403	0.00616880911962516	0.859533268420571
**CHOL**	KDM1B	cg22406543	-0.13592423586802	36	-0.799992220692074	0.429269619933112	0.0184753978963052	0.623370868811046
**CHOL**	KDM1B	cg23073439	-0.23551407804612	36	-1.41301813516715	0.166740143952378	0.0554668809579141	0.339267474226911
**CHOL**	KDM1B	cg23860886	-0.132133994693576	36	-0.777282283805782	0.442367155562218	0.0174593925536819	0.634746002300356
**CHOL**	KDM1B	cg24342628	0.0790121211977566	36	0.46216075161332	0.646909937592301	0.00624291529616898	0.792960366978898
**CHOL**	KDM2A	cg05038268	-0.442513425133559	36	-2.87732401842498	0.00688227722759335	0.195818131423434	0.0319889989032322
**CHOL**	KDM2A	cg05295919	-0.317709756928646	36	-1.95377950506444	0.0589943278151423	0.100939489647659	0.162617983773339
**CHOL**	KDM2A	cg06320126	-0.229446056195384	36	-1.37456028878595	0.178260421542065	0.0526454927036152	0.354842282271268
**CHOL**	KDM2A	cg07283932	-0.0842996741283014	36	-0.493303283646693	0.62496969770114	0.0071064350581378	0.777983415908476
**CHOL**	KDM2A	cg08836144	-0.305625957438775	15	-1.15732620413907	0.267961938651432	0.0934072258603681	0.463655826121597
**CHOL**	KDM2A	cg09993857	-0.194253514421994	36	-1.1546779415715	0.256273640265802	0.0377344278652959	0.450553720328997
**CHOL**	KDM2A	cg11637544	0.0555174862626064	36	0.324219831089836	0.747757854044195	0.00308219128091869	0.858416769537966
**CHOL**	KDM2A	cg16004548	-0.0610592898862469	36	-0.356699332776084	0.723521178148216	0.00372823688141274	0.843318498312367
**CHOL**	KDM2A	cg18132081	0.22790805266316	36	1.36483984784796	0.181268082663472	0.0519420804687137	0.358553973473064
**CHOL**	KDM2A	cg21293464	-0.434117997044738	36	-2.80990647528001	0.00815984307180895	0.188458435358135	0.0364840986222372
**CHOL**	KDM2A	cg21711868	-0.128666195100042	36	-0.756534741062683	0.454539211106585	0.0165549897615221	0.644816065298255
**CHOL**	KDM2A	cg25597833	-0.0858223021176077	36	-0.502278893282626	0.618709037808805	0.00736546754076592	0.773688979049231
**CHOL**	KDM2B	cg03486832	-0.0677464633045797	36	-0.395936004121508	0.694626015060204	0.00458958329027877	0.824961929374308
**CHOL**	KDM2B	cg05067991	-0.122312278098356	36	-0.718592441968646	0.477301708393208	0.0149602933736095	0.664992909768455
**CHOL**	KDM2B	cg07020001	-0.103028782195772	36	-0.603969974377706	0.549872574685349	0.0106149299607438	0.722723871617849
**CHOL**	KDM2B	cg07529210	-0.0831525978099068	36	-0.486543783565695	0.629703344882216	0.00691435452253612	0.781525713855941
**CHOL**	KDM2B	cg07793808	-0.0455237964615218	36	-0.265722553884622	0.792057561891368	0.00207241604427006	0.885942860816349
**CHOL**	KDM2B	cg09151742	-0.143598347659156	36	-0.846083826532934	0.403421621887342	0.0206204854504398	0.599853206414408
**CHOL**	KDM2B	cg12251659	0.122472881257931	36	0.719550354676764	0.476719119916638	0.0149996066436194	0.664444635235024
**CHOL**	KDM2B	cg12888660	-0.0906806114247228	36	-0.530941749074235	0.598910613279997	0.00822297328836156	0.759279243998893
**CHOL**	KDM2B	cg15234492	-0.0866757425024733	36	-0.507311312474005	0.615211394240552	0.00751268433835506	0.77102371729334
**CHOL**	KDM2B	cg15316703	-0.0438536157368297	36	-0.255954560397447	0.799528197823312	0.00192313961319352	0.890521821482808
**CHOL**	KDM2B	cg16837338	-0.0876969186921074	22	-0.393709425639156	0.697961512243741	0.00769074954809009	0.826728359955793
**CHOL**	KDM2B	cg17452615	-0.0672345681135474	36	-0.392930657291866	0.696823591954473	0.00452048714941525	0.826191873999401
**CHOL**	KDM2B	cg20055546	-0.0331084241970012	36	-0.193159525456403	0.847983312296816	0.00109616775280858	0.918845987149665
**CHOL**	KDM2B	cg21249371	-0.0719394678734237	36	-0.420565261169629	0.676718958219816	0.00517528703791137	0.813233561995094
**CHOL**	KDM2B	cg21781916	0.0297157188136554	36	0.17334747881346	0.86340622876775	0.000883023944612232	0.927402835053578
**CHOL**	KDM2B	cg26218726	-0.0900424753867725	36	-0.527174765332863	0.601495426370191	0.00810764737377754	0.761139404816406
**CHOL**	KDM2B	cg26509318	-0.22092693384782	36	-1.32085210101961	0.195372323690557	0.0488087100993989	0.377075806911534
**CHOL**	KDM3A	cg01744130	-0.491723168115911	36	-3.2928038056843	0.00232058987633347	0.241791674061948	0.0134052104284166
**CHOL**	KDM3A	cg02152982	-0.410994374428726	34	-2.55028375257143	0.0157511677204123	0.16891637581206	0.0605827322081359
**CHOL**	KDM3A	cg02972551	-0.408773820724822	36	-2.61171070077452	0.0133124255779947	0.167096036509969	0.0532866907854138
**CHOL**	KDM3A	cg03037033	-0.260761882961473	36	-1.57497932018531	0.124521678841639	0.0679967596056132	0.277644761378488
**CHOL**	KDM3A	cg04793821	-0.247460773735327	36	-1.48925067174081	0.145639706752144	0.0612368345376865	0.309568440314662
**CHOL**	KDM3A	cg07052513	-0.174256933400271	36	-1.03187119944357	0.309417010226674	0.0303654788380664	0.508036869851706
**CHOL**	KDM3A	cg07211384	-0.107949662487767	27	-0.542920946386238	0.591992286019701	0.0116531296312227	0.754479814852828
**CHOL**	KDM3A	cg12888358	-0.26864425972378	36	-1.6262329530682	0.113134561755614	0.0721697382825375	0.26020547827626
**CHOL**	KDM3A	cg18611949	-0.189395101340811	36	-1.12470989420701	0.26858969217559	0.0358705044118961	0.464386879936781
**CHOL**	KDM3A	cg19139691	-0.16943225659203	36	-1.00244484970191	0.323210159759207	0.0287072895738676	0.522653429124215

**CHOL**	KDM3A	cg20191795	-0.237801669178096	36	-1.42756150529809	0.162538992846673	0.0565496338638888	0.333181165437381
**CHOL**	KDM3A	cg20918903	-0.17694320438889	36	-1.04828818531036	0.301900832196551	0.0313088975794084	0.500321105617539
**CHOL**	KDM3A	cg22404242	-0.306997414748008	36	-1.88091593220731	0.0685705216126416	0.0942474126619601	0.181608114878761
**CHOL**	KDM3A	cg24749656	-0.172521855189663	36	-1.02128002767463	0.314333958895099	0.0297637905180829	0.513379576719124
**CHOL**	KDM3B	cg00435490	-0.100428956322995	36	-0.588572097160636	0.560038406863924	0.0100859752681261	0.730712083402413
**CHOL**	KDM3B	cg03315484	-0.233073111358724	36	-1.39752709358599	0.171308324774604	0.0543230752384364	0.34544973346248
**CHOL**	KDM3B	cg05191700	-0.423847810111132	36	-2.72865738223335	0.00999348561570437	0.179646966136002	0.04260222812819
**CHOL**	KDM3B	cg05708550	0.114197793874788	36	0.670266705064271	0.507215189219899	0.0130411361258685	0.689999273076852
**CHOL**	KDM3B	cg05722427	0.285001154649726	36	1.73373073724412	0.0920267441656087	0.0812256581516768	0.224367824232774
**CHOL**	KDM3B	cg15001364	-0.266791633753822	36	-1.61415548381208	0.115737678646204	0.0711777758410336	0.264022549193678
**CHOL**	KDM3B	cg18682923	0.1010392417745	36	0.592185502463603	0.557644274019243	0.0102089283783658	0.728861346703817
**CHOL**	KDM3B	cg19076659	0.0998652747954335	36	0.585235222736301	0.562253932360038	0.00997307310996744	0.732511004968715
**CHOL**	KDM3B	cg24137987	-0.373818573765904	36	-2.35009572322521	0.0247119698390126	0.139740326092375	0.0854647008827307
**CHOL**	KDM4A	cg00154982	0.233309330103541	36	1.39902497547243	0.170862380047852	0.0544332435133631	0.344818615581679
**CHOL**	KDM4A	cg00979347	0.437279069307603	36	2.83518278584408	0.00765685800048228	0.191212984454523	0.0347565416786975
**CHOL**	KDM4A	cg03587511	0.408649395190567	36	2.61075633071746	0.0133432815583376	0.166994328189616	0.0533662662870451
**CHOL**	KDM4A	cg08552254	-0.166344197086954	34	-0.954280147510314	0.347095467532033	0.0276703919045034	0.546365812128183
**CHOL**	KDM4A	cg10958452	-0.441511312338981	34	-2.78356012684273	0.00894926125953728	0.194932238923289	0.0391746581550445
**CHOL**	KDM4A	cg10968649	0.464626732932516	36	3.05950969133737	0.00430551039575328	0.215878000955543	0.0220226757661405
**CHOL**	KDM4A	cg12293275	0.523052657911905	36	3.57842727959795	0.00106377837820703	0.273584082948708	0.00706768939740692
**CHOL**	KDM4A	cg17737314	-0.366994315376024	35	-2.26636131128331	0.0301113244739264	0.134684827518317	0.0989860879222872
**CHOL**	KDM4A	cg19743044	-0.205353134258708	36	-1.22347910341055	0.229560682420713	0.0421699097498749	0.419617522785156
**CHOL**	KDM4A	cg20873046	0.169025809413522	36	0.999969296695357	0.324389353498498	0.0285697242478962	0.523787238549205
**CHOL**	KDM4A	cg21451504	0.0423892006654028	36	0.247391752107278	0.80609292683092	0.00179684433305179	0.89482296161365
**CHOL**	KDM4A	cg22094309	-0.0114860287679033	36	-0.0669788995790133	0.946990826464216	0.000131928856857102	0.971710141185216
**CHOL**	KDM4A	cg24324755	0.363468457335819	36	2.27495952228762	0.0293329969787115	0.13210931947808	0.0970786678704109
**CHOL**	KDM4A	cg24505556	0.198406357943065	36	1.18036373893069	0.246047906062732	0.0393650828722317	0.439055206997068
**CHOL**	KDM4A	cg25841161	0.241850659279553	36	1.45336489930384	0.15529094323978	0.0584917413939543	0.323194939361553
**CHOL**	KDM4B	cg01098771	-0.0749112048146436	36	-0.438034417158647	0.664131765101655	0.00561168860678149	0.804908495892039
**CHOL**	KDM4B	cg12072803	-0.0448100786275571	36	-0.261548131718208	0.79524779703741	0.00200794314660785	0.887889936747109
**CHOL**	KDM4B	cg18115693	0.0494254213901951	36	0.288549915311403	0.774677754060754	0.00244287227959836	0.875083946091106
**CHOL**	KDM4C	cg00121158	0.209191982227654	36	1.24738734099225	0.220779395136188	0.0437612854283352	0.409033395218495
**CHOL**	KDM4C	cg00667760	0.0377712475644064	36	0.220399602106257	0.82687806086244	0.00142666714257168	0.907024137996794
**CHOL**	KDM4C	cg05545111	0.242972947819515	36	1.46053121489214	0.153324089183189	0.0590358533721049	0.320507703719388
**CHOL**	KDM4C	cg14196303	-0.0721763621740802	36	-0.421957409128682	0.675712334564676	0.00520942725668399	0.812543979228896
**CHOL**	KDM4C	cg14473811	0.113626462010174	36	0.666869385994866	0.509356198578951	0.0129109728689496	0.691826635104509
**CHOL**	KDM4C	cg14497348	0.215805265459462	36	1.28871690200221	0.206196757720005	0.0465719126000291	0.390650006577129
**CHOL**	KDM4E	cg15555590	0.0471298951521199	36	0.275117870927477	0.784890712516961	0.00222122701704982	0.881152444653865
**CHOL**	KDM4E	cg19409026	-0.106647097166536	36	-0.625420908858994	0.535870517671999	0.0113736033340485	0.712153395539202
**CHOL**	KDM5B	cg00528052	0.171531259063398	36	1.01523768491283	0.317163037194696	0.0294229728358746	0.516381867847633
**CHOL**	KDM5B	cg00571448	0.0471380179550793	36	0.27516539287564	0.784854510059097	0.00222199273673338	0.881135940672079
**CHOL**	KDM5B	cg02624558	0.0753004899958835	36	0.440323663097462	0.662489487537071	0.00567016379362015	0.803694401544754
**CHOL**	KDM5B	cg04504182	0.185948764885395	36	1.1035040291022	0.277557622540444	0.034576943162404	0.474337507697795
**CHOL**	KDM5B	cg08915090	0.0686787193446021	36	0.401410106523999	0.690630121657303	0.00471676649081462	0.822212685717006
**CHOL**	KDM5B	cg09889216	-0.25803685167653	36	-1.55733965998254	0.128650483065592	0.0665830168231356	0.28401491756252
**CHOL**	KDM5B	cg11171004	0.15457963608082	36	0.912312110736381	0.368028234962559	0.0238948638908788	0.566906649866148
**CHOL**	KDM5B	cg11221497	-0.0560450633646623	36	-0.327310521936255	0.745439832577212	0.00314104912754901	0.857002825017888
**CHOL**	KDM5B	cg13134514	0.210693925088939	36	1.25675773837414	0.217407189103538	0.0443919300693834	0.404887840398368
**CHOL**	KDM5B	cg13253785	0.0198175334639899	36	0.115577782230717	0.908667104391257	0.000392734632596359	0.951500144282204
**CHOL**	KDM5B	cg13993945	-0.323757496098032	36	-1.99527997040701	0.0540759384870393	0.104818916279667	0.152796148433123
**CHOL**	KDM5B	cg14560895	-0.165208656194728	36	-0.976745541501436	0.335594015488604	0.0272939000816679	0.535173621216271
**CHOL**	KDM5B	cg21105875	0.143685640372192	25	0.696317545228087	0.493207479645507	0.020645563249167	0.678573522302364
**CHOL**	KDM5B	cg24354901	0.218100866849191	36	1.30310634151589	0.201294788029805	0.0475679881203684	0.384556536921752
**CHOL**	KDM5B	cg27531483	-0.124551312856801	36	-0.731952303432517	0.469213269853444	0.0155130295343528	0.658111358359577
**CHOL**	KDM5C	cg01859586	0.0641566901290415	36	0.374866860553187	0.710087707196065	0.00411608088831385	0.834891001188101
**CHOL**	KDM5C	cg04927657	0.163022318838606	36	0.963464154893393	0.342117518433589	0.0265762764395161	0.541301054121027
**CHOL**	KDM5C	cg04927982	-0.311729018992136	36	-1.91299968521169	0.0642004989102015	0.0971749812817993	0.172978686392831

**CHOL**	KDM5C	cg09554224	0.346808011260247	36	2.15603253085771	0.0382452758213789	0.120275796674288	0.118432278398826
**CHOL**	KDM5C	cg12234996	-0.626894805475383	36	-4.69179194977994	4.29549322375407e-05	0.392997097132018	0.000465603455272014
**CHOL**	KDM5C	cg16417926	-0.222924181388588	36	-1.33341450607085	0.191261070167922	0.0496951906477722	0.371741753720007
**CHOL**	KDM5C	cg16824069	-0.367820572463021	36	-2.30643239537025	0.0273101697391728	0.135291973527024	0.0920363604526603
**CHOL**	KDM5C	cg19903753	-0.571659145558482	36	-4.06258707983363	0.000270541031152443	0.326794178700654	0.00223874789676344
**CHOL**	KDM5C	cg24985300	-0.146020585060582	36	-0.860663989531885	0.395451964484076	0.0213220112614346	0.592184803575031
**CHOL**	KDM5C	cg27526317	-0.546815018321659	36	-3.80822805310915	0.000559030147312622	0.299006664262116	0.00412024550260901
**CHOL**	KDM6A	cg00751785	-0.392826961888591	36	-2.49078318410884	0.0177911795196577	0.154313021986621	0.0665535208478673
**CHOL**	KDM6A	cg03267667	-0.0881885872980799	36	-0.51623476599743	0.609031837848396	0.00777722692963106	0.766665302891627
**CHOL**	KDM6A	cg03478540	-0.183751449667801	36	-1.0900056752282	0.283375886043705	0.0337645952550182	0.48069449670658
**CHOL**	KDM6A	cg06877198	-0.0657881963848561	36	-0.384440656768774	0.703045990928239	0.00432808678357239	0.83025062342727
**CHOL**	KDM6A	cg07167981	-0.178970670853529	36	-1.0606949181935	0.296305524222996	0.0320305010257623	0.49427034804451
**CHOL**	KDM6A	cg11895950	-0.13803509600153	36	-0.81265527872111	0.422069750947004	0.0190536877281517	0.617178139605858
**CHOL**	KDM6A	cg14384228	-0.242140643647538	36	-1.45521598444022	0.154780990853584	0.0586320913060439	0.322461433878678
**CHOL**	KDM6A	cg15923127	-0.139416366074404	36	-0.820947621889432	0.417395258237355	0.0194369231293922	0.612842494325018
**CHOL**	KDM6A	cg17824914	-0.0864382628559273	36	-0.505910871183982	0.616183820011677	0.00747157328555039	0.771894238321257
**CHOL**	KDM6A	cg20211377	-0.0929438826310969	36	-0.544307425571141	0.589782129459861	0.00863856531854311	0.752833620595824
**CHOL**	KDM6A	cg22133177	-0.0855062476157981	36	-0.500415521172795	0.620006421247364	0.00731131838133418	0.774680667085681
**CHOL**	KDM6A	cg27306636	-0.496125635131492	36	-3.33185399816944	0.00208884193431572	0.246140645834626	0.0123152231480225
**CHOL**	KDM6B	cg01046511	-0.261423963302884	36	-1.57927117294704	0.12353360752962	0.0683424885889877	0.276289378839066
**CHOL**	KDM6B	cg02308232	-0.399876236210288	36	-2.54389874732658	0.0156764773508995	0.159901004285706	0.0603611106234346
**CHOL**	KDM6B	cg24893073	-0.289375189287311	36	-1.762750770801	0.0869303812290322	0.083738000175067	0.215465613671016
**CHOL**	KDM8	cg00636124	0.0169516170798396	36	0.0988582685765617	0.921831363864234	0.00028735732162151	0.959036978715821
**CHOL**	KDM8	cg01755539	0.0566244533420458	36	0.330705063741796	0.74289669469746	0.00320632871628552	0.855481459341793
**CHOL**	KDM8	cg06329197	-0.04022463542237	36	-0.23473789647311	0.815820136894056	0.00161802129486258	0.900888659651445
**CHOL**	KDM8	cg08572679	0.12523506901439	36	0.736034389998523	0.466757739198553	0.015683822511039	0.65596516237399
**CHOL**	KDM8	cg09140090	0.0918916788915252	36	0.538092624185411	0.594018381850782	0.00844408064950318	0.755758726973984
**CHOL**	KDM8	cg10214171	-0.130671562863507	36	-0.768529187577445	0.447478468844058	0.0170750573411914	0.639056192261231
**CHOL**	KDM8	cg10221365	-0.287515776912266	36	-1.75039971362027	0.089069592864371	0.0826653219734637	0.21921782756742
**CHOL**	KDM8	cg16752029	-0.0796275482535805	36	-0.465783412208477	0.644340688761556	0.0063405464408763	0.791364827544821
**CHOL**	KDM8	cg24206694	-0.036691634876263	36	-0.214091319453445	0.831754638261562	0.001346276069893	0.90977513882858
**CHOL**	KDM8	cg24705286	-0.0485055934011972	36	-0.283167094643961	0.778765768505522	0.00235279259120226	0.877680433072909
**CHOL**	KIAA2026	cg03495688	0.0943376927031601	36	0.552542747665238	0.584191119830266	0.00889960026455586	0.748880532183299
**CHOL**	KIAA2026	cg25608569	-0.177738619877908	36	-1.05315394145909	0.299697728999807	0.0315910169961035	0.497956563168941
**CHOL**	KMT2B	cg09284703	0.22634999384419	36	1.35500364120016	0.184351462652688	0.0512343197132649	0.362782164914238
**CHOL**	KMT2B	cg13830730	0.0172910252607072	36	0.100838211916473	0.920271194892124	0.000298979554566414	0.958068447870112
**CHOL**	KMT2B	cg15496871	-0.0584683594816962	36	-0.341510426318109	0.734821105706261	0.00341854906048085	0.850474940077555
**CHOL**	KMT2B	cg24471867	0.0527979612434451	36	0.30829237375606	0.759740834500705	0.00278764471146434	0.8662994280564
**CHOL**	L3MBTL1	cg01071811	-0.18584185533905	36	-1.10284687680001	0.277838896065659	0.0345371951958602	0.474673764922325
**CHOL**	L3MBTL1	cg01877937	-0.354847673237833	36	-2.21312045151396	0.0337035631960435	0.125916871202304	0.107639544560551
**CHOL**	L3MBTL1	cg02177317	-0.131814471783729	36	-0.77536940379141	0.443481181099506	0.0173750549716234	0.635656098259503
**CHOL**	L3MBTL1	cg02472486	-0.13563937592006	36	-0.798284198987352	0.430246431984182	0.0183980402999834	0.624264975562845
**CHOL**	L3MBTL1	cg02611863	-0.285424636520912	36	-1.73653518027542	0.0915235331019974	0.0814672231330948	0.22350520260349
**CHOL**	L3MBTL1	cg03486259	0.174515701547491	36	1.0334516047619	0.308687883054765	0.0304557300866131	0.507300107196536
**CHOL**	L3MBTL1	cg04984575	-0.207573753047441	36	-1.23730178016365	0.224452583025311	0.0430868629541999	0.413681069058046
**CHOL**	L3MBTL1	cg06362543	-0.0615149539118325	36	-0.359371328182835	0.721539733254941	0.00378408955477487	0.842220385146142
**CHOL**	L3MBTL1	cg06446163	-0.356769241350701	36	-2.2268474504936	0.0326859930926966	0.127284291573955	0.105221143203395
**CHOL**	L3MBTL1	cg06908806	0.136611895078328	36	0.804116262736455	0.426916650796617	0.018662809876892	0.621314774732266
**CHOL**	L3MBTL1	cg07678583	-0.201609804339687	36	-1.2002225372407	0.238349124840589	0.0406465132058868	0.430002361212607
**CHOL**	L3MBTL1	cg08369943	-0.239435506840693	36	-1.43796403026677	0.159585508446693	0.0573293619360594	0.329214792675927
**CHOL**	L3MBTL1	cg08633313	-0.315182860580782	36	-1.93651859022721	0.0611515566552068	0.0993402356038849	0.166902578621028
**CHOL**	L3MBTL1	cg09541000	-0.06768512409624	36	-0.395575863894037	0.694889216768775	0.0045812760239234	0.825051689218878
**CHOL**	L3MBTL1	cg10360552	-0.140190848156624	36	-0.825599297312264	0.414787062527192	0.0196534739068736	0.610349261221162
**CHOL**	L3MBTL1	cg12699433	-0.123010877454333	36	-0.722759635355302	0.474770249937003	0.0151316759720848	0.662607914505028
**CHOL**	L3MBTL1	cg14306330	0.00164560929770607	36	0.00959548164506896	0.992400113643199	2.70802996069667e-06	0.995991406089619
**CHOL**	L3MBTL1	cg15123011	0.135272697218541	36	0.79608588844827	0.43150562059974	0.0182987026127791	0.625402169222148
**CHOL**	L3MBTL1	cg15302378	-0.0549601962204849	36	-0.320955369515624	0.750208793218527	0.0030206231685942	0.86005692563356

**CHOL**	L3MBTL1	cg15330298	-0.16701628170916	36	-0.987737529633672	0.330258682646401	0.0278944383559533	0.529997193617339
**CHOL**	L3MBTL1	cg15388309	-0.252773651359445	36	-1.5233821635832	0.13691120226293	0.0638945188215864	0.296665090213425
**CHOL**	L3MBTL1	cg16862791	-0.174203320311197	36	-1.03154378946589	0.30956821082785	0.0303467968074457	0.508215021341567
**CHOL**	L3MBTL1	cg17091610	-0.21403822000984	36	-1.27765587333267	0.210026021497278	0.0458123596249809	0.395621305996737
**CHOL**	L3MBTL1	cg17774347	0.219790981492144	36	1.31371492779859	0.19773804596815	0.0483080755452801	0.380142333074307
**CHOL**	L3MBTL1	cg18911945	-0.0697675328695602	36	-0.407804835460009	0.685973591725504	0.00486750864270516	0.819232781550233
**CHOL**	L3MBTL1	cg20091959	-0.29285689115643	36	-1.78593653698263	0.0830315119742975	0.0857651586978093	0.208406576838372
**CHOL**	L3MBTL1	cg20252111	-0.0394635927809071	36	-0.230289704708538	0.819246671264833	0.00155737515517726	0.902758790196148
**CHOL**	L3MBTL1	cg20529070	-0.23588668695015	36	-1.4153852943444	0.166050588644875	0.0556425290803181	0.338337859953888
**CHOL**	L3MBTL1	cg22330467	-0.09847305001335	35	-0.568447419535643	0.573579476586594	0.00969694157893173	0.740930239303547
**CHOL**	L3MBTL1	cg22457903	-0.300557686212584	36	-1.83749656625855	0.0748892948059013	0.0903349227414622	0.19368144282348
**CHOL**	L3MBTL1	cg22601123	-0.0291512760002441	36	-0.170051958200603	0.865977121250068	0.000849796892442409	0.928790490180813
**CHOL**	L3MBTL1	cg23626798	-0.252649070643385	36	-1.52258015098801	0.137111357890253	0.063831552896966	0.296989393030901
**CHOL**	L3MBTL1	cg24343973	-0.129865823120961	36	-0.763708776448765	0.450308231857424	0.0168651320148846	0.641186135052775
**CHOL**	L3MBTL2	cg02358112	0.123954097611145	36	0.728387740824961	0.471363581187227	0.0153646183145932	0.659627312262025
**CHOL**	L3MBTL2	cg06235991	-0.148432036843724	36	-0.875194941353941	0.387608818019618	0.0220320695615767	0.585496211510809
**CHOL**	L3MBTL2	cg08053598	0.011603092076706	36	0.0676616265854991	0.946451339119757	0.000134631745740518	0.97146409831619
**CHOL**	L3MBTL2	cg09993119	-0.0661419363461441	36	-0.386516834132962	0.701522400739343	0.00437475574361738	0.829040408886651
**CHOL**	L3MBTL2	cg12821546	0.06471837692918	36	0.378162534499886	0.707660715906163	0.00418846831234742	0.833410405310706
**CHOL**	L3MBTL2	cg13843727	0.0859544878914288	36	0.503058270229693	0.618166759819532	0.00738817398867779	0.773294926889443
**CHOL**	L3MBTL2	cg14679202	-0.164987688207843	36	-0.975402554152601	0.336249832861159	0.0272209372601684	0.535776570419699
**CHOL**	L3MBTL2	cg15283028	0.121688713069883	36	0.714873749597214	0.479567215224312	0.0148081428886044	0.666891956424113
**CHOL**	L3MBTL2	cg19467356	-0.0549995853100424	36	-0.321186090743275	0.750035482156052	0.00302495438427663	0.8599976461575
**CHOL**	L3MBTL2	cg24240981	0.107507537094089	36	0.630525633783311	0.532566245295873	0.011557870532037	0.709906512869688
**CHOL**	L3MBTL2	cg26947834	0.133616732831313	36	0.786162193773359	0.437217608594815	0.0178534312925145	0.630148707629781
**CHOL**	L3MBTL2	cg27177954	0.0806070030849344	35	0.464563687261273	0.645294549653088	0.00649748894633463	0.792073656406203
**CHOL**	L3MBTL3	cg03454037	-0.143937299148871	36	-0.848123134819662	0.402300907712373	0.0207179460862717	0.598840396742879
**CHOL**	L3MBTL3	cg07826275	-0.184723828480051	36	-1.09597700729283	0.280791469382358	0.0341228928083274	0.478005417504729
**CHOL**	L3MBTL3	cg08106148	-0.267881757639276	36	-1.6212597746874	0.114200562554703	0.0717606360759077	0.261651604034299
**CHOL**	L3MBTL3	cg08358392	-0.522654415197393	36	-3.57467821810385	0.00107488528954308	0.273167637725329	0.00712687515679827
**CHOL**	L3MBTL3	cg09710740	-0.292694825291694	36	-1.78485557468748	0.0832099400490678	0.0856702607525355	0.208733063814394
**CHOL**	L3MBTL3	cg10237990	-0.22213442783959	36	-1.32844496676802	0.192879416040602	0.0493437040316221	0.373748008355858
**CHOL**	L3MBTL3	cg16487825	-0.135131722763604	36	-0.795240807744565	0.431990274644571	0.0182605824970595	0.625779778144606
**CHOL**	L3MBTL3	cg16800851	-0.364959135849476	36	-2.2857200455433	0.0286267240798586	0.133195170839996	0.0953088835516786
**CHOL**	L3MBTL3	cg18411550	-0.236176834361158	36	-1.4172290389516	0.165515058001751	0.0557794970888578	0.337621628406976
**CHOL**	L3MBTL3	cg18798995	-0.291819100052628	36	-1.77901748634681	0.0841792153835936	0.0851583871555255	0.210546824471957
**CHOL**	L3MBTL3	cg22934935	0.202582478341609	36	1.20626024625999	0.236044021504129	0.0410396605310284	0.427324561331054
**CHOL**	L3MBTL3	cg24516147	-0.225534067059028	36	-1.34985698017224	0.185980881803337	0.0508656154041863	0.364823340329044
**CHOL**	LBR	cg01017344	-0.144626895499352	36	-0.852273046557622	0.400026328673314	0.0209169389017804	0.596669499337057
**CHOL**	LBR	cg05530459	-0.00259694927704915	36	-0.0151427373703254	0.988006816337225	6.74414554756612e-06	0.993665269616916
**CHOL**	LBR	cg12994003	-0.29174897547057	35	-1.7521998586333	0.0890281743160375	0.0851174646881274	0.219150746645274
**CHOL**	LBR	cg14607642	-0.0190714772395481	36	-0.111225095652149	0.91209186445633	0.000363721244098603	0.953306398110749
**CHOL**	LBR	cg18107727	-0.20816264378397	36	-1.24097078860628	0.223111074692588	0.0433316862671321	0.411861993859782
**CHOL**	LBR	cg18289048	-0.0904996346625072	36	-0.529873357549453	0.599643183175806	0.00819018387404728	0.759811740239086
**CHOL**	LBR	cg18764107	-0.139031285401614	36	-0.818635332190821	0.418695508864587	0.019329698320425	0.614148850151061
**CHOL**	LBR	cg19322477	-0.242456044834787	36	-1.45722978554987	0.154227721903366	0.0587849336769284	0.321665012493242
**CHOL**	LBR	cg20483690	-0.451618140947658	36	-2.95150215031219	0.00569451284732972	0.203958945233019	0.0275224240386528
**CHOL**	LBR	cg26930584	-0.336632081958409	31	-1.92517994747886	0.0640650019951508	0.113321158603653	0.172718697301638
**CHOL**	MBD1	cg01719254	-0.277158926710409	36	-1.6819940173786	0.101733985524205	0.076817070655266	0.241199578076389
**CHOL**	MBD1	cg03841029	-0.261766097031398	36	-1.58148994908879	0.123025312451146	0.0685214895550513	0.275413846239553
**CHOL**	MBD1	cg05549078	-0.0687792659477171	36	-0.402000567705766	0.690199641819381	0.0047305874243068	0.821973308022809
**CHOL**	MBD1	cg12090413	-0.0236795897681198	36	-0.138113275900368	0.890965639701662	0.000560722971586443	0.942350731114736
**CHOL**	MBD1	cg13478031	0.0810366096018776	36	0.4740797610975	0.638473520123553	0.00656693209576711	0.787378476023139
**CHOL**	MBD1	cg14389183	-0.169650544859899	36	-1.00377459810197	0.322577959870932	0.0287813073712607	0.52198917961933
**CHOL**	MBD1	cg19328682	-0.39948546073208	35	-2.50329475207158	0.0174305085334624	0.159588633336322	0.0655193747995832
**CHOL**	MBD1	cg21282183	-0.091343862359679	36	-0.534857687612407	0.596229180886669	0.00834370119078398	0.757445763147444
**CHOL**	MBD2	cg15090185	0.0415139739809034	35	0.238685389330357	0.812825612399606	0.00172341003568712	0.899115368178028

**CHOL**	MBD2	cg16852837	-0.380892726912291	36	-2.402034353756	0.0219138008620708	0.145079269414681	0.078035933936644
**CHOL**	MBD2	cg17987217	-0.264458340349284	36	-1.59897188036966	0.119079838646801	0.0699382137802979	0.269309174012711
**CHOL**	MBD2	cg22224310	-0.387286814478429	36	-2.44940402607295	0.0196161267830198	0.149991076668849	0.0717517851682452
**CHOL**	MBD2	cg22836370	-0.118825466057248	36	-0.697809446973061	0.490041355488939	0.0141194913837221	0.675986828777489
**CHOL**	MBD2	cg23388714	-0.396534030906865	36	-2.51864970410706	0.0166511650147488	0.157239237667246	0.0632099122216059
**CHOL**	MBD2	cg24881765	-0.380526693432787	36	-2.39933496577429	0.0220518247364076	0.144800564414891	0.07840492018026
**CHOL**	MBD2	cg25192916	0.428517329036777	36	2.76543565750451	0.0091204582880014	0.183627101284813	0.0397376670056234
**CHOL**	MBD3	cg08660999	-0.133389008669626	36	-0.784798038064718	0.438006344801788	0.0177926276338655	0.630829687818389
**CHOL**	MBD3	cg09174855	-0.100034042317612	33	-0.559773792757158	0.579656409560423	0.0100068096224018	0.745610719842318
**CHOL**	MBD3	cg12286540	-0.0938649181736923	36	-0.549748996077323	0.58608492515911	0.00881062286375394	0.750320286227184
**CHOL**	MBD5	cg03890220	0.32589461803146	36	2.01001012819361	0.0524182000849176	0.106207302061871	0.149337927832934
**CHOL**	MBD5	cg04152151	0.224014456672224	36	1.34027962645589	0.189042615987303	0.0501824767981516	0.36880282044629
**CHOL**	MBD5	cg05218490	0.030077140959668	36	0.175457742582525	0.86176076693461	0.000904634408307736	0.92658151916918
**CHOL**	MBD5	cg06394585	0.0925691540369407	36	0.54209389559937	0.591289290324395	0.00856904827911485	0.75400787524904
**CHOL**	MBD5	cg06758980	0.353186575485937	36	2.2012800290183	0.0346039112953405	0.124740757103483	0.109899027365165
**CHOL**	MBD5	cg20595490	0.313352525092094	36	1.92404438725389	0.0627527263261249	0.0981898049815912	0.170126697745759
**CHOL**	MBD5	cg21412502	-0.103327827831819	36	-0.605741915167244	0.548708842186476	0.010676640004442	0.721880803440687
**CHOL**	MBD5	cg23120335	-0.356711467290763	36	-2.22643426242537	0.0327162168414806	0.127243070896729	0.1052966518955
**CHOL**	MBD5	cg25686162	0.113093927061896	36	0.663703369182692	0.511355882210917	0.0127902363382815	0.693558065943863
**CHOL**	MBD5	cg26664634	-0.181009851539136	36	-1.07318741623397	0.29074518496722	0.03276456635422	0.488338547569963
**CHOL**	MBTD1	cg01388767	-0.0397132089221926	36	-0.231748632826834	0.818122427682771	0.00157713896289772	0.902165910116911
**CHOL**	MBTD1	cg14577476	0.0985626263375083	36	0.577525993475099	0.567389344481735	0.00971459131054727	0.736050671434967
**CHOL**	MBTD1	cg17045523	-0.176612669805688	36	-1.04626684659994	0.302819350554338	0.031192035135893	0.501171560230847
**CHOL**	MBTD1	cg18711617	-0.279216186671035	36	-1.69553033576649	0.0991153018640317	0.0779616788991141	0.236774936072792
**CHOL**	MBTD1	cg22141797	-0.0185525671680669	36	-0.108197748996262	0.914474837084204	0.000344197748525633	0.954856000377055
**CHOL**	MBTD1	cg25259117	0.0367251862381028	36	0.214287351929676	0.831602993631681	0.00134873930422333	0.909713206794132
**CHOL**	MBTD1	cg25716740	-0.0770920376135264	36	-0.450861736108471	0.654951549528129	0.00594318226340538	0.798822583509575
**CHOL**	MBTD1	cg25855326	0.0682682035180825	36	0.398999474535978	0.69238869306947	0.00466054761158634	0.823377143731792
**CHOL**	MBTD1	cg27207041	-0.0404175267824303	36	-0.235865385191085	0.81495219019372	0.00163357647120847	0.900481367572335
**CHOL**	MBTD1	cg27214721	0.0322863208496968	36	0.188358182231546	0.851715639476365	0.00104240651400957	0.92090739909414
**CHOL**	MBTD1	cg27353579	-0.0841924377617405	36	-0.492671277100721	0.625411608998963	0.00708836657626455	0.77831534786622
**CHOL**	MLLT10	cg00879184	-0.0847396606505322	36	-0.495896562187901	0.623157902363194	0.00718081008716735	0.776728478832505
**CHOL**	MLLT10	cg03524461	0.0365508569192638	36	0.213268795862279	0.832390988197742	0.00133596514153249	0.910049949419272
**CHOL**	MLLT10	cg04188362	-0.127720917994568	36	-0.750884159903092	0.457888129369671	0.0163126328933751	0.647971715863864
**CHOL**	MLLT10	cg05913198	0.0374445247447097	36	0.21849044814176	0.828353193794881	0.00140209243335718	0.90773727952846
**CHOL**	MLLT10	cg06398735	0.166111087609857	36	0.982231862942378	0.332923836256431	0.0275928934269295	0.532594714327927
**CHOL**	MLLT10	cg11443787	-0.26144577184258	36	-1.57941258472954	0.123501160769545	0.0683538916143625	0.276240635448706
**CHOL**	MLLT10	cg15910013	-0.0165055687169227	36	-0.0962562898119208	0.923882165398008	0.000272433798669057	0.959938420497791
**CHOL**	MLLT10	cg19119538	-0.0571938725232503	36	-0.334041515013162	0.740399930230988	0.00327113905420581	0.853908234954554
**CHOL**	MLLT10	cg20569108	-0.304899491693671	36	-1.86673996267699	0.0705813095978056	0.0929637000350587	0.185487548099848
**CHOL**	MLLT10	cg23344688	-0.138001172990896	36	-0.812451685703541	0.422184920588716	0.0190443237468632	0.617280148438343
**CHOL**	MLLT10	cg26877631	-0.301247569602538	35	-1.81484228816323	0.0786471176943285	0.090750098191436	0.200441419011682
**CHOL**	MLLT10	cg27470303	-0.124860251756156	36	-0.733796565263151	0.468102952821014	0.0155900824686106	0.657131534426122
**CHOL**	MLLT10	cg27572529	-0.0867367309080642	36	-0.507670981270207	0.6149617644917	0.00752326048861794	0.770921997983552
**CHOL**	MLLT6	cg05652455	-0.283688337463583	36	-1.72504382083715	0.0936002208433515	0.0804790728128515	0.227195309194112
**CHOL**	MORF4L1	cg03589001	0.0527473014916118	36	0.307995740881307	0.759964588537216	0.00278227781464699	0.86647564615777
**CHOL**	MORF4L1	cg07757959	-0.310629220487359	36	-1.90552831591937	0.0651961214727053	0.0964905126205845	0.174994551076411
**CHOL**	MORF4L1	cg08692282	-0.0593826710165523	36	-0.346869621648451	0.730827078797054	0.00352630161706009	0.847864034448555
**CHOL**	MORF4L1	cg13747872	-0.0495454634674382	36	-0.289252453884148	0.774144685344885	0.00245475295020326	0.87490101912864
**CHOL**	MORF4L1	cg15822222	0.0271589521294709	36	0.15842098050449	0.875062307401669	0.000737608680770893	0.933880369017354
**CHOL**	MORF4L1	cg20775702	-0.178715652687155	36	-1.05913361456904	0.29700564349968	0.0319392845153957	0.495056072814837
**CHOL**	MORF4L1	cg20905746	-0.278788573590773	36	-1.69271465976272	0.0996553354057982	0.0777230687647777	0.237670360883743
**CHOL**	MORF4L1	cg23623458	-0.219651592281831	12	-0.711987203310708	0.492748623460696	0.0482468219919436	0.678170094358173
**CHOL**	MORF4L1	cg24795825	0.304656593368286	24	1.50028630446367	0.147755936090189	0.0928156398827691	0.312583909024386
**CHOL**	MPHOSPH	cg00331616	-0.0916499447413299	36	-0.536665088187194	0.594993496250387	0.00839971237108883	0.756458253073308
**CHOL**	MPHOSPH	cg03134886	-0.170787317600599	36	-1.01070194967887	0.319298136326815	0.0291683078532078	0.518641271258764
**CHOL**	MPHOSPH	cg03428000	0.23102385273392	36	1.38454352316301	0.175211974541498	0.0533720205320239	0.350802737034648

**CHOL**	MPHOSF	cg04203901	-0.1050373456667	36	-0.615874556557769	0.542078660460833	0.0110328439847059	0.716840537060714
**CHOL**	MPHOSF	cg06019692	-0.308083887426739	36	-1.8882692638171	0.0675469958339784	0.0949156816919714	0.179639473267106
**CHOL**	MPHOSF	cg09559995	-0.155130724684001	36	-0.915644631900991	0.366302211917597	0.0240655417409832	0.565208447030119
**CHOL**	MPHOSF	cg11051893	0.0317413956433	36	0.185175858417783	0.854191339789631	0.00100751619738451	0.92216741958416
**CHOL**	MPHOSF	cg19693579	0.0305388389240037	36	0.178153594932343	0.859659609294809	0.000932620682826245	0.925419417782642
**CHOL**	MPHOSF	cg20759595	-0.197871562460929	36	-1.17705229235549	0.247349199559792	0.0391531552307295	0.440639223810615
**CHOL**	MPHOSF	cg20952532	-0.18740016909695	36	-1.11242955821007	0.273757381063592	0.0351188233775654	0.470156560091421
**CHOL**	MPHOSF	cg21958444	-0.359821457149517	36	-2.248718095996	0.0311214287100033	0.129471481025202	0.101422854445326
**CHOL**	MPHOSF	cg22656705	-0.382837685400718	36	-2.41640005838636	0.0211923786745842	0.146564693362979	0.0761184980750348
**CHOL**	MSH6	cg00620552	-0.207395253745008	36	-1.23618994094096	0.224860294026135	0.0430127912759563	0.414147341027185
**CHOL**	MSH6	cg02905881	-0.0985902590045255	36	-0.577689495631167	0.56728018627072	0.00972003917057942	0.735982702557359
**CHOL**	MSH6	cg05039065	0.088317325235295	36	0.516994286805476	0.608507198708101	0.00779994993671687	0.766263216942892
**CHOL**	MSH6	cg07652213	-0.0221082118218202	36	-0.128943435502415	0.898162165654007	0.000488773029958469	0.946117945337319
**CHOL**	MSH6	cg09898070	-0.00073206173707055	36	-0.0042686179167223	0.996619093932166	5.35914386882763e-07	0.998316892964834
**CHOL**	MSH6	cg11869233	-0.30682880455208	35	-1.85192542871103	0.0730010119734594	0.0941439153028584	0.190150697296702
**CHOL**	MSH6	cg16595246	-0.291581724574321	36	-1.77743584818366	0.084443446007315	0.0850199021057351	0.211017745613474
**CHOL**	MSH6	cg18222961	-0.0249388412976794	36	-0.145462425828332	0.885204734198995	0.000621945805270839	0.939168465796141
**CHOL**	MSH6	cg19076255	-0.0820776275967816	36	-0.480210959045341	0.634152660370185	0.00673673695191596	0.784539555148944
**CHOL**	MSH6	cg19736286	-0.0447283773703751	36	-0.261070299771263	0.795613201803743	0.00200062774218668	0.888162451139064
**CHOL**	MSH6	cg21405109	-0.28258842988589	36	-1.71777385249782	0.0949343111901088	0.0798562207053726	0.22945039316256
**CHOL**	MTA1	cg01318265	-0.529011825153779	36	-3.63491629855935	0.000909263554713252	0.279853511152532	0.00620307545599195
**CHOL**	MTA1	cg01655777	-0.377205785757921	36	-2.37490422502893	0.0233373773249995	0.142284204809251	0.0818183141751083
**CHOL**	MTA1	cg05922925	-0.323969361936978	36	-1.99673873086318	0.0539097543984775	0.104956147473853	0.152428897745685
**CHOL**	MTA1	cg13052453	-0.337173836613268	36	-2.08833212617611	0.044329223671549	0.113686196096511	0.132118137549019
**CHOL**	MTA1	cg13981078	-0.371904175445776	36	-2.33612281435748	0.0255180387949187	0.138312715714002	0.0875186319829829
**CHOL**	MTA1	cg16531924	-0.375749003007387	36	-2.3642209953876	0.0239205909731103	0.141187313261045	0.0833882556870832
**CHOL**	MTA1	cg20222911	-0.326501094577522	25	-1.65663299194989	0.111174304258419	0.10660296476032	0.25685120650628
**CHOL**	MTA1	cg21517792	0.0911258838385979	36	0.533570628989156	0.597109861732485	0.00830392670536565	0.758108213791016
**CHOL**	MTA1	cg23123838	-0.359545559359382	36	-2.24673775985901	0.0312602817251052	0.129273009255051	0.101794154730968
**CHOL**	MTA1	cg23883989	-0.344290772206985	35	-2.10659037809333	0.0428397598438615	0.118536135826882	0.12874435128229
**CHOL**	MTA1	cg23966123	-0.377579048523039	36	-2.37764482723758	0.0231898558731794	0.142565937883564	0.0814534787700071
**CHOL**	MTA2	cg00501779	-0.263343424496438	36	-1.59172739269909	0.120702092144771	0.0693497592255112	0.271750096359554
**CHOL**	MTA2	cg07654871	-0.230853306033157	36	-1.38346386401407	0.175539683655155	0.0532932489064384	0.351256706886796
**CHOL**	MTA2	cg09086761	0.0258571422271728	36	0.150822180131869	0.881007265998919	0.000668591804156241	0.936887651408261
**CHOL**	MTA2	cg13708959	-0.303633608741541	36	-1.85820072122016	0.0718167737331942	0.0921933683574111	0.187801744455279
**CHOL**	MTA2	cg19570317	-0.474375408311803	36	-3.14209816029234	0.0034667635498227	0.22503202801099	0.0185051052281396
**CHOL**	MTA2	cg21002970	-0.0422872616399246	36	-0.24679574874396	0.806550394269853	0.00178821249700344	0.895130504838216
**CHOL**	MTF2	cg05396182	-0.06723110475412	36	-0.392910324904216	0.696838468532828	0.00452002144645946	0.826191873999401
**CHOL**	MTF2	cg05962718	-0.377324420425476	36	-2.37577512837117	0.0232904062774819	0.142373718249422	0.0817217926999425
**CHOL**	MTF2	cg09936581	-0.0791230058996714	36	-0.462813425866782	0.64644672454058	0.00626045006259944	0.792699933621946
**CHOL**	MTF2	cg11404133	-0.183772837556343	36	-1.09013698153923	0.283318875729343	0.0337724558235098	0.480656604917424
**CHOL**	MTF2	cg14474880	-0.361984247242913	36	-2.26426569515344	0.0300503634207074	0.131032595252018	0.0988222003699065
**CHOL**	MTF2	cg15463775	-0.328323284314467	36	-2.0267916322084	0.0505838354102715	0.107796179023038	0.145561844044783
**CHOL**	MTF2	cg16813892	0.0667480701635148	36	0.390074707090745	0.698914392782174	0.00445530487055349	0.827305522924126
**CHOL**	MTF2	cg19708337	-0.0649477396847409	36	-0.379508414537413	0.706670476385887	0.00421820889015687	0.832723242751427
**CHOL**	MTF2	cg22801127	-0.0370616812233094	36	-0.216253450823886	0.830082443789548	0.0013735682150982	0.908732407820944
**CHOL**	MTF2	cg23992808	-0.0660899300522862	36	-0.386211588636956	0.701746324892189	0.00436787885431608	0.829168936324412
**CHOL**	MTF2	cg26054395	-0.417902247012501	36	-2.68221173352134	0.0112070593264197	0.174642288058097	0.04659764688613
**CHOL**	NAP1L2	cg02379437	0.0146690617765682	31	0.0790038157649607	0.937572229907624	0.000215181373404773	0.966584424037341
**CHOL**	NAP1L2	cg04297329	-0.2912207055483	36	-1.77503105811844	0.0848465363369362	0.0848094993400498	0.211820912287655
**CHOL**	NAP1L2	cg07693661	-0.0904371997402613	31	-0.489023163828831	0.628503523175007	0.00817888709685993	0.780627089193184
**CHOL**	NAP1L2	cg20001978	-0.0914860208279752	34	-0.519702530651299	0.606847557131612	0.00836969200693672	0.765102164554343
**CHOL**	NAP1L2	cg23483095	-0.285420395969086	35	-1.71077963912403	0.0965083955020455	0.0814648024351497	0.232166637016735
**CHOL**	NCOA1	cg11380327	-0.0170207014100399	36	-0.0992612703872368	0.92151377812823	0.000289704276489735	0.958847627308334
**CHOL**	NCOA1	cg13911252	-0.169992543013874	36	-1.00585825696053	0.321589025257755	0.0288974646803237	0.521079748464631
**CHOL**	NCOA1	cg15477048	0.105819133201517	36	0.620510195755069	0.53905935555539	0.0111976889515205	0.714575597349897
**CHOL**	NCOA1	cg23006163	0.0833666798774609	24	0.39239032361642	0.698545616516654	0.00695000331379104	0.827114225505579

**CHOL**	NCOA3	cg04175879	0.155377663203182	36	0.917138192694256	0.365530361687259	0.0241422182224814	0.564442901945228
**CHOL**	NCOA3	cg09568061	-0.118438122480635	36	-0.695502333168994	0.491467276818511	0.0140275888567379	0.677102708352128
**CHOL**	NCOA3	cg12128876	0.366370904690464	36	2.29592965201473	0.0279707893742237	0.134227639803709	0.0937271971332255
**CHOL**	NCOA3	cg14341558	0.549573076894418	36	3.83571830945182	0.000517189109085716	0.302030566847198	0.00385817164187275
**CHOL**	NCOA3	cg14774045	0.239912715423263	36	1.44100482256401	0.158730221271695	0.0575581110217637	0.327957580835287
**CHOL**	NCOA3	cg18295930	0.102983427071269	36	0.603701245265791	0.550049174993149	0.0106055862513434	0.722764095659365
**CHOL**	NCOA3	cg21735648	0.215696142734215	36	1.28803345213712	0.206431818688201	0.0465248259904189	0.39098108056264
**CHOL**	NCOA3	cg22511723	0.168328836124507	36	0.995725454200479	0.326417646304367	0.0283345970710312	0.525873542655315
**CHOL**	NCOA3	cg22876277	0.29539211077128	36	1.80286818987619	0.0802787009266778	0.0872564991059124	0.203532826876873
**CHOL**	NCOA3	cg24856420	0.222311694128657	36	1.32956017844977	0.19251533222177	0.0494224893463537	0.373237744974839
**CHOL**	NCOA3	cg25373408	0.224113726227655	36	1.34090495826914	0.188841529516307	0.0502269622836444	0.368586144145109
**CHOL**	NCOR1	cg00253204	-0.138951242994273	36	-0.818154749998379	0.41896606288648	0.0193074479296534	0.61438685830893
**CHOL**	NCOR1	cg08125503	-0.16021801195072	36	-0.946450091573389	0.35059741833833	0.0256698113534409	0.549604843153506
**CHOL**	NCOR1	cg08206308	-0.32202914070225	36	-1.98339221361963	0.0554469315497505	0.103702767461429	0.155506474618981
**CHOL**	NCOR1	cg21211144	0.245029895038784	36	1.47368195347466	0.14976628683804	0.0600396494627175	0.315305648601572
**CHOL**	NCOR1	cg22291359	-0.171529566540132	36	-1.01522736374359	0.317167884534741	0.0294223921974455	0.516381867847633
**CHOL**	NCOR1	cg22625220	-0.21295957071879	36	-1.27091041103114	0.212387515038646	0.0453517787607315	0.398637534367479
**CHOL**	NCOR2	cg08976554	-0.114514865885257	36	-0.672152405536439	0.506028944680965	0.0131136545087185	0.689083621186618
**CHOL**	NCOR2	cg10641483	-0.0995136924867634	36	-0.58315421887155	0.563637855340901	0.00990297499235011	0.733493685176007
**CHOL**	NCOR2	cg13416396	0.0839525201681804	36	0.491257364982267	0.626400753072046	0.00704802564258873	0.779010457916148
**CHOL**	NCOR2	cg13419087	-0.0671307673828812	36	-0.392321279230934	0.697269507861548	0.00450653992941451	0.826414737032182
**CHOL**	NCOR2	cg13857025	0.0122822159118019	36	0.0716224125693024	0.943322060731212	0.00015085282770412	0.969774769961464
**CHOL**	NCOR2	cg16181859	-0.104656062224695	36	-0.613614141179696	0.543554114071233	0.0109528913603793	0.717854999576563
**CHOL**	NCOR2	cg19895345	-0.0760871428812638	36	-0.444950304473129	0.659175567925718	0.00578925331183385	0.801686217113268
**CHOL**	NCOR2	cg23611643	0.0429646664022925	36	0.250756453389755	0.803511626386699	0.00184596255906028	0.892964149985384
**CHOL**	NCOR2	cg24626542	-0.0743415503770532	36	-0.434684847275173	0.666537743718806	0.00552666611246394	0.806498213957701
**CHOL**	NCOR2	cg25113767	-0.0855502636761007	36	-0.500675018860023	0.619825670300993	0.00731884761505035	0.774521042331559
**CHOL**	NCOR2	cg25449924	0.128009525921122	36	0.752609149588633	0.456864252256792	0.0163864387265505	0.647112355434261
**CHOL**	ORC1	cg00298020	-0.107180643130205	36	-0.628586104455683	0.53382042661348	0.0114876902618043	0.710816395391696
**CHOL**	ORC1	cg02275620	0.0645525065736974	36	0.377189259673224	0.708377130917593	0.00416702610494724	0.833812327285156
**CHOL**	ORC1	cg04407474	-0.167310323280535	36	-0.989526532278905	0.329395785551997	0.027992744276237	0.529087565609454
**CHOL**	ORC1	cg07142859	-0.0546729788381072	36	-0.319273041722281	0.751472906478863	0.00298913461503212	0.860808435595787
**CHOL**	ORC1	cg08613513	0.224483823193069	36	1.3432367091748	0.18809316620443	0.0503929868753773	0.367564961724073
**CHOL**	ORC1	cg10586599	0.0860453809300934	36	0.503594197729982	0.61779399606975	0.00740380757940489	0.772993858506782
**CHOL**	ORC1	cg13460942	0.155512002850953	36	0.917950794546235	0.365110866821137	0.0241839830307147	0.564086246568894
**CHOL**	ORC1	cg16136434	0.293284006969568	36	1.78878616151154	0.0825626924730839	0.0860155087441257	0.207598244877051
**CHOL**	ORC1	cg16965475	-0.0394070911296552	36	-0.229959476267292	0.819501197821723	0.00155291883130095	0.902815161705527
**CHOL**	ORC1	cg17146111	-0.323134601049412	36	-1.99099309008138	0.0545668926754752	0.104415970395363	0.153814655003838
**CHOL**	ORC1	cg19776520	-0.180899516597402	36	-1.07251111406027	0.291044313838757	0.0327246351051738	0.48860967786937
**CHOL**	ORC1	cg21197365	-0.172033241110175	36	-1.01829922877844	0.315727422175692	0.0295954360468717	0.514941907013622
**CHOL**	ORC1	cg23606758	0.129632248622091	36	0.7623116839887	0.451130353209651	0.0168045198828196	0.641777936511774
**CHOL**	ORC1	cg25605769	-0.238586573035102	36	-1.43255732068901	0.16111524747499	0.0569235528326342	0.331248997358988
**CHOL**	PADI1	cg14524955	-0.411813719734878	36	-2.63508194712865	0.012577060024824	0.169590539761876	0.0510125030016244
**CHOL**	PADI1	cg21010178	-0.274858997435821	36	-1.66689064235146	0.104723472354125	0.0755474684714246	0.246185814382733
**CHOL**	PADI1	cg22193702	-0.556283824909835	36	-3.9033622572493	0.000426788425098373	0.309451693856316	0.00327916827082497
**CHOL**	PADI2	cg00569276	0.076688721092596	36	0.448489006124491	0.656645610205133	0.00588115994281798	0.79992309114153
**CHOL**	PADI2	cg01603374	0.147805567548411	36	0.871418432739576	0.389637622269544	0.021846485798308	0.587294993157346
**CHOL**	PADI2	cg07014827	0.208590118663368	28	1.08752725884256	0.286783099684129	0.0435098376039979	0.484168589961308
**CHOL**	PADI2	cg09313801	-0.0919691807466704	36	-0.538550323666207	0.593705900431373	0.00845833020721374	0.755584646765428
**CHOL**	PADI2	cg10233913	0.161426088005409	36	0.953776721532438	0.34692885395849	0.0260583818887302	0.546245223942841
**CHOL**	PADI2	cg15185794	-0.0487239865640636	36	-0.284445062520299	0.777794624478826	0.00237402686669505	0.877101948463541
**CHOL**	PADI2	cg17183414	0.0274363911794993	36	0.16004052417305	0.873796188970033	0.000752755560954506	0.933173641270863
**CHOL**	PADI2	cg18228326	-0.00014721549020503	36	-0.0008584064508634	0.999320107739589	2.16724005563073e-08	0.999676909126047
**CHOL**	PADI2	cg18879300	0.1221709120238	36	0.717749315698802	0.477814822035627	0.0149257317447271	0.665445608862305
**CHOL**	PADI2	cg19282714	0.0966548890290712	36	0.566241174740398	0.574948663326658	0.00934216757322208	0.742006330918823
**CHOL**	PADI3	cg01459162	0.369504771914023	36	2.31865873519164	0.0265588695862714	0.136533776467234	0.0901448822248865
**CHOL**	PADI3	cg12858514	-0.322210954430372	36	-1.98464169737023	0.055301421745231	0.103819899154931	0.1552359108018

**CHOL**	PADI3	cg14664242	-0.196214216467812	36	-1.16679696003016	0.251411136009401	0.0385000187440775	0.445103159115981
**CHOL**	PADI3	cg17589397	-0.312673222076495	36	-1.91942082542748	0.0633553305588805	0.0977645438036969	0.171282870043355
**CHOL**	PADI3	cg18768338	-0.247190125179368	36	-1.48751581085557	0.146094993113861	0.0611029579861915	0.310120985982856
**CHOL**	PADI3	cg21966975	-0.270001054210161	36	-1.63509037165051	0.111256261474353	0.0729005692745983	0.256964242180863
**CHOL**	PADI4	cg16091981	-0.419072309253706	36	-2.6913186553907	0.0109588286731701	0.175621600383234	0.0457956122583044
**CHOL**	PADI4	cg18333690	-0.473826753085803	36	-3.13741116285568	0.00350988174523867	0.224511791939835	0.0186852624710256
**CHOL**	PADI4	cg19159961	-0.181687960135863	33	-1.0287174814937	0.311572555211334	0.0330105148583309	0.510406975665035
**CHOL**	PADI4	cg21295838	-0.439120884562988	36	-2.84996993582775	0.00737620861982201	0.192827151259381	0.0337405052843202
**CHOL**	PADI4	cg23228178	-0.0576991629413003	36	-0.33700248438356	0.738186538997555	0.00332919340412672	0.852556245951124
**CHOL**	PADI4	cg26918754	-0.356949556654235	36	-2.22813721638486	0.0325918102725727	0.127412985995655	0.104978771461764
**CHOL**	PADI6	cg12643633	0.0612644159554125	36	0.357902155939698	0.722628972051369	0.0037533286623578	0.842839513751129
**CHOL**	PADI6	cg16017904	-0.224809284224077	36	-1.34528774257475	0.18743678905629	0.0505392142733417	0.366764533738588
**CHOL**	PARP1	cg00653565	-0.161274357812777	36	-0.95285628106585	0.347388327111632	0.0260094184879237	0.546669562963808
**CHOL**	PARP1	cg02088626	-0.121817634551352	36	-0.715642515128696	0.479098364861996	0.0148395360876867	0.666481495584459
**CHOL**	PARP1	cg03149313	0.240345769419878	36	1.44376521928971	0.157956943251047	0.0577660888780332	0.326932965552917
**CHOL**	PARP1	cg04960271	0.204898638480505	36	1.22065244601745	0.230615819378798	0.0419834520511647	0.420941507572834
**CHOL**	PARP1	cg07113065	-0.0177049341455912	36	-0.10325280362695	0.918368963206877	0.000313464693099723	0.956976694729708
**CHOL**	PARP1	cg17661881	-0.210530425209482	36	-1.25573723673739	0.217772557903108	0.0443230599388854	0.405316004821149
**CHOL**	PARP1	cg25722198	0.0431117534640853	36	0.251616499445736	0.802852182036463	0.00185862328674807	0.892511791385196
**CHOL**	PARP1	cg26464410	-0.0504087956113015	36	-0.294305422034165	0.770313911205753	0.00254104667498197	0.872714304143832
**CHOL**	PARP2	cg01813761	-0.355827279396776	12	-1.20402607280596	0.256304778011604	0.126613052762912	0.450571422763612
**CHOL**	PARP2	cg10910545	-0.343723080710052	36	-2.13427167305028	0.04011476352643	0.118145556212809	0.122729067179122
**CHOL**	PARP2	cg15078479	-0.0981212746989559	36	-0.574914703752709	0.569134130901181	0.00962778454854796	0.737426567386395
**CHOL**	PARP2	cg15365367	0.125177005313845	36	0.735687703105011	0.466965994776265	0.0156692826593425	0.656099099979281
**CHOL**	PARP2	cg18899035	0.062745031059464	5	0.10889214396533	0.920162996203211	0.0039369389226531	0.958012449101232
**CHOL**	PARP2	cg19540080	0.000717289444176271	36	0.00418248131962429	0.99668731694566	5.14504146726704e-07	0.998316892964834
**CHOL**	PARP2	cg24595957	0.0706960889955359	36	0.413259510190009	0.682011404524503	0.00499793699926473	0.816654757294874
**CHOL**	PARP2	cg26206608	0.00826318896588613	36	0.0481839023916396	0.961851612178166	6.82802918859422e-05	0.980001978725799
**CHOL**	PARP2	cg26430074	0.0356329171006016	36	0.20790585694046	0.836542850261528	0.00126970478109835	0.912470246835634
**CHOL**	PAXIP1	cg02824291	0.0387737536468885	36	0.226258034884059	0.822355470305658	0.0015034039718696	0.904567103788803
**CHOL**	PAXIP1	cg12220104	0.128624271226942	36	0.756284088492753	0.454687458629171	0.0165442031486618	0.644976434497521
**CHOL**	PAXIP1	cg22583711	-0.151632349973355	36	-0.894504122381596	0.377341031526179	0.022992369558442	0.575890534332835
**CHOL**	PAXIP1	cg24448952	-0.129095053318204	36	-0.759099016471112	0.453024215396451	0.0166655327912301	0.643400651250128
**CHOL**	PAXIP1	cg24452135	0.505309330067862	36	3.41442076721279	0.0016696453834849	0.255337519053632	0.0102654226636232
**CHOL**	PCGF1	cg03203239	-0.148647056362645	36	-0.876491379181755	0.386913903353633	0.0220959473652793	0.584862386225722
**CHOL**	PCGF1	cg08092637	-0.0850710358042761	36	-0.49784987863132	0.621794787646155	0.00723708113281243	0.775961752224085
**CHOL**	PCGF1	cg27306986	0.0620175357850844	36	0.362318709601739	0.719356335713715	0.00384617474485422	0.840832496138723
**CHOL**	PCGF2	cg09395368	0.0944583224496698	36	0.553255641534638	0.583708346071382	0.00892237468000579	0.748644086625622
**CHOL**	PCGF2	cg17902383	-0.0581066086031225	36	-0.339390278913043	0.736403257701808	0.00337637796335647	0.851356145484536
**CHOL**	PCGF2	cg20228512	-0.234301395901353	36	-1.40531860777841	0.168998578183257	0.0548971441213226	0.342318267749527
**CHOL**	PCGF2	cg25377665	-0.0769841470496542	36	-0.450226989102817	0.655404559277375	0.00592655889696279	0.799084817300086
**CHOL**	PCGF5	cg03905463	0.262884537586578	36	1.58874763564721	0.12137457034658	0.069108280102109	0.272809607510382
**CHOL**	PCGF5	cg06001881	-0.135020433642085	36	-0.794573712486208	0.43237308654191	0.0182305175008968	0.626109815585199
**CHOL**	PCGF5	cg07421809	-0.0622488093951574	36	-0.363675101912361	0.71835233260408	0.00387491427111464	0.840088112965857
**CHOL**	PCGF5	cg07424295	-0.0246579184583007	36	-0.143822866087459	0.886489426586952	0.000608012942696208	0.939769349504882
**CHOL**	PCGF5	cg08633154	0.0244394780280786	36	0.142547998186078	0.88748857685707	0.000597288086284936	0.940239916840677
**CHOL**	PCGF5	cg11113644	-0.0472789701148871	36	-0.275990033346975	0.784226372315751	0.00223530101512439	0.880983486035112
**CHOL**	PCGF5	cg12783297	0.117560915752457	36	0.690278662364198	0.494704371113729	0.0138205689125562	0.679801776141554
**CHOL**	PCGF5	cg15550435	-0.00157759179689568	36	-0.0091988733244674	0.992714229064492	2.48879587763255e-06	0.996210197699244
**CHOL**	PCGF5	cg17001035	0.0976553265610949	36	0.5721582586789	0.57097880515204	0.00953656280575409	0.738957331026415
**CHOL**	PCGF5	cg20515900	-0.0920702685824152	36	-0.539147328726203	0.593298430200637	0.00847693435683807	0.755389569385581
**CHOL**	PCGF5	cg22968329	0.0498938067288659	36	0.291291181773577	0.772598380161009	0.00248939194989742	0.873915207360894
**CHOL**	PCGF5	cg23395902	-0.0567703275147147	36	-0.331559766999845	0.742256824672928	0.00322287008612797	0.855033808038126
**CHOL**	PCGF5	cg25390656	-0.0372594496660349	36	-0.217409021884226	0.829189053043007	0.00138826658941579	0.908212836738775
**CHOL**	PCGF6	cg01917164	-0.0828239562499453	35	-0.477427754573461	0.636204599886158	0.00685980772889285	0.785915079851851
**CHOL**	PCGF6	cg06691148	0.0112206141905749	36	0.0654309806571593	0.948214077626989	0.00012590218281373	0.972477897445242
**CHOL**	PCGF6	cg11612727	-0.158316466024737	36	-0.93492659692892	0.356419221629864	0.0250641034145617	0.555234028745117

**CHOL**	PCGF6	cg11814370	0.190357327861909	36	1.13063828867771	0.266120159979514	0.0362359122707264	0.461924934746001
**CHOL**	PCGF6	cg14409890	-0.217923664355477	36	-1.30199478413774	0.201670263978228	0.0474907234861185	0.385009117178881
**CHOL**	PCGF6	cg16113748	0.107824313227467	36	0.632405329310542	0.531352243233815	0.0116260825229748	0.709083393260681
**CHOL**	PCGF6	cg16382383	0.19800479110499	36	1.17787713991073	0.247024590781843	0.0392058973005306	0.440248597139375
**CHOL**	PCGF6	cg24418712	0.163511593221283	36	0.966435085332567	0.340650957602633	0.0267360411177623	0.539947937697495
**CHOL**	PCGF6	cg25481680	0.218147791003718	36	1.30340071042572	0.201195441622316	0.0475884587198017	0.384439853698909
**CHOL**	PCGF6	cg26155979	-0.0992261225773519	36	-0.581452266291558	0.564770972528526	0.00984582340173567	0.73441750864008
**CHOL**	PCGF6	cg27217148	-0.036780023130583	36	-0.214607752165471	0.831355156069869	0.00135277010148622	0.909651953567734
**CHOL**	PHC1	cg02770112	0.0450764138173213	36	0.263105836628918	0.794056924483552	0.0020318830826304	0.887222468700516
**CHOL**	PHC1	cg08021945	-0.338187679851356	36	-2.09542105461586	0.0436544206660741	0.114370906803243	0.130592270767028
**CHOL**	PHC1	cg08867249	-0.0951736448092445	36	-0.557483537376383	0.580849200192115	0.00905802266627624	0.746527824344453
**CHOL**	PHC1	cg09059548	-0.365606825828003	36	-2.29040173337735	0.0283242417909458	0.133668351092028	0.094613597229801
**CHOL**	PHC1	cg09912793	-0.500296004175294	36	-3.36915976256446	0.00188826178302676	0.250296091793766	0.011350288761303
**CHOL**	PHC1	cg12581035	-0.022907997613824	36	-0.133610494508151	0.894498306705092	0.000524776354674965	0.944028921939992
**CHOL**	PHC1	cg14199415	-0.0933102127827468	36	-0.546471571279044	0.588310384089261	0.00870679580956149	0.75184429911192
**CHOL**	PHC2	cg06899053	-0.0283194573441868	36	-0.165195649515884	0.869768304999939	0.000801991664269213	0.930878362281635
**CHOL**	PHC2	cg13640100	-0.239598065344514	36	-1.43899973529943	0.15929378652665	0.0574072329168341	0.328785966592661
**CHOL**	PHC2	cg15586632	0.0428726805792703	36	0.250218603074156	0.803924099946558	0.00183806674005214	0.893307690776211
**CHOL**	PHC2	cg16558908	-0.220065056806973	36	-1.31543642395676	0.197165436208322	0.0484286292274562	0.379397643820872
**CHOL**	PHC2	cg17161743	-0.198334964954672	36	-1.17992161109036	0.246221357755937	0.0393367583235711	0.439284109376786
**CHOL**	PHC2	cg19746874	0.193879292434848	36	1.15236653065883	0.257208743986148	0.0375891800350373	0.451656743028721
**CHOL**	PHC2	cg21198021	0.0428499944364394	36	0.250085955906526	0.804025834883804	0.00183612202320289	0.893395215090994
**CHOL**	PHC2	cg21578869	-0.0718174095411405	36	-0.41984799343392	0.677237830432434	0.0051577403131999	0.813526103968679
**CHOL**	PHC2	cg23886755	0.126982136095338	36	0.746469393775998	0.460514655767202	0.0161244628873349	0.650278814850689
**CHOL**	PHF1	cg01097444	-0.183203195681103	36	-1.08664033490278	0.284839813339271	0.0335634109077685	0.482122417678362
**CHOL**	PHF1	cg02776641	0.0125478746328263	36	0.0731718140135666	0.942098178684307	0.000157449157801126	0.969169750632324
**CHOL**	PHF1	cg03560363	-0.114813608521124	36	-0.673929287420014	0.504912551902075	0.0131821647016418	0.688100942007841
**CHOL**	PHF1	cg03876174	0.0343906111405918	36	0.200648689320103	0.842168740115542	0.00118271413462339	0.915566814810392
**CHOL**	PHF1	cg06618123	-0.164033352752179	36	-0.969604076703756	0.339091269919334	0.0269069408151209	0.538426600260945
**CHOL**	PHF1	cg06919790	0.045826585455813	36	0.267493641398917	0.790705131353733	0.00210007593453893	0.885079137664316
**CHOL**	PHF1	cg07857404	-0.244611031127466	11	-0.756824444712414	0.468503598726369	0.0598345565492422	0.657457611756371
**CHOL**	PHF1	cg08665801	-0.123977507874803	36	-0.728527453087927	0.471279193426151	0.0153704224588468	0.659595377015071
**CHOL**	PHF1	cg08798692	-0.129205381119859	36	-0.759758770374775	0.452634909372826	0.0166940305103279	0.643076195115597
**CHOL**	PHF1	cg10033570	-0.174965861405101	36	-1.0362014458156	0.307422062114544	0.030613052657229	0.505914556021167
**CHOL**	PHF1	cg10797813	-0.0735540775188682	36	-0.430055208275845	0.669869092959502	0.00541020231965168	0.808719711085479
**CHOL**	PHF1	cg13303256	-0.132135176463435	36	-0.777289359135913	0.442363038128875	0.017459704859023	0.634746002300356
**CHOL**	PHF1	cg13528777	-0.101801633908991	36	-0.596700457171026	0.554660131982702	0.0103635726665402	0.726320197742094
**CHOL**	PHF1	cg13547657	-0.107212986539198	36	-0.628777995370401	0.533696272426583	0.0114946244826543	0.710692208095122
**CHOL**	PHF1	cg18443095	-0.00536819490862448	36	-0.0313021373025225	0.975211618622118	2.88175165769818e-05	0.986921110159736
**CHOL**	PHF1	cg20135607	-0.1045864176823	36	-0.61320128738425	0.54382382377231	0.0109383187636165	0.718089290444591
**CHOL**	PHF1	cg20872639	-0.241405350989775	36	-1.45052312506155	0.156076410538867	0.0582765434864963	0.324378410447508
**CHOL**	PHF1	cg23345642	-0.0992713213875963	36	-0.581719760988907	0.564592805497833	0.00985479525003943	0.734275969331128
**CHOL**	PHF1	cg23718418	-0.173399145488863	30	-0.931655087478992	0.359478951017257	0.030067263656268	0.558439313622387
**CHOL**	PHF1	cg24796663	0.389106032624	24	1.98120167993289	0.0602038037844562	0.151403504624389	0.165074535161708
**CHOL**	PHF1	cg26673053	-0.0876966917809353	36	-0.513332948395856	0.611038202224253	0.00769070974932036	0.768090174759202
**CHOL**	PHF10	cg04353997	-0.338992556926873	36	-2.10105472769463	0.0431246229051982	0.114915953651819	0.129375531768423
**CHOL**	PHF10	cg05243705	-0.157335909244271	36	-0.928988536477065	0.359443911608225	0.0247545883377216	0.55840282090523
**CHOL**	PHF10	cg05736120	-0.247758442937772	36	-1.49115916417146	0.145140164969298	0.0613842460469494	0.308852617583609
**CHOL**	PHF10	cg07652237	-0.261478415350363	36	-1.57962425788592	0.123452605609537	0.068370961694137	0.276163791142982
**CHOL**	PHF10	cg11442717	-0.284083449191691	36	-1.72765716380332	0.0931245076285739	0.0807034061046481	0.226320773085201
**CHOL**	PHF10	cg15342139	-0.183686917497585	36	-1.08960950365021	0.283547944224467	0.0337408836597647	0.480804062361004
**CHOL**	PHF10	cg15360169	-0.495415748433787	36	-3.32553440770697	0.00212478116262555	0.245436763796209	0.0124713464437372
**CHOL**	PHF10	cg16740543	0.32728838004819	36	2.01963521300812	0.0513591136981858	0.107117683714568	0.147129446541785
**CHOL**	PHF10	cg27124293	-0.371852974804218	36	-2.33574958487698	0.0255398917685699	0.138274634870746	0.0875611625862292
**CHOL**	PHF11	cg00092518	-0.16687942887454	36	-0.986904984471384	0.330660768344352	0.0278487437814928	0.530336133361795
**CHOL**	PHF11	cg01214340	-0.0876574897368288	36	-0.513101701810338	0.61119822151433	0.00768383550696224	0.76816748247732
**CHOL**	PHF11	cg02289189	-0.239061760884078	36	-1.43558327810515	0.160257683278844	0.0571505255169961	0.330070324137417

**CHOL**	PHF11	cg05080330	-0.0847431320361967	36	-0.495917023678706	0.623143616382736	0.00718139842730427	0.776728478832505
**CHOL**	PHF11	cg09997676	-0.0733482294341316	36	-0.428845138589882	0.670740945737834	0.005379962761122	0.809224750433912
**CHOL**	PHF11	cg16956501	-0.130674226219754	36	-0.768545123911193	0.447469131160927	0.0170757533981314	0.639056192261231
**CHOL**	PHF11	cg21320768	-0.194240197898079	36	-1.1545956820538	0.256306876771449	0.0377292544794848	0.450571422763612
**CHOL**	PHF11	cg23137039	-0.0775495545484839	36	-0.453553599281544	0.653031881545045	0.00601393341066828	0.797381334352235
**CHOL**	PHF11	cg24481868	-0.141655111068491	36	-0.834398149199546	0.409881146868665	0.0200661704918264	0.605802932508945
**CHOL**	PHF11	cg24984523	-0.0388933296679317	36	-0.226956857097599	0.821816401928296	0.00151269109265841	0.904379352283284
**CHOL**	PHF11	cg25162948	-0.190983198468533	36	-1.13449616706314	0.264521915156467	0.0364745820972709	0.460115773805613
**CHOL**	PHF12	cg01406758	-0.122592156065878	36	-0.720261802808027	0.476286690983138	0.0150288367288807	0.664090168088723
**CHOL**	PHF12	cg02058367	-0.112031026834206	36	-0.657385964031573	0.515358774206558	0.012550950973267	0.696703805380571
**CHOL**	PHF12	cg06895197	0.120696844468115	36	0.708960410336449	0.483182337595813	0.0145677282645602	0.670072711936197
**CHOL**	PHF12	cg08657247	-0.169990833233449	36	-1.00584783901764	0.321593964615315	0.0288968833834022	0.521079748464631
**CHOL**	PHF12	cg08806622	0.0195626738357826	36	0.114090843340711	0.909836854534419	0.000382698207605213	0.952116106417506
**CHOL**	PHF12	cg13564075	-0.312237072632138	36	-1.91645396892479	0.0637446358660781	0.0974919895258867	0.172042402253875
**CHOL**	PHF14	cg06715026	-0.0391050322145696	36	-0.228194106061441	0.820862209197119	0.00152920354450253	0.903776762419586
**CHOL**	PHF14	cg07287563	-0.047459358338566	36	-0.27704541858241	0.783422687982957	0.00225239069390842	0.880509600053011
**CHOL**	PHF14	cg07574654	-0.0442022928267208	36	-0.257993605806789	0.797967110823248	0.00195384269113917	0.889585472175973
**CHOL**	PHF14	cg11931558	-0.106672648728155	36	-0.625572477863749	0.535772252376402	0.0113790539866803	0.712089406367628
**CHOL**	PHF14	cg13029706	-0.251612443433949	36	-1.51590979347451	0.138785204401594	0.063308821690802	0.299410656199132
**CHOL**	PHF14	cg16800461	-0.222553930489918	36	-1.33108435196361	0.192018588049209	0.0495302519765113	0.372517131733622
**CHOL**	PHF14	cg20819769	0.123264734474354	36	0.7242741799027	0.473852115938585	0.0151941947650329	0.661819748354952
**CHOL**	PHF14	cg20964328	-0.127021836729696	36	-0.746706602140556	0.460373307308745	0.0161345470061854	0.650163947876491
**CHOL**	PHF14	cg22876561	0.0458169881390557	36	0.267437503194721	0.790747989300648	0.00209919640213437	0.885101639971434
**CHOL**	PHF14	cg23083824	-0.176468025020349	36	-1.04538240631383	0.303221859759312	0.0311409638545827	0.501666092982197
**CHOL**	PHF14	cg24491907	0.110119837388717	36	0.646032438805084	0.522595203131147	0.0121263785865175	0.702312630338149
**CHOL**	PHF19	cg11054680	0.039591494297876	6	0.0792451207785039	0.94064378811803	0.00156748642073874	0.968318554824381
**CHOL**	PHF19	cg11594303	-0.266265882913201	36	-1.61073157462224	0.116484535158892	0.0708975204035462	0.265228529185472
**CHOL**	PHF19	cg13501527	-0.288747939250099	36	-1.75858186859282	0.087647544803045	0.0833753724211791	0.216779731905221
**CHOL**	PHF19	cg13851767	0.281370863095987	36	1.70973478732395	0.0964280239179263	0.0791695625993804	0.232068208787919
**CHOL**	PHF19	cg13852822	0.400561157791677	36	2.54908790867744	0.0154827683732018	0.160449241131409	0.0598396837441956
**CHOL**	PHF19	cg14218513	-0.0819334541853609	36	-0.479361738842248	0.634750362106654	0.00671309091474462	0.784827389137052
**CHOL**	PHF19	cg27207079	0.206607179389622	36	1.23128271161837	0.226666376692795	0.0426865265753353	0.416204091293112
**CHOL**	PHF20	cg01512265	-0.194342527862606	36	-1.15522781702424	0.256051546424711	0.0377690181360278	0.450303264956358
**CHOL**	PHF20	cg02674305	-0.261707708385922	36	-1.58111124681675	0.123111947673027	0.0684909246286109	0.275560736438385
**CHOL**	PHF20	cg02957488	-0.238447081363219	36	-1.43166925640084	0.161367613265842	0.0568570106106374	0.33161107347572
**CHOL**	PHF20	cg04179482	-0.117815430608197	36	-0.691794101381957	0.493764033406445	0.0138804756893949	0.679051022943076
**CHOL**	PHF20	cg09692677	-0.378129586470473	10	-1.15528910428368	0.281309517577283	0.142981984164331	0.478530610808714
**CHOL**	PHF20	cg10372813	-0.159859548814905	36	-0.944276958352239	0.351690465794566	0.025555075347305	0.550657302928902
**CHOL**	PHF20	cg21765224	-0.0917683636583291	36	-0.537364386870512	0.594515727276652	0.00842143256852734	0.756103387927405
**CHOL**	PHF20	cg23538717	-0.353124617534425	36	-2.20083884968019	0.0346378694987759	0.124696995508834	0.109975447709892
**CHOL**	PHF20	cg23734815	0.0146627220139232	36	0.0855068189918734	0.932360022411785	0.000214995416857589	0.964101933222479
**CHOL**	PHF20	cg25561939	-0.160816172855924	36	-0.950077215606886	0.348778056806571	0.0258618414520264	0.547905740013538
**CHOL**	PHF20	cg27405321	-0.587605499368721	12	-2.29645596289377	0.0445220028833169	0.345280222888364	0.132521708684503
**CHOL**	PHF20L1	cg02898212	0.1435992551718	36	0.846089286194295	0.40341861889206	0.0206207460858957	0.599853206414408
**CHOL**	PHF20L1	cg05307234	-0.395038320239533	36	-2.50738880669962	0.0171035352869778	0.156055274457672	0.0645599027839292
**CHOL**	PHF20L1	cg10591701	-0.555162946457142	31	-3.59443838309958	0.00118822483785482	0.308205897118975	0.00775216060349532
**CHOL**	PHF20L1	cg17646969	-0.167372579025735	36	-0.989905342436686	0.329213268387549	0.0280135802097258	0.528897775869752
**CHOL**	PHF20L1	cg24511731	-0.257365963722895	36	-1.55300300160351	0.129682378977599	0.0662372392830146	0.285592822454959
**CHOL**	PHF20L1	cg26292018	0.0892488514770325	36	0.52249083315876	0.604716741895266	0.00796535748996941	0.763541748669095
**CHOL**	PHF20L1	cg27342122	-0.221213983041073	36	-1.32265652024522	0.194777668260888	0.0489356262928962	0.376282845977985
**CHOL**	PHF21B	cg01775414	-0.178116502922281	36	-1.05546629303483	0.298654684619527	0.031725488613263	0.496867355597108
**CHOL**	PHF21B	cg01995480	-0.169240289011049	36	-1.00127556477582	0.323766767469605	0.0286422754245433	0.523196818745901
**CHOL**	PHF21B	cg05064352	-0.0889205291902756	36	-0.520553383856107	0.606051557956128	0.00790686051147865	0.76452790698004
**CHOL**	PHF21B	cg10080155	-0.0947494247313076	36	-0.554976089942094	0.582544053795628	0.00897745348691373	0.74768085727211
**CHOL**	PHF21B	cg11428482	-0.0575640058751931	36	-0.336210447302013	0.738778383371514	0.00331361477239926	0.852923223305634
**CHOL**	PHF21B	cg14786713	-0.16129145939345	36	-0.952960020869291	0.347336521198775	0.026014934873269	0.546626596045776
**CHOL**	PHF21B	cg15679813	-0.130640631839038	36	-0.768344111387322	0.447586920255224	0.0170669746873031	0.639137591995722

**CHOL**	PHF21B	cg20495645	-0.136020773971555	36	-0.800571109493268	0.428938861046621	0.0185016509518208	0.623041744580843
**CHOL**	PHF21B	cg20525183	-0.107237206444964	36	-0.628921691498415	0.533603310439506	0.0114998184461198	0.710661095876377
**CHOL**	PHF21B	cg21715212	0.0306877067487987	36	0.179022857747261	0.858982323021802	0.000941735345500268	0.925074351253641
**CHOL**	PHF21B	cg23080354	-0.115325382181996	36	-0.676973680686928	0.503002953269528	0.0132999437754235	0.686530900318103
**CHOL**	PHF21B	cg25561581	-0.0954955373974577	36	-0.559386359794971	0.579564640834485	0.00911939766282925	0.745566644106922
**CHOL**	PHF21B	cg25976257	-0.148653790981497	36	-0.876531986917875	0.386892149686371	0.0220979495731707	0.584845187663011
**CHOL**	PHF23	cg00039960	-0.133309838516229	36	-0.784323807918712	0.4382807383246	0.017771513045223	0.630984440425375
**CHOL**	PHF23	cg01441172	-0.0923711378287316	36	-0.540924303426177	0.5920863949574	0.00853242710377452	0.754488509382889
**CHOL**	PHF23	cg04379155	-0.196405993485372	36	-1.16798310453951	0.250938855886748	0.0385753142769759	0.444560424368138
**CHOL**	PHF23	cg11180288	-0.00231663247569601	36	-0.0135082087717015	0.989301291673493	5.36678602744942e-06	0.99432442325613
**CHOL**	PHF23	cg13351208	0.220563349757471	36	1.31856709391628	0.196127353194283	0.0486481912562366	0.378082798027407
**CHOL**	PHF23	cg22033559	-0.285128316140084	36	-1.73457272830585	0.0918754194489634	0.0812981566648798	0.224146403882857
**CHOL**	PHF23	cg26213471	-0.0971834040109821	36	-0.569366857516857	0.572849898131565	0.00944461401516176	0.740405804636179
**CHOL**	PHF23	cg26784276	-0.0958602233864859	36	-0.56154236334986	0.578110848323537	0.00918918242770698	0.744435060905454
**CHOL**	PHIP	cg01492710	0.0847349051724418	36	0.495868531862798	0.623177473058271	0.00718000415458269	0.776728478832505
**CHOL**	PHIP	cg04480383	-0.0821302446685806	36	-0.480520895319304	0.633934581764895	0.00674537708932091	0.784452386957224
**CHOL**	PHIP	cg05801338	-0.105079394530405	36	-0.61612385744803	0.541916060751182	0.0110416791548766	0.716701540751376
**CHOL**	PHIP	cg06848288	-0.138210887149843	36	-0.813710357103966	0.421473216522938	0.0191022493267465	0.616595595873804
**CHOL**	PHIP	cg08287994	-0.348109732047515	36	-2.16523856038643	0.0374779452835079	0.121180385546193	0.116725089093811
**CHOL**	PHIP	cg08587415	-0.10200240440864	36	-0.597889614487715	0.553875522227305	0.0104044905051437	0.725731472973194
**CHOL**	PHIP	cg15564972	-0.190240943182258	36	-1.12992104918741	0.266418062373218	0.0361916164628752	0.462122382756853
**CHOL**	PHIP	cg19494004	-0.087530721385607	36	-0.512353932029908	0.611715800115275	0.00766162718628477	0.768624020063116
**CHOL**	PHIP	cg19950079	-0.12900526066284	36	-0.758562082721705	0.453341193703563	0.0166423572786873	0.64375045310629
**CHOL**	PHIP	cg21607066	0.310166694602725	36	1.90238872618224	0.0656184504957265	0.09620337844078	0.175770323595639
**CHOL**	PHIP	cg21875336	-0.159175467501013	36	-0.940130872955528	0.353782120600613	0.0253368294541659	0.552797442906864
**CHOL**	PHIP	cg26363168	-0.0812296740633775	36	-0.475216718375715	0.637671293165141	0.00659825994844255	0.786859254070434
**CHOL**	PHRF1	cg06063517	0.184955450373561	36	1.0973998769813	0.280178119485603	0.0342085186228869	0.477389592554548
**CHOL**	PHRF1	cg07530960	0.0655781534702995	36	0.383207937800433	0.70395120461677	0.00430049421257416	0.830928595453479
**CHOL**	PHRF1	cg08482978	0.259515097082632	36	1.56690365342779	0.126398310477349	0.0673480856138081	0.280618266713921
**CHOL**	PHRF1	cg12469954	0.173814734408465	36	1.02917101530965	0.310665500949586	0.0302115618974853	0.509495351130433
**CHOL**	PHRF1	cg12813709	0.115314959444317	36	0.676911673398083	0.503041807593548	0.0132975398716446	0.686530900318103
**CHOL**	PHRF1	cg15219216	0.465570630856909	36	3.06744299024796	0.00421727045786678	0.2167560123165	0.0216596248761661
**CHOL**	PHRF1	cg16143007	0.0632883847337138	36	0.369772817577956	0.713845091687753	0.00400541964220258	0.837124716669098
**CHOL**	PHRF1	cg18793109	0.176041013130694	36	1.04277182086505	0.304412104310692	0.0309904383040811	0.503017897521226
**CHOL**	PHRF1	cg20152424	0.304496656368355	36	1.86402138441271	0.0709726454690846	0.0927182137395082	0.186220310351849
**CHOL**	PHRF1	cg21006161	0.305160221075744	36	1.86850011214056	0.0703289253631191	0.0931227605269971	0.185018038608721
**CHOL**	PPARGC	cg04893087	-0.184031883497337	35	-1.07555283091484	0.289928314456114	0.0338677341435773	0.487426432148314
**CHOL**	PPARGC	cg05158538	-0.475462432079314	20	-2.29297902962653	0.034109150078731	0.226064524318776	0.108647063064521
**CHOL**	PPARGC	cg11270806	-0.377291246222195	36	-2.375531581065	0.0233035330470533	0.142348684475897	0.0817383583508033
**CHOL**	PPARGC	cg12691631	-0.360923730150623	36	-2.2566367497589	0.0305717136628378	0.13026593898584	0.100093982888334
**CHOL**	PPARGC	cg20723350	-0.334602814136504	36	-2.07039185143921	0.0460782041684464	0.111959043228068	0.135904148281776
**CHOL**	PPARGC	cg27461259	-0.426516213988407	36	-2.74964151330735	0.00948628776365555	0.181916080795005	0.0408715258244251
**CHOL**	PPARGC	cg27514608	-0.447525163546495	36	-2.91801558004268	0.00620461116547596	0.200278772007317	0.029453536860791
**CHOL**	PRDM1	cg00555933	-0.306014407754326	36	-1.8742698617629	0.0695070281869896	0.0936448177532307	0.183445167281133
**CHOL**	PRDM1	cg01761420	-0.287083252826036	36	-1.74752979367933	0.0895729586539413	0.0824167940531779	0.220130745396453
**CHOL**	PRDM1	cg05170275	-0.196855044756536	36	-1.17076104092256	0.249835313389091	0.0387519086460979	0.443443573200185
**CHOL**	PRDM1	cg08742502	-0.4473383310453	36	-2.91649252409553	0.00622880382445584	0.200111582422394	0.0295447573147807
**CHOL**	PRDM1	cg14359052	-0.412190504418213	36	-2.63798607709837	0.0124883529648914	0.169901011932541	0.0507135214569483
**CHOL**	PRDM1	cg17143179	-0.508730006278667	36	-3.44556882633787	0.00153354468281994	0.258806219288293	0.009573148375485
**CHOL**	PRDM1	cg17521665	-0.157308123898844	36	-0.928820314990974	0.359529843868794	0.0247458458445741	0.558439313622387
**CHOL**	PRDM1	cg17965230	-0.270150226290195	36	-1.63606483574106	0.111051195641077	0.0729811447646438	0.256650625759839
**CHOL**	PRDM1	cg19464016	-0.252094933204741	36	-1.51901376278206	0.13800426614897	0.0635518553475027	0.298272937159545
**CHOL**	PRDM1	cg22186515	-0.299307950397393	36	-1.82910260668586	0.0761665475399872	0.0895852491710881	0.196033229647514
**CHOL**	PRDM1	cg22196848	-0.358798548870802	36	-2.24137927016098	0.0316387841626731	0.128736398671793	0.102691404170185
**CHOL**	PRDM1	cg24040346	-0.304000787937829	36	-1.86067647553079	0.0714566889126363	0.092416479066821	0.187158019997694
**CHOL**	PRDM1	cg24793124	-0.4420857675347	36	-2.87386740336404	0.00694293503574053	0.195439825856745	0.0322035945725255
**CHOL**	PRDM1	cg24874254	-0.418906464983197	36	-2.69002685588037	0.0109937265188382	0.175482626404718	0.0458927507385096

**CHOL**	PRDM1	cg26351406	-0.300448796524347	36	-1.83676478632565	0.0749999132977049	0.0902694793329282	0.193864409060395
**CHOL**	PRDM10	cg04181189	-0.102514576800126	36	-0.600923530115862	0.551876310249714	0.0105092384565089	0.724186385526723
**CHOL**	PRDM10	cg17627813	-0.108920311898364	36	-0.638910305843932	0.527162343362801	0.0118636343440368	0.705768106072689
**CHOL**	PRDM12	cg09191327	-0.208201561032913	36	-1.24121330718992	0.223022613628023	0.0433478900165417	0.411747898630146
**CHOL**	PRDM12	cg12539975	0.1274085136773	36	0.749017159365455	0.458997812076814	0.0162329293574587	0.648907073966618
**CHOL**	PRDM12	cg13795948	0.110874264758108	36	0.650513281775609	0.51973273478342	0.012293102585651	0.700193943432239
**CHOL**	PRDM12	cg13928649	0.0841597570730394	36	0.49247867414173	0.625546308714181	0.00708286471059301	0.778395814647158
**CHOL**	PRDM12	cg23342516	0.13573377829397	36	0.798850213737394	0.429922580747776	0.0184236585699566	0.623969650691079
**CHOL**	PRDM13	cg00126261	0.150070822751221	36	0.885079048038897	0.382330824590829	0.0225212518412285	0.580730633546726
**CHOL**	PRDM13	cg00579292	-0.0434458772771993	36	-0.253570245997859	0.80135468325925	0.00188754425238547	0.891596968222158
**CHOL**	PRDM13	cg01422797	0.0201756449808668	36	0.117667166369819	0.907023770694878	0.000407056650393977	0.950841639389257
**CHOL**	PRDM13	cg01815538	0.0024690274527132	36	0.0143968241861052	0.988597543158287	6.09609656225145e-06	0.993976674970026
**CHOL**	PRDM13	cg02400433	-0.103770560005046	36	-0.608365549546596	0.54698809050358	0.0107683291237609	0.720503919223481
**CHOL**	PRDM13	cg03120185	-0.0273951872434278	36	-0.159799994872919	0.87398420745514	0.000750496284102467	0.933271940849464
**CHOL**	PRDM13	cg04910970	-0.141915712422652	36	-0.835964703875161	0.4090114870403	0.0201400694324288	0.604986112918073
**CHOL**	PRDM13	cg11809464	0.264094966020248	36	1.59660999547774	0.119606762037936	0.0697461510772362	0.270131759209731
**CHOL**	PRDM13	cg13738144	-0.129743329629101	36	-0.762976083035046	0.450739275894641	0.0168333315832454	0.641495970233763
**CHOL**	PRDM13	cg14344102	-0.200247466439852	36	-1.19177227333793	0.241603113683521	0.0400990478155795	0.433878224429693
**CHOL**	PRDM13	cg17879189	-0.184972630312048	36	-1.09750542209952	0.28013266054531	0.0342148739645577	0.477337424497544
**CHOL**	PRDM13	cg17978124	0.0822705263075419	36	0.481347231860557	0.633353313859405	0.00676843949891994	0.784034621173059
**CHOL**	PRDM13	cg25273233	0.174273581351327	33	0.985393442778653	0.332059774385015	0.0303712811570177	0.5317577160606
**CHOL**	PRDM13	cg25924096	0.0192008574401604	35	0.110320866465284	0.912823116745862	0.000368672926437363	0.953861962794036
**CHOL**	PRDM13	cg26507427	-0.17566571552272	36	-1.04047789816145	0.305460644135135	0.0308584436101092	0.503998077368528
**CHOL**	PRDM14	cg00384539		36				
**CHOL**	PRDM14	cg01295203		36				
**CHOL**	PRDM14	cg04838988		36				
**CHOL**	PRDM14	cg09050331		36				
**CHOL**	PRDM14	cg10457247		36				
**CHOL**	PRDM14	cg11229513		36				
**CHOL**	PRDM14	cg13267264		36				
**CHOL**	PRDM14	cg18035229		36				
**CHOL**	PRDM14	cg21255438		36				
**CHOL**	PRDM14	cg27029821		36				
**CHOL**	PRDM4	cg00090609	-0.0472275542948898	36	-0.275689223231272	0.78445548571027	0.00223044188467676	0.881021976229305
**CHOL**	PRDM4	cg03909902	-0.169885619925985	36	-1.0052067768989	0.321898004692768	0.0288611238576363	0.521314451907308
**CHOL**	PRDM4	cg12900929	-0.227516659665268	36	-1.36236790200527	0.182039183476489	0.0517638304252413	0.359619967273657
**CHOL**	PRDM4	cg18440127	-0.127755275785713	36	-0.751089503875453	0.457766175839101	0.0163214104910837	0.647905712350528
**CHOL**	PRDM4	cg25995916	-0.0709129284232351	36	-0.41453345843861	0.681087341852987	0.00502864341755887	0.816029421438217
**CHOL**	PRDM4	cg27140793	0.151009199478046	36	0.890742089748787	0.379327666672964	0.0228037783270003	0.577845549094473
**CHOL**	PRDM5	cg05713693	-0.312178070426613	36	-1.91605271637058	0.0637974455743321	0.0974551476552832	0.17216104068994
**CHOL**	PRDM5	cg06329345	-0.409683897857899	36	-2.61869650754586	0.0130885634931573	0.167840896164042	0.0526017310733418
**CHOL**	PRDM5	cg07070341	-0.38510325584548	36	-2.43318133927964	0.0203770504476202	0.148304517662789	0.0738544662002499
**CHOL**	PRDM5	cg10416963	-0.516070150270556	36	-3.5131552688462	0.00127395601975154	0.266328400000274	0.00820822714929199
**CHOL**	PRDM5	cg11171429	-0.390716952544076	36	-2.47498625753662	0.01846872254485	0.15265973700533	0.0684560606848704
**CHOL**	PRDM5	cg13155001	-0.327338215306481	36	-2.01997963965228	0.0513215642149791	0.107150307200032	0.147061955887344
**CHOL**	PRDM5	cg18181323	-0.394698202012983	36	-2.50483143007067	0.0172078263025494	0.155786670672281	0.064870404397456
**CHOL**	PRDM5	cg19294653	-0.356172145882731	36	-2.22257855862287	0.0329994595866477	0.126858597502709	0.105967141050263
**CHOL**	PRDM5	cg22135105	-0.446373387551615	36	-2.9086339073926	0.00635504767418104	0.199249201114304	0.0300127407731145
**CHOL**	PRDM8	cg01304890	-0.283867538851205	36	-1.72622897385225	0.0933842328152775	0.0805807796134405	0.226774871472817
**CHOL**	PRDM8	cg02895669	-0.0580285498719157	36	-0.338932810388983	0.73674479533341	0.00336731260023741	0.851561170911036
**CHOL**	PRDM8	cg04343891	-0.272989606737203	36	-1.65463738544929	0.10720202545674	0.0745233253865326	0.250350455342869
**CHOL**	PRDM8	cg05007549	-0.0762723575449628	36	-0.446039752176138	0.65839624278696	0.00581747252546664	0.801226448024112
**CHOL**	PRDM8	cg07462804	-0.173521876250589	36	-1.02738309931721	0.311494092616678	0.0301098415375247	0.510370080897681
**CHOL**	PRDM8	cg08074201	-0.204218702045521	36	-1.21642523781435	0.23220046737128	0.0417052782651573	0.422814938354933
**CHOL**	PRDM8	cg10695325	-0.251910503390511	36	-1.51782714007995	0.138302393932332	0.0634589017184606	0.298681262383318
**CHOL**	PRDM8	cg11571772	-0.181594661953104	36	-1.07677273387768	0.289163005405403	0.0329766212498623	0.486720149884811
**CHOL**	PRDM8	cg12572049	-0.128472237834644	36	-0.755375152650038	0.455225283965457	0.0165051158942412	0.645523008125881

**CHOL**	PRDM8	cg14489346	-0.173631544967911	36	-1.02805260023761	0.311183641032104	0.0301479134079438	0.510108522882852
**CHOL**	PRDM8	cg15656813	-0.33828563396145	36	-2.0961063987185	0.0435896647610973	0.1144371701447	0.130457472713333
**CHOL**	PRDM8	cg16117799	-0.218858432059496	36	-1.30785993089823	0.199695068004309	0.047899013283541	0.382552589828048
**CHOL**	PRDM8	cg18624348	-0.271626646897222	36	-1.64571639793643	0.109036909159476	0.0737810353046282	0.253335044982597
**CHOL**	PRDM8	cg19595012	-0.241277243683509	36	-1.44970577728188	0.15630290777801	0.0582147083195114	0.324675667076514
**CHOL**	PRDM8	cg20142271	-0.266589273904283	36	-1.61283744847111	0.1160247146657	0.0710698409608127	0.264524567809694
**CHOL**	PRDM8	cg26162932	-0.124498413234183	36	-0.731636532089557	0.469403528881406	0.0154998548978295	0.658223949296328
**CHOL**	PRKAA1	cg00301775	-0.386313794093666	36	-2.44216903961559	0.0199522241252677	0.149238347507043	0.0726736269126826
**CHOL**	PRKAA1	cg01883943	-0.074582716438933	24	-0.350800988767984	0.729074557827784	0.00556258159141029	0.84676402051338
**CHOL**	PRKAA1	cg03125217	0.303660917308607	36	1.85838482143483	0.071789944040577	0.0922099527007047	0.187750517941661
**CHOL**	PRKAA1	cg05874655	0.445762878025674	36	2.90366839236223	0.00643605093804215	0.198704543425732	0.0303060436216967
**CHOL**	PRKAA1	cg06971720	-0.0458424114637186	36	-0.267586213650296	0.790634459774045	0.00210152668880888	0.88502549985844
**CHOL**	PRKAA1	cg10786880	0.19061383006551	36	1.13221919985583	0.265464381131913	0.036333632212243	0.461187969042989
**CHOL**	PRKAA1	cg24728477	0.271458126393688	36	1.64461412405663	0.109265417189866	0.0736895143851716	0.253691692976435
**CHOL**	PRKCD	cg02121118	-0.245782518427795	36	-1.47849899988532	0.148479659596566	0.0604090463647093	0.313553268220947
**CHOL**	PRKCD	cg02559423	-0.237211292176243	36	-1.42380578312994	0.163615840980252	0.0562691971359228	0.334762262196038
**CHOL**	PRKCD	cg02630105	0.167194935785788	36	0.98882446132282	0.329734236298901	0.0279541465524137	0.529343326897478
**CHOL**	PRKCD	cg10531918	0.130524147519306	36	0.767647146413067	0.447995469572308	0.0170365530856414	0.639474487337764
**CHOL**	PRKCD	cg13469346	-0.0847336337846159	36	-0.495861037897751	0.623182705370938	0.0071797886943454	0.776728478832505
**CHOL**	PRKCD	cg13889007	0.284231048549213	36	1.728633659129	0.0929472746428067	0.0807872889593849	0.226033094799798
**CHOL**	PRKCD	cg13908523	-0.268585844663392	36	-1.62585184295665	0.113215961964917	0.0721383559535475	0.260292654345797
**CHOL**	PRKCD	cg14727059	0.0175346215533705	36	0.102259256447052	0.919151627953558	0.000307462953019925	0.957406991723557
**CHOL**	PRKCD	cg21903826	-0.391110616885551	36	-2.47792998149257	0.0183407028183682	0.152967514640596	0.0681059442690823
**CHOL**	PRMT1	cg07702240	-0.319966843146938	36	-1.96923651308255	0.0571188540382033	0.102378780713417	0.158839314165922
**CHOL**	PRMT1	cg08377944	-0.40429381326546	36	-2.57745746844328	0.0144620940713346	0.163453487444727	0.0567506309817635
**CHOL**	PRMT1	cg11787855	-0.194565881175341	36	-1.15660769998552	0.255494828939985	0.0378558821175369	0.449763865010799
**CHOL**	PRMT1	cg14258555	-0.249274336760275	36	-1.50088524281586	0.14261565564345	0.0621376949672748	0.305166748683203
**CHOL**	PRMT1	cg14576628	-0.249949902774433	36	-1.50522350432378	0.141501024055625	0.0624749538969484	0.3035916783525
**CHOL**	PRMT1	cg18880319	-0.179765858021435	36	-1.06556473075044	0.294129218400552	0.0323157637101826	0.491895187055325
**CHOL**	PRMT1	cg22292364	-0.286824139755912	36	-1.74581106310502	0.0898755569665249	0.0822680871467188	0.220595648482532
**CHOL**	PRMT1	cg24228123	0.200842891113879	36	1.19546466413128	0.240177268201054	0.0403378669109814	0.432127684590992
**CHOL**	PRMT1	cg25595446	-0.2109971412861	36	-1.25865058627163	0.216730714859398	0.0445197936309065	0.40402931468958
**CHOL**	PRMT1	cg26846726	-0.16525461996723	36	-0.977024916637506	0.33545769715012	0.0273090894205137	0.535018473708847
**CHOL**	PRMT2	cg04493422	0.176489699498929	36	1.04551493191951	0.30316152364063	0.0311486140292224	0.501576936837498
**CHOL**	PRMT2	cg06725035	-0.277621041745182	15	-1.04193465785027	0.316432151606623	0.0770734428196801	0.515669665083387
**CHOL**	PRMT2	cg09031657	-0.258842507614218	36	-1.56255065660477	0.127419367272616	0.0669994437480164	0.282229436182617
**CHOL**	PRMT2	cg10187059	-0.324398401562451	36	-1.99969382701898	0.0535744696275247	0.105234322936273	0.151737440936529
**CHOL**	PRMT2	cg10657630	-0.0723823482753997	36	-0.423167978356709	0.674837497477157	0.00523920434186125	0.812026237471005
**CHOL**	PRMT2	cg12162201	-0.404290233062438	36	-2.577430184267	0.0144630452186902	0.16345059254968	0.0567506309817635
**CHOL**	PRMT2	cg13271076	-0.142021843662301	36	-0.836602742123394	0.408657614375935	0.020170204077239	0.604615809069668
**CHOL**	PRMT2	cg17243659	-0.245454725637241	36	-1.47640066506785	0.149039034428014	0.0602480223376532	0.314397273287404
**CHOL**	PRMT2	cg23283320	-0.391588023867147	36	-2.48150206674153	0.0181864458620867	0.153341180436177	0.0676547613521028
**CHOL**	PRMT3	cg01769160	-0.30453359490769	36	-1.86427062224437	0.0709366908004438	0.0927407104274013	0.18615013010314
**CHOL**	PRMT3	cg04101203	-0.518064074152276	35	-3.47937103940319	0.00143382085381364	0.268390384927254	0.00905746321571892
**CHOL**	PRMT3	cg06578097	-0.086034359433181	33	-0.480801771423356	0.634033459947939	0.00740191100307777	0.784527031273401
**CHOL**	PRMT3	cg13432154	-0.170593936383072	36	-1.00952321603784	0.319854604548576	0.0291022911306715	0.519256450464356
**CHOL**	PRMT3	cg13982511	-0.0675656909617512	36	-0.394874650354417	0.695401795707054	0.00456512259513887	0.825408355437353
**CHOL**	PRMT3	cg19653624	-0.120072252872481	35	-0.694789271032808	0.492050674411757	0.0144173459098731	0.677583726444483
**CHOL**	PRMT3	cg23019633	-0.385220172371833	36	-2.43404874701451	0.0203356938600037	0.148394581202185	0.0737526791676049
**CHOL**	PRMT3	cg23071995	-0.00879039403096471	36	-0.0512583451620409	0.959419627586804	7.727102721962e-05	0.978782906150257
**CHOL**	PRMT6	cg00944433	-0.386871095523097	36	-2.44631173239448	0.0197591407407244	0.149669244551241	0.072152808098504
**CHOL**	PRMT6	cg02990361	-0.567566254944804	36	-4.01960412688754	0.000306115670654759	0.322131453752071	0.00248657851967384
**CHOL**	PRMT6	cg02993280	-0.477340412558722	36	-3.1675094041276	0.00324161948842122	0.227853869461731	0.0175355362271219
**CHOL**	PRMT6	cg09367891	-0.503531075025293	36	-3.39831393982381	0.00174450151621225	0.253543543516127	0.0106365507447461
**CHOL**	PRMT6	cg14671364	-0.560061852555405	36	-3.94192839978972	0.000382346651331366	0.313669278687792	0.00299198281206759
**CHOL**	PRMT6	cg14828511	-0.492357374344937	36	-3.29840854341376	0.002285878085152	0.24241578407184	0.0132469904466861
**CHOL**	PRMT6	cg22528358	-0.0276314011416187	36	-0.161178912185519	0.87290642771344	0.000763494329049044	0.932697166996845

**CHOL**	PRMT7	cg00894222	-0.108940346008655	36	-0.639029234259665	0.52708590498278	0.0118679989884855	0.705702204533774
**CHOL**	PRMT7	cg02477175	-0.293560520416638	36	-1.79063162432972	0.0822602745804915	0.0861777791472876	0.207112298553782
**CHOL**	PRMT7	cg06089844	-0.0703394740362279	36	-0.411164497944867	0.683532111972736	0.00494764160769318	0.817754747037522
**CHOL**	PRMT7	cg06092312	-0.0966239928001434	36	-0.566058466786921	0.575071461497442	0.00933619598464217	0.74207817351053
**CHOL**	PRMT7	cg06745885	-0.112964511655878	36	-0.662934058752616	0.511842431309171	0.012760980893651	0.693812076065178
**CHOL**	PRMT7	cg09175928	-0.151040523652477	36	-0.890931171483123	0.379227657209017	0.0228132397852144	0.577728828249016
**CHOL**	PRMT7	cg09779635	-0.125785270449917	36	-0.739319916053154	0.464786790804037	0.0158219342621587	0.654010483735652
**CHOL**	PRMT7	cg10233539	-0.0331347080589757	36	-0.193313037935474	0.84786403751537	0.00109790887815355	0.918837291000497
**CHOL**	PRMT7	cg10446007	-0.131651834403038	36	-0.774395841047784	0.44404880818922	0.017332205501685	0.63604671216277
**CHOL**	PRMT7	cg11893236	-0.175743431814522	36	-1.04095288314791	0.305243325848104	0.0308857538259457	0.503793138794072
**CHOL**	PRMT7	cg19328639	-0.141298118201199	36	-0.832252442864949	0.411074180030757	0.0199651582071999	0.606917215009959
**CHOL**	PRMT7	cg21918026	-0.0404601031327699	36	-0.236114256059146	0.814760640018298	0.00163701994551437	0.90035923221659
**CHOL**	PRMT7	cg26492318	-0.0601318641678966	36	-0.351261635540653	0.727559527861083	0.00361584108830637	0.845934658138924
**CHOL**	PRMT7	cg26561196	0.00830231423474221	36	0.0484120634309843	0.961671116317523	6.89284216524031e-05	0.979897609896988
**CHOL**	PRMT7	cg26796563	0.123877174705847	36	0.727928674248588	0.471640923794831	0.015345554413103	0.659771065179149
**CHOL**	PRMT8	cg01693063	0.238038423125773	36	1.42906810192788	0.162108591093139	0.0566622908842044	0.332691357456835
**CHOL**	PRMT8	cg06029846	-0.0378898917620187	36	-0.221092898502522	0.826342533891472	0.0014356438977375	0.906631085715365
**CHOL**	PRMT8	cg07912789	-0.087255476150808	36	-0.51073042643265	0.612840227850497	0.00761351811830422	0.769427918488776
**CHOL**	PRMT8	cg10350722	-0.334235479821905	36	-2.06783290412305	0.0463325576809139	0.111713355971779	0.136431923699952
**CHOL**	PRMT8	cg11596580	-0.0823030439887967	36	-0.481538783122078	0.633218605152833	0.0067737910498218	0.783955187619509
**CHOL**	PRMT8	cg19656577	-0.0553015096174072	36	-0.322954659610379	0.748707423127253	0.00305825696596418	0.859201692782831
**CHOL**	PRMT8	cg21667836	-0.22933513930428	36	-1.37385891285001	0.178476132092038	0.0525946061197136	0.355080871054371
**CHOL**	PRMT8	cg23739862	-0.0498826749968685	36	-0.291226030103405	0.772647780919279	0.00248828126484321	0.873945671791942
**CHOL**	PRMT8	cg24100636	-0.103406196590183	36	-0.606206302497369	0.548404063259339	0.0106928414932476	0.721589663873569
**CHOL**	PRMT8	cg27316886	-0.152680044170701	36	-0.900831638351372	0.374014770302939	0.0233111958879673	0.572645247804504
**CHOL**	PWWP2E	cg00259755	0.161234653889546	36	0.952615436715772	0.347508620527169	0.0259966136148818	0.546794879255893
**CHOL**	PWWP2E	cg05010219	-0.182215347410938	36	-1.08057928435247	0.287489839004265	0.0332024328320888	0.484982858841978
**CHOL**	PWWP2E	cg15012692	0.106103253819254	36	0.622195187686205	0.537964064615772	0.011257900471033	0.713756121750509
**CHOL**	PWWP2E	cg15278699	-0.13414175470625	36	-0.789307761179045	0.435402143129137	0.0179940103556717	0.628668331153967
**CHOL**	PWWP2E	cg19919881	0.0421378130638327	36	0.245921986892091	0.807221182548318	0.00177559528980251	0.895602297775715
**CHOL**	PWWP2E	cg22633292	-0.0672075239167875	36	-0.392771888965821	0.696939761058891	0.00451685127102556	0.826191873999401
**CHOL**	PWWP2E	cg22664157	-0.179297784678432	36	-1.06269793814245	0.295409021049201	0.0321476955905933	0.49335827450933
**CHOL**	PWWP2E	cg24520806	-0.109264639065456	36	-0.640954443676515	0.525849345386629	0.0119387613501043	0.704652977831113
**CHOL**	PYGO1	cg06364757	-0.314519961448996	36	-1.93199800373351	0.0617276831516896	0.098922806149878	0.168079922404709
**CHOL**	PYGO1	cg09275667	-0.267761418510734	36	-1.62047520101878	0.114369487780318	0.0716961772428806	0.261950840974656
**CHOL**	PYGO1	cg11480800	-0.297234619380641	36	-1.81519967353538	0.0783228801671195	0.0883484189583545	0.199831102283326
**CHOL**	PYGO1	cg12686317	-0.257480210571997	36	-1.55374132967665	0.129506223220589	0.0662960588361999	0.285356798539129
**CHOL**	PYGO1	cg13878116	0.119602167546399	36	0.702436647253957	0.487188515101133	0.0143046784817968	0.673508491528398
**CHOL**	PYGO1	cg17841803	-0.308082055794266	36	-1.88825686032923	0.067548711188728	0.0949145531024212	0.179639473267106
**CHOL**	PYGO1	cg19850348	-0.277883846159947	36	-1.68676095525026	0.100805302115394	0.0772194319566453	0.239603014288599
**CHOL**	PYGO1	cg22510134	0.151078031716042	36	0.891157584565761	0.379107924673431	0.0228245716671934	0.577657857056995
**CHOL**	PYGO1	cg23030278	-0.28103362821506	36	-1.70750975998192	0.0968449025974287	0.0789799001877207	0.232701765278736
**CHOL**	PYGO1	cg23412777	-0.00553637827612793	36	-0.0322828501621992	0.974435258722139	3.06514844163813e-05	0.986642198466054
**CHOL**	PYGO1	cg25902187	-0.302323624386114	36	-1.84937539766059	0.0731130250602112	0.0913995738619559	0.190353235325772
**CHOL**	PYGO2	cg01277844	0.263460822504532	36	1.59248989663597	0.120530498808823	0.0694116049947644	0.271507465281981
**CHOL**	PYGO2	cg02341811	-0.143649133776544	36	-0.846389362821304	0.40325358827868	0.0206350736347515	0.599742092770597
**CHOL**	PYGO2	cg04602755	-0.332994630750983	36	-2.05919665678557	0.0472001166933945	0.110885424108983	0.13834768388704
**CHOL**	PYGO2	cg10892866	-0.050143728161594	36	-0.29275394769274	0.771489502842906	0.00251439347394383	0.873319102144566
**CHOL**	PYGO2	cg14024356	0.156508786953671	36	0.923981836758295	0.362007243653549	0.0244950003937096	0.560906459384643
**CHOL**	PYGO2	cg18188585	-0.183304445957998	36	-1.087261759069	0.284569091501288	0.0336005199079686	0.481860992098597
**CHOL**	PYGO2	cg24683561	-0.20066970671101	36	-1.19439055825283	0.240591402262726	0.0402683311914829	0.432642970519093
**CHOL**	PYGO2	cg27105205	-0.356094278514583	36	-2.22202208140179	0.0330405193234619	0.126803135190822	0.106072720380051
**CHOL**	RBBP4	cg05797186	-0.149969291809307	36	-0.88446646250668	0.382656594173479	0.0224907884857852	0.580941945016978
**CHOL**	RBBP4	cg06526778	-0.0479581520718813	36	-0.279963819234993	0.781201561315844	0.00229998435014969	0.879121559128655
**CHOL**	RBBP4	cg09036220	-0.288662194856568	36	-1.75801217633745	0.0877459333158107	0.0833258627394115	0.216922862575805
**CHOL**	RBBP4	cg11229946	-0.174227756103423	36	-1.03169301532489	0.309499290784944	0.030355310996834	0.508144100388051
**CHOL**	RBBP4	cg13641837	-0.150952547963548	15	-0.550576199056229	0.591260208490158	0.0227866717366873	0.754003846684316

**CHOL**	RBBP4	cg16148416	-0.0850463133291155	36	-0.497704144355608	0.621896441082208	0.00723287541087409	0.775989024757792
**CHOL**	RBBP4	cg16396527	-0.284327524342285	36	-1.72927200083535	0.0928315691346248	0.0808421410986128	0.225827545866319
**CHOL**	RBBP4	cg18758482	-0.16226831266618	36	-0.958887171411239	0.344385130097812	0.0263310052955291	0.543647326883857
**CHOL**	RBBP4	cg20740619	0.327841315895018	36	2.02345777911913	0.0509437195769692	0.107479928407777	0.146272817626439
**CHOL**	RBBP4	cg22358606	0.235500105453269	36	1.41292938107064	0.166766041754063	0.0554602996685008	0.339297977095292
**CHOL**	RBBP4	cg22857567	-0.00396923173372318	36	-0.0231445816186851	0.981670247444302	1.57548005559951e-05	0.990519400117797
**CHOL**	RBBP4	cg23016766	-0.122535014781683	36	-0.719920963598984	0.476493830186686	0.0150148298475474	0.664248024828296
**CHOL**	RBBP4	cg26131911	-0.00343865411726765	36	-0.020050745284586	0.984120100683	1.18243421382017e-05	0.991672987016132
**CHOL**	RBBP5	cg00960017	-0.254642910944043	36	-1.53542563459244	0.133933780022549	0.0648430120940559	0.29210699879978
**CHOL**	RBBP5	cg02189925	0.273525557852406	36	1.65814829378145	0.106486935695596	0.0748162307984701	0.249054316502411
**CHOL**	RBBP5	cg03143531	-0.371826952163851	36	-2.3355599011002	0.0255510044292787	0.138255282355458	0.0875915409408764
**CHOL**	RBBP5	cg05290904	-0.0426362394484487	36	-0.248836137332787	0.804984564501318	0.00181784891430545	0.894064634736027
**CHOL**	RBBP5	cg07601985	0.488227107884642	36	3.26203114387903	0.00252033233644056	0.238365708873401	0.0143344592616652
**CHOL**	RBBP5	cg07972983	-0.146576341265137	36	-0.8640114706606	0.393636318098104	0.021484623818674	0.590417579933832
**CHOL**	RBBP5	cg14717946	-0.16543977833531	36	-0.978150405022448	0.334908903537569	0.0273703202556365	0.534493860740623
**CHOL**	RBBP5	cg18131828	-0.304062794307747	36	-1.86109465180991	0.0713960202741464	0.092454182882235	0.187040983371172
**CHOL**	RBBP5	cg21214746	0.272201709064771	35	1.62504096933966	0.113668050490936	0.0740937704177823	0.260886743831849
**CHOL**	RBBP5	cg23227967	-0.216278237071771	36	-1.29167977079217	0.205180077203952	0.0467762758308731	0.38944466222623
**CHOL**	RBBP5	cg24103461	0.0435861287395981	36	0.254390373970942	0.800726302070647	0.00189975061850482	0.891192782855972
**CHOL**	RBBP5	cg26795850	-0.202103655187468	36	-1.20328756876228	0.237176877181334	0.040845887440135	0.428587104683757
**CHOL**	RBBP7	cg01947226	-0.0397613040510967	28	-0.202904120466917	0.840789499444227	0.00158096129984376	0.914688681822802
**CHOL**	RBBP7	cg03048084	0.0350553388679557	36	0.204531705361251	0.839157487614108	0.0012288767831472	0.91388470482823
**CHOL**	RBBP7	cg03171708	-0.0511478040556314	36	-0.298631265094667	0.767039028109446	0.00261609785971327	0.870881496053606
**CHOL**	RBBP7	cg03811055	-0.0689035905488493	36	-0.402730682498426	0.68966749129774	0.00474770479052347	0.82163245163962
**CHOL**	RBBP7	cg07096248	-0.0931608503235866	36	-0.545589165046997	0.588910257761108	0.00867894403301371	0.752339019496964
**CHOL**	RBBP7	cg08363715	-0.0230142300489196	36	-0.134230420912775	0.894011810616504	0.000529654784744592	0.943822998839414
**CHOL**	RBBP7	cg14719055	-0.231226256217229	36	-1.38582502910861	0.174823617932057	0.0534655815642355	0.350277604074651
**CHOL**	RBBP7	cg15289846	-0.0775698126138932	36	-0.453672797019106	0.652946932525689	0.0060170758289545	0.79732766680119
**CHOL**	RBBP7	cg15464143	-0.0921573315739372	36	-0.539661519263502	0.592947590141274	0.0084929737628286	0.755140620896296
**CHOL**	RBBP7	cg16978043	-0.0228955566858548	36	-0.133537894946356	0.894555282962456	0.000524206515955192	0.944029649643655
**CHOL**	RBBP7	cg17878446	-0.0317994687569398	36	-0.185514993284293	0.853927436713976	0.00101120621322359	0.922107767766478
**CHOL**	RBBP7	cg17880859	0.0596657765313192	36	0.348529208692674	0.729591782911396	0.00356000488908532	0.847094356369255
**CHOL**	RBBP7	cg21122900	-0.0918483802110516	36	-0.537836921264463	0.59419299002152	0.0084361249473939	0.755881438271479
**CHOL**	RING1	cg01241018	-0.0777645277240013	36	-0.454818522666493	0.652130644554301	0.00604732177213697	0.796731077541536
**CHOL**	RING1	cg01878788	-0.148340846182902	36	-0.874645155088045	0.387903753469521	0.0220050066462594	0.585771111837253
**CHOL**	RING1	cg02475416	0.033708596225246	36	0.196664966987558	0.845260597195504	0.00113626945947667	0.91729375599117
**CHOL**	RING1	cg03037740	-0.303531313553558	36	-1.85751114488154	0.071917344832185	0.0921312583075482	0.18803945564733
**CHOL**	RING1	cg06316695	-0.130009195046653	36	-0.764566399154271	0.449804001834629	0.0169023907966786	0.640924736781158
**CHOL**	RING1	cg06671577	-0.0671574740282773	36	-0.392478063452842	0.697154769658918	0.00451012631785874	0.826379468692893
**CHOL**	RING1	cg07946923	-0.123895956321769	36	-0.728040759426101	0.471573199465957	0.0153502079928858	0.65974910897992
**CHOL**	RING1	cg08892712	-0.112439858170082	36	-0.659815603562877	0.513817275098889	0.0126427217053082	0.69536908475624
**CHOL**	RING1	cg09478665	-0.199301244123807	36	-1.18590731885098	0.243880688204476	0.0397209859092973	0.43657993552659
**CHOL**	RING1	cg09724776	0.110049766595456	36	0.645616318103478	0.522861459594768	0.0121109511277144	0.702513003174174
**CHOL**	RING1	cg09832551	-0.138895140137296	36	-0.817817912880136	0.419155756573357	0.019291859953759	0.614534270497498
**CHOL**	RING1	cg09869033	-0.142213661439567	36	-0.837755984176431	0.408018479537812	0.0202247255000477	0.604114605404525
**CHOL**	RING1	cg10265934	-0.0909546124753408	36	-0.532559407996433	0.597802231639936	0.00827274153053943	0.758529225382436
**CHOL**	RING1	cg12065531	-0.277008631973672	36	-1.68100609642916	0.101927337299507	0.0767337821879253	0.24153722119528
**CHOL**	RING1	cg12115664	-0.262386779870014	36	-1.58551678258297	0.122107171731722	0.0688468222505551	0.273913880193679
**CHOL**	RING1	cg12256228	0.147947869245506	36	0.872276167521114	0.389176241411241	0.0218885720142853	0.586815585919525
**CHOL**	RING1	cg14046664	-0.341509132161432	36	-2.11870301728709	0.0415015141528965	0.116628487349654	0.125732876081098
**CHOL**	RING1	cg14363494	-0.19900275019206	36	-1.18405788640213	0.244602146894614	0.0396020945840033	0.437478835192192
**CHOL**	RING1	cg18189601	-0.227273535562352	23	-1.06948559061809	0.296990429766018	0.0516532599670119	0.495041321017711
**CHOL**	RING1	cg20505183	0.131295648353263	36	0.772263899844888	0.445293329774163	0.0172385472765036	0.637242679429209
**CHOL**	RING1	cg24536938	-0.0577974652307402	36	-0.337578559603317	0.737756172450936	0.00334054698709863	0.852392999405237
**CHOL**	RING1	cg24550302	-0.171256450558842	36	-1.01356199754639	0.317950688980475	0.0293287718580131	0.517255816695186
**CHOL**	RNF17	cg00202166	-0.181911762610821	36	-1.07871728765035	0.288307423894847	0.0330918893761756	0.485845848160379
**CHOL**	RNF17	cg01405147	-0.0853128367731737	36	-0.499275298097507	0.620800918240392	0.00727828011828619	0.775256226560608

**CHOL**	RNF17	cg07131398	-0.358834855703393	16	-1.43843535240647	0.172289335931643	0.128762453667675	0.346780194460049
**CHOL**	RNF17	cg12020676	-0.0434668958895726	36	-0.253693152506673	0.801260503857846	0.00188937103827494	0.891519489074591
**CHOL**	RNF17	cg14855529	0.0777057168102888	36	0.454472467110513	0.652377150762929	0.00603817842500081	0.796944632179443
**CHOL**	RNF17	cg15523998	-0.193488069024129	36	-1.14995066798126	0.258188745593815	0.0374376328546861	0.45277172903186
**CHOL**	RNF17	cg17806989	-0.187643617591727	36	-1.11392740232633	0.273123297623815	0.0352101272229103	0.469419919478317
**CHOL**	RNF17	cg20477497	-0.134361575157693	36	-0.790624973915013	0.43464326477048	0.0180530328788564	0.628071170384158
**CHOL**	RNF17	cg21831927	-0.275494785625039	36	-1.67106268030623	0.10389048405679	0.075897376906586	0.244804533215589
**CHOL**	RNF17	cg23678722	-0.0418423833575095	36	-0.244194784458367	0.808547593780147	0.00175078504503679	0.896460507525587
**CHOL**	RNF17	cg24753530	-0.293650393873118	36	-1.79123154958115	0.0821621664761525	0.0862305538218376	0.206925558575733
**CHOL**	RNF20	cg04845236	-0.337225471771835	36	-2.0886929676939	0.0442946539509676	0.113721018811737	0.132040399642165
**CHOL**	RNF20	cg14625770	0.0202365807582908	36	0.118022697672804	0.906744180881419	0.000409519200786826	0.95070002936873
**CHOL**	RNF20	cg21219529	-0.0261410124782488	36	-0.152479093634132	0.879710361817866	0.000683352533387959	0.936206636482037
**CHOL**	RNF217	cg00991794	-0.344260724564246	36	-2.13805849944592	0.0397837272912182	0.1185154464775	0.122013029021217
**CHOL**	RNF217	cg02312409	-0.396328378532602	36	-2.51709998243552	0.0167127623147052	0.157076183630281	0.0634002311407883
**CHOL**	RNF217	cg03113502	-0.285162045671026	36	-1.73479608280156	0.0918353126868876	0.0813173922912842	0.224083693468532
**CHOL**	RNF217	cg06993703	-0.355736220744853	36	-2.21946391240294	0.0332298622195347	0.126548258749831	0.106544281478238
**CHOL**	RNF217	cg07439975	-0.311772096091546	36	-1.91329249812298	0.0641617475363066	0.0972018399013161	0.172892240659488
**CHOL**	RNF217	cg10621825	-0.347901889867442	36	-2.16376770519391	0.0375996166111677	0.121035724973338	0.117020412245555
**CHOL**	RNF217	cg16203203	-0.45966494525267	36	-3.01802395488582	0.00479612217666916	0.21129186189414	0.0239864692132305
**CHOL**	RNF217	cg17382198	-0.351237111028283	36	-2.187414432004	0.0356854795865756	0.123367508163495	0.112447998985663
**CHOL**	RNF217	cg18192252	-0.397123692782805	36	-2.52309567815346	0.0164756028909464	0.157707227369452	0.0627072554977269
**CHOL**	RNF217	cg19171596	-0.376453164924619	36	-2.3693823678137	0.0236371950626139	0.141716985381763	0.0826589927493028
**CHOL**	RNF217	cg19478500	-0.439575133932804	36	-2.85362373819156	0.00730836191390313	0.193226298372042	0.0335022516837573
**CHOL**	RNF217	cg20034100	0.287307198967019	36	1.74901559423276	0.0893120607459038	0.0825454265782741	0.219647820859251
**CHOL**	RNF217	cg23521835	-0.372661556314599	36	-2.34164667124564	0.0251965806526885	0.138876635554819	0.0867204705258263
**CHOL**	RNF217	cg24822696	-0.329865809073665	36	-2.03747347145638	0.0494456892556032	0.108811451995824	0.143149787873559
**CHOL**	RPH3A	cg00851732	0.211139929171979	36	1.25954208109602	0.216412657113418	0.0445800696907483	0.403683161431034
**CHOL**	RPH3A	cg00854817	0.260647795272411	36	1.57424000212097	0.124692533161695	0.0679372731803687	0.277917755822604
**CHOL**	RPH3A	cg01119407	-0.104524795748514	36	-0.612835999327718	0.544062517636652	0.0109254329262685	0.718294744026639
**CHOL**	RPH3A	cg01573562	-0.00391431813397421	36	-0.0228243755972397	0.981923794506758	1.53218864539594e-05	0.990643844758107
**CHOL**	RPH3A	cg05967001	0.157096666637934	36	0.927540158275485	0.36018422549878	0.0246793626687502	0.559095150117767
**CHOL**	RPH3A	cg08254200	0.184395071961105	36	1.09395775729919	0.281663531996306	0.0340015425635412	0.478784857197663
**CHOL**	RPH3A	cg09384610	0.144329802893249	35	0.837884547520751	0.40812338798396	0.020831092003204	0.604174971735948
**CHOL**	RPH3A	cg11596863	0.0622381888639735	36	0.363612812469154	0.71839842820951	0.00387359215306764	0.840129390639647
**CHOL**	RPH3A	cg16403344	0.105241097103949	36	0.617082596914962	0.541290986014494	0.0110756885196427	0.716313254278425
**CHOL**	RPH3A	cg17453780	0.06984242911951	36	0.408244763449669	0.68565369874329	0.00487796490531378	0.819064291938097
**CHOL**	RPH3A	cg18629535	0.0494938682674333	36	0.288950494740905	0.774373790783294	0.00244964299607404	0.874956568244405
**CHOL**	RPH3A	cg19712198	0.0690181529939143	36	0.403403484261131	0.689177255778323	0.00476350544269136	0.821290677635093
**CHOL**	RPH3A	cg20704159	-0.0431072604565313	36	-0.251590227732669	0.802872323827266	0.00185823590406722	0.892521428405521
**CHOL**	RPH3A	cg21900624	0.293513055500298	36	1.79031480645641	0.0823121250552712	0.0861499137491212	0.207169091738645
**CHOL**	RPH3A	cg23199238	0.0658291746182982	14	0.228534662963982	0.823078750335283	0.0043334802309264	0.904783002495203
**CHOL**	RPH3A	cg23549179	0.327690346881303	36	2.02241386644304	0.051056867774391	0.107380963439189	0.146500367794371
**CHOL**	RPH3A	cg23596620	0.431718314022206	36	2.790803831688	0.00856028437398469	0.186380702662176	0.0378503332152873
**CHOL**	RTF1	cg04910470	-0.329607947359836	36	-2.03568653227179	0.049634510228792	0.108641398962765	0.143506332260785
**CHOL**	RTF1	cg07091958	-0.535352144997453	36	-3.6958389428001	0.000766982566126184	0.286601919153374	0.00537392071955511
**CHOL**	RTF1	cg07129439	0.0707066487089574	36	0.413321547949907	0.681966393670223	0.00499943017165191	0.816635529388689
**CHOL**	RTF1	cg15581235	-0.0878733649415844	36	-0.514375144941736	0.610317258161656	0.00772172826615687	0.76760140775888
**CHOL**	RTF1	cg18782716	-0.108021352339373	36	-0.633574624054678	0.53059779395606	0.0116686125612269	0.708450741333648
**CHOL**	RTF1	cg22263512	0.014543496133152	36	0.0848113961778974	0.932908773245519	0.000211513279775008	0.964334497299879
**CHOL**	RTF1	cg24280607	0.204164996231564	36	1.2160914237255	0.232325946935443	0.0416833456862345	0.422931733886271
**CHOL**	RTF1	cg27576415	-0.155768018782514	36	-0.919499543875867	0.364312215774329	0.0242636756754297	0.563344620524124
**CHOL**	SATB1	cg01069104	-0.27583765340685	36	-1.67331356088293	0.103443361949291	0.0760864110369976	0.243945461970508
**CHOL**	SATB1	cg01373248	0.0531832364787545	36	0.310548390844073	0.758039780110889	0.00282845664235513	0.865069850723152
**CHOL**	SATB1	cg04004754	-0.147509608275487	36	-0.869634691849276	0.390598219830014	0.0217590845335878	0.587904116652118
**CHOL**	SATB1	cg14415616	-0.317043739313676	36	-1.94922555138206	0.0595569672483112	0.100516732637998	0.163785870691287
**CHOL**	SATB1	cg19818826	-0.23668107997285	36	-1.42043422820617	0.164587316955247	0.0560179336171145	0.336237020357576
**CHOL**	SATB1	cg26131754	-0.257667986041285	36	-1.55495499311487	0.129217080207954	0.0663927910305719	0.284942617001716

**CHOL**	SATB1	cg26768584	-0.140773052121112	36	-0.829097133859811	0.41283246925884	0.0198170522034933	0.60843985134742
**CHOL**	SCML2	cg00457389	-0.230753325552139	36	-1.38283099243976	0.175732001170955	0.0532470972533715	0.351536303301532
**CHOL**	SCML2	cg04189389	-0.212859281960688	36	-1.2702834910832	0.21260800469172	0.0453090739168198	0.39888163793531
**CHOL**	SCML2	cg05049545	-0.234544654633307	36	-1.40686254126136	0.168543798413205	0.0550111950170571	0.341686073855348
**CHOL**	SCML2	cg06131666	-0.0516758599452325	36	-0.301722581935161	0.764701400152197	0.00267039450107928	0.869445218254947
**CHOL**	SCML2	cg08300622	-0.181741357723982	36	-1.07767227193606	0.2887669970494	0.0330299211073563	0.486293749723152
**CHOL**	SCML2	cg17710066	-0.293591559205637	36	-1.79083880925424	0.0822263816166039	0.0861960036367971	0.207067173749906
**CHOL**	SCML2	cg17727916	-0.190906198697394	36	-1.13402146164571	0.264718202785502	0.0364451767010889	0.460327251295254
**CHOL**	SCML2	cg22823767	-0.224416317163601	36	-1.34281135004532	0.188229512297418	0.0503626834092741	0.367714496501063
**CHOL**	SCML2	cg26246138	-0.270531618452527	36	-1.63855685168591	0.110528194085403	0.0731873565825437	0.255873813496375
**CHOL**	SCML4	cg00977377	0.0972493592094025	36	0.569756956268492	0.572588229950671	0.0094574378666394	0.740153679850828
**CHOL**	SCML4	cg03500965	-0.361615555430751	28	-1.9777226067207	0.0586504155925993	0.130765809929491	0.161962179756028
**CHOL**	SCML4	cg05603896	-0.303663729703364	36	-1.85840378143014	0.0717871814084863	0.0922116607373576	0.187750517941661
**CHOL**	SCML4	cg08792995	-0.261292951742341	36	-1.57842171731499	0.12372866038789	0.0682740066302256	0.276510985527594
**CHOL**	SCML4	cg17388996	-0.120798935462745	36	-0.709568958379919	0.482809589309573	0.0145923828089325	0.66981166635308
**CHOL**	SCML4	cg23503775	-0.345843831278116	36	-2.14922279197877	0.0388218167901729	0.119607955633126	0.119802626656143
**CHOL**	SCML4	cg27353361	-0.13985573865396	36	-0.823586381604623	0.415914467550493	0.0195596276344449	0.611495042850848
**CHOL**	SCML4	cg27659622	-0.20044656501474	36	-1.19300678456393	0.241125706591297	0.0401788254262083	0.433321919044513
**CHOL**	SETD1A	cg26692825	0.227988096363876	36	1.3653454708806	0.181110670602092	0.0519785720836241	0.358348820986804
**CHOL**	SETD6	cg01152986	-0.300299338466095	36	-1.83576050011906	0.0751519516600968	0.0901796926831746	0.194160636559582
**CHOL**	SETD6	cg01472538	-0.268710988096148	36	-1.62666832434947	0.113041631005681	0.0722055951236084	0.260053248239176
**CHOL**	SETD6	cg01773760	-0.119771233074876	36	-0.703444028478622	0.48656867135932	0.0143451482722764	0.673034320508649
**CHOL**	SETD6	cg02549819	0.0133427802079425	31	0.0718594672525679	0.943207056221625	0.000178029783677463	0.969718299567398
**CHOL**	SETD6	cg05502081	-0.233403496206118	36	-1.39962216323527	0.170684840588546	0.0544771920412392	0.344558644646582
**CHOL**	SETD6	cg09132913	-0.232741946097763	36	-1.39542758863799	0.171934914196278	0.0541688134733741	0.346273454189692
**CHOL**	SETD6	cg19824325	-0.191354056470354	36	-1.13678282008595	0.263577867194492	0.0366163749276594	0.459007658439384
**CHOL**	SETD6	cg26666090	-0.314998420160043	36	-1.93526049827573	0.0613114253393121	0.099224004703323	0.167240301765591
**CHOL**	SETD7	cg05316864	0.0217193760710115	36	0.126674518748078	0.899944195886674	0.000471731296914028	0.947262255436596
**CHOL**	SETDB1	cg02060408	-0.417090526797318	36	-2.67590339438058	0.0113820539917743	0.173964507544064	0.0471787912814597
**CHOL**	SETDB1	cg08531528	-0.0746042701634384	36	-0.43622958707682	0.665427717920059	0.00556579712661931	0.805834453820436
**CHOL**	SETDB1	cg10589385	-0.364315757074086	36	-2.28107331076786	0.0289298176730752	0.132725970852464	0.0961180820396269
**CHOL**	SETDB1	cg10961700	-0.191871135001316	36	-1.13997189005527	0.262265313317786	0.0368145324466932	0.457653883588162
**CHOL**	SETDB1	cg12082615	-0.232186240861191	36	-1.39190570903726	0.17299002700375	0.053910450445251	0.347872683703791
**CHOL**	SETDB1	cg15448220	0.0281781368099164	36	0.164370628930894	0.870412688853789	0.000794007394078368	0.931222112890113
**CHOL**	SETDB1	cg16370685	-0.498290185072216	17	-2.22588999043199	0.0417758485329216	0.248293108539303	0.126381892310718
**CHOL**	SETDB1	cg17899625	-0.280903380506949	36	-1.70665058820736	0.0970062784682059	0.0789067091802319	0.232956947457739
**CHOL**	SETDB1	cg17918089	0.127629520128124	36	0.750337921604674	0.458212632315313	0.0162892944081352	0.648230469509683
**CHOL**	SETDB1	cg19689029	-0.407685475696653	36	-2.60336877769444	0.0135843726154836	0.166207447094007	0.0541190496875573
**CHOL**	SETDB1	cg26820223	-0.191951817871995	36	-1.1404695886222	0.262060897251137	0.0368455003843635	0.457389514510341
**CHOL**	SETMAR	cg00300090	0.171287464741583	36	1.01375109911426	0.317861735393712	0.0293393955775991	0.517158205493616
**CHOL**	SETMAR	cg02520519	0.22560508358321	36	1.35030481545328	0.185838658063359	0.0508976537385869	0.364637120797541
**CHOL**	SETMAR	cg06021447	0.0772260328845764	36	0.451650085780471	0.654389100471772	0.00596386015508968	0.798361886334212
**CHOL**	SETMAR	cg06267913	0.135886612774461	36	0.799766620854478	0.429398562519588	0.0184651715313163	0.623476646620792
**CHOL**	SETMAR	cg14751177	0.0209896303437715	36	0.122416493981607	0.9032899071357	0.000440564581968175	0.948758505509675
**CHOL**	SETMAR	cg14961031	-0.232169938854421	36	-1.39180241397696	0.173021049120811	0.0539028805076656	0.34790249154293
**CHOL**	SETMAR	cg15232450	0.219630791082284	36	1.31270890620868	0.198073260104626	0.0482376843914298	0.380617310457941
**CHOL**	SETMAR	cg15384599	-0.0996136706078514	36	-0.583745963527175	0.563244154946	0.00992288337196952	0.733216462347207
**CHOL**	SETMAR	cg16423812	-0.0982717440181559	36	-0.575804928082524	0.568539008743398	0.00965733567236997	0.736955296409305
**CHOL**	SETMAR	cg21901604	-0.245849768598104	36	-1.47892956225243	0.148365087460856	0.0604421087197411	0.313374972548392
**CHOL**	SETMAR	cg22149154	0.0784601529777557	36	0.458912088675846	0.649217687732896	0.00615599560529283	0.794782374964481
**CHOL**	SETMAR	cg23182840	-0.434063684285633	36	-2.80947330739876	0.00816872613351368	0.188411282015618	0.0365153845980648
**CHOL**	SETMAR	cg23549342	-0.351571602072856	36	-2.18979118993779	0.0354979711376538	0.123602591384075	0.112017377270941
**CHOL**	SETMAR	cg24390964	-0.00042576327038282	36	-0.0024826053732106	0.998033679027288	1.81274362407077e-07	0.99897078748674
**CHOL**	SETMAR	cg26797270	0.179817328948842	36	1.06588001856192	0.293988703996037	0.0323342717902959	0.491774788358492
**CHOL**	SETMAR	cg27265118	0.150283856945948	36	0.886364480043554	0.381647816611273	0.0225852376585501	0.580084995612859
**CHOL**	SFMBT2	cg09060610	-0.266863598926801	36	-1.61462427172137	0.115635728714065	0.0712161804321646	0.26389831845758
**CHOL**	SFMBT2	cg12328063	-0.169930523223803	36	-1.00548036726728	0.321768223509968	0.0288763827231153	0.521242679127386

**CHOL**	SFMBT2	cg20299663	-0.296305113161606	36	-1.8089759361171	0.079304867971546	0.087796720085712	0.20172785204128
**CHOL**	SFMBT2	cg25291138	-0.110329308092903	36	-0.647276454584361	0.521799648872105	0.0121725562242587	0.701679865246176
**CHOL**	SHPRH	cg00571935	-0.162025673266825	36	-0.95741466416297	0.345116791730052	0.0262523187975679	0.544363659688719
**CHOL**	SHPRH	cg02758840	0.14544595180614	36	0.857203681920407	0.397334354469189	0.0211545248967941	0.593924924517256
**CHOL**	SHPRH	cg06708562	0.0641762409786587	36	0.374981568322619	0.710003182613213	0.00411858990615088	0.834842091084057
**CHOL**	SHPRH	cg11387577	0.0669010057704688	36	0.390972474035087	0.698256892194245	0.0044757445731003	0.826967644115323
**CHOL**	SHPRH	cg11675148	0.249650131975171	36	1.5032981865188	0.141994831050286	0.0623251883952205	0.304231619893854
**CHOL**	SHPRH	cg12989205	0.00972398480545542	36	0.0567027684802914	0.955113893680904	9.45558804967278e-05	0.97610554571028
**CHOL**	SHPRH	cg14102550	-0.0854629994988022	36	-0.500160553787596	0.620184040012256	0.00730392428333226	0.774747040638364
**CHOL**	SHPRH	cg15443174	-0.0853135581867518	36	-0.499279550971595	0.620797954017186	0.00727840321048429	0.775256226560608
**CHOL**	SHPRH	cg16339435	-0.126307665810413	36	-0.742440039694588	0.462919549956526	0.0159536264424749	0.65223246765889
**CHOL**	SHPRH	cg18099523	-0.215972078568207	36	-1.28976177216054	0.205837784980843	0.0466439387210716	0.390159949437154
**CHOL**	SHPRH	cg26814539	-0.0500322974605495	36	-0.292101747806905	0.771983855188306	0.00250323078918091	0.873578006635057
**CHOL**	SHPRH	cg27398948	0.152912451372616	36	0.902235674879204	0.373279269742639	0.0233822177847827	0.57185141228637
**CHOL**	SIN3A	cg01088093	-0.230957400614333	36	-1.38412282682886	0.175339611823784	0.0533413208985297	0.350995053322512
**CHOL**	SIN3A	cg01664094	-0.220017693899121	36	-1.31513890911836	0.197264305854631	0.0484077856286875	0.379531567731493
**CHOL**	SIN3A	cg02028562	-0.38939974053168	36	-2.46514810916108	0.0189025216734515	0.15163215792614	0.0697107488763102
**CHOL**	SIN3A	cg07544511	-0.29121641599125	36	-1.77500248986026	0.0848513346947436	0.0848070009427889	0.21182489788234
**CHOL**	SIN3A	cg10483776	-0.0820737073476263	36	-0.48018786732134	0.63416890959044	0.00673609343778381	0.784539555148944
**CHOL**	SIN3A	cg11184237	-0.30499099743245	36	-1.8673576530444	0.0704926520099701	0.0930195085148406	0.185329684706442
**CHOL**	SIN3A	cg15851274	-0.219255531545304	36	-1.3103526524322	0.19886008111774	0.0480729881132139	0.381560582952457
**CHOL**	SIN3A	cg22059910	-0.0736052397336128	36	-0.430355971865932	0.669652465414965	0.00541773131624261	0.808525948201077
**CHOL**	SIN3A	cg25697800	-0.211783158698249	36	-1.26355912606004	0.214983852365108	0.0448521063082078	0.401972162930617
**CHOL**	SIN3B	cg06815056	0.0461263667641997	36	0.269247209000063	0.789366727574637	0.00212764171086546	0.884089525557562
**CHOL**	SIRT1	cg02720207	-0.145512902207208	36	-0.857606795953416	0.397114772393414	0.0211740047087646	0.593743965611023
**CHOL**	SIRT1	cg03211233	-0.138912644050025	36	-0.81792300412653	0.419096567569445	0.0192967226769689	0.614470652999442
**CHOL**	SIRT1	cg07832903	-0.130025174746303	36	-0.76466198960018	0.449747821178798	0.0169065460678067	0.640903670104894
**CHOL**	SIRT1	cg10582690	-0.203072322901266	36	-1.20930228157076	0.234888878436875	0.0412383683285159	0.425982502077541
**CHOL**	SIRT1	cg10705386	-0.214599018036779	36	-1.28116482417824	0.20880546173935	0.0460527385423498	0.394018631216975
**CHOL**	SIRT1	cg19192316	0.106685953789664	36	0.625651402496353	0.535721087671776	0.0113818927360102	0.712065190161523
**CHOL**	SIRT1	cg19681528	-0.350439167950851	36	-2.18174842676747	0.0361360481255208	0.122807610434085	0.11357794545177
**CHOL**	SIRT1	cg19980381	-0.0627206083942336	36	-0.366442329721886	0.716305603744822	0.0039338747173428	0.838715805805609
**CHOL**	SIRT1	cg25513531	-0.127540956312268	36	-0.749808639514613	0.4585271906165	0.0162666955370479	0.648494160522745
**CHOL**	SIRT4	cg04118464	-0.0254055306971435	24	-0.119200976375967	0.906197922820686	0.000645440990003499	0.950372903123735
**CHOL**	SIRT4	cg07106325	0.225682630735344	36	1.35079385924615	0.18568344325737	0.0509326498156258	0.364452069755617
**CHOL**	SIRT4	cg08264848	0.111364334195766	36	0.653424607782503	0.517877448829456	0.0124020149308663	0.698771956610918
**CHOL**	SIRT4	cg12124973	0.274053825690795	36	1.66161050407914	0.105785636586621	0.0751054993757605	0.247943352493736
**CHOL**	SIRT4	cg20010714	0.0974813869709467	36	0.571129368401467	0.571668122731241	0.00950262080577946	0.739431197455343
**CHOL**	SIRT4	cg21068152	0.303709890193906	36	1.85871498389904	0.0717418495678953	0.0922396974015947	0.1876815228599
**CHOL**	SIRT4	cg21123573	0.00417895442164364	17	0.0161851622062881	0.987299992280864	1.74636600581749e-05	0.993269806321138
**CHOL**	SIRT5	cg01064902	-0.222607685781278	36	-1.33142262036521	0.191908476425487	0.0495541817688961	0.372395310926524
**CHOL**	SIRT5	cg02206980	-0.28217038754629	36	-1.71501268596123	0.0954451571071202	0.0796201276080238	0.230312992090056
**CHOL**	SIRT5	cg02712746	-0.230116214727388	36	-1.37879919761033	0.176961042002218	0.0529534722804613	0.353105068395121
**CHOL**	SIRT5	cg06139021	0.0918811422281082	36	0.538030399012465	0.594060870401202	0.00844214429714185	0.755776687867145
**CHOL**	SIRT5	cg07534331	-0.0732999977483789	36	-0.428561618539709	0.670945288403995	0.00537288966991235	0.809408519153001
**CHOL**	SIRT5	cg07909422	-0.371135470506605	36	-2.33052190315346	0.0258477597800946	0.137741537468159	0.0883480063415061
**CHOL**	SIRT5	cg08619119	-0.19756158740914	36	-1.17513343192643	0.248105550699776	0.0390305808196194	0.441451059218206
**CHOL**	SIRT5	cg10907941	-0.293756455531396	36	-1.79193960154252	0.0820465035124177	0.0862928551663689	0.206701178608278
**CHOL**	SIRT5	cg11824369	-0.266875391766953	36	-1.61470109416482	0.115619028741432	0.0712224747307647	0.263891172453672
**CHOL**	SIRT5	cg19366958	0.0854380835870205	36	0.500013664410811	0.620286378550035	0.0072996661270227	0.774837556929868
**CHOL**	SIRT5	cg20150972	0.0922795154124647	36	0.54038315316992	0.592455375877858	0.00851550896475932	0.754748526365605
**CHOL**	SIRT7	cg11691214	-0.178683368058457	35	-1.04324716571258	0.304416446678734	0.0319277460207141	0.503017897521226
**CHOL**	SIRT7	cg12710567	-0.210157423916912	15	-0.775041945884748	0.452182836973237	0.0441661428273926	0.642680398638823
**CHOL**	SIRT7	cg17552093	0.0149677673532562	36	0.087286109488484	0.930956156501875	0.000224034059541201	0.96346991828577
**CHOL**	SIRT7	cg20397086	-0.220046043242902	17	-0.87364822728756	0.396081552777774	0.0484202611468572	0.592769825836251
**CHOL**	SMARCA	cg03460558	-0.447227802193028	36	-2.91559171918636	0.0062431541704117	0.200012707054406	0.0295947800580713
**CHOL**	SMARCA	cg06675660	-0.412898571938889	36	-2.64344803758828	0.0123230819653833	0.170485230709174	0.0502022328453618

**CHOL**	SMARCA	cg08617833	-0.564983068598764	36	-3.9927028489296	0.00033066506000866	0.319205867803275	0.00265002139469596
**CHOL**	SMARCA	cg12935118	-0.406455739694083	36	-2.59395913274489	0.0138972726853174	0.165206268330264	0.0550695003709038
**CHOL**	SMARCA	cg17420696	-0.363654258631397	35	-2.2425748131668	0.031760988792496	0.132244419820751	0.102954888208818
**CHOL**	SMARCA	cg18959966	-0.569225445666562	36	-4.03697511697097	0.000291219485941206	0.324017607994296	0.00238471360729395
**CHOL**	SMARCA	cg24447042	-0.562445485157849	36	-3.9664444107229	0.000356477693774467	0.316344923774449	0.00282372728942989
**CHOL**	SMARCA	cg26010110	-0.549630279207948	36	-3.83629035128625	0.000516351638169943	0.302093443822206	0.00385425631738558
**CHOL**	SMARCA	cg27353825	-0.202665908707257	18	-0.827843083555116	0.419936372502273	0.0410734705521384	0.615249398382017
**CHOL**	SMARCA	cg04518912	-0.144543001536931	35	-0.839148630163452	0.40742391127981	0.0208926792933052	0.603624890570989
**CHOL**	SMARCA	cg13529335	-0.242742373214285	36	-1.45905838514525	0.153726695847178	0.058923859753703	0.321029372949518
**CHOL**	SMARCA	cg14290704	0.0243476367936672	36	0.142011998020028	0.88790871132668	0.000592807417436338	0.940395126853178
**CHOL**	SMARCA	cg21210735	-0.303908724006636	36	-1.86005563653848	0.071546841055422	0.0923605125273417	0.187316246653614
**CHOL**	SMARCA	cg08405378	-0.076037893766231	36	-0.444660624978012	0.6593828522716	0.00578176128840463	0.801746041304412
**CHOL**	SMARCA	cg17218495	-0.243338125226185	36	-1.46286437857953	0.15268802005217	0.0592134431885943	0.319652836813437
**CHOL**	SMARCA	cg17747332	0.18345313762526	36	1.08817441995142	0.284171822890607	0.0336550537045525	0.481452681794366
**CHOL**	SMARCA	cg18522266	-0.0220363791833613	36	-0.12852427656743	0.898491337219018	0.000485602007512879	0.946352852298873
**CHOL**	SMARCA	cg23892580	-0.0991141045783212	36	-0.5807893392671	0.565212642226689	0.0098236057263624	0.734717643911139
**CHOL**	SMARCA	cg01278696	-0.221611269741718	36	-1.32515449965588	0.193956736790533	0.0491115548765363	0.375147238873008
**CHOL**	SMARCA	cg01575392	-0.283164190163793	36	-1.72157848666293	0.0942341623847286	0.0801819585911165	0.228233263653723
**CHOL**	SMARCA	cg02782292	0.144473006988662	36	0.851346853445966	0.400533276581449	0.0208724497483461	0.59703172482041
**CHOL**	SMARCA	cg05358385	-0.0992186169165608	36	-0.581407846791517	0.564800561234843	0.00984433394283524	0.734423121377859
**CHOL**	SMARCA	cg10878459	-0.165105754931076	36	-0.976120114935438	0.335899321249586	0.0272599103113607	0.535481296692356
**CHOL**	SMARCA	cg12253543	-0.0475103421471846	36	-0.27734371103872	0.783195579587202	0.00225723261094254	0.880365361524859
**CHOL**	SMARCA	cg13848802	-0.116405206610712	13	-0.388714953551889	0.704905576272935	0.0135501721260825	0.831604843636347
**CHOL**	SMARCA	cg13898584	-0.0445707038915872	36	-0.260148157123519	0.796318509502757	0.00198654764539155	0.888484467824802
**CHOL**	SMARCA	cg16091845	-0.106986947148234	36	-0.627436963575805	0.534564244149377	0.011446206860099	0.711271158249212
**CHOL**	SMARCA	cg16587443	-0.185380236056087	10	-0.533583156804815	0.60813173831659	0.0343658319202106	0.765951598002118
**CHOL**	SMARCA	cg19993723	0.101260751398	36	0.593497193349711	0.556776476276175	0.0102537397736876	0.728135514161798
**CHOL**	SMARCA	cg20045045	-0.161080094403619	36	-0.951677922717227	0.347977139853676	0.0259467968130788	0.547225590468751
**CHOL**	SMARCA	cg22077701	-0.0946041247742368	36	-0.554117332335625	0.583125065885751	0.00894994042429936	0.748142679566523
**CHOL**	SMARCA	cg24607315	-0.376677674057322	36	-2.37102898203264	0.0235474264155053	0.141886070133234	0.0824006338251908
**CHOL**	SMARCB	cg01745726	-0.315745557528245	36	-1.94035832522796	0.0606658603351822	0.099695257098822	0.165972918548098
**CHOL**	SMARCB	cg01912455	0.164274198026738	36	0.971067171574734	0.33837279346618	0.0269860121373279	0.537782403671406
**CHOL**	SMARCB	cg03979008	-0.0443136405156871	36	-0.258644781140293	0.797468750361187	0.00196369873575354	0.889246527135357
**CHOL**	SMARCB	cg10122452	0.0287713029657312	36	0.167833563221055	0.867708565899012	0.000827787874345895	0.929722748785177
**CHOL**	SMARCB	cg16142952	-0.0627400845977081	36	-0.366556568172612	0.716221155009725	0.00393631821532757	0.838674496518738
**CHOL**	SMARCB	cg19906397	-0.249888746121167	36	-1.50483068012728	0.141601663840835	0.062444385438009	0.303740586120548
**CHOL**	SMARCC	cg02564102	0.134006428380478	36	0.788496914941357	0.4358696882493	0.0179577228472921	0.629021751338739
**CHOL**	SMARCC	cg02627110	-0.405771632011739	36	-2.5887318215246	0.014073954863557	0.16465061734547	0.0555686567860507
**CHOL**	SMARCC	cg05592996	-0.185457753025795	23	-0.864877886757253	0.396878340146558	0.0343945781573769	0.593466904815335
**CHOL**	SMARCC	cg07143957	-0.208052461094834	36	-1.24028420231715	0.223361656082528	0.0432858265676175	0.412175130732299
**CHOL**	SMARCC	cg07304068	-0.0949890077814471	36	-0.556392161435755	0.581586595214543	0.00902291159930382	0.74699424759147
**CHOL**	SMARCC	cg07474652	-0.11921732278132	36	-0.70014377101848	0.488600985991359	0.0142127700511453	0.674633933907418
**CHOL**	SMARCC	cg12894336	-0.107461701668107	36	-0.630253670289465	0.53274201438791	0.0115480173254052	0.709951560012558
**CHOL**	SMARCC	cg16250355	-0.152002262762574	36	-0.896737845319704	0.376164634102208	0.0231046878849425	0.574692685493857
**CHOL**	SMARCC	cg20685352	-0.0438443120273806	36	-0.255900154077526	0.799569862633587	0.00192232369715431	0.890542727586256
**CHOL**	SMARCC	cg22984439	-0.245121688929971	36	-1.4742693136915	0.149608929614878	0.0600846423838816	0.315083742849318
**CHOL**	SMARCD	cg02553054	-0.105806148648393	36	-0.620433193870967	0.539109436771445	0.0111949410918058	0.714599428822647
**CHOL**	SMARCD	cg04550398	-0.106309163345966	36	-0.623416441023584	0.537170946508006	0.0113016382113193	0.713131403278169
**CHOL**	SMARCD	cg09878554	0.249450529449063	36	1.50201646844523	0.142324332921522	0.062225566642418	0.304736659213121
**CHOL**	SMARCD	cg13349421	0.000698417097080299	36	0.00407243748885274	0.99677447531004	4.87786441494071e-07	0.998352969992265
**CHOL**	SMARCD	cg17846064	-0.0748823864760576	36	-0.437864954908822	0.664253402418885	0.00560737180434966	0.804990787652399
**CHOL**	SMARCD	cg18591973	-0.0594990959148295	36	-0.347552103470241	0.730318992238727	0.00354014241468208	0.847495650950186
**CHOL**	SMARCD	cg20405918	-0.0645615499905583	36	-0.377242322775512	0.708338064898811	0.00416819373718336	0.833791575311142
**CHOL**	SMARCD	cg22402853	-0.0986766026699946	36	-0.578200398751717	0.566939162051177	0.00973707191449198	0.73578567903322
**CHOL**	SMARCD	cg25107077	0.0510625623772206	36	0.298132271382342	0.767416571315514	0.00260738527652754	0.871163449049525
**CHOL**	SMARCD	cg00945038	0.293490036764701	36	1.79016116667835	0.0823372797348728	0.0861364016801453	0.207205587930472
**CHOL**	SMARCD	cg02127607	0.09295961945642	36	0.544400388439115	0.589718872861524	0.00864149084948242	0.752803367115561

**CHOL**	SMARCD	cg02360836	-0.213903805518651	36	-1.27681502971196	0.210319301735317	0.045754838015361	0.396010740613739
**CHOL**	SMARCD	cg04367190	-0.160017907297043	36	-0.945236937438573	0.35120733561773	0.025605730655725	0.550155520660579
**CHOL**	SMARCD	cg06420487	-0.277399809695026	36	-1.6835776741496	0.10142467520015	0.0769506544188366	0.240702598612582
**CHOL**	SMARCD	cg07362569	0.343029265864921	36	2.12938839248121	0.0405452501299771	0.117669077239826	0.123581757184165
**CHOL**	SMARCD	cg11697258	-0.248099814040113	36	-1.49334840322408	0.144568827764535	0.0615535177267386	0.307956938310905
**CHOL**	SMARCD	cg12577610	-0.33202299453265	36	-2.0524425206854	0.0478885185447519	0.110239268898428	0.139817579721165
**CHOL**	SMARCD	cg15868002	-0.236861285885569	36	-1.42157998468519	0.164256672256716	0.0561032687513654	0.335729054978559
**CHOL**	SMARCD	cg19016967	0.0472652790843029	36	0.275909933262366	0.78428737900235	0.00223400660691704	0.881003759928425
**CHOL**	SMARCD	cg19143629	0.137089911573029	36	0.806983729269861	0.425285257143078	0.0187936438551008	0.620085211739465
**CHOL**	SMARCD	cg21254277	-0.321951245215045	36	-1.98285696513873	0.0555093662875309	0.103652604295518	0.155594801597255
**CHOL**	SMARCD	cg23682543	-0.28421301588467	36	-1.72851435054315	0.0929689138640092	0.0807770383982595	0.226048560248062
**CHOL**	SMARCD	cg24795630	-0.311213672186651	36	-1.90949767156767	0.0646655276626963	0.0968539497559004	0.173882342780816
**CHOL**	SMARCD	cg25945412	-0.211647960866391	36	-1.26271465702726	0.215283626799929	0.0447948593389013	0.402316297568314
**CHOL**	SMARCD	cg00864064	-0.244956061057505	36	-1.47320954366536	0.149892943842525	0.0600034718488083	0.315488533137236
**CHOL**	SMARCD	cg02917064	0.202653037097277	36	1.20669837234832	0.235877394915113	0.0410682534447504	0.427141225331403
**CHOL**	SMARCD	cg14795305	0.263791916560747	20	1.16027158991581	0.261096744297251	0.0695861752427919	0.456382513051281
**CHOL**	SMARCD	cg19786287	-0.256519371855497	36	-1.54753401426715	0.130993267964126	0.0658021881371388	0.28765205158002
**CHOL**	SMARCD	cg19902439	-0.205812830022894	36	-1.22633894187688	0.228496814907049	0.0423589210020328	0.418398993029987
**CHOL**	SMARCD	cg22750243	-0.235762794384753	36	-1.41459813996156	0.166279638290941	0.0555840952161075	0.33863604233633
**CHOL**	SMARCD	cg22787816	0.0310678206673758	36	0.181242457374801	0.857253414500059	0.000965209481020223	0.924069505294761
**CHOL**	SMNDC1	cg03999743	-0.229297470842553	36	-1.37362073157014	0.178549431679251	0.0525773301347915	0.355144958913238
**CHOL**	SMNDC1	cg08770590	-0.0126228072002702	36	-0.0736088460386667	0.941752990001304	0.000159335261615194	0.968934244198939
**CHOL**	SMNDC1	cg11559952	-0.0743220546990765	36	-0.434570220219175	0.66662014337203	0.00552376781469252	0.806517067394769
**CHOL**	SMNDC1	cg12486840	0.0460319652166993	36	0.268695000302657	0.789788128935928	0.00211894182171142	0.884332623781131
**CHOL**	SMNDC1	cg17981675	-0.0214107260885062	36	-0.124873539350267	0.901359079055952	0.000458419191637041	0.947956498370603
**CHOL**	SMNDC1	cg18209001	0.150982876176193	36	0.890583196844233	0.379411721707799	0.0227958288984356	0.577917010524867
**CHOL**	SMNDC1	cg21872391	-0.0821389932621662	36	-0.480572428539228	0.63389832497296	0.00674681421412218	0.784440122966977
**CHOL**	SMNDC1	cg23721301	-0.102774222969538	36	-0.602461760203466	0.550864103129053	0.0105625409069923	0.723346196050596
**CHOL**	SMNDC1	cg26944434	-0.0572551822807731	36	-0.334400772476495	0.740131257047153	0.00327815589800455	0.853712279976369
**CHOL**	SMYD1	cg00578637	0.21551238664302	36	1.28688267812208	0.20682806698902	0.0464455887965707	0.391569507759456
**CHOL**	SMYD1	cg00666842	-0.341466098264362	36	-2.11840079658284	0.0415288484024743	0.116599096263887	0.1257765020876
**CHOL**	SMYD1	cg03704645	-0.0816465834626597	36	-0.477672083127495	0.635940322477562	0.00666616459112505	0.785670895301218
**CHOL**	SMYD1	cg14559139	-0.119338804144977	36	-0.700867513239732	0.488154893221065	0.0142417501747531	0.67426227887647
**CHOL**	SMYD1	cg16090764	-0.158901770771353	36	-0.938472439185367	0.354621077372188	0.0252497727542717	0.553553358873608
**CHOL**	SMYD1	cg18148488	0.00835958587803329	36	0.0487460463974828	0.961406909517827	6.98826760522137e-05	0.979816870308994
**CHOL**	SMYD1	cg19074493	-0.1224093211186	36	-0.719171245282928	0.476949640042589	0.0149840418967166	0.66464523596011
**CHOL**	SMYD2	cg01770831	-0.286975122132095	36	-1.74681249797677	0.0896991408249824	0.0823547207227306	0.22032023343774
**CHOL**	SMYD2	cg04406444	-0.182102815920195	36	-1.07988905027987	0.287792723361775	0.0331614355660645	0.485283344992
**CHOL**	SMYD2	cg11523160	-0.27978815175372	36	-1.69929822777127	0.0983964560669875	0.0782814098617624	0.235361249062978
**CHOL**	SMYD2	cg14013804	0.0514909769937227	36	0.300640221211268	0.765519618794011	0.00265132071176808	0.869983167275306
**CHOL**	SMYD2	cg27570636	0.13859534947027	36	0.816018129410811	0.420170219247186	0.0192086708947862	0.615436761141634
**CHOL**	SMYD5	cg01329511	0.39540413255975	36	2.51014074302473	0.0169919571202499	0.156344428045328	0.0642215572177389
**CHOL**	SMYD5	cg05103132	-0.180120632764616	36	-1.06773810550539	0.293161565080962	0.0324434423475257	0.490836148284874
**CHOL**	SMYD5	cg06754047	-0.00654764051312598	36	-0.0381797952799345	0.969767631510232	4.28715962891287e-05	0.984061878304425
**CHOL**	SMYD5	cg08834152	-0.192910088184398	36	-1.14638259293337	0.25964108737078	0.0372143021233124	0.454542807816065
**CHOL**	SMYD5	cg15623359	-0.167452362602277	26	-0.832094726692604	0.413558768922848	0.0280402937410846	0.609153629525783
**CHOL**	SMYD5	cg17895873	-0.270138797302447	36	-1.63599017157744	0.111066896905614	0.0729749698080126	0.256664038078521
**CHOL**	SMYD5	cg20402064	-0.238030055557297	36	-1.42901484984918	0.162123788717655	0.0566583073486097	0.332703710191624
**CHOL**	SMYD5	cg25886337	-0.258296107030337	36	-1.55901614760601	0.12825335787007	0.0667168789070274	0.283414761735542
**CHOL**	SMYD5	cg26467949	0.354028244141541	36	2.20727651812771	0.0341452893713219	0.125335997649943	0.108738944588644
**CHOL**	SP100	cg05876232	0.0193506625227798	36	0.112853913250969	0.910810082617522	0.000374448140070514	0.952746161049252
**CHOL**	SP100	cg09456094	0.045936228328301	36	0.268134987799647	0.790215551136048	0.00211013707302981	0.884696609174189
**CHOL**	SP100	cg11702456	0.1244298299034	36	0.731227150124814	0.469650256642997	0.0154827825697892	0.658419590290554
**CHOL**	SP100	cg11827097	0.0454156280596406	36	0.265089868038308	0.792540849598387	0.00206257927205161	0.88626019313369
**CHOL**	SP100	cg23539753	-0.238418411892398	36	-1.4314867457968	0.161419516848222	0.0568433391292929	0.331681719326217
**CHOL**	SP100	cg27140170	0.0503682411860324	36	0.294068047664052	0.770493740174746	0.00253695972017433	0.872846712312954
**CHOL**	SP140L	cg04002454	0.0479400357359969	36	0.279857818399872	0.781282203580809	0.00229824702636866	0.879172666141421

**CHOL**	SP140L	cg05217331	-0.182668872948552	36	-1.08336151811249	0.28627123438903	0.0333679171442944	0.483559806698031
**CHOL**	SP140L	cg13278004	-0.122058704875314	36	-0.717080128754245	0.478222303100315	0.0148983274358391	0.665786639596727
**CHOL**	SP140L	cg24044052	-0.242941881552628	36	-1.46033275914145	0.153378289354785	0.0590207578123311	0.320586545637072
**CHOL**	SP140L	cg25095994	-0.265243005190212	36	-1.60407459723151	0.117947937658371	0.0703538518023351	0.267573832444747
**CHOL**	SP140L	cg26847438	0.0346697098276171	36	0.202279015319435	0.840904136923929	0.00120198877953117	0.914786486384754
**CHOL**	SSRP1	cg01511567	0.33740932795955	36	2.08997798098111	0.044171738647559	0.113845054594115	0.131774983178594
**CHOL**	SSRP1	cg01766191	-0.404156624978527	36	-2.57641207886079	0.0144985783596918	0.163342577514034	0.0568413246441496
**CHOL**	SSRP1	cg02688348	-0.0235753163635447	36	-0.137504753197007	0.891442927067832	0.000555795541641217	0.942509562080416
**CHOL**	SSRP1	cg04937561	-0.342332290343856	36	-2.1244868431556	0.0409814552730034	0.11719139701207	0.124550194612963
**CHOL**	SSRP1	cg08821673	-0.412127246396262	36	-2.63749839167096	0.0125032088545896	0.169848867222165	0.0507632498112804
**CHOL**	SSRP1	cg09381635	-0.345936505486592	36	-2.1498769880002	0.0387660968936636	0.119672065828275	0.119654409686783
**CHOL**	SSRP1	cg11948159	0.305313292547084	36	1.86953369315536	0.0701810829664016	0.0932162066059413	0.184729053232558
**CHOL**	SSRP1	cg12795368	-0.182236275970583	36	-1.08070765863963	0.287433531360977	0.0332100602796266	0.484940054917055
**CHOL**	SSRP1	cg12911972	-0.441529335363515	36	-2.86937359180189	0.00702254369521242	0.194948153986547	0.0324798908576311
**CHOL**	SSRP1	cg16313264	-0.148164454412552	36	-0.873581758142818	0.388474623060032	0.0219527055513691	0.586223518512029
**CHOL**	SSRP1	cg22636253	-0.469225623954932	36	-3.09828923286831	0.00389018842251077	0.220172686175895	0.0203150512278179
**CHOL**	SSRP1	cg26706201	-0.2659220785958	36	-1.60849341424208	0.116974878586588	0.070714551884711	0.266061833719951
**CHOL**	STK31	cg00755064	0.195864493548012	36	1.16463427550681	0.252273905758368	0.0383628998328193	0.446143268193559
**CHOL**	STK31	cg05000488	-0.833941500094698	36	-8.81153865444075	2.68364110302525e-10	0.695458425580194	9.85234431188044e-09
**CHOL**	STK31	cg05407490	-0.825490804202446	36	-8.5281084560471	5.82349461547053e-10	0.681435067822802	2.00812473455636e-08
**CHOL**	STK31	cg11755819	-0.799835533935561	36	-7.77016517309763	4.85269905925878e-09	0.639736881345984	1.40809724213345e-07
**CHOL**	STK31	cg13313214	0.0236005370342112	36	0.137651936534411	0.891327481836784	0.000556985348303173	0.942488291382577
**CHOL**	STK31	cg13514718	-0.808237644134383	36	-8.00330008716432	2.50953768549848e-09	0.653248089395898	7.71038604682736e-08
**CHOL**	STK31	cg13802883	-0.639674141054007	36	-4.85256949872301	2.66522227730157e-05	0.409183006733181	0.000307304434326957
**CHOL**	STK31	cg14898779	-0.840805867291926	36	-9.05665175327085	1.38463604134088e-10	0.706954506472527	5.37238784040259e-09
**CHOL**	STK31	cg17102984	-0.354940801074613	36	-2.21378498137171	0.0336536578299188	0.125982972267488	0.10752164347832
**CHOL**	STK31	cg17331904	0.153872849262289	36	0.908039333944291	0.370248973350265	0.0236768537400952	0.568930368558848
**CHOL**	STK31	cg21519371	-0.0708300126458107	36	-0.41404631492992	0.681440634108379	0.0050168906914057	0.816222095981819
**CHOL**	STK31	cg25570411	0.168048553756197	36	0.99401925978684	0.327235522356859	0.0282403164195493	0.526737147960119
**CHOL**	STK31	cg25612145	-0.843089713331388	36	-9.1414334813996	1.10335090042688e-10	0.710800264725201	4.38152526532969e-09
**CHOL**	SUV39H1	cg05903800	0.125587045401461	36	0.738136136120374	0.465496368634251	0.0157721059726687	0.654757162098692
**CHOL**	SUZ12	cg00160853	-0.402108021672387	36	-2.56082614499236	0.0150526750217605	0.161690861093281	0.0585382259081394
**CHOL**	SUZ12	cg01109633	-0.0461679171878756	36	-0.26949026350655	0.789181268270696	0.00213147657746654	0.883982733334658
**CHOL**	SUZ12	cg07482957	0.0107377493973719	36	0.0626149100254973	0.950439817364716	0.000115299262120761	0.973799705223446
**CHOL**	SUZ12	cg08177746	-0.0214821582323169	36	-0.125290344275835	0.90103160000345	0.000461483122318303	0.94784255044716
**CHOL**	SUZ12	cg17335595	0.0571369654095692	36	0.333708059163663	0.740649337105358	0.00326463281621431	0.854104282823391
**CHOL**	SUZ12	cg25098644	-0.152092731726097	36	-0.897284201936502	0.375877253910807	0.0231321990439065	0.574394577896378
**CHOL**	TAF3	cg00194465	-0.0239980022612541	36	-0.139971507645844	0.889508414456722	0.000575904112531158	0.941467059185646
**CHOL**	TAF3	cg02334915	-0.0119552271364117	36	-0.0697153366202487	0.944828663755398	0.000142927455883196	0.970477185404783
**CHOL**	TAF3	cg03672997	0.0422916582175469	36	0.246821453902335	0.806530662629426	0.0017885843547898	0.895130504838216
**CHOL**	TAF3	cg04179819	0.0652925532682157	36	0.381531863388378	0.705182687370724	0.00426311751228279	0.831751197484962
**CHOL**	TAF3	cg04234156	-0.206817644337043	36	-1.23259300207576	0.22618307596636	0.0427735380091238	0.415678861604702
**CHOL**	TAF3	cg05976695	0.0825651097223353	36	0.483082585132871	0.632133388217772	0.00681699734346127	0.783110147191116
**CHOL**	TAF3	cg07855525	-0.149197548039602	36	-0.87981109134369	0.385138098426857	0.0222599083410295	0.583236324560918
**CHOL**	TAF3	cg09871669	-0.0526122626578332	36	-0.307205046767065	0.760561121871148	0.00276805018197683	0.866777397267021
**CHOL**	TAF3	cg09994553	-0.703748124365124	10	-2.801747507101	0.0231354354878172	0.49526142254743	0.0813063818367931
**CHOL**	TAF3	cg11104088	-0.0221345179623164	36	-0.129096938004191	0.898041622498652	0.000489936885424106	0.946027651130161
**CHOL**	TAF3	cg12176426	0.0673499034201832	36	0.393607765877653	0.696328242168629	0.004536009490708	0.825905573382327
**CHOL**	TAF3	cg12590721	0.0295378123241503	36	0.172308747314957	0.864216399469091	0.000872482356896724	0.927885207302341
**CHOL**	TAF3	cg12777293	0.00269449593432649	36	0.0157115332093053	0.987556362403213	7.26030834010197e-06	0.993379188197206
**CHOL**	TAF3	cg27268107	0.0861583675885684	36	0.504260412258435	0.617330754639243	0.00742326430552687	0.772646707098354
**CHOL**	TCEA1	cg01677098	-0.258576871156901	36	-1.5608321298595	0.127824312933718	0.0668619982972924	0.282731926881968
**CHOL**	TCEA1	cg06047473	-0.247629356479943	36	-1.49033147706315	0.14535664083955	0.0613202981906708	0.309203317313499
**CHOL**	TCEA1	cg11530293	-0.199754115733393	36	-1.18871391340976	0.242788827464484	0.0399017067524298	0.435296474850705
**CHOL**	TCEA1	cg11787876	-0.0635617235941536	36	-0.371376309241303	0.712661563075527	0.00404009270625958	0.836505756872285
**CHOL**	TCEA1	cg12850379	-0.31785572387984	36	-1.95477799621717	0.0588715823404051	0.101032261203177	0.162377446431111
**CHOL**	TCEA1	cg23569945	-0.223754417307283	36	-1.33864176050936	0.189570082181705	0.0500660392645216	0.369507528582185

**CHOL**	TCEA1	cg26181865	-0.272069424565983	36	-1.64861333666951	0.108438237771923	0.0740217717836653	0.252411485869034
**CHOL**	TCEA1	cg26358945	-0.242501572393061	36	-1.45752051441455	0.154147977276998	0.0588070126131068	0.321596416815
**CHOL**	TDRD1	cg00337703	-0.448207325855339	36	-2.92358061807071	0.00611696348381674	0.200889806950394	0.0291315002914013
**CHOL**	TDRD1	cg02635786	-0.807822130026204	28	-6.98832160859981	2.02398779489087e-07	0.652576593760074	4.09929983118304e-06
**CHOL**	TDRD1	cg16550453	-0.968720902537975	36	-22.7624469431269	3.68530119556993e-22	0.938420187013989	5.8748836313149e-20
**CHOL**	TDRD1	cg23504215	-0.843318455360349	36	-9.15001814543757	1.07832608082957e-10	0.711186017151366	4.29310640389228e-09
**CHOL**	TDRD1	cg23825092	-0.922970655299637	36	-13.9834045349835	1.16529540189751e-15	0.851874830544242	9.82155910024e-14
**CHOL**	TDRD1	cg24203376	-0.877891284763793	36	-10.6898507573728	2.05653231651853e-12	0.770693107864223	1.11566161109653e-10
**CHOL**	TDRD1	cg26600802	-0.926043340275539	36	-14.3070172126501	5.97489992893299e-16	0.857556268068678	5.19923227149231e-14
**CHOL**	TDRD1	cg27547703	-0.909695022138366	35	-12.5838906258507	3.82909446891238e-14	0.827545033303322	2.67982133496709e-12
**CHOL**	TDRD3	cg01557749	-0.392272834276507	36	-2.48663013348457	0.0179670687991179	0.153877976511324	0.0670534972491523
**CHOL**	TDRD3	cg03447530	-0.240991344036684	36	-1.44788197594709	0.156809244640389	0.0580768279006075	0.32540595330651
**CHOL**	TDRD3	cg05666423	-0.369361921670005	36	-2.317620698753	0.0266219243726711	0.136428229179759	0.0903076782212348
**CHOL**	TDRD3	cg10765900	-0.129455826240898	36	-0.761256524321983	0.451751851386826	0.0167588109477137	0.642361241578993
**CHOL**	TDRD3	cg18522102	-0.0582484079050555	36	-0.340221319388792	0.735782957298356	0.00339287702347374	0.851001765378241
**CHOL**	TDRD3	cg19937698	-0.338623725051352	36	-2.09847247252232	0.0433667530251724	0.114666027167653	0.129966607012877
**CHOL**	TDRD3	cg20944895	-0.56284046467847	36	-3.97052074951633	0.000352345346117931	0.316789388679476	0.00279526349744018
**CHOL**	TDRD3	cg22045829	-0.00289223723486788	36	-0.0168645667214983	0.986643238353309	8.3650362227562e-06	0.993007258731262
**CHOL**	TDRD3	cg23103367	-0.17771487786805	36	-1.0530086748206	0.299763339801807	0.031582577815656	0.498030694868908
**CHOL**	TDRD3	cg23767211	-0.237541775684761	36	-1.42590796941865	0.163012407884783	0.0564260951954692	0.333790989128995
**CHOL**	TDRD3	cg26737458	-0.413740694442331	36	-2.64995160888033	0.0121289327558326	0.171181362237623	0.0495748539277699
**CHOL**	TDRD5	cg05378938	-0.387793863353556	36	-2.45317805002102	0.0194428633413695	0.150384080454677	0.071238607327548
**CHOL**	TDRD5	cg07171496	-0.540358380951128	36	-3.74455996277771	0.000668960819629889	0.291987179864124	0.00479686035600402
**CHOL**	TDRD5	cg07901138	-0.552333124262443	36	-3.86340826960801	0.000478129712402468	0.305071880157511	0.00360562454891795
**CHOL**	TDRD5	cg09656934	0.228882319654262	36	1.37099612218905	0.179358697459284	0.052387116250316	0.356199083788101
**CHOL**	TDRD5	cg11853788	0.093069938813493	36	0.545052094581678	0.589275511063627	0.00866201351074734	0.752558468483687
**CHOL**	TDRD5	cg12277666	-0.43906854537422	36	-2.84954911417237	0.00738406054591004	0.192781187537033	0.033766494598432
**CHOL**	TDRD5	cg13848905	-0.463792134462473	36	-3.05250611668572	0.0043848591582533	0.215103143989257	0.0223436805129793
**CHOL**	TDRD5	cg15545772	-0.399977987691874	36	-2.54466932435257	0.0156475719970819	0.159982390638041	0.0603065254367596
**CHOL**	TDRD7	cg24339935	-0.292439200926919	36	-1.78315092890686	0.0834919750181672	0.0855206862387746	0.20919110775108
**CHOL**	TDRD9	cg07613153	-0.399165292374687	36	-2.5385177992156	0.0158796948103473	0.15933293063657	0.0609565270934649
**CHOL**	TDRD9	cg08933517	-0.176617279195933	36	-1.04629503217717	0.302806529389394	0.0311936633105742	0.5011708007429
**CHOL**	TDRD9	cg16701547	-0.181238899662034	36	-1.07459150910703	0.29012484381593	0.032847538750705	0.487672524358084
**CHOL**	TRIM24	cg00733782	0.141237125917759	36	0.831885881786179	0.411278206672462	0.0199479257375088	0.607091491931819
**CHOL**	TRIM24	cg02308366	0.271272604566065	36	1.64340083523971	0.109517396380086	0.0735888259880565	0.254162952596653
**CHOL**	TRIM24	cg06386929	0.170518054850909	36	1.0090607214539	0.320073124444437	0.0290764070301375	0.519470273987244
**CHOL**	TRIM24	cg06650216	0.198415143992621	36	1.1804181511872	0.246026565711153	0.0393685693656126	0.439047339089612
**CHOL**	TRIM24	cg10571650	-0.266842919554978	36	-1.61448956137008	0.115665017375346	0.0712051437166244	0.263924647210354
**CHOL**	TRIM24	cg10703331	0.00150247526265322	36	0.00876087086826629	0.993061130180214	2.25743191488485e-06	0.996429783715412
**CHOL**	TRIM24	cg13935558	-0.0822652502719946	36	-0.481316152568316	0.633375171614956	0.00676757140231391	0.784049202797625
**CHOL**	TRIM24	cg26889928	0.224049315154439	36	1.34049920657765	0.188971987350567	0.0501980956211733	0.368711263423289
**CHOL**	TRIM33	cg00760200	-0.112614908352068	36	-0.660856011173673	0.513157949513654	0.0126821175831448	0.694839425695181
**CHOL**	TRIM33	cg02852257	0.0564302354056745	36	0.329567138563609	0.743748885618259	0.00318437146793984	0.85604766719286
**CHOL**	TRIM33	cg05792124	0.112698425214525	36	0.661352414098418	0.512843532057991	0.0127009350458338	0.694640966193445
**CHOL**	TRIM33	cg16036305	-0.0638883319429499	36	-0.37329240653977	0.711248249479569	0.00408171895845255	0.835586189857948
**CHOL**	TRIM33	cg17645130	0.0628010454827528	36	0.366914139400982	0.715956850303877	0.00394397131372678	0.83852542773164
**CHOL**	TRIM33	cg18199720	0.212659935661401	36	1.26903747255607	0.213046745781857	0.0452242482355111	0.399435050282066
**CHOL**	TRIM33	cg20932765	-0.156360208685864	36	-0.923082680903724	0.362468858234948	0.0244485148602869	0.561406306413332
**CHOL**	TRIM33	cg20992663	-0.142791272299325	36	-0.841229264132894	0.406097324888657	0.02038934744486	0.602428162933357
**CHOL**	TRIM33	cg21666217	0.140442481936864	36	0.827110986251377	0.413941629452574	0.0197240907325863	0.609520274107237
**CHOL**	TRIM33	cg21813112	-0.458855388515851	36	-3.01128945702418	0.00488058678107041	0.210548267570033	0.0243119912945246
**CHOL**	TRIM66	cg06492264	-0.243252153313301	36	-1.46231503391621	0.152837593802555	0.0591716100915578	0.319877529520472
**CHOL**	TRIM66	cg11245569	-0.471065373726158	16	-1.99815058261588	0.0655084039312786	0.221902586323765	0.175572272287945
**CHOL**	TRIM66	cg12608775	-0.376369681655316	36	-2.36877020170982	0.0236706475805746	0.141654137269324	0.0827484615428499
**CHOL**	TRIM66	cg13143389	-0.207425137047054	36	-1.23637606952037	0.224792002259605	0.0430251874789891	0.414125873835564
**CHOL**	TRIM66	cg27044202	-0.332857183276701	36	-2.05824077079	0.0472970124528138	0.1107939044589	0.138548171357992
**CHOL**	UBE2A	cg00142683	0.10712713757782	36	0.628268664632094	0.534025844901773	0.0114762236056171	0.710943896265741

CHOL	UBE2A	cg00428314	0.151097020472259	36	0.891272209495713	0.379047317593252	0.0228303095955943	0.57761076227399
CHOL	UBE2A	cg00935924	-0.0136444836844517	36	-0.0795677349783916	0.93704754470226	0.000186171935015269	0.966376475714444
CHOL	UBE2A	cg05206294	0.107867063034148	36	0.632659014280849	0.53118851303056	0.0116353032876128	0.708913540387013
CHOL	UBE2A	cg06206449	0.123996799898278	36	0.728642588655568	0.471209656805635	0.0153752063850135	0.65956464862963
CHOL	UBE2A	cg06273903	0.131713119188825	36	0.774762690095571	0.443834869260633	0.0173483457664497	0.635915902078637
CHOL	UBE2A	cg09080960	0.1109506638501	36	0.650967109594425	0.519443290741243	0.0123100498087779	0.700035883238185
CHOL	UBE2A	cg14625604	0.10344646579509	36	0.606444928918739	0.548247485935109	0.0107011712854948	0.721420245616303
CHOL	UBE2A	cg15661671	0.0964341320922718	36	0.564935742659258	0.575826329570276	0.00929954183238972	0.742538016884201
CHOL	UBE2A	cg16211147	0.0867066408340454	36	0.507493529434634	0.615084919919868	0.00751804156472415	0.770994531039068
CHOL	UBE2A	cg17976175	0.0425966821969384	36	0.248604851108128	0.805162016657525	0.00181447733418697	0.894185126466552
CHOL	UBE2A	cg18990292	0.132202413981935	36	0.777691919925614	0.4421288087329	0.01747747826265	0.634511428410718
CHOL	UBE2A	cg25165659	0.119390823295404	36	0.701177434369223	0.487963937421363	0.0142541686871543	0.674137214486903
CHOL	UHRF2	cg06523306	-0.0189892206586456	36	-0.110745200815187	0.912469558666187	0.000360590501222733	0.953569361600158
CHOL	UHRF2	cg08487038	-0.0907378967869948	36	-0.531279942739855	0.598678810518185	0.00823336591332731	0.75910516080956
CHOL	UHRF2	cg13428749	-0.128768229060325	36	-0.757144794360118	0.454178516293032	0.0165812568153324	0.644468909537796
CHOL	UHRF2	cg14078101	-0.291521833713305	36	-1.7770368507415	0.0845102136702121	0.084984979531568	0.211130336949179
CHOL	UHRF2	cg20595300	0.0126126810068552	36	0.0735497865884885	0.941799637298108	0.000159079722180687	0.968952323431759
CHOL	UHRF2	cg24119045	-0.185603765370089	36	-1.10138352955241	0.278465964354935	0.0344487577195549	0.475388558691826
CHOL	USP27X	cg00478198	-0.0841855989715345	36	-0.492630972700459	0.625439795394014	0.00708721507419603	0.778322791329578
CHOL	USP27X	cg01125381	-0.234496622844369	36	-1.4065576672115	0.16863352576543	0.0549886661254142	0.341828039590283
CHOL	USP27X	cg01290688	-0.346804676063235	36	-2.15600896190505	0.0382472580873121	0.120273483339326	0.118433594540634
CHOL	USP27X	cg01855997	-0.126850981149236	36	-0.745685778551848	0.460981779031985	0.0160911714185238	0.650621682505384
CHOL	USP27X	cg06455206	-0.204276114675448	36	-1.21678210468034	0.232066377926291	0.0417287310268968	0.422696600430763
CHOL	USP27X	cg06624970	-0.104258323582754	36	-0.611256463688664	0.545095275638108	0.0108697980362863	0.719109055147467
CHOL	USP27X	cg16810342	-0.14185277975412	36	-0.835586380067831	0.409221405237332	0.020122211123971	0.60522756652154
CHOL	USP27X	cg16867483	-0.109168578992314	36	-0.640384148550557	0.526215483672459	0.0119177786392011	0.70494713678181
CHOL	USP27X	cg19318186	-0.219948606727477	36	-1.31470494867206	0.197408586847333	0.0483773896013585	0.379719204988087
CHOL	USP51	cg00834229	-0.081888633009691	36	-0.479097736228377	0.634936224256645	0.00670574821619586	0.784949507983908
CHOL	USP51	cg01084827	0.222751260530197	34	1.29254623425528	0.205423628031008	0.0496181240677916	0.389675063006495
CHOL	USP51	cg02714202	-0.0511774485609358	36	-0.298804801710872	0.766907742250425	0.00261913124120723	0.870795628576031
CHOL	USP51	cg03728850	-0.119629745907066	36	-0.702600969379027	0.48708737696291	0.0143112761057893	0.673406601633844
CHOL	USP51	cg07386410	0.250532651559332	36	1.5089676076107	0.14054468037931	0.0627666094973498	0.30212309998696
CHOL	USP51	cg12064531	0.0128739774859976	36	0.0750737650104669	0.940596011059162	0.000165739296309973	0.968318554824381
CHOL	USP51	cg12517811	0.205252752576341	36	1.22285472597901	0.229793441299132	0.0421286924401646	0.419873895444022
CHOL	USP51	cg18770944	0.221254274083076	36	1.32290982257997	0.194694302324694	0.048953453800029	0.376152200856104
CHOL	USP51	cg21331369	-0.0792684580587888	36	-0.463669592538654	0.645839306782769	0.00628348844301795	0.792367257000043
CHOL	USP51	cg25424556	0.224589111305923	36	1.34390017590097	0.187880648251828	0.0504402689171842	0.367309689136076
CHOL	WDR82	cg01736403	0.0537428990737698	36	0.313825798315908	0.755570770306689	0.0028882992008534	0.863645844124048
CHOL	WDR82	cg03869063	-0.192048057591428	36	-1.14106328222176	0.261817204271232	0.0368824564246406	0.457162820654999
CHOL	WDR82	cg10989431	0.208148650999733	36	1.2408835920109	0.223142886926549	0.0433258609130086	0.411891748619384
CHOL	WDR82	cg11442381	-0.345633514121479	36	-2.147738408234	0.0389485099484934	0.119462526083963	0.120122121112159
CHOL	WDR82	cg11665333	-0.240660450918129	36	-1.44577165333989	0.157396747753334	0.057917452636117	0.326068454269357
CHOL	WDR82	cg12661343	-0.337424340525768	36	-2.09008291918343	0.0441617142910769	0.113855185579249	0.131755183185428
CHOL	WDR82	cg13539115	-0.256291774934896	36	-1.54606438810727	0.131347353950752	0.0656854738992795	0.288148559071791
CHOL	WDR82	cg15991175	-0.0163280005055639	36	-0.0952204793943943	0.924698707974331	0.000266603600509697	0.960260719610149
CHOL	WDR82	cg17632831	-0.129959088852916	36	-0.76426666820037	0.449980187401945	0.0168893647754801	0.641001972107211
CHOL	WDR82	cg24007312	-0.364658821756023	36	-2.28355058952482	0.0287678744793924	0.132976056284491	0.0956787486611988
CHOL	WDR82	cg26236504	0.293617064616546	36	1.79100906333968	0.0821985389472831	0.0862109806340372	0.207010460953867
CHOL	WDR82	cg26858096	-0.368784768366514	36	-2.31342868889058	0.0268779409623305	0.136002205379143	0.0909540449442577
CHOL	ZMYND1	cg00614684	-0.182014653218184	36	-1.07934831763708	0.288030161423642	0.0331293339861357	0.485546900792902
CHOL	ZMYND1	cg02813021	-0.0952483662294286	36	-0.557925226712028	0.580550900028598	0.00907225126937534	0.746354057324576
CHOL	ZMYND1	cg02867818	-0.364220425066573	36	-2.28038510612153	0.0289749518063641	0.132656518035675	0.0962417232750221
CHOL	ZMYND1	cg03254875	-0.131761062299861	36	-0.775049682546396	0.443667543955776	0.0173609775383878	0.635788384047726
CHOL	ZMYND1	cg05236965	-0.0241366842533513	36	-0.140780858779752	0.888873842929815	0.000582579526745974	0.941044972270409
CHOL	ZMYND1	cg08828193	-0.304735267119618	36	-1.86563154192864	0.0707406416100154	0.0928635830264648	0.185780753271539
CHOL	ZMYND1	cg12639781	0.0396734973983871	36	0.231516528515732	0.818301260174378	0.00157398639581983	0.902297996286506
CHOL	ZMYND1	cg13939078	-0.448187727788879	36	-2.92342064918544	0.00611946661132012	0.200872239340559	0.0291362865267787

**CHOL**	ZMYND1	cg14255089	0.124716118758499	36	0.732936112561541	0.468620789408628	0.0155541102781841	0.657573695729704
**CHOL**	ZMYND1	cg14694828	0.0639464298274539	36	0.373633259169938	0.710996944947377	0.00408914588767748	0.835486468375164
**CHOL**	ZMYND1	cg17632565	-0.296840031465995	36	-1.81255692316695	0.0787385863124126	0.0881140042807329	0.200615386093336
**CHOL**	ZMYND1	cg22593633	-0.104389736702861	36	-0.612035409091385	0.544585845679659	0.0108972171288926	0.718741708720199
**CHOL**	ZMYND1	cg24757747	-0.365630278646264	36	-2.29057132901377	0.0283133384451102	0.133685500662944	0.0945949838741211
**DLBC**	ACTL6A	cg03667091	-0.00794238384934036	48	-0.0538695672311749	0.957272363598649	6.30814612102626e-05	0.977547775816274
**DLBC**	ACTL6A	cg03839554	-0.215075674001421	48	-1.49366997418523	0.142090098669335	0.0462575455471655	0.304393818799424
**DLBC**	ACTL6A	cg07261848	0.0447019003455608	48	0.303486413011007	0.762887643613095	0.00199825989450445	0.868106214396798
**DLBC**	ACTL6A	cg10112003	0.183394562779176	48	1.2653027535293	0.212134797141226	0.033633565656965	0.398342524278281
**DLBC**	ACTL6A	cg20132173	-0.0504840899226842	48	-0.342836919990026	0.733282939443957	0.00254864333532166	0.849503551564433
**DLBC**	ACTL6A	cg22619386	0.132417018965138	48	0.906074726366297	0.369618069213949	0.0175342669116137	0.568340839175973
**DLBC**	ACTL6A	cg25198545	-0.0719744781351401	48	-0.4894239949021	0.626867376153788	0.00518032550282576	0.779403868697083
**DLBC**	ACTL6A	cg25575835	-0.102479358062688	48	-0.698727531646505	0.488240122682184	0.0105020188289407	0.674315197044718
**DLBC**	ACTL6B	cg05729836	-0.0386444822695339	48	-0.262295559527149	0.794264688076581	0.00149339600988032	0.887352650959022
**DLBC**	ACTL6B	cg08572611	0.111074518893617	48	0.758034697576505	0.452296419035968	0.0123375487474485	0.642712666176234
**DLBC**	ACTL6B	cg09375063	-0.121743921828657	48	-0.831895469602492	0.409765859952184	0.0148215825022221	0.605687351493828
**DLBC**	ACTL6B	cg14088161	-0.122553474971192	48	-0.837511342454911	0.406635645290171	0.0150193542275145	0.602870357327786
**DLBC**	ACTL6B	cg16024950	0.04004611583887	48	0.271824020538509	0.786973646119719	0.0016036913937802	0.882616743933992
**DLBC**	ACTL6B	cg20497359	0.0719200775802478	48	0.489052148885432	0.627128577756816	0.00517249755914886	0.779554201268935
**DLBC**	ACTL6B	cg24471254	-0.00287012928339159	48	-0.0194662440723336	0.984553357620835	8.23764210338193e-06	0.99189844047809
**DLBC**	ACTL6B	cg26990587	0.0836348794450773	48	0.569233679993029	0.571966806199555	0.00699479305979261	0.739669953479091
**DLBC**	AEBP2	cg02664216	-0.57578579916938	48	-4.7763778186023	1.85412626063979e-05	0.331529286525122	0.000224497896218229
**DLBC**	AEBP2	cg07849438	-0.344674043249418	48	-2.49029214474526	0.0164376895819653	0.118800196089901	0.0625919541526876
**DLBC**	AEBP2	cg08161947	-0.645438649351705	48	-5.7312234342832	7.30865776006321e-07	0.416591050076953	1.29279154567157e-05
**DLBC**	AEBP2	cg24304000	0.0326327000333871	48	0.221443678196811	0.825727091153529	0.00106489311146902	0.906304066305777
**DLBC**	AEBP2	cg26259627	-0.475374119213401	48	-3.66470071981357	0.000639051655715422	0.225980553217917	0.00461514895142272
**DLBC**	AFF4	cg00931577	-0.162641837515924	48	-1.11797627055385	0.269381945588533	0.0264523673105562	0.465353213197136
**DLBC**	AFF4	cg01907658	-0.500542670936379	48	-3.92144973295741	0.000291110932887953	0.250542965428124	0.00238436343578494
**DLBC**	AFF4	cg05619425	-0.211191608087624	48	-1.4654242878054	0.149607535052033	0.0446018953266368	0.315083742849318
**DLBC**	AFF4	cg06638012	-0.372274896760206	48	-2.72042877509318	0.00917127336660146	0.138588598757822	0.0399010145003856
**DLBC**	AFF4	cg13393914	-0.221394698941234	48	-1.53978271568718	0.130464767040304	0.0490156127192796	0.286891104023305
**DLBC**	AFF4	cg13565585	-0.10752471027714	48	-0.733520717105781	0.466963032954222	0.0115615633201828	0.656099099979281
**DLBC**	AICDA	cg07999668	-0.166210749701105	48	-1.14319774280796	0.258871396992804	0.0276260133162033	0.453573005803112
**DLBC**	AIRE	cg08089567	-0.0771096552867246	48	-0.524544897507772	0.602418413063425	0.00594589893843749	0.761752511717046
**DLBC**	AIRE	cg09510531	-0.190678821949911	48	-1.31741804835551	0.194223003954724	0.0363584131402059	0.37551882837816
**DLBC**	AIRE	cg11923631	-0.195766777898075	48	-1.35395322924923	0.182366157847162	0.0383246313285943	0.360079002966445
**DLBC**	AIRE	cg12843033	0.21592857348913	48	1.49988231937804	0.14047758936822	0.0466251488490506	0.302012258872773
**DLBC**	AIRE	cg15955655	0.178549944623591	48	1.23076194134763	0.224668210669864	0.0318800827250873	0.413950991920123
**DLBC**	AIRE	cg16501323	0.112119046907853	48	0.765253463610522	0.448029234463344	0.0125706806795253	0.639487339116807
**DLBC**	AIRE	cg16717549	-0.0964933918806594	48	-0.657518251568385	0.51412460544295	0.0093109746766345	0.695691813597765
**DLBC**	AIRE	cg17356252	-0.0234146077798341	48	-0.158849146392499	0.874483097619911	0.00054824385748347	0.933523436882924
**DLBC**	AIRE	cg18689454	-0.0728906311341638	48	-0.495686869851727	0.622475328322193	0.00531304410713674	0.776372494033084
**DLBC**	AIRE	cg18876487	0.120719855834302	48	0.824793933026095	0.413745287031921	0.0145732835926546	0.609345078886526
**DLBC**	AIRE	cg23272544	0.267435631042556	48	1.88240181503397	0.0661156951776	0.0715218167511303	0.176743218124677
**DLBC**	AIRE	cg27251412	-0.166156867074957	48	-1.14281661409085	0.259028010392171	0.0276081044761649	0.453765651244112
**DLBC**	ARID1A	cg04081153	-0.567969258668103	48	-4.68034264829921	2.54768503435666e-05	0.322589078791994	0.000296033770818136
**DLBC**	ARID1A	cg04360147	0.0187654860551238	48	0.127296133944067	0.899260866998159	0.000352143466885045	0.946826125537184
**DLBC**	ARID1A	cg14851949	-0.459524558759111	48	-3.5090855531053	0.00101753643352951	0.211162820102756	0.0068116538430423
**DLBC**	ARID1A	cg17385674	-0.600146010352043	48	-5.08868204551742	6.5237569721495e-06	0.360175233741474	8.98723481302282e-05
**DLBC**	ARID1A	cg20413923	-0.558754013627509	48	-4.56951802951415	3.6678654179868e-05	0.31220604774485	0.000405828363428907
**DLBC**	ARID1A	cg21294655	-0.304666060998099	48	-2.16948472743201	0.0352486736508976	0.0928214087240975	0.111432677096795
**DLBC**	ARID1A	cg24542126	-0.453896227993161	48	-3.45486591170234	0.00119396172727797	0.20602178578642	0.00778501874208888
**DLBC**	ARID1A	cg25601319	-0.412075263576049	48	-3.06736651129329	0.0036118714267664	0.16980602285127	0.0190923371457407
**DLBC**	ARID2	cg00035453	0.21571578911886	48	1.49833210514683	0.140878603966773	0.0465333016751725	0.302648572765689
**DLBC**	ARID2	cg01183821	-0.120551805627057	48	-0.823628819981217	0.414400420600609	0.0145327378399438	0.609976468187996
**DLBC**	ARID2	cg01353809	-0.258544620752214	48	-1.81525483892803	0.0760072619844533	0.0668453209199061	0.195778721237619
**DLBC**	ARID2	cg01751433	-0.137383271551118	48	-0.940698418234462	0.351773386285567	0.0188741633020882	0.550686045468613

**DLBC**	ARID2	cg06613666	-0.243499810494997	48	-1.70274714633664	0.0953644952942077	0.0592921577110995	0.230182610825864
**DLBC**	ARID2	cg07276957	-0.404376725188054	48	-2.99873041808296	0.00436350686618029	0.163520535873815	0.0222651615602538
**DLBC**	ARID2	cg23465749	-0.0440413451104195	48	-0.298993045632533	0.766292022557688	0.00193964007913507	0.870426819730062
**DLBC**	ARID4A	cg02136725	-0.343942061718823	48	-2.48429311357227	0.0166830652786835	0.118296141819394	0.0633109969057911
**DLBC**	ARID4A	cg06154646	-0.311752719018026	48	-2.22531208826534	0.0310035215558994	0.0971897578151323	0.101127377606693
**DLBC**	ARID4A	cg08066755	-0.51941065796708	48	-4.12254230292872	0.000154951102339265	0.269787431609795	0.0014005188864158
**DLBC**	ARID4A	cg08790491	-0.352539823472269	48	-2.55508608811263	0.0139886389481901	0.124284327133859	0.055318022682936
**DLBC**	ARID4A	cg10064886	-0.0992962609477066	48	-0.676804835421458	0.501919206070003	0.00985974743819505	0.685648358449986
**DLBC**	ARID4A	cg10242170	-0.0406886070106667	48	-0.276192280829643	0.783637509901864	0.00165556274046848	0.880614642404162
**DLBC**	ARID4A	cg10844118	0.193398313747227	48	1.33693202404876	0.187819304212585	0.0374029077602708	0.36721744582457
**DLBC**	ARID4A	cg12203852	-0.266054979910429	48	-1.87194145121593	0.067580628532052	0.0707852523351389	0.179689910999469
**DLBC**	ARID4A	cg14272338	-0.20365676969694	48	-1.41083520799493	0.165020629780187	0.0414760798433923	0.336883377676538
**DLBC**	ARID4A	cg14460816	-0.0731947122077826	48	-0.497765863719298	0.621020417506231	0.00535746589518012	0.775393418924016
**DLBC**	ARID4A	cg17323045	-0.441526591868788	48	-3.33751481575412	0.00168084337338727	0.194945731327268	0.0103195903550856
**DLBC**	ARID4A	cg19548283	0.416559821410203	48	3.10771130621951	0.00322866871645348	0.1735220848133	0.0174863888412348
**DLBC**	ARID4A	cg20996579	-0.155404530118028	48	-1.0669674818154	0.291554460030232	0.0241505679812052	0.489168223738948
**DLBC**	ARID4A	cg26429530	-0.184223104063818	48	-1.27121949937831	0.210041217200416	0.0339381520709083	0.395630658265452
**DLBC**	ARID4A	cg27026909	0.209075620557217	48	1.45006709283454	0.153824388651431	0.0437126151113853	0.321155321269098
**DLBC**	ARID4B	cg05652840	0.0216627136428346	48	0.146958158061729	0.883807253148069	0.000469273162371451	0.938431062699232
**DLBC**	ARID4B	cg06212607	0.32066619795554	48	2.29611668735664	0.0262750387284539	0.102826810511262	0.0894312467987187
**DLBC**	ARID4B	cg06891548	0.0286951036148527	48	0.194699836919798	0.846485312470366	0.000823408971467132	0.917965285177309
**DLBC**	ARID4B	cg07814876	-0.112867164690387	48	-0.770425306590604	0.444986612210092	0.0127389968652469	0.636897475949293
**DLBC**	ARID4B	cg10107779	0.0883122386525331	48	0.601312166146817	0.550584046204453	0.00779905149582196	0.723137129044406
**DLBC**	ARID4B	cg11732257	-0.286041294889528	48	-2.02462058248006	0.0487342807304633	0.0818196223820779	0.14155905590119
**DLBC**	ARID4B	cg22596515	-0.0511529356107267	48	-0.347390882072939	0.729882164281904	0.00261662282159515	0.847305046605083
**DLBC**	ARID4B	cg23687319	-0.188604777640647	48	-1.30255672910739	0.199209900231868	0.0355717621488778	0.381952358126828
**DLBC**	ARID4B	cg26020585	0.12420805753923	48	0.848994479666526	0.40028113517046	0.0154276415576687	0.59682741982656
**DLBC**	ASH1L	cg03119126	-0.372149245657623	48	-2.71936293040003	0.00919669933901709	0.138495061043537	0.0399780972496366
**DLBC**	ASH1L	cg08095278	-0.142084747708615	48	-0.97354272554658	0.335375497903629	0.0201880755314208	0.534920274816806
**DLBC**	ASH1L	cg09410084	-0.113157346223827	48	-0.772431728879452	0.443809507623547	0.012804585004419	0.635915902078637
**DLBC**	ASH1L	cg10558228	0.0151382550800051	48	0.10268440792237	0.9186597936302	0.000229166766867301	0.957068512771424
**DLBC**	ASH1L	cg17038116	0.0774335917221544	48	0.526761766737901	0.600890147832021	0.00599596112699329	0.760824657850589
**DLBC**	ASH1L	cg20064047	-0.572438406563706	48	-4.73501670040948	2.12652682872735e-05	0.327685729309195	0.000252527901257847
**DLBC**	ASH1L	cg21852905	-0.542190964515052	48	-4.37642947842746	6.87660334276924e-05	0.293971042001762	0.000699018006595309
**DLBC**	ASH1L	cg23821590	-0.601870012759454	48	-5.11158445786744	6.03908268592479e-06	0.362247512259066	8.4003745507183e-05
**DLBC**	ASH1L	cg24312492	-0.544248004539516	48	-4.40000274478128	6.371643596755e-05	0.296205890445245	0.000656002702443037
**DLBC**	ASH1L	cg26185843	-0.334455962644491	48	-2.40700690987938	0.0201548692169509	0.111860790948453	0.0732618505785609
**DLBC**	ASH2L	cg00590869	-0.0707428516765311	48	-0.481006484436933	0.632791998378763	0.00500455106332768	0.783604008694202
**DLBC**	ASH2L	cg01642070	-0.0573797582207692	48	-0.389810697190474	0.698475605524338	0.00329243665347392	0.827084419418615
**DLBC**	ASH2L	cg02897270	0.0491015441719115	48	0.333425055320477	0.740328555023954	0.00241096164006617	0.853863893395604
**DLBC**	ASH2L	cg03189282	0.113401269844448	48	0.774118461743987	0.442821377211216	0.0128598480023333	0.635128558012072
**DLBC**	ASH2L	cg03705346	-0.102943525962964	48	-0.701926154350507	0.486261777314962	0.0105973695376874	0.672762961107855
**DLBC**	ASH2L	cg10062221	0.0200827089741276	48	0.136235034818356	0.892229961786952	0.000403315199739507	0.942905976488584
**DLBC**	ASH2L	cg21453287	0.0227168622883428	48	0.154113026815534	0.878194781004012	0.000516055832227531	0.935341586142388
**DLBC**	ASH2L	cg24318412	-0.147298532748673	48	-1.01004474261989	0.317757492001563	0.021696857749912	0.517038827290725
**DLBC**	ASH2L	cg27255459	0.0685693752983932	48	0.466157300884026	0.643302897635695	0.00470175922881189	0.790651946705498
**DLBC**	ASXL1	cg00174466	-0.297349416557005	48	-2.1122621674504	0.0401236160597177	0.088416675526791	0.122744529576262
**DLBC**	ASXL1	cg04218270	-0.472163307277497	48	-3.63281571009027	0.000703472104700033	0.222938188739224	0.00500831965892142
**DLBC**	ASXL1	cg09065199	-0.312278869690185	48	-2.22947323254241	0.0307060430808518	0.0975180924549794	0.100409664372921
**DLBC**	ASXL1	cg10421984	-0.158550428783633	48	-1.08911770682977	0.281775022094923	0.025138238467474	0.478889960067204
**DLBC**	ASXL1	cg12592359	-0.34248532006258	48	-2.47236950058483	0.0171806564351006	0.117296194458368	0.064802520567865
**DLBC**	ASXL1	cg16926526	0.138286014368943	48	0.946999838222445	0.348587306551402	0.0191230217700475	0.547756080968339
**DLBC**	ASXL1	cg18793288	-0.293044100042965	48	-2.07878257468449	0.0432417352857848	0.0858748445699915	0.129671866271773
**DLBC**	ASXL1	cg24727216	-0.238836943966622	48	-1.66814768354016	0.10208057506326	0.0570430858033152	0.241738159617704
**DLBC**	ASXL2	cg05165989	-0.260649680709531	48	-1.8311072056551	0.0735659372224864	0.0679382560539803	0.1911805084445
**DLBC**	ASXL2	cg05496738	-0.381284420021522	48	-2.79731189875283	0.00749925174054055	0.145377808951148	0.0341667226883521
**DLBC**	ASXL2	cg06583696	-0.515933643890513	48	-4.08488862118317	0.000174530575399937	0.266187524898143	0.0015499351121761

**DLBC**	ASXL2	cg09476286	-0.310054439655343	48	-2.2118964234527	0.0319800687481078	0.0961337555499888	0.103505019927901
**DLBC**	ASXL2	cg19084174	-0.234897223385365	48	-1.63900962124201	0.108033832805328	0.0551767055541543	0.251748861408389
**DLBC**	ASXL2	cg20667782	-0.170423020914945	48	-1.173025360722	0.246824192336991	0.0290440060577757	0.440026610872973
**DLBC**	ASXL2	cg24738909	-0.387562234326905	48	-2.85143285667574	0.00649666490355488	0.150204485476463	0.0305397032755543
**DLBC**	ASXL3	cg09963640	-0.186910791573717	48	-1.29043209124648	0.20334957019039	0.0349356440067133	0.387180453810793
**DLBC**	ASXL3	cg12361352	-0.144380685146267	48	-0.989606330326241	0.327543657837493	0.0208457822433056	0.527034269368233
**DLBC**	ASXL3	cg17880622	-0.183167678384605	48	-1.26368302306963	0.212710628322292	0.0335503984048061	0.399006814711236
**DLBC**	ASXL3	cg20034091	-0.169096543315105	48	-1.16362535171426	0.250576246582627	0.0285936409611172	0.444272136350454
**DLBC**	ASXL3	cg20329085	-0.182224442163723	48	-1.25695148462779	0.215116253728996	0.0332057473218802	0.402142400216118
**DLBC**	ASXL3	cg24880056	-0.201273725821002	48	-1.39362531700716	0.170127819100492	0.0405111127058678	0.343862494715469
**DLBC**	ASXL3	cg27109877	-0.158786758673328	48	-1.09078306659092	0.281049155307359	0.0252132347299817	0.478234786910641
**DLBC**	ATAD2B	cg08192560	-0.425584674372557	48	-3.18974068568617	0.00256442892616941	0.181122315060795	0.0145298625506536
**DLBC**	ATAD2B	cg14009731	-0.388452188221022	48	-2.8591425921638	0.00636442340636616	0.150895102533701	0.03004332248027
**DLBC**	ATAD2B	cg15717719	0.126483179058644	48	0.864796049024367	0.391638326585155	0.0159979945847811	0.588690960667109
**DLBC**	ATAD2B	cg16929802	-0.114234542600841	48	-0.779881617944636	0.43945499479902	0.0130495307232233	0.632131982274971
**DLBC**	ATAD2B	cg17933838	-0.268196807538688	48	-1.88817412602033	0.0653189373842037	0.071929527573944	0.175221428098853
**DLBC**	ATAD2B	cg19614157	-0.477574897588338	48	-3.68666466675051	0.000598012820038865	0.228077782806511	0.00436210120226005
**DLBC**	ATAD2B	cg20806130	0.00672444980101062	48	0.0456084686840745	0.963819744634027	4.52182251263117e-05	0.980956279375411
**DLBC**	ATAD2B	cg27610867	-0.314430782386521	48	-2.24651569417141	0.0295139341514572	0.0988667169121994	0.0975274446497543
**DLBC**	ATAT1	cg01245721	-0.400301696821308	48	-2.96270990420053	0.00481429678510032	0.160241448478018	0.0240600387398603
**DLBC**	ATAT1	cg05093836	-0.128678210198745	48	-0.880054503315112	0.38340449193768	0.0165580817799524	0.581675391419153
**DLBC**	ATAT1	cg06814856	-0.0669981836528957	48	-0.45542709011322	0.650944525398364	0.00448875661278714	0.795924902959681
**DLBC**	ATAT1	cg07142893	-0.211710954683734	48	-1.46919683340853	0.148585713587388	0.0448215283330979	0.313684697422536
**DLBC**	ATAT1	cg07816366	-0.258008929122261	48	-1.81122519463651	0.0766386139043006	0.0665686075068161	0.196935378125378
**DLBC**	ATAT1	cg08081524	0.10004690880202	48	0.681972796752883	0.498675795126089	0.0100093839608397	0.682925194397929
**DLBC**	ATAT1	cg08188471	-0.364042656779054	48	-2.6509602875582	0.0109709872246908	0.132527055954752	0.0458233478478364
**DLBC**	ATAT1	cg08635134	0.00966870441317811	48	0.0655794092187803	0.947996868816166	9.34838450294099e-05	0.972332042304703
**DLBC**	ATAT1	cg09532691	-0.132995738495931	48	-0.910105810175907	0.367511072269373	0.0176878664580779	0.566434227360463
**DLBC**	ATAT1	cg12907138	-0.119925967757131	48	-0.819290439181823	0.416845424256694	0.0143822377424844	0.61235880607722
**DLBC**	ATAT1	cg13542073	-0.184637796905511	48	-1.2741819397406	0.208998808717476	0.0340911160461207	0.394298108822996
**DLBC**	ATAT1	cg13964084	0.499136833130528	48	3.90677455191439	0.000304672504660989	0.249137578187573	0.00247666591719927
**DLBC**	ATAT1	cg15381130	0.0175018486094316	48	0.118721497046673	0.9060129501965	0.000306314704747462	0.950242965062577
**DLBC**	ATAT1	cg15729859	-0.0866310669224498	48	-0.589777774578446	0.558225644784884	0.00750494175612198	0.729319592408896
**DLBC**	ATAT1	cg18298197	-0.198201173517462	48	-1.37147393732583	0.176880490101599	0.039283705183699	0.352971497203052
**DLBC**	ATAT1	cg20629923	0.0648191126697852	48	0.440551077005184	0.661601466337865	0.0042015173672983	0.803055694534468
**DLBC**	ATAT1	cg22759096	-0.252122224142825	48	-1.76706029917619	0.083852113687762	0.0635656159067247	0.209911128347015
**DLBC**	ATAT1	cg23328390	0.111154280452448	48	0.758585842205066	0.451969790047116	0.0123552740629015	0.642518462930127
**DLBC**	ATAT1	cg26934609	-0.180018432328605	48	-1.24122195996377	0.220816284483761	0.0324066359780484	0.409066604994448
**DLBC**	ATF7IP	cg00236831	-0.20680797450462	48	-1.43363284301612	0.158439756148959	0.0427695383187034	0.3275749432549
**DLBC**	ATF7IP	cg11409060	0.279095863611901	48	1.97125137778769	0.0547281906950866	0.0778945010852728	0.154124988605197
**DLBC**	ATF7IP	cg12526346	-0.158704181405334	48	-1.09020114264443	0.281302644595238	0.0251870171955373	0.478530610808714
**DLBC**	ATF7IP	cg12948116	-0.139541648313036	48	-0.955768550872597	0.344185344799111	0.019471871613919	0.543446833490827
**DLBC**	ATF7IP	cg17439009	-0.226825999233396	48	-1.57958005792781	0.121055744539151	0.0514500339282287	0.272305418421198
**DLBC**	ATF7IP	cg19789919	-0.225421411752083	48	-1.5692733705389	0.123438272834099	0.050814812876302	0.276139669212247
**DLBC**	ATF7IP	cg21275967	-0.187817152413007	48	-1.29691784108716	0.201127186538619	0.0352752827405309	0.384340170709539
**DLBC**	ATF7IP	cg26637901	0.0726020393950555	43	0.466109947065331	0.643603771955819	0.00527105612432119	0.790773252110986
**DLBC**	ATM	cg01756564	0.21027495966347	48	1.45876890002007	0.151423687402604	0.0442155586614741	0.31774753426478
**DLBC**	ATM	cg01800843	0.0557930782249711	48	0.378997411447035	0.706433601781405	0.00311286757781774	0.832608069342968
**DLBC**	ATM	cg01991180	-0.0136618774748436	48	-0.0926680097251818	0.926569733003454	0.00018664689613764	0.961305440023348
**DLBC**	ATM	cg02105423	-0.15787423217513	48	-1.08435377194643	0.283858688812397	0.0249242731848868	0.481126064370579
**DLBC**	ATM	cg03165700	0.365462517512082	48	2.662889990808	0.0106405073742754	0.133562851706269	0.0447441962526692
**DLBC**	ATM	cg03569616	-0.1869549597925	48	-1.29074807151299	0.203240871308345	0.0349521569910151	0.387077081782804
**DLBC**	ATM	cg04709886	-0.239980609969133	48	-1.67662248260481	0.100400575827097	0.0575906931611573	0.238891646915222
**DLBC**	ATM	cg04826772	-0.507177968084564	48	-3.99127791757186	0.0002341869136265	0.257229491310387	0.00198111535000652
**DLBC**	ATM	cg05033322	0.321692128788738	48	2.30430928786088	0.0257717097938415	0.10348582572463	0.0881384090957975
**DLBC**	ATM	cg05637308	-0.25781801201985	48	-1.80978948776508	0.0768646202936718	0.0664701273218673	0.197353254930061
**DLBC**	ATM	cg06053805	0.317293292196899	48	2.26924549320846	0.0279880506218542	0.100675033273147	0.0937684069800399

**DLBC**	ATM	cg06081482	0.195208946128178	48	1.34994211045927	0.183640169103355	0.0381065326484738	0.361902553900606
**DLBC**	ATM	cg06750635	0.0261475379875422	48	0.177401885526437	0.859971594867855	0.000683693742809963	0.925537543421688
**DLBC**	ATM	cg09808176	0.200723604949778	48	1.38965612585691	0.17132290144866	0.0402899655840346	0.345451985924756
**DLBC**	ATM	cg10610482	-0.0897499366757188	48	-0.611180207441524	0.544088887216602	0.00805505113329554	0.718305177546895
**DLBC**	ATM	cg10976287	0.0435867852471309	48	0.295901171759214	0.76863728098889	0.00189980784817951	0.871758432663882
**DLBC**	ATM	cg11588932	-0.14687509473592	48	-1.00707706244518	0.319166045202344	0.0215722934536854	0.518571497921285
**DLBC**	ATM	cg12019961	0.366083170796322	47	2.63895090035286	0.0113840099652555	0.134016887940289	0.0471820815789606
**DLBC**	ATM	cg12106634	0.12626770664331	48	0.863298922211867	0.392452143447326	0.015943533740961	0.589406909244001
**DLBC**	ATM	cg12848864	0.316020106410972	48	2.25912728508773	0.0286584117188047	0.0998687076560023	0.0953939703586793
**DLBC**	ATM	cg14761454	0.0984197478525054	48	0.670771809925279	0.50572007273673	0.00968644676735073	0.688812493234184
**DLBC**	ATM	cg15370815	-0.104635315359384	48	-0.713588366563905	0.479086769606438	0.0109485492203577	0.666477283216739
**DLBC**	ATM	cg15504467	0.1447570657804	48	0.992241239955507	0.326270800652818	0.020954608093351	0.52577753839431
**DLBC**	ATM	cg16693212	0.362585545159561	48	2.63873975214844	0.0113192665218021	0.131468277558656	0.0469662925914487
**DLBC**	ATM	cg16788234	0.127175720325647	48	0.869608748007103	0.389029385280964	0.0161736638403472	0.586666507042136
**DLBC**	ATM	cg17114932	-0.0611238881063273	48	-0.415338985201429	0.679824801834836	0.00373612969723482	0.815253204574298
**DLBC**	ATM	cg17420659	0.168435610597367	48	1.15894412246372	0.252460047675337	0.0283705549173077	0.446370984541812
**DLBC**	ATM	cg18042593	0.187120579314736	48	1.29193298893486	0.202833642628982	0.0350141112030825	0.386632372701143
**DLBC**	ATM	cg18391757	-0.193130068724568	48	-1.33500579166838	0.188444167936411	0.0372992234455562	0.368032174338995
**DLBC**	ATM	cg18457775	-0.334790604176154	48	-2.40971909495119	0.0200227647003424	0.112084748644634	0.072881526195411
**DLBC**	ATM	cg19288979	-0.345738739355099	48	-2.49902718011556	0.0160862561182697	0.119535275890853	0.0615671656235696
**DLBC**	ATM	cg20342375	-0.311770390508987	48	-2.22545180916325	0.0309934916638334	0.0972007763981263	0.101107379984747
**DLBC**	ATM	cg22477592	-0.272059690345913	48	-1.91752698923283	0.0613927518516077	0.0740164751111143	0.167384703239896
**DLBC**	ATM	cg22678707	-0.252414567901578	48	-1.76924860498362	0.0834817722647397	0.0637131140889402	0.209172281356711
**DLBC**	ATM	cg22837512	-0.214310837469942	48	-1.48810210966386	0.143547767394469	0.0459291350570681	0.306495265999324
**DLBC**	ATM	cg23275463	0.40023248790477	48	2.9620999632056	0.00482229206576985	0.160186044374442	0.0240854679767275
**DLBC**	ATM	cg23389069	-0.184439307467352	48	-1.27276390336967	0.209497296168066	0.0340178581390363	0.394979221998413
**DLBC**	ATM	cg24030675	-0.208669873345097	48	-1.44712474458709	0.15464286672522	0.0435431160418587	0.322234080838425
**DLBC**	ATM	cg24568245	0.458921407778776	48	3.50324982321777	0.00103525653362105	0.210608858517654	0.00690946695062763
**DLBC**	ATM	cg25400013	-0.0164846516003843	48	-0.111819540973005	0.911453010037627	0.000271743738386053	0.952960334060511
**DLBC**	ATM	cg25740661	-0.113632220183134	48	-0.775715614992661	0.441886923473763	0.0129122814637482	0.634354868023324
**DLBC**	ATM	cg25918541	-0.0275142292936677	48	-0.186681257598283	0.852731451857998	0.00075703281362452	0.921544397245133
**DLBC**	ATM	cg27453365	-0.0667658798811446	48	-0.453840898131339	0.652077395366485	0.00445768271630343	0.796678532762828
**DLBC**	ATM	cg27599391	-0.166771251105098	48	-1.14716297032958	0.2572460252242	0.0278126501951596	0.451712012421086
**DLBC**	ATR	cg00911981	0.091955608241437	48	0.626326958932906	0.534196410048145	0.00845583388705264	0.711033005435611
**DLBC**	ATR	cg00969988	-0.449481434942103	48	-3.41270195512046	0.00135095223016047	0.202033560357612	0.00862078400402784
**DLBC**	ATR	cg11458642	0.0091096521067024	48	0.0617872304025015	0.95099996149021	8.29857615051474e-05	0.974102246315845
**DLBC**	ATR	cg11936145	-0.378169634569936	48	-2.77062831589488	0.00804465094722674	0.143012272510759	0.0360872780410885
**DLBC**	ATR	cg15135999	-0.194920453113919	48	-1.34786822085378	0.184301542675584	0.0379939830421354	0.362737292185001
**DLBC**	ATR	cg15291273	-0.583252834990394	48	-4.86994784363756	1.35819630774815e-05	0.340183869524331	0.000171025450898283
**DLBC**	ATR	cg15480357	0.127372505723865	48	0.870976510530321	0.388289925845172	0.016223755214376	0.586069856836292
**DLBC**	ATR	cg16031872	0.0584864029614678	48	0.397354273384235	0.692944076729838	0.00342065933137119	0.823766324509693
**DLBC**	ATR	cg17945282	-0.22236487290476	48	-1.54688038615883	0.128745024957284	0.04944613670195	0.284170534019774
**DLBC**	ATR	cg20032934	-0.212514603033421	48	-1.47503712308676	0.147014694887589	0.0451624565024523	0.311520141590001
**DLBC**	ATR	cg20636078	-0.199568334821717	48	-1.38132516031325	0.173852368521263	0.0398275202635131	0.348927726171606
**DLBC**	ATR	cg24631870	-0.359313618475184	48	-2.61137963778025	0.012136059144377	0.12910627642173	0.0495987699016373
**DLBC**	ATR	cg24856922	-0.180638336328148	48	-1.24564009777639	0.219204066073914	0.0326302085514012	0.407094013133617
**DLBC**	AURKB	cg02245743	0.229260936366162	48	1.59747198924746	0.117008140675205	0.0525605769434895	0.266109200014235
**DLBC**	AURKB	cg04612488	0.110749978673269	48	0.755792308985487	0.453626762454529	0.0122655577761295	0.643909138917756
**DLBC**	AURKB	cg05617798	0.332571320687921	48	2.3917514595365	0.0209126668963109	0.110603683344108	0.075367368133587
**DLBC**	AURKB	cg05811247	0.195841524941107	48	1.35449080554141	0.18219592854156	0.0383539028912583	0.359846720561541
**DLBC**	AURKB	cg11009596	0.205651706403006	48	1.42526236425802	0.160831775977554	0.0422926243464683	0.330838700399123
**DLBC**	AURKB	cg12844497	-0.0830828642010809	48	-0.56545036519883	0.57451531891678	0.00690276232385525	0.741695758638742
**DLBC**	AURKB	cg13678641	0.00853790401983155	48	0.0579089931265521	0.954071948649182	7.28958050518557e-05	0.975658367894655
**DLBC**	AURKB	cg21724796	0.092552783897458	48	0.630429463505104	0.531533256788723	0.00856601780716956	0.709191132621375
**DLBC**	BAP1	cg00794529	-0.0843042118689293	48	-0.573821752947755	0.568883658502776	0.00710720013884131	0.737234055501298
**DLBC**	BAP1	cg02503646	0.0140546629807051	48	0.0953327783129199	0.924464602282708	0.000197533551501202	0.960171416536241
**DLBC**	BAP1	cg03075736	0.0543609281384058	48	0.369239728577689	0.713643325765107	0.00295511050806892	0.837072642741896

**DLBC**	BAP1	cg05442719	0.266965858374089	48	1.87884123279365	0.0666112785055827	0.0712707695374141	0.177750413338706
**DLBC**	BAP1	cg05908979	0.00804103905312033	48	0.0545387434854272	0.956742128322551	6.46583090538063e-05	0.977147164395112
**DLBC**	BAP1	cg09096430	0.193163161007438	48	1.33524340619382	0.188367001001558	0.0373120067703852	0.367927650796305
**DLBC**	BAP1	cg10449522	-0.290193174532667	48	-2.0566891343681	0.0454134197450838	0.0842120785453469	0.13450436212781
**DLBC**	BAP1	cg10956904	-0.42007644259413	48	-3.13953989685183	0.00295370155981295	0.176464217622539	0.0162618725508909
**DLBC**	BAP1	cg11234564	0.109962177635847	48	0.750350068044615	0.456864949707123	0.0120916805104177	0.647112355434261
**DLBC**	BAP1	cg12859382	0.182068932282919	48	1.25584201165809	0.215514678352921	0.033149096102642	0.402625990917296
**DLBC**	BAP1	cg16841708	0.100936311544473	48	0.688097562657107	0.49484684275477	0.0101881389882029	0.679906811047169
**DLBC**	BAP1	cg24291822	-0.123355760062641	48	-0.843078471246047	0.403547189557303	0.0152166435406319	0.599983756295788
**DLBC**	BAP1	cg24747396	0.114541335129845	48	0.782003903472015	0.438219155572946	0.0131197174533276	0.630960965919707
**DLBC**	BAP1	cg25749966	-0.259820941619968	48	-1.82486297507169	0.0745195525137549	0.0675069217042867	0.192956133279692
**DLBC**	BAZ1A	cg05294307	-0.0424945142293826	48	-0.288472395046046	0.774281090371847	0.0018057837395912	0.874929393396702
**DLBC**	BAZ1A	cg07414384	0.350157470230577	48	2.53539736451099	0.0146951559708652	0.122610253958278	0.0574585325491297
**DLBC**	BAZ1A	cg23422263	-0.146725041430974	48	-1.00602554447462	0.319666139518833	0.0215282377829209	0.519003392436105
**DLBC**	BAZ1A	cg26967526	-0.117067886079365	48	-0.799490397949814	0.428115338755526	0.0137048899510913	0.622410830386906
**DLBC**	BAZ1B	cg00525989	-0.385868859547434	48	-2.83678890331062	0.00675489958913522	0.148894776768438	0.0314872466077767
**DLBC**	BAZ1B	cg04742505	-0.518066738957907	48	-4.10795562502555	0.000162268698323305	0.26839314601448	0.00145583336608963
**DLBC**	BAZ1B	cg05166819	-0.396006686565278	48	-2.92497137515661	0.00533301382550767	0.15682129580441	0.0261027103020979
**DLBC**	BAZ1B	cg06059318	-0.372921177136254	48	-2.72591364160562	0.00904145220497826	0.139070204356689	0.0394819384133639
**DLBC**	BAZ1B	cg06394887	-0.330277033960699	48	-2.37322354802291	0.0218674453140429	0.109082919161877	0.0779263584216916
**DLBC**	BAZ1B	cg14093368	-0.387291246121602	48	-2.84908713205616	0.00653740364916424	0.149994509392135	0.0306865674580245
**DLBC**	BAZ1B	cg15636799	-0.592288647404267	48	-4.98568714452865	9.22272315331826e-06	0.350805841843976	0.000121977647907045
**DLBC**	BAZ1B	cg19160975	-0.480860656499196	48	-3.71962443142466	0.00054114520569488	0.231226970968838	0.00400970093645371
**DLBC**	BAZ1B	cg20612831	-0.164569493091305	48	-1.13159332663528	0.263670306109571	0.0270831180563291	0.459106669803661
**DLBC**	BAZ1B	cg21340129	-0.63518059029496	48	-5.57768911912644	1.23764428100312e-06	0.403454382287454	2.06656576940023e-05
**DLBC**	BAZ1B	cg25710357	-0.363933483862269	48	-2.65004389810936	0.0109967588721271	0.132447580676128	0.0458999985530007
**DLBC**	BAZ2A	cg14634319	-0.135356427928261	48	-0.926559110058282	0.358991494476096	0.0183213625814984	0.557967400329126
**DLBC**	BAZ2A	cg18206859	-0.0962993531028278	48	-0.656183657731441	0.514975057046899	0.00927356540802311	0.696402179382085
**DLBC**	BAZ2A	cg18955060	0.0672980793391296	48	0.457474916093956	0.649483183968732	0.00452903148273578	0.794994804715027
**DLBC**	BAZ2A	cg25683012	-0.557691209798909	48	-4.55689734709875	3.8226619377033e-05	0.311019485486971	0.000420653586201182
**DLBC**	BRD7	cg03021120	-0.0737022158621136	48	-0.50123596327891	0.618595399179612	0.00543201662298559	0.773656964145505
**DLBC**	BRD7	cg16433860	-0.0338957429434458	48	-0.23002429155641	0.819092750072884	0.00114892138968815	0.902710112188615
**DLBC**	BRD7	cg16834187	-0.16235142972764	48	-1.11592595936122	0.270249484518748	0.0263579867346088	0.466335168437137
**DLBC**	BRD8	cg01096096	-0.089626600057878	48	-0.610333502042376	0.544644643042967	0.00803292743793482	0.718766165294787
**DLBC**	BRD8	cg03204906	-0.460310546844668	48	-3.51669954462376	0.000994852107448805	0.211885799536438	0.00668799903619705
**DLBC**	BRD8	cg03684156	0.0310545412728878	48	0.210723780274042	0.834033519934978	0.000964384533669493	0.910981054572563
**DLBC**	BRD8	cg07430605	0.152948213808913	48	1.04969576154524	0.299341763511133	0.0233931561073371	0.497649124900309
**DLBC**	BRD8	cg07632946	-0.179379321696826	48	-1.23666853403073	0.222487042026089	0.0321769410524133	0.411081059932015
**DLBC**	BRD8	cg08976717	-0.348985606647216	48	-2.52573323042813	0.0150536994354168	0.121790953646926	0.0585382259081394
**DLBC**	BRD8	cg11110536	0.10071091897213	48	0.686545266530578	0.495815736840019	0.0101426892002109	0.680705376107095
**DLBC**	BRD8	cg11346339	-0.321427407920569	48	-2.30219448691191	0.0259008056044137	0.103315578562536	0.0884748955471047
**DLBC**	BRD8	cg12964860	-0.542430608272298	48	-4.37916998715444	6.81594889253622e-05	0.294230964790655	0.000693849683519972
**DLBC**	BRD8	cg14473243	0.363330275305232	48	2.64498287746982	0.0111400932950112	0.132008888953376	0.046376219713817
**DLBC**	BRD8	cg22106577	-0.109747311929331	48	-0.748865994279494	0.457750310914377	0.0120444724757139	0.647905712350528
**DLBC**	BRD8	cg22818036	0.0789941633415692	48	0.537443952679836	0.593551552400616	0.00624007784203451	0.755510704624907
**DLBC**	BRD8	cg23298160	0.437241167107583	48	3.29741778814017	0.00188673820040166	0.191179838213601	0.011342006920798
**DLBC**	BRD8	cg26008208	0.102216860694829	48	0.696918843028841	0.489360776415956	0.0104482866103061	0.67533540722185
**DLBC**	BRD8	cg26630792	-0.0666892915210038	48	-0.453317961874297	0.652451062284422	0.00444746160357343	0.79697836010071
**DLBC**	BRD8	cg27119451	-0.0988334852031562	48	-0.67361936010188	0.503924139731403	0.0097680577974025	0.687216662744583
**DLBC**	BRD9	cg02364331	-0.174528287149079	48	-1.20215897800004	0.235454056220362	0.0304601230151914	0.426659356865827
**DLBC**	BRD9	cg02822723	0.00411154451032314	48	0.027886087314655	0.977873637321223	1.69047982603683e-05	0.988527065870353
**DLBC**	BRD9	cg05193570	-0.048441685540351	48	-0.32893366030279	0.74369875311195	0.00234659689799025	0.856043754722465
**DLBC**	BRD9	cg07308931	-0.152778768986131	48	-1.04850503977473	0.299883858559811	0.0233413522529175	0.498167091454238
**DLBC**	BRD9	cg09497020	-0.232995276359272	48	-1.62497358851273	0.111001275390898	0.0542867988057335	0.256565743166638
**DLBC**	BRD9	cg10551270	-0.327546404465824	48	-2.35123337012494	0.0230513485215723	0.107286647078489	0.0810511460739976
**DLBC**	BRD9	cg10600960	-0.307362284123747	48	-2.19067749469192	0.0335802634798839	0.0944715737017672	0.107350411665125
**DLBC**	BRD9	cg13722821	-0.170405220667202	48	-1.17289917692059	0.246874288538843	0.0290379392306379	0.440064221735393

**DLBC**	BRD9	cg17977043	-0.190348485285313	48	-1.31504984037726	0.195011286090981	0.0362325458504131	0.376584534942579
**DLBC**	BRD9	cg20079193	-0.0398945003331365	48	-0.270793244941845	0.787761463909549	0.00159157115683063	0.883080181036348
**DLBC**	BRD9	cg21160852	-0.0617149508708405	48	-0.419370559295335	0.676897407094887	0.00380873516099026	0.813372621484679
**DLBC**	BRD9	cg21758832	0.00461396902534785	48	0.0312937935658515	0.97517062746148	2.12887101668694e-05	0.986918142695769
**DLBC**	BRD9	cg21910709	-0.170098743151148	48	-1.17072678025557	0.247737904986249	0.0289335824216002	0.441079383450823
**DLBC**	BRD9	cg24953233	-0.108590497784695	48	-0.740877719351928	0.462532966518494	0.0117918962091279	0.651960206870581
**DLBC**	BRD9	cg25523559	-0.0725505579377171	48	-0.493361963432809	0.624104137924801	0.00526358345707404	0.777365220186951
**DLBC**	BRD9	cg25951128	-0.0289679132391412	48	-0.196552431321464	0.8450436188458	0.000839139997430413	0.917199030115386
**DLBC**	BRD9	cg26504197	-0.242415325519483	48	-1.69468895163112	0.0968951160163163	0.0587651900467171	0.232771986381392
**DLBC**	BRDT	cg00308185	0.0939756031834395	48	0.640206785977268	0.525214419474996	0.00883141399369128	0.70420245339856
**DLBC**	BRDT	cg01515515	-0.215260395379848	48	-1.49501514225283	0.141739699963461	0.0463370378190883	0.303927734811397
**DLBC**	BRDT	cg02935097	-0.406913362594667	48	-3.02126013873842	0.00410196811602743	0.165578484658099	0.0211666561168903
**DLBC**	BRDT	cg03421440	-0.452435192686538	48	-3.44087700931875	0.00124401126096838	0.204697603581305	0.00805345330197515
**DLBC**	BRDT	cg06442199	-0.329243820502835	48	-2.3648951040435	0.0223092488726974	0.108401493339303	0.0790312252642359
**DLBC**	BRDT	cg09250199	0.188994203207897	48	1.30534572676521	0.198266722478184	0.035718808846188	0.38084795694876
**DLBC**	BRDT	cg13299824	-0.26236489019663	48	-1.84404446704883	0.0716229372453316	0.0688353356078895	0.187483925437038
**DLBC**	BRDT	cg14732540	-0.242060727423431	48	-1.69205559981695	0.0973996881127102	0.0585933957607606	0.233631723871691
**DLBC**	BRDT	cg19245011	-0.180497540489652	48	-1.2446364927443	0.21956952225041	0.0325793621228135	0.407566184994535
**DLBC**	BRPF1	cg04879506	-0.385368684090244	48	-2.83246994731401	0.00683286106331969	0.148509022677446	0.0317897011877215
**DLBC**	BRPF1	cg13694772	-0.173526948927665	48	-1.19504694087306	0.238193764906133	0.0301116020041446	0.429811786674423
**DLBC**	BRPF1	cg14712964	-0.166895828004354	48	-1.1480444360133	0.256885702700736	0.0278542174052588	0.451232079929125
**DLBC**	BRPF1	cg17738494	0.019571305332967	48	0.132764480191207	0.894958724527548	0.000383035992436222	0.944199235334988
**DLBC**	BRPF1	cg20952324	-0.163131984777747	48	-1.12143744181991	0.267921918200319	0.026612044457527	0.463622134800695
**DLBC**	BRPF1	cg25467642	-0.400744647787679	48	-2.96661507960383	0.00476339943035573	0.160596272730471	0.0238611651825559
**DLBC**	BRPF1	cg26227975	-0.639475733322366	48	-5.64135249340715	9.95032140827861e-07	0.408929213508178	1.70276133663798e-05
**DLBC**	BRPF3	cg01499736	-0.395134328259546	48	-2.91733428767484	0.0054441363522894	0.156131137369123	0.0265497206237463
**DLBC**	BRPF3	cg05882415	0.204850477932409	48	1.41946575370699	0.162504714988057	0.0419637183091362	0.3331507735515
**DLBC**	BRPF3	cg10562781	-0.490618498459746	48	-3.8187215929909	0.000399804698875711	0.240706511030895	0.00310310353630022
**DLBC**	BRPF3	cg14654948	-0.427831795239487	48	-3.21034412626039	0.00241912895936657	0.183040045017842	0.0138571326312468
**DLBC**	BRPF3	cg18779695	-0.204376853067056	48	-1.41604064342092	0.163499578617348	0.0417698980695932	0.334559582801777
**DLBC**	BRPF3	cg23517469	-0.35733083527745	48	-2.59485336694224	0.0126552038165046	0.12768532584008	0.0512733152284341
**DLBC**	BRWD3	cg03164831	0.0592745920448556	48	0.402727953146071	0.689013948145052	0.00351347726208405	0.821210939867652
**DLBC**	BRWD3	cg05835545	0.0845538105210813	48	0.575532876737853	0.567735900369559	0.00714934687363492	0.736402004691034
**DLBC**	BRWD3	cg09523186	0.0950724616440976	48	0.647746870763174	0.520368703959023	0.00903877296306842	0.700694201947504
**DLBC**	BRWD3	cg11957438	0.200187359124221	45	1.33983778777206	0.18733607708433	0.0400749787531298	0.36663201964021
**DLBC**	BRWD3	cg15511898	0.0936896913125068	48	0.638241742477969	0.526481182729791	0.00877775825823281	0.705168606576505
**DLBC**	BRWD3	cg22618269	0.165884037684462	48	1.14088696774738	0.259821981200769	0.0275175139584998	0.454798083062184
**DLBC**	BRWD3	cg25063710	0.0886890218305184	48	0.603897953711	0.548878271925038	0.00786574259325417	0.721944349047662
**DLBC**	BRWD3	cg25708850	0.157416110932033	43	1.0206803878155	0.313391018832743	0.0247798319809662	0.512469757897497
**DLBC**	CARM1	cg12317676	0.0156250026567812	48	0.105986862649104	0.916053630036371	0.00024414070802442	0.95569723999959
**DLBC**	CARM1	cg17918830	-0.489170912584509	48	-3.80390321984583	0.000418407104120517	0.239288181718761	0.00322368646706462
**DLBC**	CARM1	cg22378614	-0.470370993610523	48	-3.61509823916821	0.000741915463189414	0.221248871630151	0.00523458838593844
**DLBC**	CARM1	cg26504915	-0.471760181491664	48	-3.62882567554341	0.000711959282857606	0.222557668841047	0.00505671144441017
**DLBC**	CBX1	cg01544580	-0.228912396566735	48	-1.5949089857142	0.117581132992276	0.052400885301926	0.267037620715862
**DLBC**	CBX1	cg01553295	0.0276798091548064	48	0.187805559008666	0.851855082992787	0.000766171834846504	0.921019767710986
**DLBC**	CBX1	cg02835499	-0.132175176792017	48	-0.904390448993362	0.37050071253079	0.0174702773600009	0.569169570840296
**DLBC**	CBX1	cg04864609	-0.150612329200797	48	-1.03328934881803	0.306870573094839	0.0226840737072891	0.505352675634627
**DLBC**	CBX1	cg11729481	-0.142113660045642	48	-0.973744911399376	0.335276153854576	0.0201962923715683	0.534813525863794
**DLBC**	CBX1	cg12245530	-0.330579494745282	48	-2.37566340930974	0.0217395139769662	0.109282802346046	0.0775868319309054
**DLBC**	CBX1	cg13342109	-0.108240754163751	48	-0.738463197762909	0.463984210918316	0.0117160608619376	0.653211982051095
**DLBC**	CBX1	cg17778721	0.060252794295827	48	0.409398148379259	0.684147634860326	0.00363039922045524	0.818141839827897
**DLBC**	CBX1	cg21511817	-0.197744698377385	48	-1.368186617076	0.177899946813172	0.0391029657363628	0.354413587863525
**DLBC**	CBX4	cg04398978	-0.243874903695944	48	-1.70553582997677	0.094839476305074	0.0594749686527061	0.229278169603683
**DLBC**	CBX4	cg04925705	-0.218877714341829	48	-1.52139112390861	0.135006703550369	0.0479074538355032	0.293724627233755
**DLBC**	CBX4	cg05949524	-0.34743643039331	48	-2.51297816566959	0.0155390970422167	0.120712073164446	0.0600103512362095
**DLBC**	CBX4	cg07159673	-0.0321713135670133	48	-0.218309468280393	0.828153629365747	0.0010349934166271	0.907604297346993
**DLBC**	CBX4	cg10756274	-0.35295592404851	48	-2.5585307113959	0.0138682613778736	0.124577884320938	0.0549825464086381

DLBC	CBX4	cg21365914	-0.430108098165909	48	-3.23128942768946	0.00227941587141475	0.184992976107895	0.0132144629481212
DLBC	CBX4	cg22353329	-0.0276689407588525	48	-0.187731761197744	0.85191260102466	0.000765570282716887	0.921056353838736
DLBC	CBX4	cg27487227	-0.240401663548093	48	-1.67974443893952	0.099787460751769	0.0577929598366906	0.237891202553418
DLBC	CBX5	cg03689456	-0.475811418117502	48	-3.66905789029554	0.000630701133122041	0.226396505610988	0.0045624664264549
DLBC	CBX5	cg05754589	-0.151055795192906	48	-1.03640272314413	0.30543198922835	0.0228178532613613	0.503972175748834
DLBC	CBX5	cg06399839	-0.366762573924043	48	-2.6738319060717	0.0103454546642269	0.134514785631389	0.0437660721164211
DLBC	CBX5	cg06621757	-0.0020754846903746	48	-0.0140766523636299	0.988829703981147	4.30763669997935e-06	0.994109351796744
DLBC	CBX5	cg08288469	-0.0257750164327234	48	-0.174872764907605	0.861947055559358	0.000664351472107161	0.926681196048273
DLBC	CBX5	cg09060496	-0.294813212550713	48	-2.09252287633885	0.0419373118338844	0.0869148302944721	0.12673690280383
DLBC	CBX5	cg09158487	-0.4324272876166	48	-3.25270712417691	0.00214446577303578	0.18699335907545	0.0125631877662132
DLBC	CBX5	cg09653879	-0.309843709565184	48	-2.21023338557722	0.0321030015460539	0.0960031243571143	0.103812649219508
DLBC	CBX5	cg11005091	-0.215035209105193	48	-1.49337532534894	0.142166942500971	0.0462401411549139	0.304499741882671
DLBC	CBX5	cg11338003	-0.405178336456574	48	-3.0058411205708	0.00427932118536305	0.164169484333716	0.0219247571321215
DLBC	CBX5	cg12263485	0.182187335017451	48	1.25668673776276	0.21521127756632	0.033192225040761	0.40225832384893
DLBC	CBX5	cg12600134	-0.137120088129468	48	-0.938861768311661	0.352705594640407	0.018801918568633	0.55169154578176
DLBC	CBX5	cg13445218	-0.265403709987216	48	-1.86701147935353	0.0682805594034323	0.0704391292749781	0.181072930348128
DLBC	CBX5	cg14017196	-0.443104822943612	48	-3.3523528633552	0.00161020761937226	0.19634188411589	0.0099701123480934
DLBC	CBX5	cg14394617	0.0605616254539876	48	0.411504260608912	0.68261389551827	0.00366771047762908	0.817048733543839
DLBC	CBX5	cg15708001	-0.375662139987743	48	-2.74922709679634	0.0085083365060551	0.141122043420171	0.0376781910046722
DLBC	CBX5	cg20545544	-0.359707274638964	48	-2.61466553586273	0.0120351901109326	0.129389323428191	0.0492460330049383
DLBC	CBX5	cg20927027	0.260217816360077	48	1.82785273302713	0.0740616646202611	0.0677133119512065	0.192096593787504
DLBC	CBX5	cg21826008	-0.261765890760116	48	-1.83952428680089	0.0722968134673979	0.068521381565437	0.188738096186545
DLBC	CBX5	cg24505978	0.0227199186495173	48	0.154133772124262	0.878178516869394	0.000516194703440683	0.935337069295422
DLBC	CBX5	cg25817259	-0.131418755108013	48	-0.899123511267584	0.373269558893693	0.0172708891941399	0.57185141228637
DLBC	CBX6	cg03028106	-0.0323187441989724	48	-0.219310953001044	0.827378083980656	0.00104450122659861	0.907284687220207
DLBC	CBX6	cg04134624	-0.362456837506095	48	-2.63766137726705	0.0113504814051088	0.131374959054919	0.0470631782554649
DLBC	CBX6	cg04215348	-0.318761277701064	48	-2.28092874633174	0.0272313944984093	0.101608752161615	0.091834537177443
DLBC	CBX6	cg04801716	0.26899980582371	48	1.89426771914714	0.0644867201738294	0.0723608955331934	0.173509490858883
DLBC	CBX6	cg06060673	-0.114786026629598	48	-0.783696757866036	0.437234862069875	0.0131758319094107	0.630150225274443
DLBC	CBX6	cg09913544	-0.107804371502793	48	-0.735450936037216	0.465798392200406	0.0116217825151123	0.655042935028443
DLBC	CBX6	cg14498989	-0.363317696742176	48	-2.644877382051	0.0111430992073665	0.13199974876604	0.0463859722766092
DLBC	CBX6	cg24649346	-0.133039835588876	48	-0.910413008260055	0.367350821189305	0.0176995978535152	0.566258014068519
DLBC	CBX6	cg25836955	-0.0794860707028147	48	-0.540811901994579	0.591246608427251	0.00631803543577285	0.753998863250214
DLBC	CBX7	cg00545295	-0.296975632180977	48	-2.10934993446104	0.0403868034400402	0.0881945261092909	0.123258179161024
DLBC	CBX7	cg01525244	-0.135703959161865	48	-0.928982648939404	0.357747495470618	0.0184155645322052	0.55677920701493
DLBC	CBX7	cg02523424	-0.174646872456674	48	-1.20300148825445	0.235131035308598	0.0305015300588976	0.426302408893377
DLBC	CBX7	cg03804568	-0.179172908465409	48	-1.23519826236141	0.223028510361444	0.0321029311279539	0.411749013599558
DLBC	CBX7	cg10339854	-0.165685092470859	48	-1.13948005349135	0.26040196498683	0.0274515498670772	0.455544956650288
DLBC	CBX7	cg22268271	-0.268806843018591	48	-1.89280302311078	0.0646859278723956	0.0722571188536213	0.173931185984555
DLBC	CBX7	cg26672614	-0.237179868060418	48	-1.65588146033494	0.104553026804451	0.0562542898131573	0.245906341976826
DLBC	CBX7	cg27062573	-0.246997013636015	48	-1.72877933362275	0.0905559034214031	0.0610075247451097	0.221747802913678
DLBC	CDYL2	cg01442398	-0.224686749176061	48	-1.56388661971637	0.124698456369471	0.050484135255306	0.277917755822604
DLBC	CDYL2	cg02461391	-0.235079361525654	48	-1.64035481063162	0.107752875653505	0.055262306215309	0.251342604677184
DLBC	CDYL2	cg06919916	-0.221058400696857	48	-1.53732352354681	0.131064897705543	0.0488668165186522	0.287740815193831
DLBC	CDYL2	cg10112854	-0.255144036784324	48	-1.78970480983322	0.080085570247033	0.0650984795066006	0.203228629038286
DLBC	CECR2	cg03927893	-0.329893124809718	48	-2.3701278486014	0.0220307382762801	0.10882947379672	0.0783406890096874
DLBC	CECR2	cg12632097	-0.293890329088608	48	-2.08535211964945	0.0426137072677433	0.0863715255318101	0.128247956622926
DLBC	CECR2	cg13466481	-0.314064146484608	48	-2.24360934985961	0.0297142774511053	0.0986362881071051	0.0979778959874235
DLBC	CECR2	cg26796679	-0.255346742842777	48	-1.79122582272544	0.0798377447194463	0.0652019590804153	0.202776261016463
DLBC	CHAF1A	cg03006338	0.151036970626991	48	1.03627055137442	0.305492967226409	0.0228121664961786	0.504011872885162
DLBC	CHAF1A	cg04346879	-0.0434716014068737	48	-0.295117731316199	0.769231887281826	0.0018897801288781	0.87207265285926
DLBC	CHAF1A	cg05297461	0.0247667081676701	48	0.168027528482667	0.867298213989795	0.000613389833462537	0.929464617754607
DLBC	CHAF1A	cg07783855	0.0885930893616941	48	0.603239563622821	0.549312338356061	0.00784873548264911	0.722279523948086
DLBC	CHAF1A	cg09700233	-0.030158986838421	48	-0.204641289091772	0.838755190249611	0.000909564487120053	0.913698406530171
DLBC	CHAF1A	cg18804734	0.0873061622459091	48	0.594408936596868	0.555151082046351	0.00762236596610901	0.726657299424795
DLBC	CHAF1A	cg23811775	0.289069777038229	48	2.04799976068517	0.0462932133375846	0.0835613359969315	0.136357360129645
DLBC	CHAF1A	cg24085258	0.285043085502177	48	2.01692916708368	0.0495613981406706	0.0812495605926014	0.143356735711297

**DLBC**	CHAF1B	cg00660452	0.0305042743676058	48	0.206986378595402	0.836934041467049	0.000930510754694173	0.912726705664727
**DLBC**	CHAF1B	cg02544117	-0.0086507601894411	48	-0.0586745057231908	0.953465521803334	7.48356518552191e-05	0.975381963590045
**DLBC**	CHAF1B	cg02908490	-0.159501538037043	48	-1.09582111639673	0.278861248264333	0.0254407406361821	0.475802341261472
**DLBC**	CHAF1B	cg04634639	-0.158007524434126	48	-1.08529271745588	0.283447159142975	0.0249663777778008	0.480720869813885
**DLBC**	CHAF1B	cg07872789	-0.0983313732617816	48	-0.67016361677105	0.506104107090735	0.00966905896754782	0.689138537194859
**DLBC**	CHAF1B	cg13854874	-0.213520680782203	48	-1.48235293211163	0.145065309255258	0.0455910811216954	0.308735552983435
**DLBC**	CHAF1B	cg25942896	-0.275206747576425	48	-1.94151442609992	0.0583357781243095	0.0757387539115941	0.161311396701473
**DLBC**	CHD1	cg03255775	-0.521727322593817	48	-4.14778552862412	0.000143038210740262	0.272199399140913	0.00130726897438377
**DLBC**	CHD1	cg03833628	-0.175804499291909	48	-1.21122889531843	0.231993644179928	0.0309072219712788	0.42261359455177
**DLBC**	CHD1	cg08865521	-0.552729237656354	48	-4.49840168958145	4.62781944407674e-05	0.305509610160174	0.000495668161237037
**DLBC**	CHD1	cg11365324	-0.279703174595072	48	-1.9759044413588	0.0541814430850772	0.0782338658785614	0.153033118518698
**DLBC**	CHD1	cg12662497	0.245008060902985	48	1.71396539763436	0.093266986908243	0.0600289499074407	0.226567948399321
**DLBC**	CHD1	cg16890600	-0.267800991971494	48	-1.88517202098112	0.0657322945511915	0.0717173713009162	0.175978615589136
**DLBC**	CHD1	cg21082921	-0.23523971795761	48	-1.64153928164035	0.107505981202924	0.0553377249047761	0.250924701521159
**DLBC**	CHD4	cg00079216	-0.302146885631414	48	-2.14973555385915	0.036868767505161	0.0912927404967629	0.11528473408483
**DLBC**	CHD4	cg00824109	-0.135574975579309	48	-0.928083130141396	0.35820888977252	0.0183805740033303	0.557196320302396
**DLBC**	CHD4	cg04344119	0.147299594825271	48	1.01005218693395	0.317753963997759	0.021697170635689	0.517038827290725
**DLBC**	CHD4	cg05147525	0.0735326133942893	48	0.500076248504185	0.619405370180899	0.00540704523259401	0.774234319903801
**DLBC**	CHD4	cg11565429	0.22003390742922	48	1.52983543133986	0.132905883436837	0.0484149204185706	0.290567976170791
**DLBC**	CHD4	cg19509514	-0.0412243442187621	48	-0.279834989244498	0.780858628510665	0.00169944655626698	0.878951790519138
**DLBC**	CHD4	cg20308811	-0.0495681816939432	48	-0.336601534992949	0.737948126738378	0.00245700463644377	0.852445313103501
**DLBC**	CHRAC1	cg00296189	-0.0094468525029184	48	-0.0640745301428554	0.949188519998659	8.92430222118957e-05	0.973053945718257
**DLBC**	CHRAC1	cg07270064	-0.461948268545135	48	-3.53259817476234	0.000949033405136719	0.213396202811848	0.0064255139244612
**DLBC**	CHRAC1	cg08877624	-0.196927551433219	48	-1.36230421891441	0.17973544976883	0.0387804605134833	0.35671222732342
**DLBC**	CHRAC1	cg12002314	-0.155283950326371	48	-1.06611914811735	0.291933634186608	0.0241131052289628	0.489665483784239
**DLBC**	CHRAC1	cg18770186	-0.220332761889203	48	-1.5320192283235	0.132366862143777	0.048546525961724	0.28973046142377
**DLBC**	CHRAC1	cg19486048	-0.116702126875691	48	-0.796957978687516	0.429569859452626	0.0136193864173098	0.623608973021154
**DLBC**	CHRAC1	cg22610305	-0.278266594848319	48	-1.96490187095886	0.0554819370121652	0.0774322978084784	0.155555123809351
**DLBC**	CHRAC1	cg23548819	-0.352490158580803	48	-2.55467506078349	0.0140030663485573	0.124249311896319	0.0553616406693938
**DLBC**	CLOCK	cg05960024	-0.201677205823219	48	-1.39653734928821	0.169255147688148	0.040673695348661	0.342686280593702
**DLBC**	CLOCK	cg06085476	-0.183512936139695	48	-1.26614790543582	0.211834798985669	0.0336769977306119	0.397958859026591
**DLBC**	CLOCK	cg07236904	-0.479240977054815	48	-3.70335201095451	0.000568538543902658	0.229671914088453	0.00417844945530121
**DLBC**	CLOCK	cg07500410	-0.216334150184581	48	-1.50283771927468	0.139715589130057	0.046800464536085	0.300897916526872
**DLBC**	CLOCK	cg11774413	-0.572041415921897	48	-4.73013489333829	2.16116559996278e-05	0.327231381529928	0.000256250139740138
**DLBC**	CLOCK	cg18928811	-0.115781866742772	48	-0.790587794393228	0.433241726753148	0.0134054406664411	0.626850951786174
**DLBC**	CLOCK	cg22523077	-0.191734496995211	48	-1.32498937925366	0.191719013807417	0.0367621173380064	0.372199251823447
**DLBC**	CLOCK	cg23506964	-0.507331656704397	48	-3.99290648980239	0.000232997314945696	0.257385409894428	0.00197190960823384
**DLBC**	CREBBP	cg00039463	-0.241647584452435	48	-1.68898838448444	0.0979901246826728	0.0583935550716968	0.234627385643701
**DLBC**	CREBBP	cg00747661	-0.0666319263305741	48	-0.452926284416971	0.652730996549199	0.00443981360652305	0.797147505072823
**DLBC**	CREBBP	cg03039399	-0.579938278136323	48	-4.82818692851128	1.56090519247352e-05	0.336328406447723	0.000193173812509203
**DLBC**	CREBBP	cg03264209	-0.272643077745945	48	-1.92196862383246	0.0608165831855088	0.0743342478427813	0.166262484970954
**DLBC**	CREBBP	cg08506924	-0.43977723298818	48	-3.32111282895675	0.00176234610152501	0.19340401465474	0.010726017260369
**DLBC**	CREBBP	cg24567591	-0.39948949071721	48	-2.95555569632674	0.00490886031091498	0.159591853193496	0.0244215565023547
**DLBC**	CTCF	cg01866162	-0.0798839922622412	48	-0.543536648131058	0.589384972724844	0.00638145221975381	0.752636483390619
**DLBC**	CTCF	cg04487155	-0.587935930641356	48	-4.92958156146437	1.1129301021722e-05	0.345668658539118	0.000143843303486267
**DLBC**	CTCF	cg06241380	-0.520798951488277	48	-4.13765460834045	0.000147708254533403	0.271231547871288	0.00134442681094788
**DLBC**	CTCF	cg07967402	-0.541023129273709	48	-4.36309600236557	7.17930954903211e-05	0.292706026409116	0.000724393264665893
**DLBC**	CTCF	cg08324636	-0.164709514786099	48	-1.13258296396965	0.263258593548038	0.0271292242610723	0.458677511459408
**DLBC**	CTCF	cg10218542	-0.437100926180779	48	-3.29611033432359	0.00189383890408325	0.191057219668095	0.0113794163670543
**DLBC**	CTCF	cg10481400	-0.190629799757339	48	-1.31706657530737	0.194339842468554	0.036339720555523	0.375679589121056
**DLBC**	CTCF	cg23858565	-0.304599465319677	48	-2.16896200491787	0.0352907327573346	0.0927808342730333	0.111506732631659
**DLBC**	CXXC1	cg02159718	0.0674781780007892	48	0.4587047725964	0.64860621929256	0.0045533045063062	0.794358009063455
**DLBC**	CXXC1	cg07675399	-0.111375636144739	48	-0.760115466339135	0.451063999315442	0.0124045323266453	0.641758527428682
**DLBC**	CXXC1	cg11871295	0.106377017922487	48	0.725601195761377	0.471758878314096	0.0113160699420811	0.659870366606735
**DLBC**	CXXC1	cg13423076	-0.116474855642239	48	-0.79538457889769	0.43047504939292	0.0135663919968804	0.624468569796424
**DLBC**	CXXC1	cg15632775	0.223288665086642	48	1.5536432260202	0.127123383054688	0.0498578279561746	0.281846684375183
**DLBC**	CXXC1	cg20016845	-0.119036303844009	48	-0.813124889937615	0.420335255043346	0.0141696416328432	0.615572763803207

**DLBC**	CXXC1	cg27419474	0.0944724093918526	48	0.643621665428981	0.523016859473588	0.00892503613630181	0.702578999627156
**DLBC**	DAXX	cg00221327	0.369079277976127	46	2.63417307410243	0.0115980201987167	0.136219513431379	0.0478599821418931
**DLBC**	DAXX	cg02173004	-0.09523526037906	48	-0.64886619299284	0.519651393511586	0.00906975481946735	0.70017437111182
**DLBC**	DAXX	cg02328295	-0.188361483488304	48	-1.30081462249315	0.199800758326098	0.0354800484619144	0.382707937982481
**DLBC**	DAXX	cg03085224	-0.361322479898542	48	-2.62816468233373	0.0116288015547376	0.130553934480032	0.0479590196760459
**DLBC**	DAXX	cg03369465	0.428776633901274	47	3.18384719255404	0.0026373892303072	0.183849401779707	0.0148525450834312
**DLBC**	DAXX	cg03382121	-0.206966984016498	48	-1.43478443767048	0.158112854642235	0.0428353324728851	0.327151215863557
**DLBC**	DAXX	cg03902329	-0.052824896855695	48	-0.358776809412696	0.721403570359087	0.00279046972781482	0.842111990044188
**DLBC**	DAXX	cg04086445	-0.00286695798276654	48	-0.0194447349995369	0.984570423067523	8.2194480749488e-06	0.99189844047809
**DLBC**	DAXX	cg04152866	-0.284402569729907	48	-2.01199762129087	0.0500981003571933	0.0808848216689744	0.144479392740964
**DLBC**	DAXX	cg04271391	-0.156164746368886	48	-1.07231707612841	0.289171264135707	0.0243874280084587	0.486720149884811
**DLBC**	DAXX	cg04488476	-0.415942627896257	48	-3.10214264369653	0.00327918614805103	0.173008269701244	0.0176785174775801
**DLBC**	DAXX	cg04973788	-0.177652190540983	48	-1.22437144032905	0.227045804987947	0.0315603008040099	0.41661480689764
**DLBC**	DAXX	cg05431670	-0.0661035297099462	48	-0.449318715053818	0.655311719496256	0.00436967664011374	0.799076039050607
**DLBC**	DAXX	cg05532204	-0.1749694752037	48	-1.20529374979711	0.234253815864718	0.0306143172530581	0.425187619024263
**DLBC**	DAXX	cg06171013	-0.224140016986128	48	-1.55987965036655	0.125642556496821	0.050238747214542	0.279399623820532
**DLBC**	DAXX	cg07689821	0.193464672536221	48	1.33740858585797	0.18766495304264	0.0374285795195474	0.367017128990877
**DLBC**	DAXX	cg08375286	-0.126392906365866	48	-0.86416880897442	0.391979156531412	0.0159751667796107	0.589059876464764
**DLBC**	DAXX	cg08567382	0.408902809491487	48	3.03898841995456	0.00390657256951499	0.167201507610031	0.0203814570768459
**DLBC**	DAXX	cg09097215	0.0489467235947005	48	0.33237121420969	0.741118864132822	0.00239578175065601	0.854392899923449
**DLBC**	DAXX	cg09432361	-0.130392399083148	48	-0.891979583546694	0.377046232634125	0.0170021777386589	0.575564825194049
**DLBC**	DAXX	cg09615909	-0.546726747380016	48	-4.42855857235739	5.8083194899134e-05	0.298910136300732	0.000604727524623023
**DLBC**	DAXX	cg11111423	-0.552702303661255	48	-4.49808606823517	4.632585089816e-05	0.305479836472458	0.000496059634517751
**DLBC**	DAXX	cg12025215	-0.203569907091555	48	-1.41020744557506	0.165204805787066	0.0414407070732642	0.337162040542499
**DLBC**	DAXX	cg12832355	-0.654407433683697	48	-5.86980545251499	4.53675016302489e-07	0.428249089260482	8.45143539708712e-06
**DLBC**	DAXX	cg12923297	0.246945293432203	48	1.72839382232185	0.0906256167053002	0.0609819779483167	0.221859192308273
**DLBC**	DAXX	cg13443893	-0.1484311043933	48	-1.01798520545553	0.314009421124586	0.0220317927514147	0.513097290574599
**DLBC**	DAXX	cg13826470	0.148496224103147	48	1.01844188410429	0.313794775972178	0.0220511285728919	0.512854278492817
**DLBC**	DAXX	cg14539675	-0.225865082790929	48	-1.57252785543665	0.122681903656541	0.0510150356241531	0.274834879141189
**DLBC**	DAXX	cg14622330	-0.299266219902563	48	-2.1272131706832	0.0387959733689485	0.089560270374769	0.119732374033561
**DLBC**	DAXX	cg14746369	-0.0814486642873286	48	-0.554253202994449	0.582090295639259	0.00663388491418995	0.747320402332913
**DLBC**	DAXX	cg14910283	-0.473760697446251	48	-3.64865516208216	0.000670727836191602	0.224449198444758	0.00480555599217246
**DLBC**	DAXX	cg16922466	-0.122286246339273	48	-0.835657389475696	0.407667381488872	0.0149539260437492	0.603813115693035
**DLBC**	DAXX	cg17079757	-0.109052986672121	48	-0.744071026969601	0.460617656738872	0.0118925539021098	0.650343744684002
**DLBC**	DAXX	cg17504991	-0.181592394395172	48	-1.25244279624727	0.21673879370904	0.0329757977021716	0.404029946271471
**DLBC**	DAXX	cg18187593	-0.419882980513683	48	-3.1377844536253	0.00296827332430332	0.176301717325054	0.0163247688222589
**DLBC**	DAXX	cg18279148	0.0545481767962864	46	0.362371190887463	0.718809448669068	0.00297550359179892	0.840409915633868
**DLBC**	DAXX	cg18486541	-0.365161324929908	48	-2.66035757589375	0.0107098870889672	0.133342793224566	0.0449666944229555
**DLBC**	DAXX	cg18944910	-0.0883946994358885	48	-0.601878055727954	0.550210513862673	0.00781362288836106	0.722829585170102
**DLBC**	DAXX	cg18967278	-0.327795070461189	48	-2.35323317087222	0.0229413547154782	0.107449608218656	0.0807659474361653
**DLBC**	DAXX	cg20223162	-0.0711883964996224	48	-0.484051288540746	0.630646090502305	0.00506778779618745	0.782053566065968
**DLBC**	DAXX	cg20763184	-0.191619194918238	48	-1.32416219784148	0.191991380157504	0.0367179158611139	0.372500434656147
**DLBC**	DAXX	cg21286626	-0.170183035165865	48	-1.17132423025875	0.24750017674122	0.0289622654582661	0.44080819309477
**DLBC**	DAXX	cg24328964	-0.346044032651136	48	-2.50153390325629	0.0159866711165847	0.119746472533461	0.0612493027748627
**DLBC**	DAXX	cg24496586	-0.193578794771906	48	-1.33822820989766	0.187399715409788	0.0374727497853437	0.366716367359642
**DLBC**	DAXX	cg24513883	-0.460889395292887	48	-3.52231364875475	0.000978436505977635	0.212419034693443	0.00659645459277443
**DLBC**	DAXX	cg24728112	-0.410066142096842	48	-3.04937913821128	0.00379614233761273	0.168154240894188	0.019914835593246
**DLBC**	DAXX	cg25642825	0.374014127794867	48	2.73519981326813	0.00882551454762631	0.139886567790155	0.0387412300371313
**DLBC**	DAXX	cg26521092	0.0117906036213015	48	0.0799733235321994	0.936605392210531	0.000139018333754647	0.966176652322889
**DLBC**	DAXX	cg27052777	-0.501565838875353	48	-3.93215633035194	0.000281587050171943	0.251568290726736	0.00231782700043129
**DLBC**	DAXX	cg27393652	-0.339990209920106	48	-2.45199356445719	0.0180621767350217	0.115593342841518	0.0673147924175662
**DLBC**	DIDO1	cg00080059	-0.200495949961571	48	-1.38801396752448	0.171819232725125	0.0401986259509928	0.346076311799144
**DLBC**	DIDO1	cg01488865	-0.42513188070047	48	-3.18559785201625	0.00259462338205274	0.180737115987918	0.0146564085844053
**DLBC**	DIDO1	cg06646488	-0.266691801356462	48	-1.87676471874849	0.0669017562893066	0.0711245169107544	0.178342022025505
**DLBC**	DIDO1	cg08610063	-0.665342014123314	48	-6.04465541856031	2.48204814962525e-07	0.442679995757668	4.91216023566866e-06
**DLBC**	DIDO1	cg13815390	-0.334401638264799	48	-2.40656672126952	0.0201763837567785	0.111824455674181	0.0733150356380413
**DLBC**	DIDO1	cg20969846	-0.239517442921625	48	-1.67318943011107	0.101078363307868	0.0573686054637139	0.240085802246344

**DLBC**	DIDO1	cg21212389	-0.481536981205357	48	-3.72643389309306	0.000530062787492589	0.231877864268368	0.00393846270202469
**DLBC**	DIDO1	cg21687703	-0.147885037462784	48	-1.01415621955622	0.315813020354626	0.0218699843053689	0.515005934503045
**DLBC**	DIDO1	cg22394521	-0.603044161908277	48	-5.12724644995555	5.72828987743248e-06	0.363662261211657	8.02786133534467e-05
**DLBC**	DMAP1	cg01604592	0.31261062728939	48	2.23209815728218	0.0305196928149599	0.097725404294266	0.0999725875972459
**DLBC**	DMAP1	cg11683482	0.147417377556023	48	1.01087777256679	0.317362867596388	0.021731883205495	0.516578750143559
**DLBC**	DMAP1	cg15990015	0.15885788364208	48	1.09128430473926	0.280830941625545	0.0252358271952406	0.478010439823785
**DLBC**	DMAP1	cg18017761	0.29611686572972	48	2.10266312872222	0.0409968377793519	0.0876851981695932	0.124582343679957
**DLBC**	DMAP1	cg18167340	0.125900456728494	48	0.86074751414969	0.393841490878282	0.0158509250044434	0.590564860088378
**DLBC**	DMAP1	cg21437596	-0.287253520051476	48	-2.03397069088746	0.0477449326173025	0.0825145847819637	0.139487354965842
**DLBC**	DMAP1	cg23021241	0.329726421590958	48	2.36878402740245	0.0221019629373489	0.108719513095178	0.0785244762629652
**DLBC**	DMAP1	cg23451311	0.18744533354354	48	1.29425675998199	0.202036799284625	0.0351357530672488	0.385489611722187
**DLBC**	DMAP1	cg24485408	0.62124120440405	48	5.37692512574805	2.45684354696738e-06	0.385940634049394	3.78470667114416e-05
**DLBC**	DNMT1	cg04160663	-0.261554894083146	48	-1.83793260378811	0.0725353772701148	0.0684109626188459	0.189197557061234
**DLBC**	DNMT1	cg04762346	-0.116804730613968	48	-0.79766834640788	0.42916155355598	0.0136433450938017	0.623260475616218
**DLBC**	DNMT1	cg06128182	-0.0567081185099424	48	-0.385233089588661	0.701840363647723	0.00321581070493767	0.829242265569256
**DLBC**	DNMT1	cg06716436	-0.36469736785859	48	-2.65645852939829	0.0108175203488692	0.133004170122984	0.0453317268918298
**DLBC**	DNMT1	cg10635912	-0.354166830500491	48	-2.56856488914676	0.0135229653114936	0.125434143826763	0.053946236779772
**DLBC**	DNMT1	cg11380607	-0.301820558171168	48	-2.14718093632614	0.0370830452673466	0.0910956493347555	0.115787213466906
**DLBC**	DNMT1	cg13393778	-0.108944451286444	48	-0.743321588105206	0.461066749878228	0.0118688934661043	0.650701927481531
**DLBC**	DNMT1	cg15043801	-0.112500318763233	48	-0.767889084077476	0.446477162078919	0.0126563217218291	0.638267564900721
**DLBC**	DNMT1	cg21893394	-0.380459369635515	48	-2.79023311568838	0.00764050199317816	0.144749331943454	0.0346964690944663
**DLBC**	DNMT1	cg23401624	-0.13591641929878	48	-0.930464429943011	0.356988281560395	0.0184732730350017	0.555871127421676
**DLBC**	DNMT1	cg24477899	-0.39497731944761	48	-2.91596074354742	0.005464349528222	0.156007082878019	0.0266232609255654
**DLBC**	DNMT1	cg27429799	-0.428401208074918	48	-3.21557650786665	0.00238348751959295	0.183527595080049	0.0136953266931984
**DLBC**	DNMT3A	cg02208653	-0.15801087855674	48	-1.08531634555679	0.283436808588202	0.0249674377422728	0.480720395206039
**DLBC**	DNMT3A	cg03463641	-0.19464631407086	48	-1.34589785460482	0.184931587797868	0.0378871875813717	0.363471563152709
**DLBC**	DNMT3A	cg06112956	-0.193598776728319	48	-1.33837172578833	0.187353301813664	0.0374804863507015	0.366656505780791
**DLBC**	DNMT3A	cg07150430	0.322288884638688	48	2.30907886050806	0.0254826636233727	0.103870125161649	0.0874420331664531
**DLBC**	DNMT3A	cg12066181	-0.184801831183428	48	-1.27535394542864	0.208587479688947	0.0341517168087482	0.393788783335887
**DLBC**	DNMT3A	cg13076778	-0.283232964304937	48	-2.0029999935899	0.0510902878656549	0.0802209120689619	0.146582013539334
**DLBC**	DNMT3A	cg13344237	-0.191855101348193	48	-1.32585466033807	0.191434416593009	0.0368083799133255	0.3719096013085
**DLBC**	DNMT3A	cg15990840	-0.29353499570851	48	-2.08259290135853	0.0428765032258068	0.0861627937055951	0.128821823944093
**DLBC**	DNMT3A	cg15998962	-0.278091992416412	48	-1.96356559075138	0.0556416991004642	0.0773351562461296	0.155805569786967
**DLBC**	DNMT3A	cg21629895	-0.158751955172992	48	-1.09053780264464	0.281155974105309	0.0252021832712475	0.47838515888866
**DLBC**	DNMT3A	cg21708767	-0.236378494438683	48	-1.64995493090981	0.105765094727025	0.0558747926330983	0.2479026748778
**DLBC**	DNMT3A	cg22731525	0.0324493305566918	48	0.220198027853896	0.826691282257528	0.00105295905357745	0.906895981215869
**DLBC**	DNMT3A	cg25044635	-0.191231586633531	48	-1.32138189853851	0.192909004464571	0.0365695197263777	0.373749485550689
**DLBC**	DNMT3A	cg26470599	-0.387403713270559	48	-2.85006056276942	0.00652046913628833	0.150081637055817	0.0306293929103565
**DLBC**	DNMT3A	cg26995204	-0.181959327617169	48	-1.25506010438332	0.215795799975558	0.0331091969068923	0.402976990885489
**DLBC**	DNMT3B	cg00300969	-0.0691248566124647	48	-0.469951704395762	0.640609918813387	0.0047782458016938	0.788851397213396
**DLBC**	DNMT3B	cg13636640	0.198677982903653	48	1.37490868719053	0.175820121694814	0.0394729408906644	0.351622163734965
**DLBC**	DNMT3B	cg14224313	-0.303878638635463	48	-2.163306336428	0.035748632807084	0.0923422270189425	0.112619258518284
**DLBC**	DNMT3B	cg16523653	-0.421795273543358	48	-3.1551591680586	0.00282698299314356	0.177911252783516	0.0156986447975309
**DLBC**	DNMT3B	cg16953106	-0.425499618609939	48	-3.18896224538816	0.0025700770445907	0.181049925437203	0.0145494200397709
**DLBC**	DNMT3B	cg17475857	-0.00391859706662111	48	-0.0265774224310301	0.97891174843258	1.53554029705316e-05	0.989094294612233
**DLBC**	DNMT3B	cg17721173	-0.525044589781047	48	-4.18415161531468	0.000127428039655609	0.275671821258348	0.00118606567563633
**DLBC**	DNMT3B	cg22605822	-0.201862403124775	48	-1.3978742200113	0.168855680396268	0.0407484297953091	0.342126808197611
**DLBC**	DNMT3B	cg24403338	-0.0575714770866753	48	-0.391117467728593	0.697516185596106	0.00331447497394157	0.826595341881637
**DLBC**	DNMT3B	cg25803642	-0.26388020409493	48	-1.85548959290486	0.0699404093678611	0.0696327621131821	0.184270132636685
**DLBC**	DNMT3L	cg09247319	-0.115835637436257	48	-0.790959946593192	0.433026698284979	0.0134178949002641	0.626720972007621
**DLBC**	DNMT3L	cg15635600	0.135854767752162	48	0.930034434219525	0.357208488630937	0.0184565179209937	0.556084950863586
**DLBC**	DNMT3L	cg27187798	0.0539953155186389	48	0.366749065002416	0.715487889844445	0.00291549409795736	0.83825011882161
**DLBC**	DPF1	cg06168449	-0.191710086696511	48	-1.32481425410665	0.191776652826381	0.0367527573411836	0.372252437758327
**DLBC**	DPF1	cg06615189	0.15654434240118	48	1.07498900021348	0.287986035426557	0.0245061311378179	0.485515810780463
**DLBC**	DPF1	cg07480327	0.541069865089232	40	3.9660729018854	0.000312255630588843	0.29275659890768	0.00252695459544663
**DLBC**	DPF1	cg08057062	0.147172490475654	48	1.00916131166898	0.318176355233323	0.0216597419528065	0.517493077272504
**DLBC**	DPF1	cg13322582	0.00508749497159456	48	0.0345055162340593	0.972623341784923	2.5882605086e-05	0.985558207323099

**DLBC**	DPF1	cg13917712	-0.180061526803643	48	-1.24152905048542	0.220703941167467	0.032422153434859	0.408932558452388
**DLBC**	DPF1	cg17121541	0.277856733632379	48	1.96176542444657	0.0558575478593893	0.0772043644248545	0.156246883541524
**DLBC**	DPF1	cg22145401	0.192515571571191	48	1.33059433929255	0.189881194684249	0.0370622452973825	0.369947097579866
**DLBC**	DPF1	cg27400772	0.251754708917459	32	1.42480889181548	0.164533302421707	0.0633804334621144	0.336161984677863
**DLBC**	DPF2	cg02186298	0.0201321809153746	48	0.136570773441712	0.891966052865894	0.000405304708409373	0.942755147434878
**DLBC**	DPF2	cg14699126	-0.423067761654556	48	-3.16674909220291	0.00273628616377831	0.178986330951396	0.0153075396105976
**DLBC**	DPF2	cg15918224	-0.390961687229015	48	-2.88093344462698	0.00600405979179536	0.152851040880958	0.0286886448893144
**DLBC**	DPF2	cg15929437	-0.204419783362478	48	-1.41635105938187	0.16340921968953	0.0417874478299625	0.334436264147939
**DLBC**	DPF2	cg18224556	-0.263556168321132	48	-1.85304092200508	0.0702975397646455	0.0694618538601169	0.184954234593169
**DLBC**	DPF2	cg19517014	-0.201524788458153	48	-1.39543722192234	0.169584423009345	0.0406122403631032	0.343129697346779
**DLBC**	DPF3	cg00888802	0.0332639074142721	48	0.225731715841759	0.82241003559954	0.00110648753646526	0.904584759843209
**DLBC**	DPF3	cg05151458	0.272861266779508	48	1.92363039958954	0.0606022102104931	0.0744532709085177	0.165845440641471
**DLBC**	DPF3	cg07171024	0.0831767417488497	48	0.566093731204611	0.574081543871761	0.00691837036795484	0.741345070236027
**DLBC**	DPF3	cg10266394	-0.0293058378880431	48	-0.198847269542548	0.843258513465137	0.000858832134320262	0.916260609108509
**DLBC**	DPF3	cg12913429	0.0680055731912835	48	0.462306504789808	0.646040856417544	0.00462475798507501	0.792442915251436
**DLBC**	DPF3	cg13819165	-0.284140168585477	48	-2.00997815383016	0.0503193277783682	0.0807356354037835	0.144956185343099
**DLBC**	DPF3	cg14238615	0.134467922826234	48	0.920364625036365	0.3621838603459	0.018081622269202	0.561113060735032
**DLBC**	DPF3	cg14242696	0.254200910140009	48	1.78263133678263	0.0812465803830374	0.0646181027160092	0.205210924490844
**DLBC**	DPF3	cg15392845	-0.0852013710052206	48	-0.579972737857091	0.564763157082412	0.00725927362116925	0.73441750864008
**DLBC**	DPF3	cg15740492	0.235888225518101	48	1.64633092422023	0.106511916011191	0.0556432549380785	0.249097763172636
**DLBC**	DPF3	cg16696021	0.169454209642997	48	1.16615929067135	0.249560787388216	0.0287147291657329	0.443148411186462
**DLBC**	DPF3	cg19710444	-0.144596818685849	48	-0.991119352196124	0.326812349393087	0.0209082399740682	0.526301362301236
**DLBC**	DPF3	cg19839347	9.29105747499571e-05	48	0.00063015017959588	0.999499937847227	8.63237490036737e-09	0.999761926316662
**DLBC**	DPF3	cg20548765	-0.119077120074722	48	-0.813407710994541	0.420174785430168	0.0141793605252897	0.615436761141634
**DLBC**	DPF3	cg21035356	-0.103338838136564	48	-0.704650651406927	0.484580215048585	0.0106789154674149	0.671298405326049
**DLBC**	DPF3	cg22784589	0.0405023575158086	48	0.274925946214639	0.784604217741471	0.00164044096433837	0.881045676376207
**DLBC**	DPF3	cg23515460	0.248716177185804	48	1.74160259048522	0.0882623032347721	0.0618597367939204	0.217907890125226
**DLBC**	DPF3	cg24383902	-0.0532210550982949	48	-0.361475056314256	0.719399431462224	0.00283248070577574	0.84084113845999
**DLBC**	DPF3	cg25495195	-0.16345349700915	48	-1.12370826118758	0.266967073840402	0.0267170456845201	0.46271130643533
**DLBC**	DPF3	cg26057784	0.22335338192336	48	1.55411716585617	0.127010356650439	0.0498867332166022	0.281669158655987
**DLBC**	EED	cg00080012	0.26965868081211	48	1.89927080499695	0.0638102089625275	0.0727158041373273	0.172181494583651
**DLBC**	EED	cg01739327	0.246109100311536	48	1.72216319822181	0.0917585176558011	0.0605696892561536	0.223938452833335
**DLBC**	EED	cg02172616	0.0201916025196058	48	0.136974036235547	0.891649082593098	0.000407700812309753	0.942599420174855
**DLBC**	EED	cg04607174	-0.395237604444703	48	-2.91823793333323	0.0054308762700515	0.156212763967187	0.0265000055543114
**DLBC**	EED	cg06358433	-0.233928192126202	48	-1.63185586525812	0.109538048085416	0.0547223990714335	0.254190357293238
**DLBC**	EED	cg10086030	-0.141783712674405	48	-0.971437718957194	0.336410956195525	0.0201026211797382	0.535978412514841
**DLBC**	EED	cg10535675	-0.377162421671305	48	-2.76202335055878	0.00822820748802712	0.142251492320963	0.0367138040334719
**DLBC**	EED	cg12672785	0.0530957205897002	48	0.360621380380137	0.72003328966942	0.00281915554493951	0.841216363112428
**DLBC**	EED	cg16265078	-0.101113845231943	48	-0.689320326279442	0.494084368901556	0.0102240096975893	0.679319579982337
**DLBC**	EED	cg18574463	-0.0935422880061259	48	-0.63722871666443	0.527134857905143	0.008750159645421	0.705743453961255
**DLBC**	EED	cg19325359	-0.353761447856316	48	-2.5652040447719	0.0136377352370426	0.125147161989397	0.0542677225079534
**DLBC**	ELP3	cg01209934	0.151198766221088	48	1.03740659452435	0.304969121166984	0.0228610669067791	0.503464715999923
**DLBC**	ELP3	cg02516513	-0.0549942854113206	48	-0.373554702682431	0.710451787123398	0.00302437142790179	0.83507926881841
**DLBC**	ELP3	cg04720865	0.217412656831871	48	1.51070067382705	0.137704251693165	0.047268263350693	0.297802862121041
**DLBC**	ELP3	cg05621091	-0.312808797409468	48	-2.23366654608881	0.0304088281680481	0.0978493437367573	0.0997040667030811
**DLBC**	ELP3	cg15692687	0.143043524739081	48	0.980248875251219	0.332090875054327	0.02046144996978	0.531763734927369
**DLBC**	ELP3	cg17572663	-0.0231917748400228	48	-0.157336587954749	0.875668179498175	0.000537858420230313	0.934262228017294
**DLBC**	ELP3	cg18022570	0.0860039072065112	48	0.585476185038529	0.561089048909725	0.0073966720547862	0.731586590460064
**DLBC**	ELP3	cg18249244	-0.164213013342105	48	-1.12907413665715	0.264720418556647	0.0269659137508945	0.460327251295254
**DLBC**	ELP3	cg19781013	0.152193350089368	48	1.04439191506926	0.301761623793357	0.023162815811425	0.500169377095936
**DLBC**	ELP3	cg22414525	-0.0734977821281907	48	-0.499838083234061	0.619571769313176	0.00540192397776299	0.774330338045002
**DLBC**	ELP3	cg22487209	-0.234307835562945	48	-1.63465792250045	0.108946836468853	0.0549001618061921	0.253162979031069
**DLBC**	ELP3	cg25374552	0.0898809347023379	48	0.612079539425321	0.543498907785802	0.00807858242296593	0.71780646201872
**DLBC**	ELP4	cg04972224	-0.221704632366398	48	-1.54204962569891	0.129913512438685	0.0491529440127199	0.285995013488456
**DLBC**	ELP4	cg05195750	-0.323881709684442	48	-2.32182458566536	0.0247244089055191	0.104899361868117	0.0854796478453524
**DLBC**	ELP4	cg05635455	-0.191823204293076	48	-1.32562580748938	0.191509656709426	0.036796141705263	0.371964702620355
**DLBC**	ELP4	cg08111895	-0.236577145343381	48	-1.65142371339139	0.105463637846484	0.0559687456988233	0.24743465355659

**DLBC**	ELP4	cg08626560	-0.212831012329887	48	-1.47733739841975	0.146399540997798	0.0452970398093645	0.310614899154059
**DLBC**	ELP4	cg09616795	-0.247841092916519	48	-1.73507314643185	0.0894240604282764	0.0614252073380546	0.21985510893614
**DLBC**	ELP4	cg10583382	-0.230123507854699	48	-1.60381773993591	0.115599207472214	0.0529568288673515	0.263853673270939
**DLBC**	ELP4	cg11385473	-0.223357602533768	48	-1.55414807534011	0.12700298808297	0.0498886186096325	0.281669158655987
**DLBC**	ELP4	cg13263383	-0.211229217950186	48	-1.46569744303237	0.149533363488115	0.0446177825158472	0.315012957083904
**DLBC**	ELP4	cg19328333	-0.180260611289543	48	-1.24294781920294	0.220185460733476	0.0324938879824798	0.408350470487567
**DLBC**	ELP4	cg24001477	-0.250343817780956	48	-1.75375934360069	0.0861327821925011	0.0626720271011445	0.214090793593311
**DLBC**	ELP4	cg24531794	-0.207144019507662	48	-1.43606672164054	0.157749475587022	0.0429086448177908	0.326581757559592
**DLBC**	ELP4	cg25130734	-0.255903214857557	48	-1.79540263216359	0.0791605073082169	0.0654864553744328	0.201446271034852
**DLBC**	EP300	cg00500400	-0.0183639517206956	48	-0.12457138703372	0.901405672526119	0.000337234722800039	0.947956498370603
**DLBC**	EP300	cg02046995	-0.47572999125441	48	-3.66824630090042	0.00063224856103878	0.226319024578921	0.00457111008898242
**DLBC**	EP300	cg07519229	-0.0202514808484954	48	-0.137380400045409	0.891329692965817	0.000410122476556977	0.942488291382577
**DLBC**	EP300	cg08131204	-0.282116029633813	48	-1.99441662630573	0.0520525858782868	0.0795894541763462	0.148579249497888
**DLBC**	EP300	cg09331127	0.0312183204854001	48	0.211836202251107	0.833170640175731	0.00097458353392915	0.910498781825767
**DLBC**	EP300	cg24349919	-0.624856628204315	48	-5.42816618042425	2.06318510211087e-06	0.390445805810865	3.24642843514589e-05
**DLBC**	EPC1	cg02900516	0.217124442402667	48	1.50859885216877	0.138239625038511	0.047143023488669	0.298620314615442
**DLBC**	EPC1	cg16942190	-0.182128744843544	48	-1.25626872811083	0.215361374593424	0.0331708796982846	0.40240723121998
**DLBC**	EPC1	cg26211455	0.0338353458725826	48	0.229613952983414	0.819409714598421	0.0011448306303173	0.902798987663502
**DLBC**	EPC2	cg00066942	-0.0973047002228185	48	-0.663099231430793	0.510576393069745	0.00946820468545258	0.692851310397498
**DLBC**	EPC2	cg03664802	-0.660877933670365	48	-5.97246047949548	3.18446697560466e-07	0.436759643212411	6.14568156537307e-06
**DLBC**	EPC2	cg04098458	0.111183068694186	48	0.758784769805572	0.451851931922641	0.0123616747642561	0.642409612778061
**DLBC**	EPC2	cg05586854	-0.285089216032565	48	-2.01728445492736	0.0495229250669162	0.0812758610980625	0.14328509154346
**DLBC**	EPC2	cg10458926	-0.545314364663027	48	-4.41226731466125	6.12344338005225e-05	0.297367756307841	0.000633804090350965
**DLBC**	EPC2	cg11721798	-0.368756670997853	48	-2.69065042104765	0.00990657070607223	0.135981482405419	0.0423027643818303
**DLBC**	EPC2	cg12432635	-0.527447832531073	48	-4.21066174657352	0.000117106299128599	0.278201216041726	0.001103297573696
**DLBC**	EPC2	cg16434205	-0.166269263830577	48	-1.14361164352886	0.258701393695297	0.027645468094762	0.453377252109303
**DLBC**	EPC2	cg21347644	0.404806321800297	48	3.00254013058335	0.00431821166181984	0.163868158169486	0.0220716792391335
**DLBC**	EPC2	cg21650422	-0.211018579745409	48	-1.46416769464693	0.149949119236903	0.0445288409977695	0.315564080017195
**DLBC**	EZH1	cg00728999	-0.194285609277573	48	-1.3433057989275	0.185762929749757	0.0377468979723578	0.364538482787021
**DLBC**	EZH1	cg01716316	-0.302520948349025	48	-2.15266488461226	0.0366244003467116	0.0915189241899933	0.114728138864947
**DLBC**	EZH1	cg03623386	-0.122896477792771	48	-0.839891265375875	0.405313565072261	0.015103544253869	0.601714120453145
**DLBC**	EZH1	cg05375741	-0.164959463020123	48	-1.13434970881906	0.262524725564863	0.0272116244398873	0.457958738172791
**DLBC**	EZH1	cg05979501	-0.220723504530225	48	-1.53487515711904	0.131664580872635	0.0487188654521042	0.28863286286262
**DLBC**	EZH1	cg06975499	-0.228821788794823	48	-1.59424280333585	0.117730438439717	0.0523594110272625	0.267259672823232
**DLBC**	EZH1	cg12812489	-0.150126974587253	48	-1.02988262486863	0.308450007310758	0.0225381084987217	0.50703771513435
**DLBC**	EZH1	cg19395400	-0.161823155680587	48	-1.11219704924242	0.271832349278392	0.0261867337144235	0.468156056040873
**DLBC**	EZH1	cg22227424	0.039154385872864	48	0.265761758562661	0.791610215196459	0.00153306593308113	0.885669970353984
**DLBC**	EZH1	cg27088844	0.0494794055509458	48	0.335997203980752	0.738400808479523	0.00244821157367496	0.852689781270593
**DLBC**	EZH2	cg01893439	-0.268827015842404	48	-1.8929561346679	0.0646650792391729	0.0722679644467321	0.173882342780816
**DLBC**	EZH2	cg02303805	-0.32621675780288	48	-2.34054946862487	0.0236470402090689	0.106417373071423	0.0826897040510767
**DLBC**	EZH2	cg06816161	0.222099620216395	48	1.5449393518633	0.129213514055476	0.0493282413002668	0.284942617001716
**DLBC**	EZH2	cg13751213	-0.273685091262643	48	-1.9299077228902	0.0597982471193768	0.0749035291794413	0.164240391060754
**DLBC**	EZH2	cg18494018	-0.230187053972187	48	-1.60428539251309	0.1154959229036	0.0529860798163946	0.263672080829988
**DLBC**	EZH2	cg20101066	0.030414898897511	48	0.206379360084892	0.837405353545628	0.000925066074945817	0.912959135513882
**DLBC**	FBXL19	cg00026853	-0.121717943981204	48	-0.831715289158277	0.409866534632558	0.0148152578870115	0.605801630064887
**DLBC**	FBXL19	cg02291855	0.178257178152442	48	1.22867758788643	0.225441669690795	0.0317756215628714	0.414845521144993
**DLBC**	FBXL19	cg03181514	0.10337404302199	48	0.704893300319599	0.484430609916443	0.0106861927707122	0.671138959749247
**DLBC**	FBXL19	cg03627896	0.0897830516579597	48	0.611407546490563	0.543939717278648	0.00806099636501586	0.718191558040709
**DLBC**	FBXL19	cg04477789	0.0916210254218569	48	0.624028724810193	0.535691351000771	0.00839441229935255	0.71204013463451
**DLBC**	FBXL19	cg05643349	-0.133516834607543	48	-0.913736327460808	0.365620074281001	0.0178267451236179	0.564494331000346
**DLBC**	FBXL19	cg05768582	-0.402490527413385	48	-2.9820314118541	0.00456734460557366	0.161998624657505	0.0230587323152701
**DLBC**	FBXL19	cg06862768	-0.0101813666748953	48	-0.0690569677915872	0.945243598929388	0.00010366022736867	0.970711134015984
**DLBC**	FBXL19	cg07697662	-0.0995704785838972	48	-0.678692597056053	0.500733112249168	0.00991428020542632	0.684581064537482
**DLBC**	FBXL19	cg08535091	0.151036367682982	48	1.03626631797971	0.305494920458571	0.0228119843628691	0.504011872885162
**DLBC**	FBXL19	cg08593668	0.146845316094382	48	1.00686837910833	0.319265251396113	0.021563546858859	0.518613538391539
**DLBC**	FBXL19	cg09011601	0.0549402238012416	48	0.373186370949133	0.710724017290182	0.00301842819133051	0.835247812572851
**DLBC**	FBXL19	cg11940219	0.0182297312982368	48	0.123660602513332	0.902122770216702	0.000332323103205914	0.948281470876085

**DLBC**	FBXL19	cg26666512	-0.0850243331351024	48	-0.578758841962627	0.565575161843394	0.00722913722506888	0.734889415789574
**DLBC**	FBXL19	cg27602786	0.013099029305983	48	0.0888495621457161	0.929587176318486	0.000171584568759001	0.962682628623478
**DLBC**	FBXW9	cg02753619	0.229653109798465	48	1.6003566302239	0.11636595292472	0.0527405508401058	0.264997305167462
**DLBC**	FBXW9	cg03160342	-0.0781696314593588	48	-0.531799502990667	0.597423977247851	0.00611049128249198	0.758275077989932
**DLBC**	FBXW9	cg05868316	-0.0693687972116817	48	-0.471618161971814	0.639428734098252	0.00481203002659542	0.788156652096831
**DLBC**	FBXW9	cg06489804	0.118117873221682	48	0.806762065521602	0.423955271587808	0.0139518319744133	0.618960704200492
**DLBC**	FBXW9	cg12110584	0.2800862022792	48	1.97884042557295	0.053838884480311	0.0784482807071852	0.152286738874352
**DLBC**	FBXW9	cg14342428	0.227111107085821	48	1.58167340968107	0.12057640728112	0.0515794549617471	0.271525847431991
**DLBC**	FBXW9	cg15828427	-0.101695191720058	48	-0.693324817650341	0.491591854814442	0.0103419120189793	0.677165979085098
**DLBC**	FBXW9	cg19721801	0.117813288742617	48	0.804652389621781	0.425159680194987	0.0138799710043513	0.620015074294317
**DLBC**	FBXW9	cg21564527	0.0629680536549579	48	0.427919305712706	0.67070671733912	0.00396497578109365	0.809208554237496
**DLBC**	FBXW9	cg22303873	0.297651430055584	48	2.11461599648494	0.0399119924496938	0.0885963738141341	0.122288835786982
**DLBC**	FKBP1A	cg04613791	0.27345749355887	48	1.92817303076608	0.0600194977737162	0.0747790007834993	0.164719713508746
**DLBC**	FKBP1A	cg07516946	0.242862055822241	48	1.69800753218349	0.0962623250672958	0.0589819781582054	0.231774413998304
**DLBC**	FKBP1A	cg10996143	0.167534152678687	48	1.15256190251394	0.255044747614357	0.0280676923137657	0.44932255572147
**DLBC**	FKBP1A	cg21332426	0.0736879471944688	48	0.501138394709448	0.618663524856606	0.00542991356173482	0.773688979049231
**DLBC**	FKBP1A	cg21775445	0.209641907845136	48	1.45417493572097	0.152687396385024	0.0439497295249486	0.319652836813437
**DLBC**	FKBP1A	cg24641773	-0.174066986445917	48	-1.19888209847683	0.236713505716522	0.0302993157703632	0.428074072800017
**DLBC**	FKBP1A	cg26779912	0.21942424904593	48	1.52538192276735	0.134010570751156	0.0481470010693702	0.292186612696621
**DLBC**	FKBP2	cg03764062	0.285454533221057	48	2.02009859181624	0.0492191035197584	0.0814842905364517	0.142653065802916
**DLBC**	FKBP2	cg04446112	0.307350484254619	48	2.19058461936297	0.0335874203027103	0.0944643201715489	0.107367676488702
**DLBC**	FKBP2	cg06615912	0.187743083229238	48	1.29638768717136	0.201308157593138	0.0352474653004204	0.384563190624477
**DLBC**	FKBP2	cg09854726	-0.0856368240314225	48	-0.582958745114772	0.562768211785692	0.00733366563018882	0.732878957223432
**DLBC**	FKBP2	cg10998122	0.347738187809064	48	2.51546082944726	0.015443518127619	0.120921847260732	0.0597183144060041
**DLBC**	FKBP2	cg12423493	-0.0468359522008429	48	-0.318005864239462	0.751919708842219	0.00219360641855964	0.861105248249702
**DLBC**	FKBP2	cg15010817	0.377222659559808	48	2.76253766172774	0.00821712863231254	0.142296934885375	0.0366740553317197
**DLBC**	FKBP2	cg16977872	0.175537437170073	48	1.20933039081503	0.232714870799274	0.0308133918482373	0.423451469112313
**DLBC**	FKBP2	cg19924313	0.381454033789259	48	2.79876812238894	0.00747049572839483	0.145507179894097	0.0340689489198409
**DLBC**	FKBP2	cg21809711	0.251715776460107	48	1.76401873980811	0.0843691375533553	0.0633608321189146	0.210861520171984
**DLBC**	FKBP2	cg22074794	-0.0929028458176067	48	-0.632834653571925	0.529975158838655	0.00863093876101	0.708065445628081
**DLBC**	FKBP2	cg24677501	0.284165509658638	48	2.01017316001419	0.0502979285368637	0.0807500368795533	0.144905271732651
**DLBC**	FKBP5	cg00130530	-0.115057022021393	48	-0.78557175974535	0.436146196501752	0.0132381183164314	0.629245696679537
**DLBC**	FKBP5	cg00140191	-0.45124611696876	48	-3.42951775082616	0.00128611777370095	0.203623058079383	0.00827493557913254
**DLBC**	FKBP5	cg00610228	-0.100866495027062	48	-0.687616719455738	0.495146858079774	0.0101740498190444	0.680027093380728
**DLBC**	FKBP5	cg01294490	-0.145904537892271	48	-1.00027701270499	0.322409454354395	0.0212881341775572	0.52180230049777
**DLBC**	FKBP5	cg07485685	-0.359871737687873	48	-2.61603880463775	0.0119932619554588	0.129507667586489	0.049108048874307
**DLBC**	FKBP5	cg07843056	-0.0761172905431229	48	-0.517754655051327	0.607110684926143	0.00579384191962619	0.76529513120007
**DLBC**	FKBP5	cg08636224	-0.0831572308441897	48	-0.565960016910572	0.574171684534614	0.00691512504167385	0.741424779709904
**DLBC**	FKBP5	cg08915438	-0.16095089163251	48	-1.10604217597047	0.274459318359438	0.0259051895172999	0.470966983841494
**DLBC**	FKBP5	cg10913456	-0.267310753678298	48	-1.88145518199731	0.0662471461240109	0.0714550390320596	0.17699730348495
**DLBC**	FKBP5	cg16012111	0.24492780372275	48	1.71336811832586	0.0933776903560707	0.0599896290364502	0.226768565131921
**DLBC**	FKBP5	cg17030679	-0.316363446977796	48	-2.26185452791516	0.0284763321569918	0.100085830583673	0.0949642031818328
**DLBC**	FKBP5	cg20813374	0.0182848845170991	48	0.124034856952786	0.901828094458237	0.000334337001803649	0.94812912737731
**DLBC**	FKBP5	cg25114611	-0.421142919457962	48	-3.1492262768707	0.00287450045670486	0.177361358609576	0.0159114051891916
**DLBC**	FMR1	cg02921434	0.180953077447203	48	1.24788388633586	0.218388637252498	0.0327440162376134	0.406068233739476
**DLBC**	FMR1	cg03513471	0.191898479751971	48	1.32616589579601	0.191332127535656	0.0368250265311176	0.3718241245412
**DLBC**	FMR1	cg04552106	0.170869421655229	48	1.17619022865313	0.245570113863112	0.0291963592567923	0.438494306192001
**DLBC**	FMR1	cg04744025	0.141164583821158	48	0.967109284982208	0.338546792312461	0.0199274397254008	0.537919283619602
**DLBC**	FMR1	cg05288927	-0.0813981437857767	48	-0.553907120319411	0.582325189467327	0.00662565781176998	0.747535614749283
**DLBC**	FMR1	cg07147350	-0.0333947290759655	48	-0.226620472014342	0.821722936964354	0.00111520793005714	0.904309798011949
**DLBC**	FMR1	cg08434396	0.20483198550082	48	1.41933200258742	0.16254347587532	0.0419561422842082	0.333181575733681
**DLBC**	FMR1	cg14470409	-0.032102848194275	48	-0.217844393227183	0.82851384035029	0.00103059286218467	0.907845592656071
**DLBC**	FMR1	cg16783314	0.161508309106982	48	1.10997511962582	0.272778635648771	0.0260849339105963	0.469146967649161
**DLBC**	FMR1	cg22625568	0.0336787814748842	48	0.228550263916087	0.820231499102053	0.001134260321633	0.903338041552517
**DLBC**	FXR2	cg01807919	0.341215176931051	48	2.46198971013906	0.0176247261514854	0.116427796968089	0.0660642741750339
**DLBC**	FXR2	cg01933235	-0.121558287967986	48	-0.830607964843315	0.410485578517347	0.0147764173737077	0.606359469375257
**DLBC**	FXR2	cg05529097	-0.30696015083172	48	-2.18751298125773	0.033824876148242	0.0942245341986323	0.10795816915879

**DLBC**	FXR2	cg05913233	-0.231193580999542	48	-1.61169558446424	0.113869306762838	0.0534504718953919	0.26115415242654
**DLBC**	FXR2	cg09625173	0.0800406111826624	48	0.544609159279709	0.588652964622082	0.00640649943849415	0.752170899934468
**DLBC**	FXR2	cg17777561	-0.229741705617438	48	-1.60100841275756	0.116221247878991	0.0527812513000096	0.264857699927869
**DLBC**	FXR2	cg19178740	0.00872353378340332	48	0.0591681361296286	0.953074490418808	7.61000416701791e-05	0.975225602846894
**DLBC**	FXR2	cg22521096	0.0883813038802533	48	0.601786127480395	0.550271185081157	0.00781125487557367	0.722897083138348
**DLBC**	FXR2	cg26904212	0.198233670698222	48	1.37170800219453	0.1768080741994	0.0392965881984913	0.352881300330138
**DLBC**	G2E3	cg02259031	-0.198955410658329	48	-1.3769076366722	0.17520526822746	0.0395832554302245	0.350802737034648
**DLBC**	G2E3	cg05076167	0.0603600538081769	48	0.410129604466985	0.683614811828368	0.00364333609572601	0.817755236712911
**DLBC**	G2E3	cg07906495	-0.0221081960867506	48	-0.149981739134239	0.881434722284357	0.000488772334210215	0.937111638131926
**DLBC**	G2E3	cg08810065	-0.501709430374925	48	-3.93366065675089	0.000280273289356347	0.251712352527132	0.00230872302755058
**DLBC**	G2E3	cg09837748	0.152579332329502	48	1.04710368083975	0.30052271521943	0.0232804526541165	0.498812949023455
**DLBC**	G2E3	cg18580322	-0.0672254420519124	48	-0.45697890380577	0.64983701389554	0.00451926005907503	0.79525902806405
**DLBC**	G2E3	cg18755078	-0.275165154130267	48	-1.94119695540872	0.0583753682116742	0.0757158620475337	0.161397924251305
**DLBC**	G2E3	cg18956956	0.0751964536703846	48	0.511455225762561	0.611478803893923	0.0056545066446023	0.768425524111523
**DLBC**	G2E3	cg19554565	-0.0289524633134471	48	-0.19644751286338	0.845125252385838	0.000838245131916498	0.917249241515463
**DLBC**	G2E3	cg21061067	-0.0616903378334831	48	-0.419202667667629	0.677019215901624	0.00380569778200927	0.813443601739864
**DLBC**	G2E3	cg22926526	0.332960205312894	48	2.39489668279358	0.020754364488171	0.110862498322004	0.0749218111829593
**DLBC**	GADD45A	cg01810953	-0.0503135295735886	48	-0.341675701916248	0.734150974551251	0.00253145125815238	0.850116711533447
**DLBC**	GADD45A	cg02639608	-0.131952749114917	48	-0.902841526397314	0.37131361564361	0.0174115279989841	0.569950567538044
**DLBC**	GADD45A	cg04718306	-0.111544410868061	48	-0.761281819562624	0.45037403874106	0.0124421555959027	0.641187306147577
**DLBC**	GADD45A	cg07000785	-0.101559432970603	48	-0.692389606031854	0.492173333569627	0.0103143184253104	0.677690047642669
**DLBC**	GADD45A	cg22224769	-0.0493330467149855	48	-0.335000904369715	0.7391473037188	0.00243374949818294	0.853039953102718
**DLBC**	GADD45A	cg23369529	0.0405445205417368	48	0.275212615610503	0.784385346922998	0.00164385814595932	0.881016705439153
**DLBC**	GADD45A	cg23723105	-0.103863618732184	48	-0.708267968311962	0.482352642132291	0.0107876512961444	0.669357808536808
**DLBC**	GATAD2A	cg02887458	-0.176099043031361	48	-1.21332308254568	0.231199981816578	0.0310108729565611	0.421706437839524
**DLBC**	GATAD2A	cg08505228	-0.208597056939626	48	-1.44659678618137	0.154790090727163	0.0435127321638735	0.322471756146669
**DLBC**	GATAD2A	cg08870822	-0.167422882262368	48	-1.15177432717409	0.255365016105381	0.0280304215050387	0.449657448232277
**DLBC**	GATAD2A	cg09334331	-0.123140983356473	48	-0.841587952206204	0.40437265399073	0.0151637017819993	0.600797674647725
**DLBC**	GATAD2A	cg11795262	-0.284748904260754	48	-2.01466380242553	0.0498073171691524	0.0810819384777001	0.143880818115746
**DLBC**	GATAD2A	cg14948912	-0.294963567049769	48	-2.09369172973185	0.0418279515037754	0.0870035058867238	0.126490326891534
**DLBC**	GATAD2A	cg15264323	-0.202294509693978	48	-1.40099404541612	0.167926294742034	0.0409230686523269	0.340936351310431
**DLBC**	GATAD2A	cg22191731	-0.311932445224252	48	-2.22673322559223	0.0309016390063525	0.0973018503835808	0.10087118600454
**DLBC**	GATAD2A	cg26631329	-0.511905074959967	48	-4.04160571035192	0.00020001062269861	0.26204680576977	0.00173720429229271
**DLBC**	GATAD2B	cg02134200	-0.314821121448238	48	-2.24961116102739	0.0293018744119041	0.099112338509926	0.0970045541512262
**DLBC**	GATAD2B	cg02940454	-0.0744396115987932	48	-0.506278668071318	0.615079032351265	0.00554125577497918	0.770994531039068
**DLBC**	GATAD2B	cg09805553	-0.355147175497193	48	-2.57669933613143	0.0132488198403652	0.126129516263634	0.0531003962216058
**DLBC**	GATAD2B	cg10226922	-0.519368328076712	48	-4.12208222384446	0.000155176930654791	0.269743460209199	0.00140207132907768
**DLBC**	GATAD2B	cg10489353	-0.17047035245019	48	-1.17336089399959	0.246691018131795	0.0290601410644919	0.439890000791823
**DLBC**	GATAD2B	cg15064304	-0.613051909639327	48	-5.26290632155119	3.6197557366004e-06	0.375832643912426	5.35148760496183e-05
**DLBC**	GATAD2B	cg20773694	-0.566875520329093	48	-4.66705593543318	2.66181527908752e-05	0.32134785554838	0.000307139213618117
**DLBC**	GATAD2B	cg23046518	-0.466166659078333	48	-3.57376184544082	0.000839594222098532	0.217311354036255	0.00580023573295752
**DLBC**	GATAD2B	cg23323627	-0.404058899727495	48	-2.99591343249486	0.00439728472206963	0.163263594448993	0.0223961177267845
**DLBC**	GATAD2B	cg26398333	-0.415115859149033	48	-3.09469119680659	0.00334794742593426	0.17232117651704	0.0179831679937257
**DLBC**	GLYR1	cg00636946	-0.410953687795033	48	-3.05731859465539	0.0037137449359711	0.168882933512337	0.0195431964789918
**DLBC**	GLYR1	cg00885266	-0.575709000471481	48	-4.7754248773967	1.85999787230235e-05	0.331440853223872	0.000225173785368641
**DLBC**	GLYR1	cg01261535	-0.549702726184225	48	-4.46306230081754	5.19235993560306e-05	0.302173087174369	0.000548228079302802
**DLBC**	GLYR1	cg01565912	-0.131375964484982	48	-0.898825609509163	0.373426561264214	0.0172596440443591	0.571992319189341
**DLBC**	GLYR1	cg02454264	-0.34917165957921	48	-2.5272666702963	0.0149962821106013	0.121920847853299	0.0583631382516444
**DLBC**	GLYR1	cg06399529	-0.402925495395742	48	-2.98587826209513	0.00451961314603535	0.162348954839904	0.0228667263971562
**DLBC**	GLYR1	cg06976003	-0.0570205821598872	48	-0.38736264187687	0.700274285905191	0.00325134678985245	0.828297670635676
**DLBC**	GLYR1	cg11289281	-0.0145807607668131	48	-0.0989020447017219	0.921645789166022	0.000212598584539035	0.958959333962598
**DLBC**	GLYR1	cg15663121	0.207585232719309	48	1.43926311037356	0.156846514011023	0.0430916288431298	0.325439887738339
**DLBC**	GLYR1	cg18354686	-0.39739418855185	48	-2.93713767035995	0.00516034931562411	0.157922141094783	0.0254239527966854
**DLBC**	GTF2B	cg02705666	-0.420894939000827	48	-3.14697255645874	0.00289274738747251	0.17715254967651	0.0159851202523193
**DLBC**	GTF2B	cg04270788	-0.259760971376047	48	-1.82441129032827	0.0745889362845669	0.0674757622502274	0.193087936451462
**DLBC**	GTF2B	cg05306856	-0.371977736173343	48	-2.71790836870596	0.00923150307799341	0.138367436208645	0.0400868294975953
**DLBC**	GTF2B	cg06356032	-0.433997886689407	48	-3.26725663500078	0.00205714389250853	0.188354165650871	0.0121698442565634

**DLBC**	GTF2B	cg07167781	0.478359913973475	48	3.6945208938723	0.000583958192429864	0.22882820729671	0.00427642319701486
**DLBC**	GTF2B	cg10430205	0.297018673021397	48	2.10968522052211	0.0403564258048442	0.0882200921233918	0.123194466687414
**DLBC**	GTF2B	cg12074130	0.127934251362632	48	0.87488151540907	0.386183616797959	0.0163671726717172	0.584194148585937
**DLBC**	GTF2B	cg13868361	0.536327529450019	48	4.30984293597543	8.52340612646223e-05	0.28764721884596	0.000838373822483251
**DLBC**	GTF2B	cg14010405	0.395150752285721	48	2.9174779858029	0.00544202571215636	0.156144117031971	0.0265444196032034
**DLBC**	GTF2B	cg17811845	0.534596981495043	48	4.29035916516933	9.07411103309112e-05	0.285793932623611	0.000886266658767471
**DLBC**	GTF2B	cg22607980	-0.376755222284108	48	-2.75854775290794	0.00830343840994776	0.141944497518347	0.0369667899544166
**DLBC**	GTF2H1	cg05849260	-0.302154225338925	48	-2.14979302148597	0.0368639597510304	0.0912971758901659	0.115278967917018
**DLBC**	GTF2H1	cg07583091	-0.526395132364686	48	-4.199032302567	0.000121529947219227	0.277091835377235	0.00113893515407451
**DLBC**	GTF2H1	cg08690812	-0.330614869885741	48	-2.37594882371292	0.0217245926301023	0.109306192189566	0.0775668240474635
**DLBC**	GTF2H1	cg10797557	-0.125472985859404	48	-0.857778183749183	0.395462281545189	0.0157434701804742	0.592184803575031
**DLBC**	GTF2H1	cg11063328	-0.5081368873563	48	-4.00144756867887	0.00022685367741855	0.258203096292149	0.00193041511497469
**DLBC**	GTF2H1	cg11176342	-0.385024725058397	48	-2.82950160356336	0.00688692582391096	0.148244038906294	0.0320048690289922
**DLBC**	GTF2H1	cg11347316	-0.270114366728021	48	-1.90273268189101	0.0633456498093502	0.0729617711128798	0.171262649658323
**DLBC**	GTF2H1	cg11662851	-0.00059383230949217	48	-0.0040275673877516	0.99680389110983	3.52636811796809e-07	0.998356725052439
**DLBC**	GTF2H1	cg13777411	-0.207344449342935	48	-1.43751863086453	0.157338816583374	0.042991720673325	0.326017896262706
**DLBC**	GTF2H1	cg14132868	-0.13051514593595	48	-0.892833806148794	0.376593366494621	0.0170342033186823	0.575125456926735
**DLBC**	GTF2H1	cg17174493	0.0640160084217114	48	0.435070077837529	0.665546042990116	0.00409804933424863	0.805836605429406
**DLBC**	GTF2H1	cg18321761	-0.352255591636235	48	-2.55273411452797	0.0140713785879058	0.124084001838994	0.0555641249615525
**DLBC**	H2AFZ	cg02758870	0.433055641562084	48	3.25852360117782	0.00210914406532297	0.187537188688748	0.0124029596657143
**DLBC**	H2AFZ	cg09308803	0.104251175194238	48	0.710939781856446	0.480711015968985	0.0108683075293798	0.667936571699136
**DLBC**	H2AFZ	cg16267491	0.0200206943575927	48	0.135814177428888	0.892560796123637	0.000400828202560143	0.943086652841716
**DLBC**	H2AFZ	cg17709884	0.280864560773112	48	1.98480986155885	0.0531481494334967	0.0788849014982732	0.150854025723491
**DLBC**	H2AFZ	cg20934652	0.169898903302859	48	1.16931044909492	0.248302129425716	0.0288656373435143	0.441588730917956
**DLBC**	H2AFZ	cg23752380	-0.231298847411749	48	-1.61247088569661	0.113700201717362	0.0534991568140034	0.260920162017712
**DLBC**	H2AFZ	cg24061702	-0.0569531007474876	48	-0.386902722105627	0.700612400849271	0.00324365568475348	0.828515062587782
**DLBC**	H3F3A	cg06319713	-0.137593355759454	48	-0.942164656095298	0.351030341887521	0.0189319315491475	0.549974017262514
**DLBC**	H3F3A	cg10632229	0.122224598735153	48	0.835229722892054	0.407905609133813	0.0149388525359692	0.603993470572839
**DLBC**	H3F3A	cg10983539	0.0747812060692216	48	0.508614955513669	0.613452981975754	0.00559222878116739	0.769911450832005
**DLBC**	H3F3A	cg11978873	-0.0505695581732265	48	-0.343418820800185	0.732848088631516	0.00255728021383534	0.849202118224475
**DLBC**	H3F3A	cg15538837	0.290107776275017	48	2.05602826148446	0.0454798176646105	0.0841625218552355	0.134641660052702
**DLBC**	H3F3A	cg15624314	0.109579388592737	48	0.747706224648916	0.458442892224478	0.012007642404358	0.648445812363982
**DLBC**	H3F3A	cg16724613	-0.116399619900501	48	-0.794863749431784	0.430774938619158	0.0135488715129811	0.624763801989948
**DLBC**	H3F3A	cg16727519	-0.0319427169682129	48	-0.216756657695291	0.82935646105642	0.00102033716731136	0.908321106719928
**DLBC**	H3F3A	cg18947521	0.0137594459212692	48	0.0933299377628744	0.926046768745291	0.000189322352060332	0.961054615617427
**DLBC**	H3F3A	cg19410789	-0.15131618381298	48	-1.03823109222293	0.304589319036969	0.0228965874837236	0.503115304269104
**DLBC**	H3F3A	cg21266749	0.0934570640676903	48	0.636643036409312	0.527512974863145	0.00873422282415238	0.706103863975077
**DLBC**	H3F3A	cg21789564	0.115430311057321	48	0.788154816744242	0.434649065309396	0.0133241567107898	0.628071170384158
**DLBC**	H3F3A	cg22519151	0.1281019037316	48	0.876047125504842	0.385556300661641	0.0164100977396602	0.58356291788925
**DLBC**	H3F3A	cg27530209	0.0932638307524526	48	0.635315140930894	0.528370797111106	0.00869814212662213	0.706777821938225
**DLBC**	H3F3A	cg27560444	-0.153156492585945	48	-1.05115950696543	0.298676295914427	0.0234569112212286	0.496882591941467
**DLBC**	HAT1	cg00414306	-0.0862423199638495	48	-0.587111337624946	0.559999725564167	0.007437737752747	0.730680263166932
**DLBC**	HAT1	cg01583365	0.186995332927337	48	1.29103690877336	0.203141547998617	0.0349672545366057	0.386991712827025
**DLBC**	HAT1	cg04507121	0.322911486346003	48	2.31405828075945	0.0251839941273938	0.104271828014185	0.0866963288408918
**DLBC**	HAT1	cg04950839	0.0296979321204237	48	0.201510057687171	0.84118823037754	0.000881967172229292	0.914967592650974
**DLBC**	HAT1	cg05421799	0.16829812653117	48	1.15797055789236	0.252853102916246	0.0283242593939018	0.446853548992415
**DLBC**	HAT1	cg09545452	-0.1542294424102	48	-1.05870228118417	0.295263277855201	0.0237867209061613	0.493204169393635
**DLBC**	HAT1	cg10190161	0.046427773244477	48	0.315228405229139	0.754013870619319	0.00215553812844057	0.86253793857835
**DLBC**	HAT1	cg12941622	-0.308334674840813	48	-2.19833490454513	0.0329948106675489	0.0950702717091899	0.105967141050263
**DLBC**	HAT1	cg13293756	-0.0854953853152693	48	-0.581988828638001	0.563415825665247	0.00730946091020637	0.733329497595781
**DLBC**	HAT1	cg14446129	0.209881559114723	48	1.45591382450068	0.152208090868609	0.0440502688564272	0.318920389491288
**DLBC**	HAT1	cg16017358	0.266811411298231	48	1.87767093598862	0.0667748558809939	0.0711883291989536	0.178075532591689
**DLBC**	HDAC1	cg03762702	0.149736718507102	48	1.02714395406771	0.30972373677353	0.0224210848692753	0.508422451067924
**DLBC**	HDAC1	cg04967775	0.410982426710542	48	3.05757585049883	0.00371110336744	0.168906555064886	0.0195319379856997
**DLBC**	HDAC1	cg06473843	0.0174802293841239	48	0.118574801012303	0.906128528278683	0.000305558419321588	0.950325748610222
**DLBC**	HDAC1	cg10842753	0.222585019388031	48	1.548491622069	0.128357172477864	0.0495440908559699	0.283555810755159
**DLBC**	HDAC1	cg14172124	0.169149035552199	48	1.16399721092025	0.250427040212605	0.028611396228239	0.444083883342287

**DLBC**	HDAC1	cg20002354	0.0756513347153377	48	0.514566893712767	0.609319328703142	0.00572312444421206	0.766863842896723
**DLBC**	HDAC1	cg20793809	0.0303466248931462	48	0.205915661421582	0.837765427304089	0.000920917642405318	0.913167557706555
**DLBC**	HDAC1	cg21930229	0.378134301041112	48	2.77032625544248	0.00805102970504146	0.142985549623851	0.036110842370384
**DLBC**	HDAC1	cg23018091	-0.0146139379658679	48	-0.0991271353541809	0.921468058456582	0.000213567182870235	0.958838426887501
**DLBC**	HDAC1	cg26115470	0.0523926500579423	48	0.355832955906626	0.723592390567533	0.002744989780094	0.843376229407606
**DLBC**	HDAC10	cg00711351	0.293421054028292	48	2.08170832680355	0.0429610516585768	0.0860959149470738	0.128979316638134
**DLBC**	HDAC10	cg01120165	0.0478303823039023	48	0.324773147870493	0.746825200574703	0.00228774547133745	0.857811760316905
**DLBC**	HDAC10	cg04321608	0.085224510074788	48	0.580131399644747	0.564657067134278	0.00726321711748764	0.734318486804481
**DLBC**	HDAC10	cg04586023	0.017740000980165	48	0.120337477599946	0.904739896758545	0.000314707634776254	0.949535043014871
**DLBC**	HDAC10	cg07482373	0.183362717412316	48	1.2650753964507	0.212215554717008	0.0336218861368288	0.398385542076615
**DLBC**	HDAC10	cg07744063	0.0466381424566682	48	0.316659845415672	0.752934352049097	0.00217511633180848	0.86173387546795
**DLBC**	HDAC10	cg09151829	0.154121444520798	48	1.05794288920576	0.295605667614913	0.0237534196611774	0.493522183638208
**DLBC**	HDAC10	cg10508940	0.049021565523211	48	0.332880649308806	0.740736787194471	0.00240311388634647	0.854170102471083
**DLBC**	HDAC10	cg11645658	0.270177685251652	48	1.90321382608558	0.0632813127016827	0.0729959816079409	0.171142236756986
**DLBC**	HDAC10	cg12464820	-0.0458881019502072	48	-0.311556447584036	0.756785338906136	0.00210571790059261	0.864386413960709
**DLBC**	HDAC10	cg26943378	0.287281775169104	48	2.03418875442078	0.0477220685659394	0.0825308183443118	0.139451979641601
**DLBC**	HDAC11	cg01409735	-0.0138346647245205	48	-0.0938402422122515	0.925643619055113	0.000191397948039891	0.960857180801055
**DLBC**	HDAC11	cg01712016	0.0880192370813677	48	0.599301534849537	0.551912265437216	0.00774738609638601	0.72422135071979
**DLBC**	HDAC11	cg02762546	0.158585301008619	48	1.08936343090833	0.281667837808934	0.0251492976959944	0.478784857197663
**DLBC**	HDAC11	cg02941903	0.00572573389293437	48	0.0388344532375758	0.969190430878353	3.27840286126973e-05	0.983792974874382
**DLBC**	HDAC11	cg05987698	0.152732178954596	48	1.04817765914991	0.300033022242236	0.0233271184882186	0.498314298406215
**DLBC**	HDAC11	cg07450135	-0.0679652801017993	48	-0.462031318090175	0.646236707550409	0.00461927929931604	0.792579827008932
**DLBC**	HDAC11	cg08051076	0.0331719214865788	48	0.225106802889655	0.822893240951204	0.00110037637511175	0.904671034879323
**DLBC**	HDAC11	cg08448040	0.16454654529866	48	1.13143114424556	0.263737821554498	0.0270755655697238	0.45918749563713
**DLBC**	HDAC11	cg10455785	0.0921824626476629	48	0.627885333157051	0.533183967653219	0.00849760641978776	0.710297170690022
**DLBC**	HDAC11	cg11432034	0.0696668501739156	48	0.473654398554781	0.63798673123065	0.00485347001315481	0.787059189649381
**DLBC**	HDAC11	cg13397067	-0.0671443730359287	48	-0.456425323081473	0.650232006549149	0.00450836683038795	0.795392720372352
**DLBC**	HDAC11	cg13706066	0.0298860733872794	48	0.202787794579394	0.840195208235041	0.000893177382509848	0.914394463222514
**DLBC**	HDAC11	cg16254764	0.0126844367755282	48	0.0860369575955575	0.931810446016758	0.000160894936312373	0.963829412408657
**DLBC**	HDAC11	cg18521047	0.0753677185788549	48	0.512626745963904	0.610665366315443	0.00568029300378147	0.76781224924595
**DLBC**	HDAC11	cg19779799	0.126172323980497	48	0.862636232301514	0.392812710174951	0.0159194553386396	0.58968144755799
**DLBC**	HDAC11	cg20894727	-0.0396451850333025	48	-0.269098286664017	0.789057401355953	0.00157174069632479	0.883959728710075
**DLBC**	HDAC11	cg20922324	0.0193940718991331	48	0.131561739823616	0.895904694090765	0.000376130024828744	0.944774430962409
**DLBC**	HDAC11	cg21655280	-0.0241217514516175	48	-0.163649295442401	0.870724143410179	0.000581858893093612	0.931388810379247
**DLBC**	HDAC11	cg25127083	-0.00251855472824621	48	-0.0170817234233051	0.986445295375037	6.34311791917134e-06	0.992903494829801
**DLBC**	HDAC11	cg25150878	0.126565012176616	48	0.865364667403799	0.391329511089953	0.016018702307267	0.588442794737253
**DLBC**	HDAC11	cg27025856	0.101107489056383	48	0.689276546960109	0.494111656987648	0.0102227243432866	0.679319579982337
**DLBC**	HDAC2	cg00161229	-0.0934214580179323	48	-0.636398346897411	0.527670989461271	0.00872756881819628	0.706221525474446
**DLBC**	HDAC2	cg01129566	-0.051305942239865	48	-0.348432722226831	0.729104914099694	0.00263229970912036	0.84676402051338
**DLBC**	HDAC2	cg08198132	-0.153986504298591	48	-1.05699410559321	0.296033834766854	0.0237118435060999	0.493995473241838
**DLBC**	HDAC2	cg10999867	0.0903688321420836	48	0.615429351216396	0.541304263266887	0.00816652582272409	0.716318639437022
**DLBC**	HDAC2	cg13393754	0.0829311130980335	48	0.564410409482909	0.575216824253582	0.00687756951967882	0.742182635231008
**DLBC**	HDAC2	cg15005908	0.0802829660959048	48	0.546268862217842	0.587521043128589	0.0064453546451562	0.751349584029839
**DLBC**	HDAC2	cg15242771	-0.0819860506719347	48	-0.557934749833033	0.57959438412414	0.00672171250478104	0.745580248343172
**DLBC**	HDAC2	cg18387216	-0.0867654248999472	48	-0.590699406456255	0.55761310544447	0.00752823895806838	0.728849806356958
**DLBC**	HDAC2	cg18674783	-0.0449612824166171	48	-0.305250944873384	0.76155204434475	0.00202151691654681	0.867423823742009
**DLBC**	HDAC2	cg22867643	-0.0456212539394404	48	-0.309740898034537	0.758156850893228	0.00208129881100691	0.865178067776441
**DLBC**	HDAC2	cg24023618	-0.0234584328435554	48	-0.159146627498988	0.874250056806839	0.000550298071475597	0.933402076735167
**DLBC**	HDAC3	cg01947930	0.302538351118245	48	2.15280119445739	0.0366130640466689	0.0915294538973465	0.114711958226445
**DLBC**	HDAC3	cg01961137	0.248005047041699	48	1.73629614555489	0.0892054942176117	0.0615065033581555	0.219463401447294
**DLBC**	HDAC3	cg04284814	-0.0190634178786344	48	-0.129317890753956	0.89766991563233	0.000363413901215437	0.945879443263507
**DLBC**	HDAC3	cg08520975	0.0416273171658662	48	0.282575135319822	0.778770178339251	0.00173283353442762	0.877680433072909
**DLBC**	HDAC3	cg10414058	0.117321094534927	48	0.801243738970661	0.42711002622881	0.0137642392228733	0.621499333740677
**DLBC**	HDAC3	cg11064797	0.0685395302690357	48	0.465953446651763	0.643447715360975	0.00469766720950006	0.790729808730691
**DLBC**	HDAC3	cg11153694	0.0946077371691897	48	0.644551947207327	0.522419046099133	0.00895062393227448	0.70225120109405
**DLBC**	HDAC3	cg12082871	0.00499088347077842	48	0.0338502401951957	0.973143032627578	2.49089178188893e-05	0.985879345742617
**DLBC**	HDAC3	cg14098966	0.398192626049295	48	2.94414959403538	0.00506321989326807	0.158557367440034	0.0250344074590959

**DLBC**	HDAC3	cg17940889	0.125712197761673	48	0.859439755585154	0.394554810377602	0.0158035566660699	0.59128117197733
**DLBC**	HDAC3	cg18671773	0.301016627420633	48	2.1408909953513	0.0376153103426143	0.0906110099836924	0.117045136306281
**DLBC**	HDAC3	cg19549288	0.106606279206691	48	0.72718295181389	0.470798774470057	0.0113648987662951	0.659261720277887
**DLBC**	HDAC3	cg20642138	0.414900862034644	48	3.09275500420953	0.0033660350896859	0.172142725317091	0.0180628636108867
**DLBC**	HDAC3	cg23421926	0.278962636993048	48	1.97023097240592	0.0548487244248713	0.0778201528381153	0.154318324336652
**DLBC**	HDAC3	cg24385322	-0.0830892812262839	48	-0.565494342173533	0.574485663330916	0.0069038286547005	0.741692393751388
**DLBC**	HDAC6	cg07153944	0.0600262184253585	48	0.407853061319095	0.68527367426708	0.00360314689844885	0.818859042070768
**DLBC**	HDAC6	cg07674520	-0.119600577816402	45	-0.789943580670258	0.433895496817949	0.0143042982140172	0.627453087208259
**DLBC**	HDAC6	cg10783042	0.131918036992298	48	0.902599813650442	0.371440573971496	0.0174023684839013	0.570083731552105
**DLBC**	HDAC6	cg12510923	0.0444708606934728	48	0.301914741575394	0.764077878469211	0.00197765745081827	0.869047004411982
**DLBC**	HDAC6	cg13178935	0.151080706890419	48	1.03657763589132	0.305351305322074	0.0228253799945087	0.503903172317631
**DLBC**	HDAC6	cg17152375	0.111283895650684	48	0.759481502305003	0.45143928215675	0.0123841054311924	0.642045878646816
**DLBC**	HDAC6	cg24616736	0.18524951185778	48	1.27855313330203	0.207467772623204	0.0343173816435459	0.392417707784034
**DLBC**	HDAC6	cg26680189	0.18530694853684	48	1.27896364335227	0.207324421854618	0.0343386651760351	0.392263493991059
**DLBC**	HDAC9	cg00332048	-0.158063394459558	48	-1.08568629861105	0.283274780982377	0.0249840366680778	0.480602953939037
**DLBC**	HDAC9	cg02301805	0.041230241543555	48	0.279875089039619	0.78082805393428	0.00169993281773989	0.878951790519138
**DLBC**	HDAC9	cg03260621	-0.109674737804305	48	-0.748364750056107	0.458049565205713	0.0120285481124431	0.648105852487463
**DLBC**	HDAC9	cg07513622	-0.0209403201235712	48	-0.142055309859472	0.887656678726196	0.000438497006877639	0.940325182320004
**DLBC**	HDAC9	cg08146609	-0.114623563756782	48	-0.782572771420242	0.437888246187407	0.0131385613683052	0.630771665462888
**DLBC**	HDAC9	cg08285151	-0.260095839070165	48	-1.82693374152312	0.0742021566699662	0.0676498455016131	0.192370173975875
**DLBC**	HDAC9	cg08725366	-0.216580032464987	48	-1.50462983802627	0.139255123849946	0.0469069104625347	0.300138890252749
**DLBC**	HDAC9	cg10068437	0.188351112633297	48	1.30074036755589	0.199825972196986	0.035476141630201	0.38274680982623
**DLBC**	HDAC9	cg11321459	-0.260062448448907	48	-1.82668218894259	0.0742406522972774	0.0676324770932402	0.192426111308536
**DLBC**	HDAC9	cg11765288	-0.127949168335788	48	-0.874985223195955	0.386127776548311	0.0163709896778197	0.58416647379085
**DLBC**	HDAC9	cg12081743	-0.193583028687267	48	-1.33825861889439	0.187389880290202	0.0374743889957351	0.366709640713806
**DLBC**	HDAC9	cg16475478	-0.271907989712094	48	-1.91637238700024	0.0615432890412196	0.073933954869272	0.167730473222836
**DLBC**	HDAC9	cg18856632	0.143765539847091	48	0.985300856275325	0.329630676854722	0.0206685304475256	0.529284172536817
**DLBC**	HDAC9	cg19585556	0.0968017348521366	47	0.652429794646953	0.517443059960934	0.00937057587038337	0.698356472070243
**DLBC**	HDAC9	cg20648975	0.142010889047316	48	0.973026238670296	0.335629362693284	0.0201670926080091	0.535204481733348
**DLBC**	HDAC9	cg21433125	-0.21941768157764	48	-1.52533395813826	0.134022508123283	0.0481441189889064	0.292204450462226
**DLBC**	HDAC9	cg23074799	0.12417753526089	48	0.848782584086509	0.400397832430429	0.0154200602634695	0.596921332568564
**DLBC**	HDAC9	cg23385718	0.147490807707157	48	1.01139249571333	0.317119198002519	0.0217535383581097	0.516368293920574
**DLBC**	HDAC9	cg24350771	-0.126251017895395	48	-0.863182972080132	0.392515216450292	0.0159393195196234	0.589428216360081
**DLBC**	HDAC9	cg25620356	0.0729396900179617	48	0.496022274662138	0.622240503785576	0.00532019837991634	0.776219251476471
**DLBC**	HDGF	cg00692559	-0.0698684059198506	48	-0.475031458198031	0.637012338947196	0.00488159414578102	0.786386814606505
**DLBC**	HDGF	cg00746981	0.196200584514291	48	1.35707348307123	0.181379792679095	0.0384946693637494	0.358665089151337
**DLBC**	HDGF	cg01910231	-0.0269831672341959	48	-0.183075404113781	0.855543399111992	0.000728091313988584	0.923155807335087
**DLBC**	HDGF	cg02914429	0.208895585890727	48	1.44876144281104	0.154187162863627	0.04363736580463	0.321629113203545
**DLBC**	HDGF	cg04402095	0.0150408175328646	48	0.102023328551169	0.919181600095753	0.000226226192056926	0.957406991723557
**DLBC**	HDGF	cg05762852	0.291098676816714	48	2.06369986472085	0.0447142309208287	0.0847384396444419	0.132942958408352
**DLBC**	HDGF	cg05967123	0.354155734004467	48	2.56847287108669	0.0135260958789183	0.125426283928243	0.0539548657180674
**DLBC**	HDGF	cg06106793	0.353940918218457	48	2.56669174844391	0.013586822275065	0.125274173589324	0.0541260370783697
**DLBC**	HDGF	cg06119628	-0.161619202515131	48	-1.11075767486514	0.272445091969399	0.0261207666216268	0.468830392286083
**DLBC**	HDGF	cg07313589	0.258862259293214	48	1.81764506517201	0.0756348435944864	0.0670096692863874	0.195056189552342
**DLBC**	HDGF	cg07892339	0.127471499025229	48	0.871664605809621	0.387918252445154	0.0162489830637389	0.585771808509903
**DLBC**	HDGF	cg08597205	0.04166514933362	48	0.282832394564287	0.778574188149183	0.00173598466899285	0.877561256941966
**DLBC**	HDGF	cg08708935	0.218702712186437	48	1.52011357550003	0.135326834919423	0.0478308763177033	0.29414142649609
**DLBC**	HDGF	cg09063372	0.236332508231414	48	1.64961494993182	0.105834973860239	0.0558530544469512	0.2479992155331
**DLBC**	HDGF	cg09477492	-0.222788146879863	48	-1.54997851829449	0.12800008349926	0.0496345583901635	0.283012830044513
**DLBC**	HDGF	cg11588596	0.122712940782482	48	0.838617757030053	0.406020686313932	0.0150584658354849	0.602348931521149
**DLBC**	HDGF	cg12220178	-0.0244687725492138	48	-0.166004992474884	0.868880515121713	0.000598720830065161	0.93049145538288
**DLBC**	HDGF	cg12421458	0.332588148397348	48	2.39188753036512	0.0209057958807086	0.110614876454377	0.0753460961197055
**DLBC**	HDGF	cg13170934	0.0389906394587586	48	0.264648628258678	0.792462398551613	0.0015202699654029	0.886204554487517
**DLBC**	HDGF	cg13235992	0.0315197749504242	48	0.213883787476012	0.831582919432403	0.00099349621292539	0.909713206794132
**DLBC**	HDGF	cg14155849	0.177924788592884	48	1.22631154598399	0.226322035094658	0.0316572303958226	0.415845825318356
**DLBC**	HDGF	cg15503799	0.0746220592639712	48	0.507526470922612	0.614210320895402	0.00556845172879562	0.77057826955094
**DLBC**	HDGF	cg17170583	-0.104522108888359	48	-0.712807792633495	0.479565135234529	0.0109248712464699	0.666891956424113

**DLBC**	HDGF	cg17218029	-0.120173306856978	48	-0.821004903807453	0.41587814605602	0.0144416236809415	0.611495042850848
**DLBC**	HDGF	cg17971171	0.106361955184618	48	0.725497276616218	0.471821994787475	0.0113128655106946	0.659922755980391
**DLBC**	HDGF	cg18593717	-0.0150752797268182	48	-0.102257142042032	0.918997041144293	0.000227264058841815	0.957311834274317
**DLBC**	HDGF	cg19599803	-0.188094671926615	48	-1.29890440503024	0.200450151833144	0.0353796056071809	0.383457418718223
**DLBC**	HDGF	cg20284773	0.178650399430025	48	1.23147720799758	0.224403242344462	0.0319159652165074	0.413639129820517
**DLBC**	HDGF	cg23747104	0.0169420908193336	48	0.11492334520361	0.909006072920551	0.000287034441330549	0.951621147492173
**DLBC**	HDGF	cg26431298	0.118864092965595	48	0.811931664023812	0.421012687775972	0.0141286725965336	0.616136769245718
**DLBC**	HDGFL1	cg02685945	0.0713500892781868	48	0.48515635104853	0.629868061662924	0.00509083524000523	0.781613865436172
**DLBC**	HDGFL1	cg03930485	0.0376020392095234	48	0.255209924137667	0.799698617309175	0.00141391335271454	0.890584127620786
**DLBC**	HDGFL1	cg05276066	0.0885372697328656	48	0.602856478065934	0.549564981205077	0.00783884813175021	0.722457342343609
**DLBC**	HDGFL1	cg06566039	-0.0507079071406213	48	-0.344360769962339	0.732144363698455	0.00257129184658187	0.848679811700381
**DLBC**	HDGFL1	cg08671671	0.0493987494234774	48	0.335448155745222	0.738812161197757	0.00244023644460351	0.852924390843752
**DLBC**	HDGFL1	cg09081892	0.0430393480985518	48	0.292177799456396	0.771464449322537	0.00185238548474831	0.873318951397311
**DLBC**	HDGFL1	cg11894257	0.0757891322139385	48	0.51550957758221	0.608665804878442	0.00574399256174185	0.766409160267997
**DLBC**	HDGFL1	cg15317945	-0.209861711023454	48	-1.45576979805527	0.152247745185132	0.0440419377536917	0.318960522686887
**DLBC**	HDGFL1	cg20209681	0.142541292890419	48	0.976735673034117	0.333808929433603	0.0203180201788722	0.533405879885747
**DLBC**	HDGFL1	cg21898884	-0.0424090768964394	48	-0.287891360898128	0.774723039579389	0.00179852980320811	0.875122393835237
**DLBC**	HIST1H1	cg00667898	0.0877550289264703	48	0.597488623765124	0.55311126311285	0.00770094510188564	0.725183072429443
**DLBC**	HIST1H1	cg00700007	0.0404951868953145	48	0.274877192805062	0.784641442505756	0.00163986016168645	0.881055359742105
**DLBC**	HIST1H1	cg03821311	-0.0796892035083074	48	-0.542202810189108	0.590295945578865	0.00635036915578844	0.753305163883895
**DLBC**	HIST1H1	cg04676561	0.00236603005514335	48	0.0160472415010542	0.987266104093411	5.59809822184165e-06	0.993267023199043
**DLBC**	HIST1H1	cg07138603	0.138995747828214	48	0.95195568287039	0.346094912562571	0.0193198179143244	0.545386748797631
**DLBC**	HIST1H1	cg11206041	0.0965593400421911	48	0.657971858587609	0.513835722487252	0.0093237061493835	0.695381953155196
**DLBC**	HIST1H1	cg14334286	0.0277250378905951	48	0.188112668676868	0.851615730386695	0.000768677726034933	0.920863367754122
**DLBC**	HIST1H1	cg18798248	0.103053536247018	48	0.702684312958333	0.485793514048307	0.0106200313330155	0.672368577434732
**DLBC**	HIST1H1	cg23460835	0.055966543783918	48	0.380179444229327	0.705562054283069	0.00313225402311721	0.832072498725056
**DLBC**	HIST1H1	cg00903962	-0.0187894286446976	48	-0.127458606409602	0.899132999386901	0.000353042628794184	0.946767089256964
**DLBC**	HIST1H1	cg04290376	-0.225515844335876	48	-1.56996598088153	0.123276990279587	0.0508573960465229	0.275834393172176
**DLBC**	HIST1H1	cg07750111	0.11489526280417	48	0.784452535460829	0.436795847420536	0.0132009214148392	0.629797534088913
**DLBC**	HIST1H1	cg08283682	0.0127195007076018	48	0.0862748303186821	0.931622394067989	0.000161785698250682	0.963738834733392
**DLBC**	HIST1H1	cg10717387	-0.0441355347194881	48	-0.299633737791894	0.765806318338078	0.00194794542497514	0.870078379588329
**DLBC**	HIST1H1	cg12633985	0.172192197601327	48	1.1855727638544	0.241879426695936	0.0296501529147743	0.434196370777479
**DLBC**	HIST1H1	cg15022984	-0.083450738814572	48	-0.567971587576137	0.57281635853691	0.00696402580869791	0.740374755699148
**DLBC**	HIST1H1	cg16481032	0.116006723977318	48	0.792144100094967	0.432342922290812	0.0134575600079497	0.626092429201255
**DLBC**	HIST1H1	cg16482183	0.054274289300214	48	0.368649505219978	0.714080284329277	0.00294569847904332	0.837321559522152
**DLBC**	HIST1H1	cg17691542	0.0728453824428876	48	0.495377517697403	0.622691947979583	0.00530644974325056	0.776523807110488
**DLBC**	HIST1H1	cg20855557	0.0647161016527207	48	0.439848003009798	0.662106916917118	0.00418817381312528	0.803422139878365
**DLBC**	HIST1H1	cg23407813	-0.0429403167980787	48	-0.291504270209986	0.771976198469629	0.00184387080671936	0.873578006635057
**DLBC**	HIST1H3	cg00958605	-0.119154227193629	48	-0.813942007828829	0.419871732644629	0.0141977298581109	0.61520137057589
**DLBC**	HIST1H3	cg01043409	0.245951396603829	48	1.72098857911886	0.0919734075467451	0.0604920894913739	0.22429402089942
**DLBC**	HIST1H3	cg08041469	-0.0422706640710638	48	-0.286950069445122	0.775439169534285	0.00178680904100873	0.875638895005954
**DLBC**	HIST1H3	cg08401836	0.0621902451788967	48	0.422612810547971	0.674546802955809	0.00386762659541128	0.81181457285186
**DLBC**	HIST1H3	cg10075390	-0.00908505506820854	48	-0.0616203844585279	0.951132106542877	8.25382255923816e-05	0.974102246315845
**DLBC**	HIST1H3	cg15479139	0.0570981991045525	48	0.387891646985993	0.699885457993251	0.00326020434098312	0.828039196385421
**DLBC**	HIST1H3	cg15797414	0.281515959758047	48	1.98980887905508	0.0525755990704715	0.0792512355984941	0.149654817200449
**DLBC**	HIST1H3	cg21209604	0.331618699714769	48	2.38405263698004	0.0213047516828154	0.109970962000514	0.076419303412622
**DLBC**	HIST1H3	cg23099349	0.299280517274852	48	2.12732479566515	0.0387862078210274	0.0895688280203033	0.119706984496906
**DLBC**	HIST1H3	cg25701114	-0.0757116029985907	48	-0.514979190277518	0.609033460850447	0.00573224682861621	0.766665302891627
**DLBC**	HLTF	cg00906762	-0.124312300001783	48	-0.849718183193432	0.399882729135791	0.0154535479317334	0.596507776080798
**DLBC**	HLTF	cg02059813	-0.256297984659646	48	-1.79836688268328	0.0786828098356058	0.0656886569405962	0.200492974397351
**DLBC**	HLTF	cg02398045	-0.425484869875083	48	-3.18882727373975	0.00257105755055768	0.181037374492617	0.0145524523017635
**DLBC**	HLTF	cg02611507	-0.10700922036742	48	-0.729963272250453	0.469113861548999	0.0114509732436431	0.658028195913142
**DLBC**	HLTF	cg02710346	-0.106462345320315	48	-0.726189886477403	0.471401421084228	0.0113342309711021	0.659655031241818
**DLBC**	HLTF	cg03678609	-0.505460875334653	48	-3.9731173850237	0.000247857393596967	0.255490696494074	0.00207588545043802
**DLBC**	HLTF	cg04120413	-0.141661286904776	48	-0.970581727700678	0.336832627482973	0.0200679202075173	0.536439194745771
**DLBC**	HLTF	cg04731810	-0.128195219481681	48	-0.876695940224385	0.385207395793506	0.0164340142979565	0.583295847170024
**DLBC**	HLTF	cg04836786	-0.434380894133455	48	-3.27081024500418	0.00203633378281178	0.18868676118818	0.0120669218692916

**DLBC**	HLTF	cg05555455	-0.49356477128971	48	-3.84901040223574	0.000364231408658961	0.243606183458263	0.00287519210605939
**DLBC**	HLTF	cg07275648	-0.0749201613600498	48	-0.509565372195654	0.612792055322097	0.00561303057821591	0.769407405174747
**DLBC**	HLTF	cg09352720	-0.0558518234547813	48	-0.379397710608271	0.706138404851837	0.00311942618322406	0.832432624605237
**DLBC**	HLTF	cg09392355	-0.0829603503048486	48	-0.564610770025897	0.575081637911449	0.00688241972270319	0.74207817351053
**DLBC**	HLTF	cg15438497	-0.474349899293345	48	-3.65450929102873	0.000659000162265939	0.225007826959606	0.00473897750261753
**DLBC**	HLTF	cg16724124	-0.140942635056683	48	-0.965557886049227	0.339314503681337	0.0198648263767213	0.538584281901382
**DLBC**	HLTF	cg20117402	-0.136048670866141	48	-0.931386870698672	0.356516184245344	0.0185092408444436	0.555351715768252
**DLBC**	HLTF	cg21089667	-0.166622144064406	48	-1.14610801162261	0.257677743660266	0.0277629388926197	0.452184304218419
**DLBC**	HLTF	cg21111416	-0.079443775509243	48	-0.540522303287857	0.591444635773051	0.006311313467163	0.754115417949105
**DLBC**	HLTF	cg21835021	-0.166965660788456	48	-1.14853857485085	0.256683868672706	0.0278775318825259	0.451030309207068
**DLBC**	HLTF	cg21926402	-0.437752702825903	48	-3.30218931296123	0.00186103795987737	0.191627428831383	0.011213506858547
**DLBC**	HLTF	cg24041269	-0.473469159118129	48	-3.64576086421645	0.00067659978396506	0.224173044636028	0.00484181801065016
**DLBC**	HLTF	cg24710191	0.00903287102529853	48	0.0612664114873444	0.951412464230211	8.15927589596777e-05	0.974255380312295
**DLBC**	HLTF	cg25951608	-0.0407283976329415	48	-0.276462826195934	0.78343102278928	0.00165880237374699	0.880509600053011
**DLBC**	HLTF	cg26151310	-0.384898595172865	48	-2.82841345796728	0.00690684427902085	0.148146928566045	0.0320744414900996
**DLBC**	HLTF	cg27554556	-0.333905896880968	48	-2.40255099256913	0.0203736130077277	0.111493147971884	0.0738488887486334
**DLBC**	IDH1	cg00707300	-0.182814324031016	48	-1.26116083800874	0.213609611455651	0.0334210770709172	0.400129584725129
**DLBC**	IDH1	cg01046174	-0.243941833785857	48	-1.70603351674374	0.094746029955763	0.0595076182708066	0.229144831635225
**DLBC**	IDH1	cg02241586	0.0127199243058039	48	0.0862777039988443	0.931620122284072	0.00016179647434538	0.963738834733392
**DLBC**	IDH1	cg03448938	-0.231980528647325	48	-1.61749300922353	0.112609741238839	0.0538149656714924	0.259328090048673
**DLBC**	IDH1	cg05268564	-0.172437499685798	48	-1.18731343212754	0.241199178220673	0.0297346912978897	0.433395285148379
**DLBC**	IDH1	cg07129696	0.123421640460485	48	0.843535695706128	0.403294183089298	0.0152329013339573	0.599779534673743
**DLBC**	IDH1	cg07915542	0.226830440925046	48	1.57961266694384	0.121048265913288	0.0514520489302509	0.272297656856091
**DLBC**	IDH1	cg09406631	-0.144695035159093	48	-0.991806954953133	0.326480363758456	0.0209366531996911	0.52593788973071
**DLBC**	IDH1	cg09615745	-0.222626808917397	48	-1.54879750469446	0.128283647264964	0.0495626960487431	0.283429598341634
**DLBC**	IDH1	cg10356455	0.073246587669537	48	0.498120548732369	0.620772356239523	0.00536506260523117	0.775256226560608
**DLBC**	IDH1	cg14772913	-0.0230220967889895	48	-0.156184853121227	0.876570751218556	0.000530016940561602	0.934673990793283
**DLBC**	IDH1	cg15399923	-0.00588350460307533	48	-0.0399045603419469	0.968341908506373	3.46156264144086e-05	0.983379398362326
**DLBC**	IDH1	cg16085695	-0.260719545840227	48	-1.83163381079817	0.0734859877340912	0.067974681583134	0.19104932425846
**DLBC**	IDH1	cg16863783	-0.29705213374678	48	-2.10994588769507	0.0403328226277382	0.0882399701635147	0.123159648494276
**DLBC**	IDH1	cg24324379	-0.177147782547641	48	-1.22078228887928	0.228389253902908	0.0313813368615462	0.418295920405903
**DLBC**	IDH1	cg24830471	-0.269239820738995	48	-1.896089910292	0.0642396211101561	0.072490081071566	0.173066113195854
**DLBC**	IDH1	cg24987806	-0.0595837927499547	48	-0.404836213539591	0.687474394725089	0.00355022835846956	0.820216291381565
**DLBC**	IDH1	cg25795625	-0.206026733960676	48	-1.42797658613272	0.16005306796702	0.0424470151065033	0.329814801054946
**DLBC**	IDH1	cg26105702	-0.126392102660733	48	-0.864163224703199	0.391982191749382	0.0159749636150012	0.589059876464764
**DLBC**	ING2	cg02075087	0.24744225180268	48	1.73209869459143	0.0899574939739219	0.0612276679771808	0.220713195389261
**DLBC**	ING2	cg02552216	0.229761642808655	48	1.60115509265979	0.116188702980401	0.0527904125061319	0.264795194365236
**DLBC**	ING2	cg10348863	-0.217078112394536	48	-1.50826102634972	0.138325829929031	0.0471229068807747	0.29869870686446
**DLBC**	ING2	cg17985608	0.14612018726681	48	1.00178766944273	0.321687006398667	0.0213511091268877	0.52117399090127
**DLBC**	ING3	cg01382559	0.0210321891355694	48	0.142678807673428	0.887166991282413	0.000442352979834362	0.940058418619993
**DLBC**	ING3	cg01416111	-0.110652473036644	48	-0.755118648271386	0.454026870580995	0.0122439697891252	0.644312489647348
**DLBC**	ING3	cg10268125	0.0254074817538934	48	0.172377572597463	0.863896889800613	0.000645540129074427	0.927721413615705
**DLBC**	ING3	cg14303833	0.0232960195012882	48	0.158044183017801	0.875113746328954	0.0005427045246044	0.933881719087116
**DLBC**	ING3	cg16130600	0.120875855661126	48	0.82587556368407	0.413137662029163	0.0146109724818094	0.608682081737371
**DLBC**	ING3	cg18485114	-0.236137881148315	48	-1.64817618092352	0.10613111489401	0.0557610989132155	0.24845367133293
**DLBC**	ING3	cg19478983	0.107998728307674	48	0.736792488734452	0.464989919038796	0.0116637253160747	0.654225308574551
**DLBC**	ING3	cg19960827	-0.183966212482192	48	-1.2693846961515	0.210688784827696	0.0338435673350429	0.396447362960737
**DLBC**	ING3	cg20553930	0.194228948265362	48	1.34289867951336	0.18589376281916	0.0377248843442686	0.364717646080899
**DLBC**	ING3	cg24859794	-0.172816193347175	48	-1.19000109674815	0.240151580351607	0.0298654366830083	0.432123253016195
**DLBC**	ING4	cg00369908	0.349462381170981	48	2.52966346614886	0.0149069366847199	0.122123955853692	0.0580968003833023
**DLBC**	ING4	cg00443163	0.414163949721977	48	3.08612336093429	0.00342868572694074	0.171531777249308	0.0183282606638925
**DLBC**	ING4	cg00591160	0.116415359963223	48	0.794972710969481	0.430712189128231	0.0135525360353669	0.624707734376941
**DLBC**	ING4	cg03930474	0.406145535417705	48	3.01443171493773	0.00417964334212606	0.164954195939735	0.021500441324959
**DLBC**	ING4	cg04375284	0.160105666813939	48	1.10008063539271	0.277020796977368	0.0256338245459362	0.473805203589336
**DLBC**	ING4	cg08733494	0.228310601521244	48	1.59048518101346	0.118575480235716	0.0521257307669921	0.268551075998058
**DLBC**	ING4	cg12392916	0.134876700741841	48	0.923214263501567	0.36071300647333	0.0181917244030041	0.559632699665375
**DLBC**	ING4	cg18123790	0.197082552437984	48	1.363419797673	0.179386239574485	0.0388415324754708	0.356244686938898

**DLBC**	ING4	cg23354319	0.382355126287273	48	2.80651003051017	0.00731932360602555	0.146195442598157	0.0335355121389453
**DLBC**	ING4	cg27116036	0.297414815421334	48	2.11277181326576	0.0400777125981552	0.088455572432106	0.122651674817501
**DLBC**	INO80	cg00734724	0.0456798808443324	48	0.310139771262078	0.757855464412419	0.00208665151395241	0.864986399426337
**DLBC**	INO80	cg06380007	-0.291976035061836	48	-2.07049847353492	0.0440451722838277	0.0852500050504307	0.131495171092642
**DLBC**	INO80	cg12580230	-0.4949703566158	48	-3.86352191655192	0.00034829009229841	0.244995653928372	0.00276839179321516
**DLBC**	INO80	cg18952654	-0.33755564065751	48	-2.43216846720772	0.0189589254664352	0.113943810539702	0.0698206138863785
**DLBC**	INO80	cg21007089	-0.655264026045289	48	-5.88326350944548	4.33118941776228e-07	0.429370943829081	8.10732794911931e-06
**DLBC**	INO80	cg21598047	-0.106657814006839	48	-0.727538525074209	0.470583099905181	0.0113758892887175	0.659113461360275
**DLBC**	INO80	cg22835709	-0.692007643272661	48	-6.50157650576387	5.10974887713364e-08	0.478874578347783	1.18907509466866e-06
**DLBC**	INO80	cg26409855	-0.26917780360033	48	-1.89561904023927	0.0643033961843177	0.0724566899510977	0.173171784343065
**DLBC**	INO80	cg27441689	-0.550355768630404	48	-4.47066616170044	5.06546607786696e-05	0.302891472064763	0.000535994107809551
**DLBC**	INTS12	cg00093890	0.251263131199444	48	1.7606326440762	0.0849478592423208	0.063133161100149	0.211958232147575
**DLBC**	INTS12	cg01852585	0.155190095153455	48	1.06545886806896	0.292228991982068	0.0240839656337385	0.489942111920283
**DLBC**	INTS12	cg03607115	0.163884977801943	48	1.12675635941175	0.26568919960883	0.0268582859491433	0.461484099901087
**DLBC**	INTS12	cg19703969	0.224593868793528	48	1.5632057950881	0.124858464407676	0.0504424058996445	0.278172844382001
**DLBC**	INTS12	cg26533745	0.218802010784117	48	1.52083845488705	0.135145118519814	0.0478743199231728	0.293918908306693
**DLBC**	IWS1	cg02372421	-0.261251998123521	48	-1.8356481600417	0.0728789359249321	0.0682526065235322	0.189883580325011
**DLBC**	IWS1	cg02592214	0.0527924307005982	48	0.358555688904325	0.721567896782579	0.00278704073927747	0.842240621808666
**DLBC**	IWS1	cg10180764	-0.358602487535061	48	-2.60544779277626	0.0123201133977978	0.128595744066334	0.0501927678276134
**DLBC**	IWS1	cg10488833	-0.0750649912586097	48	-0.510556001380494	0.612103510663497	0.00563475291265516	0.768865647059922
**DLBC**	IWS1	cg15239145	-0.170287708838294	48	-1.17206617945767	0.247205181250308	0.0289979037813954	0.440443393037273
**DLBC**	IWS1	cg17144508	-0.210063356114257	48	-1.45723311080512	0.151845233978298	0.0441266135819852	0.318348734223783
**DLBC**	IWS1	cg18451256	-0.0544439949943671	48	-0.36980562581402	0.713224467580639	0.00296414859094667	0.836871557028178
**DLBC**	IWS1	cg18530950	-0.338401260877296	48	-2.43904821439363	0.0186432764386992	0.114515413363344	0.0689781721331764
**DLBC**	IWS1	cg19311602	-0.175940592817395	48	-1.2121964698596	0.23162670147807	0.0309550922009363	0.422132961973129
**DLBC**	IWS1	cg19409088	-0.0324642607836191	48	-0.220299449800878	0.826612766854171	0.00105392822822683	0.906848212331703
**DLBC**	IWS1	cg21581038	0.0223118650824452	48	0.151364112208208	0.880350372223072	0.000497819323457239	0.936521945858244
**DLBC**	IWS1	cg21679674	-0.3600573252505	48	-2.61758879983403	0.0119460987167271	0.129641277466544	0.0489643978695948
**DLBC**	IWS1	cg22409775	-0.356104808849654	48	-2.58465472522599	0.012985625708376	0.126810634885849	0.0522879796251245
**DLBC**	IWS1	cg23570261	-0.0734465705736354	48	-0.499487918029834	0.619816456090681	0.00539439872902801	0.774521042331559
**DLBC**	IWS1	cg23623107	0.0223734950410015	48	0.15178242011113	0.880022292040788	0.000500573280349718	0.936347006362666
**DLBC**	JADE1	cg09863040	0.0216469287252236	48	0.146851024192934	0.883891338382931	0.000468589523234911	0.938458198159443
**DLBC**	JADE1	cg12832492	-0.156418959302764	48	-1.07410639194153	0.288377173455162	0.0244668908293597	0.485889710679232
**DLBC**	JADE1	cg14617908	-0.0815543442390995	48	-0.554977160636826	0.581599077760016	0.00665111106426955	0.746997947056459
**DLBC**	JADE1	cg15389440	-0.0521326447440189	48	-0.354062264352455	0.724910065806287	0.00271781264800608	0.844107163370787
**DLBC**	JADE1	cg15836763	0.111756607131374	48	0.762748341138533	0.449507389381807	0.0124895392375163	0.640740731272569
**DLBC**	JADE1	cg20039387	0.127460376111672	48	0.871587289921753	0.38796000330982	0.016246147478529	0.585810568662347
**DLBC**	JADE1	cg22922695	-0.116686103824165	48	-0.796847046872649	0.429633641994753	0.0136156468256637	0.623678289705913
**DLBC**	JADE1	cg26766900	-0.00213119722563487	48	-0.0144545156696308	0.988529877825015	4.54200161455376e-06	0.993976674970026
**DLBC**	JADE2	cg04590931	-0.218107254364164	48	-1.515767775122	0.13642035323804	0.0475707744062741	0.295884462879039
**DLBC**	JADE2	cg04842522	0.0396727330515443	48	0.269285568083955	0.78891417933472	0.0015739257477791	0.883855130024739
**DLBC**	JADE2	cg17378563	0.00536113144500878	48	0.0363614850927137	0.971151463107353	2.87417303706619e-05	0.98469523123108
**DLBC**	JADE2	cg18145011	-0.0659622664871236	48	-0.448354320503118	0.656002334583294	0.0043510206001183	0.799489999006341
**DLBC**	JADE2	cg21194015	0.0068774862121101	48	0.0466464841430429	0.962996911662845	4.72998165977646e-05	0.980550489719862
**DLBC**	JADE2	cg25669900	0.0439987916855817	48	0.298703593334599	0.766511485438485	0.00193589366979121	0.870574414164559
**DLBC**	JADE2	cg26133206	-0.0462988815232309	48	-0.314351392778022	0.754675513838246	0.00214358643030217	0.862990253175548
**DLBC**	JADE3	cg10860619	-0.0780307975991333	48	-0.530849204484448	0.598077103100893	0.0060888053739569	0.758717123406078
**DLBC**	JADE3	cg17843048	-0.0352612242566647	48	-0.239302073376877	0.811934409394506	0.0012433539360788	0.898707279782236
**DLBC**	JADE3	cg19194595	-0.0136694877355108	48	-0.0927196394864645	0.926528941104275	0.000186854894951281	0.961296501047169
**DLBC**	JARID2	cg01769037	-0.369489555359605	48	-2.69684246104319	0.00974934855983743	0.136522531519839	0.0417663954674959
**DLBC**	JARID2	cg03223185	-0.14864180995719	48	-1.01946291357026	0.313315239027712	0.0220943876673494	0.512393485356468
**DLBC**	JARID2	cg05197191	-0.377824951964156	48	-2.76768228471806	0.00810706364525708	0.142751694326717	0.0362960928418007
**DLBC**	JARID2	cg06047828	-0.224049426493025	48	-1.55921586764601	0.125799507318171	0.0501981455118536	0.279628746565798
**DLBC**	JARID2	cg14862454	-0.319983430498317	48	-2.29066942273642	0.0266145401949563	0.102389395793471	0.0902944413400101
**DLBC**	JARID2	cg15109221	-0.299068834494078	48	-2.12567226909035	0.038931000248387	0.0894421677656465	0.120077639890691
**DLBC**	JARID2	cg21397480	-0.165329688168335	48	-1.13696703713592	0.261440227445881	0.0273339057898389	0.456799210204584
**DLBC**	JARID2	cg21901688	-0.300596326165324	48	-2.13760456458391	0.0378960746951554	0.0903581513040901	0.117669323654304

DLBC	JARID2	cg25338254	-0.232891929946943	48	-1.62421147693445	0.111164282091008	0.054238651034412	0.256843307424648
DLBC	JMJD6	cg02318866	-0.157535789557493	48	-1.08196996668899	0.284905361233803	0.0248175249915026	0.482139499421624
DLBC	JMJD6	cg04510639	0.341393101875518	48	2.46344281668558	0.0175619399490764	0.116549250008188	0.0658860704281659
DLBC	JMJD6	cg07312083	0.215875612955428	48	1.49949646101444	0.140577319647532	0.0466022802688818	0.302168214098698
DLBC	JMJD6	cg08857478	0.315429633573334	48	2.25443931196212	0.0289738165855593	0.099495853736208	0.0962417232750221
DLBC	JMJD6	cg10712116	-0.0769781404007059	48	-0.523644918965931	0.603039355969474	0.00592563409955079	0.762178415595023
DLBC	JMJD6	cg12504415	0.321757724408064	48	2.30483341101607	0.0257398042358406	0.103528033216256	0.0880641362794875
DLBC	JMJD6	cg15457598	0.155922880665432	48	1.07061487091316	0.289928106527092	0.0243119447150064	0.487426432148314
DLBC	JMJD6	cg21102709	0.281788860277073	48	1.9919040812357	0.0523372183412679	0.079404961776252	0.149172769240148
DLBC	JMJD6	cg21807925	0.427032138208487	48	3.20300390769049	0.00246997805135447	0.182356447062913	0.0140944379466786
DLBC	JMJD6	cg23630158	0.413216210469206	48	3.07760520228238	0.0035107674216767	0.170747636594531	0.0186886985631529
DLBC	JMJD6	cg25004679	0.37657331862259	48	2.75699574022063	0.00833723725513138	0.14180746429843	0.0370896686124373
DLBC	JMJD6	cg25908504	0.311061012376486	48	2.21984506787291	0.0313982381644966	0.0967589534206841	0.102127965036118
DLBC	JMJD6	cg26612088	0.440474352882478	48	3.32764338455074	0.00172945561731881	0.194017655547238	0.0105613682623204
DLBC	JMJD8	cg00873473	-0.318460839371182	48	-2.27853616576139	0.0273848470048867	0.101417306212998	0.0922400756796916
DLBC	JMJD8	cg02851105	-0.311447620506201	48	-2.22290020832446	0.0311771128967825	0.0969996203189743	0.101570095929155
DLBC	JMJD8	cg03899598	-0.329518681569635	48	-2.36710974991026	0.0221909916530556	0.10858256150339	0.0787270159001189
DLBC	JMJD8	cg05787952	-0.122960044913006	48	-0.840332358978682	0.40506882321684	0.0151191726450084	0.601533311062705
DLBC	JMJD8	cg06191810	-0.323581223349252	48	-2.31941842267781	0.0248659894290221	0.104704808104199	0.0858607105668032
DLBC	JMJD8	cg06646636	-0.135195170971879	48	-0.925434689220236	0.359569610731764	0.0182777342541156	0.558462528173184
DLBC	JMJD8	cg08398290	-0.309254011985901	48	-2.20558152910756	0.0324490936860651	0.0956380439293759	0.104657744370388
DLBC	JMJD8	cg26750188	-0.151222969446076	48	-1.03757654411277	0.304890807905899	0.022868386488089	0.503403478523878
DLBC	JMJD8	cg27579097	-0.0713135471862097	48	-0.484906606815689	0.63004385920556	0.00508562201227976	0.781729086317762
DLBC	KAT2B	cg00770008	-0.0340042792529776	48	-0.230761695157147	0.818523222133574	0.00115629100751448	0.902403609990944
DLBC	KAT2B	cg04371528	-0.435078785509974	48	-3.27729102877508	0.00199889453562319	0.189293549600834	0.0118921689740188
DLBC	KAT2B	cg05072819	-0.154833226466895	48	-1.06294853273254	0.293353812353879	0.0239733280181488	0.491078248401906
DLBC	KAT2B	cg12062464	-0.238231014068004	48	-1.66366060527918	0.102979384704096	0.0567540160638697	0.243268555870782
DLBC	KAT2B	cg14937543	-0.19849822067186	48	-1.37361363059754	0.176219352271941	0.0394015436098943	0.352130905721491
DLBC	KAT2B	cg18053505	-0.303993684868292	48	-2.16420872423158	0.0356752236658021	0.0924121604398026	0.112429343725234
DLBC	KAT5	cg00582417	-0.0948049433921384	48	-0.645907663655032	0.521548491666486	0.00898797729158656	0.701511893090974
DLBC	KAT5	cg02561205	-0.206748458701755	48	-1.43320184222135	0.158562239551916	0.0427449251755514	0.327723660663189
DLBC	KAT5	cg05785768	-0.226308000090464	48	-1.57577783991405	0.121930316660854	0.0512153109049455	0.273687086800002
DLBC	KAT5	cg07564927	-0.171455439335869	48	-1.18034607819475	0.243930377606954	0.029396967677856	0.436638743570073
DLBC	KAT5	cg14726348	0.0220109242135572	48	0.149321527337464	0.881952681502774	0.00048448078473496	0.937342061899078
DLBC	KAT5	cg18896539	0.177522080517689	48	1.22344553784331	0.227391819629235	0.0315140890713287	0.417059678202481
DLBC	KAT5	cg20108992	0.468160563209793	48	3.59332663655689	0.00079191821459921	0.219174312944911	0.00552178365741529
DLBC	KAT5	cg20423942	0.0315089328840376	48	0.213810143321286	0.831640011679989	0.000992812851490784	0.909713206794132
DLBC	KAT5	cg22048492	0.233727452841652	48	1.63037457595649	0.109851645076946	0.0546285222118466	0.254703113886383
DLBC	KAT6A	cg10163397	-0.292628395631134	48	-2.07555728819633	0.0435530081043676	0.0856313779296513	0.130369106640162
DLBC	KAT6A	cg12468056	-0.325423848323211	48	-2.33418573365173	0.0240083769119352	0.105900681057488	0.0835955550002607
DLBC	KAT6A	cg15251374	-0.212581626114989	48	-1.47552433600648	0.146884231264504	0.045190947761693	0.311354547329451
DLBC	KAT6A	cg20966972	-0.151077371693211	48	-1.03655421832113	0.305362106552754	0.0228243722377287	0.503910307521211
DLBC	KAT6B	cg04396765	-0.521741757907489	48	-4.14794321467172	0.000142966669042857	0.272214461944397	0.00130708062657421
DLBC	KAT6B	cg06972998	0.339154372128159	48	2.44518094975478	0.0183659121307463	0.115025688133645	0.0681724290628533
DLBC	KAT6B	cg09631472	-0.290272893173844	48	-2.05730610273066	0.0453515090840881	0.0842583525115138	0.134392629044932
DLBC	KAT6B	cg10359932	-0.252554780358467	48	-1.77029833286963	0.0833046060233228	0.0637839170819133	0.208889844023284
DLBC	KAT6B	cg10739686	-0.534726436219056	48	-4.29181403671824	9.03181799153778e-05	0.285932361591532	0.0008826900110976
DLBC	KAT6B	cg11128436	-0.252818081514904	48	-1.77226991024687	0.082972706180594	0.0639169823408767	0.208305179210642
DLBC	KAT6B	cg11348552	-0.408309496946303	48	-3.03369589494482	0.00396397170011199	0.166716645296543	0.0206159389248905
DLBC	KAT6B	cg15874302	-0.138838834470647	48	-0.950859877939403	0.346645002861766	0.0192762219571677	0.54598353785207
DLBC	KAT6B	cg15912797	-0.254511052344232	48	-1.78495680965278	0.0808633398376395	0.0647758757653682	0.204521836898671
DLBC	KAT6B	cg17147776	-0.436740055017789	48	-3.29274733002881	0.00191221978577922	0.190741875656941	0.0114683077208395
DLBC	KAT6B	cg18462764	-0.0943161622430277	48	-0.642547622693631	0.523707506771328	0.00889553846025313	0.702980492243374
DLBC	KAT6B	cg18491775	-0.232771209623461	48	-1.62332131790292	0.111354923664171	0.0541824360295691	0.257109984065496
DLBC	KAT6B	cg24671245	-0.375189569114107	48	-2.74520164979472	0.0085982641329238	0.140767212772029	0.037975097079407
DLBC	KAT6B	cg26605858	-0.0687170924422162	48	-0.467166290269102	0.642586319306067	0.00472203879371208	0.79011038135721
DLBC	KDM1A	cg00062312	-0.135890348591305	48	-0.930282595485171	0.35708139085432	0.0184661868402664	0.555940052105007

**DLBC**	KDM1A	cg00541565	-0.161506426269273	48	-1.10996183314473	0.272784301153307	0.026084325726272	0.469146967649161
**DLBC**	KDM1A	cg04886391	-0.26214047414778	48	-1.84235070686502	0.0718748206651911	0.068717628186423	0.187934585870794
**DLBC**	KDM1A	cg06958034	-0.145658672324075	48	-0.998554864651624	0.32323437734055	0.0212164488232122	0.522672589110066
**DLBC**	KDM1A	cg07118078	-0.212786867896126	48	-1.47701644201079	0.146485250853048	0.0452782511490432	0.310728952523914
**DLBC**	KDM1A	cg07785945	-0.181994033154309	48	-1.25530768451939	0.215706757202454	0.0331218281037719	0.402859274115524
**DLBC**	KDM1A	cg10499166	-0.168182716763673	48	-1.157153361533	0.253183367732689	0.0282854262180097	0.447216748844185
**DLBC**	KDM1A	cg12578844	-0.132583449505416	48	-0.907233905194748	0.36901138799311	0.0175783710827553	0.567743208215543
**DLBC**	KDM1A	cg22683154	-0.245871187044009	48	-1.72039121283063	0.0920828523940251	0.06045264061843	0.224462412080616
**DLBC**	KDM1A	cg26662347	-0.0784975593085542	48	-0.534044247317256	0.595882529684154	0.00616186681739998	0.757204340836808
**DLBC**	KDM1B	cg11007366	-0.315708712633547	48	-2.25665465660516	0.0288243865572542	0.0996719912327316	0.0958263387917377
**DLBC**	KDM1B	cg15124342	0.500252302888293	48	3.91841527346907	0.000293866364208359	0.25025236654504	0.00240338939626
**DLBC**	KDM1B	cg20677365	-0.10707781993292	44	-0.697956386190955	0.489050794815176	0.0114656595215868	0.675051323335258
**DLBC**	KDM1B	cg22406543	-0.0457528662311542	48	-0.310636338153494	0.757480314243176	0.00209332476836589	0.864735836323041
**DLBC**	KDM1B	cg23073439	-0.00847433978591335	48	-0.0574778327232822	0.954413519145063	7.18144348071139e-05	0.975799988281763
**DLBC**	KDM1B	cg23860886	-0.15854602418701	48	-1.08908667052291	0.281788562094139	0.0251368417855079	0.478894861957117
**DLBC**	KDM1B	cg24342628	-0.0711786777540357	48	-0.483984868606938	0.630692867521857	0.00506640416681286	0.782085294269472
**DLBC**	KDM2A	cg05038268	-0.243532285019368	48	-1.70298855066031	0.0953189518185666	0.0593079738467544	0.230144088695642
**DLBC**	KDM2A	cg05295919	-0.282599684793584	48	-1.99813231228595	0.0516341075256124	0.0798625818454332	0.147720791440405
**DLBC**	KDM2A	cg06320126	-0.118721426493495	48	-0.810943203605726	0.421574369469713	0.0140947771086503	0.616697188765078
**DLBC**	KDM2A	cg07283932	-0.458051779733677	48	-3.49484661562501	0.00106129147243227	0.209811432917189	0.0070541788112095
**DLBC**	KDM2A	cg08836144	-0.139577478644571	48	-0.956018840573557	0.344060237623402	0.0194818725447755	0.543328896640194
**DLBC**	KDM2A	cg09993857	-0.209912999007514	48	-1.4561419699897	0.152145292958867	0.0440634671523287	0.318816408444655
**DLBC**	KDM2A	cg11637544	-0.155893778244029	48	-1.07041006686863	0.290019260198841	0.0243028700951986	0.487526650415071
**DLBC**	KDM2A	cg16004548	-0.133759464262331	48	-0.91542701028477	0.364741606705053	0.0178915942797459	0.563672471682253
**DLBC**	KDM2A	cg18132081	-0.461644190391132	48	-3.52964279162553	0.000957395541323198	0.213115358521884	0.00647480037744039
**DLBC**	KDM2A	cg21293464	-0.250401652875188	48	-1.75419160162085	0.0860578587989532	0.0627009877626259	0.21396382345437
**DLBC**	KDM2A	cg21711868	-0.460715970827037	48	-3.52063105076487	0.000983329038415161	0.2122592057751	0.00662369895354308
**DLBC**	KDM2A	cg25597833	-0.108570207935318	48	-0.740737636920888	0.462617091316265	0.0117874900511182	0.652014815474785
**DLBC**	KDM2B	cg03486832	-0.0202253643085504	48	-0.137203160170104	0.891468995895101	0.000409065361413585	0.942513307657556
**DLBC**	KDM2B	cg05067991	-0.296735619375601	48	-2.10748050480017	0.0405565450988174	0.0880520278062217	0.123596375502915
**DLBC**	KDM2B	cg07020001	0.0722796898863243	48	0.491510300981859	0.625402756362402	0.00522435357006321	0.77831534786622
**DLBC**	KDM2B	cg07529210	-0.0453696179220329	48	-0.308028906958786	0.75945085286386	0.00205840223039125	0.86610841832602
**DLBC**	KDM2B	cg07793808	0.148790931495448	48	1.02050880993632	0.312824539921201	0.0221387412952831	0.511827712760636
**DLBC**	KDM2B	cg09151742	0.150762194698603	48	1.03434141524792	0.306383931442652	0.0227292393503396	0.504825508591724
**DLBC**	KDM2B	cg12251659	-0.330249541322647	48	-2.3730018137822	0.0218791051633962	0.109064759543818	0.0779517361300163
**DLBC**	KDM2B	cg12888660	-0.224624821843535	48	-1.56343267976589	0.124805123400014	0.0504563105882399	0.27808586153923
**DLBC**	KDM2B	cg15234492	-0.00616270845003667	48	-0.0417983160340886	0.966840380002147	3.79789754401534e-05	0.982565161163268
**DLBC**	KDM2B	cg15316703	-0.241584222312107	48	-1.68851806385403	0.0980809216529348	0.0583629364701455	0.234779753809914
**DLBC**	KDM2B	cg16837338	-0.0272445749187568	43	-0.174515178441152	0.862319519921735	0.000742266862503752	0.92682073408112
**DLBC**	KDM2B	cg17452615	0.0218228441774551	48	0.148044986795172	0.88295431985306	0.000476236527993488	0.93786761001864
**DLBC**	KDM2B	cg20055546	-0.381640213545734	48	-2.80036695464979	0.0074390412497401	0.145649252595233	0.0339580316267035
**DLBC**	KDM2B	cg21249371	0.0142348664527172	48	0.0965553446170976	0.923498973509307	0.000202631422926693	0.959803374973999
**DLBC**	KDM2B	cg21781916	-0.168539781511975	48	-1.15968183329231	0.25216250623185	0.0284056579521042	0.445996953660435
**DLBC**	KDM2B	cg26218726	-0.250289538596107	48	-1.75335368057773	0.0862031453640753	0.0626448531306519	0.214231463133018
**DLBC**	KDM2B	cg26509318	-0.0604777872141392	48	-0.410932503673254	0.68303013397699	0.0036575627463187	0.817383454744965
**DLBC**	KDM3A	cg01744130	0.0477765286475814	48	0.324406639017634	0.74710082357969	0.00228259668961317	0.858011182343122
**DLBC**	KDM3A	cg02152982	-0.0433248417651264	48	-0.294119540325362	0.769989685479596	0.00187704191397324	0.872478082084729
**DLBC**	KDM3A	cg02972551	0.0654730240909602	48	0.445014504364547	0.658396363902496	0.00428671688361546	0.801226448024112
**DLBC**	KDM3A	cg03037033	-0.152258509871977	48	-1.04484966878935	0.301552246533026	0.023182653828435	0.499953160895501
**DLBC**	KDM3A	cg04793821	-0.279607940996821	48	-1.97517461563794	0.0542668870935455	0.0781806006684817	0.153195303297849
**DLBC**	KDM3A	cg07052513	-0.132619623690219	48	-0.907485866573637	0.368879603257762	0.0175879645877352	0.567609230291242
**DLBC**	KDM3A	cg07211384	-0.099644250595052	48	-0.679200482982247	0.500414266144862	0.00992897667664953	0.684300311312534
**DLBC**	KDM3A	cg12888358	-0.154319536510395	48	-1.05933581160844	0.294977845735436	0.0238145193487831	0.492860776430384
**DLBC**	KDM3A	cg18611949	-0.0911922987879471	48	-0.621084132540284	0.537609907105922	0.00831603535823021	0.713480929294724
**DLBC**	KDM3A	cg19139691	-0.104427796743893	48	-0.712157519032722	0.479963853263875	0.0109051647327839	0.667255941970941
**DLBC**	KDM3A	cg20191795	-0.0175966407045119	48	-0.119364705432252	0.905506206939025	0.000309641764083686	0.949890773373623
**DLBC**	KDM3A	cg20918903	0.0159602734144001	48	0.10826163055588	0.914259015425482	0.000254730327462408	0.95474759413586

**DLBC**	KDM3A	cg22404242	-0.0257420519296755	48	-0.174648966070632	0.862121904851588	0.00066265323755011	0.926736375100517
**DLBC**	KDM3A	cg24749656	-0.154450341468573	48	-1.06025566387123	0.294563753558963	0.0238549079797587	0.492378441004855
**DLBC**	KDM3B	cg00435490	-0.016546895712478	48	-0.112241873869261	0.911120005154443	0.000273799757719622	0.952780078345478
**DLBC**	KDM3B	cg03315484	-0.390914382594235	48	-2.88052198358987	0.00601068479887204	0.152814054519032	0.0287132461310071
**DLBC**	KDM3B	cg05191700	-0.55311007078078	48	-4.50286659044923	4.5609146996502e-05	0.30593075039912	0.000489936202906086
**DLBC**	KDM3B	cg05708550	-0.126651765782232	48	-0.865967495457324	0.391002283185077	0.0160406697757575	0.588246371387957
**DLBC**	KDM3B	cg05722427	-0.198492440273551	48	-1.37357198941288	0.176232200685352	0.0393992488457494	0.352147534678831
**DLBC**	KDM3B	cg15001364	-0.180234581160035	48	-1.24276230741564	0.220253203559702	0.0324845042459333	0.408408678975151
**DLBC**	KDM3B	cg18682923	-0.165471422236615	48	-1.13796916514823	0.261025840572374	0.0273807915770082	0.456312232154445
**DLBC**	KDM3B	cg19076659	-0.0687387235752679	48	-0.467314045401822	0.642481413291454	0.0047250121187571	0.790041715123852
**DLBC**	KDM3B	cg24137987	-0.654436791739311	48	-5.87026604600778	4.52955706195348e-07	0.428287514382042	8.4400565767092e-06
**DLBC**	KDM4A	cg00154982	-0.218408137176787	48	-1.51796347074095	0.135866980896667	0.0477021143850342	0.294977559496409
**DLBC**	KDM4A	cg00979347	-0.347161431596606	48	-2.51071642772859	0.0156266382125986	0.120521059588205	0.0602556973041337
**DLBC**	KDM4A	cg03587511	0.0328462274437767	48	0.222894223136572	0.824604644291558	0.00107887465728831	0.905730857284295
**DLBC**	KDM4A	cg08552254	-0.249580521926829	48	-1.74805636414219	0.0871263865715982	0.0622904369252682	0.215788287709357
**DLBC**	KDM4A	cg10958452	0.0367075005393246	48	0.249130282219529	0.804369065253477	0.00134744059584452	0.893586471591677
**DLBC**	KDM4A	cg10968649	-0.371443122924987	48	-2.71337641746896	0.00934072014397771	0.137969993568267	0.040414459559544
**DLBC**	KDM4A	cg12293275	-0.254000589453014	48	-1.78112962764709	0.0814948719051218	0.0645162994424786	0.205684458520814
**DLBC**	KDM4A	cg17737314	-0.108521763328768	48	-0.740403176015454	0.462817983383869	0.0117769731159852	0.652183860438443
**DLBC**	KDM4A	cg19743044	-0.0695468224588541	48	-0.472834378574662	0.638567275538224	0.00483676051412337	0.78744416563454
**DLBC**	KDM4A	cg20873046	-0.526309412267754	48	-4.19808650930135	0.000121896778893452	0.277001597441629	0.00114182275945059
**DLBC**	KDM4A	cg21451504	-0.455823905313558	48	-3.47337649096208	0.00113068074817778	0.207775432655303	0.00743534895382437
**DLBC**	KDM4A	cg22094309	-0.288867855286713	48	-2.04643889534858	0.046452810573059	0.0834446378179456	0.136677380700475
**DLBC**	KDM4A	cg24324755	-0.433488022479682	48	-3.2625294118078	0.00208513910440268	0.187911865633345	0.0122991217067767
**DLBC**	KDM4A	cg24505556	-0.463476762090734	48	-3.54747788087354	0.00090798599192593	0.214810708998111	0.00619775925733841
**DLBC**	KDM4A	cg25841161	-0.049337733731829	48	-0.335032809679037	0.739123394156993	0.00243421196979285	0.853030373038737
**DLBC**	KDM4B	cg01098771	-0.211353191250471	48	-1.46659789221474	0.149289064036938	0.0446701714517582	0.314744239403657
**DLBC**	KDM4B	cg12072803	0.0110199019631294	48	0.0747451500931381	0.940741594441966	0.000121438239276982	0.968337610622521
**DLBC**	KDM4B	cg18115693	-0.134658684290766	48	-0.921694385779528	0.361497015942269	0.0181329612549201	0.560372598549624
**DLBC**	KDM4C	cg00121158	-0.0767502981961103	48	-0.522085821392323	0.604115763441391	0.00589060827319186	0.763043004678599
**DLBC**	KDM4C	cg00667760	0.0280913155302622	48	0.190599789672717	0.84967786052135	0.000789122008220753	0.919822691566618
**DLBC**	KDM4C	cg05545111	-0.287627078632649	48	-2.03685415872004	0.0474433670475965	0.0827293363627522	0.138877532213302
**DLBC**	KDM4C	cg14196303	0.244584790580421	48	1.71081581364148	0.0938519777497413	0.0598217197832686	0.227598140748258
**DLBC**	KDM4C	cg14473811	0.00943021855726026	48	0.063961698157195	0.949277871722859	8.89290220376958e-05	0.973118436185911
**DLBC**	KDM4C	cg14497348	-0.255711428258828	48	-1.79396289050757	0.0793934033284703	0.0653883345421696	0.201908749130407
**DLBC**	KDM4E	cg15555590	0.112023136498219	48	0.764590517019371	0.448420130297701	0.0125491831108986	0.639766527508524
**DLBC**	KDM4E	cg19409026	0.471973307431899	48	3.63093477579711	0.000707460864246195	0.222758802928206	0.00503119210834341
**DLBC**	KDM5B	cg00528052	-0.520986158246398	48	-4.13969589156045	0.000146755517757834	0.271426577084341	0.00133684997054484
**DLBC**	KDM5B	cg00571448	-0.461177682537924	48	-3.52511180182143	0.000970351738384119	0.21268485487105	0.00654762278912778
**DLBC**	KDM5B	cg02624558	-0.414443430444667	48	-3.0886376085118	0.00340480514672992	0.171763357038743	0.0182331803940736
**DLBC**	KDM5B	cg04504182	-0.442214029248299	48	-3.34397314442217	0.00164974444325333	0.195553247664015	0.0101639517873168
**DLBC**	KDM5B	cg08915090	-0.431421411855546	48	-3.24340817164297	0.00220210116186081	0.186124434607433	0.0128360750626255
**DLBC**	KDM5B	cg09889216	-0.309374238656231	48	-2.20652971413991	0.0323782832717922	0.0957124195441225	0.104477007293782
**DLBC**	KDM5B	cg11171004	-0.222289838829762	48	-1.54633127417891	0.128877420445804	0.0494127724469615	0.284341748331279
**DLBC**	KDM5B	cg11221497	-0.410604923225375	48	-3.05419750903308	0.00374593395110913	0.168596402976916	0.0196979306335395
**DLBC**	KDM5B	cg13134514	-0.23708962462277	48	-1.65521388960238	0.104688983615753	0.056211490103766	0.246136810668638
**DLBC**	KDM5B	cg13253785	-0.4680116061706	48	-3.59186260432035	0.00079539408339173	0.219034863510385	0.0055420409604457
**DLBC**	KDM5B	cg13993945	-0.358363996541426	48	-2.60345960468409	0.0123823723093868	0.128424754017143	0.050383088625788
**DLBC**	KDM5B	cg14560895	-0.37109909929173	48	-2.71046176288668	0.00941158911148042	0.137714541495133	0.0406442302286282
**DLBC**	KDM5B	cg21105875	0.106490533195605	48	0.726384363809587	0.471283366962778	0.0113402336602843	0.659595377015071
**DLBC**	KDM5B	cg24354901	-0.353986620453362	48	-2.56707064484274	0.0135738832216381	0.125306527459992	0.0540966532450877
**DLBC**	KDM5B	cg27531483	-0.392889914581632	48	-2.89772839085225	0.00573936900742049	0.154362484979962	0.0276840570785757
**DLBC**	KDM5C	cg01859586	-0.216177226341681	48	-1.5016941330292	0.140010053071716	0.0467325931887824	0.301356889817146
**DLBC**	KDM5C	cg04927657	0.0455303896704017	48	0.309122701442456	0.758624031944732	0.00207301638353862	0.865515717625705
**DLBC**	KDM5C	cg04927982	-0.532347530277201	48	-4.26514604786774	9.83847231224497e-05	0.283393892992235	0.000950775298209671
**DLBC**	KDM5C	cg09554224	0.066412564901195	48	0.451428572280429	0.653801886171023	0.00441062877675544	0.797970848817656
**DLBC**	KDM5C	cg12234996	-0.602043027387345	48	-5.11388902779482	5.99232749885437e-06	0.362455806825719	8.34489327754619e-05

DLBC	KDM5C	cg16417926	-0.322971054180887	48	-2.31453486576116	0.0251555729119364	0.104310301838713	0.0866343701189422
DLBC	KDM5C	cg16824069	-0.598870423399136	48	-5.07180799361079	6.90524108026758e-06	0.35864578402226	9.43922552448315e-05
DLBC	KDM5C	cg19903753	-0.697096047162732	48	-6.59425678054622	3.70728967930674e-08	0.485942898969906	8.95389299323155e-07
DLBC	KDM5C	cg24985300	0.23265433159855	48	1.6224595665317	0.111539735073914	0.0541280380115679	0.257404988144174
DLBC	KDM5C	cg27526317	-0.532198607746608	48	-4.26348130750087	9.89109192565044e-05	0.283235358087428	0.000954911398937764
DLBC	KDM6A	cg00751785	-0.41745007215152	48	-3.11575280522469	0.00315700911394971	0.174264562739309	0.0171709688184719
DLBC	KDM6A	cg03267667	-0.287994964160854	48	-2.03969482719975	0.0471478963666189	0.0829410993820113	0.13823628877039
DLBC	KDM6A	cg03478540	-0.403340228154483	48	-2.98954840167655	0.00447450960927058	0.16268333964771	0.022694614717929
DLBC	KDM6A	cg06877198	-0.101828552842747	48	-0.69424355066418	0.491020992930246	0.0103690541740481	0.676708639597365
DLBC	KDM6A	cg07167981	-0.573141359701583	48	-4.74367309557037	2.0664418586285e-05	0.328491018200579	0.000246597297054986
DLBC	KDM6A	cg11895950	-0.632601922141887	48	-5.53988604412101	1.40861692881817e-06	0.40018519189761	2.31283126552303e-05
DLBC	KDM6A	cg14384228	-0.165537711532397	48	-1.13843788722114	0.260832181860702	0.0274027339393832	0.45607378471312
DLBC	KDM6A	cg15923127	0.0482395635450912	48	0.327557982988053	0.744732035978757	0.00232705549102089	0.85657211982533
DLBC	KDM6A	cg17824914	0.0740992396314682	48	0.503950920016194	0.616701084042614	0.00549069731396175	0.772268630491302
DLBC	KDM6A	cg20211377	-0.035638516258335	48	-0.241865823530896	0.809959160815985	0.00127010384109561	0.897347730731703
DLBC	KDM6A	cg22133177	-0.293671795835022	48	-2.08365506343633	0.0427751727901819	0.0862431236689671	0.128614841419217
DLBC	KDM6A	cg27306636	-0.578028661990611	48	-4.80429171331922	1.68999178996126e-05	0.334117134082656	0.00020684584850259
DLBC	KDM6B	cg01046511	-0.153347833678834	48	-1.05250434383708	0.298065787941961	0.0235155580939913	0.496270223164902
DLBC	KDM6B	cg02308232	-0.0909206299918185	48	-0.619218429667967	0.538827352505009	0.00826656095810917	0.714452350996731
DLBC	KDM6B	cg24893073	-0.0535599417884561	48	-0.363783360559203	0.717686504157237	0.0028686673643828	0.839675508398125
DLBC	KDM8	cg00636124	0.0523189939451209	48	0.355331334743098	0.723965590545659	0.0027372771274296	0.843600978424491
DLBC	KDM8	cg01755539	-0.303050096325386	48	-2.1568105772482	0.0362809991147697	0.0918393608828259	0.113923069430248
DLBC	KDM8	cg06329197	-0.0398985735983555	48	-0.27082093726255	0.787740295839983	0.00159189617518339	0.883080181036348
DLBC	KDM8	cg08572679	-0.329171501586753	48	-2.3643125188669	0.0223404515059757	0.108353877456878	0.0791129408437669
DLBC	KDM8	cg09140090	-0.119244370974664	48	-0.814566658290229	0.419517599628573	0.0142192200091433	0.614856198223501
DLBC	KDM8	cg10214171	-0.235961793839654	48	-1.6468746470974	0.106399593181838	0.0556779681520273	0.248932834317474
DLBC	KDM8	cg10221365	-0.394281559194232	48	-2.90987773534323	0.0055547112151823	0.155457947920635	0.0269722991245173
DLBC	KDM8	cg16752029	-0.201707902584526	48	-1.3967589270381	0.169188887978386	0.0406860779650486	0.342607628115683
DLBC	KDM8	cg24206694	0.111429370921645	48	0.760486804585419	0.45084426577695	0.0124165047039935	0.641554983046145
DLBC	KDM8	cg24705286	0.197242648111177	48	1.36457215563661	0.179026063649359	0.0389046622339097	0.355783716835822
DLBC	KIAA202(	cg03495688	-0.469686221872426	48	-3.60834429443898	0.000757094024966425	0.220605147016794	0.00531969724434011
DLBC	KIAA202(	cg25608569	-0.446228955479881	48	-3.38183968132437	0.00147812453089717	0.199120280708666	0.00928905563911578
DLBC	KMT2B	cg09284703	-0.189084827838092	48	-1.30599485603551	0.198047685875351	0.0357530721185607	0.380606124883705
DLBC	KMT2B	cg13830730	-0.679001924160261	48	-6.27296853804509	1.12730371264648e-07	0.461043613013337	2.4212442026952e-06
DLBC	KMT2B	cg15496871	0.262462344815535	48	1.84478009716929	0.0715137726910935	0.0688864824460689	0.187244513557342
DLBC	KMT2B	cg24471867	-0.325509840124651	48	-2.33487562024965	0.0239689671613381	0.105956656017976	0.0835076282569365
DLBC	L3MBTL1	cg01071811	-0.233777097831994	48	-1.63074089424358	0.109774025384021	0.0546517314707495	0.254599026018858
DLBC	L3MBTL1	cg01877937	-0.0247580940293876	48	-0.167969050776827	0.867343955481908	0.000612963219967999	0.929485703480495
DLBC	L3MBTL1	cg02177317	-0.384351424910824	48	-2.8236950725044	0.00699383389463231	0.147726017830981	0.032371557240687
DLBC	L3MBTL1	cg02472486	-0.166394546493549	48	-1.14449787442687	0.258337656880725	0.027687145102794	0.452943873774603
DLBC	L3MBTL1	cg02611863	-0.078193502620146	48	-0.531962900908333	0.59731171007045	0.00611422385200678	0.758215993688841
DLBC	L3MBTL1	cg03486259	-0.177207547106906	48	-1.22120749598078	0.228229790705866	0.0314025147516462	0.418116718589208
DLBC	L3MBTL1	cg04984575	-0.0138126053349081	48	-0.0936905852571451	0.925761848701471	0.000190788066137931	0.960902977463105
DLBC	L3MBTL1	cg06362543	0.0890300393371394	48	0.606238513862507	0.547336593701703	0.00792634790437258	0.72070925813186
DLBC	L3MBTL1	cg06446163	0.0109951901629058	48	0.0745775160431166	0.940874243906786	0.000120894206718461	0.96840938398096
DLBC	L3MBTL1	cg06908806	-0.0849605224964765	48	-0.578321324981063	0.565867969651493	0.00721829038287429	0.735045381139579
DLBC	L3MBTL1	cg07678583	0.00264581475153036	48	0.0179448515292018	0.985760458485461	7.00033569941563e-06	0.992553031623472
DLBC	L3MBTL1	cg08369943	-0.0792358596102327	48	-0.539098728527777	0.592418532378956	0.0062783214481725	0.754738668389835
DLBC	L3MBTL1	cg08633313	-0.068031547730622	48	-0.462483902363093	0.645914615506908	0.0046282914866239	0.792382751637907
DLBC	L3MBTL1	cg09541000	-0.320264842545485	48	-2.2929141057311	0.0264741688347081	0.102569569370684	0.0899280189646007
DLBC	L3MBTL1	cg10360552	-0.251014901585365	48	-1.75877624067593	0.0852665441753651	0.0630084808179104	0.212521643521029
DLBC	L3MBTL1	cg12699433	0.0114150432911675	48	0.077425634941208	0.938620749749729	0.000130303213339227	0.967199618704618
DLBC	L3MBTL1	cg14306330	-0.224969202261672	48	-1.5659573115129	0.124212815295116	0.0506111419662531	0.277234593403191
DLBC	L3MBTL1	cg15123011	0.0290480779855238	48	0.197096822067982	0.844620074242362	0.000843790834653075	0.916969605554459
DLBC	L3MBTL1	cg15302378	-0.222055872691066	48	-1.54461925614819	0.129290903734341	0.0493088105965909	0.285076145488048
DLBC	L3MBTL1	cg15330298	0.0247508446998919	48	0.167919838203963	0.867382450105985	0.000612604313358166	0.929511334513563
DLBC	L3MBTL1	cg15388309	0.0304065489072797	48	0.20632264904339	0.837449389219664	0.000924558216450793	0.912981554765472

**DLBC**	L3MBTL1	cg16862791	-0.0709187969909493	48	-0.482208840606246	0.631944222547551	0.00502947576664348	0.783006462394026
**DLBC**	L3MBTL1	cg17091610	-0.0789803252293673	48	-0.537349212799882	0.593616451510057	0.00623789177333663	0.755530353163336
**DLBC**	L3MBTL1	cg17774347	-0.0079667700577199	48	-0.0540349782428519	0.95714129502734	6.34694251525824e-05	0.977445586722635
**DLBC**	L3MBTL1	cg18911945	-0.274015735666504	48	-1.93242843536897	0.0594779892135604	0.0750846233928555	0.163640499562622
**DLBC**	L3MBTL1	cg20091959	-0.336192489789739	48	-2.42109220420776	0.0194772638290472	0.113025390191024	0.0713377712955224
**DLBC**	L3MBTL1	cg20252111	-0.253267917108494	48	-1.7756392156881	0.0824080713215551	0.064144637836475	0.207350200543019
**DLBC**	L3MBTL1	cg20529070	-0.105270636125451	48	-0.71796951045283	0.476406836092875	0.0110819068302571	0.66416889083567
**DLBC**	L3MBTL1	cg22330467	-0.233309775039529	48	-1.62729316697755	0.110506343823711	0.0544334511289955	0.255839534113783
**DLBC**	L3MBTL1	cg22457903	-0.0135682758098899	48	-0.0920329957848839	0.927071464255214	0.000184098108453242	0.961483612813065
**DLBC**	L3MBTL1	cg22601123	-0.019135405810086	48	-0.129806403941082	0.897285560604208	0.000366163755516675	0.945641122108747
**DLBC**	L3MBTL1	cg23626798	-0.00235676844551193	48	-0.0159844256829481	0.987315945703226	5.55435750576072e-06	0.993269806321138
**DLBC**	L3MBTL1	cg24343973	-0.00399910337969279	48	-0.0271234556489535	0.978478597697258	1.59928278414703e-05	0.98883804712013
**DLBC**	L3MBTL2	cg02358112	0.092107699166829	48	0.627371735242606	0.533517530483009	0.00848382824580708	0.710583347881238
**DLBC**	L3MBTL2	cg06235991	0.143392055272741	48	0.982687363308738	0.330901861507358	0.0205612815153408	0.530640347882727
**DLBC**	L3MBTL2	cg08053598	0.224849109257427	48	1.56507684692251	0.124419124839495	0.0505571219338585	0.277488070805416
**DLBC**	L3MBTL2	cg09993119	0.239087241851986	48	1.67000181122445	0.101711061049683	0.05716270921639	0.241168895220794
**DLBC**	L3MBTL2	cg12821546	0.213044106988487	48	1.47888685722521	0.145986322856097	0.045387791522522	0.309997713338002
**DLBC**	L3MBTL2	cg13843727	0.235260764005744	48	1.64169474857336	0.107473609619655	0.0553476270805662	0.250871725230337
**DLBC**	L3MBTL2	cg14679202	-0.0628052602946009	48	-0.42680860515454	0.671509753728133	0.00394450072067257	0.809729052521642
[truncated: 945,095 more chars]
